# Systems proteomic analysis reveals that clusterin and tissue inhibitor of metalloproteinases 3 increase in leptomeningeal arteries affected by cerebral amyloid angiopathy

**DOI:** 10.1111/nan.12342

**Published:** 2016-10-05

**Authors:** A. Manousopoulou, M. Gatherer, C. Smith, J. A. R. Nicoll, C. H. Woelk, M. Johnson, R. Kalaria, J. Attems, S. D. Garbis, R. O. Carare

**Affiliations:** ^1^ Clinical and Experimental Sciences Unit Faculty of Medicine University of Southampton Southampton UK; ^2^ Institute for Life Sciences University of Southampton Southampton UK; ^3^ Pathology Department University of Edinburgh Edinburgh UK; ^4^ Institute of Neuroscience Newcastle University Newcastle upon Tyne UK; ^5^ Cancer Sciences Unit Faculty of Medicine University of Southampton Southampton UK

**Keywords:** clusterin, complement pathway, extracellular matrix remodelling, leptomeningeal arteries, proteomics, TIMP3

## Abstract

**Aims:**

Amyloid beta (Aβ) accumulation in the walls of leptomeningeal arteries as cerebral amyloid angiopathy (CAA) is a major feature of Alzheimer's disease. In this study, we used global quantitative proteomic analysis to examine the hypothesis that the leptomeningeal arteries derived from patients with CAA have a distinct endophenotypic profile compared to those from young and elderly controls.

**Methods:**

Freshly dissected leptomeningeal arteries from the Newcastle Brain Tissue Resource and Edinburgh Sudden Death Brain Bank from seven elderly (82.9 ± 7.5 years) females with severe capillary and arterial CAA, as well as seven elderly (88.3 ± 8.6 years) and five young (45.4 ± 3.9 years) females without CAA were used in this study. Arteries from four patients with CAA, two young and two elderly controls were individually analysed using quantitative proteomics. Key proteomic findings were then validated using immunohistochemistry.

**Results:**

Bioinformatics interpretation of the results showed a significant enrichment of the immune response/classical complement and extracellular matrix remodelling pathways (*P* < 0.05) in arteries affected by CAA *vs*. those from young and elderly controls. Clusterin (apolipoprotein J) and tissue inhibitor of metalloproteinases‐3 (TIMP3), validated using immunohistochemistry, were shown to co‐localize with Aβ and to be up‐regulated in leptomeningeal arteries from CAA patients compared to young and elderly controls.

**Conclusions:**

Global proteomic profiling of brain leptomeningeal arteries revealed that clusterin and TIMP3 increase in leptomeningeal arteries affected by CAA. We propose that clusterin and TIMP3 could facilitate perivascular clearance and may serve as novel candidate therapeutic targets for CAA.

## Introduction

The deposition of amyloid‐β (Aβ) peptides in the walls of cerebral arteries as cerebral amyloid angiopathy (CAA) is a major feature of Alzheimer's disease and may contribute to cognitive decline 1, 2. CAA predominantly affects the leptomeningeal and cortical arteries especially in the occipital lobe, while capillaries are less frequently and veins rarely involved 3, 4, 5. In the majority of cases there is no overproduction of Aβ in the vessel wall, suggesting that the deposition of Aβ in the walls of cerebral arteries is a result of a failure of elimination of neuronally derived Aβ 6. Increasing age and possession of at least one apolipoprotein ε4 (APOE4) allele are risk factors for CAA and both have been suggested to impair cerebral Aβ clearance systems, thereby reducing Aβ elimination from the brain 7, 8, 9, 10. We have demonstrated that Aβ and other solutes are eliminated along the basement membranes of capillaries and arteries, effectively the lymphatic drainage of the brain 11. Experimental work involving intraparenchymal injections of tracers demonstrated that the biochemical structure and morphology of the basement membranes of capillaries and arteries change with age and with possession of APOE4 genotype, resulting in failure of efficient clearance of Aβ 12, 13, 14. The exact targets for the facilitation of perivascular clearance of Aβ are not clear.

Proteomics allows the in‐depth and global assessment of gene products at the protein level as they occur in a variety of biological specimens, including cell lines, tissue, blood and proximal fluids. The advanced use of liquid chromatography combined with mass spectrometry permits the identification of thousands of proteins with ultra‐high precision and sensitivity, not available by any other analytical approach. Using stable isotope isobaric reagents allow such proteomes to be profiled in parallel across multiple biological or clinical states under identical analytical conditions, a feature referred to as the multiplex advantage 15, 16, 17, 18, 19, 20, 21, 22, 23. For example, such a strategy allows the comparison of a given *in vitro* or *in vivo* model under a given homeostatic state (that is physiological condition) relative to a perturbation state (that is pathological condition or exposure to a stimulus) under exactly the same experimental conditions.

This study employed isobaric quantitative proteomic analysis of fresh frozen human leptomeningeal arteries from young and elderly subjects and patients with CAA to test the hypothesis that leptomeningeal arteries derived from patients with CAA have a unique endophenotypic profile compared to those from young and elderly controls.

## Materials and methods

### Isolation of human leptomeningeal arteries

Human fresh frozen *post mortem* leptomeningeal arteries from the Newcastle Brain Tissue Resource and MRC Sudden Death Brain & Tissue Bank (Edinburgh) were used for this study. CAA cases were diagnosed *post mortem* by JA, according to published criteria including the neuritic Braak stages 24, Thal amyloid phases 25, CERAD scores 26, NIA‐AA scores 27 and McKeith criteria 28 and showed varying degrees of Alzheimer's disease pathology. For CAA we used a recently developed staging system, which assesses meningeal and parenchymal CAA separately and also scores capillary CAA 1, 2. All CAA cases had severe CAA as they showed widespread circumferential Aβ in meningeal and cortical arterial vessels as well as Aβ depositions in capillary walls. None of the cases was diagnosed with CAA during their lifetime. The cases from the MRC Sudden Death Brain & Tissue Bank (Edinburgh) had no neurological disease during life and no significant neuropathological changes *post mortem*. We excluded cases with arteriolosclerosis/lipohyalinosis from this cohort. Samples were collected and prepared in accordance with the National Research Ethics Service‐approved protocols. Leptomeningeal arteries in the occipital regions were removed from the frozen coronal slices from brains of young females (45.4 ± 3.9 years; *n* = 5), elderly females without CAA (88.3 ± 8.6 years; *n* = 7) and females with severe CAA (82.6 ± 7.5 years; *n* = 7) (Table 1). Only female subjects were included in the present study as it has been shown that sex‐dependent differences exist in CAA 29, 30, 31. The frozen coronal slices were placed at −20°C overnight to acclimatize from the −70°C storage prior to dissection in a cold cabinet at −12°C. Arteries were identified based on their morphology of a vessel and they were distinguished from veins by the thicker wall and leptomeningeal sheet as they penetrate the cortex. The abundant presence of vascular smooth muscle actin confirmed they were arteries. Selected vessels were eased with a micro‐scalpel from the meningeal surface of the gyri and sulci, removed and placed in pre‐cooled tubes to avoid thawing. These specimens were then snap frozen at −80°C.

**Table 1:** Details of *post mortem* samples

Sample #	Study group	Age (years)	Used in proteomic analysis	Braak stage	Thal amyloid phase	*Post mortem* delay (h)	Cause of death	Duration of dementia (years)	CAA inflammation/vasculitis
1	Young control	51	Yes	0	Not applicable	81	Metastatic carcinoma	0	Not applicable
2	Young control	46	Yes	0	Not applicable	49	Myocardial infarction; coronary artery thrombosis; coronary artery atherosclerosis	0	Not applicable
3	Young control	45	No	0	Not applicable	93	Coronary artery atherosclerosis	0	Not applicable
4	Young control	40	No	0	Not applicable	77	Bronchial asthma	0	Not applicable
5	Young control	45	No	0	Not applicable	40	Suspension by ligature	0	Not applicable
6	Elderly control	79	Yes	IV	3	9	Old age, dementia with Parkinson's disease	9	Mild, some vessels with perivascular infiltrate
7	Elderly control	88	Yes	III	0	22	Aspiration pneumonia; total anterior circulation stroke	Not available	Not remarkable
8	Elderly control	74	No	III	1	53	Heart failure and lung cancer	Not available	Not remarkable
9	Elderly control	94	No	II	1	15	Left ventricle failure; ischaemic heart disease	Not available	Not remarkable
10	Elderly control	95	No	III	0	66	Ischaemic bowel disease (inoperable)	Not available	Not remarkable
11	Elderly control	96	No	II	3	114	Stroke and left ventricular failure	2 (mild)	Not remarkable
12	Elderly control	92	No	VI	5	74	Pneumonia	>2	Not remarkable
13	CAA case	93	Yes	VI	5	53	Stroke, general deterioration	13	Mild, some vessels with perivascular infiltrate
14	CAA case	73	Yes	IV	5	47	Frontal lobe dementia	1.3	Not remarkable
15	CAA case	76	Yes	VI	3	37	not applicable	8	Not remarkable
16	CAA case	87	Yes	VI	5	54	Aspiration pneumonia secondary to stroke	8	Not remarkable
17	CAA case	86	No	VI	5	47	not applicable	6	Not remarkable
18	CAA case	77	No	VI	2	63	Aspiration pneumonia	14	Not remarkable
19	CAA case	88	No	VI	5	84	Bronchopneumonia	15	Not remarkable

CAA, cerebral amyloid angiopathy.

### Quantitative proteomic analysis on human leptomeningeal arteries

**Figure 1:**
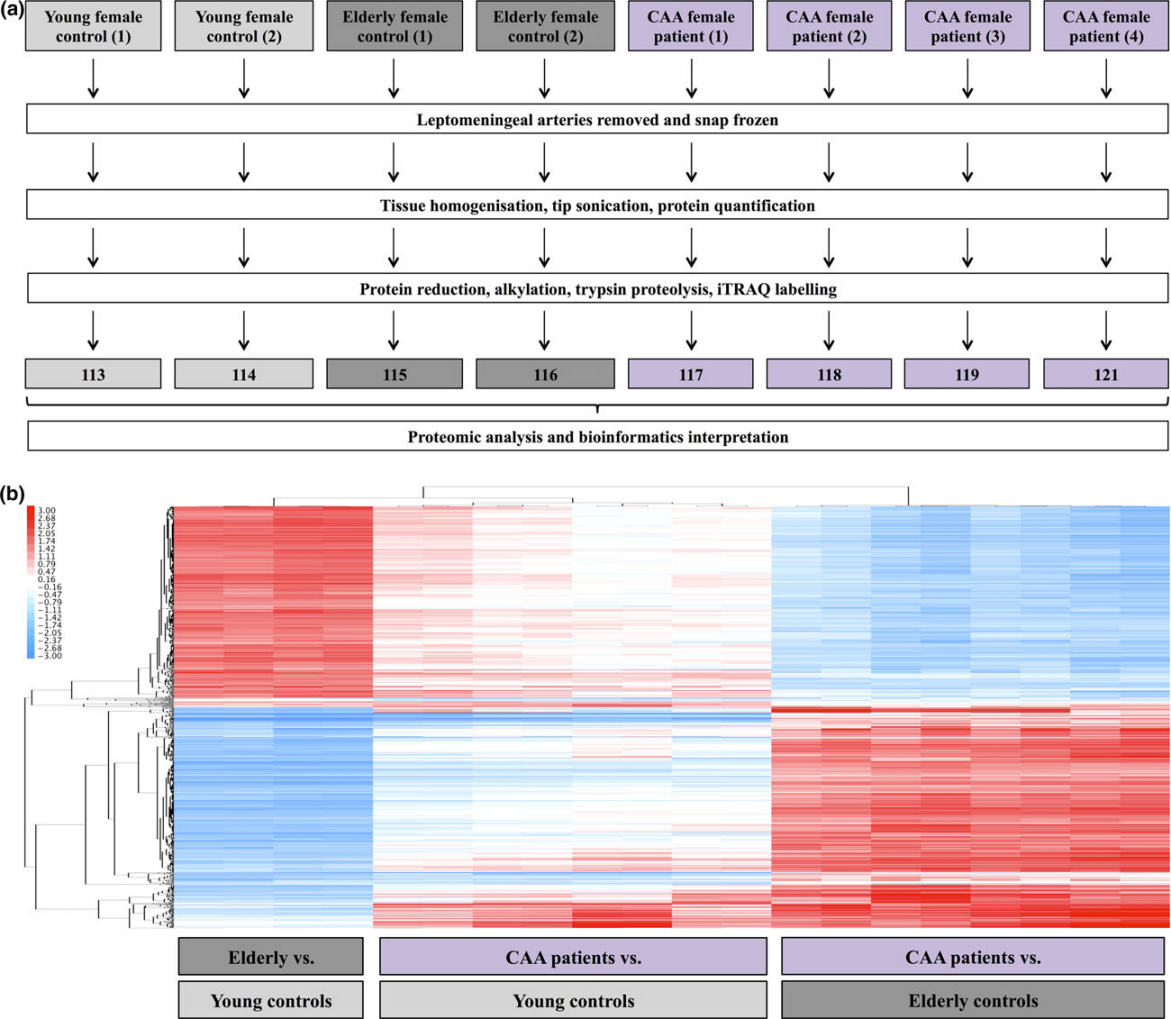
(**a**) Experimental pipeline of proteomics experiment. (**b**) Heatmap of differentially expressed proteins in leptomeningeal arteries of elderly controls compared to young controls, cerebral amyloid angiopathy (CAA) patients compared to young controls and CAA patients compared to elderly controls.

For the proteomic analysis, samples from two young and two elderly subjects and four patients with CAA were randomly selected from the cohort (Table 1). The justification for this number of CAA cases was to compensate for their innate tissue heterogeneity and to ensure a statistical power of over 0.7, factoring in a representative 30% measurement error and a fold change >1.5 between replicate observations, as reported in a recent simulation study 32. Samples were dissolved in dissolution buffer (0.5 M triethylammonium bicarbonate/0.05% sodium dodecyl sulphate), homogenized using the FastPrep system (Savant Bio, Cedex, Fr) and then subjected to pulsed probe sonication (Misonix, Farmingdale, NY, USA). Lysates were centrifuged (16 000 *g*, 10 min, 4°C) and supernatants were measured for protein content using the Direct Detect^™^ Spectroscopy system (Merck Millipore, Darmstadt, Germany) according to the manufacturer's instructions. From each lysate volume (adjusted to the highest volume of 40 μl) containing 100 μg final protein content was subjected to reduction, alkylation, trypsin proteolysis and eight‐plex isobaric tag for relative and absolute quantitation (iTRAQ AbSciex, San Hose, CA, USA) labelling per supplier's specifications (ABSciex, San Hose, CA, USA). Labelled peptides were pooled and fractionated with high‐pH reversed‐phase (RP) chromatography using the Waters, XBridge C8 column (150 × 3 mm, 3.5 μm particle) with the Shimadzu LC‐20AD HPLC (Shimadzu, Kyoto, Japan). Each resulting fraction was LC‐MS analysed with low‐pH RP capillary chromatography (PepMap C18, 50 μm ID × 50 cm L, 100 Å pore, 3.5 μm particle) and nanospray ionization FT‐MS (Ultimate 3000 UHPLC – LTQ‐Velos Pro Orbitrap Elite, Thermo Scientific, Bremen, DE) as reported previously 19, 20, 23 (Figure 1 **a**).

Unprocessed raw files were submitted to Proteome Discoverer 1.4 for target decoy searching with SequestHT for tryptic peptides as reported by the authors 19, 20, 23. Quantification ratios were normalized on the median value and log2 transformed. A protein was considered modulated in leptomeningeal arteries from elderly subjects *vs*. young controls or those affected by CAA type 1 relative to these from young and elderly controls when its log2 ratio was above or below ±1 SD across all analysed samples per category as reported previously 23.

Hierarchical clustering analysis visualized in heatmap format was generated using Gene Cluster (version 3.0) and Java Treeview (version 1.1.6r4). MetaCore (GeneGo, St. Joseph, MI, USA) and DAVID (http://david.abcc.ncifcrf.gov) were applied to identify prebuilt processed networks and gene ontology terms over‐represented in the modulated proteome. False discovery rate (FDR) and Fisher's exact corrected *P*‐values ≤0.05 were considered significant.‬‬‬‬‬‬‬‬‬‬‬‬‬‬‬‬‬‬‬

### Immunohistochemistry

The immunochemistry validation of key proteomic findings was performed in all 19 subjects (young female controls: *n* = 5, elderly female controls: *n* = 7, females with CAA type 1: *n* = 7). Three sections of occipital cortex from each of the cases were immunostained. After dewaxing in xylene and rehydration through graded alcohols, antigen retrieval was performed by immersing slides in citrate buffer, microwaving on medium power for 25 min and subsequently cooling. This was followed by incubation in pepsin for 5 min (1 mg/ml 0.2 M HCl). The tissue was blocked in 3% H_2_O_2_ and 15% goat serum. Occipital cortex from each of the cases was incubated in clusterin (Abcam: Cambridge, UK, ab42673, rabbit polyclonal, dilution 1:500), or tissue inhibitor of metalloproteinases 3 (TIMP3) (Abcam, Ab93637, rabbit polyclonal, dilution 1:100) overnight at 4°C followed by biotinylated goat anti‐rabbit antibodies (Vector BA1000 dilution 1:200) and ABC peroxidase enzyme complex (Vector PK4000, dilution 1:500). Reaction was detected using diaminobenzidine with glucose oxidase enhancement. Images were captured an Olympus: Southend‐on‐Sea, Essex, UK, BX51 microscope fitted with Olympus CC‐12 colour microscope camera.

Double immunofluorescence was performed for Aβ and TIMP3. Prior to the antigen retrieval previously described, pre‐treatment was required which consisted of 5 min in formic acid at 37°C. Tissue was blocked in 15% goat serum followed by incubation in primary antibodies overnight at 4°C. Aβ was detected using mouse monoclonal anti‐Aβ IgG2b Clone 4G8, antibody (BioLegend: London, UK, 800701; dilution 1:100). The secondary antibody for Aβ was goat anti‐mouse IgG2b, AlexaFluor 647 (A‐21242), and for TIMP 3 and clusterin was goat anti‐rabbit IgG AlexaFluor 594 (A‐27096). These were obtained from Thermo Fisher Scientific and dilution 1:200. Images were captured and examined with a Leica SP8 confocal microscope. The specificity of the immunohistochemistry staining was confirmed by omitting the primary antibody.

## Results

### Quantitative proteomic analysis

The proteomic analysis resulted in the profiling of 5957 proteins (peptide FDR confidence ≥ 99%) (Table S1). A total of 1364 proteins were differentially expressed in arteries from elderly relative to young subjects (Table S2), 280 in arteries from CAA cases relative to young controls (Table S3) and another 983 in arteries from CAA cases relative to elderly controls (Table S4). The hierarchical clustering analysis of differentially expressed proteins between groups revealed that leptomeningeal arteries derived from CAA patients compared to those from young and elderly controls had a distinct proteomic profile from arteries derived from elderly compared to young subjects (Figure 1 **b**).

**Figure 2:**
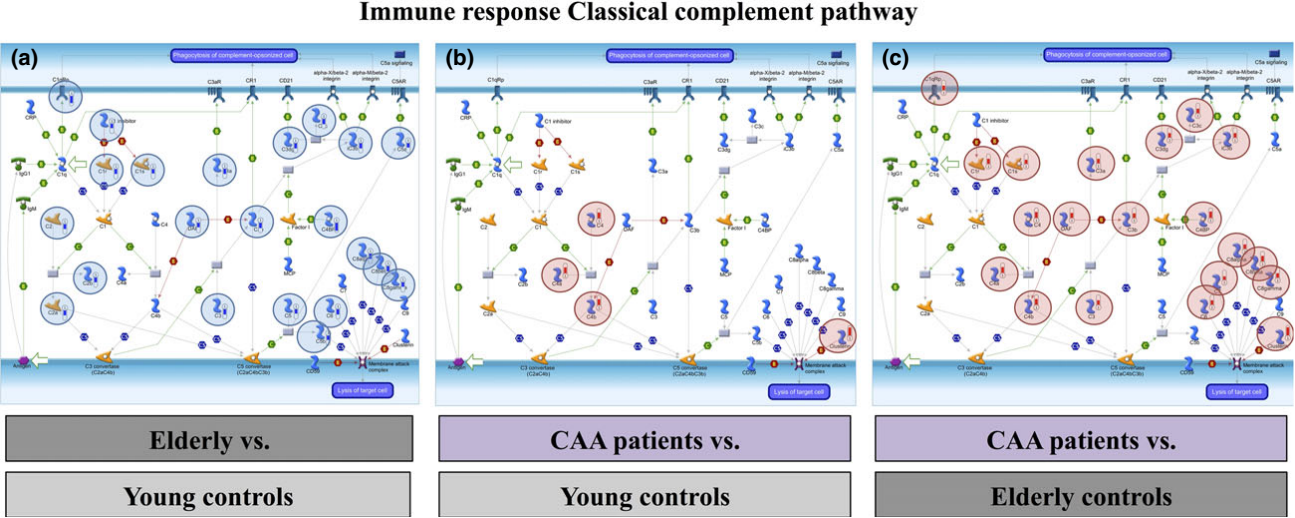
The immune response/classical complement pathway was significantly enriched in the differentially expressed proteome of leptomeningeal arteries from elderly *vs*. young controls (*P* = 5.0E‐11) (**a**), CAA patients compared to young controls (*P* = 5.007E‐2) (**b**) and cerebral amyloid angiopathy patients compared to elderly controls (*P* = 1.168E‐10) (**c**).

**Figure 3:**
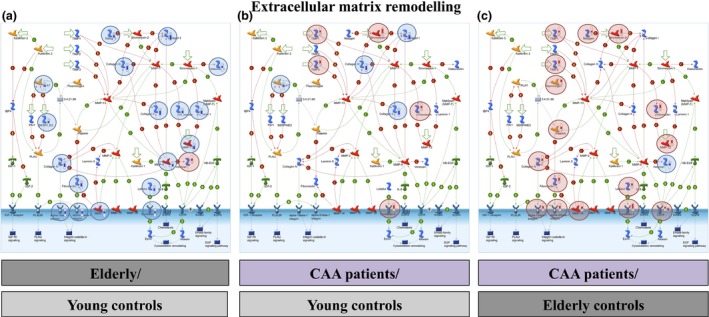
The extracellular matrix remodelling pathway was significantly enriched in the differentially expressed proteome of leptomeningeal arteries from elderly compared to young controls (*P* = 3.3E‐8) (**a**), cerebral amyloid angiopathy (CAA) patients compared to young controls (*P* = 6.349E‐6) (**b**) and CAA patients compared to elderly controls (*P* = 2.317E‐8) (**c**).

*In silico* bioinformatics analysis showed that the *immune response/classical complement pathway* (*P* = 5.0E‐11; 5.007E‐2; 1.168E‐10 in elderly *vs*. young controls; CAA *vs*. young controls; CAA *vs*. elderly controls respectively) (Figure 2) and *extracellular matrix remodelling* (*P* = 3.3E‐8; 6.349E‐6; 2.317E‐8 in elderly *vs*. young controls; CAA *vs*. young controls; CAA *vs*. elderly controls respectively) (Figure 3) were significantly over‐represented processes. For both pathways, the expression levels of most proteins were found to decrease in arteries from elderly *vs*. young controls, whereas they increased in arteries from CAA patients compared to young and elderly controls.

The expression of clusterin (apolipoprotein J) and TIMP3 from the immune response/classical complement and the extracellular matrix remodelling pathways, respectively, were up‐regulated in arteries from patients with CAA compared to both young and elderly controls [clusterin: iTRAQ mean log2 ratio (SD) = 2.30 (0.45) and 2.87 (0.44) in CAA *vs*. young and CAA *vs*. elderly controls respectively] [TIMP3: iTRAQ mean log2 ratio (SD) = 1.63 (0.89) and 2.48 (0.90) in CAA *vs*. young and CAA *vs*. elderly controls respectively].

### Immunohistochemistry

**Figure 4:**
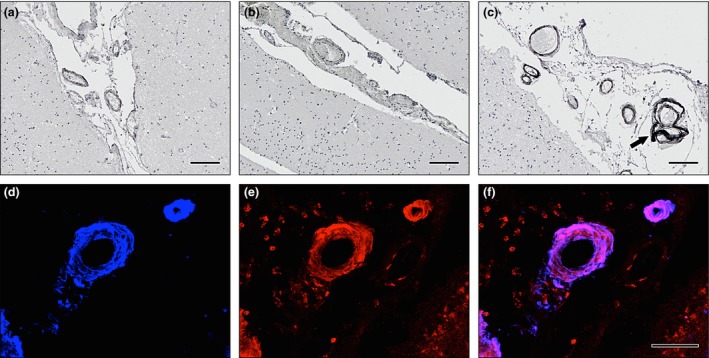
Immunohistochemistry of clusterin. DAB with haematoxylin counterstain in (**a**) young and (**b**) elderly controls and (**c**) cerebral amyloid angiopathy (CAA). The intensity of immunostaining of clusterin is increased in the leptomeningeal vessels present in the sulci in elderly control cases compared to young cases and in CAA compared to elderly control cases. Immunofluorescence for Aβ and clusterin in leptomeningeal arteries in CAA (**d**–**e**). Aβ immunofluorescence (blue) in (**d**) is present in the whole thickness of the arterial wall in a concentric manner; clusterin immunofluorescence (red) in (**e**) is also present throughout the thickness of the arterial wall; co‐localization (pink) of Aβ and clusterin occupies most of the thickness of the arterial walls in (**f**). Scale bars: (**a**–**c**) = 100 μm/(**d**–**f**) = 50 μm.

**Figure 5:**
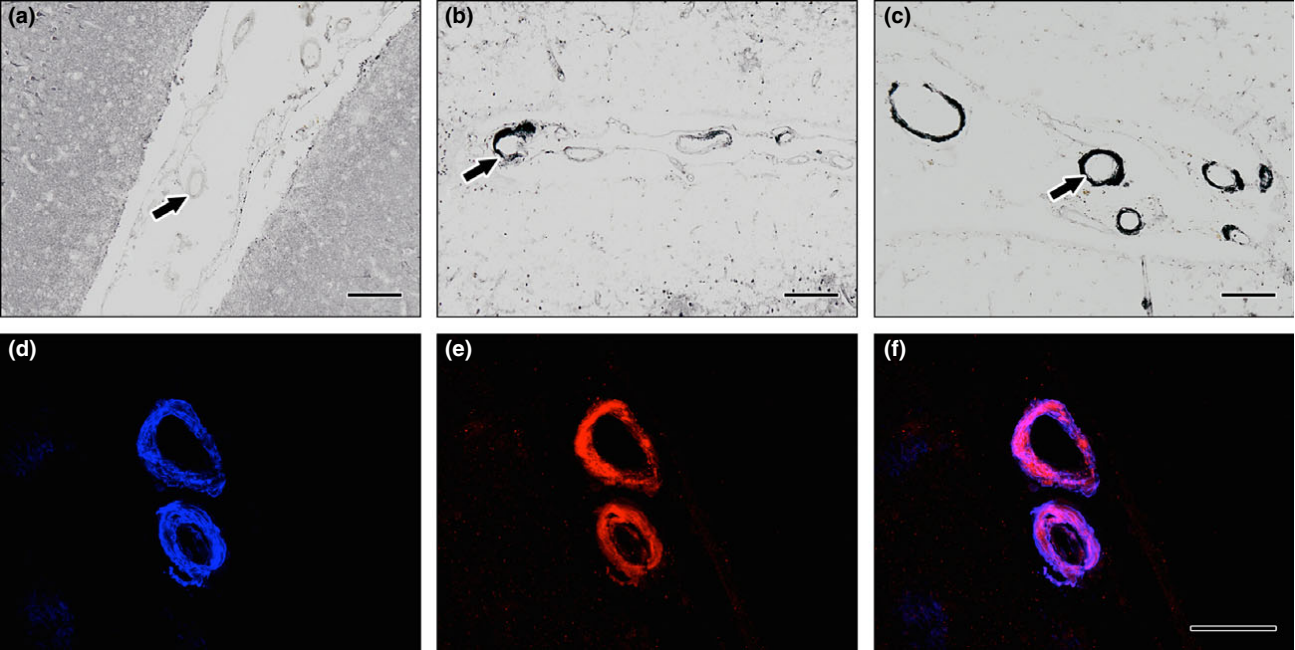
Immunohistochemistry of tissue inhibitor of metalloproteinases 3 (TIMP3) in leptomeningeal arteries. DAB with haematoxylin counterstain in (**a**) young and (**b**) elderly controls and (**c**) cerebral amyloid angiopathy (CAA). The intensity of immunostaining of TIMP3 is increased in the leptomeningeal vessels present in the sulci of elderly control cases compared to young and in CAA cases compared to elderly. Immunofluorescence for Aβ and TIMP3 in leptomeningeal arteries in CAA (**d**–**e**). Aβ immunofluorescence (blue) in (**d**) is present in the whole thickness of the arterial wall in a concentric manner; TIMP3 immunofluorescence (red) in (**e**) is also present throughout the thickness of the arterial wall; co‐localization (pink) of Aβ and TIMP3 occupies most of the thickness of the arterial walls, especially concentrated in the tunica media, with less in the endothelium and outer layers of the wall (**f**). Scale bars: (**a**–**c**) = 100 μm/(**d**–**f**) = 50 μm.

**Figure 6:**
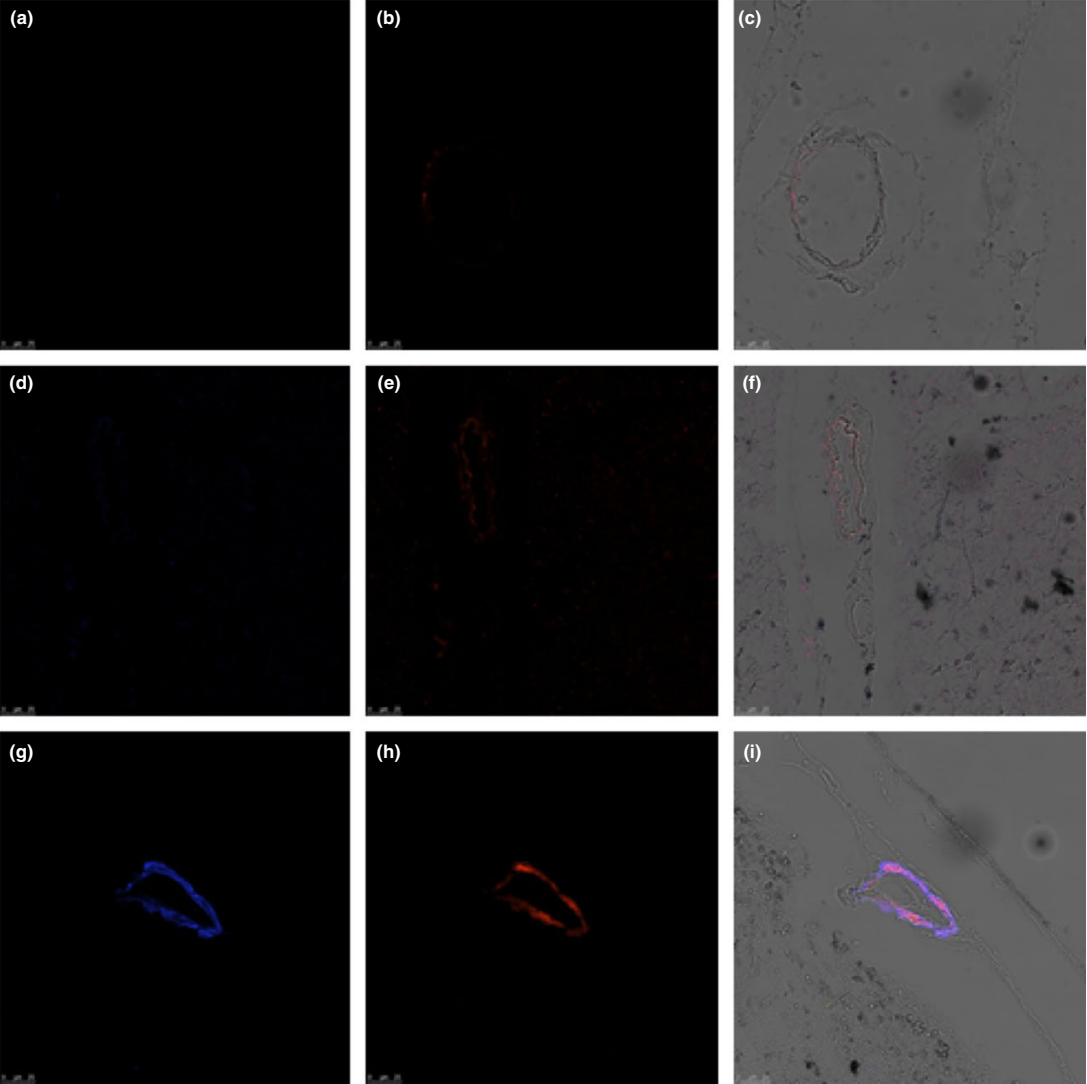
Confocal microscopy images showing distribution of tissue inhibitor of metalloproteinases 3 (TIMP3) (blue) and Aβ (red) in leptomeningeal arteries from young (**a**–**c**) and elderly females (**d**–**f**) and patients with cerebral amyloid angiopathy (CAA) (**g**–**i**). Co‐localization of Aβ and TIMP3 is observed in CAA, on transmission merged images (**c**–**i**). Images obtained with ×20 objective. False colour applied to channels.

Clusterin was found to co‐localize with Aβ in the occipital cortex of CAA cases, but not in the young or elderly controls (Figure 4). The pattern of expression for the immunocytochemistry of TIMP3 was weak in arteries from young controls, increased in elderly controls and was strong in CAA patients (Figure 5). TIMP3 and clusterin were found to co‐localize with Aβ in the leptomeningeal vessels of the occipital cortex from CAA cases (Figure 6).

## Discussion

Our study showed that the global endophenotypic profile of leptomeningeal arteries from elderly female patients with severe CAA was different from that of age‐matched and young controls. The immune response/classical complement and extracellular matrix remodelling pathways were significantly enriched in the differentially expressed proteome of arteries between patients with CAA compared to young and elderly controls. Most proteins participating in these pathways were up‐regulated in leptomeningeal arteries from patients with CAA compared to these from controls, possibly reflecting a pro‐inflammatory response in arteries affected by CAA, which could have in turn triggered tissue remodelling processes. The inflammatory profile of CAA is well characterized 33, 34 and previous studies have described an increased activation of the complement system in cerebral amyloid plaques as well as deposition of complement components in CAA affected cerebral arteries 35, 36, 37. Extracellular matrix components can influence the deposition of Aβ thus contributing to Alzheimer's disease progression 38, 39. Conversely, Aβ accumulation damages the integrity of existing extracellular matrix, which affects brain microvascular functions during the early stages of Alzheimer's disease 40, 41, 42.

The study results show that clusterin co‐localizes with Aβ within the walls of leptomeningeal arteries and its expression levels increase in leptomeningeal arteries from patients with CAA compared to those from young and elderly controls. Clusterin (apolipoprotein J or ApoJ) is a disulphide‐linked heterodimeric glycoprotein that activates microglia, initiating an inflammatory cascade 43. Genome‐wide association studies of sporadic Alzheimer's disease, in which Aβ accumulates both in cortical plaques and CAA, have highlighted the importance of common genetic variations in the gene encoding clusterin 44. Experimental work suggests that clusterin regulates Aβ fibril formation 45 and plays a major role in the clearance of Aβ42–ApoJ complexes, via LRP2 46, 47, 48. Although the predominant species of Aβ in CAA is Aβ40, with progressive failure of perivascular clearance of interstitial fluid, there is also accumulation of Aβ42 49. Clusterin appears to be sequestered with Aβ species in the vascular amyloid deposits in sporadic CAA, as well as in the white matter abnormalities in cerebral autosomal dominant arteriopathy with subcortical infarcts and leukoencephalopathy (CADASIL) 50, 51. A recent study found a significant positive correlation between clusterin concentration and regional levels of insoluble Aβ42 52. It is therefore possible that the up‐regulation of clusterin observed in the CAA arteries, is due to either entrapment of the Aβ–ApoJ complex in the perivascular drainage pathways, or a compensatory up‐regulation of ApoJ to clear the excess Aβ42 that cannot be eliminated normally.

In this study, we demonstrated that the expression of TIMP3 in the brain is restricted to the walls of leptomeningeal arteries and increases in CAA. Homeostasis of the extracellular matrix in the brain is maintained by the balanced action of matrix metalloproteinases that degrade extracellular matrix and by tissue inhibitors of metalloproteinases (TIMP) proteins. Human TIMP3 is a 25‐kDa protein that contains disulphide bonds and is expressed in normal central nervous system 53. In a study by Hoe *et al*. 54, TIMP3 expression was found to increase in human brains affected by Alzheimer's disease (AD). Furthermore, this study showed that TIMP3 prevents α‐cleavage of amyloid precursor protein (APP), whereas it promotes β‐cleavage of APP thus contributing to elevated Aβ levels in AD. TIMP3 preserves the integrity of extracellular matrix in arteries as the absence of TIMP3 in knock‐out mice results in pathological arterial vasodilation 55. Our results showed that expression of TIMP3 in the brain is restricted to the walls of leptomeningeal, thus antagonistically targeting TIMP‐3 could also facilitate perivascular drainage of Aβ. Examining this hypothesis was beyond the scope of the present study and constitutes a future objective.

In conclusion, this proteomic study demonstrates the activation of inflammatory and extracellular matrix remodelling pathways in human leptomeningeal arteries from CAA patients compared to these from cognitively normal young and elderly controls. Furthermore, we observed increased levels of clusterin and TIMP3 in leptomeningeal arteries from CAA patients compared to young and elderly controls and co‐localization of these two proteins with Aβ in the occipital cortex of the CAA cases. Future work will test the hypothesis that clusterin and TIMP3 could facilitate perivascular clearance and represent novel therapeutic targets for CAA.

## Author contributions

AM performed the proteomic experiments, interpreted the results and wrote manuscript. MG performed the immunohistochemistry experiments and interpreted the results. CHW performed the bioinformatics analysis. CS and JARN interpreted the results and edited the manuscript. MJ, RK and JA provided the samples and edited the manuscript. SDG designed the proteomic experiments, supervised their execution, interpreted the results and wrote manuscript. ROC conceived the study, funded the study, designed the immunohistochemistry experiments, interpreted the results and wrote manuscript.

## Conflicts of interest

None declared.

## Supporting information


**Table S1.** Total proteome (peptide FDR confidence > 99%) (log2 ratio).

**Table S2.** Differentially expressed proteins in leptomeningeal arteries from elderly *vs*. young controls (log2 ratio).

**Table S3.** Differentially expressed proteins in leptomeningeal arteries from CAA patients *vs*. young controls (log2 ratio).

**Table S4.** Differentially expressed proteins in leptomeningeal arteries from CAA patients *vs*. age‐matched controls (log2 ratio).
