# Supplementary material for: Systems proteomic analysis reveals that clusterin and tissue inhibitor of metalloproteinases 3 increase in leptomeningeal arteries affected by cerebral amyloid angiopathy
Source: Neuropathol Appl Neurobiol. 2016 Oct 5;43(6):492–504. doi: 10.1111/nan.12342 (PMC5638106; doi:10.1111/nan.12342)
Supplement: Supplementary file 1 — Table S1. Total proteome (peptide FDR confidence > 99%) (log2 ratio). [file NAN-43-492-s001.pdf]

Supplementary Table 1. Total proteome (peptide FDR confidence > 99%) (log2ratios)

Accession	Description	ΣCoverage	Σ# Proteins	Σ# Unique Peptides	Σ# Peptides	Σ# PSMs	OLD 1/YOUNG 1	OLD 1/YOUNG 2	OLD 2/YOUNG 1	OLD 2/YOUNG 2	CAA 1/YOUNG 1	CAA 1/YOUNG 2	CAA 2/YOUNG 1	CAA 2/YOUNG 2	CAA 3/YOUNG 1	CAA 3/YOUNG 2	CAA 4/YOUNG 1	CAA 4/YOUNG 2	CAA 1/OLD 1	CAA 1/OLD 2	CAA 2/OLD 1	CAA 2/OLD 2	CAA 3/OLD 1	CAA 3/OLD 2	CAA 4/OLD 1	CAA 4/OLD 2
P09488	Glutathione S transferase Mu 1 OS=Homo sapiens GN=GSTM1 PE=1 SV=3 - [GSTM1_HUMAN]	42.20	2	1	11	29	-1.77	-1.53	-1.70	-1.46	-0.86	-0.62	3.71	3.95	2.85	2.61	-0.96	-0.72	5.53	0.82	0.74	4.41	4.34	0.90	0.82	5.47
A8K7I4	Calcium-activated chloride channel regulator 1 OS=Homo sapiens GN=CLCA1 PE=1 SV=3 - [CLCA1_HUMAN]	0.98	1	1	1	1	-1.15	-1.20	-1.51	-1.56	4.21	4.15	3.02	2.98	2.88	2.94	-0.48	-0.53	4.23	0.68	1.03	4.12	4.48	5.34	5.70	4.60
P80511	Protein S100-A12 OS=Homo sapiens GN=S100A12 PE=1 SV=2 - [S10AC_HUMAN]	8.70	1	1	1	1	-2.50	-2.46	-2.80	-2.75	-1.43	-1.39	1.71	1.75	0.53	0.49	0.52	0.56	4.27	3.03	3.32	3.02	3.32	1.06	1.35	4.57
P30481	HLA class I histocompatibility antigen, B-44 alpha chain OS=Homo sapiens GN=HLA-B PE=1 SV=1 - [1B44_HUMAN]	35.08	6	3	9	17	0.47	0.78	0.13	0.44	0.49	0.79	4.32	3.04	3.90	3.59	1.38	1.68	3.90	0.91	1.24	3.15	3.49	0.00	0.34	4.25
P26022	Pentraxin-related protein PTX3 OS=Homo sapiens GN=PTX3 PE=1 SV=3 - [PTX3_HUMAN]	6.04	1	2	2	4	-1.77	-1.70	-1.90	-1.83	1.41	1.48	1.94	2.01	2.08	2.01	2.67	2.74	3.76	4.45	4.58	3.81	3.95	3.16	3.30	3.91
Q9H461	Frizzled-8 OS=Homo sapiens GN=FZD8 PE=1 SV=1 - [FZD8_HUMAN]	2.88	1	1	1	1	-2.60	-3.14	-3.21	-3.76	0.99	0.43	0.57	0.02	-0.26	0.29	-0.01	-0.56	3.22	2.60	3.20	2.92	3.54	3.56	4.18	3.85
P08246	Neutrophil elastase OS=Homo sapiens GN=ELANE PE=1 SV=1 - [ELNE_HUMAN]	9.36	1	3	3	10	-2.63	-2.47	-2.69	-2.55	-1.78	-1.68	0.95	1.15	0.43	0.32	-0.21	0.01	3.58	2.37	2.57	2.89	3.02	1.01	0.86	3.77
P49913	Cathelicidin antimicrobial peptide OS=Homo sapiens GN=CAMP PE=1 SV=1 - [CAMP_HUMAN]	18.24	1	3	3	4	-2.36	-2.07	-2.29	-2.00	-0.34	-0.06	1.22	1.51	0.99	0.70	0.89	1.18	3.63	3.26	3.18	3.09	3.02	2.00	1.93	3.57

Q96L94	Sorting nexin-22 OS=Homo sapiens GN=SNX22 PE=1 SV=1 - [SNX22_HUMAN]	3.63	1	1	1	1	-0.45	0.06	-2.99	-2.48	-0.36	0.14	0.46	0.98	0.64	0.14	0.59	1.10	0.97	1.04	3.58	0.62	3.16	0.07	2.61	3.52
P20160	Azurocidin OS=Homo sapiens GN=AZU1 PE=1 SV=3 - [CAP7_HUMAN]	3.98	1	1	1	2	-1.82	-2.01	-2.26	-2.46	-1.10	-1.30	1.15	0.96	-0.01	0.18	-0.66	-0.85	3.03	1.17	1.61	2.03	2.48	0.70	1.14	3.48
P02788	Lactotransferrin OS=Homo sapiens GN=LTF PE=1 SV=6 - [TRFL_HUMAN]	57.89	1	31	31	67	-2.28	-2.24	-2.34	-2.32	-1.35	-1.17	0.91	1.01	0.16	0.16	-0.30	-0.20	3.49	2.06	2.15	2.68	2.84	0.85	1.30	3.48
P21741	Midkine OS=Homo sapiens GN=MDK PE=1 SV=1 - [MK_HUMAN]	52.45	1	9	9	39	0.16	-0.34	-0.54	-0.52	2.42	2.62	2.52	2.36	2.35	2.57	2.28	2.07	3.02	2.99	3.11	3.12	3.14	3.10	3.36	3.46
Q3L8U1	Chromodomain-helicase-DNA-binding protein 9 OS=Homo sapiens GN=CHD9 PE=1 SV=2 - [CHD9_HUMAN]	0.76	3	2	2	3	-2.92	-3.23	-3.54	-3.62	-0.64	-0.73	-0.15	-0.23	-0.47	-0.38	-0.66	-0.75	0.63	0.79	2.87	0.84	3.19	1.33	2.88	3.45
P34741	Syndecan-2 OS=Homo sapiens GN=SDC2 PE=1 SV=2 - [SDC2_HUMAN]	11.94	1	2	2	3	-1.57	-1.68	-1.75	-1.85	2.81	2.70	1.61	1.50	1.62	1.73	1.26	1.14	3.24	2.83	3.00	3.33	3.51	4.37	4.54	3.42
P24158	Myeloblastin OS=Homo sapiens GN=PRTN3 PE=1 SV=3 - [PRTN3_HUMAN]	12.89	1	2	2	9	-1.23	-1.05	-1.13	-1.17	-0.67	-0.31	1.73	1.55	0.90	1.01	0.86	0.76	3.28	2.10	2.00	2.28	2.18	0.54	0.44	3.27
P05109	Protein S100-A8 OS=Homo sapiens GN=S100A8 PE=1 SV=1 - [S10A8_HUMAN]	49.46	1	7	7	44	-2.28	-2.16	-2.49	-2.41	-2.06	-2.06	0.66	0.71	0.15	0.07	-0.05	0.02	2.96	2.20	2.47	2.24	2.49	0.11	0.26	3.25
O95503	Chromobox protein homolog 6 OS=Homo sapiens GN=CBX6 PE=1 SV=1 - [CBX6_HUMAN]	4.61	1	1	1	1	-1.90	-1.87	-1.88	-1.85	0.83	0.86	1.30	1.33	0.51	0.49	-0.33	-0.30	3.26	1.58	1.55	2.42	2.40	2.72	2.70	3.24

Q4U2R8	Solute carrier family 22 member 6 OS=Homo sapiens GN=SLC22A6 PE=1 SV=1 - [S22A6_HUMAN]	9.41	1	5	5	15	-1.64	-1.85	-3.02	-3.06	0.18	0.26	0.03	0.04	0.00	-0.05	-0.24	-0.22	1.89	2.09	2.87	1.98	3.13	2.38	3.38	3.24
A5PLK6	Regulator of G-protein signaling protein-like OS=Homo sapiens GN=RGSL1 PE=2 SV=1 - [RGSL_HUMAN]	0.74	1	1	1	1	-2.83	-2.83	-3.62	-3.62	0.24	0.23	-0.48	-0.48	-0.36	-0.36	-0.73	-0.74	2.40	2.10	2.88	2.50	3.30	3.05	3.84	3.20
Q03692	Collagen alpha-1(X) chain OS=Homo sapiens GN=COL10A1 PE=1 SV=2 - [COAA1_HUMAN]	1.91	1	1	1	3	-1.57	-1.58	-1.49	-1.50	2.61	2.59	1.59	1.58	1.59	1.61	1.03	1.01	3.22	2.60	2.52	3.21	3.13	4.16	4.08	3.15
P21246	Pleiotrophin OS=Homo sapiens GN=PTN PE=1 SV=1 - [PTN_HUMAN]	40.48	1	7	7	45	-1.93	-1.76	-2.48	-2.31	1.72	1.74	0.74	0.68	0.92	0.98	-0.22	-0.45	2.23	1.74	1.94	2.93	3.18	3.66	3.65	3.12
P23141	Liver carboxylesterase 1 OS=Homo sapiens GN=CES1 PE=1 SV=2 - [EST1_HUMAN]	37.39	2	16	16	31	-1.51	-1.51	-2.17	-2.09	1.68	1.55	1.04	0.86	0.93	1.11	0.70	0.65	2.43	2.34	2.67	2.69	3.16	3.05	3.68	3.11
P59665	Neutrophil defensin 1 OS=Homo sapiens GN=DEFA1 PE=1 SV=1 - [DEF1_HUMAN]	20.21	2	3	3	15	-1.63	-1.66	-1.60	-1.29	-0.75	-0.54	1.96	1.99	1.49	1.24	1.08	1.29	3.64	2.98	2.46	3.20	2.65	0.76	0.90	3.08
Q96SJ8	Tetraspanin-18 OS=Homo sapiens GN=TSPAN18 PE=2 SV=1 - [TSN18_HUMAN]	2.82	1	1	1	1	-2.92	-2.49	-3.30	-2.87	0.09	0.51	-0.33	0.10	0.13	-0.29	0.01	0.43	2.64	2.93	3.30	2.66	3.04	2.99	3.37	3.03
O43405	Cochlin OS=Homo sapiens GN=COCH PE=1 SV=1 - [COCH_HUMAN]	14.91	1	7	7	8	-1.91	-2.00	-2.35	-2.29	-0.80	-0.76	0.58	0.81	0.18	0.03	-1.22	-1.25	3.05	0.70	1.05	2.28	2.42	1.27	1.52	2.99
P98171	Rho GTPase-activating protein 4 OS=Homo sapiens GN=ARHGAP4 PE=1 SV=2 - [RHG04_HUMAN]	1.90	1	1	2	12	-1.20	-1.31	-2.65	-2.76	0.46	0.35	0.28	0.16	0.55	0.66	0.59	0.47	1.53	1.79	3.23	1.89	3.34	1.64	3.09	2.99

P00966	Argininosuccinate synthase OS=Homo sapiens GN=ASS1 PE=1 SV=2 - [ASSY_HUMAN]	39.81	1	14	14	39	-1.24	-1.28	-1.79	-2.01	1.17	1.04	1.36	1.32	0.93	1.09	0.61	0.40	2.58	1.66	2.20	2.23	2.83	2.33	2.84	2.96
O00622	Protein CYR61 OS=Homo sapiens GN=CYR61 PE=1 SV=1 - [CYR61_HUMAN]	29.40	1	10	10	19	-0.18	0.33	-0.36	-0.43	3.27	3.07	1.99	1.75	2.09	2.34	2.05	1.82	1.95	1.33	2.26	2.20	2.82	2.67	3.87	2.85
P08311	Cathepsin G OS=Homo sapiens GN=CTSG PE=1 SV=2 - [CATG_HUMAN]	21.18	1	4	4	33	-1.62	-1.47	-1.70	-1.66	-0.12	-0.04	1.01	1.13	0.68	0.68	0.42	0.37	2.94	1.75	2.04	2.18	2.54	1.41	1.51	2.85
P04004	Vitronectin OS=Homo sapiens GN=VTN PE=1 SV=1 - [VTNC_HUMAN]	18.62	1	9	9	55	-1.72	-1.66	-1.74	-1.68	1.83	1.88	1.19	1.17	1.19	1.19	0.69	0.62	2.58	2.42	2.47	2.81	2.96	3.44	3.43	2.84
Q8N6Y2	Leucine-rich repeat-containing protein 17 OS=Homo sapiens GN=LRRC17 PE=2 SV=1 - [LRC17_HUMAN]	16.10	1	8	8	22	-3.54	-3.30	-3.61	-3.60	-0.77	-0.79	-0.79	-0.78	-0.74	-0.81	-0.59	-0.49	2.23	3.07	3.08	2.48	2.79	2.60	3.00	2.83
Q08431	Lactadherin OS=Homo sapiens GN=MFGE8 PE=1 SV=2 - [MFGM_HUMAN]	53.23	1	16	16	95	-1.11	-0.89	-1.46	-1.27	2.28	2.16	1.33	1.32	1.49	1.43	0.52	0.62	2.06	1.62	2.22	2.33	3.09	2.92	3.73	2.82
P82987	ADAMTS-like protein 3 OS=Homo sapiens GN=ADAMTSL3 PE=2 SV=4 - [ATL3_HUMAN]	15.91	1	23	23	44	-2.11	-2.08	-2.45	-2.42	0.77	0.72	0.40	0.39	0.28	0.20	-0.87	-0.79	2.46	1.34	1.65	2.26	2.68	2.84	3.17	2.82
Q96DC8	Enoyl-CoA hydratase domain-containing protein 3, mitochondrial OS=Homo sapiens GN=ECHDC3 PE=1 SV=2 - [ECHD3_HUMAN]	13.53	1	3	3	7	-0.82	-0.72	-1.40	-1.45	0.99	0.94	1.39	1.38	1.35	1.30	1.45	1.43	2.11	2.27	2.99	2.11	2.73	1.90	2.48	2.81
P05164	Myeloperoxidase OS=Homo sapiens GN=MPO PE=1 SV=1 - [PERM_HUMAN]	31.41	1	16	18	48	-1.43	-1.35	-1.64	-1.43	-0.76	-0.59	1.09	1.17	0.58	0.61	0.40	0.40	2.52	1.96	1.97	2.08	2.06	0.64	0.81	2.75

P35968	Vascular endothelial growth factor receptor 2 OS=Homo sapiens GN=KDR PE=1 SV=2 - [VGFR2_HUMAN]	7.45	5	6	7	12	-2.17	-1.53	-2.51	-2.35	0.25	0.41	0.19	0.42	0.15	0.02	0.03	0.25	2.12	1.91	1.98	1.92	2.30	2.40	2.74	2.73
P10909	Clusterin OS=Homo sapiens GN=CLU PE=1 SV=1 - [CLUS_HUMAN]	40.98	1	18	18	451	-0.28	-0.43	-0.60	-0.74	3.02	2.92	2.02	1.91	2.29	2.35	2.01	1.88	2.43	2.42	2.60	2.78	3.04	3.36	3.62	2.71
P29762	Cellular retinoic acid-binding protein 1 OS=Homo sapiens GN=CRABP1 PE=1 SV=2 - [RABP1_HUMAN]	67.88	1	9	9	87	-0.92	-0.64	-0.75	-0.73	1.60	1.57	2.01	1.88	1.28	1.41	-0.08	-0.03	2.82	0.79	0.81	2.10	1.98	2.27	2.31	2.70
Q6UWY5	Olfactomedin-like protein 1 OS=Homo sapiens GN=OLFML1 PE=1 SV=2 - [OLFL1_HUMAN]	40.55	1	14	14	65	-2.70	-2.73	-3.37	-3.25	-0.70	-0.68	-0.62	-0.59	-0.89	-0.85	-1.16	-1.23	2.01	1.75	2.15	1.94	2.43	2.25	2.58	2.70
P35555	Fibrillin-1 OS=Homo sapiens GN=FBN1 PE=1 SV=3 - [FBN1_HUMAN]	40.06	2	88	88	585	-2.21	-2.16	-2.51	-2.46	0.37	0.45	0.12	0.18	0.13	0.09	-0.12	-0.07	2.43	2.11	2.36	2.39	2.61	2.55	2.84	2.69
Q9UM07	Protein-arginine deiminase type-4 OS=Homo sapiens GN=PADI4 PE=1 SV=2 - [PADI4_HUMAN]	4.37	1	2	2	2	-1.54	-1.52	-1.71	-1.69	0.20	0.22	0.91	0.93	0.57	0.56	0.40	0.41	2.51	1.95	2.11	2.13	2.30	1.73	1.90	2.69
Q9H4F8	SPARC-related modular calcium-binding protein 1 OS=Homo sapiens GN=SMOC1 PE=1 SV=1 - [SMOC1_HUMAN]	28.57	1	12	12	32	0.09	-0.06	-0.22	-0.40	2.07	2.06	1.85	2.29	2.05	1.99	2.05	2.05	2.32	2.44	2.70	2.38	2.74	2.21	2.64	2.68
Q96L58	Beta-1,3-galactosyltransferase 6 OS=Homo sapiens GN=B3GALT6 PE=1 SV=2 - [B3GT6_HUMAN]	2.74	1	1	1	2	-1.21	-1.18	-1.60	-1.56	2.73	2.76	1.02	1.05	1.86	1.83	1.06	1.09	2.28	2.27	2.66	3.08	3.47	3.92	4.31	2.68

P18577	Blood group Rh(CE) polypeptide OS=Homo sapiens GN=RHCE PE=1 SV=2 - [RHCE_HUMAN]	4.08	2	2	2	4	-2.07	-2.14	-2.15	-2.21	0.49	0.42	0.45	0.39	0.63	0.70	1.37	1.30	2.58	3.45	3.52	2.80	2.88	2.54	2.62	2.66
P06702	Protein S100-A9 OS=Homo sapiens GN=S100A9 PE=1 SV=1 - [S10A9_HUMAN]	50.88	1	5	5	35	-1.87	-1.72	-2.00	-2.05	-1.80	-1.89	0.68	0.72	0.12	0.09	-0.08	-0.02	2.44	1.50	2.07	1.74	2.19	0.07	0.08	2.64
Q93097	Protein Wnt-2b OS=Homo sapiens GN=WNT2B PE=1 SV=2 - [WNT2B_HUMAN]	18.41	3	5	5	15	-0.86	-1.08	-1.20	-1.43	1.47	1.24	1.30	1.09	1.08	1.10	0.84	0.74	2.27	1.93	1.84	2.26	2.34	2.51	2.66	2.62
Q96NY7	Chloride intracellular channel protein 6 OS=Homo sapiens GN=CLIC6 PE=2 SV=3 - [CLIC6_HUMAN]	24.01	2	9	10	28	-0.52	-0.88	-1.43	-1.60	1.05	0.84	1.10	1.09	0.56	0.70	0.28	0.26	2.06	1.11	1.86	1.58	2.17	1.71	1.97	2.58
P14780	Matrix metalloproteinase-9 OS=Homo sapiens GN=MMP9 PE=1 SV=3 - [MMP9_HUMAN]	19.38	1	11	11	14	-1.56	-1.37	-1.34	-1.27	-0.85	-0.70	1.19	1.52	0.81	0.59	0.36	0.58	2.77	2.00	1.87	2.38	2.02	0.69	0.55	2.57
P02766	Transthyretin OS=Homo sapiens GN=TTR PE=1 SV=1 - [TTHY_HUMAN]	24.49	1	3	3	7	-1.26	-1.17	-2.18	-2.13	0.47	0.65	0.43	0.37	0.35	0.44	0.23	0.22	1.59	1.48	2.44	1.89	2.65	1.80	2.77	2.57
P07996	Thrombospondin-1 OS=Homo sapiens GN=THBS1 PE=1 SV=2 - [TSP1_HUMAN]	41.71	1	33	36	83	-1.47	-1.43	-1.73	-1.55	1.83	1.88	0.56	0.55	1.07	0.92	0.36	0.49	2.39	1.99	2.13	2.51	2.81	3.33	3.61	2.57
Q14116	Interleukin-18 OS=Homo sapiens GN=IL18 PE=1 SV=1 - [IL18_HUMAN]	22.28	1	3	3	6	-0.78	-0.72	-1.33	-1.26	1.27	1.33	1.17	1.23	1.20	1.14	0.39	0.45	2.01	1.18	1.72	1.96	2.50	2.04	2.58	2.56
P27169	Serum paraoxonase/arylesterase 1 OS=Homo sapiens GN=PON1 PE=1 SV=3 - [PON1_HUMAN]	28.45	1	7	7	14	-1.35	-1.64	-1.64	-1.66	1.23	1.05	0.73	0.76	0.97	0.79	1.32	1.31	2.52	3.03	2.99	2.72	2.76	2.92	2.78	2.56

P28330	Long-chain specific acyl-CoA dehydrogenase, mitochondrial OS=Homo sapiens GN=ACADL PE=2 SV=2 - [ACADL_HUMAN]	27.67	1	12	12	23	-2.03	-1.95	-3.02	-2.88	-0.68	-0.52	-0.03	0.03	-0.11	-0.16	-0.07	0.05	2.19	2.23	2.73	2.04	2.20	1.58	2.00	2.56
P02743	Serum amyloid P-component OS=Homo sapiens GN=APCS PE=1 SV=2 - [SAMP_HUMAN]	28.25	1	7	7	28	-0.93	-1.01	-1.67	-1.76	1.69	1.59	1.06	0.96	1.00	1.04	0.38	0.26	2.05	1.28	2.01	2.44	2.72	2.71	3.31	2.55
P80188	Neutrophil gelatinase-associated lipocalin OS=Homo sapiens GN=LCN2 PE=1 SV=2 - [NGAL_HUMAN]	39.90	1	5	5	6	-1.53	-1.59	-2.04	-1.87	-1.23	-1.19	0.63	0.88	0.74	0.35	0.43	0.69	2.28	2.15	2.53	2.29	2.50	0.39	0.86	2.54
Q8IWU5	Extracellular sulfatase Sulf 2 OS=Homo sapiens GN=SULF2 PE=1 SV=1 - [SULF2_HUMAN]	4.48	1	4	4	5	-0.19	0.00	-0.42	-0.06	3.09	3.86	2.30	2.39	2.92	2.16	1.81	1.99	2.44	2.00	2.06	2.84	2.73	3.72	3.62	2.52
P83110	Serine protease HTRA3 OS=Homo sapiens GN=HTRA3 PE=1 SV=2 - [HTRA3_HUMAN]	5.08	1	1	2	4	-1.72	-1.58	-1.96	-1.82	2.57	2.70	0.49	0.63	1.50	1.37	0.76	0.90	2.27	2.49	2.72	3.12	3.36	4.28	4.51	2.51
Q93091	Ribonuclease K6 OS=Homo sapiens GN=RNASE 6 PE=1 SV=2 - [RNAS6_HUMAN]	6.67	1	1	1	1	-1.62	-1.30	-1.84	-1.51	1.11	1.43	0.61	0.94	1.41	1.09	1.40	1.72	2.29	3.03	3.24	2.74	2.96	2.71	2.93	2.51
Q14956	Transmembrane glycoprotein NMB OS=Homo sapiens GN=GPNMB PE=1 SV=2 - [GPNMB_HUMAN]	4.72	1	2	2	5	-1.81	-1.65	-1.62	-1.45	0.46	0.62	0.83	1.00	0.82	0.66	1.57	1.73	2.70	3.39	3.18	2.50	2.31	2.25	2.06	2.51
P26678	Cardiac phospholamban OS=Homo sapiens GN=PLN PE=1 SV=1 - [PPLA_HUMAN]	21.15	1	1	1	2	-2.96	-3.13	-3.26	-3.43	-0.48	-0.65	-0.82	-0.99	-0.68	-0.51	-0.18	-0.35	2.19	2.79	3.08	2.49	2.79	2.47	2.77	2.50

Q9UBX5	Fibulin-5 OS=Homo sapiens GN=FBLN5 PE=1 SV=1 - [FBLN5_HUMAN]	20.76	1	8	8	24	-1.48	-1.33	-1.83	-2.03	1.48	1.48	0.59	0.49	0.85	0.84	0.29	0.21	2.00	1.82	2.05	2.33	2.65	2.80	3.42	2.48
O60279	Sushi domain containing protein 5 OS=Homo sapiens GN=SUSD5 PE=1 SV=3 - [SUSD5_HUMAN]	2.86	1	2	2	4	-1.98	-2.04	-1.84	-1.90	0.39	0.33	0.58	0.52	0.21	0.28	0.26	0.19	2.62	2.24	2.10	2.29	2.15	2.36	2.22	2.48
P22352	Glutathione peroxidase 3 OS=Homo sapiens GN=GPX3 PE=1 SV=2 - [GPX3_HUMAN]	24.78	1	5	5	14	-2.45	-2.28	-2.81	-2.70	0.22	0.29	-0.40	-0.35	0.10	0.14	0.64	0.65	1.96	3.18	3.52	2.58	2.86	2.61	3.05	2.48
P23276	Kell blood group glycoprotein OS=Homo sapiens GN=KEL PE=1 SV=2 - [KELL_HUMAN]	3.01	1	2	2	2	-1.28	-1.35	-1.91	-1.98	0.49	0.41	0.50	0.43	0.93	1.01	1.59	1.51	1.83	2.87	3.50	2.32	2.95	1.75	2.38	2.47
P56705	Protein Wnt-4 OS=Homo sapiens GN=WNT4 PE=1 SV=4 - [WNT4_HUMAN]	29.06	1	11	11	36	-0.31	-0.45	-0.76	-1.03	2.68	2.46	1.51	1.62	1.84	1.96	1.32	1.23	2.21	1.65	2.22	2.33	2.91	2.90	3.73	2.47
P16452	Erythrocyte membrane protein band 4.2 OS=Homo sapiens GN=EPB42 PE=1 SV=3 - [EPB42_HUMAN]	30.82	1	22	22	61	-1.80	-1.76	-1.93	-1.86	0.30	0.42	0.43	0.66	0.81	0.67	1.25	1.38	2.23	3.14	3.15	2.57	2.55	2.05	2.02	2.46
P04196	Histidine-rich glycoprotein OS=Homo sapiens GN=HRG PE=1 SV=1 - [HRG_HUMAN]	19.81	1	7	8	19	-2.02	-2.03	-2.33	-2.00	0.13	0.39	0.01	0.16	0.35	0.16	0.74	0.89	2.24	2.24	2.98	2.08	2.39	1.65	2.35	2.45
Q9UBS3	DnaJ homolog subfamily B member 9 OS=Homo sapiens GN=DNAJB9 PE=1 SV=1 - [DNJB9_HUMAN]	17.04	1	3	3	5	0.73	0.77	-0.20	-0.15	2.93	3.40	3.12	3.39	3.16	2.69	2.15	2.62	2.30	1.58	1.42	2.23	2.00	2.40	2.18	2.45

P48509	CD151 antigen OS=Homo sapiens GN=CD151 PE=1 SV=3 - [CD151_HUMAN]	7.11	1	2	2	2	-2.62	-2.57	-2.25	-2.19	0.52	0.57	0.14	0.19	0.27	0.22	-0.14	-0.10	2.81	2.48	2.10	2.87	2.50	3.13	2.75	2.45
P22003	Bone morphogenetic protein 5 OS=Homo sapiens GN=BMP5 PE=2 SV=1 - [BMP5_HUMAN]	7.71	1	3	3	7	-0.83	-1.03	-1.10	-1.30	2.93	3.13	1.21	1.41	1.79	1.66	0.94	0.74	2.57	2.59	2.45	3.89	3.75	4.55	4.41	2.44
Q8TCC7	Solute carrier family 22 member 8 OS=Homo sapiens GN=SLC22A8 PE=1 SV=1 - [S22A8_HUMAN]	11.81	1	6	6	11	-2.54	-2.52	-2.53	-2.51	0.47	0.56	-0.02	-0.04	0.29	0.33	0.42	0.44	2.42	2.97	2.95	2.37	2.36	2.55	2.54	2.42
P02730	Band 3 anion transport protein OS=Homo sapiens GN=SLC4A1 PE=1 SV=3 - [B3AT_HUMAN]	31.61	1	21	21	170	-1.59	-1.61	-1.92	-1.92	0.62	0.62	0.38	0.42	0.77	0.83	1.47	1.49	2.09	2.99	3.31	2.51	2.66	2.24	2.50	2.41
P00325	Alcohol dehydrogenase 1B OS=Homo sapiens GN=ADH1B PE=1 SV=2 - [ADH1B_HUMAN]	33.07	3	10	10	13	-1.39	-1.32	-1.16	-1.05	0.59	0.69	0.97	0.82	0.97	1.05	1.50	1.32	2.55	3.11	3.12	2.83	2.84	2.66	2.68	2.40
Q9UKG4	Solute carrier family 13 member 4 OS=Homo sapiens GN=SLC13A4 PE=2 SV=2 - [S13A4_HUMAN]	3.04	1	1	1	2	-2.45	-2.40	-2.39	-2.34	0.30	0.35	-0.06	-0.01	0.03	-0.02	0.01	0.06	2.45	2.47	2.40	2.46	2.41	2.73	2.68	2.40
P17936	Insulin-like growth factor-binding protein 3 OS=Homo sapiens GN=IGFBP3 PE=1 SV=2 - [IBP3_HUMAN]	16.84	1	3	3	10	-0.67	-0.66	-0.87	-1.00	2.73	2.88	1.15	1.39	1.70	1.70	0.94	1.00	2.15	1.68	2.01	2.65	3.04	3.63	4.02	2.39
Q9NRN5	Olfactomedin-like protein 3 OS=Homo sapiens GN=OLFML3 PE=2 SV=1 - [OLFL3_HUMAN]	45.07	1	16	16	108	-1.45	-1.64	-1.85	-1.97	1.23	1.40	0.22	0.32	0.49	0.47	-0.11	-0.11	2.03	1.59	1.80	2.35	2.61	3.33	3.64	2.39

O75084	Frizzled-7 OS=Homo sapiens GN=FZD7 PE=1 SV=2 - [FZD7_HUMAN]	4.01	2	3	3	4	-2.00	-1.70	-2.41	-2.24	0.82	1.11	0.19	0.49	0.57	0.28	0.22	0.29	2.24	1.77	2.31	2.31	2.70	2.80	2.91	2.37
Q9HD89	Resistin OS=Homo sapiens GN=RETN PE=2 SV=1 - [RETN_HUMAN]	13.89	1	1	1	2	-1.81	-1.89	-0.96	-1.04	-0.10	-0.19	1.33	1.25	0.87	0.96	0.80	0.72	3.20	2.62	1.76	2.80	1.96	1.69	0.84	2.35
Q63ZY3	KN motif and ankyrin repeat domain-containing protein 2 OS=Homo sapiens GN=KANK2 PE=1 SV=1 - [KANK2_HUMAN]	43.95	1	26	27	65	-2.06	-2.16	-2.49	-2.62	-0.25	-0.29	-0.33	-0.38	-0.37	-0.30	0.11	-0.03	1.83	2.21	2.61	1.94	2.34	1.89	2.45	2.32
P12829	Myosin light chain 4 OS=Homo sapiens GN=MYL4 PE=1 SV=3 - [MYL4_HUMAN]	30.46	1	5	5	8	-2.20	-2.08	-2.45	-2.49	-0.20	-0.30	0.05	0.04	-0.10	-0.12	-0.02	-0.04	2.18	2.02	2.17	1.76	2.21	1.88	2.11	2.32
P08670	Vimentin OS=Homo sapiens GN=VIM PE=1 SV=4 - [VIME_HUMAN]	86.70	5	44	53	2610	-2.39	-2.36	-2.61	-2.63	0.10	0.12	-0.34	-0.30	-0.18	-0.21	-0.20	-0.20	2.05	2.21	2.45	2.23	2.47	2.42	2.67	2.31
P39060	Collagen alpha-1(XVIII) chain OS=Homo sapiens GN=COL18A1 PE=1 SV=5 - [COIA1_HUMAN]	16.08	1	22	22	106	-2.35	-2.14	-2.50	-2.32	0.37	0.42	-0.15	-0.05	0.07	0.08	0.08	0.20	2.11	2.33	2.58	2.25	2.51	2.50	2.73	2.31
Q8TB73	Protein NDNF OS=Homo sapiens GN=NDNF PE=2 SV=2 - [NDNF_HUMAN]	13.91	1	7	7	9	0.12	0.05	-0.14	-0.03	1.99	1.77	2.44	2.10	1.81	2.09	1.17	0.96	2.14	0.89	1.21	1.70	2.00	1.71	1.89	2.30
Q96IZ0	PRKC apoptosis WT1 regulator protein OS=Homo sapiens GN=PAWR PE=1 SV=1 - [PAWR_HUMAN]	17.94	1	5	5	6	-2.40	-2.45	-2.88	-2.89	-0.47	-0.43	-0.63	-0.66	-0.95	-0.98	-1.05	-1.06	2.01	1.92	1.88	1.98	1.89	2.26	2.29	2.29

Q2TAA5	GDP-Man:Man(3)GlcNAc(2)-PP-Dol alpha-1,2-mannosyltransferase OS=Homo sapiens GN=ALG11 PE=1 SV=2 - [ALG11_HUMAN]	2.85	1	1	1	1	-1.09	-1.14	-2.91	-2.96	-0.01	-0.06	-0.68	-0.73	-0.23	-0.18	-0.21	-0.27	0.46	0.88	2.70	0.94	2.77	1.06	2.88	2.29
Q8IX30	Signal peptide, CUB and EGF-like domain-containing protein 3 OS=Homo sapiens GN=SCUBE3 PE=1 SV=1 - [SCUB3_HUMAN]	11.88	1	8	8	21	-0.72	-0.97	-1.02	-1.17	2.15	1.92	1.13	0.99	1.00	1.51	0.92	0.53	2.00	1.55	2.03	2.29	2.47	2.95	3.15	2.29
P17813	Endoglin OS=Homo sapiens GN=ENG PE=1 SV=2 - [EGLN_HUMAN]	8.21	1	4	4	4	-2.18	-2.20	-2.47	-2.48	0.37	0.50	-0.20	-0.26	-0.13	-0.26	-0.21	-0.08	1.99	2.14	2.13	2.11	2.11	2.68	2.68	2.29
P14207	Folate receptor beta OS=Homo sapiens GN=FOLR2 PE=1 SV=4 - [FOLR2_HUMAN]	22.35	1	6	6	15	-2.00	-1.98	-2.12	-2.14	0.23	0.31	0.09	0.09	0.17	0.10	0.34	0.42	2.16	2.35	2.43	2.16	2.13	2.18	2.35	2.28
Q9BXJ4	Complement C1q tumor necrosis factor-related protein 3 OS=Homo sapiens GN=C1QTNF3 PE=1 SV=1 - [C1QT3_HUMAN]	2.85	1	1	1	2	-2.70	-2.51	-2.96	-2.77	-0.53	-0.35	-0.75	-0.56	-0.52	-0.70	-1.26	-1.08	2.01	1.44	1.70	2.03	2.30	2.15	2.41	2.28
Q8IWU6	Extracellular sulfatase Sulf-1 OS=Homo sapiens GN=SULF1 PE=1 SV=1 - [SULF1_HUMAN]	11.02	1	7	7	16	-0.10	-0.01	-0.72	-0.81	3.49	3.30	1.17	1.09	2.21	2.31	1.65	1.54	1.66	2.04	2.63	2.86	3.30	3.99	4.43	2.27
Q9NWZ3	Interleukin-1 receptor-associated kinase 4 OS=Homo sapiens GN=IRAK4 PE=1 SV=1 - [IRAK4_HUMAN]	1.96	1	1	1	1	-1.11	-1.29	-1.72	-1.90	0.97	0.79	0.48	0.30	0.54	0.73	0.41	0.22	1.64	1.52	2.13	1.87	2.48	2.07	2.68	2.26
Q9UJH8	Meteorin OS=Homo sapiens GN=METRN PE=2 SV=2 - [METRN_HUMAN]	12.29	1	3	3	7	-1.00	-0.98	-1.31	-1.45	1.63	1.50	0.73	0.62	0.86	1.04	0.57	0.58	1.79	1.51	1.95	2.05	2.51	2.52	2.98	2.26

Q05707	Collagen alpha-1(XIV) chain OS=Homo sapiens GN=COL14A1 PE=1 SV=3 - [COEA1_HUMAN]	43.49	1	55	55	312	-3.06	-3.09	-3.32	-3.30	-0.98	-0.97	-1.13	-1.09	-1.18	-1.19	-1.43	-1.42	2.02	1.76	1.91	2.02	2.19	2.23	2.40	2.25
P27658	Collagen alpha-1(VIII) chain OS=Homo sapiens GN=COL8A1 PE=1 SV=2 - [CO8A1_HUMAN]	9.68	1	5	5	12	-0.71	-0.89	-1.08	-0.99	2.16	1.81	1.06	0.71	0.72	1.14	0.66	0.25	1.98	1.64	1.80	2.26	2.41	3.26	3.44	2.24
P09619	Platelet-derived growth factor receptor beta OS=Homo sapiens GN=PDGFRB PE=1 SV=1 - [PGFRB_HUMAN]	19.08	5	16	17	42	-1.90	-1.93	-2.05	-2.04	0.66	0.61	0.20	0.14	0.28	0.34	0.31	0.33	2.04	2.10	2.36	2.19	2.38	2.44	2.55	2.24
P09238	Stromelysin-2 OS=Homo sapiens GN=MMP10 PE=1 SV=1 - [MMP10_HUMAN]	1.89	1	1	1	2	-0.66	-0.56	-0.50	-0.54	3.96	3.81	1.67	1.58	2.58	2.62	2.06	1.69	2.39	2.72	2.55	3.31	3.15	4.60	4.44	2.24
Q8WUJ3	Cell migration-inducing and hyaluronan-binding protein OS=Homo sapiens GN=CEMIP PE=1 SV=2 - [CEMIP_HUMAN]	32.55	1	36	36	130	-0.20	-0.33	-0.77	-0.67	2.64	2.65	1.35	1.40	1.70	1.83	0.97	0.86	1.76	1.21	1.68	2.21	2.70	3.01	3.50	2.23
P54709	Sodium/potassium-transporting ATPase subunit beta-3 OS=Homo sapiens GN=ATP1B3 PE=1 SV=1 - [AT1B3_HUMAN]	31.54	1	9	9	36	-2.13	-2.15	-1.90	-2.08	0.17	0.23	0.05	0.09	0.02	-0.01	-0.03	0.04	2.32	2.11	2.12	2.24	1.98	2.30	2.38	2.23
P02549	Spectrin alpha chain, erythrocytic 1 OS=Homo sapiens GN=SPTA1 PE=1 SV=5 - [SPTA1_HUMAN]	54.57	1	107	107	359	-1.47	-1.35	-1.69	-1.57	0.32	0.38	0.48	0.55	0.69	0.64	1.25	1.33	1.90	2.69	2.92	2.10	2.30	1.74	1.93	2.22

Q16585	Beta-sarcoglycan OS=Homo sapiens GN=SGCB PE=1 SV=1 - [SGCB_HUMAN]	18.55	1	4	4	9	-1.85	-1.66	-2.32	-2.13	-0.15	0.06	-0.22	-0.03	0.07	-0.12	0.04	0.25	1.79	1.87	2.33	1.77	2.24	1.59	2.06	2.22
Q14314	Fibroleukin OS=Homo sapiens GN=FGL2 PE=1 SV=1 - [FGL2_HUMAN]	17.08	2	7	7	16	-1.26	-1.15	-1.42	-1.32	1.07	1.20	0.63	0.84	0.96	0.73	1.15	1.31	1.92	2.20	2.70	2.07	2.35	2.43	2.50	2.22
Q9BV40	Vesicle-associated membrane protein 8 OS=Homo sapiens GN=VAMP8 PE=1 SV=1 - [VAMP8_HUMAN]	17.00	1	2	2	4	-1.88	-1.65	-2.23	-1.99	0.22	0.45	-0.08	0.15	0.26	0.04	0.67	0.90	1.86	2.56	2.90	1.95	2.30	2.09	2.43	2.21
O95084	Serine protease 23 OS=Homo sapiens GN=PRSS23 PE=1 SV=1 - [PRS23_HUMAN]	23.76	1	7	7	14	-1.24	-1.25	-1.30	-1.32	1.78	1.64	0.96	0.69	0.97	1.18	0.94	0.93	1.44	2.27	2.47	2.21	2.39	2.81	3.29	2.20
Q9Y6F9	Protein Wnt-6 OS=Homo sapiens GN=WNT6 PE=1 SV=2 - [WNT6_HUMAN]	15.07	1	4	4	9	-1.33	-1.56	-1.18	-1.23	0.73	0.67	0.80	0.75	0.82	0.88	0.83	0.77	1.95	1.92	2.31	2.00	2.20	1.80	2.29	2.19
P23435	Cerebellin-1 OS=Homo sapiens GN=CBLN1 PE=1 SV=1 - [CBLN1_HUMAN]	13.99	1	3	3	3	-2.41	-2.35	-2.58	-2.51	-1.34	-1.28	-0.46	-0.39	-0.76	-0.82	-0.50	-0.44	2.01	1.91	2.07	1.62	1.79	1.05	1.22	2.18
Q15582	Transforming growth factor-beta-induced protein ig-h3 OS=Homo sapiens GN=TGFBI PE=1 SV=1 - [BGH3_HUMAN]	44.22	1	20	21	90	-2.05	-1.84	-2.02	-2.06	1.22	1.29	0.21	0.27	0.48	0.48	0.31	0.34	2.09	2.08	2.05	2.25	2.43	2.87	2.91	2.18
Q06828	Fibromodulin OS=Homo sapiens GN=FMOD PE=1 SV=2 - [FMOD_HUMAN]	29.52	1	9	9	66	-2.39	-2.48	-2.56	-2.76	-0.66	-0.68	-0.48	-0.60	-0.55	-0.56	-0.87	-0.85	2.13	1.63	1.84	1.89	2.27	1.87	2.31	2.18
Q8TBN0	Guanine nucleotide exchange factor for Rab 3A OS=Homo sapiens GN=RAB3IL1 PE=1 SV=1 - [R3GEF_HUMAN]	30.89	1	6	6	12	-1.73	-2.03	-1.90	-2.19	0.42	0.11	0.22	-0.08	-0.34	-0.04	-0.13	-0.43	2.00	1.60	1.77	1.72	1.89	2.12	2.29	2.18

Q7Z7G0	Target of Nesh-SH3 OS=Homo sapiens GN=ABI3BP PE=1 SV=1 - [TARSH_HUMAN]	15.16	1	11	11	32	-2.27	-2.27	-2.65	-2.47	-0.69	-0.67	-0.25	-0.21	-0.33	-0.39	-0.46	-0.48	1.97	1.77	1.68	1.98	1.86	1.70	1.80	2.17
Q8IYM0	Protein FAM186B OS=Homo sapiens GN=FAM186B PE=2 SV=2 - [F186B_HUMAN]	0.90	1	1	1	2	-2.20	-2.26	-2.36	-2.42	0.29	0.23	-0.27	-0.32	0.05	0.12	0.23	0.17	1.99	2.44	2.60	2.34	2.51	2.47	2.63	2.16
Q92629	Delta-sarcoglycan OS=Homo sapiens GN=SGCD PE=1 SV=2 - [SGCD_HUMAN]	29.76	1	7	7	16	-2.30	-2.32	-2.23	-2.33	0.01	-0.15	-0.17	-0.33	-0.16	-0.09	0.04	-0.08	2.17	2.50	2.22	2.34	2.11	2.51	2.27	2.15
P48061	Stromal cell-derived factor 1 OS=Homo sapiens GN=CXCL12 PE=1 SV=1 - [SDF1_HUMAN]	23.66	1	2	2	10	-0.94	-0.67	-1.06	-0.73	3.22	3.60	1.16	1.50	2.42	2.14	1.09	1.62	1.93	1.98	2.41	3.39	3.14	4.42	4.17	2.15
Q70UQ0	Inhibitor of nuclear factor kappa-B kinase-interacting protein OS=Homo sapiens GN=IKBIP PE=1 SV=1 - [IKIP_HUMAN]	6.86	1	2	2	3	-2.91	-2.90	-3.27	-3.27	-0.47	-0.47	-1.19	-1.19	-0.90	-0.90	-1.10	-1.10	1.77	1.81	2.17	2.04	2.41	2.42	2.78	2.15
Q00604	Norrin OS=Homo sapiens GN=NDP PE=1 SV=1 - [NDP_HUMAN]	9.02	1	1	1	4	0.12	-0.15	-0.55	-0.77	1.12	0.85	1.52	1.26	0.90	1.17	1.02	0.94	1.52	1.10	1.48	1.08	1.73	0.86	1.65	2.14
P06703	Protein S100-A6 OS=Homo sapiens GN=S100A6 PE=1 SV=1 - [S10A6_HUMAN]	55.56	1	6	6	23	-2.33	-1.96	-2.52	-2.50	-0.16	-0.08	-0.47	-0.42	-0.31	-0.35	-0.32	-0.35	1.80	1.62	2.12	1.77	2.13	2.05	2.23	2.13
Q9H4X1	Regulator of cell cycle RGCC OS=Homo sapiens GN=RGCC PE=1 SV=1 - [RGCC_HUMAN]	45.26	1	4	4	6	-1.14	-1.42	-1.30	-0.95	1.14	1.05	0.83	1.09	0.96	0.76	0.68	0.93	2.15	1.83	1.94	2.03	1.95	2.37	2.29	2.12
P30536	Translocator protein OS=Homo sapiens GN=TSPO PE=1 SV=3 - [TSPOA_HUMAN]	4.73	1	1	1	2	-2.73	-2.64	-2.64	-2.55	-0.16	-0.08	-0.58	-0.49	-0.42	-0.51	-0.25	-0.16	2.20	2.48	2.39	2.25	2.16	2.55	2.46	2.12

Q14766	Latent-transforming growth factor beta-binding protein 1 OS=Homo sapiens GN=LTBP1 PE=1 SV=4 - [LTBP1_HUMAN]	9.76	1	14	14	21	-1.51	-1.72	-1.85	-1.81	0.71	0.70	0.16	0.06	0.29	0.28	0.27	0.16	1.79	1.65	1.97	1.80	2.09	2.27	2.61	2.12
P02100	Hemoglobin subunit epsilon OS=Homo sapiens GN=HBE1 PE=1 SV=2 - [HBE_HUMAN]	32.65	1	3	5	6	-1.64	-1.60	-1.67	-1.62	1.58	1.62	0.38	0.43	1.24	1.19	1.52	1.57	2.08	3.17	3.19	2.87	2.90	3.20	3.23	2.11
Q96D46	60S ribosomal export protein NMD3 OS=Homo sapiens GN=NMD3 PE=1 SV=1 - [NMD3_HUMAN]	3.38	1	2	2	2	-0.45	-0.54	-0.58	-0.67	0.79	0.70	1.46	1.37	1.31	1.40	1.80	1.71	1.97	2.26	2.39	1.89	2.02	1.23	1.36	2.11
P12429	Annexin A3 OS=Homo sapiens GN=ANXA3 PE=1 SV=3 - [ANXA3_HUMAN]	53.56	1	13	14	43	-1.67	-1.37	-1.71	-1.64	-0.39	-0.44	0.20	0.22	-0.08	-0.07	-0.15	-0.15	1.65	1.34	1.54	1.57	1.58	1.01	1.27	2.10
Q9Y680	Peptidyl-prolyl cis-trans isomerase FKBP7 OS=Homo sapiens GN=FKBP7 PE=1 SV=1 - [FKBP7_HUMAN]	10.04	1	2	2	2	-1.31	-1.20	-2.71	-2.20	0.42	0.52	-0.49	-0.38	-0.10	-0.20	-0.37	-0.27	0.87	0.95	2.01	1.14	2.75	1.71	2.94	2.09
O14683	Tumor protein p53-inducible protein 11 OS=Homo sapiens GN=TP53I11 PE=1 SV=2 - [P5I11_HUMAN]	13.76	1	2	2	3	-2.96	-2.78	-2.49	-2.45	-1.32	-1.28	-0.46	-0.42	-0.52	-0.56	0.29	0.33	2.49	2.95	2.78	2.50	1.96	1.94	1.16	2.09
Q9NS98	Semaphorin-3G OS=Homo sapiens GN=SEMA3G PE=2 SV=1 - [SEM3G_HUMAN]	6.91	1	4	4	9	-1.01	-1.08	-1.09	-1.14	2.47	2.39	0.76	0.68	1.39	1.46	0.87	0.84	1.76	1.94	2.12	2.44	2.82	3.40	3.78	2.09
P02649	Apolipoprotein E OS=Homo sapiens GN=APOE PE=1 SV=1 - [APOE_HUMAN]	71.61	1	29	29	306	-0.03	0.00	-0.37	-0.21	3.99	4.07	1.77	1.76	2.81	2.75	1.40	1.46	1.84	1.51	1.82	2.83	3.06	3.91	4.22	2.08

Q9UHF1	Epidermal growth factor-like protein 7 OS=Homo sapiens GN=EGFL7 PE=1 SV=3 - [EGFL7_HUMAN]	17.58	1	4	4	12	-2.44	-2.56	-2.24	-2.35	0.20	0.33	-0.34	-0.33	-0.14	-0.09	0.23	0.36	1.99	2.56	2.63	2.28	2.35	2.41	2.60	2.08
Q14767	Latent-transforming growth factor beta-binding protein 2 OS=Homo sapiens GN=LTBP2 PE=1 SV=3 - [LTBP2_HUMAN]	5.33	1	9	9	16	-1.46	-1.46	-1.23	-1.24	1.33	1.44	0.28	0.40	0.77	0.78	0.49	0.48	1.68	2.22	1.82	2.27	2.30	2.78	2.79	2.08
Q8NES3	Beta-1,3-N-acetylglucosaminyltransferase lunatic fringe OS=Homo sapiens GN=LFNG PE=1 SV=2 - [LFNG_HUMAN]	16.09	1	5	5	9	-0.05	-0.41	-0.12	-0.60	3.50	3.00	1.32	1.09	1.95	2.10	1.43	0.87	1.86	1.35	1.58	2.54	2.84	3.84	4.04	2.07
P04430	Ig kappa chain V-I region BAN OS=Homo sapiens PE=1 SV=1 - [KV122_HUMAN]	16.67	1	1	1	1	-2.07	-2.19	-2.30	-2.42	-0.56	-0.69	-0.29	-0.41	0.30	0.43	1.17	1.04	1.84	3.24	3.46	2.53	2.76	1.49	1.72	2.07
Q99959	Plakophilin-2 OS=Homo sapiens GN=PKP2 PE=1 SV=2 - [PKP2_HUMAN]	35.98	1	25	25	85	-2.36	-2.21	-2.49	-2.58	-0.21	-0.24	-0.50	-0.52	-0.49	-0.41	-0.41	-0.47	1.89	1.91	2.15	1.95	2.10	2.12	2.31	2.07
P0C0L5	Complement C4-B OS=Homo sapiens GN=C4B PE=1 SV=2 - [CO4B_HUMAN]	35.95	1	4	50	190	-0.18	-0.23	-0.85	-0.67	2.42	2.29	1.41	1.32	1.70	1.73	1.23	1.35	1.71	1.92	1.94	2.11	2.38	2.54	2.64	2.07
Q8IUA7	ATP-binding cassette sub-family A member 9 OS=Homo sapiens GN=ABCA9 PE=1 SV=1 - [ABCA9_HUMAN]	2.83	3	1	4	7	-0.70	-0.26	-2.12	-1.67	0.67	1.11	-0.12	0.33	0.59	0.15	0.67	1.11	0.64	1.37	2.79	0.88	2.30	1.35	2.77	2.07
O94769	Extracellular matrix protein 2 OS=Homo sapiens GN=ECM2 PE=2 SV=1 - [ECM2_HUMAN]	11.02	1	7	7	11	-1.48	-1.65	-2.24	-2.41	0.88	0.71	-0.24	-0.41	0.01	0.19	-0.11	-0.29	1.29	1.37	2.12	1.69	2.46	2.34	3.10	2.06

Q8WUF5	RelA-associated inhibitor OS=Homo sapiens GN=PPP1R13L PE=1 SV=4 - [IASPP_HUMAN]	2.78	1	1	1	2	-1.05	-0.96	-1.50	-1.41	1.38	1.46	0.51	0.59	1.02	0.94	0.85	0.93	1.61	1.90	2.34	2.02	2.46	2.41	2.85	2.06
Q9NSD5	Sodium- and chloride-dependent GABA transporter 2 OS=Homo sapiens GN=SLC6A13 PE=1 SV=3 - [S6A13_HUMAN]	7.14	1	3	3	16	-1.78	-1.61	-1.92	-1.77	0.12	0.21	-0.10	0.00	0.00	0.03	-0.03	-0.10	1.68	1.82	2.08	1.78	2.05	1.88	2.13	2.05
Q99944	Epidermal growth factor-like protein 8 OS=Homo sapiens GN=EGFL8 PE=1 SV=1 - [EGFL8_HUMAN]	13.99	1	3	3	6	-1.70	-1.41	-1.77	-1.48	0.28	0.57	0.22	0.51	0.65	0.37	0.45	0.74	1.97	2.16	2.22	2.09	2.17	1.96	2.03	2.05
P20062	Transcobalamin-2 OS=Homo sapiens GN=TCN2 PE=1 SV=3 - [TCO2_HUMAN]	3.75	1	1	1	1	-2.00	-2.14	-2.07	-2.21	0.53	0.38	-0.08	-0.22	0.14	0.29	0.27	0.12	1.97	2.27	2.33	2.31	2.38	2.51	2.58	2.05
Q66K79	Carboxypeptidase Z OS=Homo sapiens GN=CPZ PE=1 SV=2 - [CBPZ_HUMAN]	28.53	1	13	13	52	-0.38	-1.04	-1.04	-1.26	1.93	2.02	0.62	0.50	0.95	1.11	0.44	0.27	1.48	1.31	1.81	1.91	2.40	2.52	3.03	2.05
Q9UHG0	Doublecortin domain-containing protein 2 OS=Homo sapiens GN=DCDC2 PE=1 SV=2 - [DCDC2_HUMAN]	2.31	1	1	1	2	-1.68	-1.61	-2.11	-2.03	-0.58	-0.51	-0.12	-0.05	-0.21	-0.28	0.04	0.11	1.61	1.72	2.14	1.43	1.86	1.09	1.51	2.05
O43866	CD5 antigen-like OS=Homo sapiens GN=CD5L PE=1 SV=1 - [CD5L_HUMAN]	29.68	1	7	7	16	-0.53	-0.45	-0.97	-1.16	0.06	0.00	1.11	1.14	0.83	0.77	1.58	1.39	1.39	1.87	2.27	1.37	1.59	0.85	0.92	2.04
P39900	Macrophage metalloelastase OS=Homo sapiens GN=MMP12 PE=1 SV=1 - [MMP12_HUMAN]	2.13	1	1	1	1	-2.56	-2.47	-2.38	-2.29	-0.07	0.02	-0.41	-0.32	-0.18	-0.27	0.05	0.14	2.20	2.61	2.43	2.32	2.15	2.48	2.30	2.04

P21810	Biglycan OS=Homo sapiens GN=BGN PE=1 SV=2 - [PGS1_HUMAN]	59.24	1	17	18	474	-2.25	-2.34	-2.44	-2.48	-0.27	-0.30	-0.50	-0.48	-0.47	-0.46	-0.53	-0.53	1.79	1.81	2.03	1.89	2.12	2.06	2.26	2.03
P78539	Sushi repeat-containing protein SRPX OS=Homo sapiens GN=SRPX PE=2 SV=1 - [SRPX_HUMAN]	28.66	1	12	12	34	-0.59	-0.72	-0.73	-0.77	2.98	2.62	1.11	1.05	1.70	1.50	0.87	0.66	1.58	1.38	1.60	2.20	2.58	3.35	3.50	2.02
Q03135	Caveolin-1 OS=Homo sapiens GN=CAV1 PE=1 SV=4 - [CAV1_HUMAN]	51.69	2	8	8	57	-2.69	-2.66	-2.73	-2.76	-0.79	-0.71	-0.83	-0.81	-0.75	-0.81	-0.35	-0.36	2.00	2.26	2.34	1.99	1.94	1.91	1.90	2.02
P35542	Serum amyloid A-4 protein OS=Homo sapiens GN=SAA4 PE=1 SV=2 - [SAA4_HUMAN]	33.08	1	4	4	11	-3.16	-3.07	-3.21	-3.13	0.01	0.09	-0.88	-0.88	-0.27	-0.35	-0.40	-0.45	2.36	2.56	2.60	2.81	2.46	3.15	2.80	2.02
P16157	Ankyrin-1 OS=Homo sapiens GN=ANK1 PE=1 SV=3 - [ANK1_HUMAN]	44.55	1	58	62	207	-1.40	-1.39	-1.52	-1.43	0.39	0.40	0.43	0.43	0.68	0.63	1.25	1.31	1.91	2.64	2.76	2.07	2.15	1.72	1.88	2.01
P53007	Tricarboxylate transport protein, mitochondrial OS=Homo sapiens GN=SLC25A1 PE=1 SV=2 - [TXTP_HUMAN]	21.22	1	7	7	14	-1.94	-1.86	-1.91	-1.83	-0.03	-0.02	0.07	0.13	-0.06	-0.11	-0.02	-0.01	2.07	1.69	1.74	1.78	1.82	1.67	1.71	2.01
P52569	Low affinity cationic amino acid transporter 2 OS=Homo sapiens GN=SLC7A2 PE=1 SV=2 - [CTR2_HUMAN]	5.32	1	4	4	6	-1.06	-1.17	-1.45	-1.56	1.00	0.89	0.49	0.38	0.55	0.67	0.74	0.62	1.61	1.80	2.19	1.75	2.15	2.04	2.43	2.00
Q9Y625	Glypican-6 OS=Homo sapiens GN=GPC6 PE=1 SV=1 - [GPC6_HUMAN]	15.68	2	6	8	13	-2.47	-2.40	-2.70	-2.81	0.43	0.33	-0.56	-0.70	0.18	0.25	0.69	0.54	1.78	3.23	3.36	2.75	2.94	2.94	3.15	2.00
P08514	Integrin alpha IIb OS=Homo sapiens GN=ITGA2B PE=1 SV=3 - [ITA2B_HUMAN]	14.92	1	12	12	19	-1.31	-1.07	-1.27	-1.03	1.17	1.33	0.75	0.83	1.00	0.91	1.22	1.32	1.79	2.28	2.43	2.07	2.16	2.41	2.41	2.00

Q6UW02	Cytochrome P450 20A1 OS=Homo sapiens GN=CYP20A1 PE=2 SV=1 - [CP20A_HUMAN]	7.58	1	3	3	4	-2.46	-2.40	-2.36	-2.30	-0.02	0.04	-0.43	-0.37	-0.11	-0.17	0.02	0.07	2.09	2.48	2.38	2.32	2.23	2.42	2.33	2.00
P13164	Interferon-induced transmembrane protein 1 OS=Homo sapiens GN=IFITM1 PE=1 SV=3 - [IFM1_HUMAN]	12.80	3	1	1	3	-3.07	-2.93	-3.14	-3.00	-0.01	0.13	-1.21	-1.07	-0.34	-0.48	0.19	0.33	1.92	3.27	3.33	2.63	2.70	3.05	3.11	2.00
Q96TA1	Niban-like protein 1 OS=Homo sapiens GN=FAM129B PE=1 SV=3 - [NIBL1_HUMAN]	16.89	1	9	9	24	-1.94	-1.77	-1.91	-1.78	0.37	0.47	0.08	0.07	0.00	-0.05	-0.24	-0.09	1.91	1.84	1.81	1.97	1.95	2.18	1.99	1.99
P21854	B-cell differentiation antigen CD72 OS=Homo sapiens GN=CD72 PE=1 SV=1 - [CD72_HUMAN]	3.90	1	1	1	1	-0.94	-1.24	-1.25	-1.55	0.24	-0.06	0.68	0.38	1.27	1.57	0.92	0.61	1.67	1.86	2.17	2.54	2.85	1.16	1.47	1.99
Q8NCH0	Carbohydrate sulfotransferase 14 OS=Homo sapiens GN=CHST14 PE=1 SV=2 - [CHSTE_HUMAN]	23.14	1	7	7	28	-0.27	-0.31	-0.60	-0.63	2.70	2.70	1.14	1.16	1.73	1.76	0.96	0.83	1.47	1.18	1.71	2.03	2.59	2.90	3.56	1.99
O95967	EGF-containing fibulin-like extracellular matrix protein 2 OS=Homo sapiens GN=EFEMP2 PE=1 SV=3 - [FBLN4_HUMAN]	14.22	1	6	6	10	-1.45	-1.33	-1.40	-1.30	1.22	1.25	0.40	0.47	0.75	0.65	0.83	0.70	1.91	1.99	2.22	2.04	2.12	2.58	2.70	1.98
Q9P299	Coatomer subunit zeta-2 OS=Homo sapiens GN=COPZ2 PE=2 SV=1 - [COPZ2_HUMAN]	18.57	1	3	3	3	-1.99	-1.90	-2.33	-2.24	-0.07	0.02	-0.41	-0.32	-0.29	-0.37	-0.70	-0.61	1.63	1.30	1.63	1.64	1.99	1.90	2.25	1.98
O94788	Retinal dehydrogenase 2 OS=Homo sapiens GN=ALDH1A2 PE=1 SV=3 - [AL1A2_HUMAN]	23.55	1	8	10	15	-1.62	-0.88	-2.11	-1.96	0.35	0.49	-0.20	-0.07	0.25	-0.07	-0.12	-0.03	1.15	1.36	1.79	1.49	2.01	1.51	2.35	1.98

Q12802	A-kinase anchor protein 13 OS=Homo sapiens GN=AKAP13 PE=1 SV=2 - [AKP13_HUMAN]	2.74	1	5	5	8	-1.30	-1.14	-1.42	-1.41	0.83	0.93	0.14	0.17	0.46	0.45	0.42	0.43	1.92	1.90	1.95	1.89	1.95	2.12	2.33	1.98
P36222	Chitinase-3-like protein 1 OS=Homo sapiens GN=CHI3L1 PE=1 SV=2 - [CH3L1_HUMAN]	27.42	1	7	7	11	-0.12	-0.26	-0.48	-0.52	2.78	2.25	1.33	1.10	1.53	2.04	2.02	1.84	1.71	2.37	2.63	2.25	2.58	2.87	3.13	1.98
Q32P28	Prolyl 3-hydroxylase 1 OS=Homo sapiens GN=LEPRE1 PE=1 SV=2 - [P3H1_HUMAN]	2.99	1	2	2	3	-2.18	-2.16	-2.52	-2.53	-0.16	-0.05	-0.61	-0.62	-0.54	-0.56	-0.24	-0.01	1.82	1.95	2.58	1.77	2.05	2.25	2.35	1.98
Q92765	Secreted frizzled-related protein 3 OS=Homo sapiens GN=FRZB PE=1 SV=2 - [SFRP3_HUMAN]	19.69	1	6	6	22	0.13	0.44	-0.52	-0.01	3.33	2.68	1.55	1.49	1.84	2.30	1.30	1.19	1.93	1.16	1.41	2.38	2.42	3.36	3.40	1.97
P00742	Coagulation factor X OS=Homo sapiens GN=F10 PE=1 SV=2 - [FA10_HUMAN]	19.06	1	9	9	15	-1.66	-1.54	-1.83	-1.77	0.60	0.69	0.07	0.13	0.24	0.12	0.02	0.00	1.81	1.80	1.85	1.93	2.09	2.34	2.42	1.97
P61626	Lysozyme C OS=Homo sapiens GN=LYZ PE=1 SV=1 - [LYSC_HUMAN]	37.16	1	4	4	19	-1.48	-1.41	-1.96	-1.81	-0.67	-0.67	-0.04	0.10	-0.11	-0.25	-0.26	-0.28	1.55	1.18	1.62	1.28	1.68	0.81	1.11	1.97
Q53TN4	Cytochrome b reductase 1 OS=Homo sapiens GN=CYBRD1 PE=1 SV=1 - [CYBR1_HUMAN]	14.34	1	3	3	28	-1.84	-1.62	-2.00	-1.81	-0.32	-0.31	0.09	0.05	-0.08	-0.06	0.29	0.33	1.93	2.42	2.22	1.88	1.92	1.81	1.59	1.97
P43356	Melanoma-associated antigen 2 OS=Homo sapiens GN=MAGEA2 PE=1 SV=1 - [MAGA2_HUMAN]	4.78	1	1	1	1	-2.47	-2.82	-2.08	-2.43	0.09	-0.26	-0.18	-0.52	-0.37	-0.02	0.58	0.23	2.35	3.06	2.66	2.49	2.10	2.55	2.16	1.97
Q16853	Membrane primary amine oxidase OS=Homo sapiens GN=AOC3 PE=1 SV=3 - [AOC3_HUMAN]	31.19	2	15	15	100	-2.38	-2.52	-2.65	-2.66	-0.07	-0.02	-0.74	-0.89	-0.47	-0.41	-0.14	-0.14	1.71	2.43	2.50	1.96	2.22	2.48	2.68	1.97

O75094	Slit homolog 3 protein OS=Homo sapiens GN=SLIT3 PE=2 SV=3 - [SLIT3_HUMAN]	18.65	1	21	21	59	0.19	0.08	-0.27	-0.23	2.75	2.92	1.73	1.73	1.86	2.02	1.34	1.24	1.78	1.26	1.57	2.29	2.37	3.30	3.21	1.96
P53814	Smoothelin OS=Homo sapiens GN=SMTN PE=1 SV=7 - [SMTN_HUMAN]	23.01	1	17	17	30	-2.70	-2.63	-3.23	-3.28	-1.21	-1.14	-1.35	-1.36	-1.28	-1.30	-1.02	-1.04	1.24	1.52	2.23	1.52	2.21	1.67	2.19	1.96
Q6UWR7	Ectonucleotide pyrophosphatase/phosphodiesterase family member 6 OS=Homo sapiens GN=ENPP6 PE=1 SV=2 - [ENPP6_HUMAN]	40.68	1	14	14	52	-1.41	-1.37	-1.36	-1.45	0.31	0.17	0.33	0.32	0.45	0.41	0.79	0.76	1.80	2.31	2.40	1.94	1.86	1.68	1.71	1.96
P26006	Integrin alpha 3 OS=Homo sapiens GN=ITGA3 PE=1 SV=5 - [ITA3_HUMAN]	9.99	1	9	9	19	-2.35	-2.34	-2.36	-2.26	0.16	0.27	-0.49	-0.35	-0.13	-0.05	0.02	0.08	2.05	2.44	2.42	2.17	2.15	2.28	2.37	1.95
Q99439	Calponin-2 OS=Homo sapiens GN=CNN2 PE=1 SV=4 - [CNN2_HUMAN]	39.16	1	8	11	19	-2.25	-2.33	-2.31	-2.39	-0.48	-0.60	-0.58	-0.62	-0.63	-0.66	-0.86	-0.83	2.18	1.47	1.47	1.77	1.73	1.89	1.87	1.95
P15153	Ras-related C3 botulinum toxin substrate 2 OS=Homo sapiens GN=RAC2 PE=1 SV=1 - [RAC2_HUMAN]	34.38	2	2	5	16	-1.07	-0.68	-1.09	-0.69	0.65	1.04	0.80	1.19	0.92	0.53	0.06	0.45	1.92	1.14	1.15	1.63	1.65	1.70	1.72	1.95
P05062	Fructose-bisphosphate aldolase B OS=Homo sapiens GN=ALDOB PE=1 SV=2 - [ALDOB_HUMAN]	5.49	1	1	2	5	-1.18	-1.13	-1.82	-1.77	-1.73	-1.69	0.07	0.12	-0.34	-0.38	-0.71	-0.66	1.30	0.48	1.11	0.83	1.47	-0.57	0.06	1.95
P04003	C4b-binding protein alpha chain OS=Homo sapiens GN=C4BPA PE=1 SV=2 - [C4BPA_HUMAN]	42.55	1	20	20	68	-2.37	-2.28	-2.60	-2.53	-0.39	-0.36	-0.66	-0.59	-0.15	-0.28	0.41	0.56	1.68	2.88	3.11	2.42	2.40	2.16	2.11	1.95

P68871	Hemoglobin subunit beta OS=Homo sapiens GN=HBB PE=1 SV=2 - [HBB_HUMAN]	86.39	1	8	15	2327	-2.13	-2.05	-2.18	-2.07	0.34	0.42	-0.33	-0.23	0.08	-0.03	0.14	0.21	1.88	2.30	2.30	2.17	2.23	2.45	2.49	1.94
P49961	Ectonucleoside triphosphate diphosphohydrolase 1 OS=Homo sapiens GN=ENTPD1 PE=1 SV=1 - [ENTP1_HUMAN]	22.75	1	9	9	23	-2.32	-2.19	-2.36	-2.35	0.00	0.05	-0.44	-0.46	-0.29	-0.28	-0.08	-0.10	1.62	2.06	2.11	1.78	1.90	1.92	2.31	1.94
Q9NQ36	Signal peptide, CUB and EGF-like domain-containing protein 2 OS=Homo sapiens GN=SCUBE2 PE=2 SV=2 - [SCUB2_HUMAN]	2.20	1	2	2	2	0.57	0.32	-0.24	-0.49	2.04	1.78	1.63	1.39	1.14	1.39	0.01	-0.24	1.12	-0.56	0.25	0.85	1.67	1.45	2.26	1.94
Q8N2S1	Latent-transforming growth factor beta-binding protein 4 OS=Homo sapiens GN=LTBP4 PE=1 SV=2 - [LTBP4_HUMAN]	9.54	1	9	9	10	-1.88	-1.75	-2.19	-2.40	0.32	0.44	-0.36	-0.40	-0.32	-0.32	-0.55	-0.48	1.57	1.15	1.44	1.48	1.81	2.14	2.38	1.93
P01619	Ig kappa chain V-III region B6 OS=Homo sapiens PE=1 SV=1 - [KV301_HUMAN]	14.81	1	1	1	3	-1.22	-1.51	-1.51	-1.80	-0.61	-0.90	0.34	0.05	0.19	0.49	1.08	0.78	1.62	2.30	2.58	1.74	2.03	0.60	0.89	1.92
Q16647	Prostacyclin synthase OS=Homo sapiens GN=PTGIS PE=1 SV=1 - [PTGIS_HUMAN]	22.40	1	8	8	17	-2.43	-2.21	-2.68	-2.39	-0.11	-0.42	-0.73	-0.78	-0.78	-0.59	-0.72	-0.88	1.68	1.70	1.78	1.79	2.12	1.89	1.96	1.91
Q92743	Serine protease HTRA1 OS=Homo sapiens GN=HTRA1 PE=1 SV=1 - [HTRA1_HUMAN]	32.29	1	10	10	22	-0.79	-0.86	-1.06	-1.07	1.38	1.50	0.35	0.61	0.67	0.74	0.23	0.21	1.75	1.00	1.21	1.92	1.99	2.27	2.47	1.90
P22897	Macrophage mannose receptor 1 OS=Homo sapiens GN=MRC1 PE=1 SV=1 - [MRC1_HUMAN]	20.88	1	28	28	51	-1.77	-1.78	-2.35	-2.32	-0.11	-0.18	-0.39	-0.33	-0.17	-0.25	-0.06	-0.01	1.49	1.67	2.32	2.01	1.92	1.78	2.24	1.90

Q96NE9	FERM domain-containing protein 6 OS=Homo sapiens GN=FRMD6 PE=1 SV=1 - [FRMD6_HUMAN]	10.61	1	5	5	6	-1.60	-1.69	-2.13	-2.14	-0.13	-0.09	-0.24	-0.19	-0.19	-0.23	-0.42	-0.40	1.59	1.00	1.36	1.51	1.83	1.58	1.87	1.90
O95425	Supervillin OS=Homo sapiens GN=SVIL PE=1 SV=2 - [SVIL_HUMAN]	22.94	1	35	35	59	-1.74	-1.81	-2.26	-2.23	-0.13	-0.18	-0.46	-0.45	-0.33	-0.39	-0.15	-0.18	1.54	1.75	2.07	1.65	1.90	1.64	1.99	1.89
Q8IWY4	Signal peptide, CUB and EGF-like domain-containing protein 1 OS=Homo sapiens GN=SCUBE1 PE=1 SV=3 - [SCUB1_HUMAN]	2.43	1	2	2	2	-1.32	-1.25	-0.90	-0.82	1.59	1.66	0.93	1.00	1.05	0.98	0.36	0.43	2.31	1.69	1.26	2.34	1.92	2.89	2.47	1.89
P05783	Keratin, type I cytoskeletal 18 OS=Homo sapiens GN=KRT18 PE=1 SV=2 - [K1C18_HUMAN]	9.77	1	3	4	5	-2.30	-1.96	-2.25	-1.91	0.62	0.96	-0.43	-0.09	0.43	0.09	0.84	1.17	1.92	3.14	3.09	2.42	2.38	2.90	2.85	1.88
P78560	Death domain-containing protein CRADD OS=Homo sapiens GN=CRADD PE=1 SV=1 - [CRADD_HUMAN]	27.64	1	4	4	6	-0.39	-0.60	-0.57	-0.77	0.53	0.33	1.24	1.04	0.14	0.34	0.19	-0.02	1.69	0.59	0.76	0.77	0.95	0.91	1.09	1.88
P55001	Microfibrillar-associated protein 2 OS=Homo sapiens GN=MFAP2 PE=2 SV=1 - [MFAP2_HUMAN]	15.85	1	3	3	10	-2.41	-2.39	-1.88	-1.68	0.94	0.78	0.48	0.37	0.46	0.62	0.28	0.15	2.71	2.51	1.60	2.66	1.89	3.15	2.31	1.88
P20851	C4b-binding protein beta chain OS=Homo sapiens GN=C4BPB PE=1 SV=1 - [C4BPB_HUMAN]	23.81	1	5	5	12	-2.47	-2.37	-2.48	-2.35	-0.64	-0.57	-0.60	-0.53	-0.29	-0.35	0.41	0.53	1.90	2.92	2.85	2.12	2.07	1.73	1.77	1.87
Q9Y6M1	Insulin-like growth factor 2 mRNA-binding protein 2 OS=Homo sapiens GN=IGF2BP2 PE=1 SV=2 - [IF2B2_HUMAN]	13.02	1	5	5	9	-0.91	-1.00	-1.83	-1.91	0.42	0.25	-0.04	-0.11	0.10	0.18	0.05	-0.22	0.95	1.02	1.87	1.31	2.13	1.34	2.19	1.86

P51911	Calponin-1 OS=Homo sapiens GN=CNN1 PE=1 SV=2 - [CNN1_HUMAN]	53.87	1	13	15	97	-3.13	-3.14	-3.31	-3.41	-1.18	-1.20	-1.62	-1.68	-1.36	-1.41	-1.39	-1.40	1.74	1.99	2.12	1.93	1.98	2.00	1.99	1.86
P51888	Prolargin OS=Homo sapiens GN=PRELP PE=1 SV=1 - [PRELP_HUMAN]	37.17	1	14	14	152	-1.97	-2.02	-2.34	-2.35	0.17	0.20	-0.60	-0.52	-0.21	-0.22	-0.70	-0.67	1.56	1.42	1.72	1.81	2.16	2.28	2.57	1.86
P11215	Integrin alpha M OS=Homo sapiens GN=ITGAM PE=1 SV=2 - [ITAM_HUMAN]	12.33	1	12	12	21	-0.59	-0.72	-0.89	-1.18	0.36	0.25	0.73	0.79	0.46	0.45	0.28	0.34	1.37	1.20	1.40	1.12	1.65	0.96	1.26	1.86
Q9NS84	Carbohydrate sulfotransferase 7 OS=Homo sapiens GN=CHST7 PE=1 SV=2 - [CHST7_HUMAN]	4.12	1	2	2	3	-0.03	0.23	-0.76	-0.01	2.29	2.76	1.64	1.39	1.66	1.92	1.90	1.66	1.24	1.44	1.81	1.56	2.18	1.87	2.49	1.86
Q96SM3	Probable carboxypeptidase X1 OS=Homo sapiens GN=CPXM1 PE=2 SV=2 - [CPXM1_HUMAN]	15.40	1	9	9	16	-3.29	-3.42	-3.51	-3.52	-1.03	-1.00	-1.67	-1.68	-1.57	-1.56	-2.40	-2.40	1.58	0.91	1.12	1.69	1.94	2.41	2.56	1.86
P02654	Apolipoprotein C-I OS=Homo sapiens GN=APOC1 PE=1 SV=1 - [APOC1_HUMAN]	19.28	1	2	2	2	-0.79	-0.63	-0.87	-0.70	1.42	1.58	0.92	1.09	1.06	0.90	0.93	1.09	1.77	1.73	1.80	1.72	1.81	2.20	2.27	1.85
Q9NR12	PDZ and LIM domain protein 7 OS=Homo sapiens GN=PDLIM7 PE=1 SV=1 - [PDLI7_HUMAN]	56.24	1	17	17	39	-3.11	-3.00	-3.32	-3.10	-1.04	-1.02	-1.44	-1.39	-1.08	-1.10	-1.09	-0.97	1.72	2.04	2.17	1.84	1.99	1.98	2.01	1.85
Q9BZQ8	Protein Niban OS=Homo sapiens GN=FAM129A PE=1 SV=1 - [NIBAN_HUMAN]	23.60	1	16	16	29	-1.78	-1.91	-2.07	-1.89	0.30	0.36	-0.30	-0.20	-0.19	-0.14	-0.17	-0.05	1.81	2.00	1.84	1.86	1.82	2.26	2.16	1.85
P08758	Annexin A5 OS=Homo sapiens GN=ANXA5 PE=1 SV=2 - [ANXA5_HUMAN]	68.75	1	20	21	317	-1.72	-1.58	-1.88	-1.82	0.69	0.75	-0.11	-0.09	0.48	0.38	0.63	0.68	1.61	2.25	2.51	2.03	2.35	2.31	2.58	1.85

Q86YB7	Enoyl-CoA hydratase domain-containing protein 2, mitochondrial OS=Homo sapiens GN=ECHDC2 PE=2 SV=2 - [ECHD2_HUMAN]	8.90	1	2	2	5	-2.43	-1.97	-2.11	-1.65	-0.27	0.19	-0.33	0.14	0.26	-0.20	0.10	0.56	2.16	2.54	2.22	2.26	1.94	2.14	1.82	1.85
Q92911	Sodium/iodide cotransporter OS=Homo sapiens GN=SLC5A5 PE=1 SV=1 - [SC5A5_HUMAN]	11.20	1	6	6	12	-1.78	-1.82	-2.31	-2.12	-0.03	0.03	-0.48	-0.31	-0.38	-0.42	-0.92	-0.93	1.34	0.89	1.38	1.15	1.74	1.45	2.01	1.85
Q9BQI4	Coiled-coil domain-containing protein 3 OS=Homo sapiens GN=CCDC3 PE=2 SV=1 - [CCDC3_HUMAN]	17.41	1	3	3	11	0.00	-0.28	-0.03	-0.05	3.85	3.72	1.75	1.54	2.55	2.59	1.79	1.76	1.88	2.07	1.82	2.87	2.65	3.99	3.86	1.84
P07099	Epoxide hydrolase 1 OS=Homo sapiens GN=EPHX1 PE=1 SV=1 - [HYEP_HUMAN]	49.67	1	17	17	55	-1.84	-1.76	-1.98	-1.85	0.33	0.39	0.07	0.07	0.13	0.14	0.20	0.20	1.88	2.07	1.89	1.83	1.87	1.98	2.09	1.84
Q8WYN0	Cysteine protease ATG4A OS=Homo sapiens GN=ATG4A PE=1 SV=1 - [ATG4A_HUMAN]	6.03	1	1	1	1	-1.53	-1.69	-1.75	-1.90	-0.45	-0.61	0.03	-0.13	-0.41	-0.25	-0.31	-0.47	1.61	1.23	1.44	1.31	1.53	1.07	1.29	1.84
Q9NZM1	Myoferlin OS=Homo sapiens GN=MYOF PE=1 SV=1 - [MYOF_HUMAN]	48.18	1	81	82	204	-2.06	-2.08	-2.15	-2.14	-0.05	-0.01	-0.44	-0.41	-0.20	-0.26	0.09	0.07	1.72	2.21	2.27	1.87	1.93	2.07	2.07	1.84
P14923	Junction plakoglobin OS=Homo sapiens GN=JUP PE=1 SV=3 - [PLAK_HUMAN]	46.98	1	22	25	91	-1.89	-1.79	-2.12	-2.24	-0.12	-0.22	-0.62	-0.65	-0.41	-0.39	0.04	-0.06	1.60	1.96	2.29	1.71	1.87	1.81	2.21	1.84
Q96D96	Voltage-gated hydrogen channel 1 OS=Homo sapiens GN=HVCN1 PE=1 SV=1 - [HVCN1_HUMAN]	5.49	1	1	1	2	-1.06	-1.02	-1.83	-1.79	0.07	0.10	-0.06	-0.02	-0.04	-0.08	0.03	0.06	1.06	1.09	1.86	1.01	1.79	1.11	1.88	1.84
P11171	Protein 4.1 OS=Homo sapiens GN=EPB41 PE=1 SV=4 - [41_HUMAN]	36.00	1	21	24	74	-1.45	-1.50	-1.37	-1.38	0.31	0.28	0.46	0.42	0.54	0.58	1.31	1.26	1.67	2.60	2.69	1.97	2.11	1.79	1.81	1.84

Q9UIF9	Bromodomain adjacent to zinc finger domain protein 2A OS=Homo sapiens GN=BAZ2A PE=1 SV=4 - [BAZ2A_HUMAN]	0.84	1	1	2	3	-1.90	-1.87	-2.16	-2.12	0.00	0.02	-0.38	-0.35	-0.53	-0.56	-0.69	-0.66	1.57	1.22	1.47	1.37	1.63	1.88	2.13	1.84
Q9UGI8	Testin OS=Homo sapiens GN=TES PE=1 SV=1 - [TES_HUMAN]	32.30	1	13	13	25	-1.61	-1.63	-1.38	-1.57	0.57	0.52	0.19	0.07	0.05	0.09	-0.88	-0.89	1.77	0.79	0.86	1.56	1.66	2.11	2.25	1.84
P55899	IgG receptor FcRn large subunit p51 OS=Homo sapiens GN=FCGRT PE=1 SV=1 - [FCGRN_HUMAN]	15.89	1	4	4	4	-2.24	-2.15	-2.17	-2.07	0.36	0.45	-0.39	-0.30	-0.05	0.05	0.78	0.86	1.90	3.02	2.94	2.33	2.26	2.58	2.51	1.83
Q9Y6C2	EMILIN-1 OS=Homo sapiens GN=EMILIN1 PE=1 SV=2 - [EMIL1_HUMAN]	30.71	1	24	24	61	-1.88	-2.00	-2.13	-2.06	0.35	0.39	-0.34	-0.19	0.05	0.02	-0.05	-0.04	1.59	1.93	2.01	1.90	2.18	2.15	2.51	1.83
Q8TER0	Sushi, nidogen and EGF-like domain-containing protein 1 OS=Homo sapiens GN=SNED1 PE=2 SV=2 - [SNED1_HUMAN]	7.86	1	9	9	17	-1.34	-1.26	-1.23	-1.25	1.04	1.30	0.43	0.61	0.86	0.79	0.74	0.97	1.87	2.08	2.03	2.17	2.11	2.57	2.07	1.83
P02042	Hemoglobin subunit delta OS=Homo sapiens GN=HBD PE=1 SV=2 - [HBD_HUMAN]	87.76	1	8	15	1186	-1.90	-1.90	-2.06	-2.01	-0.13	-0.10	-0.22	-0.15	0.08	-0.01	0.47	0.48	1.77	2.36	2.44	1.94	2.11	1.79	1.90	1.83
P15924	Desmoplakin OS=Homo sapiens GN=DSP PE=1 SV=3 - [DESP_HUMAN]	45.28	1	122	122	321	-2.60	-2.57	-2.66	-2.68	-0.62	-0.66	-0.94	-0.95	-0.89	-0.88	-0.94	-0.97	1.62	1.69	1.72	1.70	1.83	1.86	1.99	1.83
Q13797	Integrin alpha 9 OS=Homo sapiens GN=ITGA9 PE=1 SV=2 - [ITA9_HUMAN]	5.80	1	4	4	6	-0.89	-1.12	-1.69	-1.83	0.00	0.21	0.07	0.12	0.22	0.04	0.14	0.12	1.29	1.24	1.83	1.37	1.77	1.40	1.68	1.83
P11277	Spectrin beta chain, erythrocytic OS=Homo sapiens GN=SPTB PE=1 SV=5 - [SPTB1_HUMAN]	57.18	2	93	101	403	-1.20	-1.10	-1.29	-1.27	0.42	0.45	0.47	0.51	0.76	0.75	1.36	1.39	1.73	2.51	2.60	1.95	2.02	1.50	1.64	1.83

P11166	Solute carrier family 2, facilitated glucose transporter member 1 OS=Homo sapiens GN=SLC2A1 PE=1 SV=2 - [GTR1_HUMAN]	10.77	1	4	5	24	-1.50	-1.51	-1.69	-1.63	0.14	-0.05	0.01	-0.09	0.35	0.41	0.98	0.98	1.56	2.47	2.62	1.73	1.95	1.54	1.57	1.82
P02749	Beta-2-glycoprotein 1 OS=Homo sapiens GN=APOH PE=1 SV=3 - [APOH_HUMAN]	45.80	1	12	12	29	-2.20	-2.05	-2.11	-1.89	0.24	0.27	-0.17	-0.15	0.15	0.07	0.77	0.85	2.00	2.95	2.85	2.31	2.25	2.30	2.33	1.82
P35749	Myosin-11 OS=Homo sapiens GN=MYH11 PE=1 SV=3 - [MYH11_HUMAN]	51.77	10	87	114	629	-2.78	-2.80	-2.99	-3.01	-1.06	-1.05	-1.22	-1.27	-1.22	-1.19	-1.06	-1.07	1.64	1.76	1.95	1.64	1.86	1.84	1.95	1.82
P29400	Collagen alpha-5(IV) chain OS=Homo sapiens GN=COL4A5 PE=1 SV=2 - [CO4A5_HUMAN]	2.02	1	1	3	17	-2.00	-2.11	-2.38	-2.49	-0.17	-0.28	-0.63	-0.74	-0.67	-0.55	-0.58	-0.70	1.42	1.43	1.80	1.48	1.87	1.82	2.20	1.81
Q9UDX3	SEC14-like protein 4 OS=Homo sapiens GN=SEC14L4 PE=2 SV=1 - [S14L4_HUMAN]	8.37	1	2	2	4	-1.21	-1.08	-1.78	-1.65	0.49	0.61	-0.03	0.10	0.53	0.41	0.42	0.55	1.24	1.64	2.20	1.65	2.22	1.68	2.25	1.81
Q96L91	E1A-binding protein p400 OS=Homo sapiens GN=EP400 PE=1 SV=4 - [EP400_HUMAN]	0.92	1	2	2	2	-1.49	-1.49	-2.44	-2.43	-0.31	-0.32	-0.69	-0.69	-0.38	-0.38	-0.31	-0.31	0.86	1.19	2.12	1.15	2.09	1.16	2.10	1.81
P20774	Mimecan OS=Homo sapiens GN=OGN PE=1 SV=1 - [MIME_HUMAN]	46.98	1	14	14	272	-2.07	-2.12	-2.12	-2.02	0.34	0.37	-0.26	-0.25	-0.18	-0.17	-0.87	-0.86	1.86	1.24	1.22	2.01	1.96	2.45	2.44	1.81
Q14714	Sarcospan OS=Homo sapiens GN=SSPN PE=2 SV=3 - [SSPN_HUMAN]	3.70	1	1	1	1	-1.31	-1.36	-1.32	-1.36	0.19	0.15	0.42	0.38	0.31	0.36	0.33	0.29	1.79	1.65	1.65	1.70	1.71	1.49	1.49	1.81
Q96H79	Zinc finger CCCH-type antiviral protein 1-like OS=Homo sapiens GN=ZC3HAV1L PE=1 SV=2 - [ZCCHL_HUMAN]	14.67	1	3	3	4	-3.04	-2.66	-2.55	-2.17	-0.97	-0.60	-0.81	-0.43	-0.62	-0.99	-0.68	-0.30	2.29	2.37	1.87	2.08	1.59	2.05	1.56	1.81

P03952	Plasma kallikrein OS=Homo sapiens GN=KLKB1 PE=1 SV=1 - [KLKB1_HUMAN]	15.36	1	9	9	9	-1.15	-1.34	-1.57	-1.51	0.93	0.79	0.49	0.22	0.47	0.56	0.95	0.73	1.73	2.10	2.23	1.92	1.99	2.06	2.13	1.80
Q13253	Noggin OS=Homo sapiens GN=NOG PE=1 SV=1 - [NOGG_HUMAN]	5.17	1	1	1	2	-1.11	-1.22	-1.22	-1.32	1.31	1.19	0.52	0.41	0.64	0.75	0.24	0.13	1.69	1.36	1.45	1.89	2.00	2.40	2.50	1.80
P17661	Desmin OS=Homo sapiens GN=DES PE=1 SV=3 - [DESM_HUMAN]	64.26	1	24	29	301	-3.38	-3.31	-3.67	-3.59	-1.13	-1.06	-1.97	-1.93	-1.38	-1.43	-1.03	-1.01	1.58	2.41	2.75	2.05	2.39	2.46	2.61	1.80
P07355	Annexin A2 OS=Homo sapiens GN=ANXA2 PE=1 SV=2 - [ANXA2_HUMAN]	59.29	2	20	20	327	-1.74	-1.77	-2.15	-2.21	-0.26	-0.23	-0.53	-0.58	-0.27	-0.28	-0.03	-0.10	1.33	1.84	2.14	1.51	2.02	1.63	2.05	1.79
Q12805	EGF-containing fibulin-like extracellular matrix protein 1 OS=Homo sapiens GN=EFEMP1 PE=1 SV=2 - [FBLN3_HUMAN]	24.34	1	10	10	51	-0.96	-0.81	-1.43	-1.09	0.92	1.02	0.53	0.54	0.79	0.74	0.64	0.66	1.43	1.63	2.05	1.58	1.88	1.68	2.29	1.79
P35625	Metalloproteinase inhibitor 3 OS=Homo sapiens GN=TIMP3 PE=1 SV=2 - [TIMP3_HUMAN]	25.12	1	5	5	45	-0.83	-0.86	-0.94	-0.99	2.94	2.93	0.79	0.83	1.75	1.77	0.99	1.02	1.56	1.75	1.85	2.60	2.74	3.66	3.88	1.79
P50238	Cysteine-rich protein 1 OS=Homo sapiens GN=CRIP1 PE=1 SV=3 - [CRIP1_HUMAN]	9.09	1	1	1	4	-3.59	-3.46	-3.71	-3.58	-2.30	-2.18	-2.10	-1.78	-1.88	-2.12	-1.22	-1.10	1.58	2.22	2.41	1.68	1.88	1.27	1.40	1.79
O95407	Tumor necrosis factor receptor superfamily member 6B OS=Homo sapiens GN=TNFRSF6B PE=1 SV=1 - [TNF6B_HUMAN]	5.33	1	2	2	4	0.03	-0.11	-0.28	-0.41	3.38	3.24	1.44	1.31	2.20	2.34	1.79	1.66	1.47	1.77	2.07	2.34	2.65	3.34	3.64	1.78

O15244	Solute carrier family 22 member 2 OS=Homo sapiens GN=SLC22A2 PE=1 SV=2 - [S22A2_HUMAN]	9.55	1	6	6	10	-0.76	-0.78	-1.09	-1.11	0.51	0.49	0.62	0.61	0.33	0.35	0.06	0.05	1.44	0.83	1.16	1.15	1.48	1.26	1.59	1.78
Q9H3G5	Probable serine carboxypeptidase CPVL OS=Homo sapiens GN=CPVL PE=1 SV=2 - [CPVL_HUMAN]	18.49	1	8	8	17	-2.55	-2.41	-2.12	-2.04	0.11	0.20	-0.52	-0.34	-0.04	-0.19	-0.11	0.00	1.95	2.13	2.06	2.37	2.09	2.55	2.43	1.78
P19823	Inter-alpha-trypsin inhibitor heavy chain H2 OS=Homo sapiens GN=ITIH2 PE=1 SV=2 - [ITIH2_HUMAN]	28.33	1	18	18	27	-1.53	-1.53	-1.93	-2.02	0.85	0.82	-0.31	-0.36	0.24	0.22	0.38	0.42	1.16	1.90	2.21	1.73	2.17	2.15	2.52	1.78
A6NCW0	Ubiquitin carboxyl-terminal hydrolase 17-like protein 3 OS=Homo sapiens GN=USP17L3 PE=3 SV=1 - [U17L3_HUMAN]	1.32	15	1	1	6	-1.76	-1.75	-1.79	-1.74	0.16	0.25	-0.12	-0.02	-0.21	-0.31	-0.16	-0.20	1.72	1.54	1.51	1.54	1.59	1.94	1.98	1.78
Q9UBP4	Dickkopf-related protein 3 OS=Homo sapiens GN=DKK3 PE=1 SV=2 - [DKK3_HUMAN]	37.14	1	9	9	43	-0.46	-0.27	-0.58	-0.60	2.14	2.22	1.55	1.51	2.30	2.17	2.40	2.53	1.42	2.29	2.85	2.63	2.71	2.55	2.58	1.78
P14210	Hepatocyte growth factor OS=Homo sapiens GN=HGF PE=1 SV=2 - [HGF_HUMAN]	1.65	1	1	1	1	0.77	-0.38	0.74	-0.41	2.18	1.02	2.46	1.30	0.67	1.82	1.86	0.70	1.74	1.09	1.11	1.08	1.11	1.39	1.42	1.78
P07225	Vitamin K-dependent protein S OS=Homo sapiens GN=PROS1 PE=1 SV=1 - [PROS_HUMAN]	20.27	1	12	12	27	-2.30	-2.19	-2.35	-2.26	-0.25	-0.20	-0.66	-0.55	-0.15	-0.20	0.50	0.59	1.71	2.80	2.86	2.08	2.12	2.03	1.94	1.78
Q9Y6N5	Sulfide:quinone oxidoreductase, mitochondrial OS=Homo sapiens GN=SQRDL PE=1 SV=1 - [SQRD_HUMAN]	40.44	1	18	18	50	-1.95	-1.86	-1.86	-1.88	0.28	0.34	-0.17	-0.18	-0.05	-0.08	0.01	0.02	1.75	1.94	1.89	1.91	1.87	2.15	2.14	1.77

Q99538	Legumain OS=Homo sapiens GN=LGMN PE=1 SV=1 - [LGMN_HUMAN]	11.55	1	4	4	16	-2.31	-2.32	-2.46	-2.50	-0.34	-0.29	-0.69	-0.74	-0.49	-0.54	-0.15	-0.14	1.72	2.19	2.31	1.83	2.10	2.17	2.33	1.77
P26447	Protein S100-A4 OS=Homo sapiens GN=S100A4 PE=1 SV=1 - [S10A4_HUMAN]	35.64	1	4	4	34	-2.41	-2.46	-2.43	-2.29	-0.03	0.05	-0.64	-0.55	-0.22	-0.29	-0.20	-0.11	1.84	2.08	2.11	2.14	2.03	2.33	2.38	1.77
Q5TD97	Four and a half LIM domains protein 5 OS=Homo sapiens GN=FHL5 PE=1 SV=1 - [FHL5_HUMAN]	42.61	1	12	12	21	-3.41	-3.33	-3.29	-3.16	-1.07	-1.06	-1.61	-1.44	-1.29	-1.45	-1.03	-0.94	1.94	2.22	2.29	2.10	1.91	2.43	2.17	1.77
Q15113	Procollagen C-endopeptidase enhancer 1 OS=Homo sapiens GN=PCOLCE PE=1 SV=2 - [PCOC1_HUMAN]	23.83	1	8	8	17	-0.77	-0.39	-1.54	-1.41	1.03	0.90	-0.04	0.09	0.30	0.39	-0.23	-0.19	0.64	0.52	1.50	1.30	2.16	1.69	2.62	1.77
Q9HBI1	Beta-parvin OS=Homo sapiens GN=PARVB PE=1 SV=1 - [PARVB_HUMAN]	25.82	1	5	6	10	-1.17	-1.03	-1.66	-1.49	-0.40	-0.06	-0.04	0.17	0.12	-0.13	-0.36	0.00	1.13	1.01	1.37	1.02	1.72	1.01	1.44	1.77
O95833	Chloride intracellular channel protein 3 OS=Homo sapiens GN=CLIC3 PE=1 SV=2 - [CLIC3_HUMAN]	35.59	1	5	5	7	-0.82	-1.17	-2.43	-2.48	-0.14	-0.36	-0.66	-0.68	-0.52	-0.50	-1.42	-1.48	0.72	0.73	1.00	1.46	1.86	0.80	1.87	1.77
Q6NZI2	Polymerase I and transcript release factor OS=Homo sapiens GN=PTRF PE=1 SV=1 - [PTRF_HUMAN]	31.54	1	11	11	65	-2.54	-2.59	-2.65	-2.74	-0.43	-0.48	-0.80	-0.82	-0.64	-0.54	-0.26	-0.30	1.76	2.28	2.18	2.00	1.82	2.08	2.08	1.76
P04114	Apolipoprotein B-100 OS=Homo sapiens GN=APOB PE=1 SV=2 - [APOB_HUMAN]	34.54	2	123	125	251	-2.09	-2.09	-2.34	-2.28	-0.03	0.01	-0.67	-0.64	0.01	0.00	0.78	0.84	1.54	2.88	3.15	2.10	2.35	2.05	2.25	1.76
Q9HB63	Netrin-4 OS=Homo sapiens GN=NTN4 PE=2 SV=2 - [NET4_HUMAN]	14.01	1	6	6	10	-0.83	-0.80	-1.17	-1.14	0.64	0.44	-0.60	-0.57	0.33	0.18	0.00	0.10	1.80	1.83	1.74	1.80	2.00	2.14	2.05	1.75

P46734	Dual specificity mitogen-activated protein kinase kinase 3 OS=Homo sapiens GN=MAP2K3 PE=1 SV=2 - [MP2K3_HUMAN]	16.71	1	2	5	9	-1.31	-1.35	-1.44	-1.53	-0.15	-0.19	0.35	0.13	0.05	0.20	-0.02	-0.07	1.56	1.29	1.47	1.32	1.64	1.14	1.31	1.75
Q16527	Cysteine and glycine-rich protein 2 OS=Homo sapiens GN=CSRP2 PE=1 SV=3 - [CSRP2_HUMAN]	43.52	1	7	7	21	-3.17	-3.35	-3.38	-3.44	-2.38	-2.58	-1.78	-1.79	-2.17	-2.20	-1.90	-2.01	1.45	1.07	1.47	1.11	1.26	0.46	0.67	1.75
P08962	CD63 antigen OS=Homo sapiens GN=CD63 PE=1 SV=2 - [CD63_HUMAN]	4.20	1	1	1	3	-0.69	-0.79	-1.30	-1.41	1.08	0.97	0.38	0.28	0.46	0.57	0.37	0.26	1.13	1.06	1.67	1.29	1.91	1.75	2.36	1.75
Q9BXX0	EMILIN-2 OS=Homo sapiens GN=EMILIN2 PE=1 SV=3 - [EMIL2_HUMAN]	20.42	1	16	16	28	-1.33	-1.37	-1.65	-1.55	1.27	1.21	0.02	0.13	0.58	0.54	0.35	0.45	1.68	1.79	1.87	2.04	2.28	2.59	2.85	1.75
P04083	Annexin A1 OS=Homo sapiens GN=ANXA1 PE=1 SV=2 - [ANXA1_HUMAN]	43.35	1	14	14	86	-2.01	-2.01	-2.00	-2.12	-0.20	-0.31	-0.33	-0.40	-0.27	-0.23	-0.10	-0.19	1.68	1.82	1.95	1.77	1.82	1.72	1.78	1.75
Q5HYK7	SH3 domain-containing protein 19 OS=Homo sapiens GN=SH3D19 PE=1 SV=2 - [SH319_HUMAN]	8.73	1	5	5	7	-1.69	-1.80	-1.74	-1.85	0.34	0.23	0.09	-0.05	-0.06	0.05	-0.08	-0.15	1.52	1.69	1.66	1.57	1.67	1.70	1.91	1.75
O15382	Branched-chain-amino-acid aminotransferase, mitochondrial OS=Homo sapiens GN=BCAT2 PE=1 SV=2 - [BCAT2_HUMAN]	24.23	1	7	7	28	-2.09	-1.57	-2.15	-1.88	0.27	0.35	-0.42	-0.26	-0.09	-0.02	0.07	0.19	1.61	1.67	1.87	1.36	1.83	1.82	2.15	1.74
P02751	Fibronectin OS=Homo sapiens GN=FN1 PE=1 SV=4 - [FINC_HUMAN]	42.92	1	71	71	369	-2.13	-2.13	-2.21	-2.25	1.01	1.05	-0.53	-0.50	0.18	0.14	-0.04	0.02	1.68	2.10	2.21	2.32	2.40	3.06	3.24	1.74

Q96LD4	Tripartite motif-containing protein 47 OS=Homo sapiens GN=TRIM47 PE=1 SV=2 - [TRI47_HUMAN]	24.14	1	13	13	32	-1.02	-0.91	-1.38	-1.25	1.20	1.33	0.19	0.52	0.79	0.67	0.63	0.88	1.11	1.50	1.99	1.34	2.08	1.72	2.51	1.74
Q13361	Microfibrillar-associated protein 5 OS=Homo sapiens GN=MFAP5 PE=1 SV=1 - [MFAP5_HUMAN]	6.36	1	1	1	1	-1.95	-2.01	-2.10	-2.17	1.91	1.84	-0.42	-0.49	0.29	0.36	-0.74	-0.81	1.58	1.22	1.36	2.33	2.49	3.84	3.99	1.74
Q9UBX1	Cathepsin F OS=Homo sapiens GN=CTSF PE=1 SV=1 - [CATF_HUMAN]	11.78	1	6	6	9	-0.27	-0.48	-0.47	-0.21	3.69	3.51	1.21	1.25	2.18	2.36	1.28	1.17	1.31	0.92	1.55	1.91	2.59	3.01	3.77	1.74
Q99435	Protein kinase C-binding protein NELL2 OS=Homo sapiens GN=NELL2 PE=1 SV=1 - [NELL2_HUMAN]	36.40	1	22	22	59	-1.14	-0.90	-1.31	-1.12	0.53	0.64	0.46	0.48	0.94	0.90	1.69	1.81	1.53	2.75	3.01	1.97	2.35	1.62	1.72	1.74
P41214	Eukaryotic translation initiation factor 2D OS=Homo sapiens GN=EIF2D PE=1 SV=3 - [EIF2D_HUMAN]	4.97	1	1	1	1	-1.61	-1.64	-2.19	-2.22	0.09	0.05	-0.52	-0.55	-0.03	0.01	0.23	0.20	1.15	1.85	2.42	1.65	2.23	1.68	2.26	1.74
P07204	Thrombomodulin OS=Homo sapiens GN=THBD PE=1 SV=2 - [TRBM_HUMAN]	3.48	1	1	1	1	-2.03	-2.42	-2.69	-3.08	-0.09	-0.48	-1.02	-1.41	-0.69	-0.29	0.30	-0.10	1.07	2.33	2.98	1.77	2.43	1.92	2.58	1.74
Q96MW7	Tigger transposable element-derived protein 1 OS=Homo sapiens GN=TIGD1 PE=1 SV=1 - [TIGD1_HUMAN]	1.35	1	1	1	1	-2.71	-2.62	-2.32	-2.24	-0.01	0.07	-0.66	-0.57	-0.56	-0.65	-0.24	-0.16	2.11	2.47	2.08	2.09	1.71	2.68	2.29	1.73
Q8WWT9	Solute carrier family 13 member 3 OS=Homo sapiens GN=SLC13A3 PE=1 SV=1 - [S13A3_HUMAN]	9.80	1	4	4	13	-0.58	-0.68	-1.66	-2.09	-0.03	-0.12	-0.02	-0.36	-0.42	0.03	-0.17	-0.62	0.69	0.92	1.43	1.00	1.71	0.68	1.94	1.72

Q14126	Desmoglein-2 OS=Homo sapiens GN=DSG2 PE=1 SV=2 - [DSG2_HUMAN]	39.53	1	29	29	79	-2.43	-2.49	-2.63	-2.52	-0.29	-0.36	-0.76	-0.82	-0.54	-0.52	-0.17	-0.21	1.65	2.09	2.23	1.83	1.92	1.91	2.22	1.72
Q99715	Collagen alpha-1(XII) chain OS=Homo sapiens GN=COL12A1 PE=1 SV=2 - [COCA1_HUMAN]	46.82	1	105	105	416	-2.55	-2.56	-2.84	-2.87	-1.00	-0.96	-1.18	-1.14	-0.93	-0.97	-0.75	-0.73	1.49	1.92	2.09	1.70	1.91	1.76	1.98	1.72
Q5VV43	Dyslexia-associated protein KIAA0319 OS=Homo sapiens GN=KIAA0319 PE=1 SV=1 - [K0319_HUMAN]	1.68	1	1	1	1	-2.32	-2.46	-2.58	-2.72	-0.21	-0.36	-0.93	-1.07	-0.39	-0.25	-0.40	-0.54	1.44	1.93	2.18	2.10	2.37	2.09	2.35	1.72
P01210	Proenkephalin-A OS=Homo sapiens GN=PENK PE=1 SV=1 - [PENK_HUMAN]	19.48	1	4	4	6	-0.35	-0.71	-0.99	-1.34	1.93	1.73	0.66	0.31	2.00	2.36	3.62	3.26	1.07	3.98	4.60	2.74	3.38	2.42	3.05	1.71
Q10588	ADP-ribosyl cyclase 2 OS=Homo sapiens GN=BST1 PE=1 SV=2 - [BST1_HUMAN]	29.87	1	6	6	14	-1.78	-1.80	-2.10	-2.09	-0.20	-0.17	-0.48	-0.56	-0.45	-0.40	-0.45	-0.61	1.30	1.12	1.56	1.15	1.88	1.59	2.12	1.71
Q9BXJ2	Complement C1q tumor necrosis factor-related protein 7 OS=Homo sapiens GN=C1QTNF7 PE=1 SV=1 - [C1QT7_HUMAN]	10.38	1	2	2	4	-1.71	-1.68	-1.85	-1.82	1.04	1.07	-0.21	-0.17	0.34	0.30	0.32	0.35	1.56	2.04	2.17	2.05	2.19	2.73	2.87	1.71
Q8TED1	Probable glutathione peroxidase 8 OS=Homo sapiens GN=GPX8 PE=1 SV=2 - [GPX8_HUMAN]	11.96	1	3	3	4	-1.84	-1.89	-1.83	-1.87	0.15	0.10	-0.19	-0.23	-0.02	0.04	0.27	0.22	1.71	2.11	2.09	1.91	1.90	1.98	1.96	1.70
P30613	Pyruvate kinase PKLR OS=Homo sapiens GN=PKLR PE=1 SV=2 - [KPYR_HUMAN]	30.31	1	14	15	34	-1.66	-1.68	-1.77	-1.76	-0.30	-0.30	-0.21	-0.26	-0.19	-0.27	-0.18	-0.23	1.49	1.42	1.49	1.52	1.49	1.34	1.47	1.70

O43556	Epsilon-sarcoglycan OS=Homo sapiens GN=SGCE PE=1 SV=6 - [SGCE_HUMAN]	25.86	1	7	7	14	-1.65	-1.68	-1.61	-1.53	0.01	0.09	-0.04	0.05	0.00	-0.03	0.26	0.02	1.78	1.95	1.59	1.87	1.56	1.64	1.61	1.69
O60687	Sushi repeat-containing protein SRPX2 OS=Homo sapiens GN=SRPX2 PE=1 SV=1 - [SRPX2_HUMAN]	17.20	1	6	6	12	-1.57	-1.42	-1.68	-1.64	0.04	0.25	-0.37	-0.02	0.05	-0.29	-0.42	-0.22	1.26	1.50	1.65	1.31	1.63	1.77	1.71	1.68
O43323	Desert hedgehog protein OS=Homo sapiens GN=DHH PE=1 SV=1 - [DHH_HUMAN]	5.56	1	1	1	1	-0.79	-0.35	-1.27	-0.83	1.21	1.64	0.34	0.78	1.03	0.59	0.79	1.22	1.19	1.58	2.06	1.42	1.90	1.99	2.46	1.68
Q14031	Collagen alpha-6(IV) chain OS=Homo sapiens GN=COL4A6 PE=2 SV=3 - [CO4A6_HUMAN]	2.72	1	4	4	7	-1.90	-1.66	-2.17	-2.25	-0.32	-0.40	-0.67	-0.54	-0.28	-0.55	-0.04	-0.07	1.29	1.87	2.18	1.38	1.65	1.73	1.84	1.68
Q96S86	Hyaluronan and proteoglycan link protein 3 OS=Homo sapiens GN=HAPLN3 PE=2 SV=1 - [HPLN3_HUMAN]	9.72	1	2	3	3	-0.46	-0.58	-0.81	-0.93	2.50	2.37	0.80	0.68	1.32	1.44	0.20	0.07	1.32	0.67	1.01	1.94	2.29	2.95	3.29	1.68
P69905	Hemoglobin subunit alpha OS=Homo sapiens GN=HBA1 PE=1 SV=2 - [HBA_HUMAN]	67.61	1	11	12	1152	-1.83	-1.74	-1.96	-1.88	0.25	0.32	-0.36	-0.27	-0.01	-0.07	0.13	0.24	1.55	1.94	2.08	1.92	1.93	2.10	2.20	1.68
Q8N5C1	Protein FAM26E OS=Homo sapiens GN=FAM26E PE=2 SV=1 - [FA26E_HUMAN]	10.68	1	3	3	6	-1.57	-1.52	-1.73	-1.68	0.78	0.83	-0.12	-0.07	0.27	0.23	0.21	0.25	1.51	1.78	1.93	1.83	1.99	2.33	2.49	1.67
P98160	Basement membrane-specific heparan sulfate proteoglycan core protein OS=Homo sapiens GN=HSPG2 PE=1 SV=4 - [PGBM_HUMAN]	37.08	1	110	110	518	-2.01	-2.00	-2.23	-2.25	-0.12	-0.11	-0.64	-0.62	-0.48	-0.47	-0.62	-0.65	1.45	1.38	1.57	1.58	1.76	1.92	2.14	1.67

Q5T9L3	Protein wntless homolog OS=Homo sapiens GN=WLS PE=1 SV=2 - [WLS_HUMAN]	5.36	1	3	3	7	-0.94	-1.02	-1.87	-1.95	0.25	0.17	-0.26	-0.34	0.06	0.15	-0.05	-0.13	0.73	0.90	1.82	1.12	2.05	1.17	2.10	1.67
Q5BLP8	Neuropeptide like protein C4orf48 OS=Homo sapiens GN=C4orf48 PE=1 SV=3 - [CD048_HUMAN]	18.95	1	2	2	5	-0.59	-0.69	-1.12	-1.48	0.59	0.70	0.49	0.43	1.20	1.15	1.78	1.90	1.13	2.34	2.86	1.63	2.16	1.08	1.61	1.67
Q15375	Ephrin type-A receptor 7 OS=Homo sapiens GN=EPHA7 PE=1 SV=3 - [EPHA7_HUMAN]	13.03	6	5	10	16	-1.03	-1.07	-1.52	-1.59	0.16	-0.10	0.04	-0.15	-0.28	-0.05	0.07	-0.05	0.81	0.85	1.39	1.03	1.48	0.79	1.38	1.67
P15169	Carboxypeptidase N catalytic chain OS=Homo sapiens GN=CPN1 PE=1 SV=1 - [CBPN_HUMAN]	5.24	1	2	2	4	-1.40	-1.43	-1.74	-1.77	0.40	0.37	-0.14	-0.17	0.02	0.06	-0.88	-0.91	1.31	0.53	0.87	1.48	1.83	1.78	2.13	1.67
Q96AY3	Peptidyl-prolyl cis-trans isomerase FKBP10 OS=Homo sapiens GN=FKBP10 PE=1 SV=1 - [FKB10_HUMAN]	15.12	2	7	7	8	-2.10	-2.34	-1.95	-2.00	0.19	0.11	-0.30	-0.45	-0.21	-0.20	0.10	-0.07	1.43	1.83	1.98	2.24	1.78	2.22	2.11	1.67
Q641Q3	Meteorin-like protein OS=Homo sapiens GN=METRNL PE=2 SV=1 - [METRL_HUMAN]	27.65	1	6	6	15	-0.53	-0.39	-0.69	-0.67	2.44	2.61	0.91	1.00	1.54	1.38	0.58	0.73	1.34	0.87	1.18	1.92	2.24	2.78	3.10	1.67
Q9BQB6	Vitamin K epoxide reductase complex subunit 1 OS=Homo sapiens GN=VKORC1 PE=1 SV=1 - [VKOR1_HUMAN]	7.98	1	1	1	2	-1.74	-1.59	-2.16	-2.01	0.19	0.34	-0.56	-0.41	-0.09	-0.24	-0.07	0.08	1.24	1.68	2.09	1.53	1.96	1.91	2.33	1.67
P22413	Ectonucleotide pyrophosphatase/phosphodiesterase family member 1 OS=Homo sapiens GN=ENPP1 PE=1 SV=2 - [ENPP1_HUMAN]	6.59	1	5	5	6	0.54	-0.04	-0.77	-1.55	0.61	0.39	0.57	0.05	0.71	0.95	0.62	0.16	0.14	0.21	1.70	0.79	1.86	0.30	1.81	1.66

Q7Z5L7	Podocan OS=Homo sapiens GN=PODN PE=1 SV=2 - [PODN_HUMAN]	22.68	1	12	12	20	-1.62	-1.32	-2.16	-1.96	0.85	1.00	-0.59	-0.40	0.35	0.15	-0.25	-0.09	1.08	1.48	1.91	1.80	2.22	2.49	2.95	1.66
P31949	Protein S100-A11 OS=Homo sapiens GN=S100A11 PE=1 SV=2 - [S10AB_HUMAN]	36.19	1	4	4	24	-2.01	-1.80	-2.07	-1.97	-0.10	0.04	-0.50	-0.40	-0.20	-0.37	-0.04	0.06	1.58	2.05	2.05	1.73	1.79	1.97	2.02	1.66
P35908	Keratin, type II cytoskeletal 2 epidermal OS=Homo sapiens GN=KRT2 PE=1 SV=2 - [K22E_HUMAN]	28.33	2	7	16	26	-1.27	-0.59	-2.29	-2.06	-0.39	0.08	0.07	0.81	0.23	-0.37	3.64	3.68	0.62	4.91	4.79	0.85	1.95	0.85	2.20	1.66
P02745	Complement C1q subcomponent subunit A OS=Homo sapiens GN=C1QA PE=1 SV=2 - [C1QA_HUMAN]	22.86	1	3	3	5	-1.42	-1.38	-1.28	-1.24	1.04	1.07	0.37	0.41	0.74	0.70	1.07	1.10	1.84	2.49	2.23	2.15	1.81	2.44	2.19	1.65
P0C0L4	Complement C4-A OS=Homo sapiens GN=C4A PE=1 SV=2 - [CO4A_HUMAN]	33.49	1	2	48	174	-0.60	-0.64	-0.83	-1.04	1.51	1.46	0.81	0.77	1.02	1.07	0.80	0.75	1.46	1.39	1.58	1.69	1.88	2.08	2.27	1.65
Q5KU26	Collectin-12 OS=Homo sapiens GN=COLEC12 PE=1 SV=3 - [COL12_HUMAN]	23.99	1	17	17	29	-2.42	-2.35	-2.37	-2.36	-0.53	-0.46	-0.75	-0.72	-0.40	-0.44	-0.19	-0.09	1.66	2.17	2.07	1.94	1.92	1.84	1.77	1.65
A1L4H1	Soluble scavenger receptor cysteine-rich domain-containing protein SSC5D OS=Homo sapiens GN=SSC5D PE=2 SV=3 - [SRCRL_HUMAN]	7.44	1	7	7	12	-2.26	-2.35	-1.85	-1.97	-0.43	-0.61	-0.11	-0.22	-0.47	-0.40	-0.40	-0.53	2.10	1.76	1.42	1.78	1.32	1.54	1.08	1.65
O00159	Unconventional myosin-Ic OS=Homo sapiens GN=MYO1C PE=1 SV=4 - [MYO1C_HUMAN]	47.88	1	45	45	118	-2.44	-2.35	-2.44	-2.36	-0.42	-0.44	-0.71	-0.76	-0.55	-0.58	-0.53	-0.59	1.63	1.88	1.92	1.81	1.78	1.93	1.85	1.65

Q86UD1	Out at first protein homolog OS=Homo sapiens GN=OAF PE=2 SV=1 - [OAF_HUMAN]	4.03	1	1	1	1	-0.85	-0.74	-1.14	-1.02	1.58	1.69	0.45	0.57	1.05	0.95	0.74	0.85	1.36	1.60	1.88	1.83	2.12	2.41	2.70	1.65
P46721	Solute carrier organic anion transporter family member 1A2 OS=Homo sapiens GN=SLCO1A2 PE=2 SV=1 - [SO1A2_HUMAN]	1.64	1	1	1	1	-0.80	-0.68	-1.00	-0.88	0.79	0.91	0.58	0.70	0.84	0.72	0.23	0.34	1.44	1.04	1.23	1.55	1.76	1.58	1.78	1.65
P18206	Vinculin OS=Homo sapiens GN=VCL PE=1 SV=4 - [VINC_HUMAN]	60.23	1	60	60	325	-2.32	-2.29	-2.45	-2.36	-0.56	-0.52	-0.89	-0.89	-0.67	-0.71	-0.44	-0.44	1.52	1.91	1.99	1.76	1.79	1.83	1.89	1.64
Q9H4M9	EH domain-containing protein 1 OS=Homo sapiens GN=EHD1 PE=1 SV=2 - [EHD1_HUMAN]	66.48	2	20	28	95	-1.34	-1.40	-1.17	-1.27	0.18	0.12	0.41	0.36	0.18	0.22	0.09	0.04	1.79	1.45	1.36	1.51	1.41	1.46	1.35	1.64
P53708	Integrin alpha 8 OS=Homo sapiens GN=ITGA8 PE=1 SV=3 - [ITA8_HUMAN]	17.69	1	13	13	16	-1.89	-1.85	-2.25	-2.20	-0.21	-0.18	-0.83	-0.79	-0.29	-0.41	-0.22	-0.40	1.01	1.40	1.93	1.29	1.78	1.63	1.93	1.64
Q9P0K7	Ankycorbin OS=Homo sapiens GN=RAI14 PE=1 SV=2 - [RAI14_HUMAN]	21.12	1	18	18	26	-1.85	-1.86	-2.13	-2.08	-0.28	-0.20	-0.51	-0.51	-0.54	-0.52	-0.56	-0.55	1.45	1.30	1.51	1.39	1.58	1.86	1.81	1.63
P27105	Erythrocyte band 7 integral membrane protein OS=Homo sapiens GN=STOM PE=1 SV=3 - [STOM_HUMAN]	40.97	1	9	9	47	-1.31	-1.23	-1.52	-1.35	0.63	0.69	0.12	0.19	0.54	0.51	0.82	1.00	1.48	2.04	2.25	1.64	2.01	1.87	2.05	1.63
O75487	Glypican-4 OS=Homo sapiens GN=GPC4 PE=1 SV=4 - [GPC4_HUMAN]	20.50	2	6	8	10	-1.10	-1.04	-1.42	-1.30	0.91	0.97	0.15	0.22	0.63	0.63	0.81	0.93	1.22	2.01	2.23	1.77	2.11	2.04	2.31	1.63

Q969G5	Protein kinase C delta-binding protein OS=Homo sapiens GN=PRKCDBP PE=1 SV=3 - [PRDBP_HUMAN]	30.27	1	8	8	16	-2.42	-2.21	-2.60	-2.98	-0.84	-0.72	-1.10	-1.21	-0.89	-0.77	-0.28	-0.28	1.35	2.01	2.59	1.68	1.92	1.47	1.80	1.63
P29622	Kallistatin OS=Homo sapiens GN=SERPINA4 PE=1 SV=3 - [KAIN_HUMAN]	3.04	1	1	1	2	-1.20	-1.20	-1.14	-1.13	1.35	1.34	0.43	0.43	0.88	0.88	1.06	1.05	1.69	2.27	2.19	2.11	2.05	2.53	2.46	1.63
Q9NPY3	Complement component C1q receptor OS=Homo sapiens GN=CD93 PE=1 SV=3 - [C1QR1_HUMAN]	1.69	1	1	1	2	-2.10	-1.94	-2.38	-2.21	-0.52	-0.36	-0.81	-0.65	-0.49	-0.65	-0.68	-0.52	1.34	1.43	1.70	1.49	1.76	1.57	1.84	1.63
P61803	Dolichyl-diphosphooligosaccharide--protein glycosyltransferase subunit DAD1 OS=Homo sapiens GN=DAD1 PE=1 SV=3 - [DAD1_HUMAN]	19.47	1	2	2	6	-1.32	-1.21	-1.98	-1.86	0.35	0.41	-0.42	-0.40	-0.09	-0.11	0.07	0.03	0.95	1.25	1.90	1.15	1.81	1.63	2.29	1.62
Q13643	Four and a half LIM domains protein 3 OS=Homo sapiens GN=FHL3 PE=1 SV=4 - [FHL3_HUMAN]	16.79	1	5	5	9	-2.06	-1.96	-2.19	-2.08	-0.49	-0.39	-0.65	-0.52	-0.56	-0.66	-1.06	-0.96	1.50	1.01	1.21	1.51	1.67	1.54	1.70	1.62
P51884	Lumican OS=Homo sapiens GN=LUM PE=1 SV=2 - [LUM_HUMAN]	40.53	1	12	12	74	-1.96	-1.88	-2.01	-1.98	0.75	0.89	-0.33	-0.27	0.00	-0.10	-0.86	-0.68	1.58	1.09	1.18	1.80	1.82	2.76	2.63	1.62
Q3SYG4	Protein PTHB1 OS=Homo sapiens GN=BBS9 PE=1 SV=1 - [PTHB1_HUMAN]	1.92	1	1	1	1	-0.96	-0.60	-0.99	-0.63	0.63	0.99	0.56	0.92	0.53	0.16	0.47	0.83	1.58	1.44	1.46	1.16	1.19	1.58	1.61	1.62
Q9Y3Q0	N-acetylated-alpha-linked acidic dipeptidase 2 OS=Homo sapiens GN=NAALAD2 PE=1 SV=1 - [NALD2_HUMAN]	12.30	2	6	7	12	-1.37	-1.00	-1.64	-1.46	0.48	0.80	-0.08	0.13	0.36	0.15	0.35	0.67	1.35	1.73	2.07	1.51	1.75	1.79	2.04	1.61

P00915	Carbonic anhydrase 1 OS=Homo sapiens GN=CA1 PE=1 SV=2 - [CAH1_HUMAN]	53.64	1	10	10	149	-2.51	-2.50	-2.41	-2.40	-0.23	-0.18	-0.85	-0.89	-0.85	-0.93	-1.69	-1.73	1.69	0.58	0.62	1.69	1.56	2.27	2.18	1.61
Q8NEK5	Zinc finger protein 548 OS=Homo sapiens GN=ZNF548 PE=2 SV=2 - [ZN548_HUMAN]	1.31	2	1	1	1	-1.96	-2.16	-1.41	-1.60	0.33	0.13	0.14	-0.06	-0.57	-0.36	0.45	0.24	2.16	2.42	1.85	1.63	1.08	2.28	1.72	1.61
P50895	Basal cell adhesion molecule OS=Homo sapiens GN=BCAM PE=1 SV=2 - [BCAM_HUMAN]	45.70	1	21	21	105	-2.56	-2.49	-2.68	-2.63	-1.21	-1.14	-1.18	-1.15	-1.01	-1.05	-0.59	-0.58	1.38	1.95	2.08	1.49	1.59	1.34	1.53	1.61
Q93052	Lipoma-preferred partner OS=Homo sapiens GN=LPP PE=1 SV=1 - [LPP_HUMAN]	41.83	1	17	17	92	-2.16	-2.25	-2.30	-2.31	-0.82	-0.87	-0.75	-0.76	-0.76	-0.73	-0.51	-0.50	1.35	1.57	1.84	1.39	1.58	1.33	1.52	1.61
P08648	Integrin alpha 5 OS=Homo sapiens GN=ITGA5 PE=1 SV=2 - [ITA5_HUMAN]	13.25	1	10	10	26	-1.13	-1.11	-1.69	-1.32	1.35	1.35	0.11	0.17	0.72	0.74	0.76	0.96	1.26	1.72	2.33	1.74	2.20	2.12	2.71	1.61
P08174	Complement decay-accelerating factor OS=Homo sapiens GN=CD55 PE=1 SV=4 - [DAF_HUMAN]	32.81	1	9	9	27	-1.28	-1.35	-1.33	-1.36	0.86	0.71	0.28	0.28	0.41	0.58	0.63	0.49	1.56	1.74	1.83	1.80	1.90	1.98	2.16	1.60
P29373	Cellular retinoic acid-binding protein 2 OS=Homo sapiens GN=CRABP2 PE=1 SV=2 - [RABP2_HUMAN]	25.36	1	2	2	2	-1.48	-2.26	-1.24	-2.02	0.18	-0.59	0.29	-0.48	-0.17	0.61	0.21	-0.57	1.83	1.70	1.45	2.12	1.88	1.65	1.41	1.60
Q969X1	Protein lifeguard 3 OS=Homo sapiens GN=TMBIM1 PE=1 SV=2 - [LFG3_HUMAN]	4.50	1	1	1	4	0.34	0.10	-0.91	-1.15	1.94	1.69	0.62	0.38	1.05	1.29	1.11	0.87	0.34	0.78	2.02	0.98	2.24	1.58	2.84	1.60

Q13546	Receptor-interacting serine/threonine-protein kinase 1 OS=Homo sapiens GN=RIPK1 PE=1 SV=3 - [RIPK1_HUMAN]	2.68	1	1	1	1	-1.51	-1.55	-1.58	-1.61	0.42	0.39	-0.05	-0.08	0.14	0.17	-0.21	-0.24	1.52	1.31	1.37	1.72	1.79	1.92	1.98	1.60
Q9BZF9	Uveal autoantigen with coiled-coil domains and ankyrin repeats OS=Homo sapiens GN=UACA PE=1 SV=2 - [UACA_HUMAN]	21.96	1	28	28	47	-2.10	-2.08	-1.89	-1.94	-0.04	-0.11	-0.43	-0.43	-0.32	-0.27	-0.15	-0.17	1.66	1.90	1.75	1.79	1.61	1.90	1.80	1.59
P56199	Integrin alpha 1 OS=Homo sapiens GN=ITGA1 PE=1 SV=2 - [ITA1_HUMAN]	17.39	1	22	22	52	-2.08	-1.98	-2.04	-1.99	0.05	0.11	-0.51	-0.46	-0.28	-0.38	-0.50	-0.38	1.59	1.60	1.57	1.85	1.68	2.12	2.10	1.59
P98082	Disabled homolog 2 OS=Homo sapiens GN=DAB2 PE=1 SV=3 - [DAB2_HUMAN]	10.00	1	6	6	6	-0.97	-1.25	-1.32	-1.59	0.71	0.44	0.21	-0.07	0.28	0.56	0.50	0.22	1.24	1.48	1.82	1.56	1.91	1.67	2.01	1.59
P02647	Apolipoprotein A-I OS=Homo sapiens GN=APOA1 PE=1 SV=1 - [APOA1_HUMAN]	52.81	1	15	15	54	-1.77	-1.71	-1.86	-1.89	0.11	0.17	-0.36	-0.39	-0.16	-0.15	0.01	-0.06	1.55	1.89	1.94	1.93	1.74	1.87	1.95	1.59
Q9UGT4	Sushi domain containing protein 2 OS=Homo sapiens GN=SUSD2 PE=1 SV=1 - [SUSD2_HUMAN]	28.10	1	14	14	61	-1.64	-1.58	-1.86	-1.95	0.51	0.56	-0.34	-0.25	0.10	-0.01	0.17	0.22	1.31	1.78	2.19	1.66	2.07	2.31	2.32	1.59
P05546	Heparin cofactor 2 OS=Homo sapiens GN=SERPIND1 PE=1 SV=3 - [HEP2_HUMAN]	14.43	1	8	8	14	-2.98	-2.96	-3.06	-3.06	-1.10	-1.10	-1.52	-1.57	-1.00	-1.17	-1.10	-1.05	1.68	1.98	2.09	2.06	2.00	2.14	1.94	1.59
Q9H2X0	Chordin OS=Homo sapiens GN=CHRD PE=1 SV=2 - [CHRD_HUMAN]	6.60	1	5	5	8	-0.24	-0.05	-0.66	-0.46	2.55	2.74	0.86	1.06	1.89	1.69	0.92	1.11	1.16	1.17	1.58	1.97	2.39	2.77	3.19	1.58

Q9BX79	Stimulated by retinoic acid gene 6 protein homolog OS=Homo sapiens GN=STRA6 PE=1 SV=1 - [STRA6_HUMAN]	7.20	1	3	3	6	-2.35	-2.29	-2.47	-2.41	-0.72	-0.66	-0.95	-0.89	-0.69	-0.74	-0.66	-0.60	1.45	1.70	1.81	1.64	1.76	1.61	1.74	1.58
P43121	Cell surface glycoprotein MUC18 OS=Homo sapiens GN=MCAM PE=1 SV=2 - [MUC18_HUMAN]	33.59	1	17	17	71	-1.24	-1.23	-1.70	-1.64	0.06	0.02	-0.19	-0.23	-0.13	-0.19	-0.07	0.04	1.42	1.49	1.74	1.61	1.74	1.81	1.85	1.58
Q96CS2	HAUS augmin-like complex subunit 1 OS=Homo sapiens GN=HAUS1 PE=1 SV=1 - [HAUS1_HUMAN]	3.96	1	1	1	1	-0.43	-0.99	-1.92	-2.49	-0.55	-1.12	-0.41	-0.97	-0.36	0.21	-0.44	-1.01	0.08	0.00	1.49	0.66	2.16	-0.14	1.36	1.58
Q15124	Phosphoglucomutase-like protein 5 OS=Homo sapiens GN=PGM5 PE=1 SV=2 - [PGM5_HUMAN]	43.74	1	19	21	49	-2.73	-2.68	-2.90	-2.69	-0.68	-0.58	-1.45	-1.22	-0.90	-1.06	-0.78	-0.73	1.49	2.05	2.10	1.94	1.96	2.14	2.11	1.58
Q9HCE1	Putative helicase MOV-10 OS=Homo sapiens GN=MOV10 PE=1 SV=2 - [MOV10_HUMAN]	6.88	1	5	5	6	-1.04	-1.22	-1.97	-2.07	-0.30	-0.48	-0.46	-0.55	-0.40	-0.47	-0.47	-0.57	0.63	0.56	1.41	0.49	1.55	0.76	1.34	1.58
O00151	PDZ and LIM domain protein 1 OS=Homo sapiens GN=PDLIM1 PE=1 SV=4 - [PDLI1_HUMAN]	54.41	1	13	13	32	-2.22	-2.14	-2.12	-2.21	0.01	0.00	-0.46	-0.51	-0.41	-0.40	-0.43	-0.52	1.75	1.91	1.80	1.70	1.72	2.13	1.97	1.58
Q13214	Semaphorin-3B OS=Homo sapiens GN=SEMA3B PE=2 SV=1 - [SEM3B_HUMAN]	14.29	1	8	8	11	-0.71	-0.86	-0.98	-1.02	1.81	1.84	0.61	0.54	0.99	0.98	0.48	0.48	1.20	1.18	1.46	1.80	2.13	2.68	2.84	1.58
P13224	Platelet glycoprotein Ib beta chain OS=Homo sapiens GN=GP1BB PE=1 SV=1 - [GP1BB_HUMAN]	19.90	1	4	4	6	-0.97	-1.17	-1.10	-1.06	0.63	0.51	0.65	0.54	0.89	0.84	1.23	1.13	1.64	2.67	2.36	1.96	2.06	1.57	1.47	1.57

Q13576	Ras GTPase-activating-like protein IQGAP2 OS=Homo sapiens GN=IQGAP2 PE=1 SV=4 - [IQGA2_HUMAN]	18.03	1	19	24	37	-1.10	-1.10	-1.14	-1.36	0.42	0.38	0.18	0.17	0.34	0.36	0.40	0.18	1.33	1.29	1.53	1.46	1.72	1.53	1.78	1.57
Q14213	Interleukin-27 subunit beta OS=Homo sapiens GN=EBI3 PE=1 SV=2 - [IL27B_HUMAN]	4.80	1	1	1	2	-1.00	-0.63	-0.60	-0.23	2.06	2.42	0.91	1.28	1.61	1.25	0.87	1.23	1.97	1.88	1.47	2.28	1.88	3.05	2.64	1.57
O95980	Reversion-inducing cysteine-rich protein with Kazal motifs OS=Homo sapiens GN=RECK PE=1 SV=1 - [RECK_HUMAN]	3.60	1	3	3	3	-2.37	-2.37	-1.94	-1.71	-0.24	-0.02	-0.51	-0.22	-0.28	-0.26	0.04	0.26	1.66	2.15	1.98	2.12	1.48	1.88	1.68	1.57
Q13418	Integrin-linked protein kinase OS=Homo sapiens GN=ILK PE=1 SV=2 - [ILK_HUMAN]	34.51	1	14	14	33	-1.66	-1.71	-1.95	-1.93	-0.49	-0.39	-0.52	-0.44	-0.55	-0.57	-0.46	-0.32	1.36	1.28	1.44	1.25	1.36	1.29	1.39	1.57
Q96NA2	Rab-interacting lysosomal protein OS=Homo sapiens GN=RILP PE=1 SV=1 - [RILP_HUMAN]	9.48	1	2	2	3	-0.47	-0.31	-1.29	-1.13	-0.46	-0.31	0.21	0.38	0.34	0.18	0.55	0.71	0.74	1.03	1.84	0.68	1.51	-0.01	0.81	1.57
P05106	Integrin beta-3 OS=Homo sapiens GN=ITGB3 PE=1 SV=2 - [ITB3_HUMAN]	18.40	1	10	11	22	-1.47	-1.57	-1.26	-1.35	1.16	0.93	0.45	-0.03	0.25	0.73	0.89	0.66	1.34	2.50	2.28	2.20	1.99	2.70	2.49	1.56
O60487	Myelin protein zero-like protein 2 OS=Homo sapiens GN=MPZL2 PE=1 SV=1 - [MPZL2_HUMAN]	6.05	1	1	1	2	-1.87	-1.36	-1.53	-1.01	0.91	1.42	-0.03	0.49	0.64	0.14	0.50	1.01	1.90	2.38	2.03	2.04	1.70	2.77	2.42	1.56
Q08188	Protein-glutamine gamma-glutamyltransferase E OS=Homo sapiens GN=TGM3 PE=1 SV=4 - [TGM3_HUMAN]	10.53	1	4	4	8	-3.69	-3.80	-4.36	-4.40	-3.15	-3.19	-3.23	-3.21	-2.46	-2.47	-2.83	-2.97	0.36	0.98	1.51	1.09	2.29	0.51	1.91	1.56

Q86W92	Liprin-beta-1 OS=Homo sapiens GN=PPFIBP1 PE=1 SV=2 - [LIPB1_HUMAN]	34.32	2	26	26	51	-1.53	-1.45	-1.73	-1.81	0.06	0.06	-0.23	-0.28	-0.18	-0.17	-0.20	-0.18	1.22	1.26	1.52	1.33	1.51	1.44	1.57	1.56
P62736	Actin, aortic smooth muscle OS=Homo sapiens GN=ACTA2 PE=1 SV=1 - [ACTA_HUMAN]	71.09	2	3	26	1384	-2.10	-2.20	-2.56	-2.79	-0.68	-0.83	-1.20	-1.28	-1.13	-1.01	-0.57	-0.66	1.22	1.46	1.93	1.43	1.65	1.58	2.01	1.56
P26572	Alpha-1,3-mannosyl-glycoprotein 2-beta-N-acetylglucosaminyltransferase OS=Homo sapiens GN=MGAT1 PE=2 SV=2 - [MGAT1_HUMAN]	15.51	1	6	6	13	-0.93	-0.77	-0.88	-1.13	2.76	2.60	0.70	0.69	1.41	1.56	0.95	0.63	1.04	1.10	1.73	2.09	2.34	3.20	3.29	1.55
Q86UX2	Inter-alpha-trypsin inhibitor heavy chain H5 OS=Homo sapiens GN=ITIH5 PE=2 SV=2 - [ITIH5_HUMAN]	27.39	1	19	19	53	-1.56	-1.33	-1.96	-1.88	-0.21	-0.03	-0.53	-0.42	-0.29	-0.32	-0.30	-0.18	1.12	1.41	1.65	1.34	1.83	1.34	1.84	1.55
P21333	Filamin-A OS=Homo sapiens GN=FLNA PE=1 SV=4 - [FLNA_HUMAN]	62.15	1	115	126	1008	-2.34	-2.27	-2.47	-2.42	-0.89	-0.86	-1.02	-0.98	-0.83	-0.87	-0.50	-0.45	1.30	1.85	2.06	1.47	1.65	1.44	1.60	1.55
Q8WUP2	Filamin-binding LIM protein 1 OS=Homo sapiens GN=FBLIM1 PE=1 SV=2 - [FBLI1_HUMAN]	20.64	1	7	7	14	-2.30	-2.35	-2.14	-2.19	-0.45	-0.44	-0.65	-0.70	-0.80	-0.74	-1.14	-1.20	1.71	1.16	1.00	1.59	1.43	1.75	1.59	1.55
P50281	Matrix metalloproteinase-14 OS=Homo sapiens GN=MMP14 PE=1 SV=3 - [MMP14_HUMAN]	4.98	1	3	3	6	-1.39	-1.44	-1.61	-1.66	0.46	0.40	-0.12	-0.17	0.23	0.29	0.18	0.12	1.32	1.57	1.79	1.70	1.93	1.82	2.05	1.55
P08603	Complement factor H OS=Homo sapiens GN=CFH PE=1 SV=4 - [CFAH_HUMAN]	42.49	2	39	41	131	-1.81	-1.74	-1.90	-1.78	0.16	0.19	-0.47	-0.34	-0.03	-0.13	0.28	0.35	1.40	2.09	2.08	1.72	1.81	2.00	2.04	1.55

P07942	Laminin subunit beta-1 OS=Homo sapiens GN=LAMB1 PE=1 SV=2 - [LAMB1_HUMAN]	24.92	1	32	32	70	-1.97	-2.08	-2.15	-2.11	0.35	0.26	-0.57	-0.59	-0.36	-0.38	-0.39	-0.40	1.48	1.63	1.68	1.63	1.76	2.18	2.27	1.55
P16150	Leukosialin OS=Homo sapiens GN=SPN PE=1 SV=1 - [LEUK_HUMAN]	2.00	1	1	1	2	-0.11	-0.21	-0.40	-0.50	0.57	0.46	1.08	0.98	0.93	1.04	0.80	0.69	1.24	0.91	1.20	1.18	1.48	0.66	0.95	1.55
Q6UX46	Protein FAM150B OS=Homo sapiens GN=FAM150B PE=2 SV=2 - [F150B_HUMAN]	13.82	1	2	2	4	-1.49	-1.61	-1.93	-2.04	0.18	0.06	-0.45	-0.56	-0.14	-0.02	0.10	-0.02	1.10	1.60	2.03	1.51	1.95	1.66	2.10	1.54
P30504	HLA class I histocompatibility antigen, Cw-4 alpha chain OS=Homo sapiens GN=HLA-C PE=1 SV=1 - [1C04_HUMAN]	31.42	2	2	10	26	-1.45	-1.36	-1.41	-1.32	-0.34	-0.25	0.07	0.16	0.19	0.10	-0.03	0.06	1.58	1.43	1.38	1.58	1.55	1.10	1.06	1.54
P04208	Ig lambda chain V-I region WAH OS=Homo sapiens PE=1 SV=1 - [LV106_HUMAN]	11.93	2	1	1	2	-0.95	-0.65	-1.08	-0.78	-0.71	-0.41	0.40	0.70	0.47	0.18	0.45	0.75	1.41	1.41	1.53	1.16	1.29	0.23	0.36	1.54
Q06278	Aldehyde oxidase OS=Homo sapiens GN=AOX1 PE=1 SV=2 - [AOXA_HUMAN]	7.55	2	7	7	8	-0.21	-0.96	-0.54	-1.29	1.10	0.34	0.94	0.19	0.11	0.86	1.34	0.58	1.21	1.55	1.87	1.10	1.43	1.29	1.62	1.54
P01871	Ig mu chain C region OS=Homo sapiens GN=IGHM PE=1 SV=3 - [IGHM_HUMAN]	38.05	2	15	15	75	-0.82	-0.75	-0.80	-0.79	-0.47	-0.45	0.71	0.74	0.56	0.54	0.97	1.00	1.53	1.74	1.75	1.38	1.37	0.40	0.42	1.54
Q16363	Laminin subunit alpha 4 OS=Homo sapiens GN=LAMA4 PE=1 SV=4 - [LAMA4_HUMAN]	22.55	1	32	32	64	-1.77	-1.76	-2.30	-2.27	-0.06	-0.12	-0.87	-0.88	-0.61	-0.55	-0.75	-0.71	1.24	1.21	1.52	1.40	1.69	1.74	2.11	1.54
Q8IUX7	Adipocyte enhancer-binding protein 1 OS=Homo sapiens GN=AEBP1 PE=1 SV=1 - [AEBP1_HUMAN]	15.89	2	15	15	40	-2.01	-1.74	-2.32	-2.31	-0.38	-0.41	-0.96	-0.80	-0.72	-0.68	-0.90	-0.88	1.26	1.08	1.54	1.20	1.69	1.64	2.02	1.54

P05452	Tetranectin OS=Homo sapiens GN=CLEC3B PE=1 SV=3 - [TETN_HUMAN]	34.65	1	5	5	5	-2.15	-2.02	-1.85	-1.70	-0.23	0.05	-0.51	-0.24	-0.21	-0.34	-0.24	-0.10	1.38	1.91	1.92	1.69	1.70	1.76	1.77	1.54
P08493	Matrix Gla protein OS=Homo sapiens GN=MGP PE=1 SV=2 - [MGP_HUMAN]	24.27	1	3	3	30	-2.35	-2.18	-2.60	-2.35	0.97	0.95	-0.92	-0.84	0.37	0.38	0.68	0.63	1.35	3.28	3.12	2.75	2.92	3.35	3.45	1.53
Q9HB40	Retinoid-inducible serine carboxypeptidase OS=Homo sapiens GN=SCPEP1 PE=1 SV=1 - [RISC_HUMAN]	5.09	1	2	2	3	-1.78	-1.88	-1.92	-2.02	0.38	0.28	-0.46	-0.56	-0.06	0.05	0.33	0.22	1.37	2.11	2.25	1.86	2.01	2.14	2.29	1.53
Q02487	Desmocollin-2 OS=Homo sapiens GN=DSC2 PE=1 SV=1 - [DSC2_HUMAN]	15.76	1	9	10	25	-2.45	-2.42	-2.59	-2.66	-0.25	-0.27	-1.04	-1.09	-0.64	-0.74	-0.13	-0.13	1.24	2.02	2.38	1.71	1.89	2.04	2.34	1.53
P01701	Ig lambda chain V-I region NEW OS=Homo sapiens PE=1 SV=1 - [LV103_HUMAN]	11.71	1	1	1	2	-0.90	-1.91	-1.49	-2.49	-0.12	-1.14	-0.03	-1.03	-0.76	0.25	0.88	-0.13	0.93	1.79	2.37	1.18	1.77	0.76	1.35	1.53
Q9NZD4	Alpha-hemoglobin-stabilizing protein OS=Homo sapiens GN=AHSP PE=1 SV=1 - [AHSP_HUMAN]	20.59	1	2	2	3	-2.49	-2.47	-2.19	-2.17	-0.62	-0.60	-0.73	-0.70	-0.96	-0.97	-1.67	-1.65	1.82	0.83	0.52	1.55	1.25	1.86	1.55	1.53
P41218	Myeloid cell nuclear differentiation antigen OS=Homo sapiens GN=MNDA PE=1 SV=1 - [MNDA_HUMAN]	33.66	1	13	13	26	-1.27	-1.23	-1.28	-1.16	-0.67	-0.53	0.29	0.28	0.02	0.01	-0.06	-0.16	1.54	0.99	1.16	1.11	1.03	0.48	0.64	1.52
P16401	Histone H1.5 OS=Homo sapiens GN=HIST1H1B PE=1 SV=3 - [H15_HUMAN]	24.78	1	5	8	28	-1.20	-1.37	-0.99	-1.00	-0.23	-0.25	0.44	0.35	-0.06	-0.06	0.08	-0.09	1.74	1.29	1.02	1.22	1.06	1.12	0.74	1.52
Q8WX93	Palladin OS=Homo sapiens GN=PALLD PE=1 SV=3 - [PALLD_HUMAN]	20.39	2	19	19	51	-2.37	-2.43	-2.54	-2.50	-0.61	-0.59	-0.95	-1.09	-0.86	-0.85	-0.79	-0.76	1.40	1.62	1.75	1.55	1.70	1.69	1.95	1.52

Q9UER7	Death domain-associated protein 6 OS=Homo sapiens GN=DAXX PE=1 SV=2 - [DAXX_HUMAN]	3.51	1	2	2	2	-2.12	-1.71	-1.91	-1.49	-1.45	-1.04	-0.45	-0.04	-2.43	-2.83	-1.40	-1.00	1.72	0.72	0.50	-0.69	-0.89	0.65	0.44	1.52
Q9NY15	Stabilin-1 OS=Homo sapiens GN=STAB1 PE=1 SV=3 - [STAB1_HUMAN]	9.42	1	18	18	36	-1.41	-1.23	-1.54	-1.63	0.71	0.94	-0.34	-0.14	0.29	0.17	-0.02	0.11	1.19	1.45	1.61	1.68	1.88	2.29	2.56	1.52
Q07092	Collagen alpha-1(XVI) chain OS=Homo sapiens GN=COL16A1 PE=1 SV=2 - [COGA1_HUMAN]	9.04	1	8	8	27	-2.96	-3.12	-3.09	-3.29	-1.00	-1.21	-1.65	-1.82	-1.57	-1.55	-1.58	-1.72	1.36	1.34	1.25	1.71	1.69	2.03	1.97	1.51
Q96P44	Collagen alpha-1(XXI) chain OS=Homo sapiens GN=COL21A1 PE=2 SV=1 - [COLA1_HUMAN]	6.90	1	6	6	20	-2.13	-2.14	-2.45	-2.63	-1.03	-1.34	-1.12	-1.41	-1.37	-1.11	-1.07	-1.28	1.60	1.51	1.57	1.57	1.59	1.53	1.34	1.51
P07359	Platelet glycoprotein Ib alpha chain OS=Homo sapiens GN=GP1BA PE=1 SV=2 - [GP1BA_HUMAN]	7.98	1	4	4	4	-0.97	-0.85	-1.45	-1.39	0.17	0.16	0.13	0.25	0.62	0.16	0.60	0.71	1.15	1.58	1.97	1.06	2.68	1.09	1.51	1.51
Q14644	Ras GTPase-activating protein 3 OS=Homo sapiens GN=RASA3 PE=1 SV=3 - [RASA3_HUMAN]	4.32	1	3	3	4	-1.41	-1.49	-2.98	-3.06	-0.87	-0.95	-1.53	-1.61	-1.19	-1.11	-0.52	-0.60	-0.06	0.90	2.46	0.33	1.90	0.53	2.09	1.51
P04040	Catalase OS=Homo sapiens GN=CAT PE=1 SV=3 - [CATA_HUMAN]	66.79	1	27	27	119	-1.82	-1.84	-1.91	-1.86	-0.34	-0.32	-0.47	-0.45	-0.53	-0.59	-0.70	-0.68	1.43	1.20	1.14	1.37	1.28	1.52	1.45	1.51
P04626	Receptor tyrosine-protein kinase erbB-2 OS=Homo sapiens GN=ERBB2 PE=1 SV=1 - [ERBB2_HUMAN]	5.42	6	4	5	12	-1.18	-1.02	-1.94	-1.79	-0.38	-0.22	-0.58	-0.37	-0.21	-0.36	-0.24	-0.03	0.76	0.76	1.79	0.85	1.64	0.78	1.73	1.51

O00264	Membrane-associated progesterone receptor component 1 OS=Homo sapiens GN=PGRMC1 PE=1 SV=3 - [PGRC1_HUMAN]	44.10	1	7	9	30	-1.50	-1.48	-1.60	-1.55	0.30	0.18	-0.15	-0.12	-0.13	-0.07	0.15	0.16	1.44	1.69	1.72	1.72	1.47	1.82	1.60	1.51
Q12929	Epidermal growth factor receptor kinase substrate 8 OS=Homo sapiens GN=EPS8 PE=1 SV=1 - [EPS8_HUMAN]	19.10	1	12	12	24	-1.27	-1.26	-1.92	-1.93	-0.15	-0.27	-0.38	-0.51	-0.22	-0.26	-0.35	-0.48	0.74	1.02	1.51	0.95	1.60	1.11	1.63	1.51
O95810	Serum deprivation-response protein OS=Homo sapiens GN=SDPR PE=1 SV=3 - [SDPR_HUMAN]	45.41	1	15	15	51	-1.69	-1.95	-1.87	-1.85	-0.15	-0.19	-0.34	-0.29	-0.36	-0.29	-0.19	-0.23	1.53	1.68	1.65	1.63	1.52	1.61	1.66	1.50
P41222	Prostaglandin-H2 D-isomerase OS=Homo sapiens GN=PTGDS PE=1 SV=1 - [PTGDS_HUMAN]	53.16	1	8	8	40	-0.65	-0.81	-0.61	-0.71	1.55	1.45	0.77	0.59	0.81	0.92	0.76	0.52	1.51	1.41	1.31	1.71	1.42	2.30	2.01	1.50
Q86UX7	Fermitin family homolog 3 OS=Homo sapiens GN=FERMT3 PE=1 SV=1 - [URP2_HUMAN]	22.79	1	12	12	23	-0.45	-0.60	-0.88	-0.93	0.77	0.58	0.45	0.40	0.61	0.71	0.64	0.57	0.95	1.22	1.45	1.26	1.60	1.28	1.63	1.50
Q9NW15	Anoctamin-10 OS=Homo sapiens GN=ANO10 PE=1 SV=2 - [ANO10_HUMAN]	9.24	1	5	5	6	-1.58	-1.81	-1.27	-1.50	0.28	0.17	-0.02	-0.06	-0.53	-0.37	-0.22	-0.36	1.80	1.36	1.04	1.31	1.00	1.97	1.66	1.50
Q99983	Osteomodulin OS=Homo sapiens GN=OMD PE=1 SV=1 - [OMD_HUMAN]	14.73	1	5	5	9	-2.28	-2.30	-2.37	-2.39	-1.03	-1.08	-1.03	-1.08	-1.34	-1.29	-1.16	-1.19	1.39	1.14	1.21	1.31	1.41	1.48	1.58	1.50
P29536	Leiomodin-1 OS=Homo sapiens GN=LMOD1 PE=1 SV=3 - [LMOD1_HUMAN]	20.17	1	10	10	25	-2.59	-2.66	-2.60	-2.65	-1.33	-1.51	-1.09	-1.16	-0.99	-0.84	-0.30	-0.26	1.53	2.36	2.40	1.89	1.80	1.80	1.62	1.50

P09525	Annexin A4 OS=Homo sapiens GN=ANXA4 PE=1 SV=4 - [ANXA4_HUMAN]	62.70	1	18	19	93	-1.50	-1.52	-1.50	-1.62	0.36	0.43	-0.14	-0.09	0.20	0.12	0.46	0.47	1.46	1.95	1.99	1.79	1.91	1.87	1.93	1.49
Q96CM8	Acyl-CoA synthetase family member 2, mitochondrial OS=Homo sapiens GN=ACSF2 PE=1 SV=2 - [ACSF2_HUMAN]	32.85	1	16	16	43	-2.24	-2.21	-2.31	-2.45	-0.80	-0.86	-0.80	-0.93	-0.96	-0.86	-0.81	-0.94	1.36	1.41	1.50	1.48	1.48	1.43	1.59	1.49
P46977	Dolichyl-diphosphooligosaccharide--protein glycosyltransferase subunit STT3A OS=Homo sapiens GN=STT3A PE=1 SV=2 - [STT3A_HUMAN]	6.52	1	5	5	10	-2.15	-2.21	-1.83	-1.88	0.05	0.12	-0.39	-0.44	-0.24	-0.21	-0.04	0.01	1.79	2.17	1.85	2.01	1.69	2.32	1.97	1.49
Q9NPH2	Inositol-3-phosphate synthase 1 OS=Homo sapiens GN=ISYNA1 PE=1 SV=1 - [INO1_HUMAN]	22.58	1	9	9	33	-1.29	-1.25	-1.99	-1.92	-0.29	-0.37	-0.56	-0.50	-0.45	-0.45	-0.38	-0.37	0.75	1.23	1.64	1.27	1.74	1.04	1.60	1.49
P01742	Ig heavy chain V-I region EU OS=Homo sapiens PE=1 SV=1 - [HV101_HUMAN]	10.26	3	1	1	5	-1.29	-1.31	-1.39	-1.41	0.72	0.69	0.04	0.02	0.54	0.57	1.36	1.33	1.38	2.65	2.74	1.89	1.99	1.99	2.08	1.49
P07358	Complement component C8 beta chain OS=Homo sapiens GN=C8B PE=1 SV=3 - [CO8B_HUMAN]	18.10	1	9	9	15	-1.65	-1.72	-1.67	-1.85	0.24	0.10	-0.42	-0.56	-0.01	0.08	0.62	0.63	1.12	2.16	2.36	1.81	2.20	1.71	2.33	1.48
O15335	Chondroadherin OS=Homo sapiens GN=CHAD PE=2 SV=2 - [CHAD_HUMAN]	25.91	1	8	8	13	-3.49	-3.51	-4.44	-4.39	-2.72	-2.60	-2.77	-2.73	-2.74	-2.78	-2.70	-2.72	1.03	0.80	1.30	0.88	1.51	0.75	1.32	1.48
P08572	Collagen alpha-2(IV) chain OS=Homo sapiens GN=COL4A2 PE=1 SV=4 - [CO4A2_HUMAN]	14.89	1	16	16	132	-2.22	-2.37	-2.36	-2.41	0.27	0.07	-0.78	-0.82	-0.42	-0.43	-0.50	-0.59	1.28	1.63	1.74	1.72	1.81	2.19	2.40	1.48

O76074	cGMP-specific 3',5'-cyclic phosphodiesterase OS=Homo sapiens GN=PDE5A PE=1 SV=2 - [PDE5A_HUMAN]	4.80	1	3	3	4	-1.87	-1.52	-1.84	-1.99	-0.30	-0.23	-0.48	-0.58	-0.45	-0.36	-0.58	-0.74	1.45	1.61	1.37	1.54	1.58	1.52	1.75	1.48
P01877	Ig alpha-2 chain C region OS=Homo sapiens GN=IGHA2 PE=1 SV=3 - [IGHA2_HUMAN]	32.06	1	1	8	38	-1.37	-1.08	-1.30	-1.01	-0.63	-0.35	0.11	0.40	-0.13	-0.41	-0.86	-0.58	1.54	0.52	0.44	0.99	0.92	0.72	0.65	1.48
P15559	NAD(P)H dehydrogenase [quinone] 1 OS=Homo sapiens GN=NQO1 PE=1 SV=1 - [NQO1_HUMAN]	27.01	1	6	6	8	-0.41	-0.49	-0.26	-0.33	0.82	0.77	1.10	1.13	0.80	0.78	0.61	0.57	1.45	1.04	0.70	1.13	1.10	1.35	1.01	1.48
Q02388	Collagen alpha-1(VII) chain OS=Homo sapiens GN=COL7A1 PE=1 SV=2 - [CO7A1_HUMAN]	1.22	1	2	2	4	-0.55	-0.11	-1.63	-1.19	0.56	0.99	-0.21	0.23	0.17	-0.26	-0.35	0.08	0.39	0.20	1.27	0.31	1.40	1.09	2.17	1.47
Q9NR34	Mannosyl-oligosaccharide 1,2-alpha-mannosidase IC OS=Homo sapiens GN=MAN1C1 PE=2 SV=1 - [MA1C1_HUMAN]	6.19	1	3	3	5	-1.42	-1.34	-1.72	-1.64	0.05	0.13	-0.31	-0.23	0.17	0.10	0.10	0.18	1.16	1.52	1.82	1.54	1.85	1.45	1.75	1.47
Q8N6S5	ADP-ribosylation factor-like protein 6-interacting protein 6 OS=Homo sapiens GN=ARL6IP6 PE=1 SV=1 - [AR6P6_HUMAN]	10.18	1	2	2	3	-1.67	-1.67	-1.94	-1.93	-0.76	-0.75	-0.53	-0.52	-0.52	-0.52	-0.27	-0.27	1.20	1.41	1.66	1.18	1.45	0.90	1.16	1.47
P57764	Gasdermin-D OS=Homo sapiens GN=GSDMD PE=1 SV=1 - [GSDMD_HUMAN]	9.50	1	5	5	7	-0.68	-0.91	-0.79	-1.02	0.44	0.16	0.64	0.20	0.09	0.53	-0.69	-0.88	1.44	0.34	0.16	1.00	0.96	1.03	0.69	1.47

P48059	LIM and senescent cell antigen-like-containing domain protein 1 OS=Homo sapiens GN=LIMS1 PE=1 SV=4 - [LIMS1_HUMAN]	35.69	1	6	11	23	-2.04	-2.06	-2.02	-1.93	-0.48	-0.51	-0.54	-0.47	-0.49	-0.68	-0.56	-0.51	1.57	1.64	1.40	1.54	1.46	1.70	1.50	1.46
Q86SQ0	Pleckstrin homology-like domain family B member 2 OS=Homo sapiens GN=PHLDB2 PE=1 SV=2 - [PHLB2_HUMAN]	9.82	1	11	11	14	-1.99	-1.90	-2.17	-2.12	-0.28	-0.30	-0.69	-0.73	-0.46	-0.46	-0.13	-0.12	1.24	1.58	1.74	1.38	1.58	1.56	1.81	1.46
Q6ZMP0	Thrombospondin type-1 domain-containing protein 4 OS=Homo sapiens GN=THSD4 PE=2 SV=2 - [THSD4_HUMAN]	16.11	1	14	14	25	-1.61	-1.38	-1.38	-1.08	0.75	0.76	0.24	0.33	0.43	0.30	0.30	0.35	1.90	1.77	1.40	1.80	1.39	2.06	1.83	1.46
Q8TEU8	WAP, Kazal, immunoglobulin, Kunitz and NTR domain-containing protein 2 OS=Homo sapiens GN=WFIKKN2 PE=1 SV=1 - [WFKN2_HUMAN]	5.56	1	2	2	2	-0.71	-1.06	-0.97	-1.32	0.34	-0.01	0.43	0.08	0.29	0.64	1.19	0.83	1.19	1.90	2.16	1.38	1.65	1.03	1.30	1.46
P01814	Ig heavy chain V-II region OU OS=Homo sapiens PE=1 SV=1 - [HV201_HUMAN]	5.56	2	1	1	1	-0.62	-0.45	-0.34	-0.16	-0.11	0.05	1.06	1.23	0.40	0.23	-0.71	-0.54	1.73	-0.08	-0.37	0.88	0.60	0.49	0.21	1.46
Q14574	Desmocollin-3 OS=Homo sapiens GN=DSC3 PE=1 SV=3 - [DSC3_HUMAN]	17.30	1	9	10	18	-1.79	-1.95	-1.99	-2.14	-0.14	-0.28	-0.71	-0.76	-0.50	-0.32	-0.19	-0.27	1.15	1.54	1.72	1.49	1.51	1.55	1.74	1.46
P04839	Cytochrome b-245 heavy chain OS=Homo sapiens GN=CYBB PE=1 SV=2 - [CY24B_HUMAN]	11.40	2	7	7	8	-0.55	-0.38	-0.64	-0.56	0.36	0.81	1.08	1.35	0.49	0.42	0.85	0.92	1.53	1.61	1.44	1.42	1.09	0.93	1.01	1.46

Q96BW5	Phosphotriesterase-related protein OS=Homo sapiens GN=PTER PE=1 SV=1 - [PTER_HUMAN]	12.03	1	3	3	3	-1.99	-1.82	-1.29	-1.13	-0.38	-0.22	0.10	0.27	-0.07	-0.23	0.48	0.64	2.14	2.47	1.77	1.78	1.09	1.59	0.90	1.46
Q9UDR5	Alpha-aminoadipic semialdehyde synthase, mitochondrial OS=Homo sapiens GN=AASS PE=1 SV=1 - [AASS_HUMAN]	27.75	1	20	20	32	-1.78	-1.48	-1.73	-1.70	0.06	0.08	-0.22	-0.15	-0.24	-0.25	-0.33	-0.40	1.43	1.23	1.31	1.28	1.50	1.48	1.60	1.46
P05556	Integrin beta-1 OS=Homo sapiens GN=ITGB1 PE=1 SV=2 - [ITB1_HUMAN]	36.22	1	24	24	156	-1.70	-1.66	-1.87	-1.79	-0.03	0.01	-0.43	-0.37	-0.25	-0.30	-0.10	-0.06	1.32	1.62	1.82	1.61	1.64	1.77	1.75	1.46
P02774	Vitamin D-binding protein OS=Homo sapiens GN=GC PE=1 SV=1 - [VTDB_HUMAN]	48.52	1	18	19	32	-2.23	-2.12	-2.07	-2.09	-0.53	-0.49	-0.73	-0.69	-0.79	-0.72	-0.79	-0.80	1.45	1.27	1.25	1.44	1.30	1.54	1.43	1.46
Q9UP95	Solute carrier family 12 member 4 OS=Homo sapiens GN=SLC12A4 PE=1 SV=2 - [S12A4_HUMAN]	5.44	1	2	5	11	-0.71	-0.81	-1.35	-1.44	0.03	-0.07	0.05	-0.05	0.19	0.29	-0.14	-0.24	0.81	0.58	1.21	1.03	1.67	0.73	1.36	1.45
P01702	Ig lambda chain V-I region NIG-64 OS=Homo sapiens PE=1 SV=1 - [LV104_HUMAN]	7.21	2	1	1	2	-1.92	-1.90	-1.55	-1.54	-0.04	-0.03	-0.16	-0.15	-0.25	-0.26	-0.52	-0.51	1.81	1.41	1.04	1.68	1.32	1.86	1.49	1.45
Q9H488	GDP-fucose protein O-fucosyltransferase 1 OS=Homo sapiens GN=POFUT1 PE=1 SV=1 - [OFUT1_HUMAN]	19.33	1	6	6	10	-1.27	-1.15	-1.43	-1.29	0.14	0.15	0.01	-0.03	0.14	0.08	-0.03	-0.02	1.17	1.46	1.43	1.38	1.64	1.51	1.55	1.45
Q9H0U3	Magnesium transporter protein 1 OS=Homo sapiens GN=MAGT1 PE=1 SV=1 - [MAGT1_HUMAN]	12.84	1	4	4	6	-1.33	-1.53	-1.63	-1.83	-0.05	-0.26	-0.25	-0.45	-0.26	-0.06	0.13	-0.07	1.14	1.47	1.76	1.30	1.61	1.26	1.56	1.45

Q9NTU7	Cerebellin-4 OS=Homo sapiens GN=CBLN4 PE=1 SV=1 - [CBLN4_HUMAN]	9.45	1	2	2	3	-2.96	-3.01	-3.52	-3.57	-1.06	-1.12	-2.13	-2.19	-1.70	-1.64	-1.14	-1.21	0.88	1.82	2.37	1.35	1.91	1.88	2.44	1.45
Q9UDY8	Mucosa-associated lymphoid tissue lymphoma translocation protein 1 OS=Homo sapiens GN=MALT1 PE=1 SV=1 - [MALT1_HUMAN]	5.22	1	3	3	3	-0.79	0.00	-1.42	-0.63	-0.25	0.53	-0.03	0.75	0.84	0.06	0.23	1.01	0.81	1.02	1.64	0.88	1.51	0.52	1.15	1.45
Q9Y2L9	Leucine-rich repeat and calponin homology domain-containing protein 1 OS=Homo sapiens GN=LRCH1 PE=1 SV=3 - [LRCH1_HUMAN]	10.99	1	4	4	5	0.47	-0.11	0.80	0.22	2.35	1.77	2.19	1.60	0.19	0.77	1.96	1.37	1.77	1.49	1.15	0.34	0.00	1.87	1.53	1.44
Q5JTB6	Placenta-specific protein 9 OS=Homo sapiens GN=PLAC9 PE=2 SV=1 - [PLAC9_HUMAN]	21.65	1	2	2	6	-1.43	-1.66	-1.61	-1.85	0.63	0.40	-0.35	-0.47	-0.26	-0.03	-0.20	-0.31	1.14	1.49	1.66	1.43	1.61	2.05	2.23	1.44
P04920	Anion exchange protein 2 OS=Homo sapiens GN=SLC4A2 PE=1 SV=4 - [B3A2_HUMAN]	6.61	1	4	4	9	-1.39	-1.43	-1.92	-1.85	-0.01	0.01	-0.53	-0.46	-0.18	-0.21	-0.08	-0.02	1.03	1.42	1.80	1.31	1.58	1.38	1.89	1.44
P55061	Bax inhibitor 1 OS=Homo sapiens GN=TMBIM6 PE=1 SV=2 - [BI1_HUMAN]	3.80	1	1	1	2	-1.98	-2.07	-1.20	-1.28	0.04	-0.06	0.18	0.09	-0.22	-0.13	0.21	0.11	2.22	2.20	1.40	1.89	1.10	2.00	1.22	1.44
P02753	Retinol-binding protein 4 OS=Homo sapiens GN=RBP4 PE=1 SV=3 - [RET4_HUMAN]	17.91	1	3	3	5	-1.57	-1.43	-1.37	-1.35	-0.67	-0.58	-0.22	0.21	-0.29	-0.30	0.36	0.42	1.51	1.78	1.73	1.28	1.09	0.82	0.75	1.44
Q9Y5U9	Immediate early response 3-interacting protein 1 OS=Homo sapiens GN=IER3IP1 PE=1 SV=1 - [IR3IP_HUMAN]	24.39	1	1	1	2	-1.05	-1.39	-1.22	-1.56	0.42	0.08	0.16	-0.18	-0.05	0.30	0.09	-0.25	1.26	1.15	1.31	1.38	1.55	1.46	1.63	1.44

P15502	Elastin OS=Homo sapiens GN=ELN PE=1 SV=3 - [ELN_HUMAN]	12.85	1	5	5	8	-2.96	-3.07	-2.54	-2.45	0.81	0.81	-1.06	-1.14	-0.43	-0.49	-1.18	-1.24	2.15	1.82	1.17	2.75	2.08	3.98	3.41	1.44
Q96II8	Leucine-rich repeat and calponin homology domain-containing protein 3 OS=Homo sapiens GN=LRCH3 PE=1 SV=2 - [LRCH3_HUMAN]	3.86	1	2	2	2	-1.27	-1.22	-1.40	-1.35	0.12	0.17	-0.02	0.03	-0.26	-0.31	0.29	0.34	1.30	1.57	1.69	0.99	1.12	1.38	1.50	1.44
O43294	Transforming growth factor beta-1-induced transcript 1 protein OS=Homo sapiens GN=TGFB1I1 PE=1 SV=2 - [TGFI1_HUMAN]	36.88	1	11	11	35	-2.57	-2.50	-2.68	-2.68	-1.19	-1.12	-1.20	-1.15	-1.10	-1.11	-0.87	-0.92	1.57	1.62	1.75	1.48	1.62	1.44	1.46	1.44
P01344	Insulin-like growth factor II OS=Homo sapiens GN=IGF2 PE=1 SV=1 - [IGF2_HUMAN]	5.00	1	1	1	1	-0.92	-0.86	-0.80	-0.74	1.67	1.73	0.57	0.63	0.88	0.83	0.26	0.31	1.54	1.18	1.06	1.78	1.66	2.58	2.46	1.43
P61165	Transmembrane protein 258 OS=Homo sapiens GN=TMEM258 PE=1 SV=1 - [TM258_HUMAN]	10.13	1	1	1	2	-1.42	-1.05	-2.09	-1.72	-0.09	0.27	-0.72	-0.36	-0.04	-0.40	-0.50	-0.14	0.75	0.92	1.59	1.05	1.73	1.31	1.98	1.43
P00488	Coagulation factor XIII A chain OS=Homo sapiens GN=F13A1 PE=1 SV=4 - [F13A_HUMAN]	34.15	1	19	19	41	-1.57	-1.46	-1.60	-1.64	0.29	0.36	-0.27	-0.27	0.08	0.10	0.39	0.41	1.13	2.07	2.15	1.67	1.80	1.98	2.06	1.43
P50416	Carnitine O-palmitoyltransferase 1, liver isoform OS=Homo sapiens GN=CPT1A PE=1 SV=2 - [CPT1A_HUMAN]	22.77	2	16	16	30	-1.31	-1.35	-1.45	-1.38	0.00	0.16	-0.08	0.07	0.03	0.03	0.04	0.11	1.46	1.43	1.53	1.51	1.68	1.53	1.66	1.43
O00299	Chloride intracellular channel protein 1 OS=Homo sapiens GN=CLIC1 PE=1 SV=4 - [CLIC1_HUMAN]	67.22	1	11	11	30	-1.68	-1.61	-1.72	-1.70	-0.03	0.11	-0.14	-0.10	0.00	-0.10	-0.40	-0.23	1.62	1.38	1.45	1.76	1.57	1.78	1.70	1.43

Q9H239	Matrix metalloproteinase-28 OS=Homo sapiens GN=MMP28 PE=2 SV=2 - [MMP28_HUMAN]	11.92	1	6	6	8	-0.63	-0.84	-0.77	-0.87	1.89	1.62	0.55	0.46	1.04	1.14	0.92	0.66	1.24	1.40	1.68	1.58	1.80	2.33	2.56	1.43
P06280	Alpha-galactosidase A OS=Homo sapiens GN=GLA PE=1 SV=1 - [AGAL_HUMAN]	13.29	1	5	5	7	-0.83	-0.53	-1.24	-0.95	0.39	0.68	0.12	0.41	0.52	0.23	0.60	0.89	1.00	1.43	1.84	1.09	1.50	1.20	1.61	1.43
Q9H7F0	Probable cation-transporting ATPase 13A3 OS=Homo sapiens GN=ATP13A3 PE=1 SV=4 - [AT133_HUMAN]	0.57	1	1	1	1	-0.99	-0.96	-1.81	-1.79	-0.48	-0.46	-0.45	-0.43	-1.43	-1.45	-0.84	-0.82	0.59	0.15	0.97	-0.43	0.40	0.49	1.31	1.43
O76062	Delta(14)-sterol reductase OS=Homo sapiens GN=TM7SF2 PE=2 SV=3 - [ERG24_HUMAN]	7.42	1	3	3	6	-0.86	-0.80	-1.18	-1.13	0.54	0.59	0.18	0.23	0.29	0.24	0.00	0.05	1.09	0.86	1.18	1.13	1.45	1.38	1.70	1.42
P41226	Ubiquitin-like modifier-activating enzyme 7 OS=Homo sapiens GN=UBA7 PE=1 SV=2 - [UBA7_HUMAN]	5.34	1	4	4	5	-1.05	-1.06	-1.20	-1.34	0.37	0.22	-0.08	-0.11	0.06	0.21	-0.08	-0.12	1.01	0.95	1.08	1.30	1.39	1.39	1.48	1.42
P20292	Arachidonate 5-lipoxygenase-activating protein OS=Homo sapiens GN=ALOX5AP PE=1 SV=2 - [AL5AP_HUMAN]	4.97	1	1	1	2	-0.86	-0.62	-1.22	-0.97	0.33	0.57	0.14	0.38	0.67	0.43	0.56	0.80	1.06	1.43	1.78	1.32	1.68	1.18	1.53	1.42
O94813	Slit homolog 2 protein OS=Homo sapiens GN=SLIT2 PE=1 SV=1 - [SLIT2_HUMAN]	8.96	2	9	9	10	0.07	-0.26	0.03	-0.56	1.98	1.35	1.61	1.45	1.15	1.49	0.40	0.20	1.72	0.83	0.47	1.60	1.18	2.07	1.75	1.42
P67812	Signal peptidase complex catalytic subunit SEC11A OS=Homo sapiens GN=SEC11A PE=1 SV=1 - [SC11A_HUMAN]	19.55	1	4	4	10	-1.98	-2.01	-2.03	-2.00	-0.04	-0.05	-0.61	-0.61	-0.30	-0.42	-0.28	-0.19	1.42	1.75	1.75	1.65	1.64	1.98	1.97	1.42

Q92876	Kallikrein-6 OS=Homo sapiens GN=KLK6 PE=1 SV=1 - [KLK6_HUMAN]	27.87	1	5	5	8	-1.03	-0.88	-1.15	-1.03	0.32	0.47	0.22	0.30	0.38	0.23	0.53	0.64	1.37	1.58	1.49	1.60	1.48	1.54	1.49	1.41
A8MQ03	UPF0574 protein C9orf169 OS=Homo sapiens GN=C9orf169 PE=1 SV=1 - [CI169_HUMAN]	18.06	1	1	1	3	-4.33	-4.51	-3.96	-4.14	-2.22	-2.41	-2.61	-2.80	-2.93	-2.74	-2.92	-3.11	1.77	1.42	1.04	1.62	1.26	2.09	1.73	1.41
Q6ZMZ3	Nesprin-3 OS=Homo sapiens GN=SYNE3 PE=1 SV=2 - [SYNE3_HUMAN]	9.33	1	8	8	11	-1.26	-1.37	-1.38	-1.53	-0.05	0.02	-0.26	-0.21	-0.13	-0.06	-0.35	-0.25	1.15	1.10	1.29	1.41	1.63	1.35	1.36	1.41
P24941	Cyclin-dependent kinase 2 OS=Homo sapiens GN=CDK2 PE=1 SV=2 - [CDK2_HUMAN]	6.04	11	1	2	6	-0.92	-0.64	-1.47	-1.19	-0.21	0.06	-0.13	0.15	0.18	-0.10	0.07	0.34	0.85	0.99	1.54	0.85	1.41	0.69	1.25	1.41
Q96FZ2	Embryonic stem cell-specific 5-hydroxymethylcytosine-binding protein OS=Homo sapiens GN=HMCES PE=1 SV=1 - [HMCES_HUMAN]	6.50	1	2	2	2	-1.37	-1.50	-1.28	-1.42	0.79	0.64	0.07	-0.07	0.14	0.28	-0.23	-0.38	1.49	1.14	1.05	1.67	1.59	2.14	2.05	1.41
P12111	Collagen alpha-3(VI) chain OS=Homo sapiens GN=COL6A3 PE=1 SV=5 - [CO6A3_HUMAN]	46.77	1	126	126	1648	-1.90	-1.92	-1.96	-1.95	-0.24	-0.24	-0.56	-0.58	-0.24	-0.25	0.15	0.16	1.38	2.07	2.15	1.69	1.79	1.66	1.71	1.41
P17050	Alpha-N-acetylgalactosaminidase OS=Homo sapiens GN=NAGA PE=1 SV=2 - [NAGAB_HUMAN]	5.60	1	2	2	3	-1.23	-1.14	-1.47	-1.38	-0.50	-0.42	-0.12	-0.04	-0.64	-0.72	-0.58	-0.50	1.16	0.65	0.88	0.53	0.78	0.71	0.94	1.40
O95183	Vesicle-associated membrane protein 5 OS=Homo sapiens GN=VAMP5 PE=1 SV=1 - [VAMP5_HUMAN]	38.79	1	3	4	12	-1.37	-1.48	-1.64	-1.63	0.21	0.32	-0.24	-0.14	0.13	0.07	0.35	0.29	1.49	1.84	1.55	1.61	1.73	1.89	1.83	1.40

P05067	Amyloid beta A4 protein OS=Homo sapiens GN=APP PE=1 SV=3 - [A4_HUMAN]	25.06	1	15	16	193	-0.53	-0.33	-0.69	-0.55	0.39	0.57	0.38	0.43	0.59	0.71	0.69	0.84	1.44	1.57	1.54	1.51	2.13	1.35	2.49	1.40
P07738	Bisphosphoglycerate mutase OS=Homo sapiens GN=BPGM PE=1 SV=2 - [PMGE_HUMAN]	48.65	1	8	8	32	-1.30	-1.12	-1.46	-1.50	-0.42	-0.34	-0.14	-0.04	-0.27	-0.39	-0.69	-0.67	1.23	0.68	0.80	1.02	1.14	0.90	1.18	1.40
Q16610	Extracellular matrix protein 1 OS=Homo sapiens GN=ECM1 PE=1 SV=2 - [ECM1_HUMAN]	23.33	1	11	11	18	-1.80	-1.82	-1.96	-1.94	-0.21	-0.21	-0.64	-0.57	-0.25	-0.36	0.19	0.24	1.35	1.92	2.21	1.72	1.85	1.63	1.69	1.40
O95631	Netrin-1 OS=Homo sapiens GN=NTN1 PE=1 SV=2 - [NET1_HUMAN]	20.86	2	12	12	21	0.40	0.65	0.45	0.84	2.70	3.05	1.64	1.89	2.48	2.21	2.48	2.58	1.23	2.00	1.99	1.96	1.80	2.51	2.51	1.40
P31152	Mitogen-activated protein kinase 4 OS=Homo sapiens GN=MAPK4 PE=1 SV=2 - [MK04_HUMAN]	8.01	2	2	3	3	-0.80	-0.58	-1.51	-1.28	0.27	0.49	-0.18	0.05	-0.33	-0.55	-0.12	0.10	0.68	0.69	1.39	0.29	0.99	1.06	1.76	1.40
P35221	Catenin alpha-1 OS=Homo sapiens GN=CTNNA1 PE=1 SV=1 - [CTNA1_HUMAN]	39.07	1	20	29	72	-1.49	-1.54	-1.70	-1.74	-0.16	-0.17	-0.41	-0.43	-0.38	-0.31	-0.06	-0.09	1.03	1.63	1.68	1.38	1.39	1.36	1.39	1.39
P22105	Tenascin-X OS=Homo sapiens GN=TNXB PE=1 SV=3 - [TENX_HUMAN]	32.04	2	87	88	206	-2.01	-2.06	-2.35	-2.24	-0.27	-0.24	-1.01	-0.99	-0.76	-0.76	-0.92	-0.93	1.24	1.18	1.44	1.37	1.57	1.85	2.07	1.39
P07357	Complement component C8 alpha chain OS=Homo sapiens GN=C8A PE=1 SV=2 - [CO8A_HUMAN]	18.32	1	7	7	13	-1.85	-1.77	-1.65	-1.59	-0.29	-0.31	-0.65	-0.61	-0.38	-0.42	0.12	0.08	1.25	1.97	1.91	1.65	1.45	1.74	1.38	1.39
P60903	Protein S100-A10 OS=Homo sapiens GN=S100A10 PE=1 SV=2 - [S10AA_HUMAN]	17.53	1	3	3	10	-2.23	-2.23	-2.13	-2.12	-0.40	-0.31	-0.86	-0.75	-0.49	-0.56	-0.12	-0.02	1.44	2.09	2.03	1.72	1.72	1.78	1.72	1.39

Q13938	Calcyphosin OS=Homo sapiens GN=CAPS PE=1 SV=1 - [CAYP1_HUMAN]	42.86	1	8	8	26	-0.09	-0.10	-0.23	-0.25	1.72	1.70	1.16	1.09	1.42	1.41	1.38	1.40	1.31	1.50	1.55	1.41	1.66	1.70	1.99	1.39
Q9UBG0	C-type mannose receptor 2 OS=Homo sapiens GN=MRC2 PE=1 SV=2 - [MRC2_HUMAN]	8.45	1	8	8	18	-1.43	-1.42	-1.70	-1.70	0.27	0.27	-0.42	-0.38	0.11	0.16	0.87	0.89	1.23	2.37	2.56	1.67	1.84	1.59	2.01	1.39
O15230	Laminin subunit alpha 5 OS=Homo sapiens GN=LAMA5 PE=1 SV=8 - [LAMA5_HUMAN]	28.71	1	76	77	281	-1.98	-1.98	-2.08	-2.12	-0.20	-0.25	-0.76	-0.77	-0.61	-0.58	-0.74	-0.77	1.21	1.22	1.41	1.45	1.57	1.74	1.86	1.38
P02775	Platelet basic protein OS=Homo sapiens GN=PPBP PE=1 SV=3 - [CXCL7_HUMAN]	26.56	1	4	4	5	-2.18	-2.33	-1.90	-1.97	-0.15	-0.31	-0.58	-0.73	-0.75	-0.44	-0.30	-0.45	1.65	1.89	1.60	1.79	1.26	2.01	1.73	1.38
P04211	Ig lambda chain V region 4A OS=Homo sapiens PE=4 SV=1 - [LV001_HUMAN]	15.38	1	2	2	3	-1.23	-1.09	-1.18	-1.03	0.56	0.70	0.14	0.28	0.08	-0.06	0.09	0.23	1.43	1.33	1.27	1.20	1.15	1.78	1.72	1.38
P10301	Ras-related protein R-Ras OS=Homo sapiens GN=RRAS PE=1 SV=1 - [RRAS_HUMAN]	59.17	1	8	11	48	-1.86	-1.89	-1.96	-1.79	-0.14	-0.03	-0.57	-0.56	-0.34	-0.33	-0.13	-0.10	1.43	1.70	1.79	1.69	1.67	1.68	1.81	1.38
O00339	Matrilin-2 OS=Homo sapiens GN=MATN2 PE=1 SV=4 - [MATN2_HUMAN]	12.24	1	10	10	13	-1.88	-1.93	-1.92	-1.90	-0.45	-0.52	-0.57	-0.63	-0.65	-0.60	-0.37	-0.38	1.36	1.60	1.54	1.28	1.25	1.40	1.37	1.38
Q13188	Serine/threonine-protein kinase 3 OS=Homo sapiens GN=STK3 PE=1 SV=2 - [STK3_HUMAN]	9.16	1	2	4	6	-1.49	-1.35	-1.47	-1.33	0.08	0.21	0.04	-0.01	-0.25	-0.38	0.07	0.06	1.39	1.75	1.31	1.14	1.12	1.55	1.53	1.38
Q8N3D4	EH domain-binding protein 1-like protein 1 OS=Homo sapiens GN=EHBP1L1 PE=1 SV=2 - [EH1L1_HUMAN]	8.27	1	8	8	11	-1.24	-1.27	-1.91	-1.98	-0.22	-0.03	-0.68	-0.47	-0.09	-0.40	-0.08	0.12	0.74	1.22	2.10	1.13	1.38	1.15	1.83	1.38

P23327	Sarcoplasmic reticulum histidine-rich calcium-binding protein OS=Homo sapiens GN=HRC PE=2 SV=1 - [SRCH_HUMAN]	1.43	1	1	1	1	-1.96	-1.94	-1.88	-1.86	-1.23	-1.22	-0.57	-0.55	-0.66	-0.68	-0.53	-0.51	1.44	1.43	1.35	1.31	1.24	0.71	0.63	1.38
P53420	Collagen alpha-4(IV) chain OS=Homo sapiens GN=COL4A4 PE=1 SV=3 - [CO4A4_HUMAN]	1.83	1	3	3	4	-1.20	-1.06	-1.55	-1.41	-0.25	-0.12	-0.24	-0.10	-0.33	-0.47	-0.96	-0.83	1.02	0.24	0.59	0.76	1.12	0.93	1.29	1.38
B9A064	Immunoglobulin lambda-like polypeptide 5 OS=Homo sapiens GN=IGLL5 PE=2 SV=2 - [IGLL5_HUMAN]	33.64	2	3	6	44	-1.56	-1.24	-1.21	-1.26	0.52	0.44	0.14	-0.03	0.38	0.66	1.69	1.54	1.55	3.15	2.97	2.08	1.81	1.72	1.69	1.37
Q9UJS0	Calcium-binding mitochondrial carrier protein Aralar2 OS=Homo sapiens GN=SLC25A13 PE=1 SV=2 - [CMC2_HUMAN]	32.44	1	14	19	52	-1.51	-1.57	-1.36	-1.43	0.22	0.11	-0.07	-0.09	-0.10	-0.07	-0.07	-0.09	1.58	1.45	1.38	1.41	1.35	1.66	1.60	1.37
P00747	Plasminogen OS=Homo sapiens GN=PLG PE=1 SV=2 - [PLMN_HUMAN]	36.30	2	24	24	42	-0.82	-0.86	-0.96	-1.09	0.55	0.48	0.18	0.27	0.79	0.79	1.75	1.70	1.09	2.40	2.61	1.61	1.87	1.39	1.67	1.37
P30043	Flavin reductase (NADPH) OS=Homo sapiens GN=BLVRB PE=1 SV=3 - [BLVRB_HUMAN]	48.06	1	7	7	83	-1.17	-1.07	-1.27	-1.31	-0.03	-0.01	-0.05	0.03	-0.28	-0.31	-0.85	-0.76	1.22	0.44	0.38	1.07	1.16	1.23	1.35	1.36
P13671	Complement component C6 OS=Homo sapiens GN=C6 PE=1 SV=3 - [CO6_HUMAN]	10.81	1	8	8	14	-1.61	-1.55	-1.80	-1.80	0.52	0.36	-0.50	-0.50	-0.14	0.02	-0.05	-0.17	1.17	1.52	1.72	1.67	1.85	2.04	2.11	1.36

Q96QD8	Sodium-coupled neutral amino acid transporter 2 OS=Homo sapiens GN=SLC38A2 PE=1 SV=2 - [S38A2_HUMAN]	4.15	1	1	1	1	-0.84	-0.78	-1.47	-1.41	-0.60	-0.55	-0.17	-0.11	-0.35	-0.41	-0.19	-0.13	0.72	0.66	1.28	0.46	1.10	0.22	0.85	1.36
Q69YL0	Uncharacterized protein DKFZp762I1415 OS=Homo sapiens PE=4 SV=1 - [YC029_HUMAN]	11.11	1	1	1	1	-3.94	-4.27	-4.00	-4.33	-2.11	-2.44	-2.70	-3.03	-2.83	-2.50	-2.59	-2.92	1.29	1.36	1.41	1.47	1.54	1.82	1.87	1.36
Q01995	Transgelin OS=Homo sapiens GN=TAGLN PE=1 SV=4 - [TAGL_HUMAN]	60.20	1	13	13	120	-2.94	-2.90	-3.19	-3.09	-1.42	-1.35	-1.78	-1.84	-1.52	-1.63	-1.35	-1.38	1.32	1.53	1.68	1.36	1.50	1.57	1.70	1.36
Q14315	Filamin-C OS=Homo sapiens GN=FLNC PE=1 SV=3 - [FLNC_HUMAN]	36.37	1	61	74	168	-1.93	-1.85	-2.05	-1.95	-0.18	-0.12	-0.76	-0.71	-0.35	-0.43	-0.12	-0.07	1.27	1.79	1.86	1.63	1.63	1.78	1.86	1.36
Q9UKA4	A-kinase anchor protein 11 OS=Homo sapiens GN=AKAP11 PE=1 SV=1 - [AKA11_HUMAN]	1.74	1	2	2	2	-1.29	-1.84	-1.19	-1.74	0.63	0.08	0.10	-0.44	-0.11	0.45	0.01	-0.54	1.45	1.31	1.20	1.77	1.67	1.91	1.81	1.36
Q8TAM2	Tetratricopeptide repeat protein 8 OS=Homo sapiens GN=TTC8 PE=1 SV=2 - [TTC8_HUMAN]	2.96	1	1	1	2	0.80	0.89	-0.42	-0.33	0.45	0.53	0.87	0.96	0.65	0.56	0.54	0.63	0.12	-0.26	0.96	-0.21	1.02	-0.37	0.85	1.36
O60879	Protein diaphanous homolog 2 OS=Homo sapiens GN=DIAPH2 PE=1 SV=1 - [DIAP2_HUMAN]	5.27	1	5	5	6	-1.49	-1.29	-1.30	-0.98	0.02	0.26	0.11	0.36	0.22	-0.10	-0.12	0.12	1.55	1.16	1.19	1.32	1.32	1.26	1.53	1.36
P21589	5'-nucleotidase OS=Homo sapiens GN=NT5E PE=1 SV=1 - [5NTD_HUMAN]	36.76	1	14	14	47	-1.93	-1.99	-1.74	-1.64	0.37	0.42	-0.48	-0.42	0.01	-0.02	0.40	0.46	1.32	2.21	2.20	1.77	1.71	2.15	2.12	1.35

Q9Y5Y7	Lymphatic vessel endothelial hyaluronic acid receptor 1 OS=Homo sapiens GN=LYVE1 PE=1 SV=2 - [LYVE1_HUMAN]	5.28	1	2	2	3	-1.87	-1.89	-1.86	-1.87	-0.19	-0.10	-0.68	-0.57	-0.12	-0.22	0.15	0.25	1.36	2.02	2.00	1.56	1.55	1.54	1.52	1.35
P01763	Ig heavy chain V-III region WEA OS=Homo sapiens PE=1 SV=1 - [HV302_HUMAN]	26.32	2	1	2	9	-1.23	-0.88	-1.66	-1.32	-0.66	-0.44	-0.49	-0.26	0.15	-0.07	-0.10	0.24	0.66	1.13	1.78	1.06	1.47	0.41	1.19	1.35
Q14728	Major facilitator superfamily domain-containing protein 10 OS=Homo sapiens GN=MFSD10 PE=2 SV=1 - [MFS10_HUMAN]	4.62	1	2	2	4	-1.71	-1.74	-1.92	-1.95	-0.27	-0.30	-0.63	-0.66	-0.38	-0.35	-0.30	-0.33	1.13	1.42	1.62	1.39	1.61	1.42	1.63	1.35
Q96CC6	Inactive rhomboid protein 1 OS=Homo sapiens GN=RHBDF1 PE=1 SV=2 - [RHDF1_HUMAN]	4.21	1	3	3	6	-1.22	-0.84	-1.27	-0.89	0.23	0.60	0.02	0.40	0.73	0.36	-0.16	0.22	1.29	1.07	1.11	1.61	1.66	1.43	1.48	1.35
Q9ULC5	Long-chain-fatty-acid--CoA ligase 5 OS=Homo sapiens GN=ACSL5 PE=1 SV=1 - [ACSL5_HUMAN]	4.25	1	1	2	2	-0.46	-0.40	-1.24	-1.18	0.02	0.08	0.04	0.11	-0.14	-0.20	-0.31	-0.25	0.56	0.16	0.93	0.29	1.08	0.47	1.25	1.35
Q5VTJ3	Kelch domain containing protein 7A OS=Homo sapiens GN=KLHDC7A PE=2 SV=5 - [KLD7A_HUMAN]	13.77	1	5	5	7	-1.08	-1.04	-1.27	-1.26	0.47	0.51	0.05	0.06	0.25	0.28	0.37	0.31	1.15	1.13	1.22	1.23	1.55	1.53	1.72	1.35
P21731	Thromboxane A2 receptor OS=Homo sapiens GN=TBXA2R PE=1 SV=3 - [TA2R_HUMAN]	5.25	1	2	2	3	-1.62	-1.76	-1.51	-1.65	0.18	0.04	-0.23	-0.37	-0.07	0.07	0.06	-0.08	1.45	1.69	1.57	1.73	1.62	1.78	1.67	1.35
Q8NFW1	Collagen alpha-1(XXII) chain OS=Homo sapiens GN=COL22A1 PE=1 SV=2 - [COMA1_HUMAN]	2.40	1	3	3	3	-3.85	-1.10	-4.50	-1.75	-1.94	0.81	-3.21	-0.47	-0.20	-2.94	-2.93	-0.19	0.69	0.93	1.57	0.94	1.59	1.90	2.54	1.35

P02765	Alpha-2-HS-glycoprotein OS=Homo sapiens GN=AHSG PE=1 SV=1 - [FETUA_HUMAN]	26.70	1	7	7	19	-3.17	-3.04	-3.64	-3.49	-1.99	-1.97	-2.41	-2.45	-1.83	-1.88	-1.58	-1.46	1.29	1.96	2.12	1.46	1.70	1.46	1.74	1.35
P01861	Ig gamma-4 chain C region OS=Homo sapiens GN=IGHG4 PE=1 SV=1 - [IGHG4_HUMAN]	45.87	1	5	11	102	-3.30	-3.33	-3.07	-3.00	-1.52	-1.46	-1.82	-1.73	-1.64	-1.61	-1.18	-1.25	1.40	1.76	1.73	1.40	1.39	1.58	1.47	1.35
Q9H6S3	Epidermal growth factor receptor kinase substrate 8-like protein 2 OS=Homo sapiens GN=EPS8L2 PE=1 SV=2 - [ES8L2_HUMAN]	4.48	1	3	3	3	-0.97	-0.99	-1.32	-1.34	0.27	0.25	-0.04	-0.06	0.14	0.17	0.10	0.07	0.98	1.07	1.42	1.17	1.53	1.22	1.58	1.34
Q8NG11	Tetraspanin-14 OS=Homo sapiens GN=TSPAN14 PE=1 SV=1 - [TSN14_HUMAN]	10.37	1	2	2	3	-1.21	-1.27	-1.28	-1.34	0.34	0.28	0.00	-0.06	-0.03	0.04	0.17	0.10	1.26	1.38	1.45	1.28	1.35	1.53	1.60	1.34
P01591	Immunoglobulin J chain OS=Homo sapiens GN=IGJ PE=1 SV=4 - [IGJ_HUMAN]	27.67	1	4	4	8	-0.35	-0.09	-0.48	-0.34	0.04	0.12	0.80	0.89	0.77	0.64	1.18	1.29	1.09	1.50	1.67	0.93	1.16	0.38	0.50	1.34
P18440	Arylamine N-acetyltransferase 1 OS=Homo sapiens GN=NAT1 PE=1 SV=2 - [ARY1_HUMAN]	6.55	1	1	1	2	-0.94	-0.90	-1.01	-0.97	0.55	0.59	0.27	0.31	0.35	0.31	0.41	0.45	1.27	1.36	1.42	1.29	1.36	1.48	1.55	1.34
Q8N118	Cytochrome P450 4X1 OS=Homo sapiens GN=CYP4X1 PE=2 SV=1 - [CP4X1_HUMAN]	5.70	1	3	3	5	-1.43	-1.09	-2.04	-2.00	0.15	0.31	-0.31	-0.27	0.14	-0.02	0.14	0.32	0.59	1.57	2.32	1.24	2.27	1.70	2.15	1.34
P52789	Hexokinase-2 OS=Homo sapiens GN=HK2 PE=1 SV=2 - [HXK2_HUMAN]	8.83	2	4	7	25	-0.34	-0.05	-1.10	-1.28	0.97	1.25	0.18	0.47	0.64	0.35	0.33	0.61	0.57	0.67	1.26	0.72	1.65	1.29	1.92	1.34

P01613	Ig kappa chain V-I region Ni OS=Homo sapiens PE=1 SV=1 - [KV121_HUMAN]	30.36	1	1	2	13	-1.24	-1.41	-1.66	-1.92	-0.12	-0.03	-0.38	-0.21	0.71	0.67	2.00	1.71	0.92	3.25	3.66	2.16	2.66	1.07	1.58	1.34
Q16270	Insulin-like growth factor-binding protein 7 OS=Homo sapiens GN=IGFBP7 PE=1 SV=1 - [IBP7_HUMAN]	32.62	1	8	8	15	-1.95	-1.87	-1.86	-1.75	0.24	0.29	-0.66	-0.67	-0.28	-0.26	-0.63	-0.40	0.96	1.23	1.41	1.47	1.59	2.05	2.16	1.34
Q9H6R3	Acyl-CoA synthetase short-chain family member 3, mitochondrial OS=Homo sapiens GN=ACSS3 PE=1 SV=1 - [ACSS3_HUMAN]	25.36	1	15	15	30	-1.57	-1.55	-1.78	-1.80	-0.24	-0.29	-0.56	-0.49	-0.47	-0.60	-0.42	-0.44	1.14	1.25	1.32	0.98	1.31	1.41	1.33	1.34
P54802	Alpha-N-acetylglucosaminidase OS=Homo sapiens GN=NAGLU PE=1 SV=2 - [ANAG_HUMAN]	2.56	1	1	1	2	-0.81	-0.75	-1.21	-1.15	0.33	0.38	0.06	0.12	0.69	0.64	0.44	0.49	0.93	1.25	1.65	1.48	1.88	1.13	1.53	1.34
Q6P4A8	Phospholipase B-like 1 OS=Homo sapiens GN=PLBD1 PE=1 SV=2 - [PLBL1_HUMAN]	2.17	1	1	1	3	-0.24	-0.60	-0.49	-0.85	0.49	0.13	0.78	0.42	0.36	0.72	1.21	0.85	1.08	1.46	1.70	1.00	1.25	0.72	0.97	1.34
P51693	Amyloid-like protein 1 OS=Homo sapiens GN=APLP1 PE=1 SV=3 - [APLP1_HUMAN]	28.15	1	16	16	58	-0.97	-1.12	-1.18	-1.22	-0.04	0.01	0.17	0.22	0.49	0.49	1.23	1.25	1.11	2.09	2.41	1.41	1.75	1.12	1.14	1.33
P23142	Fibulin-1 OS=Homo sapiens GN=FBLN1 PE=1 SV=4 - [FBLN1_HUMAN]	31.15	1	15	15	37	-1.13	-1.15	-1.44	-1.35	0.87	0.91	-0.10	-0.24	0.20	0.20	0.45	0.46	0.94	1.73	2.03	1.23	1.75	2.05	2.35	1.33
Q9Y315	Putative deoxyribose-phosphate aldolase OS=Homo sapiens GN=DERA PE=1 SV=2 - [DEOC_HUMAN]	44.97	1	11	11	17	-1.26	-1.19	-1.36	-1.19	0.07	0.15	-0.13	-0.08	0.09	0.00	0.46	0.50	1.08	1.59	1.81	1.34	1.28	1.20	1.26	1.33

P01042	Kininogen-1 OS=Homo sapiens GN=KNG1 PE=1 SV=2 - [KNG1_HUMAN]	16.15	1	10	10	30	-1.48	-1.60	-1.62	-1.69	0.47	0.45	-0.17	-0.38	0.00	0.11	0.37	0.29	1.30	1.83	1.91	1.63	1.77	2.01	2.13	1.33
Q68EM7	Rho GTPase-activating protein 17 OS=Homo sapiens GN=ARHGAP17 PE=1 SV=1 - [RHG17_HUMAN]	12.71	1	6	7	11	-1.61	-1.36	-1.83	-1.50	-0.30	-0.12	-0.64	-0.41	-0.46	-0.67	-0.76	-0.71	1.21	1.09	1.20	1.20	1.34	1.39	1.52	1.33
Q96AM1	Mas-related G-protein coupled receptor member F OS=Homo sapiens GN=MRGPRF PE=2 SV=1 - [MRGRF_HUMAN]	4.08	1	1	1	4	-1.46	-1.44	-2.08	-1.74	-0.62	-0.40	-0.55	-0.41	-0.05	-0.37	-0.26	0.07	1.39	1.33	1.81	1.32	1.73	0.96	1.44	1.33
P00450	Ceruloplasmin OS=Homo sapiens GN=CP PE=1 SV=1 - [CERU_HUMAN]	36.62	1	26	26	72	-2.04	-2.01	-1.86	-1.66	-0.53	-0.54	-0.71	-0.71	-0.56	-0.60	-0.41	-0.40	1.40	1.54	1.53	1.34	1.34	1.40	1.38	1.33
P01612	Ig kappa chain V-I region Mev OS=Homo sapiens PE=1 SV=1 - [KV120_HUMAN]	16.51	1	1	1	2	-1.40	-1.49	-1.64	-1.73	-1.04	-1.13	-0.38	-0.46	0.19	0.28	1.06	0.97	1.08	2.47	2.70	1.71	1.96	0.35	0.59	1.33
P33241	Lymphocyte-specific protein 1 OS=Homo sapiens GN=LSP1 PE=1 SV=1 - [LSP1_HUMAN]	7.67	1	3	3	4	-1.19	-1.15	-0.94	-0.99	-0.33	-0.22	0.33	0.37	0.17	0.16	0.14	0.17	1.57	1.33	1.07	1.40	1.18	0.93	0.59	1.32
Q9UKK3	Poly [ADP-ribose] polymerase 4 OS=Homo sapiens GN=PARP4 PE=1 SV=3 - [PARP4_HUMAN]	3.83	1	4	4	4	0.24	0.25	-1.52	-1.51	0.10	0.10	-0.26	-0.25	0.42	0.41	-0.09	-0.09	-0.44	-0.33	1.43	0.20	1.96	-0.16	1.60	1.32
P04275	von Willebrand factor OS=Homo sapiens GN=VWF PE=1 SV=4 - [VWF_HUMAN]	27.20	1	65	65	138	-2.36	-2.28	-2.47	-2.36	-0.75	-0.74	-1.13	-1.10	-1.04	-1.00	-0.89	-0.89	1.16	1.26	1.46	1.19	1.35	1.35	1.57	1.32
Q9HAV4	Exportin-5 OS=Homo sapiens GN=XPO5 PE=1 SV=1 - [XPO5_HUMAN]	3.32	1	2	2	3	0.12	-0.01	-1.00	-1.12	0.48	0.35	-0.19	-0.79	0.06	-0.28	1.02	0.42	-0.10	1.06	2.47	-0.52	0.29	0.35	1.46	1.32

P00746	Complement factor D OS=Homo sapiens GN=CFD PE=1 SV=5 - [CFAD_HUMAN]	12.65	1	2	2	3	-0.86	-0.88	-1.00	-1.03	1.09	1.06	0.25	0.23	0.92	0.96	0.65	0.62	1.16	1.51	1.65	1.84	1.99	1.93	2.07	1.32
Q8WZ42	Titin OS=Homo sapiens GN=TTN PE=1 SV=4 - [TITIN_HUMAN]	0.09	1	2	3	3	-0.26	-0.39	-0.43	-0.55	-0.68	-0.82	0.83	0.70	0.34	0.47	-0.56	-0.69	1.14	-0.29	-0.13	0.77	0.93	-0.44	-0.28	1.32
P25774	Cathepsin S OS=Homo sapiens GN=CTSS PE=1 SV=3 - [CATS_HUMAN]	6.34	1	2	2	3	-0.13	-0.11	-0.87	-0.85	0.62	0.63	0.38	0.40	0.51	0.50	0.86	0.87	0.56	1.00	1.73	0.66	1.40	0.73	1.47	1.31
Q6UXI9	Nephronectin OS=Homo sapiens GN=NPNT PE=2 SV=3 - [NPNT_HUMAN]	25.66	1	11	11	27	-1.15	-1.27	-1.30	-1.24	1.01	1.16	-0.02	0.14	0.40	0.38	0.10	0.16	1.32	1.27	1.36	1.69	1.58	2.29	2.47	1.31
Q13308	Inactive tyrosine-protein kinase 7 OS=Homo sapiens GN=PTK7 PE=1 SV=2 - [PTK7_HUMAN]	17.10	1	14	14	21	-1.24	-1.24	-1.23	-1.64	0.11	0.05	-0.03	-0.21	-0.06	0.21	0.20	0.21	1.31	1.76	1.85	1.40	1.53	1.40	1.78	1.31
Q16678	Cytochrome P450 1B1 OS=Homo sapiens GN=CYP1B1 PE=1 SV=2 - [CP1B1_HUMAN]	19.52	1	10	10	19	-1.62	-1.71	-2.00	-2.00	-0.73	-0.78	-0.61	-0.67	-0.29	-0.30	0.57	0.40	1.04	2.09	2.37	1.39	1.76	1.02	1.30	1.31
Q5T7N3	KN motif and ankyrin repeat domain-containing protein 4 OS=Homo sapiens GN=KANK4 PE=2 SV=1 - [KANK4_HUMAN]	5.73	1	4	5	10	-0.45	-0.84	-1.61	-1.27	-0.34	-0.03	-0.23	-0.16	-0.04	-0.06	-0.49	-0.42	0.28	-0.03	1.12	0.41	1.39	0.46	1.23	1.31
Q9NZN4	EH domain-containing protein 2 OS=Homo sapiens GN=EHD2 PE=1 SV=2 - [EHD2_HUMAN]	58.93	1	24	26	94	-2.46	-2.49	-2.56	-2.48	-1.06	-1.05	-1.25	-1.25	-1.09	-1.03	-0.77	-0.77	1.29	1.58	1.64	1.41	1.27	1.10	1.24	1.31
Q9C0D5	Protein TANC1 OS=Homo sapiens GN=TANC1 PE=1 SV=3 - [TANC1_HUMAN]	0.91	1	1	1	1	0.80	0.46	-0.22	-0.55	1.25	0.90	1.03	0.69	0.29	0.63	1.19	0.84	0.29	0.39	1.40	-0.13	0.88	0.43	1.44	1.31

P00740	Coagulation factor IX OS=Homo sapiens GN=F9 PE=1 SV=2 - [FA9_HUMAN]	9.33	1	4	4	9	-2.51	-2.46	-2.33	-2.18	-0.60	-0.39	-0.95	-1.01	-0.78	-0.72	-0.41	-0.26	1.50	2.04	1.92	1.71	1.51	1.66	1.61	1.30
Q6NUK1	Calcium-binding mitochondrial carrier protein SCaMC-1 OS=Homo sapiens GN=SLC25A24 PE=1 SV=2 - [SCMC1_HUMAN]	32.70	1	14	14	30	-1.31	-1.29	-1.48	-1.52	-0.05	0.01	-0.15	-0.20	0.20	0.08	0.13	0.13	1.03	1.42	1.60	1.50	1.42	1.25	1.42	1.30
P28799	Granulins OS=Homo sapiens GN=GRN PE=1 SV=2 - [GRN_HUMAN]	6.24	1	3	3	4	-0.51	-0.35	-0.54	-0.38	0.25	0.63	-0.11	0.30	0.60	0.14	0.39	0.76	1.26	1.12	1.14	1.28	1.32	0.96	0.99	1.30
P21980	Protein-glutamine gamma-glutamyltransferase 2 OS=Homo sapiens GN=TGM2 PE=1 SV=2 - [TGM2_HUMAN]	52.69	1	23	23	138	-2.17	-2.11	-2.32	-2.28	-0.30	-0.25	-1.11	-1.06	-0.67	-0.60	-0.38	-0.33	0.98	1.70	1.95	1.49	1.64	1.86	2.06	1.30
O60701	UDP-glucose 6-dehydrogenase OS=Homo sapiens GN=UGDH PE=1 SV=1 - [UGDH_HUMAN]	18.22	1	6	6	8	-1.50	-1.42	-1.97	-1.76	-0.28	-0.08	-0.59	-0.55	-0.51	-0.51	-0.76	-0.68	0.79	0.72	1.25	0.97	1.56	1.17	2.01	1.30
P11047	Laminin subunit gamma-1 OS=Homo sapiens GN=LAMC1 PE=1 SV=3 - [LAMC1_HUMAN]	41.77	2	58	59	234	-2.13	-2.18	-2.13	-2.13	-0.44	-0.45	-0.89	-0.91	-0.81	-0.76	-0.76	-0.81	1.26	1.23	1.33	1.32	1.35	1.67	1.66	1.30
Q14573	Inositol 1,4,5-trisphosphate receptor type 3 OS=Homo sapiens GN=ITPR3 PE=1 SV=2 - [ITPR3_HUMAN]	4.87	1	7	11	14	-1.41	-1.45	-1.76	-1.53	-0.49	-0.55	-0.53	-0.51	-0.33	-0.27	0.06	0.07	0.94	1.45	1.80	1.16	1.29	0.75	1.07	1.30
P12109	Collagen alpha-1(VI) chain OS=Homo sapiens GN=COL6A1 PE=1 SV=3 - [CO6A1_HUMAN]	48.54	1	37	37	531	-1.54	-1.61	-1.74	-1.76	-0.27	-0.25	-0.48	-0.49	-0.21	-0.21	0.21	0.23	1.16	1.94	1.96	1.44	1.52	1.35	1.48	1.30

P09917	Arachidonate 5-lipoxygenase OS=Homo sapiens GN=ALOX5 PE=1 SV=2 - [LOX5_HUMAN]	5.64	1	3	3	4	-0.59	0.20	-0.74	0.05	0.82	1.61	0.49	1.29	1.04	0.25	0.31	1.10	1.14	0.91	1.05	0.87	1.02	1.39	1.54	1.30
A1IGU5	Rho guanine nucleotide exchange factor 37 OS=Homo sapiens GN=ARHGEF37 PE=2 SV=2 - [ARH37_HUMAN]	12.59	1	7	7	11	-0.85	-1.11	-0.95	-1.22	0.08	-0.02	0.32	0.05	0.03	0.31	0.09	-0.07	1.10	1.04	1.09	1.21	1.34	1.09	1.19	1.30
P00736	Complement C1r subcomponent OS=Homo sapiens GN=C1R PE=1 SV=2 - [C1R_HUMAN]	22.70	2	12	12	23	-1.40	-1.42	-1.57	-1.63	0.56	0.57	-0.21	-0.27	0.27	0.24	0.67	0.70	1.45	2.27	2.22	1.73	1.73	1.83	2.13	1.30
Q5BKT4	Dol-P-Glc:Glc(2)Man(9)GlcNAc(2)-PP-Dol alpha-1,2-glucosyltransferase OS=Homo sapiens GN=ALG10 PE=2 SV=1 - [AG10A_HUMAN]	3.17	2	1	1	2	-1.48	-1.41	-1.57	-1.50	0.07	0.14	-0.34	-0.27	-0.15	-0.21	-0.49	-0.43	1.20	0.99	1.08	1.30	1.39	1.53	1.62	1.29
Q9NYL2	Mitogen-activated protein kinase kinase kinase MLT OS=Homo sapiens GN=ZAK PE=1 SV=3 - [MLTK_HUMAN]	4.38	1	3	3	4	-1.78	-1.69	-2.07	-1.98	-0.64	-0.56	-0.84	-0.75	-0.54	-0.62	-0.53	-0.45	0.99	1.25	1.54	1.19	1.49	1.12	1.41	1.29
Q9BXJ0	Complement C1q tumor necrosis factor-related protein 5 OS=Homo sapiens GN=C1QTNF5 PE=1 SV=1 - [C1QT5_HUMAN]	11.52	1	2	2	4	-2.05	-2.16	-2.50	-2.61	0.11	-0.01	-1.27	-1.38	-0.65	-0.54	-0.65	-0.76	0.84	1.41	1.85	1.55	2.00	2.14	2.59	1.29

Q15628	Tumor necrosis factor receptor type 1-associated DEATH domain protein OS=Homo sapiens GN=TRADD PE=1 SV=2 - [TRADD_HUMAN]	12.82	1	4	4	6	-1.16	-0.51	-1.85	-1.20	-0.34	0.30	-0.62	0.03	0.24	-0.41	-0.66	-0.01	0.59	0.51	1.19	0.78	1.48	0.80	1.49	1.29
Q05682	Caldesmon OS=Homo sapiens GN=CALD1 PE=1 SV=3 - [CALD1_HUMAN]	21.94	1	17	17	67	-2.71	-2.75	-2.53	-2.59	-1.56	-1.57	-1.31	-1.35	-1.25	-1.20	-0.76	-0.71	1.37	1.92	1.86	1.52	1.31	1.15	1.12	1.29
P02776	Platelet factor 4 OS=Homo sapiens GN=PF4 PE=1 SV=2 - [PLF4_HUMAN]	26.73	2	3	3	7	-1.82	-1.84	-1.72	-1.75	0.21	0.18	-0.50	-0.52	-0.05	-0.02	0.38	0.35	1.37	2.20	2.10	1.82	1.74	2.00	1.91	1.29
P0CG05	Ig lambda-2 chain C regions OS=Homo sapiens GN=IGLC2 PE=1 SV=1 - [LAC2_HUMAN]	60.38	3	1	5	48	-2.04	-1.98	-1.55	-1.49	-0.08	-0.03	-0.32	-0.27	0.15	0.10	0.81	0.86	1.77	2.85	2.36	2.17	1.68	1.94	1.45	1.29
P01824	Ig heavy chain V-II region WAH OS=Homo sapiens PE=1 SV=1 - [HV206_HUMAN]	6.20	1	1	1	1	-0.69	-0.70	-1.23	-1.23	1.16	1.16	-0.01	-0.01	0.86	0.87	1.19	1.18	0.74	1.89	2.42	1.59	2.13	1.84	2.38	1.29
Q96PE1	G-protein coupled receptor 124 OS=Homo sapiens GN=GPR124 PE=1 SV=2 - [GP124_HUMAN]	3.44	1	3	3	4	-1.19	-1.22	-1.59	-1.61	0.01	-0.02	-0.37	-0.39	-0.08	-0.05	0.19	0.16	0.88	1.39	1.78	1.17	1.57	1.19	1.58	1.28
P48507	Glutamate--cysteine ligase regulatory subunit OS=Homo sapiens GN=GCLM PE=1 SV=1 - [GSH0_HUMAN]	24.82	1	5	5	13	-0.56	-0.79	-0.55	-0.59	0.33	0.14	0.56	0.54	0.52	0.57	0.59	0.52	1.25	1.25	1.14	1.26	1.15	0.98	0.84	1.28
O15247	Chloride intracellular channel protein 2 OS=Homo sapiens GN=CLIC2 PE=1 SV=3 - [CLIC2_HUMAN]	34.01	1	5	5	8	-1.42	-1.29	-0.99	-1.38	-0.03	-0.07	-0.05	-0.21	-0.29	-0.25	-0.05	-0.09	1.11	0.95	0.89	0.68	0.99	0.85	1.18	1.28

Q8N1S5	Zinc transporter ZIP11 OS=Homo sapiens GN=SLC39A11 PE=2 SV=3 - [S39AB_HUMAN]	4.97	1	1	1	2	-1.20	-0.92	-1.15	-0.88	0.88	1.15	0.06	0.34	0.18	-0.10	-0.08	0.19	1.32	1.13	1.07	1.14	1.09	2.06	2.01	1.28
Q9UHQ9	NADH-cytochrome b5 reductase 1 OS=Homo sapiens GN=CYB5R1 PE=1 SV=1 - [NB5R1_HUMAN]	38.03	1	10	10	28	-1.36	-1.46	-1.26	-1.25	-0.10	-0.18	-0.10	-0.10	-0.17	-0.22	-0.15	-0.27	1.37	1.10	1.04	1.05	1.14	1.18	1.21	1.27
Q6P1A2	Lysophospholipid acyltransferase 5 OS=Homo sapiens GN=LPCAT3 PE=1 SV=1 - [MBOA5_HUMAN]	2.05	1	1	1	2	-1.93	-1.81	-1.63	-1.51	0.31	0.42	-0.42	-0.31	0.05	-0.06	0.05	0.16	1.56	1.99	1.68	1.90	1.60	2.22	1.93	1.27
Q6XQN6	Nicotinate phosphoribosyltransferase OS=Homo sapiens GN=NAPRT1 PE=1 SV=2 - [PNCB_HUMAN]	18.59	1	7	7	13	-0.68	-0.79	-0.94	-0.90	0.88	0.74	0.51	0.40	0.52	0.61	0.51	0.40	1.10	1.15	1.38	1.10	1.42	1.03	1.63	1.27
Q07065	Cytoskeleton-associated protein 4 OS=Homo sapiens GN=CKAP4 PE=1 SV=2 - [CKAP4_HUMAN]	45.68	1	23	24	61	-1.63	-1.73	-1.76	-1.67	-0.41	-0.34	-0.48	-0.49	-0.51	-0.52	-0.58	-0.50	1.14	1.03	1.23	1.14	1.39	1.30	1.34	1.27
P36955	Pigment epithelium-derived factor OS=Homo sapiens GN=SERPINF1 PE=1 SV=4 - [PEDF_HUMAN]	28.23	1	11	11	33	-2.39	-2.44	-2.43	-2.44	-0.85	-0.86	-1.20	-1.18	-1.11	-1.09	-0.68	-0.67	1.18	1.64	1.71	1.25	1.25	1.62	1.57	1.27
Q5XPI4	E3 ubiquitin-protein ligase RNF123 OS=Homo sapiens GN=RNF123 PE=1 SV=1 - [RN123_HUMAN]	11.34	2	13	14	28	-1.03	-1.04	-1.26	-1.25	-0.41	-0.45	0.01	0.07	-0.11	-0.11	0.15	0.14	1.19	1.26	1.31	1.02	1.14	0.66	0.70	1.27
P14543	Nidogen-1 OS=Homo sapiens GN=NID1 PE=1 SV=3 - [NID1_HUMAN]	32.24	1	31	32	126	-1.81	-1.76	-1.97	-1.97	-0.16	-0.16	-0.75	-0.73	-0.48	-0.49	-0.44	-0.38	1.03	1.34	1.57	1.32	1.49	1.51	1.66	1.27

P27487	Dipeptidyl peptidase 4 OS=Homo sapiens GN=DPP4 PE=1 SV=2 - [DPP4_HUMAN]	6.14	1	5	5	5	-1.91	-1.93	-1.91	-1.92	0.66	0.65	-0.79	-0.67	0.40	0.01	-0.18	-0.15	1.24	1.37	1.78	1.97	2.36	2.38	2.52	1.27
Q15746	Myosin light chain kinase, smooth muscle OS=Homo sapiens GN=MYLK PE=1 SV=4 - [MYLK_HUMAN]	15.20	7	23	24	74	-2.28	-2.08	-2.22	-2.20	-0.74	-0.80	-0.96	-0.91	-0.97	-1.09	-1.28	-1.26	1.38	0.97	0.88	1.19	1.11	1.54	1.29	1.27
Q9NRX3	NADH dehydrogenase [ubiquinone] 1 alpha subcomplex subunit 4-like 2 OS=Homo sapiens GN=NDUFA4L2 PE=2 SV=1 - [NUA4L_HUMAN]	18.39	1	2	2	3	-1.44	-1.37	-1.62	-1.55	-0.03	0.03	-0.42	-0.36	-0.10	-0.16	0.22	0.28	1.07	1.66	1.84	1.30	1.49	1.39	1.58	1.26
Q9Y490	Talin-1 OS=Homo sapiens GN=TLN1 PE=1 SV=3 - [TLN1_HUMAN]	51.59	3	86	108	338	-1.62	-1.64	-1.78	-1.70	-0.64	-0.55	-0.58	-0.54	-0.50	-0.57	-0.35	-0.34	1.14	1.37	1.41	1.13	1.25	1.04	1.14	1.26
Q92625	Ankyrin repeat and SAM domain-containing protein 1A OS=Homo sapiens GN=ANKS1A PE=1 SV=4 - [ANS1A_HUMAN]	4.85	1	4	4	5	-1.07	-1.11	-1.21	-1.21	0.17	0.16	-0.11	-0.10	-0.10	-0.09	0.12	0.13	1.01	1.06	1.30	1.01	1.07	1.01	1.25	1.26
Q02809	Procollagen-lysine,2-oxoglutarate 5-dioxygenase 1 OS=Homo sapiens GN=PLOD1 PE=1 SV=2 - [PLOD1_HUMAN]	8.67	1	4	4	6	-0.98	-0.88	-1.26	-1.16	0.99	1.08	-0.06	0.04	0.55	0.46	0.02	0.11	0.98	1.00	1.27	1.47	1.75	1.95	2.23	1.26
Q15063	Periostin OS=Homo sapiens GN=POSTN PE=1 SV=2 - [POSTN_HUMAN]	38.16	1	23	23	54	-2.65	-2.81	-3.19	-3.18	-0.10	-0.05	-2.07	-1.91	-1.09	-1.08	-1.31	-1.25	0.83	1.25	1.72	1.78	2.26	2.66	3.36	1.26
Q96MM6	Heat shock 70 kDa protein 12B OS=Homo sapiens GN=HSPA12B PE=2 SV=2 - [HS12B_HUMAN]	28.72	1	13	14	22	-1.90	-1.98	-1.68	-1.68	-0.44	-0.47	-0.46	-0.56	-0.54	-0.41	-0.31	-0.44	1.42	1.46	1.46	1.34	1.31	1.38	1.16	1.26

Q15080	Neutrophil cytosol factor 4 OS=Homo sapiens GN=NCF4 PE=1 SV=2 - [NCF4_HUMAN]	12.98	1	4	4	6	-0.15	-0.46	-0.27	-0.58	0.39	0.07	0.93	0.62	0.53	0.84	1.02	0.71	1.13	1.18	1.29	1.02	1.15	0.52	0.64	1.26
O14494	Lipid phosphate phosphohydrolase 1 OS=Homo sapiens GN=PPAP2A PE=1 SV=1 - [LPP1_HUMAN]	11.97	1	3	3	3	-0.79	-0.67	-0.86	-0.75	0.29	0.40	-0.07	0.04	0.24	0.12	0.00	0.10	0.62	0.90	0.88	0.94	1.04	1.06	1.35	1.26
Q5HYW2	NHS-like protein 2 OS=Homo sapiens GN=NHSL2 PE=2 SV=1 - [NHSL2_HUMAN]	16.22	1	6	6	7	-0.78	0.09	-1.26	-0.39	0.51	0.70	-0.07	0.80	0.85	-0.01	-0.01	0.85	0.77	0.77	1.25	0.80	1.28	1.36	2.04	1.26
Q9HBL0	Tensin-1 OS=Homo sapiens GN=TNS1 PE=1 SV=2 - [TENS1_HUMAN]	45.24	1	49	52	171	-1.66	-1.68	-1.71	-1.62	-0.53	-0.48	-0.48	-0.47	-0.34	-0.45	-0.18	-0.14	1.21	1.55	1.61	1.25	1.31	1.07	1.08	1.26
Q86VB7	Scavenger receptor cysteine-rich type 1 protein M130 OS=Homo sapiens GN=CD163 PE=1 SV=2 - [C163A_HUMAN]	27.94	1	26	26	48	-1.19	-1.12	-1.19	-1.10	0.70	0.78	-0.08	0.08	0.62	0.51	0.88	1.03	1.18	2.13	2.10	1.58	1.67	1.77	1.81	1.25
Q6UWH4	Protein FAM198B OS=Homo sapiens GN=FAM198B PE=2 SV=1 - [F198B_HUMAN]	2.70	1	1	1	2	0.87	0.42	-0.09	-0.54	2.60	2.15	1.10	0.65	1.08	1.53	0.61	0.16	0.29	-0.25	0.70	0.69	1.65	1.72	2.67	1.25
Q5VU97	VWFA and cache domain-containing protein 1 OS=Homo sapiens GN=CACHD1 PE=2 SV=2 - [CAHD1_HUMAN]	4.40	1	5	5	7	-1.18	-0.90	-1.27	-0.99	0.33	0.60	-0.08	0.20	0.49	0.22	0.16	0.43	1.15	1.34	1.43	1.43	1.52	1.49	1.58	1.25
Q9UBC9	Small proline-rich protein 3 OS=Homo sapiens GN=SPRR3 PE=1 SV=2 - [SPRR3_HUMAN]	32.54	1	5	5	5	-4.05	-4.24	-5.52	-5.49	-2.52	-2.72	-4.05	-4.12	-3.19	-2.99	-2.82	-3.03	-0.45	-0.21	0.91	0.68	0.47	-0.56	0.64	1.25

P55268	Laminin subunit beta-2 OS=Homo sapiens GN=LAMB2 PE=1 SV=2 - [LAMB2_HUMAN]	42.99	2	60	60	266	-1.75	-1.77	-1.97	-1.93	-0.32	-0.37	-0.77	-0.80	-0.66	-0.63	-0.75	-0.81	1.01	1.00	1.21	1.23	1.38	1.45	1.61	1.25
Q8NBS9	Thioredoxin domain-containing protein 5 OS=Homo sapiens GN=TXNDC5 PE=1 SV=2 - [TXND5_HUMAN]	46.76	1	14	14	31	-1.39	-1.27	-1.44	-1.31	0.23	0.35	-0.17	-0.10	0.07	-0.11	0.04	0.14	1.22	1.40	1.49	1.31	1.41	1.57	1.65	1.25
P01743	Ig heavy chain V-I region HG3 OS=Homo sapiens PE=4 SV=1 - [HV102_HUMAN]	22.22	1	2	2	5	-1.44	-1.29	-1.65	-1.50	0.20	0.34	-0.46	-0.32	0.03	-0.11	0.26	0.41	1.03	1.71	1.91	1.35	1.57	1.62	1.83	1.25
Q6EMK4	Vasorin OS=Homo sapiens GN=VASN PE=1 SV=1 - [VASN_HUMAN]	6.84	1	4	4	6	-0.43	-0.33	-0.76	-0.69	0.39	0.46	0.34	0.52	0.52	0.51	0.83	1.10	0.88	1.27	1.59	0.79	1.15	0.68	1.01	1.25
Q9UM47	Neurogenic locus notch homolog protein 3 OS=Homo sapiens GN=NOTCH3 PE=1 SV=2 - [NOTC3_HUMAN]	3.53	1	6	6	9	-1.28	-1.37	-1.54	-1.61	0.41	0.47	-0.42	-0.34	0.02	0.03	0.15	0.10	0.84	1.20	1.45	1.35	1.75	1.40	2.08	1.25
P29279	Connective tissue growth factor OS=Homo sapiens GN=CTGF PE=1 SV=2 - [CTGF_HUMAN]	6.59	1	2	2	2	-0.26	-0.30	-0.85	-0.90	1.27	1.22	0.33	0.29	0.73	0.78	0.35	0.30	0.64	0.61	1.20	1.07	1.67	1.51	2.11	1.25
P05107	Integrin beta-2 OS=Homo sapiens GN=ITGB2 PE=1 SV=2 - [ITB2_HUMAN]	32.90	2	18	19	41	-0.21	-0.17	-0.45	-0.34	0.29	0.33	0.72	0.76	0.53	0.44	0.63	0.60	1.19	0.96	1.08	0.88	0.90	0.48	0.54	1.25
Q16873	Leukotriene C4 synthase OS=Homo sapiens GN=LTC4S PE=1 SV=1 - [LTC4S_HUMAN]	9.33	1	1	1	2	-2.21	-2.10	-2.42	-2.31	-0.68	-0.57	-1.24	-1.13	-0.75	-0.86	-0.98	-0.88	1.03	1.23	1.44	1.38	1.60	1.51	1.72	1.24
Q8TAT2	Fibroblast growth factor-binding protein 3 OS=Homo sapiens GN=FGFBP3 PE=1 SV=1 - [FGFP3_HUMAN]	2.71	1	1	1	1	-0.87	-0.94	-0.82	-0.88	1.26	1.19	0.36	0.29	0.71	0.78	1.02	0.95	1.29	1.90	1.84	1.68	1.63	2.12	2.06	1.24

Q15404	Ras suppressor protein 1 OS=Homo sapiens GN=RSU1 PE=1 SV=3 - [RSU1_HUMAN]	22.74	1	5	5	8	-2.38	-2.48	-2.04	-2.20	-0.57	-0.74	-0.86	-1.02	-0.91	-0.74	-1.59	-1.75	1.27	1.02	0.45	1.13	1.33	1.53	1.45	1.24
Q9UPY5	Cystine/glutamate transporter OS=Homo sapiens GN=SLC7A11 PE=1 SV=1 - [XCT_HUMAN]	10.78	1	4	4	9	-1.45	-1.54	-1.05	-1.27	0.34	0.11	0.16	-0.07	-0.14	0.10	0.39	0.23	1.29	1.55	1.54	1.28	1.18	1.58	1.37	1.24
P55058	Phospholipid transfer protein OS=Homo sapiens GN=PLTP PE=1 SV=1 - [PLTP_HUMAN]	15.21	1	6	6	11	-0.40	-0.34	-0.88	-0.60	2.68	3.06	0.47	0.73	1.90	1.59	0.91	1.20	1.21	1.55	2.00	2.25	2.58	3.37	3.69	1.24
Q92738	USP6 N-terminal-like protein OS=Homo sapiens GN=USP6NL PE=1 SV=3 - [US6NL_HUMAN]	6.40	1	3	3	5	-1.75	-2.10	-1.73	-2.07	-0.36	-0.71	-0.47	-0.81	-0.68	-0.33	-0.49	-0.84	1.23	1.27	1.23	1.45	1.43	1.38	1.35	1.24
P07093	Glia-derived nexin OS=Homo sapiens GN=SERPINE2 PE=1 SV=1 - [GDN_HUMAN]	31.41	1	12	12	25	-1.17	-1.18	-1.40	-1.29	1.58	1.55	-0.32	-0.35	0.47	0.57	-0.04	-0.29	0.97	0.87	1.33	1.70	2.00	3.05	2.73	1.24
P18084	Integrin beta-5 OS=Homo sapiens GN=ITGB5 PE=1 SV=1 - [ITB5_HUMAN]	8.89	2	5	6	9	-1.02	-0.93	-1.32	-1.03	0.59	0.74	-0.15	-0.08	0.49	0.18	0.18	0.18	0.93	1.12	1.65	1.54	1.54	1.65	1.90	1.24
Q13162	Peroxiredoxin-4 OS=Homo sapiens GN=PRDX4 PE=1 SV=1 - [PRDX4_HUMAN]	50.18	1	8	11	33	-0.93	-0.68	-1.29	-1.16	0.59	0.57	-0.16	-0.12	-0.02	0.13	0.07	0.09	0.79	0.93	1.41	1.31	1.42	1.50	1.84	1.24
Q0ZGT2	Nexilin OS=Homo sapiens GN=NEXN PE=1 SV=1 - [NEXN_HUMAN]	12.74	1	9	10	16	-1.79	-1.61	-2.15	-1.93	0.12	0.23	-0.87	-0.80	-0.25	-0.40	-0.50	-0.45	0.85	1.23	1.55	1.49	1.87	1.83	2.22	1.24
Q96G23	Ceramide synthase 2 OS=Homo sapiens GN=CERS2 PE=1 SV=1 - [CERS2_HUMAN]	8.95	1	3	3	5	-1.11	-1.11	-1.38	-1.38	0.21	0.24	-0.08	-0.06	0.02	-0.12	0.01	-0.13	1.26	1.30	1.15	1.19	1.29	1.14	1.57	1.24

Q68CP4	Heparan-alpha-glucosaminide N-acetyltransferase OS=Homo sapiens GN=HGSNAT PE=1 SV=2 - [HGNAT_HUMAN]	2.11	1	1	1	1	-0.91	-0.80	-1.28	-1.16	-0.12	-0.01	-0.11	0.01	0.02	-0.09	-0.08	0.03	0.86	0.84	1.20	0.85	1.22	0.78	1.14	1.24
O94851	Protein-methionine sulfoxide oxidase MICAL2 OS=Homo sapiens GN=MICAL2 PE=1 SV=1 - [MICA2_HUMAN]	1.33	1	1	1	2	-1.19	-1.30	-1.27	-1.38	0.22	0.11	-0.10	-0.21	-0.10	0.02	0.01	-0.10	1.14	1.21	1.29	1.24	1.32	1.40	1.48	1.23
Q9H2D6	TRIO and F-actin-binding protein OS=Homo sapiens GN=TRIOBP PE=1 SV=3 - [TARA_HUMAN]	7.40	1	15	17	28	-1.64	-1.58	-1.89	-1.79	-0.31	-0.28	-0.67	-0.68	-0.33	-0.34	-0.11	-0.12	0.92	1.61	1.67	1.19	1.49	1.42	1.43	1.23
O43795	Unconventional myosin-Ib OS=Homo sapiens GN=MYO1B PE=1 SV=3 - [MYO1B_HUMAN]	22.10	1	20	20	37	-1.29	-1.46	-1.36	-1.35	0.05	0.07	-0.14	-0.18	-0.13	-0.25	-0.39	-0.37	1.14	0.99	1.03	1.09	1.08	1.41	1.36	1.23
P02748	Complement component C9 OS=Homo sapiens GN=C9 PE=1 SV=2 - [CO9_HUMAN]	31.66	1	13	14	30	-1.13	-1.05	-1.38	-1.46	0.45	0.48	-0.19	-0.06	-0.08	0.01	-0.41	-0.38	1.05	0.87	1.03	0.92	1.34	1.67	1.73	1.23
Q9NVD7	Alpha-parvin OS=Homo sapiens GN=PARVA PE=1 SV=1 - [PARVA_HUMAN]	17.47	1	4	6	17	-1.70	-1.77	-1.64	-1.73	-0.21	-0.26	-0.42	-0.38	-0.32	-0.35	-0.77	-0.69	1.40	1.00	0.79	1.39	1.11	1.45	1.16	1.23
P29350	Tyrosine-protein phosphatase non-receptor type 6 OS=Homo sapiens GN=PTPN6 PE=1 SV=1 - [PTN6_HUMAN]	10.76	1	5	5	6	-1.05	-1.02	-1.06	-0.83	0.29	0.14	0.52	0.33	0.17	0.53	0.71	0.39	1.41	1.42	1.22	1.22	1.04	1.14	0.95	1.22
Q9H2A7	C-X-C motif chemokine 16 OS=Homo sapiens GN=CXCL16 PE=2 SV=4 - [CXL16_HUMAN]	5.91	1	2	2	9	-0.06	-0.28	-0.21	-0.33	2.14	2.01	0.95	0.83	1.41	1.55	1.25	1.04	1.04	1.41	1.38	1.43	1.87	2.25	2.33	1.22

P01880	Ig delta chain C region OS=Homo sapiens GN=IGHD PE=1 SV=2 - [IGHD_HUMAN]	3.39	1	1	1	2	1.11	1.25	0.02	0.16	-0.39	-0.26	1.18	1.32	1.01	0.87	0.39	0.52	0.12	-0.72	0.37	-0.21	0.89	-1.52	-0.43	1.22
Q6ZUX7	Lipoma HMGIC fusion partner-like 2 protein OS=Homo sapiens GN=LHFPL2 PE=2 SV=2 - [LHPL2_HUMAN]	10.96	1	1	1	2	-2.72	-2.43	-1.72	-1.44	-0.11	0.17	-0.56	-0.28	-0.45	-0.73	-0.30	-0.02	2.21	2.43	1.42	2.02	1.02	2.59	1.59	1.22
Q7Z304	MAM domain-containing protein 2 OS=Homo sapiens GN=MAMDC2 PE=2 SV=3 - [MAMC2_HUMAN]	16.33	1	10	10	21	-1.38	-1.41	-1.58	-1.61	-0.15	-0.24	-0.48	-0.42	-0.25	-0.15	0.42	0.39	0.78	1.56	1.84	1.25	1.39	1.10	1.22	1.22
Q96HP0	Dedicator of cytokinesis protein 6 OS=Homo sapiens GN=DOCK6 PE=1 SV=3 - [DOCK6_HUMAN]	6.01	1	8	10	12	-1.66	-1.51	-1.67	-1.76	-0.25	-0.18	-0.49	-0.66	-0.55	-0.45	-0.26	-0.36	0.89	1.41	1.44	1.24	1.25	1.47	1.35	1.22
P00751	Complement factor B OS=Homo sapiens GN=CFB PE=1 SV=2 - [CFAB_HUMAN]	30.37	1	21	21	37	-1.96	-1.94	-1.89	-2.06	-0.64	-0.59	-0.76	-0.72	-0.76	-0.83	-0.75	-0.72	1.31	1.03	0.89	1.21	0.95	1.30	1.19	1.22
Q9BQS8	FYVE and coiled-coil domain-containing protein 1 OS=Homo sapiens GN=FYCO1 PE=1 SV=3 - [FYCO1_HUMAN]	20.09	1	23	23	35	-0.97	-0.89	-1.29	-1.32	0.09	0.25	-0.08	-0.10	0.00	0.04	0.27	0.29	0.94	1.24	1.55	1.05	1.47	1.12	1.39	1.22
Q9UNL2	Translocon-associated protein subunit gamma OS=Homo sapiens GN=SSR3 PE=1 SV=1 - [SSRG_HUMAN]	7.57	1	1	1	4	-1.03	-1.05	-1.77	-1.43	-0.35	0.00	-0.62	-0.45	-0.27	-0.38	-0.22	-0.13	0.45	0.67	1.55	0.53	1.43	0.66	1.41	1.22
P01620	Ig kappa chain V-III region SIE OS=Homo sapiens PE=1 SV=1 - [KV302_HUMAN]	37.61	4	3	3	10	-0.95	-0.92	-1.73	-1.48	-0.12	-0.08	-0.60	-0.46	0.20	-0.02	0.53	0.68	0.76	1.64	2.14	1.01	1.93	0.87	1.63	1.21

Q63HR2	Tensin-like C1 domain-containing phosphatase OS=Homo sapiens GN=TENC1 PE=1 SV=2 - [TENC1_HUMAN]	29.74	1	26	28	53	-1.55	-1.44	-1.51	-1.64	-0.17	-0.24	-0.41	-0.51	-0.27	-0.25	-0.05	-0.09	1.01	1.53	1.69	1.25	1.27	1.01	1.27	1.21
Q9UGP4	LIM domain-containing protein 1 OS=Homo sapiens GN=LIMD1 PE=1 SV=1 - [LIMD1_HUMAN]	7.40	1	3	3	5	-2.10	-1.99	-2.28	-2.17	-1.27	-1.16	-1.13	-1.02	-0.91	-1.01	-0.74	-0.63	1.02	1.37	1.54	1.12	1.30	0.82	0.99	1.21
P61619	Protein transport protein Sec61 subunit alpha isoform 1 OS=Homo sapiens GN=SEC61A1 PE=1 SV=2 - [S61A1_HUMAN]	10.08	2	5	5	9	-1.70	-1.64	-1.70	-1.70	0.07	0.01	-0.56	-0.55	-0.45	-0.40	-0.20	-0.25	1.20	1.62	1.78	1.51	1.52	1.77	1.89	1.21
O95715	C-X-C motif chemokine 14 OS=Homo sapiens GN=CXCL14 PE=1 SV=2 - [CXL14_HUMAN]	6.31	1	1	1	1	0.20	-0.25	-0.19	-0.65	2.40	1.94	0.95	0.50	0.97	1.43	0.86	0.40	0.80	0.66	1.05	1.26	1.66	2.18	2.58	1.21
Q08357	Sodium-dependent phosphate transporter 2 OS=Homo sapiens GN=SLC20A2 PE=1 SV=1 - [S20A2_HUMAN]	12.88	2	6	6	13	-1.35	-1.13	-1.38	-1.35	0.03	0.03	-0.11	-0.09	-0.06	-0.08	-0.30	-0.30	1.09	0.86	1.18	1.15	1.48	1.17	1.51	1.21
P33151	Cadherin-5 OS=Homo sapiens GN=CDH5 PE=1 SV=5 - [CADH5_HUMAN]	9.69	1	6	6	16	-1.63	-1.65	-1.75	-1.87	-0.28	-0.43	-0.67	-0.68	-0.39	-0.34	-0.03	-0.09	1.06	1.41	1.47	1.25	1.28	1.48	1.43	1.21
Q9BSL1	Ubiquitin-associated domain-containing protein 1 OS=Homo sapiens GN=UBAC1 PE=1 SV=1 - [UBAC1_HUMAN]	25.43	2	8	8	12	-0.64	-1.00	-1.15	-1.32	-0.49	-0.58	0.11	-0.17	-0.39	-0.23	0.27	0.28	0.76	1.33	1.48	0.65	0.97	0.36	0.42	1.21
Q9H4B7	Tubulin beta-1 chain OS=Homo sapiens GN=TUBB1 PE=1 SV=1 - [TBB1_HUMAN]	23.95	2	5	10	106	-0.81	-0.52	-1.01	-0.79	0.46	0.63	0.04	0.37	0.86	0.63	0.83	1.31	0.93	1.76	2.03	1.44	1.71	1.30	1.57	1.21

P26599	Polypyrimidine tract-binding protein 1 OS=Homo sapiens GN=PTBP1 PE=1 SV=1 - [PTBP1_HUMAN]	14.69	1	4	6	16	-1.32	-1.51	-1.05	-1.25	0.40	0.24	-0.16	-0.35	-0.12	0.04	0.02	-0.14	1.43	1.54	1.27	1.69	1.49	1.67	1.70	1.21
Q7Z4I7	LIM and senescent cell antigen-like-containing domain protein 2 OS=Homo sapiens GN=LIMS2 PE=1 SV=1 - [LIMS2_HUMAN]	24.93	1	3	8	18	-2.03	-2.19	-1.84	-2.03	-0.09	-0.20	-0.70	-0.64	-0.49	-0.63	-0.57	-0.70	1.39	1.58	1.46	1.73	1.61	1.93	1.74	1.21
P51606	N-acylglucosamine 2-epimerase OS=Homo sapiens GN=RENBP PE=1 SV=2 - [RENBP_HUMAN]	7.49	1	3	3	5	-0.30	0.05	-0.33	-0.09	0.49	0.54	0.62	0.80	0.48	0.36	0.39	0.44	1.06	0.75	0.88	0.41	0.54	0.59	0.73	1.21
O00584	Ribonuclease T2 OS=Homo sapiens GN=RNASET2 PE=1 SV=2 - [RNT2_HUMAN]	30.08	1	7	7	14	-1.09	-0.96	-0.71	-0.52	0.03	0.19	0.44	0.56	0.33	0.28	0.26	0.44	1.48	1.21	1.22	1.06	1.02	1.07	0.82	1.21
Q13201	Multimerin-1 OS=Homo sapiens GN=MMRN1 PE=1 SV=3 - [MMRN1_HUMAN]	3.42	1	3	3	5	-0.40	-0.30	-0.62	-0.78	1.17	1.40	0.29	0.36	0.67	0.75	0.93	1.02	0.88	1.33	1.66	1.31	1.49	1.68	2.24	1.21
P13674	Prolyl 4-hydroxylase subunit alpha-1 OS=Homo sapiens GN=P4HA1 PE=1 SV=2 - [P4HA1_HUMAN]	35.02	1	13	13	17	-1.43	-1.37	-1.59	-1.54	0.14	0.24	-0.40	-0.37	-0.24	-0.26	-0.19	-0.17	1.05	1.68	1.45	1.22	1.41	1.61	1.66	1.21
P07741	Adenine phosphoribosyltransferase OS=Homo sapiens GN=APRT PE=1 SV=2 - [APT_HUMAN]	56.67	1	7	7	17	-0.60	-0.61	-0.65	-0.29	0.90	1.07	0.58	0.67	0.60	0.34	-0.37	-0.33	1.32	0.31	0.27	1.19	1.01	1.62	1.50	1.20
Q9UBU2	Dickkopf-related protein 2 OS=Homo sapiens GN=DKK2 PE=1 SV=1 - [DKK2_HUMAN]	17.76	2	5	5	12	-1.52	-1.69	-0.62	-0.87	1.97	1.75	0.30	0.23	0.77	1.06	0.63	0.73	1.88	1.83	1.11	2.13	1.40	3.52	2.51	1.20

Q6UX73	UPF0764 protein C16orf89 OS=Homo sapiens GN=C16orf89 PE=1 SV=2 - [CP089_HUMAN]	6.72	1	2	2	3	-1.24	-2.01	-0.70	-1.06	2.58	2.19	0.44	-0.10	0.55	0.80	1.12	0.70	1.74	2.36	1.82	2.08	1.54	3.80	3.26	1.20
P02462	Collagen alpha-1(IV) chain OS=Homo sapiens GN=COL4A1 PE=1 SV=3 - [CO4A1_HUMAN]	5.81	1	4	6	66	-1.28	-1.33	-1.86	-1.82	0.46	0.37	-0.63	-0.57	-0.10	-0.15	-0.30	-0.28	0.97	1.30	1.40	1.75	1.80	2.12	2.23	1.20
P04434	Ig kappa chain V-III region VH (Fragment) OS=Homo sapiens PE=4 SV=1 - [KV310_HUMAN]	15.52	1	1	1	2	-1.28	-1.23	-1.49	-1.44	-0.41	-0.37	-0.35	-0.30	-0.10	-0.14	0.54	0.58	0.99	1.83	2.03	1.17	1.37	0.86	1.06	1.20
Q96CG8	Collagen triple helix repeat-containing protein 1 OS=Homo sapiens GN=CTHRC1 PE=1 SV=1 - [CTHR1_HUMAN]	8.23	1	2	2	2	-1.14	-0.51	-1.60	-0.97	-0.10	0.53	-0.46	0.17	0.19	-0.43	-0.09	0.05	0.73	1.75	1.55	0.74	1.20	1.02	1.48	1.20
Q9P107	GEM-interacting protein OS=Homo sapiens GN=GMIP PE=1 SV=2 - [GMIP_HUMAN]	2.16	1	1	1	1	-0.03	-0.49	-0.42	-0.87	0.01	-0.45	0.71	0.26	-0.51	-0.05	-0.19	-0.64	0.80	-0.15	0.23	0.01	0.40	0.02	0.41	1.20
P01859	Ig gamma-2 chain C region OS=Homo sapiens GN=IGHG2 PE=1 SV=2 - [IGHG2_HUMAN]	40.80	1	4	10	110	-2.80	-2.69	-2.63	-2.70	-0.27	-0.15	-1.26	-1.22	-1.12	-1.15	-1.25	-1.46	1.41	1.19	1.16	1.68	1.63	2.58	2.39	1.20
Q6UVK1	Chondroitin sulfate proteoglycan 4 OS=Homo sapiens GN=CSPG4 PE=1 SV=2 - [CSPG4_HUMAN]	13.09	1	22	22	42	-0.66	-0.63	-1.12	-1.25	1.05	1.02	0.17	0.10	0.51	0.61	0.41	0.22	0.88	1.02	1.45	1.24	1.56	1.67	2.19	1.20
Q8IUI8	Cytokine receptor-like factor 3 OS=Homo sapiens GN=CRLF3 PE=1 SV=2 - [CRLF3_HUMAN]	9.50	1	3	3	7	-1.25	-1.33	-1.04	-0.96	-0.57	-0.01	0.09	0.17	-0.76	-1.05	-0.29	0.09	0.92	0.43	0.73	0.96	0.13	0.71	0.44	1.19

P12259	Coagulation factor V OS=Homo sapiens GN=F5 PE=1 SV=4 - [FA5_HUMAN]	1.93	1	3	3	4	-1.04	-0.74	-1.84	-1.53	0.22	0.52	-0.71	-0.40	-0.02	-0.32	-0.25	0.05	0.39	0.80	1.59	0.75	1.55	1.25	2.04	1.19
P50454	Serpin H1 OS=Homo sapiens GN=SERPINH1 PE=1 SV=2 - [SERPH_HUMAN]	30.86	1	12	12	26	-2.39	-2.23	-2.46	-2.41	-0.48	-0.49	-1.17	-1.08	-1.04	-1.09	-0.94	-0.85	0.98	1.34	1.43	1.30	1.45	1.68	1.84	1.19
P10153	Non-secretory ribonuclease OS=Homo sapiens GN=RNASE2 PE=1 SV=2 - [RNAS2_HUMAN]	9.32	1	1	1	2	-1.73	-1.30	-1.39	-0.95	-0.18	0.25	-0.26	0.18	0.10	-0.33	0.46	0.89	1.53	2.20	1.85	1.44	1.09	1.53	1.19	1.19
Q13164	Mitogen-activated protein kinase 7 OS=Homo sapiens GN=MAPK7 PE=1 SV=2 - [MK07_HUMAN]	0.98	1	1	1	1	-0.38	-0.35	-0.93	-0.90	0.24	0.27	0.20	0.23	0.20	0.17	-0.09	-0.07	0.64	0.30	0.83	0.59	1.13	0.61	1.15	1.19
P08294	Extracellular superoxide dismutase [Cu-Zn] OS=Homo sapiens GN=SOD3 PE=1 SV=2 - [SODE_HUMAN]	20.42	1	4	4	12	-1.36	-1.33	-1.48	-1.53	-0.05	-0.20	-0.63	-0.54	-0.66	-0.70	-0.48	-0.46	0.75	1.16	1.38	0.62	0.95	1.29	1.61	1.19
Q9Y5Q0	Fatty acid desaturase 3 OS=Homo sapiens GN=FADS3 PE=2 SV=1 - [FADS3_HUMAN]	2.70	1	1	1	1	-0.92	-0.29	-1.50	-0.87	-0.52	0.11	-0.38	0.26	0.58	-0.05	-1.47	-0.84	0.60	-0.54	0.03	0.90	1.49	0.39	0.97	1.19
P78536	Disintegrin and metalloproteinase domain-containing protein 17 OS=Homo sapiens GN=ADAM17 PE=1 SV=1 - [ADA17_HUMAN]	6.80	1	5	5	6	-1.54	-1.65	-1.41	-1.51	0.37	0.25	-0.28	-0.39	-0.08	0.04	0.66	0.55	1.31	2.21	2.07	1.61	1.48	1.89	1.75	1.19
P31327	Carbamoyl-phosphate synthase [ammonia], mitochondrial OS=Homo sapiens GN=CPS1 PE=1 SV=2 - [CPSM_HUMAN]	4.73	1	4	6	7	-0.59	-0.67	-0.93	-1.01	0.25	0.17	0.19	0.11	-0.09	0.00	0.01	-0.08	0.84	0.61	0.94	0.62	0.96	0.83	1.17	1.19

Q7L5N7	Lysophosphatidylcholine acyltransferase 2 OS=Homo sapiens GN=LPCAT2 PE=1 SV=1 - [PCAT2_HUMAN]	8.46	1	4	4	6	-0.93	-0.78	-1.06	-0.91	0.19	0.33	0.06	0.21	0.36	0.22	0.27	0.41	1.05	1.20	1.32	1.18	1.32	1.10	1.23	1.19
P12110	Collagen alpha-2(VI) chain OS=Homo sapiens GN=COL6A2 PE=1 SV=4 - [CO6A2_HUMAN]	34.74	1	31	31	507	-1.48	-1.44	-1.52	-1.48	-0.17	-0.16	-0.45	-0.40	-0.14	-0.16	0.23	0.26	1.15	1.70	1.87	1.28	1.52	1.22	1.34	1.19
Q6P589	Tumor necrosis factor alpha-induced protein 8-like protein 2 OS=Homo sapiens GN=TNFAIP8L2 PE=1 SV=1 - [TP8L2_HUMAN]	10.87	1	1	1	1	-0.94	0.60	-2.46	-0.92	-0.29	1.24	-1.34	0.20	0.08	-1.45	-2.01	-0.48	-0.34	-1.07	0.44	-0.48	1.04	0.63	2.15	1.19
P08567	Pleckstrin OS=Homo sapiens GN=PLEK PE=1 SV=3 - [PLEK_HUMAN]	8.00	1	2	2	6	-0.85	-0.95	-1.21	-1.31	0.17	0.06	-0.09	-0.19	-0.01	0.10	-0.03	-0.13	0.82	0.83	1.18	0.98	1.34	1.00	1.36	1.19
Q09666	Neuroblast differentiation associated protein AHNAK OS=Homo sapiens GN=AHNAK PE=1 SV=2 - [AHNK_HUMAN]	71.43	2	244	250	812	-1.97	-1.96	-1.89	-1.85	-0.80	-0.79	-0.71	-0.70	-0.63	-0.63	-0.09	-0.10	1.34	1.91	1.78	1.39	1.23	1.21	1.05	1.18
P13716	Delta-aminolevulinic acid dehydratase OS=Homo sapiens GN=ALAD PE=1 SV=1 - [HEM2_HUMAN]	32.42	1	9	9	45	-0.57	-0.72	-0.91	-0.80	0.20	0.25	0.45	0.41	0.30	0.31	0.37	0.43	1.01	0.97	1.32	0.92	1.26	0.84	1.06	1.18
Q687X5	Metalloreductase STEAP4 OS=Homo sapiens GN=STEAP4 PE=1 SV=1 - [STEA4_HUMAN]	10.46	1	4	4	4	-0.92	-1.21	-1.69	-1.98	1.17	0.88	-0.58	-0.86	1.11	1.40	2.35	2.06	0.40	3.28	4.04	2.35	3.13	2.08	2.85	1.18
Q9BYI3	Hyccin OS=Homo sapiens GN=FAM126A PE=1 SV=2 - [HYCCI_HUMAN]	3.45	1	2	2	3	-1.45	-1.33	-1.52	-1.40	-0.02	0.09	-0.40	-0.28	-0.40	-0.51	-0.83	-0.72	1.10	0.62	0.68	0.96	1.03	1.41	1.48	1.18

P24844	Myosin regulatory light polypeptide 9 OS=Homo sapiens GN=MYL9 PE=1 SV=4 - [MYL9_HUMAN]	55.81	1	2	7	84	-2.59	-2.56	-2.66	-2.63	-1.21	-1.18	-1.31	-1.42	-1.24	-1.17	-1.12	-1.15	1.10	1.42	1.49	1.36	1.43	0.98	1.44	1.18
P29972	Aquaporin-1 OS=Homo sapiens GN=AQP1 PE=1 SV=3 - [AQP1_HUMAN]	7.43	1	1	1	16	-0.74	-0.61	-0.90	-0.59	0.60	0.63	0.40	0.62	0.81	0.94	1.43	1.22	1.19	1.86	2.00	1.47	1.60	1.23	1.52	1.18
Q92539	Phosphatidate phosphatase LPIN2 OS=Homo sapiens GN=LPIN2 PE=1 SV=1 - [LPIN2_HUMAN]	3.79	1	3	3	3	-0.12	-0.04	-0.77	-0.69	0.04	0.12	0.35	0.43	0.08	0.00	0.08	0.16	0.52	0.21	0.85	0.16	0.81	0.15	0.79	1.18
P61952	Guanine nucleotide-binding protein G(I)/G(S)/G(O) subunit gamma-11 OS=Homo sapiens GN=GNG11 PE=1 SV=1 - [GBG11_HUMAN]	20.55	2	2	2	9	-1.71	-1.84	-1.60	-1.73	-0.32	-0.46	-0.49	-0.62	-0.51	-0.37	0.16	0.03	1.27	1.88	1.77	1.37	1.26	1.37	1.26	1.18
Q8TCT9	Minor histocompatibility antigen H13 OS=Homo sapiens GN=HM13 PE=1 SV=1 - [HM13_HUMAN]	12.73	1	5	5	8	-1.28	-1.44	-1.60	-1.35	0.63	0.54	0.08	-0.23	-0.26	-0.08	0.11	-0.11	1.04	1.39	1.53	1.23	1.41	1.55	1.87	1.17
P34096	Ribonuclease 4 OS=Homo sapiens GN=RNASE4 PE=1 SV=3 - [RNAS4_HUMAN]	33.33	1	4	4	8	-0.60	-0.14	-0.41	-0.31	2.79	2.81	1.20	1.32	1.78	1.76	1.53	1.55	1.60	2.61	1.94	2.86	2.20	3.85	3.18	1.17
P54725	UV excision repair protein RAD23 homolog A OS=Homo sapiens GN=RAD23A PE=1 SV=1 - [RD23A_HUMAN]	31.13	1	6	8	25	-1.20	-1.31	-1.37	-1.39	0.07	0.06	-0.22	-0.18	-0.12	-0.16	-0.16	-0.07	0.57	1.00	1.27	0.79	1.39	0.94	1.52	1.17
Q8TAD2	Interleukin-17D OS=Homo sapiens GN=IL17D PE=2 SV=1 - [IL17D_HUMAN]	24.26	1	4	4	7	-0.54	-0.96	-0.70	-0.97	1.53	1.17	0.53	0.35	0.93	1.08	0.71	0.46	1.04	0.92	1.16	1.60	1.69	2.06	2.22	1.17

P08253	72 kDa type IV collagenase OS=Homo sapiens GN=MMP2 PE=1 SV=2 - [MMP2_HUMAN]	1.82	1	1	1	2	-1.40	-0.63	-1.50	-0.91	-0.14	0.61	-0.37	0.20	0.31	-0.44	0.34	1.15	0.88	1.78	2.06	0.97	1.25	1.22	1.51	1.17
Q96M27	Protein PRRC1 OS=Homo sapiens GN=PRRC1 PE=1 SV=1 - [PRRC1_HUMAN]	11.91	1	5	5	9	-0.98	-1.07	-1.14	-1.14	0.55	0.53	-0.04	-0.02	0.12	0.13	0.00	0.00	1.10	0.96	1.04	1.07	1.29	1.47	1.52	1.17
Q9H7M9	Platelet receptor Gi24 OS=Homo sapiens GN=C10orf54 PE=1 SV=3 - [GI24_HUMAN]	11.25	1	2	2	2	-0.15	0.64	-1.25	-0.46	-0.34	0.44	-0.14	0.64	0.48	-0.30	-0.19	0.59	0.06	-0.04	1.05	-0.13	0.98	-0.21	0.89	1.17
Q9BXJ3	Complement C1q tumor necrosis factor-related protein 4 OS=Homo sapiens GN=C1QTNF4 PE=1 SV=2 - [C1QT4_HUMAN]	22.49	1	4	4	9	-0.25	-0.64	-0.53	-0.93	1.85	1.45	0.27	-0.12	0.69	1.08	0.47	0.07	0.58	0.73	1.00	1.37	1.05	2.09	2.17	1.17
P46939	Utrophin OS=Homo sapiens GN=UTRN PE=1 SV=2 - [UTRO_HUMAN]	31.98	4	91	94	197	-1.70	-1.75	-1.76	-1.82	-0.49	-0.52	-0.69	-0.71	-0.62	-0.57	-0.40	-0.43	1.02	1.29	1.44	1.17	1.26	1.15	1.25	1.17
Q9Y3I1	F-box only protein 7 OS=Homo sapiens GN=FBXO7 PE=1 SV=1 - [FBX7_HUMAN]	7.66	1	3	3	6	-1.01	-0.83	-1.03	-0.97	0.02	0.20	0.07	0.13	0.16	0.01	0.15	0.29	1.17	1.29	1.15	1.13	1.05	1.02	1.28	1.17
Q14393	Growth arrest specific protein 6 OS=Homo sapiens GN=GAS6 PE=1 SV=2 - [GAS6_HUMAN]	4.30	1	2	2	4	-0.87	-1.11	-1.04	-1.56	0.76	0.51	-0.22	-0.46	0.16	0.36	0.37	0.23	0.71	1.35	1.41	1.31	1.43	1.62	2.07	1.17
P53794	Sodium/myo-inositol cotransporter OS=Homo sapiens GN=SLC5A3 PE=3 SV=2 - [SC5A3_HUMAN]	3.34	1	2	2	2	-0.55	-0.73	-0.48	-0.65	1.10	0.93	0.63	0.45	0.70	0.88	0.64	0.47	1.23	1.20	1.12	1.46	1.39	1.64	1.56	1.16

Q2VPA4	Complement component receptor 1-like protein OS=Homo sapiens GN=CR1L PE=1 SV=3 - [CR1L_HUMAN]	3.87	2	1	1	1	-0.56	0.10	-1.16	-0.50	-0.72	-0.07	-0.06	0.60	0.59	-0.06	0.41	1.06	0.55	0.97	1.57	0.53	1.13	-0.18	0.42	1.16
Q8WXH0	Nesprin-2 OS=Homo sapiens GN=SYNE2 PE=1 SV=3 - [SYNE2_HUMAN]	8.71	1	46	48	73	-1.23	-1.26	-1.84	-1.70	-0.17	-0.15	-0.55	-0.54	-0.27	-0.32	-0.27	-0.24	0.64	0.90	1.25	0.97	1.36	1.08	1.35	1.16
P12107	Collagen alpha-1(XI) chain OS=Homo sapiens GN=COL11A1 PE=1 SV=4 - [COBA1_HUMAN]	4.60	1	3	6	21	-2.64	-2.78	-2.97	-3.03	-1.44	-1.51	-1.88	-1.94	-1.88	-1.73	-1.93	-2.11	0.71	0.52	0.88	0.90	1.19	1.07	1.51	1.16
Q9NZW5	MAGUK p55 subfamily member 6 OS=Homo sapiens GN=MPP6 PE=1 SV=2 - [MPP6_HUMAN]	51.67	1	25	26	59	-0.59	-0.48	-0.54	-0.52	0.68	0.64	0.63	0.58	0.43	0.45	0.28	0.25	1.09	0.81	0.81	1.14	1.01	1.13	1.13	1.16
Q9NZJ9	Diphosphoinositol polyphosphate phosphohydrolase 2 OS=Homo sapiens GN=NUDT4 PE=1 SV=2 - [NUDT4_HUMAN]	8.89	1	1	2	4	-0.28	-0.20	-0.56	-0.47	0.69	0.77	0.54	0.63	0.81	0.73	0.20	0.28	0.88	0.49	0.76	1.04	1.32	0.95	1.22	1.16
P30530	Tyrosine-protein kinase receptor UFO OS=Homo sapiens GN=AXL PE=1 SV=3 - [UFO_HUMAN]	4.25	1	3	3	5	-0.71	-0.82	-1.08	-1.20	-0.10	-0.22	0.01	-0.10	0.03	0.15	0.18	0.05	0.77	0.89	1.26	0.89	1.27	0.59	0.97	1.16
P59046	NACHT, LRR and PYD domains-containing protein 12 OS=Homo sapiens GN=NLRP12 PE=1 SV=2 - [NAL12_HUMAN]	1.60	1	1	1	1	-1.44	-1.51	-1.73	-1.80	0.17	0.09	-0.63	-0.71	-0.30	-0.22	-0.12	-0.20	0.86	1.32	1.61	1.24	1.54	1.59	1.88	1.16
Q13630	GDP-L-fucose synthase OS=Homo sapiens GN=TSTA3 PE=1 SV=1 - [FCL_HUMAN]	27.41	1	5	5	9	-0.81	-0.89	-0.79	-0.88	0.49	0.47	0.40	0.41	0.47	0.44	0.38	0.37	1.05	1.27	1.27	1.35	1.39	1.35	1.33	1.16

O75112	LIM domain-binding protein 3 OS=Homo sapiens GN=LDB3 PE=1 SV=2 - [LDB3_HUMAN]	27.10	1	13	13	22	-2.60	-2.47	-2.71	-2.57	-1.86	-1.82	-1.52	-1.38	-1.54	-1.62	-1.36	-1.31	0.85	1.24	1.64	0.96	1.34	0.48	0.85	1.16
Q9NRB3	Carbohydrate sulfotransferase 12 OS=Homo sapiens GN=CHST12 PE=1 SV=2 - [CHSTC_HUMAN]	17.15	1	5	5	10	-0.02	0.04	-0.36	-0.30	1.54	1.46	0.42	0.42	1.10	0.92	0.77	0.87	0.65	0.81	1.24	1.12	1.63	1.70	2.20	1.16
P10643	Complement component C7 OS=Homo sapiens GN=C7 PE=1 SV=2 - [CO7_HUMAN]	11.86	2	7	7	10	-0.25	-0.32	-0.51	-0.43	1.12	1.09	0.36	0.54	0.87	0.80	0.87	0.80	0.97	1.23	1.47	1.08	1.34	1.18	1.60	1.15
Q9NQ84	G-protein coupled receptor family C group 5 member C OS=Homo sapiens GN=GPRC5C PE=1 SV=2 - [GPC5C_HUMAN]	7.03	1	3	3	5	-1.13	-1.25	-1.34	-1.44	0.18	0.06	-0.34	-0.36	-0.03	-0.01	0.32	0.24	1.08	0.97	1.65	1.39	1.45	1.29	1.48	1.15
Q96KN2	Beta-Ala-His dipeptidase OS=Homo sapiens GN=CNDP1 PE=1 SV=4 - [CNDP1_HUMAN]	29.78	1	11	11	19	-0.16	-0.15	-0.05	0.05	1.21	1.20	0.99	1.02	1.02	0.91	0.65	0.73	1.22	0.88	0.70	1.09	1.06	1.29	1.28	1.15
Q7Z4H8	KDEL motif-containing protein 2 OS=Homo sapiens GN=KDELC2 PE=1 SV=2 - [KDEL2_HUMAN]	19.53	1	7	7	12	-2.47	-2.54	-2.37	-2.44	0.08	0.01	-1.16	-1.23	-0.49	-0.47	0.13	0.08	1.37	2.62	2.38	1.97	1.74	2.47	2.22	1.15
P19878	Neutrophil cytosol factor 2 OS=Homo sapiens GN=NCF2 PE=1 SV=2 - [NCF2_HUMAN]	4.18	2	1	2	3	0.24	0.12	-0.15	-0.27	-0.10	-0.23	0.94	0.82	0.70	0.82	0.58	0.46	0.75	0.35	0.73	0.62	1.01	-0.36	0.03	1.15
Q9H8L6	Multimerin-2 OS=Homo sapiens GN=MMRN2 PE=1 SV=2 - [MMRN2_HUMAN]	19.28	1	14	14	29	-1.35	-1.29	-1.46	-1.47	0.23	0.28	-0.54	-0.53	-0.18	-0.19	0.06	0.08	1.05	1.45	1.47	1.18	1.39	1.59	1.67	1.15

Q10471	Polypeptide N-acetylgalactosaminyltransferase 2 OS=Homo sapiens GN=GALNT2 PE=1 SV=1 - [GALT2_HUMAN]	15.41	2	7	7	9	-0.93	-0.66	-1.02	-1.00	0.13	0.31	-0.13	0.05	-0.03	0.16	0.03	0.16	0.72	0.83	1.27	1.09	1.31	0.90	1.44	1.15
P04792	Heat shock protein beta-1 OS=Homo sapiens GN=HSPB1 PE=1 SV=2 - [HSPB1_HUMAN]	56.59	1	10	10	85	-1.84	-1.81	-1.87	-1.75	-0.41	-0.41	-0.81	-0.84	-0.44	-0.51	-0.24	-0.28	1.04	1.67	1.76	1.49	1.58	1.58	1.53	1.15
Q9NUP1	Biogenesis of lysosome-related organelles complex 1 subunit 4 OS=Homo sapiens GN=BLOC1S4 PE=1 SV=1 - [BL1S4_HUMAN]	13.82	1	2	2	5	-0.46	-0.57	-1.05	-1.16	0.36	0.24	0.04	-0.08	0.14	0.26	0.55	0.43	0.55	1.01	1.59	0.75	1.34	0.81	1.39	1.15
Q53LP3	Ankyrin repeat domain-containing protein SOWAHC OS=Homo sapiens GN=SOWAHC PE=1 SV=1 - [SWAHC_HUMAN]	8.38	1	3	3	4	-0.40	-0.54	-0.82	-0.96	0.13	-0.02	0.27	0.13	0.14	0.28	0.43	0.28	0.72	0.83	1.24	0.71	1.13	0.51	0.93	1.15
P09486	SPARC OS=Homo sapiens GN=SPARC PE=1 SV=1 - [SPRC_HUMAN]	23.43	1	6	6	9	-1.29	-1.09	-1.36	-1.39	-0.33	-0.29	-0.27	-0.25	-0.07	-0.08	-0.05	0.11	0.74	1.44	1.52	0.97	1.30	0.72	1.04	1.14
Q8N1W1	Rho guanine nucleotide exchange factor 28 OS=Homo sapiens GN=ARHGEF28 PE=1 SV=3 - [ARG28_HUMAN]	0.47	1	1	1	1	-0.49	-0.12	-1.32	-0.95	0.41	0.77	-0.23	0.13	0.53	0.17	-0.35	0.01	0.31	0.15	0.97	0.69	1.52	0.88	1.71	1.14
Q8WV41	Sorting nexin-33 OS=Homo sapiens GN=SNX33 PE=1 SV=1 - [SNX33_HUMAN]	8.54	1	4	4	6	-0.85	-0.79	-1.18	-1.12	-0.08	-0.02	-0.19	-0.21	0.02	0.19	-0.08	-0.04	0.70	0.70	1.08	1.08	1.41	0.76	1.08	1.14
Q96FN9	Probable D-tyrosyl-tRNA(Tyr) deacylase 2 OS=Homo sapiens GN=DTD2 PE=2 SV=1 - [DTD2_HUMAN]	6.55	1	1	1	1	-1.28	-1.12	-1.04	-0.88	0.44	0.60	0.04	0.20	-0.10	-0.26	-0.21	-0.05	1.38	1.08	0.83	1.06	0.82	1.71	1.47	1.14

P35442	Thrombospondin-2 OS=Homo sapiens GN=THBS2 PE=1 SV=2 - [TSP2_HUMAN]	8.96	1	5	7	13	-1.11	-1.45	-1.51	-1.90	0.38	0.10	-0.57	-0.71	-0.62	-0.26	-0.50	-0.75	0.80	0.98	1.22	1.02	1.54	1.59	2.06	1.14
Q07157	Tight junction protein ZO-1 OS=Homo sapiens GN=TJP1 PE=1 SV=3 - [ZO1_HUMAN]	27.63	1	35	35	94	-1.29	-1.47	-1.29	-1.43	-0.03	-0.01	-0.27	-0.33	-0.10	-0.10	0.25	0.13	0.98	1.48	1.57	1.15	1.26	1.16	1.35	1.14
O43707	Alpha-actinin-4 OS=Homo sapiens GN=ACTN4 PE=1 SV=2 - [ACTN4_HUMAN]	61.14	1	38	53	387	-2.24	-2.17	-2.30	-2.22	-1.06	-0.96	-1.25	-1.17	-1.11	-1.18	-0.96	-0.92	1.07	1.28	1.37	1.10	1.14	1.22	1.24	1.14
P15144	Aminopeptidase N OS=Homo sapiens GN=ANPEP PE=1 SV=4 - [AMPN_HUMAN]	29.47	1	21	21	37	-1.24	-1.18	-1.34	-1.36	0.45	0.51	-0.29	-0.25	0.07	0.08	0.14	0.15	0.97	1.29	1.57	1.28	1.57	1.58	1.74	1.14
P46940	Ras GTPase-activating-like protein IQGAP1 OS=Homo sapiens GN=IQGAP1 PE=1 SV=1 - [IQGA1_HUMAN]	45.56	2	57	61	133	-1.52	-1.48	-1.60	-1.54	-0.16	-0.13	-0.42	-0.43	-0.33	-0.33	-0.29	-0.23	1.10	1.27	1.27	1.22	1.25	1.31	1.38	1.14
Q9UMS6	Synaptopodin-2 OS=Homo sapiens GN=SYNPO2 PE=1 SV=2 - [SYNP2_HUMAN]	34.68	1	29	29	70	-2.21	-2.15	-2.60	-2.40	-0.96	-1.02	-1.25	-1.21	-1.03	-1.08	-0.65	-0.60	1.02	1.58	1.66	1.37	1.40	1.28	1.31	1.14
P04844	Dolichyl-diphosphooligosaccharide--protein glycosyltransferase subunit 2 OS=Homo sapiens GN=RPN2 PE=1 SV=3 - [RPN2_HUMAN]	38.19	1	15	15	29	-1.34	-1.23	-1.47	-1.20	0.17	0.26	-0.38	-0.32	0.04	-0.16	-0.07	0.04	0.88	1.18	1.21	1.16	1.20	1.28	1.45	1.14
Q8IWE2	Protein NOXP20 OS=Homo sapiens GN=FAM114A1 PE=1 SV=2 - [NXP20_HUMAN]	9.95	1	4	4	4	-1.12	-0.96	-0.54	-0.43	0.64	0.94	0.67	0.97	0.86	0.56	0.02	0.22	1.71	0.83	0.33	1.37	0.79	1.73	1.15	1.14

Q8WUD6	Cholinephosphotransferase 1 OS=Homo sapiens GN=CHPT1 PE=1 SV=1 - [CHPT1_HUMAN]	2.96	1	1	1	2	-0.42	-0.29	-0.86	-0.73	0.48	0.61	0.21	0.34	0.76	0.63	0.51	0.63	0.69	0.94	1.37	1.08	1.53	0.89	1.32	1.13
Q7Z3Z3	Piwi-like protein 3 OS=Homo sapiens GN=PIWIL3 PE=2 SV=2 - [PIWL3_HUMAN]	1.47	1	1	1	2	-2.12	-0.93	-2.04	-0.84	-0.37	0.82	-0.97	0.23	0.51	-0.68	-0.65	0.53	1.21	1.47	1.38	1.48	1.39	1.74	1.65	1.13
Q9H3U1	Protein unc-45 homolog A OS=Homo sapiens GN=UNC45A PE=1 SV=1 - [UN45A_HUMAN]	23.62	2	14	15	27	-1.46	-1.45	-1.45	-1.39	-0.25	0.00	-0.45	-0.33	-0.35	-0.33	-0.39	-0.20	1.13	0.95	0.89	1.08	1.01	1.32	1.33	1.13
Q9BZZ2	Sialoadhesin OS=Homo sapiens GN=SIGLEC1 PE=1 SV=2 - [SN_HUMAN]	5.91	1	6	6	7	-0.29	-0.21	-0.47	-0.46	0.62	0.28	0.06	-0.16	0.14	0.48	1.08	0.74	0.91	2.29	2.46	1.80	2.10	1.90	2.10	1.13
P02747	Complement C1q subcomponent subunit C OS=Homo sapiens GN=C1QC PE=1 SV=3 - [C1QC_HUMAN]	17.55	1	3	3	18	-0.89	-0.69	-0.84	-0.87	1.03	1.02	0.40	0.32	0.67	0.46	0.80	0.77	1.17	1.69	1.64	1.46	1.30	1.75	1.65	1.13
O75369	Filamin-B OS=Homo sapiens GN=FLNB PE=1 SV=2 - [FLNB_HUMAN]	51.38	1	86	95	226	-1.79	-1.73	-1.86	-1.81	-0.61	-0.49	-0.81	-0.72	-0.47	-0.60	-0.17	-0.11	1.14	1.69	1.68	1.28	1.26	1.27	1.25	1.13
Q9BTT6	Leucine-rich repeat-containing protein 1 OS=Homo sapiens GN=LRRC1 PE=1 SV=1 - [LRRC1_HUMAN]	2.67	1	1	1	2	-0.64	-0.70	-0.88	-0.94	0.21	0.15	0.18	0.13	0.08	0.15	0.20	0.14	0.88	0.85	1.08	0.82	1.06	0.84	1.08	1.13
P08138	Tumor necrosis factor receptor superfamily member 16 OS=Homo sapiens GN=NGFR PE=1 SV=1 - [TNR16_HUMAN]	2.58	1	1	1	1	-1.20	-1.14	-1.50	-1.44	-0.05	0.00	-0.43	-0.37	0.09	0.03	0.28	0.34	0.82	1.48	1.78	1.26	1.57	1.13	1.43	1.13
P05090	Apolipoprotein D OS=Homo sapiens GN=APOD PE=1 SV=1 - [APOD_HUMAN]	34.92	1	7	7	46	-0.21	-0.27	-0.23	-0.23	1.32	1.41	0.78	0.88	1.00	1.07	1.28	1.27	1.00	1.30	1.45	1.13	1.14	1.52	1.60	1.13

Q9HCF6	Transient receptor potential cation channel subfamily M member 3 OS=Homo sapiens GN=TRPM3 PE=2 SV=4 - [TRPM3_HUMAN]	4.10	1	6	6	10	-0.87	-0.93	-0.88	-0.80	0.46	0.47	0.19	0.23	0.16	0.10	-0.16	0.03	1.12	0.72	0.79	0.91	0.97	1.32	1.32	1.13
Q92824	Proprotein convertase subtilisin/kexin type 5 OS=Homo sapiens GN=PCSK5 PE=1 SV=4 - [PCSK5_HUMAN]	1.72	1	3	3	4	-0.81	-1.04	-0.57	-0.79	2.95	2.72	0.50	0.27	1.45	1.68	0.77	0.54	1.36	1.58	1.34	2.52	2.28	3.74	3.50	1.13
Q13425	Beta-2-syntrophin OS=Homo sapiens GN=SNTB2 PE=1 SV=1 - [SNTB2_HUMAN]	40.56	1	16	17	40	-1.25	-1.24	-1.40	-1.40	-0.09	-0.19	-0.30	-0.33	-0.37	-0.26	-0.21	-0.25	0.89	1.14	1.22	1.08	1.07	1.05	1.17	1.13
P02763	Alpha-1-acid glycoprotein 1 OS=Homo sapiens GN=ORM1 PE=1 SV=1 - [A1AG1_HUMAN]	32.84	1	4	6	35	-1.34	-1.37	-1.32	-1.28	-0.84	-0.83	-0.26	-0.15	-0.48	-0.47	-0.52	-0.49	1.22	0.73	0.63	0.76	0.84	0.52	0.31	1.13
P49023	Paxillin OS=Homo sapiens GN=PXN PE=1 SV=3 - [PAXI_HUMAN]	32.99	1	12	12	26	-1.37	-1.70	-1.34	-1.43	-0.05	-0.15	-0.25	-0.25	-0.37	-0.21	-0.02	0.02	1.38	1.38	1.38	1.29	1.24	1.24	1.26	1.13
P09871	Complement C1s subcomponent OS=Homo sapiens GN=C1S PE=1 SV=1 - [C1S_HUMAN]	28.49	1	13	13	22	-1.49	-1.35	-1.43	-1.33	0.23	0.39	-0.45	-0.22	0.14	-0.10	0.22	0.39	1.22	1.76	1.82	1.25	1.26	1.48	1.62	1.12
P19827	Inter-alpha-trypsin inhibitor heavy chain H1 OS=Homo sapiens GN=ITIH1 PE=1 SV=3 - [ITIH1_HUMAN]	21.19	1	11	11	25	-1.36	-1.28	-1.51	-1.22	0.47	0.58	-0.45	-0.36	0.19	0.19	0.40	0.56	0.80	1.75	1.95	1.38	1.87	1.86	1.94	1.12
P04433	Ig kappa chain V-III region VG (Fragment) OS=Homo sapiens PE=1 SV=1 - [KV309_HUMAN]	7.83	1	1	1	6	-1.58	-1.64	-1.70	-1.76	-0.19	-0.25	-0.65	-0.70	-0.22	-0.15	0.41	0.34	0.99	1.99	2.11	1.46	1.58	1.37	1.49	1.12

P62304	Small nuclear ribonucleoprotein E OS=Homo sapiens GN=SNRPE PE=1 SV=1 - [RUXE_HUMAN]	40.22	1	2	2	3	0.15	-0.05	-1.24	-1.44	-0.02	-0.22	-0.19	-0.28	-0.40	-0.19	-0.30	-0.50	-0.28	-0.44	0.94	-0.31	1.08	-0.18	1.21	1.11
P09382	Galectin-1 OS=Homo sapiens GN=LGALS1 PE=1 SV=2 - [LEG1_HUMAN]	70.37	1	7	7	147	-1.22	-1.22	-1.26	-1.21	-0.38	-0.31	-0.20	-0.16	-0.23	-0.34	-0.18	-0.09	1.16	1.09	1.04	0.96	0.92	0.88	0.83	1.11
Q14554	Protein disulfide-isomerase A5 OS=Homo sapiens GN=PDIA5 PE=1 SV=1 - [PDIA5_HUMAN]	3.66	1	2	2	3	-1.20	-1.20	-1.34	-1.33	0.36	0.36	-0.29	-0.28	0.05	0.05	0.12	0.12	0.97	1.33	1.46	1.28	1.42	1.55	1.68	1.11
Q13043	Serine/threonine-protein kinase 4 OS=Homo sapiens GN=STK4 PE=1 SV=2 - [STK4_HUMAN]	11.09	1	2	4	5	-1.37	-1.44	-1.19	-1.26	-0.45	-0.53	-0.14	-0.21	-0.41	-0.33	0.26	0.18	1.28	1.63	1.45	1.07	0.89	0.90	0.72	1.11
P01833	Polymeric immunoglobulin receptor OS=Homo sapiens GN=PIGR PE=1 SV=4 - [PIGR_HUMAN]	5.63	1	3	3	4	-2.29	-2.40	-3.65	-3.66	-1.86	-1.88	-2.60	-2.62	-2.06	-2.03	-1.65	-1.77	1.35	0.64	1.85	1.89	1.65	2.02	1.77	1.11
Q93084	Sarcoplasmic/endoplasmic reticulum calcium ATPase 3 OS=Homo sapiens GN=ATP2A3 PE=1 SV=2 - [AT2A3_HUMAN]	9.49	1	3	9	20	-0.64	-0.43	-1.06	-0.85	-0.26	-0.05	-0.02	0.20	-0.06	-0.27	-0.38	-0.17	0.68	0.27	0.69	0.40	0.82	0.37	0.79	1.11
P07585	Decorin OS=Homo sapiens GN=DCN PE=1 SV=1 - [PGS2_HUMAN]	41.78	1	13	14	74	-2.13	-2.13	-2.19	-2.23	-0.63	-0.67	-1.10	-1.17	-1.02	-0.93	-1.10	-1.15	1.01	1.14	1.14	1.26	1.23	1.64	1.65	1.11
P04843	Dolichyl-diphosphooligosaccharide-protein glycosyltransferase subunit 1 OS=Homo sapiens GN=RPN1 PE=1 SV=1 - [RPN1_HUMAN]	43.33	1	20	20	65	-1.34	-1.31	-1.40	-1.40	-0.24	-0.25	-0.36	-0.35	-0.31	-0.29	-0.15	-0.12	1.04	1.25	1.26	1.01	1.11	1.10	1.09	1.11

Q9P212	1-phosphatidylinositol 4,5-bisphosphate phosphodiesterase epsilon-1 OS=Homo sapiens GN=PLCE1 PE=1 SV=3 - [PLCE1_HUMAN]	0.35	1	1	1	1	-1.59	-1.59	-1.84	-1.84	-0.52	-0.52	-0.79	-0.79	-0.82	-0.81	-0.81	-0.82	0.85	0.78	1.02	0.81	1.06	1.05	1.30	1.11
Q14624	Inter-alpha-trypsin inhibitor heavy chain H4 OS=Homo sapiens GN=ITIH4 PE=1 SV=4 - [ITIH4_HUMAN]	24.62	1	17	17	43	-1.53	-1.50	-1.70	-1.53	0.07	0.10	-0.52	-0.60	-0.04	-0.08	0.36	0.40	0.97	2.04	1.94	1.50	1.60	1.67	1.66	1.11
P48449	Lanosterol synthase OS=Homo sapiens GN=LSS PE=1 SV=1 - [ERG7_HUMAN]	12.70	1	8	8	11	-0.88	-0.92	-0.82	-0.91	0.91	0.71	0.26	0.28	0.26	0.39	0.08	-0.13	1.19	0.91	1.05	1.29	1.19	1.65	1.77	1.11
P01593	Ig kappa chain V-I region AG OS=Homo sapiens PE=1 SV=1 - [KV101_HUMAN]	31.48	9	1	2	23	-1.25	-1.33	-1.52	-1.58	-0.10	-0.16	-0.49	-0.41	0.33	0.27	1.47	1.53	0.85	2.86	2.93	1.69	1.90	1.15	1.40	1.11
Q96HU1	Small G protein signaling modulator 3 OS=Homo sapiens GN=SGSM3 PE=1 SV=1 - [SGSM3_HUMAN]	0.93	1	1	1	1	-1.38	-1.33	-1.28	-1.24	-0.14	-0.11	-0.24	-0.20	-0.27	-0.30	-0.17	-0.14	1.19	1.21	1.11	1.10	1.02	1.22	1.12	1.11
Q9BU23	Lipase maturation factor 2 OS=Homo sapiens GN=LMF2 PE=1 SV=2 - [LMF2_HUMAN]	6.65	1	4	4	8	-1.24	-1.33	-1.36	-1.48	0.11	-0.02	-0.27	-0.49	0.03	0.23	0.01	-0.08	1.02	1.54	1.46	1.51	1.39	1.53	1.47	1.10
Q15654	Thyroid receptor-interacting protein 6 OS=Homo sapiens GN=TRIP6 PE=1 SV=3 - [TRIP6_HUMAN]	38.24	1	9	9	21	-1.50	-1.60	-1.75	-1.77	-0.45	-0.27	-0.88	-0.65	-0.49	-0.69	-0.38	-0.05	0.64	1.09	1.33	1.13	1.17	1.10	1.17	1.10
P07360	Complement component C8 gamma chain OS=Homo sapiens GN=C8G PE=1 SV=3 - [CO8G_HUMAN]	40.10	1	5	5	8	-1.39	-1.46	-1.27	-1.52	0.48	0.34	-0.37	-0.33	-0.12	-0.16	0.31	0.08	1.23	1.71	1.56	1.49	1.41	1.74	1.88	1.10

Q00266	S-adenosylmethionine synthase isoform type-1 OS=Homo sapiens GN=MAT1A PE=1 SV=2 - [METK1_HUMAN]	6.58	1	1	2	2	-1.26	-1.10	-1.67	-1.51	0.48	0.63	-0.64	-0.48	0.08	-0.07	0.01	0.17	0.68	1.28	1.68	1.22	1.63	1.72	2.13	1.10
Q9UBI6	Guanine nucleotide-binding protein G(I)/G(S)/G(O) subunit gamma-12 OS=Homo sapiens GN=GNG12 PE=1 SV=3 - [GBG12_HUMAN]	66.67	1	4	4	12	-1.01	-1.03	-1.15	-1.15	-0.25	-0.05	-0.35	-0.12	-0.11	-0.15	0.01	0.19	0.78	1.25	1.45	1.01	1.23	0.99	0.94	1.10
P00167	Cytochrome b5 OS=Homo sapiens GN=CYB5A PE=1 SV=2 - [CYB5_HUMAN]	33.58	1	4	4	10	-1.80	-1.63	-1.73	-1.53	0.07	0.12	-0.69	-0.59	-0.16	-0.25	0.12	0.24	1.16	2.05	1.85	1.58	1.60	1.85	1.64	1.10
P49908	Selenoprotein P OS=Homo sapiens GN=SEPP1 PE=1 SV=3 - [SEPP1_HUMAN]	6.04	1	2	2	3	-0.02	-0.06	-0.42	0.09	0.88	1.38	0.61	1.31	1.22	0.51	1.28	1.94	0.69	1.31	1.70	0.56	0.96	0.88	1.28	1.10
Q8NHV4	Protein NEDD1 OS=Homo sapiens GN=NEDD1 PE=1 SV=1 - [NEDD1_HUMAN]	1.67	1	1	1	1	-0.02	-0.33	-1.29	-1.60	0.66	0.35	-0.26	-0.57	0.10	0.42	0.48	0.16	-0.18	0.51	1.77	0.47	1.74	0.67	1.94	1.09
P09493	Tropomyosin alpha-1 chain OS=Homo sapiens GN=TPM1 PE=1 SV=2 - [TPM1_HUMAN]	54.58	1	7	19	213	-2.45	-2.57	-2.53	-2.49	-1.26	-1.13	-1.56	-1.47	-1.17	-1.35	-0.94	-0.86	1.14	1.74	1.65	1.37	1.33	1.47	1.35	1.09
P07451	Carbonic anhydrase 3 OS=Homo sapiens GN=CA3 PE=1 SV=3 - [CAH3_HUMAN]	30.77	1	5	5	16	-0.85	-1.02	-1.14	-1.17	0.32	0.31	-0.17	-0.28	-0.20	-0.08	-0.16	-0.42	0.74	0.68	1.02	0.60	1.16	1.09	1.58	1.09
P04207	Ig kappa chain V-III region CLL OS=Homo sapiens PE=1 SV=2 - [KV308_HUMAN]	12.40	1	1	1	2	-1.46	-1.56	-2.25	-2.35	-0.55	-0.66	-1.23	-1.32	-0.13	-0.03	0.78	0.67	0.29	2.25	3.03	1.47	2.26	0.90	1.68	1.09

P08263	Glutathione S transferase A1 OS=Homo sapiens GN=GSTA1 PE=1 SV=3 - [GSTA1_HUMAN]	8.56	4	2	2	2	-1.72	-1.56	-1.43	-1.27	-1.17	-1.01	-0.40	-0.24	-0.49	-0.65	-1.34	-1.18	1.38	0.39	0.09	1.11	0.82	0.54	0.25	1.09
O75718	Cartilage-associated protein OS=Homo sapiens GN=CRTAP PE=1 SV=1 - [CRTAP_HUMAN]	10.47	1	4	4	4	-1.11	-1.08	-1.52	-1.48	0.16	0.19	-0.49	-0.46	-0.09	-0.11	-0.02	0.01	0.68	1.10	1.50	1.03	1.44	1.26	1.66	1.09
O15226	NF-kappa-B-repressing factor OS=Homo sapiens GN=NKRF PE=1 SV=2 - [NKRF_HUMAN]	1.30	1	1	1	1	-1.04	-0.98	-1.35	-1.29	0.21	0.27	-0.33	-0.27	-0.06	-0.12	-0.28	-0.22	0.77	0.77	1.07	0.95	1.26	1.24	1.55	1.09
P60660	Myosin light polypeptide 6 OS=Homo sapiens GN=MYL6 PE=1 SV=2 - [MYL6_HUMAN]	80.79	2	9	11	162	-1.95	-1.86	-1.98	-1.93	-0.73	-0.80	-0.98	-0.96	-0.87	-0.80	-0.62	-0.57	1.02	1.38	1.33	1.06	1.21	1.05	1.17	1.09
P00734	Prothrombin OS=Homo sapiens GN=F2 PE=1 SV=2 - [THRB_HUMAN]	43.73	1	22	22	61	-2.58	-2.68	-2.63	-2.58	-1.28	-1.19	-1.61	-1.59	-1.29	-1.37	-0.85	-0.79	0.97	1.77	1.95	1.51	1.44	1.33	1.45	1.09
Q9BZG1	Ras-related protein Rab-34 OS=Homo sapiens GN=RAB34 PE=1 SV=1 - [RAB34_HUMAN]	20.08	1	4	4	6	-1.26	-1.34	-0.88	-0.90	0.36	0.43	0.19	0.12	0.18	0.16	0.45	0.31	1.51	1.82	1.38	1.46	1.02	1.61	1.17	1.08
O95297	Myelin protein zero-like protein 1 OS=Homo sapiens GN=MPZL1 PE=1 SV=1 - [MPZL1_HUMAN]	17.47	1	4	4	5	-0.50	-0.63	-1.14	-1.21	0.09	-0.04	-0.12	-0.19	-0.12	-0.03	0.04	-0.05	0.48	1.09	1.17	0.55	0.95	0.88	0.97	1.08
Q96C24	Synaptotagmin-like protein 4 OS=Homo sapiens GN=SYTL4 PE=1 SV=2 - [SYTL4_HUMAN]	14.75	1	7	7	11	-1.09	-1.02	-1.04	-0.89	0.65	0.71	-0.02	0.13	0.67	0.62	0.53	0.59	1.34	1.28	1.34	1.66	1.73	1.72	2.08	1.08

P36404	ADP-ribosylation factor-like protein 2 OS=Homo sapiens GN=ARL2 PE=1 SV=4 - [ARL2_HUMAN]	38.59	1	6	6	10	-1.23	-1.43	-1.21	-1.41	-0.22	-0.28	-0.19	-0.43	-0.48	-0.43	-0.42	-0.48	1.09	0.64	0.62	0.83	0.81	0.94	0.92	1.08
P50225	Sulfotransferase 1A1 OS=Homo sapiens GN=SULT1A1 PE=1 SV=3 - [ST1A1_HUMAN]	17.29	2	2	5	10	-1.00	-0.92	-1.16	-1.13	0.57	0.62	-0.06	-0.10	0.30	0.28	0.62	0.70	0.85	1.62	1.87	1.25	1.49	1.55	1.74	1.08
Q53T59	HCLS1-binding protein 3 OS=Homo sapiens GN=HS1BP3 PE=1 SV=1 - [H1BP3_HUMAN]	11.73	1	3	3	6	-0.99	-1.11	-1.02	-1.14	0.10	-0.03	0.00	-0.12	-0.13	0.00	-0.06	-0.19	1.04	0.93	0.95	1.01	1.05	1.07	1.10	1.08
P01876	Ig alpha-1 chain C region OS=Homo sapiens GN=IGHA1 PE=1 SV=2 - [IGHA1_HUMAN]	43.34	1	4	11	46	-1.15	-1.07	-0.85	-0.68	-0.27	-0.02	0.12	0.30	0.15	-0.08	0.01	0.28	1.32	1.22	0.96	1.16	0.82	0.86	0.65	1.08
P24390	ER lumen protein retaining receptor 1 OS=Homo sapiens GN=KDELR1 PE=1 SV=1 - [ERD21_HUMAN]	3.77	2	1	1	2	-1.89	-1.75	-1.80	-1.66	0.02	0.16	-0.78	-0.64	-0.25	-0.39	-0.33	-0.20	1.16	1.56	1.47	1.53	1.45	1.89	1.80	1.08
Q16513	Serine/threonine-protein kinase N2 OS=Homo sapiens GN=PKN2 PE=1 SV=1 - [PKN2_HUMAN]	20.12	1	11	11	18	-0.82	-1.11	-1.03	-0.88	0.04	0.10	-0.01	-0.11	-0.19	-0.07	-0.07	-0.13	1.04	0.70	0.97	0.92	0.92	0.90	1.16	1.08
Q12923	Tyrosine-protein phosphatase non-receptor type 13 OS=Homo sapiens GN=PTPN13 PE=1 SV=2 - [PTN13_HUMAN]	5.79	1	9	9	18	-0.84	-1.17	-0.96	-1.27	0.13	0.01	-0.24	-0.25	0.00	-0.17	0.11	-0.22	0.76	0.75	1.16	0.81	0.97	1.36	1.43	1.08
Q13790	Apolipoprotein F OS=Homo sapiens GN=APOF PE=1 SV=2 - [APOF_HUMAN]	4.29	1	1	1	2	-1.22	-1.08	-1.25	-1.11	0.62	0.75	-0.24	-0.10	0.85	0.71	1.09	1.23	1.03	2.31	2.34	1.96	1.99	1.82	1.85	1.07

P39656	Dolichyl-diphosphooligosaccharide-protein glycosyltransferase 48 kDa subunit OS=Homo sapiens GN=DDOST PE=1 SV=4 - [OST48_HUMAN]	23.25	1	8	8	16	-1.71	-1.68	-1.32	-1.45	-0.11	-0.01	-0.31	-0.50	-0.31	-0.31	-0.22	-0.09	1.18	1.35	1.28	1.36	1.23	1.58	1.41	1.07
P17948	Vascular endothelial growth factor receptor 1 OS=Homo sapiens GN=FLT1 PE=1 SV=2 - [VGFR1_HUMAN]	7.92	5	9	10	23	-0.38	-0.69	-0.57	-0.72	1.88	1.71	0.44	0.24	0.84	0.99	0.61	0.44	0.88	1.24	1.32	1.53	1.55	2.56	2.34	1.07
Q8TD19	Serine/threonine-protein kinase Nek9 OS=Homo sapiens GN=NEK9 PE=1 SV=2 - [NEK9_HUMAN]	19.51	1	13	13	22	-1.16	-1.62	-1.07	-1.12	0.34	0.00	0.10	-0.32	0.01	0.04	-0.14	0.02	1.31	1.64	1.27	1.54	1.15	1.40	1.24	1.07
Q9NUQ6	SPATS2-like protein OS=Homo sapiens GN=SPATS2L PE=1 SV=2 - [SPS2L_HUMAN]	13.08	1	5	5	7	-0.82	-0.74	-1.11	-1.02	0.10	0.19	-0.10	-0.02	-0.15	-0.23	-0.14	-0.06	0.78	0.69	0.96	0.63	0.91	0.91	1.19	1.07
Q01955	Collagen alpha-3(IV) chain OS=Homo sapiens GN=COL4A3 PE=1 SV=3 - [CO4A3_HUMAN]	1.98	1	3	3	13	-1.05	-1.28	-1.00	-1.24	0.20	0.02	0.00	-0.23	-0.44	-0.30	-0.26	-0.42	1.10	0.84	1.17	0.42	0.86	1.29	1.25	1.07
Q53H82	Beta-lactamase-like protein 2 OS=Homo sapiens GN=LACTB2 PE=1 SV=2 - [LACB2_HUMAN]	3.13	1	1	1	1	-1.14	-1.02	-1.39	-1.26	0.01	0.13	-0.39	-0.27	0.02	-0.10	0.00	0.12	0.80	1.15	1.39	1.07	1.32	1.13	1.38	1.06
O00187	Mannan-binding lectin serine protease 2 OS=Homo sapiens GN=MASP2 PE=1 SV=4 - [MASP2_HUMAN]	5.10	1	2	2	3	-1.07	-1.08	-0.83	-0.84	1.33	1.32	0.17	0.16	0.41	0.42	0.49	0.48	1.29	1.57	1.32	1.52	1.28	2.38	2.14	1.06
P00739	Haptoglobin-related protein OS=Homo sapiens GN=HPR PE=1 SV=2 - [HPTR_HUMAN]	43.39	1	3	14	63	-1.57	-1.55	-1.32	-1.31	-0.58	-0.62	-0.11	-0.15	-0.59	-0.54	-0.63	-0.68	1.20	1.93	1.22	1.66	1.34	1.78	1.26	1.06

Q96HC4	PDZ and LIM domain protein 5 OS=Homo sapiens GN=PDLIM5 PE=1 SV=5 - [PDLI5_HUMAN]	42.62	1	21	21	73	-1.94	-1.83	-1.73	-1.73	-0.48	-0.51	-0.71	-0.76	-0.70	-0.72	-0.52	-0.53	1.22	1.38	1.30	1.28	1.15	1.46	1.34	1.06
Q9NYL9	Tropomodulin 3 OS=Homo sapiens GN=TMOD3 PE=1 SV=1 - [TMOD3_HUMAN]	54.83	1	12	14	33	-1.80	-1.86	-1.62	-1.81	-0.72	-0.75	-0.81	-0.70	-0.83	-0.76	-0.61	-0.67	0.86	1.16	1.28	1.00	1.01	0.96	1.00	1.06
P29558	RNA-binding motif, single-stranded-interacting protein 1 OS=Homo sapiens GN=RBMS1 PE=1 SV=3 - [RBMS1_HUMAN]	9.85	2	2	3	5	-2.08	-1.96	-2.04	-1.91	-0.61	-0.50	-1.04	-0.92	-0.68	-0.80	-0.58	-0.46	1.09	1.51	1.46	1.31	1.27	1.45	1.40	1.06
Q13547	Histone deacetylase 1 OS=Homo sapiens GN=HDAC1 PE=1 SV=1 - [HDAC1_HUMAN]	9.54	1	2	4	6	-1.46	-1.47	-1.67	-1.67	-0.62	-0.63	-0.68	-0.68	-0.57	-0.57	-0.37	-0.37	0.84	1.10	1.31	0.93	1.14	0.83	1.03	1.05
Q92692	Poliovirus receptor-related protein 2 OS=Homo sapiens GN=PVRL2 PE=1 SV=1 - [PVRL2_HUMAN]	10.22	1	5	5	7	-0.81	-1.08	-1.15	-1.20	0.37	0.20	-0.03	-0.21	0.03	0.15	0.56	0.50	0.75	1.29	1.71	0.92	1.27	1.00	1.37	1.05
Q9Y547	Intraflagellar transport protein 25 homolog OS=Homo sapiens GN=HSPB11 PE=1 SV=1 - [IFT25_HUMAN]	7.64	1	1	1	1	0.10	-0.12	0.08	-0.14	0.53	0.30	1.07	0.85	0.44	0.67	0.02	-0.21	1.02	-0.08	-0.07	0.60	0.62	0.41	0.43	1.05
O75095	Multiple epidermal growth factor-like domains protein 6 OS=Homo sapiens GN=MEGF6 PE=1 SV=4 - [MEGF6_HUMAN]	1.17	1	1	1	1	-1.70	-1.08	-0.89	-0.26	0.56	1.18	0.10	0.72	0.48	-0.14	0.42	1.04	1.86	2.12	1.31	1.59	0.78	2.25	1.44	1.05
Q8IWU2	Serine/threonine-protein kinase LMTK2 OS=Homo sapiens GN=LMTK2 PE=1 SV=2 - [LMTK2_HUMAN]	3.19	1	3	3	3	-0.35	-0.63	-0.97	-1.25	0.36	0.07	0.02	-0.27	-0.25	0.04	0.20	-0.09	0.42	0.55	1.17	0.42	1.04	0.69	1.31	1.05

Q96A19	Coiled-coil domain-containing protein 102A OS=Homo sapiens GN=CCDC102A PE=1 SV=2 - [C102A_HUMAN]	6.73	1	2	2	3	-0.93	-1.59	-1.75	-2.40	-0.01	-0.67	-0.76	-1.41	-0.75	-0.09	0.09	-0.57	0.23	1.03	1.83	0.87	1.69	0.91	1.72	1.05
Q92888	Rho guanine nucleotide exchange factor 1 OS=Homo sapiens GN=ARHGEF1 PE=1 SV=2 - [ARHG1_HUMAN]	14.69	1	11	11	13	-0.90	-0.52	-1.16	-0.98	0.18	0.37	-0.05	-0.04	0.19	0.06	0.05	0.21	0.78	0.97	1.32	0.94	1.30	1.02	1.47	1.05
O95786	Probable ATP-dependent RNA helicase DDX58 OS=Homo sapiens GN=DDX58 PE=1 SV=2 - [DDX58_HUMAN]	10.59	1	8	8	9	-0.59	-0.66	-0.93	-0.88	0.37	0.41	-0.09	-0.11	0.14	0.23	0.18	0.11	0.62	0.78	1.09	0.88	1.08	0.93	1.38	1.05
P30154	Serine/threonine-protein phosphatase 2A 65 kDa regulatory subunit A beta isoform OS=Homo sapiens GN=PPP2R1B PE=1 SV=3 - [2AAB_HUMAN]	4.83	1	1	2	6	-0.27	0.03	-0.65	-0.35	0.22	0.52	0.34	0.64	0.58	0.29	0.70	0.99	0.67	0.98	1.34	0.59	0.97	0.48	0.85	1.05
P13645	Keratin, type I cytoskeletal 10 OS=Homo sapiens GN=KRT10 PE=1 SV=6 - [K1C10_HUMAN]	19.01	4	6	9	31	0.86	0.87	-0.31	-0.23	-0.38	-0.34	0.87	0.85	0.33	0.07	1.12	1.05	0.12	0.34	1.18	-0.47	0.39	-0.61	-0.09	1.05
Q9HCB6	Spondin-1 OS=Homo sapiens GN=SPON1 PE=1 SV=2 - [SPON1_HUMAN]	27.26	1	15	15	27	-0.21	-0.44	-0.53	-0.43	0.81	0.97	0.62	0.61	0.59	0.59	0.81	0.78	0.68	1.29	1.24	0.95	1.08	1.03	1.33	1.05
Q13336	Urea transporter 1 OS=Homo sapiens GN=SLC14A1 PE=2 SV=2 - [UT1_HUMAN]	11.31	1	5	5	10	-0.09	-0.15	-0.21	-0.26	0.50	0.32	0.67	0.49	0.54	0.73	0.83	0.64	0.76	0.76	0.97	0.74	0.99	0.29	0.54	1.04

Q01974	Tyrosine-protein kinase transmembrane receptor ROR2 OS=Homo sapiens GN=ROR2 PE=1 SV=2 - [ROR2_HUMAN]	4.56	1	3	3	4	-0.76	-1.16	-0.97	-1.38	0.54	0.13	0.08	-0.33	0.09	0.50	0.79	0.38	0.53	1.41	1.67	1.26	1.29	1.10	1.44	1.04
P31645	Sodium-dependent serotonin transporter OS=Homo sapiens GN=SLC6A4 PE=1 SV=1 - [SC6A4_HUMAN]	2.54	1	1	1	1	-1.62	-1.45	-1.71	-1.54	-0.77	-0.60	-0.73	-0.56	-0.78	-0.95	-0.84	-0.67	0.94	0.79	0.87	0.70	0.80	0.84	0.93	1.04
Q15084	Protein disulfide-isomerase A6 OS=Homo sapiens GN=PDIA6 PE=1 SV=1 - [PDIA6_HUMAN]	39.77	1	13	14	36	-1.12	-1.16	-1.38	-1.49	-0.02	-0.11	-0.59	-0.56	-0.37	-0.29	-0.18	-0.27	0.62	1.15	1.09	0.88	1.15	0.96	1.38	1.04
P20036	HLA class II histocompatibility antigen, DP alpha 1 chain OS=Homo sapiens GN=HLA-DPA1 PE=1 SV=1 - [DPA1_HUMAN]	17.69	1	2	3	7	-0.71	-1.07	-1.31	-1.67	0.79	0.42	-0.33	-0.69	1.15	1.52	0.96	0.59	0.44	1.67	2.26	2.26	2.86	1.48	2.08	1.04
P25391	Laminin subunit alpha 1 OS=Homo sapiens GN=LAMA1 PE=1 SV=2 - [LAMA1_HUMAN]	6.11	1	14	15	26	-1.67	-1.85	-1.97	-2.09	-0.79	-0.88	-0.95	-1.11	-0.93	-0.79	-0.47	-0.59	0.72	1.24	1.51	0.78	1.29	0.90	1.24	1.04
P02746	Complement C1q subcomponent subunit B OS=Homo sapiens GN=C1QB PE=1 SV=3 - [C1QB_HUMAN]	17.00	1	4	4	39	-0.39	-0.45	-0.74	-0.73	0.79	0.78	0.20	0.25	0.66	0.72	0.76	0.81	0.57	1.45	1.54	1.21	1.28	1.37	1.50	1.04
Q13683	Integrin alpha 7 OS=Homo sapiens GN=ITGA7 PE=1 SV=3 - [ITA7_HUMAN]	19.73	1	17	17	38	-1.65	-1.63	-1.88	-1.75	-0.48	-0.35	-0.70	-0.62	-0.59	-0.69	-0.49	-0.49	0.70	1.15	1.40	0.89	1.18	0.98	1.06	1.04

P42226	Signal transducer and activator of transcription 6 OS=Homo sapiens GN=STAT6 PE=1 SV=1 - [STAT6_HUMAN]	13.70	1	12	12	18	-1.08	-1.12	-1.24	-1.39	-0.07	-0.15	-0.24	-0.40	-0.20	-0.14	-0.01	-0.19	0.76	1.03	1.20	1.15	1.18	1.08	1.31	1.04
P50224	Sulfotransferase 1A3/1A4 OS=Homo sapiens GN=SULT1A3 PE=1 SV=1 - [ST1A3_HUMAN]	25.08	2	3	6	11	-2.19	-1.77	-2.06	-1.64	-0.53	-0.11	-1.09	-0.66	-0.59	-1.01	-0.59	-0.17	1.16	1.61	1.47	1.21	1.09	1.65	1.52	1.04
Q9BXN1	Asporin OS=Homo sapiens GN=ASPN PE=1 SV=2 - [ASPN_HUMAN]	36.84	1	12	12	42	-1.88	-1.83	-1.90	-1.85	-0.13	-0.13	-1.17	-1.19	-0.90	-0.83	-1.08	-1.06	0.78	0.91	1.13	1.18	1.24	1.81	1.89	1.04
Q09328	Alpha-1,6-mannosylglycoprotein 6-beta-N-acetylglucosaminyltransferase A OS=Homo sapiens GN=MGAT5 PE=1 SV=1 - [MGT5A_HUMAN]	1.62	1	1	1	2	0.03	0.16	-0.32	-0.18	0.80	0.93	0.65	0.79	1.47	1.34	1.24	1.37	0.68	1.22	1.56	1.34	1.69	0.76	1.10	1.04
Q8TDZ2	Protein-methionine sulfoxide oxidase MICAL1 OS=Homo sapiens GN=MICAL1 PE=1 SV=2 - [MICA1_HUMAN]	14.06	1	11	11	17	-1.04	-0.85	-1.09	-1.13	-0.23	-0.20	-0.39	-0.20	-0.20	-0.22	-0.32	-0.15	0.78	0.70	0.75	0.72	1.13	0.70	0.94	1.03
O95622	Adenylate cyclase type 5 OS=Homo sapiens GN=ADCY5 PE=1 SV=3 - [ADCY5_HUMAN]	11.66	1	8	11	21	-0.76	-0.71	-0.95	-0.82	0.07	0.15	0.26	0.17	-0.02	0.24	-0.27	-0.04	0.95	0.78	0.78	0.79	0.84	0.69	1.00	1.03
P13796	Plastin-2 OS=Homo sapiens GN=LCP1 PE=1 SV=6 - [PLSL_HUMAN]	44.18	1	17	21	46	-0.48	-0.74	-0.59	-0.74	-0.25	-0.30	0.28	0.26	-0.02	-0.04	-0.23	-0.25	0.74	0.50	0.45	0.65	0.74	0.24	0.46	1.03
Q96BN8	Ubiquitin thioesterase otulin OS=Homo sapiens GN=FAM105B PE=1 SV=3 - [OTUL_HUMAN]	5.11	1	2	2	2	-0.09	0.02	-0.95	-0.84	0.17	0.34	0.01	0.13	0.37	0.26	-0.45	-0.28	0.16	0.13	0.53	0.38	1.25	0.72	1.13	1.03

P01776	Ig heavy chain V-III region WAS OS=Homo sapiens PE=1 SV=1 - [HV315_HUMAN]	23.93	3	2	2	11	-1.99	-1.83	-2.06	-1.97	-0.27	-0.19	-1.08	-0.92	-0.41	-0.54	0.21	0.29	1.19	2.23	2.38	1.63	1.69	1.70	1.80	1.03
O95479	GDH/6PGL endoplasmic bifunctional protein OS=Homo sapiens GN=H6PD PE=1 SV=2 - [G6PE_HUMAN]	11.88	1	8	8	12	-0.98	-0.91	-1.42	-1.35	-0.03	0.08	-0.25	-0.20	0.09	-0.10	-0.03	0.01	1.04	1.12	1.56	1.04	1.28	1.06	1.37	1.03
Q9BVG9	Phosphatidyl serine synthase 2 OS=Homo sapiens GN=PTDSS2 PE=1 SV=1 - [PTSS2_HUMAN]	8.42	1	3	3	4	-2.43	-2.42	-2.61	-2.60	-1.23	-1.22	-1.65	-1.63	-1.42	-1.43	-1.42	-1.41	0.84	1.02	1.19	1.04	1.22	1.18	1.36	1.03
Q99988	Growth/differentiation factor 15 OS=Homo sapiens GN=GDF15 PE=1 SV=3 - [GDF15_HUMAN]	5.19	1	1	1	1	0.18	0.02	0.72	0.57	1.79	1.63	1.69	1.54	1.07	1.23	0.94	0.78	1.57	0.76	0.21	1.08	0.54	1.60	1.05	1.03
O95302	Peptidyl-prolyl cis-trans isomerase FKBP9 OS=Homo sapiens GN=FKBP9 PE=1 SV=2 - [FKBP9_HUMAN]	17.37	2	10	10	14	-1.45	-1.48	-1.47	-1.43	-0.14	-0.07	-0.60	-0.52	-0.46	-0.37	-0.31	-0.45	0.92	1.09	1.08	1.10	1.08	1.29	1.28	1.03
P62070	Ras-related protein R-Ras2 OS=Homo sapiens GN=RRAS2 PE=1 SV=1 - [RRAS2_HUMAN]	43.14	1	5	8	24	-0.72	-0.61	-0.98	-0.67	0.35	0.36	-0.02	0.06	0.30	0.11	0.08	0.15	0.76	0.80	1.04	0.93	1.12	0.99	1.09	1.03
Q8WXX5	DnaJ homolog subfamily C member 9 OS=Homo sapiens GN=DNAJC9 PE=1 SV=1 - [DNJC9_HUMAN]	12.69	1	4	4	4	-0.80	-1.05	-0.88	-0.85	0.02	0.04	0.07	0.11	0.07	0.10	0.58	0.40	1.04	1.50	1.46	0.93	0.95	1.12	0.88	1.03
P01024	Complement C3 OS=Homo sapiens GN=C3 PE=1 SV=2 - [CO3_HUMAN]	56.34	2	78	79	354	-1.66	-1.69	-1.74	-1.71	-0.12	-0.08	-0.78	-0.76	-0.30	-0.30	0.11	0.16	1.09	1.93	1.88	1.53	1.49	1.67	1.67	1.03

O75427	Leucine-rich repeat and calponin homology domain-containing protein 4 OS=Homo sapiens GN=LRCH4 PE=1 SV=2 - [LRCH4_HUMAN]	5.42	1	2	2	4	-0.63	-0.58	-0.91	-0.87	0.06	0.10	0.05	0.09	0.02	-0.02	0.20	0.23	0.73	0.83	1.11	0.64	0.93	0.67	0.95	1.03
Q04721	Neurogenic locus notch homolog protein 2 OS=Homo sapiens GN=NOTCH2 PE=1 SV=3 - [NOTC2_HUMAN]	5.83	1	10	10	19	-1.31	-1.51	-1.20	-1.27	-0.20	-0.35	-0.42	-0.47	-0.31	-0.23	0.04	-0.11	0.97	1.41	1.41	1.16	1.11	1.05	1.15	1.03
P51153	Ras-related protein Rab-13 OS=Homo sapiens GN=RAB13 PE=1 SV=1 - [RAB13_HUMAN]	28.08	1	3	5	18	-0.77	-0.90	-1.03	-1.24	0.34	0.11	-0.07	-0.19	0.19	0.19	0.23	0.01	0.89	1.01	1.27	1.11	1.25	1.22	1.35	1.03
P16403	Histone H1.2 OS=Homo sapiens GN=HIST1H1C PE=1 SV=2 - [H12_HUMAN]	35.21	3	2	11	63	-2.80	-2.81	-2.21	-2.21	-2.25	-2.26	-1.25	-1.25	-2.48	-2.47	-2.05	-2.07	1.61	0.75	0.15	0.36	-0.23	0.54	-0.06	1.02
P22681	E3 ubiquitin-protein ligase CBL OS=Homo sapiens GN=CBL PE=1 SV=2 - [CBL_HUMAN]	11.37	2	6	6	9	-0.20	-0.29	-0.75	-0.83	0.45	0.36	0.21	0.13	0.13	0.22	0.03	-0.06	0.47	0.23	0.77	0.45	1.00	0.63	1.18	1.02
O94875	Sorbin and SH3 domain-containing protein 2 OS=Homo sapiens GN=SORBS2 PE=1 SV=3 - [SRBS2_HUMAN]	24.36	1	22	23	94	-1.93	-1.99	-2.38	-2.41	-1.43	-1.58	-1.35	-1.45	-1.38	-1.26	-1.03	-1.14	0.82	1.07	1.31	0.86	1.14	0.80	0.93	1.02
O75131	Copine-3 OS=Homo sapiens GN=CPNE3 PE=1 SV=1 - [CPNE3_HUMAN]	40.60	1	18	19	47	-1.38	-1.38	-1.13	-1.07	0.01	-0.04	-0.13	-0.16	-0.09	-0.06	-0.05	-0.07	1.36	1.21	1.16	1.25	1.10	1.15	1.13	1.02
Q15274	Nicotinate-nucleotide pyrophosphorylase [carboxylating] OS=Homo sapiens GN=QPRT PE=1 SV=3 - [NADC_HUMAN]	13.80	1	4	4	9	-0.04	-0.06	-0.25	-0.32	0.64	0.50	0.66	0.50	0.34	0.43	-0.13	0.11	0.84	0.10	0.32	0.48	0.74	0.61	0.83	1.02

Q05655	Protein kinase C delta type OS=Homo sapiens GN=PRKCD PE=1 SV=2 - [KPCD_HUMAN]	8.14	2	4	5	12	-0.85	-0.81	-1.08	-1.05	-0.01	0.02	-0.15	-0.08	-0.01	-0.04	-0.03	-0.01	0.77	0.82	1.05	0.84	1.08	0.82	1.05	1.02
P02545	Prelamin-A/C OS=Homo sapiens GN=LMNA PE=1 SV=1 - [LMNA_HUMAN]	56.48	1	40	40	263	-1.96	-1.91	-1.99	-1.98	-1.08	-1.07	-1.02	-1.02	-0.93	-0.96	-0.63	-0.62	0.98	1.29	1.35	1.03	1.04	0.90	0.87	1.02
Q13137	Calcium-binding and coiled-coil domain-containing protein 2 OS=Homo sapiens GN=CALCOCO2 PE=1 SV=1 - [CACO2_HUMAN]	12.11	1	5	5	6	-0.19	-0.43	-0.62	-0.85	0.54	0.30	0.34	0.11	0.07	0.31	0.16	-0.08	0.59	0.36	0.77	0.53	0.96	0.72	1.14	1.02
P19320	Vascular cell adhesion protein 1 OS=Homo sapiens GN=VCAM1 PE=1 SV=1 - [VCAM1_HUMAN]	19.62	1	12	13	15	-1.08	-1.11	-1.49	-1.33	0.04	0.14	-0.44	-0.38	0.05	-0.09	0.65	0.73	0.93	2.09	2.12	1.28	1.34	1.22	1.39	1.02
P01033	Metalloproteinase inhibitor 1 OS=Homo sapiens GN=TIMP1 PE=1 SV=1 - [TIMP1_HUMAN]	15.46	1	3	3	4	-0.39	-0.40	-0.32	-0.33	1.64	1.62	0.63	0.63	0.98	0.99	0.93	0.91	1.08	1.32	1.24	1.41	1.34	2.01	1.94	1.01
P19652	Alpha-1-acid glycoprotein 2 OS=Homo sapiens GN=ORM2 PE=1 SV=2 - [A1AG2_HUMAN]	34.33	1	5	7	20	-1.82	-1.67	-2.00	-1.88	-1.03	-0.88	-0.98	-0.84	-0.67	-0.87	-1.04	-0.99	0.92	0.72	0.80	1.00	1.04	0.87	0.89	1.01
Q13459	Unconventional myosin-IXb OS=Homo sapiens GN=MYO9B PE=1 SV=3 - [MYO9B_HUMAN]	2.27	1	3	3	3	-0.33	-0.54	-0.74	-0.86	0.57	0.37	0.05	0.04	0.19	0.16	0.07	0.06	0.63	0.33	0.74	0.78	1.09	0.88	1.22	1.01
Q8TE77	Protein phosphatase Slingshot homolog 3 OS=Homo sapiens GN=SSH3 PE=1 SV=2 - [SSH3_HUMAN]	22.46	1	11	11	17	-0.24	-0.50	-0.93	-1.07	0.04	-0.02	-0.15	-0.12	-0.31	-0.13	0.04	-0.18	0.32	0.81	1.12	0.51	1.02	0.53	0.97	1.01

Q9H7E9	UPF0488 protein C8orf33 OS=Homo sapiens GN=C8orf33 PE=1 SV=1 - [CH033_HUMAN]	4.80	1	1	1	2	-1.23	-1.47	-1.26	-1.50	-0.38	-0.62	-0.31	-0.55	-0.20	0.04	-0.01	-0.25	0.97	1.23	1.25	1.30	1.33	0.83	0.86	1.01
P02656	Apolipoprotein C-III OS=Homo sapiens GN=APOC3 PE=1 SV=1 - [APOC3_HUMAN]	34.34	1	3	3	7	-1.33	-1.14	-0.97	-0.74	-0.15	-0.06	-0.35	-0.21	0.83	0.62	1.79	1.93	0.97	3.21	2.62	1.90	1.62	1.22	1.06	1.01
O14907	Tax1-binding protein 3 OS=Homo sapiens GN=TAX1BP3 PE=1 SV=2 - [TX1B3_HUMAN]	28.23	1	2	2	5	-0.13	-0.31	-0.63	-0.80	0.50	0.32	0.32	0.14	0.17	0.35	0.14	-0.04	0.51	0.28	0.77	0.51	1.01	0.62	1.11	1.01
Q6DD88	Atlastin-3 OS=Homo sapiens GN=ATL3 PE=1 SV=1 - [ATLA3_HUMAN]	39.37	1	16	16	27	-1.99	-2.06	-1.60	-1.61	-0.27	-0.33	-0.62	-0.61	-0.49	-0.50	0.00	-0.04	1.32	1.84	1.57	1.56	1.23	1.75	1.29	1.01
P55042	GTP-binding protein RAD OS=Homo sapiens GN=RRAD PE=1 SV=2 - [RAD_HUMAN]	18.51	1	3	3	4	0.29	0.33	-1.77	-1.72	-0.30	-0.26	-0.86	-0.77	-0.08	-0.15	-0.86	-0.81	-1.06	-1.14	0.91	-0.47	1.59	-0.61	1.45	1.00
Q96AP7	Endothelial cell-selective adhesion molecule OS=Homo sapiens GN=ESAM PE=1 SV=1 - [ESAM_HUMAN]	18.72	1	4	4	8	-1.81	-1.48	-1.88	-1.67	-0.75	-0.53	-1.24	-0.93	-0.72	-0.57	-0.32	-0.12	0.67	1.46	1.46	1.22	1.12	0.94	1.29	1.00
Q99500	Sphingosine 1-phosphate receptor 3 OS=Homo sapiens GN=S1PR3 PE=1 SV=2 - [S1PR3_HUMAN]	5.29	1	2	2	3	-0.94	-1.05	-0.95	-1.05	0.20	0.09	-0.01	-0.11	0.27	0.38	0.51	0.40	0.99	1.46	1.46	1.36	1.36	1.13	1.13	1.00
Q8N556	Actin filament associated protein 1 OS=Homo sapiens GN=AFAP1 PE=1 SV=2 - [AFAP1_HUMAN]	3.15	1	2	2	2	-0.79	-0.94	-1.67	-1.83	-0.33	-0.49	-0.74	-0.89	-0.67	-0.51	-0.53	-0.69	0.11	0.26	1.14	0.31	1.19	0.44	1.33	1.00

Q02318	Sterol 26-hydroxylase, mitochondrial OS=Homo sapiens GN=CYP27A1 PE=1 SV=1 - [CP27A_HUMAN]	6.59	1	2	2	2	-1.48	-2.02	-1.45	-1.99	-0.41	-0.96	-0.51	-1.05	-0.82	-0.28	-0.27	-0.82	1.02	1.21	1.18	1.23	1.21	1.05	1.02	1.00
P80748	Ig lambda chain V-III region LOI OS=Homo sapiens PE=1 SV=1 - [LV302_HUMAN]	37.84	1	3	3	18	-1.12	-1.03	-1.32	-1.20	-0.04	0.08	-0.22	-0.15	0.56	0.45	1.46	1.48	0.84	2.84	2.75	1.84	1.75	1.02	1.22	1.00
P43307	Translocon-associated protein subunit alpha OS=Homo sapiens GN=SSR1 PE=1 SV=3 - [SSRA_HUMAN]	9.09	1	2	2	10	-1.29	-1.36	-1.32	-1.23	0.05	0.03	-0.26	-0.30	-0.37	-0.34	-0.23	-0.22	1.09	1.22	1.10	1.02	1.01	1.33	1.25	1.00
Q96QH2	PML-RARA-regulated adapter molecule 1 OS=Homo sapiens GN=PRAM1 PE=1 SV=2 - [PRAM_HUMAN]	5.15	1	2	2	2	0.91	1.04	-0.19	-0.06	-1.21	-1.08	0.74	0.88	0.51	0.39	0.25	0.38	-0.11	-0.65	0.44	-0.49	0.61	-2.13	-1.03	1.00
P08397	Porphobilinogen deaminase OS=Homo sapiens GN=HMBS PE=1 SV=2 - [HEM3_HUMAN]	13.02	1	4	4	6	-1.22	-1.26	-1.11	-1.15	-0.34	-0.37	-0.18	-0.21	-0.32	-0.31	-0.21	-0.26	1.10	1.01	0.89	0.98	0.87	0.88	0.77	1.00
P06331	Ig heavy chain V-II region ARH-77 OS=Homo sapiens PE=4 SV=1 - [HV209_HUMAN]	17.12	2	2	2	4	-1.90	-1.69	-1.81	-1.60	-0.23	-0.03	-0.87	-0.67	0.00	-0.20	0.43	0.63	1.08	2.34	2.24	1.73	1.65	1.65	1.56	1.00
P47712	Cytosolic phospholipase A2 OS=Homo sapiens GN=PLA2G4A PE=1 SV=2 - [PA24A_HUMAN]	3.47	1	3	3	3	-1.74	-1.63	-1.92	-1.81	-0.70	-0.59	-0.99	-0.87	-0.36	-0.47	-0.47	-0.36	0.81	1.28	1.45	1.30	1.48	1.03	1.21	1.00
P98095	Fibulin-2 OS=Homo sapiens GN=FBLN2 PE=1 SV=2 - [FBLN2_HUMAN]	16.89	1	15	15	23	-1.45	-1.38	-1.66	-1.69	0.44	0.47	-0.65	-0.74	-0.24	-0.22	-0.48	-0.40	0.67	0.98	1.30	0.88	1.21	1.65	2.01	1.00

Q8ND94	LRRN4 C-terminal-like protein OS=Homo sapiens GN=LRRN4CL PE=2 SV=1 - [LRN4L_HUMAN]	9.24	1	1	1	1	0.27	-0.23	-0.07	-0.57	1.01	0.51	0.86	0.36	0.56	1.06	1.25	0.74	0.65	0.99	1.32	0.82	1.17	0.73	1.07	1.00
P02652	Apolipoprotein A-II OS=Homo sapiens GN=APOA2 PE=1 SV=1 - [APOA2_HUMAN]	39.00	1	4	4	6	-1.43	-1.25	-1.63	-1.47	-0.12	0.03	-0.72	-0.54	-0.37	-0.52	-0.50	-0.34	0.83	0.91	1.13	0.96	1.14	1.27	1.49	1.00
O75888	Tumor necrosis factor ligand superfamily member 13 OS=Homo sapiens GN=TNFSF13 PE=1 SV=1 - [TNF13_HUMAN]	4.00	1	1	1	1	0.35	0.51	0.65	0.81	2.36	2.51	1.58	1.74	1.94	1.79	0.93	1.08	1.29	0.58	0.28	1.46	1.17	1.99	1.69	1.00
Q8N129	Protein canopy homolog 4 OS=Homo sapiens GN=CNPY4 PE=2 SV=1 - [CNPY4_HUMAN]	18.15	1	4	4	5	-1.22	-1.19	-1.48	-1.81	-0.02	-0.15	-0.56	-0.66	-0.17	-0.19	-0.30	-0.01	0.72	0.93	1.08	0.96	1.32	1.19	1.39	1.00
O00560	Syntenin-1 OS=Homo sapiens GN=SDCBP PE=1 SV=1 - [SDCB1_HUMAN]	39.60	1	7	7	21	-0.87	-0.66	-0.61	-0.39	1.39	1.32	0.61	0.69	0.80	0.80	0.95	1.02	1.05	1.52	1.32	1.17	1.23	1.42	1.70	0.99
Q86UT6	NLR family member X1 OS=Homo sapiens GN=NLRX1 PE=1 SV=1 - [NLRX1_HUMAN]	15.18	1	11	11	20	-1.29	-1.21	-1.15	-1.17	-0.14	-0.11	-0.31	-0.34	-0.16	-0.15	-0.07	-0.06	1.00	1.10	1.12	1.10	1.23	0.89	1.18	0.99
Q6ZUJ8	Phosphoinositide 3-kinase adapter protein 1 OS=Homo sapiens GN=PIK3AP1 PE=1 SV=2 - [BCAP_HUMAN]	1.99	1	1	1	2	-0.95	-0.78	-0.36	-0.76	1.11	0.65	0.49	0.17	0.41	0.50	1.20	0.69	1.50	2.16	1.46	1.48	1.20	2.04	1.40	0.99
P19256	Lymphocyte function-associated antigen 3 OS=Homo sapiens GN=CD58 PE=1 SV=1 - [LFA3_HUMAN]	4.80	1	2	2	3	-1.05	-1.08	-1.14	-1.16	-0.08	-0.12	-0.21	-0.24	-0.02	0.02	0.23	0.20	0.90	1.29	1.37	1.10	1.18	0.95	1.04	0.99
P02760	Protein AMBP OS=Homo sapiens GN=AMBP PE=1 SV=1 - [AMBP_HUMAN]	30.11	1	8	8	21	-0.57	-0.47	-1.18	-0.82	1.42	1.28	-0.11	-0.23	0.63	0.52	0.65	0.76	0.30	1.25	1.64	0.91	1.40	1.55	1.81	0.99

Q14112	Nidogen-2 OS=Homo sapiens GN=NID2 PE=1 SV=3 - [NID2_HUMAN]	32.58	2	35	36	104	-1.70	-1.46	-1.84	-1.77	-0.57	-0.44	-1.02	-0.95	-0.64	-0.72	-0.63	-0.65	0.77	1.21	1.21	1.10	1.21	1.19	1.21	0.99
Q9UNA4	DNA polymerase iota OS=Homo sapiens GN=POLI PE=1 SV=3 - [POLI_HUMAN]	1.89	1	1	1	1	-0.32	-0.04	-0.68	-0.39	0.40	0.67	0.25	0.53	0.52	0.24	0.65	0.93	0.63	0.98	1.33	0.60	0.95	0.70	1.06	0.98
P00387	NADH-cytochrome b5 reductase 3 OS=Homo sapiens GN=CYB5R3 PE=1 SV=3 - [NB5R3_HUMAN]	39.87	1	9	9	25	-1.16	-1.43	-1.34	-1.55	-0.09	-0.04	-0.48	-0.46	-0.23	-0.24	-0.04	-0.09	0.77	1.13	1.41	1.04	1.23	1.06	1.46	0.98
O75477	Erlin-1 OS=Homo sapiens GN=ERLIN1 PE=1 SV=1 - [ERLN1_HUMAN]	17.05	1	2	5	11	-1.56	-1.54	-1.35	-1.33	-0.31	-0.30	-0.43	-0.41	-0.45	-0.46	0.02	0.03	1.19	1.58	1.36	1.13	0.92	1.23	1.02	0.98
P42773	Cyclin-dependent kinase 4 inhibitor C OS=Homo sapiens GN=CDKN2C PE=1 SV=1 - [CDN2C_HUMAN]	8.93	1	2	2	2	-1.01	-0.92	-1.28	-1.19	-0.07	0.01	-0.36	-0.27	-0.04	-0.13	-0.25	-0.17	0.70	0.76	1.03	0.91	1.18	0.92	1.19	0.98
P16070	CD44 antigen OS=Homo sapiens GN=CD44 PE=1 SV=3 - [CD44_HUMAN]	10.11	1	9	9	26	-1.06	-0.85	-0.89	-0.74	0.50	0.51	0.30	0.32	0.40	0.47	0.93	0.94	1.15	1.73	1.71	1.40	1.17	1.61	1.20	0.98
P58658	Protein eva-1 homolog C OS=Homo sapiens GN=EVA1C PE=1 SV=1 - [EVA1C_HUMAN]	3.85	1	1	1	1	-0.34	0.05	-1.55	-1.16	0.06	0.45	-0.64	-0.25	0.45	0.07	-0.13	0.25	-0.24	0.22	1.42	0.44	1.65	0.39	1.60	0.98
P11413	Glucose-6-phosphate 1-dehydrogenase OS=Homo sapiens GN=G6PD PE=1 SV=4 - [G6PD_HUMAN]	43.50	1	18	18	60	-0.95	-0.89	-0.94	-0.97	0.03	0.08	-0.08	-0.11	-0.01	-0.01	0.15	0.16	1.02	1.11	0.97	1.06	0.95	0.96	1.00	0.98

Q9P291	Armadillo repeat-containing X-linked protein 1 OS=Homo sapiens GN=ARMCX1 PE=1 SV=1 - [ARMX1_HUMAN]	15.67	1	5	5	7	-0.96	-0.96	-0.97	-0.98	0.02	0.01	-0.06	-0.07	-0.05	0.09	0.37	0.08	0.96	1.18	1.18	0.90	0.93	0.96	0.97	0.98
Q9Y508	E3 ubiquitin-protein ligase RNF114 OS=Homo sapiens GN=RNF114 PE=1 SV=1 - [RN114_HUMAN]	8.77	1	1	1	1	0.21	1.00	-1.01	-0.22	-0.13	0.65	-0.10	0.69	1.00	0.21	-1.70	-0.91	-0.25	-1.90	-0.68	0.04	1.26	-0.36	0.86	0.98
P39059	Collagen alpha-1(XV) chain OS=Homo sapiens GN=COL15A1 PE=1 SV=2 - [COFA1_HUMAN]	4.68	1	5	5	7	-1.89	-1.71	-1.94	-1.94	0.30	0.48	-0.93	-0.82	-0.22	-0.46	-0.52	-0.54	0.73	1.11	1.34	1.39	1.38	2.17	2.23	0.97
P53609	Geranylgeranyl transferase type-1 subunit beta OS=Homo sapiens GN=PGGT1B PE=1 SV=2 - [PGTB1_HUMAN]	10.61	1	4	4	7	0.08	-0.11	-0.44	-0.21	0.87	0.78	0.55	0.61	0.66	0.55	0.65	0.79	0.53	0.83	1.09	0.58	0.83	0.79	1.07	0.97
P01857	Ig gamma-1 chain C region OS=Homo sapiens GN=IGHG1 PE=1 SV=1 - [IGHG1_HUMAN]	56.06	1	5	15	242	-1.10	-1.14	-0.99	-0.98	0.15	0.07	-0.08	-0.12	0.64	0.70	1.83	1.75	0.95	2.71	2.69	1.69	1.63	1.08	1.15	0.97
P11802	Cyclin-dependent kinase 4 OS=Homo sapiens GN=CDK4 PE=1 SV=2 - [CDK4_HUMAN]	8.25	11	1	2	6	-0.91	-0.89	-1.03	-1.01	0.02	0.04	-0.12	-0.10	-0.29	-0.30	-0.25	-0.23	0.85	0.67	0.78	0.64	0.76	0.91	1.03	0.97
Q13232	Nucleoside diphosphate kinase 3 OS=Homo sapiens GN=NME3 PE=1 SV=2 - [NDK3_HUMAN]	42.01	2	7	7	15	-0.93	-0.80	-1.03	-0.88	0.07	0.21	-0.20	-0.13	-0.03	-0.19	0.11	0.24	0.79	0.95	1.18	0.91	0.84	1.20	0.89	0.97
Q96JI7	Spatacsin OS=Homo sapiens GN=SPG11 PE=1 SV=3 - [SPTCS_HUMAN]	0.41	1	1	1	1	0.87	0.66	-1.15	-1.36	-0.42	-0.63	-0.24	-0.45	-0.56	-0.35	-0.03	-0.24	-1.06	-0.90	1.12	-1.19	0.84	-1.31	0.72	0.97

P29317	Ephrin type-A receptor 2 OS=Homo sapiens GN=EPHA2 PE=1 SV=2 - [EPHA2_HUMAN]	3.69	4	1	3	5	-0.59	-0.78	-1.20	-1.39	0.27	0.08	-0.29	-0.48	-0.20	-0.01	0.06	-0.14	0.35	0.65	1.26	0.61	1.23	0.84	1.45	0.97
Q96FQ6	Protein S100-A16 OS=Homo sapiens GN=S100A16 PE=1 SV=1 - [S10AG_HUMAN]	33.98	1	3	3	7	-0.79	-0.59	-1.06	-0.86	0.16	0.35	-0.25	-0.05	0.24	0.05	0.48	0.57	0.59	1.27	1.53	0.86	1.14	0.93	1.34	0.97
Q5TZA2	Rootletin OS=Homo sapiens GN=CROCC PE=1 SV=1 - [CROCC_HUMAN]	20.08	2	34	35	64	-1.07	-1.03	-1.29	-1.36	-0.36	-0.47	-0.36	-0.39	-0.27	-0.27	-0.05	-0.12	0.76	1.01	1.24	0.79	0.98	0.64	0.94	0.97
O43772	Mitochondrial carnitine/acylcarnitine carrier protein OS=Homo sapiens GN=SLC25A20 PE=1 SV=1 - [MCAT_HUMAN]	22.59	1	8	8	14	-0.59	-0.66	-0.72	-0.63	0.42	0.45	0.25	0.30	0.23	0.20	0.11	0.16	0.93	0.65	0.69	0.90	0.92	0.92	1.11	0.97
P11678	Eosinophil peroxidase OS=Homo sapiens GN=EPX PE=1 SV=2 - [PERE_HUMAN]	5.45	1	2	4	8	-2.67	-2.84	-3.06	-3.23	-2.26	-2.44	-2.15	-2.32	-2.42	-2.24	-2.65	-2.83	0.57	0.02	0.41	0.46	0.85	0.39	0.78	0.97
Q92508	Piezo-type mechanosensitive ion channel component 1 OS=Homo sapiens GN=PIEZO1 PE=1 SV=4 - [PIEZ1_HUMAN]	3.53	1	7	7	8	-1.04	-0.92	-1.06	-1.12	-0.08	0.03	-0.15	-0.21	0.01	-0.24	-0.02	0.10	1.20	1.03	1.36	0.57	0.72	1.31	0.95	0.97
Q5TDH0	Protein DDI1 homolog 2 OS=Homo sapiens GN=DDI2 PE=1 SV=1 - [DDI2_HUMAN]	43.61	2	12	12	25	-1.03	-0.98	-1.01	-1.09	0.11	0.12	0.03	0.16	0.04	0.07	0.06	0.04	1.04	0.78	0.92	1.23	1.00	0.95	0.98	0.97
Q13315	Serine-protein kinase ATM OS=Homo sapiens GN=ATM PE=1 SV=4 - [ATM_HUMAN]	0.98	1	2	2	2	-0.72	-1.06	-0.49	-0.84	0.49	0.15	0.41	0.07	-0.22	0.12	-0.37	-0.72	1.18	0.35	0.12	0.87	0.65	1.19	0.97	0.97

P49247	Ribose-5-phosphate isomerase OS=Homo sapiens GN=RPIA PE=1 SV=3 - [RPIA_HUMAN]	21.54	1	6	6	12	-1.62	-1.63	-1.67	-1.68	0.06	0.17	-0.70	-0.69	-0.39	-0.49	-0.63	-0.51	1.07	1.02	1.08	1.13	1.22	1.59	1.48	0.97
P28065	Proteasome subunit beta type-9 OS=Homo sapiens GN=PSMB9 PE=1 SV=2 - [PSB9_HUMAN]	18.26	1	3	3	6	-0.31	-0.25	-0.95	-0.89	0.25	0.31	-0.05	0.01	0.19	0.13	0.42	0.47	0.31	0.73	1.37	0.47	1.11	0.54	1.18	0.96
O43633	Charged multivesicular body protein 2a OS=Homo sapiens GN=CHMP2A PE=1 SV=1 - [CHM2A_HUMAN]	16.22	1	3	3	9	-1.04	-0.90	-0.62	-0.45	0.28	0.45	0.03	0.17	-0.02	-0.27	0.03	0.22	1.12	1.08	0.69	0.80	0.58	0.95	0.88	0.96
Q9UKV8	Protein argonaute-2 OS=Homo sapiens GN=AGO2 PE=1 SV=3 - [AGO2_HUMAN]	20.72	1	10	15	26	-1.26	-1.18	-1.25	-1.22	-0.31	-0.21	-0.37	-0.42	-0.16	-0.27	-0.02	0.08	0.90	1.26	1.23	1.12	1.16	0.87	1.05	0.96
P51571	Translocon-associated protein subunit delta OS=Homo sapiens GN=SSR4 PE=1 SV=1 - [SSRD_HUMAN]	30.64	1	4	4	10	-1.55	-1.41	-1.46	-1.32	0.34	0.11	-0.57	-0.43	-0.13	-0.26	-0.20	-0.06	0.98	1.12	1.27	1.32	1.23	1.51	1.42	0.96
Q9BRZ2	E3 ubiquitin-protein ligase TRIM56 OS=Homo sapiens GN=TRIM56 PE=1 SV=3 - [TRI56_HUMAN]	6.09	1	3	3	5	-0.54	-0.55	-0.96	-1.05	0.10	0.05	-0.06	-0.16	0.05	0.15	0.21	0.11	0.53	0.52	1.16	0.63	1.14	0.56	1.04	0.96
P50995	Annexin A11 OS=Homo sapiens GN=ANXA11 PE=1 SV=1 - [ANX11_HUMAN]	35.25	2	15	15	51	-1.08	-1.10	-1.06	-1.07	0.02	0.08	-0.15	-0.15	-0.04	-0.02	0.26	0.25	0.92	1.17	1.19	1.00	1.11	0.95	0.99	0.96
P01023	Alpha-2-macroglobulin OS=Homo sapiens GN=A2M PE=1 SV=3 - [A2MG_HUMAN]	39.62	2	42	42	154	-1.61	-1.59	-1.57	-1.52	-0.12	-0.10	-0.62	-0.60	-0.45	-0.52	-0.58	-0.54	1.01	1.01	0.94	1.15	1.06	1.43	1.33	0.96

Q6GTX8	Leukocyte-associated immunoglobulin-like receptor 1 OS=Homo sapiens GN=LAIR1 PE=1 SV=1 - [LAIR1_HUMAN]	7.67	2	2	2	2	0.32	0.49	-0.83	-0.65	0.39	0.56	0.06	0.24	0.57	0.40	0.46	0.64	-0.20	0.15	1.29	0.11	1.27	0.06	1.20	0.96
Q8IZ83	Aldehyde dehydrogenase family 16 member A1 OS=Homo sapiens GN=ALDH16A1 PE=1 SV=2 - [A16A1_HUMAN]	16.71	1	11	11	24	-1.17	-1.16	-1.04	-1.19	-0.02	-0.08	-0.21	-0.22	-0.01	0.06	0.31	0.18	1.00	1.46	1.36	1.22	1.21	1.13	1.19	0.96
P80303	Nucleobindin-2 OS=Homo sapiens GN=NUCB2 PE=1 SV=2 - [NUCB2_HUMAN]	28.57	1	10	10	23	-1.02	-0.84	-1.17	-1.10	0.05	0.09	-0.36	-0.22	-0.03	-0.19	0.03	0.08	0.80	0.94	1.06	0.72	0.98	0.91	1.07	0.96
Q9Y316	Protein MEMO1 OS=Homo sapiens GN=MEMO1 PE=1 SV=1 - [MEMO1_HUMAN]	4.38	1	1	1	3	-0.42	-0.34	-0.88	-0.80	0.68	0.75	0.01	0.09	0.31	0.23	0.29	0.37	0.49	0.72	1.17	0.69	1.15	1.08	1.54	0.95
P01137	Transforming growth factor beta-1 OS=Homo sapiens GN=TGFB1 PE=1 SV=2 - [TGFB1_HUMAN]	3.85	1	1	1	1	0.04	-0.74	-0.54	-1.33	1.15	0.35	0.35	-0.44	0.10	0.90	0.52	-0.27	0.36	0.48	1.06	0.88	1.47	1.09	1.67	0.95
O14657	Torsin-1B OS=Homo sapiens GN=TOR1B PE=1 SV=2 - [TOR1B_HUMAN]	3.27	1	1	1	2	-0.65	-0.69	-1.02	-1.05	1.05	1.00	-0.13	-0.17	0.33	0.37	0.25	0.21	0.58	0.91	1.27	1.05	1.42	1.68	2.05	0.95
Q14004	Cyclin-dependent kinase 13 OS=Homo sapiens GN=CDK13 PE=1 SV=2 - [CDK13_HUMAN]	2.18	11	1	2	5	-0.37	-0.44	-0.83	-0.90	0.60	0.52	0.06	-0.01	0.01	0.09	-0.19	-0.27	0.48	0.18	0.64	0.49	0.95	0.95	1.41	0.95
P50749	Ras association domain-containing protein 2 OS=Homo sapiens GN=RASSF2 PE=1 SV=1 - [RASF2_HUMAN]	12.27	1	3	3	3	-0.88	-0.70	-1.04	-0.86	-0.01	0.17	-0.15	0.03	0.34	0.16	-0.20	-0.03	0.78	0.68	0.84	1.07	1.23	0.85	1.01	0.95

Q13464	Rho-associated protein kinase 1 OS=Homo sapiens GN=ROCK1 PE=1 SV=1 - [ROCK1_HUMAN]	22.08	1	19	25	43	-1.29	-1.18	-1.29	-1.22	0.04	-0.04	-0.23	-0.26	-0.24	-0.20	-0.16	-0.16	0.93	1.18	1.24	1.24	1.10	1.27	1.18	0.95
P19440	Gamma-glutamyltranspeptidase 1 OS=Homo sapiens GN=GGT1 PE=1 SV=2 - [GGT1_HUMAN]	10.72	5	5	5	9	-1.05	-0.85	-1.17	-0.85	-0.23	-0.18	-0.28	0.03	-0.40	-0.33	-0.10	-0.25	0.83	0.96	1.15	0.13	0.01	0.66	0.66	0.95
Q7Z7L8	Uncharacterized protein C11orf96 OS=Homo sapiens GN=C11orf96 PE=1 SV=3 - [CK096_HUMAN]	2.07	1	1	1	4	-0.99	-0.88	-1.08	-1.23	-0.19	-0.34	-0.20	-0.34	-0.12	0.00	0.44	0.28	0.85	1.45	1.52	1.02	1.14	0.81	0.93	0.95
Q9Y303	Putative N-acetylglucosamine-6-phosphate deacetylase OS=Homo sapiens GN=AMDHD2 PE=1 SV=2 - [NAGA_HUMAN]	7.58	1	2	2	3	-0.40	-0.58	-0.51	-0.69	0.41	0.22	0.38	0.19	0.77	0.95	0.24	0.06	0.83	0.65	0.75	1.38	1.50	0.79	0.90	0.95
Q96CN9	GRIP and coiled-coil domain-containing protein 1 OS=Homo sapiens GN=GCC1 PE=1 SV=1 - [GCC1_HUMAN]	2.71	1	2	2	3	-0.20	-0.37	-0.23	-0.40	0.02	-0.16	0.65	0.48	0.37	0.54	0.66	0.48	0.91	0.87	0.89	0.77	0.81	0.20	0.23	0.95
Q32P44	Echinoderm microtubule-associated protein-like 3 OS=Homo sapiens GN=EML3 PE=1 SV=1 - [EML3_HUMAN]	12.61	1	10	10	20	-1.01	-0.96	-1.19	-0.93	0.09	0.44	-0.23	-0.06	0.22	-0.03	0.04	0.11	0.90	1.05	1.17	1.04	1.27	1.20	1.35	0.95
P48960	CD97 antigen OS=Homo sapiens GN=CD97 PE=1 SV=4 - [CD97_HUMAN]	13.65	2	6	6	12	-1.08	-1.17	-1.03	-1.10	0.98	0.94	-0.07	-0.08	0.07	0.14	-0.21	-0.16	1.14	0.77	1.08	1.28	1.27	2.10	1.89	0.94
Q8NCM8	Cytoplasmic dynein 2 heavy chain 1 OS=Homo sapiens GN=DYNC2H1 PE=1 SV=4 - [DYHC2_HUMAN]	1.18	1	3	3	6	-0.61	-0.31	-0.95	-0.79	0.63	0.92	-0.21	0.09	0.25	-0.04	-0.20	0.01	0.46	0.35	0.74	0.60	0.66	0.99	1.20	0.94

Q8NFJ9	Bardet-Biedl syndrome 1 protein OS=Homo sapiens GN=BBS1 PE=1 SV=1 - [BBS1_HUMAN]	9.44	1	4	4	4	-0.72	-0.98	-0.87	-1.13	0.16	-0.10	0.01	-0.25	-0.60	-0.34	0.02	-0.25	0.78	0.74	0.89	0.41	0.56	0.86	1.01	0.94
Q96AA3	Protein RFT1 homolog OS=Homo sapiens GN=RFT1 PE=1 SV=1 - [RFT1_HUMAN]	3.51	1	2	2	2	-1.15	-1.31	-1.65	-1.81	-0.45	-0.62	-0.77	-0.93	-0.98	-0.82	-0.26	-0.43	0.43	0.89	1.39	0.36	0.86	0.68	1.18	0.94
P51692	Signal transducer and activator of transcription 5B OS=Homo sapiens GN=STAT5B PE=1 SV=2 - [STA5B_HUMAN]	14.36	1	2	9	17	-0.60	-0.72	-0.79	-0.90	-0.15	-0.27	0.08	-0.03	-0.15	-0.04	-0.05	-0.17	0.74	0.56	0.74	0.60	0.79	0.44	0.62	0.94
Q86YV9	Hermansky-Pudlak syndrome 6 protein OS=Homo sapiens GN=HPS6 PE=1 SV=1 - [HPS6_HUMAN]	2.06	1	1	1	1	-0.65	-1.09	-0.66	-1.10	0.61	0.17	0.21	-0.23	-0.05	0.40	-0.04	-0.48	0.92	0.62	0.62	1.08	1.09	1.24	1.25	0.93
O95816	BAG family molecular chaperone regulator 2 OS=Homo sapiens GN=BAG2 PE=1 SV=1 - [BAG2_HUMAN]	20.85	1	5	5	6	-0.68	-0.80	-1.28	-1.34	0.09	0.10	-0.39	-0.45	-0.10	-0.12	0.13	0.13	0.41	0.74	1.41	0.63	1.19	0.83	1.37	0.93
Q5S007	Leucine-rich repeat serine/threonine-protein kinase 2 OS=Homo sapiens GN=LRRK2 PE=1 SV=2 - [LRRK2_HUMAN]	0.32	1	1	1	1	-0.72	-0.72	-0.53	-0.52	2.25	2.25	0.34	0.35	1.31	1.31	0.37	0.37	1.12	1.10	0.90	2.06	1.87	2.95	2.76	0.93
P13798	Acylamino-acid-releasing enzyme OS=Homo sapiens GN=APEH PE=1 SV=4 - [ACPH_HUMAN]	30.19	1	16	16	46	-0.89	-0.84	-1.08	-1.03	0.13	0.11	-0.14	-0.09	0.21	0.18	0.58	0.56	0.87	1.46	1.64	1.16	1.24	1.01	1.13	0.93
Q14192	Four and a half LIM domains protein 2 OS=Homo sapiens GN=FHL2 PE=1 SV=3 - [FHL2_HUMAN]	33.69	1	8	8	12	-2.40	-2.15	-2.08	-1.86	-0.70	-0.63	-1.15	-1.09	-0.98	-1.12	-1.42	-1.17	1.16	1.04	0.82	1.29	0.98	1.57	1.37	0.93

Q96RK4	Bardet-Biedl syndrome 4 protein OS=Homo sapiens GN=BBS4 PE=1 SV=2 - [BBS4_HUMAN]	2.50	1	1	1	2	-0.76	-1.04	-0.53	-0.80	0.41	0.13	0.33	0.06	0.00	0.28	0.27	-0.01	1.15	1.03	0.79	1.07	0.84	1.15	0.92	0.93
Q6PCB0	von Willebrand factor A domain-containing protein 1 OS=Homo sapiens GN=VWA1 PE=2 SV=1 - [VWA1_HUMAN]	33.71	1	8	8	13	-1.78	-1.79	-1.62	-1.62	-0.40	-0.51	-0.83	-0.92	-0.90	-0.81	-0.65	-0.79	1.08	1.00	0.82	0.95	0.85	1.47	1.30	0.92
Q96PE2	Rho guanine nucleotide exchange factor 17 OS=Homo sapiens GN=ARHGEF17 PE=1 SV=1 - [ARHGH_HUMAN]	8.53	1	10	10	18	-1.26	-1.26	-1.28	-1.33	-0.53	-0.47	-0.49	-0.39	-0.60	-0.45	-0.38	-0.37	0.78	0.65	0.90	0.78	0.81	0.66	0.79	0.92
P49959	Double-strand break repair protein MRE11A OS=Homo sapiens GN=MRE11A PE=1 SV=3 - [MRE11_HUMAN]	17.80	1	10	10	14	-1.20	-1.10	-1.31	-1.18	-0.16	-0.05	-0.49	-0.30	-0.17	-0.37	-0.12	0.14	0.75	0.90	1.19	0.94	0.97	0.95	1.12	0.92
Q86WG5	Myotubularin-related protein 13 OS=Homo sapiens GN=SBF2 PE=1 SV=1 - [MTMRD_HUMAN]	8.44	1	8	9	19	-0.16	-0.35	-0.49	-0.82	0.43	0.18	0.22	-0.10	-0.17	0.16	0.13	0.24	0.65	0.56	0.81	0.51	0.70	0.59	0.94	0.92
Q9GZM7	Tubulointerstitial nephritis antigen-like OS=Homo sapiens GN=TINAGL1 PE=1 SV=1 - [TINAL_HUMAN]	50.11	1	16	16	114	-1.50	-1.41	-1.67	-1.54	-0.32	-0.35	-0.79	-0.68	-0.58	-0.52	-0.67	-0.66	0.87	0.95	1.07	1.05	1.06	1.21	1.34	0.92
P33897	ATP-binding cassette sub-family D member 1 OS=Homo sapiens GN=ABCD1 PE=1 SV=2 - [ABCD1_HUMAN]	1.61	1	1	1	2	-1.21	-1.05	-1.52	-1.36	0.02	0.17	-0.67	-0.50	0.27	0.11	0.19	0.35	0.60	1.41	1.71	1.35	1.66	1.21	1.52	0.92

Q92619	Minor histocompatibility protein HA-1 OS=Homo sapiens GN=HMHA1 PE=1 SV=2 - [HMHA1_HUMAN]	5.90	1	4	4	5	-0.31	-0.26	-0.65	-0.34	0.56	0.65	0.42	0.74	0.70	0.39	0.44	0.52	0.59	0.46	0.95	1.14	1.08	0.75	1.20	0.92
Q6PD62	RNA polymerase-associated protein CTR9 homolog OS=Homo sapiens GN=CTR9 PE=1 SV=1 - [CTR9_HUMAN]	4.09	1	3	3	4	0.29	-0.14	-0.33	-0.76	-0.24	-0.68	0.52	0.09	0.56	0.99	0.52	0.09	0.29	0.24	0.86	0.73	1.36	-0.55	0.07	0.92
Q14520	Hyaluronan-binding protein 2 OS=Homo sapiens GN=HABP2 PE=1 SV=1 - [HABP2_HUMAN]	2.32	1	1	1	1	-2.31	-2.18	-2.13	-1.99	-1.25	-1.13	-1.27	-1.14	-1.08	-1.21	-0.31	-0.18	1.10	2.01	1.82	1.13	0.95	1.04	0.86	0.92
Q53RD9	Fibulin-7 OS=Homo sapiens GN=FBLN7 PE=2 SV=1 - [FBLN7_HUMAN]	14.35	1	5	5	11	-0.45	-0.27	-0.07	0.07	2.01	1.73	1.06	0.78	0.94	1.23	0.87	0.58	0.91	0.58	0.57	1.04	1.04	1.69	1.69	0.92
Q96RY7	Intraflagellar transport protein 140 homolog OS=Homo sapiens GN=IFT140 PE=1 SV=1 - [IF140_HUMAN]	1.50	1	2	2	3	-0.78	-0.50	-1.02	-0.73	0.22	0.51	-0.17	0.12	0.20	-0.08	-0.32	-0.04	0.67	0.46	0.70	0.73	0.97	0.99	1.23	0.92
Q5SW96	Low density lipoprotein receptor adapter protein 1 OS=Homo sapiens GN=LDLRAP1 PE=1 SV=3 - [ARH_HUMAN]	9.09	1	2	2	3	-0.62	-0.91	-0.93	-1.22	0.27	-0.01	-0.08	-0.36	-0.34	-0.05	-0.20	-0.49	0.60	0.42	0.73	0.61	0.92	0.88	1.19	0.92
Q13099	Intraflagellar transport protein 88 homolog OS=Homo sapiens GN=IFT88 PE=2 SV=2 - [IFT88_HUMAN]	2.88	1	2	2	2	-1.00	-1.18	-0.97	-1.16	0.06	-0.13	-0.12	-0.30	-0.25	-0.07	0.13	-0.06	0.93	1.13	1.10	0.97	0.94	1.04	1.02	0.92
P28370	Probable global transcription activator SNF2L1 OS=Homo sapiens GN=SMARCA1 PE=1 SV=2 - [SMCA1_HUMAN]	6.36	1	2	6	9	-1.22	-0.90	-1.31	-0.99	-1.26	-0.95	-0.45	-0.14	0.07	-0.25	-0.24	0.07	0.82	0.98	1.06	1.01	1.09	-0.06	0.02	0.92

P40306	Proteasome subunit beta type-10 OS=Homo sapiens GN=PSMB10 PE=1 SV=1 - [PSB10_HUMAN]	10.99	1	2	2	3	-0.94	-1.03	-1.11	-1.20	0.02	-0.08	-0.25	-0.35	-0.11	-0.01	0.38	0.28	0.74	1.32	1.48	0.95	1.13	0.94	1.10	0.92
Q96S96	Phosphatidylethanolamine binding protein 4 OS=Homo sapiens GN=PEBP4 PE=1 SV=3 - [PEBP4_HUMAN]	11.89	1	1	1	2	-0.05	-0.24	-0.76	-0.95	0.37	0.18	0.09	-0.10	-0.09	0.10	0.70	0.50	0.20	0.75	1.45	0.18	0.89	0.41	1.11	0.91
Q53GG5	PDZ and LIM domain protein 3 OS=Homo sapiens GN=PDLIM3 PE=1 SV=1 - [PDLI3_HUMAN]	15.66	1	5	5	12	-1.95	-1.74	-1.75	-1.74	-0.68	-0.70	-0.95	-0.97	-0.73	-0.74	-0.46	-0.45	1.14	1.61	1.29	1.49	1.15	1.47	1.29	0.91
P06681	Complement C2 OS=Homo sapiens GN=C2 PE=1 SV=2 - [CO2_HUMAN]	8.51	1	5	5	8	-1.38	-1.49	-1.39	-1.50	0.21	0.10	-0.54	-0.65	-0.28	-0.16	0.18	0.07	0.89	1.56	1.57	1.24	1.26	1.57	1.58	0.91
P01834	Ig kappa chain C region OS=Homo sapiens GN=IGKC PE=1 SV=1 - [IGKC_HUMAN]	84.91	1	7	7	105	-1.45	-1.42	-1.27	-1.20	-0.13	-0.01	-0.47	-0.40	0.14	0.04	0.91	0.97	0.98	2.20	2.22	1.57	1.50	1.19	1.22	0.91
Q7KYR7	Butyrophilin subfamily 2 member A1 OS=Homo sapiens GN=BTN2A1 PE=1 SV=3 - [BT2A1_HUMAN]	4.17	2	2	2	3	-1.21	-0.96	-1.09	-0.85	-0.82	-0.58	-0.25	0.00	-0.18	-0.42	-0.12	0.13	1.02	1.10	0.98	0.81	0.70	0.37	0.25	0.91
Q8IUK8	Cerebellin-2 OS=Homo sapiens GN=CBLN2 PE=2 SV=1 - [CBLN2_HUMAN]	3.13	1	1	1	2	-2.64	-2.52	-2.52	-2.39	-1.61	-1.48	-1.67	-1.54	-1.35	-1.47	-1.33	-1.20	1.03	1.32	1.19	1.20	1.08	1.02	0.89	0.91
P11532	Dystrophin OS=Homo sapiens GN=DMD PE=1 SV=3 - [DMD_HUMAN]	16.80	2	44	47	95	-0.99	-1.12	-0.76	-1.01	-0.34	-0.54	-0.17	-0.41	-0.34	-0.23	-0.26	-0.39	0.78	0.79	0.72	0.86	0.87	0.61	0.78	0.91
O60504	Vinexin OS=Homo sapiens GN=SORBS3 PE=1 SV=2 - [VINEX_HUMAN]	28.46	1	16	16	39	-1.77	-1.75	-1.80	-1.72	-0.90	-0.94	-0.82	-0.77	-0.85	-0.85	-0.44	-0.33	0.61	1.04	1.15	1.02	0.93	0.67	0.71	0.91

P28289	Tropomodulin 1 OS=Homo sapiens GN=TMOD1 PE=1 SV=1 - [TMOD1_HUMAN]	56.55	2	15	16	48	-1.37	-1.34	-1.35	-1.30	-0.17	-0.13	-0.34	-0.38	-0.14	-0.16	0.31	0.34	0.91	1.61	1.64	1.21	1.19	1.20	1.09	0.91
P18428	Lipopolysaccharide-binding protein OS=Homo sapiens GN=LBP PE=1 SV=3 - [LBP_HUMAN]	4.57	1	2	2	3	-1.15	-0.99	-1.31	-1.15	0.11	0.66	-0.47	-0.31	0.25	0.10	0.22	0.56	0.74	1.56	1.25	1.28	1.38	1.64	1.59	0.91
Q9HD40	O-phosphoseryl-tRNA(Sec) selenium transferase OS=Homo sapiens GN=SEPSECS PE=1 SV=2 - [SPCS_HUMAN]	1.80	1	1	1	1	-0.81	-0.88	-0.93	-0.99	0.12	0.05	-0.08	-0.15	-0.16	-0.09	-0.07	-0.14	0.79	0.75	0.85	0.76	0.87	0.92	1.03	0.91
P27708	CAD protein OS=Homo sapiens GN=CAD PE=1 SV=3 - [PYR1_HUMAN]	9.66	1	15	17	27	-0.68	-0.70	-0.76	-0.79	0.31	0.24	0.12	0.11	0.19	0.14	0.18	0.19	0.94	0.82	1.01	0.88	0.98	0.94	0.93	0.91
Q15208	Serine/threonine-protein kinase 38 OS=Homo sapiens GN=STK38 PE=1 SV=1 - [STK38_HUMAN]	12.90	1	3	4	8	-0.06	0.05	-0.92	-0.49	0.22	0.63	0.35	0.21	0.33	0.06	0.16	0.50	0.55	0.79	1.00	0.65	0.96	0.83	1.12	0.91
P48740	Mannan-binding lectin serine protease 1 OS=Homo sapiens GN=MASP1 PE=1 SV=3 - [MASP1_HUMAN]	5.58	1	3	3	4	-1.23	-1.23	-1.06	-1.05	0.96	0.95	-0.21	-0.21	0.37	0.38	0.91	0.91	1.07	2.15	1.97	1.64	1.46	2.17	1.99	0.91
P02458	Collagen alpha-1(II) chain OS=Homo sapiens GN=COL2A1 PE=1 SV=3 - [CO2A1_HUMAN]	9.28	1	7	10	45	-2.80	-2.98	-3.18	-3.24	-1.71	-1.82	-2.32	-2.41	-2.15	-2.03	-2.13	-2.24	0.59	0.65	1.17	1.14	1.28	1.30	1.50	0.91
Q9HC07	Transmembrane protein 165 OS=Homo sapiens GN=TMEM165 PE=1 SV=1 - [TM165_HUMAN]	7.72	1	1	1	6	-1.41	-1.47	-1.55	-1.61	-0.53	-0.59	-0.71	-0.77	-0.80	-0.74	-0.56	-0.62	0.75	0.86	0.99	0.70	0.85	0.86	1.01	0.90

Q8TCJ2	Dolichyl-diphosphooligosaccharide--protein glycosyltransferase subunit STT3B OS=Homo sapiens GN=STT3B PE=1 SV=1 - [STT3B_HUMAN]	6.17	1	6	6	8	-0.97	-0.93	-1.16	-1.25	-0.10	-0.14	-0.30	-0.36	-0.30	-0.26	-0.19	-0.28	0.62	0.79	0.98	0.83	0.93	0.82	1.04	0.90
Q9NT22	EMILIN-3 OS=Homo sapiens GN=EMILIN3 PE=2 SV=2 - [EMIL3_HUMAN]	17.62	1	9	9	12	-0.41	-0.31	-0.47	-0.59	0.03	-0.02	0.18	0.47	0.50	0.47	0.35	0.36	0.91	0.77	0.95	0.99	0.96	0.26	0.46	0.90
O60934	Nibrin OS=Homo sapiens GN=NBN PE=1 SV=1 - [NBN_HUMAN]	1.33	1	1	1	1	-1.15	-0.80	-1.18	-0.83	-0.35	0.00	-0.35	0.01	-0.56	-0.91	-0.32	0.02	0.87	0.84	0.86	0.27	0.31	0.79	0.82	0.90
Q14739	Lamin-B receptor OS=Homo sapiens GN=LBR PE=1 SV=2 - [LBR_HUMAN]	6.18	1	3	3	4	-0.81	-0.85	-0.89	-0.91	-0.46	-0.34	-0.10	-0.07	-0.11	-0.17	-0.11	-0.12	0.84	0.71	0.75	0.67	0.72	0.38	0.61	0.90
Q99584	Protein S100-A13 OS=Homo sapiens GN=S100A13 PE=1 SV=1 - [S10AD_HUMAN]	40.82	1	5	5	24	-0.31	-0.32	-0.20	-0.18	0.61	0.59	0.57	0.56	0.45	0.44	0.36	0.37	0.94	0.95	0.70	0.81	0.72	0.90	0.86	0.90
P31151	Protein S100-A7 OS=Homo sapiens GN=S100A7 PE=1 SV=4 - [S10A7_HUMAN]	21.78	2	2	2	3	-2.75	-2.72	-3.77	-3.73	-4.67	-4.50	-4.96	-4.79	-3.73	-3.76	-3.58	-3.55	-1.17	-0.83	0.18	-0.98	0.04	-0.95	1.11	0.90
P35579	Myosin-9 OS=Homo sapiens GN=MYH9 PE=1 SV=4 - [MYH9_HUMAN]	51.94	1	79	106	478	-1.83	-1.86	-1.78	-1.73	-0.66	-0.73	-0.94	-0.96	-0.90	-0.87	-0.73	-0.75	0.94	1.07	1.04	0.99	0.92	1.15	1.02	0.90
Q12882	Dihydropyrimidine dehydrogenase [NADP(+)] OS=Homo sapiens GN=DPYD PE=1 SV=2 - [DPYD_HUMAN]	1.17	1	1	1	2	-1.18	-1.70	-1.37	-1.88	-0.02	-0.54	-0.54	-1.05	-0.75	-0.23	-0.19	-0.72	0.70	0.99	1.17	0.98	1.17	1.15	1.33	0.89
Q13501	Sequestosome-1 OS=Homo sapiens GN=SQSTM1 PE=1 SV=1 - [SQSTM_HUMAN]	14.55	1	4	4	9	-0.01	0.03	-0.48	-0.38	0.25	0.28	0.46	0.47	0.18	0.19	0.54	0.49	0.54	0.63	1.08	0.47	0.93	0.61	1.00	0.89

O15511	Actin-related protein 2/3 complex subunit 5 OS=Homo sapiens GN=ARPC5 PE=1 SV=3 - [ARPC5_HUMAN]	43.71	1	5	6	22	-1.54	-1.65	-1.36	-1.40	-0.24	-0.35	-0.55	-0.70	-0.65	-0.51	-0.21	-0.20	1.05	1.35	1.03	0.97	0.80	1.15	0.90	0.89
Q96IU4	Alpha/beta hydrolase domain-containing protein 14B OS=Homo sapiens GN=ABHD14B PE=1 SV=1 - [ABHEB_HUMAN]	34.76	1	5	5	18	-0.77	-0.61	-0.63	-0.56	0.25	0.42	0.25	0.37	0.61	0.34	0.21	0.38	0.98	0.90	0.94	1.10	1.19	1.03	1.02	0.89
Q14BN4	Sarcolemmal membrane-associated protein OS=Homo sapiens GN=SLMAP PE=1 SV=1 - [SLMAP_HUMAN]	21.86	2	19	19	41	-1.88	-1.85	-1.92	-1.85	-1.01	-1.07	-1.10	-1.13	-1.04	-1.02	-0.60	-0.65	0.89	1.28	1.34	0.97	0.94	0.93	0.96	0.89
Q8IXQ3	Uncharacterized protein C9orf40 OS=Homo sapiens GN=C9orf40 PE=1 SV=1 - [CI040_HUMAN]	37.11	1	3	3	5	0.35	0.19	-0.62	-0.90	0.52	0.19	0.09	-0.19	-0.14	0.00	0.21	-0.05	-0.33	-0.20	0.86	-0.59	0.92	-0.06	1.04	0.89
O60266	Adenylate cyclase type 3 OS=Homo sapiens GN=ADCY3 PE=1 SV=3 - [ADCY3_HUMAN]	4.98	1	4	4	6	-0.53	-0.21	-0.95	-0.64	0.16	0.47	-0.13	0.19	0.16	-0.14	0.23	0.54	0.46	0.77	1.18	0.41	0.84	0.67	1.09	0.89
P17676	CCAAT/enhancer-binding protein beta OS=Homo sapiens GN=CEBPB PE=1 SV=2 - [CEBPB_HUMAN]	9.28	1	2	2	2	0.55	0.01	-0.35	-0.89	0.47	-0.07	0.47	-0.07	0.22	0.77	1.25	0.71	-0.03	0.70	1.60	0.24	1.15	-0.10	0.81	0.89
P35914	Hydroxymethylglutaryl-CoA lyase, mitochondrial OS=Homo sapiens GN=HMGCL PE=1 SV=2 - [HMGCL_HUMAN]	18.46	1	5	5	8	-1.43	-1.43	-1.38	-1.27	-0.06	-0.03	-0.63	-0.53	-0.32	-0.33	0.13	0.22	0.95	1.55	1.66	1.11	1.10	1.26	1.43	0.89

O15143	Actin-related protein 2/3 complex subunit 1B OS=Homo sapiens GN=ARPC1B PE=1 SV=3 - [ARC1B_HUMAN]	33.87	1	8	9	26	-0.38	-0.60	-1.01	-1.04	0.02	-0.03	-0.24	-0.27	-0.08	-0.07	-0.06	-0.20	0.63	0.49	0.98	0.61	1.01	0.71	0.97	0.89
Q8TAG9	Exocyst complex component 6 OS=Homo sapiens GN=EXOC6 PE=1 SV=3 - [EXOC6_HUMAN]	11.69	1	5	6	8	0.92	0.03	-1.09	-0.65	-0.33	0.11	-0.64	-0.64	0.30	-0.13	-0.22	0.22	-0.58	0.42	0.87	0.53	0.99	0.29	0.75	0.89
Q8NCN5	Pyruvate dehydrogenase phosphatase regulatory subunit, mitochondrial OS=Homo sapiens GN=PDPR PE=1 SV=2 - [PDPR_HUMAN]	17.18	1	13	13	22	-1.17	-1.16	-1.18	-1.15	-0.10	-0.02	-0.43	-0.34	-0.27	-0.28	-0.04	0.06	0.79	1.45	1.25	1.11	0.99	1.13	1.06	0.89
Q03591	Complement factor H-related protein 1 OS=Homo sapiens GN=CFHR1 PE=1 SV=2 - [FHR1_HUMAN]	26.36	2	4	7	23	-0.69	-0.72	-1.17	-1.21	0.28	0.24	-0.39	-0.33	0.02	-0.03	0.60	0.56	0.61	1.67	2.17	1.03	1.54	1.41	1.90	0.89
O75864	Protein phosphatase 1 regulatory subunit 37 OS=Homo sapiens GN=PPP1R37 PE=1 SV=4 - [PPR37_HUMAN]	2.17	1	1	1	1	1.28	1.07	0.10	-0.11	0.31	0.09	0.92	0.71	0.28	0.49	0.80	0.58	-0.30	-0.48	0.70	-0.76	0.43	-0.99	0.19	0.89
O43826	Glucose-6-phosphate translocase OS=Homo sapiens GN=SLC37A4 PE=1 SV=1 - [G6PT1_HUMAN]	5.36	1	2	2	3	-0.52	-0.60	-0.62	-0.70	0.57	0.49	0.21	0.13	0.19	0.28	0.21	0.12	0.78	0.74	0.82	0.83	0.93	1.08	1.17	0.89
Q9UBS8	E3 ubiquitin-protein ligase RNF14 OS=Homo sapiens GN=RNF14 PE=1 SV=1 - [RNF14_HUMAN]	6.54	1	2	2	3	-0.54	-0.59	-1.09	-1.14	0.48	0.42	-0.27	-0.32	0.30	0.35	0.27	0.22	0.32	0.82	1.37	0.92	1.48	1.00	1.56	0.89

P01031	Complement C5 OS=Homo sapiens GN=C5 PE=1 SV=4 - [CO5_HUMAN]	8.11	1	12	12	20	-1.34	-1.47	-1.19	-1.26	0.11	0.18	-0.54	-0.58	-0.10	-0.23	0.23	0.32	0.91	1.65	1.60	1.23	1.19	1.62	1.39	0.89
Q8IVN8	Somatomedin-B and thrombospondin type-1 domain-containing protein OS=Homo sapiens GN=SBSPON PE=1 SV=2 - [SBSPO_HUMAN]	22.35	1	4	4	13	-2.28	-2.22	-2.69	-2.48	-0.83	-0.63	-1.17	-1.12	-1.08	-1.13	-0.86	-0.84	0.84	1.89	2.11	1.80	2.21	2.06	2.46	0.89
P09874	Poly [ADP-ribose] polymerase 1 OS=Homo sapiens GN=PARP1 PE=1 SV=4 - [PARP1_HUMAN]	36.98	1	29	29	59	-1.01	-1.03	-0.99	-0.97	-0.03	-0.02	-0.08	-0.08	-0.21	-0.18	-0.39	-0.41	1.17	0.57	0.51	0.94	0.79	0.93	0.91	0.88
O14773	Tripeptidyl-peptidase 1 OS=Homo sapiens GN=TPP1 PE=1 SV=2 - [TPP1_HUMAN]	8.17	1	3	3	24	-0.47	-0.27	-0.46	-0.34	0.75	0.89	0.50	0.68	0.74	0.55	0.70	0.85	0.89	1.05	0.82	1.06	0.93	1.12	1.06	0.88
Q9UBR2	Cathepsin Z OS=Homo sapiens GN=CTSZ PE=1 SV=1 - [CATZ_HUMAN]	18.15	1	5	5	7	-0.63	-0.60	-0.59	-0.56	0.41	0.44	-0.03	0.07	0.50	0.47	0.54	0.63	0.86	1.11	1.12	0.98	1.00	0.85	0.87	0.88
Q14165	Malectin OS=Homo sapiens GN=MLEC PE=1 SV=1 - [MLEC_HUMAN]	23.29	1	5	5	9	-0.96	-0.78	-0.90	-0.77	0.05	0.12	-0.36	-0.22	0.01	-0.12	0.00	0.10	0.67	0.70	0.89	0.90	0.79	1.00	0.92	0.88
O95466	Formin-like protein 1 OS=Homo sapiens GN=FMNL1 PE=1 SV=3 - [FMNL_HUMAN]	8.18	1	5	7	8	0.39	0.01	-0.03	-0.29	0.97	0.75	0.79	0.57	1.07	1.58	0.53	0.31	0.45	0.15	0.56	1.10	1.52	0.56	0.98	0.88
P54098	DNA polymerase subunit gamma-1 OS=Homo sapiens GN=POLG PE=1 SV=1 - [DPOG1_HUMAN]	1.61	1	1	1	2	-0.83	-0.74	-0.97	-0.88	-0.02	0.06	-0.15	-0.06	0.03	-0.06	0.60	0.68	0.74	1.43	1.56	0.80	0.94	0.79	0.93	0.88
Q8NDZ4	Deleted in autism protein 1 OS=Homo sapiens GN=C3orf58 PE=1 SV=1 - [DIA1_HUMAN]	6.98	1	3	3	4	-0.20	-0.10	-0.23	-0.12	1.86	1.96	0.59	0.69	1.26	1.16	0.57	0.67	0.84	0.78	0.80	1.39	1.42	2.04	2.07	0.88

Q5VZ18	SH2 domain-containing adapter protein E OS=Homo sapiens GN=SHE PE=2 SV=1 - [SHE_HUMAN]	4.44	1	1	1	1	-0.79	-1.48	-1.20	-1.88	0.09	-0.60	-0.38	-1.06	-0.61	0.07	0.02	-0.67	0.47	0.82	1.22	0.90	1.31	0.87	1.27	0.88
P16284	Platelet endothelial cell adhesion molecule OS=Homo sapiens GN=PECAM1 PE=1 SV=1 - [PECA1_HUMAN]	18.02	1	11	11	25	-1.53	-1.38	-1.44	-1.36	0.03	0.01	-0.77	-0.54	-0.35	-0.34	-0.13	-0.09	0.85	1.45	1.29	1.28	1.11	1.66	1.50	0.88
P22792	Carboxypeptidase N subunit 2 OS=Homo sapiens GN=CPN2 PE=1 SV=3 - [CPN2_HUMAN]	5.14	1	2	2	2	-0.91	-0.76	-1.19	-1.05	-0.36	-0.22	-0.38	-0.23	-0.30	-0.44	-0.42	-0.28	0.59	0.49	0.77	0.50	0.79	0.53	0.82	0.88
P11279	Lysosome-associated membrane glycoprotein 1 OS=Homo sapiens GN=LAMP1 PE=1 SV=3 - [LAMP1_HUMAN]	6.71	1	3	3	11	-0.90	-0.88	-0.79	-0.77	0.30	0.36	-0.03	0.02	0.26	0.23	0.47	0.46	0.96	1.31	1.22	1.21	0.96	1.22	1.11	0.88
Q9Y5P4	Collagen type IV alpha-3-binding protein OS=Homo sapiens GN=COL4A3BP PE=1 SV=1 - [C43BP_HUMAN]	3.85	1	3	3	5	-0.66	-0.55	-0.82	-0.71	0.17	0.27	0.00	0.11	0.35	0.25	0.23	0.33	0.71	0.89	1.04	0.94	1.10	0.81	0.96	0.88
O14795	Protein unc-13 homolog B OS=Homo sapiens GN=UNC13B PE=1 SV=2 - [UN13B_HUMAN]	6.22	2	4	8	11	-1.39	-1.05	-1.25	-0.91	-0.24	0.09	-0.43	-0.10	-0.16	-0.49	-0.96	-0.62	1.01	0.44	0.29	0.92	0.79	1.13	0.99	0.88
P36959	GMP reductase 1 OS=Homo sapiens GN=GMPR PE=1 SV=1 - [GMPR1_HUMAN]	28.99	1	6	6	12	-0.32	-0.32	-0.57	-0.52	0.24	0.27	0.31	0.46	0.31	0.24	0.32	0.45	0.83	0.97	1.06	1.05	0.69	0.70	0.52	0.87
P04217	Alpha-1B-glycoprotein OS=Homo sapiens GN=A1BG PE=1 SV=4 - [A1BG_HUMAN]	28.48	1	9	9	21	-1.48	-1.25	-1.79	-1.44	-0.58	-0.30	-0.83	-0.69	-0.54	-0.66	-0.36	-0.24	0.50	1.06	1.51	0.87	1.16	0.70	1.05	0.87

Q9NQ76	Matrix extracellular phosphoglycoprotein OS=Homo sapiens GN=MEPE PE=1 SV=1 - [MEPE_HUMAN]	1.71	1	1	1	1	-2.41	-2.57	-3.41	-3.57	-2.60	-2.76	-2.60	-2.76	-2.58	-2.42	-2.62	-2.78	-0.13	-0.20	0.79	0.02	1.02	-0.20	0.80	0.87
O94905	Erlin-2 OS=Homo sapiens GN=ERLIN2 PE=1 SV=1 - [ERLN2_HUMAN]	45.72	1	13	15	40	-1.05	-1.14	-1.19	-1.29	-0.07	-0.13	-0.29	-0.35	-0.32	-0.21	0.03	-0.01	0.87	1.03	1.15	0.90	0.99	1.03	1.06	0.87
P98172	Ephrin-B1 OS=Homo sapiens GN=EFNB1 PE=1 SV=1 - [EFNB1_HUMAN]	9.25	1	2	2	3	-0.75	-0.76	-0.61	-0.62	0.20	0.18	0.20	0.19	0.52	0.54	0.68	0.67	1.00	1.44	1.29	1.32	1.18	0.93	0.79	0.87
Q8TER5	Rho guanine nucleotide exchange factor 40 OS=Homo sapiens GN=ARHGEF40 PE=1 SV=3 - [ARH40_HUMAN]	4.34	1	3	3	4	-0.46	-0.96	-1.04	-1.30	0.06	-0.28	-0.06	-0.47	-0.48	-0.15	-0.09	-0.44	0.53	0.54	0.86	0.41	0.67	0.56	1.07	0.87
Q9Y2H6	Fibronectin type-III domain-containing protein 3A OS=Homo sapiens GN=FNDC3A PE=1 SV=4 - [FND3A_HUMAN]	1.75	1	2	2	2	-1.36	-1.59	-1.21	-1.44	-0.28	-0.51	-0.40	-0.63	-0.43	-0.19	0.08	-0.16	1.01	1.44	1.28	1.19	1.05	1.06	0.91	0.87
P28827	Receptor-type tyrosine-protein phosphatase mu OS=Homo sapiens GN=PTPRM PE=1 SV=2 - [PTPRM_HUMAN]	4.20	1	4	5	8	-0.65	-0.32	-0.83	-0.49	-0.06	0.26	-0.02	0.31	0.10	-0.23	0.06	0.39	0.69	0.72	0.89	0.46	0.63	0.57	0.74	0.87
P11717	Cation-independent mannose-6-phosphate receptor OS=Homo sapiens GN=IGF2R PE=1 SV=3 - [MPRI_HUMAN]	10.92	1	18	18	29	-1.00	-1.00	-1.01	-0.93	-0.07	0.19	-0.34	-0.17	-0.04	-0.21	0.16	0.24	0.75	1.25	1.28	0.99	0.94	1.19	0.96	0.87
P49257	Protein ERGIC-53 OS=Homo sapiens GN=LMAN1 PE=1 SV=2 - [LMAN1_HUMAN]	24.51	1	10	10	22	-1.10	-0.93	-1.06	-0.92	0.06	0.21	-0.46	-0.28	-0.07	-0.14	0.21	0.23	0.82	1.14	1.23	1.08	1.11	1.15	1.11	0.87

Q9Y693	Lipoma HMGIC fusion partner OS=Homo sapiens GN=LHFP PE=2 SV=1 - [LHFP_HUMAN]	10.50	1	2	2	2	-0.03	0.05	-1.05	-0.97	0.14	0.21	-0.24	-0.17	0.25	0.18	0.19	0.27	-0.16	0.22	1.24	0.23	1.26	0.15	1.17	0.87
O60547	GDP-mannose 4,6 dehydratase OS=Homo sapiens GN=GMDS PE=1 SV=1 - [GMDS_HUMAN]	23.66	1	8	8	14	-0.67	-0.60	-0.83	-0.69	0.64	0.71	0.15	0.13	0.15	0.10	-0.53	-0.48	0.88	0.28	0.32	0.79	0.96	1.22	1.40	0.87
P01860	Ig gamma-3 chain C region OS=Homo sapiens GN=IGHG3 PE=1 SV=2 - [IGHG3_HUMAN]	40.58	1	4	13	151	-0.55	-0.53	-0.45	-0.29	0.13	0.27	0.21	0.29	0.32	-0.08	-0.05	-0.05	0.73	0.48	0.22	0.68	0.75	0.67	0.57	0.87
Q13740	CD166 antigen OS=Homo sapiens GN=ALCAM PE=1 SV=2 - [CD166_HUMAN]	41.17	1	21	21	48	-0.97	-0.88	-0.89	-0.82	0.21	0.31	-0.21	-0.08	0.14	0.06	0.68	0.75	0.98	1.84	1.58	1.17	1.14	1.25	1.20	0.87
Q9ULR0	Pre-mRNA-splicing factor ISY1 homolog OS=Homo sapiens GN=ISY1 PE=1 SV=3 - [ISY1_HUMAN]	11.23	1	2	2	4	-0.32	-0.22	-0.58	-0.48	0.75	0.84	0.23	0.32	0.13	0.04	0.32	0.41	0.60	0.64	0.90	0.38	0.65	1.05	1.31	0.87
P15848	Arylsulfatase B OS=Homo sapiens GN=ARSB PE=1 SV=1 - [ARSB_HUMAN]	9.57	2	4	4	6	-0.88	-0.83	-0.97	-0.92	0.23	0.27	-0.18	-0.25	-0.12	-0.16	0.05	0.10	0.73	0.86	1.02	0.75	0.87	0.99	1.11	0.87
Q9Y265	RuvB-like 1 OS=Homo sapiens GN=RUVBL1 PE=1 SV=1 - [RUVB1_HUMAN]	34.43	1	13	13	30	-1.16	-1.20	-0.98	-1.01	-0.27	-0.46	-0.20	-0.26	-0.29	-0.19	0.01	-0.08	0.98	1.01	0.99	0.66	0.87	0.70	0.69	0.86
P04271	Protein S100-B OS=Homo sapiens GN=S100B PE=1 SV=2 - [S100B_HUMAN]	79.35	1	4	4	53	0.25	0.29	0.59	0.74	0.93	1.09	1.33	1.51	1.17	1.04	0.53	0.71	1.27	0.50	-0.04	0.94	0.49	0.86	0.28	0.86
P06756	Integrin alpha V OS=Homo sapiens GN=ITGAV PE=1 SV=2 - [ITAV_HUMAN]	28.82	1	24	24	50	-0.46	-0.38	-0.46	-0.51	0.99	0.95	0.27	0.31	0.46	0.42	0.29	0.28	0.70	0.76	0.85	0.93	1.00	1.33	1.44	0.86

P17931	Galectin-3 OS=Homo sapiens GN=LGALS3 PE=1 SV=5 - [LEG3_HUMAN]	25.20	1	6	6	21	-0.84	-0.93	-0.83	-0.86	-0.03	-0.10	0.01	-0.13	-0.28	-0.19	0.09	0.06	0.91	0.97	0.86	0.68	0.69	0.72	0.75	0.86
Q06546	GA-binding protein alpha chain OS=Homo sapiens GN=GABPA PE=1 SV=1 - [GABPA_HUMAN]	1.54	1	1	1	1	-1.48	-1.49	-1.44	-1.44	-0.26	-0.27	-0.64	-0.64	-0.50	-0.49	-0.50	-0.51	0.90	0.99	0.93	1.02	0.98	1.20	1.16	0.86
P50479	PDZ and LIM domain protein 4 OS=Homo sapiens GN=PDLIM4 PE=1 SV=2 - [PDLI4_HUMAN]	43.64	1	9	9	22	-1.35	-1.17	-1.36	-1.03	-0.14	0.21	-0.49	-0.34	-0.10	-0.29	-0.28	-0.11	0.95	0.98	1.04	0.96	0.91	1.34	1.34	0.86
O43852	Calumenin OS=Homo sapiens GN=CALU PE=1 SV=2 - [CALU_HUMAN]	58.73	1	12	12	42	-0.83	-0.98	-1.03	-1.05	0.25	0.31	-0.26	-0.30	0.04	0.00	0.16	0.12	0.67	1.12	1.23	0.96	1.04	1.22	1.35	0.86
Q14149	MORC family CW-type zinc finger protein 3 OS=Homo sapiens GN=MORC3 PE=1 SV=3 - [MORC3_HUMAN]	2.02	1	1	1	3	-2.22	-2.34	-1.77	-1.89	0.02	-0.10	-0.97	-1.09	-0.86	-0.74	-0.78	-0.90	1.31	1.45	0.99	1.52	1.07	2.23	1.77	0.86
P40616	ADP-ribosylation factor-like protein 1 OS=Homo sapiens GN=ARL1 PE=1 SV=1 - [ARL1_HUMAN]	16.57	1	3	3	7	-0.67	-0.76	-0.71	-0.80	0.47	0.37	0.08	-0.01	-0.01	0.08	0.05	-0.04	0.81	0.73	0.76	0.79	0.83	1.13	1.17	0.86
Q16401	26S proteasome non-ATPase regulatory subunit 5 OS=Homo sapiens GN=PSMD5 PE=1 SV=3 - [PSMD5_HUMAN]	32.94	1	12	12	25	-0.49	-0.44	-0.56	-0.43	0.44	0.43	0.25	0.27	0.40	0.44	0.66	0.63	0.71	1.10	1.14	0.81	0.97	0.90	0.82	0.86
Q8IY81	pre-rRNA processing protein FTSJ3 OS=Homo sapiens GN=FTSJ3 PE=1 SV=2 - [SPB1_HUMAN]	2.95	1	1	1	1	0.06	-0.06	-1.64	-1.75	-0.47	-0.59	-0.84	-0.96	-1.64	-1.52	-0.79	-0.91	-0.84	-0.84	0.85	-1.54	0.15	-0.55	1.15	0.86

P05186	Alkaline phosphatase, tissue-nonspecific isozyme OS=Homo sapiens GN=ALPL PE=1 SV=4 - [PPBT_HUMAN]	34.16	1	12	12	21	-1.57	-1.48	-1.63	-1.59	0.16	0.21	-0.67	-0.66	0.25	0.19	1.16	1.26	0.89	2.86	2.78	1.79	1.78	1.65	1.63	0.86
P51648	Fatty aldehyde dehydrogenase OS=Homo sapiens GN=ALDH3A2 PE=1 SV=1 - [AL3A2_HUMAN]	25.15	1	12	12	26	-1.23	-1.28	-1.03	-1.02	0.09	0.14	-0.20	-0.12	0.06	0.07	0.35	0.48	1.22	1.56	1.25	1.32	1.17	1.45	1.24	0.86
P01598	Ig kappa chain V-I region EU OS=Homo sapiens PE=1 SV=1 - [KV106_HUMAN]	26.85	2	2	2	14	-1.23	-1.22	-1.64	-1.68	0.31	0.31	-0.87	-0.93	0.41	0.38	1.40	1.40	0.34	2.51	3.27	1.53	2.19	1.40	2.35	0.86
O15121	Sphingolipid delta(4)-desaturase DES1 OS=Homo sapiens GN=DEGS1 PE=1 SV=1 - [DEGS1_HUMAN]	4.64	1	1	1	3	-0.57	-0.49	-0.58	-0.49	0.19	0.28	0.22	0.31	0.25	0.16	0.33	0.42	0.85	0.92	0.91	0.77	0.77	0.75	0.75	0.86
P00491	Purine nucleoside phosphorylase OS=Homo sapiens GN=PNP PE=1 SV=2 - [PNPH_HUMAN]	35.99	1	9	9	25	-1.26	-1.20	-1.29	-1.26	-0.36	-0.23	-0.47	-0.45	-0.36	-0.49	-1.08	-1.12	0.62	0.27	0.26	0.83	0.99	0.92	1.04	0.86
Q9NWM8	Peptidyl-prolyl cis-trans isomerase FKBP14 OS=Homo sapiens GN=FKBP14 PE=1 SV=1 - [FKB14_HUMAN]	7.11	1	1	1	2	-0.63	0.08	-1.22	-0.50	0.10	0.82	-0.43	0.29	0.88	0.16	0.10	0.82	0.26	0.74	1.32	0.83	1.42	0.72	1.30	0.86
Q9HBG6	Intraflagellar transport protein 122 homolog OS=Homo sapiens GN=IFT122 PE=1 SV=2 - [IF122_HUMAN]	1.37	1	2	2	3	-0.16	-0.17	-0.63	-0.57	-0.03	0.03	0.16	0.22	0.08	0.02	0.35	0.40	0.19	0.53	0.98	0.48	0.69	0.74	0.59	0.86
Q6NUK4	Receptor expression-enhancing protein 3 OS=Homo sapiens GN=REEP3 PE=1 SV=1 - [REEP3_HUMAN]	7.84	1	2	2	3	0.43	0.47	-0.82	-0.77	0.16	0.20	-0.03	0.02	-0.14	-0.19	-0.35	-0.31	-0.39	-0.77	0.47	-0.58	0.66	-0.28	0.96	0.86

Q14571	Inositol 1,4,5-trisphosphate receptor type 2 OS=Homo sapiens GN=ITPR2 PE=1 SV=2 - [ITPR2_HUMAN]	12.81	1	24	27	44	-0.69	-0.66	-0.70	-0.64	0.35	0.41	0.07	0.06	0.16	0.25	0.25	0.20	0.65	0.87	0.84	0.82	0.92	0.97	1.12	0.86
Q12797	Aspartyl/asparaginyl beta-hydroxylase OS=Homo sapiens GN=ASPH PE=1 SV=3 - [ASPH_HUMAN]	19.00	1	12	12	28	-1.16	-1.21	-1.01	-1.04	0.11	0.02	-0.11	-0.26	-0.01	-0.10	0.04	-0.06	1.15	1.42	1.02	1.06	1.02	1.18	1.03	0.85
P16219	Short-chain specific acyl-CoA dehydrogenase, mitochondrial OS=Homo sapiens GN=ACADS PE=1 SV=1 - [ACADS_HUMAN]	18.45	1	7	7	14	0.12	-0.02	0.08	-0.07	0.51	0.37	0.89	0.75	0.46	0.67	0.10	-0.13	0.73	0.02	0.01	0.55	0.57	0.26	0.41	0.85
P00813	Adenosine deaminase OS=Homo sapiens GN=ADA PE=1 SV=3 - [ADA_HUMAN]	13.50	1	4	4	4	-1.04	-1.21	-1.28	-1.46	0.67	0.49	-0.50	-0.67	-0.19	-0.01	-0.58	-0.76	0.60	0.47	0.70	1.06	1.31	1.70	1.94	0.85
Q6DKJ4	Nucleoredoxin OS=Homo sapiens GN=NXN PE=1 SV=2 - [NXN_HUMAN]	19.54	1	6	6	9	-0.78	-0.88	-0.82	-0.92	0.00	-0.01	0.26	0.36	-0.20	-0.15	-0.18	-0.09	0.81	0.34	0.49	0.66	0.70	0.76	0.80	0.85
O00160	Unconventional myosin-If OS=Homo sapiens GN=MYO1F PE=1 SV=3 - [MYO1F_HUMAN]	8.65	1	5	8	12	0.02	-0.25	-0.14	-0.27	-0.20	0.02	0.65	0.47	0.43	0.25	0.24	0.31	0.66	0.62	0.64	0.46	0.48	0.06	0.20	0.85
Q96IZ2	Androgen-dependent TFPI-regulating protein OS=Homo sapiens GN=ADTRP PE=2 SV=1 - [ADTRP_HUMAN]	3.04	1	1	1	1	-1.39	-1.44	-1.19	-1.23	-0.36	-0.41	-0.40	-0.45	-0.47	-0.42	-0.28	-0.34	1.05	1.12	0.90	1.01	0.80	1.02	0.81	0.85
P28290	Sperm-specific antigen 2 OS=Homo sapiens GN=SSFA2 PE=1 SV=3 - [SSFA2_HUMAN]	3.49	1	4	4	5	-1.08	-1.16	-1.27	-1.35	0.08	0.00	-0.49	-0.56	-0.43	-0.34	0.09	0.01	0.65	1.18	1.36	0.77	0.96	1.15	1.34	0.85

Q8TBA6	Golgin subfamily A member 5 OS=Homo sapiens GN=GOLGA5 PE=1 SV=3 - [GOGA5_HUMAN]	4.51	1	2	2	2	0.31	0.68	-0.47	0.16	0.55	0.35	0.31	0.21	0.40	0.43	0.34	0.37	0.06	0.04	0.81	0.16	0.94	0.22	1.00	0.85
Q6V0I7	Protocadherin Fat 4 OS=Homo sapiens GN=FAT4 PE=1 SV=2 - [FAT4_HUMAN]	0.64	1	2	2	2	-0.93	-1.05	-1.09	-1.21	0.58	0.44	-0.31	-0.43	0.69	0.82	0.74	0.61	0.68	1.67	1.83	1.78	1.95	1.49	1.65	0.85
Q05209	Tyrosine-protein phosphatase non-receptor type 12 OS=Homo sapiens GN=PTPN12 PE=1 SV=3 - [PTN12_HUMAN]	3.85	1	3	3	3	-1.58	-1.73	-1.11	-1.26	-0.07	-0.23	-0.33	-0.48	-0.52	-0.36	-0.36	-0.51	1.31	1.23	0.75	1.25	0.78	1.49	1.02	0.85
P60468	Protein transport protein Sec61 subunit beta OS=Homo sapiens GN=SEC61B PE=1 SV=2 - [SC61B_HUMAN]	21.88	1	2	2	5	-1.81	-1.92	-1.36	-1.47	-0.86	-1.03	-0.83	-0.94	-0.83	-0.94	-0.69	-0.71	1.03	1.12	0.79	1.12	0.67	0.51	0.21	0.85
Q0VDF9	Heat shock 70 kDa protein 14 OS=Homo sapiens GN=HSPA14 PE=1 SV=1 - [HSP7E_HUMAN]	9.23	1	2	2	2	0.58	0.01	-1.31	-1.88	-0.31	-0.89	-0.52	-1.09	-0.35	0.22	-1.63	-2.21	-1.05	-2.21	-0.33	-0.33	1.56	-0.91	0.97	0.85
P13498	Cytochrome b-245 light chain OS=Homo sapiens GN=CYBA PE=1 SV=3 - [CY24A_HUMAN]	23.59	1	2	2	2	0.19	0.31	0.02	0.14	0.06	0.17	0.80	0.92	0.59	0.47	0.46	0.58	0.67	0.28	0.44	0.32	0.48	-0.15	0.02	0.84
P02452	Collagen alpha-1(I) chain OS=Homo sapiens GN=COL1A1 PE=1 SV=5 - [CO1A1_HUMAN]	37.77	1	33	34	410	-3.05	-3.20	-3.12	-3.30	-1.87	-1.98	-2.37	-2.56	-2.31	-2.17	-2.29	-2.48	0.77	0.75	0.87	1.04	1.03	1.18	1.29	0.84
O60674	Tyrosine-protein kinase JAK2 OS=Homo sapiens GN=JAK2 PE=1 SV=2 - [JAK2_HUMAN]	2.56	1	1	2	2	-0.07	-0.24	-0.61	-0.78	0.00	-0.17	0.17	0.00	-0.39	-0.22	0.28	0.11	0.29	0.35	0.89	-0.12	0.43	0.06	0.59	0.84

Q53EL6	Programmed cell death protein 4 OS=Homo sapiens GN=PDCD4 PE=1 SV=2 - [PDCD4_HUMAN]	27.93	1	9	9	15	-0.88	-0.84	-1.21	-1.03	-0.35	-0.12	-0.42	-0.33	-0.27	-0.32	-0.37	-0.33	0.47	0.47	0.66	0.62	0.85	0.57	0.88	0.84
P35249	Replication factor C subunit 4 OS=Homo sapiens GN=RFC4 PE=1 SV=2 - [RFC4_HUMAN]	18.46	1	6	6	8	-0.72	-0.62	-1.12	-1.21	-0.07	-0.27	-0.34	-0.33	-0.26	-0.11	-0.27	-0.46	0.43	0.17	0.85	0.40	1.04	0.47	0.94	0.84
Q9NTJ4	Alpha-mannosidase 2C1 OS=Homo sapiens GN=MAN2C1 PE=1 SV=1 - [MA2C1_HUMAN]	2.98	1	2	2	3	-0.67	0.03	-0.88	-0.33	-0.31	0.22	-0.08	0.45	0.41	-0.13	-0.10	0.47	0.18	0.47	0.77	0.42	0.78	0.57	0.53	0.84
P08123	Collagen alpha-2(I) chain OS=Homo sapiens GN=COL1A2 PE=1 SV=7 - [CO1A2_HUMAN]	33.75	1	30	30	538	-3.08	-3.40	-3.12	-3.43	-1.89	-2.18	-2.32	-2.62	-2.44	-2.21	-2.25	-2.54	0.76	0.81	0.85	0.91	1.01	1.21	1.29	0.84
O76024	Wolframin OS=Homo sapiens GN=WFS1 PE=1 SV=2 - [WFS1_HUMAN]	17.30	1	13	13	26	-1.35	-1.15	-1.31	-1.04	0.01	0.09	-0.47	-0.30	-0.01	-0.25	-0.14	-0.02	1.01	1.21	0.98	1.08	1.05	1.25	1.16	0.84
Q92878	DNA repair protein RAD50 OS=Homo sapiens GN=RAD50 PE=1 SV=1 - [RAD50_HUMAN]	13.80	1	15	15	21	-0.80	-0.78	-0.87	-0.83	0.18	0.13	-0.14	-0.10	-0.05	-0.09	0.27	0.20	0.71	1.01	0.89	0.83	0.76	0.87	0.89	0.84
Q9P270	SLAIN motif-containing protein 2 OS=Homo sapiens GN=SLAIN2 PE=1 SV=2 - [SLAI2_HUMAN]	2.41	1	1	1	2	-1.20	-1.00	-1.37	-1.16	-0.64	-0.44	-0.59	-0.39	-0.36	-0.56	-0.70	-0.50	0.67	0.51	0.67	0.68	0.84	0.55	0.71	0.84
Q8ND71	GTPase IMAP family member 8 OS=Homo sapiens GN=GIMAP8 PE=1 SV=2 - [GIMA8_HUMAN]	3.76	1	2	2	3	-1.34	-1.26	-1.44	-1.36	-0.40	-0.33	-0.67	-0.59	-0.59	-0.66	-0.44	-0.37	0.73	0.91	1.00	0.71	0.81	0.92	1.02	0.84
Q9HAT2	Sialate O-acetylesterase OS=Homo sapiens GN=SIAE PE=1 SV=1 - [SIAE_HUMAN]	10.71	1	5	5	5	-1.46	-0.85	-2.18	-1.57	0.13	0.73	-0.32	0.29	0.68	0.08	-0.34	0.26	0.67	1.12	1.83	1.56	2.29	1.57	2.29	0.83

O96019	Actin-like protein 6A OS=Homo sapiens GN=ACTL6A PE=1 SV=1 - [ACL6A_HUMAN]	13.75	1	4	5	7	-1.18	-1.12	-1.03	-1.02	-0.30	-0.30	-0.23	-0.25	-0.14	-0.19	-0.06	-0.09	0.93	1.03	0.93	1.02	0.71	0.80	0.61	0.83
Q9H1J7	Protein Wnt-5b OS=Homo sapiens GN=WNT5B PE=2 SV=2 - [WNT5B_HUMAN]	5.29	1	1	1	2	-0.64	-0.67	-0.51	-0.53	0.93	0.90	0.26	0.24	0.42	0.45	0.15	0.12	0.96	0.80	0.66	1.12	0.99	1.56	1.42	0.83
Q9NP58	ATP-binding cassette sub-family B member 6, mitochondrial OS=Homo sapiens GN=ABCB6 PE=1 SV=1 - [ABCB6_HUMAN]	18.41	2	9	9	20	-0.55	-0.58	-0.61	-0.72	0.15	0.14	0.05	0.10	0.15	0.17	0.59	0.50	0.71	1.40	1.25	0.70	1.13	0.79	0.92	0.83
O00203	AP-3 complex subunit beta-1 OS=Homo sapiens GN=AP3B1 PE=1 SV=3 - [AP3B1_HUMAN]	18.46	1	13	18	28	-0.90	-0.87	-0.95	-0.94	-0.03	0.03	-0.18	-0.18	-0.07	-0.10	-0.11	-0.08	0.81	0.84	0.84	0.86	0.86	1.06	0.93	0.83
O43306	Adenylate cyclase type 6 OS=Homo sapiens GN=ADCY6 PE=1 SV=2 - [ADCY6_HUMAN]	7.36	1	4	7	14	-1.40	-1.32	-1.00	-1.22	0.08	0.11	-0.29	-0.22	0.04	-0.15	0.03	-0.05	1.16	1.43	1.10	1.43	1.29	1.46	1.09	0.83
Q9Y2D5	A-kinase anchor protein 2 OS=Homo sapiens GN=AKAP2 PE=1 SV=3 - [AKAP2_HUMAN]	21.42	1	12	12	23	-1.41	-1.26	-1.52	-1.47	-0.33	-0.14	-0.70	-0.60	-0.45	-0.51	-0.54	-0.41	0.76	0.91	1.07	0.94	1.05	1.19	1.21	0.83
Q16820	Meprin A subunit beta OS=Homo sapiens GN=MEP1B PE=1 SV=3 - [MEP1B_HUMAN]	2.14	1	1	1	1	-1.46	-1.85	-1.69	-2.08	-1.73	-2.12	-0.92	-1.31	-2.33	-1.94	-1.60	-1.99	0.59	-0.14	0.09	-0.45	-0.22	-0.29	-0.06	0.83
Q29RF7	Sister chromatid cohesion protein PDS5 homolog A OS=Homo sapiens GN=PDS5A PE=1 SV=1 - [PDS5A_HUMAN]	3.29	1	3	3	4	-0.85	-0.89	-0.93	-0.91	0.09	-0.20	0.21	-0.03	-0.12	-0.02	0.18	0.06	0.87	0.92	0.75	0.79	0.61	0.64	0.69	0.83

Q9Y624	Junctional adhesion molecule A OS=Homo sapiens GN=F11R PE=1 SV=1 - [JAM1_HUMAN]	3.01	1	1	1	1	-0.96	-1.18	-1.26	-1.47	-0.32	-0.53	-0.49	-0.70	-0.59	-0.37	-0.02	-0.23	0.53	0.95	1.24	0.63	0.92	0.63	0.92	0.83
P50552	Vasodilator-stimulated phosphoprotein OS=Homo sapiens GN=VASP PE=1 SV=3 - [VASP_HUMAN]	30.53	1	10	10	18	-1.74	-1.84	-1.51	-1.57	-0.64	-0.64	-0.80	-0.71	-0.85	-0.93	-0.62	-0.72	1.10	0.88	0.90	0.93	0.93	1.14	0.69	0.83
Q9UHB6	LIM domain and actin-binding protein 1 OS=Homo sapiens GN=LIMA1 PE=1 SV=1 - [LIMA1_HUMAN]	18.18	1	11	11	22	-1.15	-1.28	-1.06	-1.03	-0.01	-0.08	-0.33	-0.32	-0.25	-0.23	0.00	-0.11	0.75	1.24	1.04	0.97	0.93	1.10	0.84	0.83
Q96N67	Dedicator of cytokinesis protein 7 OS=Homo sapiens GN=DOCK7 PE=1 SV=4 - [DOCK7_HUMAN]	7.48	1	9	11	17	-0.92	-0.71	-0.96	-0.85	-0.09	0.01	-0.29	-0.19	-0.17	-0.38	-0.06	-0.06	0.68	0.60	0.99	0.55	0.83	0.71	0.89	0.83
Q460N5	Poly [ADP-ribose] polymerase 14 OS=Homo sapiens GN=PARP14 PE=1 SV=3 - [PAR14_HUMAN]	0.50	1	1	1	1	-0.44	-0.29	-1.07	-0.92	0.61	0.76	-0.31	-0.16	0.12	-0.02	0.04	0.19	0.19	0.48	1.11	0.44	1.08	1.04	1.67	0.83
Q3B726	DNA-directed RNA polymerase I subunit RPA43 OS=Homo sapiens GN=TWISTNB PE=1 SV=1 - [RPA43_HUMAN]	2.07	1	1	1	1	-1.34	-1.29	-1.10	-1.04	0.61	0.66	-0.33	-0.28	0.22	0.17	0.24	0.29	1.07	1.59	1.34	1.54	1.30	1.94	1.69	0.83
Q9BTV4	Transmembrane protein 43 OS=Homo sapiens GN=TMEM43 PE=1 SV=1 - [TMM43_HUMAN]	33.75	1	10	10	19	-1.28	-1.30	-1.14	-1.35	0.21	0.17	-0.48	-0.59	-0.25	-0.19	0.04	-0.17	1.00	1.45	1.34	1.42	1.21	1.60	1.50	0.83
O75462	Cytokine receptor-like factor 1 OS=Homo sapiens GN=CRLF1 PE=1 SV=1 - [CRLF1_HUMAN]	7.82	1	3	3	4	-0.50	-0.65	-0.73	-0.87	2.34	2.19	0.04	-0.11	0.98	1.13	0.39	0.23	0.59	0.89	1.11	1.66	1.89	2.82	3.05	0.83

Q8WXC6	Myeloma-overexpressed gene 2 protein OS=Homo sapiens GN=MYEOV2 PE=2 SV=3 - [MYOV2_HUMAN]	35.09	1	1	1	3	1.42	1.48	0.22	0.29	0.00	0.06	0.99	1.05	0.91	0.85	0.96	1.02	-0.37	-0.45	0.74	-0.54	0.66	-1.43	-0.24	0.83
P36269	Gamma-glutamyltransferase 5 OS=Homo sapiens GN=GGT5 PE=1 SV=2 - [GGT5_HUMAN]	18.94	1	9	9	21	-1.35	-1.27	-1.48	-1.55	-0.02	-0.06	-0.79	-0.68	-0.51	-0.49	-0.25	-0.27	0.61	1.02	1.16	0.87	1.09	1.09	1.41	0.83
Q9UNF1	Melanoma-associated antigen D2 OS=Homo sapiens GN=MAGED2 PE=1 SV=2 - [MAGD2_HUMAN]	14.03	2	7	7	15	-0.61	-0.50	-0.60	-0.57	0.46	0.39	0.25	0.39	0.34	0.42	-0.03	0.13	0.92	0.72	0.63	0.98	0.78	1.17	0.97	0.82
P15151	Poliovirus receptor OS=Homo sapiens GN=PVR PE=1 SV=2 - [PVR_HUMAN]	11.75	1	5	5	9	-0.78	-0.64	-0.75	-0.58	0.83	0.81	0.10	0.14	0.39	0.34	0.68	0.66	0.82	1.34	1.37	1.02	1.07	1.49	1.53	0.82
O75165	DnaJ homolog subfamily C member 13 OS=Homo sapiens GN=DNAJC13 PE=1 SV=5 - [DJC13_HUMAN]	14.00	1	25	25	42	-0.86	-1.01	-0.95	-1.01	0.10	0.05	-0.22	-0.30	-0.09	-0.12	0.01	0.01	0.70	0.83	1.10	0.74	0.90	0.98	1.08	0.82
Q96MK3	Protein FAM20A OS=Homo sapiens GN=FAM20A PE=1 SV=4 - [FA20A_HUMAN]	2.59	1	1	1	2	-0.48	-0.56	-1.03	-1.11	0.50	0.42	-0.28	-0.35	0.01	0.09	0.53	0.45	0.26	1.02	1.57	0.60	1.15	0.96	1.51	0.82
Q8WVM7	Cohesin subunit SA-1 OS=Homo sapiens GN=STAG1 PE=1 SV=3 - [STAG1_HUMAN]	5.01	1	4	6	6	-1.38	-1.32	-1.26	-1.19	-0.23	-0.17	-0.50	-0.44	-0.43	-0.49	-0.17	-0.12	0.94	1.21	1.08	0.92	0.80	1.14	1.01	0.82
P16455	Methylated-DNA--protein-cysteine methyltransferase OS=Homo sapiens GN=MGMT PE=1 SV=1 - [MGMT_HUMAN]	28.99	1	4	4	5	-1.11	-1.02	-0.80	-0.76	-0.39	-0.32	0.02	-0.03	-0.20	-0.17	-0.21	-0.28	1.07	1.14	0.75	0.85	0.59	0.58	0.32	0.82

Q5TBB1	Ribonuclease H2 subunit B OS=Homo sapiens GN=RNASEH2B PE=1 SV=1 - [RNH2B_HUMAN]	7.05	1	1	1	1	-0.17	-0.48	-0.40	-0.71	0.94	0.63	0.35	0.05	0.19	0.50	0.15	-0.16	0.58	0.33	0.55	0.71	0.94	1.09	1.32	0.82
Q7L9L4	MOB kinase activator 1B OS=Homo sapiens GN=MOB1B PE=1 SV=3 - [MOB1B_HUMAN]	5.09	2	1	1	3	-1.13	-1.00	-1.10	-0.98	-0.29	-0.17	-0.35	-0.22	-0.57	-0.69	-0.36	-0.24	0.84	0.78	0.74	0.47	0.45	0.82	0.80	0.82
Q8NDH3	Probable aminopeptidase NPEPL1 OS=Homo sapiens GN=NPEPL1 PE=1 SV=3 - [PEPL1_HUMAN]	18.16	1	7	7	10	-1.32	-1.31	-1.02	-0.92	-0.05	-0.14	-0.22	-0.21	-0.37	-0.43	-0.14	-0.17	1.12	0.89	0.79	0.91	0.50	0.78	0.65	0.82
Q9Y6N6	Laminin subunit gamma-3 OS=Homo sapiens GN=LAMC3 PE=1 SV=3 - [LAMC3_HUMAN]	2.79	1	3	4	9	-0.82	-0.88	-1.28	-1.34	-0.51	-0.58	-0.53	-0.59	-0.46	-0.39	-0.04	-0.11	0.35	0.79	1.24	0.46	0.92	0.29	0.75	0.82
P35475	Alpha-L-iduronidase OS=Homo sapiens GN=IDUA PE=1 SV=2 - [IDUA_HUMAN]	1.23	1	1	1	1	-0.16	-0.19	-0.92	-0.96	0.71	0.67	-0.17	-0.20	0.53	0.57	-0.04	-0.08	0.04	0.12	0.88	0.76	1.52	0.85	1.61	0.82
P22692	Insulin-like growth factor-binding protein 4 OS=Homo sapiens GN=IGFBP4 PE=1 SV=2 - [IBP4_HUMAN]	4.65	1	1	1	1	-0.23	-0.26	-0.46	-0.50	1.28	1.25	0.29	0.26	0.88	0.92	1.19	1.16	0.57	1.43	1.66	1.18	1.42	1.49	1.73	0.82
Q9BVC6	Transmembrane protein 109 OS=Homo sapiens GN=TMEM109 PE=1 SV=1 - [TM109_HUMAN]	13.17	1	5	5	17	-1.09	-0.98	-1.02	-0.95	-0.47	-0.41	-0.37	-0.42	-0.30	-0.41	-0.31	-0.27	0.68	0.69	0.97	0.48	0.88	0.71	1.00	0.82
P51689	Arylsulfatase D OS=Homo sapiens GN=ARSD PE=1 SV=2 - [ARSD_HUMAN]	5.06	1	2	2	2	-0.03	-0.02	-0.50	-0.49	0.43	0.43	0.25	0.26	0.48	0.48	0.25	0.25	0.33	0.28	0.75	0.54	1.02	0.44	0.91	0.82

Q96MW5	Conserved oligomeric Golgi complex subunit 8 OS=Homo sapiens GN=COG8 PE=1 SV=2 - [COG8_HUMAN]	8.66	1	4	4	6	-0.82	-0.29	-1.08	-0.72	0.10	0.37	-0.30	0.02	0.14	-0.13	-0.03	0.45	0.38	0.75	1.05	0.36	0.78	0.58	1.01	0.82
P46937	Yorkie homolog OS=Homo sapiens GN=YAP1 PE=1 SV=2 - [YAP1_HUMAN]	25.20	1	8	8	19	-1.67	-1.48	-1.53	-1.46	-0.50	-0.47	-0.62	-0.64	-0.43	-0.34	-0.08	-0.04	0.79	1.45	1.45	1.00	1.08	1.00	0.93	0.82
O75578	Integrin alpha 10 OS=Homo sapiens GN=ITGA10 PE=2 SV=2 - [ITA10_HUMAN]	1.54	1	1	1	2	-1.47	-1.16	-1.30	-1.00	0.61	0.91	-0.55	-0.25	0.41	0.11	-0.24	0.06	0.97	1.23	1.06	1.60	1.44	2.06	1.89	0.81
Q9ULA0	Aspartyl aminopeptidase OS=Homo sapiens GN=DNPEP PE=1 SV=1 - [DNPEP_HUMAN]	25.89	1	9	9	23	-0.65	-0.60	-0.81	-0.81	-0.09	-0.14	-0.09	-0.06	-0.07	-0.15	0.01	0.08	0.53	0.68	0.86	0.50	0.81	0.50	0.76	0.81
Q2VPB7	AP-5 complex subunit beta-1 OS=Homo sapiens GN=AP5B1 PE=1 SV=4 - [AP5B1_HUMAN]	1.94	1	1	1	1	-0.12	-0.47	-0.53	-0.87	0.29	-0.06	0.22	-0.12	-0.26	0.09	-0.15	-0.50	0.40	-0.03	0.37	0.24	0.65	0.40	0.80	0.81
Q9HAS0	Protein Njmu-R1 OS=Homo sapiens GN=C17orf75 PE=1 SV=2 - [NJMU_HUMAN]	20.71	1	5	5	6	-1.69	-1.39	-1.21	-0.90	-0.16	0.14	-0.46	-0.16	-0.20	-0.50	-0.53	-0.23	1.29	1.17	0.68	1.22	0.74	1.52	1.03	0.81
Q6ZMJ2	Scavenger receptor class A member 5 OS=Homo sapiens GN=SCARA5 PE=2 SV=1 - [SCAR5_HUMAN]	6.26	1	2	2	2	-0.54	-0.44	-1.70	-1.60	-0.73	-0.63	-0.95	-0.85	-0.58	-0.68	0.13	0.22	-0.35	0.68	1.83	-0.11	1.05	-0.20	0.95	0.81
P01764	Ig heavy chain V-III region VH26 OS=Homo sapiens PE=1 SV=1 - [HV303_HUMAN]	18.80	1	2	2	9	-1.49	-1.30	-1.64	-1.46	0.20	0.35	-0.88	-0.72	-0.05	-0.22	0.46	0.62	0.65	1.86	2.01	1.29	1.42	1.57	1.64	0.81

Q13217	DnaJ homolog subfamily C member 3 OS=Homo sapiens GN=DNAJC3 PE=1 SV=1 - [DNJC3_HUMAN]	15.67	1	6	6	16	-0.63	-0.69	-1.01	-1.06	0.21	0.43	-0.20	-0.23	-0.01	-0.16	-0.04	-0.25	0.42	0.52	0.69	0.52	0.79	0.93	1.02	0.81
P68104	Elongation factor 1-alpha 1 OS=Homo sapiens GN=EEF1A1 PE=1 SV=1 - [EF1A1_HUMAN]	45.67	2	6	16	111	-1.31	-1.30	-0.80	-0.81	0.05	0.09	-0.07	-0.08	-0.21	-0.19	-0.28	-0.23	1.42	1.01	0.40	1.24	0.56	1.31	0.74	0.81
Q7Z5A7	Protein FAM19A5 OS=Homo sapiens GN=FAM19A5 PE=1 SV=2 - [F19A5_HUMAN]	8.33	1	1	1	1	-0.26	-0.77	0.11	-0.41	1.50	0.98	0.85	0.34	0.50	1.02	0.39	-0.12	1.17	0.66	0.29	1.31	0.95	1.74	1.37	0.81
P08575	Receptor-type tyrosine-protein phosphatase C OS=Homo sapiens GN=PTPRC PE=1 SV=2 - [PTPRC_HUMAN]	13.80	1	15	15	29	-0.16	-0.13	-0.54	-0.49	0.28	0.41	0.22	0.27	0.42	0.32	0.50	0.51	0.50	0.60	0.96	0.53	0.79	0.48	0.84	0.81
O75953	DnaJ homolog subfamily B member 5 OS=Homo sapiens GN=DNAJB5 PE=1 SV=1 - [DNJB5_HUMAN]	7.76	1	1	1	1	-0.70	-1.12	-0.99	-1.41	-0.33	-0.76	-0.25	-0.67	-0.75	-0.32	-0.38	-0.81	0.51	0.33	0.61	0.41	0.70	0.35	0.64	0.81
Q6ZSR9	Uncharacterized protein FLJ45252 OS=Homo sapiens PE=1 SV=2 - [YJ005_HUMAN]	17.18	1	3	3	4	-0.85	-0.28	-0.69	-0.39	-0.02	0.79	0.05	0.36	0.48	0.19	0.42	0.41	0.69	1.44	1.09	1.19	0.91	0.81	0.65	0.81
O75695	Protein XRP2 OS=Homo sapiens GN=RP2 PE=1 SV=4 - [XRP2_HUMAN]	8.57	1	3	3	4	-0.14	-0.31	-0.34	-0.52	0.50	0.32	0.40	0.22	0.22	0.40	0.80	0.62	0.59	0.94	1.14	0.57	0.78	0.62	0.83	0.81
P52895	Aldo-keto reductase family 1 member C2 OS=Homo sapiens GN=AKR1C2 PE=1 SV=3 - [AK1C2_HUMAN]	54.18	1	2	12	33	-0.75	-0.74	-0.54	-0.53	0.82	1.19	0.61	0.70	1.09	0.86	1.24	1.40	1.33	2.04	1.66	1.75	1.31	2.03	1.22	0.81

P02768	Serum albumin OS=Homo sapiens GN=ALB PE=1 SV=2 - [ALBU_HUMAN]	68.80	1	42	42	484	-1.73	-1.71	-1.56	-1.54	-0.72	-0.67	-0.85	-0.80	-0.87	-0.95	-1.48	-1.40	0.95	0.29	0.09	0.84	0.66	1.06	0.84	0.80
Q96B54	Zinc finger protein 428 OS=Homo sapiens GN=ZNF428 PE=1 SV=2 - [ZN428_HUMAN]	9.04	1	1	1	1	-1.10	-1.23	-0.58	-0.72	0.10	-0.04	0.16	0.02	-0.24	-0.10	0.34	0.20	1.31	1.44	0.92	1.03	0.52	1.18	0.66	0.80
A1A4S6	Rho GTPase-activating protein 10 OS=Homo sapiens GN=ARHGAP10 PE=1 SV=1 - [RHG10_HUMAN]	9.03	1	6	7	8	-1.24	-1.26	-1.34	-1.30	-0.26	-0.23	-0.60	-0.56	-0.19	-0.17	-0.29	-0.37	0.82	0.73	0.67	0.96	0.89	1.24	1.22	0.80
Q9Y678	Coatomer subunit gamma-1 OS=Homo sapiens GN=COPG1 PE=1 SV=1 - [COPG1_HUMAN]	29.98	1	19	20	44	-1.01	-0.96	-1.14	-1.00	0.14	0.14	-0.23	-0.26	-0.10	-0.15	-0.07	0.05	0.76	1.04	0.88	0.87	0.97	1.16	0.99	0.80
Q9H8H3	Methyltransferase-like protein 7A OS=Homo sapiens GN=METTL7A PE=1 SV=1 - [MET7A_HUMAN]	17.62	1	4	4	10	-0.74	-0.60	-0.70	-0.52	0.28	0.36	-0.04	0.10	0.13	-0.01	0.31	0.39	0.83	0.82	0.85	0.76	0.71	1.08	0.94	0.80
Q96K49	Transmembrane protein 87B OS=Homo sapiens GN=TMEM87B PE=1 SV=1 - [TM87B_HUMAN]	2.16	1	1	1	1	-1.41	-1.55	-1.28	-1.41	-0.12	-0.25	-0.54	-0.67	-0.30	-0.16	0.02	-0.12	0.93	1.43	1.29	1.28	1.15	1.28	1.14	0.80
P09105	Hemoglobin subunit theta-1 OS=Homo sapiens GN=HBQ1 PE=1 SV=2 - [HBAT_HUMAN]	41.55	1	5	5	13	-0.95	-0.89	-1.38	-1.20	0.58	0.70	-0.56	-0.35	0.31	0.26	0.76	0.90	0.77	1.71	2.20	1.24	1.49	1.57	2.01	0.80
Q8IXL7	Methionine-R sulfoxide reductase B3 OS=Homo sapiens GN=MSRB3 PE=1 SV=2 - [MSRB3_HUMAN]	6.25	1	1	1	2	-1.56	-1.20	-1.61	-1.25	-1.00	-0.65	-0.87	-0.51	-0.58	-0.93	-0.96	-0.61	0.74	0.60	0.64	0.66	0.71	0.54	0.59	0.80

Q13283	Ras GTPase-activating protein-binding protein 1 OS=Homo sapiens GN=G3BP1 PE=1 SV=1 - [G3BP1_HUMAN]	19.96	1	7	7	24	-0.86	-0.75	-1.01	-0.89	-0.03	0.06	-0.12	-0.10	-0.06	-0.17	-0.16	-0.07	0.75	0.69	0.86	0.76	0.97	0.67	0.80	0.80
Q8WUA8	Tsukushin OS=Homo sapiens GN=TSKU PE=2 SV=3 - [TSK_HUMAN]	4.25	1	1	1	1	0.51	-0.11	0.18	-0.43	2.31	1.69	0.92	0.30	0.83	1.45	0.54	-0.08	0.47	0.04	0.35	0.97	1.30	1.78	2.11	0.80
P13761	HLA class II histocompatibility antigen, DRB1-7 beta chain OS=Homo sapiens GN=HLA-DRB1 PE=1 SV=1 - [2B17_HUMAN]	21.05	3	5	5	11	-0.16	-0.11	-0.09	-0.07	-0.16	-0.04	0.66	0.78	0.78	0.66	0.07	0.18	0.97	0.23	0.17	0.86	0.79	-0.01	-0.07	0.80
P05362	Intercellular adhesion molecule 1 OS=Homo sapiens GN=ICAM1 PE=1 SV=2 - [ICAM1_HUMAN]	15.41	1	7	7	12	-0.50	-0.40	-0.60	-0.44	0.34	0.45	0.19	0.28	0.37	0.24	0.25	0.34	0.75	0.75	0.79	0.77	0.82	0.82	0.86	0.80
Q6ZW31	Rho GTPase-activating protein SYDE1 OS=Homo sapiens GN=SYDE1 PE=1 SV=1 - [SYDE1_HUMAN]	4.08	1	2	2	3	-0.35	-0.69	-1.16	-1.51	-0.06	-0.41	-0.43	-0.78	-0.77	-0.42	-0.23	-0.58	-0.03	0.12	0.93	-0.04	0.78	0.27	1.08	0.80
Q92506	Estradiol 17-beta-dehydrogenase 8 OS=Homo sapiens GN=HSD17B8 PE=1 SV=2 - [DHB8_HUMAN]	22.99	1	5	5	10	-0.79	-0.77	-0.84	-0.79	-0.07	-0.03	-0.05	-0.14	-0.23	-0.19	0.11	0.15	0.80	1.06	0.97	0.64	0.75	0.92	0.75	0.80
Q96HH9	GRAM domain-containing protein 3 OS=Homo sapiens GN=GRAMD3 PE=1 SV=1 - [GRAM3_HUMAN]	8.56	1	2	2	3	-0.79	-0.73	-0.86	-0.80	0.04	0.09	-0.12	-0.07	-0.03	-0.09	-0.39	-0.33	0.72	0.41	0.47	0.73	0.80	0.81	0.88	0.80

P40939	Trifunctional enzyme subunit alpha, mitochondrial OS=Homo sapiens GN=HADHA PE=1 SV=2 - [ECHA_HUMAN]	44.82	1	30	30	109	-1.17	-1.29	-1.03	-1.07	-0.33	-0.35	-0.25	-0.30	-0.34	-0.37	-0.23	-0.27	0.95	1.05	0.82	0.94	0.75	0.85	0.73	0.79
P22670	MHC class II regulatory factor RFX1 OS=Homo sapiens GN=RFX1 PE=1 SV=2 - [RFX1_HUMAN]	2.86	1	1	1	3	-2.02	-1.52	-1.90	-1.41	-2.03	-1.54	-1.17	-0.68	-0.66	-1.14	-1.06	-0.57	0.90	0.97	0.84	0.90	0.79	-0.03	-0.15	0.79
Q9Y4H4	G-protein-signaling modulator 3 OS=Homo sapiens GN=GPSM3 PE=1 SV=1 - [GPSM3_HUMAN]	14.37	1	1	1	3	-0.57	-0.47	-0.37	-0.27	0.33	0.43	0.36	0.46	0.42	0.32	0.47	0.57	0.98	1.05	0.84	0.92	0.72	0.89	0.69	0.79
Q9BUN8	Derlin-1 OS=Homo sapiens GN=DERL1 PE=1 SV=1 - [DERL1_HUMAN]	8.76	1	2	2	5	-1.65	-1.55	-1.57	-1.30	-0.34	-0.42	-0.82	-0.70	-0.45	-0.55	-0.64	-0.53	0.81	0.89	0.98	0.62	0.80	1.15	1.00	0.79
Q9UKX7	Nuclear pore complex protein Nup50 OS=Homo sapiens GN=NUP50 PE=1 SV=2 - [NUP50_HUMAN]	2.14	1	1	1	2	-1.64	-1.63	-1.86	-1.84	-1.13	-1.12	-1.13	-1.11	-0.83	-0.84	-0.57	-0.56	0.57	1.08	1.28	0.84	1.05	0.50	0.71	0.79
Q15836	Vesicle-associated membrane protein 3 OS=Homo sapiens GN=VAMP3 PE=1 SV=3 - [VAMP3_HUMAN]	40.00	1	1	3	16	0.19	0.06	-0.84	-0.51	-0.02	0.26	-0.15	-0.04	-0.12	-0.18	0.43	0.54	-0.28	0.25	1.05	-0.34	0.83	-0.05	0.75	0.79
O60264	SWI/SNF-related matrix associated actin-dependent regulator of chromatin subfamily A member 5 OS=Homo sapiens GN=SMARCA5 PE=1 SV=1 - [SMCA5_HUMAN]	7.89	1	4	8	8	0.01	-0.39	-0.47	-0.87	0.80	0.39	0.25	-0.15	-0.09	0.32	0.48	0.08	0.30	0.48	0.95	0.34	0.82	0.77	1.25	0.79

O60675	Transcription factor MafK OS=Homo sapiens GN=MAFK PE=1 SV=1 - [MAFK_HUMAN]	4.49	3	1	1	1	-0.45	-0.43	-0.72	-0.70	0.01	0.03	0.00	0.02	0.41	0.40	0.55	0.57	0.51	1.01	1.27	0.88	1.15	0.45	0.71	0.79
Q08J23	tRNA (cytosine(34)-C(5))-methyltransferase OS=Homo sapiens GN=NSUN2 PE=1 SV=2 - [NSUN2_HUMAN]	16.04	1	8	8	11	-0.37	-0.32	-0.76	-0.52	0.16	0.10	-0.05	-0.17	-0.26	-0.07	0.02	0.05	0.25	0.32	0.33	0.40	0.94	0.45	0.59	0.79
P01625	Ig kappa chain V-IV region Len OS=Homo sapiens PE=1 SV=2 - [KV402_HUMAN]	28.95	2	1	2	13	-0.70	-0.80	-0.65	-0.84	0.28	-0.03	-0.09	-0.25	0.25	0.27	0.65	0.64	0.44	1.20	1.41	0.99	1.28	0.95	0.85	0.79
O75891	Cytosolic 10-formyltetrahydrofolate dehydrogenase OS=Homo sapiens GN=ALDH1L1 PE=1 SV=2 - [AL1L1_HUMAN]	49.56	1	33	38	92	0.58	0.40	0.73	0.66	1.28	1.29	1.37	1.28	1.14	1.16	0.91	0.91	0.93	0.40	0.29	0.70	0.56	0.74	0.63	0.78
Q86X83	COMM domain-containing protein 2 OS=Homo sapiens GN=COMMD2 PE=1 SV=2 - [COMD2_HUMAN]	30.65	1	4	4	6	-0.83	-0.65	-0.86	-0.76	0.10	0.21	-0.22	-0.05	0.14	-0.03	0.03	0.20	0.55	0.86	0.60	0.84	0.65	1.25	0.94	0.78
P55084	Trifunctional enzyme subunit beta, mitochondrial OS=Homo sapiens GN=HADHB PE=1 SV=3 - [ECHB_HUMAN]	46.20	1	18	18	69	-1.20	-1.22	-1.04	-1.00	-0.57	-0.56	-0.33	-0.36	-0.46	-0.45	-0.42	-0.37	0.93	0.82	0.55	0.72	0.60	0.68	0.45	0.78
P31751	RAC-beta serine/threonine-protein kinase OS=Homo sapiens GN=AKT2 PE=1 SV=2 - [AKT2_HUMAN]	12.27	1	2	4	5	-0.38	-0.83	-0.44	-0.89	0.59	0.13	0.28	-0.17	0.14	0.60	0.14	-0.32	0.71	0.52	0.57	1.00	1.07	0.95	1.01	0.78
P42892	Endothelin-converting enzyme 1 OS=Homo sapiens GN=ECE1 PE=1 SV=2 - [ECE1_HUMAN]	9.35	1	5	5	8	-1.30	0.15	-1.31	-1.47	0.16	-0.01	-0.59	-0.35	-0.19	-0.02	0.40	0.23	-0.45	1.70	1.70	1.31	1.32	1.44	1.45	0.78

Q9UKU9	Angiopoietin-related protein 2 OS=Homo sapiens GN=ANGPTL2 PE=2 SV=1 - [ANGL2_HUMAN]	1.83	1	1	1	1	-1.26	-1.12	-1.28	-1.14	-0.10	0.04	-0.57	-0.42	-0.04	-0.18	-0.11	0.03	0.75	1.16	1.17	1.11	1.13	1.15	1.17	0.78
Q8NC56	LEM domain-containing protein 2 OS=Homo sapiens GN=LEMD2 PE=1 SV=1 - [LEMD2_HUMAN]	23.86	1	9	9	15	-1.09	-1.29	-1.21	-1.39	-0.20	-0.27	-0.50	-0.61	-0.36	-0.27	0.02	-0.09	0.75	1.08	1.18	0.86	0.95	1.05	1.08	0.78
P12830	Cadherin-1 OS=Homo sapiens GN=CDH1 PE=1 SV=3 - [CADH1_HUMAN]	8.62	1	5	5	10	-0.76	-1.14	-1.12	-1.13	0.19	0.24	-0.62	-0.41	0.33	-0.01	0.66	1.03	0.60	1.44	2.16	0.89	1.61	1.47	2.09	0.78
Q96T51	RUN and FYVE domain-containing protein 1 OS=Homo sapiens GN=RUFY1 PE=1 SV=2 - [RUFY1_HUMAN]	13.42	1	8	9	16	-1.20	-1.02	-1.13	-0.95	0.18	0.20	-0.26	-0.13	0.14	0.00	-0.03	0.14	0.90	1.17	1.10	1.23	1.17	1.36	1.29	0.77
P06132	Uroporphyrinogen decarboxylase OS=Homo sapiens GN=UROD PE=1 SV=2 - [DCUP_HUMAN]	14.17	1	4	4	6	-0.30	-0.18	-0.43	-0.38	0.33	0.44	0.20	0.32	0.18	0.03	0.26	0.42	0.56	0.67	0.80	0.48	0.62	0.66	0.79	0.77
Q9H3F6	BTB/POZ domain-containing adapter for CUL3-mediated RhoA degradation protein 3 OS=Homo sapiens GN=KCTD10 PE=1 SV=1 - [BACD3_HUMAN]	14.38	3	4	4	6	-0.78	-0.67	-0.91	-0.80	0.45	0.56	-0.20	-0.09	0.37	0.27	0.20	0.30	0.64	0.98	1.10	1.08	1.21	1.21	1.34	0.77
Q13822	Ectonucleotide pyrophosphatase/phosphodiesterase family member 2 OS=Homo sapiens GN=ENPP2 PE=1 SV=3 - [ENPP2_HUMAN]	3.01	1	2	2	2	0.62	0.30	-0.39	-0.70	0.79	0.47	0.32	0.01	0.19	0.50	0.41	0.09	-0.24	-0.20	0.80	-0.08	0.93	0.15	1.16	0.77

Q9UH99	SUN domain-containing protein 2 OS=Homo sapiens GN=SUN2 PE=1 SV=3 - [SUN2_HUMAN]	42.26	1	18	19	39	-0.91	-0.91	-0.90	-0.88	-0.06	0.01	-0.25	-0.17	-0.05	-0.17	0.04	0.12	0.74	1.18	0.91	0.76	0.73	0.81	0.68	0.77
P15374	Ubiquitin carboxyl-terminal hydrolase isozyme L3 OS=Homo sapiens GN=UCHL3 PE=1 SV=1 - [UCHL3_HUMAN]	37.83	1	7	7	13	-0.78	-0.93	-0.40	-0.30	0.53	0.51	0.42	0.36	0.34	0.37	0.00	-0.10	1.05	0.72	0.25	1.12	0.72	1.21	1.03	0.77
Q8TEW0	Partitioning defective 3 homolog OS=Homo sapiens GN=PARD3 PE=1 SV=2 - [PARD3_HUMAN]	9.44	1	8	8	11	-0.22	0.05	-1.04	-0.76	-0.06	0.17	-0.39	-0.27	-0.28	-0.31	-0.12	0.11	-0.06	0.29	1.07	0.19	1.01	0.32	1.13	0.77
Q9NUV9	GTPase IMAP family member 4 OS=Homo sapiens GN=GIMAP4 PE=1 SV=1 - [GIMA4_HUMAN]	9.12	1	2	2	4	-0.72	-0.72	-1.10	-0.93	-0.41	-0.40	-0.05	-0.12	-0.56	-0.43	-0.55	-0.69	0.67	0.04	0.17	0.51	0.61	0.31	0.63	0.77
O15127	Secretory carrier-associated membrane protein 2 OS=Homo sapiens GN=SCAMP2 PE=1 SV=2 - [SCAM2_HUMAN]	9.73	1	2	2	3	-0.28	0.13	-0.97	-0.56	-0.28	0.13	-0.27	0.15	0.56	0.15	0.24	0.64	0.07	0.52	1.21	0.47	1.16	-0.01	0.67	0.76
Q9GZT6	Coiled-coil domain-containing protein 90B, mitochondrial OS=Homo sapiens GN=CCDC90B PE=1 SV=2 - [CC90B_HUMAN]	25.98	1	6	6	10	-0.57	-0.57	-0.72	-0.49	0.18	0.30	0.13	0.29	0.31	0.08	0.04	0.24	1.01	0.78	0.76	0.84	0.78	0.84	0.69	0.76
Q8TDW0	Leucine-rich repeat-containing protein 8C OS=Homo sapiens GN=LRRC8C PE=2 SV=2 - [LRC8C_HUMAN]	5.35	2	2	3	5	-0.28	-0.44	-0.78	-0.94	0.36	0.20	-0.08	-0.24	0.06	0.22	-0.17	-0.33	0.25	0.12	0.61	0.53	1.04	0.62	1.12	0.76

Q9NXR1	Nuclear distribution protein nudE homolog 1 OS=Homo sapiens GN=NDE1 PE=1 SV=2 - [NDE1_HUMAN]	13.87	1	3	4	4	-0.62	-0.58	-0.70	-0.94	-0.73	-0.13	-0.75	-0.24	-0.14	-0.66	-0.63	-0.03	0.39	-0.01	0.07	0.47	0.84	-0.12	-0.04	0.76
Q8N142	Adenylosuccinate synthetase isozyme 1 OS=Homo sapiens GN=ADSSL1 PE=2 SV=1 - [PURA1_HUMAN]	10.50	1	2	3	3	-0.09	-0.31	-0.46	-0.68	0.54	0.32	0.23	0.02	0.29	0.52	0.16	-0.07	0.38	0.26	0.62	0.64	1.01	0.62	0.99	0.76
P49916	DNA ligase 3 OS=Homo sapiens GN=LIG3 PE=1 SV=2 - [DNLI3_HUMAN]	4.96	1	4	4	5	-0.71	-0.72	-0.96	-0.70	0.25	0.00	-0.02	-0.04	-0.40	-0.65	-0.07	-0.26	0.74	0.66	0.75	0.28	0.34	0.71	0.69	0.76
Q6IEU7	Olfactory receptor 5M10 OS=Homo sapiens GN=OR5M10 PE=2 SV=1 - [OR5MA_HUMAN]	4.13	1	1	1	1	3.53	3.25	-0.67	-0.95	0.38	0.10	0.02	-0.25	0.22	0.51	0.92	0.63	-3.45	-2.61	1.59	-2.99	1.21	-3.16	1.04	0.76
Q9Y6M7	Sodium bicarbonate cotransporter 3 OS=Homo sapiens GN=SLC4A7 PE=1 SV=2 - [S4A7_HUMAN]	3.79	1	1	3	3	-0.12	1.02	-0.91	0.23	-0.03	1.11	-0.22	0.93	0.98	-0.16	0.28	1.42	-0.04	0.41	1.19	-0.01	0.78	0.07	0.86	0.76
O15031	Plexin-B2 OS=Homo sapiens GN=PLXNB2 PE=1 SV=3 - [PLXB2_HUMAN]	17.25	1	23	23	37	-0.46	-0.42	-0.54	-0.49	0.27	0.26	0.19	0.20	0.22	0.15	0.37	0.35	0.70	0.71	0.88	0.84	0.76	0.74	0.81	0.75
Q9Y3C0	WASH complex subunit CCDC53 OS=Homo sapiens GN=CCDC53 PE=1 SV=1 - [CCD53_HUMAN]	23.71	1	3	3	5	-1.01	-0.96	-1.01	-0.96	-0.06	-0.01	-0.32	-0.27	-0.16	-0.20	-0.08	-0.03	0.74	0.94	0.94	0.84	0.85	0.94	0.94	0.75
Q9Y230	RuvB-like 2 OS=Homo sapiens GN=RUVBL2 PE=1 SV=3 - [RUVB2_HUMAN]	39.52	1	17	17	42	-1.03	-0.97	-1.01	-1.01	-0.19	-0.16	-0.24	-0.20	-0.27	-0.31	0.01	-0.03	0.84	0.90	0.97	0.73	0.64	0.89	0.78	0.75
Q96AC1	Fermitin family homolog 2 OS=Homo sapiens GN=FERMT2 PE=1 SV=1 - [FERM2_HUMAN]	32.21	1	16	16	33	-1.31	-1.37	-1.25	-1.32	-0.38	-0.31	-0.64	-0.61	-0.55	-0.57	-0.63	-0.56	0.70	0.81	0.62	0.78	0.72	0.93	0.86	0.75

Q00796	Sorbitol dehydrogenase OS=Homo sapiens GN=SORD PE=1 SV=4 - [DHSO_HUMAN]	24.93	1	8	8	16	-0.17	-0.34	-0.40	-0.56	0.44	0.30	0.35	0.26	0.17	0.56	0.66	0.38	0.40	0.72	0.93	0.76	0.81	0.59	0.71	0.75
O43896	Kinesin-like protein KIF1C OS=Homo sapiens GN=KIF1C PE=1 SV=3 - [KIF1C_HUMAN]	6.62	1	2	6	13	-1.71	-1.93	-1.48	-1.69	-0.24	-0.46	-0.79	-1.01	-0.86	-0.64	-0.27	-0.49	0.97	1.45	1.21	1.10	0.87	1.45	1.22	0.75
O00468	Agrin OS=Homo sapiens GN=AGRN PE=1 SV=5 - [AGRIN_HUMAN]	34.06	1	49	49	137	-1.39	-1.26	-1.45	-1.31	-0.31	-0.19	-0.73	-0.59	-0.45	-0.54	-0.41	-0.33	0.69	0.97	1.03	0.87	0.94	1.07	1.16	0.75
O76003	Glutaredoxin-3 OS=Homo sapiens GN=GLRX3 PE=1 SV=2 - [GLRX3_HUMAN]	26.87	1	8	8	26	-0.08	-0.21	-0.47	-0.46	0.17	0.03	0.21	0.22	0.20	0.31	0.22	0.29	0.60	0.23	0.75	0.32	0.78	0.34	0.59	0.75
O00461	Golgi integral membrane protein 4 OS=Homo sapiens GN=GOLIM4 PE=1 SV=1 - [GOLI4_HUMAN]	5.89	1	4	4	4	-1.01	-1.21	-1.26	-1.43	0.04	0.20	-0.63	-0.25	-0.16	-0.31	-0.10	0.27	0.51	0.91	1.16	0.60	0.86	1.03	1.28	0.74
Q7Z2K6	Endoplasmic reticulum metallopeptidase 1 OS=Homo sapiens GN=ERMP1 PE=1 SV=2 - [ERMP1_HUMAN]	16.59	1	11	11	18	-0.76	-0.89	-0.92	-1.01	0.03	-0.03	-0.26	-0.21	-0.24	-0.23	-0.09	-0.12	0.51	0.68	0.77	0.65	0.81	0.76	0.84	0.74
Q8IUW5	RELT-like protein 1 OS=Homo sapiens GN=RELL1 PE=1 SV=1 - [RELL1_HUMAN]	27.68	1	5	5	9	-0.84	-0.88	-1.18	-1.22	-0.35	-0.39	-0.27	-0.31	-0.27	-0.23	-0.35	-0.35	0.63	0.75	1.01	0.59	0.03	0.48	-0.07	0.74
Q96SK2	Transmembrane protein 209 OS=Homo sapiens GN=TMEM209 PE=1 SV=2 - [TM209_HUMAN]	3.57	1	1	1	1	-1.39	-1.27	-1.11	-0.99	-0.67	-0.55	-0.44	-0.31	-0.66	-0.78	-0.42	-0.30	1.01	0.98	0.69	0.64	0.37	0.70	0.43	0.74
Q9BX66	Sorbin and SH3 domain-containing protein 1 OS=Homo sapiens GN=SORBS1 PE=1 SV=3 - [SRBS1_HUMAN]	24.54	1	25	26	75	-1.14	-1.25	-1.14	-1.27	-0.48	-0.54	-0.55	-0.56	-0.60	-0.54	-0.40	-0.49	0.87	0.80	0.78	0.73	0.65	0.70	0.67	0.74

Q16288	NT-3 growth factor receptor OS=Homo sapiens GN=NTRK3 PE=1 SV=2 - [NTRK3_HUMAN]	5.96	2	2	5	6	-0.17	0.09	-0.66	-0.40	0.61	0.86	0.02	0.28	0.41	0.16	0.50	0.75	0.25	0.68	1.15	0.36	0.85	0.76	1.24	0.74
P14625	Endoplasmin OS=Homo sapiens GN=HSP90B1 PE=1 SV=1 - [ENPL_HUMAN]	38.11	2	25	27	145	-1.25	-1.30	-1.19	-1.14	-0.23	-0.22	-0.47	-0.49	-0.53	-0.50	-0.37	-0.39	0.87	0.85	0.80	0.82	0.72	1.04	0.86	0.74
Q9BW83	Intraflagellar transport protein 27 homolog OS=Homo sapiens GN=IFT27 PE=1 SV=1 - [IFT27_HUMAN]	39.78	1	5	5	6	-0.65	-0.87	-0.36	-0.58	0.18	0.09	0.32	0.10	0.08	0.31	0.18	-0.05	1.02	0.84	0.54	0.99	0.70	0.95	0.61	0.74
P52943	Cysteine-rich protein 2 OS=Homo sapiens GN=CRIP2 PE=1 SV=1 - [CRIP2_HUMAN]	41.83	2	7	7	33	-1.99	-1.89	-1.89	-1.88	-1.17	-1.14	-1.18	-1.20	-1.09	-1.10	-0.70	-0.73	0.87	1.28	1.19	1.00	0.83	0.91	0.73	0.74
Q8IZL8	Proline-, glutamic acid- and leucine-rich protein 1 OS=Homo sapiens GN=PELP1 PE=1 SV=2 - [PELP1_HUMAN]	1.86	1	1	1	2	-0.38	-0.92	-0.41	-0.94	-0.85	-1.39	0.27	-0.26	-0.97	-0.43	0.02	-0.52	0.71	0.41	0.42	-0.02	0.01	-0.48	-0.46	0.74
Q9NZQ8	Transient receptor potential cation channel subfamily M member 5 OS=Homo sapiens GN=TRPM5 PE=2 SV=1 - [TRPM5_HUMAN]	0.52	1	1	1	1	-0.46	-0.79	-0.73	-1.06	0.55	0.21	-0.05	-0.39	0.28	0.63	0.51	0.16	0.46	0.97	1.23	1.11	1.39	0.99	1.26	0.74
Q4KMQ2	Anoctamin-6 OS=Homo sapiens GN=ANO6 PE=1 SV=2 - [ANO6_HUMAN]	17.25	1	12	12	19	-1.18	-1.09	-1.22	-1.10	-0.23	-0.29	-0.55	-0.53	-0.39	-0.48	-0.09	-0.16	0.74	0.95	1.05	0.82	0.87	0.85	0.77	0.74
P56377	AP-1 complex subunit sigma-2 OS=Homo sapiens GN=AP1S2 PE=1 SV=1 - [AP1S2_HUMAN]	15.92	1	2	2	4	-0.96	-0.79	-1.18	-1.01	-0.84	-0.67	-0.51	-0.34	-0.06	-0.22	-0.46	-0.29	0.51	0.51	0.72	0.77	0.99	0.11	0.33	0.74

P50851	Lipopolysaccharide-responsive and beige-like anchor protein OS=Homo sapiens GN=LRBA PE=1 SV=4 - [LRBA_HUMAN]	3.56	1	6	9	17	-0.83	-0.17	-1.00	-1.08	0.11	0.09	-0.20	-0.28	-0.07	-0.02	-0.05	-0.07	0.26	0.60	1.02	0.59	1.11	0.38	0.94	0.74
P69891	Hemoglobin subunit gamma-1 OS=Homo sapiens GN=HBG1 PE=1 SV=2 - [HBG1_HUMAN]	73.47	1	1	10	44	-1.32	-1.31	-1.64	-1.62	-0.66	-0.65	-0.97	-0.95	-0.72	-0.73	-0.88	-0.87	0.41	0.45	0.76	0.63	0.94	0.65	0.96	0.74
Q96S15	WD repeat-containing protein 24 OS=Homo sapiens GN=WDR24 PE=1 SV=1 - [WDR24_HUMAN]	1.63	1	1	1	1	-0.40	-0.18	-0.36	-0.14	-0.34	-0.12	0.31	0.53	0.49	0.28	0.69	0.90	0.77	1.10	1.05	0.71	0.68	0.05	0.01	0.73
P15498	Proto-oncogene vav OS=Homo sapiens GN=VAV1 PE=1 SV=4 - [VAV_HUMAN]	1.18	2	1	1	1	-0.41	0.21	-0.80	-0.18	0.22	0.83	-0.13	0.49	0.46	-0.15	0.26	0.87	0.33	0.67	1.06	0.29	0.68	0.61	1.01	0.73
Q9BXJ8	Transmembrane protein 120A OS=Homo sapiens GN=TMEM120A PE=2 SV=1 - [T120A_HUMAN]	11.95	1	3	3	5	-0.42	-0.55	-0.50	-0.66	0.42	0.22	0.23	0.01	-0.07	0.28	0.41	0.13	0.77	0.79	0.82	0.74	0.62	0.94	0.89	0.73
Q9BW66	Cyclin-dependent kinase 2-interacting protein OS=Homo sapiens GN=CINP PE=1 SV=1 - [CINP_HUMAN]	4.25	1	1	1	1	-1.67	-1.55	-1.14	-1.02	-0.16	-0.05	-0.47	-0.35	-0.39	-0.51	-0.39	-0.28	1.25	1.28	0.75	1.19	0.67	1.49	0.96	0.73
P07951	Tropomyosin beta chain OS=Homo sapiens GN=TPM2 PE=1 SV=1 - [TPM2_HUMAN]	42.61	1	5	17	213	-1.67	-1.47	-1.85	-1.72	-0.96	-0.80	-1.10	-0.92	-0.86	-1.02	-0.61	-0.58	0.59	1.06	1.17	0.70	1.00	0.90	1.44	0.73
Q9UL46	Proteasome activator complex subunit 2 OS=Homo sapiens GN=PSME2 PE=1 SV=4 - [PSME2_HUMAN]	12.97	1	3	3	4	-1.54	-1.04	-1.18	-0.68	-0.54	-0.05	-0.51	-0.27	-0.30	-0.74	-0.28	0.16	1.09	0.90	0.52	0.84	0.47	0.98	0.62	0.73

P05155	Plasma protease C1 inhibitor OS=Homo sapiens GN=SERPING1 PE=1 SV=2 - [IC1_HUMAN]	22.60	1	11	11	28	-1.43	-1.40	-1.24	-1.19	0.21	0.20	-0.67	-0.63	0.00	-0.02	0.58	0.64	0.76	2.09	1.78	1.45	1.19	1.71	1.37	0.73
O15116	U6 snRNA-associated Sm-like protein LSm1 OS=Homo sapiens GN=LSM1 PE=1 SV=1 - [LSM1_HUMAN]	26.32	1	2	2	3	-0.22	-0.44	-0.56	-0.78	1.23	1.00	0.11	-0.11	0.25	0.48	1.07	0.84	0.39	1.30	1.62	0.73	1.07	1.43	1.77	0.73
Q9NXX6	Non-structural maintenance of chromosomes element 4 homolog A OS=Homo sapiens GN=NSMCE4A PE=1 SV=2 - [NSE4A_HUMAN]	2.60	1	1	1	1	-1.06	-0.74	-0.85	-0.52	-1.17	-0.85	-0.18	0.14	-0.51	-0.83	-0.01	0.31	0.93	1.06	0.84	0.26	0.05	-0.12	-0.33	0.73
O75844	CAAX prenyl protease 1 homolog OS=Homo sapiens GN=ZMPSTE24 PE=1 SV=2 - [FACE1_HUMAN]	11.37	1	4	4	6	-0.87	-0.81	-0.30	-0.24	0.12	0.04	0.06	-0.01	-0.22	-0.14	-0.10	-0.17	0.84	1.21	0.63	0.85	0.28	0.90	0.82	0.73
Q9BUK0	Coiled-coil-helix-coiled-coil-helix domain-containing protein 7 OS=Homo sapiens GN=CHCHD7 PE=1 SV=1 - [CHCH7_HUMAN]	21.18	1	1	1	2	-0.53	-0.46	-0.83	-0.77	-0.11	-0.05	-0.17	-0.10	0.12	0.06	0.26	0.32	0.42	0.79	1.09	0.62	0.92	0.40	0.70	0.73
O14618	Copper chaperone for superoxide dismutase OS=Homo sapiens GN=CCS PE=1 SV=1 - [CCS_HUMAN]	5.47	1	2	2	7	-0.01	0.09	-0.16	-0.18	0.17	0.31	0.44	0.49	0.30	0.12	-0.35	-0.25	0.50	-0.12	-0.11	0.41	0.52	0.36	0.47	0.73
P46531	Neurogenic locus notch homolog protein 1 OS=Homo sapiens GN=NOTCH1 PE=1 SV=4 - [NOTC1_HUMAN]	1.02	1	2	2	4	-0.40	-0.36	-0.76	-0.72	-0.53	-0.50	-0.09	-0.06	-0.20	-0.23	0.22	0.26	0.36	0.63	0.98	0.20	0.56	-0.15	0.21	0.73

Q9BYM8	RanBP-type and C3HC4-type zinc finger-containing protein 1 OS=Homo sapiens GN=RBCK1 PE=1 SV=2 - [HOIL1_HUMAN]	9.22	1	3	3	7	-1.39	-1.45	-1.14	-1.15	-0.11	-0.03	-0.43	-0.48	-0.26	-0.21	-0.17	-0.23	0.47	0.69	0.92	0.91	0.92	0.92	1.01	0.73
Q9BUF5	Tubulin beta-6 chain OS=Homo sapiens GN=TUBB6 PE=1 SV=1 - [TBB6_HUMAN]	57.85	2	7	20	329	-1.01	-0.94	-1.21	-1.08	-0.05	-0.06	-0.55	-0.45	-0.26	-0.32	-0.12	-0.06	0.63	1.49	1.47	0.91	1.24	1.08	1.35	0.73
Q16568	Cocaine- and amphetamine regulated transcript protein OS=Homo sapiens GN=CARTPT PE=1 SV=1 - [CART_HUMAN]	13.79	1	1	1	3	0.40	0.42	0.16	0.19	0.96	0.98	0.83	0.85	1.21	1.19	1.85	1.87	0.49	1.46	1.69	0.83	1.06	0.54	0.78	0.73
P23919	Thymidylate kinase OS=Homo sapiens GN=DTYMK PE=1 SV=4 - [KTHY_HUMAN]	25.00	1	6	6	11	-1.41	-1.45	-1.47	-1.46	-0.31	-0.29	-0.71	-0.69	-0.59	-0.61	-0.27	-0.32	0.74	1.04	1.10	0.83	0.77	1.08	1.01	0.73
Q9NRY6	Phospholipid scramblase 3 OS=Homo sapiens GN=PLSCR3 PE=1 SV=2 - [PLS3_HUMAN]	9.49	1	2	2	3	-1.02	-1.27	-0.83	-1.08	0.76	0.51	-0.17	-0.42	0.30	0.55	0.95	0.70	0.91	1.98	1.78	1.60	1.41	1.76	1.57	0.72
Q5VVQ6	Ubiquitin thioesterase OTU1 OS=Homo sapiens GN=YOD1 PE=1 SV=1 - [OTU1_HUMAN]	15.80	1	5	5	6	-0.62	-0.70	-0.65	-0.73	0.26	0.17	0.01	-0.07	-0.06	0.02	-0.15	-0.24	0.68	0.47	0.50	0.67	0.70	0.86	0.89	0.72
Q5VZ89	DENN domain-containing protein 4C OS=Homo sapiens GN=DENND4C PE=1 SV=2 - [DEN4C_HUMAN]	5.14	1	6	6	10	-0.58	-0.68	-0.69	-0.78	0.33	0.23	-0.03	-0.12	-0.10	0.00	0.34	0.24	0.44	0.93	1.02	0.61	0.72	0.89	1.00	0.72
Q96CW5	Gamma-tubulin complex component 3 OS=Homo sapiens GN=TUBGCP3 PE=1 SV=2 - [GCP3_HUMAN]	6.95	1	4	4	6	-0.43	-0.63	-0.59	-0.86	0.23	-0.31	0.12	-0.33	-0.45	0.09	0.06	-0.25	0.61	0.39	0.62	0.24	0.45	0.64	0.60	0.72

P43353	Aldehyde dehydrogenase family 3 member B1 OS=Homo sapiens GN=ALDH3B1 PE=1 SV=1 - [AL3B1_HUMAN]	1.92	1	1	1	1	0.50	0.76	-0.71	-0.44	0.29	0.54	-0.05	0.21	0.30	0.05	0.17	0.42	-0.49	-0.33	0.87	-0.42	0.79	-0.23	0.98	0.72
P25311	Zinc-alpha-2-glycoprotein OS=Homo sapiens GN=AZGP1 PE=1 SV=2 - [ZA2G_HUMAN]	30.87	1	8	8	10	-0.93	-1.02	-1.17	-1.15	-0.54	-0.41	-0.47	-0.51	-0.64	-0.70	-0.54	-0.46	0.49	0.60	0.68	0.42	0.53	0.65	0.69	0.72
Q6PL24	Protein TMED8 OS=Homo sapiens GN=TMED8 PE=1 SV=1 - [TMED8_HUMAN]	11.08	1	3	3	4	-1.07	-1.14	-0.96	-1.03	0.17	0.09	-0.30	-0.37	-0.40	-0.31	-0.02	-0.10	0.82	1.05	0.93	0.78	0.68	1.22	1.11	0.72
P62805	Histone H4 OS=Homo sapiens GN=HIST1H4A PE=1 SV=2 - [H4_HUMAN]	52.43	1	7	7	183	-1.03	-1.04	-1.10	-1.05	-0.30	-0.40	-0.31	-0.40	-0.31	-0.31	-0.07	-0.11	0.69	0.97	0.99	0.81	0.92	0.67	0.71	0.72
P62341	Selenoprotein T OS=Homo sapiens GN=SELT PE=2 SV=2 - [SELT_HUMAN]	6.67	1	1	1	2	-0.36	-0.31	-0.89	-0.84	0.03	0.07	-0.24	-0.19	-0.30	-0.34	-0.18	-0.14	0.18	0.18	0.71	0.05	0.58	0.37	0.90	0.72
Q9UK41	Vacuolar protein sorting-associated protein 28 homolog OS=Homo sapiens GN=VPS28 PE=1 SV=1 - [VPS28_HUMAN]	37.56	1	7	7	9	-0.27	-0.53	-0.40	-0.60	0.08	0.12	0.07	0.12	0.08	0.04	0.25	0.24	0.71	0.81	0.84	0.22	0.38	0.35	0.47	0.72
Q6WKZ4	Rab11 family-interacting protein 1 OS=Homo sapiens GN=RAB11FIP1 PE=1 SV=3 - [RFIP1_HUMAN]	2.65	1	1	2	4	0.12	-0.39	-0.70	-1.21	-0.07	-0.58	-0.04	-0.56	-0.44	0.08	0.18	-0.33	-0.11	0.06	0.88	-0.02	0.81	-0.21	0.61	0.72
Q16512	Serine/threonine-protein kinase N1 OS=Homo sapiens GN=PKN1 PE=1 SV=2 - [PKN1_HUMAN]	7.96	1	4	5	7	-1.08	-1.11	-0.65	-0.67	0.13	0.10	0.01	-0.02	0.02	0.06	-0.16	-0.19	1.14	0.93	0.49	1.17	0.73	1.19	0.76	0.72

Q8N8S7	Protein enabled homolog OS=Homo sapiens GN=ENAH PE=1 SV=2 - [ENAH_HUMAN]	29.78	1	13	13	24	-1.23	-1.42	-1.36	-1.33	-0.26	-0.22	-0.63	-0.61	-0.54	-0.50	-0.47	-0.47	0.58	0.64	0.78	0.93	0.79	0.99	1.10	0.72
Q92608	Dedicator of cytokinesis protein 2 OS=Homo sapiens GN=DOCK2 PE=1 SV=2 - [DOCK2_HUMAN]	2.51	1	2	3	4	-0.50	-0.46	-0.53	-0.48	0.48	0.52	0.13	0.17	0.21	0.17	-0.02	0.02	0.68	0.49	0.51	0.70	0.73	0.96	0.99	0.72
Q9Y5X1	Sorting nexin-9 OS=Homo sapiens GN=SNX9 PE=1 SV=1 - [SNX9_HUMAN]	21.01	1	7	7	10	-1.05	-1.15	-1.32	-1.37	0.11	-0.01	-0.38	-0.43	-0.37	-0.25	-0.11	-0.19	0.49	0.96	1.18	0.82	1.16	1.13	1.41	0.72
P33121	Long-chain-fatty-acid--CoA ligase 1 OS=Homo sapiens GN=ACSL1 PE=1 SV=1 - [ACSL1_HUMAN]	26.07	1	13	15	21	-0.59	-0.48	-0.14	-0.24	0.91	0.61	0.48	0.44	0.38	0.56	0.27	0.18	0.84	0.65	0.36	0.87	0.74	0.84	0.73	0.72
Q6UXB8	Peptidase inhibitor 16 OS=Homo sapiens GN=PI16 PE=1 SV=1 - [PI16_HUMAN]	8.21	1	4	4	7	-0.33	-0.57	-0.07	-0.31	0.82	0.72	0.58	0.34	0.64	0.79	1.08	0.98	0.97	1.48	1.36	1.07	0.87	1.05	0.90	0.71
Q99081	Transcription factor 12 OS=Homo sapiens GN=TCF12 PE=1 SV=1 - [HTF4_HUMAN]	2.79	1	1	1	1	0.00	-0.27	-0.58	-0.85	0.40	0.12	0.07	-0.20	-0.48	-0.20	0.59	0.31	0.13	0.59	1.17	-0.17	0.41	0.38	0.96	0.71
Q04771	Activin receptor type-1 OS=Homo sapiens GN=ACVR1 PE=1 SV=1 - [ACVR1_HUMAN]	4.91	1	2	2	2	-0.74	-0.89	-0.93	-1.08	0.16	0.01	-0.28	-0.43	-0.21	-0.05	-0.04	-0.20	0.51	0.70	0.89	0.72	0.91	0.89	1.08	0.71
Q8TDX7	Serine/threonine-protein kinase Nek7 OS=Homo sapiens GN=NEK7 PE=1 SV=1 - [NEK7_HUMAN]	22.52	2	6	6	12	-1.03	-1.18	-1.14	-1.13	0.05	-0.04	-0.30	-0.38	-0.36	-0.24	-0.12	-0.20	0.80	0.91	0.87	1.03	0.90	1.14	1.01	0.71
P12814	Alpha-actinin-1 OS=Homo sapiens GN=ACTN1 PE=1 SV=2 - [ACTN1_HUMAN]	63.00	1	29	47	359	-1.66	-1.68	-1.69	-1.70	-0.49	-0.60	-1.05	-1.08	-0.77	-0.72	-0.43	-0.41	0.71	1.34	1.24	0.98	0.96	1.18	1.04	0.71

P58107	Epiplakin OS=Homo sapiens GN=EPPK1 PE=1 SV=2 - [EPIPL_HUMAN]	18.82	1	9	16	75	-0.29	-0.31	-1.04	-1.14	0.01	0.03	-0.52	-0.59	-0.04	0.06	0.29	0.25	0.03	0.83	1.18	0.52	0.85	-0.07	0.84	0.71
P68133	Actin, alpha skeletal muscle OS=Homo sapiens GN=ACTA1 PE=1 SV=1 - [ACTS_HUMAN]	71.09	1	2	26	1227	-0.48	-1.20	-0.83	-0.94	-0.31	-0.43	-0.74	-1.01	-0.93	-0.61	0.18	-0.54	0.69	0.67	0.46	0.67	0.68	0.85	0.86	0.71
P29466	Caspase-1 OS=Homo sapiens GN=CASP1 PE=1 SV=1 - [CASP1_HUMAN]	17.33	2	6	6	7	-0.10	-0.22	-0.37	-0.55	0.61	0.37	0.16	-0.01	0.19	0.26	0.08	0.24	0.73	0.28	0.48	0.73	0.89	0.73	1.01	0.71
O94901	SUN domain-containing protein 1 OS=Homo sapiens GN=SUN1 PE=1 SV=3 - [SUN1_HUMAN]	16.75	1	11	12	23	-1.50	-1.43	-1.62	-1.48	-0.77	-0.72	-0.88	-0.81	-0.78	-0.77	-0.64	-0.63	0.69	0.95	0.91	0.77	0.99	0.82	0.86	0.71
Q9UHJ6	Sedoheptulokinase OS=Homo sapiens GN=SHPK PE=1 SV=3 - [SHPK_HUMAN]	14.23	1	5	5	10	-0.31	0.10	-0.33	-0.01	0.34	0.37	0.18	0.59	0.47	0.29	-0.16	0.13	0.77	0.09	0.39	0.63	0.60	0.61	0.47	0.71
Q9H330	Transmembrane protein 245 OS=Homo sapiens GN=TMEM245 PE=1 SV=2 - [TM245_HUMAN]	5.60	1	4	4	10	-0.38	-0.37	-0.71	-0.62	0.19	0.16	-0.07	-0.19	0.02	0.12	0.29	0.22	0.14	0.58	0.84	0.39	0.69	0.55	0.88	0.71
Q07890	Son of sevenless homolog 2 OS=Homo sapiens GN=SOS2 PE=1 SV=2 - [SOS2_HUMAN]	1.50	1	1	2	2	-0.54	-0.13	-1.10	-0.68	-0.15	0.26	-0.45	-0.04	-0.21	-0.62	0.19	0.60	0.15	0.74	1.28	-0.05	0.51	0.37	0.93	0.71
Q9UBY9	Heat shock protein beta-7 OS=Homo sapiens GN=HSPB7 PE=1 SV=1 - [HSPB7_HUMAN]	4.71	1	1	1	2	-1.31	-1.47	-1.87	-2.04	-0.54	-0.71	-1.23	-1.39	-0.99	-0.83	-1.10	-1.27	0.14	0.21	0.77	0.52	1.08	0.75	1.31	0.71
O75618	Death effector domain-containing protein OS=Homo sapiens GN=DEDD PE=1 SV=1 - [DEDD_HUMAN]	5.35	2	2	2	2	-1.21	-1.26	-1.00	-0.72	0.09	0.36	-0.35	-0.08	0.40	0.13	0.13	0.40	1.12	1.27	1.13	1.39	1.16	1.37	1.07	0.71

Q9UQE7	Structural maintenance of chromosomes protein 3 OS=Homo sapiens GN=SMC3 PE=1 SV=2 - [SMC3_HUMAN]	24.49	1	26	26	38	-0.86	-0.86	-0.78	-0.76	-0.05	-0.05	-0.10	-0.15	-0.16	-0.20	-0.09	-0.08	0.79	0.74	0.66	0.64	0.58	0.71	0.70	0.71
O15173	Membrane-associated progesterone receptor component 2 OS=Homo sapiens GN=PGRMC2 PE=1 SV=1 - [PGRC2_HUMAN]	44.39	1	10	12	29	-1.03	-1.01	-0.99	-0.94	-0.10	0.00	-0.33	-0.45	-0.26	-0.21	-0.15	-0.08	0.35	0.84	0.93	0.62	0.73	0.72	0.80	0.71
P09668	Pro-cathepsin H OS=Homo sapiens GN=CTSH PE=1 SV=4 - [CATH_HUMAN]	10.75	1	3	3	4	-0.91	-0.33	-0.78	-0.36	-0.19	0.22	-0.14	0.28	0.21	-0.20	0.19	0.60	0.78	0.92	0.97	0.42	0.61	0.42	0.57	0.70
Q96LW7	Bcl10-interacting CARD protein OS=Homo sapiens GN=C9orf89 PE=1 SV=1 - [BINCA_HUMAN]	10.96	1	2	2	4	-0.80	-0.65	-0.76	-0.61	-0.22	-0.07	-0.12	0.03	-0.13	-0.28	-0.28	-0.13	0.73	0.53	0.49	0.56	0.52	0.57	0.53	0.70
Q2M1Z3	Rho GTPase-activating protein 31 OS=Homo sapiens GN=ARHGAP31 PE=1 SV=2 - [RHG31_HUMAN]	1.80	1	2	2	3	-0.84	-0.95	-0.61	-0.71	0.18	0.07	0.04	-0.07	-0.08	0.04	-0.05	-0.17	0.93	0.79	0.55	0.91	0.68	1.00	0.77	0.70
Q9H223	EH domain-containing protein 4 OS=Homo sapiens GN=EHD4 PE=1 SV=1 - [EHD4_HUMAN]	29.21	2	10	14	29	-0.63	-0.77	-0.84	-0.64	0.04	0.10	-0.15	-0.21	-0.29	-0.17	-0.16	-0.06	0.69	0.55	0.59	0.68	0.56	0.68	0.66	0.70
Q8IZH2	5'-3' exoribonuclease 1 OS=Homo sapiens GN=XRN1 PE=1 SV=1 - [XRN1_HUMAN]	2.64	1	4	4	4	-0.71	-0.48	-1.11	-0.88	0.07	0.30	-0.41	-0.27	0.05	-0.01	0.03	0.09	0.29	0.65	0.96	0.59	0.95	0.76	1.17	0.70
Q9BUP0	EF-hand domain-containing protein D1 OS=Homo sapiens GN=EFHD1 PE=1 SV=1 - [EFHD1_HUMAN]	48.54	1	8	10	22	-1.79	-1.50	-1.84	-1.86	-1.02	-0.98	-1.27	-1.22	-0.92	-0.94	-0.87	-0.80	0.52	0.83	0.99	0.84	0.86	0.62	0.81	0.70

Q13496	Myotubularin OS=Homo sapiens GN=MTM1 PE=1 SV=2 - [MTM1_HUMAN]	6.14	1	4	4	5	-0.40	-0.66	-0.43	-0.69	0.27	0.00	0.21	-0.05	-0.32	-0.06	0.33	0.06	0.66	0.73	0.76	0.37	0.41	0.65	0.68	0.70
Q9UGJ0	5'-AMP-activated protein kinase subunit gamma-2 OS=Homo sapiens GN=PRKAG2 PE=1 SV=1 - [AAKG2_HUMAN]	14.41	1	5	6	11	-0.87	-0.73	-0.65	-0.45	0.22	0.46	-0.01	0.21	0.54	0.31	0.36	0.57	1.05	1.47	1.12	1.20	0.93	1.07	0.73	0.70
Q8N2H4	Protein SYS1 homolog OS=Homo sapiens GN=SYS1 PE=1 SV=1 - [SYS1_HUMAN]	14.10	1	1	1	1	-3.77	-3.54	-2.61	-2.38	-1.81	-1.58	-1.98	-1.74	-1.62	-1.85	-1.92	-1.69	1.85	1.85	0.69	1.95	0.79	1.95	0.79	0.70
Q9P266	Junctional protein associated with coronary artery disease OS=Homo sapiens GN=KIAA1462 PE=1 SV=3 - [JCAD_HUMAN]	30.91	1	29	29	48	-0.80	-0.87	-1.09	-1.24	-0.02	0.01	-0.46	-0.62	-0.45	-0.35	0.02	-0.10	0.64	1.00	1.18	0.93	0.78	1.23	1.19	0.70
Q96DR8	Mucin-like protein 1 OS=Homo sapiens GN=MUCL1 PE=1 SV=1 - [MUCL1_HUMAN]	10.00	1	1	1	1	2.79	2.64	-0.99	-1.14	0.07	-0.09	-0.36	-0.51	-0.46	-0.30	0.49	0.33	-3.09	-2.30	1.48	-3.07	0.72	-2.74	1.04	0.70
P14770	Platelet glycoprotein IX OS=Homo sapiens GN=GP9 PE=1 SV=3 - [GPIX_HUMAN]	7.34	1	1	1	2	-0.21	-0.06	-0.54	-0.39	0.49	0.63	0.10	0.25	0.47	0.33	0.51	0.65	0.36	0.72	1.04	0.57	0.90	0.68	1.01	0.70
Q9Y4G6	Talin-2 OS=Homo sapiens GN=TLN2 PE=1 SV=4 - [TLN2_HUMAN]	46.89	3	73	96	249	-0.63	-0.58	-0.62	-0.58	0.02	0.07	-0.03	0.04	0.04	-0.04	-0.15	-0.14	0.62	0.50	0.51	0.55	0.56	0.69	0.65	0.70
Q9Y6P5	Sestrin-1 OS=Homo sapiens GN=SESN1 PE=1 SV=2 - [SESN1_HUMAN]	5.49	1	2	2	2	-0.06	-0.19	-0.59	-0.72	0.36	0.23	0.05	-0.08	0.08	0.21	0.00	-0.13	0.16	0.07	0.59	0.30	0.83	0.41	0.94	0.70
P27797	Calreticulin OS=Homo sapiens GN=CALR PE=1 SV=1 - [CALR_HUMAN]	43.17	1	15	15	79	-1.04	-1.08	-0.83	-0.81	0.02	0.05	-0.19	-0.16	-0.13	-0.12	-0.08	-0.06	0.94	0.91	0.72	0.90	0.70	1.04	0.84	0.70

P28676	Grancalcin OS=Homo sapiens GN=GCA PE=1 SV=2 - [GRAN_HUMAN]	22.12	1	4	4	9	-0.30	-0.20	-0.37	-0.18	-0.19	-0.16	0.46	0.62	0.47	0.34	0.44	0.63	0.57	0.48	0.51	0.40	0.45	-0.29	0.01	0.70
A8MUH7	Putative PDZ domain-containing protein 1P OS=Homo sapiens GN=PDZK1P1 PE=5 SV=2 - [PDZ1P_HUMAN]	4.73	2	1	1	1	-0.61	-0.51	-0.65	-0.55	-0.31	-0.21	-0.02	0.08	0.04	-0.06	-0.37	-0.27	0.64	0.25	0.28	0.58	0.63	0.28	0.33	0.70
Q96MX3	Zinc finger protein 48 OS=Homo sapiens GN=ZNF48 PE=1 SV=2 - [ZNF48_HUMAN]	1.62	1	1	1	2	-1.22	-1.06	-1.64	-1.47	-0.80	-0.64	-1.01	-0.84	-0.31	-0.47	-0.27	-0.12	0.27	0.95	1.36	0.78	1.20	0.41	0.82	0.70
Q658Y4	Protein FAM91A1 OS=Homo sapiens GN=FAM91A1 PE=1 SV=3 - [F91A1_HUMAN]	3.82	1	3	3	4	-1.09	-1.19	-0.96	-1.06	0.01	-0.09	-0.33	-0.43	0.11	0.21	0.50	0.39	0.82	1.60	1.46	1.34	1.21	1.09	0.96	0.69
P62140	Serine/threonine-protein phosphatase PP1-beta catalytic subunit OS=Homo sapiens GN=PPP1CB PE=1 SV=3 - [PP1B_HUMAN]	47.71	1	4	11	31	-0.31	-0.46	-0.69	-0.67	-0.02	-0.19	-0.06	-0.23	-0.24	-0.13	-0.18	-0.30	0.31	0.20	0.29	0.25	0.21	0.25	0.28	0.69
O94763	Unconventional prefoldin RPB5 interactor 1 OS=Homo sapiens GN=URI1 PE=1 SV=3 - [RMP_HUMAN]	4.49	1	1	1	3	-0.17	-0.40	-0.56	-0.79	-0.21	-0.44	0.07	-0.16	-0.03	0.20	-0.04	-0.27	0.29	0.14	0.52	0.40	0.80	-0.05	0.34	0.69
Q13275	Semaphorin-3F OS=Homo sapiens GN=SEMA3F PE=2 SV=2 - [SEM3F_HUMAN]	3.44	1	3	3	3	-0.48	0.07	-1.12	-0.57	0.93	1.48	-0.49	0.06	0.62	0.08	-0.59	-0.04	0.04	-0.10	0.53	0.59	1.23	1.40	2.03	0.69
P60842	Eukaryotic initiation factor 4A-I OS=Homo sapiens GN=EIF4A1 PE=1 SV=1 - [IF4A1_HUMAN]	47.04	1	8	17	48	-0.62	-0.54	-0.58	-0.73	0.45	0.32	-0.02	0.05	0.01	0.02	0.35	0.20	0.86	1.08	0.96	0.57	0.63	0.89	0.88	0.69

P21802	Fibroblast growth factor receptor 2 OS=Homo sapiens GN=FGFR2 PE=1 SV=1 - [FGFR2_HUMAN]	4.14	12	1	3	7	0.74	-0.44	0.17	-1.01	1.11	-0.08	0.80	-0.38	-0.38	0.80	0.50	-0.69	0.11	-0.24	0.33	0.09	0.66	0.35	0.92	0.69
O14966	Ras-related protein Rab-7L1 OS=Homo sapiens GN=RAB7L1 PE=1 SV=1 - [RAB7L_HUMAN]	20.69	2	2	3	5	-0.77	-0.43	-0.94	-0.61	-0.21	0.12	-0.32	0.02	0.20	-0.13	-0.33	0.00	0.51	0.44	0.61	0.67	0.85	0.54	0.72	0.69
P17655	Calpain-2 catalytic subunit OS=Homo sapiens GN=CAPN2 PE=1 SV=6 - [CAN2_HUMAN]	42.43	1	22	22	45	-0.78	-0.71	-1.02	-0.91	-0.16	-0.06	-0.34	-0.25	-0.21	-0.15	0.03	0.04	0.52	0.70	0.95	0.55	0.85	0.65	0.82	0.69
Q6ZTI6	Protein FAM101A OS=Homo sapiens GN=FAM101A PE=2 SV=3 - [F101A_HUMAN]	12.50	1	2	2	3	-1.08	-1.13	-1.15	-1.20	0.28	0.22	-0.52	-0.58	0.08	0.14	0.77	0.71	0.61	1.85	1.91	1.24	1.32	1.34	1.41	0.69
O14841	5-oxoprolinase OS=Homo sapiens GN=OPLAH PE=1 SV=3 - [OPLA_HUMAN]	13.35	1	10	10	16	0.15	0.16	-0.11	-0.11	0.75	0.66	0.58	0.48	0.49	0.49	0.32	0.20	0.21	0.20	0.36	0.40	0.74	0.50	1.04	0.69
Q9Y6G5	COMM domain-containing protein 10 OS=Homo sapiens GN=COMMD10 PE=1 SV=1 - [COMDA_HUMAN]	14.85	1	3	3	5	-0.62	-0.76	-0.72	-0.86	0.13	-0.01	-0.09	-0.23	-0.22	-0.07	0.25	0.11	0.58	0.88	0.97	0.58	0.68	0.73	0.83	0.69
P19474	E3 ubiquitin-protein ligase TRIM21 OS=Homo sapiens GN=TRIM21 PE=1 SV=1 - [RO52_HUMAN]	14.11	1	6	7	10	-0.67	-0.66	-0.79	-0.81	-0.05	-0.11	-0.27	-0.26	0.17	0.28	0.28	0.19	0.46	0.96	1.17	0.79	1.02	0.53	0.69	0.69
Q27J81	Inverted formin-2 OS=Homo sapiens GN=INF2 PE=1 SV=2 - [INF2_HUMAN]	26.42	1	23	23	43	-1.05	-0.93	-1.09	-1.01	-0.18	-0.12	-0.41	-0.38	-0.20	-0.26	-0.29	-0.23	0.74	0.72	0.68	0.71	0.74	0.92	0.84	0.69

O15258	Protein RER1 OS=Homo sapiens GN=RER1 PE=1 SV=1 - [RER1_HUMAN]	13.27	1	2	2	3	-0.94	-0.95	-0.76	-0.77	-0.35	-0.37	-0.14	-0.15	-0.51	-0.50	-0.17	-0.18	0.86	0.78	0.60	0.48	0.30	0.57	0.39	0.69
Q86UP2	Kinectin OS=Homo sapiens GN=KTN1 PE=1 SV=1 - [KTN1_HUMAN]	43.18	1	50	51	95	-0.79	-0.69	-0.49	-0.48	0.10	0.12	0.14	0.18	0.05	-0.03	0.06	0.05	0.97	0.92	0.56	0.90	0.58	0.76	0.55	0.69
Q9UKK9	ADP-sugar pyrophosphatase OS=Homo sapiens GN=NUDT5 PE=1 SV=1 - [NUDT5_HUMAN]	27.40	1	6	6	12	-0.53	-0.64	-0.45	-0.39	0.14	0.08	0.12	0.23	0.04	-0.05	-0.13	0.10	0.88	0.53	0.33	0.64	0.48	0.67	0.50	0.69
P15531	Nucleoside diphosphate kinase A OS=Homo sapiens GN=NME1 PE=1 SV=1 - [NDKA_HUMAN]	48.68	1	3	8	20	-0.49	-0.35	-0.36	-0.24	0.15	0.22	0.20	0.40	0.11	-0.06	-0.30	-0.16	0.75	0.20	0.06	0.44	0.33	0.50	0.47	0.69
P42330	Aldo-keto reductase family 1 member C3 OS=Homo sapiens GN=AKR1C3 PE=1 SV=4 - [AK1C3_HUMAN]	52.01	3	5	13	30	0.40	-0.13	0.26	-0.49	1.05	0.81	1.09	0.50	0.70	1.45	1.03	0.31	0.69	0.58	0.51	0.94	1.23	0.44	0.71	0.69
Q96JB5	CDK5 regulatory subunit-associated protein 3 OS=Homo sapiens GN=CDK5RAP3 PE=1 SV=2 - [CK5P3_HUMAN]	19.96	4	10	11	23	-0.86	-0.83	-0.81	-0.83	0.03	0.04	-0.17	-0.13	0.04	0.03	0.16	0.21	0.76	1.06	1.00	0.97	0.86	0.86	0.81	0.69
P55212	Caspase-6 OS=Homo sapiens GN=CASP6 PE=1 SV=2 - [CASP6_HUMAN]	12.97	1	2	2	4	-0.84	-0.68	-1.46	-1.30	-0.40	-0.25	-0.84	-0.68	-0.61	-0.76	-0.18	-0.03	0.05	0.66	1.28	0.10	0.73	0.42	1.04	0.68
P23743	Diacylglycerol kinase alpha OS=Homo sapiens GN=DGKA PE=1 SV=3 - [DGKA_HUMAN]	14.69	1	7	7	10	-0.46	-0.78	-0.68	-0.86	-0.09	-0.19	-0.19	-0.29	-0.45	-0.15	-0.40	-0.52	0.54	0.28	0.40	0.55	0.61	0.50	0.71	0.68

P84022	Mothers against decapentaplegic homolog 3 OS=Homo sapiens GN=SMAD3 PE=1 SV=1 - [SMAD3_HUMAN]	9.65	3	4	4	7	-0.40	-0.62	-0.50	-0.72	0.34	0.12	0.12	-0.10	-0.04	0.19	0.06	-0.16	0.58	0.47	0.56	0.62	0.72	0.73	0.82	0.68
P08651	Nuclear factor 1 C-type OS=Homo sapiens GN=NFIC PE=1 SV=2 - [NFIC_HUMAN]	6.69	1	1	3	9	-1.40	-1.21	-1.87	-1.69	-0.85	-0.66	-1.25	-1.07	-1.10	-1.28	-0.72	-0.54	0.20	0.68	1.15	0.15	0.62	0.54	1.01	0.68
Q96P48	Arf-GAP with Rho-GAP domain, ANK repeat and PH domain-containing protein 1 OS=Homo sapiens GN=ARAP1 PE=1 SV=3 - [ARAP1_HUMAN]	4.83	1	5	5	13	-0.56	-0.63	-0.57	-0.63	0.21	0.12	0.13	0.08	-0.11	-0.07	0.00	-0.06	0.69	0.69	0.62	0.58	0.71	0.81	0.93	0.68
Q9Y5Q8	General transcription factor 3C polypeptide 5 OS=Homo sapiens GN=GTF3C5 PE=1 SV=2 - [TF3C5_HUMAN]	7.90	1	3	3	6	-1.39	-1.05	-1.10	-0.76	-0.39	-0.05	-0.48	-0.14	-0.25	-0.58	-0.37	-0.03	0.96	1.03	0.73	0.83	0.55	0.98	0.69	0.68
O60568	Procollagen-lysine,2-oxoglutarate 5-dioxygenase 3 OS=Homo sapiens GN=PLOD3 PE=1 SV=1 - [PLOD3_HUMAN]	6.64	1	3	4	5	-1.11	-1.14	-1.57	-1.59	0.09	0.08	-0.75	-0.68	-0.04	-0.11	-0.14	-0.36	0.22	0.55	0.99	1.01	1.46	0.74	1.19	0.68
Q86VN1	Vacuolar protein-sorting-associated protein 36 OS=Homo sapiens GN=VPS36 PE=1 SV=1 - [VPS36_HUMAN]	18.91	1	6	7	9	-0.34	-0.42	-0.73	-0.81	0.33	0.07	-0.14	-0.20	-0.11	-0.05	0.12	0.03	0.17	0.50	0.77	0.36	0.70	0.42	1.04	0.68
Q9HA77	Probable cysteine--tRNA ligase, mitochondrial OS=Homo sapiens GN=CARS2 PE=1 SV=1 - [SYCM_HUMAN]	9.04	1	5	5	9	-0.70	-0.59	-0.84	-0.71	-0.10	-0.21	-0.40	-0.34	-0.02	-0.01	0.04	-0.02	0.55	0.68	0.91	0.75	0.97	0.59	0.81	0.68

P29474	Nitric oxide synthase, endothelial OS=Homo sapiens GN=NOS3 PE=1 SV=3 - [NOS3_HUMAN]	10.89	1	8	9	14	-1.76	-1.92	-2.09	-2.05	-0.86	-0.92	-1.47	-1.47	-1.06	-1.00	-0.92	-0.95	0.53	0.72	1.17	0.85	1.15	1.22	1.46	0.68
P61009	Signal peptidase complex subunit 3 OS=Homo sapiens GN=SPCS3 PE=1 SV=1 - [SPCS3_HUMAN]	15.56	1	3	3	6	-1.08	-0.70	-1.50	-0.94	0.08	0.37	-0.53	-0.17	0.06	-0.32	-0.22	0.16	0.54	0.94	1.13	0.79	0.93	1.06	1.15	0.68
P50607	Tubby protein homolog OS=Homo sapiens GN=TUB PE=1 SV=1 - [TUB_HUMAN]	17.79	2	7	7	10	-0.48	-0.78	-0.65	-0.66	-0.06	-0.12	-0.04	-0.10	-0.08	-0.03	-0.03	-0.04	0.69	0.41	0.54	0.58	0.74	0.55	0.68	0.68
Q3SY69	Mitochondrial 10-formyltetrahydrofolate dehydrogenase OS=Homo sapiens GN=ALDH1L2 PE=1 SV=2 - [AL1L2_HUMAN]	11.70	1	5	10	14	-1.24	-0.95	-1.67	-1.37	-0.82	-0.53	-0.91	-0.75	-0.65	-0.70	-0.41	-0.23	0.25	0.83	1.25	0.34	0.76	0.40	0.83	0.68
P20810	Calpastatin OS=Homo sapiens GN=CAST PE=1 SV=4 - [ICAL_HUMAN]	26.55	1	10	10	25	-0.86	-1.17	-0.93	-1.23	-0.38	-0.61	-0.37	-0.48	-0.74	-0.54	0.03	-0.29	0.35	0.70	0.82	0.42	0.55	0.53	0.75	0.68
P12004	Proliferating cell nuclear antigen OS=Homo sapiens GN=PCNA PE=1 SV=1 - [PCNA_HUMAN]	22.22	1	5	5	7	-0.56	-0.61	-0.59	-0.50	-0.02	0.03	0.11	0.01	0.07	-0.01	-0.22	-0.35	0.65	0.21	0.11	0.49	0.46	0.49	0.65	0.68
Q6UXV4	Apolipoprotein O-like OS=Homo sapiens GN=APOOL PE=1 SV=1 - [APOOL_HUMAN]	40.67	1	8	8	14	-0.52	-0.87	-0.45	-0.73	0.03	-0.46	-0.11	-0.13	-0.15	-0.05	-0.24	-0.19	0.78	0.50	0.44	0.49	0.38	0.50	0.48	0.68
Q9H3P7	Golgi resident protein GCP60 OS=Homo sapiens GN=ACBD3 PE=1 SV=4 - [GCP60_HUMAN]	13.45	1	3	3	4	-0.43	-0.82	-0.75	-1.14	0.12	-0.28	-0.14	-0.53	-0.32	0.08	0.47	0.07	0.35	0.91	1.22	0.54	0.86	0.53	0.85	0.68

Q9NSY1	BMP-2-inducible protein kinase OS=Homo sapiens GN=BMP2K PE=1 SV=2 - [BMP2K_HUMAN]	5.25	1	5	6	8	-0.56	-0.51	-0.57	-0.52	0.14	0.18	0.10	0.14	0.14	0.10	0.25	0.29	0.70	0.85	0.82	0.68	0.70	0.89	0.78	0.67
P29353	SHC-transforming protein 1 OS=Homo sapiens GN=SHC1 PE=1 SV=4 - [SHC1_HUMAN]	2.23	1	1	1	1	-0.67	-0.65	-0.74	-0.73	-0.02	-0.02	-0.13	-0.12	-0.18	-0.19	-0.13	-0.12	0.59	0.54	0.61	0.51	0.59	0.63	0.70	0.67
Q99536	Synaptic vesicle membrane protein VAT-1 homolog OS=Homo sapiens GN=VAT1 PE=1 SV=2 - [VAT1_HUMAN]	47.33	1	11	11	30	-1.25	-1.20	-1.17	-1.28	-0.14	-0.01	-0.48	-0.54	-0.35	-0.32	-0.21	-0.22	0.70	1.13	0.90	1.01	0.81	1.14	1.02	0.67
A0M8Q6	Ig lambda-7 chain C region OS=Homo sapiens GN=IGLC7 PE=1 SV=2 - [LAC7_HUMAN]	46.23	1	1	4	28	-1.15	-1.37	-0.71	-0.93	0.57	0.34	-0.10	-0.32	0.63	0.87	1.76	1.53	1.10	2.91	2.46	2.04	1.60	1.69	1.25	0.67
Q14697	Neutral alpha glucosidase AB OS=Homo sapiens GN=GANAB PE=1 SV=3 - [GANAB_HUMAN]	31.46	1	24	24	69	-0.85	-0.80	-1.00	-0.87	-0.02	0.05	-0.26	-0.18	-0.13	-0.19	-0.07	0.03	0.63	0.80	0.87	0.70	0.79	0.82	0.83	0.67
Q9H0X9	Oxysterol-binding protein-related protein 5 OS=Homo sapiens GN=OSBPL5 PE=1 SV=1 - [OSBL5_HUMAN]	5.35	1	2	3	4	-1.58	-1.69	-1.71	-1.82	-0.70	-0.82	-1.10	-1.21	-0.81	-0.69	-0.85	-0.96	0.53	0.74	0.86	0.92	1.05	0.87	0.99	0.67
Q92954	Proteoglycan 4 OS=Homo sapiens GN=PRG4 PE=1 SV=2 - [PRG4_HUMAN]	1.57	1	2	2	3	-0.37	-0.37	-0.68	-0.67	0.50	0.50	-0.07	-0.07	0.12	0.13	-0.36	-0.37	0.35	0.01	0.31	0.52	0.84	0.85	1.16	0.67
Q96BW1	Uracil phosphoribosyltransferase homolog OS=Homo sapiens GN=UPRT PE=2 SV=1 - [UPP_HUMAN]	3.56	1	1	1	1	-0.34	0.08	-0.74	-0.31	0.21	0.63	-0.14	0.29	1.42	1.00	0.02	0.44	0.26	0.37	0.76	1.37	1.77	0.53	0.93	0.67

Q12913	Receptor-type tyrosine-protein phosphatase eta OS=Homo sapiens GN=PTPRJ PE=1 SV=3 - [PTPRJ_HUMAN]	3.22	1	3	3	5	-0.32	-0.41	-0.39	-0.48	0.13	0.04	0.21	0.12	0.10	0.19	0.19	0.10	0.59	0.52	0.59	0.55	0.62	0.44	0.50	0.67
Q8IXB1	DnaJ homolog subfamily C member 10 OS=Homo sapiens GN=DNAJC10 PE=1 SV=2 - [DJC10_HUMAN]	7.57	1	4	5	6	-0.42	-0.41	-0.63	-0.61	0.40	0.41	-0.02	-0.01	-0.03	-0.04	0.33	0.34	0.45	0.76	0.96	0.41	0.62	0.81	1.01	0.67
Q13103	Secreted phosphoprotein 24 OS=Homo sapiens GN=SPP2 PE=1 SV=1 - [SPP24_HUMAN]	5.69	1	1	1	4	-0.54	-0.64	-0.74	-0.84	0.37	0.27	-0.13	-0.23	-0.04	0.07	-0.30	-0.40	0.46	0.25	0.44	0.64	0.84	0.90	1.09	0.67
Q92575	UBX domain-containing protein 4 OS=Homo sapiens GN=UBXN4 PE=1 SV=2 - [UBXN4_HUMAN]	9.45	1	4	4	6	-1.21	-0.87	-1.32	-0.96	-0.66	-0.33	-0.72	-0.38	-0.52	-0.46	-0.40	-0.06	0.65	0.82	0.93	0.41	0.41	0.53	0.64	0.67
O15061	Synemin OS=Homo sapiens GN=SYNM PE=1 SV=2 - [SYNEM_HUMAN]	23.07	1	30	30	55	-0.98	-1.01	-1.04	-1.03	-0.16	-0.17	-0.48	-0.48	-0.32	-0.33	-0.02	-0.01	0.68	1.12	1.02	0.81	0.77	0.87	0.85	0.67
P0C0S5	Histone H2A.Z OS=Homo sapiens GN=H2AFZ PE=1 SV=2 - [H2AZ_HUMAN]	53.91	2	3	5	61	-1.27	-1.10	-1.14	-0.99	-0.55	-0.53	-0.54	-0.35	-0.58	-0.64	-0.42	-0.43	0.75	0.90	0.77	0.57	0.39	0.53	0.36	0.66
P15529	Membrane cofactor protein OS=Homo sapiens GN=CD46 PE=1 SV=3 - [MCP_HUMAN]	6.38	1	3	3	5	-1.08	-0.84	-0.81	-0.57	-0.19	0.05	-0.21	0.04	-0.03	-0.27	-0.05	0.18	0.92	1.03	0.75	0.84	0.58	0.87	0.60	0.66
Q08379	Golgin subfamily A member 2 OS=Homo sapiens GN=GOLGA2 PE=1 SV=3 - [GOGA2_HUMAN]	10.68	1	7	7	15	-0.79	-0.89	-0.80	-0.73	0.00	-0.06	-0.20	-0.23	-0.19	-0.05	0.26	-0.10	0.76	0.80	0.63	0.74	0.59	0.92	0.67	0.66

Q14258	E3 ubiquitin/ISG15 ligase TRIM25 OS=Homo sapiens GN=TRIM25 PE=1 SV=2 - [TRI25_HUMAN]	25.40	1	14	14	29	-1.01	-1.08	-0.77	-0.87	0.01	-0.06	-0.18	-0.21	-0.18	-0.12	0.05	-0.04	0.68	0.99	0.84	0.79	0.69	1.05	0.76	0.66
Q16539	Mitogen-activated protein kinase 14 OS=Homo sapiens GN=MAPK14 PE=1 SV=3 - [MK14_HUMAN]	27.78	7	6	7	18	-0.45	-0.30	-0.87	-0.58	-0.02	0.32	-0.27	0.07	0.14	-0.20	-0.05	0.12	0.66	1.12	0.82	0.90	0.71	1.13	0.83	0.66
Q5JTV8	Torsin-1A-interacting protein 1 OS=Homo sapiens GN=TOR1AIP1 PE=1 SV=2 - [TOIP1_HUMAN]	33.96	1	12	13	24	-0.74	-0.76	-1.07	-0.83	-0.41	-0.41	-0.41	-0.40	-0.23	-0.12	-0.11	-0.11	0.34	0.62	0.82	0.49	0.87	0.49	0.72	0.66
Q9UGR2	Zinc finger CCCH domain-containing protein 7B OS=Homo sapiens GN=ZC3H7B PE=1 SV=1 - [Z3H7B_HUMAN]	1.81	1	2	2	3	-0.90	-0.84	-0.92	-0.86	0.12	0.17	-0.32	-0.26	-0.09	-0.14	-0.02	0.03	0.64	0.88	0.89	0.79	0.81	1.00	1.02	0.66
Q8TDY4	Arf-GAP with SH3 domain, ANK repeat and PH domain-containing protein 3 OS=Homo sapiens GN=ASAP3 PE=1 SV=1 - [ASAP3_HUMAN]	3.77	1	2	2	2	-0.47	-0.21	-0.48	-0.22	-0.01	0.24	0.12	0.38	0.20	-0.05	0.00	0.25	0.65	0.47	0.48	0.45	0.46	0.44	0.45	0.66
P28062	Proteasome subunit beta type-8 OS=Homo sapiens GN=PSMB8 PE=1 SV=3 - [PSB8_HUMAN]	29.35	1	5	5	8	-0.82	-0.73	-0.79	-0.71	-0.15	-0.03	-0.16	-0.09	0.21	-0.04	0.37	0.28	0.64	1.02	0.98	0.94	0.92	0.63	0.70	0.66
B2RUZ4	Small integral membrane protein 1 OS=Homo sapiens GN=SMIM1 PE=1 SV=1 - [SMIM1_HUMAN]	28.21	1	2	2	3	-0.53	-0.42	-0.12	-0.01	0.60	0.70	0.48	0.58	0.46	0.36	0.41	0.51	1.06	0.94	0.53	0.92	0.51	1.12	0.71	0.66
O00501	Claudin-5 OS=Homo sapiens GN=CLDN5 PE=1 SV=1 - [CLD5_HUMAN]	7.34	1	1	1	1	-1.68	-1.84	-1.65	-1.81	-0.12	-0.28	-1.05	-1.21	-0.49	-0.33	-0.05	-0.22	0.68	1.64	1.60	1.39	1.36	1.55	1.52	0.66

Q9UQC2	GRB2-associated-binding protein 2 OS=Homo sapiens GN=GAB2 PE=1 SV=1 - [GAB2_HUMAN]	3.25	1	2	2	2	0.63	0.55	-0.05	0.29	1.97	0.46	0.54	0.06	0.17	1.20	0.80	-0.07	-0.04	0.18	0.86	0.60	1.29	1.33	2.01	0.66
Q6QNY0	Biogenesis of lysosome-related organelles complex 1 subunit 3 OS=Homo sapiens GN=BLOC1S3 PE=1 SV=1 - [BL1S3_HUMAN]	4.46	1	1	1	1	-0.39	-0.18	-0.82	-0.61	-0.21	-0.02	-0.22	-0.02	0.07	-0.12	-0.31	-0.12	0.22	0.08	0.50	0.29	0.73	0.16	0.58	0.66
P10619	Lysosomal protective protein OS=Homo sapiens GN=CTSA PE=1 SV=2 - [PPGB_HUMAN]	8.13	1	4	4	5	-0.37	-0.26	-0.39	-0.28	0.30	0.41	0.21	0.31	0.38	0.28	0.42	0.53	0.63	0.80	0.81	0.68	0.70	0.66	0.67	0.66
Q8TDB4	Protein MGARP OS=Homo sapiens GN=MGARP PE=1 SV=1 - [HUMMR_HUMAN]	43.33	1	4	4	7	-0.35	0.17	-0.93	0.04	0.57	0.65	0.57	0.11	0.37	-0.15	-0.16	0.32	-0.01	0.16	0.70	0.23	0.36	0.46	0.60	0.66
P49748	Very long-chain specific acyl-CoA dehydrogenase, mitochondrial OS=Homo sapiens GN=ACADVL PE=1 SV=1 - [ACADV_HUMAN]	38.47	1	21	21	48	-0.86	-0.72	-0.63	-0.59	-0.23	-0.23	-0.02	-0.02	-0.09	-0.11	0.02	0.01	0.70	0.88	0.74	0.77	0.60	0.57	0.38	0.66
P49755	Transmembrane emp24 domain-containing protein 10 OS=Homo sapiens GN=TMED10 PE=1 SV=2 - [TMEDA_HUMAN]	35.16	1	7	7	28	-0.82	-0.84	-0.69	-0.67	0.14	0.15	-0.21	-0.20	-0.04	0.00	0.45	0.26	0.80	1.16	1.11	1.02	0.78	0.94	0.86	0.66
P50443	Sulfate transporter OS=Homo sapiens GN=SLC26A2 PE=1 SV=2 - [S26A2_HUMAN]	2.71	1	1	1	1	-2.00	-1.88	-2.25	-2.14	-0.48	-0.37	-1.66	-1.54	-1.27	-1.38	-0.61	-0.50	0.39	1.39	1.64	0.65	0.91	1.50	1.75	0.66

Q9Y696	Chloride intracellular channel protein 4 OS=Homo sapiens GN=CLIC4 PE=1 SV=4 - [CLIC4_HUMAN]	64.03	1	13	13	31	-0.62	-0.47	-0.39	-0.33	0.26	0.48	0.17	0.24	0.20	0.10	-0.11	-0.12	0.91	0.41	0.28	0.85	0.38	1.12	0.58	0.66
Q9Y6Y8	SEC23-interacting protein OS=Homo sapiens GN=SEC23IP PE=1 SV=1 - [S23IP_HUMAN]	11.80	1	9	9	13	-0.79	-0.76	-0.69	-0.60	0.12	0.09	0.03	-0.03	-0.10	-0.13	-0.01	-0.01	0.62	0.63	0.49	0.62	0.66	0.83	0.73	0.65
O94915	Protein furry homolog-like OS=Homo sapiens GN=FRYL PE=1 SV=2 - [FRYL_HUMAN]	2.29	1	5	5	8	-0.57	-0.75	-0.55	-0.52	0.05	0.12	-0.15	-0.18	-0.03	-0.03	-0.29	0.23	0.61	0.29	0.26	0.57	0.29	0.60	0.33	0.65
P28066	Proteasome subunit alpha type-5 OS=Homo sapiens GN=PSMA5 PE=1 SV=3 - [PSA5_HUMAN]	46.89	1	9	9	33	-0.79	-0.67	-0.57	-0.56	0.18	0.25	-0.11	0.04	0.25	0.19	0.52	0.62	0.77	1.22	1.16	1.06	0.89	1.02	0.79	0.65
Q9Y5X2	Sorting nexin-8 OS=Homo sapiens GN=SNX8 PE=1 SV=1 - [SNX8_HUMAN]	8.60	1	4	4	4	-0.84	-1.14	-0.71	-0.68	0.19	-0.01	-0.05	-0.15	-0.29	-0.05	0.19	-0.07	0.84	1.02	0.90	0.79	0.73	0.83	0.70	0.65
Q8IY67	Ribonucleoprotein PTB-binding 1 OS=Homo sapiens GN=RAVER1 PE=1 SV=1 - [RAVR1_HUMAN]	4.46	1	2	2	6	-0.06	-0.11	-0.08	-0.38	0.18	0.37	-0.05	0.05	0.38	0.48	0.30	0.21	0.37	0.97	0.95	0.40	0.65	0.86	0.77	0.65
Q14515	SPARC-like protein 1 OS=Homo sapiens GN=SPARCL1 PE=1 SV=2 - [SPRL1_HUMAN]	35.39	1	17	17	63	-0.85	-0.84	-0.78	-0.53	0.09	0.19	-0.07	-0.07	0.24	0.09	0.68	0.88	0.70	1.56	1.53	0.97	0.80	0.75	0.83	0.65
P53618	Coatomer subunit beta OS=Homo sapiens GN=COPB1 PE=1 SV=3 - [COPB_HUMAN]	25.71	1	20	20	39	-0.82	-0.83	-0.82	-0.82	0.24	0.22	-0.15	-0.19	-0.01	0.02	0.11	0.19	0.76	1.04	0.92	0.91	0.79	1.06	0.92	0.65
P24821	Tenascin OS=Homo sapiens GN=TNC PE=1 SV=3 - [TENA_HUMAN]	20.54	1	34	34	56	-0.32	-0.32	-0.21	-0.17	0.98	1.01	0.44	0.42	0.59	0.49	0.13	0.08	0.63	0.33	0.23	0.89	0.76	1.33	1.20	0.65

O95210	Starch-binding domain-containing protein 1 OS=Homo sapiens GN=STBD1 PE=1 SV=1 - [STBD1_HUMAN]	10.34	1	2	2	6	-0.52	-0.76	-0.96	-1.28	-0.32	-0.51	-0.47	-0.52	-0.03	0.21	0.11	-0.19	0.25	1.00	1.20	0.88	1.29	0.60	0.99	0.65
P51570	Galactokinase OS=Homo sapiens GN=GALK1 PE=1 SV=1 - [GALK1_HUMAN]	37.24	1	11	11	23	-0.50	-0.55	-0.66	-0.63	0.09	0.10	-0.05	-0.02	-0.01	-0.15	-0.03	-0.03	0.47	0.44	0.61	0.61	0.53	0.64	0.68	0.65
Q6NUQ4	Transmembrane protein 214 OS=Homo sapiens GN=TMEM214 PE=1 SV=2 - [TM214_HUMAN]	1.60	1	1	1	1	-1.65	-1.77	-0.88	-1.00	-0.38	-0.50	-0.30	-0.41	-0.81	-0.69	0.35	0.22	1.41	2.00	1.22	0.99	0.23	1.26	0.49	0.65
Q96QD9	UAP56-interacting factor OS=Homo sapiens GN=FYTTD1 PE=1 SV=3 - [UIF_HUMAN]	8.81	1	2	2	2	-0.13	-0.29	-0.32	-0.49	0.64	0.47	0.26	0.09	0.33	0.50	0.48	0.31	0.44	0.61	0.80	0.66	0.86	0.75	0.95	0.65
Q9H0S4	Probable ATP-dependent RNA helicase DDX47 OS=Homo sapiens GN=DDX47 PE=1 SV=1 - [DDX47_HUMAN]	3.96	1	1	1	1	-0.44	-0.62	-0.81	-0.99	-0.17	-0.35	-0.23	-0.41	-0.68	-0.49	-0.30	-0.49	0.26	0.14	0.51	-0.02	0.35	0.26	0.63	0.64
Q7LBR1	Charged multivesicular body protein 1b OS=Homo sapiens GN=CHMP1B PE=1 SV=1 - [CHM1B_HUMAN]	15.08	1	4	4	5	-0.64	-0.59	-0.43	-0.37	0.34	0.39	0.15	0.21	0.27	0.22	0.21	0.26	0.85	0.86	0.64	0.90	0.69	0.96	0.75	0.64
Q9UPT8	Zinc finger CCCH domain-containing protein 4 OS=Homo sapiens GN=ZC3H4 PE=1 SV=3 - [ZC3H4_HUMAN]	4.99	1	4	4	5	-0.54	-0.90	-1.00	-1.37	0.02	-0.35	-0.43	-0.79	-0.61	-0.24	0.14	-0.23	0.17	0.69	1.14	0.33	0.80	0.54	1.00	0.64
P78329	Leukotriene-B(4) omega-hydroxylase 1 OS=Homo sapiens GN=CYP4F2 PE=1 SV=1 - [CP4F2_HUMAN]	2.31	4	1	1	1	1.31	0.95	1.11	0.75	0.82	0.45	1.69	1.33	0.85	1.22	0.85	0.48	0.43	-0.46	-0.26	-0.06	0.14	-0.51	-0.31	0.64

Q9UJ99	Cadherin-22 OS=Homo sapiens GN=CDH22 PE=2 SV=2 - [CAD22_HUMAN]	2.17	1	1	1	1	0.84	0.34	-0.14	-0.64	0.52	0.02	0.44	-0.06	-0.47	0.03	-0.17	-0.67	-0.34	-1.00	-0.03	-0.77	0.21	-0.33	0.64	0.64
O60237	Protein phosphatase 1 regulatory subunit 12B OS=Homo sapiens GN=PPP1R12B PE=1 SV=2 - [MYPT2_HUMAN]	5.30	1	5	5	8	-1.67	-1.76	-1.52	-1.68	-1.33	-1.30	-1.02	-1.10	-1.20	-1.06	-1.21	-1.22	0.65	0.48	0.44	0.36	0.17	0.32	0.17	0.64
Q69YQ0	Cytospin-A OS=Homo sapiens GN=SPECC1L PE=1 SV=2 - [CYTSA_HUMAN]	3.85	1	2	3	4	-0.48	-0.61	-0.64	-0.77	-0.68	-0.81	-0.06	-0.19	-0.71	-0.58	-0.55	-0.68	0.47	-0.06	0.09	-0.07	0.10	-0.21	-0.05	0.64
Q9UH65	Switch-associated protein 70 OS=Homo sapiens GN=SWAP70 PE=1 SV=1 - [SWP70_HUMAN]	26.32	1	13	13	21	-0.81	-0.71	-0.70	-0.62	0.03	0.06	-0.12	-0.06	-0.09	-0.14	-0.23	-0.15	0.70	0.70	0.46	0.76	0.60	0.85	0.81	0.64
Q01432	AMP deaminase 3 OS=Homo sapiens GN=AMPD3 PE=1 SV=1 - [AMPD3_HUMAN]	7.69	1	4	4	7	-0.89	-0.75	-0.94	-0.79	-0.18	-0.18	-0.25	-0.21	0.07	-0.08	0.09	0.16	0.59	0.91	0.95	0.85	0.90	0.94	0.87	0.64
P63218	Guanine nucleotide-binding protein G(I)/G(S)/G(O) subunit gamma-5 OS=Homo sapiens GN=GNG5 PE=1 SV=3 - [GBG5_HUMAN]	13.24	1	1	1	2	-1.42	-1.38	-1.04	-1.00	0.03	0.06	-0.46	-0.42	-0.02	-0.06	0.31	0.35	1.01	1.74	1.35	1.40	1.02	1.43	1.05	0.64
Q9H3N1	Thioredoxin-related transmembrane protein 1 OS=Homo sapiens GN=TMX1 PE=1 SV=1 - [TMX1_HUMAN]	17.86	1	6	6	14	-1.06	-0.98	-0.92	-0.88	-0.11	0.05	-0.23	-0.13	-0.06	-0.16	-0.10	-0.06	0.92	0.94	0.76	0.83	0.81	0.88	0.77	0.64
P52566	Rho GDP-dissociation inhibitor 2 OS=Homo sapiens GN=ARHGDIB PE=1 SV=3 - [GDIR2_HUMAN]	45.77	1	6	6	13	-0.43	-0.35	-0.31	-0.19	-0.13	-0.05	0.35	0.43	-0.04	-0.05	-0.75	-0.75	0.74	-0.42	-0.61	0.28	0.18	0.17	0.09	0.64

P48729	Casein kinase I isoform alpha OS=Homo sapiens GN=CSNK1A1 PE=1 SV=2 - [KC1A_HUMAN]	13.95	2	4	4	5	-0.41	-0.15	-0.65	-0.39	0.08	0.32	-0.07	0.18	-0.20	-0.45	-0.12	0.12	0.39	0.29	0.52	-0.01	0.23	0.47	0.71	0.64
Q9BR39	Junctophilin-2 OS=Homo sapiens GN=JPH2 PE=1 SV=2 - [JPH2_HUMAN]	7.18	2	3	3	3	-1.81	-2.06	-1.28	-1.53	-0.29	-0.75	-0.02	-0.47	-0.95	-0.49	0.02	-0.44	1.16	1.29	0.76	1.11	0.58	0.91	0.38	0.64
P49902	Cytosolic purine 5'-nucleotidase OS=Homo sapiens GN=NT5C2 PE=1 SV=1 - [5NTC_HUMAN]	17.11	1	8	8	15	-1.06	-1.32	-0.61	-0.85	-0.08	-0.32	-0.29	-0.26	-0.29	-0.20	-0.06	-0.11	1.09	1.03	0.56	0.89	0.51	0.97	0.50	0.64
Q6RW13	Type-1 angiotensin II receptor-associated protein OS=Homo sapiens GN=AGTRAP PE=1 SV=1 - [ATRAP_HUMAN]	10.69	1	1	1	2	0.40	0.24	-0.13	-0.29	0.57	0.40	0.44	0.28	0.44	0.61	1.02	0.85	0.10	0.63	1.15	0.24	0.77	0.16	0.69	0.64
P62987	Ubiquitin-60S ribosomal protein L40 OS=Homo sapiens GN=UBA52 PE=1 SV=2 - [RL40_HUMAN]	53.91	2	1	8	85	-1.23	-1.35	-0.80	-0.92	-0.46	-0.59	-0.23	-0.35	-0.81	-0.69	0.01	-0.12	1.06	1.24	0.80	0.57	0.14	0.75	0.32	0.64
Q86WV6	Stimulator of interferon genes protein OS=Homo sapiens GN=TMEM173 PE=1 SV=1 - [STING_HUMAN]	30.08	1	7	7	15	-1.97	-1.92	-2.13	-1.78	-0.48	-0.49	-1.33	-1.21	-0.64	-0.72	-0.22	-0.06	0.68	1.79	1.66	1.45	1.11	1.27	1.27	0.63
P49914	5-formyltetrahydrofolate cyclo-ligase OS=Homo sapiens GN=MTHFS PE=1 SV=2 - [MTHFS_HUMAN]	12.81	1	2	2	3	-0.41	-0.48	-0.65	-0.72	-0.38	-0.46	-0.08	-0.15	-0.19	-0.11	-0.10	-0.18	0.38	0.31	0.55	0.33	0.57	0.01	0.25	0.63
Q9UNQ0	ATP-binding cassette sub-family G member 2 OS=Homo sapiens GN=ABCG2 PE=1 SV=3 - [ABCG2_HUMAN]	2.90	1	2	2	3	-1.79	-1.75	-1.50	-1.47	-0.79	-0.77	-0.93	-0.90	-0.92	-0.95	-0.42	-0.39	0.91	1.37	1.08	0.86	0.58	0.98	0.69	0.63

P10451	Osteopontin OS=Homo sapiens GN=SPP1 PE=1 SV=1 - [OSTP_HUMAN]	24.52	1	6	6	18	-0.27	-0.31	-0.18	-0.45	0.89	0.72	0.53	0.47	0.84	0.94	1.45	1.21	0.82	1.35	1.44	1.11	0.94	1.01	0.79	0.63
P50570	Dynamin-2 OS=Homo sapiens GN=DNM2 PE=1 SV=2 - [DYN2_HUMAN]	34.94	1	13	26	114	-0.59	-0.59	-0.72	-0.68	0.14	0.14	-0.07	-0.11	-0.03	-0.19	0.07	0.02	0.47	0.66	0.65	0.46	0.56	0.87	0.88	0.63
Q9H4M7	Pleckstrin homology domain-containing family A member 4 OS=Homo sapiens GN=PLEKHA4 PE=1 SV=2 - [PKHA4_HUMAN]	2.82	1	1	1	2	-1.48	-1.62	-1.63	-1.76	-0.12	-0.26	-1.06	-1.19	-0.93	-0.78	-0.57	-0.71	0.48	0.92	1.06	0.72	0.87	1.35	1.49	0.63
Q8WWP7	GTPase IMAP family member 1 OS=Homo sapiens GN=GIMAP1 PE=1 SV=1 - [GIMA1_HUMAN]	32.35	1	8	8	14	-1.25	-1.12	-1.76	-1.72	-0.79	-0.69	-1.18	-1.08	-0.72	-0.83	-0.81	-0.72	0.09	0.45	0.93	0.43	0.80	0.52	0.86	0.63
P48065	Sodium- and chloride-dependent betaine transporter OS=Homo sapiens GN=SLC6A12 PE=2 SV=2 - [S6A12_HUMAN]	4.72	1	3	3	4	-0.74	-0.48	-0.72	-0.47	0.45	0.70	-0.15	0.10	0.40	0.15	0.03	0.28	0.64	0.77	0.75	0.92	0.90	1.17	1.16	0.63
Q9H244	P2Y purinoceptor 12 OS=Homo sapiens GN=P2RY12 PE=1 SV=1 - [P2Y12_HUMAN]	2.63	1	1	1	1	-0.30	-0.16	-0.24	-0.10	0.19	0.33	0.33	0.46	0.47	0.34	0.26	0.40	0.68	0.57	0.50	0.67	0.62	0.48	0.42	0.63
P13747	HLA class I histocompatibility antigen, alpha chain E OS=Homo sapiens GN=HLA-E PE=1 SV=3 - [HLAE_HUMAN]	18.72	1	4	6	11	-0.30	-0.24	-0.58	-0.61	0.20	0.32	0.22	-0.04	0.17	0.05	0.33	0.39	0.25	0.64	0.95	0.36	0.73	0.31	0.68	0.63
P24043	Laminin subunit alpha 2 OS=Homo sapiens GN=LAMA2 PE=1 SV=4 - [LAMA2_HUMAN]	21.91	1	56	57	99	-1.36	-1.30	-1.49	-1.44	-0.82	-0.79	-0.86	-0.80	-0.66	-0.71	-0.39	-0.38	0.54	1.04	1.01	0.59	0.70	0.54	0.62	0.63

Q13485	Mothers against decapentaplegic homolog 4 OS=Homo sapiens GN=SMAD4 PE=1 SV=1 - [SMAD4_HUMAN]	1.81	1	1	1	2	-0.75	-0.69	-0.74	-0.68	-0.25	-0.20	-0.17	-0.11	0.00	-0.05	0.12	0.18	0.63	0.88	0.86	0.73	0.72	0.48	0.47	0.63
Q9UPN7	Serine/threonine-protein phosphatase 6 regulatory subunit 1 OS=Homo sapiens GN=PPP6R1 PE=1 SV=5 - [PP6R1_HUMAN]	10.90	1	6	6	8	-0.42	0.03	-0.73	-0.11	0.07	0.51	0.20	0.49	0.76	0.50	0.20	0.55	0.65	0.89	0.91	0.92	0.94	0.74	0.59	0.63
Q13951	Core-binding factor subunit beta OS=Homo sapiens GN=CBFB PE=1 SV=2 - [PEBB_HUMAN]	3.85	1	1	1	1	-0.11	0.60	-0.63	0.08	0.24	0.94	-0.06	0.65	0.62	-0.08	0.09	0.79	0.10	0.20	0.71	0.05	0.57	0.33	0.85	0.63
Q9ULX3	RNA-binding protein NOB1 OS=Homo sapiens GN=NOB1 PE=1 SV=1 - [NOB1_HUMAN]	4.37	1	1	1	1	-0.38	0.00	-0.37	0.01	-0.61	-0.24	0.20	0.58	-0.41	-0.78	-0.39	-0.02	0.63	-0.01	-0.03	-0.38	-0.38	-0.25	-0.26	0.63
O95428	Papilin OS=Homo sapiens GN=PAPLN PE=2 SV=4 - [PPN_HUMAN]	5.48	1	4	4	6	-0.41	-0.82	-0.94	-0.96	0.36	0.29	0.04	0.03	0.26	0.35	0.46	0.31	0.26	0.69	1.20	0.47	0.79	0.45	0.95	0.63
P30566	Adenylosuccinate lyase OS=Homo sapiens GN=ADSL PE=1 SV=2 - [PUR8_HUMAN]	14.05	1	5	5	10	-0.93	-0.81	-1.02	-0.95	0.00	0.09	-0.36	-0.28	0.03	-0.03	0.39	0.51	0.48	1.29	1.41	1.15	1.11	0.87	1.01	0.63
O75954	Tetraspanin-9 OS=Homo sapiens GN=TSPAN9 PE=1 SV=1 - [TSN9_HUMAN]	15.48	1	3	3	11	-0.57	-0.78	-0.59	-0.83	0.26	0.39	0.00	-0.02	0.23	0.17	0.09	0.09	0.56	0.64	0.69	0.56	0.62	0.55	0.60	0.63
P25787	Proteasome subunit alpha type-2 OS=Homo sapiens GN=PSMA2 PE=1 SV=2 - [PSA2_HUMAN]	41.88	1	7	7	22	-0.76	-0.72	-0.57	-0.63	-0.08	-0.23	-0.01	-0.07	-0.08	-0.01	0.25	0.22	0.64	0.95	0.82	0.74	0.55	0.60	0.46	0.63

O94874	E3 UFM1-protein ligase 1 OS=Homo sapiens GN=UFL1 PE=1 SV=2 - [UFL1_HUMAN]	23.80	1	15	15	27	-0.59	-0.61	-0.82	-0.69	0.15	0.01	-0.24	-0.31	-0.07	-0.01	0.13	0.02	0.56	0.71	0.85	0.82	0.75	0.89	0.79	0.62
P42898	Methylenetetrahydrofolate reductase OS=Homo sapiens GN=MTHFR PE=1 SV=3 - [MTHR_HUMAN]	1.68	1	1	1	2	-0.94	-1.00	-0.87	-0.93	0.32	0.26	-0.31	-0.37	-0.11	-0.04	-0.01	-0.07	0.69	0.93	0.86	0.93	0.86	1.24	1.17	0.62
P43405	Tyrosine-protein kinase SYK OS=Homo sapiens GN=SYK PE=1 SV=1 - [KSYK_HUMAN]	8.66	1	4	4	5	-0.19	-0.32	-0.18	-0.31	0.39	0.26	0.38	0.25	-0.18	-0.04	0.57	0.43	0.63	0.76	0.74	0.18	0.17	0.57	0.55	0.62
P51812	Ribosomal protein S6 kinase alpha-3 OS=Homo sapiens GN=RPS6KA3 PE=1 SV=1 - [KS6A3_HUMAN]	24.32	5	8	17	29	-0.52	-0.42	-0.60	-0.73	-0.05	-0.06	0.05	-0.10	-0.08	-0.04	0.07	0.09	0.33	0.46	0.58	0.38	0.62	0.35	0.59	0.62
P30740	Leukocyte elastase inhibitor OS=Homo sapiens GN=SERPINB1 PE=1 SV=1 - [ILEU_HUMAN]	22.43	1	8	8	14	-0.81	-0.81	-0.69	-0.76	-0.46	-0.57	-0.13	-0.09	-0.44	-0.31	-0.21	-0.35	0.57	0.47	0.48	0.44	0.30	0.17	0.07	0.62
Q13158	FAS-associated death domain protein OS=Homo sapiens GN=FADD PE=1 SV=1 - [FADD_HUMAN]	6.25	1	1	1	2	-0.45	-0.43	-0.26	-0.24	0.70	0.72	0.29	0.32	0.24	0.22	0.00	0.02	0.80	0.46	0.27	0.71	0.52	1.14	0.95	0.62
Q99714	3-hydroxyacyl-CoA dehydrogenase type-2 OS=Homo sapiens GN=HSD17B10 PE=1 SV=3 - [HCD2_HUMAN]	57.85	1	12	12	34	-1.06	-0.99	-0.90	-0.85	-0.18	-0.34	-0.39	-0.45	-0.28	-0.23	-0.15	-0.32	0.68	0.73	0.51	0.63	0.66	0.84	0.54	0.62

O14735	CDP-diacylglycerol-inositol 3-phosphatidyltransferase OS=Homo sapiens GN=CDIPT PE=1 SV=1 - [CDIPT_HUMAN]	9.86	1	2	2	8	-1.00	-0.97	-0.83	-0.80	0.18	0.30	-0.28	-0.27	0.02	-0.10	-0.03	0.10	0.67	0.87	0.70	0.82	0.66	1.05	0.89	0.62
Q8N4H5	Mitochondrial import receptor subunit TOM5 homolog OS=Homo sapiens GN=TOMM5 PE=1 SV=1 - [TOM5_HUMAN]	56.86	1	4	4	5	-0.87	-0.71	-0.67	-0.51	-0.02	0.13	-0.11	0.04	0.36	0.21	0.13	0.28	0.81	1.00	0.80	1.10	0.91	0.83	0.64	0.62
O14498	Immunoglobulin superfamily containing leucine-rich repeat protein OS=Homo sapiens GN=ISLR PE=1 SV=1 - [ISLR_HUMAN]	20.09	1	6	6	10	-0.70	-0.68	-1.03	-0.78	0.16	0.24	-0.47	-0.38	-0.11	-0.04	0.39	0.34	0.29	1.00	1.10	0.62	1.04	0.91	1.30	0.62
O95747	Serine/threonine-protein kinase OSR1 OS=Homo sapiens GN=OXSR1 PE=1 SV=1 - [OXSR1_HUMAN]	28.84	1	9	11	25	-0.72	-0.72	-0.60	-0.57	0.10	0.04	0.02	0.01	0.00	-0.03	0.05	0.05	0.83	0.76	0.57	0.68	0.64	0.81	0.64	0.62
Q8NE71	ATP-binding cassette sub-family F member 1 OS=Homo sapiens GN=ABCF1 PE=1 SV=2 - [ABCF1_HUMAN]	16.92	1	10	10	17	-0.37	-0.38	-0.75	-0.82	0.10	0.13	-0.20	-0.29	-0.18	-0.19	-0.36	-0.35	0.22	0.00	0.43	0.22	0.59	0.50	0.93	0.62
Q8TEB1	DDB1- and CUL4-associated factor 11 OS=Homo sapiens GN=DCAF11 PE=1 SV=1 - [DCA11_HUMAN]	5.31	1	2	2	3	-0.67	-0.80	-0.73	-0.86	-0.30	-0.43	-0.18	-0.31	-0.28	-0.14	0.11	-0.03	0.55	0.79	0.84	0.56	0.62	0.36	0.42	0.62
O96007	Molybdopterin synthase catalytic subunit OS=Homo sapiens GN=MOCS2 PE=1 SV=1 - [MOC2B_HUMAN]	9.04	1	2	2	3	0.52	0.49	0.77	0.35	0.45	0.22	0.93	0.90	0.52	0.50	0.76	0.64	0.47	0.25	-0.01	0.06	0.21	-0.28	-0.14	0.62

Q9Y5W8	Sorting nexin-13 OS=Homo sapiens GN=SNX13 PE=1 SV=4 - [SNX13_HUMAN]	2.07	1	1	1	1	1.10	1.03	0.44	0.37	1.93	1.85	0.99	0.92	0.97	1.04	0.73	0.66	-0.05	-0.36	0.30	-0.03	0.64	0.81	1.47	0.62
P52735	Guanine nucleotide exchange factor VAV2 OS=Homo sapiens GN=VAV2 PE=1 SV=2 - [VAV2_HUMAN]	2.16	1	1	1	4	-0.14	-0.16	-0.45	-0.47	0.61	0.59	0.10	0.08	-0.08	-0.05	0.01	-0.01	0.29	0.16	0.46	0.11	0.43	0.73	1.05	0.62
Q9HD20	Probable cation-transporting ATPase 13A1 OS=Homo sapiens GN=ATP13A1 PE=1 SV=2 - [AT131_HUMAN]	7.64	1	7	7	15	-1.04	-1.06	-2.14	-2.26	-1.05	-1.18	-1.47	-1.58	-1.28	-1.15	-1.06	-1.28	0.59	0.73	0.76	0.61	0.85	0.91	1.01	0.62
Q15847	Adipogenesis regulatory factor OS=Homo sapiens GN=ADIRF PE=1 SV=1 - [ADIRF_HUMAN]	27.63	1	2	2	5	-1.72	-1.90	-2.05	-2.09	-1.21	-1.29	-1.31	-1.33	-1.49	-1.46	-1.20	-1.33	0.65	0.47	0.87	0.39	0.51	0.45	0.58	0.62
A5D8V6	Vacuolar protein sorting-associated protein 37C OS=Homo sapiens GN=VPS37C PE=1 SV=2 - [VP37C_HUMAN]	10.42	1	3	3	5	-0.81	-0.49	-0.97	-0.78	-0.21	-0.03	-0.38	-0.37	-0.08	-0.46	-0.16	0.02	0.14	0.68	0.86	0.44	0.54	0.61	0.79	0.61
Q9Y6D9	Mitotic spindle assembly checkpoint protein MAD1 OS=Homo sapiens GN=MAD1L1 PE=1 SV=2 - [MD1L1_HUMAN]	5.99	1	3	3	5	0.11	0.06	-0.05	-0.10	-0.47	0.14	-0.17	0.44	0.13	-0.47	0.24	0.18	0.08	0.13	0.29	-0.25	0.28	-0.29	0.23	0.61
P40938	Replication factor C subunit 3 OS=Homo sapiens GN=RFC3 PE=1 SV=2 - [RFC3_HUMAN]	3.93	1	1	1	2	-1.52	-1.24	-1.37	-1.09	-0.85	-0.57	-0.82	-0.54	-0.38	-0.65	-0.64	-0.37	0.76	0.88	0.73	0.90	0.75	0.66	0.51	0.61
Q6AZY7	Scavenger receptor class A member 3 OS=Homo sapiens GN=SCARA3 PE=2 SV=1 - [SCAR3_HUMAN]	4.13	1	3	3	5	-0.71	-0.50	-0.88	-0.98	0.32	0.27	-0.51	-0.38	0.35	0.98	1.17	0.89	0.59	1.40	1.57	0.88	1.21	0.76	0.93	0.61

O43147	Small G protein signaling modulator 2 OS=Homo sapiens GN=SGSM2 PE=1 SV=4 - [SGSM2_HUMAN]	5.47	1	3	4	4	-0.28	-0.17	-0.35	-0.23	0.32	0.44	0.20	0.32	0.13	0.02	0.30	0.42	0.54	0.59	0.65	0.34	0.40	0.59	0.65	0.61
Q9ULQ1	Two pore calcium channel protein 1 OS=Homo sapiens GN=TPCN1 PE=1 SV=3 - [TPC1_HUMAN]	5.27	1	3	3	6	-0.65	-0.66	-0.69	-0.77	0.17	0.15	-0.18	-0.22	-0.14	-0.09	0.42	0.32	0.50	1.05	1.10	0.56	0.66	0.81	0.91	0.61
Q99504	Eyes absent homolog 3 OS=Homo sapiens GN=EYA3 PE=1 SV=3 - [EYA3_HUMAN]	1.92	1	1	1	1	-1.41	-1.13	-1.25	-0.96	-0.12	0.16	-0.70	-0.41	-0.36	-0.64	-0.11	0.17	0.77	1.31	1.14	0.81	0.64	1.28	1.11	0.61
P04745	Alpha-amylase 1 OS=Homo sapiens GN=AMY1A PE=1 SV=2 - [AMY1_HUMAN]	18.40	3	7	7	11	-2.49	-2.25	-4.76	-4.66	-4.11	-4.07	-4.47	-4.26	-4.33	-4.43	-4.75	-4.58	-0.38	-0.62	0.33	-0.50	0.50	-0.32	0.81	0.61
Q8IUR7	Armadillo repeat-containing protein 8 OS=Homo sapiens GN=ARMC8 PE=1 SV=2 - [ARMC8_HUMAN]	6.98	1	3	3	4	-0.17	0.10	-0.66	-0.39	-0.12	0.14	-0.11	0.16	0.08	-0.19	-0.10	0.16	0.12	0.08	0.55	0.02	0.50	0.03	0.52	0.61
P48444	Coatomer subunit delta OS=Homo sapiens GN=ARCN1 PE=1 SV=1 - [COPD_HUMAN]	28.96	1	13	13	22	-0.59	-0.68	-0.62	-0.74	0.08	0.15	-0.26	-0.14	-0.09	-0.17	-0.14	-0.21	0.32	0.46	0.61	0.42	0.65	0.66	0.90	0.61
Q9NVZ3	Adaptin ear-binding coat-associated protein 2 OS=Homo sapiens GN=NECAP2 PE=1 SV=1 - [NECP2_HUMAN]	19.39	1	3	3	5	-0.48	-1.35	-0.93	-1.23	0.45	0.02	-0.38	-0.34	-0.17	0.70	0.27	0.00	0.15	0.75	1.20	1.20	1.66	0.91	1.36	0.61
Q9NZB8	Molybdenum cofactor biosynthesis protein 1 OS=Homo sapiens GN=MOCS1 PE=1 SV=3 - [MOCS1_HUMAN]	2.67	1	1	1	1	-0.55	-1.05	-0.47	-0.97	0.35	-0.15	0.07	-0.43	0.01	0.52	0.28	-0.22	0.68	0.83	0.75	1.10	1.02	0.89	0.81	0.61

Q15005	Signal peptidase complex subunit 2 OS=Homo sapiens GN=SPCS2 PE=1 SV=3 - [SPCS2_HUMAN]	29.65	1	4	4	8	-0.70	-0.95	-0.86	-1.03	-0.09	-0.43	-0.41	-0.51	-0.46	-0.53	-0.18	-0.37	0.44	0.37	0.64	0.32	0.40	0.51	0.58	0.61
Q9NUE0	Palmitoyltransferase ZDHHC18 OS=Homo sapiens GN=ZDHHC18 PE=2 SV=2 - [ZDH18_HUMAN]	6.19	1	1	1	6	-0.56	0.21	-0.83	-0.05	-0.85	-0.08	-0.20	-0.08	-0.08	-0.55	-0.49	0.67	0.34	0.02	0.78	-0.07	0.20	-0.30	-0.04	0.61
Q7Z5P9	Mucin-19 OS=Homo sapiens GN=MUC19 PE=1 SV=2 - [MUC19_HUMAN]	0.32	1	1	1	1	-1.14	-0.80	-1.18	-0.84	-0.20	0.14	-0.64	-0.29	-0.18	-0.52	-0.35	-0.01	0.56	0.79	0.83	0.65	0.69	0.92	0.96	0.61
Q9BVK6	Transmembrane emp24 domain-containing protein 9 OS=Homo sapiens GN=TMED9 PE=1 SV=2 - [TMED9_HUMAN]	24.26	1	4	5	17	-1.09	-1.07	-1.01	-1.08	0.15	0.11	-0.54	-0.56	-0.46	-0.38	0.20	0.15	0.77	1.26	1.26	1.01	0.72	1.31	1.25	0.61
Q9UBV8	Peflin OS=Homo sapiens GN=PEF1 PE=1 SV=1 - [PEF1_HUMAN]	14.79	1	4	4	8	-0.44	-0.63	-0.67	-0.69	0.25	0.29	-0.08	-0.04	0.15	0.12	0.41	0.40	0.61	1.21	1.08	0.93	0.88	0.88	0.83	0.61
P04899	Guanine nucleotide-binding protein G(i) subunit alpha 2 OS=Homo sapiens GN=GNAI2 PE=1 SV=3 - [GNAI2_HUMAN]	58.59	3	10	17	105	-0.30	-0.40	-0.46	-0.34	0.19	0.15	0.12	0.11	0.13	0.09	0.48	0.29	0.46	0.69	0.64	0.45	0.49	0.44	0.62	0.61
Q9H9S4	Calcium-binding protein 39-like OS=Homo sapiens GN=CAB39L PE=1 SV=3 - [CB39L_HUMAN]	19.29	1	5	6	13	-0.72	-0.65	-0.75	-0.62	0.25	0.44	-0.22	-0.05	0.17	0.02	0.05	0.19	0.45	0.89	0.79	0.64	0.84	0.84	1.07	0.60
Q5PRF9	Protein Smaug homolog 2 OS=Homo sapiens GN=SAMD4B PE=1 SV=1 - [SMAG2_HUMAN]	4.76	1	2	2	3	-0.13	-0.41	-0.45	-0.73	0.56	0.28	0.09	-0.19	0.01	0.30	0.09	-0.19	0.28	0.23	0.54	0.46	0.78	0.68	0.99	0.60

Q8IYM9	E3 ubiquitin-protein ligase TRIM22 OS=Homo sapiens GN=TRIM22 PE=1 SV=1 - [TRI22_HUMAN]	1.81	1	1	1	1	-0.02	-0.19	-0.16	-0.33	0.65	0.47	0.38	0.21	0.52	0.69	0.57	0.40	0.45	0.60	0.73	0.74	0.88	0.65	0.79	0.60
Q9UBQ5	Eukaryotic translation initiation factor 3 subunit K OS=Homo sapiens GN=EIF3K PE=1 SV=1 - [EIF3K_HUMAN]	34.86	1	5	5	13	-0.19	-0.16	-0.43	-0.67	0.20	0.22	-0.11	-0.12	0.20	0.18	0.12	0.08	0.09	0.26	0.76	0.41	0.93	0.37	0.88	0.60
Q9HB90	Ras-related GTP-binding protein C OS=Homo sapiens GN=RRAGC PE=1 SV=1 - [RRAGC_HUMAN]	20.05	2	4	4	5	-0.95	-1.00	-0.74	-0.79	0.13	0.08	-0.20	-0.25	-0.14	-0.09	-0.28	-0.33	0.80	0.68	0.46	0.89	0.69	1.06	0.86	0.60
P25189	Myelin protein P0 OS=Homo sapiens GN=MPZ PE=1 SV=1 - [MYP0_HUMAN]	4.84	1	1	1	2	-0.91	-1.19	-0.57	-0.84	-1.48	-1.76	-0.03	-0.31	-1.26	-0.98	-1.33	-1.61	0.94	-0.41	-0.76	-0.03	-0.38	-0.58	-0.93	0.60
P29728	2'-5'-oligoadenylate synthase 2 OS=Homo sapiens GN=OAS2 PE=1 SV=3 - [OAS2_HUMAN]	2.78	1	1	1	3	-0.09	-0.28	-0.22	-0.40	1.39	1.20	0.32	0.14	0.74	0.93	0.99	0.80	0.47	1.09	1.21	1.05	1.18	1.47	1.59	0.60
O15357	Phosphatidylinositol 3,4,5-trisphosphate 5-phosphatase 2 OS=Homo sapiens GN=INPPL1 PE=1 SV=2 - [SHIP2_HUMAN]	11.05	1	9	9	11	-1.02	-1.10	-0.68	-0.67	-0.05	-0.05	-0.14	0.01	-0.03	-0.12	0.09	0.18	0.55	0.75	0.92	0.84	0.68	0.89	0.58	0.60
Q9HAB8	Phosphopantothenate--cysteine ligase OS=Homo sapiens GN=PPCS PE=1 SV=2 - [PPCS_HUMAN]	27.97	1	8	8	13	0.11	0.23	-0.03	0.11	0.56	0.66	0.56	0.74	0.47	0.32	0.08	0.19	0.48	-0.10	0.06	0.33	0.44	0.46	0.54	0.60
O60941	Dystrobrevin beta OS=Homo sapiens GN=DTNB PE=1 SV=1 - [DTNB_HUMAN]	10.85	1	2	7	12	-0.28	-0.15	-0.38	-0.26	-0.02	0.10	0.16	0.28	0.05	-0.07	-0.03	0.09	0.49	0.25	0.35	0.23	0.34	0.24	0.34	0.60

P37802	Transgelin-2 OS=Homo sapiens GN=TAGLN2 PE=1 SV=3 - [TAGL2_HUMAN]	70.85	1	11	12	69	-2.39	-2.36	-2.29	-2.26	-1.28	-1.18	-1.84	-1.72	-1.44	-1.56	-1.72	-1.69	0.64	0.59	0.56	0.90	0.74	0.98	1.01	0.60
A6NI28	Rho GTPase-activating protein 42 OS=Homo sapiens GN=ARHGAP42 PE=1 SV=3 - [RHG42_HUMAN]	2.06	1	1	2	2	-1.14	-1.18	-1.54	-1.58	-0.39	-0.44	-1.00	-1.04	-0.55	-0.50	-0.85	-0.90	0.20	0.29	0.68	0.67	1.07	0.73	1.13	0.60
Q0JRZ9	FCH domain only protein 2 OS=Homo sapiens GN=FCHO2 PE=1 SV=2 - [FCHO2_HUMAN]	1.23	1	1	1	2	-1.24	-1.52	-0.63	-0.90	-0.72	-1.01	-0.09	-0.37	-0.86	-0.57	0.16	-0.12	1.21	1.41	0.79	0.70	0.09	0.50	-0.11	0.60
Q8IY21	Probable ATP-dependent RNA helicase DDX60 OS=Homo sapiens GN=DDX60 PE=1 SV=3 - [DDX60_HUMAN]	0.64	1	1	1	1	0.23	0.18	-0.37	-0.42	0.44	0.39	0.16	0.12	0.26	0.32	0.45	0.39	-0.01	0.22	0.82	0.12	0.72	0.19	0.79	0.60
Q96S97	Myeloid-associated differentiation marker OS=Homo sapiens GN=MYADM PE=1 SV=2 - [MYADM_HUMAN]	16.15	1	3	3	8	-0.09	0.67	-0.45	-0.06	0.54	0.40	0.08	0.30	0.31	0.25	0.08	-0.05	0.23	-0.33	-0.08	0.38	0.74	0.11	0.46	0.60
P48556	26S proteasome non-ATPase regulatory subunit 8 OS=Homo sapiens GN=PSMD8 PE=1 SV=2 - [PSMD8_HUMAN]	25.14	1	7	7	16	-0.50	-0.41	-0.53	-0.38	0.21	0.17	-0.06	-0.06	0.13	0.12	0.38	0.44	0.54	0.77	0.79	0.65	0.78	0.61	0.75	0.60
Q7Z2W4	Zinc finger CCCH-type antiviral protein 1 OS=Homo sapiens GN=ZC3HAV1 PE=1 SV=3 - [ZCCHV_HUMAN]	21.40	1	14	14	21	-0.76	-0.69	-0.90	-0.77	-0.01	-0.10	-0.27	-0.24	-0.25	-0.11	0.05	0.11	0.44	0.81	0.99	0.54	0.79	0.70	0.85	0.60
Q9BY76	Angiopoietin-related protein 4 OS=Homo sapiens GN=ANGPTL4 PE=1 SV=2 - [ANGL4_HUMAN]	6.65	1	2	2	2	-0.76	-0.84	-0.88	-0.97	0.51	0.42	-0.35	-0.43	-0.15	-0.05	-0.10	-0.19	0.46	0.66	0.79	0.73	0.86	1.25	1.37	0.60

Q7Z434	Mitochondrial antiviral-signaling protein OS=Homo sapiens GN=MAVS PE=1 SV=2 - [MAVS_HUMAN]	7.78	1	2	2	3	-0.64	-0.46	-0.86	-0.68	0.05	0.22	-0.07	0.11	0.13	-0.04	-0.50	-0.33	0.63	0.14	0.36	0.62	0.85	0.67	0.89	0.60
Q9NPD3	Exosome complex component RRP41 OS=Homo sapiens GN=EXOSC4 PE=1 SV=3 - [EXOS4_HUMAN]	15.92	1	3	3	3	-0.59	-0.24	-0.79	-0.44	-0.36	-0.01	-0.26	0.09	-0.29	-0.64	-0.58	-0.24	0.38	0.01	0.21	-0.02	0.19	0.21	0.42	0.60
Q8NFH4	Nucleoporin Nup37 OS=Homo sapiens GN=NUP37 PE=1 SV=1 - [NUP37_HUMAN]	5.21	1	1	1	3	-0.47	-0.50	-0.79	-0.82	0.09	0.06	-0.26	-0.29	0.00	0.04	0.14	0.11	0.27	0.62	0.94	0.54	0.86	0.55	0.87	0.60
O60814	Histone H2B type 1-K OS=Homo sapiens GN=HIST1H2BK PE=1 SV=3 - [H2B1K_HUMAN]	47.62	10	7	7	124	-0.95	-1.12	-0.88	-1.02	-0.47	-0.57	-0.30	-0.34	-0.40	-0.35	-0.28	-0.24	0.73	0.82	0.70	0.62	0.65	0.55	0.46	0.60
Q96H20	Vacuolar-sorting protein SNF8 OS=Homo sapiens GN=SNF8 PE=1 SV=1 - [SNF8_HUMAN]	4.65	1	1	1	1	0.16	-0.18	-0.27	-0.60	0.36	0.02	0.26	-0.07	0.08	0.41	-0.03	-0.37	0.16	-0.18	0.24	0.29	0.72	0.19	0.62	0.59
Q13228	Selenium-binding protein 1 OS=Homo sapiens GN=SELENBP1 PE=1 SV=2 - [SBP1_HUMAN]	47.67	1	18	18	60	-0.63	-0.58	-0.62	-0.57	-0.25	-0.35	-0.16	-0.07	-0.35	-0.32	-0.59	-0.72	0.52	-0.17	-0.12	0.27	0.39	0.30	0.28	0.59
P23508	Colorectal mutant cancer protein OS=Homo sapiens GN=MCC PE=1 SV=2 - [CRCM_HUMAN]	11.10	1	6	6	10	-0.41	-0.48	-0.65	-0.51	0.02	0.21	0.07	0.21	-0.08	-0.11	-0.19	-0.01	0.74	0.29	0.44	0.41	0.47	0.36	0.52	0.59
Q92530	Proteasome inhibitor PI31 subunit OS=Homo sapiens GN=PSMF1 PE=1 SV=2 - [PSMF1_HUMAN]	25.83	1	7	7	9	-0.03	-0.10	-0.03	-0.08	-0.03	-0.08	0.12	0.23	0.08	-0.02	-0.21	-0.03	0.56	0.27	0.14	0.02	0.05	-0.09	-0.03	0.59

Q9HAN9	Nicotinamide mononucleotide adenylyltransferase 1 OS=Homo sapiens GN=NMNAT1 PE=1 SV=1 - [NMNA1_HUMAN]	2.87	1	1	1	1	-1.61	-0.57	-2.22	-1.17	-0.50	0.54	-1.69	-0.65	0.60	-0.44	-0.38	0.66	-0.02	1.24	1.84	1.20	1.81	1.10	1.70	0.59
Q99735	Microsomal glutathione S-transferase 2 OS=Homo sapiens GN=MGST2 PE=1 SV=1 - [MGST2_HUMAN]	9.52	1	1	1	4	-1.49	-1.56	-1.27	-1.34	-0.39	-0.46	-0.74	-0.81	-0.55	-0.47	-0.63	-0.70	0.81	0.87	0.64	1.05	0.83	1.09	0.87	0.59
Q9BUB7	Transmembrane protein 70, mitochondrial OS=Homo sapiens GN=TMEM70 PE=1 SV=2 - [TMM70_HUMAN]	11.54	1	2	2	2	0.27	-0.10	0.07	-0.30	1.14	0.76	0.60	0.23	0.06	0.43	0.41	0.04	0.38	0.15	0.34	0.19	0.40	0.85	1.05	0.59
Q06323	Proteasome activator complex subunit 1 OS=Homo sapiens GN=PSME1 PE=1 SV=1 - [PSME1_HUMAN]	53.01	1	13	13	28	-0.87	-0.80	-0.89	-0.79	-0.23	-0.05	-0.28	-0.27	-0.23	-0.30	0.12	0.11	0.60	0.97	0.85	0.43	0.42	0.51	0.50	0.59
O00534	von Willebrand factor A domain-containing protein 5A OS=Homo sapiens GN=VWA5A PE=2 SV=2 - [VMA5A_HUMAN]	7.38	1	4	4	7	-0.40	-0.37	-0.19	-0.16	0.52	0.55	0.34	0.37	0.49	0.47	0.13	0.16	0.79	0.53	0.32	0.89	0.69	0.90	0.70	0.59
Q9Y646	Carboxypeptidase Q OS=Homo sapiens GN=CPQ PE=1 SV=1 - [CBPQ_HUMAN]	20.55	1	6	6	7	-0.90	-1.01	-0.89	-0.90	0.03	0.08	-0.26	-0.42	-0.37	-0.21	-0.11	-0.05	0.70	0.72	0.67	0.70	0.47	0.72	0.68	0.59
Q9Y5L3	Ectonucleoside triphosphate diphosphohydrolase 2 OS=Homo sapiens GN=ENTPD2 PE=1 SV=1 - [ENTP2_HUMAN]	7.68	1	3	3	10	-0.31	0.03	-0.45	-0.01	0.11	0.25	0.07	0.41	0.36	-0.21	0.16	0.76	0.38	0.53	0.60	0.19	0.27	0.29	0.46	0.59

Q9HBL7	Plasminogen receptor (KT) OS=Homo sapiens GN=PLGRKT PE=1 SV=1 - [PLRKT_HUMAN]	21.77	1	3	3	6	-1.42	-1.38	-0.99	-0.59	-0.66	-0.27	-0.46	-0.06	-0.47	-0.50	-0.49	-0.11	1.42	1.32	0.49	0.95	0.30	0.70	0.30	0.59
O95359	Transforming acidic coiled-coil-containing protein 2 OS=Homo sapiens GN=TACC2 PE=1 SV=3 - [TACC2_HUMAN]	1.93	1	2	6	7	-0.33	-0.20	-0.79	-0.66	0.27	0.39	-0.27	-0.14	0.10	-0.03	-0.46	-0.34	0.12	-0.13	0.33	0.33	0.80	0.58	1.04	0.59
Q9NXS2	Glutaminyl-peptide cyclotransferase-like protein OS=Homo sapiens GN=QPCTL PE=1 SV=2 - [QPCTL_HUMAN]	4.71	1	2	2	2	-0.29	-0.09	-0.62	-0.42	1.20	1.39	-0.09	0.11	0.63	0.43	0.07	0.27	0.25	0.37	0.69	0.75	1.08	1.47	1.80	0.59
Q08397	Lysyl oxidase homolog 1 OS=Homo sapiens GN=LOXL1 PE=1 SV=2 - [LOXL1_HUMAN]	2.09	1	1	1	1	-0.84	-0.70	-0.86	-0.71	1.69	1.83	-0.33	-0.19	0.88	0.75	0.02	0.15	0.57	0.86	0.87	1.62	1.64	2.52	2.53	0.59
Q9H5X1	MIP18 family protein FAM96A OS=Homo sapiens GN=FAM96A PE=1 SV=1 - [FA96A_HUMAN]	6.88	1	1	1	2	-0.54	-0.32	-0.84	-0.63	-0.02	0.19	-0.32	-0.11	0.40	0.19	0.05	0.26	0.27	0.60	0.90	0.76	1.06	0.50	0.80	0.59
O14933	Ubiquitin/ISG15-conjugating enzyme E2 L6 OS=Homo sapiens GN=UBE2L6 PE=1 SV=4 - [UB2L6_HUMAN]	14.38	1	1	2	3	1.13	0.72	0.49	0.09	0.88	0.47	1.01	0.61	0.40	0.81	0.78	0.37	-0.06	-0.34	0.29	-0.29	0.36	-0.27	0.37	0.59
Q6UX71	Plexin domain-containing protein 2 OS=Homo sapiens GN=PLXDC2 PE=1 SV=1 - [PXDC2_HUMAN]	10.21	1	5	5	10	-0.38	-0.42	-0.34	-0.25	0.29	0.47	0.17	0.07	0.23	0.21	0.15	0.42	0.49	0.80	0.65	0.69	0.62	0.63	0.69	0.59
Q9BRP4	Proteasomal ATPase-associated factor 1 OS=Homo sapiens GN=PAAF1 PE=1 SV=2 - [PAAF1_HUMAN]	2.04	1	1	1	1	-0.41	0.13	-0.35	0.19	-0.14	0.39	0.18	0.71	0.25	-0.28	-0.21	0.32	0.64	0.21	0.14	0.16	0.10	0.25	0.19	0.59

Q8IUZ5	5-phosphohydroxy-L-lysine phospho-lyase OS=Homo sapiens GN=PHYKPL PE=1 SV=1 - [AT2L2_HUMAN]	7.11	1	3	3	4	0.10	0.23	-0.11	0.02	0.38	0.50	0.42	0.54	0.52	0.40	0.26	0.38	0.37	0.16	0.36	0.33	0.54	0.26	0.46	0.59
Q9UKS6	Protein kinase C and casein kinase substrate in neurons protein 3 OS=Homo sapiens GN=PACSIN3 PE=1 SV=2 - [PACN3_HUMAN]	14.15	1	4	4	7	-0.91	-0.73	-1.14	-0.95	-0.06	0.12	-0.62	-0.43	-0.38	-0.56	-0.73	-0.55	0.35	0.19	0.41	0.38	0.61	0.84	1.06	0.59
P51690	Arylsulfatase E OS=Homo sapiens GN=ARSE PE=1 SV=2 - [ARSE_HUMAN]	3.74	1	1	1	3	-0.19	-0.04	-0.76	-0.62	0.41	0.55	-0.24	-0.10	-0.18	-0.32	0.06	0.20	0.00	0.25	0.82	-0.11	0.47	0.58	1.16	0.58
P17301	Integrin alpha 2 OS=Homo sapiens GN=ITGA2 PE=1 SV=1 - [ITA2_HUMAN]	4.83	1	5	5	7	-0.05	-0.28	-0.31	-0.54	0.03	-0.16	0.30	0.13	0.38	0.55	0.98	0.81	0.59	1.04	1.01	0.80	0.79	0.06	0.33	0.58
O60749	Sorting nexin-2 OS=Homo sapiens GN=SNX2 PE=1 SV=2 - [SNX2_HUMAN]	31.79	1	11	14	27	-0.73	-0.76	-0.78	-0.47	-0.05	0.05	0.02	0.20	-0.04	-0.15	-0.32	-0.23	0.69	0.57	0.25	0.61	0.59	0.67	0.67	0.58
Q9NZU5	LIM and cysteine-rich domains protein 1 OS=Homo sapiens GN=LMCD1 PE=1 SV=1 - [LMCD1_HUMAN]	35.62	1	11	11	15	-1.66	-1.49	-1.51	-1.52	-0.62	-0.21	-1.17	-1.11	-0.64	-0.75	-0.67	-0.73	0.36	0.76	0.84	0.57	0.65	0.82	0.97	0.58
Q99732	Lipopolysaccharide-induced tumor necrosis factor-alpha factor OS=Homo sapiens GN=LITAF PE=1 SV=2 - [LITAF_HUMAN]	4.35	1	1	1	2	-0.60	-0.61	-0.14	-0.14	0.24	0.23	0.38	0.38	0.15	0.16	0.28	0.27	1.04	0.88	0.41	0.79	0.33	0.82	0.36	0.58

Q8N5M1	ATP synthase mitochondrial F1 complex assembly factor 2 OS=Homo sapiens GN=ATPAF2 PE=1 SV=1 - [ATPF2_HUMAN]	7.61	1	2	2	4	-0.47	-0.78	-0.42	-0.73	0.00	-0.31	0.10	-0.21	-0.25	0.07	0.12	-0.20	0.63	0.60	0.54	0.57	0.52	0.46	0.40	0.58
P01903	HLA class II histocompatibility antigen, DR alpha chain OS=Homo sapiens GN=HLA-DRA PE=1 SV=1 - [DRA_HUMAN]	25.98	2	6	6	17	-0.56	-0.59	-0.41	-0.31	1.05	1.07	0.09	0.27	0.48	0.33	0.04	0.04	0.73	0.60	0.32	0.86	0.74	1.64	1.36	0.58
Q9UNM6	26S proteasome non-ATPase regulatory subunit 13 OS=Homo sapiens GN=PSMD13 PE=1 SV=2 - [PSD13_HUMAN]	49.73	1	15	15	32	-0.65	-0.49	-0.58	-0.58	0.39	0.56	0.03	-0.02	0.31	0.14	0.40	0.45	0.58	0.82	1.04	0.84	0.84	0.85	0.89	0.58
Q9BY12	S phase cyclin A-associated protein in the endoplasmic reticulum OS=Homo sapiens GN=SCAPER PE=1 SV=2 - [SCAPE_HUMAN]	2.36	1	2	2	4	-0.48	-0.66	-0.41	-0.59	-0.04	-0.23	0.10	-0.08	0.05	0.24	0.02	-0.17	0.64	0.50	0.43	0.74	0.68	0.42	0.36	0.58
P19388	DNA-directed RNA polymerases I, II, and III subunit RPABC1 OS=Homo sapiens GN=POLR2E PE=1 SV=4 - [RPAB1_HUMAN]	23.33	1	4	4	4	-0.74	-0.63	-1.27	-1.27	0.01	-0.01	-0.75	-0.85	-0.01	-0.23	-0.45	-0.01	-0.56	0.32	1.18	1.42	0.89	0.99	1.25	0.58
Q9ULE6	Paladin OS=Homo sapiens GN=PALD1 PE=1 SV=3 - [PALD_HUMAN]	5.96	1	5	5	7	-0.74	-0.85	-0.70	-0.53	-0.18	0.07	-0.18	-0.08	-0.26	-0.15	-0.13	0.06	0.61	0.79	0.51	0.61	0.59	0.71	0.70	0.58
P06396	Gelsolin OS=Homo sapiens GN=GSN PE=1 SV=1 - [GELS_HUMAN]	40.15	1	26	26	104	-0.82	-0.85	-0.85	-0.79	-0.37	-0.33	-0.28	-0.27	-0.30	-0.34	-0.18	-0.19	0.52	0.60	0.66	0.52	0.57	0.40	0.42	0.58

P40763	Signal transducer and activator of transcription 3 OS=Homo sapiens GN=STAT3 PE=1 SV=2 - [STAT3_HUMAN]	23.51	1	14	15	25	-1.21	-1.22	-0.97	-1.08	0.06	-0.05	-0.55	-0.56	-0.36	-0.31	-0.17	-0.12	0.59	0.96	1.02	0.89	0.86	1.04	1.07	0.58
P20618	Proteasome subunit beta type-1 OS=Homo sapiens GN=PSMB1 PE=1 SV=2 - [PSB1_HUMAN]	50.21	1	8	8	22	-0.24	-0.21	-0.36	-0.45	0.12	0.14	0.10	0.09	0.06	0.30	0.58	0.49	0.35	0.72	0.94	0.53	0.71	0.46	0.53	0.58
P12318	Low affinity immunoglobulin gamma Fc region receptor II-a OS=Homo sapiens GN=FCGR2A PE=1 SV=4 - [FCG2A_HUMAN]	5.36	3	2	2	3	0.20	0.04	0.06	-0.11	1.12	0.95	0.57	0.41	0.73	0.90	0.95	0.78	0.43	0.75	0.89	0.73	0.88	0.91	1.05	0.58
P35606	Coatomer subunit beta' OS=Homo sapiens GN=COPB2 PE=1 SV=2 - [COPB2_HUMAN]	29.91	1	21	21	44	-0.59	-0.58	-0.64	-0.62	0.12	0.22	-0.02	-0.09	0.01	0.02	0.14	0.14	0.54	0.70	0.78	0.61	0.69	0.77	0.86	0.58
Q9Y4E8	Ubiquitin carboxyl-terminal hydrolase 15 OS=Homo sapiens GN=USP15 PE=1 SV=3 - [UBP15_HUMAN]	21.81	1	18	18	28	-0.57	-0.52	-0.66	-0.56	-0.01	0.19	-0.08	-0.04	0.10	-0.02	0.18	0.25	0.63	0.78	0.86	0.66	0.67	0.57	0.65	0.58
Q9Y3B3	Transmembrane emp24 domain-containing protein 7 OS=Homo sapiens GN=TMED7 PE=1 SV=2 - [TMED7_HUMAN]	24.11	1	4	4	7	-0.74	-0.53	-0.84	-0.60	-0.05	0.18	-0.37	-0.11	0.05	-0.18	0.21	0.43	0.33	0.87	1.08	0.63	0.81	0.70	0.77	0.58
Q14166	Tubulin--tyrosine ligase-like protein 12 OS=Homo sapiens GN=TTLL12 PE=1 SV=2 - [TTL12_HUMAN]	27.02	1	12	12	31	-0.14	-0.03	-0.30	-0.17	0.30	0.41	0.20	0.33	0.30	0.34	0.08	0.21	0.54	0.26	0.41	0.39	0.53	0.35	0.51	0.58

P34897	Serine hydroxymethyltransferase, mitochondrial OS=Homo sapiens GN=SHMT2 PE=1 SV=3 - [GLYM_HUMAN]	26.19	1	11	12	21	-1.21	-1.13	-1.34	-1.35	-0.57	-0.57	-0.70	-0.63	-0.68	-0.55	-0.48	-0.36	0.56	0.73	0.80	0.48	0.68	0.52	0.75	0.58
P13667	Protein disulfide-isomerase A4 OS=Homo sapiens GN=PDIA4 PE=1 SV=2 - [PDIA4_HUMAN]	45.43	1	25	25	64	-1.44	-1.45	-1.43	-1.29	-0.57	-0.58	-0.84	-0.81	-0.69	-0.77	-0.51	-0.50	0.66	0.99	0.75	0.71	0.64	0.85	0.71	0.57
Q2YD98	UV-stimulated scaffold protein A OS=Homo sapiens GN=UVSSA PE=1 SV=2 - [UVSSA_HUMAN]	1.27	1	1	1	1	-0.91	-0.76	-0.36	-0.20	-0.36	-0.22	0.16	0.31	0.28	0.14	-0.21	-0.07	1.12	0.70	0.14	1.08	0.53	0.53	-0.03	0.57
Q9HB19	Pleckstrin homology domain-containing family A member 2 OS=Homo sapiens GN=PLEKHA2 PE=1 SV=2 - [PKHA2_HUMAN]	9.88	1	3	3	4	-0.73	-0.61	-0.56	-0.45	0.10	0.20	-0.05	0.06	0.12	0.02	-0.19	-0.08	0.73	0.55	0.37	0.77	0.61	0.81	0.64	0.57
Q15363	Transmembrane emp24 domain-containing protein 2 OS=Homo sapiens GN=TMED2 PE=1 SV=1 - [TMED2_HUMAN]	20.40	1	3	3	7	-0.48	-0.44	-0.77	-0.63	0.43	0.28	-0.26	-0.13	0.03	0.19	0.25	0.28	0.42	0.73	1.06	0.51	1.00	0.56	1.17	0.57
Q7Z6K3	Protein prenyltransferase alpha subunit repeat-containing protein 1 OS=Homo sapiens GN=PTAR1 PE=1 SV=2 - [PTAR1_HUMAN]	3.73	1	1	1	1	0.02	-0.01	0.45	0.42	1.10	1.07	0.95	0.93	0.85	0.88	0.64	0.60	0.99	0.62	0.19	0.89	0.47	1.07	0.64	0.57
Q562R1	Beta-actin-like protein 2 OS=Homo sapiens GN=ACTBL2 PE=1 SV=2 - [ACTBL_HUMAN]	24.73	1	3	12	556	-0.80	-0.79	-0.69	-0.66	0.04	0.07	-0.18	-0.15	0.32	0.29	-0.38	-0.35	0.68	0.45	0.31	1.07	1.02	0.84	0.71	0.57

Q53SF7	Cordon-bleu protein-like 1 OS=Homo sapiens GN=COBLL1 PE=1 SV=2 - [COBL1_HUMAN]	1.66	1	2	2	3	-1.50	-1.44	-1.31	-1.25	-0.48	-0.43	-0.80	-0.75	-0.66	-0.71	-0.54	-0.49	0.75	0.96	0.77	0.82	0.63	1.00	0.81	0.57
Q9Y279	V-set and immunoglobulin domain-containing protein 4 OS=Homo sapiens GN=VSIG4 PE=1 SV=1 - [VSIG4_HUMAN]	13.53	1	4	4	7	0.02	-0.47	-0.19	-0.50	1.07	0.74	0.32	0.07	0.47	0.79	1.57	1.14	0.35	1.86	1.62	1.03	0.96	1.10	1.17	0.57
Q5JTD0	Tight junction-associated protein 1 OS=Homo sapiens GN=TJAP1 PE=1 SV=1 - [TJAP1_HUMAN]	6.82	1	2	3	5	-0.43	-0.36	-0.74	-0.68	0.87	0.93	-0.23	-0.17	0.37	0.31	0.22	0.28	0.25	0.65	0.96	0.77	1.09	1.28	1.60	0.57
Q9Y3C4	EKC/KEOPS complex subunit TPRKB OS=Homo sapiens GN=TPRKB PE=1 SV=1 - [TPRKB_HUMAN]	17.14	1	2	2	2	1.34	0.80	0.51	-0.03	0.95	0.40	1.02	0.48	0.20	0.75	-0.39	-0.94	-0.27	-1.72	-0.90	-0.56	0.27	-0.41	0.42	0.57
O95376	E3 ubiquitin-protein ligase ARIH2 OS=Homo sapiens GN=ARIH2 PE=1 SV=1 - [ARI2_HUMAN]	5.48	1	2	2	4	-0.41	-0.48	-0.39	-0.46	0.29	0.21	0.12	0.05	0.22	0.30	0.50	0.43	0.58	0.91	0.89	0.74	0.72	0.68	0.66	0.57
Q9H553	Alpha-1,3/1,6-mannosyltransferase ALG2 OS=Homo sapiens GN=ALG2 PE=1 SV=1 - [ALG2_HUMAN]	8.65	1	3	3	6	-0.61	-0.58	-0.64	-0.62	0.40	0.42	-0.14	-0.11	0.16	0.14	0.16	0.18	0.53	0.78	0.81	0.78	0.82	1.00	1.03	0.57
P0CW22	40S ribosomal protein S17-like OS=Homo sapiens GN=RPS17L PE=1 SV=1 - [RS17L_HUMAN]	32.59	1	4	4	13	-1.12	-1.20	-0.89	-1.01	-0.74	-0.79	-0.46	-0.65	-0.82	-0.76	-0.47	-0.56	0.77	0.65	0.42	0.51	0.28	0.54	0.31	0.57
Q9P2X3	Protein IMPACT OS=Homo sapiens GN=IMPACT PE=1 SV=2 - [IMPCT_HUMAN]	15.94	1	4	4	10	0.36	0.03	-0.48	-0.53	0.20	-0.49	0.28	-0.02	-0.02	0.28	0.58	0.25	0.05	0.23	0.49	0.21	0.65	0.22	0.46	0.57

Q96A83	Collagen alpha-1(XXVI) chain OS=Homo sapiens GN=COL26A1 PE=1 SV=1 - [COQA1_HUMAN]	4.54	2	2	2	2	-1.69	-1.81	-1.73	-1.86	-0.70	-0.83	-1.22	-1.35	-0.92	-0.79	-0.33	-0.46	0.52	1.36	1.40	0.93	0.98	0.97	1.01	0.57
Q07954	Prolow-density lipoprotein receptor-related protein 1 OS=Homo sapiens GN=LRP1 PE=1 SV=2 - [LRP1_HUMAN]	25.40	1	86	87	208	-0.74	-0.67	-0.83	-0.77	-0.01	0.08	-0.31	-0.21	0.15	0.07	0.45	0.50	0.50	1.20	1.23	0.77	0.88	0.75	0.74	0.57
Q9H0N5	Pterin-4-alpha-carbinolamine dehydratase 2 OS=Homo sapiens GN=PCBD2 PE=1 SV=4 - [PHS2_HUMAN]	34.62	1	3	4	12	-0.73	-0.42	-0.22	-0.30	0.39	0.23	0.22	0.20	0.19	-0.02	0.36	0.28	0.86	0.83	0.59	0.70	0.41	0.66	0.58	0.57
O60711	Leupaxin OS=Homo sapiens GN=LPXN PE=1 SV=1 - [LPXN_HUMAN]	8.55	1	2	2	3	-0.20	-0.09	-1.02	-0.91	0.06	0.17	-0.52	-0.41	0.55	0.45	0.37	0.48	-0.27	0.57	1.39	0.67	1.50	0.24	1.07	0.56
P48506	Glutamate--cysteine ligase catalytic subunit OS=Homo sapiens GN=GCLC PE=1 SV=2 - [GSH1_HUMAN]	33.75	1	17	17	31	0.03	0.11	0.13	0.09	0.49	0.56	0.63	0.72	0.54	0.51	0.20	0.26	0.58	0.10	0.05	0.46	0.35	0.37	0.33	0.56
O75023	Leukocyte immunoglobulin-like receptor subfamily B member 5 OS=Homo sapiens GN=LILRB5 PE=1 SV=1 - [LIRB5_HUMAN]	6.27	1	2	2	3	0.23	0.06	-0.66	-0.83	0.14	-0.03	-0.16	-0.33	-0.30	-0.13	-0.15	-0.32	-0.33	-0.37	0.52	-0.33	0.57	-0.10	0.79	0.56
Q5BKZ1	DBIRD complex subunit ZNF326 OS=Homo sapiens GN=ZNF326 PE=1 SV=2 - [ZN326_HUMAN]	8.76	1	4	4	5	-0.71	-0.57	-1.02	-0.87	-0.55	-0.41	-0.52	-0.37	-0.70	-0.83	-0.89	-0.75	0.25	-0.18	0.13	-0.10	0.22	0.14	0.45	0.56

Q9UJ70	N-acetyl-D-glucosamine kinase OS=Homo sapiens GN=NAGK PE=1 SV=4 - [NAGK_HUMAN]	50.00	1	14	14	24	-0.46	-0.24	-0.16	-0.06	0.20	0.44	0.15	0.45	0.39	0.13	0.13	0.21	0.61	0.54	0.26	0.58	0.45	0.68	0.48	0.56
O95613	Pericentrin OS=Homo sapiens GN=PCNT PE=1 SV=4 - [PCNT_HUMAN]	1.11	1	3	3	3	1.53	1.38	0.48	0.33	0.74	0.58	0.98	0.83	0.10	0.26	0.22	0.06	-0.49	-1.30	-0.26	-1.24	-0.19	-0.81	0.24	0.56
P21964	Catechol O-methyltransferase OS=Homo sapiens GN=COMT PE=1 SV=2 - [COMT_HUMAN]	60.89	1	14	14	23	-0.87	-0.83	-0.47	-0.56	0.24	0.18	-0.05	-0.02	0.07	0.10	0.35	0.39	0.79	1.04	0.91	0.80	0.75	0.80	0.80	0.56
Q69YN4	Protein virilizer homolog OS=Homo sapiens GN=KIAA1429 PE=1 SV=2 - [VIR_HUMAN]	1.60	1	2	3	4	-0.56	-0.42	-0.64	-0.50	0.04	0.17	-0.14	0.00	-0.07	-0.20	-0.12	0.00	0.48	0.44	0.51	0.39	0.47	0.58	0.66	0.56
Q96EK6	Glucosamine 6-phosphate N-acetyltransferase OS=Homo sapiens GN=GNPNAT1 PE=1 SV=1 - [GNA1_HUMAN]	5.98	1	1	1	2	-0.02	0.38	-0.33	0.06	0.50	0.90	0.16	0.56	0.96	0.57	0.55	0.94	0.24	0.57	0.88	0.61	0.94	0.50	0.82	0.56
Q8TDG2	Actin-related protein T1 OS=Homo sapiens GN=ACTRT1 PE=2 SV=2 - [ACTT1_HUMAN]	2.93	1	1	1	1	0.18	0.17	0.13	0.12	0.92	0.91	0.63	0.62	0.92	0.93	1.03	1.02	0.50	0.86	0.90	0.78	0.83	0.73	0.78	0.56
Q3B8N2	Galectin-9B OS=Homo sapiens GN=LGALS9B PE=2 SV=3 - [LEG9B_HUMAN]	7.02	2	2	2	9	0.07	0.07	-0.05	-0.04	0.20	0.26	0.29	0.30	0.54	0.54	0.70	0.70	0.42	0.67	0.77	0.66	0.79	0.86	0.99	0.56
O15439	Multidrug resistance-associated protein 4 OS=Homo sapiens GN=ABCC4 PE=1 SV=3 - [MRP4_HUMAN]	11.17	1	11	11	14	-0.42	-0.49	-0.80	-0.70	0.10	0.15	-0.12	-0.03	0.16	0.18	-0.07	0.09	0.27	0.46	0.72	0.43	0.76	0.61	0.87	0.56

Q86Y56	HEAT repeat-containing protein 2 OS=Homo sapiens GN=HEATR2 PE=1 SV=4 - [HEAT2_HUMAN]	2.34	1	1	1	1	0.36	-0.68	0.20	-0.84	1.00	-0.04	0.70	-0.34	-0.40	0.65	0.32	-0.73	0.40	-0.04	0.11	0.32	0.48	0.63	0.78	0.56
O60216	Double-strand-break repair protein rad21 homolog OS=Homo sapiens GN=RAD21 PE=1 SV=2 - [RAD21_HUMAN]	14.42	1	6	6	9	-0.69	-0.70	-0.64	-0.82	0.03	-0.11	-0.14	-0.32	-0.25	-0.01	0.18	-0.18	0.42	0.68	0.91	0.44	0.54	0.54	0.64	0.56
Q9P0M9	39S ribosomal protein L27, mitochondrial OS=Homo sapiens GN=MRPL27 PE=1 SV=1 - [RM27_HUMAN]	7.43	1	1	1	1	0.11	0.02	0.01	-0.09	1.27	1.16	0.50	0.41	1.12	1.23	0.62	0.52	0.45	0.52	0.62	1.14	1.25	1.14	1.24	0.56
Q8NI22	Multiple coagulation factor deficiency protein 2 OS=Homo sapiens GN=MCFD2 PE=1 SV=1 - [MCFD2_HUMAN]	44.52	1	3	3	12	-1.26	-1.34	-0.95	-1.04	-0.14	-0.13	-0.38	-0.37	-0.30	-0.11	0.34	-0.05	0.96	1.48	1.08	0.91	0.59	1.22	0.81	0.56
O00233	26S proteasome non-ATPase regulatory subunit 9 OS=Homo sapiens GN=PSMD9 PE=1 SV=3 - [PSMD9_HUMAN]	35.43	1	8	8	17	-0.63	-0.69	-0.69	-0.83	0.10	0.23	-0.17	-0.13	0.26	0.02	0.19	0.35	0.49	0.77	0.91	0.61	0.87	0.70	0.63	0.56
P11172	Uridine 5'-monophosphate synthase OS=Homo sapiens GN=UMPS PE=1 SV=1 - [UMPS_HUMAN]	17.71	1	9	9	14	-0.66	-0.62	-0.56	-0.59	0.00	0.06	0.04	0.01	-0.04	-0.06	0.06	0.09	0.60	0.57	0.37	0.61	0.55	0.55	0.40	0.56
Q14693	Phosphatidate phosphatase LPIN1 OS=Homo sapiens GN=LPIN1 PE=1 SV=2 - [LPIN1_HUMAN]	2.92	1	1	1	1	0.29	0.10	-0.18	-0.37	0.54	0.34	0.32	0.12	0.69	0.90	1.12	0.92	0.08	0.83	1.30	0.63	1.10	0.23	0.70	0.56

Q86XE5	4-hydroxy-2-oxoglutarate aldolase, mitochondrial OS=Homo sapiens GN=HOGA1 PE=1 SV=1 - [HOGA1_HUMAN]	4.59	1	1	1	1	0.78	0.44	0.16	-0.18	0.13	-0.22	0.66	0.32	0.00	0.34	0.30	-0.04	-0.07	-0.47	0.14	-0.41	0.21	-0.67	-0.05	0.56
Q9NP74	Palmdelphin OS=Homo sapiens GN=PALMD PE=1 SV=1 - [PALMD_HUMAN]	8.53	1	3	3	5	-0.90	-0.96	-1.16	-1.11	-0.27	-0.23	-0.53	-0.53	-0.04	-0.08	-0.19	-0.19	0.46	0.73	1.06	0.79	1.11	0.48	0.87	0.56
Q96JC1	Vam6/Vps39-like protein OS=Homo sapiens GN=VPS39 PE=1 SV=2 - [VPS39_HUMAN]	9.37	1	7	7	9	-0.01	0.09	-0.21	-0.16	0.30	0.53	0.08	0.32	-0.05	-0.13	-0.26	0.24	0.29	0.19	0.45	0.43	0.66	0.68	0.85	0.56
Q6NUQ1	RAD50-interacting protein 1 OS=Homo sapiens GN=RINT1 PE=1 SV=1 - [RINT1_HUMAN]	2.40	1	2	2	2	-0.94	-0.80	-0.88	-0.74	0.11	0.25	-0.39	-0.25	0.01	-0.12	0.13	0.26	0.61	1.07	1.00	0.84	0.79	1.03	0.98	0.56
P53621	Coatomer subunit alpha OS=Homo sapiens GN=COPA PE=1 SV=2 - [COPA_HUMAN]	24.75	1	24	24	46	-0.65	-0.74	-0.62	-0.66	0.29	0.18	-0.04	-0.14	0.02	0.06	0.06	0.01	0.63	0.66	0.63	0.68	0.67	0.95	0.89	0.55
Q8N4T8	Carbonyl reductase family member 4 OS=Homo sapiens GN=CBR4 PE=1 SV=3 - [CBR4_HUMAN]	26.58	1	5	5	11	-0.40	-0.44	-0.19	-0.23	0.53	0.67	0.16	0.00	0.50	0.32	0.39	-0.04	0.95	0.83	0.71	0.54	-0.01	0.73	0.18	0.55
Q9P003	Protein cornichon homolog 4 OS=Homo sapiens GN=CNIH4 PE=1 SV=1 - [CNIH4_HUMAN]	14.39	1	1	1	6	-0.63	-0.41	-0.84	-0.62	0.24	0.45	-0.35	-0.13	0.06	-0.15	-0.01	0.20	0.33	0.62	0.83	0.51	0.73	0.85	1.06	0.55
Q8TB61	Adenosine 3'-phospho 5'-phosphosulfate transporter 1 OS=Homo sapiens GN=SLC35B2 PE=1 SV=1 - [S35B2_HUMAN]	2.31	1	1	1	2	-0.34	-0.31	-0.54	-0.51	0.07	0.09	-0.05	-0.02	0.02	0.00	0.04	0.07	0.34	0.39	0.58	0.37	0.57	0.38	0.59	0.55

Q9H2K8	Serine/threonine-protein kinase TAO3 OS=Homo sapiens GN=TAOK3 PE=1 SV=2 - [TAOK3_HUMAN]	5.57	1	4	5	6	-0.27	-0.21	-0.36	-0.31	-0.10	-0.05	0.14	0.19	0.09	0.04	0.09	0.14	0.40	0.37	0.45	0.34	0.44	0.15	0.24	0.55
Q96HY6	DDRGK domain-containing protein 1 OS=Homo sapiens GN=DDRGK1 PE=1 SV=2 - [DDRGK_HUMAN]	25.48	1	4	4	13	-0.32	-0.33	-0.58	-0.58	0.03	-0.01	-0.09	-0.09	0.11	0.10	0.06	0.05	0.29	0.23	0.58	0.43	0.70	0.33	0.57	0.55
P53990	IST1 homolog OS=Homo sapiens GN=IST1 PE=1 SV=1 - [IST1_HUMAN]	16.21	1	6	6	13	-0.87	-0.93	-0.50	-0.59	0.50	0.37	-0.06	-0.17	0.09	0.24	0.28	0.18	1.01	1.26	0.81	1.27	0.88	1.48	1.26	0.55
P48681	Nestin OS=Homo sapiens GN=NES PE=1 SV=2 - [NEST_HUMAN]	27.88	1	33	33	64	-1.16	-1.15	-1.34	-1.19	-0.25	-0.19	-0.82	-0.73	-0.38	-0.46	-0.39	-0.29	0.47	0.85	0.95	0.76	0.79	0.94	0.99	0.55
Q9BQI0	Allograft inflammatory factor 1-like OS=Homo sapiens GN=AIF1L PE=1 SV=1 - [AIF1L_HUMAN]	35.33	2	4	5	9	-1.95	-1.98	-1.51	-1.52	-1.46	-1.38	-1.04	-1.04	-0.92	-1.15	-0.94	-0.89	0.80	1.01	0.92	1.02	0.82	0.48	0.23	0.55
P07305	Histone H1.0 OS=Homo sapiens GN=H1F0 PE=1 SV=3 - [H10_HUMAN]	17.01	1	4	4	21	-1.44	-1.43	-1.29	-1.34	-1.13	-1.18	-0.84	-0.89	-1.06	-0.97	-0.70	-0.74	0.60	0.76	0.58	0.43	0.31	0.24	0.21	0.55
Q5EBL8	PDZ domain-containing protein 11 OS=Homo sapiens GN=PDZD11 PE=1 SV=2 - [PDZ11_HUMAN]	48.57	1	4	5	7	-0.68	-0.32	-0.49	-0.33	0.18	0.15	0.04	0.15	0.03	-0.12	0.23	0.16	0.52	0.49	0.50	0.38	0.40	0.84	0.64	0.55
O43505	N-acetyllactosaminide beta-1,3-N-acetylglucosaminyltransferase OS=Homo sapiens GN=B3GNT1 PE=1 SV=1 - [B3GN1_HUMAN]	18.80	1	6	6	9	-0.27	0.12	-0.45	-0.36	0.38	0.22	0.50	0.28	0.29	0.05	0.60	0.42	0.37	0.45	0.82	0.36	0.54	0.37	0.48	0.55

P09467	Fructose-1,6-bisphosphatase 1 OS=Homo sapiens GN=FBP1 PE=1 SV=5 - [F16P1_HUMAN]	10.95	1	2	2	2	-0.15	-0.23	0.00	-0.08	0.23	0.14	0.49	0.41	0.42	0.50	0.51	0.42	0.69	0.66	0.50	0.68	0.53	0.36	0.21	0.55
Q9Y3C8	Ubiquitin-fold modifier-conjugating enzyme 1 OS=Homo sapiens GN=UFC1 PE=1 SV=3 - [UFC1_HUMAN]	21.56	1	4	4	8	0.47	0.51	0.27	0.40	0.72	0.81	0.82	0.87	0.79	0.61	0.18	0.41	0.39	-0.01	0.02	0.42	0.43	0.29	0.41	0.55
P60604	Ubiquitin-conjugating enzyme E2 G2 OS=Homo sapiens GN=UBE2G2 PE=1 SV=1 - [UB2G2_HUMAN]	22.42	1	2	2	4	-0.12	-0.05	-0.57	-0.50	0.03	0.10	-0.09	-0.02	-0.17	-0.23	-0.08	-0.02	0.08	0.04	0.49	-0.09	0.37	0.13	0.59	0.55
P06737	Glycogen phosphorylase, liver form OS=Homo sapiens GN=PYGL PE=1 SV=4 - [PYGL_HUMAN]	32.00	1	14	22	52	-0.95	-0.71	-0.94	-1.02	0.03	0.11	-0.06	-0.22	0.46	0.44	1.17	1.29	0.60	2.18	2.22	1.35	1.41	0.95	0.98	0.55
O95071	E3 ubiquitin-protein ligase UBR5 OS=Homo sapiens GN=UBR5 PE=1 SV=2 - [UBR5_HUMAN]	0.50	1	1	1	1	-1.05	-0.97	-0.74	-0.65	-0.22	-0.13	-0.26	-0.17	-0.17	-0.25	-0.21	-0.12	0.85	0.85	0.53	0.83	0.52	0.82	0.51	0.55
Q96ND0	Protein FAM210A OS=Homo sapiens GN=FAM210A PE=1 SV=2 - [F210A_HUMAN]	8.09	1	2	2	3	1.11	1.19	0.93	1.01	-0.12	-0.05	1.41	1.49	1.04	0.96	-0.12	-0.05	0.36	-1.22	-1.05	-0.11	0.07	-1.25	-1.07	0.54
Q9H0V9	VIP36-like protein OS=Homo sapiens GN=LMAN2L PE=1 SV=1 - [LMA2L_HUMAN]	14.66	1	4	4	9	-1.10	-0.80	-0.91	-0.85	-0.11	-0.12	-0.43	-0.34	-0.03	0.02	-0.05	0.05	0.51	0.86	0.91	0.70	0.72	0.92	0.78	0.54
P12821	Angiotensin-converting enzyme OS=Homo sapiens GN=ACE PE=1 SV=1 - [ACE_HUMAN]	5.36	1	5	5	6	0.03	0.04	-0.56	-0.35	0.04	0.35	-0.30	-0.12	0.35	0.04	0.47	0.42	0.02	0.45	0.83	0.49	0.86	0.29	0.81	0.54

P26583	High mobility group protein B2 OS=Homo sapiens GN=HMGB2 PE=1 SV=2 - [HMGB2_HUMAN]	11.48	1	2	2	4	-0.80	-0.84	-0.48	-0.52	-0.88	-0.92	0.00	-0.04	-0.04	0.00	-0.19	-0.24	0.85	0.61	0.29	0.83	0.52	-0.10	-0.41	0.54
Q92616	Translational activator GCN1 OS=Homo sapiens GN=GCN1L1 PE=1 SV=6 - [GCN1L_HUMAN]	12.58	1	28	28	44	-0.49	-0.50	-0.50	-0.56	0.25	0.25	-0.13	-0.04	-0.03	0.04	0.07	-0.05	0.39	0.57	0.60	0.57	0.63	0.74	0.86	0.54
Q6ZV29	Patatin-like phospholipase domain-containing protein 7 OS=Homo sapiens GN=PNPLA7 PE=2 SV=3 - [PLPL7_HUMAN]	2.51	1	1	3	4	-0.76	-1.11	-0.60	-0.95	0.50	0.15	-0.12	-0.47	-0.31	0.05	0.56	0.21	0.69	1.32	1.16	0.84	0.68	1.24	1.08	0.54
Q13868	Exosome complex component RRP4 OS=Homo sapiens GN=EXOSC2 PE=1 SV=2 - [EXOS2_HUMAN]	11.26	1	2	2	4	-0.71	-0.59	-0.86	-0.74	-0.08	0.04	-0.38	-0.26	-0.06	-0.18	-0.31	-0.19	0.38	0.40	0.55	0.56	0.71	0.61	0.76	0.54
Q9UGP8	Translocation protein SEC63 homolog OS=Homo sapiens GN=SEC63 PE=1 SV=2 - [SEC63_HUMAN]	12.76	1	8	8	19	-0.57	-0.49	-0.56	-0.68	0.19	0.15	-0.05	-0.08	0.10	0.07	0.30	-0.01	0.39	0.75	0.79	0.66	0.67	0.58	0.73	0.54
Q86UY8	5'-nucleotidase domain-containing protein 3 OS=Homo sapiens GN=NT5DC3 PE=2 SV=1 - [NT5D3_HUMAN]	18.80	1	7	7	11	-0.22	-0.47	-0.28	-0.23	-0.01	-0.22	0.07	-0.06	-0.22	-0.19	-0.03	-0.11	0.72	0.18	0.25	0.37	0.35	0.34	0.26	0.54
P26038	Moesin OS=Homo sapiens GN=MSN PE=1 SV=3 - [MOES_HUMAN]	42.98	1	21	31	86	-0.76	-0.67	-0.78	-0.69	0.24	0.08	-0.21	-0.26	-0.18	-0.13	-0.33	-0.44	0.70	0.23	0.25	0.50	0.49	0.99	0.87	0.54
P20073	Annexin A7 OS=Homo sapiens GN=ANXA7 PE=1 SV=3 - [ANXA7_HUMAN]	20.29	1	9	9	36	-0.38	-0.41	-0.46	-0.49	0.19	0.20	0.01	0.02	0.22	0.19	0.43	0.44	0.42	0.81	0.89	0.68	0.81	0.71	0.77	0.54

Q92973	Transportin-1 OS=Homo sapiens GN=TNPO1 PE=1 SV=2 - [TNPO1_HUMAN]	12.03	1	7	9	20	-0.35	-0.30	-0.22	-0.43	0.39	0.25	0.04	0.05	0.29	0.30	0.37	0.37	0.34	0.82	0.85	0.78	0.59	0.77	0.64	0.54
P01781	Ig heavy chain V-III region GAL OS=Homo sapiens PE=1 SV=1 - [HV320_HUMAN]	31.03	2	3	3	16	-1.17	-0.81	-1.71	-1.17	0.04	0.35	-0.96	-0.61	0.21	-0.10	0.65	0.96	0.17	1.99	2.39	1.11	1.60	1.17	1.41	0.54
Q13601	KRR1 small subunit processome component homolog OS=Homo sapiens GN=KRR1 PE=1 SV=4 - [KRR1_HUMAN]	2.10	1	1	1	1	-0.82	-0.82	-0.75	-0.76	0.10	0.09	-0.28	-0.28	-0.34	-0.33	-0.34	-0.35	0.60	0.48	0.42	0.52	0.46	0.90	0.84	0.54
Q8NBF2	NHL repeat-containing protein 2 OS=Homo sapiens GN=NHLRC2 PE=1 SV=1 - [NHLC2_HUMAN]	18.46	1	9	9	15	-0.44	-0.46	-0.40	-0.55	0.06	0.05	0.11	-0.01	0.07	0.16	-0.05	-0.13	0.58	0.39	0.38	0.64	0.60	0.51	0.49	0.54
Q8WUM0	Nuclear pore complex protein Nup133 OS=Homo sapiens GN=NUP133 PE=1 SV=2 - [NU133_HUMAN]	11.25	1	8	8	11	-0.47	-0.41	-0.63	-0.34	0.22	0.01	-0.17	-0.07	-0.16	-0.17	0.02	0.17	0.24	0.37	0.62	0.32	0.35	0.19	0.38	0.54
Q06210	Glutamine--fructose-6-phosphate aminotransferase [isomerizing] 1 OS=Homo sapiens GN=GFPT1 PE=1 SV=3 - [GFPT1_HUMAN]	15.59	2	8	8	12	-0.22	0.08	-0.06	0.10	0.75	0.69	0.45	0.50	0.61	0.46	0.42	0.60	0.65	0.54	0.44	0.56	0.49	0.94	0.65	0.54
P01019	Angiotensinogen OS=Homo sapiens GN=AGT PE=1 SV=1 - [ANGT_HUMAN]	12.78	1	5	5	6	-0.61	-0.68	-0.45	-0.52	0.09	0.01	0.02	-0.05	-0.13	-0.05	0.38	0.30	0.69	0.99	0.83	0.58	0.43	0.68	0.53	0.54
P32119	Peroxiredoxin-2 OS=Homo sapiens GN=PRDX2 PE=1 SV=5 - [PRDX2_HUMAN]	54.04	1	10	11	131	-1.35	-1.34	-1.06	-1.06	-0.32	-0.39	-0.60	-0.64	-0.76	-0.63	-1.01	-1.04	0.70	0.19	0.08	0.66	0.43	0.92	0.69	0.54

Q9Y2V7	Conserved oligomeric Golgi complex subunit 6 OS=Homo sapiens GN=COG6 PE=1 SV=2 - [COG6_HUMAN]	4.11	1	2	3	5	-0.26	-0.11	-0.61	-0.47	0.24	0.38	-0.14	0.01	0.26	0.12	0.31	0.45	0.17	0.57	0.92	0.41	0.77	0.48	0.84	0.54
Q13200	26S proteasome non-ATPase regulatory subunit 2 OS=Homo sapiens GN=PSMD2 PE=1 SV=3 - [PSMD2_HUMAN]	39.21	1	26	26	57	-0.50	-0.53	-0.46	-0.37	0.11	0.14	0.02	0.10	0.12	0.05	0.27	0.27	0.61	0.78	0.79	0.59	0.61	0.67	0.53	0.54
Q96A57	Transmembrane protein 230 OS=Homo sapiens GN=TMEM230 PE=1 SV=1 - [TM230_HUMAN]	20.83	1	2	2	4	-0.63	-0.65	-0.55	-0.56	-0.46	-0.66	-0.25	-0.07	-0.43	-0.23	-0.20	-0.31	0.43	0.43	0.33	0.45	0.24	0.21	-0.01	0.54
Q99805	Transmembrane 9 superfamily member 2 OS=Homo sapiens GN=TM9SF2 PE=1 SV=1 - [TM9S2_HUMAN]	7.84	1	4	4	8	-0.07	-0.25	-0.53	-0.56	0.46	0.45	0.05	-0.08	0.33	0.53	0.46	0.33	0.17	0.67	1.08	0.59	0.91	0.52	0.97	0.54
Q8NFH5	Nucleoporin NUP53 OS=Homo sapiens GN=NUP35 PE=1 SV=1 - [NUP53_HUMAN]	9.82	1	2	2	3	-0.63	-0.51	-0.91	-0.79	-0.38	-0.26	-0.44	-0.31	-0.19	-0.30	-0.14	-0.02	0.25	0.50	0.77	0.36	0.64	0.24	0.52	0.54
P11310	Medium-chain specific acyl-CoA dehydrogenase, mitochondrial OS=Homo sapiens GN=ACADM PE=1 SV=1 - [ACADM_HUMAN]	31.59	1	13	13	29	-0.55	-0.48	-0.49	-0.45	-0.28	-0.26	0.03	0.04	-0.16	-0.20	-0.23	-0.21	0.47	0.15	0.14	0.32	0.26	0.14	0.13	0.54
P42694	Probable helicase with zinc finger domain OS=Homo sapiens GN=HELZ PE=1 SV=2 - [HELZ_HUMAN]	1.03	1	1	1	2	-1.09	-1.09	-1.25	-1.25	-0.29	-0.30	-0.78	-0.78	-0.41	-0.40	-0.35	-0.35	0.36	0.74	0.90	0.71	0.88	0.78	0.94	0.54

P18065	Insulin-like growth factor-binding protein 2 OS=Homo sapiens GN=IGFBP2 PE=1 SV=2 - [IBP2_HUMAN]	15.38	1	4	4	7	0.20	0.30	-0.08	0.17	1.10	1.31	0.34	0.59	1.20	0.89	1.47	1.74	0.13	1.24	1.75	0.72	1.22	0.78	1.37	0.54
P29590	Protein PML OS=Homo sapiens GN=PML PE=1 SV=3 - [PML_HUMAN]	10.66	1	9	9	15	-0.74	-0.76	-0.93	-0.82	0.06	0.10	-0.41	-0.33	-0.18	-0.18	0.03	0.05	0.43	0.80	0.97	0.68	0.85	0.83	0.98	0.53
O95999	B-cell lymphoma/leukemia 10 OS=Homo sapiens GN=BCL10 PE=1 SV=1 - [BCL10_HUMAN]	4.29	1	1	1	1	0.73	0.23	0.56	0.06	1.32	0.82	1.03	0.53	0.31	0.81	0.74	0.24	0.36	0.02	0.19	0.11	0.29	0.58	0.75	0.53
P23219	Prostaglandin G/H synthase 1 OS=Homo sapiens GN=PTGS1 PE=1 SV=2 - [PGH1_HUMAN]	9.85	1	3	3	10	-0.82	-0.76	-0.88	-0.51	0.03	0.01	-0.26	-0.30	0.21	-0.15	-0.19	-0.01	0.64	1.10	0.82	1.18	0.97	1.15	0.87	0.53
Q96HY7	Probable 2-oxoglutarate dehydrogenase E1 component DHKTD1, mitochondrial OS=Homo sapiens GN=DHTKD1 PE=1 SV=2 - [DHTK1_HUMAN]	6.64	1	3	3	6	-0.92	-0.85	-1.24	-1.16	-0.07	0.00	-0.77	-0.69	-0.31	-0.38	-0.52	-0.45	0.21	0.41	0.71	0.57	0.88	0.84	1.15	0.53
Q15057	Arf-GAP with coiled-coil, ANK repeat and PH domain-containing protein 2 OS=Homo sapiens GN=ACAP2 PE=1 SV=3 - [ACAP2_HUMAN]	18.25	2	10	10	12	-0.35	-0.44	-0.71	-0.70	-0.11	-0.07	-0.01	-0.07	-0.12	-0.13	-0.29	-0.27	0.53	0.28	0.31	0.39	0.57	0.29	0.62	0.53
Q14118	Dystroglycan OS=Homo sapiens GN=DAG1 PE=1 SV=2 - [DAG1_HUMAN]	19.89	1	13	13	35	-1.13	-1.14	-1.03	-1.03	-0.41	-0.36	-0.53	-0.50	-0.49	-0.44	-0.32	-0.31	0.62	0.80	0.76	0.67	0.59	0.66	0.64	0.53
Q7Z3B4	Nucleoporin p54 OS=Homo sapiens GN=NUP54 PE=1 SV=2 - [NUP54_HUMAN]	9.47	1	4	4	6	-0.39	-0.38	-0.55	-0.76	0.15	-0.05	-0.10	-0.30	-0.39	-0.11	0.09	-0.16	0.34	0.48	0.61	0.31	0.41	0.31	0.69	0.53

Q9Y5J9	Mitochondrial import inner membrane translocase subunit Tim8 B OS=Homo sapiens GN=TIMM8B PE=1 SV=1 - [TIM8B_HUMAN]	13.25	1	1	1	2	-0.51	-0.58	-0.54	-0.61	0.31	0.23	-0.07	-0.14	0.27	0.35	0.28	0.20	0.50	0.80	0.82	0.89	0.92	0.81	0.83	0.53
P24468	COUP transcription factor 2 OS=Homo sapiens GN=NR2F2 PE=1 SV=1 - [COT2_HUMAN]	2.42	2	1	1	2	-0.41	-0.55	-0.54	-0.68	-0.32	-0.46	-0.08	-0.22	-0.20	-0.05	0.26	0.12	0.39	0.68	0.81	0.39	0.52	0.07	0.21	0.53
Q12907	Vesicular integral-membrane protein VIP36 OS=Homo sapiens GN=LMAN2 PE=1 SV=1 - [LMAN2_HUMAN]	50.00	1	11	11	26	-0.67	-0.61	-0.71	-0.69	0.10	0.01	-0.26	-0.31	-0.10	0.02	0.10	0.02	0.34	0.76	0.84	0.61	0.55	0.71	0.88	0.53
Q13769	THO complex subunit 5 homolog OS=Homo sapiens GN=THOC5 PE=1 SV=2 - [THOC5_HUMAN]	1.17	1	1	1	1	-0.46	-0.27	-0.57	-0.38	0.33	0.52	-0.10	0.09	0.65	0.46	-0.14	0.05	0.42	0.33	0.43	0.96	1.06	0.78	0.89	0.53
Q8TF05	Serine/threonine-protein phosphatase 4 regulatory subunit 1 OS=Homo sapiens GN=PPP4R1 PE=1 SV=1 - [PP4R1_HUMAN]	0.74	1	1	1	1	-0.69	-0.35	-0.75	-0.41	0.05	0.39	-0.29	0.05	0.06	-0.28	0.17	0.50	0.46	0.87	0.92	0.45	0.51	0.73	0.79	0.53
Q96S66	Chloride channel CLIC like protein 1 OS=Homo sapiens GN=CLCC1 PE=1 SV=1 - [CLCC1_HUMAN]	7.08	1	3	3	4	-0.16	-0.02	-0.61	-0.48	-0.08	0.05	-0.15	-0.01	0.15	0.02	-0.01	0.12	0.07	0.15	0.60	0.21	0.67	0.06	0.52	0.53
Q9NUQ7	Ufm1-specific protease 2 OS=Homo sapiens GN=UFSP2 PE=1 SV=3 - [UFSP2_HUMAN]	9.81	1	3	3	6	-0.14	-0.78	-0.52	-1.16	0.56	-0.09	-0.06	-0.70	-0.17	0.48	0.67	0.02	0.14	0.82	1.19	0.64	1.03	0.68	1.06	0.53

Q7L5Y9	Macrophage erythroblast attacher OS=Homo sapiens GN=MAEA PE=1 SV=1 - [MAEA_HUMAN]	9.60	1	3	3	4	0.19	0.30	-0.13	-0.02	0.12	0.22	0.34	0.45	0.49	0.39	0.11	0.21	0.21	-0.08	0.23	0.23	0.55	-0.09	0.23	0.53
Q9NQT8	Kinesin-like protein KIF13B OS=Homo sapiens GN=KIF13B PE=1 SV=2 - [KI13B_HUMAN]	4.11	1	4	5	11	-0.49	-0.59	-0.40	-0.50	0.65	0.53	0.07	-0.04	-0.05	0.09	-0.01	-0.12	0.61	0.33	0.23	0.71	0.62	1.12	1.02	0.53
P08236	Beta-glucuronidase OS=Homo sapiens GN=GUSB PE=1 SV=2 - [BGLR_HUMAN]	8.14	1	5	5	10	-0.39	-0.25	-0.45	-0.34	0.26	0.25	-0.20	-0.14	0.25	0.20	0.30	0.27	0.28	0.70	0.76	0.52	0.59	0.48	0.60	0.53
Q9Y376	Calcium-binding protein 39 OS=Homo sapiens GN=CAB39 PE=1 SV=1 - [CAB39_HUMAN]	29.62	1	10	11	21	0.01	0.03	0.16	0.18	0.73	0.68	0.41	0.45	0.36	0.28	0.23	0.14	0.39	0.33	0.20	0.50	0.43	0.75	0.60	0.53
Q96AQ6	Pre-B-cell leukemia transcription factor-interacting protein 1 OS=Homo sapiens GN=PBXIP1 PE=1 SV=1 - [PBIP1_HUMAN]	28.59	1	17	17	38	0.15	0.11	0.21	0.22	0.27	0.20	0.69	0.70	0.34	0.48	0.30	0.32	0.50	0.24	0.12	0.35	0.30	-0.01	-0.06	0.53
P0CB43	Protein FAM203B OS=Homo sapiens GN=FAM203B PE=1 SV=1 - [F203B_HUMAN]	8.46	2	3	3	4	-0.35	-0.07	-0.46	-0.19	-0.60	-0.33	0.00	0.27	-0.01	-0.28	0.05	0.32	0.40	0.40	0.51	0.10	0.22	-0.27	-0.16	0.53
Q9H2P0	Activity-dependent neuroprotector homeobox protein OS=Homo sapiens GN=ADNP PE=1 SV=1 - [ADNP_HUMAN]	0.91	1	1	1	1	-0.29	0.14	-0.81	-0.38	-0.59	-0.17	-0.35	0.08	-0.14	-0.56	-0.50	-0.08	0.00	-0.20	0.31	-0.24	0.28	-0.32	0.20	0.53
Q6UB28	Methionine aminopeptidase 1D, mitochondrial OS=Homo sapiens GN=METAP1D PE=1 SV=1 - [MAP12_HUMAN]	5.67	1	1	1	1	-0.86	-1.05	-0.43	-0.62	0.30	0.10	0.04	-0.16	-0.93	-0.73	0.04	-0.16	0.95	0.90	0.46	0.15	-0.28	1.14	0.71	0.53

Q5T011	Protein SZT2 OS=Homo sapiens GN=SZT2 PE=2 SV=3 - [SZT2_HUMAN]	0.99	1	2	2	2	0.87	1.01	1.42	1.56	0.41	0.55	1.88	2.02	1.52	1.39	1.49	1.63	0.63	0.63	0.52	0.28	0.17	-0.47	-1.02	0.53
Q9H061	Transmembrane protein 126A OS=Homo sapiens GN=TMEM126A PE=1 SV=1 - [T126A_HUMAN]	6.15	1	2	2	5	-0.40	-0.53	-0.25	-0.19	0.07	-0.17	0.21	0.08	-0.10	0.02	0.21	0.10	0.66	0.61	0.46	0.46	0.31	0.46	0.28	0.52
P01034	Cystatin-C OS=Homo sapiens GN=CST3 PE=1 SV=1 - [CYTC_HUMAN]	41.78	1	5	5	32	-0.50	-0.39	-0.54	-0.46	0.99	1.16	0.00	0.02	0.63	0.56	0.87	0.89	0.41	1.54	1.45	1.14	1.12	1.63	1.65	0.52
P98170	E3 ubiquitin-protein ligase XIAP OS=Homo sapiens GN=XIAP PE=1 SV=2 - [XIAP_HUMAN]	1.61	1	1	1	2	-0.04	0.04	-0.37	-0.29	0.33	0.41	0.09	0.17	0.40	0.33	0.02	0.10	0.18	0.07	0.39	0.39	0.73	0.35	0.69	0.52
P57105	Synaptojanin-2-binding protein OS=Homo sapiens GN=SYNJ2BP PE=1 SV=2 - [SYJ2B_HUMAN]	37.24	1	5	5	12	-0.92	-0.88	-0.84	-0.73	0.01	0.07	-0.40	-0.32	0.08	0.09	0.12	0.16	0.50	1.01	0.95	1.03	0.94	0.89	0.89	0.52
Q9NXE4	Sphingomyelin phosphodiesterase 4 OS=Homo sapiens GN=SMPD4 PE=1 SV=2 - [NSMA3_HUMAN]	2.30	1	2	2	2	-0.62	-0.46	-0.72	-0.57	0.09	0.24	-0.26	-0.11	-0.10	-0.25	0.19	0.34	0.41	0.81	0.91	0.40	0.51	0.69	0.80	0.52
Q13642	Four and a half LIM domains protein 1 OS=Homo sapiens GN=FHL1 PE=1 SV=4 - [FHL1_HUMAN]	29.72	1	10	10	32	-1.85	-1.77	-1.65	-1.62	-0.81	-0.73	-1.05	-0.97	-1.01	-1.08	-0.92	-0.91	0.77	0.79	0.60	0.77	0.65	0.97	0.86	0.52
P55036	26S proteasome non-ATPase regulatory subunit 4 OS=Homo sapiens GN=PSMD4 PE=1 SV=1 - [PSMD4_HUMAN]	21.22	1	6	6	13	0.01	-0.22	-0.18	-0.17	0.14	0.11	0.09	0.06	-0.04	0.03	0.16	0.10	0.37	0.32	0.40	0.39	0.34	0.22	0.42	0.52

Q13976	cGMP-dependent protein kinase 1 OS=Homo sapiens GN=PRKG1 PE=1 SV=3 - [KGP1_HUMAN]	12.37	1	6	7	10	-1.53	-1.40	-1.41	-1.57	-0.52	-0.58	-0.70	-0.63	-0.70	-0.58	-0.57	-0.66	0.86	0.74	0.77	1.03	1.20	0.83	1.13	0.52
O94868	FCH and double SH3 domains protein 2 OS=Homo sapiens GN=FCHSD2 PE=1 SV=3 - [FCSD2_HUMAN]	8.65	1	4	4	6	-0.90	-0.59	-0.61	-0.64	-0.18	-0.40	-0.37	-0.36	-0.04	-0.10	-0.21	-0.09	0.59	0.44	0.36	0.61	0.54	0.49	0.31	0.52
Q14746	Conserved oligomeric Golgi complex subunit 2 OS=Homo sapiens GN=COG2 PE=1 SV=1 - [COG2_HUMAN]	11.38	1	4	4	6	-1.89	-1.06	-0.85	-0.94	0.19	-0.19	-0.24	-0.57	-0.53	-0.14	-0.88	-0.48	0.77	0.60	0.47	0.93	0.47	1.13	0.91	0.52
Q02127	Dihydroorotate dehydrogenase (quinone), mitochondrial OS=Homo sapiens GN=DHODH PE=1 SV=3 - [PYRD_HUMAN]	9.37	1	3	3	4	-0.75	-0.31	-0.79	-0.35	0.55	0.98	-0.33	0.10	0.39	-0.04	-0.32	0.11	0.47	0.43	0.46	0.74	0.78	1.28	1.32	0.52
Q8NAB2	Kelch repeat and BTB domain-containing protein 3 OS=Homo sapiens GN=KBTBD3 PE=2 SV=2 - [KBTB3_HUMAN]	7.40	1	2	2	3	-0.25	-0.51	-0.53	-0.78	0.46	0.21	-0.08	-0.33	-0.68	-0.43	-0.42	-0.68	0.23	-0.17	0.11	-0.14	0.14	0.70	0.98	0.52
Q9H0A8	COMM domain-containing protein 4 OS=Homo sapiens GN=COMMD4 PE=1 SV=1 - [COMD4_HUMAN]	30.15	1	4	4	7	-0.47	-0.48	-0.31	-0.34	0.49	0.46	0.15	-0.21	0.00	0.35	0.42	0.31	0.67	0.97	0.80	0.90	0.74	0.97	0.98	0.52
P00352	Retinal dehydrogenase 1 OS=Homo sapiens GN=ALDH1A1 PE=1 SV=2 - [AL1A1_HUMAN]	42.71	1	15	17	41	-0.75	-0.73	-0.74	-0.70	-0.17	-0.15	-0.16	-0.16	-0.42	-0.36	-0.81	-0.83	0.57	-0.13	-0.35	0.43	0.29	0.61	0.49	0.51

Q8N0W3	L-fucose kinase OS=Homo sapiens GN=FUK PE=2 SV=2 - [FUK_HUMAN]	14.30	1	11	11	20	-0.46	-0.31	-0.48	-0.41	0.15	0.31	-0.07	0.06	0.26	0.16	0.30	0.35	0.47	0.89	0.78	0.66	0.65	0.58	0.57	0.51
Q13469	Nuclear factor of activated T-cells, cytoplasmic 2 OS=Homo sapiens GN=NFATC2 PE=1 SV=2 - [NFAC2_HUMAN]	1.73	1	1	1	1	0.18	-0.08	0.14	-0.11	0.27	0.01	0.59	0.34	-0.20	0.06	0.32	0.07	0.47	0.15	0.18	-0.09	-0.05	0.08	0.12	0.51
O94929	Actin-binding LIM protein 3 OS=Homo sapiens GN=ABLIM3 PE=1 SV=3 - [ABLM3_HUMAN]	8.93	1	4	5	13	-0.31	-0.17	-0.30	-0.16	0.44	0.57	0.15	0.29	0.40	0.27	0.07	0.20	0.51	0.38	0.37	0.61	0.60	0.73	0.72	0.51
P31939	Bifunctional purine biosynthesis protein PURH OS=Homo sapiens GN=ATIC PE=1 SV=3 - [PUR9_HUMAN]	56.93	1	25	25	60	0.09	-0.13	0.04	0.01	0.62	0.50	0.54	0.55	0.43	0.51	0.24	0.24	0.55	0.24	0.22	0.44	0.45	0.37	0.42	0.51
Q86WA6	Valacyclovir hydrolase OS=Homo sapiens GN=BPHL PE=1 SV=1 - [BPHL_HUMAN]	13.06	1	4	4	11	-1.19	-1.05	-0.97	-0.91	-0.74	-0.70	-0.58	-0.48	-0.58	-0.64	-0.25	-0.22	0.65	0.71	0.73	0.58	0.58	0.34	0.27	0.51
O95456	Proteasome assembly chaperone 1 OS=Homo sapiens GN=PSMG1 PE=1 SV=1 - [PSMG1_HUMAN]	4.86	1	1	1	1	-0.80	-0.61	-0.57	-0.38	0.34	0.52	-0.12	0.07	0.63	0.44	0.86	1.04	0.73	1.66	1.43	1.27	1.05	1.12	0.89	0.51
Q9UIG0	Tyrosine-protein kinase BAZ1B OS=Homo sapiens GN=BAZ1B PE=1 SV=2 - [BAZ1B_HUMAN]	4.92	1	6	6	6	-0.52	-0.56	-0.90	-0.93	-0.09	-0.14	-0.30	-0.35	-0.55	-0.52	-0.06	-0.12	0.03	0.47	0.73	-0.16	0.24	0.41	0.74	0.51
Q8NBQ5	Estradiol 17-beta-dehydrogenase 11 OS=Homo sapiens GN=HSD17B11 PE=1 SV=3 - [DHB11_HUMAN]	20.00	1	6	6	8	-0.57	-0.50	-0.83	-0.48	0.16	0.27	-0.24	-0.01	0.03	0.03	-0.01	0.26	0.54	0.80	0.61	0.64	0.38	0.84	0.82	0.51

P22033	Methylmalonyl-CoA mutase, mitochondrial OS=Homo sapiens GN=MUT PE=1 SV=4 - [MUTA_HUMAN]	30.53	1	18	18	27	-0.87	-0.79	-0.67	-0.53	-0.13	-0.09	-0.28	-0.19	-0.25	-0.28	-0.19	-0.10	0.68	0.67	0.50	0.41	0.46	0.62	0.55	0.51
P04632	Calpain small subunit 1 OS=Homo sapiens GN=CAPNS1 PE=1 SV=1 - [CPNS1_HUMAN]	54.48	2	9	9	20	-0.48	-0.72	-0.59	-0.70	0.07	0.08	-0.09	-0.02	0.11	0.02	0.13	0.16	0.35	0.85	0.84	0.34	0.59	0.60	0.62	0.51
O14974	Protein phosphatase 1 regulatory subunit 12A OS=Homo sapiens GN=PPP1R12A PE=1 SV=1 - [MYPT1_HUMAN]	27.48	1	24	24	42	-1.20	-1.11	-1.13	-0.94	-0.67	-0.67	-0.55	-0.53	-0.69	-0.62	-0.44	-0.49	0.69	0.60	0.55	0.46	0.41	0.30	0.24	0.51
P23280	Carbonic anhydrase 6 OS=Homo sapiens GN=CA6 PE=1 SV=3 - [CAH6_HUMAN]	17.53	1	4	4	9	-3.25	-3.23	-3.03	-2.90	-2.28	-2.10	-2.79	-2.61	-2.45	-2.59	-2.47	-2.37	-0.15	0.33	0.66	0.30	0.13	0.36	1.10	0.51
O00443	Phosphatidylinositol 4-phosphate 3-kinase C2 domain-containing subunit alpha OS=Homo sapiens GN=PIK3C2A PE=1 SV=2 - [P3C2A_HUMAN]	3.02	1	4	4	8	-0.70	-0.61	-0.79	-0.81	0.03	0.04	-0.24	-0.15	-0.09	-0.01	0.06	-0.01	0.52	0.46	0.65	0.71	0.74	0.64	0.80	0.51
P62277	40S ribosomal protein S13 OS=Homo sapiens GN=RPS13 PE=1 SV=2 - [RS13_HUMAN]	21.19	1	3	3	9	-0.69	-0.78	-0.72	-0.80	-0.09	-0.10	-0.27	-0.32	-0.15	-0.14	0.10	-0.02	0.60	0.96	0.91	0.71	0.60	0.74	0.60	0.51
Q6DRA6	Putative histone H2B type 2-D OS=Homo sapiens GN=HIST2H2BD PE=5 SV=3 - [H2B2D_HUMAN]	16.46	2	4	4	10	-1.70	-1.58	-1.13	-1.04	-0.94	-0.92	-0.91	-0.59	-0.94	-0.96	-0.96	-0.94	0.85	0.72	0.17	0.50	0.20	0.45	-0.03	0.51

P08571	Monocyte differentiation antigen CD14 OS=Homo sapiens GN=CD14 PE=1 SV=2 - [CD14_HUMAN]	9.07	1	3	3	5	-0.26	-0.48	-0.25	-0.21	0.42	0.63	0.19	0.17	0.74	0.37	1.08	1.53	0.50	1.48	1.62	0.63	0.79	0.47	0.47	0.51
Q15532	Protein SSXT OS=Homo sapiens GN=SS18 PE=1 SV=3 - [SSXT_HUMAN]	4.31	1	1	1	1	-0.95	-0.43	-1.24	-0.72	-0.64	-0.13	-0.79	-0.27	0.03	-0.48	0.06	0.57	0.21	1.01	1.29	0.50	0.79	0.29	0.57	0.51
P07711	Cathepsin L1 OS=Homo sapiens GN=CTSL PE=1 SV=2 - [CATL1_HUMAN]	13.21	3	2	3	7	-0.54	-0.54	-0.56	-0.88	-0.59	-0.25	-0.12	0.03	0.04	0.05	-0.40	0.03	0.62	0.58	0.16	0.65	0.64	0.01	0.68	0.51
P54886	Delta-1-pyrroline-5-carboxylate synthase OS=Homo sapiens GN=ALDH18A1 PE=1 SV=2 - [P5CS_HUMAN]	19.25	1	13	13	28	-1.67	-1.57	-1.46	-1.55	-0.53	-0.80	-1.07	-1.12	-0.68	-0.59	-0.39	-0.28	0.43	1.14	1.01	0.81	0.91	0.84	0.80	0.51
P10155	60 kDa SS-A/Ro ribonucleoprotein OS=Homo sapiens GN=TROVE2 PE=1 SV=2 - [RO60_HUMAN]	23.98	1	10	10	16	-0.48	-0.58	-0.56	-0.67	-0.11	-0.11	-0.08	-0.20	-0.15	-0.06	0.01	-0.06	0.68	0.67	0.57	0.62	0.51	0.45	0.44	0.51
P17096	High mobility group protein HMG-I/HMG-Y OS=Homo sapiens GN=HMGA1 PE=1 SV=3 - [HMGA1_HUMAN]	23.36	1	2	2	3	-2.07	-2.11	-1.18	-1.23	-0.34	-0.39	-0.74	-0.78	-1.19	-1.14	-0.55	-0.60	1.38	1.52	0.63	0.96	0.08	1.71	0.82	0.51
Q9H8M7	Protein FAM188A OS=Homo sapiens GN=FAM188A PE=1 SV=1 - [F188A_HUMAN]	11.24	1	3	3	4	-0.57	-0.71	-0.40	-0.54	0.05	-0.10	0.05	-0.10	-0.22	-0.07	-0.04	-0.19	0.67	0.53	0.35	0.52	0.36	0.60	0.43	0.51
Q6ZS17	Protein FAM65A OS=Homo sapiens GN=FAM65A PE=1 SV=1 - [FA65A_HUMAN]	4.09	1	3	3	6	-0.26	-0.50	-0.82	-1.06	0.77	0.71	-0.38	-0.62	0.12	0.37	0.16	-0.09	-0.07	0.42	1.27	0.66	1.22	1.00	1.23	0.51

Q6ZXV5	Transmembrane and TPR repeat-containing protein 3 OS=Homo sapiens GN=TMTC3 PE=1 SV=2 - [TMTC3_HUMAN]	6.45	1	6	6	8	-0.65	-0.70	-0.58	-0.82	0.41	0.16	-0.25	-0.38	-0.11	0.13	-0.03	-0.14	0.42	0.57	0.72	0.74	0.76	0.96	0.97	0.51
Q14980	Nuclear mitotic apparatus protein 1 OS=Homo sapiens GN=NUMA1 PE=1 SV=2 - [NUMA1_HUMAN]	38.63	1	62	63	118	-1.02	-0.99	-1.10	-1.09	-0.58	-0.52	-0.61	-0.58	-0.48	-0.49	-0.24	-0.25	0.40	0.69	0.82	0.56	0.69	0.42	0.50	0.50
Q9NUN5	Probable lysosomal cobalamin transporter OS=Homo sapiens GN=LMBRD1 PE=1 SV=1 - [LMBD1_HUMAN]	3.33	1	1	1	5	-0.89	-0.96	-0.80	-0.87	-0.61	-0.68	-0.36	-0.43	-0.61	-0.54	-0.13	-0.20	0.58	0.77	0.67	0.38	0.30	0.27	0.18	0.50
P01036	Cystatin-S OS=Homo sapiens GN=CST4 PE=1 SV=3 - [CYTS_HUMAN]	7.80	2	1	1	2	-3.63	-3.37	-2.65	-2.39	-1.85	-1.60	-2.21	-1.95	-2.06	-2.32	-2.28	-2.02	1.48	1.36	0.37	1.34	0.36	1.76	0.78	0.50
P49590	Probable histidine--tRNA ligase, mitochondrial OS=Homo sapiens GN=HARS2 PE=1 SV=1 - [SYHM_HUMAN]	11.07	1	2	6	12	-0.29	-0.43	-0.75	-0.88	-0.49	-0.63	-0.31	-0.44	-0.53	-0.39	-0.27	-0.41	0.04	0.03	0.47	-0.06	0.39	-0.21	0.24	0.50
Q9HC35	Echinoderm microtubule-associated protein-like 4 OS=Homo sapiens GN=EML4 PE=1 SV=3 - [EMAL4_HUMAN]	10.40	1	8	8	11	-1.02	-1.12	-0.96	-1.07	-0.51	-0.69	-0.62	-0.79	-0.87	-0.79	-0.70	-0.84	0.32	0.13	0.30	0.16	0.21	0.46	0.55	0.50
P55283	Cadherin-4 OS=Homo sapiens GN=CDH4 PE=2 SV=2 - [CADH4_HUMAN]	6.88	1	2	4	6	-0.52	-0.63	-0.85	-0.96	0.13	0.02	-0.41	-0.52	-0.62	-0.51	-0.06	-0.17	0.16	0.46	0.79	0.04	0.37	0.63	0.96	0.50
Q9BXI6	TBC1 domain family member 10A OS=Homo sapiens GN=TBC1D10A PE=1 SV=1 - [TB10A_HUMAN]	2.36	1	1	1	2	-0.18	-0.48	-0.26	-0.56	0.11	-0.19	0.18	-0.12	-0.09	0.22	-0.03	-0.33	0.42	0.16	0.23	0.43	0.51	0.27	0.35	0.50

Q969N2	GPI transamidase component PIG-T OS=Homo sapiens GN=PIGT PE=1 SV=1 - [PIGT_HUMAN]	10.55	1	5	5	7	-0.11	0.07	-0.52	-0.63	0.19	0.27	-0.08	0.01	-0.02	-0.11	-0.13	0.02	-0.03	0.00	0.39	-0.02	0.34	0.17	0.68	0.50
Q9ULX6	A-kinase anchor protein 8-like OS=Homo sapiens GN=AKAP8L PE=1 SV=3 - [AKP8L_HUMAN]	5.88	1	2	2	4	-0.49	-0.33	-0.56	-0.40	0.11	0.26	-0.12	0.04	-0.10	-0.26	-0.31	-0.15	0.42	0.18	0.25	0.26	0.33	0.58	0.65	0.50
Q9BVG4	Protein PBDC1 OS=Homo sapiens GN=PBDC1 PE=1 SV=1 - [PBDC1_HUMAN]	13.30	1	3	3	4	-0.48	-0.63	-0.62	-0.77	-0.39	-0.54	-0.18	-0.33	-0.38	-0.22	-0.11	-0.26	0.35	0.38	0.51	0.29	0.43	0.08	0.21	0.50
Q00341	Vigilin OS=Homo sapiens GN=HDLBP PE=1 SV=2 - [VIGLN_HUMAN]	28.79	3	30	30	48	-0.66	-0.60	-0.58	-0.60	0.20	0.23	-0.16	-0.14	0.06	-0.03	-0.03	0.03	0.59	0.65	0.66	0.64	0.70	0.85	0.85	0.50
Q7Z4R8	UPF0669 protein C6orf120 OS=Homo sapiens GN=C6orf120 PE=1 SV=1 - [CF120_HUMAN]	14.14	1	1	1	1	-0.92	-1.14	-0.72	-0.94	-0.63	-0.86	-0.28	-0.50	-0.77	-0.55	0.12	-0.10	0.70	1.05	0.84	0.41	0.20	0.27	0.07	0.50
O15269	Serine palmitoyltransferase 1 OS=Homo sapiens GN=SPTLC1 PE=1 SV=1 - [SPTC1_HUMAN]	9.73	1	4	4	6	-0.26	-0.22	-0.62	-0.60	0.26	0.26	-0.17	-0.17	0.26	0.17	0.42	0.49	0.31	0.64	1.08	0.66	0.81	0.50	0.86	0.50
P01766	Ig heavy chain V-III region BRO OS=Homo sapiens PE=1 SV=1 - [HV305_HUMAN]	25.00	3	1	2	13	-0.35	-0.55	-0.81	-0.96	0.46	0.56	-0.16	-0.14	0.47	0.32	0.66	0.55	0.37	1.00	1.56	0.68	0.80	0.79	1.20	0.50
O43155	Leucine-rich repeat transmembrane protein FLRT2 OS=Homo sapiens GN=FLRT2 PE=1 SV=1 - [FLRT2_HUMAN]	8.33	1	4	4	5	-0.42	-0.52	-0.94	-0.90	-0.37	-0.40	-0.55	-0.46	-0.16	-0.18	-0.13	0.15	-0.10	0.06	0.82	0.27	1.04	0.03	0.80	0.50

P50993	Sodium/potassium-transporting ATPase subunit alpha 2 OS=Homo sapiens GN=ATP1A2 PE=1 SV=1 - [AT1A2_HUMAN]	53.14	1	35	55	549	-0.21	-0.22	-0.18	-0.17	0.05	0.02	0.28	0.26	0.06	0.05	-0.02	-0.04	0.50	0.24	0.14	0.27	0.21	0.18	0.12	0.50
Q9UIS9	Methyl-CpG-binding domain protein 1 OS=Homo sapiens GN=MBD1 PE=1 SV=2 - [MBD1_HUMAN]	1.98	1	1	1	2	-0.79	-0.85	-0.76	-0.82	0.32	0.25	-0.32	-0.38	-0.28	-0.22	0.49	0.42	0.52	1.29	1.25	0.60	0.57	1.09	1.06	0.50
Q08380	Galectin-3-binding protein OS=Homo sapiens GN=LGALS3BP PE=1 SV=1 - [LG3BP_HUMAN]	24.62	1	11	11	32	-0.87	-0.86	-0.74	-0.74	0.62	0.70	-0.36	-0.30	0.16	0.15	0.30	0.27	0.51	1.19	1.18	1.00	1.05	1.31	1.51	0.50
P60033	CD81 antigen OS=Homo sapiens GN=CD81 PE=1 SV=1 - [CD81_HUMAN]	18.64	1	3	3	35	0.79	0.29	0.67	0.16	1.11	0.67	0.97	0.49	0.27	0.68	0.77	0.61	0.22	0.14	0.36	-0.13	0.24	0.32	0.84	0.50
Q9Y6R7	IgGFc-binding protein OS=Homo sapiens GN=FCGBP PE=1 SV=3 - [FCGBP_HUMAN]	1.02	1	4	4	4	-0.52	0.30	-0.52	0.30	0.50	1.31	-0.08	0.73	0.90	0.09	0.35	1.16	0.49	0.87	0.86	0.63	0.64	1.00	1.00	0.50
P40189	Interleukin-6 receptor subunit beta OS=Homo sapiens GN=IL6ST PE=1 SV=2 - [IL6RB_HUMAN]	1.09	1	1	1	2	-0.04	-0.22	0.10	-0.09	0.93	0.73	0.53	0.34	0.18	0.37	0.57	0.37	0.62	0.61	0.47	0.44	0.31	0.95	0.81	0.50
Q9UHP3	Ubiquitin carboxyl-terminal hydrolase 25 OS=Homo sapiens GN=USP25 PE=1 SV=4 - [UBP25_HUMAN]	3.51	1	2	3	6	-0.47	-0.49	-0.35	-0.30	-0.06	0.12	0.18	0.24	-0.03	-0.04	-0.07	-0.03	0.78	0.51	0.22	0.60	0.26	0.65	0.36	0.50
Q9UNH6	Sorting nexin-7 OS=Homo sapiens GN=SNX7 PE=1 SV=1 - [SNX7_HUMAN]	2.84	1	1	1	2	-0.80	-0.76	-0.56	-0.52	0.18	0.22	-0.13	-0.09	-0.11	-0.15	0.26	0.29	0.73	1.07	0.82	0.69	0.45	0.97	0.73	0.50

P28070	Proteasome subunit beta type-4 OS=Homo sapiens GN=PSMB4 PE=1 SV=4 - [PSB4_HUMAN]	42.05	1	6	6	22	-0.37	-0.29	-0.67	-0.55	-0.06	0.07	-0.08	-0.01	0.17	0.08	0.03	0.17	0.38	0.62	0.89	0.48	0.88	0.40	0.61	0.50
Q9H0J9	Poly [ADP-ribose] polymerase 12 OS=Homo sapiens GN=PARP12 PE=1 SV=1 - [PAR12_HUMAN]	1.85	1	1	1	1	-0.55	-0.87	-0.41	-0.73	0.54	0.21	0.02	-0.30	-0.02	0.31	0.59	0.26	0.63	1.15	1.00	0.89	0.76	1.07	0.93	0.50
Q9HCU5	Prolactin regulatory element-binding protein OS=Homo sapiens GN=PREB PE=1 SV=2 - [PREB_HUMAN]	12.23	1	3	3	8	-0.13	0.05	-0.44	-0.37	-0.32	-0.14	-0.19	-0.05	0.09	0.02	-0.01	0.18	0.17	0.13	0.28	0.12	0.50	-0.21	0.32	0.50
Q7Z478	ATP-dependent RNA helicase DHX29 OS=Homo sapiens GN=DHX29 PE=1 SV=2 - [DHX29_HUMAN]	3.29	1	4	4	7	-0.50	-0.39	-0.40	-0.40	0.21	0.19	-0.01	0.03	-0.28	0.04	-0.06	0.11	0.45	0.51	0.53	0.58	0.33	0.52	0.60	0.50
Q6P587	Acylpyruvase FAHD1, mitochondrial OS=Homo sapiens GN=FAHD1 PE=1 SV=2 - [FAHD1_HUMAN]	33.93	1	5	5	7	1.02	0.66	0.08	-0.67	-0.12	-0.28	0.39	-0.07	-0.32	-0.29	0.03	-0.21	-0.87	-1.12	-0.03	-1.38	-0.32	-1.08	0.46	0.50
P18621	60S ribosomal protein L17 OS=Homo sapiens GN=RPL17 PE=1 SV=3 - [RL17_HUMAN]	38.04	1	6	7	23	-0.67	-0.70	-0.82	-0.81	-0.28	-0.34	-0.46	-0.47	-0.57	-0.53	-0.21	-0.35	0.44	0.57	0.32	0.28	0.36	0.24	0.31	0.49
P35659	Protein DEK OS=Homo sapiens GN=DEK PE=1 SV=1 - [DEK_HUMAN]	20.53	1	8	8	19	-0.55	-0.61	-0.62	-0.60	0.04	0.06	0.02	-0.13	-0.20	-0.04	0.00	-0.07	0.60	0.53	0.48	0.39	0.42	0.60	0.49	0.49
Q14005	Pro-interleukin-16 OS=Homo sapiens GN=IL16 PE=1 SV=4 - [IL16_HUMAN]	0.90	1	1	1	1	-0.22	0.05	-0.43	-0.15	0.31	0.58	0.00	0.28	0.01	-0.26	0.01	0.29	0.28	0.24	0.44	-0.01	0.20	0.52	0.72	0.49

P49184	Deoxyribonuclease-1-like 1 OS=Homo sapiens GN=DNASE1L1 PE=1 SV=1 - [DNSL1_HUMAN]	5.96	1	1	1	3	-0.54	-0.84	-0.47	-0.77	-0.05	-0.35	-0.04	-0.34	-0.47	-0.17	-0.01	-0.31	0.55	0.54	0.46	0.40	0.34	0.48	0.41	0.49
P54105	Methylosome subunit pICln OS=Homo sapiens GN=CLNS1A PE=1 SV=1 - [ICLN_HUMAN]	24.05	1	4	4	11	-0.16	-0.15	-0.23	-0.32	0.31	0.29	0.18	0.16	0.22	0.29	0.59	0.34	0.46	0.63	0.70	0.48	0.57	0.49	0.49	0.49
Q12846	Syntaxin-4 OS=Homo sapiens GN=STX4 PE=1 SV=2 - [STX4_HUMAN]	26.26	1	7	7	17	-0.71	-0.85	-0.47	-0.37	-0.14	-0.05	-0.10	-0.18	-0.16	-0.13	0.15	0.18	0.39	0.77	0.69	0.50	0.43	0.57	0.37	0.49
Q66K14	TBC1 domain family member 9B OS=Homo sapiens GN=TBC1D9B PE=1 SV=3 - [TBC9B_HUMAN]	13.28	1	11	11	20	0.20	0.01	-0.14	-0.33	0.29	0.16	0.31	0.17	0.16	0.25	0.30	0.12	0.17	0.13	0.37	0.17	0.42	0.15	0.53	0.49
O14786	Neuropilin-1 OS=Homo sapiens GN=NRP1 PE=1 SV=3 - [NRP1_HUMAN]	7.37	1	5	5	7	-0.95	-1.07	-1.21	-1.24	-0.03	0.28	-0.78	-0.61	-0.15	-0.16	-0.09	-0.08	0.23	0.95	1.16	0.74	0.95	0.82	1.05	0.49
Q5RI15	Cytochrome c oxidase protein 20 homolog OS=Homo sapiens GN=COX20 PE=1 SV=2 - [COX20_HUMAN]	22.03	1	2	2	5	0.36	1.00	-0.24	0.40	-0.06	0.57	0.19	0.82	0.91	0.28	-0.08	0.55	-0.12	-0.44	0.16	-0.05	0.55	-0.44	0.16	0.49
P49754	Vacuolar protein sorting-associated protein 41 homolog OS=Homo sapiens GN=VPS41 PE=1 SV=3 - [VPS41_HUMAN]	11.83	1	7	8	11	-0.29	-0.23	-0.24	-0.08	0.26	0.39	0.15	0.29	-0.07	-0.03	-0.04	0.26	0.60	0.50	0.30	0.24	-0.04	0.65	0.37	0.49
Q5T0N5	Formin-binding protein 1-like OS=Homo sapiens GN=FNBP1L PE=1 SV=2 - [FBP1L_HUMAN]	6.28	1	2	2	5	-1.04	-1.68	-0.95	-1.59	-0.89	-1.53	-0.52	-1.17	-0.97	-0.32	-0.53	-1.18	0.57	0.51	0.42	0.74	0.66	0.13	0.05	0.49

O43493	Trans-Golgi network integral membrane protein 2 OS=Homo sapiens GN=TGOLN2 PE=1 SV=2 - [TGON2_HUMAN]	4.58	1	2	2	3	-1.15	-1.04	-0.92	-0.82	-0.12	-0.01	-0.50	-0.39	-0.18	-0.28	0.05	0.15	0.71	1.20	0.97	0.90	0.68	1.02	0.79	0.49
P09038	Fibroblast growth factor 2 OS=Homo sapiens GN=FGF2 PE=1 SV=3 - [FGF2_HUMAN]	6.25	1	2	2	4	-0.53	-0.59	-0.83	-0.89	0.22	0.16	-0.41	-0.46	-0.15	-0.09	0.02	-0.04	0.18	0.56	0.85	0.47	0.77	0.74	1.04	0.49
P25490	Transcriptional repressor protein YY1 OS=Homo sapiens GN=YY1 PE=1 SV=2 - [TYY1_HUMAN]	16.18	3	4	4	6	-0.70	-0.80	-0.49	-0.58	-0.18	-0.29	-0.06	-0.16	-0.77	-0.66	-0.39	-0.49	0.69	0.32	0.10	0.07	-0.15	0.50	0.29	0.49
Q14974	Importin subunit beta-1 OS=Homo sapiens GN=KPNB1 PE=1 SV=2 - [IMB1_HUMAN]	28.88	1	22	22	52	-0.32	-0.11	-0.32	-0.18	0.10	0.26	0.10	0.22	0.17	0.12	0.00	0.08	0.41	0.33	0.32	0.39	0.53	0.47	0.45	0.49
Q7Z7N9	Transmembrane protein 179B OS=Homo sapiens GN=TMEM179B PE=1 SV=1 - [T179B_HUMAN]	7.31	1	1	1	1	-1.22	-0.62	-0.85	-0.24	0.13	0.73	-0.42	0.18	0.52	-0.08	0.38	0.98	0.86	1.61	1.22	1.17	0.80	1.34	0.96	0.49
Q969V3	Nicalin OS=Homo sapiens GN=NCLN PE=1 SV=2 - [NCLN_HUMAN]	21.49	1	10	10	20	-0.49	-0.58	-0.56	-0.62	0.30	0.28	-0.18	-0.20	0.05	-0.01	0.13	0.18	0.49	0.66	0.77	0.44	0.75	0.65	0.89	0.49
Q9NZ08	Endoplasmic reticulum aminopeptidase 1 OS=Homo sapiens GN=ERAP1 PE=1 SV=3 - [ERAP1_HUMAN]	8.29	1	7	7	8	-0.95	-0.71	-0.90	-0.67	-0.41	-0.23	-0.46	-0.27	-0.22	-0.40	0.05	0.27	0.46	0.89	0.87	0.59	0.66	0.30	0.49	0.49
Q8IXQ6	Poly [ADP-ribose] polymerase 9 OS=Homo sapiens GN=PARP9 PE=1 SV=2 - [PARP9_HUMAN]	3.04	1	2	2	2	-0.48	-0.71	-0.33	-0.56	0.88	0.65	0.09	-0.13	0.42	0.65	0.62	0.39	0.63	1.11	0.95	1.16	1.02	1.34	1.19	0.49

Q9BY49	Peroxisomal trans-2-enoyl-CoA reductase OS=Homo sapiens GN=PECR PE=1 SV=2 - [PECR_HUMAN]	4.29	1	1	1	2	-1.03	-1.09	-1.00	-1.06	-0.52	-0.58	-0.58	-0.64	-0.54	-0.47	-0.60	-0.67	0.51	0.43	0.39	0.58	0.55	0.50	0.46	0.48
Q9UJZ1	Stomatin-like protein 2, mitochondrial OS=Homo sapiens GN=STOML2 PE=1 SV=1 - [STML2_HUMAN]	29.78	1	8	8	23	-0.82	-0.88	-0.87	-0.85	-0.36	-0.29	-0.44	-0.37	-0.29	-0.28	-0.16	-0.26	0.36	0.16	0.62	0.58	0.67	0.43	0.58	0.48
Q9UN70	Protocadherin gamma-C3 OS=Homo sapiens GN=PCDHGC3 PE=1 SV=1 - [PCDGK_HUMAN]	8.24	17	3	5	8	0.22	0.31	-0.12	-0.03	0.13	0.22	0.30	0.39	0.24	0.15	0.69	0.78	0.14	0.48	0.81	-0.04	0.30	-0.10	0.24	0.48
O95865	N(G),N(G)-dimethylarginine dimethylaminohydrolase 2 OS=Homo sapiens GN=DDAH2 PE=1 SV=1 - [DDAH2_HUMAN]	49.12	1	10	11	25	-0.47	-0.24	-0.53	-0.31	-0.43	-0.18	-0.13	0.15	0.02	-0.29	-0.26	0.01	0.43	0.24	0.25	0.19	0.21	0.07	0.14	0.48
Q9HD45	Transmembrane 9 superfamily member 3 OS=Homo sapiens GN=TM9SF3 PE=1 SV=2 - [TM9S3_HUMAN]	3.40	1	3	3	4	-0.51	-0.41	-0.63	-0.53	0.27	0.37	-0.21	-0.11	-0.04	-0.14	0.05	0.14	0.35	0.56	0.67	0.41	0.53	0.76	0.88	0.48
P22531	Small proline-rich protein 2E OS=Homo sapiens GN=SPRR2E PE=2 SV=2 - [SPR2E_HUMAN]	43.06	6	2	2	2	-2.81	-2.24	-3.26	-2.91	-3.09	-2.74	-3.40	-2.82	-2.01	-2.35	-2.43	-2.09	-0.53	0.15	0.83	0.25	0.95	-0.53	0.16	0.48
O75493	Carbonic anhydrase-related protein 11 OS=Homo sapiens GN=CA11 PE=1 SV=2 - [CAH11_HUMAN]	3.35	1	1	1	2	-0.80	-0.85	-0.74	-0.79	0.76	0.71	-0.33	-0.37	0.08	0.13	-0.66	-0.71	0.53	0.15	0.08	0.96	0.90	1.55	1.48	0.48
Q8WTW3	Conserved oligomeric Golgi complex subunit 1 OS=Homo sapiens GN=COG1 PE=1 SV=1 - [COG1_HUMAN]	3.47	1	3	3	4	-0.46	-0.16	-0.72	-0.42	1.08	1.37	-0.30	0.00	1.04	0.75	0.22	0.52	0.21	0.68	0.94	1.23	1.50	1.52	1.78	0.48

Q8WVF1	Protein OSCP1 OS=Homo sapiens GN=OSCP1 PE=1 SV=4 - [OSCP1_HUMAN]	4.88	1	2	2	4	0.18	0.13	-0.04	-0.09	0.44	0.38	0.38	0.33	0.48	0.53	0.60	0.55	0.25	0.43	0.64	0.38	0.60	0.24	0.46	0.48
Q86YQ8	Copine-8 OS=Homo sapiens GN=CPNE8 PE=1 SV=2 - [CPNE8_HUMAN]	29.79	1	6	13	27	0.17	0.14	0.12	-0.03	0.31	0.01	0.38	0.28	-0.05	0.29	0.38	0.32	0.32	0.58	0.29	0.56	0.30	0.42	0.30	0.48
Q9Y4P3	Transducin beta-like protein 2 OS=Homo sapiens GN=TBL2 PE=1 SV=1 - [TBL2_HUMAN]	16.55	1	6	6	8	-1.03	-1.10	-1.00	-1.07	-0.12	-0.16	-0.49	-0.60	-0.42	-0.37	-0.32	-0.23	0.67	0.96	0.76	0.71	0.68	0.93	0.90	0.48
Q13451	Peptidyl-prolyl cis-trans isomerase FKBP5 OS=Homo sapiens GN=FKBP5 PE=1 SV=2 - [FKBP5_HUMAN]	31.29	1	16	16	28	-0.34	-0.35	-0.19	-0.36	0.17	0.15	0.31	0.17	0.13	0.12	-0.27	-0.26	0.75	0.31	0.00	0.49	0.35	0.61	0.37	0.48
Q99807	Ubiquinone biosynthesis protein COQ7 homolog OS=Homo sapiens GN=COQ7 PE=1 SV=3 - [COQ7_HUMAN]	17.97	1	4	4	6	0.12	-0.19	-0.39	-0.71	-0.02	-0.34	0.02	-0.29	-0.30	0.02	0.05	-0.27	-0.05	-0.06	0.45	-0.07	0.44	-0.16	0.36	0.48
P23083	Ig heavy chain V-I region V35 OS=Homo sapiens PE=1 SV=1 - [HV103_HUMAN]	21.37	1	2	2	3	-1.57	-1.67	-1.54	-1.64	0.51	0.40	-1.13	-1.23	0.04	0.15	0.83	0.72	0.50	2.40	2.36	1.75	1.72	2.06	2.03	0.48
Q9NYU2	UDP-glucose:glycoprotein glucosyltransferase 1 OS=Homo sapiens GN=UGGT1 PE=1 SV=3 - [UGGG1_HUMAN]	27.40	1	31	31	55	-0.85	-0.79	-0.84	-0.81	-0.22	-0.17	-0.44	-0.35	-0.30	-0.39	-0.17	-0.11	0.50	0.73	0.79	0.58	0.57	0.67	0.70	0.48
O95470	Sphingosine-1-phosphate lyase 1 OS=Homo sapiens GN=SGPL1 PE=1 SV=3 - [SGPL1_HUMAN]	8.98	1	3	3	5	-1.21	-1.20	-0.77	-0.76	-0.54	-0.54	-0.35	-0.35	-0.62	-0.61	0.07	0.07	0.91	1.28	0.84	0.62	0.18	0.65	0.21	0.47

O94804	Serine/threonine-protein kinase 10 OS=Homo sapiens GN=STK10 PE=1 SV=1 - [STK10_HUMAN]	5.27	1	4	4	5	-0.55	-0.66	-0.16	-0.31	-0.01	-0.01	-0.01	-0.11	-0.29	-0.18	0.14	0.03	0.60	0.70	0.46	0.40	0.23	0.52	0.29	0.47
Q6EEV4	DNA-directed RNA polymerase II subunit GRINL1A, isoforms 4/5 OS=Homo sapiens GN=POLR2M PE=1 SV=1 - [GL1AD_HUMAN]	26.35	1	2	2	4	0.29	0.16	0.33	-0.77	-0.41	-0.42	-0.19	-0.36	-0.57	-0.39	-0.72	-0.45	0.29	-0.75	-0.59	-0.68	0.23	-0.19	-0.02	0.47
Q9BT09	Protein canopy homolog 3 OS=Homo sapiens GN=CNPY3 PE=1 SV=1 - [CNPY3_HUMAN]	9.71	1	3	3	4	-0.93	-0.75	-0.71	-0.54	0.11	0.28	-0.30	-0.13	-0.09	-0.26	-0.05	0.12	0.68	0.88	0.66	0.70	0.48	1.02	0.81	0.47
P42566	Epidermal growth factor receptor substrate 15 OS=Homo sapiens GN=EPS15 PE=1 SV=2 - [EPS15_HUMAN]	25.78	1	17	17	37	-0.31	-0.24	-0.25	-0.39	0.16	0.18	0.12	0.06	0.05	0.08	0.18	0.18	0.43	0.52	0.40	0.33	0.44	0.49	0.53	0.47
Q86UU1	Pleckstrin homology-like domain family B member 1 OS=Homo sapiens GN=PHLDB1 PE=1 SV=1 - [PHLB1_HUMAN]	15.11	1	14	14	25	-0.37	-0.20	-0.46	-0.39	0.01	0.04	-0.18	-0.10	0.05	-0.11	0.16	0.16	0.32	0.53	0.69	0.47	0.54	0.45	0.52	0.47
Q12857	Nuclear factor 1 A-type OS=Homo sapiens GN=NFIA PE=1 SV=2 - [NFIA_HUMAN]	9.04	2	2	4	11	-1.58	-1.66	-1.25	-1.33	-1.05	-1.13	-0.84	-0.92	-0.86	-0.78	-0.47	-0.55	0.79	1.12	0.78	0.83	0.51	0.52	0.19	0.47
O14579	Coatomer subunit epsilon OS=Homo sapiens GN=COPE PE=1 SV=3 - [COPE_HUMAN]	9.42	1	2	2	4	-0.97	-0.90	-0.77	-0.70	0.03	0.10	-0.36	-0.29	-0.44	-0.51	-0.40	-0.34	0.67	0.58	0.36	0.50	0.29	0.99	0.78	0.47
P47897	Glutamine--tRNA ligase OS=Homo sapiens GN=QARS PE=1 SV=1 - [SYQ_HUMAN]	27.74	1	16	16	36	-0.54	-0.51	-0.47	-0.47	0.26	0.24	-0.14	0.00	0.04	0.07	-0.15	-0.07	0.57	0.55	0.39	0.63	0.35	0.79	0.66	0.47

Q96I99	Succinyl-CoA ligase [GDP-forming] subunit beta, mitochondrial OS=Homo sapiens GN=SUCLG2 PE=1 SV=2 - [SUCB2_HUMAN]	42.82	1	16	16	38	-1.38	-1.38	-1.18	-1.19	-0.54	-0.51	-0.71	-0.73	-0.70	-0.65	-0.37	-0.34	0.62	0.91	0.79	0.69	0.53	0.82	0.62	0.47
Q7LDG7	RAS guanyl-releasing protein 2 OS=Homo sapiens GN=RASGRP2 PE=1 SV=1 - [GRP2_HUMAN]	6.90	1	5	5	5	0.03	-0.08	-0.13	-0.06	-0.38	-0.21	0.28	0.26	0.13	0.25	0.03	0.20	0.39	0.29	0.60	0.25	0.47	-0.58	-0.27	0.47
Q14185	Dedicator of cytokinesis protein 1 OS=Homo sapiens GN=DOCK1 PE=1 SV=2 - [DOCK1_HUMAN]	12.23	1	17	18	30	-0.89	-0.71	-0.83	-0.77	-0.12	-0.16	-0.49	-0.33	-0.31	-0.41	-0.48	-0.37	0.52	0.29	0.50	0.37	0.31	0.60	0.67	0.47
Q8N3P4	Vacuolar protein sorting-associated protein 8 homolog OS=Homo sapiens GN=VPS8 PE=1 SV=3 - [VPS8_HUMAN]	3.36	2	4	4	7	0.02	-0.02	-0.06	0.05	0.07	0.02	0.16	-0.02	0.01	0.06	-0.06	-0.13	0.29	-0.01	0.00	0.07	-0.11	0.03	-0.15	0.47
Q9BQA1	Methylosome protein 50 OS=Homo sapiens GN=WDR77 PE=1 SV=1 - [MEP50_HUMAN]	23.10	1	5	5	8	-0.38	-0.45	-0.45	-0.52	0.42	0.34	-0.04	-0.11	0.29	0.37	0.92	0.84	0.39	1.30	1.36	0.78	0.85	0.78	0.85	0.47
Q9HBH5	Retinol dehydrogenase 14 OS=Homo sapiens GN=RDH14 PE=1 SV=1 - [RDH14_HUMAN]	20.83	1	6	6	19	-0.05	-0.01	-0.18	-0.18	0.15	-0.01	0.10	0.22	0.28	0.29	0.19	0.23	0.25	0.21	0.42	0.32	0.51	0.05	0.22	0.47
P52597	Heterogeneous nuclear ribonucleoprotein F OS=Homo sapiens GN=HNRNPF PE=1 SV=3 - [HNRPF_HUMAN]	34.22	1	7	9	19	-0.98	-1.04	-0.67	-0.73	-0.17	-0.27	-0.26	-0.32	-0.10	-0.02	0.07	0.01	0.71	0.98	0.73	0.78	0.63	0.61	0.42	0.47

O43854	EGF-like repeat and discoidin I-like domain-containing protein 3 OS=Homo sapiens GN=EDIL3 PE=1 SV=1 - [EDIL3_HUMAN]	26.88	1	10	10	18	0.05	-0.02	-0.01	0.04	2.19	2.26	0.55	0.52	1.30	1.21	0.85	0.70	0.42	0.73	0.62	0.96	1.17	1.82	2.10	0.47
Q96D15	Reticulocalbin-3 OS=Homo sapiens GN=RCN3 PE=1 SV=1 - [RCN3_HUMAN]	15.24	1	4	4	8	-0.42	-0.42	-0.69	-0.60	0.29	0.39	-0.03	0.10	0.09	0.15	0.70	0.68	0.58	1.07	1.17	0.64	0.81	0.88	1.08	0.47
Q96DH6	RNA-binding protein Musashi homolog 2 OS=Homo sapiens GN=MSI2 PE=1 SV=1 - [MSI2H_HUMAN]	17.68	1	3	5	8	-0.66	-0.72	-0.52	-0.59	-0.03	-0.10	-0.12	-0.19	-0.56	-0.49	-0.37	-0.45	0.59	0.29	0.15	0.20	0.06	0.61	0.47	0.47
P29692	Elongation factor 1-delta OS=Homo sapiens GN=EEF1D PE=1 SV=5 - [EF1D_HUMAN]	46.26	1	10	11	43	-0.19	-0.29	-0.35	-0.46	0.10	0.08	0.01	0.04	-0.19	-0.12	0.07	-0.01	0.33	0.32	0.55	0.25	0.31	0.23	0.46	0.47
P31937	3-hydroxyisobutyrate dehydrogenase, mitochondrial OS=Homo sapiens GN=HIBADH PE=1 SV=2 - [3HIDH_HUMAN]	32.14	1	8	8	26	-0.68	-0.60	-0.57	-0.51	-0.34	-0.29	-0.15	-0.05	-0.23	-0.31	-0.31	-0.27	0.56	0.38	0.29	0.36	0.23	0.29	0.19	0.47
Q0VF96	Cingulin-like protein 1 OS=Homo sapiens GN=CGNL1 PE=1 SV=2 - [CGNL1_HUMAN]	5.22	1	5	6	10	-0.78	-0.80	-1.19	-1.09	-0.21	-0.45	-0.65	-0.79	-0.50	-0.40	-0.17	-0.32	0.15	0.48	0.63	0.40	0.75	0.35	0.63	0.47
Q7Z6B7	SLIT-ROBO Rho GTPase-activating protein 1 OS=Homo sapiens GN=SRGAP1 PE=1 SV=1 - [SRGP1_HUMAN]	4.15	1	1	4	7	-2.72	-2.84	-3.17	-3.30	-1.79	-1.92	-2.77	-2.90	-2.03	-1.90	-2.41	-2.55	0.00	0.31	0.76	0.85	1.31	0.91	1.36	0.47

P62495	Eukaryotic peptide chain release factor subunit 1 OS=Homo sapiens GN=ETF1 PE=1 SV=3 - [ERF1_HUMAN]	20.37	1	9	9	16	-0.67	-0.45	-0.42	-0.25	-0.14	0.00	-0.20	-0.13	-0.20	-0.23	-0.27	-0.10	0.60	0.43	0.30	0.47	0.24	0.58	0.25	0.46
P02008	Hemoglobin subunit zeta OS=Homo sapiens GN=HBZ PE=1 SV=2 - [HBAZ_HUMAN]	19.01	1	2	3	26	-1.10	-1.55	-0.94	-1.39	2.81	2.35	-0.54	-0.99	0.96	1.42	1.32	0.86	0.61	2.42	2.25	2.55	2.39	3.89	3.73	0.46
P14136	Glial fibrillary acidic protein OS=Homo sapiens GN=GFAP PE=1 SV=1 - [GFAP_HUMAN]	74.31	6	38	40	1016	1.33	1.32	1.40	1.40	0.74	0.73	1.78	1.82	1.46	1.43	1.46	1.45	0.43	0.12	0.13	0.14	0.16	-0.61	-0.63	0.46
P49189	4-trimethylaminobutyraldehyde dehydrogenase OS=Homo sapiens GN=ALDH9A1 PE=1 SV=3 - [AL9A1_HUMAN]	47.77	1	19	19	51	-0.18	-0.22	-0.26	-0.32	0.16	0.11	0.30	0.15	0.03	0.01	-0.52	-0.48	0.47	-0.28	-0.31	0.27	0.36	0.32	0.32	0.46
Q16666	Gamma-interferon-inducible protein 16 OS=Homo sapiens GN=IFI16 PE=1 SV=3 - [IF16_HUMAN]	18.09	2	11	11	14	-1.08	-0.81	-0.85	-0.77	-0.18	0.06	-0.38	-0.35	-0.33	-0.39	-0.49	-0.29	0.60	0.38	0.54	0.50	0.57	0.85	0.87	0.46
P07384	Calpain-1 catalytic subunit OS=Homo sapiens GN=CAPN1 PE=1 SV=1 - [CAN1_HUMAN]	38.66	1	26	26	79	-0.57	-0.53	-0.49	-0.45	-0.04	-0.03	-0.03	-0.01	-0.05	-0.02	0.15	0.15	0.54	0.61	0.57	0.45	0.41	0.50	0.41	0.46
Q92870	Amyloid beta A4 precursor protein-binding family B member 2 OS=Homo sapiens GN=APBB2 PE=1 SV=3 - [APBB2_HUMAN]	2.64	1	1	2	3	-0.38	-0.47	-0.75	-0.83	0.02	-0.07	-0.36	-0.44	-0.28	-0.20	-0.13	-0.21	0.08	0.26	0.62	0.22	0.59	0.39	0.76	0.46

Q7Z3T8	Zinc finger FYVE domain-containing protein 16 OS=Homo sapiens GN=ZFYVE16 PE=1 SV=3 - [ZFY16_HUMAN]	1.82	1	2	2	2	0.48	0.62	0.30	0.43	0.51	0.64	0.70	0.83	0.57	0.45	0.09	0.22	0.27	-0.39	-0.21	-0.01	0.18	0.01	0.20	0.46
P07339	Cathepsin D OS=Homo sapiens GN=CTSD PE=1 SV=1 - [CATD_HUMAN]	38.35	1	12	12	68	-0.23	-0.14	-0.09	0.00	0.57	0.57	0.22	0.31	0.57	0.44	0.68	0.71	0.59	0.83	0.73	0.72	0.62	0.79	0.64	0.46
Q9BUT1	3-hydroxybutyrate dehydrogenase type 2 OS=Homo sapiens GN=BDH2 PE=1 SV=2 - [BDH2_HUMAN]	18.78	1	4	4	8	-0.21	-0.28	-0.28	-0.35	0.46	0.39	0.12	0.05	0.10	0.18	-0.25	-0.32	0.39	-0.03	0.03	0.42	0.49	0.66	0.72	0.46
O43310	CBP80/20-dependent translation initiation factor OS=Homo sapiens GN=CTIF PE=1 SV=1 - [CTIF_HUMAN]	12.21	1	5	5	6	-0.33	-0.75	-0.45	-0.87	-0.32	-0.74	-0.05	-0.47	-0.74	-0.31	-0.04	-0.47	0.33	0.29	0.40	0.05	0.17	-0.01	0.11	0.46
O15372	Eukaryotic translation initiation factor 3 subunit H OS=Homo sapiens GN=EIF3H PE=1 SV=1 - [EIF3H_HUMAN]	22.16	1	6	6	8	-0.90	-0.71	-0.59	-0.53	-0.54	-0.62	-0.12	-0.36	-0.54	-0.36	-0.35	-0.38	0.69	0.49	0.34	0.39	0.38	0.56	0.08	0.46
Q9Y2B0	Protein canopy homolog 2 OS=Homo sapiens GN=CNPY2 PE=1 SV=1 - [CNPY2_HUMAN]	52.75	1	9	9	28	-0.09	-0.06	-0.31	-0.23	0.29	0.32	0.07	0.17	0.30	0.27	0.15	0.15	0.03	0.50	0.47	0.39	0.57	0.47	0.56	0.46
P49711	Transcriptional repressor CTCF OS=Homo sapiens GN=CTCF PE=1 SV=1 - [CTCF_HUMAN]	4.40	2	2	2	3	-1.00	-1.03	-0.78	-0.81	-0.27	-0.31	-0.38	-0.42	-0.19	-0.15	-0.09	-0.13	0.67	0.91	0.69	0.87	0.66	0.71	0.48	0.46
O94885	SAM and SH3 domain-containing protein 1 OS=Homo sapiens GN=SASH1 PE=1 SV=3 - [SASH1_HUMAN]	8.58	2	6	6	9	-0.67	-0.47	-0.46	-0.45	0.03	0.28	-0.12	0.21	0.08	-0.15	-0.18	-0.12	0.61	0.45	0.33	0.62	0.30	0.60	0.48	0.46

Q53QV2	Protein LBH OS=Homo sapiens GN=LBH PE=1 SV=1 - [LBH_HUMAN]	16.19	1	1	1	1	-1.05	-0.78	-1.04	-0.77	-0.41	-0.15	-0.65	-0.38	-0.52	-0.79	-0.16	0.11	0.46	0.90	0.88	0.30	0.29	0.62	0.61	0.46
P78310	Coxsackievirus and adenovirus receptor OS=Homo sapiens GN=CXADR PE=1 SV=1 - [CXAR_HUMAN]	18.90	1	6	6	17	0.23	0.03	-0.12	-0.16	0.29	0.27	0.11	0.32	0.24	0.25	0.76	0.67	0.49	0.79	0.98	0.28	0.44	0.10	0.31	0.46
P07477	Trypsin-1 OS=Homo sapiens GN=PRSS1 PE=1 SV=1 - [TRY1_HUMAN]	15.38	4	3	3	55	-1.35	-1.11	-1.12	-0.86	-0.25	0.05	-0.87	-0.56	-0.13	-0.36	-0.35	-0.15	0.51	0.68	0.72	0.99	0.72	0.92	0.97	0.46
Q15691	Microtubule-associated protein RP/EB family member 1 OS=Homo sapiens GN=MAPRE1 PE=1 SV=3 - [MARE1_HUMAN]	46.64	1	9	11	27	-0.76	-0.86	-0.74	-0.74	-0.25	-0.15	-0.14	-0.28	-0.16	-0.19	-0.23	-0.22	0.67	0.51	0.42	0.56	0.74	0.49	0.55	0.46
Q15024	Exosome complex component RRP42 OS=Homo sapiens GN=EXOSC7 PE=1 SV=3 - [EXOS7_HUMAN]	18.21	1	3	3	4	-0.53	-0.70	-0.72	-0.98	-0.03	-0.34	-0.21	-0.54	-0.32	-0.14	-0.08	-0.25	0.39	0.45	0.64	0.42	0.70	0.92	0.67	0.46
Q15642	Cdc42-interacting protein 4 OS=Homo sapiens GN=TRIP10 PE=1 SV=3 - [CIP4_HUMAN]	8.65	1	3	3	3	-0.55	-0.74	-0.80	-0.98	-0.05	-0.24	-0.27	-0.48	-0.12	-0.02	-0.13	-0.32	0.20	0.43	0.67	0.45	0.70	0.48	0.73	0.46
Q96SW2	Protein cereblon OS=Homo sapiens GN=CRBN PE=1 SV=1 - [CRBN_HUMAN]	4.07	1	1	1	1	-0.07	0.33	-0.20	0.20	-0.20	0.20	0.19	0.59	0.00	-0.40	0.18	0.58	0.32	0.26	0.38	-0.29	-0.16	-0.14	-0.01	0.46
Q9UHG3	Prenylcysteine oxidase 1 OS=Homo sapiens GN=PCYOX1 PE=1 SV=3 - [PCYOX_HUMAN]	27.13	1	11	11	26	-0.97	-0.91	-0.98	-0.81	0.07	0.27	-0.42	-0.27	-0.10	-0.21	-0.16	0.07	0.61	0.87	0.96	0.72	0.68	0.96	0.87	0.46

Q676U5	Autophagy-related protein 16-1 OS=Homo sapiens GN=ATG16L1 PE=1 SV=2 - [A16L1_HUMAN]	7.74	1	4	4	4	-0.15	-0.38	-0.04	-0.27	0.18	-0.05	0.35	0.12	0.10	0.33	0.09	-0.14	0.56	0.25	0.14	0.51	0.41	0.32	0.20	0.46
Q01831	DNA repair protein complementing XP-C cells OS=Homo sapiens GN=XPC PE=1 SV=4 - [XPC_HUMAN]	2.98	1	2	2	6	-1.21	-1.28	-1.11	-1.07	-0.59	-0.58	-0.61	-0.62	-0.71	-0.50	-0.29	-0.05	0.69	1.14	0.84	0.73	0.64	0.74	0.45	0.46
O75083	WD repeat-containing protein 1 OS=Homo sapiens GN=WDR1 PE=1 SV=4 - [WDR1_HUMAN]	56.27	1	25	25	118	-0.99	-0.97	-0.74	-0.73	-0.09	-0.13	-0.27	-0.28	-0.17	-0.22	-0.06	-0.06	0.78	0.92	0.66	0.82	0.50	0.89	0.57	0.46
P02675	Fibrinogen beta chain OS=Homo sapiens GN=FGB PE=1 SV=2 - [FIBB_HUMAN]	64.15	1	26	26	189	-0.40	-0.39	-0.46	-0.50	0.39	0.40	-0.06	-0.06	0.23	0.25	0.77	0.71	0.38	1.19	1.20	0.68	0.75	0.81	0.86	0.45
Q969X5	Endoplasmic reticulum-Golgi intermediate compartment protein 1 OS=Homo sapiens GN=ERGIC1 PE=1 SV=1 - [ERGI1_HUMAN]	32.76	1	8	8	16	-0.74	-0.69	-0.76	-0.75	0.13	0.09	-0.29	-0.26	-0.04	-0.07	0.12	0.12	0.41	0.80	0.90	0.87	0.68	0.87	0.92	0.45
P02790	Hemopexin OS=Homo sapiens GN=HPX PE=1 SV=2 - [HEMO_HUMAN]	33.33	1	10	10	34	-1.87	-1.90	-1.98	-1.94	-0.93	-0.89	-1.58	-1.60	-1.30	-1.39	-1.92	-1.91	0.34	0.06	0.13	0.60	0.60	1.02	1.07	0.45
Q5HYI8	Rab-like protein 3 OS=Homo sapiens GN=RABL3 PE=1 SV=1 - [RABL3_HUMAN]	13.98	1	3	3	6	-0.92	-0.97	-0.51	-0.57	0.01	-0.05	-0.12	-0.18	-0.20	-0.14	0.03	-0.03	0.85	0.95	0.54	0.81	0.41	0.91	0.51	0.45
P06865	Beta-hexosaminidase subunit alpha OS=Homo sapiens GN=HEXA PE=1 SV=2 - [HEXA_HUMAN]	6.99	1	3	4	7	-0.32	-0.44	-0.22	-0.34	0.15	0.02	0.17	0.05	0.03	0.16	0.28	0.15	0.55	0.61	0.50	0.51	0.41	0.45	0.35	0.45

Q16563	Synaptophysin-like protein 1 OS=Homo sapiens GN=SYPL1 PE=1 SV=1 - [SYPL1_HUMAN]	5.79	1	1	1	4	-1.12	-0.90	-0.65	-0.43	0.12	0.34	-0.27	-0.05	0.14	-0.07	0.12	0.33	0.91	1.24	0.77	1.08	0.61	1.22	0.76	0.45
P08183	Multidrug resistance protein 1 OS=Homo sapiens GN=ABCB1 PE=1 SV=3 - [MDR1_HUMAN]	14.61	2	15	15	27	-1.11	-1.06	-1.43	-1.29	-0.88	-0.96	-1.19	-1.23	-0.69	-0.77	-0.78	-0.79	0.15	0.46	0.60	0.30	0.55	0.14	0.29	0.45
Q6UW68	Transmembrane protein 205 OS=Homo sapiens GN=TMEM205 PE=1 SV=1 - [TM205_HUMAN]	20.63	1	3	3	8	-0.83	-0.95	-0.91	-1.09	-0.10	-0.27	-0.52	-0.71	-0.41	-0.29	-0.11	-0.23	0.42	0.61	0.80	0.47	0.70	0.71	0.77	0.45
Q9UBF2	Coatomer subunit gamma-2 OS=Homo sapiens GN=COPG2 PE=1 SV=1 - [COPG2_HUMAN]	8.04	1	5	6	11	-0.34	-0.23	-0.30	-0.21	0.41	0.39	0.09	0.22	0.23	0.25	0.40	0.37	0.44	0.61	0.57	0.58	0.58	0.61	0.69	0.45
Q9Y3E7	Charged multivesicular body protein 3 OS=Homo sapiens GN=CHMP3 PE=1 SV=3 - [CHMP3_HUMAN]	26.13	1	7	7	9	-0.06	-0.07	-0.14	-0.28	0.42	0.34	0.22	0.13	0.23	0.19	0.39	0.32	0.34	0.39	0.67	0.22	0.51	0.39	0.68	0.45
P04080	Cystatin-B OS=Homo sapiens GN=CSTB PE=1 SV=2 - [CYTB_HUMAN]	45.92	1	3	3	5	0.10	-0.10	-0.12	-0.32	0.47	0.26	0.27	0.07	0.08	0.29	0.02	-0.19	0.22	-0.08	0.13	0.21	0.43	0.35	0.57	0.45
Q92982	Ninjurin-1 OS=Homo sapiens GN=NINJ1 PE=1 SV=2 - [NINJ1_HUMAN]	7.89	1	1	1	2	-0.34	-0.47	-0.28	-0.40	0.31	0.19	0.11	-0.02	0.04	0.17	0.47	0.34	0.51	0.82	0.74	0.54	0.48	0.64	0.58	0.45
Q12893	Transmembrane protein 115 OS=Homo sapiens GN=TMEM115 PE=1 SV=1 - [TM115_HUMAN]	12.82	1	2	2	2	-0.46	-0.38	-0.81	-0.74	0.18	0.25	-0.43	-0.35	-0.07	-0.14	-0.31	-0.24	0.08	0.15	0.50	0.35	0.71	0.62	0.97	0.45
P23229	Integrin alpha 6 OS=Homo sapiens GN=ITGA6 PE=1 SV=5 - [ITA6_HUMAN]	29.29	1	25	25	47	-0.85	-1.01	-1.07	-1.11	-0.50	-0.51	-0.61	-0.64	-0.44	-0.46	-0.27	-0.28	0.52	0.79	0.74	0.57	0.55	0.60	0.48	0.45

Q16134	Electron transfer flavoprotein-ubiquinone oxidoreductase, mitochondrial OS=Homo sapiens GN=ETFDH PE=1 SV=2 - [ETFD_HUMAN]	25.93	1	12	12	21	-0.49	-0.51	-0.58	-0.53	-0.10	-0.09	-0.15	-0.16	-0.19	-0.15	-0.37	-0.37	0.40	0.08	0.10	0.50	0.39	0.37	0.43	0.45
Q7KZF4	Staphylococcal nuclease domain-containing protein 1 OS=Homo sapiens GN=SND1 PE=1 SV=1 - [SND1_HUMAN]	34.84	1	23	23	59	-0.63	-0.67	-0.68	-0.72	-0.05	-0.14	-0.38	-0.43	-0.33	-0.25	-0.16	-0.16	0.31	0.49	0.61	0.47	0.50	0.62	0.60	0.45
O60684	Importin subunit alpha 7 OS=Homo sapiens GN=KPNA6 PE=1 SV=1 - [IMA7_HUMAN]	23.32	2	6	9	22	-0.43	-0.42	-0.23	-0.24	-0.03	-0.16	-0.14	-0.12	-0.22	-0.08	-0.05	-0.08	0.27	0.26	0.23	0.30	0.51	0.42	0.60	0.45
Q7Z2W9	39S ribosomal protein L21, mitochondrial OS=Homo sapiens GN=MRPL21 PE=1 SV=2 - [RM21_HUMAN]	11.22	1	2	2	3	-0.22	-0.36	-0.49	-0.64	0.55	0.39	-0.11	-0.26	0.08	0.23	-0.06	-0.21	0.16	0.16	0.43	0.48	0.76	0.74	1.02	0.45
P27824	Calnexin OS=Homo sapiens GN=CANX PE=1 SV=2 - [CALX_HUMAN]	28.38	1	19	19	93	-1.01	-1.08	-0.62	-0.64	-0.27	-0.21	-0.26	-0.26	-0.27	-0.31	-0.24	-0.27	0.75	0.82	0.41	0.72	0.32	0.75	0.39	0.45
P20700	Lamin-B1 OS=Homo sapiens GN=LMNB1 PE=1 SV=2 - [LMNB1_HUMAN]	35.32	1	17	19	35	-0.84	-0.88	-0.88	-0.89	-0.38	-0.33	-0.52	-0.47	-0.38	-0.43	-0.28	-0.23	0.42	0.53	0.44	0.39	0.39	0.39	0.42	0.44
O15260	Surfeit locus protein 4 OS=Homo sapiens GN=SURF4 PE=1 SV=3 - [SURF4_HUMAN]	10.04	1	3	3	7	-0.67	-0.51	-0.69	-0.53	0.27	0.42	-0.31	-0.15	-0.15	-0.30	0.00	0.15	0.41	0.85	0.91	0.91	0.88	0.95	0.95	0.44
Q8WVM8	Sec1 family domain-containing protein 1 OS=Homo sapiens GN=SCFD1 PE=1 SV=4 - [SCFD1_HUMAN]	33.64	1	16	16	28	-0.45	-0.43	-0.38	-0.39	-0.02	0.03	0.03	0.05	-0.04	-0.04	-0.06	-0.04	0.55	0.39	0.24	0.32	0.33	0.44	0.31	0.44

Q9UI26	Importin-11 OS=Homo sapiens GN=IPO11 PE=1 SV=1 - [IPO11_HUMAN]	4.21	1	3	3	5	0.27	0.13	-0.46	-0.59	0.29	0.16	-0.08	-0.21	0.17	0.31	0.08	-0.06	-0.29	-0.18	0.54	0.07	0.80	0.01	0.73	0.44
Q6UWP8	Suprabasin OS=Homo sapiens GN=SBSN PE=2 SV=2 - [SBSN_HUMAN]	10.85	1	3	3	4	-3.12	-3.06	-3.14	-3.08	-2.17	-2.12	-2.76	-2.71	-2.41	-2.46	-2.22	-2.17	0.41	0.90	0.92	0.69	0.72	0.94	0.96	0.44
Q6ZPD9	Probable C-mannosyltransferase DPY19L3 OS=Homo sapiens GN=DPY19L3 PE=2 SV=1 - [D19L3_HUMAN]	1.12	1	1	1	1	-1.00	-0.85	-0.86	-0.71	-0.02	0.13	-0.48	-0.33	-0.04	-0.19	-0.10	0.04	0.58	0.91	0.76	0.85	0.71	0.97	0.83	0.44
P08195	4F2 cell-surface antigen heavy chain OS=Homo sapiens GN=SLC3A2 PE=1 SV=3 - [4F2_HUMAN]	30.63	1	16	16	45	-0.33	-0.22	-0.20	-0.03	0.07	0.14	0.24	0.23	0.14	0.20	0.33	0.45	0.64	0.69	0.53	0.50	0.34	0.38	0.23	0.44
Q7KZ85	Transcription elongation factor SPT6 OS=Homo sapiens GN=SUPT6H PE=1 SV=2 - [SPT6H_HUMAN]	5.16	1	7	7	17	-0.69	-0.83	-0.52	-0.56	0.33	0.19	-0.13	-0.14	0.03	0.16	0.25	0.13	0.82	1.14	0.66	0.93	0.60	1.08	0.69	0.44
P25786	Proteasome subunit alpha type-1 OS=Homo sapiens GN=PSMA1 PE=1 SV=1 - [PSA1_HUMAN]	52.09	1	11	11	34	-0.39	-0.25	-0.74	-0.72	0.15	-0.02	-0.27	-0.17	0.15	0.09	0.43	0.13	0.32	0.61	0.92	0.41	0.74	0.43	0.73	0.44
Q06033	Inter-alpha-trypsin inhibitor heavy chain H3 OS=Homo sapiens GN=ITIH3 PE=1 SV=2 - [ITIH3_HUMAN]	13.15	1	8	8	11	0.02	-0.30	-0.44	-0.55	0.30	0.33	-0.30	-0.06	0.04	0.30	0.47	0.36	0.29	0.44	0.82	0.38	0.88	0.41	1.03	0.44
O00161	Synaptosomal-associated protein 23 OS=Homo sapiens GN=SNAP23 PE=1 SV=1 - [SNP23_HUMAN]	54.03	1	7	7	17	-0.83	-0.63	-1.05	-0.71	0.10	0.38	-0.39	-0.21	0.03	-0.18	0.20	0.48	0.22	1.03	0.89	0.69	0.78	1.32	1.26	0.44

Q6P996	Pyridoxal-dependent decarboxylase domain-containing protein 1 OS=Homo sapiens GN=PDXDC1 PE=1 SV=2 - [PDXD1_HUMAN]	5.96	1	4	4	6	-0.60	-0.72	-0.84	-0.52	0.17	0.18	-0.47	-0.14	-0.49	-0.37	-0.49	-0.27	0.63	0.46	0.35	0.26	0.29	0.88	0.63	0.44
O15084	Serine/threonine-protein phosphatase 6 regulatory ankyrin repeat subunit A OS=Homo sapiens GN=ANKRD28 PE=1 SV=5 - [ANR28_HUMAN]	6.08	1	4	4	7	-0.04	-0.25	-0.39	-0.39	-0.01	-0.05	-0.01	-0.16	-0.22	-0.24	-0.01	-0.25	0.15	0.03	0.37	0.25	0.39	0.23	0.35	0.44
Q93008	Probable ubiquitin carboxyl-terminal hydrolase FAF-X OS=Homo sapiens GN=USP9X PE=1 SV=3 - [USP9X_HUMAN]	16.89	2	37	37	65	0.00	-0.11	-0.27	-0.31	0.21	0.25	0.11	0.10	0.17	0.17	0.12	0.24	0.21	0.18	0.38	0.24	0.25	0.23	0.42	0.44
Q9UHR5	SAP30-binding protein OS=Homo sapiens GN=SAP30BP PE=1 SV=1 - [S30BP_HUMAN]	4.87	1	1	1	4	0.00	-0.42	-0.66	-1.08	-0.17	-0.59	-0.28	-0.70	-0.70	-0.28	-0.49	-0.92	-0.23	-0.49	0.17	-0.24	0.42	-0.19	0.47	0.44
P35030	Trypsin-3 OS=Homo sapiens GN=PRSS3 PE=1 SV=2 - [TRY3_HUMAN]	4.28	1	1	1	1	-0.86	-0.82	-0.86	-0.82	-0.14	-0.10	-0.49	-0.44	-0.08	-0.11	-0.23	-0.19	0.43	0.64	0.63	0.78	0.78	0.71	0.71	0.44
Q9Y6W5	Wiskott-Aldrich syndrome protein family member 2 OS=Homo sapiens GN=WASF2 PE=1 SV=3 - [WASF2_HUMAN]	17.07	1	7	7	14	-0.41	-0.48	-0.49	-0.77	-0.12	-0.27	-0.29	-0.26	-0.23	-0.08	-0.01	-0.05	0.19	0.29	0.70	0.27	0.44	0.33	0.47	0.44
P00533	Epidermal growth factor receptor OS=Homo sapiens GN=EGFR PE=1 SV=2 - [EGFR_HUMAN]	25.70	1	22	24	44	-0.31	-0.50	-0.15	-0.25	0.14	0.03	0.15	0.11	0.10	0.10	0.13	0.18	0.55	0.54	0.45	0.55	0.52	0.44	0.38	0.44

Q5SWX8	Protein odr-4 homolog OS=Homo sapiens GN=ODR4 PE=2 SV=1 - [ODR4_HUMAN]	6.39	1	3	3	4	-0.43	-0.25	-0.55	-0.40	0.24	0.21	-0.18	-0.15	-0.04	-0.18	0.28	0.34	0.16	0.61	0.87	0.25	0.40	0.51	0.58	0.44
P23284	Peptidyl-prolyl cis-trans isomerase B OS=Homo sapiens GN=PPIB PE=1 SV=2 - [PPIB_HUMAN]	47.69	1	10	11	44	-1.43	-1.36	-1.14	-1.21	-0.61	-0.58	-0.87	-0.76	-0.76	-0.81	-0.50	-0.51	0.52	0.72	0.65	0.53	0.40	0.76	0.62	0.44
Q9BZH6	WD repeat-containing protein 11 OS=Homo sapiens GN=WDR11 PE=1 SV=1 - [WDR11_HUMAN]	7.52	1	8	8	14	-0.56	-0.59	-1.12	-0.96	-0.04	0.01	-0.43	-0.27	-0.01	-0.03	-0.08	0.07	0.21	0.57	0.98	0.56	0.93	0.59	1.11	0.44
Q96RF0	Sorting nexin-18 OS=Homo sapiens GN=SNX18 PE=1 SV=2 - [SNX18_HUMAN]	14.81	1	6	6	12	-1.06	-0.72	-0.81	-1.27	0.15	0.16	-0.34	-0.61	-0.13	-0.15	-0.11	-0.05	0.46	1.32	1.07	0.71	0.75	0.91	1.14	0.44
P20908	Collagen alpha-1(V) chain OS=Homo sapiens GN=COL5A1 PE=1 SV=3 - [CO5A1_HUMAN]	6.53	1	6	8	18	-3.22	-3.17	-3.17	-3.07	-1.79	-1.91	-2.20	-2.51	-2.50	-2.39	-2.37	-2.52	0.78	1.09	0.64	1.06	0.75	1.62	1.10	0.44
Q969U7	Proteasome assembly chaperone 2 OS=Homo sapiens GN=PSMG2 PE=1 SV=1 - [PSMG2_HUMAN]	8.33	1	2	2	2	-0.31	-0.11	-0.27	-0.07	-0.63	-0.43	0.10	0.30	0.06	-0.13	0.27	0.46	0.47	0.58	0.54	0.21	0.17	-0.33	-0.38	0.44
Q8N4X5	Actin filament associated protein 1-like 2 OS=Homo sapiens GN=AFAP1L2 PE=1 SV=1 - [AF1L2_HUMAN]	6.72	1	5	5	5	-0.90	-0.74	-1.44	-1.22	-0.86	-0.64	-0.69	-0.85	-0.74	-0.96	-0.78	-0.98	-0.09	0.39	0.46	0.09	0.52	0.12	0.57	0.44
Q14683	Structural maintenance of chromosomes protein 1A OS=Homo sapiens GN=SMC1A PE=1 SV=2 - [SMC1A_HUMAN]	23.52	1	27	28	47	-0.70	-0.74	-0.67	-0.78	-0.24	-0.23	-0.22	-0.32	-0.24	-0.25	-0.06	-0.10	0.51	0.64	0.57	0.59	0.47	0.47	0.40	0.44

P13489	Ribonuclease inhibitor OS=Homo sapiens GN=RNH1 PE=1 SV=2 - [RINI_HUMAN]	35.79	1	12	12	62	0.10	0.10	-0.06	0.09	0.30	0.44	0.33	0.40	0.26	0.20	-0.31	-0.28	0.37	-0.34	-0.32	0.21	0.26	0.19	0.40	0.43
Q9UBT7	Alpha-catulin OS=Homo sapiens GN=CTNNAL1 PE=1 SV=2 - [CTNL1_HUMAN]	6.27	1	4	4	5	-1.01	-1.04	-1.18	-1.20	0.52	0.49	-0.33	-0.28	0.10	0.12	0.24	0.54	0.14	0.92	1.09	1.13	1.34	1.30	1.68	0.43
Q6GMV3	Putative peptidyl-tRNA hydrolase PTRHD1 OS=Homo sapiens GN=PTRHD1 PE=1 SV=1 - [PTRD1_HUMAN]	49.29	1	6	6	13	-0.15	-0.32	0.07	-0.06	0.43	0.30	0.33	0.33	0.26	0.43	0.44	0.42	0.68	0.78	0.49	0.66	0.41	0.72	0.35	0.43
P11586	C-1-tetrahydrofolate synthase, cytoplasmic OS=Homo sapiens GN=MTHFD1 PE=1 SV=3 - [C1TC_HUMAN]	44.92	1	37	37	81	-0.37	-0.33	-0.24	-0.17	0.02	0.03	0.21	0.24	0.08	0.04	0.08	0.04	0.49	0.34	0.27	0.34	0.35	0.39	0.23	0.43
Q96SZ5	2-aminoethanethiol dioxygenase OS=Homo sapiens GN=ADO PE=1 SV=2 - [AEDO_HUMAN]	32.96	1	6	6	11	1.02	1.11	-0.25	1.25	-0.01	0.56	0.33	0.66	0.68	0.23	0.49	0.28	-0.63	-0.78	0.74	-0.75	0.40	-1.05	0.01	0.43
Q15008	26S proteasome non-ATPase regulatory subunit 6 OS=Homo sapiens GN=PSMD6 PE=1 SV=1 - [PSMD6_HUMAN]	30.59	1	12	12	23	-0.41	-0.15	-0.44	-0.38	0.08	0.16	-0.01	0.15	0.19	0.11	0.10	0.13	0.26	0.39	0.49	0.32	0.59	0.27	0.49	0.43
Q96EP0	E3 ubiquitin-protein ligase RNF31 OS=Homo sapiens GN=RNF31 PE=1 SV=1 - [RNF31_HUMAN]	6.72	1	5	5	7	-0.60	-0.30	-0.51	-0.36	-0.01	0.12	-0.15	-0.15	0.01	-0.09	0.12	0.17	0.51	0.73	0.63	0.54	0.54	0.52	0.44	0.43
P07237	Protein disulfide-isomerase OS=Homo sapiens GN=P4HB PE=1 SV=3 - [PDIA1_HUMAN]	60.24	1	27	27	92	-1.07	-0.99	-0.87	-0.79	-0.24	-0.18	-0.49	-0.41	-0.34	-0.39	-0.12	-0.04	0.62	0.92	0.77	0.60	0.46	0.75	0.55	0.43

Q8IZ07	Ankyrin repeat domain-containing protein 13A OS=Homo sapiens GN=ANKRD13A PE=1 SV=3 - [AN13A_HUMAN]	10.68	1	5	5	6	-0.54	-0.33	-0.85	-0.41	-0.22	0.21	-0.37	-0.05	0.15	-0.23	0.07	0.04	0.38	0.29	0.91	0.60	0.64	0.46	0.61	0.43
O95848	Uridine diphosphate glucose pyrophosphatase OS=Homo sapiens GN=NUDT14 PE=1 SV=2 - [NUD14_HUMAN]	6.76	1	1	1	1	0.12	0.00	-0.36	-0.47	0.03	-0.08	0.01	-0.10	-0.05	0.07	0.14	0.02	-0.05	0.03	0.49	-0.02	0.46	-0.10	0.37	0.43
Q96BQ5	Coiled-coil domain-containing protein 127 OS=Homo sapiens GN=CCDC127 PE=1 SV=1 - [CC127_HUMAN]	17.31	1	5	5	9	0.24	0.04	-0.11	-0.12	0.25	0.27	0.22	0.25	0.15	0.09	0.17	0.21	0.05	0.22	0.37	0.15	0.36	0.30	0.40	0.43
Q7LBC6	Lysine-specific demethylase 3B OS=Homo sapiens GN=KDM3B PE=1 SV=2 - [KDM3B_HUMAN]	1.59	1	2	2	2	-0.42	-0.37	-0.75	-0.70	-0.13	-0.08	-0.38	-0.33	-0.07	-0.12	-0.18	-0.14	0.09	0.24	0.57	0.33	0.67	0.28	0.61	0.43
P84243	Histone H3.3 OS=Homo sapiens GN=H3F3A PE=1 SV=2 - [H33_HUMAN]	66.18	3	2	10	72	-1.26	-0.72	-0.89	-0.60	-0.36	0.11	-0.47	-0.03	-0.18	-0.46	-0.15	-0.02	0.93	0.99	0.56	0.63	0.34	0.70	0.54	0.43
Q96LZ7	Regulator of microtubule dynamics protein 2 OS=Homo sapiens GN=RMDN2 PE=1 SV=2 - [RMD2_HUMAN]	11.46	1	4	4	6	-0.90	-0.79	-0.29	-0.03	0.01	0.19	0.07	0.34	0.01	-0.09	-0.23	-0.05	0.89	0.75	0.24	0.79	0.37	0.97	0.46	0.43
Q9BUL8	Programmed cell death protein 10 OS=Homo sapiens GN=PDCD10 PE=1 SV=1 - [PDC10_HUMAN]	30.66	1	5	5	6	-0.74	-0.64	-0.58	-0.60	0.22	-0.16	-0.25	-0.52	-0.41	-0.29	-0.01	-0.12	0.45	0.64	0.60	0.55	0.52	0.77	0.79	0.43

P42126	Enoyl-CoA delta isomerase 1, mitochondrial OS=Homo sapiens GN=ECI1 PE=1 SV=1 - [ECI1_HUMAN]	26.49	1	6	6	16	-1.06	-1.04	-0.82	-0.83	-0.50	-0.58	-0.59	-0.54	-0.69	-0.67	-0.37	-0.45	0.72	0.97	0.48	0.66	0.33	0.71	0.35	0.43
Q8WW59	SPRY domain-containing protein 4 OS=Homo sapiens GN=SPRYD4 PE=1 SV=2 - [SPRY4_HUMAN]	25.60	1	5	5	7	-0.08	0.18	-0.30	0.06	0.14	0.50	0.19	0.34	0.05	-0.11	-0.13	0.23	0.52	0.16	0.18	0.00	-0.08	0.20	0.33	0.43
Q7L2H7	Eukaryotic translation initiation factor 3 subunit M OS=Homo sapiens GN=EIF3M PE=1 SV=1 - [EIF3M_HUMAN]	15.24	1	3	3	6	-0.85	-0.92	-0.57	-0.64	-0.09	-0.16	-0.21	-0.28	-0.43	-0.35	0.06	-0.02	0.70	0.92	0.63	0.53	0.25	0.75	0.47	0.43
Q7Z5R6	Amyloid beta A4 precursor protein-binding family B member 1-interacting protein OS=Homo sapiens GN=APBB1IP PE=1 SV=1 - [AB1IP_HUMAN]	4.05	1	2	2	3	0.72	0.61	0.78	0.68	0.88	0.77	1.15	1.04	1.05	1.16	0.71	0.60	0.49	0.00	-0.07	0.47	0.41	0.15	0.08	0.43
O43242	26S proteasome non-ATPase regulatory subunit 3 OS=Homo sapiens GN=PSMD3 PE=1 SV=2 - [PSMD3_HUMAN]	41.95	1	21	21	56	-0.37	-0.39	-0.43	-0.31	-0.08	-0.12	-0.06	0.09	-0.03	-0.10	-0.06	-0.04	0.45	0.29	0.20	0.35	0.21	0.24	0.17	0.43
Q8WYP5	Protein ELYS OS=Homo sapiens GN=AHCTF1 PE=1 SV=3 - [ELYS_HUMAN]	3.62	1	4	5	5	-0.50	-0.64	-0.30	-0.45	-0.05	-0.19	0.06	-0.08	-0.10	0.04	0.08	-0.07	0.62	0.58	0.38	0.57	0.38	0.44	0.24	0.43
O15327	Type II inositol 3,4-bisphosphate 4-phosphatase OS=Homo sapiens GN=INPP4B PE=2 SV=4 - [INP4B_HUMAN]	2.81	1	2	2	4	-0.66	-0.52	-0.82	-0.68	-0.25	-0.11	-0.46	-0.32	-0.65	-0.78	-0.99	-0.85	0.26	-0.32	-0.16	-0.09	0.07	0.40	0.56	0.43

Q9Y287	Integral membrane protein 2B OS=Homo sapiens GN=ITM2B PE=1 SV=1 - [ITM2B_HUMAN]	39.10	1	5	5	10	-0.89	-0.68	-0.51	-0.33	0.90	1.03	0.08	0.24	0.18	0.05	0.13	0.30	0.98	0.67	0.38	0.78	0.40	0.70	0.59	0.43
Q9Y619	Mitochondrial ornithine transporter 1 OS=Homo sapiens GN=SLC25A15 PE=1 SV=1 - [ORNT1_HUMAN]	2.99	1	1	1	1	-1.17	-0.96	-0.85	-0.64	0.20	0.41	-0.48	-0.27	0.18	-0.03	0.04	0.25	0.74	1.22	0.89	1.18	0.85	1.36	1.03	0.43
P01008	Antithrombin-III OS=Homo sapiens GN=SERPINC1 PE=1 SV=1 - [ANT3_HUMAN]	21.98	1	8	8	14	-0.46	-0.74	-0.35	-0.55	0.54	0.42	0.06	-0.23	-0.12	-0.09	-0.02	-0.04	0.56	0.50	0.33	0.51	0.37	1.03	0.95	0.43
Q9Y6X8	Zinc fingers and homeoboxes protein 2 OS=Homo sapiens GN=ZHX2 PE=1 SV=1 - [ZHX2_HUMAN]	1.08	2	1	1	1	0.17	0.10	-0.48	-0.55	-0.31	-0.38	-0.12	-0.19	-0.28	-0.20	0.00	-0.08	-0.23	-0.17	0.48	-0.34	0.31	-0.49	0.16	0.43
P09001	39S ribosomal protein L3, mitochondrial OS=Homo sapiens GN=MRPL3 PE=1 SV=1 - [RM03_HUMAN]	2.59	1	1	1	1	-0.21	0.27	-0.36	0.13	-0.08	0.40	0.01	0.49	0.85	0.37	0.04	0.52	0.27	0.25	0.39	0.61	0.76	0.11	0.26	0.43
P82921	28S ribosomal protein S21, mitochondrial OS=Homo sapiens GN=MRPS21 PE=1 SV=2 - [RT21_HUMAN]	16.09	1	1	1	1	-0.89	-0.82	-1.09	-1.01	-0.91	-0.85	-0.72	-0.65	-0.52	-0.59	-0.79	-0.72	0.22	0.11	0.30	0.34	0.53	-0.04	0.15	0.43
O60613	15 kDa selenoprotein OS=Homo sapiens GN=SEP15 PE=1 SV=3 - [SEP15_HUMAN]	25.31	1	3	3	6	-1.15	-0.94	-0.32	-0.39	-0.11	-0.04	0.01	-0.02	-0.05	0.03	-0.05	0.14	0.67	1.09	0.61	0.73	0.38	0.89	0.22	0.42
Q96P70	Importin-9 OS=Homo sapiens GN=IPO9 PE=1 SV=3 - [IPO9_HUMAN]	13.45	1	10	10	15	-0.34	-0.31	-0.32	-0.38	0.14	0.14	0.07	0.06	0.03	-0.02	-0.09	-0.09	0.48	0.32	0.27	0.32	0.39	0.42	0.45	0.42

Q96JY6	PDZ and LIM domain protein 2 OS=Homo sapiens GN=PDLIM2 PE=1 SV=1 - [PDLI2_HUMAN]	57.10	1	13	13	25	-1.70	-1.57	-1.60	-1.62	-1.02	-0.99	-1.25	-1.38	-0.86	-0.89	-0.42	-0.44	0.50	1.39	1.25	0.81	0.83	0.68	0.51	0.42
O75694	Nuclear pore complex protein Nup155 OS=Homo sapiens GN=NUP155 PE=1 SV=1 - [NU155_HUMAN]	6.11	1	7	7	12	-0.43	-0.21	-0.59	-0.46	0.01	0.17	-0.21	-0.09	0.21	0.05	0.07	0.16	0.21	0.45	0.69	0.49	0.73	0.36	0.63	0.42
Q9UM54	Unconventional myosin-VI OS=Homo sapiens GN=MYO6 PE=1 SV=4 - [MYO6_HUMAN]	24.88	1	28	28	40	-0.68	-0.53	-0.67	-0.62	-0.18	-0.17	-0.27	-0.28	-0.27	-0.34	-0.23	-0.24	0.44	0.46	0.44	0.35	0.42	0.50	0.48	0.42
Q13131	5'-AMP-activated protein kinase catalytic subunit alpha 1 OS=Homo sapiens GN=PRKAA1 PE=1 SV=4 - [AAPK1_HUMAN]	25.94	1	10	12	20	-0.10	-0.11	-0.35	-0.47	0.27	0.22	0.07	0.01	0.21	0.29	0.06	0.11	0.23	0.30	0.47	0.42	0.34	0.26	0.52	0.42
Q07020	60S ribosomal protein L18 OS=Homo sapiens GN=RPL18 PE=1 SV=2 - [RL18_HUMAN]	20.21	1	4	4	8	-1.10	-1.08	-1.26	-0.88	-0.54	-0.52	-0.91	-0.91	-0.46	-0.86	-0.35	-0.31	0.24	0.78	0.63	0.61	0.46	0.58	0.52	0.42
P16144	Integrin beta-4 OS=Homo sapiens GN=ITGB4 PE=1 SV=5 - [ITB4_HUMAN]	21.79	1	28	28	44	-1.12	-0.97	-1.13	-1.04	-0.67	-0.63	-0.82	-0.72	-0.67	-0.69	-0.46	-0.38	0.25	0.67	0.74	0.34	0.49	0.39	0.55	0.42
Q9H9T3	Elongator complex protein 3 OS=Homo sapiens GN=ELP3 PE=1 SV=2 - [ELP3_HUMAN]	7.31	1	3	3	7	-0.06	-0.32	-0.41	-0.67	0.06	-0.21	-0.05	-0.32	-0.16	0.11	0.52	0.25	0.06	0.58	0.93	0.20	0.55	0.10	0.45	0.42
Q32MZ4	Leucine-rich repeat flightless-interacting protein 1 OS=Homo sapiens GN=LRRFIP1 PE=1 SV=2 - [LRRF1_HUMAN]	11.26	2	5	7	10	0.18	0.20	-0.24	-0.39	0.30	0.18	0.11	-0.16	-0.02	0.23	0.06	-0.19	0.08	0.05	0.28	-0.32	0.29	0.11	0.31	0.42

P21291	Cysteine and glycine-rich protein 1 OS=Homo sapiens GN=CSRP1 PE=1 SV=3 - [CSRP1_HUMAN]	53.89	2	11	11	68	-1.14	-1.03	-1.14	-1.12	-0.96	-0.98	-0.76	-0.77	-0.69	-0.80	-0.72	-0.73	0.40	0.53	0.48	0.38	0.43	0.23	0.21	0.42
P55039	Developmentally-regulated GTP-binding protein 2 OS=Homo sapiens GN=DRG2 PE=1 SV=1 - [DRG2_HUMAN]	13.19	1	4	4	7	-0.28	-0.15	-0.27	-0.13	0.38	0.57	0.05	0.17	0.25	0.05	0.12	0.31	0.38	0.39	0.39	0.48	0.36	0.57	0.64	0.42
Q9NUP9	Protein lin-7 homolog C OS=Homo sapiens GN=LIN7C PE=1 SV=1 - [LIN7C_HUMAN]	59.90	1	5	10	35	0.18	-0.09	0.18	0.04	0.19	0.13	0.43	0.39	0.38	0.30	0.20	0.18	0.50	0.25	0.22	0.34	0.32	0.00	0.11	0.42
Q86VD7	Mitochondrial coenzyme A transporter SLC25A42 OS=Homo sapiens GN=SLC25A42 PE=2 SV=2 - [S2542_HUMAN]	3.46	1	1	1	2	-0.77	-0.69	-0.54	-0.46	-0.40	-0.33	-0.19	-0.11	-0.24	-0.31	-0.45	-0.38	0.64	0.32	0.09	0.49	0.27	0.35	0.12	0.42
O95445	Apolipoprotein M OS=Homo sapiens GN=APOM PE=1 SV=2 - [APOM_HUMAN]	4.79	1	1	1	1	-0.55	-0.58	-0.81	-0.83	-0.12	-0.15	-0.45	-0.48	-0.15	-0.12	0.74	0.71	0.16	1.30	1.55	0.46	0.72	0.42	0.67	0.42
Q7Z449	Cytochrome P450 2U1 OS=Homo sapiens GN=CYP2U1 PE=1 SV=1 - [CP2U1_HUMAN]	2.76	1	1	1	1	-0.90	-1.11	-0.63	-0.84	-0.16	-0.37	-0.28	-0.49	-0.27	-0.06	-0.24	-0.46	0.67	0.66	0.39	0.87	0.61	0.72	0.45	0.42
Q03167	Transforming growth factor beta receptor type 3 OS=Homo sapiens GN=TGFBR3 PE=1 SV=3 - [TGBR3_HUMAN]	7.52	1	5	5	8	-0.83	-0.25	-0.43	0.16	-0.30	0.28	-0.07	0.51	0.45	-0.13	0.45	1.03	0.82	1.29	0.88	0.74	0.33	0.52	0.12	0.42
P51114	Fragile X mental retardation syndrome-related protein 1 OS=Homo sapiens GN=FXR1 PE=1 SV=3 - [FXR1_HUMAN]	21.42	1	9	11	21	-0.68	-0.55	-0.50	-0.63	-0.02	-0.12	-0.37	-0.33	-0.25	-0.20	-0.34	-0.28	0.24	0.18	0.33	0.28	0.42	0.44	0.78	0.42

Q13503	Mediator of RNA polymerase II transcription subunit 21 OS=Homo sapiens GN=MED21 PE=1 SV=1 - [MED21_HUMAN]	12.50	1	1	1	1	-0.49	-0.43	-0.84	-0.78	-1.39	-1.34	-0.49	-0.43	-0.32	-0.38	0.18	0.23	0.06	0.68	1.02	0.14	0.50	-0.92	-0.57	0.42
Q99624	Sodium-coupled neutral amino acid transporter 3 OS=Homo sapiens GN=SLC38A3 PE=2 SV=1 - [S38A3_HUMAN]	4.96	1	2	2	3	-0.20	-0.29	-0.41	-0.39	0.65	0.29	0.08	-0.12	-0.02	0.23	0.30	0.19	0.21	0.73	0.82	0.57	0.78	0.83	1.03	0.42
P19838	Nuclear factor NF-kappa-B p105 subunit OS=Homo sapiens GN=NFKB1 PE=1 SV=2 - [NFKB1_HUMAN]	13.43	1	8	8	10	-0.41	-0.26	-0.57	-0.58	-0.24	0.03	-0.31	-0.07	-0.27	-0.25	-0.37	-0.24	0.12	-0.05	0.21	0.13	0.35	0.04	0.33	0.42
A2RRP1	Neuroblastoma-amplified sequence OS=Homo sapiens GN=NBAS PE=1 SV=2 - [NBAS_HUMAN]	3.21	1	6	6	8	-0.65	-0.57	-0.45	-0.36	-0.21	-0.30	-0.15	-0.24	-0.08	-0.25	-0.36	-0.31	0.39	0.29	0.26	0.55	0.22	0.30	0.22	0.42
Q12996	Cleavage stimulation factor subunit 3 OS=Homo sapiens GN=CSTF3 PE=1 SV=1 - [CSTF3_HUMAN]	3.21	1	2	2	2	-0.61	-0.52	-0.95	-0.86	-0.23	-0.14	-0.60	-0.51	-0.30	-0.38	-0.24	-0.16	0.06	0.37	0.71	0.25	0.60	0.36	0.71	0.42
Q08426	Peroxisomal bifunctional enzyme OS=Homo sapiens GN=EHHADH PE=1 SV=3 - [ECHP_HUMAN]	1.80	1	1	1	1	-0.02	0.36	-0.12	0.26	-0.55	-0.18	0.23	0.61	-0.06	-0.43	-0.21	0.17	0.31	-0.18	-0.09	-0.38	-0.28	-0.54	-0.45	0.41
P51003	Poly(A) polymerase alpha OS=Homo sapiens GN=PAPOLA PE=1 SV=4 - [PAPOA_HUMAN]	2.55	1	1	1	2	-0.78	-0.85	-0.46	-0.53	-0.53	-0.61	-0.11	-0.18	-0.48	-0.40	-0.60	-0.68	0.72	0.18	-0.14	0.41	0.09	0.23	-0.09	0.41
Q96EF6	F-box only protein 17 OS=Homo sapiens GN=FBXO17 PE=1 SV=1 - [FBX17_HUMAN]	2.88	1	1	1	2	-0.25	-0.13	-0.43	-0.30	0.25	0.36	-0.08	0.04	0.16	0.04	-0.02	0.10	0.23	0.24	0.41	0.32	0.50	0.48	0.66	0.41

Q58DX5	Inactive N-acetylated-alpha-linked acidic dipeptidase-like protein 2 OS=Homo sapiens GN=NAALADL2 PE=1 SV=3 - [NADL2_HUMAN]	3.77	1	2	2	2	-0.47	-0.62	-0.94	-1.09	-0.55	-0.71	-0.59	-0.74	-0.44	-0.28	-0.01	-0.17	-0.06	0.46	0.93	0.22	0.69	-0.10	0.37	0.41
P35219	Carbonic anhydrase-related protein OS=Homo sapiens GN=CA8 PE=1 SV=3 - [CAH8_HUMAN]	14.83	1	3	3	4	0.40	0.60	0.22	0.43	0.24	0.44	0.57	0.78	0.61	0.41	0.06	0.25	0.23	-0.34	-0.17	0.05	0.22	-0.17	0.00	0.41
Q96RT1	Protein LAP2 OS=Homo sapiens GN=ERBB2IP PE=1 SV=2 - [LAP2_HUMAN]	13.46	1	12	14	20	0.02	-0.13	-0.07	-0.27	0.28	0.18	0.22	0.22	0.24	0.33	0.27	0.24	0.21	0.14	0.50	0.35	0.49	0.03	0.56	0.41
Q8TEM1	Nuclear pore membrane glycoprotein 210 OS=Homo sapiens GN=NUP210 PE=1 SV=3 - [PO210_HUMAN]	3.87	1	6	6	9	-0.84	-0.70	-0.70	-0.55	-0.26	-0.11	-0.35	-0.20	-0.06	-0.20	-0.38	-0.24	0.55	0.47	0.31	0.67	0.53	0.57	0.42	0.41
P17858	6-phosphofructokinase, liver type OS=Homo sapiens GN=PFKL PE=1 SV=6 - [K6PL_HUMAN]	42.69	1	21	26	104	-0.53	-0.51	-0.36	-0.38	0.06	0.07	0.02	0.04	-0.01	0.00	0.09	0.12	0.56	0.54	0.54	0.51	0.42	0.54	0.40	0.41
Q9HCM4	Band 4.1-like protein 5 OS=Homo sapiens GN=EPB41L5 PE=1 SV=3 - [E41L5_HUMAN]	20.46	1	8	8	11	-0.23	-0.36	-0.15	-0.34	0.44	0.33	0.29	0.11	0.10	0.23	0.11	0.25	0.58	0.62	0.44	0.54	0.34	0.68	0.66	0.41
O00592	Podocalyxin OS=Homo sapiens GN=PODXL PE=1 SV=2 - [PODXL_HUMAN]	16.49	1	6	6	19	-1.63	-1.93	-1.83	-1.72	-0.72	-0.70	-1.45	-1.40	-1.03	-0.81	-0.42	-0.39	0.50	1.58	1.23	0.94	0.85	1.31	1.02	0.41
Q96LJ7	Dehydrogenase/reductase SDR family member 1 OS=Homo sapiens GN=DHRS1 PE=1 SV=1 - [DHRS1_HUMAN]	13.10	1	4	4	6	-0.59	-0.66	-0.82	-0.86	-0.50	-0.46	-0.35	-0.30	-0.59	-0.49	-0.75	-0.75	0.28	-0.06	-0.01	0.13	0.33	0.11	0.31	0.41

O15013	Rho guanine nucleotide exchange factor 10 OS=Homo sapiens GN=ARHGEF10 PE=1 SV=4 - [ARHGA_HUMAN]	3.43	1	4	4	4	-1.11	-0.95	-0.79	-0.73	0.07	0.36	-0.44	-0.25	0.09	-0.15	-0.26	0.05	0.72	0.86	0.53	0.88	0.61	1.16	0.84	0.41
Q9H6S0	Probable ATP-dependent RNA helicase YTHDC2 OS=Homo sapiens GN=YTHDC2 PE=1 SV=2 - [YTDC2_HUMAN]	2.10	1	2	2	2	-0.49	-0.46	-1.03	-1.01	-0.22	-0.20	-0.69	-0.66	0.37	0.35	-0.16	-0.14	-0.14	0.34	0.87	0.87	1.42	0.25	0.79	0.41
Q6PCB7	Long-chain fatty acid transport protein 1 OS=Homo sapiens GN=SLC27A1 PE=2 SV=1 - [S27A1_HUMAN]	26.16	1	11	11	18	-0.60	-0.46	-0.54	-0.32	0.01	0.04	0.03	-0.01	0.06	0.07	-0.28	-0.28	0.53	0.09	0.10	0.46	0.38	0.15	0.55	0.41
P24298	Alanine aminotransferase 1 OS=Homo sapiens GN=GPT PE=1 SV=3 - [ALAT1_HUMAN]	1.81	1	1	1	2	1.22	1.13	0.91	0.83	0.66	0.57	1.26	1.17	1.04	1.13	0.35	0.26	0.09	-0.87	-0.57	-0.06	0.25	-0.58	-0.27	0.40
P49720	Proteasome subunit beta type-3 OS=Homo sapiens GN=PSMB3 PE=1 SV=2 - [PSB3_HUMAN]	18.54	1	4	4	5	-0.55	-0.55	-0.34	-0.30	0.23	0.22	0.00	0.11	0.10	0.11	0.48	0.48	0.16	1.05	0.79	0.68	0.44	0.76	0.51	0.40
Q9UP83	Conserved oligomeric Golgi complex subunit 5 OS=Homo sapiens GN=COG5 PE=1 SV=3 - [COG5_HUMAN]	6.91	1	4	4	5	-0.64	-0.78	-0.63	-0.54	0.24	0.09	-0.13	-0.34	-0.05	0.18	0.38	0.07	0.50	0.78	0.62	0.85	0.74	0.89	0.54	0.40
O14939	Phospholipase D2 OS=Homo sapiens GN=PLD2 PE=1 SV=2 - [PLD2_HUMAN]	0.96	1	1	1	1	0.57	0.34	-0.07	-0.29	0.76	0.53	0.27	0.05	0.92	1.15	0.61	0.38	-0.24	0.05	0.68	0.62	1.25	0.18	0.81	0.40
P22102	Trifunctional purine biosynthetic protein adenosine-3 OS=Homo sapiens GN=GART PE=1 SV=1 - [PUR2_HUMAN]	15.84	1	12	12	23	0.10	0.05	0.06	0.07	0.44	0.51	0.22	0.27	0.42	0.31	0.40	0.36	0.41	0.31	0.31	0.47	0.36	0.64	0.48	0.40

P15088	Mast cell carboxypeptidase A OS=Homo sapiens GN=CPA3 PE=1 SV=2 - [CBPA3_HUMAN]	5.52	1	2	2	2	-0.21	-0.15	-0.28	-0.22	3.37	3.42	0.06	0.12	1.74	1.69	0.40	0.45	0.32	0.62	0.68	1.93	2.00	3.57	3.64	0.40
Q92688	Acidic leucine-rich nuclear phosphoprotein 32 family member B OS=Homo sapiens GN=ANP32B PE=1 SV=1 - [AN32B_HUMAN]	21.51	3	1	6	13	-1.49	-1.29	-0.86	-0.66	-0.37	-0.18	-0.52	-0.32	-0.24	-0.43	-0.24	-0.04	1.02	1.26	0.63	1.08	0.46	1.10	0.47	0.40
Q9BY43	Charged multivesicular body protein 4a OS=Homo sapiens GN=CHMP4A PE=1 SV=3 - [CHM4A_HUMAN]	22.52	1	4	5	8	-0.84	-1.15	-0.56	-0.87	0.50	0.18	-0.22	-0.53	-0.05	0.26	0.36	0.04	0.67	1.20	0.92	1.13	0.86	1.32	1.04	0.40
Q9NUY8	TBC1 domain family member 23 OS=Homo sapiens GN=TBC1D23 PE=1 SV=3 - [TBC23_HUMAN]	13.16	1	5	5	11	0.01	-0.04	0.05	-0.22	0.42	0.22	0.20	0.15	-0.04	0.00	-0.13	-0.35	0.33	-0.06	-0.51	0.43	0.33	0.46	0.01	0.40
O94916	Nuclear factor of activated T-cells 5 OS=Homo sapiens GN=NFAT5 PE=1 SV=1 - [NFAT5_HUMAN]	1.57	1	1	1	1	0.77	0.83	0.34	0.40	0.63	0.69	0.68	0.74	0.06	0.00	1.00	1.05	-0.04	0.23	0.65	-0.74	-0.31	-0.16	0.27	0.40
Q96MG8	Protein-L-isoaspartate O-methyltransferase domain-containing protein 1 OS=Homo sapiens GN=PCMTD1 PE=2 SV=2 - [PCMD1_HUMAN]	2.52	1	1	1	1	0.19	0.36	-0.40	-0.23	0.04	0.21	-0.06	0.11	0.41	0.25	0.05	0.22	-0.19	-0.13	0.45	0.09	0.68	-0.17	0.42	0.40
Q9NTJ5	Phosphatidylinositide phosphatase SAC1 OS=Homo sapiens GN=SACM1L PE=1 SV=2 - [SAC1_HUMAN]	21.12	1	12	12	30	-0.48	-0.55	-0.45	-0.52	0.23	0.09	0.00	-0.13	-0.04	0.02	-0.01	-0.04	0.45	0.51	0.45	0.56	0.53	0.54	0.63	0.40
Q15149	Plectin OS=Homo sapiens GN=PLEC PE=1 SV=3 - [PLEC_HUMAN]	54.21	1	225	235	845	-0.40	-0.37	-0.40	-0.37	0.02	0.02	-0.06	-0.04	-0.05	-0.05	0.04	0.05	0.44	0.46	0.45	0.41	0.38	0.45	0.40	0.40

O00231	26S proteasome non-ATPase regulatory subunit 11 OS=Homo sapiens GN=PSMD11 PE=1 SV=3 - [PSD11_HUMAN]	53.32	1	18	18	34	-0.35	-0.22	-0.20	-0.17	0.30	0.35	0.03	0.15	0.25	0.24	0.34	0.43	0.40	0.62	0.61	0.57	0.53	0.62	0.53	0.40
Q12873	Chromodomain-helicase-DNA-binding protein 3 OS=Homo sapiens GN=CHD3 PE=1 SV=3 - [CHD3_HUMAN]	2.75	1	2	4	5	-0.53	-0.53	-0.48	-0.48	0.14	0.14	-0.14	-0.14	-0.51	-0.50	0.31	0.31	0.45	0.85	0.79	0.06	0.01	0.66	0.60	0.40
Q96C23	Aldose 1-epimerase OS=Homo sapiens GN=GALM PE=1 SV=1 - [GALM_HUMAN]	14.04	1	3	3	3	0.06	0.15	0.07	0.25	-0.14	0.22	0.51	0.54	0.39	-0.08	-0.52	0.01	0.45	-0.39	-0.35	0.02	0.06	-0.20	-0.16	0.40
P29372	DNA-3-methyladenine glycosylase OS=Homo sapiens GN=MPG PE=1 SV=3 - [3MG_HUMAN]	4.03	1	1	1	1	-0.24	-0.41	-0.83	-1.00	-1.00	-1.18	-0.49	-0.66	-1.04	-0.86	0.25	0.07	-0.20	0.49	1.07	-0.59	0.00	-0.78	-0.19	0.40
Q99436	Proteasome subunit beta type-7 OS=Homo sapiens GN=PSMB7 PE=1 SV=1 - [PSB7_HUMAN]	7.22	1	2	2	6	-0.26	-0.33	-0.43	-0.41	-0.16	-0.24	-0.09	0.05	-0.12	-0.04	0.16	0.31	0.44	0.64	0.36	0.24	0.12	0.08	0.17	0.40
Q00059	Transcription factor A, mitochondrial OS=Homo sapiens GN=TFAM PE=1 SV=1 - [TFAM_HUMAN]	20.73	1	5	5	13	-0.17	-0.15	-0.37	-0.37	-0.21	-0.22	-0.09	-0.06	-0.11	-0.12	-0.01	-0.06	0.45	0.17	0.29	0.12	0.22	-0.12	0.12	0.40
Q8TBY9	WD repeat-containing protein 66 OS=Homo sapiens GN=WDR66 PE=2 SV=2 - [WDR66_HUMAN]	0.61	1	1	1	1	-0.81	-0.41	-0.83	-0.43	-0.37	0.03	-0.49	-0.10	-0.18	-0.57	-0.12	0.27	0.37	0.69	0.70	0.26	0.29	0.42	0.44	0.40
P30101	Protein disulfide-isomerase A3 OS=Homo sapiens GN=PDIA3 PE=1 SV=4 - [PDIA3_HUMAN]	56.04	1	27	27	98	-0.68	-0.66	-0.66	-0.62	0.05	0.02	-0.32	-0.25	-0.23	-0.27	-0.27	-0.29	0.47	0.49	0.33	0.54	0.45	0.74	0.61	0.40

Q96DX4	RING finger and SPRY domain-containing protein 1 OS=Homo sapiens GN=RSPRY1 PE=2 SV=1 - [RSPRY_HUMAN]	8.16	1	2	2	3	0.81	0.96	0.40	0.56	0.41	0.56	0.74	0.89	0.44	0.29	0.98	1.13	-0.02	0.17	0.58	-0.49	-0.08	-0.42	-0.01	0.40
Q8TAE8	Growth arrest and DNA damage-inducible proteins-interacting protein 1 OS=Homo sapiens GN=GADD45GIP1 PE=1 SV=1 - [G45IP_HUMAN]	18.02	1	3	3	4	0.47	0.08	0.53	0.13	0.79	0.19	0.86	0.43	0.58	1.06	0.70	0.17	0.36	0.17	0.24	0.52	0.60	0.05	0.18	0.40
P07686	Beta-hexosaminidase subunit beta OS=Homo sapiens GN=HEXB PE=1 SV=3 - [HEXB_HUMAN]	19.24	1	8	8	17	-0.54	-0.50	-0.36	-0.29	0.27	0.31	-0.02	-0.01	0.10	0.04	0.02	0.13	0.62	0.69	0.33	0.60	0.33	0.83	0.58	0.40
Q8NDI1	EH domain-binding protein 1 OS=Homo sapiens GN=EHBP1 PE=1 SV=3 - [EHBP1_HUMAN]	6.09	1	4	4	5	0.18	0.13	0.11	0.06	0.14	0.08	0.45	0.39	0.13	0.20	0.22	0.16	0.32	0.04	0.11	0.04	0.12	-0.06	0.01	0.40
Q8NBJ7	Sulfatase-modifying factor 2 OS=Homo sapiens GN=SUMF2 PE=1 SV=2 - [SUMF2_HUMAN]	16.94	1	5	5	12	-0.56	-0.53	-0.58	-0.60	0.18	0.28	-0.25	-0.29	0.07	0.07	0.39	0.45	0.41	0.97	1.04	0.72	0.72	0.80	0.87	0.40
O00232	26S proteasome non-ATPase regulatory subunit 12 OS=Homo sapiens GN=PSMD12 PE=1 SV=3 - [PSD12_HUMAN]	35.96	1	14	14	24	-0.21	-0.32	-0.26	-0.29	0.32	0.23	-0.06	0.05	0.22	0.18	0.16	0.14	0.44	0.52	0.49	0.62	0.59	0.61	0.72	0.40
O15018	PDZ domain-containing protein 2 OS=Homo sapiens GN=PDZD2 PE=1 SV=4 - [PDZD2_HUMAN]	2.32	1	3	3	7	0.05	0.05	-0.05	-0.05	0.09	0.08	0.24	0.33	0.12	-0.02	-0.18	-0.18	0.26	-0.22	-0.18	-0.04	0.21	0.01	0.12	0.40

Q8IY17	Neuropathy target esterase OS=Homo sapiens GN=PNPLA6 PE=1 SV=2 - [PLPL6_HUMAN]	13.84	1	13	15	22	-0.15	-0.09	-0.38	-0.34	0.24	0.28	0.01	-0.01	0.07	0.01	0.06	0.11	0.21	0.22	0.50	0.19	0.41	0.42	0.59	0.39
O00750	Phosphatidylinositol 4-phosphate 3-kinase C2 domain-containing subunit beta OS=Homo sapiens GN=PIK3C2B PE=1 SV=2 - [P3C2B_HUMAN]	0.92	1	1	1	1	0.64	0.64	0.17	0.16	0.16	0.16	0.49	0.49	-0.25	-0.24	1.21	1.20	-0.09	0.57	1.04	-0.86	-0.38	-0.50	-0.02	0.39
P06311	Ig kappa chain V-III region IARC/BL41 OS=Homo sapiens PE=1 SV=1 - [KV311_HUMAN]	24.22	1	2	2	6	-1.16	-1.11	-1.21	-1.15	0.12	0.17	-0.88	-0.82	0.72	0.67	1.79	1.84	0.34	2.95	3.00	1.86	1.91	1.27	1.31	0.39
Q86UN3	Reticulon-4 receptor-like 2 OS=Homo sapiens GN=RTN4RL2 PE=1 SV=1 - [R4RL2_HUMAN]	14.05	1	5	5	8	0.21	0.19	-0.18	-0.20	0.34	0.31	0.15	0.13	0.42	0.45	0.29	0.26	-0.01	0.08	0.47	0.26	0.66	0.10	0.49	0.39
O43592	Exportin-T OS=Homo sapiens GN=XPOT PE=1 SV=2 - [XPOT_HUMAN]	6.13	1	6	6	9	0.66	0.82	0.53	0.55	0.86	0.83	0.81	0.97	0.86	0.88	0.39	0.41	0.08	-0.40	-0.13	0.30	0.16	0.11	0.23	0.39
Q8IVN3	Musculoskeletal embryonic nuclear protein 1 OS=Homo sapiens GN=MUSTN1 PE=2 SV=2 - [MSTN1_HUMAN]	18.29	1	1	1	4	-3.16	-3.12	-2.91	-2.87	-3.19	-3.15	-2.59	-2.54	-2.91	-2.95	-2.41	-2.37	0.63	0.75	0.50	0.24	0.00	-0.05	-0.29	0.39
Q8WWX9	Selenoprotein M OS=Homo sapiens GN=SELM PE=1 SV=3 - [SELM_HUMAN]	30.34	1	3	3	9	-0.43	-0.57	-0.67	-0.64	0.26	0.32	-0.20	-0.30	-0.09	0.04	0.16	0.20	0.30	0.58	0.80	0.50	0.55	0.88	0.99	0.39
P49354	Protein farnesyltransferase/geranylgeranyltransferase type-1 subunit alpha OS=Homo sapiens GN=FNTA PE=1 SV=1 - [FNTA_HUMAN]	20.05	1	6	6	7	-0.60	-0.44	-0.48	-0.32	0.41	0.48	-0.11	-0.01	0.14	0.03	0.21	0.23	0.33	0.74	0.48	0.69	0.50	1.00	0.84	0.39

Q9BS26	Endoplasmic reticulum resident protein 44 OS=Homo sapiens GN=ERP44 PE=1 SV=1 - [ERP44_HUMAN]	36.95	1	11	12	25	-0.77	-0.82	-0.70	-0.65	-0.03	-0.08	-0.28	-0.28	-0.09	-0.06	0.10	0.13	0.29	0.82	0.79	0.50	0.64	0.77	0.77	0.39
Q04206	Transcription factor p65 OS=Homo sapiens GN=RELA PE=1 SV=2 - [TF65_HUMAN]	11.43	1	5	5	8	-0.47	-0.24	-0.52	-0.29	-0.03	0.34	-0.12	0.04	0.20	-0.04	-0.08	0.09	0.33	0.33	0.36	0.41	0.44	0.43	0.56	0.39
Q15283	Ras GTPase-activating protein 2 OS=Homo sapiens GN=RASA2 PE=1 SV=3 - [RASA2_HUMAN]	3.65	1	3	3	3	0.26	0.50	0.35	0.59	0.65	0.89	0.67	0.91	1.10	0.87	0.54	0.77	0.47	0.28	0.19	0.64	0.55	0.38	0.29	0.39
Q14789	Golgin subfamily B member 1 OS=Homo sapiens GN=GOLGB1 PE=1 SV=2 - [GOGB1_HUMAN]	10.22	1	28	29	39	-0.70	-0.51	-0.69	-0.57	0.01	0.08	-0.21	-0.10	0.06	0.00	0.24	0.29	0.25	0.70	0.93	0.53	0.57	0.45	0.72	0.39
Q9Y282	Endoplasmic reticulum-Golgi intermediate compartment protein 3 OS=Homo sapiens GN=ERGIC3 PE=1 SV=1 - [ERGI3_HUMAN]	8.62	1	3	3	6	-0.78	-0.54	-0.68	-0.44	0.21	0.21	-0.36	-0.12	-0.13	-0.34	-0.14	0.09	0.48	0.64	0.54	0.45	0.61	0.74	0.64	0.39
O95373	Importin-7 OS=Homo sapiens GN=IPO7 PE=1 SV=1 - [IPO7_HUMAN]	13.29	1	13	13	26	-0.22	-0.22	-0.11	-0.14	0.34	0.01	0.02	-0.14	0.14	0.15	0.15	0.03	0.28	0.24	0.12	0.44	0.38	0.31	0.26	0.39
P62191	26S protease regulatory subunit 4 OS=Homo sapiens GN=PSMC1 PE=1 SV=1 - [PRS4_HUMAN]	55.91	1	17	19	37	-0.50	-0.49	-0.18	-0.24	0.10	0.08	0.10	0.12	0.04	-0.04	0.21	0.23	0.59	0.78	0.46	0.56	0.37	0.57	0.34	0.39
Q04446	1,4-alpha-glucan-branching enzyme OS=Homo sapiens GN=GBE1 PE=1 SV=3 - [GLGB_HUMAN]	14.53	1	8	8	18	0.03	0.10	-0.28	-0.17	0.48	0.30	0.07	0.13	0.06	0.09	0.13	0.04	0.07	0.11	0.32	0.21	0.30	0.57	0.69	0.39

Q6NZ67	Mitotic-spindle organizing protein 2B OS=Homo sapiens GN=MZT2B PE=1 SV=1 - [MZT2B_HUMAN]	13.29	2	2	2	3	-0.94	-0.96	-0.38	-0.40	0.07	0.04	-0.06	-0.08	-0.27	-0.24	0.15	0.11	0.93	1.09	0.52	0.72	0.17	0.99	0.43	0.39
P30511	HLA class I histocompatibility antigen, alpha chain F OS=Homo sapiens GN=HLA-F PE=2 SV=3 - [HLAF_HUMAN]	14.45	1	3	5	7	0.25	0.22	-0.20	-0.23	0.15	0.11	0.12	0.09	0.13	0.17	0.16	0.12	-0.08	-0.08	0.36	-0.05	0.41	-0.12	0.33	0.38
P08754	Guanine nucleotide-binding protein G(k) subunit alpha OS=Homo sapiens GN=GNAI3 PE=1 SV=3 - [GNAI3_HUMAN]	35.31	3	4	11	79	-0.10	-0.24	-0.25	-0.29	0.00	-0.16	0.03	-0.11	-0.05	0.08	0.22	0.13	0.08	0.18	0.46	0.23	0.30	0.04	0.25	0.38
P35998	26S protease regulatory subunit 7 OS=Homo sapiens GN=PSMC2 PE=1 SV=3 - [PRS7_HUMAN]	45.96	1	17	17	51	-0.34	-0.41	-0.21	-0.28	0.14	0.11	0.09	0.05	0.00	0.07	0.21	0.11	0.46	0.50	0.44	0.45	0.35	0.32	0.26	0.38
Q15417	Calponin-3 OS=Homo sapiens GN=CNN3 PE=1 SV=1 - [CNN3_HUMAN]	47.72	1	9	12	30	-0.78	-0.68	-0.65	-0.62	-0.53	-0.49	-0.23	-0.12	-0.51	-0.71	-0.67	-0.80	0.65	0.09	-0.27	0.15	-0.06	0.38	-0.05	0.38
Q9NRC1	Suppressor of tumorigenicity 7 protein OS=Homo sapiens GN=ST7 PE=1 SV=1 - [ST7_HUMAN]	5.30	1	2	2	2	0.45	0.57	-0.24	-0.11	0.31	0.43	0.08	0.20	0.50	0.38	-0.08	0.04	-0.31	-0.52	0.16	-0.03	0.66	-0.15	0.54	0.38
Q7Z4G1	COMM domain-containing protein 6 OS=Homo sapiens GN=COMMD6 PE=1 SV=1 - [COMD6_HUMAN]	36.47	1	3	3	5	-0.04	-0.21	-0.01	0.00	0.35	0.35	0.30	0.13	0.12	0.14	0.33	0.28	0.40	0.41	0.35	0.27	0.18	0.57	0.34	0.38
P35268	60S ribosomal protein L22 OS=Homo sapiens GN=RPL22 PE=1 SV=2 - [RL22_HUMAN]	39.06	1	3	3	11	-0.86	-0.72	-0.70	-0.79	-0.18	-0.11	-0.38	-0.46	-0.34	-0.40	-0.17	-0.05	0.38	0.68	0.50	0.25	0.36	0.43	0.54	0.38

P05997	Collagen alpha-2(V) chain OS=Homo sapiens GN=COL5A2 PE=1 SV=3 - [CO5A2_HUMAN]	3.87	1	3	4	22	-3.11	-3.21	-3.21	-3.24	-2.00	-2.11	-2.48	-2.63	-2.50	-2.33	-2.31	-2.33	0.47	0.85	0.85	0.74	0.71	1.13	1.19	0.38
Q99442	Translocation protein SEC62 OS=Homo sapiens GN=SEC62 PE=1 SV=1 - [SEC62_HUMAN]	12.28	1	5	5	7	-0.84	-0.84	-0.51	-0.51	-0.27	-0.21	-0.21	-0.14	-0.26	-0.22	-0.10	-0.03	0.69	0.75	0.43	0.53	0.34	0.55	0.22	0.38
Q96BW9	Mitochondrial translocator assembly and maintenance protein 41 homolog OS=Homo sapiens GN=TAMM41 PE=1 SV=2 - [TAM41_HUMAN]	15.71	1	5	5	8	-0.06	-0.35	-0.11	-0.41	-0.01	-0.31	0.20	-0.09	-0.36	-0.06	-0.15	-0.45	0.32	-0.09	-0.04	0.03	0.09	0.03	0.09	0.38
Q96FN4	Copine-2 OS=Homo sapiens GN=CPNE2 PE=1 SV=3 - [CPNE2_HUMAN]	17.34	1	7	8	15	-0.34	-0.38	-0.21	-0.33	0.28	0.08	-0.01	-0.18	0.03	-0.01	0.08	-0.08	0.25	0.22	0.50	0.36	0.34	0.37	0.47	0.38
Q9BZV2	Thiamine transporter 2 OS=Homo sapiens GN=SLC19A3 PE=1 SV=1 - [S19A3_HUMAN]	5.44	1	1	1	1	-1.22	-0.12	-0.87	0.23	-0.31	0.79	-0.55	0.55	0.99	-0.10	-0.96	0.13	0.72	0.26	-0.10	1.14	0.80	0.89	0.54	0.38
Q9NX08	COMM domain-containing protein 8 OS=Homo sapiens GN=COMMD8 PE=1 SV=1 - [COMD8_HUMAN]	26.78	1	4	4	9	-0.17	0.24	-0.25	-0.06	0.45	0.60	0.07	0.28	0.32	-0.04	0.07	0.29	0.22	0.07	0.36	0.17	0.29	0.41	0.69	0.38
P02679	Fibrinogen gamma chain OS=Homo sapiens GN=FGG PE=1 SV=3 - [FIBG_HUMAN]	49.89	1	20	20	152	-0.60	-0.53	-0.44	-0.36	0.56	0.57	-0.09	-0.03	0.39	0.33	0.88	0.93	0.50	1.42	1.32	0.89	0.78	1.01	0.89	0.38
P43686	26S protease regulatory subunit 6B OS=Homo sapiens GN=PSMC4 PE=1 SV=2 - [PRS6B_HUMAN]	37.56	1	12	13	25	-0.32	-0.05	-0.24	-0.07	0.11	0.29	0.13	0.26	0.30	0.20	0.28	0.40	0.43	0.50	0.48	0.40	0.42	0.46	0.27	0.38

Q9BRG1	Vacuolar protein-sorting-associated protein 25 OS=Homo sapiens GN=VPS25 PE=1 SV=1 - [VPS25_HUMAN]	39.20	1	4	4	11	-0.65	-0.71	-0.43	-0.46	0.22	0.30	-0.21	-0.27	0.14	0.12	0.38	0.42	0.77	1.13	0.82	0.83	0.64	0.85	0.63	0.38
Q5QJ74	Tubulin-specific chaperone cofactor E-like protein OS=Homo sapiens GN=TBCEL PE=2 SV=2 - [TBCEL_HUMAN]	12.03	1	4	5	7	-1.07	-1.12	-0.57	-0.62	0.03	0.15	-0.26	-0.38	-0.02	-0.05	-0.09	0.02	0.70	1.16	0.64	1.14	0.64	0.79	0.58	0.38
Q08945	FACT complex subunit SSRP1 OS=Homo sapiens GN=SSRP1 PE=1 SV=1 - [SSRP1_HUMAN]	7.05	1	4	4	7	-0.19	-0.21	-0.51	-0.49	0.03	-0.05	-0.20	-0.22	0.06	0.12	-0.12	-0.15	0.05	0.14	0.57	0.41	0.77	0.28	0.64	0.38
Q9H4A4	Aminopeptidase B OS=Homo sapiens GN=RNPEP PE=1 SV=2 - [AMPB_HUMAN]	28.15	1	15	15	29	-0.09	-0.01	-0.04	-0.18	0.09	0.14	0.40	0.40	0.03	-0.17	-0.25	-0.29	0.45	-0.14	-0.25	0.11	0.15	0.23	0.10	0.37
Q9Y6F6	Protein MRVI1 OS=Homo sapiens GN=MRVI1 PE=1 SV=2 - [MRVI1_HUMAN]	12.66	1	8	9	11	-1.25	-1.08	-1.15	-1.22	-1.30	-1.14	-0.70	-0.83	-0.77	-0.61	-0.67	-0.45	0.15	0.57	0.51	0.37	0.58	-0.09	0.03	0.37
Q9UIQ6	Leucyl-cystinyl aminopeptidase OS=Homo sapiens GN=LNPEP PE=1 SV=3 - [LCAP_HUMAN]	9.07	1	8	8	13	-0.15	-0.63	-0.51	-0.75	0.17	-0.19	0.01	-0.15	-0.03	0.09	0.06	-0.36	0.12	0.22	0.37	0.28	0.63	0.41	0.54	0.37
Q8N0X7	Spartin OS=Homo sapiens GN=SPG20 PE=1 SV=1 - [SPG20_HUMAN]	9.16	1	5	5	8	-0.44	0.01	-0.24	-0.09	0.07	0.34	0.05	0.33	0.24	0.09	-0.08	0.13	0.40	0.25	0.23	0.22	0.35	0.35	0.41	0.37
P54819	Adenylate kinase 2, mitochondrial OS=Homo sapiens GN=AK2 PE=1 SV=2 - [KAD2_HUMAN]	35.56	1	7	7	22	-1.59	-1.12	-1.20	-1.10	-1.05	-0.84	-0.92	-0.86	-0.76	-0.89	-0.59	-0.45	0.50	1.03	0.59	0.59	0.32	0.62	0.16	0.37

Q9NVH6	Trimethyllysine dioxygenase, mitochondrial OS=Homo sapiens GN=TMLHE PE=1 SV=1 - [TMLH_HUMAN]	14.96	1	5	5	8	0.18	0.13	0.04	0.00	0.21	0.16	0.35	0.30	0.30	0.35	0.43	0.39	0.23	0.26	0.39	0.20	0.34	0.01	0.15	0.37
Q96FE7	Phosphoinositide-3-kinase-interacting protein 1 OS=Homo sapiens GN=PIK3IP1 PE=1 SV=2 - [P3IP1_HUMAN]	15.59	1	3	3	6	0.56	0.65	-0.22	-0.13	0.18	0.26	0.09	0.18	0.38	0.29	0.69	0.78	-0.42	0.13	0.91	-0.24	0.55	-0.40	0.38	0.37
P78371	T-complex protein 1 subunit beta OS=Homo sapiens GN=CCT2 PE=1 SV=4 - [TCPB_HUMAN]	52.34	1	20	20	59	-0.33	-0.30	-0.46	-0.31	-0.14	0.01	-0.18	-0.11	-0.10	-0.14	-0.06	-0.08	0.28	0.31	0.26	0.26	0.40	0.26	0.30	0.37
Q9NP73	Putative bifunctional UDP-N-acetylglucosamine transferase and deubiquitinase ALG13 OS=Homo sapiens GN=ALG13 PE=1 SV=2 - [ALG13_HUMAN]	2.64	1	2	2	2	0.42	0.21	-0.11	-0.31	0.23	0.02	0.20	-0.01	0.09	0.31	0.67	0.46	-0.17	0.25	0.78	-0.08	0.45	-0.20	0.32	0.37
P78333	Glypican-5 OS=Homo sapiens GN=GPC5 PE=1 SV=1 - [GPC5_HUMAN]	6.47	1	4	4	4	-0.26	-0.38	-0.36	-0.48	0.52	0.44	0.21	0.09	0.33	0.45	0.46	0.46	0.24	0.69	0.80	0.39	0.58	0.42	0.59	0.37
O60716	Catenin delta-1 OS=Homo sapiens GN=CTNND1 PE=1 SV=1 - [CTND1_HUMAN]	39.67	1	28	28	61	-0.82	-0.91	-0.75	-0.77	-0.26	-0.35	-0.43	-0.47	-0.32	-0.36	-0.07	-0.03	0.35	0.72	0.67	0.54	0.42	0.51	0.40	0.37
P11908	Ribose-phosphate pyrophosphokinase 2 OS=Homo sapiens GN=PRPS2 PE=1 SV=2 - [PRPS2_HUMAN]	24.21	1	3	7	10	-0.81	-0.76	-0.58	-0.53	-0.29	-0.15	-0.27	-0.20	-0.08	-0.24	-0.31	-0.20	0.59	0.50	0.26	0.59	0.37	0.57	0.34	0.37

Q9BVT8	Transmembrane and ubiquitin-like domain-containing protein 1 OS=Homo sapiens GN=TMUB1 PE=1 SV=1 - [TMUB1_HUMAN]	4.88	1	1	1	4	-0.50	-0.41	-0.60	-0.51	0.11	0.19	-0.30	-0.21	0.01	-0.07	0.12	0.20	0.26	0.63	0.72	0.46	0.57	0.59	0.69	0.37
P25788	Proteasome subunit alpha type-3 OS=Homo sapiens GN=PSMA3 PE=1 SV=2 - [PSA3_HUMAN]	29.02	1	8	8	17	-0.46	-0.49	-0.60	-0.55	0.29	0.38	-0.13	-0.12	0.11	0.13	0.66	0.69	0.57	1.25	1.31	0.72	0.67	0.92	0.84	0.37
Q8N668	COMM domain-containing protein 1 OS=Homo sapiens GN=COMMD1 PE=1 SV=1 - [COMD1_HUMAN]	18.95	1	2	2	3	-1.26	-1.40	-0.66	-0.28	0.08	-0.06	-0.14	0.03	0.01	-0.13	0.16	0.03	1.17	1.42	0.31	1.16	0.32	1.33	0.73	0.37
Q13439	Golgin subfamily A member 4 OS=Homo sapiens GN=GOLGA4 PE=1 SV=1 - [GOGA4_HUMAN]	6.82	1	9	9	14	-0.17	-0.34	-0.23	-0.42	0.43	-0.31	0.15	-0.11	-0.25	-0.23	0.10	-0.21	0.24	0.46	0.12	0.47	0.21	0.63	0.20	0.37
Q6PGP7	Tetratricopeptide repeat protein 37 OS=Homo sapiens GN=TTC37 PE=1 SV=1 - [TTC37_HUMAN]	9.91	1	13	13	22	-0.59	-0.60	-0.49	-0.45	0.12	0.15	-0.22	-0.27	-0.08	0.08	-0.02	0.02	0.45	0.55	0.52	0.57	0.52	0.58	0.60	0.37
Q86W42	THO complex subunit 6 homolog OS=Homo sapiens GN=THOC6 PE=1 SV=1 - [THOC6_HUMAN]	4.11	1	1	1	3	-0.42	-0.49	-0.72	-0.79	0.04	-0.04	-0.42	-0.49	-0.49	-0.41	0.07	-0.01	0.06	0.49	0.79	0.04	0.34	0.44	0.74	0.37
P49721	Proteasome subunit beta type-2 OS=Homo sapiens GN=PSMB2 PE=1 SV=1 - [PSB2_HUMAN]	28.86	1	7	7	26	-0.52	-0.51	-0.34	-0.46	0.05	0.16	-0.17	-0.11	0.23	0.20	0.36	0.54	0.45	0.96	0.92	0.78	0.42	0.72	0.56	0.37
Q96QI5	Heparan sulfate glucosamine 3-O-sulfotransferase 6 OS=Homo sapiens GN=HS3ST6 PE=1 SV=2 - [HS3S6_HUMAN]	2.05	4	1	1	1	-0.36	-0.69	0.14	-0.19	0.61	0.27	0.45	0.12	0.02	0.36	0.61	0.28	0.86	0.97	0.47	0.74	0.25	0.95	0.45	0.37

Q8TBP6	Solute carrier family 25 member 40 OS=Homo sapiens GN=SLC25A40 PE=2 SV=1 - [S2540_HUMAN]	18.93	1	3	3	3	0.35	0.39	0.13	0.18	0.68	0.72	0.43	0.48	0.45	0.41	0.98	1.02	0.14	0.63	0.85	0.09	0.31	0.32	0.54	0.37
Q9H008	Phospholysine phosphohistidine inorganic pyrophosphate phosphatase OS=Homo sapiens GN=LHPP PE=1 SV=2 - [LHPP_HUMAN]	37.04	1	6	6	16	0.90	0.81	0.69	0.49	0.47	0.41	0.98	0.81	0.79	0.83	0.69	0.66	0.13	-0.12	0.00	0.07	0.03	-0.44	-0.28	0.37
Q96GS4	Uncharacterized protein C17orf59 OS=Homo sapiens GN=C17orf59 PE=1 SV=2 - [CQ059_HUMAN]	2.80	1	1	1	1	0.24	-0.09	-0.04	-0.38	0.10	-0.24	0.26	-0.07	-0.38	-0.04	0.22	-0.12	0.07	-0.02	0.26	-0.25	0.04	-0.16	0.13	0.37
Q92522	Histone H1x OS=Homo sapiens GN=H1FX PE=1 SV=1 - [H1X_HUMAN]	36.15	1	8	8	19	-1.46	-1.63	-1.31	-1.26	-0.89	-1.01	-1.01	-0.96	-1.14	-0.94	-0.85	-0.92	0.57	0.75	0.59	0.42	0.25	0.39	0.19	0.37
O75911	Short-chain dehydrogenase/reductase 3 OS=Homo sapiens GN=DHRS3 PE=1 SV=2 - [DHRS3_HUMAN]	3.31	1	1	1	1	-0.61	-0.40	-0.54	-0.34	0.19	0.39	-0.24	-0.04	0.24	0.04	-0.38	-0.18	0.42	0.23	0.16	0.68	0.62	0.78	0.72	0.37
P60891	Ribose-phosphate pyrophosphokinase 1 OS=Homo sapiens GN=PRPS1 PE=1 SV=2 - [PRPS1_HUMAN]	43.71	2	8	12	22	-0.37	-0.24	-0.45	-0.32	0.03	0.23	-0.11	0.02	0.21	0.08	0.13	0.36	0.23	0.60	0.60	0.46	0.56	0.38	0.43	0.36
O60306	Intron-binding protein aquarius OS=Homo sapiens GN=AQR PE=1 SV=4 - [AQR_HUMAN]	4.71	1	4	4	6	0.22	0.11	-0.54	-0.65	0.09	0.25	-0.24	-0.35	-0.31	-0.19	-0.11	0.05	-0.40	0.32	0.46	-0.38	0.38	0.50	0.64	0.36

Q8WUA2	Peptidyl-prolyl cis-trans isomerase-like 4 OS=Homo sapiens GN=PPIL4 PE=1 SV=1 - [PPIL4_HUMAN]	14.43	1	5	5	8	-0.89	-0.62	-0.72	-0.48	-0.73	-0.72	-1.05	-0.67	-0.26	-0.01	-0.45	-0.43	0.06	0.19	-0.27	0.40	0.27	-0.11	0.04	0.36
Q96DE0	U8 snoRNA-decapping enzyme OS=Homo sapiens GN=NUDT16 PE=1 SV=2 - [NUD16_HUMAN]	38.46	2	6	6	10	0.27	0.08	0.03	-0.37	0.47	0.58	0.01	-0.03	0.16	0.19	0.00	-0.03	-0.04	-0.26	-0.03	-0.05	0.20	0.49	0.94	0.36
O00303	Eukaryotic translation initiation factor 3 subunit F OS=Homo sapiens GN=EIF3F PE=1 SV=1 - [EIF3F_HUMAN]	18.77	1	6	6	8	-0.77	-0.62	-0.57	-0.46	-0.04	0.06	-0.27	-0.16	-0.17	-0.29	0.05	0.22	0.49	0.83	0.65	0.49	0.31	0.58	0.51	0.36
P56545	C-terminal-binding protein 2 OS=Homo sapiens GN=CTBP2 PE=1 SV=1 - [CTBP2_HUMAN]	13.93	1	2	6	12	-0.85	-0.66	-1.03	-0.83	-0.30	-0.11	-0.73	-0.54	-0.55	-0.74	-0.62	-0.43	0.18	0.24	0.41	0.15	0.33	0.53	0.71	0.36
Q99567	Nuclear pore complex protein Nup88 OS=Homo sapiens GN=NUP88 PE=1 SV=2 - [NUP88_HUMAN]	9.04	1	6	6	8	-0.64	-0.60	-0.42	-0.45	-0.39	-0.26	-0.15	-0.08	-0.10	-0.12	0.06	0.04	0.55	0.65	0.45	0.49	0.30	0.16	0.03	0.36
Q02543	60S ribosomal protein L18a OS=Homo sapiens GN=RPL18A PE=1 SV=2 - [RL18A_HUMAN]	26.14	1	5	5	7	-0.50	-0.57	-0.56	-0.53	-0.11	-0.13	-0.24	-0.38	-0.41	-0.33	-0.20	-0.19	0.34	0.47	0.39	0.28	0.18	0.49	0.39	0.36
P56524	Histone deacetylase 4 OS=Homo sapiens GN=HDAC4 PE=1 SV=3 - [HDAC4_HUMAN]	4.06	1	2	3	5	0.85	0.61	0.51	0.28	1.65	1.41	0.81	0.58	0.84	1.08	1.47	1.23	0.02	0.63	0.96	0.27	0.60	0.79	1.12	0.36
Q9NR56	Muscleblind-like protein 1 OS=Homo sapiens GN=MBNL1 PE=1 SV=2 - [MBNL1_HUMAN]	10.57	2	1	3	7	0.14	-0.26	-0.28	-0.68	0.12	-0.29	0.02	-0.38	-0.18	0.23	0.34	-0.06	-0.06	0.21	0.62	0.12	0.54	-0.03	0.38	0.36

Q86X55	Histone-arginine methyltransferase CARM1 OS=Homo sapiens GN=CARM1 PE=1 SV=3 - [CARM1_HUMAN]	5.26	1	3	3	4	0.41	0.34	0.28	0.21	0.37	0.30	0.58	0.51	0.07	0.14	0.04	-0.04	0.23	-0.37	-0.25	-0.24	-0.11	-0.06	0.07	0.36
P62333	26S protease regulatory subunit 10B OS=Homo sapiens GN=PSMC6 PE=1 SV=1 - [PRS10_HUMAN]	52.70	1	16	16	52	-0.35	-0.35	-0.19	-0.21	0.08	0.03	0.08	0.06	0.06	0.05	0.15	0.11	0.35	0.48	0.38	0.36	0.44	0.31	0.32	0.36
P50579	Methionine aminopeptidase 2 OS=Homo sapiens GN=METAP2 PE=1 SV=1 - [MAP2_HUMAN]	9.62	1	3	3	4	-0.82	-0.59	-0.67	-0.44	-0.29	-0.06	-0.37	-0.14	-0.06	-0.28	-0.30	-0.08	0.51	0.53	0.36	0.57	0.42	0.52	0.36	0.36
O95163	Elongator complex protein 1 OS=Homo sapiens GN=IKBKAP PE=1 SV=3 - [ELP1_HUMAN]	13.89	1	12	12	18	-0.10	-0.01	-0.32	-0.41	0.21	0.14	0.07	0.04	-0.17	-0.05	0.20	0.26	0.30	0.22	0.30	0.20	0.25	0.18	0.22	0.36
Q13011	Delta(3,5)-Delta(2,4)-dienoyl-CoA isomerase, mitochondrial OS=Homo sapiens GN=ECH1 PE=1 SV=2 - [ECH1_HUMAN]	53.66	1	14	14	26	-0.73	-0.96	-0.98	-0.96	-0.09	-0.24	-0.67	-0.79	-0.36	-0.29	0.03	-0.02	0.41	0.84	0.93	0.53	0.62	0.47	0.72	0.36
Q9HAC8	Ubiquitin domain-containing protein 1 OS=Homo sapiens GN=UBTD1 PE=1 SV=1 - [UBTD1_HUMAN]	9.25	2	2	2	3	-0.31	-0.51	-0.34	-0.55	-0.05	-0.26	-0.05	-0.25	-0.27	-0.06	-0.39	-0.60	0.32	-0.08	-0.05	0.27	0.31	0.24	0.27	0.36
Q9Y5B8	Nucleoside diphosphate kinase 7 OS=Homo sapiens GN=NME7 PE=1 SV=1 - [NDK7_HUMAN]	8.78	1	3	3	4	0.17	0.38	-0.02	0.19	0.42	0.63	0.27	0.49	0.19	-0.01	0.08	0.29	0.16	-0.08	0.10	-0.15	0.04	0.23	0.42	0.36
P51572	B-cell receptor-associated protein 31 OS=Homo sapiens GN=BCAP31 PE=1 SV=3 - [BAP31_HUMAN]	39.84	1	11	11	32	-0.92	-1.06	-0.52	-0.59	-0.25	-0.36	-0.24	-0.30	-0.34	-0.21	0.03	-0.05	0.66	0.89	0.65	0.69	0.36	0.76	0.31	0.36

P14735	Insulin-degrading enzyme OS=Homo sapiens GN=IDE PE=1 SV=4 - [IDE_HUMAN]	10.50	1	9	9	13	-0.25	-0.21	-0.31	-0.13	-0.07	0.03	0.04	0.13	-0.17	-0.22	-0.33	-0.18	0.37	0.10	0.14	0.06	0.01	0.34	0.38	0.36
P02655	Apolipoprotein C-II OS=Homo sapiens GN=APOC2 PE=1 SV=1 - [APOC2_HUMAN]	17.82	1	2	2	5	-1.32	-1.15	-1.29	-1.12	-0.56	-0.42	-0.90	-0.82	0.31	0.30	1.72	1.83	0.38	3.04	3.01	1.59	1.47	0.78	0.75	0.36
Q9BXC9	Bardet-Biedl syndrome 2 protein OS=Homo sapiens GN=BBS2 PE=1 SV=1 - [BBS2_HUMAN]	8.18	1	5	5	8	-0.31	-0.31	-0.55	-0.56	-0.12	-0.08	-0.15	-0.19	0.12	0.18	-0.19	-0.05	0.20	0.27	0.43	0.44	0.79	0.41	0.41	0.36
Q9H299	SH3 domain-binding glutamic acid-rich-like protein 3 OS=Homo sapiens GN=SH3BGRL3 PE=1 SV=1 - [SH3L3_HUMAN]	87.10	1	7	7	30	-0.38	-0.38	-0.47	-0.50	0.04	0.02	-0.25	-0.19	-0.10	-0.09	-0.28	-0.16	0.16	0.24	0.26	0.32	0.35	0.45	0.47	0.36
Q9C0C2	182 kDa tankyrase-1-binding protein OS=Homo sapiens GN=TNKS1BP1 PE=1 SV=4 - [TB182_HUMAN]	19.78	1	21	21	37	-0.19	-0.36	-0.56	-0.58	-0.19	-0.22	-0.27	-0.32	-0.30	-0.20	0.23	0.27	0.15	0.44	0.77	0.30	0.43	0.26	0.32	0.36
P28068	HLA class II histocompatibility antigen, DM beta chain OS=Homo sapiens GN=HLA-DMB PE=1 SV=1 - [DMB_HUMAN]	4.56	1	1	1	1	-0.58	-0.48	-0.74	-0.65	0.24	0.34	-0.45	-0.35	-0.14	-0.23	-0.40	-0.31	0.19	0.19	0.34	0.38	0.55	0.81	0.97	0.36
Q3LXA3	Bifunctional ATP-dependent dihydroxyacetone kinase/FAD-AMP lyase (cyclizing) OS=Homo sapiens GN=DAK PE=1 SV=2 - [DHAK_HUMAN]	21.91	1	10	10	19	0.01	0.02	0.06	0.17	-0.11	0.11	0.33	0.39	0.10	-0.12	-0.09	0.11	0.20	-0.04	0.23	0.05	0.06	-0.12	0.09	0.36

Q9P0V3	SH3 domain-binding protein 4 OS=Homo sapiens GN=SH3BP4 PE=1 SV=1 - [SH3B4_HUMAN]	0.73	1	1	1	1	-0.35	-0.83	-0.25	-0.72	1.09	0.61	0.05	-0.43	-0.13	0.35	-0.02	-0.51	0.45	0.33	0.22	0.73	0.63	1.42	1.32	0.36
Q8NI27	THO complex subunit 2 OS=Homo sapiens GN=THOC2 PE=1 SV=2 - [THOC2_HUMAN]	2.26	1	4	4	4	0.04	0.54	-0.72	-0.28	-0.24	0.20	-0.42	0.12	-0.09	-0.52	-0.54	-0.10	-0.37	-0.57	0.18	-0.53	0.22	-0.29	0.46	0.36
Q9BTE3	Mini-chromosome maintenance complex-binding protein OS=Homo sapiens GN=MCMBP PE=1 SV=2 - [MCMBP_HUMAN]	4.67	1	2	2	3	0.29	0.28	-0.13	-0.14	0.17	0.16	0.16	0.15	0.09	0.10	0.00	-0.01	-0.08	-0.28	0.14	-0.16	0.27	-0.14	0.29	0.36
Q9Y5B0	RNA polymerase II subunit A C-terminal domain phosphatase OS=Homo sapiens GN=CTDP1 PE=1 SV=3 - [CTDP1_HUMAN]	3.75	1	2	2	3	-0.75	-1.01	-1.19	-1.45	-0.54	-0.81	-0.90	-1.16	-0.92	-0.65	-0.85	-1.13	-0.09	-0.10	0.33	0.12	0.57	0.19	0.63	0.35
Q4G0J3	La-related protein 7 OS=Homo sapiens GN=LARP7 PE=1 SV=1 - [LARP7_HUMAN]	6.87	1	4	4	6	-0.24	-0.14	-0.39	-0.29	-0.07	0.02	-0.10	0.00	-0.06	-0.15	-0.09	0.00	0.20	0.15	0.29	0.11	0.27	0.15	0.30	0.35
A6NHL2	Tubulin alpha chain-like 3 OS=Homo sapiens GN=TUBAL3 PE=1 SV=2 - [TBAL3_HUMAN]	6.50	1	1	4	11	0.99	0.30	0.76	0.07	0.91	0.21	1.05	0.36	0.00	0.69	0.29	-0.40	0.12	-0.69	-0.47	-0.26	-0.03	-0.10	0.13	0.35
P08133	Annexin A6 OS=Homo sapiens GN=ANXA6 PE=1 SV=3 - [ANXA6_HUMAN]	71.62	1	43	43	260	-0.48	-0.38	-0.24	-0.24	0.21	0.23	0.05	0.08	0.08	0.06	0.19	0.20	0.56	0.64	0.45	0.55	0.36	0.66	0.51	0.35
P67809	Nuclease-sensitive element-binding protein 1 OS=Homo sapiens GN=YBX1 PE=1 SV=3 - [YBOX1_HUMAN]	8.33	2	1	2	3	-2.09	-1.97	-1.47	-1.35	-1.08	-0.96	-1.18	-1.06	-1.12	-1.23	-0.65	-0.54	0.97	1.45	0.82	0.89	0.27	1.00	0.37	0.35

Q8WUY1	Protein THEM6 OS=Homo sapiens GN=THEM6 PE=1 SV=2 - [THEM6_HUMAN]	23.56	1	3	3	5	0.11	0.19	0.04	0.13	0.73	0.80	0.33	0.42	0.19	0.12	0.20	0.28	0.28	0.09	0.16	0.03	0.11	0.60	0.66	0.35
Q5T4S7	E3 ubiquitin-protein ligase UBR4 OS=Homo sapiens GN=UBR4 PE=1 SV=1 - [UBR4_HUMAN]	11.87	1	47	47	66	-0.27	-0.34	-0.17	-0.26	-0.04	-0.06	0.05	0.12	0.06	0.08	0.08	0.05	0.42	0.37	0.37	0.24	0.28	0.12	0.20	0.35
Q99816	Tumor susceptibility gene 101 protein OS=Homo sapiens GN=TSG101 PE=1 SV=2 - [TS101_HUMAN]	30.00	1	9	9	14	-0.49	-0.67	-0.28	-0.39	-0.12	-0.23	-0.01	-0.01	-0.27	-0.22	-0.09	-0.13	0.53	0.38	0.22	0.34	0.06	0.36	0.22	0.35
O95490	Latrophilin-2 OS=Homo sapiens GN=LPHN2 PE=1 SV=2 - [LPHN2_HUMAN]	4.59	1	4	4	6	-0.23	-0.03	-0.10	-0.08	0.24	0.37	0.02	0.14	-0.01	0.06	0.15	0.15	0.22	0.47	0.25	0.11	0.11	0.43	0.18	0.35
O60499	Syntaxin-10 OS=Homo sapiens GN=STX10 PE=1 SV=1 - [STX10_HUMAN]	27.71	1	3	4	6	-0.65	-0.64	-0.47	-0.46	0.10	0.11	-0.18	-0.17	0.06	0.06	0.04	0.05	0.52	0.69	0.51	0.74	0.56	0.74	0.56	0.35
Q15942	Zyxin OS=Homo sapiens GN=ZYX PE=1 SV=1 - [ZYX_HUMAN]	34.44	1	13	13	68	-1.19	-1.26	-1.02	-1.19	-0.83	-0.85	-0.64	-0.73	-0.62	-0.53	-0.40	-0.49	0.73	0.85	0.47	0.79	0.63	0.38	0.20	0.35
Q9Y5J1	U3 small nucleolar RNA-associated protein 18 homolog OS=Homo sapiens GN=UTP18 PE=1 SV=3 - [UTP18_HUMAN]	7.01	1	2	2	3	-1.02	-1.16	-0.36	-0.50	-0.07	-0.22	-0.07	-0.21	-0.07	0.07	-0.03	-0.17	1.00	1.00	0.33	1.12	0.46	0.93	0.27	0.35
Q9H1C7	Cysteine-rich and transmembrane domain-containing protein 1 OS=Homo sapiens GN=CYSTM1 PE=1 SV=1 - [CYTM1_HUMAN]	10.31	1	1	1	2	1.26	1.28	-0.23	-0.22	-0.05	-0.04	0.06	0.07	0.00	-0.01	0.14	0.15	-1.15	-1.12	0.37	-1.24	0.26	-1.33	0.16	0.35

Q96BJ3	Axin interactor, dorsalization-associated protein OS=Homo sapiens GN=AIDA PE=1 SV=1 - [AIDA_HUMAN]	21.57	1	5	5	9	0.34	-0.10	0.24	-0.14	0.50	0.18	0.52	0.16	0.00	0.30	0.19	-0.08	0.31	0.03	0.15	0.19	0.23	0.22	0.46	0.35
Q9NWU2	Glucose-induced degradation protein 8 homolog OS=Homo sapiens GN=GID8 PE=1 SV=1 - [GID8_HUMAN]	33.77	1	5	5	11	0.59	0.32	0.34	-0.30	0.60	0.09	0.37	0.02	0.27	0.57	0.38	0.69	-0.02	0.09	0.24	-0.12	0.57	-0.24	0.30	0.35
Q6UX53	Methyltransferase-like protein 7B OS=Homo sapiens GN=METTL7B PE=2 SV=2 - [MET7B_HUMAN]	5.74	1	1	1	1	0.53	0.24	0.18	-0.11	0.13	-0.17	0.47	0.18	-0.29	0.00	-0.15	-0.45	-0.01	-0.68	-0.33	-0.50	-0.14	-0.42	-0.07	0.35
Q07960	Rho GTPase-activating protein 1 OS=Homo sapiens GN=ARHGAP1 PE=1 SV=1 - [RHG01_HUMAN]	43.96	1	14	14	36	-0.86	-0.71	-0.47	-0.47	0.00	0.01	-0.19	-0.16	-0.16	-0.16	-0.29	-0.20	0.64	0.46	0.32	0.58	0.35	0.70	0.56	0.35
Q9Y6E0	Serine/threonine-protein kinase 24 OS=Homo sapiens GN=STK24 PE=1 SV=1 - [STK24_HUMAN]	26.19	1	7	9	25	0.02	0.00	-0.16	-0.10	0.32	0.28	0.19	0.23	0.14	0.18	-0.01	-0.06	0.29	-0.09	0.06	0.25	0.28	0.21	0.23	0.35
Q9BRX2	Protein pelota homolog OS=Homo sapiens GN=PELO PE=1 SV=2 - [PELO_HUMAN]	10.65	1	2	2	4	0.13	-0.06	-0.14	-0.33	0.42	0.22	0.14	-0.05	0.09	0.28	0.33	0.13	0.06	0.20	0.47	0.18	0.46	0.27	0.54	0.35
P55287	Cadherin-11 OS=Homo sapiens GN=CDH11 PE=1 SV=2 - [CAD11_HUMAN]	17.46	4	11	11	16	-0.50	-0.75	-0.82	-0.93	-0.11	-0.30	-0.54	-0.65	-0.07	0.01	0.59	0.59	-0.16	1.15	1.66	0.54	0.63	0.37	0.48	0.35
P17480	Nucleolar transcription factor 1 OS=Homo sapiens GN=UBTF PE=1 SV=1 - [UBF1_HUMAN]	13.09	1	8	8	18	-0.72	-0.51	-0.82	-0.69	-0.48	-0.21	-0.44	-0.39	-0.39	-0.38	-0.48	-0.25	0.26	0.34	0.20	0.46	0.22	0.37	0.43	0.35

P25789	Proteasome subunit alpha type-4 OS=Homo sapiens GN=PSMA4 PE=1 SV=1 - [PSA4_HUMAN]	33.33	1	8	8	18	-0.58	-0.61	-0.27	-0.33	-0.03	-0.06	-0.13	-0.19	0.03	0.02	0.37	0.34	0.42	0.90	0.64	0.67	0.32	0.54	0.28	0.35
Q9HDC9	Adipocyte plasma membrane-associated protein OS=Homo sapiens GN=APMAP PE=1 SV=2 - [APMAP_HUMAN]	35.34	1	12	12	43	-0.10	-0.09	-0.16	-0.21	0.21	0.13	0.10	0.13	0.10	0.26	0.23	0.17	0.18	0.34	0.35	0.30	0.42	0.28	0.33	0.35
P13807	Glycogen [starch] synthase, muscle OS=Homo sapiens GN=GYS1 PE=1 SV=2 - [GYS1_HUMAN]	30.39	2	15	15	25	-0.47	-0.34	-0.20	-0.19	0.29	0.30	-0.05	0.05	0.14	0.06	0.00	0.03	0.44	0.48	0.24	0.41	0.33	0.74	0.57	0.35
Q16576	Histone-binding protein RBBP7 OS=Homo sapiens GN=RBBP7 PE=1 SV=1 - [RBBP7_HUMAN]	23.29	1	3	9	22	-0.67	-0.69	-0.42	-0.44	-0.12	-0.17	-0.14	-0.17	-0.21	-0.17	-0.15	-0.20	0.58	0.61	0.23	0.65	0.36	0.62	0.25	0.35
Q69YN2	CWF19-like protein 1 OS=Homo sapiens GN=CWF19L1 PE=1 SV=2 - [C19L1_HUMAN]	3.35	1	1	1	1	-0.72	-0.97	-0.97	-1.22	-1.07	-1.33	-0.68	-0.94	-1.27	-1.01	-0.42	-0.68	0.09	0.30	0.54	-0.27	-0.01	-0.37	-0.12	0.35
Q96IG2	F-box/LRR-repeat protein 20 OS=Homo sapiens GN=FBXL20 PE=1 SV=2 - [FXL20_HUMAN]	7.80	1	3	3	6	0.20	0.07	-0.19	-0.32	0.28	0.15	0.09	-0.04	0.13	0.26	0.35	0.21	-0.05	0.15	0.53	0.09	0.48	0.07	0.45	0.35
Q99460	26S proteasome non-ATPase regulatory subunit 1 OS=Homo sapiens GN=PSMD1 PE=1 SV=2 - [PSMD1_HUMAN]	28.23	1	21	21	58	-0.58	-0.56	-0.32	-0.32	0.03	0.08	-0.03	0.01	-0.04	-0.05	0.13	0.10	0.61	0.64	0.39	0.51	0.34	0.60	0.31	0.35
Q9Y3Q8	TSC22 domain family protein 4 OS=Homo sapiens GN=TSC22D4 PE=1 SV=2 - [T22D4_HUMAN]	26.33	1	7	8	17	0.14	0.36	0.33	-0.07	0.12	-0.03	0.43	0.29	-0.02	0.22	0.25	0.09	0.35	-0.26	0.23	0.21	0.05	-0.31	0.06	0.35

P57772	Selenocysteine-specific elongation factor OS=Homo sapiens GN=EEFSEC PE=1 SV=4 - [SELB_HUMAN]	8.72	1	4	4	4	-1.06	-1.07	-0.38	-0.38	-0.22	-0.23	-0.10	-0.10	-0.26	-0.25	-0.28	-0.29	1.02	0.79	0.10	0.84	0.16	0.83	0.14	0.34
Q14108	Lysosome membrane protein 2 OS=Homo sapiens GN=SCARB2 PE=1 SV=2 - [SCRB2_HUMAN]	24.06	1	9	9	23	-0.47	-0.26	-0.24	-0.32	0.40	0.29	-0.08	-0.09	0.21	0.26	0.62	0.62	0.45	1.16	0.86	0.56	0.54	0.64	0.59	0.34
Q6NXT1	Ankyrin repeat domain-containing protein 54 OS=Homo sapiens GN=ANKRD54 PE=1 SV=2 - [ANR54_HUMAN]	12.00	1	2	2	3	0.21	0.37	-0.19	-0.03	-0.48	-0.33	0.09	0.25	0.05	-0.10	0.10	0.25	-0.06	-0.10	0.29	-0.29	0.12	-0.71	-0.31	0.34
O95394	Phosphoacetylglucosamine mutase OS=Homo sapiens GN=PGM3 PE=1 SV=1 - [AGM1_HUMAN]	9.96	1	4	4	5	0.93	0.55	0.85	0.47	1.62	1.24	1.13	0.75	0.84	1.23	0.39	0.01	0.25	-0.53	-0.46	0.33	0.42	0.67	0.76	0.34
Q13045	Protein flightless-1 homolog OS=Homo sapiens GN=FLII PE=1 SV=2 - [FLII_HUMAN]	12.61	1	14	14	19	-0.57	-0.53	-0.62	-0.51	-0.25	-0.27	-0.62	-0.58	-0.19	-0.38	-0.24	-0.11	0.28	0.52	0.64	0.33	0.46	0.43	0.46	0.34
Q8IXM3	39S ribosomal protein L41, mitochondrial OS=Homo sapiens GN=MRPL41 PE=1 SV=1 - [RM41_HUMAN]	8.03	1	1	1	2	0.10	0.20	0.18	0.28	0.27	0.37	0.46	0.56	0.19	0.09	0.35	0.45	0.42	0.26	0.17	0.03	-0.05	0.16	0.07	0.34
P19105	Myosin regulatory light chain 12A OS=Homo sapiens GN=MYL12A PE=1 SV=2 - [ML12A_HUMAN]	56.14	2	2	7	85	-0.83	-0.77	-1.19	-0.90	-0.40	-0.51	-0.84	-0.72	-0.55	-0.56	-0.56	-0.52	0.09	0.30	0.50	0.41	0.59	0.29	0.38	0.34
Q99829	Copine-1 OS=Homo sapiens GN=CPNE1 PE=1 SV=1 - [CPNE1_HUMAN]	16.57	1	8	8	15	-0.27	-0.07	-0.07	-0.03	0.85	0.90	0.20	0.29	0.45	0.37	0.29	0.47	0.50	0.62	0.41	0.64	0.54	1.06	0.94	0.34

Q9Y4X5	E3 ubiquitin-protein ligase ARIH1 OS=Homo sapiens GN=ARIH1 PE=1 SV=2 - [ARI1_HUMAN]	21.72	1	8	8	13	0.14	0.01	-0.18	0.12	0.12	0.10	-0.09	0.31	-0.09	0.00	-0.08	-0.09	-0.09	-0.10	-0.21	0.04	0.06	0.08	-0.03	0.34
P20936	Ras GTPase-activating protein 1 OS=Homo sapiens GN=RASA1 PE=1 SV=1 - [RASA1_HUMAN]	1.81	1	1	1	3	-0.58	0.32	-1.12	-0.01	-0.07	0.50	-0.60	-0.03	-0.16	-0.72	-1.09	-0.53	0.03	-0.51	0.03	-0.12	0.43	0.49	1.04	0.34
P23786	Carnitine O-palmitoyltransferase 2, mitochondrial OS=Homo sapiens GN=CPT2 PE=1 SV=2 - [CPT2_HUMAN]	36.93	1	20	20	31	-0.78	-0.84	-0.69	-0.75	-0.43	-0.62	-0.50	-0.59	-0.49	-0.45	-0.39	-0.48	0.30	0.37	0.33	0.25	0.33	0.22	0.15	0.34
P14314	Glucosidase 2 subunit beta OS=Homo sapiens GN=PRKCSH PE=1 SV=2 - [GLU2B_HUMAN]	29.36	1	13	13	55	-0.72	-0.71	-0.71	-0.72	-0.37	-0.44	-0.43	-0.44	-0.50	-0.49	-0.35	-0.30	0.46	0.55	0.37	0.49	0.25	0.43	0.29	0.34
Q8NBJ5	Procollagen galactosyltransferase 1 OS=Homo sapiens GN=COLGALT1 PE=1 SV=1 - [GT251_HUMAN]	4.18	1	2	2	2	-1.04	-0.93	-1.19	-1.08	-0.15	-0.04	-0.92	-0.80	-0.19	-0.30	-0.12	-0.01	0.18	0.93	1.07	0.77	0.92	0.88	1.02	0.34
Q13393	Phospholipase D1 OS=Homo sapiens GN=PLD1 PE=1 SV=1 - [PLD1_HUMAN]	3.91	1	4	4	7	0.00	-0.02	0.00	-0.03	0.36	0.33	0.28	0.25	0.30	0.34	0.36	0.33	0.33	0.36	0.36	0.36	0.37	0.34	0.34	0.34
Q8ND04	Protein SMG8 OS=Homo sapiens GN=SMG8 PE=1 SV=1 - [SMG8_HUMAN]	3.73	1	2	2	2	0.40	0.31	-0.13	-0.22	0.50	0.40	0.15	0.05	-0.08	0.02	0.35	0.25	-0.20	-0.05	0.48	-0.35	0.18	0.08	0.61	0.34
Q13042	Cell division cycle protein 16 homolog OS=Homo sapiens GN=CDC16 PE=1 SV=2 - [CDC16_HUMAN]	2.42	1	1	1	2	0.90	0.96	-0.26	-0.20	-0.13	-0.08	0.02	0.07	-0.16	-0.21	0.13	0.18	-0.83	-0.77	0.39	-1.08	0.08	-1.05	0.11	0.34

P24593	Insulin-like growth factor-binding protein 5 OS=Homo sapiens GN=IGFBP5 PE=1 SV=1 - [IBP5_HUMAN]	12.87	1	3	3	4	-0.25	-0.24	-0.58	-0.85	1.04	0.84	-0.31	-0.47	0.23	0.22	0.24	0.24	-0.18	0.49	0.77	0.50	0.79	1.27	1.55	0.34
Q9UBS4	DnaJ homolog subfamily B member 11 OS=Homo sapiens GN=DNAJB11 PE=1 SV=1 - [DJB11_HUMAN]	28.49	1	7	7	19	-0.42	-0.54	-0.55	-0.54	-0.15	-0.10	-0.47	-0.35	-0.31	-0.30	-0.21	-0.13	0.12	0.25	0.37	0.32	0.26	0.39	0.59	0.34
Q9Y295	Developmentally-regulated GTP-binding protein 1 OS=Homo sapiens GN=DRG1 PE=1 SV=1 - [DRG1_HUMAN]	24.80	1	7	7	12	-0.46	-0.20	-0.72	-0.43	-0.42	-0.14	-0.32	-0.16	-0.03	-0.40	-0.56	-0.58	0.09	-0.37	-0.15	-0.03	0.39	0.09	0.26	0.34
A0FGR8	Extended synaptotagmin-2 OS=Homo sapiens GN=ESYT2 PE=1 SV=1 - [ESYT2_HUMAN]	32.79	1	23	23	45	-0.59	-0.49	-0.68	-0.75	-0.17	-0.11	-0.34	-0.32	-0.31	-0.29	-0.39	-0.29	0.29	0.26	0.36	0.31	0.43	0.36	0.56	0.34
O94887	FERM, RhoGEF and pleckstrin domain-containing protein 2 OS=Homo sapiens GN=FARP2 PE=1 SV=3 - [FARP2_HUMAN]	3.98	1	3	4	6	-0.61	-0.59	-0.52	-0.50	-0.26	-0.25	-0.24	-0.23	-0.26	-0.27	0.24	0.37	0.42	1.40	1.61	0.36	0.28	0.33	0.24	0.34
Q06481	Amyloid-like protein 2 OS=Homo sapiens GN=APLP2 PE=1 SV=2 - [APLP2_HUMAN]	20.31	1	11	12	26	-0.83	-0.47	-0.58	-0.66	-0.03	0.11	-0.41	0.07	0.09	0.05	0.47	0.33	0.39	1.09	0.85	0.60	0.48	0.61	0.51	0.34
P51178	1-phosphatidylinositol 4,5-bisphosphate phosphodiesterase delta-1 OS=Homo sapiens GN=PLCD1 PE=1 SV=2 - [PLCD1_HUMAN]	34.13	1	15	15	35	0.26	0.23	0.42	0.42	0.46	0.34	0.67	0.61	0.47	0.62	0.02	0.09	0.46	-0.24	-0.29	0.24	0.17	0.16	0.07	0.34

Q7L523	Ras-related GTP-binding protein A OS=Homo sapiens GN=RRAGA PE=1 SV=1 - [RRAGA_HUMAN]	15.97	2	4	4	5	-0.27	-0.12	-0.03	0.12	0.12	0.30	0.04	0.36	0.18	-0.13	-0.18	0.14	0.57	0.36	0.11	0.52	0.28	0.58	0.34	0.34
P40222	Alpha-taxilin OS=Homo sapiens GN=TXLNA PE=1 SV=3 - [TXLNA_HUMAN]	15.93	1	5	5	7	-0.49	-0.43	-0.64	-0.55	0.03	0.26	-0.37	-0.14	0.01	-0.05	-0.07	-0.01	0.20	0.38	0.54	0.34	0.61	0.28	0.60	0.34
Q16698	2,4-dienoyl-CoA reductase, mitochondrial OS=Homo sapiens GN=DECR1 PE=1 SV=1 - [DECR_HUMAN]	43.58	1	13	13	62	-0.55	-0.61	-0.44	-0.47	-0.13	-0.13	-0.09	-0.17	-0.18	-0.20	-0.33	-0.32	0.48	0.33	0.12	0.44	0.31	0.39	0.32	0.34
Q9NR31	GTP-binding protein SAR1a OS=Homo sapiens GN=SAR1A PE=1 SV=1 - [SAR1A_HUMAN]	32.83	1	3	5	9	0.15	-0.10	0.21	-0.06	0.53	0.26	0.44	0.16	0.04	0.46	0.39	0.21	0.31	0.31	0.23	0.31	0.28	0.43	0.35	0.34
Q9HCC0	Methylcrotonoyl-CoA carboxylase beta chain, mitochondrial OS=Homo sapiens GN=MCCC2 PE=1 SV=1 - [MCCB_HUMAN]	26.29	1	10	10	21	-0.43	-0.51	-0.19	-0.29	-0.03	-0.13	0.08	-0.04	-0.22	-0.08	-0.21	-0.21	0.51	0.07	-0.03	0.23	0.01	0.34	0.06	0.33
Q9H9E3	Conserved oligomeric Golgi complex subunit 4 OS=Homo sapiens GN=COG4 PE=1 SV=3 - [COG4_HUMAN]	8.54	1	6	6	10	-0.25	-0.31	-0.33	-0.22	0.50	0.64	-0.03	0.06	0.61	0.46	0.33	0.46	0.68	0.82	0.63	1.12	0.82	1.11	0.81	0.33
P35237	Serpin B6 OS=Homo sapiens GN=SERPINB6 PE=1 SV=3 - [SPB6_HUMAN]	36.17	2	10	10	15	-0.40	-0.53	-0.43	-0.48	-0.12	-0.08	-0.22	-0.23	-0.10	-0.17	0.05	-0.02	0.20	0.58	0.51	0.28	0.32	0.37	0.29	0.33
A1L0T0	Acetolactate synthase-like protein OS=Homo sapiens GN=ILVBL PE=1 SV=2 - [ILVBL_HUMAN]	7.91	1	4	4	8	0.02	0.12	0.09	0.19	0.17	0.27	0.36	0.37	0.29	0.08	0.11	-0.10	0.39	-0.58	-0.65	0.09	0.03	0.38	0.28	0.33

Q86X76	Nitrilase homolog 1 OS=Homo sapiens GN=NIT1 PE=1 SV=2 - [NIT1_HUMAN]	24.16	1	7	7	15	-0.07	-0.06	-0.11	0.12	0.25	0.36	0.33	0.58	0.63	0.58	0.43	0.52	0.45	0.34	0.36	0.41	0.44	0.26	0.29	0.33
O43272	Proline dehydrogenase 1, mitochondrial OS=Homo sapiens GN=PRODH PE=1 SV=3 - [PROD_HUMAN]	30.00	1	13	13	45	0.26	0.23	0.15	0.08	0.95	0.92	0.41	0.31	0.47	0.57	-0.12	-0.10	0.12	-0.43	-0.34	0.27	0.38	0.72	0.76	0.33
Q96AT9	Ribulose-phosphate 3-epimerase OS=Homo sapiens GN=RPE PE=1 SV=1 - [RPE_HUMAN]	15.79	1	3	3	4	0.13	0.34	0.07	0.29	0.11	0.32	0.34	0.55	0.24	0.03	-0.33	-0.13	0.27	-0.45	-0.41	-0.07	-0.01	-0.03	0.02	0.33
Q9P0J1	[Pyruvate dehydrogenase [acetyl-transferring]]-phosphatase 1, mitochondrial OS=Homo sapiens GN=PDP1 PE=1 SV=3 - [PDP1_HUMAN]	34.64	1	12	12	29	-0.78	-0.52	-0.83	-0.63	0.00	0.05	-0.45	-0.35	-0.12	-0.26	-0.01	0.14	0.34	0.67	0.74	0.67	0.50	0.74	0.71	0.33
A8MXV4	Nucleoside diphosphate-linked moiety X motif 19, mitochondrial OS=Homo sapiens GN=NUDT19 PE=1 SV=1 - [NUD19_HUMAN]	3.47	1	1	1	1	-0.65	-0.62	-0.51	-0.47	-0.10	-0.07	-0.24	-0.21	-0.23	-0.26	-0.61	-0.58	0.47	0.04	-0.11	0.42	0.28	0.53	0.39	0.33
Q03014	Hematopoietically-expressed homeobox protein HHEX OS=Homo sapiens GN=HHEX PE=1 SV=1 - [HHEX_HUMAN]	4.07	1	1	1	1	0.08	0.15	-0.05	0.02	-0.11	-0.04	0.22	0.29	-0.01	-0.08	-0.05	0.02	0.19	-0.12	0.00	-0.13	0.00	-0.20	-0.07	0.33
P49770	Translation initiation factor eIF-2B subunit beta OS=Homo sapiens GN=EIF2B2 PE=1 SV=3 - [EI2BB_HUMAN]	7.12	1	2	2	3	-0.61	-0.94	-0.85	-1.17	-0.29	-0.62	-0.58	-0.91	-1.07	-0.73	-0.18	-0.51	0.09	0.44	0.66	-0.09	0.14	0.31	0.54	0.33

O75832	26S proteasome non-ATPase regulatory subunit 10 OS=Homo sapiens GN=PSMD10 PE=1 SV=1 - [PSD10_HUMAN]	33.19	1	5	5	17	-0.47	-0.67	-0.31	-0.40	0.46	0.07	0.00	-0.14	0.05	0.40	0.08	-0.05	0.37	0.56	0.39	0.71	0.35	0.80	0.39	0.33
P05156	Complement factor I OS=Homo sapiens GN=CFI PE=1 SV=2 - [CFAI_HUMAN]	10.46	1	5	5	7	-0.75	-0.80	-0.71	-0.75	-0.13	-0.19	-0.44	-0.49	0.06	0.11	0.21	0.16	0.37	0.97	0.92	0.90	0.85	0.60	0.55	0.33
P61221	ATP-binding cassette sub-family E member 1 OS=Homo sapiens GN=ABCE1 PE=1 SV=1 - [ABCE1_HUMAN]	21.37	1	11	11	17	-0.31	-0.29	-0.39	-0.47	0.00	0.00	-0.18	-0.18	-0.07	-0.14	-0.31	-0.20	0.16	0.16	0.13	0.01	0.23	0.16	0.42	0.33
Q13563	Polycystin-2 OS=Homo sapiens GN=PKD2 PE=1 SV=3 - [PKD2_HUMAN]	2.17	1	2	2	3	-0.51	-0.39	-0.67	-0.55	-0.38	-0.27	-0.41	-0.29	-0.23	-0.34	-0.23	-0.11	0.16	0.29	0.44	0.20	0.37	0.11	0.27	0.33
Q8N7H5	RNA polymerase II associated factor 1 homolog OS=Homo sapiens GN=PAF1 PE=1 SV=2 - [PAF1_HUMAN]	4.33	1	2	2	2	0.00	-0.46	-0.17	-0.62	0.80	0.34	0.10	-0.35	0.04	0.50	0.55	0.09	0.16	0.56	0.71	0.53	0.70	0.79	0.95	0.33
O75167	Phosphatase and actin regulator 2 OS=Homo sapiens GN=PHACTR2 PE=1 SV=2 - [PHAR2_HUMAN]	7.57	1	3	3	5	0.58	0.58	0.40	0.40	0.51	0.50	0.67	0.66	0.64	0.65	0.31	0.30	0.14	-0.27	-0.10	0.10	0.28	-0.09	0.09	0.33
Q5THJ4	Vacuolar protein sorting-associated protein 13D OS=Homo sapiens GN=VPS13D PE=1 SV=2 - [VP13D_HUMAN]	2.39	1	8	8	8	-0.27	-0.32	-0.23	-0.44	0.09	0.02	-0.30	-0.34	-0.03	0.21	0.16	0.20	0.57	0.59	0.81	0.50	0.47	0.33	0.55	0.33
P01605	Ig kappa chain V-I region Lay OS=Homo sapiens PE=1 SV=1 - [KV113_HUMAN]	16.67	1	1	1	2	-1.57	-2.00	-0.95	-1.38	-0.78	-1.22	-0.69	-1.12	-0.15	0.29	1.69	1.25	0.94	3.26	2.64	1.89	1.27	0.77	0.15	0.33

Q9C0E8	Protein lunapark OS=Homo sapiens GN=LNP PE=1 SV=2 - [LNP_HUMAN]	21.26	1	7	7	12	-0.63	-0.55	-0.54	-0.47	-0.26	-0.08	-0.26	-0.19	0.00	-0.19	-0.17	0.11	0.18	0.48	0.35	0.36	0.16	0.35	0.37	0.33
Q8IWW6	Rho GTPase-activating protein 12 OS=Homo sapiens GN=ARHGAP12 PE=1 SV=1 - [RHG12_HUMAN]	9.69	1	5	5	5	-0.37	-0.47	-0.36	-0.47	-0.30	-0.41	-0.10	-0.20	-0.23	-0.12	0.12	0.01	0.33	0.50	0.48	0.28	0.28	0.05	0.04	0.33
Q7L4I2	Arginine/serine-rich coiled coil protein 2 OS=Homo sapiens GN=RSRC2 PE=1 SV=1 - [RSRC2_HUMAN]	2.53	1	1	1	1	-0.51	-0.50	-0.58	-0.57	0.02	0.03	-0.31	-0.30	-0.14	-0.14	-0.33	-0.33	0.25	0.18	0.24	0.40	0.47	0.52	0.58	0.33
Q9UIA9	Exportin-7 OS=Homo sapiens GN=XPO7 PE=1 SV=3 - [XPO7_HUMAN]	19.87	1	18	18	32	-0.23	-0.08	-0.23	-0.09	0.04	0.21	0.11	0.15	0.22	0.06	0.06	-0.02	0.04	0.02	0.08	0.22	0.32	0.08	0.22	0.33
Q8TCD1	UPF0729 protein C18orf32 OS=Homo sapiens GN=C18orf32 PE=2 SV=1 - [CR032_HUMAN]	13.16	1	1	1	1	-0.47	-0.61	-0.29	-0.44	0.10	-0.05	-0.03	-0.18	0.12	0.27	0.32	0.17	0.49	0.80	0.62	0.76	0.59	0.55	0.37	0.32
Q96FV9	THO complex subunit 1 OS=Homo sapiens GN=THOC1 PE=1 SV=1 - [THOC1_HUMAN]	4.57	1	2	2	3	0.05	-0.46	0.03	-0.48	-0.22	-0.74	0.29	-0.22	-0.26	0.26	0.06	-0.46	0.30	0.02	0.03	0.24	0.27	-0.28	-0.26	0.32
P35520	Cystathionine beta-synthase OS=Homo sapiens GN=CBS PE=1 SV=2 - [CBS_HUMAN]	13.97	1	6	6	11	0.22	0.38	-0.17	-0.01	0.55	0.71	0.62	0.78	0.65	0.46	0.22	0.39	0.24	0.21	0.45	0.27	0.67	0.29	0.36	0.32
O60828	Polyglutamine-binding protein 1 OS=Homo sapiens GN=PQBP1 PE=1 SV=1 - [PQBP1_HUMAN]	6.79	1	1	1	4	0.04	-0.31	-0.11	-0.29	0.04	0.33	-0.46	-0.03	0.33	-0.28	-0.01	-0.19	-0.30	-0.19	0.10	0.10	0.72	-0.01	0.60	0.32

P60510	Serine/threonine-protein phosphatase 4 catalytic subunit OS=Homo sapiens GN=PPP4C PE=1 SV=1 - [PP4C_HUMAN]	18.57	1	3	4	8	-0.30	-0.19	-0.29	-0.17	-0.09	0.02	-0.03	0.09	-0.09	-0.20	-0.19	-0.08	0.33	0.11	0.10	0.13	0.12	0.19	0.18	0.32
P43246	DNA mismatch repair protein Msh2 OS=Homo sapiens GN=MSH2 PE=1 SV=1 - [MSH2_HUMAN]	3.10	1	3	3	4	-0.24	-0.43	-0.05	-0.24	0.03	-0.17	0.21	0.02	-0.06	0.14	0.28	0.08	0.50	0.52	0.33	0.41	0.23	0.25	0.07	0.32
Q3KQV9	UDP-N-acetylhexosamine pyrophosphorylase-like protein 1 OS=Homo sapiens GN=UAP1L1 PE=1 SV=2 - [UAP1L_HUMAN]	5.92	1	2	2	4	0.47	0.59	0.21	0.34	0.84	0.96	0.47	0.59	0.64	0.53	0.20	0.32	0.06	-0.26	-0.01	0.09	0.35	0.36	0.61	0.32
Q9NSK0	Kinesin light chain 4 OS=Homo sapiens GN=KLC4 PE=1 SV=3 - [KLC4_HUMAN]	23.91	2	9	12	19	0.13	0.08	-0.07	-0.04	0.23	0.21	0.16	0.12	0.12	0.11	0.17	0.20	0.03	0.17	0.31	0.07	0.29	0.05	0.20	0.32
P11766	Alcohol dehydrogenase class-3 OS=Homo sapiens GN=ADH5 PE=1 SV=4 - [ADHX_HUMAN]	32.35	1	11	11	27	0.01	-0.01	0.42	0.36	0.61	0.57	0.62	0.61	0.35	0.47	0.40	0.38	0.68	0.50	0.15	0.51	0.13	0.59	0.22	0.32
Q13107	Ubiquitin carboxyl-terminal hydrolase 4 OS=Homo sapiens GN=USP4 PE=1 SV=3 - [UBP4_HUMAN]	10.70	1	6	6	8	-0.26	-0.08	-0.34	-0.02	0.20	0.24	0.47	0.32	0.23	0.33	0.14	0.24	0.46	0.46	0.36	0.56	0.35	0.44	0.24	0.32
Q9H6U8	Alpha-1,2-mannosyltransferase ALG9 OS=Homo sapiens GN=ALG9 PE=1 SV=2 - [ALG9_HUMAN]	7.36	1	3	3	5	-0.48	-0.45	-0.44	-0.55	-0.35	-0.17	-0.16	-0.09	-0.15	-0.33	0.08	-0.11	0.25	0.51	0.51	0.23	0.29	0.27	0.37	0.32

P61326	Protein mago nashi homolog OS=Homo sapiens GN=MAGOH PE=1 SV=1 - [MGN_HUMAN]	22.60	2	3	3	4	-0.66	-0.59	-0.53	-0.46	0.08	0.15	-0.27	-0.20	0.01	-0.06	-0.22	-0.15	0.45	0.45	0.31	0.63	0.50	0.73	0.60	0.32
P31431	Syndecan-4 OS=Homo sapiens GN=SDC4 PE=1 SV=2 - [SDC4_HUMAN]	18.18	1	3	3	6	0.52	0.18	0.31	-0.01	0.67	0.27	0.47	0.15	0.25	0.57	1.16	0.83	0.01	0.65	0.85	0.08	0.37	0.09	0.16	0.32
Q9NQG5	Regulation of nuclear pre-mRNA domain-containing protein 1B OS=Homo sapiens GN=RPRD1B PE=1 SV=1 - [RPR1B_HUMAN]	26.99	1	5	5	11	-0.33	-0.37	-0.35	-0.39	-0.06	-0.15	-0.01	-0.12	-0.45	-0.43	-0.62	-0.56	0.29	-0.29	-0.37	-0.11	-0.13	0.16	0.06	0.32
A6NL28	Putative tropomyosin alpha-3 chain like protein OS=Homo sapiens PE=5 SV=2 - [TPM3L_HUMAN]	13.45	1	2	3	32	-0.69	-0.78	-0.64	-0.43	0.05	0.19	-0.27	-0.17	0.11	-0.30	0.00	0.07	0.66	-0.01	0.32	0.21	0.30	0.47	0.34	0.32
A4FU28	cTAGE family member 9 OS=Homo sapiens GN=CTAGE9 PE=2 SV=2 - [CTGE9_HUMAN]	5.02	5	1	3	8	-0.33	0.14	-0.36	-0.23	0.12	0.19	-0.05	-0.30	0.06	0.17	-0.10	0.08	0.09	0.31	0.26	0.10	0.32	0.30	0.46	0.32
Q96F15	GTPase IMAP family member 5 OS=Homo sapiens GN=GIMAP5 PE=1 SV=1 - [GIMA5_HUMAN]	7.82	1	2	2	2	-0.09	-0.48	-0.41	-0.80	-0.05	-0.44	-0.15	-0.54	-0.29	0.10	0.32	-0.08	-0.01	0.41	0.73	0.22	0.55	0.02	0.34	0.32
Q9UBF6	RING-box protein 2 OS=Homo sapiens GN=RNF7 PE=1 SV=1 - [RBX2_HUMAN]	7.96	1	1	1	1	-0.55	-0.16	-0.64	-0.26	0.24	0.62	-0.39	-0.01	0.25	-0.13	-0.04	0.34	0.21	0.51	0.61	0.45	0.55	0.77	0.87	0.32
Q9H2J4	Phosducin-like protein 3 OS=Homo sapiens GN=PDCL3 PE=1 SV=1 - [PDCL3_HUMAN]	20.50	1	4	4	5	-0.87	-0.76	-0.79	-0.67	-0.50	-0.39	-0.53	-0.42	-0.70	-0.80	-0.77	-0.66	0.39	0.11	0.02	0.10	0.02	0.36	0.27	0.32

Q8TC07	TBC1 domain family member 15 OS=Homo sapiens GN=TBC1D15 PE=1 SV=2 - [TBC15_HUMAN]	12.16	1	9	9	12	-0.57	-0.61	-0.31	-0.34	-0.18	-0.08	-0.09	-0.22	-0.13	-0.27	-0.18	0.00	0.45	0.61	0.34	0.36	0.33	0.41	0.34	0.32
P62195	26S protease regulatory subunit 8 OS=Homo sapiens GN=PSMC5 PE=1 SV=1 - [PRS8_HUMAN]	60.10	1	19	21	40	-0.39	-0.38	-0.12	-0.19	0.09	0.18	0.08	0.07	-0.01	-0.02	0.03	0.10	0.49	0.60	0.30	0.41	0.28	0.33	0.29	0.32
P17252	Protein kinase C alpha type OS=Homo sapiens GN=PRKCA PE=1 SV=4 - [KPCA_HUMAN]	35.12	2	13	21	71	0.06	0.09	0.18	0.24	0.43	0.45	0.47	0.53	0.32	0.33	0.21	0.21	0.48	0.29	0.08	0.37	0.19	0.48	0.24	0.32
Q9H0X4	Protein ITFG3 OS=Homo sapiens GN=ITFG3 PE=1 SV=1 - [ITFG3_HUMAN]	9.78	1	4	4	9	-0.28	-0.19	-0.53	-0.43	0.20	0.07	-0.19	-0.18	-0.02	-0.10	0.33	0.26	0.06	0.46	0.70	0.22	0.47	0.15	0.44	0.32
Q6UN15	Pre-mRNA 3'-end-processing factor FIP1 OS=Homo sapiens GN=FIP1L1 PE=1 SV=1 - [FIP1_HUMAN]	3.37	1	1	1	2	-0.67	-0.72	-0.19	-0.24	-0.42	-0.47	0.06	0.01	-0.42	-0.37	-0.22	-0.27	0.79	0.46	-0.03	0.33	-0.15	0.23	-0.24	0.32
P62888	60S ribosomal protein L30 OS=Homo sapiens GN=RPL30 PE=1 SV=2 - [RL30_HUMAN]	55.65	1	6	6	12	-0.77	-0.88	-0.58	-0.69	0.00	0.04	-0.35	-0.43	-0.33	-0.21	0.05	0.03	0.75	0.92	0.72	0.59	0.43	0.83	0.55	0.32
P63173	60S ribosomal protein L38 OS=Homo sapiens GN=RPL38 PE=1 SV=2 - [RL38_HUMAN]	48.57	1	4	4	7	-1.08	-1.20	-0.69	-0.84	-0.06	-0.15	-0.44	-0.54	-0.43	-0.35	-0.32	-0.46	0.70	0.75	0.38	0.69	0.44	0.83	0.63	0.32
Q15554	Telomeric repeat-binding factor 2 OS=Homo sapiens GN=TERF2 PE=1 SV=3 - [TERF2_HUMAN]	1.48	1	1	1	1	-0.39	-0.34	-0.24	-0.19	-0.34	-0.30	0.01	0.06	-0.09	-0.13	-0.21	-0.17	0.46	0.18	0.02	0.29	0.14	0.03	-0.12	0.32

O94903	Proline synthase co-transcribed bacterial homolog protein OS=Homo sapiens GN=PROSC PE=1 SV=1 - [PROSC_HUMAN]	27.27	1	7	7	19	0.33	0.47	0.14	0.18	0.33	0.21	0.39	0.43	0.24	0.33	0.01	-0.10	0.14	-0.28	-0.14	0.03	0.17	0.03	0.17	0.31
Q709C8	Vacuolar protein sorting-associated protein 13C OS=Homo sapiens GN=VPS13C PE=1 SV=1 - [VP13C_HUMAN]	11.51	1	29	29	49	-0.34	-0.24	-0.14	-0.02	0.29	0.29	0.14	0.17	0.18	0.14	0.32	0.30	0.44	0.53	0.33	0.48	0.28	0.45	0.34	0.31
Q6P1J9	Parafibromin OS=Homo sapiens GN=CDC73 PE=1 SV=1 - [CDC73_HUMAN]	10.17	1	3	3	4	-0.18	-0.55	-0.16	-0.48	0.09	-0.29	0.09	-0.17	-0.20	0.14	-0.51	-0.89	0.17	-0.31	0.24	0.36	0.32	0.23	0.43	0.31
Q7Z5K2	Wings apart-like protein homolog OS=Homo sapiens GN=WAPAL PE=1 SV=1 - [WAPL_HUMAN]	0.67	1	1	1	1	-0.35	-0.11	-0.57	-0.32	-0.48	-0.24	-0.32	-0.07	-0.34	-0.58	-0.36	-0.12	0.09	0.00	0.20	-0.20	0.02	-0.14	0.07	0.31
O95104	Splicing factor, arginine/serine-rich 15 OS=Homo sapiens GN=SCAF4 PE=1 SV=3 - [SFR15_HUMAN]	1.05	2	1	1	1	-1.03	-0.80	-1.03	-0.79	-0.42	-0.18	-0.78	-0.54	-0.36	-0.59	-0.15	0.08	0.31	0.89	0.87	0.47	0.47	0.60	0.59	0.31
P57740	Nuclear pore complex protein Nup107 OS=Homo sapiens GN=NUP107 PE=1 SV=1 - [NU107_HUMAN]	6.81	1	4	4	5	0.01	0.07	-0.01	-0.15	0.04	0.18	-0.08	0.15	-0.06	-0.28	0.27	0.12	0.18	0.36	0.28	0.15	0.15	0.10	0.25	0.31
Q9BV38	WD repeat-containing protein 18 OS=Homo sapiens GN=WDR18 PE=1 SV=2 - [WDR18_HUMAN]	4.17	1	2	2	4	-0.43	-0.41	-0.43	-0.41	-0.01	0.00	-0.18	-0.16	-0.03	-0.04	0.08	0.10	0.31	0.52	0.51	0.42	0.42	0.40	0.40	0.31
P61923	Coatomer subunit zeta-1 OS=Homo sapiens GN=COPZ1 PE=1 SV=1 - [COPZ1_HUMAN]	41.24	1	4	4	9	-0.21	-0.15	-0.29	-0.22	0.26	0.19	-0.04	0.03	-0.21	-0.07	-0.05	0.12	0.23	0.09	0.33	0.12	-0.01	0.46	0.32	0.31

P00750	Tissue-type plasminogen activator OS=Homo sapiens GN=PLAT PE=1 SV=1 - [TPA_HUMAN]	16.55	1	6	6	11	-1.64	-1.33	-1.66	-1.45	-1.05	-0.93	-1.68	-1.56	-1.22	-1.34	-1.14	-1.03	0.29	0.61	0.62	0.35	0.37	0.58	0.59	0.31
O00743	Serine/threonine-protein phosphatase 6 catalytic subunit OS=Homo sapiens GN=PPP6C PE=1 SV=1 - [PPP6_HUMAN]	13.77	1	3	3	4	-0.18	-0.09	-0.32	-0.22	-0.03	0.38	-0.07	0.02	-0.12	0.14	0.31	0.40	0.17	0.30	0.63	0.13	0.27	0.43	0.59	0.31
Q8WZ73	E3 ubiquitin-protein ligase rififylin OS=Homo sapiens GN=RFFL PE=1 SV=1 - [RFFL_HUMAN]	7.44	1	1	1	1	0.48	-0.06	0.65	0.11	0.60	0.05	0.89	0.35	0.21	0.76	0.18	-0.37	0.47	-0.30	-0.47	0.31	0.15	0.10	-0.06	0.31
Q92729	Receptor-type tyrosine-protein phosphatase U OS=Homo sapiens GN=PTPRU PE=1 SV=2 - [PTPRU_HUMAN]	6.22	1	7	7	9	-1.01	-0.69	-0.93	-0.61	0.37	0.72	-0.58	-0.47	0.39	0.04	0.31	0.63	0.28	0.96	1.01	1.10	1.11	1.15	1.20	0.31
Q96NN9	Apoptosis-inducing factor 3 OS=Homo sapiens GN=AIFM3 PE=1 SV=1 - [AIFM3_HUMAN]	9.59	1	6	6	9	0.88	0.44	0.21	-0.23	-0.17	-0.61	0.45	0.01	-0.08	0.37	0.26	-0.19	-0.37	-0.62	0.05	-0.48	0.20	-1.06	-0.39	0.31
O95365	Zinc finger and BTB domain-containing protein 7A OS=Homo sapiens GN=ZBTB7A PE=1 SV=1 - [ZBT7A_HUMAN]	2.91	1	1	1	2	0.35	0.33	-0.05	-0.08	0.30	0.27	0.19	0.17	0.39	0.43	0.49	0.46	-0.11	0.14	0.54	0.10	0.51	-0.07	0.34	0.31
Q96BP3	Peptidylprolyl isomerase domain and WD repeat-containing protein 1 OS=Homo sapiens GN=PPWD1 PE=1 SV=1 - [PPWD1_HUMAN]	3.10	1	2	2	2	0.28	-0.44	-0.06	-0.78	0.35	-0.38	0.18	-0.54	-0.64	0.08	-0.20	-0.92	-0.05	-0.48	-0.14	-0.17	0.18	0.05	0.39	0.31

Q7L576	Cytoplasmic FMR1-interacting protein 1 OS=Homo sapiens GN=CYFIP1 PE=1 SV=1 - [CYFP1_HUMAN]	22.51	1	14	25	47	-0.22	-0.01	-0.43	-0.33	-0.06	-0.10	-0.23	-0.26	-0.22	-0.19	-0.15	-0.11	0.18	0.12	0.24	0.05	0.16	0.33	0.31	0.31
Q9BYN0	Sulfiredoxin-1 OS=Homo sapiens GN=SRXN1 PE=1 SV=2 - [SRXN1_HUMAN]	6.57	1	1	1	1	0.39	0.75	0.26	0.62	0.79	1.15	0.51	0.87	0.75	0.40	0.28	0.63	0.17	-0.11	0.01	0.03	0.16	0.38	0.51	0.31
O43598	2'-deoxynucleoside 5'-phosphate N-hydrolase 1 OS=Homo sapiens GN=DNPH1 PE=1 SV=1 - [DNPH1_HUMAN]	49.43	1	5	5	12	0.28	0.12	0.04	0.14	0.49	0.66	0.26	0.47	0.51	0.44	0.33	0.41	0.26	-0.11	0.24	0.26	0.36	0.28	0.35	0.31
O00267	Transcription elongation factor SPT5 OS=Homo sapiens GN=SUPT5H PE=1 SV=1 - [SPT5H_HUMAN]	8.28	1	7	7	9	-0.49	-0.51	-0.46	-0.47	0.11	0.09	-0.21	-0.23	-0.07	-0.05	0.00	-0.02	0.33	0.50	0.45	0.48	0.44	0.59	0.55	0.31
Q5H9R7	Serine/threonine-protein phosphatase 6 regulatory subunit 3 OS=Homo sapiens GN=PPP6R3 PE=1 SV=2 - [PP6R3_HUMAN]	16.95	1	11	11	23	-0.21	-0.16	-0.27	-0.13	0.07	0.03	0.08	0.06	0.03	0.05	0.08	0.11	0.01	0.34	0.42	0.12	0.28	0.03	0.26	0.31
P18074	TFIIH basal transcription factor complex helicase XPD subunit OS=Homo sapiens GN=ERCC2 PE=1 SV=1 - [ERCC2_HUMAN]	4.34	1	2	2	3	-0.14	0.11	-0.47	-0.22	0.50	0.74	-0.23	0.02	0.54	0.29	-0.01	0.24	-0.03	0.14	0.46	0.47	0.80	0.62	0.95	0.31
Q99590	Protein SCAF11 OS=Homo sapiens GN=SCAF11 PE=1 SV=2 - [SCAFB_HUMAN]	0.96	1	1	1	1	-0.54	-0.68	-0.49	-0.64	-0.15	-0.30	-0.25	-0.40	-0.57	-0.42	-0.25	-0.40	0.34	0.29	0.24	0.14	0.10	0.37	0.32	0.31

P51798	H(+)/Cl(-) exchange transporter 7 OS=Homo sapiens GN=CLCN7 PE=1 SV=2 - [CLCN7_HUMAN]	6.21	1	3	3	4	-0.31	-0.11	-0.09	0.11	0.27	0.46	0.15	0.35	0.40	0.20	0.28	0.48	0.51	0.60	0.38	0.54	0.33	0.56	0.34	0.31
Q8WU90	Zinc finger CCCH domain-containing protein 15 OS=Homo sapiens GN=ZC3H15 PE=1 SV=1 - [ZC3HF_HUMAN]	21.36	1	7	7	15	-0.63	-0.65	-0.63	-0.84	-0.27	-0.45	-0.37	-0.64	-0.31	-0.38	-0.30	-0.39	0.24	0.40	0.31	0.40	0.35	0.28	0.31	0.31
P34931	Heat shock 70 kDa protein 1-like OS=Homo sapiens GN=HSPA1L PE=1 SV=2 - [HS71L_HUMAN]	32.61	1	1	17	131	-0.61	-0.74	-0.70	-0.82	-0.07	-0.20	-0.46	-0.58	-0.43	-0.30	-0.47	-0.60	0.21	0.15	0.22	0.34	0.43	0.53	0.61	0.30
Q9Y2L1	Exosome complex exonuclease RRP44 OS=Homo sapiens GN=DIS3 PE=1 SV=2 - [RRP44_HUMAN]	4.49	1	4	4	6	-0.56	-0.43	-0.40	-0.27	0.02	0.14	-0.16	-0.03	-0.06	-0.18	-0.05	0.08	0.46	0.52	0.35	0.41	0.25	0.56	0.40	0.30
Q15758	Neutral amino acid transporter B(0) OS=Homo sapiens GN=SLC1A5 PE=1 SV=2 - [AAAT_HUMAN]	2.03	1	1	1	1	-0.84	-0.45	0.00	0.40	0.95	1.34	0.24	0.64	0.78	0.39	0.52	0.91	1.14	1.37	0.52	1.26	0.42	1.77	0.93	0.30
Q9BV20	Methylthioribose-1-phosphate isomerase OS=Homo sapiens GN=MRI1 PE=1 SV=1 - [MTNA_HUMAN]	26.83	1	6	6	13	-0.62	-0.50	-0.52	-0.65	-0.16	-0.15	-0.35	-0.41	-0.27	-0.33	0.36	0.36	0.06	0.93	0.99	0.30	0.21	0.37	0.42	0.30
O94985	Calsyntenin-1 OS=Homo sapiens GN=CLSTN1 PE=1 SV=1 - [CSTN1_HUMAN]	15.90	1	14	14	29	-0.51	-0.41	-0.46	-0.47	0.07	0.08	-0.16	-0.11	0.00	-0.05	0.35	0.49	0.37	0.94	0.70	0.60	0.37	0.53	0.43	0.30
O14908	PDZ domain-containing protein GIPC1 OS=Homo sapiens GN=GIPC1 PE=1 SV=2 - [GIPC1_HUMAN]	43.84	3	9	9	15	-0.64	-0.72	-0.05	-0.28	0.07	0.04	0.04	-0.04	-0.45	-0.21	-0.13	-0.03	0.36	0.19	-0.04	0.24	-0.13	0.13	-0.04	0.30

Q9Y5X3	Sorting nexin-5 OS=Homo sapiens GN=SNX5 PE=1 SV=1 - [SNX5_HUMAN]	32.18	1	11	12	17	-0.61	-0.24	-0.16	-0.11	-0.09	-0.10	-0.03	0.04	-0.04	0.03	0.06	0.06	0.65	1.34	0.06	0.77	0.20	0.71	-0.27	0.30
Q9BSJ8	Extended synaptotagmin-1 OS=Homo sapiens GN=ESYT1 PE=1 SV=1 - [ESYT1_HUMAN]	31.25	1	25	25	55	-0.36	-0.35	-0.55	-0.52	0.01	-0.07	-0.34	-0.33	-0.26	-0.22	-0.21	-0.23	0.14	0.16	0.27	0.10	0.32	0.29	0.38	0.30
P08069	Insulin-like growth factor 1 receptor OS=Homo sapiens GN=IGF1R PE=1 SV=1 - [IGF1R_HUMAN]	7.17	1	5	7	10	-0.13	-0.19	0.05	-0.14	0.42	0.18	0.20	0.04	-0.05	0.27	0.51	0.40	0.50	0.75	0.87	0.54	0.66	0.75	0.58	0.30
Q9Y2R0	Cytochrome c oxidase assembly factor 3 homolog, mitochondrial OS=Homo sapiens GN=COA3 PE=1 SV=1 - [COA3_HUMAN]	18.87	1	2	2	8	-0.46	-0.52	-0.68	-0.55	-0.43	-0.30	-0.44	-0.31	-0.54	-0.48	0.03	0.00	0.70	0.36	0.55	-0.13	0.04	0.54	0.23	0.30
P16885	1-phosphatidylinositol 4,5-bisphosphate phosphodiesterase gamma 2 OS=Homo sapiens GN=PLCG2 PE=1 SV=4 - [PLCG2_HUMAN]	4.43	1	3	3	5	-0.16	-0.38	-0.57	-0.78	-0.34	-0.57	-0.33	-0.55	-0.45	-0.22	-0.40	-0.63	-0.11	-0.24	0.16	-0.03	0.37	-0.20	0.20	0.30
Q9UNZ5	Leydig cell tumor 10 kDa protein homolog OS=Homo sapiens GN=C19orf53 PE=1 SV=1 - [L10K_HUMAN]	7.07	1	1	1	1	-1.24	-1.24	-1.25	-1.26	-0.89	-0.90	-1.02	-1.02	-0.66	-0.65	-0.26	-0.27	0.28	0.98	0.99	0.62	0.64	0.33	0.34	0.30
Q9P2A4	ABI gene family member 3 OS=Homo sapiens GN=ABI3 PE=1 SV=2 - [ABI3_HUMAN]	3.01	1	1	1	1	-0.40	0.46	-0.17	0.69	0.05	0.90	0.07	0.93	1.13	0.27	0.12	0.97	0.53	0.53	0.29	0.71	0.47	0.43	0.20	0.30

Q13444	Disintegrin and metalloproteinase domain-containing protein 15 OS=Homo sapiens GN=ADAM15 PE=1 SV=4 - [ADA15_HUMAN]	7.07	1	5	5	5	-0.50	-0.42	-0.70	-0.82	-0.17	0.08	-0.46	-0.27	-0.21	-0.22	-0.34	-0.15	0.04	0.29	0.50	0.00	0.45	0.45	0.58	0.30
Q86U44	N6-adenosine-methyltransferase 70 kDa subunit OS=Homo sapiens GN=METTL3 PE=1 SV=2 - [MTA70_HUMAN]	8.28	1	3	3	4	-0.05	0.11	-0.19	-0.02	0.20	0.36	0.05	0.22	0.40	0.24	0.07	0.23	0.16	0.13	0.26	0.32	0.46	0.23	0.37	0.30
Q9UBG3	Cornulin OS=Homo sapiens GN=CRNN PE=1 SV=1 - [CRNN_HUMAN]	22.02	1	7	7	15	-3.06	-2.87	-3.83	-3.54	-3.06	-3.02	-3.41	-3.36	-3.03	-3.22	-3.13	-3.01	-0.51	-0.33	0.56	-0.33	0.50	-0.09	0.71	0.30
P30048	Thioredoxin-dependent peroxide reductase, mitochondrial OS=Homo sapiens GN=PRDX3 PE=1 SV=3 - [PRDX3_HUMAN]	43.36	1	9	9	62	-0.19	-0.23	-0.01	-0.03	0.20	0.24	0.21	0.26	0.15	0.16	0.12	0.15	0.43	0.35	0.17	0.41	0.23	0.45	0.27	0.30
Q9Y262	Eukaryotic translation initiation factor 3 subunit L OS=Homo sapiens GN=EIF3L PE=1 SV=1 - [EIF3L_HUMAN]	36.17	1	18	18	31	-0.54	-0.55	-0.56	-0.53	-0.03	-0.02	-0.21	-0.34	-0.15	-0.22	-0.18	-0.13	0.18	0.25	0.16	0.12	0.27	0.17	0.37	0.30
Q9Y484	WD repeat domain phosphoinositide-interacting protein 4 OS=Homo sapiens GN=WDR45 PE=2 SV=1 - [WIPI4_HUMAN]	7.50	1	3	3	5	0.05	0.11	-0.09	-0.03	0.33	0.38	0.14	0.20	0.29	0.23	0.06	0.11	0.15	0.02	0.15	0.22	0.36	0.26	0.40	0.30
Q5T5P2	Sickle tail protein homolog OS=Homo sapiens GN=KIAA1217 PE=1 SV=2 - [SKT_HUMAN]	17.96	1	29	29	52	-0.30	-0.36	-0.26	-0.21	-0.01	0.06	-0.08	-0.07	0.01	0.08	0.23	0.16	0.26	0.48	0.44	0.33	0.32	0.22	0.35	0.30

Q8N0U8	Vitamin K epoxide reductase complex subunit 1-like protein 1 OS=Homo sapiens GN=VKORC1L1 PE=1 SV=2 - [VKORL_HUMAN]	10.23	1	2	2	4	-0.41	-0.55	-0.60	-0.64	-0.13	0.01	-0.34	-0.18	0.06	-0.10	-0.33	-0.20	0.10	0.08	0.27	-0.02	0.46	-0.26	0.49	0.30
Q15293	Reticulocalbin-1 OS=Homo sapiens GN=RCN1 PE=1 SV=1 - [RCN1_HUMAN]	32.33	1	9	9	33	-0.67	-0.50	-0.38	-0.51	0.02	-0.02	-0.22	-0.30	-0.31	-0.21	-0.14	-0.17	0.09	0.27	0.28	0.17	0.27	0.31	0.47	0.30
Q6UVY6	DBH-like monooxygenase protein 1 OS=Homo sapiens GN=MOXD1 PE=2 SV=1 - [MOXD1_HUMAN]	4.40	1	2	2	2	-1.19	-1.21	-1.13	-1.14	-0.90	-0.92	-0.89	-0.91	-1.07	-1.05	-0.93	-0.95	0.36	0.27	0.19	0.17	0.11	0.28	0.21	0.30
Q13765	Nascent polypeptide-associated complex subunit alpha OS=Homo sapiens GN=NACA PE=1 SV=1 - [NACA_HUMAN]	12.09	3	2	2	5	-0.66	-0.68	-0.70	-0.72	-0.27	-0.29	-0.47	-0.49	-0.38	-0.35	-0.31	-0.34	0.25	0.35	0.38	0.34	0.38	0.38	0.41	0.30
Q05397	Focal adhesion kinase 1 OS=Homo sapiens GN=PTK2 PE=1 SV=2 - [FAK1_HUMAN]	16.06	3	13	13	23	-0.56	-0.49	-0.95	-1.12	-0.54	-0.56	-0.86	-0.65	-0.49	-0.69	-0.55	-0.63	0.19	0.31	0.44	0.16	0.37	0.10	0.36	0.30
Q9Y5A7	NEDD8 ultimate buster 1 OS=Homo sapiens GN=NUB1 PE=1 SV=2 - [NUB1_HUMAN]	3.25	1	2	2	2	-0.41	-0.23	-0.22	-0.04	-0.04	0.14	0.01	0.19	0.00	-0.17	-0.20	-0.02	0.47	0.21	0.02	0.26	0.08	0.35	0.17	0.30
O43491	Band 4.1-like protein 2 OS=Homo sapiens GN=EPB41L2 PE=1 SV=1 - [E41L2_HUMAN]	38.81	1	29	33	94	-0.25	-0.23	-0.02	-0.09	-0.10	-0.08	0.04	0.05	-0.10	-0.01	0.17	0.16	0.39	0.41	0.21	0.08	0.15	0.09	0.08	0.29
Q14161	ARF GTPase activating protein GIT2 OS=Homo sapiens GN=GIT2 PE=1 SV=2 - [GIT2_HUMAN]	13.44	1	6	7	12	0.12	-0.14	-0.34	-0.54	0.23	0.01	0.02	-0.24	0.12	0.05	0.10	0.04	-0.09	0.22	0.46	0.07	0.26	0.09	0.45	0.29

Q14320	Protein FAM50A OS=Homo sapiens GN=FAM50A PE=1 SV=2 - [FA50A_HUMAN]	11.80	2	4	4	5	0.21	0.31	0.19	0.29	0.08	0.18	0.42	0.52	0.52	0.43	-0.23	-0.13	0.27	-0.43	-0.42	0.25	0.27	-0.14	-0.12	0.29
Q9UBD6	Ammonium transporter Rh type C OS=Homo sapiens GN=RHCG PE=1 SV=1 - [RHCG_HUMAN]	3.97	1	1	1	2	-0.19	-0.04	-0.83	-0.68	-0.39	-0.25	-0.60	-0.45	-0.28	-0.42	-0.57	-0.43	-0.36	-0.38	0.26	-0.20	0.44	-0.22	0.42	0.29
Q92538	Golgi-specific brefeldin A-resistance guanine nucleotide exchange factor 1 OS=Homo sapiens GN=GBF1 PE=1 SV=2 - [GBF1_HUMAN]	13.02	1	15	16	26	-0.26	-0.22	-0.36	-0.57	0.13	-0.23	-0.21	-0.13	-0.22	-0.16	0.04	-0.16	0.35	0.37	0.34	0.47	0.39	0.59	0.51	0.29
Q53HV7	Single-strand selective monofunctional uracil DNA glycosylase OS=Homo sapiens GN=SMUG1 PE=1 SV=2 - [SMUG1_HUMAN]	6.30	1	1	1	2	-0.60	-0.53	-0.72	-0.65	-0.07	0.00	-0.49	-0.42	-0.35	-0.42	-0.17	-0.11	0.17	0.44	0.55	0.22	0.34	0.52	0.64	0.29
P31749	RAC-alpha serine/threonine-protein kinase OS=Homo sapiens GN=AKT1 PE=1 SV=2 - [AKT1_HUMAN]	16.46	1	4	6	8	-0.51	-0.44	-0.37	-0.30	0.17	0.22	-0.05	-0.06	0.02	-0.04	-0.16	-0.13	0.40	0.38	0.27	0.46	0.41	0.49	0.51	0.29
O60238	BCL2/adenovirus E1B 19 kDa protein-interacting protein 3-like OS=Homo sapiens GN=BNIP3L PE=1 SV=1 - [BNI3L_HUMAN]	8.22	1	1	1	1	-0.29	-0.20	-0.52	-0.43	-0.50	-0.41	-0.29	-0.20	-0.45	-0.53	0.05	0.14	0.05	0.34	0.57	-0.22	0.02	-0.23	0.00	0.29
P31947	14-3-3 protein sigma OS=Homo sapiens GN=SFN PE=1 SV=1 - [1433S_HUMAN]	23.79	1	2	5	47	-3.69	-3.59	-3.30	-3.19	-2.47	-2.37	-3.07	-2.96	-2.76	-2.85	-2.90	-2.81	0.68	0.79	0.39	0.87	0.47	1.20	0.81	0.29

Q9BSJ2	Gamma-tubulin complex component 2 OS=Homo sapiens GN=TUBGCP2 PE=1 SV=2 - [GCP2_HUMAN]	12.86	1	10	10	15	-0.56	-0.73	-0.49	-0.60	-0.16	-0.29	-0.27	-0.37	-0.31	-0.27	-0.14	-0.15	0.37	0.34	0.31	0.27	0.21	0.39	0.33	0.29
Q9Y5M8	Signal recognition particle receptor subunit beta OS=Homo sapiens GN=SRPRB PE=1 SV=3 - [SRPRB_HUMAN]	19.56	1	5	5	9	-0.89	-0.85	-0.61	-0.60	-0.05	-0.07	-0.22	-0.29	-0.32	-0.20	-0.01	-0.05	0.60	0.91	0.66	0.54	0.30	0.77	0.44	0.29
Q92820	Gamma-glutamyl hydrolase OS=Homo sapiens GN=GGH PE=1 SV=2 - [GGH_HUMAN]	26.10	1	7	7	13	-0.24	-0.41	-0.34	-0.28	0.41	0.23	0.03	-0.10	0.08	0.14	0.70	0.53	0.37	0.88	1.03	0.79	0.40	0.64	0.66	0.29
P52907	F-actin-capping protein subunit alpha-1 OS=Homo sapiens GN=CAPZA1 PE=1 SV=3 - [CAZA1_HUMAN]	51.75	1	7	9	65	-0.19	-0.14	-0.36	-0.36	0.01	-0.09	-0.01	-0.10	-0.23	0.07	0.08	-0.11	0.33	0.21	0.24	0.29	0.43	0.20	0.41	0.29
P62256	Ubiquitin-conjugating enzyme E2 H OS=Homo sapiens GN=UBE2H PE=1 SV=1 - [UBE2H_HUMAN]	26.78	1	4	4	5	-0.08	-0.12	0.19	0.07	0.57	0.52	0.51	0.43	0.22	0.27	-0.01	-0.06	0.65	0.12	-0.13	0.38	0.05	0.72	0.23	0.29
Q9NVG8	TBC1 domain family member 13 OS=Homo sapiens GN=TBC1D13 PE=1 SV=3 - [TBC13_HUMAN]	4.25	1	1	1	2	-0.19	-0.28	-0.38	-0.46	-0.07	-0.17	-0.15	-0.24	-0.01	0.09	0.14	0.05	0.10	0.34	0.52	0.31	0.50	0.10	0.29	0.29
Q96EY8	Cob(I)yrinic acid a,c-diamide adenosyltransferase, mitochondrial OS=Homo sapiens GN=MMAB PE=1 SV=1 - [MMAB_HUMAN]	20.80	1	4	4	11	-0.30	-0.39	-0.39	-0.42	0.05	-0.09	-0.01	-0.14	-0.28	-0.17	-0.39	-0.36	0.44	-0.14	-0.11	0.16	0.27	0.35	0.28	0.29

Q9UJY1	Heat shock protein beta-8 OS=Homo sapiens GN=HSPB8 PE=1 SV=1 - [HSPB8_HUMAN]	18.37	1	4	4	7	-0.66	-0.48	-0.17	-0.14	-0.28	-0.10	0.02	0.17	0.08	-0.04	-0.02	0.02	0.74	0.64	0.17	0.62	0.20	0.53	-0.09	0.29
A0AVT1	Ubiquitin-like modifier-activating enzyme 6 OS=Homo sapiens GN=UBA6 PE=1 SV=1 - [UBA6_HUMAN]	24.90	1	21	21	26	-0.02	-0.02	0.00	-0.01	0.39	0.43	0.36	0.44	0.28	0.36	0.34	0.40	0.47	0.22	0.09	0.26	0.24	0.38	0.44	0.29
Q03113	Guanine nucleotide-binding protein subunit alpha 12 OS=Homo sapiens GN=GNA12 PE=1 SV=4 - [GNA12_HUMAN]	16.01	1	4	6	19	-0.32	-0.14	-0.14	-0.01	0.02	0.22	0.18	0.38	0.12	0.02	-0.16	0.01	0.65	0.17	0.03	0.31	0.19	0.50	0.05	0.29
P02538	Keratin, type II cytoskeletal 6A OS=Homo sapiens GN=KRT6A PE=1 SV=3 - [K2C6A_HUMAN]	43.44	14	13	25	53	-3.81	-3.86	-4.62	-4.80	-3.88	-3.96	-4.40	-4.57	-3.62	-3.60	-4.10	-4.23	-0.66	-0.49	0.42	-0.33	0.80	-0.40	0.61	0.29
Q8IUD2	ELKS/Rab6-interacting/CAST family member 1 OS=Homo sapiens GN=ERC1 PE=1 SV=1 - [RB6I2_HUMAN]	26.97	1	19	27	46	-0.34	-0.45	-0.50	-0.42	-0.12	-0.29	-0.17	-0.27	-0.22	-0.20	-0.30	-0.26	0.19	0.22	0.16	0.15	0.34	0.16	0.20	0.29
P15259	Phosphoglycerate mutase 2 OS=Homo sapiens GN=PGAM2 PE=1 SV=3 - [PGAM2_HUMAN]	47.43	1	5	11	48	0.72	1.02	0.89	1.19	0.36	0.33	1.18	1.49	0.65	0.73	0.63	0.29	0.51	-0.48	-0.74	-0.34	-0.50	-0.66	-1.01	0.29
P05114	Non-histone chromosomal protein HMG-14 OS=Homo sapiens GN=HMGN1 PE=1 SV=3 - [HMGN1_HUMAN]	10.00	1	1	1	2	-2.09	-2.21	-1.24	-1.35	-1.03	-1.16	-1.01	-1.13	-1.41	-1.29	-0.99	-1.11	1.14	1.11	0.25	0.84	-0.02	1.05	0.19	0.29
Q15477	Helicase SKI2W OS=Homo sapiens GN=SKIV2L PE=1 SV=3 - [SKIV2_HUMAN]	7.87	1	8	8	16	-0.29	-0.22	-0.60	-0.60	0.16	0.26	-0.42	-0.31	0.02	0.03	-0.09	-0.05	-0.14	0.21	0.68	0.30	0.61	0.49	0.82	0.29

Q92843	Bcl-2-like protein 2 OS=Homo sapiens GN=BCL2L2 PE=1 SV=2 - [B2CL2_HUMAN]	11.40	1	1	1	2	-0.47	-0.98	-0.43	-0.94	0.43	-0.08	-0.20	-0.71	-0.63	-0.12	0.03	-0.48	0.32	0.51	0.46	0.38	0.34	0.89	0.85	0.29
Q9BS18	Anaphase-promoting complex subunit 13 OS=Homo sapiens GN=ANAPC13 PE=1 SV=1 - [APC13_HUMAN]	13.51	1	1	1	1	-0.21	1.20	-0.29	1.13	-0.82	0.59	-0.07	1.35	1.28	-0.13	-0.14	1.28	0.20	0.08	0.15	0.12	0.20	-0.62	-0.55	0.29
Q99543	DnaJ homolog subfamily C member 2 OS=Homo sapiens GN=DNAJC2 PE=1 SV=4 - [DNJC2_HUMAN]	1.93	1	1	1	1	-1.07	-0.77	-1.28	-0.98	-0.48	-0.18	-1.06	-0.75	0.06	-0.23	-0.12	0.18	0.07	0.96	1.16	0.87	1.08	0.58	0.78	0.29
P02671	Fibrinogen alpha chain OS=Homo sapiens GN=FGA PE=1 SV=2 - [FIBA_HUMAN]	32.79	1	29	29	197	0.02	0.01	-0.12	-0.03	0.70	0.73	0.12	0.13	0.66	0.62	1.22	1.19	0.33	1.34	1.34	0.80	0.74	0.93	0.83	0.29
O15320	cTAGE family member 5 OS=Homo sapiens GN=CTAGE5 PE=1 SV=4 - [CTGE5_HUMAN]	17.66	2	8	10	16	-0.33	-0.45	-0.38	-0.45	-0.14	-0.25	-0.11	-0.23	-0.42	-0.30	0.07	-0.14	0.26	0.41	0.46	-0.09	0.11	0.06	-0.10	0.29
Q9H1E5	Thioredoxin-related transmembrane protein 4 OS=Homo sapiens GN=TMX4 PE=1 SV=1 - [TMX4_HUMAN]	10.89	1	3	3	6	-0.07	0.05	-0.20	-0.14	0.07	0.11	-0.08	0.06	0.20	0.08	0.08	0.24	0.04	0.11	0.40	0.18	0.17	-0.04	0.26	0.29
P17980	26S protease regulatory subunit 6A OS=Homo sapiens GN=PSMC3 PE=1 SV=3 - [PRS6A_HUMAN]	64.01	1	23	23	50	-0.32	-0.23	-0.28	-0.24	-0.02	0.09	0.04	0.05	0.03	-0.02	0.15	0.23	0.47	0.54	0.32	0.32	0.23	0.37	0.21	0.29
Q8N3F8	MICAL-like protein 1 OS=Homo sapiens GN=MICALL1 PE=1 SV=2 - [MILK1_HUMAN]	2.67	1	2	2	2	-1.09	-0.96	-0.98	-0.85	-0.08	0.04	-0.75	-0.63	-0.44	-0.56	-0.78	-0.66	0.39	0.31	0.19	0.56	0.45	0.99	0.88	0.29

Q6NVY1	3-hydroxyisobutyryl-CoA hydrolase, mitochondrial OS=Homo sapiens GN=HIBCH PE=1 SV=2 - [HIBCH_HUMAN]	32.38	1	10	10	21	-0.84	-0.77	-0.57	-0.51	-0.05	0.00	-0.28	-0.26	-0.17	-0.14	-0.12	-0.04	0.59	0.80	0.45	0.71	0.34	0.83	0.56	0.28
P04264	Keratin, type II cytoskeletal 1 OS=Homo sapiens GN=KRT1 PE=1 SV=6 - [K2C1_HUMAN]	29.81	2	14	17	137	0.29	0.69	-0.14	-0.11	-0.10	-0.10	0.09	0.07	0.59	0.60	1.12	0.87	-0.29	0.13	1.15	0.16	0.77	-0.63	0.01	0.28
Q13541	Eukaryotic translation initiation factor 4E-binding protein 1 OS=Homo sapiens GN=EIF4EBP1 PE=1 SV=3 - [4EBP1_HUMAN]	22.03	1	1	1	2	-0.40	-0.15	-0.63	-0.38	-0.22	0.02	-0.41	-0.16	-0.17	-0.41	-0.80	-0.56	0.04	-0.40	-0.18	0.01	0.25	0.16	0.39	0.28
Q96S06	Lipase maturation factor 1 OS=Homo sapiens GN=LMF1 PE=1 SV=1 - [LMF1_HUMAN]	3.00	1	1	1	2	-0.95	-1.09	-0.46	-0.60	0.12	-0.03	-0.24	-0.38	-0.42	-0.28	-0.54	-0.69	0.77	0.42	-0.08	0.71	0.21	1.05	0.56	0.28
Q0PNE2	Elongator complex protein 6 OS=Homo sapiens GN=ELP6 PE=1 SV=1 - [ELP6_HUMAN]	5.64	1	1	1	2	0.28	0.27	-0.15	-0.15	-0.10	-0.11	0.07	0.07	-0.30	-0.29	-0.07	-0.08	-0.15	-0.34	0.07	-0.54	-0.11	-0.39	0.03	0.28
P43652	Afamin OS=Homo sapiens GN=AFM PE=1 SV=1 - [AFAM_HUMAN]	2.67	1	1	1	1	-0.63	-0.84	-0.92	-1.14	0.47	0.26	-0.71	-0.92	-0.47	-0.25	-0.50	-0.72	-0.02	0.13	0.42	0.40	0.70	1.08	1.38	0.28
O15047	Histone-lysine N-methyltransferase SETD1A OS=Homo sapiens GN=SETD1A PE=1 SV=3 - [SET1A_HUMAN]	1.99	1	2	2	2	-0.48	-0.31	-0.59	-0.42	-0.09	0.08	-0.37	-0.20	-0.18	-0.35	-0.23	-0.06	0.17	0.26	0.36	0.16	0.27	0.38	0.48	0.28
Q96KQ7	Histone-lysine N-methyltransferase EHMT2 OS=Homo sapiens GN=EHMT2 PE=1 SV=3 - [EHMT2_HUMAN]	0.99	1	1	1	1	-0.14	0.32	-0.49	-0.03	0.14	0.59	-0.27	0.18	0.49	0.04	-0.28	0.17	-0.08	-0.14	0.21	0.21	0.56	0.26	0.62	0.28

Q5U651	Ras-interacting protein 1 OS=Homo sapiens GN=RASIP1 PE=1 SV=1 - [RAIN_HUMAN]	6.96	1	5	5	8	-1.61	-1.78	-1.45	-1.65	-0.58	-0.92	-0.97	-1.31	-1.21	-0.78	-0.53	-0.97	0.57	0.86	0.55	0.96	0.64	0.96	0.62	0.28
P01717	Ig lambda chain V-IV region Hil OS=Homo sapiens PE=1 SV=1 - [LV403_HUMAN]	17.76	1	1	1	6	-1.16	-1.14	-1.15	-1.28	1.10	1.10	-0.93	-0.91	0.74	0.77	1.62	1.54	0.28	2.78	2.62	1.95	1.92	2.24	2.08	0.28
Q13409	Cytoplasmic dynein 1 intermediate chain 2 OS=Homo sapiens GN=DYNC1I2 PE=1 SV=3 - [DC1I2_HUMAN]	13.79	1	6	6	16	-0.30	-0.46	-0.27	-0.44	0.07	-0.03	-0.12	-0.18	-0.26	-0.14	-0.55	-0.68	0.38	0.03	-0.08	0.32	0.24	0.37	0.26	0.28
Q5GLZ8	Probable E3 ubiquitin-protein ligase HERC4 OS=Homo sapiens GN=HERC4 PE=1 SV=1 - [HERC4_HUMAN]	7.76	1	7	7	13	0.71	0.84	0.42	0.52	0.56	0.68	0.43	0.49	0.48	0.43	0.39	0.28	0.07	-0.16	-0.26	-0.08	0.03	-0.18	0.10	0.28
Q6ICL3	Transport and Golgi organization protein 2 homolog OS=Homo sapiens GN=TANGO2 PE=2 SV=1 - [TNG2_HUMAN]	14.13	1	3	3	5	-0.10	-0.08	-0.17	-0.15	-0.09	-0.07	0.05	0.07	-0.09	-0.11	-0.30	-0.29	0.20	-0.20	-0.14	0.02	0.09	-0.01	0.06	0.28
Q9NT99	Leucine-rich repeat-containing protein 4B OS=Homo sapiens GN=LRRC4B PE=2 SV=3 - [LRC4B_HUMAN]	14.03	3	7	7	12	0.11	0.01	-0.38	-0.16	0.77	1.00	-0.29	-0.15	0.44	0.19	0.46	0.55	0.23	0.72	0.76	0.31	0.57	0.75	0.92	0.28
Q9UDY2	Tight junction protein ZO-2 OS=Homo sapiens GN=TJP2 PE=1 SV=2 - [ZO2_HUMAN]	27.65	1	23	24	59	-0.15	-0.08	-0.17	-0.08	0.12	0.19	0.17	0.26	0.30	0.14	0.49	0.51	0.38	0.63	0.62	0.41	0.33	0.35	0.30	0.28
P23470	Receptor-type tyrosine-protein phosphatase gamma OS=Homo sapiens GN=PTPRG PE=1 SV=4 - [PTPRG_HUMAN]	3.67	1	4	4	8	-0.55	-0.52	-0.42	-0.40	-0.09	-0.07	-0.21	-0.18	0.09	0.07	-0.27	-0.25	0.40	0.28	0.15	0.65	0.53	0.44	0.32	0.28

O75367	Core histone macro-H2A.1 OS=Homo sapiens GN=H2AFY PE=1 SV=4 - [H2AY_HUMAN]	46.51	1	13	15	43	-0.89	-0.86	-0.71	-0.55	-0.40	-0.41	-0.38	-0.34	-0.34	-0.40	-0.27	-0.25	0.46	0.34	0.30	0.32	0.19	0.47	0.14	0.28
Q562E7	WD repeat-containing protein 81 OS=Homo sapiens GN=WDR81 PE=1 SV=2 - [WDR81_HUMAN]	8.91	1	11	11	17	-0.44	-0.16	-0.03	0.06	0.03	0.01	0.21	0.29	-0.07	0.03	-0.10	0.08	0.70	0.36	-0.06	0.37	-0.03	0.28	-0.13	0.28
P34910	Protein EVI2B OS=Homo sapiens GN=EVI2B PE=1 SV=2 - [EVI2B_HUMAN]	3.35	1	1	1	1	0.87	1.65	-0.45	0.34	0.45	1.23	-0.23	0.55	0.95	0.17	0.34	1.12	-1.05	-0.53	0.79	-0.67	0.65	-0.44	0.88	0.28
O15371	Eukaryotic translation initiation factor 3 subunit D OS=Homo sapiens GN=EIF3D PE=1 SV=1 - [EIF3D_HUMAN]	26.46	1	9	9	25	-0.54	-0.45	-0.40	-0.24	-0.01	-0.02	-0.07	-0.09	-0.29	-0.22	-0.27	-0.30	0.52	0.18	0.04	0.33	0.17	0.32	0.30	0.28
P25940	Collagen alpha-3(V) chain OS=Homo sapiens GN=COL5A3 PE=1 SV=3 - [CO5A3_HUMAN]	0.57	1	1	1	1	-2.20	-2.28	-2.52	-2.60	-1.26	-1.35	-2.31	-2.39	-1.77	-1.69	-1.47	-1.56	-0.05	0.73	1.05	0.54	0.87	0.92	1.24	0.28
O75886	Signal transducing adapter molecule 2 OS=Homo sapiens GN=STAM2 PE=1 SV=1 - [STAM2_HUMAN]	20.00	1	8	9	13	-0.14	0.08	-0.13	0.36	0.61	0.47	0.68	0.74	0.42	-0.14	-0.21	0.26	0.61	0.19	0.08	0.10	0.12	0.42	0.11	0.28
Q7Z3Z0	Keratin, type I cytoskeletal 25 OS=Homo sapiens GN=KRT25 PE=1 SV=1 - [K1C25_HUMAN]	6.22	4	1	3	4	0.73	0.51	0.65	0.42	0.79	0.56	0.86	0.64	0.58	0.81	0.85	0.62	0.18	0.13	0.21	0.11	0.20	0.05	0.13	0.28
P35250	Replication factor C subunit 2 OS=Homo sapiens GN=RFC2 PE=1 SV=3 - [RFC2_HUMAN]	3.67	1	1	1	1	-0.56	-0.54	-0.67	-0.65	-0.22	-0.21	-0.46	-0.44	-0.26	-0.28	-0.52	-0.50	0.16	0.05	0.15	0.32	0.43	0.32	0.43	0.28

P62310	U6 snRNA-associated Sm-like protein LSm3 OS=Homo sapiens GN=LSM3 PE=1 SV=2 - [LSM3_HUMAN]	11.76	1	1	1	4	-0.15	-0.20	-0.01	-0.06	0.46	0.41	0.20	0.16	0.05	0.11	0.43	0.38	0.41	0.59	0.44	0.29	0.15	0.60	0.46	0.28
Q92835	Phosphatidylinositol 3,4,5-trisphosphate 5-phosphatase 1 OS=Homo sapiens GN=INPP5D PE=1 SV=2 - [SHIP1_HUMAN]	4.21	1	3	3	6	0.60	0.34	0.76	0.35	1.04	0.56	0.27	0.44	0.75	0.95	1.21	0.94	0.16	0.47	0.65	0.31	0.50	0.07	0.26	0.28
Q9BR76	Coronin-1B OS=Homo sapiens GN=CORO1B PE=1 SV=1 - [COR1B_HUMAN]	14.72	1	7	7	18	-0.48	-0.44	-0.42	-0.47	-0.02	-0.13	-0.33	-0.28	-0.35	-0.29	-0.16	-0.25	0.39	0.52	0.23	0.29	0.16	0.44	0.30	0.28
P15735	Phosphorylase b kinase gamma catalytic chain, liver/testis isoform OS=Homo sapiens GN=PHKG2 PE=1 SV=1 - [PHKG2_HUMAN]	7.64	1	2	2	3	-0.53	-0.87	-0.22	-0.56	0.00	-0.34	-0.01	-0.35	-0.36	-0.01	0.00	-0.34	0.58	0.53	0.22	0.55	0.24	0.51	0.20	0.28
Q9HCE6	Rho guanine nucleotide exchange factor 10-like protein OS=Homo sapiens GN=ARHGEF10L PE=1 SV=3 - [ARGAL_HUMAN]	2.81	1	3	3	3	-0.04	-0.62	-0.06	-0.61	-0.16	-0.25	-0.13	-0.40	-0.39	-0.13	-0.07	-0.49	-0.20	-0.55	0.44	-0.06	-0.04	0.13	0.01	0.28
A6NE02	BTB/POZ domain-containing protein 17 OS=Homo sapiens GN=BTBD17 PE=2 SV=1 - [BTBDH_HUMAN]	3.56	1	1	1	2	0.13	0.20	-0.27	-0.20	0.17	0.24	-0.06	0.02	-0.26	-0.33	-0.48	-0.42	-0.13	-0.61	-0.22	-0.43	-0.03	0.02	0.42	0.28
Q9Y4B6	Protein VPRBP OS=Homo sapiens GN=VPRBP PE=1 SV=3 - [VPRBP_HUMAN]	2.65	1	4	4	5	0.01	0.00	-0.11	-0.14	0.18	0.17	0.09	-0.05	-0.04	0.10	0.43	0.29	-0.03	0.25	0.68	0.13	0.39	0.34	0.29	0.28

O00115	Deoxyribonuclease-2-alpha OS=Homo sapiens GN=DNASE2 PE=1 SV=2 - [DNS2A_HUMAN]	10.28	1	4	4	5	0.54	0.57	0.41	0.55	0.48	0.57	0.74	0.57	0.71	0.76	0.82	0.85	0.07	0.29	0.33	0.18	0.46	0.01	0.05	0.27
O95817	BAG family molecular chaperone regulator 3 OS=Homo sapiens GN=BAG3 PE=1 SV=3 - [BAG3_HUMAN]	33.91	1	12	12	21	-0.43	-0.46	-0.29	-0.34	-0.29	-0.47	-0.19	-0.17	-0.11	-0.06	0.06	0.03	0.20	0.38	0.30	0.32	0.23	-0.11	-0.17	0.27
P62330	ADP-ribosylation factor 6 OS=Homo sapiens GN=ARF6 PE=1 SV=2 - [ARF6_HUMAN]	38.86	2	5	5	9	-0.87	-0.96	-0.09	-0.18	0.20	0.34	-0.02	-0.03	-0.07	-0.26	-0.32	-0.13	0.91	0.51	0.01	0.62	0.14	0.67	0.37	0.27
Q9GZP4	PITH domain-containing protein 1 OS=Homo sapiens GN=PITHD1 PE=1 SV=1 - [PITH1_HUMAN]	45.02	1	9	9	21	-0.18	-0.14	-0.22	-0.16	0.19	0.21	0.08	0.08	-0.01	-0.01	0.11	0.14	0.37	0.12	0.08	0.33	0.28	0.41	0.36	0.27
P11234	Ras-related protein Ral-B OS=Homo sapiens GN=RALB PE=1 SV=1 - [RALB_HUMAN]	47.57	1	4	7	15	-0.33	-0.06	-0.31	0.27	-0.11	0.19	-0.26	-0.19	0.12	-0.17	-0.17	-0.19	0.08	0.14	0.25	0.21	0.49	0.01	0.19	0.27
Q99417	C-Myc-binding protein OS=Homo sapiens GN=MYCBP PE=1 SV=3 - [MYCBP_HUMAN]	35.92	1	3	3	8	-0.91	-0.89	-0.71	-0.70	-0.36	-0.35	-0.43	-0.42	-0.17	-0.18	0.18	0.04	0.26	0.56	0.54	0.30	0.29	0.28	0.27	0.27
P63241	Eukaryotic translation initiation factor 5A-1 OS=Homo sapiens GN=EIF5A PE=1 SV=2 - [IF5A1_HUMAN]	68.83	3	11	11	34	-0.85	-0.85	-0.58	-0.61	-0.54	-0.50	-0.30	-0.33	-0.53	-0.53	-0.33	-0.32	0.55	0.52	0.18	0.39	-0.02	0.35	-0.02	0.27
P41240	Tyrosine-protein kinase CSK OS=Homo sapiens GN=CSK PE=1 SV=1 - [CSK_HUMAN]	20.67	1	8	8	14	-0.32	-0.32	-0.18	-0.16	-0.11	-0.13	0.04	0.05	-0.01	0.02	-0.30	-0.32	0.44	0.43	-0.12	0.38	0.24	0.24	0.18	0.27

P26373	60S ribosomal protein L13 OS=Homo sapiens GN=RPL13 PE=1 SV=4 - [RL13_HUMAN]	24.17	1	5	5	13	-0.51	-0.53	-0.57	-0.59	-0.60	-0.57	-0.37	-0.38	-0.45	-0.43	-0.30	-0.28	0.26	0.36	0.37	0.26	0.20	0.29	0.06	0.27
Q15126	Phosphomevalonate kinase OS=Homo sapiens GN=PMVK PE=1 SV=3 - [PMVK_HUMAN]	48.44	1	10	10	21	0.14	0.11	0.13	0.10	0.32	0.39	0.24	0.19	0.24	0.32	0.37	0.21	0.20	0.08	-0.02	0.16	0.18	0.18	0.32	0.27
P08240	Signal recognition particle receptor subunit alpha OS=Homo sapiens GN=SRPR PE=1 SV=2 - [SRPR_HUMAN]	12.70	2	7	7	8	-0.08	-0.16	-0.14	-0.22	0.50	0.51	0.04	-0.07	0.29	0.19	0.33	0.28	0.14	0.41	0.57	0.36	0.55	0.60	0.77	0.27
Q92597	Protein NDRG1 OS=Homo sapiens GN=NDRG1 PE=1 SV=1 - [NDRG1_HUMAN]	34.52	1	9	9	33	-0.51	-0.62	-0.52	-0.41	-0.01	0.23	0.01	-0.17	-0.22	-0.21	0.13	0.19	0.35	0.60	0.65	0.43	0.36	0.39	0.39	0.27
O95834	Echinoderm microtubule-associated protein-like 2 OS=Homo sapiens GN=EML2 PE=2 SV=1 - [EMAL2_HUMAN]	14.02	1	7	7	10	-0.52	-0.47	-0.34	-0.32	-0.20	-0.13	-0.22	-0.25	-0.23	-0.34	-0.53	-0.48	0.28	0.07	-0.15	0.12	0.09	0.23	0.18	0.27
Q96DG6	Carboxymethylenebutenolidase homolog OS=Homo sapiens GN=CMBL PE=1 SV=1 - [CMBL_HUMAN]	32.24	1	8	8	13	0.57	0.40	0.69	0.40	0.84	0.80	1.07	0.94	0.55	0.69	0.19	0.12	0.60	-0.16	-0.50	0.30	-0.03	0.29	0.12	0.27
P30040	Endoplasmic reticulum resident protein 29 OS=Homo sapiens GN=ERP29 PE=1 SV=4 - [ERP29_HUMAN]	45.59	1	10	10	29	-0.61	-0.54	-0.46	-0.38	-0.19	-0.09	-0.31	-0.30	-0.26	-0.25	-0.33	-0.36	0.45	0.29	0.15	0.28	0.26	0.48	0.36	0.27
Q9BQ67	Glutamate-rich WD repeat-containing protein 1 OS=Homo sapiens GN=GRWD1 PE=1 SV=1 - [GRWD1_HUMAN]	6.73	1	2	2	2	-0.35	0.11	-0.87	-0.25	-0.73	0.25	-0.70	-0.05	0.08	-0.32	-0.29	0.22	-0.11	0.12	0.48	0.00	0.37	0.12	0.49	0.27

P55263	Adenosine kinase OS=Homo sapiens GN=ADK PE=1 SV=2 - [ADK_HUMAN]	29.01	1	6	6	10	-0.29	-0.29	0.57	-0.02	-0.22	-0.02	0.70	0.19	-0.08	-0.07	-1.16	-0.82	0.15	-0.99	-1.86	0.25	-0.45	0.05	-0.80	0.27
O00487	26S proteasome non-ATPase regulatory subunit 14 OS=Homo sapiens GN=PSMD14 PE=1 SV=1 - [PSDE_HUMAN]	32.90	1	9	9	14	-0.33	-0.38	0.03	-0.06	0.10	0.11	0.20	0.12	-0.02	0.04	0.19	0.23	0.56	0.52	0.16	0.40	0.04	0.39	0.05	0.27
Q9BQA9	Uncharacterized protein C17orf62 OS=Homo sapiens GN=C17orf62 PE=1 SV=1 - [CQ062_HUMAN]	16.04	1	3	3	5	-0.06	-0.09	-0.51	-0.60	0.25	0.21	-0.27	-0.37	-0.06	-0.03	-0.03	-0.11	0.07	0.04	0.49	0.07	0.50	0.30	0.74	0.27
Q14353	Guanidinoacetate N-methyltransferase OS=Homo sapiens GN=GAMT PE=1 SV=1 - [GAMT_HUMAN]	25.00	1	4	4	10	1.09	1.73	0.46	1.11	0.58	1.22	0.66	1.31	1.27	0.63	0.27	0.91	-0.37	-0.81	-0.19	-0.42	0.21	-0.52	0.10	0.27
P18583	Protein SON OS=Homo sapiens GN=SON PE=1 SV=4 - [SON_HUMAN]	7.42	1	11	11	16	-0.43	-0.30	-0.25	-0.32	-0.09	-0.04	-0.13	-0.19	-0.03	-0.10	-0.11	-0.09	0.12	0.25	0.11	0.19	0.11	0.18	0.22	0.27
Q8N5K1	CDGSH iron-sulfur domain containing protein 2 OS=Homo sapiens GN=CISD2 PE=1 SV=1 - [CISD2_HUMAN]	40.74	1	5	5	12	-0.26	-0.09	-0.08	-0.09	0.08	0.03	0.18	0.34	0.14	-0.02	0.03	0.15	0.53	0.25	0.02	0.27	-0.01	0.11	-0.05	0.27
P83436	Conserved oligomeric Golgi complex subunit 7 OS=Homo sapiens GN=COG7 PE=1 SV=1 - [COG7_HUMAN]	1.69	1	1	1	1	0.45	0.17	0.33	0.05	0.59	0.30	0.53	0.25	0.42	0.70	0.63	0.35	0.13	0.19	0.30	0.28	0.41	0.12	0.24	0.27
P78537	Biogenesis of lysosome-related organelles complex 1 subunit 1 OS=Homo sapiens GN=BLOC1S1 PE=1 SV=2 - [BL1S1_HUMAN]	18.30	1	3	3	4	-0.30	-0.27	-0.53	-0.31	0.43	0.22	-0.28	-0.42	0.07	0.04	0.23	0.28	0.14	0.55	0.54	0.37	0.39	0.47	0.51	0.27

Q92952	Small conductance calcium-activated potassium channel protein 1 OS=Homo sapiens GN=KCNN1 PE=2 SV=2 - [KCNN1_HUMAN]	1.47	3	1	1	1	0.54	0.42	0.26	0.15	0.14	0.02	0.46	0.35	0.38	0.50	0.49	0.37	-0.02	-0.05	0.22	-0.01	0.27	-0.42	-0.14	0.26
P62826	GTP-binding nuclear protein Ran OS=Homo sapiens GN=RAN PE=1 SV=3 - [RAN_HUMAN]	32.41	1	8	8	16	-0.11	-0.16	0.08	0.14	0.33	0.29	0.30	0.32	0.13	0.14	-0.27	-0.13	0.47	-0.13	-0.35	0.33	0.12	0.32	0.20	0.26
Q9NTI5	Sister chromatid cohesion protein PDS5 homolog B OS=Homo sapiens GN=PDS5B PE=1 SV=1 - [PDS5B_HUMAN]	12.72	1	13	13	24	-0.89	-0.75	-0.59	-0.61	-0.09	-0.28	-0.18	-0.24	-0.26	-0.18	-0.09	-0.21	0.54	0.50	0.47	0.50	0.22	0.55	0.44	0.26
Q7Z7H5	Transmembrane emp24 domain-containing protein 4 OS=Homo sapiens GN=TMED4 PE=1 SV=1 - [TMED4_HUMAN]	23.35	1	3	4	6	-0.93	-0.72	-0.68	-0.47	-0.60	-0.40	-0.48	-0.27	-0.04	-0.24	-0.10	0.10	0.51	0.83	0.58	0.72	0.47	0.31	0.06	0.26
P19387	DNA-directed RNA polymerase II subunit RPB3 OS=Homo sapiens GN=POLR2C PE=1 SV=2 - [RPB3_HUMAN]	20.73	1	4	4	6	0.68	0.98	-0.36	-0.07	0.29	0.59	-0.16	0.13	0.41	0.12	-0.18	0.11	-0.79	-0.85	0.18	-0.54	0.51	-0.41	0.64	0.26
P62316	Small nuclear ribonucleoprotein Sm D2 OS=Homo sapiens GN=SNRPD2 PE=1 SV=1 - [SMD2_HUMAN]	55.93	1	7	7	17	-0.09	-0.24	-0.40	-0.40	0.12	0.14	-0.23	-0.17	0.03	-0.06	-0.05	0.01	0.14	0.23	0.24	0.30	0.26	0.33	0.50	0.26
O94979	Protein transport protein Sec31A OS=Homo sapiens GN=SEC31A PE=1 SV=3 - [SC31A_HUMAN]	16.97	1	18	18	40	-0.55	-0.46	-0.47	-0.57	0.12	0.13	-0.12	-0.22	-0.06	-0.08	-0.05	-0.10	0.36	0.42	0.45	0.43	0.43	0.69	0.58	0.26

Q99729	Heterogeneous nuclear ribonucleoprotein A/B OS=Homo sapiens GN=HNRNPAB PE=1 SV=2 - [ROAA_HUMAN]	18.67	1	4	5	15	-1.01	-1.12	-0.75	-0.86	-0.63	-0.54	-0.56	-0.59	-0.61	-0.68	-0.62	-0.48	0.33	0.67	0.62	0.52	0.27	0.62	0.55	0.26
Q96AX9	E3 ubiquitin-protein ligase MIB2 OS=Homo sapiens GN=MIB2 PE=1 SV=3 - [MIB2_HUMAN]	2.67	1	1	1	1	0.36	1.12	-0.10	0.65	0.53	1.28	0.09	0.85	1.29	0.54	0.39	1.14	-0.21	0.04	0.50	0.21	0.68	0.15	0.62	0.26
Q9UBB5	Methyl-CpG-binding domain protein 2 OS=Homo sapiens GN=MBD2 PE=1 SV=1 - [MBD2_HUMAN]	3.89	1	1	1	1	0.88	1.15	0.15	0.42	0.37	0.63	0.35	0.62	0.51	0.25	0.23	0.49	-0.47	-0.64	0.08	-0.60	0.13	-0.53	0.20	0.26
Q9UHN6	Transmembrane protein 2 OS=Homo sapiens GN=TMEM2 PE=1 SV=1 - [TMEM2_HUMAN]	2.39	1	2	2	3	-0.65	-0.21	-0.99	-0.56	0.24	0.67	-0.80	-0.36	-0.10	-0.53	-0.52	-0.09	-0.09	0.14	0.48	0.15	0.50	0.87	1.22	0.26
O43736	Integral membrane protein 2A OS=Homo sapiens GN=ITM2A PE=1 SV=2 - [ITM2A_HUMAN]	13.31	1	3	3	4	-0.66	-0.43	-0.89	-0.66	0.88	1.11	-0.69	-0.46	0.29	0.07	-0.08	0.15	0.03	0.59	0.81	0.76	0.99	1.53	1.75	0.26
Q9UPU5	Ubiquitin carboxyl-terminal hydrolase 24 OS=Homo sapiens GN=USP24 PE=1 SV=3 - [UBP24_HUMAN]	7.29	1	12	12	17	0.23	0.53	0.08	0.10	0.52	0.40	0.45	0.34	0.25	0.31	0.30	0.35	0.10	-0.05	0.02	-0.03	0.21	-0.02	0.16	0.26
Q5JVF3	PCI domain-containing protein 2 OS=Homo sapiens GN=PCID2 PE=1 SV=2 - [PCID2_HUMAN]	3.01	1	1	1	1	-0.62	-0.21	-0.37	0.04	0.10	0.51	-0.17	0.24	0.28	-0.12	-0.30	0.10	0.51	0.32	0.06	0.53	0.28	0.71	0.45	0.26

Q96IX5	Up-regulated during skeletal muscle growth protein 5 OS=Homo sapiens GN=USMG5 PE=1 SV=1 - [USMG5_HUMAN]	18.97	1	1	1	1	-0.89	-1.04	-0.09	-0.23	-0.03	-0.18	0.11	-0.03	0.23	0.38	0.52	0.38	1.06	1.42	0.61	1.30	0.50	0.85	0.04	0.26
Q9NRR5	Ubiquilin-4 OS=Homo sapiens GN=UBQLN4 PE=1 SV=2 - [UBQL4_HUMAN]	12.98	1	4	6	11	-0.17	-0.49	-0.03	-0.44	-0.05	-0.19	0.16	-0.13	0.00	0.14	0.14	0.06	0.39	0.45	0.64	0.52	0.48	0.29	0.52	0.26
O43149	Zinc finger ZZ-type and EF-hand domain-containing protein 1 OS=Homo sapiens GN=ZZEF1 PE=1 SV=6 - [ZZEF1_HUMAN]	4.90	1	11	11	18	0.01	0.10	0.15	0.00	0.28	0.15	0.28	0.19	0.20	0.22	0.28	0.14	0.37	0.06	-0.07	0.40	0.10	0.02	0.06	0.26
P33176	Kinesin-1 heavy chain OS=Homo sapiens GN=KIF5B PE=1 SV=1 - [KINH_HUMAN]	44.24	1	27	35	84	-0.37	-0.35	-0.04	-0.13	0.19	0.11	-0.03	0.02	-0.03	-0.06	-0.02	-0.05	0.42	0.28	0.20	0.40	0.23	0.48	0.30	0.26
P18085	ADP-ribosylation factor 4 OS=Homo sapiens GN=ARF4 PE=1 SV=3 - [ARF4_HUMAN]	64.44	1	6	10	41	-0.03	0.13	0.02	0.12	0.59	0.66	0.25	0.30	0.33	0.17	-0.02	0.15	0.28	0.02	-0.08	0.37	0.29	0.62	0.52	0.26
Q9NQ79	Cartilage acidic protein 1 OS=Homo sapiens GN=CRTAC1 PE=1 SV=2 - [CRAC1_HUMAN]	37.37	1	17	17	57	0.19	0.23	0.01	0.07	1.50	1.36	0.18	0.31	0.73	0.72	0.72	0.80	0.10	0.39	0.69	0.49	0.66	1.27	1.39	0.25
Q06136	3-ketodihydrosphingosine reductase OS=Homo sapiens GN=KDSR PE=1 SV=1 - [KDSR_HUMAN]	8.13	1	2	2	7	-0.62	-0.46	-0.45	-0.26	0.00	0.21	-0.22	0.00	0.06	-0.07	0.02	0.17	0.56	0.75	0.38	0.69	0.30	0.71	0.39	0.25
Q8IY33	MICAL-like protein 2 OS=Homo sapiens GN=MICALL2 PE=1 SV=1 - [MILK2_HUMAN]	3.43	1	2	2	2	-0.17	-0.02	-0.47	-0.32	-0.09	0.06	-0.28	-0.13	0.22	0.07	-0.16	-0.01	-0.05	0.02	0.31	0.28	0.58	0.06	0.36	0.25

Q16881	Thioredoxin reductase 1, cytoplasmic OS=Homo sapiens GN=TXNRD1 PE=1 SV=3 - [TRXR1_HUMAN]	21.73	1	10	10	18	0.03	-0.01	0.38	0.35	0.34	0.24	0.58	0.50	0.17	0.42	0.17	-0.02	0.60	-0.03	-0.30	0.17	-0.06	0.09	-0.10	0.25
Q9Y3D8	Adenylate kinase isoenzyme 6 OS=Homo sapiens GN=AK6 PE=1 SV=1 - [KAD6_HUMAN]	15.70	1	2	2	2	0.38	0.25	0.16	0.03	-0.09	-0.23	0.35	0.22	-0.21	-0.08	0.02	-0.12	0.02	-0.36	-0.15	-0.43	-0.20	-0.49	-0.27	0.25
Q9NVH0	Exonuclease 3'-5' domain-containing protein 2 OS=Homo sapiens GN=EXD2 PE=1 SV=2 - [EXD2_HUMAN]	9.02	1	4	4	5	-0.26	-0.41	-0.18	-0.33	0.35	0.19	0.01	-0.14	-0.41	-0.24	-0.04	-0.20	0.32	0.22	0.14	0.04	-0.03	0.58	0.51	0.25
P55735	Protein SEC13 homolog OS=Homo sapiens GN=SEC13 PE=1 SV=3 - [SEC13_HUMAN]	33.85	1	9	9	17	-0.34	-0.35	-0.41	-0.29	0.01	0.18	-0.19	0.00	0.10	-0.08	0.06	0.18	0.31	0.59	0.46	0.46	0.14	0.44	0.35	0.25
O43314	Inositol hexakisphosphate and diphosphoinositol-pentakisphosphate kinase 2 OS=Homo sapiens GN=PPIP5K2 PE=1 SV=3 - [VIP2_HUMAN]	5.55	1	3	4	5	-0.37	-0.35	-0.19	-0.18	-0.03	-0.02	0.00	0.01	-0.05	-0.06	0.32	0.33	0.42	0.70	0.52	0.34	0.17	0.32	0.15	0.25
Q8NC42	E3 ubiquitin-protein ligase RNF149 OS=Homo sapiens GN=RNF149 PE=2 SV=2 - [RN149_HUMAN]	6.25	1	1	1	1	-0.73	-0.92	-1.05	-1.25	-0.40	-0.60	-0.87	-1.06	-0.98	-0.77	-0.84	-1.04	-0.08	-0.11	0.21	-0.02	0.31	0.31	0.64	0.25
Q7KZN9	Cytochrome c oxidase assembly protein COX15 homolog OS=Homo sapiens GN=COX15 PE=1 SV=1 - [COX15_HUMAN]	1.95	1	1	1	2	-0.26	-0.31	-0.08	-0.12	0.01	-0.04	0.11	0.07	-0.18	-0.13	-0.27	-0.33	0.43	-0.01	-0.20	0.16	-0.02	0.25	0.07	0.25

O00423	Echinoderm microtubule-associated protein-like 1 OS=Homo sapiens GN=EML1 PE=1 SV=3 - [EMAL1_HUMAN]	9.82	1	8	8	8	-0.39	-0.45	-0.49	-0.54	-0.80	-0.86	-0.30	-0.35	-0.81	-0.75	-0.79	-0.85	0.15	-0.39	-0.30	-0.32	-0.22	-0.43	-0.33	0.25
Q96S52	GPI transamidase component PIG-S OS=Homo sapiens GN=PIGS PE=1 SV=3 - [PIGS_HUMAN]	9.55	1	5	5	10	-0.60	-0.76	-0.51	-0.58	-0.12	-0.25	-0.29	-0.37	-0.44	-0.30	-0.30	-0.23	0.45	0.55	0.21	0.35	0.22	0.47	0.35	0.25
P23396	40S ribosomal protein S3 OS=Homo sapiens GN=RPS3 PE=1 SV=2 - [RS3_HUMAN]	69.14	1	17	17	50	-0.79	-0.72	-0.85	-0.92	-0.14	-0.16	-0.56	-0.57	-0.35	-0.37	-0.27	-0.28	0.11	0.34	0.53	0.32	0.44	0.45	0.62	0.25
Q9UI30	tRNA methyltransferase 112 homolog OS=Homo sapiens GN=TRMT112 PE=1 SV=1 - [TR112_HUMAN]	21.60	1	2	2	3	0.21	0.07	-0.24	0.00	0.45	0.30	0.34	0.19	0.20	0.36	0.37	0.22	0.18	0.17	0.22	0.17	0.24	0.22	0.28	0.25
P15311	Ezrin OS=Homo sapiens GN=EZR PE=1 SV=4 - [EZRI_HUMAN]	47.61	1	16	28	70	0.08	0.07	-0.03	-0.10	0.35	0.15	0.31	0.22	0.30	0.31	0.09	0.07	0.14	0.00	-0.12	0.09	-0.01	0.11	0.15	0.25
Q6IPR1	LYR motif-containing protein 5 OS=Homo sapiens GN=LYRM5 PE=2 SV=2 - [LYRM5_HUMAN]	10.00	1	1	1	1	0.02	0.11	-0.57	-0.49	-0.33	-0.25	-0.39	-0.30	-0.46	-0.54	0.05	0.13	-0.35	0.04	0.63	-0.53	0.06	-0.37	0.22	0.25
Q9UMX3	Bcl-2-related ovarian killer protein OS=Homo sapiens GN=BOK PE=1 SV=1 - [BOK_HUMAN]	6.60	1	1	1	1	1.42	-0.34	0.31	-1.46	1.59	-0.18	0.49	-1.27	-0.93	0.84	0.85	-0.92	-0.87	-0.56	0.54	-0.55	0.56	0.15	1.26	0.25
Q6UXH1	Cysteine-rich with EGF-like domain protein 2 OS=Homo sapiens GN=CRELD2 PE=1 SV=1 - [CREL2_HUMAN]	8.78	1	3	3	4	-1.04	-1.02	-0.38	-0.36	0.17	0.18	-0.20	-0.18	-0.24	-0.25	-0.03	-0.01	0.90	1.02	0.35	0.82	0.16	1.19	0.53	0.25

Q13263	Transcription intermediary factor 1-beta OS=Homo sapiens GN=TRIM28 PE=1 SV=5 - [TIF1B_HUMAN]	29.10	1	14	14	38	-0.37	-0.39	-0.40	-0.43	-0.30	-0.36	-0.42	-0.43	-0.39	-0.36	-0.11	-0.21	0.04	0.19	0.28	0.11	0.01	0.01	0.11	0.25
Q13586	Stromal interaction molecule 1 OS=Homo sapiens GN=STIM1 PE=1 SV=3 - [STIM1_HUMAN]	13.28	1	6	7	9	-0.32	-0.07	-0.01	-0.04	0.06	0.31	0.08	0.06	0.11	-0.13	-0.15	0.09	0.49	0.48	0.17	0.21	0.23	0.38	0.21	0.25
Q16625	Occludin OS=Homo sapiens GN=OCLN PE=1 SV=1 - [OCLN_HUMAN]	8.43	1	3	3	4	-0.33	-0.52	-0.88	-1.06	-0.21	-0.40	-0.70	-0.88	0.26	0.45	-0.04	-0.23	-0.31	0.30	0.84	0.81	1.36	0.11	0.66	0.25
P13929	Beta-enolase OS=Homo sapiens GN=ENO3 PE=1 SV=5 - [ENOB_HUMAN]	19.35	1	1	5	74	0.32	0.19	0.23	0.13	0.25	0.29	0.33	0.31	0.32	0.45	-0.05	-0.19	0.08	-0.32	-0.31	0.16	0.26	-0.01	0.15	0.25
Q08999	Retinoblastoma-like protein 2 OS=Homo sapiens GN=RBL2 PE=1 SV=3 - [RBL2_HUMAN]	1.76	1	1	1	1	-0.46	-0.23	-1.11	-0.88	-0.55	-0.32	-0.92	-0.69	-0.67	-0.89	-1.00	-0.77	-0.41	-0.53	0.11	-0.40	0.25	-0.10	0.54	0.25
O43914	TYRO protein tyrosine kinase-binding protein OS=Homo sapiens GN=TYROBP PE=1 SV=1 - [TYOBP_HUMAN]	13.27	1	1	1	4	0.33	0.83	0.65	1.16	0.74	1.24	0.84	1.34	0.65	0.14	1.01	1.51	0.57	0.69	0.36	-0.15	-0.48	0.40	0.07	0.25
Q8TEQ8	GPI ethanolamine phosphate transferase 3 OS=Homo sapiens GN=PIGO PE=1 SV=3 - [PIGO_HUMAN]	1.74	1	2	2	5	-0.40	-0.42	-0.20	-0.22	0.10	0.07	-0.02	-0.04	0.05	0.07	0.17	0.14	0.44	0.58	0.36	0.51	0.31	0.48	0.28	0.25
Q9BW27	Nuclear pore complex protein Nup85 OS=Homo sapiens GN=NUP85 PE=1 SV=1 - [NUP85_HUMAN]	5.79	1	3	3	4	-0.44	-0.44	-0.36	-0.49	-0.16	-0.15	0.04	0.02	-0.32	-0.69	-0.02	-0.16	0.39	0.42	0.34	0.15	0.12	0.26	0.19	0.25

Q01970	1-phosphatidylinositol 4,5-bisphosphate phosphodiesterase beta-3 OS=Homo sapiens GN=PLCB3 PE=1 SV=2 - [PLCB3_HUMAN]	11.51	1	10	10	19	-0.14	-0.31	-0.31	-0.42	0.04	-0.15	-0.10	-0.29	-0.22	0.08	0.01	-0.13	-0.06	0.03	0.06	0.13	0.30	0.16	0.24	0.24
Q8TD55	Pleckstrin homology domain-containing family O member 2 OS=Homo sapiens GN=PLEKHO2 PE=2 SV=1 - [PKHO2_HUMAN]	23.47	1	9	9	17	0.18	0.19	-0.34	0.02	-0.02	0.04	0.08	0.16	0.08	-0.08	-0.10	0.02	-0.13	-0.25	0.19	-0.13	0.14	-0.08	0.22	0.24
Q9UHA4	Ragulator complex protein LAMTOR3 OS=Homo sapiens GN=LAMTOR3 PE=1 SV=1 - [LTOR3_HUMAN]	43.55	1	3	3	8	-0.26	-0.03	-0.40	-0.11	0.01	0.13	-0.16	0.06	0.37	0.10	-0.08	0.26	0.15	0.35	0.34	0.34	0.52	0.44	0.39	0.24
Q969V5	Mitochondrial ubiquitin ligase activator of NFKB 1 OS=Homo sapiens GN=MUL1 PE=1 SV=1 - [MUL1_HUMAN]	5.68	1	1	1	4	0.35	0.30	-0.09	-0.13	0.36	0.32	0.09	0.05	0.22	0.27	0.69	0.64	-0.20	0.34	0.77	-0.04	0.39	0.00	0.43	0.24
O60610	Protein diaphanous homolog 1 OS=Homo sapiens GN=DIAPH1 PE=1 SV=2 - [DIAP1_HUMAN]	12.50	1	12	12	23	-0.19	-0.19	-0.15	0.09	0.11	0.16	0.22	0.35	0.21	0.12	0.26	0.26	0.52	0.32	0.17	0.31	0.28	0.18	0.07	0.24
Q9P0M6	Core histone macro-H2A.2 OS=Homo sapiens GN=H2AFY2 PE=1 SV=3 - [H2AW_HUMAN]	48.39	1	14	16	41	-1.08	-1.09	-0.93	-0.94	-0.46	-0.64	-0.59	-0.70	-0.70	-0.57	-0.14	-0.10	0.45	1.04	0.80	0.62	0.26	0.47	0.23	0.24
P10746	Uroporphyrinogen-III synthase OS=Homo sapiens GN=UROS PE=1 SV=1 - [HEM4_HUMAN]	40.75	1	7	7	9	0.05	0.03	-0.29	-0.20	-0.35	-0.27	-0.04	-0.03	-0.08	-0.15	-0.31	-0.33	-0.07	-0.29	0.02	-0.11	0.01	-0.26	-0.08	0.24

Q9BSQ5	Malcavernin OS=Homo sapiens GN=CCM2 PE=1 SV=1 - [CCM2_HUMAN]	2.93	1	1	1	2	-0.32	-0.26	0.06	0.12	0.25	0.30	0.24	0.30	0.07	0.01	0.13	0.18	0.61	0.45	0.06	0.36	-0.02	0.55	0.17	0.24
Q5T0F9	Coiled-coil and C2 domain-containing protein 1B OS=Homo sapiens GN=CC2D1B PE=1 SV=1 - [C2D1B_HUMAN]	2.10	1	2	2	3	0.69	0.70	0.25	0.26	0.60	0.61	0.43	0.44	0.39	0.38	0.23	0.24	-0.21	-0.46	-0.02	-0.28	0.17	-0.11	0.34	0.24
Q9BZI7	Regulator of nonsense transcripts 3B OS=Homo sapiens GN=UPF3B PE=1 SV=1 - [REN3B_HUMAN]	12.22	1	4	4	5	0.17	0.32	0.00	0.15	-0.22	-0.08	0.18	0.32	0.22	0.08	0.10	0.24	0.06	-0.07	0.10	-0.06	0.11	-0.41	-0.24	0.24
Q6UX98	Probable palmitoyltransferase ZDHHC24 OS=Homo sapiens GN=ZDHHC24 PE=2 SV=1 - [ZDH24_HUMAN]	2.46	1	1	1	1	-0.42	-0.44	-0.23	-0.25	0.52	0.50	-0.05	-0.07	0.23	0.25	0.58	0.56	0.42	1.01	0.81	0.70	0.51	0.93	0.73	0.24
Q6R327	Rapamycin-insensitive companion of mTOR OS=Homo sapiens GN=RICTOR PE=1 SV=1 - [RICTR_HUMAN]	5.85	1	6	6	8	0.02	0.15	-0.22	-0.09	0.31	0.43	-0.04	0.09	0.45	0.32	0.48	0.60	-0.01	0.46	0.70	0.21	0.57	0.27	0.51	0.24
O75923	Dysferlin OS=Homo sapiens GN=DYSF PE=1 SV=1 - [DYSF_HUMAN]	2.50	1	3	4	7	-0.85	-0.64	-0.72	-0.51	-0.95	-1.12	-0.54	-0.33	-0.83	-1.03	-1.07	-0.87	0.36	-0.22	-0.35	-0.15	-0.28	-0.33	-0.21	0.24
Q969M7	NEDD8-conjugating enzyme UBE2F OS=Homo sapiens GN=UBE2F PE=1 SV=1 - [UBE2F_HUMAN]	26.49	1	3	3	6	0.54	0.51	0.15	0.24	0.42	0.21	0.31	0.64	-0.18	0.09	0.13	0.69	0.44	0.19	0.26	-0.65	-0.38	-0.21	-0.04	0.24
Q9P2C4	Transmembrane protein 181 OS=Homo sapiens GN=TMEM181 PE=1 SV=2 - [TM181_HUMAN]	3.92	1	2	2	4	-0.21	0.05	-0.30	0.23	0.29	0.78	-0.15	-0.13	0.14	0.07	0.38	0.57	0.11	0.76	0.68	0.35	0.40	0.74	0.57	0.24

A9UHW6	MIF4G domain-containing protein OS=Homo sapiens GN=MIF4GD PE=1 SV=1 - [MI4GD_HUMAN]	17.12	1	3	3	3	-1.14	-0.61	-0.12	0.41	-0.52	0.00	0.06	0.58	-0.19	-0.71	0.40	0.92	1.25	1.54	0.52	0.46	-0.56	0.60	-0.42	0.24
Q8IY95	Transmembrane protein 192 OS=Homo sapiens GN=TMEM192 PE=1 SV=1 - [TM192_HUMAN]	11.44	1	3	3	3	-0.35	-0.45	-0.06	-0.16	-0.13	-0.24	0.12	0.02	0.20	0.31	0.20	0.10	0.52	0.55	0.26	0.68	0.40	0.20	-0.09	0.24
P13646	Keratin, type I cytoskeletal 13 OS=Homo sapiens GN=KRT13 PE=1 SV=4 - [K1C13_HUMAN]	53.71	9	16	21	81	-3.73	-3.63	-3.82	-3.81	-3.51	-3.58	-3.72	-3.76	-3.69	-3.69	-3.95	-4.04	-0.08	0.02	0.43	0.25	0.50	0.06	0.44	0.24
Q9BTX1	Nucleoporin NDC1 OS=Homo sapiens GN=NDC1 PE=1 SV=2 - [NDC1_HUMAN]	1.48	1	1	1	1	0.69	0.62	-0.53	-0.60	0.00	-0.07	-0.35	-0.42	-0.97	-0.90	0.07	-0.01	-0.98	-0.61	0.60	-1.56	-0.34	-0.71	0.51	0.24
Q9Y679	Ancient ubiquitous protein 1 OS=Homo sapiens GN=AUP1 PE=1 SV=1 - [AUP1_HUMAN]	10.29	1	3	3	5	-0.27	-0.41	-0.04	-0.12	0.24	0.52	0.31	0.02	0.10	0.12	0.02	0.01	0.62	0.29	0.06	0.45	0.23	0.92	0.63	0.24
Q9H6K4	Optic atrophy 3 protein OS=Homo sapiens GN=OPA3 PE=1 SV=1 - [OPA3_HUMAN]	8.38	1	2	2	7	-0.07	0.04	-0.24	-0.24	-0.29	-0.20	-0.11	-0.06	-0.11	-0.13	-0.08	0.01	0.01	0.09	0.16	-0.12	0.19	-0.07	-0.07	0.24
Q8N5G0	Small integral membrane protein 20 OS=Homo sapiens GN=SMIM20 PE=1 SV=2 - [SMI20_HUMAN]	8.33	1	1	1	3	-0.39	-0.13	-0.45	-0.19	-0.03	0.22	-0.27	-0.02	0.00	-0.25	-0.05	0.20	0.17	0.34	0.39	0.17	0.23	0.35	0.40	0.24
Q13884	Beta-1-syntrophin OS=Homo sapiens GN=SNTB1 PE=1 SV=3 - [SNTB1_HUMAN]	24.54	1	9	10	19	-0.73	-0.74	-0.83	-0.62	-1.00	-0.81	-0.71	-0.49	-0.52	-0.59	-0.69	-0.55	0.28	-0.03	0.17	0.18	0.14	-0.06	-0.24	0.24

Q9BXW7	Cat eye syndrome critical region protein 5 OS=Homo sapiens GN=CECR5 PE=1 SV=1 - [CECR5_HUMAN]	5.20	1	2	2	3	-0.16	-0.53	-0.46	-0.82	-0.39	-0.77	-0.28	-0.65	-0.80	-0.43	-0.16	-0.53	-0.06	0.01	0.29	-0.24	0.06	-0.25	0.04	0.24
O14818	Proteasome subunit alpha type-7 OS=Homo sapiens GN=PSMA7 PE=1 SV=1 - [PSA7_HUMAN]	52.02	2	10	10	27	-0.32	-0.31	-0.14	-0.13	-0.16	-0.31	0.02	0.04	-0.05	-0.02	0.25	0.23	0.41	0.53	0.41	0.36	0.10	0.02	-0.19	0.24
Q15631	Translin OS=Homo sapiens GN=TSN PE=1 SV=1 - [TSN_HUMAN]	38.60	1	6	6	12	-0.41	-0.39	-0.11	-0.08	-0.10	-0.08	0.10	0.11	0.05	0.03	-0.15	-0.13	0.54	0.16	-0.23	0.47	-0.02	0.30	-0.15	0.24
Q712K3	Ubiquitin-conjugating enzyme E2 R2 OS=Homo sapiens GN=UBE2R2 PE=1 SV=1 - [UB2R2_HUMAN]	13.03	1	3	3	5	0.43	0.36	0.23	0.17	0.11	0.04	0.41	0.34	-0.18	-0.11	-0.07	-0.14	0.03	-0.50	-0.30	-0.51	-0.31	-0.34	-0.14	0.24
O94919	Endonuclease domain-containing 1 protein OS=Homo sapiens GN=ENDOD1 PE=1 SV=2 - [ENDD1_HUMAN]	22.80	1	9	9	37	0.06	0.10	-0.03	-0.06	0.14	0.10	0.04	-0.11	0.02	0.19	0.41	0.28	-0.15	0.23	0.34	0.12	0.27	0.13	0.08	0.24
Q9NQW7	Xaa-Pro aminopeptidase 1 OS=Homo sapiens GN=XPNPEP1 PE=1 SV=3 - [XPP1_HUMAN]	37.88	1	16	16	26	0.02	0.07	0.12	0.13	0.38	0.46	0.26	0.26	0.32	0.22	-0.03	0.11	0.26	-0.02	-0.09	0.20	0.14	0.12	0.24	0.24
P61224	Ras-related protein Rap-1b OS=Homo sapiens GN=RAP1B PE=1 SV=1 - [RAP1B_HUMAN]	69.02	2	3	8	48	-0.55	-0.59	-0.29	-0.19	-0.48	-0.36	-0.28	-0.16	-0.31	-0.37	-0.31	-0.30	0.12	0.03	-0.05	0.35	0.27	0.03	0.06	0.24
P35568	Insulin receptor substrate 1 OS=Homo sapiens GN=IRS1 PE=1 SV=1 - [IRS1_HUMAN]	2.25	1	2	2	3	0.36	0.23	0.07	-0.22	-0.04	-0.18	-0.33	-0.05	0.04	0.18	0.38	0.24	-0.64	0.02	0.31	-0.15	0.14	-0.42	-0.13	0.24

P54619	5'-AMP-activated protein kinase subunit gamma-1 OS=Homo sapiens GN=PRKAG1 PE=1 SV=1 - [AAKG1_HUMAN]	26.59	1	6	7	13	0.04	-0.02	-0.19	0.00	0.14	0.07	-0.19	0.07	0.38	0.20	0.15	0.33	-0.17	0.20	0.34	-0.02	0.43	0.21	0.03	0.24
Q96EC8	Protein YIPF6 OS=Homo sapiens GN=YIPF6 PE=1 SV=2 - [YIPF6_HUMAN]	5.08	1	1	1	3	-0.01	-0.20	-0.16	-0.34	0.23	0.03	0.02	-0.17	0.07	0.26	0.35	0.16	0.08	0.37	0.51	0.31	0.45	0.22	0.37	0.24
Q8N5X7	Eukaryotic translation initiation factor 4E type 3 OS=Homo sapiens GN=EIF4E3 PE=2 SV=4 - [IF4E3_HUMAN]	4.91	1	1	1	1	0.22	0.02	0.04	-0.16	0.44	0.24	0.21	0.01	-0.02	0.18	0.32	0.12	0.05	0.11	0.28	0.00	0.18	0.20	0.38	0.23
P62899	60S ribosomal protein L31 OS=Homo sapiens GN=RPL31 PE=1 SV=1 - [RL31_HUMAN]	33.60	1	5	5	12	-0.58	-0.66	-0.61	-0.62	-0.13	-0.38	-0.51	-0.49	-0.40	-0.52	-0.33	-0.37	0.33	0.17	0.16	0.21	0.21	0.16	0.24	0.23
P41743	Protein kinase C iota type OS=Homo sapiens GN=PRKCI PE=1 SV=2 - [KPCI_HUMAN]	2.35	1	1	1	2	0.22	0.52	0.27	0.56	0.16	0.45	0.44	0.73	0.51	0.23	0.11	0.40	0.27	-0.11	-0.16	0.03	-0.01	-0.08	-0.12	0.23
Q2TAZ0	Autophagy-related protein 2 homolog A OS=Homo sapiens GN=ATG2A PE=1 SV=3 - [ATG2A_HUMAN]	1.50	1	2	2	5	-0.41	-0.79	-0.39	-0.77	-0.20	-0.58	-0.22	-0.60	-0.23	0.15	0.14	-0.24	0.25	0.56	0.53	0.59	0.57	0.20	0.18	0.23
P50402	Emerin OS=Homo sapiens GN=EMD PE=1 SV=1 - [EMD_HUMAN]	27.17	1	7	7	19	-0.83	-0.67	-0.79	-0.62	-0.58	-0.55	-0.53	-0.44	-0.39	-0.52	-0.15	-0.07	0.35	0.62	0.41	0.36	0.29	0.18	0.20	0.23
Q9Y5Y2	Cytosolic Fe-S cluster assembly factor NUBP2 OS=Homo sapiens GN=NUBP2 PE=1 SV=1 - [NUBP2_HUMAN]	23.25	1	4	4	5	0.04	-0.04	-0.38	0.29	0.28	0.44	0.36	0.46	0.60	0.43	-0.16	0.59	0.55	0.64	0.31	0.42	0.84	0.22	0.64	0.23

P11182	Lipoamide acyltransferase component of branched-chain alpha-keto acid dehydrogenase complex, mitochondrial OS=Homo sapiens GN=DBT PE=1 SV=3 - [ODB2_HUMAN]	31.54	1	14	14	24	-0.82	-0.90	-0.68	-0.74	-0.43	-0.45	-0.57	-0.59	-0.52	-0.53	-0.48	-0.61	0.29	0.36	0.14	0.51	0.22	0.45	0.29	0.23
Q9NZ43	Vesicle transport protein USE1 OS=Homo sapiens GN=USE1 PE=1 SV=2 - [USE1_HUMAN]	10.04	1	2	2	5	-0.52	-0.60	-0.36	-0.57	-0.05	-0.47	-0.23	-0.31	-0.12	0.21	0.15	0.02	0.29	0.63	0.46	0.51	0.48	0.18	0.16	0.23
P16402	Histone H1.3 OS=Homo sapiens GN=HIST1H1D PE=1 SV=2 - [H13_HUMAN]	30.77	3	2	11	63	-1.00	-1.04	-0.83	-0.87	-0.90	-0.94	-0.66	-0.70	-1.12	-1.08	-0.79	-0.84	0.39	0.21	0.04	-0.05	-0.22	0.08	-0.09	0.23
P61313	60S ribosomal protein L15 OS=Homo sapiens GN=RPL15 PE=1 SV=2 - [RL15_HUMAN]	42.16	1	8	8	19	-0.74	-0.84	-0.62	-0.61	-0.44	-0.45	-0.47	-0.47	-0.37	-0.36	-0.22	-0.17	0.55	0.62	0.34	0.49	0.32	0.41	0.14	0.23
P30876	DNA-directed RNA polymerase II subunit RPB2 OS=Homo sapiens GN=POLR2B PE=1 SV=1 - [RPB2_HUMAN]	4.94	1	4	4	7	-0.22	-0.35	-0.37	-0.22	0.00	0.04	-0.20	-0.05	0.18	0.04	0.04	0.04	0.08	0.13	0.41	0.29	0.44	0.34	0.35	0.23
P57087	Junctional adhesion molecule B OS=Homo sapiens GN=JAM2 PE=1 SV=1 - [JAM2_HUMAN]	29.19	1	7	7	10	-0.50	-0.76	-0.61	-0.48	-0.49	-0.56	-0.04	-0.04	-0.11	0.09	0.76	0.55	0.77	1.32	1.01	0.48	0.41	-0.17	-0.09	0.23
Q92621	Nuclear pore complex protein Nup205 OS=Homo sapiens GN=NUP205 PE=1 SV=3 - [NU205_HUMAN]	5.17	1	8	8	11	-0.70	-0.64	-0.82	-0.76	-0.30	-0.24	-0.60	-0.58	-0.39	-0.45	-0.67	-0.37	0.14	0.35	0.31	0.29	0.41	0.30	0.60	0.23

P0DI81	Trafficking protein particle complex subunit 2 OS=Homo sapiens GN=TRAPPC2 PE=1 SV=1 - [TPC2A_HUMAN]	10.00	1	1	1	1	-0.57	-0.77	-0.08	-0.27	-0.28	-0.49	0.09	-0.10	-0.38	-0.18	-0.12	-0.32	0.72	0.45	-0.05	0.42	-0.07	0.27	-0.22	0.23
Q6P1N9	Putative deoxyribonuclease TATDN1 OS=Homo sapiens GN=TATDN1 PE=1 SV=2 - [TATD1_HUMAN]	10.44	1	3	3	5	-0.14	-0.04	-0.17	-0.06	-0.05	0.05	-0.06	0.04	-0.11	-0.21	-0.24	-0.14	0.26	-0.09	-0.17	-0.02	-0.05	0.07	0.10	0.23
Q9NY33	Dipeptidyl peptidase 3 OS=Homo sapiens GN=DPP3 PE=1 SV=2 - [DPP3_HUMAN]	25.24	1	15	15	20	-0.52	-0.47	-0.57	-0.50	-0.14	-0.06	-0.26	-0.06	-0.20	-0.35	-0.16	-0.12	0.31	0.38	0.39	0.19	0.10	0.30	0.20	0.23
Q14344	Guanine nucleotide-binding protein subunit alpha 13 OS=Homo sapiens GN=GNA13 PE=1 SV=2 - [GNA13_HUMAN]	45.89	2	14	16	45	-0.16	-0.19	-0.07	0.08	0.00	0.10	0.08	0.16	0.08	0.09	0.11	0.21	0.39	0.33	0.01	0.31	0.16	0.15	0.16	0.23
Q8NF37	Lysophosphatidylcholine acyltransferase 1 OS=Homo sapiens GN=LPCAT1 PE=1 SV=2 - [PCAT1_HUMAN]	8.61	1	4	4	6	0.03	0.12	-0.31	-0.22	-0.16	0.04	-0.04	0.09	-0.13	-0.20	-0.05	0.03	-0.10	-0.08	0.15	-0.07	0.14	-0.15	0.04	0.23
Q5T2E6	UPF0668 protein C10orf76 OS=Homo sapiens GN=C10orf76 PE=2 SV=1 - [CJ076_HUMAN]	4.64	1	3	3	4	-0.17	-0.35	-0.11	-0.29	-0.07	-0.26	0.06	-0.12	-0.31	-0.12	0.11	-0.08	0.28	0.28	0.22	0.08	0.02	0.08	0.02	0.23
P50135	Histamine N-methyltransferase OS=Homo sapiens GN=HNMT PE=1 SV=1 - [HNMT_HUMAN]	3.42	1	1	1	3	0.09	-0.04	0.24	0.12	-0.24	-0.37	0.41	0.28	-0.14	-0.02	-0.31	-0.44	0.38	-0.39	-0.55	-0.07	-0.22	-0.34	-0.50	0.23
P80108	Phosphatidylinositol-glycan-specific phospholipase D OS=Homo sapiens GN=GPLD1 PE=1 SV=3 - [PHLD_HUMAN]	1.79	1	1	1	1	-0.25	-0.58	-0.37	-0.70	0.36	0.03	-0.20	-0.53	-0.20	0.13	0.30	-0.04	0.10	0.56	0.67	0.41	0.54	0.60	0.72	0.23

Q9H307	Pinin OS=Homo sapiens GN=PNN PE=1 SV=4 - [PININ_HUMAN]	6.28	1	4	4	4	-0.10	-0.27	-0.54	-0.71	-0.38	-0.56	-0.37	-0.54	-0.51	-0.33	-0.42	-0.60	-0.22	-0.32	0.12	-0.21	0.24	-0.30	0.14	0.23
P60900	Proteasome subunit alpha type-6 OS=Homo sapiens GN=PSMA6 PE=1 SV=1 - [PSA6_HUMAN]	41.87	1	9	9	23	0.11	0.06	-0.15	-0.16	-0.05	-0.16	-0.02	-0.01	0.30	-0.06	0.29	0.47	0.13	0.43	0.46	0.26	0.19	-0.13	0.05	0.23
Q8NBZ7	UDP-glucuronic acid decarboxylase 1 OS=Homo sapiens GN=UXS1 PE=1 SV=1 - [UXS1_HUMAN]	4.29	1	1	1	2	-0.75	-0.71	-0.35	-0.31	0.02	0.05	-0.18	-0.14	-0.44	-0.47	0.46	0.49	0.63	1.22	0.81	0.31	-0.09	0.75	0.35	0.23
P23443	Ribosomal protein S6 kinase beta-1 OS=Homo sapiens GN=RPS6KB1 PE=1 SV=2 - [KS6B1_HUMAN]	4.19	1	2	2	5	0.44	-0.21	0.09	-0.55	0.53	-0.13	0.26	-0.39	-0.13	0.52	0.20	-0.45	-0.12	-0.23	0.10	0.12	0.46	0.07	0.42	0.23
P22415	Upstream stimulatory factor 1 OS=Homo sapiens GN=USF1 PE=1 SV=1 - [USF1_HUMAN]	6.13	1	1	1	1	0.86	-0.04	0.29	-0.60	0.00	-0.90	0.46	-0.44	-0.97	-0.07	0.18	-0.72	-0.34	-0.67	-0.12	-0.90	-0.33	-0.87	-0.31	0.23
P13647	Keratin, type II cytoskeletal 5 OS=Homo sapiens GN=KRT5 PE=1 SV=3 - [K2C5_HUMAN]	36.78	9	11	21	48	-2.82	-2.71	-3.22	-3.11	-2.36	-2.21	-3.57	-3.66	-3.11	-3.22	-2.45	-2.28	-0.59	0.22	1.35	0.10	0.54	0.62	0.47	0.23
P49641	Alpha-mannosidase 2x OS=Homo sapiens GN=MAN2A2 PE=2 SV=3 - [MA2A2_HUMAN]	10.17	1	9	9	14	-0.10	-0.15	0.07	-0.04	1.10	1.04	0.13	0.11	0.72	0.79	1.07	1.01	0.18	1.17	1.02	0.89	0.69	1.18	1.01	0.23
Q9H7D0	Dedicator of cytokinesis protein 5 OS=Homo sapiens GN=DOCK5 PE=1 SV=3 - [DOCK5_HUMAN]	4.17	1	6	8	9	-0.63	-0.55	-0.34	-0.40	0.10	0.15	-0.46	-0.42	-0.17	-0.17	-0.10	-0.07	0.26	0.57	0.43	0.36	0.27	0.69	0.51	0.23

O75436	Vacuolar protein sorting-associated protein 26A OS=Homo sapiens GN=VPS26A PE=1 SV=2 - [VP26A_HUMAN]	39.14	1	10	11	23	-0.12	0.02	0.07	0.08	0.25	0.30	0.25	0.15	0.15	0.18	0.27	0.19	0.49	0.44	0.10	0.41	0.19	0.31	0.13	0.23
Q8IX05	CD302 antigen OS=Homo sapiens GN=CD302 PE=1 SV=1 - [CD302_HUMAN]	8.19	1	1	1	1	0.04	0.24	-0.22	-0.01	0.10	0.30	-0.05	0.15	-0.11	-0.31	0.17	0.36	-0.03	0.13	0.38	-0.31	-0.06	0.05	0.30	0.23
Q14669	E3 ubiquitin-protein ligase TRIP12 OS=Homo sapiens GN=TRIP12 PE=1 SV=1 - [TRIPC_HUMAN]	6.58	1	10	10	13	-0.26	-0.22	-0.48	-0.51	-0.27	-0.20	-0.27	-0.23	-0.16	-0.37	-0.10	-0.01	0.14	0.24	0.39	0.07	0.41	0.12	0.33	0.23
P60981	Destrin OS=Homo sapiens GN=DSTN PE=1 SV=3 - [DEST_HUMAN]	71.52	1	10	12	48	-0.46	-0.57	-0.19	-0.19	0.15	0.21	-0.14	-0.09	-0.21	-0.12	-0.40	-0.38	0.48	0.12	-0.14	0.44	0.08	0.57	0.28	0.23
Q12965	Unconventional myosin-Ie OS=Homo sapiens GN=MYO1E PE=1 SV=2 - [MYO1E_HUMAN]	13.90	1	10	11	17	-0.33	-0.33	-0.25	-0.21	0.01	0.08	-0.04	0.01	-0.14	-0.20	-0.09	-0.09	0.43	0.29	0.09	0.33	0.40	0.58	0.39	0.23
Q9Y5B6	PAX3- and PAX7-binding protein 1 OS=Homo sapiens GN=PAXBP1 PE=1 SV=2 - [PAXB1_HUMAN]	1.53	1	1	1	2	0.20	-0.04	0.21	-0.03	0.45	0.21	0.37	0.14	0.15	0.39	0.64	0.40	0.23	0.45	0.43	0.22	0.21	0.24	0.23	0.23
O43488	Aflatoxin B1 aldehyde reductase member 2 OS=Homo sapiens GN=AKR7A2 PE=1 SV=3 - [ARK72_HUMAN]	34.82	1	9	10	28	0.34	0.16	0.01	0.04	0.13	0.08	0.07	0.10	-0.06	0.00	-0.12	-0.20	0.19	-0.35	-0.13	0.03	0.02	-0.15	0.11	0.23
Q01518	Adenylyl cyclase-associated protein 1 OS=Homo sapiens GN=CAP1 PE=1 SV=5 - [CAP1_HUMAN]	44.63	1	16	18	38	-0.54	-0.51	-0.46	-0.44	-0.16	-0.14	-0.23	-0.17	-0.20	-0.22	-0.24	-0.14	0.37	0.48	0.28	0.36	0.33	0.42	0.48	0.23

P20645	Cation-dependent mannose-6-phosphate receptor OS=Homo sapiens GN=M6PR PE=1 SV=1 - [MPRD_HUMAN]	20.58	1	4	5	10	-0.46	-0.48	-1.03	-0.84	-0.18	-0.10	-0.87	-0.67	-0.15	-0.20	0.06	0.20	-0.19	0.74	0.69	0.22	0.35	0.20	0.28	0.23
Q16769	Glutaminyl-peptide cyclotransferase OS=Homo sapiens GN=QPCT PE=1 SV=1 - [QPCT_HUMAN]	12.47	1	4	4	5	-0.83	-0.72	-0.78	-0.67	0.92	1.03	-0.62	-0.50	0.32	0.21	0.34	0.45	0.27	1.18	1.12	1.07	1.02	1.73	1.68	0.23
Q13136	Liprin-alpha-1 OS=Homo sapiens GN=PPFIA1 PE=1 SV=1 - [LIPA1_HUMAN]	15.56	1	8	14	23	-0.09	-0.06	-0.21	-0.19	-0.07	0.06	-0.08	0.02	0.01	-0.10	-0.18	-0.01	-0.01	-0.09	0.19	0.02	0.12	-0.01	0.25	0.23
P18077	60S ribosomal protein L35a OS=Homo sapiens GN=RPL35A PE=1 SV=2 - [RL35A_HUMAN]	20.91	1	3	3	5	-0.74	-0.86	-0.52	-0.64	-0.05	-0.17	-0.35	-0.47	-0.16	-0.04	0.08	-0.04	0.44	0.83	0.60	0.73	0.51	0.68	0.45	0.23
P55145	Mesencephalic astrocyte-derived neurotrophic factor OS=Homo sapiens GN=MANF PE=1 SV=3 - [MANF_HUMAN]	25.82	1	4	4	7	-1.01	-0.92	-0.69	-0.69	-0.74	-0.86	-0.57	-0.56	-0.94	-0.75	-0.67	-0.78	0.47	0.31	-0.02	0.20	-0.16	0.21	-0.08	0.23
P14868	Aspartate--tRNA ligase, cytoplasmic OS=Homo sapiens GN=DARS PE=1 SV=2 - [SYDC_HUMAN]	45.51	1	20	20	54	0.09	0.08	0.08	0.06	0.30	0.29	0.17	0.14	0.21	0.24	0.28	0.25	0.19	0.18	0.16	0.22	0.27	0.27	0.32	0.23
O75828	Carbonyl reductase [NADPH] 3 OS=Homo sapiens GN=CBR3 PE=1 SV=3 - [CBR3_HUMAN]	38.63	1	5	10	30	0.74	0.63	0.42	0.31	0.52	0.40	0.38	0.49	0.03	0.19	0.18	0.05	-0.20	-0.81	-0.34	-0.46	-0.04	-0.66	-0.02	0.23
Q8NEQ5	Transmembrane protein C1orf162 OS=Homo sapiens GN=C1orf162 PE=2 SV=1 - [CA162_HUMAN]	14.84	1	1	1	1	-2.57	-2.64	-2.91	-2.98	-1.88	-1.96	-2.74	-2.82	-1.80	-1.72	-1.85	-1.93	-0.12	0.73	1.06	0.87	1.22	0.67	1.01	0.23

P61011	Signal recognition particle 54 kDa protein OS=Homo sapiens GN=SRP54 PE=1 SV=1 - [SRP54_HUMAN]	23.81	1	9	9	21	-0.19	-0.24	0.00	0.00	0.02	-0.03	0.09	0.02	-0.16	-0.14	-0.24	-0.29	0.34	-0.18	-0.27	0.22	-0.14	0.17	-0.01	0.23
Q96K76	Ubiquitin carboxyl-terminal hydrolase 47 OS=Homo sapiens GN=USP47 PE=1 SV=3 - [UBP47_HUMAN]	8.80	1	9	9	13	-0.03	0.15	0.20	0.26	0.29	0.46	0.28	0.33	0.14	0.10	0.31	0.27	0.28	0.18	0.00	0.22	0.09	0.26	0.36	0.23
Q13724	Mannosyl-oligosaccharide glucosidase OS=Homo sapiens GN=MOGS PE=1 SV=5 - [MOGS_HUMAN]	21.15	1	13	13	21	-0.69	-0.47	-0.56	-0.52	-0.04	-0.08	-0.44	-0.37	-0.19	-0.27	-0.09	-0.05	0.35	0.49	0.42	0.48	0.30	0.46	0.44	0.23
P15586	N-acetylglucosamine-6-sulfatase OS=Homo sapiens GN=GNS PE=1 SV=3 - [GNS_HUMAN]	9.78	1	4	4	7	0.31	0.32	0.02	0.07	0.32	0.31	0.15	0.13	0.15	0.16	0.20	0.19	0.07	0.06	0.20	0.12	0.31	0.18	0.14	0.23
P14550	Alcohol dehydrogenase [NADP(+)] OS=Homo sapiens GN=AKR1A1 PE=1 SV=3 - [AK1A1_HUMAN]	44.31	1	13	13	32	0.88	0.86	1.05	0.91	0.84	0.89	1.13	1.12	0.93	0.85	0.18	0.19	0.33	-0.61	-0.86	0.02	-0.20	0.08	-0.09	0.23
P36969	Phospholipid hydroperoxide glutathione peroxidase, mitochondrial OS=Homo sapiens GN=GPX4 PE=1 SV=3 - [GPX4_HUMAN]	49.24	1	10	10	26	-0.08	0.02	0.27	0.31	0.68	0.77	0.39	0.45	0.50	0.51	0.60	0.69	0.43	0.70	0.39	0.57	0.15	0.60	0.31	0.22
P23458	Tyrosine-protein kinase JAK1 OS=Homo sapiens GN=JAK1 PE=1 SV=2 - [JAK1_HUMAN]	5.72	1	5	6	8	-0.38	-0.24	-0.21	0.01	0.23	0.39	-0.03	0.04	0.03	-0.25	0.01	0.14	0.52	0.39	0.15	0.34	0.05	0.71	0.33	0.22

Q5VUD6	Protein FAM69B OS=Homo sapiens GN=FAM69B PE=2 SV=3 - [FA69B_HUMAN]	2.78	1	1	1	1	0.08	-0.12	0.15	-0.04	0.57	0.37	0.31	0.12	0.48	0.68	0.55	0.35	0.29	0.48	0.40	0.63	0.56	0.48	0.40	0.22
Q9GZT4	Serine racemase OS=Homo sapiens GN=SRR PE=1 SV=1 - [SRR_HUMAN]	15.59	1	4	4	7	-0.10	-0.13	-0.13	-0.20	0.27	0.39	-0.19	-0.13	-0.16	-0.08	-0.14	-0.25	0.03	-0.02	-0.09	0.05	0.08	0.20	0.38	0.22
Q99996	A-kinase anchor protein 9 OS=Homo sapiens GN=AKAP9 PE=1 SV=3 - [AKAP9_HUMAN]	2.20	1	6	6	7	-0.03	-0.09	-0.13	-0.25	0.09	0.07	-0.02	-0.19	-0.12	0.06	-0.17	-0.17	-0.10	-0.16	0.05	0.06	0.04	0.12	0.26	0.22
O14613	Cdc42 effector protein 2 OS=Homo sapiens GN=CDC42EP2 PE=1 SV=1 - [BORG1_HUMAN]	9.52	1	1	1	1	0.05	0.28	-0.45	-0.21	1.71	1.94	-0.29	-0.05	0.90	0.67	0.28	0.51	-0.28	0.24	0.73	0.65	1.15	1.65	2.14	0.22
Q8TEQ6	Gem-associated protein 5 OS=Homo sapiens GN=GEMIN5 PE=1 SV=3 - [GEMI5_HUMAN]	5.77	1	5	5	6	-0.20	-0.09	-0.02	-0.25	0.21	0.12	0.08	-0.02	0.01	0.13	0.13	0.12	0.07	0.27	0.41	0.13	0.21	0.24	0.35	0.22
Q8IV36	Protein HID1 OS=Homo sapiens GN=HID1 PE=1 SV=1 - [HID1_HUMAN]	2.79	1	1	1	1	-0.28	0.42	-0.33	0.36	0.53	1.22	-0.18	0.52	0.72	0.03	-0.07	0.62	0.16	0.21	0.26	0.34	0.40	0.79	0.85	0.22
P63244	Guanine nucleotide-binding protein subunit beta-2-like 1 OS=Homo sapiens GN=GNB2L1 PE=1 SV=3 - [GBLP_HUMAN]	57.10	1	13	13	30	-0.92	-0.73	-0.75	-0.63	-0.27	-0.22	-0.54	-0.41	-0.28	-0.38	-0.33	-0.23	0.48	0.41	0.33	0.47	0.21	0.56	0.36	0.22
P01621	Ig kappa chain V-III region NG9 (Fragment) OS=Homo sapiens PE=1 SV=1 - [KV303_HUMAN]	38.00	1	2	2	3	-2.92	-2.18	-1.97	-1.24	-0.95	-0.22	-1.82	-1.08	-0.46	-1.19	-0.69	0.04	1.16	2.23	1.28	1.76	0.81	1.96	1.01	0.22

Q9Y2W2	WW domain-binding protein 11 OS=Homo sapiens GN=WBP11 PE=1 SV=1 - [WBP11_HUMAN]	9.05	1	4	4	9	-1.40	-1.52	-1.07	-1.40	-0.05	-0.46	-0.91	-1.06	-0.91	-0.49	-0.38	-0.59	0.48	1.01	0.57	0.29	0.40	0.88	0.80	0.22
Q9UKA2	F-box/LRR-repeat protein 4 OS=Homo sapiens GN=FBXL4 PE=1 SV=2 - [FBXL4_HUMAN]	3.22	1	1	1	1	-1.11	0.44	-1.92	-0.37	-1.34	0.20	-1.76	-0.21	-0.06	-1.61	-1.05	0.50	-0.60	0.07	0.87	-0.47	0.34	-0.25	0.56	0.22
O75351	Vacuolar protein sorting-associated protein 4B OS=Homo sapiens GN=VPS4B PE=1 SV=2 - [VPS4B_HUMAN]	33.78	2	6	11	21	-0.57	-0.32	-0.56	-0.40	0.13	0.08	-0.34	-0.33	-0.07	-0.10	-0.15	-0.15	0.69	0.50	0.38	0.72	0.42	0.85	0.51	0.22
Q7L1Q6	Basic leucine zipper and W2 domain-containing protein 1 OS=Homo sapiens GN=BZW1 PE=1 SV=1 - [BZW1_HUMAN]	23.39	1	8	9	16	-0.32	-0.42	-0.09	-0.16	0.32	0.20	0.12	0.04	0.10	0.12	0.21	0.15	0.46	0.49	0.28	0.48	0.19	0.65	0.36	0.22
P46781	40S ribosomal protein S9 OS=Homo sapiens GN=RPS9 PE=1 SV=3 - [RS9_HUMAN]	34.54	1	9	9	21	-0.75	-0.80	-0.70	-0.66	-0.23	-0.30	-0.53	-0.49	-0.33	-0.30	-0.15	-0.18	0.35	0.64	0.54	0.43	0.45	0.35	0.41	0.22
O94992	Protein HEXIM1 OS=Homo sapiens GN=HEXIM1 PE=1 SV=1 - [HEXI1_HUMAN]	10.86	1	3	3	4	-0.54	-0.72	-0.04	-0.46	0.08	0.05	0.06	-0.20	-0.13	0.07	0.18	-0.14	0.65	0.50	0.23	0.63	0.27	0.60	0.40	0.22
Q96QC0	Serine/threonine-protein phosphatase 1 regulatory subunit 10 OS=Homo sapiens GN=PPP1R10 PE=1 SV=1 - [PP1RA_HUMAN]	0.85	1	1	1	1	-0.71	-0.78	-0.70	-0.78	-0.01	-0.09	-0.54	-0.62	-0.15	-0.07	0.03	-0.05	0.22	0.74	0.73	0.67	0.66	0.69	0.68	0.22

O15211	Ral guanine nucleotide dissociation stimulator-like 2 OS=Homo sapiens GN=RGL2 PE=1 SV=1 - [RGL2_HUMAN]	3.35	1	2	2	2	0.28	0.74	-0.64	-0.18	-0.29	0.16	-0.48	-0.03	0.01	-0.44	-0.07	0.38	-0.71	-0.34	0.57	-0.69	0.23	-0.59	0.33	0.22
P56192	Methionine--tRNA ligase, cytoplasmic OS=Homo sapiens GN=MARS PE=1 SV=2 - [SYMC_HUMAN]	26.67	1	18	18	39	0.08	0.06	0.07	-0.02	0.36	0.20	0.20	0.22	0.18	0.19	0.14	0.06	0.16	-0.07	-0.04	0.06	0.27	0.19	0.27	0.22
Q9BUP3	Oxidoreductase HTATIP2 OS=Homo sapiens GN=HTATIP2 PE=1 SV=2 - [HTAI2_HUMAN]	4.55	1	1	1	1	-0.41	0.22	-0.56	0.08	-0.01	0.63	-0.40	0.24	0.43	-0.20	-0.50	0.13	0.07	-0.08	0.05	0.24	0.39	0.39	0.53	0.22
O14972	Down syndrome critical region protein 3 OS=Homo sapiens GN=DSCR3 PE=2 SV=1 - [DSCR3_HUMAN]	20.20	1	4	4	5	0.21	0.54	0.42	0.47	0.18	0.23	0.40	0.44	0.51	0.28	0.03	0.28	0.42	-0.33	-0.41	0.00	0.07	-0.28	-0.27	0.22
P26885	Peptidyl-prolyl cis-trans isomerase FKBP2 OS=Homo sapiens GN=FKBP2 PE=1 SV=2 - [FKBP2_HUMAN]	54.93	1	7	7	13	-0.41	-0.18	-0.29	-0.07	-0.06	0.17	-0.02	0.14	-0.04	-0.14	-0.20	-0.11	0.37	0.32	0.03	0.32	0.04	0.41	0.22	0.22
P23469	Receptor-type tyrosine-protein phosphatase epsilon OS=Homo sapiens GN=PTPRE PE=1 SV=1 - [PTPRE_HUMAN]	5.00	1	1	4	6	1.32	1.02	0.49	0.19	0.94	0.63	0.65	0.35	0.74	1.05	1.32	1.01	-0.62	0.00	0.82	-0.24	0.58	-0.40	0.43	0.22
P62854	40S ribosomal protein S26 OS=Homo sapiens GN=RPS26 PE=1 SV=3 - [RS26_HUMAN]	20.87	2	2	2	7	-0.28	-0.27	-0.42	-0.47	-0.03	-0.04	-0.37	-0.35	0.01	-0.19	-0.06	0.06	-0.03	0.22	0.40	0.22	0.32	0.21	0.50	0.22

Q9UNX3	60S ribosomal protein L26-like 1 OS=Homo sapiens GN=RPL26L1 PE=1 SV=1 - [RL26L_HUMAN]	28.28	2	5	5	12	-0.77	-0.72	-0.53	-0.61	-0.36	-0.32	-0.45	-0.46	-0.54	-0.53	-0.27	-0.29	0.37	0.50	0.33	0.26	0.04	0.34	0.29	0.22
P31942	Heterogeneous nuclear ribonucleoprotein H3 OS=Homo sapiens GN=HNRNPH3 PE=1 SV=2 - [HNRH3_HUMAN]	41.33	1	9	10	80	-0.41	-0.50	-0.48	-0.58	-0.31	-0.45	-0.35	-0.44	-0.45	-0.42	-0.40	-0.34	0.11	0.12	0.10	0.05	0.14	0.06	0.17	0.22
P78344	Eukaryotic translation initiation factor 4 gamma 2 OS=Homo sapiens GN=EIF4G2 PE=1 SV=1 - [IF4G2_HUMAN]	17.09	1	13	14	26	-0.02	-0.01	0.06	0.06	0.17	0.26	0.10	0.15	0.18	0.17	-0.01	0.01	0.20	0.01	-0.04	0.23	0.22	0.25	0.28	0.22
Q9NZN8	CCR4-NOT transcription complex subunit 2 OS=Homo sapiens GN=CNOT2 PE=1 SV=1 - [CNOT2_HUMAN]	8.15	1	3	3	7	-0.22	-0.24	-0.70	-0.73	-0.23	-0.25	-0.14	-0.15	-0.30	-0.28	0.25	0.00	-0.28	0.20	0.68	-0.19	0.30	-0.09	0.40	0.22
Q15036	Sorting nexin-17 OS=Homo sapiens GN=SNX17 PE=1 SV=1 - [SNX17_HUMAN]	24.04	1	9	9	15	-0.11	0.20	-0.20	0.07	0.31	0.59	0.03	0.33	0.21	0.09	0.06	0.18	0.12	-0.08	0.07	0.06	0.13	0.31	0.29	0.22
P67936	Tropomyosin alpha-4 chain OS=Homo sapiens GN=TPM4 PE=1 SV=3 - [TPM4_HUMAN]	48.39	2	7	16	197	-0.83	-0.75	-0.84	-0.83	-0.27	-0.27	-0.63	-0.55	-0.50	-0.57	-0.47	-0.48	0.21	0.38	0.47	0.21	0.20	0.57	0.79	0.22
Q9GZQ3	COMM domain-containing protein 5 OS=Homo sapiens GN=COMMD5 PE=1 SV=1 - [COMD5_HUMAN]	22.32	1	5	5	8	0.22	0.19	0.05	0.03	0.33	0.31	0.19	0.18	0.43	0.33	0.39	0.47	0.04	0.29	0.45	0.27	0.44	0.10	0.27	0.22
P60228	Eukaryotic translation initiation factor 3 subunit E OS=Homo sapiens GN=EIF3E PE=1 SV=1 - [EIF3E_HUMAN]	28.09	1	9	9	14	-0.69	-0.69	-0.51	-0.49	-0.06	-0.05	-0.45	-0.33	-0.32	-0.26	-0.13	-0.13	0.39	0.81	0.33	0.69	0.25	0.99	0.53	0.21

P50440	Glycine amidinotransferase, mitochondrial OS=Homo sapiens GN=GATM PE=1 SV=1 - [GATM_HUMAN]	36.88	1	12	12	19	0.07	-0.16	0.25	0.27	-0.11	-0.22	0.28	0.28	0.07	0.07	0.52	0.54	0.41	0.46	0.38	0.10	-0.20	-0.14	-0.39	0.21
Q8IV08	Phospholipase D3 OS=Homo sapiens GN=PLD3 PE=1 SV=1 - [PLD3_HUMAN]	21.63	1	8	8	16	0.28	0.33	0.56	0.68	0.87	0.96	0.66	0.78	0.95	0.64	0.52	0.62	0.23	0.40	0.28	0.45	0.28	0.60	0.47	0.21
Q53GQ0	Estradiol 17-beta-dehydrogenase 12 OS=Homo sapiens GN=HSD17B12 PE=1 SV=2 - [DHB12_HUMAN]	17.31	1	7	7	14	-0.89	-0.83	-0.46	-0.60	-0.19	-0.20	-0.31	-0.47	-0.41	-0.36	-0.09	-0.25	0.50	0.64	0.37	0.54	0.19	0.72	0.45	0.21
O60486	Plexin-C1 OS=Homo sapiens GN=PLXNC1 PE=1 SV=1 - [PLXC1_HUMAN]	2.81	1	4	4	4	0.46	0.35	-0.25	-0.27	0.25	0.13	-0.10	-0.12	0.45	0.49	0.92	0.89	-0.25	0.71	0.92	0.14	0.36	-0.22	0.79	0.21
Q9UQN3	Charged multivesicular body protein 2b OS=Homo sapiens GN=CHMP2B PE=1 SV=1 - [CHM2B_HUMAN]	25.82	1	7	7	8	-0.25	-0.26	0.09	-0.08	0.25	0.29	0.19	0.07	0.11	0.18	0.21	0.17	0.56	0.42	0.23	0.39	0.22	0.54	0.26	0.21
P46778	60S ribosomal protein L21 OS=Homo sapiens GN=RPL21 PE=1 SV=2 - [RL21_HUMAN]	25.62	1	4	4	13	-0.92	-0.95	-0.65	-0.65	-0.05	-0.06	-0.47	-0.55	-0.27	-0.24	0.06	0.05	0.45	0.84	0.68	0.54	0.26	0.66	0.20	0.21
P49585	Choline-phosphate cytidylyltransferase A OS=Homo sapiens GN=PCYT1A PE=1 SV=2 - [PCY1A_HUMAN]	21.80	2	7	7	10	0.50	0.53	0.29	0.32	0.45	0.50	0.44	0.49	0.53	0.51	0.59	0.63	-0.08	0.02	0.17	0.06	0.25	-0.05	0.15	0.21
Q9UHI5	Large neutral amino acids transporter small subunit 2 OS=Homo sapiens GN=SLC7A8 PE=1 SV=1 - [LAT2_HUMAN]	2.06	1	1	1	2	0.90	1.52	0.78	1.39	0.22	0.83	0.92	1.54	1.18	0.57	-0.06	0.55	0.08	-0.95	-0.83	-0.30	-0.17	-0.70	-0.57	0.21

P00918	Carbonic anhydrase 2 OS=Homo sapiens GN=CA2 PE=1 SV=2 - [CAH2_HUMAN]	60.77	1	14	14	85	-0.65	-0.68	-0.50	-0.42	-0.47	-0.43	-0.30	-0.25	-0.55	-0.46	-0.52	-0.51	0.50	0.14	-0.04	0.15	0.00	0.28	0.01	0.21
P01714	Ig lambda chain V-III region SH OS=Homo sapiens PE=1 SV=1 - [LV301_HUMAN]	25.00	1	2	2	6	-2.08	-2.23	-2.06	-2.17	-0.79	-0.87	-1.87	-1.82	-0.66	-0.74	0.46	0.31	0.37	2.55	2.53	1.46	1.54	1.39	1.28	0.21
Q04864	Proto-oncogene c-Rel OS=Homo sapiens GN=REL PE=1 SV=1 - [REL_HUMAN]	4.36	1	2	2	3	0.02	0.21	-0.17	0.02	-0.13	0.06	-0.02	0.17	0.18	-0.01	0.25	0.44	0.02	0.24	0.42	0.01	0.20	-0.16	0.03	0.21
Q9UNI6	Dual specificity protein phosphatase 12 OS=Homo sapiens GN=DUSP12 PE=1 SV=1 - [DUS12_HUMAN]	3.24	1	1	1	1	0.39	-0.16	0.25	-0.31	0.28	-0.28	0.40	-0.16	-0.35	0.21	-0.04	-0.60	0.06	-0.43	-0.29	-0.16	-0.01	-0.13	0.01	0.21
P46019	Phosphorylase b kinase regulatory subunit alpha, liver isoform OS=Homo sapiens GN=PHKA2 PE=1 SV=1 - [KPB2_HUMAN]	3.48	1	3	4	6	0.59	0.78	0.35	0.55	0.82	1.01	0.50	0.69	0.40	0.22	0.79	0.98	-0.03	0.21	0.44	-0.34	-0.11	0.22	0.45	0.21
Q8WWH5	Probable tRNA pseudouridine synthase 1 OS=Homo sapiens GN=TRUB1 PE=1 SV=1 - [TRUB1_HUMAN]	3.44	1	1	1	1	-0.74	-0.14	-0.16	0.44	-0.26	0.33	-0.01	0.59	0.17	-0.42	-0.67	-0.08	0.79	0.07	-0.52	0.35	-0.23	0.46	-0.12	0.21
P23258	Tubulin gamma-1 chain OS=Homo sapiens GN=TUBG1 PE=1 SV=2 - [TBG1_HUMAN]	29.05	2	10	10	16	-0.35	-0.23	-0.54	-0.37	-0.32	-0.18	-0.29	-0.19	-0.01	-0.02	-0.12	-0.11	0.09	0.33	0.45	0.13	0.32	0.04	0.37	0.21
Q00013	55 kDa erythrocyte membrane protein OS=Homo sapiens GN=MPP1 PE=1 SV=2 - [EM55_HUMAN]	48.93	1	14	14	28	0.13	0.23	0.29	0.25	0.12	0.20	0.39	0.40	0.25	0.24	0.45	0.50	0.03	0.27	0.16	0.15	0.03	-0.03	-0.09	0.21

Q53FT3	Protein Hikeshi OS=Homo sapiens GN=C11orf73 PE=1 SV=2 - [HIKES_HUMAN]	12.69	1	2	2	3	-0.06	-0.46	-0.02	-0.41	-0.01	-0.41	0.13	-0.27	-0.39	0.02	-0.06	-0.46	0.25	0.01	-0.04	0.11	0.06	0.03	-0.01	0.21
Q7Z3U7	Protein MON2 homolog OS=Homo sapiens GN=MON2 PE=1 SV=2 - [MON2_HUMAN]	3.08	1	5	5	5	-0.68	-0.75	-0.22	-0.29	0.22	0.15	-0.08	-0.15	-0.11	-0.03	-0.02	-0.09	0.66	0.67	0.20	0.68	0.22	0.89	0.42	0.21
P05165	Propionyl-CoA carboxylase alpha chain, mitochondrial OS=Homo sapiens GN=PCCA PE=1 SV=4 - [PCCA_HUMAN]	36.13	1	21	21	48	-0.13	-0.12	-0.22	-0.21	-0.07	-0.14	-0.14	-0.15	-0.10	-0.10	-0.08	-0.09	0.08	0.04	0.06	0.14	0.13	0.01	-0.02	0.21
Q92542	Nicastrin OS=Homo sapiens GN=NCSTN PE=1 SV=2 - [NICA_HUMAN]	12.27	1	7	7	20	-0.44	-0.58	-0.53	-0.73	0.24	0.27	-0.22	-0.32	0.17	0.24	0.57	0.00	0.26	0.78	0.56	0.25	0.43	0.54	0.28	0.21
Q9NT62	Ubiquitin-like-conjugating enzyme ATG3 OS=Homo sapiens GN=ATG3 PE=1 SV=1 - [ATG3_HUMAN]	21.66	1	6	6	13	-0.13	0.05	-0.13	-0.07	-0.05	-0.02	0.07	0.05	-0.04	-0.10	-0.01	0.03	0.02	0.03	0.06	0.15	0.10	0.04	0.03	0.21
Q92896	Golgi apparatus protein 1 OS=Homo sapiens GN=GLG1 PE=1 SV=2 - [GSLG1_HUMAN]	19.51	1	21	21	35	-0.44	-0.59	-0.58	-0.55	-0.03	-0.05	-0.46	-0.47	-0.19	-0.06	0.13	0.05	0.14	0.64	0.69	0.41	0.46	0.52	0.52	0.21
P55072	Transitional endoplasmic reticulum ATPase OS=Homo sapiens GN=VCP PE=1 SV=4 - [TERA_HUMAN]	55.09	5	33	33	110	-0.37	-0.34	-0.32	-0.34	-0.25	-0.20	-0.14	-0.15	-0.13	-0.14	0.06	0.12	0.32	0.50	0.38	0.28	0.21	0.20	0.08	0.21
P54136	Arginine--tRNA ligase, cytoplasmic OS=Homo sapiens GN=RARS PE=1 SV=2 - [SYRC_HUMAN]	34.70	1	19	19	29	-0.09	-0.05	-0.23	-0.19	-0.01	0.06	-0.07	0.06	-0.01	-0.09	-0.08	-0.05	0.17	-0.01	0.02	0.23	0.18	0.22	0.15	0.21

Q9Y2V2	Calcium-regulated heat stable protein 1 OS=Homo sapiens GN=CARHSP1 PE=1 SV=2 - [CHSP1_HUMAN]	21.77	1	4	4	7	-0.49	-0.23	-0.61	-0.46	-0.45	-0.30	-0.46	-0.31	-0.35	-0.50	-0.68	-0.53	0.09	-0.18	-0.07	0.12	0.15	-0.06	0.14	0.21
Q9NWX6	Probable tRNA(His) guanylyltransferase OS=Homo sapiens GN=THG1L PE=1 SV=2 - [THG1_HUMAN]	3.69	1	1	1	1	-0.73	-0.59	-0.78	-0.63	-0.49	-0.35	-0.63	-0.49	-0.55	-0.69	-0.76	-0.62	0.16	-0.02	0.01	0.07	0.12	0.23	0.27	0.21
P52435	DNA-directed RNA polymerase II subunit RPB11-a OS=Homo sapiens GN=POLR2J PE=1 SV=1 - [RPB11_HUMAN]	22.22	3	2	2	3	-1.05	-1.19	-0.24	-0.38	0.02	-0.13	-0.10	-0.24	-0.30	-0.15	0.09	-0.05	1.01	1.15	0.33	0.93	0.12	1.06	0.24	0.21
P83881	60S ribosomal protein L36a OS=Homo sapiens GN=RPL36A PE=1 SV=2 - [RL36A_HUMAN]	37.74	1	2	5	17	-0.90	-0.85	-0.77	-0.69	-0.41	-0.42	-0.69	-0.55	-0.40	-0.53	-0.42	-0.28	0.37	0.59	0.42	0.38	0.29	0.58	0.26	0.21
P11021	78 kDa glucose-regulated protein OS=Homo sapiens GN=HSPA5 PE=1 SV=2 - [GRP78_HUMAN]	55.96	1	35	38	211	-0.63	-0.71	-0.60	-0.61	-0.25	-0.29	-0.44	-0.41	-0.35	-0.36	-0.17	-0.24	0.28	0.53	0.40	0.36	0.29	0.42	0.35	0.21
O00505	Importin subunit alpha 4 OS=Homo sapiens GN=KPNA3 PE=1 SV=2 - [IMA4_HUMAN]	11.32	1	3	5	10	-0.30	-0.17	-0.05	0.08	-0.15	-0.02	0.09	0.22	-0.10	-0.22	-0.48	-0.35	0.44	-0.17	-0.43	0.11	-0.14	0.14	-0.11	0.20
O00170	AH receptor-interacting protein OS=Homo sapiens GN=AIP PE=1 SV=2 - [AIP_HUMAN]	33.64	1	9	9	22	0.03	0.17	0.08	0.27	0.02	0.06	0.17	0.17	0.12	-0.18	-0.11	0.10	0.32	-0.06	-0.20	-0.02	-0.16	-0.33	-0.14	0.20
Q9UNZ2	NSFL1 cofactor p47 OS=Homo sapiens GN=NSFL1C PE=1 SV=2 - [NSF1C_HUMAN]	40.00	1	12	12	50	-0.10	-0.01	-0.09	0.06	-0.18	-0.04	0.06	0.12	0.01	-0.03	-0.17	-0.06	0.22	0.05	-0.04	0.01	0.03	-0.01	-0.02	0.20

Q5ZPR3	CD276 antigen OS=Homo sapiens GN=CD276 PE=1 SV=1 - [CD276_HUMAN]	5.24	1	2	2	4	-0.56	-0.62	-0.31	-0.36	-0.08	-0.14	-0.17	-0.22	-0.17	-0.11	0.02	-0.04	0.45	0.58	0.32	0.48	0.23	0.47	0.21	0.20
Q96GZ6	Solute carrier family 41 member 3 OS=Homo sapiens GN=SLC41A3 PE=2 SV=2 - [S41A3_HUMAN]	3.55	1	1	1	4	-0.82	-0.88	-0.52	-0.57	-0.28	-0.33	-0.38	-0.43	-0.55	-0.50	-0.08	-0.13	0.50	0.75	0.45	0.36	0.06	0.53	0.23	0.20
P18124	60S ribosomal protein L7 OS=Homo sapiens GN=RPL7 PE=1 SV=1 - [RL7_HUMAN]	46.37	1	13	13	38	-0.90	-0.81	-0.58	-0.53	-0.42	-0.42	-0.43	-0.43	-0.40	-0.41	-0.25	-0.23	0.43	0.61	0.37	0.51	0.21	0.37	0.17	0.20
Q9H1Y0	Autophagy protein 5 OS=Homo sapiens GN=ATG5 PE=1 SV=2 - [ATG5_HUMAN]	24.73	1	6	6	9	0.48	0.65	0.11	0.48	0.34	0.10	0.37	0.25	0.12	-0.16	0.48	0.23	0.23	-0.23	-0.24	-0.45	-0.27	-0.44	-0.39	0.20
O43175	D-3-phosphoglycerate dehydrogenase OS=Homo sapiens GN=PHGDH PE=1 SV=4 - [SERA_HUMAN]	41.84	1	19	19	61	0.36	0.31	0.41	0.41	0.20	0.13	0.55	0.54	0.25	0.31	0.15	0.08	0.27	-0.21	-0.29	0.03	-0.10	0.03	-0.13	0.20
P46779	60S ribosomal protein L28 OS=Homo sapiens GN=RPL28 PE=1 SV=3 - [RL28_HUMAN]	43.80	1	7	7	12	-0.86	-0.71	-0.72	-0.74	-0.43	-0.44	-0.48	-0.54	-0.38	-0.35	-0.34	-0.35	0.22	0.34	0.38	0.39	0.26	0.35	0.28	0.20
Q13619	Cullin-4A OS=Homo sapiens GN=CUL4A PE=1 SV=3 - [CUL4A_HUMAN]	15.02	1	3	11	19	-0.13	-0.01	-0.30	-0.18	-0.26	-0.14	-0.16	-0.04	-0.07	-0.19	-0.16	-0.05	0.02	-0.03	0.14	-0.03	0.14	-0.14	0.03	0.20
Q9BWS9	Chitinase domain-containing protein 1 OS=Homo sapiens GN=CHID1 PE=1 SV=1 - [CHID1_HUMAN]	19.59	1	6	6	9	-0.60	-0.69	-1.00	-0.99	-0.54	-0.55	-0.91	-0.79	-0.72	-0.68	-0.53	-0.40	-0.22	0.08	0.52	0.04	0.42	0.13	0.45	0.20
Q02878	60S ribosomal protein L6 OS=Homo sapiens GN=RPL6 PE=1 SV=3 - [RL6_HUMAN]	27.08	1	9	9	27	-0.59	-0.65	-0.52	-0.52	-0.28	-0.28	-0.42	-0.44	-0.34	-0.31	-0.22	-0.18	0.26	0.68	0.37	0.31	0.23	0.28	0.26	0.20

O60462	Neuropilin-2 OS=Homo sapiens GN=NRP2 PE=1 SV=2 - [NRP2_HUMAN]	7.20	1	5	5	9	0.42	0.25	0.20	0.06	0.83	0.66	0.36	0.14	0.51	0.62	0.91	0.66	-0.19	0.42	0.76	0.38	0.51	0.48	0.63	0.20
Q9Y263	Phospholipase A-2-activating protein OS=Homo sapiens GN=PLAA PE=1 SV=2 - [PLAP_HUMAN]	21.64	1	12	12	19	0.45	0.26	0.08	0.20	0.32	0.34	0.26	0.26	0.35	0.32	0.29	0.15	-0.03	-0.03	-0.22	-0.08	0.29	0.18	0.21	0.20
Q13596	Sorting nexin-1 OS=Homo sapiens GN=SNX1 PE=1 SV=3 - [SNX1_HUMAN]	36.59	1	14	16	37	-0.24	-0.24	-0.21	-0.15	0.07	0.17	0.11	0.10	-0.01	-0.03	-0.03	-0.01	0.26	0.32	0.22	0.30	0.25	0.37	0.23	0.20
Q9UBU8	Mortality factor 4-like protein 1 OS=Homo sapiens GN=MORF4L1 PE=1 SV=2 - [MO4L1_HUMAN]	7.46	1	2	2	3	0.39	0.59	-0.35	-0.15	-0.37	-0.17	-0.21	-0.01	-0.04	-0.24	-0.10	0.10	-0.55	-0.48	0.25	-0.60	0.14	-0.78	-0.04	0.20
P07948	Tyrosine-protein kinase Lyn OS=Homo sapiens GN=LYN PE=1 SV=3 - [LYN_HUMAN]	23.24	11	8	12	24	0.04	-0.14	0.11	0.04	0.45	0.36	0.35	0.22	0.18	0.37	0.34	0.23	0.33	0.36	0.09	0.29	0.17	0.32	0.24	0.20
Q8TEX9	Importin-4 OS=Homo sapiens GN=IPO4 PE=1 SV=2 - [IPO4_HUMAN]	1.57	1	1	1	1	0.17	0.26	0.38	0.48	0.96	1.05	0.51	0.61	0.52	0.43	0.12	0.22	0.40	-0.04	-0.26	0.29	0.08	0.78	0.56	0.20
Q8N2U9	PQ-loop repeat-containing protein 1 OS=Homo sapiens GN=PQLC1 PE=2 SV=1 - [PQLC1_HUMAN]	4.80	1	1	1	2	-0.50	-0.46	-0.13	-0.08	-0.17	-0.13	0.01	0.05	-0.33	-0.37	-0.32	-0.28	0.56	0.19	-0.19	0.17	-0.20	0.31	-0.06	0.20
Q9UM22	Mammalian ependymin-related protein 1 OS=Homo sapiens GN=EPDR1 PE=1 SV=2 - [EPDR1_HUMAN]	18.75	1	4	4	10	-0.35	-0.26	0.04	0.09	0.23	0.41	0.00	0.12	0.27	0.12	0.50	0.46	0.43	0.60	0.50	0.34	0.27	0.35	0.24	0.20
P35443	Thrombospondin-4 OS=Homo sapiens GN=THBS4 PE=1 SV=2 - [TSP4_HUMAN]	3.12	1	2	2	3	-1.57	-2.06	-0.51	-0.48	0.46	0.49	0.34	-0.35	0.31	0.31	0.52	0.03	1.97	2.10	1.03	1.92	0.83	2.02	0.97	0.20

P56537	Eukaryotic translation initiation factor 6 OS=Homo sapiens GN=EIF6 PE=1 SV=1 - [IF6_HUMAN]	24.08	1	4	4	7	-0.36	-0.37	-0.33	-0.65	0.45	0.43	-0.42	-0.52	0.37	0.47	0.48	0.46	0.00	0.65	0.81	0.47	0.84	0.78	0.84	0.20
Q9UI10	Translation initiation factor eIF-2B subunit delta OS=Homo sapiens GN=EIF2B4 PE=1 SV=2 - [EI2BD_HUMAN]	18.93	1	7	7	9	-0.05	-0.12	-0.03	-0.09	0.17	0.17	0.01	-0.05	0.00	0.04	0.22	0.20	0.16	0.21	0.31	0.16	0.20	0.17	0.17	0.20
P42766	60S ribosomal protein L35 OS=Homo sapiens GN=RPL35 PE=1 SV=2 - [RL35_HUMAN]	8.13	1	1	1	4	-0.38	-0.60	-1.09	-0.97	-0.51	-0.73	-0.84	-0.73	-0.67	-0.61	-0.42	-0.62	-0.18	-0.01	0.36	0.12	0.35	-0.11	0.24	0.20
P62847	40S ribosomal protein S24 OS=Homo sapiens GN=RPS24 PE=1 SV=1 - [RS24_HUMAN]	19.55	1	2	2	7	-0.97	-1.08	-0.68	-0.81	-0.67	-0.83	-0.54	-0.59	-0.67	-0.56	-0.44	-0.55	0.48	0.52	0.23	0.45	0.15	0.18	-0.06	0.20
P05198	Eukaryotic translation initiation factor 2 subunit 1 OS=Homo sapiens GN=EIF2S1 PE=1 SV=3 - [IF2A_HUMAN]	46.03	1	13	13	25	-0.72	-0.63	-0.33	-0.37	-0.26	-0.27	-0.23	-0.27	-0.34	-0.36	-0.37	-0.32	0.37	0.33	0.03	0.32	0.10	0.31	0.09	0.19
P09429	High mobility group protein B1 OS=Homo sapiens GN=HMGB1 PE=1 SV=3 - [HMGB1_HUMAN]	26.05	2	8	9	28	-1.37	-1.53	-0.78	-0.97	-0.63	-0.77	-0.72	-0.90	-0.89	-0.71	-0.07	-0.28	0.73	1.54	0.75	0.81	0.13	0.82	0.11	0.19
Q8NEU8	DCC-interacting protein 13-beta OS=Homo sapiens GN=APPL2 PE=1 SV=3 - [DP13B_HUMAN]	15.81	1	7	7	12	-0.02	-0.06	0.07	0.25	0.75	0.81	0.22	0.52	0.39	0.43	0.39	0.37	0.38	0.49	0.10	0.49	0.42	0.73	0.53	0.19
Q9NP85	Podocin OS=Homo sapiens GN=NPHS2 PE=1 SV=1 - [PODO_HUMAN]	2.09	1	1	1	1	1.14	1.08	0.72	0.67	1.38	1.32	0.85	0.79	1.21	1.27	0.87	0.81	-0.23	-0.26	0.15	0.16	0.59	0.22	0.64	0.19

Q9UPZ6	Thrombospondin type-1 domain-containing protein 7A OS=Homo sapiens GN=THSD7A PE=1 SV=4 - [THS7A_HUMAN]	5.37	1	7	8	10	-0.33	-0.39	-0.48	-0.29	-0.25	-0.12	-0.36	-0.11	-0.41	-0.46	-0.44	-0.40	0.26	-0.04	0.15	0.04	0.08	0.27	0.06	0.19
P35240	Merlin OS=Homo sapiens GN=NF2 PE=1 SV=1 - [MERL_HUMAN]	1.51	1	1	1	1	-0.78	-0.74	-0.16	-0.12	0.12	0.16	-0.03	0.01	-0.09	-0.13	-0.26	-0.22	0.81	0.53	-0.09	0.69	0.07	0.89	0.27	0.19
Q68CQ7	Glycosyltransferase 8 domain-containing protein 1 OS=Homo sapiens GN=GLT8D1 PE=1 SV=2 - [GL8D1_HUMAN]	3.77	1	1	1	1	-1.29	-0.66	-1.16	-0.53	-0.02	0.60	-1.03	-0.40	0.10	-0.53	0.19	0.81	0.32	1.48	1.34	0.79	0.66	1.25	1.12	0.19
Q9BYD1	39S ribosomal protein L13, mitochondrial OS=Homo sapiens GN=MRPL13 PE=1 SV=1 - [RM13_HUMAN]	22.47	1	3	3	6	0.11	0.35	0.03	0.19	0.06	0.24	0.20	0.32	0.42	0.25	0.08	0.21	0.02	-0.14	0.02	0.17	0.25	-0.07	0.09	0.19
Q10570	Cleavage and polyadenylation specificity factor subunit 1 OS=Homo sapiens GN=CPSF1 PE=1 SV=2 - [CPSF1_HUMAN]	2.49	1	3	3	5	-0.24	-0.26	-0.40	-0.53	-0.15	-0.37	-0.13	-0.34	-0.28	-0.39	-0.01	-0.23	0.08	0.23	0.33	0.01	0.24	-0.02	0.07	0.19
Q9BQ39	ATP-dependent RNA helicase DDX50 OS=Homo sapiens GN=DDX50 PE=1 SV=1 - [DDX50_HUMAN]	5.02	1	2	2	3	-0.99	-0.61	-0.85	-0.46	-0.76	-0.38	-0.72	-0.33	-0.34	-0.72	-0.62	-0.24	0.33	0.38	0.23	0.31	0.16	0.21	0.07	0.19
Q9Y5B9	FACT complex subunit SPT16 OS=Homo sapiens GN=SUPT16H PE=1 SV=1 - [SP16H_HUMAN]	14.04	1	12	13	19	-0.19	-0.09	-0.10	-0.10	0.04	0.16	0.03	-0.09	-0.12	0.01	-0.03	0.00	0.07	-0.01	0.05	0.07	0.14	0.13	0.32	0.19

Q15147	1-phosphatidylinositol 4,5-bisphosphate phosphodiesterase beta-4 OS=Homo sapiens GN=PLCB4 PE=1 SV=3 - [PLCB4_HUMAN]	6.55	1	6	6	8	-0.06	-0.01	0.25	0.30	-0.03	0.00	0.26	0.34	0.08	0.07	-0.03	0.03	0.51	0.15	-0.22	0.29	-0.02	0.14	-0.22	0.19
Q9NRG9	Aladin OS=Homo sapiens GN=AAAS PE=1 SV=1 - [AAAS_HUMAN]	6.96	1	2	2	2	-0.88	-0.82	-0.43	-0.37	-0.33	-0.28	-0.31	-0.25	-0.13	-0.18	-0.28	-0.22	0.63	0.61	0.16	0.72	0.28	0.53	0.09	0.19
Q9Y4W2	Ribosomal biogenesis protein LAS1L OS=Homo sapiens GN=LAS1L PE=1 SV=2 - [LAS1L_HUMAN]	5.45	1	2	2	2	-0.29	-0.14	-0.26	-0.10	0.43	0.59	-0.13	0.03	0.16	0.01	0.21	0.36	0.22	0.51	0.46	0.33	0.30	0.71	0.67	0.19
Q03519	Antigen peptide transporter 2 OS=Homo sapiens GN=TAP2 PE=1 SV=1 - [TAP2_HUMAN]	12.24	1	6	6	10	-0.85	-0.47	-0.82	-0.40	0.08	0.29	-0.46	-0.23	-0.07	-0.45	-0.03	0.16	0.25	0.70	0.66	0.34	0.25	0.91	0.77	0.19
P62995	Transformer-2 protein homolog beta OS=Homo sapiens GN=TRA2B PE=1 SV=1 - [TRA2B_HUMAN]	13.54	1	2	3	6	-0.30	-0.50	-0.09	-0.29	0.16	-0.04	0.04	-0.16	-0.31	-0.10	0.05	-0.15	0.40	0.36	0.14	0.23	0.02	0.45	0.24	0.19
P0DJI8	Serum amyloid A-1 protein OS=Homo sapiens GN=SAA1 PE=1 SV=1 - [SAA1_HUMAN]	43.44	1	4	5	19	-0.12	-0.07	-0.13	0.00	0.76	0.96	-0.26	-0.04	0.21	0.20	0.29	0.55	-0.14	0.11	0.52	0.35	0.52	0.82	0.95	0.19
Q13057	Bifunctional coenzyme A synthase OS=Homo sapiens GN=COASY PE=1 SV=4 - [COASY_HUMAN]	11.17	1	4	4	8	-0.06	-0.04	0.13	0.07	0.06	-0.07	0.04	0.00	-0.12	-0.09	-0.17	-0.21	0.18	0.01	-0.21	-0.01	-0.11	0.16	0.07	0.19
Q15427	Splicing factor 3B subunit 4 OS=Homo sapiens GN=SF3B4 PE=1 SV=1 - [SF3B4_HUMAN]	8.96	1	2	2	4	-0.20	-0.13	-0.29	-0.23	-0.23	-0.17	-0.16	-0.10	-0.32	-0.38	-0.43	-0.27	0.09	0.17	-0.01	-0.15	-0.06	-0.05	0.05	0.19

O60524	Nuclear export mediator factor NEMF OS=Homo sapiens GN=NEMF PE=1 SV=4 - [NEMF_HUMAN]	8.64	1	8	8	9	0.21	-0.11	-0.05	-0.24	0.37	0.29	-0.09	-0.16	0.09	0.17	-0.01	-0.18	-0.38	-0.21	0.06	-0.19	0.38	-0.16	0.40	0.19
P16930	Fumarylacetoacetase OS=Homo sapiens GN=FAH PE=1 SV=2 - [FAAA_HUMAN]	19.57	1	6	6	10	-0.06	-0.05	0.02	0.17	0.18	0.14	-0.04	0.29	0.07	-0.14	-0.71	-0.56	0.45	-0.64	-0.84	0.38	0.12	0.23	-0.04	0.19
O60826	Coiled-coil domain-containing protein 22 OS=Homo sapiens GN=CCDC22 PE=1 SV=1 - [CCD22_HUMAN]	27.59	1	12	12	26	-0.05	0.23	-0.24	0.08	0.26	0.32	0.04	0.07	0.13	0.13	-0.03	0.05	0.00	-0.08	-0.02	0.00	0.22	-0.03	0.22	0.19
P28072	Proteasome subunit beta type-6 OS=Homo sapiens GN=PSMB6 PE=1 SV=4 - [PSB6_HUMAN]	20.50	1	4	4	6	-0.17	-0.31	0.07	-0.13	0.47	0.49	0.08	0.10	0.35	0.33	0.69	0.56	0.42	0.86	0.69	0.33	0.29	0.63	0.59	0.19
O14980	Exportin-1 OS=Homo sapiens GN=XPO1 PE=1 SV=1 - [XPO1_HUMAN]	16.53	1	15	15	34	-0.13	-0.28	-0.20	-0.08	0.03	0.03	0.04	0.02	0.06	0.10	0.25	0.17	0.24	0.46	0.30	0.28	0.24	0.19	0.08	0.19
Q9UL54	Serine/threonine-protein kinase TAO2 OS=Homo sapiens GN=TAOK2 PE=1 SV=2 - [TAOK2_HUMAN]	5.18	1	3	4	7	0.58	-0.12	0.72	0.02	0.19	-0.51	0.84	0.14	-0.38	0.33	0.79	0.09	0.31	-0.09	0.30	-0.23	-0.36	-0.41	-0.54	0.19
P53367	Arfaptin-1 OS=Homo sapiens GN=ARFIP1 PE=1 SV=2 - [ARFP1_HUMAN]	3.22	1	1	1	1	-0.45	-0.38	0.03	0.10	0.03	0.09	0.15	0.22	-0.02	-0.08	-0.01	0.06	0.66	0.45	-0.03	0.40	-0.07	0.47	-0.01	0.19
Q6PJG6	BRCA1-associated ATM activator 1 OS=Homo sapiens GN=BRAT1 PE=1 SV=2 - [BRAT1_HUMAN]	2.80	1	1	1	1	-0.63	-0.91	-0.18	-0.47	1.07	0.77	-0.06	-0.34	0.42	0.71	0.15	-0.14	0.62	0.78	0.33	1.37	0.93	1.67	1.23	0.19

Q6Y7W6	PERQ amino acid-rich with GYF domain-containing protein 2 OS=Homo sapiens GN=GIGYF2 PE=1 SV=1 - [PERQ2_HUMAN]	10.93	1	10	10	13	-0.21	-0.07	-0.27	-0.10	-0.01	0.03	0.05	-0.03	0.07	-0.01	-0.20	0.01	-0.01	-0.19	-0.07	-0.10	0.16	-0.04	0.17	0.19
O95340	Bifunctional 3'-phosphoadenosine 5'-phosphosulfate synthase 2 OS=Homo sapiens GN=PAPSS2 PE=1 SV=2 - [PAPS2_HUMAN]	14.66	1	7	7	11	-0.09	-0.02	-0.21	-0.39	0.01	0.17	-0.29	-0.31	-0.26	-0.18	0.10	0.03	-0.07	0.20	0.42	0.09	0.30	0.57	0.73	0.19
Q5JRA6	Melanoma inhibitory activity protein 3 OS=Homo sapiens GN=MIA3 PE=1 SV=1 - [MIA3_HUMAN]	16.20	1	23	23	33	-0.25	-0.20	-0.37	-0.28	0.05	0.16	-0.22	-0.09	0.06	-0.01	-0.02	0.12	0.05	0.18	0.17	0.30	0.36	0.28	0.22	0.19
Q92947	Glutaryl-CoA dehydrogenase, mitochondrial OS=Homo sapiens GN=GCDH PE=1 SV=1 - [GCDH_HUMAN]	23.74	1	7	7	24	0.07	-0.01	-0.13	0.01	-0.22	-0.15	0.03	0.01	-0.19	-0.29	-0.36	-0.15	0.07	-0.35	-0.16	-0.11	-0.09	0.27	0.12	0.19
Q9NR50	Translation initiation factor eIF-2B subunit gamma OS=Homo sapiens GN=EIF2B3 PE=1 SV=1 - [EI2BG_HUMAN]	13.27	1	4	4	6	0.01	-0.01	-0.01	-0.03	0.33	0.29	0.11	0.09	0.11	0.14	0.13	0.09	0.07	0.06	0.15	0.10	0.18	0.39	0.41	0.19
Q03518	Antigen peptide transporter 1 OS=Homo sapiens GN=TAP1 PE=1 SV=2 - [TAP1_HUMAN]	10.27	1	5	5	6	-0.23	-0.45	-0.36	-0.63	-0.02	-0.21	-0.35	-0.55	-0.41	-0.28	-0.21	-0.28	0.03	0.13	0.21	0.06	0.21	0.24	0.32	0.19
Q5VT52	Regulation of nuclear pre-mRNA domain-containing protein 2 OS=Homo sapiens GN=RPRD2 PE=1 SV=1 - [RPRD2_HUMAN]	2.53	1	2	2	2	-0.16	-0.14	-0.17	-0.15	0.26	0.28	-0.05	-0.02	-0.01	-0.03	0.07	0.09	0.17	0.23	0.24	0.16	0.17	0.41	0.42	0.19

Q13049	E3 ubiquitin-protein ligase TRIM32 OS=Homo sapiens GN=TRIM32 PE=1 SV=2 - [TRI32_HUMAN]	5.36	1	3	3	4	0.20	0.24	0.00	0.08	0.29	0.33	0.16	0.20	0.11	0.10	-0.12	0.20	0.01	0.24	-0.11	0.03	0.12	0.07	0.24	0.19
Q9NXL9	DNA helicase MCM9 OS=Homo sapiens GN=MCM9 PE=1 SV=4 - [MCM9_HUMAN]	1.22	1	1	1	1	0.43	0.40	0.68	0.65	-0.23	-0.27	0.80	0.77	0.24	0.28	-0.29	-0.33	0.43	-0.71	-0.97	-0.12	-0.37	-0.68	-0.93	0.19
Q8IWE4	DCN1-like protein 3 OS=Homo sapiens GN=DCUN1D3 PE=1 SV=1 - [DCNL3_HUMAN]	7.24	1	2	2	2	-0.37	-0.40	0.09	0.05	0.57	0.53	0.21	0.17	0.31	0.35	0.59	0.55	0.63	0.97	0.51	0.74	0.30	0.92	0.47	0.19
P14384	Carboxypeptidase M OS=Homo sapiens GN=CPM PE=1 SV=2 - [CBPM_HUMAN]	16.70	1	5	5	8	0.20	0.33	0.36	0.50	0.70	0.83	0.48	0.62	0.70	0.57	0.37	0.50	0.34	0.18	0.00	0.40	0.24	0.48	0.32	0.18
Q12866	Tyrosine-protein kinase Mer OS=Homo sapiens GN=MERTK PE=1 SV=2 - [MERTK_HUMAN]	2.70	1	2	3	4	1.10	0.88	0.78	0.56	0.68	0.45	0.90	0.68	0.59	0.82	1.14	0.91	-0.15	0.05	0.36	-0.26	0.07	-0.44	-0.11	0.18
Q02040	A-kinase anchor protein 17A OS=Homo sapiens GN=AKAP17A PE=1 SV=2 - [AK17A_HUMAN]	1.01	1	1	1	1	3.39	2.64	-2.19	-2.94	-1.10	-1.86	-2.07	-2.82	-2.58	-1.82	-1.75	-2.51	-5.40	-5.13	0.44	-5.18	0.40	-4.50	1.07	0.18
P16220	Cyclic AMP-responsive element-binding protein 1 OS=Homo sapiens GN=CREB1 PE=1 SV=2 - [CREB1_HUMAN]	4.11	1	1	1	2	-0.41	-0.10	-0.41	-0.10	-0.27	0.03	-0.29	0.02	0.13	-0.17	0.05	0.35	0.18	0.47	0.46	0.27	0.27	0.12	0.12	0.18
Q5UCC4	ER membrane protein complex subunit 10 OS=Homo sapiens GN=EMC10 PE=1 SV=1 - [EMC10_HUMAN]	3.82	1	1	1	2	-0.28	-0.04	-0.35	-0.10	-0.16	0.08	-0.23	0.02	0.15	-0.09	0.15	0.39	0.11	0.44	0.50	0.22	0.29	0.10	0.17	0.18

Q12981	Vesicle transport protein SEC20 OS=Homo sapiens GN=BNIP1 PE=1 SV=3 - [SEC20_HUMAN]	17.98	1	4	4	5	0.28	0.45	-0.37	-0.04	0.28	0.86	-0.22	0.04	0.42	0.15	0.48	0.70	-0.31	0.26	0.74	0.02	0.51	0.41	0.64	0.18
O14744	Protein arginine N-methyltransferase 5 OS=Homo sapiens GN=PRMT5 PE=1 SV=4 - [ANM5_HUMAN]	24.18	1	13	13	23	-0.17	-0.20	-0.04	-0.19	0.26	0.00	0.11	-0.02	0.10	0.18	0.49	0.41	0.42	0.57	0.65	0.28	0.28	0.17	0.27	0.18
Q641Q2	WASH complex subunit FAM21A OS=Homo sapiens GN=FAM21A PE=2 SV=3 - [FA21A_HUMAN]	17.45	4	14	14	19	-0.21	-0.35	0.06	-0.23	-0.06	-0.28	-0.14	-0.48	-0.43	-0.18	0.06	0.03	0.12	0.28	0.26	0.09	0.08	0.24	0.34	0.18
Q9BZJ0	Crooked neck-like protein 1 OS=Homo sapiens GN=CRNKL1 PE=1 SV=4 - [CRNL1_HUMAN]	1.53	1	1	1	2	0.01	-0.05	-0.15	-0.21	0.04	-0.03	-0.03	-0.09	-0.03	0.03	-0.05	-0.12	0.02	-0.05	0.10	0.05	0.21	0.01	0.17	0.18
O00471	Exocyst complex component 5 OS=Homo sapiens GN=EXOC5 PE=1 SV=1 - [EXOC5_HUMAN]	20.62	1	12	12	24	-0.32	-0.20	-0.24	-0.13	-0.03	-0.01	-0.08	-0.05	0.20	0.06	0.08	0.21	0.17	0.05	0.34	0.16	0.31	0.14	0.33	0.18
Q9Y5U2	Protein TSSC4 OS=Homo sapiens GN=TSSC4 PE=1 SV=3 - [TSSC4_HUMAN]	6.08	1	2	2	3	-0.23	-0.34	-0.42	-0.53	0.00	-0.11	-0.30	-0.41	-0.38	-0.26	0.41	0.29	-0.02	0.64	0.83	-0.01	0.19	0.21	0.40	0.18
O14545	TRAF-type zinc finger domain-containing protein 1 OS=Homo sapiens GN=TRAFD1 PE=1 SV=1 - [TRAD1_HUMAN]	5.33	1	2	2	2	-0.22	-0.43	-0.27	-0.48	-0.11	-0.32	-0.15	-0.36	-0.53	-0.32	0.11	-0.11	0.12	0.33	0.37	-0.07	-0.01	0.09	0.14	0.18
Q9NPQ8	Synembryn-A OS=Homo sapiens GN=RIC8A PE=1 SV=3 - [RIC8A_HUMAN]	18.08	2	7	7	20	-0.29	-0.17	-0.41	-0.24	-0.34	-0.24	-0.15	-0.11	-0.15	-0.15	-0.19	-0.08	0.06	0.29	0.25	0.17	0.19	-0.06	0.14	0.18

Q9GZM5	Protein YIPF3 OS=Homo sapiens GN=YIPF3 PE=1 SV=1 - [YIPF3_HUMAN]	5.43	1	2	2	3	-0.40	-0.41	-0.25	-0.25	0.03	0.02	-0.13	-0.14	-0.10	-0.09	0.47	0.46	0.33	0.88	0.72	0.34	0.19	0.41	0.26	0.18
Q8IWA5	Choline transporter-like protein 2 OS=Homo sapiens GN=SLC44A2 PE=1 SV=3 - [CTL2_HUMAN]	22.66	1	15	15	28	0.01	-0.07	0.03	-0.03	0.40	0.39	0.17	0.01	0.22	0.27	0.32	0.32	0.09	0.47	0.38	0.25	0.27	0.41	0.36	0.18
O60256	Phosphoribosyl pyrophosphate synthase-associated protein 2 OS=Homo sapiens GN=PRPSAP2 PE=1 SV=1 - [KPRB_HUMAN]	34.96	1	8	9	18	0.14	0.04	-0.03	-0.15	0.17	0.17	0.12	0.11	0.15	0.19	0.36	0.23	0.04	0.19	0.39	0.09	0.24	0.03	0.09	0.18
Q9NPA8	Transcription and mRNA export factor ENY2 OS=Homo sapiens GN=ENY2 PE=1 SV=1 - [ENY2_HUMAN]	16.83	1	1	1	1	-1.26	-1.58	-0.02	-0.34	1.02	0.69	0.10	-0.23	-0.19	0.14	0.36	0.03	1.41	1.63	0.38	1.43	0.19	2.26	1.02	0.18
Q53GL7	Poly [ADP-ribose] polymerase 10 OS=Homo sapiens GN=PARP10 PE=1 SV=2 - [PAR10_HUMAN]	10.34	1	6	6	9	-0.58	-0.78	-0.30	0.07	0.10	0.29	0.12	0.19	0.23	0.32	0.04	0.27	0.58	0.85	0.02	0.69	0.55	0.97	-0.01	0.18
O76082	Solute carrier family 22 member 5 OS=Homo sapiens GN=SLC22A5 PE=1 SV=1 - [S22A5_HUMAN]	2.15	1	1	1	2	0.07	0.03	-0.03	-0.08	-0.06	-0.11	0.08	0.04	0.02	0.07	0.19	0.14	0.06	0.12	0.22	0.03	0.14	-0.15	-0.04	0.18
P00846	ATP synthase subunit a OS=Homo sapiens GN=MT-ATP6 PE=1 SV=1 - [ATP6_HUMAN]	8.41	1	2	2	4	-0.39	-0.33	-0.19	-0.12	0.05	0.02	-0.08	-0.06	-0.91	-0.43	-1.01	-1.48	0.31	-0.62	-0.31	-0.02	0.05	0.32	0.22	0.18

Q9P253	Vacuolar protein sorting-associated protein 18 homolog OS=Homo sapiens GN=VPS18 PE=1 SV=2 - [VPS18_HUMAN]	11.00	1	9	9	17	0.59	0.47	0.41	0.29	0.57	0.22	0.53	0.22	0.43	0.78	0.77	0.43	-0.01	0.35	0.53	0.26	0.34	0.00	-0.01	0.18
P60866	40S ribosomal protein S20 OS=Homo sapiens GN=RPS20 PE=1 SV=1 - [RS20_HUMAN]	25.21	1	3	3	13	-0.43	-0.57	-0.58	-0.63	-0.21	-0.35	-0.40	-0.45	-0.53	-0.43	-0.36	-0.39	0.15	0.14	0.22	0.05	0.14	0.20	0.23	0.18
O43264	Centromere/kinetochore protein zw10 homolog OS=Homo sapiens GN=ZW10 PE=1 SV=3 - [ZW10_HUMAN]	7.19	1	5	5	9	0.07	-0.13	-0.04	-0.50	0.72	0.15	-0.06	-0.07	0.07	0.43	0.49	0.08	0.11	0.25	0.41	0.29	0.50	0.34	0.75	0.18
P49207	60S ribosomal protein L34 OS=Homo sapiens GN=RPL34 PE=1 SV=3 - [RL34_HUMAN]	35.90	1	5	5	16	-0.79	-0.66	-0.43	-0.47	-0.28	-0.24	-0.41	-0.36	-0.22	-0.34	-0.02	-0.04	0.36	0.71	0.36	0.45	0.15	0.49	0.15	0.18
P62841	40S ribosomal protein S15 OS=Homo sapiens GN=RPS15 PE=1 SV=2 - [RS15_HUMAN]	28.28	1	3	3	8	-1.50	-1.50	-0.58	-0.63	-0.91	-0.97	-0.67	-0.66	-0.78	-0.73	-0.57	-0.63	0.88	0.75	0.11	0.43	-0.18	0.41	-0.22	0.18
Q9GZS3	WD repeat-containing protein 61 OS=Homo sapiens GN=WDR61 PE=1 SV=1 - [WDR61_HUMAN]	37.05	1	9	9	12	-0.34	-0.30	-0.22	-0.14	0.34	0.34	-0.19	-0.12	0.11	0.01	0.23	0.30	0.31	0.59	0.56	0.44	0.41	0.64	0.60	0.18
O00410	Importin-5 OS=Homo sapiens GN=IPO5 PE=1 SV=4 - [IPO5_HUMAN]	24.34	1	20	22	36	0.31	0.21	0.01	0.20	0.17	0.14	0.35	0.39	0.08	0.10	-0.08	-0.11	0.30	-0.23	-0.22	-0.05	0.05	0.07	0.07	0.18
Q92643	GPI-anchor transamidase OS=Homo sapiens GN=PIGK PE=1 SV=2 - [GPI8_HUMAN]	10.38	1	3	3	3	-0.18	-0.09	0.03	0.12	-0.05	0.04	0.15	0.24	-0.23	-0.31	0.12	0.20	0.38	0.31	0.08	-0.10	-0.31	0.12	-0.10	0.18

Q15751	Probable E3 ubiquitin-protein ligase HERC1 OS=Homo sapiens GN=HERC1 PE=1 SV=2 - [HERC1_HUMAN]	0.72	1	2	2	3	0.76	0.56	0.06	-0.07	0.07	0.45	0.17	-0.04	0.30	-0.20	-0.18	0.28	-0.53	-0.93	-0.24	-0.92	-0.22	-0.71	-0.01	0.18
P12268	Inosine-5'-monophosphate dehydrogenase 2 OS=Homo sapiens GN=IMPDH2 PE=1 SV=2 - [IMDH2_HUMAN]	19.26	1	8	9	22	-0.40	-0.41	-0.18	-0.20	0.04	0.11	-0.03	-0.08	0.01	-0.06	0.13	0.26	0.51	0.60	0.28	0.36	0.02	0.41	0.22	0.18
Q9ULS5	Transmembrane and coiled-coil domains protein 3 OS=Homo sapiens GN=TMCC3 PE=2 SV=3 - [TMCC3_HUMAN]	1.89	1	1	1	2	-0.68	-0.70	-0.62	-0.64	-0.10	-0.13	-0.51	-0.53	-0.14	-0.12	-0.43	-0.45	0.22	0.26	0.20	0.59	0.54	0.56	0.50	0.18
Q9BRK5	45 kDa calcium-binding protein OS=Homo sapiens GN=SDF4 PE=1 SV=1 - [CAB45_HUMAN]	12.98	1	4	4	8	-0.43	-0.45	-0.72	-0.75	-0.12	-0.16	-0.56	-0.62	-0.21	-0.12	0.00	-0.04	-0.01	0.59	0.71	0.17	0.38	0.32	0.58	0.17
P40121	Macrophage-capping protein OS=Homo sapiens GN=CAPG PE=1 SV=2 - [CAPG_HUMAN]	22.99	1	7	7	27	0.31	0.50	0.26	0.31	0.17	0.26	0.38	0.44	0.21	0.19	0.10	0.19	-0.05	-0.19	-0.18	-0.08	-0.22	-0.20	-0.12	0.17
P41227	N-alpha-acetyltransferase 10 OS=Homo sapiens GN=NAA10 PE=1 SV=1 - [NAA10_HUMAN]	22.13	2	4	4	7	-0.13	-0.10	-0.28	-0.16	0.01	-0.06	-0.05	-0.03	-0.16	-0.09	0.20	0.13	0.13	0.05	-0.16	0.04	-0.16	-0.07	-0.27	0.17
P30419	Glycylpeptide N-tetradecanoyltransferase 1 OS=Homo sapiens GN=NMT1 PE=1 SV=2 - [NMT1_HUMAN]	7.66	1	2	3	3	0.37	-0.04	0.03	-0.38	1.07	0.67	0.14	-0.27	-0.33	0.08	-0.16	-0.57	-0.17	-0.52	-0.19	-0.26	0.08	0.69	1.03	0.17
Q8WUX9	Charged multivesicular body protein 7 OS=Homo sapiens GN=CHMP7 PE=1 SV=1 - [CHMP7_HUMAN]	9.27	1	4	4	5	1.17	0.93	0.87	0.63	0.93	0.90	0.98	0.74	0.28	0.52	0.68	0.65	-0.14	-0.34	-0.25	-0.62	-0.31	-0.11	-0.02	0.17

P21953	2-oxoisovalerate dehydrogenase subunit beta, mitochondrial OS=Homo sapiens GN=BCKDHB PE=1 SV=2 - [ODBB_HUMAN]	15.56	1	6	6	9	-0.45	-0.51	-0.11	-0.11	0.02	-0.08	0.00	-0.04	-0.28	-0.03	-0.11	-0.27	0.46	0.25	-0.11	0.36	-0.02	0.42	0.07	0.17
Q12768	WASH complex subunit strumpellin OS=Homo sapiens GN=KIAA0196 PE=1 SV=1 - [STRUM_HUMAN]	16.05	1	16	16	25	0.04	0.06	-0.23	-0.16	0.15	0.12	-0.06	-0.03	0.12	0.10	0.11	0.07	-0.05	0.08	0.36	0.02	0.29	-0.04	0.25	0.17
Q13492	Phosphatidylinositol-binding clathrin assembly protein OS=Homo sapiens GN=PICALM PE=1 SV=2 - [PICAL_HUMAN]	31.75	1	13	13	22	-0.56	-0.71	-0.33	-0.56	-0.04	-0.20	-0.33	-0.43	-0.47	-0.29	-0.14	-0.24	0.26	0.46	0.11	0.37	0.13	0.51	0.38	0.17
P61970	Nuclear transport factor 2 OS=Homo sapiens GN=NUTF2 PE=1 SV=1 - [NTF2_HUMAN]	51.18	1	4	4	25	-0.21	-0.14	0.01	0.01	0.24	0.21	0.16	0.13	0.02	0.11	-0.35	-0.37	0.36	-0.09	-0.32	0.27	0.11	0.42	0.15	0.17
P05388	60S acidic ribosomal protein P0 OS=Homo sapiens GN=RPLP0 PE=1 SV=1 - [RLA0_HUMAN]	30.60	2	9	9	19	-0.94	-1.02	-0.67	-0.73	-0.34	-0.36	-0.62	-0.57	-0.42	-0.48	-0.19	-0.15	0.50	0.89	0.58	0.52	0.29	0.64	0.32	0.17
Q53H96	Pyrroline-5-carboxylate reductase 3 OS=Homo sapiens GN=PYCRL PE=1 SV=2 - [P5CR3_HUMAN]	20.80	1	4	4	9	0.08	0.31	-0.28	-0.15	0.14	0.30	0.03	0.14	0.18	-0.04	-0.16	-0.06	-0.19	-0.43	-0.09	-0.17	0.23	-0.09	0.28	0.17
P06312	Ig kappa chain V-IV region (Fragment) OS=Homo sapiens GN=IGKV4-1 PE=4 SV=1 - [KV401_HUMAN]	22.31	4	1	2	4	-1.63	-1.63	-1.15	-1.14	-0.71	-0.70	-1.05	-1.04	-0.98	-0.98	-0.41	-0.41	0.64	1.23	0.74	0.68	0.20	0.91	0.43	0.17

O75340	Programmed cell death protein 6 OS=Homo sapiens GN=PDCD6 PE=1 SV=1 - [PDCD6_HUMAN]	32.98	1	6	6	17	-0.10	-0.05	-0.14	-0.16	0.16	0.16	-0.08	-0.02	0.07	0.15	0.30	0.28	0.14	0.39	0.42	0.31	0.27	0.31	0.27	0.17
Q9BVV7	Mitochondrial import inner membrane translocase subunit Tim21 OS=Homo sapiens GN=TIMM21 PE=1 SV=1 - [TIM21_HUMAN]	8.87	1	2	2	3	-0.01	-0.09	-0.01	-0.09	0.18	0.09	0.10	0.01	-0.02	0.07	0.03	-0.06	0.16	0.04	0.04	0.11	0.12	0.17	0.17	0.17
P10644	cAMP-dependent protein kinase type I-alpha regulatory subunit OS=Homo sapiens GN=PRKAR1A PE=1 SV=1 - [KAP0_HUMAN]	29.13	1	9	11	22	-0.07	-0.08	-0.12	-0.03	0.28	0.37	0.12	0.25	0.10	0.01	0.08	0.03	0.31	0.37	0.13	0.32	0.04	0.47	0.42	0.17
Q13637	Ras-related protein Rab-32 OS=Homo sapiens GN=RAB32 PE=1 SV=3 - [RAB32_HUMAN]	15.11	2	2	3	8	0.31	0.10	0.00	-0.21	0.28	0.06	0.11	-0.10	-0.06	0.16	0.12	-0.10	-0.14	-0.18	0.12	-0.12	0.19	-0.05	0.26	0.17
Q13595	Transformer-2 protein homolog alpha OS=Homo sapiens GN=TRA2A PE=1 SV=1 - [TRA2A_HUMAN]	11.35	1	2	3	6	0.03	-0.14	-0.13	-0.31	0.36	0.17	-0.03	-0.21	0.17	0.35	0.29	0.11	-0.01	0.26	0.42	0.35	0.52	0.30	0.47	0.17
P21399	Cytoplasmic aconitate hydratase OS=Homo sapiens GN=ACO1 PE=1 SV=3 - [ACOC_HUMAN]	19.24	1	14	14	21	0.63	0.34	0.58	0.41	0.43	0.28	0.66	0.57	0.28	0.29	0.35	0.40	0.13	0.01	-0.19	0.20	-0.09	0.10	-0.08	0.17
O75531	Barrier-to-autointegration factor OS=Homo sapiens GN=BANF1 PE=1 SV=1 - [BAF_HUMAN]	35.96	1	2	2	9	-0.19	-0.14	-0.20	-0.19	-0.58	-0.58	-0.24	0.02	-0.60	-0.59	-0.13	-0.13	0.21	0.50	0.23	0.17	-0.02	0.05	-0.30	0.17
P06727	Apolipoprotein A-IV OS=Homo sapiens GN=APOA4 PE=1 SV=3 - [APOA4_HUMAN]	55.05	1	20	21	94	-0.10	0.06	-0.51	-0.29	3.88	4.15	-0.20	-0.09	2.44	2.31	-0.03	0.14	-0.07	0.11	0.39	2.57	2.73	4.06	4.29	0.17

Q06830	Peroxiredoxin-1 OS=Homo sapiens GN=PRDX1 PE=1 SV=1 - [PRDX1_HUMAN]	62.81	1	9	13	119	-0.09	-0.13	0.19	0.20	0.11	0.13	0.32	0.34	0.02	0.02	0.13	0.15	0.53	0.24	-0.09	0.27	-0.08	0.27	-0.10	0.17
Q9NVK5	FGFR1 oncogene partner 2 OS=Homo sapiens GN=FGFR1OP2 PE=2 SV=1 - [FGOP2_HUMAN]	26.09	1	4	4	5	0.25	0.21	-0.06	-0.09	0.14	0.10	0.04	0.01	-0.19	-0.16	-0.11	-0.15	-0.15	-0.35	-0.05	-0.37	-0.06	-0.13	0.18	0.17
Q9UNP9	Peptidyl-prolyl cis-trans isomerase E OS=Homo sapiens GN=PPIE PE=1 SV=1 - [PPIE_HUMAN]	11.96	1	3	3	4	-0.66	-0.41	-0.45	-0.20	0.28	0.31	0.07	0.22	0.45	0.20	-0.17	-0.07	0.68	0.35	0.19	0.82	0.46	0.79	0.28	0.17
O75400	Pre-mRNA-processing factor 40 homolog A OS=Homo sapiens GN=PRPF40A PE=1 SV=2 - [PR40A_HUMAN]	8.67	2	8	8	13	-0.25	-0.05	-0.26	-0.07	-0.49	-0.26	-0.17	-0.09	-0.16	-0.35	-0.20	-0.34	0.19	-0.11	0.05	0.09	-0.01	-0.10	-0.11	0.17
Q13347	Eukaryotic translation initiation factor 3 subunit I OS=Homo sapiens GN=EIF3I PE=1 SV=1 - [EIF3I_HUMAN]	28.31	1	8	8	15	-0.08	-0.37	-0.06	-0.13	0.09	0.04	-0.03	-0.14	-0.14	-0.04	0.13	0.03	0.13	0.37	0.29	0.18	0.05	0.31	0.12	0.17
Q9BRT3	Migration and invasion enhancer 1 OS=Homo sapiens GN=MIEN1 PE=1 SV=1 - [MIEN1_HUMAN]	15.65	1	2	2	6	0.41	0.58	0.37	0.54	0.38	0.54	0.48	0.65	0.28	0.12	0.17	0.33	0.13	-0.23	-0.21	-0.26	-0.22	-0.05	-0.01	0.17
Q8IUX4	DNA dC->dU-editing enzyme APOBEC-3F OS=Homo sapiens GN=APOBEC3F PE=1 SV=3 - [ABC3F_HUMAN]	4.02	2	1	1	1	-0.01	-0.28	-0.08	-0.35	0.11	-0.17	0.03	-0.25	-0.07	0.21	0.18	-0.10	0.09	0.20	0.26	0.24	0.32	0.10	0.17	0.17
Q96I24	Far upstream element-binding protein 3 OS=Homo sapiens GN=FUBP3 PE=1 SV=2 - [FUBP3_HUMAN]	32.34	1	11	12	23	-0.44	-0.23	-0.53	-0.35	-0.39	-0.26	-0.45	-0.26	-0.14	-0.35	-0.20	0.02	0.02	0.22	0.33	0.10	0.28	0.01	0.24	0.17

Q9BZX2	Uridine-cytidine kinase 2 OS=Homo sapiens GN=UCK2 PE=1 SV=1 - [UCK2_HUMAN]	6.90	1	1	1	1	0.20	-0.14	0.13	-0.20	0.34	0.00	0.23	-0.10	-0.48	-0.14	-0.49	-0.83	0.09	-0.68	-0.62	-0.31	-0.24	0.13	0.19	0.16
Q9H7V2	Synapse differentiation inducing gene protein 1 OS=Homo sapiens GN=SYNDIG1 PE=2 SV=1 - [SYNG1_HUMAN]	3.88	1	1	1	1	-0.56	-0.56	-0.63	-0.63	0.03	0.02	-0.53	-0.53	-0.38	-0.37	-0.34	-0.35	0.08	0.23	0.29	0.22	0.29	0.57	0.64	0.16
P23193	Transcription elongation factor A protein 1 OS=Homo sapiens GN=TCEA1 PE=1 SV=2 - [TCEA1_HUMAN]	5.65	1	2	2	3	-0.10	-0.19	-0.32	-0.41	-0.01	-0.11	-0.21	-0.31	-0.12	-0.02	-0.32	-0.42	-0.06	-0.22	-0.01	0.11	0.32	0.07	0.29	0.16
Q9P2E9	Ribosome-binding protein 1 OS=Homo sapiens GN=RRBP1 PE=1 SV=4 - [RRBP1_HUMAN]	20.57	4	22	24	39	-0.99	-0.95	-0.95	-0.95	-0.28	-0.38	-0.97	-0.85	-0.49	-0.49	-0.23	-0.23	0.34	0.74	0.75	0.52	0.52	0.76	0.58	0.16
Q9BSB4	Autophagy-related protein 101 OS=Homo sapiens GN=ATG101 PE=1 SV=1 - [ATGA1_HUMAN]	7.34	1	1	1	1	0.49	0.27	0.25	0.03	0.17	-0.06	0.35	0.13	-0.03	0.21	0.14	-0.09	-0.08	-0.35	-0.11	-0.26	-0.01	-0.33	-0.09	0.16
Q9P2R3	Ankyrin repeat and FYVE domain-containing protein 1 OS=Homo sapiens GN=ANKFY1 PE=1 SV=2 - [ANFY1_HUMAN]	27.72	1	22	22	35	-0.01	-0.11	0.07	-0.02	-0.02	-0.06	0.17	0.05	0.02	0.13	0.24	0.18	0.30	0.40	0.12	0.29	0.08	0.22	-0.11	0.16
Q9Y5S9	RNA-binding protein 8A OS=Homo sapiens GN=RBM8A PE=1 SV=1 - [RBM8A_HUMAN]	10.92	1	2	2	2	-0.19	0.36	-0.83	-0.29	-0.52	0.02	-0.73	-0.19	0.40	-0.14	-0.63	-0.10	-0.49	-0.44	0.19	0.08	0.72	-0.35	0.29	0.16
P28074	Proteasome subunit beta type-5 OS=Homo sapiens GN=PSMB5 PE=1 SV=3 - [PSB5_HUMAN]	29.28	1	7	7	17	-0.04	0.00	-0.03	-0.16	0.11	0.11	0.10	0.08	0.11	0.15	0.36	0.38	0.15	0.44	0.39	0.21	0.16	0.18	0.07	0.16

O43318	Mitogen-activated protein kinase kinase kinase 7 OS=Homo sapiens GN=MAP3K7 PE=1 SV=1 - [M3K7_HUMAN]	13.53	1	7	7	8	-0.29	-0.34	-0.23	-0.20	0.00	-0.04	-0.13	-0.12	-0.10	-0.14	-0.07	0.06	0.38	0.43	0.18	0.34	0.03	0.27	0.02	0.16
Q9H4A6	Golgi phosphoprotein 3 OS=Homo sapiens GN=GOLPH3 PE=1 SV=1 - [GOLP3_HUMAN]	17.79	1	4	4	5	-0.61	-0.54	-0.48	-0.60	-0.01	-0.10	-0.37	-0.38	-0.16	-0.14	0.12	0.10	0.29	0.61	0.62	0.46	0.48	0.59	0.48	0.16
P05387	60S acidic ribosomal protein P2 OS=Homo sapiens GN=RPLP2 PE=1 SV=1 - [RLA2_HUMAN]	32.17	1	5	5	32	-0.88	-0.84	-0.65	-0.60	-0.23	-0.26	-0.49	-0.48	-0.44	-0.48	-0.06	-0.07	0.36	0.79	0.57	0.49	0.20	0.56	0.43	0.16
Q9BX67	Junctional adhesion molecule C OS=Homo sapiens GN=JAM3 PE=1 SV=1 - [JAM3_HUMAN]	20.32	1	5	5	17	-0.02	0.20	-0.27	-0.08	0.11	0.17	-0.26	-0.03	0.20	0.10	0.55	0.71	-0.13	0.59	0.83	-0.01	0.29	0.17	0.39	0.16
O00571	ATP-dependent RNA helicase DDX3X OS=Homo sapiens GN=DDX3X PE=1 SV=3 - [DDX3X_HUMAN]	41.99	2	24	25	55	-0.23	-0.16	-0.12	-0.14	0.09	0.07	-0.03	-0.08	-0.02	0.02	0.15	0.06	0.27	0.33	0.20	0.23	0.24	0.35	0.19	0.16
P42785	Lysosomal Pro-X carboxypeptidase OS=Homo sapiens GN=PRCP PE=1 SV=1 - [PCP_HUMAN]	17.14	1	6	6	10	0.79	0.81	0.84	0.91	0.96	0.94	0.95	1.19	0.96	0.75	0.88	0.95	0.29	0.12	0.11	0.15	0.13	0.15	0.13	0.16
P18433	Receptor-type tyrosine-protein phosphatase alpha OS=Homo sapiens GN=PTPRA PE=1 SV=2 - [PTPRA_HUMAN]	15.96	1	10	12	22	0.00	0.17	0.07	0.33	0.16	0.27	0.09	0.26	0.30	0.07	0.14	0.22	0.06	0.15	0.20	0.02	0.08	-0.05	0.01	0.16
Q9Y5P6	Mannose-1-phosphate guanyltransferase beta OS=Homo sapiens GN=GMPPB PE=1 SV=2 - [GMPPB_HUMAN]	11.67	1	3	3	10	0.21	0.08	0.18	0.03	0.69	0.51	0.30	0.13	0.24	0.42	0.08	-0.08	0.10	-0.16	-0.11	0.20	0.26	0.43	0.48	0.16

Q567U6	Coiled-coil domain-containing protein 93 OS=Homo sapiens GN=CCDC93 PE=1 SV=2 - [CCD93_HUMAN]	13.95	1	7	7	12	0.24	0.19	0.01	-0.01	0.04	0.00	0.21	0.18	0.03	0.12	0.07	-0.04	-0.04	-0.17	0.10	-0.13	0.06	-0.18	0.00	0.16
P30453	HLA class I histocompatibility antigen, A-34 alpha chain OS=Homo sapiens GN=HLA-A PE=1 SV=1 - [1A34_HUMAN]	47.12	7	3	14	38	-0.31	-0.16	-0.28	-0.13	0.37	0.47	-0.03	0.14	0.53	0.37	0.28	0.36	0.08	0.46	0.42	0.57	0.54	0.78	0.72	0.16
Q96KP1	Exocyst complex component 2 OS=Homo sapiens GN=EXOC2 PE=1 SV=1 - [EXOC2_HUMAN]	17.64	1	15	15	21	0.03	-0.04	-0.02	-0.04	0.16	0.12	0.05	0.07	0.04	0.00	0.11	0.02	0.09	0.11	0.04	-0.02	-0.16	0.05	0.00	0.16
P30533	Alpha-2-macroglobulin receptor-associated protein OS=Homo sapiens GN=LRPAP1 PE=1 SV=1 - [AMRP_HUMAN]	25.77	1	8	8	19	-0.28	-0.32	-0.41	-0.32	-0.20	-0.05	-0.23	-0.14	-0.16	-0.11	-0.11	-0.03	0.08	0.26	0.32	0.20	0.21	0.25	0.24	0.16
A8MWY0	UPF0577 protein KIAA1324-like OS=Homo sapiens GN=KIAA1324L PE=2 SV=2 - [K132L_HUMAN]	5.25	1	4	4	7	0.14	0.28	0.30	0.30	0.53	0.71	0.26	0.28	0.55	0.47	0.49	0.50	0.20	0.39	0.27	0.26	0.25	0.37	0.21	0.16
Q15436	Protein transport protein Sec23A OS=Homo sapiens GN=SEC23A PE=1 SV=2 - [SC23A_HUMAN]	21.96	1	13	14	34	-0.21	-0.12	-0.24	-0.22	0.24	0.20	-0.22	-0.26	0.09	-0.01	-0.06	-0.11	0.05	0.14	0.13	0.30	0.24	0.36	0.41	0.16
Q9NRA0	Sphingosine kinase 2 OS=Homo sapiens GN=SPHK2 PE=1 SV=2 - [SPHK2_HUMAN]	6.27	1	2	2	3	1.00	0.24	0.52	-0.24	0.83	0.06	0.62	-0.15	0.12	0.89	0.75	-0.02	-0.33	-0.25	0.23	-0.08	0.40	-0.19	0.29	0.16
P61916	Epididymal secretory protein E1 OS=Homo sapiens GN=NPC2 PE=1 SV=1 - [NPC2_HUMAN]	33.77	1	5	5	8	0.15	-0.10	0.32	0.29	0.47	0.47	0.58	0.54	0.40	0.51	0.59	0.59	0.75	0.57	0.21	0.63	0.00	0.59	0.08	0.16

Q9Y3P9	Rab GTPase-activating protein 1 OS=Homo sapiens GN=RABGAP1 PE=1 SV=3 - [RBGP1_HUMAN]	16.74	2	11	12	17	-0.01	0.27	0.16	0.11	0.10	0.32	0.08	0.36	0.09	-0.07	-0.24	-0.01	0.13	-0.27	-0.15	-0.05	-0.03	-0.09	-0.04	0.16
Q01130	Serine/arginine-rich splicing factor 2 OS=Homo sapiens GN=SRSF2 PE=1 SV=4 - [SRSF2_HUMAN]	11.76	2	2	2	2	-0.69	-0.13	-0.40	0.16	-0.60	-0.04	-0.31	0.26	-0.28	-0.84	-0.59	-0.03	0.44	0.10	-0.19	-0.12	-0.40	0.08	-0.21	0.16
P30047	GTP cyclohydrolase 1 feedback regulatory protein OS=Homo sapiens GN=GCHFR PE=1 SV=3 - [GFRP_HUMAN]	19.05	1	1	1	2	0.01	-0.35	-0.14	-0.50	0.48	0.11	-0.04	-0.41	0.08	0.45	0.26	-0.10	0.00	0.26	0.40	0.47	0.62	0.45	0.60	0.16
Q6YN16	Hydroxysteroid dehydrogenase-like protein 2 OS=Homo sapiens GN=HSDL2 PE=1 SV=1 - [HSDL2_HUMAN]	42.82	1	13	13	49	-0.13	-0.10	-0.22	-0.11	-0.17	-0.20	-0.25	-0.25	-0.09	-0.06	-0.16	-0.19	-0.02	0.04	0.08	0.08	0.06	0.04	0.22	0.16
P27635	60S ribosomal protein L10 OS=Homo sapiens GN=RPL10 PE=1 SV=4 - [RL10_HUMAN]	36.45	2	6	6	26	-0.47	-0.43	-0.56	-0.69	-0.44	-0.52	-0.46	-0.59	-0.41	-0.39	-0.13	-0.25	0.21	0.42	0.50	0.15	0.26	0.17	0.20	0.16
Q6Y1H2	Very-long-chain (3R)-3-hydroxyacyl-[acyl-carrier protein] dehydratase 2 OS=Homo sapiens GN=PTPLB PE=1 SV=1 - [HACD2_HUMAN]	3.54	1	1	1	2	-0.96	-0.77	-0.80	-0.60	-0.73	-0.54	-0.70	-0.51	-0.47	-0.65	-0.21	-0.03	0.31	0.75	0.58	0.34	0.17	0.22	0.05	0.16
Q5TAQ9	DDB1- and CUL4-associated factor 8 OS=Homo sapiens GN=DCAF8 PE=1 SV=1 - [DCAF8_HUMAN]	9.05	1	3	3	4	-0.31	-0.51	-0.07	-0.27	0.41	0.21	0.02	-0.18	-0.13	0.07	0.24	0.03	0.39	0.56	0.31	0.42	0.18	0.71	0.47	0.16

Q5T4F4	Protrudin OS=Homo sapiens GN=ZFYVE27 PE=1 SV=1 - [ZFY27_HUMAN]	6.08	1	1	1	2	-0.05	0.10	-0.53	-0.37	0.30	0.45	-0.44	-0.28	0.10	-0.05	-0.14	0.00	-0.33	-0.09	0.38	0.03	0.51	0.33	0.81	0.16
Q71H61	Immunoglobulin-like domain-containing receptor 2 OS=Homo sapiens GN=ILDR2 PE=2 SV=1 - [ILDR2_HUMAN]	8.29	1	4	4	6	0.16	0.32	-0.33	-0.16	0.30	0.46	-0.27	-0.07	0.44	0.17	0.27	0.43	-0.34	0.12	0.60	0.37	0.69	0.12	0.61	0.16
P03886	NADH-ubiquinone oxidoreductase chain 1 OS=Homo sapiens GN=MT-ND1 PE=1 SV=1 - [NU1M_HUMAN]	6.29	1	1	1	5	-0.67	-0.21	-0.44	0.02	0.13	0.58	-0.35	0.11	0.33	-0.12	0.16	0.62	0.38	0.84	0.60	0.58	0.35	0.78	0.55	0.15
P26440	Isovaleryl-CoA dehydrogenase, mitochondrial OS=Homo sapiens GN=IVD PE=1 SV=1 - [IVD_HUMAN]	32.15	1	13	13	27	-0.43	-0.71	-0.43	-0.45	-0.21	-0.33	-0.22	-0.31	-0.36	-0.32	-0.25	-0.39	0.37	0.36	0.21	0.42	0.17	0.31	0.23	0.15
Q13895	Bystin OS=Homo sapiens GN=BYSL PE=1 SV=3 - [BYST_HUMAN]	1.83	1	1	1	2	-0.63	-0.45	-0.66	-0.48	-0.23	-0.05	-0.57	-0.39	-0.04	-0.21	-0.18	-0.01	0.12	0.45	0.47	0.45	0.48	0.38	0.41	0.15
P62750	60S ribosomal protein L23a OS=Homo sapiens GN=RPL23A PE=1 SV=1 - [RL23A_HUMAN]	51.28	1	10	10	18	-1.20	-1.13	-0.70	-0.65	-0.59	-0.60	-0.55	-0.54	-0.59	-0.58	-0.37	-0.46	0.65	0.66	0.26	0.40	0.14	0.42	0.29	0.15
P46063	ATP-dependent DNA helicase Q1 OS=Homo sapiens GN=RECQL PE=1 SV=3 - [RECQ1_HUMAN]	17.26	1	7	7	12	-0.70	-0.60	-0.79	-1.03	0.01	-0.10	-0.70	-0.72	-0.18	-0.35	-0.32	-0.35	-0.18	0.29	0.52	0.46	0.69	0.43	0.99	0.15
Q9GZN8	UPF0687 protein C20orf27 OS=Homo sapiens GN=C20orf27 PE=1 SV=3 - [CT027_HUMAN]	8.05	1	1	1	1	-0.90	-1.05	-0.16	-0.31	-0.22	-0.38	-0.07	-0.23	-0.19	-0.03	0.00	-0.16	0.88	0.91	0.16	0.90	0.16	0.67	-0.07	0.15

Q9P2I0	Cleavage and polyadenylation specificity factor subunit 2 OS=Homo sapiens GN=CPSF2 PE=1 SV=2 - [CPSF2_HUMAN]	9.46	1	6	6	7	-0.28	-0.31	-0.34	-0.24	-0.08	-0.01	-0.26	-0.20	-0.31	-0.28	0.01	0.04	0.07	0.36	0.25	0.03	-0.14	0.18	0.42	0.15
Q5VYK3	Proteasome-associated protein ECM29 homolog OS=Homo sapiens GN=ECM29 PE=1 SV=2 - [ECM29_HUMAN]	11.33	1	15	15	23	0.05	-0.12	0.10	0.05	0.27	0.20	0.25	0.14	0.16	0.17	0.07	0.02	0.42	0.03	-0.08	0.14	0.10	0.31	0.22	0.15
O75600	2-amino-3-ketobutyrate coenzyme A ligase, mitochondrial OS=Homo sapiens GN=GCAT PE=1 SV=1 - [KBL_HUMAN]	10.02	1	3	3	3	-0.21	-0.09	-0.12	0.01	0.09	0.21	-0.03	0.09	0.21	0.10	-0.33	-0.22	0.24	-0.12	-0.22	0.34	-0.14	0.29	0.19	0.15
Q9UL26	Ras-related protein Rab-22A OS=Homo sapiens GN=RAB22A PE=1 SV=2 - [RB22A_HUMAN]	42.27	1	5	7	15	0.04	0.85	-0.07	0.03	0.17	0.32	0.02	0.29	0.39	0.29	0.26	0.78	-0.42	0.05	0.33	0.05	0.40	0.00	0.22	0.15
P15170	Eukaryotic peptide chain release factor GTP-binding subunit ERF3A OS=Homo sapiens GN=GSPT1 PE=1 SV=1 - [ERF3A_HUMAN]	15.43	1	3	6	12	0.07	0.05	0.26	0.00	0.32	0.45	0.35	0.14	0.01	0.13	-0.23	-0.34	0.35	-0.40	-0.34	-0.03	0.04	0.37	0.43	0.15
P47895	Aldehyde dehydrogenase family 1 member A3 OS=Homo sapiens GN=ALDH1A3 PE=1 SV=2 - [AL1A3_HUMAN]	7.03	1	2	3	7	0.55	-0.15	0.00	0.08	-0.04	0.30	0.09	-0.06	0.08	0.30	-0.10	-0.28	-0.41	-0.64	-0.10	-0.22	0.33	0.44	0.21	0.15
Q68CZ2	Tensin-3 OS=Homo sapiens GN=TNS3 PE=1 SV=2 - [TENS3_HUMAN]	9.20	1	8	11	17	-0.64	-0.59	-0.84	-0.79	-0.08	0.00	-0.55	-0.54	0.03	-0.20	-0.27	-0.27	0.16	0.47	0.52	0.67	0.85	0.72	0.77	0.15

Q8WUY3	Protein prune homolog 2 OS=Homo sapiens GN=PRUNE 2 PE=1 SV=3 - [PRUN2_HUMAN]	4.86	1	8	8	13	0.27	0.74	-0.10	0.61	0.24	-0.10	0.71	0.62	0.36	0.70	0.69	0.35	-0.06	-0.38	-0.26	-0.34	-0.21	-0.85	-0.73	0.15		
P82675	28S ribosomal protein S5, mitochondrial OS=Homo sapiens GN=MRPS5 PE=1 SV=2 - [RT05_HUMAN]	2.09	1	1	1	1	-0.01	-0.28	-0.03	-0.31	-0.17	-0.45	0.06	-0.22	-0.16	0.12	0.01	-0.27	0.12	0.03	0.04	0.16	0.18	-0.18	-0.15	0.15		
Q8TD16	Protein bicaudal D homolog 2 OS=Homo sapiens GN=BICD2 PE=1 SV=1 - [BICD2_HUMAN]	1.09	1	1	1	2	-0.28	-0.31	-0.39	-0.42	-0.10	-0.14	-0.31	-0.34	-0.16	-0.13	-0.26	-0.30	0.03	0.02	0.13	0.18	0.30	0.16	0.27	0.15		
P52948	Nuclear pore complex protein Nup98-Nup96 OS=Homo sapiens GN=NUP98 PE=1 SV=4 - [NUP98_HUMAN]	10.07	1	15	15	25	-0.59	-0.77	-0.46	-0.52	-0.38	-0.46	-0.38	-0.57	-0.47	-0.44	-0.27	-0.37	0.18	0.26	0.28	0.18	0.18	-0.06	-0.12	0.15		
Q9Y5K6	CD2-associated protein OS=Homo sapiens GN=CD2AP PE=1 SV=1 - [CD2AP_HUMAN]	9.08	1	5	5	9	-0.91	-0.95	-0.40	-0.45	-0.66	-0.71	-0.32	-0.36	-0.49	-0.44	-0.30	-0.35	0.64	0.61	0.10	0.50	0.00	0.23	-0.27	0.15		
Q5TGY3	AT-hook DNA binding motif-containing protein 1 OS=Homo sapiens GN=AHDC1 PE=1 SV=1 - [AHDC1_HUMAN]	1.12	1	1	1	1	-1.49	-1.39	-1.08	-0.98	-0.89	-0.79	-0.99	-0.89	-0.26	-0.35	-0.41	-0.31	0.55	1.09	0.67	1.17	0.76	0.59	0.18	0.15		
P19013	Keratin, type II cytoskeletal 4 OS=Homo sapiens GN=KRT4 PE=1 SV=4 - [K2C4_HUMAN]	43.82	3	17	22	157	-3.20	-3.35	-3.69	-3.71	-3.42	-3.54	-3.87	-4.11	-3.35	-3.18	-2.97	-3.12	-0.27	-0.21	0.35	0.03	0.35	-0.14	0.53	0.15		
Q16762	Thiosulfate sulfurtransferase OS=Homo sapiens GN=TST PE=1 SV=4 - [THTR_HUMAN]	44.78	2	10	10	18	0.12	0.11	-0.13	-0.11	0.59	0.63	0.04	0.04	0.06	0.14	0.50	0.43	-0.07	0.32	0.44	0.05	0.32	0.45	0.58	0.15		

Q9ULC3	Ras-related protein Rab-23 OS=Homo sapiens GN=RAB23 PE=1 SV=1 - [RAB23_HUMAN]	40.93	1	8	8	11	0.00	0.06	0.14	0.14	0.60	0.33	0.60	0.46	0.58	0.50	0.28	0.52	0.21	0.40	0.23	0.34	0.28	0.26	0.01	0.15
Q96M96	FYVE, RhoGEF and PH domain-containing protein 4 OS=Homo sapiens GN=FGD4 PE=1 SV=2 - [FGD4_HUMAN]	12.53	1	5	5	9	-0.36	-0.50	0.15	0.01	-0.17	-0.32	0.23	0.09	-0.16	-0.02	0.04	-0.10	0.21	0.05	-0.11	0.17	-0.13	0.18	-0.34	0.15
Q5BJF6	Outer dense fiber protein 2 OS=Homo sapiens GN=ODF2 PE=1 SV=1 - [ODFP2_HUMAN]	1.45	1	1	1	2	-0.57	-0.34	-0.59	-0.36	-1.09	-0.86	-0.50	-0.27	-0.32	-0.55	-0.22	0.01	0.12	0.35	0.37	0.05	0.07	-0.54	-0.52	0.15
Q9Y3B8	Oligoribonuclease, mitochondrial OS=Homo sapiens GN=REXO2 PE=1 SV=3 - [ORN_HUMAN]	16.88	1	3	3	4	-0.16	-0.20	-0.45	-0.49	-0.16	0.03	-0.36	-0.40	-0.38	-0.33	-0.45	-0.49	-0.15	-0.29	0.00	-0.15	0.15	0.17	-0.09	0.15
P51809	Vesicle-associated membrane protein 7 OS=Homo sapiens GN=VAMP7 PE=1 SV=3 - [VAMP7_HUMAN]	4.09	1	1	1	1	-0.09	0.31	-0.11	0.29	-0.35	0.04	-0.02	0.38	0.19	-0.20	-0.27	0.13	0.12	-0.18	-0.16	-0.08	-0.06	-0.28	-0.26	0.15
Q99613	Eukaryotic translation initiation factor 3 subunit C OS=Homo sapiens GN=EIF3C PE=1 SV=1 - [EIF3C_HUMAN]	26.83	2	20	20	39	-0.35	-0.32	-0.43	-0.29	-0.24	-0.12	-0.25	-0.25	-0.26	-0.23	-0.17	-0.14	0.20	0.19	0.04	0.22	0.00	0.17	0.09	0.15
P23368	NAD-dependent malic enzyme, mitochondrial OS=Homo sapiens GN=ME2 PE=1 SV=1 - [MAOM_HUMAN]	26.20	1	11	12	20	-0.06	-0.02	-0.21	-0.16	0.16	0.15	-0.03	-0.10	-0.01	-0.08	0.05	0.04	0.04	0.18	0.22	0.03	0.16	0.21	0.33	0.15
P51580	Thiopurine S-methyltransferase OS=Homo sapiens GN=TPMT PE=1 SV=1 - [TPMT_HUMAN]	7.35	1	2	2	3	0.58	0.27	0.56	0.25	0.50	0.19	0.65	0.34	0.12	0.44	-0.29	-0.60	0.12	-0.86	-0.85	-0.11	-0.09	-0.10	-0.08	0.15

P00519	Tyrosine-protein kinase ABL1 OS=Homo sapiens GN=ABL1 PE=1 SV=4 - [ABL1_HUMAN]	3.27	1	2	2	2	-1.58	-1.82	-0.79	-1.04	-0.37	-0.62	-0.71	-0.95	-0.94	-0.68	-0.31	-0.56	0.93	1.27	0.48	0.92	0.14	1.19	0.40	0.15
Q9BTZ2	Dehydrogenase/reductase SDR family member 4 OS=Homo sapiens GN=DHRS4 PE=1 SV=3 - [DHRS4_HUMAN]	7.91	3	3	3	4	-1.87	-1.38	-1.84	-0.89	-1.15	-0.05	-1.45	-0.80	-1.08	-0.99	-1.11	-0.85	0.63	0.55	0.04	0.91	0.88	1.32	0.82	0.15
Q9BSD7	Cancer-related nucleoside-triphosphatase OS=Homo sapiens GN=NTPCR PE=1 SV=1 - [NTPCR_HUMAN]	34.21	1	5	6	10	-0.23	-0.20	-0.21	-0.17	-0.20	-0.17	-0.13	-0.09	-0.11	-0.14	-0.25	-0.22	0.16	-0.01	-0.04	0.12	0.10	0.01	-0.01	0.14
Q8IYI6	Exocyst complex component 8 OS=Homo sapiens GN=EXOC8 PE=1 SV=2 - [EXOC8_HUMAN]	16.83	1	12	12	17	-0.16	-0.01	0.04	0.14	0.23	0.17	0.12	0.27	0.24	0.08	0.04	0.31	0.22	0.22	0.04	0.29	0.08	0.42	0.10	0.14
P13639	Elongation factor 2 OS=Homo sapiens GN=EEF2 PE=1 SV=4 - [EF2_HUMAN]	48.14	1	35	37	122	-0.44	-0.45	-0.32	-0.23	-0.43	-0.43	-0.21	-0.19	-0.39	-0.43	-0.47	-0.48	0.25	-0.05	-0.18	-0.05	-0.10	0.03	-0.18	0.14
Q9BPZ3	Polyadenylate-binding protein-interacting protein 2 OS=Homo sapiens GN=PAIP2 PE=1 SV=1 - [PAIP2_HUMAN]	6.30	1	1	1	1	-0.85	-0.85	-0.08	-0.08	0.06	0.06	0.00	0.00	-0.15	-0.15	-0.14	-0.14	0.91	0.72	-0.06	0.73	-0.04	0.90	0.12	0.14
P46976	Glycogenin-1 OS=Homo sapiens GN=GYG1 PE=1 SV=4 - [GLYG_HUMAN]	14.00	1	4	4	9	0.58	0.12	0.63	-0.30	0.79	0.10	0.52	-0.30	0.18	0.87	0.72	0.25	0.18	0.14	0.08	0.13	0.28	-0.01	-0.06	0.14
O43660	Pleiotropic regulator 1 OS=Homo sapiens GN=PLRG1 PE=1 SV=1 - [PLRG1_HUMAN]	8.17	1	3	3	3	-0.13	-0.02	-0.37	-0.26	0.03	0.14	-0.29	-0.18	-0.11	-0.21	-0.15	-0.04	-0.11	-0.01	0.23	-0.05	0.19	0.14	0.38	0.14

Q9BZL4	Protein phosphatase 1 regulatory subunit 12C OS=Homo sapiens GN=PPP1R12C PE=1 SV=1 - [PP12C_HUMAN]	23.27	1	13	13	21	-0.75	-0.71	-0.85	-0.89	-0.68	-0.75	-0.71	-0.76	-0.52	-0.63	-0.40	-0.39	0.00	0.36	0.43	0.15	0.36	-0.03	0.20	0.14
Q6IAA8	Ragulator complex protein LAMTOR1 OS=Homo sapiens GN=LAMTOR1 PE=1 SV=2 - [LTOR1_HUMAN]	40.37	1	5	5	14	-0.41	-0.53	-0.23	-0.31	0.07	-0.02	-0.01	-0.09	-0.06	0.03	0.29	0.29	0.46	0.87	0.56	0.45	0.19	0.45	0.24	0.14
P05160	Coagulation factor XIII B chain OS=Homo sapiens GN=F13B PE=1 SV=3 - [F13B_HUMAN]	3.63	1	1	1	2	-0.81	-0.89	-0.36	-0.44	0.62	0.54	-0.28	-0.36	-0.36	-0.27	0.59	0.51	0.59	1.41	0.95	0.57	0.12	1.42	0.96	0.14
P30837	Aldehyde dehydrogenase X, mitochondrial OS=Homo sapiens GN=ALDH1B1 PE=1 SV=3 - [AL1B1_HUMAN]	24.76	1	9	10	20	-1.83	-1.67	-1.59	-1.63	-0.31	-0.25	-1.55	-1.60	-1.16	-1.13	-1.32	-1.28	0.29	0.63	0.29	0.64	0.56	1.44	1.37	0.14
P01771	Ig heavy chain V-III region HIL OS=Homo sapiens PE=1 SV=1 - [HV310_HUMAN]	9.09	1	1	1	1	-0.44	-0.47	-0.38	-0.40	0.44	0.42	-0.30	-0.33	0.10	0.13	0.75	0.72	0.20	1.20	1.13	0.60	0.54	0.87	0.81	0.14
Q9Y276	Mitochondrial chaperone BCS1 OS=Homo sapiens GN=BCS1L PE=1 SV=1 - [BCS1_HUMAN]	30.55	1	10	10	23	-0.01	-0.02	-0.07	0.02	0.15	0.17	0.09	0.18	0.21	0.19	0.27	0.29	0.19	0.23	0.27	0.08	0.20	0.17	0.28	0.14
P03915	NADH-ubiquinone oxidoreductase chain 5 OS=Homo sapiens GN=MT-ND5 PE=1 SV=2 - [NU5M_HUMAN]	8.46	1	3	3	5	-0.08	0.04	-0.50	-0.37	0.23	0.34	-0.42	-0.29	0.01	-0.11	-0.40	-0.29	-0.28	-0.32	0.09	0.01	0.42	0.29	0.70	0.14
Q14139	Ubiquitin conjugation factor E4 A OS=Homo sapiens GN=UBE4A PE=1 SV=2 - [UBE4A_HUMAN]	15.29	1	12	12	25	-0.06	-0.14	0.03	0.04	0.31	0.32	0.21	0.19	0.15	0.22	0.32	0.34	0.38	0.50	0.37	0.33	0.33	0.41	0.21	0.14

P50914	60S ribosomal protein L14 OS=Homo sapiens GN=RPL14 PE=1 SV=4 - [RL14_HUMAN]	20.93	1	4	4	14	-0.55	-0.51	-0.57	-0.56	-0.28	-0.34	-0.56	-0.47	-0.26	-0.35	-0.18	-0.20	0.12	0.38	0.41	0.33	0.35	0.26	0.22	0.14
O94776	Metastasis-associated protein MTA2 OS=Homo sapiens GN=MTA2 PE=1 SV=1 - [MTA2_HUMAN]	10.33	1	6	6	10	-0.08	-0.06	0.05	-0.13	0.05	0.03	-0.20	-0.05	-0.15	-0.22	-0.06	-0.06	-0.20	-0.16	0.06	-0.21	0.01	-0.18	0.30	0.14
P62913	60S ribosomal protein L11 OS=Homo sapiens GN=RPL11 PE=1 SV=2 - [RL11_HUMAN]	28.65	1	5	5	18	-0.78	-0.88	-0.64	-0.54	-0.24	-0.27	-0.55	-0.46	-0.37	-0.41	-0.14	-0.14	0.37	0.64	0.37	0.36	0.19	0.52	0.22	0.14
P52294	Importin subunit alpha 5 OS=Homo sapiens GN=KPNA1 PE=1 SV=3 - [IMA5_HUMAN]	14.31	1	3	6	13	-0.02	-0.37	-0.15	-0.40	-0.10	-0.36	-0.07	-0.33	-0.25	0.01	0.03	-0.23	0.59	0.32	0.18	0.36	0.04	0.28	0.03	0.14
Q5VWZ2	Lysophospholipase-like protein 1 OS=Homo sapiens GN=LYPLAL1 PE=1 SV=3 - [LYPL1_HUMAN]	21.10	1	5	5	8	0.24	0.23	0.22	0.31	0.27	0.37	0.30	0.26	0.31	0.35	0.45	0.50	-0.09	0.15	0.23	-0.01	0.16	0.15	0.08	0.14
P61247	40S ribosomal protein S3a OS=Homo sapiens GN=RPS3A PE=1 SV=2 - [RS3A_HUMAN]	40.15	1	11	11	29	-0.63	-0.75	-0.66	-0.64	-0.29	-0.39	-0.53	-0.53	-0.40	-0.50	-0.26	-0.30	0.19	0.48	0.42	0.21	0.28	0.23	0.20	0.14
Q9NRF8	CTP synthase 2 OS=Homo sapiens GN=CTPS2 PE=1 SV=1 - [PYRG2_HUMAN]	15.19	1	6	7	12	-0.12	-0.05	-0.13	-0.06	0.05	0.46	0.13	0.42	0.43	-0.04	0.12	0.28	0.07	0.31	0.27	0.39	0.32	0.12	0.36	0.14
Q8TAD8	Smad nuclear-interacting protein 1 OS=Homo sapiens GN=SNIP1 PE=1 SV=1 - [SNIP1_HUMAN]	4.80	1	1	1	2	-0.14	-0.45	-0.11	-0.42	-0.14	-0.46	-0.04	-0.35	-0.12	0.20	0.34	0.02	0.16	0.49	0.45	0.37	0.34	-0.02	-0.05	0.14

O75410	Transforming acidic coiled-coil-containing protein 1 OS=Homo sapiens GN=TACC1 PE=1 SV=2 - [TACC1_HUMAN]	25.47	1	16	19	36	-0.05	0.00	-0.14	-0.15	0.09	0.15	-0.08	-0.03	0.02	-0.04	-0.09	-0.01	0.07	0.05	0.03	0.15	0.13	0.24	0.28	0.14
O95674	Phosphatidate cytidylyltransferase 2 OS=Homo sapiens GN=CDS2 PE=1 SV=1 - [CDS2_HUMAN]	8.76	1	3	3	6	-0.35	-0.30	-0.25	-0.25	-0.18	-0.01	-0.18	-0.17	-0.06	-0.17	-0.13	0.03	0.16	0.34	0.29	0.17	0.22	0.27	0.30	0.14
Q9UPT5	Exocyst complex component 7 OS=Homo sapiens GN=EXOC7 PE=1 SV=3 - [EXOC7_HUMAN]	26.67	1	13	13	25	0.02	0.12	-0.14	0.08	-0.05	0.11	0.03	0.24	0.10	-0.02	-0.11	0.12	0.17	-0.02	0.09	0.04	0.16	-0.11	0.04	0.14
P46736	Lys-63-specific deubiquitinase BRCC36 OS=Homo sapiens GN=BRCC3 PE=1 SV=2 - [BRCC3_HUMAN]	9.49	1	3	3	6	0.13	0.13	-0.10	-0.06	-0.11	-0.10	0.00	0.00	-0.03	-0.09	0.15	0.07	-0.07	0.01	0.21	-0.13	0.09	-0.24	0.04	0.14
Q8WZA0	Protein LZIC OS=Homo sapiens GN=LZIC PE=1 SV=1 - [LZIC_HUMAN]	32.63	1	6	6	11	-0.25	-0.52	0.04	-0.19	-0.09	-0.18	0.14	0.12	-0.36	-0.31	-0.58	-0.64	0.44	-0.29	-0.62	0.27	-0.05	0.12	0.02	0.14
P61457	Pterin-4-alpha-carbinolamine dehydratase OS=Homo sapiens GN=PCBD1 PE=1 SV=2 - [PHS_HUMAN]	36.54	1	3	4	10	0.58	0.52	0.48	0.45	0.17	0.10	0.53	0.39	0.30	0.44	0.17	0.03	0.05	-0.26	-0.17	-0.08	0.02	-0.27	-0.17	0.14
Q14558	Phosphoribosyl pyrophosphate synthase-associated protein 1 OS=Homo sapiens GN=PRPSAP1 PE=1 SV=2 - [KPRA_HUMAN]	23.60	1	5	6	17	-0.31	-0.22	-0.22	-0.40	-0.16	-0.23	-0.15	-0.09	-0.27	-0.26	-0.24	0.02	-0.04	0.18	0.02	0.19	0.05	0.33	0.18	0.14
P20702	Integrin alpha X OS=Homo sapiens GN=ITGAX PE=1 SV=3 - [ITAX_HUMAN]	1.03	1	1	1	1	0.33	1.54	0.29	1.50	0.54	1.76	0.36	1.58	1.78	0.57	0.48	1.69	0.09	0.16	0.19	0.28	0.32	0.20	0.24	0.14

O43143	Putative pre-mRNA-splicing factor ATP-dependent RNA helicase DHX15 OS=Homo sapiens GN=DHX15 PE=1 SV=2 - [DHX15_HUMAN]	25.28	1	16	17	31	-0.13	-0.18	-0.24	-0.23	-0.04	-0.15	-0.10	-0.21	-0.30	-0.22	-0.34	-0.30	0.12	-0.13	0.01	-0.04	-0.02	0.07	0.23	0.14
Q96F10	Diamine acetyltransferase 2 OS=Homo sapiens GN=SAT2 PE=1 SV=1 - [SAT2_HUMAN]	10.59	1	2	2	3	0.56	0.48	0.17	0.10	0.36	0.29	0.24	0.17	-0.06	0.02	-0.32	-0.39	-0.26	-0.87	-0.49	-0.51	-0.12	-0.21	0.17	0.14
Q7Z6Z7	E3 ubiquitin-protein ligase HUWE1 OS=Homo sapiens GN=HUWE1 PE=1 SV=3 - [HUWE1_HUMAN]	17.51	1	50	50	86	0.03	0.02	0.02	0.06	0.09	0.12	0.04	0.10	0.01	-0.02	-0.07	0.01	0.09	-0.04	0.07	0.03	-0.04	-0.06	0.05	0.14
P78347	General transcription factor II-I OS=Homo sapiens GN=GTF2I PE=1 SV=2 - [GTF2I_HUMAN]	27.66	3	21	21	41	-0.26	-0.29	-0.28	-0.33	-0.17	-0.26	-0.15	-0.27	-0.35	-0.21	-0.10	-0.20	0.18	0.03	0.16	-0.07	-0.04	0.05	0.03	0.13
P49757	Protein numb homolog OS=Homo sapiens GN=NUMB PE=1 SV=2 - [NUMB_HUMAN]	9.22	1	3	6	9	0.06	-0.65	0.29	-0.36	-0.45	-0.78	-0.14	-0.45	-0.60	-0.20	0.40	-0.32	0.09	0.34	0.19	0.16	0.21	-0.07	-0.03	0.13
Q9BVP2	Guanine nucleotide-binding protein-like 3 OS=Homo sapiens GN=GNL3 PE=1 SV=2 - [GNL3_HUMAN]	4.55	1	2	2	2	0.14	0.97	-0.23	0.60	-0.23	0.60	-0.16	0.67	0.52	-0.30	-0.02	0.81	-0.24	-0.15	0.21	-0.41	-0.04	-0.38	-0.01	0.13
Q14839	Chromodomain-helicase-DNA-binding protein 4 OS=Homo sapiens GN=CHD4 PE=1 SV=2 - [CHD4_HUMAN]	8.89	1	10	12	19	-0.17	-0.28	-0.37	-0.42	-0.08	-0.30	-0.31	-0.40	-0.42	-0.26	-0.16	-0.32	-0.04	0.13	0.16	0.21	0.07	0.14	0.11	0.13

Q9NP80	Calcium-independent phospholipase A2-gamma OS=Homo sapiens GN=PNPLA8 PE=1 SV=1 - [PLPL8_HUMAN]	10.36	1	7	7	10	-0.23	-0.40	-0.16	-0.39	-0.09	-0.33	-0.09	-0.30	-0.24	-0.07	-0.20	-0.34	0.21	0.15	-0.03	0.19	-0.03	-0.05	-0.12	0.13
Q9BV23	Monoacylglycerol lipase ABHD6 OS=Homo sapiens GN=ABHD6 PE=2 SV=1 - [ABHD6_HUMAN]	3.86	1	1	1	1	0.07	0.22	0.48	0.62	0.26	0.40	0.55	0.69	-0.39	-0.53	0.60	0.73	0.53	0.53	0.12	-0.57	-0.98	0.17	-0.23	0.13
A6NKF9	Putative Golgi pH regulator C OS=Homo sapiens GN=GPR89C PE=5 SV=2 - [GPHRC_HUMAN]	7.81	2	2	2	3	-0.21	-0.04	0.01	0.18	0.29	0.46	0.08	0.25	0.36	0.19	0.20	0.37	0.35	0.42	0.19	0.43	0.21	0.49	0.26	0.13
Q13523	Serine/threonine-protein kinase PRP4 homolog OS=Homo sapiens GN=PRPF4B PE=1 SV=3 - [PRP4B_HUMAN]	3.28	1	3	3	6	0.01	0.02	0.20	0.22	-0.12	-0.15	0.04	0.05	-0.14	0.00	0.07	0.06	0.09	0.17	-0.03	-0.23	-0.26	-0.14	-0.21	0.13
Q6IAN0	Dehydrogenase/reductase SDR family member 7B OS=Homo sapiens GN=DHRS7B PE=1 SV=2 - [DRS7B_HUMAN]	11.08	1	3	3	4	0.54	0.80	0.58	0.85	0.71	0.97	0.65	0.92	0.99	0.73	0.78	1.04	0.17	0.25	0.19	0.22	0.18	0.16	0.11	0.13
O76021	Ribosomal L1 domain-containing protein 1 OS=Homo sapiens GN=RSL1D1 PE=1 SV=3 - [RL1D1_HUMAN]	7.35	1	3	3	5	-0.24	0.05	-0.56	-0.27	-0.69	-0.41	-0.49	-0.20	-0.25	-0.53	-0.30	-0.02	-0.20	-0.06	0.26	-0.27	0.06	-0.47	-0.15	0.13
P83111	Serine beta-lactamase-like protein LACTB, mitochondrial OS=Homo sapiens GN=LACTB PE=1 SV=2 - [LACTB_HUMAN]	20.84	1	9	9	16	-0.05	-0.14	-0.21	-0.06	0.19	0.35	-0.10	0.02	0.03	-0.19	-0.04	0.06	0.19	0.37	0.17	0.38	0.12	0.41	0.29	0.13

P27144	Adenylate kinase 4, mitochondrial OS=Homo sapiens GN=AK4 PE=1 SV=1 - [KAD4_HUMAN]	52.91	1	9	9	24	-1.13	-1.13	-0.79	-0.91	-0.15	-0.24	-0.73	-0.86	-0.68	-0.58	-0.33	-0.46	0.29	0.67	0.41	0.57	0.25	0.70	0.65	0.13
P13284	Gamma-interferon-inducible lysosomal thiol reductase OS=Homo sapiens GN=IFI30 PE=1 SV=3 - [GILT_HUMAN]	4.40	1	1	1	2	0.36	0.68	0.39	0.71	0.77	1.09	0.45	0.78	0.64	0.31	0.18	0.50	0.15	-0.17	-0.21	-0.01	-0.04	0.40	0.36	0.13
Q9BTC0	Death-inducer obliterator 1 OS=Homo sapiens GN=DIDO1 PE=1 SV=5 - [DIDO1_HUMAN]	0.71	1	1	1	1	-1.45	-0.97	-1.03	-0.56	-1.19	-0.72	-0.96	-0.49	-0.01	-0.48	-0.51	-0.04	0.54	0.94	0.52	0.99	0.58	0.24	-0.18	0.13
P62081	40S ribosomal protein S7 OS=Homo sapiens GN=RPS7 PE=1 SV=1 - [RS7_HUMAN]	18.04	1	4	4	9	-0.55	-0.48	-0.44	-0.53	-0.24	-0.26	-0.37	-0.36	-0.22	-0.22	-0.10	-0.03	0.13	0.37	0.40	0.25	0.09	0.20	0.25	0.13
Q6I9Y2	THO complex subunit 7 homolog OS=Homo sapiens GN=THOC7 PE=1 SV=3 - [THOC7_HUMAN]	10.78	1	2	2	4	-0.75	-0.78	-0.76	-0.78	-0.35	-0.38	-0.69	-0.72	-0.45	-0.42	-0.19	-0.22	0.11	0.56	0.57	0.36	0.37	0.38	0.39	0.13
Q96A65	Exocyst complex component 4 OS=Homo sapiens GN=EXOC4 PE=1 SV=1 - [EXOC4_HUMAN]	19.20	1	16	16	22	-0.24	-0.35	-0.19	-0.14	0.00	0.11	0.07	-0.10	-0.23	-0.13	0.02	-0.13	0.37	0.36	0.22	0.32	-0.03	0.35	0.20	0.13
O75822	Eukaryotic translation initiation factor 3 subunit J OS=Homo sapiens GN=EIF3J PE=1 SV=2 - [EIF3J_HUMAN]	24.42	1	5	5	8	-0.36	-0.45	-0.15	-0.23	-0.51	-0.59	-0.10	-0.09	-0.29	-0.23	-0.23	-0.24	0.39	0.35	0.05	0.28	-0.05	-0.02	-0.29	0.13
O43719	HIV Tat-specific factor 1 OS=Homo sapiens GN=HTATSF1 PE=1 SV=1 - [HTSF1_HUMAN]	20.26	1	8	8	13	0.01	-0.19	0.02	-0.21	-0.07	-0.17	-0.12	-0.22	-0.34	0.18	-0.07	-0.24	0.14	0.02	-0.08	0.09	0.20	0.07	-0.03	0.13

Q9NRY5	Protein FAM114A2 OS=Homo sapiens GN=FAM114A2 PE=1 SV=4 - [F1142_HUMAN]	15.84	1	6	6	12	-0.18	-0.18	0.08	0.09	0.20	0.13	0.20	0.13	0.12	0.19	0.18	0.11	0.17	0.38	-0.03	0.28	-0.01	0.26	0.11	0.13
Q5F1R6	DnaJ homolog subfamily C member 21 OS=Homo sapiens GN=DNAJC21 PE=1 SV=2 - [DJC21_HUMAN]	3.01	1	1	1	1	-1.06	-1.10	-1.27	-1.30	-0.70	-0.74	-1.20	-1.24	-1.02	-0.98	0.15	0.11	-0.09	1.22	1.42	0.11	0.32	0.35	0.55	0.13
Q96IJ6	Mannose-1-phosphate guanyltransferase alpha OS=Homo sapiens GN=GMPPA PE=1 SV=1 - [GMPPA_HUMAN]	8.10	1	3	3	5	-0.17	-0.20	0.08	0.05	0.12	0.09	0.14	0.11	0.19	0.22	0.17	0.14	0.37	0.35	0.09	0.43	0.18	0.27	0.02	0.13
Q9Y2D4	Exocyst complex component 6B OS=Homo sapiens GN=EXOC6B PE=1 SV=3 - [EXC6B_HUMAN]	14.80	1	9	10	14	0.05	0.02	-0.16	-0.10	-0.04	0.08	-0.01	0.01	-0.06	0.04	-0.02	-0.14	0.00	0.04	0.04	0.17	0.01	0.05	0.05	0.13
Q02338	D-beta-hydroxybutyrate dehydrogenase, mitochondrial OS=Homo sapiens GN=BDH1 PE=1 SV=3 - [BDH_HUMAN]	27.11	1	8	8	15	1.32	1.39	1.25	1.33	0.89	0.95	1.39	1.39	1.18	1.01	0.71	0.84	0.13	-0.55	-0.44	-0.12	-0.12	-0.40	-0.35	0.13
P17987	T-complex protein 1 subunit alpha OS=Homo sapiens GN=TCP1 PE=1 SV=1 - [TCPA_HUMAN]	53.78	1	25	25	72	-0.35	-0.20	-0.22	-0.14	-0.17	-0.07	-0.17	-0.12	-0.11	-0.18	-0.26	-0.11	0.30	0.06	0.00	0.13	0.05	0.09	0.03	0.13
P30626	Sorcin OS=Homo sapiens GN=SRI PE=1 SV=1 - [SORCN_HUMAN]	56.06	1	11	11	49	-0.26	-0.20	-0.09	0.13	0.19	0.12	0.08	0.30	0.31	0.22	0.40	0.47	0.34	0.65	0.35	0.36	0.24	0.31	0.12	0.13
Q8IXK0	Polyhomeotic like protein 2 OS=Homo sapiens GN=PHC2 PE=1 SV=1 - [PHC2_HUMAN]	4.31	2	3	3	3	0.22	0.39	0.09	0.26	0.13	0.30	0.15	0.33	0.27	0.10	0.18	0.35	-0.01	-0.03	0.09	-0.09	0.05	-0.11	0.02	0.13

Q9BU02	Thiamine-triphosphatase OS=Homo sapiens GN=THTPA PE=1 SV=3 - [THTPA_HUMAN]	12.17	1	2	2	3	0.39	0.15	0.27	0.04	0.41	0.17	0.34	0.11	0.19	0.43	0.08	-0.16	0.01	-0.30	-0.20	0.07	0.19	0.01	0.12	0.13
O76094	Signal recognition particle subunit SRP72 OS=Homo sapiens GN=SRP72 PE=1 SV=3 - [SRP72_HUMAN]	21.46	1	10	10	20	-0.38	-0.31	-0.38	-0.32	0.04	0.02	-0.16	-0.14	-0.05	-0.11	-0.15	-0.14	0.30	0.30	0.25	0.39	0.35	0.49	0.48	0.13
P47813	Eukaryotic translation initiation factor 1A, X-chromosomal OS=Homo sapiens GN=EIF1AX PE=1 SV=2 - [IF1AX_HUMAN]	14.58	2	2	2	3	-0.31	-0.38	0.04	-0.03	0.20	0.12	0.11	0.03	0.38	0.46	0.36	0.28	0.47	0.67	0.31	0.80	0.45	0.49	0.14	0.13
Q8N1F7	Nuclear pore complex protein Nup93 OS=Homo sapiens GN=NUP93 PE=1 SV=2 - [NUP93_HUMAN]	18.07	1	13	13	24	-0.56	-0.35	-0.49	-0.37	-0.27	-0.16	-0.40	-0.27	-0.17	-0.21	-0.06	0.05	0.17	0.50	0.48	0.28	0.28	0.20	0.20	0.13
Q9NV70	Exocyst complex component 1 OS=Homo sapiens GN=EXOC1 PE=1 SV=4 - [EXOC1_HUMAN]	21.25	1	17	17	25	-0.33	-0.42	-0.11	-0.16	-0.12	-0.05	-0.01	0.01	-0.14	-0.07	-0.04	-0.07	0.36	0.44	0.07	0.30	0.02	0.21	0.05	0.13
P62424	60S ribosomal protein L7a OS=Homo sapiens GN=RPL7A PE=1 SV=2 - [RL7A_HUMAN]	30.08	1	10	10	24	-0.85	-0.83	-0.55	-0.58	-0.38	-0.45	-0.52	-0.59	-0.38	-0.45	-0.22	-0.26	0.33	0.62	0.42	0.42	0.19	0.35	0.12	0.12
Q86XP3	ATP-dependent RNA helicase DDX42 OS=Homo sapiens GN=DDX42 PE=1 SV=1 - [DDX42_HUMAN]	21.32	1	14	14	27	-0.20	-0.15	-0.02	0.05	-0.15	-0.13	-0.16	-0.18	-0.27	-0.25	-0.10	-0.24	0.05	-0.12	-0.25	-0.16	-0.34	0.01	-0.18	0.12

Q9H6T3	RNA polymerase II associated protein 3 OS=Homo sapiens GN=RPAP3 PE=1 SV=2 - [RPAP3_HUMAN]	7.67	1	4	4	5	-0.04	0.32	-0.38	-0.02	0.22	0.57	-0.32	0.04	0.32	-0.04	0.02	0.37	-0.23	0.06	0.40	0.03	0.37	0.24	0.58	0.12
Q7L1V2	Vacuolar fusion protein MON1 homolog B OS=Homo sapiens GN=MON1B PE=1 SV=1 - [MON1B_HUMAN]	2.93	1	1	1	1	0.56	0.01	0.50	-0.04	0.27	-0.29	0.57	0.02	-0.26	0.30	0.66	0.10	0.06	0.10	0.15	-0.23	-0.17	-0.31	-0.26	0.12
Q16637	Survival motor neuron protein OS=Homo sapiens GN=SMN1 PE=1 SV=1 - [SMN_HUMAN]	10.54	1	2	2	2	0.27	0.21	0.04	-0.02	0.86	0.79	0.10	0.04	0.16	0.23	0.55	0.48	-0.11	0.28	0.50	-0.01	0.22	0.57	0.80	0.12
Q96HD9	N-acyl-aromatic-L-amino acid amidohydrolase (carboxylate-forming) OS=Homo sapiens GN=ACY3 PE=1 SV=1 - [ACY3_HUMAN]	5.96	1	1	1	1	0.25	0.27	0.54	0.56	0.39	0.41	0.60	0.62	-0.11	-0.13	0.08	0.09	0.40	-0.17	-0.46	-0.35	-0.64	0.12	-0.17	0.12
P55196	Afadin OS=Homo sapiens GN=MLLT4 PE=1 SV=3 - [AFAD_HUMAN]	14.36	1	20	20	36	-0.19	-0.41	-0.23	-0.37	-0.04	-0.16	-0.22	-0.26	-0.25	-0.12	0.02	-0.09	0.16	0.23	0.30	0.11	0.13	0.06	0.05	0.12
Q9BVL4	Selenoprotein O OS=Homo sapiens GN=SELO PE=2 SV=3 - [SELO_HUMAN]	3.74	1	2	2	3	0.35	0.21	0.33	0.19	0.25	0.11	0.39	0.24	0.29	0.44	0.50	0.35	0.09	0.16	0.17	0.12	0.15	-0.11	-0.09	0.12
P46060	Ran GTPase-activating protein 1 OS=Homo sapiens GN=RANGAP1 PE=1 SV=1 - [RAGP1_HUMAN]	24.19	1	13	13	22	-0.04	-0.03	0.02	-0.02	0.11	0.17	0.15	0.20	0.06	0.08	0.26	0.18	0.16	0.19	0.07	0.08	0.00	0.20	-0.02	0.12
Q02108	Guanylate cyclase soluble subunit alpha 3 OS=Homo sapiens GN=GUCY1A3 PE=1 SV=2 - [GCYA3_HUMAN]	14.64	1	8	8	10	-0.45	-0.53	-0.61	-0.47	-0.39	-0.28	-0.21	-0.25	-0.54	-0.35	-0.33	-0.15	0.27	0.20	-0.01	0.13	-0.03	0.13	-0.13	0.12

Q9Y3B7	39S ribosomal protein L11, mitochondrial OS=Homo sapiens GN=MRPL11 PE=1 SV=1 - [RM11_HUMAN]	26.04	1	4	4	5	0.36	0.22	0.29	0.35	0.17	0.06	0.51	0.41	0.44	0.56	0.43	0.59	0.17	0.38	0.14	0.19	0.13	-0.24	-0.14	0.12
P56277	Cx9C motif-containing protein 4 OS=Homo sapiens GN=CMC4 PE=1 SV=1 - [CMC4_HUMAN]	38.24	1	2	2	3	-0.18	-0.11	-0.51	-0.44	-0.96	-0.89	-0.45	-0.38	-1.09	-1.16	-0.77	-0.70	-0.22	-0.58	-0.26	-0.95	-0.62	-0.80	-0.47	0.12
P07203	Glutathione peroxidase 1 OS=Homo sapiens GN=GPX1 PE=1 SV=4 - [GPX1_HUMAN]	53.69	1	8	8	31	0.08	0.04	0.30	0.35	0.01	0.01	0.16	0.22	0.06	-0.14	0.08	0.08	0.23	-0.02	-0.08	-0.09	-0.09	-0.16	-0.02	0.12
P69849	Nodal modulator 3 OS=Homo sapiens GN=NOMO3 PE=2 SV=2 - [NOMO3_HUMAN]	21.85	3	19	19	32	-0.46	-0.27	-0.51	-0.25	-0.04	0.04	-0.34	-0.21	-0.16	-0.25	-0.18	-0.12	0.25	0.28	0.25	0.27	0.20	0.40	0.47	0.12
Q96SB4	SRSF protein kinase 1 OS=Homo sapiens GN=SRPK1 PE=1 SV=2 - [SRPK1_HUMAN]	3.51	2	1	2	3	0.62	-0.11	0.49	-0.24	-0.02	-0.76	0.55	-0.18	-0.40	0.34	0.18	-0.56	-0.02	-0.44	-0.31	-0.26	-0.12	-0.66	-0.53	0.12
Q96MD2	UPF0536 protein C12orf66 OS=Homo sapiens GN=C12orf66 PE=2 SV=4 - [CL066_HUMAN]	3.15	1	1	1	1	-0.41	-0.66	-0.24	-0.49	0.13	-0.12	-0.19	-0.43	-0.31	-0.05	0.34	0.09	0.28	0.76	0.58	0.39	0.22	0.53	0.36	0.12
P41091	Eukaryotic translation initiation factor 2 subunit 3 OS=Homo sapiens GN=EIF2S3 PE=1 SV=3 - [IF2G_HUMAN]	32.20	2	11	11	17	-0.68	-0.68	-0.40	-0.44	-0.35	-0.33	-0.36	-0.43	-0.44	-0.40	-0.32	-0.29	0.17	0.43	0.21	0.29	0.06	0.29	0.09	0.12
O75592	E3 ubiquitin-protein ligase MYCBP2 OS=Homo sapiens GN=MYCBP2 PE=1 SV=3 - [MYCB2_HUMAN]	2.82	1	8	8	10	0.45	0.31	0.20	-0.02	0.28	-0.05	0.10	-0.07	0.11	0.13	0.30	-0.04	-0.26	-0.28	-0.02	-0.19	0.05	-0.22	-0.07	0.12

P08865	40S ribosomal protein SA OS=Homo sapiens GN=RPSA PE=1 SV=4 - [RSSA_HUMAN]	42.03	1	9	9	27	-0.77	-0.73	-0.45	-0.60	-0.02	-0.07	-0.49	-0.41	-0.51	-0.56	-0.26	-0.26	0.31	0.31	0.37	0.27	0.37	0.73	0.61	0.12
O43396	Thioredoxin-like protein 1 OS=Homo sapiens GN=TXNL1 PE=1 SV=3 - [TXNL1_HUMAN]	38.75	1	8	8	18	-0.21	-0.28	0.07	0.14	0.00	0.14	0.17	0.23	0.12	-0.11	-0.40	-0.21	0.52	-0.11	-0.31	0.05	-0.01	0.31	-0.05	0.12
Q6ZT07	TBC1 domain family member 9 OS=Homo sapiens GN=TBC1D9 PE=2 SV=2 - [TBCD9_HUMAN]	2.37	1	2	2	2	-0.03	0.58	-0.28	0.34	-0.35	0.25	-0.22	0.39	-0.06	-0.66	-1.07	-0.46	-0.13	-1.03	-0.79	-0.60	-0.35	-0.34	-0.10	0.12
Q96GG9	DCN1-like protein 1 OS=Homo sapiens GN=DCUN1D1 PE=1 SV=1 - [DCNL1_HUMAN]	18.15	1	4	4	6	-0.55	-0.31	-0.24	-0.27	-0.51	-0.48	-0.04	-0.05	-0.13	-0.15	-0.09	-0.01	0.45	0.31	-0.01	0.32	-0.01	0.03	-0.38	0.12
Q9Y605	MORF4 family-associated protein 1 OS=Homo sapiens GN=MRFAP1 PE=1 SV=1 - [MOFA1_HUMAN]	7.87	1	1	1	1	0.18	0.70	0.19	0.71	0.55	1.06	0.25	0.77	0.62	0.11	0.33	0.84	0.12	0.15	0.13	-0.04	-0.05	0.35	0.33	0.12
O00291	Huntingtin-interacting protein 1 OS=Homo sapiens GN=HIP1 PE=1 SV=5 - [HIP1_HUMAN]	18.51	1	13	14	19	-0.37	-0.89	-0.58	-0.47	-0.15	-0.23	-0.52	-0.58	-0.26	-0.29	-0.37	-0.43	0.04	0.22	0.02	0.24	0.28	0.46	0.18	0.12
P55884	Eukaryotic translation initiation factor 3 subunit B OS=Homo sapiens GN=EIF3B PE=1 SV=3 - [EIF3B_HUMAN]	32.80	1	19	19	36	-0.34	-0.36	-0.30	-0.38	-0.14	-0.22	-0.23	-0.36	-0.11	-0.14	-0.07	-0.01	0.14	0.23	0.22	0.16	0.16	0.09	0.15	0.12
Q9P000	COMM domain-containing protein 9 OS=Homo sapiens GN=COMMD9 PE=1 SV=2 - [COMD9_HUMAN]	34.85	1	5	5	6	0.35	0.29	0.20	0.24	0.23	0.12	0.25	0.19	0.09	0.20	0.27	0.40	0.16	0.31	0.29	0.04	0.15	0.13	0.01	0.12

Q9Y4L1	Hypoxia up-regulated protein 1 OS=Homo sapiens GN=HYOU1 PE=1 SV=1 - [HYOU1_HUMAN]	47.85	2	34	34	77	-0.30	-0.23	-0.22	-0.20	0.09	0.19	-0.18	-0.10	-0.05	-0.17	-0.22	-0.15	0.22	0.10	-0.01	0.18	0.11	0.47	0.36	0.12
P12270	Nucleoprotein TPR OS=Homo sapiens GN=TPR PE=1 SV=3 - [TPR_HUMAN]	19.76	1	41	42	71	-0.34	-0.28	-0.43	-0.36	-0.60	-0.47	-0.40	-0.30	-0.36	-0.42	-0.20	-0.08	0.08	0.11	0.22	-0.05	0.06	-0.11	-0.11	0.12
Q5SSJ5	Heterochromatin protein 1-binding protein 3 OS=Homo sapiens GN=HP1BP3 PE=1 SV=1 - [HP1B3_HUMAN]	30.92	1	18	18	41	-0.75	-0.85	-0.51	-0.58	-0.62	-0.74	-0.43	-0.51	-0.68	-0.57	-0.34	-0.40	0.39	0.46	0.14	0.22	-0.05	0.10	-0.18	0.12
Q9H0W8	Protein SMG9 OS=Homo sapiens GN=SMG9 PE=1 SV=1 - [SMG9_HUMAN]	5.96	1	2	2	3	0.05	0.34	-0.69	-0.39	-0.41	-0.12	-0.63	-0.34	0.05	-0.24	-0.12	0.16	-0.63	-0.17	0.56	-0.26	0.48	-0.48	0.26	0.12
Q969Q0	60S ribosomal protein L36a-like OS=Homo sapiens GN=RPL36AL PE=1 SV=3 - [RL36L_HUMAN]	37.74	1	1	4	14	-0.38	-0.39	-0.25	-0.25	0.16	0.15	-0.19	-0.20	0.91	0.92	0.24	0.23	0.25	0.63	0.49	1.33	1.19	0.53	0.39	0.12
O14656	Torsin-1A OS=Homo sapiens GN=TOR1A PE=1 SV=1 - [TOR1A_HUMAN]	9.34	1	3	3	7	-0.28	-0.24	-0.32	-0.27	0.20	0.33	-0.20	-0.16	0.06	0.05	0.20	0.36	0.15	0.61	0.68	0.33	0.47	0.55	0.69	0.12
Q7Z4W1	L-xylulose reductase OS=Homo sapiens GN=DCXR PE=1 SV=2 - [DCXR_HUMAN]	48.77	1	9	9	23	0.14	-0.05	0.15	0.07	0.11	0.03	0.20	0.12	0.05	0.06	-0.10	-0.19	0.06	-0.24	-0.42	-0.08	-0.11	-0.10	-0.06	0.12
Q9BV81	ER membrane protein complex subunit 6 OS=Homo sapiens GN=EMC6 PE=1 SV=1 - [EMC6_HUMAN]	20.00	1	2	2	4	0.45	0.69	-0.02	0.22	0.26	0.49	0.04	0.27	0.29	0.05	0.32	0.55	-0.36	-0.13	0.34	-0.37	0.10	-0.21	0.26	0.12
Q9BY42	Protein RTF2 homolog OS=Homo sapiens GN=RTFDC1 PE=1 SV=3 - [RTF2_HUMAN]	13.40	1	3	3	5	-0.36	-0.37	-0.15	-0.16	0.08	0.06	-0.10	-0.11	0.00	0.01	-0.15	-0.16	0.31	0.22	0.00	0.40	0.20	0.42	0.21	0.12

P14316	Interferon regulatory factor 2 OS=Homo sapiens GN=IRF2 PE=1 SV=2 - [IRF2_HUMAN]	5.16	1	1	1	1	0.06	0.36	-0.17	0.13	0.41	0.71	-0.12	0.19	0.61	0.32	0.01	0.31	-0.12	-0.05	0.18	0.29	0.52	0.34	0.56	0.12
Q9UHD9	Ubiquilin-2 OS=Homo sapiens GN=UBQLN2 PE=1 SV=2 - [UBQL2_HUMAN]	20.03	1	5	8	20	0.22	-0.12	-0.05	-0.10	0.30	0.09	0.09	0.19	-0.03	0.00	0.19	0.10	0.12	0.36	0.13	-0.01	0.15	0.20	0.19	0.12
Q9Y608	Leucine-rich repeat flightless-interacting protein 2 OS=Homo sapiens GN=LRRFIP2 PE=1 SV=1 - [LRRF2_HUMAN]	22.19	1	9	10	27	-0.25	-0.73	-0.37	-0.61	-0.15	-0.46	-0.34	-0.58	-0.51	-0.44	-0.15	-0.37	-0.35	0.38	0.25	-0.15	0.16	0.17	0.21	0.12
P60709	Actin, cytoplasmic 1 OS=Homo sapiens GN=ACTB PE=1 SV=1 - [ACTB_HUMAN]	73.87	3	9	28	1359	-0.41	-0.40	-0.38	-0.42	-0.06	-0.09	-0.33	-0.33	-0.22	-0.18	-0.14	-0.14	0.08	0.19	0.28	0.16	0.23	0.36	0.36	0.12
Q9ULT8	E3 ubiquitin-protein ligase HECTD1 OS=Homo sapiens GN=HECTD1 PE=1 SV=3 - [HECD1_HUMAN]	4.33	1	8	8	10	0.27	0.03	-0.15	-0.60	0.25	0.01	-0.06	-0.29	-0.17	0.07	0.07	-0.28	-0.27	-0.25	0.10	0.00	0.40	0.26	0.67	0.11
Q9H8Y8	Golgi reassembly-stacking protein 2 OS=Homo sapiens GN=GORASP2 PE=1 SV=3 - [GORS2_HUMAN]	22.35	2	7	7	12	0.48	0.46	0.77	0.49	0.76	0.69	0.69	0.51	0.46	0.38	0.22	0.19	0.23	-0.04	-0.44	0.20	-0.01	0.38	0.15	0.11
Q5VYS8	Terminal uridylyltransferase 7 OS=Homo sapiens GN=ZCCHC6 PE=1 SV=1 - [TUT7_HUMAN]	0.80	1	1	1	1	0.62	0.38	0.21	-0.03	0.31	0.06	0.26	0.02	0.06	0.31	0.41	0.16	-0.30	-0.21	0.19	-0.28	0.12	-0.32	0.08	0.11
Q14152	Eukaryotic translation initiation factor 3 subunit A OS=Homo sapiens GN=EIF3A PE=1 SV=1 - [EIF3A_HUMAN]	23.30	1	30	30	52	-0.43	-0.25	-0.45	-0.33	-0.27	-0.15	-0.31	-0.32	-0.15	-0.30	-0.21	-0.21	0.11	0.15	0.26	0.19	0.33	0.11	0.17	0.11

Q9HD33	39S ribosomal protein L47, mitochondrial OS=Homo sapiens GN=MRPL47 PE=1 SV=2 - [RM47_HUMAN]	11.20	1	3	3	3	-0.02	0.30	0.04	0.35	-0.32	-0.01	-0.13	0.18	0.01	-0.30	-0.14	0.17	-0.22	-0.35	-0.03	-0.25	-0.31	-0.32	-0.38	0.11
Q9UQR1	Zinc finger protein 148 OS=Homo sapiens GN=ZNF148 PE=1 SV=2 - [ZN148_HUMAN]	1.76	1	1	1	1	-1.22	-1.11	-0.48	-0.37	-0.55	-0.45	-0.43	-0.33	-0.99	-1.10	-0.61	-0.51	0.84	0.61	-0.13	0.15	-0.58	0.65	-0.09	0.11
Q9UIJ7	GTP:AMP phosphotransferase AK3, mitochondrial OS=Homo sapiens GN=AK3 PE=1 SV=4 - [KAD3_HUMAN]	70.48	1	16	16	35	-0.82	-0.84	-0.39	-0.43	-0.21	-0.32	-0.39	-0.45	-0.49	-0.46	-0.43	-0.41	0.31	0.36	0.00	0.29	0.07	0.42	0.16	0.11
Q12972	Nuclear inhibitor of protein phosphatase 1 OS=Homo sapiens GN=PPP1R8 PE=1 SV=2 - [PP1R8_HUMAN]	15.10	1	3	3	5	-0.44	-0.57	-0.24	-0.76	0.06	-0.08	-0.19	-0.32	-0.21	-0.08	0.08	-0.05	0.30	0.52	0.32	0.39	0.19	0.48	0.28	0.11
O95721	Synaptosomal-associated protein 29 OS=Homo sapiens GN=SNAP29 PE=1 SV=1 - [SNP29_HUMAN]	45.35	1	9	9	19	-0.29	-0.19	-0.04	-0.15	-0.22	-0.15	-0.02	0.02	-0.22	-0.04	0.14	0.13	0.24	0.37	0.13	-0.13	0.03	0.11	0.00	0.11
Q9H3P2	Negative elongation factor A OS=Homo sapiens GN=NELFA PE=1 SV=3 - [NELFA_HUMAN]	2.65	1	1	1	2	-0.57	-0.60	-0.65	-0.68	-0.18	-0.21	-0.60	-0.64	-0.21	-0.18	-0.14	-0.18	0.02	0.43	0.51	0.42	0.51	0.37	0.46	0.11
P81877	Single-stranded DNA-binding protein 2 OS=Homo sapiens GN=SSBP2 PE=1 SV=2 - [SSBP2_HUMAN]	2.77	3	1	1	2	-0.01	-0.22	-0.17	-0.37	0.35	0.14	-0.12	-0.33	-0.14	0.07	0.16	-0.05	-0.05	0.17	0.32	0.12	0.27	0.35	0.50	0.11
P62241	40S ribosomal protein S8 OS=Homo sapiens GN=RPS8 PE=1 SV=2 - [RS8_HUMAN]	51.44	1	10	10	23	-0.54	-0.64	-0.46	-0.62	-0.45	-0.36	-0.34	-0.45	-0.55	-0.35	-0.31	-0.27	0.26	0.35	0.24	0.18	0.18	0.22	0.15	0.11

Q15046	Lysine--tRNA ligase OS=Homo sapiens GN=KARS PE=1 SV=3 - [SYK_HUMAN]	28.31	1	15	15	25	0.04	0.08	-0.11	0.10	0.30	0.28	0.06	0.04	-0.03	0.02	-0.07	-0.17	0.20	-0.01	-0.04	0.13	0.24	0.21	0.46	0.11
Q8IWZ6	Bardet-Biedl syndrome 7 protein OS=Homo sapiens GN=BBS7 PE=1 SV=2 - [BBS7_HUMAN]	6.01	1	3	3	3	0.75	0.14	-0.03	-0.78	0.22	-0.52	0.01	-0.73	-0.70	0.04	0.14	-0.61	-0.66	-0.65	0.17	-0.49	0.11	-0.42	0.24	0.11
Q15750	TGF-beta-activated kinase 1 and MAP3K7-binding protein 1 OS=Homo sapiens GN=TAB1 PE=1 SV=1 - [TAB1_HUMAN]	21.03	1	8	8	13	-0.07	0.17	-0.03	-0.02	0.03	0.11	-0.03	0.00	0.13	-0.07	0.17	0.06	-0.01	0.08	0.11	0.13	0.28	0.19	0.16	0.11
Q9HAU0	Pleckstrin homology domain-containing family A member 5 OS=Homo sapiens GN=PLEKHA5 PE=1 SV=1 - [PKHA5_HUMAN]	25.63	1	21	21	39	0.01	-0.03	0.00	0.03	0.12	0.09	0.12	0.08	0.03	0.07	0.17	0.16	0.21	0.19	0.12	0.14	0.13	0.09	-0.03	0.11
Q9C0C4	Semaphorin-4C OS=Homo sapiens GN=SEMA4C PE=1 SV=2 - [SEM4C_HUMAN]	3.48	1	2	2	3	0.60	0.51	0.07	-0.02	0.43	0.34	0.12	0.03	0.04	0.13	0.24	0.15	-0.43	-0.35	0.17	-0.44	0.09	-0.18	0.34	0.11
Q8N4N3	Kelch-like protein 36 OS=Homo sapiens GN=KLHL36 PE=1 SV=1 - [KLH36_HUMAN]	2.27	1	1	1	1	-0.76	-0.16	-0.93	-0.32	-0.47	0.13	-0.88	-0.27	0.14	-0.46	-0.59	0.01	-0.06	0.17	0.33	0.33	0.50	0.28	0.44	0.11
O00148	ATP-dependent RNA helicase DDX39A OS=Homo sapiens GN=DDX39A PE=1 SV=2 - [DX39A_HUMAN]	27.87	1	4	12	30	-0.41	-0.17	0.06	0.30	0.29	0.53	0.10	0.34	0.20	-0.03	-0.26	-0.03	0.56	0.15	-0.32	0.40	-0.06	0.68	0.22	0.11
Q9BT40	Inositol polyphosphate 5-phosphatase K OS=Homo sapiens GN=INPP5K PE=1 SV=3 - [INP5K_HUMAN]	7.81	1	3	3	5	-0.39	-0.10	-0.39	-0.10	-0.25	0.04	-0.35	-0.06	-0.47	-0.76	-0.79	-0.50	0.10	-0.39	-0.40	-0.34	-0.34	0.13	0.12	0.11

Q8WWY3	U4/U6 small nuclear ribonucleoprotein Prp31 OS=Homo sapiens GN=PRPF31 PE=1 SV=2 - [PRP31_HUMAN]	12.63	1	4	5	8	-0.32	-0.47	-0.33	-0.38	-0.19	-0.29	-0.25	-0.34	-0.45	-0.31	-0.29	-0.41	0.22	0.05	-0.01	0.04	-0.09	0.22	0.17	0.11
Q9BY44	Eukaryotic translation initiation factor 2A OS=Homo sapiens GN=EIF2A PE=1 SV=3 - [EIF2A_HUMAN]	21.54	1	9	9	15	0.05	-0.11	-0.04	-0.11	0.24	0.08	0.05	-0.04	-0.15	-0.03	0.29	0.13	0.05	0.48	0.11	-0.05	0.04	0.30	0.04	0.11
Q8NB90	Spermatogenesis-associated protein 5 OS=Homo sapiens GN=SPATA5 PE=1 SV=3 - [SPAT5_HUMAN]	5.15	1	2	3	5	-0.90	-1.41	-0.95	-1.46	-0.16	-0.68	-0.91	-1.42	-0.51	0.01	0.36	-0.15	0.05	1.27	1.31	0.94	0.99	0.72	0.77	0.10
Q03252	Lamin-B2 OS=Homo sapiens GN=LMNB2 PE=1 SV=3 - [LMNB2_HUMAN]	45.17	1	25	27	90	-0.66	-0.57	-0.57	-0.52	-0.58	-0.54	-0.57	-0.47	-0.51	-0.55	-0.20	-0.18	0.11	0.39	0.37	0.07	0.14	-0.04	-0.08	0.10
P35244	Replication protein A 14 kDa subunit OS=Homo sapiens GN=RPA3 PE=1 SV=1 - [RFA3_HUMAN]	54.55	1	4	4	9	0.00	0.05	0.14	0.04	0.35	0.36	0.02	0.02	0.32	0.42	0.37	0.49	0.02	0.82	0.78	0.28	0.31	0.44	0.45	0.10
Q99598	Translin-associated protein X OS=Homo sapiens GN=TSNAX PE=1 SV=1 - [TSNAX_HUMAN]	25.17	1	5	5	6	0.51	0.01	0.25	0.06	-0.24	-0.90	0.19	-0.12	-0.52	-0.01	-0.10	-0.76	-0.19	-0.66	-0.36	-0.49	-0.53	-0.77	-0.51	0.10
Q02818	Nucleobindin-1 OS=Homo sapiens GN=NUCB1 PE=1 SV=4 - [NUCB1_HUMAN]	53.80	1	20	20	39	-0.37	-0.36	-0.56	-0.32	-0.05	-0.07	-0.37	-0.33	-0.08	-0.19	-0.22	-0.18	0.02	0.23	0.30	0.29	0.29	0.21	0.31	0.10
Q13610	Periodic tryptophan protein 1 homolog OS=Homo sapiens GN=PWP1 PE=1 SV=1 - [PWP1_HUMAN]	12.77	1	4	4	7	0.19	-0.10	0.34	0.06	0.25	-0.05	0.38	0.09	-0.24	0.05	0.30	0.01	0.25	0.12	-0.04	-0.11	-0.26	0.04	-0.11	0.10

P35658	Nuclear pore complex protein Nup214 OS=Homo sapiens GN=NUP214 PE=1 SV=2 - [NU214_HUMAN]	5.74	1	8	8	11	-0.73	-0.55	-0.76	-0.69	-0.88	-0.71	-0.83	-0.65	-0.30	-0.48	-0.17	-0.03	-0.04	0.57	0.61	0.19	0.45	-0.17	0.00	0.10
O60763	General vesicular transport factor p115 OS=Homo sapiens GN=USO1 PE=1 SV=2 - [USO1_HUMAN]	35.76	1	25	25	51	0.25	0.10	0.28	0.33	0.53	0.56	0.36	0.40	0.32	0.35	0.22	0.20	0.14	0.04	-0.11	0.22	0.11	0.36	0.26	0.10
Q9H3M7	Thioredoxin-interacting protein OS=Homo sapiens GN=TXNIP PE=1 SV=1 - [TXNIP_HUMAN]	5.88	1	1	1	1	-1.83	-1.73	-0.49	-0.39	-0.03	0.06	-0.45	-0.35	0.83	0.74	0.13	0.22	1.44	1.97	0.62	2.60	1.26	1.79	0.44	0.10
P02787	Serotransferrin OS=Homo sapiens GN=TF PE=1 SV=3 - [TRFE_HUMAN]	55.87	1	36	36	109	-0.94	-0.89	-0.72	-0.65	-0.49	-0.47	-0.67	-0.60	-0.64	-0.72	-0.52	-0.49	0.25	0.35	0.22	0.29	0.10	0.41	0.21	0.10
P05455	Lupus La protein OS=Homo sapiens GN=SSB PE=1 SV=2 - [LA_HUMAN]	30.39	1	13	13	27	-0.42	-0.26	0.03	-0.09	-0.46	-0.31	-0.05	-0.06	-0.33	-0.40	-0.45	-0.41	0.20	-0.21	-0.50	-0.08	-0.28	-0.03	-0.27	0.10
Q2M389	WASH complex subunit 7 OS=Homo sapiens GN=KIAA1033 PE=1 SV=2 - [WASH7_HUMAN]	10.66	1	9	9	13	0.16	0.71	0.12	0.19	-0.12	-0.03	0.32	0.27	0.76	0.14	0.00	0.23	0.04	0.02	-0.24	0.11	0.05	-0.28	-0.23	0.10
Q6DT37	Serine/threonine-protein kinase MRCK gamma OS=Homo sapiens GN=CDC42BPG PE=1 SV=2 - [MRCKG_HUMAN]	1.87	1	1	1	2	0.52	-0.16	-2.07	-2.75	-2.79	-3.47	-2.03	-2.71	-2.66	-1.98	-1.79	-2.47	-2.50	-2.30	0.28	-2.47	0.12	-3.33	-0.74	0.10
Q8IWA4	Mitofusin-1 OS=Homo sapiens GN=MFN1 PE=1 SV=2 - [MFN1_HUMAN]	1.75	1	1	1	2	-0.28	-0.10	0.01	0.20	0.05	0.23	0.05	0.24	0.08	-0.10	0.29	0.47	0.39	0.58	0.27	0.21	-0.08	0.31	0.02	0.10

O75379	Vesicle-associated membrane protein 4 OS=Homo sapiens GN=VAMP4 PE=1 SV=2 - [VAMP4_HUMAN]	9.93	1	1	1	1	-0.77	-0.59	-0.97	-0.78	-0.89	-0.71	-0.93	-0.75	-0.72	-0.90	-0.63	-0.46	-0.10	0.14	0.33	-0.10	0.10	-0.14	0.06	0.10
Q9NY27	Serine/threonine-protein phosphatase 4 regulatory subunit 2 OS=Homo sapiens GN=PPP4R2 PE=1 SV=3 - [PP4R2_HUMAN]	11.27	1	3	3	6	0.22	0.27	-0.11	0.16	0.58	0.50	-0.08	0.20	0.30	0.37	0.55	0.47	-0.03	0.26	0.27	0.18	0.23	0.34	0.39	0.10
P61225	Ras-related protein Rap-2b OS=Homo sapiens GN=RAP2B PE=1 SV=1 - [RAP2B_HUMAN]	58.47	1	5	8	17	-0.06	-0.19	0.44	0.47	0.00	-0.09	0.39	0.26	0.44	0.40	0.64	0.59	0.54	0.67	0.21	0.41	0.14	-0.04	-0.28	0.10
Q8NE86	Calcium uniporter protein, mitochondrial OS=Homo sapiens GN=MCU PE=1 SV=1 - [MCU_HUMAN]	25.36	1	7	7	16	-0.40	-0.35	-0.08	0.09	-0.23	-0.13	0.01	0.00	-0.19	-0.26	-0.21	-0.29	0.51	0.25	-0.12	0.20	-0.19	0.15	-0.09	0.10
O75044	SLIT-ROBO Rho GTPase-activating protein 2 OS=Homo sapiens GN=SRGAP2 PE=1 SV=2 - [SRGP2_HUMAN]	15.13	2	13	14	23	0.04	-0.09	0.18	0.10	0.33	0.21	0.30	0.28	0.23	0.42	0.16	-0.01	0.17	0.04	-0.01	0.04	0.16	0.28	0.08	0.10
Q7Z7M0	Multiple epidermal growth factor-like domains protein 8 OS=Homo sapiens GN=MEGF8 PE=1 SV=2 - [MEGF8_HUMAN]	1.55	1	2	2	2	-0.73	-0.53	-0.48	-0.28	-0.12	0.08	-0.45	-0.24	-0.25	-0.45	-0.44	-0.24	0.34	0.30	0.05	0.32	0.07	0.60	0.35	0.10
P16234	Platelet-derived growth factor receptor alpha OS=Homo sapiens GN=PDGFRA PE=1 SV=1 - [PGFRA_HUMAN]	3.76	5	2	3	7	0.45	0.12	-0.16	-0.49	0.63	0.30	-0.13	-0.45	0.46	0.79	0.53	0.20	-0.52	0.09	0.69	0.38	0.99	0.17	0.77	0.10

P61962	DDB1- and CUL4-associated factor 7 OS=Homo sapiens GN=DCAF7 PE=1 SV=1 - [DCAF7_HUMAN]	12.28	1	3	3	5	-0.13	-0.40	0.01	-0.14	0.18	0.18	0.02	-0.23	-0.02	0.18	0.11	-0.08	0.23	0.23	0.09	0.26	0.13	0.27	0.15	0.10
Q5VW32	BRO1 domain-containing protein BROX OS=Homo sapiens GN=BROX PE=1 SV=1 - [BROX_HUMAN]	27.74	1	8	8	11	0.31	0.29	0.23	0.19	0.16	0.25	0.30	0.32	-0.01	0.07	0.03	0.11	-0.02	-0.23	-0.13	-0.20	0.07	0.26	0.50	0.10
Q99848	Probable rRNA-processing protein EBP2 OS=Homo sapiens GN=EBNA1BP2 PE=1 SV=2 - [EBP2_HUMAN]	3.27	1	1	1	2	-0.02	-0.15	-0.66	-0.79	-0.13	-0.27	-0.62	-0.75	-0.68	-0.54	0.11	-0.03	-0.55	0.13	0.76	-0.49	0.15	-0.13	0.51	0.10
P07814	Bifunctional glutamate/proline--tRNA ligase OS=Homo sapiens GN=EPRS PE=1 SV=5 - [SYEP_HUMAN]	32.28	1	37	37	86	-0.28	-0.26	-0.18	-0.12	-0.04	0.06	-0.20	-0.09	-0.08	-0.15	-0.17	-0.11	0.20	0.16	0.03	0.18	0.07	0.25	0.15	0.10
Q14160	Protein scribble homolog OS=Homo sapiens GN=SCRIB PE=1 SV=4 - [SCRIB_HUMAN]	11.17	1	14	14	23	-0.13	-0.20	-0.15	-0.17	-0.13	-0.14	-0.14	-0.18	-0.23	-0.21	0.03	-0.01	-0.06	0.21	0.34	0.04	0.06	-0.04	-0.03	0.10
Q03426	Mevalonate kinase OS=Homo sapiens GN=MVK PE=1 SV=1 - [KIME_HUMAN]	11.36	1	2	2	3	0.55	0.47	0.12	0.04	0.34	0.25	0.15	0.07	0.42	0.51	0.66	0.58	-0.35	0.11	0.55	-0.01	0.43	-0.23	0.21	0.10
O95819	Mitogen-activated protein kinase kinase kinase kinase 4 OS=Homo sapiens GN=MAP4K4 PE=1 SV=2 - [M4K4_HUMAN]	7.83	1	3	7	12	-0.28	-0.14	-0.30	-0.16	-0.46	-0.33	-0.26	-0.13	-0.19	-0.32	-0.33	-0.20	0.07	-0.05	-0.04	-0.01	0.01	-0.21	-0.18	0.10

Q5SRE7	Phytanoyl-CoA dioxygenase domain-containing protein 1 OS=Homo sapiens GN=PHYHD1 PE=1 SV=2 - [PHYD1_HUMAN]	31.62	1	6	6	11	0.71	0.72	0.41	0.10	0.89	0.69	0.69	0.79	0.60	0.62	0.61	0.70	0.12	-0.06	0.21	0.11	0.07	0.08	0.24	0.10
O94829	Importin-13 OS=Homo sapiens GN=IPO13 PE=1 SV=3 - [IPO13_HUMAN]	5.92	1	4	4	5	0.64	0.76	0.47	0.64	1.00	1.11	0.52	0.82	0.65	0.53	0.05	0.39	0.11	-0.35	-0.20	-0.07	0.05	0.35	0.46	0.10
P26012	Integrin beta-8 OS=Homo sapiens GN=ITGB8 PE=1 SV=1 - [ITB8_HUMAN]	11.44	1	6	6	7	1.00	0.88	1.08	0.97	0.32	0.20	0.88	0.76	0.59	0.71	0.42	0.29	-0.30	-0.88	-0.35	-0.87	0.20	-1.08	-0.53	0.10
Q99459	Cell division cycle 5-like protein OS=Homo sapiens GN=CDC5L PE=1 SV=2 - [CDC5L_HUMAN]	16.46	1	9	9	15	-0.34	-0.31	-0.19	-0.10	-0.23	0.06	-0.27	-0.28	-0.23	-0.04	-0.06	-0.02	0.07	0.29	0.08	0.15	0.11	0.16	0.14	0.10
P15880	40S ribosomal protein S2 OS=Homo sapiens GN=RPS2 PE=1 SV=2 - [RS2_HUMAN]	46.76	1	13	13	29	-0.71	-0.72	-0.64	-0.59	-0.44	-0.45	-0.64	-0.64	-0.58	-0.59	-0.26	-0.33	0.08	0.35	0.33	0.21	0.17	0.39	0.13	0.10
Q96S55	ATPase WRNIP1 OS=Homo sapiens GN=WRNIP1 PE=1 SV=2 - [WRIP1_HUMAN]	4.66	1	3	3	4	0.45	0.23	0.04	-0.18	0.11	-0.11	0.07	-0.14	-0.09	0.13	0.24	0.02	-0.32	-0.20	0.20	-0.29	0.12	-0.36	0.05	0.10
Q14498	RNA-binding protein 39 OS=Homo sapiens GN=RBM39 PE=1 SV=2 - [RBM39_HUMAN]	27.55	1	11	11	29	0.23	-0.20	-0.07	-0.45	-0.05	-0.18	-0.07	-0.29	-0.36	-0.08	-0.08	-0.34	-0.16	-0.23	0.01	-0.23	0.10	0.28	0.24	0.10
Q9BV47	Dual specificity protein phosphatase 26 OS=Homo sapiens GN=DUSP26 PE=1 SV=1 - [DUS26_HUMAN]	9.95	1	1	1	2	0.77	0.21	0.83	0.27	0.85	0.28	0.86	0.30	0.21	0.78	-0.26	-0.82	0.15	-1.02	-1.09	0.04	-0.02	0.06	0.00	0.10

P49792	E3 SUMO-protein ligase RanBP2 OS=Homo sapiens GN=RANBP2 PE=1 SV=2 - [RBP2_HUMAN]	16.04	7	32	33	56	-0.50	-0.59	-0.38	-0.35	-0.13	-0.10	-0.20	-0.04	-0.28	-0.13	-0.12	-0.16	0.53	0.39	0.16	0.35	0.05	0.26	0.05	0.10
O15305	Phosphomannomutase 2 OS=Homo sapiens GN=PMM2 PE=1 SV=1 - [PMM2_HUMAN]	10.57	1	2	3	3	-0.11	-0.43	0.07	-0.24	0.39	0.07	0.11	-0.21	-0.10	0.23	-0.05	-0.37	0.27	0.07	-0.13	0.37	0.19	0.49	0.30	0.10
P20042	Eukaryotic translation initiation factor 2 subunit 2 OS=Homo sapiens GN=EIF2S2 PE=1 SV=2 - [IF2B_HUMAN]	37.84	1	10	10	25	-0.30	-0.38	-0.16	-0.31	-0.19	-0.56	-0.26	-0.20	-0.08	-0.12	-0.17	-0.36	0.19	0.04	-0.06	0.17	0.15	-0.06	-0.44	0.10
Q9Y2Q9	28S ribosomal protein S28, mitochondrial OS=Homo sapiens GN=MRPS28 PE=1 SV=1 - [RT28_HUMAN]	9.09	1	2	2	3	-0.11	0.00	-0.16	-0.05	0.51	0.61	-0.13	-0.02	0.25	0.15	0.07	0.17	0.04	0.19	0.22	0.29	0.34	0.60	0.65	0.09
P10620	Microsomal glutathione S-transferase 1 OS=Homo sapiens GN=MGST1 PE=1 SV=1 - [MGST1_HUMAN]	9.03	1	2	2	3	1.01	0.76	1.45	1.19	0.96	0.70	1.48	1.23	0.83	1.09	1.22	0.97	0.52	0.22	-0.22	0.11	-0.32	-0.07	-0.50	0.09
P62910	60S ribosomal protein L32 OS=Homo sapiens GN=RPL32 PE=1 SV=2 - [RL32_HUMAN]	39.26	1	6	6	12	-1.09	-1.07	-0.86	-0.88	-0.49	-0.48	-0.68	-0.70	-1.00	-0.97	-0.57	-0.52	0.36	0.48	0.25	0.04	-0.08	0.33	0.22	0.09
Q9Y394	Dehydrogenase/reductase SDR family member 7 OS=Homo sapiens GN=DHRS7 PE=1 SV=1 - [DHRS7_HUMAN]	25.96	1	8	8	25	-0.48	-0.45	-0.35	-0.45	-0.04	-0.15	-0.31	-0.38	-0.32	-0.24	0.16	0.03	0.14	0.45	0.51	0.15	0.17	0.21	0.32	0.09
P09012	U1 small nuclear ribonucleoprotein A OS=Homo sapiens GN=SNRPA PE=1 SV=3 - [SNRPA_HUMAN]	19.15	1	3	5	10	-0.31	-0.05	-0.14	-0.03	0.10	-0.36	-0.43	-0.48	-0.11	-0.05	-0.32	-0.04	-0.17	0.01	0.38	-0.01	-0.04	0.12	0.06	0.09

O75879	Glutamyl-tRNA(Gln) amidotransferase subunit B, mitochondrial OS=Homo sapiens GN=PET112 PE=1 SV=1 - [GATB_HUMAN]	6.64	1	3	3	5	-0.22	-0.04	-0.51	-0.33	0.73	0.91	-0.48	-0.30	0.02	-0.16	-0.13	0.05	-0.20	0.10	0.38	0.10	0.39	0.93	1.22	0.09
Q96JJ3	Engulfment and cell motility protein 2 OS=Homo sapiens GN=ELMO2 PE=1 SV=2 - [ELMO2_HUMAN]	12.78	1	5	8	11	-0.06	-0.29	-0.12	-0.35	0.16	-0.07	-0.09	-0.32	-0.38	-0.15	-0.11	-0.34	0.02	-0.04	0.01	-0.06	0.01	0.21	0.27	0.09
Q13685	Angio-associated migratory cell protein OS=Homo sapiens GN=AAMP PE=1 SV=2 - [AAMP_HUMAN]	11.75	1	4	4	7	0.31	0.50	0.26	0.29	0.22	0.25	0.23	0.37	0.31	0.13	-0.13	-0.10	-0.08	-0.22	-0.40	-0.15	-0.04	-0.05	-0.05	0.09
Q8WTV0	Scavenger receptor class B member 1 OS=Homo sapiens GN=SCARB1 PE=1 SV=1 - [SCRB1_HUMAN]	2.17	1	1	1	2	0.18	0.11	-0.07	-0.14	0.53	0.45	-0.04	-0.11	0.21	0.29	0.20	0.12	-0.16	0.03	0.27	0.14	0.39	0.34	0.58	0.09
Q9NZ09	Ubiquitin-associated protein 1 OS=Homo sapiens GN=UBAP1 PE=1 SV=1 - [UBAP1_HUMAN]	8.17	1	3	3	3	0.06	0.30	-0.21	0.03	-0.34	-0.11	-0.18	0.06	-0.20	-0.43	0.07	0.31	-0.19	0.01	0.28	-0.46	-0.19	-0.42	-0.15	0.09
P30050	60S ribosomal protein L12 OS=Homo sapiens GN=RPL12 PE=1 SV=1 - [RL12_HUMAN]	63.03	1	8	8	23	-0.77	-0.59	-0.59	-0.57	-0.44	-0.68	-0.49	-0.56	-0.57	-0.45	-0.25	-0.24	0.12	0.39	0.41	0.33	0.25	0.30	0.12	0.09
P17302	Gap junction alpha-1 protein OS=Homo sapiens GN=GJA1 PE=1 SV=2 - [CXA1_HUMAN]	37.96	1	9	9	40	0.52	0.43	0.14	0.28	0.14	0.06	0.33	0.23	0.27	0.39	0.55	0.56	-0.20	0.17	0.48	-0.16	-0.13	-0.42	-0.44	0.09
Q6WCQ1	Myosin phosphatase Rho-interacting protein OS=Homo sapiens GN=MPRIP PE=1 SV=3 - [MPRIP_HUMAN]	24.49	1	20	21	35	-0.79	-0.82	-0.73	-0.75	-0.63	-0.69	-0.71	-0.66	-0.74	-0.78	-0.80	-0.82	0.20	0.12	-0.07	0.09	0.06	0.14	0.11	0.09

Q96S59	Ran-binding protein 9 OS=Homo sapiens GN=RANBP9 PE=1 SV=1 - [RANB9_HUMAN]	7.41	1	4	4	6	0.36	0.27	0.32	0.32	-0.05	0.12	0.07	-0.03	0.07	0.05	0.09	0.04	-0.26	-0.45	-0.20	-0.33	-0.24	-0.16	-0.40	0.09
P46439	Glutathione S transferase Mu 5 OS=Homo sapiens GN=GSTM5 PE=1 SV=3 - [GSTM5_HUMAN]	68.81	1	8	16	55	0.55	0.68	0.29	0.41	0.55	0.54	0.43	0.63	0.42	0.31	-0.25	-0.35	0.03	-1.00	-0.82	-0.18	-0.17	-0.03	-0.15	0.09
Q9H3S3	Transmembrane protease serine 5 OS=Homo sapiens GN=TMPRSS5 PE=1 SV=2 - [TMPS5_HUMAN]	2.41	1	1	1	1	-0.34	-0.21	-0.43	-0.29	-0.11	0.02	-0.40	-0.27	-0.15	-0.28	-0.93	-0.80	0.00	-0.58	-0.50	0.10	0.18	0.22	0.30	0.09
Q8WTS6	Histone-lysine N-methyltransferase SETD7 OS=Homo sapiens GN=SETD7 PE=1 SV=1 - [SETD7_HUMAN]	27.60	1	6	6	12	-0.47	-0.22	-0.35	-0.13	-0.10	0.10	-0.22	-0.08	-0.19	-0.32	-0.30	-0.20	0.36	0.18	0.00	0.20	-0.04	0.22	0.17	0.09
O00213	Amyloid beta A4 precursor protein-binding family B member 1 OS=Homo sapiens GN=APBB1 PE=1 SV=2 - [APBB1_HUMAN]	16.76	1	7	8	11	-0.05	0.18	-0.22	-0.14	-0.22	0.15	-0.32	-0.02	0.16	-0.27	0.03	0.12	-0.14	-0.01	0.19	-0.13	0.27	0.00	0.22	0.09
Q99424	Peroxisomal acyl-coenzyme A oxidase 2 OS=Homo sapiens GN=ACOX2 PE=1 SV=1 - [ACOX2_HUMAN]	4.70	1	2	2	3	0.18	-0.11	0.09	-0.19	0.35	0.06	0.12	-0.17	0.37	0.66	0.81	0.52	0.00	0.64	0.72	0.52	0.60	0.16	0.24	0.09
P10586	Receptor-type tyrosine-protein phosphatase F OS=Homo sapiens GN=PTPRF PE=1 SV=2 - [PTPRF_HUMAN]	16.15	1	15	21	36	-0.14	0.11	0.01	0.01	0.22	0.16	-0.18	0.03	0.19	0.06	-0.15	-0.01	0.02	-0.03	-0.09	0.02	0.00	0.20	0.11	0.09

Q96EK5	KIF1-binding protein OS=Homo sapiens GN=KIAA1279 PE=1 SV=1 - [KBP_HUMAN]	12.72	1	5	5	9	0.55	0.28	0.44	0.36	0.36	0.27	0.47	0.25	-0.10	-0.01	0.22	0.17	-0.12	-0.52	-0.22	-0.57	-0.20	-0.27	-0.10	0.09
Q6PI48	Aspartate--tRNA ligase, mitochondrial OS=Homo sapiens GN=DARS2 PE=1 SV=1 - [SYDM_HUMAN]	10.39	1	5	5	11	-1.06	-1.14	-0.91	-0.99	-0.93	-1.02	-0.57	-0.80	-0.84	-0.75	-0.39	-0.48	0.23	0.97	0.59	0.34	0.19	0.42	0.04	0.09
Q9Y2K6	Ubiquitin carboxyl-terminal hydrolase 20 OS=Homo sapiens GN=USP20 PE=1 SV=2 - [UBP20_HUMAN]	3.39	1	2	2	4	-0.14	0.28	-0.30	0.13	-0.70	-0.29	-0.27	0.15	-0.35	-0.77	-0.48	-0.07	-0.08	-0.34	-0.19	-0.60	-0.44	-0.58	-0.43	0.09
Q9NR28	Diablo homolog, mitochondrial OS=Homo sapiens GN=DIABLO PE=1 SV=1 - [DBLOH_HUMAN]	27.62	1	6	6	8	-0.35	-0.36	-0.37	-0.35	-0.01	-0.17	-0.21	-0.39	-0.17	-0.01	-0.08	-0.23	0.13	0.17	0.08	0.33	0.25	0.29	0.24	0.09
P52272	Heterogeneous nuclear ribonucleoprotein M OS=Homo sapiens GN=HNRNPM PE=1 SV=3 - [HNRPM_HUMAN]	44.66	1	29	29	121	-0.28	-0.41	-0.33	-0.36	-0.25	-0.32	-0.27	-0.32	-0.26	-0.19	-0.02	-0.10	0.10	0.35	0.27	0.16	0.19	0.08	0.04	0.09
Q14696	LDLR chaperone MESD OS=Homo sapiens GN=MESDC2 PE=1 SV=2 - [MESD_HUMAN]	17.95	1	4	4	5	-0.80	-0.53	-0.59	-0.32	-0.27	-0.01	-0.56	-0.30	-0.20	-0.46	-0.16	0.10	0.29	0.65	0.43	0.37	0.16	0.51	0.30	0.09
O60551	Glycylpeptide N-tetradecanoyltransferase 2 OS=Homo sapiens GN=NMT2 PE=1 SV=1 - [NMT2_HUMAN]	11.65	1	4	5	7	-0.07	0.44	-0.47	0.15	-0.82	-0.28	-0.21	0.10	-0.45	-0.52	-0.70	-0.40	-0.08	-0.69	-0.88	-0.86	-0.59	-0.53	-0.37	0.09
Q12792	Twinfilin-1 OS=Homo sapiens GN=TWF1 PE=1 SV=3 - [TWF1_HUMAN]	23.43	1	6	8	18	-0.01	-0.18	0.11	-0.04	0.26	0.25	0.13	0.12	0.09	0.24	0.11	0.02	0.02	-0.28	-0.14	0.20	0.17	0.15	0.11	0.09

P68400	Casein kinase II subunit alpha OS=Homo sapiens GN=CSNK2A1 PE=1 SV=1 - [CSK21_HUMAN]	50.38	2	15	16	36	0.03	0.09	0.23	0.14	0.02	0.04	0.22	0.25	0.05	0.03	-0.01	-0.05	0.04	0.02	-0.25	0.00	-0.08	-0.03	-0.17	0.09
P47914	60S ribosomal protein L29 OS=Homo sapiens GN=RPL29 PE=1 SV=2 - [RL29_HUMAN]	14.47	1	2	2	4	-1.27	-1.26	-0.71	-0.73	-0.63	-0.64	-0.81	-0.86	-0.82	-0.83	-0.36	-0.47	0.52	0.80	0.26	0.48	0.01	0.61	0.07	0.08
P16435	NADPH--cytochrome P450 reductase OS=Homo sapiens GN=POR PE=1 SV=2 - [NCPR_HUMAN]	26.14	1	15	15	30	-0.06	-0.10	0.02	-0.08	0.05	0.08	-0.19	-0.13	-0.05	-0.15	0.09	0.13	-0.03	0.31	0.24	0.08	0.04	0.11	0.16	0.08
P48728	Aminomethyltransferase, mitochondrial OS=Homo sapiens GN=AMT PE=1 SV=1 - [GCST_HUMAN]	10.17	1	4	4	8	-0.62	-0.69	-0.42	-0.48	-0.46	-0.54	-0.40	-0.46	-0.74	-0.67	-0.44	-0.51	0.28	0.18	-0.03	-0.02	-0.22	0.14	-0.07	0.08
P62266	40S ribosomal protein S23 OS=Homo sapiens GN=RPS23 PE=1 SV=3 - [RS23_HUMAN]	48.25	1	5	5	13	-0.66	-0.72	-0.44	-0.49	-0.30	-0.39	-0.56	-0.59	-0.50	-0.45	-0.38	-0.44	0.38	0.35	0.08	0.19	-0.06	0.34	-0.04	0.08
Q96CT7	Coiled-coil domain-containing protein 124 OS=Homo sapiens GN=CCDC124 PE=1 SV=1 - [CC124_HUMAN]	24.22	1	4	4	6	-0.21	0.23	-0.48	-0.03	-0.68	-0.24	-0.45	-0.01	-0.14	-0.58	-0.48	-0.03	-0.19	-0.26	0.00	-0.34	-0.08	-0.49	-0.23	0.08
P06213	Insulin receptor OS=Homo sapiens GN=INSR PE=1 SV=4 - [INSR_HUMAN]	11.58	1	10	12	15	-0.21	-0.19	-0.14	-0.01	0.24	0.26	-0.14	-0.18	0.08	0.06	0.17	0.27	0.11	0.37	0.29	0.33	0.29	0.44	0.41	0.08
Q13615	Myotubularin-related protein 3 OS=Homo sapiens GN=MTMR3 PE=1 SV=3 - [MTMR3_HUMAN]	3.51	2	4	4	5	0.05	0.20	-0.02	-0.16	-0.12	-0.27	0.00	0.10	0.21	0.11	0.00	0.17	0.01	-0.04	0.07	0.09	0.17	-0.19	-0.12	0.08

Q63HN8	E3 ubiquitin-protein ligase RNF213 OS=Homo sapiens GN=RNF213 PE=1 SV=3 - [RN213_HUMAN]	1.56	1	6	6	8	-0.24	-0.02	-0.17	-0.08	0.31	0.58	-0.14	-0.06	0.27	0.01	0.08	0.33	0.34	0.51	0.24	0.18	0.21	0.32	0.48	0.08
Q96KR1	Zinc finger RNA-binding protein OS=Homo sapiens GN=ZFR PE=1 SV=2 - [ZFR_HUMAN]	9.59	1	8	8	12	-0.46	-0.30	-0.21	-0.36	-0.31	-0.39	-0.29	-0.46	-0.26	-0.13	-0.52	-0.34	0.17	-0.06	-0.15	0.19	0.39	0.26	0.03	0.08
Q15393	Splicing factor 3B subunit 3 OS=Homo sapiens GN=SF3B3 PE=1 SV=4 - [SF3B3_HUMAN]	19.80	1	18	19	38	-0.44	-0.29	-0.40	-0.37	-0.24	-0.22	-0.38	-0.31	-0.30	-0.32	-0.23	-0.18	0.06	0.27	0.24	0.06	0.18	0.13	0.06	0.08
O15145	Actin-related protein 2/3 complex subunit 3 OS=Homo sapiens GN=ARPC3 PE=1 SV=3 - [ARPC3_HUMAN]	7.30	1	1	1	2	-1.50	-1.23	-1.08	-0.81	-0.56	-0.30	-1.06	-0.79	-0.66	-0.93	-0.22	0.04	0.49	1.28	0.86	0.60	0.18	0.92	0.50	0.08
Q9NX24	H/ACA ribonucleoprotein complex subunit 2 OS=Homo sapiens GN=NHP2 PE=1 SV=1 - [NHP2_HUMAN]	12.42	1	1	1	2	-0.84	-0.87	-0.73	-0.76	-0.22	-0.25	-0.71	-0.74	-0.58	-0.54	-0.39	-0.43	0.18	0.45	0.34	0.33	0.22	0.61	0.50	0.08
P11387	DNA topoisomerase 1 OS=Homo sapiens GN=TOP1 PE=1 SV=2 - [TOP1_HUMAN]	14.51	2	10	10	15	-0.58	-0.65	-0.40	-0.47	-0.45	-0.57	-0.27	-0.39	-0.52	-0.45	-0.64	-0.62	0.15	-0.21	-0.43	0.17	-0.01	0.04	-0.14	0.08
O60645	Exocyst complex component 3 OS=Homo sapiens GN=EXOC3 PE=1 SV=2 - [EXOC3_HUMAN]	15.48	1	11	11	18	-0.03	-0.04	0.13	0.18	-0.01	-0.08	0.15	0.12	0.16	0.36	0.06	0.00	0.21	0.05	-0.07	0.19	0.07	-0.03	-0.20	0.08
P52657	Transcription initiation factor IIA subunit 2 OS=Homo sapiens GN=GTF2A2 PE=1 SV=1 - [T2AG_HUMAN]	8.26	1	1	1	2	-0.16	-0.22	-0.19	-0.26	-0.11	-0.18	-0.17	-0.24	-0.33	-0.26	0.02	-0.05	0.04	0.19	0.21	-0.07	-0.04	0.03	0.06	0.08

Q9HBF4	Zinc finger FYVE domain-containing protein 1 OS=Homo sapiens GN=ZFYVE1 PE=1 SV=1 - [ZFYV1_HUMAN]	6.95	1	4	4	5	-0.08	-0.16	-0.23	-0.30	-0.09	-0.18	-0.21	-0.29	-0.21	-0.13	-0.01	-0.10	-0.08	0.07	0.21	-0.02	0.13	-0.03	0.11	0.08
O43677	NADH dehydrogenase [ubiquinone] 1 subunit C1, mitochondrial OS=Homo sapiens GN=NDUFC1 PE=2 SV=1 - [NDUC1_HUMAN]	27.63	1	2	2	3	-0.19	-0.02	0.25	0.42	0.11	0.27	0.27	0.43	0.34	0.18	0.32	0.48	0.51	0.51	0.06	0.39	-0.04	0.28	-0.16	0.08
Q86WC4	Osteopetrosis-associated transmembrane protein 1 OS=Homo sapiens GN=OSTM1 PE=1 SV=1 - [OSTM1_HUMAN]	2.10	1	1	1	2	0.46	0.62	0.43	0.59	0.54	0.69	0.45	0.60	0.76	0.61	0.29	0.44	0.04	-0.17	-0.14	0.18	0.22	0.06	0.09	0.08
Q9BYC9	39S ribosomal protein L20, mitochondrial OS=Homo sapiens GN=MRPL20 PE=1 SV=1 - [RM20_HUMAN]	5.37	1	1	1	2	0.32	0.23	0.16	0.07	0.11	0.02	0.18	0.09	0.15	0.24	0.23	0.13	-0.09	-0.09	0.07	-0.05	0.11	-0.22	-0.07	0.08
Q9Y2Q3	Glutathione S transferase kappa 1 OS=Homo sapiens GN=GSTK1 PE=1 SV=3 - [GSTK1_HUMAN]	49.56	1	8	8	21	-0.44	-0.49	-0.41	-0.43	-0.08	-0.13	-0.39	-0.42	-0.37	-0.27	-0.08	-0.10	0.07	0.42	0.28	0.40	0.13	0.48	0.13	0.08
P36578	60S ribosomal protein L4 OS=Homo sapiens GN=RPL4 PE=1 SV=5 - [RL4_HUMAN]	31.15	1	14	14	41	-0.79	-0.67	-0.52	-0.39	-0.35	-0.46	-0.43	-0.42	-0.36	-0.42	-0.13	-0.09	0.36	0.56	0.42	0.24	0.06	0.39	0.02	0.08
Q6P1X6	UPF0598 protein C8orf82 OS=Homo sapiens GN=C8orf82 PE=1 SV=2 - [CH082_HUMAN]	24.54	1	4	4	10	-0.33	-0.41	-0.34	-0.42	-0.62	-0.48	-0.35	-0.48	-0.37	-0.45	-0.46	-0.24	0.32	0.28	0.05	0.07	0.06	-0.08	-0.14	0.08

O95487	Protein transport protein Sec24B OS=Homo sapiens GN=SEC24B PE=1 SV=2 - [SC24B_HUMAN]	9.46	1	8	9	15	-0.18	0.01	0.00	0.07	0.20	0.19	0.07	-0.06	-0.08	0.00	0.12	0.15	0.15	0.20	0.07	0.06	-0.07	0.30	0.11	0.08
O60437	Periplakin OS=Homo sapiens GN=PPL PE=1 SV=4 - [PEPL_HUMAN]	13.50	1	18	18	24	-1.42	-1.33	-1.63	-1.61	-1.36	-1.28	-1.70	-1.61	-1.47	-1.49	-1.11	-1.15	-0.13	0.24	0.56	-0.06	0.28	0.15	0.38	0.08
Q9BYJ9	YTH domain-containing family protein 1 OS=Homo sapiens GN=YTHDF1 PE=1 SV=1 - [YTHD1_HUMAN]	4.29	2	1	2	2	0.07	-0.17	0.11	-0.13	0.20	-0.05	0.13	-0.12	0.15	0.40	0.15	-0.10	0.11	0.09	0.04	0.36	0.32	0.11	0.07	0.08
Q92900	Regulator of nonsense transcripts 1 OS=Homo sapiens GN=UPF1 PE=1 SV=2 - [RENT1_HUMAN]	36.23	2	32	32	60	-0.34	-0.36	-0.24	-0.13	-0.08	0.00	-0.19	-0.14	-0.11	-0.17	-0.14	-0.09	0.19	0.19	0.05	0.22	0.03	0.24	0.07	0.08
Q53S33	BolA-like protein 3 OS=Homo sapiens GN=BOLA3 PE=1 SV=1 - [BOLA3_HUMAN]	12.15	1	1	1	1	-0.36	0.27	-0.64	0.00	-0.55	0.08	-0.63	0.01	-0.23	-0.86	-1.54	-0.90	-0.21	-1.17	-0.90	-0.47	-0.19	-0.20	0.07	0.08
Q14764	Major vault protein OS=Homo sapiens GN=MVP PE=1 SV=4 - [MVP_HUMAN]	45.80	1	29	29	69	-0.97	-0.86	-0.89	-0.82	-0.32	-0.22	-0.86	-0.78	-0.61	-0.64	-0.30	-0.27	0.08	0.59	0.58	0.31	0.29	0.61	0.56	0.08
Q9UKM9	RNA-binding protein Raly OS=Homo sapiens GN=RALY PE=1 SV=1 - [RALY_HUMAN]	40.20	1	11	12	24	-0.76	-0.83	-0.61	-0.65	-0.38	-0.42	-0.35	-0.52	-0.54	-0.42	-0.39	-0.53	0.17	0.22	-0.03	0.22	0.02	0.35	-0.01	0.08
P00738	Haptoglobin OS=Homo sapiens GN=HP PE=1 SV=1 - [HPT_HUMAN]	50.99	1	11	21	129	-1.78	-1.69	-1.72	-1.66	-1.38	-1.19	-1.59	-1.53	-1.18	-1.33	-0.80	-0.76	0.24	1.02	0.90	0.41	0.29	0.27	0.08	0.08

Q9NUB1	Acetyl-coenzyme A synthetase 2-like, mitochondrial OS=Homo sapiens GN=ACSS1 PE=1 SV=2 - [ACS2L_HUMAN]	15.67	1	7	7	12	-0.44	-0.24	-0.34	-0.59	-0.70	-0.55	-0.52	-0.55	-0.78	-0.68	-0.53	-0.78	-0.03	-0.09	-0.19	-0.24	-0.20	-0.19	-0.23	0.08
P49790	Nuclear pore complex protein Nup153 OS=Homo sapiens GN=NUP153 PE=1 SV=2 - [NU153_HUMAN]	2.92	1	3	3	4	-1.32	-1.40	-1.24	-1.33	-0.78	-0.87	-1.23	-1.32	-0.88	-0.78	-0.11	-0.20	0.14	1.21	1.13	0.56	0.49	0.52	0.45	0.08
Q4G0X4	BTB/POZ domain-containing protein KCTD21 OS=Homo sapiens GN=KCTD21 PE=2 SV=1 - [KCD21_HUMAN]	6.54	1	1	1	2	0.03	0.04	-0.44	-0.42	-0.24	-0.23	-0.43	-0.41	-0.10	-0.11	-0.22	-0.21	-0.40	-0.24	0.22	-0.10	0.37	-0.28	0.18	0.08
Q96ME7	Zinc finger protein 512 OS=Homo sapiens GN=ZNF512 PE=1 SV=2 - [ZN512_HUMAN]	13.58	1	6	6	11	-0.58	-0.55	-0.54	-0.49	-0.36	-0.37	-0.35	-0.43	-0.27	-0.24	-0.21	-0.22	0.11	0.26	0.35	0.24	0.25	0.08	0.04	0.07
Q9NQ86	E3 ubiquitin-protein ligase TRIM36 OS=Homo sapiens GN=TRIM36 PE=2 SV=2 - [TRI36_HUMAN]	6.73	1	4	4	5	-0.17	-0.06	-0.36	-0.24	-0.06	0.05	-0.35	-0.23	-0.24	-0.35	-0.12	-0.01	-0.12	0.06	0.24	-0.15	0.04	0.10	0.28	0.07
Q15599	Na(+)/H(+) exchange regulatory cofactor NHE-RF2 OS=Homo sapiens GN=SLC9A3R2 PE=1 SV=2 - [NHRF2_HUMAN]	18.69	1	6	6	27	-1.16	-0.80	-1.07	-0.83	-0.73	-0.59	-1.01	-0.82	-0.80	-0.91	-0.81	-0.60	0.19	0.42	0.39	0.22	0.09	0.19	0.17	0.07
Q15643	Thyroid receptor-interacting protein 11 OS=Homo sapiens GN=TRIP11 PE=1 SV=3 - [TRIPB_HUMAN]	1.21	1	2	2	3	0.20	0.38	-0.09	0.09	0.14	0.32	-0.08	0.10	0.28	0.11	0.04	0.21	-0.22	-0.16	0.12	-0.06	0.23	-0.07	0.21	0.07

Q96RS6	NudC domain-containing protein 1 OS=Homo sapiens GN=NUDCD1 PE=1 SV=2 - [NUDC1_HUMAN]	15.61	1	6	6	7	-0.12	-0.35	-0.22	-0.53	0.18	-0.06	-0.20	-0.37	-0.13	0.21	0.05	-0.19	-0.03	0.18	0.15	0.16	0.46	0.28	0.45	0.07
Q96CW9	Netrin-G2 OS=Homo sapiens GN=NTNG2 PE=1 SV=2 - [NTNG2_HUMAN]	1.70	1	1	1	1	0.24	0.53	0.29	0.58	0.72	1.01	0.30	0.59	0.24	-0.04	-0.10	0.18	0.12	-0.34	-0.40	-0.25	-0.30	0.46	0.41	0.07
P51452	Dual specificity protein phosphatase 3 OS=Homo sapiens GN=DUSP3 PE=1 SV=1 - [DUS3_HUMAN]	44.32	1	6	6	21	0.66	0.37	0.43	0.46	0.29	0.22	0.40	0.45	0.36	0.49	0.13	-0.03	0.09	-0.29	-0.19	0.03	0.16	-0.20	-0.27	0.07
Q53GS9	U4/U6.U5 tri-snRNP-associated protein 2 OS=Homo sapiens GN=USP39 PE=1 SV=2 - [SNUT2_HUMAN]	6.90	1	3	3	4	-0.68	-0.67	-0.67	-0.51	-0.27	-0.33	-0.41	-0.50	-0.30	-0.24	-0.13	-0.19	0.22	0.29	0.32	0.16	0.38	0.38	0.45	0.07
O15042	U2 snRNP-associated SURP motif-containing protein OS=Homo sapiens GN=U2SURP PE=1 SV=2 - [SR140_HUMAN]	6.90	1	6	6	10	-0.10	0.20	-0.10	-0.13	0.44	0.33	-0.09	-0.11	-0.27	-0.05	0.18	0.01	0.02	0.04	0.02	-0.45	-0.13	0.20	0.04	0.07
Q86VU5	Catechol O-methyltransferase domain-containing protein 1 OS=Homo sapiens GN=COMTD1 PE=1 SV=1 - [CMTD1_HUMAN]	4.20	1	1	1	3	0.07	0.12	0.15	0.20	0.21	0.25	0.16	0.21	0.04	0.00	0.15	0.19	0.14	0.09	0.00	-0.04	-0.12	0.12	0.04	0.07
Q8WVH0	Complexin-3 OS=Homo sapiens GN=CPLX3 PE=2 SV=1 - [CPLX3_HUMAN]	12.03	1	1	1	2	1.31	0.86	0.78	0.33	0.14	-0.32	0.95	0.49	0.20	0.67	-0.62	-1.08	-0.29	-1.15	-0.80	-0.62	-0.08	-1.19	-0.66	0.07
Q13098	COP9 signalosome complex subunit 1 OS=Homo sapiens GN=GPS1 PE=1 SV=4 - [CSN1_HUMAN]	24.64	1	9	9	13	-0.32	-0.03	0.07	0.00	0.01	-0.06	0.08	0.07	0.14	0.08	-0.12	-0.12	0.12	-0.24	-0.31	-0.08	-0.01	-0.14	-0.21	0.07

P42285	Superkiller viralicidic activity 2-like 2 OS=Homo sapiens GN=SKIV2L2 PE=1 SV=3 - [SK2L2_HUMAN]	7.01	1	4	4	5	-0.74	-0.34	-0.75	-0.41	-0.28	-0.02	-0.66	-0.34	-0.17	-0.04	-0.15	0.03	0.06	0.53	0.53	0.14	0.15	0.43	0.50	0.07
P38919	Eukaryotic initiation factor 4A-III OS=Homo sapiens GN=EIF4A3 PE=1 SV=4 - [IF4A3_HUMAN]	31.87	1	12	14	21	-0.52	-0.61	-0.27	-0.30	-0.20	-0.18	-0.22	-0.26	-0.28	-0.33	-0.16	-0.24	0.36	0.38	0.17	0.29	0.10	0.31	0.15	0.07
P62829	60S ribosomal protein L23 OS=Homo sapiens GN=RPL23 PE=1 SV=1 - [RL23_HUMAN]	62.86	1	7	7	17	-0.97	-1.10	-0.57	-0.69	-0.47	-0.50	-0.52	-0.54	-0.57	-0.61	-0.57	-0.70	0.43	0.19	-0.04	0.28	0.09	0.44	0.03	0.07
Q9H0Q3	FXYD domain-containing ion transport regulator 6 OS=Homo sapiens GN=FXYD6 PE=2 SV=1 - [FXYD6_HUMAN]	26.32	1	2	2	21	-0.18	-0.18	0.21	0.21	0.17	0.16	-0.42	-0.42	-0.11	-0.10	0.74	0.73	-0.13	0.84	0.43	0.11	-0.28	0.33	-0.06	0.07
P39019	40S ribosomal protein S19 OS=Homo sapiens GN=RPS19 PE=1 SV=2 - [RS19_HUMAN]	48.28	1	9	9	23	-0.06	0.00	-0.18	-0.15	-0.36	-0.40	-0.26	-0.22	-0.47	-0.37	-0.35	-0.40	-0.11	-0.22	-0.09	-0.20	-0.03	-0.10	-0.08	0.07
Q96EE3	Nucleoporin SEH1 OS=Homo sapiens GN=SEH1L PE=1 SV=3 - [SEH1_HUMAN]	18.33	1	4	4	8	-0.02	-0.11	-0.07	-0.20	0.07	0.06	-0.09	-0.12	0.01	0.02	0.06	0.05	0.07	0.17	0.01	0.16	-0.03	0.16	0.24	0.07
Q9Y2T2	AP-3 complex subunit mu-1 OS=Homo sapiens GN=AP3M1 PE=1 SV=1 - [AP3M1_HUMAN]	37.80	1	9	12	17	-0.46	-0.40	-0.14	-0.31	-0.02	0.00	-0.13	-0.09	0.05	0.02	-0.06	0.03	0.39	0.43	0.29	0.52	0.19	0.63	0.30	0.07
P23378	Glycine dehydrogenase (decarboxylating), mitochondrial OS=Homo sapiens GN=GLDC PE=1 SV=2 - [GCSP_HUMAN]	6.86	1	4	4	6	-0.35	-0.48	-0.22	-0.35	-0.23	-0.36	-0.21	-0.34	-0.32	-0.18	-0.39	-0.52	0.19	-0.03	-0.17	0.20	0.07	0.10	-0.03	0.07

P84098	60S ribosomal protein L19 OS=Homo sapiens GN=RPL19 PE=1 SV=1 - [RL19_HUMAN]	17.35	1	4	4	25	-0.34	-0.36	-0.41	-0.58	-0.37	-0.53	-0.55	-0.59	-0.58	-0.39	-0.34	-0.50	-0.15	-0.07	0.15	-0.04	0.12	-0.08	0.00	0.07
Q9H9P8	L-2-hydroxyglutarate dehydrogenase, mitochondrial OS=Homo sapiens GN=L2HGDH PE=1 SV=3 - [L2HDH_HUMAN]	19.87	1	7	7	14	0.07	0.00	0.36	0.46	0.20	0.10	0.38	0.35	0.30	0.31	0.01	-0.09	0.09	-0.07	-0.56	0.15	0.09	0.01	-0.34	0.07
P40227	T-complex protein 1 subunit zeta OS=Homo sapiens GN=CCT6A PE=1 SV=3 - [TCPZ_HUMAN]	43.31	2	20	20	55	-0.26	-0.30	-0.10	-0.10	-0.16	-0.15	-0.14	-0.16	-0.17	-0.09	-0.10	-0.11	0.19	0.23	0.03	0.19	0.04	0.12	-0.03	0.07
O75521	Enoyl-CoA delta isomerase 2, mitochondrial OS=Homo sapiens GN=ECI2 PE=1 SV=4 - [ECI2_HUMAN]	32.99	1	10	10	15	-0.43	-0.13	-0.35	-0.25	-0.27	-0.20	-0.32	-0.27	-0.31	-0.45	-0.37	-0.27	0.01	0.05	-0.18	-0.19	-0.19	-0.08	-0.05	0.07
Q93100	Phosphorylase b kinase regulatory subunit beta OS=Homo sapiens GN=PHKB PE=1 SV=3 - [KPBB_HUMAN]	10.98	1	10	10	14	-0.09	-0.25	0.18	-0.01	-0.12	-0.14	0.18	0.13	-0.26	-0.07	0.00	-0.09	0.23	0.02	-0.19	-0.10	-0.36	-0.04	-0.25	0.07
P50990	T-complex protein 1 subunit theta OS=Homo sapiens GN=CCT8 PE=1 SV=4 - [TCPQ_HUMAN]	57.66	1	30	30	95	-0.31	-0.24	-0.08	-0.06	-0.06	0.01	-0.11	-0.12	-0.15	-0.14	-0.04	-0.05	0.23	0.23	0.04	0.21	0.00	0.25	0.04	0.07
Q9BVJ7	Dual specificity protein phosphatase 23 OS=Homo sapiens GN=DUSP23 PE=1 SV=1 - [DUS23_HUMAN]	24.00	1	3	3	6	0.14	0.10	-0.12	-0.15	0.98	0.95	-0.12	-0.15	0.51	0.54	0.79	0.75	-0.20	0.66	0.91	0.44	0.70	0.83	1.09	0.07

O14681	Etoposide-induced protein 2.4 homolog OS=Homo sapiens GN=EI24 PE=1 SV=4 - [EI24_HUMAN]	2.35	1	1	1	2	-0.27	-0.41	-0.19	-0.32	0.04	-0.11	-0.18	-0.32	-0.23	-0.09	0.02	-0.13	0.14	0.29	0.20	0.21	0.13	0.29	0.20	0.07
Q92797	Symplekin OS=Homo sapiens GN=SYMPK PE=1 SV=2 - [SYMPK_HUMAN]	2.04	1	2	2	2	0.04	0.63	-0.85	-0.26	-0.49	0.09	-0.85	-0.26	-0.01	-0.59	-0.31	0.27	-0.83	-0.35	0.54	-0.60	0.29	-0.55	0.34	0.07
O95154	Aflatoxin B1 aldehyde reductase member 3 OS=Homo sapiens GN=AKR7A3 PE=1 SV=2 - [ARK73_HUMAN]	11.48	1	2	3	5	0.32	-0.15	0.18	-0.29	0.78	0.31	0.01	-0.46	-0.24	0.23	0.39	-0.08	-0.05	0.08	0.21	0.01	0.12	0.44	0.58	0.07
Q8IVM0	Coiled-coil domain-containing protein 50 OS=Homo sapiens GN=CCDC50 PE=1 SV=1 - [CCD50_HUMAN]	15.69	1	4	4	7	-1.21	-0.98	-1.19	-0.97	-0.71	-0.49	-1.19	-0.96	-0.64	-0.86	-0.49	-0.27	0.08	0.73	0.70	0.38	0.36	0.48	0.46	0.07
Q9BTT0	Acidic leucine-rich nuclear phosphoprotein 32 family member E OS=Homo sapiens GN=ANP32E PE=1 SV=1 - [AN32E_HUMAN]	32.84	1	6	6	13	-0.08	0.29	-0.13	-0.02	-0.21	-0.17	-0.18	-0.02	-0.01	-0.08	0.10	0.12	-0.26	0.11	0.15	0.07	0.05	-0.44	-0.19	0.07
Q8NBX0	Saccharopine dehydrogenase-like oxidoreductase OS=Homo sapiens GN=SCCPDH PE=1 SV=1 - [SCPDL_HUMAN]	27.27	1	8	8	21	-0.13	0.05	-0.06	-0.09	-0.18	0.01	-0.16	-0.05	-0.04	-0.13	-0.12	0.05	0.00	0.34	0.10	0.20	0.08	0.06	-0.09	0.07
P24928	DNA-directed RNA polymerase II subunit RPB1 OS=Homo sapiens GN=POLR2A PE=1 SV=2 - [RPB1_HUMAN]	6.24	1	8	8	12	-0.37	-0.33	-0.35	-0.41	-0.23	-0.32	-0.26	-0.29	-0.35	-0.35	-0.34	-0.33	0.13	0.14	0.10	0.08	0.16	0.11	0.01	0.07

Q9Y617	Phosphoserine aminotransferase OS=Homo sapiens GN=PSAT1 PE=1 SV=2 - [SERC_HUMAN]	40.54	1	13	13	36	0.42	0.52	0.67	0.87	0.33	0.43	0.66	0.89	0.62	0.33	0.04	0.19	0.14	-0.53	-0.68	0.00	-0.24	-0.19	-0.47	0.07
Q8WVC6	Dephospho-CoA kinase domain-containing protein OS=Homo sapiens GN=DCAKD PE=1 SV=1 - [DCAKD_HUMAN]	27.27	1	5	5	12	0.18	0.15	0.04	0.30	0.12	0.39	0.05	0.22	0.29	0.15	0.24	0.41	-0.14	0.15	0.11	-0.12	0.03	-0.24	-0.10	0.07
Q8NBI6	Xyloside xylosyltransferase 1 OS=Homo sapiens GN=XXYLT1 PE=1 SV=1 - [XXLT1_HUMAN]	2.54	1	1	1	1	-1.10	-0.94	-0.96	-0.79	-0.39	-0.23	-0.95	-0.79	-0.56	-0.72	-0.77	-0.61	0.20	0.34	0.19	0.41	0.27	0.69	0.55	0.07
Q15165	Serum paraoxonase/arylesterase 2 OS=Homo sapiens GN=PON2 PE=1 SV=3 - [PON2_HUMAN]	47.46	1	8	8	16	-0.57	-0.41	-0.16	0.02	0.22	0.16	-0.18	0.04	0.07	-0.06	-0.21	-0.20	0.36	0.16	-0.12	0.41	0.14	0.73	0.39	0.07
Q9UD71	Protein phosphatase 1 regulatory subunit 1B OS=Homo sapiens GN=PPP1R1B PE=1 SV=2 - [PPR1B_HUMAN]	38.73	1	5	5	23	-0.21	-0.07	-0.41	-0.32	-0.74	-0.72	-0.42	-0.37	-0.40	-0.30	0.13	0.24	-0.03	0.21	0.38	-0.08	0.21	-0.65	-0.52	0.07
Q15007	Pre-mRNA-splicing regulator WTAP OS=Homo sapiens GN=WTAP PE=1 SV=2 - [FL2D_HUMAN]	6.06	1	2	2	3	0.03	0.09	-0.47	-0.41	-0.10	-0.04	-0.47	-0.40	0.03	-0.02	0.06	0.12	-0.44	0.03	0.53	-0.02	0.48	-0.14	0.36	0.07
Q08170	Serine/arginine-rich splicing factor 4 OS=Homo sapiens GN=SRSF4 PE=1 SV=2 - [SRSF4_HUMAN]	20.85	1	6	9	20	-0.23	-0.53	-0.23	-0.42	-0.26	-0.55	-0.24	-0.44	-0.78	-0.44	-0.38	-0.64	0.27	-0.15	-0.10	-0.10	-0.25	0.01	0.01	0.07
Q9NVE7	Pantothenate kinase 4 OS=Homo sapiens GN=PANK4 PE=1 SV=1 - [PANK4_HUMAN]	31.31	1	18	18	36	0.24	0.04	0.15	0.05	0.03	-0.02	0.11	0.03	0.03	0.06	0.19	0.08	0.04	-0.06	-0.07	0.04	0.07	-0.06	-0.09	0.07

O60830	Mitochondrial import inner membrane translocase subunit Tim17-B OS=Homo sapiens GN=TIMM17B PE=1 SV=1 - [TI17B_HUMAN]	12.79	1	1	1	1	0.62	0.37	0.61	0.36	0.42	0.16	0.61	0.36	0.30	0.56	0.50	0.24	0.04	-0.12	-0.11	-0.03	-0.01	-0.22	-0.20	0.07
Q8WUH1	Protein Churchill OS=Homo sapiens GN=CHURC1 PE=1 SV=2 - [CHUR_HUMAN]	9.35	1	1	1	1	-0.36	-0.46	-0.73	-0.82	-0.18	-0.28	-0.73	-0.82	-0.61	-0.51	-0.83	-0.93	-0.31	-0.46	-0.10	-0.12	0.25	0.16	0.53	0.06
Q86UF1	Tetraspanin-33 OS=Homo sapiens GN=TSPAN33 PE=2 SV=1 - [TSN33_HUMAN]	3.18	1	1	1	1	0.41	0.37	-0.73	-0.76	0.24	0.20	-0.73	-0.76	-0.14	-0.10	-0.36	-0.40	-1.08	-0.77	0.37	-0.48	0.66	-0.19	0.95	0.06
Q9BUQ8	Probable ATP-dependent RNA helicase DDX23 OS=Homo sapiens GN=DDX23 PE=1 SV=3 - [DDX23_HUMAN]	9.88	1	7	7	12	-0.08	-0.24	-0.06	-0.17	-0.01	0.24	-0.02	-0.28	-0.38	-0.12	-0.03	-0.14	0.02	0.15	0.03	-0.01	-0.03	0.35	0.32	0.06
Q99470	Stromal cell-derived factor 2 OS=Homo sapiens GN=SDF2 PE=1 SV=2 - [SDF2_HUMAN]	13.74	1	2	2	10	-0.35	-0.59	-0.39	-0.59	0.02	-0.08	-0.16	-0.32	-0.24	-0.04	0.15	-0.01	0.24	0.50	0.26	0.72	0.39	0.74	0.30	0.06
O95263	High affinity cAMP-specific and IBMX-insensitive 3',5'-cyclic phosphodiesterase 8B OS=Homo sapiens GN=PDE8B PE=1 SV=2 - [PDE8B_HUMAN]	3.28	1	2	2	2	0.10	-0.07	-0.35	-0.10	0.10	-0.13	-0.20	-0.10	-0.19	0.04	0.26	-0.43	0.03	-0.35	-0.33	-0.08	-0.05	-0.08	-0.05	0.06
Q8IXI1	Mitochondrial Rho GTPase 2 OS=Homo sapiens GN=RHOT2 PE=1 SV=2 - [MIRO2_HUMAN]	7.77	1	2	3	7	-0.18	-0.11	-0.21	-0.14	0.51	0.57	-0.21	-0.14	-0.17	-0.24	-0.15	-0.08	0.02	0.03	0.06	-0.03	0.00	0.67	0.70	0.06

P78527	DNA-dependent protein kinase catalytic subunit OS=Homo sapiens GN=PRKDC PE=1 SV=3 - [PRKDC_HUMAN]	19.26	1	68	68	122	-0.26	-0.33	-0.26	-0.31	-0.16	-0.12	-0.26	-0.26	-0.29	-0.20	-0.21	-0.11	0.03	0.10	0.10	0.00	0.08	0.15	0.16	0.06
Q86V88	Magnesium-dependent phosphatase 1 OS=Homo sapiens GN=MDP1 PE=1 SV=1 - [MGDP1_HUMAN]	26.14	1	3	3	5	0.91	1.08	0.70	0.79	0.64	0.79	0.69	0.86	0.93	0.77	0.42	0.58	-0.19	-0.45	-0.35	0.01	0.12	-0.19	-0.01	0.06
Q9BZ29	Dedicator of cytokinesis protein 9 OS=Homo sapiens GN=DOCK9 PE=1 SV=2 - [DOCK9_HUMAN]	5.90	1	8	8	12	-0.06	-0.55	-0.08	-0.37	0.13	-0.41	-0.17	-0.47	-0.28	-0.08	-0.23	-0.60	-0.08	-0.15	0.00	-0.14	0.03	0.17	0.19	0.06
Q9UJC3	Protein Hook homolog 1 OS=Homo sapiens GN=HOOK1 PE=1 SV=2 - [HOOK1_HUMAN]	1.10	1	1	1	1	0.44	0.88	0.02	0.47	-0.07	0.37	0.02	0.47	0.29	-0.15	-0.37	0.07	-0.36	-0.80	-0.39	-0.56	-0.14	-0.52	-0.11	0.06
Q8N983	39S ribosomal protein L43, mitochondrial OS=Homo sapiens GN=MRPL43 PE=1 SV=1 - [RM43_HUMAN]	16.74	1	4	4	5	0.31	0.58	0.06	0.33	0.21	0.47	0.05	0.32	0.26	-0.01	0.26	0.52	-0.20	-0.05	0.20	-0.29	-0.03	-0.12	0.13	0.06
O75533	Splicing factor 3B subunit 1 OS=Homo sapiens GN=SF3B1 PE=1 SV=3 - [SF3B1_HUMAN]	17.64	1	16	16	27	-0.53	-0.52	-0.25	-0.21	-0.22	-0.29	-0.31	-0.28	-0.50	-0.34	-0.23	-0.28	0.32	0.26	0.16	0.27	0.06	0.28	0.07	0.06
O00214	Galectin-8 OS=Homo sapiens GN=LGALS8 PE=1 SV=4 - [LEG8_HUMAN]	13.25	1	4	4	5	-0.31	0.17	0.09	0.43	0.01	0.47	-0.23	0.36	0.55	0.20	-0.29	0.18	-0.02	0.02	-0.24	0.28	0.27	0.10	0.03	0.06
P34896	Serine hydroxymethyltransferase, cytosolic OS=Homo sapiens GN=SHMT1 PE=1 SV=1 - [GLYC_HUMAN]	19.05	1	6	7	9	0.27	0.11	-0.13	0.00	0.61	0.50	0.23	0.07	0.22	0.14	0.40	0.19	0.04	0.02	0.58	0.07	0.15	0.26	0.25	0.06

Q9H7C9	Mth938 domain-containing protein OS=Homo sapiens GN=AAMDC PE=1 SV=1 - [AAMDC_HUMAN]	24.59	1	3	3	7	0.45	0.57	0.33	0.45	0.82	0.93	0.24	0.35	0.52	0.41	0.29	0.40	-0.04	-0.15	-0.04	-0.01	0.12	0.35	0.48	0.06
Q96GA7	Serine dehydratase-like OS=Homo sapiens GN=SDSL PE=1 SV=1 - [SDSL_HUMAN]	13.37	1	3	3	4	0.47	-0.74	0.59	-0.35	0.78	0.16	0.59	0.46	0.11	1.10	1.34	0.36	0.18	1.10	0.71	0.89	0.50	0.30	0.17	0.06
Q6P9F7	Leucine-rich repeat-containing protein 8B OS=Homo sapiens GN=LRRC8B PE=2 SV=2 - [LRC8B_HUMAN]	4.98	2	1	3	4	-0.63	-0.28	-0.81	-0.47	-0.06	0.28	-0.82	-0.47	0.28	-0.06	0.08	0.42	-0.13	0.72	0.89	0.60	0.78	0.55	0.74	0.06
Q9NRX4	14 kDa phosphohistidine phosphatase OS=Homo sapiens GN=PHPT1 PE=1 SV=1 - [PHP14_HUMAN]	45.60	1	4	4	15	0.67	0.49	0.64	0.44	0.52	0.36	0.63	0.77	0.52	0.68	0.29	0.16	0.21	-0.25	-0.37	-0.01	0.07	-0.14	-0.14	0.06
Q9BY89	Uncharacterized protein KIAA1671 OS=Homo sapiens GN=KIAA1671 PE=1 SV=2 - [K1671_HUMAN]	5.43	1	7	7	9	-0.98	-1.13	-0.83	-0.96	-0.55	-0.80	-0.78	-0.97	-1.03	-0.78	-0.81	-0.95	0.22	0.06	0.00	0.12	0.11	0.19	0.16	0.06
Q8TDN6	Ribosome biogenesis protein BRX1 homolog OS=Homo sapiens GN=BRIX1 PE=1 SV=2 - [BRX1_HUMAN]	2.27	1	1	1	1	-0.70	-0.62	-0.46	-0.38	-0.31	-0.24	-0.46	-0.39	-0.27	-0.34	-0.22	-0.15	0.29	0.49	0.24	0.39	0.15	0.38	0.13	0.06
O94911	ATP-binding cassette sub-family A member 8 OS=Homo sapiens GN=ABCA8 PE=1 SV=3 - [ABCA8_HUMAN]	12.40	3	12	15	23	-1.14	-1.10	-1.16	-0.93	-0.67	-0.53	-0.90	-0.87	-0.01	-0.19	0.28	0.35	0.19	1.15	1.14	0.78	0.65	0.46	0.21	0.06
P22304	Iduronate 2-sulfatase OS=Homo sapiens GN=IDS PE=1 SV=1 - [IDS_HUMAN]	4.91	1	1	1	1	0.65	0.85	0.83	1.02	1.11	1.30	0.82	1.02	0.86	0.67	-0.94	-0.75	0.22	-1.59	-1.77	0.05	-0.13	0.44	0.27	0.06

P07947	Tyrosine-protein kinase Yes OS=Homo sapiens GN=YES1 PE=1 SV=3 - [YES_HUMAN]	16.94	12	1	10	20	0.30	0.38	0.11	0.20	-0.20	-0.12	0.11	0.20	0.26	0.18	0.08	0.16	-0.13	-0.21	-0.04	-0.09	0.10	-0.52	-0.34	0.06
P51532	Transcription activator BRG1 OS=Homo sapiens GN=SMARCA4 PE=1 SV=2 - [SMCA4_HUMAN]	5.89	1	2	9	12	-0.21	-0.13	-0.28	-0.20	0.07	0.14	-0.29	-0.21	0.40	0.33	-0.05	0.03	-0.03	0.17	0.24	0.57	0.64	0.26	0.33	0.06
P14621	Acylphosphatase-2 OS=Homo sapiens GN=ACYP2 PE=1 SV=2 - [ACYP2_HUMAN]	46.46	1	5	5	12	0.73	0.79	0.71	0.84	0.62	0.71	0.76	0.89	0.66	0.52	0.47	0.49	-0.02	-0.34	-0.21	-0.23	-0.09	-0.08	0.09	0.06
Q9UBI9	Headcase protein homolog OS=Homo sapiens GN=HECA PE=1 SV=1 - [HDC_HUMAN]	3.87	1	2	2	2	-0.45	-0.51	-1.30	-1.36	0.10	0.04	-0.37	-0.43	-0.75	-0.68	-0.05	-0.12	-0.07	0.26	0.38	0.02	0.15	0.48	0.60	0.06
Q9P016	Thymocyte nuclear protein 1 OS=Homo sapiens GN=THYN1 PE=1 SV=1 - [THYN1_HUMAN]	20.44	1	5	5	7	-0.49	-0.42	-0.21	-0.36	-0.15	-0.09	-0.18	-0.33	-0.40	-0.40	-0.30	-0.34	0.37	0.39	0.10	0.11	0.04	0.32	0.12	0.06
Q8NAT1	Protein O-linked-mannose beta-1,4-N-acetylglucosaminyltransferase 2 OS=Homo sapiens GN=POMGNT2 PE=1 SV=1 - [PMGT2_HUMAN]	7.59	1	4	4	8	0.13	0.25	0.14	0.31	0.06	0.18	0.18	0.32	0.14	0.01	-0.15	0.00	0.11	-0.25	-0.32	-0.13	-0.18	-0.17	-0.22	0.06
P28907	ADP-ribosyl cyclase 1 OS=Homo sapiens GN=CD38 PE=1 SV=2 - [CD38_HUMAN]	11.67	1	3	3	4	0.08	0.48	-0.42	-0.03	-0.53	-0.14	-0.43	-0.04	-0.19	-0.58	-0.09	0.30	-0.46	-0.17	0.33	-0.63	-0.12	-0.63	-0.12	0.06
Q8N9N2	Activating signal cointegrator 1 complex subunit 1 OS=Homo sapiens GN=ASCC1 PE=1 SV=1 - [ASCC1_HUMAN]	2.25	1	1	1	2	-0.47	-0.60	-0.37	-0.50	0.44	0.30	-0.38	-0.51	-0.01	0.12	0.49	0.36	0.15	0.97	0.86	0.62	0.53	0.89	0.79	0.06

Q92859	Neogenin OS=Homo sapiens GN=NEO1 PE=1 SV=2 - [NEO1_HUMAN]	13.76	1	14	14	33	-0.30	-0.24	-0.19	-0.37	0.17	0.28	-0.09	-0.06	0.05	0.05	0.24	0.22	0.17	0.56	0.58	0.33	0.27	0.38	0.25	0.06
Q9C0I1	Myotubularin-related protein 12 OS=Homo sapiens GN=MTMR12 PE=1 SV=2 - [MTMRC_HUMAN]	4.42	1	3	3	3	-0.07	0.21	0.06	0.33	0.30	0.22	0.14	0.12	0.19	0.27	0.36	0.15	0.21	-0.09	-0.17	0.04	-0.12	0.14	-0.02	0.06
O43414	ERI1 exoribonuclease 3 OS=Homo sapiens GN=ERI3 PE=1 SV=2 - [ERI3_HUMAN]	7.12	1	3	3	5	0.19	0.19	0.30	0.30	0.21	0.21	0.29	0.29	0.07	0.07	-0.33	-0.34	0.16	-0.52	-0.63	-0.09	-0.20	0.01	-0.11	0.06
C4AMC7	Putative WAS protein family homolog 3 OS=Homo sapiens GN=WASH3P PE=2 SV=2 - [WASH3_HUMAN]	10.15	5	3	3	4	-0.05	0.17	0.02	0.25	0.09	0.31	0.02	0.24	0.31	0.10	0.25	0.47	0.12	0.30	0.23	0.17	0.11	0.12	0.05	0.06
P49356	Protein farnesyltransferase subunit beta OS=Homo sapiens GN=FNTB PE=1 SV=1 - [FNTB_HUMAN]	5.72	1	2	2	2	-0.13	-0.39	-0.02	-0.28	0.26	0.00	-0.03	-0.29	-0.13	0.13	0.23	-0.03	0.16	0.37	0.25	0.30	0.18	0.38	0.27	0.05
Q9BYD2	39S ribosomal protein L9, mitochondrial OS=Homo sapiens GN=MRPL9 PE=1 SV=2 - [RM09_HUMAN]	8.99	1	2	2	2	-0.09	-0.41	-0.13	-0.45	0.11	-0.21	-0.14	-0.46	-0.32	0.00	-0.01	-0.33	0.01	0.09	0.12	0.12	0.16	0.19	0.23	0.05
Q8N766	ER membrane protein complex subunit 1 OS=Homo sapiens GN=EMC1 PE=1 SV=1 - [EMC1_HUMAN]	20.34	1	15	15	29	0.01	0.04	0.13	0.14	0.09	0.13	-0.04	-0.10	0.00	0.12	0.06	-0.09	0.11	-0.09	-0.11	0.11	-0.03	0.17	-0.01	0.05
Q8IUR0	Trafficking protein particle complex subunit 5 OS=Homo sapiens GN=TRAPPC5 PE=1 SV=1 - [TPPC5_HUMAN]	7.98	1	2	2	4	0.56	0.60	0.18	0.25	0.30	0.20	0.22	0.12	0.19	0.12	0.11	0.10	-0.29	-0.44	-0.14	-0.42	-0.02	-0.28	0.09	0.05

P35251	Replication factor C subunit 1 OS=Homo sapiens GN=RFC1 PE=1 SV=4 - [RFC1_HUMAN]	2.87	1	2	2	2	-0.12	0.49	-0.57	0.03	-0.18	0.42	-0.58	0.02	-0.40	-1.00	-0.37	0.23	-0.41	-0.24	0.20	-0.85	-0.40	-0.08	0.37	0.05
P40429	60S ribosomal protein L13a OS=Homo sapiens GN=RPL13A PE=1 SV=2 - [RL13A_HUMAN]	35.96	2	8	8	16	-0.85	-0.86	-0.65	-0.76	-0.39	-0.45	-0.55	-0.66	-0.70	-0.64	-0.29	-0.40	0.25	0.56	0.26	0.33	0.04	0.42	0.30	0.05
Q9NZK5	Adenosine deaminase CECR1 OS=Homo sapiens GN=CECR1 PE=1 SV=2 - [CECR1_HUMAN]	3.72	1	1	1	1	0.73	0.68	0.36	0.30	0.51	0.46	0.34	0.29	0.86	0.92	1.26	1.20	-0.33	0.53	0.90	0.22	0.60	-0.23	0.14	0.05
O95232	Luc7-like protein 3 OS=Homo sapiens GN=LUC7L3 PE=1 SV=2 - [LC7L3_HUMAN]	11.34	1	3	4	5	-0.72	-0.84	-0.21	-0.33	-0.39	-0.37	-0.22	-0.34	-0.44	-0.32	-0.13	-0.25	0.55	0.60	0.08	0.31	-0.07	0.32	0.00	0.05
Q9P0J7	E3 ubiquitin-protein ligase KCMF1 OS=Homo sapiens GN=KCMF1 PE=1 SV=2 - [KCMF1_HUMAN]	4.46	1	1	1	2	0.64	0.35	0.65	0.36	0.36	0.06	0.64	0.35	0.02	0.32	0.82	0.52	0.05	0.18	0.16	-0.29	-0.30	-0.30	-0.31	0.05
Q86WW8	Cytochrome c oxidase assembly factor 5 OS=Homo sapiens GN=COA5 PE=1 SV=1 - [COA5_HUMAN]	20.27	1	1	1	1	-0.30	-0.21	0.39	0.48	-0.46	-0.37	0.38	0.47	-0.06	-0.15	-0.60	-0.51	0.74	-0.29	-0.99	0.18	-0.51	-0.18	-0.87	0.05
P50453	Serpin B9 OS=Homo sapiens GN=SERPINB9 PE=1 SV=1 - [SPB9_HUMAN]	14.10	1	4	4	6	0.70	0.30	0.14	-0.25	0.23	-0.17	0.13	-0.26	-0.01	0.39	0.48	0.08	-0.51	-0.21	0.34	-0.28	0.28	-0.48	0.07	0.05
Q9H8M5	Metal transporter CNNM2 OS=Homo sapiens GN=CNNM2 PE=1 SV=2 - [CNNM2_HUMAN]	4.80	2	3	4	7	-0.03	-0.26	-0.21	-0.43	-0.18	-0.41	-0.23	-0.45	-0.83	-0.61	-0.80	-1.02	-0.14	-0.76	-0.59	-0.54	-0.36	-0.16	0.01	0.05
Q8ND90	Paraneoplastic antigen Ma1 OS=Homo sapiens GN=PNMA1 PE=1 SV=2 - [PNMA1_HUMAN]	21.53	2	5	5	8	0.14	0.05	0.24	-0.16	0.53	0.44	0.29	0.14	0.11	0.20	0.41	0.32	0.20	0.28	0.17	0.10	0.00	0.38	0.27	0.05

P16278	Beta-galactosidase OS=Homo sapiens GN=GLB1 PE=1 SV=2 - [BGAL_HUMAN]	11.08	2	6	6	9	0.27	0.29	-0.03	-0.01	0.40	0.31	0.10	0.07	0.30	0.29	0.40	0.33	-0.09	0.02	0.14	0.13	0.20	0.00	0.16	0.05
Q9Y512	Sorting and assembly machinery component 50 homolog OS=Homo sapiens GN=SAMM50 PE=1 SV=3 - [SAM50_HUMAN]	38.59	1	14	14	29	0.38	0.22	0.35	0.11	0.06	-0.12	0.22	0.22	-0.05	-0.06	-0.05	-0.07	0.02	-0.38	-0.34	-0.13	-0.12	-0.47	-0.26	0.05
O60841	Eukaryotic translation initiation factor 5B OS=Homo sapiens GN=EIF5B PE=1 SV=4 - [IF2P_HUMAN]	14.43	1	14	14	22	-0.34	-0.47	-0.28	-0.43	-0.23	0.06	-0.17	-0.20	-0.19	-0.15	0.08	0.10	0.18	0.31	0.35	0.22	0.04	0.17	0.08	0.05
Q9NX95	Syntabulin OS=Homo sapiens GN=SYBU PE=1 SV=2 - [SYBU_HUMAN]	10.26	1	5	7	8	-0.08	-0.04	-0.09	-0.20	0.27	0.18	-0.10	-0.04	0.13	0.03	0.52	0.40	-0.19	0.41	0.58	0.10	0.24	0.28	0.28	0.05
Q7LG56	Ribonucleoside-diphosphate reductase subunit M2 B OS=Homo sapiens GN=RRM2B PE=1 SV=1 - [RIR2B_HUMAN]	7.69	1	3	3	4	0.45	0.39	0.23	0.18	0.50	0.44	0.22	0.16	0.29	0.36	0.25	0.19	-0.17	-0.19	0.01	-0.06	0.15	0.04	0.25	0.05
Q8IU81	Interferon regulatory factor 2-binding protein 1 OS=Homo sapiens GN=IRF2BP1 PE=1 SV=1 - [I2BP1_HUMAN]	7.19	1	2	2	2	-0.35	-0.46	-0.43	-0.54	0.17	0.05	-0.45	-0.56	-0.13	-0.02	0.20	0.09	-0.04	0.56	0.63	0.37	0.45	0.50	0.58	0.05
A6NGN9	IgLON family member 5 OS=Homo sapiens GN=IGLON5 PE=2 SV=4 - [IGLO5_HUMAN]	25.60	1	6	6	12	0.48	0.57	0.27	0.63	0.02	0.33	0.25	0.53	0.41	0.06	0.03	0.47	-0.20	-0.50	-0.20	-0.23	0.10	-0.50	-0.23	0.05
Q8WZ82	Ovarian cancer-associated gene 2 protein OS=Homo sapiens GN=OVCA2 PE=1 SV=1 - [OVCA2_HUMAN]	9.69	1	1	1	2	0.63	0.47	0.41	0.25	0.76	0.60	0.39	0.23	-0.03	0.13	0.00	-0.17	-0.18	-0.63	-0.41	-0.47	-0.24	0.12	0.34	0.05

Q9HD26	Golgi-associated PDZ and coiled-coil motif-containing protein OS=Homo sapiens GN=GOPC PE=1 SV=1 - [GOPC_HUMAN]	19.48	1	6	6	12	0.33	0.23	0.22	0.20	0.75	0.61	0.31	0.22	0.34	0.29	0.29	0.25	-0.07	0.14	0.15	-0.05	0.06	0.17	0.28	0.05
Q15906	Vacuolar protein sorting-associated protein 72 homolog OS=Homo sapiens GN=VPS72 PE=1 SV=1 - [VPS72_HUMAN]	8.79	1	1	1	1	4.76	4.20	0.29	-0.27	-0.31	-0.87	0.28	-0.28	-0.29	0.27	-0.29	-0.85	-4.43	-5.05	-0.58	-4.46	0.02	-5.09	-0.62	0.05
P02461	Collagen alpha-1(III) chain OS=Homo sapiens GN=COL3A1 PE=1 SV=4 - [CO3A1_HUMAN]	13.98	1	13	14	36	-2.66	-2.85	-2.54	-2.83	-1.56	-1.71	-2.39	-2.51	-2.34	-2.10	-2.60	-2.68	0.16	0.04	-0.03	0.40	0.35	1.21	1.01	0.05
A6NHR9	Structural maintenance of chromosomes flexible hinge domain containing protein 1 OS=Homo sapiens GN=SMCHD1 PE=1 SV=2 - [SMHD1_HUMAN]	1.90	1	4	4	6	-0.21	-0.05	-0.04	0.13	0.17	0.33	-0.05	0.11	0.17	0.00	-0.10	0.06	0.22	0.12	-0.06	0.25	0.07	0.37	0.19	0.05
O60231	Putative pre-mRNA-splicing factor ATP-dependent RNA helicase DHX16 OS=Homo sapiens GN=DHX16 PE=1 SV=2 - [DHX16_HUMAN]	1.73	1	1	2	2	0.60	0.52	-0.23	-0.32	0.11	0.02	-0.25	-0.33	0.13	0.22	0.13	0.04	-0.80	-0.47	0.36	-0.36	0.48	-0.51	0.33	0.05
Q86VX2	COMM domain-containing protein 7 OS=Homo sapiens GN=COMMD7 PE=1 SV=2 - [COMD7_HUMAN]	7.50	1	1	1	2	-0.50	-0.22	-0.20	0.08	0.29	0.56	-0.22	0.06	0.32	0.04	0.64	0.91	0.34	1.15	0.84	0.58	0.28	0.77	0.47	0.05
Q9BWF3	RNA-binding protein 4 OS=Homo sapiens GN=RBM4 PE=1 SV=1 - [RBM4_HUMAN]	25.27	1	2	8	15	0.99	1.07	0.28	0.36	-0.33	-0.26	0.26	0.34	-0.35	-0.42	0.23	0.30	-0.67	-0.76	-0.05	-1.38	-0.67	-1.34	-0.63	0.05

Q08257	Quinone oxidoreductase OS=Homo sapiens GN=CRYZ PE=1 SV=1 - [QOR_HUMAN]	45.29	1	12	12	38	-0.14	-0.31	-0.20	-0.13	-0.06	-0.02	-0.10	-0.04	-0.08	-0.27	-0.43	-0.30	0.22	-0.06	-0.16	0.25	-0.02	0.34	0.18	0.05
Q99832	T-complex protein 1 subunit eta OS=Homo sapiens GN=CCT7 PE=1 SV=2 - [TCPH_HUMAN]	51.93	1	24	24	47	-0.40	-0.47	-0.18	-0.27	-0.24	-0.18	-0.21	-0.21	-0.27	-0.22	-0.13	-0.18	0.13	0.19	0.09	0.16	-0.08	0.27	0.05	0.05
P82912	28S ribosomal protein S11, mitochondrial OS=Homo sapiens GN=MRPS11 PE=1 SV=2 - [RT11_HUMAN]	16.49	1	2	2	2	-0.36	-0.42	-0.52	-0.58	0.01	-0.05	-0.54	-0.60	-0.21	-0.15	0.39	0.33	-0.12	0.76	0.91	0.24	0.40	0.36	0.52	0.04
P61158	Actin-related protein 3 OS=Homo sapiens GN=ACTR3 PE=1 SV=3 - [ARP3_HUMAN]	42.34	1	14	17	63	-0.41	-0.26	-0.24	-0.09	-0.11	-0.13	-0.19	-0.12	-0.28	-0.24	-0.18	-0.12	0.18	0.32	0.09	0.18	-0.03	0.27	0.13	0.04
Q9H7N4	Splicing factor, arginine/serine-rich 19 OS=Homo sapiens GN=SCAF1 PE=1 SV=3 - [SFR19_HUMAN]	1.37	1	1	1	1	-1.07	-0.89	-0.55	-0.36	-0.56	-0.39	-0.56	-0.38	0.09	-0.08	-0.22	-0.05	0.57	0.86	0.32	1.02	0.49	0.50	-0.03	0.04
O75312	Zinc finger protein ZPR1 OS=Homo sapiens GN=ZNF259 PE=1 SV=1 - [ZPR1_HUMAN]	8.50	1	3	3	4	0.12	0.02	0.21	0.12	-0.07	-0.18	0.20	0.10	0.01	0.12	0.00	-0.11	0.14	-0.11	-0.22	0.03	-0.06	-0.21	-0.31	0.04
P49593	Protein phosphatase 1F OS=Homo sapiens GN=PPM1F PE=1 SV=3 - [PPM1F_HUMAN]	23.57	1	7	7	12	0.44	0.51	0.57	0.52	0.23	0.23	0.54	0.51	0.31	0.27	-0.14	-0.06	0.08	-0.66	-0.75	-0.14	-0.19	-0.31	-0.30	0.04
P83731	60S ribosomal protein L24 OS=Homo sapiens GN=RPL24 PE=1 SV=1 - [RL24_HUMAN]	25.48	1	4	4	7	-0.65	-0.43	-0.58	-0.51	-0.57	-0.46	-0.45	-0.49	-0.51	-0.63	-0.38	-0.10	0.04	0.48	0.38	0.12	-0.04	0.10	-0.01	0.04

Q9UHB9	Signal recognition particle subunit SRP68 OS=Homo sapiens GN=SRP68 PE=1 SV=2 - [SRP68_HUMAN]	13.88	1	8	8	12	-0.25	-0.36	-0.12	-0.14	-0.06	-0.05	-0.14	-0.12	-0.01	-0.02	-0.22	-0.34	0.46	0.17	-0.11	0.43	0.17	0.28	0.07	0.04
O00142	Thymidine kinase 2, mitochondrial OS=Homo sapiens GN=TK2 PE=1 SV=4 - [KITM_HUMAN]	20.38	1	5	5	9	0.50	0.43	0.33	0.26	-0.06	-0.14	0.31	0.24	0.11	0.19	0.16	0.08	-0.14	-0.34	-0.17	-0.29	-0.11	-0.58	-0.41	0.04
Q13123	Protein Red OS=Homo sapiens GN=IK PE=1 SV=3 - [RED_HUMAN]	11.67	1	5	5	6	-0.14	-0.19	-0.12	-0.19	-0.07	-0.23	-0.14	-0.28	-0.21	-0.04	0.04	0.02	-0.02	0.16	0.29	0.03	0.11	-0.08	-0.08	0.04
Q86VR2	Protein FAM134C OS=Homo sapiens GN=FAM134C PE=1 SV=1 - [F134C_HUMAN]	8.15	1	3	3	6	0.18	0.13	0.04	-0.01	0.36	0.14	0.12	0.08	0.21	0.32	0.25	0.20	-0.08	0.08	0.22	0.05	0.17	-0.12	0.00	0.04
O15540	Fatty acid-binding protein, brain OS=Homo sapiens GN=FABP7 PE=1 SV=3 - [FABP7_HUMAN]	51.52	1	5	5	18	0.27	0.24	0.94	0.73	0.60	0.51	0.73	0.67	0.25	0.39	0.13	0.09	0.27	-0.37	-0.76	-0.09	-0.29	0.25	-0.36	0.04
Q9UNH7	Sorting nexin-6 OS=Homo sapiens GN=SNX6 PE=1 SV=1 - [SNX6_HUMAN]	28.08	1	9	11	24	-0.06	-0.10	0.12	-0.08	-0.14	-0.10	-0.08	-0.20	-0.20	-0.12	-0.41	-0.46	0.16	-0.50	-0.29	-0.12	-0.12	0.28	-0.09	0.04
P23297	Protein S100-A1 OS=Homo sapiens GN=S100A1 PE=1 SV=2 - [S10A1_HUMAN]	15.96	1	1	1	11	1.41	1.72	1.76	2.07	1.16	1.47	1.56	1.87	1.59	1.28	0.88	1.18	0.21	-0.53	-0.88	-0.10	-0.44	-0.26	-0.61	0.04
P51665	26S proteasome non-ATPase regulatory subunit 7 OS=Homo sapiens GN=PSMD7 PE=1 SV=2 - [PSMD7_HUMAN]	12.96	1	5	5	8	-0.46	-0.45	-0.08	-0.07	-0.07	-0.06	-0.10	-0.06	-0.16	-0.17	-0.10	-0.04	0.26	0.34	-0.01	0.25	0.01	0.29	0.06	0.04

P51398	28S ribosomal protein S29, mitochondrial OS=Homo sapiens GN=DAP3 PE=1 SV=1 - [RT29_HUMAN]	5.53	1	2	2	2	-0.11	-0.50	0.12	-0.27	0.64	0.25	0.09	-0.29	0.02	0.41	0.45	0.06	0.26	0.57	0.33	0.55	0.33	0.74	0.51	0.04
Q9NUU7	ATP-dependent RNA helicase DDX19A OS=Homo sapiens GN=DDX19A PE=1 SV=1 - [DD19A_HUMAN]	13.18	2	5	5	11	-0.18	0.20	0.13	0.22	0.08	0.43	-0.12	0.10	-0.19	-0.15	-0.21	-0.02	0.11	-0.13	-0.15	0.12	-0.25	0.23	0.14	0.04
Q9UJV9	Probable ATP-dependent RNA helicase DDX41 OS=Homo sapiens GN=DDX41 PE=1 SV=2 - [DDX41_HUMAN]	2.09	1	1	1	1	-0.59	-0.68	-0.37	-0.46	-0.22	-0.31	-0.39	-0.48	-0.49	-0.39	-0.16	-0.26	0.25	0.43	0.21	0.23	0.01	0.35	0.13	0.04
Q13490	Baculoviral IAP repeat-containing protein 2 OS=Homo sapiens GN=BIRC2 PE=1 SV=2 - [BIRC2_HUMAN]	2.75	1	1	1	1	0.17	0.59	0.08	0.50	0.27	0.69	0.05	0.48	0.66	0.24	0.00	0.41	-0.06	-0.17	-0.08	0.10	0.19	0.09	0.18	0.04
O60343	TBC1 domain family member 4 OS=Homo sapiens GN=TBC1D4 PE=1 SV=2 - [TBCD4_HUMAN]	5.39	1	4	4	8	0.32	0.23	0.22	0.22	-0.55	-0.57	-0.26	-0.36	-0.33	-0.28	-0.26	-0.39	-0.53	-0.44	-0.48	-0.63	-0.47	-0.82	-0.80	0.04
Q9P258	Protein RCC2 OS=Homo sapiens GN=RCC2 PE=1 SV=2 - [RCC2_HUMAN]	8.24	1	3	3	6	-0.28	-0.14	-0.30	-0.24	0.05	0.02	-0.18	-0.20	-0.11	-0.09	-0.12	0.01	-0.18	0.16	0.26	0.11	0.25	0.21	0.33	0.04
Q14847	LIM and SH3 domain protein 1 OS=Homo sapiens GN=LASP1 PE=1 SV=2 - [LASP1_HUMAN]	30.65	1	9	9	24	-0.78	-0.78	-0.82	-0.80	-0.79	-0.87	-0.75	-0.77	-0.87	-0.80	-0.87	-0.94	0.04	-0.05	-0.14	-0.04	-0.02	-0.07	-0.04	0.04
Q92556	Engulfment and cell motility protein 1 OS=Homo sapiens GN=ELMO1 PE=1 SV=2 - [ELMO1_HUMAN]	14.58	1	7	11	18	0.23	0.21	0.28	0.25	0.34	0.04	0.32	0.22	0.08	0.28	0.30	0.09	0.07	-0.24	-0.29	-0.10	-0.14	-0.18	-0.27	0.04

P10645	Chromogranin-A OS=Homo sapiens GN=CHGA PE=1 SV=7 - [CMGA_HUMAN]	37.86	1	11	11	57	-0.24	-0.24	0.01	0.00	1.60	1.44	-0.02	-0.01	0.71	0.82	1.40	1.30	0.27	1.26	1.26	0.96	0.69	1.49	1.33	0.04
O15231	Zinc finger protein 185 OS=Homo sapiens GN=ZNF185 PE=1 SV=3 - [ZN185_HUMAN]	7.84	1	2	2	2	-1.39	-1.21	-0.81	-0.63	-1.60	-1.57	-0.83	-0.66	-1.31	-1.34	-0.97	-0.94	0.61	2.27	1.97	1.93	1.64	1.62	1.32	0.04
O15270	Serine palmitoyltransferase 2 OS=Homo sapiens GN=SPTLC2 PE=1 SV=1 - [SPTC2_HUMAN]	8.19	1	3	3	4	0.76	0.22	0.32	-0.22	0.74	0.20	0.29	-0.25	0.09	0.64	0.78	0.23	-0.42	0.02	0.46	-0.10	0.35	-0.04	0.41	0.04
P55060	Exportin-2 OS=Homo sapiens GN=CSE1L PE=1 SV=3 - [XPO2_HUMAN]	20.29	1	16	16	24	0.49	0.55	0.47	0.47	0.24	0.25	0.36	0.35	0.23	0.19	0.22	0.19	-0.03	-0.10	-0.17	-0.05	-0.10	-0.12	-0.16	0.04
P46087	Putative ribosomal RNA methyltransferase NOP2 OS=Homo sapiens GN=NOP2 PE=1 SV=2 - [NOP2_HUMAN]	2.09	1	2	2	2	0.29	0.25	0.06	0.03	0.12	0.08	0.03	0.00	0.32	0.36	0.17	0.12	-0.20	-0.11	0.10	0.11	0.34	-0.18	0.04	0.04
P62314	Small nuclear ribonucleoprotein Sm D1 OS=Homo sapiens GN=SNRPD1 PE=1 SV=1 - [SMD1_HUMAN]	36.97	1	4	4	12	-0.18	-0.04	-0.24	-0.20	0.09	0.13	-0.29	-0.21	0.03	0.00	-0.02	-0.04	-0.03	-0.01	0.20	0.15	0.34	0.28	0.32	0.03
Q99766	ATP synthase subunit s, mitochondrial OS=Homo sapiens GN=ATP5S PE=1 SV=3 - [ATP5S_HUMAN]	18.60	1	4	4	7	0.17	-0.09	0.04	-0.01	0.16	0.04	0.07	-0.11	0.03	0.14	0.34	0.22	-0.09	0.24	0.31	0.09	-0.11	0.07	0.16	0.03
P15289	Arylsulfatase A OS=Homo sapiens GN=ARSA PE=1 SV=3 - [ARSA_HUMAN]	9.27	1	3	3	6	0.52	0.78	0.51	0.76	0.24	0.49	0.48	0.73	0.63	0.38	0.03	0.28	0.01	-0.49	-0.48	-0.11	-0.09	-0.30	-0.28	0.03

Q8IWX8	Calcium homeostasis endoplasmic reticulum protein OS=Homo sapiens GN=CHERP PE=1 SV=3 - [CHERP_HUMAN]	5.35	1	5	5	5	-0.48	-0.66	-0.26	-0.49	0.21	0.05	-0.45	-0.68	-0.23	-0.06	-0.40	-0.50	0.23	0.30	0.05	0.16	0.20	0.42	0.53	0.03
O95452	Gap junction beta-6 protein OS=Homo sapiens GN=GJB6 PE=1 SV=2 - [CXB6_HUMAN]	5.75	1	1	1	2	0.28	0.48	0.59	0.79	1.36	1.55	0.56	0.76	0.98	0.79	0.76	0.96	0.33	0.48	0.17	0.53	0.23	1.06	0.75	0.03
Q9P2X0	Dolichol-phosphate mannosyltransferase subunit 3 OS=Homo sapiens GN=DPM3 PE=1 SV=2 - [DPM3_HUMAN]	23.91	1	2	2	3	0.19	0.30	-0.16	-0.04	0.01	0.13	-0.19	-0.07	0.08	-0.03	0.08	0.19	-0.32	-0.10	0.24	-0.19	0.16	-0.19	0.16	0.03
Q9NWU5	39S ribosomal protein L22, mitochondrial OS=Homo sapiens GN=MRPL22 PE=1 SV=1 - [RM22_HUMAN]	11.65	1	2	2	5	0.70	0.93	0.28	0.51	1.09	1.31	0.25	0.48	0.85	0.63	0.23	0.46	-0.39	-0.46	-0.05	-0.04	0.38	0.37	0.79	0.03
O75191	Xylulose kinase OS=Homo sapiens GN=XYLB PE=1 SV=3 - [XYLB_HUMAN]	3.36	1	1	1	1	0.05	-0.15	0.45	0.26	0.96	0.75	0.42	0.23	0.56	0.77	0.65	0.44	0.43	0.60	0.19	0.75	0.35	0.89	0.48	0.03
Q8WUY8	N-acetyltransferase 14 OS=Homo sapiens GN=NAT14 PE=1 SV=1 - [NAT14_HUMAN]	14.56	1	3	3	5	0.39	0.22	0.29	0.43	0.30	0.26	0.31	0.40	0.27	0.47	0.29	0.38	0.08	0.10	0.04	0.09	0.06	0.00	-0.01	0.03
Q96A26	Protein FAM162A OS=Homo sapiens GN=FAM162A PE=1 SV=2 - [F162A_HUMAN]	27.27	1	4	4	9	-0.58	-0.59	-0.39	-0.21	-0.36	-0.45	-0.34	-0.23	-0.38	-0.34	-0.09	-0.17	0.42	0.52	0.57	0.24	0.31	0.34	0.22	0.03
P49459	Ubiquitin-conjugating enzyme E2 A OS=Homo sapiens GN=UBE2A PE=1 SV=2 - [UBE2A_HUMAN]	17.76	1	2	2	4	-0.52	-0.42	-0.29	-0.19	0.00	0.09	-0.32	-0.22	-0.13	-0.23	-0.15	-0.05	0.25	0.38	0.14	0.32	0.09	0.50	0.27	0.03

Q9Y3U8	60S ribosomal protein L36 OS=Homo sapiens GN=RPL36 PE=1 SV=3 - [RL36_HUMAN]	29.52	1	4	4	15	-0.62	-0.62	-0.43	-0.43	-0.28	-0.34	-0.59	-0.71	-0.38	-0.36	-0.08	-0.26	0.09	0.55	0.36	0.35	0.22	0.36	0.07	0.03
A6NJZ7	RIMS-binding protein 3C OS=Homo sapiens GN=RIMBP3C PE=1 SV=3 - [RIM3C_HUMAN]	0.49	2	1	1	1	2.34	2.41	1.50	1.58	0.91	0.99	1.47	1.55	1.47	1.40	1.22	1.29	-0.81	-1.11	-0.29	-0.91	-0.08	-1.44	-0.61	0.03
P53396	ATP-citrate synthase OS=Homo sapiens GN=ACLY PE=1 SV=3 - [ACLY_HUMAN]	38.51	1	34	34	91	-0.16	-0.14	0.02	-0.02	-0.13	-0.11	-0.10	-0.05	-0.14	-0.18	-0.17	-0.18	0.16	-0.02	-0.21	-0.01	-0.13	0.07	-0.14	0.03
Q6PJT7	Zinc finger CCCH domain-containing protein 14 OS=Homo sapiens GN=ZC3H14 PE=1 SV=1 - [ZC3HE_HUMAN]	2.17	1	1	1	2	0.76	1.15	0.48	0.88	0.08	0.47	0.45	0.84	0.47	0.08	0.57	0.96	-0.25	-0.19	0.09	-0.65	-0.37	-0.70	-0.42	0.03
Q14919	Dr1-associated corepressor OS=Homo sapiens GN=DRAP1 PE=1 SV=3 - [NC2A_HUMAN]	15.61	1	2	2	7	-0.43	-0.66	-0.02	-1.02	-0.51	-0.74	-0.51	-0.75	-0.76	-0.47	-0.54	-0.67	-0.02	-0.16	-0.07	-0.10	0.36	-0.10	0.27	0.03
Q86XL3	Ankyrin repeat and LEM domain-containing protein 2 OS=Homo sapiens GN=ANKLE2 PE=1 SV=4 - [ANKL2_HUMAN]	3.94	1	3	3	4	0.75	0.48	0.76	0.49	0.44	0.16	0.73	0.45	0.32	0.60	0.53	0.25	0.03	-0.22	-0.23	-0.12	-0.12	-0.33	-0.34	0.03
O14558	Heat shock protein beta-6 OS=Homo sapiens GN=HSPB6 PE=1 SV=2 - [HSPB6_HUMAN]	21.25	1	3	3	10	-0.96	-1.07	-0.93	-1.04	-1.12	-1.25	-1.02	-1.14	-0.80	-0.66	0.07	-0.04	0.02	1.04	1.01	0.13	0.24	-0.35	-0.39	0.03
P30531	Sodium- and chloride-dependent GABA transporter 1 OS=Homo sapiens GN=SLC6A1 PE=1 SV=2 - [SC6A1_HUMAN]	12.69	1	4	4	47	0.22	0.08	0.38	0.28	-0.04	-0.08	0.40	0.34	0.10	0.25	0.54	0.44	0.46	0.50	0.14	0.34	-0.03	-0.06	-0.37	0.03

Q13505	Metaxin-1 OS=Homo sapiens GN=MTX1 PE=1 SV=2 - [MTX1_HUMAN]	9.87	1	6	6	11	0.32	0.28	0.20	0.15	-0.01	0.01	0.16	0.16	0.18	0.16	0.04	0.03	-0.10	-0.28	-0.20	-0.05	-0.06	-0.27	-0.17	0.03
Q9H444	Charged multivesicular body protein 4b OS=Homo sapiens GN=CHMP4B PE=1 SV=1 - [CHM4B_HUMAN]	45.54	2	9	9	28	0.09	0.00	0.10	-0.04	0.24	0.34	-0.08	-0.20	0.03	0.11	0.17	0.14	-0.13	0.12	0.19	0.05	0.15	0.22	0.28	0.03
Q9NUQ8	ATP-binding cassette sub-family F member 3 OS=Homo sapiens GN=ABCF3 PE=1 SV=2 - [ABCF3_HUMAN]	11.28	1	6	6	8	0.24	-0.07	-0.06	-0.18	0.22	-0.06	-0.08	-0.13	-0.15	-0.02	-0.13	-0.31	-0.02	-0.24	-0.08	-0.14	-0.01	0.13	0.19	0.03
P06746	DNA polymerase beta OS=Homo sapiens GN=POLB PE=1 SV=3 - [DPOLB_HUMAN]	2.99	1	1	1	1	0.20	0.12	-0.12	-0.20	-0.15	-0.24	-0.15	-0.24	-0.37	-0.28	-0.22	-0.31	-0.30	-0.42	-0.10	-0.46	-0.13	-0.37	-0.05	0.03
Q9NZB2	Constitutive coactivator of PPAR-gamma-like protein 1 OS=Homo sapiens GN=FAM120A PE=1 SV=2 - [F120A_HUMAN]	19.86	1	14	15	25	-0.38	-0.38	-0.26	-0.53	-0.22	-0.34	-0.30	-0.56	-0.38	-0.32	-0.43	-0.41	0.19	0.38	0.19	0.36	0.10	0.39	-0.02	0.03
P62244	40S ribosomal protein S15a OS=Homo sapiens GN=RPS15A PE=1 SV=2 - [RS15A_HUMAN]	30.00	1	4	4	7	-0.42	-0.20	-1.02	-0.52	-0.65	-0.24	-0.92	-0.55	-0.24	-0.64	-0.40	-0.19	-0.34	0.02	0.33	-0.11	0.27	-0.06	0.25	0.03
Q02252	Methylmalonate-semialdehyde dehydrogenase [acylating], mitochondrial OS=Homo sapiens GN=ALDH6A1 PE=1 SV=2 - [MMSA_HUMAN]	52.34	2	25	25	80	-0.22	-0.22	-0.12	-0.06	-0.30	-0.30	-0.12	-0.08	-0.28	-0.31	-0.47	-0.45	0.20	-0.23	-0.47	0.07	-0.23	0.05	-0.28	0.03

Q9BUJ2	Heterogeneous nuclear ribonucleoprotein U-like protein 1 OS=Homo sapiens GN=HNRNPUL1 PE=1 SV=2 - [HNRL1_HUMAN]	16.36	1	12	12	21	-0.58	-0.56	-0.32	-0.33	-0.44	-0.39	-0.40	-0.36	-0.43	-0.47	-0.32	-0.28	0.26	0.27	0.04	0.14	-0.12	0.03	-0.13	0.03
P62280	40S ribosomal protein S11 OS=Homo sapiens GN=RPS11 PE=1 SV=3 - [RS11_HUMAN]	24.05	1	5	5	10	-0.55	-0.75	-0.33	-0.54	-0.24	-0.45	-0.37	-0.57	-0.43	-0.22	0.02	-0.18	0.23	0.58	0.36	0.36	0.14	0.29	0.08	0.03
Q9UI43	Putative ribosomal RNA methyltransferase 2 OS=Homo sapiens GN=FTSJ2 PE=1 SV=1 - [RRMJ2_HUMAN]	3.25	1	1	1	2	-0.18	-0.51	-0.43	-0.75	-1.07	-1.41	-0.46	-0.79	-0.98	-0.65	-0.39	-0.72	-0.23	-0.21	0.03	-0.44	-0.19	-0.91	-0.67	0.03
P16333	Cytoplasmic protein NCK1 OS=Homo sapiens GN=NCK1 PE=1 SV=1 - [NCK1_HUMAN]	20.69	1	6	7	9	-0.11	-0.27	0.05	0.10	0.37	0.21	0.10	0.09	0.06	0.22	0.06	-0.09	0.20	0.09	0.01	0.31	0.24	0.32	0.14	0.03
Q07812	Apoptosis regulator BAX OS=Homo sapiens GN=BAX PE=1 SV=1 - [BAX_HUMAN]	11.98	1	2	2	4	0.11	0.00	0.15	0.03	0.30	0.18	0.11	-0.01	-0.08	0.05	0.13	0.01	0.05	0.02	-0.02	-0.04	-0.07	0.17	0.14	0.03
P46777	60S ribosomal protein L5 OS=Homo sapiens GN=RPL5 PE=1 SV=3 - [RL5_HUMAN]	34.34	1	12	12	30	-0.65	-0.69	-0.51	-0.49	-0.44	-0.53	-0.49	-0.57	-0.63	-0.47	-0.35	-0.45	0.21	0.27	0.08	0.18	0.07	0.22	0.04	0.03
Q9BVL2	Nucleoporin p58/p45 OS=Homo sapiens GN=NUPL1 PE=1 SV=1 - [NUPL1_HUMAN]	7.51	1	3	3	6	0.00	-0.14	0.23	-0.04	-0.63	-0.18	0.17	-0.10	-0.03	-0.05	-0.23	-0.36	0.10	-0.10	-0.34	0.02	0.05	-0.20	-0.16	0.03
Q8IZA0	Dyslexia-associated protein KIAA0319-like protein OS=Homo sapiens GN=KIAA0319L PE=1 SV=2 - [K319L_HUMAN]	3.05	1	3	3	5	0.32	0.23	0.20	0.11	0.70	0.61	0.16	0.08	0.05	0.14	0.37	0.28	-0.10	0.05	0.17	-0.15	-0.02	0.36	0.48	0.03

Q08211	ATP-dependent RNA helicase A OS=Homo sapiens GN=DHX9 PE=1 SV=4 - [DHX9_HUMAN]	30.47	1	30	30	80	-0.12	-0.18	-0.13	-0.14	-0.25	-0.18	-0.25	-0.17	-0.24	-0.27	-0.28	-0.27	0.06	-0.01	-0.09	0.01	-0.17	0.08	0.02	0.03
Q8WVT3	Trafficking protein particle complex subunit 12 OS=Homo sapiens GN=TRAPPC12 PE=1 SV=3 - [TPC12_HUMAN]	13.88	1	7	7	9	0.13	0.07	-0.04	-0.02	0.09	0.16	-0.09	0.03	0.15	0.11	0.15	0.16	0.07	0.09	0.16	-0.16	0.13	-0.08	0.01	0.03
A6NIH7	Protein unc-119 homolog B OS=Homo sapiens GN=UNC119B PE=1 SV=1 - [U119B_HUMAN]	15.14	1	2	2	4	0.11	0.16	0.02	0.06	0.11	0.14	-0.02	0.03	0.38	0.35	-0.02	0.02	-0.07	-0.13	-0.04	0.26	0.36	-0.02	0.07	0.03
P18031	Tyrosine-protein phosphatase non-receptor type 1 OS=Homo sapiens GN=PTPN1 PE=1 SV=1 - [PTN1_HUMAN]	6.67	1	3	3	4	0.14	0.15	0.07	0.09	0.24	0.26	0.03	0.05	0.29	0.28	0.20	0.21	-0.05	0.07	0.13	0.18	0.25	0.09	0.16	0.03
P05230	Fibroblast growth factor 1 OS=Homo sapiens GN=FGF1 PE=1 SV=1 - [FGF1_HUMAN]	22.58	1	3	3	5	0.19	0.20	0.39	0.25	0.08	0.24	0.44	0.61	0.28	0.27	0.50	0.38	0.35	0.13	0.10	0.16	-0.23	-0.08	-0.37	0.02
Q9UK59	Lariat debranching enzyme OS=Homo sapiens GN=DBR1 PE=1 SV=2 - [DBR1_HUMAN]	2.02	1	1	1	2	-0.09	-0.31	-0.24	-0.46	0.50	0.27	-0.27	-0.50	-0.13	0.10	-0.06	-0.29	-0.13	0.03	0.17	0.22	0.37	0.57	0.72	0.02
Q9Y3D2	Methionine-R sulfoxide reductase B2, mitochondrial OS=Homo sapiens GN=MSRB2 PE=2 SV=2 - [MSRB2_HUMAN]	21.43	1	3	3	3	0.12	-0.11	0.21	-0.02	0.05	-0.18	0.17	-0.06	-0.17	0.06	-0.64	-0.87	0.11	-0.75	-0.85	-0.03	-0.12	-0.09	-0.18	0.02

P46020	Phosphorylase b kinase regulatory subunit alpha, skeletal muscle isoform OS=Homo sapiens GN=PHKA1 PE=1 SV=2 - [KPB1_HUMAN]	2.13	1	2	2	3	0.40	0.58	0.37	0.56	0.11	0.28	0.33	0.52	0.27	0.09	0.35	0.52	-0.01	-0.05	-0.03	-0.28	-0.25	-0.31	-0.28	0.02
Q96EV2	RNA-binding protein 33 OS=Homo sapiens GN=RBM33 PE=1 SV=3 - [RBM33_HUMAN]	1.03	1	1	1	1	-0.55	-1.01	-0.41	-0.87	0.11	-0.36	-0.45	-0.91	-0.10	0.36	0.07	-0.40	0.16	0.62	0.47	0.94	0.80	0.64	0.49	0.02
Q86VM9	Zinc finger CCCH domain-containing protein 18 OS=Homo sapiens GN=ZC3H18 PE=1 SV=2 - [ZCH18_HUMAN]	6.72	1	4	4	7	-0.67	-0.87	-0.08	-0.27	-0.16	-0.37	-0.12	-0.32	-0.44	-0.23	-0.06	-0.26	0.60	0.62	0.02	0.47	-0.12	0.49	-0.10	0.02
Q5BJE1	Coiled-coil domain-containing protein 178 OS=Homo sapiens GN=CCDC178 PE=2 SV=3 - [CC178_HUMAN]	1.04	1	1	1	2	-0.61	-1.03	-0.19	-0.61	3.94	3.51	-0.23	-0.65	1.97	2.40	-0.36	-0.79	0.43	0.25	-0.17	3.04	2.62	4.53	4.11	0.02
Q5VWJ9	Sorting nexin-30 OS=Homo sapiens GN=SNX30 PE=1 SV=1 - [SNX30_HUMAN]	23.57	1	6	6	15	0.36	0.52	0.42	0.52	0.16	0.30	0.17	0.44	0.59	0.26	0.16	0.31	-0.22	-0.18	-0.31	0.05	-0.15	-0.23	-0.06	0.02
O75179	Ankyrin repeat domain-containing protein 17 OS=Homo sapiens GN=ANKRD17 PE=1 SV=3 - [ANR17_HUMAN]	2.00	1	1	3	3	-0.46	-0.66	-0.36	-0.57	0.00	-0.21	-0.41	-0.61	-0.45	-0.25	0.16	-0.04	0.10	0.62	0.53	0.24	0.15	0.44	0.35	0.02
Q5VTL8	Pre-mRNA-splicing factor 38B OS=Homo sapiens GN=PRPF38B PE=1 SV=1 - [PR38B_HUMAN]	2.38	1	1	1	1	-0.10	-0.05	-0.07	-0.02	0.03	0.07	-0.11	-0.07	-0.28	-0.32	0.17	0.21	0.04	0.28	0.24	-0.19	-0.21	0.11	0.08	0.02

Q9Y3E5	Peptidyl-tRNA hydrolase 2, mitochondrial OS=Homo sapiens GN=PTRH2 PE=1 SV=1 - [PTH2_HUMAN]	18.99	1	2	2	6	-0.01	-0.32	0.13	-0.07	0.32	0.12	0.12	-0.15	-0.22	-0.02	0.22	-0.08	0.22	0.15	0.13	0.01	-0.13	0.31	0.18	0.02
P62917	60S ribosomal protein L8 OS=Homo sapiens GN=RPL8 PE=1 SV=2 - [RL8_HUMAN]	21.79	1	5	5	13	-0.57	-0.52	-0.56	-0.41	-0.49	-0.41	-0.59	-0.47	-0.50	-0.58	-0.19	-0.05	0.11	0.49	0.38	0.15	0.07	0.35	0.07	0.02
P05166	Propionyl-CoA carboxylase beta chain, mitochondrial OS=Homo sapiens GN=PCCB PE=1 SV=3 - [PCCB_HUMAN]	42.30	1	17	17	49	0.01	0.02	-0.02	0.02	-0.30	-0.29	-0.16	-0.15	-0.18	-0.19	-0.28	-0.23	-0.05	-0.15	-0.18	-0.07	-0.11	-0.39	-0.26	0.02
Q6P2Q9	Pre-mRNA-processing-splicing factor 8 OS=Homo sapiens GN=PRPF8 PE=1 SV=2 - [PRP8_HUMAN]	20.51	1	37	37	64	-0.20	-0.34	-0.16	-0.21	-0.19	-0.32	-0.20	-0.25	-0.26	-0.21	-0.08	-0.13	-0.03	0.19	0.01	0.03	-0.07	-0.02	-0.16	0.02
Q53F19	Uncharacterized protein C17orf85 OS=Homo sapiens GN=C17orf85 PE=1 SV=2 - [CQ085_HUMAN]	1.61	1	1	1	1	-0.33	-0.32	-0.36	-0.36	-0.57	-0.57	-0.40	-0.40	-0.41	-0.41	-0.57	-0.58	-0.02	-0.24	-0.21	-0.05	-0.01	-0.26	-0.22	0.02
Q71UM5	40S ribosomal protein S27-like OS=Homo sapiens GN=RPS27L PE=1 SV=3 - [RS27L_HUMAN]	39.29	1	1	4	9	-0.89	-0.81	-1.16	-1.08	-1.11	-1.04	-1.20	-1.12	-0.64	-0.71	-0.88	-0.81	-0.25	0.02	0.28	0.21	0.48	-0.24	0.03	0.02
Q9Y342	Plasmolipin OS=Homo sapiens GN=PLLP PE=1 SV=1 - [PLLP_HUMAN]	8.79	1	1	1	3	1.03	0.19	0.23	-0.61	1.01	0.17	0.19	-0.66	-0.63	0.22	0.09	-0.76	-0.79	-0.93	-0.14	-0.78	0.02	-0.03	0.77	0.02
A4D1P6	WD repeat-containing protein 91 OS=Homo sapiens GN=WDR91 PE=1 SV=2 - [WDR91_HUMAN]	2.95	1	2	2	3	-0.89	-0.52	-0.49	-0.12	-0.52	-0.15	-0.54	-0.16	-0.43	-0.80	-0.34	0.03	0.41	0.55	0.15	0.12	-0.27	0.35	-0.04	0.02

Q8N9V3	WD repeat, SAM and U-box domain-containing protein 1 OS=Homo sapiens GN=WDSUB1 PE=1 SV=3 - [WSDU1_HUMAN]	5.04	1	2	2	2	-0.75	-1.07	0.17	-0.15	0.41	0.08	0.13	-0.19	0.03	0.36	0.36	0.03	0.93	1.11	0.19	1.14	0.22	1.14	0.22	0.02
O43399	Tumor protein D54 OS=Homo sapiens GN=TPD52L2 PE=1 SV=2 - [TPD54_HUMAN]	46.12	1	10	10	26	-0.09	0.00	0.11	0.06	0.23	0.41	0.06	-0.05	0.10	-0.06	-0.23	-0.18	0.08	0.00	-0.21	0.12	0.00	0.30	0.16	0.02
Q6PJI9	WD repeat-containing protein 59 OS=Homo sapiens GN=WDR59 PE=1 SV=2 - [WDR59_HUMAN]	1.03	1	1	1	1	0.26	0.06	0.14	-0.05	0.45	0.25	0.10	-0.10	-0.35	-0.14	0.20	0.00	-0.11	-0.06	0.05	-0.37	-0.25	0.17	0.29	0.02
Q8N357	Solute carrier family 35 member F6 OS=Homo sapiens GN=SLC35F6 PE=1 SV=1 - [S35F6_HUMAN]	3.77	1	1	1	1	-0.26	-0.03	-0.24	-0.01	0.03	0.26	-0.28	-0.05	0.18	-0.04	-0.02	0.21	0.03	0.25	0.22	0.25	0.23	0.28	0.26	0.02
P57076	UPF0769 protein C21orf59 OS=Homo sapiens GN=C21orf59 PE=1 SV=1 - [CU059_HUMAN]	16.55	1	2	2	3	0.82	0.32	1.49	0.61	1.36	1.29	0.98	0.56	1.41	0.84	1.89	1.00	0.21	0.69	0.40	0.05	0.84	0.52	0.67	0.02
Q9H3S7	Tyrosine-protein phosphatase non-receptor type 23 OS=Homo sapiens GN=PTPN23 PE=1 SV=1 - [PTN23_HUMAN]	19.01	1	24	24	43	0.40	0.25	0.24	0.39	0.12	0.21	0.23	0.30	0.17	0.15	0.22	0.27	-0.03	-0.16	-0.13	-0.13	-0.02	-0.05	-0.12	0.02
Q6L8Q7	2',5'-phosphodiesterase 12 OS=Homo sapiens GN=PDE12 PE=1 SV=2 - [PDE12_HUMAN]	22.50	1	9	9	15	0.41	0.50	0.32	0.16	0.09	0.11	0.17	0.21	0.16	0.18	0.15	0.22	-0.10	-0.22	-0.17	-0.26	-0.08	-0.15	-0.01	0.02
P09913	Interferon-induced protein with tetratricopeptide repeats 2 OS=Homo sapiens GN=IFIT2 PE=1 SV=1 - [IFIT2_HUMAN]	8.26	1	3	3	5	-0.26	-0.19	-0.39	-0.32	0.10	0.16	-0.44	-0.37	-0.22	-0.29	-0.09	-0.03	-0.12	0.18	0.30	0.01	0.14	0.34	0.47	0.02

P46109	Crk-like protein OS=Homo sapiens GN=CRKL PE=1 SV=1 - [CRKL_HUMAN]	61.06	1	13	13	23	0.31	0.46	0.33	0.47	0.30	0.45	0.34	0.46	0.30	0.23	0.07	0.17	-0.05	-0.29	-0.40	-0.20	-0.14	0.10	-0.03	0.01
Q8NBM4	Ubiquitin-associated domain-containing protein 2 OS=Homo sapiens GN=UBAC2 PE=2 SV=1 - [UBAC2_HUMAN]	8.43	1	2	2	4	-0.33	0.30	-0.83	-0.19	-0.39	0.24	-0.33	-0.24	0.70	0.36	-0.09	0.54	-0.49	0.24	0.73	0.43	0.93	-0.08	0.42	0.01
P37198	Nuclear pore glycoprotein p62 OS=Homo sapiens GN=NUP62 PE=1 SV=3 - [NUP62_HUMAN]	22.99	1	8	8	22	-0.37	-0.20	-0.30	-0.24	-0.34	-0.17	-0.31	-0.23	-0.19	-0.22	-0.06	0.06	-0.04	0.31	0.24	0.11	0.07	0.02	0.08	0.01
P02786	Transferrin receptor protein 1 OS=Homo sapiens GN=TFRC PE=1 SV=2 - [TFR1_HUMAN]	18.82	2	11	11	20	-0.45	-0.32	-0.32	-0.25	0.12	0.02	-0.35	-0.30	-0.13	-0.16	0.32	0.32	0.30	0.93	0.62	0.49	0.14	0.58	0.28	0.01
O95825	Quinone oxidoreductase-like protein 1 OS=Homo sapiens GN=CRYZL1 PE=1 SV=2 - [QORL1_HUMAN]	28.94	1	7	7	8	-0.25	-0.18	0.25	-0.05	-0.20	0.04	0.04	0.11	-0.10	-0.12	-0.06	0.01	0.34	0.20	-0.13	0.16	-0.29	0.01	-0.43	0.01
Q96ST3	Paired amphipathic helix protein Sin3a OS=Homo sapiens GN=SIN3A PE=1 SV=2 - [SIN3A_HUMAN]	7.15	1	6	6	9	-0.32	-0.31	-0.25	-0.24	-0.79	-0.76	-0.63	-0.56	-0.68	-0.53	-0.46	-0.46	-0.04	0.00	0.04	0.00	-0.08	-0.08	-0.12	0.01
Q7L5N1	COP9 signalosome complex subunit 6 OS=Homo sapiens GN=COPS6 PE=1 SV=1 - [CSN6_HUMAN]	36.39	1	8	8	15	-0.02	0.15	-0.02	0.06	-0.14	0.07	-0.09	0.12	0.00	0.00	-0.24	-0.04	0.10	-0.09	-0.34	-0.04	0.11	-0.13	-0.01	0.01
O43169	Cytochrome b5 type B OS=Homo sapiens GN=CYB5B PE=1 SV=2 - [CYB5B_HUMAN]	50.68	1	4	4	11	-0.09	-0.13	-0.17	-0.20	0.28	0.19	-0.04	-0.19	0.06	0.14	0.08	0.06	-0.07	-0.08	-0.02	0.10	0.19	0.09	0.16	0.01

O43293	Death-associated protein kinase 3 OS=Homo sapiens GN=DAPK3 PE=1 SV=1 - [DAPK3_HUMAN]	14.76	1	5	5	5	-0.45	-0.44	-0.66	-0.55	-0.56	-0.46	-0.49	-0.56	-0.50	-0.57	-0.73	-0.61	-0.38	-0.15	0.00	0.07	0.08	-0.08	-0.03	0.01
Q9H845	Acyl-CoA dehydrogenase family member 9, mitochondrial OS=Homo sapiens GN=ACAD9 PE=1 SV=1 - [ACAD9_HUMAN]	15.46	1	9	9	14	-0.33	-0.31	-0.14	-0.34	-0.53	-0.56	-0.23	-0.28	-0.42	-0.31	-0.12	-0.16	0.09	0.20	-0.09	-0.06	-0.22	-0.29	-0.31	0.01
O15027	Protein transport protein Sec16A OS=Homo sapiens GN=SEC16A PE=1 SV=3 - [SC16A_HUMAN]	5.23	1	8	8	16	-0.04	0.14	-0.09	-0.14	0.05	0.00	-0.27	-0.19	-0.16	-0.04	0.16	0.05	-0.15	-0.01	0.22	-0.19	0.06	-0.18	0.06	0.01
P29992	Guanine nucleotide-binding protein subunit alpha 11 OS=Homo sapiens GN=GNA11 PE=1 SV=2 - [GNA11_HUMAN]	44.01	2	8	14	29	0.41	0.60	0.32	0.36	0.27	0.24	0.32	0.31	0.13	0.24	0.14	0.21	-0.04	-0.36	-0.06	0.08	0.00	0.15	0.17	0.01
Q9Y3Z3	Deoxynucleoside triphosphate triphosphohydrolase SAMHD1 OS=Homo sapiens GN=SAMHD1 PE=1 SV=2 - [SAMH1_HUMAN]	43.93	1	22	22	36	0.12	0.12	0.22	0.27	0.43	0.35	0.18	0.26	0.22	0.26	0.27	0.22	0.08	0.17	-0.04	0.20	0.12	0.26	0.19	0.01
Q9NWV8	BRISC and BRCA1-A complex member 1 OS=Homo sapiens GN=BABAM1 PE=1 SV=1 - [BABA1_HUMAN]	11.55	1	3	3	4	0.08	-0.14	-0.10	-0.22	-0.11	-0.02	-0.24	-0.15	-0.11	-0.04	0.14	-0.02	-0.11	0.08	0.21	-0.01	0.14	0.19	0.03	0.01
P45984	Mitogen-activated protein kinase 9 OS=Homo sapiens GN=MAPK9 PE=1 SV=2 - [MK09_HUMAN]	26.65	7	5	8	21	-0.15	-0.40	0.23	-0.09	0.10	0.10	0.19	0.18	-0.24	-0.28	-0.13	-0.07	0.42	0.32	-0.09	0.11	-0.26	0.50	0.12	0.01

Q12904	Aminoacyl tRNA synthase complex-interacting multifunctional protein 1 OS=Homo sapiens GN=AIMP1 PE=1 SV=2 - [AIMP1_HUMAN]	37.50	1	8	8	21	-0.19	-0.46	-0.22	-0.29	0.48	0.22	-0.03	-0.16	0.06	0.34	0.20	0.12	0.24	0.73	0.37	0.48	0.70	0.57	0.61	0.01
P51531	Probable global transcription activator SNF2L2 OS=Homo sapiens GN=SMARCA2 PE=1 SV=2 - [SMCA2_HUMAN]	10.82	1	6	13	21	-0.48	-0.07	-0.24	-0.08	-0.29	-0.07	-0.55	-0.17	-0.20	-0.53	-0.45	-0.13	-0.01	0.20	-0.01	-0.02	0.19	0.16	0.35	0.01
Q8IYB3	Serine/arginine repetitive matrix protein 1 OS=Homo sapiens GN=SRRM1 PE=1 SV=2 - [SRRM1_HUMAN]	9.96	1	8	8	9	-0.99	-1.01	-0.40	-0.38	-0.64	-0.67	-0.56	-0.54	-0.31	-0.29	-0.38	-0.41	0.46	0.80	0.71	0.73	0.71	0.63	0.54	0.01
Q9Y2H1	Serine/threonine-protein kinase 38-like OS=Homo sapiens GN=STK38L PE=1 SV=3 - [ST38L_HUMAN]	23.92	1	6	7	10	0.13	0.27	0.09	0.23	0.11	0.25	0.03	0.17	0.12	-0.01	-0.41	-0.27	-0.04	-0.54	-0.50	-0.12	-0.07	-0.04	0.00	0.01
Q8IYB8	ATP-dependent RNA helicase SUPV3L1, mitochondrial OS=Homo sapiens GN=SUPV3L1 PE=1 SV=1 - [SUV3_HUMAN]	9.41	1	5	5	7	0.00	-0.19	0.22	0.03	0.51	0.02	0.16	-0.03	0.06	0.26	-0.11	-0.08	0.24	-0.01	-0.02	0.24	0.21	0.20	0.27	0.01
P35232	Prohibitin OS=Homo sapiens GN=PHB PE=1 SV=1 - [PHB_HUMAN]	49.26	1	12	12	54	0.25	0.26	0.28	0.24	0.26	0.20	0.18	0.16	0.22	0.26	0.31	0.23	0.04	0.09	0.02	0.22	0.02	0.06	-0.07	0.01
Q07817	Bcl-2-like protein 1 OS=Homo sapiens GN=BCL2L1 PE=1 SV=1 - [B2CL1_HUMAN]	4.29	1	1	1	1	-0.91	-0.91	-0.29	-0.29	-0.13	-0.13	-0.34	-0.34	-0.31	-0.31	0.08	0.08	0.62	1.00	0.37	0.63	0.01	0.76	0.14	0.01

Q8WX92	Negative elongation factor B OS=Homo sapiens GN=NELFB PE=1 SV=1 - [NELFB_HUMAN]	3.10	1	1	2	2	0.31	0.41	-0.10	0.00	-0.33	-0.23	-0.15	-0.06	0.24	0.15	-0.09	0.00	-0.41	-0.39	0.01	-0.13	0.28	-0.65	-0.25	0.01
Q00403	Transcription initiation factor IIB OS=Homo sapiens GN=GTF2B PE=1 SV=1 - [TF2B_HUMAN]	5.06	1	1	1	2	0.00	0.21	-0.14	0.07	0.10	0.30	-0.20	0.02	0.10	-0.11	-0.02	0.19	-0.14	-0.01	0.12	-0.08	0.07	0.08	0.22	0.01
O60927	Protein phosphatase 1 regulatory subunit 11 OS=Homo sapiens GN=PPP1R11 PE=1 SV=1 - [PP1RB_HUMAN]	12.70	1	2	2	3	0.66	0.63	0.47	0.45	0.02	-0.01	0.42	0.39	0.00	0.03	-0.09	-0.12	-0.18	-0.74	-0.56	-0.59	-0.41	-0.65	-0.47	0.01
P61353	60S ribosomal protein L27 OS=Homo sapiens GN=RPL27 PE=1 SV=2 - [RL27_HUMAN]	36.03	1	5	5	17	-0.35	-0.39	-0.50	-0.51	-0.13	-0.18	-0.37	-0.51	-0.18	-0.15	-0.02	-0.12	-0.04	0.30	0.23	0.10	0.21	0.20	0.31	0.01
Q15291	Retinoblastoma-binding protein 5 OS=Homo sapiens GN=RBBP5 PE=1 SV=2 - [RBBP5_HUMAN]	7.25	1	3	3	6	-0.03	0.02	-0.04	-0.19	-0.17	-0.32	-0.03	-0.31	-0.38	-0.12	-0.14	-0.13	-0.27	-0.06	-0.10	-0.17	-0.15	-0.16	-0.27	0.01
O75175	CCR4-NOT transcription complex subunit 3 OS=Homo sapiens GN=CNOT3 PE=1 SV=1 - [CNOT3_HUMAN]	2.66	1	2	2	3	0.07	0.13	-0.17	-0.11	-0.36	-0.30	-0.23	-0.17	0.00	-0.06	-0.09	-0.03	-0.24	-0.15	0.09	-0.09	0.15	-0.44	-0.20	0.01
P16083	Ribosyldihydronicotinamide dehydrogenase [quinone] OS=Homo sapiens GN=NQO2 PE=1 SV=5 - [NQO2_HUMAN]	31.17	1	5	5	17	0.60	0.72	0.82	0.91	-0.27	-0.16	0.77	0.88	0.22	0.06	-0.93	-0.77	0.23	-1.49	-1.81	-0.46	-0.60	-0.82	-0.94	0.01
Q5T1M5	FK506-binding protein 15 OS=Homo sapiens GN=FKBP15 PE=1 SV=2 - [FKB15_HUMAN]	7.71	1	6	7	18	0.27	-0.07	0.07	-0.33	-0.15	-0.41	-0.07	-0.35	-0.35	-0.08	0.06	-0.06	-0.23	-0.21	0.04	-0.03	0.18	-0.10	-0.19	0.01

Q49A26	Putative oxidoreductase GLYR1 OS=Homo sapiens GN=GLYR1 PE=1 SV=3 - [GLYR1_HUMAN]	17.72	1	8	8	12	-0.50	-0.60	-0.58	-0.58	-0.03	-0.28	-0.55	-0.56	-0.32	-0.40	0.07	-0.33	-0.01	0.33	0.22	0.10	0.14	0.39	0.17	0.01
Q9Y6K9	NF-kappa-B essential modulator OS=Homo sapiens GN=IKBKG PE=1 SV=2 - [NEMO_HUMAN]	16.95	1	5	5	7	0.29	0.32	-0.21	-0.19	0.15	0.23	-0.01	-0.05	0.54	0.50	0.67	0.71	-0.19	0.54	0.68	0.34	0.45	0.19	0.20	0.01
Q9ULC4	Malignant T-cell-amplified sequence 1 OS=Homo sapiens GN=MCTS1 PE=1 SV=1 - [MCTS1_HUMAN]	41.99	1	6	6	15	-0.43	-0.44	-0.30	-0.24	-0.01	0.07	-0.36	-0.37	-0.31	-0.29	-0.35	-0.27	0.18	0.18	0.03	0.27	-0.05	0.50	0.36	0.00
Q9H3K2	Growth hormone-inducible transmembrane protein OS=Homo sapiens GN=GHITM PE=1 SV=2 - [GHITM_HUMAN]	15.36	1	5	5	10	-0.38	-0.39	-0.35	-0.29	0.06	0.08	-0.39	-0.33	-0.09	-0.27	0.10	0.15	0.06	0.26	0.23	0.12	0.09	0.23	0.36	0.00
P48643	T-complex protein 1 subunit epsilon OS=Homo sapiens GN=CCT5 PE=1 SV=1 - [TCPE_HUMAN]	60.07	1	29	30	76	-0.46	-0.43	-0.16	-0.08	-0.16	-0.12	-0.20	-0.16	-0.24	-0.26	-0.12	-0.07	0.26	0.30	-0.02	0.20	-0.10	0.28	-0.07	0.00
Q96A33	Coiled-coil domain-containing protein 47 OS=Homo sapiens GN=CCDC47 PE=1 SV=1 - [CCD47_HUMAN]	22.36	1	9	9	14	-0.25	-0.29	-0.12	-0.27	0.05	0.13	-0.14	-0.26	-0.04	-0.03	0.25	0.06	0.24	0.54	0.25	0.25	0.06	0.28	0.12	0.00
Q6P4E1	Protein CASC4 OS=Homo sapiens GN=CASC4 PE=1 SV=1 - [CASC4_HUMAN]	9.01	1	3	3	4	-0.12	-0.07	-0.02	0.03	0.07	-0.06	-0.08	-0.03	0.14	0.16	0.18	0.41	0.09	0.49	0.39	0.31	0.21	0.17	0.07	0.00
Q9P2T1	GMP reductase 2 OS=Homo sapiens GN=GMPR2 PE=1 SV=1 - [GMPR2_HUMAN]	27.01	1	6	6	12	0.27	0.40	0.24	0.35	0.29	0.38	0.12	0.28	0.18	0.12	0.33	0.59	-0.08	0.21	0.16	-0.07	-0.09	0.05	-0.03	0.00

P45974	Ubiquitin carboxyl-terminal hydrolase 5 OS=Homo sapiens GN=USP5 PE=1 SV=2 - [UBP5_HUMAN]	41.61	1	25	25	63	-0.17	-0.08	0.08	-0.03	-0.08	-0.14	0.03	-0.06	-0.04	-0.11	0.00	-0.07	0.11	0.10	-0.10	0.02	-0.08	-0.14	-0.24	0.00
Q92841	Probable ATP-dependent RNA helicase DDX17 OS=Homo sapiens GN=DDX17 PE=1 SV=2 - [DDX17_HUMAN]	39.64	1	18	25	74	-0.23	-0.28	0.02	-0.14	-0.22	-0.32	-0.10	-0.18	-0.40	-0.30	-0.42	-0.57	0.10	-0.30	-0.35	-0.15	-0.11	-0.06	-0.23	0.00
Q15020	Squamous cell carcinoma antigen recognized by T-cells 3 OS=Homo sapiens GN=SART3 PE=1 SV=1 - [SART3_HUMAN]	18.90	1	13	13	24	-0.07	-0.16	0.02	-0.07	-0.21	-0.27	-0.11	-0.20	-0.05	0.02	-0.01	0.04	0.07	0.04	-0.06	0.13	0.10	-0.13	-0.13	0.00
Q5JSL3	Dedicator of cytokinesis protein 11 OS=Homo sapiens GN=DOCK11 PE=1 SV=2 - [DOC11_HUMAN]	1.93	1	3	3	4	-0.28	0.38	-0.60	0.05	-0.46	0.19	-0.67	-0.01	-0.06	-0.71	-0.62	0.03	-0.33	-0.34	-0.01	-0.40	-0.07	-0.20	0.12	0.00
O75110	Probable phospholipid-transporting ATPase IIA OS=Homo sapiens GN=ATP9A PE=1 SV=3 - [ATP9A_HUMAN]	10.12	1	6	7	15	0.50	0.80	0.42	0.85	-0.29	0.11	0.19	0.61	0.35	-0.01	-0.08	0.23	-0.01	-0.78	-0.73	-0.39	-0.40	-0.50	-0.60	0.00
P04181	Ornithine aminotransferase, mitochondrial OS=Homo sapiens GN=OAT PE=1 SV=1 - [OAT_HUMAN]	14.81	1	6	6	11	-0.34	-0.01	-0.24	-0.23	-0.21	-0.10	-0.50	-0.24	0.06	-0.31	-0.24	-0.18	-0.19	0.03	0.10	-0.04	0.13	0.01	0.13	0.00
P98194	Calcium-transporting ATPase type 2C member 1 OS=Homo sapiens GN=ATP2C1 PE=1 SV=3 - [AT2C1_HUMAN]	12.30	1	8	8	11	0.60	0.55	0.65	0.33	0.33	-0.01	0.23	0.04	0.09	0.15	-0.11	-0.28	-0.39	-0.52	-0.33	-0.40	-0.07	-0.29	-0.30	0.00

P05386	60S acidic ribosomal protein P1 OS=Homo sapiens GN=RPLP1 PE=1 SV=1 - [RLA1_HUMAN]	14.04	1	1	1	2	-1.40	-1.34	-0.62	-0.56	-0.13	-0.08	-0.68	-0.63	-0.33	-0.38	-0.03	0.02	0.77	1.38	0.59	1.05	0.28	1.26	0.47	0.00
Q16836	Hydroxyacyl-coenzyme A dehydrogenase, mitochondrial OS=Homo sapiens GN=HADH PE=1 SV=3 - [HCDH_HUMAN]	25.16	1	6	6	14	-0.72	-0.63	-0.35	-0.52	-0.86	-0.92	-0.48	-0.58	-0.78	-0.76	-0.84	-0.78	0.08	-0.14	-0.33	-0.16	-0.14	-0.45	-0.42	0.00
O00186	Syntaxin-binding protein 3 OS=Homo sapiens GN=STXBP3 PE=1 SV=2 - [STXB3_HUMAN]	23.31	1	9	9	12	0.10	-0.04	0.14	0.14	0.16	0.25	0.21	0.25	0.17	0.17	0.11	0.21	0.27	0.07	0.07	0.12	0.10	0.15	0.13	0.00
P50991	T-complex protein 1 subunit delta OS=Homo sapiens GN=CCT4 PE=1 SV=4 - [TCPD_HUMAN]	59.55	1	23	24	83	-0.13	-0.04	-0.09	-0.11	-0.14	-0.19	-0.23	-0.22	-0.23	-0.24	-0.19	-0.22	-0.07	-0.08	-0.12	0.00	-0.11	-0.10	-0.12	0.00
Q09161	Nuclear cap-binding protein subunit 1 OS=Homo sapiens GN=NCBP1 PE=1 SV=1 - [NCBP1_HUMAN]	8.23	1	5	5	6	0.00	-0.43	0.36	-0.06	-0.26	-0.68	0.30	-0.13	-0.41	0.02	-0.04	-0.46	0.36	-0.03	-0.40	0.05	-0.31	-0.27	-0.63	0.00
Q9NVM4	Protein arginine N-methyltransferase 7 OS=Homo sapiens GN=PRMT7 PE=1 SV=1 - [ANM7_HUMAN]	1.88	1	1	1	1	0.99	0.57	0.69	0.28	0.54	0.12	0.62	0.21	0.54	0.95	0.34	-0.08	-0.31	-0.64	-0.35	0.00	0.30	-0.46	-0.17	0.00
P53370	Nucleoside diphosphate-linked moiety X motif 6 OS=Homo sapiens GN=NUDT6 PE=1 SV=2 - [NUDT6_HUMAN]	12.34	1	2	2	3	0.20	0.40	0.34	0.54	0.61	0.81	0.27	0.47	0.48	0.28	0.45	0.64	0.13	0.25	0.11	0.11	-0.03	0.39	0.25	0.00

Q86VW0	SEC14 domain and spectrin repeat-containing protein 1 OS=Homo sapiens GN=SESTD1 PE=1 SV=2 - [SESD1_HUMAN]	12.21	1	6	6	12	-0.60	-0.75	-0.38	-0.35	-0.17	-0.19	-0.22	-0.28	-0.37	-0.29	-0.39	-0.50	0.40	0.26	-0.05	0.34	0.17	0.42	0.14	0.00
Q9Y224	UPF0568 protein C14orf166 OS=Homo sapiens GN=C14orf166 PE=1 SV=1 - [CN166_HUMAN]	45.90	1	11	11	19	-0.20	-0.26	0.02	0.03	0.03	0.14	-0.11	-0.05	0.01	0.00	0.09	0.17	0.20	0.31	0.24	0.21	-0.03	0.36	0.20	0.00
Q14966	Zinc finger protein 638 OS=Homo sapiens GN=ZNF638 PE=1 SV=2 - [ZN638_HUMAN]	5.81	1	8	8	11	0.06	-0.12	-0.01	0.00	-0.06	-0.26	0.04	-0.05	-0.17	0.02	-0.16	-0.35	0.13	0.53	0.03	0.52	-0.06	0.22	-0.09	0.00
O75396	Vesicle-trafficking protein SEC22b OS=Homo sapiens GN=SEC22B PE=1 SV=4 - [SC22B_HUMAN]	43.72	1	7	7	24	0.09	-0.11	0.04	0.01	-0.01	-0.03	-0.04	-0.05	-0.15	-0.14	0.08	0.06	-0.12	0.14	-0.02	0.08	-0.09	-0.01	-0.11	0.00
Q99698	Lysosomal-trafficking regulator OS=Homo sapiens GN=LYST PE=1 SV=3 - [LYST_HUMAN]	0.87	1	2	2	3	1.52	0.83	-0.01	-0.11	-0.36	0.14	-0.48	-0.17	0.07	-0.51	-0.20	0.62	-0.95	-1.71	-0.19	-0.73	0.21	-0.71	0.23	0.00
Q13948	Protein CASP OS=Homo sapiens GN=CUX1 PE=1 SV=2 - [CASP_HUMAN]	1.77	2	1	1	2	0.11	0.26	-0.11	0.04	-0.22	-0.08	-0.17	-0.03	-0.13	-0.27	-0.01	0.12	-0.23	-0.12	0.09	-0.35	-0.13	-0.35	-0.13	0.00
Q9Y3L3	SH3 domain-binding protein 1 OS=Homo sapiens GN=SH3BP1 PE=1 SV=3 - [3BP1_HUMAN]	5.56	1	3	3	3	0.68	0.67	0.10	0.10	0.44	0.43	0.03	0.03	0.31	0.32	0.84	0.83	-0.59	0.17	0.74	-0.32	0.26	-0.26	0.32	0.00
P01617	Ig kappa chain V-II region TEW OS=Homo sapiens PE=1 SV=1 - [KV204_HUMAN]	32.74	4	2	2	4	-1.30	-1.21	-0.88	-1.23	0.21	0.15	-1.15	-1.26	-0.62	-0.85	-0.44	-0.56	0.10	0.79	0.67	0.76	0.35	1.48	1.29	0.00

Q9H0D6	5'-3' exoribonuclease 2 OS=Homo sapiens GN=XRN2 PE=1 SV=1 - [XRN2_HUMAN]	7.05	1	6	6	8	-0.47	-0.93	-0.26	-0.58	0.35	0.03	0.02	-0.06	-0.58	-0.37	0.05	0.11	0.12	0.25	0.11	0.01	-0.11	0.37	0.27	0.00
Q9UJY4	ADP-ribosylation factor-binding protein GGA2 OS=Homo sapiens GN=GGA2 PE=1 SV=3 - [GGA2_HUMAN]	8.97	1	3	4	6	0.32	0.52	-0.08	0.14	0.23	-0.02	0.08	0.24	-0.09	0.16	0.45	-0.05	-0.19	-0.09	0.06	-0.25	0.15	-0.23	0.16	0.00
Q96PE7	Methylmalonyl-CoA epimerase, mitochondrial OS=Homo sapiens GN=MCEE PE=1 SV=1 - [MCEE_HUMAN]	28.98	1	5	5	9	-0.44	-0.59	-0.28	-0.25	-0.28	-0.46	-0.48	-0.42	-0.65	-0.44	-0.28	-0.66	0.06	0.03	0.02	-0.15	-0.29	0.10	-0.04	0.00
A5YKK6	CCR4-NOT transcription complex subunit 1 OS=Homo sapiens GN=CNOT1 PE=1 SV=2 - [CNOT1_HUMAN]	9.30	1	19	19	29	-0.02	-0.10	-0.17	-0.04	-0.19	-0.10	-0.41	-0.31	-0.29	-0.18	-0.11	-0.13	-0.18	-0.02	-0.04	-0.08	0.02	-0.08	-0.04	-0.01
P02741	C-reactive protein OS=Homo sapiens GN=CRP PE=1 SV=1 - [CRP_HUMAN]	18.75	1	4	4	6	-0.95	-0.83	-0.72	-0.42	-0.06	0.05	-0.61	-0.49	-0.24	-0.37	-0.06	0.38	0.00	0.89	0.74	0.33	0.22	0.78	0.55	-0.01
O60493	Sorting nexin-3 OS=Homo sapiens GN=SNX3 PE=1 SV=3 - [SNX3_HUMAN]	66.05	1	9	11	23	0.41	0.16	0.39	0.20	-0.02	0.06	0.16	0.34	-0.03	-0.08	0.04	0.02	-0.07	-0.10	-0.27	-0.11	-0.21	-0.04	-0.19	-0.01
O60831	PRA1 family protein 2 OS=Homo sapiens GN=PRAF2 PE=1 SV=1 - [PRAF2_HUMAN]	26.97	1	5	5	11	0.29	0.34	-0.04	-0.12	0.18	0.22	-0.16	-0.11	0.13	0.15	-0.18	-0.13	-0.25	-0.25	-0.07	-0.27	0.22	-0.05	0.24	-0.01
P01602	Ig kappa chain V-I region HK102 (Fragment) OS=Homo sapiens GN=IGKV1-5 PE=4 SV=1 - [KV110_HUMAN]	13.68	1	1	1	2	-1.78	-1.64	-1.57	-1.43	-0.21	-0.07	-1.64	-1.50	0.85	0.72	2.12	2.25	0.20	3.91	3.69	2.53	2.32	1.56	1.35	-0.01

Q9UPM8	AP-4 complex subunit epsilon-1 OS=Homo sapiens GN=AP4E1 PE=1 SV=2 - [AP4E1_HUMAN]	1.23	1	1	1	1	0.36	0.51	-0.26	-0.11	-0.42	-0.28	-0.33	-0.18	0.59	0.45	-0.87	-0.72	-0.64	-1.23	-0.61	0.11	0.74	-0.80	-0.18	-0.01
P25325	3-mercaptopyruvate sulfurtransferase OS=Homo sapiens GN=MPST PE=1 SV=3 - [THTM_HUMAN]	12.12	1	3	3	5	-0.11	-0.04	0.00	0.07	0.12	0.19	-0.07	0.00	-0.17	-0.24	-0.30	-0.23	0.09	-0.18	-0.29	-0.10	-0.20	0.22	0.11	-0.01
O95453	Poly(A)-specific ribonuclease PARN OS=Homo sapiens GN=PARN PE=1 SV=1 - [PARN_HUMAN]	3.76	1	2	2	3	-0.37	-0.37	-0.73	-0.73	-0.71	-0.71	-0.80	-0.80	-0.58	-0.58	-0.31	-0.31	-0.38	0.06	0.42	-0.18	0.18	-0.36	0.00	-0.01
Q9UBI1	COMM domain-containing protein 3 OS=Homo sapiens GN=COMMD3 PE=1 SV=1 - [COMD3_HUMAN]	16.92	1	2	2	2	-0.76	-0.60	-0.45	-0.29	-0.09	0.06	-0.52	-0.37	-0.29	-0.44	-0.14	0.01	0.29	0.62	0.31	0.35	0.04	0.65	0.34	-0.01
P42166	Lamina-associated polypeptide 2, isoform alpha OS=Homo sapiens GN=TMPO PE=1 SV=2 - [LAP2A_HUMAN]	15.56	1	1	8	19	-0.64	-0.30	-0.26	0.09	0.07	0.41	-0.33	0.01	0.13	-0.21	-0.38	-0.04	0.37	0.26	-0.12	0.46	0.08	0.69	0.31	-0.01
Q14376	UDP-glucose 4-epimerase OS=Homo sapiens GN=GALE PE=1 SV=2 - [GALE_HUMAN]	17.53	1	5	5	10	0.72	0.62	0.71	0.65	0.44	0.53	0.58	0.39	0.40	0.41	0.09	0.11	-0.03	-0.58	-0.58	-0.28	-0.27	-0.21	-0.20	-0.01
P16152	Carbonyl reductase [NADPH] 1 OS=Homo sapiens GN=CBR1 PE=1 SV=3 - [CBR1_HUMAN]	71.84	1	14	18	69	0.88	0.75	1.05	0.91	0.77	0.82	0.93	0.71	0.70	0.77	0.40	0.33	0.06	-0.46	-0.67	-0.03	-0.10	-0.03	-0.31	-0.01
Q5SRE5	Nucleoporin NUP188 homolog OS=Homo sapiens GN=NUP188 PE=1 SV=1 - [NU188_HUMAN]	2.74	1	4	4	6	-0.36	-0.50	-0.33	-0.33	-0.22	-0.48	-0.14	-0.20	-0.25	0.02	-0.27	-0.29	0.32	0.20	-0.08	0.29	0.12	0.00	-0.17	-0.01

Q8TC12	Retinol dehydrogenase 11 OS=Homo sapiens GN=RDH11 PE=1 SV=2 - [RDH11_HUMAN]	16.98	2	5	5	7	-0.54	-0.31	-0.19	-0.18	0.06	0.23	-0.24	-0.38	0.09	-0.10	-0.08	0.16	-0.02	0.43	0.11	0.36	0.12	0.74	0.23	-0.01
O94927	HAUS augmin-like complex subunit 5 OS=Homo sapiens GN=HAUS5 PE=1 SV=2 - [HAUS5_HUMAN]	2.37	1	1	1	1	1.03	1.71	0.79	1.47	1.21	1.88	0.72	1.39	1.63	0.96	-0.66	0.01	-0.26	-1.69	-1.46	-0.05	0.20	0.16	0.40	-0.01
O43516	WAS/WASL-interacting protein family member 1 OS=Homo sapiens GN=WIPF1 PE=1 SV=3 - [WIPF1_HUMAN]	7.75	1	3	3	5	-1.00	-0.99	-0.75	-0.73	-0.34	-0.33	-0.82	-0.81	-0.72	-0.73	-0.53	-0.52	0.24	0.48	0.22	0.30	0.05	0.65	0.39	-0.01
P84157	Matrix-remodeling-associated protein 7 OS=Homo sapiens GN=MXRA7 PE=1 SV=1 - [MXRA7_HUMAN]	15.20	1	3	3	5	-0.59	-0.71	-0.64	-0.62	-0.47	-0.50	-0.61	-0.69	-0.40	-0.33	0.39	0.39	0.10	1.10	1.21	0.26	0.33	0.12	0.08	-0.01
Q9BTW9	Tubulin-specific chaperone D OS=Homo sapiens GN=TBCD PE=1 SV=2 - [TBCD_HUMAN]	21.14	1	17	17	29	0.35	0.29	0.30	0.19	0.15	0.11	0.05	0.10	0.18	0.26	0.12	0.12	-0.14	-0.16	-0.01	-0.09	0.01	-0.32	-0.18	-0.01
O60573	Eukaryotic translation initiation factor 4E type 2 OS=Homo sapiens GN=EIF4E2 PE=1 SV=1 - [IF4E2_HUMAN]	13.88	1	2	2	3	0.09	0.16	0.01	0.08	0.27	0.34	-0.07	0.01	0.12	0.05	0.24	0.31	-0.10	0.16	0.23	-0.01	0.08	0.17	0.25	-0.01
P13473	Lysosome-associated membrane glycoprotein 2 OS=Homo sapiens GN=LAMP2 PE=1 SV=2 - [LAMP2_HUMAN]	6.83	1	2	3	5	0.80	0.84	0.80	0.84	0.37	0.40	0.73	0.76	0.67	0.64	0.94	0.97	-0.02	0.14	0.14	-0.13	-0.12	-0.45	-0.45	-0.01

Q9Y383	Putative RNA binding protein Luc7-like 2 OS=Homo sapiens GN=LUC7L2 PE=1 SV=2 - [LC7L2_HUMAN]	26.02	1	7	10	19	-0.17	-0.09	0.04	-0.01	-0.23	-0.36	-0.05	-0.11	-0.12	-0.14	-0.08	-0.23	0.23	-0.01	-0.09	0.10	-0.07	-0.12	-0.34	-0.01
P49368	T-complex protein 1 subunit gamma OS=Homo sapiens GN=CCT3 PE=1 SV=4 - [TCPG_HUMAN]	55.05	2	28	28	89	-0.20	-0.27	-0.12	-0.15	-0.35	-0.34	-0.25	-0.23	-0.32	-0.33	-0.22	-0.21	0.08	0.05	-0.13	-0.05	-0.19	-0.10	-0.28	-0.01
Q15386	Ubiquitin-protein ligase E3C OS=Homo sapiens GN=UBE3C PE=1 SV=3 - [UBE3C_HUMAN]	12.74	1	10	10	17	0.16	0.08	0.16	0.18	0.26	0.18	0.13	0.14	0.17	0.27	0.05	0.00	0.00	-0.10	-0.13	0.10	0.01	0.12	0.05	-0.01
Q13873	Bone morphogenetic protein receptor type-2 OS=Homo sapiens GN=BMPR2 PE=1 SV=2 - [BMPR2_HUMAN]	6.55	1	5	5	7	0.24	0.07	0.35	0.18	0.14	-0.04	0.28	0.10	-0.01	0.17	0.11	-0.07	0.09	-0.12	-0.24	-0.05	-0.15	-0.12	-0.23	-0.01
P22748	Carbonic anhydrase 4 OS=Homo sapiens GN=CA4 PE=1 SV=2 - [CAH4_HUMAN]	18.59	1	5	5	8	-0.33	-0.45	-0.27	-0.32	-1.64	-1.61	-0.27	-0.39	-0.64	-0.58	-0.50	-0.63	0.11	-0.16	-0.18	-0.16	-0.29	-0.69	-1.06	-0.01
Q96L92	Sorting nexin-27 OS=Homo sapiens GN=SNX27 PE=1 SV=2 - [SNX27_HUMAN]	29.02	1	11	11	22	0.60	0.51	0.40	0.30	0.37	0.33	0.40	0.35	0.25	0.37	0.23	0.14	-0.05	-0.31	-0.27	-0.11	-0.14	-0.14	-0.07	-0.01
Q9UQ80	Proliferation-associated protein 2G4 OS=Homo sapiens GN=PA2G4 PE=1 SV=3 - [PA2G4_HUMAN]	38.32	1	13	13	34	-0.33	-0.31	-0.15	-0.17	-0.15	-0.26	-0.15	-0.16	-0.25	-0.19	-0.10	-0.16	0.19	0.25	0.00	0.14	-0.02	0.04	-0.11	-0.01

Q9P035	Very-long-chain (3R)-3-hydroxyacyl-[acyl-carrier protein] dehydratase 3 OS=Homo sapiens GN=PTPLAD1 PE=1 SV=2 - [HACD3_HUMAN]	24.31	1	6	6	23	0.35	0.22	0.21	0.23	0.21	0.13	0.29	0.17	0.09	0.14	0.13	0.09	-0.02	-0.21	-0.06	-0.07	-0.10	-0.18	-0.22	-0.01
P11117	Lysosomal acid phosphatase OS=Homo sapiens GN=ACP2 PE=1 SV=3 - [PPAL_HUMAN]	10.87	1	3	3	6	0.10	0.19	0.28	0.36	0.35	0.44	0.20	0.29	0.76	0.68	0.78	0.86	0.15	0.68	0.50	0.61	0.44	0.24	0.06	-0.01
O43237	Cytoplasmic dynein 1 light intermediate chain 2 OS=Homo sapiens GN=DYNC1LI2 PE=1 SV=1 - [DC1L2_HUMAN]	42.48	1	13	14	34	0.37	0.40	0.11	0.40	-0.16	0.01	0.04	0.23	-0.03	-0.04	0.02	-0.05	0.00	-0.57	-0.44	-0.40	-0.38	-0.62	-0.47	-0.01
P49406	39S ribosomal protein L19, mitochondrial OS=Homo sapiens GN=MRPL19 PE=1 SV=2 - [RM19_HUMAN]	6.51	1	3	3	4	0.56	0.44	0.42	0.31	0.34	0.22	0.20	0.09	0.15	0.13	0.13	0.01	-0.26	-0.42	-0.30	-0.26	-0.13	-0.23	-0.09	-0.01
P54578	Ubiquitin carboxyl-terminal hydrolase 14 OS=Homo sapiens GN=USP14 PE=1 SV=3 - [UBP14_HUMAN]	37.25	1	17	17	30	-0.21	-0.42	0.08	0.14	-0.36	-0.43	0.10	0.12	-0.30	-0.16	-0.68	-0.69	0.41	-0.18	-0.73	0.11	-0.28	0.10	-0.34	-0.01
Q9UBL6	Copine-7 OS=Homo sapiens GN=CPNE7 PE=2 SV=1 - [CPNE7_HUMAN]	2.84	1	1	2	5	0.86	0.89	0.45	0.48	0.23	0.25	0.37	0.40	0.15	0.13	-0.13	-0.11	-0.43	-0.99	-0.59	-0.70	-0.29	-0.64	-0.23	-0.01
P13010	X-ray repair cross-complementing protein 5 OS=Homo sapiens GN=XRCC5 PE=1 SV=3 - [XRCC5_HUMAN]	38.80	1	20	21	62	-0.24	-0.21	0.04	0.11	-0.09	-0.08	-0.12	-0.03	-0.25	-0.28	-0.27	-0.22	0.24	-0.03	-0.24	0.01	-0.19	0.14	-0.11	-0.01

P62249	40S ribosomal protein S16 OS=Homo sapiens GN=RPS16 PE=1 SV=2 - [RS16_HUMAN]	54.79	1	9	9	21	-0.56	-0.70	-0.50	-0.52	-0.49	-0.52	-0.62	-0.60	-0.38	-0.45	-0.18	-0.29	-0.15	0.37	0.30	0.12	0.18	0.06	0.09	-0.01
O75935	Dynactin subunit 3 OS=Homo sapiens GN=DCTN3 PE=1 SV=1 - [DCTN3_HUMAN]	34.95	1	6	6	12	0.25	0.28	0.30	0.35	0.44	0.41	0.29	0.28	0.33	0.40	0.73	0.59	0.01	0.51	0.26	0.09	0.08	0.12	0.02	-0.01
Q5EBL4	RILP-like protein 1 OS=Homo sapiens GN=RILPL1 PE=1 SV=1 - [RIPL1_HUMAN]	23.33	1	7	7	9	-0.24	-0.20	0.13	0.17	-0.18	-0.15	-0.03	0.01	0.06	0.02	0.11	0.15	0.10	0.36	-0.02	0.29	-0.07	0.04	-0.33	-0.01
Q14C86	GTPase-activating protein and VPS9 domain-containing protein 1 OS=Homo sapiens GN=GAPVD1 PE=1 SV=2 - [GAPD1_HUMAN]	16.10	1	16	16	29	0.33	0.38	0.14	0.24	0.08	0.16	0.23	0.19	0.18	0.12	0.23	0.22	-0.15	-0.05	0.06	-0.15	0.01	-0.31	-0.09	-0.01
Q04637	Eukaryotic translation initiation factor 4 gamma 1 OS=Homo sapiens GN=EIF4G1 PE=1 SV=4 - [IF4G1_HUMAN]	16.95	1	18	22	36	0.16	0.20	0.40	0.30	0.15	0.03	0.32	0.36	0.08	0.12	0.05	0.00	0.09	-0.20	-0.28	0.04	-0.27	-0.11	-0.31	-0.01
Q9H0C8	Integrin-linked kinase-associated serine/threonine phosphatase 2C OS=Homo sapiens GN=ILKAP PE=1 SV=1 - [ILKAP_HUMAN]	8.93	1	3	3	5	0.02	0.24	-0.20	0.01	-0.30	-0.09	-0.28	-0.06	-0.08	-0.29	-0.51	-0.30	-0.25	-0.53	-0.31	-0.28	-0.05	-0.34	-0.11	-0.01
Q9BRJ6	Uncharacterized protein C7orf50 OS=Homo sapiens GN=C7orf50 PE=1 SV=1 - [CG050_HUMAN]	10.82	1	1	1	1	-0.59	-0.72	-0.41	-0.55	-0.26	-0.40	-0.49	-0.62	-0.39	-0.25	-0.31	-0.45	0.15	0.28	0.10	0.37	0.19	0.31	0.13	-0.01

Q6SZW1	Sterile alpha and TIR motif containing protein 1 OS=Homo sapiens GN=SARM1 PE=1 SV=1 - [SARM1_HUMAN]	7.87	1	4	4	7	0.20	0.00	0.13	0.05	0.11	-0.17	0.00	-0.30	-0.34	-0.32	-0.16	-0.38	-0.22	-0.60	-0.47	-0.69	-0.41	-0.35	-0.07	-0.02
P49006	MARCKS-related protein OS=Homo sapiens GN=MARCKSL1 PE=1 SV=2 - [MRP_HUMAN]	11.79	1	2	2	6	0.41	-0.34	0.00	-0.75	0.35	-0.21	-0.08	-0.83	-0.43	0.27	0.31	-0.33	-0.41	-0.09	0.31	-0.11	0.30	-0.05	0.36	-0.02
Q05519	Serine/arginine-rich splicing factor 11 OS=Homo sapiens GN=SRSF11 PE=1 SV=1 - [SRS11_HUMAN]	5.79	1	2	2	2	-0.27	0.08	-0.26	0.09	-0.64	-0.29	-0.34	0.01	0.04	-0.31	-0.23	0.12	-0.01	0.05	0.03	-0.01	-0.02	-0.39	-0.40	-0.02
Q15800	Methylsterol monooxygenase 1 OS=Homo sapiens GN=MSMO1 PE=1 SV=1 - [MSMO1_HUMAN]	4.78	1	1	1	1	0.59	0.20	0.55	0.16	0.53	0.14	0.47	0.08	0.09	0.49	0.39	0.00	-0.06	-0.19	-0.16	-0.07	-0.03	-0.08	-0.04	-0.02
P30038	Delta-1-pyrroline-5-carboxylate dehydrogenase, mitochondrial OS=Homo sapiens GN=ALDH4A1 PE=1 SV=3 - [AL4A1_HUMAN]	50.80	1	23	23	57	0.20	0.26	0.34	0.46	-0.18	-0.01	0.23	0.36	0.11	-0.03	-0.25	-0.24	0.06	-0.50	-0.57	-0.14	-0.30	-0.30	-0.54	-0.02
P41252	Isoleucine--tRNA ligase, cytoplasmic OS=Homo sapiens GN=IARS PE=1 SV=2 - [SYIC_HUMAN]	19.02	1	20	20	31	0.20	0.25	0.07	0.13	0.12	0.26	-0.04	-0.01	0.08	-0.15	-0.07	0.02	-0.15	-0.17	-0.11	-0.14	-0.02	-0.04	0.17	-0.02
Q9H0R6	Glutamyl-tRNA(Gln) amidotransferase subunit A, mitochondrial OS=Homo sapiens GN=QRSL1 PE=1 SV=2 - [GATA_HUMAN]	3.60	1	2	2	2	-0.10	0.02	-0.22	-0.09	-0.55	-0.43	-0.30	-0.17	-0.46	-0.58	-0.41	-0.29	-0.14	-0.30	-0.19	-0.44	-0.33	-0.47	-0.35	-0.02

P62753	40S ribosomal protein S6 OS=Homo sapiens GN=RPS6 PE=1 SV=1 - [RS6_HUMAN]	28.92	1	6	6	12	-0.74	-0.70	-0.36	-0.47	-0.19	-0.27	-0.35	-0.43	-0.51	-0.34	-0.13	-0.21	0.35	0.62	0.19	0.21	-0.01	0.36	0.04	-0.02
P49419	Alpha-aminoadipic semialdehyde dehydrogenase OS=Homo sapiens GN=ALDH7A1 PE=1 SV=5 - [AL7A1_HUMAN]	53.80	1	21	21	71	-0.15	-0.07	0.11	0.16	0.44	0.43	0.10	0.14	0.20	0.19	0.30	0.33	0.23	0.39	0.07	0.30	0.02	0.56	0.24	-0.02
P63220	40S ribosomal protein S21 OS=Homo sapiens GN=RPS21 PE=1 SV=1 - [RS21_HUMAN]	37.35	1	3	3	12	-0.45	-0.32	-0.43	-0.27	-0.49	-0.21	-0.51	-0.36	-0.14	-0.27	-0.42	-0.22	0.18	0.21	0.09	0.21	0.19	0.22	0.08	-0.02
Q7Z2Z2	Elongation factor Tu GTP-binding domain-containing protein 1 OS=Homo sapiens GN=EFTUD1 PE=1 SV=2 - [ETUD1_HUMAN]	5.89	1	5	5	6	-0.17	0.29	-0.25	0.22	0.00	0.49	-0.22	0.21	0.48	0.03	-0.20	0.18	-0.04	-0.14	-0.08	0.60	0.59	0.52	0.59	-0.02
Q9NX05	Constitutive coactivator of PPAR-gamma-like protein 2 OS=Homo sapiens GN=FAM120C PE=2 SV=3 - [F120C_HUMAN]	3.56	1	2	3	6	0.22	0.46	0.12	0.35	0.24	0.47	0.04	0.27	0.15	-0.07	-0.03	0.20	-0.13	-0.25	-0.15	-0.27	-0.16	0.00	0.10	-0.02
Q9NRW4	Dual specificity protein phosphatase 22 OS=Homo sapiens GN=DUSP22 PE=1 SV=1 - [DUS22_HUMAN]	5.98	1	1	1	1	-0.02	0.05	-0.22	-0.15	-0.25	-0.19	-0.31	-0.23	-0.01	-0.08	0.27	0.34	-0.23	0.30	0.49	-0.03	0.17	-0.25	-0.05	-0.02
Q9H270	Vacuolar protein sorting-associated protein 11 homolog OS=Homo sapiens GN=VPS11 PE=1 SV=1 - [VPS11_HUMAN]	19.45	1	14	15	22	0.17	0.23	-0.02	0.09	0.09	0.16	-0.06	0.22	0.29	0.13	0.23	0.21	0.04	0.10	0.14	0.10	0.12	0.03	0.18	-0.02

Q14DG7	Transmembrane protein 132B OS=Homo sapiens GN=TMEM132B PE=2 SV=2 - [T132B_HUMAN]	2.04	1	2	2	3	0.58	0.82	0.16	0.40	0.54	0.78	0.08	0.32	0.44	0.20	-0.12	0.11	-0.44	-0.69	-0.29	-0.34	0.07	-0.05	0.36	-0.02
Q13838	Spliceosome RNA helicase DDX39B OS=Homo sapiens GN=DDX39B PE=1 SV=1 - [DX39B_HUMAN]	32.48	1	6	14	43	-0.24	-0.22	-0.04	0.12	-0.29	-0.24	-0.15	-0.21	-0.21	-0.31	0.05	-0.21	0.08	0.14	-0.06	0.05	-0.28	0.00	-0.38	-0.02
O95870	Abhydrolase domain-containing protein 16A OS=Homo sapiens GN=ABHD16A PE=1 SV=3 - [ABHGA_HUMAN]	22.76	1	11	11	15	0.38	0.33	0.18	0.06	-0.07	-0.13	-0.08	-0.06	-0.14	-0.03	0.06	-0.01	-0.26	-0.13	-0.14	-0.41	-0.03	-0.45	-0.19	-0.02
P61289	Proteasome activator complex subunit 3 OS=Homo sapiens GN=PSME3 PE=1 SV=1 - [PSME3_HUMAN]	12.20	1	3	3	3	-0.56	-0.15	-0.65	-0.25	-0.20	0.20	-0.74	-0.33	-0.18	-0.58	-0.71	-0.31	-0.12	-0.15	-0.06	0.01	0.11	0.34	0.44	-0.02
Q9Y3A5	Ribosome maturation protein SBDS OS=Homo sapiens GN=SBDS PE=1 SV=4 - [SBDS_HUMAN]	42.00	1	10	10	23	-0.85	-0.82	-0.50	-0.41	-0.56	-0.42	-0.50	-0.51	-0.65	-0.71	-0.50	-0.47	0.33	0.31	0.01	0.14	-0.17	0.27	-0.09	-0.02
Q9BXM0	Periaxin OS=Homo sapiens GN=PRX PE=1 SV=2 - [PRAX_HUMAN]	4.93	1	1	1	1	-0.45	-0.36	-0.34	-0.24	0.41	0.51	-0.42	-0.32	-0.03	-0.12	-0.30	-0.21	0.09	0.16	0.03	0.36	0.25	0.85	0.73	-0.02
Q8WXA3	RUN and FYVE domain-containing protein 2 OS=Homo sapiens GN=RUFY2 PE=1 SV=2 - [RUFY2_HUMAN]	4.12	1	1	3	4	-1.39	-1.26	-1.34	-1.21	-0.14	-0.02	-1.43	-1.30	-0.52	-0.64	-0.54	-0.42	0.02	0.85	0.80	0.78	0.73	1.23	1.18	-0.02
Q08345	Epithelial discoidin domain-containing receptor 1 OS=Homo sapiens GN=DDR1 PE=1 SV=1 - [DDR1_HUMAN]	6.68	1	4	4	5	-0.31	-0.47	-0.33	-0.42	0.02	-0.04	-0.30	-0.44	-0.12	-0.07	-0.33	-0.25	0.07	0.13	-0.01	0.28	0.33	0.40	0.37	-0.02

Q9BYT8	Neurolysin, mitochondrial OS=Homo sapiens GN=NLN PE=1 SV=1 - [NEUL_HUMAN]	6.11	1	4	4	4	0.80	0.19	0.57	-0.03	2.43	1.82	0.49	-0.12	0.84	1.45	0.81	0.19	-0.26	0.01	0.23	0.68	0.91	1.62	1.84	-0.02
Q9UL33	Trafficking protein particle complex subunit 2-like protein OS=Homo sapiens GN=TRAPPC2L PE=1 SV=1 - [TPC2L_HUMAN]	5.71	1	1	1	2	0.32	0.20	0.18	0.06	0.24	0.11	0.10	-0.03	0.35	0.48	0.30	0.17	-0.17	-0.02	0.12	0.18	0.33	-0.10	0.04	-0.02
Q9BZE1	39S ribosomal protein L37, mitochondrial OS=Homo sapiens GN=MRPL37 PE=1 SV=2 - [RM37_HUMAN]	13.95	1	4	4	5	0.00	-0.10	-0.04	-0.05	0.08	0.06	-0.02	-0.03	-0.15	-0.14	-0.12	-0.13	0.14	0.12	-0.08	0.16	-0.07	0.12	-0.01	-0.02
P39023	60S ribosomal protein L3 OS=Homo sapiens GN=RPL3 PE=1 SV=2 - [RL3_HUMAN]	39.95	2	18	18	41	-0.76	-0.75	-0.48	-0.44	-0.48	-0.43	-0.53	-0.54	-0.41	-0.40	-0.27	-0.15	0.37	0.48	0.20	0.17	-0.12	0.19	-0.08	-0.02
P49407	Beta-arrestin-1 OS=Homo sapiens GN=ARRB1 PE=1 SV=2 - [ARRB1_HUMAN]	35.65	3	12	13	19	0.03	-0.02	0.40	0.43	0.25	0.30	0.15	0.28	0.12	0.07	0.08	0.05	0.13	-0.03	-0.23	0.01	-0.20	0.42	-0.02	-0.02
Q96G03	Phosphoglucomutase-2 OS=Homo sapiens GN=PGM2 PE=1 SV=4 - [PGM2_HUMAN]	19.93	1	11	12	19	-0.35	-0.37	-0.35	-0.22	-0.47	-0.48	-0.50	-0.43	-0.41	-0.50	-0.52	-0.43	-0.10	-0.09	-0.06	-0.01	0.05	0.04	-0.19	-0.02
P49137	MAP kinase-activated protein kinase 2 OS=Homo sapiens GN=MAPKAPK2 PE=1 SV=1 - [MAPK2_HUMAN]	5.75	2	2	2	2	-0.42	-0.16	-0.75	-0.49	-0.28	-0.02	-0.84	-0.58	-0.08	-0.34	-0.95	-0.70	-0.36	-0.53	-0.20	0.11	0.45	0.13	0.46	-0.02
P35241	Radixin OS=Homo sapiens GN=RDX PE=1 SV=1 - [RADI_HUMAN]	38.42	1	13	24	57	0.05	0.03	0.22	0.27	0.13	0.13	0.21	0.15	0.13	0.12	-0.02	-0.06	0.19	-0.13	-0.32	0.13	0.01	0.07	0.14	-0.02

Q15067	Peroxisomal acyl-coenzyme A oxidase 1 OS=Homo sapiens GN=ACOX1 PE=1 SV=3 - [ACOX1_HUMAN]	15.30	1	7	7	11	0.19	0.32	0.20	0.12	0.05	0.07	0.12	0.16	0.22	0.19	0.44	0.32	0.12	0.08	-0.17	0.00	-0.25	-0.13	-0.29	-0.02
O43347	RNA-binding protein Musashi homolog 1 OS=Homo sapiens GN=MSI1 PE=1 SV=1 - [MSI1H_HUMAN]	10.77	1	1	3	8	-0.91	-1.21	-0.89	-0.81	-0.68	-0.43	-1.05	-0.98	-0.71	-0.77	-0.71	-0.65	0.22	0.57	0.17	0.54	0.23	0.58	0.19	-0.03
Q53GT1	Kelch-like protein 22 OS=Homo sapiens GN=KLHL22 PE=1 SV=2 - [KLH22_HUMAN]	5.36	1	3	3	4	0.59	0.52	0.62	0.56	0.54	0.47	0.53	0.47	0.54	0.61	0.53	0.46	0.00	-0.05	-0.09	0.05	0.02	-0.07	-0.10	-0.03
P20648	Potassium-transporting ATPase alpha chain 1 OS=Homo sapiens GN=ATP4A PE=2 SV=5 - [ATP4A_HUMAN]	5.12	1	1	4	19	1.99	1.66	0.44	0.12	0.49	0.16	0.35	0.03	0.36	0.69	0.78	0.45	-1.58	-1.20	0.33	-1.27	0.28	-1.52	0.02	-0.03
P62851	40S ribosomal protein S25 OS=Homo sapiens GN=RPS25 PE=1 SV=1 - [RS25_HUMAN]	24.00	1	4	4	9	-0.70	-0.69	-0.69	-0.69	-0.52	-0.56	-0.79	-0.81	-0.96	-0.93	-0.91	-0.95	-0.02	-0.31	-0.23	-0.39	-0.21	-0.06	0.15	-0.03
Q9UIV1	CCR4-NOT transcription complex subunit 7 OS=Homo sapiens GN=CNOT7 PE=1 SV=3 - [CNOT7_HUMAN]	9.12	1	2	2	4	-0.51	-0.12	-0.56	-0.30	-0.88	-0.74	-0.61	-0.25	-0.04	-0.36	-0.50	-0.11	-0.08	0.01	0.25	0.11	0.16	0.00	-0.16	-0.03
O60832	H/ACA ribonucleoprotein complex subunit 4 OS=Homo sapiens GN=DKC1 PE=1 SV=3 - [DKC1_HUMAN]	10.51	1	5	5	8	-0.96	-0.84	-0.51	-0.25	-0.60	-0.27	-0.68	-0.35	-0.38	-0.71	-0.66	-0.38	0.56	0.33	-0.14	0.65	-0.08	0.73	-0.02	-0.03
P10636	Microtubule-associated protein tau OS=Homo sapiens GN=MAPT PE=1 SV=5 - [TAU_HUMAN]	27.04	1	16	16	84	1.02	0.93	1.22	1.12	0.29	0.28	1.07	1.01	0.67	0.84	0.21	0.09	0.12	-1.06	-1.32	-0.30	-0.52	-0.84	-1.08	-0.03

Q8N3E9	1-phosphatidylinositol 4,5-bisphosphate phosphodiesterase delta-3 OS=Homo sapiens GN=PLCD3 PE=1 SV=3 - [PLCD3_HUMAN]	32.19	1	16	16	28	0.54	0.62	0.41	0.42	0.02	0.14	0.11	0.40	0.35	0.20	0.19	0.28	-0.27	-0.38	-0.19	-0.26	-0.01	-0.53	-0.42	-0.03
Q14677	Clathrin interactor 1 OS=Homo sapiens GN=CLINT1 PE=1 SV=1 - [EPN4_HUMAN]	5.60	1	3	3	5	-0.24	-0.23	-0.05	-0.03	1.54	1.79	-0.14	-0.12	1.60	1.35	0.92	0.93	0.16	1.17	0.97	1.57	1.37	1.72	1.51	-0.03
Q9Y2L5	Trafficking protein particle complex subunit 8 OS=Homo sapiens GN=TRAPPC8 PE=1 SV=2 - [TPPC8_HUMAN]	7.25	1	9	9	12	0.12	0.10	0.08	0.02	0.27	0.36	-0.03	0.15	0.12	0.09	0.12	0.12	0.14	0.11	0.08	0.15	0.14	0.29	0.23	-0.03
P19174	1-phosphatidylinositol 4,5-bisphosphate phosphodiesterase gamma 1 OS=Homo sapiens GN=PLCG1 PE=1 SV=1 - [PLCG1_HUMAN]	11.63	1	11	11	17	0.35	0.49	0.04	0.23	0.00	-0.03	-0.08	0.17	0.00	0.18	0.03	0.13	-0.33	-0.22	-0.12	-0.32	-0.05	-0.39	-0.20	-0.03
Q9Y2W6	Tudor and KH domain-containing protein OS=Homo sapiens GN=TDRKH PE=1 SV=2 - [TDRKH_HUMAN]	6.42	1	2	2	3	0.77	1.14	0.56	0.94	0.17	0.54	0.47	0.84	1.14	0.77	0.69	0.40	-0.24	0.44	0.71	0.03	0.24	-0.61	-0.41	-0.03
Q09028	Histone-binding protein RBBP4 OS=Homo sapiens GN=RBBP4 PE=1 SV=3 - [RBBP4_HUMAN]	22.35	1	3	9	21	-0.61	-0.53	-0.02	0.03	-0.55	-0.42	-0.55	-0.40	-0.33	-0.47	-0.30	-0.15	0.12	0.31	0.10	0.17	-0.14	0.04	-0.46	-0.03
P62906	60S ribosomal protein L10a OS=Homo sapiens GN=RPL10A PE=1 SV=2 - [RL10A_HUMAN]	40.09	1	8	8	22	-0.84	-0.87	-0.42	-0.50	-0.56	-0.52	-0.47	-0.56	-0.58	-0.58	-0.43	-0.37	0.45	0.39	0.12	0.28	-0.09	0.29	-0.03	-0.03

Q9Y4C2	Protein FAM115A OS=Homo sapiens GN=FAM115A PE=1 SV=3 - [F115A_HUMAN]	2.28	1	2	2	2	0.58	0.53	0.24	0.18	0.76	0.70	0.15	0.09	0.69	0.75	0.09	0.03	-0.38	-0.49	-0.15	0.20	0.54	0.16	0.50	-0.03
P16035	Metalloproteinase inhibitor 2 OS=Homo sapiens GN=TIMP2 PE=1 SV=2 - [TIMP2_HUMAN]	10.45	1	2	2	2	0.24	0.00	0.00	-0.24	0.11	-0.14	-0.09	-0.33	-0.11	0.14	0.08	-0.16	-0.28	-0.15	0.08	-0.08	0.17	-0.15	0.09	-0.03
Q08AG7	Mitotic-spindle organizing protein 1 OS=Homo sapiens GN=MZT1 PE=1 SV=2 - [MZT1_HUMAN]	13.41	1	1	1	2	-0.35	-0.18	-0.45	-0.28	-0.72	-0.56	-0.54	-0.38	-0.27	-0.43	0.13	0.29	-0.14	0.48	0.58	-0.05	0.05	-0.39	-0.29	-0.03
Q9UN37	Vacuolar protein sorting-associated protein 4A OS=Homo sapiens GN=VPS4A PE=1 SV=1 - [VPS4A_HUMAN]	37.07	2	10	15	23	0.29	0.27	0.34	0.40	0.27	0.39	0.27	0.30	0.29	0.28	0.40	0.38	0.08	0.06	0.03	-0.01	-0.12	0.00	-0.02	-0.03
Q15738	Sterol-4-alpha-carboxylate 3 dehydrogenase, decarboxylating OS=Homo sapiens GN=NSDHL PE=1 SV=2 - [NSDHL_HUMAN]	13.67	1	4	4	7	-0.03	0.01	-0.18	-0.17	0.29	0.32	-0.24	-0.20	0.04	0.11	0.28	0.31	-0.06	0.38	0.37	0.12	0.35	0.30	0.32	-0.03
Q8NHG8	E3 ubiquitin-protein ligase ZNRF2 OS=Homo sapiens GN=ZNRF2 PE=1 SV=1 - [ZNRF2_HUMAN]	14.05	1	2	2	2	0.49	-0.01	0.55	0.05	0.90	0.40	0.46	-0.05	0.55	1.05	1.25	0.74	0.02	0.76	0.70	0.59	0.54	0.39	0.34	-0.03
O15533	Tapasin OS=Homo sapiens GN=TAPBP PE=1 SV=1 - [TPSN_HUMAN]	4.91	1	2	2	4	-0.09	0.03	-0.54	-0.42	-0.09	0.03	-0.64	-0.52	-0.16	-0.28	-0.23	-0.12	-0.49	-0.14	0.31	-0.16	0.30	-0.01	0.44	-0.03
P67870	Casein kinase II subunit beta OS=Homo sapiens GN=CSNK2B PE=1 SV=1 - [CSK2B_HUMAN]	45.58	1	7	7	17	0.12	0.01	0.14	0.07	0.22	0.14	0.31	0.15	0.01	0.12	0.24	0.22	0.18	0.32	0.25	0.02	-0.03	0.08	0.01	-0.03

Q8TCS8	Polyribonucleotide nucleotidyltransferase 1, mitochondrial OS=Homo sapiens GN=PNPT1 PE=1 SV=2 - [PNPT1_HUMAN]	15.33	1	11	11	23	-0.36	-0.60	-0.23	-0.40	-0.49	-0.55	-0.37	-0.51	-0.53	-0.33	-0.34	-0.41	0.14	-0.11	-0.06	0.06	-0.04	-0.05	-0.18	-0.03
Q96CP6	GRAM domain-containing protein 1A OS=Homo sapiens GN=GRAMD1A PE=1 SV=2 - [GRM1A_HUMAN]	4.70	1	2	2	3	-0.18	-0.27	-0.18	-0.27	-0.43	-0.53	-0.28	-0.37	-0.35	-0.25	-0.25	-0.34	-0.04	-0.06	-0.07	-0.05	-0.04	-0.27	-0.27	-0.03
Q92696	Geranylgeranyl transferase type-2 subunit alpha OS=Homo sapiens GN=RABGGTA PE=1 SV=2 - [PGTA_HUMAN]	16.05	1	8	8	12	0.80	0.79	0.81	0.89	0.58	0.59	0.69	0.78	0.60	0.61	0.57	0.64	0.07	-0.09	-0.37	-0.05	-0.32	-0.08	-0.37	-0.03
P23610	Factor VIII intron 22 protein OS=Homo sapiens GN=F8A1 PE=1 SV=2 - [F8I2_HUMAN]	2.96	1	1	1	4	0.02	0.03	0.01	0.01	-0.07	-0.07	-0.09	-0.08	0.08	0.08	0.31	0.31	-0.05	0.29	0.30	0.09	0.10	-0.10	-0.09	-0.03
P02750	Leucine-rich alpha-2-glycoprotein OS=Homo sapiens GN=LRG1 PE=1 SV=2 - [A2GL_HUMAN]	16.14	1	5	5	8	-0.60	-0.77	-0.48	-0.53	-0.58	-0.63	-0.71	-0.69	-1.04	-0.89	-1.37	-1.46	0.16	-0.65	-0.95	0.03	-0.23	0.13	-0.03	-0.03
O15239	NADH dehydrogenase [ubiquinone] 1 alpha subcomplex subunit 1 OS=Homo sapiens GN=NDUFA1 PE=1 SV=1 - [NDUA1_HUMAN]	18.57	1	1	1	1	-0.19	0.51	-0.56	0.14	0.29	0.99	-0.66	0.04	0.59	-0.11	-0.17	0.53	-0.41	0.03	0.39	0.11	0.48	0.47	0.83	-0.04
Q9UNF0	Protein kinase C and casein kinase substrate in neurons protein 2 OS=Homo sapiens GN=PACSIN2 PE=1 SV=2 - [PACN2_HUMAN]	33.54	1	12	12	23	-0.81	-0.56	-0.36	-0.40	-0.23	-0.30	-0.48	-0.47	-0.14	-0.36	-0.25	-0.18	0.19	0.33	0.24	0.15	0.11	0.14	0.07	-0.04

Q9BUH6	Uncharacterized protein C9orf142 OS=Homo sapiens GN=C9orf142 PE=1 SV=2 - [CI142_HUMAN]	16.18	1	2	2	2	-0.59	-0.36	-0.41	-0.18	-0.62	-0.39	-0.51	-0.28	-0.76	-0.98	-1.79	-1.54	0.14	-0.38	-1.09	-0.36	-0.54	-0.05	-0.23	-0.04
Q9Y6X9	MORC family CW-type zinc finger protein 2 OS=Homo sapiens GN=MORC2 PE=1 SV=2 - [MORC2_HUMAN]	2.03	1	1	1	1	-1.60	-1.59	-1.99	-1.98	-1.07	-1.07	-2.09	-2.08	-1.36	-1.36	-1.41	-1.40	-0.44	0.19	0.58	0.27	0.66	0.50	0.90	-0.04
Q13144	Translation initiation factor eIF-2B subunit epsilon OS=Homo sapiens GN=EIF2B5 PE=1 SV=3 - [EI2BE_HUMAN]	9.57	1	5	5	7	0.04	-0.04	0.10	0.03	0.25	0.18	0.00	-0.07	0.28	0.35	0.12	0.04	0.02	0.09	0.02	0.35	0.28	0.20	0.13	-0.04
Q96LT7	Protein C9orf72 OS=Homo sapiens GN=C9orf72 PE=1 SV=2 - [CI072_HUMAN]	3.53	1	2	2	3	1.02	1.09	0.68	0.75	0.37	0.44	0.58	0.65	0.51	0.44	0.49	0.56	-0.38	-0.52	-0.19	-0.54	-0.20	-0.66	-0.33	-0.04
Q6ICB0	Desumoylating isopeptidase 1 OS=Homo sapiens GN=DESI1 PE=1 SV=1 - [DESI1_HUMAN]	4.76	1	1	1	1	0.15	-0.01	0.32	0.16	0.28	0.11	0.22	0.05	0.24	0.41	0.37	0.21	0.12	0.23	0.05	0.29	0.12	0.11	-0.06	-0.04
Q9UKV5	E3 ubiquitin-protein ligase AMFR OS=Homo sapiens GN=AMFR PE=1 SV=2 - [AMFR_HUMAN]	9.33	1	2	2	4	-0.53	-0.51	-0.28	-0.26	-1.14	-1.12	-0.38	-0.37	-0.88	-0.89	0.01	0.02	0.20	0.54	0.29	-0.33	-0.57	-0.63	-0.87	-0.04
O95396	Adenylyltransferase and sulfurtransferase MOCS3 OS=Homo sapiens GN=MOCS3 PE=1 SV=1 - [MOCS3_HUMAN]	11.96	1	5	5	7	0.04	0.09	0.15	0.20	0.29	0.33	0.05	0.10	0.08	0.04	0.07	0.12	0.07	0.04	-0.08	0.02	-0.08	0.23	0.12	-0.04

Q9H4A3	Serine/threonine-protein kinase WNK1 OS=Homo sapiens GN=WNK1 PE=1 SV=2 - [WNK1_HUMAN]	6.05	3	10	14	20	0.80	0.84	0.60	0.48	0.15	0.07	0.31	0.24	0.16	0.23	0.15	0.03	-0.20	-0.48	-0.18	-0.19	-0.18	-0.51	-0.26	-0.04
P51610	Host cell factor 1 OS=Homo sapiens GN=HCFC1 PE=1 SV=2 - [HCFC1_HUMAN]	10.91	2	17	17	22	-0.43	-0.30	-0.53	-0.25	-0.41	-0.20	-0.56	-0.39	-0.22	-0.33	-0.33	-0.20	-0.20	0.14	0.25	-0.02	0.29	-0.11	0.31	-0.04
P62263	40S ribosomal protein S14 OS=Homo sapiens GN=RPS14 PE=1 SV=3 - [RS14_HUMAN]	37.75	1	5	5	18	-0.41	-0.47	-0.37	-0.36	-0.27	-0.28	-0.33	-0.44	-0.18	-0.13	-0.19	-0.21	0.15	0.29	0.22	0.37	0.13	0.32	0.07	-0.04
P46108	Adapter molecule crk OS=Homo sapiens GN=CRK PE=1 SV=2 - [CRK_HUMAN]	44.74	1	11	11	28	-0.06	0.19	0.09	0.03	-0.05	0.12	-0.13	0.00	-0.01	-0.10	-0.29	-0.19	-0.24	-0.28	-0.42	-0.10	-0.03	-0.03	-0.16	-0.04
Q9BTE1	Dynactin subunit 5 OS=Homo sapiens GN=DCTN5 PE=1 SV=1 - [DCTN5_HUMAN]	23.63	1	4	4	6	0.44	0.43	0.29	0.29	0.19	0.18	0.19	0.19	0.14	0.15	-0.01	-0.03	-0.19	-0.44	-0.31	-0.26	-0.11	-0.26	-0.12	-0.04
Q9UL25	Ras-related protein Rab-21 OS=Homo sapiens GN=RAB21 PE=1 SV=3 - [RAB21_HUMAN]	28.89	1	6	6	21	-0.02	0.23	-0.14	0.05	-0.14	-0.12	-0.22	0.03	-0.09	-0.06	-0.10	0.19	-0.26	-0.13	0.10	-0.25	0.02	-0.28	-0.10	-0.04
A1L188	Uncharacterized protein C17orf89 OS=Homo sapiens GN=C17orf89 PE=2 SV=1 - [CQ089_HUMAN]	25.68	1	2	2	2	0.58	0.59	0.43	0.43	0.50	0.50	0.32	0.33	0.56	0.56	0.77	0.77	-0.20	0.20	0.34	0.01	0.17	-0.10	0.06	-0.04
Q9UL18	Protein argonaute-1 OS=Homo sapiens GN=AGO1 PE=1 SV=3 - [AGO1_HUMAN]	17.85	2	6	12	15	-0.41	-0.51	-0.40	-0.68	-0.19	-0.29	-0.53	-0.69	-0.57	-0.49	-0.32	-0.41	-0.06	0.10	0.32	-0.04	0.09	0.20	0.38	-0.04
Q68E01	Integrator complex subunit 3 OS=Homo sapiens GN=INTS3 PE=1 SV=1 - [INT3_HUMAN]	2.68	1	1	1	1	-0.80	-0.47	-0.62	-0.29	-0.98	-0.66	-0.72	-0.39	-0.53	-0.86	-0.65	-0.32	0.13	0.16	-0.03	-0.03	-0.21	-0.20	-0.38	-0.04

Q96IV0	Peptide-N(4)-(N-acetyl-beta-glucosaminyl)asparagine amidase OS=Homo sapiens GN=NGLY1 PE=1 SV=1 - [NGLY1_HUMAN]	10.55	1	5	5	8	0.54	0.54	0.34	0.34	0.24	0.24	0.24	0.24	0.13	0.13	0.17	0.17	-0.25	-0.36	-0.17	-0.38	-0.17	-0.32	-0.12	-0.04
A5PLN9	Trafficking protein particle complex subunit 13 OS=Homo sapiens GN=TRAPPC13 PE=1 SV=2 - [TPC13_HUMAN]	6.00	1	2	2	2	0.08	0.41	-0.12	0.21	0.29	0.62	-0.23	0.11	0.46	0.14	0.14	0.47	-0.25	0.06	0.26	0.08	0.29	0.20	0.40	-0.04
Q9NPL8	Complex I assembly factor TIMMDC1, mitochondrial OS=Homo sapiens GN=TIMMDC1 PE=1 SV=2 - [TIDC1_HUMAN]	5.96	1	1	1	2	-0.55	-0.19	-0.30	0.06	-0.01	0.34	-0.40	-0.04	0.26	-0.09	-0.14	0.21	0.21	0.41	0.15	0.48	0.23	0.52	0.26	-0.04
Q9BYD3	39S ribosomal protein L4, mitochondrial OS=Homo sapiens GN=MRPL4 PE=1 SV=1 - [RM04_HUMAN]	11.25	1	2	2	3	1.53	0.48	0.59	0.03	0.15	-0.17	0.37	-0.08	0.33	0.59	0.55	0.21	-0.51	-0.26	0.19	-0.12	0.35	-0.66	-0.21	-0.04
Q7Z5L9	Interferon regulatory factor 2-binding protein 2 OS=Homo sapiens GN=IRF2BP2 PE=1 SV=2 - [I2BP2_HUMAN]	5.28	1	2	3	4	0.22	-0.01	0.09	-0.13	0.06	-0.18	-0.01	-0.24	-0.38	-0.15	-0.52	-0.75	-0.18	-0.73	-0.61	-0.34	-0.21	-0.18	-0.06	-0.04
Q9NRQ2	Phospholipid scramblase 4 OS=Homo sapiens GN=PLSCR4 PE=1 SV=2 - [PLS4_HUMAN]	17.02	1	4	4	13	0.08	0.23	0.16	0.31	-0.19	-0.04	0.05	0.20	0.06	-0.08	-0.05	0.09	0.03	-0.13	-0.21	-0.13	-0.21	-0.29	-0.36	-0.04
Q8N3U4	Cohesin subunit SA-2 OS=Homo sapiens GN=STAG2 PE=1 SV=3 - [STAG2_HUMAN]	3.90	1	2	4	5	-0.19	-0.55	-0.14	-0.50	0.17	-0.20	-0.24	-0.60	-0.14	0.22	-0.06	-0.42	0.00	0.14	0.08	0.44	0.39	0.34	0.29	-0.04

P42336	Phosphatidylinositol 4,5-bisphosphate 3-kinase catalytic subunit alpha isoform OS=Homo sapiens GN=PIK3CA PE=1 SV=2 - [PK3CA_HUMAN]	6.65	1	6	6	11	0.13	0.18	0.10	0.15	0.20	0.23	0.02	0.00	0.03	0.04	0.06	0.06	-0.08	0.08	0.09	0.04	0.07	0.04	0.16	-0.04
Q9Y450	HBS1-like protein OS=Homo sapiens GN=HBS1L PE=1 SV=1 - [HBS1L_HUMAN]	33.92	1	15	15	26	-0.11	-0.14	0.20	0.20	0.09	0.11	0.29	0.20	0.04	0.11	-0.08	-0.03	0.20	-0.15	-0.38	0.16	-0.05	0.04	-0.21	-0.04
O43172	U4/U6 small nuclear ribonucleoprotein Prp4 OS=Homo sapiens GN=PRPF4 PE=1 SV=2 - [PRP4_HUMAN]	13.22	1	5	5	8	0.40	0.31	-0.13	-0.07	-0.13	-0.15	-0.26	-0.17	-0.05	-0.01	-0.19	-0.20	-0.41	-0.56	-0.10	-0.21	0.29	-0.35	0.01	-0.04
Q92609	TBC1 domain family member 5 OS=Homo sapiens GN=TBC1D5 PE=1 SV=1 - [TBCD5_HUMAN]	8.18	1	5	5	7	-0.30	-0.02	-0.48	-0.27	-0.06	0.09	-0.55	-0.38	0.01	-0.14	-0.30	-0.11	-0.31	0.12	0.31	0.22	0.43	0.24	0.46	-0.04
O60762	Dolichol-phosphate mannosyltransferase subunit 1 OS=Homo sapiens GN=DPM1 PE=1 SV=1 - [DPM1_HUMAN]	23.08	1	6	6	11	-0.20	-0.30	-0.15	-0.27	-0.09	-0.12	-0.25	-0.34	0.02	0.04	0.03	-0.02	0.01	0.19	0.14	0.46	0.07	0.27	-0.12	-0.04
Q6NXT6	Transmembrane anterior posterior transformation protein 1 homolog OS=Homo sapiens GN=TAPT1 PE=1 SV=1 - [TAPT1_HUMAN]	6.35	1	3	3	3	-0.51	-0.50	-0.10	-0.09	-0.35	-0.34	-0.21	-0.20	-0.36	-0.36	-0.25	-0.24	0.35	0.26	-0.14	0.17	-0.22	0.14	-0.26	-0.04
Q86Y37	CDK2-associated and cullin domain-containing protein 1 OS=Homo sapiens GN=CACUL1 PE=1 SV=1 - [CACL1_HUMAN]	3.52	1	1	1	1	-0.17	-0.36	0.09	-0.10	0.33	0.14	-0.02	-0.21	-0.20	-0.01	-0.18	-0.38	0.21	0.00	-0.28	0.20	-0.06	0.49	0.22	-0.04

Q6P179	Endoplasmic reticulum aminopeptidase 2 OS=Homo sapiens GN=ERAP2 PE=1 SV=2 - [ERAP2_HUMAN]	1.35	1	1	1	1	0.04	0.10	-0.22	-0.16	0.66	0.72	-0.33	-0.27	0.04	-0.01	-0.95	-0.89	-0.31	-0.98	-0.73	-0.02	0.24	0.61	0.86	-0.04
Q96PK6	RNA-binding protein 14 OS=Homo sapiens GN=RBM14 PE=1 SV=2 - [RBM14_HUMAN]	26.31	1	15	15	30	-0.32	-0.44	-0.29	-0.38	-0.30	-0.26	-0.37	-0.36	-0.38	-0.25	-0.20	-0.24	0.00	0.12	0.09	0.10	0.02	-0.04	-0.09	-0.04
Q13148	TAR DNA-binding protein 43 OS=Homo sapiens GN=TARDBP PE=1 SV=1 - [TADBP_HUMAN]	25.85	1	7	7	22	-0.18	-0.01	0.07	0.17	-0.21	-0.06	0.01	0.00	0.00	-0.06	-0.22	-0.17	0.18	-0.20	-0.34	-0.02	-0.10	-0.12	-0.27	-0.04
Q9NXC5	WD repeat-containing protein mio OS=Homo sapiens GN=MIOS PE=1 SV=2 - [MIO_HUMAN]	9.26	1	6	6	10	0.36	0.16	0.28	0.21	0.44	0.14	0.14	0.10	0.13	0.16	0.30	0.34	0.00	0.14	0.23	0.02	0.21	0.05	0.00	-0.04
Q9Y371	Endophilin-B1 OS=Homo sapiens GN=SH3GLB1 PE=1 SV=1 - [SHLB1_HUMAN]	13.15	1	4	5	7	-0.17	-0.14	-0.05	-0.02	-0.16	-0.14	-0.15	-0.13	-0.30	-0.31	-0.22	-0.21	0.07	-0.05	-0.18	-0.12	-0.24	-0.01	-0.13	-0.04
P27361	Mitogen-activated protein kinase 3 OS=Homo sapiens GN=MAPK3 PE=1 SV=4 - [MK03_HUMAN]	52.24	8	12	18	61	0.27	0.39	0.42	0.40	0.36	0.31	0.19	0.29	0.24	0.18	0.11	0.17	-0.03	-0.17	-0.20	-0.16	-0.17	-0.17	-0.15	-0.04
Q5HYI7	Metaxin-3 OS=Homo sapiens GN=MTX3 PE=1 SV=2 - [MTX3_HUMAN]	24.68	1	7	7	12	0.10	0.40	0.24	0.32	-0.11	0.03	0.14	0.30	0.14	0.09	-0.05	0.12	0.03	-0.51	-0.47	-0.16	-0.14	-0.28	-0.49	-0.04
P57088	Transmembrane protein 33 OS=Homo sapiens GN=TMEM33 PE=1 SV=2 - [TMM33_HUMAN]	10.53	1	3	3	4	-0.59	-0.52	-0.50	-0.23	0.30	0.41	-0.09	-0.02	0.18	0.12	-0.07	0.20	0.50	0.48	0.43	0.73	0.18	0.96	0.62	-0.05
Q9HA64	Ketosamine-3-kinase OS=Homo sapiens GN=FN3KRP PE=1 SV=2 - [KT3K_HUMAN]	23.95	1	8	8	15	0.10	0.19	0.18	0.20	-0.04	-0.02	0.11	0.24	-0.07	0.00	0.04	-0.05	0.09	-0.21	-0.27	-0.24	-0.48	-0.16	-0.36	-0.05

Q96AG4	Leucine-rich repeat-containing protein 59 OS=Homo sapiens GN=LRRC59 PE=1 SV=1 - [LRC59_HUMAN]	24.10	1	7	7	18	-0.41	-0.26	-0.32	-0.32	-0.43	-0.47	-0.52	-0.55	-0.49	-0.41	-0.04	-0.06	0.05	0.41	0.33	0.14	-0.04	0.03	-0.05	-0.05
Q8NBJ4	Golgi membrane protein 1 OS=Homo sapiens GN=GOLM1 PE=1 SV=1 - [GOLM1_HUMAN]	4.99	1	2	2	3	-0.03	-0.09	-0.04	-0.10	0.18	0.12	-0.15	-0.21	-0.02	0.05	0.19	0.12	-0.06	0.23	0.23	0.11	0.12	0.20	0.20	-0.05
Q9NQX7	Integral membrane protein 2C OS=Homo sapiens GN=ITM2C PE=1 SV=1 - [ITM2C_HUMAN]	9.36	1	2	2	2	0.23	0.18	0.25	0.21	0.66	0.61	0.14	0.10	0.16	0.21	-0.04	-0.09	-0.03	-0.26	-0.29	0.01	-0.02	0.42	0.39	-0.05
P17900	Ganglioside GM2 activator OS=Homo sapiens GN=GM2A PE=1 SV=4 - [SAP3_HUMAN]	27.46	1	5	5	8	0.34	0.43	0.66	0.75	0.31	0.47	0.53	0.65	0.58	0.42	0.50	0.62	0.24	0.20	-0.12	0.05	-0.26	-0.22	-0.45	-0.05
Q5R372	Rab GTPase-activating protein 1-like OS=Homo sapiens GN=RABGAP1L PE=1 SV=1 - [RBG1L_HUMAN]	1.96	1	1	2	2	0.49	0.52	0.25	0.28	0.12	0.14	0.14	0.17	0.21	0.18	0.08	0.10	-0.29	-0.40	-0.17	-0.27	-0.03	-0.39	-0.15	-0.05
Q13247	Serine/arginine-rich splicing factor 6 OS=Homo sapiens GN=SRSF6 PE=1 SV=2 - [SRSF6_HUMAN]	18.02	1	3	6	16	-0.23	-0.29	-0.11	-0.50	-0.11	-0.23	-0.28	-0.52	-0.37	-0.20	-0.32	-0.42	-0.20	-0.12	-0.21	-0.06	0.16	0.04	0.18	-0.05
Q07507	Dermatopontin OS=Homo sapiens GN=DPT PE=2 SV=2 - [DERM_HUMAN]	23.38	1	4	4	10	-1.25	-1.12	-1.56	-1.43	0.13	0.59	-2.43	-2.16	-0.53	-0.66	-1.30	-1.02	-0.49	-0.29	0.42	0.39	0.69	0.94	1.24	-0.05
Q9Y2S2	Lambda-crystallin homolog OS=Homo sapiens GN=CRYL1 PE=1 SV=3 - [CRYL1_HUMAN]	46.08	1	12	12	20	0.78	0.85	0.61	0.82	0.27	0.36	0.46	0.68	0.64	0.49	0.19	0.47	-0.06	-0.48	-0.34	-0.26	-0.17	-0.50	-0.25	-0.05

Q99720	Sigma non-opioid intracellular receptor 1 OS=Homo sapiens GN=SIGMAR1 PE=1 SV=1 - [SGMR1_HUMAN]	29.60	1	4	4	6	-0.22	-0.08	-0.80	-0.72	-0.13	-0.04	-1.01	-0.89	-0.36	-0.43	0.04	0.08	-0.72	0.26	0.84	-0.10	0.46	0.21	0.66	-0.05
P29144	Tripeptidyl-peptidase 2 OS=Homo sapiens GN=TPP2 PE=1 SV=4 - [TPP2_HUMAN]	29.62	1	30	30	50	-0.27	-0.31	-0.12	-0.09	-0.08	-0.13	-0.28	-0.13	-0.17	-0.25	-0.13	-0.15	0.14	0.25	-0.09	0.04	-0.01	0.07	-0.07	-0.05
P51116	Fragile X mental retardation syndrome-related protein 2 OS=Homo sapiens GN=FXR2 PE=1 SV=2 - [FXR2_HUMAN]	17.98	1	5	8	13	0.04	0.04	0.26	0.26	0.37	0.36	0.14	0.14	0.15	0.16	0.14	0.14	0.16	0.11	-0.11	0.15	-0.07	0.31	0.09	-0.05
Q9NUQ2	1-acyl-sn-glycerol-3-phosphate acyltransferase epsilon OS=Homo sapiens GN=AGPAT5 PE=1 SV=3 - [PLCE_HUMAN]	9.07	1	3	3	5	0.17	0.37	0.09	0.29	-0.01	0.18	-0.02	0.18	-0.04	-0.23	-0.06	0.13	-0.13	-0.23	-0.16	-0.37	-0.29	-0.19	-0.12	-0.05
P62993	Growth factor receptor-bound protein 2 OS=Homo sapiens GN=GRB2 PE=1 SV=1 - [GRB2_HUMAN]	44.70	1	10	10	25	0.26	0.60	0.37	0.64	0.35	0.57	0.16	0.43	0.39	0.16	0.20	0.43	-0.03	-0.07	-0.19	-0.21	-0.12	-0.03	0.03	-0.05
Q02539	Histone H1.1 OS=Homo sapiens GN=HIST1H1A PE=1 SV=3 - [H11_HUMAN]	22.79	2	1	7	36	-1.34	-1.33	-0.68	-0.66	-1.12	-1.11	-0.79	-0.78	-1.34	-1.35	-1.72	-1.71	0.60	-0.38	-1.04	0.02	-0.64	0.21	-0.45	-0.05
Q8N6C5	Immunoglobulin superfamily member 1 OS=Homo sapiens GN=IGSF1 PE=1 SV=3 - [IGSF1_HUMAN]	3.07	1	3	3	4	0.16	0.13	0.17	0.09	-0.10	-0.16	0.06	0.11	0.04	0.04	0.44	0.33	0.11	0.28	0.40	0.03	-0.15	-0.22	-0.29	-0.05
Q13424	Alpha-1-syntrophin OS=Homo sapiens GN=SNTA1 PE=1 SV=1 - [SNTA1_HUMAN]	36.83	1	13	13	33	0.54	0.56	0.47	0.49	0.16	0.12	0.36	0.36	0.25	0.34	0.15	0.20	-0.15	-0.48	-0.39	-0.33	-0.11	-0.58	-0.32	-0.05

P57721	Poly(rC)-binding protein 3 OS=Homo sapiens GN=PCBP3 PE=2 SV=2 - [PCBP3_HUMAN]	19.68	2	1	6	27	0.16	-0.06	0.33	0.16	-0.23	0.10	0.03	0.05	-0.09	-0.01	-0.36	-0.38	0.17	-0.49	-0.72	-0.15	-0.31	0.05	-0.30	-0.05
Q9BRX8	Redox-regulatory protein FAM213A OS=Homo sapiens GN=FAM213A PE=1 SV=3 - [F213A_HUMAN]	44.98	1	11	11	19	-0.04	-0.15	0.34	0.15	0.29	0.16	0.27	0.11	-0.07	-0.06	0.08	-0.01	0.15	0.20	-0.14	0.08	-0.26	0.41	-0.04	-0.05
Q9P246	Stromal interaction molecule 2 OS=Homo sapiens GN=STIM2 PE=1 SV=2 - [STIM2_HUMAN]	6.03	1	2	3	6	0.28	0.47	0.27	0.26	-0.14	-0.12	-0.05	0.00	0.11	0.08	-0.13	-0.12	-0.27	-0.40	-0.23	-0.16	-0.15	-0.44	-0.22	-0.05
O14522	Receptor-type tyrosine-protein phosphatase T OS=Homo sapiens GN=PTPRT PE=1 SV=6 - [PTPRT_HUMAN]	7.84	1	7	7	9	1.05	1.21	0.73	0.89	0.22	0.38	0.62	0.78	0.43	0.28	0.23	0.39	-0.38	-0.81	-0.50	-0.74	-0.42	-0.84	-0.53	-0.05
Q9UEY8	Gamma-adducin OS=Homo sapiens GN=ADD3 PE=1 SV=1 - [ADDG_HUMAN]	26.35	1	16	16	45	0.26	0.17	0.25	0.19	-0.11	-0.23	0.12	0.18	-0.04	-0.01	0.10	0.00	-0.04	-0.18	-0.15	-0.25	-0.20	-0.36	-0.38	-0.05
P47756	F-actin-capping protein subunit beta OS=Homo sapiens GN=CAPZB PE=1 SV=4 - [CAPZB_HUMAN]	46.21	1	14	14	57	0.04	0.09	0.14	0.21	-0.17	-0.22	0.05	0.05	-0.12	-0.09	-0.15	-0.13	-0.02	-0.16	-0.29	-0.09	-0.36	-0.27	-0.38	-0.05
P00390	Glutathione reductase, mitochondrial OS=Homo sapiens GN=GSR PE=1 SV=2 - [GSHR_HUMAN]	30.27	1	12	12	25	-0.24	-0.28	0.04	0.05	0.00	0.05	0.00	-0.02	-0.16	-0.12	-0.24	-0.29	0.32	0.04	-0.20	0.23	0.15	0.27	0.07	-0.05
Q9NZ01	Very-long-chain enoyl-CoA reductase OS=Homo sapiens GN=TECR PE=1 SV=1 - [TECR_HUMAN]	12.99	1	4	4	8	-0.21	-0.36	-0.10	-0.09	-0.28	-0.27	-0.22	-0.12	-0.26	-0.13	0.08	0.06	0.02	0.38	0.17	0.20	0.00	0.07	-0.17	-0.05

Q9NPJ3	Acyl-coenzyme A thioesterase 13 OS=Homo sapiens GN=ACOT13 PE=1 SV=1 - [ACO13_HUMAN]	37.86	1	4	4	10	-0.40	-0.27	0.11	0.29	0.10	0.10	0.09	0.09	0.06	0.06	0.14	0.14	0.44	0.52	0.01	0.48	-0.08	0.50	-0.06	-0.05
O75494	Serine/arginine-rich splicing factor 10 OS=Homo sapiens GN=SRSF10 PE=1 SV=1 - [SRS10_HUMAN]	18.70	2	4	4	10	-0.15	-0.27	-0.12	-0.26	-0.03	-0.19	-0.30	-0.42	-0.28	-0.13	-0.06	-0.20	-0.10	0.09	0.11	0.02	0.01	0.08	0.06	-0.05
Q9C0B0	RING finger protein unkempt homolog OS=Homo sapiens GN=UNK PE=1 SV=2 - [UNK_HUMAN]	3.58	1	2	2	4	0.57	0.54	0.44	0.42	0.06	0.03	0.33	0.30	0.01	0.04	0.31	0.27	-0.19	-0.26	-0.14	-0.50	-0.37	-0.53	-0.40	-0.05
Q24JP5	Transmembrane protein 132A OS=Homo sapiens GN=TMEM132A PE=2 SV=1 - [T132A_HUMAN]	6.45	1	5	5	9	-0.26	-0.17	-0.16	-0.19	-0.35	-0.09	-0.28	-0.11	-0.02	-0.20	-0.21	0.27	0.04	0.06	0.10	0.09	-0.01	-0.11	-0.21	-0.05
Q6P1M3	Lethal(2) giant larvae protein homolog 2 OS=Homo sapiens GN=LLGL2 PE=1 SV=2 - [L2GL2_HUMAN]	3.04	1	2	2	4	-1.50	-1.63	-1.32	-1.44	-0.95	-1.25	-1.55	-1.68	-1.61	-1.48	-1.60	-1.84	0.00	-0.20	-0.11	0.03	0.07	0.02	0.03	-0.05
P09601	Heme oxygenase 1 OS=Homo sapiens GN=HMOX1 PE=1 SV=1 - [HMOX1_HUMAN]	5.56	1	1	1	2	-0.48	-0.13	-0.44	-0.09	-0.32	0.02	-0.56	-0.21	0.09	-0.25	0.45	0.79	-0.03	0.93	0.89	0.26	0.22	0.13	0.10	-0.05
Q9P0I2	ER membrane protein complex subunit 3 OS=Homo sapiens GN=EMC3 PE=1 SV=3 - [EMC3_HUMAN]	32.18	1	5	5	6	0.42	0.37	0.23	0.18	-0.18	-0.23	0.11	0.06	-0.33	-0.28	-0.49	-0.55	-0.25	-0.90	-0.72	-0.67	-0.48	-0.61	-0.43	-0.05

O75208	Ubiquinone biosynthesis protein COQ9, mitochondrial OS=Homo sapiens GN=COQ9 PE=1 SV=1 - [COQ9_HUMAN]	9.12	1	3	3	6	-0.09	0.12	0.13	0.31	-0.32	-0.31	0.01	0.19	-0.12	-0.30	-0.10	0.10	0.16	-0.01	-0.20	-0.18	-0.39	-0.20	-0.41	-0.05
Q86VS8	Protein Hook homolog 3 OS=Homo sapiens GN=HOOK3 PE=1 SV=2 - [HOOK3_HUMAN]	24.23	1	13	13	27	0.02	-0.04	0.03	0.09	-0.29	-0.21	0.00	-0.13	-0.19	-0.18	-0.28	-0.19	-0.08	-0.17	-0.26	-0.13	-0.20	-0.15	-0.30	-0.05
Q7Z3C6	Autophagy-related protein 9A OS=Homo sapiens GN=ATG9A PE=1 SV=3 - [ATG9A_HUMAN]	6.08	1	4	4	5	0.46	0.29	0.45	0.50	0.01	0.36	0.05	0.19	0.19	0.02	0.35	0.17	-0.34	-0.11	0.10	-0.14	0.14	-0.45	-0.18	-0.05
O43504	Ragulator complex protein LAMTOR5 OS=Homo sapiens GN=LAMTOR5 PE=1 SV=1 - [LTOR5_HUMAN]	59.34	1	3	3	6	-0.28	-0.22	-0.25	-0.19	-0.26	-0.20	-0.37	-0.31	-0.28	-0.33	0.05	0.11	-0.04	0.34	0.30	-0.02	-0.05	0.00	-0.03	-0.06
Q15029	116 kDa U5 small nuclear ribonucleoprotein component OS=Homo sapiens GN=EFTUD2 PE=1 SV=1 - [U5S1_HUMAN]	36.21	1	26	27	46	-0.24	-0.09	-0.11	-0.03	-0.38	-0.31	-0.25	-0.14	-0.25	-0.32	-0.12	-0.02	-0.08	0.01	-0.01	-0.05	-0.14	-0.24	-0.33	-0.06
P11498	Pyruvate carboxylase, mitochondrial OS=Homo sapiens GN=PC PE=1 SV=2 - [PYC_HUMAN]	35.74	1	35	35	81	-0.23	-0.42	-0.28	-0.45	-0.38	-0.58	-0.43	-0.57	-0.61	-0.36	-0.32	-0.53	-0.16	-0.14	-0.07	-0.18	-0.06	-0.18	-0.10	-0.06
Q96QK1	Vacuolar protein sorting-associated protein 35 OS=Homo sapiens GN=VPS35 PE=1 SV=2 - [VPS35_HUMAN]	29.15	1	21	21	63	0.29	0.29	0.33	0.35	0.23	0.21	0.27	0.27	0.17	0.24	0.15	0.13	0.09	-0.18	-0.21	-0.10	-0.11	-0.12	-0.12	-0.06

Q9BWH2	FUN14 domain-containing protein 2 OS=Homo sapiens GN=FUNDC2 PE=1 SV=2 - [FUND2_HUMAN]	17.99	1	5	5	6	0.04	0.08	0.18	0.22	-0.79	-0.67	0.20	0.23	0.14	0.11	-0.41	-0.38	0.06	-0.48	-0.60	-0.22	-0.26	-0.60	-0.77	-0.06
Q96C90	Protein phosphatase 1 regulatory subunit 14B OS=Homo sapiens GN=PPP1R14B PE=1 SV=3 - [PP14B_HUMAN]	16.33	1	1	1	1	-0.24	0.05	-1.26	-0.98	0.01	0.29	-1.38	-1.10	-0.06	-0.33	-0.28	0.00	-1.09	-0.04	0.98	-0.07	0.96	0.23	1.25	-0.06
Q9Y6M5	Zinc transporter 1 OS=Homo sapiens GN=SLC30A1 PE=1 SV=3 - [ZNT1_HUMAN]	2.17	1	1	1	1	0.42	-0.02	0.25	-0.19	0.42	-0.03	0.13	-0.31	-0.14	0.31	0.18	-0.27	-0.24	-0.24	-0.07	-0.08	0.09	-0.02	0.15	-0.06
Q9Y6D6	Brefeldin A-inhibited guanine nucleotide-exchange protein 1 OS=Homo sapiens GN=ARFGEF1 PE=1 SV=2 - [BIG1_HUMAN]	4.33	1	3	7	9	0.42	0.42	0.24	0.24	-0.01	-0.02	0.12	0.11	-0.39	-0.37	-0.19	-0.20	-0.25	-0.61	-0.43	-0.77	-0.58	-0.45	-0.27	-0.06
Q8IWV7	E3 ubiquitin-protein ligase UBR1 OS=Homo sapiens GN=UBR1 PE=1 SV=1 - [UBR1_HUMAN]	3.77	1	4	4	5	-0.01	0.23	0.10	0.34	0.00	0.23	-0.02	0.22	0.31	0.08	-0.14	0.10	0.04	-0.12	-0.24	0.11	0.01	-0.01	-0.12	-0.06
P05161	Ubiquitin-like protein ISG15 OS=Homo sapiens GN=ISG15 PE=1 SV=5 - [ISG15_HUMAN]	23.03	1	3	3	4	0.05	0.18	0.22	0.35	0.45	0.57	0.10	0.22	0.30	0.18	0.22	0.34	0.10	0.17	0.00	0.15	-0.01	0.39	0.22	-0.06
Q96EK9	Protein KTI12 homolog OS=Homo sapiens GN=KTI12 PE=1 SV=1 - [KTI12_HUMAN]	2.82	1	1	1	2	0.56	0.48	0.24	0.17	0.47	0.39	0.12	0.04	0.58	0.66	-0.06	-0.14	-0.38	-0.62	-0.30	0.13	0.45	-0.10	0.22	-0.06
Q96PX1	RING finger protein 157 OS=Homo sapiens GN=RNF157 PE=1 SV=3 - [RN157_HUMAN]	2.50	1	1	1	1	0.44	0.34	0.70	0.60	0.54	0.43	0.58	0.48	0.31	0.42	0.16	0.06	0.20	-0.27	-0.54	0.01	-0.25	0.08	-0.18	-0.06

Q5JTH9	RRP12-like protein OS=Homo sapiens GN=RRP12 PE=1 SV=2 - [RRP12_HUMAN]	1.62	1	2	2	2	0.06	0.21	-0.21	-0.05	0.58	0.72	-0.33	-0.18	0.28	0.13	-0.08	0.07	-0.33	-0.13	0.13	0.10	0.37	0.50	0.77	-0.06
Q96FZ7	Charged multivesicular body protein 6 OS=Homo sapiens GN=CHMP6 PE=1 SV=3 - [CHMP6_HUMAN]	26.87	1	4	5	11	0.88	1.01	0.73	0.59	0.02	-0.12	0.45	0.31	0.15	0.29	0.51	0.36	-0.38	-0.56	-0.23	-0.58	-0.26	-0.82	-0.73	-0.06
Q15006	ER membrane protein complex subunit 2 OS=Homo sapiens GN=EMC2 PE=1 SV=1 - [EMC2_HUMAN]	20.88	1	5	5	7	0.25	0.30	0.14	0.24	0.21	0.15	0.20	-0.10	0.06	0.28	0.11	0.28	-0.31	-0.01	0.16	-0.07	0.18	-0.06	-0.02	-0.06
Q9NWV4	UPF0587 protein C1orf123 OS=Homo sapiens GN=C1orf123 PE=1 SV=1 - [CA123_HUMAN]	41.88	1	6	6	11	0.06	-0.04	0.52	0.28	-0.22	-0.30	0.43	0.37	0.06	0.14	-0.20	-0.27	0.41	-0.50	-0.82	-0.09	-0.41	-0.34	-0.81	-0.06
Q674X7	Kazrin OS=Homo sapiens GN=KAZN PE=1 SV=2 - [KAZRN_HUMAN]	5.55	1	4	4	8	0.60	0.50	0.27	0.25	-0.14	-0.04	-0.09	-0.11	-0.17	-0.15	-0.27	-0.14	-0.27	-0.27	-0.18	-0.51	-0.38	-0.46	-0.21	-0.06
Q15233	Non-POU domain-containing octamer-binding protein OS=Homo sapiens GN=NONO PE=1 SV=4 - [NONO_HUMAN]	30.57	1	11	13	43	-0.37	-0.14	-0.31	-0.36	-0.41	-0.38	-0.36	-0.43	-0.47	-0.43	-0.44	-0.46	-0.05	-0.22	0.01	-0.14	-0.16	-0.18	-0.14	-0.06
O15144	Actin-related protein 2/3 complex subunit 2 OS=Homo sapiens GN=ARPC2 PE=1 SV=1 - [ARPC2_HUMAN]	45.67	1	12	12	41	-0.27	-0.19	-0.10	-0.03	-0.12	-0.10	-0.21	-0.14	-0.23	-0.26	-0.33	-0.32	0.05	-0.14	-0.33	-0.05	-0.16	0.14	-0.02	-0.06
P61964	WD repeat-containing protein 5 OS=Homo sapiens GN=WDR5 PE=1 SV=1 - [WDR5_HUMAN]	4.19	1	1	1	1	-0.14	-0.35	-0.14	-0.35	-0.29	-0.51	-0.26	-0.47	-0.59	-0.38	-0.09	-0.31	-0.06	0.06	0.05	-0.20	-0.20	-0.16	-0.17	-0.06

Q8WVJ2	NudC domain-containing protein 2 OS=Homo sapiens GN=NUDCD2 PE=1 SV=1 - [NUDC2_HUMAN]	41.40	1	5	5	8	0.74	0.85	0.71	0.78	0.93	0.87	0.63	0.65	0.62	0.67	0.22	0.24	-0.05	-0.58	-0.54	-0.11	-0.06	-0.04	0.00	-0.06
Q9Y5L0	Transportin-3 OS=Homo sapiens GN=TNPO3 PE=1 SV=3 - [TNPO3_HUMAN]	15.82	1	10	10	16	0.29	0.34	0.23	0.37	0.39	0.54	0.10	-0.03	0.27	0.18	0.05	0.38	-0.04	0.00	0.02	0.06	-0.07	0.16	0.18	-0.06
P54727	UV excision repair protein RAD23 homolog B OS=Homo sapiens GN=RAD23B PE=1 SV=1 - [RD23B_HUMAN]	17.11	1	6	8	25	-0.24	0.02	-0.01	0.13	-0.28	-0.09	-0.20	-0.13	-0.12	-0.26	-0.17	-0.01	-0.07	-0.08	-0.10	-0.09	-0.05	-0.19	-0.27	-0.06
Q13325	Interferon-induced protein with tetratricopeptide repeats 5 OS=Homo sapiens GN=IFIT5 PE=1 SV=1 - [IFIT5_HUMAN]	19.92	1	7	7	7	-0.20	-0.41	-0.23	-0.21	0.12	-0.26	-0.32	-0.07	-0.23	-0.35	-0.03	-0.17	0.40	0.17	-0.14	0.15	-0.33	-0.11	-0.23	-0.06
P51608	Methyl-CpG-binding protein 2 OS=Homo sapiens GN=MECP2 PE=1 SV=1 - [MECP2_HUMAN]	33.13	1	13	13	32	-0.49	-0.46	-0.49	-0.46	-0.63	-0.66	-0.52	-0.48	-0.66	-0.61	-0.40	-0.37	0.06	0.40	0.35	0.00	0.06	0.04	-0.09	-0.06
P22392	Nucleoside diphosphate kinase B OS=Homo sapiens GN=NME2 PE=1 SV=1 - [NDKB_HUMAN]	64.47	2	5	10	28	-0.08	-0.01	0.77	0.72	0.26	0.21	0.48	0.44	0.17	0.22	0.27	0.16	0.52	0.18	-0.50	0.15	-0.52	0.23	-0.49	-0.06
O00629	Importin subunit alpha 3 OS=Homo sapiens GN=KPNA4 PE=1 SV=1 - [IMA3_HUMAN]	18.23	1	4	6	24	-0.19	-0.34	0.13	0.02	-0.10	-0.30	-0.10	-0.15	-0.29	0.01	-0.20	-0.25	0.21	0.16	-0.18	0.24	-0.16	0.27	-0.18	-0.06
P54760	Ephrin type-B receptor 4 OS=Homo sapiens GN=EPHB4 PE=1 SV=2 - [EPHB4_HUMAN]	3.44	5	1	3	4	0.31	0.01	0.44	0.15	0.15	-0.15	0.31	0.02	0.28	0.58	-0.16	-0.46	0.06	-0.46	-0.60	0.31	0.18	-0.17	-0.31	-0.06

O43760	Synaptogyrin-2 OS=Homo sapiens GN=SYNGR2 PE=1 SV=1 - [SNG2_HUMAN]	4.46	1	1	1	1	-0.51	-0.22	0.35	0.64	0.70	0.98	0.23	0.51	0.79	0.51	0.34	0.62	0.79	0.86	-0.01	1.05	0.19	1.19	0.33	-0.06
O43681	ATPase ASNA1 OS=Homo sapiens GN=ASNA1 PE=1 SV=2 - [ASNA_HUMAN]	38.51	1	9	9	29	0.23	0.16	0.35	0.33	0.62	0.53	0.25	0.23	0.34	0.42	0.37	0.29	-0.06	0.03	0.01	0.09	0.03	0.29	0.28	-0.06
Q9NSE4	Isoleucine--tRNA ligase, mitochondrial OS=Homo sapiens GN=IARS2 PE=1 SV=2 - [SYIM_HUMAN]	28.95	1	24	24	61	-0.52	-0.61	-0.38	-0.48	-0.61	-0.70	-0.50	-0.66	-0.73	-0.49	-0.51	-0.57	0.06	0.14	-0.12	0.00	-0.10	-0.05	-0.23	-0.06
Q9UBT2	SUMO-activating enzyme subunit 2 OS=Homo sapiens GN=UBA2 PE=1 SV=2 - [SAE2_HUMAN]	25.31	1	13	13	25	-0.07	-0.02	0.23	0.22	-0.18	-0.21	0.09	0.03	-0.16	-0.10	-0.35	-0.51	0.03	-0.43	-0.43	-0.02	-0.13	-0.12	-0.24	-0.06
Q86TU7	Histone-lysine N-methyltransferase setd3 OS=Homo sapiens GN=SETD3 PE=1 SV=1 - [SETD3_HUMAN]	10.27	1	5	5	6	0.90	0.38	0.46	-0.05	0.60	0.25	0.46	-0.05	0.18	0.71	0.44	0.00	-0.38	-0.45	-0.03	-0.16	0.28	-0.22	0.21	-0.07
Q9UHD2	Serine/threonine-protein kinase TBK1 OS=Homo sapiens GN=TBK1 PE=1 SV=1 - [TBK1_HUMAN]	16.19	1	9	9	13	0.28	0.24	0.16	0.16	0.20	0.06	0.11	0.31	-0.02	0.12	-0.14	-0.09	-0.06	-0.41	-0.40	-0.28	-0.13	-0.22	-0.25	-0.07
Q9H5N1	Rab GTPase-binding effector protein 2 OS=Homo sapiens GN=RABEP2 PE=1 SV=2 - [RABE2_HUMAN]	11.25	1	6	6	8	0.45	0.74	0.37	0.54	0.34	0.61	0.26	0.54	0.34	-0.04	-0.05	0.24	-0.18	-0.58	-0.29	-0.32	-0.15	-0.24	0.05	-0.07
P51805	Plexin-A3 OS=Homo sapiens GN=PLXNA3 PE=1 SV=2 - [PLXA3_HUMAN]	4.06	1	1	8	13	-1.20	-0.81	-0.66	-0.27	-0.06	0.32	-0.78	-0.40	0.20	-0.18	-0.35	0.03	0.47	0.85	0.30	1.04	0.51	1.11	0.57	-0.07

Q15459	Splicing factor 3A subunit 1 OS=Homo sapiens GN=SF3A1 PE=1 SV=1 - [SF3A1_HUMAN]	24.46	1	15	15	29	-0.35	-0.37	-0.23	-0.22	-0.39	-0.54	-0.43	-0.51	-0.49	-0.41	-0.16	-0.27	-0.24	0.12	0.00	-0.09	-0.21	-0.19	-0.26	-0.07
Q9BQG0	Myb-binding protein 1A OS=Homo sapiens GN=MYBBP1A PE=1 SV=2 - [MBB1A_HUMAN]	3.09	1	2	2	3	0.38	-0.08	0.33	-0.13	0.11	-0.35	0.20	-0.26	-0.74	-0.28	0.56	0.10	-0.13	0.19	0.23	-0.63	-0.57	-0.29	-0.23	-0.07
O14874	[3-methyl-2-oxobutanoate dehydrogenase [lipoamide]] kinase, mitochondrial OS=Homo sapiens GN=BCKDK PE=1 SV=2 - [BCKD_HUMAN]	10.68	1	4	4	5	-0.20	-0.17	-0.25	-0.21	-0.36	-0.33	-0.38	-0.35	-0.31	-0.34	-0.06	-0.03	-0.12	0.14	0.18	-0.11	-0.06	-0.18	-0.13	-0.07
Q9UHY1	Nuclear receptor-binding protein OS=Homo sapiens GN=NRBP1 PE=1 SV=1 - [NRBP_HUMAN]	15.14	1	4	4	7	-0.07	0.10	0.11	0.20	-0.50	-0.36	-0.18	0.03	-0.37	-0.64	-0.32	-0.12	0.03	0.00	-0.11	0.06	0.06	-0.11	-0.21	-0.07
O43157	Plexin-B1 OS=Homo sapiens GN=PLXNB1 PE=1 SV=3 - [PLXB1_HUMAN]	9.56	2	13	13	19	0.66	0.72	0.57	0.59	0.57	0.89	0.48	0.75	0.68	0.35	0.38	0.73	0.03	-0.14	-0.02	-0.10	-0.08	-0.13	-0.09	-0.07
O43395	U4/U6 small nuclear ribonucleoprotein Prp3 OS=Homo sapiens GN=PRPF3 PE=1 SV=2 - [PRPF3_HUMAN]	6.30	1	4	4	5	-0.41	-0.53	-0.38	-0.50	-0.68	-0.81	-0.51	-0.63	-0.64	-0.51	-0.52	-0.64	-0.05	-0.10	-0.14	-0.08	-0.10	-0.29	-0.32	-0.07
O60664	Perilipin-3 OS=Homo sapiens GN=PLIN3 PE=1 SV=3 - [PLIN3_HUMAN]	24.65	1	8	8	14	-0.20	-0.14	-0.08	-0.11	-0.20	-0.13	-0.02	0.00	-0.32	-0.38	-0.26	-0.33	0.02	-0.44	-0.41	-0.29	-0.43	-0.23	-0.35	-0.07
P07737	Profilin-1 OS=Homo sapiens GN=PFN1 PE=1 SV=2 - [PROF1_HUMAN]	62.86	1	8	8	38	-0.21	-0.16	-0.08	-0.25	-0.32	-0.32	-0.30	-0.21	-0.51	-0.42	-0.76	-0.75	0.08	-0.48	-0.53	-0.18	-0.36	-0.12	-0.32	-0.07

P63208	S-phase kinase-associated protein 1 OS=Homo sapiens GN=SKP1 PE=1 SV=2 - [SKP1_HUMAN]	50.92	1	8	8	26	0.03	0.01	0.38	0.39	-0.05	0.04	0.15	0.20	0.06	-0.06	-0.14	0.00	0.30	-0.15	-0.49	0.01	-0.25	0.03	-0.35	-0.07
Q96SU4	Oxysterol-binding protein-related protein 9 OS=Homo sapiens GN=OSBPL9 PE=1 SV=2 - [OSBL9_HUMAN]	6.93	1	3	3	3	0.75	0.62	0.66	0.54	0.81	0.68	0.53	0.40	0.10	0.24	0.06	-0.07	-0.16	-0.68	-0.60	-0.48	-0.39	0.05	0.13	-0.07
Q8IWZ3	Ankyrin repeat and KH domain-containing protein 1 OS=Homo sapiens GN=ANKHD1 PE=1 SV=1 - [ANKH1_HUMAN]	3.15	1	3	5	6	-0.48	-0.09	-0.52	-0.13	-0.12	0.26	-0.65	-0.26	0.08	-0.30	-0.24	0.14	-0.12	0.24	0.27	0.20	0.25	0.34	0.38	-0.07
Q9NP66	High mobility group protein 20A OS=Homo sapiens GN=HMG20A PE=1 SV=1 - [HM20A_HUMAN]	5.76	1	1	1	2	0.24	-0.13	-0.13	-0.50	0.40	0.03	-0.26	-0.63	0.21	0.58	0.32	-0.06	-0.45	0.08	0.45	0.38	0.75	0.15	0.51	-0.07
P53004	Biliverdin reductase A OS=Homo sapiens GN=BLVRA PE=1 SV=2 - [BIEA_HUMAN]	15.20	1	5	5	7	-0.16	0.21	0.10	0.12	-0.19	0.01	-0.07	0.09	-0.31	-0.44	-0.51	-0.31	0.13	-0.27	-0.62	-0.43	-0.42	-0.16	-0.18	-0.07
Q8IUE6	Histone H2A type 2-B OS=Homo sapiens GN=HIST2H2AB PE=1 SV=3 - [H2A2B_HUMAN]	34.62	1	1	3	55	0.19	0.15	-0.07	-0.10	-0.49	-0.53	-0.20	-0.24	-0.56	-0.52	-0.11	-0.15	-0.34	-0.29	-0.04	-0.68	-0.42	-0.69	-0.44	-0.07
Q9H1A4	Anaphase-promoting complex subunit 1 OS=Homo sapiens GN=ANAPC1 PE=1 SV=1 - [APC1_HUMAN]	0.51	1	1	1	1	0.04	0.48	-0.18	0.26	0.43	0.87	-0.31	0.13	0.49	0.06	-0.05	0.39	-0.29	-0.08	0.13	0.05	0.27	0.38	0.59	-0.07
O75874	Isocitrate dehydrogenase [NADP] cytoplasmic OS=Homo sapiens GN=IDH1 PE=1 SV=2 - [IDHC_HUMAN]	36.71	1	14	14	24	-0.20	-0.21	0.26	0.19	-0.23	-0.33	0.10	0.04	-0.20	-0.14	-0.21	-0.29	0.32	-0.03	-0.48	0.01	-0.39	-0.05	-0.57	-0.07

O60307	Microtubule-associated serine/threonine-protein kinase 3 OS=Homo sapiens GN=MAST3 PE=1 SV=2 - [MAST3_HUMAN]	5.42	4	3	4	7	0.84	0.87	0.09	0.23	0.73	0.97	0.56	0.80	0.78	0.53	0.37	0.61	-0.49	-0.47	0.28	-0.27	0.48	-0.13	0.62	-0.07
O75934	Pre-mRNA-splicing factor SPF27 OS=Homo sapiens GN=BCAS2 PE=1 SV=1 - [SPF27_HUMAN]	17.33	1	4	4	4	-0.59	-0.69	-0.41	-0.52	-0.47	-0.58	-0.55	-0.65	-0.52	-0.41	-0.20	-0.31	0.09	0.40	0.21	0.20	0.03	0.10	-0.08	-0.07
P35222	Catenin beta-1 OS=Homo sapiens GN=CTNNB1 PE=1 SV=1 - [CTNB1_HUMAN]	43.28	1	23	26	81	0.03	0.04	0.18	0.20	-0.20	-0.11	-0.07	-0.02	-0.13	-0.14	0.04	0.11	-0.08	0.07	-0.12	-0.13	-0.18	-0.18	-0.27	-0.07
Q9HAU5	Regulator of nonsense transcripts 2 OS=Homo sapiens GN=UPF2 PE=1 SV=1 - [RENT2_HUMAN]	2.91	1	4	4	4	-0.17	0.01	0.29	0.29	-0.35	-0.27	-0.02	0.16	-0.40	-0.57	-0.44	-0.26	0.21	-0.26	-0.11	0.14	0.33	0.22	0.41	-0.07
O60739	Eukaryotic translation initiation factor 1b OS=Homo sapiens GN=EIF1B PE=1 SV=2 - [EIF1B_HUMAN]	47.79	2	3	3	16	0.36	0.07	0.13	-0.20	-0.44	-0.60	-0.30	-0.37	-0.51	-0.49	-0.32	-0.32	-0.42	-0.55	-0.44	-0.65	-0.32	-0.71	-0.55	-0.07
Q5VZK9	Leucine-rich repeat-containing protein 16A OS=Homo sapiens GN=LRRC16A PE=1 SV=1 - [LR16A_HUMAN]	7.59	1	8	8	13	0.83	0.74	0.79	0.65	0.26	0.18	0.56	0.39	0.46	0.64	0.19	0.01	-0.38	-0.87	-0.57	-0.35	-0.04	-0.81	-0.36	-0.07
Q9Y2K2	Serine/threonine-protein kinase SIK3 OS=Homo sapiens GN=SIK3 PE=1 SV=3 - [SIK3_HUMAN]	2.38	1	3	3	3	0.30	0.19	0.31	0.21	0.02	-0.09	0.17	0.07	-0.32	-0.21	-0.07	-0.18	-0.07	-0.36	-0.38	-0.48	-0.49	-0.30	-0.31	-0.07
Q92673	Sortilin-related receptor OS=Homo sapiens GN=SORL1 PE=1 SV=2 - [SORL_HUMAN]	8.63	1	15	15	21	0.60	0.67	0.31	0.45	-0.12	-0.15	0.23	0.30	0.17	0.12	-0.15	-0.13	-0.36	-0.80	-0.46	-0.37	-0.18	-0.57	-0.33	-0.07

P26639	Threonine--tRNA ligase, cytoplasmic OS=Homo sapiens GN=TARS PE=1 SV=3 - [SYTC_HUMAN]	30.15	1	17	18	31	0.21	0.15	0.17	0.10	-0.08	-0.38	0.07	-0.08	-0.24	-0.13	-0.26	-0.34	-0.23	-0.44	-0.44	-0.33	-0.26	-0.43	-0.41	-0.07
Q9H446	RWD domain-containing protein 1 OS=Homo sapiens GN=RWDD1 PE=1 SV=1 - [RWDD1_HUMAN]	3.70	1	1	1	2	-0.26	0.10	0.02	0.38	0.24	0.60	-0.12	0.24	0.26	-0.10	0.00	0.35	0.20	0.26	-0.03	0.19	-0.09	0.49	0.20	-0.08
P57737	Coronin-7 OS=Homo sapiens GN=CORO7 PE=1 SV=2 - [CORO7_HUMAN]	16.43	1	9	9	13	0.08	0.32	0.15	0.39	-0.14	-0.20	0.01	0.23	0.05	-0.03	0.17	0.33	-0.04	0.06	0.06	0.00	-0.09	-0.18	-0.42	-0.08
Q9H2G2	STE20-like serine/threonine-protein kinase OS=Homo sapiens GN=SLK PE=1 SV=1 - [SLK_HUMAN]	20.40	1	19	19	32	0.17	-0.02	0.31	0.00	0.02	0.03	0.21	0.00	0.00	-0.01	0.09	-0.02	0.03	0.04	-0.12	-0.23	-0.17	0.00	-0.04	-0.08
Q969E2	Secretory carrier-associated membrane protein 4 OS=Homo sapiens GN=SCAMP4 PE=2 SV=1 - [SCAM4_HUMAN]	8.73	1	2	2	10	0.45	0.38	0.40	0.37	0.01	0.10	0.14	0.18	-0.01	0.09	-0.06	-0.01	-0.14	-0.39	-0.28	-0.27	-0.26	-0.24	-0.23	-0.08
P58549	FXYD domain-containing ion transport regulator 7 OS=Homo sapiens GN=FXYD7 PE=2 SV=1 - [FXYD7_HUMAN]	16.25	1	1	1	2	1.46	1.85	-0.44	-0.06	0.28	0.66	-0.58	-0.20	-0.06	-0.44	-0.48	0.13	-1.99	-2.04	0.43	-1.87	0.03	-1.20	0.70	-0.08
P35527	Keratin, type I cytoskeletal 9 OS=Homo sapiens GN=KRT9 PE=1 SV=3 - [K1C9_HUMAN]	9.15	1	4	4	7	0.96	0.08	0.72	-0.16	0.32	-0.56	0.58	-0.30	0.56	1.44	1.52	0.64	-0.32	0.57	0.80	0.51	0.75	-0.65	-0.41	-0.08
Q9BZF1	Oxysterol-binding protein-related protein 8 OS=Homo sapiens GN=OSBPL8 PE=1 SV=3 - [OSBL8_HUMAN]	14.06	1	6	7	13	-0.28	-0.11	-0.35	-0.14	-0.38	-0.12	0.03	-0.06	-0.25	-0.21	-0.22	-0.40	0.10	-0.01	-0.26	0.22	-0.02	0.30	0.05	-0.08

P19525	Interferon-induced, double-stranded RNA-activated protein kinase OS=Homo sapiens GN=EIF2AK2 PE=1 SV=2 - [E2AK2_HUMAN]	18.87	1	9	9	12	-0.37	-0.21	0.15	0.13	-0.02	0.13	-0.14	-0.02	-0.06	-0.19	-0.04	0.08	0.21	0.14	-0.13	0.19	-0.08	0.20	-0.08	-0.08
P16112	Aggrecan core protein OS=Homo sapiens GN=ACAN PE=1 SV=2 - [PGCA_HUMAN]	7.41	1	13	13	22	0.78	0.68	1.08	0.84	2.16	2.11	1.18	1.19	1.29	1.20	0.61	0.67	0.25	-0.35	-0.72	0.24	0.03	1.05	0.93	-0.08
Q8IWE5	Pleckstrin homology domain-containing family M member 2 OS=Homo sapiens GN=PLEKHM2 PE=1 SV=2 - [PKHM2_HUMAN]	3.04	1	2	2	4	-0.10	-0.02	0.02	0.10	-0.07	0.00	-0.12	-0.05	-0.05	-0.12	-0.14	-0.08	0.03	-0.04	-0.17	0.01	-0.11	0.01	-0.11	-0.08
P12694	2-oxoisovalerate dehydrogenase subunit alpha, mitochondrial OS=Homo sapiens GN=BCKDHA PE=1 SV=2 - [ODBA_HUMAN]	33.93	1	12	12	21	-0.08	-0.04	-0.12	-0.18	-0.23	-0.14	-0.29	-0.23	-0.12	-0.15	-0.13	-0.07	-0.08	0.15	0.14	-0.05	-0.02	0.01	0.12	-0.08
Q99623	Prohibitin-2 OS=Homo sapiens GN=PHB2 PE=1 SV=2 - [PHB2_HUMAN]	65.22	1	18	18	71	0.17	0.13	0.27	0.19	-0.19	-0.22	0.05	0.04	-0.06	0.00	-0.13	-0.14	-0.04	-0.25	-0.37	-0.10	-0.28	-0.32	-0.47	-0.08
Q9UQ35	Serine/arginine repetitive matrix protein 2 OS=Homo sapiens GN=SRRM2 PE=1 SV=2 - [SRRM2_HUMAN]	8.25	1	15	15	25	-0.21	-0.43	-0.24	-0.30	-0.14	-0.24	-0.33	-0.45	-0.29	-0.15	0.16	-0.02	0.07	0.31	0.39	0.22	0.12	0.18	0.24	-0.08
Q13190	Syntaxin-5 OS=Homo sapiens GN=STX5 PE=1 SV=2 - [STX5_HUMAN]	20.56	1	5	5	7	0.03	0.46	-0.29	0.14	-0.11	0.12	-0.43	0.00	-0.04	-0.29	0.14	0.18	-0.35	0.12	0.30	-0.29	0.03	-0.35	-0.04	-0.08
P58401	Neurexin-2-beta OS=Homo sapiens GN=NRXN2 PE=2 SV=1 - [NRX2B_HUMAN]	8.86	1	1	4	5	0.15	0.05	-0.68	-0.78	1.03	0.93	-0.82	-0.92	-0.08	0.03	0.09	-0.01	-0.92	-0.06	0.77	-0.10	0.74	0.86	1.69	-0.08

O94832	Unconventional myosin-Id OS=Homo sapiens GN=MYO1D PE=1 SV=2 - [MYO1D_HUMAN]	24.16	2	21	21	37	-0.23	-0.16	0.13	0.19	0.24	0.17	-0.07	-0.12	0.00	0.03	-0.25	-0.21	-0.05	-0.17	-0.35	0.05	-0.05	0.23	0.13	-0.08
Q5VT25	Serine/threonine-protein kinase MRCK alpha OS=Homo sapiens GN=CDC42BPA PE=1 SV=1 - [MRCKA_HUMAN]	14.03	1	15	20	34	-0.43	-0.39	-0.28	-0.26	0.03	-0.05	-0.22	-0.28	-0.10	-0.10	-0.14	-0.14	0.09	0.29	-0.19	0.19	0.05	0.37	0.08	-0.08
O95336	6-phosphogluconolactonase OS=Homo sapiens GN=PGLS PE=1 SV=2 - [6PGL_HUMAN]	51.16	1	9	9	25	0.50	0.55	0.63	0.75	0.15	0.24	0.51	0.59	0.28	0.25	0.09	0.13	-0.02	-0.34	-0.50	-0.18	-0.19	-0.20	-0.34	-0.08
Q9Y4J8	Dystrobrevin alpha OS=Homo sapiens GN=DTNA PE=1 SV=2 - [DTNA_HUMAN]	22.21	1	10	15	27	0.27	0.32	0.47	0.47	0.05	0.00	0.34	0.30	0.16	0.21	0.33	0.24	-0.12	-0.14	-0.21	-0.17	-0.11	-0.30	-0.44	-0.08
O15355	Protein phosphatase 1G OS=Homo sapiens GN=PPM1G PE=1 SV=1 - [PPM1G_HUMAN]	18.32	1	5	5	7	0.28	0.25	0.30	0.35	-0.05	-0.02	0.08	0.06	-0.14	-0.11	-0.13	-0.10	-0.14	-0.34	-0.60	-0.35	-0.43	-0.31	-0.37	-0.08
Q9P2N5	RNA-binding protein 27 OS=Homo sapiens GN=RBM27 PE=1 SV=2 - [RBM27_HUMAN]	2.74	1	1	2	3	0.10	-0.34	0.06	-0.38	-0.19	-0.64	-0.08	-0.52	-0.53	-0.09	-0.01	-0.46	-0.13	-0.11	-0.08	-0.16	-0.12	-0.31	-0.27	-0.08
Q4G0N4	NAD kinase 2, mitochondrial OS=Homo sapiens GN=NADK2 PE=1 SV=2 - [NAKD2_HUMAN]	29.19	1	11	11	23	-0.02	-0.01	0.24	0.28	-0.34	-0.28	0.07	0.06	-0.18	-0.26	-0.08	-0.10	0.15	-0.21	-0.43	-0.18	-0.40	-0.42	-0.50	-0.08
P03923	NADH-ubiquinone oxidoreductase chain 6 OS=Homo sapiens GN=MT-ND6 PE=1 SV=2 - [NU6M_HUMAN]	8.05	1	1	1	3	1.03	1.13	0.64	0.74	0.72	0.82	0.50	0.60	0.41	0.32	0.33	0.42	-0.48	-0.70	-0.32	-0.69	-0.29	-0.33	0.07	-0.08

Q9NX46	Poly(ADP-ribose) glycohydrolase ARH3 OS=Homo sapiens GN=ADPRHL2 PE=1 SV=1 - [ARHL2_HUMAN]	28.10	1	7	7	16	0.31	0.29	0.12	0.01	0.05	0.00	-0.02	-0.18	-0.48	-0.27	-0.25	-0.14	-0.33	-0.48	-0.28	-0.58	-0.12	-0.36	-0.17	-0.08
Q16531	DNA damage-binding protein 1 OS=Homo sapiens GN=DDB1 PE=1 SV=1 - [DDB1_HUMAN]	29.47	1	31	31	74	-0.18	-0.12	-0.02	0.01	-0.07	-0.06	-0.13	-0.13	-0.06	-0.10	0.07	0.11	0.01	0.30	0.10	0.06	-0.06	0.03	-0.06	-0.08
Q9NRZ5	1-acyl-sn-glycerol-3-phosphate acyltransferase delta OS=Homo sapiens GN=AGPAT4 PE=1 SV=1 - [PLCD_HUMAN]	2.38	1	1	1	1	0.73	0.48	0.33	0.08	0.09	-0.16	0.18	-0.06	0.06	0.31	0.10	-0.15	-0.49	-0.62	-0.23	-0.39	0.02	-0.65	-0.25	-0.08
Q8IWV2	Contactin-4 OS=Homo sapiens GN=CNTN4 PE=1 SV=1 - [CNTN4_HUMAN]	3.02	2	2	2	3	0.08	0.17	0.23	0.32	0.49	0.58	0.09	0.18	0.45	0.37	0.55	0.63	0.07	0.47	0.31	0.32	0.17	0.40	0.24	-0.08
Q01081	Splicing factor U2AF 35 kDa subunit OS=Homo sapiens GN=U2AF1 PE=1 SV=3 - [U2AF1_HUMAN]	24.17	2	5	5	14	0.22	0.07	0.04	-0.11	0.05	-0.21	-0.16	-0.41	-0.12	0.03	-0.03	-0.29	-0.32	-0.36	-0.19	-0.23	-0.05	-0.29	-0.10	-0.08
Q9HA65	TBC1 domain family member 17 OS=Homo sapiens GN=TBC1D17 PE=1 SV=2 - [TBC17_HUMAN]	16.05	1	9	9	16	0.48	0.45	0.31	0.27	0.21	0.13	0.11	0.11	0.27	0.27	0.45	0.25	-0.31	-0.34	-0.10	-0.08	0.04	-0.24	-0.09	-0.08
Q99627	COP9 signalosome complex subunit 8 OS=Homo sapiens GN=COPS8 PE=1 SV=1 - [CSN8_HUMAN]	49.28	1	6	6	14	0.51	0.65	0.46	0.47	-0.05	0.01	0.26	0.33	0.13	0.11	0.12	0.18	-0.12	-0.30	-0.31	-0.32	-0.29	-0.33	-0.45	-0.08
Q9NZN5	Rho guanine nucleotide exchange factor 12 OS=Homo sapiens GN=ARHGEF12 PE=1 SV=1 - [ARHGC_HUMAN]	16.71	1	22	22	42	0.05	0.06	0.08	0.15	-0.01	0.07	0.00	-0.06	-0.02	0.09	-0.03	0.04	-0.06	-0.08	-0.04	0.01	-0.17	-0.01	-0.23	-0.08

Q8WWM7	Ataxin-2-like protein OS=Homo sapiens GN=ATXN2L PE=1 SV=2 - [ATX2L_HUMAN]	7.44	1	5	6	10	0.26	-0.15	0.15	-0.59	-0.27	-0.42	-0.20	-0.64	-0.32	-0.24	0.32	0.12	-0.05	0.28	0.36	-0.16	0.05	-0.28	-0.14	-0.08
Q96EM0	Trans-L-3-hydroxyproline dehydratase OS=Homo sapiens GN=L3HYPDH PE=1 SV=2 - [T3HPD_HUMAN]	8.47	1	2	2	3	-0.38	-0.35	-0.50	-0.47	-0.21	-0.19	-0.64	-0.62	-0.15	-0.17	0.44	0.46	-0.21	0.82	0.94	0.24	0.36	0.15	0.27	-0.08
Q15345	Leucine-rich repeat-containing protein 41 OS=Homo sapiens GN=LRRC41 PE=1 SV=3 - [LRC41_HUMAN]	2.46	1	1	1	1	-1.05	-1.55	-0.40	-0.90	-0.08	-0.58	-0.55	-1.05	-1.02	-0.52	-0.24	-0.74	0.55	0.81	0.16	0.56	-0.08	0.95	0.31	-0.08
Q9Y6I9	Testis-expressed sequence 264 protein OS=Homo sapiens GN=TEX264 PE=1 SV=1 - [TX264_HUMAN]	5.11	1	1	1	1	-0.21	-0.58	0.03	-0.34	1.17	0.80	-0.12	-0.49	-0.21	0.16	1.51	1.14	0.15	1.73	1.49	0.41	0.17	1.37	1.13	-0.08
Q9BTU6	Phosphatidylinositol 4-kinase type 2-alpha OS=Homo sapiens GN=PI4K2A PE=1 SV=1 - [P4K2A_HUMAN]	15.03	1	6	6	10	0.11	0.32	0.32	0.12	0.37	0.57	0.19	0.18	0.29	0.24	0.16	0.26	-0.10	0.00	0.06	0.12	0.14	0.27	-0.15	-0.09
Q96K17	Transcription factor BTF3 homolog 4 OS=Homo sapiens GN=BTF3L4 PE=1 SV=1 - [BT3L4_HUMAN]	12.03	1	2	2	3	-0.07	0.02	-0.02	0.07	0.01	0.10	-0.17	-0.08	-0.09	-0.17	-0.32	-0.24	-0.04	-0.25	-0.31	-0.07	-0.12	0.07	0.02	-0.09
Q8NHH9	Atlastin-2 OS=Homo sapiens GN=ATL2 PE=1 SV=2 - [ATLA2_HUMAN]	21.44	1	9	10	12	-0.08	0.02	0.30	0.32	-0.06	0.03	0.02	0.09	0.05	-0.07	0.13	0.22	0.16	0.07	-0.09	-0.18	-0.32	-0.33	-0.31	-0.09
O95372	Acyl-protein thioesterase 2 OS=Homo sapiens GN=LYPLA2 PE=1 SV=1 - [LYPA2_HUMAN]	36.36	1	6	6	11	0.11	0.29	0.48	0.62	0.15	0.24	0.31	0.50	0.28	0.15	0.25	0.32	0.18	0.14	-0.20	-0.02	-0.30	-0.02	-0.35	-0.09

Q96T37	Putative RNA binding protein 15 OS=Homo sapiens GN=RBM15 PE=1 SV=2 - [RBM15_HUMAN]	4.20	1	3	3	4	-0.41	-0.77	-0.29	-0.65	-0.29	-0.66	-0.44	-0.80	-0.76	-0.39	-0.22	-0.59	0.02	0.20	0.07	0.05	-0.06	0.10	-0.02	-0.09
Q5T013	Putative hydroxypyruvate isomerase OS=Homo sapiens GN=HYI PE=1 SV=2 - [HYI_HUMAN]	11.55	1	3	3	4	0.39	0.66	0.21	0.49	-0.33	-0.06	0.06	0.34	0.12	-0.15	-0.34	-0.07	-0.27	-0.72	-0.55	-0.51	-0.34	-0.73	-0.56	-0.09
O14787	Transportin-2 OS=Homo sapiens GN=TNPO2 PE=1 SV=3 - [TNPO2_HUMAN]	12.71	1	5	7	11	0.12	-0.06	0.55	0.32	0.24	-0.03	0.40	0.17	-0.01	0.20	0.00	0.19	0.28	-0.11	-0.04	0.08	-0.28	0.04	-0.33	-0.09
Q9Y5S1	Transient receptor potential cation channel subfamily V member 2 OS=Homo sapiens GN=TRPV2 PE=1 SV=1 - [TRPV2_HUMAN]	5.89	1	3	3	4	0.41	0.14	0.36	0.09	0.61	0.34	0.21	-0.06	-0.05	0.23	0.58	0.30	-0.15	0.17	0.22	-0.16	-0.10	0.18	0.24	-0.09
Q5T440	Putative transferase CAF17, mitochondrial OS=Homo sapiens GN=IBA57 PE=1 SV=1 - [CAF17_HUMAN]	16.29	1	5	5	7	0.03	-0.05	-0.27	-0.35	-0.40	-0.48	-0.42	-0.50	-0.43	-0.34	-0.40	-0.49	-0.40	-0.43	-0.14	-0.34	-0.04	-0.44	-0.15	-0.09
P07900	Heat shock protein HSP 90-alpha OS=Homo sapiens GN=HSP90AA1 PE=1 SV=5 - [HS90A_HUMAN]	53.01	4	26	44	416	-0.02	0.04	0.19	0.18	-0.05	-0.06	0.06	0.16	-0.02	-0.10	-0.13	-0.12	0.12	-0.25	-0.40	-0.09	-0.28	-0.11	-0.22	-0.09
P63146	Ubiquitin-conjugating enzyme E2 B OS=Homo sapiens GN=UBE2B PE=1 SV=1 - [UBE2B_HUMAN]	11.18	1	1	1	1	0.04	0.14	0.58	0.67	-0.24	-0.15	0.43	0.52	0.14	0.05	-0.25	-0.16	0.44	-0.28	-0.82	0.04	-0.49	-0.30	-0.83	-0.09

Q8NHP8	Putative phospholipase B-like 2 OS=Homo sapiens GN=PLBD2 PE=1 SV=2 - [PLBL2_HUMAN]	5.60	1	3	3	5	1.18	0.72	0.66	0.59	0.44	0.37	0.84	0.61	0.85	0.91	0.73	0.68	-0.05	0.03	-0.09	0.25	0.20	0.03	-0.23	-0.09
O95400	CD2 antigen cytoplasmic tail-binding protein 2 OS=Homo sapiens GN=CD2BP2 PE=1 SV=1 - [CD2B2_HUMAN]	30.79	1	6	6	8	0.22	-0.03	0.42	0.11	0.44	-0.07	0.14	-0.08	0.06	0.32	0.38	0.18	0.04	0.38	0.18	0.22	0.13	0.07	-0.13	-0.09
Q9NZC7	WW domain-containing oxidoreductase OS=Homo sapiens GN=WWOX PE=1 SV=1 - [WWOX_HUMAN]	6.76	1	2	2	4	0.03	0.11	-0.12	-0.04	-0.15	-0.08	-0.27	-0.20	-0.11	-0.18	-0.20	-0.13	-0.25	-0.23	-0.08	-0.19	-0.03	-0.20	-0.05	-0.09
P22314	Ubiquitin-like modifier-activating enzyme 1 OS=Homo sapiens GN=UBA1 PE=1 SV=3 - [UBA1_HUMAN]	40.08	1	31	31	139	0.82	0.89	0.90	0.90	0.57	0.55	0.78	0.74	0.59	0.55	0.05	0.08	-0.07	-0.79	-0.85	-0.25	-0.30	-0.36	-0.44	-0.09
Q9UG63	ATP-binding cassette sub-family F member 2 OS=Homo sapiens GN=ABCF2 PE=1 SV=2 - [ABCF2_HUMAN]	6.58	1	4	4	6	0.63	0.71	0.31	0.39	0.24	0.32	0.16	0.24	0.19	0.11	0.16	0.24	-0.42	-0.47	-0.15	-0.49	-0.17	-0.40	-0.08	-0.09
P27816	Microtubule-associated protein 4 OS=Homo sapiens GN=MAP4 PE=1 SV=3 - [MAP4_HUMAN]	40.45	1	39	39	88	-0.41	-0.33	-0.06	-0.11	-0.61	-0.50	-0.22	-0.27	-0.37	-0.34	0.02	-0.06	0.20	0.36	0.08	0.04	-0.17	-0.24	-0.48	-0.09
Q9P2J5	Leucine--tRNA ligase, cytoplasmic OS=Homo sapiens GN=LARS PE=1 SV=2 - [SYLC_HUMAN]	25.17	1	26	26	35	0.07	0.27	0.13	0.27	0.18	0.20	0.01	0.10	0.06	-0.01	-0.18	-0.10	-0.13	-0.11	-0.35	-0.02	-0.15	0.08	-0.17	-0.09

P54920	Alpha-soluble NSF attachment protein OS=Homo sapiens GN=NAPA PE=1 SV=3 - [SNAA_HUMAN]	54.92	1	12	14	42	0.46	0.22	0.37	0.56	0.11	0.10	0.21	0.29	0.30	0.29	0.41	0.43	-0.15	-0.10	-0.15	-0.09	-0.14	-0.23	-0.36	-0.09
P08727	Keratin, type I cytoskeletal 19 OS=Homo sapiens GN=KRT19 PE=1 SV=4 - [K1C19_HUMAN]	12.25	1	2	5	13	-2.51	-2.66	-2.82	-2.96	-2.45	-2.60	-2.97	-3.11	-2.61	-2.46	-2.63	-2.78	-0.40	-0.11	0.19	0.14	0.38	0.05	0.35	-0.09
Q8NDH6	Islet cell autoantigen 1 like protein OS=Homo sapiens GN=ICA1L PE=2 SV=1 - [ICA1L_HUMAN]	8.71	1	2	3	5	1.06	0.90	0.98	0.82	0.38	0.22	0.37	0.21	-0.10	0.06	0.00	-0.17	-0.73	-1.06	-0.98	-0.77	-0.13	-0.69	-0.61	-0.09
Q9H1B7	Interferon regulatory factor 2-binding protein-like OS=Homo sapiens GN=IRF2BPL PE=1 SV=1 - [I2BPL_HUMAN]	4.65	1	2	3	5	-0.61	-0.44	-0.61	-0.44	-0.74	-0.57	-0.77	-0.60	-0.50	-0.67	-0.50	-0.33	-0.11	0.12	0.12	-0.03	-0.02	-0.15	-0.14	-0.09
Q9UJ68	Mitochondrial peptide methionine sulfoxide reductase OS=Homo sapiens GN=MSRA PE=1 SV=1 - [MSRA_HUMAN]	15.74	1	3	3	6	1.21	1.00	0.85	0.75	0.60	0.46	0.73	0.60	0.44	0.71	0.18	0.08	-0.35	-0.83	-0.78	-0.47	-0.22	-0.55	-0.38	-0.09
P41219	Peripherin OS=Homo sapiens GN=PRPH PE=1 SV=2 - [PERI_HUMAN]	21.28	1	6	11	326	-0.40	-0.46	-0.84	-1.09	-1.27	-1.29	-0.73	-0.65	-1.26	-1.03	-0.81	-0.59	-0.35	-0.17	-0.15	-0.76	-0.16	-0.60	-0.24	-0.09
Q9NX20	39S ribosomal protein L16, mitochondrial OS=Homo sapiens GN=MRPL16 PE=1 SV=1 - [RM16_HUMAN]	6.37	1	1	1	2	0.20	0.05	0.29	0.14	0.01	-0.15	0.14	-0.01	-0.34	-0.19	-0.50	-0.65	-0.01	-0.69	-0.79	-0.36	-0.45	-0.21	-0.30	-0.09
Q96RL7	Vacuolar protein sorting-associated protein 13A OS=Homo sapiens GN=VPS13A PE=1 SV=2 - [VP13A_HUMAN]	5.04	1	13	13	18	0.25	-0.02	0.35	0.16	0.04	0.30	0.09	0.16	0.16	-0.21	-0.20	0.12	-0.10	-0.48	-0.47	-0.37	-0.09	-0.17	-0.13	-0.09

P08913	Alpha-2A adrenergic receptor OS=Homo sapiens GN=ADRA2A PE=1 SV=3 - [ADA2A_HUMAN]	7.56	1	2	2	4	-0.05	-0.09	-0.46	-0.50	-0.36	-0.41	-0.61	-0.65	-0.64	-0.59	-0.73	-0.78	-0.51	-0.68	-0.28	-0.52	-0.11	-0.33	0.08	-0.09
P40855	Peroxisomal biogenesis factor 19 OS=Homo sapiens GN=PEX19 PE=1 SV=1 - [PEX19_HUMAN]	53.18	1	11	11	15	-0.01	0.04	0.19	0.22	-0.26	-0.18	0.03	0.07	-0.19	-0.14	0.31	0.09	0.03	0.13	-0.09	-0.10	-0.36	-0.20	-0.37	-0.09
Q6UWE0	E3 ubiquitin-protein ligase LRSAM1 OS=Homo sapiens GN=LRSAM1 PE=1 SV=1 - [LRSM1_HUMAN]	11.20	1	6	6	8	0.91	0.67	0.55	0.62	-0.02	0.04	0.21	0.39	0.12	0.05	0.27	0.39	-0.64	0.00	0.37	-0.36	-0.53	-0.68	-0.30	-0.09
P22087	rRNA 2'-O-methyltransferase fibrillarin OS=Homo sapiens GN=FBL PE=1 SV=2 - [FBRL_HUMAN]	28.35	1	6	7	16	-0.23	-0.31	0.05	-0.19	-0.10	-0.26	-0.11	-0.21	-0.35	-0.27	-0.14	-0.22	0.15	0.10	-0.28	-0.09	-0.28	-0.01	-0.09	-0.09
Q9H2U2	Inorganic pyrophosphatase 2, mitochondrial OS=Homo sapiens GN=PPA2 PE=1 SV=2 - [IPYR2_HUMAN]	49.70	1	14	15	30	-0.73	-0.51	-0.22	-0.16	-0.23	-0.22	-0.39	-0.37	-0.20	-0.32	-0.17	-0.02	0.31	0.54	0.16	0.28	-0.01	0.28	-0.07	-0.09
Q9NXR7	BRCA1-A complex subunit BRE OS=Homo sapiens GN=BRE PE=1 SV=2 - [BRE_HUMAN]	6.27	1	2	2	3	0.33	0.17	0.26	0.10	0.18	0.02	0.10	-0.05	-0.12	0.04	0.01	-0.15	-0.17	-0.32	-0.25	-0.26	-0.18	-0.17	-0.09	-0.09
Q86VH4	Leucine-rich repeat transmembrane neuronal protein 4 OS=Homo sapiens GN=LRRTM4 PE=2 SV=2 - [LRRT4_HUMAN]	3.22	1	2	2	4	-1.34	-1.17	-1.14	-0.96	-0.44	-0.28	-1.29	-1.12	-0.69	-0.85	-1.22	-1.05	0.11	0.13	-0.08	0.52	0.32	0.88	0.67	-0.09
O75431	Metaxin-2 OS=Homo sapiens GN=MTX2 PE=1 SV=1 - [MTX2_HUMAN]	23.95	1	5	5	10	0.25	0.20	0.47	0.36	0.05	-0.05	0.24	0.21	-0.05	0.14	0.10	-0.12	0.07	-0.05	-0.21	-0.08	-0.30	-0.21	-0.43	-0.09

Q9ULZ3	Apoptosis-associated speck-like protein containing a CARD OS=Homo sapiens GN=PYCARD PE=1 SV=2 - [ASC_HUMAN]	17.95	1	3	3	4	-0.19	-0.03	-0.16	0.00	-0.01	0.15	0.05	0.21	0.54	0.41	0.28	0.46	-0.17	0.48	0.44	0.26	0.24	-0.44	-0.37	-0.09
Q9BV73	Centrosome-associated protein CEP250 OS=Homo sapiens GN=CEP250 PE=1 SV=2 - [CP250_HUMAN]	0.90	1	1	2	3	2.70	3.13	2.54	2.97	0.72	1.15	2.39	2.82	2.29	1.86	2.20	2.62	-0.26	-0.50	-0.35	-0.81	-0.65	-2.00	-1.83	-0.09
O95630	STAM-binding protein OS=Homo sapiens GN=STAMBP PE=1 SV=1 - [STABP_HUMAN]	15.09	1	5	5	12	-0.11	-0.12	0.26	0.10	0.44	0.30	0.26	0.03	0.25	0.39	0.70	0.61	0.14	0.61	0.26	0.35	0.16	0.39	0.23	-0.09
Q5TBA9	Protein furry homolog OS=Homo sapiens GN=FRY PE=1 SV=1 - [FRY_HUMAN]	2.42	1	5	5	7	0.78	0.22	-0.24	0.51	-0.17	-0.35	-0.40	-0.22	-0.39	-0.34	0.44	0.02	-1.13	-0.35	0.68	-1.10	-0.06	-0.98	0.05	-0.09
Q13362	Serine/threonine-protein phosphatase 2A 56 kDa regulatory subunit gamma isoform OS=Homo sapiens GN=PPP2R5C PE=1 SV=3 - [2A5G_HUMAN]	6.49	1	1	3	9	0.48	0.86	0.70	1.08	0.46	0.83	0.54	0.92	0.56	0.19	0.72	1.09	0.12	0.25	0.02	-0.26	-0.47	-0.04	-0.26	-0.09
P30041	Peroxiredoxin-6 OS=Homo sapiens GN=PRDX6 PE=1 SV=3 - [PRDX6_HUMAN]	54.02	1	13	13	41	0.32	0.24	0.37	0.49	-0.48	-0.45	0.17	0.19	-0.21	-0.23	-0.80	-0.78	-0.05	-0.92	-1.25	-0.21	-0.56	-0.72	-0.88	-0.09
P27482	Calmodulin-like protein 3 OS=Homo sapiens GN=CALML3 PE=1 SV=2 - [CALL3_HUMAN]	44.30	2	3	5	15	-2.97	-3.00	-3.43	-3.46	-2.25	-2.06	-2.72	-2.51	-2.85	-2.90	-3.19	-3.19	-0.19	0.00	-0.09	-0.04	0.24	0.20	0.29	-0.09

Q13084	39S ribosomal protein L28, mitochondrial OS=Homo sapiens GN=MRPL28 PE=1 SV=4 - [RM28_HUMAN]	3.52	1	1	1	1	0.38	0.26	0.27	0.15	0.04	-0.09	0.12	-0.01	0.09	0.22	0.15	0.02	-0.21	-0.23	-0.12	-0.13	-0.01	-0.37	-0.25	-0.09
P17844	Probable ATP-dependent RNA helicase DDX5 OS=Homo sapiens GN=DDX5 PE=1 SV=1 - [DDX5_HUMAN]	27.20	1	8	15	47	0.46	0.57	0.29	0.46	0.03	0.27	0.19	0.30	0.33	0.21	0.32	0.34	-0.20	-0.07	-0.14	-0.23	-0.11	-0.08	-0.15	-0.09
Q13435	Splicing factor 3B subunit 2 OS=Homo sapiens GN=SF3B2 PE=1 SV=2 - [SF3B2_HUMAN]	18.44	1	12	12	22	-0.70	-0.73	-0.30	-0.41	-0.44	-0.51	-0.45	-0.50	-0.58	-0.45	-0.34	-0.37	0.23	0.31	0.03	0.03	-0.01	0.17	-0.11	-0.09
Q13003	Glutamate receptor ionotropic, kainate 3 OS=Homo sapiens GN=GRIK3 PE=2 SV=3 - [GRIK3_HUMAN]	2.61	1	1	1	2	0.80	0.74	0.17	0.12	0.03	-0.03	0.01	-0.04	-0.16	-0.10	-0.64	-0.71	-0.73	-1.44	-0.82	-0.87	-0.24	-0.79	-0.16	-0.09
P04233	HLA class II histocompatibility antigen gamma chain OS=Homo sapiens GN=CD74 PE=1 SV=3 - [HG2A_HUMAN]	13.51	1	4	4	8	-0.64	-0.49	-0.86	-0.71	-0.15	-0.10	-0.56	-0.70	-0.24	-0.34	-0.11	-0.01	-0.01	0.54	0.75	0.29	0.51	0.47	0.70	-0.09
Q969M3	Protein YIPF5 OS=Homo sapiens GN=YIPF5 PE=1 SV=1 - [YIPF5_HUMAN]	8.17	1	2	2	3	0.09	0.04	0.02	-0.03	-0.05	-0.10	-0.14	-0.19	0.00	0.06	0.59	0.54	-0.18	0.50	0.57	-0.01	0.07	-0.15	-0.08	-0.09
Q9P287	BRCA2 and CDKN1A-interacting protein OS=Homo sapiens GN=BCCIP PE=1 SV=1 - [BCCIP_HUMAN]	12.10	1	2	2	2	0.25	-0.05	0.55	0.25	0.68	0.37	0.39	0.09	-0.13	0.18	0.33	0.03	0.19	0.09	-0.21	-0.04	-0.33	0.41	0.11	-0.10

Q9NRA8	Eukaryotic translation initiation factor 4E transporter OS=Homo sapiens GN=EIF4ENIF1 PE=1 SV=2 - [4ET_HUMAN]	2.84	1	2	2	2	0.28	0.00	0.37	0.09	0.24	-0.04	0.22	-0.06	-0.36	-0.08	0.18	-0.11	-0.01	-0.09	-0.20	-0.32	-0.42	-0.05	-0.15	-0.10
Q15287	RNA-binding protein with serine-rich domain 1 OS=Homo sapiens GN=RNPS1 PE=1 SV=1 - [RNPS1_HUMAN]	11.48	1	3	3	5	0.13	-0.15	0.14	-0.15	-0.01	-0.43	-0.08	-0.36	-0.59	-0.03	0.12	-0.12	-0.15	-0.01	0.05	-0.07	-0.07	-0.16	-0.17	-0.10
Q9Y3D0	Mitotic spindle-associated MMXD complex subunit MIP18 OS=Homo sapiens GN=FAM96B PE=1 SV=1 - [MIP18_HUMAN]	17.18	1	2	2	3	0.92	0.91	0.63	0.63	0.21	0.19	0.48	0.47	0.18	0.20	0.30	0.29	-0.38	-0.61	-0.33	-0.69	-0.41	-0.73	-0.45	-0.10
O95793	Double-stranded RNA-binding protein Staufen homolog 1 OS=Homo sapiens GN=STAU1 PE=1 SV=2 - [STAU1_HUMAN]	15.08	1	6	6	11	-0.01	-0.05	0.09	0.05	0.09	0.05	-0.26	-0.25	-0.05	-0.19	0.30	0.26	0.00	0.32	0.21	-0.14	-0.24	0.08	-0.02	-0.10
Q8IW45	ATP-dependent (S)-NAD(P)H-hydrate dehydratase OS=Homo sapiens GN=CARKD PE=1 SV=1 - [NNRD_HUMAN]	34.87	1	9	9	28	0.14	0.28	0.28	0.23	-0.06	-0.10	0.06	0.17	0.03	-0.09	-0.25	-0.15	-0.19	-0.38	-0.41	-0.32	-0.25	-0.33	-0.26	-0.10
Q8N8N7	Prostaglandin reductase 2 OS=Homo sapiens GN=PTGR2 PE=1 SV=1 - [PTGR2_HUMAN]	17.38	1	5	5	6	0.71	0.70	0.62	0.61	0.05	-0.15	0.46	0.45	0.31	0.61	-0.03	-0.05	-0.19	-0.74	-0.66	-0.41	-0.16	-0.80	-0.28	-0.10
P62701	40S ribosomal protein S4, X isoform OS=Homo sapiens GN=RPS4X PE=1 SV=2 - [RS4X_HUMAN]	36.88	3	9	9	20	-0.40	-0.28	-0.59	-0.46	-0.58	-0.45	-0.56	-0.60	-0.44	-0.45	-0.43	-0.32	-0.09	0.08	0.25	-0.08	0.03	0.03	0.10	-0.10

Q00839	Heterogeneous nuclear ribonucleoprotein U OS=Homo sapiens GN=HNRNPU PE=1 SV=6 - [HNRPU_HUMAN]	25.82	1	18	18	58	-0.19	-0.28	-0.04	0.06	-0.25	-0.34	-0.08	-0.17	-0.31	-0.24	-0.26	-0.33	0.18	-0.02	-0.24	0.02	-0.21	-0.02	-0.35	-0.10
Q16629	Serine/arginine-rich splicing factor 7 OS=Homo sapiens GN=SRSF7 PE=1 SV=1 - [SRSF7_HUMAN]	24.79	1	5	6	11	-0.07	-0.06	-0.01	-0.08	0.06	-0.14	-0.09	-0.28	-0.24	-0.12	0.21	0.02	0.02	0.35	0.14	0.13	-0.06	0.19	-0.03	-0.10
Q8IVF2	Protein AHNAK2 OS=Homo sapiens GN=AHNAK2 PE=1 SV=2 - [AHNK2_HUMAN]	18.31	1	29	31	44	-0.24	-0.11	-0.16	-0.09	-0.59	-0.50	-0.48	-0.38	-0.24	-0.38	0.00	0.07	-0.05	0.30	0.19	-0.11	-0.23	-0.39	-0.44	-0.10
P24666	Low molecular weight phosphotyrosine protein phosphatase OS=Homo sapiens GN=ACP1 PE=1 SV=3 - [PPAC_HUMAN]	43.67	1	6	6	18	0.18	0.27	-0.20	0.10	-0.19	-0.07	-0.25	-0.14	-0.31	-0.16	-0.49	-0.39	-0.35	-0.57	-0.49	-0.47	-0.39	-0.23	-0.13	-0.10
Q9UBQ0	Vacuolar protein sorting-associated protein 29 OS=Homo sapiens GN=VPS29 PE=1 SV=1 - [VPS29_HUMAN]	25.82	1	5	5	15	0.06	0.22	0.40	0.48	0.14	0.15	0.22	0.27	0.14	0.05	0.01	0.31	0.04	-0.10	-0.34	-0.09	-0.26	-0.02	-0.13	-0.10
O43583	Density-regulated protein OS=Homo sapiens GN=DENR PE=1 SV=2 - [DENR_HUMAN]	12.12	1	2	2	6	-0.15	-0.29	-0.35	-0.38	-0.06	-0.48	-0.51	-0.44	-0.74	-0.81	-0.56	-0.48	0.11	-0.02	-0.08	-0.36	-0.31	-0.28	-0.23	-0.10
O75385	Serine/threonine-protein kinase ULK1 OS=Homo sapiens GN=ULK1 PE=1 SV=2 - [ULK1_HUMAN]	2.86	1	2	2	4	0.61	0.41	-0.40	-0.60	-0.38	-0.59	-0.56	-0.76	-0.53	-0.32	-0.71	-0.91	-1.12	-1.31	-0.31	-0.90	0.11	-1.01	0.00	-0.10

Q96JB2	Conserved oligomeric Golgi complex subunit 3 OS=Homo sapiens GN=COG3 PE=1 SV=3 - [COG3_HUMAN]	5.07	1	3	3	3	0.20	-0.12	0.23	-0.10	1.14	0.81	0.07	-0.26	-0.03	0.30	0.39	0.06	-0.08	0.19	0.16	0.13	0.11	0.92	0.90	-0.10
Q15365	Poly(rC)-binding protein 1 OS=Homo sapiens GN=PCBP1 PE=1 SV=2 - [PCBP1_HUMAN]	40.45	2	6	10	41	0.21	0.29	0.32	0.40	0.26	0.19	0.16	0.06	-0.02	0.03	-0.10	-0.12	-0.02	-0.47	-0.59	-0.14	-0.22	-0.12	-0.22	-0.10
Q9NWW6	Nicotinamide riboside kinase 1 OS=Homo sapiens GN=NMRK1 PE=1 SV=1 - [NRK1_HUMAN]	16.58	1	2	2	2	0.79	0.59	0.79	0.59	0.73	0.53	0.62	0.43	0.12	0.32	0.92	0.72	-0.11	0.13	0.13	-0.44	-0.43	-0.08	-0.07	-0.10
Q9BYN8	28S ribosomal protein S26, mitochondrial OS=Homo sapiens GN=MRPS26 PE=1 SV=1 - [RT26_HUMAN]	24.39	1	5	5	6	0.35	0.79	0.53	0.72	0.00	0.13	0.05	-0.03	-0.08	-0.07	-0.04	-0.04	0.04	-0.47	-0.65	-0.45	-0.62	-0.37	-0.55	-0.10
Q13404	Ubiquitin-conjugating enzyme E2 variant 1 OS=Homo sapiens GN=UBE2V1 PE=1 SV=2 - [UB2V1_HUMAN]	59.86	1	3	8	50	-0.12	0.05	-0.20	-0.11	-0.05	0.14	-0.55	-0.36	-0.20	-0.24	-0.59	-0.43	-0.35	-0.52	-0.19	-0.06	-0.05	0.08	0.13	-0.10
Q9BXR0	Queuine tRNA-ribosyltransferase OS=Homo sapiens GN=QTRT1 PE=1 SV=3 - [TGT_HUMAN]	5.21	1	2	2	2	0.54	0.13	0.44	0.04	0.15	-0.27	0.28	-0.13	-0.24	0.18	0.38	-0.04	-0.20	-0.16	-0.07	-0.33	-0.23	-0.41	-0.32	-0.10
Q9Y3A3	MOB-like protein phocein OS=Homo sapiens GN=MOB4 PE=1 SV=1 - [PHOCN_HUMAN]	22.67	1	4	4	6	0.28	0.32	0.27	0.16	-0.11	0.02	-0.04	-0.15	-0.18	-0.11	0.02	0.06	-0.61	-0.30	-0.20	-0.33	-0.14	-0.97	-0.53	-0.10
Q16775	Hydroxyacylglutathione hydrolase, mitochondrial OS=Homo sapiens GN=HAGH PE=1 SV=2 - [GLO2_HUMAN]	37.99	1	9	9	23	-0.12	0.07	0.07	0.26	0.19	0.18	-0.02	0.08	0.02	-0.03	-0.30	-0.15	-0.20	-0.08	-0.39	-0.12	0.01	0.19	0.21	-0.10

Q9UIG8	Solute carrier organic anion transporter family member 3A1 OS=Homo sapiens GN=SLCO3A1 PE=1 SV=3 - [SO3A1_HUMAN]	2.25	1	1	1	1	0.77	0.47	0.39	0.09	0.70	0.40	0.22	-0.07	-0.02	0.28	0.47	0.17	-0.49	-0.30	0.08	-0.46	-0.08	-0.09	0.30	-0.10
Q13033	Striatin-3 OS=Homo sapiens GN=STRN3 PE=1 SV=3 - [STRN3_HUMAN]	11.92	1	6	7	9	0.09	0.21	-0.57	-0.09	-0.06	0.14	-0.74	-0.38	-0.12	-0.42	-0.17	0.06	-0.57	-0.14	0.14	-0.25	0.01	0.10	0.19	-0.10
Q96D05	Uncharacterized protein C10orf35 OS=Homo sapiens GN=C10orf35 PE=1 SV=1 - [CJ035_HUMAN]	22.31	1	2	2	2	1.19	1.35	0.39	0.55	-0.55	-0.39	0.22	0.39	0.27	0.11	0.15	0.30	-0.91	-1.04	-0.24	-1.05	-0.25	-1.76	-0.96	-0.10
Q9NRS6	Sorting nexin-15 OS=Homo sapiens GN=SNX15 PE=1 SV=1 - [SNX15_HUMAN]	38.89	1	8	8	11	0.14	-0.07	-0.47	-0.80	0.19	-0.10	-0.64	-0.86	-0.50	-0.16	-0.08	-0.15	-0.18	-0.14	0.37	-0.16	0.34	0.04	0.69	-0.10
P62318	Small nuclear ribonucleoprotein Sm D3 OS=Homo sapiens GN=SNRPD3 PE=1 SV=1 - [SMD3_HUMAN]	31.75	1	3	3	5	-0.33	-0.42	-0.50	-0.59	0.10	0.00	-0.66	-0.76	-0.27	-0.17	-0.01	-0.11	-0.28	0.32	0.49	0.19	0.36	0.41	0.58	-0.10
Q96EK7	Constitutive coactivator of peroxisome proliferator-activated receptor gamma OS=Homo sapiens GN=FAM120B PE=1 SV=1 - [F120B_HUMAN]	10.00	1	6	6	14	0.25	0.37	0.27	0.33	0.01	0.01	0.10	0.07	0.02	0.08	0.20	0.23	-0.10	0.04	-0.12	-0.15	-0.05	-0.26	-0.43	-0.10
P28161	Glutathione S transferase Mu 2 OS=Homo sapiens GN=GSTM2 PE=1 SV=2 - [GSTM2_HUMAN]	73.39	1	9	16	56	0.72	0.66	0.93	0.63	0.66	0.57	0.68	0.58	0.30	0.43	-0.10	-0.15	-0.05	-0.70	-0.92	-0.51	-0.42	-0.23	-0.20	-0.10

P43490	Nicotinamide phosphoribosyltransferase OS=Homo sapiens GN=NAMPT PE=1 SV=1 - [NAMPT_HUMAN]	46.44	1	18	18	39	-0.38	-0.41	-0.19	-0.24	-0.22	-0.24	-0.31	-0.33	-0.42	-0.40	-0.22	-0.25	0.10	0.08	0.00	0.09	-0.18	0.22	-0.12	-0.10
Q9Y657	Spindlin-1 OS=Homo sapiens GN=SPIN1 PE=1 SV=3 - [SPIN1_HUMAN]	13.74	1	3	3	4	-0.08	-0.31	-0.01	-0.24	0.27	0.03	-0.18	-0.41	-0.31	-0.07	0.10	-0.14	-0.05	0.18	0.11	0.04	-0.03	0.33	0.26	-0.10
Q9BZL1	Ubiquitin-like protein 5 OS=Homo sapiens GN=UBL5 PE=1 SV=1 - [UBL5_HUMAN]	39.73	1	3	3	4	0.44	0.41	0.41	0.38	0.26	0.23	0.24	0.21	0.02	0.06	-0.05	-0.09	-0.15	-0.49	-0.46	-0.36	-0.32	-0.20	-0.16	-0.10
Q06203	Amidophosphoribosyltransferase OS=Homo sapiens GN=PPAT PE=1 SV=1 - [PUR1_HUMAN]	14.31	1	7	7	12	0.42	0.16	0.35	0.29	-0.10	-0.11	0.15	-0.05	0.01	0.27	0.27	0.04	-0.09	-0.24	-0.10	0.05	-0.06	-0.26	-0.38	-0.10
O15111	Inhibitor of nuclear factor kappa-B kinase subunit alpha OS=Homo sapiens GN=CHUK PE=1 SV=2 - [IKKA_HUMAN]	5.10	1	2	2	3	0.42	0.09	0.31	-0.02	0.86	0.53	0.52	0.19	0.21	0.54	0.33	0.00	-0.29	-0.09	0.08	0.15	0.27	0.42	0.53	-0.10
Q6P4A7	Sideroflexin-4 OS=Homo sapiens GN=SFXN4 PE=1 SV=1 - [SFXN4_HUMAN]	12.17	1	3	3	6	0.87	0.89	0.52	0.54	0.32	0.33	0.35	0.37	0.13	0.12	0.28	0.29	-0.47	-0.58	-0.24	-0.72	-0.37	-0.57	-0.22	-0.10
Q9Y2W1	Thyroid hormone receptor-associated protein 3 OS=Homo sapiens GN=THRAP3 PE=1 SV=2 - [TR150_HUMAN]	9.32	1	7	7	10	-0.58	-0.56	-0.28	-0.20	-0.33	-0.35	-0.30	-0.43	-0.55	-0.39	-0.19	-0.23	0.10	0.29	-0.07	-0.07	-0.22	0.24	-0.07	-0.11
Q9BU61	NADH dehydrogenase [ubiquinone] 1 alpha subcomplex assembly factor 3 OS=Homo sapiens GN=NDUFAF3 PE=1 SV=1 - [NDUF3_HUMAN]	11.41	1	2	2	5	0.15	0.11	0.00	0.25	0.26	0.25	-0.19	0.17	0.12	0.17	0.03	0.13	-0.07	-0.01	-0.07	-0.21	-0.09	0.13	0.06	-0.11

Q96GS6	Alpha/beta hydrolase domain-containing protein 17A OS=Homo sapiens GN=ABHD17A PE=1 SV=1 - [AB17A_HUMAN]	10.97	1	2	2	3	0.70	1.52	0.31	1.13	-0.87	-0.06	0.14	0.96	0.73	-0.09	-0.26	0.56	-0.51	-0.95	-0.57	-0.76	-0.36	-1.59	-1.20	-0.11
O60477	BMP/retinoic acid-inducible neural-specific protein 1 OS=Homo sapiens GN=BRINP1 PE=1 SV=2 - [BRNP1_HUMAN]	3.02	1	2	2	3	0.14	0.07	-0.03	-0.10	0.22	0.14	-0.20	-0.27	0.43	0.50	0.23	0.16	-0.28	0.10	0.26	0.39	0.56	0.06	0.23	-0.11
Q8NCW5	NAD(P)H-hydrate epimerase OS=Homo sapiens GN=APOA1BP PE=1 SV=2 - [NNRE_HUMAN]	40.63	1	9	9	25	0.43	0.44	0.51	0.70	0.23	0.18	0.50	0.53	0.45	0.30	0.14	0.07	0.16	-0.32	-0.63	0.04	-0.26	-0.09	-0.34	-0.11
P53992	Protein transport protein Sec24C OS=Homo sapiens GN=SEC24C PE=1 SV=3 - [SC24C_HUMAN]	17.64	1	17	17	26	-0.18	-0.05	-0.20	-0.14	-0.02	0.02	-0.27	-0.21	-0.12	-0.23	-0.22	-0.24	-0.14	-0.08	-0.08	0.02	0.02	-0.01	0.12	-0.11
Q08AM6	Protein VAC14 homolog OS=Homo sapiens GN=VAC14 PE=1 SV=1 - [VAC14_HUMAN]	19.95	1	9	9	14	0.14	0.37	0.22	0.36	0.02	0.09	0.06	0.09	0.27	-0.05	0.23	0.36	-0.29	-0.06	0.01	-0.13	-0.20	-0.30	-0.23	-0.11
O95218	Zinc finger Ran-binding domain-containing protein 2 OS=Homo sapiens GN=ZRANB2 PE=1 SV=2 - [ZRAB2_HUMAN]	5.45	1	2	2	3	-0.55	-0.58	-0.65	-0.68	-0.92	-0.96	-0.82	-0.85	-0.78	-0.74	-0.38	-0.42	-0.21	0.17	0.26	-0.16	-0.06	-0.39	-0.29	-0.11
Q2LD37	Uncharacterized protein KIAA1109 OS=Homo sapiens GN=KIAA1109 PE=1 SV=2 - [K1109_HUMAN]	1.86	1	6	6	9	0.40	0.66	0.32	0.67	0.32	0.39	0.35	0.59	0.48	0.21	0.35	-0.19	-0.18	-0.10	0.08	-0.11	-0.07	-0.28	0.06	-0.11
P09960	Leukotriene A-4 hydrolase OS=Homo sapiens GN=LTA4H PE=1 SV=2 - [LKHA4_HUMAN]	36.33	1	17	17	42	0.05	0.12	0.23	0.17	-0.12	-0.02	0.07	0.07	0.36	0.35	0.89	0.88	-0.06	0.83	0.81	0.32	0.29	-0.17	-0.27	-0.11

P45954	Short/branched chain specific acyl-CoA dehydrogenase, mitochondrial OS=Homo sapiens GN=ACADSB PE=1 SV=1 - [ACDSB_HUMAN]	21.76	1	7	7	13	0.12	-0.05	0.16	0.10	0.22	0.04	-0.02	-0.08	-0.21	-0.04	0.06	0.07	-0.08	-0.02	-0.03	-0.12	-0.25	0.01	-0.06	-0.11
P52815	39S ribosomal protein L12, mitochondrial OS=Homo sapiens GN=MRPL12 PE=1 SV=2 - [RM12_HUMAN]	17.68	1	3	3	5	0.23	0.10	0.26	0.26	0.38	-0.48	0.31	0.12	-0.50	-0.17	0.17	-0.15	-0.35	-0.15	-0.25	-0.47	-0.70	0.02	-0.04	-0.11
Q13185	Chromobox protein homolog 3 OS=Homo sapiens GN=CBX3 PE=1 SV=4 - [CBX3_HUMAN]	29.51	1	2	4	6	-0.40	-0.54	-0.43	0.12	-0.37	-0.52	-0.54	-0.15	0.10	-0.51	-0.47	-0.41	0.11	0.13	-0.18	-0.08	-0.20	0.00	-0.29	-0.11
P61513	60S ribosomal protein L37a OS=Homo sapiens GN=RPL37A PE=1 SV=2 - [RL37A_HUMAN]	19.57	1	1	1	2	-0.96	-0.77	-0.82	-0.63	-0.42	-0.23	-1.00	-0.80	-0.32	-0.51	-0.15	0.04	0.02	0.82	0.67	0.49	0.35	0.53	0.39	-0.11
Q9NXW2	DnaJ homolog subfamily B member 12 OS=Homo sapiens GN=DNAJB12 PE=1 SV=4 - [DJB12_HUMAN]	12.53	1	3	3	3	-0.75	-0.58	-0.61	-0.44	-0.20	-0.04	-0.78	-0.62	-0.12	-0.29	-0.34	-0.17	0.02	0.42	0.27	0.49	0.36	0.53	0.39	-0.11
P55010	Eukaryotic translation initiation factor 5 OS=Homo sapiens GN=EIF5 PE=1 SV=2 - [IF5_HUMAN]	20.65	2	8	9	13	0.92	0.88	0.44	0.26	0.20	0.02	0.49	0.25	-0.03	0.27	-0.06	-0.19	-0.37	-0.98	-0.61	-0.57	-0.42	-0.69	-0.25	-0.11
P31943	Heterogeneous nuclear ribonucleoprotein H OS=Homo sapiens GN=HNRNPH1 PE=1 SV=4 - [HNRH1_HUMAN]	40.53	1	7	13	76	0.17	0.24	0.05	0.11	-0.26	-0.13	0.00	-0.06	-0.09	-0.04	-0.18	-0.20	-0.25	-0.53	-0.28	-0.38	-0.12	-0.52	-0.32	-0.11

Q9GZZ1	N-alpha-acetyltransferase 50 OS=Homo sapiens GN=NAA50 PE=1 SV=1 - [NAA50_HUMAN]	12.43	1	2	2	4	-0.44	-0.44	-0.07	-0.08	-0.03	-0.04	-0.25	-0.25	-0.27	-0.26	-0.52	-0.53	0.25	-0.08	-0.45	0.21	-0.16	0.39	0.02	-0.11
Q99496	E3 ubiquitin-protein ligase RING2 OS=Homo sapiens GN=RNF2 PE=1 SV=1 - [RING2_HUMAN]	4.17	2	1	1	2	-0.44	-0.54	0.04	-0.06	-0.46	-0.55	-0.14	-0.23	-0.58	-0.48	-0.29	-0.39	0.36	0.16	-0.33	-0.01	-0.48	-0.03	-0.51	-0.11
O60488	Long-chain-fatty-acid--CoA ligase 4 OS=Homo sapiens GN=ACSL4 PE=1 SV=2 - [ACSL4_HUMAN]	8.44	1	3	4	5	-0.04	0.09	-0.45	-0.31	0.47	0.60	-0.62	-0.49	-0.08	-0.21	0.39	0.53	-0.52	0.44	0.84	-0.13	0.27	0.50	0.90	-0.11
Q969G3	SWI/SNF-related matrix associated actin-dependent regulator of chromatin subfamily E member 1 OS=Homo sapiens GN=SMARCE1 PE=1 SV=2 - [SMCE1_HUMAN]	22.87	1	6	7	11	-0.07	0.17	-0.16	0.11	-0.40	-0.31	-0.13	-0.09	-0.21	-0.29	-0.02	-0.08	-0.03	-0.18	-0.18	-0.04	-0.05	-0.34	-0.43	-0.11
Q15052	Rho guanine nucleotide exchange factor 6 OS=Homo sapiens GN=ARHGEF6 PE=1 SV=2 - [ARHG6_HUMAN]	13.27	1	8	11	14	0.02	-0.09	0.34	0.31	0.04	0.06	0.07	0.05	0.13	0.09	0.21	0.12	0.25	0.19	-0.20	0.08	-0.24	0.05	-0.26	-0.11
P05091	Aldehyde dehydrogenase, mitochondrial OS=Homo sapiens GN=ALDH2 PE=1 SV=2 - [ALDH2_HUMAN]	49.90	1	18	21	86	-0.17	-0.13	0.05	0.10	0.04	0.00	-0.04	-0.14	-0.04	0.02	0.35	0.35	0.03	0.47	0.29	0.04	0.01	0.11	-0.08	-0.11
P54252	Ataxin-3 OS=Homo sapiens GN=ATXN3 PE=1 SV=4 - [ATX3_HUMAN]	11.26	1	3	3	4	0.44	0.05	0.69	0.05	0.18	0.23	0.24	-0.13	0.27	0.19	0.26	-0.04	-0.14	-0.16	-0.09	-0.22	-0.13	-0.14	0.09	-0.11
Q9UMX0	Ubiquilin-1 OS=Homo sapiens GN=UBQLN1 PE=1 SV=2 - [UBQL1_HUMAN]	13.24	1	2	6	15	0.02	0.02	0.37	0.40	0.10	0.32	-0.08	0.22	0.12	-0.15	-0.28	0.24	0.26	0.31	0.35	0.14	-0.24	0.39	0.01	-0.11

Q96MU7	YTH domain-containing protein 1 OS=Homo sapiens GN=YTHDC1 PE=1 SV=3 - [YTDC1_HUMAN]	3.03	1	2	2	2	-0.76	-0.72	-0.40	-0.35	-0.07	-0.03	-0.57	-0.53	-0.20	-0.24	-0.17	-0.13	0.24	0.60	0.23	0.55	0.19	0.67	0.31	-0.11
Q969G2	LIM/homeobox protein Lhx4 OS=Homo sapiens GN=LHX4 PE=1 SV=2 - [LHX4_HUMAN]	1.54	4	1	1	1	0.46	0.60	-0.23	-0.09	-0.37	-0.23	-0.41	-0.26	-0.20	-0.33	-0.03	0.10	-0.81	-0.48	0.19	-0.76	-0.07	-0.84	-0.16	-0.11
Q9UJU6	Drebrin-like protein OS=Homo sapiens GN=DBNL PE=1 SV=1 - [DBNL_HUMAN]	33.26	1	10	10	32	0.32	0.29	0.46	0.28	0.04	0.01	0.33	0.21	0.07	0.20	-0.27	-0.33	0.12	-0.50	-0.74	-0.20	-0.34	-0.38	-0.45	-0.11
P43003	Excitatory amino acid transporter 1 OS=Homo sapiens GN=SLC1A3 PE=1 SV=1 - [EAA1_HUMAN]	16.97	2	5	7	58	1.26	1.08	1.53	1.42	-0.01	0.10	1.23	0.91	0.78	0.78	0.69	0.44	-0.06	-0.70	-0.71	-0.53	-0.60	-1.43	-1.52	-0.11
Q9P0V9	Septin-10 OS=Homo sapiens GN=SEPT10 PE=1 SV=2 - [SEP10_HUMAN]	31.28	1	9	10	29	-0.44	-0.52	-0.51	-0.60	-0.35	-0.24	-0.50	-0.66	-0.46	-0.42	-0.45	-0.39	0.09	0.06	-0.05	0.05	-0.07	0.33	0.17	-0.11
P63272	Transcription elongation factor SPT4 OS=Homo sapiens GN=SUPT4H1 PE=1 SV=1 - [SPT4H_HUMAN]	13.68	1	1	1	1	-0.42	-0.51	-0.10	-0.19	-0.16	-0.25	-0.28	-0.37	-0.44	-0.35	-0.24	-0.33	0.19	0.19	-0.13	0.10	-0.21	0.24	-0.07	-0.11
Q9NVJ2	ADP-ribosylation factor-like protein 8B OS=Homo sapiens GN=ARL8B PE=1 SV=1 - [ARL8B_HUMAN]	39.78	1	2	6	17	0.07	0.60	0.33	0.50	0.12	0.30	0.20	0.43	0.61	-0.07	-0.27	0.28	0.18	-0.33	-0.64	-0.16	-0.38	-0.32	-0.23	-0.11
P37108	Signal recognition particle 14 kDa protein OS=Homo sapiens GN=SRP14 PE=1 SV=2 - [SRP14_HUMAN]	22.79	1	3	3	6	-0.54	-0.56	-0.26	-0.29	-0.23	-0.26	-0.44	-0.46	-0.33	-0.30	-0.23	-0.26	0.15	0.31	0.03	0.27	0.00	0.29	0.01	-0.11

P20340	Ras-related protein Rab-6A OS=Homo sapiens GN=RAB6A PE=1 SV=3 - [RAB6A_HUMAN]	41.83	4	5	11	37	0.37	0.55	0.62	0.64	0.24	0.30	0.33	0.37	0.20	0.31	0.39	0.28	-0.17	-0.32	-0.32	-0.23	-0.30	-0.26	-0.27	-0.11
Q9P2B2	Prostaglandin F2 receptor negative regulator OS=Homo sapiens GN=PTGFRN PE=1 SV=2 - [FPRP_HUMAN]	3.41	1	3	3	5	0.01	0.01	0.03	0.03	-0.17	-0.17	-0.15	-0.15	0.16	0.16	0.14	0.14	-0.10	0.14	0.11	0.19	0.17	-0.19	-0.21	-0.12
Q9Y6G3	39S ribosomal protein L42, mitochondrial OS=Homo sapiens GN=MRPL42 PE=1 SV=1 - [RM42_HUMAN]	10.56	1	1	1	1	0.01	0.05	-0.05	-0.01	-0.03	0.01	-0.23	-0.19	0.00	-0.04	-0.26	-0.22	-0.18	-0.26	-0.21	-0.02	0.04	-0.06	0.00	-0.12
Q6UX04	Peptidyl-prolyl cis-trans isomerase CWC27 homolog OS=Homo sapiens GN=CWC27 PE=1 SV=1 - [CWC27_HUMAN]	5.51	1	2	2	2	0.34	0.29	0.14	0.09	0.44	0.38	-0.04	-0.09	-0.29	-0.24	-0.18	-0.23	-0.32	-0.51	-0.32	-0.55	-0.35	0.08	0.28	-0.12
Q9P0U4	CXXC-type zinc finger protein 1 OS=Homo sapiens GN=CXXC1 PE=1 SV=2 - [CXXC1_HUMAN]	3.51	1	2	2	4	-0.96	-0.97	-0.25	-0.31	-0.39	-0.45	-0.43	-0.49	-0.08	-0.02	0.00	-0.02	0.77	0.96	-0.12	0.75	0.27	0.60	-0.15	-0.12
P07992	DNA excision repair protein ERCC-1 OS=Homo sapiens GN=ERCC1 PE=1 SV=1 - [ERCC1_HUMAN]	3.03	1	1	1	1	0.12	0.44	-0.02	0.29	-0.23	0.09	-0.20	0.11	0.34	0.03	-0.22	0.09	-0.27	-0.34	-0.20	-0.06	0.08	-0.36	-0.22	-0.12
Q9H0U4	Ras-related protein Rab-1B OS=Homo sapiens GN=RAB1B PE=1 SV=1 - [RAB1B_HUMAN]	71.64	5	4	12	46	0.17	0.08	0.57	0.54	0.15	0.08	0.35	0.18	0.09	0.16	-0.01	0.00	0.23	-0.07	-0.44	0.00	-0.38	0.03	-0.42	-0.12

Q8NC51	Plasminogen activator inhibitor 1 RNA-binding protein OS=Homo sapiens GN=SERBP1 PE=1 SV=2 - [PAIRB_HUMAN]	19.36	1	6	6	14	-1.24	-1.29	-0.82	-0.93	-1.05	-0.90	-1.04	-1.13	-1.13	-0.99	-0.64	-0.73	0.18	0.47	0.02	0.07	-0.22	0.13	-0.13	-0.12
Q07666	KH domain-containing, RNA-binding, signal transduction-associated protein 1 OS=Homo sapiens GN=KHDRBS1 PE=1 SV=1 - [KHDR1_HUMAN]	22.80	2	6	7	16	0.03	-0.05	0.23	0.33	0.17	0.14	-0.20	-0.01	-0.14	-0.21	0.00	-0.04	-0.17	0.06	-0.30	-0.06	-0.37	0.07	-0.21	-0.12
Q5TCZ1	SH3 and PX domain-containing protein 2A OS=Homo sapiens GN=SH3PXD2A PE=1 SV=1 - [SPD2A_HUMAN]	6.53	1	4	4	6	0.39	0.79	0.29	0.69	-0.25	0.14	0.11	0.51	0.25	-0.14	-0.02	0.38	-0.23	-0.40	-0.31	-0.50	-0.40	-0.66	-0.56	-0.12
Q86TI0	TBC1 domain family member 1 OS=Homo sapiens GN=TBC1D1 PE=1 SV=2 - [TBCD1_HUMAN]	6.76	1	6	6	9	0.14	-0.15	0.12	-0.11	0.26	0.19	-0.05	-0.30	-0.18	-0.11	0.12	-0.17	-0.10	-0.01	-0.04	0.03	-0.18	0.29	0.13	-0.12
Q9BT78	COP9 signalosome complex subunit 4 OS=Homo sapiens GN=COPS4 PE=1 SV=1 - [CSN4_HUMAN]	49.01	1	15	15	27	0.35	0.38	0.37	0.49	0.00	0.05	0.18	0.24	0.09	0.12	0.11	0.16	-0.07	-0.31	-0.19	-0.12	-0.13	-0.31	-0.23	-0.12
O75355	Ectonucleoside triphosphate diphosphohydrolase 3 OS=Homo sapiens GN=ENTPD3 PE=1 SV=2 - [ENTP3_HUMAN]	4.54	1	2	2	3	0.79	0.41	0.97	0.60	0.38	0.00	0.78	0.41	-0.20	0.18	0.26	-0.12	0.05	-0.52	-0.71	-0.58	-0.76	-0.42	-0.60	-0.12
Q16777	Histone H2A type 2-C OS=Homo sapiens GN=HIST2H2AC PE=1 SV=4 - [H2A2C_HUMAN]	55.04	3	2	5	71	-1.01	-0.96	-0.74	-0.69	-0.49	-0.45	-0.92	-0.87	-0.54	-0.58	-0.55	-0.51	0.14	0.46	0.18	0.45	0.19	0.50	0.23	-0.12

Q9BV86	N-terminal Xaa-Pro-Lys N-methyltransferase 1 OS=Homo sapiens GN=NTMT1 PE=1 SV=3 - [NTM1A_HUMAN]	29.60	1	4	4	6	1.12	0.65	1.16	0.50	1.07	0.55	0.98	0.13	0.48	1.07	0.53	-0.03	-0.09	-0.93	-1.18	-0.40	-0.35	-0.07	-0.11	-0.12
P62861	40S ribosomal protein S30 OS=Homo sapiens GN=FAU PE=1 SV=1 - [RS30_HUMAN]	16.95	1	1	1	4	-0.94	-1.02	-0.62	-0.70	-0.57	-0.65	-0.81	-0.89	-0.87	-0.79	-0.52	-0.60	0.19	0.43	0.11	0.19	-0.13	0.36	0.04	-0.12
P32121	Beta-arrestin-2 OS=Homo sapiens GN=ARRB2 PE=1 SV=2 - [ARRB2_HUMAN]	4.65	2	1	2	4	1.05	1.30	0.75	1.00	0.19	0.44	0.56	0.82	1.27	1.02	0.24	0.49	-0.43	-0.80	-0.50	0.01	0.31	-0.87	-0.57	-0.12
Q08378	Golgin subfamily A member 3 OS=Homo sapiens GN=GOLGA3 PE=1 SV=2 - [GOGA3_HUMAN]	13.82	1	15	15	30	0.13	0.00	-0.08	-0.16	-0.01	-0.07	-0.28	-0.33	-0.16	0.08	-0.03	-0.18	-0.30	-0.20	0.04	-0.15	0.00	-0.04	-0.01	-0.12
Q8N8A2	Serine/threonine-protein phosphatase 6 regulatory ankyrin repeat subunit B OS=Homo sapiens GN=ANKRD44 PE=1 SV=3 - [ANR44_HUMAN]	7.05	1	5	5	11	0.34	0.38	-0.02	0.02	0.05	0.16	-0.15	-0.10	0.09	0.06	-0.05	-0.06	-0.44	-0.30	0.01	-0.13	0.28	-0.19	0.33	-0.12
Q86TB9	Protein PAT1 homolog 1 OS=Homo sapiens GN=PATL1 PE=1 SV=2 - [PATL1_HUMAN]	3.12	1	2	2	3	0.09	0.19	-0.07	0.03	-0.24	-0.15	-0.25	-0.16	0.07	-0.02	0.07	0.16	-0.29	-0.02	0.13	-0.08	0.08	-0.34	-0.19	-0.12
Q14204	Cytoplasmic dynein 1 heavy chain 1 OS=Homo sapiens GN=DYNC1H1 PE=1 SV=5 - [DYHC1_HUMAN]	43.03	1	176	176	507	0.13	0.14	0.24	0.23	0.08	0.10	0.05	0.05	0.01	0.02	-0.03	-0.03	-0.03	-0.17	-0.26	-0.12	-0.16	-0.07	-0.15	-0.12
P12931	Proto-oncogene tyrosine-protein kinase Src OS=Homo sapiens GN=SRC PE=1 SV=3 - [SRC_HUMAN]	22.57	4	6	11	21	0.75	0.64	0.65	0.68	0.17	0.12	0.41	0.44	0.30	0.32	0.11	0.18	-0.15	-0.58	-0.45	-0.40	-0.26	-0.46	-0.47	-0.12

Q9BSH4	Translational activator of cytochrome c oxidase 1 OS=Homo sapiens GN=TACO1 PE=1 SV=1 - [TACO1_HUMAN]	11.45	1	2	2	2	0.41	0.06	0.25	-0.10	0.77	0.42	0.06	-0.29	-0.26	0.09	0.31	-0.05	-0.29	-0.10	0.06	-0.29	-0.12	0.34	0.51	-0.12
P55087	Aquaporin-4 OS=Homo sapiens GN=AQP4 PE=1 SV=2 - [AQP4_HUMAN]	8.05	1	2	2	10	1.08	1.23	1.70	1.82	0.03	0.24	1.71	1.70	1.04	0.90	1.21	1.44	0.47	-0.12	-0.54	-0.15	-0.57	-1.05	-1.59	-0.12
Q2TAY7	WD40 repeat-containing protein SMU1 OS=Homo sapiens GN=SMU1 PE=1 SV=2 - [SMU1_HUMAN]	8.97	1	3	3	3	-0.25	0.23	-0.02	0.26	-0.31	-0.20	-0.40	-0.15	0.01	-0.31	-0.02	-0.02	-0.10	0.24	-0.01	-0.03	-0.24	-0.08	-0.15	-0.12
P50897	Palmitoyl-protein thioesterase 1 OS=Homo sapiens GN=PPT1 PE=1 SV=1 - [PPT1_HUMAN]	30.72	1	9	9	20	0.59	0.57	0.90	0.70	0.45	0.33	0.72	0.47	0.33	0.43	0.56	0.38	-0.26	-0.49	-0.39	-0.19	-0.25	-0.34	-0.30	-0.12
Q13405	39S ribosomal protein L49, mitochondrial OS=Homo sapiens GN=MRPL49 PE=1 SV=1 - [RM49_HUMAN]	27.71	1	4	4	5	0.41	0.38	0.46	0.43	0.20	0.17	0.30	0.29	0.40	0.37	0.36	0.32	-0.04	-0.05	-0.14	0.02	-0.23	-0.20	-0.27	-0.12
Q9NXG2	THUMP domain-containing protein 1 OS=Homo sapiens GN=THUMPD1 PE=1 SV=2 - [THUM1_HUMAN]	9.07	1	4	4	5	-0.32	-0.47	-0.44	-0.59	-0.26	-0.55	-0.62	-0.87	-0.60	-0.44	-0.69	-0.85	-0.25	-0.11	-0.26	-0.09	0.03	0.05	0.17	-0.12
P56945	Breast cancer anti-estrogen resistance protein 1 OS=Homo sapiens GN=BCAR1 PE=1 SV=2 - [BCAR1_HUMAN]	4.48	1	2	2	3	-0.52	-0.45	-0.46	-0.39	-0.17	-0.11	-0.65	-0.58	-0.25	-0.32	-0.85	-0.79	-0.07	-0.33	-0.40	0.23	0.18	0.33	0.27	-0.13

P07910	Heterogeneous nuclear ribonucleoproteins C1/C2 OS=Homo sapiens GN=HNRNPC PE=1 SV=4 - [HNRPC_HUMAN]	36.93	5	10	12	33	-0.46	-0.54	-0.16	-0.25	-0.28	-0.34	-0.39	-0.43	-0.48	-0.41	-0.22	-0.24	0.22	0.26	0.01	0.17	-0.18	0.21	-0.11	-0.13
P06732	Creatine kinase M-type OS=Homo sapiens GN=CKM PE=1 SV=2 - [KCRM_HUMAN]	5.77	1	1	2	3	0.85	1.28	0.32	0.75	-0.12	0.30	0.13	0.56	-0.02	-0.44	-0.55	-0.13	-0.67	-1.40	-0.87	-1.26	-0.73	-0.99	-0.46	-0.13
P24752	Acetyl-CoA acetyltransferase, mitochondrial OS=Homo sapiens GN=ACAT1 PE=1 SV=1 - [THIL_HUMAN]	53.16	1	19	19	91	-0.30	-0.27	-0.25	-0.16	-0.51	-0.53	-0.41	-0.40	-0.49	-0.49	-0.54	-0.59	-0.05	-0.27	-0.28	-0.26	-0.34	-0.25	-0.33	-0.13
P42765	3-ketoacyl-CoA thiolase, mitochondrial OS=Homo sapiens GN=ACAA2 PE=1 SV=2 - [THIM_HUMAN]	26.70	1	9	9	27	-0.08	-0.09	-0.01	0.01	-0.46	-0.47	-0.17	-0.04	-0.16	-0.29	-0.43	-0.30	-0.09	-0.03	-0.20	-0.37	-0.28	-0.45	-0.42	-0.13
P33240	Cleavage stimulation factor subunit 2 OS=Homo sapiens GN=CSTF2 PE=1 SV=1 - [CSTF2_HUMAN]	11.61	2	5	5	8	-0.05	-0.25	-0.29	-0.50	-0.25	-0.33	-0.43	-0.58	-0.17	-0.02	0.00	-0.15	-0.33	0.06	0.30	0.24	0.43	-0.22	0.03	-0.13
Q9BRJ2	39S ribosomal protein L45, mitochondrial OS=Homo sapiens GN=MRPL45 PE=1 SV=2 - [RM45_HUMAN]	12.42	1	4	4	6	-0.10	0.00	-0.14	0.07	0.00	0.07	-0.36	-0.04	0.12	-0.08	0.04	0.37	0.15	0.56	0.25	0.15	0.09	-0.05	0.10	-0.13
Q9UHX1	Poly(U)-binding-splicing factor PUF60 OS=Homo sapiens GN=PUF60 PE=1 SV=1 - [PUF60_HUMAN]	15.38	1	8	8	15	-0.41	-0.17	-0.09	0.01	-0.57	-0.39	-0.25	-0.23	-0.24	-0.36	-0.47	-0.43	-0.17	-0.11	-0.38	-0.14	-0.20	-0.16	-0.37	-0.13

O43617	Trafficking protein particle complex subunit 3 OS=Homo sapiens GN=TRAPPC3 PE=1 SV=1 - [TPPC3_HUMAN]	20.56	1	4	4	9	0.46	0.38	0.22	0.06	0.05	-0.04	-0.09	-0.14	-0.01	0.02	0.25	0.28	-0.49	-0.17	0.26	-0.31	-0.28	-0.43	-0.11	-0.13
O95989	Diphosphoinositol polyphosphate phosphohydrolase 1 OS=Homo sapiens GN=NUDT3 PE=1 SV=1 - [NUDT3_HUMAN]	32.56	1	4	4	7	0.68	0.58	0.80	0.71	0.11	0.24	0.58	0.55	0.32	0.24	0.37	0.29	-0.10	-0.28	-0.30	-0.23	-0.38	-0.52	-0.73	-0.13
P35052	Glypican-1 OS=Homo sapiens GN=GPC1 PE=1 SV=2 - [GPC1_HUMAN]	36.74	1	14	14	24	-0.34	-0.32	-0.22	-0.28	-0.14	-0.11	-0.42	-0.37	-0.21	-0.28	0.10	0.24	0.01	0.63	0.55	0.18	0.16	0.17	0.18	-0.13
P19784	Casein kinase II subunit alpha' OS=Homo sapiens GN=CSNK2A2 PE=1 SV=1 - [CSK22_HUMAN]	41.14	1	11	12	23	0.31	0.23	0.27	0.15	0.13	0.09	0.14	0.08	0.13	0.17	-0.01	-0.01	-0.16	-0.22	-0.08	-0.07	0.07	-0.14	-0.09	-0.13
P17152	Transmembrane protein 11, mitochondrial OS=Homo sapiens GN=TMEM11 PE=1 SV=1 - [TMM11_HUMAN]	8.85	1	2	2	2	0.30	0.31	0.41	0.43	-0.02	-0.01	0.22	0.23	-0.15	-0.16	0.00	0.01	-0.02	-0.29	-0.41	-0.43	-0.54	-0.33	-0.45	-0.13
O75643	U5 small nuclear ribonucleoprotein 200 kDa helicase OS=Homo sapiens GN=SNRNP200 PE=1 SV=2 - [U520_HUMAN]	20.13	2	35	35	61	-0.01	0.09	0.01	0.03	-0.14	0.00	-0.23	-0.15	-0.20	-0.25	-0.13	-0.09	-0.24	-0.15	-0.07	-0.09	-0.07	-0.11	0.00	-0.13
Q5W0V3	Protein FAM160B1 OS=Homo sapiens GN=FAM160B1 PE=2 SV=1 - [F16B1_HUMAN]	6.93	1	5	5	9	0.13	0.40	0.37	0.46	0.17	0.21	0.12	0.23	0.26	0.27	0.25	0.34	0.06	0.18	-0.08	0.29	0.05	0.06	0.02	-0.13
Q9UBE0	SUMO-activating enzyme subunit 1 OS=Homo sapiens GN=SAE1 PE=1 SV=1 - [SAE1_HUMAN]	35.84	1	11	11	18	0.05	0.00	0.51	0.27	0.47	0.44	0.32	0.29	0.14	0.17	0.00	-0.04	0.21	0.07	-0.30	0.02	-0.34	0.04	-0.38	-0.13

P26641	Elongation factor 1-gamma OS=Homo sapiens GN=EEF1G PE=1 SV=3 - [EF1G_HUMAN]	23.57	1	10	10	19	-0.01	-0.09	0.37	0.42	-0.22	-0.26	0.09	0.10	-0.20	-0.06	-0.21	-0.22	0.11	-0.24	-0.55	-0.10	-0.47	-0.25	-0.50	-0.13
P50542	Peroxisomal targeting signal 1 receptor OS=Homo sapiens GN=PEX5 PE=1 SV=3 - [PEX5_HUMAN]	21.91	1	9	9	13	-0.33	-0.33	-0.20	-0.12	-0.39	-0.29	-0.13	-0.07	-0.23	-0.25	-0.09	-0.26	-0.17	0.19	-0.16	-0.23	-0.28	-0.14	-0.42	-0.13
P53582	Methionine aminopeptidase 1 OS=Homo sapiens GN=METAP1 PE=1 SV=2 - [MAP11_HUMAN]	31.61	1	8	8	24	0.06	-0.40	-0.14	-0.27	0.02	0.06	-0.24	-0.23	0.17	-0.13	-0.43	-0.58	-0.33	-0.10	-0.19	0.31	-0.03	0.20	0.15	-0.13
Q765P7	MTSS1-like protein OS=Homo sapiens GN=MTSS1L PE=1 SV=1 - [MTSSL_HUMAN]	14.73	1	7	8	15	0.16	0.12	0.39	0.48	-0.01	0.05	0.36	0.37	0.13	0.13	0.39	0.41	0.21	0.02	-0.28	-0.04	-0.39	0.01	-0.42	-0.13
Q8TB22	Spermatogenesis-associated protein 20 OS=Homo sapiens GN=SPATA20 PE=2 SV=3 - [SPT20_HUMAN]	12.34	1	8	8	12	-0.03	0.00	0.20	0.19	-0.57	-0.58	-0.07	-0.09	-0.29	-0.35	-0.05	-0.03	0.02	-0.01	-0.16	-0.33	-0.34	-0.65	-0.98	-0.13
Q8WUK0	Phosphatidylglycerophosphatase and protein-tyrosine phosphatase 1 OS=Homo sapiens GN=PTPMT1 PE=1 SV=1 - [PTPM1_HUMAN]	5.97	1	1	1	2	0.13	0.14	0.07	0.08	0.32	0.32	-0.12	-0.12	0.07	0.07	-0.21	-0.21	-0.20	-0.34	-0.29	-0.03	0.03	0.17	0.23	-0.13
Q92844	TRAF family member-associated NF-kappa-B activator OS=Homo sapiens GN=TANK PE=1 SV=2 - [TANK_HUMAN]	6.35	1	2	2	3	-0.25	-0.26	-0.19	-0.20	-0.44	-0.46	-0.39	-0.40	-0.27	-0.25	0.24	0.22	-0.08	0.49	0.43	0.03	-0.03	-0.21	-0.27	-0.13
O00506	Serine/threonine-protein kinase 25 OS=Homo sapiens GN=STK25 PE=1 SV=1 - [STK25_HUMAN]	14.32	1	3	5	9	-0.67	-0.74	-0.07	-0.19	-0.16	-0.31	-0.49	-0.32	-0.15	-0.41	-0.24	-0.04	0.28	0.71	0.18	0.62	0.24	0.41	-0.11	-0.13

Q05048	Cleavage stimulation factor subunit 1 OS=Homo sapiens GN=CSTF1 PE=1 SV=1 - [CSTF1_HUMAN]	4.18	1	1	1	2	-0.13	0.03	-0.46	-0.30	-0.38	-0.23	-0.66	-0.50	-0.25	-0.40	-0.13	0.02	-0.47	0.00	0.32	-0.24	0.09	-0.27	0.06	-0.13
Q8WUG5	Solute carrier family 22 member 17 OS=Homo sapiens GN=SLC22A17 PE=2 SV=1 - [S22AH_HUMAN]	1.86	1	1	1	1	0.18	0.26	0.23	0.31	0.34	0.40	0.03	0.11	0.16	0.09	-0.19	-0.13	-0.09	-0.37	-0.43	-0.06	-0.11	0.14	0.09	-0.14
O15085	Rho guanine nucleotide exchange factor 11 OS=Homo sapiens GN=ARHGEF11 PE=1 SV=1 - [ARHGB_HUMAN]	16.49	1	15	15	23	0.41	0.36	0.19	0.12	0.21	0.34	-0.21	-0.04	0.10	0.12	0.05	0.07	-0.55	-0.57	-0.21	-0.39	0.12	-0.37	0.21	-0.14
Q9Y5Z4	Heme-binding protein 2 OS=Homo sapiens GN=HEBP2 PE=1 SV=1 - [HEBP2_HUMAN]	22.44	1	4	4	9	0.42	0.47	0.64	0.76	0.22	0.36	0.45	0.56	0.35	0.21	0.04	0.11	0.12	-0.32	-0.68	-0.12	-0.47	-0.15	-0.52	-0.14
P27986	Phosphatidylinositol 3-kinase regulatory subunit alpha OS=Homo sapiens GN=PIK3R1 PE=1 SV=2 - [P85A_HUMAN]	16.99	2	8	10	14	0.25	0.37	0.44	0.46	0.15	0.19	0.34	0.22	0.15	0.21	0.07	0.06	0.06	-0.39	-0.36	-0.05	-0.12	-0.11	-0.26	-0.14
Q8N122	Regulatory-associated protein of mTOR OS=Homo sapiens GN=RPTOR PE=1 SV=1 - [RPTOR_HUMAN]	7.04	1	7	7	11	0.11	0.09	0.36	0.01	-0.05	-0.18	-0.07	-0.19	-0.11	-0.22	0.02	-0.07	-0.20	-0.17	-0.16	-0.26	-0.34	-0.37	-0.33	-0.14
Q16850	Lanosterol 14 alpha demethylase OS=Homo sapiens GN=CYP51A1 PE=1 SV=3 - [CP51A_HUMAN]	7.95	1	2	2	2	0.12	-0.23	0.56	0.15	-0.16	0.00	-0.07	-0.05	0.10	-0.08	0.19	0.44	0.23	0.67	0.29	0.37	0.00	0.21	-0.16	-0.14
Q15048	Leucine-rich repeat-containing protein 14 OS=Homo sapiens GN=LRRC14 PE=2 SV=1 - [LRC14_HUMAN]	1.42	1	1	1	1	0.32	0.11	0.54	0.34	0.82	0.61	0.34	0.13	0.37	0.58	0.35	0.14	0.08	0.04	-0.19	0.29	0.07	0.49	0.26	-0.14

Q9Y3D6	Mitochondrial fission 1 protein OS=Homo sapiens GN=FIS1 PE=1 SV=2 - [FIS1_HUMAN]	32.24	1	5	5	15	0.05	-0.07	0.45	0.28	-0.28	-0.34	0.20	-0.02	-0.28	-0.14	-0.13	-0.28	0.07	-0.08	-0.52	-0.07	-0.51	-0.32	-0.80	-0.14
Q9H269	Vacuolar protein sorting-associated protein 16 homolog OS=Homo sapiens GN=VPS16 PE=1 SV=2 - [VPS16_HUMAN]	16.33	1	9	9	17	-0.08	0.14	0.25	0.14	0.04	-0.04	-0.21	-0.16	0.02	0.13	0.13	0.02	-0.21	0.06	0.01	-0.21	-0.05	-0.21	0.04	-0.14
O95278	Laforin OS=Homo sapiens GN=EPM2A PE=1 SV=2 - [EPM2A_HUMAN]	6.34	1	2	2	2	0.31	0.21	0.31	0.21	0.00	-0.11	0.10	0.01	-0.13	-0.03	0.45	0.39	-0.15	-0.29	-0.40	-0.30	-0.30	-0.33	-0.33	-0.14
P12956	X-ray repair cross-complementing protein 6 OS=Homo sapiens GN=XRCC6 PE=1 SV=2 - [XRCC6_HUMAN]	37.27	1	19	19	52	-0.03	-0.06	0.16	0.18	0.02	-0.02	0.03	0.04	-0.21	-0.16	-0.22	-0.22	0.16	-0.07	-0.43	-0.12	-0.33	0.03	-0.16	-0.14
Q8IX12	Cell division cycle and apoptosis regulator protein 1 OS=Homo sapiens GN=CCAR1 PE=1 SV=2 - [CCAR1_HUMAN]	9.22	1	9	9	12	-0.38	0.04	-0.10	0.05	-0.25	-0.12	-0.27	-0.25	-0.28	-0.49	0.02	0.09	-0.28	0.33	0.18	-0.13	-0.06	-0.17	-0.18	-0.14
P09211	Glutathione S transferase P OS=Homo sapiens GN=GSTP1 PE=1 SV=2 - [GSTP1_HUMAN]	63.81	1	11	11	47	-0.42	-0.20	0.11	0.20	-0.23	-0.17	-0.06	0.02	-0.23	-0.36	-0.36	-0.33	0.42	-0.09	-0.45	0.03	-0.25	-0.04	-0.28	-0.14
Q9P227	Rho GTPase-activating protein 23 OS=Homo sapiens GN=ARHGAP23 PE=1 SV=2 - [RHG23_HUMAN]	5.90	1	6	6	10	0.66	0.52	0.46	0.38	0.02	-0.03	0.26	0.05	0.00	0.09	-0.05	-0.29	-0.25	-0.73	-0.66	-0.64	-0.34	-0.58	-0.40	-0.14

Q8NBU5	ATPase family AAA domain-containing protein 1 OS=Homo sapiens GN=ATAD1 PE=1 SV=1 - [ATAD1_HUMAN]	29.36	1	8	8	14	0.17	0.10	0.32	0.28	0.05	-0.07	0.21	0.13	-0.16	0.01	-0.05	-0.16	0.04	-0.27	-0.46	-0.10	-0.39	-0.12	-0.32	-0.14
Q9UBW8	COP9 signalosome complex subunit 7a OS=Homo sapiens GN=COPS7A PE=1 SV=1 - [CSN7A_HUMAN]	29.09	1	7	7	9	0.49	0.56	0.51	0.69	0.03	-0.04	0.35	0.44	0.23	0.20	0.00	-0.09	-0.14	-0.47	-0.50	-0.46	-0.29	-0.45	-0.43	-0.14
P30414	NK-tumor recognition protein OS=Homo sapiens GN=NKTR PE=1 SV=2 - [NKTR_HUMAN]	0.48	1	1	1	1	-0.25	-0.23	-0.07	-0.05	0.26	0.27	-0.28	-0.26	0.10	0.09	-0.06	-0.05	0.03	0.19	0.01	0.37	0.20	0.49	0.31	-0.14
Q8N511	Transmembrane protein 199 OS=Homo sapiens GN=TMEM199 PE=1 SV=1 - [TM199_HUMAN]	5.77	1	1	1	1	0.20	0.04	0.20	0.03	0.41	0.24	-0.01	-0.18	0.17	0.34	0.25	0.08	-0.16	0.05	0.05	0.16	0.18	0.18	0.19	-0.14
Q9BXP5	Serrate RNA effector molecule homolog OS=Homo sapiens GN=SRRT PE=1 SV=1 - [SRRT_HUMAN]	12.21	1	9	9	15	-0.27	-0.05	-0.31	0.06	-0.14	-0.06	-0.20	-0.27	-0.19	-0.33	-0.33	-0.27	-0.17	-0.08	-0.08	0.02	0.03	0.21	0.17	-0.14
Q58A45	PAB-dependent poly(A)-specific ribonuclease subunit PAN3 OS=Homo sapiens GN=PAN3 PE=1 SV=3 - [PAN3_HUMAN]	2.93	1	1	1	1	-1.85	-1.97	-1.38	-1.50	-1.09	-1.22	-1.59	-1.71	-1.70	-1.57	-1.57	-1.70	0.32	0.28	-0.19	0.31	-0.16	0.75	0.28	-0.14
Q5VSL9	Striatin-interacting protein 1 OS=Homo sapiens GN=STRIP1 PE=1 SV=1 - [STRP1_HUMAN]	12.43	1	7	7	15	0.05	0.28	-0.09	0.09	0.18	0.11	-0.23	-0.02	-0.07	-0.24	0.14	0.06	-0.13	-0.19	-0.27	0.00	-0.22	-0.29	0.01	-0.14
P46783	40S ribosomal protein S10 OS=Homo sapiens GN=RPS10 PE=1 SV=1 - [RS10_HUMAN]	23.03	2	3	3	10	-0.43	-0.54	-0.40	-0.37	-0.26	-0.36	-0.59	-0.70	-0.77	-0.73	-0.30	-0.38	-0.25	-0.18	0.17	-0.04	-0.24	0.21	0.18	-0.14

Q9C0D3	Protein zyg-11 homolog B OS=Homo sapiens GN=ZYG11B PE=1 SV=2 - [ZY11B_HUMAN]	3.23	1	2	2	2	0.95	0.91	0.74	0.71	0.48	0.44	0.53	0.50	0.18	0.23	0.00	-0.04	-0.36	-0.94	-0.75	-0.69	-0.48	-0.49	-0.28	-0.14
Q6PML9	Zinc transporter 9 OS=Homo sapiens GN=SLC30A9 PE=1 SV=1 - [ZNT9_HUMAN]	11.97	1	6	6	12	0.77	0.83	0.68	0.78	0.20	0.17	0.22	0.34	0.18	0.12	0.46	0.40	-0.23	-0.04	-0.12	-0.62	-0.56	-0.64	-0.35	-0.14
P69892	Hemoglobin subunit gamma-2 OS=Homo sapiens GN=HBG2 PE=1 SV=2 - [HBG2_HUMAN]	73.47	1	1	10	44	-0.99	-0.88	-0.91	-0.80	1.05	1.15	-1.12	-1.01	0.36	0.26	-0.23	-0.12	-0.07	0.77	0.68	1.28	1.20	2.02	1.94	-0.14
Q86SZ2	Trafficking protein particle complex subunit 6B OS=Homo sapiens GN=TRAPPC6B PE=1 SV=1 - [TPC6B_HUMAN]	39.24	1	7	7	9	-0.08	0.03	-0.06	0.07	0.04	0.19	-0.28	-0.13	0.15	-0.05	0.14	0.19	-0.06	0.30	0.21	0.21	-0.01	0.15	0.13	-0.14
O00192	Armadillo repeat protein deleted in velo-cardio-facial syndrome OS=Homo sapiens GN=ARVCF PE=1 SV=1 - [ARVC_HUMAN]	16.84	1	11	11	22	0.30	0.50	0.30	0.50	0.06	0.10	0.03	0.15	0.17	0.00	-0.09	0.05	-0.28	-0.49	-0.45	-0.20	-0.22	-0.30	-0.15	-0.15
P63165	Small ubiquitin-related modifier 1 OS=Homo sapiens GN=SUMO1 PE=1 SV=1 - [SUMO1_HUMAN]	15.84	1	2	2	3	0.60	0.52	0.36	0.27	0.10	0.01	0.15	0.06	-0.05	0.04	-0.21	-0.30	-0.40	-0.81	-0.57	-0.53	-0.29	-0.52	-0.27	-0.15
Q9UNK0	Syntaxin-8 OS=Homo sapiens GN=STX8 PE=1 SV=2 - [STX8_HUMAN]	21.61	1	4	4	8	0.60	0.61	0.40	0.45	0.31	0.25	0.31	0.21	0.22	0.33	0.39	0.35	-0.33	-0.26	-0.01	-0.32	-0.14	-0.37	-0.11	-0.15
P30520	Adenylosuccinate synthetase isozyme 2 OS=Homo sapiens GN=ADSS PE=1 SV=3 - [PURA2_HUMAN]	32.02	1	10	11	20	0.54	0.69	0.72	0.73	0.28	0.44	0.48	0.56	0.36	0.25	0.03	0.01	-0.26	-0.70	-0.85	-0.39	-0.33	-0.40	-0.38	-0.15

P84103	Serine/arginine-rich splicing factor 3 OS=Homo sapiens GN=SRSF3 PE=1 SV=1 - [SRSF3_HUMAN]	24.39	1	3	4	19	-0.05	-0.13	0.27	0.28	-0.15	-0.15	0.07	0.01	-0.10	-0.11	-0.09	-0.08	0.05	-0.09	-0.32	-0.10	-0.27	-0.22	-0.39	-0.15
P82663	28S ribosomal protein S25, mitochondrial OS=Homo sapiens GN=MRPS25 PE=1 SV=1 - [RT25_HUMAN]	23.70	1	3	3	4	0.02	0.09	-0.03	0.04	-0.75	-0.68	-0.25	-0.17	0.22	0.15	-0.22	-0.15	-0.21	-0.23	-0.19	0.16	0.22	-0.79	-0.73	-0.15
Q96GK7	Fumarylacetoacetate hydrolase domain-containing protein 2A OS=Homo sapiens GN=FAHD2A PE=1 SV=1 - [FAH2A_HUMAN]	42.36	2	9	9	17	0.28	0.40	0.29	0.31	-0.47	-0.47	0.03	0.08	-0.17	-0.25	0.05	0.12	-0.31	-0.20	-0.49	-0.47	-0.30	-0.71	-0.65	-0.15
P52630	Signal transducer and activator of transcription 2 OS=Homo sapiens GN=STAT2 PE=1 SV=1 - [STAT2_HUMAN]	10.81	1	6	7	16	-0.38	-0.12	-0.35	-0.01	-0.10	-0.05	-0.49	-0.37	-0.25	-0.34	-0.17	-0.13	-0.12	0.03	0.14	-0.04	0.03	0.11	-0.11	-0.15
Q8IZD0	Sterile alpha motif domain-containing protein 14 OS=Homo sapiens GN=SAMD14 PE=2 SV=2 - [SAM14_HUMAN]	4.80	1	1	1	1	-0.50	-0.31	-0.21	-0.01	-0.52	-0.34	-0.42	-0.23	-0.31	-0.49	-0.38	-0.19	0.14	0.13	-0.18	0.04	-0.26	-0.03	-0.33	-0.15
P08107	Heat shock 70 kDa protein 1A/1B OS=Homo sapiens GN=HSPA1A PE=1 SV=5 - [HSP71_HUMAN]	65.52	2	18	32	210	-0.27	-0.31	-0.13	-0.13	-0.74	-0.82	-0.26	-0.39	-0.51	-0.50	-0.31	-0.33	-0.08	0.04	-0.15	-0.16	-0.33	-0.37	-0.68	-0.15
Q13427	Peptidyl-prolyl cis-trans isomerase G OS=Homo sapiens GN=PPIG PE=1 SV=2 - [PPIG_HUMAN]	6.10	1	4	4	7	0.24	-0.05	0.61	0.09	0.09	-0.17	0.34	-0.11	-0.28	-0.10	0.32	-0.18	-0.01	-0.12	-0.17	-0.32	-0.38	-0.17	-0.40	-0.15

Q8NEY1	Neuron navigator 1 OS=Homo sapiens GN=NAV1 PE=1 SV=2 - [NAV1_HUMAN]	1.44	3	2	2	2	0.69	0.91	0.28	0.51	0.51	0.73	0.07	0.29	0.73	0.51	0.19	0.41	-0.56	-0.49	-0.09	-0.15	0.26	-0.19	0.21	-0.15
Q9NPA0	ER membrane protein complex subunit 7 OS=Homo sapiens GN=EMC7 PE=1 SV=1 - [EMC7_HUMAN]	22.31	1	3	3	7	1.17	0.65	0.17	-0.35	0.01	-0.52	-0.05	-0.57	-0.45	0.07	0.41	-0.12	-1.16	-0.76	0.24	-1.06	-0.06	-1.18	-0.18	-0.15
Q9GZL7	Ribosome biogenesis protein WDR12 OS=Homo sapiens GN=WDR12 PE=1 SV=2 - [WDR12_HUMAN]	4.96	1	1	1	2	1.00	0.85	0.44	0.30	0.41	0.26	0.23	0.09	-0.03	0.12	0.49	0.34	-0.71	-0.50	0.05	-0.84	-0.29	-0.61	-0.05	-0.15
P18754	Regulator of chromosome condensation OS=Homo sapiens GN=RCC1 PE=1 SV=1 - [RCC1_HUMAN]	19.48	1	6	6	8	0.33	0.32	0.24	0.23	-0.17	-0.20	0.03	0.01	-0.23	-0.21	-0.36	-0.38	-0.25	-0.69	-0.60	-0.51	-0.42	-0.52	-0.44	-0.15
Q14195	Dihydropyrimidinase-related protein 3 OS=Homo sapiens GN=DPYSL3 PE=1 SV=1 - [DPYL3_HUMAN]	54.56	1	18	21	135	-0.16	-0.08	0.19	0.10	-0.24	-0.26	-0.05	-0.04	-0.29	-0.19	-0.39	-0.41	0.08	-0.40	-0.69	-0.10	-0.53	-0.19	-0.51	-0.15
P27694	Replication protein A 70 kDa DNA-binding subunit OS=Homo sapiens GN=RPA1 PE=1 SV=2 - [RFA1_HUMAN]	26.62	1	12	12	23	-0.13	-0.07	0.15	0.14	0.16	0.13	0.03	0.00	0.09	0.03	-0.12	-0.19	0.14	-0.16	-0.43	0.02	-0.14	0.13	0.06	-0.15
Q9UBX3	Mitochondrial dicarboxylate carrier OS=Homo sapiens GN=SLC25A10 PE=1 SV=2 - [DIC_HUMAN]	6.62	1	2	2	3	1.22	1.22	0.76	0.77	0.74	0.74	0.54	0.55	-0.15	-0.15	0.29	0.30	-0.62	-0.92	-0.46	-1.34	-0.88	-0.49	-0.03	-0.15

O43924	Retinal rod rhodopsin-sensitive cGMP 3',5'-cyclic phosphodiesterase subunit delta OS=Homo sapiens GN=PDE6D PE=1 SV=1 - [PDE6D_HUMAN]	8.00	1	1	1	3	0.51	0.22	0.55	0.26	0.20	-0.09	0.33	0.05	-0.06	0.23	0.48	0.19	-0.12	-0.02	-0.07	-0.25	-0.29	-0.33	-0.37	-0.15
P48637	Glutathione synthetase OS=Homo sapiens GN=GSS PE=1 SV=1 - [GSHB_HUMAN]	51.48	1	19	19	34	0.37	0.42	0.54	0.58	0.18	0.09	0.12	0.15	0.04	0.11	-0.19	-0.21	-0.23	-0.71	-0.78	-0.18	-0.29	-0.47	-0.30	-0.15
P08238	Heat shock protein HSP 90-beta OS=Homo sapiens GN=HSP90AB1 PE=1 SV=4 - [HS90B_HUMAN]	45.99	4	14	31	374	0.13	0.14	0.37	0.36	-0.10	-0.13	0.15	0.09	-0.07	-0.09	-0.27	-0.21	-0.04	-0.44	-0.53	-0.18	-0.32	-0.32	-0.45	-0.15
P62269	40S ribosomal protein S18 OS=Homo sapiens GN=RPS18 PE=1 SV=3 - [RS18_HUMAN]	32.89	1	6	6	16	-0.71	-0.68	-0.48	-0.45	-0.57	-0.61	-0.58	-0.62	-0.58	-0.63	-0.31	-0.39	-0.02	0.30	0.06	0.13	-0.04	0.01	-0.04	-0.15
Q96AX1	Vacuolar protein sorting-associated protein 33A OS=Homo sapiens GN=VPS33A PE=1 SV=1 - [VP33A_HUMAN]	16.44	1	9	9	14	0.23	0.39	0.31	0.42	0.03	0.22	0.08	0.20	0.29	0.06	0.05	0.14	-0.14	-0.28	-0.26	-0.14	-0.12	-0.18	-0.29	-0.15
Q969Z0	Protein TBRG4 OS=Homo sapiens GN=TBRG4 PE=1 SV=1 - [TBRG4_HUMAN]	3.33	1	1	1	6	-0.67	-0.88	-0.18	-0.40	-0.84	-1.06	-0.40	-0.61	-0.83	-0.61	-0.37	-0.59	0.32	0.30	-0.19	0.09	-0.40	-0.19	-0.68	-0.15
Q9H000	Probable E3 ubiquitin-protein ligase makorin-2 OS=Homo sapiens GN=MKRN2 PE=1 SV=2 - [MKRN2_HUMAN]	9.13	1	4	4	5	-0.09	0.04	0.07	0.09	-0.23	-0.23	-0.15	-0.11	-0.16	-0.26	-0.16	-0.08	-0.17	-0.19	-0.24	-0.13	-0.21	-0.32	-0.30	-0.15
Q13310	Polyadenylate-binding protein 4 OS=Homo sapiens GN=PABPC4 PE=1 SV=1 - [PABP4_HUMAN]	18.79	1	3	9	19	-0.52	-0.45	-0.15	-0.12	-0.30	-0.18	-0.35	-0.27	-0.17	-0.26	-0.33	-0.34	0.27	0.27	-0.25	0.14	-0.10	0.27	-0.04	-0.15

Q8N5N7	39S ribosomal protein L50, mitochondrial OS=Homo sapiens GN=MRPL50 PE=1 SV=2 - [RM50_HUMAN]	20.89	1	2	2	4	0.31	0.60	0.47	0.26	0.18	0.30	-0.40	-0.22	-0.07	-0.19	0.74	0.75	-0.50	0.19	0.33	-0.18	-0.25	-0.09	0.01	-0.15
Q15768	Ephrin-B3 OS=Homo sapiens GN=EFNB3 PE=1 SV=1 - [EFNB3_HUMAN]	4.12	1	1	1	1	0.17	-0.19	-0.21	-0.57	0.89	0.53	-0.43	-0.79	1.30	1.67	0.82	0.46	-0.54	0.66	1.03	1.53	1.91	0.71	1.09	-0.16
Q12905	Interleukin enhancer-binding factor 2 OS=Homo sapiens GN=ILF2 PE=1 SV=2 - [ILF2_HUMAN]	37.69	1	11	11	24	-0.61	-0.67	-0.08	-0.23	-0.15	-0.35	-0.32	-0.52	-0.48	-0.43	-0.07	-0.21	0.09	0.47	0.04	0.30	-0.08	0.38	0.01	-0.16
Q9BWJ5	Splicing factor 3B subunit 5 OS=Homo sapiens GN=SF3B5 PE=1 SV=1 - [SF3B5_HUMAN]	43.02	1	3	3	5	-0.23	-0.30	-0.24	0.01	-0.18	0.07	-0.26	-0.18	-0.22	-0.47	-0.01	-0.18	0.30	0.27	-0.24	-0.37	-0.20	-0.12	0.05	-0.16
Q92499	ATP-dependent RNA helicase DDX1 OS=Homo sapiens GN=DDX1 PE=1 SV=2 - [DDX1_HUMAN]	43.78	1	25	26	54	0.02	0.08	0.18	0.17	0.00	0.05	-0.04	-0.02	-0.16	-0.12	-0.04	-0.06	0.04	0.01	-0.26	-0.07	-0.36	0.01	-0.14	-0.16
Q12824	SWI/SNF-related matrix associated actin-dependent regulator of chromatin subfamily B member 1 OS=Homo sapiens GN=SMARCB1 PE=1 SV=2 - [SNF5_HUMAN]	3.38	1	1	1	1	-0.45	-0.58	-0.60	-0.73	-0.43	-0.56	-0.82	-0.95	-0.76	-0.62	-0.24	-0.37	-0.32	0.22	0.36	-0.14	0.01	0.00	0.15	-0.16
O43166	Signal-induced proliferation-associated 1-like protein 1 OS=Homo sapiens GN=SIPA1L1 PE=1 SV=4 - [SI1L1_HUMAN]	5.43	3	7	7	10	0.85	0.80	0.26	-0.04	-0.02	-0.30	0.14	-0.16	-0.30	0.01	-0.12	-0.40	-0.65	-0.43	-0.51	-0.81	-0.42	-0.74	-0.30	-0.16

Q96PX8	SLIT and NTRK-like protein 1 OS=Homo sapiens GN=SLITRK1 PE=1 SV=2 - [SLIK1_HUMAN]	5.60	1	2	2	2	0.09	0.34	-0.32	-0.07	-0.08	0.16	-0.54	-0.29	-0.25	-0.50	-0.15	0.09	-0.57	-0.24	0.16	-0.56	-0.15	-0.19	0.22	-0.16
Q12829	Ras-related protein Rab-40B OS=Homo sapiens GN=RAB40B PE=2 SV=1 - [RB40B_HUMAN]	6.12	1	1	1	1	2.42	2.16	1.08	0.83	0.66	0.40	0.86	0.60	0.11	0.37	0.94	0.68	-1.51	-1.48	-0.14	-2.02	-0.67	-1.78	-0.44	-0.16
P21912	Succinate dehydrogenase [ubiquinone] iron-sulfur subunit, mitochondrial OS=Homo sapiens GN=SDHB PE=1 SV=3 - [SDHB_HUMAN]	32.14	1	9	9	37	0.20	0.20	0.44	0.54	-0.25	-0.19	0.25	0.24	0.05	-0.01	-0.10	-0.01	-0.06	-0.33	-0.51	-0.16	-0.37	-0.50	-0.63	-0.16
O00258	Tail-anchored protein insertion receptor WRB OS=Homo sapiens GN=WRB PE=1 SV=2 - [WRB_HUMAN]	12.07	1	2	2	3	0.77	0.65	0.82	0.70	1.40	1.27	0.60	0.48	0.93	1.06	0.34	0.21	-0.12	-0.43	-0.49	0.32	0.27	0.61	0.56	-0.16
P30622	CAP-Gly domain-containing linker protein 1 OS=Homo sapiens GN=CLIP1 PE=1 SV=2 - [CLIP1_HUMAN]	23.44	1	25	30	56	-0.01	0.16	0.19	0.24	-0.07	0.08	-0.14	-0.04	-0.19	-0.31	-0.51	-0.54	-0.14	-0.55	-0.72	-0.33	-0.35	-0.28	-0.30	-0.16
Q15435	Protein phosphatase 1 regulatory subunit 7 OS=Homo sapiens GN=PPP1R7 PE=1 SV=1 - [PP1R7_HUMAN]	40.00	1	12	12	31	0.08	0.12	0.11	-0.04	0.05	0.05	-0.08	-0.13	-0.18	-0.16	-0.36	-0.41	-0.11	-0.33	-0.31	-0.09	-0.20	-0.08	-0.13	-0.16
Q9C0C6	CLOCK-interacting pacemaker OS=Homo sapiens GN=CIPC PE=2 SV=2 - [CIPC_HUMAN]	2.76	1	1	1	1	-0.01	-0.08	0.07	0.00	0.21	0.14	-0.16	-0.22	-0.16	-0.08	-0.05	-0.12	-0.09	-0.03	-0.12	-0.05	-0.12	0.20	0.12	-0.16

Q96CS3	FAS-associated factor 2 OS=Homo sapiens GN=FAF2 PE=1 SV=2 - [FAF2_HUMAN]	22.47	1	6	6	15	-0.09	-0.46	0.18	-0.12	-0.10	-0.16	-0.14	-0.22	-0.06	0.21	0.50	0.35	0.23	0.59	0.31	0.47	0.08	0.53	-0.01	-0.16
Q15907	Ras-related protein Rab-11B OS=Homo sapiens GN=RAB11B PE=1 SV=4 - [RB11B_HUMAN]	55.50	3	11	11	32	0.36	0.40	0.33	0.38	0.45	0.46	0.15	0.20	0.26	0.14	0.20	0.23	-0.11	-0.13	-0.14	-0.11	-0.13	0.11	0.13	-0.16
P31040	Succinate dehydrogenase [ubiquinone] flavoprotein subunit, mitochondrial OS=Homo sapiens GN=SDHA PE=1 SV=2 - [SDHA_HUMAN]	48.49	1	21	21	156	0.30	0.33	0.44	0.50	0.05	0.02	0.25	0.32	0.18	0.08	0.15	0.21	-0.03	-0.23	-0.30	-0.20	-0.36	-0.27	-0.41	-0.16
Q9ULB1	Neurexin-1 OS=Homo sapiens GN=NRXN1 PE=2 SV=1 - [NRX1A_HUMAN]	21.87	2	20	26	43	1.03	1.10	1.24	1.11	0.21	0.21	0.83	0.78	0.62	0.48	0.69	0.69	-0.39	-0.34	-0.41	-0.38	-0.40	-0.82	-0.70	-0.16
Q7Z6K5	Arpin OS=Homo sapiens GN=ARPIN PE=1 SV=1 - [ARPIN_HUMAN]	28.32	1	4	4	10	0.38	0.46	0.47	0.57	-0.03	0.17	0.30	0.41	0.24	-0.01	-0.14	-0.02	-0.21	-0.42	-0.59	-0.18	-0.32	-0.30	-0.41	-0.16
Q9NP77	RNA polymerase II subunit A C-terminal domain phosphatase SSU72 OS=Homo sapiens GN=SSU72 PE=1 SV=1 - [SSU72_HUMAN]	19.59	1	3	3	3	0.00	0.03	-0.12	-0.08	-0.23	-0.20	-0.34	-0.31	-0.24	-0.26	-0.45	-0.43	-0.28	-0.45	-0.34	-0.23	-0.11	-0.24	-0.13	-0.16
Q9BZC7	ATP-binding cassette sub-family A member 2 OS=Homo sapiens GN=ABCA2 PE=1 SV=3 - [ABCA2_HUMAN]	3.20	1	5	5	7	0.65	0.55	0.51	0.43	0.19	0.09	0.36	0.28	-0.14	-0.04	-0.26	-0.36	-0.34	-0.84	-0.58	-0.66	-0.40	-0.47	-0.28	-0.16
Q13428	Treacle protein OS=Homo sapiens GN=TCOF1 PE=1 SV=3 - [TCOF_HUMAN]	9.61	1	12	12	16	-0.56	-0.86	-0.31	-0.46	-0.28	-0.68	-0.58	-0.77	-0.78	-0.46	0.13	-0.15	0.10	0.43	0.16	0.32	-0.02	0.31	0.02	-0.16

Q9H0R3	Transmembrane protein 222 OS=Homo sapiens GN=TMEM222 PE=1 SV=2 - [TM222_HUMAN]	28.85	1	3	3	6	-0.19	-0.40	0.16	0.24	-0.43	-0.65	-0.06	-0.17	-0.41	-0.26	-0.34	-0.69	0.12	-0.28	-0.24	0.32	-0.52	-0.26	-0.66	-0.16
Q9UPQ3	Arf-GAP with GTPase, ANK repeat and PH domain-containing protein 1 OS=Homo sapiens GN=AGAP1 PE=1 SV=4 - [AGAP1_HUMAN]	7.35	1	2	4	5	0.44	0.63	0.43	0.63	-0.08	0.11	0.21	0.41	0.47	0.28	0.10	0.29	-0.17	-0.33	-0.34	-0.12	-0.12	-0.53	-0.53	-0.16
O75420	PERQ amino acid-rich with GYF domain-containing protein 1 OS=Homo sapiens GN=GIGYF1 PE=1 SV=2 - [PERQ1_HUMAN]	1.06	1	1	1	1	0.41	0.16	0.24	-0.01	0.55	0.29	0.01	-0.24	-0.08	0.17	-0.22	-0.48	-0.34	-0.62	-0.46	-0.20	-0.03	0.12	0.29	-0.16
Q9GZU7	Carboxy-terminal domain RNA polymerase II polypeptide A small phosphatase 1 OS=Homo sapiens GN=CTDSP1 PE=1 SV=1 - [CTDS1_HUMAN]	12.64	1	2	2	4	-0.21	-0.02	0.16	0.29	-0.11	0.02	-0.07	0.07	0.23	0.07	0.39	0.52	0.09	0.60	0.23	0.28	-0.06	0.13	-0.28	-0.16
Q14694	Ubiquitin carboxyl-terminal hydrolase 10 OS=Homo sapiens GN=USP10 PE=1 SV=2 - [UBP10_HUMAN]	8.77	1	5	5	7	0.49	0.59	0.31	0.40	-0.08	0.01	0.08	0.17	-0.04	-0.12	0.09	0.18	-0.36	-0.40	-0.22	-0.59	-0.40	-0.59	-0.41	-0.16
Q9Y312	Protein AAR2 homolog OS=Homo sapiens GN=AAR2 PE=1 SV=2 - [AAR2_HUMAN]	5.21	1	1	1	1	-0.38	0.08	-0.61	-0.15	0.69	1.15	-0.83	-0.38	1.14	0.69	0.77	1.22	-0.40	1.15	1.38	1.10	1.33	1.06	1.28	-0.16
Q7L014	Probable ATP-dependent RNA helicase DDX46 OS=Homo sapiens GN=DDX46 PE=1 SV=2 - [DDX46_HUMAN]	17.85	1	15	15	22	0.10	0.14	0.21	0.23	0.12	0.24	0.07	0.10	-0.10	0.03	-0.15	-0.11	-0.07	-0.23	-0.29	-0.18	-0.24	-0.13	-0.10	-0.16

P13987	CD59 glycoprotein OS=Homo sapiens GN=CD59 PE=1 SV=1 - [CD59_HUMAN]	25.00	1	4	4	36	0.22	0.31	0.36	0.49	0.13	0.20	0.08	0.25	0.15	0.01	0.22	0.33	-0.06	0.00	-0.08	-0.12	-0.25	-0.14	-0.18	-0.16
Q6BDS2	UHRF1-binding protein 1 OS=Homo sapiens GN=UHRF1BP1 PE=1 SV=1 - [URFB1_HUMAN]	1.60	1	1	2	2	-0.47	-0.50	-0.16	-0.19	-0.62	-0.66	-0.39	-0.42	-0.38	-0.34	-0.16	-0.20	0.13	0.31	0.00	0.16	-0.15	-0.17	-0.48	-0.17
P06753	Tropomyosin alpha-3 chain OS=Homo sapiens GN=TPM3 PE=1 SV=2 - [TPM3_HUMAN]	50.18	1	6	17	175	0.12	0.38	0.17	0.25	0.11	0.25	0.05	0.17	0.13	0.04	0.16	0.22	-0.20	-0.24	-0.11	-0.25	-0.34	-0.15	-0.23	-0.17
P46199	Translation initiation factor IF-2, mitochondrial OS=Homo sapiens GN=MTIF2 PE=1 SV=2 - [IF2M_HUMAN]	3.99	1	2	2	3	-0.03	-0.14	-0.20	-0.31	-0.80	-0.91	-0.62	-0.73	-0.85	-0.74	-0.61	-0.72	0.13	-0.05	-0.35	-0.22	-0.52	-0.79	-0.61	-0.17
P22234	Multifunctional protein ADE2 OS=Homo sapiens GN=PAICS PE=1 SV=3 - [PUR6_HUMAN]	25.88	1	9	9	22	0.52	0.27	0.33	0.52	-0.25	-0.33	0.22	0.30	0.04	-0.04	-0.29	-0.30	-0.15	-0.74	-0.61	-0.65	-0.58	-0.76	-0.69	-0.17
Q7L311	Armadillo repeat-containing X-linked protein 2 OS=Homo sapiens GN=ARMCX2 PE=2 SV=1 - [ARMX2_HUMAN]	3.48	1	2	2	3	-0.58	-0.08	-0.29	0.21	-0.68	-0.19	-0.52	-0.02	-0.38	-0.88	-0.59	-0.10	0.11	-0.01	-0.30	-0.27	-0.56	-0.12	-0.41	-0.17
P27338	Amine oxidase [flavin-containing] B OS=Homo sapiens GN=MAOB PE=1 SV=3 - [AOFB_HUMAN]	42.88	1	16	20	50	1.43	1.45	1.54	1.40	0.42	0.24	1.23	1.22	0.85	0.91	0.71	0.56	-0.18	-0.86	-0.87	-0.54	-0.54	-1.20	-1.16	-0.17
O15118	Niemann-Pick C1 protein OS=Homo sapiens GN=NPC1 PE=1 SV=2 - [NPC1_HUMAN]	1.33	1	2	2	2	0.32	0.41	0.23	0.33	0.56	0.65	0.00	0.10	0.60	0.51	0.58	0.67	-0.26	0.26	0.34	0.22	0.31	0.22	0.31	-0.17

P49840	Glycogen synthase kinase-3 alpha OS=Homo sapiens GN=GSK3A PE=1 SV=2 - [GSK3A_HUMAN]	24.64	1	5	8	14	0.35	0.78	0.06	0.77	-0.66	-0.23	-0.12	0.27	0.17	-0.31	-0.19	-0.20	-0.19	-0.76	-0.75	-0.31	-0.30	-0.56	-0.55	-0.17
P49768	Presenilin-1 OS=Homo sapiens GN=PSEN1 PE=1 SV=1 - [PSN1_HUMAN]	6.00	2	2	2	4	-0.13	0.01	0.04	0.17	-0.11	0.01	-0.19	-0.06	0.09	-0.04	-0.12	0.01	-0.01	0.02	-0.16	0.12	-0.04	0.00	-0.17	-0.17
P25208	Nuclear transcription factor Y subunit beta OS=Homo sapiens GN=NFYB PE=1 SV=2 - [NFYB_HUMAN]	6.28	1	1	1	3	0.24	0.00	0.19	-0.05	-0.03	-0.27	-0.05	-0.28	-0.49	-0.25	0.07	-0.17	-0.23	-0.16	-0.12	-0.46	-0.40	-0.28	-0.23	-0.17
Q9NRH2	SNF-related serine/threonine-protein kinase OS=Homo sapiens GN=SNRK PE=1 SV=2 - [SNRK_HUMAN]	9.02	1	3	3	7	0.28	0.33	0.59	0.56	0.40	-0.40	0.36	0.02	0.14	0.16	0.52	0.14	-0.38	-0.02	-0.13	-0.17	-0.39	-0.66	-0.29	-0.17
Q16891	Mitochondrial inner membrane protein OS=Homo sapiens GN=IMMT PE=1 SV=1 - [IMMT_HUMAN]	57.39	1	37	37	110	0.13	0.18	0.35	0.41	-0.27	-0.26	0.09	0.14	-0.05	-0.02	-0.04	0.07	0.07	-0.15	-0.40	-0.26	-0.36	-0.38	-0.70	-0.17
Q9UJW0	Dynactin subunit 4 OS=Homo sapiens GN=DCTN4 PE=1 SV=1 - [DCTN4_HUMAN]	33.70	1	10	10	23	0.17	0.32	0.44	0.41	0.10	0.17	0.17	0.33	0.10	0.13	0.08	0.21	-0.16	-0.20	-0.20	-0.11	-0.10	-0.17	-0.28	-0.17
O95260	Arginyl-tRNA--protein transferase 1 OS=Homo sapiens GN=ATE1 PE=1 SV=2 - [ATE1_HUMAN]	28.96	1	9	9	11	0.21	0.29	0.52	0.55	0.17	0.20	0.36	0.37	0.08	-0.04	0.01	0.17	0.14	-0.09	-0.28	-0.19	-0.43	-0.01	-0.28	-0.17
Q9Y217	Myotubularin-related protein 6 OS=Homo sapiens GN=MTMR6 PE=1 SV=3 - [MTMR6_HUMAN]	7.25	1	4	4	6	0.16	-0.04	0.24	0.09	-0.02	-0.23	-0.07	-0.27	-0.19	0.02	0.14	-0.06	-0.17	-0.13	-0.01	-0.11	-0.16	-0.20	-0.24	-0.17

Q99653	Calcineurin B homologous protein 1 OS=Homo sapiens GN=CHP1 PE=1 SV=3 - [CHP1_HUMAN]	67.18	1	10	10	17	0.19	0.14	0.37	0.31	0.06	-0.07	0.14	0.07	0.06	0.17	0.38	0.33	-0.11	-0.07	-0.01	-0.17	-0.21	-0.14	-0.33	-0.17
P61201	COP9 signalosome complex subunit 2 OS=Homo sapiens GN=COPS2 PE=1 SV=1 - [CSN2_HUMAN]	22.57	1	8	8	18	0.24	0.27	0.55	0.41	-0.26	-0.29	0.16	0.21	-0.10	-0.06	-0.10	-0.22	0.15	-0.14	-0.68	-0.20	-0.55	-0.46	-0.81	-0.17
Q9HB07	UPF0160 protein MYG1, mitochondrial OS=Homo sapiens GN=C12orf10 PE=1 SV=2 - [MYG1_HUMAN]	14.36	1	4	4	6	0.72	0.80	0.73	0.70	0.46	0.39	0.65	0.45	0.24	0.21	0.07	0.11	-0.13	-0.67	-0.78	-0.47	-0.67	-0.29	-0.60	-0.17
P59998	Actin-related protein 2/3 complex subunit 4 OS=Homo sapiens GN=ARPC4 PE=1 SV=3 - [ARPC4_HUMAN]	47.02	1	6	6	20	-0.02	-0.03	0.03	-0.02	-0.07	-0.11	-0.23	-0.25	-0.15	-0.16	-0.24	-0.28	-0.18	-0.19	-0.29	-0.16	-0.09	-0.08	-0.14	-0.17
Q8TAQ2	SWI/SNF complex subunit SMARCC2 OS=Homo sapiens GN=SMARCC2 PE=1 SV=1 - [SMRC2_HUMAN]	15.24	2	14	14	20	-0.34	-0.34	-0.24	-0.28	-0.29	-0.40	-0.43	-0.44	-0.36	-0.17	-0.12	-0.09	-0.06	0.25	0.15	0.10	0.00	0.01	-0.14	-0.17
O00178	GTP-binding protein 1 OS=Homo sapiens GN=GTPBP1 PE=1 SV=3 - [GTPB1_HUMAN]	22.57	1	9	9	19	0.23	0.65	0.39	0.67	0.08	0.25	0.24	0.24	0.33	0.30	0.00	0.00	-0.42	-0.30	-0.38	-0.06	-0.19	0.05	-0.37	-0.17
P08579	U2 small nuclear ribonucleoprotein B'' OS=Homo sapiens GN=SNRPB2 PE=1 SV=1 - [RU2B_HUMAN]	19.56	1	2	4	7	-0.80	-0.74	-0.74	-0.68	-0.46	-0.41	-0.97	-0.92	-0.48	-0.53	-0.68	-0.63	-0.12	0.12	0.06	0.30	0.24	0.32	0.26	-0.17
O00442	RNA 3'-terminal phosphate cyclase OS=Homo sapiens GN=RTCA PE=1 SV=1 - [RTCA_HUMAN]	14.48	1	4	4	9	0.07	0.23	0.21	0.18	0.05	0.11	0.06	0.11	0.04	0.06	-0.23	-0.21	-0.09	-0.39	-0.53	-0.14	-0.20	-0.12	-0.17	-0.17

Q6XUX3	Dual serine/threonine and tyrosine protein kinase OS=Homo sapiens GN=DSTYK PE=1 SV=2 - [DUSTY_HUMAN]	1.18	1	1	1	1	-0.24	-0.35	0.33	0.22	0.02	-0.09	0.10	-0.01	-0.05	0.07	0.02	-0.10	0.39	0.27	-0.31	0.34	-0.23	0.25	-0.33	-0.17
Q9Y3B4	Pre-mRNA branch site protein p14 OS=Homo sapiens GN=SF3B14 PE=1 SV=1 - [PM14_HUMAN]	28.00	1	3	3	7	-0.76	-0.81	-0.47	-0.52	-0.57	-0.63	-0.71	-0.76	-0.80	-0.74	-0.73	-0.79	0.11	0.03	-0.26	0.05	-0.24	0.17	-0.12	-0.17
Q9GZY8	Mitochondrial fission factor OS=Homo sapiens GN=MFF PE=1 SV=1 - [MFF_HUMAN]	40.06	1	7	7	10	0.45	0.54	0.50	0.57	0.22	0.29	0.15	0.20	-0.01	0.05	-0.01	0.06	-0.33	-0.41	-0.57	-0.51	-0.60	-0.11	-0.27	-0.17
Q7Z5G4	Golgin subfamily A member 7 OS=Homo sapiens GN=GOLGA7 PE=1 SV=2 - [GOGA7_HUMAN]	32.12	1	4	4	6	0.64	0.73	0.39	0.54	0.00	0.00	0.11	0.25	0.23	0.09	0.17	0.16	-0.29	-0.25	-0.15	-0.19	-0.12	-0.59	-0.48	-0.17
Q9HD34	LYR motif-containing protein 4 OS=Homo sapiens GN=LYRM4 PE=1 SV=1 - [LYRM4_HUMAN]	13.19	1	1	1	2	0.28	0.23	0.56	0.51	0.03	-0.03	0.32	0.27	0.13	0.19	0.00	-0.05	0.09	-0.27	-0.56	-0.06	-0.34	-0.27	-0.55	-0.17
Q9BV57	1,2-dihydroxy 3-keto-5-methylthiopentene dioxygenase OS=Homo sapiens GN=ADI1 PE=1 SV=1 - [MTND_HUMAN]	21.79	1	3	3	6	0.76	0.91	0.94	1.09	0.43	0.57	0.49	0.63	0.50	0.36	0.38	0.52	-0.22	-0.55	-0.56	-0.56	-0.55	-0.46	-0.52	-0.17
P13726	Tissue factor OS=Homo sapiens GN=F3 PE=1 SV=1 - [TF_HUMAN]	22.03	1	4	4	11	0.24	0.64	0.82	0.98	0.01	0.10	0.70	1.01	0.46	0.43	0.49	0.81	0.09	0.07	-0.04	0.06	-0.39	-0.23	-0.89	-0.17
O76071	Probable cytosolic iron-sulfur protein assembly protein CIAO1 OS=Homo sapiens GN=CIAO1 PE=1 SV=1 - [CIAO1_HUMAN]	5.31	1	3	3	4	-0.02	0.00	0.24	0.26	-0.33	-0.25	0.03	0.02	-0.04	-0.12	0.29	0.28	0.08	0.29	0.16	-0.07	0.05	-0.25	-0.14	-0.17

Q15018	BRISC complex subunit Abro1 OS=Homo sapiens GN=FAM175B PE=1 SV=2 - [F175B_HUMAN]	8.92	1	3	3	3	0.50	0.38	0.45	0.33	0.34	0.21	0.21	0.09	0.14	0.27	0.29	0.17	-0.23	-0.20	-0.16	-0.20	-0.15	-0.18	-0.13	-0.18
Q15172	Serine/threonine-protein phosphatase 2A 56 kDa regulatory subunit alpha isoform OS=Homo sapiens GN=PPP2R5A PE=1 SV=1 - [2A5A_HUMAN]	8.23	1	3	3	4	-0.24	-0.12	-0.01	0.12	-0.50	-0.38	-0.25	-0.12	-0.76	-0.88	-0.61	-0.49	0.05	-0.36	-0.60	-0.61	-0.84	-0.28	-0.51	-0.18
Q9UHD1	Cysteine and histidine-rich domain-containing protein 1 OS=Homo sapiens GN=CHORDC1 PE=1 SV=2 - [CHRD1_HUMAN]	18.98	1	4	4	5	-0.19	-0.21	0.27	0.25	-0.36	-0.39	0.03	0.01	-0.30	-0.28	-0.24	-0.27	0.28	-0.05	-0.52	-0.06	-0.52	-0.19	-0.65	-0.18
Q96RQ3	Methylcrotonoyl-CoA carboxylase subunit alpha, mitochondrial OS=Homo sapiens GN=MCCC1 PE=1 SV=3 - [MCCA_HUMAN]	20.55	1	10	10	18	-0.31	-0.29	-0.27	-0.40	-0.53	-0.57	-0.58	-0.64	-0.62	-0.47	-0.58	-0.65	-0.29	-0.28	-0.45	-0.59	-0.14	-0.70	-0.43	-0.18
Q15154	Pericentriolar material 1 protein OS=Homo sapiens GN=PCM1 PE=1 SV=4 - [PCM1_HUMAN]	4.10	1	5	6	7	-0.17	-0.14	-0.26	-0.34	-0.29	-0.13	-0.57	-0.34	-0.17	-0.27	-0.46	-0.42	-0.26	-0.28	-0.19	0.00	0.09	-0.06	0.02	-0.18
P42167	Lamina-associated polypeptide 2, isoforms beta/gamma OS=Homo sapiens GN=TMPO PE=1 SV=2 - [LAP2B_HUMAN]	38.33	1	6	13	24	-0.20	-0.29	-0.36	-0.39	-0.62	-0.71	-0.48	-0.57	-0.74	-0.65	-0.27	-0.37	-0.26	-0.20	-0.33	-0.50	-0.55	-0.62	-0.60	-0.18
P47755	F-actin-capping protein subunit alpha 2 OS=Homo sapiens GN=CAPZA2 PE=1 SV=3 - [CAZA2_HUMAN]	69.93	1	10	12	71	0.25	0.29	0.19	0.26	0.11	0.14	0.06	0.03	0.06	0.16	0.09	0.11	-0.26	-0.27	-0.07	-0.20	-0.08	-0.37	-0.18	-0.18

Q15785	Mitochondrial import receptor subunit TOM34 OS=Homo sapiens GN=TOMM34 PE=1 SV=2 - [TOM34_HUMAN]	18.45	2	3	4	5	0.46	0.85	0.30	0.69	0.02	0.40	0.06	0.44	0.32	-0.06	-0.34	0.04	-0.35	-0.80	-0.64	-0.49	-0.32	-0.46	-0.30	-0.18
P31146	Coronin-1A OS=Homo sapiens GN=CORO1A PE=1 SV=4 - [COR1A_HUMAN]	37.09	1	15	16	28	0.30	0.51	0.80	0.74	-0.59	-0.56	0.20	0.25	-0.24	-0.18	-0.36	-0.38	-0.03	-0.98	-1.19	-0.48	-0.80	-0.88	-1.05	-0.18
Q92905	COP9 signalosome complex subunit 5 OS=Homo sapiens GN=COPS5 PE=1 SV=4 - [CSN5_HUMAN]	18.26	1	5	5	10	0.80	0.84	0.52	0.56	-0.11	-0.12	0.17	0.19	0.25	0.22	-0.17	-0.14	-0.53	-0.83	-0.70	-0.44	-0.47	-0.80	-0.65	-0.18
P82930	28S ribosomal protein S34, mitochondrial OS=Homo sapiens GN=MRPS34 PE=1 SV=2 - [RT34_HUMAN]	28.90	1	3	3	7	0.29	0.06	0.25	0.02	0.43	0.20	0.01	-0.22	-0.31	-0.07	0.04	-0.19	-0.23	-0.24	-0.21	-0.33	-0.29	0.12	0.16	-0.18
P49321	Nuclear autoantigenic sperm protein OS=Homo sapiens GN=NASP PE=1 SV=2 - [NASP_HUMAN]	16.24	1	8	8	10	0.07	0.10	0.04	0.10	-0.41	-0.28	-0.11	-0.04	-0.22	-0.29	-0.57	-0.41	0.05	-0.38	-0.68	-0.21	-0.40	-0.49	-0.46	-0.18
Q13616	Cullin-1 OS=Homo sapiens GN=CUL1 PE=1 SV=2 - [CUL1_HUMAN]	16.62	1	12	12	20	0.42	0.52	0.46	0.47	-0.03	0.09	0.19	0.16	0.17	0.04	0.33	0.43	-0.24	-0.10	-0.02	-0.14	-0.21	-0.38	-0.39	-0.18
Q9Y320	Thioredoxin-related transmembrane protein 2 OS=Homo sapiens GN=TMX2 PE=1 SV=1 - [TMX2_HUMAN]	10.47	1	3	3	4	0.22	0.27	0.16	0.22	-0.18	0.01	0.00	-0.08	-0.17	-0.21	-0.33	-0.37	-0.29	-0.56	-0.62	-0.41	-0.21	-0.41	-0.10	-0.18
Q96GQ5	UPF0420 protein C16orf58 OS=Homo sapiens GN=C16orf58 PE=1 SV=2 - [CP058_HUMAN]	14.10	1	5	5	9	-0.23	0.27	0.19	0.38	0.00	0.10	-0.25	-0.11	-0.11	-0.05	0.07	0.13	0.02	0.01	-0.18	0.00	-0.05	0.16	0.03	-0.18

Q9UNN5	FAS-associated factor 1 OS=Homo sapiens GN=FAF1 PE=1 SV=2 - [FAF1_HUMAN]	21.23	1	10	10	20	0.37	0.40	0.43	0.38	0.10	0.15	0.27	0.22	-0.05	0.11	0.13	0.03	-0.22	-0.48	-0.45	-0.36	-0.39	-0.21	-0.39	-0.18
Q9UNS2	COP9 signalosome complex subunit 3 OS=Homo sapiens GN=COPS3 PE=1 SV=3 - [CSN3_HUMAN]	17.26	1	7	7	16	0.47	0.61	0.46	0.72	0.08	0.19	0.36	0.36	0.28	0.17	-0.04	0.13	-0.26	-0.54	-0.58	-0.41	-0.50	-0.47	-0.32	-0.18
P20290	Transcription factor BTF3 OS=Homo sapiens GN=BTF3 PE=1 SV=1 - [BTF3_HUMAN]	30.10	1	4	4	9	-0.15	-0.35	-0.19	-0.22	-0.04	-0.26	-0.38	-0.51	-0.19	-0.58	-0.32	-0.51	-0.09	-0.13	-0.28	-0.01	-0.33	0.10	0.07	-0.18
P46782	40S ribosomal protein S5 OS=Homo sapiens GN=RPS5 PE=1 SV=4 - [RS5_HUMAN]	34.80	1	6	6	28	-0.29	-0.52	-0.33	-0.48	-0.68	-0.76	-0.52	-0.55	-0.72	-0.66	-0.63	-0.71	-0.07	0.03	-0.37	-0.19	-0.25	-0.22	-0.49	-0.18
Q9UDX4	SEC14-like protein 3 OS=Homo sapiens GN=SEC14L3 PE=2 SV=1 - [S14L3_HUMAN]	6.25	1	1	2	2	-0.91	-0.84	-0.49	-0.42	-0.36	-0.30	-0.73	-0.66	-0.70	-0.76	-0.94	-0.88	0.23	-0.03	-0.46	0.18	-0.24	0.53	0.11	-0.18
Q16186	Proteasomal ubiquitin receptor ADRM1 OS=Homo sapiens GN=ADRM1 PE=1 SV=2 - [ADRM1_HUMAN]	9.34	1	3	3	8	-0.32	-0.76	-0.08	-0.20	-0.46	-0.54	-0.29	-0.77	-0.51	-0.43	-0.32	-0.40	0.05	0.37	-0.19	0.29	-0.27	0.21	-0.35	-0.18
Q02880	DNA topoisomerase 2-beta OS=Homo sapiens GN=TOP2B PE=1 SV=3 - [TOP2B_HUMAN]	14.02	1	17	17	24	-0.20	-0.34	-0.18	-0.23	-0.32	-0.41	-0.32	-0.39	-0.37	-0.30	-0.33	-0.41	-0.10	-0.10	-0.19	-0.19	-0.28	-0.22	-0.20	-0.18
P53602	Diphosphomevalonate decarboxylase OS=Homo sapiens GN=MVD PE=1 SV=1 - [MVD1_HUMAN]	15.00	1	4	4	8	0.32	0.75	0.24	0.63	0.69	0.61	-0.09	0.38	0.35	0.32	0.22	0.60	-0.32	-0.16	-0.39	-0.23	-0.04	-0.15	-0.03	-0.18

Q5T447	E3 ubiquitin-protein ligase HECTD3 OS=Homo sapiens GN=HECTD3 PE=1 SV=1 - [HECD3_HUMAN]	9.41	1	6	6	8	0.09	0.23	0.21	0.43	0.03	0.19	-0.05	-0.12	0.14	0.04	0.00	0.19	-0.29	-0.27	-0.22	-0.05	-0.15	-0.10	-0.14	-0.18
Q96I51	Williams-Beuren syndrome chromosomal region 16 protein OS=Homo sapiens GN=WBSCR16 PE=1 SV=2 - [WBS16_HUMAN]	8.62	1	2	2	4	-0.05	-0.25	-0.02	-0.22	0.03	-0.18	-0.27	-0.47	-0.27	-0.07	0.06	-0.15	-0.16	0.11	0.08	0.01	-0.01	0.06	0.03	-0.18
Q8TAT6	Nuclear protein localization protein 4 homolog OS=Homo sapiens GN=NPLOC4 PE=1 SV=3 - [NPL4_HUMAN]	23.85	1	11	11	24	0.20	0.41	0.15	0.49	-0.31	-0.21	0.23	0.33	0.08	0.01	-0.02	0.10	-0.04	-0.19	-0.35	-0.30	-0.42	-0.40	-0.47	-0.18
P98175	RNA-binding protein 10 OS=Homo sapiens GN=RBM10 PE=1 SV=3 - [RBM10_HUMAN]	8.06	1	6	6	7	0.11	0.08	-0.12	-0.21	0.30	0.47	-0.48	-0.28	-0.12	-0.22	-0.14	-0.10	-0.38	-0.17	0.12	-0.16	0.09	0.40	0.67	-0.18
Q86UD5	Mitochondrial sodium/hydrogen exchanger 9B2 OS=Homo sapiens GN=SLC9B2 PE=1 SV=2 - [SL9B2_HUMAN]	2.79	1	1	1	1	-0.84	-0.79	-0.61	-0.56	-0.38	-0.33	-0.86	-0.81	0.32	0.27	1.19	1.24	0.04	2.04	1.81	1.15	0.92	0.45	0.22	-0.18
Q07866	Kinesin light chain 1 OS=Homo sapiens GN=KLC1 PE=1 SV=2 - [KLC1_HUMAN]	31.76	1	13	19	41	0.40	0.32	0.54	0.51	-0.04	-0.02	0.28	0.26	-0.08	-0.02	-0.17	-0.14	0.05	-0.45	-0.65	-0.17	-0.47	-0.38	-0.56	-0.18
Q99595	Mitochondrial import inner membrane translocase subunit Tim17-A OS=Homo sapiens GN=TIMM17A PE=1 SV=1 - [TI17A_HUMAN]	12.87	1	1	1	1	0.61	0.63	0.43	0.45	0.77	0.79	0.18	0.20	0.23	0.21	0.74	0.76	-0.37	0.14	0.31	-0.36	-0.18	0.15	0.33	-0.18

Q96JP5	E3 ubiquitin-protein ligase ZFP91 OS=Homo sapiens GN=ZFP91 PE=1 SV=1 - [ZFP91_HUMAN]	2.11	1	1	1	1	-0.83	-0.85	-0.69	-0.71	-0.13	-0.15	-0.94	-0.96	-0.59	-0.56	-0.08	-0.10	-0.06	0.76	0.61	0.29	0.16	0.68	0.55	-0.18
Q7L0Y3	Mitochondrial ribonuclease P protein 1 OS=Homo sapiens GN=TRMT10C PE=1 SV=2 - [MRRP1_HUMAN]	10.17	1	4	4	7	0.45	0.43	0.35	0.33	-0.15	-0.18	0.10	0.08	0.22	0.25	0.05	0.02	-0.29	-0.39	-0.30	-0.17	-0.07	-0.61	-0.52	-0.18
Q13330	Metastasis-associated protein MTA1 OS=Homo sapiens GN=MTA1 PE=1 SV=2 - [MTA1_HUMAN]	8.95	1	3	4	5	-0.62	-0.84	-1.04	-0.70	-0.15	-0.31	-0.65	-0.74	-0.95	-0.75	-0.29	-0.32	0.70	0.53	0.64	-0.09	-0.21	0.38	0.59	-0.18
P51687	Sulfite oxidase, mitochondrial OS=Homo sapiens GN=SUOX PE=1 SV=2 - [SUOX_HUMAN]	17.98	1	7	7	14	0.21	-0.21	-0.20	-0.42	0.19	0.22	-0.37	-0.52	-0.03	0.08	0.00	-0.21	-0.27	0.23	0.29	0.21	0.30	0.58	0.33	-0.19
P30273	High affinity immunoglobulin epsilon receptor subunit gamma OS=Homo sapiens GN=FCER1G PE=1 SV=1 - [FCERG_HUMAN]	23.26	1	2	2	6	0.28	0.18	0.29	0.19	0.19	0.09	0.04	-0.06	0.24	0.34	0.75	0.65	-0.18	0.48	0.46	0.09	0.08	-0.10	-0.11	-0.19
Q12888	Tumor suppressor p53-binding protein 1 OS=Homo sapiens GN=TP53BP1 PE=1 SV=2 - [TP53B_HUMAN]	16.48	1	23	23	38	-0.16	0.08	-0.17	-0.18	-0.03	-0.15	-0.32	-0.40	-0.36	-0.17	0.03	-0.01	-0.27	0.07	0.26	0.04	-0.07	-0.07	-0.07	-0.19
P49591	Serine--tRNA ligase, cytoplasmic OS=Homo sapiens GN=SARS PE=1 SV=3 - [SYSC_HUMAN]	22.76	1	8	8	16	-0.11	0.08	0.07	0.44	0.00	0.23	-0.14	0.05	-0.05	-0.25	-0.39	-0.15	-0.04	-0.21	-0.36	-0.11	-0.24	-0.09	-0.16	-0.19

Q9NQG6	Mitochondrial dynamics protein MID51 OS=Homo sapiens GN=MIEF1 PE=1 SV=1 - [MID51_HUMAN]	4.54	1	2	2	3	0.92	0.73	0.50	0.32	0.32	0.13	0.25	0.07	0.06	0.25	-0.20	-0.39	-0.61	-1.11	-0.71	-0.64	-0.22	-0.62	-0.20	-0.19
Q9NSY0	Nuclear receptor-binding protein 2 OS=Homo sapiens GN=NRBP2 PE=1 SV=2 - [NRBP2_HUMAN]	8.38	1	4	4	8	0.70	0.28	0.97	0.56	0.84	0.42	0.72	0.31	-0.02	0.40	0.81	0.39	0.08	0.12	-0.16	-0.27	-0.54	0.13	-0.14	-0.19
Q8NF91	Nesprin-1 OS=Homo sapiens GN=SYNE1 PE=1 SV=4 - [SYNE1_HUMAN]	10.00	1	66	67	98	0.44	0.37	0.23	0.18	-0.12	-0.24	0.04	-0.05	-0.17	-0.09	-0.16	-0.15	-0.42	-0.64	-0.48	-0.50	-0.40	-0.62	-0.50	-0.19
Q9NP61	ADP-ribosylation factor GTPase-activating protein 3 OS=Homo sapiens GN=ARFGAP3 PE=1 SV=1 - [ARFG3_HUMAN]	5.43	1	3	3	4	-0.16	-0.36	-0.10	-0.30	-0.42	-0.62	-0.35	-0.55	-0.41	-0.21	-0.59	-0.80	-0.14	-0.43	-0.49	-0.02	-0.07	-0.27	-0.33	-0.19
P43487	Ran-specific GTPase-activating protein OS=Homo sapiens GN=RANBP1 PE=1 SV=1 - [RANG_HUMAN]	23.88	2	4	4	8	0.19	0.37	0.41	0.62	-0.05	0.19	0.12	0.24	0.03	-0.21	0.02	0.02	-0.10	-0.31	-0.39	-0.37	-0.42	-0.43	-0.30	-0.19
Q9NR19	Acetyl-coenzyme A synthetase, cytoplasmic OS=Homo sapiens GN=ACSS2 PE=1 SV=1 - [ACSA_HUMAN]	10.98	1	6	6	7	0.52	0.30	0.40	0.30	0.26	-0.06	0.14	0.05	-0.15	0.19	0.07	0.12	-0.19	-0.12	-0.13	-0.37	-0.32	-0.34	-0.25	-0.19
P11216	Glycogen phosphorylase, brain form OS=Homo sapiens GN=PYGB PE=1 SV=5 - [PYGB_HUMAN]	52.55	1	28	39	152	0.60	0.59	0.69	0.74	0.18	0.19	0.52	0.53	0.13	0.17	-0.35	-0.38	-0.10	-0.92	-1.04	-0.54	-0.59	-0.46	-0.63	-0.19

Q969Y2	tRNA modification GTPase GTPBP3, mitochondrial OS=Homo sapiens GN=GTPBP3 PE=1 SV=2 - [GTPB3_HUMAN]	2.85	1	1	1	2	-0.03	-0.19	0.18	0.02	-0.14	-0.31	-0.07	-0.23	-0.34	-0.17	-0.05	-0.22	0.01	-0.02	-0.23	-0.11	-0.32	-0.13	-0.34	-0.19
Q9HAV0	Guanine nucleotide-binding protein subunit beta-4 OS=Homo sapiens GN=GNB4 PE=1 SV=3 - [GBB4_HUMAN]	24.71	1	4	7	29	0.20	0.16	0.07	0.03	-0.27	-0.31	-0.18	-0.22	-0.26	-0.22	-0.03	-0.08	-0.33	-0.22	-0.10	-0.39	-0.25	0.17	0.17	-0.19
Q9H871	Protein RMD5 homolog A OS=Homo sapiens GN=RMND5A PE=1 SV=1 - [RMD5A_HUMAN]	6.39	1	2	2	4	-0.04	-0.04	0.10	0.11	0.37	0.37	-0.15	-0.15	-0.09	-0.08	0.04	0.04	-0.05	0.08	-0.07	-0.01	-0.15	0.39	0.25	-0.19
Q9Y6G9	Cytoplasmic dynein 1 light intermediate chain 1 OS=Homo sapiens GN=DYNC1LI1 PE=1 SV=3 - [DC1L1_HUMAN]	31.93	1	10	11	18	-0.37	0.05	0.14	0.17	-0.28	-0.05	-0.11	0.06	0.05	-0.22	0.06	0.13	0.11	0.23	-0.25	0.06	-0.46	-0.18	-0.44	-0.19
Q9UKU7	Isobutyryl-CoA dehydrogenase, mitochondrial OS=Homo sapiens GN=ACAD8 PE=1 SV=1 - [ACAD8_HUMAN]	29.88	1	10	10	20	0.15	0.20	-0.13	0.12	0.12	0.09	0.01	0.13	0.22	0.07	0.14	0.06	-0.30	-0.34	-0.43	-0.14	-0.18	-0.24	-0.04	-0.19
P48723	Heat shock 70 kDa protein 13 OS=Homo sapiens GN=HSPA13 PE=1 SV=1 - [HSP13_HUMAN]	12.10	1	5	5	7	0.64	0.73	0.58	0.68	0.18	0.26	0.21	0.30	0.22	0.18	0.23	0.32	-0.25	-0.40	-0.41	-0.42	-0.50	-0.25	-0.30	-0.19
Q5J8M3	ER membrane protein complex subunit 4 OS=Homo sapiens GN=EMC4 PE=1 SV=2 - [EMC4_HUMAN]	15.85	1	2	2	4	-0.03	-0.30	-0.04	-0.32	-0.33	-0.61	-0.30	-0.57	-0.44	-0.16	-0.16	-0.44	-0.22	-0.13	-0.12	-0.10	-0.08	-0.32	-0.31	-0.19

Q9NXD2	Myotubularin-related protein 10 OS=Homo sapiens GN=MTMR10 PE=1 SV=3 - [MTMRA_HUMAN]	2.83	1	2	2	3	0.23	0.35	0.49	0.60	0.30	0.42	0.23	0.35	0.16	0.05	-0.17	-0.06	0.06	-0.39	-0.66	-0.30	-0.53	0.24	0.00	-0.19
Q9NYF8	Bcl-2-associated transcription factor 1 OS=Homo sapiens GN=BCLAF1 PE=1 SV=2 - [BCLF1_HUMAN]	8.26	1	7	7	14	0.14	-0.06	0.30	0.16	0.08	-0.05	0.08	-0.05	-0.14	0.01	-0.03	-0.21	0.06	-0.10	-0.28	0.05	-0.33	0.08	-0.27	-0.19
P14678	Small nuclear ribonucleoprotein-associated proteins B and B' OS=Homo sapiens GN=SNRPB PE=1 SV=2 - [RSMB_HUMAN]	15.00	2	3	3	5	-0.49	-0.42	-0.29	-0.07	0.12	0.11	-0.44	-0.45	-0.40	-0.19	0.10	0.10	0.03	0.54	0.30	0.04	-0.19	0.52	0.29	-0.19
Q9UBK7	Rab-like protein 2A OS=Homo sapiens GN=RABL2A PE=2 SV=1 - [RBL2A_HUMAN]	16.23	2	3	3	3	0.75	0.61	0.58	0.44	0.00	-0.13	0.32	0.19	0.04	0.18	0.27	0.13	-0.37	-0.48	-0.31	-0.54	-0.36	-0.76	-0.59	-0.19
O60825	6-phosphofructo-2-kinase/fructose-2,6-bisphosphatase 2 OS=Homo sapiens GN=PFKFB2 PE=1 SV=2 - [F262_HUMAN]	39.01	4	17	17	33	0.22	0.31	0.27	0.33	0.22	0.24	0.04	0.11	0.31	0.14	0.29	0.34	-0.29	0.01	0.05	-0.06	-0.03	-0.09	-0.16	-0.19
P50336	Protoporphyrinogen oxidase OS=Homo sapiens GN=PPOX PE=1 SV=1 - [PPOX_HUMAN]	17.40	1	7	7	13	-0.06	0.27	-0.19	-0.28	-0.38	-0.68	-0.35	-0.34	-0.44	-0.50	-0.31	-0.50	-0.27	-0.50	-0.39	-0.37	-0.26	-0.55	-0.49	-0.19
Q9UIW2	Plexin-A1 OS=Homo sapiens GN=PLXNA1 PE=1 SV=3 - [PLXA1_HUMAN]	11.81	1	9	20	33	0.28	0.51	0.29	0.57	-0.05	0.09	0.05	0.28	-0.10	-0.29	-0.12	-0.10	-0.18	-0.43	-0.63	-0.54	-0.55	-0.35	-0.36	-0.19
Q9GZU1	Mucolipin-1 OS=Homo sapiens GN=MCOLN1 PE=1 SV=1 - [MCLN1_HUMAN]	3.45	1	1	1	1	0.34	0.26	0.56	0.48	0.10	0.01	0.31	0.22	-0.01	0.08	-0.27	-0.36	0.02	-0.61	-0.84	-0.23	-0.45	-0.26	-0.48	-0.19

P27449	V-type proton ATPase 16 kDa proteolipid subunit OS=Homo sapiens GN=ATP6V0C PE=1 SV=1 - [VATL_HUMAN]	11.61	1	1	1	9	-0.10	0.21	0.20	0.51	-0.28	0.03	-0.06	0.25	0.14	-0.17	-0.26	0.05	0.10	-0.15	-0.46	-0.03	-0.33	-0.20	-0.50	-0.19
Q96HN2	Putative adenosylhomocysteinase 3 OS=Homo sapiens GN=AHCYL2 PE=1 SV=1 - [SAHH3_HUMAN]	35.52	2	7	22	66	0.03	0.07	0.06	0.02	-0.19	-0.36	-0.09	-0.01	-0.07	-0.03	0.30	0.32	-0.20	0.25	0.30	-0.02	-0.10	-0.46	-0.33	-0.20
P61026	Ras-related protein Rab-10 OS=Homo sapiens GN=RAB10 PE=1 SV=1 - [RAB10_HUMAN]	52.00	1	8	12	37	0.37	0.13	0.39	0.55	0.08	-0.18	0.05	0.03	-0.07	-0.02	0.02	-0.25	-0.22	-0.15	-0.30	-0.23	-0.37	-0.28	-0.32	-0.20
Q8TDY2	RB1-inducible coiled-coil protein 1 OS=Homo sapiens GN=RB1CC1 PE=1 SV=3 - [RBCC1_HUMAN]	5.46	1	7	7	15	0.21	0.28	0.06	0.35	-0.28	-0.32	-0.29	-0.29	0.33	0.23	-0.30	-0.04	-0.51	-0.47	-0.22	0.08	0.20	-0.47	0.11	-0.20
O95147	Dual specificity protein phosphatase 14 OS=Homo sapiens GN=DUSP14 PE=1 SV=1 - [DUS14_HUMAN]	21.72	1	3	3	3	0.54	0.63	0.03	0.12	-0.19	-0.11	-0.22	-0.14	-0.07	-0.15	-0.18	-0.11	-0.71	-0.72	-0.22	-0.66	-0.15	-0.75	-0.24	-0.20
Q969R2	Oxysterol-binding protein 2 OS=Homo sapiens GN=OSBP2 PE=1 SV=2 - [OSBP2_HUMAN]	5.68	1	3	3	4	-0.19	-0.61	0.62	-0.03	-0.24	-0.11	0.50	0.08	0.01	0.27	-0.25	-0.68	0.74	-0.06	-1.01	0.52	-0.31	0.02	-0.07	-0.20
O75051	Plexin-A2 OS=Homo sapiens GN=PLXNA2 PE=1 SV=4 - [PLXA2_HUMAN]	10.35	1	8	16	28	0.63	0.38	0.58	0.40	0.37	0.18	0.11	-0.14	0.30	0.33	0.20	0.01	-0.47	-0.61	-0.36	-0.06	0.04	-0.09	0.07	-0.20
Q9UKM7	Endoplasmic reticulum mannosyl-oligosaccharide 1,2-alpha-mannosidase OS=Homo sapiens GN=MAN1B1 PE=1 SV=2 - [MA1B1_HUMAN]	6.44	1	2	3	3	0.24	-0.19	-0.13	-0.55	-0.52	-0.96	-0.39	-0.82	-0.05	0.38	-0.20	-0.64	-0.57	-0.44	-0.08	0.17	0.54	-0.78	-0.42	-0.20

Q9H267	Vacuolar protein sorting-associated protein 33B OS=Homo sapiens GN=VPS33B PE=1 SV=2 - [VP33B_HUMAN]	18.96	1	10	11	21	0.23	0.22	0.14	0.23	0.06	0.11	-0.01	0.04	0.01	0.00	-0.11	-0.12	-0.13	-0.29	-0.32	-0.27	-0.25	-0.22	-0.24	-0.20
P43378	Tyrosine-protein phosphatase non-receptor type 9 OS=Homo sapiens GN=PTPN9 PE=1 SV=1 - [PTN9_HUMAN]	4.55	1	2	2	3	0.25	-0.18	0.27	-0.15	-0.24	-0.67	0.01	-0.42	-0.32	0.11	0.00	-0.44	-0.19	-0.25	-0.28	-0.11	-0.13	-0.51	-0.53	-0.20
Q9BY32	Inosine triphosphate pyrophosphatase OS=Homo sapiens GN=ITPA PE=1 SV=2 - [ITPA_HUMAN]	11.34	1	3	3	7	-0.45	-0.64	0.26	-0.06	0.38	0.12	0.02	-0.30	-0.21	-0.07	-0.47	-0.53	0.31	-0.02	-0.74	0.41	-0.29	0.75	0.10	-0.20
Q5VUB5	Protein FAM171A1 OS=Homo sapiens GN=FAM171A1 PE=1 SV=1 - [F1711_HUMAN]	16.85	1	11	11	19	0.61	0.51	0.60	0.44	0.24	0.05	0.53	0.43	0.26	0.28	0.32	0.28	-0.16	-0.25	-0.52	-0.41	-0.62	-0.47	-0.56	-0.20
Q86UR5	Regulating synaptic membrane exocytosis protein 1 OS=Homo sapiens GN=RIMS1 PE=1 SV=1 - [RIMS1_HUMAN]	7.39	1	7	8	11	-0.35	-0.14	-0.61	-0.61	0.20	0.32	-0.60	-0.38	0.34	0.22	0.01	0.14	-0.19	-0.14	0.71	-0.13	0.67	0.61	0.89	-0.20
P49427	Ubiquitin-conjugating enzyme E2 R1 OS=Homo sapiens GN=CDC34 PE=1 SV=2 - [UB2R1_HUMAN]	10.17	1	2	2	2	0.49	0.07	0.17	-0.25	0.31	-0.11	-0.09	-0.51	0.30	0.73	0.36	-0.07	-0.53	-0.13	0.18	0.26	0.59	-0.20	0.12	-0.20
Q96CX2	BTB/POZ domain-containing protein KCTD12 OS=Homo sapiens GN=KCTD12 PE=1 SV=1 - [KCD12_HUMAN]	40.00	1	8	9	31	-0.95	-0.85	-1.07	-1.09	-1.04	-0.92	-1.22	-1.13	-0.74	-0.81	-0.68	-0.60	-0.24	0.22	0.28	0.11	0.14	-0.16	-0.16	-0.20
O95140	Mitofusin-2 OS=Homo sapiens GN=MFN2 PE=1 SV=3 - [MFN2_HUMAN]	25.23	1	15	15	32	0.63	0.68	0.59	0.53	0.23	0.16	0.30	0.25	0.16	0.13	0.23	0.16	-0.36	-0.50	-0.44	-0.39	-0.52	-0.38	-0.53	-0.20

Q96LR5	Ubiquitin-conjugating enzyme E2 E2 OS=Homo sapiens GN=UBE2E2 PE=1 SV=1 - [UB2E2_HUMAN]	21.89	3	3	3	3	0.92	0.79	0.43	0.30	0.65	0.51	0.17	0.03	0.16	0.30	0.46	0.32	-0.70	-0.39	0.25	-0.53	0.13	-0.12	0.65	-0.20
P09234	U1 small nuclear ribonucleoprotein C OS=Homo sapiens GN=SNRPC PE=1 SV=1 - [RU1C_HUMAN]	13.21	1	2	2	3	-0.49	-0.44	-0.61	-0.57	-1.14	-1.10	-0.88	-0.84	-0.82	-0.86	-0.31	-0.27	-0.34	0.18	0.30	-0.34	-0.21	-0.67	-0.54	-0.20
Q9UPR0	Inactive phospholipase C-like protein 2 OS=Homo sapiens GN=PLCL2 PE=1 SV=2 - [PLCL2_HUMAN]	4.88	1	4	4	6	1.27	1.25	0.54	0.52	-0.09	-0.11	0.28	0.26	0.20	0.22	0.29	0.26	-0.94	-0.98	-0.26	-1.02	-0.29	-1.38	-0.65	-0.20
Q8NI37	Protein phosphatase PTC7 homolog OS=Homo sapiens GN=PPTC7 PE=2 SV=1 - [PPTC7_HUMAN]	13.82	1	3	3	4	0.29	0.31	0.09	0.10	0.30	0.31	-0.18	-0.17	0.21	0.20	0.16	0.17	-0.42	-0.13	0.07	-0.06	0.15	-0.01	0.20	-0.20
Q96BY9	Store-operated calcium entry-associated regulatory factor OS=Homo sapiens GN=TMEM66 PE=1 SV=1 - [SARAF_HUMAN]	2.36	1	1	1	1	0.47	0.29	0.55	0.37	0.75	0.56	0.29	0.11	0.35	0.54	0.67	0.49	-0.13	0.21	0.12	0.10	0.02	0.26	0.17	-0.20
Q14642	Type I inositol 1,4,5-trisphosphate 5-phosphatase OS=Homo sapiens GN=INPP5A PE=1 SV=1 - [I5P1_HUMAN]	18.93	1	7	7	9	0.25	0.22	0.68	0.49	0.23	0.16	0.29	0.16	-0.07	0.00	0.05	-0.01	0.09	-0.13	-0.59	-0.12	-0.52	0.07	-0.38	-0.20
Q8IYB7	DIS3-like exonuclease 2 OS=Homo sapiens GN=DIS3L2 PE=1 SV=4 - [DI3L2_HUMAN]	4.29	1	3	3	4	0.38	0.34	0.80	0.27	-0.20	0.08	0.11	0.00	0.02	-0.46	-0.34	-0.04	-0.29	-0.71	-1.14	-0.29	-0.20	-0.28	-0.20	-0.20
Q86TI2	Dipeptidyl peptidase 9 OS=Homo sapiens GN=DPP9 PE=1 SV=3 - [DPP9_HUMAN]	10.78	1	5	6	7	-0.11	0.17	-0.20	-0.75	-0.09	-0.30	-0.46	-0.67	0.15	-0.08	0.30	0.07	-0.30	-0.09	0.82	0.01	0.94	0.00	0.09	-0.20

Q15019	Septin-2 OS=Homo sapiens GN=SEPT2 PE=1 SV=1 - [SEPT2_HUMAN]	73.13	1	19	19	70	0.20	0.14	0.28	0.30	-0.16	-0.09	-0.06	-0.05	-0.21	-0.16	-0.14	-0.13	-0.08	-0.17	-0.32	-0.21	-0.35	-0.18	-0.28	-0.20
P15056	Serine/threonine-protein kinase B-raf OS=Homo sapiens GN=BRAF PE=1 SV=4 - [BRAF_HUMAN]	11.75	2	5	7	21	0.50	0.68	0.45	0.64	0.47	0.56	0.23	0.37	0.23	0.09	-0.03	-0.10	-0.26	-0.96	-0.84	-0.40	-0.24	-0.12	0.00	-0.20
P17812	CTP synthase 1 OS=Homo sapiens GN=CTPS1 PE=1 SV=2 - [PYRG1_HUMAN]	22.00	1	11	12	22	0.56	0.31	0.62	0.38	0.31	0.15	0.23	0.07	0.00	0.22	0.21	-0.05	-0.23	-0.35	-0.47	-0.29	-0.39	-0.21	-0.25	-0.20
O00764	Pyridoxal kinase OS=Homo sapiens GN=PDXK PE=1 SV=1 - [PDXK_HUMAN]	39.42	1	11	11	31	0.51	0.43	0.49	0.50	0.17	0.15	0.18	0.18	0.31	0.18	0.02	0.06	-0.29	-0.38	-0.37	-0.25	-0.20	-0.38	-0.37	-0.20
Q14012	Calcium/calmodulin-dependent protein kinase type 1 OS=Homo sapiens GN=CAMK1 PE=1 SV=1 - [KCC1A_HUMAN]	45.95	1	7	11	18	-0.28	-0.31	0.31	0.08	-0.18	-0.14	-0.04	-0.20	-0.21	-0.11	0.02	-0.15	0.12	0.23	-0.08	0.20	-0.02	0.03	-0.19	-0.20
Q8N2G8	GH3 domain-containing protein OS=Homo sapiens GN=GHDC PE=1 SV=2 - [GHDC_HUMAN]	9.62	1	5	5	6	0.52	0.49	0.24	0.36	0.27	0.36	0.04	0.06	0.19	0.16	0.40	0.48	-0.35	0.01	0.07	-0.33	-0.09	-0.12	0.07	-0.20
P46776	60S ribosomal protein L27a OS=Homo sapiens GN=RPL27A PE=1 SV=2 - [RL27A_HUMAN]	20.95	1	3	3	8	-0.51	-0.16	-0.50	-0.19	-0.55	-0.36	-0.76	-0.48	-0.25	-0.60	-0.24	0.02	-0.25	0.28	0.21	-0.06	-0.02	-0.16	-0.07	-0.20
O60936	Nucleolar protein 3 OS=Homo sapiens GN=NOL3 PE=1 SV=1 - [NOL3_HUMAN]	21.92	1	3	3	6	0.30	0.33	0.25	0.17	-0.09	-0.04	0.00	-0.10	-0.19	-0.23	0.48	0.28	-0.38	0.18	0.23	-0.51	-0.32	-0.41	-0.23	-0.21
Q99569	Plakophilin-4 OS=Homo sapiens GN=PKP4 PE=1 SV=2 - [PKP4_HUMAN]	25.76	1	22	23	43	0.36	0.28	0.16	0.15	-0.05	-0.14	-0.02	-0.19	-0.19	-0.08	0.06	-0.09	-0.23	-0.10	0.05	-0.22	-0.25	-0.25	-0.07	-0.21

P36405	ADP-ribosylation factor-like protein 3 OS=Homo sapiens GN=ARL3 PE=1 SV=2 - [ARL3_HUMAN]	57.69	1	8	8	21	0.77	0.60	0.76	0.77	0.59	0.58	0.47	0.41	0.40	0.40	0.06	0.07	-0.09	-0.76	-0.75	-0.11	-0.44	-0.02	-0.31	-0.21
O43402	ER membrane protein complex subunit 8 OS=Homo sapiens GN=EMC8 PE=1 SV=1 - [EMC8_HUMAN]	36.19	1	6	6	10	-0.22	-0.20	-0.08	-0.03	-0.23	-0.06	-0.35	-0.30	-0.12	-0.20	0.07	0.24	-0.07	0.03	0.11	-0.03	-0.08	-0.07	-0.12	-0.21
Q13867	Bleomycin hydrolase OS=Homo sapiens GN=BLMH PE=1 SV=1 - [BLMH_HUMAN]	15.60	1	5	5	7	-0.18	-0.27	0.05	-0.04	-0.34	-0.43	-0.22	-0.31	-0.14	-0.05	-0.78	-0.87	0.02	-0.59	-0.83	0.16	-0.07	-0.18	-0.41	-0.21
P06748	Nucleophosmin OS=Homo sapiens GN=NPM1 PE=1 SV=2 - [NPM_HUMAN]	37.76	2	9	9	21	-0.48	-0.36	-0.03	0.05	-0.14	-0.23	-0.28	-0.28	-0.36	-0.31	-0.37	-0.36	0.33	0.04	-0.33	0.26	-0.14	0.39	-0.14	-0.21
P49458	Signal recognition particle 9 kDa protein OS=Homo sapiens GN=SRP9 PE=1 SV=2 - [SRP09_HUMAN]	19.77	1	2	2	6	-0.73	-0.76	-0.17	-0.15	-0.33	-0.46	-0.43	-0.42	-0.48	-0.56	-0.23	-0.24	0.32	0.55	-0.06	0.17	-0.30	0.35	-0.28	-0.21
Q9BY77	Polymerase delta-interacting protein 3 OS=Homo sapiens GN=POLDIP3 PE=1 SV=2 - [PDIP3_HUMAN]	19.24	1	6	6	7	0.65	0.43	0.24	0.45	0.22	0.31	0.04	0.39	0.18	0.44	0.40	0.22	-0.11	0.04	0.04	-0.25	0.05	-0.32	-0.08	-0.21
Q96N66	Lysophospholipid acyltransferase 7 OS=Homo sapiens GN=MBOAT7 PE=1 SV=2 - [MBOA7_HUMAN]	13.14	1	6	6	14	-0.56	-0.60	-0.21	-0.31	-0.19	-0.32	-0.53	-0.55	-0.40	-0.31	0.05	-0.10	0.02	0.51	0.16	0.10	-0.06	0.11	-0.10	-0.21
Q9NX40	OCIA domain-containing protein 1 OS=Homo sapiens GN=OCIAD1 PE=1 SV=1 - [OCAD1_HUMAN]	22.45	1	7	7	16	0.60	0.58	0.50	0.51	-0.02	-0.09	0.12	0.19	0.03	0.15	0.09	0.05	-0.32	-0.53	-0.47	-0.38	-0.28	-0.52	-0.54	-0.21

Q9NTZ6	RNA-binding protein 12 OS=Homo sapiens GN=RBM12 PE=1 SV=1 - [RBM12_HUMAN]	11.37	1	9	9	14	-0.31	-0.37	-0.23	-0.17	-0.54	-0.47	-0.41	-0.51	-0.52	-0.49	-0.51	-0.48	-0.15	-0.21	-0.25	-0.18	-0.31	-0.09	-0.22	-0.21
Q9H6Q4	Cytosolic Fe-S cluster assembly factor NARFL OS=Homo sapiens GN=NARFL PE=1 SV=1 - [NARFL_HUMAN]	22.48	1	6	6	16	0.86	0.85	0.66	0.41	0.40	-0.03	0.59	0.08	0.23	0.32	0.59	0.28	-0.26	-0.56	-0.13	-0.40	-0.15	-0.89	-0.46	-0.21
Q8N2Y8	Iporin OS=Homo sapiens GN=RUSC2 PE=1 SV=3 - [RUSC2_HUMAN]	0.99	1	1	1	1	0.19	0.02	0.54	0.37	0.16	-0.02	0.27	0.10	-0.24	-0.06	0.22	0.05	0.14	0.04	-0.32	-0.22	-0.57	-0.05	-0.40	-0.21
Q99873	Protein arginine N-methyltransferase 1 OS=Homo sapiens GN=PRMT1 PE=1 SV=2 - [ANM1_HUMAN]	24.65	2	7	7	13	0.15	0.01	0.36	0.13	0.08	-0.11	0.09	-0.14	-0.14	0.05	0.13	-0.01	-0.10	-0.01	-0.14	-0.07	-0.24	0.00	-0.41	-0.21
Q9BXB4	Oxysterol-binding protein-related protein 11 OS=Homo sapiens GN=OSBPL11 PE=1 SV=2 - [OSB11_HUMAN]	2.01	1	1	1	1	0.79	0.17	0.48	-0.14	0.68	0.06	0.20	-0.42	-0.17	0.45	-0.06	-0.69	-0.53	-0.85	-0.54	-0.31	0.01	-0.12	0.19	-0.21
P45973	Chromobox protein homolog 5 OS=Homo sapiens GN=CBX5 PE=1 SV=1 - [CBX5_HUMAN]	39.27	1	6	7	9	-0.45	-0.71	0.05	-0.25	-0.31	-0.65	-0.28	-0.54	-0.50	-0.25	-0.37	-0.55	-0.04	-0.03	-0.34	0.25	-0.24	-0.02	-0.39	-0.21
Q8NDA8	Maestro heat-like repeat-containing protein family member 1 OS=Homo sapiens GN=MROH1 PE=2 SV=3 - [MROH1_HUMAN]	4.02	1	6	6	8	0.26	0.51	0.21	0.40	0.13	0.09	0.11	0.13	0.12	0.28	0.05	-0.08	-0.33	-0.40	-0.30	-0.25	-0.13	-0.49	-0.38	-0.21
P36542	ATP synthase subunit gamma, mitochondrial OS=Homo sapiens GN=ATP5C1 PE=1 SV=1 - [ATPG_HUMAN]	47.32	1	12	13	34	0.37	0.35	0.57	0.46	-0.11	-0.15	0.34	0.22	0.05	0.09	-0.04	-0.18	0.05	-0.31	-0.62	-0.18	-0.52	-0.42	-0.59	-0.21

Q8TEA7	TBC domain-containing protein kinase-like protein OS=Homo sapiens GN=TBCK PE=1 SV=4 - [TBCK_HUMAN]	6.94	1	6	6	9	0.20	0.12	0.53	0.41	0.01	-0.06	0.20	0.14	-0.09	-0.08	0.01	-0.14	0.06	-0.17	-0.52	-0.32	-0.63	-0.15	-0.56	-0.21
Q9H1K1	Iron-sulfur cluster assembly enzyme ISCU, mitochondrial OS=Homo sapiens GN=ISCU PE=1 SV=2 - [ISCU_HUMAN]	25.75	1	5	5	12	0.37	0.34	0.11	0.06	-0.38	-0.37	-0.22	-0.18	-0.38	-0.39	-0.36	-0.34	-0.39	-0.82	-0.68	-0.59	-0.38	-0.85	-0.64	-0.21
Q9Y485	DmX-like protein 1 OS=Homo sapiens GN=DMXL1 PE=1 SV=3 - [DMXL1_HUMAN]	3.67	1	5	7	8	0.62	0.13	0.36	-0.18	0.13	-0.26	0.01	-0.25	-0.20	0.19	0.05	-0.18	-0.53	-0.30	0.01	-0.39	-0.13	-0.37	-0.11	-0.21
P13797	Plastin-3 OS=Homo sapiens GN=PLS3 PE=1 SV=4 - [PLST_HUMAN]	39.21	2	16	20	51	-0.27	-0.32	-0.09	-0.19	-0.23	-0.21	-0.37	-0.39	-0.54	-0.53	-0.75	-0.71	0.06	-0.32	-0.49	-0.06	-0.34	0.23	-0.11	-0.21
P31153	S-adenosylmethionine synthase isoform type-2 OS=Homo sapiens GN=MAT2A PE=1 SV=1 - [METK2_HUMAN]	37.72	1	12	13	28	0.56	0.67	0.82	0.95	-0.07	-0.04	0.50	0.51	0.26	0.24	0.11	0.18	-0.08	-0.54	-0.70	-0.35	-0.56	-0.59	-0.75	-0.21
Q6P3W7	SCY1-like protein 2 OS=Homo sapiens GN=SCYL2 PE=1 SV=1 - [SCYL2_HUMAN]	6.14	1	4	4	5	0.21	0.23	0.32	0.33	-0.19	-0.18	0.04	0.06	-0.19	-0.21	-0.27	-0.26	-0.11	-0.48	-0.59	-0.39	-0.49	-0.41	-0.52	-0.21
P43304	Glycerol-3-phosphate dehydrogenase, mitochondrial OS=Homo sapiens GN=GPD2 PE=1 SV=3 - [GPDM_HUMAN]	43.05	1	24	24	57	0.66	0.68	0.62	0.66	0.20	0.21	0.40	0.46	0.40	0.35	0.48	0.49	-0.26	-0.24	-0.28	-0.25	-0.25	-0.40	-0.41	-0.21
Q9Y6C9	Mitochondrial carrier homolog 2 OS=Homo sapiens GN=MTCH2 PE=1 SV=1 - [MTCH2_HUMAN]	20.79	1	5	5	15	0.32	0.33	0.40	0.51	0.22	0.01	0.30	0.20	0.10	0.26	0.10	-0.03	0.27	-0.36	-0.52	-0.18	-0.34	-0.29	-0.52	-0.21

Q9P2W9	Syntaxin-18 OS=Homo sapiens GN=STX18 PE=1 SV=1 - [STX18_HUMAN]	17.61	1	5	5	5	-0.72	-0.67	-0.51	-0.46	-0.25	-0.20	-0.78	-0.73	-0.61	-0.65	-0.22	-0.17	-0.01	0.50	0.29	0.10	-0.11	0.46	0.24	-0.21
Q99570	Phosphoinositide 3-kinase regulatory subunit 4 OS=Homo sapiens GN=PIK3R4 PE=1 SV=3 - [PI3R4_HUMAN]	7.95	1	9	9	13	0.68	0.78	0.55	0.60	0.18	0.15	0.17	0.25	0.17	0.08	-0.02	0.08	-0.35	-0.67	-0.45	-0.51	-0.38	-0.24	-0.10	-0.21
P42345	Serine/threonine-protein kinase mTOR OS=Homo sapiens GN=MTOR PE=1 SV=1 - [MTOR_HUMAN]	11.93	1	23	23	35	0.27	0.33	0.33	0.32	0.02	-0.01	0.10	0.09	0.13	0.17	0.15	0.15	-0.19	-0.09	-0.30	-0.11	-0.20	-0.29	-0.40	-0.21
O00273	DNA fragmentation factor subunit alpha OS=Homo sapiens GN=DFFA PE=1 SV=1 - [DFFA_HUMAN]	3.32	1	1	1	1	-0.12	-0.21	0.31	0.22	-0.68	-0.77	0.03	-0.06	-0.27	-0.18	-0.40	-0.50	0.20	-0.28	-0.71	-0.03	-0.45	-0.58	-1.00	-0.21
P51149	Ras-related protein Rab-7a OS=Homo sapiens GN=RAB7A PE=1 SV=1 - [RAB7A_HUMAN]	62.80	1	13	13	38	0.14	0.18	0.35	0.42	-0.15	-0.17	0.02	0.09	0.04	0.01	0.12	0.19	0.04	-0.01	-0.25	0.03	-0.35	-0.19	-0.46	-0.21
P51659	Peroxisomal multifunctional enzyme type 2 OS=Homo sapiens GN=HSD17B4 PE=1 SV=3 - [DHB4_HUMAN]	42.80	1	23	23	55	0.01	0.06	0.04	0.06	0.06	0.06	-0.17	-0.10	-0.08	-0.10	0.00	0.05	-0.10	0.02	-0.08	-0.01	-0.15	0.13	0.00	-0.21
Q05086	Ubiquitin-protein ligase E3A OS=Homo sapiens GN=UBE3A PE=1 SV=4 - [UBE3A_HUMAN]	10.06	1	7	7	11	0.32	0.33	0.52	0.60	0.30	0.35	0.16	0.35	0.23	0.08	0.04	0.16	-0.07	-0.27	-0.44	-0.23	-0.40	-0.09	-0.33	-0.21
Q9ULV4	Coronin-1C OS=Homo sapiens GN=CORO1C PE=1 SV=1 - [COR1C_HUMAN]	26.16	1	11	12	23	-0.45	-0.50	-0.49	-0.38	-0.54	-0.49	-0.75	-0.76	-0.48	-0.49	-0.34	-0.37	-0.22	-0.01	-0.04	-0.04	-0.14	-0.25	-0.54	-0.22

O95352	Ubiquitin-like modifier-activating enzyme ATG7 OS=Homo sapiens GN=ATG7 PE=1 SV=1 - [ATG7_HUMAN]	10.95	1	6	6	7	0.28	0.64	0.34	0.47	0.04	0.16	0.20	0.25	-0.07	-0.14	-0.03	0.07	-0.28	-0.66	-0.35	-0.54	-0.45	-0.61	-0.25	-0.22
P62837	Ubiquitin-conjugating enzyme E2 D2 OS=Homo sapiens GN=UBE2D2 PE=1 SV=1 - [UB2D2_HUMAN]	24.49	1	2	3	14	0.40	0.37	0.43	0.40	0.10	0.07	0.15	0.12	-0.13	-0.10	-0.44	-0.47	-0.20	-0.83	-0.87	-0.47	-0.49	-0.32	-0.35	-0.22
P61160	Actin-related protein 2 OS=Homo sapiens GN=ACTR2 PE=1 SV=1 - [ARP2_HUMAN]	39.85	1	11	12	57	-0.16	-0.14	0.10	0.08	-0.28	-0.35	-0.20	-0.24	-0.37	-0.44	-0.35	-0.35	0.03	-0.16	-0.34	-0.16	-0.32	-0.09	-0.27	-0.22
Q9NZD2	Glycolipid transfer protein OS=Homo sapiens GN=GLTP PE=1 SV=3 - [GLTP_HUMAN]	13.40	1	3	3	9	0.31	0.43	0.48	0.61	-0.07	0.16	0.18	0.31	-0.03	-0.26	-0.41	-0.18	-0.07	-0.78	-0.98	-0.53	-0.64	-0.46	-0.67	-0.22
Q8ND56	Protein LSM14 homolog A OS=Homo sapiens GN=LSM14A PE=1 SV=3 - [LS14A_HUMAN]	10.58	1	5	5	7	-0.01	-0.18	-0.18	0.07	-0.45	-0.55	-0.50	-0.25	-0.55	-0.59	-0.43	-0.17	-0.28	-0.23	-0.06	-0.55	-0.37	-0.18	-0.13	-0.22
P51148	Ras-related protein Rab-5C OS=Homo sapiens GN=RAB5C PE=1 SV=2 - [RAB5C_HUMAN]	48.15	1	5	7	18	-0.08	0.22	0.33	0.61	0.00	0.28	-0.06	0.17	0.05	-0.15	-0.24	-0.12	0.06	-0.03	-0.15	0.04	-0.14	0.07	-0.22	-0.22
Q96FV2	Secernin-2 OS=Homo sapiens GN=SCRN2 PE=1 SV=3 - [SCRN2_HUMAN]	9.65	1	3	3	6	0.55	0.75	0.68	0.87	-0.30	-0.11	0.40	0.59	0.17	-0.02	-0.67	-0.48	-0.10	-1.21	-1.35	-0.54	-0.66	-0.87	-0.99	-0.22
P32969	60S ribosomal protein L9 OS=Homo sapiens GN=RPL9 PE=1 SV=1 - [RL9_HUMAN]	41.15	1	5	5	18	-0.77	-0.75	-0.48	-0.52	0.19	0.05	-0.65	-0.71	-0.34	-0.44	-0.12	-0.03	-0.10	0.58	0.69	0.42	0.35	0.38	0.40	-0.22

O95155	Ubiquitin conjugation factor E4 B OS=Homo sapiens GN=UBE4B PE=1 SV=1 - [UBE4B_HUMAN]	4.76	1	4	4	4	0.52	0.66	0.43	0.55	0.08	-0.02	0.15	0.12	0.05	-0.08	0.15	0.02	-0.32	-0.77	-0.43	-0.57	-0.47	-0.82	-0.48	-0.22
Q99447	Ethanolamine-phosphate cytidylyltransferase OS=Homo sapiens GN=PCYT2 PE=1 SV=1 - [PCY2_HUMAN]	25.19	1	8	8	13	0.93	0.86	1.00	0.93	0.71	0.81	0.69	0.80	0.61	0.61	0.31	0.25	-0.19	-0.98	-1.02	-0.52	-0.55	-0.59	-0.63	-0.22
Q9H9S5	Fukutin-related protein OS=Homo sapiens GN=FKRP PE=1 SV=1 - [FKRP_HUMAN]	2.63	1	1	1	1	0.25	-0.05	0.06	-0.23	1.02	0.72	-0.22	-0.51	-0.12	0.18	0.20	-0.09	-0.41	-0.04	0.14	-0.04	0.15	0.75	0.94	-0.22
P52803	Ephrin-A5 OS=Homo sapiens GN=EFNA5 PE=1 SV=1 - [EFNA5_HUMAN]	6.14	1	1	1	1	0.42	0.07	0.19	-0.16	-0.01	-0.37	-0.09	-0.44	-0.57	-0.21	-0.14	-0.50	-0.46	-0.55	-0.33	-0.60	-0.37	-0.45	-0.22	-0.22
Q96TC7	Regulator of microtubule dynamics protein 3 OS=Homo sapiens GN=RMDN3 PE=1 SV=2 - [RMD3_HUMAN]	32.55	1	13	13	24	0.32	0.18	0.39	0.36	-0.12	-0.37	0.10	0.01	-0.02	0.03	0.11	0.09	-0.13	0.03	-0.26	-0.31	-0.35	-0.39	-0.56	-0.22
Q9Y2J8	Protein-arginine deiminase type-2 OS=Homo sapiens GN=PADI2 PE=1 SV=2 - [PADI2_HUMAN]	26.47	1	15	15	36	1.49	1.54	1.54	1.34	0.83	0.70	1.24	1.12	0.98	1.08	1.06	0.91	-0.07	-0.41	-0.55	-0.31	-0.48	-0.59	-0.88	-0.22
Q8WXI9	Transcriptional repressor p66-beta OS=Homo sapiens GN=GATAD2B PE=1 SV=1 - [P66B_HUMAN]	6.41	1	3	3	3	-0.17	0.05	-0.29	-0.16	-0.13	0.54	-0.60	-0.46	-0.03	-0.46	0.21	0.49	-0.37	0.52	0.65	-0.04	0.06	0.53	0.21	-0.22
Q8WUM4	Programmed cell death 6-interacting protein OS=Homo sapiens GN=PDCD6IP PE=1 SV=1 - [PDC6I_HUMAN]	39.86	1	27	27	61	-0.06	-0.16	0.14	0.18	-0.04	0.00	-0.13	-0.14	-0.20	-0.15	-0.22	-0.19	-0.01	-0.21	-0.42	-0.14	-0.20	0.00	-0.21	-0.22

Q12851	Mitogen-activated protein kinase kinase kinase kinase 2 OS=Homo sapiens GN=MAP4K2 PE=1 SV=2 - [M4K2_HUMAN]	7.68	2	4	4	4	0.96	0.87	0.63	0.54	-0.04	-0.13	0.35	0.26	0.21	0.30	-0.07	-0.17	-0.56	-1.03	-0.71	-0.63	-0.30	-1.02	-0.69	-0.22
Q8TBF8	Protein FAM81A OS=Homo sapiens GN=FAM81A PE=2 SV=3 - [FA81A_HUMAN]	11.68	1	3	3	3	1.69	2.03	1.32	1.66	-0.23	0.10	0.32	0.66	0.52	0.19	-0.15	0.18	-0.57	-1.84	-1.47	-0.60	-0.25	-0.74	-0.40	-0.22
Q7L3T8	Probable proline--tRNA ligase, mitochondrial OS=Homo sapiens GN=PARS2 PE=1 SV=1 - [SYPM_HUMAN]	1.68	1	1	1	1	-0.06	0.47	-0.21	0.32	-0.39	0.14	-0.49	0.03	0.03	-0.50	-0.51	0.02	-0.38	-0.44	-0.30	-0.41	-0.25	-0.35	-0.20	-0.22
O14776	Transcription elongation regulator 1 OS=Homo sapiens GN=TCERG1 PE=1 SV=2 - [TCRG1_HUMAN]	8.74	1	9	9	16	0.19	0.02	0.09	0.04	-0.04	-0.22	-0.16	-0.30	-0.37	-0.28	-0.21	-0.14	-0.25	-0.07	-0.09	-0.25	-0.23	-0.09	-0.08	-0.22
Q9NUT2	ATP-binding cassette sub-family B member 8, mitochondrial OS=Homo sapiens GN=ABCB8 PE=1 SV=3 - [ABCB8_HUMAN]	10.61	1	5	5	10	0.67	0.70	0.50	0.53	0.05	0.08	0.21	0.24	0.11	0.08	0.01	0.04	-0.40	-0.65	-0.49	-0.55	-0.38	-0.63	-0.46	-0.22
Q96CV9	Optineurin OS=Homo sapiens GN=OPTN PE=1 SV=2 - [OPTN_HUMAN]	35.36	1	20	21	37	0.05	0.04	0.18	0.23	-0.18	-0.11	-0.19	-0.08	-0.13	-0.14	-0.11	0.00	-0.13	-0.04	-0.22	-0.07	-0.31	-0.14	-0.37	-0.22
P61106	Ras-related protein Rab-14 OS=Homo sapiens GN=RAB14 PE=1 SV=4 - [RAB14_HUMAN]	74.88	5	10	13	57	0.44	0.59	0.64	0.54	0.09	0.04	0.19	0.25	0.08	0.13	0.13	0.19	-0.28	-0.41	-0.52	-0.40	-0.50	-0.30	-0.51	-0.23

P63092	Guanine nucleotide-binding protein G(s) subunit alpha isoforms short OS=Homo sapiens GN=GNAS PE=1 SV=1 - [GNAS2_HUMAN]	42.39	2	10	14	59	0.48	0.53	0.55	0.53	0.09	0.11	0.29	0.35	0.14	0.34	0.26	0.18	-0.19	-0.31	-0.32	-0.45	-0.43	-0.39	-0.42	-0.23
Q14671	Pumilio homolog 1 OS=Homo sapiens GN=PUM1 PE=1 SV=3 - [PUM1_HUMAN]	2.87	2	2	3	5	0.30	0.32	0.14	0.17	-0.02	0.00	-0.15	-0.12	0.08	0.06	0.24	0.26	-0.39	-0.05	0.10	-0.21	-0.04	-0.34	-0.18	-0.23
Q16620	BDNF/NT-3 growth factors receptor OS=Homo sapiens GN=NTRK2 PE=1 SV=1 - [NTRK2_HUMAN]	13.02	2	5	8	42	0.70	0.17	0.88	0.38	-0.26	-0.49	0.14	0.17	0.05	0.06	-0.19	-0.29	0.21	-0.78	-1.23	-0.20	-0.59	-0.95	-1.34	-0.23
Q8NB37	Parkinson disease 7 domain-containing protein 1 OS=Homo sapiens GN=PDDC1 PE=1 SV=1 - [PDDC1_HUMAN]	19.55	1	3	3	4	0.44	0.25	0.74	0.48	0.08	0.14	0.17	0.18	-0.17	0.27	0.16	-0.10	-0.01	-0.34	-0.57	-0.28	-0.65	-0.28	-0.36	-0.23
Q96I59	Probable asparagine--tRNA ligase, mitochondrial OS=Homo sapiens GN=NARS2 PE=1 SV=3 - [SYNM_HUMAN]	3.14	1	1	1	4	0.15	0.28	-0.24	-0.10	-0.10	0.04	-0.53	-0.39	0.10	-0.04	-0.58	-0.44	-0.62	-0.72	-0.34	-0.15	0.24	-0.26	0.13	-0.23
Q6PHR2	Serine/threonine-protein kinase ULK3 OS=Homo sapiens GN=ULK3 PE=1 SV=2 - [ULK3_HUMAN]	6.99	1	3	3	5	0.35	0.63	0.33	0.62	-0.16	0.12	0.04	0.33	0.05	-0.23	-0.46	-0.18	-0.25	-0.81	-0.80	-0.55	-0.53	-0.52	-0.51	-0.23
Q8N4V1	Membrane magnesium transporter 1 OS=Homo sapiens GN=MMGT1 PE=1 SV=1 - [MMGT1_HUMAN]	18.32	1	1	1	1	-0.13	0.05	-0.09	0.09	-0.31	-0.14	-0.38	-0.21	-0.60	-0.77	-0.49	-0.32	-0.20	-0.36	-0.40	-0.61	-0.65	-0.19	-0.23	-0.23

Q8NFF5	FAD synthase OS=Homo sapiens GN=FLAD1 PE=1 SV=1 - [FAD1_HUMAN]	2.39	1	1	1	2	0.14	0.35	0.04	0.25	-0.24	-0.04	-0.25	-0.05	0.12	-0.08	-0.16	0.04	-0.34	-0.30	-0.20	-0.19	-0.09	-0.40	-0.30	-0.23
Q9Y4P1	Cysteine protease ATG4B OS=Homo sapiens GN=ATG4B PE=1 SV=2 - [ATG4B_HUMAN]	21.63	1	5	5	9	0.72	0.92	0.78	0.98	0.22	0.32	0.59	0.79	0.32	0.13	0.34	0.54	-0.35	-0.72	-0.46	-0.55	-0.61	-0.73	-0.68	-0.23
Q8N135	Leucine-rich repeat LGI family member 4 OS=Homo sapiens GN=LGI4 PE=2 SV=1 - [LGI4_HUMAN]	11.36	1	7	7	9	0.93	0.93	0.75	0.75	2.28	2.34	0.67	0.67	1.41	1.41	0.53	0.52	-0.43	-0.19	-0.05	0.41	0.56	0.86	1.06	-0.23
Q9BTE6	Alanyl-tRNA editing protein Aarsd1 OS=Homo sapiens GN=AARSD1 PE=1 SV=2 - [AASD1_HUMAN]	38.35	1	10	10	18	0.76	0.71	0.24	0.66	0.17	0.26	0.22	0.17	0.31	0.29	0.11	0.11	-0.61	-0.76	-0.41	-0.20	-0.27	-0.45	-0.20	-0.23
Q86TX2	Acyl-coenzyme A thioesterase 1 OS=Homo sapiens GN=ACOT1 PE=1 SV=1 - [ACOT1_HUMAN]	36.34	2	12	12	23	0.32	0.29	0.44	0.61	-0.06	0.09	0.23	0.21	0.18	0.13	0.05	0.09	-0.06	-0.12	-0.53	-0.16	-0.34	-0.27	-0.61	-0.23
P07195	L-lactate dehydrogenase B chain OS=Homo sapiens GN=LDHB PE=1 SV=2 - [LDHB_HUMAN]	63.17	3	18	19	100	0.46	0.46	0.85	0.85	0.25	0.26	0.64	0.61	0.36	0.29	0.19	0.24	0.07	-0.26	-0.61	-0.17	-0.48	-0.26	-0.65	-0.23
Q13614	Myotubularin-related protein 2 OS=Homo sapiens GN=MTMR2 PE=1 SV=4 - [MTMR2_HUMAN]	9.64	1	5	6	7	0.82	0.78	0.58	0.54	0.26	0.21	0.29	0.25	0.20	0.25	0.09	0.04	-0.48	-0.73	-0.50	-0.54	-0.30	-0.58	-0.34	-0.23
Q9UKB1	F-box/WD repeat-containing protein 11 OS=Homo sapiens GN=FBXW11 PE=1 SV=1 - [FBW1B_HUMAN]	2.40	2	1	1	1	1.48	0.62	1.16	0.31	0.29	-0.57	0.87	0.01	-0.03	0.83	1.26	0.40	-0.55	-0.21	0.09	-0.62	-0.30	-1.20	-0.89	-0.23

O43447	Peptidyl-prolyl cis-trans isomerase H OS=Homo sapiens GN=PPIH PE=1 SV=1 - [PPIH_HUMAN]	31.07	1	4	5	5	0.49	0.18	0.43	0.46	0.12	0.07	0.05	0.03	-0.13	-0.07	0.26	0.20	-0.30	0.04	-0.25	-0.27	-0.55	-0.12	-0.40	-0.23
Q9UBF8	Phosphatidylinositol 4-kinase beta OS=Homo sapiens GN=PI4KB PE=1 SV=1 - [PI4KB_HUMAN]	2.94	1	2	2	4	0.71	1.13	0.49	0.92	-1.34	-0.92	0.20	0.62	0.30	-0.12	-0.21	0.21	-0.40	-0.91	-0.71	-0.80	-0.58	-2.06	-1.85	-0.23
P50502	Hsc70-interacting protein OS=Homo sapiens GN=ST13 PE=1 SV=2 - [F10A1_HUMAN]	27.91	3	9	9	33	-0.24	-0.16	-0.01	-0.08	-0.48	-0.49	-0.31	-0.31	-0.42	-0.42	-0.07	-0.08	0.04	0.14	-0.15	-0.15	-0.42	-0.19	-0.52	-0.23
Q9UMS4	Pre-mRNA-processing factor 19 OS=Homo sapiens GN=PRPF19 PE=1 SV=1 - [PRP19_HUMAN]	24.21	1	8	8	18	-0.13	-0.20	-0.15	0.13	-0.69	-0.39	-0.24	-0.28	-0.31	-0.46	-0.21	0.07	-0.06	0.25	-0.06	0.01	-0.29	-0.21	-0.69	-0.23
Q8WW22	DnaJ homolog subfamily A member 4 OS=Homo sapiens GN=DNAJA4 PE=1 SV=1 - [DNJA4_HUMAN]	22.92	1	8	8	13	0.28	0.40	0.47	0.53	-0.10	-0.02	0.23	0.23	0.05	0.12	0.05	0.04	-0.10	-0.28	-0.43	-0.35	-0.27	-0.43	-0.59	-0.23
O00194	Ras-related protein Rab-27B OS=Homo sapiens GN=RAB27B PE=1 SV=4 - [RB27B_HUMAN]	24.31	1	4	4	7	0.37	0.51	0.49	0.63	0.38	0.51	0.19	0.33	0.37	0.24	0.12	0.25	-0.13	-0.25	-0.37	-0.10	-0.22	-0.01	-0.13	-0.23
Q969Q5	Ras-related protein Rab-24 OS=Homo sapiens GN=RAB24 PE=1 SV=1 - [RAB24_HUMAN]	25.62	1	4	4	9	0.33	0.27	0.76	0.63	0.40	0.22	0.28	0.21	0.17	0.43	0.39	0.13	0.00	0.00	-0.38	0.06	-0.54	0.06	-0.51	-0.23
Q9C005	Protein dpy-30 homolog OS=Homo sapiens GN=DPY30 PE=1 SV=1 - [DPY30_HUMAN]	11.11	1	2	2	3	-0.13	-0.20	0.09	0.02	0.15	0.07	-0.20	-0.28	-0.14	-0.05	0.45	0.36	-0.02	0.58	0.35	0.10	-0.12	0.26	0.04	-0.23

P54577	Tyrosine--tRNA ligase, cytoplasmic OS=Homo sapiens GN=YARS PE=1 SV=4 - [SYYC_HUMAN]	39.77	1	20	20	42	-0.25	-0.16	-0.14	-0.02	-0.43	-0.46	-0.28	-0.28	-0.51	-0.45	-0.64	-0.66	-0.06	-0.64	-0.60	-0.37	-0.33	-0.35	-0.32	-0.23
P05496	ATP synthase F(0) complex subunit C1, mitochondrial OS=Homo sapiens GN=ATP5G1 PE=2 SV=2 - [AT5G1_HUMAN]	5.15	3	1	1	1	-0.85	-0.86	-0.28	-0.29	-0.21	-0.22	-0.58	-0.59	-0.46	-0.44	-0.29	-0.30	0.32	0.56	-0.01	0.43	-0.13	0.62	0.06	-0.23
P61966	AP-1 complex subunit sigma-1A OS=Homo sapiens GN=AP1S1 PE=1 SV=1 - [AP1S1_HUMAN]	17.72	1	2	2	2	0.74	0.48	0.65	0.39	0.25	-0.01	0.35	0.09	0.13	0.40	0.33	0.06	-0.34	-0.41	-0.32	-0.31	-0.22	-0.51	-0.41	-0.23
Q86V81	THO complex subunit 4 OS=Homo sapiens GN=ALYREF PE=1 SV=3 - [THOC4_HUMAN]	19.84	1	2	2	5	0.13	0.16	-0.37	-0.34	-0.27	-0.23	-0.67	-0.63	-0.24	-0.27	0.13	0.16	-0.74	0.01	0.50	-0.36	0.14	-0.41	0.09	-0.23
P14415	Sodium/potassium-transporting ATPase subunit beta-2 OS=Homo sapiens GN=ATP1B2 PE=1 SV=3 - [AT1B2_HUMAN]	29.66	1	6	6	27	0.66	0.90	0.71	0.90	0.00	0.21	0.46	0.52	0.35	0.29	0.15	0.19	-0.28	-0.72	-0.47	-0.38	-0.54	-0.61	-0.64	-0.23
Q9Y6U3	Adseverin OS=Homo sapiens GN=SCIN PE=1 SV=4 - [ADSV_HUMAN]	24.76	1	12	12	15	0.95	0.91	0.99	0.97	0.17	0.45	0.84	0.91	0.75	0.51	0.21	0.35	-0.09	-0.64	-0.71	-0.42	-0.58	-0.86	-1.06	-0.23
Q8IYU2	E3 ubiquitin-protein ligase HACE1 OS=Homo sapiens GN=HACE1 PE=1 SV=2 - [HACE1_HUMAN]	4.40	1	3	3	4	0.86	0.72	0.80	0.67	1.22	1.08	0.51	0.37	0.49	0.63	0.80	0.66	-0.30	-0.05	-0.01	-0.20	-0.14	0.34	0.40	-0.24
O95881	Thioredoxin domain-containing protein 12 OS=Homo sapiens GN=TXNDC12 PE=1 SV=1 - [TXD12_HUMAN]	36.05	1	5	5	12	-0.07	0.18	0.36	0.61	0.21	0.45	0.06	0.31	0.65	0.41	0.41	0.65	0.19	0.48	0.04	0.51	0.08	0.27	-0.17	-0.24

Q9NWH9	SAFB-like transcription modulator OS=Homo sapiens GN=SLTM PE=1 SV=2 - [SLTM_HUMAN]	6.96	1	5	5	6	0.02	-0.05	-0.09	-0.21	-0.10	-0.24	-0.60	-0.64	-0.22	-0.05	0.28	0.16	-0.41	0.18	0.29	-0.13	-0.01	-0.14	-0.12	-0.24
Q13510	Acid ceramidase OS=Homo sapiens GN=ASAH1 PE=1 SV=5 - [ASAH1_HUMAN]	36.46	1	14	14	32	0.57	0.64	0.77	0.83	0.16	0.23	0.54	0.58	0.50	0.40	0.57	0.59	-0.02	0.12	-0.25	-0.14	-0.35	-0.51	-0.67	-0.24
Q16740	Putative ATP-dependent Clp protease proteolytic subunit, mitochondrial OS=Homo sapiens GN=CLPP PE=1 SV=1 - [CLPP_HUMAN]	25.99	1	5	5	15	-0.34	-0.14	-0.06	0.02	-0.31	-0.03	-0.33	-0.18	-0.30	-0.39	-0.16	-0.04	-0.20	0.19	-0.08	-0.15	-0.19	-0.16	-0.22	-0.24
O76041	Nebulette OS=Homo sapiens GN=NEBL PE=1 SV=1 - [NEBL_HUMAN]	7.89	2	6	6	34	0.19	0.20	0.17	0.17	-0.48	-0.48	-0.03	0.03	-0.11	-0.16	-0.12	-0.07	-0.22	-0.24	-0.26	-0.26	-0.29	-0.66	-0.64	-0.24
Q9H3K6	BolA-like protein 2 OS=Homo sapiens GN=BOLA2 PE=1 SV=1 - [BOLA2_HUMAN]	18.60	1	2	2	2	-0.29	-0.23	0.15	0.21	0.39	0.45	-0.15	-0.09	0.13	0.07	-0.18	-0.12	0.19	0.12	-0.33	0.39	-0.05	0.66	0.22	-0.24
Q3V6T2	Girdin OS=Homo sapiens GN=CCDC88A PE=1 SV=2 - [GRDN_HUMAN]	4.22	1	8	8	10	0.19	0.40	-0.11	0.11	-0.20	0.01	-0.41	-0.20	0.06	-0.15	-0.30	-0.09	-0.54	-0.76	-0.43	-0.82	-0.48	-0.81	-0.47	-0.24
Q9Y2Z0	Suppressor of G2 allele of SKP1 homolog OS=Homo sapiens GN=SUGT1 PE=1 SV=3 - [SUGT1_HUMAN]	52.33	1	15	15	34	0.47	0.34	0.61	0.54	0.08	0.07	0.19	0.08	-0.23	-0.09	-0.46	-0.52	-0.18	-0.89	-0.96	-0.49	-0.48	-0.50	-0.42	-0.24
Q13636	Ras-related protein Rab-31 OS=Homo sapiens GN=RAB31 PE=1 SV=1 - [RAB31_HUMAN]	41.24	1	5	7	15	1.43	1.42	1.05	1.05	0.68	0.68	0.75	0.75	0.79	0.80	0.39	0.38	-0.62	-1.03	-0.67	-0.60	-0.22	-0.76	-0.39	-0.24

O43290	U4/U6.U5 tri-snRNP-associated protein 1 OS=Homo sapiens GN=SART1 PE=1 SV=1 - [SNUT1_HUMAN]	15.75	1	9	9	16	-0.10	0.31	-0.30	0.00	-0.46	-0.31	-0.61	-0.25	-0.29	-0.48	-0.25	-0.16	-0.48	-0.34	-0.10	-0.26	-0.02	-0.14	0.03	-0.24
Q13608	Peroxisome assembly factor 2 OS=Homo sapiens GN=PEX6 PE=1 SV=2 - [PEX6_HUMAN]	3.37	1	2	2	3	0.64	0.26	0.26	-0.12	0.36	-0.03	-0.04	-0.42	-0.22	0.16	-0.02	-0.40	-0.62	-0.65	-0.28	-0.44	-0.07	-0.30	0.08	-0.24
Q04323	UBX domain-containing protein 1 OS=Homo sapiens GN=UBXN1 PE=1 SV=2 - [UBXN1_HUMAN]	21.89	1	4	4	8	0.57	0.41	0.79	0.58	0.29	0.22	0.40	0.29	0.10	0.26	0.16	0.01	-0.19	-0.42	-0.64	-0.27	-0.50	-0.30	-0.57	-0.24
P08621	U1 small nuclear ribonucleoprotein 70 kDa OS=Homo sapiens GN=SNRNP70 PE=1 SV=2 - [RU17_HUMAN]	22.43	1	10	10	21	-0.25	-0.21	-0.15	-0.20	-0.23	-0.25	-0.42	-0.31	-0.26	-0.30	0.02	0.01	-0.08	0.27	0.21	-0.08	-0.11	0.00	-0.09	-0.24
Q96BM9	ADP-ribosylation factor-like protein 8A OS=Homo sapiens GN=ARL8A PE=1 SV=1 - [ARL8A_HUMAN]	32.26	1	1	5	12	0.65	0.28	1.01	0.63	0.73	0.35	0.70	0.33	0.02	0.41	0.54	0.16	0.10	-0.11	-0.47	-0.22	-0.57	0.06	-0.29	-0.24
Q9H063	Repressor of RNA polymerase III transcription MAF1 homolog OS=Homo sapiens GN=MAF1 PE=1 SV=2 - [MAF1_HUMAN]	10.16	1	1	1	1	-0.08	-0.47	0.13	-0.26	0.61	0.21	-0.17	-0.57	-0.58	-0.18	-0.68	-1.08	-0.04	-0.59	-0.81	-0.07	-0.28	0.67	0.46	-0.24
P55081	Microfibrillar-associated protein 1 OS=Homo sapiens GN=MFAP1 PE=1 SV=2 - [MFAP1_HUMAN]	25.06	1	5	6	9	0.69	-0.04	0.03	-0.13	0.47	0.26	0.03	-0.44	-0.01	-0.05	0.08	-0.13	-0.34	-0.08	0.00	0.07	0.16	-0.23	0.42	-0.24
O75915	PRA1 family protein 3 OS=Homo sapiens GN=ARL6IP5 PE=1 SV=1 - [PRAF3_HUMAN]	23.94	1	5	5	28	0.30	0.24	0.20	0.18	0.03	-0.05	-0.15	-0.13	-0.04	0.05	0.12	-0.02	-0.25	-0.05	-0.16	-0.24	-0.23	-0.31	-0.21	-0.24

Q9P2D3	HEAT repeat-containing protein 5B OS=Homo sapiens GN=HEATR5B PE=1 SV=2 - [HTR5B_HUMAN]	2.70	1	3	3	6	0.28	0.47	0.37	0.56	0.02	0.21	0.06	0.25	0.32	0.13	-0.19	-0.01	-0.16	-0.47	-0.56	-0.12	-0.20	-0.28	-0.37	-0.24
O75937	DnaJ homolog subfamily C member 8 OS=Homo sapiens GN=DNAJC8 PE=1 SV=2 - [DNJC8_HUMAN]	13.04	1	2	2	3	0.11	-0.17	0.55	0.28	-0.01	-0.29	0.25	-0.03	-0.25	0.03	-0.01	-0.29	0.19	-0.11	-0.56	-0.05	-0.49	-0.14	-0.58	-0.24
Q9BUK6	Protein misato homolog 1 OS=Homo sapiens GN=MSTO1 PE=1 SV=1 - [MSTO1_HUMAN]	5.96	1	2	2	3	-0.09	-0.03	0.08	0.14	-0.09	-0.03	-0.23	-0.17	-0.20	-0.25	-0.21	-0.16	-0.09	-0.12	-0.29	-0.13	-0.30	-0.02	-0.18	-0.24
Q9UEU0	Vesicle transport through interaction with t-SNAREs homolog 1B OS=Homo sapiens GN=VTI1B PE=1 SV=3 - [VTI1B_HUMAN]	37.93	1	7	7	9	0.14	0.17	0.05	0.11	-0.28	-0.31	-0.14	-0.14	-0.08	0.04	0.18	0.07	-0.31	-0.32	-0.27	-0.39	-0.33	-0.51	-0.63	-0.24
O94906	Pre-mRNA-processing factor 6 OS=Homo sapiens GN=PRPF6 PE=1 SV=1 - [PRP6_HUMAN]	12.75	1	10	10	15	0.05	-0.09	0.00	-0.14	-0.20	-0.63	-0.36	-0.45	-0.33	-0.30	-0.14	-0.21	-0.18	-0.15	-0.15	-0.29	-0.17	-0.31	-0.36	-0.24
Q15311	RalA-binding protein 1 OS=Homo sapiens GN=RALBP1 PE=1 SV=3 - [RBP1_HUMAN]	10.38	1	6	6	8	0.35	0.27	0.45	0.37	0.32	0.23	0.14	0.06	0.21	0.30	0.04	-0.05	-0.16	-0.31	-0.41	-0.03	-0.12	-0.05	-0.15	-0.24
P01009	Alpha-1-antitrypsin OS=Homo sapiens GN=SERPINA1 PE=1 SV=3 - [A1AT_HUMAN]	49.28	3	19	19	95	-1.50	-1.49	-1.17	-1.17	-0.99	-1.04	-1.42	-1.43	-1.60	-1.50	-1.51	-1.52	-0.01	-0.08	-0.32	-0.05	-0.33	0.41	0.11	-0.24
Q3ZAQ7	Vacuolar ATPase assembly integral membrane protein VMA21 OS=Homo sapiens GN=VMA21 PE=1 SV=1 - [VMA21_HUMAN]	11.88	1	1	1	5	-0.27	-0.14	-0.09	0.04	-0.49	-0.37	-0.40	-0.27	-0.07	-0.19	0.21	0.34	-0.07	0.49	0.30	0.11	-0.07	-0.24	-0.42	-0.24

P22307	Non-specific lipid-transfer protein OS=Homo sapiens GN=SCP2 PE=1 SV=2 - [NLTP_HUMAN]	31.81	1	14	14	26	-0.45	-0.50	-0.16	-0.07	0.07	0.02	-0.31	-0.32	-0.22	-0.13	0.18	0.23	0.14	0.60	0.24	0.23	-0.13	0.46	0.08	-0.24
P36873	Serine/threonine-protein phosphatase PP1-gamma catalytic subunit OS=Homo sapiens GN=PPP1CC PE=1 SV=1 - [PP1G_HUMAN]	39.32	1	2	10	23	0.97	1.11	0.76	0.90	0.46	0.60	0.45	0.60	0.69	0.55	0.64	0.78	-0.46	-0.33	-0.12	-0.39	-0.17	-0.53	-0.32	-0.24
Q8NHP6	Motile sperm domain-containing protein 2 OS=Homo sapiens GN=MOSPD2 PE=1 SV=1 - [MSPD2_HUMAN]	13.71	1	6	6	10	0.00	0.04	0.37	0.41	-0.19	-0.15	-0.24	0.03	0.09	0.10	0.22	0.18	-0.17	0.14	-0.19	0.14	-0.06	-0.20	-0.57	-0.25
Q86SK9	Stearoyl-CoA desaturase 5 OS=Homo sapiens GN=SCD5 PE=1 SV=2 - [SCD5_HUMAN]	9.70	1	2	2	5	0.14	0.21	0.45	0.52	-0.28	-0.27	-0.05	-0.03	-0.16	-0.21	-0.46	-0.39	-0.05	-0.59	-0.91	-0.25	-0.56	-0.36	-0.56	-0.25
O14936	Peripheral plasma membrane protein CASK OS=Homo sapiens GN=CASK PE=1 SV=3 - [CSKP_HUMAN]	28.83	1	20	20	35	0.46	0.45	0.41	0.33	-0.02	0.01	0.09	0.26	0.05	-0.07	-0.03	-0.04	-0.32	-0.41	-0.46	-0.38	-0.27	-0.49	-0.45	-0.25
Q13224	Glutamate receptor ionotropic, NMDA 2B OS=Homo sapiens GN=GRIN2B PE=1 SV=3 - [NMDE2_HUMAN]	3.77	1	5	5	7	1.26	1.60	1.44	1.78	-0.48	-0.31	1.13	1.20	0.32	0.43	-0.06	-0.30	-0.11	-1.31	-1.50	-0.48	-0.66	-2.38	-2.30	-0.25
P06576	ATP synthase subunit beta, mitochondrial OS=Homo sapiens GN=ATP5B PE=1 SV=3 - [ATPB_HUMAN]	70.13	1	26	26	382	0.50	0.46	0.57	0.51	0.17	0.15	0.19	0.23	0.11	0.11	0.16	0.13	-0.17	-0.29	-0.33	-0.30	-0.43	-0.33	-0.33	-0.25

Q9Y4P8	WD repeat domain phosphoinositide-interacting protein 2 OS=Homo sapiens GN=WIPI2 PE=1 SV=1 - [WIPI2_HUMAN]	12.33	1	4	4	7	0.42	0.49	0.65	0.63	-0.04	-0.14	0.31	0.40	0.03	0.06	-0.06	-0.04	-0.06	-0.57	-0.81	-0.28	-0.56	-0.45	-0.82	-0.25
Q13617	Cullin-2 OS=Homo sapiens GN=CUL2 PE=1 SV=2 - [CUL2_HUMAN]	17.58	1	11	11	19	-0.01	-0.22	0.49	0.12	-0.30	-0.46	0.14	-0.02	-0.28	-0.12	0.19	-0.06	0.21	0.00	0.10	-0.03	-0.49	-0.40	-0.51	-0.25
Q00613	Heat shock factor protein 1 OS=Homo sapiens GN=HSF1 PE=1 SV=1 - [HSF1_HUMAN]	3.02	1	1	1	2	1.17	0.77	0.63	0.23	0.67	0.27	0.32	-0.08	-0.07	0.33	0.43	0.03	-0.79	-0.73	-0.20	-0.80	-0.27	-0.51	0.02	-0.25
Q9Y296	Trafficking protein particle complex subunit 4 OS=Homo sapiens GN=TRAPPC4 PE=1 SV=1 - [TPPC4_HUMAN]	11.42	1	2	2	3	0.20	0.30	0.15	0.26	-0.15	-0.05	-0.16	-0.05	0.10	0.00	-0.03	0.08	-0.30	-0.22	-0.18	-0.17	-0.12	-0.37	-0.32	-0.25
P14317	Hematopoietic lineage cell-specific protein OS=Homo sapiens GN=HCLS1 PE=1 SV=3 - [HCLS1_HUMAN]	23.46	1	8	8	14	0.66	0.58	0.42	0.40	0.19	0.22	0.05	0.14	-0.13	0.02	-0.22	-0.12	-0.50	-0.69	-0.76	-0.66	-0.53	-0.66	-0.41	-0.25
Q13371	Phosducin-like protein OS=Homo sapiens GN=PDCL PE=1 SV=3 - [PHLP_HUMAN]	9.63	1	2	2	6	0.44	0.75	0.27	0.58	0.29	0.59	-0.04	0.26	0.42	0.12	0.17	0.47	-0.43	-0.27	-0.10	-0.29	-0.11	-0.17	0.00	-0.25
Q86VP6	Cullin-associated NEDD8-dissociated protein 1 OS=Homo sapiens GN=CAND1 PE=1 SV=2 - [CAND1_HUMAN]	30.08	1	28	30	68	0.24	0.29	0.42	0.38	0.07	0.08	-0.02	0.00	0.02	0.04	0.14	0.18	-0.18	-0.02	-0.22	-0.10	-0.24	-0.11	-0.20	-0.25
Q9P015	39S ribosomal protein L15, mitochondrial OS=Homo sapiens GN=MRPL15 PE=1 SV=1 - [RM15_HUMAN]	12.84	1	3	3	5	-0.28	-0.04	0.09	0.33	-0.23	0.01	-0.23	0.02	0.14	-0.10	0.04	0.28	0.11	0.33	-0.05	0.21	-0.16	0.04	-0.33	-0.25

O43741	5'-AMP-activated protein kinase subunit beta-2 OS=Homo sapiens GN=PRKAB2 PE=1 SV=1 - [AAKB2_HUMAN]	10.66	1	2	2	3	-0.20	-0.21	0.60	0.60	-0.14	-0.15	0.29	0.28	0.11	0.12	-0.06	-0.07	0.54	0.14	-0.67	0.35	-0.45	0.04	-0.76	-0.25
Q8NBN7	Retinol dehydrogenase 13 OS=Homo sapiens GN=RDH13 PE=1 SV=2 - [RDH13_HUMAN]	22.96	1	6	6	8	-0.57	-0.20	-0.18	-0.02	-0.56	-0.42	-0.49	-0.33	-0.49	-0.33	-0.69	-0.59	-0.10	-0.16	-0.39	-0.09	-0.31	-0.01	-0.30	-0.25
O95831	Apoptosis-inducing factor 1, mitochondrial OS=Homo sapiens GN=AIFM1 PE=1 SV=1 - [AIFM1_HUMAN]	23.49	1	12	12	21	0.02	0.13	0.05	0.06	-0.51	-0.50	-0.18	-0.27	-0.28	-0.24	0.02	-0.05	-0.13	0.02	-0.19	-0.22	-0.26	-0.36	-0.51	-0.25
Q96DZ1	Endoplasmic reticulum lectin 1 OS=Homo sapiens GN=ERLEC1 PE=1 SV=1 - [ERLEC_HUMAN]	8.49	1	3	3	3	0.67	0.34	0.43	-0.18	0.87	0.81	0.23	-0.50	-0.01	0.51	0.69	0.63	-0.78	0.03	0.26	-0.32	0.21	0.18	0.42	-0.25
Q8N9N7	Leucine-rich repeat-containing protein 57 OS=Homo sapiens GN=LRRC57 PE=1 SV=1 - [LRC57_HUMAN]	29.71	1	7	7	7	-0.38	-0.25	0.17	0.23	0.14	0.18	-0.17	-0.16	0.23	0.15	0.31	0.46	-0.03	0.62	0.35	0.48	0.16	0.41	0.07	-0.25
Q99943	1-acyl-sn-glycerol-3-phosphate acyltransferase alpha OS=Homo sapiens GN=AGPAT1 PE=1 SV=2 - [PLCA_HUMAN]	18.02	1	3	3	5	0.01	-0.26	0.41	0.14	0.06	-0.21	0.10	-0.17	0.20	-0.08	0.14	-0.14	0.15	0.14	-0.27	0.50	0.09	0.04	-0.36	-0.25
P28482	Mitogen-activated protein kinase 1 OS=Homo sapiens GN=MAPK1 PE=1 SV=3 - [MK01_HUMAN]	54.17	6	11	19	63	0.07	0.10	0.32	0.19	0.14	0.23	0.05	0.07	0.04	-0.02	0.03	0.00	0.00	0.07	-0.26	0.06	-0.19	0.14	-0.05	-0.25
Q9ULP9	TBC1 domain family member 24 OS=Homo sapiens GN=TBC1D24 PE=1 SV=2 - [TBC24_HUMAN]	12.16	1	6	6	12	0.61	0.60	0.83	0.89	-0.17	-0.11	0.40	0.50	-0.12	-0.03	-0.34	-0.35	-0.28	-0.98	-1.21	-0.68	-0.96	-0.78	-0.86	-0.25

Q8IWB7	WD repeat and FYVE domain-containing protein 1 OS=Homo sapiens GN=WDFY1 PE=1 SV=1 - [WDFY1_HUMAN]	11.46	1	4	4	5	-0.15	0.02	0.03	0.20	-0.06	0.06	-0.29	-0.04	0.08	-0.09	0.00	0.01	-0.01	0.19	-0.12	0.00	-0.08	-0.02	-0.11	-0.25
P23246	Splicing factor, proline and glutamine-rich OS=Homo sapiens GN=SFPQ PE=1 SV=2 - [SFPQ_HUMAN]	27.02	1	16	17	40	-0.28	-0.25	-0.24	-0.24	-0.59	-0.63	-0.59	-0.63	-0.77	-0.74	-0.41	-0.52	-0.22	-0.23	-0.18	-0.43	-0.46	-0.41	-0.42	-0.25
Q9HD42	Charged multivesicular body protein 1a OS=Homo sapiens GN=CHMP1A PE=1 SV=1 - [CHM1A_HUMAN]	15.82	1	3	4	7	-0.40	-0.43	-0.06	-0.09	0.14	0.08	-0.39	-0.43	-0.17	-0.11	-0.11	-0.16	0.08	0.28	-0.06	0.19	-0.15	0.35	0.01	-0.25
Q5TCY1	Tau-tubulin kinase 1 OS=Homo sapiens GN=TTBK1 PE=1 SV=2 - [TTBK1_HUMAN]	2.35	1	2	2	3	1.46	0.31	1.08	0.46	0.53	-0.16	0.67	0.14	0.56	1.73	0.47	-0.21	-0.12	-0.99	-0.61	0.28	0.14	-0.95	-0.57	-0.25
Q13017	Rho GTPase-activating protein 5 OS=Homo sapiens GN=ARHGAP5 PE=1 SV=2 - [RHG05_HUMAN]	15.45	1	14	14	20	0.40	0.44	0.54	0.66	0.30	0.35	0.41	0.34	0.30	0.25	0.02	0.11	-0.17	-0.47	-0.46	-0.19	-0.47	-0.29	-0.43	-0.25
Q86WU2	Probable D-lactate dehydrogenase, mitochondrial OS=Homo sapiens GN=LDHD PE=1 SV=1 - [LDHD_HUMAN]	10.45	1	4	4	5	0.82	0.82	0.71	0.70	-0.03	-0.04	0.39	0.38	0.35	0.36	0.22	0.21	-0.38	-0.60	-0.49	-0.43	-0.31	-0.87	-0.75	-0.26
Q9Y3L5	Ras-related protein Rap-2c OS=Homo sapiens GN=RAP2C PE=1 SV=1 - [RAP2C_HUMAN]	49.18	1	1	6	15	1.14	1.36	1.04	1.26	0.35	0.57	0.72	0.94	0.45	0.24	0.51	0.73	-0.37	-0.63	-0.53	-0.87	-0.77	-0.81	-0.70	-0.26

Q8TB45	DEP domain-containing mTOR-interacting protein OS=Homo sapiens GN=DEPTOR PE=1 SV=2 - [DPTOR_HUMAN]	7.33	1	2	2	2	0.10	0.15	0.37	0.43	-0.05	0.00	0.05	0.11	-0.13	-0.18	-0.20	-0.16	0.01	-0.29	-0.58	-0.25	-0.52	-0.16	-0.44	-0.26
O14807	Ras-related protein M-Ras OS=Homo sapiens GN=MRAS PE=1 SV=2 - [RASM_HUMAN]	50.00	1	8	8	12	0.59	0.70	0.98	1.03	0.43	0.19	0.78	0.98	0.94	0.76	0.72	0.51	-0.04	-0.33	-0.69	-0.06	-0.41	-0.61	-0.94	-0.26
P00338	L-lactate dehydrogenase A chain OS=Homo sapiens GN=LDHA PE=1 SV=2 - [LDHA_HUMAN]	48.49	3	15	16	51	0.03	0.03	0.30	0.35	0.11	0.04	-0.05	0.01	-0.08	-0.01	0.18	0.15	0.17	0.14	-0.06	-0.09	-0.21	0.11	-0.17	-0.26
P82673	28S ribosomal protein S35, mitochondrial OS=Homo sapiens GN=MRPS35 PE=1 SV=1 - [RT35_HUMAN]	18.27	1	4	4	5	0.13	-0.10	0.58	0.34	0.18	-0.06	0.26	0.02	-0.18	0.06	0.23	-0.01	0.18	0.10	-0.35	-0.04	-0.48	0.03	-0.42	-0.26
Q6NSW5	Protein FAM45B OS=Homo sapiens GN=FAM45B PE=2 SV=1 - [FA45B_HUMAN]	9.80	2	3	3	5	0.01	0.06	0.21	0.21	0.60	0.56	-0.05	0.04	0.19	0.10	0.16	0.20	-0.04	0.15	0.00	-0.06	-0.32	0.50	0.21	-0.26
Q13442	28 kDa heat- and acid-stable phosphoprotein OS=Homo sapiens GN=PDAP1 PE=1 SV=1 - [HAP28_HUMAN]	13.26	1	2	2	3	-0.63	-0.77	-0.40	-0.54	-0.42	-0.56	-0.72	-0.86	-0.86	-0.72	-0.72	-0.86	-0.04	-0.08	-0.31	-0.06	-0.28	0.19	-0.03	-0.26
Q8NAN2	Protein FAM73A OS=Homo sapiens GN=FAM73A PE=2 SV=1 - [FA73A_HUMAN]	3.32	1	1	1	2	0.57	0.28	0.19	-0.09	0.92	0.64	-0.13	-0.41	0.37	0.66	0.25	-0.03	-0.64	-0.31	0.06	0.12	0.50	0.34	0.71	-0.26
Q8WVV9	Heterogeneous nuclear ribonucleoprotein L-like OS=Homo sapiens GN=HNRNPLL PE=1 SV=1 - [HNRLL_HUMAN]	21.03	1	7	8	16	0.30	0.16	0.28	0.11	-0.22	-0.27	0.05	-0.05	-0.03	-0.10	-0.23	-0.16	-0.21	-0.45	-0.51	-0.30	-0.33	-0.34	-0.46	-0.26

Q9Y6B6	GTP-binding protein SAR1b OS=Homo sapiens GN=SAR1B PE=1 SV=1 - [SAR1B_HUMAN]	32.83	1	3	5	10	0.76	0.82	0.73	0.79	0.65	0.70	0.41	0.47	0.32	0.26	0.17	0.22	-0.30	-0.59	-0.56	-0.47	-0.43	-0.13	-0.10	-0.26
Q13618	Cullin-3 OS=Homo sapiens GN=CUL3 PE=1 SV=2 - [CUL3_HUMAN]	30.34	1	19	19	28	0.29	0.27	0.41	0.38	-0.19	0.04	0.07	0.10	0.06	0.06	0.20	0.14	-0.09	-0.11	-0.12	-0.16	-0.29	-0.45	-0.51	-0.26
P09661	U2 small nuclear ribonucleoprotein A' OS=Homo sapiens GN=SNRPA1 PE=1 SV=2 - [RU2A_HUMAN]	30.98	1	5	5	8	-0.15	-0.09	-0.37	-0.35	-0.47	-0.44	-0.71	-0.63	-0.40	-0.64	-0.58	-0.37	-0.35	-0.44	-0.02	-0.19	0.01	-0.43	-0.04	-0.26
Q15366	Poly(rC)-binding protein 2 OS=Homo sapiens GN=PCBP2 PE=1 SV=1 - [PCBP2_HUMAN]	35.89	2	4	9	39	0.42	0.43	0.35	0.34	0.04	-0.05	0.10	0.13	-0.22	-0.13	-0.25	-0.36	-0.21	-0.64	-0.72	-0.45	-0.45	-0.39	-0.33	-0.26
P22059	Oxysterol-binding protein 1 OS=Homo sapiens GN=OSBP PE=1 SV=1 - [OSBP1_HUMAN]	29.49	1	16	17	38	-0.09	-0.20	-0.07	-0.17	-0.34	-0.44	-0.52	-0.57	-0.56	-0.46	-0.24	-0.31	-0.40	-0.20	-0.18	-0.38	-0.18	-0.48	-0.28	-0.26
Q8WZA9	Immunity-related GTPase family Q protein OS=Homo sapiens GN=IRGQ PE=1 SV=1 - [IRGQ_HUMAN]	8.99	1	5	5	10	0.50	0.63	0.15	0.33	-0.28	0.10	-0.04	0.12	0.06	-0.06	-0.50	-0.39	-0.67	-0.86	-0.72	-0.41	-0.23	-0.26	-0.24	-0.26
P48047	ATP synthase subunit O, mitochondrial OS=Homo sapiens GN=ATP5O PE=1 SV=1 - [ATPO_HUMAN]	48.83	1	9	9	21	-0.05	0.00	0.48	0.28	-0.06	-0.11	0.13	0.07	-0.05	0.04	-0.05	-0.07	0.06	-0.40	-0.57	-0.07	-0.39	-0.46	-0.65	-0.26
Q12906	Interleukin enhancer-binding factor 3 OS=Homo sapiens GN=ILF3 PE=1 SV=3 - [ILF3_HUMAN]	31.66	1	19	21	47	-0.28	-0.21	-0.13	-0.07	-0.36	-0.35	-0.43	-0.42	-0.37	-0.40	-0.26	-0.18	-0.10	-0.06	-0.15	-0.11	-0.21	-0.10	-0.13	-0.26

O00567	Nucleolar protein 56 OS=Homo sapiens GN=NOP56 PE=1 SV=4 - [NOP56_HUMAN]	19.36	1	9	9	17	0.39	0.44	0.31	0.35	0.38	0.18	0.25	0.08	-0.01	0.05	0.08	-0.09	0.05	-0.26	-0.27	-0.30	-0.32	-0.11	0.11	-0.26
P13861	cAMP-dependent protein kinase type II-alpha regulatory subunit OS=Homo sapiens GN=PRKAR2A PE=1 SV=2 - [KAP2_HUMAN]	51.24	1	14	16	35	0.29	0.25	0.28	0.21	-0.13	-0.09	-0.10	-0.15	0.05	0.03	0.12	0.13	-0.33	-0.04	-0.06	-0.12	-0.07	-0.26	-0.31	-0.26
P31689	DnaJ homolog subfamily A member 1 OS=Homo sapiens GN=DNAJA1 PE=1 SV=2 - [DNJA1_HUMAN]	32.24	1	9	9	23	-0.06	0.04	0.28	0.34	0.13	0.12	-0.23	-0.04	-0.05	-0.02	0.16	0.05	0.13	0.25	-0.18	-0.01	-0.48	0.22	-0.16	-0.26
Q9NTX5	Ethylmalonyl-CoA decarboxylase OS=Homo sapiens GN=ECHDC1 PE=1 SV=2 - [ECHD1_HUMAN]	37.46	1	9	9	19	-0.36	-0.28	-0.18	-0.12	-0.08	0.02	-0.44	-0.36	-0.13	-0.19	0.27	0.26	-0.03	0.55	0.32	0.16	0.02	0.26	0.11	-0.26
Q8NFX7	Syntaxin-binding protein 6 OS=Homo sapiens GN=STXBP6 PE=1 SV=2 - [STXB6_HUMAN]	37.62	1	7	7	12	0.53	0.53	0.79	0.56	0.40	0.44	0.34	0.24	0.22	0.31	0.14	0.15	-0.25	-0.34	-0.61	-0.30	-0.51	-0.15	-0.37	-0.26
O96005	Cleft lip and palate transmembrane protein 1 OS=Homo sapiens GN=CLPTM1 PE=1 SV=1 - [CLPT1_HUMAN]	9.27	1	4	4	5	0.12	-0.01	0.35	0.22	-0.30	-0.41	-0.09	-0.15	-0.60	-0.49	-0.11	-0.17	-0.20	-0.12	-0.35	-0.03	-0.26	0.15	-0.07	-0.26
O75064	DENN domain-containing protein 4B OS=Homo sapiens GN=DENND4B PE=1 SV=4 - [DEN4B_HUMAN]	6.02	1	5	5	6	1.25	0.25	1.04	0.43	-0.10	-0.10	0.15	0.10	-0.19	-0.12	-0.16	-0.48	-0.10	-0.73	-0.90	-0.40	-0.57	-0.42	-0.60	-0.26

Q9UBL3	Set1/Ash2 histone methyltransferase complex subunit ASH2 OS=Homo sapiens GN=ASH2L PE=1 SV=1 - [ASH2L_HUMAN]	4.14	1	2	2	2	-0.72	-0.35	-0.32	0.05	-0.84	-0.73	-0.65	-0.27	-0.09	-0.46	-0.50	-0.13	0.13	0.23	-0.18	0.29	-0.11	-0.46	-0.54	-0.26
P53701	Cytochrome c-type heme lyase OS=Homo sapiens GN=HCCS PE=1 SV=1 - [CCHL_HUMAN]	19.40	1	5	5	6	0.09	0.62	0.19	0.72	-0.69	-0.17	-0.13	0.40	0.33	-0.19	-0.12	0.40	-0.17	-0.20	-0.31	-0.25	-0.35	-0.80	-0.90	-0.26
Q14699	Raftlin OS=Homo sapiens GN=RFTN1 PE=1 SV=4 - [RFTN1_HUMAN]	2.60	1	2	2	3	-0.35	-0.20	0.16	0.30	0.28	0.42	-0.17	-0.02	0.04	-0.10	-0.46	-0.32	0.23	-0.10	-0.61	0.28	-0.22	0.61	0.11	-0.26
Q6UWP2	Dehydrogenase/reductase SDR family member 11 OS=Homo sapiens GN=DHRS11 PE=1 SV=1 - [DHR11_HUMAN]	15.00	1	3	3	5	0.29	0.14	0.44	-0.15	0.78	0.27	0.03	-0.48	-0.76	-0.24	0.26	-0.26	-0.56	-0.02	-0.18	-0.50	-0.64	0.48	0.33	-0.26
Q15661	Tryptase alpha/beta-1 OS=Homo sapiens GN=TPSAB1 PE=1 SV=1 - [TRYB1_HUMAN]	22.91	2	5	5	10	-0.95	-0.65	-1.72	-1.43	1.69	2.09	-1.84	-1.55	0.45	0.28	-1.59	-1.30	-0.46	-0.12	-0.02	1.38	2.08	2.85	3.51	-0.26
Q9H019	Mitochondrial fission regulator 1-like OS=Homo sapiens GN=MTFR1L PE=1 SV=2 - [MFR1L_HUMAN]	30.14	1	6	6	12	0.78	0.82	0.69	0.78	0.05	0.14	0.36	0.45	0.18	0.22	0.32	0.41	-0.36	-0.63	-0.48	-0.53	-0.43	-0.69	-0.66	-0.26
Q9Y2U8	Inner nuclear membrane protein Man1 OS=Homo sapiens GN=LEMD3 PE=1 SV=2 - [MAN1_HUMAN]	9.11	1	6	6	11	-0.88	-1.02	-0.35	-0.48	-0.74	-0.81	-0.75	-0.83	-0.65	-0.86	-0.38	-0.52	0.18	0.51	0.02	0.41	-0.14	0.13	-0.28	-0.26
Q9NZT2	Opioid growth factor receptor OS=Homo sapiens GN=OGFR PE=1 SV=3 - [OGFR_HUMAN]	3.69	1	2	2	4	0.67	0.73	0.56	0.62	0.12	0.17	0.23	0.29	0.30	0.25	0.26	0.31	-0.38	-0.41	-0.30	-0.39	-0.28	-0.57	-0.46	-0.27

A6NDB9	Paralemmin-3 OS=Homo sapiens GN=PALM3 PE=1 SV=2 - [PALM3_HUMAN]	2.82	1	1	2	3	1.13	1.12	1.07	1.06	-0.39	-0.41	0.74	0.73	0.15	0.16	0.16	0.14	-0.34	-0.97	-0.91	-0.94	-0.87	-1.54	-1.48	-0.27
P34913	Bifunctional epoxide hydrolase 2 OS=Homo sapiens GN=EPHX2 PE=1 SV=2 - [HYES_HUMAN]	14.05	1	5	5	6	0.82	1.08	0.68	1.12	0.12	0.41	0.49	0.79	0.45	0.16	0.09	0.38	-0.24	-0.69	-0.74	-0.59	-0.63	-0.68	-0.72	-0.27
P98196	Probable phospholipid-transporting ATPase IH OS=Homo sapiens GN=ATP11A PE=1 SV=3 - [AT11A_HUMAN]	1.15	1	1	1	2	0.32	0.46	0.20	0.34	0.15	0.29	-0.13	0.01	0.18	0.05	0.28	0.42	-0.40	-0.03	0.08	-0.25	-0.12	-0.19	-0.07	-0.27
O94830	Phospholipase DDHD2 OS=Homo sapiens GN=DDHD2 PE=1 SV=2 - [DDHD2_HUMAN]	14.21	1	8	8	10	0.89	0.62	0.80	0.41	0.15	0.35	0.44	0.42	0.34	0.14	0.09	0.29	-0.36	-0.53	-0.45	-0.39	-0.22	-0.55	-0.47	-0.27
A4D126	Isoprenoid synthase domain-containing protein OS=Homo sapiens GN=ISPD PE=1 SV=2 - [ISPD_HUMAN]	7.54	1	2	3	3	-0.09	0.37	-0.01	0.45	-0.13	0.33	-0.34	0.12	0.71	0.25	-0.15	0.31	-0.19	-0.05	-0.14	0.38	0.30	-0.05	-0.14	-0.27
O14562	Ubiquitin domain-containing protein UBFD1 OS=Homo sapiens GN=UBFD1 PE=1 SV=2 - [UBFD1_HUMAN]	11.00	1	3	3	5	-0.01	-0.04	0.25	0.59	-0.64	-0.41	-0.08	0.35	-0.35	-0.51	-0.93	-0.72	0.08	-0.81	-1.18	-0.37	-0.87	-0.55	-1.01	-0.27
Q92552	28S ribosomal protein S27, mitochondrial OS=Homo sapiens GN=MRPS27 PE=1 SV=3 - [RT27_HUMAN]	10.39	1	4	4	7	0.17	0.19	0.00	0.20	0.28	0.22	-0.25	-0.26	-0.07	-0.09	0.60	0.59	-0.28	0.15	0.19	-0.21	-0.21	-0.19	-0.20	-0.27

Q6PCB6	Alpha/beta hydrolase domain-containing protein 17C OS=Homo sapiens GN=ABHD17C PE=2 SV=2 - [AB17C_HUMAN]	3.34	1	1	1	1	1.87	1.18	1.37	0.67	0.87	0.17	1.04	0.34	0.27	0.97	1.33	0.63	-0.78	-0.53	-0.04	-0.87	-0.37	-1.02	-0.52	-0.27
Q9HAD4	WD repeat-containing protein 41 OS=Homo sapiens GN=WDR41 PE=2 SV=3 - [WDR41_HUMAN]	6.32	1	2	2	2	0.91	0.86	0.84	0.79	-0.25	-0.30	0.50	0.46	-0.07	-0.02	-0.27	-0.32	-0.35	-1.18	-1.11	-0.89	-0.82	-1.18	-1.11	-0.27
O00422	Histone deacetylase complex subunit SAP18 OS=Homo sapiens GN=SAP18 PE=1 SV=1 - [SAP18_HUMAN]	22.88	1	4	4	7	-0.27	-0.05	-0.09	0.05	-0.28	-0.32	-0.39	-0.36	-0.25	-0.34	-0.27	-0.22	-0.10	-0.12	-0.26	-0.08	-0.26	-0.33	-0.32	-0.27
Q16798	NADP-dependent malic enzyme, mitochondrial OS=Homo sapiens GN=ME3 PE=2 SV=2 - [MAON_HUMAN]	33.44	1	15	16	33	0.84	0.66	0.88	0.83	-0.03	-0.09	0.51	0.47	0.01	-0.01	-0.30	-0.28	-0.19	-1.07	-1.13	-0.65	-0.76	-0.73	-0.90	-0.27
P13051	Uracil-DNA glycosylase OS=Homo sapiens GN=UNG PE=1 SV=2 - [UNG_HUMAN]	7.67	1	1	1	2	-0.56	-0.73	-0.10	-0.27	-0.26	-0.44	-0.43	-0.60	-0.84	-0.66	-0.17	-0.35	0.18	0.39	-0.08	-0.08	-0.53	0.28	-0.18	-0.27
Q14156	Protein EFR3 homolog A OS=Homo sapiens GN=EFR3A PE=1 SV=2 - [EFR3A_HUMAN]	11.94	1	7	8	11	0.12	0.15	0.50	0.51	-0.02	-0.07	0.09	0.03	-0.12	-0.23	-0.26	-0.25	0.02	-0.44	-0.59	-0.09	-0.17	-0.15	-0.47	-0.27
Q99733	Nucleosome assembly protein 1-like 4 OS=Homo sapiens GN=NAP1L4 PE=1 SV=1 - [NP1L4_HUMAN]	28.00	1	6	7	27	0.57	0.56	0.51	0.47	0.46	0.34	0.41	0.27	0.42	0.46	0.28	0.20	-0.15	-0.27	-0.40	-0.13	-0.13	-0.29	-0.16	-0.27
O60271	C-Jun-amino-terminal kinase-interacting protein 4 OS=Homo sapiens GN=SPAG9 PE=1 SV=4 - [JIP4_HUMAN]	22.71	1	19	21	36	-0.03	-0.17	0.26	0.11	-0.06	-0.13	0.05	-0.15	-0.27	-0.18	0.05	0.00	-0.05	-0.06	-0.21	-0.20	-0.42	-0.17	-0.44	-0.27

Q9H9C1	Spermatogenesis-defective protein 39 homolog OS=Homo sapiens GN=VIPAS39 PE=1 SV=1 - [SPE39_HUMAN]	18.26	1	8	8	11	0.23	0.02	0.06	0.46	0.07	-0.06	0.14	0.10	-0.07	0.12	-0.13	-0.03	0.22	-0.06	-0.32	-0.03	-0.53	-0.14	-0.36	-0.27
Q9NUJ3	T-complex protein 11-like protein 1 OS=Homo sapiens GN=TCP11L1 PE=1 SV=1 - [T11L1_HUMAN]	11.00	1	5	5	8	0.63	0.80	0.50	0.55	0.25	0.25	0.07	0.28	0.37	0.09	0.04	0.30	-0.36	-0.47	-0.33	-0.48	-0.25	-0.49	-0.47	-0.27
Q8N3R9	MAGUK p55 subfamily member 5 OS=Homo sapiens GN=MPP5 PE=1 SV=3 - [MPP5_HUMAN]	8.30	1	4	4	8	0.43	0.79	0.55	0.95	0.19	0.17	-0.10	0.62	0.28	-0.11	0.05	0.25	-0.03	0.02	-0.41	-0.56	-0.54	-0.21	-0.22	-0.27
P37840	Alpha-synuclein OS=Homo sapiens GN=SNCA PE=1 SV=1 - [SYUA_HUMAN]	57.14	1	5	8	34	-0.39	-0.25	0.04	0.16	-0.48	-0.38	-0.21	-0.15	-0.55	-0.62	-1.04	-0.95	0.12	-0.82	-1.27	-0.19	-0.57	-0.29	-0.63	-0.27
Q8NHV1	GTPase IMAP family member 7 OS=Homo sapiens GN=GIMAP7 PE=1 SV=1 - [GIMA7_HUMAN]	9.67	1	2	2	2	-0.19	0.10	-0.21	0.08	-0.93	-0.65	-0.55	-0.26	-0.26	-0.54	-0.68	-0.39	-0.30	-0.48	-0.47	-0.32	-0.30	-0.75	-0.74	-0.27
Q9ULF5	Zinc transporter ZIP10 OS=Homo sapiens GN=SLC39A10 PE=1 SV=2 - [S39AA_HUMAN]	7.58	1	4	4	8	0.03	0.53	-0.22	0.58	-0.72	-0.36	-0.51	0.25	-0.06	-0.55	-0.08	0.26	-0.52	-0.12	0.12	-0.59	-0.35	-1.04	-0.81	-0.27
Q9GZT8	Putative GTP cyclohydrolase 1 type 2 NIF3L1 OS=Homo sapiens GN=NIF3L1 PE=1 SV=2 - [GTPC1_HUMAN]	25.73	1	8	8	12	0.40	0.44	0.71	0.80	0.27	0.34	0.29	0.29	0.31	0.35	0.32	0.27	-0.11	-0.11	-0.50	-0.18	-0.46	-0.34	-0.48	-0.27
Q9H2U1	Probable ATP-dependent RNA helicase DHX36 OS=Homo sapiens GN=DHX36 PE=1 SV=2 - [DHX36_HUMAN]	9.52	2	7	7	9	-0.21	0.48	0.05	0.18	-0.27	0.22	-0.28	-0.04	0.04	-0.26	0.09	0.00	-0.49	0.30	-0.05	-0.21	-0.42	-0.08	-0.18	-0.27

O60890	Oligophrenin-1 OS=Homo sapiens GN=OPHN1 PE=1 SV=1 - [OPHN1_HUMAN]	5.49	1	3	3	5	0.26	0.45	0.32	0.82	-0.08	0.02	0.38	0.48	0.28	-0.34	-0.09	0.01	0.08	-0.43	-0.80	-0.56	-0.62	-0.44	-0.81	-0.27
Q99755	Phosphatidylinositol 4-phosphate 5-kinase type-1 alpha OS=Homo sapiens GN=PIP5K1A PE=1 SV=1 - [PI51A_HUMAN]	8.72	2	4	5	7	1.00	0.82	0.70	0.73	-0.02	0.31	0.08	0.36	0.54	0.28	0.12	0.38	-0.30	-0.43	-0.42	-0.26	-0.24	-0.48	-0.40	-0.27
P11441	Ubiquitin-like protein 4A OS=Homo sapiens GN=UBL4A PE=1 SV=1 - [UBL4A_HUMAN]	51.59	1	7	7	14	0.27	0.36	0.36	0.40	0.11	0.01	0.08	0.21	0.06	-0.04	0.13	0.15	-0.15	-0.30	-0.40	-0.51	-0.37	-0.40	-0.22	-0.27
Q96PU8	Protein quaking OS=Homo sapiens GN=QKI PE=1 SV=1 - [QKI_HUMAN]	29.62	1	8	8	14	0.06	0.28	0.40	0.47	-0.11	-0.12	0.07	0.09	-0.27	-0.12	-0.04	0.04	-0.07	-0.32	-0.38	-0.27	-0.50	-0.53	-0.51	-0.28
O15040	Tectonin beta propeller repeat-containing protein 2 OS=Homo sapiens GN=TECPR2 PE=1 SV=4 - [TCPR2_HUMAN]	0.78	1	1	1	1	0.97	0.78	0.53	0.35	-0.27	-0.46	0.19	0.01	-0.18	0.01	-0.04	-0.23	-0.72	-1.00	-0.57	-0.93	-0.49	-1.25	-0.82	-0.28
O43765	Small glutamine-rich tetratricopeptide repeat-containing protein alpha OS=Homo sapiens GN=SGTA PE=1 SV=1 - [SGTA_HUMAN]	22.04	1	6	6	9	-0.07	-0.02	0.64	0.39	0.04	-0.09	0.30	0.14	0.02	0.06	0.13	0.03	0.00	0.07	-0.51	0.16	-0.49	0.11	-0.58	-0.28
Q96LW4	DNA-directed primase/polymerase protein OS=Homo sapiens GN=PRIMPOL PE=1 SV=2 - [PRIPO_HUMAN]	1.25	1	1	1	1	0.08	-0.13	0.47	0.26	-0.10	-0.33	0.13	-0.08	-0.49	-0.26	0.02	-0.20	0.11	-0.06	-0.46	-0.32	-0.71	-0.20	-0.60	-0.28

O43747	AP-1 complex subunit gamma-1 OS=Homo sapiens GN=AP1G1 PE=1 SV=5 - [AP1G1_HUMAN]	14.84	2	10	10	21	0.45	0.42	0.38	0.31	0.12	0.22	0.19	0.11	0.06	0.14	-0.06	0.04	-0.09	-0.37	-0.63	-0.24	-0.27	-0.25	-0.22	-0.28
O75936	Gamma-butyrobetaine dioxygenase OS=Homo sapiens GN=BBOX1 PE=1 SV=1 - [BODG_HUMAN]	17.31	1	6	6	8	1.90	1.94	1.87	1.88	0.82	0.82	1.48	1.47	1.14	1.14	1.21	1.22	-0.32	-0.60	-0.55	-0.73	-0.67	-1.02	-0.96	-0.28
P55769	NHP2-like protein 1 OS=Homo sapiens GN=NHP2L1 PE=1 SV=3 - [NH2L1_HUMAN]	15.63	1	2	2	7	0.14	0.06	-0.10	-0.18	-0.20	-0.28	-0.44	-0.52	-0.53	-0.45	-0.48	-0.56	-0.52	-0.61	-0.38	-0.56	-0.32	-0.35	-0.12	-0.28
P38117	Electron transfer flavoprotein subunit beta OS=Homo sapiens GN=ETFB PE=1 SV=3 - [ETFB_HUMAN]	45.88	1	12	12	34	0.22	0.12	0.17	0.15	-0.16	-0.17	-0.16	-0.20	-0.32	-0.22	-0.22	-0.29	-0.30	-0.45	-0.35	-0.37	-0.38	-0.33	-0.46	-0.28
Q9Y6X5	Bis(5'-adenosyl)-triphosphatase ENPP4 OS=Homo sapiens GN=ENPP4 PE=1 SV=3 - [ENPP4_HUMAN]	5.08	1	2	2	3	0.10	0.07	0.39	0.36	0.02	-0.02	0.05	0.02	-0.11	-0.07	0.26	0.23	0.01	0.17	-0.13	-0.14	-0.43	-0.10	-0.39	-0.28
A0JNW5	UHRF1-binding protein 1-like OS=Homo sapiens GN=UHRF1BP1L PE=1 SV=2 - [UH1BL_HUMAN]	3.14	1	2	3	5	0.94	1.13	0.94	0.58	0.13	0.61	-0.05	0.24	0.49	0.16	-0.18	-0.10	-0.84	-1.11	-1.12	-0.61	-0.05	-0.54	0.02	-0.28
P46100	Transcriptional regulator ATRX OS=Homo sapiens GN=ATRX PE=1 SV=5 - [ATRX_HUMAN]	1.08	1	2	2	2	0.31	-1.02	-0.24	-1.57	-0.46	-1.80	-0.58	-1.91	-1.36	-0.02	-0.15	-1.49	-0.83	-0.46	0.08	-0.30	0.25	-0.79	-0.24	-0.28
Q9Y520	Protein PRRC2C OS=Homo sapiens GN=PRRC2C PE=1 SV=4 - [PRC2C_HUMAN]	2.18	1	5	5	6	-0.38	-0.41	-0.13	-0.16	-0.32	-0.35	-0.47	-0.50	-0.38	-0.34	0.08	0.04	-0.03	0.47	0.21	0.07	-0.18	0.05	-0.21	-0.28

Q7Z699	Sprouty-related, EVH1 domain-containing protein 1 OS=Homo sapiens GN=SPRED1 PE=1 SV=2 - [SPRE1_HUMAN]	6.08	1	1	2	4	1.05	0.90	0.70	0.55	0.42	0.27	0.36	0.21	0.28	0.44	0.20	0.04	-0.64	-0.85	-0.50	-0.58	-0.23	-0.65	-0.30	-0.28
P62136	Serine/threonine-protein phosphatase PP1-alpha catalytic subunit OS=Homo sapiens GN=PPP1CA PE=1 SV=1 - [PP1A_HUMAN]	41.82	1	3	11	24	0.04	0.16	0.15	0.27	-0.17	-0.06	-0.19	-0.07	-0.10	-0.21	-0.32	-0.21	-0.18	-0.36	-0.47	-0.22	-0.33	-0.23	-0.34	-0.28
Q9HBL8	NmrA-like family domain-containing protein 1 OS=Homo sapiens GN=NMRAL1 PE=1 SV=1 - [NMRL1_HUMAN]	20.40	1	6	6	8	0.10	0.40	0.01	0.31	0.25	0.55	-0.34	-0.03	0.12	-0.18	0.07	0.37	-0.38	-0.02	0.06	-0.25	-0.16	0.13	0.22	-0.28
Q14240	Eukaryotic initiation factor 4A-II OS=Homo sapiens GN=EIF4A2 PE=1 SV=2 - [IF4A2_HUMAN]	60.69	1	12	21	51	-0.13	-0.13	0.35	0.30	-0.02	-0.04	0.10	0.07	-0.04	-0.01	-0.27	-0.28	0.06	-0.06	-0.66	0.09	-0.45	0.17	-0.40	-0.28
P04406	Glyceraldehyde-3-phosphate dehydrogenase OS=Homo sapiens GN=GAPDH PE=1 SV=3 - [G3P_HUMAN]	67.46	1	23	23	481	0.40	0.36	0.64	0.65	0.10	0.09	0.26	0.30	0.02	0.00	-0.10	-0.14	-0.02	-0.49	-0.75	-0.30	-0.62	-0.33	-0.60	-0.28
Q86YR5	G-protein-signaling modulator 1 OS=Homo sapiens GN=GPSM1 PE=1 SV=2 - [GPSM1_HUMAN]	9.33	1	5	5	8	0.91	0.94	0.10	0.13	0.03	0.05	-0.24	-0.21	-0.08	-0.11	-0.47	-0.44	-1.10	-1.38	-0.57	-0.99	-0.18	-0.90	-0.09	-0.28
O96011	Peroxisomal membrane protein 11B OS=Homo sapiens GN=PEX11B PE=1 SV=1 - [PX11B_HUMAN]	9.27	1	2	2	3	0.52	0.33	0.60	0.41	0.19	-0.01	0.26	0.06	-0.06	0.14	0.10	-0.10	-0.21	-0.42	-0.51	-0.35	-0.43	-0.35	-0.43	-0.28

Q6ZSZ5	Rho guanine nucleotide exchange factor 18 OS=Homo sapiens GN=ARHGEF18 PE=1 SV=3 - [ARHGI_HUMAN]	1.71	1	1	1	2	-0.10	-0.06	-0.10	-0.05	-0.21	-0.17	-0.44	-0.40	-0.36	-0.40	-0.44	-0.40	-0.28	-0.33	-0.34	-0.26	-0.27	-0.12	-0.13	-0.28
P51858	Hepatoma-derived growth factor OS=Homo sapiens GN=HDGF PE=1 SV=1 - [HDGF_HUMAN]	32.50	1	6	7	12	-0.22	-0.49	-0.33	-0.33	-0.32	-0.32	-0.67	-0.68	-0.62	-0.61	-0.61	-0.62	-0.31	-0.11	-0.29	-0.31	-0.25	0.11	-0.03	-0.28
Q13557	Calcium/calmodulin-dependent protein kinase type II subunit delta OS=Homo sapiens GN=CAMK2D PE=1 SV=3 - [KCC2D_HUMAN]	39.68	1	8	15	56	0.09	0.00	0.34	0.27	-0.10	-0.22	-0.07	-0.11	-0.15	-0.09	-0.13	-0.39	0.02	-0.33	-0.57	0.03	-0.30	-0.06	-0.30	-0.28
Q52LJ0	Protein FAM98B OS=Homo sapiens GN=FAM98B PE=1 SV=1 - [FA98B_HUMAN]	28.18	1	6	7	11	0.74	0.68	0.38	0.50	0.12	0.06	0.02	0.08	0.11	0.02	-0.11	-0.17	-0.69	-0.84	-0.65	-0.77	-0.49	-0.87	-0.47	-0.28
P42261	Glutamate receptor 1 OS=Homo sapiens GN=GRIA1 PE=1 SV=2 - [GRIA1_HUMAN]	9.60	1	2	7	13	0.36	0.22	0.30	0.16	0.77	0.57	-0.21	-0.36	0.26	-0.07	0.71	0.39	-0.37	-0.17	-0.10	-0.21	-0.13	-0.02	0.06	-0.28
P52594	Arf-GAP domain and FG repeat-containing protein 1 OS=Homo sapiens GN=AGFG1 PE=1 SV=2 - [AGFG1_HUMAN]	10.68	1	4	4	6	0.20	0.29	0.49	0.44	0.06	0.18	0.12	0.30	0.11	0.03	0.12	-0.12	0.05	-0.06	-0.23	-0.21	-0.55	-0.25	-0.57	-0.28
Q9UKZ1	CCR4-NOT transcription complex subunit 11 OS=Homo sapiens GN=CNOT11 PE=1 SV=1 - [CNO11_HUMAN]	8.24	1	2	2	2	-0.87	-0.44	-0.80	-0.38	-0.35	0.07	-1.15	-0.72	-0.36	-0.78	-0.37	0.05	-0.23	0.51	0.43	0.12	0.06	0.50	0.43	-0.28

Q13155	Aminoacyl tRNA synthase complex-interacting multifunctional protein 2 OS=Homo sapiens GN=AIMP2 PE=1 SV=2 - [AIMP2_HUMAN]	28.13	1	7	7	11	-0.37	-0.28	0.05	0.08	0.19	0.28	-0.17	-0.26	-0.07	-0.15	-0.16	-0.10	0.25	0.19	-0.24	0.25	-0.17	0.27	0.12	-0.28
Q96DI7	U5 small nuclear ribonucleoprotein 40 kDa protein OS=Homo sapiens GN=SNRNP40 PE=1 SV=1 - [SNR40_HUMAN]	11.76	1	2	2	5	0.31	0.19	0.15	0.03	0.29	0.12	0.05	-0.12	-0.05	0.18	0.34	0.30	-0.45	-0.37	-0.28	-0.26	-0.14	-0.01	0.10	-0.28
P42338	Phosphatidylinositol 4,5-bisphosphate 3-kinase catalytic subunit beta isoform OS=Homo sapiens GN=PIK3CB PE=1 SV=1 - [PK3CB_HUMAN]	4.30	1	3	3	5	0.62	0.20	0.72	0.31	0.45	0.03	0.38	-0.04	-0.42	0.01	0.12	-0.53	-0.18	-1.00	-1.00	-0.58	-0.69	-0.18	-0.29	-0.28
P07437	Tubulin beta chain OS=Homo sapiens GN=TUBB PE=1 SV=2 - [TBB5_HUMAN]	72.30	3	4	25	892	0.12	0.13	0.29	0.32	0.20	0.11	0.00	0.01	-0.04	0.08	0.03	0.02	-0.19	-0.26	-0.22	-0.13	-0.15	-0.09	-0.17	-0.28
Q5T8P6	RNA-binding protein 26 OS=Homo sapiens GN=RBM26 PE=1 SV=3 - [RBM26_HUMAN]	3.18	1	2	3	4	0.06	-0.16	-0.28	-0.50	-0.50	-0.72	-0.63	-0.84	-0.93	-0.71	-0.57	-0.80	-0.63	-0.63	-0.30	-0.74	-0.40	-0.58	-0.24	-0.29
Q7Z3J2	UPF0505 protein C16orf62 OS=Homo sapiens GN=C16orf62 PE=1 SV=2 - [CP062_HUMAN]	11.53	1	8	8	9	0.57	0.61	0.17	0.21	0.17	0.09	0.18	0.24	0.17	0.27	0.23	0.26	-0.39	-0.66	0.06	-0.41	0.00	-0.38	-0.04	-0.29
Q9BZ71	Membrane-associated phosphatidylinositol transfer protein 3 OS=Homo sapiens GN=PITPNM3 PE=1 SV=2 - [PITM3_HUMAN]	3.90	1	2	2	3	1.39	1.07	0.61	0.07	-0.53	-0.47	-0.35	-0.28	0.27	0.20	0.51	0.57	-1.29	-0.49	0.50	-0.77	0.24	-1.55	-0.55	-0.29

Q92572	AP-3 complex subunit sigma-1 OS=Homo sapiens GN=AP3S1 PE=1 SV=1 - [AP3S1_HUMAN]	24.35	1	3	3	6	0.23	-0.03	0.19	-0.34	0.29	0.03	-0.18	-0.24	-0.08	0.09	-0.02	-0.36	-0.16	-0.17	-0.32	-0.55	-0.46	-0.06	0.10	-0.29
Q96AJ9	Vesicle transport through interaction with t-SNAREs homolog 1A OS=Homo sapiens GN=VTI1A PE=1 SV=2 - [VTI1A_HUMAN]	8.76	1	2	2	3	0.88	0.76	0.73	0.60	0.07	-0.06	0.38	0.25	0.40	0.53	0.28	0.15	-0.45	-0.60	-0.44	-0.32	-0.16	-0.83	-0.67	-0.29
Q9Y2I1	Nischarin OS=Homo sapiens GN=NISCH PE=1 SV=3 - [NISCH_HUMAN]	0.93	1	1	1	1	0.35	0.51	0.60	0.75	-0.73	-0.58	0.25	0.40	0.02	-0.13	-0.24	-0.09	-0.05	-0.59	-0.84	-0.45	-0.69	-1.10	-1.35	-0.29
Q6PKG0	La-related protein 1 OS=Homo sapiens GN=LARP1 PE=1 SV=2 - [LARP1_HUMAN]	18.25	2	13	13	19	-0.15	-0.03	0.10	0.16	-0.20	-0.29	-0.31	-0.31	-0.20	-0.15	-0.04	-0.04	-0.20	-0.08	-0.21	-0.13	-0.35	-0.25	-0.46	-0.29
Q9NQE9	Histidine triad nucleotide-binding protein 3 OS=Homo sapiens GN=HINT3 PE=1 SV=1 - [HINT3_HUMAN]	37.36	1	5	5	7	0.33	0.25	0.13	0.20	0.10	0.32	0.00	-0.07	0.13	0.11	0.21	0.23	-0.26	-0.32	-0.12	-0.17	0.04	-0.18	0.02	-0.29
Q15464	SH2 domain-containing adapter protein B OS=Homo sapiens GN=SHB PE=1 SV=2 - [SHB_HUMAN]	3.93	1	1	1	1	0.17	1.44	-0.63	0.64	-0.73	0.53	-0.98	0.29	0.52	-0.75	-0.38	0.89	-1.10	-0.55	0.25	-0.89	-0.08	-0.92	-0.11	-0.29
O14879	Interferon-induced protein with tetratricopeptide repeats 3 OS=Homo sapiens GN=IFIT3 PE=1 SV=1 - [IFIT3_HUMAN]	31.43	1	11	12	20	-0.04	0.18	0.06	0.28	0.11	0.33	-0.26	-0.09	-0.01	-0.14	0.17	0.28	-0.27	0.13	0.03	-0.10	-0.22	0.06	0.02	-0.29

P45880	Voltage-dependent anion-selective channel protein 2 OS=Homo sapiens GN=VDAC2 PE=1 SV=2 - [VDAC2_HUMAN]	64.97	1	14	15	165	0.56	0.54	0.70	0.59	0.19	0.24	0.35	0.31	0.17	0.29	0.22	0.01	-0.24	-0.33	-0.54	-0.27	-0.37	-0.30	-0.39	-0.29
Q8IXS8	Protein FAM126B OS=Homo sapiens GN=FAM126B PE=2 SV=1 - [F126B_HUMAN]	8.87	1	3	3	4	0.94	1.24	0.39	0.69	-0.24	-0.05	-0.01	0.34	0.22	-0.07	-0.27	0.02	-0.84	-1.11	-0.67	-0.74	-0.38	-1.20	-0.66	-0.29
O94766	Galactosylgalactosylxylosylprotein 3-beta-glucuronosyltransferase 3 OS=Homo sapiens GN=B3GAT3 PE=1 SV=2 - [B3GA3_HUMAN]	14.03	1	3	3	6	0.51	0.34	0.16	-0.02	0.03	-0.16	-0.20	-0.37	-0.64	-0.46	-0.19	-0.37	-0.66	-0.69	-0.34	-0.94	-0.58	-0.50	-0.15	-0.29
Q8IZQ1	WD repeat and FYVE domain-containing protein 3 OS=Homo sapiens GN=WDFY3 PE=1 SV=2 - [WDFY3_HUMAN]	3.09	1	7	7	11	0.54	0.85	0.29	0.65	0.06	0.34	0.27	0.30	0.23	0.32	0.13	0.15	-0.49	-0.67	-0.63	-0.56	-0.48	-0.50	-0.45	-0.29
Q8N2F6	Armadillo repeat-containing protein 10 OS=Homo sapiens GN=ARMC10 PE=1 SV=1 - [ARM10_HUMAN]	27.99	1	7	7	12	0.22	0.44	0.48	0.61	0.24	0.31	0.23	0.26	0.21	0.12	0.20	0.28	0.01	-0.10	-0.38	-0.28	-0.47	-0.15	-0.46	-0.29
Q96TA2	ATP-dependent zinc metalloprotease YME1L1 OS=Homo sapiens GN=YME1L1 PE=1 SV=2 - [YMEL1_HUMAN]	4.14	1	2	2	2	0.22	-0.20	0.71	0.29	0.35	-0.07	0.35	-0.06	0.04	0.46	0.31	-0.11	0.19	0.10	-0.40	0.27	-0.21	0.11	-0.38	-0.29
Q9H425	Uncharacterized protein C1orf198 OS=Homo sapiens GN=C1orf198 PE=1 SV=1 - [CA198_HUMAN]	27.22	1	6	6	11	0.04	0.10	0.09	0.14	-0.59	-0.59	-0.12	-0.16	-0.40	-0.37	-0.33	-0.37	-0.08	-0.32	-0.47	-0.34	-0.48	-0.48	-0.74	-0.29

O14495	Lipid phosphate phosphohydrolase 3 OS=Homo sapiens GN=PPAP2B PE=1 SV=1 - [LPP3_HUMAN]	18.33	1	7	7	16	0.50	0.57	0.62	0.59	-0.43	-0.28	0.39	0.41	0.20	0.06	0.27	0.23	-0.22	-0.45	-0.74	-0.49	-0.61	-0.97	-1.26	-0.29
P27448	MAP/microtubule affinity-regulating kinase 3 OS=Homo sapiens GN=MARK3 PE=1 SV=4 - [MARK3_HUMAN]	11.42	2	3	6	9	-0.14	0.19	0.21	0.54	-0.02	0.05	-0.15	0.18	0.20	-0.13	-0.32	0.00	0.05	-0.18	-0.53	0.04	-0.30	-0.61	-0.68	-0.29
Q9UJV8	Purine-rich element-binding protein gamma OS=Homo sapiens GN=PURG PE=2 SV=1 - [PURG_HUMAN]	4.90	1	1	1	1	0.28	-0.39	0.26	-0.41	0.78	0.11	-0.09	-0.76	-0.08	0.59	0.40	-0.28	-0.32	0.12	0.13	0.34	0.36	0.48	0.50	-0.29
P78318	Immunoglobulin-binding protein 1 OS=Homo sapiens GN=IGBP1 PE=1 SV=1 - [IGBP1_HUMAN]	28.91	1	8	8	17	0.06	0.03	0.33	0.23	-0.18	-0.15	0.01	-0.02	-0.19	-0.09	0.14	0.10	-0.12	-0.04	-0.20	-0.16	-0.54	-0.38	-0.46	-0.29
P52655	Transcription initiation factor IIA subunit 1 OS=Homo sapiens GN=GTF2A1 PE=1 SV=1 - [TF2AA_HUMAN]	5.32	1	2	2	4	0.39	0.74	0.17	0.52	-0.54	-0.19	-0.18	0.17	0.40	0.05	-0.17	0.18	-0.52	-0.55	-0.34	-0.31	-0.09	-0.94	-0.73	-0.29
Q8WY22	BRI3-binding protein OS=Homo sapiens GN=BRI3BP PE=1 SV=1 - [BRI3B_HUMAN]	17.13	1	3	3	3	0.34	0.49	0.09	0.25	-0.54	-0.39	-0.27	-0.11	-0.12	-0.27	-0.65	-0.50	-0.55	-0.98	-0.74	-0.57	-0.33	-0.89	-0.65	-0.29
Q14203	Dynactin subunit 1 OS=Homo sapiens GN=DCTN1 PE=1 SV=3 - [DCTN1_HUMAN]	43.58	1	43	43	105	0.46	0.59	0.60	0.70	-0.03	-0.02	0.36	0.37	0.15	0.09	0.19	0.23	-0.14	-0.32	-0.49	-0.34	-0.56	-0.54	-0.66	-0.29
O75506	Heat shock factor-binding protein 1 OS=Homo sapiens GN=HSBP1 PE=1 SV=1 - [HSBP1_HUMAN]	64.47	1	4	5	12	0.10	-0.04	0.02	0.17	-0.61	-0.58	-0.13	-0.09	-0.19	-0.23	0.10	0.13	0.02	0.19	-0.03	-0.42	-0.32	-0.39	-0.53	-0.29

O75955	Flotillin-1 OS=Homo sapiens GN=FLOT1 PE=1 SV=3 - [FLOT1_HUMAN]	53.40	1	20	20	53	0.58	0.71	0.77	0.72	-0.02	-0.04	0.30	0.39	0.32	0.16	0.44	0.52	-0.18	-0.14	-0.22	-0.34	-0.49	-0.60	-0.73	-0.29
O14672	Disintegrin and metalloproteinase domain-containing protein 10 OS=Homo sapiens GN=ADAM10 PE=1 SV=1 - [ADA10_HUMAN]	16.98	1	11	11	26	0.63	0.60	0.63	0.60	-0.14	-0.18	0.19	0.16	-0.16	-0.09	0.11	0.12	-0.19	-0.41	-0.38	-0.54	-0.45	-0.68	-0.70	-0.29
Q9BQ70	Transcription factor 25 OS=Homo sapiens GN=TCF25 PE=1 SV=1 - [TCF25_HUMAN]	18.05	1	5	5	9	0.31	0.36	0.48	0.49	0.23	0.34	0.13	0.24	0.20	0.15	0.20	0.31	-0.13	-0.10	-0.29	-0.14	-0.25	-0.09	-0.27	-0.29
P82932	28S ribosomal protein S6, mitochondrial OS=Homo sapiens GN=MRPS6 PE=1 SV=3 - [RT06_HUMAN]	16.00	1	2	2	4	0.70	0.73	0.55	0.58	0.22	0.25	0.19	0.23	0.42	0.40	0.42	0.45	-0.45	-0.27	-0.13	-0.27	-0.12	-0.50	-0.35	-0.30
O95169	NADH dehydrogenase [ubiquinone] 1 beta subcomplex subunit 8, mitochondrial OS=Homo sapiens GN=NDUFB8 PE=1 SV=1 - [NDUB8_HUMAN]	36.02	1	5	5	39	0.60	0.78	0.37	0.75	0.08	0.11	0.09	0.38	0.33	0.04	0.19	0.35	-0.32	-0.48	-0.32	-0.56	-0.51	-0.76	-0.51	-0.30
Q06124	Tyrosine-protein phosphatase non-receptor type 11 OS=Homo sapiens GN=PTPN11 PE=1 SV=2 - [PTN11_HUMAN]	47.91	1	24	24	42	0.53	0.47	0.51	0.50	0.20	0.20	0.20	0.16	0.00	0.12	-0.08	-0.07	-0.21	-0.56	-0.64	-0.33	-0.46	-0.16	-0.36	-0.30
Q96F86	Enhancer of mRNA-decapping protein 3 OS=Homo sapiens GN=EDC3 PE=1 SV=1 - [EDC3_HUMAN]	7.87	1	3	3	4	0.48	0.04	0.58	0.14	0.38	-0.06	0.22	-0.22	-0.36	0.08	0.40	-0.04	-0.20	-0.07	-0.18	-0.37	-0.47	-0.12	-0.22	-0.30

Q08752	Peptidyl-prolyl cis-trans isomerase D OS=Homo sapiens GN=PPID PE=1 SV=3 - [PPID_HUMAN]	26.22	1	9	10	16	0.76	0.78	0.85	0.82	0.24	0.07	0.44	0.45	0.14	0.17	-0.26	-0.05	-0.27	-0.82	-0.86	-0.62	-0.59	-0.50	-0.64	-0.30
P62714	Serine/threonine-protein phosphatase 2A catalytic subunit beta isoform OS=Homo sapiens GN=PPP2CB PE=1 SV=1 - [PP2AB_HUMAN]	47.25	1	1	11	34	0.71	0.61	0.97	0.88	0.35	0.25	0.61	0.52	0.16	0.26	0.16	0.06	-0.04	-0.54	-0.82	-0.41	-0.68	-0.38	-0.64	-0.30
Q86X10	Ral GTPase-activating protein subunit beta OS=Homo sapiens GN=RALGAPB PE=1 SV=1 - [RLGPB_HUMAN]	10.37	1	8	8	11	-0.13	-0.18	0.15	0.07	-0.01	0.04	-0.18	-0.06	-0.17	-0.03	-0.18	0.01	-0.20	-0.07	0.00	0.13	-0.07	0.10	-0.03	-0.30
Q96AG3	Solute carrier family 25 member 46 OS=Homo sapiens GN=SLC25A46 PE=1 SV=1 - [S2546_HUMAN]	17.94	1	5	5	8	0.53	0.34	0.61	0.63	0.15	-0.10	0.25	0.27	0.02	0.07	0.45	0.14	-0.52	-0.18	-0.48	-0.28	-0.57	-0.29	-0.32	-0.30
O75821	Eukaryotic translation initiation factor 3 subunit G OS=Homo sapiens GN=EIF3G PE=1 SV=2 - [EIF3G_HUMAN]	19.38	1	6	6	8	-0.24	-0.33	-0.17	-0.15	-0.25	-0.28	-0.34	-0.27	-0.31	-0.42	-0.18	-0.16	-0.05	0.19	0.05	-0.01	-0.13	-0.01	-0.14	-0.30
Q14457	Beclin-1 OS=Homo sapiens GN=BECN1 PE=1 SV=2 - [BECN1_HUMAN]	11.11	1	4	4	6	0.14	0.17	0.09	0.48	-0.70	-0.13	-0.26	-0.03	-0.07	-0.21	-0.12	0.39	-0.34	-0.26	0.00	-0.32	-0.64	-0.86	-0.94	-0.30
Q9NQ50	39S ribosomal protein L40, mitochondrial OS=Homo sapiens GN=MRPL40 PE=1 SV=1 - [RM40_HUMAN]	6.80	1	1	1	1	0.76	0.48	0.64	0.35	0.49	0.20	0.28	-0.01	0.43	0.72	0.47	0.18	-0.43	-0.29	-0.17	-0.02	0.11	-0.29	-0.17	-0.30

P02792	Ferritin light chain OS=Homo sapiens GN=FTL PE=1 SV=2 - [FRIL_HUMAN]	23.43	1	4	4	5	1.54	1.57	1.69	1.95	-0.03	-0.40	1.03	0.96	0.58	0.72	0.85	0.30	-0.30	-0.75	-1.13	-0.85	-1.05	-1.66	-2.29	-0.30
P20339	Ras-related protein Rab-5A OS=Homo sapiens GN=RAB5A PE=1 SV=2 - [RAB5A_HUMAN]	48.37	1	5	7	12	0.50	0.75	0.67	0.95	0.19	0.29	0.09	0.24	0.03	0.07	-0.18	0.13	-0.49	-0.73	-0.69	-0.58	-0.70	-0.52	-0.58	-0.30
O43252	Bifunctional 3'-phosphoadenosine 5'-phosphosulfate synthase 1 OS=Homo sapiens GN=PAPSS1 PE=1 SV=2 - [PAPS1_HUMAN]	3.85	1	2	2	2	-0.21	-0.12	-0.17	-0.08	-0.46	-0.37	-0.53	-0.44	-0.33	-0.42	0.00	0.09	-0.26	0.22	0.17	-0.17	-0.22	-0.26	-0.31	-0.30
Q9NUL5	UPF0515 protein C19orf66 OS=Homo sapiens GN=C19orf66 PE=1 SV=2 - [CS066_HUMAN]	12.71	1	3	3	7	0.25	0.20	0.52	0.47	0.24	0.18	0.20	0.17	0.13	0.15	0.49	0.43	0.03	0.31	-0.03	0.06	-0.38	-0.03	-0.30	-0.30
P0CG48	Polyubiquitin-C OS=Homo sapiens GN=UBC PE=1 SV=3 - [UBC_HUMAN]	79.85	3	1	8	87	1.39	0.85	1.87	1.33	2.60	2.06	1.50	0.97	1.29	1.83	1.51	0.97	0.17	0.12	-0.36	0.47	0.00	1.19	0.71	-0.30
P30153	Serine/threonine-protein phosphatase 2A 65 kDa regulatory subunit A alpha isoform OS=Homo sapiens GN=PPP2R1A PE=1 SV=4 - [2AAA_HUMAN]	37.69	1	18	19	65	0.64	0.51	0.78	0.72	0.12	0.10	0.38	0.37	0.12	0.19	-0.04	0.04	-0.15	-0.56	-0.70	-0.42	-0.55	-0.47	-0.65	-0.30
Q9H0W9	Ester hydrolase C11orf54 OS=Homo sapiens GN=C11orf54 PE=1 SV=1 - [CK054_HUMAN]	20.32	1	4	4	6	0.18	0.04	0.47	0.31	-0.02	-0.22	0.05	-0.20	-0.16	0.02	-0.12	-0.30	0.03	-0.20	-0.52	-0.11	-0.40	-0.19	-0.46	-0.30

O14745	Na(+)/H(+) exchange regulatory cofactor NHE-RF1 OS=Homo sapiens GN=SLC9A3R1 PE=1 SV=4 - [NHRF1_HUMAN]	39.39	1	14	14	44	0.61	0.61	1.03	0.97	0.09	0.16	0.63	0.63	0.30	0.24	0.31	0.44	0.09	-0.22	-0.53	-0.31	-0.55	-0.47	-0.86	-0.30
P09651	Heterogeneous nuclear ribonucleoprotein A1 OS=Homo sapiens GN=HNRNPA1 PE=1 SV=5 - [ROA1_HUMAN]	43.82	2	11	14	62	0.34	0.34	0.53	0.50	-0.04	-0.11	0.12	0.05	-0.03	-0.01	-0.04	-0.10	-0.18	-0.33	-0.53	-0.27	-0.48	-0.44	-0.56	-0.30
Q7Z392	Trafficking protein particle complex subunit 11 OS=Homo sapiens GN=TRAPPC11 PE=1 SV=2 - [TPC11_HUMAN]	7.41	1	5	5	10	-0.31	-0.27	0.07	0.26	-0.21	0.08	-0.20	0.14	-0.12	-0.45	-0.34	-0.01	0.49	0.30	-0.40	0.21	-0.35	0.36	-0.32	-0.30
Q9UFG5	UPF0449 protein C19orf25 OS=Homo sapiens GN=C19orf25 PE=1 SV=2 - [CS025_HUMAN]	31.36	1	2	2	4	-0.13	-0.19	-0.23	-0.21	-0.24	0.02	-0.71	-0.45	-0.28	-0.54	-0.45	-0.19	-0.14	-0.32	-0.22	-0.14	-0.27	-0.13	-0.03	-0.30
Q7L2E3	Putative ATP-dependent RNA helicase DHX30 OS=Homo sapiens GN=DHX30 PE=1 SV=1 - [DHX30_HUMAN]	21.19	1	19	19	33	0.24	0.18	0.50	0.43	0.07	-0.02	0.10	-0.06	-0.12	0.09	0.27	0.06	-0.14	-0.15	-0.39	-0.25	-0.43	-0.20	-0.44	-0.30
Q9NZZ3	Charged multivesicular body protein 5 OS=Homo sapiens GN=CHMP5 PE=1 SV=1 - [CHMP5_HUMAN]	42.92	1	6	6	11	0.52	0.58	0.60	0.48	0.44	0.47	0.21	0.18	0.21	0.15	0.27	0.23	-0.32	-0.32	-0.24	-0.34	-0.49	0.08	0.01	-0.30
Q9Y4F1	FERM, RhoGEF and pleckstrin domain-containing protein 1 OS=Homo sapiens GN=FARP1 PE=1 SV=1 - [FARP1_HUMAN]	26.22	1	20	21	51	-0.28	-0.22	0.15	0.03	-0.07	-0.07	-0.22	-0.27	-0.32	-0.23	-0.39	-0.55	-0.25	-0.11	-0.45	-0.16	-0.24	0.08	-0.06	-0.30

O95749	Geranylgeranyl pyrophosphate synthase OS=Homo sapiens GN=GGPS1 PE=1 SV=1 - [GGPPS_HUMAN]	7.67	1	2	2	4	0.12	-0.09	0.53	0.14	-0.06	-0.21	0.13	-0.08	-0.79	-0.55	-0.24	-0.44	0.06	-0.65	-0.57	-0.66	-1.05	-0.26	-0.36	-0.30
Q6ZMI0	Protein phosphatase 1 regulatory subunit 21 OS=Homo sapiens GN=PPP1R21 PE=1 SV=1 - [PPR21_HUMAN]	22.05	1	14	14	24	0.39	0.24	0.49	0.10	0.09	0.08	0.04	0.14	0.00	0.08	0.00	-0.15	-0.20	-0.29	-0.53	-0.22	-0.41	-0.29	-0.45	-0.30
O95777	U6 snRNA-associated Sm-like protein LSm8 OS=Homo sapiens GN=LSM8 PE=1 SV=3 - [LSM8_HUMAN]	59.38	1	4	4	10	-0.13	-0.41	0.07	0.01	0.11	0.22	-0.30	-0.35	-0.28	-0.15	-0.23	-0.37	0.11	0.04	-0.30	0.09	-0.18	0.79	0.37	-0.30
P05154	Plasma serine protease inhibitor OS=Homo sapiens GN=SERPINA5 PE=1 SV=3 - [IPSP_HUMAN]	1.48	1	1	1	1	0.94	1.04	1.31	1.41	0.80	0.89	0.94	1.04	0.67	0.58	0.79	0.88	0.06	-0.15	-0.52	-0.33	-0.70	-0.16	-0.53	-0.30
P09972	Fructose-bisphosphate aldolase C OS=Homo sapiens GN=ALDOC PE=1 SV=2 - [ALDOC_HUMAN]	76.10	1	20	25	290	0.78	0.77	0.82	0.87	0.60	0.57	0.49	0.51	0.46	0.46	0.16	0.19	-0.24	-0.65	-0.76	-0.38	-0.46	-0.28	-0.37	-0.30
Q969E8	Pre-rRNA-processing protein TSR2 homolog OS=Homo sapiens GN=TSR2 PE=1 SV=1 - [TSR2_HUMAN]	10.99	1	2	2	4	1.03	1.08	0.85	0.89	0.26	0.30	0.48	0.53	0.59	0.55	0.12	0.17	-0.50	-0.90	-0.72	-0.45	-0.26	-0.79	-0.61	-0.30
Q6ZVK8	8-oxo-dGDP phosphatase NUDT18 OS=Homo sapiens GN=NUDT18 PE=1 SV=3 - [NUD18_HUMAN]	8.36	1	2	2	4	0.78	0.74	0.77	0.73	0.75	0.70	0.41	0.36	0.47	0.52	0.49	0.44	-0.32	-0.29	-0.29	-0.23	-0.22	-0.05	-0.04	-0.30
P62834	Ras-related protein Rap-1A OS=Homo sapiens GN=RAP1A PE=1 SV=1 - [RAP1A_HUMAN]	54.35	1	3	8	45	-0.16	-0.14	0.28	0.22	-0.52	-0.38	-0.03	0.09	-0.15	-0.23	-0.16	-0.03	0.10	-0.13	-0.48	-0.31	-0.57	-0.44	-0.85	-0.30

P26640	Valine--tRNA ligase OS=Homo sapiens GN=VARS PE=1 SV=4 - [SYVC_HUMAN]	24.37	1	25	25	55	0.49	0.37	0.57	0.48	0.21	0.09	0.13	0.05	0.08	0.12	0.09	0.04	-0.31	-0.48	-0.47	-0.30	-0.31	-0.27	-0.34	-0.30
Q15102	Platelet-activating factor acetylhydrolase IB subunit gamma OS=Homo sapiens GN=PAFAH1B3 PE=1 SV=1 - [PA1B3_HUMAN]	13.42	1	4	4	6	1.42	1.38	1.44	1.41	0.68	0.64	1.07	1.04	0.85	0.89	0.63	0.59	-0.29	-0.78	-0.81	-0.50	-0.52	-0.76	-0.78	-0.30
P61769	Beta-2-microglobulin OS=Homo sapiens GN=B2M PE=1 SV=1 - [B2MG_HUMAN]	35.29	1	3	3	10	-0.37	-0.49	-0.03	0.14	0.11	0.27	-0.23	-0.27	0.25	0.19	0.61	0.63	0.25	1.27	0.71	0.63	0.15	0.95	0.47	-0.31
Q9UID3	Vacuolar protein sorting-associated protein 51 homolog OS=Homo sapiens GN=VPS51 PE=1 SV=2 - [VPS51_HUMAN]	19.95	1	13	13	24	0.61	0.52	0.66	0.62	0.02	0.01	0.17	0.15	-0.01	0.02	0.11	0.02	-0.35	-0.58	-0.36	-0.52	-0.53	-0.57	-0.58	-0.31
Q9UJ41	Rab5 GDP/GTP exchange factor OS=Homo sapiens GN=RABGEF1 PE=1 SV=2 - [RABX5_HUMAN]	22.32	1	13	14	22	0.35	0.38	0.46	0.40	0.13	0.18	0.16	-0.05	0.09	-0.02	0.04	0.10	-0.35	-0.31	-0.47	-0.23	-0.60	-0.30	-0.35	-0.31
O75153	Clustered mitochondria protein homolog OS=Homo sapiens GN=CLUH PE=1 SV=2 - [CLU_HUMAN]	3.28	1	3	3	4	0.77	0.26	0.28	0.58	-0.15	0.36	-0.42	0.21	0.50	0.32	-0.23	-0.16	0.00	-0.41	-0.73	0.27	-0.04	0.09	-0.23	-0.31
Q8N1B4	Vacuolar protein sorting-associated protein 52 homolog OS=Homo sapiens GN=VPS52 PE=1 SV=1 - [VPS52_HUMAN]	26.28	1	14	14	26	0.48	0.58	0.17	0.34	-0.01	-0.08	-0.05	0.01	-0.05	0.01	0.06	-0.07	-0.44	-0.45	-0.31	-0.44	-0.18	-0.32	-0.23	-0.31

P19404	NADH dehydrogenase [ubiquinone] flavoprotein 2, mitochondrial OS=Homo sapiens GN=NDUFV2 PE=1 SV=2 - [NDUV2_HUMAN]	52.61	1	13	13	69	0.65	0.71	0.83	0.81	0.04	0.18	0.36	0.47	0.24	0.22	0.26	0.29	-0.18	-0.41	-0.46	-0.38	-0.58	-0.58	-0.65	-0.31
Q9Y3I0	tRNA-splicing ligase RtcB homolog OS=Homo sapiens GN=RTCB PE=1 SV=1 - [RTCB_HUMAN]	47.13	1	20	20	48	-0.09	-0.04	0.19	0.24	-0.20	-0.27	-0.09	-0.11	-0.21	-0.17	-0.15	-0.15	0.02	-0.11	-0.30	-0.16	-0.42	-0.14	-0.52	-0.31
Q15042	Rab3 GTPase-activating protein catalytic subunit OS=Homo sapiens GN=RAB3GAP1 PE=1 SV=3 - [RB3GP_HUMAN]	11.42	1	9	9	12	0.26	0.03	0.32	0.15	-0.10	-0.12	-0.19	-0.06	-0.33	-0.09	0.03	-0.11	-0.42	-0.49	-0.41	-0.51	-0.63	-0.50	-0.42	-0.31
Q969L2	Protein MAL2 OS=Homo sapiens GN=MAL2 PE=1 SV=1 - [MAL2_HUMAN]	6.25	1	1	1	4	0.60	0.65	0.22	0.27	0.11	0.16	-0.16	-0.11	-0.20	-0.24	0.33	0.37	-0.70	-0.27	0.11	-0.82	-0.43	-0.51	-0.12	-0.31
Q86U42	Polyadenylate-binding protein 2 OS=Homo sapiens GN=PABPN1 PE=1 SV=3 - [PABP2_HUMAN]	19.28	1	4	4	6	0.00	-0.03	0.25	0.22	-0.01	-0.04	-0.12	-0.15	-0.22	-0.18	-0.24	-0.28	-0.07	-0.24	-0.50	-0.15	-0.40	-0.03	-0.28	-0.31
O75970	Multiple PDZ domain protein OS=Homo sapiens GN=MPDZ PE=1 SV=2 - [MPDZ_HUMAN]	6.52	1	7	7	9	0.04	0.31	0.15	0.59	-0.15	0.11	-0.08	0.18	-0.30	-0.49	-0.29	-0.17	0.34	-0.35	-0.48	-0.39	-0.49	-0.20	-0.39	-0.31
O43674	NADH dehydrogenase [ubiquinone] 1 beta subcomplex subunit 5, mitochondrial OS=Homo sapiens GN=NDUFB5 PE=1 SV=1 - [NDUB5_HUMAN]	16.93	1	3	3	5	0.68	0.69	0.79	0.79	-0.04	-0.05	0.37	0.37	0.02	0.02	0.33	0.33	-0.29	-0.35	-0.46	-0.63	-0.73	-0.74	-0.85	-0.31

O15160	DNA-directed RNA polymerases I and III subunit RPAC1 OS=Homo sapiens GN=POLR1C PE=1 SV=1 - [RPAC1_HUMAN]	8.96	1	2	2	4	-0.07	0.16	0.00	0.24	-0.54	-0.31	-0.37	-0.14	-0.31	-0.54	-0.25	-0.03	-0.24	-0.18	-0.26	-0.44	-0.51	-0.49	-0.56	-0.31
P15882	N-chimaerin OS=Homo sapiens GN=CHN1 PE=1 SV=3 - [CHIN_HUMAN]	18.95	1	6	6	9	1.13	0.79	1.33	0.83	1.08	0.79	0.76	0.43	0.15	0.43	0.23	-0.12	-0.19	-0.91	-1.02	-0.44	-0.51	0.05	-0.09	-0.31
P22830	Ferrochelatase, mitochondrial OS=Homo sapiens GN=FECH PE=1 SV=2 - [HEMH_HUMAN]	30.50	1	12	12	21	0.12	0.10	0.40	0.41	-0.40	-0.29	0.00	0.01	-0.25	-0.38	-0.34	-0.42	-0.09	-0.66	-0.80	-0.46	-0.57	-0.60	-0.86	-0.31
O75964	ATP synthase subunit g, mitochondrial OS=Homo sapiens GN=ATP5L PE=1 SV=3 - [ATP5L_HUMAN]	44.66	2	4	5	19	0.16	0.09	0.36	0.45	-0.14	-0.12	0.19	0.16	-0.03	-0.05	-0.14	-0.14	0.09	-0.36	-0.67	-0.37	-0.34	-0.39	-0.35	-0.31
O15400	Syntaxin-7 OS=Homo sapiens GN=STX7 PE=1 SV=4 - [STX7_HUMAN]	45.59	1	9	9	23	0.40	0.44	0.25	0.34	-0.28	-0.14	0.03	0.07	0.07	-0.01	-0.23	-0.09	-0.27	-0.37	-0.46	-0.31	-0.39	-0.60	-0.70	-0.31
P55786	Puromycin-sensitive aminopeptidase OS=Homo sapiens GN=NPEPPS PE=1 SV=2 - [PSA_HUMAN]	35.04	2	29	29	80	0.81	0.82	0.88	1.06	0.08	0.06	0.55	0.69	0.23	0.12	-0.01	0.04	-0.07	-0.76	-0.82	-0.59	-0.71	-0.75	-0.86	-0.31
O95219	Sorting nexin-4 OS=Homo sapiens GN=SNX4 PE=1 SV=1 - [SNX4_HUMAN]	33.11	1	11	11	15	0.68	0.78	0.27	0.48	0.18	0.16	0.19	0.20	0.13	0.08	-0.04	0.15	-0.33	-0.38	-0.43	-0.68	-0.60	-0.66	-0.51	-0.31
P08237	6-phosphofructokinase, muscle type OS=Homo sapiens GN=PFKM PE=1 SV=2 - [K6PF_HUMAN]	43.08	1	22	26	103	0.07	0.01	0.37	0.27	-0.05	-0.09	-0.07	-0.14	-0.27	-0.19	-0.20	-0.30	-0.10	-0.30	-0.50	-0.35	-0.50	-0.26	-0.45	-0.31

O15254	Peroxisomal acyl-coenzyme A oxidase 3 OS=Homo sapiens GN=ACOX3 PE=1 SV=2 - [ACOX3_HUMAN]	6.29	1	4	4	5	0.28	0.21	0.32	0.19	0.27	-0.17	-0.26	-0.16	-0.01	0.26	0.22	0.05	-0.28	0.01	-0.04	0.06	0.02	-0.12	-0.17	-0.31
Q13085	Acetyl-CoA carboxylase 1 OS=Homo sapiens GN=ACACA PE=1 SV=2 - [ACACA_HUMAN]	7.33	2	14	14	20	0.62	0.53	0.61	0.54	-0.04	-0.08	0.22	0.21	-0.02	0.04	0.05	0.01	-0.32	-0.56	-0.46	-0.49	-0.42	-0.64	-0.40	-0.31
Q9BSF0	Small membrane A-kinase anchor protein OS=Homo sapiens GN=C2orf88 PE=1 SV=2 - [SMAKA_HUMAN]	26.32	1	2	2	4	1.52	1.40	1.45	1.33	0.77	0.65	1.07	0.96	1.09	1.21	1.09	0.96	-0.39	-0.43	-0.36	-0.28	-0.20	-0.77	-0.70	-0.31
Q96JA1	Leucine-rich repeats and immunoglobulin-like domains protein 1 OS=Homo sapiens GN=LRIG1 PE=1 SV=2 - [LRIG1_HUMAN]	2.38	1	2	2	3	1.07	1.05	1.12	1.10	0.47	0.44	0.41	0.39	0.42	0.45	0.69	0.66	-0.46	-0.37	-0.43	-0.59	-0.64	-0.61	-0.66	-0.31
P30711	Glutathione S transferase theta-1 OS=Homo sapiens GN=GSTT1 PE=1 SV=4 - [GSTT1_HUMAN]	27.92	1	4	5	9	0.53	0.71	0.91	1.04	0.45	0.62	0.44	0.62	0.63	0.51	0.38	0.40	-0.04	-0.14	-0.43	0.01	-0.24	-0.07	-0.34	-0.31
A8MVW0	Protein FAM171A2 OS=Homo sapiens GN=FAM171A2 PE=1 SV=1 - [F1712_HUMAN]	7.38	1	4	4	5	1.26	0.99	1.20	0.94	0.13	-0.15	0.83	0.56	-0.06	0.22	0.14	-0.13	-0.37	-1.11	-1.07	-1.01	-0.95	-1.15	-1.09	-0.31
P53680	AP-2 complex subunit sigma OS=Homo sapiens GN=AP2S1 PE=1 SV=2 - [AP2S1_HUMAN]	37.32	1	5	5	16	0.91	0.70	0.78	0.83	0.12	-0.03	0.24	0.19	0.08	0.12	0.11	0.10	-0.32	-0.75	-0.70	-0.68	-0.60	-0.63	-0.82	-0.31
O60506	Heterogeneous nuclear ribonucleoprotein Q OS=Homo sapiens GN=SYNCRIP PE=1 SV=2 - [HNRPQ_HUMAN]	30.82	1	11	16	40	-0.36	-0.32	-0.03	0.01	-0.37	-0.29	-0.39	-0.36	-0.38	-0.38	-0.30	-0.29	0.02	-0.05	-0.30	0.00	-0.40	0.08	-0.43	-0.31

Q9NQC7	Ubiquitin carboxyl-terminal hydrolase CYLD OS=Homo sapiens GN=CYLD PE=1 SV=1 - [CYLD_HUMAN]	14.02	1	10	10	14	1.15	1.05	1.09	0.99	0.43	0.29	0.40	0.27	0.21	0.34	0.40	0.31	-0.36	-0.43	-0.36	-0.48	-0.40	-0.10	-0.02	-0.31
O15228	Dihydroxyacetone phosphate acyltransferase OS=Homo sapiens GN=GNPAT PE=1 SV=1 - [GNPAT_HUMAN]	4.12	1	3	3	3	-0.18	0.12	0.10	0.39	-0.86	-0.57	-0.28	0.02	-0.11	-0.40	-0.22	0.07	-0.04	-0.03	-0.32	-0.19	-0.47	-0.70	-0.98	-0.31
P61019	Ras-related protein Rab-2A OS=Homo sapiens GN=RAB2A PE=1 SV=1 - [RAB2A_HUMAN]	66.51	1	5	11	32	0.89	0.81	0.89	0.75	0.05	0.17	0.08	0.13	0.27	0.00	0.34	0.24	-0.56	-0.16	-0.25	-0.58	-0.43	-0.61	-0.80	-0.31
Q99614	Tetratricopeptide repeat protein 1 OS=Homo sapiens GN=TTC1 PE=1 SV=1 - [TTC1_HUMAN]	26.37	1	7	7	9	0.26	0.53	0.53	0.76	-0.32	-0.01	0.24	0.38	-0.02	-0.32	-0.38	-0.04	-0.01	-0.75	-0.92	-0.41	-0.74	-0.50	-0.64	-0.31
Q96JM3	Chromosome alignment-maintaining phosphoprotein 1 OS=Homo sapiens GN=CHAMP1 PE=1 SV=2 - [CHAP1_HUMAN]	5.42	1	4	4	5	-0.16	-0.30	0.04	-0.09	-0.18	-0.33	-0.33	-0.47	-0.40	-0.26	0.12	-0.03	-0.12	0.28	0.07	-0.07	-0.27	-0.04	-0.24	-0.31
Q9BXJ9	N-alpha-acetyltransferase 15, NatA auxiliary subunit OS=Homo sapiens GN=NAA15 PE=1 SV=1 - [NAA15_HUMAN]	13.05	2	10	10	14	0.37	0.38	0.37	0.54	0.07	0.17	0.10	0.15	0.08	-0.09	-0.05	0.08	-0.21	-0.35	-0.47	-0.41	-0.48	-0.30	-0.40	-0.31
P54803	Galactocerebrosidase OS=Homo sapiens GN=GALC PE=1 SV=2 - [GALC_HUMAN]	3.80	1	2	2	4	0.84	0.75	0.68	0.60	-0.34	-0.43	0.30	0.22	0.29	0.38	0.76	0.68	-0.48	-0.07	0.08	-0.43	-0.27	-1.19	-1.04	-0.31

Q8IX01	SURP and G-patch domain containing protein 2 OS=Homo sapiens GN=SUGP2 PE=1 SV=2 - [SUGP2_HUMAN]	11.28	1	9	9	12	0.29	0.68	0.46	0.57	-0.16	-0.06	-0.04	-0.03	-0.14	-0.20	0.03	0.10	-0.47	-0.52	-0.70	-0.42	-0.56	-0.58	-0.75	-0.31
P10599	Thioredoxin OS=Homo sapiens GN=TXN PE=1 SV=3 - [THIO_HUMAN]	40.95	1	4	4	15	0.04	-0.03	0.29	0.26	-0.23	-0.21	-0.11	-0.14	-0.26	-0.28	-0.35	-0.27	-0.05	-0.23	-0.53	-0.15	-0.45	-0.22	-0.51	-0.31
Q9NRY4	Rho GTPase-activating protein 35 OS=Homo sapiens GN=ARHGAP35 PE=1 SV=3 - [RHG35_HUMAN]	13.48	1	15	16	25	0.45	0.50	0.55	0.49	-0.08	0.12	0.21	0.02	0.17	0.17	-0.14	0.11	-0.47	-0.61	-0.58	-0.46	-0.52	-0.52	-0.53	-0.31
Q86Y82	Syntaxin-12 OS=Homo sapiens GN=STX12 PE=1 SV=1 - [STX12_HUMAN]	55.80	1	9	9	22	0.98	0.54	0.92	0.65	0.28	0.14	0.30	0.14	0.27	0.52	0.50	0.32	-0.55	-0.23	-0.26	-0.21	-0.38	-0.62	-0.57	-0.32
P04075	Fructose-bisphosphate aldolase A OS=Homo sapiens GN=ALDOA PE=1 SV=2 - [ALDOA_HUMAN]	75.82	1	18	23	307	0.68	0.58	0.60	0.53	0.13	0.06	0.16	0.13	-0.04	0.04	-0.19	-0.35	-0.32	-0.84	-0.77	-0.58	-0.64	-0.59	-0.54	-0.32
P53384	Cytosolic Fe-S cluster assembly factor NUBP1 OS=Homo sapiens GN=NUBP1 PE=1 SV=2 - [NUBP1_HUMAN]	9.06	1	2	2	2	-0.17	0.28	-0.15	0.30	-0.26	0.19	-0.53	-0.08	-0.12	-0.46	-0.27	0.06	-0.31	-0.37	-0.41	-0.53	-0.57	-0.10	-0.12	-0.32
P15927	Replication protein A 32 kDa subunit OS=Homo sapiens GN=RPA2 PE=1 SV=1 - [RFA2_HUMAN]	17.78	1	3	3	9	0.32	0.12	0.37	0.24	-0.16	-0.07	0.04	-0.14	-0.11	-0.16	-0.07	-0.14	-0.21	-0.12	-0.42	-0.31	-0.48	-0.46	-0.55	-0.32

Q1KMD3	Heterogeneous nuclear ribonucleoprotein U-like protein 2 OS=Homo sapiens GN=HNRNPUL2 PE=1 SV=1 - [HNRL2_HUMAN]	30.52	1	18	18	42	0.11	0.10	0.25	0.18	-0.11	-0.06	-0.26	-0.29	-0.24	-0.23	-0.05	-0.09	-0.19	-0.16	-0.39	-0.20	-0.41	-0.16	-0.36	-0.32
Q15334	Lethal(2) giant larvae protein homolog 1 OS=Homo sapiens GN=LLGL1 PE=1 SV=3 - [L2GL1_HUMAN]	24.91	1	17	17	43	0.61	0.64	0.77	0.82	0.32	0.29	0.36	0.43	0.29	0.34	0.36	0.20	-0.21	-0.38	-0.31	-0.26	-0.24	-0.36	-0.40	-0.32
O75027	ATP-binding cassette sub-family B member 7, mitochondrial OS=Homo sapiens GN=ABCB7 PE=1 SV=2 - [ABCB7_HUMAN]	10.37	1	6	6	10	0.45	0.62	0.27	0.26	-0.20	0.22	-0.03	0.00	0.12	0.15	0.01	0.43	-0.69	-0.35	-0.18	-0.45	-0.08	-0.58	-0.40	-0.32
Q6IQ23	Pleckstrin homology domain-containing family A member 7 OS=Homo sapiens GN=PLEKHA7 PE=1 SV=2 - [PKHA7_HUMAN]	1.78	1	1	1	1	0.93	1.61	0.34	1.01	-0.72	-0.05	-0.05	0.63	0.45	-0.22	-0.60	0.07	-0.92	-1.53	-0.94	-1.12	-0.53	-1.67	-1.07	-0.32
Q9HCD5	Nuclear receptor coactivator 5 OS=Homo sapiens GN=NCOA5 PE=1 SV=2 - [NCOA5_HUMAN]	5.18	1	3	3	5	0.34	0.37	0.17	0.20	-0.51	-0.49	-0.22	-0.19	-0.21	-0.23	-0.18	-0.15	-0.50	-0.51	-0.34	-0.54	-0.36	-0.86	-0.69	-0.32
Q9BZ23	Pantothenate kinase 2, mitochondrial OS=Homo sapiens GN=PANK2 PE=1 SV=3 - [PANK2_HUMAN]	7.19	2	4	4	6	0.32	0.38	0.57	0.62	0.02	0.05	0.04	0.12	0.17	0.12	0.01	0.06	-0.04	-0.31	-0.56	-0.18	-0.42	-0.18	-0.48	-0.32
O15498	Synaptobrevin homolog YKT6 OS=Homo sapiens GN=YKT6 PE=1 SV=1 - [YKT6_HUMAN]	36.36	1	7	7	14	0.45	0.52	0.47	0.63	0.04	0.22	0.14	0.32	0.26	0.11	-0.02	0.31	-0.13	-0.50	-0.43	-0.28	-0.32	-0.35	-0.68	-0.32

Q08828	Adenylate cyclase type 1 OS=Homo sapiens GN=ADCY1 PE=1 SV=2 - [ADCY1_HUMAN]	4.56	1	3	3	7	0.91	0.78	0.66	0.52	0.21	-0.02	0.20	0.14	0.14	0.22	-0.23	-0.21	-0.58	-0.82	-0.58	-0.49	-0.23	-0.51	-0.26	-0.32
Q6NYC1	Bifunctional arginine demethylase and lysyl-hydroxylase JMJD6 OS=Homo sapiens GN=JMJD6 PE=1 SV=1 - [JMJD6_HUMAN]	3.47	1	1	1	2	1.33	0.91	1.09	0.67	0.43	0.01	0.71	0.28	0.40	0.82	0.75	0.32	-0.57	-0.58	-0.34	-0.48	-0.23	-0.91	-0.67	-0.32
O75140	DEP domain-containing protein 5 OS=Homo sapiens GN=DEPDC5 PE=1 SV=2 - [DEPD5_HUMAN]	2.25	1	2	2	2	-0.47	0.05	-0.35	0.04	0.05	0.29	-0.73	-0.14	0.19	0.01	0.00	0.24	-0.20	0.20	0.20	0.51	0.39	0.23	0.23	-0.32
P42858	Huntingtin OS=Homo sapiens GN=HTT PE=1 SV=2 - [HD_HUMAN]	7.10	1	18	18	27	0.76	0.67	0.64	0.62	0.11	0.29	0.25	0.24	0.42	0.27	0.17	0.35	-0.35	-0.52	-0.24	-0.47	-0.40	-0.78	-0.63	-0.32
Q9NWS1	PCNA-interacting partner OS=Homo sapiens GN=PARPBP PE=1 SV=3 - [PARI_HUMAN]	1.21	1	1	1	1	0.24	0.23	0.65	0.64	0.18	0.17	0.26	0.26	0.06	0.07	0.13	0.12	0.08	-0.10	-0.52	-0.14	-0.54	-0.07	-0.48	-0.32
P36957	Dihydrolipoyllysine-residue succinyltransferase component of 2-oxoglutarate dehydrogenase complex, mitochondrial OS=Homo sapiens GN=DLST PE=1 SV=4 - [ODO2_HUMAN]	23.62	1	9	9	40	0.53	0.48	0.54	0.47	-0.08	-0.08	0.18	0.14	0.07	0.07	0.00	-0.06	-0.27	-0.51	-0.60	-0.44	-0.50	-0.55	-0.56	-0.32
P21359	Neurofibromin OS=Homo sapiens GN=NF1 PE=1 SV=2 - [NF1_HUMAN]	3.28	1	9	9	13	0.34	0.41	0.13	0.15	-0.15	-0.12	-0.30	-0.14	-0.10	-0.21	-0.22	-0.16	-0.50	-0.29	-0.26	-0.47	-0.26	-0.45	-0.26	-0.32

P23434	Glycine cleavage system H protein, mitochondrial OS=Homo sapiens GN=GCSH PE=1 SV=2 - [GCSH_HUMAN]	38.73	1	3	3	8	0.41	0.29	0.51	-0.17	-0.33	-0.42	0.00	-0.11	-0.58	-0.41	-0.73	-0.76	-0.32	-1.11	-1.37	-0.37	-0.90	-0.72	-0.87	-0.32
Q99797	Mitochondrial intermediate peptidase OS=Homo sapiens GN=MIPEP PE=1 SV=2 - [MIPEP_HUMAN]	2.81	1	1	1	3	0.39	0.72	0.50	0.83	-0.37	-0.04	0.11	0.44	0.07	-0.25	-0.08	0.25	-0.22	-0.46	-0.58	-0.61	-0.72	-0.77	-0.88	-0.32
Q9NXC2	Glucose-fructose oxidoreductase domain-containing protein 1 OS=Homo sapiens GN=GFOD1 PE=1 SV=1 - [GFOD1_HUMAN]	8.21	1	3	3	3	0.98	0.88	0.80	0.71	0.19	0.09	0.42	0.32	0.24	0.34	0.22	0.12	-0.50	-0.75	-0.58	-0.61	-0.43	-0.80	-0.63	-0.32
Q9H0M0	NEDD4-like E3 ubiquitin-protein ligase WWP1 OS=Homo sapiens GN=WWP1 PE=1 SV=1 - [WWP1_HUMAN]	1.41	1	1	1	1	0.11	0.27	-0.44	-0.27	0.00	0.17	-0.82	-0.65	0.06	-0.10	-0.13	0.03	-0.87	-0.23	0.30	-0.18	0.37	-0.12	0.42	-0.32
O75326	Semaphorin-7A OS=Homo sapiens GN=SEMA7A PE=1 SV=1 - [SEM7A_HUMAN]	28.98	1	17	17	25	0.60	0.70	0.52	0.59	0.97	0.99	0.03	0.03	0.71	0.65	1.05	1.03	-0.47	0.37	0.64	0.12	0.24	0.36	0.47	-0.32
Q01085	Nucleolysin TIAR OS=Homo sapiens GN=TIAL1 PE=1 SV=1 - [TIAR_HUMAN]	19.73	2	7	7	13	0.00	0.09	0.15	0.15	-0.16	-0.07	-0.43	-0.14	-0.29	-0.30	-0.39	-0.36	-0.24	-0.50	-0.51	-0.37	-0.38	-0.34	-0.34	-0.32
P15954	Cytochrome c oxidase subunit 7C, mitochondrial OS=Homo sapiens GN=COX7C PE=1 SV=1 - [COX7C_HUMAN]	14.29	1	1	1	2	0.62	0.76	0.78	0.93	0.29	0.43	0.40	0.54	0.32	0.19	0.35	0.48	-0.16	-0.27	-0.44	-0.40	-0.56	-0.34	-0.51	-0.32

O75438	NADH dehydrogenase [ubiquinone] 1 beta subcomplex subunit 1 OS=Homo sapiens GN=NDUFB1 PE=1 SV=1 - [NDUB1_HUMAN]	20.69	1	2	2	5	0.88	0.85	0.79	0.89	-0.10	0.17	0.31	0.59	0.45	0.40	0.04	0.06	-0.48	-0.76	-0.75	-0.45	-0.58	-1.00	-0.73	-0.32
Q9UN36	Protein NDRG2 OS=Homo sapiens GN=NDRG2 PE=1 SV=2 - [NDRG2_HUMAN]	51.21	1	10	10	75	0.54	0.57	0.76	0.63	-0.11	-0.05	0.39	0.32	0.22	0.13	0.03	0.17	-0.07	-0.51	-0.64	-0.56	-0.55	-0.65	-0.84	-0.32
P24539	ATP synthase F(0) complex subunit B1, mitochondrial OS=Homo sapiens GN=ATP5F1 PE=1 SV=2 - [AT5F1_HUMAN]	41.41	1	14	14	45	0.40	0.45	0.58	0.63	0.20	0.25	0.26	0.26	0.11	0.11	0.11	0.17	-0.12	-0.20	-0.48	-0.27	-0.51	-0.19	-0.44	-0.32
Q92665	28S ribosomal protein S31, mitochondrial OS=Homo sapiens GN=MRPS31 PE=1 SV=3 - [RT31_HUMAN]	10.13	1	3	3	4	0.38	0.55	0.18	0.08	0.22	0.39	-0.12	0.06	0.10	-0.08	0.10	0.27	-0.47	-0.44	-0.14	-0.55	-0.39	-0.36	0.12	-0.33
Q8N6R0	Methyltransferase-like protein 13 OS=Homo sapiens GN=METTL13 PE=1 SV=1 - [MET13_HUMAN]	6.29	1	3	3	5	0.38	0.29	0.28	0.19	0.25	0.15	-0.11	-0.20	-0.08	0.01	0.29	0.19	-0.43	-0.09	0.00	-0.34	-0.23	-0.15	-0.05	-0.33
Q92882	Osteoclast-stimulating factor 1 OS=Homo sapiens GN=OSTF1 PE=1 SV=2 - [OSTF1_HUMAN]	26.17	1	4	4	8	0.38	0.43	0.50	0.55	-0.38	-0.34	-0.02	0.08	0.35	0.22	-0.26	-0.24	-0.26	-0.44	-0.69	-0.41	-0.30	-0.62	-0.90	-0.33
P50395	Rab GDP dissociation inhibitor beta OS=Homo sapiens GN=GDI2 PE=1 SV=2 - [GDIB_HUMAN]	66.07	1	19	27	89	0.31	0.32	0.41	0.48	-0.05	-0.02	0.04	0.04	-0.10	-0.17	-0.53	-0.59	-0.15	-0.85	-0.95	-0.46	-0.73	-0.29	-0.60	-0.33
P35580	Myosin-10 OS=Homo sapiens GN=MYH10 PE=1 SV=3 - [MYH10_HUMAN]	47.01	1	63	86	264	-0.24	-0.27	-0.06	-0.08	-0.47	-0.51	-0.38	-0.41	-0.58	-0.53	-0.63	-0.69	-0.06	-0.31	-0.56	-0.24	-0.48	-0.24	-0.50	-0.33

Q8N1F8	Serine/threonine-protein kinase 11-interacting protein OS=Homo sapiens GN=STK11IP PE=1 SV=3 - [S11IP_HUMAN]	4.09	1	3	3	3	0.14	-0.06	0.16	-0.03	0.24	0.04	-0.23	-0.43	-0.07	0.13	0.15	-0.05	-0.31	0.02	-0.01	0.02	0.00	0.09	0.06	-0.33
P63151	Serine/threonine-protein phosphatase 2A 55 kDa regulatory subunit B alpha isoform OS=Homo sapiens GN=PPP2R2A PE=1 SV=1 - [2ABA_HUMAN]	35.35	1	8	12	27	0.86	0.47	0.82	0.63	0.25	-0.07	0.36	0.25	0.05	0.25	0.18	-0.08	-0.27	-0.52	-0.75	-0.45	-0.59	-0.68	-0.72	-0.33
P41586	Pituitary adenylate cyclase-activating polypeptide type I receptor OS=Homo sapiens GN=ADCYAP1R1 PE=1 SV=1 - [PACR_HUMAN]	1.71	1	1	1	1	0.06	0.08	0.38	0.40	-0.30	-0.29	-0.01	0.01	-0.09	-0.10	0.10	0.11	-0.01	0.05	-0.29	-0.13	-0.46	-0.38	-0.70	-0.33
O75489	NADH dehydrogenase [ubiquinone] iron-sulfur protein 3, mitochondrial OS=Homo sapiens GN=NDUFS3 PE=1 SV=1 - [NDUS3_HUMAN]	42.42	1	11	11	33	0.59	0.60	0.95	0.67	0.14	0.18	0.49	0.47	0.36	0.36	0.49	0.43	-0.24	-0.14	-0.53	-0.31	-0.49	-0.50	-0.72	-0.33
O75882	Attractin OS=Homo sapiens GN=ATRN PE=1 SV=2 - [ATRN_HUMAN]	3.71	1	4	4	5	0.68	0.78	0.50	0.60	0.12	0.21	0.11	0.21	0.30	0.20	0.16	0.25	-0.52	-0.52	-0.34	-0.44	-0.26	-0.58	-0.40	-0.33
Q96PU5	E3 ubiquitin-protein ligase NEDD4-like OS=Homo sapiens GN=NEDD4L PE=1 SV=2 - [NED4L_HUMAN]	11.49	1	8	8	13	0.69	0.51	0.58	0.47	0.18	0.25	0.09	0.09	0.26	0.22	0.30	0.31	-0.27	-0.17	-0.32	-0.42	-0.36	-0.48	-0.32	-0.33

O43684	Mitotic checkpoint protein BUB3 OS=Homo sapiens GN=BUB3 PE=1 SV=1 - [BUB3_HUMAN]	22.26	1	6	6	10	-0.08	0.00	-0.12	-0.16	-0.31	-0.19	-0.49	-0.50	-0.58	-0.62	-0.45	-0.42	-0.26	-0.19	-0.14	-0.46	-0.36	-0.09	0.00	-0.33
Q9Y5S2	Serine/threonine-protein kinase MRCK beta OS=Homo sapiens GN=CDC42BPB PE=1 SV=2 - [MRCKB_HUMAN]	20.69	1	26	31	52	0.36	0.35	0.37	0.33	-0.05	-0.03	-0.04	-0.01	-0.09	-0.07	-0.16	-0.21	-0.26	-0.51	-0.55	-0.38	-0.54	-0.39	-0.44	-0.33
Q01105	Protein SET OS=Homo sapiens GN=SET PE=1 SV=3 - [SET_HUMAN]	22.76	1	5	5	25	-0.11	-0.08	0.07	0.01	-0.70	-0.52	-0.49	-0.34	-0.66	-0.61	-0.43	-0.45	-0.21	-0.46	-0.62	-0.57	-0.68	-0.46	-0.72	-0.33
Q96ER9	Coiled-coil domain-containing protein 51 OS=Homo sapiens GN=CCDC51 PE=1 SV=2 - [CCD51_HUMAN]	11.68	1	3	3	7	0.48	0.63	0.39	0.63	0.10	0.47	0.32	0.56	0.49	0.18	0.20	0.50	-0.23	-0.19	-0.19	-0.44	-0.47	-0.46	-0.45	-0.33
Q9UK17	Potassium voltage-gated channel subfamily D member 3 OS=Homo sapiens GN=KCND3 PE=1 SV=3 - [KCND3_HUMAN]	8.24	1	4	4	4	0.37	0.91	0.28	0.82	-0.57	-0.04	-0.11	0.43	0.12	-0.42	-0.19	0.35	-0.43	-0.55	-0.47	-0.76	-0.67	-0.96	-0.87	-0.33
Q6P1N0	Coiled-coil and C2 domain-containing protein 1A OS=Homo sapiens GN=CC2D1A PE=1 SV=1 - [C2D1A_HUMAN]	14.20	1	12	12	22	0.18	0.27	0.12	0.24	-0.04	-0.03	0.04	0.08	-0.14	-0.16	-0.14	-0.11	-0.16	-0.36	-0.36	-0.28	-0.32	-0.23	-0.29	-0.33
Q5VU43	Myomegalin OS=Homo sapiens GN=PDE4DIP PE=1 SV=1 - [MYOME_HUMAN]	1.71	1	3	3	5	0.22	0.65	0.17	0.60	0.15	0.58	-0.23	0.21	0.43	0.00	-0.23	0.20	-0.39	-0.44	-0.40	-0.19	-0.14	-0.08	-0.03	-0.33
O75534	Cold shock domain-containing protein E1 OS=Homo sapiens GN=CSDE1 PE=1 SV=2 - [CSDE1_HUMAN]	29.07	1	19	19	33	0.32	0.42	0.48	0.64	0.05	0.15	0.13	0.09	0.02	-0.03	-0.23	-0.08	-0.10	-0.49	-0.66	-0.30	-0.57	-0.15	-0.44	-0.33

Q9UKD2	mRNA turnover protein 4 homolog OS=Homo sapiens GN=MRTO4 PE=1 SV=2 - [MRT4_HUMAN]	10.04	1	1	1	2	-0.54	-0.66	-0.23	-0.35	-0.05	-0.18	-0.63	-0.75	-0.35	-0.22	0.30	0.18	-0.03	0.85	0.53	0.35	0.04	0.47	0.16	-0.33
O75962	Triple functional domain protein OS=Homo sapiens GN=TRIO PE=1 SV=2 - [TRIO_HUMAN]	10.43	1	16	22	31	0.81	0.70	0.58	0.35	-0.08	0.01	-0.12	-0.05	0.04	0.18	0.02	-0.22	-0.60	-0.52	-0.52	-0.35	-0.27	-0.63	-0.39	-0.33
Q8TEH3	DENN domain-containing protein 1A OS=Homo sapiens GN=DENND1A PE=1 SV=2 - [DEN1A_HUMAN]	7.73	1	6	6	12	0.30	0.33	0.03	-0.04	-0.07	-0.22	-0.36	-0.57	-0.09	-0.01	-0.30	-0.32	-0.63	-0.67	-0.38	-0.35	0.12	-0.39	-0.20	-0.33
Q7Z6G3	N-terminal EF-hand calcium-binding protein 2 OS=Homo sapiens GN=NECAB2 PE=1 SV=1 - [NECA2_HUMAN]	20.21	1	5	5	7	0.33	0.43	0.39	0.63	-0.28	-0.04	-0.20	0.08	0.18	-0.04	-0.18	-0.04	-0.44	-0.53	-0.41	-0.33	-0.36	-0.44	-0.64	-0.33
Q7L775	EPM2A-interacting protein 1 OS=Homo sapiens GN=EPM2AIP1 PE=1 SV=1 - [EPMIP_HUMAN]	20.10	1	9	9	15	0.72	0.64	0.57	0.55	0.14	0.06	0.20	0.22	0.24	0.40	0.33	0.29	-0.41	-0.22	-0.23	-0.47	-0.22	-0.62	-0.27	-0.33
Q92734	Protein TFG OS=Homo sapiens GN=TFG PE=1 SV=2 - [TFG_HUMAN]	39.25	2	12	12	34	-0.14	-0.09	-0.08	0.15	0.05	-0.20	-0.27	-0.25	0.04	-0.06	0.26	0.18	-0.08	0.18	0.37	0.16	0.16	0.03	0.01	-0.33
Q8TBM8	DnaJ homolog subfamily B member 14 OS=Homo sapiens GN=DNAJB14 PE=2 SV=1 - [DJB14_HUMAN]	5.54	1	2	2	2	0.68	0.64	0.31	0.27	-0.19	-0.24	-0.09	-0.13	-0.41	-0.37	-0.27	-0.31	-0.71	-0.94	-0.57	-1.02	-0.64	-0.89	-0.51	-0.33
Q9Y2A7	Nck-associated protein 1 OS=Homo sapiens GN=NCKAP1 PE=1 SV=1 - [NCKP1_HUMAN]	25.09	1	27	28	64	0.25	0.20	0.46	0.43	-0.23	-0.20	-0.01	0.06	-0.15	-0.15	-0.17	-0.20	-0.13	-0.37	-0.68	-0.36	-0.60	-0.50	-0.73	-0.33

Q15788	Nuclear receptor coactivator 1 OS=Homo sapiens GN=NCOA1 PE=1 SV=3 - [NCOA1_HUMAN]	1.60	1	1	1	1	-0.33	-0.26	0.15	0.23	-0.11	-0.05	-0.24	-0.17	0.04	-0.03	0.18	0.25	0.15	0.52	0.03	0.33	-0.15	0.21	-0.28	-0.33
Q9NZM3	Intersectin-2 OS=Homo sapiens GN=ITSN2 PE=1 SV=3 - [ITSN2_HUMAN]	8.07	1	9	12	20	0.42	0.35	0.43	0.48	0.33	0.26	-0.04	-0.01	-0.05	-0.09	0.03	0.07	-0.41	-0.42	-0.40	-0.48	-0.52	-0.11	-0.11	-0.33
P49789	Bis(5'-adenosyl)-triphosphatase OS=Homo sapiens GN=FHIT PE=1 SV=3 - [FHIT_HUMAN]	37.41	1	5	5	8	0.75	1.00	0.69	0.54	0.19	0.43	0.30	0.35	-0.63	-0.08	-0.18	-0.16	-0.56	-1.14	-0.87	-1.10	-1.07	-0.57	-0.66	-0.33
Q96A49	Synapse-associated protein 1 OS=Homo sapiens GN=SYAP1 PE=1 SV=1 - [SYAP1_HUMAN]	25.85	1	6	6	9	0.36	0.89	0.91	0.46	0.41	0.33	0.51	0.03	0.52	0.28	0.21	0.13	-0.12	-0.14	-0.70	-0.22	-0.44	0.03	-0.52	-0.33
Q9BXV9	Uncharacterized protein C14orf142 OS=Homo sapiens GN=C14orf142 PE=1 SV=2 - [CN142_HUMAN]	18.00	1	1	1	2	1.10	0.65	1.05	0.60	0.33	-0.12	0.65	0.21	0.55	1.00	0.52	0.06	-0.39	-0.57	-0.54	-0.06	-0.01	-0.78	-0.73	-0.33
O43809	Cleavage and polyadenylation specificity factor subunit 5 OS=Homo sapiens GN=NUDT21 PE=1 SV=1 - [CPSF5_HUMAN]	24.67	1	6	6	13	-0.42	-0.17	-0.03	0.17	-0.21	0.02	-0.28	-0.07	-0.27	-0.28	-0.11	-0.09	0.05	0.19	-0.21	-0.07	-0.30	-0.04	-0.35	-0.33
P49756	RNA-binding protein 25 OS=Homo sapiens GN=RBM25 PE=1 SV=3 - [RBM25_HUMAN]	6.52	1	4	4	6	-0.44	-0.58	-0.22	-0.36	-0.20	-0.35	-0.62	-0.76	-0.68	-0.53	-0.20	-0.34	-0.12	0.25	0.02	-0.06	-0.28	0.22	0.00	-0.33
Q6P2E9	Enhancer of mRNA-decapping protein 4 OS=Homo sapiens GN=EDC4 PE=1 SV=1 - [EDC4_HUMAN]	10.49	1	10	10	14	0.19	0.07	0.25	0.04	-0.16	-0.32	-0.29	-0.37	-0.27	0.01	-0.11	-0.14	-0.26	-0.18	-0.22	-0.22	-0.17	-0.34	-0.39	-0.33

P85037	Forkhead box protein K1 OS=Homo sapiens GN=FOXK1 PE=1 SV=1 - [FOXK1_HUMAN]	3.82	1	2	2	2	-0.16	0.27	-0.27	0.16	-0.85	-0.42	-0.67	-0.24	-0.34	-0.77	-0.22	0.21	-0.46	-0.05	0.05	-0.58	-0.47	-0.70	-0.59	-0.34
Q9NS69	Mitochondrial import receptor subunit TOM22 homolog OS=Homo sapiens GN=TOMM22 PE=1 SV=3 - [TOM22_HUMAN]	16.20	1	2	2	5	0.77	0.69	0.08	0.43	0.00	-0.04	-0.33	-0.06	0.12	0.18	-0.13	-0.28	-0.76	-0.77	-0.68	-0.56	0.10	-0.79	-0.21	-0.34
P48067	Sodium- and chloride-dependent glycine transporter 1 OS=Homo sapiens GN=SLC6A9 PE=2 SV=3 - [SC6A9_HUMAN]	4.96	1	2	2	3	0.19	0.41	0.25	0.47	0.05	0.26	-0.15	0.07	0.24	0.03	0.32	0.53	-0.28	0.14	0.07	-0.13	-0.19	-0.16	-0.22	-0.34
Q9NWT6	Hypoxia-inducible factor 1-alpha inhibitor OS=Homo sapiens GN=HIF1AN PE=1 SV=2 - [HIF1N_HUMAN]	4.58	1	1	1	2	0.17	0.36	0.29	0.48	-0.89	-0.71	-0.11	0.08	-0.07	-0.26	-0.57	-0.39	-0.23	-0.74	-0.86	-0.40	-0.51	-1.08	-1.20	-0.34
Q15049	Membrane protein MLC1 OS=Homo sapiens GN=MLC1 PE=1 SV=5 - [MLC1_HUMAN]	14.06	1	4	4	14	1.55	1.30	1.69	1.62	0.06	-0.05	0.91	1.21	0.72	0.53	0.79	0.98	-0.06	-0.20	-0.52	-0.26	-0.57	-1.13	-1.45	-0.34
Q96HE7	ERO1-like protein alpha OS=Homo sapiens GN=ERO1L PE=1 SV=2 - [ERO1A_HUMAN]	24.36	1	7	7	19	0.20	0.25	0.20	0.21	-0.09	0.05	-0.11	-0.19	0.05	-0.03	0.18	0.31	-0.47	0.02	0.06	-0.25	-0.11	-0.21	-0.09	-0.34
Q16851	UTP--glucose-1-phosphate uridylyltransferase OS=Homo sapiens GN=UGP2 PE=1 SV=5 - [UGPA_HUMAN]	32.28	1	16	16	39	0.28	0.28	0.49	0.44	-0.06	-0.15	0.16	0.09	-0.12	-0.09	-0.47	-0.45	-0.14	-0.70	-0.96	-0.30	-0.49	-0.43	-0.54	-0.34
P19075	Tetraspanin-8 OS=Homo sapiens GN=TSPAN8 PE=1 SV=1 - [TSN8_HUMAN]	14.35	1	3	3	6	0.15	0.23	0.46	0.54	0.14	0.22	0.06	0.14	0.09	0.01	0.26	0.34	-0.04	0.12	-0.20	-0.11	-0.42	-0.02	-0.33	-0.34

Q9H7B4	Histone-lysine N-methyltransferase SMYD3 OS=Homo sapiens GN=SMYD3 PE=1 SV=4 - [SMYD3_HUMAN]	6.07	1	2	2	2	0.32	0.13	0.43	0.24	-0.47	-0.66	0.03	-0.16	-0.09	0.11	-0.32	-0.51	-0.24	-0.63	-0.75	-0.19	-0.29	-0.81	-0.92	-0.34
Q9UHQ4	B-cell receptor-associated protein 29 OS=Homo sapiens GN=BCAP29 PE=1 SV=2 - [BAP29_HUMAN]	19.09	1	4	4	9	-0.10	-0.22	0.08	0.05	-0.59	-0.68	-0.35	-0.33	-0.70	-0.61	-0.54	-0.58	-0.15	-0.33	-0.59	-0.43	-0.62	-0.55	-0.67	-0.34
O75688	Protein phosphatase 1B OS=Homo sapiens GN=PPM1B PE=1 SV=1 - [PPM1B_HUMAN]	18.37	1	4	7	13	1.27	1.26	1.03	1.02	0.29	0.27	0.63	0.62	0.20	0.25	0.18	0.16	-0.53	-0.97	-0.85	-0.80	-0.70	-0.81	-0.76	-0.34
Q9H2M9	Rab3 GTPase-activating protein non-catalytic subunit OS=Homo sapiens GN=RAB3GAP2 PE=1 SV=1 - [RBGPR_HUMAN]	15.72	1	18	19	30	0.22	0.21	0.31	0.39	-0.05	-0.04	-0.08	-0.10	-0.17	-0.15	-0.04	0.03	-0.19	-0.11	-0.41	-0.30	-0.52	-0.29	-0.41	-0.34
P83916	Chromobox protein homolog 1 OS=Homo sapiens GN=CBX1 PE=1 SV=1 - [CBX1_HUMAN]	36.76	1	3	4	11	-0.17	-0.09	-0.15	0.01	-0.95	-0.88	-0.57	-0.57	-0.60	-0.76	-0.70	-0.40	-0.33	-0.55	-0.56	-0.59	-0.53	-0.80	-0.75	-0.34
Q8IZP0	Abl interactor 1 OS=Homo sapiens GN=ABI1 PE=1 SV=4 - [ABI1_HUMAN]	31.10	1	10	13	37	0.52	0.20	0.62	0.40	-0.13	-0.28	0.16	-0.06	-0.10	0.06	-0.08	-0.26	-0.20	-0.50	-0.62	-0.41	-0.63	-0.59	-0.63	-0.34
Q7Z7H8	39S ribosomal protein L10, mitochondrial OS=Homo sapiens GN=MRPL10 PE=1 SV=3 - [RM10_HUMAN]	3.07	1	1	1	1	0.14	-0.02	0.15	-0.01	-0.30	-0.46	-0.25	-0.41	-0.22	-0.06	-0.03	-0.20	-0.34	-0.17	-0.19	-0.17	-0.18	-0.45	-0.47	-0.34

Q9UMS0	NFU1 iron-sulfur cluster scaffold homolog, mitochondrial OS=Homo sapiens GN=NFU1 PE=1 SV=2 - [NFU1_HUMAN]	13.78	1	3	3	8	0.18	0.24	0.40	0.46	0.04	0.09	0.00	-0.11	0.15	0.10	0.00	0.01	-0.13	-0.25	-0.47	-0.05	-0.27	-0.16	-0.38	-0.34
O75096	Low-density lipoprotein receptor-related protein 4 OS=Homo sapiens GN=LRP4 PE=1 SV=4 - [LRP4_HUMAN]	3.62	1	4	4	6	0.66	0.51	0.40	0.33	0.18	0.34	-0.42	0.25	0.41	0.29	-0.08	0.36	-0.45	-0.49	-0.24	-0.30	-0.08	-0.45	-0.19	-0.34
Q9NP97	Dynein light chain roadblock-type 1 OS=Homo sapiens GN=DYNLRB1 PE=1 SV=3 - [DLRB1_HUMAN]	51.04	2	3	3	12	0.19	-0.01	0.20	0.02	-0.35	-0.33	-0.40	-0.46	-0.45	-0.25	-0.29	-0.17	-0.40	-0.14	-0.50	-0.38	-0.42	-0.36	-0.51	-0.34
Q15276	Rab GTPase-binding effector protein 1 OS=Homo sapiens GN=RABEP1 PE=1 SV=2 - [RABE1_HUMAN]	21.35	1	16	16	27	0.56	0.42	0.55	0.49	0.09	-0.01	0.24	0.27	0.18	0.10	0.05	0.09	-0.20	-0.52	-0.65	-0.37	-0.67	-0.45	-0.49	-0.34
O43752	Syntaxin-6 OS=Homo sapiens GN=STX6 PE=1 SV=1 - [STX6_HUMAN]	40.00	1	6	7	14	0.65	0.90	0.65	0.90	0.30	0.65	0.09	0.41	0.47	0.31	0.11	0.24	-0.24	-0.47	-0.55	-0.32	-0.28	-0.54	-0.54	-0.34
P55795	Heterogeneous nuclear ribonucleoprotein H2 OS=Homo sapiens GN=HNRNPH2 PE=1 SV=1 - [HNRH2_HUMAN]	39.20	1	6	11	49	0.60	0.49	0.51	0.50	-0.19	-0.29	0.05	0.04	-0.11	-0.04	0.02	0.19	-0.44	-0.62	-0.49	-0.56	-0.57	-0.63	-0.75	-0.34
Q8N4Q1	Mitochondrial intermembrane space import and assembly protein 40 OS=Homo sapiens GN=CHCHD4 PE=1 SV=1 - [MIA40_HUMAN]	24.65	1	3	3	6	0.43	0.35	0.45	0.37	-0.21	-0.30	0.04	-0.04	-0.02	0.06	-0.13	-0.22	-0.34	-0.56	-0.58	-0.34	-0.36	-0.66	-0.68	-0.35

P35573	Glycogen debranching enzyme OS=Homo sapiens GN=AGL PE=1 SV=3 - [GDE_HUMAN]	25.59	1	32	32	60	0.68	0.68	0.83	0.95	0.44	0.40	0.44	0.61	0.40	0.34	0.18	0.24	-0.17	-0.41	-0.58	-0.33	-0.47	-0.32	-0.51	-0.35
P10809	60 kDa heat shock protein, mitochondrial OS=Homo sapiens GN=HSPD1 PE=1 SV=2 - [CH60_HUMAN]	68.76	1	32	32	296	0.20	0.18	0.47	0.48	-0.31	-0.28	0.08	0.09	-0.10	-0.14	-0.36	-0.35	-0.05	-0.57	-0.88	-0.37	-0.65	-0.59	-0.83	-0.35
Q9NP72	Ras-related protein Rab-18 OS=Homo sapiens GN=RAB18 PE=1 SV=1 - [RAB18_HUMAN]	47.57	1	8	8	16	0.69	0.63	0.55	0.63	0.45	0.58	0.22	0.31	0.20	0.12	0.01	0.23	-0.09	-0.20	-0.52	-0.15	-0.38	0.02	-0.10	-0.35
Q9H469	F-box/LRR-repeat protein 15 OS=Homo sapiens GN=FBXL15 PE=1 SV=2 - [FXL15_HUMAN]	13.67	1	2	2	2	1.16	1.50	0.09	0.43	0.14	0.48	-0.03	0.31	0.42	0.08	-0.11	0.22	-0.94	-1.27	-0.20	-0.72	-0.13	-0.66	-0.08	-0.35
Q12874	Splicing factor 3A subunit 3 OS=Homo sapiens GN=SF3A3 PE=1 SV=1 - [SF3A3_HUMAN]	23.75	1	9	9	14	-0.54	-0.50	-0.25	-0.16	-0.87	-0.89	-0.61	-0.53	-0.66	-0.71	-0.75	-0.73	0.02	-0.27	-0.57	-0.04	-0.46	-0.36	-0.71	-0.35
O95873	Uncharacterized protein C6orf47 OS=Homo sapiens GN=C6orf47 PE=2 SV=2 - [CF047_HUMAN]	4.42	1	1	1	1	-0.03	0.32	-0.46	-0.10	-1.06	-0.71	-0.87	-0.51	-0.63	-0.98	-0.51	-0.16	-0.78	-0.47	-0.05	-0.92	-0.49	-1.04	-0.62	-0.35
P12081	Histidine--tRNA ligase, cytoplasmic OS=Homo sapiens GN=HARS PE=1 SV=2 - [SYHC_HUMAN]	27.31	1	10	14	29	0.03	0.17	0.29	0.44	-0.30	-0.25	-0.23	-0.15	-0.30	-0.27	-0.47	-0.34	-0.22	-0.49	-0.69	-0.42	-0.70	-0.35	-0.63	-0.35
P13804	Electron transfer flavoprotein subunit alpha, mitochondrial OS=Homo sapiens GN=ETFA PE=1 SV=1 - [ETFA_HUMAN]	39.64	1	10	10	23	-0.40	-0.42	-0.03	-0.16	-0.37	-0.49	-0.42	-0.39	-0.57	-0.51	-0.18	-0.20	0.10	0.23	-0.13	-0.09	-0.29	-0.13	-0.39	-0.35

Q5JPI3	Uncharacterized protein C3orf38 OS=Homo sapiens GN=C3orf38 PE=1 SV=1 - [CC038_HUMAN]	2.43	1	1	1	1	-0.66	-0.53	-0.03	0.10	-0.24	-0.11	-0.44	-0.31	0.04	-0.09	-0.34	-0.22	0.27	0.32	-0.31	0.60	-0.02	0.40	-0.22	-0.35
O95433	Activator of 90 kDa heat shock protein ATPase homolog 1 OS=Homo sapiens GN=AHSA1 PE=1 SV=1 - [AHSA1_HUMAN]	49.41	1	12	12	25	0.57	0.42	0.83	0.57	-0.12	-0.15	0.36	0.29	0.06	0.07	-0.24	-0.21	-0.34	-0.83	-0.86	-0.60	-0.67	-0.65	-0.66	-0.35
Q155Q3	Dixin OS=Homo sapiens GN=DIXDC1 PE=1 SV=2 - [DIXC1_HUMAN]	4.10	1	2	2	5	0.09	-0.58	0.29	-0.16	0.38	-0.96	-0.28	-0.64	-0.31	0.28	-0.08	-0.45	0.03	-0.45	-0.28	0.14	0.19	0.27	0.15	-0.35
Q9Y399	28S ribosomal protein S2, mitochondrial OS=Homo sapiens GN=MRPS2 PE=1 SV=1 - [RT02_HUMAN]	9.12	1	2	2	4	0.31	0.02	0.25	-0.04	0.21	-0.08	-0.17	-0.45	-0.22	0.08	0.29	0.00	-0.42	-0.01	0.04	-0.20	-0.14	-0.11	-0.05	-0.35
Q92879	CUGBP Elav-like family member 1 OS=Homo sapiens GN=CELF1 PE=1 SV=2 - [CELF1_HUMAN]	6.38	1	2	2	2	0.32	0.12	0.37	0.18	-0.07	-0.27	-0.04	-0.24	0.18	0.38	0.04	-0.16	-0.30	-0.27	-0.33	0.10	0.04	-0.40	-0.46	-0.35
Q96DD7	Protein shisa-4 OS=Homo sapiens GN=SHISA4 PE=2 SV=3 - [SHSA4_HUMAN]	4.57	1	1	1	3	0.63	0.82	0.76	0.96	0.24	0.43	0.35	0.54	0.64	0.45	0.30	0.49	-0.22	-0.32	-0.46	-0.14	-0.28	-0.40	-0.54	-0.35
P26196	Probable ATP-dependent RNA helicase DDX6 OS=Homo sapiens GN=DDX6 PE=1 SV=2 - [DDX6_HUMAN]	43.89	4	14	15	37	0.27	0.35	0.34	0.38	0.07	0.10	-0.15	-0.03	-0.02	-0.06	-0.13	-0.10	-0.25	-0.15	-0.34	-0.27	-0.33	-0.10	-0.31	-0.35
Q9NP92	28S ribosomal protein S30, mitochondrial OS=Homo sapiens GN=MRPS30 PE=1 SV=2 - [RT30_HUMAN]	2.73	1	1	1	1	0.04	0.07	0.27	0.30	0.11	0.13	-0.14	-0.11	-0.09	-0.11	-0.17	-0.14	-0.12	-0.20	-0.44	-0.12	-0.35	0.06	-0.18	-0.35

Q96KP4	Cytosolic non specific dipeptidase OS=Homo sapiens GN=CNDP2 PE=1 SV=2 - [CNDP2_HUMAN]	66.11	1	21	21	75	1.11	1.02	1.43	1.36	0.46	0.45	1.02	0.93	0.69	0.69	0.63	0.58	-0.01	-0.47	-0.80	-0.47	-0.64	-0.65	-0.95	-0.35
P07858	Cathepsin B OS=Homo sapiens GN=CTSB PE=1 SV=3 - [CATB_HUMAN]	28.32	1	7	7	24	0.33	0.22	0.38	0.40	0.14	0.15	0.11	-0.07	0.07	0.10	0.22	0.14	-0.20	-0.29	-0.25	-0.25	-0.23	-0.18	-0.22	-0.35
P07998	Ribonuclease pancreatic OS=Homo sapiens GN=RNASE1 PE=1 SV=4 - [RNAS1_HUMAN]	51.28	1	4	4	17	1.12	0.94	0.40	0.49	0.58	0.78	0.33	0.57	0.82	0.81	0.32	0.52	-0.30	-0.40	0.01	-0.08	-0.16	-0.16	0.32	-0.35
O60888	Protein CutA OS=Homo sapiens GN=CUTA PE=1 SV=2 - [CUTA_HUMAN]	15.64	1	3	3	4	-0.01	0.21	0.20	0.32	0.09	-0.38	0.05	-0.09	-0.12	-0.04	-0.19	-0.29	-0.08	-0.48	-0.87	-0.20	-0.58	-0.23	-0.85	-0.35
Q7Z6B0	Coiled-coil domain-containing protein 91 OS=Homo sapiens GN=CCDC91 PE=1 SV=2 - [CCD91_HUMAN]	4.76	1	2	2	6	0.76	0.67	1.11	1.07	0.40	0.34	0.68	0.62	0.40	0.45	0.33	0.23	-0.06	-0.38	-0.77	-0.24	-0.63	-0.38	-0.73	-0.35
Q9BYH1	Seizure 6-like protein OS=Homo sapiens GN=SEZ6L PE=1 SV=1 - [SE6L1_HUMAN]	5.96	1	5	5	7	0.15	0.25	0.04	0.41	0.43	0.59	-0.18	-0.01	0.38	0.22	0.49	0.77	-0.29	0.37	0.30	0.10	0.12	0.07	0.16	-0.35
Q00325	Phosphate carrier protein, mitochondrial OS=Homo sapiens GN=SLC25A3 PE=1 SV=2 - [MPCP_HUMAN]	23.20	1	9	9	58	0.25	0.30	0.60	0.75	-0.17	0.03	0.14	0.29	0.04	0.00	-0.09	-0.07	-0.05	-0.38	-0.70	-0.29	-0.51	-0.42	-0.71	-0.35
Q8IV20	Laccase domain-containing protein 1 OS=Homo sapiens GN=LACC1 PE=1 SV=1 - [LACC1_HUMAN]	2.56	1	1	1	1	0.11	0.05	0.57	0.51	0.20	0.14	0.15	0.09	0.17	0.23	0.36	0.29	0.10	0.25	-0.21	0.15	-0.31	0.07	-0.38	-0.35

Q5SQN1	Synaptosomal-associated protein 47 OS=Homo sapiens GN=SNAP47 PE=1 SV=3 - [SNP47_HUMAN]	12.28	1	5	5	9	1.12	0.68	1.15	0.71	0.91	0.46	0.74	0.29	0.00	0.45	-0.07	-0.52	-0.33	-1.18	-1.22	-0.64	-0.67	-0.23	-0.26	-0.35
Q9BV44	THUMP domain-containing protein 3 OS=Homo sapiens GN=THUMPD3 PE=1 SV=1 - [THUM3_HUMAN]	4.73	1	2	2	3	-0.11	0.20	0.05	0.37	-0.13	0.18	-0.36	-0.05	-0.14	-0.45	0.04	0.35	-0.19	0.16	-0.02	-0.31	-0.47	-0.03	-0.20	-0.35
Q9BXK5	Bcl-2-like protein 13 OS=Homo sapiens GN=BCL2L13 PE=1 SV=1 - [B2L13_HUMAN]	16.29	1	5	5	13	0.45	0.45	0.58	0.51	-0.06	0.14	0.16	0.16	-0.24	-0.28	-0.12	0.02	-0.21	-0.47	-0.73	-0.70	-0.82	-0.61	-0.74	-0.35
Q6UXN9	WD repeat-containing protein 82 OS=Homo sapiens GN=WDR82 PE=1 SV=1 - [WDR82_HUMAN]	24.28	1	6	6	7	-0.74	-0.99	-0.29	-0.55	0.38	0.12	-0.71	-0.97	-0.71	-0.45	-0.43	-0.69	0.08	0.31	-0.14	0.32	-0.12	1.10	0.66	-0.35
Q13332	Receptor-type tyrosine-protein phosphatase S OS=Homo sapiens GN=PTPRS PE=1 SV=3 - [PTPRS_HUMAN]	17.40	1	16	24	47	0.56	0.66	0.43	0.66	-0.05	0.15	0.01	0.20	0.11	-0.11	0.34	0.32	-0.45	-0.30	-0.40	-0.44	-0.41	-0.68	-0.55	-0.35
O15347	High mobility group protein B3 OS=Homo sapiens GN=HMGB3 PE=1 SV=4 - [HMGB3_HUMAN]	20.50	1	3	3	5	-0.28	-0.36	0.04	-0.04	-0.37	-0.45	-0.38	-0.45	-0.59	-0.52	-0.33	-0.41	-0.04	-0.05	-0.37	-0.20	-0.52	-0.10	-0.42	-0.35
Q5SGD2	Protein phosphatase 1L OS=Homo sapiens GN=PPM1L PE=1 SV=1 - [PPM1L_HUMAN]	6.94	1	2	2	3	-0.04	0.88	0.11	1.04	-0.63	0.29	-0.30	0.62	0.15	-0.77	-0.24	0.68	-0.20	-0.19	-0.35	-0.70	-0.86	-0.61	-0.77	-0.35
P40818	Ubiquitin carboxyl-terminal hydrolase 8 OS=Homo sapiens GN=USP8 PE=1 SV=1 - [UBP8_HUMAN]	2.68	1	2	2	2	0.09	0.14	0.31	0.37	-0.10	-0.05	-0.11	-0.05	0.09	0.04	-0.04	0.01	-0.14	-0.12	-0.36	-0.01	-0.24	-0.20	-0.43	-0.35

Q96Q11	CCA tRNA nucleotidyltransferase 1, mitochondrial OS=Homo sapiens GN=TRNT1 PE=1 SV=2 - [TRNT1_HUMAN]	17.97	1	6	6	11	0.04	0.05	0.21	0.18	-0.25	-0.28	-0.20	-0.24	-0.43	-0.40	-0.42	-0.46	-0.21	-0.31	-0.67	-0.35	-0.68	-0.24	-0.47	-0.36
P46379	Large proline-rich protein BAG6 OS=Homo sapiens GN=BAG6 PE=1 SV=2 - [BAG6_HUMAN]	20.67	1	17	17	40	0.55	0.55	0.45	0.47	0.12	0.14	0.14	0.15	0.23	0.21	0.05	0.19	-0.32	-0.60	-0.41	-0.47	-0.22	-0.35	-0.36	-0.36
Q96DB5	Regulator of microtubule dynamics protein 1 OS=Homo sapiens GN=RMDN1 PE=1 SV=1 - [RMD1_HUMAN]	16.24	1	4	5	5	0.40	0.36	0.62	0.58	0.38	0.33	0.20	0.16	0.15	0.20	0.55	0.51	-0.15	0.15	-0.07	-0.18	-0.39	-0.04	-0.26	-0.36
P51688	N-sulphoglucosamine sulphohydrolase OS=Homo sapiens GN=SGSH PE=1 SV=1 - [SPHM_HUMAN]	2.39	1	1	1	1	0.22	0.16	0.16	0.10	-0.08	-0.15	-0.26	-0.32	-0.26	-0.19	0.12	0.05	-0.42	-0.10	-0.05	-0.39	-0.32	-0.32	-0.26	-0.36
Q8TAB3	Protocadherin-19 OS=Homo sapiens GN=PCDH19 PE=1 SV=3 - [PCD19_HUMAN]	3.05	1	2	2	2	0.02	-0.21	0.11	-0.12	0.12	-0.11	-0.31	-0.54	-0.41	-0.17	-0.17	-0.41	-0.28	-0.18	-0.28	-0.16	-0.25	0.09	0.00	-0.36
P0DJI9	Serum amyloid A-2 protein OS=Homo sapiens GN=SAA2 PE=1 SV=1 - [SAA2_HUMAN]	24.59	1	2	3	14	0.03	0.17	-0.29	-0.05	-0.06	-0.02	-0.67	-0.62	-0.23	-0.31	-0.31	-0.22	-0.69	-0.33	-0.02	-0.32	0.01	-0.20	0.12	-0.36
Q93034	Cullin-5 OS=Homo sapiens GN=CUL5 PE=1 SV=4 - [CUL5_HUMAN]	23.21	1	17	17	26	0.23	0.58	0.51	0.67	-0.40	-0.29	0.18	0.30	0.13	0.06	0.17	0.05	-0.03	-0.21	-0.51	-0.26	-0.52	-0.73	-0.96	-0.36
Q9UBV2	Protein sel-1 homolog 1 OS=Homo sapiens GN=SEL1L PE=1 SV=3 - [SE1L1_HUMAN]	10.33	1	6	6	9	0.25	0.32	0.38	0.46	0.12	0.19	-0.04	0.04	-0.01	-0.08	0.17	0.24	-0.23	-0.07	-0.21	-0.30	-0.43	-0.15	-0.28	-0.36

Q15070	Mitochondrial inner membrane protein OXA1L OS=Homo sapiens GN=OXA1L PE=1 SV=3 - [OXA1L_HUMAN]	1.84	1	1	1	2	0.73	0.80	0.49	0.56	0.17	0.23	0.07	0.14	0.26	0.20	-0.03	0.04	-0.60	-0.75	-0.52	-0.50	-0.26	-0.57	-0.34	-0.36
P01116	GTPase KRas OS=Homo sapiens GN=KRAS PE=1 SV=1 - [RASK_HUMAN]	51.32	1	2	7	23	0.98	0.97	0.44	0.43	0.65	0.32	0.02	0.00	-0.12	-0.02	-0.38	-0.52	-0.91	-0.69	-0.35	-1.05	-0.51	-0.85	-0.29	-0.36
Q9NPI6	mRNA-decapping enzyme 1A OS=Homo sapiens GN=DCP1A PE=1 SV=2 - [DCP1A_HUMAN]	6.19	1	3	3	6	0.12	0.02	0.19	0.01	-0.37	-0.46	-0.47	-0.41	-0.39	-0.28	-0.33	-0.43	-0.71	-0.45	-0.50	-0.38	-0.42	-0.61	-0.48	-0.36
Q14738	Serine/threonine-protein phosphatase 2A 56 kDa regulatory subunit delta isoform OS=Homo sapiens GN=PPP2R5D PE=1 SV=1 - [2A5D_HUMAN]	26.91	1	11	14	22	0.50	0.69	0.75	0.75	0.19	0.26	0.44	0.50	0.38	0.18	0.22	0.19	-0.20	-0.44	-0.61	-0.46	-0.68	-0.49	-0.60	-0.36
O00541	Pescadillo homolog OS=Homo sapiens GN=PES1 PE=1 SV=1 - [PESC_HUMAN]	3.06	1	1	1	1	0.48	0.42	-0.13	-0.18	-0.91	-0.97	-0.55	-0.60	-0.04	0.02	-0.13	-0.19	-0.97	-0.60	-0.01	-0.42	0.18	-1.40	-0.80	-0.36
Q9Y365	PCTP-like protein OS=Homo sapiens GN=STARD10 PE=1 SV=2 - [PCTL_HUMAN]	15.46	1	3	3	3	1.36	1.34	1.09	1.40	0.52	0.94	0.67	0.88	1.27	0.51	0.49	0.70	-0.41	-0.66	-0.60	-0.02	-0.21	-0.40	-0.60	-0.36
Q9BX68	Histidine triad nucleotide-binding protein 2, mitochondrial OS=Homo sapiens GN=HINT2 PE=1 SV=1 - [HINT2_HUMAN]	47.24	1	5	5	11	0.16	0.10	0.41	0.48	-0.18	-0.16	-0.04	0.09	0.00	-0.14	0.28	0.39	0.09	0.15	0.05	0.02	-0.42	-0.19	-0.61	-0.36
P32189	Glycerol kinase OS=Homo sapiens GN=GK PE=1 SV=3 - [GLPK_HUMAN]	17.17	3	8	8	13	0.37	0.38	0.45	0.78	0.16	0.13	0.25	0.38	0.18	0.21	0.02	0.04	0.02	-0.50	-0.77	-0.23	-0.51	-0.22	-0.55	-0.36

Q9NPE3	H/ACA ribonucleoprotein complex subunit 3 OS=Homo sapiens GN=NOP10 PE=1 SV=1 - [NOP10_HUMAN]	20.31	1	1	1	2	0.50	0.45	0.27	0.22	0.01	-0.05	-0.15	-0.21	0.12	0.18	0.48	0.41	-0.60	-0.02	0.20	-0.29	-0.06	-0.51	-0.28	-0.36
P62258	14-3-3 protein epsilon OS=Homo sapiens GN=YWHAE PE=1 SV=1 - [1433E_HUMAN]	71.37	1	18	20	336	0.39	0.43	0.58	0.64	0.13	0.15	0.21	0.23	0.10	0.09	0.16	0.10	-0.13	-0.29	-0.48	-0.22	-0.47	-0.32	-0.44	-0.36
O75306	NADH dehydrogenase [ubiquinone] iron-sulfur protein 2, mitochondrial OS=Homo sapiens GN=NDUFS2 PE=1 SV=2 - [NDUS2_HUMAN]	49.03	1	15	15	44	0.88	0.76	0.81	0.73	0.23	0.27	0.42	0.37	0.33	0.38	0.65	0.49	-0.43	-0.29	-0.18	-0.45	-0.36	-0.50	-0.60	-0.36
Q9UMX5	Neudesin OS=Homo sapiens GN=NENF PE=1 SV=1 - [NENF_HUMAN]	55.23	1	6	6	11	-0.20	-0.10	0.08	-0.10	0.13	0.41	-0.25	0.09	0.11	0.16	0.29	0.18	0.16	0.36	0.27	0.03	-0.34	0.48	-0.14	-0.36
Q96DV4	39S ribosomal protein L38, mitochondrial OS=Homo sapiens GN=MRPL38 PE=1 SV=2 - [RM38_HUMAN]	5.53	1	2	2	4	0.23	0.45	0.17	0.39	0.10	0.32	-0.26	-0.04	0.22	0.01	-0.03	0.19	-0.43	-0.25	-0.19	-0.19	-0.12	-0.14	-0.08	-0.36
P21397	Amine oxidase [flavin-containing] A OS=Homo sapiens GN=MAOA PE=1 SV=1 - [AOFA_HUMAN]	34.91	1	12	16	43	0.21	0.19	0.04	0.39	-0.44	-0.35	-0.24	-0.18	-0.23	-0.18	-0.25	-0.17	-0.29	-0.35	-0.37	-0.34	-0.44	-0.65	-0.68	-0.36
Q8WXE0	Caskin-2 OS=Homo sapiens GN=CASKIN2 PE=1 SV=2 - [CSKI2_HUMAN]	5.07	1	3	3	3	-0.15	-0.06	0.02	0.11	-0.20	-0.11	-0.41	-0.31	-0.60	-0.69	-0.14	-0.05	-0.20	0.02	-0.16	-0.51	-0.68	-0.07	-0.24	-0.36
P25398	40S ribosomal protein S12 OS=Homo sapiens GN=RPS12 PE=1 SV=3 - [RS12_HUMAN]	34.09	1	4	4	10	-0.68	-0.54	-0.36	-0.22	-0.87	-0.80	-0.76	-0.65	-0.97	-1.21	-1.36	-1.28	-0.04	-0.82	-0.96	-0.39	-0.74	-0.37	-0.54	-0.36

Q15717	ELAV-like protein 1 OS=Homo sapiens GN=ELAVL1 PE=1 SV=2 - [ELAV1_HUMAN]	29.45	1	8	9	22	-0.03	-0.01	-0.05	-0.12	-0.29	-0.28	-0.47	-0.46	-0.49	-0.53	-0.21	-0.20	-0.35	-0.14	-0.10	-0.42	-0.43	-0.29	-0.32	-0.36
P56378	6.8 kDa mitochondrial proteolipid OS=Homo sapiens GN=MP68 PE=1 SV=1 - [68MP_HUMAN]	32.76	1	2	2	6	0.33	0.48	0.60	0.75	-0.17	-0.01	0.18	0.32	0.19	-0.03	-0.29	-0.12	-0.11	-0.62	-0.92	-0.33	-0.65	-0.51	-0.78	-0.36
Q969K3	E3 ubiquitin-protein ligase RNF34 OS=Homo sapiens GN=RNF34 PE=1 SV=1 - [RNF34_HUMAN]	15.32	1	4	4	8	0.99	0.72	0.83	0.53	0.35	-0.03	0.34	-0.01	-0.15	0.14	0.36	0.05	-0.64	-0.69	-0.49	-1.01	-0.79	-0.61	-0.49	-0.36
Q12962	Transcription initiation factor TFIID subunit 10 OS=Homo sapiens GN=TAF10 PE=1 SV=1 - [TAF10_HUMAN]	21.10	1	2	2	2	-0.21	-0.72	-0.27	-0.78	-1.40	-1.92	-0.70	-1.21	-1.29	-0.77	-0.58	-1.10	-0.43	-0.37	-0.31	-0.53	-0.47	-1.21	-1.15	-0.37
Q92990	Glomulin OS=Homo sapiens GN=GLMN PE=1 SV=2 - [GLMN_HUMAN]	4.04	1	2	2	2	0.90	0.69	0.72	0.52	0.47	0.26	0.30	0.09	0.14	0.35	0.47	0.26	-0.55	-0.43	-0.26	-0.52	-0.34	-0.45	-0.27	-0.37
Q7Z739	YTH domain-containing family protein 3 OS=Homo sapiens GN=YTHDF3 PE=1 SV=1 - [YTHD3_HUMAN]	3.59	1	1	2	3	-0.94	-0.81	-0.58	-0.45	-0.51	-0.39	-1.01	-0.88	-0.60	-0.72	-0.68	-0.56	-0.01	0.27	-0.10	0.25	-0.11	0.41	0.05	-0.37
P61163	Alpha-centractin OS=Homo sapiens GN=ACTR1A PE=1 SV=1 - [ACTZ_HUMAN]	25.53	1	3	7	30	0.75	0.56	0.63	0.58	0.06	-0.06	0.06	0.01	-0.09	-0.04	0.07	-0.02	-0.54	-0.65	-0.52	-0.47	-0.42	-0.60	-0.41	-0.37
P35611	Alpha-adducin OS=Homo sapiens GN=ADD1 PE=1 SV=2 - [ADDA_HUMAN]	44.50	1	23	24	113	0.50	0.52	0.68	0.63	-0.02	-0.02	0.22	0.28	0.07	0.07	0.22	0.29	-0.26	-0.18	-0.32	-0.39	-0.57	-0.47	-0.58	-0.37
Q99574	Neuroserpin OS=Homo sapiens GN=SERPINI1 PE=1 SV=1 - [NEUS_HUMAN]	2.44	1	1	1	1	-0.64	-0.11	-0.28	0.25	-1.14	-0.61	-0.71	-0.18	-0.18	-0.71	-0.89	-0.36	-0.01	-0.24	-0.61	-0.03	-0.39	-0.51	-0.87	-0.37

Q9UKV3	Apoptotic chromatin condensation inducer in the nucleus OS=Homo sapiens GN=ACIN1 PE=1 SV=2 - [ACINU_HUMAN]	7.16	1	7	7	10	0.54	0.04	0.04	0.04	-0.05	-0.36	-0.16	-0.47	-0.36	-0.03	0.09	-0.11	-0.08	0.40	-0.21	0.26	-0.35	0.30	-0.38	-0.37
Q9UPN3	Microtubule-actin cross-linking factor 1, isoforms 1/2/3/5 OS=Homo sapiens GN=MACF1 PE=1 SV=4 - [MACF1_HUMAN]	22.10	1	125	129	238	0.48	0.44	0.53	0.54	-0.01	-0.05	0.14	0.12	0.03	0.07	0.06	0.03	-0.36	-0.41	-0.54	-0.41	-0.49	-0.52	-0.59	-0.37
Q9BY67	Cell adhesion molecule 1 OS=Homo sapiens GN=CADM1 PE=1 SV=2 - [CADM1_HUMAN]	12.22	1	4	4	13	1.28	1.33	1.34	1.19	0.66	0.43	0.68	0.70	0.58	0.55	0.67	0.52	-0.55	-0.64	-0.66	-0.70	-0.64	-0.67	-0.46	-0.37
Q6YP21	Kynurenine--oxoglutarate transaminase 3 OS=Homo sapiens GN=CCBL2 PE=1 SV=1 - [KAT3_HUMAN]	22.03	1	8	9	17	0.21	0.04	0.44	0.31	0.24	0.13	0.19	0.01	-0.19	-0.13	-0.38	-0.52	-0.01	-0.76	-1.00	-0.46	-0.96	-0.19	-0.63	-0.37
P55209	Nucleosome assembly protein 1-like 1 OS=Homo sapiens GN=NAP1L1 PE=1 SV=1 - [NP1L1_HUMAN]	19.69	1	5	6	20	0.36	0.80	0.48	0.55	0.32	0.22	0.12	0.27	0.09	-0.09	-0.14	-0.07	0.03	-0.23	-0.70	-0.08	-0.49	0.21	-0.30	-0.37
Q01469	Fatty acid-binding protein, epidermal OS=Homo sapiens GN=FABP5 PE=1 SV=3 - [FABP5_HUMAN]	42.96	2	5	5	18	0.76	0.71	0.96	1.17	0.31	0.22	0.68	0.73	0.33	0.35	-0.31	-0.31	0.00	-0.96	-1.42	-0.27	-0.83	-0.52	-0.95	-0.37
P10109	Adrenodoxin, mitochondrial OS=Homo sapiens GN=FDX1 PE=1 SV=1 - [ADX_HUMAN]	19.57	1	3	3	4	0.03	0.26	0.04	0.48	0.08	0.16	-0.26	-0.28	0.15	-0.13	-0.05	0.02	-0.16	0.04	-0.08	-0.08	-0.29	-0.12	-0.33	-0.37

P49750	YLP motif-containing protein 1 OS=Homo sapiens GN=YLPM1 PE=1 SV=3 - [YLPM1_HUMAN]	8.10	1	10	10	18	0.11	-0.06	0.23	0.36	-0.21	-0.37	-0.09	-0.26	-0.37	-0.29	-0.08	-0.24	0.13	-0.16	-0.36	-0.33	-0.63	-0.14	-0.76	-0.37
Q9Y3D7	Mitochondrial import inner membrane translocase subunit TIM16 OS=Homo sapiens GN=PAM16 PE=1 SV=2 - [TIM16_HUMAN]	8.80	1	1	1	1	0.07	0.06	0.22	0.21	0.07	0.06	-0.22	-0.22	0.01	0.02	0.53	0.52	-0.23	0.47	0.31	-0.02	-0.16	-0.01	-0.16	-0.37
Q9UJQ1	Lysosome-associated membrane glycoprotein 5 OS=Homo sapiens GN=LAMP5 PE=1 SV=1 - [LAMP5_HUMAN]	5.36	1	1	1	1	0.29	0.40	0.69	0.80	0.43	0.53	0.26	0.36	0.66	0.56	-0.08	0.03	0.02	-0.36	-0.77	0.30	-0.10	0.12	-0.28	-0.37
Q9NTK5	Obg-like ATPase 1 OS=Homo sapiens GN=OLA1 PE=1 SV=2 - [OLA1_HUMAN]	38.38	1	13	13	36	0.35	0.32	0.29	0.45	-0.48	-0.38	-0.09	-0.07	-0.31	-0.32	-0.63	-0.55	-0.33	-0.85	-0.86	-0.56	-0.45	-0.72	-0.77	-0.37
Q9P0N9	TBC1 domain family member 7 OS=Homo sapiens GN=TBC1D7 PE=1 SV=1 - [TBCD7_HUMAN]	6.14	1	1	1	1	0.99	1.87	0.59	1.47	-0.10	0.77	0.16	1.04	1.03	0.16	-0.58	0.30	-0.78	-1.56	-1.17	-0.80	-0.40	-1.11	-0.71	-0.37
P49903	Selenide, water dikinase 1 OS=Homo sapiens GN=SEPHS1 PE=1 SV=2 - [SPS1_HUMAN]	12.76	1	3	3	10	0.13	0.06	0.34	0.28	0.37	0.30	-0.10	-0.16	0.01	0.08	0.11	0.04	-0.17	-0.01	-0.23	-0.02	-0.23	0.23	0.01	-0.37
P49327	Fatty acid synthase OS=Homo sapiens GN=FASN PE=1 SV=3 - [FAS_HUMAN]	35.09	2	64	64	140	0.80	0.81	0.97	0.96	0.43	0.43	0.47	0.38	0.33	0.31	0.15	0.20	-0.28	-0.63	-0.78	-0.41	-0.51	-0.40	-0.50	-0.37
Q96JJ7	Protein disulfide-isomerase TMX3 OS=Homo sapiens GN=TMX3 PE=1 SV=2 - [TMX3_HUMAN]	24.89	1	9	9	14	0.19	0.03	0.22	0.18	0.36	0.28	-0.01	-0.05	0.02	0.18	0.27	0.24	-0.06	0.17	0.07	0.02	-0.05	0.25	0.10	-0.37

Q9NZJ6	Hexaprenyldihydroxybenzoate methyltransferase, mitochondrial OS=Homo sapiens GN=COQ3 PE=1 SV=3 - [COQ3_HUMAN]	20.05	1	5	5	16	0.62	0.69	0.72	0.87	0.14	0.23	0.08	0.23	0.28	0.16	0.11	0.23	-0.21	-0.38	-0.62	-0.36	-0.56	-0.47	-0.60	-0.37
Q9H2H8	Peptidyl-prolyl cis-trans isomerase-like 3 OS=Homo sapiens GN=PPIL3 PE=1 SV=1 - [PPIL3_HUMAN]	43.48	1	7	7	12	0.38	0.52	0.26	0.51	0.09	0.46	-0.01	0.01	0.13	-0.03	-0.49	-0.27	-0.22	-0.78	-0.79	-0.32	-0.50	-0.15	-0.39	-0.37
P82664	28S ribosomal protein S10, mitochondrial OS=Homo sapiens GN=MRPS10 PE=1 SV=2 - [RT10_HUMAN]	14.93	1	2	2	3	-0.40	-0.28	0.43	0.54	-0.27	-0.17	-0.01	0.11	-0.30	-0.40	0.01	0.12	0.44	0.41	-0.42	0.02	-0.80	0.10	-0.72	-0.37
Q9UBB4	Ataxin-10 OS=Homo sapiens GN=ATXN10 PE=1 SV=1 - [ATX10_HUMAN]	24.63	1	10	10	15	0.48	0.40	0.61	0.59	-0.03	-0.05	0.02	0.23	0.08	0.01	0.10	0.07	-0.28	-0.37	-0.61	-0.43	-0.68	-0.54	-0.73	-0.37
Q9NVA1	Ubiquinol-cytochrome-c reductase complex assembly factor 1 OS=Homo sapiens GN=UQCC1 PE=1 SV=3 - [UQCC1_HUMAN]	12.37	1	3	3	4	0.66	0.90	0.54	0.78	-0.10	0.14	0.10	0.34	0.29	0.05	-0.01	0.23	-0.50	-0.66	-0.55	-0.57	-0.45	-0.78	-0.66	-0.38
P04062	Glucosylceramidase OS=Homo sapiens GN=GBA PE=1 SV=3 - [GLCM_HUMAN]	15.11	1	7	7	8	0.65	0.69	0.50	0.64	0.71	0.74	0.09	0.22	0.33	0.18	0.31	0.50	-0.51	-0.25	-0.20	-0.49	-0.20	0.04	0.19	-0.38
P36507	Dual specificity mitogen-activated protein kinase kinase 2 OS=Homo sapiens GN=MAP2K2 PE=1 SV=1 - [MP2K2_HUMAN]	50.25	1	13	20	31	0.43	0.38	0.62	0.68	0.16	0.27	0.25	0.28	0.24	0.09	0.12	0.19	-0.15	-0.26	-0.41	-0.30	-0.35	-0.43	-0.51	-0.38

Q9NTG7	NAD-dependent protein deacetylase sirtuin-3, mitochondrial OS=Homo sapiens GN=SIRT3 PE=1 SV=2 - [SIR3_HUMAN]	14.79	1	4	4	9	0.56	0.51	0.44	0.35	0.19	0.24	-0.10	-0.06	0.22	0.18	0.30	0.29	-0.60	-0.25	-0.14	-0.48	-0.33	-0.44	-0.32	-0.38
P30085	UMP-CMP kinase OS=Homo sapiens GN=CMPK1 PE=1 SV=3 - [KCY_HUMAN]	47.45	1	9	9	26	0.56	0.51	1.12	1.05	0.48	0.38	0.86	0.71	0.30	0.29	-0.12	-0.12	0.05	-0.74	-1.18	-0.52	-0.75	-0.20	-0.73	-0.38
Q96FJ2	Dynein light chain 2, cytoplasmic OS=Homo sapiens GN=DYNLL2 PE=1 SV=1 - [DYL2_HUMAN]	32.58	1	2	2	25	0.96	0.44	0.76	0.56	0.34	0.32	0.32	0.36	-0.08	0.03	0.48	0.06	-0.07	-0.14	-0.15	-0.30	-0.72	0.17	-0.19	-0.38
Q5XKP0	Protein QIL1 OS=Homo sapiens GN=QIL1 PE=1 SV=1 - [QIL1_HUMAN]	24.58	1	2	2	4	0.27	0.19	0.35	0.45	-0.48	-0.61	0.14	0.01	-0.36	-0.19	-0.13	-0.26	-0.16	-0.44	-0.87	-0.05	-0.55	-0.82	-1.08	-0.38
O43813	LanC-like protein 1 OS=Homo sapiens GN=LANCL1 PE=1 SV=1 - [LANC1_HUMAN]	28.07	1	10	10	26	0.78	0.88	0.89	1.01	0.49	0.52	0.38	0.50	0.42	0.35	0.13	0.34	-0.31	-0.57	-0.65	-0.35	-0.54	-0.29	-0.50	-0.38
O75976	Carboxypeptidase D OS=Homo sapiens GN=CPD PE=1 SV=2 - [CBPD_HUMAN]	6.16	1	5	5	9	0.74	0.80	0.44	0.33	0.14	0.21	0.13	0.18	0.33	0.36	0.15	0.14	-0.45	-0.44	-0.09	-0.18	-0.37	-0.60	-0.32	-0.38
Q9P0P8	Uncharacterized protein C6orf203 OS=Homo sapiens GN=C6orf203 PE=1 SV=1 - [CF203_HUMAN]	3.75	1	1	1	1	-0.27	-0.33	0.30	0.24	-0.41	-0.47	-0.14	-0.20	-0.34	-0.28	-0.16	-0.22	0.18	0.12	-0.46	0.02	-0.54	-0.15	-0.72	-0.38
A6NEL2	Ankyrin repeat domain-containing protein SOWAHB OS=Homo sapiens GN=SOWAHB PE=2 SV=1 - [SWAHB_HUMAN]	2.52	1	1	1	1	0.45	-0.28	0.64	-0.09	0.78	0.05	0.20	-0.53	-1.11	-0.38	0.38	-0.36	-0.20	-0.07	-0.26	-0.79	-0.98	0.32	0.13	-0.38

P28288	ATP-binding cassette sub-family D member 3 OS=Homo sapiens GN=ABCD3 PE=1 SV=1 - [ABCD3_HUMAN]	17.45	1	9	9	13	0.35	0.23	0.05	0.48	0.03	0.10	0.11	0.11	0.07	0.10	0.19	0.13	-0.19	-0.13	0.07	-0.23	-0.16	-0.30	-0.24	-0.38
Q9H9G7	Protein argonaute-3 OS=Homo sapiens GN=AGO3 PE=1 SV=2 - [AGO3_HUMAN]	10.12	1	2	7	10	-0.76	-0.68	-0.36	-0.28	-0.51	-0.43	-0.81	-0.72	-0.58	-0.67	-0.66	-0.58	0.01	0.11	-0.30	0.13	-0.27	0.24	-0.16	-0.38
Q9UJF2	Ras GTPase-activating protein nGAP OS=Homo sapiens GN=RASAL2 PE=1 SV=2 - [NGAP_HUMAN]	7.73	1	5	6	9	0.02	-0.10	-0.08	-0.14	-0.16	-0.28	-0.29	-0.41	-0.35	-0.28	-0.26	-0.33	-0.26	-0.52	-0.46	-0.41	-0.49	-0.19	-0.53	-0.38
O96000	NADH dehydrogenase [ubiquinone] 1 beta subcomplex subunit 10 OS=Homo sapiens GN=NDUFB10 PE=1 SV=3 - [NDUBA_HUMAN]	47.09	1	9	9	26	0.85	0.69	0.90	0.82	-0.11	-0.30	0.55	0.33	0.12	0.31	0.23	0.07	-0.36	-0.69	-0.83	-0.54	-0.60	-0.98	-0.85	-0.38
Q8NEB9	Phosphatidylinositol 3-kinase catalytic subunit type 3 OS=Homo sapiens GN=PIK3C3 PE=1 SV=1 - [PK3C3_HUMAN]	18.04	1	13	13	15	0.45	0.51	0.36	0.47	-0.07	-0.06	0.04	-0.03	0.06	0.09	-0.13	-0.15	-0.19	-0.53	-0.68	-0.28	-0.36	-0.09	-0.42	-0.38
Q15382	GTP-binding protein Rheb OS=Homo sapiens GN=RHEB PE=1 SV=1 - [RHEB_HUMAN]	21.74	1	4	4	11	0.72	0.78	1.09	1.05	0.25	0.20	0.58	0.61	0.20	0.20	0.41	0.40	-0.11	-0.46	-0.72	-0.52	-0.73	-0.54	-0.86	-0.38
Q15424	Scaffold attachment factor B1 OS=Homo sapiens GN=SAFB PE=1 SV=4 - [SAFB1_HUMAN]	10.71	1	3	8	13	0.02	-0.21	0.20	-0.03	-0.08	-0.31	-0.25	-0.48	-0.31	-0.08	0.29	0.06	-0.21	0.27	0.09	-0.07	-0.24	-0.12	-0.29	-0.38

Q96EP5	DAZ-associated protein 1 OS=Homo sapiens GN=DAZAP1 PE=1 SV=1 - [DAZP1_HUMAN]	14.50	1	3	3	5	0.42	0.32	0.53	0.43	0.24	0.14	0.09	-0.01	-0.07	0.04	-0.04	-0.14	-0.28	-0.45	-0.57	-0.35	-0.46	-0.19	-0.31	-0.38
P52788	Spermine synthase OS=Homo sapiens GN=SMS PE=1 SV=2 - [SPSY_HUMAN]	22.68	1	7	7	13	0.85	0.78	1.20	1.17	0.24	0.21	0.72	0.71	0.28	0.35	0.27	0.24	-0.17	-0.71	-0.91	-0.62	-0.70	-0.67	-0.96	-0.38
O94819	Kelch repeat and BTB domain-containing protein 11 OS=Homo sapiens GN=KBTBD11 PE=1 SV=1 - [KBTBB_HUMAN]	43.98	1	20	20	50	0.92	0.98	0.92	1.00	0.33	0.27	0.43	0.44	0.35	0.37	0.30	0.30	-0.52	-0.66	-0.60	-0.63	-0.50	-0.71	-0.52	-0.38
P53999	Activated RNA polymerase II transcriptional coactivator p15 OS=Homo sapiens GN=SUB1 PE=1 SV=3 - [TCP4_HUMAN]	28.35	1	3	3	16	-0.29	-0.37	-0.06	-0.07	-0.29	-0.43	-0.37	-0.49	-0.63	-0.44	-0.30	-0.48	-0.02	-0.04	-0.40	-0.22	-0.41	-0.12	-0.26	-0.38
Q9UBU6	Protein FAM8A1 OS=Homo sapiens GN=FAM8A1 PE=1 SV=1 - [FA8A1_HUMAN]	4.12	1	1	1	1	-0.15	-0.07	-0.69	-0.60	-1.18	-1.10	-1.13	-1.05	-0.85	-0.93	-0.10	-0.03	-0.92	0.06	0.58	-0.74	-0.21	-1.04	-0.51	-0.38
P21453	Sphingosine 1-phosphate receptor 1 OS=Homo sapiens GN=S1PR1 PE=1 SV=2 - [S1PR1_HUMAN]	5.24	1	2	2	4	0.49	0.61	0.40	0.52	-0.24	-0.13	-0.05	0.07	0.09	-0.03	-0.08	0.03	-0.48	-0.56	-0.49	-0.48	-0.39	-0.74	-0.66	-0.38
P16104	Histone H2AX OS=Homo sapiens GN=H2AFX PE=1 SV=2 - [H2AX_HUMAN]	42.66	2	1	5	68	-0.40	-0.49	0.19	0.10	-0.85	-0.94	-0.26	-0.35	-0.91	-0.81	-0.44	-0.54	0.19	-0.04	-0.63	-0.39	-0.97	-0.47	-1.05	-0.38
P18669	Phosphoglycerate mutase 1 OS=Homo sapiens GN=PGAM1 PE=1 SV=2 - [PGAM1_HUMAN]	71.65	2	9	15	113	0.95	0.84	1.27	1.05	0.55	0.44	0.82	0.67	0.33	0.39	0.28	0.19	-0.14	-0.54	-0.89	-0.49	-0.69	-0.36	-0.64	-0.39

Q6PI78	Transmembrane protein 65 OS=Homo sapiens GN=TMEM65 PE=1 SV=2 - [TMM65_HUMAN]	23.33	1	5	5	13	0.42	0.42	0.80	0.75	-0.14	-0.25	0.26	0.20	-0.01	0.01	-0.09	-0.13	-0.12	-0.52	-0.67	-0.48	-0.54	-0.71	-0.88	-0.39
Q9BUR5	Apolipoprotein O OS=Homo sapiens GN=APOO PE=1 SV=1 - [APOO_HUMAN]	31.82	1	5	5	7	0.40	0.53	0.64	0.53	-0.12	-0.16	0.15	0.16	-0.10	-0.02	-0.05	-0.12	-0.22	-0.34	-0.44	-0.45	-0.59	-0.40	-0.76	-0.39
Q9ULH1	Arf-GAP with SH3 domain, ANK repeat and PH domain-containing protein 1 OS=Homo sapiens GN=ASAP1 PE=1 SV=4 - [ASAP1_HUMAN]	14.88	1	12	12	25	0.54	0.43	0.53	0.64	-0.64	-0.43	0.04	0.22	-0.10	-0.03	-0.30	-0.26	-0.43	-0.76	-0.84	-0.75	-0.83	-1.14	-1.15	-0.39
Q96RD7	Pannexin-1 OS=Homo sapiens GN=PANX1 PE=1 SV=4 - [PANX1_HUMAN]	2.11	1	1	1	1	0.33	0.24	0.78	0.69	0.70	0.61	0.33	0.24	0.00	0.10	-0.01	-0.10	0.05	-0.33	-0.78	-0.20	-0.65	0.36	-0.09	-0.39
P19634	Sodium/hydrogen exchanger 1 OS=Homo sapiens GN=SLC9A1 PE=1 SV=2 - [SL9A1_HUMAN]	11.53	1	7	7	18	0.32	0.47	0.43	0.32	-0.06	0.11	-0.27	-0.19	0.19	0.19	-0.01	-0.02	-0.47	-0.36	-0.40	-0.35	-0.23	-0.20	0.03	-0.39
P62633	Cellular nucleic acid-binding protein OS=Homo sapiens GN=CNBP PE=1 SV=1 - [CNBP_HUMAN]	15.25	1	2	2	3	0.21	0.40	0.11	0.30	-0.65	-0.46	-0.34	-0.15	-0.02	-0.21	0.05	0.23	-0.49	-0.16	-0.07	-0.39	-0.29	-0.87	-0.77	-0.39
Q9NX55	Huntingtin-interacting protein K OS=Homo sapiens GN=HYPK PE=1 SV=2 - [HYPK_HUMAN]	13.18	1	1	1	2	0.41	-0.12	0.43	-0.10	-0.77	-1.31	-0.02	-0.55	-0.68	-0.14	-0.04	-0.58	-0.37	-0.44	-0.47	-0.52	-0.54	-1.20	-1.22	-0.39
O00533	Neural cell adhesion molecule L1-like protein OS=Homo sapiens GN=CHL1 PE=1 SV=4 - [CHL1_HUMAN]	25.25	1	22	22	51	0.11	0.19	0.36	0.34	-0.44	-0.49	-0.09	-0.16	-0.29	-0.29	-0.25	-0.29	-0.11	-0.36	-0.55	-0.32	-0.59	-0.58	-0.81	-0.39

Q15814	Tubulin-specific chaperone C OS=Homo sapiens GN=TBCC PE=1 SV=2 - [TBCC_HUMAN]	15.03	1	4	4	7	0.37	0.25	0.29	0.39	-0.02	0.00	0.27	0.12	0.08	0.02	0.03	0.00	-0.32	-0.34	-0.11	-0.72	-0.86	-0.18	-0.32	-0.39
Q9BSF4	Uncharacterized protein C19orf52 OS=Homo sapiens GN=C19orf52 PE=1 SV=2 - [CS052_HUMAN]	18.08	1	3	3	4	0.29	0.24	0.41	0.37	0.01	-0.04	-0.04	-0.08	-0.24	-0.19	-0.03	-0.08	-0.27	-0.31	-0.44	-0.45	-0.57	-0.29	-0.41	-0.39
P84095	Rho-related GTP-binding protein RhoG OS=Homo sapiens GN=RHOG PE=1 SV=1 - [RHOG_HUMAN]	60.21	2	8	9	21	0.50	0.33	0.77	0.70	0.14	-0.05	0.15	0.12	0.09	0.14	0.07	0.12	-0.31	-0.35	-0.47	-0.32	-0.34	-0.46	-0.61	-0.39
Q9NVH1	DnaJ homolog subfamily C member 11 OS=Homo sapiens GN=DNAJC11 PE=1 SV=2 - [DJC11_HUMAN]	29.52	1	13	13	22	0.72	0.58	0.73	0.53	-0.11	-0.04	0.23	0.15	-0.12	-0.07	0.00	0.12	-0.47	-0.59	-0.59	-0.66	-0.64	-0.92	-0.59	-0.39
Q9Y6M9	NADH dehydrogenase [ubiquinone] 1 beta subcomplex subunit 9 OS=Homo sapiens GN=NDUFB9 PE=1 SV=3 - [NDUB9_HUMAN]	39.66	1	5	5	14	0.79	0.70	0.79	0.72	0.06	0.13	0.26	0.32	0.12	0.12	-0.07	-0.14	-0.38	-0.79	-0.99	-0.58	-0.78	-0.66	-0.46	-0.39
P23946	Chymase OS=Homo sapiens GN=CMA1 PE=1 SV=1 - [CMA1_HUMAN]	5.67	1	1	1	1	0.45	0.27	0.60	0.42	1.94	1.76	0.15	-0.03	0.73	0.91	0.09	-0.09	-0.25	-0.35	-0.51	0.49	0.35	1.48	1.33	-0.39
P23677	Inositol-trisphosphate 3-kinase A OS=Homo sapiens GN=ITPKA PE=1 SV=1 - [IP3KA_HUMAN]	21.91	1	7	8	13	1.05	0.75	1.05	0.86	0.06	-0.18	0.34	0.04	0.31	0.45	0.28	0.16	-0.90	-0.92	-1.23	-0.80	-0.80	-1.34	-1.53	-0.39
Q00765	Receptor expression-enhancing protein 5 OS=Homo sapiens GN=REEP5 PE=1 SV=3 - [REEP5_HUMAN]	20.11	1	5	5	19	-0.04	-0.03	0.30	0.25	-0.04	-0.06	-0.31	-0.24	-0.04	-0.17	0.18	0.27	-0.14	0.13	-0.21	0.01	-0.33	0.00	-0.34	-0.39

Q9Y6K5	2'-5'-oligoadenylate synthase 3 OS=Homo sapiens GN=OAS3 PE=1 SV=3 - [OAS3_HUMAN]	3.59	1	3	3	5	0.98	1.04	0.97	1.03	1.34	1.39	0.52	0.58	0.79	0.74	0.59	0.64	-0.41	-0.39	-0.39	-0.21	-0.20	0.34	0.35	-0.39
O95563	Mitochondrial pyruvate carrier 2 OS=Homo sapiens GN=MPC2 PE=1 SV=1 - [MPC2_HUMAN]	9.45	1	1	1	4	-0.02	-0.01	0.88	0.85	-0.46	-0.34	0.40	0.45	-0.12	-0.24	-0.07	-0.09	0.52	-0.04	-0.89	-0.09	-0.96	-0.36	-1.23	-0.39
O14617	AP-3 complex subunit delta-1 OS=Homo sapiens GN=AP3D1 PE=1 SV=1 - [AP3D1_HUMAN]	22.55	1	19	19	31	0.27	0.60	0.47	0.87	0.01	0.00	0.18	0.21	-0.02	-0.02	0.01	0.02	-0.36	-0.51	-0.69	-0.52	-0.67	-0.62	-0.42	-0.39
Q8TEA8	D-tyrosyl-tRNA(Tyr) deacylase 1 OS=Homo sapiens GN=DTD1 PE=1 SV=2 - [DTD1_HUMAN]	25.84	1	3	3	7	0.33	0.20	0.28	0.16	0.46	0.33	-0.17	-0.29	-0.06	0.07	0.04	-0.08	-0.44	-0.28	-0.24	-0.22	-0.18	0.12	0.16	-0.39
P20337	Ras-related protein Rab-3B OS=Homo sapiens GN=RAB3B PE=1 SV=2 - [RAB3B_HUMAN]	39.73	1	3	7	27	0.56	0.69	0.58	0.72	-0.40	-0.27	0.13	0.27	0.54	0.40	-0.06	0.08	-0.37	-0.61	-0.64	-0.12	-0.15	-0.97	-1.00	-0.39
Q8IWT6	Leucine-rich repeat-containing protein 8A OS=Homo sapiens GN=LRRC8A PE=1 SV=1 - [LRC8A_HUMAN]	16.79	2	10	11	19	0.57	0.41	0.78	0.60	0.20	0.01	0.43	0.27	0.06	0.05	0.25	0.11	-0.15	-0.46	-0.43	-0.35	-0.52	-0.44	-0.60	-0.39
P11274	Breakpoint cluster region protein OS=Homo sapiens GN=BCR PE=1 SV=2 - [BCR_HUMAN]	9.13	1	9	10	20	0.63	0.56	0.37	0.30	0.08	0.10	-0.14	-0.07	-0.07	0.08	-0.19	-0.18	-0.55	-0.70	-0.71	-0.43	-0.26	-0.36	-0.16	-0.39
A1A5C7	Solute carrier family 22 member 23 OS=Homo sapiens GN=SLC22A23 PE=2 SV=2 - [S22AN_HUMAN]	7.43	1	3	3	5	0.68	0.74	0.77	0.77	0.07	0.10	0.31	0.27	0.37	0.31	0.49	0.41	-0.31	-0.49	-0.36	-0.34	-0.42	-0.82	-0.68	-0.39

O60869	Endothelial differentiation related factor 1 OS=Homo sapiens GN=EDF1 PE=1 SV=1 - [EDF1_HUMAN]	27.03	1	4	4	11	-0.29	-0.30	-0.01	-0.04	-0.48	-0.37	-0.67	-0.50	-0.61	-0.65	-0.56	-0.55	-0.07	-0.26	-0.52	-0.24	-0.50	-0.08	-0.33	-0.39
Q10713	Mitochondrial processing peptidase subunit alpha OS=Homo sapiens GN=PMPCA PE=1 SV=2 - [MPPA_HUMAN]	12.95	1	6	6	10	0.09	0.18	0.36	0.48	-0.15	-0.24	-0.25	-0.24	-0.33	-0.24	0.11	0.03	-0.26	-0.23	-0.51	-0.42	-0.70	-0.24	-0.46	-0.39
P82650	28S ribosomal protein S22, mitochondrial OS=Homo sapiens GN=MRPS22 PE=1 SV=1 - [RT22_HUMAN]	21.94	1	6	6	7	0.52	0.67	0.19	0.38	-0.04	0.15	-0.11	-0.08	0.04	-0.14	-0.03	0.05	-0.55	-0.54	-0.32	-0.46	-0.30	-0.57	-0.22	-0.39
Q9NRZ7	1-acyl-sn-glycerol-3-phosphate acyltransferase gamma OS=Homo sapiens GN=AGPAT3 PE=1 SV=1 - [PLCC_HUMAN]	8.51	1	3	3	5	-0.01	-0.03	0.43	0.41	-0.37	-0.39	-0.03	-0.05	-0.27	-0.25	-0.20	-0.23	0.04	-0.19	-0.63	-0.21	-0.64	-0.38	-0.82	-0.39
Q15369	Transcription elongation factor B polypeptide 1 OS=Homo sapiens GN=TCEB1 PE=1 SV=1 - [ELOC_HUMAN]	58.04	1	5	5	8	0.52	0.61	0.51	0.60	0.02	0.11	0.05	0.14	0.11	0.03	0.29	0.38	-0.41	-0.22	-0.22	-0.46	-0.45	-0.51	-0.51	-0.39
P11217	Glycogen phosphorylase, muscle form OS=Homo sapiens GN=PYGM PE=1 SV=6 - [PYGM_HUMAN]	50.24	1	26	38	127	0.77	0.83	0.84	0.87	-0.10	-0.07	0.39	0.37	0.01	0.11	0.01	-0.05	-0.45	-0.79	-0.91	-0.55	-0.62	-0.92	-0.92	-0.39
A6NCE7	Microtubule-associated proteins 1A/1B light chain 3 beta 2 OS=Homo sapiens GN=MAP1LC3B2 PE=2 SV=1 - [MP3B2_HUMAN]	11.20	2	1	1	4	0.27	0.45	0.75	0.93	0.64	0.81	0.29	0.47	0.50	0.33	0.31	0.49	0.08	0.05	-0.44	0.09	-0.39	0.35	-0.13	-0.39

Q9Y5U8	Mitochondrial pyruvate carrier 1 OS=Homo sapiens GN=MPC1 PE=1 SV=1 - [MPC1_HUMAN]	8.26	1	2	2	3	0.62	0.46	0.58	0.43	0.62	0.46	0.12	-0.03	0.40	0.56	1.09	0.93	-0.44	0.48	0.51	-0.03	0.01	-0.02	0.02	-0.40
Q96F24	Nuclear receptor-binding factor 2 OS=Homo sapiens GN=NRBF2 PE=1 SV=1 - [NRBF2_HUMAN]	4.53	1	1	2	2	0.08	-0.09	0.26	0.08	-0.25	-0.44	-0.20	-0.38	-0.49	-0.31	-0.21	-0.40	-0.23	-0.29	-0.47	-0.36	-0.53	-0.35	-0.53	-0.40
O43815	Striatin OS=Homo sapiens GN=STRN PE=1 SV=4 - [STRN_HUMAN]	21.92	1	11	12	25	0.49	0.45	0.40	0.35	0.15	0.20	0.10	0.02	0.03	0.03	0.12	0.17	-0.39	-0.22	-0.09	-0.43	-0.36	-0.34	-0.41	-0.40
Q12767	Uncharacterized protein KIAA0195 OS=Homo sapiens GN=KIAA0195 PE=1 SV=1 - [K0195_HUMAN]	3.39	1	4	4	9	0.70	0.68	0.72	0.70	0.16	0.32	0.30	0.04	0.51	0.82	0.51	0.35	-0.58	-0.51	-0.36	-0.32	0.13	-0.37	-0.50	-0.40
O14880	Microsomal glutathione S-transferase 3 OS=Homo sapiens GN=MGST3 PE=1 SV=1 - [MGST3_HUMAN]	30.92	1	5	5	32	0.45	0.58	0.70	0.63	-0.17	-0.16	0.18	0.22	-0.12	0.00	-0.09	-0.13	-0.31	-0.62	-0.79	-0.57	-0.74	-0.89	-0.91	-0.40
Q8N7R7	Cyclin-Y-like protein 1 OS=Homo sapiens GN=CCNYL1 PE=1 SV=2 - [CCYL1_HUMAN]	4.46	1	1	2	3	0.38	-0.23	0.31	-0.30	0.71	0.10	-0.15	-0.76	0.02	0.63	0.68	0.07	-0.48	0.31	0.37	0.28	0.36	0.31	0.39	-0.40
P19971	Thymidine phosphorylase OS=Homo sapiens GN=TYMP PE=1 SV=2 - [TYPH_HUMAN]	14.11	1	5	5	6	0.80	0.88	0.81	0.88	0.50	0.57	0.07	0.15	0.64	0.57	0.22	0.29	-0.43	-0.58	-0.59	-0.21	-0.21	-0.45	-0.29	-0.40
Q9UHD8	Septin-9 OS=Homo sapiens GN=SEPT9 PE=1 SV=2 - [SEPT9_HUMAN]	41.81	1	20	20	67	0.32	0.41	0.53	0.52	-0.37	-0.39	0.04	0.09	-0.29	-0.25	-0.35	-0.41	-0.28	-0.72	-0.81	-0.61	-0.71	-0.83	-0.85	-0.40

P22626	Heterogeneous nuclear ribonucleoproteins A2/B1 OS=Homo sapiens GN=HNRNPA2B1 PE=1 SV=2 - [ROA2_HUMAN]	55.81	1	17	20	123	0.38	0.37	0.52	0.44	-0.10	-0.09	-0.01	-0.06	-0.12	-0.14	-0.16	-0.21	-0.30	-0.54	-0.53	-0.43	-0.46	-0.41	-0.53	-0.40
Q9GZT3	SRA stem-loop-interacting RNA-binding protein, mitochondrial OS=Homo sapiens GN=SLIRP PE=1 SV=1 - [SLIRP_HUMAN]	48.62	1	5	5	7	0.27	-0.08	-0.04	-0.65	-0.20	-0.43	-0.23	-0.63	-0.55	-0.43	-0.09	-0.48	-0.62	-0.58	-0.56	-0.75	-0.34	-0.73	-0.50	-0.40
Q96PY5	Formin-like protein 2 OS=Homo sapiens GN=FMNL2 PE=1 SV=3 - [FMNL2_HUMAN]	21.73	2	20	22	36	0.44	0.54	0.60	0.71	-0.21	-0.12	0.22	0.15	-0.08	-0.06	0.04	0.09	-0.23	-0.53	-0.73	-0.41	-0.70	-0.71	-0.88	-0.40
Q96RW7	Hemicentin-1 OS=Homo sapiens GN=HMCN1 PE=1 SV=2 - [HMCN1_HUMAN]	0.21	1	1	1	1	-0.63	-0.85	-0.06	-0.28	-0.21	-0.44	-0.52	-0.74	-0.69	-0.47	-0.33	-0.55	0.17	0.31	-0.27	0.19	-0.37	0.41	-0.17	-0.40
Q9Y3D9	28S ribosomal protein S23, mitochondrial OS=Homo sapiens GN=MRPS23 PE=1 SV=2 - [RT23_HUMAN]	18.95	1	3	3	4	0.38	0.43	0.15	0.20	0.19	-0.10	-0.25	-0.27	0.27	0.33	-0.08	-0.03	-0.57	-0.41	-0.23	-0.12	-0.02	-0.21	-0.31	-0.40
Q5T8D3	Acyl-CoA-binding domain-containing protein 5 OS=Homo sapiens GN=ACBD5 PE=1 SV=1 - [ACBD5_HUMAN]	9.93	1	4	4	7	1.02	1.13	0.61	0.71	0.12	0.22	0.14	0.25	0.41	0.31	0.43	0.53	-0.83	-0.59	-0.18	-0.68	-0.26	-0.92	-0.50	-0.40
Q02153	Guanylate cyclase soluble subunit beta-1 OS=Homo sapiens GN=GUCY1B3 PE=1 SV=1 - [GCYB1_HUMAN]	28.11	1	14	14	27	0.24	0.40	0.31	0.45	-0.21	-0.17	-0.23	-0.14	-0.05	-0.29	-0.06	0.02	-0.43	-0.26	-0.57	-0.48	-0.59	-0.76	-0.81	-0.40

Q96CN4	EVI5-like protein OS=Homo sapiens GN=EVI5L PE=1 SV=1 - [EVI5L_HUMAN]	3.78	1	3	3	4	0.43	0.58	0.25	0.41	-0.25	-0.10	-0.21	-0.05	-0.07	-0.22	-0.12	0.03	-0.58	-0.54	-0.38	-0.61	-0.44	-0.69	-0.52	-0.40
P04424	Argininosuccinate lyase OS=Homo sapiens GN=ASL PE=1 SV=4 - [ARLY_HUMAN]	9.91	1	4	4	7	0.37	0.15	0.83	0.62	-0.53	-0.75	0.37	0.16	-0.46	-0.24	-0.08	-0.30	0.06	-0.44	-0.92	-0.57	-1.04	-0.92	-1.38	-0.40
Q9Y5J6	Mitochondrial import inner membrane translocase subunit Tim10 B OS=Homo sapiens GN=TIMM10B PE=1 SV=1 - [T10B_HUMAN]	31.07	1	3	3	4	0.16	0.71	0.06	0.27	0.04	0.25	-0.40	0.09	0.35	0.14	0.16	0.37	-0.56	0.00	0.10	0.01	0.12	-0.14	-0.03	-0.40
P61328	Fibroblast growth factor 12 OS=Homo sapiens GN=FGF12 PE=1 SV=1 - [FGF12_HUMAN]	23.87	2	4	4	6	0.57	0.48	0.88	0.83	0.67	0.68	0.28	0.25	0.77	0.61	0.04	0.09	-0.17	-0.42	-0.73	-0.11	-0.14	0.04	-0.09	-0.40
Q5EBM0	UMP-CMP kinase 2, mitochondrial OS=Homo sapiens GN=CMPK2 PE=1 SV=3 - [CMPK2_HUMAN]	16.70	1	6	6	9	0.88	0.83	0.62	0.58	0.20	0.27	0.13	0.08	0.36	0.32	0.80	0.75	-0.53	-0.04	0.18	-0.75	-0.25	-0.55	-0.42	-0.40
P55008	Allograft inflammatory factor 1 OS=Homo sapiens GN=AIF1 PE=1 SV=1 - [AIF1_HUMAN]	44.90	2	7	8	17	0.19	0.21	0.32	0.65	-0.33	-0.11	-0.18	-0.06	-0.19	-0.38	-0.48	-0.44	-0.19	-0.68	-0.84	-0.48	-0.73	-0.51	-0.61	-0.40
Q6IBS0	Twinfilin-2 OS=Homo sapiens GN=TWF2 PE=1 SV=2 - [TWF2_HUMAN]	38.97	3	8	11	23	0.48	0.59	0.53	0.48	0.22	-0.03	0.02	0.17	-0.02	0.00	-0.22	-0.24	-0.28	-0.60	-1.01	-0.38	-0.46	-0.40	-0.38	-0.40
Q9Y4D8	Probable E3 ubiquitin-protein ligase HECTD4 OS=Homo sapiens GN=HECTD4 PE=1 SV=5 - [HECD4_HUMAN]	2.18	1	6	6	6	0.46	0.47	0.46	0.67	-0.24	-0.10	0.09	-0.03	0.09	0.19	0.09	0.05	-0.45	-0.81	-0.59	-0.47	-0.45	-1.02	-0.93	-0.40

Q9NRW7	Vacuolar protein sorting-associated protein 45 OS=Homo sapiens GN=VPS45 PE=1 SV=1 - [VPS45_HUMAN]	19.47	1	10	10	17	0.81	0.90	0.74	0.80	-0.08	-0.10	0.20	0.37	0.42	0.31	-0.10	0.10	-0.49	-1.02	-0.90	-0.80	-0.63	-1.02	-0.92	-0.40
P02795	Metallothionein-2 OS=Homo sapiens GN=MT2A PE=1 SV=1 - [MT2_HUMAN]	13.11	5	1	1	1	1.85	1.80	1.62	1.57	1.13	1.08	1.16	1.11	1.21	1.26	1.31	1.25	-0.64	-0.54	-0.32	-0.56	-0.33	-0.74	-0.51	-0.40
Q9H6U6	Breast carcinoma-amplified sequence 3 OS=Homo sapiens GN=BCAS3 PE=1 SV=3 - [BCAS3_HUMAN]	7.44	1	6	6	13	0.80	0.74	0.84	0.53	0.21	0.03	0.24	0.10	0.08	0.07	0.00	-0.16	-0.54	-0.74	-0.62	-0.59	-0.52	-0.59	-0.43	-0.40
Q9ULX7	Carbonic anhydrase 14 OS=Homo sapiens GN=CA14 PE=1 SV=1 - [CAH14_HUMAN]	4.15	1	1	1	1	0.92	1.98	0.68	1.74	-0.24	0.81	0.22	1.28	0.56	-0.49	0.41	1.47	-0.65	-0.51	-0.27	-1.38	-1.14	-1.18	-0.94	-0.40
Q86TM6	E3 ubiquitin-protein ligase synoviolin OS=Homo sapiens GN=SYVN1 PE=1 SV=2 - [SYVN1_HUMAN]	1.30	1	1	1	1	0.56	0.63	0.50	0.58	0.10	0.17	0.04	0.12	0.09	0.01	0.20	0.27	-0.46	-0.35	-0.31	-0.51	-0.46	-0.48	-0.43	-0.40
Q9UK99	F-box only protein 3 OS=Homo sapiens GN=FBXO3 PE=1 SV=3 - [FBX3_HUMAN]	17.41	1	6	6	9	1.04	1.21	0.69	0.86	-0.27	-0.15	0.23	0.40	0.17	0.01	0.36	0.35	-0.61	-0.79	-0.73	-0.95	-0.66	-1.42	-1.10	-0.40
Q8N3V7	Synaptopodin OS=Homo sapiens GN=SYNPO PE=1 SV=2 - [SYNPO_HUMAN]	20.24	1	12	12	27	-0.30	-0.44	-0.25	-0.40	-0.60	-0.53	-0.90	-1.05	-0.82	-0.79	-0.60	-0.63	-0.47	-0.17	-0.32	-0.25	-0.49	-0.19	-0.48	-0.40
Q14444	Caprin-1 OS=Homo sapiens GN=CAPRIN1 PE=1 SV=2 - [CAPR1_HUMAN]	17.91	1	8	8	19	-0.31	-0.19	0.37	0.18	-0.02	-0.10	-0.19	-0.29	-0.20	-0.11	-0.22	-0.20	0.04	-0.16	-0.57	-0.15	-0.55	-0.08	-0.38	-0.40

O60220	Mitochondrial import inner membrane translocase subunit Tim8 A OS=Homo sapiens GN=TIMM8A PE=1 SV=1 - [TIM8A_HUMAN]	22.68	1	2	2	7	0.87	0.74	0.87	0.64	0.07	-0.08	0.40	0.25	0.04	0.18	-0.03	-0.17	-0.34	-0.90	-0.83	-0.66	-0.56	-0.73	-0.81	-0.40
Q14684	Ribosomal RNA processing protein 1 homolog B OS=Homo sapiens GN=RRP1B PE=1 SV=3 - [RRP1B_HUMAN]	3.96	1	2	2	2	-0.34	-0.22	-0.24	-0.13	-0.18	-0.08	-0.71	-0.60	-0.35	-0.45	-0.28	-0.17	-0.32	0.06	-0.04	-0.09	-0.18	0.14	0.04	-0.40
P19447	TFIIH basal transcription factor complex helicase XPB subunit OS=Homo sapiens GN=ERCC3 PE=1 SV=1 - [ERCC3_HUMAN]	4.60	1	2	2	3	-0.52	-0.58	-0.26	-0.31	-0.31	-0.37	-0.72	-0.78	-0.55	-0.48	-0.57	-0.63	-0.14	-0.04	-0.31	0.07	-0.19	0.20	-0.07	-0.40
O14964	Hepatocyte growth factor-regulated tyrosine kinase substrate OS=Homo sapiens GN=HGS PE=1 SV=1 - [HGS_HUMAN]	22.78	1	14	14	25	0.50	0.47	0.62	0.62	0.19	0.06	0.16	0.04	-0.03	-0.01	-0.05	-0.25	-0.33	-0.57	-0.88	-0.46	-0.72	-0.39	-0.55	-0.40
P49815	Tuberin OS=Homo sapiens GN=TSC2 PE=1 SV=2 - [TSC2_HUMAN]	10.90	1	14	14	23	0.21	0.25	0.41	0.25	0.22	0.03	0.07	-0.17	-0.08	-0.10	-0.07	-0.07	-0.37	-0.35	-0.40	-0.57	-0.33	-0.28	-0.27	-0.40
Q96I15	Selenocysteine lyase OS=Homo sapiens GN=SCLY PE=1 SV=4 - [SCLY_HUMAN]	24.94	1	7	7	14	0.34	0.68	0.60	0.76	0.00	0.15	0.10	0.34	0.05	-0.11	-0.11	0.05	-0.29	-0.58	-0.71	-0.47	-0.67	-0.42	-0.62	-0.41
Q96JH7	Deubiquitinating protein VCIP135 OS=Homo sapiens GN=VCPIP1 PE=1 SV=2 - [VCIP1_HUMAN]	15.06	1	14	14	19	0.91	0.67	0.84	0.62	0.00	0.09	0.19	0.16	0.21	0.10	0.10	0.15	-0.49	-0.38	-0.55	-0.67	-0.50	-0.86	-0.81	-0.41
O75787	Renin receptor OS=Homo sapiens GN=ATP6AP2 PE=1 SV=2 - [RENR_HUMAN]	12.29	1	3	3	6	0.77	0.92	0.62	0.64	-0.10	0.11	0.13	0.26	0.05	0.17	0.14	0.35	-0.47	-0.53	-0.86	-0.54	-0.55	-0.68	-0.54	-0.41

Q9H479	Fructosamine 3-kinase OS=Homo sapiens GN=FN3K PE=1 SV=1 - [FN3K_HUMAN]	33.66	1	9	9	24	0.67	0.71	0.75	0.61	-0.18	-0.44	0.25	0.18	-0.07	-0.10	-0.12	-0.24	-0.38	-0.81	-0.72	-0.64	-0.65	-1.08	-1.04	-0.41
Q15075	Early endosome antigen 1 OS=Homo sapiens GN=EEA1 PE=1 SV=2 - [EEA1_HUMAN]	34.37	1	43	44	79	0.22	0.25	0.37	0.39	-0.37	-0.42	-0.09	-0.09	-0.22	-0.23	-0.24	-0.23	-0.24	-0.37	-0.60	-0.38	-0.55	-0.55	-0.69	-0.41
O15484	Calpain-5 OS=Homo sapiens GN=CAPN5 PE=1 SV=2 - [CAN5_HUMAN]	13.28	1	6	6	9	0.56	0.47	0.57	0.77	0.01	0.00	0.15	0.23	0.38	0.26	0.26	0.18	-0.19	-0.28	-0.38	-0.25	-0.38	-0.53	-0.63	-0.41
P24534	Elongation factor 1-beta OS=Homo sapiens GN=EEF1B2 PE=1 SV=3 - [EF1B_HUMAN]	30.67	1	5	6	17	0.27	0.13	0.69	0.64	-0.24	-0.21	0.21	0.17	0.07	-0.03	-0.21	-0.14	0.21	-0.26	-0.93	-0.17	-0.69	-0.40	-1.01	-0.41
Q9Y2X3	Nucleolar protein 58 OS=Homo sapiens GN=NOP58 PE=1 SV=1 - [NOP58_HUMAN]	21.36	1	9	9	16	0.08	-0.02	0.30	0.11	-0.06	-0.26	-0.17	-0.31	-0.29	-0.18	-0.04	-0.23	-0.12	-0.30	-0.34	-0.23	-0.26	-0.21	-0.32	-0.41
O43464	Serine protease HTRA2, mitochondrial OS=Homo sapiens GN=HTRA2 PE=1 SV=2 - [HTRA2_HUMAN]	32.10	1	8	9	16	0.00	0.15	-0.20	0.09	-0.33	-0.18	-0.26	-0.10	-0.13	-0.28	-0.07	0.21	-0.19	-0.06	0.12	-0.24	-0.31	-0.33	-0.48	-0.41
Q9H9Q2	COP9 signalosome complex subunit 7b OS=Homo sapiens GN=COPS7B PE=1 SV=1 - [CSN7B_HUMAN]	16.29	1	3	3	3	0.91	0.69	0.55	0.33	-0.43	-0.65	0.08	-0.14	-0.51	-0.28	-0.03	-0.26	-0.78	-0.94	-0.58	-1.17	-0.80	-1.36	-0.99	-0.41
P17568	NADH dehydrogenase [ubiquinone] 1 beta subcomplex subunit 7 OS=Homo sapiens GN=NDUFB7 PE=1 SV=4 - [NDUB7_HUMAN]	51.82	1	6	6	25	0.92	0.95	1.12	1.20	0.17	0.05	0.70	0.72	0.54	0.37	0.43	0.27	-0.25	-0.58	-0.73	-0.51	-0.70	-0.69	-0.95	-0.41

Q9NUM4	Transmembrane protein 106B OS=Homo sapiens GN=TMEM106B PE=1 SV=2 - [T106B_HUMAN]	8.39	1	2	2	3	0.08	-0.02	0.51	0.41	0.29	0.19	0.03	-0.07	0.43	0.53	0.68	0.57	0.01	0.61	0.17	0.48	0.06	0.19	-0.24	-0.41
O94822	E3 ubiquitin-protein ligase listerin OS=Homo sapiens GN=LTN1 PE=1 SV=6 - [LTN1_HUMAN]	1.19	1	2	2	3	0.36	0.21	0.67	0.52	0.32	0.17	0.19	0.05	-0.25	-0.10	0.07	-0.08	-0.11	-0.28	-0.60	-0.43	-0.74	-0.06	-0.37	-0.41
Q9GZZ9	Ubiquitin-like modifier-activating enzyme 5 OS=Homo sapiens GN=UBA5 PE=1 SV=1 - [UBA5_HUMAN]	31.93	1	8	8	15	0.72	0.52	0.80	0.59	0.35	0.21	0.47	0.27	0.18	0.26	0.37	0.27	-0.31	-0.48	-0.54	-0.28	-0.38	-0.39	-0.38	-0.41
Q76NI1	Protein very KIND OS=Homo sapiens GN=KNDC1 PE=2 SV=2 - [VKIND_HUMAN]	4.69	1	6	6	10	-0.28	0.25	0.22	0.35	-0.91	-0.99	-0.35	-0.49	-0.44	-0.37	-1.22	-1.16	-0.35	-0.92	-1.00	-0.78	-0.62	-0.65	-0.72	-0.41
Q8TB96	T-cell immunomodulatory protein OS=Homo sapiens GN=ITFG1 PE=1 SV=1 - [TIP_HUMAN]	9.80	1	5	5	7	-0.05	0.40	0.77	0.76	-0.06	0.03	-0.14	0.14	0.00	-0.18	0.04	0.00	-0.07	0.12	-0.38	-0.19	-0.56	-0.06	-0.68	-0.41
Q9NP79	Vacuolar protein sorting-associated protein VTA1 homolog OS=Homo sapiens GN=VTA1 PE=1 SV=1 - [VTA1_HUMAN]	26.06	1	6	6	10	0.45	0.50	0.88	0.88	0.24	0.32	0.38	0.43	0.46	0.42	0.24	0.25	-0.27	-0.31	-0.71	0.01	-0.42	-0.22	-0.60	-0.41
Q96E09	Protein FAM122A OS=Homo sapiens GN=FAM122A PE=1 SV=1 - [F122A_HUMAN]	13.94	1	2	2	3	0.65	1.05	-0.14	0.26	0.03	0.42	-0.62	-0.22	0.26	-0.13	-0.08	0.31	-1.21	-0.72	0.06	-0.75	0.04	-0.64	0.15	-0.41
P14866	Heterogeneous nuclear ribonucleoprotein L OS=Homo sapiens GN=HNRNPL PE=1 SV=2 - [HNRPL_HUMAN]	36.16	1	15	16	60	-0.45	-0.35	-0.18	-0.03	-0.74	-0.74	-0.66	-0.56	-0.55	-0.75	-0.49	-0.44	-0.20	-0.15	-0.28	-0.24	-0.57	-0.27	-0.61	-0.42

Q15120	[Pyruvate dehydrogenase (acetyl-transferring)] kinase isozyme 3, mitochondrial OS=Homo sapiens GN=PDK3 PE=1 SV=1 - [PDK3_HUMAN]	18.97	1	6	7	18	0.37	0.45	0.28	0.35	-0.09	0.01	-0.26	-0.13	0.00	-0.14	-0.19	-0.08	-0.47	-0.59	-0.35	-0.36	-0.23	-0.50	-0.41	-0.42
Q13627	Dual specificity tyrosine-phosphorylation-regulated kinase 1A OS=Homo sapiens GN=DYRK1A PE=1 SV=2 - [DYR1A_HUMAN]	6.68	1	3	3	4	-0.55	-0.44	0.25	0.36	-0.56	-0.45	-0.23	-0.12	-0.06	-0.17	0.30	0.41	0.37	0.86	0.06	0.41	-0.38	-0.03	-0.82	-0.42
Q96KG9	N-terminal kinase-like protein OS=Homo sapiens GN=SCYL1 PE=1 SV=1 - [NTKL_HUMAN]	25.25	1	13	13	19	0.25	0.09	0.51	0.32	0.21	0.17	0.05	-0.09	0.02	0.15	-0.02	-0.03	-0.18	-0.07	-0.41	-0.14	-0.27	-0.24	-0.36	-0.42
Q9BRF8	Calcineurin-like phosphoesterase domain-containing protein 1 OS=Homo sapiens GN=CPPED1 PE=1 SV=3 - [CPPED_HUMAN]	19.43	1	5	5	8	0.30	0.35	0.59	0.64	0.02	-0.07	0.03	0.09	0.00	-0.04	-0.33	-0.43	-0.23	-0.84	-1.04	-0.55	-0.59	-0.98	-0.73	-0.42
P22570	NADPH:adrenodoxin oxidoreductase, mitochondrial OS=Homo sapiens GN=FDXR PE=1 SV=3 - [ADRO_HUMAN]	17.92	1	8	8	15	0.19	0.41	-0.07	0.15	-0.34	-0.35	-0.59	-0.56	-0.40	-0.55	-0.31	-0.24	-0.67	-0.50	-0.30	-0.59	-0.37	-0.59	-0.33	-0.42
Q15904	V-type proton ATPase subunit S1 OS=Homo sapiens GN=ATP6AP1 PE=1 SV=2 - [VAS1_HUMAN]	13.62	1	6	6	10	0.90	0.86	1.05	1.02	0.66	0.75	0.68	0.61	0.82	0.78	0.50	0.69	-0.33	-0.36	-0.54	-0.38	-0.40	-0.25	-0.33	-0.42
Q86TV6	Tetratricopeptide repeat protein 7B OS=Homo sapiens GN=TTC7B PE=1 SV=3 - [TTC7B_HUMAN]	13.17	1	9	9	13	0.93	0.69	0.77	0.60	-0.26	-0.28	0.30	0.18	0.06	0.08	-0.16	-0.32	-0.32	-0.99	-0.90	-0.70	-0.66	-0.99	-0.95	-0.42

P25705	ATP synthase subunit alpha, mitochondrial OS=Homo sapiens GN=ATP5A1 PE=1 SV=1 - [ATPA_HUMAN]	53.53	1	29	29	352	0.66	0.65	0.67	0.68	-0.19	-0.24	0.13	0.14	-0.09	-0.07	-0.18	-0.24	-0.38	-0.76	-0.84	-0.58	-0.74	-0.83	-0.88	-0.42
O95741	Copine-6 OS=Homo sapiens GN=CPNE6 PE=1 SV=3 - [CPNE6_HUMAN]	36.62	1	15	16	45	1.35	1.44	1.37	1.38	0.19	0.13	0.95	0.93	0.69	0.65	0.52	0.46	-0.52	-0.93	-0.89	-0.76	-0.76	-1.35	-1.35	-0.42
Q9NSK7	Protein C19orf12 OS=Homo sapiens GN=C19orf12 PE=1 SV=3 - [CS012_HUMAN]	5.92	1	1	1	1	0.59	0.55	0.34	0.31	0.25	0.21	-0.14	-0.18	-0.16	-0.11	0.37	0.33	-0.67	-0.21	0.03	-0.67	-0.42	-0.35	-0.11	-0.42
P30049	ATP synthase subunit delta, mitochondrial OS=Homo sapiens GN=ATP5D PE=1 SV=2 - [ATPD_HUMAN]	34.52	1	3	3	25	0.62	0.88	0.67	0.63	0.17	0.21	0.21	0.27	0.47	0.24	0.03	0.17	-0.37	-0.80	-0.71	-0.71	-0.59	-0.73	-0.57	-0.42
Q8IYD1	Eukaryotic peptide chain release factor GTP-binding subunit ERF3B OS=Homo sapiens GN=GSPT2 PE=1 SV=2 - [ERF3B_HUMAN]	8.44	1	2	5	9	-0.22	-0.18	0.23	0.28	-0.17	-0.13	-0.25	-0.21	-0.14	-0.17	-0.30	-0.26	0.03	-0.07	-0.53	0.08	-0.37	0.04	-0.42	-0.42
Q7L7X3	Serine/threonine-protein kinase TAO1 OS=Homo sapiens GN=TAOK1 PE=1 SV=1 - [TAOK1_HUMAN]	7.79	1	6	7	11	0.62	0.52	0.77	0.78	0.09	-0.03	0.03	-0.14	0.26	0.13	0.14	0.18	-0.53	-0.20	-0.04	-0.15	-0.13	-0.33	-0.36	-0.42
Q9P2K6	Kelch-like protein 42 OS=Homo sapiens GN=KLHL42 PE=1 SV=2 - [KLH42_HUMAN]	3.17	1	1	1	2	0.17	0.04	-0.30	-0.44	-0.64	-0.78	-0.79	-0.92	-0.63	-0.49	-0.17	-0.32	-0.90	-0.34	0.13	-0.63	-0.15	-0.83	-0.36	-0.42
O15068	Guanine nucleotide exchange factor DBS OS=Homo sapiens GN=MCF2L PE=1 SV=2 - [MCF2L_HUMAN]	2.29	1	2	2	3	0.31	0.49	0.24	0.43	-0.27	-0.09	-0.24	-0.06	-0.19	-0.37	-0.62	-0.44	-0.49	-0.92	-0.86	-0.64	-0.58	-0.59	-0.53	-0.42

Q15437	Protein transport protein Sec23B OS=Homo sapiens GN=SEC23B PE=1 SV=2 - [SC23B_HUMAN]	4.04	1	1	2	8	0.14	0.50	0.36	0.72	0.65	1.01	-0.12	0.23	-0.16	-0.51	0.24	0.59	-0.21	0.11	-0.12	-0.62	-0.84	0.50	0.28	-0.42
Q86TP1	Protein prune homolog OS=Homo sapiens GN=PRUNE PE=1 SV=2 - [PRUNE_HUMAN]	10.38	1	4	4	8	0.96	0.88	1.12	1.07	0.49	0.52	0.64	0.58	0.41	0.41	0.32	0.35	-0.25	-0.50	-0.85	-0.46	-0.65	-0.36	-0.70	-0.42
O00625	Pirin OS=Homo sapiens GN=PIR PE=1 SV=1 - [PIR_HUMAN]	6.55	1	2	2	2	0.75	0.87	1.08	1.21	0.41	0.53	0.60	0.72	0.53	0.41	0.35	0.47	-0.10	-0.40	-0.74	-0.30	-0.64	-0.36	-0.69	-0.42
Q15526	Surfeit locus protein 1 OS=Homo sapiens GN=SURF1 PE=1 SV=1 - [SURF1_HUMAN]	17.00	1	4	4	7	0.30	0.26	0.45	0.41	0.26	0.22	-0.04	-0.07	0.29	0.33	0.53	0.48	-0.28	0.23	0.08	0.06	-0.09	-0.05	-0.20	-0.42
O95208	Epsin-2 OS=Homo sapiens GN=EPN2 PE=1 SV=3 - [EPN2_HUMAN]	8.42	2	4	4	5	0.68	0.91	0.66	0.67	-0.26	-0.11	-0.02	0.28	-0.10	-0.38	0.25	0.01	-0.45	-0.43	-0.61	-1.03	-0.69	-1.11	-0.79	-0.42
Q9BSA4	Protein tweety homolog 2 OS=Homo sapiens GN=TTYH2 PE=1 SV=3 - [TTYH2_HUMAN]	5.06	1	2	2	2	1.98	1.83	1.63	1.48	0.77	0.61	1.14	0.99	0.50	0.67	0.44	0.28	-0.78	-0.89	-0.32	-1.07	-0.48	-1.23	-0.88	-0.42
P37235	Hippocalcin-like protein 1 OS=Homo sapiens GN=HPCAL1 PE=1 SV=3 - [HPCL1_HUMAN]	57.51	1	5	11	31	0.85	0.94	0.90	0.97	0.30	0.29	0.38	0.46	0.51	0.42	0.29	0.35	-0.49	-0.68	-0.63	-0.41	-0.56	-0.46	-0.59	-0.42
Q4G0F5	Vacuolar protein sorting-associated protein 26B OS=Homo sapiens GN=VPS26B PE=1 SV=2 - [VP26B_HUMAN]	29.76	1	7	8	20	0.92	1.08	0.70	0.79	0.14	0.03	0.21	0.25	0.41	0.38	0.00	0.22	-0.57	-0.85	-0.57	-0.55	-0.34	-0.40	-0.52	-0.42
Q96MW1	Coiled-coil domain-containing protein 43 OS=Homo sapiens GN=CCDC43 PE=1 SV=2 - [CCD43_HUMAN]	29.46	1	4	4	7	0.74	0.79	0.86	0.95	0.51	0.39	0.57	0.42	0.40	0.26	0.30	0.24	-0.31	-0.43	-0.76	-0.38	-0.54	-0.36	-0.56	-0.42

Q8IXI2	Mitochondrial Rho GTPase 1 OS=Homo sapiens GN=RHOT1 PE=1 SV=2 - [MIRO1_HUMAN]	23.95	1	10	11	21	0.73	0.78	0.64	0.75	0.20	-0.07	0.33	0.26	0.13	0.25	0.13	0.06	-0.30	-0.69	-0.61	-0.24	-0.34	-0.49	-0.57	-0.42
Q9H074	Polyadenylate-binding protein-interacting protein 1 OS=Homo sapiens GN=PAIP1 PE=1 SV=1 - [PAIP1_HUMAN]	10.02	1	5	5	10	0.76	0.92	0.61	0.79	0.10	0.31	0.12	0.28	0.23	-0.12	-0.14	0.02	-0.58	-0.84	-0.73	-0.65	-0.54	-0.62	-0.53	-0.43
Q9NUJ1	Mycophenolic acid acyl-glucuronide esterase, mitochondrial OS=Homo sapiens GN=ABHD10 PE=1 SV=1 - [ABHDA_HUMAN]	49.02	1	11	11	26	0.37	0.33	0.74	0.50	0.07	-0.16	0.21	0.23	-0.03	0.21	0.32	0.04	-0.02	-0.43	-0.59	-0.46	-0.42	-0.64	-0.61	-0.43
Q92769	Histone deacetylase 2 OS=Homo sapiens GN=HDAC2 PE=1 SV=2 - [HDAC2_HUMAN]	10.86	1	3	5	8	-0.01	-0.10	0.25	0.16	-0.79	-0.89	-0.24	-0.33	-0.69	-0.59	-0.28	-0.38	-0.17	-0.27	-0.54	-0.55	-0.81	-0.80	-1.06	-0.43
O75439	Mitochondrial-processing peptidase subunit beta OS=Homo sapiens GN=PMPCB PE=1 SV=2 - [MPPB_HUMAN]	12.88	1	5	5	10	0.52	0.12	0.30	0.28	-0.35	-0.32	-0.11	-0.29	-0.44	0.09	0.20	-0.19	-0.37	-0.06	-0.27	-0.32	-0.20	-0.22	-0.54	-0.43
Q5JR12	Protein phosphatase 1J OS=Homo sapiens GN=PPM1J PE=1 SV=1 - [PPM1J_HUMAN]	4.95	1	1	2	6	0.29	0.16	0.61	0.48	0.38	0.25	0.12	-0.01	-0.08	0.06	0.10	-0.04	-0.12	-0.19	-0.51	-0.21	-0.52	0.07	-0.24	-0.43
Q9Y4D1	Disheveled-associated activator of morphogenesis 1 OS=Homo sapiens GN=DAAM1 PE=1 SV=2 - [DAAM1_HUMAN]	10.76	1	8	11	22	0.92	1.07	0.74	0.86	-0.15	-0.04	0.39	0.49	0.35	0.23	0.23	0.39	-0.65	-0.90	-0.72	-0.70	-0.42	-1.13	-1.02	-0.43

Q02978	Mitochondrial 2-oxoglutarate/malate carrier protein OS=Homo sapiens GN=SLC25A11 PE=1 SV=3 - [M2OM_HUMAN]	13.06	1	4	4	13	0.40	0.43	0.55	0.56	0.04	-0.06	0.10	0.11	-0.14	-0.21	-0.27	-0.32	-0.19	-0.44	-0.75	-0.70	-0.82	-0.23	-0.53	-0.43
O60884	DnaJ homolog subfamily A member 2 OS=Homo sapiens GN=DNAJA2 PE=1 SV=1 - [DNJA2_HUMAN]	40.78	1	12	12	31	0.55	0.29	0.78	0.65	-0.09	-0.11	0.16	0.04	-0.01	0.07	-0.01	-0.20	-0.39	-0.53	-0.59	-0.49	-0.53	-0.50	-0.71	-0.43
Q96Q05	Trafficking protein particle complex subunit 9 OS=Homo sapiens GN=TRAPPC9 PE=1 SV=2 - [TPPC9_HUMAN]	6.18	1	7	7	13	-0.09	0.05	0.33	0.47	-0.43	-0.29	-0.16	-0.02	0.03	-0.11	-0.11	0.03	-0.02	-0.02	-0.44	0.01	-0.41	-0.36	-0.78	-0.43
Q17RN3	Protein FAM98C OS=Homo sapiens GN=FAM98C PE=2 SV=1 - [FA98C_HUMAN]	11.17	1	2	2	6	0.63	0.52	-0.08	-0.20	0.64	0.51	0.27	0.16	0.72	0.84	0.90	0.78	-0.41	0.38	0.60	0.71	0.92	0.56	0.70	-0.43
Q93009	Ubiquitin carboxyl-terminal hydrolase 7 OS=Homo sapiens GN=USP7 PE=1 SV=2 - [UBP7_HUMAN]	22.78	1	21	21	36	0.14	0.01	0.26	0.22	-0.35	-0.43	-0.19	-0.16	-0.38	-0.37	-0.17	-0.22	-0.27	-0.37	-0.61	-0.44	-0.58	-0.48	-0.59	-0.43
O43390	Heterogeneous nuclear ribonucleoprotein R OS=Homo sapiens GN=HNRNPR PE=1 SV=1 - [HNRPR_HUMAN]	40.92	1	17	22	53	0.09	0.19	0.35	0.32	-0.17	-0.12	-0.16	-0.19	-0.14	-0.11	-0.15	-0.21	-0.39	-0.38	-0.47	-0.35	-0.41	-0.34	-0.45	-0.43
Q93075	Putative deoxyribonuclease TATDN2 OS=Homo sapiens GN=TATDN2 PE=2 SV=2 - [TATD2_HUMAN]	1.31	1	1	1	2	0.22	0.17	0.46	0.60	-0.29	0.55	-0.04	0.35	0.37	-0.67	-0.09	0.91	-0.20	-0.30	-0.55	-0.86	-1.10	-0.53	-0.77	-0.43
O43567	E3 ubiquitin-protein ligase RNF13 OS=Homo sapiens GN=RNF13 PE=1 SV=1 - [RNF13_HUMAN]	6.30	1	1	1	2	0.24	0.32	0.07	0.14	-0.43	-0.36	-0.43	-0.35	-0.20	-0.27	0.03	0.10	-0.61	-0.20	-0.03	-0.48	-0.30	-0.69	-0.51	-0.43

Q02790	Peptidyl-prolyl cis-trans isomerase FKBP4 OS=Homo sapiens GN=FKBP4 PE=1 SV=3 - [FKBP4_HUMAN]	36.17	1	13	13	30	0.40	0.35	0.53	0.49	-0.31	-0.34	-0.01	0.03	-0.26	-0.31	-0.51	-0.47	-0.31	-0.90	-1.01	-0.75	-0.75	-0.71	-0.93	-0.43
O95628	CCR4-NOT transcription complex subunit 4 OS=Homo sapiens GN=CNOT4 PE=1 SV=3 - [CNOT4_HUMAN]	2.61	1	1	1	1	0.71	0.90	0.47	0.66	-0.30	-0.11	-0.02	0.17	0.57	0.39	-0.85	-0.67	-0.68	-1.56	-1.32	-0.29	-0.05	-1.03	-0.79	-0.43
P43250	G protein-coupled receptor kinase 6 OS=Homo sapiens GN=GRK6 PE=1 SV=2 - [GRK6_HUMAN]	1.56	1	1	1	1	1.03	1.16	0.93	1.07	0.03	0.16	0.44	0.58	0.41	0.28	0.62	0.75	-0.53	-0.40	-0.32	-0.72	-0.62	-1.02	-0.92	-0.43
Q9HAR2	Latrophilin-3 OS=Homo sapiens GN=LPHN3 PE=1 SV=2 - [LPHN3_HUMAN]	9.40	1	11	11	14	0.78	0.84	0.78	0.86	-0.13	-0.25	0.22	0.34	0.27	0.13	0.23	0.17	-0.49	-0.75	-0.68	-0.52	-0.45	-1.08	-0.79	-0.43
Q6UWP7	Lysocardiolipin acyltransferase 1 OS=Homo sapiens GN=LCLAT1 PE=1 SV=1 - [LCLT1_HUMAN]	4.59	1	2	2	2	-0.25	-0.29	0.46	0.42	-0.33	-0.38	-0.03	-0.08	0.05	0.10	-0.13	-0.18	0.27	0.12	-0.60	0.38	-0.33	-0.10	-0.81	-0.43
Q9NZL9	Methionine adenosyltransferase 2 subunit beta OS=Homo sapiens GN=MAT2B PE=1 SV=1 - [MAT2B_HUMAN]	30.84	1	8	8	19	0.69	0.70	0.57	0.56	0.19	0.20	0.17	0.24	0.22	0.23	0.20	0.25	-0.28	-0.41	-0.40	-0.45	-0.52	-0.58	-0.64	-0.43
P29401	Transketolase OS=Homo sapiens GN=TKT PE=1 SV=3 - [TKT_HUMAN]	46.23	1	21	21	76	0.40	0.53	0.69	0.74	0.08	0.17	0.17	0.12	0.20	0.09	0.11	0.23	-0.30	-0.26	-0.55	-0.27	-0.52	-0.41	-0.65	-0.43
Q9P2B4	CTTNBP2 N-terminal-like protein OS=Homo sapiens GN=CTTNBP2NL PE=1 SV=2 - [CT2NL_HUMAN]	5.16	1	3	3	3	0.46	0.59	0.37	0.49	-0.20	-0.08	-0.13	0.00	-0.18	-0.29	-0.12	0.00	-0.54	-0.58	-0.49	-0.73	-0.63	-0.68	-0.59	-0.43

O75129	Astrotactin-2 OS=Homo sapiens GN=ASTN2 PE=2 SV=2 - [ASTN2_HUMAN]	7.09	1	7	7	12	0.52	0.38	0.33	0.43	-0.32	-0.10	-0.11	-0.08	-0.02	-0.17	-0.34	-0.26	-0.41	-0.48	-0.70	-0.29	-0.40	-0.77	-0.52	-0.43
Q9UJ14	Gamma-glutamyltransferase 7 OS=Homo sapiens GN=GGT7 PE=1 SV=2 - [GGT7_HUMAN]	13.44	1	6	6	9	0.54	0.94	0.78	1.06	-0.04	-0.08	0.10	0.56	0.39	0.20	0.20	0.46	-0.51	-0.68	-0.46	-0.50	-0.64	-0.53	-0.18	-0.44
Q16773	Kynurenine--oxoglutarate transaminase 1 OS=Homo sapiens GN=CCBL1 PE=1 SV=1 - [KAT1_HUMAN]	10.66	1	3	4	7	0.61	0.67	0.40	0.47	-0.02	0.05	0.15	0.21	-0.40	-0.48	-0.36	-0.26	-0.41	-0.45	-0.61	-0.57	-0.72	-0.56	-0.71	-0.44
Q58WW2	DDB1- and CUL4-associated factor 6 OS=Homo sapiens GN=DCAF6 PE=1 SV=1 - [DCAF6_HUMAN]	5.70	1	3	3	6	0.69	0.30	0.82	0.39	-0.05	-0.29	0.32	0.24	0.33	0.69	0.51	0.27	-0.31	-0.17	-0.31	0.03	-0.10	-0.75	-0.89	-0.44
O43572	A-kinase anchor protein 10, mitochondrial OS=Homo sapiens GN=AKAP10 PE=1 SV=2 - [AKA10_HUMAN]	17.07	1	7	7	11	0.83	0.69	1.17	1.18	0.20	0.21	0.32	0.18	0.30	0.29	0.19	0.20	-0.11	-0.17	-0.80	-0.22	-0.83	-0.34	-0.95	-0.44
Q8NFH8	RalBP1-associated Eps domain-containing protein 2 OS=Homo sapiens GN=REPS2 PE=1 SV=2 - [REPS2_HUMAN]	20.61	2	10	11	17	0.04	0.30	0.54	0.78	-0.19	-0.41	0.13	-0.06	-0.16	0.12	-0.17	-0.06	-0.04	-0.31	-0.79	-0.11	-0.56	-0.72	-0.92	-0.44
Q68D91	Metallo-beta-lactamase domain-containing protein 2 OS=Homo sapiens GN=MBLAC2 PE=1 SV=3 - [MBLC2_HUMAN]	49.46	1	11	11	23	0.91	0.63	0.66	0.78	0.15	0.11	0.32	0.17	0.26	0.43	0.52	0.32	-0.53	-0.49	-0.23	-0.45	-0.40	-0.64	-0.56	-0.44

Q9BRA2	Thioredoxin domain-containing protein 17 OS=Homo sapiens GN=TXNDC17 PE=1 SV=1 - [TXD17_HUMAN]	18.70	1	2	2	4	1.03	0.93	1.10	1.00	-0.59	-0.69	0.60	0.50	0.06	0.16	-0.15	-0.25	-0.38	-1.18	-1.25	-0.84	-0.90	-1.64	-1.70	-0.44
P43034	Platelet-activating factor acetylhydrolase IB subunit alpha OS=Homo sapiens GN=PAFAH1B1 PE=1 SV=2 - [LIS1_HUMAN]	41.71	1	14	15	45	0.67	0.62	1.03	0.87	0.29	0.32	0.58	0.62	0.18	0.23	0.32	0.17	-0.05	-0.47	-0.72	-0.49	-0.73	-0.37	-0.66	-0.44
Q6VY07	Phosphofurin acidic cluster sorting protein 1 OS=Homo sapiens GN=PACS1 PE=1 SV=2 - [PACS1_HUMAN]	12.88	1	9	9	19	0.33	0.41	0.66	0.69	0.04	-0.08	0.08	0.04	0.01	0.20	0.22	0.10	-0.27	-0.31	-0.52	-0.42	-0.58	-0.31	-0.57	-0.44
O95167	NADH dehydrogenase [ubiquinone] 1 alpha subcomplex subunit 3 OS=Homo sapiens GN=NDUFA3 PE=1 SV=1 - [NDUA3_HUMAN]	50.00	1	4	4	9	0.94	0.97	0.86	1.01	0.06	0.08	0.31	0.42	0.15	0.11	0.20	0.32	-0.53	-0.64	-0.69	-0.79	-0.76	-0.94	-0.90	-0.44
Q8TF61	F-box only protein 41 OS=Homo sapiens GN=FBXO41 PE=2 SV=5 - [FBX41_HUMAN]	8.80	1	6	6	8	0.98	0.38	0.74	0.68	-0.46	-0.27	0.29	-0.20	-0.08	0.03	-0.07	-0.11	-0.35	0.29	0.18	-0.78	-0.68	-0.41	-0.51	-0.44
Q9H078	Caseinolytic peptidase B protein homolog OS=Homo sapiens GN=CLPB PE=1 SV=1 - [CLPB_HUMAN]	10.33	1	6	6	9	0.57	0.30	0.67	0.26	-0.12	-0.23	0.05	-0.21	-0.06	-0.10	-0.28	-0.52	-0.54	-0.82	-0.95	-0.52	-0.43	-0.87	-0.81	-0.44
Q7Z417	Nuclear fragile X mental retardation-interacting protein 2 OS=Homo sapiens GN=NUFIP2 PE=1 SV=1 - [NUFP2_HUMAN]	4.17	1	2	2	2	-0.10	-0.17	0.50	0.43	0.01	-0.07	-0.01	-0.07	0.00	0.07	0.16	0.08	0.15	0.27	-0.34	0.20	-0.40	0.09	-0.51	-0.44

Q96T76	MMS19 nucleotide excision repair protein homolog OS=Homo sapiens GN=MMS19 PE=1 SV=2 - [MMS19_HUMAN]	6.80	1	7	7	13	0.65	0.41	0.78	0.52	0.18	-0.05	0.20	0.03	-0.08	0.06	0.16	0.07	-0.35	-0.48	-0.65	-0.55	-0.66	-0.45	-0.58	-0.44
O95671	N-acetylserotonin O-methyltransferase-like protein OS=Homo sapiens GN=ASMTL PE=1 SV=3 - [ASML_HUMAN]	20.77	1	10	10	25	0.05	0.11	0.16	0.24	0.31	0.37	-0.23	-0.23	0.04	-0.03	0.10	0.18	-0.30	0.05	-0.02	-0.08	-0.19	0.26	0.15	-0.44
Q6S8J3	POTE ankyrin domain family member E OS=Homo sapiens GN=POTEE PE=1 SV=3 - [POTEE_HUMAN]	10.33	5	1	11	689	-0.16	-0.17	-0.25	-0.22	-0.04	-0.28	-0.64	-0.73	-0.36	-0.11	-0.33	-0.57	-0.26	-0.34	-0.54	-0.28	-0.42	-0.16	-0.36	-0.44
Q86T03	Type 1 phosphatidylinositol 4,5-bisphosphate 4-phosphatase OS=Homo sapiens GN=TMEM55B PE=1 SV=1 - [TM55B_HUMAN]	18.41	1	4	4	5	0.80	0.85	0.52	0.58	0.13	0.17	0.02	0.07	0.29	0.25	0.37	0.41	-0.73	-0.43	-0.16	-0.53	-0.25	-0.69	-0.41	-0.44
Q9Y426	C2 domain-containing protein 2 OS=Homo sapiens GN=C2CD2 PE=1 SV=2 - [CU025_HUMAN]	11.06	1	5	5	7	0.99	0.93	0.53	0.48	0.22	0.16	0.02	-0.03	0.22	0.28	0.02	-0.04	-0.91	-0.96	-0.51	-0.68	-0.22	-0.78	-0.33	-0.44
Q8WU79	Stromal membrane-associated protein 2 OS=Homo sapiens GN=SMAP2 PE=1 SV=1 - [SMAP2_HUMAN]	11.42	1	4	5	9	0.82	0.52	0.88	0.44	0.48	0.33	0.24	0.09	0.34	0.57	0.27	0.10	-0.49	-0.52	-0.49	-0.25	-0.22	-0.32	-0.28	-0.44
P02511	Alpha-crystallin B chain OS=Homo sapiens GN=CRYAB PE=1 SV=2 - [CRYAB_HUMAN]	44.00	1	7	7	58	0.42	0.39	0.54	0.46	-0.05	-0.13	0.01	-0.04	-0.20	-0.16	-0.11	-0.11	-0.46	-0.45	-0.71	-0.38	-0.65	-0.52	-0.59	-0.44

Q16718	NADH dehydrogenase [ubiquinone] 1 alpha subcomplex subunit 5 OS=Homo sapiens GN=NDUFA5 PE=1 SV=3 - [NDUA5_HUMAN]	69.83	1	7	7	21	0.52	0.73	0.94	0.85	0.06	0.02	0.42	0.48	0.32	0.27	0.36	0.26	-0.03	-0.46	-0.75	-0.28	-0.73	-0.40	-0.92	-0.44
P14618	Pyruvate kinase PKM OS=Homo sapiens GN=PKM PE=1 SV=4 - [KPYM_HUMAN]	63.65	1	29	30	320	0.63	0.65	0.77	0.79	0.18	0.20	0.24	0.26	0.04	0.02	-0.49	-0.48	-0.31	-1.05	-1.21	-0.65	-0.72	-0.52	-0.61	-0.44
Q8N465	D-2-hydroxyglutarate dehydrogenase, mitochondrial OS=Homo sapiens GN=D2HGDH PE=1 SV=3 - [D2HDH_HUMAN]	15.74	1	5	5	6	0.65	0.65	0.95	0.93	0.20	0.48	0.40	0.65	0.70	0.27	0.60	0.70	-0.32	0.00	-0.46	0.28	-0.59	-0.35	-0.73	-0.44
Q4VCS5	Angiomotin OS=Homo sapiens GN=AMOT PE=1 SV=1 - [AMOT_HUMAN]	5.17	2	4	4	4	0.16	0.07	-0.21	-0.14	0.46	-0.12	-0.23	-0.65	-0.30	0.05	-0.13	-0.56	-0.66	-0.62	-0.41	-0.33	-0.12	0.28	0.65	-0.44
P21266	Glutathione S transferase Mu 3 OS=Homo sapiens GN=GSTM3 PE=1 SV=3 - [GSTM3_HUMAN]	66.22	1	14	15	45	1.27	1.40	1.33	1.47	0.64	0.74	0.89	1.04	1.02	0.84	0.92	0.91	-0.45	-0.59	-0.54	-0.39	-0.44	-0.71	-0.67	-0.44
Q96CN7	Isochorismatase domain-containing protein 1 OS=Homo sapiens GN=ISOC1 PE=1 SV=3 - [ISOC1_HUMAN]	18.79	1	4	4	7	0.17	0.63	0.46	0.64	-0.11	0.02	-0.06	0.13	-0.04	-0.13	-0.19	-0.02	-0.34	-0.61	-0.48	-0.59	-0.64	-0.45	-0.51	-0.44
P19021	Peptidyl-glycine alpha-amidating monooxygenase OS=Homo sapiens GN=PAM PE=1 SV=2 - [AMD_HUMAN]	8.02	1	5	5	8	0.07	0.00	0.05	0.24	0.08	0.08	-0.25	-0.27	-0.15	-0.07	0.40	0.33	-0.15	0.34	0.42	0.23	-0.35	0.06	0.01	-0.45

Q3ZCQ8	Mitochondrial import inner membrane translocase subunit TIM50 OS=Homo sapiens GN=TIMM50 PE=1 SV=2 - [TIM50_HUMAN]	22.66	1	5	5	13	0.62	0.90	0.30	0.45	0.15	0.34	0.10	0.15	0.35	0.02	0.00	0.10	-0.59	-0.67	-0.42	-0.65	-0.37	-0.50	-0.29	-0.45
Q96B86	Repulsive guidance molecule A OS=Homo sapiens GN=RGMA PE=1 SV=3 - [RGMA_HUMAN]	6.67	2	2	2	3	0.69	0.72	0.61	0.63	0.16	0.17	0.10	0.12	0.04	0.03	-0.13	-0.11	-0.54	-0.82	-0.74	-0.64	-0.55	-0.55	-0.47	-0.45
Q9BXW6	Oxysterol-binding protein-related protein 1 OS=Homo sapiens GN=OSBPL1A PE=1 SV=2 - [OSBL1_HUMAN]	8.21	1	6	6	11	0.59	0.61	1.12	1.11	0.07	0.19	0.42	0.60	0.25	0.24	-0.35	-0.10	-0.13	-0.89	-1.52	-0.32	-0.63	-0.54	-1.05	-0.45
Q7L3B6	Hsp90 co-chaperone Cdc37-like 1 OS=Homo sapiens GN=CDC37L1 PE=1 SV=1 - [CD37L_HUMAN]	6.53	1	1	1	1	1.78	1.63	1.45	1.31	0.81	0.65	0.94	0.80	0.81	0.96	0.91	0.75	-0.78	-0.87	-0.55	-0.79	-0.46	-0.99	-0.67	-0.45
Q9H0R4	Haloacid dehalogenase-like hydrolase domain-containing protein 2 OS=Homo sapiens GN=HDHD2 PE=1 SV=1 - [HDHD2_HUMAN]	54.05	1	9	9	37	0.97	0.85	1.04	0.94	0.28	0.13	0.47	0.46	0.59	0.77	0.94	0.78	-0.21	0.02	-0.14	-0.05	-0.36	-0.68	-0.80	-0.45
P52565	Rho GDP-dissociation inhibitor 1 OS=Homo sapiens GN=ARHGDIA PE=1 SV=3 - [GDIR1_HUMAN]	33.82	1	6	6	23	0.66	0.49	0.97	0.87	0.07	-0.12	0.46	0.34	-0.10	0.03	-0.52	-0.58	-0.05	-0.88	-1.39	-0.55	-0.94	-0.58	-1.01	-0.45
Q8TCU6	Phosphatidylinositol 3,4,5-trisphosphate-dependent Rac exchanger 1 protein OS=Homo sapiens GN=PREX1 PE=1 SV=3 - [PREX1_HUMAN]	14.35	1	16	16	25	0.75	0.87	0.59	0.75	0.00	0.01	0.23	0.40	0.25	0.10	-0.12	0.01	-0.59	-0.95	-0.96	-0.71	-0.49	-0.95	-0.60	-0.45

Q96P16	Regulation of nuclear pre-mRNA domain-containing protein 1A OS=Homo sapiens GN=RPRD1A PE=1 SV=1 - [RPR1A_HUMAN]	8.33	2	3	3	3	1.26	1.25	1.00	1.00	0.42	0.41	0.49	0.49	0.38	0.39	0.14	0.13	-0.71	-1.11	-0.86	-0.84	-0.58	-0.85	-0.60	-0.45
Q15173	Serine/threonine-protein phosphatase 2A 56 kDa regulatory subunit beta isoform OS=Homo sapiens GN=PPP2R5B PE=1 SV=1 - [2A5B_HUMAN]	10.66	1	3	3	4	0.66	0.74	0.75	0.83	0.29	0.36	0.24	0.32	0.09	0.01	0.04	0.11	-0.50	-0.62	-0.73	-0.62	-0.54	-0.39	-0.45	-0.45
Q9BV79	Trans-2-enoyl-CoA reductase, mitochondrial OS=Homo sapiens GN=MECR PE=1 SV=2 - [MECR_HUMAN]	8.04	1	3	3	7	0.60	0.65	0.59	0.66	-0.19	-0.13	0.08	0.15	0.09	0.03	-0.22	0.03	-0.19	-0.46	-0.82	-0.19	-0.54	-0.46	-0.80	-0.45
Q9BXS5	AP-1 complex subunit mu-1 OS=Homo sapiens GN=AP1M1 PE=1 SV=3 - [AP1M1_HUMAN]	26.48	2	9	9	17	0.51	0.48	0.54	0.55	-0.01	-0.12	0.08	0.07	-0.15	-0.08	-0.05	-0.12	-0.32	-0.59	-0.65	-0.53	-0.59	-0.50	-0.58	-0.45
P06730	Eukaryotic translation initiation factor 4E OS=Homo sapiens GN=EIF4E PE=1 SV=2 - [IF4E_HUMAN]	18.43	1	3	3	6	0.28	0.36	0.26	0.46	-0.43	-0.32	-0.31	-0.23	-0.31	-0.39	-0.77	-0.60	-0.52	-1.05	-1.02	-0.54	-0.49	-0.73	-0.80	-0.45
Q9P032	NADH dehydrogenase [ubiquinone] 1 alpha subcomplex assembly factor 4 OS=Homo sapiens GN=NDUFAF4 PE=1 SV=1 - [NDUF4_HUMAN]	20.57	1	4	4	6	0.09	-0.06	0.44	0.29	-1.03	-1.18	-0.09	-0.24	-0.70	-0.54	-0.66	-0.81	-0.05	-0.74	-1.10	-0.60	-0.95	-1.14	-1.49	-0.45

P48735	Isocitrate dehydrogenase [NADP], mitochondrial OS=Homo sapiens GN=IDH2 PE=1 SV=2 - [IDHP_HUMAN]	48.89	1	22	22	82	-0.39	-0.49	-0.23	-0.26	-0.99	-1.04	-0.74	-0.78	-0.91	-0.84	-0.85	-0.93	-0.20	-0.39	-0.65	-0.40	-0.65	-0.49	-0.71	-0.45
P27701	CD82 antigen OS=Homo sapiens GN=CD82 PE=1 SV=1 - [CD82_HUMAN]	11.24	1	3	3	13	0.84	0.87	0.95	0.98	0.85	0.97	0.44	0.47	0.57	0.40	0.24	0.26	-0.35	-0.68	-0.85	-0.26	-0.40	0.09	-0.06	-0.45
Q9NWB6	Arginine and glutamate-rich protein 1 OS=Homo sapiens GN=ARGLU1 PE=1 SV=1 - [ARGL1_HUMAN]	5.86	1	2	2	4	0.23	0.08	0.26	0.12	-0.21	-0.36	-0.26	-0.40	-0.44	-0.30	-0.49	-0.64	-0.43	-0.71	-0.75	-0.49	-0.52	-0.45	-0.49	-0.45
P78406	mRNA export factor OS=Homo sapiens GN=RAE1 PE=1 SV=1 - [RAE1L_HUMAN]	34.51	1	9	9	28	-0.02	0.07	-0.03	0.04	-0.37	-0.47	-0.16	-0.32	-0.34	-0.25	-0.25	-0.24	-0.27	-0.29	-0.28	-0.40	-0.49	-0.57	-0.61	-0.45
Q13242	Serine/arginine-rich splicing factor 9 OS=Homo sapiens GN=SRSF9 PE=1 SV=1 - [SRSF9_HUMAN]	26.24	1	5	5	7	0.07	0.02	0.20	0.13	-0.33	-0.41	-0.30	-0.30	-0.18	-0.21	-0.04	-0.06	-0.36	-0.07	-0.19	-0.28	-0.38	-0.44	-0.54	-0.45
P36915	Guanine nucleotide-binding protein-like 1 OS=Homo sapiens GN=GNL1 PE=1 SV=2 - [GNL1_HUMAN]	24.71	1	14	14	25	0.69	0.76	0.73	0.65	0.13	0.14	0.17	0.13	0.09	0.17	0.15	0.16	-0.54	-0.38	-0.50	-0.66	-0.50	-0.57	-0.57	-0.45
Q9Y4D7	Plexin-D1 OS=Homo sapiens GN=PLXND1 PE=1 SV=3 - [PLXD1_HUMAN]	3.38	1	4	4	6	1.07	1.69	0.86	1.48	0.22	0.84	0.34	0.97	0.80	0.19	0.14	0.76	-0.67	-0.93	-0.72	-0.85	-0.64	-0.86	-0.65	-0.45
O95081	Arf-GAP domain and FG repeat-containing protein 2 OS=Homo sapiens GN=AGFG2 PE=1 SV=2 - [AGFG2_HUMAN]	8.52	1	3	3	5	1.19	1.09	0.99	0.89	0.61	0.50	0.47	0.37	0.35	0.46	0.20	0.09	-0.66	-0.99	-0.79	-0.70	-0.50	-0.60	-0.40	-0.45

P09622	Dihydrolipoyl dehydrogenase, mitochondrial OS=Homo sapiens GN=DLD PE=1 SV=2 - [DLDH_HUMAN]	44.01	1	15	15	57	0.11	0.10	0.37	0.42	-0.24	-0.25	0.04	-0.09	-0.33	-0.24	-0.33	-0.41	-0.17	-0.62	-0.89	-0.49	-0.69	-0.48	-0.68	-0.46
Q12789	General transcription factor 3C polypeptide 1 OS=Homo sapiens GN=GTF3C1 PE=1 SV=4 - [TF3C1_HUMAN]	1.09	1	2	2	2	-0.22	-0.27	-0.53	-0.57	-0.59	-0.63	-1.05	-1.09	-0.55	-0.50	-0.58	-0.62	-0.77	-0.34	-0.05	-0.25	0.06	-0.38	-0.08	-0.46
P27348	14-3-3 protein theta OS=Homo sapiens GN=YWHAQ PE=1 SV=1 - [1433T_HUMAN]	43.27	1	7	11	88	0.42	0.39	0.49	0.44	-0.21	-0.24	-0.13	-0.02	-0.11	-0.23	-0.20	-0.22	-0.37	-0.55	-0.60	-0.43	-0.81	-0.52	-0.61	-0.46
P06733	Alpha-enolase OS=Homo sapiens GN=ENO1 PE=1 SV=2 - [ENOA_HUMAN]	76.04	1	26	30	333	0.81	0.83	1.12	1.14	-0.08	-0.03	0.59	0.62	0.19	0.17	-0.25	-0.22	-0.17	-1.06	-1.38	-0.60	-0.88	-0.84	-1.18	-0.46
P51668	Ubiquitin-conjugating enzyme E2 D1 OS=Homo sapiens GN=UBE2D1 PE=1 SV=1 - [UB2D1_HUMAN]	24.49	2	2	3	15	0.71	0.70	0.85	0.84	0.33	0.31	0.33	0.32	0.04	0.06	-0.29	-0.31	-0.32	-0.99	-1.14	-0.62	-0.76	-0.40	-0.54	-0.46
Q3SXM5	Inactive hydroxysteroid dehydrogenase-like protein 1 OS=Homo sapiens GN=HSDL1 PE=1 SV=3 - [HSDL1_HUMAN]	15.15	1	5	5	8	0.71	0.77	1.03	1.01	0.35	0.45	0.55	0.51	0.44	0.39	0.21	0.31	-0.19	-0.44	-0.82	-0.29	-0.57	-0.32	-0.70	-0.46
Q13625	Apoptosis-stimulating of p53 protein 2 OS=Homo sapiens GN=TP53BP2 PE=1 SV=2 - [ASPP2_HUMAN]	6.56	1	4	4	5	0.97	0.90	1.18	1.10	0.73	0.65	0.66	0.58	0.31	0.39	0.92	0.84	-0.26	-0.05	-0.26	-0.56	-0.76	-0.26	-0.47	-0.46

Q9Y2Y0	ADP-ribosylation factor-like protein 2-binding protein OS=Homo sapiens GN=ARL2BP PE=1 SV=1 - [AR2BP_HUMAN]	14.72	1	2	2	2	0.36	0.29	0.49	0.42	0.05	-0.02	-0.03	-0.10	-0.08	0.00	0.22	0.14	-0.33	-0.13	-0.27	-0.33	-0.46	-0.32	-0.46	-0.46
Q07955	Serine/arginine-rich splicing factor 1 OS=Homo sapiens GN=SRSF1 PE=1 SV=2 - [SRSF1_HUMAN]	37.50	1	9	9	31	0.26	0.16	0.45	0.32	-0.32	-0.47	-0.19	-0.25	-0.41	-0.35	-0.26	-0.40	-0.37	-0.57	-0.76	-0.54	-0.68	-0.64	-0.88	-0.46
Q9UL15	BAG family molecular chaperone regulator 5 OS=Homo sapiens GN=BAG5 PE=1 SV=1 - [BAG5_HUMAN]	11.41	1	4	4	6	0.66	0.75	0.71	0.80	0.00	0.08	0.19	0.27	0.00	-0.07	0.18	0.26	-0.42	-0.47	-0.53	-0.71	-0.75	-0.68	-0.73	-0.46
Q9BQD3	KxDL motif-containing protein 1 OS=Homo sapiens GN=KXD1 PE=1 SV=2 - [KXDL1_HUMAN]	3.98	1	1	1	2	0.13	0.26	0.14	0.26	0.27	0.39	-0.38	-0.26	0.11	-0.01	0.22	0.34	-0.46	0.09	0.08	-0.11	-0.11	0.12	0.11	-0.46
Q9Y277	Voltage-dependent anion-selective channel protein 3 OS=Homo sapiens GN=VDAC3 PE=1 SV=1 - [VDAC3_HUMAN]	55.48	1	12	14	77	0.89	0.75	1.19	1.08	0.25	0.32	0.55	0.59	0.43	0.40	0.50	0.41	-0.32	-0.38	-0.54	-0.29	-0.66	-0.57	-0.87	-0.46
Q86U28	Iron-sulfur cluster assembly 2 homolog, mitochondrial OS=Homo sapiens GN=ISCA2 PE=1 SV=2 - [ISCA2_HUMAN]	23.38	1	3	3	5	0.57	0.64	0.56	0.62	0.07	0.11	0.05	0.09	0.11	0.06	0.04	0.18	-0.47	-0.40	-0.54	-0.37	-0.46	-0.51	-0.51	-0.46
Q9BPY8	Homeodomain-only protein OS=Homo sapiens GN=HOPX PE=1 SV=1 - [HOP_HUMAN]	49.32	1	4	4	6	-0.26	0.09	-0.19	0.16	-0.14	0.20	-0.72	-0.37	0.13	-0.21	-0.20	0.14	-0.40	0.06	-0.01	0.08	0.01	0.10	0.03	-0.46

Q9NY35	Claudin domain-containing protein 1 OS=Homo sapiens GN=CLDND1 PE=1 SV=1 - [CLDN1_HUMAN]	12.25	1	3	3	7	0.72	0.82	0.82	0.92	0.29	0.39	0.38	0.48	0.33	0.23	0.28	0.38	-0.17	-0.65	-0.57	-0.44	-0.73	-0.60	-0.58	-0.46
Q5VIR6	Vacuolar protein sorting-associated protein 53 homolog OS=Homo sapiens GN=VPS53 PE=1 SV=1 - [VPS53_HUMAN]	22.17	1	12	12	19	0.84	0.63	0.74	0.64	0.13	0.06	0.28	0.22	0.18	0.27	0.11	0.01	-0.55	-0.63	-0.66	-0.46	-0.38	-0.68	-0.63	-0.46
O00217	NADH dehydrogenase [ubiquinone] iron-sulfur protein 8, mitochondrial OS=Homo sapiens GN=NDUFS8 PE=1 SV=1 - [NDUS8_HUMAN]	25.24	1	4	4	30	0.67	0.57	1.16	1.06	0.03	0.24	0.46	0.49	-0.15	-0.09	0.27	0.23	-0.22	-0.36	-0.83	-0.44	-0.88	-0.43	-0.84	-0.46
P48426	Phosphatidylinositol 5-phosphate 4-kinase type-2 alpha OS=Homo sapiens GN=PIP4K2A PE=1 SV=2 - [PI42A_HUMAN]	27.09	1	5	10	28	0.89	0.65	0.71	0.77	0.18	-0.05	0.19	0.11	-0.12	0.09	-0.03	-0.03	-0.65	-0.93	-0.76	-0.74	-0.58	-0.66	-0.60	-0.46
Q08462	Adenylate cyclase type 2 OS=Homo sapiens GN=ADCY2 PE=1 SV=5 - [ADCY2_HUMAN]	3.30	1	3	3	5	0.60	0.55	0.65	0.60	0.26	0.20	0.12	0.07	0.09	0.15	0.21	0.15	-0.42	-0.38	-0.44	-0.42	-0.46	-0.36	-0.41	-0.46
P63010	AP-2 complex subunit beta OS=Homo sapiens GN=AP2B1 PE=1 SV=1 - [AP2B1_HUMAN]	50.91	1	23	38	126	0.50	0.65	0.75	0.77	0.33	0.12	0.26	0.25	0.05	0.12	0.00	-0.04	-0.23	-0.47	-0.81	-0.34	-0.64	-0.35	-0.40	-0.46
Q8N5J2	Protein FAM63A OS=Homo sapiens GN=FAM63A PE=1 SV=2 - [FA63A_HUMAN]	21.11	1	5	5	9	0.10	-0.15	0.17	-0.07	0.08	-0.17	-0.36	-0.60	-0.43	-0.18	-0.12	-0.36	-0.40	-0.70	-0.67	-0.25	-0.32	-0.04	-0.11	-0.46

Q9P0T7	Transmembrane protein 9 OS=Homo sapiens GN=TMEM9 PE=1 SV=1 - [TMEM9_HUMAN]	7.10	1	1	1	2	0.31	0.02	0.36	0.07	0.22	-0.07	-0.16	-0.45	-0.09	0.21	0.39	0.10	-0.42	0.09	0.03	-0.07	-0.12	-0.54	-0.51	-0.46
P62312	U6 snRNA-associated Sm-like protein LSm6 OS=Homo sapiens GN=LSM6 PE=1 SV=1 - [LSM6_HUMAN]	37.50	1	3	3	5	-0.20	-0.41	0.22	0.01	-0.29	-0.50	-0.30	-0.51	-0.88	-0.66	-0.49	-0.71	-0.05	-0.29	-0.71	-0.43	-0.85	-0.11	-0.53	-0.46
Q16822	Phosphoenolpyruvate carboxykinase [GTP], mitochondrial OS=Homo sapiens GN=PCK2 PE=1 SV=3 - [PCKGM_HUMAN]	26.56	2	13	13	17	0.24	0.33	0.21	0.16	-0.13	-0.21	-0.35	-0.56	-0.08	-0.07	0.25	0.19	-0.66	-0.30	-0.07	-0.37	-0.19	-0.67	-0.36	-0.46
Q86UE4	Protein LYRIC OS=Homo sapiens GN=MTDH PE=1 SV=2 - [LYRIC_HUMAN]	20.79	1	9	9	13	-0.43	-0.35	-0.43	-0.34	-0.94	-0.77	-0.87	-0.74	-0.47	-0.66	-0.09	0.12	-0.34	0.47	0.36	-0.09	-0.39	-0.32	-0.53	-0.46
Q14254	Flotillin-2 OS=Homo sapiens GN=FLOT2 PE=1 SV=2 - [FLOT2_HUMAN]	46.73	1	21	21	53	0.48	0.51	0.75	0.74	0.02	-0.03	0.24	0.22	0.27	0.29	0.69	0.68	-0.21	0.13	-0.06	-0.22	-0.40	-0.53	-0.80	-0.46
P60484	Phosphatidylinositol 3,4,5-trisphosphate 3-phosphatase and dual-specificity protein phosphatase PTEN OS=Homo sapiens GN=PTEN PE=1 SV=1 - [PTEN_HUMAN]	15.14	1	5	5	7	1.24	1.37	0.56	0.70	-0.36	-0.23	0.04	0.17	0.11	-0.03	-0.20	-0.07	-1.14	-1.43	-0.77	-1.23	-0.55	-1.61	-0.94	-0.46
P04156	Major prion protein OS=Homo sapiens GN=PRNP PE=1 SV=1 - [PRIO_HUMAN]	20.16	1	3	3	17	0.85	0.85	0.76	0.84	0.07	0.16	0.26	0.26	0.03	0.05	0.26	0.29	-0.71	-0.60	-0.46	-0.78	-0.77	-0.81	-0.73	-0.46
P61978	Heterogeneous nuclear ribonucleoprotein K OS=Homo sapiens GN=HNRNPK PE=1 SV=1 - [HNRPK_HUMAN]	37.37	1	15	15	80	0.35	0.17	0.28	0.16	-0.38	-0.43	-0.25	-0.41	-0.40	-0.40	-0.33	-0.40	-0.54	-0.55	-0.53	-0.70	-0.54	-0.69	-0.73	-0.46

P28331	NADH-ubiquinone oxidoreductase 75 kDa subunit, mitochondrial OS=Homo sapiens GN=NDUFS1 PE=1 SV=3 - [NDUS1_HUMAN]	51.86	1	29	29	105	0.67	0.59	0.78	0.69	0.05	-0.04	0.35	0.18	0.18	0.22	0.36	0.27	-0.35	-0.50	-0.50	-0.55	-0.49	-0.72	-0.63	-0.46
Q9BQ95	Evolutionarily conserved signaling intermediate in Toll pathway, mitochondrial OS=Homo sapiens GN=ECSIT PE=1 SV=1 - [ECSIT_HUMAN]	10.21	1	2	2	2	1.35	1.25	1.30	1.20	0.71	0.61	0.77	0.67	0.52	0.62	0.85	0.75	-0.52	-0.50	-0.45	-0.69	-0.64	-0.65	-0.60	-0.46
Q9NRF2	SH2B adapter protein 1 OS=Homo sapiens GN=SH2B1 PE=1 SV=3 - [SH2B1_HUMAN]	4.23	1	2	2	4	0.30	0.13	0.33	0.16	-0.26	-0.43	-0.20	-0.37	-0.30	-0.13	-0.14	-0.31	-0.45	-0.44	-0.47	-0.40	-0.42	-0.58	-0.60	-0.46
Q53HC9	Protein TSSC1 OS=Homo sapiens GN=TSSC1 PE=1 SV=2 - [TSSC1_HUMAN]	12.92	1	4	4	8	0.85	0.72	1.08	0.95	0.31	0.18	0.55	0.42	0.44	0.57	0.37	0.23	-0.24	-0.48	-0.71	-0.25	-0.47	-0.55	-0.78	-0.47
Q12974	Protein tyrosine phosphatase type IVA 2 OS=Homo sapiens GN=PTP4A2 PE=1 SV=1 - [TP4A2_HUMAN]	17.96	2	3	3	4	0.62	0.45	0.90	0.74	0.14	-0.03	0.37	0.21	-0.04	0.13	-0.05	-0.22	-0.19	-0.66	-0.95	-0.46	-0.74	-0.49	-0.78	-0.47
Q6NXE6	Armadillo repeat-containing protein 6 OS=Homo sapiens GN=ARMC6 PE=1 SV=2 - [ARMC6_HUMAN]	10.18	1	4	4	4	1.22	1.24	1.22	1.24	0.47	0.49	0.89	0.91	0.71	0.69	0.56	0.57	-0.69	-0.78	-0.57	-0.83	-0.62	-1.19	-0.98	-0.47
Q9UDY4	DnaJ homolog subfamily B member 4 OS=Homo sapiens GN=DNAJB4 PE=1 SV=1 - [DNJB4_HUMAN]	34.42	1	7	8	13	0.27	0.19	0.37	0.18	-0.32	-0.38	-0.16	-0.15	-0.16	-0.15	0.00	-0.20	-0.40	-0.17	-0.51	-0.10	-0.43	-0.49	-0.77	-0.47

P34932	Heat shock 70 kDa protein 4 OS=Homo sapiens GN=HSPA4 PE=1 SV=4 - [HSP74_HUMAN]	47.14	1	31	33	76	0.47	0.38	0.60	0.58	-0.25	-0.30	0.19	0.15	-0.19	-0.13	-0.53	-0.47	-0.21	-0.83	-1.07	-0.59	-0.74	-0.67	-0.91	-0.47
O95139	NADH dehydrogenase [ubiquinone] 1 beta subcomplex subunit 6 OS=Homo sapiens GN=NDUFB6 PE=1 SV=3 - [NDUB6_HUMAN]	25.78	1	3	3	9	0.01	0.22	0.23	0.44	-0.28	-0.30	0.13	0.11	0.11	0.13	-0.21	-0.09	-0.26	-0.12	-0.36	-0.48	-0.67	-0.36	-0.59	-0.47
Q75T13	GPI inositol-deacylase OS=Homo sapiens GN=PGAP1 PE=2 SV=1 - [PGAP1_HUMAN]	1.19	1	1	1	4	1.91	1.34	-0.19	-0.76	0.36	-0.22	-0.72	-1.30	-0.76	-0.17	0.25	-0.34	-2.58	-1.66	0.43	-2.06	0.05	-1.57	0.53	-0.47
Q9UHW9	Solute carrier family 12 member 6 OS=Homo sapiens GN=SLC12A6 PE=1 SV=2 - [S12A6_HUMAN]	7.22	1	5	7	10	0.84	0.74	0.34	0.24	-0.16	-0.27	-0.19	-0.29	-0.26	-0.15	-0.32	-0.42	-0.97	-1.15	-0.66	-0.96	-0.46	-1.02	-0.52	-0.47
Q9NZ32	Actin-related protein 10 OS=Homo sapiens GN=ACTR10 PE=1 SV=1 - [ARP10_HUMAN]	18.23	1	5	5	11	0.59	0.58	0.83	0.82	-0.04	-0.14	0.17	0.24	-0.04	-0.06	-0.02	0.00	-0.20	-0.49	-0.85	-0.34	-0.86	-0.90	-1.03	-0.47
P35080	Profilin-2 OS=Homo sapiens GN=PFN2 PE=1 SV=3 - [PROF2_HUMAN]	32.14	1	5	5	52	0.58	0.43	0.79	0.59	0.11	-0.03	0.27	0.21	0.09	0.17	-0.30	-0.30	-0.13	-0.66	-0.94	-0.34	-0.55	-0.43	-0.65	-0.47
Q5T1J5	Putative coiled-coil-helix-coiled-coil-helix domain-containing protein CHCHD2P9, mitochondrial OS=Homo sapiens GN=CHCHD2P9 PE=5 SV=1 - [CHCH9_HUMAN]	8.61	2	1	1	1	0.01	-0.23	-0.14	-0.38	-1.12	-1.36	-0.68	-0.91	-1.02	-0.78	0.10	-0.14	-0.63	0.10	0.24	-0.76	-0.61	-1.14	-0.99	-0.47

Q14157	Ubiquitin-associated protein 2-like OS=Homo sapiens GN=UBAP2L PE=1 SV=2 - [UBP2L_HUMAN]	13.52	1	9	9	25	-0.14	-0.24	0.20	0.21	-0.46	-0.49	-0.50	-0.48	-0.43	-0.41	-0.12	-0.06	-0.02	0.16	-0.18	-0.06	-0.42	-0.12	-0.40	-0.47
P51151	Ras-related protein Rab-9A OS=Homo sapiens GN=RAB9A PE=1 SV=1 - [RAB9A_HUMAN]	40.30	1	4	6	12	0.35	0.37	0.49	0.67	0.35	0.41	-0.31	0.10	0.25	0.13	-0.19	0.21	-0.23	-0.27	-0.57	-0.18	-0.28	0.06	-0.28	-0.47
Q9UG56	Phosphatidylserine decarboxylase proenzyme OS=Homo sapiens GN=PISD PE=2 SV=4 - [PISD_HUMAN]	3.18	1	1	1	1	0.89	1.01	0.90	1.02	0.33	0.44	0.36	0.48	0.39	0.27	0.50	0.62	-0.48	-0.39	-0.40	-0.59	-0.59	-0.58	-0.59	-0.47
Q13573	SNW domain-containing protein 1 OS=Homo sapiens GN=SNW1 PE=1 SV=1 - [SNW1_HUMAN]	5.41	1	2	2	3	-0.82	-0.30	-0.29	0.23	-0.20	0.31	-0.54	-0.67	-0.07	-0.58	-0.16	0.35	-0.22	0.66	0.13	0.26	-0.26	0.60	0.07	-0.47
Q01650	Large neutral amino acids transporter small subunit 1 OS=Homo sapiens GN=SLC7A5 PE=1 SV=2 - [LAT1_HUMAN]	2.76	1	2	2	3	0.19	0.11	0.48	0.40	-0.52	-0.60	-0.05	-0.13	-0.32	-0.23	0.12	0.03	-0.19	-0.07	-0.37	-0.39	-0.68	-0.72	-1.02	-0.47
Q04609	Glutamate carboxypeptidase 2 OS=Homo sapiens GN=FOLH1 PE=1 SV=1 - [FOLH1_HUMAN]	4.13	2	1	2	4	1.21	1.23	1.49	1.51	0.00	0.02	0.95	0.97	0.19	0.17	0.03	0.05	-0.20	-1.17	-1.45	-1.00	-1.28	-1.22	-1.50	-0.47
P27695	DNA-(apurinic or apyrimidinic site) lyase OS=Homo sapiens GN=APEX1 PE=1 SV=2 - [APEX1_HUMAN]	16.04	1	4	4	12	-0.12	0.01	-0.08	0.00	-0.29	-0.25	-0.52	-0.32	-0.23	-0.27	-0.27	-0.22	-0.39	-0.39	-0.49	-0.25	-0.34	-0.36	-0.45	-0.47
P45381	Aspartoacylase OS=Homo sapiens GN=ASPA PE=1 SV=1 - [ACY2_HUMAN]	13.10	1	4	4	7	1.19	1.30	1.54	1.55	0.57	0.59	0.90	0.82	0.54	0.61	0.08	0.09	-0.17	-1.14	-1.46	-0.55	-0.89	-0.96	-0.98	-0.47

Q02952	A-kinase anchor protein 12 OS=Homo sapiens GN=AKAP12 PE=1 SV=4 - [AKA12_HUMAN]	29.91	1	37	37	131	0.47	0.53	0.77	0.76	-0.02	-0.04	0.21	0.07	0.05	0.13	0.15	0.21	-0.31	-0.23	-0.54	-0.33	-0.67	-0.57	-0.78	-0.47
Q13126	S-methyl-5'-thioadenosine phosphorylase OS=Homo sapiens GN=MTAP PE=1 SV=2 - [MTAP_HUMAN]	29.68	1	6	6	10	0.04	0.31	0.32	0.58	-0.32	-0.13	-0.37	-0.17	0.00	-0.20	-0.46	-0.35	-0.45	-0.18	-0.83	-0.09	-0.25	-0.36	-0.62	-0.47
Q9UBC2	Epidermal growth factor receptor substrate 15-like 1 OS=Homo sapiens GN=EPS15L1 PE=1 SV=1 - [EP15R_HUMAN]	45.37	1	29	29	53	0.25	0.43	0.52	0.62	-0.15	-0.10	0.00	0.15	0.14	0.00	0.01	0.20	-0.32	-0.08	-0.39	-0.31	-0.46	-0.42	-0.70	-0.48
P00367	Glutamate dehydrogenase 1, mitochondrial OS=Homo sapiens GN=GLUD1 PE=1 SV=2 - [DHE3_HUMAN]	45.16	3	24	24	94	0.54	0.44	0.54	0.55	-0.36	-0.36	-0.02	-0.01	-0.32	-0.21	-0.30	-0.28	-0.37	-0.75	-0.81	-0.64	-0.69	-0.87	-0.78	-0.48
Q9NXA8	NAD-dependent protein deacylase sirtuin-5, mitochondrial OS=Homo sapiens GN=SIRT5 PE=1 SV=2 - [SIR5_HUMAN]	30.97	1	7	7	13	0.47	0.49	0.68	0.48	-0.35	-0.42	0.08	-0.13	-0.30	-0.10	-0.25	-0.25	-0.44	-0.72	-0.73	-0.62	-0.74	-0.98	-0.91	-0.48
P23468	Receptor-type tyrosine-protein phosphatase delta OS=Homo sapiens GN=PTPRD PE=1 SV=2 - [PTPRD_HUMAN]	15.69	1	15	24	54	1.36	1.35	1.05	1.01	0.23	0.20	0.47	0.25	0.31	0.43	0.51	0.31	-0.76	-0.92	-0.57	-0.85	-0.51	-0.99	-0.60	-0.48
Q9UQ90	Paraplegin OS=Homo sapiens GN=SPG7 PE=1 SV=2 - [SPG7_HUMAN]	13.21	1	8	8	11	0.46	0.72	0.59	0.73	-0.22	0.02	0.06	0.17	0.19	-0.02	-0.10	0.04	-0.23	-0.50	-0.56	-0.45	-0.42	-0.72	-0.87	-0.48
Q8N6L0	Protein KASH5 OS=Homo sapiens GN=CCDC155 PE=2 SV=2 - [KASH5_HUMAN]	1.25	1	1	1	1	0.33	0.25	0.31	0.23	-0.30	-0.39	-0.23	-0.31	-0.38	-0.29	-0.19	-0.28	-0.51	-0.52	-0.50	-0.59	-0.57	-0.65	-0.63	-0.48

Q66K74	Microtubule-associated protein 1S OS=Homo sapiens GN=MAP1S PE=1 SV=2 - [MAP1S_HUMAN]	17.56	1	13	13	25	0.68	0.52	0.83	0.76	0.11	-0.09	0.23	0.11	0.04	0.19	0.23	0.15	-0.44	-0.50	-0.63	-0.35	-0.70	-0.49	-0.66	-0.48
Q15185	Prostaglandin E synthase 3 OS=Homo sapiens GN=PTGES3 PE=1 SV=1 - [TEBP_HUMAN]	31.87	1	4	4	17	0.22	0.17	0.67	0.51	-0.12	-0.17	0.04	0.00	-0.10	-0.23	0.02	0.04	-0.06	-0.27	-0.71	-0.28	-0.56	-0.46	-0.85	-0.48
P18859	ATP synthase-coupling factor 6, mitochondrial OS=Homo sapiens GN=ATP5J PE=1 SV=1 - [ATP5J_HUMAN]	47.22	1	5	5	31	0.42	0.39	0.77	0.68	-0.06	-0.05	0.25	0.23	0.04	0.00	0.01	-0.04	-0.16	-0.46	-0.61	-0.54	-0.68	-0.70	-0.87	-0.48
Q9UKG1	DCC-interacting protein 13-alpha OS=Homo sapiens GN=APPL1 PE=1 SV=1 - [DP13A_HUMAN]	31.73	1	17	17	28	0.65	0.62	0.60	0.66	-0.17	-0.23	-0.03	0.09	-0.08	-0.08	-0.46	-0.49	-0.42	-1.11	-1.14	-0.70	-0.62	-0.83	-0.82	-0.48
Q9BYV8	Centrosomal protein of 41 kDa OS=Homo sapiens GN=CEP41 PE=1 SV=1 - [CEP41_HUMAN]	12.87	1	3	3	6	0.58	0.46	0.47	0.35	-0.32	-0.54	0.24	0.12	0.13	0.33	0.15	-0.03	-0.51	-0.71	-0.60	-1.03	-0.92	-1.17	-1.06	-0.48
P30042	ES1 protein homolog, mitochondrial OS=Homo sapiens GN=C21orf33 PE=1 SV=3 - [ES1_HUMAN]	35.07	1	8	8	34	0.28	0.19	0.54	0.48	-0.13	-0.18	0.02	-0.05	-0.13	-0.06	-0.09	-0.14	-0.27	-0.40	-0.55	-0.28	-0.53	-0.31	-0.52	-0.48
Q9P2P5	E3 ubiquitin-protein ligase HECW2 OS=Homo sapiens GN=HECW2 PE=1 SV=2 - [HECW2_HUMAN]	3.94	1	4	5	5	0.95	1.49	0.51	1.05	-0.95	-0.41	-0.03	0.51	0.57	0.03	0.25	0.79	-0.93	-0.70	-0.26	-0.89	-0.44	-1.92	-1.48	-0.48
Q9UPY6	Wiskott-Aldrich syndrome protein family member 3 OS=Homo sapiens GN=WASF3 PE=1 SV=2 - [WASF3_HUMAN]	22.91	1	7	7	13	1.00	0.88	1.32	1.29	0.04	0.23	0.76	0.73	0.35	0.18	-0.02	0.10	0.06	-0.64	-1.13	-0.29	-0.76	-0.67	-1.15	-0.48

O94952	F-box only protein 21 OS=Homo sapiens GN=FBXO21 PE=2 SV=2 - [FBX21_HUMAN]	6.69	1	2	2	4	0.84	0.60	0.81	0.57	0.02	-0.23	0.26	0.02	0.30	0.55	0.28	0.03	-0.52	-0.55	-0.53	-0.26	-0.23	-0.84	-0.81	-0.48
Q9BWD1	Acetyl-CoA acetyltransferase, cytosolic OS=Homo sapiens GN=ACAT2 PE=1 SV=2 - [THIC_HUMAN]	22.17	1	7	7	21	0.97	1.11	0.94	1.11	0.08	0.21	0.48	0.57	0.37	0.33	0.09	0.15	-0.58	-0.84	-0.95	-0.74	-0.72	-0.95	-0.99	-0.48
P63104	14-3-3 protein zeta/delta OS=Homo sapiens GN=YWHAZ PE=1 SV=1 - [1433Z_HUMAN]	66.94	1	12	17	106	0.14	0.31	0.53	0.60	0.02	0.04	0.06	0.04	-0.10	-0.02	-0.07	-0.02	-0.09	-0.34	-0.62	-0.33	-0.60	-0.26	-0.58	-0.48
Q9Y3C6	Peptidyl-prolyl cis-trans isomerase-like 1 OS=Homo sapiens GN=PPIL1 PE=1 SV=1 - [PPIL1_HUMAN]	36.14	1	6	6	20	0.92	0.90	1.19	1.05	0.32	0.48	0.62	0.68	0.31	0.34	0.28	0.26	-0.18	-0.60	-0.87	-0.58	-0.81	-0.48	-0.77	-0.48
Q9UJ83	2-hydroxyacyl-CoA lyase 1 OS=Homo sapiens GN=HACL1 PE=1 SV=2 - [HACL1_HUMAN]	3.81	1	1	1	1	-0.67	-0.50	0.10	0.28	-0.03	0.15	-0.45	-0.27	0.00	-0.17	-0.30	-0.12	0.28	0.38	-0.40	0.53	-0.24	0.63	-0.14	-0.48
Q9Y5Y5	Peroxisomal membrane protein PEX16 OS=Homo sapiens GN=PEX16 PE=1 SV=2 - [PEX16_HUMAN]	3.27	1	1	1	1	0.53	0.74	0.65	0.86	0.22	0.42	0.10	0.31	0.57	0.37	0.31	0.51	-0.38	-0.22	-0.34	-0.13	-0.25	-0.33	-0.45	-0.48
P49589	Cysteine--tRNA ligase, cytoplasmic OS=Homo sapiens GN=CARS PE=1 SV=3 - [SYCC_HUMAN]	23.13	1	13	13	24	0.31	0.55	0.20	0.58	-0.28	0.06	-0.08	0.03	-0.13	-0.29	-0.40	-0.26	-0.34	-0.64	-0.82	-0.55	-0.69	-0.58	-0.58	-0.49
P78362	SRSF protein kinase 2 OS=Homo sapiens GN=SRPK2 PE=1 SV=3 - [SRPK2_HUMAN]	13.08	2	5	6	13	0.82	0.51	0.66	0.30	-0.38	-0.59	-0.22	-0.22	-0.63	-0.27	-0.36	-0.66	-0.54	-0.87	-0.75	-0.66	-0.49	-1.03	-0.90	-0.49

O75155	Cullin-associated NEDD8-dissociated protein 2 OS=Homo sapiens GN=CAND2 PE=1 SV=3 - [CAND2_HUMAN]	12.38	1	8	10	15	1.60	1.10	0.06	0.09	0.51	0.66	-0.95	-0.47	0.02	-0.59	-0.48	-0.18	-0.98	-0.45	0.00	-1.05	0.48	-0.44	0.55	-0.49
Q9C0C9	Ubiquitin-conjugating enzyme E2 O OS=Homo sapiens GN=UBE2O PE=1 SV=3 - [UBE2O_HUMAN]	13.78	1	13	13	20	1.06	0.87	1.16	1.09	0.06	-0.06	0.66	0.53	0.28	0.40	0.20	0.23	-0.23	-0.58	-0.82	-0.48	-0.83	-0.95	-1.15	-0.49
P38646	Stress-70 protein, mitochondrial OS=Homo sapiens GN=HSPA9 PE=1 SV=2 - [GRP75_HUMAN]	47.57	1	27	27	117	0.35	0.37	0.63	0.54	-0.28	-0.34	0.04	-0.03	-0.24	-0.22	-0.12	-0.09	-0.25	-0.51	-0.67	-0.54	-0.71	-0.69	-0.88	-0.49
P86790	Vacuolar fusion protein CCZ1 homolog B OS=Homo sapiens GN=CCZ1B PE=1 SV=1 - [CCZ1B_HUMAN]	13.28	1	5	5	8	0.42	0.35	0.65	0.60	0.18	0.24	-0.04	0.10	0.19	0.27	0.29	0.32	-0.27	0.01	-0.27	-0.03	-0.35	-0.04	-0.33	-0.49
Q9NQ48	Leucine zipper transcription factor-like protein 1 OS=Homo sapiens GN=LZTFL1 PE=1 SV=1 - [LZTL1_HUMAN]	38.13	1	10	10	18	0.91	1.04	0.95	1.13	-0.11	0.15	0.57	0.61	0.37	0.28	-0.02	0.05	-0.25	-1.02	-1.15	-0.69	-0.81	-0.96	-1.24	-0.49
O14828	Secretory carrier-associated membrane protein 3 OS=Homo sapiens GN=SCAMP3 PE=1 SV=3 - [SCAM3_HUMAN]	24.50	1	5	5	9	0.76	0.40	0.52	0.34	-0.11	-0.48	0.15	-0.09	0.37	0.23	0.67	0.30	-0.55	0.16	0.31	-0.31	-0.01	-0.54	-0.59	-0.49
P68371	Tubulin beta-4B chain OS=Homo sapiens GN=TUBB4B PE=1 SV=1 - [TBB4B_HUMAN]	73.48	4	1	26	975	-0.12	-0.15	0.12	0.46	-0.54	-0.70	-0.43	-0.05	-0.51	-0.65	-0.35	-0.38	-0.19	-0.41	-0.52	-0.47	-0.72	-0.40	-0.70	-0.49

Q8N0X4	Citrate lyase subunit beta-like protein, mitochondrial OS=Homo sapiens GN=CLYBL PE=2 SV=2 - [CLYBL_HUMAN]	20.00	1	5	5	7	-0.88	-0.55	-0.53	-0.56	-1.10	-0.86	-0.94	-0.75	-1.15	-1.27	-1.32	-1.30	-0.15	-0.81	-0.97	-0.40	-0.74	-0.18	-0.31	-0.49
Q92667	A-kinase anchor protein 1, mitochondrial OS=Homo sapiens GN=AKAP1 PE=1 SV=1 - [AKAP1_HUMAN]	7.64	1	5	5	7	-0.33	-0.27	0.08	0.18	-0.81	-0.76	-0.41	-0.35	-0.48	-0.53	0.06	0.11	-0.40	0.33	0.02	-0.17	-0.53	-0.50	-0.87	-0.49
Q96AE4	Far upstream element-binding protein 1 OS=Homo sapiens GN=FUBP1 PE=1 SV=3 - [FUBP1_HUMAN]	20.81	1	9	12	27	0.15	0.16	0.21	0.26	-0.18	-0.19	-0.42	-0.37	-0.41	-0.40	-0.54	-0.54	-0.48	-0.68	-0.54	-0.52	-0.57	-0.42	-0.38	-0.49
Q9Y6N8	Cadherin-10 OS=Homo sapiens GN=CDH10 PE=1 SV=2 - [CAD10_HUMAN]	10.79	1	4	6	8	1.40	1.58	0.79	0.86	-0.29	-0.33	0.20	0.27	0.07	0.06	-0.31	-0.34	-1.15	-1.56	-0.72	-1.27	-0.43	-1.48	-0.75	-0.49
Q92930	Ras-related protein Rab-8B OS=Homo sapiens GN=RAB8B PE=1 SV=2 - [RAB8B_HUMAN]	36.23	6	2	9	34	0.01	0.14	0.21	0.35	-0.39	-0.26	-0.35	-0.21	-0.27	-0.40	-0.47	-0.34	-0.30	-0.47	-0.68	-0.38	-0.58	-0.41	-0.62	-0.49
P10176	Cytochrome c oxidase subunit 8A, mitochondrial OS=Homo sapiens GN=COX8A PE=1 SV=2 - [COX8A_HUMAN]	13.04	1	1	1	3	0.25	0.32	0.23	0.31	0.28	0.35	-0.32	-0.25	0.32	0.25	0.16	0.22	-0.51	-0.09	-0.08	0.03	0.05	0.02	0.03	-0.49
Q9NP90	Ras-related protein Rab-9B OS=Homo sapiens GN=RAB9B PE=1 SV=1 - [RAB9B_HUMAN]	31.84	1	3	5	8	1.03	1.08	0.78	0.50	0.14	-0.28	0.47	0.11	0.06	0.45	0.02	-0.12	-0.80	-0.85	-0.61	-0.70	-0.45	-0.96	-0.71	-0.49

Q8IZR5	CKLF-like MARVEL transmembrane domain-containing protein 4 OS=Homo sapiens GN=CMTM4 PE=1 SV=1 - [CKLF4_HUMAN]	7.26	1	2	2	2	1.22	1.28	1.08	1.13	0.16	0.20	0.53	0.58	0.49	0.45	0.69	0.74	-0.64	-0.53	-0.39	-0.75	-0.60	-1.08	-0.94	-0.49
P55822	SH3 domain-binding glutamic acid-rich protein OS=Homo sapiens GN=SH3BGR PE=1 SV=3 - [SH3BG_HUMAN]	5.44	1	1	1	1	-0.26	-0.35	0.55	0.46	-0.30	-0.39	0.00	-0.09	-0.80	-0.71	-0.70	-0.79	0.31	-0.43	-1.25	-0.42	-1.23	-0.05	-0.87	-0.49
P33316	Deoxyuridine 5'-triphosphate nucleotidohydrolase, mitochondrial OS=Homo sapiens GN=DUT PE=1 SV=4 - [DUT_HUMAN]	40.08	1	7	7	17	-0.15	0.08	-0.03	0.12	-0.83	-0.74	-0.53	-0.51	-0.68	-0.67	-0.56	-0.55	-0.38	-0.08	-0.47	-0.47	-0.73	-0.72	-0.83	-0.49
P42677	40S ribosomal protein S27 OS=Homo sapiens GN=RPS27 PE=1 SV=3 - [RS27_HUMAN]	39.29	1	1	4	11	-0.18	-0.06	0.05	0.18	-0.83	-0.71	-0.50	-0.38	-0.51	-0.62	-0.65	-0.53	-0.27	-0.46	-0.70	-0.41	-0.64	-0.66	-0.89	-0.49
O60232	Sjoegren syndrome/scleroderma autoantigen 1 OS=Homo sapiens GN=SSSCA1 PE=1 SV=1 - [SSA27_HUMAN]	4.02	1	1	1	2	-0.07	-0.01	0.01	0.07	-0.54	-0.48	-0.55	-0.48	-0.47	-0.52	-0.21	-0.15	-0.42	-0.13	-0.22	-0.42	-0.50	-0.48	-0.56	-0.49
Q9Y305	Acyl-coenzyme A thioesterase 9, mitochondrial OS=Homo sapiens GN=ACOT9 PE=1 SV=2 - [ACOT9_HUMAN]	35.31	1	15	15	38	0.25	0.05	0.26	0.26	-0.29	-0.36	-0.16	-0.18	-0.30	-0.25	-0.13	0.03	-0.34	-0.39	-0.40	-0.49	-0.53	-0.54	-0.68	-0.49

Q16816	Phosphorylase b kinase gamma catalytic chain, skeletal muscle/heart isoform OS=Homo sapiens GN=PHKG1 PE=2 SV=3 - [PHKG1_HUMAN]	10.59	1	2	2	4	1.06	1.47	0.93	1.44	-0.12	0.39	0.47	0.88	0.96	0.55	0.39	0.80	-0.54	-0.67	-0.64	-0.48	-0.44	-1.19	-1.16	-0.49
Q9Y3D3	28S ribosomal protein S16, mitochondrial OS=Homo sapiens GN=MRPS16 PE=1 SV=1 - [RT16_HUMAN]	18.98	1	2	2	4	0.18	0.48	0.24	0.54	-0.24	0.05	-0.32	-0.02	-0.19	-0.48	-0.10	0.19	-0.44	-0.28	-0.35	-0.63	-0.69	-0.44	-0.50	-0.49
Q9H1K0	Rabenosyn-5 OS=Homo sapiens GN=ZFYVE20 PE=1 SV=2 - [RBNS5_HUMAN]	9.44	1	6	6	9	0.83	0.90	0.74	0.76	0.10	0.07	0.18	0.21	0.18	0.19	0.25	0.25	-0.72	-0.64	-0.47	-0.68	-0.38	-0.84	-0.66	-0.49
P16870	Carboxypeptidase E OS=Homo sapiens GN=CPE PE=1 SV=1 - [CBPE_HUMAN]	34.87	1	13	13	21	0.77	0.71	0.77	0.74	0.01	-0.18	0.13	0.09	0.17	0.15	0.11	0.06	-0.44	-0.65	-0.82	-0.75	-0.61	-0.85	-0.92	-0.49
Q96P47	Arf-GAP with GTPase, ANK repeat and PH domain-containing protein 3 OS=Homo sapiens GN=AGAP3 PE=1 SV=2 - [AGAP3_HUMAN]	15.31	1	7	9	14	0.78	1.53	0.32	1.07	-0.68	0.07	-0.24	0.22	0.28	-0.44	-1.00	-0.25	-0.96	-1.77	-1.32	-1.19	-0.73	-1.48	-1.02	-0.49
Q96QG7	Myotubularin-related protein 9 OS=Homo sapiens GN=MTMR9 PE=1 SV=1 - [MTMR9_HUMAN]	3.10	1	2	2	6	0.21	0.16	0.27	0.07	-0.05	0.05	-0.33	-0.44	-0.03	-0.05	-0.09	-0.06	-0.53	-0.22	-0.13	-0.15	-0.06	-0.46	-0.41	-0.49
Q08AD1	Calmodulin-regulated spectrin-associated protein 2 OS=Homo sapiens GN=CAMSAP2 PE=1 SV=3 - [CAMP2_HUMAN]	8.53	1	7	7	9	1.10	1.31	0.81	0.93	-0.02	0.15	0.38	0.58	0.67	0.67	0.12	0.32	-0.54	-0.83	-1.06	-0.74	-0.11	-1.17	-0.85	-0.50

Q8ND24	RING finger protein 214 OS=Homo sapiens GN=RNF214 PE=1 SV=2 - [RN214_HUMAN]	7.25	1	5	5	9	0.39	0.41	0.43	0.41	0.06	0.03	-0.20	-0.10	0.00	-0.11	0.04	0.05	-0.55	-0.35	-0.28	-0.48	-0.35	-0.36	-0.30	-0.50
Q8N183	Mimitin, mitochondrial OS=Homo sapiens GN=NDUFAF2 PE=1 SV=1 - [MIMIT_HUMAN]	53.25	1	9	9	15	0.44	0.55	0.66	0.77	-0.22	-0.18	0.12	0.14	-0.11	-0.19	-0.14	-0.11	-0.35	-0.57	-0.85	-0.61	-0.81	-0.59	-0.91	-0.50
Q9NVI7	ATPase family AAA domain-containing protein 3A OS=Homo sapiens GN=ATAD3A PE=1 SV=2 - [ATD3A_HUMAN]	25.55	2	5	14	36	0.39	0.53	0.30	0.32	-0.22	-0.17	-0.31	-0.23	-0.13	-0.20	-0.24	-0.14	-0.52	-0.61	-0.58	-0.55	-0.43	-0.58	-0.59	-0.50
Q86UL8	Membrane-associated guanylate kinase, WW and PDZ domain-containing protein 2 OS=Homo sapiens GN=MAGI2 PE=1 SV=3 - [MAGI2_HUMAN]	13.20	1	13	14	21	0.95	0.67	0.74	0.57	0.39	0.41	0.29	0.19	0.17	0.31	0.36	0.18	-0.54	-0.63	-0.47	-0.63	-0.52	-0.41	0.24	-0.50
O60443	Non-syndromic hearing impairment protein 5 OS=Homo sapiens GN=DFNA5 PE=1 SV=2 - [DFNA5_HUMAN]	8.47	1	2	2	6	0.28	0.33	0.43	0.48	0.33	0.26	-0.13	-0.08	-0.01	0.06	0.12	0.05	-0.36	-0.41	-0.58	-0.45	-0.61	-0.22	-0.38	-0.50
O43148	mRNA cap guanine-N7 methyltransferase OS=Homo sapiens GN=RNMT PE=1 SV=1 - [MCES_HUMAN]	8.40	1	3	3	3	0.54	0.86	0.67	0.99	0.23	0.54	0.11	0.43	0.39	0.08	-0.47	-0.16	-0.38	-1.01	-1.14	-0.43	-0.56	-0.33	-0.46	-0.50
Q96AX2	Ras-related protein Rab-37 OS=Homo sapiens GN=RAB37 PE=1 SV=3 - [RAB37_HUMAN]	17.04	4	2	4	16	1.69	1.12	1.55	0.98	1.66	1.08	0.99	0.41	0.49	1.07	1.37	0.79	-0.65	-0.32	-0.19	-0.60	-0.45	-0.05	0.09	-0.50
Q7Z7L7	Protein zer-1 homolog OS=Homo sapiens GN=ZER1 PE=1 SV=1 - [ZER1_HUMAN]	5.61	1	3	3	6	1.17	0.98	1.02	0.83	-0.06	-0.26	0.46	0.26	-0.21	-0.01	-0.19	-0.38	-0.66	-1.35	-1.20	-1.15	-1.00	-1.24	-1.09	-0.50

O14976	Cyclin-G-associated kinase OS=Homo sapiens GN=GAK PE=1 SV=2 - [GAK_HUMAN]	11.67	1	12	12	20	0.86	0.69	0.87	0.79	0.03	-0.02	0.27	0.31	-0.01	0.17	0.19	0.13	-0.46	-0.55	-0.56	-0.51	-0.68	-0.65	-0.72	-0.50
Q9UMY4	Sorting nexin-12 OS=Homo sapiens GN=SNX12 PE=1 SV=3 - [SNX12_HUMAN]	58.14	1	6	8	15	1.06	1.16	1.08	1.11	0.26	0.36	0.48	0.51	0.25	0.18	-0.35	-0.35	-0.38	-1.29	-1.48	-0.76	-0.90	-0.82	-0.68	-0.50
Q5T3F8	Transmembrane protein 63B OS=Homo sapiens GN=TMEM63B PE=1 SV=1 - [TM63B_HUMAN]	0.96	1	1	1	1	0.94	0.45	0.82	0.34	0.10	-0.39	0.26	-0.23	-0.15	0.34	0.34	-0.15	-0.62	-0.59	-0.48	-0.57	-0.45	-0.86	-0.74	-0.50
P10768	S-formylglutathione hydrolase OS=Homo sapiens GN=ESD PE=1 SV=2 - [ESTD_HUMAN]	37.23	1	7	7	8	0.33	0.34	0.15	0.30	-0.07	0.00	0.04	-0.11	0.07	0.39	-0.03	-0.03	-0.46	-0.57	-1.01	-0.45	-0.54	-0.63	-0.72	-0.50
Q9Y375	Complex I intermediate-associated protein 30, mitochondrial OS=Homo sapiens GN=NDUFAF1 PE=1 SV=2 - [CIA30_HUMAN]	5.81	1	1	1	2	-0.32	-0.25	0.69	0.77	-0.45	-0.39	0.13	0.20	0.00	-0.06	0.21	0.27	0.51	0.53	-0.49	0.29	-0.72	-0.15	-1.17	-0.50
Q5R3I4	Tetratricopeptide repeat protein 38 OS=Homo sapiens GN=TTC38 PE=1 SV=1 - [TTC38_HUMAN]	2.35	1	1	1	1	0.06	0.31	-0.03	0.22	-0.30	-0.06	-0.59	-0.35	0.29	0.05	-0.11	0.13	-0.60	-0.17	-0.08	0.01	0.11	-0.38	-0.29	-0.50
Q16774	Guanylate kinase OS=Homo sapiens GN=GUK1 PE=1 SV=2 - [KGUA_HUMAN]	44.16	1	7	7	23	0.38	0.39	0.57	0.63	0.15	0.20	0.15	0.19	0.27	0.44	0.26	0.23	-0.33	-0.20	-0.50	0.03	-0.10	-0.32	-0.39	-0.50
Q9UPW5	Cytosolic carboxypeptidase 1 OS=Homo sapiens GN=AGTPBP1 PE=1 SV=3 - [CBPC1_HUMAN]	1.22	1	1	1	1	1.09	1.08	0.68	0.67	0.54	0.52	0.12	0.11	-0.10	-0.08	-0.71	-0.73	-0.92	-1.79	-1.40	-1.14	-0.73	-0.57	-0.16	-0.50

Q7RTV0	PHD finger-like domain-containing protein 5A OS=Homo sapiens GN=PHF5A PE=1 SV=1 - [PHF5A_HUMAN]	33.64	1	4	4	5	0.22	0.14	0.21	0.20	-0.72	-0.67	-0.27	-0.28	-0.49	-0.54	-0.72	-0.74	-0.26	-0.84	-0.89	-0.36	-0.61	-0.58	-0.81	-0.50
Q99685	Monoglyceride lipase OS=Homo sapiens GN=MGLL PE=1 SV=2 - [MGLL_HUMAN]	25.41	1	6	6	11	0.25	0.38	0.45	0.47	-0.27	-0.62	0.10	-0.21	-0.04	0.11	-0.28	-0.24	-0.20	-1.16	-0.74	-0.30	-0.55	-0.73	-1.17	-0.50
O15078	Centrosomal protein of 290 kDa OS=Homo sapiens GN=CEP290 PE=1 SV=2 - [CE290_HUMAN]	0.56	2	2	2	3	0.90	1.18	0.90	1.17	-0.09	0.18	0.33	0.61	0.43	0.16	-0.25	0.01	-0.51	-1.15	-1.16	-0.71	-0.71	-1.01	-1.01	-0.50
Q4G176	Acyl-CoA synthetase family member 3, mitochondrial OS=Homo sapiens GN=ACSF3 PE=1 SV=3 - [ACSF3_HUMAN]	14.93	1	8	8	11	0.05	0.15	0.22	0.10	-0.87	-1.00	-0.34	-0.49	-0.65	-0.48	-0.40	-0.56	-0.52	-0.66	-0.65	-0.66	-0.66	-1.06	-1.17	-0.50
O75323	Protein NipSnap homolog 2 OS=Homo sapiens GN=GBAS PE=1 SV=1 - [NIPS2_HUMAN]	27.62	1	7	7	14	0.32	0.30	0.73	0.73	-0.11	-0.27	0.16	0.13	-0.20	-0.08	-0.21	-0.26	-0.18	-0.67	-0.77	-0.50	-0.70	-0.47	-0.79	-0.50
O60503	Adenylate cyclase type 9 OS=Homo sapiens GN=ADCY9 PE=1 SV=4 - [ADCY9_HUMAN]	6.73	1	8	8	13	0.83	0.50	1.05	0.78	0.21	0.21	0.22	0.12	0.01	0.15	0.12	-0.01	-0.36	-0.65	-0.71	-0.50	-0.69	-0.60	-0.64	-0.50
Q9H6V9	UPF0554 protein C2orf43 OS=Homo sapiens GN=C2orf43 PE=1 SV=1 - [CB043_HUMAN]	10.77	1	3	3	3	0.48	0.53	0.37	0.42	0.71	0.76	-0.20	-0.15	0.62	0.58	1.04	1.08	-0.63	0.56	0.66	0.13	0.24	0.21	0.33	-0.51
P00395	Cytochrome c oxidase subunit 1 OS=Homo sapiens GN=MT-CO1 PE=1 SV=1 - [COX1_HUMAN]	7.60	1	2	2	2	0.58	0.84	0.68	0.94	0.12	0.37	0.11	0.37	0.69	0.44	0.04	0.29	-0.42	-0.54	-0.65	-0.12	-0.21	-0.48	-0.58	-0.51

P61020	Ras-related protein Rab-5B OS=Homo sapiens GN=RAB5B PE=1 SV=1 - [RAB5B_HUMAN]	37.21	1	4	6	16	0.66	0.59	0.65	0.63	0.09	-0.11	-0.09	0.12	0.00	0.19	0.10	0.15	-0.38	-0.66	-0.56	-0.56	-0.52	-0.68	-0.75	-0.51
Q9Y676	28S ribosomal protein S18b, mitochondrial OS=Homo sapiens GN=MRPS18B PE=1 SV=1 - [RT18B_HUMAN]	15.89	1	2	2	4	0.07	0.17	0.56	0.66	0.19	0.28	-0.01	0.09	-0.03	-0.13	0.06	0.15	-0.02	0.00	-0.51	-0.16	-0.66	0.10	-0.39	-0.51
Q8NI60	Chaperone activity of bc1 complex-like, mitochondrial OS=Homo sapiens GN=ADCK3 PE=1 SV=1 - [ADCK3_HUMAN]	5.56	1	3	3	4	0.89	0.66	0.53	0.31	-0.17	-0.40	-0.04	-0.27	-0.58	-0.35	-0.31	-0.54	-0.88	-1.19	-0.84	-1.21	-0.85	-1.07	-0.71	-0.51
P52429	Diacylglycerol kinase epsilon OS=Homo sapiens GN=DGKE PE=1 SV=1 - [DGKE_HUMAN]	6.53	1	3	3	5	0.68	0.50	1.15	0.98	0.34	0.16	0.58	0.41	0.20	0.38	0.28	0.10	-0.04	-0.39	-0.88	-0.27	-0.74	-0.35	-0.83	-0.51
Q8N6H7	ADP-ribosylation factor GTPase-activating protein 2 OS=Homo sapiens GN=ARFGAP2 PE=1 SV=1 - [ARFG2_HUMAN]	19.39	1	9	9	15	0.68	0.75	0.65	0.68	0.10	0.04	0.23	0.14	0.16	0.16	0.00	-0.05	-0.55	-0.88	-0.79	-0.61	-0.61	-0.61	-0.61	-0.51
P62820	Ras-related protein Rab-1A OS=Homo sapiens GN=RAB1A PE=1 SV=3 - [RAB1A_HUMAN]	68.78	4	5	13	60	0.51	0.50	0.47	0.50	0.18	0.15	0.07	0.05	-0.06	-0.10	0.11	0.10	-0.30	-0.26	-0.49	-0.11	-0.52	-0.27	-0.36	-0.51
Q9NXW9	Alpha-ketoglutarate-dependent dioxygenase alkB homolog 4 OS=Homo sapiens GN=ALKBH4 PE=1 SV=1 - [ALKB4_HUMAN]	5.63	1	1	1	2	1.54	0.39	1.39	0.24	0.73	-0.42	0.81	-0.33	-1.14	0.01	0.20	-0.95	-0.67	-1.34	-1.19	-1.50	-1.35	-0.83	-0.68	-0.51

Q8N9T8	Protein KRI1 homolog OS=Homo sapiens GN=KRI1 PE=1 SV=3 - [KRI1_HUMAN]	1.85	1	1	1	1	0.72	0.61	0.47	0.35	-0.26	-0.38	-0.11	-0.22	0.07	0.19	0.52	0.40	-0.78	-0.20	0.05	-0.50	-0.24	-1.00	-0.75	-0.51
Q8N163	Cell cycle and apoptosis regulator protein 2 OS=Homo sapiens GN=CCAR2 PE=1 SV=2 - [CCAR2_HUMAN]	21.24	1	14	14	21	0.04	0.07	0.31	0.27	-0.30	-0.34	-0.36	-0.33	-0.15	-0.22	-0.20	-0.18	-0.24	-0.10	-0.43	-0.18	-0.34	-0.32	-0.53	-0.51
Q9BZF3	Oxysterol-binding protein-related protein 6 OS=Homo sapiens GN=OSBPL6 PE=1 SV=1 - [OSBL6_HUMAN]	3.43	1	1	2	3	0.58	0.42	0.72	0.56	0.05	-0.11	0.14	-0.01	0.01	0.17	0.19	0.03	-0.38	-0.38	-0.53	-0.38	-0.51	-0.54	-0.68	-0.51
P30084	Enoyl-CoA hydratase, mitochondrial OS=Homo sapiens GN=ECHS1 PE=1 SV=4 - [ECHM_HUMAN]	63.79	1	18	18	51	0.08	0.15	0.43	0.31	-0.56	-0.53	-0.17	-0.27	-0.42	-0.37	-0.26	-0.29	-0.24	-0.25	-0.68	-0.30	-0.73	-0.85	-0.96	-0.51
P99999	Cytochrome c OS=Homo sapiens GN=CYCS PE=1 SV=2 - [CYC_HUMAN]	57.14	1	7	7	44	0.26	0.61	0.92	1.17	0.03	0.12	0.29	0.47	0.42	0.11	-0.03	0.16	0.00	-0.41	-0.93	-0.26	-0.78	-0.43	-0.99	-0.51
Q96EY7	Pentatricopeptide repeat domain-containing protein 3, mitochondrial OS=Homo sapiens GN=PTCD3 PE=1 SV=3 - [PTCD3_HUMAN]	14.37	1	7	7	9	-0.02	-0.05	0.51	0.33	0.02	-0.12	-0.31	-0.41	-0.10	0.02	0.50	0.32	-0.39	0.37	-0.02	0.09	-0.07	0.02	-0.20	-0.51
Q92574	Hamartin OS=Homo sapiens GN=TSC1 PE=1 SV=2 - [TSC1_HUMAN]	1.46	1	1	1	2	0.68	0.73	0.74	0.80	0.28	0.33	0.17	0.22	-0.01	-0.06	0.08	0.12	-0.46	-0.60	-0.67	-0.71	-0.77	-0.42	-0.48	-0.51
P22061	Protein-L-isoaspartate(D-aspartate) O-methyltransferase OS=Homo sapiens GN=PCMT1 PE=1 SV=4 - [PIMT_HUMAN]	66.08	1	14	14	57	0.46	0.72	0.80	0.96	0.49	0.68	0.15	0.36	0.43	0.23	0.23	0.36	-0.24	-0.28	-0.60	-0.28	-0.40	-0.10	-0.19	-0.51

Q15119	[Pyruvate dehydrogenase (acetyl-transferring)] kinase isozyme 2, mitochondrial OS=Homo sapiens GN=PDK2 PE=1 SV=2 - [PDK2_HUMAN]	21.38	1	5	7	16	0.51	0.52	0.60	0.61	-0.34	-0.26	-0.07	0.03	-0.27	-0.25	-0.22	-0.03	-0.42	-0.48	-0.64	-0.73	-0.84	-0.93	-0.96	-0.51
Q6DKK2	Tetratricopeptide repeat protein 19, mitochondrial OS=Homo sapiens GN=TTC19 PE=1 SV=4 - [TTC19_HUMAN]	20.79	1	7	7	12	0.65	0.45	0.69	0.60	0.28	0.26	-0.01	0.00	0.11	0.21	0.30	0.23	-0.40	-0.22	-0.26	-0.27	-0.26	-0.20	-0.35	-0.51
O75368	SH3 domain-binding glutamic acid-rich-like protein OS=Homo sapiens GN=SH3BGRL PE=1 SV=1 - [SH3L1_HUMAN]	82.46	1	7	7	37	0.38	0.43	0.60	0.56	-0.34	-0.24	0.00	-0.01	-0.02	-0.21	-0.16	-0.13	-0.43	-0.45	-0.64	-0.42	-0.61	-0.65	-0.75	-0.51
Q86T65	Disheveled-associated activator of morphogenesis 2 OS=Homo sapiens GN=DAAM2 PE=2 SV=3 - [DAAM2_HUMAN]	11.99	1	8	11	22	0.81	0.88	0.44	0.52	-0.32	-0.26	-0.14	0.09	0.12	-0.04	-0.04	0.09	-0.87	-0.78	-0.44	-0.75	-0.40	-0.90	-0.56	-0.51
P08247	Synaptophysin OS=Homo sapiens GN=SYP PE=1 SV=3 - [SYPH_HUMAN]	16.93	1	5	6	22	1.63	1.42	1.66	1.62	-0.11	-0.11	0.89	0.78	0.33	0.47	0.41	0.42	-0.73	-1.06	-1.26	-1.01	-0.90	-1.73	-1.67	-0.51
Q8TCB0	Interferon-induced protein 44 OS=Homo sapiens GN=IFI44 PE=2 SV=2 - [IFI44_HUMAN]	1.80	1	1	1	1	0.97	0.65	0.81	0.49	1.37	1.04	0.24	-0.09	1.04	1.37	1.30	0.97	-0.68	0.33	0.48	0.42	0.59	0.37	0.53	-0.51
Q5RKV6	Exosome complex component MTR3 OS=Homo sapiens GN=EXOSC6 PE=1 SV=1 - [EXOS6_HUMAN]	20.22	1	3	3	4	0.54	0.54	-0.16	-0.15	-0.01	-0.01	-0.74	-0.73	-0.31	-0.31	-0.15	-0.15	-1.22	-0.68	0.01	-0.81	-0.12	-0.56	0.13	-0.52

Q15031	Probable leucine--tRNA ligase, mitochondrial OS=Homo sapiens GN=LARS2 PE=1 SV=2 - [SYLM_HUMAN]	8.42	1	6	6	7	0.19	-0.17	0.10	-0.13	-0.65	-0.83	-0.57	-0.75	-0.66	-0.45	-0.61	-0.75	-0.71	-0.62	-0.41	-0.63	-0.49	-0.76	-0.69	-0.52
Q9Y4Z0	U6 snRNA-associated Sm-like protein LSm4 OS=Homo sapiens GN=LSM4 PE=1 SV=1 - [LSM4_HUMAN]	15.83	1	2	2	4	-0.29	-0.22	0.05	0.13	-0.21	-0.14	-0.53	-0.45	-0.50	-0.57	-0.67	-0.60	-0.18	-0.38	-0.73	-0.25	-0.59	0.07	-0.28	-0.52
Q00577	Transcriptional activator protein Pur-alpha OS=Homo sapiens GN=PURA PE=1 SV=2 - [PURA_HUMAN]	34.47	1	9	9	25	0.22	0.31	0.41	0.61	-0.31	-0.24	-0.05	-0.12	-0.29	-0.42	-0.23	-0.38	-0.25	-0.49	-0.79	-0.53	-0.78	-0.77	-1.03	-0.52
Q7Z794	Keratin, type II cytoskeletal 1b OS=Homo sapiens GN=KRT77 PE=2 SV=3 - [K2C1B_HUMAN]	4.84	1	1	3	5	0.38	0.11	1.00	0.73	0.12	-0.15	0.42	0.15	-0.13	0.14	0.24	-0.04	0.09	-0.14	-0.76	-0.21	-0.82	-0.28	-0.90	-0.52
Q9H467	CUE domain-containing protein 2 OS=Homo sapiens GN=CUEDC2 PE=1 SV=1 - [CUED2_HUMAN]	10.45	1	2	2	3	0.83	0.96	0.96	1.10	0.19	0.32	0.38	0.52	0.23	0.10	0.06	0.19	-0.39	-0.77	-0.91	-0.69	-0.83	-0.65	-0.79	-0.52
P35270	Sepiapterin reductase OS=Homo sapiens GN=SPR PE=1 SV=1 - [SPRE_HUMAN]	27.97	1	5	5	7	0.59	0.53	0.89	0.87	0.15	0.09	0.23	0.22	0.03	0.12	-0.24	-0.32	-0.23	-0.70	-1.05	-0.31	-0.65	-0.33	-0.68	-0.52
O43678	NADH dehydrogenase [ubiquinone] 1 alpha subcomplex subunit 2 OS=Homo sapiens GN=NDUFA2 PE=1 SV=3 - [NDUA2_HUMAN]	41.41	1	4	4	19	0.83	0.87	0.94	1.08	-0.01	-0.12	0.38	0.50	0.14	0.27	0.14	0.27	-0.16	-0.77	-0.78	-0.54	-0.85	-1.17	-1.11	-0.52

O60883	Prosaposin receptor GPR37L1 OS=Homo sapiens GN=GPR37L1 PE=1 SV=2 - [ETBR2_HUMAN]	6.24	1	3	3	6	1.68	1.68	1.95	1.95	0.05	0.05	1.32	1.32	0.99	1.00	0.79	0.78	-0.33	-0.41	-1.00	-0.65	-0.66	-0.80	-1.39	-0.52
Q9BTX7	Alpha-tocopherol transfer protein-like OS=Homo sapiens GN=TTPAL PE=2 SV=2 - [TTPAL_HUMAN]	4.97	1	1	1	3	0.92	0.83	1.11	1.01	0.61	0.51	0.52	0.43	0.55	0.65	0.32	0.22	-0.34	-0.60	-0.79	-0.24	-0.42	-0.32	-0.51	-0.52
O00399	Dynactin subunit 6 OS=Homo sapiens GN=DCTN6 PE=1 SV=1 - [DCTN6_HUMAN]	21.58	1	3	3	6	0.18	0.31	0.59	0.53	0.02	-0.11	-0.10	-0.20	-0.16	-0.08	-0.10	-0.07	-0.21	-0.37	-0.70	-0.40	-0.65	-0.18	-0.59	-0.52
Q9UBB9	Tuftelin-interacting protein 11 OS=Homo sapiens GN=TFIP11 PE=1 SV=1 - [TFP11_HUMAN]	8.36	1	3	3	7	-0.95	-0.89	-0.22	-0.17	-0.59	-0.54	-0.81	-0.75	-1.03	-1.08	-0.26	-0.21	0.20	0.70	-0.03	-0.11	-0.83	0.35	-0.38	-0.52
O15240	Neurosecretory protein VGF OS=Homo sapiens GN=VGF PE=1 SV=2 - [VGF_HUMAN]	7.64	1	4	4	7	-0.17	-0.15	-0.08	-0.09	-0.48	-0.43	-0.60	-0.81	-0.81	-0.67	-0.74	-0.81	-0.82	-0.70	-0.67	-0.68	-0.56	-0.40	-0.43	-0.52
P56385	ATP synthase subunit e, mitochondrial OS=Homo sapiens GN=ATP5I PE=1 SV=2 - [ATP5I_HUMAN]	31.88	1	2	2	12	0.83	0.90	1.06	1.08	-0.20	-0.12	0.54	0.59	0.14	-0.06	-0.09	0.02	-0.33	-0.87	-1.15	-0.78	-1.02	-0.86	-1.20	-0.52
P28838	Cytosol aminopeptidase OS=Homo sapiens GN=LAP3 PE=1 SV=3 - [AMPL_HUMAN]	44.89	1	19	19	46	0.65	0.59	0.73	0.74	-0.05	-0.08	0.23	0.10	-0.02	0.02	0.04	-0.07	-0.41	-0.60	-0.95	-0.61	-0.78	-0.62	-0.82	-0.52
Q96PY6	Serine/threonine-protein kinase Nek1 OS=Homo sapiens GN=NEK1 PE=1 SV=2 - [NEK1_HUMAN]	2.46	1	2	2	2	-1.29	-1.51	-0.35	-0.57	-0.13	-0.35	-0.94	-1.15	-0.51	-0.29	-0.38	-0.60	0.41	0.92	-0.03	1.03	0.10	1.15	0.21	-0.52

Q15813	Tubulin-specific chaperone E OS=Homo sapiens GN=TBCE PE=1 SV=1 - [TBCE_HUMAN]	3.98	2	2	2	2	-0.30	0.28	0.38	0.96	-0.17	0.40	-0.21	0.37	0.19	-0.38	-0.23	0.34	0.14	0.07	-0.61	-0.05	-0.72	0.10	-0.57	-0.52
Q99426	Tubulin-folding cofactor B OS=Homo sapiens GN=TBCB PE=1 SV=2 - [TBCB_HUMAN]	42.21	1	9	9	25	1.36	1.44	1.41	1.42	0.13	0.09	0.73	0.77	0.37	0.36	-0.21	-0.14	-0.59	-1.63	-1.48	-1.09	-1.02	-1.23	-1.18	-0.52
O94955	Rho-related BTB domain-containing protein 3 OS=Homo sapiens GN=RHOBTB3 PE=1 SV=2 - [RHBT3_HUMAN]	3.11	1	1	1	2	-0.46	-0.28	0.53	0.72	-0.31	-0.14	-0.05	0.13	-0.08	-0.25	-0.33	-0.15	0.46	0.13	-0.86	0.23	-0.75	0.13	-0.86	-0.52
Q96RP9	Elongation factor G, mitochondrial OS=Homo sapiens GN=GFM1 PE=1 SV=2 - [EFGM_HUMAN]	23.57	1	14	14	18	0.36	0.30	0.47	0.26	-0.47	-0.67	-0.31	-0.35	-0.29	-0.17	-0.19	-0.27	-0.59	-0.54	-0.60	-0.66	-0.46	-0.81	-0.93	-0.52
Q96B45	UPF0693 protein C10orf32 OS=Homo sapiens GN=C10orf32 PE=1 SV=1 - [CJ032_HUMAN]	41.90	1	3	3	6	1.14	0.86	0.47	0.19	0.23	-0.06	-0.12	-0.40	-0.23	0.05	0.04	-0.25	-1.21	-1.10	-0.43	-1.06	-0.38	-0.94	-0.26	-0.52
P53611	Geranylgeranyl transferase type-2 subunit beta OS=Homo sapiens GN=RABGGTB PE=1 SV=2 - [PGTB2_HUMAN]	7.25	1	2	2	3	-0.14	-0.54	0.31	-0.10	-0.67	-1.08	-0.28	-0.68	-0.95	-0.54	-0.37	-0.78	-0.08	-0.23	-0.68	-0.37	-0.82	-0.55	-1.00	-0.52
Q00587	Cdc42 effector protein 1 OS=Homo sapiens GN=CDC42EP1 PE=1 SV=1 - [BORG5_HUMAN]	16.11	1	4	4	6	-0.37	-0.28	-0.06	0.11	-0.46	-0.33	-0.57	-0.41	-0.65	-0.69	-0.27	-0.18	-0.08	0.11	-0.13	-0.29	-0.68	-0.09	-0.54	-0.52
Q14657	EKC/KEOPS complex subunit LAGE3 OS=Homo sapiens GN=LAGE3 PE=1 SV=2 - [LAGE3_HUMAN]	16.08	1	1	1	2	-0.36	0.08	-0.14	0.29	-0.85	-0.42	-0.73	-0.30	-0.35	-0.78	-0.71	-0.28	-0.32	-0.35	-0.57	-0.39	-0.60	-0.51	-0.72	-0.52

P48553	Trafficking protein particle complex subunit 10 OS=Homo sapiens GN=TRAPPC10 PE=1 SV=2 - [TPC10_HUMAN]	6.35	1	6	6	9	0.24	0.36	0.67	0.62	0.00	0.11	0.08	0.20	-0.06	0.02	0.16	0.05	-0.11	-0.30	-0.57	-0.21	-0.59	-0.26	-0.68	-0.53
O15530	3-phosphoinositide-dependent protein kinase 1 OS=Homo sapiens GN=PDPK1 PE=1 SV=1 - [PDPK1_HUMAN]	33.09	2	12	12	21	0.57	0.72	0.85	0.95	0.00	0.14	0.31	0.33	0.29	0.17	0.04	0.19	-0.35	-0.70	-0.89	-0.29	-0.66	-0.74	-1.00	-0.53
Q9Y4H2	Insulin receptor substrate 2 OS=Homo sapiens GN=IRS2 PE=1 SV=2 - [IRS2_HUMAN]	2.91	1	3	3	4	0.15	0.21	-0.16	-0.09	-0.94	-0.88	-0.75	-0.68	-0.17	-0.23	-0.49	-0.43	-0.84	-0.64	-0.34	-0.35	-0.04	-1.11	-0.80	-0.53
O15294	UDP-N-acetylglucosamine--peptide N-acetylglucosaminyltransferase 110 kDa subunit OS=Homo sapiens GN=OGT PE=1 SV=3 - [OGT1_HUMAN]	15.87	1	13	13	18	0.90	0.93	1.09	1.04	0.08	-0.03	0.47	0.36	0.11	0.30	-0.07	-0.10	-0.30	-0.84	-1.08	-0.48	-0.79	-0.75	-1.00	-0.53
Q9UDW1	Cytochrome b-c1 complex subunit 9 OS=Homo sapiens GN=UQCR10 PE=1 SV=3 - [QCR9_HUMAN]	26.98	1	1	1	9	0.51	0.76	0.70	0.92	-0.49	-0.13	0.18	0.14	-0.02	-0.09	-0.41	-0.20	-0.37	-0.73	-1.05	-0.67	-0.76	-0.93	-1.17	-0.53
P53803	DNA-directed RNA polymerases I, II, and III subunit RPABC4 OS=Homo sapiens GN=POLR2K PE=2 SV=1 - [RPAB4_HUMAN]	12.07	1	1	1	1	0.22	0.23	0.21	0.23	0.00	0.01	-0.38	-0.36	-0.03	-0.04	-0.15	-0.14	-0.54	-0.36	-0.36	-0.22	-0.22	-0.23	-0.23	-0.53
Q8TB37	Iron-sulfur protein NUBPL OS=Homo sapiens GN=NUBPL PE=1 SV=3 - [NUBPL_HUMAN]	21.32	1	5	5	7	0.27	0.31	0.19	0.37	-0.50	-0.48	-0.50	-0.58	-0.38	-0.25	-0.59	-0.65	-0.67	-0.93	-0.87	-0.50	-0.84	-0.80	-0.69	-0.53

O95573	Long-chain-fatty-acid--CoA ligase 3 OS=Homo sapiens GN=ACSL3 PE=1 SV=3 - [ACSL3_HUMAN]	29.17	1	14	15	23	0.33	0.29	0.57	0.54	0.07	0.07	-0.01	-0.08	-0.16	-0.03	0.06	0.08	-0.51	-0.50	-0.48	-0.60	-0.53	-0.55	-0.59	-0.53
Q02218	2-oxoglutarate dehydrogenase, mitochondrial OS=Homo sapiens GN=OGDH PE=1 SV=3 - [ODO1_HUMAN]	39.39	1	27	30	82	0.33	0.30	0.52	0.48	-0.46	-0.57	-0.06	-0.09	-0.29	-0.20	-0.12	-0.19	-0.46	-0.55	-0.55	-0.66	-0.56	-0.82	-0.90	-0.53
O60303	Uncharacterized protein KIAA0556 OS=Homo sapiens GN=KIAA0556 PE=1 SV=4 - [K0556_HUMAN]	0.74	1	1	1	1	4.07	4.56	1.77	2.25	0.91	1.39	1.17	1.66	2.23	1.75	0.88	1.37	-2.84	-3.18	-0.88	-2.29	0.01	-3.17	-0.87	-0.53
Q15118	[Pyruvate dehydrogenase (acetyl-transferring)] kinase isozyme 1, mitochondrial OS=Homo sapiens GN=PDK1 PE=1 SV=1 - [PDK1_HUMAN]	8.94	1	2	3	4	0.94	0.85	0.81	0.71	0.38	0.28	0.21	0.12	0.46	0.56	0.28	0.18	-0.67	-0.66	-0.53	-0.35	-0.21	-0.57	-0.44	-0.53
O95782	AP-2 complex subunit alpha-1 OS=Homo sapiens GN=AP2A1 PE=1 SV=3 - [AP2A1_HUMAN]	49.33	1	28	39	106	0.81	0.79	0.88	0.92	0.06	0.13	0.26	0.33	0.11	0.09	0.05	0.15	-0.50	-0.78	-0.93	-0.74	-0.90	-0.79	-0.90	-0.53
Q9Y4W6	AFG3-like protein 2 OS=Homo sapiens GN=AFG3L2 PE=1 SV=2 - [AFG32_HUMAN]	31.37	1	22	23	42	0.65	0.59	0.70	0.61	-0.20	-0.20	0.13	0.09	-0.24	-0.15	-0.29	-0.38	-0.41	-0.86	-0.89	-0.67	-0.79	-0.83	-0.89	-0.53
Q9Y5J7	Mitochondrial import inner membrane translocase subunit Tim9 OS=Homo sapiens GN=TIMM9 PE=1 SV=1 - [TIM9_HUMAN]	25.84	1	2	2	11	0.52	0.55	0.51	0.48	-0.05	0.07	-0.12	-0.03	0.12	-0.03	0.19	0.34	-0.63	-0.32	-0.24	-0.58	-0.50	-0.58	-0.49	-0.53

Q9BV68	E3 ubiquitin-protein ligase RNF126 OS=Homo sapiens GN=RNF126 PE=1 SV=1 - [RN126_HUMAN]	4.29	1	1	1	2	1.43	1.39	0.88	0.84	0.09	0.04	0.29	0.25	-0.15	-0.10	0.21	0.17	-1.09	-1.21	-0.67	-1.50	-0.95	-1.36	-0.81	-0.53
Q9H832	Ubiquitin-conjugating enzyme E2 Z OS=Homo sapiens GN=UBE2Z PE=1 SV=2 - [UBE2Z_HUMAN]	20.06	1	6	6	12	0.91	0.89	0.92	0.78	0.11	0.02	0.30	0.16	0.14	0.25	0.09	-0.05	-0.41	-0.58	-0.70	-0.64	-0.60	-0.78	-0.76	-0.53
Q6P6C2	RNA demethylase ALKBH5 OS=Homo sapiens GN=ALKBH5 PE=1 SV=2 - [ALKB5_HUMAN]	1.78	1	1	1	2	0.68	0.64	0.46	0.43	-0.22	-0.26	-0.13	-0.17	-0.36	-0.32	-0.07	-0.11	-0.76	-0.74	-0.54	-0.97	-0.75	-0.91	-0.70	-0.53
Q6ZT12	E3 ubiquitin-protein ligase UBR3 OS=Homo sapiens GN=UBR3 PE=2 SV=2 - [UBR3_HUMAN]	1.91	1	3	3	3	0.52	0.54	0.53	0.64	0.23	0.59	-0.35	0.04	-0.04	0.01	0.14	0.28	-0.44	-0.25	-0.36	-0.48	-0.48	-0.15	-0.25	-0.53
P23526	Adenosylhomocysteinase OS=Homo sapiens GN=AHCY PE=1 SV=4 - [SAHH_HUMAN]	35.65	1	14	15	35	0.26	0.32	0.54	0.44	-0.33	-0.40	-0.12	-0.08	-0.42	-0.46	-0.52	-0.63	-0.37	-1.09	-1.15	-0.70	-0.92	-0.65	-0.85	-0.53
P52209	6-phosphogluconate dehydrogenase, decarboxylating OS=Homo sapiens GN=PGD PE=1 SV=3 - [6PGD_HUMAN]	41.61	1	16	16	48	0.64	0.61	0.72	0.65	-0.53	-0.56	0.07	0.15	-0.12	-0.16	-0.33	-0.42	-0.40	-0.95	-1.12	-0.78	-0.90	-1.11	-1.01	-0.54
Q14247	Src substrate cortactin OS=Homo sapiens GN=CTTN PE=1 SV=2 - [SRC8_HUMAN]	34.55	1	15	15	33	0.13	0.10	0.48	0.40	-0.27	-0.28	0.01	-0.13	-0.24	-0.13	0.01	-0.04	-0.22	-0.16	-0.43	-0.33	-0.56	-0.54	-0.86	-0.54
O94779	Contactin-5 OS=Homo sapiens GN=CNTN5 PE=1 SV=2 - [CNTN5_HUMAN]	3.55	1	2	2	4	1.16	0.39	1.14	0.36	1.26	0.48	0.54	-0.24	-0.36	0.43	0.76	-0.02	-0.57	-0.40	-0.38	-0.74	-0.48	0.08	0.11	-0.54

O95861	3'(2'),5'-bisphosphate nucleotidase 1 OS=Homo sapiens GN=BPNT1 PE=1 SV=1 - [BPNT1_HUMAN]	30.52	1	7	7	9	0.62	0.64	0.83	0.88	-0.07	0.02	0.39	0.41	-0.04	-0.15	-0.59	-0.45	-0.17	-1.08	-1.37	-0.74	-0.94	-0.63	-0.87	-0.54
Q9NRD5	PRKCA-binding protein OS=Homo sapiens GN=PICK1 PE=1 SV=2 - [PICK1_HUMAN]	14.46	1	5	5	6	0.52	0.56	0.63	0.49	-0.33	-0.29	0.07	0.11	-0.01	0.24	0.16	0.20	-0.39	-0.35	-0.38	-0.24	-0.36	-0.86	-0.80	-0.54
O75116	Rho-associated protein kinase 2 OS=Homo sapiens GN=ROCK2 PE=1 SV=4 - [ROCK2_HUMAN]	32.93	1	31	37	67	0.57	0.65	0.74	0.70	-0.21	-0.22	0.12	0.10	-0.13	-0.12	-0.14	-0.05	-0.48	-0.76	-0.74	-0.65	-0.82	-0.90	-0.97	-0.54
P07311	Acylphosphatase-1 OS=Homo sapiens GN=ACYP1 PE=1 SV=2 - [ACYP1_HUMAN]	39.39	1	4	4	8	0.15	0.30	0.48	0.31	0.27	0.37	-0.14	-0.12	0.01	-0.13	-0.27	-0.24	-0.06	-0.24	-0.69	-0.21	-0.57	0.06	-0.10	-0.54
Q7KZI7	Serine/threonine-protein kinase MARK2 OS=Homo sapiens GN=MARK2 PE=1 SV=2 - [MARK2_HUMAN]	15.61	2	8	11	14	0.67	0.72	0.68	0.62	-0.08	-0.07	0.05	0.03	-0.02	-0.10	-0.10	-0.09	-0.69	-0.98	-0.92	-0.83	-0.86	-0.99	-0.91	-0.54
Q14232	Translation initiation factor eIF-2B subunit alpha OS=Homo sapiens GN=EIF2B1 PE=1 SV=1 - [EI2BA_HUMAN]	14.75	1	4	4	6	-0.23	-0.34	0.03	-0.08	0.29	0.17	-0.10	-0.22	-0.25	-0.21	-0.52	-0.08	-0.26	-0.28	-0.55	0.12	-0.13	0.50	0.24	-0.54
Q9UBQ7	Glyoxylate reductase/hydroxypyruvate reductase OS=Homo sapiens GN=GRHPR PE=1 SV=1 - [GRHPR_HUMAN]	41.16	1	10	10	17	0.30	0.23	0.43	0.45	-0.05	0.03	-0.24	-0.09	0.08	-0.02	-0.26	-0.20	-0.36	-0.43	-0.52	-0.40	-0.40	-0.46	-0.51	-0.54
P08574	Cytochrome c1, heme protein, mitochondrial OS=Homo sapiens GN=CYC1 PE=1 SV=3 - [CY1_HUMAN]	36.92	1	8	8	86	1.02	1.05	1.04	1.02	-0.04	-0.04	0.45	0.43	0.18	0.28	0.07	0.01	-0.52	-0.92	-0.96	-0.86	-0.66	-1.15	-1.07	-0.54

P31948	Stress-induced-phosphoprotein 1 OS=Homo sapiens GN=STIP1 PE=1 SV=1 - [STIP1_HUMAN]	53.22	1	31	31	80	0.23	0.24	0.49	0.43	-0.50	-0.46	-0.11	-0.10	-0.29	-0.34	-0.25	-0.22	-0.30	-0.51	-0.69	-0.55	-0.70	-0.74	-0.93	-0.54
O95822	Malonyl-CoA decarboxylase, mitochondrial OS=Homo sapiens GN=MLYCD PE=1 SV=3 - [DCMC_HUMAN]	4.46	1	2	2	2	0.77	0.87	0.75	0.85	0.34	0.43	0.15	0.25	0.08	-0.01	0.22	0.31	-0.57	-0.55	-0.53	-0.75	-0.73	-0.45	-0.43	-0.54
Q9UQ13	Leucine-rich repeat protein SHOC-2 OS=Homo sapiens GN=SHOC2 PE=1 SV=2 - [SHOC2_HUMAN]	3.44	1	1	1	1	-0.01	0.16	0.53	0.70	-0.14	0.03	-0.07	0.10	-0.04	-0.21	-0.48	-0.32	0.00	-0.47	-1.02	-0.17	-0.71	-0.14	-0.69	-0.54
Q8NFD5	AT-rich interactive domain-containing protein 1B OS=Homo sapiens GN=ARID1B PE=1 SV=2 - [ARI1B_HUMAN]	2.59	1	4	4	5	0.05	0.60	0.61	0.39	0.56	0.46	0.01	-0.35	-0.62	0.01	-0.08	-0.57	0.02	-0.12	-0.69	-1.19	-0.98	0.50	-0.06	-0.54
P52757	Beta-chimaerin OS=Homo sapiens GN=CHN2 PE=1 SV=2 - [CHIO_HUMAN]	3.63	1	1	1	1	1.11	1.08	1.11	1.07	0.64	0.60	0.51	0.47	0.25	0.30	-0.02	-0.06	-0.55	-1.12	-1.13	-0.79	-0.78	-0.48	-0.48	-0.54
P52797	Ephrin-A3 OS=Homo sapiens GN=EFNA3 PE=1 SV=1 - [EFNA3_HUMAN]	6.72	1	1	1	1	1.47	0.96	1.25	0.75	1.23	0.72	0.64	0.14	-0.03	0.48	0.02	-0.48	-0.77	-1.44	-1.23	-0.96	-0.74	-0.26	-0.04	-0.54
Q12791	Calcium-activated potassium channel subunit alpha 1 OS=Homo sapiens GN=KCNMA1 PE=1 SV=2 - [KCMA1_HUMAN]	6.39	1	5	5	10	0.72	0.69	0.31	0.28	-0.17	-0.21	-0.44	-0.45	-0.33	-0.29	0.10	0.06	-0.74	-0.35	-0.19	-0.67	-0.48	-0.66	-0.47	-0.54

Q9NX14	NADH dehydrogenase [ubiquinone] 1 beta subcomplex subunit 11, mitochondrial OS=Homo sapiens GN=NDUFB11 PE=1 SV=1 - [NDUBB_HUMAN]	33.33	1	3	3	10	0.73	0.92	1.05	1.00	0.08	-0.08	0.46	0.47	0.27	0.25	0.54	0.29	-0.38	-0.42	-0.74	-0.47	-0.78	-0.66	-0.94	-0.54
P25685	DnaJ homolog subfamily B member 1 OS=Homo sapiens GN=DNAJB1 PE=1 SV=4 - [DNJB1_HUMAN]	33.82	1	11	12	22	0.49	0.56	0.71	0.90	-0.02	0.00	0.18	0.24	0.16	0.06	0.14	0.21	-0.18	-0.37	-0.49	-0.40	-0.75	-0.58	-0.64	-0.54
Q8NCA5	Protein FAM98A OS=Homo sapiens GN=FAM98A PE=1 SV=1 - [FA98A_HUMAN]	10.60	1	3	4	10	0.00	0.07	0.21	0.24	-0.08	-0.15	-0.32	-0.29	-0.29	-0.30	-0.21	-0.23	-0.26	-0.14	-0.41	-0.29	-0.48	-0.05	-0.31	-0.54
Q6IQ20	N-acyl-phosphatidylethanolamine hydrolyzing phospholipase D OS=Homo sapiens GN=NAPEPLD PE=1 SV=2 - [NAPEP_HUMAN]	6.36	1	2	2	2	0.99	1.07	1.16	1.24	-0.03	0.04	0.55	0.63	0.19	0.11	-0.22	-0.14	-0.38	-1.20	-1.37	-0.85	-1.01	-1.04	-1.21	-0.54
Q96GX9	Methylthioribulose-1-phosphate dehydratase OS=Homo sapiens GN=APIP PE=1 SV=1 - [MTNB_HUMAN]	12.81	1	2	2	3	0.57	0.39	1.01	0.84	0.32	0.14	0.41	0.23	0.30	0.48	-0.06	-0.24	-0.10	-0.62	-1.08	-0.06	-0.50	-0.26	-0.71	-0.54
Q16795	NADH dehydrogenase [ubiquinone] 1 alpha subcomplex subunit 9, mitochondrial OS=Homo sapiens GN=NDUFA9 PE=1 SV=2 - [NDUA9_HUMAN]	29.71	1	10	10	31	0.57	0.65	0.86	0.84	-0.13	-0.20	0.25	0.18	-0.03	0.14	-0.04	-0.02	-0.48	-0.81	-0.92	-0.68	-0.68	-1.05	-1.24	-0.54

P29966	Myristoylated alanine-rich C-kinase substrate OS=Homo sapiens GN=MARCKS PE=1 SV=4 - [MARCS_HUMAN]	22.89	1	4	4	31	0.66	0.66	1.13	1.09	-0.31	-0.34	0.49	0.43	0.00	0.08	-0.12	-0.21	-0.08	-0.81	-1.26	-0.57	-1.03	-0.93	-1.52	-0.54
Q92845	Kinesin-associated protein 3 OS=Homo sapiens GN=KIFAP3 PE=1 SV=2 - [KIFA3_HUMAN]	8.71	1	4	4	5	0.68	0.64	1.01	0.97	0.03	0.08	0.22	0.36	0.11	-0.04	0.00	0.03	-0.22	-0.53	-0.87	-0.63	-0.82	-0.57	-0.90	-0.54
Q5TC12	ATP synthase mitochondrial F1 complex assembly factor 1 OS=Homo sapiens GN=ATPAF1 PE=1 SV=1 - [ATPF1_HUMAN]	7.32	1	2	2	2	0.46	0.40	0.81	0.75	0.47	0.41	0.20	0.15	-0.17	-0.11	0.03	-0.03	-0.20	-0.42	-0.78	-0.54	-0.89	0.00	-0.35	-0.54
Q6P1Q0	LETM1 domain-containing protein 1 OS=Homo sapiens GN=LETMD1 PE=1 SV=1 - [LTMD1_HUMAN]	10.28	1	3	3	6	0.83	0.67	0.68	0.58	0.07	-0.08	0.27	0.17	-0.02	0.05	0.11	0.07	-0.55	-0.69	-0.70	-0.76	-0.76	-0.87	-0.88	-0.54
O15514	DNA-directed RNA polymerase II subunit RPB4 OS=Homo sapiens GN=POLR2D PE=1 SV=1 - [RPB4_HUMAN]	13.38	1	1	1	1	-1.91	-1.70	-0.57	-0.35	-0.03	0.18	-1.18	-0.96	0.21	0.00	0.08	0.29	0.79	2.00	0.65	1.94	0.60	1.87	0.52	-0.54
Q9NS86	LanC-like protein 2 OS=Homo sapiens GN=LANCL2 PE=1 SV=1 - [LANC2_HUMAN]	25.78	1	9	9	22	0.85	1.04	0.72	0.88	0.03	0.00	0.52	0.44	0.09	0.24	0.19	0.13	-0.51	-0.80	-0.80	-0.75	-0.64	-1.03	-0.98	-0.54
Q13491	Neuronal membrane glycoprotein M6-b OS=Homo sapiens GN=GPM6B PE=1 SV=2 - [GPM6B_HUMAN]	24.53	1	4	5	19	0.86	0.75	1.00	1.02	0.02	0.18	0.32	0.34	0.45	0.37	0.33	0.56	-0.36	-0.60	-0.73	-0.44	-0.62	-1.11	-1.09	-0.54

Q9Y2G3	Probable phospholipid-transporting ATPase IF OS=Homo sapiens GN=ATP11B PE=1 SV=2 - [AT11B_HUMAN]	1.53	2	1	2	2	-0.18	-0.70	-0.63	-1.15	-0.79	-1.31	-1.24	-1.76	-0.94	-0.42	-0.40	-0.92	-1.00	-0.22	0.23	-0.21	0.24	-0.62	-0.17	-0.55
Q96BY7	Autophagy-related protein 2 homolog B OS=Homo sapiens GN=ATG2B PE=1 SV=5 - [ATG2B_HUMAN]	3.18	1	4	4	4	0.88	0.93	0.68	0.45	-0.07	-0.20	0.08	-0.05	-0.12	0.01	-0.25	-0.26	-0.75	-1.05	-0.93	-0.83	-0.64	-0.96	-0.77	-0.55
P12236	ADP/ATP translocase 3 OS=Homo sapiens GN=SLC25A6 PE=1 SV=4 - [ADT3_HUMAN]	50.34	2	3	16	86	0.43	0.37	0.81	0.79	-0.42	-0.48	0.35	0.30	-0.31	-0.20	-0.29	-0.40	-0.04	-0.58	-0.96	-0.63	-0.95	-0.81	-1.13	-0.55
P19022	Cadherin-2 OS=Homo sapiens GN=CDH2 PE=1 SV=4 - [CADH2_HUMAN]	28.26	1	14	15	49	1.05	1.05	1.05	0.97	0.15	0.02	0.41	0.41	0.11	0.27	0.26	0.20	-0.60	-0.77	-0.81	-0.84	-0.82	-1.23	-1.13	-0.55
P61088	Ubiquitin-conjugating enzyme E2 N OS=Homo sapiens GN=UBE2N PE=1 SV=1 - [UBE2N_HUMAN]	46.05	2	7	7	24	0.12	0.37	0.44	0.59	-0.08	0.08	-0.11	0.05	-0.06	-0.22	-0.31	-0.01	-0.27	-0.47	-0.87	-0.51	-0.90	-0.42	-0.61	-0.55
Q8IVT5	Kinase suppressor of Ras 1 OS=Homo sapiens GN=KSR1 PE=1 SV=2 - [KSR1_HUMAN]	2.82	1	2	2	3	1.00	0.67	0.70	0.37	0.13	-0.20	0.09	-0.24	-0.17	0.16	0.26	-0.07	-0.86	-0.74	-0.44	-0.81	-0.50	-0.89	-0.58	-0.55
Q8WUW1	Protein BRICK1 OS=Homo sapiens GN=BRK1 PE=1 SV=1 - [BRK1_HUMAN]	36.00	1	3	3	11	0.41	0.47	0.61	0.63	0.16	-0.03	0.03	0.00	0.14	0.20	0.13	-0.05	-0.36	-0.34	-0.50	-0.18	-0.43	-0.32	-0.75	-0.55
Q9BZ72	Membrane-associated phosphatidylinositol transfer protein 2 OS=Homo sapiens GN=PITPNM2 PE=1 SV=1 - [PITM2_HUMAN]	3.71	1	3	3	3	1.02	1.17	0.52	0.67	0.19	0.34	0.28	0.43	0.26	0.11	0.55	0.69	-1.01	-0.47	0.02	-1.02	-0.24	-0.84	-0.35	-0.55

Q13433	Zinc transporter ZIP6 OS=Homo sapiens GN=SLC39A6 PE=1 SV=3 - [S39A6_HUMAN]	1.59	1	1	1	1	1.39	1.30	1.09	1.00	0.46	0.36	0.47	0.38	0.50	0.59	0.56	0.46	-0.86	-0.83	-0.53	-0.77	-0.46	-0.95	-0.64	-0.55
P21926	CD9 antigen OS=Homo sapiens GN=CD9 PE=1 SV=4 - [CD9_HUMAN]	12.72	1	5	5	14	0.28	0.40	0.70	0.81	0.59	0.61	0.20	0.24	0.23	0.16	0.03	0.11	-0.04	-0.24	-0.73	-0.14	-0.62	0.06	-0.37	-0.55
Q01968	Inositol polyphosphate 5-phosphatase OCRL-1 OS=Homo sapiens GN=OCRL PE=1 SV=3 - [OCRL_HUMAN]	8.10	1	8	8	13	1.18	0.97	1.17	1.06	0.36	0.29	0.66	0.53	0.31	0.38	0.35	0.26	-0.54	-0.94	-0.89	-0.76	-0.73	-0.73	-0.77	-0.55
P09417	Dihydropteridine reductase OS=Homo sapiens GN=QDPR PE=1 SV=2 - [DHPR_HUMAN]	48.36	1	8	8	62	0.64	1.02	0.82	0.85	0.32	0.47	0.26	0.26	0.40	0.35	0.27	0.31	-0.68	-0.79	-0.81	-0.51	-0.63	-0.48	-0.42	-0.55
Q6GYQ0	Ral GTPase-activating protein subunit alpha 1 OS=Homo sapiens GN=RALGAPA1 PE=1 SV=1 - [RGPA1_HUMAN]	5.11	1	8	8	14	-0.20	0.04	0.28	0.52	-0.43	0.09	-0.34	-0.10	-0.22	-0.55	-0.27	0.20	-0.08	-0.06	-0.33	-0.31	-0.48	-0.27	-0.45	-0.55
Q9UMZ2	Synergin gamma OS=Homo sapiens GN=SYNRG PE=1 SV=2 - [SYNRG_HUMAN]	5.56	1	5	5	6	0.44	0.47	0.87	0.90	0.27	0.15	0.26	0.26	0.16	-0.10	0.47	0.27	-0.13	-0.80	-0.87	-0.60	-0.85	-0.33	-0.76	-0.55
Q15257	Serine/threonine-protein phosphatase 2A activator OS=Homo sapiens GN=PPP2R4 PE=1 SV=3 - [PTPA_HUMAN]	28.77	1	6	6	12	1.28	1.41	1.50	1.74	-0.35	-0.20	0.77	0.91	0.42	0.26	-0.25	-0.22	-0.46	-1.27	-1.23	-0.96	-1.24	-1.58	-1.53	-0.55
Q99683	Mitogen-activated protein kinase kinase kinase 5 OS=Homo sapiens GN=MAP3K5 PE=1 SV=1 - [M3K5_HUMAN]	3.49	1	2	4	5	0.25	0.75	0.41	0.92	0.27	0.77	-0.20	0.30	0.88	0.39	0.28	0.51	-0.39	-0.29	-0.14	0.17	0.01	0.01	-0.16	-0.55

Q15811	Intersectin-1 OS=Homo sapiens GN=ITSN1 PE=1 SV=3 - [ITSN1_HUMAN]	18.94	1	24	27	52	0.79	0.78	0.90	1.01	0.17	0.12	0.33	0.27	0.05	0.13	-0.02	-0.03	-0.40	-0.66	-0.87	-0.63	-0.70	-0.70	-0.69	-0.55
P42025	Beta-centractin OS=Homo sapiens GN=ACTR1B PE=1 SV=1 - [ACTY_HUMAN]	26.60	1	4	7	23	1.62	1.66	1.94	1.87	0.75	0.97	1.31	1.19	1.22	0.92	0.79	0.97	-0.35	-0.64	-0.82	-0.65	-0.95	-0.82	-0.97	-0.55
P60953	Cell division control protein 42 homolog OS=Homo sapiens GN=CDC42 PE=1 SV=2 - [CDC42_HUMAN]	31.41	2	4	5	20	0.16	0.27	0.72	0.70	-0.30	-0.30	0.07	0.06	-0.05	-0.15	-0.14	-0.13	0.02	-0.25	-0.85	-0.25	-0.63	-0.38	-0.94	-0.55
Q9NX00	Transmembrane protein 160 OS=Homo sapiens GN=TMEM160 PE=1 SV=1 - [TM160_HUMAN]	10.64	1	2	2	3	1.25	1.16	0.62	0.53	-0.10	-0.19	0.00	-0.09	-0.13	-0.04	0.14	0.04	-1.19	-1.11	-0.48	-1.26	-0.62	-1.37	-0.74	-0.55
Q9H3Q1	Cdc42 effector protein 4 OS=Homo sapiens GN=CDC42EP4 PE=1 SV=1 - [BORG4_HUMAN]	31.74	1	7	7	18	1.09	0.82	1.16	0.96	0.01	-0.12	0.47	-0.26	0.01	0.14	0.41	0.06	-0.54	-0.82	-0.98	-0.65	-0.73	-1.01	-1.18	-0.55
Q9Y3Y2	Chromatin target of PRMT1 protein OS=Homo sapiens GN=CHTOP PE=1 SV=2 - [CHTOP_HUMAN]	29.03	1	5	5	13	-0.30	-0.36	-0.18	-0.40	-0.77	-0.76	-0.79	-0.85	-0.95	-0.89	-0.31	-0.57	-0.47	-0.03	-0.10	-0.34	-0.55	-0.49	-0.44	-0.55
P03928	ATP synthase protein 8 OS=Homo sapiens GN=MT-ATP8 PE=1 SV=1 - [ATP8_HUMAN]	32.35	1	3	3	9	0.46	0.56	0.66	0.62	-0.14	-0.22	0.04	0.26	-0.29	-0.17	-0.42	-0.39	-0.41	-0.92	-1.10	-0.50	-0.79	-0.82	-0.76	-0.55
O75947	ATP synthase subunit d, mitochondrial OS=Homo sapiens GN=ATP5H PE=1 SV=3 - [ATP5H_HUMAN]	80.12	1	11	11	39	0.69	0.51	0.98	0.85	0.01	-0.06	0.36	0.20	-0.06	-0.04	0.12	-0.02	-0.36	-0.57	-0.80	-0.64	-0.89	-0.72	-0.90	-0.55

Q8N490	Probable hydrolase PNKD OS=Homo sapiens GN=PNKD PE=1 SV=2 - [PNKD_HUMAN]	12.21	1	3	3	5	1.10	1.31	0.82	1.03	-0.27	-0.07	0.21	0.41	0.04	-0.15	-0.04	0.15	-0.84	-1.14	-0.87	-1.23	-0.94	-1.39	-1.11	-0.55
Q7Z4V5	Hepatoma-derived growth factor-related protein 2 OS=Homo sapiens GN=HDGFRP2 PE=1 SV=1 - [HDGR2_HUMAN]	5.37	1	2	3	7	0.14	0.08	0.39	0.28	-0.21	-0.26	0.09	-0.02	-0.31	-0.25	-0.09	-0.28	-0.08	-0.19	-0.79	-0.33	-0.65	-0.36	-0.68	-0.55
Q9NRX5	Serine incorporator 1 OS=Homo sapiens GN=SERINC1 PE=1 SV=1 - [SERC1_HUMAN]	3.75	1	1	1	2	0.79	1.03	0.85	1.10	0.17	0.41	0.24	0.48	0.90	0.66	0.53	0.77	-0.50	-0.25	-0.32	-0.09	-0.15	-0.63	-0.70	-0.55
Q9UJC5	SH3 domain-binding glutamic acid-rich-like protein 2 OS=Homo sapiens GN=SH3BGRL2 PE=1 SV=2 - [SH3L2_HUMAN]	59.81	1	5	5	31	0.39	0.47	0.74	0.83	-0.29	-0.19	0.01	0.06	-0.05	-0.04	-0.02	0.03	-0.25	-0.44	-0.70	-0.38	-0.56	-0.69	-1.01	-0.55
Q6P1M0	Long-chain fatty acid transport protein 4 OS=Homo sapiens GN=SLC27A4 PE=1 SV=1 - [S27A4_HUMAN]	18.97	1	9	9	14	1.30	0.93	0.92	0.97	0.05	0.04	0.36	0.10	-0.03	0.09	0.11	0.04	-0.78	-1.03	-0.89	-0.92	-0.84	-1.01	-1.01	-0.55
P11233	Ras-related protein Ral-A OS=Homo sapiens GN=RALA PE=1 SV=1 - [RALA_HUMAN]	50.00	1	4	7	20	0.62	0.79	0.96	0.96	-0.51	-0.17	0.23	0.35	0.18	0.07	-0.09	-0.03	-0.25	-0.74	-0.98	-0.61	-1.07	-1.39	-1.14	-0.55
Q96S19	UPF0585 protein C16orf13 OS=Homo sapiens GN=C16orf13 PE=1 SV=2 - [CP013_HUMAN]	36.27	1	6	6	9	0.68	0.84	0.87	0.83	0.51	0.56	0.26	0.24	0.25	0.18	0.10	-0.03	-0.51	-0.76	-0.64	-0.73	-0.68	-0.43	-0.27	-0.55

Q16630	Cleavage and polyadenylation specificity factor subunit 6 OS=Homo sapiens GN=CPSF6 PE=1 SV=2 - [CPSF6_HUMAN]	15.43	1	8	8	11	-0.11	-0.12	0.34	0.21	-0.14	-0.23	-0.41	-0.58	-0.19	-0.14	-0.03	-0.18	-0.34	0.10	-0.35	-0.15	-0.32	0.11	-0.26	-0.55
P25054	Adenomatous polyposis coli protein OS=Homo sapiens GN=APC PE=1 SV=2 - [APC_HUMAN]	2.60	1	5	5	8	0.52	0.83	0.53	0.67	0.26	0.45	-0.13	-0.38	0.04	-0.40	-0.15	0.14	-0.50	-0.59	-0.68	-0.71	-0.79	-0.68	-0.69	-0.55
O15067	Phosphoribosylformylglycinamidine synthase OS=Homo sapiens GN=PFAS PE=1 SV=4 - [PUR4_HUMAN]	6.28	1	7	7	9	0.80	0.65	0.58	0.62	-0.31	-0.27	-0.06	-0.02	0.01	-0.03	-0.38	-0.35	-0.70	-1.13	-1.08	-0.91	-0.69	-1.14	-1.03	-0.56
P27987	Inositol-trisphosphate 3-kinase B OS=Homo sapiens GN=ITPKB PE=1 SV=5 - [IP3KB_HUMAN]	8.14	1	5	6	8	0.98	0.49	0.87	0.38	0.07	-0.26	0.17	-0.23	-0.16	0.21	0.07	-0.28	-0.66	-0.91	-0.81	-0.72	-0.63	-1.11	-1.01	-0.56
P49915	GMP synthase [glutamine-hydrolyzing] OS=Homo sapiens GN=GMPS PE=1 SV=1 - [GUAA_HUMAN]	25.40	1	13	13	22	0.29	0.49	0.59	0.47	-0.22	-0.15	-0.04	-0.05	-0.33	-0.30	-0.50	-0.54	-0.51	-0.96	-1.13	-0.50	-0.80	-0.66	-0.54	-0.56
O14733	Dual specificity mitogen-activated protein kinase kinase 7 OS=Homo sapiens GN=MAP2K7 PE=1 SV=2 - [MP2K7_HUMAN]	2.63	1	1	1	2	0.45	0.68	0.22	0.45	-0.53	-0.31	-0.40	-0.17	-0.17	-0.39	-0.32	-0.10	-0.80	-0.77	-0.54	-0.82	-0.58	-1.00	-0.77	-0.56
P46926	Glucosamine-6-phosphate isomerase 1 OS=Homo sapiens GN=GNPDA1 PE=1 SV=1 - [GNPI1_HUMAN]	32.18	1	4	6	13	0.90	1.21	0.83	0.94	0.09	0.08	0.09	0.29	0.25	0.03	-0.16	-0.12	-0.97	-1.28	-1.13	-0.88	-0.83	-1.21	-1.06	-0.56

Q8TBC4	NEDD8-activating enzyme E1 catalytic subunit OS=Homo sapiens GN=UBA3 PE=1 SV=2 - [UBA3_HUMAN]	31.32	1	10	10	18	0.92	0.83	0.77	0.96	0.07	0.01	0.20	0.34	0.13	0.09	-0.06	0.01	-0.49	-0.85	-0.86	-0.83	-0.82	-0.83	-0.78	-0.56
P53041	Serine/threonine-protein phosphatase 5 OS=Homo sapiens GN=PPP5C PE=1 SV=1 - [PPP5_HUMAN]	37.07	1	13	13	43	0.80	0.82	0.74	0.69	0.14	0.07	0.14	0.13	0.10	0.10	-0.17	-0.04	-0.59	-0.76	-0.87	-0.50	-0.51	-0.54	-0.67	-0.56
Q9Y3F4	Serine-threonine kinase receptor-associated protein OS=Homo sapiens GN=STRAP PE=1 SV=1 - [STRAP_HUMAN]	45.71	1	11	11	23	0.29	0.29	0.73	0.73	-0.12	-0.10	0.07	0.09	-0.17	-0.18	-0.13	-0.15	-0.19	-0.44	-0.86	-0.58	-0.91	-0.43	-0.85	-0.56
Q8N335	Glycerol-3-phosphate dehydrogenase 1-like protein OS=Homo sapiens GN=GPD1L PE=1 SV=1 - [GPD1L_HUMAN]	36.47	1	13	13	40	0.70	0.71	0.87	0.91	-0.09	-0.03	0.25	0.30	-0.01	-0.01	-0.17	-0.14	-0.38	-0.87	-1.06	-0.71	-0.87	-0.76	-0.98	-0.56
Q04725	Transducin-like enhancer protein 2 OS=Homo sapiens GN=TLE2 PE=1 SV=2 - [TLE2_HUMAN]	4.17	4	2	2	2	0.43	0.45	0.41	0.43	-0.21	-0.20	-0.22	-0.20	-0.15	-0.16	-0.04	-0.03	-0.59	-0.47	-0.45	-0.56	-0.54	-0.66	-0.64	-0.56
O60783	28S ribosomal protein S14, mitochondrial OS=Homo sapiens GN=MRPS14 PE=1 SV=1 - [RT14_HUMAN]	11.72	1	1	1	2	0.57	0.45	0.46	0.35	-0.04	-0.16	-0.16	-0.28	-0.12	0.00	0.23	0.11	-0.68	-0.33	-0.24	-0.54	-0.43	-0.62	-0.52	-0.56
O15075	Serine/threonine-protein kinase DCLK1 OS=Homo sapiens GN=DCLK1 PE=1 SV=2 - [DCLK1_HUMAN]	40.00	2	20	21	57	0.69	0.73	0.80	0.94	0.02	0.02	0.19	0.31	0.17	0.13	-0.13	-0.02	-0.37	-0.78	-1.08	-0.57	-0.88	-0.66	-0.88	-0.56

P62068	Ubiquitin carboxyl-terminal hydrolase 46 OS=Homo sapiens GN=USP46 PE=1 SV=1 - [UBP46_HUMAN]	8.47	2	2	2	2	0.33	-0.82	1.25	-0.17	1.13	-0.52	0.42	-0.79	-0.74	0.88	1.36	-0.28	0.08	0.55	-0.11	0.12	-0.53	0.78	-0.13	-0.56
P48730	Casein kinase I isoform delta OS=Homo sapiens GN=CSNK1D PE=1 SV=2 - [KC1D_HUMAN]	14.70	1	2	6	10	1.32	1.27	1.17	1.12	0.26	0.20	0.54	0.49	0.59	0.65	0.21	0.15	-0.72	-1.10	-0.96	-0.64	-0.49	-1.08	-0.93	-0.56
P31321	cAMP-dependent protein kinase type I-beta regulatory subunit OS=Homo sapiens GN=PRKAR1B PE=1 SV=4 - [KAP1_HUMAN]	8.14	1	2	4	5	0.71	0.79	0.75	0.83	0.18	0.25	0.12	0.20	0.26	0.18	-0.04	0.04	-0.53	-0.74	-0.78	-0.50	-0.53	-0.55	-0.59	-0.56
Q969T9	WW domain-binding protein 2 OS=Homo sapiens GN=WBP2 PE=1 SV=1 - [WBP2_HUMAN]	33.33	1	7	7	21	0.49	0.57	0.44	0.54	-0.06	-0.14	-0.11	-0.10	-0.12	-0.14	-0.28	-0.20	-0.50	-0.69	-0.80	-0.44	-0.64	-0.50	-0.63	-0.56
O43676	NADH dehydrogenase [ubiquinone] 1 beta subcomplex subunit 3 OS=Homo sapiens GN=NDUFB3 PE=1 SV=3 - [NDUB3_HUMAN]	28.57	1	3	3	7	0.89	0.85	1.01	0.95	-0.18	-0.15	0.40	0.32	0.00	0.19	0.13	0.03	-0.48	-0.95	-1.05	-0.80	-0.87	-1.27	-1.37	-0.57
Q70E73	Ras-associated and pleckstrin homology domains-containing protein 1 OS=Homo sapiens GN=RAPH1 PE=1 SV=3 - [RAPH1_HUMAN]	2.88	1	2	2	2	0.22	0.40	0.47	0.64	0.01	0.18	-0.16	0.01	0.01	-0.16	-0.04	0.13	-0.33	-0.26	-0.51	-0.36	-0.59	-0.23	-0.48	-0.57
Q96RU3	Formin-binding protein 1 OS=Homo sapiens GN=FNBP1 PE=1 SV=2 - [FNBP1_HUMAN]	12.64	1	8	8	9	0.24	0.06	0.26	0.08	-0.03	-0.16	-0.25	-0.25	-0.24	-0.20	-0.21	-0.28	-0.59	-0.49	-0.52	-0.41	-0.43	-0.44	-0.46	-0.57

O14734	Acyl-coenzyme A thioesterase 8 OS=Homo sapiens GN=ACOT8 PE=1 SV=1 - [ACOT8_HUMAN]	8.78	1	2	2	3	0.58	1.10	0.38	0.89	0.01	0.52	-0.25	0.26	0.65	-0.07	-1.05	-0.33	-0.78	-1.74	-1.60	-0.73	-0.59	-0.59	-0.39	-0.57
Q6PIL6	Kv channel-interacting protein 4 OS=Homo sapiens GN=KCNIP4 PE=1 SV=1 - [KCIP4_HUMAN]	30.80	1	5	5	7	1.35	1.94	0.74	1.33	-0.58	-0.21	0.12	0.62	0.31	-0.18	-0.22	0.23	-1.10	-1.29	-1.25	-1.51	-1.42	-1.55	-0.94	-0.57
P55265	Double-stranded RNA-specific adenosine deaminase OS=Homo sapiens GN=ADAR PE=1 SV=4 - [DSRAD_HUMAN]	17.05	1	19	19	35	0.32	0.35	0.51	0.61	-0.11	-0.02	-0.10	-0.05	0.00	0.05	-0.09	-0.09	-0.54	-0.51	-0.68	-0.45	-0.57	-0.51	-0.69	-0.57
Q9UHV9	Prefoldin subunit 2 OS=Homo sapiens GN=PFDN2 PE=1 SV=1 - [PFD2_HUMAN]	48.70	1	6	6	16	0.78	0.87	0.80	0.81	0.04	0.05	0.15	0.21	-0.07	-0.15	-0.17	-0.06	-0.53	-0.65	-0.94	-0.56	-0.86	-0.58	-0.86	-0.57
Q9BQE5	Apolipoprotein L2 OS=Homo sapiens GN=APOL2 PE=1 SV=1 - [APOL2_HUMAN]	19.88	1	7	7	14	0.91	1.10	0.65	0.85	0.68	0.85	0.05	0.29	0.50	0.27	0.02	0.23	-0.76	-0.75	-0.66	-0.57	-0.34	-0.26	-0.15	-0.57
O75251	NADH dehydrogenase [ubiquinone] iron-sulfur protein 7, mitochondrial OS=Homo sapiens GN=NDUFS7 PE=1 SV=3 - [NDUS7_HUMAN]	18.78	1	5	5	9	0.80	0.88	0.98	0.95	-0.33	-0.41	0.34	0.29	-0.06	-0.06	-0.14	-0.23	-0.66	-1.22	-1.13	-0.86	-1.01	-1.15	-1.33	-0.57
Q01082	Spectrin beta chain, non-erythrocytic 1 OS=Homo sapiens GN=SPTBN1 PE=1 SV=2 - [SPTB2_HUMAN]	66.16	1	122	136	825	0.64	0.71	0.78	0.83	-0.18	-0.13	0.15	0.19	0.02	-0.03	0.06	0.08	-0.49	-0.61	-0.73	-0.65	-0.78	-0.83	-0.96	-0.57

O00562	Membrane-associated phosphatidylinositol transfer protein 1 OS=Homo sapiens GN=PITPNM1 PE=1 SV=4 - [PITM1_HUMAN]	6.59	1	5	5	6	1.22	0.93	0.80	0.39	0.92	0.34	0.23	-0.32	0.00	0.11	0.34	0.07	-0.82	-1.05	-0.51	-0.96	-0.48	-0.61	-0.07	-0.57
Q16143	Beta-synuclein OS=Homo sapiens GN=SNCB PE=1 SV=1 - [SYUB_HUMAN]	51.49	1	3	7	29	0.92	0.11	1.36	0.65	0.58	0.42	0.45	0.21	-0.25	0.09	0.12	-0.01	-0.09	-0.32	-0.75	-0.30	-0.64	0.29	-0.13	-0.57
Q9NY12	H/ACA ribonucleoprotein complex subunit 1 OS=Homo sapiens GN=GAR1 PE=1 SV=1 - [GAR1_HUMAN]	21.66	1	3	3	5	0.13	0.06	0.42	0.35	0.23	0.16	-0.21	-0.28	-0.23	-0.15	0.44	0.36	-0.29	0.31	0.01	-0.25	-0.54	0.09	-0.21	-0.57
O14957	Cytochrome b-c1 complex subunit 10 OS=Homo sapiens GN=UQCR11 PE=2 SV=1 - [QCR10_HUMAN]	21.43	1	1	1	2	0.29	0.17	0.78	0.67	0.17	0.06	0.15	0.04	-0.02	0.10	0.10	-0.02	-0.08	-0.18	-0.68	-0.16	-0.65	-0.13	-0.63	-0.57
P27544	Ceramide synthase 1 OS=Homo sapiens GN=CERS1 PE=1 SV=1 - [CERS1_HUMAN]	10.00	1	3	3	5	1.56	1.31	1.52	1.24	-0.28	-0.02	0.93	0.60	0.34	0.43	0.44	0.28	-0.66	-1.03	-0.96	-0.94	-0.86	-1.35	-1.27	-0.57
Q7Z7K0	COX assembly mitochondrial protein homolog OS=Homo sapiens GN=CMC1 PE=1 SV=1 - [COXM1_HUMAN]	25.47	1	2	2	3	0.07	0.01	0.13	0.07	-0.19	-0.25	-0.51	-0.56	-1.03	-0.97	-0.32	-0.06	-0.52	-0.96	-0.47	-1.01	-1.07	-0.27	-0.33	-0.57
P10253	Lysosomal alpha-glucosidase OS=Homo sapiens GN=GAA PE=1 SV=4 - [LYAG_HUMAN]	14.08	1	9	9	29	0.94	0.68	0.84	0.88	0.34	0.29	0.36	0.21	0.32	0.26	0.42	0.45	-0.53	-0.54	-0.50	-0.37	-0.42	-0.52	-0.67	-0.57

Q9NY47	Voltage-dependent calcium channel subunit alpha 2/delta-2 OS=Homo sapiens GN=CACNA2D2 PE=1 SV=2 - [CA2D2_HUMAN]	10.00	1	10	11	20	0.80	0.87	0.79	0.81	0.14	0.26	0.26	0.25	0.26	0.26	0.21	0.29	-0.63	-0.60	-0.62	-0.66	-0.69	-0.63	-0.68	-0.57
Q86TW2	Uncharacterized aarF domain-containing protein kinase 1 OS=Homo sapiens GN=ADCK1 PE=2 SV=2 - [ADCK1_HUMAN]	6.98	1	2	2	2	-0.07	0.59	0.20	0.86	-0.01	0.64	-0.44	0.22	0.56	-0.09	-0.27	0.38	-0.32	-0.20	-0.47	0.01	-0.26	0.04	-0.23	-0.57
P54289	Voltage-dependent calcium channel subunit alpha 2/delta-1 OS=Homo sapiens GN=CACNA2D1 PE=1 SV=3 - [CA2D1_HUMAN]	34.27	1	30	31	73	0.63	0.62	0.72	0.73	0.13	0.12	0.11	0.09	0.06	0.02	0.08	0.13	-0.48	-0.47	-0.53	-0.55	-0.59	-0.49	-0.52	-0.57
Q13564	NEDD8-activating enzyme E1 regulatory subunit OS=Homo sapiens GN=NAE1 PE=1 SV=1 - [ULA1_HUMAN]	10.30	1	4	4	6	0.47	0.41	0.98	0.93	-0.17	-0.22	0.29	0.24	-0.06	-0.01	-0.25	-0.32	-0.11	-0.71	-1.09	-0.43	-0.95	-0.65	-1.16	-0.58
Q13151	Heterogeneous nuclear ribonucleoprotein A0 OS=Homo sapiens GN=HNRNPA0 PE=1 SV=1 - [ROA0_HUMAN]	30.49	1	5	7	15	0.26	0.76	0.70	0.60	0.24	-0.05	0.06	-0.15	-0.26	0.14	0.20	-0.08	-0.15	-0.65	-0.80	-0.58	-0.82	-0.35	-0.67	-0.58
O43150	Arf-GAP with SH3 domain, ANK repeat and PH domain-containing protein 2 OS=Homo sapiens GN=ASAP2 PE=1 SV=3 - [ASAP2_HUMAN]	8.85	1	6	6	11	0.44	0.54	0.35	0.41	-0.20	-0.37	-0.29	-0.16	-0.35	-0.40	-0.48	-0.31	-0.67	-0.81	-0.83	-0.74	-0.65	-0.44	-0.87	-0.58

Q14914	Prostaglandin reductase 1 OS=Homo sapiens GN=PTGR1 PE=1 SV=2 - [PTGR1_HUMAN]	29.79	1	8	8	16	0.58	0.57	0.91	1.02	-0.36	-0.36	0.22	0.32	-0.13	-0.19	-0.91	-0.85	-0.13	-1.36	-1.68	-0.62	-1.07	-0.75	-1.28	-0.58
Q9BQ04	RNA-binding protein 4B OS=Homo sapiens GN=RBM4B PE=1 SV=1 - [RBM4B_HUMAN]	22.01	1	1	7	13	0.34	0.52	0.32	0.51	-0.23	-0.05	-0.32	-0.13	-0.33	-0.51	0.08	0.26	-0.60	-0.25	-0.24	-0.82	-0.80	-0.59	-0.57	-0.58
Q6V1X1	Dipeptidyl peptidase 8 OS=Homo sapiens GN=DPP8 PE=1 SV=1 - [DPP8_HUMAN]	8.24	1	3	4	5	0.28	0.43	0.48	0.63	-0.42	-0.28	-0.16	-0.02	-0.18	-0.32	-0.09	0.04	-0.39	-0.37	-0.58	-0.57	-0.77	-0.72	-0.92	-0.58
Q99828	Calcium and integrin-binding protein 1 OS=Homo sapiens GN=CIB1 PE=1 SV=4 - [CIB1_HUMAN]	6.28	1	1	1	2	0.80	0.88	0.69	0.77	-0.35	-0.28	0.05	0.13	0.25	0.18	0.50	0.57	-0.69	-0.30	-0.19	-0.59	-0.48	-1.16	-1.06	-0.58
P52888	Thimet oligopeptidase OS=Homo sapiens GN=THOP1 PE=1 SV=2 - [THOP1_HUMAN]	13.50	1	8	8	14	1.37	1.18	1.14	1.07	0.49	0.42	0.43	0.38	0.27	0.37	-0.02	-0.09	-0.88	-1.25	-1.10	-0.97	-0.76	-0.82	-0.67	-0.58
Q04837	Single-stranded DNA-binding protein, mitochondrial OS=Homo sapiens GN=SSBP1 PE=1 SV=1 - [SSBP_HUMAN]	24.32	1	4	4	9	-0.07	0.08	0.29	0.31	-0.09	-0.09	-0.37	-0.35	-0.39	-0.31	-0.23	-0.18	-0.22	-0.17	-0.49	-0.21	-0.57	0.04	-0.33	-0.58
P12955	Xaa-Pro dipeptidase OS=Homo sapiens GN=PEPD PE=1 SV=3 - [PEPD_HUMAN]	23.33	1	9	9	21	1.18	0.90	0.90	0.83	-0.05	-0.03	0.43	0.22	0.12	0.15	-0.38	-0.36	-0.62	-1.28	-1.18	-0.88	-0.90	-0.88	-0.84	-0.58
P62857	40S ribosomal protein S28 OS=Homo sapiens GN=RPS28 PE=1 SV=1 - [RS28_HUMAN]	30.43	1	2	2	11	-0.23	-0.12	-0.25	-0.11	-0.88	-0.70	-0.92	-0.76	-0.61	-0.78	-0.67	-0.59	-0.72	-0.35	-0.32	-0.45	-0.49	-0.66	-0.77	-0.58

Q8WYK1	Contactin-associated protein-like 5 OS=Homo sapiens GN=CNTNAP5 PE=2 SV=1 - [CNTP5_HUMAN]	3.68	1	4	4	7	1.39	1.28	1.33	1.22	0.94	0.77	0.40	0.30	0.46	0.64	0.35	0.24	-0.59	-0.96	-1.09	-0.53	-0.46	-0.26	-0.26	-0.58
Q9NRV9	Heme-binding protein 1 OS=Homo sapiens GN=HEBP1 PE=1 SV=1 - [HEBP1_HUMAN]	40.74	1	6	6	14	0.66	0.53	0.88	0.82	0.07	-0.05	0.24	0.10	-0.14	-0.02	-0.30	-0.44	-0.32	-0.99	-1.34	-0.76	-1.06	-0.69	-1.02	-0.58
P61018	Ras-related protein Rab-4B OS=Homo sapiens GN=RAB4B PE=1 SV=1 - [RAB4B_HUMAN]	41.78	4	3	7	23	1.36	1.26	1.27	1.17	0.39	0.29	0.62	0.53	0.35	0.45	0.38	0.28	-0.68	-0.97	-0.89	-0.87	-0.78	-0.98	-0.89	-0.58
O75348	V-type proton ATPase subunit G 1 OS=Homo sapiens GN=ATP6V1G1 PE=1 SV=3 - [VATG1_HUMAN]	27.12	1	2	2	3	0.64	0.99	0.70	1.05	0.31	0.66	0.05	0.40	0.60	0.26	0.54	0.88	-0.53	-0.09	-0.16	-0.35	-0.41	-0.34	-0.40	-0.58
Q96EY1	DnaJ homolog subfamily A member 3, mitochondrial OS=Homo sapiens GN=DNAJA3 PE=1 SV=2 - [DNJA3_HUMAN]	11.67	1	4	4	9	0.95	0.95	1.02	1.00	-0.15	-0.11	0.39	0.33	0.14	0.19	0.15	0.09	-0.52	-0.81	-0.88	-0.73	-0.80	-1.08	-1.13	-0.58
Q71RG4	Transmembrane and ubiquitin-like domain-containing protein 2 OS=Homo sapiens GN=TMUB2 PE=2 SV=2 - [TMUB2_HUMAN]	8.72	1	1	1	1	-0.09	-0.14	0.04	-0.02	-0.92	-0.98	-0.61	-0.66	-0.07	-0.01	0.75	0.70	-0.47	0.85	0.72	0.10	-0.01	-0.85	-0.98	-0.58
Q9HCU4	Cadherin EGF LAG seven-pass G-type receptor 2 OS=Homo sapiens GN=CELSR2 PE=1 SV=1 - [CELR2_HUMAN]	3.11	1	5	5	6	0.91	0.93	0.64	0.66	0.08	-0.09	-0.12	0.02	-0.40	-0.09	-0.25	-0.43	-0.86	-0.89	-0.80	-0.76	-0.56	-0.83	-0.73	-0.58

O43865	Putative adenosylhomocysteinase 2 OS=Homo sapiens GN=AHCYL1 PE=1 SV=2 - [SAHH2_HUMAN]	40.57	2	10	24	69	0.95	0.86	0.91	0.95	0.34	0.20	0.31	0.30	0.26	0.44	0.33	0.21	-0.50	-0.71	-0.58	-0.65	-0.64	-0.72	-0.74	-0.58
Q13561	Dynactin subunit 2 OS=Homo sapiens GN=DCTN2 PE=1 SV=4 - [DCTN2_HUMAN]	50.87	1	18	18	45	0.64	0.77	0.80	0.87	0.02	0.07	0.24	0.25	0.22	0.12	0.30	0.35	-0.44	-0.34	-0.54	-0.51	-0.67	-0.69	-0.91	-0.58
P19623	Spermidine synthase OS=Homo sapiens GN=SRM PE=1 SV=1 - [SPEE_HUMAN]	14.57	1	4	4	6	0.68	0.54	0.75	1.04	0.06	-0.22	-0.01	0.28	0.14	0.16	-0.42	-0.44	-0.35	-0.91	-1.42	-0.45	-0.95	-0.77	-1.22	-0.58
Q13825	Methylglutaconyl-CoA hydratase, mitochondrial OS=Homo sapiens GN=AUH PE=1 SV=1 - [AUHM_HUMAN]	25.66	1	8	8	25	0.29	0.27	0.66	0.65	-0.34	-0.33	0.09	0.14	-0.23	-0.22	-0.08	0.10	-0.17	-0.47	-0.77	-0.46	-0.94	-0.62	-1.09	-0.58
Q9HCY8	Protein S100-A14 OS=Homo sapiens GN=S100A14 PE=1 SV=1 - [S10AE_HUMAN]	8.65	1	1	1	1	-1.47	-1.37	-1.27	-1.17	-2.02	-1.93	-1.91	-1.82	-1.83	-1.92	-1.79	-1.70	-0.39	-0.32	-0.52	-0.42	-0.61	-0.57	-0.77	-0.58
P56556	NADH dehydrogenase [ubiquinone] 1 alpha subcomplex subunit 6 OS=Homo sapiens GN=NDUFA6 PE=1 SV=3 - [NDUA6_HUMAN]	32.47	1	4	4	9	0.44	0.40	0.98	0.91	-0.30	-0.35	0.37	0.26	-0.37	-0.25	-0.13	-0.04	-0.13	-0.75	-1.19	-0.62	-1.31	-0.54	-1.23	-0.58
Q9P265	Disco-interacting protein 2 homolog B OS=Homo sapiens GN=DIP2B PE=1 SV=3 - [DIP2B_HUMAN]	15.42	1	16	18	28	0.47	0.48	0.58	0.57	-0.29	-0.09	-0.20	-0.05	-0.18	-0.25	-0.23	-0.11	-0.56	-0.70	-0.69	-0.59	-0.63	-0.63	-0.62	-0.58
Q15819	Ubiquitin-conjugating enzyme E2 variant 2 OS=Homo sapiens GN=UBE2V2 PE=1 SV=4 - [UB2V2_HUMAN]	64.14	1	3	8	55	0.71	0.59	0.55	0.52	0.24	0.10	-0.01	-0.13	-0.17	-0.03	-0.27	-0.31	-0.60	-0.88	-0.86	-0.89	-0.86	-0.38	-0.37	-0.58

P42356	Phosphatidylinositol 4-kinase alpha OS=Homo sapiens GN=PI4KA PE=1 SV=3 - [PI4KA_HUMAN]	11.40	3	23	23	45	0.90	0.78	0.93	0.86	-0.20	-0.10	0.32	0.21	0.13	0.09	-0.13	-0.20	-0.59	-1.19	-1.12	-0.96	-0.83	-1.14	-1.12	-0.59
P19338	Nucleolin OS=Homo sapiens GN=NCL PE=1 SV=3 - [NUCL_HUMAN]	28.31	1	21	21	50	0.10	-0.01	0.49	0.30	-0.28	-0.33	-0.27	-0.43	-0.42	-0.31	-0.03	-0.10	-0.21	-0.09	-0.46	-0.27	-0.66	-0.33	-0.66	-0.59
Q9H9A6	Leucine-rich repeat-containing protein 40 OS=Homo sapiens GN=LRRC40 PE=1 SV=1 - [LRC40_HUMAN]	19.60	1	10	10	14	0.79	0.77	0.68	0.78	0.08	0.11	0.30	0.45	0.19	0.00	0.01	0.06	-0.50	-0.73	-0.65	-0.63	-0.49	-0.67	-0.60	-0.59
P05141	ADP/ATP translocase 2 OS=Homo sapiens GN=SLC25A5 PE=1 SV=7 - [ADT2_HUMAN]	47.65	2	4	15	79	0.81	0.67	0.85	0.75	-0.31	-0.39	0.20	0.17	-0.08	-0.11	-0.32	-0.36	-0.44	-1.03	-1.17	-0.71	-0.81	-1.08	-1.18	-0.59
Q12931	Heat shock protein 75 kDa, mitochondrial OS=Homo sapiens GN=TRAP1 PE=1 SV=3 - [TRAP1_HUMAN]	30.40	1	16	17	46	0.62	0.60	0.66	0.63	-0.49	-0.64	-0.03	-0.12	-0.25	-0.29	-0.20	-0.23	-0.52	-0.85	-0.93	-0.84	-0.88	-1.19	-1.20	-0.59
P55285	Cadherin-6 OS=Homo sapiens GN=CDH6 PE=1 SV=1 - [CADH6_HUMAN]	6.33	1	2	3	5	0.19	0.26	0.20	0.27	-0.29	-0.23	-0.45	-0.38	-0.36	-0.43	-0.07	-0.01	-0.59	-0.26	-0.27	-0.58	-0.59	-0.50	-0.51	-0.59
Q86VP3	Phosphofurin acidic cluster sorting protein 2 OS=Homo sapiens GN=PACS2 PE=1 SV=3 - [PACS2_HUMAN]	4.95	1	3	3	4	1.10	0.90	0.73	0.65	-0.02	0.12	0.10	0.15	0.18	0.30	0.06	0.12	-0.91	-1.02	-0.66	-0.59	-0.46	-0.97	-0.60	-0.59
O14561	Acyl carrier protein, mitochondrial OS=Homo sapiens GN=NDUFAB1 PE=1 SV=3 - [ACPM_HUMAN]	15.38	1	3	3	7	0.64	0.70	0.98	1.17	0.01	0.17	0.41	0.64	0.46	0.31	0.01	0.30	-0.17	-0.33	-0.78	-0.33	-0.79	-0.45	-0.88	-0.59

O95168	NADH dehydrogena se [ubiquinone] 1 beta subcomplex subunit 4 OS=Homo sapiens GN=NDUFB 4 PE=1 SV=3 - [NDUB4_HU MAN]	41.09	1	5	5	16	0.64	0.67	0.66	0.79	-0.25	-0.29	-0.01	0.15	-0.20	-0.18	-0.16	-0.29	-0.44	-0.75	-0.96	-0.76	-0.87	-0.96	-1.08	-0.59
P43243	Matrin-3 OS=Homo sapiens GN=MATR3 PE=1 SV=2 - [MATR3_HU MAN]	22.43	1	14	14	42	0.30	0.25	0.36	0.24	-0.08	-0.25	-0.26	-0.31	-0.15	-0.03	-0.10	-0.14	-0.55	-0.39	-0.50	-0.42	-0.45	-0.51	-0.62	-0.59
Q9NQR4	Omega-amidase NIT2 OS=Homo sapiens GN=NIT2 PE=1 SV=1 - [NIT2_HUMA N]	36.59	1	8	8	12	0.10	0.21	0.21	0.32	0.11	-0.17	-0.27	-0.27	-0.24	-0.31	-0.58	-0.56	-0.42	-0.80	-0.95	-0.44	-0.66	-0.29	-0.42	-0.59
P48169	Gamma-aminobutyric acid receptor subunit alpha 4 OS=Homo sapiens GN=GABRA 4 PE=2 SV=2 - [GBRA4_HU MAN]	6.86	1	3	3	3	0.03	0.17	0.20	0.35	-0.33	-0.19	-0.45	-0.31	0.22	0.08	-0.51	-0.37	-0.42	-0.53	-0.71	0.09	-0.09	-0.37	-0.55	-0.59
Q66LE6	Serine/threon ine-protein phosphatase 2A 55 kDa regulatory subunit B delta isoform OS=Homo sapiens GN=PPP2R2 D PE=2 SV=1 - [2ABD_HUM AN]	21.85	1	4	8	20	0.58	0.86	0.70	0.78	-0.29	-0.22	0.06	0.11	0.10	0.07	-0.23	-0.08	-0.67	-0.96	-0.90	-0.67	-0.68	-1.00	-0.95	-0.59
Q2TAL8	Glutamine-rich protein 1 OS=Homo sapiens GN=QRICH1 PE=1 SV=1 - [QRIC1_HU MAN]	3.87	1	3	3	5	0.26	-0.35	-0.11	-0.10	0.09	-0.48	-0.23	-0.40	-0.32	-0.08	0.24	0.20	-0.35	0.56	0.17	-0.49	-0.28	0.03	0.06	-0.59
Q96QR8	Transcription al activator protein Pur-beta OS=Homo sapiens GN=PURB PE=1 SV=3 - [PURB_HUM AN]	20.83	1	5	5	7	0.26	0.28	0.34	0.37	-0.33	-0.32	-0.31	-0.29	-0.50	-0.51	-0.37	-0.36	-0.52	-0.63	-0.72	-0.74	-0.82	-0.61	-0.70	-0.59

Q00688	Peptidyl-prolyl cis-trans isomerase FKBP3 OS=Homo sapiens GN=FKBP3 PE=1 SV=1 - [FKBP3_HUMAN]	21.43	1	5	5	9	-0.22	-0.27	0.05	0.00	-0.32	-0.38	-0.71	-0.76	-0.89	-0.83	-0.33	-0.43	-0.22	-0.12	-0.26	-0.46	-0.81	-0.12	-0.50	-0.59
Q00978	Interferon regulatory factor 9 OS=Homo sapiens GN=IRF9 PE=1 SV=1 - [IRF9_HUMAN]	7.38	1	3	3	4	0.42	0.78	0.19	0.55	0.19	0.54	-0.46	-0.11	0.55	0.19	0.11	0.46	-0.83	-0.31	-0.09	-0.20	0.03	-0.25	-0.02	-0.59
O60359	Voltage-dependent calcium channel gamma-3 subunit OS=Homo sapiens GN=CACNG3 PE=2 SV=1 - [CCG3_HUMAN]	13.02	1	4	4	7	3.41	2.25	2.46	1.24	1.11	0.52	2.11	0.58	0.03	0.62	1.33	0.75	-1.61	-2.08	-1.13	-2.76	-1.81	-2.32	-1.37	-0.59
P14406	Cytochrome c oxidase subunit 7A2, mitochondrial OS=Homo sapiens GN=COX7A2 PE=1 SV=1 - [CX7A2_HUMAN]	56.63	1	3	3	10	0.70	0.89	0.87	1.12	-0.18	-0.02	0.18	0.52	0.18	0.06	-0.17	0.07	-0.33	-0.86	-1.05	-0.64	-0.78	-0.89	-1.08	-0.60
Q9Y6M4	Casein kinase I isoform gamma-3 OS=Homo sapiens GN=CSNK1G3 PE=1 SV=2 - [KC1G3_HUMAN]	8.50	2	1	3	4	0.30	0.42	0.03	0.16	0.36	0.48	-0.63	-0.50	-0.33	-0.45	0.14	0.26	-0.87	-0.15	0.11	-0.72	-0.45	0.05	0.31	-0.60
P39687	Acidic leucine-rich nuclear phosphoprotein 32 family member A OS=Homo sapiens GN=ANP32A PE=1 SV=1 - [AN32A_HUMAN]	26.10	3	3	8	25	0.32	0.47	0.42	0.49	-0.22	-0.13	-0.23	-0.13	-0.18	-0.27	-0.21	-0.12	-0.68	-0.65	-0.63	-0.75	-0.69	-0.64	-0.57	-0.60
Q86V48	Leucine zipper protein 1 OS=Homo sapiens GN=LUZP1 PE=1 SV=2 - [LUZP1_HUMAN]	14.22	1	13	13	17	0.51	0.64	0.48	0.56	0.06	-0.04	0.03	-0.29	-0.24	-0.02	-0.11	0.02	-0.46	-0.62	-0.59	-0.66	-0.57	-0.74	-0.73	-0.60

P59190	Ras-related protein Rab-15 OS=Homo sapiens GN=RAB15 PE=1 SV=1 - [RAB15_HUMAN]	15.09	1	1	3	21	0.45	-0.51	0.70	-0.26	1.57	0.61	0.04	-0.92	0.32	1.28	0.63	-0.33	-0.35	0.19	-0.07	0.86	0.61	1.11	0.86	-0.60
Q9H8T0	AKT-interacting protein OS=Homo sapiens GN=AKTIP PE=1 SV=1 - [AKTIP_HUMAN]	7.53	1	1	1	2	-0.11	-0.33	0.45	0.23	-0.48	-0.70	-0.21	-0.43	-0.62	-0.40	-0.62	-0.84	-0.05	-0.50	-1.07	-0.26	-0.82	-0.38	-0.94	-0.60
Q9NZL4	Hsp70-binding protein 1 OS=Homo sapiens GN=HSPBP1 PE=1 SV=1 - [HPBP1_HUMAN]	6.91	1	3	3	5	1.03	0.86	0.80	0.64	-0.12	-0.28	0.14	-0.02	0.01	0.18	-0.04	-0.21	-0.83	-1.06	-0.84	-0.82	-0.59	-1.16	-0.93	-0.60
O95298	NADH dehydrogenase [ubiquinone] 1 subunit C2 OS=Homo sapiens GN=NDUFC2 PE=1 SV=1 - [NDUC2_HUMAN]	30.25	2	5	5	13	0.49	0.45	1.03	1.05	-0.33	-0.35	0.38	0.31	0.02	0.04	0.20	0.01	-0.07	-0.38	-0.91	-0.50	-1.01	-0.91	-1.34	-0.60
O75146	Huntingtin-interacting protein 1-related protein OS=Homo sapiens GN=HIP1R PE=1 SV=2 - [HIP1R_HUMAN]	20.51	1	16	17	29	0.84	0.86	0.84	0.79	0.21	0.15	0.22	0.27	0.20	0.19	0.04	0.04	-0.47	-0.70	-0.83	-0.67	-0.61	-0.72	-0.74	-0.60
P61586	Transforming protein RhoA OS=Homo sapiens GN=RHOA PE=1 SV=1 - [RHOA_HUMAN]	60.62	1	2	8	53	0.26	0.47	0.81	1.02	-0.34	-0.13	0.15	0.36	0.23	0.02	0.02	0.23	-0.06	-0.23	-0.79	-0.21	-0.76	-0.61	-1.17	-0.60
Q8WV93	Lactation elevated protein 1 OS=Homo sapiens GN=LACE1 PE=2 SV=2 - [LACE1_HUMAN]	8.11	1	3	3	4	0.13	-0.13	0.38	-0.53	-0.88	-1.34	-0.43	-1.20	-0.83	-0.76	-0.79	-1.24	-1.01	-0.91	-1.16	-0.66	-0.26	-1.03	-1.28	-0.60
O00116	Alkyldihydroxyacetonephosphate synthase, peroxisomal OS=Homo sapiens GN=AGPS PE=1 SV=1 - [ADAS_HUMAN]	22.64	1	9	9	15	0.63	0.40	0.66	0.52	-0.23	-0.40	0.07	-0.16	-0.22	-0.05	0.26	0.19	-0.44	-0.18	-0.30	-0.49	-0.68	-0.72	-0.91	-0.60

Q08495	Dematin OS=Homo sapiens GN=DMTN PE=1 SV=3 - [DEMA_HUMAN]	50.12	1	16	16	49	0.71	0.68	0.80	0.79	0.02	0.05	0.23	0.29	0.20	0.20	0.41	0.43	-0.49	-0.35	-0.40	-0.41	-0.65	-0.70	-1.00	-0.60
P31150	Rab GDP dissociation inhibitor alpha OS=Homo sapiens GN=GDI1 PE=1 SV=2 - [GDIA_HUMAN]	60.40	1	14	22	113	0.96	1.08	1.19	1.31	0.30	0.27	0.42	0.57	0.34	0.28	0.20	0.13	-0.49	-0.90	-0.96	-0.69	-0.87	-0.67	-0.89	-0.60
Q8TDN4	CDK5 and ABL1 enzyme substrate 1 OS=Homo sapiens GN=CABLES1 PE=1 SV=2 - [CABL1_HUMAN]	2.53	1	1	1	2	1.23	1.01	1.35	1.14	0.17	-0.05	0.69	0.47	0.30	0.52	-0.23	-0.45	-0.48	-1.45	-1.58	-0.68	-0.80	-1.08	-1.20	-0.60
Q9P0J0	NADH dehydrogenase [ubiquinone] 1 alpha subcomplex subunit 13 OS=Homo sapiens GN=NDUFA13 PE=1 SV=3 - [NDUAD_HUMAN]	52.78	1	7	7	39	0.76	0.76	0.92	0.89	-0.40	-0.29	0.24	0.29	-0.07	0.05	-0.10	-0.06	-0.42	-0.71	-1.12	-0.74	-0.80	-1.04	-1.32	-0.60
O75380	NADH dehydrogenase [ubiquinone] iron-sulfur protein 6, mitochondrial OS=Homo sapiens GN=NDUFS6 PE=1 SV=1 - [NDUS6_HUMAN]	50.81	1	7	7	18	0.62	0.69	0.90	0.85	-0.09	-0.07	0.25	0.25	0.08	0.12	0.18	0.22	-0.23	-0.46	-0.77	-0.58	-0.76	-0.75	-1.09	-0.60
P07954	Fumarate hydratase, mitochondrial OS=Homo sapiens GN=FH PE=1 SV=3 - [FUMH_HUMAN]	43.53	2	14	15	60	0.79	0.93	0.91	1.03	-0.02	0.04	0.39	0.38	0.07	0.06	0.13	0.23	-0.42	-0.60	-0.80	-0.71	-0.88	-0.94	-0.99	-0.60
Q6FI81	Anamorsin OS=Homo sapiens GN=CIAPIN1 PE=1 SV=2 - [CPIN1_HUMAN]	13.14	1	5	5	8	0.69	0.84	0.87	0.99	0.14	0.25	0.26	0.40	0.05	0.05	-0.15	-0.15	-0.41	-0.78	-1.02	-0.57	-0.80	-0.57	-0.64	-0.60
Q01813	6-phosphofructokinase type C OS=Homo sapiens GN=PFKP PE=1 SV=2 - [K6PP_HUMAN]	43.49	1	24	29	89	0.39	0.46	0.53	0.59	-0.10	-0.11	-0.02	0.00	-0.19	-0.16	-0.13	-0.20	-0.38	-0.58	-0.85	-0.54	-0.74	-0.63	-0.71	-0.60

Q70EL1	Inactive ubiquitin carboxyl-terminal hydrolase 54 OS=Homo sapiens GN=USP54 PE=1 SV=4 - [UBP54_HUMAN]	0.53	1	1	1	2	0.04	0.11	0.52	0.58	-0.32	-0.26	-0.15	-0.09	-0.19	-0.25	0.00	0.06	-0.14	-0.04	-0.52	-0.26	-0.73	-0.37	-0.85	-0.60
Q99757	Thioredoxin, mitochondrial OS=Homo sapiens GN=TXN2 PE=1 SV=2 - [THIOM_HUMAN]	21.69	1	3	3	5	-0.27	-0.28	0.40	0.40	-0.42	-0.43	-0.27	-0.27	-0.40	-0.40	-0.44	-0.44	0.06	-0.03	-0.83	-0.09	-0.77	0.01	-0.56	-0.61
Q9UK45	U6 snRNA-associated Sm-like protein LSm7 OS=Homo sapiens GN=LSM7 PE=1 SV=1 - [LSM7_HUMAN]	25.24	1	2	2	3	-0.03	-0.01	0.41	0.00	-0.65	-0.04	-0.22	-0.67	-0.17	-0.52	-0.52	-0.59	-0.60	-0.48	-0.93	-0.12	-0.13	-0.04	-0.06	-0.61
Q8N0Z6	Tetratricopeptide repeat protein 5 OS=Homo sapiens GN=TTC5 PE=1 SV=2 - [TTC5_HUMAN]	4.09	1	2	2	2	0.57	0.66	0.72	0.81	-0.40	-0.31	0.05	0.14	0.05	-0.03	-0.19	-0.11	-0.47	-0.76	-0.91	-0.58	-0.72	-0.99	-1.14	-0.61
Q9Y5L4	Mitochondrial import inner membrane translocase subunit Tim13 OS=Homo sapiens GN=TIMM13 PE=1 SV=1 - [TIM13_HUMAN]	50.53	1	4	4	11	0.76	0.84	0.59	0.57	0.24	0.26	0.14	0.16	0.11	0.09	0.03	0.14	-0.51	-0.63	-0.71	-0.57	-0.67	-0.73	-0.67	-0.61
P11940	Polyadenylate-binding protein 1 OS=Homo sapiens GN=PABPC1 PE=1 SV=2 - [PABP1_HUMAN]	23.74	4	9	14	28	0.26	0.07	0.36	0.34	-0.34	-0.36	-0.19	-0.27	-0.38	-0.31	-0.41	-0.43	-0.50	-0.41	-0.52	-0.50	-0.47	-0.44	-0.62	-0.61
Q8N1G4	Leucine-rich repeat-containing protein 47 OS=Homo sapiens GN=LRRC47 PE=1 SV=1 - [LRC47_HUMAN]	38.94	1	20	20	43	0.77	0.86	0.75	0.82	-0.06	0.07	0.05	0.14	0.04	0.05	0.17	0.15	-0.65	-0.55	-0.56	-0.66	-0.64	-0.77	-0.78	-0.61

Q9Y285	Phenylalanine--tRNA ligase alpha subunit OS=Homo sapiens GN=FARSA PE=1 SV=3 - [SYFA_HUMAN]	25.98	1	9	9	19	0.32	0.35	0.61	0.64	-0.33	-0.22	-0.21	0.02	0.03	-0.05	-0.25	-0.23	-0.58	-0.89	-0.94	-0.62	-0.66	-0.63	-0.79	-0.61
Q86UN2	Reticulon-4 receptor-like 1 OS=Homo sapiens GN=RTN4RL1 PE=2 SV=1 - [R4RL1_HUMAN]	3.17	1	1	1	1	0.79	0.42	0.78	0.42	0.20	-0.17	0.11	-0.26	-0.39	-0.01	-0.28	-0.66	-0.62	-1.06	-1.07	-0.77	-0.76	-0.60	-0.60	-0.61
O43639	Cytoplasmic protein NCK2 OS=Homo sapiens GN=NCK2 PE=1 SV=2 - [NCK2_HUMAN]	29.74	1	8	9	14	0.51	0.67	0.82	0.97	0.09	0.41	0.03	0.30	0.15	-0.06	0.20	0.31	-0.42	-0.70	-1.01	-0.77	-1.13	-0.85	-1.07	-0.61
Q04760	Lactoylglutathione lyase OS=Homo sapiens GN=GLO1 PE=1 SV=4 - [LGUL_HUMAN]	46.20	1	8	8	22	0.74	0.83	0.93	1.02	0.11	0.03	0.34	0.32	0.09	0.05	-0.21	-0.21	-0.46	-1.12	-1.15	-0.67	-0.93	-0.81	-0.97	-0.61
P06744	Glucose-6-phosphate isomerase OS=Homo sapiens GN=GPI PE=1 SV=4 - [G6PI_HUMAN]	35.30	1	21	21	108	1.04	1.05	1.43	1.36	0.42	0.43	0.75	0.69	0.57	0.55	0.64	0.75	-0.28	-0.26	-0.66	-0.48	-0.83	-0.69	-1.08	-0.61
Q5T5Y3	Calmodulin-regulated spectrin-associated protein 1 OS=Homo sapiens GN=CAMSAP1 PE=1 SV=2 - [CAMP1_HUMAN]	4.37	1	5	5	5	0.98	1.57	0.86	1.46	-0.53	0.07	0.18	0.78	0.40	-0.20	-0.28	0.31	-0.74	-1.25	-1.14	-1.14	-1.02	-1.52	-1.40	-0.61
Q14155	Rho guanine nucleotide exchange factor 7 OS=Homo sapiens GN=ARHGEF7 PE=1 SV=2 - [ARHG7_HUMAN]	15.44	1	9	12	22	0.73	0.71	0.76	0.79	-0.15	-0.19	-0.04	-0.01	0.05	-0.11	-0.12	-0.17	-0.60	-0.76	-0.82	-0.59	-0.67	-0.82	-0.81	-0.61
Q8TCD5	5'(3')-deoxyribonucleotidase, cytosolic type OS=Homo sapiens GN=NT5C PE=1 SV=2 - [NT5C_HUMAN]	31.84	1	5	5	8	0.70	0.36	0.84	0.19	0.14	-0.22	0.15	-0.41	-0.01	0.68	0.08	-0.27	-0.59	-0.62	-0.46	-0.34	-0.87	-0.92	-1.17	-0.61

Q92804	TATA-binding protein-associated factor 2N OS=Homo sapiens GN=TAF15 PE=1 SV=1 - [RBP56_HUMAN]	14.70	1	3	5	7	-0.70	-0.49	-0.05	0.16	-0.98	-0.77	-0.73	-0.52	-0.58	-0.79	-0.70	-0.49	0.03	0.01	-0.65	-0.06	-0.71	-0.29	-0.94	-0.61
P78563	Double-stranded RNA-specific editase 1 OS=Homo sapiens GN=ADARB1 PE=1 SV=1 - [RED1_HUMAN]	7.29	1	4	4	5	0.37	0.29	0.39	0.31	-0.53	-0.61	-0.28	-0.37	-0.04	0.04	-0.25	-0.33	-0.60	-0.61	-0.64	-0.29	-0.32	-0.91	-0.94	-0.61
Q13620	Cullin-4B OS=Homo sapiens GN=CUL4B PE=1 SV=4 - [CUL4B_HUMAN]	10.08	1	2	10	18	1.40	1.07	1.00	0.67	0.06	-0.28	0.32	-0.01	0.10	0.44	0.15	-0.19	-1.03	-1.25	-0.85	-0.94	-0.53	-1.36	-0.96	-0.62
Q9H0Q0	Protein FAM49A OS=Homo sapiens GN=FAM49A PE=1 SV=1 - [FA49A_HUMAN]	45.82	1	8	10	31	1.52	1.78	1.43	1.77	-0.17	0.23	0.34	0.45	0.42	0.49	0.21	0.25	-1.04	-1.25	-0.90	-0.86	-0.55	-1.49	-1.48	-0.62
Q9BVC4	Target of rapamycin complex subunit LST8 OS=Homo sapiens GN=MLST8 PE=1 SV=1 - [LST8_HUMAN]	7.36	1	2	2	3	-1.22	-1.06	-0.24	-0.09	-0.35	-0.19	-0.92	-0.77	-0.38	-0.53	-1.49	-1.34	0.35	-0.27	-1.25	0.72	-0.25	0.86	-0.12	-0.62
P53816	HRAS-like suppressor 3 OS=Homo sapiens GN=PLA2G16 PE=1 SV=2 - [HRSL3_HUMAN]	16.05	1	2	2	4	0.40	0.41	0.45	0.46	-0.06	-0.05	-0.23	-0.22	0.14	0.13	0.02	0.03	-0.58	-0.37	-0.43	-0.24	-0.29	-0.48	-0.53	-0.62
P26368	Splicing factor U2AF 65 kDa subunit OS=Homo sapiens GN=U2AF2 PE=1 SV=4 - [U2AF2_HUMAN]	21.89	1	7	7	20	-0.19	-0.21	0.23	0.01	-0.58	-0.61	-0.58	-0.68	-0.41	-0.34	-0.25	-0.27	-0.30	-0.05	-0.53	-0.02	-0.16	-0.41	-0.82	-0.62
Q9NX63	Coiled-coil-helix-coiled-coil-helix domain-containing protein 3, mitochondrial OS=Homo sapiens GN=CHCHD3 PE=1 SV=1 - [CHCH3_HUMAN]	39.65	1	10	10	48	0.26	0.28	0.55	0.70	-0.65	-0.71	-0.16	-0.14	-0.36	-0.43	-0.47	-0.44	-0.28	-0.78	-1.14	-0.71	-0.98	-0.85	-1.21	-0.62

Q8WUD1	Ras-related protein Rab-2B OS=Homo sapiens GN=RAB2B PE=1 SV=1 - [RAB2B_HUMAN]	52.78	1	3	9	21	0.63	0.49	0.92	0.84	0.06	0.00	0.31	0.19	0.12	0.28	0.27	0.11	-0.64	-0.60	-0.73	-0.54	-0.71	-0.53	-0.88	-0.62
O75880	Protein SCO1 homolog, mitochondrial OS=Homo sapiens GN=SCO1 PE=1 SV=1 - [SCO1_HUMAN]	5.32	1	2	2	5	1.11	1.09	0.98	0.96	0.10	0.07	0.30	0.28	0.28	0.31	0.17	0.14	-0.76	-0.94	-0.82	-0.77	-0.63	-1.03	-0.90	-0.62
Q86WG3	Caytaxin OS=Homo sapiens GN=ATCAY PE=1 SV=2 - [ATCAY_HUMAN]	12.67	1	3	3	8	1.59	1.73	1.44	1.58	-0.43	-0.30	0.20	0.34	-0.08	-0.21	0.19	0.32	-1.01	-1.39	-1.25	-1.77	-1.62	-2.04	-1.90	-0.62
Q96J02	E3 ubiquitin-protein ligase Itchy homolog OS=Homo sapiens GN=ITCH PE=1 SV=2 - [ITCH_HUMAN]	11.52	1	8	8	13	0.19	0.42	0.29	0.29	0.05	0.01	-0.34	-0.16	-0.30	-0.34	-0.19	-0.19	-0.51	-0.55	-0.36	-0.63	-0.56	-0.56	-0.50	-0.62
O15066	Kinesin-like protein KIF3B OS=Homo sapiens GN=KIF3B PE=1 SV=1 - [KIF3B_HUMAN]	21.69	4	11	14	29	0.84	0.59	0.75	0.53	-0.01	-0.23	0.18	0.09	-0.21	-0.04	-0.04	0.04	-0.40	-0.59	-0.49	-0.53	-0.78	-0.60	-0.77	-0.62
Q9BWM7	Sideroflexin-3 OS=Homo sapiens GN=SFXN3 PE=1 SV=2 - [SFXN3_HUMAN]	37.85	1	9	10	25	0.68	0.66	0.68	0.57	0.09	-0.10	-0.07	-0.16	-0.10	-0.01	0.02	-0.08	-0.83	-0.68	-0.51	-0.69	-0.80	-0.59	-0.53	-0.62
Q9UBM7	7-dehydrocholesterol reductase OS=Homo sapiens GN=DHCR7 PE=1 SV=1 - [DHCR7_HUMAN]	6.32	1	3	3	4	1.42	1.21	1.37	1.16	0.78	0.57	0.68	0.48	0.71	0.92	1.04	0.83	-0.68	-0.37	-0.33	-0.46	-0.41	-0.65	-0.60	-0.62
P13693	Translationally-controlled tumor protein OS=Homo sapiens GN=TPT1 PE=1 SV=1 - [TCTP_HUMAN]	47.09	2	6	6	14	0.32	0.62	0.84	1.03	0.03	0.31	0.25	0.31	0.09	0.01	-0.33	-0.16	-0.41	-0.73	-1.36	-0.48	-0.85	-0.40	-0.93	-0.62

Q6N022	Teneurin-4 OS=Homo sapiens GN=TENM4 PE=1 SV=2 - [TEN4_HUMAN]	3.18	2	7	7	10	1.35	1.40	1.66	1.19	0.92	1.17	0.55	0.32	0.52	0.55	0.16	0.28	-0.76	-1.12	-1.24	-0.85	-0.71	-0.47	-0.46	-0.62
P60983	Glia maturation factor beta OS=Homo sapiens GN=GMFB PE=1 SV=2 - [GMFB_HUMAN]	42.96	1	5	6	21	0.20	0.12	0.68	0.68	-0.31	-0.26	0.02	0.04	-0.33	-0.32	-0.62	-0.58	-0.12	-0.71	-1.37	-0.47	-0.98	-0.59	-1.15	-0.62
Q7Z460	CLIP-associating protein 1 OS=Homo sapiens GN=CLASP1 PE=1 SV=1 - [CLAP1_HUMAN]	15.02	1	17	19	36	0.76	0.74	0.82	0.80	0.26	0.19	0.22	0.16	0.01	0.03	-0.06	-0.18	-0.53	-0.86	-0.77	-0.69	-0.62	-0.55	-0.63	-0.62
P14324	Farnesyl pyrophosphate synthase OS=Homo sapiens GN=FDPS PE=1 SV=4 - [FPPS_HUMAN]	14.32	1	5	5	13	0.76	0.62	0.87	0.74	-0.18	-0.30	0.15	0.08	-0.05	0.13	-0.32	-0.45	-0.56	-1.18	-1.20	-0.70	-0.74	-0.95	-1.11	-0.62
P00568	Adenylate kinase isoenzyme 1 OS=Homo sapiens GN=AK1 PE=1 SV=3 - [KAD1_HUMAN]	43.30	1	6	7	16	0.65	0.53	1.07	1.12	-0.08	-0.27	0.40	0.45	-0.11	0.03	-0.43	-0.56	-0.27	-1.13	-1.54	-0.59	-0.91	-0.93	-1.24	-0.62
P63279	SUMO-conjugating enzyme UBC9 OS=Homo sapiens GN=UBE2I PE=1 SV=1 - [UBC9_HUMAN]	20.25	1	4	4	9	-0.31	-0.26	0.14	0.19	-0.36	-0.32	-0.54	-0.50	-0.66	-0.70	-0.56	-0.52	-0.18	-0.25	-0.70	-0.37	-0.81	-0.07	-0.52	-0.62
Q05469	Hormone-sensitive lipase OS=Homo sapiens GN=LIPE PE=1 SV=4 - [LIPS_HUMAN]	1.21	1	1	1	1	0.75	0.75	0.59	0.59	-0.54	-0.54	-0.10	-0.09	1.42	1.42	-0.64	-0.64	-0.79	-1.38	-1.22	0.71	0.87	-1.30	-1.14	-0.62
O95502	Neuronal pentraxin receptor OS=Homo sapiens GN=NPTXR PE=2 SV=2 - [NPTXR_HUMAN]	14.40	1	6	6	11	0.12	0.27	0.57	0.72	-0.20	-0.05	-0.55	-0.34	-0.29	-0.49	-0.44	-0.23	-0.37	-0.57	-0.93	-0.73	-1.07	-0.69	-0.91	-0.62

Q8WZA2	Rap guanine nucleotide exchange factor 4 OS=Homo sapiens GN=RAPGEF4 PE=1 SV=1 - [RPGF4_HUMAN]	8.61	3	8	8	12	1.15	1.08	1.00	1.36	0.28	0.27	0.27	0.25	0.39	0.38	-0.03	-0.06	-0.83	-0.98	-1.31	-0.71	-0.98	-0.82	-0.58	-0.62
Q969S9	Ribosome-releasing factor 2, mitochondrial OS=Homo sapiens GN=GFM2 PE=1 SV=1 - [RRF2M_HUMAN]	5.13	1	3	3	5	0.29	0.16	-0.44	-0.23	-1.37	-1.52	-0.49	-1.01	-1.56	-1.41	-1.33	-1.49	-0.72	-1.64	-0.89	-1.69	-0.93	-1.70	-0.95	-0.62
Q9BZE9	Tether containing UBX domain for GLUT4 OS=Homo sapiens GN=ASPSCR1 PE=1 SV=1 - [ASPC1_HUMAN]	17.90	1	6	6	10	0.79	0.59	0.72	0.57	-0.45	-0.58	-0.03	-0.21	-0.45	-0.36	0.09	-0.07	-0.92	-0.59	-0.81	-0.91	-0.79	-1.12	-0.82	-0.62
Q9NZJ7	Mitochondrial carrier homolog 1 OS=Homo sapiens GN=MTCH1 PE=1 SV=1 - [MTCH1_HUMAN]	11.31	1	5	5	9	0.03	0.25	0.51	0.49	-0.59	-0.55	-0.20	0.03	-0.59	-0.49	-0.64	-0.35	-0.09	-0.54	-1.08	-0.52	-1.07	-0.58	-1.22	-0.62
P58546	Myotrophin OS=Homo sapiens GN=MTPN PE=1 SV=2 - [MTPN_HUMAN]	34.75	1	4	4	7	0.26	0.39	0.48	0.70	-0.39	-0.02	-0.14	0.19	0.01	-0.12	-0.83	-0.46	-0.14	-0.97	-1.40	-0.34	-0.97	-0.45	-1.25	-0.62
Q5TEU4	NADH dehydrogenase [ubiquinone] 1 alpha subcomplex assembly factor 5 OS=Homo sapiens GN=NDUFAF5 PE=1 SV=1 - [NDUF5_HUMAN]	5.51	1	2	2	2	1.11	1.10	1.15	1.14	-0.19	-0.20	0.46	0.45	0.12	0.14	0.03	0.02	-0.60	-1.08	-1.12	-0.94	-0.98	-1.32	-1.36	-0.63
Q13423	NAD(P) transhydrogenase, mitochondrial OS=Homo sapiens GN=NNT PE=1 SV=3 - [NNTM_HUMAN]	34.44	1	32	32	91	0.54	0.62	0.80	0.85	-0.19	-0.12	0.13	0.20	-0.03	-0.12	-0.09	-0.01	-0.35	-0.60	-0.89	-0.56	-0.85	-0.80	-1.06	-0.63
Q9H9B4	Sideroflexin-1 OS=Homo sapiens GN=SFXN1 PE=1 SV=4 - [SFXN1_HUMAN]	38.82	1	10	11	47	1.14	1.15	1.17	1.11	0.12	0.11	0.47	0.49	0.30	0.28	0.15	0.10	-0.74	-1.02	-0.98	-0.80	-0.74	-1.01	-1.03	-0.63

Q53GL0	Pleckstrin homology domain-containing family O member 1 OS=Homo sapiens GN=PLEKHO1 PE=1 SV=2 - [PKHO1_HUMAN]	8.31	1	2	2	3	-0.56	-1.81	-0.50	-1.74	-0.54	-1.79	-1.19	-2.44	-1.85	-0.61	-0.59	-1.84	-0.57	-0.02	-0.09	-0.01	-0.07	0.01	-0.06	-0.63
Q96LD8	Sentrin-specific protease 8 OS=Homo sapiens GN=SENP8 PE=1 SV=1 - [SENP8_HUMAN]	3.30	1	1	1	2	1.69	2.11	1.41	1.84	-0.01	0.07	0.72	1.14	0.77	0.35	-0.52	-0.10	-0.92	-2.20	-1.93	-1.31	-1.03	-2.28	-1.92	-0.63
Q13542	Eukaryotic translation initiation factor 4E-binding protein 2 OS=Homo sapiens GN=EIF4EBP2 PE=1 SV=1 - [4EBP2_HUMAN]	50.00	1	2	2	4	1.35	1.16	0.43	-0.11	0.23	0.34	-0.27	-1.30	-0.17	-0.01	0.61	0.12	-1.56	-0.74	0.18	-1.33	-0.41	-0.83	0.44	-0.63
Q15286	Ras-related protein Rab-35 OS=Homo sapiens GN=RAB35 PE=1 SV=1 - [RAB35_HUMAN]	53.23	4	8	11	51	0.67	0.59	1.03	0.97	0.05	0.05	0.31	0.21	0.07	0.04	-0.01	-0.01	-0.49	-0.80	-1.14	-0.65	-0.90	-0.69	-0.94	-0.63
P51991	Heterogeneous nuclear ribonucleoprotein A3 OS=Homo sapiens GN=HNRNPA3 PE=1 SV=2 - [ROA3_HUMAN]	28.31	1	8	10	41	0.44	0.55	0.65	0.68	-0.22	-0.29	-0.06	-0.20	-0.41	-0.19	-0.29	-0.36	-0.54	-0.56	-1.00	-0.73	-0.88	-0.69	-0.94	-0.63
O75569	Interferon-inducible double-stranded RNA-dependent protein kinase activator A OS=Homo sapiens GN=PRKRA PE=1 SV=1 - [PRKRA_HUMAN]	35.78	1	10	10	16	0.78	0.86	0.93	1.03	0.07	0.09	0.26	0.27	0.07	-0.01	0.05	0.01	-0.49	-0.73	-1.08	-0.64	-0.84	-0.58	-0.95	-0.63
O14514	Brain-specific angiogenesis inhibitor 1 OS=Homo sapiens GN=BAI1 PE=1 SV=2 - [BAI1_HUMAN]	3.35	1	3	4	6	1.27	1.54	1.27	1.54	1.02	1.29	0.57	0.85	0.91	0.64	0.87	1.14	-0.64	-0.39	-0.40	-0.60	-0.60	-0.27	-0.27	-0.63

P49588	Alanine--tRNA ligase, cytoplasmic OS=Homo sapiens GN=AARS PE=1 SV=2 - [SYAC_HUMAN]	48.45	1	34	34	102	1.27	1.27	1.39	1.44	0.30	0.28	0.75	0.74	0.42	0.44	0.24	0.21	-0.50	-0.90	-1.13	-0.71	-0.83	-0.91	-1.16	-0.63
P61604	10 kDa heat shock protein, mitochondrial OS=Homo sapiens GN=HSPE1 PE=1 SV=2 - [CH10_HUMAN]	73.53	1	9	9	29	0.42	0.44	0.80	0.71	-0.12	-0.12	0.13	0.12	-0.03	-0.05	-0.10	-0.05	-0.24	-0.55	-0.88	-0.41	-0.81	-0.60	-0.83	-0.63
A6NDN8	Putative ubiquitin-like protein FUBI-like protein ENSP00000310146 OS=Homo sapiens PE=4 SV=2 - [UBIML_HUMAN]	17.65	1	1	1	1	0.91	1.06	1.15	1.30	0.09	0.23	0.46	0.61	0.45	0.31	0.62	0.76	-0.40	-0.29	-0.54	-0.58	-0.81	-0.84	-1.08	-0.63
Q2KHT3	Protein CLEC16A OS=Homo sapiens GN=CLEC16A PE=2 SV=2 - [CL16A_HUMAN]	3.04	1	2	2	2	0.03	0.72	0.10	0.79	0.14	0.49	-0.60	0.09	0.64	-0.05	0.35	0.70	-0.57	-0.31	-0.31	-0.05	-0.11	-0.55	-0.54	-0.63
Q9Y2H5	Pleckstrin homology domain-containing family A member 6 OS=Homo sapiens GN=PLEKHA6 PE=1 SV=4 - [PKHA6_HUMAN]	15.36	1	13	13	24	0.51	0.54	0.33	0.47	0.24	0.13	-0.20	-0.12	-0.07	-0.01	0.39	0.45	-0.82	-0.34	-0.08	-0.55	-0.25	-0.60	-0.49	-0.63
Q9Y2G0	Protein EFR3 homolog B OS=Homo sapiens GN=EFR3B PE=1 SV=2 - [EFR3B_HUMAN]	15.79	1	9	10	18	1.54	1.14	1.47	1.24	0.06	-0.12	0.53	0.36	0.09	0.39	0.16	-0.30	-0.72	-1.42	-1.46	-0.93	-1.03	-1.38	-1.47	-0.63
Q8NEV8	Exophilin-5 OS=Homo sapiens GN=EXPH5 PE=2 SV=3 - [EXPH5_HUMAN]	0.35	1	1	1	3	1.76	1.59	1.81	1.64	0.53	0.35	1.12	0.94	0.43	0.61	0.79	0.61	-0.59	-0.96	-1.02	-1.12	-1.17	-1.25	-1.30	-0.63
Q99572	P2X purinoceptor 7 OS=Homo sapiens GN=P2RX7 PE=1 SV=4 - [P2RX7_HUMAN]	14.45	1	7	7	12	1.08	1.06	0.82	0.80	-0.26	-0.20	0.18	0.17	-0.05	-0.03	0.38	0.35	-0.90	-0.76	-0.45	-1.00	-0.74	-1.41	-1.19	-0.63

Q9Y2R5	28S ribosomal protein S17, mitochondrial OS=Homo sapiens GN=MRPS17 PE=2 SV=1 - [RT17_HUMAN]	22.31	1	1	1	1	-0.93	-0.27	1.17	1.84	0.93	1.59	0.48	1.14	0.90	0.25	0.27	0.93	1.46	1.21	-0.90	1.21	-0.89	1.85	-0.26	-0.63
Q9Y6X4	Soluble lamin associated protein of 75 kDa OS=Homo sapiens GN=FAM169A PE=1 SV=2 - [F169A_HUMAN]	14.33	1	5	5	12	0.62	0.60	0.57	0.34	-0.12	-0.03	-0.15	-0.17	-0.11	-0.09	-0.27	-0.32	-0.71	-0.78	-0.59	-0.48	-0.43	-0.74	-0.69	-0.63
Q96CQ1	Solute carrier family 25 member 36 OS=Homo sapiens GN=SLC25A36 PE=2 SV=1 - [S2536_HUMAN]	6.75	2	2	2	3	0.53	0.17	0.55	0.02	0.09	-0.19	0.07	-0.68	-0.19	0.11	0.15	0.17	-0.79	-0.37	-0.40	-0.33	-0.17	-0.37	-0.22	-0.63
Q7RTP6	Protein-methionine sulfoxide oxidase MICAL3 OS=Homo sapiens GN=MICAL3 PE=1 SV=2 - [MICA3_HUMAN]	13.09	1	21	21	34	0.88	1.10	0.83	1.01	0.01	0.05	0.19	0.25	0.20	0.15	0.08	0.10	-0.57	-0.89	-0.77	-0.87	-0.77	-0.99	-0.88	-0.64
Q12800	Alpha-globin transcription factor CP2 OS=Homo sapiens GN=TFCP2 PE=1 SV=2 - [TFCP2_HUMAN]	6.37	1	1	2	4	0.18	0.25	0.28	0.35	0.23	0.29	-0.42	-0.35	0.20	0.14	-0.43	-0.37	-0.54	-0.61	-0.72	-0.01	-0.11	0.03	-0.07	-0.64
O75190	DnaJ homolog subfamily B member 6 OS=Homo sapiens GN=DNAJB6 PE=1 SV=2 - [DNJB6_HUMAN]	13.50	2	3	3	8	1.14	1.15	1.12	0.98	0.05	-0.24	0.26	0.16	0.21	0.12	0.07	-0.16	-0.74	-0.87	-0.90	-0.81	-0.90	-1.13	-1.08	-0.64
P61204	ADP-ribosylation factor 3 OS=Homo sapiens GN=ARF3 PE=1 SV=2 - [ARF3_HUMAN]	58.56	2	5	10	75	1.07	1.21	1.05	1.28	0.50	0.57	0.35	0.48	0.40	0.30	-0.13	-0.05	-0.66	-1.41	-1.31	-0.89	-0.87	-0.76	-0.66	-0.64
Q8TD22	Sideroflexin-5 OS=Homo sapiens GN=SFXN5 PE=2 SV=1 - [SFXN5_HUMAN]	14.71	1	4	4	7	1.52	1.38	1.30	1.11	0.20	-0.01	0.51	0.19	-0.39	0.16	0.30	0.30	-0.88	-0.96	-0.92	-1.32	-1.11	-1.11	-1.03	-0.64

Q969Z3	Mitochondrial amidoxime reducing component 2 OS=Homo sapiens GN=MARC2 PE=1 SV=1 - [MARC2_HUMAN]	21.79	1	7	8	12	0.12	0.29	0.36	0.42	-0.65	-0.65	-0.26	-0.09	-0.41	-0.41	-0.51	-0.51	-0.37	-0.78	-0.92	-0.73	-0.86	-0.99	-1.13	-0.64
Q5R3F8	Protein phosphatase 1 regulatory subunit 29 OS=Homo sapiens GN=ELFN2 PE=1 SV=1 - [PPR29_HUMAN]	1.10	1	1	1	2	1.26	1.57	1.10	1.41	0.18	0.48	0.40	0.71	0.31	0.01	0.48	0.78	-0.81	-0.78	-0.62	-1.23	-1.06	-1.10	-0.94	-0.64
P84090	Enhancer of rudimentary homolog OS=Homo sapiens GN=ERH PE=1 SV=1 - [ERH_HUMAN]	33.65	1	3	3	7	-0.14	-0.16	0.30	0.29	-0.56	-0.55	-0.46	-0.41	-0.49	-0.48	-0.32	-0.28	-0.30	-0.19	-0.50	-0.32	-0.74	-0.42	-0.84	-0.64
Q9UBJ2	ATP-binding cassette sub-family D member 2 OS=Homo sapiens GN=ABCD2 PE=1 SV=1 - [ABCD2_HUMAN]	1.62	1	1	1	1	1.59	1.53	1.55	1.48	0.60	0.52	0.85	0.78	0.50	0.57	0.58	0.51	-0.69	-1.00	-0.97	-0.99	-0.95	-1.01	-0.97	-0.64
Q9P0L2	Serine/threonine-protein kinase MARK1 OS=Homo sapiens GN=MARK1 PE=1 SV=2 - [MARK1_HUMAN]	9.81	2	4	5	6	1.14	0.78	1.06	0.70	-0.32	-0.68	0.35	-0.01	-0.69	-0.33	-0.09	-0.46	-0.73	-1.22	-1.15	-1.43	-1.35	-1.47	-1.39	-0.64
A8MWD9	Small nuclear ribonucleoprotein G-like protein OS=Homo sapiens PE=3 SV=2 - [RUXGL_HUMAN]	25.00	2	2	2	7	-0.12	-0.21	0.12	0.04	-0.37	-0.47	-0.58	-0.67	-0.51	-0.41	-0.41	-0.51	-0.41	-0.29	-0.54	-0.26	-0.50	-0.27	-0.51	-0.64
O75122	CLIP-associating protein 2 OS=Homo sapiens GN=CLASP2 PE=1 SV=2 - [CLAP2_HUMAN]	27.74	1	27	29	50	0.92	0.93	1.06	1.08	0.08	0.02	0.32	0.37	0.14	0.14	0.07	0.06	-0.56	-0.79	-1.04	-0.83	-0.88	-0.81	-0.89	-0.64
Q99700	Ataxin-2 OS=Homo sapiens GN=ATXN2 PE=1 SV=2 - [ATX2_HUMAN]	5.79	1	5	6	9	0.06	-0.05	0.35	0.24	-0.28	-0.40	-0.36	-0.47	-0.30	-0.18	-0.21	-0.32	-0.37	-0.27	-0.55	-0.22	-0.50	-0.36	-0.64	-0.64

Q9BZV1	UBX domain-containing protein 6 OS=Homo sapiens GN=UBXN6 PE=1 SV=1 - [UBXN6_HUMAN]	32.20	1	11	11	23	0.19	0.18	0.71	0.74	-0.09	-0.14	0.14	0.17	-0.13	-0.05	-0.13	0.01	-0.12	-0.25	-0.80	-0.42	-0.82	-0.39	-0.81	-0.64
Q9Y6R1	Electrogenic sodium bicarbonate cotransporter 1 OS=Homo sapiens GN=SLC4A4 PE=1 SV=1 - [S4A4_HUMAN]	19.37	2	13	14	34	0.46	0.49	0.99	0.91	-0.68	-0.71	0.31	-0.07	-0.01	0.18	0.94	0.79	-0.18	0.34	-0.14	-0.23	-0.75	-1.00	-1.60	-0.64
P06307	Cholecystokinin OS=Homo sapiens GN=CCK PE=1 SV=1 - [CCKN_HUMAN]	8.70	1	1	1	4	1.36	1.42	1.09	1.15	0.62	0.68	0.38	0.45	0.56	0.50	0.48	0.54	-0.92	-0.87	-0.61	-0.83	-0.56	-0.76	-0.49	-0.64
Q9NYB0	Telomeric repeat-binding factor 2-interacting protein 1 OS=Homo sapiens GN=TERF2IP PE=1 SV=1 - [TE2IP_HUMAN]	26.07	1	7	7	11	0.90	0.87	0.73	0.82	0.05	-0.06	0.19	0.12	-0.02	0.10	0.13	-0.05	-0.50	-0.79	-0.80	-0.60	-0.56	-0.88	-0.92	-0.64
Q14257	Reticulocalbin-2 OS=Homo sapiens GN=RCN2 PE=1 SV=1 - [RCN2_HUMAN]	57.41	1	13	14	32	0.50	0.55	0.42	0.67	0.08	0.05	-0.08	0.06	0.16	0.11	-0.02	0.09	-0.49	-0.42	-0.63	-0.41	-0.51	-0.35	-0.45	-0.64
Q96QZ7	Membrane-associated guanylate kinase, WW and PDZ domain-containing protein 1 OS=Homo sapiens GN=MAGI1 PE=1 SV=3 - [MAGI1_HUMAN]	6.71	1	6	7	10	0.03	0.02	0.28	0.27	-0.33	-0.34	-0.43	-0.44	-0.63	-0.61	-0.50	-0.52	-0.41	-0.53	-0.78	-0.61	-0.85	-0.38	-0.62	-0.64
Q5W0Z9	Probable palmitoyltransferase ZDHHC20 OS=Homo sapiens GN=ZDHHC20 PE=1 SV=1 - [ZDH20_HUMAN]	3.01	1	1	1	1	0.22	0.64	0.38	0.80	-0.49	-0.08	-0.33	0.09	0.32	-0.09	0.87	1.28	-0.49	0.66	0.49	-0.28	-0.44	-0.72	-0.88	-0.64

Q9NVT9	Armadillo repeat-containing protein 1 OS=Homo sapiens GN=ARMC1 PE=1 SV=1 - [ARMC1_HUMAN]	14.89	1	4	4	7	0.94	0.87	0.54	0.64	0.01	-0.02	-0.07	-0.10	-0.13	0.03	0.00	0.02	-0.94	-0.56	-0.62	-0.88	-0.73	-0.94	-0.86	-0.64
Q9H4G4	Golgi-associated plant pathogenesis-related protein 1 OS=Homo sapiens GN=GLIPR2 PE=1 SV=3 - [GAPR1_HUMAN]	47.40	1	5	5	12	0.53	0.37	0.32	0.28	-0.68	-0.74	-0.31	-0.36	-0.54	-0.32	-0.18	-0.25	-0.68	-0.64	-0.44	-0.87	-0.81	-0.92	-1.01	-0.64
Q9ULH7	MKL/myocardin-like protein 2 OS=Homo sapiens GN=MKL2 PE=1 SV=3 - [MKL2_HUMAN]	3.77	1	2	2	3	0.21	0.17	1.09	1.05	0.08	0.04	0.38	0.34	-0.16	-0.12	0.30	0.26	0.22	0.10	-0.79	-0.30	-1.17	-0.15	-1.03	-0.65
P10114	Ras-related protein Rap-2a OS=Homo sapiens GN=RAP2A PE=1 SV=1 - [RAP2A_HUMAN]	54.10	1	3	7	16	1.55	1.47	1.67	1.55	0.36	0.23	0.93	0.85	0.36	0.51	0.54	0.42	-0.55	-1.06	-1.29	-1.11	-1.19	-1.37	-1.45	-0.65
Q6P995	Protein FAM171B OS=Homo sapiens GN=FAM171B PE=2 SV=3 - [F171B_HUMAN]	13.08	1	8	8	15	1.21	1.22	1.26	1.27	0.17	0.17	0.38	0.38	0.54	0.54	0.40	0.40	-0.80	-0.80	-0.86	-0.62	-0.63	-1.06	-1.11	-0.65
Q9NPF4	Probable tRNA N6-adenosine threonylcarbamoyltransferase OS=Homo sapiens GN=OSGEP PE=1 SV=1 - [OSGEP_HUMAN]	9.85	1	2	2	3	0.75	0.69	0.66	0.61	-0.17	-0.23	-0.05	-0.10	-0.20	-0.14	-0.27	-0.33	-0.74	-1.01	-0.93	-0.85	-0.76	-0.94	-0.85	-0.65
P10915	Hyaluronan and proteoglycan link protein 1 OS=Homo sapiens GN=HAPLN1 PE=2 SV=2 - [HPLN1_HUMAN]	33.33	1	6	7	18	1.15	1.47	1.39	1.25	-0.56	-0.08	0.64	0.41	-0.14	0.00	-0.49	-0.49	-0.45	-1.52	-1.73	-1.12	-1.40	-1.52	-1.72	-0.65
Q08722	Leukocyte surface antigen CD47 OS=Homo sapiens GN=CD47 PE=1 SV=1 - [CD47_HUMAN]	12.07	1	5	5	22	0.75	0.85	1.40	1.49	0.19	0.30	0.77	0.78	0.49	0.48	0.35	0.39	-0.04	-0.46	-0.93	-0.51	-0.96	-0.74	-1.14	-0.65

Q8TAF3	WD repeat-containing protein 48 OS=Homo sapiens GN=WDR48 PE=1 SV=1 - [WDR48_HUMAN]	16.25	1	9	9	14	0.52	0.63	0.79	0.88	-0.33	-0.19	0.14	0.11	0.07	-0.08	-0.21	-0.13	-0.37	-0.74	-0.96	-0.71	-0.87	-0.84	-1.00	-0.65
Q96C19	EF-hand domain-containing protein D2 OS=Homo sapiens GN=EFHD2 PE=1 SV=1 - [EFHD2_HUMAN]	35.83	1	8	10	28	1.12	0.89	1.35	1.39	-0.17	-0.07	0.20	0.23	0.07	-0.09	-0.36	-0.24	-0.48	-1.09	-1.54	-0.95	-0.96	-0.87	-1.26	-0.65
Q92890	Ubiquitin fusion degradation protein 1 homolog OS=Homo sapiens GN=UFD1L PE=1 SV=3 - [UFD1_HUMAN]	17.92	1	4	4	6	0.03	-0.16	0.09	0.07	-0.35	-0.46	-0.50	-0.44	-0.49	-0.33	-0.23	-0.32	0.12	-0.01	-0.62	-0.29	-0.52	-0.36	-0.54	-0.65
Q14689	Disco-interacting protein 2 homolog A OS=Homo sapiens GN=DIP2A PE=1 SV=2 - [DIP2A_HUMAN]	5.41	1	4	6	9	0.45	1.04	0.76	0.96	0.14	0.43	0.33	0.25	0.52	0.12	0.11	0.45	-0.66	-0.54	-0.46	-0.48	-0.61	-0.33	-0.54	-0.65
O14827	Ras-specific guanine nucleotide-releasing factor 2 OS=Homo sapiens GN=RASGRF2 PE=1 SV=2 - [RGRF2_HUMAN]	10.99	1	8	8	17	0.46	0.96	0.79	0.74	-0.26	-0.08	0.24	0.21	0.08	-0.03	-0.15	-0.12	-0.51	-1.01	-0.83	-0.33	-0.65	-1.08	-1.04	-0.65
O43300	Leucine-rich repeat transmembrane neuronal protein 2 OS=Homo sapiens GN=LRRTM2 PE=2 SV=3 - [LRRT2_HUMAN]	1.55	1	1	1	2	0.92	0.78	0.89	0.75	0.48	0.33	0.18	0.03	0.31	0.46	0.29	0.14	-0.69	-0.62	-0.60	-0.43	-0.39	-0.46	-0.43	-0.65
Q99726	Zinc transporter 3 OS=Homo sapiens GN=SLC30A3 PE=2 SV=2 - [ZNT3_HUMAN]	12.63	1	3	3	6	0.17	1.91	0.40	2.14	-0.39	1.35	-0.32	1.43	1.85	0.11	-0.59	1.15	-0.43	-0.75	-0.99	-0.02	-0.25	-0.58	-0.81	-0.65
P53667	LIM domain kinase 1 OS=Homo sapiens GN=LIMK1 PE=1 SV=3 - [LIMK1_HUMAN]	7.26	1	4	4	5	0.58	0.91	0.69	1.08	-0.20	-0.25	0.08	0.22	0.27	0.20	-0.43	-0.24	-0.64	-1.03	-1.12	-0.68	-0.69	-1.17	-1.34	-0.65

Q9BVA1	Tubulin beta-2B chain OS=Homo sapiens GN=TUBB2B PE=1 SV=1 - [TBB2B_HUMAN]	70.34	2	1	24	924	1.43	1.64	1.72	1.93	0.45	0.65	0.82	1.03	0.71	0.55	0.89	0.44	-0.36	-0.88	-1.21	-0.84	-1.16	-1.00	-1.29	-0.65
Q16540	39S ribosomal protein L23, mitochondrial OS=Homo sapiens GN=MRPL23 PE=1 SV=1 - [RM23_HUMAN]	15.03	1	2	2	3	0.62	0.48	0.42	0.27	0.17	0.02	-0.30	-0.44	0.05	0.20	0.57	0.42	-0.87	-0.05	0.15	-0.39	-0.18	-0.47	-0.26	-0.65
P09110	3-ketoacyl-CoA thiolase, peroxisomal OS=Homo sapiens GN=ACAA1 PE=1 SV=2 - [THIK_HUMAN]	42.69	1	11	11	20	0.90	0.56	0.99	0.88	0.01	-0.05	0.21	0.09	0.12	0.17	0.10	0.07	-0.39	-0.57	-0.89	-0.71	-0.83	-0.52	-0.88	-0.65
P49069	Calcium signal-modulating cyclophilin ligand OS=Homo sapiens GN=CAMLG PE=1 SV=1 - [CAMLG_HUMAN]	6.42	1	2	2	4	1.04	0.89	1.02	0.87	0.04	-0.12	0.30	0.15	-0.11	0.05	0.00	-0.15	-0.69	-1.03	-1.01	-0.97	-0.94	-1.02	-1.00	-0.65
O95248	Myotubularin-related protein 5 OS=Homo sapiens GN=SBF1 PE=1 SV=3 - [MTMR5_HUMAN]	18.69	1	26	28	48	1.13	1.03	1.10	1.07	-0.18	-0.14	0.32	0.23	0.10	-0.02	0.01	0.06	-0.63	-0.90	-0.90	-0.76	-0.96	-1.06	-1.07	-0.65
Q10567	AP-1 complex subunit beta-1 OS=Homo sapiens GN=AP1B1 PE=1 SV=2 - [AP1B1_HUMAN]	38.78	1	15	30	75	0.36	0.33	0.92	0.90	-0.04	-0.07	0.18	0.13	0.16	-0.06	0.04	-0.14	-0.24	-0.47	-0.94	-0.54	-0.75	-0.48	-0.92	-0.65
Q9BRQ6	Coiled-coil-helix-coiled-coil-helix domain-containing protein 6, mitochondrial OS=Homo sapiens GN=CHCHD6 PE=1 SV=1 - [CHCH6_HUMAN]	28.09	1	5	5	15	0.78	0.73	0.94	1.07	-0.55	-0.33	-0.05	0.15	-0.17	-0.39	-0.63	-0.41	-0.66	-1.12	-1.37	-0.87	-1.20	-1.22	-1.32	-0.65

Q9UEW8	STE20/SPS1-related proline-alanine-rich protein kinase OS=Homo sapiens GN=STK39 PE=1 SV=3 - [STK39_HUMAN]	22.02	1	8	10	15	0.70	1.06	0.75	0.98	0.08	0.05	0.31	0.22	0.20	-0.12	0.19	0.06	-0.68	-0.76	-0.90	-0.80	-0.79	-0.88	-0.99	-0.65
Q16595	Frataxin, mitochondrial OS=Homo sapiens GN=FXN PE=1 SV=2 - [FRDA_HUMAN]	17.62	1	4	4	5	0.08	0.17	0.60	0.53	-0.43	-0.48	-0.12	-0.17	-0.26	-0.29	-0.44	-0.46	-0.26	-0.57	-0.98	-0.37	-0.83	-0.59	-1.05	-0.65
Q00610	Clathrin heavy chain 1 OS=Homo sapiens GN=CLTC PE=1 SV=5 - [CLH1_HUMAN]	52.60	2	70	70	426	0.87	0.79	0.97	1.02	0.07	0.17	0.30	0.28	0.21	0.20	0.18	0.21	-0.45	-0.62	-0.75	-0.57	-0.69	-0.74	-0.92	-0.65
Q99523	Sortilin OS=Homo sapiens GN=SORT1 PE=1 SV=3 - [SORT_HUMAN]	14.44	1	9	9	21	0.90	1.02	0.92	0.88	-0.06	0.08	0.25	0.18	0.37	0.20	0.00	0.10	-0.58	-0.89	-1.01	-0.64	-0.80	-0.77	-1.01	-0.65
Q7Z406	Myosin-14 OS=Homo sapiens GN=MYH14 PE=1 SV=2 - [MYH14_HUMAN]	25.46	1	32	45	116	1.00	0.96	0.84	1.02	-0.31	-0.41	0.22	0.24	0.04	0.19	0.26	0.26	-0.68	-0.77	-0.63	-0.79	-0.78	-1.27	-1.22	-0.65
P31930	Cytochrome b-c1 complex subunit 1, mitochondrial OS=Homo sapiens GN=UQCRC1 PE=1 SV=3 - [QCR1_HUMAN]	46.46	1	16	16	98	1.06	1.23	1.20	1.23	0.08	0.18	0.49	0.58	0.34	0.31	0.23	0.27	-0.49	-0.89	-0.95	-0.76	-0.86	-0.84	-1.15	-0.66
O94973	AP-2 complex subunit alpha-2 OS=Homo sapiens GN=AP2A2 PE=1 SV=2 - [AP2A2_HUMAN]	30.99	1	15	26	69	0.79	0.85	0.95	0.96	-0.27	-0.25	0.12	0.14	-0.09	0.07	-0.11	-0.18	-0.57	-1.02	-0.97	-0.88	-0.79	-1.09	-1.07	-0.66
Q9H3H3	UPF0696 protein C11orf68 OS=Homo sapiens GN=C11orf68 PE=1 SV=2 - [CK068_HUMAN]	36.65	1	7	7	14	0.66	0.87	0.87	0.98	-0.01	0.19	0.02	0.20	0.04	-0.06	-0.22	-0.11	-0.66	-0.93	-0.98	-0.78	-0.89	-0.72	-0.79	-0.66

Q86US8	Telomerase-binding protein EST1A OS=Homo sapiens GN=SMG6 PE=1 SV=2 - [EST1A_HUMAN]	2.68	1	2	2	2	0.80	0.86	0.58	0.20	0.28	-0.06	-0.14	-0.16	0.03	0.37	0.61	0.27	-0.89	-0.57	0.07	-0.79	-0.13	-0.93	-0.27	-0.66
Q6PCE3	Glucose 1,6-bisphosphate synthase OS=Homo sapiens GN=PGM2L1 PE=1 SV=3 - [PGM2L_HUMAN]	23.47	1	14	14	22	1.09	1.03	0.99	1.08	0.21	0.12	0.15	0.23	0.16	0.18	-0.10	0.00	-0.58	-1.17	-1.04	-0.62	-0.78	-0.72	-0.88	-0.66
Q9H1E3	Nuclear ubiquitous casein and cyclin-dependent kinase substrate 1 OS=Homo sapiens GN=NUCKS1 PE=1 SV=1 - [NUCKS_HUMAN]	15.64	1	3	3	9	-0.35	-0.13	-0.12	-0.03	-0.73	-0.64	-0.90	-0.76	-0.74	-0.76	-0.26	-0.38	-0.58	-0.13	-0.12	-0.59	-0.76	-0.54	-0.70	-0.66
Q96K21	Zinc finger FYVE domain-containing protein 19 OS=Homo sapiens GN=ZFYVE19 PE=1 SV=3 - [ZFY19_HUMAN]	10.62	1	2	3	3	0.64	0.48	0.87	0.71	1.02	0.86	0.15	-0.01	0.55	0.72	0.95	0.79	-0.44	0.31	0.08	0.11	-0.12	0.36	0.13	-0.66
P48739	Phosphatidylinositol transfer protein beta isoform OS=Homo sapiens GN=PITPNB PE=1 SV=2 - [PIPNB_HUMAN]	26.94	1	7	9	16	0.44	0.63	0.68	0.86	-0.38	-0.29	0.05	0.08	-0.25	-0.33	-0.35	-0.43	-0.39	-0.92	-1.21	-0.76	-0.99	-1.00	-1.24	-0.66
Q96F07	Cytoplasmic FMR1-interacting protein 2 OS=Homo sapiens GN=CYFIP2 PE=1 SV=2 - [CYFP2_HUMAN]	21.44	1	14	25	48	1.28	1.17	1.30	1.24	-0.13	-0.06	0.51	0.45	0.14	0.01	-0.23	-0.08	-0.83	-1.28	-1.27	-1.02	-1.04	-1.35	-1.37	-0.66
Q9BQT9	Calsyntenin-3 OS=Homo sapiens GN=CLSTN3 PE=1 SV=1 - [CSTN3_HUMAN]	4.81	1	2	2	2	0.33	0.80	0.68	1.15	0.01	0.47	-0.04	0.43	0.12	-0.35	-0.10	0.36	-0.32	-0.43	-0.79	-0.65	-0.99	-0.34	-0.69	-0.66
O60229	Kalirin OS=Homo sapiens GN=KALRN PE=1 SV=2 - [KALRN_HUMAN]	3.32	1	3	9	13	0.60	0.77	0.79	1.07	0.14	0.10	0.28	0.12	0.20	-0.04	-0.26	-0.23	-0.58	-0.86	-1.01	-0.72	-0.84	-0.58	-0.87	-0.66

Q14008	Cytoskeleton-associated protein 5 OS=Homo sapiens GN=CKAP5 PE=1 SV=3 - [CKAP5_HUMAN]	20.62	1	38	38	74	0.77	0.76	0.94	0.86	-0.05	0.05	0.26	0.26	-0.04	0.01	-0.01	-0.10	-0.51	-0.79	-1.01	-0.64	-0.92	-0.87	-0.98	-0.66
O94876	Transmembrane and coiled-coil domains protein 1 OS=Homo sapiens GN=TMCC1 PE=1 SV=3 - [TMCC1_HUMAN]	4.44	1	1	2	5	0.89	1.13	1.03	1.27	0.30	0.53	0.30	0.54	0.25	0.01	-0.19	0.04	-0.53	-1.08	-1.22	-0.84	-0.98	-0.61	-0.75	-0.66
Q8WVY7	Ubiquitin-like domain-containing CTD phosphatase 1 OS=Homo sapiens GN=UBLCP1 PE=1 SV=2 - [UBCP1_HUMAN]	12.26	1	4	4	9	1.00	0.88	0.91	0.78	0.38	0.39	0.44	0.31	0.29	0.37	0.14	0.06	-0.43	-0.75	-1.10	-0.68	-0.93	-0.62	-0.79	-0.66
P20338	Ras-related protein Rab-4A OS=Homo sapiens GN=RAB4A PE=1 SV=3 - [RAB4A_HUMAN]	22.48	4	1	5	20	1.31	1.38	1.00	1.07	0.25	0.32	0.27	0.35	0.39	0.32	0.33	0.39	-0.98	-0.98	-0.68	-0.96	-0.65	-1.07	-0.77	-0.66
P16615	Sarcoplasmic/endoplasmic reticulum calcium ATPase 2 OS=Homo sapiens GN=ATP2A2 PE=1 SV=1 - [AT2A2_HUMAN]	32.82	2	23	29	89	0.53	0.55	0.74	0.89	-0.20	-0.24	-0.04	0.01	-0.12	-0.13	-0.20	-0.16	-0.52	-0.68	-0.99	-0.72	-0.86	-0.79	-1.07	-0.66
P49821	NADH dehydrogenase [ubiquinone] flavoprotein 1, mitochondrial OS=Homo sapiens GN=NDUFV1 PE=1 SV=4 - [NDUV1_HUMAN]	39.01	1	14	14	37	0.78	0.75	0.93	0.90	-0.40	-0.30	0.15	0.08	0.03	0.02	-0.07	-0.08	-0.53	-0.76	-0.96	-0.75	-0.82	-0.90	-1.29	-0.66
O95182	NADH dehydrogenase [ubiquinone] 1 alpha subcomplex subunit 7 OS=Homo sapiens GN=NDUFA7 PE=1 SV=3 - [NDUA7_HUMAN]	61.06	1	8	8	21	0.58	0.56	1.23	1.15	-0.25	-0.21	0.20	0.38	-0.01	-0.13	0.22	0.35	-0.52	-0.52	-0.75	-0.66	-1.06	-0.89	-1.30	-0.66

Q9H4Z3	Phosphorylated CTD-interacting factor 1 OS=Homo sapiens GN=PCIF1 PE=1 SV=1 - [PCIF1_HUMAN]	3.55	1	2	2	4	0.73	0.57	0.93	0.77	0.10	-0.06	0.20	0.05	0.02	0.18	-0.07	-0.23	-0.47	-0.79	-1.00	-0.51	-0.71	-0.64	-0.85	-0.66
Q9Y606	tRNA pseudouridine synthase A, mitochondrial OS=Homo sapiens GN=PUS1 PE=1 SV=3 - [TRUA_HUMAN]	11.24	1	4	4	6	0.13	0.28	-0.15	0.00	-0.16	-0.39	-0.11	0.05	-0.61	-0.77	-0.47	-0.69	-0.51	-0.49	-0.67	-0.86	-0.72	-0.21	-0.38	-0.67
Q14832	Metabotropic glutamate receptor 3 OS=Homo sapiens GN=GRM3 PE=1 SV=2 - [GRM3_HUMAN]	8.42	1	5	6	11	0.71	0.90	0.92	1.01	-0.63	-0.70	0.30	0.28	0.05	-0.04	-0.21	-0.12	-0.36	-1.17	-1.13	-0.98	-0.93	-1.54	-1.49	-0.67
Q5JSH3	WD repeat-containing protein 44 OS=Homo sapiens GN=WDR44 PE=1 SV=1 - [WDR44_HUMAN]	24.42	1	17	17	25	0.85	0.67	0.92	0.90	-0.32	-0.30	0.16	0.06	-0.01	0.05	-0.18	-0.14	-0.71	-1.00	-1.16	-0.56	-0.70	-1.04	-0.96	-0.67
Q52LD8	Raftlin-2 OS=Homo sapiens GN=RFTN2 PE=2 SV=3 - [RFTN2_HUMAN]	12.18	1	4	4	6	0.93	0.59	1.11	0.91	0.44	0.10	0.48	0.44	0.24	0.37	0.29	0.02	0.00	-0.63	-0.89	-0.31	-0.92	-0.60	-0.83	-0.67
P78356	Phosphatidylinositol 5-phosphate 4-kinase type-2 beta OS=Homo sapiens GN=PIP4K2B PE=1 SV=1 - [PI42B_HUMAN]	27.16	1	5	10	28	1.20	0.97	1.25	1.01	-0.08	-0.14	0.46	0.32	0.29	0.28	-0.06	-0.32	-0.67	-1.38	-1.41	-0.61	-0.85	-1.18	-1.42	-0.67
Q8IYQ7	Threonine synthase-like 1 OS=Homo sapiens GN=THNSL1 PE=1 SV=2 - [THNS1_HUMAN]	18.57	1	11	11	16	0.64	0.42	0.71	0.56	-0.55	-0.54	-0.01	-0.23	-0.40	-0.38	-0.56	-0.61	-0.52	-1.04	-1.02	-0.60	-0.86	-0.91	-1.06	-0.67
Q9NXU5	ADP-ribosylation factor-like protein 15 OS=Homo sapiens GN=ARL15 PE=1 SV=1 - [ARL15_HUMAN]	21.08	1	4	4	7	0.48	0.28	0.57	0.33	0.07	-0.17	0.02	-0.22	0.05	0.20	0.17	-0.06	-0.45	-0.03	-0.23	-0.20	-0.39	-0.46	-0.51	-0.67

Q9UK22	F-box only protein 2 OS=Homo sapiens GN=FBXO2 PE=1 SV=2 - [FBX2_HUMAN]	31.42	1	6	6	12	0.86	0.45	0.81	0.81	-0.13	-0.28	0.08	-0.07	-0.08	0.08	0.30	-0.21	-0.30	-0.65	-0.66	-0.87	-0.87	-1.06	-1.16	-0.67
P25686	DnaJ homolog subfamily B member 2 OS=Homo sapiens GN=DNAJB2 PE=1 SV=3 - [DNJB2_HUMAN]	31.79	1	7	7	13	1.03	0.83	1.24	0.99	-0.16	-0.18	0.44	0.19	-0.03	0.23	0.24	0.02	-0.56	-0.81	-1.08	-0.81	-0.94	-0.95	-1.18	-0.67
O15212	Prefoldin subunit 6 OS=Homo sapiens GN=PFDN6 PE=1 SV=1 - [PFD6_HUMAN]	38.76	1	6	6	11	0.53	0.74	0.88	1.12	-0.22	-0.05	0.06	0.34	0.15	0.05	-0.31	-0.24	-0.26	-0.96	-1.11	-0.55	-0.90	-0.79	-1.15	-0.67
Q13574	Diacylglycerol kinase zeta OS=Homo sapiens GN=DGKZ PE=1 SV=3 - [DGKZ_HUMAN]	9.85	1	7	7	13	0.63	0.81	0.74	0.93	0.09	0.02	-0.02	0.02	0.12	-0.03	-0.09	-0.10	-0.52	-0.98	-0.97	-0.79	-0.78	-0.99	-0.68	-0.67
P21796	Voltage-dependent anion-selective channel protein 1 OS=Homo sapiens GN=VDAC1 PE=1 SV=2 - [VDAC1_HUMAN]	73.50	1	15	17	300	0.76	0.91	1.01	1.03	0.11	-0.01	0.35	0.40	0.19	0.27	0.08	0.12	-0.51	-0.77	-0.89	-0.62	-0.62	-0.84	-1.12	-0.67
Q9H3Z4	DnaJ homolog subfamily C member 5 OS=Homo sapiens GN=DNAJC5 PE=1 SV=1 - [DNJC5_HUMAN]	35.86	1	4	4	20	0.54	0.22	0.93	0.37	-0.27	-0.59	0.19	-0.12	-0.32	0.00	0.34	0.01	-0.29	-0.20	-0.59	-0.51	-0.90	-0.83	-1.22	-0.67
O76031	ATP-dependent Clp protease ATP-binding subunit clpX-like, mitochondrial OS=Homo sapiens GN=CLPX PE=1 SV=2 - [CLPX_HUMAN]	5.85	1	3	3	4	-0.40	-0.62	-0.15	-0.37	-0.27	-0.49	-0.89	-1.11	-0.66	-0.43	-0.41	-0.64	-0.43	-0.01	-0.26	-0.01	-0.25	0.11	-0.14	-0.67

P82979	SAP domain-containing ribonucleoprotein OS=Homo sapiens GN=SARNP PE=1 SV=3 - [SARNP_HUMAN]	11.43	1	2	2	4	-0.08	0.04	0.34	0.45	-0.31	-0.20	-0.40	-0.28	-0.30	-0.42	-0.07	0.04	-0.27	0.02	-0.41	-0.31	-0.72	-0.25	-0.66	-0.67
Q9NRN7	L-aminoadipate semialdehyde dehydrogenase-phosphopantetheinyl transferase OS=Homo sapiens GN=AASDHPPT PE=1 SV=2 - [ADPPT_HUMAN]	18.45	1	6	6	11	0.79	0.87	1.31	1.39	0.40	0.47	0.57	0.65	0.35	0.27	0.18	0.26	-0.17	-0.60	-1.12	-0.49	-1.00	-0.41	-0.93	-0.67
Q8WV99	AN1-type zinc finger protein 2B OS=Homo sapiens GN=ZFAND2B PE=1 SV=1 - [ZFN2B_HUMAN]	3.11	1	1	1	1	0.74	0.93	0.74	0.93	0.44	0.63	0.00	0.19	0.51	0.32	-0.56	-0.37	-0.68	-1.29	-1.30	-0.39	-0.39	-0.31	-0.31	-0.67
P81605	Dermcidin OS=Homo sapiens GN=DCD PE=1 SV=2 - [DCD_HUMAN]	10.00	1	1	1	2	1.42	1.25	1.46	1.29	0.44	0.26	0.72	0.55	0.21	0.39	0.08	-0.09	-0.64	-1.33	-1.37	-1.00	-1.04	-1.00	-1.04	-0.67
P11142	Heat shock cognate 71 kDa protein OS=Homo sapiens GN=HSPA8 PE=1 SV=1 - [HSP7C_HUMAN]	57.28	1	20	34	308	0.56	0.60	0.92	0.90	-0.12	-0.11	0.12	0.19	0.00	-0.05	-0.06	0.00	-0.35	-0.71	-0.94	-0.64	-0.89	-0.81	-0.99	-0.67
O96008	Mitochondrial import receptor subunit TOM40 homolog OS=Homo sapiens GN=TOMM40 PE=1 SV=1 - [TOM40_HUMAN]	35.18	1	7	7	14	0.55	0.70	0.79	0.79	-0.29	-0.22	0.11	0.20	-0.09	-0.02	0.09	-0.06	-0.41	-0.87	-0.65	-0.67	-0.83	-0.82	-1.09	-0.67
P42684	Abelson tyrosine-protein kinase 2 OS=Homo sapiens GN=ABL2 PE=1 SV=1 - [ABL2_HUMAN]	2.20	1	2	2	4	1.08	1.07	1.16	1.15	0.35	0.34	0.42	0.41	0.13	0.15	0.07	0.06	-0.61	-1.00	-1.09	-0.90	-0.98	-0.75	-0.82	-0.68

Q9Y2H0	Disks large-associated protein 4 OS=Homo sapiens GN=DLGAP4 PE=1 SV=3 - [DLGP4_HUMAN]	9.17	1	6	6	7	0.19	0.31	0.03	0.34	-0.49	-0.49	-0.34	-0.08	-0.30	-0.77	-0.39	-0.30	-0.74	-0.96	-0.91	-1.43	-1.37	-1.11	-1.05	-0.68
Q9NT68	Teneurin-2 OS=Homo sapiens GN=TENM2 PE=1 SV=3 - [TEN2_HUMAN]	1.23	2	3	3	4	1.83	1.30	1.57	0.98	0.41	-0.20	0.83	-0.43	-0.40	0.36	0.51	0.17	-0.58	-1.16	-1.07	-1.07	-1.18	-1.28	-1.19	-0.68
O94772	Lymphocyte antigen 6H OS=Homo sapiens GN=LY6H PE=2 SV=1 - [LY6H_HUMAN]	22.14	1	3	3	7	0.63	0.50	0.57	0.44	-0.73	-0.64	0.31	0.17	-0.34	-0.44	-0.52	-0.43	-0.27	-1.15	-1.34	-1.04	-1.23	-1.38	-1.57	-0.68
Q92614	Unconventional myosin-XVIIIa OS=Homo sapiens GN=MYO18A PE=1 SV=3 - [MY18A_HUMAN]	22.30	3	35	36	61	1.02	1.09	1.05	0.96	0.07	0.03	0.30	0.29	0.02	0.11	0.07	0.02	-0.67	-0.77	-0.93	-0.83	-0.82	-0.82	-0.94	-0.68
Q5HYK3	2-methoxy-6-polyprenyl-1,4-benzoquinol methylase, mitochondrial OS=Homo sapiens GN=COQ5 PE=1 SV=2 - [COQ5_HUMAN]	18.96	1	5	5	7	0.43	0.30	0.69	0.56	-0.16	-0.30	-0.06	-0.19	0.01	0.14	-0.11	-0.25	-0.43	-0.54	-0.80	-0.26	-0.51	-0.61	-0.86	-0.68
Q96T49	Protein phosphatase 1 regulatory inhibitor subunit 16B OS=Homo sapiens GN=PPP1R16B PE=1 SV=1 - [PP16B_HUMAN]	2.12	1	1	1	2	1.36	1.25	1.16	1.04	0.25	0.13	0.41	0.30	0.44	0.56	0.46	0.34	-0.89	-0.90	-0.70	-0.77	-0.56	-1.13	-0.93	-0.68
P55290	Cadherin-13 OS=Homo sapiens GN=CDH13 PE=1 SV=1 - [CAD13_HUMAN]	17.67	1	9	9	28	0.73	0.57	0.69	0.72	0.64	0.64	-0.04	0.01	0.15	0.09	-0.38	-0.30	-0.71	-0.89	-1.10	-0.59	-0.60	-0.01	-0.10	-0.68
P07602	Prosaposin OS=Homo sapiens GN=PSAP PE=1 SV=2 - [SAP_HUMAN]	24.62	1	10	10	55	0.69	0.76	1.00	1.07	-0.10	0.06	0.26	0.32	0.38	0.19	0.38	0.47	-0.33	-0.14	-0.53	-0.35	-0.80	-0.65	-1.07	-0.68

Q9NX18	Succinate dehydrogenase assembly factor 2, mitochondrial OS=Homo sapiens GN=SDHAF2 PE=1 SV=1 - [SDHF2_HUMAN]	7.23	1	1	1	2	1.92	1.62	2.25	1.95	0.85	0.55	1.51	1.21	0.78	1.09	1.24	0.93	-0.36	-0.68	-1.02	-0.80	-1.13	-1.09	-1.42	-0.68
Q6PII5	Hydroxyacylglutathione hydrolase-like protein OS=Homo sapiens GN=HAGHL PE=2 SV=1 - [HAGHL_HUMAN]	2.41	1	1	1	1	0.55	0.48	1.00	0.93	-0.23	-0.30	0.26	0.19	0.48	0.55	0.37	0.30	-0.23	-0.17	-0.63	0.04	-0.42	-0.79	-1.25	-0.68
P50151	Guanine nucleotide-binding protein G(I)/G(S)/G(O) subunit gamma-10 OS=Homo sapiens GN=GNG10 PE=1 SV=1 - [GBG10_HUMAN]	30.88	1	1	2	4	0.93	0.87	1.10	1.04	0.14	0.08	0.35	0.30	0.55	0.61	0.72	0.66	-0.52	-0.21	-0.38	-0.29	-0.46	-0.81	-0.98	-0.68
Q8IVD9	NudC domain-containing protein 3 OS=Homo sapiens GN=NUDCD3 PE=1 SV=3 - [NUDC3_HUMAN]	28.25	1	8	8	13	1.44	1.37	2.02	1.88	-0.16	-0.20	1.00	0.99	0.34	0.44	0.04	-0.04	-0.45	-1.54	-1.91	-0.95	-1.41	-1.57	-2.16	-0.68
Q15370	Transcription elongation factor B polypeptide 2 OS=Homo sapiens GN=TCEB2 PE=1 SV=1 - [ELOB_HUMAN]	20.34	1	3	3	5	0.39	0.31	0.59	0.51	0.10	0.02	-0.16	-0.24	-0.16	-0.08	0.21	0.13	-0.49	-0.17	-0.37	-0.43	-0.63	-0.30	-0.50	-0.68
P26992	Ciliary neurotrophic factor receptor subunit alpha OS=Homo sapiens GN=CNTFR PE=1 SV=2 - [CNTFR_HUMAN]	16.40	1	4	4	8	1.24	1.35	1.41	0.98	0.19	0.27	0.56	0.36	0.65	0.97	1.05	0.70	-0.57	-0.47	-0.36	-0.55	-0.45	-1.04	-1.40	-0.68
Q9UHY7	Enolase-phosphatase E1 OS=Homo sapiens GN=ENOPH1 PE=1 SV=1 - [ENOPH_HUMAN]	32.57	1	6	6	15	0.93	0.87	1.00	0.99	0.21	0.25	0.23	0.32	0.33	0.36	0.08	0.18	-0.56	-0.65	-0.95	-0.55	-0.68	-0.53	-0.67	-0.68

P41208	Centrin-2 OS=Homo sapiens GN=CETN2 PE=1 SV=1 - [CETN2_HUMAN]	6.98	1	1	1	2	-0.42	-0.36	0.40	0.46	-0.78	-0.73	-0.34	-0.29	-0.46	-0.51	-0.52	-0.47	0.13	-0.10	-0.92	-0.06	-0.88	-0.38	-1.20	-0.68
O60925	Prefoldin subunit 1 OS=Homo sapiens GN=PFDN1 PE=1 SV=2 - [PFD1_HUMAN]	35.25	1	5	5	9	0.68	0.59	0.88	0.93	-0.29	-0.28	0.07	0.18	-0.17	-0.16	-0.21	-0.24	-0.60	-0.89	-1.04	-0.80	-0.94	-0.91	-1.11	-0.68
Q6GQQ9	OTU domain-containing protein 7B OS=Homo sapiens GN=OTUD7B PE=1 SV=1 - [OTU7B_HUMAN]	4.51	1	2	2	2	0.68	0.78	0.98	1.09	0.47	0.58	0.23	0.34	0.58	0.48	0.49	0.59	-0.39	-0.18	-0.49	-0.17	-0.47	-0.22	-0.52	-0.68
Q8WUI4	Histone deacetylase 7 OS=Homo sapiens GN=HDAC7 PE=1 SV=2 - [HDAC7_HUMAN]	2.73	1	1	1	1	-0.82	-0.78	-1.26	-1.22	-0.15	-0.12	-2.00	-1.96	-1.67	-1.70	-0.71	-0.68	-1.13	0.11	0.54	-0.85	-0.41	0.65	1.09	-0.68
Q8WWV3	Reticulon-4-interacting protein 1, mitochondrial OS=Homo sapiens GN=RTN4IP1 PE=1 SV=2 - [RT4I1_HUMAN]	22.22	1	6	6	11	0.26	0.43	0.60	0.74	-0.28	-0.19	0.00	0.03	-0.13	-0.24	-0.26	-0.12	-0.37	-0.51	-1.09	-0.55	-0.93	-0.67	-1.08	-0.69
Q14019	Coactosin-like protein OS=Homo sapiens GN=COTL1 PE=1 SV=3 - [COTL1_HUMAN]	42.25	1	6	6	25	0.83	0.81	1.30	1.52	0.02	0.15	0.55	0.75	0.15	0.11	-0.38	-0.22	-0.51	-1.32	-1.59	-0.50	-1.12	-0.73	-1.09	-0.69
P31946	14-3-3 protein beta/alpha OS=Homo sapiens GN=YWHAB PE=1 SV=3 - [1433B_HUMAN]	60.98	1	6	13	98	0.33	0.43	0.51	0.75	-0.10	0.04	-0.12	0.06	0.11	-0.03	-0.06	-0.07	-0.50	-0.48	-0.71	-0.47	-0.55	-0.51	-0.63	-0.69
Q12959	Disks large homolog 1 OS=Homo sapiens GN=DLG1 PE=1 SV=2 - [DLG1_HUMAN]	28.43	1	12	17	38	1.30	1.29	1.26	1.37	0.66	0.43	0.55	0.80	0.66	0.80	0.78	0.82	-0.66	-0.46	-0.58	-0.59	-0.48	-0.72	-0.70	-0.69
O14949	Cytochrome b-c1 complex subunit 8 OS=Homo sapiens GN=UQCRQ PE=1 SV=4 - [QCR8_HUMAN]	46.34	1	4	4	18	0.61	0.76	1.15	1.28	-0.22	-0.22	0.38	0.42	0.16	-0.01	-0.11	0.00	-0.27	-0.72	-1.35	-0.74	-1.13	-0.99	-1.60	-0.69

Q9NZ72	Stathmin-3 OS=Homo sapiens GN=STMN3 PE=1 SV=3 - [STMN3_HUMAN]	10.56	1	2	2	3	0.68	0.86	0.64	0.82	-0.03	0.14	-0.11	0.06	0.25	0.08	-0.53	-0.35	-0.74	-1.20	-1.16	-0.57	-0.52	-0.73	-0.69	-0.69
P11177	Pyruvate dehydrogenase E1 component subunit beta, mitochondrial OS=Homo sapiens GN=PDHB PE=1 SV=3 - [ODPB_HUMAN]	44.01	1	11	11	52	0.56	0.42	0.60	0.57	-0.11	-0.15	0.08	-0.04	-0.27	-0.07	-0.10	-0.13	-0.39	-0.59	-0.68	-0.54	-0.55	-0.72	-0.82	-0.69
Q15121	Astrocytic phosphoprotein PEA-15 OS=Homo sapiens GN=PEA15 PE=1 SV=2 - [PEA15_HUMAN]	45.38	1	5	5	12	1.03	0.88	1.42	1.48	-0.32	-0.43	0.76	0.69	-0.29	0.05	-0.40	-0.60	-0.23	-1.21	-1.71	-1.16	-1.38	-1.53	-1.74	-0.69
Q92806	G protein-activated inward rectifier potassium channel 3 OS=Homo sapiens GN=KCNJ9 PE=2 SV=2 - [IRK9_HUMAN]	2.29	1	1	1	1	0.70	0.66	0.35	0.32	-0.15	-0.19	-0.40	-0.43	-0.40	-0.36	-0.40	-0.44	-1.04	-1.09	-0.76	-1.03	-0.68	-0.87	-0.52	-0.69
O00429	Dynamin-1-like protein OS=Homo sapiens GN=DNM1L PE=1 SV=2 - [DNM1L_HUMAN]	41.71	1	27	27	66	1.33	1.32	1.33	1.41	0.06	0.07	0.61	0.65	0.16	0.19	-0.15	-0.11	-0.64	-1.42	-1.55	-1.03	-1.21	-1.34	-1.43	-0.69
Q9UQ26	Regulating synaptic membrane exocytosis protein 2 OS=Homo sapiens GN=RIMS2 PE=1 SV=2 - [RIMS2_HUMAN]	3.40	1	2	3	6	1.10	1.01	0.96	0.93	0.06	0.05	-0.04	0.18	0.26	0.18	-0.94	-0.28	-0.78	-0.74	-0.67	-0.72	-0.64	-0.72	-1.26	-0.69
Q9UGV2	Protein NDRG3 OS=Homo sapiens GN=NDRG3 PE=1 SV=2 - [NDRG3_HUMAN]	18.93	1	5	5	22	0.54	0.89	1.03	1.13	0.17	0.34	0.18	0.49	0.13	0.01	0.14	0.12	-0.47	-0.82	-1.06	-0.66	-0.89	-0.76	-1.08	-0.69
P12271	Retinaldehyde-binding protein 1 OS=Homo sapiens GN=RLBP1 PE=1 SV=2 - [RLBP1_HUMAN]	17.35	1	3	3	5	0.80	0.29	0.94	0.43	0.00	-0.09	0.19	-0.21	0.24	0.31	0.69	0.45	-0.56	0.09	-0.06	-0.45	-0.36	-0.72	-0.85	-0.69

Q92520	Protein FAM3C OS=Homo sapiens GN=FAM3C PE=1 SV=1 - [FAM3C_HUMAN]	33.04	1	5	5	8	0.13	0.09	0.35	0.31	0.24	0.20	-0.40	-0.44	-0.10	-0.05	0.56	0.51	-0.48	0.43	0.21	-0.15	-0.37	0.09	-0.13	-0.69
O75223	Gamma-glutamylcyclotransferase OS=Homo sapiens GN=GGCT PE=1 SV=1 - [GGCT_HUMAN]	25.53	1	4	4	9	0.56	0.73	0.92	1.02	-0.09	0.36	0.10	0.44	-0.41	-0.31	-0.70	-0.62	-0.34	-1.54	-1.86	-0.87	-1.27	-0.78	-0.95	-0.69
Q9BZZ5	Apoptosis inhibitor 5 OS=Homo sapiens GN=API5 PE=1 SV=3 - [API5_HUMAN]	25.76	1	11	11	21	-0.49	-0.36	-0.02	-0.09	-0.41	-0.32	-0.71	-0.69	-0.50	-0.51	-0.23	-0.31	-0.22	0.08	-0.45	-0.35	-0.47	-0.15	-0.48	-0.69
Q96LL9	DnaJ homolog subfamily C member 30 OS=Homo sapiens GN=DNAJC30 PE=1 SV=3 - [DJC30_HUMAN]	3.98	1	1	1	1	0.69	0.72	0.57	0.60	-0.02	0.01	-0.19	-0.15	-0.05	-0.08	-0.09	-0.07	-0.82	-0.78	-0.66	-0.73	-0.61	-0.72	-0.60	-0.69
Q9H0E2	Toll-interacting protein OS=Homo sapiens GN=TOLLIP PE=1 SV=1 - [TOLIP_HUMAN]	46.35	1	10	10	21	1.42	1.35	1.39	1.38	0.30	0.34	0.48	0.50	0.26	0.21	0.13	0.17	-0.81	-1.15	-1.17	-1.09	-0.93	-1.15	-1.23	-0.69
Q9BT88	Synaptotagmin-11 OS=Homo sapiens GN=SYT11 PE=1 SV=2 - [SYT11_HUMAN]	12.30	1	3	3	4	0.90	0.95	0.81	0.85	0.15	0.19	0.05	0.10	0.68	0.63	0.64	0.68	-0.80	-0.26	-0.17	-0.24	-0.14	-0.77	-0.68	-0.69
Q9Y5K5	Ubiquitin carboxyl-terminal hydrolase isozyme L5 OS=Homo sapiens GN=UCHL5 PE=1 SV=3 - [UCHL5_HUMAN]	23.40	1	7	7	15	0.91	1.01	1.02	1.20	-0.08	0.01	0.26	0.41	0.34	0.18	0.22	0.25	-0.55	-0.76	-0.89	-0.65	-0.78	-1.00	-1.11	-0.69
Q9Y570	Protein phosphatase methylesterase 1 OS=Homo sapiens GN=PPME1 PE=1 SV=3 - [PPME1_HUMAN]	23.58	1	7	7	11	0.83	0.75	1.28	1.09	0.04	-0.10	0.47	0.43	0.14	0.11	-0.26	-0.31	-0.29	-1.09	-1.44	-0.63	-1.13	-0.93	-1.23	-0.69

P51784	Ubiquitin carboxyl-terminal hydrolase 11 OS=Homo sapiens GN=USP11 PE=1 SV=3 - [UBP11_HUMAN]	9.76	1	7	7	14	1.08	0.62	1.01	0.41	-0.01	-0.26	0.16	0.13	0.02	0.09	0.27	0.28	-0.66	-0.48	-0.70	-0.57	-0.77	-0.68	-0.92	-0.69
Q8N684	Cleavage and polyadenylation specificity factor subunit 7 OS=Homo sapiens GN=CPSF7 PE=1 SV=1 - [CPSF7_HUMAN]	11.46	1	5	5	8	0.10	0.23	0.55	0.68	-0.20	-0.08	-0.21	-0.08	-0.09	-0.21	-0.05	0.08	-0.25	0.08	-0.15	-0.28	-0.73	-0.32	-0.77	-0.69
Q86UK7	Zinc finger protein 598 OS=Homo sapiens GN=ZNF598 PE=1 SV=1 - [ZN598_HUMAN]	2.10	1	1	1	2	0.41	-0.21	0.41	-0.21	0.48	-0.15	-0.35	-0.97	-0.81	-0.19	0.42	-0.21	-0.70	0.02	0.01	-0.56	-0.56	0.05	0.05	-0.69
P47985	Cytochrome b-c1 complex subunit Rieske, mitochondrial OS=Homo sapiens GN=UQCRFS1 PE=1 SV=2 - [UCRI_HUMAN]	28.83	2	7	7	18	0.77	1.07	1.10	1.38	-0.07	-0.21	0.21	0.23	0.09	-0.07	-0.26	-0.16	-0.39	-1.04	-1.25	-0.99	-1.17	-0.98	-1.26	-0.69
Q92747	Actin-related protein 2/3 complex subunit 1A OS=Homo sapiens GN=ARPC1A PE=1 SV=2 - [ARC1A_HUMAN]	29.19	1	8	9	18	0.34	0.51	0.44	0.61	-0.77	-0.28	-0.26	-0.15	-0.24	-0.32	-0.39	-0.34	-0.61	-0.62	-0.93	-0.52	-0.75	-0.68	-0.87	-0.69
P23381	Tryptophan--tRNA ligase, cytoplasmic OS=Homo sapiens GN=WARS PE=1 SV=2 - [SYWC_HUMAN]	25.05	1	10	10	20	0.25	0.32	0.33	0.60	-0.59	-0.11	-0.32	-0.04	-0.55	-0.64	-0.63	-0.33	-0.53	-0.74	-1.17	-0.79	-1.10	-0.75	-0.83	-0.69
P50148	Guanine nucleotide-binding protein G(q) subunit alpha OS=Homo sapiens GN=GNAQ PE=1 SV=4 - [GNAQ_HUMAN]	37.60	1	6	12	42	1.20	1.26	1.09	1.22	0.16	0.29	0.33	0.44	0.42	0.45	0.16	0.08	-0.80	-1.04	-1.18	-0.67	-0.60	-0.78	-1.03	-0.69

Q96GW7	Brevican core protein OS=Homo sapiens GN=BCAN PE=1 SV=2 - [PGCB_HUMAN]	18.99	1	13	13	62	1.36	1.72	1.36	1.52	0.15	0.17	0.74	0.85	0.44	0.50	0.37	0.46	-0.87	-1.31	-1.17	-1.15	-0.98	-1.56	-1.37	-0.70
Q12933	TNF receptor-associated factor 2 OS=Homo sapiens GN=TRAF2 PE=1 SV=2 - [TRAF2_HUMAN]	4.79	1	2	2	3	0.77	0.37	0.77	0.37	0.31	-0.09	0.01	-0.39	-0.05	0.35	0.22	-0.18	-0.70	-0.55	-0.55	-0.39	-0.39	-0.48	-0.48	-0.70
P05129	Protein kinase C gamma type OS=Homo sapiens GN=PRKCG PE=1 SV=3 - [KPCG_HUMAN]	24.25	1	9	12	23	0.78	0.75	1.01	1.00	-0.15	-0.23	0.23	0.21	0.06	0.03	-0.05	0.02	-0.43	-0.97	-1.06	-0.78	-0.82	-0.98	-1.26	-0.70
Q9NVA2	Septin-11 OS=Homo sapiens GN=SEPT11 PE=1 SV=3 - [SEP11_HUMAN]	49.88	2	8	19	82	0.66	0.72	0.72	0.72	-0.10	-0.34	0.02	-0.08	-0.16	-0.06	-0.19	-0.51	-0.72	-1.14	-1.05	-0.97	-0.81	-1.02	-0.83	-0.70
P35613	Basigin OS=Homo sapiens GN=BSG PE=1 SV=2 - [BASI_HUMAN]	33.25	1	9	9	32	0.84	0.90	0.95	0.90	0.10	0.23	0.22	0.39	0.29	0.32	0.59	0.65	-0.60	-0.38	-0.29	-0.73	-0.67	-0.74	-0.97	-0.70
P48147	Prolyl endopeptidase OS=Homo sapiens GN=PREP PE=1 SV=2 - [PPCE_HUMAN]	41.69	1	22	22	37	1.46	1.37	1.70	1.71	0.29	0.35	0.96	0.82	0.48	0.62	-0.07	0.03	-0.45	-1.54	-1.77	-0.99	-1.13	-1.20	-1.37	-0.70
Q69YW2	Uncharacterized membrane protein C1orf95 OS=Homo sapiens GN=C1orf95 PE=2 SV=1 - [CA095_HUMAN]	21.28	1	2	2	5	1.10	0.77	0.64	0.43	-0.58	-0.77	-0.01	-0.33	-0.93	-0.60	-0.20	-0.39	-1.05	-1.04	-0.74	-1.32	-0.76	-1.41	-1.10	-0.70
Q9Y4C0	Neurexin-3 OS=Homo sapiens GN=NRXN3 PE=1 SV=4 - [NRX3A_HUMAN]	16.01	2	14	19	33	0.80	0.85	0.80	0.68	0.07	-0.04	0.22	0.18	0.14	0.13	0.05	0.22	-0.67	-0.78	-0.96	-0.60	-0.74	-0.73	-0.71	-0.70
Q13813	Spectrin alpha chain, non-erythrocytic 1 OS=Homo sapiens GN=SPTAN1 PE=1 SV=3 - [SPTN1_HUMAN]	70.79	1	159	159	933	0.73	0.72	0.88	0.89	-0.22	-0.19	0.13	0.16	-0.04	-0.05	0.02	0.04	-0.51	-0.70	-0.85	-0.73	-0.90	-0.92	-1.14	-0.70

Q4L180	Filamin A-interacting protein 1-like OS=Homo sapiens GN=FILIP1L PE=1 SV=2 - [FIL1L_HUMAN]	4.49	1	4	5	5	0.24	0.72	0.68	1.16	-0.29	0.18	-0.09	0.39	0.26	-0.22	0.38	0.86	-0.27	0.14	-0.30	-0.43	-0.86	-0.55	-0.99	-0.70
Q9HB71	Calcyclin-binding protein OS=Homo sapiens GN=CACYBP PE=1 SV=2 - [CYBP_HUMAN]	45.61	1	8	8	13	-0.15	-0.22	0.36	0.37	-0.31	-0.26	-0.48	-0.21	-0.32	-0.56	-0.55	-0.45	-0.25	-0.39	-0.90	-0.47	-0.74	-0.26	-0.75	-0.70
O43768	Alpha-endosulfine OS=Homo sapiens GN=ENSA PE=1 SV=1 - [ENSA_HUMAN]	28.93	1	2	3	6	-0.10	-0.06	0.11	0.15	-0.65	-0.61	-0.66	-0.62	-0.80	-0.83	-0.74	-0.71	-0.50	-0.64	-0.86	-0.70	-0.91	-0.56	-0.78	-0.70
Q8N568	Serine/threonine-protein kinase DCLK2 OS=Homo sapiens GN=DCLK2 PE=2 SV=4 - [DCLK2_HUMAN]	25.59	2	16	17	32	1.14	1.05	1.24	1.19	-0.04	0.03	0.33	0.36	0.29	0.12	0.15	0.24	-0.68	-0.87	-0.91	-0.81	-0.84	-1.02	-1.00	-0.70
Q01433	AMP deaminase 2 OS=Homo sapiens GN=AMPD2 PE=1 SV=2 - [AMPD2_HUMAN]	18.20	1	15	15	25	0.90	1.03	0.96	1.14	0.10	0.33	0.33	0.49	0.14	0.05	0.28	0.38	-0.54	-0.66	-0.81	-0.62	-0.92	-0.70	-0.83	-0.70
Q9UL51	Potassium/sodium hyperpolarization-activated cyclic nucleotide-gated channel 2 OS=Homo sapiens GN=HCN2 PE=1 SV=3 - [HCN2_HUMAN]	6.64	3	4	4	8	0.84	1.03	0.73	0.92	-0.54	-0.34	-0.04	0.16	-0.08	-0.27	-0.31	-0.12	-0.82	-1.14	-1.04	-1.08	-0.97	-1.39	-1.28	-0.70
Q9Y2E4	Disco-interacting protein 2 homolog C OS=Homo sapiens GN=DIP2C PE=2 SV=2 - [DIP2C_HUMAN]	8.68	1	8	10	16	1.03	1.07	1.07	0.97	0.16	0.01	0.15	0.20	0.12	0.14	-0.15	-0.17	-0.63	-1.14	-1.14	-0.85	-0.84	-0.88	-0.95	-0.70

Q9BPX5	Actin-related protein 2/3 complex subunit 5-like protein OS=Homo sapiens GN=ARPC5L PE=1 SV=1 - [ARP5L_HUMAN]	50.98	1	6	7	20	0.84	0.93	1.06	1.12	0.14	0.16	0.19	0.27	-0.01	-0.16	-0.09	0.00	-0.60	-0.90	-1.01	-0.84	-1.03	-0.72	-0.96	-0.71
Q8IVS2	Malonyl-CoA-acyl carrier protein transacylase, mitochondrial OS=Homo sapiens GN=MCAT PE=1 SV=2 - [FABD_HUMAN]	5.90	1	1	1	2	0.78	0.68	0.48	0.39	-0.52	-0.62	-0.28	-0.38	0.01	0.11	-0.25	-0.35	-1.00	-1.02	-0.73	-0.64	-0.34	-1.31	-1.02	-0.71
P54652	Heat shock-related 70 kDa protein 2 OS=Homo sapiens GN=HSPA2 PE=1 SV=1 - [HSP72_HUMAN]	55.87	1	15	30	158	0.79	0.83	1.07	0.90	-0.11	-0.18	0.11	0.06	-0.12	0.06	0.12	0.01	-0.52	-0.67	-1.02	-0.81	-0.90	-0.96	-1.23	-0.71
Q07021	Complement component 1 Q subcomponent-binding protein, mitochondrial OS=Homo sapiens GN=C1QBP PE=1 SV=1 - [C1QBP_HUMAN]	53.19	1	9	9	45	0.75	0.73	1.10	1.16	-0.25	-0.33	0.28	0.42	0.06	-0.08	0.05	0.05	-0.23	-0.66	-1.05	-0.62	-0.89	-1.11	-1.42	-0.71
Q96I25	Splicing factor 45 OS=Homo sapiens GN=RBM17 PE=1 SV=1 - [SPF45_HUMAN]	5.24	1	2	2	3	-0.47	-0.59	0.44	0.31	-0.41	-0.54	-0.34	-0.46	-0.48	-0.36	-0.25	-0.38	0.19	0.22	-0.69	0.14	-0.76	0.04	-0.87	-0.71
Q9NQP4	Prefoldin subunit 4 OS=Homo sapiens GN=PFDN4 PE=1 SV=1 - [PFD4_HUMAN]	29.10	1	4	4	9	0.69	0.62	1.09	1.03	-0.39	-0.45	0.32	0.27	-0.07	0.04	0.07	0.03	-0.29	-0.50	-1.01	-0.81	-1.03	-1.11	-1.51	-0.71
P51797	Chloride transport protein 6 OS=Homo sapiens GN=CLCN6 PE=1 SV=2 - [CLCN6_HUMAN]	8.29	1	4	4	6	1.65	1.65	1.62	1.62	0.21	0.90	0.85	0.85	0.67	0.67	1.25	1.52	-0.91	-0.36	-0.34	-0.95	-0.92	-0.71	-0.58	-0.71

P26232	Catenin alpha-2 OS=Homo sapiens GN=CTNNA2 PE=1 SV=5 - [CTNA2_HUMAN]	44.49	1	26	34	73	1.34	1.15	1.47	1.29	0.04	0.13	1.00	0.90	0.56	0.62	0.44	0.40	-0.63	-0.79	-1.10	-0.85	-1.00	-1.32	-1.41	-0.71
Q9NNW7	Thioredoxin reductase 2, mitochondrial OS=Homo sapiens GN=TXNRD2 PE=1 SV=3 - [TRXR2_HUMAN]	7.82	1	4	4	8	1.08	0.88	1.22	1.01	0.61	0.11	0.68	0.37	0.26	0.57	0.60	0.29	-0.52	-0.60	-0.89	-0.76	-0.96	-1.29	-1.42	-0.71
P22695	Cytochrome b-c1 complex subunit 2, mitochondrial OS=Homo sapiens GN=UQCRC2 PE=1 SV=3 - [QCR2_HUMAN]	63.36	1	22	22	104	1.00	1.01	1.07	1.03	0.03	0.00	0.30	0.35	0.14	0.14	0.04	0.02	-0.68	-0.95	-1.09	-0.97	-0.99	-1.01	-1.06	-0.71
Q6IQ22	Ras-related protein Rab-12 OS=Homo sapiens GN=RAB12 PE=1 SV=3 - [RAB12_HUMAN]	31.56	1	6	7	22	0.71	1.05	0.82	0.94	0.10	0.34	0.04	0.32	0.18	0.06	0.00	0.17	-0.71	-0.75	-0.86	-0.72	-0.66	-0.48	-0.74	-0.71
Q9UI09	NADH dehydrogenase [ubiquinone] 1 alpha subcomplex subunit 12 OS=Homo sapiens GN=NDUFA12 PE=1 SV=1 - [NDUAC_HUMAN]	71.72	1	8	8	24	0.73	0.65	0.92	0.85	-0.39	-0.37	0.05	0.11	-0.24	-0.21	-0.05	-0.15	-0.38	-0.73	-1.14	-0.65	-1.03	-0.76	-1.09	-0.71
P53365	Arfaptin-2 OS=Homo sapiens GN=ARFIP2 PE=1 SV=1 - [ARFP2_HUMAN]	14.37	1	4	4	5	1.33	0.93	1.40	0.91	0.33	0.07	0.59	0.41	0.24	0.53	0.55	0.19	-0.68	-0.57	-0.65	-0.72	-0.72	-0.91	-0.92	-0.71
Q9NQ88	Fructose-2,6-bisphosphatase TIGAR OS=Homo sapiens GN=TIGAR PE=1 SV=1 - [TIGAR_HUMAN]	8.89	1	2	2	3	1.21	1.12	1.46	1.37	0.50	0.41	0.68	0.59	0.39	0.49	0.35	0.25	-0.47	-0.85	-1.11	-0.69	-0.94	-0.72	-0.97	-0.71
O94826	Mitochondrial import receptor subunit TOM70 OS=Homo sapiens GN=TOMM70A PE=1 SV=1 - [TOM70_HUMAN]	48.36	1	24	24	61	0.82	0.79	0.96	1.01	-0.21	-0.24	0.17	0.24	-0.06	0.01	0.02	-0.01	-0.45	-0.78	-0.97	-0.76	-1.04	-0.88	-1.20	-0.71

Q9Y3S1	Serine/threonine-protein kinase WNK2 OS=Homo sapiens GN=WNK2 PE=1 SV=4 - [WNK2_HUMAN]	9.66	1	10	14	26	1.35	1.17	1.00	0.75	-0.15	-0.37	0.08	0.13	0.11	0.26	-0.13	-0.27	-1.01	-1.41	-0.90	-1.05	-0.74	-1.23	-1.20	-0.71
Q9NR46	Endophilin-B2 OS=Homo sapiens GN=SH3GLB2 PE=1 SV=1 - [SHLB2_HUMAN]	27.34	1	11	12	25	1.22	1.32	1.26	1.26	-0.32	-0.44	0.48	0.43	0.00	0.04	-0.13	-0.12	-0.63	-1.30	-1.29	-1.02	-1.12	-1.49	-1.54	-0.71
P28845	Corticosteroid 11-beta-dehydrogenase isozyme 1 OS=Homo sapiens GN=HSD11B1 PE=1 SV=3 - [DHI1_HUMAN]	5.82	1	2	2	3	0.16	0.23	0.17	0.23	-0.20	-0.14	-0.61	-0.54	0.05	-0.01	0.63	0.69	-0.72	0.47	0.46	-0.14	-0.14	-0.38	-0.39	-0.71
P62158	Calmodulin OS=Homo sapiens GN=CALM1 PE=1 SV=2 - [CALM_HUMAN]	82.55	2	8	10	184	0.75	0.53	1.11	0.96	-0.12	-0.22	0.29	0.26	0.01	0.08	-0.15	-0.23	-0.36	-0.74	-1.29	-0.54	-0.91	-0.88	-1.30	-0.71
Q9H1P3	Oxysterol-binding protein-related protein 2 OS=Homo sapiens GN=OSBPL2 PE=1 SV=1 - [OSBL2_HUMAN]	11.25	1	4	4	7	1.21	1.17	1.33	1.37	0.10	-0.07	0.51	0.51	0.17	0.35	0.04	-0.03	-0.68	-1.14	-1.22	-0.86	-0.87	-1.16	-1.13	-0.71
P09455	Retinol-binding protein 1 OS=Homo sapiens GN=RBP1 PE=1 SV=2 - [RET1_HUMAN]	51.85	1	6	6	16	1.46	1.57	1.32	1.49	0.24	0.10	0.84	0.82	0.39	0.38	0.16	0.17	-0.74	-1.36	-1.20	-1.17	-0.97	-1.24	-1.07	-0.71
Q9H1Z4	WD repeat-containing protein 13 OS=Homo sapiens GN=WDR13 PE=1 SV=2 - [WDR13_HUMAN]	26.19	1	9	9	19	0.86	0.88	0.76	0.69	-0.19	-0.30	0.01	-0.09	-0.10	-0.09	0.02	0.03	-0.81	-0.82	-0.79	-0.88	-0.79	-1.06	-0.97	-0.71
Q8WWI5	Choline transporter-like protein 1 OS=Homo sapiens GN=SLC44A1 PE=1 SV=1 - [CTL1_HUMAN]	7.00	1	4	4	17	1.06	0.76	0.77	0.91	0.07	-0.17	-0.01	0.02	0.04	-0.07	0.42	0.33	-0.69	-1.04	-0.79	-0.84	-0.83	-1.03	-1.21	-0.72

Q96FW1	Ubiquitin thioesterase OTUB1 OS=Homo sapiens GN=OTUB1 PE=1 SV=2 - [OTUB1_HUMAN]	42.07	1	8	8	34	1.33	1.36	1.33	1.51	0.33	0.35	0.47	0.53	0.41	0.44	-0.05	0.11	-0.52	-1.28	-1.30	-0.79	-0.88	-0.90	-0.97	-0.72
P63027	Vesicle-associated membrane protein 2 OS=Homo sapiens GN=VAMP2 PE=1 SV=3 - [VAMP2_HUMAN]	35.34	1	2	4	32	1.41	1.72	1.42	1.44	-0.23	-0.02	0.46	0.71	0.65	0.27	0.19	0.50	-0.80	-1.29	-1.36	-1.15	-1.03	-1.76	-1.57	-0.72
Q14289	Protein-tyrosine kinase 2-beta OS=Homo sapiens GN=PTK2B PE=1 SV=2 - [FAK2_HUMAN]	27.45	1	23	23	39	1.10	1.20	1.13	0.96	0.26	0.35	0.35	0.30	0.16	0.16	0.24	0.21	-0.71	-1.03	-0.83	-0.77	-0.52	-0.82	-0.71	-0.72
O60518	Ran-binding protein 6 OS=Homo sapiens GN=RANBP6 PE=1 SV=2 - [RNBP6_HUMAN]	9.77	1	6	8	10	0.68	0.84	0.98	1.19	0.14	0.34	0.25	0.46	0.37	0.16	0.06	0.27	-0.53	-0.70	-0.78	-0.65	-0.76	-0.64	-0.78	-0.72
Q08AE8	Protein spire homolog 1 OS=Homo sapiens GN=SPIRE1 PE=1 SV=3 - [SPIR1_HUMAN]	7.28	1	4	4	4	0.35	1.30	0.67	1.35	0.16	0.75	-0.12	0.57	0.45	0.16	0.06	0.56	-0.68	-0.86	-0.91	-0.20	-0.71	-0.21	-0.54	-0.72
Q9NZ45	CDGSH iron-sulfur domain containing protein 1 OS=Homo sapiens GN=CISD1 PE=1 SV=1 - [CISD1_HUMAN]	44.44	1	5	5	25	1.02	0.98	1.46	1.39	0.24	0.11	0.67	0.57	0.21	0.32	0.33	0.22	-0.34	-0.72	-1.18	-0.72	-1.11	-0.87	-1.30	-0.72
P43007	Neutral amino acid transporter A OS=Homo sapiens GN=SLC1A4 PE=1 SV=1 - [SATT_HUMAN]	18.80	1	4	6	31	1.65	1.77	1.51	1.61	0.17	0.13	0.73	0.90	0.42	0.57	0.30	0.79	-0.85	-1.24	-1.22	-1.10	-0.85	-1.41	-1.29	-0.72
Q3ZCW2	Galectin-related protein OS=Homo sapiens GN=LGALSL PE=1 SV=2 - [LEGL_HUMAN]	48.84	1	6	6	20	0.86	1.49	1.35	1.37	0.47	0.82	0.55	0.50	0.66	0.46	0.14	0.45	-0.87	-0.74	-0.88	-0.71	-0.65	-0.65	-0.60	-0.72

O00139	Kinesin-like protein KIF2A OS=Homo sapiens GN=KIF2A PE=1 SV=3 - [KIF2A_HUMAN]	33.85	2	17	18	31	1.25	1.34	1.33	1.30	0.07	0.14	0.54	0.47	0.55	0.43	0.33	0.40	-0.69	-1.18	-1.00	-0.66	-0.90	-1.21	-1.32	-0.72
P23471	Receptor-type tyrosine-protein phosphatase zeta OS=Homo sapiens GN=PTPRZ1 PE=1 SV=4 - [PTPRZ_HUMAN]	10.63	1	21	21	84	1.55	1.34	1.63	1.64	0.20	0.12	0.90	0.84	0.52	0.63	0.55	0.38	-0.52	-0.91	-1.23	-0.90	-1.07	-1.22	-1.61	-0.72
P49619	Diacylglycerol kinase gamma OS=Homo sapiens GN=DGKG PE=2 SV=3 - [DGKG_HUMAN]	7.84	1	4	4	5	1.54	1.23	1.56	1.32	0.71	0.45	0.70	0.43	0.39	0.66	0.46	0.45	-0.78	-0.59	-0.69	-0.84	-0.72	-0.70	-0.80	-0.72
Q6PFW1	Inositol hexakisphosphate and diphosphoinositol-pentakisphosphate kinase 1 OS=Homo sapiens GN=PPIP5K1 PE=1 SV=1 - [VIP1_HUMAN]	1.88	1	1	2	4	0.60	0.25	1.03	0.67	0.25	-0.11	0.24	-0.11	-0.31	0.05	0.10	-0.25	-0.30	-0.49	-0.92	-0.52	-0.95	-0.37	-0.80	-0.72
Q8TBX8	Phosphatidylinositol 5-phosphate 4-kinase type-2 gamma OS=Homo sapiens GN=PIP4K2C PE=1 SV=3 - [PI42C_HUMAN]	15.20	1	8	8	17	0.87	0.89	1.09	1.06	0.20	0.20	0.30	0.35	0.05	0.06	0.01	0.15	-0.52	-0.81	-0.89	-0.89	-0.97	-0.75	-0.85	-0.72
P67775	Serine/threonine-protein phosphatase 2A catalytic subunit alpha isoform OS=Homo sapiens GN=PPP2CA PE=1 SV=1 - [PP2AA_HUMAN]	49.84	1	2	12	38	0.84	0.80	1.22	1.14	-0.25	-0.17	0.33	0.35	0.07	0.03	-0.15	-0.24	-0.38	-1.15	-1.55	-0.78	-1.12	-1.04	-1.32	-0.72
O43759	Synaptogyrin-1 OS=Homo sapiens GN=SYNGR1 PE=1 SV=2 - [SNG1_HUMAN]	5.15	1	1	1	3	1.62	1.50	1.53	1.41	0.09	-0.04	0.74	0.62	0.27	0.40	0.39	0.27	-0.82	-1.22	-1.14	-1.19	-1.10	-1.55	-1.46	-0.72

Q9Y6D5	Brefeldin A-inhibited guanine nucleotide-exchange protein 2 OS=Homo sapiens GN=ARFGEF2 PE=1 SV=3 - [BIG2_HUMAN]	15.13	1	17	21	32	0.86	0.78	0.93	0.83	-0.16	-0.23	0.24	0.26	-0.01	0.07	0.13	0.05	-0.61	-0.81	-0.98	-0.72	-0.73	-0.95	-1.06	-0.72
Q96C86	m7GpppX diphosphatase OS=Homo sapiens GN=DCPS PE=1 SV=2 - [DCPS_HUMAN]	14.84	1	4	4	6	0.80	0.53	0.75	0.94	0.16	-0.07	-0.03	-0.01	-0.11	-0.27	-0.06	-0.01	-0.78	-0.75	-0.69	-1.22	-1.16	-0.83	-1.00	-0.72
Q99798	Aconitate hydratase, mitochondrial OS=Homo sapiens GN=ACO2 PE=1 SV=2 - [ACON_HUMAN]	52.05	1	37	37	162	0.87	1.03	1.05	1.16	-0.31	-0.16	0.20	0.35	0.08	-0.02	-0.06	0.06	-0.60	-1.00	-1.12	-0.85	-0.95	-1.24	-1.33	-0.72
O94910	Latrophilin-1 OS=Homo sapiens GN=LPHN1 PE=1 SV=1 - [LPHN1_HUMAN]	6.24	1	7	7	10	1.16	0.81	0.89	0.49	-0.10	-0.03	0.03	-0.31	-0.04	0.24	-0.07	-0.10	-1.07	-1.52	-1.04	-1.32	-0.68	-1.71	-1.08	-0.72
Q9Y266	Nuclear migration protein nudC OS=Homo sapiens GN=NUDC PE=1 SV=1 - [NUDC_HUMAN]	39.88	1	13	13	26	0.43	0.56	0.80	0.83	-0.24	-0.29	0.18	0.26	-0.24	-0.22	-0.44	-0.34	-0.24	-0.92	-1.28	-0.79	-1.11	-0.85	-1.20	-0.72
Q12849	G-rich sequence factor 1 OS=Homo sapiens GN=GRSF1 PE=1 SV=3 - [GRSF1_HUMAN]	13.13	1	4	4	6	0.75	0.44	0.58	0.26	-0.40	-0.73	-0.22	-0.54	-0.57	-0.25	-0.32	-0.64	-0.89	-1.00	-1.03	-0.94	-0.83	-1.17	-1.01	-0.73
Q9P1Z2	Calcium-binding and coiled-coil domain-containing protein 1 OS=Homo sapiens GN=CALCOCO1 PE=1 SV=2 - [CACO1_HUMAN]	12.45	1	7	7	13	1.01	0.91	0.87	0.83	0.15	0.09	-0.15	-0.06	0.20	0.31	0.62	0.57	-0.84	-0.37	-0.18	-0.65	-0.34	-0.77	-0.47	-0.73
Q15111	Inactive phospholipase C-like protein 1 OS=Homo sapiens GN=PLCL1 PE=1 SV=3 - [PLCL1_HUMAN]	11.78	1	9	9	13	1.24	1.47	1.32	1.76	0.17	0.32	0.67	0.82	0.40	0.31	0.12	0.46	-0.49	-0.98	-1.23	-0.68	-0.95	-0.93	-1.25	-0.73

Q14643	Inositol 1,4,5-trisphosphate receptor type 1 OS=Homo sapiens GN=ITPR1 PE=1 SV=3 - [ITPR1_HUMAN]	13.45	1	28	31	47	0.60	0.65	0.68	0.72	-0.27	-0.21	-0.14	-0.01	-0.13	-0.22	-0.16	-0.21	-0.69	-0.84	-0.99	-0.85	-0.94	-0.84	-1.03	-0.73
Q684P5	Rap1 GTPase-activating protein 2 OS=Homo sapiens GN=RAP1GAP2 PE=1 SV=2 - [RPGP2_HUMAN]	20.41	1	9	9	12	1.70	1.52	1.08	1.04	0.05	-0.14	0.49	0.39	-0.03	0.31	0.33	0.29	-0.98	-0.99	-0.81	-1.16	-0.78	-1.19	-0.95	-0.73
Q9UEE9	Craniofacial development protein 1 OS=Homo sapiens GN=CFDP1 PE=1 SV=1 - [CFDP1_HUMAN]	7.02	1	1	1	2	0.25	0.07	0.89	0.71	0.26	0.08	0.10	-0.08	-0.04	0.14	-0.43	-0.61	-0.09	-0.67	-1.32	-0.07	-0.71	0.00	-0.64	-0.73
Q9NPB8	Glycerophosphocholine phosphodiesterase GPCPD1 OS=Homo sapiens GN=GPCPD1 PE=1 SV=2 - [GPCP1_HUMAN]	3.13	1	2	2	2	0.77	0.47	0.50	0.20	0.05	-0.26	-0.29	-0.59	-0.86	-0.55	-0.60	-0.91	-1.01	-1.37	-1.11	-1.30	-1.02	-0.74	-0.47	-0.73
Q8NFW8	N-acylneuraminate cytidylyltransferase OS=Homo sapiens GN=CMAS PE=1 SV=2 - [NEUA_HUMAN]	35.71	1	13	13	23	0.43	0.39	0.49	0.47	-0.26	-0.36	-0.21	-0.21	-0.39	-0.31	-0.34	-0.50	-0.46	-0.77	-0.99	-0.80	-0.78	-0.68	-0.80	-0.73
Q96D71	RalBP1-associated Eps domain-containing protein 1 OS=Homo sapiens GN=REPS1 PE=1 SV=3 - [REPS1_HUMAN]	5.78	1	3	4	7	0.38	0.39	0.73	0.73	-0.16	-0.16	-0.07	-0.06	-0.33	-0.32	-0.33	-0.33	-0.40	-0.71	-1.06	-0.68	-1.02	-0.56	-0.90	-0.73
P00403	Cytochrome c oxidase subunit 2 OS=Homo sapiens GN=MT-CO2 PE=1 SV=1 - [COX2_HUMAN]	27.75	1	6	6	30	0.94	1.19	0.95	1.13	0.11	0.22	0.23	0.39	0.30	0.12	0.25	0.34	-0.70	-0.77	-0.69	-0.78	-0.78	-1.05	-0.85	-0.73

Q92783	Signal transducing adapter molecule 1 OS=Homo sapiens GN=STAM PE=1 SV=3 - [STAM1_HUMAN]	26.48	1	9	10	16	0.64	0.95	0.78	1.07	0.11	0.30	0.24	0.44	0.31	0.08	-0.01	0.29	-0.28	-0.46	-0.75	-0.50	-0.64	-0.67	-0.81	-0.73
O75170	Serine/threonine-protein phosphatase 6 regulatory subunit 2 OS=Homo sapiens GN=PPP6R2 PE=1 SV=2 - [PP6R2_HUMAN]	18.01	1	12	12	20	0.76	0.76	0.87	0.79	-0.03	-0.08	0.02	0.01	0.09	0.23	0.37	0.27	-0.75	-0.40	-0.56	-0.48	-0.35	-0.97	-1.04	-0.73
Q9BW30	Tubulin polymerization-promoting protein family member 3 OS=Homo sapiens GN=TPPP3 PE=1 SV=1 - [TPPP3_HUMAN]	48.30	1	9	9	16	0.03	0.14	0.48	0.62	-0.56	-0.54	-0.29	-0.15	-0.38	-0.46	-0.43	-0.53	-0.30	-0.82	-1.41	-0.42	-0.92	-0.72	-1.21	-0.73
P60520	Gamma-aminobutyric acid receptor-associated protein-like 2 OS=Homo sapiens GN=GABARAPL2 PE=1 SV=1 - [GBRL2_HUMAN]	39.32	3	5	5	13	0.95	0.98	1.28	1.47	0.67	0.86	0.44	0.69	0.51	0.33	0.22	0.49	-0.24	-0.64	-0.97	-0.48	-0.83	-0.20	-0.58	-0.73
Q9P0L0	Vesicle-associated membrane protein-associated protein A OS=Homo sapiens GN=VAPA PE=1 SV=3 - [VAPA_HUMAN]	51.81	1	12	13	40	0.27	0.25	0.79	0.85	-0.28	-0.34	-0.09	-0.08	-0.29	-0.28	-0.02	-0.01	-0.22	-0.27	-0.72	-0.50	-0.98	-0.58	-1.03	-0.73
O60313	Dynamin-like 120 kDa protein, mitochondrial OS=Homo sapiens GN=OPA1 PE=1 SV=3 - [OPA1_HUMAN]	44.17	1	35	35	80	1.00	1.15	1.26	1.27	-0.22	-0.24	0.43	0.47	0.13	0.08	0.09	0.13	-0.63	-1.09	-1.21	-0.97	-1.02	-1.37	-1.54	-0.73

A2RTX5	Probable threonine--tRNA ligase 2, cytoplasmic OS=Homo sapiens GN=TARSL2 PE=1 SV=1 - [SYTC2_HUMAN]	5.24	1	3	4	7	0.69	0.56	0.73	0.60	-0.14	-0.27	-0.07	-0.20	-0.12	0.01	0.03	-0.10	-0.70	-0.66	-0.70	-0.65	-0.68	-0.85	-0.89	-0.73
Q5T0D9	Tumor protein p63-regulated gene 1-like protein OS=Homo sapiens GN=TPRG1L PE=1 SV=1 - [TPRGL_HUMAN]	42.65	1	7	7	15	1.69	1.64	1.60	1.64	0.22	0.28	0.77	0.82	0.38	0.41	0.02	0.02	-0.76	-1.69	-1.70	-1.19	-1.15	-1.42	-1.34	-0.73
Q5JTZ9	Alanine--tRNA ligase, mitochondrial OS=Homo sapiens GN=AARS2 PE=1 SV=1 - [SYAM_HUMAN]	6.19	1	5	5	6	0.38	0.21	-0.35	-0.51	-0.99	-1.15	-1.14	-1.31	-0.90	-0.73	-0.79	-0.96	-1.47	-1.16	-0.44	-1.08	-0.35	-1.38	-0.66	-0.73
P78417	Glutathione S transferase omega-1 OS=Homo sapiens GN=GSTO1 PE=1 SV=2 - [GSTO1_HUMAN]	34.02	1	8	8	18	0.49	0.83	0.66	0.87	-0.15	-0.15	-0.12	0.07	-0.25	-0.22	-0.79	-0.84	-0.64	-1.22	-1.23	-0.79	-0.92	-0.52	-0.56	-0.73
P55011	Solute carrier family 12 member 2 OS=Homo sapiens GN=SLC12A2 PE=1 SV=1 - [S12A2_HUMAN]	17.00	2	15	15	27	0.89	1.10	0.95	1.08	-0.06	-0.06	0.23	0.41	0.24	0.10	0.26	0.37	-0.57	-0.68	-0.76	-0.63	-0.82	-1.24	-1.20	-0.73
P13591	Neural cell adhesion molecule 1 OS=Homo sapiens GN=NCAM1 PE=1 SV=3 - [NCAM1_HUMAN]	41.03	1	28	28	164	1.28	1.26	1.56	1.57	0.31	0.26	0.78	0.81	0.47	0.49	0.63	0.64	-0.40	-0.73	-1.07	-0.81	-1.09	-1.06	-1.33	-0.73
O15217	Glutathione S transferase A4 OS=Homo sapiens GN=GSTA4 PE=1 SV=1 - [GSTA4_HUMAN]	3.60	1	1	1	1	0.70	1.13	0.89	1.33	0.28	0.71	0.10	0.53	0.53	0.11	-0.40	0.02	-0.55	-1.10	-1.30	-0.57	-0.75	-0.44	-0.63	-0.73

O95571	Persulfide dioxygenase ETHE1, mitochondrial OS=Homo sapiens GN=ETHE1 PE=1 SV=2 - [ETHE1_HUMAN]	5.51	1	1	1	3	0.07	-0.05	0.14	0.02	-0.22	-0.35	-0.66	-0.78	-0.57	-0.44	-0.48	-0.61	-0.68	-0.55	-0.62	-0.48	-0.55	-0.31	-0.38	-0.73
Q9Y250	Leucine zipper putative tumor suppressor 1 OS=Homo sapiens GN=LZTS1 PE=1 SV=3 - [LZTS1_HUMAN]	10.91	1	4	5	8	0.56	0.74	0.21	0.40	-0.56	-0.38	-0.59	-0.40	-0.45	-0.63	-0.93	-0.75	-1.09	-1.49	-1.15	-1.15	-0.80	-1.14	-0.79	-0.73
Q9Y2I8	WD repeat-containing protein 37 OS=Homo sapiens GN=WDR37 PE=1 SV=2 - [WDR37_HUMAN]	25.10	1	10	10	16	1.20	1.38	1.41	1.34	0.12	0.02	0.57	0.56	0.36	0.36	0.11	0.16	-0.74	-1.19	-1.18	-1.02	-0.97	-1.36	-1.28	-0.73
Q8TF74	WAS/WASL-interacting protein family member 2 OS=Homo sapiens GN=WIPF2 PE=1 SV=1 - [WIPF2_HUMAN]	29.09	1	8	8	13	0.80	0.39	0.70	0.47	0.25	0.00	0.00	-0.32	-0.49	-0.13	-0.01	-0.34	-0.94	-0.94	-0.81	-0.85	-0.80	-0.71	-0.48	-0.73
Q96HR9	Receptor expression-enhancing protein 6 OS=Homo sapiens GN=REEP6 PE=1 SV=1 - [REEP6_HUMAN]	11.41	1	2	2	3	1.05	1.29	0.78	1.03	-0.13	0.11	-0.02	0.22	0.05	-0.19	-0.09	0.15	-1.01	-1.13	-0.87	-1.20	-0.94	-1.19	-0.93	-0.74
Q9NP78	ATP-binding cassette sub-family B member 9 OS=Homo sapiens GN=ABCB9 PE=1 SV=1 - [ABCB9_HUMAN]	1.83	1	1	1	1	1.52	0.48	1.48	0.44	1.17	0.12	0.68	-0.36	-0.41	0.64	0.12	-0.93	-0.78	-1.40	-1.37	-0.85	-0.81	-0.36	-0.33	-0.74
P08842	Steryl-sulfatase OS=Homo sapiens GN=STS PE=1 SV=2 - [STS_HUMAN]	8.92	1	6	6	9	0.78	0.77	0.90	0.89	0.11	0.10	0.10	0.09	0.06	0.07	0.05	0.03	-0.63	-0.73	-0.85	-0.68	-0.80	-0.69	-0.81	-0.74

Q16204	Coiled-coil domain-containing protein 6 OS=Homo sapiens GN=CCDC6 PE=1 SV=2 - [CCDC6_HUMAN]	33.54	1	15	15	27	0.90	0.89	0.87	0.92	-0.36	-0.18	-0.01	0.05	-0.06	-0.12	-0.11	-0.14	-0.78	-0.88	-0.94	-1.08	-1.05	-1.10	-1.17	-0.74
Q9Y2Z4	Tyrosine--tRNA ligase, mitochondrial OS=Homo sapiens GN=YARS2 PE=1 SV=2 - [SYYM_HUMAN]	20.55	1	7	7	11	0.34	0.21	0.39	0.24	-0.60	-0.54	-0.20	-0.57	-0.86	-0.49	-0.40	-0.48	-0.79	-0.67	-0.78	-0.84	-0.94	-1.10	-0.87	-0.74
Q9UKF7	Cytoplasmic phosphatidylinositol transfer protein 1 OS=Homo sapiens GN=PITPNC1 PE=1 SV=3 - [PITC1_HUMAN]	6.33	1	2	2	4	0.92	1.40	1.23	1.71	-0.10	0.38	0.43	0.91	0.42	-0.06	-0.48	0.00	-0.43	-1.39	-1.71	-0.94	-1.25	-1.03	-1.35	-0.74
Q9NPG2	Neuroglobin OS=Homo sapiens GN=NGB PE=1 SV=1 - [NGB_HUMAN]	12.58	1	1	1	2	1.55	1.32	1.12	0.89	0.77	0.53	0.32	0.09	-0.13	0.11	0.00	-0.24	-1.17	-1.55	-1.13	-1.41	-0.98	-0.80	-0.38	-0.74
Q8WWC4	Uncharacterized protein C2orf47, mitochondrial OS=Homo sapiens GN=C2orf47 PE=1 SV=1 - [CB047_HUMAN]	17.87	1	5	5	7	0.55	0.59	0.62	0.79	0.20	-0.37	-0.18	-0.35	0.08	0.00	-0.04	-0.21	-0.68	-0.60	-0.67	-0.52	-0.74	-0.55	-0.74	-0.74
B4DS77	Protein shisa-9 OS=Homo sapiens GN=SHISA9 PE=2 SV=3 - [SHSA9_HUMAN]	2.36	1	1	1	1	0.89	1.47	0.89	1.47	0.09	0.66	0.09	0.66	0.48	-0.09	0.28	0.85	-0.75	-0.60	-0.61	-0.95	-0.95	-0.82	-0.82	-0.74
Q6KCM7	Calcium-binding mitochondrial carrier protein SCaMC-2 OS=Homo sapiens GN=SLC25A25 PE=1 SV=1 - [SCMC2_HUMAN]	27.72	1	10	10	14	1.37	1.19	1.28	1.24	0.16	0.13	0.50	0.64	0.30	0.23	0.31	0.28	-0.60	-0.88	-1.11	-0.92	-0.90	-1.25	-1.20	-0.74
P61960	Ubiquitin-fold modifier 1 OS=Homo sapiens GN=UFM1 PE=1 SV=1 - [UFM1_HUMAN]	27.06	1	2	2	3	0.56	0.63	0.60	0.67	-0.06	0.01	-0.20	-0.13	-0.09	-0.16	-0.20	-0.14	-0.70	-0.75	-0.81	-0.69	-0.73	-0.63	-0.68	-0.74

Q96S38	Ribosomal protein S6 kinase delta-1 OS=Homo sapiens GN=RPS6KC1 PE=1 SV=2 - [KS6C1_HUMAN]	3.19	1	2	2	3	0.59	0.91	0.37	0.69	-0.33	-0.01	-0.44	-0.11	-0.05	-0.36	-0.38	-0.06	-0.97	-0.96	-0.75	-0.92	-0.70	-0.94	-0.72	-0.74
O43776	Asparagine--tRNA ligase, cytoplasmic OS=Homo sapiens GN=NARS PE=1 SV=1 - [SYNC_HUMAN]	35.58	1	14	14	31	0.80	0.78	0.89	0.77	0.02	0.04	0.16	0.08	0.04	0.05	-0.14	-0.03	-0.72	-0.80	-0.84	-0.78	-0.89	-0.80	-0.86	-0.74
O00401	Neural Wiskott-Aldrich syndrome protein OS=Homo sapiens GN=WASL PE=1 SV=2 - [WASL_HUMAN]	21.19	1	7	7	17	0.57	0.61	0.59	0.64	-0.05	-0.05	-0.22	-0.06	-0.31	-0.29	-0.40	-0.25	-0.69	-0.85	-0.97	-0.82	-0.83	-0.76	-0.88	-0.74
O43687	A-kinase anchor protein 7 isoforms alpha and beta OS=Homo sapiens GN=AKAP7 PE=1 SV=4 - [AKA7A_HUMAN]	28.85	2	3	3	4	1.03	0.90	1.49	1.36	0.13	0.00	0.68	0.55	0.23	0.37	0.65	0.51	-0.30	-0.38	-0.84	-0.63	-1.08	-0.92	-1.37	-0.74
Q3YEC7	Rab-like protein 6 OS=Homo sapiens GN=RABL6 PE=1 SV=2 - [RABL6_HUMAN]	18.79	1	9	9	13	1.09	0.86	1.10	1.06	0.12	0.13	0.26	0.30	0.06	0.17	-0.10	-0.08	-0.68	-1.18	-1.31	-0.84	-0.90	-0.96	-1.18	-0.74
Q15223	Poliovirus receptor-related protein 1 OS=Homo sapiens GN=PVRL1 PE=1 SV=3 - [PVRL1_HUMAN]	21.47	1	7	7	13	0.50	0.96	0.89	1.22	-0.42	-0.50	0.10	0.21	-0.27	0.05	0.16	-0.37	-0.62	-0.70	-1.00	-0.82	-0.83	-0.76	-1.15	-0.74
P36776	Lon protease homolog, mitochondrial OS=Homo sapiens GN=LONP1 PE=1 SV=2 - [LONM_HUMAN]	29.61	1	20	20	42	0.18	0.11	0.27	0.25	-0.78	-0.79	-0.46	-0.59	-0.67	-0.69	-0.65	-0.69	-0.59	-0.89	-1.02	-0.87	-0.96	-0.89	-1.01	-0.74

Q9Y697	Cysteine desulfurase, mitochondrial OS=Homo sapiens GN=NFS1 PE=1 SV=3 - [NFS1_HUMAN]	28.45	1	11	11	21	0.70	0.45	0.67	0.56	-0.16	-0.42	-0.02	-0.16	-0.18	-0.07	-0.05	-0.10	-0.67	-0.69	-0.64	-0.48	-0.69	-1.00	-1.06	-0.74
P40926	Malate dehydrogenase, mitochondrial OS=Homo sapiens GN=MDH2 PE=1 SV=3 - [MDHM_HUMAN]	50.00	1	14	14	110	0.72	0.64	1.16	0.98	-0.15	-0.10	0.21	0.23	0.02	-0.04	-0.29	-0.24	-0.51	-0.89	-1.25	-0.83	-1.06	-0.93	-1.18	-0.75
Q8NE01	Metal transporter CNNM3 OS=Homo sapiens GN=CNNM3 PE=1 SV=1 - [CNNM3_HUMAN]	2.69	1	2	2	3	0.75	0.90	1.05	1.20	0.03	0.17	0.24	0.39	0.22	0.08	0.34	0.48	-0.45	-0.40	-0.71	-0.64	-0.94	-0.74	-1.04	-0.75
Q96B97	SH3 domain-containing kinase-binding protein 1 OS=Homo sapiens GN=SH3KBP1 PE=1 SV=2 - [SH3K1_HUMAN]	16.84	1	8	8	12	0.30	0.30	0.91	0.87	-0.22	-0.30	0.19	0.16	-0.11	-0.10	-0.17	-0.15	-0.29	-0.20	-0.93	-0.33	-0.86	-0.34	-1.10	-0.75
P56181	NADH dehydrogenase [ubiquinone] flavoprotein 3, mitochondrial OS=Homo sapiens GN=NDUFV3 PE=1 SV=2 - [NDUV3_HUMAN]	34.26	1	2	2	7	0.75	0.65	1.55	1.51	0.18	-0.02	0.74	0.54	-0.03	0.20	0.56	0.32	-0.01	-0.23	-1.05	-0.63	-1.48	-0.65	-1.46	-0.75
P10515	Dihydrolipoyllysine-residue acetyltransferase component of pyruvate dehydrogenase complex, mitochondrial OS=Homo sapiens GN=DLAT PE=1 SV=3 - [ODP2_HUMAN]	22.87	1	12	12	23	0.58	0.63	0.84	0.82	-0.44	-0.43	-0.02	0.00	-0.08	-0.16	-0.02	-0.11	-0.59	-0.80	-0.91	-0.80	-0.87	-1.01	-1.23	-0.75

Q9NSD9	Phenylalanine--tRNA ligase beta subunit OS=Homo sapiens GN=FARSB PE=1 SV=3 - [SYFB_HUMAN]	40.24	1	20	20	40	0.73	0.57	0.87	0.78	-0.11	-0.15	0.09	0.10	-0.21	-0.18	-0.41	-0.48	-0.54	-0.98	-1.24	-0.88	-1.11	-0.83	-0.93	-0.75
P55789	FAD-linked sulfhydryl oxidase ALR OS=Homo sapiens GN=GFER PE=1 SV=2 - [ALR_HUMAN]	11.71	1	2	2	5	1.15	1.29	1.00	1.14	-0.60	-0.47	0.19	0.33	0.12	-0.01	-0.13	0.01	-0.90	-1.27	-1.13	-1.13	-0.98	-1.77	-1.62	-0.75
Q9BRT2	Ubiquinol-cytochrome-c reductase complex assembly factor 2 OS=Homo sapiens GN=UQCC2 PE=1 SV=1 - [UQCC2_HUMAN]	46.03	1	5	5	10	0.65	0.76	0.94	1.28	0.03	0.26	0.22	0.52	0.64	0.12	0.24	0.42	-0.29	-0.35	-0.62	-0.40	-0.69	-0.64	-1.08	-0.75
Q8WXF7	Atlastin-1 OS=Homo sapiens GN=ATL1 PE=1 SV=1 - [ATLA1_HUMAN]	37.63	1	17	18	43	0.70	0.80	0.87	1.11	-0.43	-0.39	0.21	0.31	-0.10	-0.18	-0.07	-0.09	-0.59	-1.01	-0.82	-0.98	-1.02	-1.25	-1.43	-0.75
Q7Z403	Transmembrane channel-like protein 6 OS=Homo sapiens GN=TMC6 PE=2 SV=2 - [TMC6_HUMAN]	4.84	1	2	2	2	0.97	-0.09	0.66	-0.39	0.02	-1.04	-0.15	-1.20	-1.11	-0.05	0.52	-0.54	-1.06	-0.44	-0.15	-0.99	-0.68	-0.96	-0.66	-0.75
P50583	Bis(5'-nucleosyl)-tetraphosphatase [asymmetrical] OS=Homo sapiens GN=NUDT2 PE=1 SV=3 - [AP4A_HUMAN]	4.76	1	1	1	1	0.94	0.97	0.88	0.92	0.20	0.23	0.07	0.10	0.04	0.01	0.18	0.21	-0.82	-0.75	-0.70	-0.90	-0.84	-0.76	-0.70	-0.75
P38159	RNA-binding motif protein, X chromosome OS=Homo sapiens GN=RBMX PE=1 SV=3 - [RBMX_HUMAN]	21.23	3	3	7	17	-0.38	-0.34	0.07	0.11	-0.63	-0.60	-0.74	-0.71	-0.58	-0.62	-0.16	-0.12	-0.31	0.23	-0.23	-0.21	-0.66	-0.27	-0.72	-0.75
Q9H7D7	WD repeat-containing protein 26 OS=Homo sapiens GN=WDR26 PE=1 SV=3 - [WDR26_HUMAN]	3.48	1	2	2	4	0.13	-0.12	-0.04	-0.28	0.14	-0.11	-0.86	-1.10	-0.46	-0.21	0.09	-0.16	-0.93	-0.03	0.13	-0.31	-0.14	-0.01	0.16	-0.75

O95292	Vesicle-associated membrane protein-associated protein B/C OS=Homo sapiens GN=VAPB PE=1 SV=3 - [VAPB_HUMAN]	42.39	1	8	10	20	0.46	0.62	0.77	0.95	-0.36	-0.36	-0.04	0.13	-0.25	-0.31	-0.03	0.14	-0.57	-0.45	-0.72	-0.75	-1.10	-0.96	-1.31	-0.75
Q16555	Dihydropyrimidinase-related protein 2 OS=Homo sapiens GN=DPYSL2 PE=1 SV=1 - [DPYL2_HUMAN]	65.21	1	25	29	601	1.54	1.44	1.67	1.56	0.19	0.17	0.80	0.71	0.31	0.39	0.23	0.11	-0.69	-1.27	-1.49	-1.08	-1.02	-1.28	-1.44	-0.75
P32455	Interferon-induced guanylate-binding protein 1 OS=Homo sapiens GN=GBP1 PE=1 SV=2 - [GBP1_HUMAN]	8.61	2	5	5	8	-0.09	0.14	0.28	0.20	-0.43	-0.39	-0.63	-0.55	-0.54	-0.44	-0.59	-0.55	-0.56	-0.56	-0.66	-0.44	-0.69	-0.31	-0.52	-0.75
Q9UGM6	Tryptophan--tRNA ligase, mitochondrial OS=Homo sapiens GN=WARS2 PE=1 SV=1 - [SYWM_HUMAN]	14.72	1	4	4	5	-0.23	-0.49	-0.19	-0.45	-0.33	-0.59	-1.01	-1.27	-1.09	-0.82	-0.29	-0.56	-0.72	-0.06	-0.11	-0.56	-0.60	-0.11	-0.16	-0.75
O95202	LETM1 and EF-hand domain-containing protein 1, mitochondrial OS=Homo sapiens GN=LETM1 PE=1 SV=1 - [LETM1_HUMAN]	27.33	1	17	17	34	1.09	0.88	1.35	1.12	-0.06	-0.14	0.51	0.31	0.07	0.32	0.09	-0.03	-0.53	-1.05	-1.10	-0.82	-1.03	-1.11	-1.16	-0.75
Q96MT3	Prickle-like protein 1 OS=Homo sapiens GN=PRICKLE1 PE=1 SV=2 - [PRIC1_HUMAN]	1.56	1	1	1	2	0.73	0.73	0.63	0.63	-0.51	-0.52	-0.19	-0.19	-0.02	-0.01	-0.01	-0.02	-0.87	-0.74	-0.64	-0.71	-0.61	-1.26	-1.15	-0.76
Q9Y6I3	Epsin-1 OS=Homo sapiens GN=EPN1 PE=1 SV=2 - [EPN1_HUMAN]	14.58	2	6	6	17	0.86	1.34	1.32	1.19	-0.11	-0.12	0.13	0.44	0.20	0.26	-0.17	-0.01	-0.68	-1.17	-1.37	-0.96	-0.95	-1.12	-1.43	-0.76
Q96E17	Ras-related protein Rab-3C OS=Homo sapiens GN=RAB3C PE=2 SV=1 - [RAB3C_HUMAN]	50.22	1	5	10	38	0.92	1.30	1.09	1.27	0.05	0.05	0.24	0.51	0.16	0.20	-0.02	-0.06	-0.62	-1.51	-1.54	-1.20	-1.35	-1.43	-1.46	-0.76

Q2NKQ1	Small G protein signaling modulator 1 OS=Homo sapiens GN=SGSM1 PE=1 SV=2 - [SGSM1_HUMAN]	3.40	1	2	3	4	1.38	1.24	1.10	0.96	-0.03	-0.17	0.28	0.14	0.28	0.42	-0.23	-0.37	-1.04	-1.60	-1.33	-0.93	-0.65	-1.43	-1.15	-0.76
Q92823	Neuronal cell adhesion molecule OS=Homo sapiens GN=NRCAM PE=1 SV=3 - [NRCAM_HUMAN]	31.06	1	27	27	77	1.24	1.33	1.45	1.50	0.09	0.04	0.69	0.66	0.42	0.35	0.24	0.26	-0.61	-1.09	-1.29	-1.00	-1.06	-1.34	-1.51	-0.76
Q2M2I8	AP2-associated protein kinase 1 OS=Homo sapiens GN=AAK1 PE=1 SV=3 - [AAK1_HUMAN]	42.14	1	22	23	41	1.04	1.19	0.99	1.14	-0.26	-0.42	0.17	0.29	-0.11	-0.05	-0.04	-0.12	-0.88	-1.33	-1.32	-1.20	-1.28	-1.48	-1.66	-0.76
Q00169	Phosphatidylinositol transfer protein alpha isoform OS=Homo sapiens GN=PITPNA PE=1 SV=2 - [PIPNA_HUMAN]	37.04	1	6	8	16	1.40	1.16	1.46	1.23	0.08	-0.10	0.45	0.36	0.33	0.35	-0.05	-0.21	-0.76	-1.44	-1.54	-0.61	-0.86	-1.18	-1.33	-0.76
Q8ND76	Cyclin-Y OS=Homo sapiens GN=CCNY PE=1 SV=2 - [CCNY_HUMAN]	18.18	1	4	5	10	0.53	0.22	0.51	0.18	-0.26	-0.59	-0.21	-0.35	-0.24	-0.28	-0.21	-0.17	-0.87	-0.94	-0.98	-1.21	-0.97	-0.95	-0.92	-0.76
Q9Y4E6	WD repeat-containing protein 7 OS=Homo sapiens GN=WDR7 PE=1 SV=2 - [WDR7_HUMAN]	10.20	1	12	12	19	1.18	0.94	1.34	0.96	0.05	0.02	0.42	0.34	0.18	0.31	0.13	0.04	-0.70	-0.89	-1.07	-0.76	-0.82	-1.14	-1.20	-0.76
Q8N612	FTS and Hook-interacting protein OS=Homo sapiens GN=FAM160A2 PE=1 SV=3 - [F16A2_HUMAN]	2.78	1	2	2	4	1.12	1.12	0.83	0.84	-0.27	-0.27	0.01	0.01	0.92	0.92	0.53	0.53	-1.05	-0.58	-0.30	-0.16	0.13	-1.40	-1.12	-0.76
Q15493	Regucalcin OS=Homo sapiens GN=RGN PE=1 SV=1 - [RGN_HUMAN]	28.76	1	7	7	13	1.50	1.27	1.62	1.20	0.49	-0.01	0.56	0.33	0.67	0.82	-0.13	-0.08	-0.81	-1.45	-1.82	-0.56	-0.84	-0.85	-1.22	-0.76

Q9C0E4	Glutamate receptor-interacting protein 2 OS=Homo sapiens GN=GRIP2 PE=1 SV=3 - [GRIP2_HUMAN]	0.77	2	1	1	1	1.12	1.29	1.34	1.52	-0.55	-0.38	0.52	0.70	0.02	-0.15	-0.54	-0.37	-0.54	-1.66	-1.89	-1.24	-1.46	-1.69	-1.91	-0.76
P43155	Carnitine O-acetyltransferase OS=Homo sapiens GN=CRAT PE=1 SV=5 - [CACP_HUMAN]	19.01	1	11	11	28	0.55	0.61	0.58	0.60	-0.73	-0.70	-0.25	-0.11	-0.25	-0.30	-0.40	-0.27	-0.63	-0.85	-1.04	-0.80	-0.89	-1.29	-1.29	-0.76
Q9HCM2	Plexin-A4 OS=Homo sapiens GN=PLXNA4 PE=1 SV=4 - [PLXA4_HUMAN]	23.18	1	23	33	56	1.40	1.34	1.51	1.20	0.10	-0.07	0.44	0.52	0.47	0.31	0.25	0.38	-1.00	-1.12	-1.07	-0.95	-0.84	-1.37	-1.33	-0.76
Q9H0B6	Kinesin light chain 2 OS=Homo sapiens GN=KLC2 PE=1 SV=1 - [KLC2_HUMAN]	32.96	1	11	15	29	1.00	1.18	1.45	1.29	0.21	0.20	0.45	0.46	0.36	0.23	0.34	0.43	-0.57	-0.70	-1.09	-0.66	-0.96	-0.88	-1.19	-0.76
Q02413	Desmoglein-1 OS=Homo sapiens GN=DSG1 PE=1 SV=2 - [DSG1_HUMAN]	0.95	1	1	1	1	0.91	0.88	1.02	0.99	-0.36	-0.40	0.19	0.17	-0.13	-0.10	0.15	0.11	-0.66	-0.75	-0.87	-0.98	-1.09	-1.29	-1.40	-0.76
Q8TB92	3-hydroxymethyl-3-methylglutaryl-CoA lyase, cytoplasmic OS=Homo sapiens GN=HMGCL L1 PE=1 SV=3 - [HMGC2_HUMAN]	2.43	1	1	1	1	0.86	1.05	1.23	1.42	0.72	0.90	0.40	0.60	0.87	0.69	0.56	0.75	-0.40	-0.29	-0.67	-0.14	-0.51	-0.16	-0.53	-0.76
P05771	Protein kinase C beta type OS=Homo sapiens GN=PRKCB PE=1 SV=4 - [KPCB_HUMAN]	39.49	2	17	25	68	0.88	0.93	1.22	1.40	0.11	0.23	0.33	0.39	0.22	0.08	-0.03	0.00	-0.53	-0.93	-1.25	-0.73	-0.96	-0.79	-0.92	-0.76
Q96ME1	F-box/LRR-repeat protein 18 OS=Homo sapiens GN=FBXL18 PE=1 SV=2 - [FXL18_HUMAN]	6.58	1	4	4	5	0.99	0.39	0.97	0.37	0.15	-0.46	0.14	-0.46	0.03	0.64	0.49	-0.11	-0.80	-0.49	-0.48	-0.33	-0.30	-0.86	-0.84	-0.76

P35609	Alpha-actinin-2 OS=Homo sapiens GN=ACTN2 PE=1 SV=1 - [ACTN2_HUMAN]	34.68	2	14	29	108	0.40	0.36	0.63	0.46	-0.86	-0.90	-0.17	-0.16	-0.35	-0.28	-0.68	-0.72	-0.64	-1.07	-1.12	-0.70	-0.90	-1.27	-1.50	-0.76
O95858	Tetraspanin-15 OS=Homo sapiens GN=TSPAN15 PE=2 SV=1 - [TSN15_HUMAN]	4.42	1	1	1	1	1.21	0.93	1.68	1.40	0.54	0.25	0.85	0.57	0.34	0.63	0.54	0.25	-0.30	-0.67	-1.14	-0.55	-1.01	-0.69	-1.15	-0.76
Q7L804	Rab11 family-interacting protein 2 OS=Homo sapiens GN=RAB11FIP2 PE=1 SV=1 - [RFIP2_HUMAN]	13.87	1	4	5	6	0.72	1.04	0.73	1.07	0.10	0.29	-0.02	0.22	0.15	-0.01	0.17	0.30	-0.83	-0.59	-0.98	-0.74	-0.88	-0.74	-1.11	-0.76
Q9H7Z7	Prostaglandin E synthase 2 OS=Homo sapiens GN=PTGES2 PE=1 SV=1 - [PGES2_HUMAN]	41.11	1	11	11	19	0.20	0.31	0.55	0.58	0.22	0.11	-0.15	0.12	0.06	0.03	-0.12	0.29	-0.25	-0.20	-0.66	-0.12	-0.52	-0.24	-0.53	-0.76
P61421	V-type proton ATPase subunit d 1 OS=Homo sapiens GN=ATP6V0D1 PE=1 SV=1 - [VA0D1_HUMAN]	31.62	2	8	8	28	1.27	0.98	1.36	1.37	0.11	0.05	0.64	0.68	0.36	0.29	0.11	0.18	-0.41	-0.95	-1.35	-1.03	-1.15	-1.07	-1.26	-0.77
P42704	Leucine-rich PPR motif-containing protein, mitochondrial OS=Homo sapiens GN=LRPPRC PE=1 SV=3 - [LPPRC_HUMAN]	40.82	1	48	48	95	-0.11	-0.13	0.13	0.08	-0.80	-0.92	-0.74	-0.70	-0.78	-0.68	-0.55	-0.60	-0.54	-0.54	-0.66	-0.64	-0.81	-0.80	-0.93	-0.77
P30044	Peroxiredoxin-5, mitochondrial OS=Homo sapiens GN=PRDX5 PE=1 SV=4 - [PRDX5_HUMAN]	52.80	1	10	10	40	0.93	1.12	1.33	1.46	-0.02	-0.07	0.49	0.69	0.24	0.12	-0.16	-0.09	-0.34	-0.97	-1.40	-0.81	-1.22	-1.00	-1.50	-0.77
Q5VZF2	Muscleblind-like protein 2 OS=Homo sapiens GN=MBNL2 PE=1 SV=2 - [MBNL2_HUMAN]	10.99	2	1	3	5	1.74	0.58	1.84	0.68	1.34	0.17	1.02	-0.15	0.17	1.34	1.51	0.34	-0.67	-0.23	-0.33	-0.37	-0.47	-0.42	-0.52	-0.77

P35637	RNA-binding protein FUS OS=Homo sapiens GN=FUS PE=1 SV=1 - [FUS_HUMAN]	17.30	1	7	9	22	-0.42	-0.21	0.06	-0.04	-0.80	-0.91	-0.78	-0.98	-0.85	-0.73	-0.40	-0.49	-0.51	-0.36	-0.47	-0.38	-0.53	-0.39	-0.50	-0.77
Q99471	Prefoldin subunit 5 OS=Homo sapiens GN=PFDN5 PE=1 SV=2 - [PFD5_HUMAN]	36.36	1	5	5	8	0.67	0.73	0.97	1.03	-0.33	-0.28	0.20	0.13	-0.08	-0.13	-0.25	-0.25	-0.53	-0.90	-1.29	-0.77	-1.07	-1.02	-1.32	-0.77
Q8NFU3	Thiosulfate sulfurtransferase/rhodanese-like domain-containing protein 1 OS=Homo sapiens GN=TSTD1 PE=1 SV=3 - [TSTD1_HUMAN]	20.00	1	2	2	3	1.44	1.80	1.54	1.89	0.16	0.52	0.70	1.06	0.77	0.42	0.37	0.72	-0.68	-1.07	-1.17	-1.00	-1.09	-1.30	-1.39	-0.77
Q9Y5I4	Protocadherin alpha-C2 OS=Homo sapiens GN=PCDHAC2 PE=2 SV=1 - [PCDC2_HUMAN]	1.89	1	1	1	1	1.06	1.07	0.85	0.86	0.41	0.42	0.02	0.03	0.01	0.01	-0.05	-0.04	-0.98	-1.10	-0.90	-1.02	-0.81	-0.66	-0.46	-0.77
Q9UI12	V-type proton ATPase subunit H OS=Homo sapiens GN=ATP6V1H PE=1 SV=1 - [VATH_HUMAN]	35.40	1	14	14	30	1.57	1.27	1.74	1.59	-0.02	-0.13	0.70	0.58	0.34	0.35	0.27	0.14	-0.76	-1.03	-1.35	-1.00	-1.20	-1.37	-1.69	-0.77
P49441	Inositol polyphosphate 1-phosphatase OS=Homo sapiens GN=INPP1 PE=1 SV=1 - [INPP_HUMAN]	18.05	1	5	5	8	1.19	1.29	1.40	1.50	-0.03	0.28	0.61	0.69	0.36	0.05	-0.12	-0.02	-0.62	-1.52	-1.68	-1.08	-1.16	-1.32	-1.60	-0.77
Q9BWU0	Kanadaptin OS=Homo sapiens GN=SLC4A1AP PE=1 SV=1 - [NADAP_HUMAN]	2.64	1	1	1	2	0.51	0.35	0.38	0.22	-0.25	-0.41	-0.46	-0.62	-0.28	-0.12	-0.07	-0.23	-0.91	-0.57	-0.45	-0.60	-0.46	-0.78	-0.64	-0.77
Q5TFE4	5'-nucleotidase domain-containing protein 1 OS=Homo sapiens GN=NT5DC1 PE=1 SV=1 - [NT5D1_HUMAN]	10.55	1	3	3	5	1.02	0.66	1.34	1.30	0.06	0.05	0.29	0.25	-0.15	0.21	-0.12	-0.17	-0.13	-1.08	-1.47	-0.78	-1.42	-0.62	-1.27	-0.77

Q12879	Glutamate receptor ionotropic, NMDA 2A OS=Homo sapiens GN=GRIN2A PE=1 SV=1 - [NMDE1_HUMAN]	2.12	1	3	3	4	0.95	0.99	0.74	0.79	-0.06	-0.01	-0.09	-0.04	-0.30	-0.34	-0.61	-0.57	-0.98	-1.56	-1.36	-1.26	-1.05	-1.02	-0.82	-0.77
Q96F85	CB1 cannabinoid receptor-interacting protein 1 OS=Homo sapiens GN=CNRIP1 PE=1 SV=1 - [CNRP1_HUMAN]	39.63	1	5	5	17	0.63	0.75	1.12	1.26	0.23	0.22	0.29	0.40	0.30	0.14	0.10	0.20	-0.34	-0.55	-0.96	-0.42	-0.89	-0.63	-0.93	-0.77
Q9H492	Microtubule-associated proteins 1A/1B light chain 3A OS=Homo sapiens GN=MAP1LC3A PE=1 SV=2 - [MLP3A_HUMAN]	11.57	1	1	1	4	0.53	0.48	0.98	0.93	-0.25	-0.31	0.15	0.10	-0.23	-0.18	-0.27	-0.33	-0.33	-0.80	-1.25	-0.68	-1.13	-0.80	-1.25	-0.77
Q9Y3E1	Hepatoma-derived growth factor-related protein 3 OS=Homo sapiens GN=HDGFRP3 PE=1 SV=1 - [HDGR3_HUMAN]	19.21	1	3	4	8	0.12	0.14	0.63	0.66	-0.69	-0.74	-0.16	-0.13	-0.64	-0.62	-0.71	-0.76	-0.47	-0.78	-1.29	-0.76	-1.27	-0.79	-1.26	-0.77
Q9C0B5	Palmitoyltransferase ZDHHC5 OS=Homo sapiens GN=ZDHHC5 PE=1 SV=2 - [ZDHC5_HUMAN]	23.22	1	11	11	15	0.59	0.58	0.60	0.67	-0.26	-0.29	-0.29	-0.35	0.05	-0.01	-0.01	-0.03	-0.67	-0.58	-0.66	-0.42	-0.49	-0.68	-0.88	-0.77
P48163	NADP-dependent malic enzyme OS=Homo sapiens GN=ME1 PE=1 SV=1 - [MAOX_HUMAN]	22.73	1	11	11	21	1.02	0.93	1.04	1.04	-0.18	-0.30	0.20	0.32	0.01	0.06	-0.25	-0.38	-0.59	-1.27	-1.30	-0.94	-1.07	-1.21	-1.41	-0.77
P54764	Ephrin type-A receptor 4 OS=Homo sapiens GN=EPHA4 PE=1 SV=1 - [EPHA4_HUMAN]	15.11	1	9	11	18	1.38	1.27	1.37	1.14	-0.04	-0.03	0.65	0.53	0.04	0.11	0.30	0.16	-0.68	-1.03	-0.98	-1.24	-1.34	-1.40	-1.50	-0.77
Q9UKY7	Protein CDV3 homolog OS=Homo sapiens GN=CDV3 PE=1 SV=1 - [CDV3_HUMAN]	55.81	1	7	7	13	-0.11	-0.04	0.17	0.26	-0.56	-0.41	-0.68	-0.59	-0.50	-0.53	-0.66	-0.52	-0.48	-0.35	-0.59	-0.40	-0.58	-0.43	-0.55	-0.77

Q8NFV4	Alpha/beta hydrolase domain-containing protein 11 OS=Homo sapiens GN=ABHD11 PE=2 SV=1 - [ABHDB_HUMAN]	17.46	1	5	5	11	0.29	0.28	0.30	0.16	-0.60	-0.82	-0.46	-0.64	-0.89	-0.74	-0.44	-0.66	-0.87	-0.74	-0.81	-0.92	-1.01	-0.90	-0.99	-0.77
P62879	Guanine nucleotide-binding protein G(I)/G(S)/G(T) subunit beta-2 OS=Homo sapiens GN=GNB2 PE=1 SV=3 - [GBB2_HUMAN]	47.06	2	6	12	78	1.24	1.38	1.26	1.30	0.26	0.15	0.43	0.62	0.58	0.11	0.10	0.20	-0.55	-0.86	-1.06	-0.75	-1.09	-0.74	-0.92	-0.77
Q15349	Ribosomal protein S6 kinase alpha-2 OS=Homo sapiens GN=RPS6KA2 PE=1 SV=2 - [KS6A2_HUMAN]	26.19	4	9	17	27	0.89	0.94	1.26	1.15	0.27	0.33	0.33	0.31	0.42	0.34	0.23	0.14	-0.43	-0.79	-1.04	-0.70	-0.98	-0.84	-0.99	-0.77
P21283	V-type proton ATPase subunit C 1 OS=Homo sapiens GN=ATP6V1C1 PE=1 SV=4 - [VATC1_HUMAN]	34.82	2	14	14	43	1.41	1.38	1.48	1.44	-0.14	-0.09	0.53	0.60	0.24	0.18	0.06	0.05	-0.63	-1.28	-1.46	-1.03	-1.21	-1.41	-1.59	-0.78
O00193	Small acidic protein OS=Homo sapiens GN=SMAP PE=1 SV=1 - [SMAP_HUMAN]	9.29	1	2	2	4	0.89	1.06	0.57	0.83	-0.71	-0.45	-0.27	-0.01	-0.20	-0.37	-0.36	-0.10	-0.69	-0.55	-0.94	-1.22	-0.90	-1.36	-1.30	-0.78
P61081	NEDD8-conjugating enzyme Ubc12 OS=Homo sapiens GN=UBE2M PE=1 SV=1 - [UBC12_HUMAN]	31.15	1	6	6	17	0.82	0.81	0.87	0.82	-0.15	-0.15	0.03	0.02	-0.22	-0.15	-0.43	-0.48	-0.71	-1.21	-1.24	-0.92	-1.11	-0.95	-1.34	-0.78
Q96E11	Ribosome-recycling factor, mitochondrial OS=Homo sapiens GN=MRRF PE=1 SV=1 - [RRFM_HUMAN]	14.12	1	2	2	3	0.81	0.63	0.80	0.62	0.00	-0.18	-0.04	-0.22	-0.16	0.03	0.00	-0.19	-0.80	-0.81	-0.81	-0.75	-0.74	-0.82	-0.81	-0.78

Q8N8R5	UPF0565 protein C2orf69 OS=Homo sapiens GN=C2orf69 PE=1 SV=1 - [CB069_HUMAN]	4.16	1	1	1	2	1.32	1.43	1.20	1.31	0.03	0.13	0.36	0.47	0.33	0.23	-0.10	0.00	-0.90	-1.42	-1.31	-1.06	-0.94	-1.31	-1.19	-0.78
Q2TAL6	Brorin OS=Homo sapiens GN=VWC2 PE=2 SV=1 - [VWC2_HUMAN]	4.62	1	1	1	1	0.77	0.54	0.77	0.54	0.30	0.06	-0.08	-0.31	-0.12	0.12	0.50	0.27	-0.79	-0.26	-0.27	-0.62	-0.62	-0.49	-0.49	-0.78
Q9BV35	Calcium-binding mitochondrial carrier protein SCaMC-3 OS=Homo sapiens GN=SLC25A23 PE=1 SV=2 - [SCMC3_HUMAN]	14.96	2	5	5	9	1.03	1.20	1.14	1.17	-0.17	-0.09	0.30	0.33	0.00	-0.03	-0.02	0.08	-0.64	-0.91	-0.91	-0.79	-0.77	-1.12	-1.16	-0.78
Q92974	Rho guanine nucleotide exchange factor 2 OS=Homo sapiens GN=ARHGEF2 PE=1 SV=4 - [ARHG2_HUMAN]	30.53	1	24	24	45	1.00	0.98	0.92	0.95	-0.14	-0.04	-0.12	-0.09	0.01	-0.04	-0.10	-0.07	-0.84	-1.10	-1.02	-0.91	-0.86	-1.09	-1.05	-0.78
Q96SB3	Neurabin-2 OS=Homo sapiens GN=PPP1R9B PE=1 SV=2 - [NEB2_HUMAN]	13.99	1	9	10	27	0.51	1.07	0.83	0.94	-0.19	0.05	-0.08	0.29	0.09	-0.24	-0.36	-0.15	-0.52	-1.20	-1.04	-0.77	-0.87	-0.73	-0.93	-0.78
Q96CW1	AP-2 complex subunit mu OS=Homo sapiens GN=AP2M1 PE=1 SV=2 - [AP2M1_HUMAN]	38.39	1	15	15	57	0.93	0.85	1.09	1.04	-0.11	-0.10	0.22	0.17	0.06	0.09	-0.09	-0.09	-0.69	-1.06	-1.12	-0.89	-1.01	-1.07	-1.25	-0.78
Q8IYB5	Stromal membrane-associated protein 1 OS=Homo sapiens GN=SMAP1 PE=1 SV=2 - [SMAP1_HUMAN]	9.42	1	3	4	10	1.45	1.30	1.31	1.22	0.14	0.02	0.48	0.40	0.25	0.16	0.16	0.12	-0.85	-1.33	-1.25	-1.14	-1.05	-1.19	-1.10	-0.78
Q03001	Dystonin OS=Homo sapiens GN=DST PE=1 SV=4 - [DYST_HUMAN]	14.68	1	91	95	149	0.88	1.03	1.12	1.14	-0.03	-0.01	0.29	0.38	0.13	0.06	0.10	0.12	-0.62	-0.87	-1.03	-0.83	-0.94	-1.00	-1.07	-0.78
Q08623	Pseudouridine-5'-monophosphatase OS=Homo sapiens GN=HDHD1 PE=1 SV=3 - [HDHD1_HUMAN]	11.40	1	3	3	5	2.49	1.42	2.44	1.37	1.28	0.59	1.60	0.53	0.42	1.49	1.27	0.19	-0.83	-0.96	-1.18	-0.96	-1.12	-0.86	-1.18	-0.78

O75475	PC4 and SFRS1-interacting protein OS=Homo sapiens GN=PSIP1 PE=1 SV=1 - [PSIP1_HUMAN]	25.09	1	11	12	21	0.02	-0.22	0.47	0.33	-0.41	-0.47	-0.29	-0.39	-0.52	-0.47	-0.40	-0.51	-0.40	-0.34	-0.90	-0.65	-1.04	-0.53	-1.15	-0.78
Q9NR45	Sialic acid synthase OS=Homo sapiens GN=NANS PE=1 SV=2 - [SIAS_HUMAN]	47.08	1	12	12	25	0.99	0.92	1.30	1.24	0.08	0.33	0.64	0.38	0.14	0.21	-0.36	-0.09	-0.42	-1.36	-1.65	-0.71	-1.13	-0.88	-1.25	-0.78
Q9BVA0	Katanin p80 WD40 repeat-containing subunit B1 OS=Homo sapiens GN=KATNB1 PE=1 SV=1 - [KTNB1_HUMAN]	10.08	1	5	5	8	1.17	0.73	1.06	0.63	0.47	0.03	0.22	-0.22	-0.05	0.38	0.47	0.03	-0.90	-0.69	-0.59	-0.75	-0.64	-0.72	-0.61	-0.78
Q5VST6	Alpha/beta hydrolase domain-containing protein 17B OS=Homo sapiens GN=ABHD17B PE=2 SV=1 - [AB17B_HUMAN]	3.82	1	1	1	1	1.53	1.41	1.69	1.57	0.31	0.19	0.85	0.73	0.33	0.46	0.49	0.36	-0.63	-1.03	-1.20	-1.04	-1.20	-1.23	-1.40	-0.78
P52564	Dual specificity mitogen-activated protein kinase kinase 6 OS=Homo sapiens GN=MAP2K6 PE=1 SV=1 - [MP2K6_HUMAN]	26.35	1	4	7	11	1.34	1.30	1.08	1.00	-0.31	-0.29	0.17	0.10	-0.26	-0.18	-0.16	-0.20	-0.87	-1.44	-1.15	-1.05	-0.97	-1.33	-1.25	-0.78
Q7L1I2	Synaptic vesicle glycoprotein 2B OS=Homo sapiens GN=SV2B PE=2 SV=1 - [SV2B_HUMAN]	6.15	2	4	4	13	1.40	1.74	1.75	1.85	0.23	0.19	0.77	0.88	0.71	0.54	0.36	0.25	-0.56	-1.77	-1.51	-1.10	-1.17	-1.03	-1.31	-0.78
Q16658	Fascin OS=Homo sapiens GN=FSCN1 PE=1 SV=3 - [FSCN1_HUMAN]	54.77	1	19	19	60	1.21	1.07	1.30	1.20	0.13	0.15	0.24	0.26	0.15	0.19	-0.14	-0.04	-0.69	-1.09	-1.15	-0.79	-0.89	-0.67	-0.82	-0.78
A4D161	Protein FAM221A OS=Homo sapiens GN=FAM221A PE=2 SV=1 - [F221A_HUMAN]	4.03	1	1	1	1	2.20	2.25	2.00	2.05	0.34	0.39	1.15	1.20	0.12	0.08	0.75	0.79	-1.00	-1.45	-1.25	-2.09	-1.89	-1.87	-1.67	-0.78

O14594	Neurocan core protein OS=Homo sapiens GN=NCAN PE=1 SV=3 - [NCAN_HUMAN]	15.44	1	17	17	43	1.41	1.41	1.23	1.30	0.42	0.47	0.33	0.45	0.44	0.35	0.22	0.23	-1.08	-1.10	-1.03	-0.95	-0.82	-1.13	-0.80	-0.78
O60784	Target of Myb protein 1 OS=Homo sapiens GN=TOM1 PE=1 SV=2 - [TOM1_HUMAN]	20.33	1	8	8	21	1.14	1.07	1.19	1.09	0.03	0.02	0.23	0.20	0.16	0.14	-0.01	-0.01	-0.76	-1.06	-1.03	-0.99	-0.99	-0.86	-0.96	-0.79
Q12765	Secernin-1 OS=Homo sapiens GN=SCRN1 PE=1 SV=2 - [SCRN1_HUMAN]	58.21	1	16	16	102	1.38	1.39	1.59	1.55	0.35	0.34	0.70	0.70	0.50	0.47	0.29	0.38	-0.67	-1.01	-1.17	-0.83	-1.01	-1.06	-1.23	-0.79
P52306	Rap1 GTPase-GDP dissociation stimulator 1 OS=Homo sapiens GN=RAP1GDS1 PE=1 SV=3 - [GDS1_HUMAN]	32.13	1	16	16	37	1.37	1.54	1.55	1.71	-0.12	-0.07	0.56	0.83	0.41	0.33	-0.02	0.06	-0.62	-1.45	-1.51	-1.03	-1.19	-1.56	-1.76	-0.79
Q14183	Double C2-like domain-containing protein alpha OS=Homo sapiens GN=DOC2A PE=1 SV=5 - [DOC2A_HUMAN]	11.50	1	3	3	5	1.25	0.91	0.84	0.51	0.25	-0.09	-0.01	-0.34	-0.25	0.08	0.01	-0.33	-1.20	-1.23	-0.84	-1.13	-0.73	-1.01	-0.61	-0.79
P54687	Branched-chain-amino-acid aminotransferase, cytosolic OS=Homo sapiens GN=BCAT1 PE=1 SV=3 - [BCAT1_HUMAN]	11.40	1	4	4	5	1.07	1.22	0.98	1.12	0.29	0.31	0.26	0.34	0.32	0.25	0.10	0.08	-0.87	-1.12	-1.03	-1.05	-1.04	-0.97	-0.87	-0.79
P25098	Beta-adrenergic receptor kinase 1 OS=Homo sapiens GN=ADRBK1 PE=1 SV=2 - [ARBK1_HUMAN]	16.98	2	10	10	19	1.08	0.95	1.00	0.99	-0.17	-0.27	0.08	0.13	-0.10	0.07	-0.13	-0.21	-0.94	-1.12	-1.09	-0.93	-0.88	-1.18	-1.20	-0.79
Q9BXF6	Rab11 family-interacting protein 5 OS=Homo sapiens GN=RAB11FIP5 PE=1 SV=1 - [RFIP5_HUMAN]	33.23	1	15	15	26	0.94	1.07	1.03	1.14	0.04	0.10	0.20	0.20	-0.03	0.09	0.21	0.23	-0.78	-0.84	-0.88	-0.71	-0.84	-0.85	-0.85	-0.79

P01112	GTPase HRas OS=Homo sapiens GN=HRAS PE=1 SV=1 - [RASH_HUMAN]	66.14	1	3	9	28	0.90	1.23	1.51	1.66	0.25	0.24	0.59	0.76	0.74	0.53	0.27	0.26	-0.60	-0.96	-1.42	-0.32	-0.88	-1.01	-1.46	-0.79
Q9Y291	28S ribosomal protein S33, mitochondrial OS=Homo sapiens GN=MRPS33 PE=1 SV=1 - [RT33_HUMAN]	8.49	1	1	1	1	-0.40	-0.31	-0.02	0.08	-0.39	-0.30	-0.87	-0.78	-0.13	-0.22	0.00	0.09	-0.42	0.41	0.02	0.21	-0.17	-0.01	-0.39	-0.79
O75157	TSC22 domain family protein 2 OS=Homo sapiens GN=TSC22D2 PE=1 SV=3 - [T22D2_HUMAN]	3.85	1	1	3	9	1.27	1.03	1.33	1.10	-0.04	-0.29	0.48	0.24	0.09	0.33	0.44	0.20	-0.74	-0.82	-0.90	-0.91	-0.97	-1.33	-1.40	-0.79
P34949	Mannose-6-phosphate isomerase OS=Homo sapiens GN=MPI PE=1 SV=2 - [MPI_HUMAN]	25.53	1	7	7	11	1.02	1.10	1.59	1.21	0.34	-0.15	0.35	0.47	0.37	0.64	0.29	-0.18	-0.58	-0.85	-1.00	-0.51	-0.89	-1.00	-1.27	-0.79
Q9Y2T3	Guanine deaminase OS=Homo sapiens GN=GDA PE=1 SV=1 - [GUAD_HUMAN]	27.75	1	11	11	22	1.26	1.23	1.47	1.38	0.01	0.01	0.63	0.44	0.23	0.35	-0.22	-0.25	-0.56	-1.68	-1.85	-0.96	-1.09	-1.27	-1.46	-0.79
Q9HCJ6	Synaptic vesicle membrane protein VAT-1 homolog-like OS=Homo sapiens GN=VAT1L PE=1 SV=2 - [VAT1L_HUMAN]	14.56	1	6	6	9	0.84	0.79	1.34	1.14	0.45	0.12	0.34	0.41	0.26	0.17	0.06	-0.03	-0.32	-0.92	-1.28	-0.57	-1.01	-0.76	-1.04	-0.80
Q96PE3	Type I inositol 3,4-bisphosphate 4-phosphatase OS=Homo sapiens GN=INPP4A PE=1 SV=1 - [INP4A_HUMAN]	20.27	1	15	15	29	1.37	1.01	1.24	0.80	-0.20	-0.10	0.50	0.24	0.06	0.17	-0.21	-0.27	-0.99	-1.52	-1.38	-1.23	-0.98	-1.46	-1.31	-0.80

P48454	Serine/threonine-protein phosphatase 2B catalytic subunit gamma isoform OS=Homo sapiens GN=PPP3CC PE=1 SV=3 - [PP2BC_HUMAN]	12.89	1	3	6	21	0.96	0.79	0.74	0.57	-0.10	-0.28	-0.12	-0.29	-0.16	0.02	-0.07	-0.25	-1.02	-1.02	-0.81	-0.91	-0.69	-1.08	-0.86	-0.80
Q13177	Serine/threonine-protein kinase PAK 2 OS=Homo sapiens GN=PAK2 PE=1 SV=3 - [PAK2_HUMAN]	30.34	1	7	13	25	0.60	0.73	0.95	1.08	-0.04	0.07	0.23	0.35	-0.13	-0.24	-0.10	-0.10	-0.37	-0.73	-1.16	-0.73	-1.00	-0.62	-0.91	-0.80
P23634	Plasma membrane calcium-transporting ATPase 4 OS=Homo sapiens GN=ATP2B4 PE=1 SV=2 - [AT2B4_HUMAN]	32.07	2	18	34	168	0.98	0.98	1.02	1.14	0.02	-0.05	0.23	0.26	0.03	0.07	0.16	0.19	-0.72	-0.65	-0.81	-0.87	-0.93	-1.13	-1.16	-0.80
Q9BQI7	PH and SEC7 domain-containing protein 2 OS=Homo sapiens GN=PSD2 PE=2 SV=3 - [PSD2_HUMAN]	11.54	1	6	6	12	1.28	1.13	1.13	0.96	0.38	0.07	0.76	0.60	0.29	0.59	0.29	0.05	-0.78	-1.19	-0.91	-1.29	-1.03	-1.19	-0.91	-0.80
Q14831	Metabotropic glutamate receptor 7 OS=Homo sapiens GN=GRM7 PE=1 SV=1 - [GRM7_HUMAN]	1.20	1	1	1	1	0.62	0.43	0.66	0.47	-0.17	-0.37	-0.20	-0.40	-0.43	-0.23	0.36	0.16	-0.77	-0.26	-0.30	-0.82	-0.86	-0.81	-0.85	-0.80
Q9H6Z4	Ran-binding protein 3 OS=Homo sapiens GN=RANBP3 PE=1 SV=1 - [RANB3_HUMAN]	8.11	1	4	4	6	0.13	-0.35	0.14	-0.33	-0.70	-1.18	-0.72	-1.20	-1.17	-0.69	-0.39	-0.87	-0.79	-0.51	-0.53	-0.78	-0.79	-0.84	-0.86	-0.80
P00505	Aspartate aminotransferase, mitochondrial OS=Homo sapiens GN=GOT2 PE=1 SV=3 - [AATM_HUMAN]	42.79	1	19	19	92	1.13	1.07	1.29	1.33	-0.06	0.02	0.41	0.53	0.30	0.20	0.20	0.17	-0.63	-1.02	-1.09	-0.86	-0.97	-1.17	-1.18	-0.80

Q09019	Dystrophia myotonica WD repeat-containing protein OS=Homo sapiens GN=DMWD PE=1 SV=3 - [DMWD_HUMAN]	12.76	1	3	3	5	1.20	1.23	1.06	1.10	-0.37	-0.34	0.20	0.23	0.05	0.02	-0.32	-0.29	-0.95	-1.51	-1.38	-1.15	-1.01	-1.59	-1.45	-0.80
Q8N5B7	Ceramide synthase 5 OS=Homo sapiens GN=CERS5 PE=2 SV=1 - [CERS5_HUMAN]	1.79	1	1	1	1	1.13	0.92	1.04	0.83	0.19	-0.03	0.18	-0.03	-0.06	0.16	-0.17	-0.38	-0.90	-1.30	-1.21	-0.95	-0.85	-0.96	-0.87	-0.80
P41594	Metabotropic glutamate receptor 5 OS=Homo sapiens GN=GRM5 PE=1 SV=2 - [GRM5_HUMAN]	6.35	1	9	9	13	1.73	1.57	1.70	1.48	-0.12	-0.09	0.30	0.13	0.20	0.43	0.25	0.18	-1.09	-1.35	-1.32	-1.20	-1.06	-1.30	-1.42	-0.80
Q9Y698	Voltage-dependent calcium channel gamma-2 subunit OS=Homo sapiens GN=CACNG2 PE=1 SV=1 - [CCG2_HUMAN]	15.48	1	3	3	7	1.33	0.46	1.31	0.14	-0.35	-0.36	0.33	-0.30	-0.74	0.15	0.03	0.02	-0.93	-1.30	-1.28	-1.24	-0.90	-1.70	-1.68	-0.80
Q14249	Endonuclease G, mitochondrial OS=Homo sapiens GN=ENDOG PE=1 SV=4 - [NUCG_HUMAN]	6.73	1	2	2	2	0.37	0.89	0.34	0.86	0.72	1.23	-0.53	-0.01	0.76	0.25	0.55	1.07	-0.84	0.18	0.21	-0.09	-0.06	0.33	0.36	-0.80
Q17RY0	Cytoplasmic polyadenylation element-binding protein 4 OS=Homo sapiens GN=CPEB4 PE=1 SV=1 - [CPEB4_HUMAN]	5.08	2	1	2	4	0.96	0.70	1.07	0.81	-0.28	-0.54	0.20	-0.05	-0.26	0.00	0.13	-0.13	-0.70	-0.83	-0.94	-0.93	-1.03	-1.26	-1.37	-0.80
Q9ULR3	Protein phosphatase 1H OS=Homo sapiens GN=PPM1H PE=1 SV=2 - [PPM1H_HUMAN]	17.51	1	7	8	15	0.55	0.90	0.91	1.25	-0.17	0.00	0.05	0.21	0.10	0.17	-0.07	0.02	-0.63	-1.04	-1.21	-0.72	-0.67	-0.90	-0.88	-0.80
O76054	SEC14-like protein 2 OS=Homo sapiens GN=SEC14L2 PE=1 SV=1 - [S14L2_HUMAN]	25.81	1	7	8	13	1.55	1.46	1.35	1.32	0.34	0.24	0.45	0.39	0.34	0.34	0.32	0.23	-1.00	-1.32	-1.36	-1.03	-1.16	-1.23	-1.37	-0.81

Q9NVS9	Pyridoxine-5'-phosphate oxidase OS=Homo sapiens GN=PNPO PE=1 SV=1 - [PNPO_HUMAN]	27.97	1	5	5	12	1.90	1.82	1.51	1.61	0.29	0.18	0.60	0.47	0.56	0.54	0.10	0.30	-1.21	-1.28	-1.07	-1.15	-0.93	-1.63	-1.36	-0.81
Q86UX6	Serine/threonine-protein kinase 32C OS=Homo sapiens GN=STK32C PE=1 SV=1 - [ST32C_HUMAN]	9.05	1	3	3	7	0.16	0.27	0.25	0.36	-0.28	-0.18	-0.62	-0.51	-0.46	-0.56	-0.52	-0.42	-0.72	-0.68	-0.78	-0.69	-0.78	-0.46	-0.55	-0.81
P63167	Dynein light chain 1, cytoplasmic OS=Homo sapiens GN=DYNLL1 PE=1 SV=1 - [DYL1_HUMAN]	32.58	1	2	2	13	0.22	0.40	0.66	0.84	-0.57	-0.40	-0.26	-0.08	-0.37	-0.54	-0.46	-0.50	-0.69	-0.66	-1.09	-0.73	-1.17	-0.81	-1.25	-0.81
P07108	Acyl-CoA-binding protein OS=Homo sapiens GN=DBI PE=1 SV=2 - [ACBP_HUMAN]	64.37	1	5	5	18	1.06	0.81	1.43	1.29	-0.07	-0.24	0.72	0.61	-0.04	0.10	-0.11	-0.36	-0.27	-1.13	-1.60	-0.71	-1.29	-0.96	-1.58	-0.81
P40123	Adenylyl cyclase-associated protein 2 OS=Homo sapiens GN=CAP2 PE=1 SV=1 - [CAP2_HUMAN]	39.20	1	13	15	31	0.56	0.67	0.80	0.87	-0.15	-0.20	0.01	-0.01	-0.17	-0.12	-0.21	-0.27	-0.71	-0.87	-1.04	-1.02	-0.83	-0.78	-0.93	-0.81
P08559	Pyruvate dehydrogenase E1 component subunit alpha, somatic form, mitochondrial OS=Homo sapiens GN=PDHA1 PE=1 SV=3 - [ODPA_HUMAN]	57.18	2	23	23	65	0.74	0.83	0.92	1.03	-0.39	-0.30	0.10	0.13	-0.11	-0.19	-0.16	-0.16	-0.67	-0.95	-1.14	-0.91	-1.03	-1.18	-1.29	-0.81
Q86XE3	Calcium uptake protein 3, mitochondrial OS=Homo sapiens GN=MICU3 PE=2 SV=1 - [MICU3_HUMAN]	14.72	1	7	7	10	1.31	0.97	1.23	0.98	-0.11	-0.73	0.09	0.11	-0.12	0.22	-0.49	-0.61	-0.80	-1.72	-1.35	-1.02	-0.81	-1.31	-1.36	-0.81

Q6QNY1	Biogenesis of lysosome-related organelles complex 1 subunit 2 OS=Homo sapiens GN=BLOC1S2 PE=1 SV=1 - [BL1S2_HUMAN]	34.51	1	4	4	7	-0.16	0.22	0.11	0.49	0.03	0.40	-0.76	-0.39	0.18	-0.19	0.20	0.57	-0.55	0.36	0.09	0.00	-0.27	0.17	-0.10	-0.81
O95847	Mitochondrial uncoupling protein 4 OS=Homo sapiens GN=SLC25A27 PE=1 SV=1 - [UCP4_HUMAN]	8.36	1	2	2	3	1.30	1.23	1.45	1.38	0.05	-0.03	0.58	0.51	0.32	0.40	-0.02	-0.10	-0.67	-1.32	-1.48	-0.87	-1.02	-1.27	-1.42	-0.81
Q16537	Serine/threonine-protein phosphatase 2A 56 kDa regulatory subunit epsilon isoform OS=Homo sapiens GN=PPP2R5E PE=1 SV=1 - [2A5E_HUMAN]	13.06	1	5	5	9	1.65	1.49	1.68	1.52	0.23	0.31	0.81	0.64	0.26	0.43	-0.06	-0.12	-0.92	-1.60	-1.67	-1.19	-1.22	-1.40	-1.45	-0.81
P37837	Transaldolase OS=Homo sapiens GN=TALDO1 PE=1 SV=2 - [TALDO_HUMAN]	39.76	1	14	14	43	0.44	0.38	0.61	0.70	-0.42	-0.28	-0.20	-0.13	-0.42	-0.53	-0.79	-0.78	-0.46	-1.39	-1.48	-1.03	-1.12	-0.84	-1.15	-0.81
Q5T5C0	Syntaxin-binding protein 5 OS=Homo sapiens GN=STXBP5 PE=1 SV=1 - [STXB5_HUMAN]	11.21	1	7	8	13	1.72	1.64	1.41	1.92	-0.40	-0.25	0.56	0.86	0.72	0.24	0.18	0.36	-0.85	-1.62	-1.88	-1.49	-1.13	-1.90	-2.19	-0.81
O60241	Brain-specific angiogenesis inhibitor 2 OS=Homo sapiens GN=BAI2 PE=2 SV=2 - [BAI2_HUMAN]	3.60	1	3	3	4	0.80	0.76	0.98	0.94	-0.04	-0.09	0.11	0.06	-0.47	-0.42	-0.65	-0.70	-0.64	-1.45	-1.63	-1.19	-1.37	-0.86	-1.04	-0.81
Q8N3J5	Protein phosphatase 1K, mitochondrial OS=Homo sapiens GN=PPM1K PE=1 SV=1 - [PPM1K_HUMAN]	3.76	1	1	1	1	0.02	-0.01	0.67	0.65	-0.32	-0.35	-0.20	-0.23	-0.03	0.00	0.09	0.06	-0.16	0.08	-0.58	0.02	-0.64	-0.35	-1.01	-0.81

Q9NZQ3	NCK-interacting protein with SH3 domain OS=Homo sapiens GN=NCKIPSD PE=1 SV=1 - [SPN90_HUMAN]	27.56	1	13	13	31	1.48	1.71	1.25	1.57	0.34	0.33	0.32	0.51	0.30	0.15	0.34	0.30	-1.10	-1.29	-1.41	-1.36	-1.38	-1.49	-1.40	-0.81
Q99490	Arf-GAP with GTPase, ANK repeat and PH domain-containing protein 2 OS=Homo sapiens GN=AGAP2 PE=1 SV=2 - [AGAP2_HUMAN]	8.47	1	6	7	9	1.97	1.45	1.43	1.15	0.36	0.07	0.60	0.38	0.16	0.55	0.22	-0.07	-1.13	-1.46	-1.21	-0.96	-0.98	-1.50	-1.09	-0.81
O14639	Actin-binding LIM protein 1 OS=Homo sapiens GN=ABLIM1 PE=1 SV=3 - [ABLM1_HUMAN]	33.55	1	20	20	47	0.69	0.69	0.76	0.88	-0.25	-0.34	-0.06	-0.06	-0.15	-0.17	-0.03	0.07	-0.64	-0.56	-0.61	-0.72	-0.80	-0.95	-1.03	-0.81
Q01844	RNA-binding protein EWS OS=Homo sapiens GN=EWSR1 PE=1 SV=1 - [EWS_HUMAN]	12.20	1	6	6	17	-0.01	0.05	0.36	0.42	-0.50	-0.50	-0.45	-0.48	-0.28	-0.37	-0.29	-0.23	-0.44	-0.27	-0.65	-0.33	-0.70	-0.46	-0.85	-0.81
P35813	Protein phosphatase 1A OS=Homo sapiens GN=PPM1A PE=1 SV=1 - [PPM1A_HUMAN]	35.60	1	9	11	26	1.27	1.16	1.41	1.39	0.11	0.15	0.49	0.47	0.01	0.03	0.09	0.02	-0.70	-1.27	-1.31	-1.08	-1.14	-0.95	-1.24	-0.82
Q12979	Active breakpoint cluster region related protein OS=Homo sapiens GN=ABR PE=2 SV=2 - [ABR_HUMAN]	25.84	1	17	18	36	0.84	0.68	1.11	0.99	-0.21	-0.32	0.22	0.08	-0.02	-0.14	-0.17	-0.21	-0.44	-1.01	-1.26	-0.92	-1.21	-1.25	-1.45	-0.82
Q9BPW8	Protein NipSnap homolog 1 OS=Homo sapiens GN=NIPSNAP1 PE=1 SV=1 - [NIPS1_HUMAN]	35.92	1	9	9	27	1.03	1.20	1.29	1.25	-0.41	-0.39	0.31	0.42	0.11	-0.01	-0.19	-0.13	-0.66	-1.15	-1.30	-1.00	-1.22	-1.36	-1.64	-0.82

Q96HD1	Cysteine-rich with EGF-like domain protein 1 OS=Homo sapiens GN=CRELD1 PE=1 SV=3 - [CREL1_HUMAN]	7.86	1	3	3	5	0.02	0.59	-0.05	0.60	-0.56	-0.26	-0.48	-0.28	-0.29	-0.99	-0.88	0.03	-0.82	-0.56	-0.57	-0.85	-0.85	-0.60	-0.53	-0.82
P35754	Glutaredoxin-1 OS=Homo sapiens GN=GLRX PE=1 SV=2 - [GLRX1_HUMAN]	38.68	1	3	3	10	0.80	0.91	0.83	0.94	0.17	0.18	0.03	-0.05	0.16	0.33	-0.28	0.10	-0.91	-1.07	-0.89	-0.56	-0.46	-0.72	-0.67	-0.82
Q9Y2X7	ARF GTPase activating protein GIT1 OS=Homo sapiens GN=GIT1 PE=1 SV=2 - [GIT1_HUMAN]	37.71	1	17	18	42	0.92	0.92	1.17	1.08	-0.01	0.01	0.24	0.16	0.12	0.08	-0.11	-0.05	-0.74	-0.89	-1.06	-0.69	-0.91	-0.98	-1.02	-0.82
P51970	NADH dehydrogenase [ubiquinone] 1 alpha subcomplex subunit 8 OS=Homo sapiens GN=NDUFA8 PE=1 SV=3 - [NDUA8_HUMAN]	25.00	1	4	4	9	0.86	0.99	1.11	1.56	-0.78	-0.49	0.32	0.45	0.02	-0.18	-0.31	-0.12	-0.42	-1.08	-1.73	-0.98	-1.59	-1.56	-2.07	-0.82
Q9Y6K8	Adenylate kinase isoenzyme 5 OS=Homo sapiens GN=AK5 PE=1 SV=2 - [KAD5_HUMAN]	13.35	1	5	6	13	1.07	0.74	1.11	0.94	-0.12	-0.36	0.15	0.01	-0.21	0.00	-0.15	-0.32	-0.79	-1.10	-1.13	-0.92	-0.94	-1.10	-1.23	-0.82
Q9Y5K8	V-type proton ATPase subunit D OS=Homo sapiens GN=ATP6V1D PE=1 SV=1 - [VATD_HUMAN]	42.91	1	8	8	17	1.46	1.40	1.44	1.35	0.13	0.15	0.66	0.64	0.56	0.31	0.08	0.45	-0.97	-1.59	-1.31	-1.15	-1.10	-1.62	-1.53	-0.82
Q8IUS5	Epoxide hydrolase 4 OS=Homo sapiens GN=EPHX4 PE=2 SV=2 - [EPHX4_HUMAN]	4.97	1	2	2	3	1.13	1.05	0.78	0.70	-0.25	-0.33	-0.10	-0.18	0.12	0.20	-0.21	-0.29	-1.17	-1.33	-0.99	-0.89	-0.55	-1.39	-1.05	-0.82

Q9H8M1	Coenzyme Q-binding protein COQ10 homolog B, mitochondrial OS=Homo sapiens GN=COQ10B PE=2 SV=1 - [CQ10B_HUMAN]	12.18	1	3	3	5	1.07	1.67	1.07	1.76	0.02	0.01	0.34	0.72	0.53	0.16	-0.05	0.13	-0.74	-1.48	-1.15	-1.16	-0.88	-1.17	-1.25	-0.82
Q08174	Protocadherin-1 OS=Homo sapiens GN=PCDH1 PE=1 SV=2 - [PCDH1_HUMAN]	24.43	1	18	18	36	0.43	0.39	0.33	0.44	-0.31	-0.30	-0.43	-0.44	-0.19	-0.23	-0.21	-0.28	-0.87	-0.70	-0.51	-0.77	-0.56	-0.66	-0.47	-0.82
O95057	GTP-binding protein Di-Ras1 OS=Homo sapiens GN=DIRAS1 PE=1 SV=1 - [DIRA1_HUMAN]	44.95	1	7	7	11	0.78	0.57	1.82	1.61	-0.23	-0.07	0.93	0.72	0.49	0.34	0.16	0.08	-0.50	-0.51	-1.55	-0.41	-1.44	-1.42	-1.19	-0.82
P23588	Eukaryotic translation initiation factor 4B OS=Homo sapiens GN=EIF4B PE=1 SV=2 - [IF4B_HUMAN]	18.33	1	7	7	14	0.08	0.30	0.52	0.70	-0.59	-0.53	-0.27	-0.24	-0.42	-0.34	-0.19	-0.20	-0.53	-0.14	-0.94	-0.49	-1.00	-0.62	-1.25	-0.82
Q9HBT6	Cadherin-20 OS=Homo sapiens GN=CDH20 PE=2 SV=2 - [CAD20_HUMAN]	6.49	1	4	4	6	1.46	1.24	1.37	1.22	0.45	0.48	0.64	0.28	0.28	0.44	0.46	0.30	-0.90	-0.90	-0.92	-0.88	-0.90	-0.92	-0.94	-0.82
O95319	CUGBP Elav-like family member 2 OS=Homo sapiens GN=CELF2 PE=1 SV=1 - [CELF2_HUMAN]	13.19	1	6	6	9	1.29	1.36	1.13	1.27	0.16	0.35	0.08	0.16	0.23	0.16	-0.21	-0.15	-1.02	-1.16	-0.98	-0.76	-0.71	-0.88	-0.69	-0.82
P13073	Cytochrome c oxidase subunit 4 isoform 1, mitochondrial OS=Homo sapiens GN=COX4I1 PE=1 SV=1 - [COX41_HUMAN]	39.05	1	7	7	29	0.80	1.11	1.17	1.36	-0.20	-0.10	0.26	0.44	0.21	0.15	0.06	0.15	-0.52	-0.90	-1.21	-0.81	-1.13	-1.14	-1.31	-0.82
O43432	Eukaryotic translation initiation factor 4 gamma 3 OS=Homo sapiens GN=EIF4G3 PE=1 SV=2 - [IF4G3_HUMAN]	7.70	1	6	10	14	0.98	1.05	0.99	0.91	0.31	0.36	0.18	0.24	0.33	0.28	0.09	0.13	-0.83	-1.04	-1.00	-0.67	-0.53	-0.84	-0.70	-0.83

O43761	Synaptogyrin-3 OS=Homo sapiens GN=SYNGR3 PE=1 SV=2 - [SNG3_HUMAN]	20.09	1	4	4	11	1.33	1.35	1.11	1.07	-0.27	-0.14	0.21	0.21	0.27	0.40	0.08	0.21	-1.06	-1.64	-1.43	-1.29	-1.16	-1.68	-1.37	-0.83
Q9NRL3	Striatin-4 OS=Homo sapiens GN=STRN4 PE=1 SV=2 - [STRN4_HUMAN]	9.16	1	5	6	7	0.60	0.12	0.71	0.23	-0.17	-0.31	-0.20	-0.54	-0.17	0.11	-0.07	-0.44	-0.75	-0.59	-0.71	-0.26	-0.36	-0.79	-0.90	-0.83
Q2TAA2	Isoamyl acetate-hydrolyzing esterase 1 homolog OS=Homo sapiens GN=IAH1 PE=1 SV=1 - [IAH1_HUMAN]	20.56	1	5	5	7	0.81	0.73	1.20	1.12	0.08	0.06	0.31	0.46	0.27	0.29	0.03	0.08	-0.25	-0.71	-1.22	-0.50	-0.94	-0.75	-1.20	-0.83
Q9BSH5	Haloacid dehalogenase-like hydrolase domain-containing protein 3 OS=Homo sapiens GN=HDHD3 PE=1 SV=1 - [HDHD3_HUMAN]	51.79	1	9	9	14	0.87	0.96	0.68	0.94	-0.02	0.06	-0.45	0.01	0.05	-0.07	-0.23	-0.20	-1.11	-1.01	-0.78	-0.81	-0.70	-0.96	-0.61	-0.83
P41217	OX-2 membrane glycoprotein OS=Homo sapiens GN=CD200 PE=1 SV=4 - [OX2G_HUMAN]	15.11	1	3	3	6	0.59	1.07	1.12	1.60	0.29	0.08	-0.30	-0.27	0.08	0.19	0.20	0.38	-0.31	-0.39	-0.92	-0.37	-0.90	-1.00	-1.53	-0.83
Q9H598	Vesicular inhibitory amino acid transporter OS=Homo sapiens GN=SLC32A1 PE=1 SV=2 - [VIAAT_HUMAN]	15.62	1	7	7	12	1.21	0.84	1.17	1.64	-0.36	-0.26	0.57	0.75	0.22	0.30	-0.12	-0.23	-0.31	-1.34	-1.31	-0.82	-1.00	-1.10	-1.21	-0.83
Q8NFZ8	Cell adhesion molecule 4 OS=Homo sapiens GN=CADM4 PE=1 SV=1 - [CADM4_HUMAN]	40.46	1	10	10	33	1.22	0.89	1.24	0.89	-0.05	-0.18	0.26	-0.05	-0.24	0.04	-0.14	-0.43	-0.96	-1.32	-1.30	-1.28	-1.14	-1.27	-1.30	-0.83
Q9C0B1	Alpha-ketoglutarate-dependent dioxygenase FTO OS=Homo sapiens GN=FTO PE=1 SV=3 - [FTO_HUMAN]	19.60	1	6	6	13	0.89	0.69	1.19	0.96	0.57	0.51	0.23	0.24	0.26	0.39	0.54	0.28	-0.66	-0.42	-0.64	-0.62	-0.67	-0.63	-0.79	-0.83

Q59EK9	RUN domain-containing protein 3A OS=Homo sapiens GN=RUNDC3A PE=2 SV=2 - [RUN3A_HUMAN]	10.09	1	4	4	5	1.54	1.47	1.39	1.33	0.47	0.40	0.50	0.44	0.47	0.53	0.39	0.32	-0.98	-1.14	-1.01	-0.98	-0.83	-1.09	-0.94	-0.83
Q9NYB9	Abl interactor 2 OS=Homo sapiens GN=ABI2 PE=1 SV=1 - [ABI2_HUMAN]	17.54	1	5	8	19	1.21	1.22	1.10	1.06	-0.20	-0.18	0.15	0.09	0.11	0.07	-0.04	-0.08	-0.95	-1.11	-1.00	-1.01	-0.90	-1.20	-1.28	-0.83
O75663	TIP41-like protein OS=Homo sapiens GN=TIPRL PE=1 SV=2 - [TIPRL_HUMAN]	26.84	1	6	6	15	0.74	1.03	1.13	1.22	0.01	-0.01	0.18	0.21	-0.07	-0.10	-0.25	-0.25	-0.71	-1.27	-1.47	-0.66	-1.08	-0.63	-1.02	-0.83
P62937	Peptidyl-prolyl cis-trans isomerase A OS=Homo sapiens GN=PPIA PE=1 SV=2 - [PPIA_HUMAN]	71.52	4	14	15	170	0.96	0.94	1.09	1.15	-0.33	-0.17	0.23	0.28	-0.14	-0.20	-0.46	-0.45	-0.62	-1.28	-1.56	-1.02	-1.28	-1.17	-1.42	-0.83
Q16181	Septin-7 OS=Homo sapiens GN=SEPT7 PE=1 SV=2 - [SEPT7_HUMAN]	50.80	2	20	21	142	0.85	0.90	1.16	1.03	-0.18	-0.21	0.24	0.23	0.00	0.02	-0.08	-0.10	-0.59	-0.91	-1.21	-0.91	-1.03	-1.15	-1.34	-0.83
Q01581	Hydroxymethylglutaryl-CoA synthase, cytoplasmic OS=Homo sapiens GN=HMGCS1 PE=1 SV=2 - [HMCS1_HUMAN]	5.77	1	2	2	7	0.78	0.01	0.77	-0.06	0.16	0.14	-0.12	-0.89	0.01	-0.08	0.38	-0.35	-0.84	-0.28	-0.35	-0.54	-0.96	-0.51	-0.75	-0.83
Q96DZ9	CKLF-like MARVEL transmembrane domain-containing protein 5 OS=Homo sapiens GN=CMTM5 PE=2 SV=2 - [CKLF5_HUMAN]	11.66	1	2	2	3	1.13	1.88	1.33	2.08	-1.47	-0.72	0.43	1.18	0.66	-0.08	-0.03	0.71	-0.65	-1.16	-1.36	-1.18	-1.38	-2.62	-2.81	-0.83
Q92529	SHC-transforming protein 3 OS=Homo sapiens GN=SHC3 PE=1 SV=1 - [SHC3_HUMAN]	10.61	2	3	4	7	1.15	1.47	1.26	1.48	0.06	0.11	0.27	0.61	0.18	0.04	-0.03	0.02	-0.83	-1.43	-1.46	-1.43	-1.45	-1.37	-1.38	-0.84

Q9NQC3	Reticulon-4 OS=Homo sapiens GN=RTN4 PE=1 SV=2 - [RTN4_HUMAN]	45.47	1	39	39	138	1.23	0.92	1.48	1.23	-0.16	-0.25	0.47	0.27	0.06	0.12	0.09	-0.04	-0.59	-1.15	-1.34	-0.87	-0.91	-1.48	-1.67	-0.84
P01011	Alpha-1-antichymotrypsin OS=Homo sapiens GN=SERPINA3 PE=1 SV=2 - [AACT_HUMAN]	35.46	1	13	13	26	-1.02	-1.06	-0.72	-0.69	-0.81	-0.85	-1.53	-1.65	-0.69	-0.68	0.31	0.35	-0.46	1.30	1.04	0.30	-0.01	0.22	-0.09	-0.84
P61086	Ubiquitin-conjugating enzyme E2 K OS=Homo sapiens GN=UBE2K PE=1 SV=3 - [UBE2K_HUMAN]	35.00	1	5	5	13	1.02	0.88	1.02	0.88	-0.27	-0.41	-0.06	-0.02	-0.36	-0.22	-0.51	-0.46	-0.85	-1.59	-1.64	-1.21	-1.20	-1.31	-1.07	-0.84
Q07002	Cyclin-dependent kinase 18 OS=Homo sapiens GN=CDK18 PE=1 SV=3 - [CDK18_HUMAN]	6.36	12	1	3	7	0.60	0.95	0.87	1.23	-0.65	-0.29	-0.03	0.33	-0.64	-0.99	-0.53	-0.18	-0.57	-1.12	-1.40	-1.56	-1.83	-1.26	-1.54	-0.84
Q9Y2S7	Polymerase delta-interacting protein 2 OS=Homo sapiens GN=POLDIP2 PE=1 SV=1 - [PDIP2_HUMAN]	2.45	1	1	1	1	-0.52	-0.45	-0.31	-0.24	-1.37	-1.30	-1.21	-1.14	-1.19	-1.26	-1.16	-1.09	-0.64	-0.63	-0.85	-0.71	-0.91	-0.87	-1.08	-0.84
P54750	Calcium/calmodulin-dependent 3',5'-cyclic nucleotide phosphodiesterase 1A OS=Homo sapiens GN=PDE1A PE=2 SV=2 - [PDE1A_HUMAN]	20.37	1	6	9	12	2.02	2.20	1.48	1.59	0.01	0.16	0.44	0.58	0.10	-0.15	0.03	0.30	-1.36	-1.97	-1.45	-1.88	-1.47	-2.05	-1.51	-0.84
Q9Y496	Kinesin-like protein KIF3A OS=Homo sapiens GN=KIF3A PE=1 SV=4 - [KIF3A_HUMAN]	8.44	1	5	5	9	0.79	0.79	0.87	0.88	-0.04	-0.08	0.15	0.17	0.28	0.22	-0.08	-0.20	-0.78	-0.94	-0.95	-0.69	-0.75	-0.54	-0.92	-0.84
O60282	Kinesin heavy chain isoform 5C OS=Homo sapiens GN=KIF5C PE=1 SV=1 - [KIF5C_HUMAN]	31.14	1	17	26	63	1.34	1.25	1.59	1.50	0.00	-0.19	0.75	0.60	0.01	0.12	-0.04	-0.08	-0.60	-1.40	-1.58	-1.10	-1.35	-1.44	-1.77	-0.84

P09914	Interferon-induced protein with tetratricopeptide repeats 1 OS=Homo sapiens GN=IFIT1 PE=1 SV=2 - [IFIT1_HUMAN]	11.51	1	4	4	7	0.58	0.20	0.45	0.07	0.33	-0.06	-0.46	-0.84	0.00	0.39	0.61	0.23	-0.98	0.04	0.17	-0.16	-0.03	-0.27	-0.14	-0.84
O14737	Programmed cell death protein 5 OS=Homo sapiens GN=PDCD5 PE=1 SV=3 - [PDCD5_HUMAN]	28.00	1	3	3	8	0.38	0.67	0.40	0.65	-0.22	0.05	-0.56	-0.22	0.10	-0.32	-0.49	-0.10	-0.72	-0.86	-0.78	-0.67	-0.53	-0.63	-0.76	-0.84
Q3KQU3	MAP7 domain-containing protein 1 OS=Homo sapiens GN=MAP7D1 PE=1 SV=1 - [MA7D1_HUMAN]	8.44	1	5	5	6	0.34	0.28	0.40	0.30	-0.58	-0.53	-0.51	-0.61	-0.30	-0.19	-0.19	-0.31	-0.77	-0.58	-0.63	-0.74	-0.56	-1.02	-0.84	-0.84
Q96BS2	Calcineurin B homologous protein 3 OS=Homo sapiens GN=TESC PE=1 SV=3 - [CHP3_HUMAN]	33.18	1	6	6	11	1.40	1.46	1.11	1.17	-0.16	-0.11	0.29	0.30	0.00	0.06	0.04	-0.01	-1.00	-1.15	-1.21	-1.10	-1.01	-1.58	-1.29	-0.84
Q14938	Nuclear factor 1 X-type OS=Homo sapiens GN=NFIX PE=1 SV=2 - [NFIX_HUMAN]	9.96	1	2	4	10	0.12	0.35	0.17	0.41	-1.10	-0.87	-0.73	-0.50	-1.09	-1.32	-0.84	-0.61	-0.79	-0.95	-1.01	-1.41	-1.46	-1.23	-1.29	-0.84
O15034	RIMS-binding protein 2 OS=Homo sapiens GN=RIMBP2 PE=1 SV=3 - [RIMB2_HUMAN]	4.56	1	4	4	6	0.92	1.24	1.23	1.38	0.09	-0.27	0.20	0.25	0.02	0.09	-0.11	-0.17	-0.93	-1.25	-1.75	-0.80	-1.23	-1.36	-1.38	-0.84
Q96DA2	Ras-related protein Rab-39B OS=Homo sapiens GN=RAB39B PE=1 SV=1 - [RB39B_HUMAN]	24.41	5	3	6	21	1.80	1.55	1.96	1.50	0.03	-0.26	0.95	0.67	0.18	0.54	0.34	0.74	-0.82	-0.82	-0.76	-1.30	-1.23	-1.79	-1.77	-0.84
Q5SW79	Centrosomal protein of 170 kDa OS=Homo sapiens GN=CEP170 PE=1 SV=1 - [CE170_HUMAN]	16.73	2	20	20	28	1.40	1.40	1.33	1.43	0.10	0.27	0.30	0.45	0.31	0.08	0.20	0.38	-0.94	-1.06	-1.10	-1.24	-1.29	-1.37	-1.30	-0.85

P30460	HLA class I histocompatibility antigen, B-8 alpha chain OS=Homo sapiens GN=HLA-B PE=1 SV=1 - [1B08_HUMAN]	34.53	6	2	11	23	-0.73	-0.88	-0.37	-0.53	0.16	0.00	-1.28	-1.44	-0.54	-0.38	-1.46	-1.62	-0.50	-0.72	-1.09	0.38	0.03	0.87	0.51	-0.85
P06241	Tyrosine-protein kinase Fyn OS=Homo sapiens GN=FYN PE=1 SV=3 - [FYN_HUMAN]	25.14	12	6	13	27	1.05	1.05	1.60	1.51	0.01	0.20	0.71	0.71	0.42	0.36	0.21	0.28	-0.50	-0.76	-1.13	-0.59	-1.05	-0.86	-1.32	-0.85
Q96S82	Ubiquitin-like protein 7 OS=Homo sapiens GN=UBL7 PE=1 SV=2 - [UBL7_HUMAN]	3.16	1	1	1	2	1.41	1.25	1.34	1.19	0.12	-0.04	0.43	0.28	0.11	0.27	0.29	0.13	-0.92	-1.11	-1.06	-1.11	-1.04	-1.31	-1.25	-0.85
O43581	Synaptotagmin-7 OS=Homo sapiens GN=SYT7 PE=1 SV=3 - [SYT7_HUMAN]	35.48	1	12	12	25	0.88	0.92	1.30	0.97	-0.20	-0.16	0.45	0.32	-0.17	0.09	-0.03	-0.01	-0.58	-1.02	-0.99	-0.89	-1.24	-0.86	-1.07	-0.85
Q9UI08	Ena/VASP-like protein OS=Homo sapiens GN=EVL PE=1 SV=2 - [EVL_HUMAN]	23.56	1	9	9	17	0.88	0.80	1.06	1.00	-0.20	-0.28	0.27	0.15	-0.27	-0.30	-0.30	-0.34	-0.55	-0.99	-1.35	-1.05	-1.33	-1.06	-1.31	-0.85
O14979	Heterogeneous nuclear ribonucleoprotein D-like OS=Homo sapiens GN=HNRNPDL PE=1 SV=3 - [HNRDL_HUMAN]	25.95	1	7	8	24	0.09	0.20	0.52	0.73	-0.60	-0.69	-0.19	-0.18	-0.50	-0.48	-0.46	-0.55	-0.55	-0.59	-0.99	-0.60	-1.09	-0.96	-1.18	-0.85
Q9UN86	Ras GTPase-activating protein-binding protein 2 OS=Homo sapiens GN=G3BP2 PE=1 SV=2 - [G3BP2_HUMAN]	22.82	1	9	9	13	0.50	0.68	0.52	0.88	0.00	0.01	-0.03	0.23	-0.03	-0.21	-0.24	-0.26	-0.63	-0.78	-1.35	-0.74	-1.02	-0.80	-1.09	-0.85
P42224	Signal transducer and activator of transcription 1-alpha/beta OS=Homo sapiens GN=STAT1 PE=1 SV=2 - [STAT1_HUMAN]	38.13	1	24	24	58	0.57	0.64	0.72	0.75	0.09	0.18	-0.18	-0.10	0.11	0.07	0.41	0.39	-0.77	-0.16	-0.35	-0.45	-0.63	-0.27	-0.48	-0.85

P23763	Vesicle-associated membrane protein 1 OS=Homo sapiens GN=VAMP1 PE=1 SV=1 - [VAMP1_HUMAN]	34.75	1	4	4	13	2.52	2.88	2.90	3.04	0.14	0.37	1.50	2.06	1.16	1.00	0.82	1.20	-0.93	-1.75	-1.88	-1.49	-1.85	-2.79	-2.68	-0.85
Q14CZ8	Hepatocyte cell adhesion molecule OS=Homo sapiens GN=HEPACAM PE=1 SV=1 - [HECAM_HUMAN]	33.41	1	11	11	24	2.15	2.11	2.20	2.53	-0.02	-0.01	1.20	1.48	1.11	0.74	1.12	1.33	-0.60	-0.92	-1.08	-0.98	-1.22	-1.98	-2.21	-0.86
Q96JJ6	Junctophilin-4 OS=Homo sapiens GN=JPH4 PE=1 SV=2 - [JPH4_HUMAN]	4.94	1	1	1	2	0.71	0.72	0.70	0.71	-0.99	-0.98	0.05	0.06	-0.63	-0.64	-1.30	-1.29	-0.77	-2.00	-2.00	-1.32	-1.31	-1.71	-1.70	-0.86
Q16650	T-box brain protein 1 OS=Homo sapiens GN=TBR1 PE=1 SV=1 - [TBR1_HUMAN]	5.57	1	2	2	2	0.66	0.57	0.64	0.56	-0.09	0.15	-0.28	-0.36	0.36	0.12	0.19	0.43	-0.88	0.05	0.58	0.00	0.55	-0.25	0.29	-0.86
Q9P2Q2	FERM domain-containing protein 4A OS=Homo sapiens GN=FRMD4A PE=1 SV=3 - [FRM4A_HUMAN]	1.25	1	1	1	2	1.19	0.76	0.97	0.54	0.41	-0.03	0.05	-0.38	-0.23	0.21	0.45	0.01	-1.08	-0.74	-0.53	-0.95	-0.73	-0.80	-0.58	-0.86
O60733	85/88 kDa calcium-independent phospholipase A2 OS=Homo sapiens GN=PLA2G6 PE=1 SV=2 - [PLPL9_HUMAN]	12.78	1	6	6	7	1.06	0.99	1.02	0.89	0.03	-0.06	-0.02	0.09	0.16	0.15	0.36	0.51	-0.89	-0.59	-0.34	-0.90	-0.60	-1.19	-0.89	-0.86
Q99622	Protein C10 OS=Homo sapiens GN=C12orf57 PE=1 SV=1 - [C10_HUMAN]	22.22	1	2	2	3	0.61	0.63	0.92	0.94	-0.43	-0.42	0.00	0.02	-0.34	-0.35	-0.45	-0.44	-0.56	-1.06	-1.37	-0.93	-1.24	-1.06	-1.37	-0.86
Q96GR2	Long-chain-fatty-acid--CoA ligase ACSBG1 OS=Homo sapiens GN=ACSBG1 PE=1 SV=2 - [ACBG1_HUMAN]	14.50	1	8	8	10	1.65	0.95	1.55	1.20	-0.01	0.09	0.36	0.28	-0.09	0.29	-0.14	-0.13	-0.74	-1.11	-1.32	-1.01	-1.23	-1.53	-1.28	-0.86

Q96AB3	Isochorismatase domain-containing protein 2, mitochondrial OS=Homo sapiens GN=ISOC2 PE=1 SV=1 - [ISOC2_HUMAN]	35.12	1	3	3	4	0.65	0.46	1.00	0.82	0.30	0.11	0.08	-0.11	0.15	0.34	0.65	0.46	-0.51	0.01	-0.35	-0.27	-0.63	-0.36	-0.72	-0.86
Q8WWI1	LIM domain only protein 7 OS=Homo sapiens GN=LMO7 PE=1 SV=3 - [LMO7_HUMAN]	12.12	1	17	17	28	0.24	0.20	0.44	0.46	-0.37	-0.58	-0.54	-0.63	-0.71	-0.68	-0.79	-0.80	-0.73	-1.01	-1.20	-0.76	-0.98	-0.68	-0.82	-0.86
Q15056	Eukaryotic translation initiation factor 4H OS=Homo sapiens GN=EIF4H PE=1 SV=5 - [IF4H_HUMAN]	14.52	1	3	3	17	1.38	1.26	1.20	1.28	-0.02	-0.04	0.37	0.44	0.15	-0.01	-0.18	-0.20	-0.83	-1.49	-1.51	-1.44	-1.15	-1.39	-1.33	-0.86
O94967	WD repeat-containing protein 47 OS=Homo sapiens GN=WDR47 PE=1 SV=1 - [WDR47_HUMAN]	23.29	1	17	17	25	1.45	1.25	1.45	1.11	0.15	-0.05	0.61	0.29	0.24	0.49	0.05	-0.10	-0.79	-0.95	-1.24	-0.89	-1.03	-1.28	-1.42	-0.86
P12235	ADP/ATP translocase 1 OS=Homo sapiens GN=SLC25A4 PE=1 SV=4 - [ADT1_HUMAN]	45.30	2	4	13	70	0.98	1.08	1.41	1.55	-0.15	-0.32	0.52	0.56	0.17	0.20	-0.26	-0.35	-0.45	-1.32	-1.79	-0.83	-1.23	-1.26	-1.55	-0.86
Q53H12	Acylglycerol kinase, mitochondrial OS=Homo sapiens GN=AGK PE=1 SV=2 - [AGK_HUMAN]	35.55	1	15	15	26	0.91	0.71	1.01	0.68	-0.22	-0.19	0.17	0.05	-0.12	-0.09	-0.17	-0.37	-0.63	-0.94	-1.08	-0.72	-0.94	-1.01	-1.04	-0.86
Q8WXG6	MAP kinase-activating death domain protein OS=Homo sapiens GN=MADD PE=1 SV=2 - [MADD_HUMAN]	16.64	1	17	17	30	1.29	1.41	1.51	1.40	-0.14	-0.42	0.72	0.44	0.17	0.34	0.18	0.07	-0.83	-1.31	-1.13	-1.02	-0.96	-1.74	-1.63	-0.86

O60641	Clathrin coat assembly protein AP180 OS=Homo sapiens GN=SNAP91 PE=1 SV=2 - [AP180_HUMAN]	20.51	1	14	14	65	1.20	1.17	1.25	1.46	0.73	0.64	0.54	0.48	0.42	0.60	0.14	0.04	-0.82	-1.26	-1.28	-0.80	-0.83	-0.53	-0.69	-0.86
Q9UNE7	E3 ubiquitin-protein ligase CHIP OS=Homo sapiens GN=STUB1 PE=1 SV=2 - [CHIP_HUMAN]	48.84	1	13	13	22	0.79	1.03	0.98	1.38	-0.25	-0.27	0.12	0.29	0.18	0.04	0.12	0.16	-0.62	-0.90	-1.15	-0.80	-1.00	-1.22	-1.47	-0.86
Q5T6V5	UPF0553 protein C9orf64 OS=Homo sapiens GN=C9orf64 PE=1 SV=1 - [CI064_HUMAN]	9.09	1	3	3	7	1.12	1.08	0.98	0.94	-0.27	-0.32	0.06	0.02	-0.27	-0.23	-0.23	-0.28	-1.01	-1.34	-1.21	-1.32	-1.18	-1.41	-1.28	-0.86
P51674	Neuronal membrane glycoprotein M6-a OS=Homo sapiens GN=GPM6A PE=1 SV=2 - [GPM6A_HUMAN]	18.35	1	5	6	16	1.57	1.17	1.45	1.07	0.12	-0.21	0.63	0.39	0.27	0.39	0.44	0.20	-0.77	-1.16	-1.18	-1.26	-1.17	-1.80	-1.56	-0.86
P47929	Galectin-7 OS=Homo sapiens GN=LGALS7 PE=1 SV=2 - [LEG7_HUMAN]	19.12	1	2	2	3	-1.42	-1.57	-2.08	-2.22	-2.73	-2.88	-3.01	-3.15	-2.85	-2.70	-2.38	-2.52	-1.53	-0.95	-0.29	-1.25	-0.58	-1.32	-0.67	-0.87
Q9HCG7	Non-lysosomal glucosylceramidase OS=Homo sapiens GN=GBA2 PE=1 SV=2 - [GBA2_HUMAN]	3.45	1	2	2	4	1.93	1.45	1.03	0.56	0.79	0.31	-0.26	-0.74	-0.10	0.38	0.08	-0.40	-1.00	-1.84	-0.95	-1.52	-0.62	-1.15	-0.26	-0.87
Q8IWB9	Testis-expressed sequence 2 protein OS=Homo sapiens GN=TEX2 PE=1 SV=2 - [TEX2_HUMAN]	4.70	1	4	4	4	1.02	0.38	0.96	0.32	0.21	-0.44	0.03	-0.61	-0.44	0.20	0.21	-0.44	-0.94	-0.81	-0.75	-0.79	-0.72	-0.83	-0.77	-0.87
O14662	Syntaxin-16 OS=Homo sapiens GN=STX16 PE=1 SV=3 - [STX16_HUMAN]	8.62	1	2	2	4	1.46	1.54	1.29	1.37	0.48	0.03	0.62	0.22	0.49	0.89	0.70	0.27	-1.47	-1.00	-0.62	-1.45	-0.82	-1.98	-1.35	-0.87

Q96FE5	Leucine-rich repeat and immunoglobulin-like domain-containing nogo receptor-interacting protein 1 OS=Homo sapiens GN=LINGO1 PE=1 SV=2 - [LIGO1_HUMAN]	12.10	1	5	5	9	1.21	0.92	1.09	0.84	-0.27	-0.42	0.22	0.02	0.05	0.38	-0.01	-0.12	-0.91	-1.38	-1.15	-0.94	-0.67	-1.59	-1.27	-0.87
P46527	Cyclin-dependent kinase inhibitor 1B OS=Homo sapiens GN=CDKN1B PE=1 SV=1 - [CDN1B_HUMAN]	32.32	1	5	5	7	0.77	0.53	0.81	0.52	-0.50	-0.75	-0.12	-0.31	-0.79	-0.47	-0.20	-0.50	-0.88	-0.81	-0.87	-0.86	-0.91	-1.29	-1.34	-0.87
P07919	Cytochrome b-c1 complex subunit 6, mitochondrial OS=Homo sapiens GN=UQCRH PE=1 SV=2 - [QCR6_HUMAN]	38.46	1	4	4	16	1.47	1.47	1.57	1.57	-0.15	0.04	0.66	0.66	0.19	0.21	0.22	0.35	-0.77	-1.23	-1.32	-1.20	-1.31	-1.56	-1.68	-0.87
Q99497	Protein DJ-1 OS=Homo sapiens GN=PARK7 PE=1 SV=2 - [PARK7_HUMAN]	70.90	1	12	12	71	0.97	0.96	1.33	1.23	-0.06	-0.03	0.34	0.22	-0.13	-0.07	-0.38	-0.39	-0.72	-1.52	-1.65	-1.10	-1.26	-1.21	-1.24	-0.87
Q9NZ94	Neuroligin-3 OS=Homo sapiens GN=NLGN3 PE=1 SV=2 - [NLGN3_HUMAN]	5.66	1	2	4	6	-0.02	0.81	0.05	0.88	0.25	1.07	-0.89	-0.06	0.49	-0.33	-0.43	0.39	-0.81	-0.41	-0.49	-0.28	-0.34	0.25	0.19	-0.87
Q9GZM8	Nuclear distribution protein nudE-like 1 OS=Homo sapiens GN=NDEL1 PE=1 SV=1 - [NDEL1_HUMAN]	13.33	1	3	4	6	1.59	0.80	1.57	0.94	-0.12	-0.41	0.39	-0.10	-0.33	0.24	-0.58	-0.44	-1.02	-1.22	-1.37	-1.25	-1.16	-1.68	-1.54	-0.87
P61981	14-3-3 protein gamma OS=Homo sapiens GN=YWHAG PE=1 SV=2 - [1433G_HUMAN]	61.54	1	11	15	122	0.95	1.10	1.27	1.24	-0.11	-0.23	0.31	0.40	-0.14	-0.18	-0.06	-0.04	-0.55	-1.16	-1.41	-1.03	-1.33	-1.25	-1.58	-0.87

Q16281	Cyclic nucleotide-gated cation channel alpha-3 OS=Homo sapiens GN=CNGA3 PE=1 SV=2 - [CNGA3_HUMAN]	2.45	1	1	1	1	1.14	2.19	0.81	1.87	-0.48	0.57	-0.12	0.93	0.41	-0.64	-1.02	0.03	-1.21	-2.15	-1.83	-1.75	-1.42	-1.63	-1.31	-0.87
Q9H6K5	Putative uncharacterized protein FLJ22184 OS=Homo sapiens PE=2 SV=1 - [YS027_HUMAN]	4.38	1	2	2	3	1.08	1.49	1.18	1.59	-0.03	0.38	0.24	0.65	0.66	0.25	0.04	0.45	-0.78	-1.03	-1.14	-0.80	-0.89	-1.12	-1.22	-0.87
Q5SQI0	Alpha-tubulin N-acetyltransferase 1 OS=Homo sapiens GN=ATAT1 PE=1 SV=1 - [ATAT_HUMAN]	17.81	1	6	6	10	1.06	0.59	1.04	0.68	-0.24	-0.48	0.37	-0.01	-0.07	0.37	0.23	-0.04	-0.82	-0.71	-0.98	-0.62	-1.14	-1.19	-1.24	-0.87
P49411	Elongation factor Tu, mitochondrial OS=Homo sapiens GN=TUFM PE=1 SV=2 - [EFTU_HUMAN]	54.20	1	21	21	59	-0.04	-0.26	0.12	0.04	-1.11	-1.28	-0.66	-0.81	-1.00	-0.84	-0.91	-1.05	-0.53	-0.76	-1.15	-0.77	-0.92	-1.17	-1.32	-0.87
P50213	Isocitrate dehydrogenase [NAD] subunit alpha, mitochondrial OS=Homo sapiens GN=IDH3A PE=1 SV=1 - [IDH3A_HUMAN]	39.62	1	15	15	43	1.17	1.22	1.55	1.57	-0.08	-0.04	0.51	0.55	0.25	0.14	0.17	0.18	-0.71	-1.17	-1.36	-0.89	-1.26	-1.26	-1.76	-0.88
Q9NV96	Cell cycle control protein 50A OS=Homo sapiens GN=TMEM30A PE=1 SV=1 - [CC50A_HUMAN]	22.71	2	7	7	18	1.29	1.09	1.97	1.84	0.19	0.09	0.85	0.70	0.40	0.41	0.70	0.62	-0.34	-0.63	-1.27	-0.65	-1.23	-0.93	-1.76	-0.88
Q9NZC3	Glycerophosphodiester phosphodiesterase 1 OS=Homo sapiens GN=GDE1 PE=1 SV=1 - [GDE1_HUMAN]	22.36	1	4	4	8	1.05	1.41	0.89	1.25	-0.02	0.33	-0.05	0.31	0.23	-0.12	-0.13	0.23	-1.04	-1.17	-1.01	-1.14	-0.98	-1.09	-0.93	-0.88

Q96JG6	Coiled-coil domain-containing protein 132 OS=Homo sapiens GN=CCDC132 PE=1 SV=3 - [CC132_HUMAN]	13.28	1	9	9	13	0.65	0.64	0.53	0.72	-0.30	0.09	0.08	0.12	0.37	0.33	-0.07	0.21	-0.86	-0.74	-0.63	-0.54	-0.83	-0.99	-0.85	-0.88
Q6NT16	MFS-type transporter SLC18B1 OS=Homo sapiens GN=SLC18B1 PE=1 SV=1 - [S18B1_HUMAN]	6.80	1	2	2	5	2.09	1.96	1.85	1.75	0.08	0.05	0.86	0.67	0.43	0.51	0.41	0.21	-1.18	-1.67	-1.39	-1.45	-1.31	-2.23	-1.79	-0.88
Q16890	Tumor protein D53 OS=Homo sapiens GN=TPD52L1 PE=1 SV=1 - [TPD53_HUMAN]	18.63	1	3	3	7	2.03	1.56	2.43	1.89	0.94	0.54	1.37	1.08	0.50	1.00	0.86	0.62	-0.48	-1.17	-1.58	-1.02	-1.40	-1.03	-1.21	-0.88
P10606	Cytochrome c oxidase subunit 5B, mitochondrial OS=Homo sapiens GN=COX5B PE=1 SV=2 - [COX5B_HUMAN]	61.24	1	7	7	28	0.88	0.99	1.21	1.44	0.04	0.10	0.28	0.26	0.21	0.14	0.28	0.20	-0.52	-0.60	-1.16	-0.73	-1.18	-0.84	-1.39	-0.88
P41250	Glycine--tRNA ligase OS=Homo sapiens GN=GARS PE=1 SV=3 - [SYG_HUMAN]	24.76	1	17	17	31	1.25	1.34	1.45	1.60	0.04	0.20	0.46	0.52	0.45	0.19	0.02	0.17	-0.86	-1.28	-1.39	-0.89	-1.03	-1.12	-1.43	-0.88
P11836	B-lymphocyte antigen CD20 OS=Homo sapiens GN=MS4A1 PE=1 SV=1 - [CD20_HUMAN]	2.36	1	1	1	2	0.79	0.95	1.06	1.22	-0.63	-0.47	0.12	0.28	-0.51	-0.66	-0.80	-0.65	-0.62	-1.59	-1.86	-1.42	-1.69	-1.43	-1.70	-0.88
Q969T7	7-methylguanosine phosphate-specific 5'-nucleotidase OS=Homo sapiens GN=NT5C3B PE=1 SV=4 - [5NT3B_HUMAN]	3.00	1	1	1	1	1.42	1.14	1.52	1.23	-0.32	-0.61	0.57	0.29	0.15	0.43	-0.30	-0.59	-0.79	-1.72	-1.82	-0.96	-1.05	-1.75	-1.85	-0.88
O00408	cGMP-dependent 3',5'-cyclic phosphodiesterase OS=Homo sapiens GN=PDE2A PE=1 SV=1 - [PDE2A_HUMAN]	25.82	1	17	17	30	1.90	1.90	1.81	1.79	0.01	0.06	0.73	0.70	0.22	0.46	0.50	0.26	-0.97	-1.10	-1.07	-1.11	-1.15	-1.84	-1.65	-0.88

Q53FP2	Transmembrane protein 35 OS=Homo sapiens GN=TMEM35 PE=2 SV=2 - [TMM35_HUMAN]	13.77	1	2	2	4	0.58	0.31	1.37	1.03	-1.09	-1.22	0.46	0.10	-0.54	-0.17	-0.47	-0.60	-0.35	-1.06	-1.78	-1.01	-1.66	-1.59	-2.31	-0.88
Q6QEF8	Coronin-6 OS=Homo sapiens GN=CORO6 PE=2 SV=2 - [CORO6_HUMAN]	8.26	1	2	4	6	0.61	0.59	0.91	0.90	0.25	0.23	-0.03	-0.04	-0.12	-0.10	-0.06	-0.08	-0.58	-0.67	-0.98	-0.68	-0.98	-0.37	-0.68	-0.88
Q8N3F0	Maturin OS=Homo sapiens GN=MTURN PE=2 SV=2 - [MTURN_HUMAN]	43.51	1	2	2	5	1.91	1.54	1.68	1.32	0.68	0.31	0.74	0.37	0.20	0.57	0.14	-0.23	-1.12	-1.77	-1.55	-1.31	-1.08	-1.25	-1.02	-0.88
Q8NE62	Choline dehydrogenase, mitochondrial OS=Homo sapiens GN=CHDH PE=2 SV=2 - [CHDH_HUMAN]	6.57	1	3	3	6	1.04	1.23	0.81	1.00	0.04	0.23	-0.13	0.06	0.25	0.06	0.38	0.57	-1.12	-0.65	-0.43	-0.95	-0.71	-1.02	-0.79	-0.88
Q86WK6	Amphoterin-induced protein 1 OS=Homo sapiens GN=AMIGO1 PE=1 SV=1 - [AMGO1_HUMAN]	9.74	1	4	4	5	1.02	0.60	1.13	0.71	-0.22	-0.65	0.18	-0.24	-0.52	-0.09	0.06	-0.36	-0.78	-0.95	-1.06	-1.08	-1.19	-1.26	-1.37	-0.88
Q6DN90	IQ motif and SEC7 domain-containing protein 1 OS=Homo sapiens GN=IQSEC1 PE=1 SV=1 - [IQEC1_HUMAN]	31.36	1	21	26	60	1.31	1.03	1.13	1.03	-0.17	-0.29	0.33	0.25	-0.04	0.19	0.06	-0.04	-0.92	-1.16	-1.06	-1.15	-1.09	-1.57	-1.47	-0.88
Q9Y2K9	Syntaxin-binding protein 5-like OS=Homo sapiens GN=STXBP5L PE=1 SV=2 - [STB5L_HUMAN]	8.85	1	6	7	10	0.75	0.23	0.99	0.47	-0.31	-0.21	-0.24	-0.49	-0.26	-0.35	-0.49	-0.40	-0.51	-1.32	-1.71	-1.15	-1.26	-1.29	-1.43	-0.88
Q96GD0	Pyridoxal phosphate phosphatase OS=Homo sapiens GN=PDXP PE=1 SV=2 - [PLPP_HUMAN]	40.20	1	9	9	23	1.36	1.20	1.30	1.25	0.38	0.35	0.34	0.23	0.32	0.28	0.15	0.14	-0.95	-1.08	-1.04	-0.85	-0.79	-0.91	-0.89	-0.88

Q9UMF0	Intercellular adhesion molecule 5 OS=Homo sapiens GN=ICAM5 PE=1 SV=3 - [ICAM5_HUMAN]	23.05	1	15	15	36	0.69	0.81	0.52	0.57	0.27	0.30	-0.39	-0.36	-0.04	-0.03	0.31	0.28	-1.09	-0.41	-0.25	-0.72	-0.47	-0.48	-0.24	-0.89
Q9P2U8	Vesicular glutamate transporter 2 OS=Homo sapiens GN=SLC17A6 PE=1 SV=1 - [VGLU2_HUMAN]	5.33	1	3	3	5	2.33	2.47	2.83	2.95	-1.32	-0.56	1.29	1.43	0.16	-0.37	0.37	0.02	-0.73	-2.00	-2.32	-1.64	-1.86	-2.03	-2.13	-0.89
Q9UJY5	ADP-ribosylation factor-binding protein GGA1 OS=Homo sapiens GN=GGA1 PE=1 SV=1 - [GGA1_HUMAN]	5.95	1	3	4	4	0.88	0.99	1.04	1.10	0.15	0.20	0.09	0.15	0.15	0.04	-0.20	-0.09	-0.56	-1.07	-1.49	-0.76	-0.86	-0.80	-0.91	-0.89
P30405	Peptidyl-prolyl cis-trans isomerase F, mitochondrial OS=Homo sapiens GN=PPIF PE=1 SV=1 - [PPIF_HUMAN]	43.96	1	6	7	20	0.75	0.65	0.91	0.91	-0.14	-0.35	0.04	-0.01	-0.24	-0.24	-0.14	-0.26	-0.64	-0.95	-1.26	-0.82	-1.17	-0.71	-1.05	-0.89
P78324	Tyrosine-protein phosphatase non-receptor type substrate 1 OS=Homo sapiens GN=SIRPA PE=1 SV=2 - [SHPS1_HUMAN]	33.33	4	13	13	81	1.61	1.52	1.80	1.71	0.07	-0.02	0.69	0.77	0.33	0.46	0.08	0.14	-0.78	-1.53	-1.66	-1.18	-1.31	-1.49	-1.57	-0.89
A3KMH1	von Willebrand factor A domain-containing protein 8 OS=Homo sapiens GN=VWA8 PE=1 SV=2 - [VWA8_HUMAN]	9.19	1	13	13	18	0.42	0.26	0.36	0.24	-0.53	-0.58	-0.61	-0.75	-0.72	-0.37	-0.75	-0.93	-1.19	-0.95	-1.13	-0.57	-0.72	-0.55	-0.93	-0.89
Q04917	14-3-3 protein eta OS=Homo sapiens GN=YWHAH PE=1 SV=4 - [1433F_HUMAN]	51.63	1	10	13	105	0.93	0.89	1.18	1.16	-0.20	-0.30	0.23	0.25	0.09	0.02	-0.11	-0.13	-0.63	-1.03	-1.36	-0.95	-1.18	-1.32	-1.56	-0.89

O75382	Tripartite motif-containing protein 3 OS=Homo sapiens GN=TRIM3 PE=1 SV=2 - [TRIM3_HUMAN]	22.85	1	13	14	33	1.05	0.78	1.13	0.84	-0.13	-0.39	0.14	-0.09	-0.08	0.24	0.00	-0.18	-0.87	-0.73	-0.86	-0.72	-0.74	-0.93	-1.13	-0.89
Q99615	DnaJ homolog subfamily C member 7 OS=Homo sapiens GN=DNAJC7 PE=1 SV=2 - [DNJC7_HUMAN]	9.31	1	4	4	5	0.60	0.31	0.39	0.10	0.15	-0.15	-0.56	-0.85	-0.11	0.19	0.14	-0.15	-1.10	-0.45	-0.25	-0.38	-0.17	-0.47	-0.26	-0.89
Q5JPH6	Probable glutamate--tRNA ligase, mitochondrial OS=Homo sapiens GN=EARS2 PE=1 SV=2 - [SYEM_HUMAN]	3.06	1	2	2	3	0.35	0.38	-0.02	0.02	-0.91	-0.88	-0.97	-0.93	-0.87	-0.90	-0.51	-0.48	-1.26	-0.86	-0.50	-1.21	-0.85	-1.28	-0.91	-0.89
O95299	NADH dehydrogenase [ubiquinone] 1 alpha subcomplex subunit 10, mitochondrial OS=Homo sapiens GN=NDUFA10 PE=1 SV=1 - [NDUAA_HUMAN]	34.37	1	13	13	31	1.01	0.93	1.14	1.08	-0.40	-0.45	0.23	0.10	-0.31	-0.14	-0.15	-0.17	-0.64	-1.06	-1.27	-1.00	-1.25	-1.20	-1.42	-0.89
Q9BW91	ADP-ribose pyrophosphatase, mitochondrial OS=Homo sapiens GN=NUDT9 PE=1 SV=1 - [NUDT9_HUMAN]	16.86	1	3	3	6	1.16	1.17	1.41	1.42	0.39	0.39	0.46	0.47	0.08	0.07	0.01	0.01	-0.64	-1.15	-1.41	-1.05	-1.31	-0.79	-1.04	-0.89
Q8TDJ6	DmX-like protein 2 OS=Homo sapiens GN=DMXL2 PE=1 SV=2 - [DMXL2_HUMAN]	14.79	1	33	36	59	1.28	1.59	1.36	1.34	-0.04	0.19	0.39	0.36	0.32	0.23	0.09	0.08	-0.97	-1.28	-1.21	-0.97	-1.06	-1.28	-1.24	-0.89
Q8WW12	PEST proteolytic signal-containing nuclear protein OS=Homo sapiens GN=PCNP PE=1 SV=2 - [PCNP_HUMAN]	7.30	1	1	1	1	-0.30	-0.32	-0.40	-0.41	-1.45	-1.47	-1.35	-1.36	-1.17	-1.15	-1.22	-1.24	-0.99	-0.91	-0.82	-0.82	-0.72	-1.16	-1.07	-0.89

Q9UPQ0	LIM and calponin homology domains-containing protein 1 OS=Homo sapiens GN=LIMCH1 PE=1 SV=4 - [LIMC1_HUMAN]	22.62	1	18	18	26	0.88	0.68	0.77	0.64	0.12	-0.02	-0.10	-0.38	-0.17	-0.06	0.05	-0.03	-0.90	-0.77	-0.72	-0.80	-0.96	-0.68	-0.68	-0.89
P05060	Secretogranin-1 OS=Homo sapiens GN=CHGB PE=1 SV=2 - [SCG1_HUMAN]	12.56	1	6	6	15	0.45	0.71	0.58	0.83	-0.10	-0.30	-0.35	-0.29	0.03	0.08	0.31	0.38	-0.70	-0.19	-0.09	-0.60	-0.99	-0.61	-0.86	-0.89
O60331	Phosphatidylinositol 4-phosphate 5-kinase type-1 gamma OS=Homo sapiens GN=PIP5K1C PE=1 SV=2 - [PI51C_HUMAN]	27.40	2	10	11	27	1.36	0.79	1.60	1.34	0.13	-0.37	0.68	0.32	-0.02	0.34	0.34	0.00	-0.45	-0.96	-1.34	-0.82	-1.24	-1.23	-1.53	-0.89
P61956	Small ubiquitin-related modifier 2 OS=Homo sapiens GN=SUMO2 PE=1 SV=3 - [SUMO2_HUMAN]	23.16	2	2	2	8	0.48	0.58	0.73	0.83	-0.40	-0.22	-0.28	-0.14	-0.33	-0.53	-0.49	-0.35	-0.65	-0.82	-1.07	-0.87	-1.11	-0.80	-1.04	-0.89
P51553	Isocitrate dehydrogenase [NAD] subunit gamma, mitochondrial OS=Homo sapiens GN=IDH3G PE=1 SV=1 - [IDH3G_HUMAN]	29.52	1	10	10	35	1.39	1.17	1.31	1.13	-0.37	-0.25	0.24	0.17	0.08	-0.03	-0.05	-0.15	-0.99	-1.29	-1.25	-1.19	-1.17	-1.17	-0.99	-0.90
P08243	Asparagine synthetase [glutamine-hydrolyzing] OS=Homo sapiens GN=ASNS PE=1 SV=4 - [ASNS_HUMAN]	3.74	1	2	2	3	0.99	1.45	1.17	1.64	0.46	0.92	0.21	0.68	1.06	0.59	0.31	0.77	-0.72	-0.68	-0.86	-0.36	-0.54	-0.54	-0.73	-0.90
P22676	Calretinin OS=Homo sapiens GN=CALB2 PE=1 SV=2 - [CALB2_HUMAN]	46.13	1	11	12	29	1.54	1.62	1.88	1.89	0.23	0.20	0.89	0.88	0.73	0.60	0.18	0.08	-0.58	-1.48	-1.71	-1.04	-1.25	-1.53	-1.77	-0.90
Q5M775	Cytospin-B OS=Homo sapiens GN=SPECC1 PE=1 SV=1 - [CYTSB_HUMAN]	14.23	1	13	13	18	1.21	1.41	1.03	1.13	-0.31	-0.14	0.02	0.16	0.08	-0.05	-0.04	-0.13	-1.02	-1.25	-1.27	-1.18	-1.21	-1.31	-1.26	-0.90

P82909	28S ribosomal protein S36, mitochondrial OS=Homo sapiens GN=MRPS36 PE=1 SV=2 - [RT36_HUMAN]	40.78	1	3	3	6	0.75	0.82	0.63	0.83	-0.33	-0.22	-0.29	-0.15	-0.29	-0.42	-0.55	-0.60	-1.08	-1.25	-1.24	-1.05	-1.07	-1.06	-1.14	-0.90
Q92777	Synapsin-2 OS=Homo sapiens GN=SYN2 PE=1 SV=3 - [SYN2_HUMAN]	40.72	1	15	16	111	1.38	1.51	1.41	1.65	-0.44	-0.32	0.58	0.67	0.07	0.05	-0.40	-0.21	-0.74	-1.66	-1.60	-1.13	-1.31	-1.76	-1.58	-0.90
Q9UM82	Spermatogenesis-associated protein 2 OS=Homo sapiens GN=SPATA2 PE=1 SV=2 - [SPAT2_HUMAN]	5.96	1	2	2	2	0.90	0.76	1.18	1.04	0.15	0.01	0.22	0.08	-0.11	0.03	0.15	0.01	-0.63	-0.74	-1.02	-0.84	-1.11	-0.77	-1.05	-0.90
Q9H098	Protein FAM107B OS=Homo sapiens GN=FAM107B PE=1 SV=1 - [F107B_HUMAN]	25.19	1	3	3	4	0.13	0.39	0.19	0.46	-1.22	-1.21	-0.76	-0.50	-0.89	-1.15	-0.64	-0.39	-0.83	-0.76	-0.84	-1.03	-1.18	-1.27	-1.11	-0.90
Q96E39	RNA binding motif protein, X-linked-like-1 OS=Homo sapiens GN=RBMXL1 PE=1 SV=1 - [RMXL1_HUMAN]	18.72	2	2	6	15	-0.13	0.01	0.11	0.25	-0.65	-0.51	-0.85	-0.71	-0.68	-0.82	-0.48	-0.35	-0.66	-0.35	-0.60	-0.66	-0.90	-0.53	-0.78	-0.90
Q6ZVL6	UPF0606 protein KIAA1549L OS=Homo sapiens GN=KIAA1549L PE=2 SV=2 - [K154L_HUMAN]	7.46	1	8	8	14	0.82	0.47	0.77	0.91	0.51	0.25	0.15	0.22	0.26	0.45	0.46	0.30	-0.99	-0.40	-0.28	-0.55	-0.65	-0.27	-0.60	-0.90
Q92599	Septin-8 OS=Homo sapiens GN=SEPT8 PE=1 SV=4 - [SEPT8_HUMAN]	49.69	2	12	16	66	1.20	1.08	1.12	1.07	-0.09	-0.07	0.21	0.13	-0.01	0.07	-0.20	-0.25	-0.92	-1.22	-1.45	-1.13	-1.16	-1.36	-1.11	-0.90
Q9BQE3	Tubulin alpha 1C chain OS=Homo sapiens GN=TUBA1C PE=1 SV=1 - [TBA1C_HUMAN]	54.34	2	7	23	933	0.98	0.95	1.07	1.19	-0.03	0.02	0.12	0.15	0.06	0.09	0.08	0.07	-0.87	-1.09	-1.07	-0.90	-1.12	-0.95	-1.20	-0.90
Q9UQ03	Coronin-2B OS=Homo sapiens GN=CORO2B PE=2 SV=4 - [COR2B_HUMAN]	34.17	1	11	11	18	1.75	1.58	2.00	1.79	0.29	0.20	0.59	0.64	0.43	0.36	0.14	0.31	-0.78	-0.85	-1.19	-1.25	-1.68	-1.42	-1.39	-0.90

P62072	Mitochondrial import inner membrane translocase subunit Tim10 OS=Homo sapiens GN=TIMM10 PE=1 SV=1 - [TIM10_HUMAN]	37.78	1	3	3	16	1.33	1.27	1.36	1.34	0.02	0.07	0.28	0.34	0.36	0.28	0.40	0.46	-0.81	-0.92	-0.85	-0.90	-0.80	-0.97	-1.06	-0.90
Q8WU20	Fibroblast growth factor receptor substrate 2 OS=Homo sapiens GN=FRS2 PE=1 SV=4 - [FRS2_HUMAN]	4.13	1	1	1	1	1.30	0.42	1.87	0.98	-0.12	-1.00	0.90	0.02	-0.01	0.87	0.57	-0.32	-0.34	-0.72	-1.29	-0.40	-0.96	-1.43	-2.00	-0.90
Q14894	Ketimine reductase mu crystallin OS=Homo sapiens GN=CRYM PE=1 SV=1 - [CRYM_HUMAN]	41.08	1	10	10	32	1.29	1.41	1.38	1.38	-0.22	-0.23	0.45	0.44	0.02	0.01	-0.20	-0.37	-0.89	-1.53	-1.48	-0.91	-0.96	-1.15	-1.61	-0.90
Q8WXD9	Caskin-1 OS=Homo sapiens GN=CASKIN1 PE=1 SV=1 - [CSKI1_HUMAN]	21.80	1	17	17	43	1.30	0.83	1.31	0.82	0.05	-0.09	0.29	0.17	0.28	0.52	0.22	0.01	-0.77	-1.07	-1.13	-1.03	-1.08	-1.15	-1.16	-0.90
Q86YS7	C2 domain-containing protein 5 OS=Homo sapiens GN=C2CD5 PE=1 SV=1 - [C2CD5_HUMAN]	11.60	1	9	9	12	1.09	1.14	0.77	0.92	0.37	0.34	0.36	0.32	0.26	0.22	0.31	0.35	-0.74	-0.78	-0.79	-0.84	-0.66	-0.81	-0.47	-0.90
Q9P260	LisH domain and HEAT repeat-containing protein KIAA1468 OS=Homo sapiens GN=KIAA1468 PE=1 SV=2 - [K1468_HUMAN]	11.68	1	10	10	17	0.82	0.96	1.11	1.18	-0.12	-0.01	0.15	0.28	0.03	-0.04	-0.01	0.12	-0.89	-0.83	-1.09	-1.13	-1.12	-1.16	-1.25	-0.90
P55083	Microfibril-associated glycoprotein 4 OS=Homo sapiens GN=MFAP4 PE=1 SV=2 - [MFAP4_HUMAN]	13.73	1	3	3	5	-0.65	-0.84	-0.10	-0.31	0.18	-0.01	-1.13	-1.32	-0.88	-0.76	-1.12	-1.25	-0.41	-0.39	-0.90	0.12	-0.34	0.81	0.39	-0.90
Q9NZJ4	Sacsin OS=Homo sapiens GN=SACS PE=1 SV=2 - [SACS_HUMAN]	1.27	1	4	4	5	0.85	0.59	1.00	0.58	-0.32	-0.76	0.18	-0.39	-0.50	-0.21	-0.32	-0.61	-0.87	-1.16	-1.32	-1.03	-1.18	-1.31	-1.35	-0.90

Q4V328	GRIP1-associated protein 1 OS=Homo sapiens GN=GRIPAP1 PE=1 SV=1 - [GRAP1_HUMAN]	39.95	2	27	27	59	1.05	1.12	1.09	1.07	-0.12	-0.16	0.11	0.14	0.01	-0.04	-0.01	-0.09	-0.93	-1.17	-1.17	-1.09	-1.12	-1.24	-1.27	-0.90
P48634	Protein PRRC2A OS=Homo sapiens GN=PRRC2A PE=1 SV=3 - [PRC2A_HUMAN]	2.92	1	3	3	3	0.46	0.49	0.49	0.52	-0.46	-0.43	-0.48	-0.45	-0.43	-0.46	-0.27	-0.24	-0.88	-0.72	-0.75	-0.89	-0.91	-0.93	-0.96	-0.91
Q14318	Peptidyl-prolyl cis-trans isomerase FKBP8 OS=Homo sapiens GN=FKBP8 PE=1 SV=2 - [FKBP8_HUMAN]	22.82	1	7	7	14	0.71	0.74	1.08	0.85	-0.04	0.17	0.10	0.10	0.16	0.20	0.22	0.28	-0.76	-0.64	-0.94	-0.55	-0.89	-0.84	-1.27	-0.91
Q13796	Protein Shroom2 OS=Homo sapiens GN=SHROOM2 PE=1 SV=1 - [SHRM2_HUMAN]	2.72	1	3	3	5	1.00	0.65	1.08	0.98	0.30	-0.29	0.00	0.00	-0.03	0.01	-0.27	-0.35	-0.72	-1.00	-1.34	-0.70	-0.93	-0.95	-0.77	-0.91
Q6NXP0	EF-hand calcium-binding domain-containing protein 12 OS=Homo sapiens GN=EFCAB12 PE=2 SV=1 - [EFC12_HUMAN]	1.92	1	1	1	1	2.08	1.56	1.94	1.42	0.15	-0.38	0.97	0.45	0.02	0.55	-0.39	-0.92	-1.05	-2.47	-2.34	-1.50	-1.36	-1.95	-1.82	-0.91
P12277	Creatine kinase B-type OS=Homo sapiens GN=CKB PE=1 SV=1 - [KCRB_HUMAN]	54.59	1	15	16	390	1.69	1.71	1.71	1.73	-0.19	-0.17	0.58	0.56	0.35	0.36	0.26	0.26	-0.97	-1.41	-1.43	-1.21	-1.20	-1.89	-1.91	-0.91
Q16543	Hsp90 co-chaperone Cdc37 OS=Homo sapiens GN=CDC37 PE=1 SV=1 - [CDC37_HUMAN]	23.81	1	7	7	22	-0.14	0.12	0.50	0.76	-0.80	-0.65	-0.25	-0.15	-0.53	-0.74	-0.66	-0.65	-0.27	-0.75	-1.26	-0.73	-1.30	-0.70	-1.37	-0.91
Q9BXM9	FSD1-like protein OS=Homo sapiens GN=FSD1L PE=1 SV=2 - [FSD1L_HUMAN]	12.26	1	5	5	8	1.54	1.81	1.55	1.66	0.52	0.56	0.43	0.54	0.65	0.45	0.14	0.26	-0.91	-1.39	-1.41	-1.06	-0.97	-1.12	-0.97	-0.91

Q8N9B5	Junction-mediating and -regulatory protein OS=Homo sapiens GN=JMY PE=1 SV=2 - [JMY_HUMAN]	1.42	1	1	1	2	0.63	0.90	0.79	1.06	-0.13	0.14	-0.18	0.08	0.12	-0.14	0.32	0.58	-0.76	-0.31	-0.48	-0.75	-0.90	-0.78	-0.94	-0.91
P62942	Peptidyl-prolyl cis-trans isomerase FKBP1A OS=Homo sapiens GN=FKBP1A PE=1 SV=2 - [FKB1A_HUMAN]	58.33	1	4	4	19	0.54	0.66	0.59	0.65	-0.13	-0.04	-0.37	-0.25	-0.15	-0.32	-0.73	-0.57	-0.89	-1.23	-1.32	-0.83	-0.77	-0.88	-0.78	-0.91
Q9BSU1	UPF0183 protein C16orf70 OS=Homo sapiens GN=C16orf70 PE=1 SV=1 - [CP070_HUMAN]	9.48	1	3	3	4	1.23	1.33	1.40	1.50	-0.38	-0.29	0.43	0.53	0.34	0.25	0.16	0.25	-0.75	-1.07	-1.25	-0.95	-1.13	-1.63	-1.81	-0.91
P09104	Gamma-enolase OS=Homo sapiens GN=ENO2 PE=1 SV=3 - [ENOG_HUMAN]	73.73	1	18	21	366	1.74	1.75	1.76	1.86	0.65	0.71	0.82	0.85	0.56	0.55	0.02	-0.02	-0.86	-1.59	-1.55	-1.09	-1.24	-1.19	-1.20	-0.91
P62745	Rho-related GTP-binding protein RhoB OS=Homo sapiens GN=RHOB PE=1 SV=1 - [RHOB_HUMAN]	47.45	1	6	7	42	1.85	1.95	1.99	1.98	0.41	0.42	0.89	0.88	0.89	0.82	0.44	0.55	-0.91	-1.16	-1.55	-1.00	-1.14	-1.52	-1.65	-0.91
Q9H4G0	Band 4.1-like protein 1 OS=Homo sapiens GN=EPB41L1 PE=1 SV=2 - [E41L1_HUMAN]	41.32	1	27	29	92	1.52	1.50	1.72	1.77	0.28	0.28	0.71	0.77	0.48	0.48	0.40	0.40	-0.59	-1.13	-1.33	-0.98	-1.23	-1.29	-1.41	-0.91
Q92563	Testican-2 OS=Homo sapiens GN=SPOCK2 PE=1 SV=1 - [TICN2_HUMAN]	6.60	1	3	3	5	1.20	0.91	1.35	1.07	0.16	-0.13	0.37	0.09	0.41	0.70	0.77	0.49	-0.77	-0.42	-0.57	-0.47	-0.62	-1.05	-1.21	-0.92
Q92854	Semaphorin-4D OS=Homo sapiens GN=SEMA4D PE=1 SV=1 - [SEM4D_HUMAN]	9.51	1	6	6	9	1.35	1.57	1.20	1.41	0.20	0.51	0.31	0.43	0.40	0.15	0.21	0.44	-0.98	-1.14	-1.00	-1.18	-1.03	-1.08	-0.97	-0.92

Q8IWP9	Coiled-coil domain-containing protein 28A OS=Homo sapiens GN=CCDC28A PE=2 SV=1 - [CC28A_HUMAN]	12.41	1	2	2	4	0.47	0.55	0.96	1.04	-0.02	0.06	-0.02	0.06	0.05	-0.02	0.64	0.71	-0.43	0.18	-0.32	-0.46	-0.95	-0.50	-0.99	-0.92
Q9HC38	Glyoxalase domain-containing protein 4 OS=Homo sapiens GN=GLOD4 PE=1 SV=1 - [GLOD4_HUMAN]	44.41	1	12	12	20	0.67	0.48	0.83	0.78	-0.18	-0.27	-0.18	-0.03	-0.42	-0.54	-0.67	-0.81	-0.72	-1.46	-1.77	-1.22	-1.45	-0.97	-1.17	-0.92
Q8N350	Protein Dos OS=Homo sapiens GN=DOS PE=1 SV=2 - [DOS_HUMAN]	4.55	1	2	2	4	1.24	0.95	0.94	0.65	-0.17	-0.47	-0.04	-0.33	-0.62	-0.32	-0.01	-0.31	-1.22	-1.25	-0.96	-1.53	-1.23	-1.43	-1.14	-0.92
P39748	Flap endonuclease 1 OS=Homo sapiens GN=FEN1 PE=1 SV=1 - [FEN1_HUMAN]	2.89	1	1	1	1	0.83	0.40	1.57	1.15	-0.28	-0.72	0.59	0.17	-0.25	0.18	0.39	-0.04	-0.18	-0.43	-1.18	-0.62	-1.36	-1.13	-1.87	-0.92
Q9H115	Beta-soluble NSF attachment protein OS=Homo sapiens GN=NAPB PE=1 SV=2 - [SNAB_HUMAN]	56.04	1	13	15	64	1.57	1.59	1.80	1.78	-0.04	-0.05	0.80	0.81	0.45	0.44	0.31	0.23	-0.74	-1.25	-1.57	-1.13	-1.28	-1.74	-1.82	-0.92
O43920	NADH dehydrogenase [ubiquinone] iron-sulfur protein 5 OS=Homo sapiens GN=NDUFS5 PE=1 SV=3 - [NDUS5_HUMAN]	50.94	1	5	5	11	1.42	1.65	1.49	1.66	0.09	0.10	0.47	0.72	0.24	0.07	0.17	0.28	-0.90	-1.20	-1.09	-1.18	-1.06	-1.29	-1.39	-0.92
P30519	Heme oxygenase 2 OS=Homo sapiens GN=HMOX2 PE=1 SV=2 - [HMOX2_HUMAN]	54.43	1	12	12	18	0.74	0.73	0.99	1.02	-0.01	-0.08	0.29	0.08	0.09	0.04	0.04	0.07	-0.60	-0.59	-1.23	-0.57	-1.05	-0.96	-1.26	-0.92
P45985	Dual specificity mitogen-activated protein kinase kinase 4 OS=Homo sapiens GN=MAP2K4 PE=1 SV=1 - [MP2K4_HUMAN]	20.80	1	7	7	12	1.17	1.19	1.16	1.25	0.09	0.26	0.32	0.44	0.37	0.30	0.17	-0.10	-0.67	-1.49	-1.47	-0.54	-0.53	-1.15	-1.09	-0.92

Q16643	Drebrin OS=Homo sapiens GN=DBN1 PE=1 SV=4 - [DREB_HUMAN]	31.12	1	16	16	44	1.08	0.89	1.03	0.96	-0.07	-0.13	0.10	0.04	-0.03	0.13	0.36	0.20	-0.87	-0.68	-0.74	-0.96	-0.93	-1.12	-1.08	-0.92
Q96C01	Protein FAM136A OS=Homo sapiens GN=FAM136A PE=1 SV=1 - [F136A_HUMAN]	42.75	1	5	5	8	0.76	0.62	1.14	0.95	-0.05	-0.23	0.11	-0.03	-0.19	0.00	0.18	-0.01	-0.60	-0.58	-0.97	-0.73	-1.11	-0.83	-1.21	-0.92
Q5T848	Probable G-protein coupled receptor 158 OS=Homo sapiens GN=GPR158 PE=1 SV=1 - [GP158_HUMAN]	7.49	1	8	8	15	1.29	1.27	1.38	1.43	0.28	0.27	0.53	0.49	0.29	0.52	0.22	0.23	-0.84	-1.16	-1.23	-0.95	-0.87	-1.16	-1.24	-0.92
O94760	N(G),N(G)-dimethylarginine dimethylaminohydrolase 1 OS=Homo sapiens GN=DDAH1 PE=1 SV=3 - [DDAH1_HUMAN]	51.93	1	12	13	67	1.43	1.66	1.67	1.74	0.20	0.33	0.76	0.88	0.47	0.43	0.26	0.37	-0.57	-1.12	-1.31	-1.08	-1.16	-1.14	-1.32	-0.92
Q9NRW1	Ras-related protein Rab-6B OS=Homo sapiens GN=RAB6B PE=1 SV=1 - [RAB6B_HUMAN]	40.38	4	3	9	29	1.11	1.15	1.69	1.54	0.06	-0.15	0.71	0.63	0.01	0.18	-0.03	-0.03	-0.35	-1.05	-1.63	-0.99	-1.36	-1.25	-1.42	-0.92
Q9Y216	Myotubularin-related protein 7 OS=Homo sapiens GN=MTMR7 PE=1 SV=3 - [MTMR7_HUMAN]	3.79	1	2	2	2	1.50	1.80	1.45	1.76	0.17	0.48	0.46	0.77	0.23	-0.07	0.41	0.71	-0.98	-1.08	-1.04	-1.54	-1.49	-1.34	-1.29	-0.92
Q14197	Peptidyl-tRNA hydrolase ICT1, mitochondrial OS=Homo sapiens GN=ICT1 PE=1 SV=1 - [ICT1_HUMAN]	6.80	1	1	1	2	0.25	0.04	-0.06	-0.27	0.52	0.31	-1.04	-1.25	0.32	0.53	0.35	0.14	-1.24	0.10	0.41	0.31	0.62	0.25	0.56	-0.92
P38606	V-type proton ATPase catalytic subunit A OS=Homo sapiens GN=ATP6V1A PE=1 SV=2 - [VATA_HUMAN]	49.92	1	28	28	166	1.54	1.62	1.64	1.74	0.15	0.16	0.67	0.72	0.41	0.43	0.26	0.26	-0.79	-1.22	-1.46	-1.12	-1.23	-1.52	-1.66	-0.92

Q8N9R8	Protein SCAI OS=Homo sapiens GN=SCAI PE=1 SV=2 - [SCAI_HUMAN]	31.85	1	16	16	37	1.36	1.37	1.31	1.35	-0.15	-0.08	0.48	0.61	0.38	0.35	0.17	0.28	-0.83	-1.16	-1.28	-1.09	-1.23	-1.49	-1.73	-0.92
O43301	Heat shock 70 kDa protein 12A OS=Homo sapiens GN=HSPA12A PE=1 SV=2 - [HS12A_HUMAN]	41.93	1	22	23	75	1.15	1.16	1.38	1.48	-0.22	-0.19	0.26	0.36	0.01	0.00	-0.22	-0.21	-0.81	-1.22	-1.52	-1.18	-1.45	-1.42	-1.56	-0.92
Q8N461	F-box/LRR-repeat protein 16 OS=Homo sapiens GN=FBXL16 PE=1 SV=2 - [FXL16_HUMAN]	6.26	1	3	3	5	1.71	1.24	1.71	1.23	-0.38	-0.13	0.55	0.24	0.05	0.15	-0.28	-0.33	-0.94	-1.78	-1.78	-1.25	-1.24	-1.38	-1.38	-0.92
Q8N128	Protein FAM177A1 OS=Homo sapiens GN=FAM177A1 PE=1 SV=1 - [F177A_HUMAN]	22.54	1	3	3	6	0.97	0.90	1.09	1.03	-0.04	-0.11	0.11	0.04	0.07	0.14	0.06	-0.02	-0.80	-0.90	-1.04	-0.80	-0.92	-1.02	-1.15	-0.92
Q9UI47	Catenin alpha-3 OS=Homo sapiens GN=CTNNA3 PE=1 SV=2 - [CTNA3_HUMAN]	6.03	1	2	3	6	0.39	0.34	0.60	0.55	-1.15	-1.20	-0.39	-0.44	-0.83	-0.78	-0.52	-0.57	-0.73	-0.91	-1.12	-1.14	-1.34	-1.56	-1.76	-0.92
O43615	Mitochondrial import inner membrane translocase subunit TIM44 OS=Homo sapiens GN=TIMM44 PE=1 SV=2 - [TIM44_HUMAN]	26.55	1	12	12	28	0.93	1.12	1.02	0.95	-0.14	-0.24	0.00	0.01	-0.09	-0.09	-0.25	-0.27	-1.12	-1.48	-1.25	-1.05	-1.03	-1.11	-1.11	-0.92
Q8IV38	Ankyrin repeat and MYND domain-containing protein 2 OS=Homo sapiens GN=ANKMY2 PE=1 SV=1 - [ANKY2_HUMAN]	42.40	1	13	13	20	0.35	0.04	0.72	1.00	-0.41	-0.52	-0.21	-0.41	-0.67	-0.40	-0.33	-0.41	-0.52	-0.63	-1.05	-0.58	-0.83	-0.76	-1.37	-0.93
Q9H477	Ribokinase OS=Homo sapiens GN=RBKS PE=1 SV=1 - [RBSK_HUMAN]	3.11	1	1	1	2	1.06	0.71	0.89	0.54	-0.09	-0.45	-0.10	-0.45	-0.48	-0.13	-0.84	-1.19	-1.10	-1.89	-1.73	-1.16	-0.99	-1.17	-1.00	-0.93

Q7Z4S6	Kinesin-like protein KIF21A OS=Homo sapiens GN=KIF21A PE=1 SV=2 - [KI21A_HUMAN]	15.23	2	18	18	38	1.30	1.39	1.48	1.57	-0.01	-0.02	0.43	0.52	0.25	0.17	0.21	0.13	-0.78	-1.15	-1.34	-1.07	-1.28	-1.30	-1.40	-0.93
Q9Y243	RAC-gamma serine/threonine-protein kinase OS=Homo sapiens GN=AKT3 PE=1 SV=1 - [AKT3_HUMAN]	17.33	1	4	6	9	1.00	0.62	1.20	0.86	0.14	-0.32	0.40	-0.08	-0.16	0.09	-0.13	-0.31	-0.65	-1.13	-1.26	-0.88	-1.01	-0.96	-1.16	-0.93
Q96DB2	Histone deacetylase 11 OS=Homo sapiens GN=HDAC11 PE=1 SV=1 - [HDA11_HUMAN]	14.99	1	3	3	7	1.34	1.54	1.19	1.27	-0.50	-0.23	0.92	0.13	-0.29	-0.09	0.00	-0.43	-0.86	-1.60	-1.69	-1.62	-1.44	-1.71	-1.66	-0.93
P43897	Elongation factor Ts, mitochondrial OS=Homo sapiens GN=TSFM PE=1 SV=2 - [EFTS_HUMAN]	30.46	1	8	8	16	0.33	0.22	0.69	0.46	-0.70	-0.82	-0.30	-0.47	-0.77	-0.57	-0.60	-0.70	-0.54	-1.12	-1.23	-0.90	-1.19	-1.20	-1.35	-0.93
Q96DA6	Mitochondrial import inner membrane translocase subunit TIM14 OS=Homo sapiens GN=DNAJC19 PE=1 SV=3 - [TIM14_HUMAN]	26.72	1	3	3	6	1.12	1.04	1.22	1.15	-0.29	-0.35	0.29	0.21	-0.16	-0.08	0.07	-0.10	-0.78	-1.23	-1.42	-1.27	-1.42	-1.38	-1.53	-0.93
P14649	Myosin light chain 6B OS=Homo sapiens GN=MYL6B PE=1 SV=1 - [MYL6B_HUMAN]	38.94	1	6	9	31	1.14	1.14	1.16	1.15	0.22	0.19	0.30	0.33	0.50	0.47	-0.22	-0.20	-0.89	-1.44	-1.51	-0.87	-0.78	-0.94	-1.09	-0.93
Q7Z4H3	HD domain-containing protein 2 OS=Homo sapiens GN=HDDC2 PE=1 SV=1 - [HDDC2_HUMAN]	20.10	1	3	3	5	1.11	1.04	0.98	0.91	-0.40	-0.48	-0.02	-0.09	-0.34	-0.27	-0.39	-0.47	-1.07	-1.50	-1.37	-1.35	-1.21	-1.53	-1.40	-0.93
P59780	AP-3 complex subunit sigma-2 OS=Homo sapiens GN=AP3S2 PE=1 SV=1 - [AP3S2_HUMAN]	10.88	1	2	2	3	1.03	1.13	0.95	1.06	-0.17	-0.07	-0.04	0.06	0.16	0.06	-0.03	0.08	-1.02	-1.05	-0.98	-0.94	-0.86	-1.22	-1.14	-0.93

Q13363	C-terminal-binding protein 1 OS=Homo sapiens GN=CTBP1 PE=1 SV=2 - [CTBP1_HUMAN]	32.50	1	7	11	31	1.28	1.21	1.25	1.24	-0.08	-0.06	0.14	0.29	0.18	0.09	-0.08	-0.09	-0.88	-1.21	-1.38	-0.96	-0.95	-1.29	-1.25	-0.93
Q8WXS3	Brain and acute leukemia cytoplasmic protein OS=Homo sapiens GN=BAALC PE=2 SV=3 - [BAALC_HUMAN]	8.33	1	1	1	2	1.20	1.01	1.16	0.97	-0.47	-0.67	0.17	-0.03	-0.02	0.18	0.15	-0.05	-0.98	-1.05	-1.01	-0.99	-0.94	-1.69	-1.65	-0.93
P00558	Phosphoglycerate kinase 1 OS=Homo sapiens GN=PGK1 PE=1 SV=3 - [PGK1_HUMAN]	72.90	1	27	27	126	0.83	0.88	1.04	1.21	-0.04	-0.01	0.10	0.23	-0.10	-0.13	-0.51	-0.46	-0.75	-1.39	-1.60	-0.91	-1.08	-0.92	-1.09	-0.93
Q9Y478	5'-AMP-activated protein kinase subunit beta-1 OS=Homo sapiens GN=PRKAB1 PE=1 SV=4 - [AAKB1_HUMAN]	12.59	1	2	2	4	-0.72	-0.40	-0.04	0.28	-0.27	0.04	-1.03	-0.72	-0.44	-0.75	-0.44	-0.13	-0.26	0.28	-0.41	-0.01	-0.68	0.43	-0.25	-0.93
Q99961	Endophilin-A2 OS=Homo sapiens GN=SH3GL1 PE=1 SV=1 - [SH3G1_HUMAN]	36.14	1	9	13	25	0.44	0.66	0.63	0.87	-0.30	-0.02	-0.16	-0.06	-0.16	-0.22	-0.42	-0.39	-0.47	-0.80	-1.25	-0.73	-0.98	-0.67	-1.05	-0.93
P36543	V-type proton ATPase subunit E 1 OS=Homo sapiens GN=ATP6V1E1 PE=1 SV=1 - [VATE1_HUMAN]	50.44	2	13	13	84	1.59	1.48	1.80	1.67	-0.06	-0.01	0.70	0.65	0.31	0.37	0.28	0.22	-0.85	-1.30	-1.48	-1.15	-1.36	-1.64	-1.83	-0.93
O43427	Acidic fibroblast growth factor intracellular-binding protein OS=Homo sapiens GN=FIBP PE=1 SV=3 - [FIBP_HUMAN]	18.96	1	5	5	13	1.25	1.21	1.34	1.48	0.03	0.31	0.32	0.48	0.34	0.13	-0.16	0.12	-0.83	-1.38	-1.40	-1.05	-1.11	-1.31	-1.25	-0.93

Q86UW8	Hyaluronan and proteoglycan link protein 4 OS=Homo sapiens GN=HAPLN4 PE=2 SV=1 - [HPLN4_HUMAN]	29.60	1	9	9	17	1.92	1.79	1.73	1.61	0.06	0.10	0.68	0.78	0.46	0.37	0.41	0.28	-1.00	-1.59	-1.32	-1.52	-1.33	-1.71	-1.52	-0.94
O14924	Regulator of G-protein signaling 12 OS=Homo sapiens GN=RGS12 PE=1 SV=1 - [RGS12_HUMAN]	3.52	1	3	3	4	0.91	0.88	1.01	0.98	-0.31	-0.34	0.00	-0.02	-0.22	-0.18	-0.01	-0.04	-0.85	-0.92	-1.02	-1.04	-0.97	-1.24	-1.33	-0.94
O43426	Synaptojanin-1 OS=Homo sapiens GN=SYNJ1 PE=1 SV=2 - [SYNJ1_HUMAN]	26.76	1	33	33	81	1.33	1.36	1.66	1.73	0.23	0.22	0.49	0.51	0.27	0.28	-0.13	-0.12	-0.74	-1.52	-1.68	-1.12	-1.47	-1.19	-1.40	-0.94
Q8IZD9	Dedicator of cytokinesis protein 3 OS=Homo sapiens GN=DOCK3 PE=1 SV=1 - [DOCK3_HUMAN]	2.02	1	3	4	4	1.33	2.30	1.05	2.03	-0.50	0.47	0.05	1.02	0.84	-0.12	-0.34	0.62	-1.23	-1.67	-1.40	-1.43	-1.15	-1.85	-1.57	-0.94
Q9BTV5	Fibronectin type III and SPRY domain-containing protein 1 OS=Homo sapiens GN=FSD1 PE=1 SV=1 - [FSD1_HUMAN]	25.00	1	9	9	13	1.62	1.81	1.59	1.58	-0.44	-0.38	0.78	0.83	0.24	0.00	-0.12	0.01	-0.78	-1.95	-2.06	-1.45	-1.57	-2.21	-1.97	-0.94
Q9Y566	SH3 and multiple ankyrin repeat domains protein 1 OS=Homo sapiens GN=SHANK1 PE=1 SV=2 - [SHAN1_HUMAN]	1.30	1	2	2	3	1.74	1.25	1.75	1.26	-0.09	-0.59	0.74	0.25	-0.06	0.44	0.40	-0.09	-0.94	-1.33	-1.35	-1.27	-1.28	-1.85	-1.86	-0.94
P50150	Guanine nucleotide-binding protein G(I)/G(S)/G(O) subunit gamma-4 OS=Homo sapiens GN=GNG4 PE=1 SV=1 - [GBG4_HUMAN]	30.67	1	2	2	4	1.63	1.71	1.69	1.77	-0.62	-0.55	0.68	0.76	0.40	0.32	0.21	0.28	-0.89	-1.42	-1.48	-1.28	-1.33	-2.27	-2.33	-0.94

Q86V21	Acetoacetyl-CoA synthetase OS=Homo sapiens GN=AACS PE=1 SV=1 - [AACS_HUMAN]	1.34	1	1	1	1	2.22	2.38	1.88	2.04	0.79	0.94	0.87	1.03	1.07	0.92	0.78	0.93	-1.29	-1.43	-1.10	-1.28	-0.93	-1.45	-1.11	-0.95
Q14003	Potassium voltage-gated channel subfamily C member 3 OS=Homo sapiens GN=KCNC3 PE=1 SV=3 - [KCNC3_HUMAN]	5.55	3	3	4	6	2.10	2.24	2.17	2.31	-0.03	0.10	1.16	1.30	0.99	0.85	0.10	0.23	-0.88	-1.99	-2.07	-1.22	-1.29	-2.15	-2.22	-0.95
Q96G97	Seipin OS=Homo sapiens GN=BSCL2 PE=1 SV=3 - [BSCL2_HUMAN]	2.26	1	1	1	4	1.26	1.65	1.16	1.55	-0.24	0.15	0.14	0.54	0.28	-0.11	-0.21	0.18	-1.06	-1.46	-1.37	-1.33	-1.23	-1.52	-1.42	-0.95
P16298	Serine/threonine-protein phosphatase 2B catalytic subunit beta isoform OS=Homo sapiens GN=PPP3CB PE=1 SV=2 - [PP2BB_HUMAN]	25.00	1	7	12	37	1.29	1.05	1.31	1.33	0.37	0.23	0.40	0.29	0.09	0.23	0.01	0.09	-0.93	-1.54	-1.35	-1.05	-1.26	-1.27	-1.29	-0.95
Q9NYI0	PH and SEC7 domain-containing protein 3 OS=Homo sapiens GN=PSD3 PE=1 SV=2 - [PSD3_HUMAN]	11.74	1	13	13	29	1.80	1.63	1.95	1.94	-0.31	-0.44	0.92	0.85	0.37	0.35	0.24	0.08	-0.76	-1.55	-1.73	-1.40	-1.63	-2.14	-2.40	-0.95
Q92752	Tenascin-R OS=Homo sapiens GN=TNR PE=1 SV=3 - [TENR_HUMAN]	29.68	1	28	28	102	1.66	1.70	1.64	1.75	-0.03	0.01	0.58	0.70	0.46	0.39	0.21	0.32	-0.95	-1.38	-1.39	-1.30	-1.24	-1.59	-1.54	-0.95
O00244	Copper transport protein ATOX1 OS=Homo sapiens GN=ATOX1 PE=1 SV=1 - [ATOX1_HUMAN]	13.24	1	2	2	3	0.41	0.33	0.95	0.88	0.10	0.02	-0.06	-0.14	-0.16	-0.08	-0.34	-0.43	-0.41	-0.74	-1.30	-0.46	-1.00	-0.32	-0.87	-0.95
Q9UDT6	CAP-Gly domain-containing linker protein 2 OS=Homo sapiens GN=CLIP2 PE=1 SV=1 - [CLIP2_HUMAN]	30.50	1	25	30	65	1.49	1.28	1.60	1.45	-0.12	-0.13	0.50	0.51	0.12	0.05	-0.07	-0.15	-0.85	-1.28	-1.54	-1.18	-1.31	-1.44	-1.61	-0.95

Q96A00	Protein phosphatase 1 regulatory subunit 14A OS=Homo sapiens GN=PPP1R14A PE=1 SV=1 - [PP14A_HUMAN]	34.69	1	3	3	8	0.89	0.93	0.16	0.11	-1.69	-1.72	-1.38	-1.40	-1.14	-1.08	-0.46	-0.52	-1.04	-0.89	-0.62	-0.97	-1.21	-1.15	-1.34	-0.95
Q93050	V-type proton ATPase 116 kDa subunit a isoform 1 OS=Homo sapiens GN=ATP6V0A1 PE=2 SV=3 - [VPP1_HUMAN]	24.01	1	19	19	93	1.55	1.52	1.59	1.65	-0.22	-0.06	0.56	0.56	0.32	0.21	0.12	0.05	-0.88	-1.32	-1.55	-1.21	-1.33	-1.61	-1.67	-0.95
O75335	Liprin-alpha-4 OS=Homo sapiens GN=PPFIA4 PE=2 SV=3 - [LIPA4_HUMAN]	17.30	2	8	15	23	1.39	1.76	1.37	1.36	-0.33	-0.01	0.24	0.32	0.40	0.25	-0.09	0.06	-1.16	-1.54	-1.29	-1.08	-0.82	-1.70	-1.38	-0.95
Q13508	Ecto-ADP-ribosyltransferase 3 OS=Homo sapiens GN=ART3 PE=1 SV=2 - [NAR3_HUMAN]	9.77	1	3	3	4	1.39	0.98	1.48	1.07	0.22	-0.19	0.46	0.05	-0.12	0.29	0.96	0.55	-0.88	-0.43	-0.52	-1.07	-1.16	-1.19	-1.28	-0.96
O00451	GDNF family receptor alpha-2 OS=Homo sapiens GN=GFRA2 PE=2 SV=2 - [GFRA2_HUMAN]	11.42	1	4	4	6	1.11	1.20	0.90	1.00	0.81	0.90	-0.12	-0.02	0.56	0.47	0.49	0.58	-1.17	-0.61	-0.41	-0.61	-0.40	-0.31	-0.11	-0.96
P20674	Cytochrome c oxidase subunit 5A, mitochondrial OS=Homo sapiens GN=COX5A PE=1 SV=2 - [COX5A_HUMAN]	52.67	1	7	7	64	1.37	1.52	1.65	1.68	0.35	0.33	0.55	0.61	0.56	0.49	0.48	0.43	-0.70	-1.00	-1.17	-0.86	-1.01	-1.16	-1.26	-0.96
Q15555	Microtubule-associated protein RP/EB family member 2 OS=Homo sapiens GN=MAPRE2 PE=1 SV=1 - [MARE2_HUMAN]	37.92	1	9	10	30	1.18	0.90	1.59	1.41	-0.09	-0.41	0.42	0.31	-0.16	0.03	-0.16	-0.35	-0.76	-1.32	-1.61	-1.07	-1.64	-1.32	-1.76	-0.96

O75390	Citrate synthase, mitochondrial OS=Homo sapiens GN=CS PE=1 SV=2 - [CISY_HUMAN]	37.55	1	14	14	58	1.02	1.06	1.40	1.39	-0.15	-0.15	0.42	0.39	-0.01	-0.05	-0.12	-0.14	-0.63	-1.17	-1.53	-1.06	-1.41	-1.31	-1.53	-0.96
P54284	Voltage-dependent L-type calcium channel subunit beta-3 OS=Homo sapiens GN=CACNB3 PE=1 SV=1 - [CACB3_HUMAN]	10.33	1	2	5	9	2.14	2.30	1.14	1.30	-0.05	0.10	0.12	0.27	0.12	-0.03	0.19	0.34	-1.97	-1.95	-0.95	-2.14	-1.14	-2.21	-1.21	-0.96
Q96NT1	Nucleosome assembly protein 1-like 5 OS=Homo sapiens GN=NAP1L5 PE=2 SV=1 - [NP1L5_HUMAN]	18.13	1	3	3	4	0.90	0.79	1.24	1.13	-0.17	-0.29	0.22	0.11	-0.18	-0.06	-0.10	-0.22	-0.63	-1.00	-1.35	-0.93	-1.27	-1.09	-1.43	-0.96
Q5TCQ9	Membrane-associated guanylate kinase, WW and PDZ domain-containing protein 3 OS=Homo sapiens GN=MAGI3 PE=1 SV=2 - [MAGI3_HUMAN]	3.25	1	3	4	5	1.24	1.27	1.30	1.33	0.33	0.35	0.25	0.28	0.25	0.23	0.18	0.20	-0.94	-1.06	-1.12	-0.99	-1.04	-0.93	-0.98	-0.96
Q96GY0	Zinc finger C2HC domain-containing protein 1A OS=Homo sapiens GN=ZC2HC1A PE=1 SV=2 - [ZC21A_HUMAN]	18.46	1	6	6	10	0.49	0.15	1.09	0.69	-0.16	-0.42	0.06	-0.37	-0.34	0.20	-0.07	-0.47	-0.46	-0.50	-0.92	-0.45	-0.87	-0.60	-1.08	-0.97
Q9UBS5	Gamma-aminobutyric acid type B receptor subunit 1 OS=Homo sapiens GN=GABBR1 PE=1 SV=1 - [GABR1_HUMAN]	9.26	1	7	7	14	1.11	1.09	1.40	1.32	0.14	-0.15	0.36	0.36	0.01	0.21	0.17	0.09	-0.69	-1.18	-1.25	-1.46	-1.49	-1.10	-1.28	-0.97
Q9NZH0	G-protein coupled receptor family C group 5 member B OS=Homo sapiens GN=GPRC5B PE=2 SV=2 - [GPC5B_HUMAN]	8.68	1	3	3	10	1.75	1.55	1.81	1.65	0.20	0.12	0.77	0.62	0.31	0.67	0.82	0.63	-0.84	-0.84	-0.87	-1.07	-1.09	-1.43	-1.55	-0.97

O43837	Isocitrate dehydrogenase [NAD] subunit beta, mitochondrial OS=Homo sapiens GN=IDH3B PE=1 SV=2 - [IDH3B_HUMAN]	41.82	1	14	14	39	1.22	1.23	1.64	1.61	-0.24	-0.33	0.55	0.50	0.09	0.14	0.06	-0.01	-0.64	-1.15	-1.58	-0.99	-1.41	-1.55	-1.73	-0.97
Q9ULP0	Protein NDRG4 OS=Homo sapiens GN=NDRG4 PE=1 SV=2 - [NDRG4_HUMAN]	29.55	1	6	6	16	1.61	1.31	2.00	1.65	0.30	0.35	0.89	0.82	0.50	0.58	0.22	0.01	-0.65	-1.08	-1.40	-0.83	-1.14	-0.84	-1.10	-0.97
Q9P2U7	Vesicular glutamate transporter 1 OS=Homo sapiens GN=SLC17A7 PE=1 SV=1 - [VGLU1_HUMAN]	10.00	1	6	6	18	1.69	1.78	1.53	1.66	-0.38	-0.47	0.66	0.61	0.33	0.38	-0.11	-0.04	-0.83	-1.62	-1.82	-1.51	-1.30	-1.84	-1.85	-0.97
Q9Y2C4	Nuclease EXOG, mitochondrial OS=Homo sapiens GN=EXOG PE=1 SV=2 - [EXOG_HUMAN]	20.11	1	4	4	12	1.31	1.37	1.40	1.68	0.33	0.47	0.42	0.66	0.56	0.24	0.30	0.61	-1.01	-1.13	-1.11	-1.31	-1.15	-1.47	-1.36	-0.97
P09497	Clathrin light chain B OS=Homo sapiens GN=CLTB PE=1 SV=1 - [CLCB_HUMAN]	28.82	1	7	7	29	1.21	1.06	1.30	1.25	-0.14	-0.24	0.23	0.20	0.02	0.07	-0.06	0.00	-0.89	-1.24	-1.29	-1.13	-1.22	-1.32	-1.59	-0.98
Q9NQ66	1-phosphatidylinositol 4,5-bisphosphate phosphodiesterase beta-1 OS=Homo sapiens GN=PLCB1 PE=1 SV=1 - [PLCB1_HUMAN]	34.87	1	35	35	66	1.16	1.15	1.28	1.31	-0.03	-0.10	0.28	0.17	-0.02	0.02	-0.37	-0.49	-0.81	-1.53	-1.64	-1.07	-1.27	-1.19	-1.55	-0.98
Q8N2K0	Monoacylglycerol lipase ABHD12 OS=Homo sapiens GN=ABHD12 PE=1 SV=2 - [ABD12_HUMAN]	17.84	1	7	7	12	0.57	0.70	0.77	0.73	0.06	-0.25	-0.20	-0.27	-0.12	-0.11	-0.16	-0.12	-0.69	-0.73	-0.81	-0.72	-0.66	-0.97	-0.73	-0.98

O95886	Disks large-associated protein 3 OS=Homo sapiens GN=DLGAP3 PE=1 SV=3 - [DLGP3_HUMAN]	11.34	1	7	8	11	0.72	0.76	0.51	0.71	0.02	0.08	-0.28	-0.48	0.12	0.08	0.20	0.25	-1.28	-0.78	-0.56	-0.61	-0.24	-0.98	-0.74	-0.98
P14927	Cytochrome b-c1 complex subunit 7 OS=Homo sapiens GN=UQCRB PE=1 SV=2 - [QCR7_HUMAN]	43.24	1	7	7	26	1.09	1.19	1.36	1.38	-0.41	-0.23	0.51	0.50	0.00	-0.04	-0.18	-0.17	-0.62	-1.16	-1.62	-1.03	-1.52	-1.35	-1.63	-0.98
Q92796	Disks large homolog 3 OS=Homo sapiens GN=DLG3 PE=1 SV=2 - [DLG3_HUMAN]	28.03	1	14	15	27	1.83	1.36	1.74	1.66	0.04	0.11	0.25	0.29	0.10	0.40	0.12	0.17	-0.86	-1.41	-1.57	-1.45	-1.45	-1.43	-1.47	-0.98
O15021	Microtubule-associated serine/threonine-protein kinase 4 OS=Homo sapiens GN=MAST4 PE=1 SV=3 - [MAST4_HUMAN]	2.59	5	3	5	6	-0.88	-0.81	-0.45	-0.38	-0.47	-0.41	-0.78	-0.71	-0.40	-0.46	-0.07	-0.01	-0.64	-0.01	-0.38	-0.43	-0.78	-0.23	-0.59	-0.98
Q969J3	Loss of heterozygosity 12 chromosomal region 1 protein OS=Homo sapiens GN=LOH12CR1 PE=1 SV=1 - [L12R1_HUMAN]	20.92	1	4	4	5	-0.07	-0.36	0.35	0.07	-0.56	-0.85	-0.69	-0.98	-0.78	-0.49	-0.15	-0.44	-0.57	-0.08	-0.51	-0.38	-0.81	-0.51	-0.94	-0.98
P35612	Beta-adducin OS=Homo sapiens GN=ADD2 PE=1 SV=3 - [ADDB_HUMAN]	45.59	1	24	25	76	1.29	1.32	1.61	1.60	0.00	0.07	0.63	0.57	0.18	0.31	0.41	0.37	-0.67	-0.84	-1.21	-0.96	-1.32	-1.18	-1.48	-0.99
P84085	ADP-ribosylation factor 5 OS=Homo sapiens GN=ARF5 PE=1 SV=2 - [ARF5_HUMAN]	51.11	1	3	8	45	1.72	1.89	1.78	1.77	0.52	0.13	0.69	0.67	0.28	0.27	0.12	-0.12	-1.05	-1.61	-1.94	-1.43	-1.34	-1.48	-1.55	-0.99
Q05084	Islet cell autoantigen 1 OS=Homo sapiens GN=ICA1 PE=1 SV=2 - [ICA69_HUMAN]	14.08	1	5	6	14	0.46	0.91	1.02	1.26	-1.07	-0.37	-0.03	0.48	-0.04	0.01	0.09	0.17	-0.44	-0.72	-0.69	-0.77	-0.76	-1.42	-1.91	-0.99

Q99747	Gamma-soluble NSF attachment protein OS=Homo sapiens GN=NAPG PE=1 SV=1 - [SNAG_HUMAN]	53.85	1	15	15	36	1.17	1.11	1.29	1.44	-0.14	-0.13	0.44	0.40	0.03	0.04	-0.01	-0.01	-0.66	-1.03	-1.36	-0.84	-1.24	-1.06	-1.43	-0.99
Q9GZN7	Protein rogdi homolog OS=Homo sapiens GN=ROGDI PE=1 SV=1 - [ROGDI_HUMAN]	7.32	1	2	2	3	1.28	1.24	1.50	1.46	-0.32	-0.36	0.44	0.41	-0.09	-0.04	-0.11	-0.15	-0.78	-1.39	-1.61	-1.30	-1.51	-1.61	-1.83	-0.99
P68366	Tubulin alpha 4A chain OS=Homo sapiens GN=TUBA4A PE=1 SV=1 - [TBA4A_HUMAN]	58.26	2	7	23	929	1.07	1.06	1.36	1.24	0.00	-0.10	0.44	0.48	0.31	0.38	0.26	0.28	-0.77	-1.05	-1.23	-0.97	-1.09	-0.79	-0.98	-0.99
P09669	Cytochrome c oxidase subunit 6C OS=Homo sapiens GN=COX6C PE=1 SV=2 - [COX6C_HUMAN]	37.33	1	4	4	9	1.18	1.41	1.73	1.71	-0.36	-0.28	0.45	0.70	0.47	0.31	0.22	0.33	-0.83	-1.01	-1.40	-1.15	-1.36	-1.77	-2.06	-0.99
P31323	cAMP-dependent protein kinase type II-beta regulatory subunit OS=Homo sapiens GN=PRKAR2B PE=1 SV=3 - [KAP3_HUMAN]	46.17	1	11	13	35	1.29	1.34	1.41	1.47	-0.29	-0.07	0.53	0.73	0.21	0.23	0.28	0.31	-0.75	-0.99	-0.92	-0.97	-1.15	-1.41	-1.49	-0.99
P63000	Ras-related C3 botulinum toxin substrate 1 OS=Homo sapiens GN=RAC1 PE=1 SV=1 - [RAC1_HUMAN]	38.54	3	6	9	35	0.64	0.65	1.25	1.06	-0.06	0.05	0.27	0.13	-0.11	-0.21	-0.05	-0.19	-0.39	-0.89	-1.25	-0.86	-1.35	-0.82	-1.34	-0.99
Q8N584	Tetratricopeptide repeat protein 39C OS=Homo sapiens GN=TTC39C PE=2 SV=2 - [TT39C_HUMAN]	4.29	1	2	2	5	2.07	2.65	2.18	2.75	-0.23	0.34	1.12	1.69	0.97	0.40	0.07	0.64	-0.90	-1.99	-2.11	-1.64	-1.74	-2.32	-2.42	-0.99
P05408	Neuroendocrine protein 7B2 OS=Homo sapiens GN=SCG5 PE=1 SV=2 - [7B2_HUMAN]	21.70	1	3	3	10	1.58	1.85	1.23	1.63	0.01	-0.08	0.51	0.74	0.37	0.33	0.37	0.35	-0.96	-1.07	-1.14	-0.90	-1.03	-1.52	-1.45	-0.99

Q9BUW7	UPF0184 protein C9orf16 OS=Homo sapiens GN=C9orf16 PE=1 SV=1 - [CI016_HUMAN]	10.84	1	1	1	2	2.17	1.83	2.05	1.71	0.68	0.33	1.00	0.65	0.34	0.69	0.57	0.22	-1.12	-1.60	-1.49	-1.46	-1.33	-1.51	-1.39	-0.99
Q5T5U3	Rho GTPase-activating protein 21 OS=Homo sapiens GN=ARHGAP21 PE=1 SV=1 - [RHG21_HUMAN]	7.00	1	9	9	13	1.09	1.15	1.25	1.23	-0.16	-0.08	0.20	0.07	0.05	0.09	0.01	0.04	-1.02	-1.08	-1.21	-1.13	-1.17	-1.24	-1.28	-1.00
Q92581	Sodium/hydrogen exchanger 6 OS=Homo sapiens GN=SLC9A6 PE=1 SV=2 - [SL9A6_HUMAN]	7.62	1	4	4	7	1.67	1.68	1.66	1.70	0.13	-0.07	0.60	0.63	0.25	0.22	0.20	0.23	-0.99	-1.32	-1.17	-1.28	-1.12	-1.76	-1.83	-1.00
Q92598	Heat shock protein 105 kDa OS=Homo sapiens GN=HSPH1 PE=1 SV=1 - [HS105_HUMAN]	40.79	1	25	28	66	1.23	1.33	1.51	1.66	-0.13	-0.05	0.54	0.64	0.14	0.09	-0.34	-0.28	-0.68	-1.62	-1.92	-1.18	-1.42	-1.41	-1.72	-1.00
Q9BY50	Signal peptidase complex catalytic subunit SEC11C OS=Homo sapiens GN=SEC11C PE=1 SV=3 - [SC11C_HUMAN]	10.42	1	1	1	3	-0.27	0.32	-0.20	0.39	-0.51	0.08	-1.26	-0.67	-0.31	-0.89	-0.62	-0.04	-0.94	-0.35	-0.43	-0.59	-0.66	-0.26	-0.33	-1.00
P19367	Hexokinase-1 OS=Homo sapiens GN=HK1 PE=1 SV=3 - [HXK1_HUMAN]	42.97	2	36	39	155	1.71	1.75	1.77	1.83	0.06	0.13	0.64	0.70	0.32	0.30	0.14	0.13	-1.01	-1.51	-1.66	-1.37	-1.44	-1.54	-1.68	-1.00
Q9UPX0	Protein turtle homolog B OS=Homo sapiens GN=IGSF9B PE=2 SV=2 - [TUTLB_HUMAN]	1.70	1	2	2	4	1.88	1.65	1.71	1.48	0.02	-0.21	0.64	0.41	0.28	0.52	0.32	0.08	-1.18	-1.56	-1.39	-1.33	-1.16	-1.87	-1.70	-1.00
Q9NUI1	Peroxisomal 2,4-dienoyl-CoA reductase OS=Homo sapiens GN=DECR2 PE=1 SV=1 - [DECR2_HUMAN]	7.88	1	2	2	2	1.49	1.54	1.36	1.40	0.58	0.62	0.29	0.34	0.27	0.23	0.42	0.45	-1.14	-1.07	-0.95	-1.23	-1.10	-0.93	-0.80	-1.00

Q96A73	Putative monooxygenase p33MONOX OS=Homo sapiens GN=KIAA1191 PE=1 SV=1 - [P33MX_HUMAN]	13.11	1	2	2	2	-0.16	-0.15	0.33	0.34	-0.78	-0.77	-0.74	-0.73	-0.33	-0.33	-1.26	-1.26	-0.52	-1.10	-1.59	-0.14	-0.63	-0.64	-1.12	-1.00
P63096	Guanine nucleotide-binding protein G(i) subunit alpha 1 OS=Homo sapiens GN=GNAI1 PE=1 SV=2 - [GNAI1_HUMAN]	54.80	3	7	14	82	1.66	1.69	1.65	1.55	-0.01	-0.12	0.49	0.54	0.25	0.37	0.27	0.20	-1.04	-1.32	-1.29	-1.18	-1.27	-1.60	-1.70	-1.00
Q4KMP7	TBC1 domain family member 10B OS=Homo sapiens GN=TBC1D10B PE=1 SV=3 - [TB10B_HUMAN]	9.16	1	5	5	10	1.27	1.15	1.39	1.30	-0.16	-0.28	0.12	0.13	-0.01	0.02	0.11	-0.01	-1.14	-1.22	-1.24	-1.18	-1.23	-1.41	-1.57	-1.01
P36871	Phosphoglucomutase-1 OS=Homo sapiens GN=PGM1 PE=1 SV=3 - [PGM1_HUMAN]	50.36	1	24	24	58	1.38	1.30	1.62	1.60	-0.06	0.01	0.48	0.50	0.13	0.23	-0.24	-0.20	-0.84	-1.60	-1.82	-1.13	-1.41	-1.31	-1.57	-1.01
O60939	Sodium channel subunit beta-2 OS=Homo sapiens GN=SCN2B PE=1 SV=1 - [SCN2B_HUMAN]	29.30	1	5	5	12	1.10	1.16	1.64	1.61	-0.15	-0.28	0.57	0.39	0.05	0.18	-0.16	-0.24	-0.63	-1.25	-1.91	-0.88	-1.54	-1.26	-1.93	-1.01
Q8N4C8	Misshapen-like kinase 1 OS=Homo sapiens GN=MINK1 PE=1 SV=2 - [MINK1_HUMAN]	13.59	1	11	15	23	1.27	1.18	1.40	1.29	-0.02	0.01	0.36	0.18	0.11	0.26	0.25	0.20	-0.98	-1.00	-0.96	-1.10	-1.01	-1.29	-1.37	-1.01
O43670	BUB3-interacting and GLEBS motif-containing protein ZNF207 OS=Homo sapiens GN=ZNF207 PE=1 SV=1 - [ZN207_HUMAN]	11.30	1	4	4	11	0.02	0.32	0.03	0.05	-0.52	-0.45	-0.95	-0.91	-0.77	-0.82	-0.26	-0.39	-1.21	-0.56	-0.32	-0.78	-0.66	-0.84	-0.68	-1.01

Q9P121	Neurotrimin OS=Homo sapiens GN=NTM PE=1 SV=1 - [NTRI_HUMAN]	46.80	1	8	11	65	1.65	1.79	1.83	1.80	0.28	0.19	0.71	0.83	0.38	0.47	0.46	0.31	-0.97	-1.31	-1.32	-1.32	-1.34	-1.67	-1.52	-1.01
P61758	Prefoldin subunit 3 OS=Homo sapiens GN=VBP1 PE=1 SV=3 - [PFD3_HUMAN]	17.77	2	4	4	9	1.35	0.94	1.17	1.10	-0.10	-0.07	0.18	-0.12	-0.21	0.11	-0.20	-0.29	-1.11	-1.50	-1.38	-1.13	-1.03	-1.49	-1.28	-1.01
P17612	cAMP-dependent protein kinase catalytic subunit alpha OS=Homo sapiens GN=PRKACA PE=1 SV=2 - [KAPCA_HUMAN]	31.91	2	5	9	17	0.50	0.62	1.30	1.18	-0.26	-0.52	0.00	0.08	-0.29	-0.56	0.02	0.14	-0.47	-0.47	-1.41	-0.86	-1.43	-1.16	-1.74	-1.01
Q8N1A0	Keratin-like protein KRT222 OS=Homo sapiens GN=KRT222 PE=2 SV=1 - [KT222_HUMAN]	9.49	1	2	3	9	1.25	1.05	1.15	0.95	0.33	0.12	0.08	-0.13	0.02	0.24	0.15	-0.06	-1.12	-1.09	-1.00	-0.99	-0.88	-0.94	-0.84	-1.01
Q9NZV8	Potassium voltage-gated channel subfamily D member 2 OS=Homo sapiens GN=KCND2 PE=1 SV=2 - [KCND2_HUMAN]	3.33	1	1	1	1	2.10	2.17	1.93	2.00	-0.03	0.04	0.85	0.93	0.79	0.72	-0.19	-0.13	-1.19	-2.28	-2.12	-1.34	-1.17	-2.14	-1.98	-1.01
P14854	Cytochrome c oxidase subunit 6B1 OS=Homo sapiens GN=COX6B1 PE=1 SV=2 - [CX6B1_HUMAN]	75.58	1	7	7	30	1.55	1.87	1.61	2.00	0.01	0.28	0.52	0.74	0.49	0.22	0.25	0.37	-1.06	-1.34	-1.27	-1.33	-1.31	-1.30	-1.59	-1.01
Q8N0W4	Neuroligin-4, X-linked OS=Homo sapiens GN=NLGN4X PE=1 SV=1 - [NLGNX_HUMAN]	7.97	2	2	5	9	1.33	0.82	1.76	1.25	0.47	0.05	0.66	0.18	0.06	0.46	0.19	-0.22	-0.73	-1.55	-1.98	-1.02	-1.44	-1.14	-1.36	-1.01
Q7L266	Isoaspartyl peptidase/L-asparaginase OS=Homo sapiens GN=ASRGL1 PE=1 SV=2 - [ASGL1_HUMAN]	29.55	1	7	7	12	1.71	1.14	2.07	1.64	0.23	0.18	0.70	0.53	0.33	0.51	0.67	0.31	-0.55	-1.31	-1.40	-1.14	-1.35	-1.02	-1.34	-1.01

Q8TCZ2	CD99 antigen-like protein 2 OS=Homo sapiens GN=CD99L2 PE=1 SV=1 - [C99L2_HUMAN]	29.01	1	4	4	12	1.41	1.09	1.85	1.54	0.61	0.49	0.68	0.44	0.51	0.62	0.67	0.62	-0.67	-0.78	-1.24	-1.13	-1.39	-0.68	-1.12	-1.01
Q9Y6T7	Diacylglycerol kinase beta OS=Homo sapiens GN=DGKB PE=2 SV=2 - [DGKB_HUMAN]	17.41	1	9	9	18	1.17	1.27	1.41	1.37	-0.28	-0.07	0.38	0.50	0.37	0.21	0.27	0.09	-0.65	-1.09	-1.03	-0.79	-1.03	-1.24	-1.58	-1.01
Q14103	Heterogeneous nuclear ribonucleoprotein D0 OS=Homo sapiens GN=HNRNPD PE=1 SV=1 - [HNRPD_HUMAN]	22.25	1	7	8	25	-0.08	0.04	0.57	0.55	-0.46	-0.56	-0.55	-0.55	-0.61	-0.55	-0.30	-0.39	-0.35	-0.23	-0.89	-0.58	-1.09	-0.44	-0.98	-1.01
P17600	Synapsin-1 OS=Homo sapiens GN=SYN1 PE=1 SV=3 - [SYN1_HUMAN]	64.11	1	31	32	419	1.43	1.35	1.70	1.57	0.10	0.04	0.56	0.49	0.15	0.29	-0.07	-0.13	-0.86	-1.41	-1.66	-1.16	-1.36	-1.44	-1.54	-1.02
P53597	Succinyl-CoA ligase [ADP/GDP-forming] subunit alpha, mitochondrial OS=Homo sapiens GN=SUCLG1 PE=1 SV=4 - [SUCA_HUMAN]	26.88	1	8	8	23	0.39	0.26	0.76	0.63	-0.70	-0.79	-0.27	-0.39	-0.57	-0.53	-0.40	-0.40	-0.66	-0.79	-1.20	-0.94	-1.27	-1.14	-1.47	-1.02
Q96FC7	Phytanoyl-CoA hydroxylase-interacting protein-like OS=Homo sapiens GN=PHYHIPL PE=1 SV=3 - [PHIPL_HUMAN]	39.10	1	11	12	22	1.43	1.55	1.76	1.75	-0.44	-0.49	0.60	0.59	0.14	0.08	0.24	0.24	-0.93	-1.56	-1.49	-1.33	-1.38	-1.93	-1.65	-1.02
Q9BQ69	O-acetyl-ADP-ribose deacetylase MACROD1 OS=Homo sapiens GN=MACROD1 PE=1 SV=2 - [MACD1_HUMAN]	17.85	1	4	4	14	1.32	1.12	1.55	1.03	0.14	-0.26	0.46	0.15	0.25	0.59	0.26	-0.06	-0.95	-1.17	-1.34	-0.76	-0.74	-1.30	-1.43	-1.02

Q8NBF6	Late secretory pathway protein AVL9 homolog OS=Homo sapiens GN=AVL9 PE=1 SV=1 - [AVL9_HUMAN]	4.63	1	3	3	4	1.57	1.93	1.40	1.76	-0.38	-0.02	0.32	0.68	0.54	0.18	0.12	0.47	-1.20	-1.45	-1.29	-1.36	-1.19	-1.96	-1.79	-1.02
P47736	Rap1 GTPase-activating protein 1 OS=Homo sapiens GN=RAP1GAP PE=1 SV=2 - [RPGP1_HUMAN]	13.73	1	7	7	10	2.04	1.89	1.91	1.76	-0.08	-0.23	0.73	0.58	0.23	0.34	0.24	0.14	-1.18	-1.92	-1.74	-1.74	-1.61	-2.11	-2.03	-1.02
Q96QE2	Proton myo-inositol cotransporter OS=Homo sapiens GN=SLC2A13 PE=1 SV=3 - [MYCT_HUMAN]	5.86	1	3	3	6	1.48	1.80	1.75	2.24	-0.09	0.14	0.52	0.91	0.44	0.27	0.01	0.45	-0.88	-1.44	-1.65	-1.20	-1.48	-1.59	-2.03	-1.02
Q5VT66	Mitochondrial amidoxime-reducing component 1 OS=Homo sapiens GN=MARC1 PE=1 SV=1 - [MARC1_HUMAN]	9.50	1	1	2	4	1.13	1.14	1.72	1.74	-0.21	-0.20	0.64	0.65	0.69	0.68	-0.05	-0.04	-0.43	-1.17	-1.77	-0.41	-1.01	-1.35	-1.95	-1.02
Q9P2E7	Protocadherin-10 OS=Homo sapiens GN=PCDH10 PE=2 SV=2 - [PCD10_HUMAN]	2.31	1	2	2	4	0.30	0.61	0.25	0.56	-0.62	-0.32	-0.83	-0.52	-0.30	-0.61	-0.66	-0.35	-1.08	-0.95	-0.91	-0.88	-0.83	-0.94	-0.89	-1.02
Q9UHG2	ProSAAS OS=Homo sapiens GN=PCSK1N PE=1 SV=1 - [PCSK1_HUMAN]	41.15	1	7	7	27	1.68	1.37	1.50	1.40	0.37	0.14	0.71	0.37	0.24	0.52	0.31	0.09	-1.04	-1.33	-1.27	-1.13	-1.18	-1.42	-1.34	-1.02
Q96HU8	GTP-binding protein Di-Ras2 OS=Homo sapiens GN=DIRAS2 PE=1 SV=1 - [DIRA2_HUMAN]	23.12	1	4	4	8	1.34	1.65	1.82	2.01	-0.13	-0.15	0.72	0.71	0.38	0.39	0.21	0.28	-0.72	-1.45	-1.81	-1.24	-1.59	-1.81	-2.17	-1.02
P21579	Synaptotagmin-1 OS=Homo sapiens GN=SYT1 PE=1 SV=1 - [SYT1_HUMAN]	43.36	2	10	18	83	1.49	1.53	1.72	1.62	0.09	0.03	0.66	0.50	0.30	0.39	0.23	0.16	-1.03	-1.33	-1.48	-1.20	-1.25	-1.49	-1.56	-1.03

Q92623	Tetratricopeptide repeat protein 9A OS=Homo sapiens GN=TTC9 PE=2 SV=3 - [TTC9A_HUMAN]	30.63	1	4	4	6	1.30	0.84	1.27	0.81	0.09	-0.38	-0.20	-0.67	-0.53	-0.06	-0.07	-0.54	-1.19	-1.37	-1.34	-1.33	-1.30	-1.23	-1.20	-1.03
Q8IYB4	PEX5-related protein OS=Homo sapiens GN=PEX5L PE=1 SV=2 - [PEX5R_HUMAN]	17.57	1	8	8	14	1.12	1.11	1.33	1.25	-0.21	-0.49	0.14	0.05	-0.16	-0.05	-0.18	-0.42	-0.93	-1.23	-1.50	-0.92	-0.97	-1.81	-1.39	-1.03
P52824	Diacylglycerol kinase theta OS=Homo sapiens GN=DGKQ PE=1 SV=2 - [DGKQ_HUMAN]	0.74	1	1	1	1	1.62	1.55	1.57	1.50	0.13	0.05	0.48	0.41	0.14	0.22	-0.31	-0.38	-1.09	-1.92	-1.88	-1.37	-1.32	-1.51	-1.46	-1.03
Q15438	Cytohesin-1 OS=Homo sapiens GN=CYTH1 PE=1 SV=1 - [CYH1_HUMAN]	12.56	1	1	4	5	1.19	0.92	0.96	0.69	-0.20	-0.47	-0.14	-0.40	-0.13	0.15	-0.10	-0.37	-1.27	-1.29	-1.06	-1.01	-0.78	-1.40	-1.17	-1.03
Q9Y2Q0	Probable phospholipid-transporting ATPase IA OS=Homo sapiens GN=ATP8A1 PE=1 SV=1 - [AT8A1_HUMAN]	21.39	2	20	20	48	2.17	1.99	2.09	1.85	0.14	0.13	1.06	0.85	0.69	0.66	0.66	0.55	-0.92	-1.40	-1.30	-1.23	-1.16	-1.94	-1.90	-1.03
O75582	Ribosomal protein S6 kinase alpha-5 OS=Homo sapiens GN=RPS6KA5 PE=1 SV=1 - [KS6A5_HUMAN]	6.98	1	4	5	8	1.46	1.45	1.77	1.80	0.25	0.23	0.69	0.71	0.19	0.16	0.45	0.43	-0.66	-0.92	-1.17	-0.97	-1.45	-1.23	-1.57	-1.03
P55809	Succinyl-CoA:3-ketoacid coenzyme A transferase 1, mitochondrial OS=Homo sapiens GN=OXCT1 PE=1 SV=1 - [SCOT1_HUMAN]	41.35	2	16	16	53	1.35	1.26	1.39	1.23	-0.29	-0.25	0.33	0.35	-0.01	0.01	-0.13	-0.21	-0.85	-1.58	-1.71	-1.26	-1.37	-1.64	-1.72	-1.03

Q13526	Peptidyl-prolyl cis-trans isomerase NIMA-interacting 1 OS=Homo sapiens GN=PIN1 PE=1 SV=1 - [PIN1_HUMAN]	47.24	1	5	5	20	1.38	1.13	1.78	1.75	0.17	0.08	0.75	0.56	0.27	0.35	0.31	0.14	-0.65	-1.20	-1.62	-0.98	-1.43	-1.16	-1.70	-1.03
A0MZ66	Shootin-1 OS=Homo sapiens GN=KIAA1598 PE=1 SV=4 - [SHOT1_HUMAN]	21.08	1	11	11	26	1.51	1.51	1.82	1.76	0.08	0.14	0.59	0.57	0.35	0.29	-0.02	0.01	-0.81	-1.49	-1.60	-1.25	-1.47	-1.48	-1.71	-1.03
Q8IVF5	T-lymphoma invasion and metastasis-inducing protein 2 OS=Homo sapiens GN=TIAM2 PE=2 SV=4 - [TIAM2_HUMAN]	4.29	1	4	4	5	1.11	0.94	2.01	1.58	-0.29	0.09	0.49	0.49	0.00	-0.05	-0.68	-0.61	-0.40	-1.49	-1.81	-0.93	-1.24	-1.14	-1.73	-1.03
Q9P2R7	Succinyl-CoA ligase [ADP-forming] subunit beta, mitochondrial OS=Homo sapiens GN=SUCLA2 PE=1 SV=3 - [SUCB1_HUMAN]	40.60	1	20	20	58	0.63	0.63	0.95	0.96	-0.83	-0.82	-0.09	-0.16	-0.48	-0.41	-0.42	-0.50	-0.70	-1.00	-1.41	-0.96	-1.39	-1.46	-1.82	-1.03
Q5VWQ8	Disabled homolog 2-interacting protein OS=Homo sapiens GN=DAB2IP PE=1 SV=2 - [DAB2P_HUMAN]	4.79	1	4	6	8	0.51	0.57	1.26	1.19	-0.31	-0.39	0.17	0.10	-0.27	-0.19	-0.08	-0.09	-0.43	-0.64	-1.13	-0.94	-1.42	-1.04	-1.57	-1.03
Q643R3	Lysophospholipid acyltransferase LPCAT4 OS=Homo sapiens GN=LPCAT4 PE=1 SV=1 - [LPCT4_HUMAN]	2.48	1	1	1	2	1.20	1.21	1.27	1.28	-0.58	-0.57	0.18	0.19	-0.27	-0.27	-0.29	-0.29	-0.96	-1.48	-1.57	-1.44	-1.52	-1.79	-1.87	-1.03
Q9P1A6	Disks large-associated protein 2 OS=Homo sapiens GN=DLGAP2 PE=1 SV=4 - [DLGP2_HUMAN]	5.98	1	5	6	9	1.44	1.34	1.41	1.45	0.14	0.37	0.36	0.59	0.29	0.06	-0.21	-0.15	-1.16	-1.70	-1.82	-1.50	-1.36	-1.47	-1.33	-1.03

Q6ZVM7	TOM1-like protein 2 OS=Homo sapiens GN=TOM1L2 PE=1 SV=1 - [TM1L2_HUMAN]	50.10	1	15	15	35	1.28	1.43	1.37	1.40	0.08	0.21	0.26	0.29	0.05	0.06	-0.08	-0.06	-0.97	-1.36	-1.32	-1.29	-1.27	-1.25	-1.21	-1.03
P49841	Glycogen synthase kinase-3 beta OS=Homo sapiens GN=GSK3B PE=1 SV=2 - [GSK3B_HUMAN]	29.52	1	5	8	14	0.94	0.71	1.40	1.16	-0.33	-0.53	0.31	0.09	-0.22	-0.13	-0.28	-0.45	-0.65	-1.21	-1.68	-1.04	-1.49	-1.43	-1.72	-1.03
Q6UUV9	CREB-regulated transcription coactivator 1 OS=Homo sapiens GN=CRTC1 PE=1 SV=2 - [CRTC1_HUMAN]	8.20	1	3	3	8	1.50	1.22	1.63	1.06	0.01	0.25	0.00	0.42	0.62	-0.32	0.06	0.93	-0.76	-0.93	-0.93	-0.84	-0.90	-0.96	-1.21	-1.03
O43295	SLIT-ROBO Rho GTPase-activating protein 3 OS=Homo sapiens GN=SRGAP3 PE=1 SV=3 - [SRGP3_HUMAN]	14.74	1	10	12	19	1.08	1.08	1.38	1.38	0.02	0.04	0.49	0.16	-0.06	0.03	-0.04	0.08	-0.65	-0.89	-1.17	-1.10	-1.42	-1.24	-1.41	-1.04
Q4J6C6	Prolyl endopeptidase-like OS=Homo sapiens GN=PREPL PE=1 SV=1 - [PPCEL_HUMAN]	22.28	1	12	12	21	1.38	1.39	1.46	1.58	0.05	0.18	0.58	0.53	0.34	0.23	0.22	0.40	-0.73	-1.23	-1.17	-0.96	-1.26	-1.32	-1.62	-1.04
A7KAX9	Rho GTPase-activating protein 32 OS=Homo sapiens GN=ARHGAP32 PE=1 SV=1 - [RHG32_HUMAN]	7.62	3	12	12	27	1.68	1.42	1.52	1.40	0.15	0.09	0.16	0.30	0.23	0.50	0.25	-0.03	-1.31	-1.34	-1.34	-1.17	-0.96	-1.52	-1.33	-1.04
Q86YM7	Homer protein homolog 1 OS=Homo sapiens GN=HOMER1 PE=1 SV=2 - [HOME1_HUMAN]	31.07	1	9	10	22	1.87	1.64	1.84	1.48	-0.01	-0.54	0.64	0.24	-0.05	0.28	-0.01	-0.20	-1.03	-1.83	-1.79	-1.62	-1.49	-1.63	-1.85	-1.04
Q9UNX4	WD repeat-containing protein 3 OS=Homo sapiens GN=WDR3 PE=1 SV=1 - [WDR3_HUMAN]	2.97	1	2	2	2	-0.37	-0.40	-0.12	-0.14	-0.69	-0.72	-1.22	-1.25	-0.78	-0.75	0.37	0.34	-0.79	0.74	0.48	-0.35	-0.60	-0.34	-0.59	-1.04

O43739	Cytohesin-3 OS=Homo sapiens GN=CYTH3 PE=1 SV=2 - [CYH3_HUMAN]	20.50	1	3	7	9	0.61	0.73	0.97	1.09	-0.34	-0.23	-0.14	-0.01	0.03	-0.09	-0.16	-0.04	-0.69	-0.77	-1.13	-0.67	-1.02	-0.97	-1.33	-1.04
Q8WWN9	Interactor protein for cytohesin exchange factors 1 OS=Homo sapiens GN=IPCEF1 PE=1 SV=1 - [ICEF1_HUMAN]	8.70	1	3	3	3	1.17	0.86	1.19	1.01	0.27	0.16	0.08	0.10	-0.03	0.42	-0.19	-0.28	-0.70	-1.09	-1.30	-0.94	-0.74	-1.01	-0.86	-1.04
Q9UHC6	Contactin-associated protein-like 2 OS=Homo sapiens GN=CNTNAP2 PE=1 SV=1 - [CNTP2_HUMAN]	27.72	1	30	30	63	1.34	1.42	1.67	1.60	0.31	0.27	0.34	0.37	0.31	0.26	0.25	0.17	-0.97	-1.26	-1.29	-1.07	-1.21	-1.07	-1.35	-1.04
Q9UII2	ATPase inhibitor, mitochondrial OS=Homo sapiens GN=ATPIF1 PE=1 SV=1 - [ATIF1_HUMAN]	17.92	1	3	3	4	0.94	0.84	1.27	1.17	-0.23	-0.34	0.16	0.06	-0.39	-0.29	-0.33	-0.44	-0.73	-1.27	-1.60	-1.20	-1.52	-1.19	-1.52	-1.04
Q13613	Myotubularin-related protein 1 OS=Homo sapiens GN=MTMR1 PE=1 SV=4 - [MTMR1_HUMAN]	10.23	1	4	5	6	1.51	1.45	1.37	1.31	-0.12	-0.18	0.19	0.13	0.16	0.23	0.20	0.13	-1.26	-1.31	-1.17	-1.23	-1.07	-1.60	-1.49	-1.04
Q12840	Kinesin heavy chain isoform 5A OS=Homo sapiens GN=KIF5A PE=1 SV=2 - [KIF5A_HUMAN]	11.24	1	3	10	22	1.58	1.67	1.41	1.50	-0.10	-0.02	0.31	0.39	0.32	0.24	-0.13	-0.06	-1.22	-1.71	-1.55	-1.31	-1.14	-1.70	-1.53	-1.04
P27707	Deoxycytidine kinase OS=Homo sapiens GN=DCK PE=1 SV=1 - [DCK_HUMAN]	10.77	1	2	2	3	0.86	0.58	0.82	0.54	0.55	0.27	-0.29	-0.57	0.31	0.59	0.37	0.09	-1.09	-0.48	-0.45	-0.23	-0.19	-0.33	-0.28	-1.04
O60502	Bifunctional protein NCOAT OS=Homo sapiens GN=MGEA5 PE=1 SV=2 - [NCOAT_HUMAN]	21.83	1	14	14	18	0.76	0.72	1.17	1.28	-0.26	-0.12	0.20	0.36	0.14	0.01	-0.03	0.17	-0.52	-0.64	-1.15	-0.79	-1.25	-1.07	-1.51	-1.04

O14576	Cytoplasmic dynein 1 intermediate chain 1 OS=Homo sapiens GN=DYNC1I1 PE=1 SV=2 - [DC1I1_HUMAN]	20.31	1	8	8	23	1.35	1.45	1.40	1.45	-0.23	-0.05	0.19	0.33	0.18	0.06	-0.15	-0.03	-1.01	-1.35	-1.38	-1.14	-1.09	-1.49	-1.43	-1.04
Q9Y639	Neuroplastin OS=Homo sapiens GN=NPTN PE=1 SV=2 - [NPTN_HUMAN]	32.66	1	13	13	54	1.34	1.33	1.53	1.56	0.16	-0.01	0.53	0.60	0.24	0.33	0.12	0.19	-0.79	-1.21	-1.49	-1.15	-1.39	-1.24	-1.68	-1.04
Q6MZT1	Regulator of G-protein signaling 7-binding protein OS=Homo sapiens GN=RGS7BP PE=2 SV=3 - [R7BP_HUMAN]	33.07	1	4	4	5	1.31	1.20	1.71	1.36	0.35	0.23	0.42	0.48	0.28	0.48	0.48	0.36	-0.65	-0.83	-1.14	-0.89	-1.07	-0.97	-1.38	-1.05
O14523	C2 domain-containing protein 2-like OS=Homo sapiens GN=C2CD2L PE=1 SV=3 - [C2C2L_HUMAN]	15.58	1	7	7	16	1.49	1.48	1.40	1.36	-0.30	-0.32	0.23	0.36	0.09	0.12	0.01	-0.07	-1.15	-1.14	-1.39	-1.18	-1.34	-1.52	-1.73	-1.05
Q8N9I9	Probable E3 ubiquitin-protein ligase DTX3 OS=Homo sapiens GN=DTX3 PE=1 SV=2 - [DTX3_HUMAN]	9.80	1	2	2	3	1.01	0.77	1.30	1.18	-0.51	-0.58	0.18	0.05	-0.10	0.08	-0.11	-0.75	-0.77	-1.11	-1.40	-0.90	-1.19	-1.53	-1.82	-1.05
O15126	Secretory carrier-associated membrane protein 1 OS=Homo sapiens GN=SCAMP1 PE=1 SV=2 - [SCAM1_HUMAN]	21.89	1	5	5	14	1.10	1.37	1.50	1.57	0.13	0.05	0.37	0.16	0.05	-0.05	0.12	0.17	-0.99	-1.19	-1.42	-1.19	-1.34	-1.31	-1.35	-1.05
A6NHQ2	rRNA/tRNA 2'-O-methyltransferase fibrillarin-like protein 1 OS=Homo sapiens GN=FBLL1 PE=3 SV=1 - [FBLL1_HUMAN]	11.41	1	2	3	12	1.61	1.77	1.90	2.05	0.09	0.24	0.79	0.94	0.50	0.36	0.50	0.64	-0.77	-1.11	-1.40	-1.23	-1.51	-1.54	-1.82	-1.05

P52758	Ribonuclease UK114 OS=Homo sapiens GN=HRSP12 PE=1 SV=1 - [UK114_HUMAN]	43.07	1	5	5	8	1.23	1.11	1.55	1.43	0.49	0.37	0.43	0.31	-0.17	-0.04	-0.32	-0.45	-0.74	-1.54	-1.87	-1.24	-1.55	-0.75	-1.07	-1.05
Q8IXS6	Paralemmin-2 OS=Homo sapiens GN=PALM2 PE=2 SV=3 - [PALM2_HUMAN]	12.93	1	3	5	7	1.01	0.99	1.02	1.01	-0.43	-0.45	-0.09	-0.11	-0.04	-0.01	-0.10	-0.12	-1.04	-1.10	-1.12	-0.99	-1.00	-1.45	-1.47	-1.05
P55327	Tumor protein D52 OS=Homo sapiens GN=TPD52 PE=1 SV=2 - [TPD52_HUMAN]	46.88	1	9	9	24	1.27	1.06	1.67	1.63	-0.05	-0.04	0.50	0.49	0.00	0.04	-0.07	0.02	-0.68	-1.20	-1.60	-1.15	-1.42	-1.29	-1.65	-1.05
Q99259	Glutamate decarboxylase 1 OS=Homo sapiens GN=GAD1 PE=1 SV=1 - [DCE1_HUMAN]	17.68	1	8	9	13	1.62	1.74	2.03	2.20	-0.15	0.19	0.59	0.88	0.66	0.30	-0.22	0.12	-0.64	-1.72	-2.09	-1.21	-1.55	-1.67	-2.07	-1.05
Q9H254	Spectrin beta chain, non-erythrocytic 4 OS=Homo sapiens GN=SPTBN4 PE=1 SV=2 - [SPTN4_HUMAN]	18.88	1	33	37	74	1.45	1.56	1.71	1.57	0.12	0.15	0.61	0.59	0.23	0.40	0.16	0.24	-1.20	-1.38	-1.54	-1.23	-1.21	-1.45	-1.57	-1.05
Q8N573	Oxidation resistance protein 1 OS=Homo sapiens GN=OXR1 PE=1 SV=2 - [OXR1_HUMAN]	39.47	1	25	25	50	1.21	1.47	1.58	1.49	0.20	0.10	0.46	0.57	0.34	0.36	-0.12	-0.10	-0.94	-1.43	-1.57	-0.88	-1.04	-1.54	-1.43	-1.05
Q8IZS8	Voltage-dependent calcium channel subunit alpha 2/delta-3 OS=Homo sapiens GN=CACNA2D3 PE=1 SV=1 - [CA2D3_HUMAN]	5.87	1	4	4	7	1.92	1.69	1.74	1.51	0.44	0.21	0.63	0.40	0.57	0.81	0.48	0.24	-1.24	-1.44	-1.27	-1.08	-0.90	-1.49	-1.32	-1.06
Q9BWQ8	Protein lifeguard 2 OS=Homo sapiens GN=FAIM2 PE=1 SV=1 - [LFG2_HUMAN]	11.71	1	3	3	6	0.35	0.53	0.93	1.11	-0.63	-0.46	-0.19	-0.01	-0.56	-0.74	-0.63	-0.46	-0.48	-0.97	-1.56	-1.05	-1.63	-0.99	-1.58	-1.06

Q9BX40	Protein LSM14 homolog B OS=Homo sapiens GN=LSM14B PE=1 SV=1 - [LS14B_HUMAN]	2.86	1	1	1	2	0.62	0.67	0.98	1.03	-0.19	-0.14	-0.14	-0.10	0.00	-0.04	0.07	0.11	-0.71	-0.55	-0.91	-0.63	-0.98	-0.83	-1.18	-1.06
Q96PQ0	VPS10 domain-containing receptor SorCS2 OS=Homo sapiens GN=SORCS2 PE=1 SV=3 - [SORC2_HUMAN]	2.93	1	3	3	3	2.12	1.83	1.76	1.48	0.46	0.17	0.64	0.36	0.23	0.52	0.43	0.14	-1.42	-1.68	-1.33	-1.57	-1.20	-1.67	-1.32	-1.06
O15394	Neural cell adhesion molecule 2 OS=Homo sapiens GN=NCAM2 PE=1 SV=2 - [NCAM2_HUMAN]	29.15	1	20	20	49	1.72	1.94	1.93	2.03	0.17	0.21	1.04	0.87	0.53	0.67	0.61	0.66	-0.88	-1.19	-1.38	-1.23	-1.39	-1.65	-1.54	-1.06
Q8WXI2	Connector enhancer of kinase suppressor of ras 2 OS=Homo sapiens GN=CNKSR2 PE=1 SV=1 - [CNKR2_HUMAN]	13.73	1	10	10	12	1.53	1.61	1.65	1.69	0.12	-0.27	0.80	0.50	0.46	0.29	0.34	0.30	-1.20	-1.49	-1.42	-1.70	-1.50	-2.08	-2.01	-1.06
Q12860	Contactin-1 OS=Homo sapiens GN=CNTN1 PE=1 SV=1 - [CNTN1_HUMAN]	53.05	1	41	41	137	1.61	1.67	1.77	1.71	0.19	0.10	0.59	0.60	0.41	0.42	0.28	0.31	-0.90	-1.45	-1.56	-1.28	-1.25	-1.64	-1.67	-1.06
Q9Y2H9	Microtubule-associated serine/threonine-protein kinase 1 OS=Homo sapiens GN=MAST1 PE=1 SV=2 - [MAST1_HUMAN]	5.73	4	5	7	8	1.19	1.27	1.34	1.34	0.32	-0.09	0.25	0.17	-0.07	0.16	-0.22	-0.68	-1.11	-1.41	-1.57	-1.31	-1.37	-1.37	-1.44	-1.06
Q92932	Receptor-type tyrosine-protein phosphatase N2 OS=Homo sapiens GN=PTPRN2 PE=1 SV=2 - [PTPR2_HUMAN]	9.26	1	6	6	8	1.45	1.80	1.51	1.86	0.20	0.73	0.24	0.59	0.16	0.22	0.04	0.34	-1.09	-1.46	-1.32	-1.14	-1.10	-1.52	-1.62	-1.06
Q8N1I0	Dedicator of cytokinesis protein 4 OS=Homo sapiens GN=DOCK4 PE=1 SV=3 - [DOCK4_HUMAN]	3.92	1	5	6	8	1.01	0.65	1.28	0.92	0.13	0.17	0.15	-0.21	-0.26	0.11	-0.13	-0.49	-0.81	-1.13	-1.40	-0.87	-1.14	-1.00	-1.21	-1.07

O00305	Voltage-dependent L-type calcium channel subunit beta-4 OS=Homo sapiens GN=CACNB4 PE=1 SV=2 - [CACB4_HUMAN]	20.77	2	5	9	13	1.34	1.56	1.60	1.82	-0.32	-0.13	0.50	0.51	0.29	0.05	-0.13	0.01	-0.82	-1.30	-1.69	-1.08	-1.46	-1.72	-1.99	-1.07
O00478	Butyrophilin subfamily 3 member A3 OS=Homo sapiens GN=BTN3A3 PE=1 SV=1 - [BT3A3_HUMAN]	10.62	3	5	5	7	1.26	0.88	1.24	0.85	0.24	-0.15	0.10	-0.28	-0.52	-0.13	-0.07	-0.46	-1.11	-1.33	-1.31	-1.37	-1.34	-1.04	-1.02	-1.07
P15121	Aldose reductase OS=Homo sapiens GN=AKR1B1 PE=1 SV=3 - [ALDR_HUMAN]	40.82	2	10	10	21	1.26	1.24	1.49	1.63	-0.10	-0.15	0.37	0.28	-0.01	0.04	-0.36	-0.44	-0.83	-1.78	-1.87	-1.31	-1.62	-1.44	-1.73	-1.07
O75508	Claudin-11 OS=Homo sapiens GN=CLDN11 PE=1 SV=2 - [CLD11_HUMAN]	14.98	1	3	3	20	1.14	1.02	1.42	1.30	-0.37	-0.36	0.29	0.16	-0.04	-0.15	-0.14	-0.09	-0.86	-1.08	-1.27	-1.18	-1.46	-1.40	-1.68	-1.07
Q9BYC5	Alpha-(1,6)-fucosyltransferase OS=Homo sapiens GN=FUT8 PE=1 SV=2 - [FUT8_HUMAN]	4.35	1	1	1	1	-0.16	-0.10	-0.09	-0.02	-0.57	-0.50	-1.23	-1.16	-0.48	-0.54	-0.01	0.05	-1.01	0.16	0.08	-0.35	-0.42	-0.42	-0.49	-1.07
Q7Z2K8	G protein-regulated inducer of neurite outgrowth 1 OS=Homo sapiens GN=GPRIN1 PE=2 SV=2 - [GRIN1_HUMAN]	20.63	1	16	16	24	0.97	0.80	1.36	1.15	-0.13	0.01	-0.29	-0.40	0.01	-0.01	-0.01	-0.15	-0.98	-1.37	-1.50	-1.30	-1.43	-1.24	-1.41	-1.07
Q8IUQ0	Clavesin-1 OS=Homo sapiens GN=CLVS1 PE=1 SV=1 - [CLVS1_HUMAN]	11.02	1	1	3	6	1.90	1.90	1.88	1.88	0.49	0.49	0.74	0.74	0.32	0.33	0.04	0.04	-1.11	-1.85	-1.83	-1.55	-1.52	-1.43	-1.40	-1.07
Q15637	Splicing factor 1 OS=Homo sapiens GN=SF1 PE=1 SV=4 - [SF01_HUMAN]	3.91	1	1	1	2	0.14	0.46	0.49	0.82	-0.17	0.15	-0.65	-0.32	0.15	-0.17	0.21	0.53	-0.73	0.08	-0.28	-0.28	-0.63	-0.32	-0.68	-1.07

Q13555	Calcium/calmodulin-dependent protein kinase type II subunit gamma OS=Homo sapiens GN=CAMK2G PE=1 SV=3 - [KCC2G_HUMAN]	43.91	1	9	16	65	1.34	1.70	1.71	1.79	0.02	-0.05	0.62	0.62	0.26	0.30	0.27	0.34	-0.84	-1.43	-1.66	-1.19	-1.45	-1.41	-1.68	-1.07
Q9NWS8	Required for meiotic nuclear division protein 1 homolog OS=Homo sapiens GN=RMND1 PE=1 SV=2 - [RMND1_HUMAN]	12.03	1	5	5	8	1.10	0.96	1.00	1.04	-0.22	-0.27	-0.31	-0.24	-0.19	-0.19	-0.29	-0.34	-1.09	-1.25	-1.35	-1.18	-1.16	-1.35	-1.22	-1.07
Q8TBG9	Synaptoporin OS=Homo sapiens GN=SYNPR PE=2 SV=1 - [SYNPR_HUMAN]	10.57	1	2	3	5	1.27	2.03	1.65	2.42	-0.82	-0.06	0.51	1.28	0.65	-0.11	-0.46	0.30	-0.70	-1.73	-2.12	-1.35	-1.72	-2.11	-2.49	-1.08
O00519	Fatty-acid amide hydrolase 1 OS=Homo sapiens GN=FAAH PE=1 SV=2 - [FAAH1_HUMAN]	9.33	1	5	5	6	1.57	1.53	1.67	1.64	0.20	0.20	0.53	0.50	0.30	0.34	-0.14	-0.15	-0.98	-2.08	-2.05	-1.20	-1.30	-1.75	-1.72	-1.08
Q8TDC3	Serine/threonine-protein kinase BRSK1 OS=Homo sapiens GN=BRSK1 PE=1 SV=2 - [BRSK1_HUMAN]	11.70	1	7	7	16	2.68	2.74	1.75	1.51	0.14	0.18	0.61	0.57	0.26	0.23	-0.07	-0.15	-1.69	-1.90	-1.82	-2.38	-1.57	-2.33	-2.00	-1.08
Q99784	Noelin OS=Homo sapiens GN=OLFM1 PE=1 SV=4 - [NOE1_HUMAN]	20.00	1	7	7	11	1.18	1.32	1.61	1.39	0.28	0.46	0.33	0.46	0.63	0.13	0.07	0.36	-0.80	-0.85	-1.16	-0.66	-0.91	-0.91	-1.34	-1.08
Q8N9I0	Synaptotagmin-2 OS=Homo sapiens GN=SYT2 PE=1 SV=2 - [SYT2_HUMAN]	37.95	2	10	18	49	2.43	2.48	2.27	2.58	0.13	0.23	1.39	1.47	1.16	1.16	0.70	0.67	-0.78	-1.92	-2.09	-1.34	-1.78	-2.14	-2.45	-1.08
P56381	ATP synthase subunit epsilon, mitochondrial OS=Homo sapiens GN=ATP5E PE=1 SV=2 - [ATP5E_HUMAN]	29.41	2	2	2	9	1.08	1.11	1.16	1.19	-0.36	-0.29	0.19	0.10	-0.05	-0.12	-0.33	-0.25	-0.94	-1.47	-1.54	-1.18	-1.14	-1.49	-1.43	-1.08

Q5FWE3	Proline-rich transmembrane protein 3 OS=Homo sapiens GN=PRRT3 PE=1 SV=3 - [PRRT3_HUMAN]	10.19	1	6	6	11	2.11	1.81	2.25	2.04	0.29	0.35	0.85	0.74	0.01	0.26	0.54	0.58	-0.87	-1.10	-1.21	-1.69	-1.81	-1.30	-1.41	-1.08
Q9H1V8	Sodium-dependent neutral amino acid transporter SLC6A17 OS=Homo sapiens GN=SLC6A17 PE=2 SV=3 - [S6A17_HUMAN]	9.22	1	5	5	11	1.98	2.20	1.82	2.10	-0.28	-0.11	0.70	0.95	0.46	0.31	0.34	0.44	-1.10	-1.78	-1.85	-1.27	-1.25	-2.44	-2.39	-1.08
Q9NZR1	Tropomodulin 2 OS=Homo sapiens GN=TMOD2 PE=1 SV=1 - [TMOD2_HUMAN]	50.43	1	15	16	54	1.63	1.52	1.91	1.79	0.16	0.02	0.75	0.65	0.35	0.51	0.14	0.08	-0.75	-1.50	-1.80	-1.18	-1.67	-1.46	-1.73	-1.08
P26378	ELAV-like protein 4 OS=Homo sapiens GN=ELAVL4 PE=1 SV=2 - [ELAV4_HUMAN]	35.00	1	2	12	24	1.43	1.10	1.76	1.42	1.05	0.71	0.61	0.27	0.07	0.41	0.96	0.62	-0.77	-0.47	-0.80	-0.99	-1.31	-0.40	-0.73	-1.08
Q16864	V-type proton ATPase subunit F OS=Homo sapiens GN=ATP6V1F PE=1 SV=2 - [VATF_HUMAN]	63.03	1	6	6	15	1.82	1.40	1.90	1.84	-0.13	0.02	0.69	0.54	0.46	0.23	0.19	0.30	-0.96	-1.09	-1.53	-1.07	-1.50	-1.41	-1.85	-1.09
P30086	Phosphatidylethanolamine binding protein 1 OS=Homo sapiens GN=PEBP1 PE=1 SV=3 - [PEBP1_HUMAN]	82.35	1	11	11	116	1.59	1.40	1.92	1.80	0.60	0.44	0.76	0.64	0.54	0.56	0.20	0.10	-0.71	-1.26	-1.63	-1.01	-1.25	-0.92	-1.32	-1.09
Q9H993	UPF0364 protein C6orf211 OS=Homo sapiens GN=C6orf211 PE=1 SV=1 - [CF211_HUMAN]	3.63	1	2	2	2	1.99	2.05	2.18	2.25	0.40	0.46	1.04	1.10	0.54	0.48	0.27	0.33	-0.89	-1.71	-1.92	-1.48	-1.68	-1.61	-1.81	-1.09
Q8ND83	SLAIN motif-containing protein 1 OS=Homo sapiens GN=SLAIN1 PE=1 SV=3 - [SLAI1_HUMAN]	1.58	1	1	1	1	0.64	1.14	0.92	1.42	-0.51	-0.01	-0.23	0.27	-0.09	-0.59	-0.33	0.16	-0.81	-0.97	-1.26	-1.20	-1.48	-1.17	-1.45	-1.09

P19086	Guanine nucleotide-binding protein G(z) subunit alpha OS=Homo sapiens GN=GNAZ PE=2 SV=3 - [GNAZ_HUMAN]	38.31	1	10	11	29	2.07	1.52	1.89	1.89	0.10	0.07	0.80	0.72	0.51	0.53	0.29	0.22	-1.09	-1.47	-1.50	-1.39	-1.45	-1.93	-1.76	-1.09
P49773	Histidine triad nucleotide-binding protein 1 OS=Homo sapiens GN=HINT1 PE=1 SV=2 - [HINT1_HUMAN]	47.62	1	4	4	8	1.87	2.10	1.81	2.15	0.50	0.27	0.67	1.00	0.86	0.89	0.84	0.60	-1.06	-1.03	-0.97	-1.31	-1.24	-1.87	-1.81	-1.09
O75608	Acyl-protein thioesterase 1 OS=Homo sapiens GN=LYPLA1 PE=1 SV=1 - [LYPA1_HUMAN]	5.65	1	1	1	3	0.78	0.52	0.82	0.56	0.26	-0.01	-0.33	-0.59	-0.36	-0.10	-0.08	-0.35	-1.06	-0.86	-0.91	-0.85	-0.89	-0.54	-0.58	-1.09
Q7L0J3	Synaptic vesicle glycoprotein 2A OS=Homo sapiens GN=SV2A PE=1 SV=1 - [SV2A_HUMAN]	12.53	1	10	10	34	2.03	2.20	2.14	2.21	-0.15	-0.18	1.09	1.11	0.51	0.64	0.31	0.24	-0.77	-1.68	-1.96	-1.36	-1.69	-1.95	-2.04	-1.09
P00441	Superoxide dismutase [Cu-Zn] OS=Homo sapiens GN=SOD1 PE=1 SV=2 - [SODC_HUMAN]	68.83	1	7	7	31	0.94	1.10	1.29	1.35	-0.11	-0.09	0.24	0.27	-0.20	-0.24	-0.87	-0.82	-0.82	-1.85	-2.12	-1.34	-1.47	-1.01	-1.38	-1.09
Q8WXD2	Secretogranin-3 OS=Homo sapiens GN=SCG3 PE=1 SV=3 - [SCG3_HUMAN]	14.96	1	7	7	12	1.05	1.07	1.01	1.34	-0.06	0.19	0.02	0.28	0.17	0.02	0.04	0.30	-0.67	-0.66	-1.09	-0.87	-1.29	-1.29	-1.28	-1.09
O76070	Gamma-synuclein OS=Homo sapiens GN=SNCG PE=1 SV=2 - [SYUG_HUMAN]	50.39	1	5	6	14	0.69	0.63	1.13	1.01	-0.47	-0.45	-0.45	-0.02	-0.49	-0.27	-0.58	-0.53	-0.94	-1.23	-1.68	-1.08	-1.39	-1.04	-1.38	-1.09
P21695	Glycerol-3-phosphate dehydrogenase [NAD(+)], cytoplasmic OS=Homo sapiens GN=GPD1 PE=1 SV=4 - [GPDA_HUMAN]	33.52	1	8	9	14	1.70	1.67	1.94	1.63	0.34	0.29	0.73	0.57	0.49	0.44	0.04	-0.14	-1.04	-1.56	-1.61	-1.23	-1.18	-1.23	-1.34	-1.09

Q9NZN3	EH domain-containing protein 3 OS=Homo sapiens GN=EHD3 PE=1 SV=2 - [EHD3_HUMAN]	65.79	2	15	25	57	1.09	0.99	1.55	1.61	0.01	-0.06	0.62	0.49	0.34	0.31	0.35	0.35	-0.80	-1.01	-1.50	-1.02	-1.34	-1.60	-1.92	-1.09
Q02156	Protein kinase C epsilon type OS=Homo sapiens GN=PRKCE PE=1 SV=1 - [KPCE_HUMAN]	32.70	2	19	20	50	1.53	1.61	1.51	1.62	0.08	0.00	0.45	0.41	0.27	0.29	0.23	0.09	-0.99	-1.38	-1.39	-1.34	-1.41	-1.67	-1.72	-1.09
O75746	Calcium-binding mitochondrial carrier protein Aralar1 OS=Homo sapiens GN=SLC25A12 PE=1 SV=2 - [CMC1_HUMAN]	50.88	1	23	28	101	1.57	1.43	1.71	1.55	0.06	0.03	0.48	0.47	0.22	0.28	0.19	0.13	-0.96	-1.32	-1.49	-1.25	-1.23	-1.39	-1.64	-1.09
O75334	Liprin-alpha-2 OS=Homo sapiens GN=PPFIA2 PE=1 SV=2 - [LIPA2_HUMAN]	18.77	2	10	18	30	1.51	1.41	1.58	1.79	0.18	-0.08	0.45	0.29	0.17	0.22	-0.02	0.03	-1.01	-1.61	-1.57	-1.41	-1.34	-1.41	-1.29	-1.10
Q99767	Amyloid beta A4 precursor protein-binding family A member 2 OS=Homo sapiens GN=APBA2 PE=1 SV=3 - [APBA2_HUMAN]	5.61	1	2	3	4	1.07	1.25	1.39	1.57	-0.15	0.02	0.23	0.41	0.27	0.10	0.06	0.24	-0.79	-1.00	-1.32	-0.94	-1.26	-1.24	-1.55	-1.10
Q96KG7	Multiple epidermal growth factor-like domains protein 10 OS=Homo sapiens GN=MEGF10 PE=1 SV=1 - [MEG10_HUMAN]	2.19	1	2	2	2	0.28	0.48	0.09	0.28	0.28	0.47	-1.07	-0.88	0.68	0.49	-0.25	-0.06	-1.30	-0.53	-0.34	0.24	0.43	-0.02	0.17	-1.10
P30046	D-dopachrome decarboxylase OS=Homo sapiens GN=DDT PE=1 SV=3 - [DOPD_HUMAN]	33.05	2	4	4	9	1.58	1.75	2.11	2.01	0.95	0.77	0.95	0.83	0.63	0.98	0.42	0.32	-0.57	-1.16	-1.70	-1.08	-1.34	-0.77	-1.20	-1.10
Q9HC56	Protocadherin-9 OS=Homo sapiens GN=PCDH9 PE=1 SV=2 - [PCDH9_HUMAN]	9.46	1	8	8	12	0.98	1.25	1.36	1.43	-0.34	-0.31	0.25	0.33	0.16	0.15	-0.05	0.05	-0.91	-1.42	-1.55	-1.07	-1.25	-1.24	-1.59	-1.10

P62079	Tetraspanin-5 OS=Homo sapiens GN=TSPAN5 PE=2 SV=1 - [TSN5_HUMAN]	5.97	1	1	1	1	0.78	1.08	0.64	0.94	0.13	0.42	-0.52	-0.22	0.52	0.22	0.30	0.59	-1.24	-0.47	-0.34	-0.52	-0.38	-0.66	-0.53	-1.10
Q8NC96	Adaptin ear-binding coat-associated protein 1 OS=Homo sapiens GN=NECAP1 PE=1 SV=2 - [NECP1_HUMAN]	32.36	1	7	7	16	1.66	1.57	1.72	1.62	0.06	0.00	0.38	0.30	0.21	0.27	0.17	0.04	-1.15	-1.39	-1.29	-1.28	-1.42	-1.39	-1.43	-1.10
O14531	Dihydropyrimidinase-related protein 4 OS=Homo sapiens GN=DPYSL4 PE=1 SV=2 - [DPYL4_HUMAN]	37.76	1	14	14	44	1.42	1.21	1.62	1.43	-0.24	-0.01	0.64	0.50	0.02	0.06	-0.35	-0.20	-1.06	-1.81	-2.05	-1.34	-1.53	-1.75	-2.05	-1.10
Q92945	Far upstream element-binding protein 2 OS=Homo sapiens GN=KHSRP PE=1 SV=4 - [FUBP2_HUMAN]	36.01	1	15	17	48	0.57	0.47	0.65	0.48	-0.31	-0.27	-0.49	-0.45	-0.41	-0.37	-0.50	-0.36	-0.91	-0.78	-1.04	-0.85	-1.05	-0.86	-0.94	-1.10
O00483	NADH dehydrogenase [ubiquinone] 1 alpha subcomplex subunit 4 OS=Homo sapiens GN=NDUFA4 PE=1 SV=1 - [NDUA4_HUMAN]	55.56	1	5	5	16	1.32	1.32	1.59	1.66	-0.13	-0.12	0.36	0.51	0.21	0.23	0.03	0.04	-0.83	-1.19	-1.57	-1.10	-1.45	-1.40	-1.77	-1.10
Q9UPW8	Protein unc-13 homolog A OS=Homo sapiens GN=UNC13A PE=2 SV=4 - [UN13A_HUMAN]	6.46	2	6	10	13	1.96	2.02	1.87	1.97	-0.05	0.34	0.66	0.80	0.62	0.69	0.07	0.30	-1.00	-1.52	-1.67	-1.37	-1.15	-2.03	-2.12	-1.11
Q8TC26	Transmembrane protein 163 OS=Homo sapiens GN=TMEM163 PE=2 SV=1 - [TM163_HUMAN]	21.80	1	5	5	16	1.69	1.58	1.76	1.56	-0.22	-0.26	0.28	0.39	0.13	-0.14	-0.36	-0.20	-1.24	-2.04	-1.94	-1.78	-1.64	-2.07	-2.13	-1.11
P33402	Guanylate cyclase soluble subunit alpha 2 OS=Homo sapiens GN=GUCY1A2 PE=1 SV=1 - [GCYA2_HUMAN]	9.56	1	5	5	7	0.98	1.45	1.19	1.66	-0.30	0.00	0.11	0.57	0.32	0.03	-0.34	0.09	-1.15	-1.32	-1.53	-0.94	-1.13	-1.30	-1.51	-1.11

Q7Z408	CUB and sushi domain-containing protein 2 OS=Homo sapiens GN=CSMD2 PE=1 SV=2 - [CSMD2_HUMAN]	0.29	1	1	1	1	1.18	0.58	1.05	0.44	0.17	-0.43	-0.13	-0.73	-0.87	-0.26	0.37	-0.24	-1.25	-0.80	-0.67	-1.41	-1.27	-1.02	-0.89	-1.11
P32004	Neural cell adhesion molecule L1 OS=Homo sapiens GN=L1CAM PE=1 SV=2 - [L1CAM_HUMAN]	25.38	1	25	25	57	2.38	2.28	2.40	2.50	0.79	0.68	1.00	1.08	0.68	0.77	0.77	0.76	-1.06	-1.65	-1.70	-1.48	-1.56	-1.54	-1.57	-1.11
Q5TH69	Brefeldin A-inhibited guanine nucleotide-exchange protein 3 OS=Homo sapiens GN=ARFGEF3 PE=1 SV=3 - [BIG3_HUMAN]	6.11	1	10	10	12	1.32	1.20	1.31	1.41	-0.08	0.10	0.31	0.36	0.14	-0.09	0.17	0.10	-1.05	-1.28	-1.36	-1.29	-1.41	-1.04	-1.28	-1.11
Q9NZG7	Ninjurin-2 OS=Homo sapiens GN=NINJ2 PE=2 SV=1 - [NINJ2_HUMAN]	17.61	1	2	2	8	1.31	1.47	1.02	1.05	0.30	0.23	-0.18	-0.13	0.15	0.00	-0.24	-0.14	-1.60	-1.66	-1.18	-1.28	-1.06	-1.21	-0.86	-1.11
P36551	Oxygen-dependent coproporphyrinogen-III oxidase, mitochondrial OS=Homo sapiens GN=CPOX PE=1 SV=3 - [HEM6_HUMAN]	13.88	1	6	6	10	0.15	0.50	0.46	0.67	-0.21	-0.18	-0.55	-0.46	-0.37	-0.15	-0.08	-0.16	-1.03	-0.71	-0.87	-0.73	-1.04	-0.56	-0.83	-1.11
Q99962	Endophilin-A1 OS=Homo sapiens GN=SH3GL2 PE=1 SV=1 - [SH3G2_HUMAN]	34.09	1	7	11	33	1.03	1.47	1.02	1.45	-0.19	-0.07	0.07	0.16	-0.15	-0.33	-0.49	-0.32	-0.86	-1.63	-1.72	-1.37	-1.43	-1.21	-1.39	-1.11
P53677	AP-3 complex subunit mu-2 OS=Homo sapiens GN=AP3M2 PE=1 SV=1 - [AP3M2_HUMAN]	24.16	1	6	9	16	1.62	1.17	1.82	1.30	0.50	0.22	0.41	0.37	0.13	0.29	0.04	-0.09	-1.22	-1.17	-1.32	-1.11	-1.11	-0.95	-0.92	-1.11
Q9UM19	Hippocalcin-like protein 4 OS=Homo sapiens GN=HPCAL4 PE=2 SV=3 - [HPCL4_HUMAN]	59.69	1	6	9	24	1.78	1.74	2.13	2.02	-0.31	-0.19	0.79	0.56	0.22	0.08	0.48	0.29	-0.93	-1.48	-1.54	-1.29	-1.41	-2.08	-2.30	-1.11

Q86SX6	Glutaredoxin-related protein 5, mitochondrial OS=Homo sapiens GN=GLRX5 PE=1 SV=2 - [GLRX5_HUMAN]	28.03	1	3	3	4	0.62	0.60	0.87	0.69	0.11	-0.08	-0.16	-0.37	-0.18	-0.05	-0.03	-0.05	-1.04	-0.65	-0.74	-0.74	-0.83	-0.69	-0.78	-1.11
Q96RR4	Calcium/calmodulin-dependent protein kinase kinase 2 OS=Homo sapiens GN=CAMKK2 PE=1 SV=2 - [KKCC2_HUMAN]	27.38	1	11	11	16	1.03	0.90	1.30	1.17	-0.62	-0.86	-0.09	-0.10	-0.83	-0.30	-0.45	-0.59	-0.94	-1.41	-1.45	-1.40	-1.54	-1.40	-1.54	-1.11
P18507	Gamma-aminobutyric acid receptor subunit gamma-2 OS=Homo sapiens GN=GABRG2 PE=1 SV=2 - [GBRG2_HUMAN]	11.56	1	4	4	5	0.86	0.64	1.26	1.03	-0.18	-0.27	0.15	-0.07	0.04	0.08	0.03	-0.08	-0.71	-0.77	-0.85	-0.83	-1.05	-0.76	-1.07	-1.12
Q6BCY4	NADH-cytochrome b5 reductase 2 OS=Homo sapiens GN=CYB5R2 PE=1 SV=1 - [NB5R2_HUMAN]	33.70	1	8	8	10	1.27	1.39	1.52	1.38	0.56	0.58	0.34	0.25	0.16	0.19	0.00	-0.07	-1.12	-1.48	-1.63	-1.12	-1.13	-0.86	-0.75	-1.12
Q9UKE5	TRAF2 and NCK-interacting protein kinase OS=Homo sapiens GN=TNIK PE=1 SV=1 - [TNIK_HUMAN]	9.71	1	6	10	16	1.48	1.56	1.40	1.40	-0.04	0.10	0.15	0.23	0.00	0.04	-0.03	0.11	-1.27	-1.55	-1.28	-1.30	-1.36	-1.70	-1.32	-1.12
Q9H936	Mitochondrial glutamate carrier 1 OS=Homo sapiens GN=SLC25A22 PE=1 SV=1 - [GHC1_HUMAN]	39.94	2	12	12	25	1.37	1.41	1.42	1.34	-0.13	-0.15	0.39	0.45	0.10	0.13	-0.06	-0.02	-1.04	-1.34	-1.56	-1.38	-1.37	-1.52	-1.54	-1.12
Q9BR01	Sulfotransferase 4A1 OS=Homo sapiens GN=SULT4A1 PE=1 SV=2 - [ST4A1_HUMAN]	25.35	1	6	6	11	1.61	1.45	1.81	1.72	0.68	0.73	0.74	0.82	0.57	0.85	0.47	0.30	-0.84	-1.14	-1.41	-0.69	-0.88	-0.89	-1.15	-1.12

Q9NZU7	Calcium-binding protein 1 OS=Homo sapiens GN=CABP1 PE=1 SV=5 - [CABP1_HUMAN]	11.62	1	4	4	5	1.02	1.10	0.84	0.93	-0.41	-0.33	-0.34	-0.26	-0.16	-0.24	-0.63	-0.55	-1.30	-1.64	-1.48	-1.23	-1.05	-1.45	-1.27	-1.12
Q99719	Septin-5 OS=Homo sapiens GN=SEPT5 PE=1 SV=1 - [SEPT5_HUMAN]	63.69	2	15	18	48	1.40	1.43	1.74	1.82	-0.25	-0.19	0.35	0.51	0.22	0.27	-0.09	0.08	-0.86	-1.37	-1.82	-1.23	-1.59	-1.65	-2.03	-1.12
P09496	Clathrin light chain A OS=Homo sapiens GN=CLTA PE=1 SV=1 - [CLCA_HUMAN]	19.35	2	6	6	38	1.75	1.77	1.84	1.82	-0.21	-0.19	0.60	0.62	0.26	0.21	0.27	0.26	-1.11	-1.53	-1.42	-1.49	-1.41	-2.02	-2.03	-1.12
O75896	Tumor suppressor candidate 2 OS=Homo sapiens GN=TUSC2 PE=1 SV=3 - [TUSC2_HUMAN]	31.82	1	2	2	3	1.61	1.17	1.98	1.54	1.33	-0.01	0.79	0.35	-0.19	1.15	1.06	0.62	-0.76	-0.54	-0.92	-1.02	-1.21	-0.89	-1.08	-1.12
P38405	Guanine nucleotide-binding protein G(olf) subunit alpha OS=Homo sapiens GN=GNAL PE=1 SV=1 - [GNAL_HUMAN]	23.10	1	2	6	27	1.08	0.53	0.84	0.29	0.14	-0.41	-0.35	-0.89	-0.80	-0.25	-0.03	-0.59	-1.37	-1.11	-0.88	-1.29	-1.05	-0.95	-0.72	-1.12
Q8NDY3	[Protein ADP-ribosylarginine] hydrolase-like protein 1 OS=Homo sapiens GN=ADPRHL1 PE=2 SV=1 - [ARHL1_HUMAN]	3.39	1	1	1	1	2.14	2.18	2.07	2.11	0.33	0.37	0.88	0.92	0.59	0.55	1.01	1.05	-1.21	-1.13	-1.06	-1.56	-1.48	-1.82	-1.75	-1.12
Q9Y2J0	Rabphilin-3A OS=Homo sapiens GN=RPH3A PE=1 SV=1 - [RP3A_HUMAN]	36.74	2	22	22	58	1.22	1.12	1.63	1.45	0.51	0.33	0.47	0.35	0.00	0.11	-0.10	-0.16	-0.83	-1.27	-1.68	-0.92	-1.27	-0.78	-1.15	-1.12
Q8IU85	Calcium/calmodulin-dependent protein kinase type 1D OS=Homo sapiens GN=CAMK1D PE=1 SV=1 - [KCC1D_HUMAN]	40.78	1	9	13	21	1.34	1.35	1.46	1.65	0.01	-0.02	0.28	0.27	0.08	0.12	0.14	0.21	-1.02	-1.18	-1.25	-1.22	-1.20	-1.38	-1.36	-1.13

Q8N2Q7	Neuroligin-1 OS=Homo sapiens GN=NLGN1 PE=1 SV=2 - [NLGN1_HUMAN]	3.21	1	1	2	3	0.56	0.35	0.80	0.60	0.02	-0.18	-0.39	-0.59	-0.75	-0.54	-0.20	-0.41	-0.89	-0.75	-1.01	-1.07	-1.31	-0.55	-0.80	-1.13
O60669	Monocarboxylate transporter 2 OS=Homo sapiens GN=SLC16A7 PE=1 SV=2 - [MOT2_HUMAN]	2.30	1	1	1	2	1.24	1.33	1.15	1.24	-0.43	-0.35	-0.04	0.05	-0.26	-0.34	-0.07	0.01	-1.22	-1.31	-1.23	-1.55	-1.46	-1.69	-1.60	-1.13
Q9C0H5	Rho GTPase-activating protein 39 OS=Homo sapiens GN=ARHGAP39 PE=1 SV=2 - [RHG39_HUMAN]	9.14	1	7	7	10	1.48	1.40	1.38	1.48	0.68	0.71	0.30	0.29	0.48	0.45	0.26	0.69	-1.07	-1.26	-1.23	-0.89	-0.87	-0.73	-0.63	-1.13
P01303	Pro-neuropeptide Y OS=Homo sapiens GN=NPY PE=1 SV=1 - [NPY_HUMAN]	29.90	1	3	3	5	3.03	2.67	3.19	2.68	0.68	0.81	1.83	1.99	1.51	1.39	0.78	0.68	-0.94	-1.78	-1.99	-1.48	-1.68	-2.14	-2.34	-1.13
O00330	Pyruvate dehydrogenase protein X component, mitochondrial OS=Homo sapiens GN=PDHX PE=1 SV=3 - [ODPX_HUMAN]	15.17	1	7	7	17	0.94	0.78	1.13	1.10	-0.28	-0.31	0.02	-0.22	-0.24	-0.05	-0.01	-0.06	-0.79	-0.94	-1.35	-0.91	-1.30	-1.06	-1.46	-1.13
Q14123	Calcium/calmodulin-dependent 3',5'-cyclic nucleotide phosphodiesterase 1C OS=Homo sapiens GN=PDE1C PE=1 SV=1 - [PDE1C_HUMAN]	10.72	1	2	5	7	1.71	1.50	1.79	1.59	0.14	-0.07	0.60	0.40	-0.33	-0.12	0.11	-0.10	-1.05	-1.59	-1.68	-1.79	-1.88	-1.58	-1.67	-1.13
Q6PIU2	Neutral cholesterol ester hydrolase 1 OS=Homo sapiens GN=NCEH1 PE=1 SV=3 - [NCEH1_HUMAN]	25.49	1	9	9	25	1.71	1.63	1.90	1.84	-0.20	0.04	0.71	0.61	0.37	0.24	0.12	0.08	-0.97	-1.52	-1.70	-1.40	-1.46	-1.75	-2.01	-1.13

O14578	Citron Rho-interacting kinase OS=Homo sapiens GN=CIT PE=1 SV=2 - [CTRO_HUMAN]	12.48	2	24	24	41	1.67	1.56	1.59	1.68	-0.04	0.01	0.51	0.67	0.39	0.34	0.03	0.14	-1.01	-1.45	-1.61	-1.30	-1.19	-1.39	-1.43	-1.13
Q9UHL4	Dipeptidyl peptidase 2 OS=Homo sapiens GN=DPP7 PE=1 SV=3 - [DPP2_HUMAN]	19.72	1	7	7	11	-0.03	-0.26	-0.32	-0.36	-0.14	-0.11	-1.37	-1.33	-0.93	-0.97	-0.72	-0.55	-1.42	-0.46	-0.36	-0.76	-0.45	0.02	0.27	-1.14
O14525	Astrotactin-1 OS=Homo sapiens GN=ASTN1 PE=2 SV=3 - [ASTN1_HUMAN]	7.14	1	6	6	9	0.47	0.67	0.77	1.03	-0.40	-0.35	-0.19	0.02	-0.03	-0.23	0.03	0.07	-0.84	-0.78	-1.13	-0.67	-0.91	-0.97	-1.24	-1.14
Q13449	Limbic system-associated membrane protein OS=Homo sapiens GN=LSAMP PE=1 SV=2 - [LSAMP_HUMAN]	37.87	1	11	11	101	1.96	1.80	2.14	1.94	0.15	0.07	0.75	0.75	0.40	0.45	0.34	0.31	-0.95	-1.58	-1.74	-1.38	-1.56	-1.76	-1.93	-1.14
Q99963	Endophilin-A3 OS=Homo sapiens GN=SH3GL3 PE=1 SV=1 - [SH3G3_HUMAN]	37.46	1	11	11	24	1.53	1.54	1.77	1.69	-0.38	-0.46	0.50	0.51	-0.05	-0.02	-0.48	-0.52	-0.98	-2.02	-2.29	-1.52	-1.83	-1.83	-2.12	-1.14
Q13554	Calcium/calmodulin-dependent protein kinase type II subunit beta OS=Homo sapiens GN=CAMK2B PE=1 SV=3 - [KCC2B_HUMAN]	39.79	1	11	19	82	1.88	1.83	1.96	2.02	0.20	0.03	0.95	0.93	0.32	0.48	0.41	0.28	-0.87	-1.46	-1.64	-1.35	-1.60	-1.63	-2.05	-1.14
O14490	Disks large-associated protein 1 OS=Homo sapiens GN=DLGAP1 PE=1 SV=1 - [DLGP1_HUMAN]	14.33	1	11	12	14	1.64	1.23	1.89	1.77	-0.33	-0.27	0.69	0.62	-0.21	0.08	-0.23	-0.67	-0.85	-1.83	-2.09	-1.38	-1.70	-1.66	-1.87	-1.14
Q14978	Nucleolar and coiled-body phosphoprotein 1 OS=Homo sapiens GN=NOLC1 PE=1 SV=2 - [NOLC1_HUMAN]	10.30	1	6	6	10	-0.01	-0.19	0.65	0.47	-0.71	-0.75	-0.65	-0.72	-0.66	-0.62	-0.43	-0.47	-0.44	-0.26	-0.98	-0.61	-1.32	-0.56	-1.28	-1.14

Q8NFP9	Neurobeachin OS=Homo sapiens GN=NBEA PE=1 SV=3 - [NBEA_HUMAN]	8.45	1	15	18	36	1.19	1.27	1.22	1.25	-0.20	-0.14	0.09	0.19	0.01	-0.03	-0.18	-0.02	-0.94	-1.08	-1.21	-1.35	-1.07	-1.40	-1.41	-1.14
Q9BQ61	Uncharacterized protein C19orf43 OS=Homo sapiens GN=C19orf43 PE=1 SV=1 - [CS043_HUMAN]	13.64	1	1	1	2	-0.38	-0.41	0.03	-0.01	-0.93	-0.97	-1.18	-1.21	-0.79	-0.75	-0.63	-0.67	-0.74	-0.25	-0.66	-0.34	-0.75	-0.57	-0.97	-1.14
Q76N89	E3 ubiquitin-protein ligase HECW1 OS=Homo sapiens GN=HECW1 PE=1 SV=3 - [HECW1_HUMAN]	3.24	1	3	4	9	0.66	1.52	0.66	1.26	-0.17	0.47	-0.74	-0.04	-0.45	-0.58	-1.06	-0.47	-1.39	-1.68	-1.52	-1.23	-1.22	-0.83	-0.75	-1.14
Q86XD5	Protein FAM131B OS=Homo sapiens GN=FAM131B PE=1 SV=3 - [F131B_HUMAN]	18.67	1	5	5	15	1.55	1.45	1.70	1.80	0.39	0.42	0.59	0.54	0.52	0.50	0.31	0.37	-1.12	-1.07	-1.46	-0.96	-1.01	-1.26	-1.14	-1.14
P49418	Amphiphysin OS=Homo sapiens GN=AMPH PE=1 SV=1 - [AMPH_HUMAN]	47.48	2	22	24	86	1.60	1.53	1.87	2.01	0.19	0.14	0.64	0.65	0.22	0.14	-0.05	-0.09	-0.80	-1.65	-1.98	-1.37	-1.52	-1.32	-1.51	-1.14
O43251	RNA binding protein fox-1 homolog 2 OS=Homo sapiens GN=RBFOX2 PE=1 SV=3 - [RFOX2_HUMAN]	9.23	2	1	3	4	0.90	1.07	0.88	1.05	-0.72	-0.56	-0.32	-0.15	-0.21	-0.37	-0.62	-0.45	-1.17	-1.51	-1.50	-1.24	-1.22	-1.64	-1.62	-1.14
Q15878	Voltage-dependent R-type calcium channel subunit alpha 1E OS=Homo sapiens GN=CACNA1E PE=1 SV=3 - [CAC1E_HUMAN]	5.62	1	7	8	11	1.67	1.53	1.60	1.50	-0.24	-0.12	0.24	0.21	0.11	0.12	0.33	0.47	-1.29	-1.31	-1.09	-1.26	-1.21	-1.40	-1.54	-1.14
P22694	cAMP-dependent protein kinase catalytic subunit beta OS=Homo sapiens GN=PRKACB PE=1 SV=2 - [KAPCB_HUMAN]	24.50	1	4	8	21	1.11	1.53	1.51	1.82	-0.18	-0.05	0.16	0.52	0.54	-0.06	-0.26	0.19	-0.71	-1.15	-1.51	-0.73	-1.14	-1.12	-1.62	-1.14

Q7RTN6	STE20-related kinase adapter protein alpha OS=Homo sapiens GN=STRADA PE=1 SV=1 - [STRAA_HUMAN]	3.25	1	1	1	1	2.25	2.01	2.29	2.05	0.20	-0.04	1.08	0.85	0.40	0.64	0.79	0.55	-1.11	-1.46	-1.50	-1.58	-1.61	-2.07	-2.10	-1.14
P80404	4-aminobutyrate aminotransferase, mitochondrial OS=Homo sapiens GN=ABAT PE=1 SV=3 - [GABT_HUMAN]	48.80	1	19	19	61	1.81	1.82	2.21	2.17	0.31	0.30	0.99	0.93	0.58	0.57	0.22	0.26	-0.80	-1.60	-1.88	-1.28	-1.52	-1.62	-1.83	-1.14
Q9NP81	Serine--tRNA ligase, mitochondrial OS=Homo sapiens GN=SARS2 PE=1 SV=1 - [SYSM_HUMAN]	30.69	1	12	12	21	0.99	1.15	1.02	1.16	-0.62	-0.41	-0.17	-0.07	-0.34	-0.37	-0.16	-0.19	-1.17	-1.26	-1.31	-1.33	-1.30	-1.51	-1.42	-1.14
O60268	Uncharacterized protein KIAA0513 OS=Homo sapiens GN=KIAA0513 PE=2 SV=1 - [K0513_HUMAN]	24.57	1	10	10	18	1.49	1.51	1.77	1.74	-0.39	-0.36	0.49	0.42	-0.09	-0.10	-0.08	-0.15	-0.96	-1.65	-1.98	-1.45	-1.81	-2.02	-2.33	-1.14
O15354	Prosaposin receptor GPR37 OS=Homo sapiens GN=GPR37 PE=1 SV=2 - [GPR37_HUMAN]	1.31	1	1	1	2	1.69	1.90	1.85	2.06	0.04	0.25	0.64	0.86	0.47	0.27	0.43	0.63	-0.99	-1.26	-1.43	-1.39	-1.55	-1.66	-1.83	-1.14
Q8IV01	Synaptotagmin-12 OS=Homo sapiens GN=SYT12 PE=2 SV=1 - [SYT12_HUMAN]	27.55	1	9	9	15	2.17	2.05	2.05	1.93	0.10	-0.11	0.81	0.76	0.34	0.56	0.28	0.09	-1.18	-1.94	-1.81	-1.69	-1.52	-2.15	-2.05	-1.14
P62873	Guanine nucleotide-binding protein G(I)/G(S)/G(T) subunit beta-1 OS=Homo sapiens GN=GNB1 PE=1 SV=3 - [GBB1_HUMAN]	47.06	2	7	12	142	1.62	1.68	1.70	1.70	-0.03	0.00	0.51	0.57	0.22	0.25	0.28	0.33	-0.99	-1.51	-1.47	-1.30	-1.25	-1.50	-1.77	-1.14

Q9UI40	Sodium/potassium/calcium exchanger 2 OS=Homo sapiens GN=SLC24A2 PE=1 SV=1 - [NCKX2_HUMAN]	4.69	1	2	2	5	2.15	2.45	2.30	2.54	-0.40	-0.10	0.86	1.18	0.87	0.73	0.03	0.31	-1.07	-2.11	-2.22	-1.34	-1.41	-2.57	-2.75	-1.14
P42658	Dipeptidyl aminopeptidase-like protein 6 OS=Homo sapiens GN=DPP6 PE=1 SV=2 - [DPP6_HUMAN]	23.24	1	15	16	34	1.39	1.56	1.60	1.78	-0.04	-0.11	0.31	0.55	0.36	0.24	0.12	0.21	-0.86	-1.18	-1.33	-1.09	-1.32	-1.82	-2.15	-1.14
Q8NI08	Nuclear receptor coactivator 7 OS=Homo sapiens GN=NCOA7 PE=1 SV=2 - [NCOA7_HUMAN]	9.24	1	6	6	8	1.69	1.85	1.75	2.03	0.24	0.49	0.60	0.75	0.71	0.57	0.46	0.65	-0.94	-1.21	-1.36	-1.15	-1.28	-1.49	-1.55	-1.15
Q9UPY8	Microtubule-associated protein RP/EB family member 3 OS=Homo sapiens GN=MAPRE3 PE=1 SV=1 - [MARE3_HUMAN]	36.30	1	7	10	17	1.47	1.84	1.24	1.84	0.14	0.57	0.59	0.67	0.66	0.16	0.11	0.39	-1.10	-1.41	-1.41	-1.14	-0.89	-1.15	-1.08	-1.15
Q8N608	Inactive dipeptidyl peptidase 10 OS=Homo sapiens GN=DPP10 PE=1 SV=2 - [DPP10_HUMAN]	12.56	1	10	11	17	2.06	1.99	2.06	1.99	-0.02	-0.16	0.73	0.72	0.37	0.44	0.51	0.42	-1.15	-1.67	-1.73	-1.58	-1.58	-2.10	-2.12	-1.15
P42262	Glutamate receptor 2 OS=Homo sapiens GN=GRIA2 PE=1 SV=3 - [GRIA2_HUMAN]	24.92	1	14	19	27	1.77	1.24	2.12	1.67	-0.26	-0.10	0.64	0.48	0.28	0.31	-0.15	-0.19	-0.97	-1.47	-1.66	-0.97	-1.33	-1.36	-1.78	-1.15
Q8IWT1	Sodium channel subunit beta-4 OS=Homo sapiens GN=SCN4B PE=1 SV=1 - [SCN4B_HUMAN]	10.96	1	3	3	5	1.55	1.61	1.97	2.03	-0.33	-0.28	0.76	0.82	0.30	0.25	-0.30	-0.25	-0.74	-1.85	-2.27	-1.27	-1.69	-1.90	-2.32	-1.15
Q8TDQ7	Glucosamine-6-phosphate isomerase 2 OS=Homo sapiens GN=GNPDA2 PE=1 SV=1 - [GNPI2_HUMAN]	28.99	1	3	5	8	1.10	0.94	0.97	0.81	-0.02	-0.19	-0.24	-0.40	-0.75	-0.58	-0.25	-0.41	-1.28	-1.34	-1.22	-1.65	-1.52	-1.13	-1.01	-1.15

P43004	Excitatory amino acid transporter 2 OS=Homo sapiens GN=SLC1A2 PE=1 SV=2 - [EAA2_HUMAN]	20.91	1	12	12	58	2.43	2.38	2.65	2.73	0.02	0.08	1.33	1.37	0.65	0.66	0.17	0.26	-0.96	-2.37	-2.67	-1.72	-2.05	-2.70	-3.13	-1.15
P05023	Sodium/potassium-transporting ATPase subunit alpha 1 OS=Homo sapiens GN=ATP1A1 PE=1 SV=1 - [AT1A1_HUMAN]	47.61	1	27	49	471	1.83	1.91	2.03	2.08	-0.05	-0.01	0.73	0.81	0.33	0.28	0.18	0.19	-0.94	-1.68	-1.76	-1.51	-1.61	-1.94	-2.01	-1.15
P78369	Claudin-10 OS=Homo sapiens GN=CLDN10 PE=1 SV=2 - [CLD10_HUMAN]	9.21	1	2	2	8	1.27	1.96	1.22	1.91	-0.45	0.24	0.00	0.69	0.73	0.05	-0.03	0.65	-1.21	-1.30	-1.25	-1.20	-1.14	-1.74	-1.68	-1.15
Q5TF21	Protein SOGA3 OS=Homo sapiens GN=SOGA3 PE=2 SV=1 - [SOGA3_HUMAN]	20.91	1	16	18	31	1.92	1.73	2.10	1.84	0.09	-0.01	1.02	0.55	0.42	0.53	0.38	0.34	-0.99	-1.39	-1.47	-1.34	-1.25	-2.00	-1.80	-1.15
Q07343	cAMP-specific 3',5'-cyclic phosphodiesterase 4B OS=Homo sapiens GN=PDE4B PE=1 SV=1 - [PDE4B_HUMAN]	3.13	1	1	2	2	1.72	1.45	1.75	1.48	-0.05	-0.33	0.53	0.26	0.01	0.29	0.58	0.31	-1.13	-1.13	-1.17	-1.40	-1.43	-1.79	-1.82	-1.15
O43236	Septin-4 OS=Homo sapiens GN=SEPT4 PE=1 SV=1 - [SEPT4_HUMAN]	41.84	2	15	18	35	1.39	1.61	1.69	1.91	-0.23	0.06	0.51	0.74	0.29	0.22	0.19	0.22	-0.83	-1.40	-1.46	-1.27	-1.48	-1.67	-1.94	-1.15
P51513	RNA-binding protein Nova-1 OS=Homo sapiens GN=NOVA1 PE=1 SV=1 - [NOVA1_HUMAN]	11.37	1	2	5	9	2.87	2.04	2.51	1.68	0.90	0.06	1.29	0.46	0.54	1.38	1.27	0.44	-1.53	-1.59	-1.24	-1.47	-1.10	-1.99	-1.63	-1.16
P01911	HLA class II histocompatibility antigen, DRB1-15 beta chain OS=Homo sapiens GN=HLA-DRB1 PE=1 SV=2 - [2B1F_HUMAN]	14.66	6	3	3	7	-0.83	-0.91	-0.78	-0.85	1.10	1.16	-1.40	-1.25	0.04	0.12	-0.29	-0.37	-1.52	0.55	0.49	0.88	0.94	1.87	2.04	-1.16

Q9BUE6	Iron-sulfur cluster assembly 1 homolog, mitochondrial OS=Homo sapiens GN=ISCA1 PE=2 SV=1 - [ISCA1_HUMAN]	9.30	1	1	1	2	1.12	0.82	1.56	1.26	-0.14	-0.45	0.34	0.03	-0.32	-0.02	-0.42	-0.73	-0.73	-1.53	-1.98	-1.10	-1.54	-1.28	-1.72	-1.16
P12532	Creatine kinase U-type, mitochondrial OS=Homo sapiens GN=CKMT1A PE=1 SV=1 - [KCRU_HUMAN]	46.76	1	13	16	82	2.15	2.15	1.87	2.08	0.14	0.16	0.83	0.64	0.24	0.43	0.15	0.06	-1.23	-2.13	-2.00	-1.75	-1.66	-1.87	-1.82	-1.16
Q99576	TSC22 domain family protein 3 OS=Homo sapiens GN=TSC22D3 PE=1 SV=2 - [T22D3_HUMAN]	19.40	1	2	3	7	0.67	0.40	1.10	0.79	-0.30	-0.44	0.07	-0.44	-0.43	-0.19	-0.14	-0.58	-0.79	-0.80	-1.24	-0.86	-1.32	-0.99	-1.42	-1.16
P20336	Ras-related protein Rab-3A OS=Homo sapiens GN=RAB3A PE=1 SV=1 - [RAB3A_HUMAN]	53.64	2	5	10	49	1.75	1.66	2.03	1.87	-0.23	-0.11	0.90	0.76	0.18	0.36	-0.12	-0.28	-0.82	-1.83	-2.33	-1.44	-1.69	-1.95	-2.25	-1.16
Q8N4Q0	Zinc-binding alcohol dehydrogenase domain-containing protein 2 OS=Homo sapiens GN=ZADH2 PE=1 SV=1 - [ZADH2_HUMAN]	18.04	1	4	4	5	0.89	0.70	1.01	0.82	-0.84	-1.04	-0.22	-0.41	-0.49	-0.29	-0.42	-0.62	-1.06	-1.31	-1.43	-1.16	-1.27	-1.75	-1.87	-1.16
P50750	Cyclin-dependent kinase 9 OS=Homo sapiens GN=CDK9 PE=1 SV=3 - [CDK9_HUMAN]	8.06	11	1	2	6	0.37	0.76	0.65	1.04	-1.45	-0.41	-0.58	-0.19	-0.01	-0.39	-2.43	-1.38	-0.90	-2.29	-2.45	-0.73	-1.01	-1.33	-1.49	-1.17
Q12756	Kinesin-like protein KIF1A OS=Homo sapiens GN=KIF1A PE=1 SV=2 - [KIF1A_HUMAN]	12.54	1	13	17	28	1.85	2.00	1.67	1.87	0.06	0.37	0.34	0.42	0.58	0.41	0.03	0.02	-1.26	-1.79	-1.73	-1.50	-1.48	-1.60	-1.47	-1.17
Q9P2K5	Myelin expression factor 2 OS=Homo sapiens GN=MYEF2 PE=1 SV=3 - [MYEF2_HUMAN]	10.33	1	6	6	12	0.73	0.58	0.94	0.96	-0.26	-0.38	-0.29	-0.34	-0.31	-0.20	-0.02	-0.11	-1.06	-0.81	-0.77	-0.97	-1.05	-1.16	-1.14	-1.17

Q9UNA1	Rho GTPase-activating protein 26 OS=Homo sapiens GN=ARHGAP26 PE=1 SV=1 - [RHG26_HUMAN]	11.43	1	8	8	14	1.45	1.22	1.37	1.16	-0.12	-0.29	0.25	0.05	0.03	-0.03	-0.01	0.15	-1.21	-1.28	-1.23	-1.37	-1.28	-1.69	-1.61	-1.17
Q9NZ56	Formin-2 OS=Homo sapiens GN=FMN2 PE=1 SV=4 - [FMN2_HUMAN]	10.16	1	9	9	22	1.69	1.13	1.29	1.28	0.36	-0.11	0.31	-0.22	0.61	0.45	0.45	-0.29	-1.33	-1.18	-1.12	-1.15	-1.17	-1.23	-1.16	-1.17
P21281	V-type proton ATPase subunit B, brain isoform OS=Homo sapiens GN=ATP6V1B2 PE=1 SV=3 - [VATB2_HUMAN]	62.04	2	23	23	90	1.62	1.77	1.96	1.91	0.04	0.04	0.67	0.74	0.37	0.40	0.23	0.19	-1.07	-1.58	-1.77	-1.45	-1.57	-1.73	-2.05	-1.17
P02794	Ferritin heavy chain OS=Homo sapiens GN=FTH1 PE=1 SV=2 - [FRIH_HUMAN]	46.45	1	7	7	22	2.36	2.29	2.89	2.75	0.48	0.50	1.70	1.54	1.40	1.29	0.96	1.19	-0.92	-1.36	-1.73	-1.03	-1.27	-1.76	-2.18	-1.17
Q9BW62	Katanin p60 ATPase-containing subunit A-like 1 OS=Homo sapiens GN=KATNAL1 PE=1 SV=1 - [KATL1_HUMAN]	15.71	2	6	6	8	1.22	1.12	1.44	1.47	-0.25	-0.08	0.25	0.32	0.16	-0.02	-0.11	0.11	-0.75	-1.32	-1.56	-0.92	-1.34	-1.21	-1.56	-1.18
Q14011	Cold-inducible RNA-binding protein OS=Homo sapiens GN=CIRBP PE=1 SV=1 - [CIRBP_HUMAN]	14.53	1	2	2	10	0.58	0.67	0.94	0.98	-0.21	-0.18	-0.33	-0.28	-0.26	-0.30	0.15	0.17	-0.85	-0.50	-0.80	-0.89	-1.20	-0.89	-1.17	-1.18
Q5SYC1	Clavesin-2 OS=Homo sapiens GN=CLVS2 PE=2 SV=1 - [CLVS2_HUMAN]	26.30	1	4	6	11	1.47	1.71	1.75	2.11	-0.18	0.07	0.79	1.09	0.87	0.43	0.48	0.92	-0.95	-1.58	-1.42	-1.11	-1.46	-1.91	-1.91	-1.18
Q9UH03	Neuronal-specific septin-3 OS=Homo sapiens GN=SEPT3 PE=1 SV=3 - [SEPT3_HUMAN]	32.96	1	12	12	41	1.47	1.35	2.02	1.90	-0.17	-0.36	0.69	0.66	0.15	0.25	-0.08	-0.12	-0.73	-1.46	-1.89	-1.23	-1.69	-1.58	-1.98	-1.18

Q9UPT6	C-Jun-amino-terminal kinase-interacting protein 3 OS=Homo sapiens GN=MAPK8IP3 PE=1 SV=3 - [JIP3_HUMAN]	13.77	1	12	14	24	1.82	1.66	1.80	1.74	0.11	0.03	0.79	0.66	0.10	0.25	0.35	0.24	-1.06	-1.29	-1.36	-1.62	-1.69	-1.76	-1.67	-1.18
Q9C040	Tripartite motif-containing protein 2 OS=Homo sapiens GN=TRIM2 PE=1 SV=1 - [TRIM2_HUMAN]	23.66	1	12	13	38	1.65	1.66	1.75	1.79	0.13	0.22	0.45	0.77	0.46	0.38	-0.01	0.18	-0.86	-1.58	-1.69	-1.21	-1.35	-1.09	-1.30	-1.18
P42263	Glutamate receptor 3 OS=Homo sapiens GN=GRIA3 PE=1 SV=2 - [GRIA3_HUMAN]	16.67	1	6	12	16	1.70	1.67	1.58	1.54	-0.41	-0.39	0.27	0.29	0.09	0.18	-0.09	-0.10	-1.33	-1.20	-1.05	-1.49	-1.41	-1.86	-1.80	-1.18
P46821	Microtubule-associated protein 1B OS=Homo sapiens GN=MAP1B PE=1 SV=2 - [MAP1B_HUMAN]	46.68	3	81	82	308	0.89	0.84	1.21	1.22	-0.46	-0.40	-0.03	0.01	-0.20	-0.24	-0.19	-0.18	-0.87	-1.05	-1.37	-1.07	-1.39	-1.31	-1.67	-1.18
P61266	Syntaxin-1B OS=Homo sapiens GN=STX1B PE=1 SV=1 - [STX1B_HUMAN]	59.03	1	16	17	133	1.90	1.85	1.88	2.12	-0.03	-0.14	0.75	0.87	0.24	0.36	0.27	0.29	-1.08	-1.76	-2.02	-1.54	-1.61	-1.87	-1.94	-1.18
Q9Y4G8	Rap guanine nucleotide exchange factor 2 OS=Homo sapiens GN=RAPGEF2 PE=1 SV=1 - [RPGF2_HUMAN]	14.68	2	14	14	27	1.11	1.40	1.35	1.48	-0.23	-0.07	0.22	0.41	0.33	0.07	-0.07	0.28	-1.01	-1.33	-1.57	-1.24	-1.47	-1.44	-1.74	-1.19
P04179	Superoxide dismutase [Mn], mitochondrial OS=Homo sapiens GN=SOD2 PE=1 SV=2 - [SODM_HUMAN]	44.59	1	9	9	41	0.27	0.41	0.77	0.83	-0.27	-0.13	-0.42	-0.26	-0.20	-0.36	-0.30	-0.14	-0.67	-0.62	-1.13	-0.56	-1.06	-0.59	-1.14	-1.19
Q9UQB3	Catenin delta-2 OS=Homo sapiens GN=CTNND2 PE=1 SV=3 - [CTND2_HUMAN]	22.61	1	21	22	52	1.18	1.24	1.07	1.38	-0.37	-0.33	0.10	0.12	0.03	-0.02	0.08	-0.15	-1.13	-1.51	-1.60	-1.42	-1.58	-1.69	-1.78	-1.19

Q8N126	Cell adhesion molecule 3 OS=Homo sapiens GN=CADM3 PE=1 SV=1 - [CADM3_HUMAN]	21.86	1	6	6	19	1.64	1.34	1.99	1.56	-0.53	-0.59	0.50	0.31	0.17	0.29	0.31	0.29	-0.95	-1.36	-1.56	-1.34	-1.59	-2.15	-2.40	-1.19
Q9UGC6	Regulator of G-protein signaling 17 OS=Homo sapiens GN=RGS17 PE=1 SV=2 - [RGS17_HUMAN]	3.81	1	1	1	1	1.27	1.17	1.10	1.00	-0.24	-0.35	-0.15	-0.25	0.35	0.46	0.27	0.16	-1.37	-1.00	-0.83	-0.78	-0.61	-1.53	-1.36	-1.19
P46459	Vesicle-fusing ATPase OS=Homo sapiens GN=NSF PE=1 SV=3 - [NSF_HUMAN]	47.04	1	30	31	121	1.66	1.62	2.02	1.96	-0.23	-0.29	0.74	0.69	0.28	0.27	0.11	0.15	-0.85	-1.56	-1.85	-1.34	-1.59	-1.95	-2.31	-1.19
Q9NQR7	Coiled-coil domain-containing protein 177 OS=Homo sapiens GN=CCDC177 PE=2 SV=3 - [CC177_HUMAN]	12.45	1	7	7	11	1.93	1.79	1.55	1.48	-0.04	0.25	0.29	0.14	0.32	0.16	-0.34	-0.07	-2.18	-2.23	-1.46	-1.77	-1.15	-1.98	-1.80	-1.19
P13611	Versican core protein OS=Homo sapiens GN=VCAN PE=1 SV=3 - [CSPG2_HUMAN]	8.57	1	23	23	91	1.43	1.61	1.60	1.86	0.32	0.42	0.55	0.75	0.49	0.38	0.39	0.39	-1.10	-1.35	-1.56	-1.26	-1.21	-1.29	-1.32	-1.19
Q16623	Syntaxin-1A OS=Homo sapiens GN=STX1A PE=1 SV=1 - [STX1A_HUMAN]	52.08	1	13	14	68	1.32	1.24	1.40	1.58	-0.35	-0.26	0.39	0.49	0.05	0.11	-0.21	-0.10	-0.86	-1.53	-1.77	-1.34	-1.60	-1.64	-1.90	-1.19
Q8TB36	Ganglioside-induced differentiation associated protein 1 OS=Homo sapiens GN=GDAP1 PE=1 SV=3 - [GDAP1_HUMAN]	40.22	1	12	12	23	1.67	1.00	1.99	1.36	-0.26	-0.55	0.67	0.17	-0.21	0.04	-0.05	-0.34	-0.97	-1.59	-2.04	-1.48	-1.53	-1.88	-2.20	-1.19
Q9UPR5	Sodium/calcium exchanger 2 OS=Homo sapiens GN=SLC8A2 PE=2 SV=2 - [NAC2_HUMAN]	26.28	1	16	16	41	2.14	1.76	2.34	2.03	0.14	-0.01	1.06	0.67	0.52	0.57	0.51	0.29	-0.97	-1.58	-1.81	-1.29	-1.45	-1.73	-2.05	-1.20

O14910	Protein lin-7 homolog A OS=Homo sapiens GN=LIN7A PE=1 SV=2 - [LIN7A_HUMAN]	51.07	2	6	11	23	1.75	1.83	1.78	1.65	0.02	0.21	0.18	0.05	0.27	0.17	0.34	0.32	-1.35	-1.40	-1.33	-1.44	-1.37	-1.67	-1.69	-1.20
Q9P0K9	DOMON domain-containing protein FRRS1L OS=Homo sapiens GN=FRRS1L PE=2 SV=2 - [FRS1L_HUMAN]	14.53	1	3	3	5	1.60	1.93	1.77	2.10	0.34	0.66	0.51	0.84	0.87	0.54	0.48	0.80	-1.04	-1.12	-1.30	-1.03	-1.20	-1.28	-1.45	-1.20
Q96P71	N-terminal EF-hand calcium-binding protein 3 OS=Homo sapiens GN=NECAB3 PE=1 SV=2 - [NECA3_HUMAN]	2.27	1	1	1	2	1.90	2.01	1.72	1.83	0.06	0.16	0.45	0.56	0.41	0.31	0.24	0.35	-1.39	-1.65	-1.48	-1.56	-1.38	-1.86	-1.68	-1.20
P17677	Neuromodulin OS=Homo sapiens GN=GAP43 PE=1 SV=1 - [NEUM_HUMAN]	34.87	1	5	5	14	0.74	0.99	1.64	1.66	-0.38	-0.48	0.35	0.30	-0.31	-0.46	-0.84	-0.63	-0.47	-1.57	-2.30	-1.09	-1.82	-1.30	-1.90	-1.20
Q02641	Voltage-dependent L-type calcium channel subunit beta-1 OS=Homo sapiens GN=CACNB1 PE=1 SV=3 - [CACB1_HUMAN]	12.21	2	2	6	11	1.89	1.52	2.07	1.70	0.21	-0.17	0.80	0.43	0.30	0.67	0.58	0.21	-1.04	-1.31	-1.49	-1.19	-1.36	-1.70	-1.88	-1.20
O43526	Potassium voltage-gated channel subfamily KQT member 2 OS=Homo sapiens GN=KCNQ2 PE=1 SV=2 - [KCNQ2_HUMAN]	7.00	1	5	5	9	1.25	1.03	1.31	1.24	0.12	-0.05	0.32	0.37	0.06	0.22	-0.02	-0.06	-0.93	-1.06	-1.13	-1.05	-1.23	-1.19	-1.38	-1.20
O00499	Myc box-dependent-interacting protein 1 OS=Homo sapiens GN=BIN1 PE=1 SV=1 - [BIN1_HUMAN]	44.01	2	21	23	81	1.52	1.46	1.68	1.74	-0.03	-0.05	0.35	0.38	0.00	-0.08	-0.26	-0.22	-1.10	-1.66	-1.83	-1.40	-1.69	-1.51	-1.75	-1.21

P48547	Potassium voltage-gated channel subfamily C member 1 OS=Homo sapiens GN=KCNC1 PE=2 SV=1 - [KCNC1_HUMAN]	7.83	3	3	4	7	2.93	2.63	2.77	2.48	0.27	-0.03	1.50	1.20	0.76	1.06	0.67	0.37	-1.38	-2.25	-2.10	-1.84	-1.68	-2.68	-2.52	-1.21
O75914	Serine/threonine-protein kinase PAK 3 OS=Homo sapiens GN=PAK3 PE=1 SV=2 - [PAK3_HUMAN]	22.00	1	3	11	22	1.02	1.07	1.30	1.35	-0.26	-0.21	0.03	0.08	-0.44	-0.48	-0.91	-0.86	-0.94	-1.92	-2.21	-1.47	-1.75	-1.29	-1.58	-1.21
O95670	V-type proton ATPase subunit G 2 OS=Homo sapiens GN=ATP6V1G2 PE=1 SV=1 - [VATG2_HUMAN]	16.95	1	1	1	5	2.88	2.40	2.31	2.11	-0.13	-0.26	0.87	0.38	0.38	0.54	0.54	0.03	-1.79	-2.35	-2.08	-2.32	-1.89	-1.81	-1.25	-1.21
Q9UQ16	Dynamin-3 OS=Homo sapiens GN=DNM3 PE=1 SV=4 - [DYN3_HUMAN]	34.87	1	17	28	106	1.86	1.80	2.01	2.02	-0.25	-0.19	0.67	0.69	0.39	0.44	0.11	0.13	-1.09	-1.81	-1.89	-1.45	-1.61	-2.14	-2.25	-1.21
Q01484	Ankyrin-2 OS=Homo sapiens GN=ANK2 PE=1 SV=4 - [ANK2_HUMAN]	40.81	1	104	116	385	1.74	1.81	1.92	1.90	0.04	0.01	0.66	0.67	0.37	0.37	0.21	0.24	-1.01	-1.58	-1.73	-1.31	-1.50	-1.71	-1.87	-1.22
O15020	Spectrin beta chain, non-erythrocytic 2 OS=Homo sapiens GN=SPTBN2 PE=1 SV=3 - [SPTN2_HUMAN]	39.83	1	68	79	194	1.59	1.66	1.84	1.80	-0.27	-0.29	0.48	0.47	0.23	0.17	0.04	0.07	-1.20	-1.69	-1.75	-1.49	-1.58	-2.02	-2.09	-1.22
O75689	Arf-GAP with dual PH domain-containing protein 1 OS=Homo sapiens GN=ADAP1 PE=1 SV=2 - [ADAP1_HUMAN]	14.44	1	6	6	9	1.36	1.56	1.39	1.64	-0.47	-0.21	0.14	0.35	0.41	0.25	-0.17	0.09	-1.16	-1.52	-1.56	-1.16	-1.19	-1.84	-1.87	-1.22
O95757	Heat shock 70 kDa protein 4L OS=Homo sapiens GN=HSPA4L PE=1 SV=3 - [HS74L_HUMAN]	42.79	1	22	26	59	1.61	1.46	1.64	1.57	0.14	-0.01	0.57	0.50	0.21	0.31	0.09	-0.05	-1.09	-1.58	-1.71	-1.30	-1.50	-1.44	-1.62	-1.22

Q6H8Q1	Actin-binding LIM protein 2 OS=Homo sapiens GN=ABLIM2 PE=1 SV=2 - [ABLM2_HUMAN]	23.40	1	10	10	20	1.38	1.71	1.68	1.84	-0.08	-0.11	0.44	0.57	0.27	0.36	-0.18	0.11	-1.30	-1.76	-1.93	-1.59	-1.57	-1.95	-2.06	-1.22
Q9H9H5	MAP6 domain-containing protein 1 OS=Homo sapiens GN=MAP6D1 PE=1 SV=1 - [MA6D1_HUMAN]	20.10	1	2	2	5	1.56	1.65	1.87	1.77	-0.09	-0.27	0.33	0.49	0.01	0.36	0.38	0.26	-1.25	-1.33	-1.50	-1.51	-1.42	-1.94	-1.99	-1.22
Q17R89	Rho GTPase-activating protein 44 OS=Homo sapiens GN=ARHGAP44 PE=1 SV=1 - [RHG44_HUMAN]	6.60	1	4	5	8	1.73	1.78	1.99	1.97	-0.49	-0.33	0.57	0.62	0.34	0.19	-0.27	-0.22	-1.11	-1.99	-2.31	-1.41	-1.71	-2.09	-2.31	-1.22
O60245	Protocadherin-7 OS=Homo sapiens GN=PCDH7 PE=1 SV=2 - [PCDH7_HUMAN]	10.66	1	7	7	9	1.27	1.48	1.63	1.43	0.54	0.48	0.16	0.26	0.19	0.25	-0.46	-0.32	-1.22	-1.21	-1.08	-0.98	-1.05	-0.73	-0.88	-1.22
Q15714	TSC22 domain family protein 1 OS=Homo sapiens GN=TSC22D1 PE=1 SV=3 - [T22D1_HUMAN]	4.10	1	3	5	18	1.27	1.48	1.46	1.34	0.23	-0.24	0.57	0.07	0.08	0.00	-0.40	-0.17	-1.21	-1.51	-1.66	-1.36	-1.25	-1.46	-1.37	-1.22
Q6UXD5	Seizure 6-like protein 2 OS=Homo sapiens GN=SEZ6L2 PE=1 SV=2 - [SE6L2_HUMAN]	5.05	1	3	3	6	1.82	2.08	1.89	2.15	0.28	0.90	0.60	0.86	1.05	0.80	0.75	1.37	-1.09	-0.75	-0.76	-0.99	-1.06	-1.23	-1.24	-1.22
P47870	Gamma-aminobutyric acid receptor subunit beta-2 OS=Homo sapiens GN=GABRB2 PE=1 SV=2 - [GBRB2_HUMAN]	14.26	2	4	5	12	2.00	1.72	1.72	1.71	-0.47	-0.46	0.43	0.58	0.13	-0.05	-0.02	-0.03	-1.22	-1.90	-1.92	-1.74	-1.76	-2.18	-2.17	-1.23
P60174	Triosephosphate isomerase OS=Homo sapiens GN=TPI1 PE=1 SV=3 - [TPIS_HUMAN]	74.13	1	18	18	94	1.30	1.24	1.66	1.61	-0.13	-0.18	0.26	0.23	-0.11	-0.11	-0.50	-0.52	-0.87	-1.72	-2.06	-1.27	-1.65	-1.37	-1.62	-1.23

Q7Z6G8	Ankyrin repeat and sterile alpha motif domain-containing protein 1B OS=Homo sapiens GN=ANKS1B PE=1 SV=2 - [ANS1B_HUMAN]	2.16	1	2	2	2	1.61	1.51	1.58	1.79	-1.70	-1.99	0.41	0.50	0.16	0.01	0.08	-0.21	-0.95	-1.53	-1.51	-1.31	-1.60	-3.32	-3.30	-1.23
P62166	Neuronal calcium sensor 1 OS=Homo sapiens GN=NCS1 PE=1 SV=2 - [NCS1_HUMAN]	65.26	1	9	9	11	1.79	1.78	2.16	2.18	0.47	0.45	0.92	0.94	0.59	0.61	0.46	0.30	-0.79	-1.23	-1.56	-1.39	-1.49	-1.19	-1.70	-1.23
Q15818	Neuronal pentraxin-1 OS=Homo sapiens GN=NPTX1 PE=2 SV=2 - [NPTX1_HUMAN]	15.05	1	8	8	16	0.92	0.99	1.22	1.26	0.16	0.04	-0.03	-0.16	-0.20	-0.04	0.00	-0.14	-1.17	-1.10	-1.29	-1.11	-1.25	-0.68	-1.06	-1.23
P05937	Calbindin OS=Homo sapiens GN=CALB1 PE=1 SV=2 - [CALB1_HUMAN]	52.87	1	11	12	21	1.99	1.80	2.24	2.10	0.84	0.46	0.96	0.81	0.33	0.59	-0.31	-0.38	-0.97	-2.18	-2.48	-1.42	-1.54	-1.34	-1.48	-1.23
Q96MC5	Uncharacterized protein C16orf45 OS=Homo sapiens GN=C16orf45 PE=2 SV=1 - [CP045_HUMAN]	18.14	1	3	3	5	1.35	1.23	1.53	1.25	-0.10	-0.09	0.28	0.22	-0.14	-0.09	0.39	0.08	-1.20	-0.95	-1.18	-1.66	-1.70	-1.57	-1.62	-1.23
Q9UPV7	Protein KIAA1045 OS=Homo sapiens GN=KIAA1045 PE=1 SV=2 - [K1045_HUMAN]	24.25	1	10	10	22	1.78	1.84	1.69	1.74	-0.32	-0.36	0.40	0.53	0.26	0.03	-0.18	0.05	-1.15	-1.67	-1.66	-1.41	-1.44	-1.95	-2.11	-1.23
O94856	Neurofascin OS=Homo sapiens GN=NFASC PE=1 SV=4 - [NFASC_HUMAN]	39.64	1	42	42	123	1.59	1.65	1.68	1.75	-0.25	-0.31	0.43	0.45	0.09	0.16	-0.03	-0.06	-1.17	-1.68	-1.62	-1.46	-1.54	-1.83	-1.95	-1.23
Q9Y6A2	Cholesterol 24-hydroxylase OS=Homo sapiens GN=CYP46A1 PE=1 SV=1 - [CP46A_HUMAN]	9.60	1	4	4	4	2.17	2.57	1.94	2.33	-0.26	0.13	0.59	0.99	0.64	0.25	-0.05	0.34	-1.35	-2.22	-1.99	-1.90	-1.65	-2.45	-2.22	-1.24
Q7L099	Protein RUFY3 OS=Homo sapiens GN=RUFY3 PE=1 SV=1 - [RUFY3_HUMAN]	42.43	1	17	18	63	2.01	1.87	2.01	1.81	0.02	-0.05	0.79	0.60	0.34	0.43	0.21	0.15	-1.04	-1.45	-1.69	-1.46	-1.48	-1.88	-1.94	-1.24

Q9Y2J2	Band 4.1-like protein 3 OS=Homo sapiens GN=EPB41L3 PE=1 SV=2 - [E41L3_HUMAN]	45.63	2	32	38	143	1.70	1.77	2.02	1.99	0.13	0.05	0.72	0.70	0.26	0.42	0.14	0.18	-0.97	-1.60	-1.92	-1.44	-1.60	-1.71	-2.00	-1.24
P60880	Synaptosomal-associated protein 25 OS=Homo sapiens GN=SNAP25 PE=1 SV=1 - [SNP25_HUMAN]	64.08	1	15	15	179	1.56	1.63	1.46	1.54	-0.28	-0.22	0.37	0.43	0.18	0.14	0.11	0.07	-1.20	-1.59	-1.51	-1.53	-1.49	-1.76	-1.73	-1.24
P53805	Calcipressin-1 OS=Homo sapiens GN=RCAN1 PE=1 SV=4 - [RCAN1_HUMAN]	8.33	1	2	2	2	1.68	1.98	1.73	2.03	0.11	0.41	0.43	0.73	0.52	0.23	0.38	0.67	-1.20	-1.30	-1.35	-1.42	-1.47	-1.58	-1.63	-1.24
Q08209	Serine/threonine-protein phosphatase 2B catalytic subunit alpha isoform OS=Homo sapiens GN=PPP3CA PE=1 SV=1 - [PP2BA_HUMAN]	37.24	1	10	15	95	1.83	1.74	1.96	1.93	0.29	0.29	0.69	0.57	0.37	0.38	0.31	0.21	-1.20	-1.55	-1.48	-1.30	-1.38	-1.48	-1.50	-1.25
P40925	Malate dehydrogenase, cytoplasmic OS=Homo sapiens GN=MDH1 PE=1 SV=4 - [MDHC_HUMAN]	47.90	1	16	16	96	1.78	1.69	1.92	1.91	0.16	0.01	0.64	0.66	0.16	0.26	-0.20	-0.25	-1.07	-2.11	-2.36	-1.43	-1.72	-1.70	-1.99	-1.25
Q9H305	Cell death-inducing p53-target protein 1 OS=Homo sapiens GN=CDIP1 PE=2 SV=1 - [CDIP1_HUMAN]	3.37	1	1	1	1	1.91	1.86	2.42	2.37	-0.06	-0.11	1.10	1.05	0.44	0.50	0.57	0.52	-0.76	-1.34	-1.85	-1.39	-1.89	-1.99	-2.50	-1.25
Q8N6N7	Acyl-CoA-binding domain-containing protein 7 OS=Homo sapiens GN=ACBD7 PE=1 SV=1 - [ACBD7_HUMAN]	52.27	1	4	4	7	1.79	1.81	2.33	2.34	0.14	0.11	0.76	0.87	0.64	0.66	0.19	0.29	-0.79	-1.59	-1.89	-1.13	-1.66	-1.70	-2.24	-1.26

Q13303	Voltage-gated potassium channel subunit beta-2 OS=Homo sapiens GN=KCNAB2 PE=1 SV=2 - [KCAB2_HUMAN]	35.42	1	11	12	27	1.69	1.68	1.56	1.45	-0.32	-0.20	0.37	0.35	0.12	0.06	-0.29	-0.31	-1.38	-2.23	-2.07	-1.64	-1.57	-2.20	-2.13	-1.26
Q9Y6V0	Protein piccolo OS=Homo sapiens GN=PCLO PE=1 SV=4 - [PCLO_HUMAN]	11.33	1	41	43	65	1.53	1.60	1.81	1.82	-0.15	-0.24	0.60	0.56	0.34	0.35	0.05	0.05	-1.00	-1.52	-1.85	-1.37	-1.67	-1.56	-1.88	-1.26
Q9NQX3	Gephyrin OS=Homo sapiens GN=GPHN PE=1 SV=1 - [GEPH_HUMAN]	35.60	1	20	20	34	1.41	1.59	1.68	1.79	-0.16	0.01	0.54	0.52	0.21	0.12	0.06	0.08	-1.11	-1.48	-1.70	-1.47	-1.66	-1.63	-1.78	-1.26
Q7Z3B1	Neuronal growth regulator 1 OS=Homo sapiens GN=NEGR1 PE=1 SV=3 - [NEGR1_HUMAN]	40.11	1	10	10	61	1.64	1.43	1.84	1.78	0.41	0.36	0.64	0.47	0.11	0.25	0.35	0.21	-0.96	-1.19	-1.55	-1.50	-1.64	-1.30	-1.70	-1.26
P13637	Sodium/potassium-transporting ATPase subunit alpha 3 OS=Homo sapiens GN=ATP1A3 PE=1 SV=3 - [AT1A3_HUMAN]	54.49	1	30	54	506	1.98	1.90	2.13	2.02	-0.22	-0.08	0.72	0.81	0.43	0.36	0.08	0.21	-1.17	-1.76	-1.81	-1.54	-1.71	-2.00	-2.23	-1.26
Q7Z7K6	Centromere protein V OS=Homo sapiens GN=CENPV PE=1 SV=1 - [CENPV_HUMAN]	21.45	1	4	4	6	0.24	0.02	0.22	0.04	-0.87	-1.08	-0.48	-0.78	-1.17	-0.40	0.07	-0.51	-0.85	-0.70	-0.88	-1.07	-1.22	-1.12	-1.29	-1.26
Q9BRK0	Receptor expression-enhancing protein 2 OS=Homo sapiens GN=REEP2 PE=2 SV=2 - [REEP2_HUMAN]	14.29	1	2	2	3	1.80	1.62	1.76	1.59	-0.30	-0.49	0.44	0.26	-0.12	0.06	-0.06	-0.24	-1.30	-1.85	-1.83	-1.71	-1.67	-2.12	-2.09	-1.26
Q9Y4I1	Unconventional myosin-Va OS=Homo sapiens GN=MYO5A PE=1 SV=2 - [MYO5A_HUMAN]	34.77	2	57	58	115	1.63	1.73	1.79	1.86	-0.14	-0.10	0.54	0.63	0.20	0.23	0.02	0.08	-1.07	-1.67	-1.84	-1.38	-1.67	-1.82	-2.04	-1.26

Q9H313	Protein tweety homolog 1 OS=Homo sapiens GN=TTYH1 PE=2 SV=1 - [TTYH1_HUMAN]	14.00	1	5	5	9	2.46	2.53	2.32	2.33	-0.52	-0.51	0.99	0.97	0.55	0.64	0.31	0.40	-1.41	-2.12	-1.92	-1.88	-1.68	-3.01	-2.87	-1.26
Q13509	Tubulin beta-3 chain OS=Homo sapiens GN=TUBB3 PE=1 SV=2 - [TBB3_HUMAN]	53.78	2	4	20	819	1.31	1.39	1.77	1.79	-0.35	-0.36	0.32	0.37	0.20	0.07	-0.08	0.18	-1.09	-1.51	-1.77	-1.41	-1.66	-1.87	-2.04	-1.27
O75899	Gamma-aminobutyric acid type B receptor subunit 2 OS=Homo sapiens GN=GABBR2 PE=1 SV=1 - [GABR2_HUMAN]	12.75	1	9	9	19	1.88	2.25	2.23	2.67	-0.39	0.25	0.62	1.02	0.87	0.27	-0.04	0.47	-1.13	-1.82	-2.01	-1.38	-1.62	-2.00	-2.47	-1.27
Q8WZ74	Cortactin-binding protein 2 OS=Homo sapiens GN=CTTNBP2 PE=1 SV=1 - [CTTB2_HUMAN]	1.86	1	3	3	6	1.62	1.39	1.49	1.26	0.13	-0.11	0.16	-0.08	-0.17	0.07	0.06	-0.18	-1.41	-1.56	-1.43	-1.52	-1.39	-1.51	-1.37	-1.27
O95970	Leucine-rich glioma-inactivated protein 1 OS=Homo sapiens GN=LGI1 PE=1 SV=1 - [LGI1_HUMAN]	22.26	1	12	12	23	1.70	1.76	2.10	2.16	0.56	0.51	0.74	0.81	0.52	0.52	0.25	0.15	-1.01	-1.44	-1.96	-1.33	-1.59	-1.37	-1.50	-1.27
O43181	NADH dehydrogenase [ubiquinone] iron-sulfur protein 4, mitochondrial OS=Homo sapiens GN=NDUFS4 PE=1 SV=1 - [NDUS4_HUMAN]	31.43	1	6	6	21	1.56	1.39	1.69	1.63	0.23	0.05	0.03	0.13	0.05	0.15	0.46	0.20	-1.33	-1.22	-1.38	-1.43	-1.54	-1.40	-1.60	-1.27
Q5U5X0	Complex III assembly factor LYRM7 OS=Homo sapiens GN=LYRM7 PE=1 SV=1 - [LYRM7_HUMAN]	23.08	1	2	2	2	1.55	1.17	2.13	1.75	-0.16	-0.54	0.79	0.41	-0.03	0.35	0.11	-0.28	-0.70	-1.44	-2.02	-1.17	-1.74	-1.73	-2.30	-1.27
Q8TBB6	Probable cationic amino acid transporter OS=Homo sapiens GN=SLC7A14 PE=2 SV=3 - [S7A14_HUMAN]	11.54	1	5	5	10	1.53	1.39	1.97	1.83	-0.06	-0.20	0.72	0.58	0.53	0.67	0.44	0.30	-0.75	-1.09	-1.53	-0.86	-1.30	-1.61	-2.04	-1.27

A6NL88	Protein shisa-7 OS=Homo sapiens GN=SHISA7 PE=2 SV=3 - [SHSA7_HUMAN]	1.30	1	1	1	1	1.50	1.28	1.41	1.19	-0.74	-0.97	0.07	-0.15	-0.72	-0.49	-0.64	-0.87	-1.38	-2.14	-2.05	-1.96	-1.86	-2.26	-2.17	-1.27
O75781	Paralemmin-1 OS=Homo sapiens GN=PALM PE=1 SV=2 - [PALM_HUMAN]	39.02	1	12	13	39	1.79	1.69	1.74	1.72	0.10	0.06	0.37	0.34	0.19	0.06	-0.25	-0.17	-1.26	-1.79	-1.78	-1.58	-1.69	-1.75	-1.70	-1.27
Q9BQI5	SH3-containing GRB2-like protein 3-interacting protein 1 OS=Homo sapiens GN=SGIP1 PE=1 SV=2 - [SGIP1_HUMAN]	35.87	1	19	19	39	1.41	1.40	1.56	1.40	-0.01	0.01	0.02	0.20	0.11	0.05	-0.14	-0.02	-1.11	-1.43	-1.59	-0.96	-1.22	-1.24	-1.51	-1.27
Q00535	Cyclin-dependent kinase 5 OS=Homo sapiens GN=CDK5 PE=1 SV=3 - [CDK5_HUMAN]	31.16	11	7	8	16	1.66	1.56	2.18	2.08	-0.02	-0.08	0.69	0.72	0.22	0.23	0.05	0.01	-0.52	-1.51	-2.15	-1.27	-2.02	-1.66	-2.33	-1.27
Q9NVX2	Notchless protein homolog 1 OS=Homo sapiens GN=NLE1 PE=1 SV=4 - [NLE1_HUMAN]	4.74	1	1	1	2	0.31	0.16	0.02	-0.13	0.01	-0.14	-1.32	-1.47	0.00	0.15	0.16	0.01	-1.58	-0.15	0.14	-0.13	0.16	-0.32	-0.03	-1.28
Q96HS1	Serine/threonine-protein phosphatase PGAM5, mitochondrial OS=Homo sapiens GN=PGAM5 PE=1 SV=2 - [PGAM5_HUMAN]	11.42	1	4	4	6	1.74	1.63	1.68	1.62	0.20	0.01	0.18	0.28	0.11	0.28	0.22	0.17	-1.29	-1.54	-1.52	-1.45	-1.43	-1.58	-1.56	-1.28
O15197	Ephrin type-B receptor 6 OS=Homo sapiens GN=EPHB6 PE=1 SV=4 - [EPHB6_HUMAN]	5.00	1	4	4	5	1.19	2.37	1.18	2.21	-0.15	0.36	-0.15	0.86	0.10	-0.48	0.22	0.73	-1.39	-1.00	-0.99	-1.66	-1.71	-1.63	-1.62	-1.28
Q02750	Dual specificity mitogen-activated protein kinase kinase 1 OS=Homo sapiens GN=MAP2K1 PE=1 SV=2 - [MP2K1_HUMAN]	41.73	1	7	14	46	1.76	1.83	2.24	2.26	0.60	0.41	0.93	0.87	0.62	0.59	0.38	0.47	-0.85	-1.38	-1.80	-1.16	-1.67	-1.34	-1.61	-1.28

Q8WXF1	Paraspeckle component 1 OS=Homo sapiens GN=PSPC1 PE=1 SV=1 - [PSPC1_HUMAN]	28.11	1	12	13	25	0.73	0.84	1.03	0.88	-0.67	-0.57	-0.31	-0.28	-0.54	-0.68	-0.44	-0.46	-1.09	-1.39	-1.34	-1.40	-1.53	-1.22	-1.56	-1.28
Q05193	Dynamin-1 OS=Homo sapiens GN=DNM1 PE=1 SV=2 - [DYN1_HUMAN]	61.34	1	30	43	219	2.07	2.07	2.19	2.12	-0.30	-0.26	0.75	0.81	0.44	0.39	0.23	0.27	-1.33	-1.77	-1.87	-1.67	-1.71	-2.36	-2.21	-1.28
Q02246	Contactin-2 OS=Homo sapiens GN=CNTN2 PE=1 SV=1 - [CNTN2_HUMAN]	24.90	1	19	19	38	1.74	1.56	1.62	1.53	0.41	0.32	0.48	0.34	0.46	0.53	0.10	0.12	-1.26	-1.34	-1.45	-1.18	-1.11	-1.30	-1.30	-1.28
P61601	Neurocalcin-delta OS=Homo sapiens GN=NCALD PE=2 SV=2 - [NCALD_HUMAN]	65.80	1	8	11	32	1.30	1.58	1.30	1.73	-0.17	0.20	0.25	0.48	0.15	-0.04	0.16	0.23	-1.16	-1.37	-1.51	-1.37	-1.43	-1.51	-1.60	-1.28
Q9HCD6	Protein TANC2 OS=Homo sapiens GN=TANC2 PE=1 SV=3 - [TANC2_HUMAN]	3.47	1	4	4	7	1.29	1.04	1.67	1.34	-0.62	-0.82	0.32	0.07	-0.20	-0.16	-0.16	-0.42	-0.92	-1.45	-1.83	-1.42	-1.50	-1.93	-2.17	-1.28
Q14244	Ensconsin OS=Homo sapiens GN=MAP7 PE=1 SV=1 - [MAP7_HUMAN]	2.00	1	2	2	2	-0.11	-0.22	0.06	-0.05	-0.89	-1.01	-1.29	-1.40	-1.22	-1.11	-0.78	-0.90	-1.13	-0.67	-0.84	-0.97	-1.13	-0.80	-0.97	-1.28
Q9Y4F5	Centrosomal protein of 170 kDa protein B OS=Homo sapiens GN=CEP170B PE=1 SV=4 - [C170B_HUMAN]	15.04	1	17	17	24	1.81	1.57	1.78	1.53	-0.20	-0.35	0.21	0.05	-0.06	0.14	0.06	-0.03	-1.40	-1.65	-1.66	-1.50	-1.39	-1.83	-1.75	-1.29
Q9NQW6	Actin-binding protein anillin OS=Homo sapiens GN=ANLN PE=1 SV=2 - [ANLN_HUMAN]	9.88	1	10	10	15	1.78	1.57	2.22	1.99	-0.29	-0.47	0.99	0.78	0.19	0.40	0.26	0.05	-0.81	-1.56	-2.15	-1.36	-1.86	-2.05	-2.65	-1.29
P20591	Interferon-induced GTP-binding protein Mx1 OS=Homo sapiens GN=MX1 PE=1 SV=4 - [MX1_HUMAN]	24.47	2	12	13	22	0.38	0.51	0.57	0.44	0.44	0.50	-0.86	-0.90	-0.16	-0.22	0.32	0.36	-1.13	-0.03	-0.18	-0.37	-0.52	0.09	0.01	-1.29

Q5JVS0	Intracellular hyaluronan-binding protein 4 OS=Homo sapiens GN=HABP4 PE=1 SV=1 - [HABP4_HUMAN]	11.38	1	3	3	5	0.53	0.50	0.52	0.50	-0.73	-0.63	-0.83	-0.85	-0.69	-0.77	-0.84	-0.66	-0.93	-0.86	-1.49	-1.16	-1.15	-1.14	-1.29	-1.29
Q7Z3D6	UPF0317 protein C14orf159, mitochondrial OS=Homo sapiens GN=C14orf159 PE=1 SV=2 - [CN159_HUMAN]	21.43	1	10	10	23	0.58	0.56	0.56	0.53	-1.06	-1.10	-0.91	-0.75	-1.16	-1.00	-1.66	-1.79	-1.24	-2.07	-2.09	-1.60	-1.66	-1.44	-1.59	-1.29
Q5JTJ3	Cytochrome c oxidase assembly factor 6 homolog OS=Homo sapiens GN=COA6 PE=1 SV=1 - [COA6_HUMAN]	23.20	1	3	3	4	0.44	0.86	0.87	1.30	-0.81	-1.02	-0.57	-0.49	0.06	-0.52	-0.70	-0.29	-1.29	-1.14	-1.58	-0.76	-1.27	-1.60	-1.42	-1.29
Q9UBB6	Neurochondrin OS=Homo sapiens GN=NCDN PE=1 SV=1 - [NCDN_HUMAN]	25.10	1	15	15	45	2.64	2.54	2.64	2.56	0.11	0.11	1.10	0.94	0.55	0.65	0.24	0.09	-1.40	-2.40	-2.34	-1.91	-1.93	-2.43	-2.45	-1.29
Q6ZWB6	BTB/POZ domain-containing protein KCTD8 OS=Homo sapiens GN=KCTD8 PE=2 SV=1 - [KCTD8_HUMAN]	6.34	1	2	3	5	2.55	2.42	2.71	2.58	0.02	0.08	1.36	1.23	0.64	0.78	0.42	0.28	-1.14	-2.12	-2.29	-1.74	-1.90	-2.53	-2.51	-1.29
Q14982	Opioid-binding protein/cell adhesion molecule OS=Homo sapiens GN=OPCML PE=1 SV=1 - [OPCM_HUMAN]	38.55	1	10	13	50	1.76	1.69	2.00	2.10	-0.03	-0.02	0.57	0.71	0.24	0.25	0.18	0.25	-0.97	-1.64	-1.99	-1.43	-1.56	-1.67	-1.92	-1.29
Q12926	ELAV-like protein 2 OS=Homo sapiens GN=ELAVL2 PE=1 SV=2 - [ELAV2_HUMAN]	35.38	1	3	12	24	0.98	0.89	1.25	1.05	0.15	0.22	0.01	0.13	0.28	0.17	0.04	-0.15	-0.71	-1.01	-1.20	-0.66	-1.17	-0.78	-1.00	-1.29
Q8N111	Cell cycle exit and neuronal differentiation protein 1 OS=Homo sapiens GN=CEND1 PE=2 SV=1 - [CEND_HUMAN]	51.68	1	6	6	29	1.96	1.83	2.28	2.41	-0.55	-0.55	1.22	1.10	0.39	0.46	0.07	0.11	-0.87	-1.73	-2.24	-1.40	-1.90	-2.31	-2.81	-1.29

Q96SI9	Spermatid perinuclear RNA-binding protein OS=Homo sapiens GN=STRBP PE=1 SV=1 - [STRBP_HUMAN]	5.36	1	1	3	5	2.08	1.61	1.69	1.22	0.41	-0.06	0.33	-0.13	-0.08	0.39	0.25	-0.23	-1.69	-1.82	-1.44	-1.66	-1.27	-1.68	-1.29	-1.29
P62328	Thymosin beta-4 OS=Homo sapiens GN=TMSB4X PE=1 SV=2 - [TYB4_HUMAN]	45.45	1	2	2	3	-0.34	-0.59	0.82	0.57	-1.49	-1.74	-0.54	-0.78	-1.39	-1.14	-1.04	-1.30	-0.14	-0.70	-1.86	-0.77	-1.92	-1.16	-2.33	-1.29
Q9ULU8	Calcium-dependent secretion activator 1 OS=Homo sapiens GN=CADPS PE=1 SV=3 - [CAPS1_HUMAN]	31.71	1	24	31	57	1.90	1.78	1.99	1.94	-0.36	-0.42	0.76	0.73	0.23	0.28	-0.05	-0.02	-0.99	-1.77	-1.84	-1.55	-1.50	-2.02	-2.17	-1.29
Q6ZSY5	Protein phosphatase 1 regulatory subunit 3F OS=Homo sapiens GN=PPP1R3F PE=1 SV=3 - [PPR3F_HUMAN]	9.64	1	4	4	5	1.81	1.63	1.64	1.46	0.88	0.33	0.28	0.10	-0.01	0.18	0.22	0.03	-1.47	-1.59	-1.43	-1.60	-1.43	-1.35	-1.39	-1.30
O00555	Voltage-dependent P/Q-type calcium channel subunit alpha 1A OS=Homo sapiens GN=CACNA1A PE=1 SV=2 - [CAC1A_HUMAN]	3.99	1	6	6	9	1.15	1.11	1.12	1.10	-0.45	-0.45	-0.26	-0.26	-0.52	-0.52	-0.12	-0.12	-1.40	-1.35	-1.25	-1.60	-1.59	-1.58	-1.57	-1.30
Q8N336	ELMO domain-containing protein 1 OS=Homo sapiens GN=ELMOD1 PE=2 SV=3 - [ELMD1_HUMAN]	10.78	1	3	3	6	1.72	1.73	1.92	1.93	-0.09	0.02	0.57	0.58	0.30	0.18	0.03	0.07	-1.10	-2.05	-2.31	-1.87	-2.11	-1.61	-1.80	-1.30
Q8N9F7	Glycerophosphodiester phosphodiesterase domain containing protein 1 OS=Homo sapiens GN=GDPD1 PE=1 SV=2 - [GDPD1_HUMAN]	17.20	1	4	4	6	1.13	1.38	1.32	1.57	-0.36	-0.12	-0.05	0.20	0.59	0.34	-0.21	0.04	-1.12	-1.33	-1.52	-0.76	-0.94	-1.51	-1.69	-1.30

Q8N7J2	APC membrane recruitment protein 2 OS=Homo sapiens GN=AMER2 PE=1 SV=3 - [AMER2_HUMAN]	5.81	1	3	3	4	1.36	1.39	1.96	1.73	-0.81	-0.31	-0.02	0.36	0.10	-0.49	-0.38	0.13	-0.97	-1.25	-1.59	-1.25	-1.59	-2.19	-2.79	-1.30
P05026	Sodium/potassium-transporting ATPase subunit beta-1 OS=Homo sapiens GN=ATP1B1 PE=1 SV=1 - [AT1B1_HUMAN]	46.20	1	12	12	84	1.95	1.92	2.41	2.28	0.09	0.07	1.05	0.95	0.44	0.59	0.40	0.30	-0.88	-1.69	-2.06	-1.20	-1.73	-1.88	-2.20	-1.30
Q00975	Voltage-dependent N-type calcium channel subunit alpha 1B OS=Homo sapiens GN=CACNA1B PE=1 SV=1 - [CAC1B_HUMAN]	3.12	1	5	6	10	1.98	1.92	1.80	1.73	0.38	0.30	0.73	0.57	0.62	0.78	0.42	0.28	-1.35	-1.68	-1.79	-1.32	-1.26	-1.67	-1.61	-1.30
Q9P1Y5	Calmodulin-regulated spectrin-associated protein 3 OS=Homo sapiens GN=CAMSAP3 PE=1 SV=2 - [CAMP3_HUMAN]	11.21	1	8	8	11	1.63	1.40	1.53	1.39	0.41	0.32	0.64	0.42	0.22	0.35	0.24	0.00	-1.22	-1.52	-1.60	-1.21	-1.28	-1.13	-1.20	-1.30
Q8N6T3	ADP-ribosylation factor GTPase-activating protein 1 OS=Homo sapiens GN=ARFGAP1 PE=1 SV=2 - [ARFG1_HUMAN]	32.76	1	10	10	17	1.66	1.63	1.92	1.80	-0.33	-0.46	0.57	0.58	0.03	-0.01	0.07	-0.11	-1.10	-1.56	-1.93	-1.44	-1.59	-1.88	-2.17	-1.30
Q15700	Disks large homolog 2 OS=Homo sapiens GN=DLG2 PE=1 SV=3 - [DLG2_HUMAN]	33.68	1	15	20	37	1.72	1.67	1.72	1.60	-0.15	-0.29	0.35	0.27	0.15	0.23	-0.10	-0.17	-1.11	-1.78	-2.14	-1.39	-1.56	-1.91	-1.98	-1.30
O94925	Glutaminase kidney isoform, mitochondrial OS=Homo sapiens GN=GLS PE=1 SV=1 - [GLSK_HUMAN]	42.00	2	21	21	56	1.27	1.34	1.52	1.44	-0.40	-0.54	0.26	0.13	-0.10	0.02	-0.23	-0.37	-1.12	-1.78	-1.84	-1.36	-1.50	-1.79	-1.79	-1.30

Q9UQB8	Brain-specific angiogenesis inhibitor 1-associated protein 2 OS=Homo sapiens GN=BAIAP2 PE=1 SV=1 - [BAIP2_HUMAN]	48.55	1	21	21	46	1.24	1.30	1.54	1.40	0.01	-0.10	0.15	0.05	-0.01	0.04	-0.01	-0.10	-1.14	-1.19	-1.57	-1.32	-1.58	-1.15	-1.44	-1.30
Q9UIC8	Leucine carboxyl methyltransferase 1 OS=Homo sapiens GN=LCMT1 PE=1 SV=2 - [LCMT1_HUMAN]	16.47	1	5	5	6	1.97	2.07	2.16	2.29	0.37	0.11	0.63	0.94	0.30	0.44	0.30	0.11	-1.20	-1.72	-1.89	-1.68	-1.57	-1.80	-1.62	-1.31
A6NDG6	Phosphoglycolate phosphatase OS=Homo sapiens GN=PGP PE=1 SV=1 - [PGP_HUMAN]	18.69	1	5	5	9	1.17	1.12	1.56	1.50	0.23	0.17	0.19	0.13	0.01	0.07	-0.10	-0.16	-0.93	-1.26	-1.66	-1.07	-1.46	-0.96	-1.34	-1.31
P53779	Mitogen-activated protein kinase 10 OS=Homo sapiens GN=MAPK10 PE=1 SV=2 - [MK10_HUMAN]	27.16	8	6	9	21	1.68	1.72	2.13	2.15	-0.28	-0.25	0.56	0.76	0.52	0.36	0.01	0.25	-0.99	-1.66	-1.95	-1.38	-1.81	-1.88	-2.14	-1.31
Q9NY59	Sphingomyelin phosphodiesterase 3 OS=Homo sapiens GN=SMPD3 PE=1 SV=1 - [NSMA2_HUMAN]	2.90	1	2	2	4	1.49	1.42	1.55	1.48	0.41	0.33	0.18	0.10	0.06	0.14	0.22	0.14	-1.26	-1.26	-1.33	-1.32	-1.38	-1.10	-1.16	-1.31
P13521	Secretogranin-2 OS=Homo sapiens GN=SCG2 PE=1 SV=2 - [SCG2_HUMAN]	26.74	1	12	12	26	1.85	1.73	1.78	1.88	-0.11	-0.11	0.55	0.68	0.36	0.30	0.03	0.10	-1.20	-1.83	-1.82	-1.36	-1.46	-1.90	-2.10	-1.31
Q14416	Metabotropic glutamate receptor 2 OS=Homo sapiens GN=GRM2 PE=1 SV=2 - [GRM2_HUMAN]	3.44	2	2	3	6	2.83	2.79	2.55	2.52	-0.51	-0.55	1.17	1.14	0.82	0.86	0.55	0.51	-1.60	-2.27	-2.00	-1.93	-1.66	-3.35	-3.08	-1.32
P56211	cAMP-regulated phosphoprotein 19 OS=Homo sapiens GN=ARPP19 PE=1 SV=2 - [ARP19_HUMAN]	42.86	1	4	5	11	0.47	0.44	0.86	0.77	0.00	-0.18	-0.52	-0.61	-0.35	-0.26	-0.72	-0.76	-0.92	-1.19	-1.59	-0.81	-1.35	-0.69	-1.03	-1.32

A6NGB9	WAS/WASL-interacting protein family member 3 OS=Homo sapiens GN=WIPF3 PE=2 SV=4 - [WIPF3_HUMAN]	15.32	1	5	5	7	1.37	1.52	0.93	1.07	-0.09	0.05	-0.32	-0.17	-0.18	-0.32	-0.69	-0.55	-1.35	-2.27	-1.75	-1.66	-1.21	-1.72	-1.03	-1.32
Q96EQ0	Small glutamine-rich tetratricopeptide repeat-containing protein beta OS=Homo sapiens GN=SGTB PE=1 SV=1 - [SGTB_HUMAN]	13.82	1	4	4	5	1.48	1.61	1.62	1.84	-0.22	-0.14	0.23	0.41	0.07	-0.01	-0.24	-0.24	-1.15	-1.73	-2.08	-1.51	-1.79	-1.66	-1.85	-1.32
O14994	Synapsin-3 OS=Homo sapiens GN=SYN3 PE=1 SV=2 - [SYN3_HUMAN]	34.48	1	10	10	43	1.78	1.71	2.09	1.99	-0.19	-0.08	0.71	0.51	0.36	0.33	0.08	0.00	-1.15	-1.81	-2.11	-1.57	-1.86	-2.06	-2.52	-1.32
P61764	Syntaxin-binding protein 1 OS=Homo sapiens GN=STXBP1 PE=1 SV=1 - [STXB1_HUMAN]	59.93	1	32	34	267	1.90	1.87	2.12	2.08	-0.09	-0.07	0.76	0.72	0.20	0.36	0.20	0.21	-1.18	-1.68	-1.91	-1.59	-1.78	-1.99	-2.16	-1.32
Q16799	Reticulon-1 OS=Homo sapiens GN=RTN1 PE=1 SV=1 - [RTN1_HUMAN]	50.77	1	24	25	58	1.20	1.23	1.49	1.58	-0.38	-0.26	0.24	0.38	0.09	-0.04	0.18	0.11	-0.86	-1.07	-1.38	-1.29	-1.53	-1.45	-1.88	-1.32
Q86UW7	Calcium-dependent secretion activator 2 OS=Homo sapiens GN=CADPS2 PE=1 SV=2 - [CAPS2_HUMAN]	13.43	1	9	16	32	1.74	1.60	1.99	1.89	0.12	0.26	0.51	0.41	0.20	0.03	0.05	0.13	-1.17	-1.80	-1.85	-1.54	-1.58	-1.78	-1.70	-1.33
Q15181	Inorganic pyrophosphatase OS=Homo sapiens GN=PPA1 PE=1 SV=2 - [IPYR_HUMAN]	67.13	1	13	14	35	0.81	0.59	1.46	1.22	-0.31	-0.45	0.04	-0.05	-0.28	-0.23	-0.50	-0.56	-0.77	-1.21	-1.80	-1.10	-1.47	-1.11	-1.83	-1.33
Q96ID5	Immunoglobulin superfamily member 21 OS=Homo sapiens GN=IGSF21 PE=2 SV=1 - [IGS21_HUMAN]	20.13	1	8	8	16	1.64	1.58	1.58	1.52	0.18	0.14	0.36	0.25	0.07	0.42	0.30	0.11	-1.35	-1.47	-1.62	-1.15	-1.28	-1.49	-1.31	-1.33

O43819	Protein SCO2 homolog, mitochondrial OS=Homo sapiens GN=SCO2 PE=1 SV=3 - [SCO2_HUMAN]	13.16	1	3	3	5	0.39	-0.23	-0.21	-0.84	0.46	-0.17	-0.87	-1.50	-0.30	0.33	0.24	-0.11	-1.40	-1.42	-1.24	-1.00	-0.81	-0.22	-0.03	-1.33
Q15025	TNFAIP3-interacting protein 1 OS=Homo sapiens GN=TNIP1 PE=1 SV=2 - [TNIP1_HUMAN]	1.26	1	1	1	1	2.03	2.21	1.92	2.10	0.32	0.49	0.53	0.71	0.37	0.20	0.13	0.30	-1.45	-1.90	-1.80	-1.80	-1.69	-1.73	-1.62	-1.33
Q6UB35	Monofunctional C1-tetrahydrofolate synthase, mitochondrial OS=Homo sapiens GN=MTHFD1L PE=1 SV=1 - [C1TM_HUMAN]	6.95	1	6	6	7	1.36	1.14	1.61	1.36	-0.14	-0.26	0.00	-0.14	-0.23	0.01	0.32	-0.03	-1.22	-1.04	-1.20	-1.33	-1.54	-1.21	-1.37	-1.33
Q9BY11	Protein kinase C and casein kinase substrate in neurons protein 1 OS=Homo sapiens GN=PACSIN1 PE=1 SV=1 - [PACN1_HUMAN]	59.68	1	21	21	106	1.24	1.23	1.57	1.53	-0.03	0.04	-0.01	0.08	-0.14	-0.21	-0.33	-0.10	-1.02	-1.33	-1.63	-1.49	-1.71	-1.28	-1.62	-1.33
Q9NPD7	Neuritin OS=Homo sapiens GN=NRN1 PE=1 SV=1 - [NRN1_HUMAN]	16.20	1	2	2	2	1.14	1.12	1.44	1.43	0.04	0.02	0.05	0.03	-0.22	-0.20	-0.46	-0.48	-1.04	-1.60	-1.91	-1.31	-1.61	-1.11	-1.42	-1.34
P15104	Glutamine synthetase OS=Homo sapiens GN=GLUL PE=1 SV=4 - [GLNA_HUMAN]	32.17	1	12	12	86	1.71	1.39	1.88	1.65	0.35	0.22	0.58	0.30	0.20	0.25	0.08	-0.02	-1.10	-1.51	-1.62	-1.25	-1.49	-1.30	-1.50	-1.34
Q16720	Plasma membrane calcium-transporting ATPase 3 OS=Homo sapiens GN=ATP2B3 PE=1 SV=3 - [AT2B3_HUMAN]	25.49	2	12	26	87	2.31	2.01	2.47	2.38	-0.34	-0.52	0.94	0.99	0.51	0.48	0.36	0.28	-1.13	-1.75	-2.05	-1.70	-1.89	-2.47	-2.60	-1.34
Q5JU85	IQ motif and SEC7 domain-containing protein 2 OS=Homo sapiens GN=IQSEC2 PE=1 SV=1 - [IQEC2_HUMAN]	19.69	1	15	19	38	1.74	1.56	1.52	1.79	-0.06	-0.31	0.36	0.29	0.28	0.16	0.22	0.12	-1.35	-1.68	-1.70	-1.53	-1.35	-1.99	-1.85	-1.34

P25713	Metallothionein-3 OS=Homo sapiens GN=MT3 PE=1 SV=1 - [MT3_HUMAN]	17.65	1	1	1	2	1.17	1.09	1.69	1.60	1.15	1.06	0.28	0.20	0.36	0.45	0.46	0.37	-0.83	-0.71	-1.23	-0.69	-1.21	-0.03	-0.55	-1.34
O94805	Actin-like protein 6B OS=Homo sapiens GN=ACTL6B PE=1 SV=1 - [ACL6B_HUMAN]	10.33	1	3	4	4	1.65	0.96	1.24	0.55	0.74	0.05	-0.16	-0.85	-0.29	0.40	-0.02	-0.72	-1.76	-1.66	-1.26	-1.21	-0.80	-0.92	-0.52	-1.34
P32418	Sodium/calcium exchanger 1 OS=Homo sapiens GN=SLC8A1 PE=1 SV=3 - [NAC1_HUMAN]	17.68	1	11	12	27	2.06	1.90	2.47	2.25	-0.04	-0.01	0.88	0.91	0.49	0.48	0.48	0.46	-1.08	-1.63	-1.98	-1.57	-1.76	-1.99	-2.36	-1.34
P41732	Tetraspanin-7 OS=Homo sapiens GN=TSPAN7 PE=1 SV=2 - [TSN7_HUMAN]	6.02	1	1	1	4	1.71	1.64	1.42	1.35	-0.72	-0.80	0.01	-0.05	0.00	0.07	-0.71	-0.78	-1.64	-2.41	-2.13	-1.61	-1.32	-2.45	-2.16	-1.34
P63098	Calcineurin subunit B type 1 OS=Homo sapiens GN=PPP3R1 PE=1 SV=2 - [CANB1_HUMAN]	72.94	2	12	12	94	1.71	1.67	2.21	2.21	0.12	0.25	0.73	0.82	0.42	0.32	0.07	0.11	-0.98	-1.69	-2.22	-1.35	-1.93	-1.52	-2.05	-1.34
Q13367	AP-3 complex subunit beta-2 OS=Homo sapiens GN=AP3B2 PE=1 SV=2 - [AP3B2_HUMAN]	24.03	1	17	22	40	1.90	1.69	2.00	1.99	-0.18	-0.22	0.63	0.52	0.23	0.31	0.04	-0.02	-1.14	-1.72	-1.99	-1.54	-1.85	-1.67	-2.02	-1.35
P23528	Cofilin-1 OS=Homo sapiens GN=CFL1 PE=1 SV=3 - [COF1_HUMAN]	69.28	1	10	14	57	1.02	0.89	1.34	1.31	-0.20	-0.39	-0.17	-0.24	-0.51	-0.42	-0.65	-0.62	-1.04	-1.61	-1.93	-1.30	-1.67	-1.22	-1.59	-1.35
Q05329	Glutamate decarboxylase 2 OS=Homo sapiens GN=GAD2 PE=1 SV=1 - [DCE2_HUMAN]	21.71	1	9	10	20	2.08	2.55	2.25	2.71	-0.08	-0.17	0.84	1.27	0.73	0.53	0.00	0.02	-1.18	-1.70	-1.91	-1.52	-1.69	-1.90	-2.16	-1.35
P17658	Potassium voltage-gated channel subfamily A member 6 OS=Homo sapiens GN=KCNA6 PE=1 SV=1 - [KCNA6_HUMAN]	10.21	1	3	3	4	1.00	0.74	1.22	1.08	-0.38	-0.46	0.13	-0.18	-0.34	0.16	0.01	-0.19	-0.90	-0.85	-1.21	-0.81	-1.03	-1.40	-1.62	-1.35

O75347	Tubulin-specific chaperone A OS=Homo sapiens GN=TBCA PE=1 SV=3 - [TBCA_HUMAN]	39.81	1	5	5	10	0.96	1.04	1.15	1.11	-0.59	-0.53	-0.37	-0.29	-0.40	-0.47	-1.08	-1.03	-1.22	-1.89	-2.20	-1.56	-1.57	-1.65	-1.70	-1.35
Q8N6D5	Ankyrin repeat domain-containing protein 29 OS=Homo sapiens GN=ANKRD29 PE=2 SV=2 - [ANR29_HUMAN]	20.93	1	6	6	8	1.52	1.29	1.65	1.76	-0.11	-0.34	0.51	0.33	-0.19	-0.02	-0.11	-0.52	-0.90	-1.71	-2.13	-1.22	-1.71	-1.62	-2.11	-1.35
O75061	Putative tyrosine-protein phosphatase auxilin OS=Homo sapiens GN=DNAJC6 PE=1 SV=3 - [AUXI_HUMAN]	21.47	1	15	15	31	1.61	1.37	1.81	1.64	-0.33	-0.15	0.44	0.46	0.15	0.16	0.07	0.02	-0.96	-1.66	-1.86	-1.47	-1.87	-1.85	-2.22	-1.35
P05413	Fatty acid-binding protein, heart OS=Homo sapiens GN=FABP3 PE=1 SV=4 - [FABPH_HUMAN]	41.35	1	5	6	14	1.06	1.15	1.38	1.48	-0.73	-0.60	-0.04	-0.01	-0.59	-0.71	-0.81	-0.69	-1.04	-1.83	-2.16	-1.35	-1.76	-1.39	-1.82	-1.35
Q8WY91	THAP domain-containing protein 4 OS=Homo sapiens GN=THAP4 PE=1 SV=2 - [THAP4_HUMAN]	5.20	1	2	2	3	3.38	2.53	3.39	2.55	1.50	0.65	1.98	1.13	1.36	2.21	1.82	0.97	-1.35	-1.55	-1.58	-1.14	-1.15	-1.89	-1.91	-1.35
P09936	Ubiquitin carboxyl-terminal hydrolase isozyme L1 OS=Homo sapiens GN=UCHL1 PE=1 SV=2 - [UCHL1_HUMAN]	66.37	1	13	13	126	1.72	1.80	2.15	2.21	-0.10	-0.04	0.76	0.80	0.27	0.26	0.08	0.05	-0.99	-1.80	-2.11	-1.57	-1.89	-1.96	-2.30	-1.36
Q9C0D0	Phosphatase and actin regulator 1 OS=Homo sapiens GN=PHACTR1 PE=2 SV=3 - [PHAR1_HUMAN]	11.38	1	6	6	8	1.63	1.28	1.42	1.07	-0.62	-0.98	0.00	-0.35	-0.48	-0.13	-0.20	-0.56	-1.57	-1.83	-1.62	-1.73	-1.52	-2.27	-2.06	-1.36

A1Z1Q3	O-acetyl-ADP-ribose deacetylase MACROD2 OS=Homo sapiens GN=MACROD2 PE=1 SV=1 - [MACD2_HUMAN]	2.46	1	1	1	2	1.95	1.96	1.85	1.86	0.77	0.77	0.43	0.44	0.89	0.89	0.47	0.47	-1.47	-1.48	-1.39	-1.04	-0.93	-1.20	-1.10	-1.36
P09471	Guanine nucleotide-binding protein G(o) subunit alpha OS=Homo sapiens GN=GNAO1 PE=1 SV=4 - [GNAO_HUMAN]	58.19	1	17	19	159	2.42	2.36	2.57	2.42	0.05	-0.08	1.07	1.00	0.54	0.61	0.45	0.34	-1.26	-1.99	-2.11	-1.76	-1.87	-2.51	-2.59	-1.36
Q969P0	Immunoglobulin superfamily member 8 OS=Homo sapiens GN=IGSF8 PE=1 SV=1 - [IGSF8_HUMAN]	34.26	1	14	14	72	1.58	1.58	1.72	1.70	0.07	-0.01	0.50	0.44	0.25	0.29	0.18	0.27	-1.29	-1.48	-1.49	-1.57	-1.62	-1.79	-1.75	-1.36
Q6ICH7	Aspartate beta-hydroxylase domain-containing protein 2 OS=Homo sapiens GN=ASPHD2 PE=2 SV=1 - [ASPH2_HUMAN]	2.71	1	1	1	1	1.64	2.07	1.80	2.23	0.53	0.96	0.38	0.81	0.86	0.43	0.00	0.43	-1.20	-1.63	-1.80	-1.17	-1.34	-1.12	-1.29	-1.36
Q9HAV7	GrpE protein homolog 1, mitochondrial OS=Homo sapiens GN=GRPEL1 PE=1 SV=2 - [GRPE1_HUMAN]	17.51	1	4	4	6	0.63	0.61	1.17	1.15	-0.69	-0.72	-0.26	-0.28	0.03	0.06	0.03	0.01	-0.83	-0.60	-1.14	-0.55	-1.08	-1.43	-1.46	-1.36
P68036	Ubiquitin-conjugating enzyme E2 L3 OS=Homo sapiens GN=UBE2L3 PE=1 SV=1 - [UB2L3_HUMAN]	51.95	1	6	6	18	0.65	0.70	1.03	0.97	-0.43	-0.26	-0.45	-0.33	-0.56	-0.57	-0.98	-0.81	-1.02	-1.57	-1.68	-1.34	-1.61	-1.40	-1.48	-1.37
P00492	Hypoxanthine-guanine phosphoribosyltransferase OS=Homo sapiens GN=HPRT1 PE=1 SV=2 - [HPRT_HUMAN]	62.39	2	10	10	23	1.75	1.63	1.75	1.73	0.00	-0.11	0.25	0.20	0.03	-0.11	-0.34	-0.24	-1.17	-1.40	-1.74	-1.17	-1.50	-1.20	-1.48	-1.37

Q9Y6R0	Numb-like protein OS=Homo sapiens GN=NUMBL PE=1 SV=1 - [NUMBL_HUMAN]	16.75	1	6	9	14	0.47	0.77	1.36	1.17	-0.44	-0.72	-0.08	-0.11	-0.54	-0.28	-0.09	-0.12	-0.58	-0.55	-1.44	-0.94	-1.70	-1.51	-1.90	-1.37
Q96MZ0	Ganglioside-induced differentiation associated protein 1-like 1 OS=Homo sapiens GN=GDAP1L1 PE=2 SV=2 - [GD1L1_HUMAN]	20.16	1	6	6	11	2.51	2.77	2.72	2.91	-0.67	-0.39	1.27	1.38	0.82	0.57	0.24	0.55	-1.44	-2.12	-2.09	-1.89	-1.97	-2.82	-2.93	-1.37
Q8TAC9	Secretory carrier-associated membrane protein 5 OS=Homo sapiens GN=SCAMP5 PE=1 SV=1 - [SCAM5_HUMAN]	8.51	1	2	2	12	1.93	1.77	2.31	2.07	-0.14	-0.33	0.76	0.74	0.41	0.31	0.20	0.14	-1.05	-1.93	-2.23	-1.56	-1.94	-2.12	-2.42	-1.37
Q9C0H9	SRC kinase signaling inhibitor 1 OS=Homo sapiens GN=SRCIN1 PE=1 SV=3 - [SRCN1_HUMAN]	32.89	1	25	25	41	1.59	1.52	1.82	1.75	-0.29	-0.33	0.40	0.41	0.13	0.09	-0.04	-0.02	-1.05	-1.67	-1.96	-1.43	-1.63	-1.84	-2.02	-1.37
P78352	Disks large homolog 4 OS=Homo sapiens GN=DLG4 PE=1 SV=3 - [DLG4_HUMAN]	43.37	1	21	23	79	1.83	1.92	1.92	1.99	-0.13	-0.13	0.57	0.50	0.22	0.25	0.05	0.14	-1.24	-1.80	-1.95	-1.67	-1.77	-2.19	-2.10	-1.38
Q01064	Calcium/calmodulin-dependent 3',5'-cyclic nucleotide phosphodiesterase 1B OS=Homo sapiens GN=PDE1B PE=1 SV=2 - [PDE1B_HUMAN]	27.43	1	11	13	24	2.31	2.10	2.12	2.35	-0.29	-0.22	0.81	0.75	0.60	0.45	0.33	0.36	-1.36	-1.99	-1.89	-1.77	-1.68	-2.66	-2.58	-1.38
Q16849	Receptor-type tyrosine-protein phosphatase-like N OS=Homo sapiens GN=PTPRN PE=1 SV=1 - [PTPRN_HUMAN]	7.15	1	4	4	5	1.15	1.89	1.57	2.05	-0.36	0.38	0.67	1.08	0.78	0.04	0.05	0.37	-0.90	-1.03	-1.52	-1.23	-1.36	-1.64	-2.00	-1.38
Q15843	NEDD8 OS=Homo sapiens GN=NEDD8 PE=1 SV=1 - [NEDD8_HUMAN]	17.28	1	2	2	13	1.22	0.99	1.64	1.45	-0.40	-0.45	0.18	0.03	-0.24	-0.01	-0.11	-0.36	-1.07	-1.46	-1.76	-1.17	-1.53	-1.44	-1.81	-1.38

Q9UPA5	Protein bassoon OS=Homo sapiens GN=BSN PE=2 SV=4 - [BSN_HUMAN]	25.17	1	64	65	126	1.75	1.79	1.77	1.91	-0.06	-0.05	0.37	0.24	0.21	0.13	-0.14	-0.21	-1.35	-1.97	-1.99	-1.57	-1.69	-1.85	-1.91	-1.38
O43708	Maleylacetoacetate isomerase OS=Homo sapiens GN=GSTZ1 PE=1 SV=3 - [MAAI_HUMAN]	7.41	1	2	2	4	0.77	0.85	1.78	1.86	0.16	0.24	0.33	0.41	0.29	0.21	0.06	0.14	-0.39	-0.71	-1.72	-0.53	-1.54	-0.63	-1.64	-1.39
Q96PV0	Ras/Rap GTPase-activating protein SynGAP OS=Homo sapiens GN=SYNGAP1 PE=1 SV=4 - [SYGP1_HUMAN]	21.97	1	23	24	49	2.06	2.06	2.05	2.02	-0.08	-0.27	0.66	0.60	0.24	0.36	0.21	0.02	-1.38	-2.13	-2.07	-1.87	-1.79	-2.12	-2.12	-1.39
O14548	Cytochrome c oxidase subunit 7A-related protein, mitochondrial OS=Homo sapiens GN=COX7A2L PE=1 SV=2 - [COX7R_HUMAN]	34.21	1	5	5	11	1.54	1.63	1.74	1.99	0.05	-0.05	0.40	0.49	0.12	-0.18	-0.03	0.15	-1.09	-1.33	-1.65	-1.60	-1.72	-1.50	-1.80	-1.39
Q92558	Wiskott-Aldrich syndrome protein family member 1 OS=Homo sapiens GN=WASF1 PE=1 SV=1 - [WASF1_HUMAN]	14.31	1	7	7	15	2.34	2.62	2.29	2.52	-0.25	-0.08	0.77	1.06	0.52	0.38	0.10	0.24	-1.51	-2.48	-2.33	-1.85	-1.80	-2.61	-2.56	-1.39
Q9NS61	Kv channel-interacting protein 2 OS=Homo sapiens GN=KCNIP2 PE=1 SV=3 - [KCIP2_HUMAN]	6.67	1	1	1	2	2.37	2.16	2.49	2.28	0.84	0.63	1.03	0.82	0.28	0.50	0.50	0.29	-1.28	-1.86	-1.99	-1.84	-1.96	-1.54	-1.67	-1.40
Q86XE0	Sorting nexin-32 OS=Homo sapiens GN=SNX32 PE=2 SV=1 - [SNX32_HUMAN]	4.71	1	1	2	3	2.68	1.73	2.34	1.39	0.88	-0.07	0.88	-0.07	-0.10	0.85	0.34	-0.62	-1.74	-2.33	-2.01	-1.79	-1.46	-1.81	-1.48	-1.40
Q8NCB2	CaM kinase-like vesicle-associated protein OS=Homo sapiens GN=CAMKV PE=1 SV=2 - [CAMKV_HUMAN]	51.10	1	15	15	39	1.93	1.75	2.08	2.24	-0.25	-0.37	0.70	0.59	0.18	0.20	0.27	0.23	-1.06	-1.64	-1.75	-1.66	-2.08	-1.72	-1.93	-1.40

Q9BYB0	SH3 and multiple ankyrin repeat domains protein 3 OS=Homo sapiens GN=SHANK3 PE=1 SV=3 - [SHAN3_HUMAN]	15.71	1	18	18	31	1.84	1.59	1.76	1.68	0.03	-0.05	0.00	-0.04	-0.07	0.03	-0.14	-0.47	-1.37	-1.81	-1.83	-1.67	-1.70	-1.70	-1.73	-1.41
P24588	A-kinase anchor protein 5 OS=Homo sapiens GN=AKAP5 PE=1 SV=3 - [AKAP5_HUMAN]	26.93	1	7	7	13	1.32	1.35	1.92	2.05	-0.08	-0.12	0.45	0.46	0.28	0.19	0.09	0.08	-0.91	-1.74	-2.18	-1.26	-1.72	-1.42	-2.18	-1.41
Q9UJD0	Regulating synaptic membrane exocytosis protein 3 OS=Homo sapiens GN=RIMS3 PE=1 SV=1 - [RIMS3_HUMAN]	44.16	1	7	9	14	1.95	1.93	1.80	1.78	-0.19	0.02	0.21	0.24	0.11	0.13	0.03	-0.09	-1.44	-2.01	-1.83	-1.79	-1.63	-1.68	-1.94	-1.41
Q9BQG1	Synaptotagmin-3 OS=Homo sapiens GN=SYT3 PE=2 SV=1 - [SYT3_HUMAN]	3.05	1	1	1	2	2.01	1.91	2.23	2.13	0.01	-0.09	0.75	0.66	0.49	0.59	0.41	0.31	-1.20	-1.59	-1.82	-1.39	-1.60	-2.02	-2.24	-1.41
O14775	Guanine nucleotide-binding protein subunit beta-5 OS=Homo sapiens GN=GNB5 PE=1 SV=2 - [GBB5_HUMAN]	26.08	1	9	9	16	1.36	1.24	1.74	1.62	-0.08	-0.25	0.46	0.36	-0.07	0.12	-0.07	-0.17	-0.91	-1.10	-1.55	-1.39	-1.63	-1.55	-1.86	-1.42
P63215	Guanine nucleotide-binding protein G(I)/G(S)/G(O) subunit gamma-3 OS=Homo sapiens GN=GNG3 PE=2 SV=1 - [GBG3_HUMAN]	72.00	1	4	4	38	2.00	1.92	2.14	2.31	-0.25	-0.20	0.58	0.70	0.05	0.18	0.13	0.26	-1.16	-1.83	-2.07	-1.75	-1.90	-2.36	-2.44	-1.42
Q9UQM7	Calcium/calmodulin-dependent protein kinase type II subunit alpha OS=Homo sapiens GN=CAMK2A PE=1 SV=2 - [KCC2A_HUMAN]	37.24	1	10	15	80	2.08	2.10	2.16	2.06	-0.32	0.01	0.71	0.66	0.44	0.37	0.40	0.31	-1.17	-1.74	-1.77	-1.64	-1.75	-2.27	-2.24	-1.42

O60262	Guanine nucleotide-binding protein G(I)/G(S)/G(O) subunit gamma-7 OS=Homo sapiens GN=GNG7 PE=1 SV=1 - [GBG7_HUMAN]	47.06	1	3	3	19	1.47	1.63	1.95	1.92	-0.12	-0.10	0.44	0.44	0.27	0.15	0.04	0.32	-1.00	-1.21	-1.73	-1.40	-1.59	-1.71	-2.04	-1.42
Q99946	Proline-rich transmembrane protein 1 OS=Homo sapiens GN=PRRT1 PE=2 SV=2 - [PRRT1_HUMAN]	8.50	1	2	2	4	1.60	1.53	1.24	1.16	0.05	-0.03	-0.25	-0.32	-0.01	0.07	0.17	0.09	-1.79	-1.42	-1.07	-1.50	-1.14	-1.56	-1.20	-1.42
O00154	Cytosolic acyl coenzyme A thioester hydrolase OS=Homo sapiens GN=ACOT7 PE=1 SV=3 - [BACH_HUMAN]	27.11	2	8	8	30	2.55	2.33	2.51	2.32	0.10	0.04	1.02	0.74	0.47	0.66	0.02	0.08	-1.37	-2.24	-2.35	-1.69	-1.85	-2.24	-2.36	-1.42
O75381	Peroxisomal membrane protein PEX14 OS=Homo sapiens GN=PEX14 PE=1 SV=1 - [PEX14_HUMAN]	12.73	1	4	4	8	1.44	1.44	1.88	1.89	0.12	0.05	0.37	0.38	0.33	0.24	0.42	0.44	-1.02	-0.99	-1.51	-1.11	-1.61	-1.32	-1.82	-1.42
Q93045	Stathmin-2 OS=Homo sapiens GN=STMN2 PE=1 SV=3 - [STMN2_HUMAN]	21.23	2	2	5	51	1.50	1.67	1.78	1.95	0.19	0.35	0.29	0.46	0.36	0.20	-0.27	-0.10	-1.16	-1.76	-2.05	-1.27	-1.55	-1.33	-1.61	-1.42
P84074	Neuron-specific calcium-binding protein hippocalcin OS=Homo sapiens GN=HPCA PE=1 SV=2 - [HPCA_HUMAN]	49.74	1	5	11	30	1.86	1.69	1.83	1.67	0.11	-0.06	0.57	0.40	0.24	0.41	-0.77	-0.94	-1.22	-2.63	-2.61	-1.42	-1.39	-1.77	-1.74	-1.42
Q13522	Protein phosphatase 1 regulatory subunit 1A OS=Homo sapiens GN=PPP1R1A PE=1 SV=2 - [PPR1A_HUMAN]	12.28	1	1	1	2	1.01	0.48	1.17	0.64	0.81	0.27	-0.32	-0.85	-0.03	0.51	0.61	0.07	-1.27	-0.40	-0.57	-0.48	-0.64	-0.22	-0.39	-1.43

Q8N3J6	Cell adhesion molecule 2 OS=Homo sapiens GN=CADM2 PE=2 SV=1 - [CADM2_HUMAN]	18.39	1	6	6	18	1.66	1.62	2.00	2.14	0.09	0.04	0.54	0.77	0.50	0.44	0.37	0.48	-0.87	-1.22	-1.72	-1.40	-1.72	-1.83	-2.23	-1.43
Q8N9F0	N-acetylaspartate synthetase OS=Homo sapiens GN=NAT8L PE=1 SV=3 - [NAT8L_HUMAN]	18.87	1	2	2	2	2.29	1.53	2.30	1.54	0.22	-0.54	0.81	0.05	-0.56	0.20	0.65	-0.12	-1.43	-1.64	-1.66	-2.06	-2.07	-2.09	-2.10	-1.43
Q9P1U1	Actin-related protein 3B OS=Homo sapiens GN=ACTR3B PE=2 SV=1 - [ARP3B_HUMAN]	30.14	2	6	9	24	1.55	1.61	1.66	1.84	-0.15	0.04	0.34	0.34	0.20	0.25	0.12	0.21	-1.26	-1.42	-1.87	-1.57	-1.76	-1.42	-1.37	-1.43
P80723	Brain acid soluble protein 1 OS=Homo sapiens GN=BASP1 PE=1 SV=2 - [BASP1_HUMAN]	53.30	1	10	10	79	1.38	1.18	2.07	2.02	-0.63	-0.64	0.58	0.39	-0.32	-0.01	-0.37	-0.56	-0.72	-1.87	-2.74	-1.49	-2.25	-2.05	-2.74	-1.44
P49802	Regulator of G-protein signaling 7 OS=Homo sapiens GN=RGS7 PE=1 SV=3 - [RGS7_HUMAN]	19.80	1	5	7	10	1.71	1.56	2.17	2.02	0.11	-0.04	0.77	0.62	0.17	0.32	0.28	0.12	-0.95	-1.42	-1.89	-1.35	-1.81	-1.61	-2.07	-1.44
P54829	Tyrosine-protein phosphatase non-receptor type 5 OS=Homo sapiens GN=PTPN5 PE=1 SV=4 - [PTN5_HUMAN]	1.42	1	1	1	2	2.15	2.30	2.11	2.26	-0.14	0.01	0.61	0.76	0.47	0.32	-0.03	0.11	-1.49	-2.18	-2.14	-1.80	-1.75	-2.30	-2.26	-1.44
O75145	Liprin-alpha-3 OS=Homo sapiens GN=PPFIA3 PE=1 SV=3 - [LIPA3_HUMAN]	28.06	1	22	24	58	1.87	1.96	2.01	2.09	-0.31	-0.21	0.46	0.66	0.33	0.32	0.19	0.24	-1.44	-1.88	-1.95	-1.70	-1.86	-2.02	-2.09	-1.44
P68402	Platelet-activating factor acetylhydrolase IB subunit beta OS=Homo sapiens GN=PAFAH1B2 PE=1 SV=1 - [PA1B2_HUMAN]	5.24	1	1	1	1	0.51	0.61	1.76	1.85	-0.47	-0.39	0.25	0.35	-0.20	-0.29	-0.54	-0.46	-0.20	-1.05	-2.30	-0.77	-2.01	-1.01	-2.25	-1.44

Q9UNW9	RNA-binding protein Nova-2 OS=Homo sapiens GN=NOVA2 PE=1 SV=1 - [NOVA2_HUMAN]	21.14	1	5	8	14	1.25	0.68	1.62	1.13	-0.40	-0.64	-0.11	-0.51	-0.74	-0.43	-0.28	-0.77	-0.99	-1.18	-1.59	-1.27	-1.67	-1.38	-1.74	-1.44
Q96BR5	Cytochrome c oxidase assembly factor 7 OS=Homo sapiens GN=COA7 PE=1 SV=2 - [COA7_HUMAN]	17.32	1	4	4	5	1.46	1.27	1.91	1.71	-0.10	-0.30	0.32	0.27	0.32	0.34	0.12	0.07	-1.09	-1.33	-1.78	-1.09	-1.53	-1.58	-2.03	-1.44
O15083	ERC protein 2 OS=Homo sapiens GN=ERC2 PE=1 SV=3 - [ERC2_HUMAN]	17.76	1	10	17	22	2.30	2.10	2.23	2.03	-0.80	-0.77	0.88	0.69	0.58	0.52	-0.18	-0.16	-1.38	-2.14	-2.29	-2.15	-2.19	-2.55	-2.69	-1.44
Q9H2X9	Solute carrier family 12 member 5 OS=Homo sapiens GN=SLC12A5 PE=2 SV=3 - [S12A5_HUMAN]	14.66	1	12	15	44	2.43	2.33	2.41	2.56	-0.06	-0.08	0.82	0.96	0.43	0.46	0.22	0.24	-1.36	-2.16	-2.08	-1.83	-1.94	-2.38	-2.49	-1.45
Q13885	Tubulin beta-2A chain OS=Homo sapiens GN=TUBB2A PE=1 SV=1 - [TBB2A_HUMAN]	71.24	2	3	25	936	1.34	1.52	1.86	1.76	-0.41	-0.48	0.12	0.22	-0.12	-0.05	-0.14	-0.18	-1.12	-1.24	-1.89	-1.38	-1.73	-1.68	-1.71	-1.46
P49758	Regulator of G-protein signaling 6 OS=Homo sapiens GN=RGS6 PE=1 SV=5 - [RGS6_HUMAN]	22.67	1	7	8	16	1.56	1.64	1.82	1.93	-0.34	-0.27	0.26	0.38	0.06	0.02	0.00	0.08	-1.16	-1.55	-1.82	-1.40	-1.79	-1.52	-2.18	-1.46
Q01814	Plasma membrane calcium-transporting ATPase 2 OS=Homo sapiens GN=ATP2B2 PE=1 SV=2 - [AT2B2_HUMAN]	35.56	1	26	38	134	2.11	2.06	2.16	2.14	-0.07	-0.09	0.85	0.83	0.37	0.45	0.09	0.16	-1.28	-2.17	-2.18	-1.67	-1.89	-2.32	-2.38	-1.46
P20020	Plasma membrane calcium-transporting ATPase 1 OS=Homo sapiens GN=ATP2B1 PE=1 SV=3 - [AT2B1_HUMAN]	29.81	1	13	31	129	1.96	1.82	2.11	1.93	0.08	-0.06	0.61	0.52	0.12	0.30	0.24	0.24	-1.31	-1.67	-1.70	-1.57	-1.76	-1.87	-1.98	-1.46

O15079	Syntaphilin OS=Homo sapiens GN=SNPH PE=1 SV=2 - [SNPH_HUMAN]	15.79	1	4	6	12	2.62	2.55	2.94	2.88	0.28	0.08	1.41	1.35	0.54	0.76	0.60	0.32	-1.16	-1.99	-2.31	-1.72	-2.08	-1.94	-2.25	-1.46
P11137	Microtubule-associated protein 2 OS=Homo sapiens GN=MAP2 PE=1 SV=4 - [MTAP2_HUMAN]	51.12	1	67	67	193	1.48	1.47	1.83	1.77	-0.35	-0.42	0.35	0.28	-0.06	0.00	-0.37	-0.36	-1.14	-1.77	-2.15	-1.48	-1.76	-1.78	-2.10	-1.46
Q6U841	Sodium-driven chloride bicarbonate exchanger OS=Homo sapiens GN=SLC4A10 PE=2 SV=1 - [S4A10_HUMAN]	16.91	2	12	15	18	1.89	1.76	1.89	1.87	0.05	-0.13	0.48	0.55	0.12	0.29	0.30	0.05	-1.38	-2.23	-2.50	-1.70	-1.69	-1.91	-2.13	-1.46
Q12955	Ankyrin-3 OS=Homo sapiens GN=ANK3 PE=1 SV=3 - [ANK3_HUMAN]	17.64	1	43	55	102	1.78	1.74	1.96	1.82	-0.19	-0.24	0.41	0.34	0.04	0.11	-0.08	-0.18	-1.31	-1.79	-1.98	-1.71	-1.76	-1.81	-1.98	-1.47
O75525	KH domain-containing, RNA-binding, signal transduction-associated protein 3 OS=Homo sapiens GN=KHDRBS3 PE=1 SV=1 - [KHDR3_HUMAN]	13.29	1	5	6	13	1.73	1.57	1.96	1.90	-0.44	-0.04	0.09	0.15	0.09	-0.03	-0.15	0.14	-1.58	-1.67	-1.57	-1.45	-1.36	-1.71	-1.60	-1.47
Q9UKU0	Long-chain-fatty-acid--CoA ligase 6 OS=Homo sapiens GN=ACSL6 PE=2 SV=4 - [ACSL6_HUMAN]	40.89	1	21	22	41	1.85	1.71	2.06	1.86	-0.14	-0.15	0.80	0.56	0.33	0.48	0.29	0.19	-1.18	-1.65	-1.91	-1.49	-1.72	-2.20	-2.23	-1.47
P78559	Microtubule-associated protein 1A OS=Homo sapiens GN=MAP1A PE=1 SV=6 - [MAP1A_HUMAN]	34.53	1	76	77	218	1.44	1.50	1.59	1.66	-0.26	-0.20	0.08	0.13	-0.07	-0.11	-0.14	-0.06	-1.27	-1.59	-1.74	-1.55	-1.66	-1.69	-1.93	-1.48
Q9Y281	Cofilin-2 OS=Homo sapiens GN=CFL2 PE=1 SV=1 - [COF2_HUMAN]	63.25	1	7	11	36	0.77	0.69	1.15	1.23	-0.45	-0.38	-0.21	-0.19	-0.28	-0.28	-0.52	-0.53	-0.91	-1.31	-1.72	-0.93	-1.33	-1.42	-1.53	-1.49

P04350	Tubulin beta-4A chain OS=Homo sapiens GN=TUBB4A PE=1 SV=2 - [TBB4A_HUMAN]	74.55	4	4	25	826	2.18	2.14	2.28	2.23	-0.14	-0.18	0.75	0.55	0.30	0.45	0.44	0.29	-1.43	-1.87	-2.10	-1.79	-1.87	-2.23	-2.43	-1.49
O95197	Reticulon-3 OS=Homo sapiens GN=RTN3 PE=1 SV=2 - [RTN3_HUMAN]	30.23	1	25	26	57	1.74	1.92	2.10	1.99	-0.24	-0.13	0.55	0.50	0.20	0.07	0.05	0.03	-1.23	-1.81	-2.04	-1.68	-1.91	-2.06	-2.28	-1.49
O15056	Synaptojanin-2 OS=Homo sapiens GN=SYNJ2 PE=1 SV=3 - [SYNJ2_HUMAN]	1.87	1	1	1	2	1.02	0.88	1.22	1.08	0.65	0.51	-0.33	-0.47	-0.79	-0.64	-0.13	-0.28	-1.30	-1.15	-1.36	-1.64	-1.83	-0.39	-0.59	-1.49
Q9UPX8	SH3 and multiple ankyrin repeat domains protein 2 OS=Homo sapiens GN=SHANK2 PE=1 SV=3 - [SHAN2_HUMAN]	7.96	1	8	8	18	1.36	1.46	1.96	2.05	-0.15	-0.06	0.41	0.49	-0.04	-0.14	-0.17	0.04	-1.08	-1.57	-2.01	-1.47	-1.65	-1.65	-2.11	-1.50
O60861	Growth arrest specific protein 7 OS=Homo sapiens GN=GAS7 PE=1 SV=3 - [GAS7_HUMAN]	32.77	1	12	13	22	1.70	1.81	1.41	1.58	-0.44	-0.46	0.09	0.01	-0.21	-0.09	-0.49	-0.42	-1.62	-2.06	-1.86	-2.12	-1.73	-2.56	-2.36	-1.50
Q16566	Calcium/calmodulin-dependent protein kinase type IV OS=Homo sapiens GN=CAMK4 PE=1 SV=1 - [KCC4_HUMAN]	23.04	2	9	10	16	1.68	1.15	1.69	1.51	-0.17	-0.23	0.44	0.18	-0.11	0.09	-0.05	-0.19	-1.24	-1.78	-1.88	-1.56	-1.58	-1.62	-1.71	-1.50
Q9P0K1	Disintegrin and metalloproteinase domain-containing protein 22 OS=Homo sapiens GN=ADAM22 PE=1 SV=1 - [ADA22_HUMAN]	18.65	1	12	12	24	2.11	2.03	2.24	2.51	-0.13	0.10	0.83	0.94	0.52	0.53	0.01	0.24	-1.11	-1.93	-2.34	-1.40	-2.00	-2.10	-2.30	-1.50
P35498	Sodium channel protein type 1 subunit alpha OS=Homo sapiens GN=SCN1A PE=1 SV=2 - [SCN1A_HUMAN]	8.36	4	10	17	29	1.62	1.74	2.11	2.26	0.20	0.32	0.52	0.77	0.46	0.30	0.15	0.25	-1.18	-1.81	-2.12	-1.58	-1.92	-1.85	-2.27	-1.51

Q96JN2	Coiled-coil domain-containing protein 136 OS=Homo sapiens GN=CCDC136 PE=2 SV=3 - [CC136_HUMAN]	8.06	1	6	6	15	2.98	2.55	2.90	2.51	-0.06	-0.33	1.13	1.00	0.59	0.84	0.51	0.25	-1.68	-1.92	-1.66	-1.73	-1.60	-2.64	-2.59	-1.51
Q8IWQ3	Serine/threonine-protein kinase BRSK2 OS=Homo sapiens GN=BRSK2 PE=1 SV=3 - [BRSK2_HUMAN]	20.11	1	9	9	16	1.50	1.67	2.36	2.14	0.03	0.13	0.62	0.54	0.01	0.05	0.24	0.36	-0.95	-1.44	-1.75	-1.58	-1.87	-1.65	-1.98	-1.52
Q8NHG7	Small VCP/p97-interacting protein OS=Homo sapiens GN=SVIP PE=3 SV=1 - [SVIP_HUMAN]	32.47	1	2	2	7	0.98	1.33	1.46	1.50	-0.63	-0.93	-0.12	-0.27	-0.33	-0.48	-0.16	-0.46	-0.96	-1.10	-1.62	-1.43	-1.60	-1.54	-2.12	-1.52
Q14194	Dihydropyrimidinase-related protein 1 OS=Homo sapiens GN=CRMP1 PE=1 SV=1 - [DPYL1_HUMAN]	62.06	1	24	26	116	1.45	1.67	1.84	1.96	-0.29	-0.20	0.34	0.53	0.34	0.17	0.18	0.23	-1.25	-1.51	-1.73	-1.44	-1.67	-1.74	-1.89	-1.52
Q9ULJ8	Neurabin-1 OS=Homo sapiens GN=PPP1R9A PE=1 SV=2 - [NEB1_HUMAN]	7.38	1	5	6	9	0.71	1.52	0.42	1.13	-0.47	-0.29	-0.44	-0.47	-0.21	-0.28	-0.77	-0.87	-1.33	-1.47	-1.18	-1.60	-1.30	-1.82	-1.43	-1.53
Q13972	Ras-specific guanine nucleotide-releasing factor 1 OS=Homo sapiens GN=RASGRF1 PE=1 SV=2 - [RGRF1_HUMAN]	3.38	1	3	3	3	1.80	1.93	1.94	2.33	-0.08	0.21	0.35	0.74	0.68	0.40	-0.10	0.18	-1.33	-1.69	-1.55	-1.39	-1.40	-1.92	-1.93	-1.53
Q9BPU6	Dihydropyrimidinase-related protein 5 OS=Homo sapiens GN=DPYSL5 PE=1 SV=1 - [DPYL5_HUMAN]	37.77	1	15	15	33	2.04	2.24	2.09	2.29	-0.04	0.06	0.48	0.41	0.27	0.33	-0.22	0.00	-1.46	-2.36	-2.38	-1.68	-1.86	-1.93	-2.21	-1.53
Q9UK76	Hematological and neurological expressed 1 protein OS=Homo sapiens GN=HN1 PE=1 SV=3 - [HN1_HUMAN]	51.95	1	3	3	9	1.50	1.32	1.28	1.28	-0.32	-0.56	-0.37	-0.52	-0.58	-0.66	-0.47	-0.82	-1.96	-2.08	-1.75	-2.18	-1.69	-2.03	-1.64	-1.53

Q5JRX3	Presequence protease, mitochondrial OS=Homo sapiens GN=PITRM1 PE=1 SV=3 - [PREP_HUMAN]	30.57	1	24	24	42	1.21	1.08	1.20	1.23	-0.24	-0.12	-0.31	-0.30	-0.14	-0.16	0.30	0.24	-1.28	-0.78	-1.00	-1.36	-1.49	-1.37	-1.54	-1.53
O75077	Disintegrin and metalloproteinase domain-containing protein 23 OS=Homo sapiens GN=ADAM23 PE=1 SV=1 - [ADA23_HUMAN]	14.42	2	11	11	29	2.36	2.50	2.42	2.66	-0.11	0.11	0.70	1.01	0.59	0.52	0.36	0.37	-1.43	-1.76	-1.13	-1.76	-1.93	-2.11	-2.57	-1.53
P48066	Sodium- and chloride-dependent GABA transporter 3 OS=Homo sapiens GN=SLC6A11 PE=2 SV=1 - [S6A11_HUMAN]	9.18	1	6	6	15	2.28	2.28	2.26	2.26	-0.49	-0.49	0.50	0.50	0.11	0.11	-0.37	-0.38	-1.37	-2.33	-2.38	-1.85	-1.88	-2.54	-2.58	-1.53
Q9ULD0	2-oxoglutarate dehydrogenase-like, mitochondrial OS=Homo sapiens GN=OGDHL PE=1 SV=3 - [OGDHL_HUMAN]	34.75	1	24	27	54	1.93	1.83	2.05	2.01	-0.62	-0.66	0.66	0.51	0.13	0.16	-0.03	-0.18	-1.37	-2.10	-2.15	-1.88	-1.85	-2.15	-2.43	-1.54
Q15773	Myeloid leukemia factor 2 OS=Homo sapiens GN=MLF2 PE=1 SV=1 - [MLF2_HUMAN]	23.39	1	4	4	20	1.62	1.57	1.64	1.65	-0.47	-0.33	-0.14	-0.30	0.01	-0.18	-0.23	-0.47	-1.42	-1.70	-1.74	-1.63	-1.35	-1.83	-1.71	-1.55
Q99250	Sodium channel protein type 2 subunit alpha OS=Homo sapiens GN=SCN2A PE=1 SV=3 - [SCN2A_HUMAN]	11.12	7	10	18	36	1.87	1.73	2.07	1.98	-0.48	-0.75	0.44	0.36	-0.09	-0.01	-0.16	-0.39	-1.28	-1.79	-2.38	-1.78	-2.23	-2.08	-2.53	-1.55
Q9P2W3	Guanine nucleotide-binding protein G(I)/G(S)/G(O) subunit gamma-13 OS=Homo sapiens GN=GNG13 PE=2 SV=1 - [GBG13_HUMAN]	37.31	1	4	4	8	2.74	2.86	2.62	2.86	0.60	0.47	1.30	1.25	0.88	1.02	0.76	0.62	-1.38	-1.78	-2.23	-1.56	-1.99	-1.96	-2.41	-1.55

P59768	Guanine nucleotide-binding protein G(I)/G(S)/G(O) subunit gamma-2 OS=Homo sapiens GN=GNG2 PE=1 SV=2 - [GBG2_HUMAN]	50.70	1	2	3	11	2.03	2.03	2.50	2.44	0.00	-0.11	1.06	0.73	0.49	0.69	0.64	0.52	-1.07	-1.50	-1.91	-1.31	-1.85	-2.05	-2.69	-1.55
P16389	Potassium voltage-gated channel subfamily A member 2 OS=Homo sapiens GN=KCNA2 PE=1 SV=2 - [KCNA2_HUMAN]	19.24	1	5	6	15	1.74	2.48	1.86	2.66	0.20	0.23	0.92	1.05	0.65	0.33	0.52	0.58	-1.38	-1.89	-2.07	-1.79	-1.90	-1.77	-1.93	-1.55
P62760	Visinin-like protein 1 OS=Homo sapiens GN=VSNL1 PE=1 SV=2 - [VISL1_HUMAN]	55.50	1	9	12	74	1.96	2.20	2.50	2.70	0.19	0.26	0.80	0.97	0.38	0.46	0.46	0.40	-0.97	-1.61	-2.10	-1.65	-2.06	-1.72	-2.21	-1.55
Q9UPP5	Uncharacterized protein KIAA1107 OS=Homo sapiens GN=KIAA1107 PE=1 SV=2 - [K1107_HUMAN]	5.46	1	7	7	11	1.76	1.69	1.74	1.67	0.33	0.30	0.05	0.16	0.18	0.21	0.43	0.53	-1.42	-1.43	-1.36	-1.65	-1.62	-1.40	-1.38	-1.56
Q14168	MAGUK p55 subfamily member 2 OS=Homo sapiens GN=MPP2 PE=1 SV=3 - [MPP2_HUMAN]	43.58	1	22	22	44	2.23	2.19	2.31	2.30	0.22	0.05	0.52	0.45	0.34	0.45	0.23	0.18	-1.65	-1.87	-2.01	-1.67	-1.56	-1.96	-2.02	-1.56
P29323	Ephrin type-B receptor 2 OS=Homo sapiens GN=EPHB2 PE=1 SV=5 - [EPHB2_HUMAN]	5.40	5	2	5	8	2.29	2.72	2.17	2.60	-0.32	0.11	0.55	0.98	0.50	0.08	0.12	0.55	-1.68	-2.16	-2.05	-2.18	-2.06	-2.62	-2.51	-1.56
Q9UF11	Pleckstrin homology domain-containing family B member 1 OS=Homo sapiens GN=PLEKHB1 PE=1 SV=1 - [PKHB1_HUMAN]	23.87	1	4	4	23	1.90	1.64	1.95	1.77	-0.30	-0.27	0.32	0.12	-0.04	0.01	-0.37	-0.77	-1.45	-2.06	-2.11	-1.98	-2.12	-2.29	-2.34	-1.56
Q14141	Septin-6 OS=Homo sapiens GN=SEPT6 PE=1 SV=4 - [SEPT6_HUMAN]	38.02	2	5	14	52	1.80	1.15	1.76	1.55	-0.25	-0.87	0.34	0.08	-0.05	0.31	-0.13	-0.16	-1.01	-1.27	-1.89	-1.14	-1.25	-1.45	-1.56	-1.56

Q8NFZ4	Neuroligin-2 OS=Homo sapiens GN=NLGN2 PE=1 SV=1 - [NLGN2_HUMAN]	13.17	1	7	9	15	1.63	1.81	1.84	1.66	0.31	0.30	0.11	0.24	0.52	0.30	0.19	0.45	-1.73	-1.31	-1.59	-1.54	-1.37	-1.34	-1.40	-1.57
P17174	Aspartate aminotransferase, cytoplasmic OS=Homo sapiens GN=GOT1 PE=1 SV=3 - [AATC_HUMAN]	53.51	1	19	19	65	1.88	1.91	2.00	2.27	0.32	0.34	0.63	0.70	0.41	0.31	-0.04	0.04	-1.15	-2.05	-2.23	-1.66	-1.86	-1.59	-1.95	-1.59
Q9NZN1	Interleukin-1 receptor accessory protein-like 1 OS=Homo sapiens GN=IL1RAPL1 PE=1 SV=2 - [IRPL1_HUMAN]	4.17	1	2	2	3	3.44	2.29	3.52	2.24	0.83	-0.05	1.71	0.59	0.68	1.52	1.17	0.33	-1.68	-1.95	-2.05	-1.89	-1.52	-2.62	-2.30	-1.59
P02689	Myelin P2 protein OS=Homo sapiens GN=PMP2 PE=1 SV=3 - [MYP2_HUMAN]	37.12	2	4	5	10	2.25	1.95	2.40	2.16	-0.01	-0.17	0.64	0.38	0.18	0.17	0.19	-0.12	-1.57	-1.97	-2.13	-2.13	-2.31	-2.06	-2.26	-1.59
Q53HC0	Coiled-coil domain-containing protein 92 OS=Homo sapiens GN=CCDC92 PE=1 SV=2 - [CCD92_HUMAN]	24.77	1	5	5	8	1.38	1.40	1.32	1.34	-0.46	-0.44	-0.34	-0.32	-0.12	-0.13	-0.30	-0.28	-1.67	-1.67	-1.61	-1.48	-1.42	-1.86	-1.79	-1.60
C9J069	Uncharacterized protein C9orf172 OS=Homo sapiens GN=C9orf172 PE=3 SV=1 - [CI172_HUMAN]	6.05	1	3	3	4	2.51	2.43	2.57	2.49	0.49	0.41	0.90	0.83	0.56	0.64	0.60	0.52	-1.55	-1.90	-1.97	-1.84	-1.89	-2.03	-2.09	-1.60
O60299	Leucine zipper putative tumor suppressor 3 OS=Homo sapiens GN=LZTS3 PE=2 SV=1 - [LZTS3_HUMAN]	6.09	1	3	3	5	2.25	2.38	2.17	2.30	-0.66	-0.53	0.50	0.64	0.15	0.02	-0.40	-0.27	-1.69	-2.65	-2.57	-2.20	-2.12	-2.93	-2.85	-1.60
Q8TAE6	Protein phosphatase 1 regulatory subunit 14C OS=Homo sapiens GN=PPP1R14C PE=1 SV=3 - [PP14C_HUMAN]	10.30	1	1	1	2	0.94	0.93	1.73	1.72	-0.28	-0.29	0.06	0.05	-0.65	-0.64	-0.68	-0.69	-0.82	-1.62	-2.41	-1.55	-2.33	-1.23	-2.02	-1.61

Q9BZC1	CUGBP Elav-like family member 4 OS=Homo sapiens GN=CELF4 PE=1 SV=1 - [CELF4_HUMAN]	3.91	2	2	2	2	1.60	1.76	2.27	2.43	-0.71	-0.56	0.60	0.76	-0.04	-0.19	-0.09	0.06	-0.95	-1.69	-2.36	-1.77	-2.43	-2.33	-3.00	-1.61
O95294	RasGAP-activating-like protein 1 OS=Homo sapiens GN=RASAL1 PE=1 SV=3 - [RASL1_HUMAN]	2.49	1	2	2	3	1.77	1.79	1.64	1.66	-0.74	-0.73	-0.03	-0.01	0.07	0.06	-0.32	-0.31	-1.74	-2.09	-1.97	-1.68	-1.55	-2.53	-2.40	-1.61
A5YM72	Carnosine synthase 1 OS=Homo sapiens GN=CARNS1 PE=1 SV=3 - [CRNS1_HUMAN]	8.59	1	6	6	7	1.32	2.13	1.38	2.03	-0.23	-0.05	0.05	0.11	0.05	-0.20	-0.32	-0.22	-1.88	-1.93	-1.96	-2.04	-1.94	-2.20	-1.88	-1.63
A5PKW4	PH and SEC7 domain-containing protein 1 OS=Homo sapiens GN=PSD PE=1 SV=2 - [PSD1_HUMAN]	2.25	1	2	2	3	1.79	1.64	1.75	1.61	0.20	0.04	0.06	-0.09	0.35	0.50	-0.26	-0.41	-1.68	-2.04	-2.01	-1.26	-1.22	-1.61	-1.58	-1.63
Q9UI15	Transgelin-3 OS=Homo sapiens GN=TAGLN3 PE=1 SV=2 - [TAGL3_HUMAN]	66.83	1	11	12	43	1.35	1.38	1.57	1.61	0.16	0.15	-0.12	-0.15	-0.17	-0.22	-0.43	-0.45	-1.27	-1.70	-1.97	-1.50	-1.80	-1.26	-1.51	-1.64
Q9NZ53	Podocalyxin-like protein 2 OS=Homo sapiens GN=PODXL2 PE=1 SV=1 - [PDXL2_HUMAN]	4.79	1	2	2	3	1.39	1.54	1.52	1.67	0.10	0.25	-0.18	-0.03	0.39	0.24	0.50	0.65	-1.52	-0.88	-1.01	-1.12	-1.24	-1.31	-1.44	-1.64
Q8IXJ6	NAD-dependent protein deacetylase sirtuin-2 OS=Homo sapiens GN=SIRT2 PE=1 SV=2 - [SIR2_HUMAN]	42.93	1	13	13	38	2.40	2.63	2.59	2.54	-0.32	-0.42	0.77	0.83	0.31	0.20	-0.20	-0.07	-1.57	-2.44	-2.53	-2.17	-2.08	-2.81	-3.00	-1.65
P29218	Inositol monophosphatase 1 OS=Homo sapiens GN=IMPA1 PE=1 SV=1 - [IMPA1_HUMAN]	42.24	1	11	11	24	1.89	1.93	2.05	2.10	0.13	0.26	0.52	0.52	0.33	0.09	-0.24	-0.17	-1.41	-2.03	-2.35	-1.58	-1.95	-1.68	-1.97	-1.65

P11169	Solute carrier family 2, facilitated glucose transporter member 3 OS=Homo sapiens GN=SLC2A3 PE=1 SV=1 - [GTR3_HUMAN]	10.69	2	5	6	12	1.25	0.85	1.52	1.23	0.15	-0.04	-0.11	-0.28	-0.28	-0.05	0.27	0.12	-1.32	-0.85	-1.14	-1.35	-1.59	-1.04	-1.45	-1.65
Q13153	Serine/threonine-protein kinase PAK 1 OS=Homo sapiens GN=PAK1 PE=1 SV=2 - [PAK1_HUMAN]	34.13	1	7	15	36	1.15	1.13	1.43	1.11	-0.64	-1.00	-0.61	-0.55	-1.04	-0.88	-1.04	-1.32	-1.53	-2.18	-2.47	-2.00	-2.21	-1.87	-2.31	-1.65
Q9C026	E3 ubiquitin-protein ligase TRIM9 OS=Homo sapiens GN=TRIM9 PE=1 SV=1 - [TRIM9_HUMAN]	4.37	1	3	3	4	1.68	1.74	1.54	1.60	0.80	0.98	0.03	0.09	0.13	0.07	-0.36	-0.30	-1.31	-2.03	-1.90	-1.89	-2.14	-0.21	-0.15	-1.65
Q05586	Glutamate receptor ionotropic, NMDA 1 OS=Homo sapiens GN=GRIN1 PE=1 SV=1 - [NMDZ1_HUMAN]	7.25	1	7	7	13	2.14	1.91	2.27	1.95	-0.34	-0.11	0.28	0.65	0.30	0.51	0.59	0.08	-1.45	-1.59	-1.87	-1.51	-1.92	-1.67	-2.08	-1.65
Q05639	Elongation factor 1-alpha 2 OS=Homo sapiens GN=EEF1A2 PE=1 SV=1 - [EF1A2_HUMAN]	41.25	1	5	15	71	2.14	2.25	2.62	2.60	-0.12	0.15	0.91	0.89	0.60	0.48	0.33	0.23	-1.53	-1.98	-2.24	-1.38	-1.83	-2.20	-2.21	-1.66
Q8TD30	Alanine aminotransferase 2 OS=Homo sapiens GN=GPT2 PE=1 SV=1 - [ALAT2_HUMAN]	3.63	1	2	2	3	1.90	2.14	1.60	1.85	0.43	0.67	-0.11	0.13	0.88	0.64	0.42	0.66	-1.96	-1.47	-1.19	-1.23	-0.93	-1.48	-1.19	-1.66
Q8N5V2	Ephexin-1 OS=Homo sapiens GN=NGEF PE=2 SV=2 - [NGEF_HUMAN]	13.66	1	8	8	8	1.60	1.44	1.78	1.44	-0.29	-0.47	0.17	-0.27	-0.24	0.13	-0.29	-0.36	-1.38	-1.95	-1.95	-1.52	-1.73	-1.91	-1.92	-1.68
Q8N5S9	Calcium/calmodulin-dependent protein kinase kinase 1 OS=Homo sapiens GN=CAMKK1 PE=1 SV=2 - [KKCC1_HUMAN]	20.00	1	8	8	14	2.03	2.03	2.45	2.38	-0.03	-0.06	0.53	0.64	0.36	0.45	0.13	0.05	-1.34	-1.68	-2.09	-1.52	-1.90	-2.05	-2.35	-1.68

Q92736	Ryanodine receptor 2 OS=Homo sapiens GN=RYR2 PE=1 SV=3 - [RYR2_HUMAN]	11.94	2	42	44	68	1.95	1.92	2.12	2.19	-0.25	-0.31	0.42	0.38	0.07	0.23	0.10	0.05	-1.59	-1.82	-2.07	-1.68	-1.86	-2.24	-2.49	-1.69
Q16653	Myelin-oligodendrocyte glycoprotein OS=Homo sapiens GN=MOG PE=1 SV=2 - [MOG_HUMAN]	27.94	1	8	8	55	2.74	2.40	2.70	2.29	-0.28	-0.43	0.77	0.55	0.16	0.38	0.33	0.04	-1.83	-2.35	-2.13	-2.07	-2.01	-2.65	-2.95	-1.69
Q92561	Phytanoyl-CoA hydroxylase-interacting protein OS=Homo sapiens GN=PHYHIP PE=1 SV=1 - [PHYIP_HUMAN]	34.24	1	8	9	22	2.73	2.75	3.03	3.16	-0.14	0.13	1.29	1.38	0.67	0.67	0.70	0.49	-1.46	-2.27	-2.47	-2.08	-2.40	-2.91	-3.11	-1.69
P09543	2',3'-cyclic-nucleotide 3'-phosphodiesterase OS=Homo sapiens GN=CNP PE=1 SV=2 - [CN37_HUMAN]	64.37	1	32	33	238	2.17	2.10	2.38	2.37	-0.28	-0.35	0.65	0.58	0.21	0.30	0.06	0.00	-1.40	-2.17	-2.34	-1.93	-2.04	-2.37	-2.62	-1.70
Q13009	T-lymphoma invasion and metastasis-inducing protein 1 OS=Homo sapiens GN=TIAM1 PE=1 SV=2 - [TIAM1_HUMAN]	3.71	1	4	4	7	1.75	1.61	1.69	1.55	0.03	-0.11	-0.07	-0.21	-0.39	-0.24	-0.31	-0.46	-1.77	-2.05	-2.01	-1.96	-1.90	-1.73	-1.68	-1.70
Q96Q04	Serine/threonine-protein kinase LMTK3 OS=Homo sapiens GN=LMTK3 PE=2 SV=2 - [LMTK3_HUMAN]	2.12	1	2	2	3	1.49	1.26	1.60	1.37	0.03	-0.21	-0.18	-0.41	-0.30	-0.06	-0.10	-0.34	-1.61	-1.58	-1.70	-1.52	-1.63	-1.48	-1.59	-1.72
Q96JE9	Microtubule-associated protein 6 OS=Homo sapiens GN=MAP6 PE=1 SV=2 - [MAP6_HUMAN]	70.97	1	45	45	131	2.01	1.88	2.27	2.19	0.29	0.27	0.51	0.46	0.22	0.27	0.10	0.01	-1.45	-2.10	-2.32	-1.77	-2.04	-1.67	-2.06	-1.72

P14867	Gamma-aminobutyric acid receptor subunit alpha 1 OS=Homo sapiens GN=GABRA1 PE=1 SV=3 - [GBRA1_HUMAN]	26.54	3	9	12	37	1.74	1.75	1.81	1.92	-0.27	-0.50	0.39	0.22	-0.41	-0.25	-0.17	-0.30	-1.50	-2.15	-2.31	-1.99	-2.16	-2.61	-2.47	-1.72
Q9H6F2	Trimeric intracellular cation channel type A OS=Homo sapiens GN=TMEM38A PE=1 SV=1 - [TM38A_HUMAN]	3.34	1	1	1	1	1.42	1.44	1.86	1.88	-0.35	-0.33	0.07	0.09	-0.16	-0.18	-0.38	-0.37	-1.29	-1.80	-2.24	-1.56	-2.00	-1.78	-2.22	-1.73
Q9HCH3	Copine-5 OS=Homo sapiens GN=CPNE5 PE=1 SV=2 - [CPNE5_HUMAN]	26.14	1	6	13	28	2.09	1.86	2.49	2.31	-0.18	-0.37	0.47	0.25	0.01	0.24	0.44	0.24	-1.82	-1.42	-1.86	-1.82	-1.98	-2.48	-2.47	-1.73
Q9NUQ9	Protein FAM49B OS=Homo sapiens GN=FAM49B PE=1 SV=1 - [FA49B_HUMAN]	43.21	1	7	10	30	1.93	2.41	2.32	2.39	0.11	0.29	0.50	0.67	0.32	0.07	0.01	0.03	-1.73	-2.08	-2.05	-1.95	-2.15	-2.06	-2.45	-1.73
Q9NY65	Tubulin alpha 8 chain OS=Homo sapiens GN=TUBA8 PE=1 SV=1 - [TBA8_HUMAN]	43.88	2	5	17	669	2.22	2.42	2.53	2.71	-0.06	-0.19	0.64	1.01	0.57	0.42	0.00	0.16	-1.61	-2.18	-2.49	-1.87	-2.13	-2.59	-3.12	-1.75
Q09470	Potassium voltage-gated channel subfamily A member 1 OS=Homo sapiens GN=KCNA1 PE=1 SV=2 - [KCNA1_HUMAN]	12.53	2	4	5	7	1.71	1.32	1.98	1.59	0.22	-0.17	0.38	0.07	0.08	0.47	0.08	0.10	-1.43	-1.62	-1.90	-1.46	-1.73	-0.93	-1.04	-1.76
P28472	Gamma-aminobutyric acid receptor subunit beta-3 OS=Homo sapiens GN=GABRB3 PE=1 SV=1 - [GBRB3_HUMAN]	9.09	1	2	3	5	1.67	1.75	1.50	1.59	-0.20	-0.12	-0.29	-0.21	0.29	0.21	-0.34	-0.26	-1.89	-2.00	-1.84	-1.63	-1.52	-1.88	-1.72	-1.77
P24310	Cytochrome c oxidase subunit 7A1, mitochondrial OS=Homo sapiens GN=COX7A1 PE=1 SV=2 - [CX7A1_HUMAN]	16.46	1	1	1	3	1.58	1.49	1.68	1.59	-0.01	-0.11	-0.16	-0.25	-0.29	-0.19	0.27	0.17	-1.68	-1.31	-1.42	-1.74	-1.84	-1.61	-1.71	-1.78

P60201	Myelin proteolipid protein OS=Homo sapiens GN=PLP1 PE=1 SV=2 - [MYPR_HUMAN]	23.47	1	7	7	76	2.05	1.96	2.42	2.22	-0.53	-0.70	0.57	0.38	-0.04	0.07	-0.34	-0.41	-1.50	-2.38	-2.74	-2.10	-2.35	-2.65	-3.12	-1.78
Q8N987	N-terminal EF-hand calcium-binding protein 1 OS=Homo sapiens GN=NECAB1 PE=1 SV=1 - [NECA1_HUMAN]	15.67	1	5	5	9	1.19	1.58	1.59	1.64	-0.22	-0.15	-0.04	-0.06	0.04	-0.12	-0.17	0.05	-0.89	-1.36	-1.78	-1.28	-1.75	-1.55	-1.90	-1.79
Q68DU8	BTB/POZ domain-containing protein KCTD16 OS=Homo sapiens GN=KCTD16 PE=2 SV=1 - [KCD16_HUMAN]	30.84	1	13	13	22	2.42	2.37	2.69	2.61	0.07	0.24	0.61	0.85	0.56	0.45	0.48	0.56	-1.35	-2.18	-2.13	-1.82	-1.91	-2.37	-2.51	-1.79
P23515	Oligodendrocyte-myelin glycoprotein OS=Homo sapiens GN=OMG PE=1 SV=2 - [OMGP_HUMAN]	18.18	1	8	8	21	2.24	2.27	2.48	2.59	-0.14	-0.14	0.73	0.79	0.51	0.34	0.40	0.43	-1.35	-1.73	-2.07	-1.70	-1.84	-2.41	-2.80	-1.79
P78357	Contactin-associated protein 1 OS=Homo sapiens GN=CNTNAP1 PE=1 SV=1 - [CNTP1_HUMAN]	22.18	1	28	28	77	2.53	2.41	2.54	2.57	0.11	0.05	0.81	0.76	0.55	0.45	0.46	0.56	-1.66	-1.79	-2.06	-1.80	-1.72	-2.37	-2.39	-1.80
P07197	Neurofilament medium polypeptide OS=Homo sapiens GN=NEFM PE=1 SV=3 - [NFM_HUMAN]	56.55	1	46	50	385	2.04	1.98	2.42	2.40	-0.46	-0.57	0.51	0.51	-0.06	-0.03	-0.20	-0.27	-1.43	-2.18	-2.48	-2.05	-2.37	-2.52	-2.81	-1.80
Q14576	ELAV-like protein 3 OS=Homo sapiens GN=ELAVL3 PE=1 SV=3 - [ELAV3_HUMAN]	31.88	1	5	10	16	2.05	2.16	2.66	2.96	0.16	0.43	0.79	1.07	0.74	0.74	0.75	0.77	-0.92	-1.27	-2.18	-1.52	-2.18	-1.69	-2.58	-1.80
P16949	Stathmin OS=Homo sapiens GN=STMN1 PE=1 SV=3 - [STMN1_HUMAN]	46.98	2	6	9	75	1.86	1.75	2.18	2.28	-0.38	-0.52	0.18	0.29	-0.39	-0.38	-0.83	-0.61	-1.36	-2.56	-2.88	-1.96	-2.23	-1.90	-2.38	-1.81

Q96PE5	Opalin OS=Homo sapiens GN=OPALIN PE=2 SV=1 - [OPALI_HUMAN]	22.70	1	2	2	10	2.24	1.78	2.32	1.90	-0.56	-0.35	0.70	0.26	-0.63	-0.05	-0.01	-0.34	-1.60	-1.91	-1.94	-2.23	-2.23	-2.83	-2.69	-1.82
O94811	Tubulin polymerization-promoting protein OS=Homo sapiens GN=TPPP PE=1 SV=1 - [TPPP_HUMAN]	43.84	1	8	8	43	1.68	1.80	1.77	1.96	0.03	-0.03	0.12	0.10	0.06	-0.01	-0.05	0.04	-1.54	-1.81	-2.00	-1.77	-1.74	-1.80	-1.76	-1.84
Q6NV74	Uncharacterized protein KIAA1211-like OS=Homo sapiens GN=KIAA1211L PE=2 SV=3 - [K121L_HUMAN]	13.10	1	6	6	12	1.96	2.05	2.12	2.37	-0.06	-0.03	0.26	0.47	-0.28	-0.07	-0.21	-0.17	-1.42	-2.14	-2.47	-2.25	-2.55	-2.13	-2.35	-1.84
Q7Z6L0	Proline-rich transmembrane protein 2 OS=Homo sapiens GN=PRRT2 PE=1 SV=1 - [PRRT2_HUMAN]	42.94	1	6	6	34	2.51	2.42	2.76	2.54	0.10	-0.04	0.79	0.92	0.58	0.57	0.40	0.27	-1.62	-2.22	-2.29	-1.99	-2.25	-2.66	-2.73	-1.84
Q9UFN0	Protein NipSnap homolog 3A OS=Homo sapiens GN=NIPSNAP3A PE=1 SV=2 - [NPS3A_HUMAN]	16.60	2	4	4	7	1.60	1.91	1.95	2.25	-0.69	-0.39	0.01	0.31	-0.47	-0.77	-0.94	-0.42	-1.54	-2.21	-3.13	-2.35	-2.69	-2.31	-2.66	-1.88
Q9GZV7	Hyaluronan and proteoglycan link protein 2 OS=Homo sapiens GN=HAPLN2 PE=1 SV=1 - [HPLN2_HUMAN]	36.47	1	10	10	26	2.53	2.47	2.75	2.68	-0.02	-0.11	0.80	0.79	0.38	0.46	0.00	-0.25	-1.74	-2.57	-2.98	-1.98	-2.41	-2.60	-2.86	-1.88
P04216	Thy-1 membrane glycoprotein OS=Homo sapiens GN=THY1 PE=1 SV=2 - [THY1_HUMAN]	9.94	1	2	2	5	1.52	1.61	1.79	1.88	0.15	0.24	-0.17	-0.07	-0.12	-0.21	-0.06	0.03	-1.63	-1.57	-1.85	-1.70	-1.97	-1.38	-1.65	-1.89
O75363	Breast carcinoma-amplified sequence 1 OS=Homo sapiens GN=BCAS1 PE=1 SV=2 - [BCAS1_HUMAN]	23.29	1	9	9	23	1.98	1.67	2.52	2.07	0.02	-0.25	0.87	0.49	-0.06	-0.07	-0.21	-0.28	-1.48	-2.05	-2.50	-2.04	-2.45	-2.89	-3.36	-1.90

Q96A23	Copine-4 OS=Homo sapiens GN=CPNE4 PE=2 SV=1 - [CPNE4_HUMAN]	15.44	1	6	7	13	2.39	2.32	2.58	2.29	0.13	0.11	0.65	0.91	0.54	0.52	0.43	0.70	-1.64	-1.83	-1.97	-1.91	-2.19	-2.54	-2.85	-1.90
Q09428	ATP-binding cassette sub-family C member 8 OS=Homo sapiens GN=ABCC8 PE=1 SV=6 - [ABCC8_HUMAN]	0.63	1	1	1	1	1.77	1.65	1.84	1.72	-0.10	-0.22	-0.12	-0.24	-0.03	0.10	0.45	0.33	-1.83	-1.31	-1.39	-1.64	-1.71	-1.88	-1.96	-1.90
O14810	Complexin-1 OS=Homo sapiens GN=CPLX1 PE=1 SV=1 - [CPLX1_HUMAN]	50.75	1	4	4	18	1.85	2.02	2.25	2.23	-0.11	-0.45	0.48	0.43	-0.38	-0.32	-0.46	-0.52	-1.49	-2.31	-2.71	-1.94	-2.57	-2.17	-2.64	-1.90
Q96T17	MAP7 domain-containing protein 2 OS=Homo sapiens GN=MAP7D2 PE=1 SV=2 - [MA7D2_HUMAN]	5.60	1	3	4	5	1.01	1.01	1.47	1.47	-0.02	-0.02	-0.44	-0.42	-0.39	-0.45	-0.56	-0.51	-1.37	-1.46	-1.93	-1.17	-1.76	-1.20	-1.51	-1.91
P20472	Parvalbumin alpha OS=Homo sapiens GN=PVALB PE=1 SV=2 - [PRVA_HUMAN]	57.27	1	5	5	9	2.22	2.21	2.78	2.61	0.54	0.67	0.64	0.62	0.75	0.61	0.09	0.47	-1.33	-1.88	-2.69	-1.47	-1.98	-1.38	-2.10	-1.91
P20916	Myelin-associated glycoprotein OS=Homo sapiens GN=MAG PE=1 SV=1 - [MAG_HUMAN]	24.60	1	13	13	30	2.59	2.77	2.64	2.75	0.07	0.15	0.83	0.75	0.46	0.55	0.10	0.09	-1.96	-2.60	-2.68	-2.08	-2.16	-2.36	-2.47	-1.92
O00746	Nucleoside diphosphate kinase, mitochondrial OS=Homo sapiens GN=NME4 PE=1 SV=1 - [NDKM_HUMAN]	4.28	1	1	1	1	1.82	1.87	1.75	1.80	-0.78	-0.73	-0.24	-0.19	-0.23	-0.27	-0.20	-0.15	-2.00	-2.01	-1.95	-2.06	-1.99	-2.61	-2.54	-1.93
P07196	Neurofilament light polypeptide OS=Homo sapiens GN=NEFL PE=1 SV=3 - [NFL_HUMAN]	59.67	1	29	31	342	2.35	2.37	2.48	2.58	-0.46	-0.43	0.52	0.54	0.10	0.04	-0.20	-0.14	-1.73	-2.54	-2.72	-2.41	-2.40	-2.42	-2.75	-1.94

Q8N145	Leucine-rich repeat LGI family member 3 OS=Homo sapiens GN=LGI3 PE=2 SV=1 - [LGI3_HUMAN]	25.73	1	12	12	21	2.19	2.29	2.49	2.57	-0.27	-0.01	0.40	0.48	0.06	0.06	-0.16	-0.16	-1.66	-2.42	-2.54	-1.99	-2.30	-2.48	-2.60	-1.95
Q8TAM6	Ermin OS=Homo sapiens GN=ERMN PE=2 SV=1 - [ERMIN_HUMAN]	29.23	1	7	7	27	2.41	2.61	2.54	2.93	-0.75	-0.75	0.47	0.46	0.05	0.15	0.19	0.04	-1.92	-2.33	-2.55	-2.35	-2.67	-2.04	-2.49	-1.95
Q6F5E8	Leucine-rich repeat-containing protein 16C OS=Homo sapiens GN=RLTPR PE=1 SV=2 - [LR16C_HUMAN]	4.39	1	4	4	6	0.88	1.02	1.25	1.16	0.02	0.28	-0.30	-0.38	0.03	0.36	-0.02	0.00	-1.29	-0.73	-1.19	-1.00	-1.16	-1.03	-0.89	-1.96
P11229	Muscarinic acetylcholine receptor M1 OS=Homo sapiens GN=CHRM1 PE=1 SV=2 - [ACM1_HUMAN]	2.83	1	1	1	2	1.77	1.63	2.40	2.26	-0.85	-0.99	0.35	0.21	0.39	0.54	0.07	-0.08	-1.36	-1.70	-2.33	-1.21	-1.83	-2.64	-3.26	-1.98
Q13875	Myelin-associated oligodendrocyte basic protein OS=Homo sapiens GN=MOBP PE=2 SV=2 - [MOBP_HUMAN]	6.01	1	1	1	10	2.24	2.03	2.09	2.08	-0.85	-0.65	-0.12	-0.09	-0.35	-0.62	-0.83	-0.91	-2.07	-2.68	-2.54	-2.49	-2.27	-2.69	-2.74	-1.99
P12036	Neurofilament heavy polypeptide OS=Homo sapiens GN=NEFH PE=1 SV=4 - [NFH_HUMAN]	36.16	1	26	30	233	2.15	2.07	2.25	2.43	-0.38	-0.50	0.53	0.57	0.15	0.25	-0.32	-0.31	-1.71	-2.73	-2.63	-2.39	-2.35	-2.45	-2.58	-2.01
Q16352	Alpha-internexin OS=Homo sapiens GN=INA PE=1 SV=2 - [AINX_HUMAN]	56.31	1	24	29	321	2.36	2.34	2.38	2.36	-0.70	-0.67	0.36	0.41	0.02	0.02	-0.17	-0.17	-2.01	-2.69	-2.70	-2.39	-2.35	-2.86	-2.82	-2.06
P48539	Purkinje cell protein 4 OS=Homo sapiens GN=PCP4 PE=1 SV=3 - [PCP4_HUMAN]	27.42	1	1	1	5	1.55	1.73	1.75	1.93	0.00	0.17	-0.39	-0.22	0.13	-0.04	-0.68	-0.51	-1.89	-2.23	-2.44	-1.56	-1.76	-1.57	-1.77	-2.08

P02686	Myelin basic protein OS=Homo sapiens GN=MBP PE=1 SV=3 - [MBP_HUMAN]	41.45	1	12	12	338	2.23	2.17	2.33	2.30	-0.29	-0.29	0.31	0.28	-0.06	-0.09	-0.24	-0.29	-1.88	-2.47	-2.57	-2.21	-2.29	-2.49	-2.47	-2.11
P51649	Succinate-semialdehyde dehydrogenase, mitochondrial OS=Homo sapiens GN=ALDH5A1 PE=1 SV=2 - [SSDH_HUMAN]	27.48	1	12	12	32	1.38	1.38	1.70	1.54	-0.38	-0.35	-0.48	-0.45	-0.62	-0.67	-0.47	-0.61	-1.73	-2.00	-2.29	-1.98	-2.26	-1.85	-2.00	-2.18
O43525	Potassium voltage-gated channel subfamily KQT member 3 OS=Homo sapiens GN=KCNQ3 PE=1 SV=2 - [KCNQ3_HUMAN]	1.49	1	1	1	1	1.47	1.67	1.43	1.63	0.29	0.48	-0.82	-0.63	0.09	-0.11	-1.07	-0.88	-2.24	-2.54	-2.51	-1.55	-1.51	-1.20	-1.16	-2.19
Q6PUV4	Complexin-2 OS=Homo sapiens GN=CPLX2 PE=1 SV=2 - [CPLX2_HUMAN]	37.31	1	4	4	9	2.91	1.09	3.56	2.22	0.18	-0.70	1.00	0.25	-0.21	0.09	-0.48	-0.73	-1.66	-3.39	-4.05	-2.46	-3.11	-2.74	-3.40	-2.23
P04439	HLA class I histocompatibility antigen, A-3 alpha chain OS=Homo sapiens GN=HLA-A PE=1 SV=2 - [1A03_HUMAN]	50.68	5	5	14	33	-0.34	-0.30	-0.04	0.20	-1.18	-0.94	-2.45	-2.31	-0.96	-1.09	-0.23	0.02	-2.09	-0.26	-0.25	-1.05	-1.17	-0.70	-1.21	-2.38
Q92686	Neurogranin OS=Homo sapiens GN=NRGN PE=1 SV=1 - [NEUG_HUMAN]	19.23	1	1	1	13	1.21	1.10	2.00	1.77	0.11	-0.11	-0.31	-0.52	0.20	-0.07	-0.08	-0.09	-1.88	-1.28	-2.04	-1.47	-1.84	-1.37	-1.84	-2.40
Q7Z7J9	Calcium/calmodulin-dependent protein kinase II inhibitor 1 OS=Homo sapiens GN=CAMK2N1 PE=1 SV=1 - [CK2N1_HUMAN]	16.67	2	1	1	1	2.01	1.86	2.66	2.50	-0.08	-0.24	-0.10	-0.26	-1.17	-1.00	-0.33	-0.49	-2.06	-2.34	-2.99	-2.99	-3.62	-2.11	-2.75	-2.70
