# Supplementary material for: Systems proteomic analysis reveals that clusterin and tissue inhibitor of metalloproteinases 3 increase in leptomeningeal arteries affected by cerebral amyloid angiopathy
Source: Neuropathol Appl Neurobiol. 2016 Oct 5;43(6):492–504. doi: 10.1111/nan.12342 (PMC5638106; doi:10.1111/nan.12342)
Supplement: Supplementary file 2 — Table S2. Differentially expressed proteins in leptomeningeal arteries from elderly vs. young controls (log2 ratio). [file NAN-43-492-s002.pdf]

Supplementary Table 2. Differentially expressed proteins in leptomeningeal arteries from elderly vs. young controls (log2ratios)

Accession	Description	OLD 1/ YOUNG 1	OLD 1/ YOUNG 2	OLD 2/ YOUNG 1	OLD 2/ YOUNG 2	CAA 1/YOUNG 1	CAA 1/YOUNG 2	CAA 2/YOUNG 1	CAA 2/YOUNG 2	CAA 3/YOUNG 1	CAA 3/YOUNG 2	CAA 4/YOUNG 1	CAA 4/YOUNG 2	CAA 1/OLD 1	CAA 1/OLD 2	CAA 2/OLD 1	CAA 2/OLD 2	CAA 3/OLD 1	CAA 3/OLD 2	CAA 4/OLD 1	CAA 4/OLD 2
Q09666	Neuroblast differentiation-associated protein AHNAK OS=Homo sapiens GN=AHNAK PE=1 SV=2 - [AHNK_HUMAN]	-1.97	-1.96	-1.89	-1.85	-0.80	-0.79	-0.71	-0.70	-0.63	-0.63	-0.09	-0.10	1.34	1.91	1.78	1.39	1.23	1.21	1.05	1.18
P12111	Collagen alpha-3(VI) chain OS=Homo sapiens GN=COL6A3 PE=1 SV=5 - [CO6A3_HUMAN]	-1.90	-1.92	-1.96	-1.95	-0.24	-0.24	-0.56	-0.58	-0.24	-0.25	0.15	0.16	1.38	2.07	2.15	1.69	1.79	1.66	1.71	1.41
P04114	Apolipoprotein B-100 OS=Homo sapiens GN=APOB PE=1 SV=2 - [APOB_HUMAN]	-2.09	-2.09	-2.34	-2.28	-0.03	0.01	-0.67	-0.64	0.01	0.00	0.78	0.84	1.54	2.88	3.15	2.10	2.35	2.05	2.25	1.76
P15924	Desmoplakin OS=Homo sapiens GN=DSP PE=1 SV=3 - [DESP_HUMAN]	-2.60	-2.57	-2.66	-2.68	-0.62	-0.66	-0.94	-0.95	-0.89	-0.88	-0.94	-0.97	1.62	1.69	1.72	1.70	1.83	1.86	1.99	1.83
P21333	Filamin-A OS=Homo sapiens GN=FLNA PE=1 SV=4 - [FLNA_HUMAN]	-2.34	-2.27	-2.47	-2.42	-0.89	-0.86	-1.02	-0.98	-0.83	-0.87	-0.50	-0.45	1.30	1.85	2.06	1.47	1.65	1.44	1.60	1.55
P98160	Basement membrane-specific heparan sulfate proteoglycan core protein OS=Homo sapiens GN=HSPG2 PE=1 SV=4 - [PGBM_HUMAN]	-2.01	-2.00	-2.23	-2.25	-0.12	-0.11	-0.64	-0.62	-0.48	-0.47	-0.62	-0.65	1.45	1.38	1.57	1.58	1.76	1.92	2.14	1.67
P02549	Spectrin alpha chain, erythrocytic 1 OS=Homo sapiens GN=SPTA1 PE=1 SV=5 - [SPTA1_HUMAN]	-1.47	-1.35	-1.69	-1.57	0.32	0.38	0.48	0.55	0.69	0.64	1.25	1.33	1.90	2.69	2.92	2.10	2.30	1.74	1.93	2.22
Q99715	Collagen alpha-1(XII) chain OS=Homo sapiens GN=COL12A1 PE=1 SV=2 - [COCA1_HUMAN]	-2.55	-2.56	-2.84	-2.87	-1.00	-0.96	-1.18	-1.14	-0.93	-0.97	-0.75	-0.73	1.49	1.92	2.09	1.70	1.91	1.76	1.98	1.72
Q01484	Ankyrin-2 OS=Homo sapiens GN=ANK2 PE=1 SV=4 - [ANK2_HUMAN]	1.74	1.81	1.92	1.90	0.04	0.01	0.66	0.67	0.37	0.37	0.21	0.24	-1.01	-1.58	-1.73	-1.31	-1.50	-1.71	-1.87	-1.22

P11277	Spectrin beta chain, erythrocytic OS=Homo sapiens GN=SPTB PE=1 SV=5 - [SPTB1_HUMAN]	-1.20	-1.10	-1.29	-1.27	0.42	0.45	0.47	0.51	0.76	0.75	1.36	1.39	1.73	2.51	2.60	1.95	2.02	1.50	1.64	1.83
P46939	Utrophin OS=Homo sapiens GN=UTRN PE=1 SV=2 - [UTRO_HUMAN]	-1.70	-1.75	-1.76	-1.82	-0.49	-0.52	-0.69	-0.71	-0.62	-0.57	-0.40	-0.43	1.02	1.29	1.44	1.17	1.26	1.15	1.25	1.17
P35555	Fibrillin-1 OS=Homo sapiens GN=FBN1 PE=1 SV=3 - [FBN1_HUMAN]	-2.21	-2.16	-2.51	-2.46	0.37	0.45	0.12	0.18	0.13	0.09	-0.12	-0.07	2.43	2.11	2.36	2.39	2.61	2.55	2.84	2.69
P35749	Myosin-11 OS=Homo sapiens GN=MYH11 PE=1 SV=3 - [MYH11_HUMAN]	-2.78	-2.80	-2.99	-3.01	-1.06	-1.05	-1.22	-1.27	-1.22	-1.19	-1.06	-1.07	1.64	1.76	1.95	1.64	1.86	1.84	1.95	1.82
P22105	Tenascin-X OS=Homo sapiens GN=TNXB PE=1 SV=3 - [TENX_HUMAN]	-2.01	-2.06	-2.35	-2.24	-0.27	-0.24	-1.01	-0.99	-0.76	-0.76	-0.92	-0.93	1.24	1.18	1.44	1.37	1.57	1.85	2.07	1.39
O75369	Filamin-B OS=Homo sapiens GN=FLNB PE=1 SV=2 - [FLNB_HUMAN]	-1.79	-1.73	-1.86	-1.81	-0.61	-0.49	-0.81	-0.72	-0.47	-0.60	-0.17	-0.11	1.14	1.69	1.68	1.28	1.26	1.27	1.25	1.13
Q9Y490	Talin-1 OS=Homo sapiens GN=TLN1 PE=1 SV=3 - [TLN1_HUMAN]	-1.62	-1.64	-1.78	-1.70	-0.64	-0.55	-0.58	-0.54	-0.50	-0.57	-0.35	-0.34	1.14	1.37	1.41	1.13	1.25	1.04	1.14	1.26
Q9NZM1	Myoferlin OS=Homo sapiens GN=MYOF PE=1 SV=1 - [MYOF_HUMAN]	-2.06	-2.08	-2.15	-2.14	-0.05	-0.01	-0.44	-0.41	-0.20	-0.26	0.09	0.07	1.72	2.21	2.27	1.87	1.93	2.07	2.07	1.84
P35579	Myosin-9 OS=Homo sapiens GN=MYH9 PE=1 SV=4 - [MYH9_HUMAN]	-1.83	-1.86	-1.78	-1.73	-0.66	-0.73	-0.94	-0.96	-0.90	-0.87	-0.73	-0.75	0.94	1.07	1.04	0.99	0.92	1.15	1.02	0.90
P01024	Complement C3 OS=Homo sapiens GN=C3 PE=1 SV=2 - [CO3_HUMAN]	-1.66	-1.69	-1.74	-1.71	-0.12	-0.08	-0.78	-0.76	-0.30	-0.30	0.11	0.16	1.09	1.93	1.88	1.53	1.49	1.67	1.67	1.03
P78559	Microtubule-associated protein 1A OS=Homo sapiens GN=MAP1A PE=1 SV=6 - [MAP1A_HUMAN]	1.44	1.50	1.59	1.66	-0.26	-0.20	0.08	0.13	-0.07	-0.11	-0.14	-0.06	-1.27	-1.59	-1.74	-1.55	-1.66	-1.69	-1.93	-1.48
O15230	Laminin subunit alpha-5 OS=Homo sapiens GN=LAMA5 PE=1 SV=8 - [LAMA5_HUMAN]	-1.98	-1.98	-2.08	-2.12	-0.20	-0.25	-0.76	-0.77	-0.61	-0.58	-0.74	-0.77	1.21	1.22	1.41	1.45	1.57	1.74	1.86	1.38

P02751	Fibronectin OS=Homo sapiens GN=FN1 PE=1 SV=4 - [FINC_HUMAN]	-2.13	-2.13	-2.21	-2.25	1.01	1.05	-0.53	-0.50	0.18	0.14	-0.04	0.02	1.68	2.10	2.21	2.32	2.40	3.06	3.24	1.74
O15020	Spectrin beta chain, non-erythrocytic 2 OS=Homo sapiens GN=SPTBN2 PE=1 SV=3 - [SPTN2_HUMAN]	1.59	1.66	1.84	1.80	-0.27	-0.29	0.48	0.47	0.23	0.17	0.04	0.07	-1.20	-1.69	-1.75	-1.49	-1.58	-2.02	-2.09	-1.22
P11137	Microtubule-associated protein 2 OS=Homo sapiens GN=MAP2 PE=1 SV=4 - [MTAP2_HUMAN]	1.48	1.47	1.83	1.77	-0.35	-0.42	0.35	0.28	-0.06	0.00	-0.37	-0.36	-1.14	-1.77	-2.15	-1.48	-1.76	-1.78	-2.10	-1.46
P04275	von Willebrand factor OS=Homo sapiens GN=VWF PE=1 SV=4 - [VWF_HUMAN]	-2.36	-2.28	-2.47	-2.36	-0.75	-0.74	-1.13	-1.10	-1.04	-1.00	-0.89	-0.89	1.16	1.26	1.46	1.19	1.35	1.35	1.57	1.32
Q9UPA5	Protein bassoon OS=Homo sapiens GN=BSN PE=2 SV=4 - [BSN_HUMAN]	1.75	1.79	1.77	1.91	-0.06	-0.05	0.37	0.24	0.21	0.13	-0.14	-0.21	-1.35	-1.97	-1.99	-1.57	-1.69	-1.85	-1.91	-1.38
Q14315	Filamin-C OS=Homo sapiens GN=FLNC PE=1 SV=3 - [FLNC_HUMAN]	-1.93	-1.85	-2.05	-1.95	-0.18	-0.12	-0.76	-0.71	-0.35	-0.43	-0.12	-0.07	1.27	1.79	1.86	1.63	1.63	1.78	1.86	1.36
P18206	Vinculin OS=Homo sapiens GN=VCL PE=1 SV=4 - [VINC_HUMAN]	-2.32	-2.29	-2.45	-2.36	-0.56	-0.52	-0.89	-0.89	-0.67	-0.71	-0.44	-0.44	1.52	1.91	1.99	1.76	1.79	1.83	1.89	1.64
P55268	Laminin subunit beta-2 OS=Homo sapiens GN=LAMB2 PE=1 SV=2 - [LAMB2_HUMAN]	-1.75	-1.77	-1.97	-1.93	-0.32	-0.37	-0.77	-0.80	-0.66	-0.63	-0.75	-0.81	1.01	1.00	1.21	1.23	1.38	1.45	1.61	1.25
P11047	Laminin subunit gamma-1 OS=Homo sapiens GN=LAMC1 PE=1 SV=3 - [LAMC1_HUMAN]	-2.13	-2.18	-2.13	-2.13	-0.44	-0.45	-0.89	-0.91	-0.81	-0.76	-0.76	-0.81	1.26	1.23	1.33	1.32	1.35	1.67	1.66	1.30
P16157	Ankyrin-1 OS=Homo sapiens GN=ANK1 PE=1 SV=3 - [ANK1_HUMAN]	-1.40	-1.39	-1.52	-1.43	0.39	0.40	0.43	0.43	0.68	0.63	1.25	1.31	1.91	2.64	2.76	2.07	2.15	1.72	1.88	2.01
Q9Y4I1	Unconventional myosin-Va OS=Homo sapiens GN=MYO5A PE=1 SV=2 - [MYO5A_HUMAN]	1.63	1.73	1.79	1.86	-0.14	-0.10	0.54	0.63	0.20	0.23	0.02	0.08	-1.07	-1.67	-1.84	-1.38	-1.67	-1.82	-2.04	-1.26

P46940	Ras GTPase-activating-like protein IQGAP1 OS=Homo sapiens GN=IQGAP1 PE=1 SV=1 - [IQGA1_HUMAN]	-1.52	-1.48	-1.60	-1.54	-0.16	-0.13	-0.42	-0.43	-0.33	-0.33	-0.29	-0.23	1.10	1.27	1.27	1.22	1.25	1.31	1.38	1.14
P24043	Laminin subunit alpha-2 OS=Homo sapiens GN=LAMA2 PE=1 SV=4 - [LAMA2_HUMAN]	-1.36	-1.30	-1.49	-1.44	-0.82	-0.79	-0.86	-0.80	-0.66	-0.71	-0.39	-0.38	0.54	1.04	1.01	0.59	0.70	0.54	0.62	0.63
Q05707	Collagen alpha-1(XIV) chain OS=Homo sapiens GN=COL14A1 PE=1 SV=3 - [COEA1_HUMAN]	-3.06	-3.09	-3.32	-3.30	-0.98	-0.97	-1.13	-1.09	-1.18	-1.19	-1.43	-1.42	2.02	1.76	1.91	2.02	2.19	2.23	2.40	2.25
Q9HBL0	Tensin-1 OS=Homo sapiens GN=TNS1 PE=1 SV=2 - [TENS1_HUMAN]	-1.66	-1.68	-1.71	-1.62	-0.53	-0.48	-0.48	-0.47	-0.34	-0.45	-0.18	-0.14	1.21	1.55	1.61	1.25	1.31	1.07	1.08	1.26
O00468	Agrin OS=Homo sapiens GN=AGRN PE=1 SV=5 - [AGRIN_HUMAN]	-1.39	-1.26	-1.45	-1.31	-0.31	-0.19	-0.73	-0.59	-0.45	-0.54	-0.41	-0.33	0.69	0.97	1.03	0.87	0.94	1.07	1.16	0.75
P07197	Neurofilament medium polypeptide OS=Homo sapiens GN=NEFM PE=1 SV=3 - [NFM_HUMAN]	2.04	1.98	2.42	2.40	-0.46	-0.57	0.51	0.51	-0.06	-0.03	-0.20	-0.27	-1.43	-2.18	-2.48	-2.05	-2.37	-2.52	-2.81	-1.80
Q8WXH0	Nesprin-2 OS=Homo sapiens GN=SYNE2 PE=1 SV=3 - [SYNE2_HUMAN]	-1.23	-1.26	-1.84	-1.70	-0.17	-0.15	-0.55	-0.54	-0.27	-0.32	-0.27	-0.24	0.64	0.90	1.25	0.97	1.36	1.08	1.35	1.16
Q96JE9	Microtubule-associated protein 6 OS=Homo sapiens GN=MAP6 PE=1 SV=2 - [MAP6_HUMAN]	2.01	1.88	2.27	2.19	0.29	0.27	0.51	0.46	0.22	0.27	0.10	0.01	-1.45	-2.10	-2.32	-1.77	-2.04	-1.67	-2.06	-1.72
O00159	Unconventional myosin-Ic OS=Homo sapiens GN=MYO1C PE=1 SV=4 - [MYO1C_HUMAN]	-2.44	-2.35	-2.44	-2.36	-0.42	-0.44	-0.71	-0.76	-0.55	-0.58	-0.53	-0.59	1.63	1.88	1.92	1.81	1.78	1.93	1.85	1.65
P08670	Vimentin OS=Homo sapiens GN=VIM PE=1 SV=4 - [VIME_HUMAN]	-2.39	-2.36	-2.61	-2.63	0.10	0.12	-0.34	-0.30	-0.18	-0.21	-0.20	-0.20	2.05	2.21	2.45	2.23	2.47	2.42	2.67	2.31
Q12955	Ankyrin-3 OS=Homo sapiens GN=ANK3 PE=1 SV=3 - [ANK3_HUMAN]	1.78	1.74	1.96	1.82	-0.19	-0.24	0.41	0.34	0.04	0.11	-0.08	-0.18	-1.31	-1.79	-1.98	-1.71	-1.76	-1.81	-1.98	-1.47

Q92736	Ryanodine receptor 2 OS=Homo sapiens GN=RYR2 PE=1 SV=3 - [RYR2_HUMAN]	1.95	1.92	2.12	2.19	-0.25	-0.31	0.42	0.38	0.07	0.23	0.10	0.05	-1.59	-1.82	-2.07	-1.68	-1.86	-2.24	-2.49	-1.69
O94856	Neurofascin OS=Homo sapiens GN=NFASC PE=1 SV=4 - [NFASC_HUMAN]	1.59	1.65	1.68	1.75	-0.25	-0.31	0.43	0.45	0.09	0.16	-0.03	-0.06	-1.17	-1.68	-1.62	-1.46	-1.54	-1.83	-1.95	-1.23
P02768	Serum albumin OS=Homo sapiens GN=ALB PE=1 SV=2 - [ALBU_HUMAN]	-1.73	-1.71	-1.56	-1.54	-0.72	-0.67	-0.85	-0.80	-0.87	-0.95	-1.48	-1.40	0.95	0.29	0.09	0.84	0.66	1.06	0.84	0.80
P01023	Alpha-2-macroglobulin OS=Homo sapiens GN=A2M PE=1 SV=3 - [A2MG_HUMAN]	-1.61	-1.59	-1.57	-1.52	-0.12	-0.10	-0.62	-0.60	-0.45	-0.52	-0.58	-0.54	1.01	1.01	0.94	1.15	1.06	1.43	1.33	0.96
Q9Y6V0	Protein piccolo OS=Homo sapiens GN=PCLO PE=1 SV=4 - [PCLO_HUMAN]	1.53	1.60	1.81	1.82	-0.15	-0.24	0.60	0.56	0.34	0.35	0.05	0.05	-1.00	-1.52	-1.85	-1.37	-1.67	-1.56	-1.88	-1.26
Q12860	Contactin-1 OS=Homo sapiens GN=CNTN1 PE=1 SV=1 - [CNTN1_HUMAN]	1.61	1.67	1.77	1.71	0.19	0.10	0.59	0.60	0.41	0.42	0.28	0.31	-0.90	-1.45	-1.56	-1.28	-1.25	-1.64	-1.67	-1.06
P02545	Prelamin-A/C OS=Homo sapiens GN=LMNA PE=1 SV=1 - [LMNA_HUMAN]	-1.96	-1.91	-1.99	-1.98	-1.08	-1.07	-1.02	-1.02	-0.93	-0.96	-0.63	-0.62	0.98	1.29	1.35	1.03	1.04	0.90	0.87	1.02
P08603	Complement factor H OS=Homo sapiens GN=CFH PE=1 SV=4 - [CFAH_HUMAN]	-1.81	-1.74	-1.90	-1.78	0.16	0.19	-0.47	-0.34	-0.03	-0.13	0.28	0.35	1.40	2.09	2.08	1.72	1.81	2.00	2.04	1.55
P14136	Glial fibrillary acidic protein OS=Homo sapiens GN=GFAP PE=1 SV=1 - [GFAP_HUMAN]	1.33	1.32	1.40	1.40	0.74	0.73	1.78	1.82	1.46	1.43	1.46	1.45	0.43	0.12	0.13	0.14	0.16	-0.61	-0.63	0.46
O43707	Alpha-actinin-4 OS=Homo sapiens GN=ACTN4 PE=1 SV=2 - [ACTN4_HUMAN]	-2.24	-2.17	-2.30	-2.22	-1.06	-0.96	-1.25	-1.17	-1.11	-1.18	-0.96	-0.92	1.07	1.28	1.37	1.10	1.14	1.22	1.24	1.14
P12109	Collagen alpha-1(VI) chain OS=Homo sapiens GN=COL6A1 PE=1 SV=3 - [CO6A1_HUMAN]	-1.54	-1.61	-1.74	-1.76	-0.27	-0.25	-0.48	-0.49	-0.21	-0.21	0.21	0.23	1.16	1.94	1.96	1.44	1.52	1.35	1.48	1.30

P19367	Hexokinase-1 OS=Homo sapiens GN=HK1 PE=1 SV=3 - [HXK1_HUMAN]	1.71	1.75	1.77	1.83	0.06	0.13	0.64	0.70	0.32	0.30	0.14	0.13	-1.01	-1.51	-1.66	-1.37	-1.44	-1.54	-1.68	-1.00
Q9NQ66	1-phosphatidylinositol 4,5-bisphosphate phosphodiesterase beta-1 OS=Homo sapiens GN=PLCB1 PE=1 SV=1 - [PLCB1_HUMAN]	1.16	1.15	1.28	1.31	-0.03	-0.10	0.28	0.17	-0.02	0.02	-0.37	-0.49	-0.81	-1.53	-1.64	-1.07	-1.27	-1.19	-1.55	-0.98
O95425	Supervillin OS=Homo sapiens GN=SVIL PE=1 SV=2 - [SVIL_HUMAN]	-1.74	-1.81	-2.26	-2.23	-0.13	-0.18	-0.46	-0.45	-0.33	-0.39	-0.15	-0.18	1.54	1.75	2.07	1.65	1.90	1.64	1.99	1.89
Q14112	Nidogen-2 OS=Homo sapiens GN=NID2 PE=1 SV=3 - [NID2_HUMAN]	-1.70	-1.46	-1.84	-1.77	-0.57	-0.44	-1.02	-0.95	-0.64	-0.72	-0.63	-0.65	0.77	1.21	1.21	1.10	1.21	1.19	1.21	0.99
Q07157	Tight junction protein ZO-1 OS=Homo sapiens GN=TJP1 PE=1 SV=3 - [ZO1_HUMAN]	-1.29	-1.47	-1.29	-1.43	-0.03	-0.01	-0.27	-0.33	-0.10	-0.10	0.25	0.13	0.98	1.48	1.57	1.15	1.26	1.16	1.35	1.14
P49588	Alanine--tRNA ligase, cytoplasmic OS=Homo sapiens GN=AARS PE=1 SV=2 - [SYAC_HUMAN]	1.27	1.27	1.39	1.44	0.30	0.28	0.75	0.74	0.42	0.44	0.24	0.21	-0.50	-0.90	-1.13	-0.71	-0.83	-0.91	-1.16	-0.63
O43426	Synaptojanin-1 OS=Homo sapiens GN=SYNJ1 PE=1 SV=2 - [SYNJ1_HUMAN]	1.33	1.36	1.66	1.73	0.23	0.22	0.49	0.51	0.27	0.28	-0.13	-0.12	-0.74	-1.52	-1.68	-1.12	-1.47	-1.19	-1.40	-0.94
Q9H254	Spectrin beta chain, non-erythrocytic 4 OS=Homo sapiens GN=SPTBN4 PE=1 SV=2 - [SPTN4_HUMAN]	1.45	1.56	1.71	1.57	0.12	0.15	0.61	0.59	0.23	0.40	0.16	0.24	-1.20	-1.38	-1.54	-1.23	-1.21	-1.45	-1.57	-1.05
Q8TDJ6	DmX-like protein 2 OS=Homo sapiens GN=DMXL2 PE=1 SV=2 - [DMXL2_HUMAN]	1.28	1.59	1.36	1.34	-0.04	0.19	0.39	0.36	0.32	0.23	0.09	0.08	-0.97	-1.28	-1.21	-0.97	-1.06	-1.28	-1.24	-0.89
P02452	Collagen alpha-1(I) chain OS=Homo sapiens GN=COL1A1 PE=1 SV=5 - [CO1A1_HUMAN]	-3.05	-3.20	-3.12	-3.30	-1.87	-1.98	-2.37	-2.56	-2.31	-2.17	-2.29	-2.48	0.77	0.75	0.87	1.04	1.03	1.18	1.29	0.84
P07996	Thrombospondin-1 OS=Homo sapiens GN=THBS1 PE=1 SV=2 - [TSP1_HUMAN]	-1.47	-1.43	-1.73	-1.55	1.83	1.88	0.56	0.55	1.07	0.92	0.36	0.49	2.39	1.99	2.13	2.51	2.81	3.33	3.61	2.57

P48681	Nestin OS=Homo sapiens GN=NES PE=1 SV=2 - [NEST_HUMAN]	-1.16	-1.15	-1.34	-1.19	-0.25	-0.19	-0.82	-0.73	-0.38	-0.46	-0.39	-0.29	0.47	0.85	0.95	0.76	0.79	0.94	0.99	0.55
P09543	2',3'-cyclic-nucleotide 3'-phosphodiesterase OS=Homo sapiens GN=CNP PE=1 SV=2 - [CN37_HUMAN]	2.17	2.10	2.38	2.37	-0.28	-0.35	0.65	0.58	0.21	0.30	0.06	0.00	-1.40	-2.17	-2.34	-1.93	-2.04	-2.37	-2.62	-1.70
P61764	Syntaxin-binding protein 1 OS=Homo sapiens GN=STXBP1 PE=1 SV=1 - [STXB1_HUMAN]	1.90	1.87	2.12	2.08	-0.09	-0.07	0.76	0.72	0.20	0.36	0.20	0.21	-1.18	-1.68	-1.91	-1.59	-1.78	-1.99	-2.16	-1.32
Q9Y2J2	Band 4.1-like protein 3 OS=Homo sapiens GN=EPB41L3 PE=1 SV=2 - [E41L3_HUMAN]	1.70	1.77	2.02	1.99	0.13	0.05	0.72	0.70	0.26	0.42	0.14	0.18	-0.97	-1.60	-1.92	-1.44	-1.60	-1.71	-2.00	-1.24
Q16363	Laminin subunit alpha-4 OS=Homo sapiens GN=LAMA4 PE=1 SV=4 - [LAMA4_HUMAN]	-1.77	-1.76	-2.30	-2.27	-0.06	-0.12	-0.87	-0.88	-0.61	-0.55	-0.75	-0.71	1.24	1.21	1.52	1.40	1.69	1.74	2.11	1.54
P07942	Laminin subunit beta-1 OS=Homo sapiens GN=LAMB1 PE=1 SV=2 - [LAMB1_HUMAN]	-1.97	-2.08	-2.15	-2.11	0.35	0.26	-0.57	-0.59	-0.36	-0.38	-0.39	-0.40	1.48	1.63	1.68	1.63	1.76	2.18	2.27	1.55
P17600	Synapsin-1 OS=Homo sapiens GN=SYN1 PE=1 SV=3 - [SYN1_HUMAN]	1.43	1.35	1.70	1.57	0.10	0.04	0.56	0.49	0.15	0.29	-0.07	-0.13	-0.86	-1.41	-1.66	-1.16	-1.36	-1.44	-1.54	-1.02
P02788	Lactotransferrin OS=Homo sapiens GN=LTF PE=1 SV=6 - [TRFL_HUMAN]	-2.28	-2.24	-2.34	-2.32	-1.35	-1.17	0.91	1.01	0.16	0.16	-0.30	-0.20	3.49	2.06	2.15	2.68	2.84	0.85	1.30	3.48
P14543	Nidogen-1 OS=Homo sapiens GN=NID1 PE=1 SV=3 - [NID1_HUMAN]	-1.81	-1.76	-1.97	-1.97	-0.16	-0.16	-0.75	-0.73	-0.48	-0.49	-0.44	-0.38	1.03	1.34	1.57	1.32	1.49	1.51	1.66	1.27
P12110	Collagen alpha-2(VI) chain OS=Homo sapiens GN=COL6A2 PE=1 SV=4 - [CO6A2_HUMAN]	-1.48	-1.44	-1.52	-1.48	-0.17	-0.16	-0.45	-0.40	-0.14	-0.16	0.23	0.26	1.15	1.70	1.87	1.28	1.52	1.22	1.34	1.19
Q05193	Dynamin-1 OS=Homo sapiens GN=DNM1 PE=1 SV=2 - [DYN1_HUMAN]	2.07	2.07	2.19	2.12	-0.30	-0.26	0.75	0.81	0.44	0.39	0.23	0.27	-1.33	-1.77	-1.87	-1.67	-1.71	-2.36	-2.21	-1.28

P13637	Sodium/potassium-transporting ATPase subunit alpha-3 OS=Homo sapiens GN=ATP1A3 PE=1 SV=3 - [AT1A3_HUMAN]	1.98	1.90	2.13	2.02	-0.22	-0.08	0.72	0.81	0.43	0.36	0.08	0.21	-1.17	-1.76	-1.81	-1.54	-1.71	-2.00	-2.23	-1.26
P46459	Vesicle-fusing ATPase OS=Homo sapiens GN=NSF PE=1 SV=3 - [NSF_HUMAN]	1.66	1.62	2.02	1.96	-0.23	-0.29	0.74	0.69	0.28	0.27	0.11	0.15	-0.85	-1.56	-1.85	-1.34	-1.59	-1.95	-2.31	-1.19
Q9UHC6	Contactin-associated protein-like 2 OS=Homo sapiens GN=CNTNAP2 PE=1 SV=1 - [CNTP2_HUMAN]	1.34	1.42	1.67	1.60	0.31	0.27	0.34	0.37	0.31	0.26	0.25	0.17	-0.97	-1.26	-1.29	-1.07	-1.21	-1.07	-1.35	-1.04
P08123	Collagen alpha-2(I) chain OS=Homo sapiens GN=COL1A2 PE=1 SV=7 - [CO1A2_HUMAN]	-3.08	-3.40	-3.12	-3.43	-1.89	-2.18	-2.32	-2.62	-2.44	-2.21	-2.25	-2.54	0.76	0.81	0.85	0.91	1.01	1.21	1.29	0.84
P07196	Neurofilament light polypeptide OS=Homo sapiens GN=NEFL PE=1 SV=3 - [NFL_HUMAN]	2.35	2.37	2.48	2.58	-0.46	-0.43	0.52	0.54	0.10	0.04	-0.20	-0.14	-1.73	-2.54	-2.72	-2.41	-2.40	-2.42	-2.75	-1.94
Q14126	Desmoglein-2 OS=Homo sapiens GN=DSG2 PE=1 SV=2 - [DSG2_HUMAN]	-2.43	-2.49	-2.63	-2.52	-0.29	-0.36	-0.76	-0.82	-0.54	-0.52	-0.17	-0.21	1.65	2.09	2.23	1.83	1.92	1.91	2.22	1.72
Q9UMS6	Synaptopodin-2 OS=Homo sapiens GN=SYNPO2 PE=1 SV=2 - [SYNP2_HUMAN]	-2.21	-2.15	-2.60	-2.40	-0.96	-1.02	-1.25	-1.21	-1.03	-1.08	-0.65	-0.60	1.02	1.58	1.66	1.37	1.40	1.28	1.31	1.14
P12814	Alpha-actinin-1 OS=Homo sapiens GN=ACTN1 PE=1 SV=2 - [ACTN1_HUMAN]	-1.66	-1.68	-1.69	-1.70	-0.49	-0.60	-1.05	-1.08	-0.77	-0.72	-0.43	-0.41	0.71	1.34	1.24	0.98	0.96	1.18	1.04	0.71
P78357	Contactin-associated protein 1 OS=Homo sapiens GN=CNTNAP1 PE=1 SV=1 - [CNTP1_HUMAN]	2.53	2.41	2.54	2.57	0.11	0.05	0.81	0.76	0.55	0.45	0.46	0.56	-1.66	-1.79	-2.06	-1.80	-1.72	-2.37	-2.39	-1.80
Q92752	Tenascin-R OS=Homo sapiens GN=TNR PE=1 SV=3 - [TENR_HUMAN]	1.66	1.70	1.64	1.75	-0.03	0.01	0.58	0.70	0.46	0.39	0.21	0.32	-0.95	-1.38	-1.39	-1.30	-1.24	-1.59	-1.54	-0.95

P38606	V-type proton ATPase catalytic subunit A OS=Homo sapiens GN=ATP6V1A PE=1 SV=2 - [VATA_HUMAN]	1.54	1.62	1.64	1.74	0.15	0.16	0.67	0.72	0.41	0.43	0.26	0.26	-0.79	-1.22	-1.46	-1.12	-1.23	-1.52	-1.66	-0.92
P13591	Neural cell adhesion molecule 1 OS=Homo sapiens GN=NCAM1 PE=1 SV=3 - [NCAM1_HUMAN]	1.28	1.26	1.56	1.57	0.31	0.26	0.78	0.81	0.47	0.49	0.63	0.64	-0.40	-0.73	-1.07	-0.81	-1.09	-1.06	-1.33	-0.73
P22897	Macrophage mannose receptor 1 OS=Homo sapiens GN=MRC1 PE=1 SV=1 - [MRC1_HUMAN]	-1.77	-1.78	-2.35	-2.32	-0.11	-0.18	-0.39	-0.33	-0.17	-0.25	-0.06	-0.01	1.49	1.67	2.32	2.01	1.92	1.78	2.24	1.90
Q9BZF9	Uveal autoantigen with coiled-coil domains and ankyrin repeats OS=Homo sapiens GN=UACA PE=1 SV=2 - [UACA_HUMAN]	-2.10	-2.08	-1.89	-1.94	-0.04	-0.11	-0.43	-0.43	-0.32	-0.27	-0.15	-0.17	1.66	1.90	1.75	1.79	1.61	1.90	1.80	1.59
P05023	Sodium/potassium-transporting ATPase subunit alpha-1 OS=Homo sapiens GN=ATP1A1 PE=1 SV=1 - [AT1A1_HUMAN]	1.83	1.91	2.03	2.08	-0.05	-0.01	0.73	0.81	0.33	0.28	0.18	0.19	-0.94	-1.68	-1.76	-1.51	-1.61	-1.94	-2.01	-1.15
Q9H4G0	Band 4.1-like protein 1 OS=Homo sapiens GN=EPB41L1 PE=1 SV=2 - [E41L1_HUMAN]	1.52	1.50	1.72	1.77	0.28	0.28	0.71	0.77	0.48	0.48	0.40	0.40	-0.59	-1.13	-1.33	-0.98	-1.23	-1.29	-1.41	-0.91
Q92823	Neuronal cell adhesion molecule OS=Homo sapiens GN=NRCAM PE=1 SV=3 - [NRCAM_HUMAN]	1.24	1.33	1.45	1.50	0.09	0.04	0.69	0.66	0.42	0.35	0.24	0.26	-0.61	-1.09	-1.29	-1.00	-1.06	-1.34	-1.51	-0.76
O00429	Dynamin-1-like protein OS=Homo sapiens GN=DNM1L PE=1 SV=2 - [DNM1L_HUMAN]	1.33	1.32	1.33	1.41	0.06	0.07	0.61	0.65	0.16	0.19	-0.15	-0.11	-0.64	-1.42	-1.55	-1.03	-1.21	-1.34	-1.43	-0.69
Q4V328	GRIP1-associated protein 1 OS=Homo sapiens GN=GRIPAP1 PE=1 SV=1 - [GRAP1_HUMAN]	1.05	1.12	1.09	1.07	-0.12	-0.16	0.11	0.14	0.01	-0.04	-0.01	-0.09	-0.93	-1.17	-1.17	-1.09	-1.12	-1.24	-1.27	-0.90

P04040	Catalase OS=Homo sapiens GN=CAT PE=1 SV=3 - [CATA_HUMAN]	-1.82	-1.84	-1.91	-1.86	-0.34	-0.32	-0.47	-0.45	-0.53	-0.59	-0.70	-0.68	1.43	1.20	1.14	1.37	1.28	1.52	1.45	1.51
P12036	Neurofilament heavy polypeptide OS=Homo sapiens GN=NEFH PE=1 SV=4 - [NFH_HUMAN]	2.15	2.07	2.25	2.43	-0.38	-0.50	0.53	0.57	0.15	0.25	-0.32	-0.31	-1.71	-2.73	-2.63	-2.39	-2.35	-2.45	-2.58	-2.01
Q01814	Plasma membrane calcium-transporting ATPase 2 OS=Homo sapiens GN=ATP2B2 PE=1 SV=2 - [AT2B2_HUMAN]	2.11	2.06	2.16	2.14	-0.07	-0.09	0.85	0.83	0.37	0.45	0.09	0.16	-1.28	-2.17	-2.18	-1.67	-1.89	-2.32	-2.38	-1.46
P26232	Catenin alpha-2 OS=Homo sapiens GN=CTNNA2 PE=1 SV=5 - [CTNA2_HUMAN]	1.34	1.15	1.47	1.29	0.04	0.13	1.00	0.90	0.56	0.62	0.44	0.40	-0.63	-0.79	-1.10	-0.85	-1.00	-1.32	-1.41	-0.71
O95248	Myotubularin-related protein 5 OS=Homo sapiens GN=SBF1 PE=1 SV=3 - [MTMR5_HUMAN]	1.13	1.03	1.10	1.07	-0.18	-0.14	0.32	0.23	0.10	-0.02	0.01	0.06	-0.63	-0.90	-0.90	-0.76	-0.96	-1.06	-1.07	-0.65
Q63ZY3	KN motif and ankyrin repeat domain-containing protein 2 OS=Homo sapiens GN=KANK2 PE=1 SV=1 - [KANK2_HUMAN]	-2.06	-2.16	-2.49	-2.62	-0.25	-0.29	-0.33	-0.38	-0.37	-0.30	0.11	-0.03	1.83	2.21	2.61	1.94	2.34	1.89	2.45	2.32
Q86W92	Liprin-beta-1 OS=Homo sapiens GN=PPFIBP1 PE=1 SV=2 - [LIPB1_HUMAN]	-1.53	-1.45	-1.73	-1.81	0.06	0.06	-0.23	-0.28	-0.18	-0.17	-0.20	-0.18	1.22	1.26	1.52	1.33	1.51	1.44	1.57	1.56
P00450	Ceruloplasmin OS=Homo sapiens GN=CP PE=1 SV=1 - [CERU_HUMAN]	-2.04	-2.01	-1.86	-1.66	-0.53	-0.54	-0.71	-0.71	-0.56	-0.60	-0.41	-0.40	1.40	1.54	1.53	1.34	1.34	1.40	1.38	1.33
Q63HR2	Tensin-like C1 domain-containing phosphatase OS=Homo sapiens GN=TENC1 PE=1 SV=2 - [TENC1_HUMAN]	-1.55	-1.44	-1.51	-1.64	-0.17	-0.24	-0.41	-0.51	-0.27	-0.25	-0.05	-0.09	1.01	1.53	1.69	1.25	1.27	1.01	1.27	1.21
P32004	Neural cell adhesion molecule L1 OS=Homo sapiens GN=L1CAM PE=1 SV=2 - [L1CAM_HUMAN]	2.38	2.28	2.40	2.50	0.79	0.68	1.00	1.08	0.68	0.77	0.77	0.76	-1.06	-1.65	-1.70	-1.48	-1.56	-1.54	-1.57	-1.11

O95197	Reticulon-3 OS=Homo sapiens GN=RTN3 PE=1 SV=2 - [RTN3_HUMAN]	1.74	1.92	2.10	1.99	-0.24	-0.13	0.55	0.50	0.20	0.07	0.05	0.03	-1.23	-1.81	-2.04	-1.68	-1.91	-2.06	-2.28	-1.49
Q9C0H9	SRC kinase signaling inhibitor 1 OS=Homo sapiens GN=SRCIN1 PE=1 SV=3 - [SRCN1_HUMAN]	1.59	1.52	1.82	1.75	-0.29	-0.33	0.40	0.41	0.13	0.09	-0.04	-0.02	-1.05	-1.67	-1.96	-1.43	-1.63	-1.84	-2.02	-1.37
Q92598	Heat shock protein 105 kDa OS=Homo sapiens GN=HSPH1 PE=1 SV=1 - [HS105_HUMAN]	1.23	1.33	1.51	1.66	-0.13	-0.05	0.54	0.64	0.14	0.09	-0.34	-0.28	-0.68	-1.62	-1.92	-1.18	-1.42	-1.41	-1.72	-1.00
Q16555	Dihydropyrimidinase-related protein 2 OS=Homo sapiens GN=DPYSL2 PE=1 SV=1 - [DPYL2_HUMAN]	1.54	1.44	1.67	1.56	0.19	0.17	0.80	0.71	0.31	0.39	0.23	0.11	-0.69	-1.27	-1.49	-1.08	-1.02	-1.28	-1.44	-0.75
Q8N573	Oxidation resistance protein 1 OS=Homo sapiens GN=OXR1 PE=1 SV=2 - [OXR1_HUMAN]	1.21	1.47	1.58	1.49	0.20	0.10	0.46	0.57	0.34	0.36	-0.12	-0.10	-0.94	-1.43	-1.57	-0.88	-1.04	-1.54	-1.43	-1.05
Q9UDT6	CAP-Gly domain-containing linker protein 2 OS=Homo sapiens GN=CLIP2 PE=1 SV=1 - [CLIP2_HUMAN]	1.49	1.28	1.60	1.45	-0.12	-0.13	0.50	0.51	0.12	0.05	-0.07	-0.15	-0.85	-1.28	-1.54	-1.18	-1.31	-1.44	-1.61	-0.95
Q99959	Plakophilin-2 OS=Homo sapiens GN=PKP2 PE=1 SV=2 - [PKP2_HUMAN]	-2.36	-2.21	-2.49	-2.58	-0.21	-0.24	-0.50	-0.52	-0.49	-0.41	-0.41	-0.47	1.89	1.91	2.15	1.95	2.10	2.12	2.31	2.07
P13667	Protein disulfide-isomerase A4 OS=Homo sapiens GN=PDIA4 PE=1 SV=2 - [PDIA4_HUMAN]	-1.44	-1.45	-1.43	-1.29	-0.57	-0.58	-0.84	-0.81	-0.69	-0.77	-0.51	-0.50	0.66	0.99	0.75	0.71	0.64	0.85	0.71	0.57
Q9BX66	Sorbin and SH3 domain-containing protein 1 OS=Homo sapiens GN=SORBS1 PE=1 SV=3 - [SRBS1_HUMAN]	-1.14	-1.25	-1.14	-1.27	-0.48	-0.54	-0.55	-0.56	-0.60	-0.54	-0.40	-0.49	0.87	0.80	0.78	0.73	0.65	0.70	0.67	0.74
P14625	Endoplasmin OS=Homo sapiens GN=HSP90B1 PE=1 SV=1 - [ENPL_HUMAN]	-1.25	-1.30	-1.19	-1.14	-0.23	-0.22	-0.47	-0.49	-0.53	-0.50	-0.37	-0.39	0.87	0.85	0.80	0.82	0.72	1.04	0.86	0.74

Q16352	Alpha-internexin OS=Homo sapiens GN=INA PE=1 SV=2 - [AINX_HUMAN]	2.36	2.34	2.38	2.36	-0.70	-0.67	0.36	0.41	0.02	0.02	-0.17	-0.17	-2.01	-2.69	-2.70	-2.39	-2.35	-2.86	-2.82	-2.06
Q9ULD0	2-oxoglutarate dehydrogenase like, mitochondrial OS=Homo sapiens GN=OGDHL PE=1 SV=3 - [OGDHL_HUMAN]	1.93	1.83	2.05	2.01	-0.62	-0.66	0.66	0.51	0.13	0.16	-0.03	-0.18	-1.37	-2.10	-2.15	-1.88	-1.85	-2.15	-2.43	-1.54
Q14194	Dihydropyrimidinase-related protein 1 OS=Homo sapiens GN=CRMP1 PE=1 SV=1 - [DPYL1_HUMAN]	1.45	1.67	1.84	1.96	-0.29	-0.20	0.34	0.53	0.34	0.17	0.18	0.23	-1.25	-1.51	-1.73	-1.44	-1.67	-1.74	-1.89	-1.52
Q9ULU8	Calcium-dependent secretion activator 1 OS=Homo sapiens GN=CADPS PE=1 SV=3 - [CAPS1_HUMAN]	1.90	1.78	1.99	1.94	-0.36	-0.42	0.76	0.73	0.23	0.28	-0.05	-0.02	-0.99	-1.77	-1.84	-1.55	-1.50	-2.02	-2.17	-1.29
O14578	Citron Rho-interacting kinase OS=Homo sapiens GN=CIT PE=1 SV=2 - [CTRO_HUMAN]	1.67	1.56	1.59	1.68	-0.04	0.01	0.51	0.67	0.39	0.34	0.03	0.14	-1.01	-1.45	-1.61	-1.30	-1.19	-1.39	-1.43	-1.13
P36871	Phosphoglucomutase-1 OS=Homo sapiens GN=PGM1 PE=1 SV=3 - [PGM1_HUMAN]	1.38	1.30	1.62	1.60	-0.06	0.01	0.48	0.50	0.13	0.23	-0.24	-0.20	-0.84	-1.60	-1.82	-1.13	-1.41	-1.31	-1.57	-1.01
P35612	Beta-adducin OS=Homo sapiens GN=ADD2 PE=1 SV=3 - [ADDB_HUMAN]	1.29	1.32	1.61	1.60	0.00	0.07	0.63	0.57	0.18	0.31	0.41	0.37	-0.67	-0.84	-1.21	-0.96	-1.32	-1.18	-1.48	-0.99
Q16799	Reticulon-1 OS=Homo sapiens GN=RTN1 PE=1 SV=1 - [RTN1_HUMAN]	1.20	1.23	1.49	1.58	-0.38	-0.26	0.24	0.38	0.09	-0.04	0.18	0.11	-0.86	-1.07	-1.38	-1.29	-1.53	-1.45	-1.88	-1.32
Q5JRX3	Presequence protease, mitochondrial OS=Homo sapiens GN=PITRM1 PE=1 SV=3 - [PREP_HUMAN]	1.21	1.08	1.20	1.23	-0.24	-0.12	-0.31	-0.30	-0.14	-0.16	0.30	0.24	-1.28	-0.78	-1.00	-1.36	-1.49	-1.37	-1.54	-1.53
P17661	Desmin OS=Homo sapiens GN=DES PE=1 SV=3 - [DESM_HUMAN]	-3.38	-3.31	-3.67	-3.59	-1.13	-1.06	-1.97	-1.93	-1.38	-1.43	-1.03	-1.01	1.58	2.41	2.75	2.05	2.39	2.46	2.61	1.80

Q9NZN4	EH domain-containing protein 2 OS=Homo sapiens GN=EHD2 PE=1 SV=2 - [EHD2_HUMAN]	-2.46	-2.49	-2.56	-2.48	-1.06	-1.05	-1.25	-1.25	-1.09	-1.03	-0.77	-0.77	1.29	1.58	1.64	1.41	1.27	1.10	1.24	1.31
Q9Y6C2	EMILIN-1 OS=Homo sapiens GN=EMILIN1 PE=1 SV=2 - [EMIL1_HUMAN]	-1.88	-2.00	-2.13	-2.06	0.35	0.39	-0.34	-0.19	0.05	0.02	-0.05	-0.04	1.59	1.93	2.01	1.90	2.18	2.15	2.51	1.83
P05556	Integrin beta-1 OS=Homo sapiens GN=ITGB1 PE=1 SV=2 - [ITB1_HUMAN]	-1.70	-1.66	-1.87	-1.79	-0.03	0.01	-0.43	-0.37	-0.25	-0.30	-0.10	-0.06	1.32	1.62	1.82	1.61	1.64	1.77	1.75	1.46
Q96PV0	Ras/Rap GTPase-activating protein SynGAP OS=Homo sapiens GN=SYNGAP1 PE=1 SV=4 - [SYGP1_HUMAN]	2.06	2.06	2.05	2.02	-0.08	-0.27	0.66	0.60	0.24	0.36	0.21	0.02	-1.38	-2.13	-2.07	-1.87	-1.79	-2.12	-2.12	-1.39
P21281	V-type proton ATPase subunit B, brain isoform OS=Homo sapiens GN=ATP6V1B2 PE=1 SV=3 - [VATB2_HUMAN]	1.62	1.77	1.96	1.91	0.04	0.04	0.67	0.74	0.37	0.40	0.23	0.19	-1.07	-1.58	-1.77	-1.45	-1.57	-1.73	-2.05	-1.17
P13611	Versican core protein OS=Homo sapiens GN=VCAN PE=1 SV=3 - [CSPG2_HUMAN]	1.43	1.61	1.60	1.86	0.32	0.42	0.55	0.75	0.49	0.38	0.39	0.39	-1.10	-1.35	-1.56	-1.26	-1.21	-1.29	-1.32	-1.19
O75746	Calcium-binding mitochondrial carrier protein Aralar1 OS=Homo sapiens GN=SLC25A12 PE=1 SV=2 - [CMC1_HUMAN]	1.57	1.43	1.71	1.55	0.06	0.03	0.48	0.47	0.22	0.28	0.19	0.13	-0.96	-1.32	-1.49	-1.25	-1.23	-1.39	-1.64	-1.09
Q9HCM2	Plexin-A4 OS=Homo sapiens GN=PLXNA4 PE=1 SV=4 - [PLXA4_HUMAN]	1.40	1.34	1.51	1.20	0.10	-0.07	0.44	0.52	0.47	0.31	0.25	0.38	-1.00	-1.12	-1.07	-0.95	-0.84	-1.37	-1.33	-0.76
Q15063	Periostin OS=Homo sapiens GN=POSTN PE=1 SV=2 - [POSTN_HUMAN]	-2.65	-2.81	-3.19	-3.18	-0.10	-0.05	-2.07	-1.91	-1.09	-1.08	-1.31	-1.25	0.83	1.25	1.72	1.78	2.26	2.66	3.36	1.26
P82987	ADAMTS-like protein 3 OS=Homo sapiens GN=ADAMTSL3 PE=2 SV=4 - [ATL3_HUMAN]	-2.11	-2.08	-2.45	-2.42	0.77	0.72	0.40	0.39	0.28	0.20	-0.87	-0.79	2.46	1.34	1.65	2.26	2.68	2.84	3.17	2.82

P21980	Protein-glutamine gamma-glutamyltransferase 2 OS=Homo sapiens GN=TGM2 PE=1 SV=2 - [TGM2_HUMAN]	-2.17	-2.11	-2.32	-2.28	-0.30	-0.25	-1.11	-1.06	-0.67	-0.60	-0.38	-0.33	0.98	1.70	1.95	1.49	1.64	1.86	2.06	1.30
Q15746	Myosin light chain kinase, smooth muscle OS=Homo sapiens GN=MYLK PE=1 SV=4 - [MYLK_HUMAN]	-2.28	-2.08	-2.22	-2.20	-0.74	-0.80	-0.96	-0.91	-0.97	-1.09	-1.28	-1.26	1.38	0.97	0.88	1.19	1.11	1.54	1.29	1.27
Q07065	Cytoskeleton-associated protein 4 OS=Homo sapiens GN=CKAP4 PE=1 SV=2 - [CKAP4_HUMAN]	-1.63	-1.73	-1.76	-1.67	-0.41	-0.34	-0.48	-0.49	-0.51	-0.52	-0.58	-0.50	1.14	1.03	1.23	1.14	1.39	1.30	1.34	1.27
Q14168	MAGUK p55 subfamily member 2 OS=Homo sapiens GN=MPP2 PE=1 SV=3 - [MPP2_HUMAN]	2.23	2.19	2.31	2.30	0.22	0.05	0.52	0.45	0.34	0.45	0.23	0.18	-1.65	-1.87	-2.01	-1.67	-1.56	-1.96	-2.02	-1.56
O75145	Liprin-alpha-3 OS=Homo sapiens GN=PPFIA3 PE=1 SV=3 - [LIPA3_HUMAN]	1.87	1.96	2.01	2.09	-0.31	-0.21	0.46	0.66	0.33	0.32	0.19	0.24	-1.44	-1.88	-1.95	-1.70	-1.86	-2.02	-2.09	-1.44
P49418	Amphiphysin OS=Homo sapiens GN=AMPH PE=1 SV=1 - [AMPH_HUMAN]	1.60	1.53	1.87	2.01	0.19	0.14	0.64	0.65	0.22	0.14	-0.05	-0.09	-0.80	-1.65	-1.98	-1.37	-1.52	-1.32	-1.51	-1.14
P48147	Prolyl endopeptidase OS=Homo sapiens GN=PREP PE=1 SV=2 - [PPCE_HUMAN]	1.46	1.37	1.70	1.71	0.29	0.35	0.96	0.82	0.48	0.62	-0.07	0.03	-0.45	-1.54	-1.77	-0.99	-1.13	-1.20	-1.37	-0.70
O95757	Heat shock 70 kDa protein 4L OS=Homo sapiens GN=HSPA4L PE=1 SV=3 - [HS74L_HUMAN]	1.61	1.46	1.64	1.57	0.14	-0.01	0.57	0.50	0.21	0.31	0.09	-0.05	-1.09	-1.58	-1.71	-1.30	-1.50	-1.44	-1.62	-1.22
O43301	Heat shock 70 kDa protein 12A OS=Homo sapiens GN=HSPA12A PE=1 SV=2 - [HS12A_HUMAN]	1.15	1.16	1.38	1.48	-0.22	-0.19	0.26	0.36	0.01	0.00	-0.22	-0.21	-0.81	-1.22	-1.52	-1.18	-1.45	-1.42	-1.56	-0.92
Q9Y2J0	Rabphilin-3A OS=Homo sapiens GN=RPH3A PE=1 SV=1 - [RP3A_HUMAN]	1.22	1.12	1.63	1.45	0.51	0.33	0.47	0.35	0.00	0.11	-0.10	-0.16	-0.83	-1.27	-1.68	-0.92	-1.27	-0.78	-1.15	-1.12

P00734	Prothrombin OS=Homo sapiens GN=F2 PE=1 SV=2 - [THRB_HUMAN]	-2.58	-2.68	-2.63	-2.58	-1.28	-1.19	-1.61	-1.59	-1.29	-1.37	-0.85	-0.79	0.97	1.77	1.95	1.51	1.44	1.33	1.45	1.09
O94875	Sorbin and SH3 domain-containing protein 2 OS=Homo sapiens GN=SORBS2 PE=1 SV=3 - [SRBS2_HUMAN]	-1.93	-1.99	-2.38	-2.41	-1.43	-1.58	-1.35	-1.45	-1.38	-1.26	-1.03	-1.14	0.82	1.07	1.31	0.86	1.14	0.80	0.93	1.02
P39060	Collagen alpha-1(XVIII) chain OS=Homo sapiens GN=COL18A1 PE=1 SV=5 - [COIA1_HUMAN]	-2.35	-2.14	-2.50	-2.32	0.37	0.42	-0.15	-0.05	0.07	0.08	0.08	0.20	2.11	2.33	2.58	2.25	2.51	2.50	2.73	2.31
P14923	Junction plakoglobin OS=Homo sapiens GN=JUP PE=1 SV=3 - [PLAK_HUMAN]	-1.89	-1.79	-2.12	-2.24	-0.12	-0.22	-0.62	-0.65	-0.41	-0.39	0.04	-0.06	1.60	1.96	2.29	1.71	1.87	1.81	2.21	1.84
P56199	Integrin alpha-1 OS=Homo sapiens GN=ITGA1 PE=1 SV=2 - [ITA1_HUMAN]	-2.08	-1.98	-2.04	-1.99	0.05	0.11	-0.51	-0.46	-0.28	-0.38	-0.50	-0.38	1.59	1.60	1.57	1.85	1.68	2.12	2.10	1.59
P16452	Erythrocyte membrane protein band 4.2 OS=Homo sapiens GN=EPB42 PE=1 SV=3 - [EPB42_HUMAN]	-1.80	-1.76	-1.93	-1.86	0.30	0.42	0.43	0.66	0.81	0.67	1.25	1.38	2.23	3.14	3.15	2.57	2.55	2.05	2.02	2.46
P78352	Disks large homolog 4 OS=Homo sapiens GN=DLG4 PE=1 SV=3 - [DLG4_HUMAN]	1.83	1.92	1.92	1.99	-0.13	-0.13	0.57	0.50	0.22	0.25	0.05	0.14	-1.24	-1.80	-1.95	-1.67	-1.77	-2.19	-2.10	-1.38
Q9UKU0	Long-chain-fatty-acid--CoA ligase 6 OS=Homo sapiens GN=ACSL6 PE=2 SV=4 - [ACSL6_HUMAN]	1.85	1.71	2.06	1.86	-0.14	-0.15	0.80	0.56	0.33	0.48	0.29	0.19	-1.18	-1.65	-1.91	-1.49	-1.72	-2.20	-2.23	-1.47
O00499	Myc box-dependent-interacting protein 1 OS=Homo sapiens GN=BIN1 PE=1 SV=1 - [BIN1_HUMAN]	1.52	1.46	1.68	1.74	-0.03	-0.05	0.35	0.38	0.00	-0.08	-0.26	-0.22	-1.10	-1.66	-1.83	-1.40	-1.69	-1.51	-1.75	-1.21
P23471	Receptor-type tyrosine-protein phosphatase zeta OS=Homo sapiens GN=PTPRZ1 PE=1 SV=4 - [PTPRZ_HUMAN]	1.55	1.34	1.63	1.64	0.20	0.12	0.90	0.84	0.52	0.63	0.55	0.38	-0.52	-0.91	-1.23	-0.90	-1.07	-1.22	-1.61	-0.72

Q9BY11	Protein kinase C and casein kinase substrate in neurons protein 1 OS=Homo sapiens GN=PACSIN1 PE=1 SV=1 - [PACN1_HUMAN]	1.24	1.23	1.57	1.53	-0.03	0.04	-0.01	0.08	-0.14	-0.21	-0.33	-0.10	-1.02	-1.33	-1.63	-1.49	-1.71	-1.28	-1.62	-1.33
O94925	Glutaminase kidney isoform, mitochondrial OS=Homo sapiens GN=GLS PE=1 SV=1 - [GLSK_HUMAN]	1.27	1.34	1.52	1.44	-0.40	-0.54	0.26	0.13	-0.10	0.02	-0.23	-0.37	-1.12	-1.78	-1.84	-1.36	-1.50	-1.79	-1.79	-1.30
Q9UQB8	Brain-specific angiogenesis inhibitor 1-associated protein 2 OS=Homo sapiens GN=BAIAP2 PE=1 SV=1 - [BAIP2_HUMAN]	1.24	1.30	1.54	1.40	0.01	-0.10	0.15	0.05	-0.01	0.04	-0.01	-0.10	-1.14	-1.19	-1.57	-1.32	-1.58	-1.15	-1.44	-1.30
Q9UQB3	Catenin delta-2 OS=Homo sapiens GN=CTNND2 PE=1 SV=3 - [CTND2_HUMAN]	1.18	1.24	1.07	1.38	-0.37	-0.33	0.10	0.12	0.03	-0.02	0.08	-0.15	-1.13	-1.51	-1.60	-1.42	-1.58	-1.69	-1.78	-1.19
P06744	Glucose-6-phosphate isomerase OS=Homo sapiens GN=GPI PE=1 SV=4 - [G6PI_HUMAN]	1.04	1.05	1.43	1.36	0.42	0.43	0.75	0.69	0.57	0.55	0.64	0.75	-0.28	-0.26	-0.66	-0.48	-0.83	-0.69	-1.08	-0.61
Q96KP4	Cytosolic non-specific dipeptidase OS=Homo sapiens GN=CNDP2 PE=1 SV=2 - [CNDP2_HUMAN]	1.11	1.02	1.43	1.36	0.46	0.45	1.02	0.93	0.69	0.69	0.63	0.58	-0.01	-0.47	-0.80	-0.47	-0.64	-0.65	-0.95	-0.35
P50895	Basal cell adhesion molecule OS=Homo sapiens GN=BCAM PE=1 SV=2 - [BCAM_HUMAN]	-2.56	-2.49	-2.68	-2.63	-1.21	-1.14	-1.18	-1.15	-1.01	-1.05	-0.59	-0.58	1.38	1.95	2.08	1.49	1.59	1.34	1.53	1.61
P00751	Complement factor B OS=Homo sapiens GN=CFB PE=1 SV=2 - [CFAB_HUMAN]	-1.96	-1.94	-1.89	-2.06	-0.64	-0.59	-0.76	-0.72	-0.76	-0.83	-0.75	-0.72	1.31	1.03	0.89	1.21	0.95	1.30	1.19	1.22
P02730	Band 3 anion transport protein OS=Homo sapiens GN=SLC4A1 PE=1 SV=3 - [B3AT_HUMAN]	-1.59	-1.61	-1.92	-1.92	0.62	0.62	0.38	0.42	0.77	0.83	1.47	1.49	2.09	2.99	3.31	2.51	2.66	2.24	2.50	2.41

Q96HC4	PDZ and LIM domain protein 5 OS=Homo sapiens GN=PDLIM5 PE=1 SV=5 - [PDLI5_HUMAN]	-1.94	-1.83	-1.73	-1.73	-0.48	-0.51	-0.71	-0.76	-0.70	-0.72	-0.52	-0.53	1.22	1.38	1.30	1.28	1.15	1.46	1.34	1.06
P11171	Protein 4.1 OS=Homo sapiens GN=EPB41 PE=1 SV=4 - [41_HUMAN]	-1.45	-1.50	-1.37	-1.38	0.31	0.28	0.46	0.42	0.54	0.58	1.31	1.26	1.67	2.60	2.69	1.97	2.11	1.79	1.81	1.84
P15144	Aminopeptidase N OS=Homo sapiens GN=ANPEP PE=1 SV=4 - [AMPN_HUMAN]	-1.24	-1.18	-1.34	-1.36	0.45	0.51	-0.29	-0.25	0.07	0.08	0.14	0.15	0.97	1.29	1.57	1.28	1.57	1.58	1.74	1.14
O15394	Neural cell adhesion molecule 2 OS=Homo sapiens GN=NCAM2 PE=1 SV=2 - [NCAM2_HUMAN]	1.72	1.94	1.93	2.03	0.17	0.21	1.04	0.87	0.53	0.67	0.61	0.66	-0.88	-1.19	-1.38	-1.23	-1.39	-1.65	-1.54	-1.06
Q9Y2Q0	Probable phospholipid-transporting ATPase IA OS=Homo sapiens GN=ATP8A1 PE=1 SV=1 - [AT8A1_HUMAN]	2.17	1.99	2.09	1.85	0.14	0.13	1.06	0.85	0.69	0.66	0.66	0.55	-0.92	-1.40	-1.30	-1.23	-1.16	-1.94	-1.90	-1.03
Q9NQX3	Gephyrin OS=Homo sapiens GN=GPHN PE=1 SV=1 - [GEPH_HUMAN]	1.41	1.59	1.68	1.79	-0.16	0.01	0.54	0.52	0.21	0.12	0.06	0.08	-1.11	-1.48	-1.70	-1.47	-1.66	-1.63	-1.78	-1.26
Q5SW79	Centrosomal protein of 170 kDa OS=Homo sapiens GN=CEP170 PE=1 SV=1 - [CE170_HUMAN]	1.40	1.40	1.33	1.43	0.10	0.27	0.30	0.45	0.31	0.08	0.20	0.38	-0.94	-1.06	-1.10	-1.24	-1.29	-1.37	-1.30	-0.85
Q9ULB1	Neurexin-1 OS=Homo sapiens GN=NRXN1 PE=2 SV=1 - [NRX1A_HUMAN]	1.03	1.10	1.24	1.11	0.21	0.21	0.83	0.78	0.62	0.48	0.69	0.69	-0.39	-0.34	-0.41	-0.38	-0.40	-0.82	-0.70	-0.16
P04003	C4b-binding protein alpha chain OS=Homo sapiens GN=C4BPA PE=1 SV=2 - [C4BPA_HUMAN]	-2.37	-2.28	-2.60	-2.53	-0.39	-0.36	-0.66	-0.59	-0.15	-0.28	0.41	0.56	1.68	2.88	3.11	2.42	2.40	2.16	2.11	1.95
P07355	Annexin A2 OS=Homo sapiens GN=ANXA2 PE=1 SV=2 - [ANXA2_HUMAN]	-1.74	-1.77	-2.15	-2.21	-0.26	-0.23	-0.53	-0.58	-0.27	-0.28	-0.03	-0.10	1.33	1.84	2.14	1.51	2.02	1.63	2.05	1.79
Q15582	Transforming growth factor-beta-induced protein ig-h3 OS=Homo sapiens GN=TGFBI PE=1 SV=1 - [BGH3_HUMAN]	-2.05	-1.84	-2.02	-2.06	1.22	1.29	0.21	0.27	0.48	0.48	0.31	0.34	2.09	2.08	2.05	2.25	2.43	2.87	2.91	2.18

P08758	Annexin A5 OS=Homo sapiens GN=ANXA5 PE=1 SV=2 - [ANXA5_HUMAN]	-1.72	-1.58	-1.88	-1.82	0.69	0.75	-0.11	-0.09	0.48	0.38	0.63	0.68	1.61	2.25	2.51	2.03	2.35	2.31	2.58	1.85
P35221	Catenin alpha-1 OS=Homo sapiens GN=CTNNA1 PE=1 SV=1 - [CTNA1_HUMAN]	-1.49	-1.54	-1.70	-1.74	-0.16	-0.17	-0.41	-0.43	-0.38	-0.31	-0.06	-0.09	1.03	1.63	1.68	1.38	1.39	1.36	1.39	1.39
Q9UDR5	Alpha-aminoadipic semialdehyde synthase, mitochondrial OS=Homo sapiens GN=AASS PE=1 SV=1 - [AASS_HUMAN]	-1.78	-1.48	-1.73	-1.70	0.06	0.08	-0.22	-0.15	-0.24	-0.25	-0.33	-0.40	1.43	1.23	1.31	1.28	1.50	1.48	1.60	1.46
P04843	Dolichyl-diphosphooligosaccharide--protein glycosyltransferase subunit 1 OS=Homo sapiens GN=RPN1 PE=1 SV=1 - [RPN1_HUMAN]	-1.34	-1.31	-1.40	-1.40	-0.24	-0.25	-0.36	-0.35	-0.31	-0.29	-0.15	-0.12	1.04	1.25	1.26	1.01	1.11	1.10	1.09	1.11
O43795	Unconventional myosin-Ib OS=Homo sapiens GN=MYO1B PE=1 SV=3 - [MYO1B_HUMAN]	-1.29	-1.46	-1.36	-1.35	0.05	0.07	-0.14	-0.18	-0.13	-0.25	-0.39	-0.37	1.14	0.99	1.03	1.09	1.08	1.41	1.36	1.23
Q9H4M9	EH domain-containing protein 1 OS=Homo sapiens GN=EHD1 PE=1 SV=2 - [EHD1_HUMAN]	-1.34	-1.40	-1.17	-1.27	0.18	0.12	0.41	0.36	0.18	0.22	0.09	0.04	1.79	1.45	1.36	1.51	1.41	1.46	1.35	1.64
P17174	Aspartate aminotransferase, cytoplasmic OS=Homo sapiens GN=GOT1 PE=1 SV=3 - [AATC_HUMAN]	1.88	1.91	2.00	2.27	0.32	0.34	0.63	0.70	0.41	0.31	-0.04	0.04	-1.15	-2.05	-2.23	-1.66	-1.86	-1.59	-1.95	-1.59
P80404	4-aminobutyrate aminotransferase, mitochondrial OS=Homo sapiens GN=ABAT PE=1 SV=3 - [GABT_HUMAN]	1.81	1.82	2.21	2.17	0.31	0.30	0.99	0.93	0.58	0.57	0.22	0.26	-0.80	-1.60	-1.88	-1.28	-1.52	-1.62	-1.83	-1.14
Q93050	V-type proton ATPase 116 kDa subunit a isoform 1 OS=Homo sapiens GN=ATP6V0A1 PE=2 SV=3 - [VPP1_HUMAN]	1.55	1.52	1.59	1.65	-0.22	-0.06	0.56	0.56	0.32	0.21	0.12	0.05	-0.88	-1.32	-1.55	-1.21	-1.33	-1.61	-1.67	-0.95

Q02156	Protein kinase C epsilon type OS=Homo sapiens GN=PRKCE PE=1 SV=1 - [KPCE_HUMAN]	1.53	1.61	1.51	1.62	0.08	0.00	0.45	0.41	0.27	0.29	0.23	0.09	-0.99	-1.38	-1.39	-1.34	-1.41	-1.67	-1.72	-1.09
Q02246	Contactin-2 OS=Homo sapiens GN=CNTN2 PE=1 SV=1 - [CNTN2_HUMAN]	1.74	1.56	1.62	1.53	0.41	0.32	0.48	0.34	0.46	0.53	0.10	0.12	-1.26	-1.34	-1.45	-1.18	-1.11	-1.30	-1.30	-1.28
Q9BQI5	SH3-containing GRB2-like protein 3-interacting protein 1 OS=Homo sapiens GN=SGIP1 PE=1 SV=2 - [SGIP1_HUMAN]	1.41	1.40	1.56	1.40	-0.01	0.01	0.02	0.20	0.11	0.05	-0.14	-0.02	-1.11	-1.43	-1.59	-0.96	-1.22	-1.24	-1.51	-1.27
P00505	Aspartate aminotransferase, mitochondrial OS=Homo sapiens GN=GOT2 PE=1 SV=3 - [AATM_HUMAN]	1.13	1.07	1.29	1.33	-0.06	0.02	0.41	0.53	0.30	0.20	0.20	0.17	-0.63	-1.02	-1.09	-0.86	-0.97	-1.17	-1.18	-0.80
Q16658	Fascin OS=Homo sapiens GN=FSCN1 PE=1 SV=3 - [FSCN1_HUMAN]	1.21	1.07	1.30	1.20	0.13	0.15	0.24	0.26	0.15	0.19	-0.14	-0.04	-0.69	-1.09	-1.15	-0.79	-0.89	-0.67	-0.82	-0.78
Q15124	Phosphoglucomutase-like protein 5 OS=Homo sapiens GN=PGM5 PE=1 SV=2 - [PGM5_HUMAN]	-2.73	-2.68	-2.90	-2.69	-0.68	-0.58	-1.45	-1.22	-0.90	-1.06	-0.78	-0.73	1.49	2.05	2.10	1.94	1.96	2.14	2.11	1.58
Q8WX93	Palladin OS=Homo sapiens GN=PALLD PE=1 SV=3 - [PALLD_HUMAN]	-2.37	-2.43	-2.54	-2.50	-0.61	-0.59	-0.95	-1.09	-0.86	-0.85	-0.79	-0.76	1.40	1.62	1.75	1.55	1.70	1.69	1.95	1.52
Q86UX2	Inter-alpha-trypsin inhibitor heavy chain H5 OS=Homo sapiens GN=ITIH5 PE=2 SV=2 - [ITIH5_HUMAN]	-1.56	-1.33	-1.96	-1.88	-0.21	-0.03	-0.53	-0.42	-0.29	-0.32	-0.30	-0.18	1.12	1.41	1.65	1.34	1.83	1.34	1.84	1.55
Q14BN4	Sarcolemmal membrane-associated protein OS=Homo sapiens GN=SLMAP PE=1 SV=1 - [SLMAP_HUMAN]	-1.88	-1.85	-1.92	-1.85	-1.01	-1.07	-1.10	-1.13	-1.04	-1.02	-0.60	-0.65	0.89	1.28	1.34	0.97	0.94	0.93	0.96	0.89
P00488	Coagulation factor XIII A chain OS=Homo sapiens GN=F13A1 PE=1 SV=4 - [F13A_HUMAN]	-1.57	-1.46	-1.60	-1.64	0.29	0.36	-0.27	-0.27	0.08	0.10	0.39	0.41	1.13	2.07	2.15	1.67	1.80	1.98	2.06	1.43

Q13576	Ras GTPase-activating-like protein IQGAP2 OS=Homo sapiens GN=IQGAP2 PE=1 SV=4 - [IQGA2_HUMAN]	-1.10	-1.10	-1.14	-1.36	0.42	0.38	0.18	0.17	0.34	0.36	0.40	0.18	1.33	1.29	1.53	1.46	1.72	1.53	1.78	1.57
Q13464	Rho-associated protein kinase 1 OS=Homo sapiens GN=ROCK1 PE=1 SV=1 - [ROCK1_HUMAN]	-1.29	-1.18	-1.29	-1.22	0.04	-0.04	-0.23	-0.26	-0.24	-0.20	-0.16	-0.16	0.93	1.18	1.24	1.24	1.10	1.27	1.18	0.95
P01009	Alpha-1-antitrypsin OS=Homo sapiens GN=SERPINA1 PE=1 SV=3 - [A1AT_HUMAN]	-1.50	-1.49	-1.17	-1.17	-0.99	-1.04	-1.42	-1.43	-1.60	-1.50	-1.51	-1.52	-0.01	-0.08	-0.32	-0.05	-0.33	0.41	0.11	-0.24
P09104	Gamma-enolase OS=Homo sapiens GN=ENO2 PE=1 SV=3 - [ENOG_HUMAN]	1.74	1.75	1.76	1.86	0.65	0.71	0.82	0.85	0.56	0.55	0.02	-0.02	-0.86	-1.59	-1.55	-1.09	-1.24	-1.19	-1.20	-0.91
Q9BYB0	SH3 and multiple ankyrin repeat domains protein 3 OS=Homo sapiens GN=SHANK3 PE=1 SV=3 - [SHAN3_HUMAN]	1.84	1.59	1.76	1.68	0.03	-0.05	0.00	-0.04	-0.07	0.03	-0.14	-0.47	-1.37	-1.81	-1.83	-1.67	-1.70	-1.70	-1.73	-1.41
P60174	Triosephosphate isomerase OS=Homo sapiens GN=TPI1 PE=1 SV=3 - [TPIS_HUMAN]	1.30	1.24	1.66	1.61	-0.13	-0.18	0.26	0.23	-0.11	-0.11	-0.50	-0.52	-0.87	-1.72	-2.06	-1.27	-1.65	-1.37	-1.62	-1.23
Q7Z4S6	Kinesin-like protein KIF21A OS=Homo sapiens GN=KIF21A PE=1 SV=2 - [KI21A_HUMAN]	1.30	1.39	1.48	1.57	-0.01	-0.02	0.43	0.52	0.25	0.17	0.21	0.13	-0.78	-1.15	-1.34	-1.07	-1.28	-1.30	-1.40	-0.93
P02774	Vitamin D-binding protein OS=Homo sapiens GN=GC PE=1 SV=1 - [VTDB_HUMAN]	-2.23	-2.12	-2.07	-2.09	-0.53	-0.49	-0.73	-0.69	-0.79	-0.72	-0.79	-0.80	1.45	1.27	1.25	1.44	1.30	1.54	1.43	1.46
Q9P0K7	Ankycorbin OS=Homo sapiens GN=RAI14 PE=1 SV=2 - [RAI14_HUMAN]	-1.85	-1.86	-2.13	-2.08	-0.28	-0.20	-0.51	-0.51	-0.54	-0.52	-0.56	-0.55	1.45	1.30	1.51	1.39	1.58	1.86	1.81	1.63

P19823	Inter-alpha-trypsin inhibitor heavy chain H2 OS=Homo sapiens GN=ITIH2 PE=1 SV=2 - [ITIH2_HUMAN]	-1.53	-1.53	-1.93	-2.02	0.85	0.82	-0.31	-0.36	0.24	0.22	0.38	0.42	1.16	1.90	2.21	1.73	2.17	2.15	2.52	1.78
Q9Y6N5	Sulfide:quinone oxidoreductase, mitochondrial OS=Homo sapiens GN=SQRDL PE=1 SV=1 - [SQRD_HUMAN]	-1.95	-1.86	-1.86	-1.88	0.28	0.34	-0.17	-0.18	-0.05	-0.08	0.01	0.02	1.75	1.94	1.89	1.91	1.87	2.15	2.14	1.77
Q9NY15	Stabilin-1 OS=Homo sapiens GN=STAB1 PE=1 SV=3 - [STAB1_HUMAN]	-1.41	-1.23	-1.54	-1.63	0.71	0.94	-0.34	-0.14	0.29	0.17	-0.02	0.11	1.19	1.45	1.61	1.68	1.88	2.29	2.56	1.52
P09525	Annexin A4 OS=Homo sapiens GN=ANXA4 PE=1 SV=4 - [ANXA4_HUMAN]	-1.50	-1.52	-1.50	-1.62	0.36	0.43	-0.14	-0.09	0.20	0.12	0.46	0.47	1.46	1.95	1.99	1.79	1.91	1.87	1.93	1.49
O60437	Periplakin OS=Homo sapiens GN=PPL PE=1 SV=4 - [PEPL_HUMAN]	-1.42	-1.33	-1.63	-1.61	-1.36	-1.28	-1.70	-1.61	-1.47	-1.49	-1.11	-1.15	-0.13	0.24	0.56	-0.06	0.28	0.15	0.38	0.08
P09471	Guanine nucleotide-binding protein G(o) subunit alpha OS=Homo sapiens GN=GNAO1 PE=1 SV=4 - [GNAO_HUMAN]	2.42	2.36	2.57	2.42	0.05	-0.08	1.07	1.00	0.54	0.61	0.45	0.34	-1.26	-1.99	-2.11	-1.76	-1.87	-2.51	-2.59	-1.36
Q9UQ16	Dynamin-3 OS=Homo sapiens GN=DNM3 PE=1 SV=4 - [DYN3_HUMAN]	1.86	1.80	2.01	2.02	-0.25	-0.19	0.67	0.69	0.39	0.44	0.11	0.13	-1.09	-1.81	-1.89	-1.45	-1.61	-2.14	-2.25	-1.21
Q13367	AP-3 complex subunit beta-2 OS=Homo sapiens GN=AP3B2 PE=1 SV=2 - [AP3B2_HUMAN]	1.90	1.69	2.00	1.99	-0.18	-0.22	0.63	0.52	0.23	0.31	0.04	-0.02	-1.14	-1.72	-1.99	-1.54	-1.85	-1.67	-2.02	-1.35
Q7L099	Protein RUFY3 OS=Homo sapiens GN=RUFY3 PE=1 SV=1 - [RUFY3_HUMAN]	2.01	1.87	2.01	1.81	0.02	-0.05	0.79	0.60	0.34	0.43	0.21	0.15	-1.04	-1.45	-1.69	-1.46	-1.48	-1.88	-1.94	-1.24
O00408	cGMP-dependent 3',5'-cyclic phosphodiesterase OS=Homo sapiens GN=PDE2A PE=1 SV=1 - [PDE2A_HUMAN]	1.90	1.90	1.81	1.79	0.01	0.06	0.73	0.70	0.22	0.46	0.50	0.26	-0.97	-1.10	-1.07	-1.11	-1.15	-1.84	-1.65	-0.88

P41250	Glycine--tRNA ligase OS=Homo sapiens GN=GARS PE=1 SV=3 - [SYG_HUMAN]	1.25	1.34	1.45	1.60	0.04	0.20	0.46	0.52	0.45	0.19	0.02	0.17	-0.86	-1.28	-1.39	-0.89	-1.03	-1.12	-1.43	-0.88
Q9Y4F5	Centrosomal protein of 170 kDa protein B OS=Homo sapiens GN=CEP170B PE=1 SV=4 - [C170B_HUMAN]	1.81	1.57	1.78	1.53	-0.20	-0.35	0.21	0.05	-0.06	0.14	0.06	-0.03	-1.40	-1.65	-1.66	-1.50	-1.39	-1.83	-1.75	-1.29
O60282	Kinesin heavy chain isoform 5C OS=Homo sapiens GN=KIF5C PE=1 SV=1 - [KIF5C_HUMAN]	1.34	1.25	1.59	1.50	0.00	-0.19	0.75	0.60	0.01	0.12	-0.04	-0.08	-0.60	-1.40	-1.58	-1.10	-1.35	-1.44	-1.77	-0.84
Q8WXG6	MAP kinase-activating death domain protein OS=Homo sapiens GN=MADD PE=1 SV=2 - [MADD_HUMAN]	1.29	1.41	1.51	1.40	-0.14	-0.42	0.72	0.44	0.17	0.34	0.18	0.07	-0.83	-1.31	-1.13	-1.02	-0.96	-1.74	-1.63	-0.86
O14594	Neurocan core protein OS=Homo sapiens GN=NCAN PE=1 SV=3 - [NCAN_HUMAN]	1.41	1.41	1.23	1.30	0.42	0.47	0.33	0.45	0.44	0.35	0.22	0.23	-1.08	-1.10	-1.03	-0.95	-0.82	-1.13	-0.80	-0.78
O00139	Kinesin-like protein KIF2A OS=Homo sapiens GN=KIF2A PE=1 SV=3 - [KIF2A_HUMAN]	1.25	1.34	1.33	1.30	0.07	0.14	0.54	0.47	0.55	0.43	0.33	0.40	-0.69	-1.18	-1.00	-0.66	-0.90	-1.21	-1.32	-0.72
O94967	WD repeat-containing protein 47 OS=Homo sapiens GN=WDR47 PE=1 SV=1 - [WDR47_HUMAN]	1.45	1.25	1.45	1.11	0.15	-0.05	0.61	0.29	0.24	0.49	0.05	-0.10	-0.79	-0.95	-1.24	-0.89	-1.03	-1.28	-1.42	-0.86
P19013	Keratin, type II cytoskeletal 4 OS=Homo sapiens GN=KRT4 PE=1 SV=4 - [K2C4_HUMAN]	-3.20	-3.35	-3.69	-3.71	-3.42	-3.54	-3.87	-4.11	-3.35	-3.18	-2.97	-3.12	-0.27	-0.21	0.35	0.03	0.35	-0.14	0.53	0.15
P53814	Smoothelin OS=Homo sapiens GN=SMTN PE=1 SV=7 - [SMTN_HUMAN]	-2.70	-2.63	-3.23	-3.28	-1.21	-1.14	-1.35	-1.36	-1.28	-1.30	-1.02	-1.04	1.24	1.52	2.23	1.52	2.21	1.67	2.19	1.96
Q9NR12	PDZ and LIM domain protein 7 OS=Homo sapiens GN=PDLIM7 PE=1 SV=1 - [PDLI7_HUMAN]	-3.11	-3.00	-3.32	-3.10	-1.04	-1.02	-1.44	-1.39	-1.08	-1.10	-1.09	-0.97	1.72	2.04	2.17	1.84	1.99	1.98	2.01	1.85

Q05682	Caldesmon OS=Homo sapiens GN=CALD1 PE=1 SV=3 - [CALD1_HUMAN]	-2.71	-2.75	-2.53	-2.59	-1.56	-1.57	-1.31	-1.35	-1.25	-1.20	-0.76	-0.71	1.37	1.92	1.86	1.52	1.31	1.15	1.12	1.29
P21810	Biglycan OS=Homo sapiens GN=BGN PE=1 SV=2 - [PGS1_HUMAN]	-2.25	-2.34	-2.44	-2.48	-0.27	-0.30	-0.50	-0.48	-0.47	-0.46	-0.53	-0.53	1.79	1.81	2.03	1.89	2.12	2.06	2.26	2.03
Q5KU26	Collectin-12 OS=Homo sapiens GN=COLEC12 PE=1 SV=3 - [COL12_HUMAN]	-2.42	-2.35	-2.37	-2.36	-0.53	-0.46	-0.75	-0.72	-0.40	-0.44	-0.19	-0.09	1.66	2.17	2.07	1.94	1.92	1.84	1.77	1.65
Q93052	Lipoma-preferred partner OS=Homo sapiens GN=LPP PE=1 SV=1 - [LPP_HUMAN]	-2.16	-2.25	-2.30	-2.31	-0.82	-0.87	-0.75	-0.76	-0.76	-0.73	-0.51	-0.50	1.35	1.57	1.84	1.39	1.58	1.33	1.52	1.61
P07099	Epoxide hydrolase 1 OS=Homo sapiens GN=EPHX1 PE=1 SV=1 - [HYEP_HUMAN]	-1.84	-1.76	-1.98	-1.85	0.33	0.39	0.07	0.07	0.13	0.14	0.20	0.20	1.88	2.07	1.89	1.83	1.87	1.98	2.09	1.84
Q13683	Integrin alpha-7 OS=Homo sapiens GN=ITGA7 PE=1 SV=3 - [ITA7_HUMAN]	-1.65	-1.63	-1.88	-1.75	-0.48	-0.35	-0.70	-0.62	-0.59	-0.69	-0.49	-0.49	0.70	1.15	1.40	0.89	1.18	0.98	1.06	1.04
P43121	Cell surface glycoprotein MUC18 OS=Homo sapiens GN=MCAM PE=1 SV=2 - [MUC18_HUMAN]	-1.24	-1.23	-1.70	-1.64	0.06	0.02	-0.19	-0.23	-0.13	-0.19	-0.07	0.04	1.42	1.49	1.74	1.61	1.74	1.81	1.85	1.58
Q14624	Inter-alpha-trypsin inhibitor heavy chain H4 OS=Homo sapiens GN=ITIH4 PE=1 SV=4 - [ITIH4_HUMAN]	-1.53	-1.50	-1.70	-1.53	0.07	0.10	-0.52	-0.60	-0.04	-0.08	0.36	0.40	0.97	2.04	1.94	1.50	1.60	1.67	1.66	1.11
P61266	Syntaxin-1B OS=Homo sapiens GN=STX1B PE=1 SV=1 - [STX1B_HUMAN]	1.90	1.85	1.88	2.12	-0.03	-0.14	0.75	0.87	0.24	0.36	0.27	0.29	-1.08	-1.76	-2.02	-1.54	-1.61	-1.87	-1.94	-1.18
Q9UPR5	Sodium/calcium exchanger 2 OS=Homo sapiens GN=SLC8A2 PE=2 SV=2 - [NAC2_HUMAN]	2.14	1.76	2.34	2.03	0.14	-0.01	1.06	0.67	0.52	0.57	0.51	0.29	-0.97	-1.58	-1.81	-1.29	-1.45	-1.73	-2.05	-1.20

P40925	Malate dehydrogenase, cytoplasmic OS=Homo sapiens GN=MDH1 PE=1 SV=4 - [MDHC_HUMAN]	1.78	1.69	1.92	1.91	0.16	0.01	0.64	0.66	0.16	0.26	-0.20	-0.25	-1.07	-2.11	-2.36	-1.43	-1.72	-1.70	-1.99	-1.25
Q5TF21	Protein SOGA3 OS=Homo sapiens GN=SOGA3 PE=2 SV=1 - [SOGA3_HUMAN]	1.92	1.73	2.10	1.84	0.09	-0.01	1.02	0.55	0.42	0.53	0.38	0.34	-0.99	-1.39	-1.47	-1.34	-1.25	-2.00	-1.80	-1.15
P52306	Rap1 GTPase-GDP dissociation stimulator 1 OS=Homo sapiens GN=RAP1GDS1 PE=1 SV=3 - [GDS1_HUMAN]	1.37	1.54	1.55	1.71	-0.12	-0.07	0.56	0.83	0.41	0.33	-0.02	0.06	-0.62	-1.45	-1.51	-1.03	-1.19	-1.56	-1.76	-0.79
Q12765	Secernin-1 OS=Homo sapiens GN=SCRN1 PE=1 SV=2 - [SCRN1_HUMAN]	1.38	1.39	1.59	1.55	0.35	0.34	0.70	0.70	0.50	0.47	0.29	0.38	-0.67	-1.01	-1.17	-0.83	-1.01	-1.06	-1.23	-0.79
P27338	Amine oxidase [flavin-containing] B OS=Homo sapiens GN=MAOB PE=1 SV=3 - [AOFB_HUMAN]	1.43	1.45	1.54	1.40	0.42	0.24	1.23	1.22	0.85	0.91	0.71	0.56	-0.18	-0.86	-0.87	-0.54	-0.54	-1.20	-1.16	-0.17
Q8N9R8	Protein SCAI OS=Homo sapiens GN=SCAI PE=1 SV=2 - [SCAI_HUMAN]	1.36	1.37	1.31	1.35	-0.15	-0.08	0.48	0.61	0.38	0.35	0.17	0.28	-0.83	-1.16	-1.28	-1.09	-1.23	-1.49	-1.73	-0.92
P31930	Cytochrome b-c1 complex subunit 1, mitochondrial OS=Homo sapiens GN=UQCRC1 PE=1 SV=3 - [QCR1_HUMAN]	1.06	1.23	1.20	1.23	0.08	0.18	0.49	0.58	0.34	0.31	0.23	0.27	-0.49	-0.89	-0.95	-0.76	-0.86	-0.84	-1.15	-0.66
P55809	Succinyl-CoA:3-ketoacid coenzyme A transferase 1, mitochondrial OS=Homo sapiens GN=OXCT1 PE=1 SV=1 - [SCOT1_HUMAN]	1.35	1.26	1.39	1.23	-0.29	-0.25	0.33	0.35	-0.01	0.01	-0.13	-0.21	-0.85	-1.58	-1.71	-1.26	-1.37	-1.64	-1.72	-1.03
Q8N568	Serine/threonine-protein kinase DCLK2 OS=Homo sapiens GN=DCLK2 PE=2 SV=4 - [DCLK2_HUMAN]	1.14	1.05	1.24	1.19	-0.04	0.03	0.33	0.36	0.29	0.12	0.15	0.24	-0.68	-0.87	-0.91	-0.81	-0.84	-1.02	-1.00	-0.70

P13646	Keratin, type I cytoskeletal 13 OS=Homo sapiens GN=KRT13 PE=1 SV=4 - [K1C13_HUMAN]	-3.73	-3.63	-3.82	-3.81	-3.51	-3.58	-3.72	-3.76	-3.69	-3.69	-3.95	-4.04	-0.08	0.02	0.43	0.25	0.50	0.06	0.44	0.24
Q96CM8	Acyl-CoA synthetase family member 2, mitochondrial OS=Homo sapiens GN=ACSF2 PE=1 SV=2 - [ACSF2_HUMAN]	-2.24	-2.21	-2.31	-2.45	-0.80	-0.86	-0.80	-0.93	-0.96	-0.86	-0.81	-0.94	1.36	1.41	1.50	1.48	1.48	1.43	1.59	1.49
P08572	Collagen alpha-2(IV) chain OS=Homo sapiens GN=COL4A2 PE=1 SV=4 - [CO4A2_HUMAN]	-2.22	-2.37	-2.36	-2.41	0.27	0.07	-0.78	-0.82	-0.42	-0.43	-0.50	-0.59	1.28	1.63	1.74	1.72	1.81	2.19	2.40	1.48
P23141	Liver carboxylesterase 1 OS=Homo sapiens GN=CES1 PE=1 SV=2 - [EST1_HUMAN]	-1.51	-1.51	-2.17	-2.09	1.68	1.55	1.04	0.86	0.93	1.11	0.70	0.65	2.43	2.34	2.67	2.69	3.16	3.05	3.68	3.11
P09619	Platelet-derived growth factor receptor beta OS=Homo sapiens GN=PDGFRB PE=1 SV=1 - [PGFRB_HUMAN]	-1.90	-1.93	-2.05	-2.04	0.66	0.61	0.20	0.14	0.28	0.34	0.31	0.33	2.04	2.10	2.36	2.19	2.38	2.44	2.55	2.24
Q9NRN5	Olfactomedin-like protein 3 OS=Homo sapiens GN=OLFML3 PE=2 SV=1 - [OLFL3_HUMAN]	-1.45	-1.64	-1.85	-1.97	1.23	1.40	0.22	0.32	0.49	0.47	-0.11	-0.11	2.03	1.59	1.80	2.35	2.61	3.33	3.64	2.39
Q9BZQ8	Protein Niban OS=Homo sapiens GN=FAM129A PE=1 SV=1 - [NIBAN_HUMAN]	-1.78	-1.91	-2.07	-1.89	0.30	0.36	-0.30	-0.20	-0.19	-0.14	-0.17	-0.05	1.81	2.00	1.84	1.86	1.82	2.26	2.16	1.85
O60504	Vinexin OS=Homo sapiens GN=SORBS3 PE=1 SV=2 - [VINEX_HUMAN]	-1.77	-1.75	-1.80	-1.72	-0.90	-0.94	-0.82	-0.77	-0.85	-0.85	-0.44	-0.33	0.61	1.04	1.15	1.02	0.93	0.67	0.71	0.91
Q6DD88	Atlastin-3 OS=Homo sapiens GN=ATL3 PE=1 SV=1 - [ATLA3_HUMAN]	-1.99	-2.06	-1.60	-1.61	-0.27	-0.33	-0.62	-0.61	-0.49	-0.50	0.00	-0.04	1.32	1.84	1.57	1.56	1.23	1.75	1.29	1.01
Q9BXX0	EMILIN-2 OS=Homo sapiens GN=EMILIN2 PE=1 SV=3 - [EMIL2_HUMAN]	-1.33	-1.37	-1.65	-1.55	1.27	1.21	0.02	0.13	0.58	0.54	0.35	0.45	1.68	1.79	1.87	2.04	2.28	2.59	2.85	1.75

Q9GZM7	Tubulointerstitial nephritis antigen-like OS=Homo sapiens GN=TINAGL1 PE=1 SV=1 - [TINAL_HUMAN]	-1.50	-1.41	-1.67	-1.54	-0.32	-0.35	-0.79	-0.68	-0.58	-0.52	-0.67	-0.66	0.87	0.95	1.07	1.05	1.06	1.21	1.34	0.92
P05164	Myeloperoxidase OS=Homo sapiens GN=MPO PE=1 SV=1 - [PERM_HUMAN]	-1.43	-1.35	-1.64	-1.43	-0.76	-0.59	1.09	1.17	0.58	0.61	0.40	0.40	2.52	1.96	1.97	2.08	2.06	0.64	0.81	2.75
Q13425	Beta-2-syntrophin OS=Homo sapiens GN=SNTB2 PE=1 SV=1 - [SNTB2_HUMAN]	-1.25	-1.24	-1.40	-1.40	-0.09	-0.19	-0.30	-0.33	-0.37	-0.26	-0.21	-0.25	0.89	1.14	1.22	1.08	1.07	1.05	1.17	1.13
P50416	Carnitine O-palmitoyltransferase 1, liver isoform OS=Homo sapiens GN=CPT1A PE=1 SV=2 - [CPT1A_HUMAN]	-1.31	-1.35	-1.45	-1.38	0.00	0.16	-0.08	0.07	0.03	0.03	0.04	0.11	1.46	1.43	1.53	1.51	1.68	1.53	1.66	1.43
Q96AC1	Fermitin family homolog 2 OS=Homo sapiens GN=FERMT2 PE=1 SV=1 - [FERM2_HUMAN]	-1.31	-1.37	-1.25	-1.32	-0.38	-0.31	-0.64	-0.61	-0.55	-0.57	-0.63	-0.56	0.70	0.81	0.62	0.78	0.72	0.93	0.86	0.75
Q96I99	Succinyl-CoA ligase [GDP-forming] subunit beta, mitochondrial OS=Homo sapiens GN=SUCLG2 PE=1 SV=2 - [SUCB2_HUMAN]	-1.38	-1.38	-1.18	-1.19	-0.54	-0.51	-0.71	-0.73	-0.70	-0.65	-0.37	-0.34	0.62	0.91	0.79	0.69	0.53	0.82	0.62	0.47
Q9UBB6	Neurochondrin OS=Homo sapiens GN=NCDN PE=1 SV=1 - [NCDN_HUMAN]	2.64	2.54	2.64	2.56	0.11	0.11	1.10	0.94	0.55	0.65	0.24	0.09	-1.40	-2.40	-2.34	-1.91	-1.93	-2.43	-2.45	-1.29
Q9BPU6	Dihydropyrimidinase-related protein 5 OS=Homo sapiens GN=DPYSL5 PE=1 SV=1 - [DPYL5_HUMAN]	2.04	2.24	2.09	2.29	-0.04	0.06	0.48	0.41	0.27	0.33	-0.22	0.00	-1.46	-2.36	-2.38	-1.68	-1.86	-1.93	-2.21	-1.53
Q8NCB2	CaM kinase-like vesicle-associated protein OS=Homo sapiens GN=CAMKV PE=1 SV=2 - [CAMKV_HUMAN]	1.93	1.75	2.08	2.24	-0.25	-0.37	0.70	0.59	0.18	0.20	0.27	0.23	-1.06	-1.64	-1.75	-1.66	-2.08	-1.72	-1.93	-1.40
O43236	Septin-4 OS=Homo sapiens GN=SEPT4 PE=1 SV=1 - [SEPT4_HUMAN]	1.39	1.61	1.69	1.91	-0.23	0.06	0.51	0.74	0.29	0.22	0.19	0.22	-0.83	-1.40	-1.46	-1.27	-1.48	-1.67	-1.94	-1.15

Q99719	Septin-5 OS=Homo sapiens GN=SEPT5 PE=1 SV=1 - [SEPT5_HUMAN]	1.40	1.43	1.74	1.82	-0.25	-0.19	0.35	0.51	0.22	0.27	-0.09	0.08	-0.86	-1.37	-1.82	-1.23	-1.59	-1.65	-2.03	-1.12
Q5JU85	IQ motif and SEC7 domain-containing protein 2 OS=Homo sapiens GN=IQSEC2 PE=1 SV=1 - [IQEC2_HUMAN]	1.74	1.56	1.52	1.79	-0.06	-0.31	0.36	0.29	0.28	0.16	0.22	0.12	-1.35	-1.68	-1.70	-1.53	-1.35	-1.99	-1.85	-1.34
Q9NZR1	Tropomodulin-2 OS=Homo sapiens GN=TMOD2 PE=1 SV=1 - [TMOD2_HUMAN]	1.63	1.52	1.91	1.79	0.16	0.02	0.75	0.65	0.35	0.51	0.14	0.08	-0.75	-1.50	-1.80	-1.18	-1.67	-1.46	-1.73	-1.08
P42658	Dipeptidyl aminopeptidase-like protein 6 OS=Homo sapiens GN=DPP6 PE=1 SV=2 - [DPP6_HUMAN]	1.39	1.56	1.60	1.78	-0.04	-0.11	0.31	0.55	0.36	0.24	0.12	0.21	-0.86	-1.18	-1.33	-1.09	-1.32	-1.82	-2.15	-1.14
P12277	Creatine kinase B-type OS=Homo sapiens GN=CKB PE=1 SV=1 - [KCRB_HUMAN]	1.69	1.71	1.71	1.73	-0.19	-0.17	0.58	0.56	0.35	0.36	0.26	0.26	-0.97	-1.41	-1.43	-1.21	-1.20	-1.89	-1.91	-0.91
Q92777	Synapsin-2 OS=Homo sapiens GN=SYN2 PE=1 SV=3 - [SYN2_HUMAN]	1.38	1.51	1.41	1.65	-0.44	-0.32	0.58	0.67	0.07	0.05	-0.40	-0.21	-0.74	-1.66	-1.60	-1.13	-1.31	-1.76	-1.58	-0.90
O75061	Putative tyrosine-protein phosphatase auxilin OS=Homo sapiens GN=DNAJC6 PE=1 SV=3 - [AUXI_HUMAN]	1.61	1.37	1.81	1.64	-0.33	-0.15	0.44	0.46	0.15	0.16	0.07	0.02	-0.96	-1.66	-1.86	-1.47	-1.87	-1.85	-2.22	-1.35
Q15700	Disks large homolog 2 OS=Homo sapiens GN=DLG2 PE=1 SV=3 - [DLG2_HUMAN]	1.72	1.67	1.72	1.60	-0.15	-0.29	0.35	0.27	0.15	0.23	-0.10	-0.17	-1.11	-1.78	-2.14	-1.39	-1.56	-1.91	-1.98	-1.30
P50213	Isocitrate dehydrogenase [NAD] subunit alpha, mitochondrial OS=Homo sapiens GN=IDH3A PE=1 SV=1 - [IDH3A_HUMAN]	1.17	1.22	1.55	1.57	-0.08	-0.04	0.51	0.55	0.25	0.14	0.17	0.18	-0.71	-1.17	-1.36	-0.89	-1.26	-1.26	-1.76	-0.88
P60880	Synaptosomal-associated protein 25 OS=Homo sapiens GN=SNAP25 PE=1 SV=1 - [SNP25_HUMAN]	1.56	1.63	1.46	1.54	-0.28	-0.22	0.37	0.43	0.18	0.14	0.11	0.07	-1.20	-1.59	-1.51	-1.53	-1.49	-1.76	-1.73	-1.24

Q99747	Gamma-soluble NSF attachment protein OS=Homo sapiens GN=NAPG PE=1 SV=1 - [SNAG_HUMAN]	1.17	1.11	1.29	1.44	-0.14	-0.13	0.44	0.40	0.03	0.04	-0.01	-0.01	-0.66	-1.03	-1.36	-0.84	-1.24	-1.06	-1.43	-0.99
Q6ZVM7	TOM1-like protein 2 OS=Homo sapiens GN=TOM1L2 PE=1 SV=1 - [TM1L2_HUMAN]	1.28	1.43	1.37	1.40	0.08	0.21	0.26	0.29	0.05	0.06	-0.08	-0.06	-0.97	-1.36	-1.32	-1.29	-1.27	-1.25	-1.21	-1.03
O95741	Copine-6 OS=Homo sapiens GN=CPNE6 PE=1 SV=3 - [CPNE6_HUMAN]	1.35	1.44	1.37	1.38	0.19	0.13	0.95	0.93	0.69	0.65	0.52	0.46	-0.52	-0.93	-0.89	-0.76	-0.76	-1.35	-1.35	-0.42
Q9Y2J8	Protein-arginine deiminase type-2 OS=Homo sapiens GN=PADI2 PE=1 SV=2 - [PADI2_HUMAN]	1.49	1.54	1.54	1.34	0.83	0.70	1.24	1.12	0.98	1.08	1.06	0.91	-0.07	-0.41	-0.55	-0.31	-0.48	-0.59	-0.88	-0.22
Q8NFP9	Neurobeachin OS=Homo sapiens GN=NBEA PE=1 SV=3 - [NBEA_HUMAN]	1.19	1.27	1.22	1.25	-0.20	-0.14	0.09	0.19	0.01	-0.03	-0.18	-0.02	-0.94	-1.08	-1.21	-1.35	-1.07	-1.40	-1.41	-1.14
Q16853	Membrane primary amine oxidase OS=Homo sapiens GN=AOC3 PE=1 SV=3 - [AOC3_HUMAN]	-2.38	-2.52	-2.65	-2.66	-0.07	-0.02	-0.74	-0.89	-0.47	-0.41	-0.14	-0.14	1.71	2.43	2.50	1.96	2.22	2.48	2.68	1.97
Q8IUX7	Adipocyte enhancer-binding protein 1 OS=Homo sapiens GN=AEBP1 PE=1 SV=1 - [AEBP1_HUMAN]	-2.01	-1.74	-2.32	-2.31	-0.38	-0.41	-0.96	-0.80	-0.72	-0.68	-0.90	-0.88	1.26	1.08	1.54	1.20	1.69	1.64	2.02	1.54
P02647	Apolipoprotein A-I OS=Homo sapiens GN=APOA1 PE=1 SV=1 - [APOA1_HUMAN]	-1.77	-1.71	-1.86	-1.89	0.11	0.17	-0.36	-0.39	-0.16	-0.15	0.01	-0.06	1.55	1.89	1.94	1.93	1.74	1.87	1.95	1.59
O95810	Serum deprivation-response protein OS=Homo sapiens GN=SDPR PE=1 SV=3 - [SDPR_HUMAN]	-1.69	-1.95	-1.87	-1.85	-0.15	-0.19	-0.34	-0.29	-0.36	-0.29	-0.19	-0.23	1.53	1.68	1.65	1.63	1.52	1.61	1.66	1.50

Q9H6R3	Acyl-CoA synthetase short-chain family member 3, mitochondrial OS=Homo sapiens GN=ACSS3 PE=1 SV=1 - [ACSS3_HUMAN]	-1.57	-1.55	-1.78	-1.80	-0.24	-0.29	-0.56	-0.49	-0.47	-0.60	-0.42	-0.44	1.14	1.25	1.32	0.98	1.31	1.41	1.33	1.34
Q9H2D6	TRIO and F-actin-binding protein OS=Homo sapiens GN=TRIOBP PE=1 SV=3 - [TARA_HUMAN]	-1.64	-1.58	-1.89	-1.79	-0.31	-0.28	-0.67	-0.68	-0.33	-0.34	-0.11	-0.12	0.92	1.61	1.67	1.19	1.49	1.42	1.43	1.23
P98095	Fibulin-2 OS=Homo sapiens GN=FBLN2 PE=1 SV=2 - [FBLN2_HUMAN]	-1.45	-1.38	-1.66	-1.69	0.44	0.47	-0.65	-0.74	-0.24	-0.22	-0.48	-0.40	0.67	0.98	1.30	0.88	1.21	1.65	2.01	1.00
P23142	Fibulin-1 OS=Homo sapiens GN=FBLN1 PE=1 SV=4 - [FBLN1_HUMAN]	-1.13	-1.15	-1.44	-1.35	0.87	0.91	-0.10	-0.24	0.20	0.20	0.45	0.46	0.94	1.73	2.03	1.23	1.75	2.05	2.35	1.33
P28289	Tropomodulin-1 OS=Homo sapiens GN=TMOD1 PE=1 SV=1 - [TMOD1_HUMAN]	-1.37	-1.34	-1.35	-1.30	-0.17	-0.13	-0.34	-0.38	-0.14	-0.16	0.31	0.34	0.91	1.61	1.64	1.21	1.19	1.20	1.09	0.91
P08183	Multidrug resistance protein 1 OS=Homo sapiens GN=ABCB1 PE=1 SV=3 - [MDR1_HUMAN]	-1.11	-1.06	-1.43	-1.29	-0.88	-0.96	-1.19	-1.23	-0.69	-0.77	-0.78	-0.79	0.15	0.46	0.60	0.30	0.55	0.14	0.29	0.45
P04844	Dolichyl-diphosphooligosaccharide--protein glycosyltransferase subunit 2 OS=Homo sapiens GN=RPN2 PE=1 SV=3 - [RPN2_HUMAN]	-1.34	-1.23	-1.47	-1.20	0.17	0.26	-0.38	-0.32	0.04	-0.16	-0.07	0.04	0.88	1.18	1.21	1.16	1.20	1.28	1.45	1.14
Q969P0	Immunoglobulin superfamily member 8 OS=Homo sapiens GN=IGSF8 PE=1 SV=1 - [IGSF8_HUMAN]	1.58	1.58	1.72	1.70	0.07	-0.01	0.50	0.44	0.25	0.29	0.18	0.27	-1.29	-1.48	-1.49	-1.57	-1.62	-1.79	-1.75	-1.36
P42262	Glutamate receptor 2 OS=Homo sapiens GN=GRIA2 PE=1 SV=3 - [GRIA2_HUMAN]	1.77	1.24	2.12	1.67	-0.26	-0.10	0.64	0.48	0.28	0.31	-0.15	-0.19	-0.97	-1.47	-1.66	-0.97	-1.33	-1.36	-1.78	-1.15
Q92796	Disks large homolog 3 OS=Homo sapiens GN=DLG3 PE=1 SV=2 - [DLG3_HUMAN]	1.83	1.36	1.74	1.66	0.04	0.11	0.25	0.29	0.10	0.40	0.12	0.17	-0.86	-1.41	-1.57	-1.45	-1.45	-1.43	-1.47	-0.98

O43837	Isocitrate dehydrogenase [NAD] subunit beta, mitochondrial OS=Homo sapiens GN=IDH3B PE=1 SV=2 - [IDH3B_HUMAN]	1.22	1.23	1.64	1.61	-0.24	-0.33	0.55	0.50	0.09	0.14	0.06	-0.01	-0.64	-1.15	-1.58	-0.99	-1.41	-1.55	-1.73	-0.97
Q9UI12	V-type proton ATPase subunit H OS=Homo sapiens GN=ATP6V1H PE=1 SV=1 - [VATH_HUMAN]	1.57	1.27	1.74	1.59	-0.02	-0.13	0.70	0.58	0.34	0.35	0.27	0.14	-0.76	-1.03	-1.35	-1.00	-1.20	-1.37	-1.69	-0.77
Q9Y4G8	Rap guanine nucleotide exchange factor 2 OS=Homo sapiens GN=RAPGEF2 PE=1 SV=1 - [RPGF2_HUMAN]	1.11	1.40	1.35	1.48	-0.23	-0.07	0.22	0.41	0.33	0.07	-0.07	0.28	-1.01	-1.33	-1.57	-1.24	-1.47	-1.44	-1.74	-1.19
P21266	Glutathione S-transferase Mu 3 OS=Homo sapiens GN=GSTM3 PE=1 SV=3 - [GSTM3_HUMAN]	1.27	1.40	1.33	1.47	0.64	0.74	0.89	1.04	1.02	0.84	0.92	0.91	-0.45	-0.59	-0.54	-0.39	-0.44	-0.71	-0.67	-0.44
O60641	Clathrin coat assembly protein AP180 OS=Homo sapiens GN=SNAP91 PE=1 SV=2 - [AP180_HUMAN]	1.20	1.17	1.25	1.46	0.73	0.64	0.54	0.48	0.42	0.60	0.14	0.04	-0.82	-1.26	-1.28	-0.80	-0.83	-0.53	-0.69	-0.86
P21283	V-type proton ATPase subunit C 1 OS=Homo sapiens GN=ATP6V1C1 PE=1 SV=4 - [VATC1_HUMAN]	1.41	1.38	1.48	1.44	-0.14	-0.09	0.53	0.60	0.24	0.18	0.06	0.05	-0.63	-1.28	-1.46	-1.03	-1.21	-1.41	-1.59	-0.78
O14531	Dihydropyrimidinase-related protein 4 OS=Homo sapiens GN=DPYSL4 PE=1 SV=2 - [DPYL4_HUMAN]	1.42	1.21	1.62	1.43	-0.24	-0.01	0.64	0.50	0.02	0.06	-0.35	-0.20	-1.06	-1.81	-2.05	-1.34	-1.53	-1.75	-2.05	-1.10
O75390	Citrate synthase, mitochondrial OS=Homo sapiens GN=CS PE=1 SV=2 - [CISY_HUMAN]	1.02	1.06	1.40	1.39	-0.15	-0.15	0.42	0.39	-0.01	-0.05	-0.12	-0.14	-0.63	-1.17	-1.53	-1.06	-1.41	-1.31	-1.53	-0.96
Q96F07	Cytoplasmic FMR1-interacting protein 2 OS=Homo sapiens GN=CYFIP2 PE=1 SV=2 - [CYFP2_HUMAN]	1.28	1.17	1.30	1.24	-0.13	-0.06	0.51	0.45	0.14	0.01	-0.23	-0.08	-0.83	-1.28	-1.27	-1.02	-1.04	-1.35	-1.37	-0.66

Q6UWY5	Olfactomedin-like protein 1 OS=Homo sapiens GN=OLFML1 PE=1 SV=2 - [OLFL1_HUMAN]	-2.70	-2.73	-3.37	-3.25	-0.70	-0.68	-0.62	-0.59	-0.89	-0.85	-1.16	-1.23	2.01	1.75	2.15	1.94	2.43	2.25	2.58	2.70
P51888	Prolargin OS=Homo sapiens GN=PRELP PE=1 SV=1 - [PRELP_HUMAN]	-1.97	-2.02	-2.34	-2.35	0.17	0.20	-0.60	-0.52	-0.21	-0.22	-0.70	-0.67	1.56	1.42	1.72	1.81	2.16	2.28	2.57	1.86
P04083	Annexin A1 OS=Homo sapiens GN=ANXA1 PE=1 SV=2 - [ANXA1_HUMAN]	-2.01	-2.01	-2.00	-2.12	-0.20	-0.31	-0.33	-0.40	-0.27	-0.23	-0.10	-0.19	1.68	1.82	1.95	1.77	1.82	1.72	1.78	1.75
P25391	Laminin subunit alpha-1 OS=Homo sapiens GN=LAMA1 PE=1 SV=2 - [LAMA1_HUMAN]	-1.67	-1.85	-1.97	-2.09	-0.79	-0.88	-0.95	-1.11	-0.93	-0.79	-0.47	-0.59	0.72	1.24	1.51	0.78	1.29	0.90	1.24	1.04
P20774	Mimecan OS=Homo sapiens GN=OGN PE=1 SV=1 - [MIME_HUMAN]	-2.07	-2.12	-2.12	-2.02	0.34	0.37	-0.26	-0.25	-0.18	-0.17	-0.87	-0.86	1.86	1.24	1.22	2.01	1.96	2.45	2.44	1.81
P00966	Argininosuccinate synthase OS=Homo sapiens GN=ASS1 PE=1 SV=2 - [ASSY_HUMAN]	-1.24	-1.28	-1.79	-2.01	1.17	1.04	1.36	1.32	0.93	1.09	0.61	0.40	2.58	1.66	2.20	2.23	2.83	2.33	2.84	2.96
Q9UGT4	Sushi domain-containing protein 2 OS=Homo sapiens GN=SUSD2 PE=1 SV=1 - [SUSD2_HUMAN]	-1.64	-1.58	-1.86	-1.95	0.51	0.56	-0.34	-0.25	0.10	-0.01	0.17	0.22	1.31	1.78	2.19	1.66	2.07	2.31	2.32	1.59
Q13418	Integrin-linked protein kinase OS=Homo sapiens GN=ILK PE=1 SV=2 - [ILK_HUMAN]	-1.66	-1.71	-1.95	-1.93	-0.49	-0.39	-0.52	-0.44	-0.55	-0.57	-0.46	-0.32	1.36	1.28	1.44	1.25	1.36	1.29	1.39	1.57
Q14766	Latent-transforming growth factor beta-binding protein 1 OS=Homo sapiens GN=LTBP1 PE=1 SV=4 - [LTBP1_HUMAN]	-1.51	-1.72	-1.85	-1.81	0.71	0.70	0.16	0.06	0.29	0.28	0.27	0.16	1.79	1.65	1.97	1.80	2.09	2.27	2.61	2.12
P30613	Pyruvate kinase PKLR OS=Homo sapiens GN=PKLR PE=1 SV=2 - [KPYR_HUMAN]	-1.66	-1.68	-1.77	-1.76	-0.30	-0.30	-0.21	-0.26	-0.19	-0.27	-0.18	-0.23	1.49	1.42	1.49	1.52	1.49	1.34	1.47	1.70

Q13308	Inactive tyrosine-protein kinase 7 OS=Homo sapiens GN=PTK7 PE=1 SV=2 - [PTK7_HUMAN]	-1.24	-1.24	-1.23	-1.64	0.11	0.05	-0.03	-0.21	-0.06	0.21	0.20	0.21	1.31	1.76	1.85	1.40	1.53	1.40	1.78	1.31
P21589	5'-nucleotidase OS=Homo sapiens GN=NT5E PE=1 SV=1 - [5NTD_HUMAN]	-1.93	-1.99	-1.74	-1.64	0.37	0.42	-0.48	-0.42	0.01	-0.02	0.40	0.46	1.32	2.21	2.20	1.77	1.71	2.15	2.12	1.35
Q6NUK1	Calcium-binding mitochondrial carrier protein SCaMC-1 OS=Homo sapiens GN=SLC25A24 PE=1 SV=2 - [SCMC1_HUMAN]	-1.31	-1.29	-1.48	-1.52	-0.05	0.01	-0.15	-0.20	0.20	0.08	0.13	0.13	1.03	1.42	1.60	1.50	1.42	1.25	1.42	1.30
Q9H8L6	Multimerin-2 OS=Homo sapiens GN=MMRN2 PE=1 SV=2 - [MMRN2_HUMAN]	-1.35	-1.29	-1.46	-1.47	0.23	0.28	-0.54	-0.53	-0.18	-0.19	0.06	0.08	1.05	1.45	1.47	1.18	1.39	1.59	1.67	1.15
Q6UWR7	Ectonucleotide pyrophosphatase/phosphodiesterase family member 6 OS=Homo sapiens GN=ENPP6 PE=1 SV=2 - [ENPP6_HUMAN]	-1.41	-1.37	-1.36	-1.45	0.31	0.17	0.33	0.32	0.45	0.41	0.79	0.76	1.80	2.31	2.40	1.94	1.86	1.68	1.71	1.96
Q9UJS0	Calcium-binding mitochondrial carrier protein Aralar2 OS=Homo sapiens GN=SLC25A13 PE=1 SV=2 - [CMC2_HUMAN]	-1.51	-1.57	-1.36	-1.43	0.22	0.11	-0.07	-0.09	-0.10	-0.07	-0.07	-0.09	1.58	1.45	1.38	1.41	1.35	1.66	1.60	1.37
Q9H3U1	Protein unc-45 homolog A OS=Homo sapiens GN=UNC45A PE=1 SV=1 - [UN45A_HUMAN]	-1.46	-1.45	-1.45	-1.39	-0.25	0.00	-0.45	-0.33	-0.35	-0.33	-0.39	-0.20	1.13	0.95	0.89	1.08	1.01	1.32	1.33	1.13
Q8NBS9	Thioredoxin domain-containing protein 5 OS=Homo sapiens GN=TXNDC5 PE=1 SV=2 - [TXND5_HUMAN]	-1.39	-1.27	-1.44	-1.31	0.23	0.35	-0.17	-0.10	0.07	-0.11	0.04	0.14	1.22	1.40	1.49	1.31	1.41	1.57	1.65	1.25
P20916	Myelin-associated glycoprotein OS=Homo sapiens GN=MAG PE=1 SV=1 - [MAG_HUMAN]	2.59	2.77	2.64	2.75	0.07	0.15	0.83	0.75	0.46	0.55	0.10	0.09	-1.96	-2.60	-2.68	-2.08	-2.16	-2.36	-2.47	-1.92

Q68DU8	BTB/POZ domain-containing protein KCTD16 OS=Homo sapiens GN=KCTD16 PE=2 SV=1 - [KCD16_HUMAN]	2.42	2.37	2.69	2.61	0.07	0.24	0.61	0.85	0.56	0.45	0.48	0.56	-1.35	-2.18	-2.13	-1.82	-1.91	-2.37	-2.51	-1.79
Q8IXJ6	NAD-dependent protein deacetylase sirtuin-2 OS=Homo sapiens GN=SIRT2 PE=1 SV=2 - [SIR2_HUMAN]	2.40	2.63	2.59	2.54	-0.32	-0.42	0.77	0.83	0.31	0.20	-0.20	-0.07	-1.57	-2.44	-2.53	-2.17	-2.08	-2.81	-3.00	-1.65
P09936	Ubiquitin carboxyl-terminal hydrolase isozyme L1 OS=Homo sapiens GN=UCHL1 PE=1 SV=2 - [UCHL1_HUMAN]	1.72	1.80	2.15	2.21	-0.10	-0.04	0.76	0.80	0.27	0.26	0.08	0.05	-0.99	-1.80	-2.11	-1.57	-1.89	-1.96	-2.30	-1.36
P12532	Creatine kinase U-type, mitochondrial OS=Homo sapiens GN=CKMT1A PE=1 SV=1 - [KCRU_HUMAN]	2.15	2.15	1.87	2.08	0.14	0.16	0.83	0.64	0.24	0.43	0.15	0.06	-1.23	-2.13	-2.00	-1.75	-1.66	-1.87	-1.82	-1.16
Q9NYI0	PH and SEC7 domain-containing protein 3 OS=Homo sapiens GN=PSD3 PE=1 SV=2 - [PSD3_HUMAN]	1.80	1.63	1.95	1.94	-0.31	-0.44	0.92	0.85	0.37	0.35	0.24	0.08	-0.76	-1.55	-1.73	-1.40	-1.63	-2.14	-2.40	-0.95
P20020	Plasma membrane calcium-transporting ATPase 1 OS=Homo sapiens GN=ATP2B1 PE=1 SV=3 - [AT2B1_HUMAN]	1.96	1.82	2.11	1.93	0.08	-0.06	0.61	0.52	0.12	0.30	0.24	0.24	-1.31	-1.67	-1.70	-1.57	-1.76	-1.87	-1.98	-1.46
Q12756	Kinesin-like protein KIF1A OS=Homo sapiens GN=KIF1A PE=1 SV=2 - [KIF1A_HUMAN]	1.85	2.00	1.67	1.87	0.06	0.37	0.34	0.42	0.58	0.41	0.03	0.02	-1.26	-1.79	-1.73	-1.50	-1.48	-1.60	-1.47	-1.17
Q9H115	Beta-soluble NSF attachment protein OS=Homo sapiens GN=NAPB PE=1 SV=2 - [SNAB_HUMAN]	1.57	1.59	1.80	1.78	-0.04	-0.05	0.80	0.81	0.45	0.44	0.31	0.23	-0.74	-1.25	-1.57	-1.13	-1.28	-1.74	-1.82	-0.92

P78324	Tyrosine-protein phosphatase non-receptor type substrate 1 OS=Homo sapiens GN=SIRPA PE=1 SV=2 - [SHPS1_HUMAN]	1.61	1.52	1.80	1.71	0.07	-0.02	0.69	0.77	0.33	0.46	0.08	0.14	-0.78	-1.53	-1.66	-1.18	-1.31	-1.49	-1.57	-0.89
P36543	V-type proton ATPase subunit E 1 OS=Homo sapiens GN=ATP6V1E1 PE=1 SV=1 - [VATE1_HUMAN]	1.59	1.48	1.80	1.67	-0.06	-0.01	0.70	0.65	0.31	0.37	0.28	0.22	-0.85	-1.30	-1.48	-1.15	-1.36	-1.64	-1.83	-0.93
Q16623	Syntaxin-1A OS=Homo sapiens GN=STX1A PE=1 SV=1 - [STX1A_HUMAN]	1.32	1.24	1.40	1.58	-0.35	-0.26	0.39	0.49	0.05	0.11	-0.21	-0.10	-0.86	-1.53	-1.77	-1.34	-1.60	-1.64	-1.90	-1.19
Q9NZQ3	NCK-interacting protein with SH3 domain OS=Homo sapiens GN=NCKIPSD PE=1 SV=1 - [SPN90_HUMAN]	1.48	1.71	1.25	1.57	0.34	0.33	0.32	0.51	0.30	0.15	0.34	0.30	-1.10	-1.29	-1.41	-1.36	-1.38	-1.49	-1.40	-0.81
Q9Y639	Neuroplastin OS=Homo sapiens GN=NPTN PE=1 SV=2 - [NPTN_HUMAN]	1.34	1.33	1.53	1.56	0.16	-0.01	0.53	0.60	0.24	0.33	0.12	0.19	-0.79	-1.21	-1.49	-1.15	-1.39	-1.24	-1.68	-1.04
Q96GW7	Brevican core protein OS=Homo sapiens GN=BCAN PE=1 SV=2 - [PGCB_HUMAN]	1.36	1.72	1.36	1.52	0.15	0.17	0.74	0.85	0.44	0.50	0.37	0.46	-0.87	-1.31	-1.17	-1.15	-0.98	-1.56	-1.37	-0.70
P02538	Keratin, type II cytoskeletal 6A OS=Homo sapiens GN=KRT6A PE=1 SV=3 - [K2C6A_HUMAN]	-3.81	-3.86	-4.62	-4.80	-3.88	-3.96	-4.40	-4.57	-3.62	-3.60	-4.10	-4.23	-0.66	-0.49	0.42	-0.33	0.80	-0.40	0.61	0.29
P51911	Calponin-1 OS=Homo sapiens GN=CNN1 PE=1 SV=2 - [CNN1_HUMAN]	-3.13	-3.14	-3.31	-3.41	-1.18	-1.20	-1.62	-1.68	-1.36	-1.41	-1.39	-1.40	1.74	1.99	2.12	1.93	1.98	2.00	1.99	1.86
Q01995	Transgelin OS=Homo sapiens GN=TAGLN PE=1 SV=4 - [TAGL_HUMAN]	-2.94	-2.90	-3.19	-3.09	-1.42	-1.35	-1.78	-1.84	-1.52	-1.63	-1.35	-1.38	1.32	1.53	1.68	1.36	1.50	1.57	1.70	1.36
P02461	Collagen alpha-1(III) chain OS=Homo sapiens GN=COL3A1 PE=1 SV=4 - [CO3A1_HUMAN]	-2.66	-2.85	-2.54	-2.83	-1.56	-1.71	-2.39	-2.51	-2.34	-2.10	-2.60	-2.68	0.16	0.04	-0.03	0.40	0.35	1.21	1.01	0.05

O75112	LIM domain-binding protein 3 OS=Homo sapiens GN=LDB3 PE=1 SV=2 - [LDB3_HUMAN]	-2.60	-2.47	-2.71	-2.57	-1.86	-1.82	-1.52	-1.38	-1.54	-1.62	-1.36	-1.31	0.85	1.24	1.64	0.96	1.34	0.48	0.85	1.16
P07585	Decorin OS=Homo sapiens GN=DCN PE=1 SV=1 - [PGS2_HUMAN]	-2.13	-2.13	-2.19	-2.23	-0.63	-0.67	-1.10	-1.17	-1.02	-0.93	-1.10	-1.15	1.01	1.14	1.14	1.26	1.23	1.64	1.65	1.11
O00151	PDZ and LIM domain protein 1 OS=Homo sapiens GN=PDLIM1 PE=1 SV=4 - [PDLI1_HUMAN]	-2.22	-2.14	-2.12	-2.21	0.01	0.00	-0.46	-0.51	-0.41	-0.40	-0.43	-0.52	1.75	1.91	1.80	1.70	1.72	2.13	1.97	1.58
P53708	Integrin alpha-8 OS=Homo sapiens GN=ITGA8 PE=1 SV=3 - [ITA8_HUMAN]	-1.89	-1.85	-2.25	-2.20	-0.21	-0.18	-0.83	-0.79	-0.29	-0.41	-0.22	-0.40	1.01	1.40	1.93	1.29	1.78	1.63	1.93	1.64
Q96MM6	Heat shock 70 kDa protein 12B OS=Homo sapiens GN=HSPA12B PE=2 SV=2 - [HS12B_HUMAN]	-1.90	-1.98	-1.68	-1.68	-0.44	-0.47	-0.46	-0.56	-0.54	-0.41	-0.31	-0.44	1.42	1.46	1.46	1.34	1.31	1.38	1.16	1.26
P12429	Annexin A3 OS=Homo sapiens GN=ANXA3 PE=1 SV=3 - [ANXA3_HUMAN]	-1.67	-1.37	-1.71	-1.64	-0.39	-0.44	0.20	0.22	-0.08	-0.07	-0.15	-0.15	1.65	1.34	1.54	1.57	1.58	1.01	1.27	2.10
Q96JY6	PDZ and LIM domain protein 2 OS=Homo sapiens GN=PDLIM2 PE=1 SV=1 - [PDLI2_HUMAN]	-1.70	-1.57	-1.60	-1.62	-1.02	-0.99	-1.25	-1.38	-0.86	-0.89	-0.42	-0.44	0.50	1.39	1.25	0.81	0.83	0.68	0.51	0.42
Q9UGI8	Testin OS=Homo sapiens GN=TES PE=1 SV=1 - [TES_HUMAN]	-1.61	-1.63	-1.38	-1.57	0.57	0.52	0.19	0.07	0.05	0.09	-0.88	-0.89	1.77	0.79	0.86	1.56	1.66	2.11	2.25	1.84
P54886	Delta-1-pyrroline-5-carboxylate synthase OS=Homo sapiens GN=ALDH18A1 PE=1 SV=2 - [P5CS_HUMAN]	-1.67	-1.57	-1.46	-1.55	-0.53	-0.80	-1.07	-1.12	-0.68	-0.59	-0.39	-0.28	0.43	1.14	1.01	0.81	0.91	0.84	0.80	0.51
P13674	Prolyl 4-hydroxylase subunit alpha-1 OS=Homo sapiens GN=P4HA1 PE=1 SV=2 - [P4HA1_HUMAN]	-1.43	-1.37	-1.59	-1.54	0.14	0.24	-0.40	-0.37	-0.24	-0.26	-0.19	-0.17	1.05	1.68	1.45	1.22	1.41	1.61	1.66	1.21

Q15084	Protein disulfide-isomerase A6 OS=Homo sapiens GN=PDIA6 PE=1 SV=1 - [PDIA6_HUMAN]	-1.12	-1.16	-1.38	-1.49	-0.02	-0.11	-0.59	-0.56	-0.37	-0.29	-0.18	-0.27	0.62	1.15	1.09	0.88	1.15	0.96	1.38	1.04
Q8N8S7	Protein enabled homolog OS=Homo sapiens GN=ENAH PE=1 SV=2 - [ENAH_HUMAN]	-1.23	-1.42	-1.36	-1.33	-0.26	-0.22	-0.63	-0.61	-0.54	-0.50	-0.47	-0.47	0.58	0.64	0.78	0.93	0.79	0.99	1.10	0.72
P09871	Complement C1s subcomponent OS=Homo sapiens GN=C1S PE=1 SV=1 - [C1S_HUMAN]	-1.49	-1.35	-1.43	-1.33	0.23	0.39	-0.45	-0.22	0.14	-0.10	0.22	0.39	1.22	1.76	1.82	1.25	1.26	1.48	1.62	1.12
P41218	Myeloid cell nuclear differentiation antigen OS=Homo sapiens GN=MNDA PE=1 SV=1 - [MNDA_HUMAN]	-1.27	-1.23	-1.28	-1.16	-0.67	-0.53	0.29	0.28	0.02	0.01	-0.06	-0.16	1.54	0.99	1.16	1.11	1.03	0.48	0.64	1.52
Q8NCN5	Pyruvate dehydrogenase phosphatase regulatory subunit, mitochondrial OS=Homo sapiens GN=PDPR PE=1 SV=2 - [PDPR_HUMAN]	-1.17	-1.16	-1.18	-1.15	-0.10	-0.02	-0.43	-0.34	-0.27	-0.28	-0.04	0.06	0.79	1.45	1.25	1.11	0.99	1.13	1.06	0.89
P43004	Excitatory amino acid transporter 2 OS=Homo sapiens GN=SLC1A2 PE=1 SV=2 - [EAA2_HUMAN]	2.43	2.38	2.65	2.73	0.02	0.08	1.33	1.37	0.65	0.66	0.17	0.26	-0.96	-2.37	-2.67	-1.72	-2.05	-2.70	-3.13	-1.15
Q8N145	Leucine-rich repeat LGI family member 3 OS=Homo sapiens GN=LGI3 PE=2 SV=1 - [LGI3_HUMAN]	2.19	2.29	2.49	2.57	-0.27	-0.01	0.40	0.48	0.06	0.06	-0.16	-0.16	-1.66	-2.42	-2.54	-1.99	-2.30	-2.48	-2.60	-1.95
Q9H2X9	Solute carrier family 12 member 5 OS=Homo sapiens GN=SLC12A5 PE=2 SV=3 - [S12A5_HUMAN]	2.43	2.33	2.41	2.56	-0.06	-0.08	0.82	0.96	0.43	0.46	0.22	0.24	-1.36	-2.16	-2.08	-1.83	-1.94	-2.38	-2.49	-1.45
Q9P0K1	Disintegrin and metalloproteinase domain-containing protein 22 OS=Homo sapiens GN=ADAM22 PE=1 SV=1 - [ADA22_HUMAN]	2.11	2.03	2.24	2.51	-0.13	0.10	0.83	0.94	0.52	0.53	0.01	0.24	-1.11	-1.93	-2.34	-1.40	-2.00	-2.10	-2.30	-1.50

Q16720	Plasma membrane calcium-transporting ATPase 3 OS=Homo sapiens GN=ATP2B3 PE=1 SV=3 - [AT2B3_HUMAN]	2.31	2.01	2.47	2.38	-0.34	-0.52	0.94	0.99	0.51	0.48	0.36	0.28	-1.13	-1.75	-2.05	-1.70	-1.89	-2.47	-2.60	-1.34
P02686	Myelin basic protein OS=Homo sapiens GN=MBP PE=1 SV=3 - [MBP_HUMAN]	2.23	2.17	2.33	2.30	-0.29	-0.29	0.31	0.28	-0.06	-0.09	-0.24	-0.29	-1.88	-2.47	-2.57	-2.21	-2.29	-2.49	-2.47	-2.11
P05026	Sodium/potassium-transporting ATPase subunit beta-1 OS=Homo sapiens GN=ATP1B1 PE=1 SV=1 - [AT1B1_HUMAN]	1.95	1.92	2.41	2.28	0.09	0.07	1.05	0.95	0.44	0.59	0.40	0.30	-0.88	-1.69	-2.06	-1.20	-1.73	-1.88	-2.20	-1.30
P63098	Calcineurin subunit B type 1 OS=Homo sapiens GN=PPP3R1 PE=1 SV=2 - [CANB1_HUMAN]	1.71	1.67	2.21	2.21	0.12	0.25	0.73	0.82	0.42	0.32	0.07	0.11	-0.98	-1.69	-2.22	-1.35	-1.93	-1.52	-2.05	-1.34
O95970	Leucine-rich glioma-inactivated protein 1 OS=Homo sapiens GN=LGI1 PE=1 SV=1 - [LGI1_HUMAN]	1.70	1.76	2.10	2.16	0.56	0.51	0.74	0.81	0.52	0.52	0.25	0.15	-1.01	-1.44	-1.96	-1.33	-1.59	-1.37	-1.50	-1.27
Q9UH03	Neuronal-specific septin-3 OS=Homo sapiens GN=SEPT3 PE=1 SV=3 - [SEPT3_HUMAN]	1.47	1.35	2.02	1.90	-0.17	-0.36	0.69	0.66	0.15	0.25	-0.08	-0.12	-0.73	-1.46	-1.89	-1.23	-1.69	-1.58	-1.98	-1.18
P13521	Secretogranin-2 OS=Homo sapiens GN=SCG2 PE=1 SV=2 - [SCG2_HUMAN]	1.85	1.73	1.78	1.88	-0.11	-0.11	0.55	0.68	0.36	0.30	0.03	0.10	-1.20	-1.83	-1.82	-1.36	-1.46	-1.90	-2.10	-1.31
Q6U841	Sodium-driven chloride bicarbonate exchanger OS=Homo sapiens GN=SLC4A10 PE=2 SV=1 - [S4A10_HUMAN]	1.89	1.76	1.89	1.87	0.05	-0.13	0.48	0.55	0.12	0.29	0.30	0.05	-1.38	-2.23	-2.50	-1.70	-1.69	-1.91	-2.13	-1.46
Q9C040	Tripartite motif-containing protein 2 OS=Homo sapiens GN=TRIM2 PE=1 SV=1 - [TRIM2_HUMAN]	1.65	1.66	1.75	1.79	0.13	0.22	0.45	0.77	0.46	0.38	-0.01	0.18	-0.86	-1.58	-1.69	-1.21	-1.35	-1.09	-1.30	-1.18

O94760	N(G),N(G)-dimethylarginine dimethylaminohydrolase 1 OS=Homo sapiens GN=DDAH1 PE=1 SV=3 - [DDAH1_HUMAN]	1.43	1.66	1.67	1.74	0.20	0.33	0.76	0.88	0.47	0.43	0.26	0.37	-0.57	-1.12	-1.31	-1.08	-1.16	-1.14	-1.32	-0.92
Q9UPT6	C-Jun-amino-terminal kinase-interacting protein 3 OS=Homo sapiens GN=MAPK8IP3 PE=1 SV=3 - [JIP3_HUMAN]	1.82	1.66	1.80	1.74	0.11	0.03	0.79	0.66	0.10	0.25	0.35	0.24	-1.06	-1.29	-1.36	-1.62	-1.69	-1.76	-1.67	-1.18
O75781	Paralemmin-1 OS=Homo sapiens GN=PALM PE=1 SV=2 - [PALM_HUMAN]	1.79	1.69	1.74	1.72	0.10	0.06	0.37	0.34	0.19	0.06	-0.25	-0.17	-1.26	-1.79	-1.78	-1.58	-1.69	-1.75	-1.70	-1.27
P15104	Glutamine synthetase OS=Homo sapiens GN=GLUL PE=1 SV=4 - [GLNA_HUMAN]	1.71	1.39	1.88	1.65	0.35	0.22	0.58	0.30	0.20	0.25	0.08	-0.02	-1.10	-1.51	-1.62	-1.25	-1.49	-1.30	-1.50	-1.34
Q4J6C6	Prolyl endopeptidase-like OS=Homo sapiens GN=PREPL PE=1 SV=1 - [PPCEL_HUMAN]	1.38	1.39	1.46	1.58	0.05	0.18	0.58	0.53	0.34	0.23	0.22	0.40	-0.73	-1.23	-1.17	-0.96	-1.26	-1.32	-1.62	-1.04
O60861	Growth arrest-specific protein 7 OS=Homo sapiens GN=GAS7 PE=1 SV=3 - [GAS7_HUMAN]	1.70	1.81	1.41	1.58	-0.44	-0.46	0.09	0.01	-0.21	-0.09	-0.49	-0.42	-1.62	-2.06	-1.86	-2.12	-1.73	-2.56	-2.36	-1.50
P51649	Succinate-semialdehyde dehydrogenase, mitochondrial OS=Homo sapiens GN=ALDH5A1 PE=1 SV=2 - [SSDH_HUMAN]	1.38	1.38	1.70	1.54	-0.38	-0.35	-0.48	-0.45	-0.62	-0.67	-0.47	-0.61	-1.73	-2.00	-2.29	-1.98	-2.26	-1.85	-2.00	-2.18
A7KAX9	Rho GTPase-activating protein 32 OS=Homo sapiens GN=ARHGAP32 PE=1 SV=1 - [RHG32_HUMAN]	1.68	1.42	1.52	1.40	0.15	0.09	0.16	0.30	0.23	0.50	0.25	-0.03	-1.31	-1.34	-1.34	-1.17	-0.96	-1.52	-1.33	-1.04
Q12959	Disks large homolog 1 OS=Homo sapiens GN=DLG1 PE=1 SV=2 - [DLG1_HUMAN]	1.30	1.29	1.26	1.37	0.66	0.43	0.55	0.80	0.66	0.80	0.78	0.82	-0.66	-0.46	-0.58	-0.59	-0.48	-0.72	-0.70	-0.69

Q8TB36	Ganglioside-induced differentiation-associated protein 1 OS=Homo sapiens GN=GDAP1 PE=1 SV=3 - [GDAP1_HUMAN]	1.67	1.00	1.99	1.36	-0.26	-0.55	0.67	0.17	-0.21	0.04	-0.05	-0.34	-0.97	-1.59	-2.04	-1.48	-1.53	-1.88	-2.20	-1.19
Q9H936	Mitochondrial glutamate carrier 1 OS=Homo sapiens GN=SLC25A22 PE=1 SV=1 - [GHC1_HUMAN]	1.37	1.41	1.42	1.34	-0.13	-0.15	0.39	0.45	0.10	0.13	-0.06	-0.02	-1.04	-1.34	-1.56	-1.38	-1.37	-1.52	-1.54	-1.12
Q92599	Septin-8 OS=Homo sapiens GN=SEPT8 PE=1 SV=4 - [SEPT8_HUMAN]	1.20	1.08	1.12	1.07	-0.09	-0.07	0.21	0.13	-0.01	0.07	-0.20	-0.25	-0.92	-1.22	-1.45	-1.13	-1.16	-1.36	-1.11	-0.90
Q5TD97	Four and a half LIM domains protein 5 OS=Homo sapiens GN=FHL5 PE=1 SV=1 - [FHL5_HUMAN]	-3.41	-3.33	-3.29	-3.16	-1.07	-1.06	-1.61	-1.44	-1.29	-1.45	-1.03	-0.94	1.94	2.22	2.29	2.10	1.91	2.43	2.17	1.77
P28330	Long-chain specific acyl-CoA dehydrogenase, mitochondrial OS=Homo sapiens GN=ACADL PE=2 SV=2 - [ACADL_HUMAN]	-2.03	-1.95	-3.02	-2.88	-0.68	-0.52	-0.03	0.03	-0.11	-0.16	-0.07	0.05	2.19	2.23	2.73	2.04	2.20	1.58	2.00	2.56
P50454	Serpin H1 OS=Homo sapiens GN=SERPINH1 PE=1 SV=2 - [SERPH_HUMAN]	-2.39	-2.23	-2.46	-2.41	-0.48	-0.49	-1.17	-1.08	-1.04	-1.09	-0.94	-0.85	0.98	1.34	1.43	1.30	1.45	1.68	1.84	1.19
P07225	Vitamin K-dependent protein S OS=Homo sapiens GN=PROS1 PE=1 SV=1 - [PROS_HUMAN]	-2.30	-2.19	-2.35	-2.26	-0.25	-0.20	-0.66	-0.55	-0.15	-0.20	0.50	0.59	1.71	2.80	2.86	2.08	2.12	2.03	1.94	1.78
P51884	Lumican OS=Homo sapiens GN=LUM PE=1 SV=2 - [LUM_HUMAN]	-1.96	-1.88	-2.01	-1.98	0.75	0.89	-0.33	-0.27	0.00	-0.10	-0.86	-0.68	1.58	1.09	1.18	1.80	1.82	2.76	2.63	1.62
Q7Z5L7	Podocan OS=Homo sapiens GN=PODN PE=1 SV=2 - [PODN_HUMAN]	-1.62	-1.32	-2.16	-1.96	0.85	1.00	-0.59	-0.40	0.35	0.15	-0.25	-0.09	1.08	1.48	1.91	1.80	2.22	2.49	2.95	1.66

Q12929	Epidermal growth factor receptor kinase substrate 8 OS=Homo sapiens GN=EPS8 PE=1 SV=1 - [EPS8_HUMAN]	-1.27	-1.26	-1.92	-1.93	-0.15	-0.27	-0.38	-0.51	-0.22	-0.26	-0.35	-0.48	0.74	1.02	1.51	0.95	1.60	1.11	1.63	1.51
P02749	Beta-2-glycoprotein 1 OS=Homo sapiens GN=APOH PE=1 SV=3 - [APOH_HUMAN]	-2.20	-2.05	-2.11	-1.89	0.24	0.27	-0.17	-0.15	0.15	0.07	0.77	0.85	2.00	2.95	2.85	2.31	2.25	2.30	2.33	1.82
Q9BXN1	Asporin OS=Homo sapiens GN=ASPN PE=1 SV=2 - [ASPN_HUMAN]	-1.88	-1.83	-1.90	-1.85	-0.13	-0.13	-1.17	-1.19	-0.90	-0.83	-1.08	-1.06	0.78	0.91	1.13	1.18	1.24	1.81	1.89	1.04
Q9NYL9	Tropomodulin-3 OS=Homo sapiens GN=TMOD3 PE=1 SV=1 - [TMOD3_HUMAN]	-1.80	-1.86	-1.62	-1.81	-0.72	-0.75	-0.81	-0.70	-0.83	-0.76	-0.61	-0.67	0.86	1.16	1.28	1.00	1.01	0.96	1.00	1.06
P00736	Complement C1r subcomponent OS=Homo sapiens GN=C1R PE=1 SV=2 - [C1R_HUMAN]	-1.40	-1.42	-1.57	-1.63	0.56	0.57	-0.21	-0.27	0.27	0.24	0.67	0.70	1.45	2.27	2.22	1.73	1.73	1.83	2.13	1.30
P05186	Alkaline phosphatase, tissue-nonspecific isozyme OS=Homo sapiens GN=ALPL PE=1 SV=4 - [PPBT_HUMAN]	-1.57	-1.48	-1.63	-1.59	0.16	0.21	-0.67	-0.66	0.25	0.19	1.16	1.26	0.89	2.86	2.78	1.79	1.78	1.65	1.63	0.86
Q9Y2D5	A-kinase anchor protein 2 OS=Homo sapiens GN=AKAP2 PE=1 SV=3 - [AKAP2_HUMAN]	-1.41	-1.26	-1.52	-1.47	-0.33	-0.14	-0.70	-0.60	-0.45	-0.51	-0.54	-0.41	0.76	0.91	1.07	0.94	1.05	1.19	1.21	0.83
P49023	Paxillin OS=Homo sapiens GN=PXN PE=1 SV=3 - [PAXI_HUMAN]	-1.37	-1.70	-1.34	-1.43	-0.05	-0.15	-0.25	-0.25	-0.37	-0.21	-0.02	0.02	1.38	1.38	1.38	1.29	1.24	1.24	1.26	1.13
P42226	Signal transducer and activator of transcription 6 OS=Homo sapiens GN=STAT6 PE=1 SV=1 - [STAT6_HUMAN]	-1.08	-1.12	-1.24	-1.39	-0.07	-0.15	-0.24	-0.40	-0.20	-0.14	-0.01	-0.19	0.76	1.03	1.20	1.15	1.18	1.08	1.31	1.04
P19320	Vascular cell adhesion protein 1 OS=Homo sapiens GN=VCAM1 PE=1 SV=1 - [VCAM1_HUMAN]	-1.08	-1.11	-1.49	-1.33	0.04	0.14	-0.44	-0.38	0.05	-0.09	0.65	0.73	0.93	2.09	2.12	1.28	1.34	1.22	1.39	1.02

P07093	Glia-derived nexin OS=Homo sapiens GN=SERPINE2 PE=1 SV=1 - [GDN_HUMAN]	-1.17	-1.18	-1.40	-1.29	1.58	1.55	-0.32	-0.35	0.47	0.57	-0.04	-0.29	0.97	0.87	1.33	1.70	2.00	3.05	2.73	1.24
P01031	Complement C5 OS=Homo sapiens GN=C5 PE=1 SV=4 - [CO5_HUMAN]	-1.34	-1.47	-1.19	-1.26	0.11	0.18	-0.54	-0.58	-0.10	-0.23	0.23	0.32	0.91	1.65	1.60	1.23	1.19	1.62	1.39	0.89
O75077	Disintegrin and metalloproteinase domain-containing protein 23 OS=Homo sapiens GN=ADAM23 PE=1 SV=1 - [ADA23_HUMAN]	2.36	2.50	2.42	2.66	-0.11	0.11	0.70	1.01	0.59	0.52	0.36	0.37	-1.43	-1.76	-1.13	-1.76	-1.93	-2.11	-2.57	-1.53
Q14CZ8	Hepatocyte cell adhesion molecule OS=Homo sapiens GN=HEPACAM PE=1 SV=1 - [HECAM_HUMAN]	2.15	2.11	2.20	2.53	-0.02	-0.01	1.20	1.48	1.11	0.74	1.12	1.33	-0.60	-0.92	-1.08	-0.98	-1.22	-1.98	-2.21	-0.86
Q01064	Calcium/calmodulin-dependent 3',5'-cyclic nucleotide phosphodiesterase 1B OS=Homo sapiens GN=PDE1B PE=1 SV=2 - [PDE1B_HUMAN]	2.31	2.10	2.12	2.35	-0.29	-0.22	0.81	0.75	0.60	0.45	0.33	0.36	-1.36	-1.99	-1.89	-1.77	-1.68	-2.66	-2.58	-1.38
P32418	Sodium/calcium exchanger 1 OS=Homo sapiens GN=SLC8A1 PE=1 SV=3 - [NAC1_HUMAN]	2.06	1.90	2.47	2.25	-0.04	-0.01	0.88	0.91	0.49	0.48	0.48	0.46	-1.08	-1.63	-1.98	-1.57	-1.76	-1.99	-2.36	-1.34
P29218	Inositol monophosphatase 1 OS=Homo sapiens GN=IMPA1 PE=1 SV=1 - [IMPA1_HUMAN]	1.89	1.93	2.05	2.10	0.13	0.26	0.52	0.52	0.33	0.09	-0.24	-0.17	-1.41	-2.03	-2.35	-1.58	-1.95	-1.68	-1.97	-1.65
P05937	Calbindin OS=Homo sapiens GN=CALB1 PE=1 SV=2 - [CALB1_HUMAN]	1.99	1.80	2.24	2.10	0.84	0.46	0.96	0.81	0.33	0.59	-0.31	-0.38	-0.97	-2.18	-2.48	-1.42	-1.54	-1.34	-1.48	-1.23
Q13554	Calcium/calmodulin-dependent protein kinase type II subunit beta OS=Homo sapiens GN=CAMK2B PE=1 SV=3 - [KCC2B_HUMAN]	1.88	1.83	1.96	2.02	0.20	0.03	0.95	0.93	0.32	0.48	0.41	0.28	-0.87	-1.46	-1.64	-1.35	-1.60	-1.63	-2.05	-1.14

Q13449	Limbic system-associated membrane protein OS=Homo sapiens GN=LSAMP PE=1 SV=2 - [LSAMP_HUMAN]	1.96	1.80	2.14	1.94	0.15	0.07	0.75	0.75	0.40	0.45	0.34	0.31	-0.95	-1.58	-1.74	-1.38	-1.56	-1.76	-1.93	-1.14
P22676	Calretinin OS=Homo sapiens GN=CALB2 PE=1 SV=2 - [CALB2_HUMAN]	1.54	1.62	1.88	1.89	0.23	0.20	0.89	0.88	0.73	0.60	0.18	0.08	-0.58	-1.48	-1.71	-1.04	-1.25	-1.53	-1.77	-0.90
P30086	Phosphatidylethanolamine-binding protein 1 OS=Homo sapiens GN=PEBP1 PE=1 SV=3 - [PEBP1_HUMAN]	1.59	1.40	1.92	1.80	0.60	0.44	0.76	0.64	0.54	0.56	0.20	0.10	-0.71	-1.26	-1.63	-1.01	-1.25	-0.92	-1.32	-1.09
Q9UQ03	Coronin-2B OS=Homo sapiens GN=CORO2B PE=2 SV=4 - [COR2B_HUMAN]	1.75	1.58	2.00	1.79	0.29	0.20	0.59	0.64	0.43	0.36	0.14	0.31	-0.78	-0.85	-1.19	-1.25	-1.68	-1.42	-1.39	-0.90
O14490	Disks large-associated protein 1 OS=Homo sapiens GN=DLGAP1 PE=1 SV=1 - [DLGP1_HUMAN]	1.64	1.23	1.89	1.77	-0.33	-0.27	0.69	0.62	-0.21	0.08	-0.23	-0.67	-0.85	-1.83	-2.09	-1.38	-1.70	-1.66	-1.87	-1.14
A0MZ66	Shootin-1 OS=Homo sapiens GN=KIAA1598 PE=1 SV=4 - [SHOT1_HUMAN]	1.51	1.51	1.82	1.76	0.08	0.14	0.59	0.57	0.35	0.29	-0.02	0.01	-0.81	-1.49	-1.60	-1.25	-1.47	-1.48	-1.71	-1.03
Q96FC7	Phytanoyl-CoA hydroxylase-interacting protein-like OS=Homo sapiens GN=PHYHIPL PE=1 SV=3 - [PHIPL_HUMAN]	1.43	1.55	1.76	1.75	-0.44	-0.49	0.60	0.59	0.14	0.08	0.24	0.24	-0.93	-1.56	-1.49	-1.33	-1.38	-1.93	-1.65	-1.02
Q99963	Endophilin-A3 OS=Homo sapiens GN=SH3GL3 PE=1 SV=1 - [SH3G3_HUMAN]	1.53	1.54	1.77	1.69	-0.38	-0.46	0.50	0.51	-0.05	-0.02	-0.48	-0.52	-0.98	-2.02	-2.29	-1.52	-1.83	-1.83	-2.12	-1.14
Q9UI15	Transgelin-3 OS=Homo sapiens GN=TAGLN3 PE=1 SV=2 - [TAGL3_HUMAN]	1.35	1.38	1.57	1.61	0.16	0.15	-0.12	-0.15	-0.17	-0.22	-0.43	-0.45	-1.27	-1.70	-1.97	-1.50	-1.80	-1.26	-1.51	-1.64

P31323	cAMP-dependent protein kinase type II-beta regulatory subunit OS=Homo sapiens GN=PRKAR2B PE=1 SV=3 - [KAP3_HUMAN]	1.29	1.34	1.41	1.47	-0.29	-0.07	0.53	0.73	0.21	0.23	0.28	0.31	-0.75	-0.99	-0.92	-0.97	-1.15	-1.41	-1.49	-0.99
Q13303	Voltage-gated potassium channel subunit beta-2 OS=Homo sapiens GN=KCNAB2 PE=1 SV=2 - [KCAB2_HUMAN]	1.69	1.68	1.56	1.45	-0.32	-0.20	0.37	0.35	0.12	0.06	-0.29	-0.31	-1.38	-2.23	-2.07	-1.64	-1.57	-2.20	-2.13	-1.26
Q9Y2T3	Guanine deaminase OS=Homo sapiens GN=GDA PE=1 SV=1 - [GUAD_HUMAN]	1.26	1.23	1.47	1.38	0.01	0.01	0.63	0.44	0.23	0.35	-0.22	-0.25	-0.56	-1.68	-1.85	-0.96	-1.09	-1.27	-1.46	-0.79
Q8N4C8	Misshapen-like kinase 1 OS=Homo sapiens GN=MINK1 PE=1 SV=2 - [MINK1_HUMAN]	1.27	1.18	1.40	1.29	-0.02	0.01	0.36	0.18	0.11	0.26	0.25	0.20	-0.98	-1.00	-0.96	-1.10	-1.01	-1.29	-1.37	-1.01
Q9NR46	Endophilin-B2 OS=Homo sapiens GN=SH3GLB2 PE=1 SV=1 - [SHLB2_HUMAN]	1.22	1.32	1.26	1.26	-0.32	-0.44	0.48	0.43	0.00	0.04	-0.13	-0.12	-0.63	-1.30	-1.29	-1.02	-1.12	-1.49	-1.54	-0.71
P13647	Keratin, type II cytoskeletal 5 OS=Homo sapiens GN=KRT5 PE=1 SV=3 - [K2C5_HUMAN]	-2.82	-2.71	-3.22	-3.11	-2.36	-2.21	-3.57	-3.66	-3.11	-3.22	-2.45	-2.28	-0.59	0.22	1.35	0.10	0.54	0.62	0.47	0.23
Q6NZI2	Polymerase I and transcript release factor OS=Homo sapiens GN=PTRF PE=1 SV=1 - [PTRF_HUMAN]	-2.54	-2.59	-2.65	-2.74	-0.43	-0.48	-0.80	-0.82	-0.64	-0.54	-0.26	-0.30	1.76	2.28	2.18	2.00	1.82	2.08	2.08	1.76
O43294	Transforming growth factor beta-1-induced transcript 1 protein OS=Homo sapiens GN=TGFB1I1 PE=1 SV=2 - [TGFI1_HUMAN]	-2.57	-2.50	-2.68	-2.68	-1.19	-1.12	-1.20	-1.15	-1.10	-1.11	-0.87	-0.92	1.57	1.62	1.75	1.48	1.62	1.44	1.46	1.44
Q7Z7G0	Target of Nesh-SH3 OS=Homo sapiens GN=ABI3BP PE=1 SV=1 - [TARSH_HUMAN]	-2.27	-2.27	-2.65	-2.47	-0.69	-0.67	-0.25	-0.21	-0.33	-0.39	-0.46	-0.48	1.97	1.77	1.68	1.98	1.86	1.70	1.80	2.17

P36955	Pigment epithelium-derived factor OS=Homo sapiens GN=SERPINF1 PE=1 SV=4 - [PEDF_HUMAN]	-2.39	-2.44	-2.43	-2.44	-0.85	-0.86	-1.20	-1.18	-1.11	-1.09	-0.68	-0.67	1.18	1.64	1.71	1.25	1.25	1.62	1.57	1.27
P37802	Transgelin-2 OS=Homo sapiens GN=TAGLN2 PE=1 SV=3 - [TAGL2_HUMAN]	-2.39	-2.36	-2.29	-2.26	-1.28	-1.18	-1.84	-1.72	-1.44	-1.56	-1.72	-1.69	0.64	0.59	0.56	0.90	0.74	0.98	1.01	0.60
Q86SQ0	Pleckstrin homology-like domain family B member 2 OS=Homo sapiens GN=PHLDB2 PE=1 SV=2 - [PHLB2_HUMAN]	-1.99	-1.90	-2.17	-2.12	-0.28	-0.30	-0.69	-0.73	-0.46	-0.46	-0.13	-0.12	1.24	1.58	1.74	1.38	1.58	1.56	1.81	1.46
Q16610	Extracellular matrix protein 1 OS=Homo sapiens GN=ECM1 PE=1 SV=2 - [ECM1_HUMAN]	-1.80	-1.82	-1.96	-1.94	-0.21	-0.21	-0.64	-0.57	-0.25	-0.36	0.19	0.24	1.35	1.92	2.21	1.72	1.85	1.63	1.69	1.40
P69905	Hemoglobin subunit alpha OS=Homo sapiens GN=HBA1 PE=1 SV=2 - [HBA_HUMAN]	-1.83	-1.74	-1.96	-1.88	0.25	0.32	-0.36	-0.27	-0.01	-0.07	0.13	0.24	1.55	1.94	2.08	1.92	1.93	2.10	2.20	1.68
O00299	Chloride intracellular channel protein 1 OS=Homo sapiens GN=CLIC1 PE=1 SV=4 - [CLIC1_HUMAN]	-1.68	-1.61	-1.72	-1.70	-0.03	0.11	-0.14	-0.10	0.00	-0.10	-0.40	-0.23	1.62	1.38	1.45	1.76	1.57	1.78	1.70	1.43
P00738	Haptoglobin OS=Homo sapiens GN=HP PE=1 SV=1 - [HPT_HUMAN]	-1.78	-1.69	-1.72	-1.66	-1.38	-1.19	-1.59	-1.53	-1.18	-1.33	-0.80	-0.76	0.24	1.02	0.90	0.41	0.29	0.27	0.08	0.08
Q9NZU5	LIM and cysteine-rich domains protein 1 OS=Homo sapiens GN=LMCD1 PE=1 SV=1 - [LMCD1_HUMAN]	-1.66	-1.49	-1.51	-1.52	-0.62	-0.21	-1.17	-1.11	-0.64	-0.75	-0.67	-0.73	0.36	0.76	0.84	0.57	0.65	0.82	0.97	0.58
O94901	SUN domain-containing protein 1 OS=Homo sapiens GN=SUN1 PE=1 SV=3 - [SUN1_HUMAN]	-1.50	-1.43	-1.62	-1.48	-0.77	-0.72	-0.88	-0.81	-0.78	-0.77	-0.64	-0.63	0.69	0.95	0.91	0.77	0.99	0.82	0.86	0.71
P16284	Platelet endothelial cell adhesion molecule OS=Homo sapiens GN=PECAM1 PE=1 SV=1 - [PECA1_HUMAN]	-1.53	-1.38	-1.44	-1.36	0.03	0.01	-0.77	-0.54	-0.35	-0.34	-0.13	-0.09	0.85	1.45	1.29	1.28	1.11	1.66	1.50	0.88

P34897	Serine hydroxymethyltransferase, mitochondrial OS=Homo sapiens GN=SHMT2 PE=1 SV=3 - [GLYM_HUMAN]	-1.21	-1.13	-1.34	-1.35	-0.57	-0.57	-0.70	-0.63	-0.68	-0.55	-0.48	-0.36	0.56	0.73	0.80	0.48	0.68	0.52	0.75	0.58
Q99536	Synaptic vesicle membrane protein VAT-1 homolog OS=Homo sapiens GN=VAT1 PE=1 SV=2 - [VAT1_HUMAN]	-1.25	-1.20	-1.17	-1.28	-0.14	-0.01	-0.48	-0.54	-0.35	-0.32	-0.21	-0.22	0.70	1.13	0.90	1.01	0.81	1.14	1.02	0.67
P14780	Matrix metalloproteinase-9 OS=Homo sapiens GN=MMP9 PE=1 SV=3 - [MMP9_HUMAN]	-1.56	-1.37	-1.34	-1.27	-0.85	-0.70	1.19	1.52	0.81	0.59	0.36	0.58	2.77	2.00	1.87	2.38	2.02	0.69	0.55	2.57
Q6UXI9	Nephronectin OS=Homo sapiens GN=NPNT PE=2 SV=3 - [NPNT_HUMAN]	-1.15	-1.27	-1.30	-1.24	1.01	1.16	-0.02	0.14	0.40	0.38	0.10	0.16	1.32	1.27	1.36	1.69	1.58	2.29	2.47	1.31
P19827	Inter-alpha-trypsin inhibitor heavy chain H1 OS=Homo sapiens GN=ITIH1 PE=1 SV=3 - [ITIH1_HUMAN]	-1.36	-1.28	-1.51	-1.22	0.47	0.58	-0.45	-0.36	0.19	0.19	0.40	0.56	0.80	1.75	1.95	1.38	1.87	1.86	1.94	1.12
P05155	Plasma protease C1 inhibitor OS=Homo sapiens GN=SERPING1 PE=1 SV=2 - [IC1_HUMAN]	-1.43	-1.40	-1.24	-1.19	0.21	0.20	-0.67	-0.63	0.00	-0.02	0.58	0.64	0.76	2.09	1.78	1.45	1.19	1.71	1.37	0.73
Q9Y315	Putative deoxyribose-phosphate aldolase OS=Homo sapiens GN=DERA PE=1 SV=2 - [DEOC_HUMAN]	-1.26	-1.19	-1.36	-1.19	0.07	0.15	-0.13	-0.08	0.09	0.00	0.46	0.50	1.08	1.59	1.81	1.34	1.28	1.20	1.26	1.33
Q86UT6	NLR family member X1 OS=Homo sapiens GN=NLRX1 PE=1 SV=1 - [NLRX1_HUMAN]	-1.29	-1.21	-1.15	-1.17	-0.14	-0.11	-0.31	-0.34	-0.16	-0.15	-0.07	-0.06	1.00	1.10	1.12	1.10	1.23	0.89	1.18	0.99
Q9GZV7	Hyaluronan and proteoglycan link protein 2 OS=Homo sapiens GN=HAPLN2 PE=1 SV=1 - [HPLN2_HUMAN]	2.53	2.47	2.75	2.68	-0.02	-0.11	0.80	0.79	0.38	0.46	0.00	-0.25	-1.74	-2.57	-2.98	-1.98	-2.41	-2.60	-2.86	-1.88

Q8N9I0	Synaptotagmin-2 OS=Homo sapiens GN=SYT2 PE=1 SV=2 - [SYT2_HUMAN]	2.43	2.48	2.27	2.58	0.13	0.23	1.39	1.47	1.16	1.16	0.70	0.67	-0.78	-1.92	-2.09	-1.34	-1.78	-2.14	-2.45	-1.08
P35498	Sodium channel protein type 1 subunit alpha OS=Homo sapiens GN=SCN1A PE=1 SV=2 - [SCN1A_HUMAN]	1.62	1.74	2.11	2.26	0.20	0.32	0.52	0.77	0.46	0.30	0.15	0.25	-1.18	-1.81	-2.12	-1.58	-1.92	-1.85	-2.27	-1.51
Q7L0J3	Synaptic vesicle glycoprotein 2A OS=Homo sapiens GN=SV2A PE=1 SV=1 - [SV2A_HUMAN]	2.03	2.20	2.14	2.21	-0.15	-0.18	1.09	1.11	0.51	0.64	0.31	0.24	-0.77	-1.68	-1.96	-1.36	-1.69	-1.95	-2.04	-1.09
Q14982	Opioid-binding protein/cell adhesion molecule OS=Homo sapiens GN=OPCML PE=1 SV=1 - [OPCM_HUMAN]	1.76	1.69	2.00	2.10	-0.03	-0.02	0.57	0.71	0.24	0.25	0.18	0.25	-0.97	-1.64	-1.99	-1.43	-1.56	-1.67	-1.92	-1.29
Q9UQM7	Calcium/calmodulin-dependent protein kinase type II subunit alpha OS=Homo sapiens GN=CAMK2A PE=1 SV=2 - [KCC2A_HUMAN]	2.08	2.10	2.16	2.06	-0.32	0.01	0.71	0.66	0.44	0.37	0.40	0.31	-1.17	-1.74	-1.77	-1.64	-1.75	-2.27	-2.24	-1.42
O15083	ERC protein 2 OS=Homo sapiens GN=ERC2 PE=1 SV=3 - [ERC2_HUMAN]	2.30	2.10	2.23	2.03	-0.80	-0.77	0.88	0.69	0.58	0.52	-0.18	-0.16	-1.38	-2.14	-2.29	-2.15	-2.19	-2.55	-2.69	-1.44
P80723	Brain acid soluble protein 1 OS=Homo sapiens GN=BASP1 PE=1 SV=2 - [BASP1_HUMAN]	1.38	1.18	2.07	2.02	-0.63	-0.64	0.58	0.39	-0.32	-0.01	-0.37	-0.56	-0.72	-1.87	-2.74	-1.49	-2.25	-2.05	-2.74	-1.44
Q8N608	Inactive dipeptidyl peptidase 10 OS=Homo sapiens GN=DPP10 PE=1 SV=2 - [DPP10_HUMAN]	2.06	1.99	2.06	1.99	-0.02	-0.16	0.73	0.72	0.37	0.44	0.51	0.42	-1.15	-1.67	-1.73	-1.58	-1.58	-2.10	-2.12	-1.15
O14994	Synapsin-3 OS=Homo sapiens GN=SYN3 PE=1 SV=2 - [SYN3_HUMAN]	1.78	1.71	2.09	1.99	-0.19	-0.08	0.71	0.51	0.36	0.33	0.08	0.00	-1.15	-1.81	-2.11	-1.57	-1.86	-2.06	-2.52	-1.32
Q9NQW6	Actin-binding protein anillin OS=Homo sapiens GN=ANLN PE=1 SV=2 - [ANLN_HUMAN]	1.78	1.57	2.22	1.99	-0.29	-0.47	0.99	0.78	0.19	0.40	0.26	0.05	-0.81	-1.56	-2.15	-1.36	-1.86	-2.05	-2.65	-1.29

Q99250	Sodium channel protein type 2 subunit alpha OS=Homo sapiens GN=SCN2A PE=1 SV=3 - [SCN2A_HUMAN]	1.87	1.73	2.07	1.98	-0.48	-0.75	0.44	0.36	-0.09	-0.01	-0.16	-0.39	-1.28	-1.79	-2.38	-1.78	-2.23	-2.08	-2.53	-1.55
Q08209	Serine/threonine-protein phosphatase 2B catalytic subunit alpha isoform OS=Homo sapiens GN=PPP3CA PE=1 SV=1 - [PP2BA_HUMAN]	1.83	1.74	1.96	1.93	0.29	0.29	0.69	0.57	0.37	0.38	0.31	0.21	-1.20	-1.55	-1.48	-1.30	-1.38	-1.48	-1.50	-1.25
P19086	Guanine nucleotide-binding protein G(z) subunit alpha OS=Homo sapiens GN=GNAZ PE=2 SV=3 - [GNAZ_HUMAN]	2.07	1.52	1.89	1.89	0.10	0.07	0.80	0.72	0.51	0.53	0.29	0.22	-1.09	-1.47	-1.50	-1.39	-1.45	-1.93	-1.76	-1.09
Q6H8Q1	Actin-binding LIM protein 2 OS=Homo sapiens GN=ABLIM2 PE=1 SV=2 - [ABLM2_HUMAN]	1.38	1.71	1.68	1.84	-0.08	-0.11	0.44	0.57	0.27	0.36	-0.18	0.11	-1.30	-1.76	-1.93	-1.59	-1.57	-1.95	-2.06	-1.22
Q8N6T3	ADP-ribosylation factor GTPase-activating protein 1 OS=Homo sapiens GN=ARFGAP1 PE=1 SV=2 - [ARFG1_HUMAN]	1.66	1.63	1.92	1.80	-0.33	-0.46	0.57	0.58	0.03	-0.01	0.07	-0.11	-1.10	-1.56	-1.93	-1.44	-1.59	-1.88	-2.17	-1.30
O75334	Liprin-alpha-2 OS=Homo sapiens GN=PPFIA2 PE=1 SV=2 - [LIPA2_HUMAN]	1.51	1.41	1.58	1.79	0.18	-0.08	0.45	0.29	0.17	0.22	-0.02	0.03	-1.01	-1.61	-1.57	-1.41	-1.34	-1.41	-1.29	-1.10
Q7Z3B1	Neuronal growth regulator 1 OS=Homo sapiens GN=NEGR1 PE=1 SV=3 - [NEGR1_HUMAN]	1.64	1.43	1.84	1.78	0.41	0.36	0.64	0.47	0.11	0.25	0.35	0.21	-0.96	-1.19	-1.55	-1.50	-1.64	-1.30	-1.70	-1.26
Q9UPV7	Protein KIAA1045 OS=Homo sapiens GN=KIAA1045 PE=1 SV=2 - [K1045_HUMAN]	1.78	1.84	1.69	1.74	-0.32	-0.36	0.40	0.53	0.26	0.03	-0.18	0.05	-1.15	-1.67	-1.66	-1.41	-1.44	-1.95	-2.11	-1.23
O60268	Uncharacterized protein KIAA0513 OS=Homo sapiens GN=KIAA0513 PE=2 SV=1 - [K0513_HUMAN]	1.49	1.51	1.77	1.74	-0.39	-0.36	0.49	0.42	-0.09	-0.10	-0.08	-0.15	-0.96	-1.65	-1.98	-1.45	-1.81	-2.02	-2.33	-1.14

P00492	Hypoxanthine-guanine phosphoribosyltransferase OS=Homo sapiens GN=HPRT1 PE=1 SV=2 - [HPRT_HUMAN]	1.75	1.63	1.75	1.73	0.00	-0.11	0.25	0.20	0.03	-0.11	-0.34	-0.24	-1.17	-1.40	-1.74	-1.17	-1.50	-1.20	-1.48	-1.37
Q8WXI2	Connector enhancer of kinase suppressor of ras 2 OS=Homo sapiens GN=CNKSR2 PE=1 SV=1 - [CNKR2_HUMAN]	1.53	1.61	1.65	1.69	0.12	-0.27	0.80	0.50	0.46	0.29	0.34	0.30	-1.20	-1.49	-1.42	-1.70	-1.50	-2.08	-2.01	-1.06
P15121	Aldose reductase OS=Homo sapiens GN=AKR1B1 PE=1 SV=3 - [ALDR_HUMAN]	1.26	1.24	1.49	1.63	-0.10	-0.15	0.37	0.28	-0.01	0.04	-0.36	-0.44	-0.83	-1.78	-1.87	-1.31	-1.62	-1.44	-1.73	-1.07
P21579	Synaptotagmin-1 OS=Homo sapiens GN=SYT1 PE=1 SV=1 - [SYT1_HUMAN]	1.49	1.53	1.72	1.62	0.09	0.03	0.66	0.50	0.30	0.39	0.23	0.16	-1.03	-1.33	-1.48	-1.20	-1.25	-1.49	-1.56	-1.03
Q5TH69	Brefeldin A-inhibited guanine nucleotide-exchange protein 3 OS=Homo sapiens GN=ARFGEF3 PE=1 SV=3 - [BIG3_HUMAN]	1.32	1.20	1.31	1.41	-0.08	0.10	0.31	0.36	0.14	-0.09	0.17	0.10	-1.05	-1.28	-1.36	-1.29	-1.41	-1.04	-1.28	-1.11
O43295	SLIT-ROBO Rho GTPase-activating protein 3 OS=Homo sapiens GN=SRGAP3 PE=1 SV=3 - [SRGP3_HUMAN]	1.08	1.08	1.38	1.38	0.02	0.04	0.49	0.16	-0.06	0.03	-0.04	0.08	-0.65	-0.89	-1.17	-1.10	-1.42	-1.24	-1.41	-1.04
Q14894	Ketimine reductase mu-crystallin OS=Homo sapiens GN=CRYM PE=1 SV=1 - [CRYM_HUMAN]	1.29	1.41	1.38	1.38	-0.22	-0.23	0.45	0.44	0.02	0.01	-0.20	-0.37	-0.89	-1.53	-1.48	-0.91	-0.96	-1.15	-1.61	-0.90
Q9H0E2	Toll-interacting protein OS=Homo sapiens GN=TOLLIP PE=1 SV=1 - [TOLIP_HUMAN]	1.42	1.35	1.39	1.38	0.30	0.34	0.48	0.50	0.26	0.21	0.13	0.17	-0.81	-1.15	-1.17	-1.09	-0.93	-1.15	-1.23	-0.69
Q9Y2I8	WD repeat-containing protein 37 OS=Homo sapiens GN=WDR37 PE=1 SV=2 - [WDR37_HUMAN]	1.20	1.38	1.41	1.34	0.12	0.02	0.57	0.56	0.36	0.36	0.11	0.16	-0.74	-1.19	-1.18	-1.02	-0.97	-1.36	-1.28	-0.73

Q6KCM7	Calcium-binding mitochondrial carrier protein SCaMC-2 OS=Homo sapiens GN=SLC25A25 PE=1 SV=1 - [SCMC2_HUMAN]	1.37	1.19	1.28	1.24	0.16	0.13	0.50	0.64	0.30	0.23	0.31	0.28	-0.60	-0.88	-1.11	-0.92	-0.90	-1.25	-1.20	-0.74
P51553	Isocitrate dehydrogenase [NAD] subunit gamma, mitochondrial OS=Homo sapiens GN=IDH3G PE=1 SV=1 - [IDH3G_HUMAN]	1.39	1.17	1.31	1.13	-0.37	-0.25	0.24	0.17	0.08	-0.03	-0.05	-0.15	-0.99	-1.29	-1.25	-1.19	-1.17	-1.17	-0.99	-0.90
Q9H9B4	Sideroflexin-1 OS=Homo sapiens GN=SFXN1 PE=1 SV=4 - [SFXN1_HUMAN]	1.14	1.15	1.17	1.11	0.12	0.11	0.47	0.49	0.30	0.28	0.15	0.10	-0.74	-1.02	-0.98	-0.80	-0.74	-1.01	-1.03	-0.63
P29536	Leiomodin-1 OS=Homo sapiens GN=LMOD1 PE=1 SV=3 - [LMOD1_HUMAN]	-2.59	-2.66	-2.60	-2.65	-1.33	-1.51	-1.09	-1.16	-0.99	-0.84	-0.30	-0.26	1.53	2.36	2.40	1.89	1.80	1.80	1.62	1.50
P00915	Carbonic anhydrase 1 OS=Homo sapiens GN=CA1 PE=1 SV=2 - [CAH1_HUMAN]	-2.51	-2.50	-2.41	-2.40	-0.23	-0.18	-0.85	-0.89	-0.85	-0.93	-1.69	-1.73	1.69	0.58	0.62	1.69	1.56	2.27	2.18	1.61
Q16678	Cytochrome P450 1B1 OS=Homo sapiens GN=CYP1B1 PE=1 SV=2 - [CP1B1_HUMAN]	-1.62	-1.71	-2.00	-2.00	-0.73	-0.78	-0.61	-0.67	-0.29	-0.30	0.57	0.40	1.04	2.09	2.37	1.39	1.76	1.02	1.30	1.31
P02790	Hemopexin OS=Homo sapiens GN=HPX PE=1 SV=2 - [HEMO_HUMAN]	-1.87	-1.90	-1.98	-1.94	-0.93	-0.89	-1.58	-1.60	-1.30	-1.39	-1.92	-1.91	0.34	0.06	0.13	0.60	0.60	1.02	1.07	0.45
O00339	Matrilin-2 OS=Homo sapiens GN=MATN2 PE=1 SV=4 - [MATN2_HUMAN]	-1.88	-1.93	-1.92	-1.90	-0.45	-0.52	-0.57	-0.63	-0.65	-0.60	-0.37	-0.38	1.36	1.60	1.54	1.28	1.25	1.40	1.37	1.38
P04792	Heat shock protein beta-1 OS=Homo sapiens GN=HSPB1 PE=1 SV=2 - [HSPB1_HUMAN]	-1.84	-1.81	-1.87	-1.75	-0.41	-0.41	-0.81	-0.84	-0.44	-0.51	-0.24	-0.28	1.04	1.67	1.76	1.49	1.58	1.58	1.53	1.15
P01042	Kininogen-1 OS=Homo sapiens GN=KNG1 PE=1 SV=2 - [KNG1_HUMAN]	-1.48	-1.60	-1.62	-1.69	0.47	0.45	-0.17	-0.38	0.00	0.11	0.37	0.29	1.30	1.83	1.91	1.63	1.77	2.01	2.13	1.33

Q13642	Four and a half LIM domains protein 1 OS=Homo sapiens GN=FHL1 PE=1 SV=4 - [FHL1_HUMAN]	-1.85	-1.77	-1.65	-1.62	-0.81	-0.73	-1.05	-0.97	-1.01	-1.08	-0.92	-0.91	0.77	0.79	0.60	0.77	0.65	0.97	0.86	0.52
Q7Z304	MAM domain-containing protein 2 OS=Homo sapiens GN=MAMDC2 PE=2 SV=3 - [MAMC2_HUMAN]	-1.38	-1.41	-1.58	-1.61	-0.15	-0.24	-0.48	-0.42	-0.25	-0.15	0.42	0.39	0.78	1.56	1.84	1.25	1.39	1.10	1.22	1.22
P50552	Vasodilator-stimulated phosphoprotein OS=Homo sapiens GN=VASP PE=1 SV=3 - [VASP_HUMAN]	-1.74	-1.84	-1.51	-1.57	-0.64	-0.64	-0.80	-0.71	-0.85	-0.93	-0.62	-0.72	1.10	0.88	0.90	0.93	0.93	1.14	0.69	0.83
O95302	Peptidyl-prolyl cis-trans isomerase FKBP9 OS=Homo sapiens GN=FKBP9 PE=1 SV=2 - [FKBP9_HUMAN]	-1.45	-1.48	-1.47	-1.43	-0.14	-0.07	-0.60	-0.52	-0.46	-0.37	-0.31	-0.45	0.92	1.09	1.08	1.10	1.08	1.29	1.28	1.03
P05106	Integrin beta-3 OS=Homo sapiens GN=ITGB3 PE=1 SV=2 - [ITB3_HUMAN]	-1.47	-1.57	-1.26	-1.35	1.16	0.93	0.45	-0.03	0.25	0.73	0.89	0.66	1.34	2.50	2.28	2.20	1.99	2.70	2.49	1.56
Q9BTV4	Transmembrane protein 43 OS=Homo sapiens GN=TMEM43 PE=1 SV=1 - [TMM43_HUMAN]	-1.28	-1.30	-1.14	-1.35	0.21	0.17	-0.48	-0.59	-0.25	-0.19	0.04	-0.17	1.00	1.45	1.34	1.42	1.21	1.60	1.50	0.83
Q96PE2	Rho guanine nucleotide exchange factor 17 OS=Homo sapiens GN=ARHGEF17 PE=1 SV=1 - [ARHGH_HUMAN]	-1.26	-1.26	-1.28	-1.33	-0.53	-0.47	-0.49	-0.39	-0.60	-0.45	-0.38	-0.37	0.78	0.65	0.90	0.78	0.81	0.66	0.79	0.92
P08648	Integrin alpha-5 OS=Homo sapiens GN=ITGA5 PE=1 SV=2 - [ITA5_HUMAN]	-1.13	-1.11	-1.69	-1.32	1.35	1.35	0.11	0.17	0.72	0.74	0.76	0.96	1.26	1.72	2.33	1.74	2.20	2.12	2.71	1.61
Q04721	Neurogenic locus notch homolog protein 2 OS=Homo sapiens GN=NOTCH2 PE=1 SV=3 - [NOTC2_HUMAN]	-1.31	-1.51	-1.20	-1.27	-0.20	-0.35	-0.42	-0.47	-0.31	-0.23	0.04	-0.11	0.97	1.41	1.41	1.16	1.11	1.05	1.15	1.03

Q9UHQ9	NADH-cytochrome b5 reductase 1 OS=Homo sapiens GN=CYB5R1 PE=1 SV=1 - [NB5R1_HUMAN]	-1.36	-1.46	-1.26	-1.25	-0.10	-0.18	-0.10	-0.10	-0.17	-0.22	-0.15	-0.27	1.37	1.10	1.04	1.05	1.14	1.18	1.21	1.27
Q9UKV8	Protein argonaute-2 OS=Homo sapiens GN=AGO2 PE=1 SV=3 - [AGO2_HUMAN]	-1.26	-1.18	-1.25	-1.22	-0.31	-0.21	-0.37	-0.42	-0.16	-0.27	-0.02	0.08	0.90	1.26	1.23	1.12	1.16	0.87	1.05	0.96
P23284	Peptidyl-prolyl cis-trans isomerase B OS=Homo sapiens GN=PPIB PE=1 SV=2 - [PPIB_HUMAN]	-1.43	-1.36	-1.14	-1.21	-0.61	-0.58	-0.87	-0.76	-0.76	-0.81	-0.50	-0.51	0.52	0.72	0.65	0.53	0.40	0.76	0.62	0.44
P49959	Double-strand break repair protein MRE11A OS=Homo sapiens GN=MRE11A PE=1 SV=3 - [MRE11_HUMAN]	-1.20	-1.10	-1.31	-1.18	-0.16	-0.05	-0.49	-0.30	-0.17	-0.37	-0.12	0.14	0.75	0.90	1.19	0.94	0.97	0.95	1.12	0.92
Q05329	Glutamate decarboxylase 2 OS=Homo sapiens GN=GAD2 PE=1 SV=1 - [DCE2_HUMAN]	2.08	2.55	2.25	2.71	-0.08	-0.17	0.84	1.27	0.73	0.53	0.00	0.02	-1.18	-1.70	-1.91	-1.52	-1.69	-1.90	-2.16	-1.35
P62760	Visinin-like protein 1 OS=Homo sapiens GN=VSNL1 PE=1 SV=2 - [VISL1_HUMAN]	1.96	2.20	2.50	2.70	0.19	0.26	0.80	0.97	0.38	0.46	0.46	0.40	-0.97	-1.61	-2.10	-1.65	-2.06	-1.72	-2.21	-1.55
O75899	Gamma-aminobutyric acid type B receptor subunit 2 OS=Homo sapiens GN=GABBR2 PE=1 SV=1 - [GABR2_HUMAN]	1.88	2.25	2.23	2.67	-0.39	0.25	0.62	1.02	0.87	0.27	-0.04	0.47	-1.13	-1.82	-2.01	-1.38	-1.62	-2.00	-2.47	-1.27
P62166	Neuronal calcium sensor 1 OS=Homo sapiens GN=NCS1 PE=1 SV=2 - [NCS1_HUMAN]	1.79	1.78	2.16	2.18	0.47	0.45	0.92	0.94	0.59	0.61	0.46	0.30	-0.79	-1.23	-1.56	-1.39	-1.49	-1.19	-1.70	-1.23
Q8IWQ3	Serine/threonine-protein kinase BRSK2 OS=Homo sapiens GN=BRSK2 PE=1 SV=3 - [BRSK2_HUMAN]	1.50	1.67	2.36	2.14	0.03	0.13	0.62	0.54	0.01	0.05	0.24	0.36	-0.95	-1.44	-1.75	-1.58	-1.87	-1.65	-1.98	-1.52

O75363	Breast carcinoma-amplified sequence 1 OS=Homo sapiens GN=BCAS1 PE=1 SV=2 - [BCAS1_HUMAN]	1.98	1.67	2.52	2.07	0.02	-0.25	0.87	0.49	-0.06	-0.07	-0.21	-0.28	-1.48	-2.05	-2.50	-2.04	-2.45	-2.89	-3.36	-1.90
Q8IV01	Synaptotagmin-12 OS=Homo sapiens GN=SYT12 PE=2 SV=1 - [SYT12_HUMAN]	2.17	2.05	2.05	1.93	0.10	-0.11	0.81	0.76	0.34	0.56	0.28	0.09	-1.18	-1.94	-1.81	-1.69	-1.52	-2.15	-2.05	-1.14
P14867	Gamma-aminobutyric acid receptor subunit alpha-1 OS=Homo sapiens GN=GABRA1 PE=1 SV=3 - [GBRA1_HUMAN]	1.74	1.75	1.81	1.92	-0.27	-0.50	0.39	0.22	-0.41	-0.25	-0.17	-0.30	-1.50	-2.15	-2.31	-1.99	-2.16	-2.61	-2.47	-1.72
Q86UW7	Calcium-dependent secretion activator 2 OS=Homo sapiens GN=CADPS2 PE=1 SV=2 - [CAPS2_HUMAN]	1.74	1.60	1.99	1.89	0.12	0.26	0.51	0.41	0.20	0.03	0.05	0.13	-1.17	-1.80	-1.85	-1.54	-1.58	-1.78	-1.70	-1.33
Q6PIU2	Neutral cholesterol ester hydrolase 1 OS=Homo sapiens GN=NCEH1 PE=1 SV=3 - [NCEH1_HUMAN]	1.71	1.63	1.90	1.84	-0.20	0.04	0.71	0.61	0.37	0.24	0.12	0.08	-0.97	-1.52	-1.70	-1.40	-1.46	-1.75	-2.01	-1.13
Q13555	Calcium/calmodulin-dependent protein kinase type II subunit gamma OS=Homo sapiens GN=CAMK2G PE=1 SV=3 - [KCC2G_HUMAN]	1.34	1.70	1.71	1.79	0.02	-0.05	0.62	0.62	0.26	0.30	0.27	0.34	-0.84	-1.43	-1.66	-1.19	-1.45	-1.41	-1.68	-1.07
Q15111	Inactive phospholipase C-like protein 1 OS=Homo sapiens GN=PLCL1 PE=1 SV=3 - [PLCL1_HUMAN]	1.24	1.47	1.32	1.76	0.17	0.32	0.67	0.82	0.40	0.31	0.12	0.46	-0.49	-0.98	-1.23	-0.68	-0.95	-0.93	-1.25	-0.73
Q8IU85	Calcium/calmodulin-dependent protein kinase type 1D OS=Homo sapiens GN=CAMK1D PE=1 SV=1 - [KCC1D_HUMAN]	1.34	1.35	1.46	1.65	0.01	-0.02	0.28	0.27	0.08	0.12	0.14	0.21	-1.02	-1.18	-1.25	-1.22	-1.20	-1.38	-1.36	-1.13

P55327	Tumor protein D52 OS=Homo sapiens GN=TPD52 PE=1 SV=2 - [TPD52_HUMAN]	1.27	1.06	1.67	1.63	-0.05	-0.04	0.50	0.49	0.00	0.04	-0.07	0.02	-0.68	-1.20	-1.60	-1.15	-1.42	-1.29	-1.65	-1.05
O14775	Guanine nucleotide-binding protein subunit beta-5 OS=Homo sapiens GN=GNB5 PE=1 SV=2 - [GBB5_HUMAN]	1.36	1.24	1.74	1.62	-0.08	-0.25	0.46	0.36	-0.07	0.12	-0.07	-0.17	-0.91	-1.10	-1.55	-1.39	-1.63	-1.55	-1.86	-1.42
Q86UW8	Hyaluronan and proteoglycan link protein 4 OS=Homo sapiens GN=HAPLN4 PE=2 SV=1 - [HPLN4_HUMAN]	1.92	1.79	1.73	1.61	0.06	0.10	0.68	0.78	0.46	0.37	0.41	0.28	-1.00	-1.59	-1.32	-1.52	-1.33	-1.71	-1.52	-0.94
Q9BTV5	Fibronectin type III and SPRY domain-containing protein 1 OS=Homo sapiens GN=FSD1 PE=1 SV=1 - [FSD1_HUMAN]	1.62	1.81	1.59	1.58	-0.44	-0.38	0.78	0.83	0.24	0.00	-0.12	0.01	-0.78	-1.95	-2.06	-1.45	-1.57	-2.21	-1.97	-0.94
Q16566	Calcium/calmodulin-dependent protein kinase type IV OS=Homo sapiens GN=CAMK4 PE=1 SV=1 - [KCC4_HUMAN]	1.68	1.15	1.69	1.51	-0.17	-0.23	0.44	0.18	-0.11	0.09	-0.05	-0.19	-1.24	-1.78	-1.88	-1.56	-1.58	-1.62	-1.71	-1.50
P41594	Metabotropic glutamate receptor 5 OS=Homo sapiens GN=GRM5 PE=1 SV=2 - [GRM5_HUMAN]	1.73	1.57	1.70	1.48	-0.12	-0.09	0.30	0.13	0.20	0.43	0.25	0.18	-1.09	-1.35	-1.32	-1.20	-1.06	-1.30	-1.42	-0.80
Q86YM7	Homer protein homolog 1 OS=Homo sapiens GN=HOMER1 PE=1 SV=2 - [HOME1_HUMAN]	1.87	1.64	1.84	1.48	-0.01	-0.54	0.64	0.24	-0.05	0.28	-0.01	-0.20	-1.03	-1.83	-1.79	-1.62	-1.49	-1.63	-1.85	-1.04
Q99426	Tubulin-folding cofactor B OS=Homo sapiens GN=TBCB PE=1 SV=2 - [TBCB_HUMAN]	1.36	1.44	1.41	1.42	0.13	0.09	0.73	0.77	0.37	0.36	-0.21	-0.14	-0.59	-1.63	-1.48	-1.09	-1.02	-1.23	-1.18	-0.52
P35813	Protein phosphatase 1A OS=Homo sapiens GN=PPM1A PE=1 SV=1 - [PPM1A_HUMAN]	1.27	1.16	1.41	1.39	0.11	0.15	0.49	0.47	0.01	0.03	0.09	0.02	-0.70	-1.27	-1.31	-1.08	-1.14	-0.95	-1.24	-0.82

Q9Y6T7	Diacylglycerol kinase beta OS=Homo sapiens GN=DGKB PE=2 SV=2 - [DGKB_HUMAN]	1.17	1.27	1.41	1.37	-0.28	-0.07	0.38	0.50	0.37	0.21	0.27	0.09	-0.65	-1.09	-1.03	-0.79	-1.03	-1.24	-1.58	-1.01
Q9NZ56	Formin-2 OS=Homo sapiens GN=FMN2 PE=1 SV=4 - [FMN2_HUMAN]	1.69	1.13	1.29	1.28	0.36	-0.11	0.31	-0.22	0.61	0.45	0.45	-0.29	-1.33	-1.18	-1.12	-1.15	-1.17	-1.23	-1.16	-1.17
Q9BPW8	Protein NipSnap homolog 1 OS=Homo sapiens GN=NIPSNAP1 PE=1 SV=1 - [NIPS1_HUMAN]	1.03	1.20	1.29	1.25	-0.41	-0.39	0.31	0.42	0.11	-0.01	-0.19	-0.13	-0.66	-1.15	-1.30	-1.00	-1.22	-1.36	-1.64	-0.82
Q96GD0	Pyridoxal phosphate phosphatase OS=Homo sapiens GN=PDXP PE=1 SV=2 - [PLPP_HUMAN]	1.36	1.20	1.30	1.25	0.38	0.35	0.34	0.23	0.32	0.28	0.15	0.14	-0.95	-1.08	-1.04	-0.85	-0.79	-0.91	-0.89	-0.88
Q9Y2G0	Protein EFR3 homolog B OS=Homo sapiens GN=EFR3B PE=1 SV=2 - [EFR3B_HUMAN]	1.54	1.14	1.47	1.24	0.06	-0.12	0.53	0.36	0.09	0.39	0.16	-0.30	-0.72	-1.42	-1.46	-0.93	-1.03	-1.38	-1.47	-0.63
Q5T5U3	Rho GTPase-activating protein 21 OS=Homo sapiens GN=ARHGAP21 PE=1 SV=1 - [RHG21_HUMAN]	1.09	1.15	1.25	1.23	-0.16	-0.08	0.20	0.07	0.05	0.09	0.01	0.04	-1.02	-1.08	-1.21	-1.13	-1.17	-1.24	-1.28	-1.00
P54764	Ephrin type-A receptor 4 OS=Homo sapiens GN=EPHA4 PE=1 SV=1 - [EPHA4_HUMAN]	1.38	1.27	1.37	1.14	-0.04	-0.03	0.65	0.53	0.04	0.11	0.30	0.16	-0.68	-1.03	-0.98	-1.24	-1.34	-1.40	-1.50	-0.77
Q96SM3	Probable carboxypeptidase X1 OS=Homo sapiens GN=CPXM1 PE=2 SV=2 - [CPXM1_HUMAN]	-3.29	-3.42	-3.51	-3.52	-1.03	-1.00	-1.67	-1.68	-1.57	-1.56	-2.40	-2.40	1.58	0.91	1.12	1.69	1.94	2.41	2.56	1.86
Q06828	Fibromodulin OS=Homo sapiens GN=FMOD PE=1 SV=2 - [FMOD_HUMAN]	-2.39	-2.48	-2.56	-2.76	-0.66	-0.68	-0.48	-0.60	-0.55	-0.56	-0.87	-0.85	2.13	1.63	1.84	1.89	2.27	1.87	2.31	2.18
Q02487	Desmocollin-2 OS=Homo sapiens GN=DSC2 PE=1 SV=1 - [DSC2_HUMAN]	-2.45	-2.42	-2.59	-2.66	-0.25	-0.27	-1.04	-1.09	-0.64	-0.74	-0.13	-0.13	1.24	2.02	2.38	1.71	1.89	2.04	2.34	1.53

Q8N2S1	Latent-transforming growth factor beta-binding protein 4 OS=Homo sapiens GN=LTBP4 PE=1 SV=2 - [LTBP4_HUMAN]	-1.88	-1.75	-2.19	-2.40	0.32	0.44	-0.36	-0.40	-0.32	-0.32	-0.55	-0.48	1.57	1.15	1.44	1.48	1.81	2.14	2.38	1.93
P49961	Ectonucleoside triphosphate diphosphohydrolase 1 OS=Homo sapiens GN=ENTPD1 PE=1 SV=1 - [ENTP1_HUMAN]	-2.32	-2.19	-2.36	-2.35	0.00	0.05	-0.44	-0.46	-0.29	-0.28	-0.08	-0.10	1.62	2.06	2.11	1.78	1.90	1.92	2.31	1.94
P26006	Integrin alpha-3 OS=Homo sapiens GN=ITGA3 PE=1 SV=5 - [ITA3_HUMAN]	-2.35	-2.34	-2.36	-2.26	0.16	0.27	-0.49	-0.35	-0.13	-0.05	0.02	0.08	2.05	2.44	2.42	2.17	2.15	2.28	2.37	1.95
Q14574	Desmocollin-3 OS=Homo sapiens GN=DSC3 PE=1 SV=3 - [DSC3_HUMAN]	-1.79	-1.95	-1.99	-2.14	-0.14	-0.28	-0.71	-0.76	-0.50	-0.32	-0.19	-0.27	1.15	1.54	1.72	1.49	1.51	1.55	1.74	1.46
P54709	Sodium/potassium-transporting ATPase subunit beta-3 OS=Homo sapiens GN=ATP1B3 PE=1 SV=1 - [AT1B3_HUMAN]	-2.13	-2.15	-1.90	-2.08	0.17	0.23	0.05	0.09	0.02	-0.01	-0.03	0.04	2.32	2.11	2.12	2.24	1.98	2.30	2.38	2.23
P60660	Myosin light polypeptide 6 OS=Homo sapiens GN=MYL6 PE=1 SV=2 - [MYL6_HUMAN]	-1.95	-1.86	-1.98	-1.93	-0.73	-0.80	-0.98	-0.96	-0.87	-0.80	-0.62	-0.57	1.02	1.38	1.33	1.06	1.21	1.05	1.17	1.09
Q0ZGT2	Nexilin OS=Homo sapiens GN=NEXN PE=1 SV=1 - [NEXN_HUMAN]	-1.79	-1.61	-2.15	-1.93	0.12	0.23	-0.87	-0.80	-0.25	-0.40	-0.50	-0.45	0.85	1.23	1.55	1.49	1.87	1.83	2.22	1.24
Q9NPH2	Inositol-3-phosphate synthase 1 OS=Homo sapiens GN=ISYNA1 PE=1 SV=1 - [INO1_HUMAN]	-1.29	-1.25	-1.99	-1.92	-0.29	-0.37	-0.56	-0.50	-0.45	-0.45	-0.38	-0.37	0.75	1.23	1.64	1.27	1.74	1.04	1.60	1.49
P07358	Complement component C8 beta chain OS=Homo sapiens GN=C8B PE=1 SV=3 - [CO8B_HUMAN]	-1.65	-1.72	-1.67	-1.85	0.24	0.10	-0.42	-0.56	-0.01	0.08	0.62	0.63	1.12	2.16	2.36	1.81	2.20	1.71	2.33	1.48
Q96TA1	Niban-like protein 1 OS=Homo sapiens GN=FAM129B PE=1 SV=3 - [NIBL1_HUMAN]	-1.94	-1.77	-1.91	-1.78	0.37	0.47	0.08	0.07	0.00	-0.05	-0.24	-0.09	1.91	1.84	1.81	1.97	1.95	2.18	1.99	1.99

P00742	Coagulation factor X OS=Homo sapiens GN=F10 PE=1 SV=2 - [FA10_HUMAN]	-1.66	-1.54	-1.83	-1.77	0.60	0.69	0.07	0.13	0.24	0.12	0.02	0.00	1.81	1.80	1.85	1.93	2.09	2.34	2.42	1.97
Q15654	Thyroid receptor-interacting protein 6 OS=Homo sapiens GN=TRIP6 PE=1 SV=3 - [TRIP6_HUMAN]	-1.50	-1.60	-1.75	-1.77	-0.45	-0.27	-0.88	-0.65	-0.49	-0.69	-0.38	-0.05	0.64	1.09	1.33	1.13	1.17	1.10	1.17	1.10
P04004	Vitronectin OS=Homo sapiens GN=VTN PE=1 SV=1 - [VTNC_HUMAN]	-1.72	-1.66	-1.74	-1.68	1.83	1.88	1.19	1.17	1.19	1.19	0.69	0.62	2.58	2.42	2.47	2.81	2.96	3.44	3.43	2.84
P30837	Aldehyde dehydrogenase X, mitochondrial OS=Homo sapiens GN=ALDH1B1 PE=1 SV=3 - [AL1B1_HUMAN]	-1.83	-1.67	-1.59	-1.63	-0.31	-0.25	-1.55	-1.60	-1.16	-1.13	-1.32	-1.28	0.29	0.63	0.29	0.64	0.56	1.44	1.37	0.14
P00387	NADH-cytochrome b5 reductase 3 OS=Homo sapiens GN=CYB5R3 PE=1 SV=3 - [NB5R3_HUMAN]	-1.16	-1.43	-1.34	-1.55	-0.09	-0.04	-0.48	-0.46	-0.23	-0.24	-0.04	-0.09	0.77	1.13	1.41	1.04	1.23	1.06	1.46	0.98
P36269	Gamma-glutamyltransferase 5 OS=Homo sapiens GN=GGT5 PE=1 SV=2 - [GGT5_HUMAN]	-1.35	-1.27	-1.48	-1.55	-0.02	-0.06	-0.79	-0.68	-0.51	-0.49	-0.25	-0.27	0.61	1.02	1.16	0.87	1.09	1.09	1.41	0.83
P03952	Plasma kallikrein OS=Homo sapiens GN=KLKB1 PE=1 SV=1 - [KLKB1_HUMAN]	-1.15	-1.34	-1.57	-1.51	0.93	0.79	0.49	0.22	0.47	0.56	0.95	0.73	1.73	2.10	2.23	1.92	1.99	2.06	2.13	1.80
P04217	Alpha-1B-glycoprotein OS=Homo sapiens GN=A1BG PE=1 SV=4 - [A1BG_HUMAN]	-1.48	-1.25	-1.79	-1.44	-0.58	-0.30	-0.83	-0.69	-0.54	-0.66	-0.36	-0.24	0.50	1.06	1.51	0.87	1.16	0.70	1.05	0.87
Q8NC56	LEM domain-containing protein 2 OS=Homo sapiens GN=LEMD2 PE=1 SV=1 - [LEMD2_HUMAN]	-1.09	-1.29	-1.21	-1.39	-0.20	-0.27	-0.50	-0.61	-0.36	-0.27	0.02	-0.09	0.75	1.08	1.18	0.86	0.95	1.05	1.08	0.78
P08174	Complement decay-accelerating factor OS=Homo sapiens GN=CD55 PE=1 SV=4 - [DAF_HUMAN]	-1.28	-1.35	-1.33	-1.36	0.86	0.71	0.28	0.28	0.41	0.58	0.63	0.49	1.56	1.74	1.83	1.80	1.90	1.98	2.16	1.60

P27105	Erythrocyte band 7 integral membrane protein OS=Homo sapiens GN=STOM PE=1 SV=3 - [STOM_HUMAN]	-1.31	-1.23	-1.52	-1.35	0.63	0.69	0.12	0.19	0.54	0.51	0.82	1.00	1.48	2.04	2.25	1.64	2.01	1.87	2.05	1.63
P00491	Purine nucleoside phosphorylase OS=Homo sapiens GN=PNP PE=1 SV=2 - [PNPH_HUMAN]	-1.26	-1.20	-1.29	-1.26	-0.36	-0.23	-0.47	-0.45	-0.36	-0.49	-1.08	-1.12	0.62	0.27	0.26	0.83	0.99	0.92	1.04	0.86
Q8TER0	Sushi, nidogen and EGF-like domain-containing protein 1 OS=Homo sapiens GN=SNED1 PE=2 SV=2 - [SNED1_HUMAN]	-1.34	-1.26	-1.23	-1.25	1.04	1.30	0.43	0.61	0.86	0.79	0.74	0.97	1.87	2.08	2.03	2.17	2.11	2.57	2.07	1.83
Q14767	Latent-transforming growth factor beta-binding protein 2 OS=Homo sapiens GN=LTBP2 PE=1 SV=3 - [LTBP2_HUMAN]	-1.46	-1.46	-1.23	-1.24	1.33	1.44	0.28	0.40	0.77	0.78	0.49	0.48	1.68	2.22	1.82	2.27	2.30	2.78	2.79	2.08
Q92561	Phytanoyl-CoA hydroxylase-interacting protein OS=Homo sapiens GN=PHYHIP PE=1 SV=1 - [PHYIP_HUMAN]	2.73	2.75	3.03	3.16	-0.14	0.13	1.29	1.38	0.67	0.67	0.70	0.49	-1.46	-2.27	-2.47	-2.08	-2.40	-2.91	-3.11	-1.69
P23515	Oligodendrocyte-myelin glycoprotein OS=Homo sapiens GN=OMG PE=1 SV=2 - [OMGP_HUMAN]	2.24	2.27	2.48	2.59	-0.14	-0.14	0.73	0.79	0.51	0.34	0.40	0.43	-1.35	-1.73	-2.07	-1.70	-1.84	-2.41	-2.80	-1.79
Q8N5S9	Calcium/calmodulin-dependent protein kinase kinase 1 OS=Homo sapiens GN=CAMKK1 PE=1 SV=2 - [KKCC1_HUMAN]	2.03	2.03	2.45	2.38	-0.03	-0.06	0.53	0.64	0.36	0.45	0.13	0.05	-1.34	-1.68	-2.09	-1.52	-1.90	-2.05	-2.35	-1.68
O00154	Cytosolic acyl coenzyme A thioester hydrolase OS=Homo sapiens GN=ACOT7 PE=1 SV=3 - [BACH_HUMAN]	2.55	2.33	2.51	2.32	0.10	0.04	1.02	0.74	0.47	0.66	0.02	0.08	-1.37	-2.24	-2.35	-1.69	-1.85	-2.24	-2.36	-1.42

Q16653	Myelin-oligodendrocyte glycoprotein OS=Homo sapiens GN=MOG PE=1 SV=2 - [MOG_HUMAN]	2.74	2.40	2.70	2.29	-0.28	-0.43	0.77	0.55	0.16	0.38	0.33	0.04	-1.83	-2.35	-2.13	-2.07	-2.01	-2.65	-2.95	-1.69
Q99259	Glutamate decarboxylase 1 OS=Homo sapiens GN=GAD1 PE=1 SV=1 - [DCE1_HUMAN]	1.62	1.74	2.03	2.20	-0.15	0.19	0.59	0.88	0.66	0.30	-0.22	0.12	-0.64	-1.72	-2.09	-1.21	-1.55	-1.67	-2.07	-1.05
Q9UPX8	SH3 and multiple ankyrin repeat domains protein 2 OS=Homo sapiens GN=SHANK2 PE=1 SV=3 - [SHAN2_HUMAN]	1.36	1.46	1.96	2.05	-0.15	-0.06	0.41	0.49	-0.04	-0.14	-0.17	0.04	-1.08	-1.57	-2.01	-1.47	-1.65	-1.65	-2.11	-1.50
O94811	Tubulin polymerization-promoting protein OS=Homo sapiens GN=TPPP PE=1 SV=1 - [TPPP_HUMAN]	1.68	1.80	1.77	1.96	0.03	-0.03	0.12	0.10	0.06	-0.01	-0.05	0.04	-1.54	-1.81	-2.00	-1.77	-1.74	-1.80	-1.76	-1.84
Q8IVD9	NudC domain-containing protein 3 OS=Homo sapiens GN=NUDCD3 PE=1 SV=3 - [NUDC3_HUMAN]	1.44	1.37	2.02	1.88	-0.16	-0.20	1.00	0.99	0.34	0.44	0.04	-0.04	-0.45	-1.54	-1.91	-0.95	-1.41	-1.57	-2.16	-0.68
Q9P121	Neurotrimin OS=Homo sapiens GN=NTM PE=1 SV=1 - [NTRI_HUMAN]	1.65	1.79	1.83	1.80	0.28	0.19	0.71	0.83	0.38	0.47	0.46	0.31	-0.97	-1.31	-1.32	-1.32	-1.34	-1.67	-1.52	-1.01
Q9H0Q0	Protein FAM49A OS=Homo sapiens GN=FAM49A PE=1 SV=1 - [FA49A_HUMAN]	1.52	1.78	1.43	1.77	-0.17	0.23	0.34	0.45	0.42	0.49	0.21	0.25	-1.04	-1.25	-0.90	-0.86	-0.55	-1.49	-1.48	-0.62
P61601	Neurocalcin-delta OS=Homo sapiens GN=NCALD PE=2 SV=2 - [NCALD_HUMAN]	1.30	1.58	1.30	1.73	-0.17	0.20	0.25	0.48	0.15	-0.04	0.16	0.23	-1.16	-1.37	-1.51	-1.37	-1.43	-1.51	-1.60	-1.28
P21695	Glycerol-3-phosphate dehydrogenase [NAD(+)], cytoplasmic OS=Homo sapiens GN=GPD1 PE=1 SV=4 - [GPDA_HUMAN]	1.70	1.67	1.94	1.63	0.34	0.29	0.73	0.57	0.49	0.44	0.04	-0.14	-1.04	-1.56	-1.61	-1.23	-1.18	-1.23	-1.34	-1.09

Q96ID5	Immunoglobulin superfamily member 21 OS=Homo sapiens GN=IGSF21 PE=2 SV=1 - [IGS21_HUMAN]	1.64	1.58	1.58	1.52	0.18	0.14	0.36	0.25	0.07	0.42	0.30	0.11	-1.35	-1.47	-1.62	-1.15	-1.28	-1.49	-1.31	-1.33
Q96FW1	Ubiquitin thioesterase OTUB1 OS=Homo sapiens GN=OTUB1 PE=1 SV=2 - [OTUB1_HUMAN]	1.33	1.36	1.33	1.51	0.33	0.35	0.47	0.53	0.41	0.44	-0.05	0.11	-0.52	-1.28	-1.30	-0.79	-0.88	-0.90	-0.97	-0.72
O14576	Cytoplasmic dynein 1 intermediate chain 1 OS=Homo sapiens GN=DYNC1I1 PE=1 SV=2 - [DC1I1_HUMAN]	1.35	1.45	1.40	1.45	-0.23	-0.05	0.19	0.33	0.18	0.06	-0.15	-0.03	-1.01	-1.35	-1.38	-1.14	-1.09	-1.49	-1.43	-1.04
Q8N5V2	Ephexin-1 OS=Homo sapiens GN=NGEF PE=2 SV=2 - [NGEF_HUMAN]	1.60	1.44	1.78	1.44	-0.29	-0.47	0.17	-0.27	-0.24	0.13	-0.29	-0.36	-1.38	-1.95	-1.95	-1.52	-1.73	-1.91	-1.92	-1.68
Q5T848	Probable G-protein coupled receptor 158 OS=Homo sapiens GN=GPR158 PE=1 SV=1 - [GP158_HUMAN]	1.29	1.27	1.38	1.43	0.28	0.27	0.53	0.49	0.29	0.52	0.22	0.23	-0.84	-1.16	-1.23	-0.95	-0.87	-1.16	-1.24	-0.92
Q9P1Y5	Calmodulin-regulated spectrin-associated protein 3 OS=Homo sapiens GN=CAMSAP3 PE=1 SV=2 - [CAMP3_HUMAN]	1.63	1.40	1.53	1.39	0.41	0.32	0.64	0.42	0.22	0.35	0.24	0.00	-1.22	-1.52	-1.60	-1.21	-1.28	-1.13	-1.20	-1.30
Q6BCY4	NADH-cytochrome b5 reductase 2 OS=Homo sapiens GN=CYB5R2 PE=1 SV=1 - [NB5R2_HUMAN]	1.27	1.39	1.52	1.38	0.56	0.58	0.34	0.25	0.16	0.19	0.00	-0.07	-1.12	-1.48	-1.63	-1.12	-1.13	-0.86	-0.75	-1.12
O75335	Liprin-alpha-4 OS=Homo sapiens GN=PPFIA4 PE=2 SV=3 - [LIPA4_HUMAN]	1.39	1.76	1.37	1.36	-0.33	-0.01	0.24	0.32	0.40	0.25	-0.09	0.06	-1.16	-1.54	-1.29	-1.08	-0.82	-1.70	-1.38	-0.95
Q9Y5K8	V-type proton ATPase subunit D OS=Homo sapiens GN=ATP6V1D PE=1 SV=1 - [VATD_HUMAN]	1.46	1.40	1.44	1.35	0.13	0.15	0.66	0.64	0.56	0.31	0.08	0.45	-0.97	-1.59	-1.31	-1.15	-1.10	-1.62	-1.53	-0.82

Q02338	D-beta-hydroxybutyrate dehydrogenase, mitochondrial OS=Homo sapiens GN=BDH1 PE=1 SV=3 - [BDH_HUMAN]	1.32	1.39	1.25	1.33	0.89	0.95	1.39	1.39	1.18	1.01	0.71	0.84	0.13	-0.55	-0.44	-0.12	-0.12	-0.40	-0.35	0.13
Q6P995	Protein FAM171B OS=Homo sapiens GN=FAM171B PE=2 SV=3 - [F171B_HUMAN]	1.21	1.22	1.26	1.27	0.17	0.17	0.38	0.38	0.54	0.54	0.40	0.40	-0.80	-0.80	-0.86	-0.62	-0.63	-1.06	-1.11	-0.65
Q8IYB4	PEX5-related protein OS=Homo sapiens GN=PEX5L PE=1 SV=2 - [PEX5R_HUMAN]	1.12	1.11	1.33	1.25	-0.21	-0.49	0.14	0.05	-0.16	-0.05	-0.18	-0.42	-0.93	-1.23	-1.50	-0.92	-0.97	-1.81	-1.39	-1.03
Q9UNA1	Rho GTPase-activating protein 26 OS=Homo sapiens GN=ARHGAP26 PE=1 SV=1 - [RHG26_HUMAN]	1.45	1.22	1.37	1.16	-0.12	-0.29	0.25	0.05	0.03	-0.03	-0.01	0.15	-1.21	-1.28	-1.23	-1.37	-1.28	-1.69	-1.61	-1.17
O60784	Target of Myb protein 1 OS=Homo sapiens GN=TOM1 PE=1 SV=2 - [TOM1_HUMAN]	1.14	1.07	1.19	1.09	0.03	0.02	0.23	0.20	0.16	0.14	-0.01	-0.01	-0.76	-1.06	-1.03	-0.99	-0.99	-0.86	-0.96	-0.79
P52888	Thimet oligopeptidase OS=Homo sapiens GN=THOP1 PE=1 SV=2 - [THOP1_HUMAN]	1.37	1.18	1.14	1.07	0.49	0.42	0.43	0.38	0.27	0.37	-0.02	-0.09	-0.88	-1.25	-1.10	-0.97	-0.76	-0.82	-0.67	-0.58
O15335	Chondroadherin OS=Homo sapiens GN=CHAD PE=2 SV=2 - [CHAD_HUMAN]	-3.49	-3.51	-4.44	-4.39	-2.72	-2.60	-2.77	-2.73	-2.74	-2.78	-2.70	-2.72	1.03	0.80	1.30	0.88	1.51	0.75	1.32	1.48
Q8N6Y2	Leucine-rich repeat-containing protein 17 OS=Homo sapiens GN=LRRC17 PE=2 SV=1 - [LRC17_HUMAN]	-3.54	-3.30	-3.61	-3.60	-0.77	-0.79	-0.79	-0.78	-0.74	-0.81	-0.59	-0.49	2.23	3.07	3.08	2.48	2.79	2.60	3.00	2.83
Q07092	Collagen alpha-1(XVI) chain OS=Homo sapiens GN=COL16A1 PE=1 SV=2 - [COGA1_HUMAN]	-2.96	-3.12	-3.09	-3.29	-1.00	-1.21	-1.65	-1.82	-1.57	-1.55	-1.58	-1.72	1.36	1.34	1.25	1.71	1.69	2.03	1.97	1.51
P05546	Heparin cofactor 2 OS=Homo sapiens GN=SERPIND1 PE=1 SV=3 - [HEP2_HUMAN]	-2.98	-2.96	-3.06	-3.06	-1.10	-1.10	-1.52	-1.57	-1.00	-1.17	-1.10	-1.05	1.68	1.98	2.09	2.06	2.00	2.14	1.94	1.59

Q969G5	Protein kinase C delta-binding protein OS=Homo sapiens GN=PRKCDBP PE=1 SV=3 - [PRDBP_HUMAN]	-2.42	-2.21	-2.60	-2.98	-0.84	-0.72	-1.10	-1.21	-0.89	-0.77	-0.28	-0.28	1.35	2.01	2.59	1.68	1.92	1.47	1.80	1.63
Q03135	Caveolin-1 OS=Homo sapiens GN=CAV1 PE=1 SV=4 - [CAV1_HUMAN]	-2.69	-2.66	-2.73	-2.76	-0.79	-0.71	-0.83	-0.81	-0.75	-0.81	-0.35	-0.36	2.00	2.26	2.34	1.99	1.94	1.91	1.90	2.02
Q16647	Prostacyclin synthase OS=Homo sapiens GN=PTGIS PE=1 SV=1 - [PTGIS_HUMAN]	-2.43	-2.21	-2.68	-2.39	-0.11	-0.42	-0.73	-0.78	-0.78	-0.59	-0.72	-0.88	1.68	1.70	1.78	1.79	2.12	1.89	1.96	1.91
Q99439	Calponin-2 OS=Homo sapiens GN=CNN2 PE=1 SV=4 - [CNN2_HUMAN]	-2.25	-2.33	-2.31	-2.39	-0.48	-0.60	-0.58	-0.62	-0.63	-0.66	-0.86	-0.83	2.18	1.47	1.47	1.77	1.73	1.89	1.87	1.95
P68871	Hemoglobin subunit beta OS=Homo sapiens GN=HBB PE=1 SV=2 - [HBB_HUMAN]	-2.13	-2.05	-2.18	-2.07	0.34	0.42	-0.33	-0.23	0.08	-0.03	0.14	0.21	1.88	2.30	2.30	2.17	2.23	2.45	2.49	1.94
P29474	Nitric oxide synthase, endothelial OS=Homo sapiens GN=NOS3 PE=1 SV=3 - [NOS3_HUMAN]	-1.76	-1.92	-2.09	-2.05	-0.86	-0.92	-1.47	-1.47	-1.06	-1.00	-0.92	-0.95	0.53	0.72	1.17	0.85	1.15	1.22	1.46	0.68
Q9H3G5	Probable serine carboxypeptidase CPVL OS=Homo sapiens GN=CPVL PE=1 SV=2 - [CPVL_HUMAN]	-2.55	-2.41	-2.12	-2.04	0.11	0.20	-0.52	-0.34	-0.04	-0.19	-0.11	0.00	1.95	2.13	2.06	2.37	2.09	2.55	2.43	1.78
Q9UBX5	Fibulin-5 OS=Homo sapiens GN=FBLN5 PE=1 SV=1 - [FBLN5_HUMAN]	-1.48	-1.33	-1.83	-2.03	1.48	1.48	0.59	0.49	0.85	0.84	0.29	0.21	2.00	1.82	2.05	2.33	2.65	2.80	3.42	2.48
P02042	Hemoglobin subunit delta OS=Homo sapiens GN=HBD PE=1 SV=2 - [HBD_HUMAN]	-1.90	-1.90	-2.06	-2.01	-0.13	-0.10	-0.22	-0.15	0.08	-0.01	0.47	0.48	1.77	2.36	2.44	1.94	2.11	1.79	1.90	1.83
Q8N3D4	EH domain-binding protein 1-like protein 1 OS=Homo sapiens GN=EHBP1L1 PE=1 SV=2 - [EH1L1_HUMAN]	-1.24	-1.27	-1.91	-1.98	-0.22	-0.03	-0.68	-0.47	-0.09	-0.40	-0.08	0.12	0.74	1.22	2.10	1.13	1.38	1.15	1.83	1.38

Q9BUP0	EF-hand domain-containing protein D1 OS=Homo sapiens GN=EFHD1 PE=1 SV=1 - [EFHD1_HUMAN]	-1.79	-1.50	-1.84	-1.86	-1.02	-0.98	-1.27	-1.22	-0.92	-0.94	-0.87	-0.80	0.52	0.83	0.99	0.84	0.86	0.62	0.81	0.70
Q14192	Four and a half LIM domains protein 2 OS=Homo sapiens GN=FHL2 PE=1 SV=3 - [FHL2_HUMAN]	-2.40	-2.15	-2.08	-1.86	-0.70	-0.63	-1.15	-1.09	-0.98	-1.12	-1.42	-1.17	1.16	1.04	0.82	1.29	0.98	1.57	1.37	0.93
P13671	Complement component C6 OS=Homo sapiens GN=C6 PE=1 SV=3 - [CO6_HUMAN]	-1.61	-1.55	-1.80	-1.80	0.52	0.36	-0.50	-0.50	-0.14	0.02	-0.05	-0.17	1.17	1.52	1.72	1.67	1.85	2.04	2.11	1.36
P10301	Ras-related protein R-Ras OS=Homo sapiens GN=RRAS PE=1 SV=1 - [RRAS_HUMAN]	-1.86	-1.89	-1.96	-1.79	-0.14	-0.03	-0.57	-0.56	-0.34	-0.33	-0.13	-0.10	1.43	1.70	1.79	1.69	1.67	1.68	1.81	1.38
Q96HP0	Dedicator of cytokinesis protein 6 OS=Homo sapiens GN=DOCK6 PE=1 SV=3 - [DOCK6_HUMAN]	-1.66	-1.51	-1.67	-1.76	-0.25	-0.18	-0.49	-0.66	-0.55	-0.45	-0.26	-0.36	0.89	1.41	1.44	1.24	1.25	1.47	1.35	1.22
Q16270	Insulin-like growth factor-binding protein 7 OS=Homo sapiens GN=IGFBP7 PE=1 SV=1 - [IBP7_HUMAN]	-1.95	-1.87	-1.86	-1.75	0.24	0.29	-0.66	-0.67	-0.28	-0.26	-0.63	-0.40	0.96	1.23	1.41	1.47	1.59	2.05	2.16	1.34
Q8WWP7	GTPase IMAP family member 1 OS=Homo sapiens GN=GIMAP1 PE=1 SV=1 - [GIMA1_HUMAN]	-1.25	-1.12	-1.76	-1.72	-0.79	-0.69	-1.18	-1.08	-0.72	-0.83	-0.81	-0.72	0.09	0.45	0.93	0.43	0.80	0.52	0.86	0.63
Q9UBG0	C-type mannose receptor 2 OS=Homo sapiens GN=MRC2 PE=1 SV=2 - [MRC2_HUMAN]	-1.43	-1.42	-1.70	-1.70	0.27	0.27	-0.42	-0.38	0.11	0.16	0.87	0.89	1.23	2.37	2.56	1.67	1.84	1.59	2.01	1.39
Q6PCB0	von Willebrand factor A domain containing protein 1 OS=Homo sapiens GN=VWA1 PE=2 SV=1 - [VWA1_HUMAN]	-1.78	-1.79	-1.62	-1.62	-0.40	-0.51	-0.83	-0.92	-0.90	-0.81	-0.65	-0.79	1.08	1.00	0.82	0.95	0.85	1.47	1.30	0.92

Q6ZMZ3	Nesprin-3 OS=Homo sapiens GN=SYNE3 PE=1 SV=2 - [SYNE3_HUMAN]	-1.26	-1.37	-1.38	-1.53	-0.05	0.02	-0.26	-0.21	-0.13	-0.06	-0.35	-0.25	1.15	1.10	1.29	1.41	1.63	1.35	1.36	1.41
P07738	Bisphosphoglycerate mutase OS=Homo sapiens GN=BPGM PE=1 SV=2 - [PMGE_HUMAN]	-1.30	-1.12	-1.46	-1.50	-0.42	-0.34	-0.14	-0.04	-0.27	-0.39	-0.69	-0.67	1.23	0.68	0.80	1.02	1.14	0.90	1.18	1.40
P46937	Yorkie homolog OS=Homo sapiens GN=YAP1 PE=1 SV=2 - [YAP1_HUMAN]	-1.67	-1.48	-1.53	-1.46	-0.50	-0.47	-0.62	-0.64	-0.43	-0.34	-0.08	-0.04	0.79	1.45	1.45	1.00	1.08	1.00	0.93	0.82
P39656	Dolichyl-diphosphooligosaccharide--protein glycosyltransferase 48 kDa subunit OS=Homo sapiens GN=DDOST PE=1 SV=4 - [OST48_HUMAN]	-1.71	-1.68	-1.32	-1.45	-0.11	-0.01	-0.31	-0.50	-0.31	-0.31	-0.22	-0.09	1.18	1.35	1.28	1.36	1.23	1.58	1.41	1.07
Q92522	Histone H1x OS=Homo sapiens GN=H1FX PE=1 SV=1 - [H1X_HUMAN]	-1.46	-1.63	-1.31	-1.26	-0.89	-1.01	-1.01	-0.96	-1.14	-0.94	-0.85	-0.92	0.57	0.75	0.59	0.42	0.25	0.39	0.19	0.37
Q9Y6F6	Protein MRVI1 OS=Homo sapiens GN=MRVI1 PE=1 SV=2 - [MRVI1_HUMAN]	-1.25	-1.08	-1.15	-1.22	-1.30	-1.14	-0.70	-0.83	-0.77	-0.61	-0.67	-0.45	0.15	0.57	0.51	0.37	0.58	-0.09	0.03	0.37
Q8TAM6	Ermin OS=Homo sapiens GN=ERMN PE=2 SV=1 - [ERMIN_HUMAN]	2.41	2.61	2.54	2.93	-0.75	-0.75	0.47	0.46	0.05	0.15	0.19	0.04	-1.92	-2.33	-2.55	-2.35	-2.67	-2.04	-2.49	-1.95
P02794	Ferritin heavy chain OS=Homo sapiens GN=FTH1 PE=1 SV=2 - [FRIH_HUMAN]	2.36	2.29	2.89	2.75	0.48	0.50	1.70	1.54	1.40	1.29	0.96	1.19	-0.92	-1.36	-1.73	-1.03	-1.27	-1.76	-2.18	-1.17
Q92558	Wiskott-Aldrich syndrome protein family member 1 OS=Homo sapiens GN=WASF1 PE=1 SV=1 - [WASF1_HUMAN]	2.34	2.62	2.29	2.52	-0.25	-0.08	0.77	1.06	0.52	0.38	0.10	0.24	-1.51	-2.48	-2.33	-1.85	-1.80	-2.61	-2.56	-1.39
Q9NUQ9	Protein FAM49B OS=Homo sapiens GN=FAM49B PE=1 SV=1 - [FA49B_HUMAN]	1.93	2.41	2.32	2.39	0.11	0.29	0.50	0.67	0.32	0.07	0.01	0.03	-1.73	-2.08	-2.05	-1.95	-2.15	-2.06	-2.45	-1.73

Q02750	Dual specificity mitogen-activated protein kinase kinase 1 OS=Homo sapiens GN=MAP2K1 PE=1 SV=2 - [MP2K1_HUMAN]	1.76	1.83	2.24	2.26	0.60	0.41	0.93	0.87	0.62	0.59	0.38	0.47	-0.85	-1.38	-1.80	-1.16	-1.67	-1.34	-1.61	-1.28
P60201	Myelin proteolipid protein OS=Homo sapiens GN=PLP1 PE=1 SV=2 - [MYPR_HUMAN]	2.05	1.96	2.42	2.22	-0.53	-0.70	0.57	0.38	-0.04	0.07	-0.34	-0.41	-1.50	-2.38	-2.74	-2.10	-2.35	-2.65	-3.12	-1.78
Q00535	Cyclin-dependent kinase 5 OS=Homo sapiens GN=CDK5 PE=1 SV=3 - [CDK5_HUMAN]	1.66	1.56	2.18	2.08	-0.02	-0.08	0.69	0.72	0.22	0.23	0.05	0.01	-0.52	-1.51	-2.15	-1.27	-2.02	-1.66	-2.33	-1.27
P24588	A-kinase anchor protein 5 OS=Homo sapiens GN=AKAP5 PE=1 SV=3 - [AKAP5_HUMAN]	1.32	1.35	1.92	2.05	-0.08	-0.12	0.45	0.46	0.28	0.19	0.09	0.08	-0.91	-1.74	-2.18	-1.26	-1.72	-1.42	-2.18	-1.41
P14854	Cytochrome c oxidase subunit 6B1 OS=Homo sapiens GN=COX6B1 PE=1 SV=2 - [CX6B1_HUMAN]	1.55	1.87	1.61	2.00	0.01	0.28	0.52	0.74	0.49	0.22	0.25	0.37	-1.06	-1.34	-1.27	-1.33	-1.31	-1.30	-1.59	-1.01
Q05586	Glutamate receptor ionotropic, NMDA 1 OS=Homo sapiens GN=GRIN1 PE=1 SV=1 - [NMDZ1_HUMAN]	2.14	1.91	2.27	1.95	-0.34	-0.11	0.28	0.65	0.30	0.51	0.59	0.08	-1.45	-1.59	-1.87	-1.51	-1.92	-1.67	-2.08	-1.65
P49758	Regulator of G-protein signaling 6 OS=Homo sapiens GN=RGS6 PE=1 SV=5 - [RGS6_HUMAN]	1.56	1.64	1.82	1.93	-0.34	-0.27	0.26	0.38	0.06	0.02	0.00	0.08	-1.16	-1.55	-1.82	-1.40	-1.79	-1.52	-2.18	-1.46
Q5T5C0	Syntaxin-binding protein 5 OS=Homo sapiens GN=STXBP5 PE=1 SV=1 - [STXB5_HUMAN]	1.72	1.64	1.41	1.92	-0.40	-0.25	0.56	0.86	0.72	0.24	0.18	0.36	-0.85	-1.62	-1.88	-1.49	-1.13	-1.90	-2.19	-0.81
Q9NV96	Cell cycle control protein 50A OS=Homo sapiens GN=TMEM30A PE=1 SV=1 - [CC50A_HUMAN]	1.29	1.09	1.97	1.84	0.19	0.09	0.85	0.70	0.40	0.41	0.70	0.62	-0.34	-0.63	-1.27	-0.65	-1.23	-0.93	-1.76	-0.88

Q9UPY8	Microtubule-associated protein RP/EB family member 3 OS=Homo sapiens GN=MAPRE3 PE=1 SV=1 - [MARE3_HUMAN]	1.47	1.84	1.24	1.84	0.14	0.57	0.59	0.67	0.66	0.16	0.11	0.39	-1.10	-1.41	-1.41	-1.14	-0.89	-1.15	-1.08	-1.15
Q9UJD0	Regulating synaptic membrane exocytosis protein 3 OS=Homo sapiens GN=RIMS3 PE=1 SV=1 - [RIMS3_HUMAN]	1.95	1.93	1.80	1.78	-0.19	0.02	0.21	0.24	0.11	0.13	0.03	-0.09	-1.44	-2.01	-1.83	-1.79	-1.63	-1.68	-1.94	-1.41
P47736	Rap1 GTPase-activating protein 1 OS=Homo sapiens GN=RAP1GAP PE=1 SV=2 - [RPGP1_HUMAN]	2.04	1.89	1.91	1.76	-0.08	-0.23	0.73	0.58	0.23	0.34	0.24	0.14	-1.18	-1.92	-1.74	-1.74	-1.61	-2.11	-2.03	-1.02
P62873	Guanine nucleotide-binding protein G(I)/G(S)/G(T) subunit beta-1 OS=Homo sapiens GN=GNB1 PE=1 SV=3 - [GBB1_HUMAN]	1.62	1.68	1.70	1.70	-0.03	0.00	0.51	0.57	0.22	0.25	0.28	0.33	-0.99	-1.51	-1.47	-1.30	-1.25	-1.50	-1.77	-1.14
P20674	Cytochrome c oxidase subunit 5A, mitochondrial OS=Homo sapiens GN=COX5A PE=1 SV=2 - [COX5A_HUMAN]	1.37	1.52	1.65	1.68	0.35	0.33	0.55	0.61	0.56	0.49	0.48	0.43	-0.70	-1.00	-1.17	-0.86	-1.01	-1.16	-1.26	-0.96
Q9UPP5	Uncharacterized protein KIAA1107 OS=Homo sapiens GN=KIAA1107 PE=1 SV=2 - [K1107_HUMAN]	1.76	1.69	1.74	1.67	0.33	0.30	0.05	0.16	0.18	0.21	0.43	0.53	-1.42	-1.43	-1.36	-1.65	-1.62	-1.40	-1.38	-1.56
Q8NFZ4	Neuroligin-2 OS=Homo sapiens GN=NLGN2 PE=1 SV=1 - [NLGN2_HUMAN]	1.63	1.81	1.84	1.66	0.31	0.30	0.11	0.24	0.52	0.30	0.19	0.45	-1.73	-1.31	-1.59	-1.54	-1.37	-1.34	-1.40	-1.57
Q7L266	Isoaspartyl peptidase/L-asparaginase OS=Homo sapiens GN=ASRGL1 PE=1 SV=2 - [ASGL1_HUMAN]	1.71	1.14	2.07	1.64	0.23	0.18	0.70	0.53	0.33	0.51	0.67	0.31	-0.55	-1.31	-1.40	-1.14	-1.35	-1.02	-1.34	-1.01

Q5T0D9	Tumor protein p63-regulated gene 1-like protein OS=Homo sapiens GN=TPRG1L PE=1 SV=1 - [TPRGL_HUMAN]	1.69	1.64	1.60	1.64	0.22	0.28	0.77	0.82	0.38	0.41	0.02	0.02	-0.76	-1.69	-1.70	-1.19	-1.15	-1.42	-1.34	-0.73
Q8NC96	Adaptin ear-binding coat-associated protein 1 OS=Homo sapiens GN=NECAP1 PE=1 SV=2 - [NECP1_HUMAN]	1.66	1.57	1.72	1.62	0.06	0.00	0.38	0.30	0.21	0.27	0.17	0.04	-1.15	-1.39	-1.29	-1.28	-1.42	-1.39	-1.43	-1.10
P63096	Guanine nucleotide-binding protein G(i) subunit alpha-1 OS=Homo sapiens GN=GNAI1 PE=1 SV=2 - [GNAI1_HUMAN]	1.66	1.69	1.65	1.55	-0.01	-0.12	0.49	0.54	0.25	0.37	0.27	0.20	-1.04	-1.32	-1.29	-1.18	-1.27	-1.60	-1.70	-1.00
Q8TDC3	Serine/threonine-protein kinase BRSK1 OS=Homo sapiens GN=BRSK1 PE=1 SV=2 - [BRSK1_HUMAN]	2.68	2.74	1.75	1.51	0.14	0.18	0.61	0.57	0.26	0.23	-0.07	-0.15	-1.69	-1.90	-1.82	-2.38	-1.57	-2.33	-2.00	-1.08
Q15878	Voltage-dependent R-type calcium channel subunit alpha-1E OS=Homo sapiens GN=CACNA1E PE=1 SV=3 - [CAC1E_HUMAN]	1.67	1.53	1.60	1.50	-0.24	-0.12	0.24	0.21	0.11	0.12	0.33	0.47	-1.29	-1.31	-1.09	-1.26	-1.21	-1.40	-1.54	-1.14
Q9NQR7	Coiled-coil domain-containing protein 177 OS=Homo sapiens GN=CCDC177 PE=2 SV=3 - [CC177_HUMAN]	1.93	1.79	1.55	1.48	-0.04	0.25	0.29	0.14	0.32	0.16	-0.34	-0.07	-2.18	-2.23	-1.46	-1.77	-1.15	-1.98	-1.80	-1.19
Q9C0H5	Rho GTPase-activating protein 39 OS=Homo sapiens GN=ARHGAP39 PE=1 SV=2 - [RHG39_HUMAN]	1.48	1.40	1.38	1.48	0.68	0.71	0.30	0.29	0.48	0.45	0.26	0.69	-1.07	-1.26	-1.23	-0.89	-0.87	-0.73	-0.63	-1.13
O60245	Protocadherin-7 OS=Homo sapiens GN=PCDH7 PE=1 SV=2 - [PCDH7_HUMAN]	1.27	1.48	1.63	1.43	0.54	0.48	0.16	0.26	0.19	0.25	-0.46	-0.32	-1.22	-1.21	-1.08	-0.98	-1.05	-0.73	-0.88	-1.22
Q9UHG2	ProSAAS OS=Homo sapiens GN=PCSK1N PE=1 SV=1 - [PCSK1_HUMAN]	1.68	1.37	1.50	1.40	0.37	0.14	0.71	0.37	0.24	0.52	0.31	0.09	-1.04	-1.33	-1.27	-1.13	-1.18	-1.42	-1.34	-1.02

Q99784	Noelin OS=Homo sapiens GN=OLFM1 PE=1 SV=4 - [NOE1_HUMAN]	1.18	1.32	1.61	1.39	0.28	0.46	0.33	0.46	0.63	0.13	0.07	0.36	-0.80	-0.85	-1.16	-0.66	-0.91	-0.91	-1.34	-1.08
P14927	Cytochrome b-c1 complex subunit 7 OS=Homo sapiens GN=UQCRB PE=1 SV=2 - [QCR7_HUMAN]	1.09	1.19	1.36	1.38	-0.41	-0.23	0.51	0.50	0.00	-0.04	-0.18	-0.17	-0.62	-1.16	-1.62	-1.03	-1.52	-1.35	-1.63	-0.98
O14523	C2 domain-containing protein 2-like OS=Homo sapiens GN=C2CD2L PE=1 SV=3 - [C2C2L_HUMAN]	1.49	1.48	1.40	1.36	-0.30	-0.32	0.23	0.36	0.09	0.12	0.01	-0.07	-1.15	-1.14	-1.39	-1.18	-1.34	-1.52	-1.73	-1.05
P16298	Serine/threonine-protein phosphatase 2B catalytic subunit beta isoform OS=Homo sapiens GN=PPP3CB PE=1 SV=2 - [PP2BB_HUMAN]	1.29	1.05	1.31	1.33	0.37	0.23	0.40	0.29	0.09	0.23	0.01	0.09	-0.93	-1.54	-1.35	-1.05	-1.26	-1.27	-1.29	-0.95
Q9UBS5	Gamma-aminobutyric acid type B receptor subunit 1 OS=Homo sapiens GN=GABBR1 PE=1 SV=1 - [GABR1_HUMAN]	1.11	1.09	1.40	1.32	0.14	-0.15	0.36	0.36	0.01	0.21	0.17	0.09	-0.69	-1.18	-1.25	-1.46	-1.49	-1.10	-1.28	-0.97
O76054	SEC14-like protein 2 OS=Homo sapiens GN=SEC14L2 PE=1 SV=1 - [S14L2_HUMAN]	1.55	1.46	1.35	1.32	0.34	0.24	0.45	0.39	0.34	0.34	0.32	0.23	-1.00	-1.32	-1.36	-1.03	-1.16	-1.23	-1.37	-0.81
P09497	Clathrin light chain B OS=Homo sapiens GN=CLTB PE=1 SV=1 - [CLCB_HUMAN]	1.21	1.06	1.30	1.25	-0.14	-0.24	0.23	0.20	0.02	0.07	-0.06	0.00	-0.89	-1.24	-1.29	-1.13	-1.22	-1.32	-1.59	-0.98
P45985	Dual specificity mitogen-activated protein kinase kinase 4 OS=Homo sapiens GN=MAP2K4 PE=1 SV=1 - [MP2K4_HUMAN]	1.17	1.19	1.16	1.25	0.09	0.26	0.32	0.44	0.37	0.30	0.17	-0.10	-0.67	-1.49	-1.47	-0.54	-0.53	-1.15	-1.09	-0.92
Q13363	C-terminal-binding protein 1 OS=Homo sapiens GN=CTBP1 PE=1 SV=2 - [CTBP1_HUMAN]	1.28	1.21	1.25	1.24	-0.08	-0.06	0.14	0.29	0.18	0.09	-0.08	-0.09	-0.88	-1.21	-1.38	-0.96	-0.95	-1.29	-1.25	-0.93

P68366	Tubulin alpha-4A chain OS=Homo sapiens GN=TUBA4A PE=1 SV=1 - [TBA4A_HUMAN]	1.07	1.06	1.36	1.24	0.00	-0.10	0.44	0.48	0.31	0.38	0.26	0.28	-0.77	-1.05	-1.23	-0.97	-1.09	-0.79	-0.98	-0.99
P34949	Mannose-6-phosphate isomerase OS=Homo sapiens GN=MPI PE=1 SV=2 - [MPI_HUMAN]	1.02	1.10	1.59	1.21	0.34	-0.15	0.35	0.47	0.37	0.64	0.29	-0.18	-0.58	-0.85	-1.00	-0.51	-0.89	-1.00	-1.27	-0.79
Q15493	Regucalcin OS=Homo sapiens GN=RGN PE=1 SV=1 - [RGN_HUMAN]	1.50	1.27	1.62	1.20	0.49	-0.01	0.56	0.33	0.67	0.82	-0.13	-0.08	-0.81	-1.45	-1.82	-0.56	-0.84	-0.85	-1.22	-0.76
Q6N022	Teneurin-4 OS=Homo sapiens GN=TENM4 PE=1 SV=2 - [TEN4_HUMAN]	1.35	1.40	1.66	1.19	0.92	1.17	0.55	0.32	0.52	0.55	0.16	0.28	-0.76	-1.12	-1.24	-0.85	-0.71	-0.47	-0.46	-0.62
Q13153	Serine/threonine-protein kinase PAK 1 OS=Homo sapiens GN=PAK1 PE=1 SV=2 - [PAK1_HUMAN]	1.15	1.13	1.43	1.11	-0.64	-1.00	-0.61	-0.55	-1.04	-0.88	-1.04	-1.32	-1.53	-2.18	-2.47	-2.00	-2.21	-1.87	-2.31	-1.65
P04745	Alpha-amylase 1 OS=Homo sapiens GN=AMY1A PE=1 SV=2 - [AMY1_HUMAN]	-2.49	-2.25	-4.76	-4.66	-4.11	-4.07	-4.47	-4.26	-4.33	-4.43	-4.75	-4.58	-0.38	-0.62	0.33	-0.50	0.50	-0.32	0.81	0.61
Q9UBG3	Cornulin OS=Homo sapiens GN=CRNN PE=1 SV=1 - [CRNN_HUMAN]	-3.06	-2.87	-3.83	-3.54	-3.06	-3.02	-3.41	-3.36	-3.03	-3.22	-3.13	-3.01	-0.51	-0.33	0.56	-0.33	0.50	-0.09	0.71	0.30
P02765	Alpha-2-HS-glycoprotein OS=Homo sapiens GN=AHSG PE=1 SV=1 - [FETUA_HUMAN]	-3.17	-3.04	-3.64	-3.49	-1.99	-1.97	-2.41	-2.45	-1.83	-1.88	-1.58	-1.46	1.29	1.96	2.12	1.46	1.70	1.46	1.74	1.35
Q16527	Cysteine and glycine-rich protein 2 OS=Homo sapiens GN=CSRP2 PE=1 SV=3 - [CSRP2_HUMAN]	-3.17	-3.35	-3.38	-3.44	-2.38	-2.58	-1.78	-1.79	-2.17	-2.20	-1.90	-2.01	1.45	1.07	1.47	1.11	1.26	0.46	0.67	1.75
P02458	Collagen alpha-1(II) chain OS=Homo sapiens GN=COL2A1 PE=1 SV=3 - [CO2A1_HUMAN]	-2.80	-2.98	-3.18	-3.24	-1.71	-1.82	-2.32	-2.41	-2.15	-2.03	-2.13	-2.24	0.59	0.65	1.17	1.14	1.28	1.30	1.50	0.91
P09493	Tropomyosin alpha-1 chain OS=Homo sapiens GN=TPM1 PE=1 SV=2 - [TPM1_HUMAN]	-2.45	-2.57	-2.53	-2.49	-1.26	-1.13	-1.56	-1.47	-1.17	-1.35	-0.94	-0.86	1.14	1.74	1.65	1.37	1.33	1.47	1.35	1.09

Q7Z4H8	KDEL motif-containing protein 2 OS=Homo sapiens GN=KDELC2 PE=1 SV=2 - [KDEL2_HUMAN]	-2.47	-2.54	-2.37	-2.44	0.08	0.01	-1.16	-1.23	-0.49	-0.47	0.13	0.08	1.37	2.62	2.38	1.97	1.74	2.47	2.22	1.15
P05109	Protein S100-A8 OS=Homo sapiens GN=S100A8 PE=1 SV=1 - [S10A8_HUMAN]	-2.28	-2.16	-2.49	-2.41	-2.06	-2.06	0.66	0.71	0.15	0.07	-0.05	0.02	2.96	2.20	2.47	2.24	2.49	0.11	0.26	3.25
O94769	Extracellular matrix protein 2 OS=Homo sapiens GN=ECM2 PE=2 SV=1 - [ECM2_HUMAN]	-1.48	-1.65	-2.24	-2.41	0.88	0.71	-0.24	-0.41	0.01	0.19	-0.11	-0.29	1.29	1.37	2.12	1.69	2.46	2.34	3.10	2.06
Q92629	Delta-sarcoglycan OS=Homo sapiens GN=SGCD PE=1 SV=2 - [SGCD_HUMAN]	-2.30	-2.32	-2.23	-2.33	0.01	-0.15	-0.17	-0.33	-0.16	-0.09	0.04	-0.08	2.17	2.50	2.22	2.34	2.11	2.51	2.27	2.15
P21246	Pleiotrophin OS=Homo sapiens GN=PTN PE=1 SV=1 - [PTN_HUMAN]	-1.93	-1.76	-2.48	-2.31	1.72	1.74	0.74	0.68	0.92	0.98	-0.22	-0.45	2.23	1.74	1.94	2.93	3.18	3.66	3.65	3.12
O43405	Cochlin OS=Homo sapiens GN=COCH PE=1 SV=1 - [COCH_HUMAN]	-1.91	-2.00	-2.35	-2.29	-0.80	-0.76	0.58	0.81	0.18	0.03	-1.22	-1.25	3.05	0.70	1.05	2.28	2.42	1.27	1.52	2.99
Q8WUP2	Filamin-binding LIM protein 1 OS=Homo sapiens GN=FBLIM1 PE=1 SV=2 - [FBLI1_HUMAN]	-2.30	-2.35	-2.14	-2.19	-0.45	-0.44	-0.65	-0.70	-0.80	-0.74	-1.14	-1.20	1.71	1.16	1.00	1.59	1.43	1.75	1.59	1.55
P04196	Histidine-rich glycoprotein OS=Homo sapiens GN=HRG PE=1 SV=1 - [HRG_HUMAN]	-2.02	-2.03	-2.33	-2.00	0.13	0.39	0.01	0.16	0.35	0.16	0.74	0.89	2.24	2.24	2.98	2.08	2.39	1.65	2.35	2.45
Q96AY3	Peptidyl-prolyl cis-trans isomerase FKBP10 OS=Homo sapiens GN=FKBP10 PE=1 SV=1 - [FKB10_HUMAN]	-2.10	-2.34	-1.95	-2.00	0.19	0.11	-0.30	-0.45	-0.21	-0.20	0.10	-0.07	1.43	1.83	1.98	2.24	1.78	2.22	2.11	1.67
A1L4H1	Soluble scavenger receptor cysteine-rich domain-containing protein SSC5D OS=Homo sapiens GN=SSC5D PE=2 SV=3 - [SRCRL_HUMAN]	-2.26	-2.35	-1.85	-1.97	-0.43	-0.61	-0.11	-0.22	-0.47	-0.40	-0.40	-0.53	2.10	1.76	1.42	1.78	1.32	1.54	1.08	1.65

P52943	Cysteine-rich protein 2 OS=Homo sapiens GN=CRIP2 PE=1 SV=1 - [CRIP2_HUMAN]	-1.99	-1.89	-1.89	-1.88	-1.17	-1.14	-1.18	-1.20	-1.09	-1.10	-0.70	-0.73	0.87	1.28	1.19	1.00	0.83	0.91	0.73	0.74
O15382	Branched-chain amino-acid aminotransferase, mitochondrial OS=Homo sapiens GN=BCAT2 PE=1 SV=2 - [BCAT2_HUMAN]	-2.09	-1.57	-2.15	-1.88	0.27	0.35	-0.42	-0.26	-0.09	-0.02	0.07	0.19	1.61	1.67	1.87	1.36	1.83	1.82	2.15	1.74
P53007	Tricarboxylate transport protein, mitochondrial OS=Homo sapiens GN=SLC25A1 PE=1 SV=2 - [TXTP_HUMAN]	-1.94	-1.86	-1.91	-1.83	-0.03	-0.02	0.07	0.13	-0.06	-0.11	-0.02	-0.01	2.07	1.69	1.74	1.78	1.82	1.67	1.71	2.01
Q86WV6	Stimulator of interferon genes protein OS=Homo sapiens GN=TMEM173 PE=1 SV=1 - [STING_HUMAN]	-1.97	-1.92	-2.13	-1.78	-0.48	-0.49	-1.33	-1.21	-0.64	-0.72	-0.22	-0.06	0.68	1.79	1.66	1.45	1.11	1.27	1.27	0.63
P27169	Serum paraoxonase/arylesterase 1 OS=Homo sapiens GN=PON1 PE=1 SV=3 - [PON1_HUMAN]	-1.35	-1.64	-1.64	-1.66	1.23	1.05	0.73	0.76	0.97	0.79	1.32	1.31	2.52	3.03	2.99	2.72	2.76	2.92	2.78	2.56
P07357	Complement component C8 alpha chain OS=Homo sapiens GN=C8A PE=1 SV=2 - [CO8A_HUMAN]	-1.85	-1.77	-1.65	-1.59	-0.29	-0.31	-0.65	-0.61	-0.38	-0.42	0.12	0.08	1.25	1.97	1.91	1.65	1.45	1.74	1.38	1.39
O00264	Membrane-associated progesterone receptor component 1 OS=Homo sapiens GN=PGRMC1 PE=1 SV=3 - [PGRC1_HUMAN]	-1.50	-1.48	-1.60	-1.55	0.30	0.18	-0.15	-0.12	-0.13	-0.07	0.15	0.16	1.44	1.69	1.72	1.72	1.47	1.82	1.60	1.51
Q14573	Inositol 1,4,5-trisphosphate receptor type 3 OS=Homo sapiens GN=ITPR3 PE=1 SV=2 - [ITPR3_HUMAN]	-1.41	-1.45	-1.76	-1.53	-0.49	-0.55	-0.53	-0.51	-0.33	-0.27	0.06	0.07	0.94	1.45	1.80	1.16	1.29	0.75	1.07	1.30
O43556	Epsilon-sarcoglycan OS=Homo sapiens GN=SGCE PE=1 SV=6 - [SGCE_HUMAN]	-1.65	-1.68	-1.61	-1.53	0.01	0.09	-0.04	0.05	0.00	-0.03	0.26	0.02	1.78	1.95	1.59	1.87	1.56	1.64	1.61	1.69

O95084	Serine protease 23 OS=Homo sapiens GN=PRSS23 PE=1 SV=1 - [PRS23_HUMAN]	-1.24	-1.25	-1.30	-1.32	1.78	1.64	0.96	0.69	0.97	1.18	0.94	0.93	1.44	2.27	2.47	2.21	2.39	2.81	3.29	2.20
Q14314	Fibroleukin OS=Homo sapiens GN=FGL2 PE=1 SV=1 - [FGL2_HUMAN]	-1.26	-1.15	-1.42	-1.32	1.07	1.20	0.63	0.84	0.96	0.73	1.15	1.31	1.92	2.20	2.70	2.07	2.35	2.43	2.50	2.22
P30043	Flavin reductase (NADPH) OS=Homo sapiens GN=BLVRB PE=1 SV=3 - [BLVRB_HUMAN]	-1.17	-1.07	-1.27	-1.31	-0.03	-0.01	-0.05	0.03	-0.28	-0.31	-0.85	-0.76	1.22	0.44	0.38	1.07	1.16	1.23	1.35	1.36
P09382	Galectin-1 OS=Homo sapiens GN=LGALS1 PE=1 SV=2 - [LEG1_HUMAN]	-1.22	-1.22	-1.26	-1.21	-0.38	-0.31	-0.20	-0.16	-0.23	-0.34	-0.18	-0.09	1.16	1.09	1.04	0.96	0.92	0.88	0.83	1.11
P01834	Ig kappa chain C region OS=Homo sapiens GN=IGKC PE=1 SV=1 - [IGKC_HUMAN]	-1.45	-1.42	-1.27	-1.20	-0.13	-0.01	-0.47	-0.40	0.14	0.04	0.91	0.97	0.98	2.20	2.22	1.57	1.50	1.19	1.22	0.91
Q96MZ0	Ganglioside-induced differentiation-associated protein 1-like 1 OS=Homo sapiens GN=GDAP1L1 PE=2 SV=2 - [GD1L1_HUMAN]	2.51	2.77	2.72	2.91	-0.67	-0.39	1.27	1.38	0.82	0.57	0.24	0.55	-1.44	-2.12	-2.09	-1.89	-1.97	-2.82	-2.93	-1.37
Q7Z6L0	Proline-rich transmembrane protein 2 OS=Homo sapiens GN=PRRT2 PE=1 SV=1 - [PRRT2_HUMAN]	2.51	2.42	2.76	2.54	0.10	-0.04	0.79	0.92	0.58	0.57	0.40	0.27	-1.62	-2.22	-2.29	-1.99	-2.25	-2.66	-2.73	-1.84
Q96JN2	Coiled-coil domain-containing protein 136 OS=Homo sapiens GN=CCDC136 PE=2 SV=3 - [CC136_HUMAN]	2.98	2.55	2.90	2.51	-0.06	-0.33	1.13	1.00	0.59	0.84	0.51	0.25	-1.68	-1.92	-1.66	-1.73	-1.60	-2.64	-2.59	-1.51
Q8N111	Cell cycle exit and neuronal differentiation protein 1 OS=Homo sapiens GN=CEND1 PE=2 SV=1 - [CEND_HUMAN]	1.96	1.83	2.28	2.41	-0.55	-0.55	1.22	1.10	0.39	0.46	0.07	0.11	-0.87	-1.73	-2.24	-1.40	-1.90	-2.31	-2.81	-1.29

Q6NV74	Uncharacterized protein KIAA1211-like OS=Homo sapiens GN=KIAA1211L PE=2 SV=3 - [K121L_HUMAN]	1.96	2.05	2.12	2.37	-0.06	-0.03	0.26	0.47	-0.28	-0.07	-0.21	-0.17	-1.42	-2.14	-2.47	-2.25	-2.55	-2.13	-2.35	-1.84
Q9HCH3	Copine-5 OS=Homo sapiens GN=CPNE5 PE=1 SV=2 - [CPNE5_HUMAN]	2.09	1.86	2.49	2.31	-0.18	-0.37	0.47	0.25	0.01	0.24	0.44	0.24	-1.82	-1.42	-1.86	-1.82	-1.98	-2.48	-2.47	-1.73
Q96A23	Copine-4 OS=Homo sapiens GN=CPNE4 PE=2 SV=1 - [CPNE4_HUMAN]	2.39	2.32	2.58	2.29	0.13	0.11	0.65	0.91	0.54	0.52	0.43	0.70	-1.64	-1.83	-1.97	-1.91	-2.19	-2.54	-2.85	-1.90
P16949	Stathmin OS=Homo sapiens GN=STMN1 PE=1 SV=3 - [STMN1_HUMAN]	1.86	1.75	2.18	2.28	-0.38	-0.52	0.18	0.29	-0.39	-0.38	-0.83	-0.61	-1.36	-2.56	-2.88	-1.96	-2.23	-1.90	-2.38	-1.81
P48066	Sodium- and chloride-dependent GABA transporter 3 OS=Homo sapiens GN=SLC6A11 PE=2 SV=1 - [S6A11_HUMAN]	2.28	2.28	2.26	2.26	-0.49	-0.49	0.50	0.50	0.11	0.11	-0.37	-0.38	-1.37	-2.33	-2.38	-1.85	-1.88	-2.54	-2.58	-1.53
P53779	Mitogen-activated protein kinase 10 OS=Homo sapiens GN=MAPK10 PE=1 SV=2 - [MK10_HUMAN]	1.68	1.72	2.13	2.15	-0.28	-0.25	0.56	0.76	0.52	0.36	0.01	0.25	-0.99	-1.66	-1.95	-1.38	-1.81	-1.88	-2.14	-1.31
Q8N3J6	Cell adhesion molecule 2 OS=Homo sapiens GN=CADM2 PE=2 SV=1 - [CADM2_HUMAN]	1.66	1.62	2.00	2.14	0.09	0.04	0.54	0.77	0.50	0.44	0.37	0.48	-0.87	-1.22	-1.72	-1.40	-1.72	-1.83	-2.23	-1.43
Q5FWE3	Proline-rich transmembrane protein 3 OS=Homo sapiens GN=PRRT3 PE=1 SV=3 - [PRRT3_HUMAN]	2.11	1.81	2.25	2.04	0.29	0.35	0.85	0.74	0.01	0.26	0.54	0.58	-0.87	-1.10	-1.21	-1.69	-1.81	-1.30	-1.41	-1.08
Q8NI08	Nuclear receptor coactivator 7 OS=Homo sapiens GN=NCOA7 PE=1 SV=2 - [NCOA7_HUMAN]	1.69	1.85	1.75	2.03	0.24	0.49	0.60	0.75	0.71	0.57	0.46	0.65	-0.94	-1.21	-1.36	-1.15	-1.28	-1.49	-1.55	-1.15
A5YM72	Carnosine synthase 1 OS=Homo sapiens GN=CARNS1 PE=1 SV=3 - [CRNS1_HUMAN]	1.32	2.13	1.38	2.03	-0.23	-0.05	0.05	0.11	0.05	-0.20	-0.32	-0.22	-1.88	-1.93	-1.96	-2.04	-1.94	-2.20	-1.88	-1.63

Q9UM19	Hippocalcin-like protein 4 OS=Homo sapiens GN=HPCAL4 PE=2 SV=3 - [HPCL4_HUMAN]	1.78	1.74	2.13	2.02	-0.31	-0.19	0.79	0.56	0.22	0.08	0.48	0.29	-0.93	-1.48	-1.54	-1.29	-1.41	-2.08	-2.30	-1.11
P62745	Rho-related GTP-binding protein RhoB OS=Homo sapiens GN=RHOB PE=1 SV=1 - [RHOB_HUMAN]	1.85	1.95	1.99	1.98	0.41	0.42	0.89	0.88	0.89	0.82	0.44	0.55	-0.91	-1.16	-1.55	-1.00	-1.14	-1.52	-1.65	-0.91
Q9UPW8	Protein unc-13 homolog A OS=Homo sapiens GN=UNC13A PE=2 SV=4 - [UN13A_HUMAN]	1.96	2.02	1.87	1.97	-0.05	0.34	0.66	0.80	0.62	0.69	0.07	0.30	-1.00	-1.52	-1.67	-1.37	-1.15	-2.03	-2.12	-1.11
O75936	Gamma-butyrobetaine dioxygenase OS=Homo sapiens GN=BBOX1 PE=1 SV=1 - [BODG_HUMAN]	1.90	1.94	1.87	1.88	0.82	0.82	1.48	1.47	1.14	1.14	1.21	1.22	-0.32	-0.60	-0.55	-0.73	-0.67	-1.02	-0.96	-0.28
Q92932	Receptor-type tyrosine-protein phosphatase N2 OS=Homo sapiens GN=PTPRN2 PE=1 SV=2 - [PTPR2_HUMAN]	1.45	1.80	1.51	1.86	0.20	0.73	0.24	0.59	0.16	0.22	0.04	0.34	-1.09	-1.46	-1.32	-1.14	-1.10	-1.52	-1.62	-1.06
Q9P1U1	Actin-related protein 3B OS=Homo sapiens GN=ACTR3B PE=2 SV=1 - [ARP3B_HUMAN]	1.55	1.61	1.66	1.84	-0.15	0.04	0.34	0.34	0.20	0.25	0.12	0.21	-1.26	-1.42	-1.87	-1.57	-1.76	-1.42	-1.37	-1.43
Q16864	V-type proton ATPase subunit F OS=Homo sapiens GN=ATP6V1F PE=1 SV=2 - [VATF_HUMAN]	1.82	1.40	1.90	1.84	-0.13	0.02	0.69	0.54	0.46	0.23	0.19	0.30	-0.96	-1.09	-1.53	-1.07	-1.50	-1.41	-1.85	-1.09
P09496	Clathrin light chain A OS=Homo sapiens GN=CLTA PE=1 SV=1 - [CLCA_HUMAN]	1.75	1.77	1.84	1.82	-0.21	-0.19	0.60	0.62	0.26	0.21	0.27	0.26	-1.11	-1.53	-1.42	-1.49	-1.41	-2.02	-2.03	-1.12
Q8N6D5	Ankyrin repeat domain-containing protein 29 OS=Homo sapiens GN=ANKRD29 PE=2 SV=2 - [ANR29_HUMAN]	1.52	1.29	1.65	1.76	-0.11	-0.34	0.51	0.33	-0.19	-0.02	-0.11	-0.52	-0.90	-1.71	-2.13	-1.22	-1.71	-1.62	-2.11	-1.35

Q15257	Serine/threonine-protein phosphatase 2A activator OS=Homo sapiens GN=PPP2R4 PE=1 SV=3 - [PTPA_HUMAN]	1.28	1.41	1.50	1.74	-0.35	-0.20	0.77	0.91	0.42	0.26	-0.25	-0.22	-0.46	-1.27	-1.23	-0.96	-1.24	-1.58	-1.53	-0.55
Q9BR01	Sulfotransferase 4A1 OS=Homo sapiens GN=SULT4A1 PE=1 SV=2 - [ST4A1_HUMAN]	1.61	1.45	1.81	1.72	0.68	0.73	0.74	0.82	0.57	0.85	0.47	0.30	-0.84	-1.14	-1.41	-0.69	-0.88	-0.89	-1.15	-1.12
Q9P2U7	Vesicular glutamate transporter 1 OS=Homo sapiens GN=SLC17A7 PE=1 SV=1 - [VGLU1_HUMAN]	1.69	1.78	1.53	1.66	-0.38	-0.47	0.66	0.61	0.33	0.38	-0.11	-0.04	-0.83	-1.62	-1.82	-1.51	-1.30	-1.84	-1.85	-0.97
Q9ULP0	Protein NDRG4 OS=Homo sapiens GN=NDRG4 PE=1 SV=2 - [NDRG4_HUMAN]	1.61	1.31	2.00	1.65	0.30	0.35	0.89	0.82	0.50	0.58	0.22	0.01	-0.65	-1.08	-1.40	-0.83	-1.14	-0.84	-1.10	-0.97
O14910	Protein lin-7 homolog A OS=Homo sapiens GN=LIN7A PE=1 SV=2 - [LIN7A_HUMAN]	1.75	1.83	1.78	1.65	0.02	0.21	0.18	0.05	0.27	0.17	0.34	0.32	-1.35	-1.40	-1.33	-1.44	-1.37	-1.67	-1.69	-1.20
O75689	Arf-GAP with dual PH domain-containing protein 1 OS=Homo sapiens GN=ADAP1 PE=1 SV=2 - [ADAP1_HUMAN]	1.36	1.56	1.39	1.64	-0.47	-0.21	0.14	0.35	0.41	0.25	-0.17	0.09	-1.16	-1.52	-1.56	-1.16	-1.19	-1.84	-1.87	-1.22
O43181	NADH dehydrogenase [ubiquinone] iron-sulfur protein 4, mitochondrial OS=Homo sapiens GN=NDUFS4 PE=1 SV=1 - [NDUS4_HUMAN]	1.56	1.39	1.69	1.63	0.23	0.05	0.03	0.13	0.05	0.15	0.46	0.20	-1.33	-1.22	-1.38	-1.43	-1.54	-1.40	-1.60	-1.27
P54750	Calcium/calmodulin-dependent 3',5'-cyclic nucleotide phosphodiesterase 1A OS=Homo sapiens GN=PDE1A PE=2 SV=2 - [PDE1A_HUMAN]	2.02	2.20	1.48	1.59	0.01	0.16	0.44	0.58	0.10	-0.15	0.03	0.30	-1.36	-1.97	-1.45	-1.88	-1.47	-2.05	-1.51	-0.84
Q8N126	Cell adhesion molecule 3 OS=Homo sapiens GN=CADM3 PE=1 SV=1 - [CADM3_HUMAN]	1.64	1.34	1.99	1.56	-0.53	-0.59	0.50	0.31	0.17	0.29	0.31	0.29	-0.95	-1.36	-1.56	-1.34	-1.59	-2.15	-2.40	-1.19

P42263	Glutamate receptor 3 OS=Homo sapiens GN=GRIA3 PE=1 SV=2 - [GRIA3_HUMAN]	1.70	1.67	1.58	1.54	-0.41	-0.39	0.27	0.29	0.09	0.18	-0.09	-0.10	-1.33	-1.20	-1.05	-1.49	-1.41	-1.86	-1.80	-1.18
P06241	Tyrosine-protein kinase Fyn OS=Homo sapiens GN=FYN PE=1 SV=3 - [FYN_HUMAN]	1.05	1.05	1.60	1.51	0.01	0.20	0.71	0.71	0.42	0.36	0.21	0.28	-0.50	-0.76	-1.13	-0.59	-1.05	-0.86	-1.32	-0.85
P09455	Retinol-binding protein 1 OS=Homo sapiens GN=RBP1 PE=1 SV=2 - [RET1_HUMAN]	1.46	1.57	1.32	1.49	0.24	0.10	0.84	0.82	0.39	0.38	0.16	0.17	-0.74	-1.36	-1.20	-1.17	-0.97	-1.24	-1.07	-0.71
Q9BW62	Katanin p60 ATPase-containing subunit A-like 1 OS=Homo sapiens GN=KATNAL1 PE=1 SV=1 - [KATL1_HUMAN]	1.22	1.12	1.44	1.47	-0.25	-0.08	0.25	0.32	0.16	-0.02	-0.11	0.11	-0.75	-1.32	-1.56	-0.92	-1.34	-1.21	-1.56	-1.18
Q92854	Semaphorin-4D OS=Homo sapiens GN=SEMA4D PE=1 SV=1 - [SEM4D_HUMAN]	1.35	1.57	1.20	1.41	0.20	0.51	0.31	0.43	0.40	0.15	0.21	0.44	-0.98	-1.14	-1.00	-1.18	-1.03	-1.08	-0.97	-0.92
Q9UKE5	TRAF2 and NCK-interacting protein kinase OS=Homo sapiens GN=TNIK PE=1 SV=1 - [TNIK_HUMAN]	1.48	1.56	1.40	1.40	-0.04	0.10	0.15	0.23	0.00	0.04	-0.03	0.11	-1.27	-1.55	-1.28	-1.30	-1.36	-1.70	-1.32	-1.12
Q6UB35	Monofunctional C1-tetrahydrofolate synthase, mitochondrial OS=Homo sapiens GN=MTHFD1L PE=1 SV=1 - [C1TM_HUMAN]	1.36	1.14	1.61	1.36	-0.14	-0.26	0.00	-0.14	-0.23	0.01	0.32	-0.03	-1.22	-1.04	-1.20	-1.33	-1.54	-1.21	-1.37	-1.33
P62879	Guanine nucleotide-binding protein G(I)/G(S)/G(T) subunit beta-2 OS=Homo sapiens GN=GNB2 PE=1 SV=3 - [GBB2_HUMAN]	1.24	1.38	1.26	1.30	0.26	0.15	0.43	0.62	0.58	0.11	0.10	0.20	-0.55	-0.86	-1.06	-0.75	-1.09	-0.74	-0.92	-0.77
P53677	AP-3 complex subunit mu-2 OS=Homo sapiens GN=AP3M2 PE=1 SV=1 - [AP3M2_HUMAN]	1.62	1.17	1.82	1.30	0.50	0.22	0.41	0.37	0.13	0.29	0.04	-0.09	-1.22	-1.17	-1.32	-1.11	-1.11	-0.95	-0.92	-1.11

O95319	CUGBP Elav-like family member 2 OS=Homo sapiens GN=CELF2 PE=1 SV=1 - [CELF2_HUMAN]	1.29	1.36	1.13	1.27	0.16	0.35	0.08	0.16	0.23	0.16	-0.21	-0.15	-1.02	-1.16	-0.98	-0.76	-0.71	-0.88	-0.69	-0.82
P10915	Hyaluronan and proteoglycan link protein 1 OS=Homo sapiens GN=HAPLN1 PE=2 SV=2 - [HPLN1_HUMAN]	1.15	1.47	1.39	1.25	-0.56	-0.08	0.64	0.41	-0.14	0.00	-0.49	-0.49	-0.45	-1.52	-1.73	-1.12	-1.40	-1.52	-1.72	-0.65
Q00169	Phosphatidylinositol transfer protein alpha isoform OS=Homo sapiens GN=PITPNA PE=1 SV=2 - [PIPNA_HUMAN]	1.40	1.16	1.46	1.23	0.08	-0.10	0.45	0.36	0.33	0.35	-0.05	-0.21	-0.76	-1.44	-1.54	-0.61	-0.86	-1.18	-1.33	-0.76
P50148	Guanine nucleotide-binding protein G(q) subunit alpha OS=Homo sapiens GN=GNAQ PE=1 SV=4 - [GNAQ_HUMAN]	1.20	1.26	1.09	1.22	0.16	0.29	0.33	0.44	0.42	0.45	0.16	0.08	-0.80	-1.04	-1.18	-0.67	-0.60	-0.78	-1.03	-0.69
Q96BS2	Calcineurin B homologous protein 3 OS=Homo sapiens GN=TESC PE=1 SV=3 - [CHP3_HUMAN]	1.40	1.46	1.11	1.17	-0.16	-0.11	0.29	0.30	0.00	0.06	0.04	-0.01	-1.00	-1.15	-1.21	-1.10	-1.01	-1.58	-1.29	-0.84
Q99490	Arf-GAP with GTPase, ANK repeat and PH domain-containing protein 2 OS=Homo sapiens GN=AGAP2 PE=1 SV=2 - [AGAP2_HUMAN]	1.97	1.45	1.43	1.15	0.36	0.07	0.60	0.38	0.16	0.55	0.22	-0.07	-1.13	-1.46	-1.21	-0.96	-0.98	-1.50	-1.09	-0.81
P14649	Myosin light chain 6B OS=Homo sapiens GN=MYL6B PE=1 SV=1 - [MYL6B_HUMAN]	1.14	1.14	1.16	1.15	0.22	0.19	0.30	0.33	0.50	0.47	-0.22	-0.20	-0.89	-1.44	-1.51	-0.87	-0.78	-0.94	-1.09	-0.93
Q9UMY4	Sorting nexin-12 OS=Homo sapiens GN=SNX12 PE=1 SV=3 - [SNX12_HUMAN]	1.06	1.16	1.08	1.11	0.26	0.36	0.48	0.51	0.25	0.18	-0.35	-0.35	-0.38	-1.29	-1.48	-0.76	-0.90	-0.82	-0.68	-0.50
O00555	Voltage-dependent P/Q-type calcium channel subunit alpha-1A OS=Homo sapiens GN=CACNA1A PE=1 SV=2 - [CAC1A_HUMAN]	1.15	1.11	1.12	1.10	-0.45	-0.45	-0.26	-0.26	-0.52	-0.52	-0.12	-0.12	-1.40	-1.35	-1.25	-1.60	-1.59	-1.58	-1.57	-1.30

Q9C0D0	Phosphatase and actin regulator 1 OS=Homo sapiens GN=PHACTR1 PE=2 SV=3 - [PHAR1_HUMAN]	1.63	1.28	1.42	1.07	-0.62	-0.98	0.00	-0.35	-0.48	-0.13	-0.20	-0.56	-1.57	-1.83	-1.62	-1.73	-1.52	-2.27	-2.06	-1.36
P20908	Collagen alpha-1(V) chain OS=Homo sapiens GN=COL5A1 PE=1 SV=3 - [CO5A1_HUMAN]	-3.22	-3.17	-3.17	-3.07	-1.79	-1.91	-2.20	-2.51	-2.50	-2.39	-2.37	-2.52	0.78	1.09	0.64	1.06	0.75	1.62	1.10	0.44
Q9Y625	Glypican-6 OS=Homo sapiens GN=GPC6 PE=1 SV=1 - [GPC6_HUMAN]	-2.47	-2.40	-2.70	-2.81	0.43	0.33	-0.56	-0.70	0.18	0.25	0.69	0.54	1.78	3.23	3.36	2.75	2.94	2.94	3.15	2.00
Q96P44	Collagen alpha-1(XXI) chain OS=Homo sapiens GN=COL21A1 PE=2 SV=1 - [COLA1_HUMAN]	-2.13	-2.14	-2.45	-2.63	-1.03	-1.34	-1.12	-1.41	-1.37	-1.11	-1.07	-1.28	1.60	1.51	1.57	1.57	1.59	1.53	1.34	1.51
Q8TCC7	Solute carrier family 22 member 8 OS=Homo sapiens GN=SLC22A8 PE=1 SV=1 - [S22A8_HUMAN]	-2.54	-2.52	-2.53	-2.51	0.47	0.56	-0.02	-0.04	0.29	0.33	0.42	0.44	2.42	2.97	2.95	2.37	2.36	2.55	2.54	2.42
P06703	Protein S100-A6 OS=Homo sapiens GN=S100A6 PE=1 SV=1 - [S10A6_HUMAN]	-2.33	-1.96	-2.52	-2.50	-0.16	-0.08	-0.47	-0.42	-0.31	-0.35	-0.32	-0.35	1.80	1.62	2.12	1.77	2.13	2.05	2.23	2.13
P35968	Vascular endothelial growth factor receptor 2 OS=Homo sapiens GN=KDR PE=1 SV=2 - [VGFR2_HUMAN]	-2.17	-1.53	-2.51	-2.35	0.25	0.41	0.19	0.42	0.15	0.02	0.03	0.25	2.12	1.91	1.98	1.92	2.30	2.40	2.74	2.73
Q8TBN0	Guanine nucleotide exchange factor for Rab-3A OS=Homo sapiens GN=RAB3IL1 PE=1 SV=1 - [R3GEF_HUMAN]	-1.73	-2.03	-1.90	-2.19	0.42	0.11	0.22	-0.08	-0.34	-0.04	-0.13	-0.43	2.00	1.60	1.77	1.72	1.89	2.12	2.29	2.18
P14207	Folate receptor beta OS=Homo sapiens GN=FOLR2 PE=1 SV=4 - [FOLR2_HUMAN]	-2.00	-1.98	-2.12	-2.14	0.23	0.31	0.09	0.09	0.17	0.10	0.34	0.42	2.16	2.35	2.43	2.16	2.13	2.18	2.35	2.28
Q92911	Sodium/iodide cotransporter OS=Homo sapiens GN=SLC5A5 PE=1 SV=1 - [SC5A5_HUMAN]	-1.78	-1.82	-2.31	-2.12	-0.03	0.03	-0.48	-0.31	-0.38	-0.42	-0.92	-0.93	1.34	0.89	1.38	1.15	1.74	1.45	2.01	1.85

Q10588	ADP-ribosyl cyclase 2 OS=Homo sapiens GN=BST1 PE=1 SV=2 - [BST1_HUMAN]	-1.78	-1.80	-2.10	-2.09	-0.20	-0.17	-0.48	-0.56	-0.45	-0.40	-0.45	-0.61	1.30	1.12	1.56	1.15	1.88	1.59	2.12	1.71
P48059	LIM and senescent cell antigen-like-containing domain protein 1 OS=Homo sapiens GN=LIMS1 PE=1 SV=4 - [LIMS1_HUMAN]	-2.04	-2.06	-2.02	-1.93	-0.48	-0.51	-0.54	-0.47	-0.49	-0.68	-0.56	-0.51	1.57	1.64	1.40	1.54	1.46	1.70	1.50	1.46
P33151	Cadherin-5 OS=Homo sapiens GN=CDH5 PE=1 SV=5 - [CADH5_HUMAN]	-1.63	-1.65	-1.75	-1.87	-0.28	-0.43	-0.67	-0.68	-0.39	-0.34	-0.03	-0.09	1.06	1.41	1.47	1.25	1.28	1.48	1.43	1.21
O60701	UDP-glucose 6-dehydrogenase OS=Homo sapiens GN=UGDH PE=1 SV=1 - [UGDH_HUMAN]	-1.50	-1.42	-1.97	-1.76	-0.28	-0.08	-0.59	-0.55	-0.51	-0.51	-0.76	-0.68	0.79	0.72	1.25	0.97	1.56	1.17	2.01	1.30
O00592	Podocalyxin OS=Homo sapiens GN=PODXL PE=1 SV=2 - [PODXL_HUMAN]	-1.63	-1.93	-1.83	-1.72	-0.72	-0.70	-1.45	-1.40	-1.03	-0.81	-0.42	-0.39	0.50	1.58	1.23	0.94	0.85	1.31	1.02	0.41
P49247	Ribose-5-phosphate isomerase OS=Homo sapiens GN=RPIA PE=1 SV=3 - [RPIA_HUMAN]	-1.62	-1.63	-1.67	-1.68	0.06	0.17	-0.70	-0.69	-0.39	-0.49	-0.63	-0.51	1.07	1.02	1.08	1.13	1.22	1.59	1.48	0.97
O60687	Sushi repeat-containing protein SRPX2 OS=Homo sapiens GN=SRPX2 PE=1 SV=1 - [SRPX2_HUMAN]	-1.57	-1.42	-1.68	-1.64	0.04	0.25	-0.37	-0.02	0.05	-0.29	-0.42	-0.22	1.26	1.50	1.65	1.31	1.63	1.77	1.71	1.68
Q9UM47	Neurogenic locus notch homolog protein 3 OS=Homo sapiens GN=NOTCH3 PE=1 SV=2 - [NOTC3_HUMAN]	-1.28	-1.37	-1.54	-1.61	0.41	0.47	-0.42	-0.34	0.02	0.03	0.15	0.10	0.84	1.20	1.45	1.35	1.75	1.40	2.08	1.25
Q13976	cGMP-dependent protein kinase 1 OS=Homo sapiens GN=PRKG1 PE=1 SV=3 - [KGP1_HUMAN]	-1.53	-1.40	-1.41	-1.57	-0.52	-0.58	-0.70	-0.63	-0.70	-0.58	-0.57	-0.66	0.86	0.74	0.77	1.03	1.20	0.83	1.13	0.52

Q68EM7	Rho GTPase-activating protein 17 OS=Homo sapiens GN=ARHGAP17 PE=1 SV=1 - [RHG17_HUMAN]	-1.61	-1.36	-1.83	-1.50	-0.30	-0.12	-0.64	-0.41	-0.46	-0.67	-0.76	-0.71	1.21	1.09	1.20	1.20	1.34	1.39	1.52	1.33
P23919	Thymidylate kinase OS=Homo sapiens GN=DTYMK PE=1 SV=4 - [KTHY_HUMAN]	-1.41	-1.45	-1.47	-1.46	-0.31	-0.29	-0.71	-0.69	-0.59	-0.61	-0.27	-0.32	0.74	1.04	1.10	0.83	0.77	1.08	1.01	0.73
P00750	Tissue-type plasminogen activator OS=Homo sapiens GN=PLAT PE=1 SV=1 - [TPA_HUMAN]	-1.64	-1.33	-1.66	-1.45	-1.05	-0.93	-1.68	-1.56	-1.22	-1.34	-1.14	-1.03	0.29	0.61	0.62	0.35	0.37	0.58	0.59	0.31
P36404	ADP-ribosylation factor-like protein 2 OS=Homo sapiens GN=ARL2 PE=1 SV=4 - [ARL2_HUMAN]	-1.23	-1.43	-1.21	-1.41	-0.22	-0.28	-0.19	-0.43	-0.48	-0.43	-0.42	-0.48	1.09	0.64	0.62	0.83	0.81	0.94	0.92	1.08
P09486	SPARC OS=Homo sapiens GN=SPARC PE=1 SV=1 - [SPRC_HUMAN]	-1.29	-1.09	-1.36	-1.39	-0.33	-0.29	-0.27	-0.25	-0.07	-0.08	-0.05	0.11	0.74	1.44	1.52	0.97	1.30	0.72	1.04	1.14
P54725	UV excision repair protein RAD23 homolog A OS=Homo sapiens GN=RAD23A PE=1 SV=1 - [RD23A_HUMAN]	-1.20	-1.31	-1.37	-1.39	0.07	0.06	-0.22	-0.18	-0.12	-0.16	-0.16	-0.07	0.57	1.00	1.27	0.79	1.39	0.94	1.52	1.17
Q08357	Sodium-dependent phosphate transporter 2 OS=Homo sapiens GN=SLC20A2 PE=1 SV=1 - [S20A2_HUMAN]	-1.35	-1.13	-1.38	-1.35	0.03	0.03	-0.11	-0.09	-0.06	-0.08	-0.30	-0.30	1.09	0.86	1.18	1.15	1.48	1.17	1.51	1.21
A1A4S6	Rho GTPase-activating protein 10 OS=Homo sapiens GN=ARHGAP10 PE=1 SV=1 - [RHG10_HUMAN]	-1.24	-1.26	-1.34	-1.30	-0.26	-0.23	-0.60	-0.56	-0.19	-0.17	-0.29	-0.37	0.82	0.73	0.67	0.96	0.89	1.24	1.22	0.80
O95967	EGF-containing fibulin-like extracellular matrix protein 2 OS=Homo sapiens GN=EFEMP2 PE=1 SV=3 - [FBLN4_HUMAN]	-1.45	-1.33	-1.40	-1.30	1.22	1.25	0.40	0.47	0.75	0.65	0.83	0.70	1.91	1.99	2.22	2.04	2.12	2.58	2.70	1.98

Q9H488	GDP-fucose protein O-fucosyltransferase 1 OS=Homo sapiens GN=POFUT1 PE=1 SV=1 - [OFUT1_HUMAN]	-1.27	-1.15	-1.43	-1.29	0.14	0.15	0.01	-0.03	0.14	0.08	-0.03	-0.02	1.17	1.46	1.43	1.38	1.64	1.51	1.55	1.45
Q14576	ELAV-like protein 3 OS=Homo sapiens GN=ELAVL3 PE=1 SV=3 - [ELAV3_HUMAN]	2.05	2.16	2.66	2.96	0.16	0.43	0.79	1.07	0.74	0.74	0.75	0.77	-0.92	-1.27	-2.18	-1.52	-2.18	-1.69	-2.58	-1.80
Q9NY65	Tubulin alpha-8 chain OS=Homo sapiens GN=TUBA8 PE=1 SV=1 - [TBA8_HUMAN]	2.22	2.42	2.53	2.71	-0.06	-0.19	0.64	1.01	0.57	0.42	0.00	0.16	-1.61	-2.18	-2.49	-1.87	-2.13	-2.59	-3.12	-1.75
P16389	Potassium voltage-gated channel subfamily A member 2 OS=Homo sapiens GN=KCNA2 PE=1 SV=2 - [KCNA2_HUMAN]	1.74	2.48	1.86	2.66	0.20	0.23	0.92	1.05	0.65	0.33	0.52	0.58	-1.38	-1.89	-2.07	-1.79	-1.90	-1.77	-1.93	-1.55
P20472	Parvalbumin alpha OS=Homo sapiens GN=PVALB PE=1 SV=2 - [PRVA_HUMAN]	2.22	2.21	2.78	2.61	0.54	0.67	0.64	0.62	0.75	0.61	0.09	0.47	-1.33	-1.88	-2.69	-1.47	-1.98	-1.38	-2.10	-1.91
Q05639	Elongation factor 1-alpha 2 OS=Homo sapiens GN=EEF1A2 PE=1 SV=1 - [EF1A2_HUMAN]	2.14	2.25	2.62	2.60	-0.12	0.15	0.91	0.89	0.60	0.48	0.33	0.23	-1.53	-1.98	-2.24	-1.38	-1.83	-2.20	-2.21	-1.66
Q9H313	Protein tweety homolog 1 OS=Homo sapiens GN=TTYH1 PE=2 SV=1 - [TTYH1_HUMAN]	2.46	2.53	2.32	2.33	-0.52	-0.51	0.99	0.97	0.55	0.64	0.31	0.40	-1.41	-2.12	-1.92	-1.88	-1.68	-3.01	-2.87	-1.26
Q9UIC8	Leucine carboxyl methyltransferase 1 OS=Homo sapiens GN=LCMT1 PE=1 SV=2 - [LCMT1_HUMAN]	1.97	2.07	2.16	2.29	0.37	0.11	0.63	0.94	0.30	0.44	0.30	0.11	-1.20	-1.72	-1.89	-1.68	-1.57	-1.80	-1.62	-1.31
Q9H1V8	Sodium-dependent neutral amino acid transporter SLC6A17 OS=Homo sapiens GN=SLC6A17 PE=2 SV=3 - [S6A17_HUMAN]	1.98	2.20	1.82	2.10	-0.28	-0.11	0.70	0.95	0.46	0.31	0.34	0.44	-1.10	-1.78	-1.85	-1.27	-1.25	-2.44	-2.39	-1.08

P49802	Regulator of G-protein signaling 7 OS=Homo sapiens GN=RGS7 PE=1 SV=3 - [RGS7_HUMAN]	1.71	1.56	2.17	2.02	0.11	-0.04	0.77	0.62	0.17	0.32	0.28	0.12	-0.95	-1.42	-1.89	-1.35	-1.81	-1.61	-2.07	-1.44
O14548	Cytochrome c oxidase subunit 7A-related protein, mitochondrial OS=Homo sapiens GN=COX7A2L PE=1 SV=2 - [COX7R_HUMAN]	1.54	1.63	1.74	1.99	0.05	-0.05	0.40	0.49	0.12	-0.18	-0.03	0.15	-1.09	-1.33	-1.65	-1.60	-1.72	-1.50	-1.80	-1.39
O75525	KH domain-containing, RNA-binding, signal transduction-associated protein 3 OS=Homo sapiens GN=KHDRBS3 PE=1 SV=1 - [KHDR3_HUMAN]	1.73	1.57	1.96	1.90	-0.44	-0.04	0.09	0.15	0.09	-0.03	-0.15	0.14	-1.58	-1.67	-1.57	-1.45	-1.36	-1.71	-1.60	-1.47
P20336	Ras-related protein Rab-3A OS=Homo sapiens GN=RAB3A PE=1 SV=1 - [RAB3A_HUMAN]	1.75	1.66	2.03	1.87	-0.23	-0.11	0.90	0.76	0.18	0.36	-0.12	-0.28	-0.82	-1.83	-2.33	-1.44	-1.69	-1.95	-2.25	-1.16
Q8TBB6	Probable cationic amino acid transporter OS=Homo sapiens GN=SLC7A14 PE=2 SV=3 - [S7A14_HUMAN]	1.53	1.39	1.97	1.83	-0.06	-0.20	0.72	0.58	0.53	0.67	0.44	0.30	-0.75	-1.09	-1.53	-0.86	-1.30	-1.61	-2.04	-1.27
O00305	Voltage-dependent L-type calcium channel subunit beta-4 OS=Homo sapiens GN=CACNB4 PE=1 SV=2 - [CACB4_HUMAN]	1.34	1.56	1.60	1.82	-0.32	-0.13	0.50	0.51	0.29	0.05	-0.13	0.01	-0.82	-1.30	-1.69	-1.08	-1.46	-1.72	-1.99	-1.07
Q86XD5	Protein FAM131B OS=Homo sapiens GN=FAM131B PE=1 SV=3 - [F131B_HUMAN]	1.55	1.45	1.70	1.80	0.39	0.42	0.59	0.54	0.52	0.50	0.31	0.37	-1.12	-1.07	-1.46	-0.96	-1.01	-1.26	-1.14	-1.14
Q13224	Glutamate receptor ionotropic, NMDA 2B OS=Homo sapiens GN=GRIN2B PE=1 SV=3 - [NMDE2_HUMAN]	1.26	1.60	1.44	1.78	-0.48	-0.31	1.13	1.20	0.32	0.43	-0.06	-0.30	-0.11	-1.31	-1.50	-0.48	-0.66	-2.38	-2.30	-0.25

Q13526	Peptidyl-prolyl cis-trans isomerase NIMA-interacting 1 OS=Homo sapiens GN=PIN1 PE=1 SV=1 - [PIN1_HUMAN]	1.38	1.13	1.78	1.75	0.17	0.08	0.75	0.56	0.27	0.35	0.31	0.14	-0.65	-1.20	-1.62	-0.98	-1.43	-1.16	-1.70	-1.03
Q00975	Voltage-dependent N-type calcium channel subunit alpha-1B OS=Homo sapiens GN=CACNA1B PE=1 SV=1 - [CAC1B_HUMAN]	1.98	1.92	1.80	1.73	0.38	0.30	0.73	0.57	0.62	0.78	0.42	0.28	-1.35	-1.68	-1.79	-1.32	-1.26	-1.67	-1.61	-1.30
P84074	Neuron-specific calcium-binding protein hippocalcin OS=Homo sapiens GN=HPCA PE=1 SV=2 - [HPCA_HUMAN]	1.86	1.69	1.83	1.67	0.11	-0.06	0.57	0.40	0.24	0.41	-0.77	-0.94	-1.22	-2.63	-2.61	-1.42	-1.39	-1.77	-1.74	-1.42
O43920	NADH dehydrogenase [ubiquinone] iron-sulfur protein 5 OS=Homo sapiens GN=NDUFS5 PE=1 SV=3 - [NDUS5_HUMAN]	1.42	1.65	1.49	1.66	0.09	0.10	0.47	0.72	0.24	0.07	0.17	0.28	-0.90	-1.20	-1.09	-1.18	-1.06	-1.29	-1.39	-0.92
O00483	NADH dehydrogenase [ubiquinone] 1 alpha subcomplex subunit 4 OS=Homo sapiens GN=NDUFA4 PE=1 SV=1 - [NDUA4_HUMAN]	1.32	1.32	1.59	1.66	-0.13	-0.12	0.36	0.51	0.21	0.23	0.03	0.04	-0.83	-1.19	-1.57	-1.10	-1.45	-1.40	-1.77	-1.10
Q9BXM9	FSD1-like protein OS=Homo sapiens GN=FSD1L PE=1 SV=2 - [FSD1L_HUMAN]	1.54	1.81	1.55	1.66	0.52	0.56	0.43	0.54	0.65	0.45	0.14	0.26	-0.91	-1.39	-1.41	-1.06	-0.97	-1.12	-0.97	-0.91
Q8N987	N-terminal EF-hand calcium-binding protein 1 OS=Homo sapiens GN=NECAB1 PE=1 SV=1 - [NECA1_HUMAN]	1.19	1.58	1.59	1.64	-0.22	-0.15	-0.04	-0.06	0.04	-0.12	-0.17	0.05	-0.89	-1.36	-1.78	-1.28	-1.75	-1.55	-1.90	-1.79
O00519	Fatty-acid amide hydrolase 1 OS=Homo sapiens GN=FAAH PE=1 SV=2 - [FAAH1_HUMAN]	1.57	1.53	1.67	1.64	0.20	0.20	0.53	0.50	0.30	0.34	-0.14	-0.15	-0.98	-2.08	-2.05	-1.20	-1.30	-1.75	-1.72	-1.08

P08247	Synaptophysin OS=Homo sapiens GN=SYP PE=1 SV=3 - [SYPH_HUMAN]	1.63	1.42	1.66	1.62	-0.11	-0.11	0.89	0.78	0.33	0.47	0.41	0.42	-0.73	-1.06	-1.26	-1.01	-0.90	-1.73	-1.67	-0.51
Q9NVS9	Pyridoxine-5'-phosphate oxidase OS=Homo sapiens GN=PNPO PE=1 SV=1 - [PNPO_HUMAN]	1.90	1.82	1.51	1.61	0.29	0.18	0.60	0.47	0.56	0.54	0.10	0.30	-1.21	-1.28	-1.07	-1.15	-0.93	-1.63	-1.36	-0.81
O60939	Sodium channel subunit beta-2 OS=Homo sapiens GN=SCN2B PE=1 SV=1 - [SCN2B_HUMAN]	1.10	1.16	1.64	1.61	-0.15	-0.28	0.57	0.39	0.05	0.18	-0.16	-0.24	-0.63	-1.25	-1.91	-0.88	-1.54	-1.26	-1.93	-1.01
O15126	Secretory carrier-associated membrane protein 1 OS=Homo sapiens GN=SCAMP1 PE=1 SV=2 - [SCAM1_HUMAN]	1.10	1.37	1.50	1.57	0.13	0.05	0.37	0.16	0.05	-0.05	0.12	0.17	-0.99	-1.19	-1.42	-1.19	-1.34	-1.31	-1.35	-1.05
Q8TC26	Transmembrane protein 163 OS=Homo sapiens GN=TMEM163 PE=2 SV=1 - [TM163_HUMAN]	1.69	1.58	1.76	1.56	-0.22	-0.26	0.28	0.39	0.13	-0.14	-0.36	-0.20	-1.24	-2.04	-1.94	-1.78	-1.64	-2.07	-2.13	-1.11
Q14141	Septin-6 OS=Homo sapiens GN=SEPT6 PE=1 SV=4 - [SEPT6_HUMAN]	1.80	1.15	1.76	1.55	-0.25	-0.87	0.34	0.08	-0.05	0.31	-0.13	-0.16	-1.01	-1.27	-1.89	-1.14	-1.25	-1.45	-1.56	-1.56
Q16537	Serine/threonine-protein phosphatase 2A 56 kDa regulatory subunit epsilon isoform OS=Homo sapiens GN=PPP2R5E PE=1 SV=1 - [2A5E_HUMAN]	1.65	1.49	1.68	1.52	0.23	0.31	0.81	0.64	0.26	0.43	-0.06	-0.12	-0.92	-1.60	-1.67	-1.19	-1.22	-1.40	-1.45	-0.81
A6NDG6	Phosphoglycolate phosphatase OS=Homo sapiens GN=PGP PE=1 SV=1 - [PGP_HUMAN]	1.17	1.12	1.56	1.50	0.23	0.17	0.19	0.13	0.01	0.07	-0.10	-0.16	-0.93	-1.26	-1.66	-1.07	-1.46	-0.96	-1.34	-1.31
P49441	Inositol polyphosphate 1-phosphatase OS=Homo sapiens GN=INPP1 PE=1 SV=1 - [INPP_HUMAN]	1.19	1.29	1.40	1.50	-0.03	0.28	0.61	0.69	0.36	0.05	-0.12	-0.02	-0.62	-1.52	-1.68	-1.08	-1.16	-1.32	-1.60	-0.77

O43427	Acidic fibroblast growth factor intracellular-binding protein OS=Homo sapiens GN=FIBP PE=1 SV=3 - [FIBP_HUMAN]	1.25	1.21	1.34	1.48	0.03	0.31	0.32	0.48	0.34	0.13	-0.16	0.12	-0.83	-1.38	-1.40	-1.05	-1.11	-1.31	-1.25	-0.93
P05413	Fatty acid-binding protein, heart OS=Homo sapiens GN=FABP3 PE=1 SV=4 - [FABPH_HUMAN]	1.06	1.15	1.38	1.48	-0.73	-0.60	-0.04	-0.01	-0.59	-0.71	-0.81	-0.69	-1.04	-1.83	-2.16	-1.35	-1.76	-1.39	-1.82	-1.35
Q9P1A6	Disks large-associated protein 2 OS=Homo sapiens GN=DLGAP2 PE=1 SV=4 - [DLGP2_HUMAN]	1.44	1.34	1.41	1.45	0.14	0.37	0.36	0.59	0.29	0.06	-0.21	-0.15	-1.16	-1.70	-1.82	-1.50	-1.36	-1.47	-1.33	-1.03
P52758	Ribonuclease UK114 OS=Homo sapiens GN=HRSP12 PE=1 SV=1 - [UK114_HUMAN]	1.23	1.11	1.55	1.43	0.49	0.37	0.43	0.31	-0.17	-0.04	-0.32	-0.45	-0.74	-1.54	-1.87	-1.24	-1.55	-0.75	-1.07	-1.05
P43003	Excitatory amino acid transporter 1 OS=Homo sapiens GN=SLC1A3 PE=1 SV=1 - [EAA1_HUMAN]	1.26	1.08	1.53	1.42	-0.01	0.10	1.23	0.91	0.78	0.78	0.69	0.44	-0.06	-0.70	-0.71	-0.53	-0.60	-1.43	-1.52	-0.11
Q9Y2H9	Microtubule-associated serine/threonine-protein kinase 1 OS=Homo sapiens GN=MAST1 PE=1 SV=2 - [MAST1_HUMAN]	1.19	1.27	1.34	1.34	0.32	-0.09	0.25	0.17	-0.07	0.16	-0.22	-0.68	-1.11	-1.41	-1.57	-1.31	-1.37	-1.37	-1.44	-1.06
Q53HC0	Coiled-coil domain-containing protein 92 OS=Homo sapiens GN=CCDC92 PE=1 SV=2 - [CCD92_HUMAN]	1.38	1.40	1.32	1.34	-0.46	-0.44	-0.34	-0.32	-0.12	-0.13	-0.30	-0.28	-1.67	-1.67	-1.61	-1.48	-1.42	-1.86	-1.79	-1.60
Q4KMP7	TBC1 domain family member 10B OS=Homo sapiens GN=TBC1D10B PE=1 SV=3 - [TB10B_HUMAN]	1.27	1.15	1.39	1.30	-0.16	-0.28	0.12	0.13	-0.01	0.02	0.11	-0.01	-1.14	-1.22	-1.24	-1.18	-1.23	-1.41	-1.57	-1.01
O43526	Potassium voltage-gated channel subfamily KQT member 2 OS=Homo sapiens GN=KCNQ2 PE=1 SV=2 - [KCNQ2_HUMAN]	1.25	1.03	1.31	1.24	0.12	-0.05	0.32	0.37	0.06	0.22	-0.02	-0.06	-0.93	-1.06	-1.13	-1.05	-1.23	-1.19	-1.38	-1.20

Q9BV35	Calcium-binding mitochondrial carrier protein SCaMC-3 OS=Homo sapiens GN=SLC25A23 PE=1 SV=2 - [SCMC3_HUMAN]	1.03	1.20	1.14	1.17	-0.17	-0.09	0.30	0.33	0.00	-0.03	-0.02	0.08	-0.64	-0.91	-0.91	-0.79	-0.77	-1.12	-1.16	-0.78
P51674	Neuronal membrane glycoprotein M6-a OS=Homo sapiens GN=GPM6A PE=1 SV=2 - [GPM6A_HUMAN]	1.57	1.17	1.45	1.07	0.12	-0.21	0.63	0.39	0.27	0.39	0.44	0.20	-0.77	-1.16	-1.18	-1.26	-1.17	-1.80	-1.56	-0.86
Q9NYB9	Abl interactor 2 OS=Homo sapiens GN=ABI2 PE=1 SV=1 - [ABI2_HUMAN]	1.21	1.22	1.10	1.06	-0.20	-0.18	0.15	0.09	0.11	0.07	-0.04	-0.08	-0.95	-1.11	-1.00	-1.01	-0.90	-1.20	-1.28	-0.83
Q9UBC9	Small proline-rich protein 3 OS=Homo sapiens GN=SPRR3 PE=1 SV=2 - [SPRR3_HUMAN]	-4.05	-4.24	-5.52	-5.49	-2.52	-2.72	-4.05	-4.12	-3.19	-2.99	-2.82	-3.03	-0.45	-0.21	0.91	0.68	0.47	-0.56	0.64	1.25
Q4U2R8	Solute carrier family 22 member 6 OS=Homo sapiens GN=SLC22A6 PE=1 SV=1 - [S22A6_HUMAN]	-1.64	-1.85	-3.02	-3.06	0.18	0.26	0.03	0.04	0.00	-0.05	-0.24	-0.22	1.89	2.09	2.87	1.98	3.13	2.38	3.38	3.24
P01861	Ig gamma-4 chain C region OS=Homo sapiens GN=IGHG4 PE=1 SV=1 - [IGHG4_HUMAN]	-3.30	-3.33	-3.07	-3.00	-1.52	-1.46	-1.82	-1.73	-1.64	-1.61	-1.18	-1.25	1.40	1.76	1.73	1.40	1.39	1.58	1.47	1.35
Q96IZ0	PRKC apoptosis WT1 regulator protein OS=Homo sapiens GN=PAWR PE=1 SV=1 - [PAWR_HUMAN]	-2.40	-2.45	-2.88	-2.89	-0.47	-0.43	-0.63	-0.66	-0.95	-0.98	-1.05	-1.06	2.01	1.92	1.88	1.98	1.89	2.26	2.29	2.29
P22352	Glutathione peroxidase 3 OS=Homo sapiens GN=GPX3 PE=1 SV=2 - [GPX3_HUMAN]	-2.45	-2.28	-2.81	-2.70	0.22	0.29	-0.40	-0.35	0.10	0.14	0.64	0.65	1.96	3.18	3.52	2.58	2.86	2.61	3.05	2.48
P12829	Myosin light chain 4 OS=Homo sapiens GN=MYL4 PE=1 SV=3 - [MYL4_HUMAN]	-2.20	-2.08	-2.45	-2.49	-0.20	-0.30	0.05	0.04	-0.10	-0.12	-0.02	-0.04	2.18	2.02	2.17	1.76	2.21	1.88	2.11	2.32
P15502	Elastin OS=Homo sapiens GN=ELN PE=1 SV=3 - [ELN_HUMAN]	-2.96	-3.07	-2.54	-2.45	0.81	0.81	-1.06	-1.14	-0.43	-0.49	-1.18	-1.24	2.15	1.82	1.17	2.75	2.08	3.98	3.41	1.44

Q99983	Osteomodulin OS=Homo sapiens GN=OMD PE=1 SV=1 - [OMD_HUMAN]	-2.28	-2.30	-2.37	-2.39	-1.03	-1.08	-1.03	-1.08	-1.34	-1.29	-1.16	-1.19	1.39	1.14	1.21	1.31	1.41	1.48	1.58	1.50
P20851	C4b-binding protein beta chain OS=Homo sapiens GN=C4BPB PE=1 SV=1 - [C4BPB_HUMAN]	-2.47	-2.37	-2.48	-2.35	-0.64	-0.57	-0.60	-0.53	-0.29	-0.35	0.41	0.53	1.90	2.92	2.85	2.12	2.07	1.73	1.77	1.87
Q15404	Ras suppressor protein 1 OS=Homo sapiens GN=RSU1 PE=1 SV=3 - [RSU1_HUMAN]	-2.38	-2.48	-2.04	-2.20	-0.57	-0.74	-0.86	-1.02	-0.91	-0.74	-1.59	-1.75	1.27	1.02	0.45	1.13	1.33	1.53	1.45	1.24
Q96NE9	FERM domain-containing protein 6 OS=Homo sapiens GN=FRMD6 PE=1 SV=1 - [FRMD6_HUMAN]	-1.60	-1.69	-2.13	-2.14	-0.13	-0.09	-0.24	-0.19	-0.19	-0.23	-0.42	-0.40	1.59	1.00	1.36	1.51	1.83	1.58	1.87	1.90
Q13643	Four and a half LIM domains protein 3 OS=Homo sapiens GN=FHL3 PE=1 SV=4 - [FHL3_HUMAN]	-2.06	-1.96	-2.19	-2.08	-0.49	-0.39	-0.65	-0.52	-0.56	-0.66	-1.06	-0.96	1.50	1.01	1.21	1.51	1.67	1.54	1.70	1.62
P06702	Protein S100-A9 OS=Homo sapiens GN=S100A9 PE=1 SV=1 - [S10A9_HUMAN]	-1.87	-1.72	-2.00	-2.05	-1.80	-1.89	0.68	0.72	0.12	0.09	-0.08	-0.02	2.44	1.50	2.07	1.74	2.19	0.07	0.08	2.64
P39059	Collagen alpha-1(XV) chain OS=Homo sapiens GN=COL15A1 PE=1 SV=2 - [COFA1_HUMAN]	-1.89	-1.71	-1.94	-1.94	0.30	0.48	-0.93	-0.82	-0.22	-0.46	-0.52	-0.54	0.73	1.11	1.34	1.39	1.38	2.17	2.23	0.97
P27487	Dipeptidyl peptidase 4 OS=Homo sapiens GN=DPP4 PE=1 SV=2 - [DPP4_HUMAN]	-1.91	-1.93	-1.91	-1.92	0.66	0.65	-0.79	-0.67	0.40	0.01	-0.18	-0.15	1.24	1.37	1.78	1.97	2.36	2.38	2.52	1.27
P35442	Thrombospondin-2 OS=Homo sapiens GN=THBS2 PE=1 SV=2 - [TSP2_HUMAN]	-1.11	-1.45	-1.51	-1.90	0.38	0.10	-0.57	-0.71	-0.62	-0.26	-0.50	-0.75	0.80	0.98	1.22	1.02	1.54	1.59	2.06	1.14

P46977	Dolichyl-diphosphooligosaccharide--protein glycosyltransferase subunit STT3A OS=Homo sapiens GN=STT3A PE=1 SV=2 - [STT3A_HUMAN]	-2.15	-2.21	-1.83	-1.88	0.05	0.12	-0.39	-0.44	-0.24	-0.21	-0.04	0.01	1.79	2.17	1.85	2.01	1.69	2.32	1.97	1.49
P19652	Alpha-1-acid glycoprotein 2 OS=Homo sapiens GN=ORM2 PE=1 SV=2 - [A1AG2_HUMAN]	-1.82	-1.67	-2.00	-1.88	-1.03	-0.88	-0.98	-0.84	-0.67	-0.87	-1.04	-0.99	0.92	0.72	0.80	1.00	1.04	0.87	0.89	1.01
P80188	Neutrophil gelatinase-associated lipocalin OS=Homo sapiens GN=LCN2 PE=1 SV=2 - [NGAL_HUMAN]	-1.53	-1.59	-2.04	-1.87	-1.23	-1.19	0.63	0.88	0.74	0.35	0.43	0.69	2.28	2.15	2.53	2.29	2.50	0.39	0.86	2.54
Q5HYK7	SH3 domain-containing protein 19 OS=Homo sapiens GN=SH3D19 PE=1 SV=2 - [SH319_HUMAN]	-1.69	-1.80	-1.74	-1.85	0.34	0.23	0.09	-0.05	-0.06	0.05	-0.08	-0.15	1.52	1.69	1.66	1.57	1.67	1.70	1.91	1.75
Q53GG5	PDZ and LIM domain protein 3 OS=Homo sapiens GN=PDLIM3 PE=1 SV=1 - [PDLI3_HUMAN]	-1.95	-1.74	-1.75	-1.74	-0.68	-0.70	-0.95	-0.97	-0.73	-0.74	-0.46	-0.45	1.14	1.61	1.29	1.49	1.15	1.47	1.29	0.91
P07951	Tropomyosin beta chain OS=Homo sapiens GN=TPM2 PE=1 SV=1 - [TPM2_HUMAN]	-1.67	-1.47	-1.85	-1.72	-0.96	-0.80	-1.10	-0.92	-0.86	-1.02	-0.61	-0.58	0.59	1.06	1.17	0.70	1.00	0.90	1.44	0.73
P61619	Protein transport protein Sec61 subunit alpha isoform 1 OS=Homo sapiens GN=SEC61A1 PE=1 SV=2 - [S61A1_HUMAN]	-1.70	-1.64	-1.70	-1.70	0.07	0.01	-0.56	-0.55	-0.45	-0.40	-0.20	-0.25	1.20	1.62	1.78	1.51	1.52	1.77	1.89	1.21
P05452	Tetranectin OS=Homo sapiens GN=CLEC3B PE=1 SV=3 - [TETN_HUMAN]	-2.15	-2.02	-1.85	-1.70	-0.23	0.05	-0.51	-0.24	-0.21	-0.34	-0.24	-0.10	1.38	1.91	1.92	1.69	1.70	1.76	1.77	1.54
O60237	Protein phosphatase 1 regulatory subunit 12B OS=Homo sapiens GN=PPP1R12B PE=1 SV=2 - [MYPT2_HUMAN]	-1.67	-1.76	-1.52	-1.68	-1.33	-1.30	-1.02	-1.10	-1.20	-1.06	-1.21	-1.22	0.65	0.48	0.44	0.36	0.17	0.32	0.17	0.64

Q5U651	Ras-interacting protein 1 OS=Homo sapiens GN=RASIP1 PE=1 SV=1 - [RAIN_HUMAN]	-1.61	-1.78	-1.45	-1.65	-0.58	-0.92	-0.97	-1.31	-1.21	-0.78	-0.53	-0.97	0.57	0.86	0.55	0.96	0.64	0.96	0.62	0.28
P07360	Complement component C8 gamma chain OS=Homo sapiens GN=C8G PE=1 SV=3 - [CO8G_HUMAN]	-1.39	-1.46	-1.27	-1.52	0.48	0.34	-0.37	-0.33	-0.12	-0.16	0.31	0.08	1.23	1.71	1.56	1.49	1.41	1.74	1.88	1.10
P78536	Disintegrin and metalloproteinase domain-containing protein 17 OS=Homo sapiens GN=ADAM17 PE=1 SV=1 - [ADA17_HUMAN]	-1.54	-1.65	-1.41	-1.51	0.37	0.25	-0.28	-0.39	-0.08	0.04	0.66	0.55	1.31	2.21	2.07	1.61	1.48	1.89	1.75	1.19
P06681	Complement C2 OS=Homo sapiens GN=C2 PE=1 SV=2 - [CO2_HUMAN]	-1.38	-1.49	-1.39	-1.50	0.21	0.10	-0.54	-0.65	-0.28	-0.16	0.18	0.07	0.89	1.56	1.57	1.24	1.26	1.57	1.58	0.91
Q9NW15	Anoctamin-10 OS=Homo sapiens GN=ANO10 PE=1 SV=2 - [ANO10_HUMAN]	-1.58	-1.81	-1.27	-1.50	0.28	0.17	-0.02	-0.06	-0.53	-0.37	-0.22	-0.36	1.80	1.36	1.04	1.31	1.00	1.97	1.66	1.50
Q12802	A-kinase anchor protein 13 OS=Homo sapiens GN=AKAP13 PE=1 SV=2 - [AKP13_HUMAN]	-1.30	-1.14	-1.42	-1.41	0.83	0.93	0.14	0.17	0.46	0.45	0.42	0.43	1.92	1.90	1.95	1.89	1.95	2.12	2.33	1.98
O15511	Actin-related protein 2/3 complex subunit 5 OS=Homo sapiens GN=ARPC5 PE=1 SV=3 - [ARPC5_HUMAN]	-1.54	-1.65	-1.36	-1.40	-0.24	-0.35	-0.55	-0.70	-0.65	-0.51	-0.21	-0.20	1.05	1.35	1.03	0.97	0.80	1.15	0.90	0.89
Q8TCT9	Minor histocompatibility antigen H13 OS=Homo sapiens GN=HM13 PE=1 SV=1 - [HM13_HUMAN]	-1.28	-1.44	-1.60	-1.35	0.63	0.54	0.08	-0.23	-0.26	-0.08	0.11	-0.11	1.04	1.39	1.53	1.23	1.41	1.55	1.87	1.17
P35914	Hydroxymethylglutaryl-CoA lyase, mitochondrial OS=Homo sapiens GN=HMGCL PE=1 SV=2 - [HMGCL_HUMAN]	-1.43	-1.43	-1.38	-1.27	-0.06	-0.03	-0.63	-0.53	-0.32	-0.33	0.13	0.22	0.95	1.55	1.66	1.11	1.10	1.26	1.43	0.89

P23763	Vesicle-associated membrane protein 1 OS=Homo sapiens GN=VAMP1 PE=1 SV=1 - [VAMP1_HUMAN]	2.52	2.88	2.90	3.04	0.14	0.37	1.50	2.06	1.16	1.00	0.82	1.20	-0.93	-1.75	-1.88	-1.49	-1.85	-2.79	-2.68	-0.85
O15079	Syntaphilin OS=Homo sapiens GN=SNPH PE=1 SV=2 - [SNPH_HUMAN]	2.62	2.55	2.94	2.88	0.28	0.08	1.41	1.35	0.54	0.76	0.60	0.32	-1.16	-1.99	-2.31	-1.72	-2.08	-1.94	-2.25	-1.46
Q9P2W3	Guanine nucleotide-binding protein G(I)/G(S)/G(O) subunit gamma-13 OS=Homo sapiens GN=GNG13 PE=2 SV=1 - [GBG13_HUMAN]	2.74	2.86	2.62	2.86	0.60	0.47	1.30	1.25	0.88	1.02	0.76	0.62	-1.38	-1.78	-2.23	-1.56	-1.99	-1.96	-2.41	-1.55
Q8N6N7	Acyl-CoA-binding domain-containing protein 7 OS=Homo sapiens GN=ACBD7 PE=1 SV=1 - [ACBD7_HUMAN]	1.79	1.81	2.33	2.34	0.14	0.11	0.76	0.87	0.64	0.66	0.19	0.29	-0.79	-1.59	-1.89	-1.13	-1.66	-1.70	-2.24	-1.26
Q9Y6A2	Cholesterol 24-hydroxylase OS=Homo sapiens GN=CYP46A1 PE=1 SV=1 - [CP46A_HUMAN]	2.17	2.57	1.94	2.33	-0.26	0.13	0.59	0.99	0.64	0.25	-0.05	0.34	-1.35	-2.22	-1.99	-1.90	-1.65	-2.45	-2.22	-1.24
P63215	Guanine nucleotide-binding protein G(I)/G(S)/G(O) subunit gamma-3 OS=Homo sapiens GN=GNG3 PE=2 SV=1 - [GBG3_HUMAN]	2.00	1.92	2.14	2.31	-0.25	-0.20	0.58	0.70	0.05	0.18	0.13	0.26	-1.16	-1.83	-2.07	-1.75	-1.90	-2.36	-2.44	-1.42
Q9UFN0	Protein NipSnap homolog 3A OS=Homo sapiens GN=NIPSNAP3A PE=1 SV=2 - [NPS3A_HUMAN]	1.60	1.91	1.95	2.25	-0.69	-0.39	0.01	0.31	-0.47	-0.77	-0.94	-0.42	-1.54	-2.21	-3.13	-2.35	-2.69	-2.31	-2.66	-1.88
P04350	Tubulin beta-4A chain OS=Homo sapiens GN=TUBB4A PE=1 SV=2 - [TBB4A_HUMAN]	2.18	2.14	2.28	2.23	-0.14	-0.18	0.75	0.55	0.30	0.45	0.44	0.29	-1.43	-1.87	-2.10	-1.79	-1.87	-2.23	-2.43	-1.49
O14810	Complexin-1 OS=Homo sapiens GN=CPLX1 PE=1 SV=1 - [CPLX1_HUMAN]	1.85	2.02	2.25	2.23	-0.11	-0.45	0.48	0.43	-0.38	-0.32	-0.46	-0.52	-1.49	-2.31	-2.71	-1.94	-2.57	-2.17	-2.64	-1.90

Q6PUV4	Complexin-2 OS=Homo sapiens GN=CPLX2 PE=1 SV=2 - [CPLX2_HUMAN]	2.91	1.09	3.56	2.22	0.18	-0.70	1.00	0.25	-0.21	0.09	-0.48	-0.73	-1.66	-3.39	-4.05	-2.46	-3.11	-2.74	-3.40	-2.23
O15197	Ephrin type-B receptor 6 OS=Homo sapiens GN=EPHB6 PE=1 SV=4 - [EPHB6_HUMAN]	1.19	2.37	1.18	2.21	-0.15	0.36	-0.15	0.86	0.10	-0.48	0.22	0.73	-1.39	-1.00	-0.99	-1.66	-1.71	-1.63	-1.62	-1.28
P02689	Myelin P2 protein OS=Homo sapiens GN=PMP2 PE=1 SV=3 - [MYP2_HUMAN]	2.25	1.95	2.40	2.16	-0.01	-0.17	0.64	0.38	0.18	0.17	0.19	-0.12	-1.57	-1.97	-2.13	-2.13	-2.31	-2.06	-2.26	-1.59
P49773	Histidine triad nucleotide-binding protein 1 OS=Homo sapiens GN=HINT1 PE=1 SV=2 - [HINT1_HUMAN]	1.87	2.10	1.81	2.15	0.50	0.27	0.67	1.00	0.86	0.89	0.84	0.60	-1.06	-1.03	-0.97	-1.31	-1.24	-1.87	-1.81	-1.09
Q5SYC1	Clavesin-2 OS=Homo sapiens GN=CLVS2 PE=2 SV=1 - [CLVS2_HUMAN]	1.47	1.71	1.75	2.11	-0.18	0.07	0.79	1.09	0.87	0.43	0.48	0.92	-0.95	-1.58	-1.42	-1.11	-1.46	-1.91	-1.91	-1.18
Q16849	Receptor-type tyrosine-protein phosphatase-like N OS=Homo sapiens GN=PTPRN PE=1 SV=1 - [PTPRN_HUMAN]	1.15	1.89	1.57	2.05	-0.36	0.38	0.67	1.08	0.78	0.04	0.05	0.37	-0.90	-1.03	-1.52	-1.23	-1.36	-1.64	-2.00	-1.38
Q96HU8	GTP-binding protein Di-Ras2 OS=Homo sapiens GN=DIRAS2 PE=1 SV=1 - [DIRA2_HUMAN]	1.34	1.65	1.82	2.01	-0.13	-0.15	0.72	0.71	0.38	0.39	0.21	0.28	-0.72	-1.45	-1.81	-1.24	-1.59	-1.81	-2.17	-1.02
P30046	D-dopachrome decarboxylase OS=Homo sapiens GN=DDT PE=1 SV=3 - [DOPD_HUMAN]	1.58	1.75	2.11	2.01	0.95	0.77	0.95	0.83	0.63	0.98	0.42	0.32	-0.57	-1.16	-1.70	-1.08	-1.34	-0.77	-1.20	-1.10
Q17R89	Rho GTPase-activating protein 44 OS=Homo sapiens GN=ARHGAP44 PE=1 SV=1 - [RHG44_HUMAN]	1.73	1.78	1.99	1.97	-0.49	-0.33	0.57	0.62	0.34	0.19	-0.27	-0.22	-1.11	-1.99	-2.31	-1.41	-1.71	-2.09	-2.31	-1.22
P02792	Ferritin light chain OS=Homo sapiens GN=FTL PE=1 SV=2 - [FRIL_HUMAN]	1.54	1.57	1.69	1.95	-0.03	-0.40	1.03	0.96	0.58	0.72	0.85	0.30	-0.30	-0.75	-1.13	-0.85	-1.05	-1.66	-2.29	-0.30

O75381	Peroxisomal membrane protein PEX14 OS=Homo sapiens GN=PEX14 PE=1 SV=1 - [PEX14_HUMAN]	1.44	1.44	1.88	1.89	0.12	0.05	0.37	0.38	0.33	0.24	0.42	0.44	-1.02	-0.99	-1.51	-1.11	-1.61	-1.32	-1.82	-1.42
P42025	Beta-centractin OS=Homo sapiens GN=ACTR1B PE=1 SV=1 - [ACTY_HUMAN]	1.62	1.66	1.94	1.87	0.75	0.97	1.31	1.19	1.22	0.92	0.79	0.97	-0.35	-0.64	-0.82	-0.65	-0.95	-0.82	-0.97	-0.55
Q7L1I2	Synaptic vesicle glycoprotein 2B OS=Homo sapiens GN=SV2B PE=2 SV=1 - [SV2B_HUMAN]	1.40	1.74	1.75	1.85	0.23	0.19	0.77	0.88	0.71	0.54	0.36	0.25	-0.56	-1.77	-1.51	-1.10	-1.17	-1.03	-1.31	-0.78
Q96EQ0	Small glutamine-rich tetratricopeptide repeat-containing protein beta OS=Homo sapiens GN=SGTB PE=1 SV=1 - [SGTB_HUMAN]	1.48	1.61	1.62	1.84	-0.22	-0.14	0.23	0.41	0.07	-0.01	-0.24	-0.24	-1.15	-1.73	-2.08	-1.51	-1.79	-1.66	-1.85	-1.32
P22694	cAMP-dependent protein kinase catalytic subunit beta OS=Homo sapiens GN=PRKACB PE=1 SV=2 - [KAPCB_HUMAN]	1.11	1.53	1.51	1.82	-0.18	-0.05	0.16	0.52	0.54	-0.06	-0.26	0.19	-0.71	-1.15	-1.51	-0.73	-1.14	-1.12	-1.62	-1.14
O75582	Ribosomal protein S6 kinase alpha-5 OS=Homo sapiens GN=RPS6KA5 PE=1 SV=1 - [KS6A5_HUMAN]	1.46	1.45	1.77	1.80	0.25	0.23	0.69	0.71	0.19	0.16	0.45	0.43	-0.66	-0.92	-1.17	-0.97	-1.45	-1.23	-1.57	-1.03
Q13509	Tubulin beta-3 chain OS=Homo sapiens GN=TUBB3 PE=1 SV=2 - [TBB3_HUMAN]	1.31	1.39	1.77	1.79	-0.35	-0.36	0.32	0.37	0.20	0.07	-0.08	0.18	-1.09	-1.51	-1.77	-1.41	-1.66	-1.87	-2.04	-1.27
Q9UF11	Pleckstrin homology domain-containing family B member 1 OS=Homo sapiens GN=PLEKHB1 PE=1 SV=1 - [PKHB1_HUMAN]	1.90	1.64	1.95	1.77	-0.30	-0.27	0.32	0.12	-0.04	0.01	-0.37	-0.77	-1.45	-2.06	-2.11	-1.98	-2.12	-2.29	-2.34	-1.56

Q96BR5	Cytochrome c oxidase assembly factor 7 OS=Homo sapiens GN=COA7 PE=1 SV=2 - [COA7_HUMAN]	1.46	1.27	1.91	1.71	-0.10	-0.30	0.32	0.27	0.32	0.34	0.12	0.07	-1.09	-1.33	-1.78	-1.09	-1.53	-1.58	-2.03	-1.44
P47870	Gamma-aminobutyric acid receptor subunit beta-2 OS=Homo sapiens GN=GABRB2 PE=1 SV=2 - [GBRB2_HUMAN]	2.00	1.72	1.72	1.71	-0.47	-0.46	0.43	0.58	0.13	-0.05	-0.02	-0.03	-1.22	-1.90	-1.92	-1.74	-1.76	-2.18	-2.17	-1.23
P09669	Cytochrome c oxidase subunit 6C OS=Homo sapiens GN=COX6C PE=1 SV=2 - [COX6C_HUMAN]	1.18	1.41	1.73	1.71	-0.36	-0.28	0.45	0.70	0.47	0.31	0.22	0.33	-0.83	-1.01	-1.40	-1.15	-1.36	-1.77	-2.06	-0.99
Q92581	Sodium/hydrogen exchanger 6 OS=Homo sapiens GN=SLC9A6 PE=1 SV=2 - [SL9A6_HUMAN]	1.67	1.68	1.66	1.70	0.13	-0.07	0.60	0.63	0.25	0.22	0.20	0.23	-0.99	-1.32	-1.17	-1.28	-1.12	-1.76	-1.83	-1.00
Q9Y2C4	Nuclease EXOG, mitochondrial OS=Homo sapiens GN=EXOG PE=1 SV=2 - [EXOG_HUMAN]	1.31	1.37	1.40	1.68	0.33	0.47	0.42	0.66	0.56	0.24	0.30	0.61	-1.01	-1.13	-1.11	-1.31	-1.15	-1.47	-1.36	-0.97
Q15773	Myeloid leukemia factor 2 OS=Homo sapiens GN=MLF2 PE=1 SV=1 - [MLF2_HUMAN]	1.62	1.57	1.64	1.65	-0.47	-0.33	-0.14	-0.30	0.01	-0.18	-0.23	-0.47	-1.42	-1.70	-1.74	-1.63	-1.35	-1.83	-1.71	-1.55
P51797	Chloride transport protein 6 OS=Homo sapiens GN=CLCN6 PE=1 SV=2 - [CLCN6_HUMAN]	1.65	1.65	1.62	1.62	0.21	0.90	0.85	0.85	0.67	0.67	1.25	1.52	-0.91	-0.36	-0.34	-0.95	-0.92	-0.71	-0.58	-0.71
Q96HS1	Serine/threonine-protein phosphatase PGAM5, mitochondrial OS=Homo sapiens GN=PGAM5 PE=1 SV=2 - [PGAM5_HUMAN]	1.74	1.63	1.68	1.62	0.20	0.01	0.18	0.28	0.11	0.28	0.22	0.17	-1.29	-1.54	-1.52	-1.45	-1.43	-1.58	-1.56	-1.28
Q15049	Membrane protein MLC1 OS=Homo sapiens GN=MLC1 PE=1 SV=5 - [MLC1_HUMAN]	1.55	1.30	1.69	1.62	0.06	-0.05	0.91	1.21	0.72	0.53	0.79	0.98	-0.06	-0.20	-0.52	-0.26	-0.57	-1.13	-1.45	-0.34

P43007	Neutral amino acid transporter A OS=Homo sapiens GN=SLC1A4 PE=1 SV=1 - [SATT_HUMAN]	1.65	1.77	1.51	1.61	0.17	0.13	0.73	0.90	0.42	0.57	0.30	0.79	-0.85	-1.24	-1.22	-1.10	-0.85	-1.41	-1.29	-0.72
Q09470	Potassium voltage-gated channel subfamily A member 1 OS=Homo sapiens GN=KCNA1 PE=1 SV=2 - [KCNA1_HUMAN]	1.71	1.32	1.98	1.59	0.22	-0.17	0.38	0.07	0.08	0.47	0.08	0.10	-1.43	-1.62	-1.90	-1.46	-1.73	-0.93	-1.04	-1.76
P07919	Cytochrome b-c1 complex subunit 6, mitochondrial OS=Homo sapiens GN=UQCRH PE=1 SV=2 - [QCR6_HUMAN]	1.47	1.47	1.57	1.57	-0.15	0.04	0.66	0.66	0.19	0.21	0.22	0.35	-0.77	-1.23	-1.32	-1.20	-1.31	-1.56	-1.68	-0.87
Q8N9F7	Glycerophosphodiester phosphodiesterase domain-containing protein 1 OS=Homo sapiens GN=GDPD1 PE=1 SV=2 - [GDPD1_HUMAN]	1.13	1.38	1.32	1.57	-0.36	-0.12	-0.05	0.20	0.59	0.34	-0.21	0.04	-1.12	-1.33	-1.52	-0.76	-0.94	-1.51	-1.69	-1.30
Q13009	T-lymphoma invasion and metastasis-inducing protein 1 OS=Homo sapiens GN=TIAM1 PE=1 SV=2 - [TIAM1_HUMAN]	1.75	1.61	1.69	1.55	0.03	-0.11	-0.07	-0.21	-0.39	-0.24	-0.31	-0.46	-1.77	-2.05	-2.01	-1.96	-1.90	-1.73	-1.68	-1.70
P45381	Aspartoacylase OS=Homo sapiens GN=ASPA PE=1 SV=1 - [ACY2_HUMAN]	1.19	1.30	1.54	1.55	0.57	0.59	0.90	0.82	0.54	0.61	0.08	0.09	-0.17	-1.14	-1.46	-0.55	-0.89	-0.96	-0.98	-0.47
Q8TCZ2	CD99 antigen-like protein 2 OS=Homo sapiens GN=CD99L2 PE=1 SV=1 - [C99L2_HUMAN]	1.41	1.09	1.85	1.54	0.61	0.49	0.68	0.44	0.51	0.62	0.67	0.62	-0.67	-0.78	-1.24	-1.13	-1.39	-0.68	-1.12	-1.01
Q8IZS8	Voltage-dependent calcium channel subunit alpha-2/delta-3 OS=Homo sapiens GN=CACNA2D3 PE=1 SV=1 - [CA2D3_HUMAN]	1.92	1.69	1.74	1.51	0.44	0.21	0.63	0.40	0.57	0.81	0.48	0.24	-1.24	-1.44	-1.27	-1.08	-0.90	-1.49	-1.32	-1.06

Q6ZSY5	Protein phosphatase 1 regulatory subunit 3F OS=Homo sapiens GN=PPP1R3F PE=1 SV=3 - [PPR3F_HUMAN]	1.81	1.63	1.64	1.46	0.88	0.33	0.28	0.10	-0.01	0.18	0.22	0.03	-1.47	-1.59	-1.43	-1.60	-1.43	-1.35	-1.39	-1.30
Q15102	Platelet-activating factor acetylhydrolase IB subunit gamma OS=Homo sapiens GN=PAFAH1B3 PE=1 SV=1 - [PA1B3_HUMAN]	1.42	1.38	1.44	1.41	0.68	0.64	1.07	1.04	0.85	0.89	0.63	0.59	-0.29	-0.78	-0.81	-0.50	-0.52	-0.76	-0.78	-0.30
Q9H1P3	Oxysterol-binding protein-related protein 2 OS=Homo sapiens GN=OSBPL2 PE=1 SV=1 - [OSBL2_HUMAN]	1.21	1.17	1.33	1.37	0.10	-0.07	0.51	0.51	0.17	0.35	0.04	-0.03	-0.68	-1.14	-1.22	-0.86	-0.87	-1.16	-1.13	-0.71
Q6MZT1	Regulator of G-protein signaling 7-binding protein OS=Homo sapiens GN=RGS7BP PE=2 SV=3 - [R7BP_HUMAN]	1.31	1.20	1.71	1.36	0.35	0.23	0.42	0.48	0.28	0.48	0.48	0.36	-0.65	-0.83	-1.14	-0.89	-1.07	-0.97	-1.38	-1.05
Q9HCD6	Protein TANC2 OS=Homo sapiens GN=TANC2 PE=1 SV=3 - [TANC2_HUMAN]	1.29	1.04	1.67	1.34	-0.62	-0.82	0.32	0.07	-0.20	-0.16	-0.16	-0.42	-0.92	-1.45	-1.83	-1.42	-1.50	-1.93	-2.17	-1.28
Q59EK9	RUN domain-containing protein 3A OS=Homo sapiens GN=RUNDC3A PE=2 SV=2 - [RUN3A_HUMAN]	1.54	1.47	1.39	1.33	0.47	0.40	0.50	0.44	0.47	0.53	0.39	0.32	-0.98	-1.14	-1.01	-0.98	-0.83	-1.09	-0.94	-0.83
P49619	Diacylglycerol kinase gamma OS=Homo sapiens GN=DGKG PE=2 SV=3 - [DGKG_HUMAN]	1.54	1.23	1.56	1.32	0.71	0.45	0.70	0.43	0.39	0.66	0.46	0.45	-0.78	-0.59	-0.69	-0.84	-0.72	-0.70	-0.80	-0.72
Q13613	Myotubularin-related protein 1 OS=Homo sapiens GN=MTMR1 PE=1 SV=4 - [MTMR1_HUMAN]	1.51	1.45	1.37	1.31	-0.12	-0.18	0.19	0.13	0.16	0.23	0.20	0.13	-1.26	-1.31	-1.17	-1.23	-1.07	-1.60	-1.49	-1.04

Q6NXE6	Armadillo repeat-containing protein 6 OS=Homo sapiens GN=ARMC6 PE=1 SV=2 - [ARMC6_HUMAN]	1.22	1.24	1.22	1.24	0.47	0.49	0.89	0.91	0.71	0.69	0.56	0.57	-0.69	-0.78	-0.57	-0.83	-0.62	-1.19	-0.98	-0.47
O60359	Voltage-dependent calcium channel gamma-3 subunit OS=Homo sapiens GN=CACNG3 PE=2 SV=1 - [CCG3_HUMAN]	3.41	2.25	2.46	1.24	1.11	0.52	2.11	0.58	0.03	0.62	1.33	0.75	-1.61	-2.08	-1.13	-2.76	-1.81	-2.32	-1.37	-0.59
Q8WYK1	Contactin-associated protein-like 5 OS=Homo sapiens GN=CNTNAP5 PE=2 SV=1 - [CNTP5_HUMAN]	1.39	1.28	1.33	1.22	0.94	0.77	0.40	0.30	0.46	0.64	0.35	0.24	-0.59	-0.96	-1.09	-0.53	-0.46	-0.26	-0.26	-0.58
Q9HBT6	Cadherin-20 OS=Homo sapiens GN=CDH20 PE=2 SV=2 - [CAD20_HUMAN]	1.46	1.24	1.37	1.22	0.45	0.48	0.64	0.28	0.28	0.44	0.46	0.30	-0.90	-0.90	-0.92	-0.88	-0.90	-0.92	-0.94	-0.82
Q9BY67	Cell adhesion molecule 1 OS=Homo sapiens GN=CADM1 PE=1 SV=2 - [CADM1_HUMAN]	1.28	1.33	1.34	1.19	0.66	0.43	0.68	0.70	0.58	0.55	0.67	0.52	-0.55	-0.64	-0.66	-0.70	-0.64	-0.67	-0.46	-0.37
Q8TD22	Sideroflexin-5 OS=Homo sapiens GN=SFXN5 PE=2 SV=1 - [SFXN5_HUMAN]	1.52	1.38	1.30	1.11	0.20	-0.01	0.51	0.19	-0.39	0.16	0.30	0.30	-0.88	-0.96	-0.92	-1.32	-1.11	-1.11	-1.03	-0.64
O43761	Synaptogyrin-3 OS=Homo sapiens GN=SYNGR3 PE=1 SV=2 - [SNG3_HUMAN]	1.33	1.35	1.11	1.07	-0.27	-0.14	0.21	0.21	0.27	0.40	0.08	0.21	-1.06	-1.64	-1.43	-1.29	-1.16	-1.68	-1.37	-0.83
Q08188	Protein-glutamine gamma-glutamyltransferase E OS=Homo sapiens GN=TGM3 PE=1 SV=4 - [TGM3_HUMAN]	-3.69	-3.80	-4.36	-4.40	-3.15	-3.19	-3.23	-3.21	-2.46	-2.47	-2.83	-2.97	0.36	0.98	1.51	1.09	2.29	0.51	1.91	1.56
P35542	Serum amyloid A-4 protein OS=Homo sapiens GN=SAA4 PE=1 SV=2 - [SAA4_HUMAN]	-3.16	-3.07	-3.21	-3.13	0.01	0.09	-0.88	-0.88	-0.27	-0.35	-0.40	-0.45	2.36	2.56	2.60	2.81	2.46	3.15	2.80	2.02
P23280	Carbonic anhydrase 6 OS=Homo sapiens GN=CA6 PE=1 SV=3 - [CAH6_HUMAN]	-3.25	-3.23	-3.03	-2.90	-2.28	-2.10	-2.79	-2.61	-2.45	-2.59	-2.47	-2.37	-0.15	0.33	0.66	0.30	0.13	0.36	1.10	0.51

P01859	Ig gamma-2 chain C region OS=Homo sapiens GN=IGHG2 PE=1 SV=2 - [IGHG2_HUMAN]	-2.80	-2.69	-2.63	-2.70	-0.27	-0.15	-1.26	-1.22	-1.12	-1.15	-1.25	-1.46	1.41	1.19	1.16	1.68	1.63	2.58	2.39	1.20
Q99538	Legumain OS=Homo sapiens GN=LGMN PE=1 SV=1 - [LGMN_HUMAN]	-2.31	-2.32	-2.46	-2.50	-0.34	-0.29	-0.69	-0.74	-0.49	-0.54	-0.15	-0.14	1.72	2.19	2.31	1.83	2.10	2.17	2.33	1.77
P17813	Endoglin OS=Homo sapiens GN=ENG PE=1 SV=2 - [EGLN_HUMAN]	-2.18	-2.20	-2.47	-2.48	0.37	0.50	-0.20	-0.26	-0.13	-0.26	-0.21	-0.08	1.99	2.14	2.13	2.11	2.11	2.68	2.68	2.29
Q8IVN8	Somatomedin-B and thrombospondin type-1 domain-containing protein OS=Homo sapiens GN=SBSPON PE=1 SV=2 - [SBSPO_HUMAN]	-2.28	-2.22	-2.69	-2.48	-0.83	-0.63	-1.17	-1.12	-1.08	-1.13	-0.86	-0.84	0.84	1.89	2.11	1.80	2.21	2.06	2.46	0.89
Q9UHF1	Epidermal growth factor-like protein 7 OS=Homo sapiens GN=EGFL7 PE=1 SV=3 - [EGFL7_HUMAN]	-2.44	-2.56	-2.24	-2.35	0.20	0.33	-0.34	-0.33	-0.14	-0.09	0.23	0.36	1.99	2.56	2.63	2.28	2.35	2.41	2.60	2.08
P26447	Protein S100-A4 OS=Homo sapiens GN=S100A4 PE=1 SV=1 - [S10A4_HUMAN]	-2.41	-2.46	-2.43	-2.29	-0.03	0.05	-0.64	-0.55	-0.22	-0.29	-0.20	-0.11	1.84	2.08	2.11	2.14	2.03	2.33	2.38	1.77
Q14031	Collagen alpha-6(IV) chain OS=Homo sapiens GN=COL4A6 PE=2 SV=3 - [CO4A6_HUMAN]	-1.90	-1.66	-2.17	-2.25	-0.32	-0.40	-0.67	-0.54	-0.28	-0.55	-0.04	-0.07	1.29	1.87	2.18	1.38	1.65	1.73	1.84	1.68
P00740	Coagulation factor IX OS=Homo sapiens GN=F9 PE=1 SV=2 - [FA9_HUMAN]	-2.51	-2.46	-2.33	-2.18	-0.60	-0.39	-0.95	-1.01	-0.78	-0.72	-0.41	-0.26	1.50	2.04	1.92	1.71	1.51	1.66	1.61	1.30
Q16585	Beta-sarcoglycan OS=Homo sapiens GN=SGCB PE=1 SV=1 - [SGCB_HUMAN]	-1.85	-1.66	-2.32	-2.13	-0.15	0.06	-0.22	-0.03	0.07	-0.12	0.04	0.25	1.79	1.87	2.33	1.77	2.24	1.59	2.06	2.22
P55899	IgG receptor FcRn large subunit p51 OS=Homo sapiens GN=FCGRT PE=1 SV=1 - [FCGRN_HUMAN]	-2.24	-2.15	-2.17	-2.07	0.36	0.45	-0.39	-0.30	-0.05	0.05	0.78	0.86	1.90	3.02	2.94	2.33	2.26	2.58	2.51	1.83

P67812	Signal peptidase complex catalytic subunit SEC11A OS=Homo sapiens GN=SEC11A PE=1 SV=1 - [SC11A_HUMAN]	-1.98	-2.01	-2.03	-2.00	-0.04	-0.05	-0.61	-0.61	-0.30	-0.42	-0.28	-0.19	1.42	1.75	1.75	1.65	1.64	1.98	1.97	1.42
P31949	Protein S100-A11 OS=Homo sapiens GN=S100A11 PE=1 SV=2 - [S10AB_HUMAN]	-2.01	-1.80	-2.07	-1.97	-0.10	0.04	-0.50	-0.40	-0.20	-0.37	-0.04	0.06	1.58	2.05	2.05	1.73	1.79	1.97	2.02	1.66
P02775	Platelet basic protein OS=Homo sapiens GN=PPBP PE=1 SV=3 - [CXCL7_HUMAN]	-2.18	-2.33	-1.90	-1.97	-0.15	-0.31	-0.58	-0.73	-0.75	-0.44	-0.30	-0.45	1.65	1.89	1.60	1.79	1.26	2.01	1.73	1.38
P04920	Anion exchange protein 2 OS=Homo sapiens GN=SLC4A2 PE=1 SV=4 - [B3A2_HUMAN]	-1.39	-1.43	-1.92	-1.85	-0.01	0.01	-0.53	-0.46	-0.18	-0.21	-0.08	-0.02	1.03	1.42	1.80	1.31	1.58	1.38	1.89	1.44
Q9H0U3	Magnesium transporter protein 1 OS=Homo sapiens GN=MAGT1 PE=1 SV=1 - [MAGT1_HUMAN]	-1.33	-1.53	-1.63	-1.83	-0.05	-0.26	-0.25	-0.45	-0.26	-0.06	0.13	-0.07	1.14	1.47	1.76	1.30	1.61	1.26	1.56	1.45
P02462	Collagen alpha-1(IV) chain OS=Homo sapiens GN=COL4A1 PE=1 SV=3 - [CO4A1_HUMAN]	-1.28	-1.33	-1.86	-1.82	0.46	0.37	-0.63	-0.57	-0.10	-0.15	-0.30	-0.28	0.97	1.30	1.40	1.75	1.80	2.12	2.23	1.20
P61626	Lysozyme C OS=Homo sapiens GN=LYZ PE=1 SV=1 - [LYSC_HUMAN]	-1.48	-1.41	-1.96	-1.81	-0.67	-0.67	-0.04	0.10	-0.11	-0.25	-0.26	-0.28	1.55	1.18	1.62	1.28	1.68	0.81	1.11	1.97
Q8N129	Protein canopy homolog 4 OS=Homo sapiens GN=CNPY4 PE=2 SV=1 - [CNPY4_HUMAN]	-1.22	-1.19	-1.48	-1.81	-0.02	-0.15	-0.56	-0.66	-0.17	-0.19	-0.30	-0.01	0.72	0.93	1.08	0.96	1.32	1.19	1.39	1.00
Q9NVD7	Alpha-parvin OS=Homo sapiens GN=PARVA PE=1 SV=1 - [PARVA_HUMAN]	-1.70	-1.77	-1.64	-1.73	-0.21	-0.26	-0.42	-0.38	-0.32	-0.35	-0.77	-0.69	1.40	1.00	0.79	1.39	1.11	1.45	1.16	1.23
Q96AP7	Endothelial cell-selective adhesion molecule OS=Homo sapiens GN=ESAM PE=1 SV=1 - [ESAM_HUMAN]	-1.81	-1.48	-1.88	-1.67	-0.75	-0.53	-1.24	-0.93	-0.72	-0.57	-0.32	-0.12	0.67	1.46	1.46	1.22	1.12	0.94	1.29	1.00

P08311	Cathepsin G OS=Homo sapiens GN=CTSG PE=1 SV=2 - [CATG_HUMAN]	-1.62	-1.47	-1.70	-1.66	-0.12	-0.04	1.01	1.13	0.68	0.68	0.42	0.37	2.94	1.75	2.04	2.18	2.54	1.41	1.51	2.85
P11166	Solute carrier family 2, facilitated glucose transporter member 1 OS=Homo sapiens GN=SLC2A1 PE=1 SV=2 - [GTR1_HUMAN]	-1.50	-1.51	-1.69	-1.63	0.14	-0.05	0.01	-0.09	0.35	0.41	0.98	0.98	1.56	2.47	2.62	1.73	1.95	1.54	1.57	1.82
P00167	Cytochrome b5 OS=Homo sapiens GN=CYB5A PE=1 SV=2 - [CYB5_HUMAN]	-1.80	-1.63	-1.73	-1.53	0.07	0.12	-0.69	-0.59	-0.16	-0.25	0.12	0.24	1.16	2.05	1.85	1.58	1.60	1.85	1.64	1.10
P08294	Extracellular superoxide dismutase [Cu-Zn] OS=Homo sapiens GN=SOD3 PE=1 SV=2 - [SODE_HUMAN]	-1.36	-1.33	-1.48	-1.53	-0.05	-0.20	-0.63	-0.54	-0.66	-0.70	-0.48	-0.46	0.75	1.16	1.38	0.62	0.95	1.29	1.61	1.19
Q9BQI0	Allograft inflammatory factor 1-like OS=Homo sapiens GN=AIF1L PE=1 SV=1 - [AIF1L_HUMAN]	-1.95	-1.98	-1.51	-1.52	-1.46	-1.38	-1.04	-1.04	-0.92	-1.15	-0.94	-0.89	0.80	1.01	0.92	1.02	0.82	0.48	0.23	0.55
Q9BU23	Lipase maturation factor 2 OS=Homo sapiens GN=LMF2 PE=1 SV=2 - [LMF2_HUMAN]	-1.24	-1.33	-1.36	-1.48	0.11	-0.02	-0.27	-0.49	0.03	0.23	0.01	-0.08	1.02	1.54	1.46	1.51	1.39	1.53	1.47	1.10
O75718	Cartilage-associated protein OS=Homo sapiens GN=CRTAP PE=1 SV=1 - [CRTAP_HUMAN]	-1.11	-1.08	-1.52	-1.48	0.16	0.19	-0.49	-0.46	-0.09	-0.11	-0.02	0.01	0.68	1.10	1.50	1.03	1.44	1.26	1.66	1.09
P02652	Apolipoprotein A-II OS=Homo sapiens GN=APOA2 PE=1 SV=1 - [APOA2_HUMAN]	-1.43	-1.25	-1.63	-1.47	-0.12	0.03	-0.72	-0.54	-0.37	-0.52	-0.50	-0.34	0.83	0.91	1.13	0.96	1.14	1.27	1.49	1.00
Q07507	Dermatopontin OS=Homo sapiens GN=DPT PE=2 SV=2 - [DERM_HUMAN]	-1.25	-1.12	-1.56	-1.43	0.13	0.59	-2.43	-2.16	-0.53	-0.66	-1.30	-1.02	-0.49	-0.29	0.42	0.39	0.69	0.94	1.24	-0.05
P28290	Sperm-specific antigen 2 OS=Homo sapiens GN=SSFA2 PE=1 SV=3 - [SSFA2_HUMAN]	-1.08	-1.16	-1.27	-1.35	0.08	0.00	-0.49	-0.56	-0.43	-0.34	0.09	0.01	0.65	1.18	1.36	0.77	0.96	1.15	1.34	0.85

P07305	Histone H1.0 OS=Homo sapiens GN=H1F0 PE=1 SV=3 - [H10_HUMAN]	-1.44	-1.43	-1.29	-1.34	-1.13	-1.18	-0.84	-0.89	-1.06	-0.97	-0.70	-0.74	0.60	0.76	0.58	0.43	0.31	0.24	0.21	0.55
P51571	Translocon-associated protein subunit delta OS=Homo sapiens GN=SSR4 PE=1 SV=1 - [SSRD_HUMAN]	-1.55	-1.41	-1.46	-1.32	0.34	0.11	-0.57	-0.43	-0.13	-0.26	-0.20	-0.06	0.98	1.12	1.27	1.32	1.23	1.51	1.42	0.96
P02763	Alpha-1-acid glycoprotein 1 OS=Homo sapiens GN=ORM1 PE=1 SV=1 - [A1AG1_HUMAN]	-1.34	-1.37	-1.32	-1.28	-0.84	-0.83	-0.26	-0.15	-0.48	-0.47	-0.52	-0.49	1.22	0.73	0.63	0.76	0.84	0.52	0.31	1.13
Q9Y6F9	Protein Wnt-6 OS=Homo sapiens GN=WNT6 PE=1 SV=2 - [WNT6_HUMAN]	-1.33	-1.56	-1.18	-1.23	0.73	0.67	0.80	0.75	0.82	0.88	0.83	0.77	1.95	1.92	2.31	2.00	2.20	1.80	2.29	2.19
Q92625	Ankyrin repeat and SAM domain-containing protein 1A OS=Homo sapiens GN=ANKS1A PE=1 SV=4 - [ANS1A_HUMAN]	-1.07	-1.11	-1.21	-1.21	0.17	0.16	-0.11	-0.10	-0.10	-0.09	0.12	0.13	1.01	1.06	1.30	1.01	1.07	1.01	1.25	1.26
Q8WVM7	Cohesin subunit SA-1 OS=Homo sapiens GN=STAG1 PE=1 SV=3 - [STAG1_HUMAN]	-1.38	-1.32	-1.26	-1.19	-0.23	-0.17	-0.50	-0.44	-0.43	-0.49	-0.17	-0.12	0.94	1.21	1.08	0.92	0.80	1.14	1.01	0.82
Q9P2U8	Vesicular glutamate transporter 2 OS=Homo sapiens GN=SLC17A6 PE=1 SV=1 - [VGLU2_HUMAN]	2.33	2.47	2.83	2.95	-1.32	-0.56	1.29	1.43	0.16	-0.37	0.37	0.02	-0.73	-2.00	-2.32	-1.64	-1.86	-2.03	-2.13	-0.89
P01303	Pro-neuropeptide Y OS=Homo sapiens GN=NPY PE=1 SV=1 - [NPY_HUMAN]	3.03	2.67	3.19	2.68	0.68	0.81	1.83	1.99	1.51	1.39	0.78	0.68	-0.94	-1.78	-1.99	-1.48	-1.68	-2.14	-2.34	-1.13
C9J069	Uncharacterized protein C9orf172 OS=Homo sapiens GN=C9orf172 PE=3 SV=1 - [CI172_HUMAN]	2.51	2.43	2.57	2.49	0.49	0.41	0.90	0.83	0.56	0.64	0.60	0.52	-1.55	-1.90	-1.97	-1.84	-1.89	-2.03	-2.09	-1.60
P48547	Potassium voltage-gated channel subfamily C member 1 OS=Homo sapiens GN=KCNC1 PE=2 SV=1 - [KCNC1_HUMAN]	2.93	2.63	2.77	2.48	0.27	-0.03	1.50	1.20	0.76	1.06	0.67	0.37	-1.38	-2.25	-2.10	-1.84	-1.68	-2.68	-2.52	-1.21

Q13972	Ras-specific guanine nucleotide-releasing factor 1 OS=Homo sapiens GN=RASGRF1 PE=1 SV=2 - [RGRF1_HUMAN]	1.80	1.93	1.94	2.33	-0.08	0.21	0.35	0.74	0.68	0.40	-0.10	0.18	-1.33	-1.69	-1.55	-1.39	-1.40	-1.92	-1.93	-1.53
Q14003	Potassium voltage-gated channel subfamily C member 3 OS=Homo sapiens GN=KCNC3 PE=1 SV=3 - [KCNC3_HUMAN]	2.10	2.24	2.17	2.31	-0.03	0.10	1.16	1.30	0.99	0.85	0.10	0.23	-0.88	-1.99	-2.07	-1.22	-1.29	-2.15	-2.22	-0.95
O60299	Leucine zipper putative tumor suppressor 3 OS=Homo sapiens GN=LZTS3 PE=2 SV=1 - [LZTS3_HUMAN]	2.25	2.38	2.17	2.30	-0.66	-0.53	0.50	0.64	0.15	0.02	-0.40	-0.27	-1.69	-2.65	-2.57	-2.20	-2.12	-2.93	-2.85	-1.60
Q96QE2	Proton myo-inositol cotransporter OS=Homo sapiens GN=SLC2A13 PE=1 SV=3 - [MYCT_HUMAN]	1.48	1.80	1.75	2.24	-0.09	0.14	0.52	0.91	0.44	0.27	0.01	0.45	-0.88	-1.44	-1.65	-1.20	-1.48	-1.59	-2.03	-1.02
Q6UXD5	Seizure 6-like protein 2 OS=Homo sapiens GN=SEZ6L2 PE=1 SV=2 - [SE6L2_HUMAN]	1.82	2.08	1.89	2.15	0.28	0.90	0.60	0.86	1.05	0.80	0.75	1.37	-1.09	-0.75	-0.76	-0.99	-1.06	-1.23	-1.24	-1.22
Q9P0K9	DOMON domain-containing protein FRRS1L OS=Homo sapiens GN=FRRS1L PE=2 SV=2 - [FRS1L_HUMAN]	1.60	1.93	1.77	2.10	0.34	0.66	0.51	0.84	0.87	0.54	0.48	0.80	-1.04	-1.12	-1.30	-1.03	-1.20	-1.28	-1.45	-1.20
Q8IWT1	Sodium channel subunit beta-4 OS=Homo sapiens GN=SCN4B PE=1 SV=1 - [SCN4B_HUMAN]	1.55	1.61	1.97	2.03	-0.33	-0.28	0.76	0.82	0.30	0.25	-0.30	-0.25	-0.74	-1.85	-2.27	-1.27	-1.69	-1.90	-2.32	-1.15
O60883	Prosaposin receptor GPR37L1 OS=Homo sapiens GN=GPR37L1 PE=1 SV=2 - [ETBR2_HUMAN]	1.68	1.68	1.95	1.95	0.05	0.05	1.32	1.32	0.99	1.00	0.79	0.78	-0.33	-0.41	-1.00	-0.65	-0.66	-0.80	-1.39	-0.52

Q8N336	ELMO domain-containing protein 1 OS=Homo sapiens GN=ELMOD1 PE=2 SV=3 - [ELMD1_HUMAN]	1.72	1.73	1.92	1.93	-0.09	0.02	0.57	0.58	0.30	0.18	0.03	0.07	-1.10	-2.05	-2.31	-1.87	-2.11	-1.61	-1.80	-1.30
O60262	Guanine nucleotide-binding protein G(I)/G(S)/G(O) subunit gamma-7 OS=Homo sapiens GN=GNG7 PE=1 SV=1 - [GBG7_HUMAN]	1.47	1.63	1.95	1.92	-0.12	-0.10	0.44	0.44	0.27	0.15	0.04	0.32	-1.00	-1.21	-1.73	-1.40	-1.59	-1.71	-2.04	-1.42
Q16890	Tumor protein D53 OS=Homo sapiens GN=TPD52L1 PE=1 SV=1 - [TPD53_HUMAN]	2.03	1.56	2.43	1.89	0.94	0.54	1.37	1.08	0.50	1.00	0.86	0.62	-0.48	-1.17	-1.58	-1.02	-1.40	-1.03	-1.21	-0.88
P84085	ADP-ribosylation factor 5 OS=Homo sapiens GN=ARF5 PE=1 SV=2 - [ARF5_HUMAN]	1.72	1.89	1.78	1.77	0.52	0.13	0.69	0.67	0.28	0.27	0.12	-0.12	-1.05	-1.61	-1.94	-1.43	-1.34	-1.48	-1.55	-0.99
Q8NBF6	Late secretory pathway protein AVL9 homolog OS=Homo sapiens GN=AVL9 PE=1 SV=1 - [AVL9_HUMAN]	1.57	1.93	1.40	1.76	-0.38	-0.02	0.32	0.68	0.54	0.18	0.12	0.47	-1.20	-1.45	-1.29	-1.36	-1.19	-1.96	-1.79	-1.02
Q13885	Tubulin beta-2A chain OS=Homo sapiens GN=TUBB2A PE=1 SV=1 - [TBB2A_HUMAN]	1.34	1.52	1.86	1.76	-0.41	-0.48	0.12	0.22	-0.12	-0.05	-0.14	-0.18	-1.12	-1.24	-1.89	-1.38	-1.73	-1.68	-1.71	-1.46
Q9H8M1	Coenzyme Q-binding protein COQ10 homolog B, mitochondrial OS=Homo sapiens GN=COQ10B PE=2 SV=1 - [CQ10B_HUMAN]	1.07	1.67	1.07	1.76	0.02	0.01	0.34	0.72	0.53	0.16	-0.05	0.13	-0.74	-1.48	-1.15	-1.16	-0.88	-1.17	-1.25	-0.82
Q8N7J2	APC membrane recruitment protein 2 OS=Homo sapiens GN=AMER2 PE=1 SV=3 - [AMER2_HUMAN]	1.36	1.39	1.96	1.73	-0.81	-0.31	-0.02	0.36	0.10	-0.49	-0.38	0.13	-0.97	-1.25	-1.59	-1.25	-1.59	-2.19	-2.79	-1.30
Q8TBF8	Protein FAM81A OS=Homo sapiens GN=FAM81A PE=2 SV=3 - [FA81A_HUMAN]	1.69	2.03	1.32	1.66	-0.23	0.10	0.32	0.66	0.52	0.19	-0.15	0.18	-0.57	-1.84	-1.47	-0.60	-0.25	-0.74	-0.40	-0.22

Q9NZH0	G-protein coupled receptor family C group 5 member B OS=Homo sapiens GN=GPRC5B PE=2 SV=2 - [GPC5B_HUMAN]	1.75	1.55	1.81	1.65	0.20	0.12	0.77	0.62	0.31	0.67	0.82	0.63	-0.84	-0.84	-0.87	-1.07	-1.09	-1.43	-1.55	-0.97
P05408	Neuroendocrine protein 7B2 OS=Homo sapiens GN=SCG5 PE=1 SV=2 - [7B2_HUMAN]	1.58	1.85	1.23	1.63	0.01	-0.08	0.51	0.74	0.37	0.33	0.37	0.35	-0.96	-1.07	-1.14	-0.90	-1.03	-1.52	-1.45	-0.99
Q9C026	E3 ubiquitin-protein ligase TRIM9 OS=Homo sapiens GN=TRIM9 PE=1 SV=1 - [TRIM9_HUMAN]	1.68	1.74	1.54	1.60	0.80	0.98	0.03	0.09	0.13	0.07	-0.36	-0.30	-1.31	-2.03	-1.90	-1.89	-2.14	-0.21	-0.15	-1.65
Q86WG3	Caytaxin OS=Homo sapiens GN=ATCAY PE=1 SV=2 - [ATCAY_HUMAN]	1.59	1.73	1.44	1.58	-0.43	-0.30	0.20	0.34	-0.08	-0.21	0.19	0.32	-1.01	-1.39	-1.25	-1.77	-1.62	-2.04	-1.90	-0.62
P10114	Ras-related protein Rap-2a OS=Homo sapiens GN=RAP2A PE=1 SV=1 - [RAP2A_HUMAN]	1.55	1.47	1.67	1.55	0.36	0.23	0.93	0.85	0.36	0.51	0.54	0.42	-0.55	-1.06	-1.29	-1.11	-1.19	-1.37	-1.45	-0.65
O14514	Brain-specific angiogenesis inhibitor 1 OS=Homo sapiens GN=BAI1 PE=1 SV=2 - [BAI1_HUMAN]	1.27	1.54	1.27	1.54	1.02	1.29	0.57	0.85	0.91	0.64	0.87	1.14	-0.64	-0.39	-0.40	-0.60	-0.60	-0.27	-0.27	-0.63
Q9NRW1	Ras-related protein Rab-6B OS=Homo sapiens GN=RAB6B PE=1 SV=1 - [RAB6B_HUMAN]	1.11	1.15	1.69	1.54	0.06	-0.15	0.71	0.63	0.01	0.18	-0.03	-0.03	-0.35	-1.05	-1.63	-0.99	-1.36	-1.25	-1.42	-0.92
Q9BSU1	UPF0183 protein C16orf70 OS=Homo sapiens GN=C16orf70 PE=1 SV=1 - [CP070_HUMAN]	1.23	1.33	1.40	1.50	-0.38	-0.29	0.43	0.53	0.34	0.25	0.16	0.25	-0.75	-1.07	-1.25	-0.95	-1.13	-1.63	-1.81	-0.91
Q96DA2	Ras-related protein Rab-39B OS=Homo sapiens GN=RAB39B PE=1 SV=1 - [RB39B_HUMAN]	1.80	1.55	1.96	1.50	0.03	-0.26	0.95	0.67	0.18	0.54	0.34	0.74	-0.82	-0.82	-0.76	-1.30	-1.23	-1.79	-1.77	-0.84

Q12840	Kinesin heavy chain isoform 5A OS=Homo sapiens GN=KIF5A PE=1 SV=2 - [KIF5A_HUMAN]	1.58	1.67	1.41	1.50	-0.10	-0.02	0.31	0.39	0.32	0.24	-0.13	-0.06	-1.22	-1.71	-1.55	-1.31	-1.14	-1.70	-1.53	-1.04
Q92529	SHC-transforming protein 3 OS=Homo sapiens GN=SHC3 PE=1 SV=1 - [SHC3_HUMAN]	1.15	1.47	1.26	1.48	0.06	0.11	0.27	0.61	0.18	0.04	-0.03	0.02	-0.83	-1.43	-1.46	-1.43	-1.45	-1.37	-1.38	-0.84
Q96PQ0	VPS10 domain-containing receptor SorCS2 OS=Homo sapiens GN=SORCS2 PE=1 SV=3 - [SORC2_HUMAN]	2.12	1.83	1.76	1.48	0.46	0.17	0.64	0.36	0.23	0.52	0.43	0.14	-1.42	-1.68	-1.33	-1.57	-1.20	-1.67	-1.32	-1.06
Q9BW91	ADP-ribose pyrophosphatase, mitochondrial OS=Homo sapiens GN=NUDT9 PE=1 SV=1 - [NUDT9_HUMAN]	1.16	1.17	1.41	1.42	0.39	0.39	0.46	0.47	0.08	0.07	0.01	0.01	-0.64	-1.15	-1.41	-1.05	-1.31	-0.79	-1.04	-0.89
Q9Y365	PCTP-like protein OS=Homo sapiens GN=STARD10 PE=1 SV=2 - [PCTL_HUMAN]	1.36	1.34	1.09	1.40	0.52	0.94	0.67	0.88	1.27	0.51	0.49	0.70	-0.41	-0.66	-0.60	-0.02	-0.21	-0.40	-0.60	-0.36
Q08623	Pseudouridine-5'-monophosphatase OS=Homo sapiens GN=HDHD1 PE=1 SV=3 - [HDHD1_HUMAN]	2.49	1.42	2.44	1.37	1.28	0.59	1.60	0.53	0.42	1.49	1.27	0.19	-0.83	-0.96	-1.18	-0.96	-1.12	-0.86	-1.18	-0.78
O75914	Serine/threonine-protein kinase PAK 3 OS=Homo sapiens GN=PAK3 PE=1 SV=2 - [PAK3_HUMAN]	1.02	1.07	1.30	1.35	-0.26	-0.21	0.03	0.08	-0.44	-0.48	-0.91	-0.86	-0.94	-1.92	-2.21	-1.47	-1.75	-1.29	-1.58	-1.21
Q15714	TSC22 domain family protein 1 OS=Homo sapiens GN=TSC22D1 PE=1 SV=3 - [T22D1_HUMAN]	1.27	1.48	1.46	1.34	0.23	-0.24	0.57	0.07	0.08	0.00	-0.40	-0.17	-1.21	-1.51	-1.66	-1.36	-1.25	-1.46	-1.37	-1.22
P62072	Mitochondrial import inner membrane translocase subunit Tim10 OS=Homo sapiens GN=TIMM10 PE=1 SV=1 - [TIM10_HUMAN]	1.33	1.27	1.36	1.34	0.02	0.07	0.28	0.34	0.36	0.28	0.40	0.46	-0.81	-0.92	-0.85	-0.90	-0.80	-0.97	-1.06	-0.90

Q5TCQ9	Membrane-associated guanylate kinase, WW and PDZ domain-containing protein 3 OS=Homo sapiens GN=MAGI3 PE=1 SV=2 - [MAGI3_HUMAN]	1.24	1.27	1.30	1.33	0.33	0.35	0.25	0.28	0.25	0.23	0.18	0.20	-0.94	-1.06	-1.12	-0.99	-1.04	-0.93	-0.98	-0.96
O75508	Claudin-11 OS=Homo sapiens GN=CLDN11 PE=1 SV=2 - [CLD11_HUMAN]	1.14	1.02	1.42	1.30	-0.37	-0.36	0.29	0.16	-0.04	-0.15	-0.14	-0.09	-0.86	-1.08	-1.27	-1.18	-1.46	-1.40	-1.68	-1.07
Q9UK76	Hematological and neurological expressed 1 protein OS=Homo sapiens GN=HN1 PE=1 SV=3 - [HN1_HUMAN]	1.50	1.32	1.28	1.28	-0.32	-0.56	-0.37	-0.52	-0.58	-0.66	-0.47	-0.82	-1.96	-2.08	-1.75	-2.18	-1.69	-2.03	-1.64	-1.53
Q15056	Eukaryotic translation initiation factor 4H OS=Homo sapiens GN=EIF4H PE=1 SV=5 - [IF4H_HUMAN]	1.38	1.26	1.20	1.28	-0.02	-0.04	0.37	0.44	0.15	-0.01	-0.18	-0.20	-0.83	-1.49	-1.51	-1.44	-1.15	-1.39	-1.33	-0.86
Q96DB2	Histone deacetylase 11 OS=Homo sapiens GN=HDAC11 PE=1 SV=1 - [HDA11_HUMAN]	1.34	1.54	1.19	1.27	-0.50	-0.23	0.92	0.13	-0.29	-0.09	0.00	-0.43	-0.86	-1.60	-1.69	-1.62	-1.44	-1.71	-1.66	-0.93
Q8WZ74	Cortactin-binding protein 2 OS=Homo sapiens GN=CTTNBP2 PE=1 SV=1 - [CTTB2_HUMAN]	1.62	1.39	1.49	1.26	0.13	-0.11	0.16	-0.08	-0.17	0.07	0.06	-0.18	-1.41	-1.56	-1.43	-1.52	-1.39	-1.51	-1.37	-1.27
Q96MC5	Uncharacterized protein C16orf45 OS=Homo sapiens GN=C16orf45 PE=2 SV=1 - [CP045_HUMAN]	1.35	1.23	1.53	1.25	-0.10	-0.09	0.28	0.22	-0.14	-0.09	0.39	0.08	-1.20	-0.95	-1.18	-1.66	-1.70	-1.57	-1.62	-1.23
P27544	Ceramide synthase 1 OS=Homo sapiens GN=CERS1 PE=1 SV=1 - [CERS1_HUMAN]	1.56	1.31	1.52	1.24	-0.28	-0.02	0.93	0.60	0.34	0.43	0.44	0.28	-0.66	-1.03	-0.96	-0.94	-0.86	-1.35	-1.27	-0.57
Q8N461	F-box/LRR-repeat protein 16 OS=Homo sapiens GN=FBXL16 PE=1 SV=2 - [FXL16_HUMAN]	1.71	1.24	1.71	1.23	-0.38	-0.13	0.55	0.24	0.05	0.15	-0.28	-0.33	-0.94	-1.78	-1.78	-1.25	-1.24	-1.38	-1.38	-0.92

Q8IYB5	Stromal membrane-associated protein 1 OS=Homo sapiens GN=SMAP1 PE=1 SV=2 - [SMAP1_HUMAN]	1.45	1.30	1.31	1.22	0.14	0.02	0.48	0.40	0.25	0.16	0.16	0.12	-0.85	-1.33	-1.25	-1.14	-1.05	-1.19	-1.10	-0.78
P61018	Ras-related protein Rab-4B OS=Homo sapiens GN=RAB4B PE=1 SV=1 - [RAB4B_HUMAN]	1.36	1.26	1.27	1.17	0.39	0.29	0.62	0.53	0.35	0.45	0.38	0.28	-0.68	-0.97	-0.89	-0.87	-0.78	-0.98	-0.89	-0.58
Q9UBM7	7-dehydrocholesterol reductase OS=Homo sapiens GN=DHCR7 PE=1 SV=1 - [DHCR7_HUMAN]	1.42	1.21	1.37	1.16	0.78	0.57	0.68	0.48	0.71	0.92	1.04	0.83	-0.68	-0.37	-0.33	-0.46	-0.41	-0.65	-0.60	-0.62
Q96DA6	Mitochondrial import inner membrane translocase subunit TIM14 OS=Homo sapiens GN=DNAJC19 PE=1 SV=3 - [TIM14_HUMAN]	1.12	1.04	1.22	1.15	-0.29	-0.35	0.29	0.21	-0.16	-0.08	0.07	-0.10	-0.78	-1.23	-1.42	-1.27	-1.42	-1.38	-1.53	-0.93
Q6UUV9	CREB-regulated transcription coactivator 1 OS=Homo sapiens GN=CRTC1 PE=1 SV=2 - [CRTC1_HUMAN]	1.50	1.22	1.63	1.06	0.01	0.25	0.00	0.42	0.62	-0.32	0.06	0.93	-0.76	-0.93	-0.93	-0.84	-0.90	-0.96	-1.21	-1.03
P01833	Polymeric immunoglobulin receptor OS=Homo sapiens GN=PIGR PE=1 SV=4 - [PIGR_HUMAN]	-2.29	-2.40	-3.65	-3.66	-1.86	-1.88	-2.60	-2.62	-2.06	-2.03	-1.65	-1.77	1.35	0.64	1.85	1.89	1.65	2.02	1.77	1.11
P27482	Calmodulin-like protein 3 OS=Homo sapiens GN=CALML3 PE=1 SV=2 - [CALL3_HUMAN]	-2.97	-3.00	-3.43	-3.46	-2.25	-2.06	-2.72	-2.51	-2.85	-2.90	-3.19	-3.19	-0.19	0.00	-0.09	-0.04	0.24	0.20	0.29	-0.09
P05997	Collagen alpha-2(V) chain OS=Homo sapiens GN=COL5A2 PE=1 SV=3 - [CO5A2_HUMAN]	-3.11	-3.21	-3.21	-3.24	-2.00	-2.11	-2.48	-2.63	-2.50	-2.33	-2.31	-2.33	0.47	0.85	0.85	0.74	0.71	1.13	1.19	0.38
Q6UWP8	Suprabasin OS=Homo sapiens GN=SBSN PE=2 SV=2 - [SBSN_HUMAN]	-3.12	-3.06	-3.14	-3.08	-2.17	-2.12	-2.76	-2.71	-2.41	-2.46	-2.22	-2.17	0.41	0.90	0.92	0.69	0.72	0.94	0.96	0.44

Q14644	Ras GTPase-activating protein 3 OS=Homo sapiens GN=RASA3 PE=1 SV=3 - [RASA3_HUMAN]	-1.41	-1.49	-2.98	-3.06	-0.87	-0.95	-1.53	-1.61	-1.19	-1.11	-0.52	-0.60	-0.06	0.90	2.46	0.33	1.90	0.53	2.09	1.51
P12107	Collagen alpha-1(XI) chain OS=Homo sapiens GN=COL11A1 PE=1 SV=4 - [COBA1_HUMAN]	-2.64	-2.78	-2.97	-3.03	-1.44	-1.51	-1.88	-1.94	-1.88	-1.73	-1.93	-2.11	0.71	0.52	0.88	0.90	1.19	1.07	1.51	1.16
P62736	Actin, aortic smooth muscle OS=Homo sapiens GN=ACTA2 PE=1 SV=1 - [ACTA_HUMAN]	-2.10	-2.20	-2.56	-2.79	-0.68	-0.83	-1.20	-1.28	-1.13	-1.01	-0.57	-0.66	1.22	1.46	1.93	1.43	1.65	1.58	2.01	1.56
Q9BVG9	Phosphatidylserine synthase 2 OS=Homo sapiens GN=PTDSS2 PE=1 SV=1 - [PTSS2_HUMAN]	-2.43	-2.42	-2.61	-2.60	-1.23	-1.22	-1.65	-1.63	-1.42	-1.43	-1.42	-1.41	0.84	1.02	1.19	1.04	1.22	1.18	1.36	1.03
P08246	Neutrophil elastase OS=Homo sapiens GN=ELANE PE=1 SV=1 - [ELNE_HUMAN]	-2.63	-2.47	-2.69	-2.55	-1.78	-1.68	0.95	1.15	0.43	0.32	-0.21	0.01	3.58	2.37	2.57	2.89	3.02	1.01	0.86	3.77
P23435	Cerebellin-1 OS=Homo sapiens GN=CBLN1 PE=1 SV=1 - [CBLN1_HUMAN]	-2.41	-2.35	-2.58	-2.51	-1.34	-1.28	-0.46	-0.39	-0.76	-0.82	-0.50	-0.44	2.01	1.91	2.07	1.62	1.79	1.05	1.22	2.18
Q9BX79	Stimulated by retinoic acid gene 6 protein homolog OS=Homo sapiens GN=STRA6 PE=1 SV=1 - [STRA6_HUMAN]	-2.35	-2.29	-2.47	-2.41	-0.72	-0.66	-0.95	-0.89	-0.69	-0.74	-0.66	-0.60	1.45	1.70	1.81	1.64	1.76	1.61	1.74	1.58
P08493	Matrix Gla protein OS=Homo sapiens GN=MGP PE=1 SV=2 - [MGP_HUMAN]	-2.35	-2.18	-2.60	-2.35	0.97	0.95	-0.92	-0.84	0.37	0.38	0.68	0.63	1.35	3.28	3.12	2.75	2.92	3.35	3.45	1.53
Q6UW02	Cytochrome P450 20A1 OS=Homo sapiens GN=CYP20A1 PE=2 SV=1 - [CP20A_HUMAN]	-2.46	-2.40	-2.36	-2.30	-0.02	0.04	-0.43	-0.37	-0.11	-0.17	0.02	0.07	2.09	2.48	2.38	2.32	2.23	2.42	2.33	2.00
O75084	Frizzled-7 OS=Homo sapiens GN=FZD7 PE=1 SV=2 - [FZD7_HUMAN]	-2.00	-1.70	-2.41	-2.24	0.82	1.11	0.19	0.49	0.57	0.28	0.22	0.29	2.24	1.77	2.31	2.31	2.70	2.80	2.91	2.37

Q9P299	Coatomer subunit zeta-2 OS=Homo sapiens GN=COPZ2 PE=2 SV=1 - [COPZ2_HUMAN]	-1.99	-1.90	-2.33	-2.24	-0.07	0.02	-0.41	-0.32	-0.29	-0.37	-0.70	-0.61	1.63	1.30	1.63	1.64	1.99	1.90	2.25	1.98
Q9UGP4	LIM domain-containing protein 1 OS=Homo sapiens GN=LIMD1 PE=1 SV=1 - [LIMD1_HUMAN]	-2.10	-1.99	-2.28	-2.17	-1.27	-1.16	-1.13	-1.02	-0.91	-1.01	-0.74	-0.63	1.02	1.37	1.54	1.12	1.30	0.82	0.99	1.21
Q96H79	Zinc finger CCCH-type antiviral protein 1-like OS=Homo sapiens GN=ZC3HAV1L PE=1 SV=2 - [ZCCHL_HUMAN]	-3.04	-2.66	-2.55	-2.17	-0.97	-0.60	-0.81	-0.43	-0.62	-0.99	-0.68	-0.30	2.29	2.37	1.87	2.08	1.59	2.05	1.56	1.81
P02766	Transthyretin OS=Homo sapiens GN=TTR PE=1 SV=1 - [TTHY_HUMAN]	-1.26	-1.17	-2.18	-2.13	0.47	0.65	0.43	0.37	0.35	0.44	0.23	0.22	1.59	1.48	2.44	1.89	2.65	1.80	2.77	2.57
P60903	Protein S100-A10 OS=Homo sapiens GN=S100A10 PE=1 SV=2 - [S10AA_HUMAN]	-2.23	-2.23	-2.13	-2.12	-0.40	-0.31	-0.86	-0.75	-0.49	-0.56	-0.12	-0.02	1.44	2.09	2.03	1.72	1.72	1.78	1.72	1.39
Q92738	USP6 N-terminal-like protein OS=Homo sapiens GN=USP6NL PE=1 SV=3 - [US6NL_HUMAN]	-1.75	-2.10	-1.73	-2.07	-0.36	-0.71	-0.47	-0.81	-0.68	-0.33	-0.49	-0.84	1.23	1.27	1.23	1.45	1.43	1.38	1.35	1.24
Q7Z4I7	LIM and senescent cell antigen-like-containing domain protein 2 OS=Homo sapiens GN=LIMS2 PE=1 SV=1 - [LIMS2_HUMAN]	-2.03	-2.19	-1.84	-2.03	-0.09	-0.20	-0.70	-0.64	-0.49	-0.63	-0.57	-0.70	1.39	1.58	1.46	1.73	1.61	1.93	1.74	1.21
Q8N118	Cytochrome P450 4X1 OS=Homo sapiens GN=CYP4X1 PE=2 SV=1 - [CP4X1_HUMAN]	-1.43	-1.09	-2.04	-2.00	0.15	0.31	-0.31	-0.27	0.14	-0.02	0.14	0.32	0.59	1.57	2.32	1.24	2.27	1.70	2.15	1.34
P49913	Cathelicidin antimicrobial peptide OS=Homo sapiens GN=CAMP PE=1 SV=1 - [CAMP_HUMAN]	-2.36	-2.07	-2.29	-2.00	-0.34	-0.06	1.22	1.51	0.99	0.70	0.89	1.18	3.63	3.26	3.18	3.09	3.02	2.00	1.93	3.57

O76074	cGMP-specific 3',5'-cyclic phosphodiesterase OS=Homo sapiens GN=PDE5A PE=1 SV=2 - [PDE5A_HUMAN]	-1.87	-1.52	-1.84	-1.99	-0.30	-0.23	-0.48	-0.58	-0.45	-0.36	-0.58	-0.74	1.45	1.61	1.37	1.54	1.58	1.52	1.75	1.48
Q9NYL2	Mitogen-activated protein kinase kinase kinase MLT OS=Homo sapiens GN=ZAK PE=1 SV=3 - [MLTK_HUMAN]	-1.78	-1.69	-2.07	-1.98	-0.64	-0.56	-0.84	-0.75	-0.54	-0.62	-0.53	-0.45	0.99	1.25	1.54	1.19	1.49	1.12	1.41	1.29
P05783	Keratin, type I cytoskeletal 18 OS=Homo sapiens GN=KRT18 PE=1 SV=2 - [K1C18_HUMAN]	-2.30	-1.96	-2.25	-1.91	0.62	0.96	-0.43	-0.09	0.43	0.09	0.84	1.17	1.92	3.14	3.09	2.42	2.38	2.90	2.85	1.88
Q8TED1	Probable glutathione peroxidase 8 OS=Homo sapiens GN=GPX8 PE=1 SV=2 - [GPX8_HUMAN]	-1.84	-1.89	-1.83	-1.87	0.15	0.10	-0.19	-0.23	-0.02	0.04	0.27	0.22	1.71	2.11	2.09	1.91	1.90	1.98	1.96	1.70
Q53TN4	Cytochrome b reductase 1 OS=Homo sapiens GN=CYBRD1 PE=1 SV=1 - [CYBR1_HUMAN]	-1.84	-1.62	-2.00	-1.81	-0.32	-0.31	0.09	0.05	-0.08	-0.06	0.29	0.33	1.93	2.42	2.22	1.88	1.92	1.81	1.59	1.97
P47712	Cytosolic phospholipase A2 OS=Homo sapiens GN=PLA2G4A PE=1 SV=2 - [PA24A_HUMAN]	-1.74	-1.63	-1.92	-1.81	-0.70	-0.59	-0.99	-0.87	-0.36	-0.47	-0.47	-0.36	0.81	1.28	1.45	1.30	1.48	1.03	1.21	1.00
Q9NSD5	Sodium- and chloride-dependent GABA transporter 2 OS=Homo sapiens GN=SLC6A13 PE=1 SV=3 - [S6A13_HUMAN]	-1.78	-1.61	-1.92	-1.77	0.12	0.21	-0.10	0.00	0.00	0.03	-0.03	-0.10	1.68	1.82	2.08	1.78	2.05	1.88	2.13	2.05
Q8NFW1	Collagen alpha-1(XXII) chain OS=Homo sapiens GN=COL22A1 PE=1 SV=2 - [COMA1_HUMAN]	-3.85	-1.10	-4.50	-1.75	-1.94	0.81	-3.21	-0.47	-0.20	-2.94	-2.93	-0.19	0.69	0.93	1.57	0.94	1.59	1.90	2.54	1.35
P02776	Platelet factor 4 OS=Homo sapiens GN=PF4 PE=1 SV=2 - [PLF4_HUMAN]	-1.82	-1.84	-1.72	-1.75	0.21	0.18	-0.50	-0.52	-0.05	-0.02	0.38	0.35	1.37	2.20	2.10	1.82	1.74	2.00	1.91	1.29

O95980	Reversion-inducing cysteine-rich protein with Kazal motifs OS=Homo sapiens GN=RECK PE=1 SV=1 - [RECK_HUMAN]	-2.37	-2.37	-1.94	-1.71	-0.24	-0.02	-0.51	-0.22	-0.28	-0.26	0.04	0.26	1.66	2.15	1.98	2.12	1.48	1.88	1.68	1.57
Q8N5C1	Protein FAM26E OS=Homo sapiens GN=FAM26E PE=2 SV=1 - [FA26E_HUMAN]	-1.57	-1.52	-1.73	-1.68	0.78	0.83	-0.12	-0.07	0.27	0.23	0.21	0.25	1.51	1.78	1.93	1.83	1.99	2.33	2.49	1.67
P55001	Microfibrillar-associated protein 2 OS=Homo sapiens GN=MFAP2 PE=2 SV=1 - [MFAP2_HUMAN]	-2.41	-2.39	-1.88	-1.68	0.94	0.78	0.48	0.37	0.46	0.62	0.28	0.15	2.71	2.51	1.60	2.66	1.89	3.15	2.31	1.88
P50281	Matrix metalloproteinase-14 OS=Homo sapiens GN=MMP14 PE=1 SV=3 - [MMP14_HUMAN]	-1.39	-1.44	-1.61	-1.66	0.46	0.40	-0.12	-0.17	0.23	0.29	0.18	0.12	1.32	1.57	1.79	1.70	1.93	1.82	2.05	1.55
Q9NR34	Mannosyl-oligosaccharide 1,2-alpha-mannosidase IC OS=Homo sapiens GN=MAN1C1 PE=2 SV=1 - [MA1C1_HUMAN]	-1.42	-1.34	-1.72	-1.64	0.05	0.13	-0.31	-0.23	0.17	0.10	0.10	0.18	1.16	1.52	1.82	1.54	1.85	1.45	1.75	1.47
P50224	Sulfotransferase 1A3/1A4 OS=Homo sapiens GN=SULT1A3 PE=1 SV=1 - [ST1A3_HUMAN]	-2.19	-1.77	-2.06	-1.64	-0.53	-0.11	-1.09	-0.66	-0.59	-1.01	-0.59	-0.17	1.16	1.61	1.47	1.21	1.09	1.65	1.52	1.04
O95183	Vesicle-associated membrane protein 5 OS=Homo sapiens GN=VAMP5 PE=1 SV=1 - [VAMP5_HUMAN]	-1.37	-1.48	-1.64	-1.63	0.21	0.32	-0.24	-0.14	0.13	0.07	0.35	0.29	1.49	1.84	1.55	1.61	1.73	1.89	1.83	1.40
P02100	Hemoglobin subunit epsilon OS=Homo sapiens GN=HBE1 PE=1 SV=2 - [HBE_HUMAN]	-1.64	-1.60	-1.67	-1.62	1.58	1.62	0.38	0.43	1.24	1.19	1.52	1.57	2.08	3.17	3.19	2.87	2.90	3.20	3.23	2.11
Q96PE1	G-protein coupled receptor 124 OS=Homo sapiens GN=GPR124 PE=1 SV=2 - [GP124_HUMAN]	-1.19	-1.22	-1.59	-1.61	0.01	-0.02	-0.37	-0.39	-0.08	-0.05	0.19	0.16	0.88	1.39	1.78	1.17	1.57	1.19	1.58	1.28

O60568	Procollagen-lysine,2-oxoglutarate 5-dioxygenase 3 OS=Homo sapiens GN=PLOD3 PE=1 SV=1 - [PLOD3_HUMAN]	-1.11	-1.14	-1.57	-1.59	0.09	0.08	-0.75	-0.68	-0.04	-0.11	-0.14	-0.36	0.22	0.55	0.99	1.01	1.46	0.74	1.19	0.68
Q9BR39	Junctophilin-2 OS=Homo sapiens GN=JPH2 PE=1 SV=2 - [JPH2_HUMAN]	-1.81	-2.06	-1.28	-1.53	-0.29	-0.75	-0.02	-0.47	-0.95	-0.49	0.02	-0.44	1.16	1.29	0.76	1.11	0.58	0.91	0.38	0.64
Q99944	Epidermal growth factor-like protein 8 OS=Homo sapiens GN=EGFL8 PE=1 SV=1 - [EGFL8_HUMAN]	-1.70	-1.41	-1.77	-1.48	0.28	0.57	0.22	0.51	0.65	0.37	0.45	0.74	1.97	2.16	2.22	2.09	2.17	1.96	2.03	2.05
Q9NQ84	G-protein coupled receptor family C group 5 member C OS=Homo sapiens GN=GPRC5C PE=1 SV=2 - [GPC5C_HUMAN]	-1.13	-1.25	-1.34	-1.44	0.18	0.06	-0.34	-0.36	-0.03	-0.01	0.32	0.24	1.08	0.97	1.65	1.39	1.45	1.29	1.48	1.15
P53420	Collagen alpha-4(IV) chain OS=Homo sapiens GN=COL4A4 PE=1 SV=3 - [CO4A4_HUMAN]	-1.20	-1.06	-1.55	-1.41	-0.25	-0.12	-0.24	-0.10	-0.33	-0.47	-0.96	-0.83	1.02	0.24	0.59	0.76	1.12	0.93	1.29	1.38
Q96G23	Ceramide synthase 2 OS=Homo sapiens GN=CERS2 PE=1 SV=1 - [CERS2_HUMAN]	-1.11	-1.11	-1.38	-1.38	0.21	0.24	-0.08	-0.06	0.02	-0.12	0.01	-0.13	1.26	1.30	1.15	1.19	1.29	1.14	1.57	1.24
P02753	Retinol-binding protein 4 OS=Homo sapiens GN=RBP4 PE=1 SV=3 - [RET4_HUMAN]	-1.57	-1.43	-1.37	-1.35	-0.67	-0.58	-0.22	0.21	-0.29	-0.30	0.36	0.42	1.51	1.78	1.73	1.28	1.09	0.82	0.75	1.44
P49790	Nuclear pore complex protein Nup153 OS=Homo sapiens GN=NUP153 PE=1 SV=2 - [NU153_HUMAN]	-1.32	-1.40	-1.24	-1.33	-0.78	-0.87	-1.23	-1.32	-0.88	-0.78	-0.11	-0.20	0.14	1.21	1.13	0.56	0.49	0.52	0.45	0.08
P00739	Haptoglobin-related protein OS=Homo sapiens GN=HPR PE=1 SV=2 - [HPTR_HUMAN]	-1.57	-1.55	-1.32	-1.31	-0.58	-0.62	-0.11	-0.15	-0.59	-0.54	-0.63	-0.68	1.20	1.93	1.22	1.66	1.34	1.78	1.26	1.06

P59665	Neutrophil defensin 1 OS=Homo sapiens GN=DEFA1 PE=1 SV=1 - [DEF1_HUMAN]	-1.63	-1.66	-1.60	-1.29	-0.75	-0.54	1.96	1.99	1.49	1.24	1.08	1.29	3.64	2.98	2.46	3.20	2.65	0.76	0.90	3.08
B9A064	Immunoglobulin lambda-like polypeptide 5 OS=Homo sapiens GN=IGLL5 PE=2 SV=2 - [IGLL5_HUMAN]	-1.56	-1.24	-1.21	-1.26	0.52	0.44	0.14	-0.03	0.38	0.66	1.69	1.54	1.55	3.15	2.97	2.08	1.81	1.72	1.69	1.37
P02745	Complement C1q subcomponent subunit A OS=Homo sapiens GN=C1QA PE=1 SV=2 - [C1QA_HUMAN]	-1.42	-1.38	-1.28	-1.24	1.04	1.07	0.37	0.41	0.74	0.70	1.07	1.10	1.84	2.49	2.23	2.15	1.81	2.44	2.19	1.65
Q9BYM8	RanBP-type and C3HC4-type zinc finger-containing protein 1 OS=Homo sapiens GN=RBCK1 PE=1 SV=2 - [HOIL1_HUMAN]	-1.39	-1.45	-1.14	-1.15	-0.11	-0.03	-0.43	-0.48	-0.26	-0.21	-0.17	-0.23	0.47	0.69	0.92	0.91	0.92	0.92	1.01	0.73
Q96BW5	Phosphotriesterase-related protein OS=Homo sapiens GN=PTER PE=1 SV=1 - [PTER_HUMAN]	-1.99	-1.82	-1.29	-1.13	-0.38	-0.22	0.10	0.27	-0.07	-0.23	0.48	0.64	2.14	2.47	1.77	1.78	1.09	1.59	0.90	1.46
Q8N584	Tetratricopeptide repeat protein 39C OS=Homo sapiens GN=TTC39C PE=2 SV=2 - [TT39C_HUMAN]	2.07	2.65	2.18	2.75	-0.23	0.34	1.12	1.69	0.97	0.40	0.07	0.64	-0.90	-1.99	-2.11	-1.64	-1.74	-2.32	-2.42	-0.99
P29323	Ephrin type-B receptor 2 OS=Homo sapiens GN=EPHB2 PE=1 SV=5 - [EPHB2_HUMAN]	2.29	2.72	2.17	2.60	-0.32	0.11	0.55	0.98	0.50	0.08	0.12	0.55	-1.68	-2.16	-2.05	-2.18	-2.06	-2.62	-2.51	-1.56
Q6ZWB6	BTB/POZ domain-containing protein KCTD8 OS=Homo sapiens GN=KCTD8 PE=2 SV=1 - [KCTD8_HUMAN]	2.55	2.42	2.71	2.58	0.02	0.08	1.36	1.23	0.64	0.78	0.42	0.28	-1.14	-2.12	-2.29	-1.74	-1.90	-2.53	-2.51	-1.29
Q8WY91	THAP domain-containing protein 4 OS=Homo sapiens GN=THAP4 PE=1 SV=2 - [THAP4_HUMAN]	3.38	2.53	3.39	2.55	1.50	0.65	1.98	1.13	1.36	2.21	1.82	0.97	-1.35	-1.55	-1.58	-1.14	-1.15	-1.89	-1.91	-1.35

Q9UI40	Sodium/potassium/calcium exchanger 2 OS=Homo sapiens GN=SLC24A2 PE=1 SV=1 - [NCKX2_HUMAN]	2.15	2.45	2.30	2.54	-0.40	-0.10	0.86	1.18	0.87	0.73	0.03	0.31	-1.07	-2.11	-2.22	-1.34	-1.41	-2.57	-2.75	-1.14
Q14416	Metabotropic glutamate receptor 2 OS=Homo sapiens GN=GRM2 PE=1 SV=2 - [GRM2_HUMAN]	2.83	2.79	2.55	2.52	-0.51	-0.55	1.17	1.14	0.82	0.86	0.55	0.51	-1.60	-2.27	-2.00	-1.93	-1.66	-3.35	-3.08	-1.32
P59768	Guanine nucleotide-binding protein G(I)/G(S)/G(O) subunit gamma-2 OS=Homo sapiens GN=GNG2 PE=1 SV=2 - [GBG2_HUMAN]	2.03	2.03	2.50	2.44	0.00	-0.11	1.06	0.73	0.49	0.69	0.64	0.52	-1.07	-1.50	-1.91	-1.31	-1.85	-2.05	-2.69	-1.55
Q9BZC1	CUGBP Elav-like family member 4 OS=Homo sapiens GN=CELF4 PE=1 SV=1 - [CELF4_HUMAN]	1.60	1.76	2.27	2.43	-0.71	-0.56	0.60	0.76	-0.04	-0.19	-0.09	0.06	-0.95	-1.69	-2.36	-1.77	-2.43	-2.33	-3.00	-1.61
Q8TBG9	Synaptoporin OS=Homo sapiens GN=SYNPR PE=2 SV=1 - [SYNPR_HUMAN]	1.27	2.03	1.65	2.42	-0.82	-0.06	0.51	1.28	0.65	-0.11	-0.46	0.30	-0.70	-1.73	-2.12	-1.35	-1.72	-2.11	-2.49	-1.08
Q9H993	UPF0364 protein C6orf211 OS=Homo sapiens GN=C6orf211 PE=1 SV=1 - [CF211_HUMAN]	1.99	2.05	2.18	2.25	0.40	0.46	1.04	1.10	0.54	0.48	0.27	0.33	-0.89	-1.71	-1.92	-1.48	-1.68	-1.61	-1.81	-1.09
Q9NZN1	Interleukin-1 receptor accessory protein-like 1 OS=Homo sapiens GN=IL1RAPL1 PE=1 SV=2 - [IRPL1_HUMAN]	3.44	2.29	3.52	2.24	0.83	-0.05	1.71	0.59	0.68	1.52	1.17	0.33	-1.68	-1.95	-2.05	-1.89	-1.52	-2.62	-2.30	-1.59
Q96DZ9	CKLF-like MARVEL transmembrane domain-containing protein 5 OS=Homo sapiens GN=CMTM5 PE=2 SV=2 - [CKLF5_HUMAN]	1.13	1.88	1.33	2.08	-1.47	-0.72	0.43	1.18	0.66	-0.08	-0.03	0.71	-0.65	-1.16	-1.36	-1.18	-1.38	-2.62	-2.81	-0.83

Q8TAC9	Secretory carrier-associated membrane protein 5 OS=Homo sapiens GN=SCAMP5 PE=1 SV=1 - [SCAM5_HUMAN]	1.93	1.77	2.31	2.07	-0.14	-0.33	0.76	0.74	0.41	0.31	0.20	0.14	-1.05	-1.93	-2.23	-1.56	-1.94	-2.12	-2.42	-1.37
A6NHQ2	rRNA/tRNA 2'-O-methyltransferase fibrillarin-like protein 1 OS=Homo sapiens GN=FBLL1 PE=3 SV=1 - [FBLL1_HUMAN]	1.61	1.77	1.90	2.05	0.09	0.24	0.79	0.94	0.50	0.36	0.50	0.64	-0.77	-1.11	-1.40	-1.23	-1.51	-1.54	-1.82	-1.05
P53805	Calcipressin-1 OS=Homo sapiens GN=RCAN1 PE=1 SV=4 - [RCAN1_HUMAN]	1.68	1.98	1.73	2.03	0.11	0.41	0.43	0.73	0.52	0.23	0.38	0.67	-1.20	-1.30	-1.35	-1.42	-1.47	-1.58	-1.63	-1.24
Q93045	Stathmin-2 OS=Homo sapiens GN=STMN2 PE=1 SV=3 - [STMN2_HUMAN]	1.50	1.67	1.78	1.95	0.19	0.35	0.29	0.46	0.36	0.20	-0.27	-0.10	-1.16	-1.76	-2.05	-1.27	-1.55	-1.33	-1.61	-1.42
P78369	Claudin-10 OS=Homo sapiens GN=CLDN10 PE=1 SV=2 - [CLD10_HUMAN]	1.27	1.96	1.22	1.91	-0.45	0.24	0.00	0.69	0.73	0.05	-0.03	0.65	-1.21	-1.30	-1.25	-1.20	-1.14	-1.74	-1.68	-1.15
Q96PE5	Opalin OS=Homo sapiens GN=OPALIN PE=2 SV=1 - [OPALI_HUMAN]	2.24	1.78	2.32	1.90	-0.56	-0.35	0.70	0.26	-0.63	-0.05	-0.01	-0.34	-1.60	-1.91	-1.94	-2.23	-2.23	-2.83	-2.69	-1.82
Q8NFU3	Thiosulfate sulfurtransferase/rhodanese-like domain-containing protein 1 OS=Homo sapiens GN=TSTD1 PE=1 SV=3 - [TSTD1_HUMAN]	1.44	1.80	1.54	1.89	0.16	0.52	0.70	1.06	0.77	0.42	0.37	0.72	-0.68	-1.07	-1.17	-1.00	-1.09	-1.30	-1.39	-0.77
P04216	Thy-1 membrane glycoprotein OS=Homo sapiens GN=THY1 PE=1 SV=2 - [THY1_HUMAN]	1.52	1.61	1.79	1.88	0.15	0.24	-0.17	-0.07	-0.12	-0.21	-0.06	0.03	-1.63	-1.57	-1.85	-1.70	-1.97	-1.38	-1.65	-1.89
Q8TD30	Alanine aminotransferase 2 OS=Homo sapiens GN=GPT2 PE=1 SV=1 - [ALAT2_HUMAN]	1.90	2.14	1.60	1.85	0.43	0.67	-0.11	0.13	0.88	0.64	0.42	0.66	-1.96	-1.47	-1.19	-1.23	-0.93	-1.48	-1.19	-1.66
P55087	Aquaporin-4 OS=Homo sapiens GN=AQP4 PE=1 SV=2 - [AQP4_HUMAN]	1.08	1.23	1.70	1.82	0.03	0.24	1.71	1.70	1.04	0.90	1.21	1.44	0.47	-0.12	-0.54	-0.15	-0.57	-1.05	-1.59	-0.12

Q7Z6G8	Ankyrin repeat and sterile alpha motif domain-containing protein 1B OS=Homo sapiens GN=ANKS1B PE=1 SV=2 - [ANS1B_HUMAN]	1.61	1.51	1.58	1.79	-1.70	-1.99	0.41	0.50	0.16	0.01	0.08	-0.21	-0.95	-1.53	-1.51	-1.31	-1.60	-3.32	-3.30	-1.23
Q9H9H5	MAP6 domain-containing protein 1 OS=Homo sapiens GN=MAP6D1 PE=1 SV=1 - [MA6D1_HUMAN]	1.56	1.65	1.87	1.77	-0.09	-0.27	0.33	0.49	0.01	0.36	0.38	0.26	-1.25	-1.33	-1.50	-1.51	-1.42	-1.94	-1.99	-1.22
P50150	Guanine nucleotide-binding protein G(I)/G(S)/G(O) subunit gamma-4 OS=Homo sapiens GN=GNG4 PE=1 SV=1 - [GBG4_HUMAN]	1.63	1.71	1.69	1.77	-0.62	-0.55	0.68	0.76	0.40	0.32	0.21	0.28	-0.89	-1.42	-1.48	-1.28	-1.33	-2.27	-2.33	-0.94
Q9Y216	Myotubularin-related protein 7 OS=Homo sapiens GN=MTMR7 PE=1 SV=3 - [MTMR7_HUMAN]	1.50	1.80	1.45	1.76	0.17	0.48	0.46	0.77	0.23	-0.07	0.41	0.71	-0.98	-1.08	-1.04	-1.54	-1.49	-1.34	-1.29	-0.92
Q5U5X0	Complex III assembly factor LYRM7 OS=Homo sapiens GN=LYRM7 PE=1 SV=1 - [LYRM7_HUMAN]	1.55	1.17	2.13	1.75	-0.16	-0.54	0.79	0.41	-0.03	0.35	0.11	-0.28	-0.70	-1.44	-2.02	-1.17	-1.74	-1.73	-2.30	-1.27
Q6NT16	MFS-type transporter SLC18B1 OS=Homo sapiens GN=SLC18B1 PE=1 SV=1 - [S18B1_HUMAN]	2.09	1.96	1.85	1.75	0.08	0.05	0.86	0.67	0.43	0.51	0.41	0.21	-1.18	-1.67	-1.39	-1.45	-1.31	-2.23	-1.79	-0.88
Q02641	Voltage-dependent L-type calcium channel subunit beta-1 OS=Homo sapiens GN=CACNB1 PE=1 SV=3 - [CACB1_HUMAN]	1.89	1.52	2.07	1.70	0.21	-0.17	0.80	0.43	0.30	0.67	0.58	0.21	-1.04	-1.31	-1.49	-1.19	-1.36	-1.70	-1.88	-1.20
P51513	RNA-binding protein Nova-1 OS=Homo sapiens GN=NOVA1 PE=1 SV=1 - [NOVA1_HUMAN]	2.87	2.04	2.51	1.68	0.90	0.06	1.29	0.46	0.54	1.38	1.27	0.44	-1.53	-1.59	-1.24	-1.47	-1.10	-1.99	-1.63	-1.16
Q9NZ53	Podocalyxin-like protein 2 OS=Homo sapiens GN=PODXL2 PE=1 SV=1 - [PDXL2_HUMAN]	1.39	1.54	1.52	1.67	0.10	0.25	-0.18	-0.03	0.39	0.24	0.50	0.65	-1.52	-0.88	-1.01	-1.12	-1.24	-1.31	-1.44	-1.64

O95294	RasGAP-activating-like protein 1 OS=Homo sapiens GN=RASAL1 PE=1 SV=3 - [RASL1_HUMAN]	1.77	1.79	1.64	1.66	-0.74	-0.73	-0.03	-0.01	0.07	0.06	-0.32	-0.31	-1.74	-2.09	-1.97	-1.68	-1.55	-2.53	-2.40	-1.61
A5PKW4	PH and SEC7 domain-containing protein 1 OS=Homo sapiens GN=PSD PE=1 SV=2 - [PSD1_HUMAN]	1.79	1.64	1.75	1.61	0.20	0.04	0.06	-0.09	0.35	0.50	-0.26	-0.41	-1.68	-2.04	-2.01	-1.26	-1.22	-1.61	-1.58	-1.63
Q9BRK0	Receptor expression-enhancing protein 2 OS=Homo sapiens GN=REEP2 PE=2 SV=2 - [REEP2_HUMAN]	1.80	1.62	1.76	1.59	-0.30	-0.49	0.44	0.26	-0.12	0.06	-0.06	-0.24	-1.30	-1.85	-1.83	-1.71	-1.67	-2.12	-2.09	-1.26
Q14123	Calcium/calmodulin-dependent 3',5'-cyclic nucleotide phosphodiesterase 1C OS=Homo sapiens GN=PDE1C PE=1 SV=1 - [PDE1C_HUMAN]	1.71	1.50	1.79	1.59	0.14	-0.07	0.60	0.40	-0.33	-0.12	0.11	-0.10	-1.05	-1.59	-1.68	-1.79	-1.88	-1.58	-1.67	-1.13
Q9H6K5	Putative uncharacterized protein FLJ22184 OS=Homo sapiens PE=2 SV=1 - [YS027_HUMAN]	1.08	1.49	1.18	1.59	-0.03	0.38	0.24	0.65	0.66	0.25	0.04	0.45	-0.78	-1.03	-1.14	-0.80	-0.89	-1.12	-1.22	-0.87
P28472	Gamma-aminobutyric acid receptor subunit beta-3 OS=Homo sapiens GN=GABRB3 PE=1 SV=1 - [GBRB3_HUMAN]	1.67	1.75	1.50	1.59	-0.20	-0.12	-0.29	-0.21	0.29	0.21	-0.34	-0.26	-1.89	-2.00	-1.84	-1.63	-1.52	-1.88	-1.72	-1.77
Q99767	Amyloid beta A4 precursor protein-binding family A member 2 OS=Homo sapiens GN=APBA2 PE=1 SV=3 - [APBA2_HUMAN]	1.07	1.25	1.39	1.57	-0.15	0.02	0.23	0.41	0.27	0.10	0.06	0.24	-0.79	-1.00	-1.32	-0.94	-1.26	-1.24	-1.55	-1.10
Q8N9F0	N-acetylaspartate synthetase OS=Homo sapiens GN=NAT8L PE=1 SV=3 - [NAT8L_HUMAN]	2.29	1.53	2.30	1.54	0.22	-0.54	0.81	0.05	-0.56	0.20	0.65	-0.12	-1.43	-1.64	-1.66	-2.06	-2.07	-2.09	-2.10	-1.43
O75896	Tumor suppressor candidate 2 OS=Homo sapiens GN=TUSC2 PE=1 SV=3 - [TUSC2_HUMAN]	1.61	1.17	1.98	1.54	1.33	-0.01	0.79	0.35	-0.19	1.15	1.06	0.62	-0.76	-0.54	-0.92	-1.02	-1.21	-0.89	-1.08	-1.12

Q9UPX0	Protein turtle homolog B OS=Homo sapiens GN=IGSF9B PE=2 SV=2 - [TUTLB_HUMAN]	1.88	1.65	1.71	1.48	0.02	-0.21	0.64	0.41	0.28	0.52	0.32	0.08	-1.18	-1.56	-1.39	-1.33	-1.16	-1.87	-1.70	-1.00
Q9NY59	Sphingomyelin phosphodiesterase 3 OS=Homo sapiens GN=SMPD3 PE=1 SV=1 - [NSMA2_HUMAN]	1.49	1.42	1.55	1.48	0.41	0.33	0.18	0.10	0.06	0.14	0.22	0.14	-1.26	-1.26	-1.33	-1.32	-1.38	-1.10	-1.16	-1.31
Q9BSA4	Protein tweety homolog 2 OS=Homo sapiens GN=TTYH2 PE=1 SV=3 - [TTYH2_HUMAN]	1.98	1.83	1.63	1.48	0.77	0.61	1.14	0.99	0.50	0.67	0.44	0.28	-0.78	-0.89	-0.32	-1.07	-0.48	-1.23	-0.88	-0.42
Q9GZN7	Protein rogdi homolog OS=Homo sapiens GN=ROGDI PE=1 SV=1 - [ROGDI_HUMAN]	1.28	1.24	1.50	1.46	-0.32	-0.36	0.44	0.41	-0.09	-0.04	-0.11	-0.15	-0.78	-1.39	-1.61	-1.30	-1.51	-1.61	-1.83	-0.99
P63027	Vesicle-associated membrane protein 2 OS=Homo sapiens GN=VAMP2 PE=1 SV=3 - [VAMP2_HUMAN]	1.41	1.72	1.42	1.44	-0.23	-0.02	0.46	0.71	0.65	0.27	0.19	0.50	-0.80	-1.29	-1.36	-1.15	-1.03	-1.76	-1.57	-0.72
Q9NPD7	Neuritin OS=Homo sapiens GN=NRN1 PE=1 SV=1 - [NRN1_HUMAN]	1.14	1.12	1.44	1.43	0.04	0.02	0.05	0.03	-0.22	-0.20	-0.46	-0.48	-1.04	-1.60	-1.91	-1.31	-1.61	-1.11	-1.42	-1.34
P26378	ELAV-like protein 4 OS=Homo sapiens GN=ELAVL4 PE=1 SV=2 - [ELAV4_HUMAN]	1.43	1.10	1.76	1.42	1.05	0.71	0.61	0.27	0.07	0.41	0.96	0.62	-0.77	-0.47	-0.80	-0.99	-1.31	-0.40	-0.73	-1.08
Q9NUI1	Peroxisomal 2,4-dienoyl-CoA reductase OS=Homo sapiens GN=DECR2 PE=1 SV=1 - [DECR2_HUMAN]	1.49	1.54	1.36	1.40	0.58	0.62	0.29	0.34	0.27	0.23	0.42	0.45	-1.14	-1.07	-0.95	-1.23	-1.10	-0.93	-0.80	-1.00
O95847	Mitochondrial uncoupling protein 4 OS=Homo sapiens GN=SLC25A27 PE=1 SV=1 - [UCP4_HUMAN]	1.30	1.23	1.45	1.38	0.05	-0.03	0.58	0.51	0.32	0.40	-0.02	-0.10	-0.67	-1.32	-1.48	-0.87	-1.02	-1.27	-1.42	-0.81
Q96Q04	Serine/threonine-protein kinase LMTK3 OS=Homo sapiens GN=LMTK3 PE=2 SV=2 - [LMTK3_HUMAN]	1.49	1.26	1.60	1.37	0.03	-0.21	-0.18	-0.41	-0.30	-0.06	-0.10	-0.34	-1.61	-1.58	-1.70	-1.52	-1.63	-1.48	-1.59	-1.72

Q9NQ88	Fructose-2,6-bisphosphatase TIGAR OS=Homo sapiens GN=TIGAR PE=1 SV=1 - [TIGAR_HUMAN]	1.21	1.12	1.46	1.37	0.50	0.41	0.68	0.59	0.39	0.49	0.35	0.25	-0.47	-0.85	-1.11	-0.69	-0.94	-0.72	-0.97	-0.71
O14662	Syntaxin-16 OS=Homo sapiens GN=STX16 PE=1 SV=3 - [STX16_HUMAN]	1.46	1.54	1.29	1.37	0.48	0.03	0.62	0.22	0.49	0.89	0.70	0.27	-1.47	-1.00	-0.62	-1.45	-0.82	-1.98	-1.35	-0.87
Q9BSF0	Small membrane A-kinase anchor protein OS=Homo sapiens GN=C2orf88 PE=1 SV=2 - [SMAKA_HUMAN]	1.52	1.40	1.45	1.33	0.77	0.65	1.07	0.96	1.09	1.21	1.09	0.96	-0.39	-0.43	-0.36	-0.28	-0.20	-0.77	-0.70	-0.31
Q8N3F0	Maturin OS=Homo sapiens GN=MTURN PE=2 SV=2 - [MTURN_HUMAN]	1.91	1.54	1.68	1.32	0.68	0.31	0.74	0.37	0.20	0.57	0.14	-0.23	-1.12	-1.77	-1.55	-1.31	-1.08	-1.25	-1.02	-0.88
P54284	Voltage-dependent L-type calcium channel subunit beta-3 OS=Homo sapiens GN=CACNB3 PE=1 SV=1 - [CACB3_HUMAN]	2.14	2.30	1.14	1.30	-0.05	0.10	0.12	0.27	0.12	-0.03	0.19	0.34	-1.97	-1.95	-0.95	-2.14	-1.14	-2.21	-1.21	-0.96
Q9Y566	SH3 and multiple ankyrin repeat domains protein 1 OS=Homo sapiens GN=SHANK1 PE=1 SV=2 - [SHAN1_HUMAN]	1.74	1.25	1.75	1.26	-0.09	-0.59	0.74	0.25	-0.06	0.44	0.40	-0.09	-0.94	-1.33	-1.35	-1.27	-1.28	-1.85	-1.86	-0.94
Q9BQ95	Evolutionarily conserved signaling intermediate in Toll pathway, mitochondrial OS=Homo sapiens GN=ECSIT PE=1 SV=1 - [ECSIT_HUMAN]	1.35	1.25	1.30	1.20	0.71	0.61	0.77	0.67	0.52	0.62	0.85	0.75	-0.52	-0.50	-0.45	-0.69	-0.64	-0.65	-0.60	-0.46
P56381	ATP synthase subunit epsilon, mitochondrial OS=Homo sapiens GN=ATP5E PE=1 SV=2 - [ATP5E_HUMAN]	1.08	1.11	1.16	1.19	-0.36	-0.29	0.19	0.10	-0.05	-0.12	-0.33	-0.25	-0.94	-1.47	-1.54	-1.18	-1.14	-1.49	-1.43	-1.08
Q99946	Proline-rich transmembrane protein 1 OS=Homo sapiens GN=PRRT1 PE=2 SV=2 - [PRRT1_HUMAN]	1.60	1.53	1.24	1.16	0.05	-0.03	-0.25	-0.32	-0.01	0.07	0.17	0.09	-1.79	-1.42	-1.07	-1.50	-1.14	-1.56	-1.20	-1.42

P42684	Abelson tyrosine-protein kinase 2 OS=Homo sapiens GN=ABL2 PE=1 SV=1 - [ABL2_HUMAN]	1.08	1.07	1.16	1.15	0.35	0.34	0.42	0.41	0.13	0.15	0.07	0.06	-0.61	-1.00	-1.09	-0.90	-0.98	-0.75	-0.82	-0.68
Q5TEU4	NADH dehydrogenase [ubiquinone] 1 alpha subcomplex assembly factor 5 OS=Homo sapiens GN=NDUFAF5 PE=1 SV=1 - [NDUF5_HUMAN]	1.11	1.10	1.15	1.14	-0.19	-0.20	0.46	0.45	0.12	0.14	0.03	0.02	-0.60	-1.08	-1.12	-0.94	-0.98	-1.32	-1.36	-0.63
Q8IZR5	CKLF-like MARVEL transmembrane domain-containing protein 4 OS=Homo sapiens GN=CMTM4 PE=1 SV=1 - [CKLF4_HUMAN]	1.22	1.28	1.08	1.13	0.16	0.20	0.53	0.58	0.49	0.45	0.69	0.74	-0.64	-0.53	-0.39	-0.75	-0.60	-1.08	-0.94	-0.49
P48730	Casein kinase I isoform delta OS=Homo sapiens GN=CSNK1D PE=1 SV=2 - [KC1D_HUMAN]	1.32	1.27	1.17	1.12	0.26	0.20	0.54	0.49	0.59	0.65	0.21	0.15	-0.72	-1.10	-0.96	-0.64	-0.49	-1.08	-0.93	-0.56
Q96JA1	Leucine-rich repeats and immunoglobulin like domains protein 1 OS=Homo sapiens GN=LRIG1 PE=1 SV=2 - [LRIG1_HUMAN]	1.07	1.05	1.12	1.10	0.47	0.44	0.41	0.39	0.42	0.45	0.69	0.66	-0.46	-0.37	-0.43	-0.59	-0.64	-0.61	-0.66	-0.31
P31151	Protein S100-A7 OS=Homo sapiens GN=S100A7 PE=1 SV=4 - [S10A7_HUMAN]	-2.75	-2.72	-3.77	-3.73	-4.67	-4.50	-4.96	-4.79	-3.73	-3.76	-3.58	-3.55	-1.17	-0.83	0.18	-0.98	0.04	-0.95	1.11	0.90
Q3L8U1	Chromodomain-helicase-DNA-binding protein 9 OS=Homo sapiens GN=CHD9 PE=1 SV=2 - [CHD9_HUMAN]	-2.92	-3.23	-3.54	-3.62	-0.64	-0.73	-0.15	-0.23	-0.47	-0.38	-0.66	-0.75	0.63	0.79	2.87	0.84	3.19	1.33	2.88	3.45
Q9NTU7	Cerebellin-4 OS=Homo sapiens GN=CBLN4 PE=1 SV=1 - [CBLN4_HUMAN]	-2.96	-3.01	-3.52	-3.57	-1.06	-1.12	-2.13	-2.19	-1.70	-1.64	-1.14	-1.21	0.88	1.82	2.37	1.35	1.91	1.88	2.44	1.45

Q70UQ0	Inhibitor of nuclear factor kappa-B kinase-interacting protein OS=Homo sapiens GN=IKBIP PE=1 SV=1 - [IKIP_HUMAN]	-2.91	-2.90	-3.27	-3.27	-0.47	-0.47	-1.19	-1.19	-0.90	-0.90	-1.10	-1.10	1.77	1.81	2.17	2.04	2.41	2.42	2.78	2.15
P11678	Eosinophil peroxidase OS=Homo sapiens GN=EPX PE=1 SV=2 - [PERE_HUMAN]	-2.67	-2.84	-3.06	-3.23	-2.26	-2.44	-2.15	-2.32	-2.42	-2.24	-2.65	-2.83	0.57	0.02	0.41	0.46	0.85	0.39	0.78	0.97
P31947	14-3-3 protein sigma OS=Homo sapiens GN=SFN PE=1 SV=1 - [1433S_HUMAN]	-3.69	-3.59	-3.30	-3.19	-2.47	-2.37	-3.07	-2.96	-2.76	-2.85	-2.90	-2.81	0.68	0.79	0.39	0.87	0.47	1.20	0.81	0.29
P08727	Keratin, type I cytoskeletal 19 OS=Homo sapiens GN=KRT19 PE=1 SV=4 - [K1C19_HUMAN]	-2.51	-2.66	-2.82	-2.96	-2.45	-2.60	-2.97	-3.11	-2.61	-2.46	-2.63	-2.78	-0.40	-0.11	0.19	0.14	0.38	0.05	0.35	-0.09
P22531	Small proline-rich protein 2E OS=Homo sapiens GN=SPRR2E PE=2 SV=2 - [SPR2E_HUMAN]	-2.81	-2.24	-3.26	-2.91	-3.09	-2.74	-3.40	-2.82	-2.01	-2.35	-2.43	-2.09	-0.53	0.15	0.83	0.25	0.95	-0.53	0.16	0.48
P24844	Myosin regulatory light polypeptide 9 OS=Homo sapiens GN=MYL9 PE=1 SV=4 - [MYL9_HUMAN]	-2.59	-2.56	-2.66	-2.63	-1.21	-1.18	-1.31	-1.42	-1.24	-1.17	-1.12	-1.15	1.10	1.42	1.49	1.36	1.43	0.98	1.44	1.18
Q9BXJ0	Complement C1q tumor necrosis factor-related protein 5 OS=Homo sapiens GN=C1QTNF5 PE=1 SV=1 - [C1QT5_HUMAN]	-2.05	-2.16	-2.50	-2.61	0.11	-0.01	-1.27	-1.38	-0.65	-0.54	-0.65	-0.76	0.84	1.41	1.85	1.55	2.00	2.14	2.59	1.29
Q32P28	Prolyl 3-hydroxylase 1 OS=Homo sapiens GN=LEPRE1 PE=1 SV=2 - [P3H1_HUMAN]	-2.18	-2.16	-2.52	-2.53	-0.16	-0.05	-0.61	-0.62	-0.54	-0.56	-0.24	-0.01	1.82	1.95	2.58	1.77	2.05	2.25	2.35	1.98
O14683	Tumor protein p53-inducible protein 11 OS=Homo sapiens GN=TP53I11 PE=1 SV=2 - [P5I11_HUMAN]	-2.96	-2.78	-2.49	-2.45	-1.32	-1.28	-0.46	-0.42	-0.52	-0.56	0.29	0.33	2.49	2.95	2.78	2.50	1.96	1.94	1.16	2.09

Q96L91	E1A-binding protein p400 OS=Homo sapiens GN=EP400 PE=1 SV=4 - [EP400_HUMAN]	-1.49	-1.49	-2.44	-2.43	-0.31	-0.32	-0.69	-0.69	-0.38	-0.38	-0.31	-0.31	0.86	1.19	2.12	1.15	2.09	1.16	2.10	1.81
P47929	Galectin-7 OS=Homo sapiens GN=LGALS7 PE=1 SV=2 - [LEG7_HUMAN]	-1.42	-1.57	-2.08	-2.22	-2.73	-2.88	-3.01	-3.15	-2.85	-2.70	-2.38	-2.52	-1.53	-0.95	-0.29	-1.25	-0.58	-1.32	-0.67	-0.87
P16403	Histone H1.2 OS=Homo sapiens GN=HIST1H1C PE=1 SV=2 - [H12_HUMAN]	-2.80	-2.81	-2.21	-2.21	-2.25	-2.26	-1.25	-1.25	-2.48	-2.47	-2.05	-2.07	1.61	0.75	0.15	0.36	-0.23	0.54	-0.06	1.02
P18577	Blood group Rh(CE) polypeptide OS=Homo sapiens GN=RHCE PE=1 SV=2 - [RHCE_HUMAN]	-2.07	-2.14	-2.15	-2.21	0.49	0.42	0.45	0.39	0.63	0.70	1.37	1.30	2.58	3.45	3.52	2.80	2.88	2.54	2.62	2.66
Q9Y680	Peptidyl-prolyl cis-trans isomerase FKBP7 OS=Homo sapiens GN=FKBP7 PE=1 SV=1 - [FKBP7_HUMAN]	-1.31	-1.20	-2.71	-2.20	0.42	0.52	-0.49	-0.38	-0.10	-0.20	-0.37	-0.27	0.87	0.95	2.01	1.14	2.75	1.71	2.94	2.09
P48509	CD151 antigen OS=Homo sapiens GN=CD151 PE=1 SV=3 - [CD151_HUMAN]	-2.62	-2.57	-2.25	-2.19	0.52	0.57	0.14	0.19	0.27	0.22	-0.14	-0.10	2.81	2.48	2.10	2.87	2.50	3.13	2.75	2.45
P01714	Ig lambda chain V-III region SH OS=Homo sapiens PE=1 SV=1 - [LV301_HUMAN]	-2.08	-2.23	-2.06	-2.17	-0.79	-0.87	-1.87	-1.82	-0.66	-0.74	0.46	0.31	0.37	2.55	2.53	1.46	1.54	1.39	1.28	0.21
Q9NZD4	Alpha-hemoglobin-stabilizing protein OS=Homo sapiens GN=AHSP PE=1 SV=1 - [AHSP_HUMAN]	-2.49	-2.47	-2.19	-2.17	-0.62	-0.60	-0.73	-0.70	-0.96	-0.97	-1.67	-1.65	1.82	0.83	0.52	1.55	1.25	1.86	1.55	1.53
Q15847	Adipogenesis regulatory factor OS=Homo sapiens GN=ADIRF PE=1 SV=1 - [ADIRF_HUMAN]	-1.72	-1.90	-2.05	-2.09	-1.21	-1.29	-1.31	-1.33	-1.49	-1.46	-1.20	-1.33	0.65	0.47	0.87	0.39	0.51	0.45	0.58	0.62
Q6UX46	Protein FAM150B OS=Homo sapiens GN=FAM150B PE=2 SV=2 - [F150B_HUMAN]	-1.49	-1.61	-1.93	-2.04	0.18	0.06	-0.45	-0.56	-0.14	-0.02	0.10	-0.02	1.10	1.60	2.03	1.51	1.95	1.66	2.10	1.54

Q9HB40	Retinoid-inducible serine carboxypeptidase OS=Homo sapiens GN=SCPEP1 PE=1 SV=1 - [RISC_HUMAN]	-1.78	-1.88	-1.92	-2.02	0.38	0.28	-0.46	-0.56	-0.06	0.05	0.33	0.22	1.37	2.11	2.25	1.86	2.01	2.14	2.29	1.53
P29373	Cellular retinoic acid-binding protein 2 OS=Homo sapiens GN=CRABP2 PE=1 SV=2 - [RABP2_HUMAN]	-1.48	-2.26	-1.24	-2.02	0.18	-0.59	0.29	-0.48	-0.17	0.61	0.21	-0.57	1.83	1.70	1.45	2.12	1.88	1.65	1.41	1.60
Q9BV40	Vesicle-associated membrane protein 8 OS=Homo sapiens GN=VAMP8 PE=1 SV=1 - [VAMP8_HUMAN]	-1.88	-1.65	-2.23	-1.99	0.22	0.45	-0.08	0.15	0.26	0.04	0.67	0.90	1.86	2.56	2.90	1.95	2.30	2.09	2.43	2.21
Q02318	Sterol 26-hydroxylase, mitochondrial OS=Homo sapiens GN=CYP27A1 PE=1 SV=1 - [CP27A_HUMAN]	-1.48	-2.02	-1.45	-1.99	-0.41	-0.96	-0.51	-1.05	-0.82	-0.28	-0.27	-0.82	1.02	1.21	1.18	1.23	1.21	1.05	1.02	1.00
P23276	Kell blood group glycoprotein OS=Homo sapiens GN=KEL PE=1 SV=2 - [KELL_HUMAN]	-1.28	-1.35	-1.91	-1.98	0.49	0.41	0.50	0.43	0.93	1.01	1.59	1.51	1.83	2.87	3.50	2.32	2.95	1.75	2.38	2.47
P01776	Ig heavy chain V-III region WAS OS=Homo sapiens PE=1 SV=1 - [HV315_HUMAN]	-1.99	-1.83	-2.06	-1.97	-0.27	-0.19	-1.08	-0.92	-0.41	-0.54	0.21	0.29	1.19	2.23	2.38	1.63	1.69	1.70	1.80	1.03
Q14728	Major facilitator superfamily domain-containing protein 10 OS=Homo sapiens GN=MFSD10 PE=2 SV=1 - [MFS10_HUMAN]	-1.71	-1.74	-1.92	-1.95	-0.27	-0.30	-0.63	-0.66	-0.38	-0.35	-0.30	-0.33	1.13	1.42	1.62	1.39	1.61	1.42	1.63	1.35
Q8N6S5	ADP-ribosylation factor-like protein 6-interacting protein 6 OS=Homo sapiens GN=ARL6IP6 PE=1 SV=1 - [AR6P6_HUMAN]	-1.67	-1.67	-1.94	-1.93	-0.76	-0.75	-0.53	-0.52	-0.52	-0.52	-0.27	-0.27	1.20	1.41	1.66	1.18	1.45	0.90	1.16	1.47

P29558	RNA-binding motif, single-stranded-interacting protein 1 OS=Homo sapiens GN=RBMS1 PE=1 SV=3 - [RBMS1_HUMAN]	-2.08	-1.96	-2.04	-1.91	-0.61	-0.50	-1.04	-0.92	-0.68	-0.80	-0.58	-0.46	1.09	1.51	1.46	1.31	1.27	1.45	1.40	1.06
O60279	Sushi domain-containing protein 5 OS=Homo sapiens GN=SUSD5 PE=1 SV=3 - [SUSD5_HUMAN]	-1.98	-2.04	-1.84	-1.90	0.39	0.33	0.58	0.52	0.21	0.28	0.26	0.19	2.62	2.24	2.10	2.29	2.15	2.36	2.22	2.48
Q9Y5Y7	Lymphatic vessel endothelial hyaluronic acid receptor 1 OS=Homo sapiens GN=LYVE1 PE=1 SV=2 - [LYVE1_HUMAN]	-1.87	-1.89	-1.86	-1.87	-0.19	-0.10	-0.68	-0.57	-0.12	-0.22	0.15	0.25	1.36	2.02	2.00	1.56	1.55	1.54	1.52	1.35
P61803	Dolichyl-diphosphooligosaccharide--protein glycosyltransferase subunit DAD1 OS=Homo sapiens GN=DAD1 PE=1 SV=3 - [DAD1_HUMAN]	-1.32	-1.21	-1.98	-1.86	0.35	0.41	-0.42	-0.40	-0.09	-0.11	0.07	0.03	0.95	1.25	1.90	1.15	1.81	1.63	2.29	1.62
Q96A83	Collagen alpha-1(XXVI) chain OS=Homo sapiens GN=COL26A1 PE=1 SV=1 - [COQA1_HUMAN]	-1.69	-1.81	-1.73	-1.86	-0.70	-0.83	-1.22	-1.35	-0.92	-0.79	-0.33	-0.46	0.52	1.36	1.40	0.93	0.98	0.97	1.01	0.57
Q5JTB6	Placenta-specific protein 9 OS=Homo sapiens GN=PLAC9 PE=2 SV=1 - [PLAC9_HUMAN]	-1.43	-1.66	-1.61	-1.85	0.63	0.40	-0.35	-0.47	-0.26	-0.03	-0.20	-0.31	1.14	1.49	1.66	1.43	1.61	2.05	2.23	1.44
P34741	Syndecan-2 OS=Homo sapiens GN=SDC2 PE=1 SV=2 - [SDC2_HUMAN]	-1.57	-1.68	-1.75	-1.85	2.81	2.70	1.61	1.50	1.62	1.73	1.26	1.14	3.24	2.83	3.00	3.33	3.51	4.37	4.54	3.42
P26022	Pentraxin-related protein PTX3 OS=Homo sapiens GN=PTX3 PE=1 SV=3 - [PTX3_HUMAN]	-1.77	-1.70	-1.90	-1.83	1.41	1.48	1.94	2.01	2.08	2.01	2.67	2.74	3.76	4.45	4.58	3.81	3.95	3.16	3.30	3.91
Q9H0X9	Oxysterol-binding protein-related protein 5 OS=Homo sapiens GN=OSBPL5 PE=1 SV=1 - [OSBL5_HUMAN]	-1.58	-1.69	-1.71	-1.82	-0.70	-0.82	-1.10	-1.21	-0.81	-0.69	-0.85	-0.96	0.53	0.74	0.86	0.92	1.05	0.87	0.99	0.67

Q9BXJ2	Complement C1q tumor necrosis factor-related protein 7 OS=Homo sapiens GN=C1QTNF7 PE=1 SV=1 - [C1QT7_HUMAN]	-1.71	-1.68	-1.85	-1.82	1.04	1.07	-0.21	-0.17	0.34	0.30	0.32	0.35	1.56	2.04	2.17	2.05	2.19	2.73	2.87	1.71
Q96AA3	Protein RFT1 homolog OS=Homo sapiens GN=RFT1 PE=1 SV=1 - [RFT1_HUMAN]	-1.15	-1.31	-1.65	-1.81	-0.45	-0.62	-0.77	-0.93	-0.98	-0.82	-0.26	-0.43	0.43	0.89	1.39	0.36	0.86	0.68	1.18	0.94
P15169	Carboxypeptidase N catalytic chain OS=Homo sapiens GN=CPN1 PE=1 SV=1 - [CBPN_HUMAN]	-1.40	-1.43	-1.74	-1.77	0.40	0.37	-0.14	-0.17	0.02	0.06	-0.88	-0.91	1.31	0.53	0.87	1.48	1.83	1.78	2.13	1.67
Q9UKA4	A-kinase anchor protein 11 OS=Homo sapiens GN=AKAP11 PE=1 SV=1 - [AKA11_HUMAN]	-1.29	-1.84	-1.19	-1.74	0.63	0.08	0.10	-0.44	-0.11	0.45	0.01	-0.54	1.45	1.31	1.20	1.77	1.67	1.91	1.81	1.36
P61952	Guanine nucleotide-binding protein G(I)/G(S)/G(O) subunit gamma-11 OS=Homo sapiens GN=GNG11 PE=1 SV=1 - [GBG11_HUMAN]	-1.71	-1.84	-1.60	-1.73	-0.32	-0.46	-0.49	-0.62	-0.51	-0.37	0.16	0.03	1.27	1.88	1.77	1.37	1.26	1.37	1.26	1.18
Q9UM07	Protein-arginine deiminase type-4 OS=Homo sapiens GN=PADI4 PE=1 SV=2 - [PADI4_HUMAN]	-1.54	-1.52	-1.71	-1.69	0.20	0.22	0.91	0.93	0.57	0.56	0.40	0.41	2.51	1.95	2.11	2.13	2.30	1.73	1.90	2.69
O43896	Kinesin-like protein KIF1C OS=Homo sapiens GN=KIF1C PE=1 SV=3 - [KIF1C_HUMAN]	-1.71	-1.93	-1.48	-1.69	-0.24	-0.46	-0.79	-1.01	-0.86	-0.64	-0.27	-0.49	0.97	1.45	1.21	1.10	0.87	1.45	1.22	0.75
P01598	Ig kappa chain V-I region EU OS=Homo sapiens PE=1 SV=1 - [KV106_HUMAN]	-1.23	-1.22	-1.64	-1.68	0.31	0.31	-0.87	-0.93	0.41	0.38	1.40	1.40	0.34	2.51	3.27	1.53	2.19	1.40	2.35	0.86
Q13547	Histone deacetylase 1 OS=Homo sapiens GN=HDAC1 PE=1 SV=1 - [HDAC1_HUMAN]	-1.46	-1.47	-1.67	-1.67	-0.62	-0.63	-0.68	-0.68	-0.57	-0.57	-0.37	-0.37	0.84	1.10	1.31	0.93	1.14	0.83	1.03	1.05

P21731	Thromboxane A2 receptor OS=Homo sapiens GN=TBXA2R PE=1 SV=3 - [TA2R_HUMAN]	-1.62	-1.76	-1.51	-1.65	0.18	0.04	-0.23	-0.37	-0.07	0.07	0.06	-0.08	1.45	1.69	1.57	1.73	1.62	1.78	1.67	1.35
Q86YB7	Enoyl-CoA hydratase domain-containing protein 2, mitochondrial OS=Homo sapiens GN=ECHDC2 PE=2 SV=2 - [ECHD2_HUMAN]	-2.43	-1.97	-2.11	-1.65	-0.27	0.19	-0.33	0.14	0.26	-0.20	0.10	0.56	2.16	2.54	2.22	2.26	1.94	2.14	1.82	1.85
Q9UDX3	SEC14-like protein 4 OS=Homo sapiens GN=SEC14L4 PE=2 SV=1 - [S14L4_HUMAN]	-1.21	-1.08	-1.78	-1.65	0.49	0.61	-0.03	0.10	0.53	0.41	0.42	0.55	1.24	1.64	2.20	1.65	2.22	1.68	2.25	1.81
P23083	Ig heavy chain V-I region V35 OS=Homo sapiens PE=1 SV=1 - [HV103_HUMAN]	-1.57	-1.67	-1.54	-1.64	0.51	0.40	-1.13	-1.23	0.04	0.15	0.83	0.72	0.50	2.40	2.36	1.75	1.72	2.06	2.03	0.48
P06331	Ig heavy chain V-II region ARH 77 OS=Homo sapiens PE=4 SV=1 - [HV209_HUMAN]	-1.90	-1.69	-1.81	-1.60	-0.23	-0.03	-0.87	-0.67	0.00	-0.20	0.43	0.63	1.08	2.34	2.24	1.73	1.65	1.65	1.56	1.00
Q9NRX3	NADH dehydrogenase [ubiquinone] 1 alpha subcomplex subunit 4-like 2 OS=Homo sapiens GN=NDUFA4L2 PE=2 SV=1 - [NUA4L_HUMAN]	-1.44	-1.37	-1.62	-1.55	-0.03	0.03	-0.42	-0.36	-0.10	-0.16	0.22	0.28	1.07	1.66	1.84	1.30	1.49	1.39	1.58	1.26
P46734	Dual specificity mitogen-activated protein kinase kinase 3 OS=Homo sapiens GN=MAP2K3 PE=1 SV=2 - [MP2K3_HUMAN]	-1.31	-1.35	-1.44	-1.53	-0.15	-0.19	0.35	0.13	0.05	0.20	-0.02	-0.07	1.56	1.29	1.47	1.32	1.64	1.14	1.31	1.75
P01743	Ig heavy chain V-I region HG3 OS=Homo sapiens PE=4 SV=1 - [HV102_HUMAN]	-1.44	-1.29	-1.65	-1.50	0.20	0.34	-0.46	-0.32	0.03	-0.11	0.26	0.41	1.03	1.71	1.91	1.35	1.57	1.62	1.83	1.25
Q9UER7	Death domain-associated protein 6 OS=Homo sapiens GN=DAXX PE=1 SV=2 - [DAXX_HUMAN]	-2.12	-1.71	-1.91	-1.49	-1.45	-1.04	-0.45	-0.04	-2.43	-2.83	-1.40	-1.00	1.72	0.72	0.50	-0.69	-0.89	0.65	0.44	1.52

P60468	Protein transport protein Sec61 subunit beta OS=Homo sapiens GN=SEC61B PE=1 SV=2 - [SC61B_HUMAN]	-1.81	-1.92	-1.36	-1.47	-0.86	-1.03	-0.83	-0.94	-0.83	-0.94	-0.69	-0.71	1.03	1.12	0.79	1.12	0.67	0.51	0.21	0.85
Q9UNQ0	ATP-binding cassette sub-family G member 2 OS=Homo sapiens GN=ABCG2 PE=1 SV=3 - [ABCG2_HUMAN]	-1.79	-1.75	-1.50	-1.47	-0.79	-0.77	-0.93	-0.90	-0.92	-0.95	-0.42	-0.39	0.91	1.37	1.08	0.86	0.58	0.98	0.69	0.63
P01764	Ig heavy chain V-III region VH26 OS=Homo sapiens PE=1 SV=1 - [HV303_HUMAN]	-1.49	-1.30	-1.64	-1.46	0.20	0.35	-0.88	-0.72	-0.05	-0.22	0.46	0.62	0.65	1.86	2.01	1.29	1.42	1.57	1.64	0.81
Q14956	Transmembrane glycoprotein NMB OS=Homo sapiens GN=GPNMB PE=1 SV=2 - [GPNMB_HUMAN]	-1.81	-1.65	-1.62	-1.45	0.46	0.62	0.83	1.00	0.82	0.66	1.57	1.73	2.70	3.39	3.18	2.50	2.31	2.25	2.06	2.51
Q6P1M3	Lethal(2) giant larvae protein homolog 2 OS=Homo sapiens GN=LLGL2 PE=1 SV=2 - [L2GL2_HUMAN]	-1.50	-1.63	-1.32	-1.44	-0.95	-1.25	-1.55	-1.68	-1.61	-1.48	-1.60	-1.84	0.00	-0.20	-0.11	0.03	0.07	0.02	0.03	-0.05
Q9Y2H6	Fibronectin type-III domain-containing protein 3A OS=Homo sapiens GN=FNDC3A PE=1 SV=4 - [FND3A_HUMAN]	-1.36	-1.59	-1.21	-1.44	-0.28	-0.51	-0.40	-0.63	-0.43	-0.19	0.08	-0.16	1.01	1.44	1.28	1.19	1.05	1.06	0.91	0.87
Q96FZ2	Embryonic stem cell-specific 5-hydroxymethylcytosine-binding protein OS=Homo sapiens GN=HMCES PE=1 SV=1 - [HMCES_HUMAN]	-1.37	-1.50	-1.28	-1.42	0.79	0.64	0.07	-0.07	0.14	0.28	-0.23	-0.38	1.49	1.14	1.05	1.67	1.59	2.14	2.05	1.41
Q9BYI3	Hyccin OS=Homo sapiens GN=FAM126A PE=1 SV=2 - [HYCCI_HUMAN]	-1.45	-1.33	-1.52	-1.40	-0.02	0.09	-0.40	-0.28	-0.40	-0.51	-0.83	-0.72	1.10	0.62	0.68	0.96	1.03	1.41	1.48	1.18
P17050	Alpha-N-acetylgalactosaminidase OS=Homo sapiens GN=NAGA PE=1 SV=2 - [NAGAB_HUMAN]	-1.23	-1.14	-1.47	-1.38	-0.50	-0.42	-0.12	-0.04	-0.64	-0.72	-0.58	-0.50	1.16	0.65	0.88	0.53	0.78	0.71	0.94	1.40

Q8ND71	GTPase IMAP family member 8 OS=Homo sapiens GN=GIMAP8 PE=1 SV=2 - [GIMA8_HUMAN]	-1.34	-1.26	-1.44	-1.36	-0.40	-0.33	-0.67	-0.59	-0.59	-0.66	-0.44	-0.37	0.73	0.91	1.00	0.71	0.81	0.92	1.02	0.84
Q96II8	Leucine-rich repeat and calponin homology domain-containing protein 3 OS=Homo sapiens GN=LRCH3 PE=1 SV=2 - [LRCH3_HUMAN]	-1.27	-1.22	-1.40	-1.35	0.12	0.17	-0.02	0.03	-0.26	-0.31	0.29	0.34	1.30	1.57	1.69	0.99	1.12	1.38	1.50	1.44
Q8NG11	Tetraspanin-14 OS=Homo sapiens GN=TSPAN14 PE=1 SV=1 - [TSN14_HUMAN]	-1.21	-1.27	-1.28	-1.34	0.34	0.28	0.00	-0.06	-0.03	0.04	0.17	0.10	1.26	1.38	1.45	1.28	1.35	1.53	1.60	1.34
Q14554	Protein disulfide-isomerase A5 OS=Homo sapiens GN=PDIA5 PE=1 SV=1 - [PDIA5_HUMAN]	-1.20	-1.20	-1.34	-1.33	0.36	0.36	-0.29	-0.28	0.05	0.05	0.12	0.12	0.97	1.33	1.46	1.28	1.42	1.55	1.68	1.11
Q12857	Nuclear factor 1 A-type OS=Homo sapiens GN=NFIA PE=1 SV=2 - [NFIA_HUMAN]	-1.58	-1.66	-1.25	-1.33	-1.05	-1.13	-0.84	-0.92	-0.86	-0.78	-0.47	-0.55	0.79	1.12	0.78	0.83	0.51	0.52	0.19	0.47
O75477	Erlin-1 OS=Homo sapiens GN=ERLIN1 PE=1 SV=1 - [ERLN1_HUMAN]	-1.56	-1.54	-1.35	-1.33	-0.31	-0.30	-0.43	-0.41	-0.45	-0.46	0.02	0.03	1.19	1.58	1.36	1.13	0.92	1.23	1.02	0.98
Q13188	Serine/threonine-protein kinase 3 OS=Homo sapiens GN=STK3 PE=1 SV=2 - [STK3_HUMAN]	-1.49	-1.35	-1.47	-1.33	0.08	0.21	0.04	-0.01	-0.25	-0.38	0.07	0.06	1.39	1.75	1.31	1.14	1.12	1.55	1.53	1.38
P30504	HLA class I histocompatibility antigen, Cw-4 alpha chain OS=Homo sapiens GN=HLA-C PE=1 SV=1 - [1C04_HUMAN]	-1.45	-1.36	-1.41	-1.32	-0.34	-0.25	0.07	0.16	0.19	0.10	-0.03	0.06	1.58	1.43	1.38	1.58	1.55	1.10	1.06	1.54
Q9BUN8	Derlin-1 OS=Homo sapiens GN=DERL1 PE=1 SV=1 - [DERL1_HUMAN]	-1.65	-1.55	-1.57	-1.30	-0.34	-0.42	-0.82	-0.70	-0.45	-0.55	-0.64	-0.53	0.81	0.89	0.98	0.62	0.80	1.15	1.00	0.79
P08263	Glutathione S-transferase A1 OS=Homo sapiens GN=GSTA1 PE=1 SV=3 - [GSTA1_HUMAN]	-1.72	-1.56	-1.43	-1.27	-1.17	-1.01	-0.40	-0.24	-0.49	-0.65	-1.34	-1.18	1.38	0.39	0.09	1.11	0.82	0.54	0.25	1.09

Q13043	Serine/threonine-protein kinase 4 OS=Homo sapiens GN=STK4 PE=1 SV=2 - [STK4_HUMAN]	-1.37	-1.44	-1.19	-1.26	-0.45	-0.53	-0.14	-0.21	-0.41	-0.33	0.26	0.18	1.28	1.63	1.45	1.07	0.89	0.90	0.72	1.11
Q53SF7	Cordon-bleu protein-like 1 OS=Homo sapiens GN=COBLL1 PE=1 SV=2 - [COBL1_HUMAN]	-1.50	-1.44	-1.31	-1.25	-0.48	-0.43	-0.80	-0.75	-0.66	-0.71	-0.54	-0.49	0.75	0.96	0.77	0.82	0.63	1.00	0.81	0.57
P01621	Ig kappa chain V-III region NG9 (Fragment) OS=Homo sapiens PE=1 SV=1 - [KV303_HUMAN]	-2.92	-2.18	-1.97	-1.24	-0.95	-0.22	-1.82	-1.08	-0.46	-1.19	-0.69	0.04	1.16	2.23	1.28	1.76	0.81	1.96	1.01	0.22
P43307	Translocon-associated protein subunit alpha OS=Homo sapiens GN=SSR1 PE=1 SV=3 - [SSRA_HUMAN]	-1.29	-1.36	-1.32	-1.23	0.05	0.03	-0.26	-0.30	-0.37	-0.34	-0.23	-0.22	1.09	1.22	1.10	1.02	1.01	1.33	1.25	1.00
P17096	High mobility group protein HMG-I/HMG-Y OS=Homo sapiens GN=HMGA1 PE=1 SV=3 - [HMGA1_HUMAN]	-2.07	-2.11	-1.18	-1.23	-0.34	-0.39	-0.74	-0.78	-1.19	-1.14	-0.55	-0.60	1.38	1.52	0.63	0.96	0.08	1.71	0.82	0.51
Q6ZTI6	Protein FAM101A OS=Homo sapiens GN=FAM101A PE=2 SV=3 - [F101A_HUMAN]	-1.08	-1.13	-1.15	-1.20	0.28	0.22	-0.52	-0.58	0.08	0.14	0.77	0.71	0.61	1.85	1.91	1.24	1.32	1.34	1.41	0.69
P06311	Ig kappa chain V-III region IARC/BL41 OS=Homo sapiens PE=1 SV=1 - [KV311_HUMAN]	-1.16	-1.11	-1.21	-1.15	0.12	0.17	-0.88	-0.82	0.72	0.67	1.79	1.84	0.34	2.95	3.00	1.86	1.91	1.27	1.31	0.39
Q9BV73	Centrosome-associated protein CEP250 OS=Homo sapiens GN=CEP250 PE=1 SV=2 - [CP250_HUMAN]	2.70	3.13	2.54	2.97	0.72	1.15	2.39	2.82	2.29	1.86	2.20	2.62	-0.26	-0.50	-0.35	-0.81	-0.65	-2.00	-1.83	-0.09
Q7Z7J9	Calcium/calmodulin-dependent protein kinase II inhibitor 1 OS=Homo sapiens GN=CAMK2N1 PE=1 SV=1 - [CK2N1_HUMAN]	2.01	1.86	2.66	2.50	-0.08	-0.24	-0.10	-0.26	-1.17	-1.00	-0.33	-0.49	-2.06	-2.34	-2.99	-2.99	-3.62	-2.11	-2.75	-2.70

Q9H305	Cell death-inducing p53-target protein 1 OS=Homo sapiens GN=CDIP1 PE=2 SV=1 - [CDIP1_HUMAN]	1.91	1.86	2.42	2.37	-0.06	-0.11	1.10	1.05	0.44	0.50	0.57	0.52	-0.76	-1.34	-1.85	-1.39	-1.89	-1.99	-2.50	-1.25
Q9NS61	Kv channel-interacting protein 2 OS=Homo sapiens GN=KCNIP2 PE=1 SV=3 - [KCIP2_HUMAN]	2.37	2.16	2.49	2.28	0.84	0.63	1.03	0.82	0.28	0.50	0.50	0.29	-1.28	-1.86	-1.99	-1.84	-1.96	-1.54	-1.67	-1.40
P54829	Tyrosine-protein phosphatase non-receptor type 5 OS=Homo sapiens GN=PTPN5 PE=1 SV=4 - [PTN5_HUMAN]	2.15	2.30	2.11	2.26	-0.14	0.01	0.61	0.76	0.47	0.32	-0.03	0.11	-1.49	-2.18	-2.14	-1.80	-1.75	-2.30	-2.26	-1.44
P11229	Muscarinic acetylcholine receptor M1 OS=Homo sapiens GN=CHRM1 PE=1 SV=2 - [ACM1_HUMAN]	1.77	1.63	2.40	2.26	-0.85	-0.99	0.35	0.21	0.39	0.54	0.07	-0.08	-1.36	-1.70	-2.33	-1.21	-1.83	-2.64	-3.26	-1.98
O60303	Uncharacterized protein KIAA0556 OS=Homo sapiens GN=KIAA0556 PE=1 SV=4 - [K0556_HUMAN]	4.07	4.56	1.77	2.25	0.91	1.39	1.17	1.66	2.23	1.75	0.88	1.37	-2.84	-3.18	-0.88	-2.29	0.01	-3.17	-0.87	-0.53
Q6ICH7	Aspartate beta-hydroxylase domain-containing protein 2 OS=Homo sapiens GN=ASPHD2 PE=2 SV=1 - [ASPH2_HUMAN]	1.64	2.07	1.80	2.23	0.53	0.96	0.38	0.81	0.86	0.43	0.00	0.43	-1.20	-1.63	-1.80	-1.17	-1.34	-1.12	-1.29	-1.36
Q9BQG1	Synaptotagmin-3 OS=Homo sapiens GN=SYT3 PE=2 SV=1 - [SYT3_HUMAN]	2.01	1.91	2.23	2.13	0.01	-0.09	0.75	0.66	0.49	0.59	0.41	0.31	-1.20	-1.59	-1.82	-1.39	-1.60	-2.02	-2.24	-1.41
O95670	V-type proton ATPase subunit G 2 OS=Homo sapiens GN=ATP6V1G2 PE=1 SV=1 - [VATG2_HUMAN]	2.88	2.40	2.31	2.11	-0.13	-0.26	0.87	0.38	0.38	0.54	0.54	0.03	-1.79	-2.35	-2.08	-2.32	-1.89	-1.81	-1.25	-1.21
Q8NDY3	[Protein ADP-ribosylarginine] hydrolase-like protein 1 OS=Homo sapiens GN=ADPRHL1 PE=2 SV=1 - [ARHL1_HUMAN]	2.14	2.18	2.07	2.11	0.33	0.37	0.88	0.92	0.59	0.55	1.01	1.05	-1.21	-1.13	-1.06	-1.56	-1.48	-1.82	-1.75	-1.12

Q15025	TNFAIP3-interacting protein 1 OS=Homo sapiens GN=TNIP1 PE=1 SV=2 - [TNIP1_HUMAN]	2.03	2.21	1.92	2.10	0.32	0.49	0.53	0.71	0.37	0.20	0.13	0.30	-1.45	-1.90	-1.80	-1.80	-1.69	-1.73	-1.62	-1.33
Q13875	Myelin-associated oligodendrocyte basic protein OS=Homo sapiens GN=MOBP PE=2 SV=2 - [MOBP_HUMAN]	2.24	2.03	2.09	2.08	-0.85	-0.65	-0.12	-0.09	-0.35	-0.62	-0.83	-0.91	-2.07	-2.68	-2.54	-2.49	-2.27	-2.69	-2.74	-1.99
P23297	Protein S100-A1 OS=Homo sapiens GN=S100A1 PE=1 SV=2 - [S10A1_HUMAN]	1.41	1.72	1.76	2.07	1.16	1.47	1.56	1.87	1.59	1.28	0.88	1.18	0.21	-0.53	-0.88	-0.10	-0.44	-0.26	-0.61	0.04
O15354	Prosaposin receptor GPR37 OS=Homo sapiens GN=GPR37 PE=1 SV=2 - [GPR37_HUMAN]	1.69	1.90	1.85	2.06	0.04	0.25	0.64	0.86	0.47	0.27	0.43	0.63	-0.99	-1.26	-1.43	-1.39	-1.55	-1.66	-1.83	-1.14
Q7RTN6	STE20-related kinase adapter protein alpha OS=Homo sapiens GN=STRADA PE=1 SV=1 - [STRAA_HUMAN]	2.25	2.01	2.29	2.05	0.20	-0.04	1.08	0.85	0.40	0.64	0.79	0.55	-1.11	-1.46	-1.50	-1.58	-1.61	-2.07	-2.10	-1.14
A4D161	Protein FAM221A OS=Homo sapiens GN=FAM221A PE=2 SV=1 - [F221A_HUMAN]	2.20	2.25	2.00	2.05	0.34	0.39	1.15	1.20	0.12	0.08	0.75	0.79	-1.00	-1.45	-1.25	-2.09	-1.89	-1.87	-1.67	-0.78
Q86V21	Acetoacetyl-CoA synthetase OS=Homo sapiens GN=AACS PE=1 SV=1 - [AACS_HUMAN]	2.22	2.38	1.88	2.04	0.79	0.94	0.87	1.03	1.07	0.92	0.78	0.93	-1.29	-1.43	-1.10	-1.28	-0.93	-1.45	-1.11	-0.95
Q9NZV8	Potassium voltage-gated channel subfamily D member 2 OS=Homo sapiens GN=KCND2 PE=1 SV=2 - [KCND2_HUMAN]	2.10	2.17	1.93	2.00	-0.03	0.04	0.85	0.93	0.79	0.72	-0.19	-0.13	-1.19	-2.28	-2.12	-1.34	-1.17	-2.14	-1.98	-1.01
Q9NX18	Succinate dehydrogenase assembly factor 2, mitochondrial OS=Homo sapiens GN=SDHAF2 PE=1 SV=1 - [SDHF2_HUMAN]	1.92	1.62	2.25	1.95	0.85	0.55	1.51	1.21	0.78	1.09	1.24	0.93	-0.36	-0.68	-1.02	-0.80	-1.13	-1.09	-1.42	-0.68

P48539	Purkinje cell protein 4 OS=Homo sapiens GN=PCP4 PE=1 SV=3 - [PCP4_HUMAN]	1.55	1.73	1.75	1.93	0.00	0.17	-0.39	-0.22	0.13	-0.04	-0.68	-0.51	-1.89	-2.23	-2.44	-1.56	-1.76	-1.57	-1.77	-2.08
Q9BVA1	Tubulin beta-2B chain OS=Homo sapiens GN=TUBB2B PE=1 SV=1 - [TBB2B_HUMAN]	1.43	1.64	1.72	1.93	0.45	0.65	0.82	1.03	0.71	0.55	0.89	0.44	-0.36	-0.88	-1.21	-0.84	-1.16	-1.00	-1.29	-0.65
Q9H6F2	Trimeric intracellular cation channel type A OS=Homo sapiens GN=TMEM38A PE=1 SV=1 - [TM38A_HUMAN]	1.42	1.44	1.86	1.88	-0.35	-0.33	0.07	0.09	-0.16	-0.18	-0.38	-0.37	-1.29	-1.80	-2.24	-1.56	-2.00	-1.78	-2.22	-1.73
Q8IUQ0	Clavesin-1 OS=Homo sapiens GN=CLVS1 PE=1 SV=1 - [CLVS1_HUMAN]	1.90	1.90	1.88	1.88	0.49	0.49	0.74	0.74	0.32	0.33	0.04	0.04	-1.11	-1.85	-1.83	-1.55	-1.52	-1.43	-1.40	-1.07
A1Z1Q3	O-acetyl-ADP-ribose deacetylase MACROD2 OS=Homo sapiens GN=MACROD2 PE=1 SV=1 - [MACD2_HUMAN]	1.95	1.96	1.85	1.86	0.77	0.77	0.43	0.44	0.89	0.89	0.47	0.47	-1.47	-1.48	-1.39	-1.04	-0.93	-1.20	-1.10	-1.36
Q96LD8	Sentrin-specific protease 8 OS=Homo sapiens GN=SENP8 PE=1 SV=1 - [SENP8_HUMAN]	1.69	2.11	1.41	1.84	-0.01	0.07	0.72	1.14	0.77	0.35	-0.52	-0.10	-0.92	-2.20	-1.93	-1.31	-1.03	-2.28	-1.92	-0.63
Q96P71	N-terminal EF-hand calcium-binding protein 3 OS=Homo sapiens GN=NECAB3 PE=1 SV=2 - [NECA3_HUMAN]	1.90	2.01	1.72	1.83	0.06	0.16	0.45	0.56	0.41	0.31	0.24	0.35	-1.39	-1.65	-1.48	-1.56	-1.38	-1.86	-1.68	-1.20
O00746	Nucleoside diphosphate kinase, mitochondrial OS=Homo sapiens GN=NME4 PE=1 SV=1 - [NDKM_HUMAN]	1.82	1.87	1.75	1.80	-0.78	-0.73	-0.24	-0.19	-0.23	-0.27	-0.20	-0.15	-2.00	-2.01	-1.95	-2.06	-1.99	-2.61	-2.54	-1.93
Q92686	Neurogranin OS=Homo sapiens GN=NRGN PE=1 SV=1 - [NEUG_HUMAN]	1.21	1.10	2.00	1.77	0.11	-0.11	-0.31	-0.52	0.20	-0.07	-0.08	-0.09	-1.88	-1.28	-2.04	-1.47	-1.84	-1.37	-1.84	-2.40
Q5VT66	Mitochondrial amidoxime-reducing component 1 OS=Homo sapiens GN=MARC1 PE=1 SV=1 - [MARC1_HUMAN]	1.13	1.14	1.72	1.74	-0.21	-0.20	0.64	0.65	0.69	0.68	-0.05	-0.04	-0.43	-1.17	-1.77	-0.41	-1.01	-1.35	-1.95	-1.02

Q09428	ATP-binding cassette sub-family C member 8 OS=Homo sapiens GN=ABCC8 PE=1 SV=6 - [ABCC8_HUMAN]	1.77	1.65	1.84	1.72	-0.10	-0.22	-0.12	-0.24	-0.03	0.10	0.45	0.33	-1.83	-1.31	-1.39	-1.64	-1.71	-1.88	-1.96	-1.90
Q9BUW7	UPF0184 protein C9orf16 OS=Homo sapiens GN=C9orf16 PE=1 SV=1 - [CI016_HUMAN]	2.17	1.83	2.05	1.71	0.68	0.33	1.00	0.65	0.34	0.69	0.57	0.22	-1.12	-1.60	-1.49	-1.46	-1.33	-1.51	-1.39	-0.99
Q8NEV8	Exophilin-5 OS=Homo sapiens GN=EXPH5 PE=2 SV=3 - [EXPH5_HUMAN]	1.76	1.59	1.81	1.64	0.53	0.35	1.12	0.94	0.43	0.61	0.79	0.61	-0.59	-0.96	-1.02	-1.12	-1.17	-1.25	-1.30	-0.63
O43525	Potassium voltage-gated channel subfamily KQT member 3 OS=Homo sapiens GN=KCNQ3 PE=1 SV=2 - [KCNQ3_HUMAN]	1.47	1.67	1.43	1.63	0.29	0.48	-0.82	-0.63	0.09	-0.11	-1.07	-0.88	-2.24	-2.54	-2.51	-1.55	-1.51	-1.20	-1.16	-2.19
P25713	Metallothionein-3 OS=Homo sapiens GN=MT3 PE=1 SV=1 - [MT3_HUMAN]	1.17	1.09	1.69	1.60	1.15	1.06	0.28	0.20	0.36	0.45	0.46	0.37	-0.83	-0.71	-1.23	-0.69	-1.21	-0.03	-0.55	-1.34
P24310	Cytochrome c oxidase subunit 7A1, mitochondrial OS=Homo sapiens GN=COX7A1 PE=1 SV=2 - [CX7A1_HUMAN]	1.58	1.49	1.68	1.59	-0.01	-0.11	-0.16	-0.25	-0.29	-0.19	0.27	0.17	-1.68	-1.31	-1.42	-1.74	-1.84	-1.61	-1.71	-1.78
A6NJZ7	RIMS-binding protein 3C OS=Homo sapiens GN=RIMBP3C PE=1 SV=3 - [RIM3C_HUMAN]	2.34	2.41	1.50	1.58	0.91	0.99	1.47	1.55	1.47	1.40	1.22	1.29	-0.81	-1.11	-0.29	-0.91	-0.08	-1.44	-0.61	0.03
P02795	Metallothionein-2 OS=Homo sapiens GN=MT2A PE=1 SV=1 - [MT2_HUMAN]	1.85	1.80	1.62	1.57	1.13	1.08	1.16	1.11	1.21	1.26	1.31	1.25	-0.64	-0.54	-0.32	-0.56	-0.33	-0.74	-0.51	-0.40
Q5VST6	Alpha/beta hydrolase domain-containing protein 17B OS=Homo sapiens GN=ABHD17B PE=2 SV=1 - [AB17B_HUMAN]	1.53	1.41	1.69	1.57	0.31	0.19	0.85	0.73	0.33	0.46	0.49	0.36	-0.63	-1.03	-1.20	-1.04	-1.20	-1.23	-1.40	-0.78
Q96G97	Seipin OS=Homo sapiens GN=BSCL2 PE=1 SV=3 - [BSCL2_HUMAN]	1.26	1.65	1.16	1.55	-0.24	0.15	0.14	0.54	0.28	-0.11	-0.21	0.18	-1.06	-1.46	-1.37	-1.33	-1.23	-1.52	-1.42	-0.95

Q9C0E4	Glutamate receptor-interacting protein 2 OS=Homo sapiens GN=GRIP2 PE=1 SV=3 - [GRIP2_HUMAN]	1.12	1.29	1.34	1.52	-0.55	-0.38	0.52	0.70	0.02	-0.15	-0.54	-0.37	-0.54	-1.66	-1.89	-1.24	-1.46	-1.69	-1.91	-0.76
Q04609	Glutamate carboxypeptidase 2 OS=Homo sapiens GN=FOLH1 PE=1 SV=1 - [FOLH1_HUMAN]	1.21	1.23	1.49	1.51	0.00	0.02	0.95	0.97	0.19	0.17	0.03	0.05	-0.20	-1.17	-1.45	-1.00	-1.28	-1.22	-1.50	-0.47
P52824	Diacylglycerol kinase theta OS=Homo sapiens GN=DGKQ PE=1 SV=2 - [DGKQ_HUMAN]	1.62	1.55	1.57	1.50	0.13	0.05	0.48	0.41	0.14	0.22	-0.31	-0.38	-1.09	-1.92	-1.88	-1.37	-1.32	-1.51	-1.46	-1.03
Q9UBJ2	ATP-binding cassette sub-family D member 2 OS=Homo sapiens GN=ABCD2 PE=1 SV=1 - [ABCD2_HUMAN]	1.59	1.53	1.55	1.48	0.60	0.52	0.85	0.78	0.50	0.57	0.58	0.51	-0.69	-1.00	-0.97	-0.99	-0.95	-1.01	-0.97	-0.64
Q07343	cAMP-specific 3',5'-cyclic phosphodiesterase 4B OS=Homo sapiens GN=PDE4B PE=1 SV=1 - [PDE4B_HUMAN]	1.72	1.45	1.75	1.48	-0.05	-0.33	0.53	0.26	0.01	0.29	0.58	0.31	-1.13	-1.13	-1.17	-1.40	-1.43	-1.79	-1.82	-1.15
Q6NXP0	EF-hand calcium-binding domain-containing protein 12 OS=Homo sapiens GN=EFCAB12 PE=2 SV=1 - [EFC12_HUMAN]	2.08	1.56	1.94	1.42	0.15	-0.38	0.97	0.45	0.02	0.55	-0.39	-0.92	-1.05	-2.47	-2.34	-1.50	-1.36	-1.95	-1.82	-0.91
Q5R3F8	Protein phosphatase 1 regulatory subunit 29 OS=Homo sapiens GN=ELFN2 PE=1 SV=1 - [PPR29_HUMAN]	1.26	1.57	1.10	1.41	0.18	0.48	0.40	0.71	0.31	0.01	0.48	0.78	-0.81	-0.78	-0.62	-1.23	-1.06	-1.10	-0.94	-0.64
O43759	Synaptogyrin-1 OS=Homo sapiens GN=SYNGR1 PE=1 SV=2 - [SNG1_HUMAN]	1.62	1.50	1.53	1.41	0.09	-0.04	0.74	0.62	0.27	0.40	0.39	0.27	-0.82	-1.22	-1.14	-1.19	-1.10	-1.55	-1.46	-0.72
Q86XE0	Sorting nexin-32 OS=Homo sapiens GN=SNX32 PE=2 SV=1 - [SNX32_HUMAN]	2.68	1.73	2.34	1.39	0.88	-0.07	0.88	-0.07	-0.10	0.85	0.34	-0.62	-1.74	-2.33	-2.01	-1.79	-1.46	-1.81	-1.48	-1.40

P41732	Tetraspanin-7 OS=Homo sapiens GN=TSPAN7 PE=1 SV=2 - [TSN7_HUMAN]	1.71	1.64	1.42	1.35	-0.72	-0.80	0.01	-0.05	0.00	0.07	-0.71	-0.78	-1.64	-2.41	-2.13	-1.61	-1.32	-2.45	-2.16	-1.34
Q8N8R5	UPF0565 protein C2orf69 OS=Homo sapiens GN=C2orf69 PE=1 SV=1 - [CB069_HUMAN]	1.32	1.43	1.20	1.31	0.03	0.13	0.36	0.47	0.33	0.23	-0.10	0.00	-0.90	-1.42	-1.31	-1.06	-0.94	-1.31	-1.19	-0.78
Q7L3B6	Hsp90 co-chaperone Cdc37-like 1 OS=Homo sapiens GN=CDC37L1 PE=1 SV=1 - [CD37L_HUMAN]	1.78	1.63	1.45	1.31	0.81	0.65	0.94	0.80	0.81	0.96	0.91	0.75	-0.78	-0.87	-0.55	-0.79	-0.46	-0.99	-0.67	-0.45
P81605	Dermcidin OS=Homo sapiens GN=DCD PE=1 SV=2 - [DCD_HUMAN]	1.42	1.25	1.46	1.29	0.44	0.26	0.72	0.55	0.21	0.39	0.08	-0.09	-0.64	-1.33	-1.37	-1.00	-1.04	-1.00	-1.04	-0.67
Q643R3	Lysophospholipid acyltransferase LPCAT4 OS=Homo sapiens GN=LPCAT4 PE=1 SV=1 - [LPCT4_HUMAN]	1.20	1.21	1.27	1.28	-0.58	-0.57	0.18	0.19	-0.27	-0.27	-0.29	-0.29	-0.96	-1.48	-1.57	-1.44	-1.52	-1.79	-1.87	-1.03
O60669	Monocarboxylate transporter 2 OS=Homo sapiens GN=SLC16A7 PE=1 SV=2 - [MOT2_HUMAN]	1.24	1.33	1.15	1.24	-0.43	-0.35	-0.04	0.05	-0.26	-0.34	-0.07	0.01	-1.22	-1.31	-1.23	-1.55	-1.46	-1.69	-1.60	-1.13
Q969T7	7-methylguanosine phosphate-specific 5'-nucleotidase OS=Homo sapiens GN=NT5C3B PE=1 SV=4 - [5NT3B_HUMAN]	1.42	1.14	1.52	1.23	-0.32	-0.61	0.57	0.29	0.15	0.43	-0.30	-0.59	-0.79	-1.72	-1.82	-0.96	-1.05	-1.75	-1.85	-0.88
Q96SI9	Spermatid perinuclear RNA-binding protein OS=Homo sapiens GN=STRBP PE=1 SV=1 - [STRBP_HUMAN]	2.08	1.61	1.69	1.22	0.41	-0.06	0.33	-0.13	-0.08	0.39	0.25	-0.23	-1.69	-1.82	-1.44	-1.66	-1.27	-1.68	-1.29	-1.29
Q96S82	Ubiquitin-like protein 7 OS=Homo sapiens GN=UBL7 PE=1 SV=2 - [UBL7_HUMAN]	1.41	1.25	1.34	1.19	0.12	-0.04	0.43	0.28	0.11	0.27	0.29	0.13	-0.92	-1.11	-1.06	-1.11	-1.04	-1.31	-1.25	-0.85
A6NL88	Protein shisa-7 OS=Homo sapiens GN=SHISA7 PE=2 SV=3 - [SHSA7_HUMAN]	1.50	1.28	1.41	1.19	-0.74	-0.97	0.07	-0.15	-0.72	-0.49	-0.64	-0.87	-1.38	-2.14	-2.05	-1.96	-1.86	-2.26	-2.17	-1.27

P06307	Cholecystokinin OS=Homo sapiens GN=CCK PE=1 SV=1 - [CCKN_HUMAN]	1.36	1.42	1.09	1.15	0.62	0.68	0.38	0.45	0.56	0.50	0.48	0.54	-0.92	-0.87	-0.61	-0.83	-0.56	-0.76	-0.49	-0.64
Q8TDN4	CDK5 and ABL1 enzyme substrate 1 OS=Homo sapiens GN=CABLES1 PE=1 SV=2 - [CABL1_HUMAN]	1.23	1.01	1.35	1.14	0.17	-0.05	0.69	0.47	0.30	0.52	-0.23	-0.45	-0.48	-1.45	-1.58	-0.68	-0.80	-1.08	-1.20	-0.60
O75157	TSC22 domain family protein 2 OS=Homo sapiens GN=TSC22D2 PE=1 SV=3 - [T22D2_HUMAN]	1.27	1.03	1.33	1.10	-0.04	-0.29	0.48	0.24	0.09	0.33	0.44	0.20	-0.74	-0.82	-0.90	-0.91	-0.97	-1.33	-1.40	-0.79
P52757	Beta-chimaerin OS=Homo sapiens GN=CHN2 PE=1 SV=2 - [CHIO_HUMAN]	1.11	1.08	1.11	1.07	0.64	0.60	0.51	0.47	0.25	0.30	-0.02	-0.06	-0.55	-1.12	-1.13	-0.79	-0.78	-0.48	-0.48	-0.54
A6NDB9	Paralemmin-3 OS=Homo sapiens GN=PALM3 PE=1 SV=2 - [PALM3_HUMAN]	1.13	1.12	1.07	1.06	-0.39	-0.41	0.74	0.73	0.15	0.16	0.16	0.14	-0.34	-0.97	-0.91	-0.94	-0.87	-1.54	-1.48	-0.27
Q69YL0	Uncharacterized protein DKFZp762I1415 OS=Homo sapiens PE=4 SV=1 - [YC029_HUMAN]	-3.94	-4.27	-4.00	-4.33	-2.11	-2.44	-2.70	-3.03	-2.83	-2.50	-2.59	-2.92	1.29	1.36	1.41	1.47	1.54	1.82	1.87	1.36
A8MQ03	UPF0574 protein C9orf169 OS=Homo sapiens GN=C9orf169 PE=1 SV=1 - [CI169_HUMAN]	-4.33	-4.51	-3.96	-4.14	-2.22	-2.41	-2.61	-2.80	-2.93	-2.74	-2.92	-3.11	1.77	1.42	1.04	1.62	1.26	2.09	1.73	1.41
Q9H461	Frizzled-8 OS=Homo sapiens GN=FZD8 PE=1 SV=1 - [FZD8_HUMAN]	-2.60	-3.14	-3.21	-3.76	0.99	0.43	0.57	0.02	-0.26	0.29	-0.01	-0.56	3.22	2.60	3.20	2.92	3.54	3.56	4.18	3.85
A5PLK6	Regulator of G-protein signaling protein-like OS=Homo sapiens GN=RGSL1 PE=2 SV=1 - [RGSL_HUMAN]	-2.83	-2.83	-3.62	-3.62	0.24	0.23	-0.48	-0.48	-0.36	-0.36	-0.73	-0.74	2.40	2.10	2.88	2.50	3.30	3.05	3.84	3.20
P50238	Cysteine-rich protein 1 OS=Homo sapiens GN=CRIP1 PE=1 SV=3 - [CRIP1_HUMAN]	-3.59	-3.46	-3.71	-3.58	-2.30	-2.18	-2.10	-1.78	-1.88	-2.12	-1.22	-1.10	1.58	2.22	2.41	1.68	1.88	1.27	1.40	1.79

Q9NQ76	Matrix extracellular phosphoglycoprotein OS=Homo sapiens GN=MEPE PE=1 SV=1 - [MEPE_HUMAN]	-2.41	-2.57	-3.41	-3.57	-2.60	-2.76	-2.60	-2.76	-2.58	-2.42	-2.62	-2.78	-0.13	-0.20	0.79	0.02	1.02	-0.20	0.80	0.87
P26678	Cardiac phospholamban OS=Homo sapiens GN=PLN PE=1 SV=1 - [PPLA_HUMAN]	-2.96	-3.13	-3.26	-3.43	-0.48	-0.65	-0.82	-0.99	-0.68	-0.51	-0.18	-0.35	2.19	2.79	3.08	2.49	2.79	2.47	2.77	2.50
Q7Z6B7	SLIT-ROBO Rho GTPase-activating protein 1 OS=Homo sapiens GN=SRGAP1 PE=1 SV=1 - [SRGP1_HUMAN]	-2.72	-2.84	-3.17	-3.30	-1.79	-1.92	-2.77	-2.90	-2.03	-1.90	-2.41	-2.55	0.00	0.31	0.76	0.85	1.31	0.91	1.36	0.47
P07204	Thrombomodulin OS=Homo sapiens GN=THBD PE=1 SV=2 - [TRBM_HUMAN]	-2.03	-2.42	-2.69	-3.08	-0.09	-0.48	-1.02	-1.41	-0.69	-0.29	0.30	-0.10	1.07	2.33	2.98	1.77	2.43	1.92	2.58	1.74
P13164	Interferon-induced transmembrane protein 1 OS=Homo sapiens GN=IFITM1 PE=1 SV=3 - [IFM1_HUMAN]	-3.07	-2.93	-3.14	-3.00	-0.01	0.13	-1.21	-1.07	-0.34	-0.48	0.19	0.33	1.92	3.27	3.33	2.63	2.70	3.05	3.11	2.00
Q8NEQ5	Transmembrane protein C1orf162 OS=Homo sapiens GN=C1orf162 PE=2 SV=1 - [CA162_HUMAN]	-2.57	-2.64	-2.91	-2.98	-1.88	-1.96	-2.74	-2.82	-1.80	-1.72	-1.85	-1.93	-0.12	0.73	1.06	0.87	1.22	0.67	1.01	0.23
Q2TAA5	GDP-Man:Man(3)GlcNAc(2)-PP-Dol alpha-1,2-mannosyltransferase OS=Homo sapiens GN=ALG11 PE=1 SV=2 - [ALG11_HUMAN]	-1.09	-1.14	-2.91	-2.96	-0.01	-0.06	-0.68	-0.73	-0.23	-0.18	-0.21	-0.27	0.46	0.88	2.70	0.94	2.77	1.06	2.88	2.29
Q96SJ8	Tetraspanin-18 OS=Homo sapiens GN=TSPAN18 PE=2 SV=1 - [TSN18_HUMAN]	-2.92	-2.49	-3.30	-2.87	0.09	0.51	-0.33	0.10	0.13	-0.29	0.01	0.43	2.64	2.93	3.30	2.66	3.04	2.99	3.37	3.03
Q8IVN3	Musculoskeletal embryonic nuclear protein 1 OS=Homo sapiens GN=MUSTN1 PE=2 SV=2 - [MSTN1_HUMAN]	-3.16	-3.12	-2.91	-2.87	-3.19	-3.15	-2.59	-2.54	-2.91	-2.95	-2.41	-2.37	0.63	0.75	0.50	0.24	0.00	-0.05	-0.29	0.39

Q9BXJ4	Complement C1q tumor necrosis factor-related protein 3 OS=Homo sapiens GN=C1QTNF3 PE=1 SV=1 - [C1QT3_HUMAN]	-2.70	-2.51	-2.96	-2.77	-0.53	-0.35	-0.75	-0.56	-0.52	-0.70	-1.26	-1.08	2.01	1.44	1.70	2.03	2.30	2.15	2.41	2.28
P98171	Rho GTPase-activating protein 4 OS=Homo sapiens GN=ARHGAP4 PE=1 SV=2 - [RHG04_HUMAN]	-1.20	-1.31	-2.65	-2.76	0.46	0.35	0.28	0.16	0.55	0.66	0.59	0.47	1.53	1.79	3.23	1.89	3.34	1.64	3.09	2.99
P80511	Protein S100-A12 OS=Homo sapiens GN=S100A12 PE=1 SV=2 - [S10AC_HUMAN]	-2.50	-2.46	-2.80	-2.75	-1.43	-1.39	1.71	1.75	0.53	0.49	0.52	0.56	4.27	3.03	3.32	3.02	3.32	1.06	1.35	4.57
Q5VV43	Dyslexia-associated protein KIAA0319 OS=Homo sapiens GN=KIAA0319 PE=1 SV=1 - [K0319_HUMAN]	-2.32	-2.46	-2.58	-2.72	-0.21	-0.36	-0.93	-1.07	-0.39	-0.25	-0.40	-0.54	1.44	1.93	2.18	2.10	2.37	2.09	2.35	1.72
P25940	Collagen alpha-3(V) chain OS=Homo sapiens GN=COL5A3 PE=1 SV=3 - [CO5A3_HUMAN]	-2.20	-2.28	-2.52	-2.60	-1.26	-1.35	-2.31	-2.39	-1.77	-1.69	-1.47	-1.56	-0.05	0.73	1.05	0.54	0.87	0.92	1.24	0.28
P30536	Translocator protein OS=Homo sapiens GN=TSPO PE=1 SV=3 - [TSPOA_HUMAN]	-2.73	-2.64	-2.64	-2.55	-0.16	-0.08	-0.58	-0.49	-0.42	-0.51	-0.25	-0.16	2.20	2.48	2.39	2.25	2.16	2.55	2.46	2.12
P29400	Collagen alpha-5(IV) chain OS=Homo sapiens GN=COL4A5 PE=1 SV=2 - [CO4A5_HUMAN]	-2.00	-2.11	-2.38	-2.49	-0.17	-0.28	-0.63	-0.74	-0.67	-0.55	-0.58	-0.70	1.42	1.43	1.80	1.48	1.87	1.82	2.20	1.81
P20160	Azurocidin OS=Homo sapiens GN=AZU1 PE=1 SV=3 - [CAP7_HUMAN]	-1.82	-2.01	-2.26	-2.46	-1.10	-1.30	1.15	0.96	-0.01	0.18	-0.66	-0.85	3.03	1.17	1.61	2.03	2.48	0.70	1.14	3.48
P43356	Melanoma-associated antigen 2 OS=Homo sapiens GN=MAGEA2 PE=1 SV=1 - [MAGA2_HUMAN]	-2.47	-2.82	-2.08	-2.43	0.09	-0.26	-0.18	-0.52	-0.37	-0.02	0.58	0.23	2.35	3.06	2.66	2.49	2.10	2.55	2.16	1.97

Q8IYM0	Protein FAM186B OS=Homo sapiens GN=FAM186B PE=2 SV=2 - [F186B_HUMAN]	-2.20	-2.26	-2.36	-2.42	0.29	0.23	-0.27	-0.32	0.05	0.12	0.23	0.17	1.99	2.44	2.60	2.34	2.51	2.47	2.63	2.16
P04430	Ig kappa chain V-I region BAN OS=Homo sapiens PE=1 SV=1 - [KV122_HUMAN]	-2.07	-2.19	-2.30	-2.42	-0.56	-0.69	-0.29	-0.41	0.30	0.43	1.17	1.04	1.84	3.24	3.46	2.53	2.76	1.49	1.72	2.07
P01036	Cystatin-S OS=Homo sapiens GN=CST4 PE=1 SV=3 - [CYTS_HUMAN]	-3.63	-3.37	-2.65	-2.39	-1.85	-1.60	-2.21	-1.95	-2.06	-2.32	-2.28	-2.02	1.48	1.36	0.37	1.34	0.36	1.76	0.78	0.50
Q8IUK8	Cerebellin-2 OS=Homo sapiens GN=CBLN2 PE=2 SV=1 - [CBLN2_HUMAN]	-2.64	-2.52	-2.52	-2.39	-1.61	-1.48	-1.67	-1.54	-1.35	-1.47	-1.33	-1.20	1.03	1.32	1.19	1.20	1.08	1.02	0.89	0.91
Q8N2H4	Protein SYS1 homolog OS=Homo sapiens GN=SYS1 PE=1 SV=1 - [SYS1_HUMAN]	-3.77	-3.54	-2.61	-2.38	-1.81	-1.58	-1.98	-1.74	-1.62	-1.85	-1.92	-1.69	1.85	1.85	0.69	1.95	0.79	1.95	0.79	0.70
P04207	Ig kappa chain V-III region CLL OS=Homo sapiens PE=1 SV=2 - [KV308_HUMAN]	-1.46	-1.56	-2.25	-2.35	-0.55	-0.66	-1.23	-1.32	-0.13	-0.03	0.78	0.67	0.29	2.25	3.03	1.47	2.26	0.90	1.68	1.09
Q9UKG4	Solute carrier family 13 member 4 OS=Homo sapiens GN=SLC13A4 PE=2 SV=2 - [S13A4_HUMAN]	-2.45	-2.40	-2.39	-2.34	0.30	0.35	-0.06	-0.01	0.03	-0.02	0.01	0.06	2.45	2.47	2.40	2.46	2.41	2.73	2.68	2.40
Q16873	Leukotriene C4 synthase OS=Homo sapiens GN=LTC4S PE=1 SV=1 - [LTC4S_HUMAN]	-2.21	-2.10	-2.42	-2.31	-0.68	-0.57	-1.24	-1.13	-0.75	-0.86	-0.98	-0.88	1.03	1.23	1.44	1.38	1.60	1.51	1.72	1.24
P39900	Macrophage metalloelastase OS=Homo sapiens GN=MMP12 PE=1 SV=1 - [MMP12_HUMAN]	-2.56	-2.47	-2.38	-2.29	-0.07	0.02	-0.41	-0.32	-0.18	-0.27	0.05	0.14	2.20	2.61	2.43	2.32	2.15	2.48	2.30	2.04
Q96MW7	Tigger transposable element-derived protein 1 OS=Homo sapiens GN=TIGD1 PE=1 SV=1 - [TIGD1_HUMAN]	-2.71	-2.62	-2.32	-2.24	-0.01	0.07	-0.66	-0.57	-0.56	-0.65	-0.24	-0.16	2.11	2.47	2.08	2.09	1.71	2.68	2.29	1.73

P41214	Eukaryotic translation initiation factor 2D OS=Homo sapiens GN=EIF2D PE=1 SV=3 - [EIF2D_HUMAN]	-1.61	-1.64	-2.19	-2.22	0.09	0.05	-0.52	-0.55	-0.03	0.01	0.23	0.20	1.15	1.85	2.42	1.65	2.23	1.68	2.26	1.74
Q9NPY3	Complement component C1q receptor OS=Homo sapiens GN=CD93 PE=1 SV=3 - [C1QR1_HUMAN]	-2.10	-1.94	-2.38	-2.21	-0.52	-0.36	-0.81	-0.65	-0.49	-0.65	-0.68	-0.52	1.34	1.43	1.70	1.49	1.76	1.57	1.84	1.63
P20062	Transcobalamin-2 OS=Homo sapiens GN=TCN2 PE=1 SV=3 - [TCO2_HUMAN]	-2.00	-2.14	-2.07	-2.21	0.53	0.38	-0.08	-0.22	0.14	0.29	0.27	0.12	1.97	2.27	2.33	2.31	2.38	2.51	2.58	2.05
Q13361	Microfibrillar-associated protein 5 OS=Homo sapiens GN=MFAP5 PE=1 SV=1 - [MFAP5_HUMAN]	-1.95	-2.01	-2.10	-2.17	1.91	1.84	-0.42	-0.49	0.29	0.36	-0.74	-0.81	1.58	1.22	1.36	2.33	2.49	3.84	3.99	1.74
P50443	Sulfate transporter OS=Homo sapiens GN=SLC26A2 PE=1 SV=2 - [S26A2_HUMAN]	-2.00	-1.88	-2.25	-2.14	-0.48	-0.37	-1.66	-1.54	-1.27	-1.38	-0.61	-0.50	0.39	1.39	1.64	0.65	0.91	1.50	1.75	0.66
Q9UIF9	Bromodomain adjacent to zinc finger domain protein 2A OS=Homo sapiens GN=BAZ2A PE=1 SV=4 - [BAZ2A_HUMAN]	-1.90	-1.87	-2.16	-2.12	0.00	0.02	-0.38	-0.35	-0.53	-0.56	-0.69	-0.66	1.57	1.22	1.47	1.37	1.63	1.88	2.13	1.84
Q16820	Meprin A subunit beta OS=Homo sapiens GN=MEP1B PE=1 SV=3 - [MEP1B_HUMAN]	-1.46	-1.85	-1.69	-2.08	-1.73	-2.12	-0.92	-1.31	-2.33	-1.94	-1.60	-1.99	0.59	-0.14	0.09	-0.45	-0.22	-0.29	-0.06	0.83
Q9UBY9	Heat shock protein beta-7 OS=Homo sapiens GN=HSPB7 PE=1 SV=1 - [HSPB7_HUMAN]	-1.31	-1.47	-1.87	-2.04	-0.54	-0.71	-1.23	-1.39	-0.99	-0.83	-1.10	-1.27	0.14	0.21	0.77	0.52	1.08	0.75	1.31	0.71
Q9UHG0	Doublecortin domain-containing protein 2 OS=Homo sapiens GN=DCDC2 PE=1 SV=2 - [DCDC2_HUMAN]	-1.68	-1.61	-2.11	-2.03	-0.58	-0.51	-0.12	-0.05	-0.21	-0.28	0.04	0.11	1.61	1.72	2.14	1.43	1.86	1.09	1.51	2.05

Q9BQB6	Vitamin K epoxide reductase complex subunit 1 OS=Homo sapiens GN=VKORC1 PE=1 SV=1 - [VKOR1_HUMAN]	-1.74	-1.59	-2.16	-2.01	0.19	0.34	-0.56	-0.41	-0.09	-0.24	-0.07	0.08	1.24	1.68	2.09	1.53	1.96	1.91	2.33	1.67
Q14520	Hyaluronan-binding protein 2 OS=Homo sapiens GN=HABP2 PE=1 SV=1 - [HABP2_HUMAN]	-2.31	-2.18	-2.13	-1.99	-1.25	-1.13	-1.27	-1.14	-1.08	-1.21	-0.31	-0.18	1.10	2.01	1.82	1.13	0.95	1.04	0.86	0.92
Q9Y6X9	MORC family CW-type zinc finger protein 2 OS=Homo sapiens GN=MORC2 PE=1 SV=2 - [MORC2_HUMAN]	-1.60	-1.59	-1.99	-1.98	-1.07	-1.07	-2.09	-2.08	-1.36	-1.36	-1.41	-1.40	-0.44	0.19	0.58	0.27	0.66	0.50	0.90	-0.04
P01613	Ig kappa chain V-I region Ni OS=Homo sapiens PE=1 SV=1 - [KV121_HUMAN]	-1.24	-1.41	-1.66	-1.92	-0.12	-0.03	-0.38	-0.21	0.71	0.67	2.00	1.71	0.92	3.25	3.66	2.16	2.66	1.07	1.58	1.34
Q8WYN0	Cysteine protease ATG4A OS=Homo sapiens GN=ATG4A PE=1 SV=1 - [ATG4A_HUMAN]	-1.53	-1.69	-1.75	-1.90	-0.45	-0.61	0.03	-0.13	-0.41	-0.25	-0.31	-0.47	1.61	1.23	1.44	1.31	1.53	1.07	1.29	1.84
Q9NWZ3	Interleukin-1 receptor-associated kinase 4 OS=Homo sapiens GN=IRAK4 PE=1 SV=1 - [IRAK4_HUMAN]	-1.11	-1.29	-1.72	-1.90	0.97	0.79	0.48	0.30	0.54	0.73	0.41	0.22	1.64	1.52	2.13	1.87	2.48	2.07	2.68	2.26
Q14149	MORC family CW-type zinc finger protein 3 OS=Homo sapiens GN=MORC3 PE=1 SV=3 - [MORC3_HUMAN]	-2.22	-2.34	-1.77	-1.89	0.02	-0.10	-0.97	-1.09	-0.86	-0.74	-0.78	-0.90	1.31	1.45	0.99	1.52	1.07	2.23	1.77	0.86
Q12882	Dihydropyrimidine dehydrogenase [NADP(+)] OS=Homo sapiens GN=DPYD PE=1 SV=2 - [DPYD_HUMAN]	-1.18	-1.70	-1.37	-1.88	-0.02	-0.54	-0.54	-1.05	-0.75	-0.23	-0.19	-0.72	0.70	0.99	1.17	0.98	1.17	1.15	1.33	0.89
P23327	Sarcoplasmic reticulum histidine-rich calcium-binding protein OS=Homo sapiens GN=HRC PE=2 SV=1 - [SRCH_HUMAN]	-1.96	-1.94	-1.88	-1.86	-1.23	-1.22	-0.57	-0.55	-0.66	-0.68	-0.53	-0.51	1.44	1.43	1.35	1.31	1.24	0.71	0.63	1.38

O95503	Chromobox protein homolog 6 OS=Homo sapiens GN=CBX6 PE=1 SV=1 - [CBX6_HUMAN]	-1.90	-1.87	-1.88	-1.85	0.83	0.86	1.30	1.33	0.51	0.49	-0.33	-0.30	3.26	1.58	1.55	2.42	2.40	2.72	2.70	3.24
Q9UKX7	Nuclear pore complex protein Nup50 OS=Homo sapiens GN=NUP50 PE=1 SV=2 - [NUP50_HUMAN]	-1.64	-1.63	-1.86	-1.84	-1.13	-1.12	-1.13	-1.11	-0.83	-0.84	-0.57	-0.56	0.57	1.08	1.28	0.84	1.05	0.50	0.71	0.79
Q9P212	1-phosphatidylinositol 4,5-bisphosphate phosphodiesterase epsilon-1 OS=Homo sapiens GN=PLCE1 PE=1 SV=3 - [PLCE1_HUMAN]	-1.59	-1.59	-1.84	-1.84	-0.52	-0.52	-0.79	-0.79	-0.82	-0.81	-0.81	-0.82	0.85	0.78	1.02	0.81	1.06	1.05	1.30	1.11
P83110	Serine protease HTRA3 OS=Homo sapiens GN=HTRA3 PE=1 SV=2 - [HTRA3_HUMAN]	-1.72	-1.58	-1.96	-1.82	2.57	2.70	0.49	0.63	1.50	1.37	0.76	0.90	2.27	2.49	2.72	3.12	3.36	4.28	4.51	2.51
O00501	Claudin-5 OS=Homo sapiens GN=CLDN5 PE=1 SV=1 - [CLD5_HUMAN]	-1.68	-1.84	-1.65	-1.81	-0.12	-0.28	-1.05	-1.21	-0.49	-0.33	-0.05	-0.22	0.68	1.64	1.60	1.39	1.36	1.55	1.52	0.66
P59046	NACHT, LRR and PYD domains-containing protein 12 OS=Homo sapiens GN=NLRP12 PE=1 SV=2 - [NAL12_HUMAN]	-1.44	-1.51	-1.73	-1.80	0.17	0.09	-0.63	-0.71	-0.30	-0.22	-0.12	-0.20	0.86	1.32	1.61	1.24	1.54	1.59	1.88	1.16
P01619	Ig kappa chain V-III region B6 OS=Homo sapiens PE=1 SV=1 - [KV301_HUMAN]	-1.22	-1.51	-1.51	-1.80	-0.61	-0.90	0.34	0.05	0.19	0.49	1.08	0.78	1.62	2.30	2.58	1.74	2.03	0.60	0.89	1.92
P05062	Fructose-bisphosphate aldolase B OS=Homo sapiens GN=ALDOB PE=1 SV=2 - [ALDOB_HUMAN]	-1.18	-1.13	-1.82	-1.77	-1.73	-1.69	0.07	0.12	-0.34	-0.38	-0.71	-0.66	1.30	0.48	1.11	0.83	1.47	-0.57	0.06	1.95
Q9H4M7	Pleckstrin homology domain-containing family A member 4 OS=Homo sapiens GN=PLEKHA4 PE=1 SV=2 - [PKHA4_HUMAN]	-1.48	-1.62	-1.63	-1.76	-0.12	-0.26	-1.06	-1.19	-0.93	-0.78	-0.57	-0.71	0.48	0.92	1.06	0.72	0.87	1.35	1.49	0.63

P04433	Ig kappa chain V-III region VG (Fragment) OS=Homo sapiens PE=1 SV=1 - [KV309_HUMAN]	-1.58	-1.64	-1.70	-1.76	-0.19	-0.25	-0.65	-0.70	-0.22	-0.15	0.41	0.34	0.99	1.99	2.11	1.46	1.58	1.37	1.49	1.12
Q96AM1	Mas-related G-protein coupled receptor member F OS=Homo sapiens GN=MRGPRF PE=2 SV=1 - [MRGRF_HUMAN]	-1.46	-1.44	-2.08	-1.74	-0.62	-0.40	-0.55	-0.41	-0.05	-0.37	-0.26	0.07	1.39	1.33	1.81	1.32	1.73	0.96	1.44	1.33
A6NCW0	Ubiquitin carboxyl-terminal hydrolase 17-like protein 3 OS=Homo sapiens GN=USP17L3 PE=3 SV=1 - [U17L3_HUMAN]	-1.76	-1.75	-1.79	-1.74	0.16	0.25	-0.12	-0.02	-0.21	-0.31	-0.16	-0.20	1.72	1.54	1.51	1.54	1.59	1.94	1.98	1.78
P01612	Ig kappa chain V-I region Mev OS=Homo sapiens PE=1 SV=1 - [KV120_HUMAN]	-1.40	-1.49	-1.64	-1.73	-1.04	-1.13	-0.38	-0.46	0.19	0.28	1.06	0.97	1.08	2.47	2.70	1.71	1.96	0.35	0.59	1.33
P08651	Nuclear factor 1 C-type OS=Homo sapiens GN=NFIC PE=1 SV=2 - [NFIC_HUMAN]	-1.40	-1.21	-1.87	-1.69	-0.85	-0.66	-1.25	-1.07	-1.10	-1.28	-0.72	-0.54	0.20	0.68	1.15	0.15	0.62	0.54	1.01	0.68
P24390	ER lumen protein retaining receptor 1 OS=Homo sapiens GN=KDELR1 PE=1 SV=1 - [ERD21_HUMAN]	-1.89	-1.75	-1.80	-1.66	0.02	0.16	-0.78	-0.64	-0.25	-0.39	-0.33	-0.20	1.16	1.56	1.47	1.53	1.45	1.89	1.80	1.08
P69891	Hemoglobin subunit gamma-1 OS=Homo sapiens GN=HBG1 PE=1 SV=2 - [HBG1_HUMAN]	-1.32	-1.31	-1.64	-1.62	-0.66	-0.65	-0.97	-0.95	-0.72	-0.73	-0.88	-0.87	0.41	0.45	0.76	0.63	0.94	0.65	0.96	0.74
Q13546	Receptor-interacting serine/threonine-protein kinase 1 OS=Homo sapiens GN=RIPK1 PE=1 SV=3 - [RIPK1_HUMAN]	-1.51	-1.55	-1.58	-1.61	0.42	0.39	-0.05	-0.08	0.14	0.17	-0.21	-0.24	1.52	1.31	1.37	1.72	1.79	1.92	1.98	1.60
Q9HC07	Transmembrane protein 165 OS=Homo sapiens GN=TMEM165 PE=1 SV=1 - [TM165_HUMAN]	-1.41	-1.47	-1.55	-1.61	-0.53	-0.59	-0.71	-0.77	-0.80	-0.74	-0.56	-0.62	0.75	0.86	0.99	0.70	0.85	0.86	1.01	0.90

Q8NEK5	Zinc finger protein 548 OS=Homo sapiens GN=ZNF548 PE=2 SV=2 - [ZN548_HUMAN]	-1.96	-2.16	-1.41	-1.60	0.33	0.13	0.14	-0.06	-0.57	-0.36	0.45	0.24	2.16	2.42	1.85	1.63	1.08	2.28	1.72	1.61
P01593	Ig kappa chain V-I region AG OS=Homo sapiens PE=1 SV=1 - [KV101_HUMAN]	-1.25	-1.33	-1.52	-1.58	-0.10	-0.16	-0.49	-0.41	0.33	0.27	1.47	1.53	0.85	2.86	2.93	1.69	1.90	1.15	1.40	1.11
A6NI28	Rho GTPase-activating protein 42 OS=Homo sapiens GN=ARHGAP42 PE=1 SV=3 - [RHG42_HUMAN]	-1.14	-1.18	-1.54	-1.58	-0.39	-0.44	-1.00	-1.04	-0.55	-0.50	-0.85	-0.90	0.20	0.29	0.68	0.67	1.07	0.73	1.13	0.60
Q96L58	Beta-1,3-galactosyltransferase 6 OS=Homo sapiens GN=B3GALT6 PE=1 SV=2 - [B3GT6_HUMAN]	-1.21	-1.18	-1.60	-1.56	2.73	2.76	1.02	1.05	1.86	1.83	1.06	1.09	2.28	2.27	2.66	3.08	3.47	3.92	4.31	2.68
A8K7I4	Calcium-activated chloride channel regulator 1 OS=Homo sapiens GN=CLCA1 PE=1 SV=3 - [CLCA1_HUMAN]	-1.15	-1.20	-1.51	-1.56	4.21	4.15	3.02	2.98	2.88	2.94	-0.48	-0.53	4.23	0.68	1.03	4.12	4.48	5.34	5.70	4.60
P31645	Sodium-dependent serotonin transporter OS=Homo sapiens GN=SLC6A4 PE=1 SV=1 - [SC6A4_HUMAN]	-1.62	-1.45	-1.71	-1.54	-0.77	-0.60	-0.73	-0.56	-0.78	-0.95	-0.84	-0.67	0.94	0.79	0.87	0.70	0.80	0.84	0.93	1.04
P01702	Ig lambda chain V-I region NIG-64 OS=Homo sapiens PE=1 SV=1 - [LV104_HUMAN]	-1.92	-1.90	-1.55	-1.54	-0.04	-0.03	-0.16	-0.15	-0.25	-0.26	-0.52	-0.51	1.81	1.41	1.04	1.68	1.32	1.86	1.49	1.45
Q6P1A2	Lysophospholipid acyltransferase 5 OS=Homo sapiens GN=LPCAT3 PE=1 SV=1 - [MBOA5_HUMAN]	-1.93	-1.81	-1.63	-1.51	0.31	0.42	-0.42	-0.31	0.05	-0.06	0.05	0.16	1.56	1.99	1.68	1.90	1.60	2.22	1.93	1.27
Q93091	Ribonuclease K6 OS=Homo sapiens GN=RNASE6 PE=1 SV=2 - [RNAS6_HUMAN]	-1.62	-1.30	-1.84	-1.51	1.11	1.43	0.61	0.94	1.41	1.09	1.40	1.72	2.29	3.03	3.24	2.74	2.96	2.71	2.93	2.51

Q00266	S-adenosylmethionine synthase isoform type-1 OS=Homo sapiens GN=MAT1A PE=1 SV=2 - [METK1_HUMAN]	-1.26	-1.10	-1.67	-1.51	0.48	0.63	-0.64	-0.48	0.08	-0.07	0.01	0.17	0.68	1.28	1.68	1.22	1.63	1.72	2.13	1.10
Q58A45	PAB-dependent poly(A)-specific ribonuclease subunit PAN3 OS=Homo sapiens GN=PAN3 PE=1 SV=3 - [PAN3_HUMAN]	-1.85	-1.97	-1.38	-1.50	-1.09	-1.22	-1.59	-1.71	-1.70	-1.57	-1.57	-1.70	0.32	0.28	-0.19	0.31	-0.16	0.75	0.28	-0.14
Q03692	Collagen alpha-1(X) chain OS=Homo sapiens GN=COL10A1 PE=1 SV=2 - [COAA1_HUMAN]	-1.57	-1.58	-1.49	-1.50	2.61	2.59	1.59	1.58	1.59	1.61	1.03	1.01	3.22	2.60	2.52	3.21	3.13	4.16	4.08	3.15
Q5BKT4	Dol-P-Glc:Glc(2)Man(9)GlcNAc(2)-PP-Dol alpha-1,2-glucosyltransferase OS=Homo sapiens GN=ALG10 PE=2 SV=1 - [AG10A_HUMAN]	-1.48	-1.41	-1.57	-1.50	0.07	0.14	-0.34	-0.27	-0.15	-0.21	-0.49	-0.43	1.20	0.99	1.08	1.30	1.39	1.53	1.62	1.29
Q9H7E9	UPF0488 protein C8orf33 OS=Homo sapiens GN=C8orf33 PE=1 SV=1 - [CH033_HUMAN]	-1.23	-1.47	-1.26	-1.50	-0.38	-0.62	-0.31	-0.55	-0.20	0.04	-0.01	-0.25	0.97	1.23	1.25	1.30	1.33	0.83	0.86	1.01
P0CG05	Ig lambda-2 chain C regions OS=Homo sapiens GN=IGLC2 PE=1 SV=1 - [LAC2_HUMAN]	-2.04	-1.98	-1.55	-1.49	-0.08	-0.03	-0.32	-0.27	0.15	0.10	0.81	0.86	1.77	2.85	2.36	2.17	1.68	1.94	1.45	1.29
Q96MX3	Zinc finger protein 48 OS=Homo sapiens GN=ZNF48 PE=1 SV=2 - [ZNF48_HUMAN]	-1.22	-1.06	-1.64	-1.47	-0.80	-0.64	-1.01	-0.84	-0.31	-0.47	-0.27	-0.12	0.27	0.95	1.36	0.78	1.20	0.41	0.82	0.70
P09488	Glutathione S-transferase Mu 1 OS=Homo sapiens GN=GSTM1 PE=1 SV=3 - [GSTM1_HUMAN]	-1.77	-1.53	-1.70	-1.46	-0.86	-0.62	3.71	3.95	2.85	2.61	-0.96	-0.72	5.53	0.82	0.74	4.41	4.34	0.90	0.82	5.47
Q06546	GA-binding protein alpha chain OS=Homo sapiens GN=GABPA PE=1 SV=1 - [GABPA_HUMAN]	-1.48	-1.49	-1.44	-1.44	-0.26	-0.27	-0.64	-0.64	-0.50	-0.49	-0.50	-0.51	0.90	0.99	0.93	1.02	0.98	1.20	1.16	0.86

P08138	Tumor necrosis factor receptor superfamily member 16 OS=Homo sapiens GN=NGFR PE=1 SV=1 - [TNR16_HUMAN]	-1.20	-1.14	-1.50	-1.44	-0.05	0.00	-0.43	-0.37	0.09	0.03	0.28	0.34	0.82	1.48	1.78	1.26	1.57	1.13	1.43	1.13
P04434	Ig kappa chain V-III region VH (Fragment) OS=Homo sapiens PE=4 SV=1 - [KV310_HUMAN]	-1.28	-1.23	-1.49	-1.44	-0.41	-0.37	-0.35	-0.30	-0.10	-0.14	0.54	0.58	0.99	1.83	2.03	1.17	1.37	0.86	1.06	1.20
Q6ZUX7	Lipoma HMGIC fusion partner-like 2 protein OS=Homo sapiens GN=LHFPL2 PE=2 SV=2 - [LHPL2_HUMAN]	-2.72	-2.43	-1.72	-1.44	-0.11	0.17	-0.56	-0.28	-0.45	-0.73	-0.30	-0.02	2.21	2.43	1.42	2.02	1.02	2.59	1.59	1.22
P01602	Ig kappa chain V-I region HK102 (Fragment) OS=Homo sapiens GN=IGKV1-5 PE=4 SV=1 - [KV110_HUMAN]	-1.78	-1.64	-1.57	-1.43	-0.21	-0.07	-1.64	-1.50	0.85	0.72	2.12	2.25	0.20	3.91	3.69	2.53	2.32	1.56	1.35	-0.01
Q96K49	Transmembrane protein 87B OS=Homo sapiens GN=TMEM87B PE=1 SV=1 - [TM87B_HUMAN]	-1.41	-1.55	-1.28	-1.41	-0.12	-0.25	-0.54	-0.67	-0.30	-0.16	0.02	-0.12	0.93	1.43	1.29	1.28	1.15	1.28	1.14	0.80
P01742	Ig heavy chain V-I region EU OS=Homo sapiens PE=1 SV=1 - [HV101_HUMAN]	-1.29	-1.31	-1.39	-1.41	0.72	0.69	0.04	0.02	0.54	0.57	1.36	1.33	1.38	2.65	2.74	1.89	1.99	1.99	2.08	1.49
P22670	MHC class II regulatory factor RFX1 OS=Homo sapiens GN=RFX1 PE=1 SV=2 - [RFX1_HUMAN]	-2.02	-1.52	-1.90	-1.41	-2.03	-1.54	-1.17	-0.68	-0.66	-1.14	-1.06	-0.57	0.90	0.97	0.84	0.90	0.79	-0.03	-0.15	0.79
O94851	Protein-methionine sulfoxide oxidase MICAL2 OS=Homo sapiens GN=MICAL2 PE=1 SV=1 - [MICA2_HUMAN]	-1.19	-1.30	-1.27	-1.38	0.22	0.11	-0.10	-0.21	-0.10	0.02	0.01	-0.10	1.14	1.21	1.29	1.24	1.32	1.40	1.48	1.23
Q14714	Sarcospan OS=Homo sapiens GN=SSPN PE=2 SV=3 - [SSPN_HUMAN]	-1.31	-1.36	-1.32	-1.36	0.19	0.15	0.42	0.38	0.31	0.36	0.33	0.29	1.79	1.65	1.65	1.70	1.71	1.49	1.49	1.81

P05114	Non-histone chromosomal protein HMG-14 OS=Homo sapiens GN=HMGN1 PE=1 SV=3 - [HMGN1_HUMAN]	-2.09	-2.21	-1.24	-1.35	-1.03	-1.16	-1.01	-1.13	-1.41	-1.29	-0.99	-1.11	1.14	1.11	0.25	0.84	-0.02	1.05	0.19	0.29
P67809	Nuclease-sensitive element-binding protein 1 OS=Homo sapiens GN=YBX1 PE=1 SV=3 - [YBOX1_HUMAN]	-2.09	-1.97	-1.47	-1.35	-1.08	-0.96	-1.18	-1.06	-1.12	-1.23	-0.65	-0.54	0.97	1.45	0.82	0.89	0.27	1.00	0.37	0.35
Q99735	Microsomal glutathione S-transferase 2 OS=Homo sapiens GN=MGST2 PE=1 SV=1 - [MGST2_HUMAN]	-1.49	-1.56	-1.27	-1.34	-0.39	-0.46	-0.74	-0.81	-0.55	-0.47	-0.63	-0.70	0.81	0.87	0.64	1.05	0.83	1.09	0.87	0.59
Q13253	Noggin OS=Homo sapiens GN=NOG PE=1 SV=1 - [NOGG_HUMAN]	-1.11	-1.22	-1.22	-1.32	1.31	1.19	0.52	0.41	0.64	0.75	0.24	0.13	1.69	1.36	1.45	1.89	2.00	2.40	2.50	1.80
P01717	Ig lambda chain V-IV region Hil OS=Homo sapiens PE=1 SV=1 - [LV403_HUMAN]	-1.16	-1.14	-1.15	-1.28	1.10	1.10	-0.93	-0.91	0.74	0.77	1.62	1.54	0.28	2.78	2.62	1.95	1.92	2.24	2.08	0.28
P55061	Bax inhibitor 1 OS=Homo sapiens GN=TMBIM6 PE=1 SV=2 - [BI1_HUMAN]	-1.98	-2.07	-1.20	-1.28	0.04	-0.06	0.18	0.09	-0.22	-0.13	0.21	0.11	2.22	2.20	1.40	1.89	1.10	2.00	1.22	1.44
Q9UNZ5	Leydig cell tumor 10 kDa protein homolog OS=Homo sapiens GN=C19orf53 PE=1 SV=1 - [L10K_HUMAN]	-1.24	-1.24	-1.25	-1.26	-0.89	-0.90	-1.02	-1.02	-0.66	-0.65	-0.26	-0.27	0.28	0.98	0.99	0.62	0.64	0.33	0.34	0.30
P42694	Probable helicase with zinc finger domain OS=Homo sapiens GN=HELZ PE=1 SV=2 - [HELZ_HUMAN]	-1.09	-1.09	-1.25	-1.25	-0.29	-0.30	-0.78	-0.78	-0.41	-0.40	-0.35	-0.35	0.36	0.74	0.90	0.71	0.88	0.78	0.94	0.54
Q8IXL7	Methionine-R-sulfoxide reductase B3 OS=Homo sapiens GN=MSRB3 PE=1 SV=2 - [MSRB3_HUMAN]	-1.56	-1.20	-1.61	-1.25	-1.00	-0.65	-0.87	-0.51	-0.58	-0.93	-0.96	-0.61	0.74	0.60	0.64	0.66	0.71	0.54	0.59	0.80
Q96HU1	Small G protein signaling modulator 3 OS=Homo sapiens GN=SGSM3 PE=1 SV=1 - [SGSM3_HUMAN]	-1.38	-1.33	-1.28	-1.24	-0.14	-0.11	-0.24	-0.20	-0.27	-0.30	-0.17	-0.14	1.19	1.21	1.11	1.10	1.02	1.22	1.12	1.11

Q96IZ2	Androgen-dependent TFPI-regulating protein OS=Homo sapiens GN=ADTRP PE=2 SV=1 - [ADTRP_HUMAN]	-1.39	-1.44	-1.19	-1.23	-0.36	-0.41	-0.40	-0.45	-0.47	-0.42	-0.28	-0.34	1.05	1.12	0.90	1.01	0.80	1.02	0.81	0.85
Q8WXA3	RUN and FYVE domain-containing protein 2 OS=Homo sapiens GN=RUFY2 PE=1 SV=2 - [RUFY2_HUMAN]	-1.39	-1.26	-1.34	-1.21	-0.14	-0.02	-1.43	-1.30	-0.52	-0.64	-0.54	-0.42	0.02	0.85	0.80	0.78	0.73	1.23	1.18	-0.02
Q9HCY8	Protein S100-A14 OS=Homo sapiens GN=S100A14 PE=1 SV=1 - [S10AE_HUMAN]	-1.47	-1.37	-1.27	-1.17	-2.02	-1.93	-1.91	-1.82	-1.83	-1.92	-1.79	-1.70	-0.39	-0.32	-0.52	-0.42	-0.61	-0.57	-0.77	-0.58
P06312	Ig kappa chain V-IV region (Fragment) OS=Homo sapiens GN=IGKV4-1 PE=4 SV=1 - [KV401_HUMAN]	-1.63	-1.63	-1.15	-1.14	-0.71	-0.70	-1.05	-1.04	-0.98	-0.98	-0.41	-0.41	0.64	1.23	0.74	0.68	0.20	0.91	0.43	0.17
Q9UKU9	Angiopoietin-related protein 2 OS=Homo sapiens GN=ANGPTL2 PE=2 SV=1 - [ANGL2_HUMAN]	-1.26	-1.12	-1.28	-1.14	-0.10	0.04	-0.57	-0.42	-0.04	-0.18	-0.11	0.03	0.75	1.16	1.17	1.11	1.13	1.15	1.17	0.78
P29622	Kallistatin OS=Homo sapiens GN=SERPINA4 PE=1 SV=3 - [KAIN_HUMAN]	-1.20	-1.20	-1.14	-1.13	1.35	1.34	0.43	0.43	0.88	0.88	1.06	1.05	1.69	2.27	2.19	2.11	2.05	2.53	2.46	1.63
