# Supplementary material for: Systems proteomic analysis reveals that clusterin and tissue inhibitor of metalloproteinases 3 increase in leptomeningeal arteries affected by cerebral amyloid angiopathy
Source: Neuropathol Appl Neurobiol. 2016 Oct 5;43(6):492–504. doi: 10.1111/nan.12342 (PMC5638106; doi:10.1111/nan.12342)
Supplement: Supplementary file 3 — Table S3. Differentially expressed proteins in leptomeningeal arteries from CAA patients vs. young controls (log2 ratio). [file NAN-43-492-s003.pdf]

Supplementary Table 3. Differentially expressed proteins in leptomeningeal arteries from CAA patients vs. young controls (log2ratios)

Accession	Description	OLD 1/ YOUNG 1	OLD 1/ YOUNG 2	OLD 2/ YOUNG 1	OLD 2/ YOUNG 2	CAA 1/YOUNG 1	CAA 1/YOUNG 2	CAA 2/YOUNG 1	CAA 2/YOUNG 2	CAA 3/YOUNG 1	CAA 3/YOUNG 2	CAA 4/YOUNG 1	CAA 4/YOUNG 2	CAA 1/OLD 1	CAA 1/OLD 2	CAA 2/OLD 1	CAA 2/OLD 2	CAA 3/OLD 1	CAA 3/OLD 2	CAA 4/OLD 1	CAA 4/OLD 2
P26022	Pentraxin-related protein PTX3 OS=Homo sapiens GN=PTX3 PE=1 SV=3 [PTX3_HUMAN]	-1.77	-1.70	-1.90	-1.83	1.41	1.48	1.94	2.01	2.08	2.01	2.67	2.74	3.76	4.45	4.58	3.81	3.95	3.16	3.30	3.91
Q9UBS3	DnaJ homolog subfamily B member 9 OS=Homo sapiens GN=DNAJB9 PE=1 SV=1 - [DNJB9_HUMAN]	0.73	0.77	-0.20	-0.15	2.93	3.40	3.12	3.39	3.16	2.69	2.15	2.62	2.30	1.58	1.42	2.23	2.00	2.40	2.18	2.45
Q9BV73	Centrosome associated protein CEP250 OS=Homo sapiens GN=CEP250 PE=1 SV=2 - [CP250_HUMAN]	2.70	3.13	2.54	2.97	0.72	1.15	2.39	2.82	2.29	1.86	2.20	2.62	-0.26	-0.50	-0.35	-0.81	-0.65	-2.00	-1.83	-0.09
O95631	Netrin-1 OS=Homo sapiens GN=NTN1 PE=1 SV=2 [NET1_HUMAN]	0.40	0.65	0.45	0.84	2.70	3.05	1.64	1.89	2.48	2.21	2.48	2.58	1.23	2.00	1.99	1.96	1.80	2.51	2.51	1.40
Q9UBP4	Dickkopf-related protein 3 OS=Homo sapiens GN=DKK3 PE=1 SV=2 [DKK3_HUMAN]	-0.46	-0.27	-0.58	-0.60	2.14	2.22	1.55	1.51	2.30	2.17	2.40	2.53	1.42	2.29	2.85	2.63	2.71	2.55	2.58	1.78
P21741	Midkine OS=Homo sapiens GN=MDK PE=1 SV=1 [MK_HUMAN]	0.16	-0.34	-0.54	-0.52	2.42	2.62	2.52	2.36	2.35	2.57	2.28	2.07	3.02	2.99	3.11	3.12	3.14	3.10	3.36	3.46
Q9H4F8	SPARC-related modular calcium-binding protein 1 OS=Homo sapiens GN=SMOC1 PE=1 SV=1 - [SMOC1_HUMAN]	0.09	-0.06	-0.22	-0.40	2.07	2.06	1.85	2.29	2.05	1.99	2.05	2.05	2.32	2.44	2.70	2.38	2.74	2.21	2.64	2.68
Q8IWU5	Extracellular sulfatase Sulf-2 OS=Homo sapiens GN=SULF2 PE=1 SV=1 [SULF2_HUMAN]	-0.19	0.00	-0.42	-0.06	3.09	3.86	2.30	2.39	2.92	2.16	1.81	1.99	2.44	2.00	2.06	2.84	2.73	3.72	3.62	2.52

P10909	Clusterin OS=Homo sapiens GN=CLU PE=1 SV=1 [CLUS_HUMAN]	-0.28	-0.43	-0.60	-0.74	3.02	2.92	2.02	1.91	2.29	2.35	2.01	1.88	2.43	2.42	2.60	2.78	3.04	3.36	3.62	2.71
Q16568	Cocaine- and amphetamine-regulated transcript protein OS=Homo sapiens GN=CARTPT PE=1 SV=1 - [CART_HUMAN]	0.40	0.42	0.16	0.19	0.96	0.98	0.83	0.85	1.21	1.19	1.85	1.87	0.49	1.46	1.69	0.83	1.06	0.54	0.78	0.73
P36222	Chitinase-3-like protein 1 OS=Homo sapiens GN=CHI3L1 PE=1 SV=2 [CH3L1_HUMAN]	-0.12	-0.26	-0.48	-0.52	2.78	2.25	1.33	1.10	1.53	2.04	2.02	1.84	1.71	2.37	2.63	2.25	2.58	2.87	3.13	1.98
O00622	Protein CYR61 OS=Homo sapiens GN=CYR61 PE=1 SV=1 [CYR61_HUMAN]	-0.18	0.33	-0.36	-0.43	3.27	3.07	1.99	1.75	2.09	2.34	2.05	1.82	1.95	1.33	2.26	2.20	2.82	2.67	3.87	2.85
Q9BQI4	Coiled-coil domain-containing protein 3 OS=Homo sapiens GN=CCDC3 PE=2 SV=1 [CCDC3_HUMAN]	0.00	-0.28	-0.03	-0.05	3.85	3.72	1.75	1.54	2.55	2.59	1.79	1.76	1.88	2.07	1.82	2.87	2.65	3.99	3.86	1.84
Q93091	Ribonuclease K6 OS=Homo sapiens GN=RNASE6 PE=1 SV=2 - [RNAS6_HUMAN]	-1.62	-1.30	-1.84	-1.51	1.11	1.43	0.61	0.94	1.41	1.09	1.40	1.72	2.29	3.03	3.24	2.74	2.96	2.71	2.93	2.51
Q96D46	60S ribosomal export protein NMD3 OS=Homo sapiens GN=NMD3 PE=1 SV=1 [NMD3_HUMAN]	-0.45	-0.54	-0.58	-0.67	0.79	0.70	1.46	1.37	1.31	1.40	1.80	1.71	1.97	2.26	2.39	1.89	2.02	1.23	1.36	2.11
P09238	Stromelysin-2 OS=Homo sapiens GN=MMP10 PE=1 SV=1 [MMP10_HUMAN]	-0.66	-0.56	-0.50	-0.54	3.96	3.81	1.67	1.58	2.58	2.62	2.06	1.69	2.39	2.72	2.55	3.31	3.15	4.60	4.44	2.24

Q9NS84	Carbohydrate sulfotransferase 7 OS=Homo sapiens GN=CHST7 PE=1 SV=2 [CHST7_HUMAN]	-0.03	0.23	-0.76	-0.01	2.29	2.76	1.64	1.39	1.66	1.92	1.90	1.66	1.24	1.44	1.81	1.56	2.18	1.87	2.49	1.86
O95407	Tumor necrosis factor receptor superfamily member 6B OS=Homo sapiens GN=TNFRSF6B PE=1 SV=1 - [TNF6B_HUMAN]	0.03	-0.11	-0.28	-0.41	3.38	3.24	1.44	1.31	2.20	2.34	1.79	1.66	1.47	1.77	2.07	2.34	2.65	3.34	3.64	1.78
P48061	Stromal cell-derived factor 1 OS=Homo sapiens GN=CXCL12 PE=1 SV=1 - [SDF1_HUMAN]	-0.94	-0.67	-1.06	-0.73	3.22	3.60	1.16	1.50	2.42	2.14	1.09	1.62	1.93	1.98	2.41	3.39	3.14	4.42	4.17	2.15
P34096	Ribonuclease 4 OS=Homo sapiens GN=RNASE4 PE=1 SV=3 - [RNAS4_HUMAN]	-0.60	-0.14	-0.41	-0.31	2.79	2.81	1.20	1.32	1.78	1.76	1.53	1.55	1.60	2.61	1.94	2.86	2.20	3.85	3.18	1.17
Q8IWU6	Extracellular sulfatase Sulf-1 OS=Homo sapiens GN=SULF1 PE=1 SV=1 [SULF1_HUMAN]	-0.10	-0.01	-0.72	-0.81	3.49	3.30	1.17	1.09	2.21	2.31	1.65	1.54	1.66	2.04	2.63	2.86	3.30	3.99	4.43	2.27
P02649	Apolipoprotein E OS=Homo sapiens GN=APOE PE=1 SV=1 [APOE_HUMAN]	-0.03	0.00	-0.37	-0.21	3.99	4.07	1.77	1.76	2.81	2.75	1.40	1.46	1.84	1.51	1.82	2.83	3.06	3.91	4.22	2.08
P14136	Glial fibrillary acidic protein OS=Homo sapiens GN=GFAP PE=1 SV=1 [GFAP_HUMAN]	1.33	1.32	1.40	1.40	0.74	0.73	1.78	1.82	1.46	1.43	1.46	1.45	0.43	0.12	0.13	0.14	0.16	-0.61	-0.63	0.46

Q96DC8	Enoyl-CoA hydratase domain-containing protein 3, mitochondrial OS=Homo sapiens GN=ECHDC3 PE=1 SV=2 - [ECHD3_HUMAN]	-0.82	-0.72	-1.40	-1.45	0.99	0.94	1.39	1.38	1.35	1.30	1.45	1.43	2.11	2.27	2.99	2.11	2.73	1.90	2.48	2.81
Q13938	Calcyphosin OS=Homo sapiens GN=CAPS PE=1 SV=1 [CAYP1_HUMAN]	-0.09	-0.10	-0.23	-0.25	1.72	1.70	1.16	1.09	1.42	1.41	1.38	1.40	1.31	1.50	1.55	1.41	1.66	1.70	1.99	1.39
P52895	Aldo-keto reductase family 1 member C2 OS=Homo sapiens GN=AKR1C2 PE=1 SV=3 - [AK1C2_HUMAN]	-0.75	-0.74	-0.54	-0.53	0.82	1.19	0.61	0.70	1.09	0.86	1.24	1.40	1.33	2.04	1.66	1.75	1.31	2.03	1.22	0.81
Q09328	Alpha-1,6-mannosylglycoprotein 6 beta-N-acetylglucosaminyltransferase A OS=Homo sapiens GN=MGAT5 PE=1 SV=1 [MGT5A_HUMAN]	0.03	0.16	-0.32	-0.18	0.80	0.93	0.65	0.79	1.47	1.34	1.24	1.37	0.68	1.22	1.56	1.34	1.69	0.76	1.10	1.04
O60303	Uncharacterized protein KIAA0556 OS=Homo sapiens GN=KIAA0556 PE=1 SV=4 - [K0556_HUMAN]	4.07	4.56	1.77	2.25	0.91	1.39	1.17	1.66	2.23	1.75	0.88	1.37	-2.84	-3.18	-0.88	-2.29	0.01	-3.17	-0.87	-0.53
P0C0L5	Complement C4-B OS=Homo sapiens GN=C4B PE=1 SV=2 [CO4B_HUMAN]	-0.18	-0.23	-0.85	-0.67	2.42	2.29	1.41	1.32	1.70	1.73	1.23	1.35	1.71	1.92	1.94	2.11	2.38	2.54	2.64	2.07
P08514	Integrin alpha-IIb OS=Homo sapiens GN=ITGA2B PE=1 SV=3 - [ITA2B_HUMAN]	-1.31	-1.07	-1.27	-1.03	1.17	1.33	0.75	0.83	1.00	0.91	1.22	1.32	1.79	2.28	2.43	2.07	2.16	2.41	2.41	2.00

Q14314	Fibroleukin OS=Homo sapiens GN=FGL2 PE=1 SV=1 [FGL2_HUMAN]	-1.26	-1.15	-1.42	-1.32	1.07	1.20	0.63	0.84	0.96	0.73	1.15	1.31	1.92	2.20	2.70	2.07	2.35	2.43	2.50	2.22
P27169	Serum paraoxonase/arylesterase 1 OS=Homo sapiens GN=PON1 PE=1 SV=3 [PON1_HUMAN]	-1.35	-1.64	-1.64	-1.66	1.23	1.05	0.73	0.76	0.97	0.79	1.32	1.31	2.52	3.03	2.99	2.72	2.76	2.92	2.78	2.56
A6NJZ7	RIMS-binding protein 3C OS=Homo sapiens GN=RIMBP3C PE=1 SV=3 - [RIM3C_HUMAN]	2.34	2.41	1.50	1.58	0.91	0.99	1.47	1.55	1.47	1.40	1.22	1.29	-0.81	-1.11	-0.29	-0.91	-0.08	-1.44	-0.61	0.03
P05090	Apolipoprotein D OS=Homo sapiens GN=APOD PE=1 SV=1 [APOD_HUMAN]	-0.21	-0.27	-0.23	-0.23	1.32	1.41	0.78	0.88	1.00	1.07	1.28	1.27	1.00	1.30	1.45	1.13	1.14	1.52	1.60	1.13
P02795	Metallothionein-2 OS=Homo sapiens GN=MT2A PE=1 SV=1 [MT2_HUMAN]	1.85	1.80	1.62	1.57	1.13	1.08	1.16	1.11	1.21	1.26	1.31	1.25	-0.64	-0.54	-0.32	-0.56	-0.33	-0.74	-0.51	-0.40
O75094	Slit homolog 3 protein OS=Homo sapiens GN=SLIT3 PE=2 SV=3 [SLIT3_HUMAN]	0.19	0.08	-0.27	-0.23	2.75	2.92	1.73	1.73	1.86	2.02	1.34	1.24	1.78	1.26	1.57	2.29	2.37	3.30	3.21	1.96
P56705	Protein Wnt-4 OS=Homo sapiens GN=WNT4 PE=1 SV=4 [WNT4_HUMAN]	-0.31	-0.45	-0.76	-1.03	2.68	2.46	1.51	1.62	1.84	1.96	1.32	1.23	2.21	1.65	2.22	2.33	2.91	2.90	3.73	2.47
Q14213	Interleukin-27 subunit beta OS=Homo sapiens GN=EBI3 PE=1 SV=2 [IL27B_HUMAN]	-1.00	-0.63	-0.60	-0.23	2.06	2.42	0.91	1.28	1.61	1.25	0.87	1.23	1.97	1.88	1.47	2.28	1.88	3.05	2.64	1.57
P56524	Histone deacetylase 4 OS=Homo sapiens GN=HDAC4 PE=1 SV=3 [HDAC4_HUMAN]	0.85	0.61	0.51	0.28	1.65	1.41	0.81	0.58	0.84	1.08	1.47	1.23	0.02	0.63	0.96	0.27	0.60	0.79	1.12	0.36

O75936	Gamma-butyrobetaine dioxygenase OS=Homo sapiens GN=BBOX1 PE=1 SV=1 [BODG_HUMAN]	1.90	1.94	1.87	1.88	0.82	0.82	1.48	1.47	1.14	1.14	1.21	1.22	-0.32	-0.60	-0.55	-0.73	-0.67	-1.02	-0.96	-0.28
Q92765	Secreted frizzled-related protein 3 OS=Homo sapiens GN=FRZB PE=1 SV=2 [SFRP3_HUMAN]	0.13	0.44	-0.52	-0.01	3.33	2.68	1.55	1.49	1.84	2.30	1.30	1.19	1.93	1.16	1.41	2.38	2.42	3.36	3.40	1.97
P23297	Protein S100-A1 OS=Homo sapiens GN=S100A1 PE=1 SV=2 - [S10A1_HUMAN]	1.41	1.72	1.76	2.07	1.16	1.47	1.56	1.87	1.59	1.28	0.88	1.18	0.21	-0.53	-0.88	-0.10	-0.44	-0.26	-0.61	0.04
Q9UBX1	Cathepsin F OS=Homo sapiens GN=CTSF PE=1 SV=1 [CATF_HUMAN]	-0.27	-0.48	-0.47	-0.21	3.69	3.51	1.21	1.25	2.18	2.36	1.28	1.17	1.31	0.92	1.55	1.91	2.59	3.01	3.77	1.74
P34741	Syndecan-2 OS=Homo sapiens GN=SDC2 PE=1 SV=2 [SDC2_HUMAN]	-1.57	-1.68	-1.75	-1.85	2.81	2.70	1.61	1.50	1.62	1.73	1.26	1.14	3.24	2.83	3.00	3.33	3.51	4.37	4.54	3.42
O14514	Brain-specific angiogenesis inhibitor 1 OS=Homo sapiens GN=BAI1 PE=1 SV=2 [BAI1_HUMAN]	1.27	1.54	1.27	1.54	1.02	1.29	0.57	0.85	0.91	0.64	0.87	1.14	-0.64	-0.39	-0.40	-0.60	-0.60	-0.27	-0.27	-0.63
Q9H2X0	Chordin OS=Homo sapiens GN=CHRD PE=1 SV=2 [CHRD_HUMAN]	-0.24	-0.05	-0.66	-0.46	2.55	2.74	0.86	1.06	1.89	1.69	0.92	1.11	1.16	1.17	1.58	1.97	2.39	2.77	3.19	1.58
P02654	Apolipoprotein C-I OS=Homo sapiens GN=APOC1 PE=1 SV=1 [APOC1_HUMAN]	-0.79	-0.63	-0.87	-0.70	1.42	1.58	0.92	1.09	1.06	0.90	0.93	1.09	1.77	1.73	1.80	1.72	1.81	2.20	2.27	1.85
Q96L58	Beta-1,3-galactosyltransferase 6 OS=Homo sapiens GN=B3GALT6 PE=1 SV=2 - [B3GT6_HUMAN]	-1.21	-1.18	-1.60	-1.56	2.73	2.76	1.02	1.05	1.86	1.83	1.06	1.09	2.28	2.27	2.66	3.08	3.47	3.92	4.31	2.68

O75888	Tumor necrosis factor ligand superfamily member 13 OS=Homo sapiens GN=TNFSF13 PE=1 SV=1 - [TNF13_HUMAN]	0.35	0.51	0.65	0.81	2.36	2.51	1.58	1.74	1.94	1.79	0.93	1.08	1.29	0.58	0.28	1.46	1.17	1.99	1.69	1.00
Q9H2A7	C-X-C motif chemokine 16 OS=Homo sapiens GN=CXCL16 PE=2 SV=4 - [CXL16_HUMAN]	-0.06	-0.28	-0.21	-0.33	2.14	2.01	0.95	0.83	1.41	1.55	1.25	1.04	1.04	1.41	1.38	1.43	1.87	2.25	2.33	1.22
Q6IAN0	Dehydrogenase/reductase SDR family member 7B OS=Homo sapiens GN=DHRS7B PE=1 SV=2 - [DRS7B_HUMAN]	0.54	0.80	0.58	0.85	0.71	0.97	0.65	0.92	0.99	0.73	0.78	1.04	0.17	0.25	0.19	0.22	0.18	0.16	0.11	0.13
O00560	Syntenin-1 OS=Homo sapiens GN=SDCBP PE=1 SV=1 [SDCB1_HUMAN]	-0.87	-0.66	-0.61	-0.39	1.39	1.32	0.61	0.69	0.80	0.80	0.95	1.02	1.05	1.52	1.32	1.17	1.23	1.42	1.70	0.99
P35625	Metalloproteinase inhibitor 3 OS=Homo sapiens GN=TIMP3 PE=1 SV=2 [TIMP3_HUMAN]	-0.83	-0.86	-0.94	-0.99	2.94	2.93	0.79	0.83	1.75	1.77	0.99	1.02	1.56	1.75	1.85	2.60	2.74	3.66	3.88	1.79
Q8TDG2	Actin-related protein T1 OS=Homo sapiens GN=ACTRT1 PE=2 SV=2 - [ACTT1_HUMAN]	0.18	0.17	0.13	0.12	0.92	0.91	0.63	0.62	0.92	0.93	1.03	1.02	0.50	0.86	0.90	0.78	0.83	0.73	0.78	0.56
Q03692	Collagen alpha-1(X) chain OS=Homo sapiens GN=COL10A1 PE=1 SV=2 - [COAA1_HUMAN]	-1.57	-1.58	-1.49	-1.50	2.61	2.59	1.59	1.58	1.59	1.61	1.03	1.01	3.22	2.60	2.52	3.21	3.13	4.16	4.08	3.15
P17936	Insulin-like growth factor-binding protein 3 OS=Homo sapiens GN=IGFBP3 PE=1 SV=2 - [IBP3_HUMAN]	-0.67	-0.66	-0.87	-1.00	2.73	2.88	1.15	1.39	1.70	1.70	0.94	1.00	2.15	1.68	2.01	2.65	3.04	3.63	4.02	2.39

P57076	UPF0769 protein C21orf59 OS=Homo sapiens GN=C21orf59 PE=1 SV=1 - [CU059_HUMAN]	0.82	0.32	1.49	0.61	1.36	1.29	0.98	0.56	1.41	0.84	1.89	1.00	0.21	0.69	0.40	0.05	0.84	0.52	0.67	0.02
P42025	Beta-centractin OS=Homo sapiens GN=ACTR1B PE=1 SV=1 - [ACTY_HUMAN]	1.62	1.66	1.94	1.87	0.75	0.97	1.31	1.19	1.22	0.92	0.79	0.97	-0.35	-0.64	-0.82	-0.65	-0.95	-0.82	-0.97	-0.55
P0CG48	Polyubiquitin-C OS=Homo sapiens GN=UBC PE=1 SV=3 [UBC_HUMAN]	1.39	0.85	1.87	1.33	2.60	2.06	1.50	0.97	1.29	1.83	1.51	0.97	0.17	0.12	-0.36	0.47	0.00	1.19	0.71	-0.30
Q8WY91	THAP domain-containing protein 4 OS=Homo sapiens GN=THAP4 PE=1 SV=2 [THAP4_HUMAN]	3.38	2.53	3.39	2.55	1.50	0.65	1.98	1.13	1.36	2.21	1.82	0.97	-1.35	-1.55	-1.58	-1.14	-1.15	-1.89	-1.91	-1.35
P10620	Microsomal glutathione S-transferase 1 OS=Homo sapiens GN=MGST1 PE=1 SV=1 [MGST1_HUMAN]	1.01	0.76	1.45	1.19	0.96	0.70	1.48	1.23	0.83	1.09	1.22	0.97	0.52	0.22	-0.22	0.11	-0.32	-0.07	-0.50	0.09
Q8TB73	Protein NDNF OS=Homo sapiens GN=NDNF PE=2 SV=2 [NDNF_HUMAN]	0.12	0.05	-0.14	-0.03	1.99	1.77	2.44	2.10	1.81	2.09	1.17	0.96	2.14	0.89	1.21	1.70	2.00	1.71	1.89	2.30
Q9BSF0	Small membrane A-kinase anchor protein OS=Homo sapiens GN=C2orf88 PE=1 SV=2 - [SMAKA_HUMAN]	1.52	1.40	1.45	1.33	0.77	0.65	1.07	0.96	1.09	1.21	1.09	0.96	-0.39	-0.43	-0.36	-0.28	-0.20	-0.77	-0.70	-0.31
O95452	Gap junction beta-6 protein OS=Homo sapiens GN=GJB6 PE=1 SV=2 [CXB6_HUMAN]	0.28	0.48	0.59	0.79	1.36	1.55	0.56	0.76	0.98	0.79	0.76	0.96	0.33	0.48	0.17	0.53	0.23	1.06	0.75	0.03

P42785	Lysosomal Pro-X carboxypeptidase OS=Homo sapiens GN=PRCP PE=1 SV=1 [PCP_HUMAN]	0.79	0.81	0.84	0.91	0.96	0.94	0.95	1.19	0.96	0.75	0.88	0.95	0.29	0.12	0.11	0.15	0.13	0.15	0.13	0.16
Q00604	Norrin OS=Homo sapiens GN=NDP PE=1 SV=1 [NDP_HUMAN]	0.12	-0.15	-0.55	-0.77	1.12	0.85	1.52	1.26	0.90	1.17	1.02	0.94	1.52	1.10	1.48	1.08	1.73	0.86	1.65	2.14
Q86V21	Acetoacetyl-CoA synthetase OS=Homo sapiens GN=AACS PE=1 SV=1 [AACS_HUMAN]	2.22	2.38	1.88	2.04	0.79	0.94	0.87	1.03	1.07	0.92	0.78	0.93	-1.29	-1.43	-1.10	-1.28	-0.93	-1.45	-1.11	-0.95
O95084	Serine protease 23 OS=Homo sapiens GN=PRSS23 PE=1 SV=1 - [PRS23_HUMAN]	-1.24	-1.25	-1.30	-1.32	1.78	1.64	0.96	0.69	0.97	1.18	0.94	0.93	1.44	2.27	2.47	2.21	2.39	2.81	3.29	2.20
Q9H4X1	Regulator of cell cycle RGCC OS=Homo sapiens GN=RGCC PE=1 SV=1 [RGCC_HUMAN]	-1.14	-1.42	-1.30	-0.95	1.14	1.05	0.83	1.09	0.96	0.76	0.68	0.93	2.15	1.83	1.94	2.03	1.95	2.37	2.29	2.12
P01033	Metalloproteinase inhibitor 1 OS=Homo sapiens GN=TIMP1 PE=1 SV=1 [TIMP1_HUMAN]	-0.39	-0.40	-0.32	-0.33	1.64	1.62	0.63	0.63	0.98	0.99	0.93	0.91	1.08	1.32	1.24	1.41	1.34	2.01	1.94	1.01
Q9Y2J8	Protein-arginine deiminase type-2 OS=Homo sapiens GN=PADI2 PE=1 SV=2 [PADI2_HUMAN]	1.49	1.54	1.54	1.34	0.83	0.70	1.24	1.12	0.98	1.08	1.06	0.91	-0.07	-0.41	-0.55	-0.31	-0.48	-0.59	-0.88	-0.22
O75891	Cytosolic 10 formyltetrahydrofolate dehydrogenase OS=Homo sapiens GN=ALDH1L1 PE=1 SV=2 - [AL1L1_HUMAN]	0.58	0.40	0.73	0.66	1.28	1.29	1.37	1.28	1.14	1.16	0.91	0.91	0.93	0.40	0.29	0.70	0.56	0.74	0.63	0.78

P21266	Glutathione S-transferase Mu 3 OS=Homo sapiens GN=GSTM3 PE=1 SV=3 [GSTM3_HUMAN]	1.27	1.40	1.33	1.47	0.64	0.74	0.89	1.04	1.02	0.84	0.92	0.91	-0.45	-0.59	-0.54	-0.39	-0.44	-0.71	-0.67	-0.44
P05154	Plasma serine protease inhibitor OS=Homo sapiens GN=SERPINA5 PE=1 SV=3 - [IPSP_HUMAN]	0.94	1.04	1.31	1.41	0.80	0.89	0.94	1.04	0.67	0.58	0.79	0.88	0.06	-0.15	-0.52	-0.33	-0.70	-0.16	-0.53	-0.30
Q8NES3	Beta-1,3-N-acetylglucosaminyltransferase lunatic fringe OS=Homo sapiens GN=LFNG PE=1 SV=2 [LFNG_HUMAN]	-0.05	-0.41	-0.12	-0.60	3.50	3.00	1.32	1.09	1.95	2.10	1.43	0.87	1.86	1.35	1.58	2.54	2.84	3.84	4.04	2.07
Q8WUJ3	Cell migration-inducing and hyaluronan-binding protein OS=Homo sapiens GN=CEMIP PE=1 SV=2 [CEMIP_HUMAN]	-0.20	-0.33	-0.77	-0.67	2.64	2.65	1.35	1.40	1.70	1.83	0.97	0.86	1.76	1.21	1.68	2.21	2.70	3.01	3.50	2.23
Q02338	D-beta-hydroxybutyrate dehydrogenase, mitochondrial OS=Homo sapiens GN=BDH1 PE=1 SV=3 [BDH_HUMAN]	1.32	1.39	1.25	1.33	0.89	0.95	1.39	1.39	1.18	1.01	0.71	0.84	0.13	-0.55	-0.44	-0.12	-0.12	-0.40	-0.35	0.13
Q9NS98	Semaphorin-3G OS=Homo sapiens GN=SEMA3G PE=2 SV=1 - [SEM3G_HUMAN]	-1.01	-1.08	-1.09	-1.14	2.47	2.39	0.76	0.68	1.39	1.46	0.87	0.84	1.76	1.94	2.12	2.44	2.82	3.40	3.78	2.09
Q8NCH0	Carbohydrate sulfotransferase 14 OS=Homo sapiens GN=CHST14 PE=1 SV=2 - [CHSTE_HUMAN]	-0.27	-0.31	-0.60	-0.63	2.70	2.70	1.14	1.16	1.73	1.76	0.96	0.83	1.47	1.18	1.71	2.03	2.59	2.90	3.56	1.99

Q9NP85	Podocin OS=Homo sapiens GN=NPHS2 PE=1 SV=1 [PODO_HUMAN]	1.14	1.08	0.72	0.67	1.38	1.32	0.85	0.79	1.21	1.27	0.87	0.81	-0.23	-0.26	0.15	0.16	0.59	0.22	0.64	0.19
P53609	Geranylgeranyl transferase type-1 subunit beta OS=Homo sapiens GN=PGGT1B PE=1 SV=2 - [PGTB1_HUMAN]	0.08	-0.11	-0.44	-0.21	0.87	0.78	0.55	0.61	0.66	0.55	0.65	0.79	0.53	0.83	1.09	0.58	0.83	0.79	1.07	0.97
Q99988	Growth/differentiation factor 15 OS=Homo sapiens GN=GDF15 PE=1 SV=3 [GDF15_HUMAN]	0.18	0.02	0.72	0.57	1.79	1.63	1.69	1.54	1.07	1.23	0.94	0.78	1.57	0.76	0.21	1.08	0.54	1.60	1.05	1.03
Q9HCB6	Spondin-1 OS=Homo sapiens GN=SPON1 PE=1 SV=2 [SPON1_HUMAN]	-0.21	-0.44	-0.53	-0.43	0.81	0.97	0.62	0.61	0.59	0.59	0.81	0.78	0.68	1.29	1.24	0.95	1.08	1.03	1.33	1.05
Q15283	Ras GTPase-activating protein 2 OS=Homo sapiens GN=RASA2 PE=1 SV=3 [RASA2_HUMAN]	0.26	0.50	0.35	0.59	0.65	0.89	0.67	0.91	1.10	0.87	0.54	0.77	0.47	0.28	0.19	0.64	0.55	0.38	0.29	0.39
Q9Y6F9	Protein Wnt-6 OS=Homo sapiens GN=WNT6 PE=1 SV=2 [WNT6_HUMAN]	-1.33	-1.56	-1.18	-1.23	0.73	0.67	0.80	0.75	0.82	0.88	0.83	0.77	1.95	1.92	2.31	2.00	2.20	1.80	2.29	2.19
P32004	Neural cell adhesion molecule L1 OS=Homo sapiens GN=L1CAM PE=1 SV=2 [L1CAM_HUMAN]	2.38	2.28	2.40	2.50	0.79	0.68	1.00	1.08	0.68	0.77	0.77	0.76	-1.06	-1.65	-1.70	-1.48	-1.56	-1.54	-1.57	-1.11
Q7L3B6	Hsp90 co-chaperone Cdc37-like 1 OS=Homo sapiens GN=CDC37L1 PE=1 SV=1 - [CD37L_HUMAN]	1.78	1.63	1.45	1.31	0.81	0.65	0.94	0.80	0.81	0.96	0.91	0.75	-0.78	-0.87	-0.55	-0.79	-0.46	-0.99	-0.67	-0.45

P0C0L4	Complement C4-A OS=Homo sapiens GN=C4A PE=1 SV=2 [CO4A_HUMAN]	-0.60	-0.64	-0.83	-1.04	1.51	1.46	0.81	0.77	1.02	1.07	0.80	0.75	1.46	1.39	1.58	1.69	1.88	2.08	2.27	1.65
Q9BQ95	Evolutionarily conserved signaling intermediate in Toll pathway, mitochondrial OS=Homo sapiens GN=ECSIT PE=1 SV=1 [ECSIT_HUMAN]	1.35	1.25	1.30	1.20	0.71	0.61	0.77	0.67	0.52	0.62	0.85	0.75	-0.52	-0.50	-0.45	-0.69	-0.64	-0.65	-0.60	-0.46
Q93097	Protein Wnt-2b OS=Homo sapiens GN=WNT2B PE=1 SV=2 - [WNT2B_HUMAN]	-0.86	-1.08	-1.20	-1.43	1.47	1.24	1.30	1.09	1.08	1.10	0.84	0.74	2.27	1.93	1.84	2.26	2.34	2.51	2.66	2.62
P22003	Bone morphogenetic protein 5 OS=Homo sapiens GN=BMP5 PE=2 SV=1 [BMP5_HUMAN]	-0.83	-1.03	-1.10	-1.30	2.93	3.13	1.21	1.41	1.79	1.66	0.94	0.74	2.57	2.59	2.45	3.89	3.75	4.55	4.41	2.44
Q641Q3	Meteorin-like protein OS=Homo sapiens GN=METRNL PE=2 SV=1 - [METRL_HUMAN]	-0.53	-0.39	-0.69	-0.67	2.44	2.61	0.91	1.00	1.54	1.38	0.58	0.73	1.34	0.87	1.18	1.92	2.24	2.78	3.10	1.67
Q96KN2	Beta-Ala-His dipeptidase OS=Homo sapiens GN=CNDP1 PE=1 SV=4 [CNDP1_HUMAN]	-0.16	-0.15	-0.05	0.05	1.21	1.20	0.99	1.02	1.02	0.91	0.65	0.73	1.22	0.88	0.70	1.09	1.06	1.29	1.28	1.15
P04271	Protein S100-B OS=Homo sapiens GN=S100B PE=1 SV=2 [S100B_HUMAN]	0.25	0.29	0.59	0.74	0.93	1.09	1.33	1.51	1.17	1.04	0.53	0.71	1.27	0.50	-0.04	0.94	0.49	0.86	0.28	0.86
Q5SRE7	Phytanoyl-CoA dioxygenase domain-containing protein 1 OS=Homo sapiens GN=PHYHD1 PE=1 SV=2 - [PHYD1_HUMAN]	0.71	0.72	0.41	0.10	0.89	0.69	0.69	0.79	0.60	0.62	0.61	0.70	0.12	-0.06	0.21	0.11	0.07	0.08	0.24	0.10

P14210	Hepatocyte growth factor OS=Homo sapiens GN=HGF PE=1 SV=2 [HGF_HUMAN]	0.77	-0.38	0.74	-0.41	2.18	1.02	2.46	1.30	0.67	1.82	1.86	0.70	1.74	1.09	1.11	1.08	1.11	1.39	1.42	1.78
P01303	Pro-neuropeptide Y OS=Homo sapiens GN=NPY PE=1 SV=1 [NPY_HUMAN]	3.03	2.67	3.19	2.68	0.68	0.81	1.83	1.99	1.51	1.39	0.78	0.68	-0.94	-1.78	-1.99	-1.48	-1.68	-2.14	-2.34	-1.13
Q8NDZ4	Deleted in autism protein 1 OS=Homo sapiens GN=C3orf58 PE=1 SV=1 - [DIA1_HUMAN]	-0.20	-0.10	-0.23	-0.12	1.86	1.96	0.59	0.69	1.26	1.16	0.57	0.67	0.84	0.78	0.80	1.39	1.42	2.04	2.07	0.88
P16112	Aggrecan core protein OS=Homo sapiens GN=ACAN PE=1 SV=2 [PGCA_HUMAN]	0.78	0.68	1.08	0.84	2.16	2.11	1.18	1.19	1.29	1.20	0.61	0.67	0.25	-0.35	-0.72	0.24	0.03	1.05	0.93	-0.08
P78539	Sushi repeat-containing protein SRPX OS=Homo sapiens GN=SRPX PE=2 SV=1 [SRPX_HUMAN]	-0.59	-0.72	-0.73	-0.77	2.98	2.62	1.11	1.05	1.70	1.50	0.87	0.66	1.58	1.38	1.60	2.20	2.58	3.35	3.50	2.02
Q9Y5W8	Sorting nexin-13 OS=Homo sapiens GN=SNX13 PE=1 SV=4 [SNX13_HUMAN]	1.10	1.03	0.44	0.37	1.93	1.85	0.99	0.92	0.97	1.04	0.73	0.66	-0.05	-0.36	0.30	-0.03	0.64	0.81	1.47	0.62
P23141	Liver carboxylesterase 1 OS=Homo sapiens GN=CES1 PE=1 SV=2 [EST1_HUMAN]	-1.51	-1.51	-2.17	-2.09	1.68	1.55	1.04	0.86	0.93	1.11	0.70	0.65	2.43	2.34	2.67	2.69	3.16	3.05	3.68	3.11
P26572	Alpha-1,3-mannosyl-glycoprotein 2-beta-N-acetylglucosaminyltransferase OS=Homo sapiens GN=MGAT1 PE=2 SV=2 [MGAT1_HUMAN]	-0.93	-0.77	-0.88	-1.13	2.76	2.60	0.70	0.69	1.41	1.56	0.95	0.63	1.04	1.10	1.73	2.09	2.34	3.20	3.29	1.55

P04004	Vitronectin OS=Homo sapiens GN=VTN PE=1 SV=1 [VTNC_HUMAN]	-1.72	-1.66	-1.74	-1.68	1.83	1.88	1.19	1.17	1.19	1.19	0.69	0.62	2.58	2.42	2.47	2.81	2.96	3.44	3.43	2.84
Q8IV08	Phospholipase D3 OS=Homo sapiens GN=PLD3 PE=1 SV=1 [PLD3_HUMAN]	0.28	0.33	0.56	0.68	0.87	0.96	0.66	0.78	0.95	0.64	0.52	0.62	0.23	0.40	0.28	0.45	0.28	0.60	0.47	0.21
Q08431	Lactadherin OS=Homo sapiens GN=MFGE8 PE=1 SV=2 [MFGM_HUMAN]	-1.11	-0.89	-1.46	-1.27	2.28	2.16	1.33	1.32	1.49	1.43	0.52	0.62	2.06	1.62	2.22	2.33	3.09	2.92	3.73	2.82
Q7Z6K3	Protein prenyltransferase alpha subunit repeat-containing protein 1 OS=Homo sapiens GN=PTAR1 PE=1 SV=2 [PTAR1_HUMAN]	0.02	-0.01	0.45	0.42	1.10	1.07	0.95	0.93	0.85	0.88	0.64	0.60	0.99	0.62	0.19	0.89	0.47	1.07	0.64	0.57
Q7Z5R6	Amyloid beta A4 precursor protein-binding family B member 1-interacting protein OS=Homo sapiens GN=APBB1IP PE=1 SV=1 - [AB1IP_HUMAN]	0.72	0.61	0.78	0.68	0.88	0.77	1.15	1.04	1.05	1.16	0.71	0.60	0.49	0.00	-0.07	0.47	0.41	0.15	0.08	0.43
Q15102	Platelet-activating factor acetylhydrolase IB subunit gamma OS=Homo sapiens GN=PAFAH1B3 PE=1 SV=1 - [PA1B3_HUMAN]	1.42	1.38	1.44	1.41	0.68	0.64	1.07	1.04	0.85	0.89	0.63	0.59	-0.29	-0.78	-0.81	-0.50	-0.52	-0.76	-0.78	-0.30
Q9UJH8	Meteorin OS=Homo sapiens GN=METRN PE=2 SV=2 - [METRN_HUMAN]	-1.00	-0.98	-1.31	-1.45	1.63	1.50	0.73	0.62	0.86	1.04	0.57	0.58	1.79	1.51	1.95	2.05	2.51	2.52	2.98	2.26
Q53RD9	Fibulin-7 OS=Homo sapiens GN=FBLN7 PE=2 SV=1 [FBLN7_HUMAN]	-0.45	-0.27	-0.07	0.07	2.01	1.73	1.06	0.78	0.94	1.23	0.87	0.58	0.91	0.58	0.57	1.04	1.04	1.69	1.69	0.92

P15559	NAD(P)H dehydrogenase [quinone] 1 OS=Homo sapiens GN=NQO1 PE=1 SV=1 [NQO1_HUMAN]	-0.41	-0.49	-0.26	-0.33	0.82	0.77	1.10	1.13	0.80	0.78	0.61	0.57	1.45	1.04	0.70	1.13	1.10	1.35	1.01	1.48
Q8IX30	Signal peptide, CUB and EGF-like domain-containing protein 3 OS=Homo sapiens GN=SCUBE3 PE=1 SV=1 - [SCUB3_HUMAN]	-0.72	-0.97	-1.02	-1.17	2.15	1.92	1.13	0.99	1.00	1.51	0.92	0.53	2.00	1.55	2.03	2.29	2.47	2.95	3.15	2.29
Q8N135	Leucine-rich repeat LGI family member 4 OS=Homo sapiens GN=LGI4 PE=2 SV=1 [LGI4_HUMAN]	0.93	0.93	0.75	0.75	2.28	2.34	0.67	0.67	1.41	1.41	0.53	0.52	-0.43	-0.19	-0.05	0.41	0.56	0.86	1.06	-0.23
P41222	Prostaglandin-H2 D-isomerase OS=Homo sapiens GN=PTGDS PE=1 SV=1 [PTGDS_HUMAN]	-0.65	-0.81	-0.61	-0.71	1.55	1.45	0.77	0.59	0.81	0.92	0.76	0.52	1.51	1.41	1.31	1.71	1.42	2.30	2.01	1.50
P04745	Alpha-amylase 1 OS=Homo sapiens GN=AMY1A PE=1 SV=2 [AMY1_HUMAN]	-2.49	-2.25	-4.76	-4.66	-4.11	-4.07	-4.47	-4.26	-4.33	-4.43	-4.75	-4.58	-0.38	-0.62	0.33	-0.50	0.50	-0.32	0.81	0.61
P02538	Keratin, type II cytoskeletal 6A OS=Homo sapiens GN=KRT6A PE=1 SV=3 [K2C6A_HUMAN]	-3.81	-3.86	-4.62	-4.80	-3.88	-3.96	-4.40	-4.57	-3.62	-3.60	-4.10	-4.23	-0.66	-0.49	0.42	-0.33	0.80	-0.40	0.61	0.29
P13646	Keratin, type I cytoskeletal 13 OS=Homo sapiens GN=KRT13 PE=1 SV=4 [K1C13_HUMAN]	-3.73	-3.63	-3.82	-3.81	-3.51	-3.58	-3.72	-3.76	-3.69	-3.69	-3.95	-4.04	-0.08	0.02	0.43	0.25	0.50	0.06	0.44	0.24
P31151	Protein S100-A7 OS=Homo sapiens GN=S100A7 PE=1 SV=4 - [S10A7_HUMAN]	-2.75	-2.72	-3.77	-3.73	-4.67	-4.50	-4.96	-4.79	-3.73	-3.76	-3.58	-3.55	-1.17	-0.83	0.18	-0.98	0.04	-0.95	1.11	0.90

P27482	Calmodulin-like protein 3 OS=Homo sapiens GN=CALML3 PE=1 SV=2 - [CALL3_HUMAN]	-2.97	-3.00	-3.43	-3.46	-2.25	-2.06	-2.72	-2.51	-2.85	-2.90	-3.19	-3.19	-0.19	0.00	-0.09	-0.04	0.24	0.20	0.29	-0.09
P19013	Keratin, type II cytoskeletal 4 OS=Homo sapiens GN=KRT4 PE=1 SV=4 [K2C4_HUMAN]	-3.20	-3.35	-3.69	-3.71	-3.42	-3.54	-3.87	-4.11	-3.35	-3.18	-2.97	-3.12	-0.27	-0.21	0.35	0.03	0.35	-0.14	0.53	0.15
A8MQ03	UPF0574 protein C9orf169 OS=Homo sapiens GN=C9orf169 PE=1 SV=1 - [CI169_HUMAN]	-4.33	-4.51	-3.96	-4.14	-2.22	-2.41	-2.61	-2.80	-2.93	-2.74	-2.92	-3.11	1.77	1.42	1.04	1.62	1.26	2.09	1.73	1.41
Q9UBC9	Small proline-rich protein 3 OS=Homo sapiens GN=SPRR3 PE=1 SV=2 [SPRR3_HUMAN]	-4.05	-4.24	-5.52	-5.49	-2.52	-2.72	-4.05	-4.12	-3.19	-2.99	-2.82	-3.03	-0.45	-0.21	0.91	0.68	0.47	-0.56	0.64	1.25
Q9UBG3	Cornulin OS=Homo sapiens GN=CRNN PE=1 SV=1 [CRNN_HUMAN]	-3.06	-2.87	-3.83	-3.54	-3.06	-3.02	-3.41	-3.36	-3.03	-3.22	-3.13	-3.01	-0.51	-0.33	0.56	-0.33	0.50	-0.09	0.71	0.30
Q08188	Protein-glutamine gamma-glutamyltransferase E OS=Homo sapiens GN=TGM3 PE=1 SV=4 [TGM3_HUMAN]	-3.69	-3.80	-4.36	-4.40	-3.15	-3.19	-3.23	-3.21	-2.46	-2.47	-2.83	-2.97	0.36	0.98	1.51	1.09	2.29	0.51	1.91	1.56
Q69YL0	Uncharacterized protein DKFZp762I1415 OS=Homo sapiens PE=4 SV=1 [YC029_HUMAN]	-3.94	-4.27	-4.00	-4.33	-2.11	-2.44	-2.70	-3.03	-2.83	-2.50	-2.59	-2.92	1.29	1.36	1.41	1.47	1.54	1.82	1.87	1.36
P11678	Eosinophil peroxidase OS=Homo sapiens GN=EPX PE=1 SV=2 [PERE_HUMAN]	-2.67	-2.84	-3.06	-3.23	-2.26	-2.44	-2.15	-2.32	-2.42	-2.24	-2.65	-2.83	0.57	0.02	0.41	0.46	0.85	0.39	0.78	0.97

P31947	14-3-3 protein sigma OS=Homo sapiens GN=SFN PE=1 SV=1 [1433S_HUMAN]	-3.69	-3.59	-3.30	-3.19	-2.47	-2.37	-3.07	-2.96	-2.76	-2.85	-2.90	-2.81	0.68	0.79	0.39	0.87	0.47	1.20	0.81	0.29
Q9NQ76	Matrix extracellular phosphoglycoprotein OS=Homo sapiens GN=MEPE PE=1 SV=1 [MEPE_HUMAN]	-2.41	-2.57	-3.41	-3.57	-2.60	-2.76	-2.60	-2.76	-2.58	-2.42	-2.62	-2.78	-0.13	-0.20	0.79	0.02	1.02	-0.20	0.80	0.87
P08727	Keratin, type I cytoskeletal 19 OS=Homo sapiens GN=KRT19 PE=1 SV=4 [K1C19_HUMAN]	-2.51	-2.66	-2.82	-2.96	-2.45	-2.60	-2.97	-3.11	-2.61	-2.46	-2.63	-2.78	-0.40	-0.11	0.19	0.14	0.38	0.05	0.35	-0.09
O15335	Chondroadherin OS=Homo sapiens GN=CHAD PE=2 SV=2 [CHAD_HUMAN]	-3.49	-3.51	-4.44	-4.39	-2.72	-2.60	-2.77	-2.73	-2.74	-2.78	-2.70	-2.72	1.03	0.80	1.30	0.88	1.51	0.75	1.32	1.48
P02461	Collagen alpha-1(III) chain OS=Homo sapiens GN=COL3A1 PE=1 SV=4 - [CO3A1_HUMAN]	-2.66	-2.85	-2.54	-2.83	-1.56	-1.71	-2.39	-2.51	-2.34	-2.10	-2.60	-2.68	0.16	0.04	-0.03	0.40	0.35	1.21	1.01	0.05
Q7Z6B7	SLIT-ROBO Rho GTPase-activating protein 1 OS=Homo sapiens GN=SRGAP1 PE=1 SV=1 - [SRGP1_HUMAN]	-2.72	-2.84	-3.17	-3.30	-1.79	-1.92	-2.77	-2.90	-2.03	-1.90	-2.41	-2.55	0.00	0.31	0.76	0.85	1.31	0.91	1.36	0.47
P08123	Collagen alpha-2(I) chain OS=Homo sapiens GN=COL1A2 PE=1 SV=7 - [CO1A2_HUMAN]	-3.08	-3.40	-3.12	-3.43	-1.89	-2.18	-2.32	-2.62	-2.44	-2.21	-2.25	-2.54	0.76	0.81	0.85	0.91	1.01	1.21	1.29	0.84
P20908	Collagen alpha-1(V) chain OS=Homo sapiens GN=COL5A1 PE=1 SV=3 - [CO5A1_HUMAN]	-3.22	-3.17	-3.17	-3.07	-1.79	-1.91	-2.20	-2.51	-2.50	-2.39	-2.37	-2.52	0.78	1.09	0.64	1.06	0.75	1.62	1.10	0.44

P47929	Galectin-7 OS=Homo sapiens GN=LGALS7 PE=1 SV=2 - [LEG7_HUMAN]	-1.42	-1.57	-2.08	-2.22	-2.73	-2.88	-3.01	-3.15	-2.85	-2.70	-2.38	-2.52	-1.53	-0.95	-0.29	-1.25	-0.58	-1.32	-0.67	-0.87
Q02040	A-kinase anchor protein 17A OS=Homo sapiens GN=AKAP17A PE=1 SV=2 - [AK17A_HUMAN]	3.39	2.64	-2.19	-2.94	-1.10	-1.86	-2.07	-2.82	-2.58	-1.82	-1.75	-2.51	-5.40	-5.13	0.44	-5.18	0.40	-4.50	1.07	0.18
P02452	Collagen alpha-1(I) chain OS=Homo sapiens GN=COL1A1 PE=1 SV=5 - [CO1A1_HUMAN]	-3.05	-3.20	-3.12	-3.30	-1.87	-1.98	-2.37	-2.56	-2.31	-2.17	-2.29	-2.48	0.77	0.75	0.87	1.04	1.03	1.18	1.29	0.84
Q6DT37	Serine/threonine-protein kinase MRCK gamma OS=Homo sapiens GN=CDC42BPG PE=1 SV=2 - [MRCKG_HUMAN]	0.52	-0.16	-2.07	-2.75	-2.79	-3.47	-2.03	-2.71	-2.66	-1.98	-1.79	-2.47	-2.50	-2.30	0.28	-2.47	0.12	-3.33	-0.74	0.10
Q96SM3	Probable carboxypeptidase X1 OS=Homo sapiens GN=CPXM1 PE=2 SV=2 [CPXM1_HUMAN]	-3.29	-3.42	-3.51	-3.52	-1.03	-1.00	-1.67	-1.68	-1.57	-1.56	-2.40	-2.40	1.58	0.91	1.12	1.69	1.94	2.41	2.56	1.86
Q8IVN3	Musculoskeletal embryonic nuclear protein 1 OS=Homo sapiens GN=MUSTN1 PE=2 SV=2 - [MSTN1_HUMAN]	-3.16	-3.12	-2.91	-2.87	-3.19	-3.15	-2.59	-2.54	-2.91	-2.95	-2.41	-2.37	0.63	0.75	0.50	0.24	0.00	-0.05	-0.29	0.39
P23280	Carbonic anhydrase 6 OS=Homo sapiens GN=CA6 PE=1 SV=3 [CAH6_HUMAN]	-3.25	-3.23	-3.03	-2.90	-2.28	-2.10	-2.79	-2.61	-2.45	-2.59	-2.47	-2.37	-0.15	0.33	0.66	0.30	0.13	0.36	1.10	0.51
P05997	Collagen alpha-2(V) chain OS=Homo sapiens GN=COL5A2 PE=1 SV=3 - [CO5A2_HUMAN]	-3.11	-3.21	-3.21	-3.24	-2.00	-2.11	-2.48	-2.63	-2.50	-2.33	-2.31	-2.33	0.47	0.85	0.85	0.74	0.71	1.13	1.19	0.38

P13647	Keratin, type II cytoskeletal 5 OS=Homo sapiens GN=KRT5 PE=1 SV=3 [K2C5_HUMAN]	-2.82	-2.71	-3.22	-3.11	-2.36	-2.21	-3.57	-3.66	-3.11	-3.22	-2.45	-2.28	-0.59	0.22	1.35	0.10	0.54	0.62	0.47	0.23
P02458	Collagen alpha-1(II) chain OS=Homo sapiens GN=COL2A1 PE=1 SV=3 - [CO2A1_HUMAN]	-2.80	-2.98	-3.18	-3.24	-1.71	-1.82	-2.32	-2.41	-2.15	-2.03	-2.13	-2.24	0.59	0.65	1.17	1.14	1.28	1.30	1.50	0.91
Q6UWP8	Suprabasin OS=Homo sapiens GN=SBSN PE=2 SV=2 [SBSN_HUMAN]	-3.12	-3.06	-3.14	-3.08	-2.17	-2.12	-2.76	-2.71	-2.41	-2.46	-2.22	-2.17	0.41	0.90	0.92	0.69	0.72	0.94	0.96	0.44
P12107	Collagen alpha-1(XI) chain OS=Homo sapiens GN=COL11A1 PE=1 SV=4 - [COBA1_HUMAN]	-2.64	-2.78	-2.97	-3.03	-1.44	-1.51	-1.88	-1.94	-1.88	-1.73	-1.93	-2.11	0.71	0.52	0.88	0.90	1.19	1.07	1.51	1.16
P22531	Small proline-rich protein 2E OS=Homo sapiens GN=SPRR2E PE=2 SV=2 - [SPR2E_HUMAN]	-2.81	-2.24	-3.26	-2.91	-3.09	-2.74	-3.40	-2.82	-2.01	-2.35	-2.43	-2.09	-0.53	0.15	0.83	0.25	0.95	-0.53	0.16	0.48
P16403	Histone H1.2 OS=Homo sapiens GN=HIST1H1C PE=1 SV=2 - [H12_HUMAN]	-2.80	-2.81	-2.21	-2.21	-2.25	-2.26	-1.25	-1.25	-2.48	-2.47	-2.05	-2.07	1.61	0.75	0.15	0.36	-0.23	0.54	-0.06	1.02
P01036	Cystatin-S OS=Homo sapiens GN=CST4 PE=1 SV=3 [CYTS_HUMAN]	-3.63	-3.37	-2.65	-2.39	-1.85	-1.60	-2.21	-1.95	-2.06	-2.32	-2.28	-2.02	1.48	1.36	0.37	1.34	0.36	1.76	0.78	0.50
Q16527	Cysteine and glycine-rich protein 2 OS=Homo sapiens GN=CSRP2 PE=1 SV=3 [CSRP2_HUMAN]	-3.17	-3.35	-3.38	-3.44	-2.38	-2.58	-1.78	-1.79	-2.17	-2.20	-1.90	-2.01	1.45	1.07	1.47	1.11	1.26	0.46	0.67	1.75

Q16820	Meprin A subunit beta OS=Homo sapiens GN=MEP1B PE=1 SV=3 [MEP1B_HUMAN]	-1.46	-1.85	-1.69	-2.08	-1.73	-2.12	-0.92	-1.31	-2.33	-1.94	-1.60	-1.99	0.59	-0.14	0.09	-0.45	-0.22	-0.29	-0.06	0.83
Q8NEQ5	Transmembrane protein C1orf162 OS=Homo sapiens GN=C1orf162 PE=2 SV=1 - [CA162_HUMAN]	-2.57	-2.64	-2.91	-2.98	-1.88	-1.96	-2.74	-2.82	-1.80	-1.72	-1.85	-1.93	-0.12	0.73	1.06	0.87	1.22	0.67	1.01	0.23
P02790	Hemopexin OS=Homo sapiens GN=HPX PE=1 SV=2 [HEMO_HUMAN]	-1.87	-1.90	-1.98	-1.94	-0.93	-0.89	-1.58	-1.60	-1.30	-1.39	-1.92	-1.91	0.34	0.06	0.13	0.60	0.60	1.02	1.07	0.45
Q6P1M3	Lethal(2) giant larvae protein homolog 2 OS=Homo sapiens GN=LLGL2 PE=1 SV=2 [L2GL2_HUMAN]	-1.50	-1.63	-1.32	-1.44	-0.95	-1.25	-1.55	-1.68	-1.61	-1.48	-1.60	-1.84	0.00	-0.20	-0.11	0.03	0.07	0.02	0.03	-0.05
Q7Z3D6	UPF0317 protein C14orf159, mitochondrial OS=Homo sapiens GN=C14orf159 PE=1 SV=2 - [CN159_HUMAN]	0.58	0.56	0.56	0.53	-1.06	-1.10	-0.91	-0.75	-1.16	-1.00	-1.66	-1.79	-1.24	-2.07	-2.09	-1.60	-1.66	-1.44	-1.59	-1.29
P01833	Polymeric immunoglobulin receptor OS=Homo sapiens GN=PIGR PE=1 SV=4 [PIGR_HUMAN]	-2.29	-2.40	-3.65	-3.66	-1.86	-1.88	-2.60	-2.62	-2.06	-2.03	-1.65	-1.77	1.35	0.64	1.85	1.89	1.65	2.02	1.77	1.11
Q15404	Ras suppressor protein 1 OS=Homo sapiens GN=RSU1 PE=1 SV=3 [RSU1_HUMAN]	-2.38	-2.48	-2.04	-2.20	-0.57	-0.74	-0.86	-1.02	-0.91	-0.74	-1.59	-1.75	1.27	1.02	0.45	1.13	1.33	1.53	1.45	1.24
Q07092	Collagen alpha-1(XVI) chain OS=Homo sapiens GN=COL16A1 PE=1 SV=2 - [COGA1_HUMAN]	-2.96	-3.12	-3.09	-3.29	-1.00	-1.21	-1.65	-1.82	-1.57	-1.55	-1.58	-1.72	1.36	1.34	1.25	1.71	1.69	2.03	1.97	1.51

Q02539	Histone H1.1 OS=Homo sapiens GN=HIST1H1A PE=1 SV=3 - [H11_HUMAN]	-1.34	-1.33	-0.68	-0.66	-1.12	-1.11	-0.79	-0.78	-1.34	-1.35	-1.72	-1.71	0.60	-0.38	-1.04	0.02	-0.64	0.21	-0.45	-0.05
Q9HCY8	Protein S100-A14 OS=Homo sapiens GN=S100A14 PE=1 SV=1 - [S10AE_HUMAN]	-1.47	-1.37	-1.27	-1.17	-2.02	-1.93	-1.91	-1.82	-1.83	-1.92	-1.79	-1.70	-0.39	-0.32	-0.52	-0.42	-0.61	-0.57	-0.77	-0.58
Q58A45	PAB-dependent poly(A)-specific ribonuclease subunit PAN3 OS=Homo sapiens GN=PAN3 PE=1 SV=3 [PAN3_HUMAN]	-1.85	-1.97	-1.38	-1.50	-1.09	-1.22	-1.59	-1.71	-1.70	-1.57	-1.57	-1.70	0.32	0.28	-0.19	0.31	-0.16	0.75	0.28	-0.14
Q8N2H4	Protein SYS1 homolog OS=Homo sapiens GN=SYS1 PE=1 SV=1 [SYS1_HUMAN]	-3.77	-3.54	-2.61	-2.38	-1.81	-1.58	-1.98	-1.74	-1.62	-1.85	-1.92	-1.69	1.85	1.85	0.69	1.95	0.79	1.95	0.79	0.70
P37802	Transgelin-2 OS=Homo sapiens GN=TAGLN2 PE=1 SV=3 - [TAGL2_HUMAN]	-2.39	-2.36	-2.29	-2.26	-1.28	-1.18	-1.84	-1.72	-1.44	-1.56	-1.72	-1.69	0.64	0.59	0.56	0.90	0.74	0.98	1.01	0.60
Q9NZD4	Alpha-hemoglobin-stabilizing protein OS=Homo sapiens GN=AHSP PE=1 SV=1 [AHSP_HUMAN]	-2.49	-2.47	-2.19	-2.17	-0.62	-0.60	-0.73	-0.70	-0.96	-0.97	-1.67	-1.65	1.82	0.83	0.52	1.55	1.25	1.86	1.55	1.53
P25940	Collagen alpha-3(V) chain OS=Homo sapiens GN=COL5A3 PE=1 SV=3 - [CO5A3_HUMAN]	-2.20	-2.28	-2.52	-2.60	-1.26	-1.35	-2.31	-2.39	-1.77	-1.69	-1.47	-1.56	-0.05	0.73	1.05	0.54	0.87	0.92	1.24	0.28
P01009	Alpha-1-antitrypsin OS=Homo sapiens GN=SERPINA1 PE=1 SV=3 - [A1AT_HUMAN]	-1.50	-1.49	-1.17	-1.17	-0.99	-1.04	-1.42	-1.43	-1.60	-1.50	-1.51	-1.52	-0.01	-0.08	-0.32	-0.05	-0.33	0.41	0.11	-0.24

P02750	Leucine-rich alpha-2-glycoprotein OS=Homo sapiens GN=LRG1 PE=1 SV=2 [A2GL_HUMAN]	-0.60	-0.77	-0.48	-0.53	-0.58	-0.63	-0.71	-0.69	-1.04	-0.89	-1.37	-1.46	0.16	-0.65	-0.95	0.03	-0.23	0.13	-0.03	-0.03
P02765	Alpha-2-HS-glycoprotein OS=Homo sapiens GN=AHSG PE=1 SV=1 [FETUA_HUMAN]	-3.17	-3.04	-3.64	-3.49	-1.99	-1.97	-2.41	-2.45	-1.83	-1.88	-1.58	-1.46	1.29	1.96	2.12	1.46	1.70	1.46	1.74	1.35
Q05707	Collagen alpha-1(XIV) chain OS=Homo sapiens GN=COL14A1 PE=1 SV=3 - [COEA1_HUMAN]	-3.06	-3.09	-3.32	-3.30	-0.98	-0.97	-1.13	-1.09	-1.18	-1.19	-1.43	-1.42	2.02	1.76	1.91	2.02	2.19	2.23	2.40	2.25
Q9BVG9	Phosphatidylserine synthase 2 OS=Homo sapiens GN=PTDSS2 PE=1 SV=1 - [PTSS2_HUMAN]	-2.43	-2.42	-2.61	-2.60	-1.23	-1.22	-1.65	-1.63	-1.42	-1.43	-1.42	-1.41	0.84	1.02	1.19	1.04	1.22	1.18	1.36	1.03
P02768	Serum albumin OS=Homo sapiens GN=ALB PE=1 SV=2 [ALBU_HUMAN]	-1.73	-1.71	-1.56	-1.54	-0.72	-0.67	-0.85	-0.80	-0.87	-0.95	-1.48	-1.40	0.95	0.29	0.09	0.84	0.66	1.06	0.84	0.80
Q9Y6X9	MORC family CW-type zinc finger protein 2 OS=Homo sapiens GN=MORC2 PE=1 SV=2 - [MORC2_HUMAN]	-1.60	-1.59	-1.99	-1.98	-1.07	-1.07	-2.09	-2.08	-1.36	-1.36	-1.41	-1.40	-0.44	0.19	0.58	0.27	0.66	0.50	0.90	-0.04
P51911	Calponin-1 OS=Homo sapiens GN=CNN1 PE=1 SV=2 [CNN1_HUMAN]	-3.13	-3.14	-3.31	-3.41	-1.18	-1.20	-1.62	-1.68	-1.36	-1.41	-1.39	-1.40	1.74	1.99	2.12	1.93	1.98	2.00	1.99	1.86
Q01995	Transgelin OS=Homo sapiens GN=TAGLN PE=1 SV=4 [TAGL_HUMAN]	-2.94	-2.90	-3.19	-3.09	-1.42	-1.35	-1.78	-1.84	-1.52	-1.63	-1.35	-1.38	1.32	1.53	1.68	1.36	1.50	1.57	1.70	1.36

Q15847	Adipogenesis regulatory factor OS=Homo sapiens GN=ADIRF PE=1 SV=1 [ADIRF_HUMAN]	-1.72	-1.90	-2.05	-2.09	-1.21	-1.29	-1.31	-1.33	-1.49	-1.46	-1.20	-1.33	0.65	0.47	0.87	0.39	0.51	0.45	0.58	0.62
O75112	LIM domain-binding protein 3 OS=Homo sapiens GN=LDB3 PE=1 SV=2 [LDB3_HUMAN]	-2.60	-2.47	-2.71	-2.57	-1.86	-1.82	-1.52	-1.38	-1.54	-1.62	-1.36	-1.31	0.85	1.24	1.64	0.96	1.34	0.48	0.85	1.16
Q8N0X4	Citrate lyase subunit beta like protein, mitochondrial OS=Homo sapiens GN=CLYBL PE=2 SV=2 [CLYBL_HUMAN]	-0.88	-0.55	-0.53	-0.56	-1.10	-0.86	-0.94	-0.75	-1.15	-1.27	-1.32	-1.30	-0.15	-0.81	-0.97	-0.40	-0.74	-0.18	-0.31	-0.49
Q9HD20	Probable cation-transporting ATPase 13A1 OS=Homo sapiens GN=ATP13A1 PE=1 SV=2 - [AT131_HUMAN]	-1.04	-1.06	-2.14	-2.26	-1.05	-1.18	-1.47	-1.58	-1.28	-1.15	-1.06	-1.28	0.59	0.73	0.76	0.61	0.85	0.91	1.01	0.62
P25398	40S ribosomal protein S12 OS=Homo sapiens GN=RPS12 PE=1 SV=3 [RS12_HUMAN]	-0.68	-0.54	-0.36	-0.22	-0.87	-0.80	-0.76	-0.65	-0.97	-1.21	-1.36	-1.28	-0.04	-0.82	-0.96	-0.39	-0.74	-0.37	-0.54	-0.36
Q96P44	Collagen alpha-1(XXI) chain OS=Homo sapiens GN=COL21A1 PE=2 SV=1 - [COLA1_HUMAN]	-2.13	-2.14	-2.45	-2.63	-1.03	-1.34	-1.12	-1.41	-1.37	-1.11	-1.07	-1.28	1.60	1.51	1.57	1.57	1.59	1.53	1.34	1.51
Q15746	Myosin light chain kinase, smooth muscle OS=Homo sapiens GN=MYLK PE=1 SV=4 [MYLK_HUMAN]	-2.28	-2.08	-2.22	-2.20	-0.74	-0.80	-0.96	-0.91	-0.97	-1.09	-1.28	-1.26	1.38	0.97	0.88	1.19	1.11	1.54	1.29	1.27

P01861	Ig gamma-4 chain C region OS=Homo sapiens GN=IGHG4 PE=1 SV=1 [IGHG4_HUMAN]	-3.30	-3.33	-3.07	-3.00	-1.52	-1.46	-1.82	-1.73	-1.64	-1.61	-1.18	-1.25	1.40	1.76	1.73	1.40	1.39	1.58	1.47	1.35
Q8WW12	PEST proteolytic signal-containing nuclear protein OS=Homo sapiens GN=PCNP PE=1 SV=2 [PCNP_HUMAN]	-0.30	-0.32	-0.40	-0.41	-1.45	-1.47	-1.35	-1.36	-1.17	-1.15	-1.22	-1.24	-0.99	-0.91	-0.82	-0.82	-0.72	-1.16	-1.07	-0.89
Q6UWY5	Olfactomedin-like protein 1 OS=Homo sapiens GN=OLFML1 PE=1 SV=2 - [OLFL1_HUMAN]	-2.70	-2.73	-3.37	-3.25	-0.70	-0.68	-0.62	-0.59	-0.89	-0.85	-1.16	-1.23	2.01	1.75	2.15	1.94	2.43	2.25	2.58	2.70
O60237	Protein phosphatase 1 regulatory subunit 12B OS=Homo sapiens GN=PPP1R12B PE=1 SV=2 - [MYPT2_HUMAN]	-1.67	-1.76	-1.52	-1.68	-1.33	-1.30	-1.02	-1.10	-1.20	-1.06	-1.21	-1.22	0.65	0.48	0.44	0.36	0.17	0.32	0.17	0.64
Q9NTU7	Cerebellin-4 OS=Homo sapiens GN=CBLN4 PE=1 SV=1 [CBLN4_HUMAN]	-2.96	-3.01	-3.52	-3.57	-1.06	-1.12	-2.13	-2.19	-1.70	-1.64	-1.14	-1.21	0.88	1.82	2.37	1.35	1.91	1.88	2.44	1.45
Q8IUK8	Cerebellin-2 OS=Homo sapiens GN=CBLN2 PE=2 SV=1 [CBLN2_HUMAN]	-2.64	-2.52	-2.52	-2.39	-1.61	-1.48	-1.67	-1.54	-1.35	-1.47	-1.33	-1.20	1.03	1.32	1.19	1.20	1.08	1.02	0.89	0.91
Q99983	Osteomodulin OS=Homo sapiens GN=OMD PE=1 SV=1 [OMD_HUMAN]	-2.28	-2.30	-2.37	-2.39	-1.03	-1.08	-1.03	-1.08	-1.34	-1.29	-1.16	-1.19	1.39	1.14	1.21	1.31	1.41	1.48	1.58	1.50
Q14192	Four and a half LIM domains protein 2 OS=Homo sapiens GN=FHL2 PE=1 SV=3 [FHL2_HUMAN]	-2.40	-2.15	-2.08	-1.86	-0.70	-0.63	-1.15	-1.09	-0.98	-1.12	-1.42	-1.17	1.16	1.04	0.82	1.29	0.98	1.57	1.37	0.93

P07585	Decorin OS=Homo sapiens GN=DCN PE=1 SV=1 [PGS2_HUMAN]	-2.13	-2.13	-2.19	-2.23	-0.63	-0.67	-1.10	-1.17	-1.02	-0.93	-1.10	-1.15	1.01	1.14	1.14	1.26	1.23	1.64	1.65	1.11
O60437	Periplakin OS=Homo sapiens GN=PPL PE=1 SV=4 [PEPL_HUMAN]	-1.42	-1.33	-1.63	-1.61	-1.36	-1.28	-1.70	-1.61	-1.47	-1.49	-1.11	-1.15	-0.13	0.24	0.56	-0.06	0.28	0.15	0.38	0.08
P24844	Myosin regulatory light polypeptide 9 OS=Homo sapiens GN=MYL9 PE=1 SV=4 [MYL9_HUMAN]	-2.59	-2.56	-2.66	-2.63	-1.21	-1.18	-1.31	-1.42	-1.24	-1.17	-1.12	-1.15	1.10	1.42	1.49	1.36	1.43	0.98	1.44	1.18
O94875	Sorbin and SH3 domain containing protein 2 OS=Homo sapiens GN=SORBS2 PE=1 SV=3 - [SRBS2_HUMAN]	-1.93	-1.99	-2.38	-2.41	-1.43	-1.58	-1.35	-1.45	-1.38	-1.26	-1.03	-1.14	0.82	1.07	1.31	0.86	1.14	0.80	0.93	1.02
P05114	Non-histone chromosomal protein HMG-14 OS=Homo sapiens GN=HMGN1 PE=1 SV=3 - [HMGN1_HUMAN]	-2.09	-2.21	-1.24	-1.35	-1.03	-1.16	-1.01	-1.13	-1.41	-1.29	-0.99	-1.11	1.14	1.11	0.25	0.84	-0.02	1.05	0.19	0.29
P50238	Cysteine-rich protein 1 OS=Homo sapiens GN=CRIP1 PE=1 SV=3 [CRIP1_HUMAN]	-3.59	-3.46	-3.71	-3.58	-2.30	-2.18	-2.10	-1.78	-1.88	-2.12	-1.22	-1.10	1.58	2.22	2.41	1.68	1.88	1.27	1.40	1.79
Q12962	Transcription initiation factor TFIID subunit 10 OS=Homo sapiens GN=TAF10 PE=1 SV=1 [TAF10_HUMAN]	-0.21	-0.72	-0.27	-0.78	-1.40	-1.92	-0.70	-1.21	-1.29	-0.77	-0.58	-1.10	-0.43	-0.37	-0.31	-0.53	-0.47	-1.21	-1.15	-0.37
Q9Y2S7	Polymerase delta-interacting protein 2 OS=Homo sapiens GN=POLDIP2 PE=1 SV=1 - [PDIP2_HUMAN]	-0.52	-0.45	-0.31	-0.24	-1.37	-1.30	-1.21	-1.14	-1.19	-1.26	-1.16	-1.09	-0.64	-0.63	-0.85	-0.71	-0.91	-0.87	-1.08	-0.84

P35749	Myosin-11 OS=Homo sapiens GN=MYH11 PE=1 SV=3 [MYH11_HUMAN]	-2.78	-2.80	-2.99	-3.01	-1.06	-1.05	-1.22	-1.27	-1.22	-1.19	-1.06	-1.07	1.64	1.76	1.95	1.64	1.86	1.84	1.95	1.82
P49411	Elongation factor Tu, mitochondrial OS=Homo sapiens GN=TUFM PE=1 SV=2 [EFTU_HUMAN]	-0.04	-0.26	0.12	0.04	-1.11	-1.28	-0.66	-0.81	-1.00	-0.84	-0.91	-1.05	-0.53	-0.76	-1.15	-0.77	-0.92	-1.17	-1.32	-0.87
P05546	Heparin cofactor 2 OS=Homo sapiens GN=SERPIND1 PE=1 SV=3 - [HEP2_HUMAN]	-2.98	-2.96	-3.06	-3.06	-1.10	-1.10	-1.52	-1.57	-1.00	-1.17	-1.10	-1.05	1.68	1.98	2.09	2.06	2.00	2.14	1.94	1.59
P53814	Smoothelin OS=Homo sapiens GN=SMTN PE=1 SV=7 [SMTN_HUMAN]	-2.70	-2.63	-3.23	-3.28	-1.21	-1.14	-1.35	-1.36	-1.28	-1.30	-1.02	-1.04	1.24	1.52	2.23	1.52	2.21	1.67	2.19	1.96
P00750	Tissue-type plasminogen activator OS=Homo sapiens GN=PLAT PE=1 SV=1 [TPA_HUMAN]	-1.64	-1.33	-1.66	-1.45	-1.05	-0.93	-1.68	-1.56	-1.22	-1.34	-1.14	-1.03	0.29	0.61	0.62	0.35	0.37	0.58	0.59	0.31
P17661	Desmin OS=Homo sapiens GN=DES PE=1 SV=3 [DESM_HUMAN]	-3.38	-3.31	-3.67	-3.59	-1.13	-1.06	-1.97	-1.93	-1.38	-1.43	-1.03	-1.01	1.58	2.41	2.75	2.05	2.39	2.46	2.61	1.80
P19652	Alpha-1-acid glycoprotein 2 OS=Homo sapiens GN=ORM2 PE=1 SV=2 [A1AG2_HUMAN]	-1.82	-1.67	-2.00	-1.88	-1.03	-0.88	-0.98	-0.84	-0.67	-0.87	-1.04	-0.99	0.92	0.72	0.80	1.00	1.04	0.87	0.89	1.01
Q8N4X5	Actin filament-associated protein 1-like 2 OS=Homo sapiens GN=AFAP1L2 PE=1 SV=1 - [AF1L2_HUMAN]	-0.90	-0.74	-1.44	-1.22	-0.86	-0.64	-0.69	-0.85	-0.74	-0.96	-0.78	-0.98	-0.09	0.39	0.46	0.09	0.52	0.12	0.57	0.44
P15924	Desmoplakin OS=Homo sapiens GN=DSP PE=1 SV=3 [DESP_HUMAN]	-2.60	-2.57	-2.66	-2.68	-0.62	-0.66	-0.94	-0.95	-0.89	-0.88	-0.94	-0.97	1.62	1.69	1.72	1.70	1.83	1.86	1.99	1.83

Q5U651	Ras-interacting protein 1 OS=Homo sapiens GN=RASIP 1 PE=1 SV=1 - [RAIN_HUMAN]	-1.61	-1.78	-1.45	-1.65	-0.58	-0.92	-0.97	-1.31	-1.21	-0.78	-0.53	-0.97	0.57	0.86	0.55	0.96	0.64	0.96	0.62	0.28
Q9NR12	PDZ and LIM domain protein 7 OS=Homo sapiens GN=PDLIM 7 PE=1 SV=1 - [PDLI7_HUMAN]	-3.11	-3.00	-3.32	-3.10	-1.04	-1.02	-1.44	-1.39	-1.08	-1.10	-1.09	-0.97	1.72	2.04	2.17	1.84	1.99	1.98	2.01	1.85
Q9H0X9	Oxysterol-binding protein-related protein 5 OS=Homo sapiens GN=OSBPL 5 PE=1 SV=1 - [OSBL5_HUMAN]	-1.58	-1.69	-1.71	-1.82	-0.70	-0.82	-1.10	-1.21	-0.81	-0.69	-0.85	-0.96	0.53	0.74	0.86	0.92	1.05	0.87	0.99	0.67
Q5JTZ9	Alanine--tRNA ligase, mitochondrial OS=Homo sapiens GN=AARS2 PE=1 SV=1 [SYAM_HUMAN]	0.38	0.21	-0.35	-0.51	-0.99	-1.15	-1.14	-1.31	-0.90	-0.73	-0.79	-0.96	-1.47	-1.16	-0.44	-1.08	-0.35	-1.38	-0.66	-0.73
Q6UVY6	DBH-like monooxygenase protein 1 OS=Homo sapiens GN=MOXD 1 PE=2 SV=1 - [MOXD1_HUMAN]	-1.19	-1.21	-1.13	-1.14	-0.90	-0.92	-0.89	-0.91	-1.07	-1.05	-0.93	-0.95	0.36	0.27	0.19	0.17	0.11	0.28	0.21	0.30
P29474	Nitric oxide synthase, endothelial OS=Homo sapiens GN=NOS3 PE=1 SV=3 [NOS3_HUMAN]	-1.76	-1.92	-2.09	-2.05	-0.86	-0.92	-1.47	-1.47	-1.06	-1.00	-0.92	-0.95	0.53	0.72	1.17	0.85	1.15	1.22	1.46	0.68
Q96CM8	Acyl-CoA synthetase family member 2, mitochondrial OS=Homo sapiens GN=ACSF2 PE=1 SV=2 [ACSF2_HUMAN]	-2.24	-2.21	-2.31	-2.45	-0.80	-0.86	-0.80	-0.93	-0.96	-0.86	-0.81	-0.94	1.36	1.41	1.50	1.48	1.48	1.43	1.59	1.49

Q6DRA6	Putative histone H2B type 2-D OS=Homo sapiens GN=HIST2 H2BD PE=5 SV=3 - [H2B2D_HU MAN]	-1.70	-1.58	-1.13	-1.04	-0.94	-0.92	-0.91	-0.59	-0.94	-0.96	-0.96	-0.94	0.85	0.72	0.17	0.50	0.20	0.45	-0.03	0.51
O15231	Zinc finger protein 185 OS=Homo sapiens GN=ZNF18 5 PE=1 SV=3 - [ZN185_HU MAN]	-1.39	-1.21	-0.81	-0.63	-1.60	-1.57	-0.83	-0.66	-1.31	-1.34	-0.97	-0.94	0.61	2.27	1.97	1.93	1.64	1.62	1.32	0.04
Q14847	LIM and SH3 domain protein 1 OS=Homo sapiens GN=LASP1 PE=1 SV=2 [LASP1_HU MAN]	-0.78	-0.78	-0.82	-0.80	-0.79	-0.87	-0.75	-0.77	-0.87	-0.80	-0.87	-0.94	0.04	-0.05	-0.14	-0.04	-0.02	-0.07	-0.04	0.04
Q5TD97	Four and a half LIM domains protein 5 OS=Homo sapiens GN=FHL5 PE=1 SV=1 [FHL5_HUM AN]	-3.41	-3.33	-3.29	-3.16	-1.07	-1.06	-1.61	-1.44	-1.29	-1.45	-1.03	-0.94	1.94	2.22	2.29	2.10	1.91	2.43	2.17	1.77
P48735	Isocitrate dehydrogen ase [NADP], mitochondri al OS=Homo sapiens GN=IDH2 PE=1 SV=2 [IDHP_HUM AN]	-0.39	-0.49	-0.23	-0.26	-0.99	-1.04	-0.74	-0.78	-0.91	-0.84	-0.85	-0.93	-0.20	-0.39	-0.65	-0.40	-0.65	-0.49	-0.71	-0.45
O43707	Alpha- actinin-4 OS=Homo sapiens GN=ACTN4 PE=1 SV=2 [ACTN4_HU MAN]	-2.24	-2.17	-2.30	-2.22	-1.06	-0.96	-1.25	-1.17	-1.11	-1.18	-0.96	-0.92	1.07	1.28	1.37	1.10	1.14	1.22	1.24	1.14
Q92522	Histone H1x OS=Homo sapiens GN=H1FX PE=1 SV=1 [H1X_HUM AN]	-1.46	-1.63	-1.31	-1.26	-0.89	-1.01	-1.01	-0.96	-1.14	-0.94	-0.85	-0.92	0.57	0.75	0.59	0.42	0.25	0.39	0.19	0.37
O43294	Transformin g growth factor beta- 1-induced transcript 1 protein OS=Homo sapiens GN=TGFB1 I1 PE=1 SV=2 - [TGFI1_HU MAN]	-2.57	-2.50	-2.68	-2.68	-1.19	-1.12	-1.20	-1.15	-1.10	-1.11	-0.87	-0.92	1.57	1.62	1.75	1.48	1.62	1.44	1.46	1.44

Q13642	Four and a half LIM domains protein 1 OS=Homo sapiens GN=FHL1 PE=1 SV=4 [FHL1_HUMAN]	-1.85	-1.77	-1.65	-1.62	-0.81	-0.73	-1.05	-0.97	-1.01	-1.08	-0.92	-0.91	0.77	0.79	0.60	0.77	0.65	0.97	0.86	0.52
Q14244	Ensconsin OS=Homo sapiens GN=MAP7 PE=1 SV=1 [MAP7_HUMAN]	-0.11	-0.22	0.06	-0.05	-0.89	-1.01	-1.29	-1.40	-1.22	-1.11	-0.78	-0.90	-1.13	-0.67	-0.84	-0.97	-1.13	-0.80	-0.97	-1.28
P04275	von Willebrand factor OS=Homo sapiens GN=VWF PE=1 SV=4 [VWF_HUMAN]	-2.36	-2.28	-2.47	-2.36	-0.75	-0.74	-1.13	-1.10	-1.04	-1.00	-0.89	-0.89	1.16	1.26	1.46	1.19	1.35	1.35	1.57	1.32
Q9BQI0	Allograft inflammatory factor 1-like OS=Homo sapiens GN=AIF1L PE=1 SV=1 [AIF1L_HUMAN]	-1.95	-1.98	-1.51	-1.52	-1.46	-1.38	-1.04	-1.04	-0.92	-1.15	-0.94	-0.89	0.80	1.01	0.92	1.02	0.82	0.48	0.23	0.55
Q16873	Leukotriene C4 synthase OS=Homo sapiens GN=LTC4S PE=1 SV=1 [LTC4S_HUMAN]	-2.21	-2.10	-2.42	-2.31	-0.68	-0.57	-1.24	-1.13	-0.75	-0.86	-0.98	-0.88	1.03	1.23	1.44	1.38	1.60	1.51	1.72	1.24
P69891	Hemoglobin subunit gamma-1 OS=Homo sapiens GN=HBG1 PE=1 SV=2 [HBG1_HUMAN]	-1.32	-1.31	-1.64	-1.62	-0.66	-0.65	-0.97	-0.95	-0.72	-0.73	-0.88	-0.87	0.41	0.45	0.76	0.63	0.94	0.65	0.96	0.74
P09493	Tropomyosin alpha-1 chain OS=Homo sapiens GN=TPM1 PE=1 SV=2 [TPM1_HUMAN]	-2.45	-2.57	-2.53	-2.49	-1.26	-1.13	-1.56	-1.47	-1.17	-1.35	-0.94	-0.86	1.14	1.74	1.65	1.37	1.33	1.47	1.35	1.09
Q8IVN8	Somatomedin-B and thrombospondin type-1 domain-containing protein OS=Homo sapiens GN=SBSPON PE=1 SV=2 - [SBSPO_HUMAN]	-2.28	-2.22	-2.69	-2.48	-0.83	-0.63	-1.17	-1.12	-1.08	-1.13	-0.86	-0.84	0.84	1.89	2.11	1.80	2.21	2.06	2.46	0.89

P16402	Histone H1.3 OS=Homo sapiens GN=HIST1H1D PE=1 SV=2 - [H13_HUMAN]	-1.00	-1.04	-0.83	-0.87	-0.90	-0.94	-0.66	-0.70	-1.12	-1.08	-0.79	-0.84	0.39	0.21	0.04	-0.05	-0.22	0.08	-0.09	0.23
Q6WCQ1	Myosin phosphatase Rho-interacting protein OS=Homo sapiens GN=MPRIP PE=1 SV=3 [MPRIP_HUMAN]	-0.79	-0.82	-0.73	-0.75	-0.63	-0.69	-0.71	-0.66	-0.74	-0.78	-0.80	-0.82	0.20	0.12	-0.07	0.09	0.06	0.14	0.11	0.09
Q71UM5	40S ribosomal protein S27-like OS=Homo sapiens GN=RPS27L PE=1 SV=3 - [RS27L_HUMAN]	-0.89	-0.81	-1.16	-1.08	-1.11	-1.04	-1.20	-1.12	-0.64	-0.71	-0.88	-0.81	-0.25	0.02	0.28	0.21	0.48	-0.24	0.03	0.02
Q9BUP0	EF-hand domain-containing protein D1 OS=Homo sapiens GN=EFHD1 PE=1 SV=1 [EFHD1_HUMAN]	-1.79	-1.50	-1.84	-1.86	-1.02	-0.98	-1.27	-1.22	-0.92	-0.94	-0.87	-0.80	0.52	0.83	0.99	0.84	0.86	0.62	0.81	0.70
P00734	Prothrombin OS=Homo sapiens GN=F2 PE=1 SV=2 [THRB_HUMAN]	-2.58	-2.68	-2.63	-2.58	-1.28	-1.19	-1.61	-1.59	-1.29	-1.37	-0.85	-0.79	0.97	1.77	1.95	1.51	1.44	1.33	1.45	1.09
P08183	Multidrug resistance protein 1 OS=Homo sapiens GN=ABCB1 PE=1 SV=3 [MDR1_HUMAN]	-1.11	-1.06	-1.43	-1.29	-0.88	-0.96	-1.19	-1.23	-0.69	-0.77	-0.78	-0.79	0.15	0.46	0.60	0.30	0.55	0.14	0.29	0.45
Q9Y3B4	Pre-mRNA branch site protein p14 OS=Homo sapiens GN=SF3B14 PE=1 SV=1 - [PM14_HUMAN]	-0.76	-0.81	-0.47	-0.52	-0.57	-0.63	-0.71	-0.76	-0.80	-0.74	-0.73	-0.79	0.11	0.03	-0.26	0.05	-0.24	0.17	-0.12	-0.17
Q9NZN4	EH domain-containing protein 2 OS=Homo sapiens GN=EHD2 PE=1 SV=2 [EHD2_HUMAN]	-2.46	-2.49	-2.56	-2.48	-1.06	-1.05	-1.25	-1.25	-1.09	-1.03	-0.77	-0.77	1.29	1.58	1.64	1.41	1.27	1.10	1.24	1.31

Q8WX93	Palladin OS=Homo sapiens GN=PALLD PE=1 SV=3 [PALLD_HUMAN]	-2.37	-2.43	-2.54	-2.50	-0.61	-0.59	-0.95	-1.09	-0.86	-0.85	-0.79	-0.76	1.40	1.62	1.75	1.55	1.70	1.69	1.95	1.52
P00738	Haptoglobin OS=Homo sapiens GN=HP PE=1 SV=1 [HPT_HUMAN]	-1.78	-1.69	-1.72	-1.66	-1.38	-1.19	-1.59	-1.53	-1.18	-1.33	-0.80	-0.76	0.24	1.02	0.90	0.41	0.29	0.27	0.08	0.08
P35579	Myosin-9 OS=Homo sapiens GN=MYH9 PE=1 SV=4 [MYH9_HUMAN]	-1.83	-1.86	-1.78	-1.73	-0.66	-0.73	-0.94	-0.96	-0.90	-0.87	-0.73	-0.75	0.94	1.07	1.04	0.99	0.92	1.15	1.02	0.90
P07305	Histone H1.0 OS=Homo sapiens GN=H1F0 PE=1 SV=3 [H10_HUMAN]	-1.44	-1.43	-1.29	-1.34	-1.13	-1.18	-0.84	-0.89	-1.06	-0.97	-0.70	-0.74	0.60	0.76	0.58	0.43	0.31	0.24	0.21	0.55
P52943	Cysteine-rich protein 2 OS=Homo sapiens GN=CRIP2 PE=1 SV=1 [CRIP2_HUMAN]	-1.99	-1.89	-1.89	-1.88	-1.17	-1.14	-1.18	-1.20	-1.09	-1.10	-0.70	-0.73	0.87	1.28	1.19	1.00	0.83	0.91	0.73	0.74
Q8NC51	Plasminogen activator inhibitor 1 RNA-binding protein OS=Homo sapiens GN=SERBP1 PE=1 SV=2 - [PAIRB_HUMAN]	-1.24	-1.29	-0.82	-0.93	-1.05	-0.90	-1.04	-1.13	-1.13	-0.99	-0.64	-0.73	0.18	0.47	0.02	0.07	-0.22	0.13	-0.13	-0.12
Q15124	Phosphoglucomutase-like protein 5 OS=Homo sapiens GN=PGM5 PE=1 SV=2 [PGM5_HUMAN]	-2.73	-2.68	-2.90	-2.69	-0.68	-0.58	-1.45	-1.22	-0.90	-1.06	-0.78	-0.73	1.49	2.05	2.10	1.94	1.96	2.14	2.11	1.58
Q99715	Collagen alpha-1(XII) chain OS=Homo sapiens GN=COL12A1 PE=1 SV=2 - [COCA1_HUMAN]	-2.55	-2.56	-2.84	-2.87	-1.00	-0.96	-1.18	-1.14	-0.93	-0.97	-0.75	-0.73	1.49	1.92	2.09	1.70	1.91	1.76	1.98	1.72

P21291	Cysteine and glycine-rich protein 1 OS=Homo sapiens GN=CSRP1 PE=1 SV=3 [CSRP1_HUMAN]	-1.14	-1.03	-1.14	-1.12	-0.96	-0.98	-0.76	-0.77	-0.69	-0.80	-0.72	-0.73	0.40	0.53	0.48	0.38	0.43	0.23	0.21	0.42
P46199	Translation initiation factor IF-2, mitochondrial OS=Homo sapiens GN=MTIF2 PE=1 SV=2 [IF2M_HUMAN]	-0.03	-0.14	-0.20	-0.31	-0.80	-0.91	-0.62	-0.73	-0.85	-0.74	-0.61	-0.72	0.13	-0.05	-0.35	-0.22	-0.52	-0.79	-0.61	-0.17
P00751	Complement factor B OS=Homo sapiens GN=CFB PE=1 SV=2 [CFAB_HUMAN]	-1.96	-1.94	-1.89	-2.06	-0.64	-0.59	-0.76	-0.72	-0.76	-0.83	-0.75	-0.72	1.31	1.03	0.89	1.21	0.95	1.30	1.19	1.22
P50552	Vasodilator-stimulated phosphoprotein OS=Homo sapiens GN=VASP PE=1 SV=3 [VASP_HUMAN]	-1.74	-1.84	-1.51	-1.57	-0.64	-0.64	-0.80	-0.71	-0.85	-0.93	-0.62	-0.72	1.10	0.88	0.90	0.93	0.93	1.14	0.69	0.83
Q8WWP7	GTPase IMAP family member 1 OS=Homo sapiens GN=GIMAP1 PE=1 SV=1 - [GIMA1_HUMAN]	-1.25	-1.12	-1.76	-1.72	-0.79	-0.69	-1.18	-1.08	-0.72	-0.83	-0.81	-0.72	0.09	0.45	0.93	0.43	0.80	0.52	0.86	0.63
P60468	Protein transport protein Sec61 subunit beta OS=Homo sapiens GN=SEC61B PE=1 SV=2 - [SC61B_HUMAN]	-1.81	-1.92	-1.36	-1.47	-0.86	-1.03	-0.83	-0.94	-0.83	-0.94	-0.69	-0.71	1.03	1.12	0.79	1.12	0.67	0.51	0.21	0.85
Q05682	Caldesmon OS=Homo sapiens GN=CALD1 PE=1 SV=3 [CALD1_HUMAN]	-2.71	-2.75	-2.53	-2.59	-1.56	-1.57	-1.31	-1.35	-1.25	-1.20	-0.76	-0.71	1.37	1.92	1.86	1.52	1.31	1.15	1.12	1.29
O43768	Alpha-endosulfine OS=Homo sapiens GN=ENSA PE=1 SV=1 [ENSA_HUMAN]	-0.10	-0.06	0.11	0.15	-0.65	-0.61	-0.66	-0.62	-0.80	-0.83	-0.74	-0.71	-0.50	-0.64	-0.86	-0.70	-0.91	-0.56	-0.78	-0.70

Q9BQ61	Uncharacterized protein C19orf43 OS=Homo sapiens GN=C19orf43 PE=1 SV=1 - [CS043_HUMAN]	-0.38	-0.41	0.03	-0.01	-0.93	-0.97	-1.18	-1.21	-0.79	-0.75	-0.63	-0.67	-0.74	-0.25	-0.66	-0.34	-0.75	-0.57	-0.97	-1.14
Q9NYL9	Tropomodulin-3 OS=Homo sapiens GN=TMOD3 PE=1 SV=1 - [TMOD3_HUMAN]	-1.80	-1.86	-1.62	-1.81	-0.72	-0.75	-0.81	-0.70	-0.83	-0.76	-0.61	-0.67	0.86	1.16	1.28	1.00	1.01	0.96	1.00	1.06
P36955	Pigment epithelium-derived factor OS=Homo sapiens GN=SERPINF1 PE=1 SV=4 - [PEDF_HUMAN]	-2.39	-2.44	-2.43	-2.44	-0.85	-0.86	-1.20	-1.18	-1.11	-1.09	-0.68	-0.67	1.18	1.64	1.71	1.25	1.25	1.62	1.57	1.27
Q5JVS0	Intracellular hyaluronan-binding protein 4 OS=Homo sapiens GN=HABP4 PE=1 SV=1 [HABP4_HUMAN]	0.53	0.50	0.52	0.50	-0.73	-0.63	-0.83	-0.85	-0.69	-0.77	-0.84	-0.66	-0.93	-0.86	-1.49	-1.16	-1.15	-1.14	-1.29	-1.29
P62736	Actin, aortic smooth muscle OS=Homo sapiens GN=ACTA2 PE=1 SV=1 [ACTA_HUMAN]	-2.10	-2.20	-2.56	-2.79	-0.68	-0.83	-1.20	-1.28	-1.13	-1.01	-0.57	-0.66	1.22	1.46	1.93	1.43	1.65	1.58	2.01	1.56
Q14BN4	Sarcolemmal membrane-associated protein OS=Homo sapiens GN=SLMAP PE=1 SV=1 [SLMAP_HUMAN]	-1.88	-1.85	-1.92	-1.85	-1.01	-1.07	-1.10	-1.13	-1.04	-1.02	-0.60	-0.65	0.89	1.28	1.34	0.97	0.94	0.93	0.96	0.89
Q9UGP4	LIM domain-containing protein 1 OS=Homo sapiens GN=LIMD1 PE=1 SV=1 [LIMD1_HUMAN]	-2.10	-1.99	-2.28	-2.17	-1.27	-1.16	-1.13	-1.02	-0.91	-1.01	-0.74	-0.63	1.02	1.37	1.54	1.12	1.30	0.82	0.99	1.21
O94901	SUN domain-containing protein 1 OS=Homo sapiens GN=SUN1 PE=1 SV=3 [SUN1_HUMAN]	-1.50	-1.43	-1.62	-1.48	-0.77	-0.72	-0.88	-0.81	-0.78	-0.77	-0.64	-0.63	0.69	0.95	0.91	0.77	0.99	0.82	0.86	0.71

P62841	40S ribosomal protein S15 OS=Homo sapiens GN=RPS15 PE=1 SV=2 [RS15_HUMAN]	-1.50	-1.50	-0.58	-0.63	-0.91	-0.97	-0.67	-0.66	-0.78	-0.73	-0.57	-0.63	0.88	0.75	0.11	0.43	-0.18	0.41	-0.22	0.18
P02545	Prelamin-A/C OS=Homo sapiens GN=LMNA PE=1 SV=1 [LMNA_HUMAN]	-1.96	-1.91	-1.99	-1.98	-1.08	-1.07	-1.02	-1.02	-0.93	-0.96	-0.63	-0.62	0.98	1.29	1.35	1.03	1.04	0.90	0.87	1.02
Q9BX79	Stimulated by retinoic acid gene 6 protein homolog OS=Homo sapiens GN=STRA6 PE=1 SV=1 [STRA6_HUMAN]	-2.35	-2.29	-2.47	-2.41	-0.72	-0.66	-0.95	-0.89	-0.69	-0.74	-0.66	-0.60	1.45	1.70	1.81	1.64	1.76	1.61	1.74	1.58
Q15599	Na(+)/H(+) exchange regulatory cofactor NHE-RF2 OS=Homo sapiens GN=SLC9A3R2 PE=1 SV=2 - [NHRF2_HUMAN]	-1.16	-0.80	-1.07	-0.83	-0.73	-0.59	-1.01	-0.82	-0.80	-0.91	-0.81	-0.60	0.19	0.42	0.39	0.22	0.09	0.19	0.17	0.07
Q14644	Ras GTPase-activating protein 3 OS=Homo sapiens GN=RASA3 PE=1 SV=3 [RASA3_HUMAN]	-1.41	-1.49	-2.98	-3.06	-0.87	-0.95	-1.53	-1.61	-1.19	-1.11	-0.52	-0.60	-0.06	0.90	2.46	0.33	1.90	0.53	2.09	1.51
P62861	40S ribosomal protein S30 OS=Homo sapiens GN=FAU PE=1 SV=1 [RS30_HUMAN]	-0.94	-1.02	-0.62	-0.70	-0.57	-0.65	-0.81	-0.89	-0.87	-0.79	-0.52	-0.60	0.19	0.43	0.11	0.19	-0.13	0.36	0.04	-0.12
P42704	Leucine-rich PPR motif-containing protein, mitochondrial OS=Homo sapiens GN=LRPPRC PE=1 SV=3 - [LPPRC_HUMAN]	-0.11	-0.13	0.13	0.08	-0.80	-0.92	-0.74	-0.70	-0.78	-0.68	-0.55	-0.60	-0.54	-0.54	-0.66	-0.64	-0.81	-0.80	-0.93	-0.77

Q96CX2	BTB/POZ domain-containing protein KCTD12 OS=Homo sapiens GN=KCTD12 PE=1 SV=1 - [KCD12_HUMAN]	-0.95	-0.85	-1.07	-1.09	-1.04	-0.92	-1.22	-1.13	-0.74	-0.81	-0.68	-0.60	-0.24	0.22	0.28	0.11	0.14	-0.16	-0.16	-0.20
Q9UMS6	Synaptopodin-2 OS=Homo sapiens GN=SYNPO2 PE=1 SV=2 - [SYNP2_HUMAN]	-2.21	-2.15	-2.60	-2.40	-0.96	-1.02	-1.25	-1.21	-1.03	-1.08	-0.65	-0.60	1.02	1.58	1.66	1.37	1.40	1.28	1.31	1.14
P62857	40S ribosomal protein S28 OS=Homo sapiens GN=RPS28 PE=1 SV=1 [RS28_HUMAN]	-0.23	-0.12	-0.25	-0.11	-0.88	-0.70	-0.92	-0.76	-0.61	-0.78	-0.67	-0.59	-0.72	-0.35	-0.32	-0.45	-0.49	-0.66	-0.77	-0.58
P41219	Peripherin OS=Homo sapiens GN=PRPH PE=1 SV=2 [PERI_HUMAN]	-0.40	-0.46	-0.84	-1.09	-1.27	-1.29	-0.73	-0.65	-1.26	-1.03	-0.81	-0.59	-0.35	-0.17	-0.15	-0.76	-0.16	-0.60	-0.24	-0.09
P07951	Tropomyosin beta chain OS=Homo sapiens GN=TPM2 PE=1 SV=1 [TPM2_HUMAN]	-1.67	-1.47	-1.85	-1.72	-0.96	-0.80	-1.10	-0.92	-0.86	-1.02	-0.61	-0.58	0.59	1.06	1.17	0.70	1.00	0.90	1.44	0.73
P50895	Basal cell adhesion molecule OS=Homo sapiens GN=BCAM PE=1 SV=2 [BCAM_HUMAN]	-2.56	-2.49	-2.68	-2.63	-1.21	-1.14	-1.18	-1.15	-1.01	-1.05	-0.59	-0.58	1.38	1.95	2.08	1.49	1.59	1.34	1.53	1.61
P22670	MHC class II regulatory factor RFX1 OS=Homo sapiens GN=RFX1 PE=1 SV=2 [RFX1_HUMAN]	-2.02	-1.52	-1.90	-1.41	-2.03	-1.54	-1.17	-0.68	-0.66	-1.14	-1.06	-0.57	0.90	0.97	0.84	0.90	0.79	-0.03	-0.15	0.79
P60660	Myosin light polypeptide 6 OS=Homo sapiens GN=MYL6 PE=1 SV=2 [MYL6_HUMAN]	-1.95	-1.86	-1.98	-1.93	-0.73	-0.80	-0.98	-0.96	-0.87	-0.80	-0.62	-0.57	1.02	1.38	1.33	1.06	1.21	1.05	1.17	1.09

Q9UKX7	Nuclear pore complex protein Nup50 OS=Homo sapiens GN=NUP50 PE=1 SV=2 [NUP50_HUMAN]	-1.64	-1.63	-1.86	-1.84	-1.13	-1.12	-1.13	-1.11	-0.83	-0.84	-0.57	-0.56	0.57	1.08	1.28	0.84	1.05	0.50	0.71	0.79
P08651	Nuclear factor 1 C-type OS=Homo sapiens GN=NFIC PE=1 SV=2 [NFIC_HUMAN]	-1.40	-1.21	-1.87	-1.69	-0.85	-0.66	-1.25	-1.07	-1.10	-1.28	-0.72	-0.54	0.20	0.68	1.15	0.15	0.62	0.54	1.01	0.68
P67809	Nuclease-sensitive element-binding protein 1 OS=Homo sapiens GN=YBX1 PE=1 SV=3 [YBOX1_HUMAN]	-2.09	-1.97	-1.47	-1.35	-1.08	-0.96	-1.18	-1.06	-1.12	-1.23	-0.65	-0.54	0.97	1.45	0.82	0.89	0.27	1.00	0.37	0.35
