# Supplementary material for: Systems proteomic analysis reveals that clusterin and tissue inhibitor of metalloproteinases 3 increase in leptomeningeal arteries affected by cerebral amyloid angiopathy
Source: Neuropathol Appl Neurobiol. 2016 Oct 5;43(6):492–504. doi: 10.1111/nan.12342 (PMC5638106; doi:10.1111/nan.12342)
Supplement: Supplementary file 4 — Table S4. Differentially expressed proteins in leptomeningeal arteries from CAA patients vs. age‐matched controls (log2 ratio). [file NAN-43-492-s004.pdf]

Supplementary Table 4. Differentially expressed proteins in leptomeningeal arteries from CAA patients vs. age-matched controls (log2ratios)

Accession	Description	OLD 1/ YOUNG 1	OLD 1/ YOUNG 2	OLD 2/ YOUNG 1	OLD 2/ YOUNG 2	CAA 1/YOUNG 1	CAA 1/YOUNG 2	CAA 2/YOUNG 1	CAA 2/YOUNG 2	CAA 3/YOUNG 1	CAA 3/YOUNG 2	CAA 4/YOUNG 1	CAA 4/YOUNG 2	CAA 1/OLD 1	CAA 1/OLD 2	CAA 2/OLD 1	CAA 2/OLD 2	CAA 3/OLD 1	CAA 3/OLD 2	CAA 4/OLD 1	CAA 4/OLD 2
Q8NDY3	[Protein ADP-ribosylarginine] hydrolase-like protein 1 OS=Homo sapiens GN=ADPRHL1 PE=2 SV=1 - [ARHL1_HUMAN]	2.14	2.18	2.07	2.11	0.33	0.37	0.88	0.92	0.59	0.55	1.01	1.05	-1.21	-1.13	-1.06	-1.56	-1.48	-1.82	-1.75	-1.12
Q9NQ66	1-phosphatidylinositol 4,5-bisphosphate phosphodiesterase beta-1 OS=Homo sapiens GN=PLCB1 PE=1 SV=1 - [PLCB1_HUMAN]	1.16	1.15	1.28	1.31	-0.03	-0.10	0.28	0.17	-0.02	0.02	-0.37	-0.49	-0.81	-1.53	-1.64	-1.07	-1.27	-1.19	-1.55	-0.98
Q9ULD0	2-oxoglutarate dehydrogenase-like, mitochondrial OS=Homo sapiens GN=OGDHL PE=1 SV=3 - [OGDHL_HUMAN]	1.93	1.83	2.05	2.01	-0.62	-0.66	0.66	0.51	0.13	0.16	-0.03	-0.18	-1.37	-2.10	-2.15	-1.88	-1.85	-2.15	-2.43	-1.54
P09543	2',3'-cyclic-nucleotide 3'-phosphodiesterase OS=Homo sapiens GN=CNP PE=1 SV=2 - [CN37_HUMAN]	2.17	2.10	2.38	2.37	-0.28	-0.35	0.65	0.58	0.21	0.30	0.06	0.00	-1.40	-2.17	-2.34	-1.93	-2.04	-2.37	-2.62	-1.70
P82909	28S ribosomal protein S36, mitochondrial OS=Homo sapiens GN=MRPS36 PE=1 SV=2 - [RT36_HUMAN]	0.75	0.82	0.63	0.83	-0.33	-0.22	-0.29	-0.15	-0.29	-0.42	-0.55	-0.60	-1.08	-1.25	-1.24	-1.05	-1.07	-1.06	-1.14	-0.90
P80404	4-aminobutyrate aminotransferase, mitochondrial OS=Homo sapiens GN=ABAT PE=1 SV=3 - [GABT_HUMAN]	1.81	1.82	2.21	2.17	0.31	0.30	0.99	0.93	0.58	0.57	0.22	0.26	-0.80	-1.60	-1.88	-1.28	-1.52	-1.62	-1.83	-1.14
P21589	5'-nucleotidase OS=Homo sapiens GN=NT5E PE=1 SV=1 - [5NTD_HUMAN]	-1.93	-1.99	-1.74	-1.64	0.37	0.42	-0.48	-0.42	0.01	-0.02	0.40	0.46	1.32	2.21	2.20	1.77	1.71	2.15	2.12	1.35

Q96D46	60S ribosomal export protein NMD3 OS=Homo sapiens GN=NMD3 PE=1 SV=1 - [NMD3_HUMAN]	-0.45	-0.54	-0.58	-0.67	0.79	0.70	1.46	1.37	1.31	1.40	1.80	1.71	1.97	2.26	2.39	1.89	2.02	1.23	1.36	2.11
Q969T7	7-methylguanosine phosphate-specific 5'-nucleotidase OS=Homo sapiens GN=NT5C3B PE=1 SV=4 - [5NT3B_HUMAN]	1.42	1.14	1.52	1.23	-0.32	-0.61	0.57	0.29	0.15	0.43	-0.30	-0.59	-0.79	-1.72	-1.82	-0.96	-1.05	-1.75	-1.85	-0.88
Q9UKA4	A-kinase anchor protein 11 OS=Homo sapiens GN=AKAP11 PE=1 SV=1 - [AKA11_HUMAN]	-1.29	-1.84	-1.19	-1.74	0.63	0.08	0.10	-0.44	-0.11	0.45	0.01	-0.54	1.45	1.31	1.20	1.77	1.67	1.91	1.81	1.36
Q12802	A-kinase anchor protein 13 OS=Homo sapiens GN=AKAP13 PE=1 SV=2 - [AKP13_HUMAN]	-1.30	-1.14	-1.42	-1.41	0.83	0.93	0.14	0.17	0.46	0.45	0.42	0.43	1.92	1.90	1.95	1.89	1.95	2.12	2.33	1.98
P24588	A-kinase anchor protein 5 OS=Homo sapiens GN=AKAP5 PE=1 SV=3 - [AKAP5_HUMAN]	1.32	1.35	1.92	2.05	-0.08	-0.12	0.45	0.46	0.28	0.19	0.09	0.08	-0.91	-1.74	-2.18	-1.26	-1.72	-1.42	-2.18	-1.41
O43427	Acidic fibroblast growth factor intracellular-binding protein OS=Homo sapiens GN=FIBP PE=1 SV=3 - [FIBP_HUMAN]	1.25	1.21	1.34	1.48	0.03	0.31	0.32	0.48	0.34	0.13	-0.16	0.12	-0.83	-1.38	-1.40	-1.05	-1.11	-1.31	-1.25	-0.93
Q6H8Q1	Actin-binding LIM protein 2 OS=Homo sapiens GN=ABLIM2 PE=1 SV=2 - [ABLM2_HUMAN]	1.38	1.71	1.68	1.84	-0.08	-0.11	0.44	0.57	0.27	0.36	-0.18	0.11	-1.30	-1.76	-1.93	-1.59	-1.57	-1.95	-2.06	-1.22
Q9NQW6	Actin-binding protein anillin OS=Homo sapiens GN=ANLN PE=1 SV=2 - [ANLN_HUMAN]	1.78	1.57	2.22	1.99	-0.29	-0.47	0.99	0.78	0.19	0.40	0.26	0.05	-0.81	-1.56	-2.15	-1.36	-1.86	-2.05	-2.65	-1.29

Q9P1U1	Actin-related protein 3B OS=Homo sapiens GN=ACTR3B PE=2 SV=1 - [ARP3B_HUMAN]	1.55	1.61	1.66	1.84	-0.15	0.04	0.34	0.34	0.20	0.25	0.12	0.21	-1.26	-1.42	-1.87	-1.57	-1.76	-1.42	-1.37	-1.43
P62736	Actin, aortic smooth muscle OS=Homo sapiens GN=ACTA2 PE=1 SV=1 - [ACTA_HUMAN]	-2.10	-2.20	-2.56	-2.79	-0.68	-0.83	-1.20	-1.28	-1.13	-1.01	-0.57	-0.66	1.22	1.46	1.93	1.43	1.65	1.58	2.01	1.56
Q96CM8	Acyl-CoA synthetase family member 2, mitochondrial OS=Homo sapiens GN=ACSF2 PE=1 SV=2 - [ACSF2_HUMAN]	-2.24	-2.21	-2.31	-2.45	-0.80	-0.86	-0.80	-0.93	-0.96	-0.86	-0.81	-0.94	1.36	1.41	1.50	1.48	1.48	1.43	1.59	1.49
Q8N6N7	Acyl-CoA-binding domain-containing protein 7 OS=Homo sapiens GN=ACBD7 PE=1 SV=1 - [ACBD7_HUMAN]	1.79	1.81	2.33	2.34	0.14	0.11	0.76	0.87	0.64	0.66	0.19	0.29	-0.79	-1.59	-1.89	-1.13	-1.66	-1.70	-2.24	-1.26
P82987	ADAMTS-like protein 3 OS=Homo sapiens GN=ADAMTSL3 PE=2 SV=4 - [ATL3_HUMAN]	-2.11	-2.08	-2.45	-2.42	0.77	0.72	0.40	0.39	0.28	0.20	-0.87	-0.79	2.46	1.34	1.65	2.26	2.68	2.84	3.17	2.82
Q8NC96	Adaptin ear-binding coat-associated protein 1 OS=Homo sapiens GN=NECAP1 PE=1 SV=2 - [NECP1_HUMAN]	1.66	1.57	1.72	1.62	0.06	0.00	0.38	0.30	0.21	0.27	0.17	0.04	-1.15	-1.39	-1.29	-1.28	-1.42	-1.39	-1.43	-1.10
Q9Y6K8	Adenylate kinase isoenzyme 5 OS=Homo sapiens GN=AK5 PE=1 SV=2 - [KAD5_HUMAN]	1.07	0.74	1.11	0.94	-0.12	-0.36	0.15	0.01	-0.21	0.00	-0.15	-0.32	-0.79	-1.10	-1.13	-0.92	-0.94	-1.10	-1.23	-0.82
Q8IUX7	Adipocyte enhancer-binding protein 1 OS=Homo sapiens GN=AEBP1 PE=1 SV=1 - [AEBP1_HUMAN]	-2.01	-1.74	-2.32	-2.31	-0.38	-0.41	-0.96	-0.80	-0.72	-0.68	-0.90	-0.88	1.26	1.08	1.54	1.20	1.69	1.64	2.02	1.54
Q10588	ADP-ribosyl cyclase 2 OS=Homo sapiens GN=BST1 PE=1 SV=2 - [BST1_HUMAN]	-1.78	-1.80	-2.10	-2.09	-0.20	-0.17	-0.48	-0.56	-0.45	-0.40	-0.45	-0.61	1.30	1.12	1.56	1.15	1.88	1.59	2.12	1.71

P84085	ADP-ribosylation factor 5 OS=Homo sapiens GN=ARF5 PE=1 SV=2 - [ARF5_HUMAN]	1.72	1.89	1.78	1.77	0.52	0.13	0.69	0.67	0.28	0.27	0.12	-0.12	-1.05	-1.61	-1.94	-1.43	-1.34	-1.48	-1.55	-0.99
Q8N6T3	ADP-ribosylation factor GTPase activating protein 1 OS=Homo sapiens GN=ARFGAP1 PE=1 SV=2 - [ARFG1_HUMAN]	1.66	1.63	1.92	1.80	-0.33	-0.46	0.57	0.58	0.03	-0.01	0.07	-0.11	-1.10	-1.56	-1.93	-1.44	-1.59	-1.88	-2.17	-1.30
P00325	Alcohol dehydrogenase 1B OS=Homo sapiens GN=ADH1B PE=1 SV=2 - [ADH1B_HUMAN]	-1.39	-1.32	-1.16	-1.05	0.59	0.69	0.97	0.82	0.97	1.05	1.50	1.32	2.55	3.11	3.12	2.83	2.84	2.66	2.68	2.40
Q8IZ83	Aldehyde dehydrogenase family 16 member A1 OS=Homo sapiens GN=ALDH16A1 PE=1 SV=2 - [A16A1_HUMAN]	-1.17	-1.16	-1.04	-1.19	-0.02	-0.08	-0.21	-0.22	-0.01	0.06	0.31	0.18	1.00	1.46	1.36	1.22	1.21	1.13	1.19	0.96
Q06278	Aldehyde oxidase OS=Homo sapiens GN=AOX1 PE=1 SV=2 - [AOXA_HUMAN]	-0.21	-0.96	-0.54	-1.29	1.10	0.34	0.94	0.19	0.11	0.86	1.34	0.58	1.21	1.55	1.87	1.10	1.43	1.29	1.62	1.54
P15121	Aldose reductase OS=Homo sapiens GN=AKR1B1 PE=1 SV=3 - [ALDR_HUMAN]	1.26	1.24	1.49	1.63	-0.10	-0.15	0.37	0.28	-0.01	0.04	-0.36	-0.44	-0.83	-1.78	-1.87	-1.31	-1.62	-1.44	-1.73	-1.07
P26572	Alpha-1,3-mannosyl-glycoprotein 2-beta-N-acetylglucosaminyltransferase OS=Homo sapiens GN=MGAT1 PE=2 SV=2 - [MGAT1_HUMAN]	-0.93	-0.77	-0.88	-1.13	2.76	2.60	0.70	0.69	1.41	1.56	0.95	0.63	1.04	1.10	1.73	2.09	2.34	3.20	3.29	1.55
P02765	Alpha-2-HS-glycoprotein OS=Homo sapiens GN=AHSG PE=1 SV=1 - [FETUA_HUMAN]	-3.17	-3.04	-3.64	-3.49	-1.99	-1.97	-2.41	-2.45	-1.83	-1.88	-1.58	-1.46	1.29	1.96	2.12	1.46	1.70	1.46	1.74	1.35
O43707	Alpha-actinin-4 OS=Homo sapiens GN=ACTN4 PE=1 SV=2 - [ACTN4_HUMAN]	-2.24	-2.17	-2.30	-2.22	-1.06	-0.96	-1.25	-1.17	-1.11	-1.18	-0.96	-0.92	1.07	1.28	1.37	1.10	1.14	1.22	1.24	1.14

Q9UDR5	Alpha-aminoadipic semialdehyde synthase, mitochondrial OS=Homo sapiens GN=AASS PE=1 SV=1 - [AASS_HUMAN]	-1.78	-1.48	-1.73	-1.70	0.06	0.08	-0.22	-0.15	-0.24	-0.25	-0.33	-0.40	1.43	1.23	1.31	1.28	1.50	1.48	1.60	1.46
P06280	Alpha-galactosidase A OS=Homo sapiens GN=GLA PE=1 SV=1 - [AGAL_HUMAN]	-0.83	-0.53	-1.24	-0.95	0.39	0.68	0.12	0.41	0.52	0.23	0.60	0.89	1.00	1.43	1.84	1.09	1.50	1.20	1.61	1.43
Q16352	Alpha-internexin OS=Homo sapiens GN=INA PE=1 SV=2 - [AINX_HUMAN]	2.36	2.34	2.38	2.36	-0.70	-0.67	0.36	0.41	0.02	0.02	-0.17	-0.17	-2.01	-2.69	-2.70	-2.39	-2.35	-2.86	-2.82	-2.06
P54802	Alpha-N-acetylglucosaminidase OS=Homo sapiens GN=NAGLU PE=1 SV=2 - [ANAG_HUMAN]	-0.81	-0.75	-1.21	-1.15	0.33	0.38	0.06	0.12	0.69	0.64	0.44	0.49	0.93	1.25	1.65	1.48	1.88	1.13	1.53	1.34
P15144	Aminopeptidase N OS=Homo sapiens GN=ANPEP PE=1 SV=4 - [AMPN_HUMAN]	-1.24	-1.18	-1.34	-1.36	0.45	0.51	-0.29	-0.25	0.07	0.08	0.14	0.15	0.97	1.29	1.57	1.28	1.57	1.58	1.74	1.14
P49418	Amphiphysin OS=Homo sapiens GN=AMPH PE=1 SV=1 - [AMPH_HUMAN]	1.60	1.53	1.87	2.01	0.19	0.14	0.64	0.65	0.22	0.14	-0.05	-0.09	-0.80	-1.65	-1.98	-1.37	-1.52	-1.32	-1.51	-1.14
Q99767	Amyloid beta A4 precursor protein-binding family A member 2 OS=Homo sapiens GN=APBA2 PE=1 SV=3 - [APBA2_HUMAN]	1.07	1.25	1.39	1.57	-0.15	0.02	0.23	0.41	0.27	0.10	0.06	0.24	-0.79	-1.00	-1.32	-0.94	-1.26	-1.24	-1.55	-1.10
P05067	Amyloid beta A4 protein OS=Homo sapiens GN=APP PE=1 SV=3 - [A4_HUMAN]	-0.53	-0.33	-0.69	-0.55	0.39	0.57	0.38	0.43	0.59	0.71	0.69	0.84	1.44	1.57	1.54	1.51	2.13	1.35	2.49	1.40
P04920	Anion exchange protein 2 OS=Homo sapiens GN=SLC4A2 PE=1 SV=4 - [B3A2_HUMAN]	-1.39	-1.43	-1.92	-1.85	-0.01	0.01	-0.53	-0.46	-0.18	-0.21	-0.08	-0.02	1.03	1.42	1.80	1.31	1.58	1.38	1.89	1.44

Q9P0K7	Ankycorbin OS=Homo sapiens GN=RAI14 PE=1 SV=2 - [RAI14_HUMAN]	-1.85	-1.86	-2.13	-2.08	-0.28	-0.20	-0.51	-0.51	-0.54	-0.52	-0.56	-0.55	1.45	1.30	1.51	1.39	1.58	1.86	1.81	1.63
Q7Z6G8	Ankyrin repeat and sterile alpha motif domain-containing protein 1B OS=Homo sapiens GN=ANKS1B PE=1 SV=2 - [ANS1B_HUMAN]	1.61	1.51	1.58	1.79	-1.70	-1.99	0.41	0.50	0.16	0.01	0.08	-0.21	-0.95	-1.53	-1.51	-1.31	-1.60	-3.32	-3.30	-1.23
Q8N6D5	Ankyrin repeat domain-containing protein 29 OS=Homo sapiens GN=ANKRD29 PE=2 SV=2 - [ANR29_HUMAN]	1.52	1.29	1.65	1.76	-0.11	-0.34	0.51	0.33	-0.19	-0.02	-0.11	-0.52	-0.90	-1.71	-2.13	-1.22	-1.71	-1.62	-2.11	-1.35
P16157	Ankyrin-1 OS=Homo sapiens GN=ANK1 PE=1 SV=3 - [ANK1_HUMAN]	-1.40	-1.39	-1.52	-1.43	0.39	0.40	0.43	0.43	0.68	0.63	1.25	1.31	1.91	2.64	2.76	2.07	2.15	1.72	1.88	2.01
Q01484	Ankyrin-2 OS=Homo sapiens GN=ANK2 PE=1 SV=4 - [ANK2_HUMAN]	1.74	1.81	1.92	1.90	0.04	0.01	0.66	0.67	0.37	0.37	0.21	0.24	-1.01	-1.58	-1.73	-1.31	-1.50	-1.71	-1.87	-1.22
Q12955	Ankyrin-3 OS=Homo sapiens GN=ANK3 PE=1 SV=3 - [ANK3_HUMAN]	1.78	1.74	1.96	1.82	-0.19	-0.24	0.41	0.34	0.04	0.11	-0.08	-0.18	-1.31	-1.79	-1.98	-1.71	-1.76	-1.81	-1.98	-1.47
P04083	Annexin A1 OS=Homo sapiens GN=ANXA1 PE=1 SV=2 - [ANXA1_HUMAN]	-2.01	-2.01	-2.00	-2.12	-0.20	-0.31	-0.33	-0.40	-0.27	-0.23	-0.10	-0.19	1.68	1.82	1.95	1.77	1.82	1.72	1.78	1.75
P07355	Annexin A2 OS=Homo sapiens GN=ANXA2 PE=1 SV=2 - [ANXA2_HUMAN]	-1.74	-1.77	-2.15	-2.21	-0.26	-0.23	-0.53	-0.58	-0.27	-0.28	-0.03	-0.10	1.33	1.84	2.14	1.51	2.02	1.63	2.05	1.79
P09525	Annexin A4 OS=Homo sapiens GN=ANXA4 PE=1 SV=4 - [ANXA4_HUMAN]	-1.50	-1.52	-1.50	-1.62	0.36	0.43	-0.14	-0.09	0.20	0.12	0.46	0.47	1.46	1.95	1.99	1.79	1.91	1.87	1.93	1.49
P08758	Annexin A5 OS=Homo sapiens GN=ANXA5 PE=1 SV=2 - [ANXA5_HUMAN]	-1.72	-1.58	-1.88	-1.82	0.69	0.75	-0.11	-0.09	0.48	0.38	0.63	0.68	1.61	2.25	2.51	2.03	2.35	2.31	2.58	1.85
Q13367	AP-3 complex subunit beta-2 OS=Homo sapiens GN=AP3B2 PE=1 SV=2 - [AP3B2_HUMAN]	1.90	1.69	2.00	1.99	-0.18	-0.22	0.63	0.52	0.23	0.31	0.04	-0.02	-1.14	-1.72	-1.99	-1.54	-1.85	-1.67	-2.02	-1.35

Q8N7J2	APC membrane recruitment protein 2 OS=Homo sapiens GN=AMER2 PE=1 SV=3 - [AMER2_HUMAN]	1.36	1.39	1.96	1.73	-0.81	-0.31	-0.02	0.36	0.10	-0.49	-0.38	0.13	-0.97	-1.25	-1.59	-1.25	-1.59	-2.19	-2.79	-1.30
P02647	Apolipoprotein A-I OS=Homo sapiens GN=APOA1 PE=1 SV=1 - [APOA1_HUMAN]	-1.77	-1.71	-1.86	-1.89	0.11	0.17	-0.36	-0.39	-0.16	-0.15	0.01	-0.06	1.55	1.89	1.94	1.93	1.74	1.87	1.95	1.59
P04114	Apolipoprotein B-100 OS=Homo sapiens GN=APOB PE=1 SV=2 - [APOB_HUMAN]	-2.09	-2.09	-2.34	-2.28	-0.03	0.01	-0.67	-0.64	0.01	0.00	0.78	0.84	1.54	2.88	3.15	2.10	2.35	2.05	2.25	1.76
P02654	Apolipoprotein C-I OS=Homo sapiens GN=APOC1 PE=1 SV=1 - [APOC1_HUMAN]	-0.79	-0.63	-0.87	-0.70	1.42	1.58	0.92	1.09	1.06	0.90	0.93	1.09	1.77	1.73	1.80	1.72	1.81	2.20	2.27	1.85
P05090	Apolipoprotein D OS=Homo sapiens GN=APOD PE=1 SV=1 - [APOD_HUMAN]	-0.21	-0.27	-0.23	-0.23	1.32	1.41	0.78	0.88	1.00	1.07	1.28	1.27	1.00	1.30	1.45	1.13	1.14	1.52	1.60	1.13
P02649	Apolipoprotein E OS=Homo sapiens GN=APOE PE=1 SV=1 - [APOE_HUMAN]	-0.03	0.00	-0.37	-0.21	3.99	4.07	1.77	1.76	2.81	2.75	1.40	1.46	1.84	1.51	1.82	2.83	3.06	3.91	4.22	2.08
Q13790	Apolipoprotein F OS=Homo sapiens GN=APOF PE=1 SV=2 - [APOF_HUMAN]	-1.22	-1.08	-1.25	-1.11	0.62	0.75	-0.24	-0.10	0.85	0.71	1.09	1.23	1.03	2.31	2.34	1.96	1.99	1.82	1.85	1.07
P29972	Aquaporin-1 OS=Homo sapiens GN=AQP1 PE=1 SV=3 - [AQP1_HUMAN]	-0.74	-0.61	-0.90	-0.59	0.60	0.63	0.40	0.62	0.81	0.94	1.43	1.22	1.19	1.86	2.00	1.47	1.60	1.23	1.52	1.18
P20292	Arachidonate 5-lipoxygenase-activating protein OS=Homo sapiens GN=ALOX5AP PE=1 SV=2 - [AL5AP_HUMAN]	-0.86	-0.62	-1.22	-0.97	0.33	0.57	0.14	0.38	0.67	0.43	0.56	0.80	1.06	1.43	1.78	1.32	1.68	1.18	1.53	1.42
O75689	Arf-GAP with dual PH domain-containing protein 1 OS=Homo sapiens GN=ADAP1 PE=1 SV=2 - [ADAP1_HUMAN]	1.36	1.56	1.39	1.64	-0.47	-0.21	0.14	0.35	0.41	0.25	-0.17	0.09	-1.16	-1.52	-1.56	-1.16	-1.19	-1.84	-1.87	-1.22

P00966	Argininosuccinate synthase OS=Homo sapiens GN=ASS1 PE=1 SV=2 - [ASSY_HUMAN]	-1.24	-1.28	-1.79	-2.01	1.17	1.04	1.36	1.32	0.93	1.09	0.61	0.40	2.58	1.66	2.20	2.23	2.83	2.33	2.84	2.96
P18440	Arylamine N-acetyltransferase 1 OS=Homo sapiens GN=NAT1 PE=1 SV=2 - [ARY1_HUMAN]	-0.94	-0.90	-1.01	-0.97	0.55	0.59	0.27	0.31	0.35	0.31	0.41	0.45	1.27	1.36	1.42	1.29	1.36	1.48	1.55	1.34
P17174	Aspartate aminotransferase, cytoplasmic OS=Homo sapiens GN=GOT1 PE=1 SV=3 - [AATC_HUMAN]	1.88	1.91	2.00	2.27	0.32	0.34	0.63	0.70	0.41	0.31	-0.04	0.04	-1.15	-2.05	-2.23	-1.66	-1.86	-1.59	-1.95	-1.59
Q6ICH7	Aspartate beta hydroxylase domain-containing protein 2 OS=Homo sapiens GN=ASPHD2 PE=2 SV=1 - [ASPH2_HUMAN]	1.64	2.07	1.80	2.23	0.53	0.96	0.38	0.81	0.86	0.43	0.00	0.43	-1.20	-1.63	-1.80	-1.17	-1.34	-1.12	-1.29	-1.36
Q6DD88	Atlastin-3 OS=Homo sapiens GN=ATL3 PE=1 SV=1 - [ATLA3_HUMAN]	-1.99	-2.06	-1.60	-1.61	-0.27	-0.33	-0.62	-0.61	-0.49	-0.50	0.00	-0.04	1.32	1.84	1.57	1.56	1.23	1.75	1.29	1.01
P56381	ATP synthase subunit epsilon, mitochondrial OS=Homo sapiens GN=ATP5E PE=1 SV=2 - [ATP5E_HUMAN]	1.08	1.11	1.16	1.19	-0.36	-0.29	0.19	0.10	-0.05	-0.12	-0.33	-0.25	-0.94	-1.47	-1.54	-1.18	-1.14	-1.49	-1.43	-1.08
Q09428	ATP-binding cassette sub-family C member 8 OS=Homo sapiens GN=ABCC8 PE=1 SV=6 - [ABCC8_HUMAN]	1.77	1.65	1.84	1.72	-0.10	-0.22	-0.12	-0.24	-0.03	0.10	0.45	0.33	-1.83	-1.31	-1.39	-1.64	-1.71	-1.88	-1.96	-1.90
P21854	B-cell differentiation antigen CD72 OS=Homo sapiens GN=CD72 PE=1 SV=1 - [CD72_HUMAN]	-0.94	-1.24	-1.25	-1.55	0.24	-0.06	0.68	0.38	1.27	1.57	0.92	0.61	1.67	1.86	2.17	2.54	2.85	1.16	1.47	1.99
P02730	Band 3 anion transport protein OS=Homo sapiens GN=SLC4A1 PE=1 SV=3 - [B3AT_HUMAN]	-1.59	-1.61	-1.92	-1.92	0.62	0.62	0.38	0.42	0.77	0.83	1.47	1.49	2.09	2.99	3.31	2.51	2.66	2.24	2.50	2.41

Q9Y2J2	Band 4.1-like protein 3 OS=Homo sapiens GN=EPB41L3 PE=1 SV=2 - [E41L3_HUMAN]	1.70	1.77	2.02	1.99	0.13	0.05	0.72	0.70	0.26	0.42	0.14	0.18	-0.97	-1.60	-1.92	-1.44	-1.60	-1.71	-2.00	-1.24
P50895	Basal cell adhesion molecule OS=Homo sapiens GN=BCAM PE=1 SV=2 - [BCAM_HUMAN]	-2.56	-2.49	-2.68	-2.63	-1.21	-1.14	-1.18	-1.15	-1.01	-1.05	-0.59	-0.58	1.38	1.95	2.08	1.49	1.59	1.34	1.53	1.61
P98160	Basement membrane-specific heparan sulfate proteoglycan core protein OS=Homo sapiens GN=HSPG2 PE=1 SV=4 - [PGBM_HUMAN]	-2.01	-2.00	-2.23	-2.25	-0.12	-0.11	-0.64	-0.62	-0.48	-0.47	-0.62	-0.65	1.45	1.38	1.57	1.58	1.76	1.92	2.14	1.67
P55061	Bax inhibitor 1 OS=Homo sapiens GN=TMBIM6 PE=1 SV=2 - [BI1_HUMAN]	-1.98	-2.07	-1.20	-1.28	0.04	-0.06	0.18	0.09	-0.22	-0.13	0.21	0.11	2.22	2.20	1.40	1.89	1.10	2.00	1.22	1.44
Q96L58	Beta-1,3-galactosyltransferase 6 OS=Homo sapiens GN=B3GALT6 PE=1 SV=2 - [B3GT6_HUMAN]	-1.21	-1.18	-1.60	-1.56	2.73	2.76	1.02	1.05	1.86	1.83	1.06	1.09	2.28	2.27	2.66	3.08	3.47	3.92	4.31	2.68
Q8NES3	Beta-1,3-N-acetylglucosaminyltransferase lunatic fringe OS=Homo sapiens GN=LFNG PE=1 SV=2 - [LFNG_HUMAN]	-0.05	-0.41	-0.12	-0.60	3.50	3.00	1.32	1.09	1.95	2.10	1.43	0.87	1.86	1.35	1.58	2.54	2.84	3.84	4.04	2.07
P02749	Beta-2-glycoprotein 1 OS=Homo sapiens GN=APOH PE=1 SV=3 - [APOH_HUMAN]	-2.20	-2.05	-2.11	-1.89	0.24	0.27	-0.17	-0.15	0.15	0.07	0.77	0.85	2.00	2.95	2.85	2.31	2.25	2.30	2.33	1.82
Q16585	Beta-sarcoglycan OS=Homo sapiens GN=SGCB PE=1 SV=1 - [SGCB_HUMAN]	-1.85	-1.66	-2.32	-2.13	-0.15	0.06	-0.22	-0.03	0.07	-0.12	0.04	0.25	1.79	1.87	2.33	1.77	2.24	1.59	2.06	2.22
P21810	Biglycan OS=Homo sapiens GN=BGN PE=1 SV=2 - [PGS1_HUMAN]	-2.25	-2.34	-2.44	-2.48	-0.27	-0.30	-0.50	-0.48	-0.47	-0.46	-0.53	-0.53	1.79	1.81	2.03	1.89	2.12	2.06	2.26	2.03

P18577	Blood group Rh(CE) polypeptide OS=Homo sapiens GN=RHCE PE=1 SV=2 - [RHCE_HUMAN]	-2.07	-2.14	-2.15	-2.21	0.49	0.42	0.45	0.39	0.63	0.70	1.37	1.30	2.58	3.45	3.52	2.80	2.88	2.54	2.62	2.66
P22003	Bone morphogenetic protein 5 OS=Homo sapiens GN=BMP5 PE=2 SV=1 - [BMP5_HUMAN]	-0.83	-1.03	-1.10	-1.30	2.93	3.13	1.21	1.41	1.79	1.66	0.94	0.74	2.57	2.59	2.45	3.89	3.75	4.55	4.41	2.44
Q9UQB8	Brain-specific angiogenesis inhibitor 1-associated protein 2 OS=Homo sapiens GN=BAIAP2 PE=1 SV=1 - [BAIP2_HUMAN]	1.24	1.30	1.54	1.40	0.01	-0.10	0.15	0.05	-0.01	0.04	-0.01	-0.10	-1.14	-1.19	-1.57	-1.32	-1.58	-1.15	-1.44	-1.30
O15382	Branched-chain-amino-acid aminotransferase, mitochondrial OS=Homo sapiens GN=BCAT2 PE=1 SV=2 - [BCAT2_HUMAN]	-2.09	-1.57	-2.15	-1.88	0.27	0.35	-0.42	-0.26	-0.09	-0.02	0.07	0.19	1.61	1.67	1.87	1.36	1.83	1.82	2.15	1.74
O75363	Breast carcinoma-amplified sequence 1 OS=Homo sapiens GN=BCAS1 PE=1 SV=2 - [BCAS1_HUMAN]	1.98	1.67	2.52	2.07	0.02	-0.25	0.87	0.49	-0.06	-0.07	-0.21	-0.28	-1.48	-2.05	-2.50	-2.04	-2.45	-2.89	-3.36	-1.90
Q5TH69	Brefeldin A-inhibited guanine nucleotide-exchange protein 3 OS=Homo sapiens GN=ARFGEF3 PE=1 SV=3 - [BIG3_HUMAN]	1.32	1.20	1.31	1.41	-0.08	0.10	0.31	0.36	0.14	-0.09	0.17	0.10	-1.05	-1.28	-1.36	-1.29	-1.41	-1.04	-1.28	-1.11
Q9UIF9	Bromodomain adjacent to zinc finger domain protein 2A OS=Homo sapiens GN=BAZ2A PE=1 SV=4 - [BAZ2A_HUMAN]	-1.90	-1.87	-2.16	-2.12	0.00	0.02	-0.38	-0.35	-0.53	-0.56	-0.69	-0.66	1.57	1.22	1.47	1.37	1.63	1.88	2.13	1.84
Q68DU8	BTB/POZ domain-containing protein KCTD16 OS=Homo sapiens GN=KCTD16 PE=2 SV=1 - [KCD16_HUMAN]	2.42	2.37	2.69	2.61	0.07	0.24	0.61	0.85	0.56	0.45	0.48	0.56	-1.35	-2.18	-2.13	-1.82	-1.91	-2.37	-2.51	-1.79

Q6ZWB6	BTB/POZ domain-containing protein KCTD8 OS=Homo sapiens GN=KCTD8 PE=2 SV=1 - [KCTD8_HUMAN]	2.55	2.42	2.71	2.58	0.02	0.08	1.36	1.23	0.64	0.78	0.42	0.28	-1.14	-2.12	-2.29	-1.74	-1.90	-2.53	-2.51	-1.29
Q9UPT6	C-Jun-amino-terminal kinase-interacting protein 3 OS=Homo sapiens GN=MAPK8IP3 PE=1 SV=3 - [JIP3_HUMAN]	1.82	1.66	1.80	1.74	0.11	0.03	0.79	0.66	0.10	0.25	0.35	0.24	-1.06	-1.29	-1.36	-1.62	-1.69	-1.76	-1.67	-1.18
Q13363	C-terminal-binding protein 1 OS=Homo sapiens GN=CTBP1 PE=1 SV=2 - [CTBP1_HUMAN]	1.28	1.21	1.25	1.24	-0.08	-0.06	0.14	0.29	0.18	0.09	-0.08	-0.09	-0.88	-1.21	-1.38	-0.96	-0.95	-1.29	-1.25	-0.93
Q9UBG0	C-type mannose receptor 2 OS=Homo sapiens GN=MRC2 PE=1 SV=2 - [MRC2_HUMAN]	-1.43	-1.42	-1.70	-1.70	0.27	0.27	-0.42	-0.38	0.11	0.16	0.87	0.89	1.23	2.37	2.56	1.67	1.84	1.59	2.01	1.39
Q9H2A7	C-X-C motif chemokine 16 OS=Homo sapiens GN=CXCL16 PE=2 SV=4 - [CXL16_HUMAN]	-0.06	-0.28	-0.21	-0.33	2.14	2.01	0.95	0.83	1.41	1.55	1.25	1.04	1.04	1.41	1.38	1.43	1.87	2.25	2.33	1.22
O14523	C2 domain-containing protein 2-like OS=Homo sapiens GN=C2CD2L PE=1 SV=3 - [C2C2L_HUMAN]	1.49	1.48	1.40	1.36	-0.30	-0.32	0.23	0.36	0.09	0.12	0.01	-0.07	-1.15	-1.14	-1.39	-1.18	-1.34	-1.52	-1.73	-1.05
P04003	C4b-binding protein alpha chain OS=Homo sapiens GN=C4BPA PE=1 SV=2 - [C4BPA_HUMAN]	-2.37	-2.28	-2.60	-2.53	-0.39	-0.36	-0.66	-0.59	-0.15	-0.28	0.41	0.56	1.68	2.88	3.11	2.42	2.40	2.16	2.11	1.95
P20851	C4b-binding protein beta chain OS=Homo sapiens GN=C4BPB PE=1 SV=1 - [C4BPB_HUMAN]	-2.47	-2.37	-2.48	-2.35	-0.64	-0.57	-0.60	-0.53	-0.29	-0.35	0.41	0.53	1.90	2.92	2.85	2.12	2.07	1.73	1.77	1.87
P33151	Cadherin-5 OS=Homo sapiens GN=CDH5 PE=1 SV=5 - [CADH5_HUMAN]	-1.63	-1.65	-1.75	-1.87	-0.28	-0.43	-0.67	-0.68	-0.39	-0.34	-0.03	-0.09	1.06	1.41	1.47	1.25	1.28	1.48	1.43	1.21

P05937	Calbindin OS=Homo sapiens GN=CALB1 PE=1 SV=2 - [CALB1_HUMAN]	1.99	1.80	2.24	2.10	0.84	0.46	0.96	0.81	0.33	0.59	-0.31	-0.38	-0.97	-2.18	-2.48	-1.42	-1.54	-1.34	-1.48	-1.23
Q96BS2	Calcineurin B homologous protein 3 OS=Homo sapiens GN=TESC PE=1 SV=3 - [CHP3_HUMAN]	1.40	1.46	1.11	1.17	-0.16	-0.11	0.29	0.30	0.00	0.06	0.04	-0.01	-1.00	-1.15	-1.21	-1.10	-1.01	-1.58	-1.29	-0.84
P63098	Calcineurin subunit B type 1 OS=Homo sapiens GN=PPP3R1 PE=1 SV=2 - [CANB1_HUMAN]	1.71	1.67	2.21	2.21	0.12	0.25	0.73	0.82	0.42	0.32	0.07	0.11	-0.98	-1.69	-2.22	-1.35	-1.93	-1.52	-2.05	-1.34
P53805	Calcipressin-1 OS=Homo sapiens GN=RCAN1 PE=1 SV=4 - [RCAN1_HUMAN]	1.68	1.98	1.73	2.03	0.11	0.41	0.43	0.73	0.52	0.23	0.38	0.67	-1.20	-1.30	-1.35	-1.42	-1.47	-1.58	-1.63	-1.24
O75746	Calcium-binding mitochondrial carrier protein Aralar1 OS=Homo sapiens GN=SLC25A12 PE=1 SV=2 - [CMC1_HUMAN]	1.57	1.43	1.71	1.55	0.06	0.03	0.48	0.47	0.22	0.28	0.19	0.13	-0.96	-1.32	-1.49	-1.25	-1.23	-1.39	-1.64	-1.09
Q9UJS0	Calcium-binding mitochondrial carrier protein Aralar2 OS=Homo sapiens GN=SLC25A13 PE=1 SV=2 - [CMC2_HUMAN]	-1.51	-1.57	-1.36	-1.43	0.22	0.11	-0.07	-0.09	-0.10	-0.07	-0.07	-0.09	1.58	1.45	1.38	1.41	1.35	1.66	1.60	1.37
Q6NUK1	Calcium-binding mitochondrial carrier protein SCaMC-1 OS=Homo sapiens GN=SLC25A24 PE=1 SV=2 - [SCMC1_HUMAN]	-1.31	-1.29	-1.48	-1.52	-0.05	0.01	-0.15	-0.20	0.20	0.08	0.13	0.13	1.03	1.42	1.60	1.50	1.42	1.25	1.42	1.30
Q9NZU7	Calcium-binding protein 1 OS=Homo sapiens GN=CABP1 PE=1 SV=5 - [CABP1_HUMAN]	1.02	1.10	0.84	0.93	-0.41	-0.33	-0.34	-0.26	-0.16	-0.24	-0.63	-0.55	-1.30	-1.64	-1.48	-1.23	-1.05	-1.45	-1.27	-1.12

Q9ULU8	Calcium-dependent secretion activator 1 OS=Homo sapiens GN=CADPS PE=1 SV=3 - [CAPS1_HUMAN]	1.90	1.78	1.99	1.94	-0.36	-0.42	0.76	0.73	0.23	0.28	-0.05	-0.02	-0.99	-1.77	-1.84	-1.55	-1.50	-2.02	-2.17	-1.29
Q86UW7	Calcium-dependent secretion activator 2 OS=Homo sapiens GN=CADPS2 PE=1 SV=2 - [CAPS2_HUMAN]	1.74	1.60	1.99	1.89	0.12	0.26	0.51	0.41	0.20	0.03	0.05	0.13	-1.17	-1.80	-1.85	-1.54	-1.58	-1.78	-1.70	-1.33
P54750	Calcium/calmodulin-dependent 3',5'-cyclic nucleotide phosphodiesterase 1A OS=Homo sapiens GN=PDE1A PE=2 SV=2 - [PDE1A_HUMAN]	2.02	2.20	1.48	1.59	0.01	0.16	0.44	0.58	0.10	-0.15	0.03	0.30	-1.36	-1.97	-1.45	-1.88	-1.47	-2.05	-1.51	-0.84
Q01064	Calcium/calmodulin-dependent 3',5'-cyclic nucleotide phosphodiesterase 1B OS=Homo sapiens GN=PDE1B PE=1 SV=2 - [PDE1B_HUMAN]	2.31	2.10	2.12	2.35	-0.29	-0.22	0.81	0.75	0.60	0.45	0.33	0.36	-1.36	-1.99	-1.89	-1.77	-1.68	-2.66	-2.58	-1.38
Q14123	Calcium/calmodulin-dependent 3',5'-cyclic nucleotide phosphodiesterase 1C OS=Homo sapiens GN=PDE1C PE=1 SV=1 - [PDE1C_HUMAN]	1.71	1.50	1.79	1.59	0.14	-0.07	0.60	0.40	-0.33	-0.12	0.11	-0.10	-1.05	-1.59	-1.68	-1.79	-1.88	-1.58	-1.67	-1.13
Q7Z7J9	Calcium/calmodulin-dependent protein kinase II inhibitor 1 OS=Homo sapiens GN=CAMK2N1 PE=1 SV=1 - [CK2N1_HUMAN]	2.01	1.86	2.66	2.50	-0.08	-0.24	-0.10	-0.26	-1.17	-1.00	-0.33	-0.49	-2.06	-2.34	-2.99	-2.99	-3.62	-2.11	-2.75	-2.70
Q8N5S9	Calcium/calmodulin-dependent protein kinase kinase 1 OS=Homo sapiens GN=CAMKK1 PE=1 SV=2 - [KKCC1_HUMAN]	2.03	2.03	2.45	2.38	-0.03	-0.06	0.53	0.64	0.36	0.45	0.13	0.05	-1.34	-1.68	-2.09	-1.52	-1.90	-2.05	-2.35	-1.68

Q96RR4	Calcium/calmodulin-dependent protein kinase kinase 2 OS=Homo sapiens GN=CAMKK2 PE=1 SV=2 - [KKCC2_HUMAN]	1.03	0.90	1.30	1.17	-0.62	-0.86	-0.09	-0.10	-0.83	-0.30	-0.45	-0.59	-0.94	-1.41	-1.45	-1.40	-1.54	-1.40	-1.54	-1.11
Q8IU85	Calcium/calmodulin-dependent protein kinase type 1D OS=Homo sapiens GN=CAMK1D PE=1 SV=1 - [KCC1D_HUMAN]	1.34	1.35	1.46	1.65	0.01	-0.02	0.28	0.27	0.08	0.12	0.14	0.21	-1.02	-1.18	-1.25	-1.22	-1.20	-1.38	-1.36	-1.13
Q9UQM7	Calcium/calmodulin-dependent protein kinase type II subunit alpha OS=Homo sapiens GN=CAMK2A PE=1 SV=2 - [KCC2A_HUMAN]	2.08	2.10	2.16	2.06	-0.32	0.01	0.71	0.66	0.44	0.37	0.40	0.31	-1.17	-1.74	-1.77	-1.64	-1.75	-2.27	-2.24	-1.42
Q13554	Calcium/calmodulin-dependent protein kinase type II subunit beta OS=Homo sapiens GN=CAMK2B PE=1 SV=3 - [KCC2B_HUMAN]	1.88	1.83	1.96	2.02	0.20	0.03	0.95	0.93	0.32	0.48	0.41	0.28	-0.87	-1.46	-1.64	-1.35	-1.60	-1.63	-2.05	-1.14
Q13555	Calcium/calmodulin-dependent protein kinase type II subunit gamma OS=Homo sapiens GN=CAMK2G PE=1 SV=3 - [KCC2G_HUMAN]	1.34	1.70	1.71	1.79	0.02	-0.05	0.62	0.62	0.26	0.30	0.27	0.34	-0.84	-1.43	-1.66	-1.19	-1.45	-1.41	-1.68	-1.07
Q16566	Calcium/calmodulin-dependent protein kinase type IV OS=Homo sapiens GN=CAMK4 PE=1 SV=1 - [KCC4_HUMAN]	1.68	1.15	1.69	1.51	-0.17	-0.23	0.44	0.18	-0.11	0.09	-0.05	-0.19	-1.24	-1.78	-1.88	-1.56	-1.58	-1.62	-1.71	-1.50
Q13938	Calcyphosin OS=Homo sapiens GN=CAPS PE=1 SV=1 - [CAYP1_HUMAN]	-0.09	-0.10	-0.23	-0.25	1.72	1.70	1.16	1.09	1.42	1.41	1.38	1.40	1.31	1.50	1.55	1.41	1.66	1.70	1.99	1.39

Q9P1Y5	Calmodulin-regulated spectrin-associated protein 3 OS=Homo sapiens GN=CAMSAP3 PE=1 SV=2 - [CAMP3_HUMAN]	1.63	1.40	1.53	1.39	0.41	0.32	0.64	0.42	0.22	0.35	0.24	0.00	-1.22	-1.52	-1.60	-1.21	-1.28	-1.13	-1.20	-1.30
P51911	Calponin-1 OS=Homo sapiens GN=CNN1 PE=1 SV=2 - [CNN1_HUMAN]	-3.13	-3.14	-3.31	-3.41	-1.18	-1.20	-1.62	-1.68	-1.36	-1.41	-1.39	-1.40	1.74	1.99	2.12	1.93	1.98	2.00	1.99	1.86
Q99439	Calponin-2 OS=Homo sapiens GN=CNN2 PE=1 SV=4 - [CNN2_HUMAN]	-2.25	-2.33	-2.31	-2.39	-0.48	-0.60	-0.58	-0.62	-0.63	-0.66	-0.86	-0.83	2.18	1.47	1.47	1.77	1.73	1.89	1.87	1.95
Q8NCB2	CaM kinase-like vesicle-associated protein OS=Homo sapiens GN=CAMKV PE=1 SV=2 - [CAMKV_HUMAN]	1.93	1.75	2.08	2.24	-0.25	-0.37	0.70	0.59	0.18	0.20	0.27	0.23	-1.06	-1.64	-1.75	-1.66	-2.08	-1.72	-1.93	-1.40
Q07343	cAMP-specific 3',5'-cyclic phosphodiesterase 4B OS=Homo sapiens GN=PDE4B PE=1 SV=1 - [PDE4B_HUMAN]	1.72	1.45	1.75	1.48	-0.05	-0.33	0.53	0.26	0.01	0.29	0.58	0.31	-1.13	-1.13	-1.17	-1.40	-1.43	-1.79	-1.82	-1.15
Q9UDT6	CAP-Gly domain-containing linker protein 2 OS=Homo sapiens GN=CLIP2 PE=1 SV=1 - [CLIP2_HUMAN]	1.49	1.28	1.60	1.45	-0.12	-0.13	0.50	0.51	0.12	0.05	-0.07	-0.15	-0.85	-1.28	-1.54	-1.18	-1.31	-1.44	-1.61	-0.95
Q8NCH0	Carbohydrate sulfotransferase 14 OS=Homo sapiens GN=CHST14 PE=1 SV=2 - [CHSTE_HUMAN]	-0.27	-0.31	-0.60	-0.63	2.70	2.70	1.14	1.16	1.73	1.76	0.96	0.83	1.47	1.18	1.71	2.03	2.59	2.90	3.56	1.99
Q9NS84	Carbohydrate sulfotransferase 7 OS=Homo sapiens GN=CHST7 PE=1 SV=2 - [CHST7_HUMAN]	-0.03	0.23	-0.76	-0.01	2.29	2.76	1.64	1.39	1.66	1.92	1.90	1.66	1.24	1.44	1.81	1.56	2.18	1.87	2.49	1.86
Q66K79	Carboxypeptidase Z OS=Homo sapiens GN=CPZ PE=1 SV=2 - [CBPZ_HUMAN]	-0.38	-1.04	-1.04	-1.26	1.93	2.02	0.62	0.50	0.95	1.11	0.44	0.27	1.48	1.31	1.81	1.91	2.40	2.52	3.03	2.05

P26678	Cardiac phospholamban OS=Homo sapiens GN=PLN PE=1 SV=1 - [PPLA_HUMAN]	-2.96	-3.13	-3.26	-3.43	-0.48	-0.65	-0.82	-0.99	-0.68	-0.51	-0.18	-0.35	2.19	2.79	3.08	2.49	2.79	2.47	2.77	2.50
P50416	Carnitine O-palmitoyltransferase 1, liver isoform OS=Homo sapiens GN=CPT1A PE=1 SV=2 - [CPT1A_HUMAN]	-1.31	-1.35	-1.45	-1.38	0.00	0.16	-0.08	0.07	0.03	0.03	0.04	0.11	1.46	1.43	1.53	1.51	1.68	1.53	1.66	1.43
A5YM72	Carnosine synthase 1 OS=Homo sapiens GN=CARNS1 PE=1 SV=3 - [CRNS1_HUMAN]	1.32	2.13	1.38	2.03	-0.23	-0.05	0.05	0.11	0.05	-0.20	-0.32	-0.22	-1.88	-1.93	-1.96	-2.04	-1.94	-2.20	-1.88	-1.63
Q8WXD9	Caskin-1 OS=Homo sapiens GN=CASKIN1 PE=1 SV=1 - [CSKI1_HUMAN]	1.30	0.83	1.31	0.82	0.05	-0.09	0.29	0.17	0.28	0.52	0.22	0.01	-0.77	-1.07	-1.13	-1.03	-1.08	-1.15	-1.16	-0.90
P04040	Catalase OS=Homo sapiens GN=CAT PE=1 SV=3 - [CATA_HUMAN]	-1.82	-1.84	-1.91	-1.86	-0.34	-0.32	-0.47	-0.45	-0.53	-0.59	-0.70	-0.68	1.43	1.20	1.14	1.37	1.28	1.52	1.45	1.51
P35221	Catenin alpha-1 OS=Homo sapiens GN=CTNNA1 PE=1 SV=1 - [CTNA1_HUMAN]	-1.49	-1.54	-1.70	-1.74	-0.16	-0.17	-0.41	-0.43	-0.38	-0.31	-0.06	-0.09	1.03	1.63	1.68	1.38	1.39	1.36	1.39	1.39
Q9UQB3	Catenin delta-2 OS=Homo sapiens GN=CTNND2 PE=1 SV=3 - [CTND2_HUMAN]	1.18	1.24	1.07	1.38	-0.37	-0.33	0.10	0.12	0.03	-0.02	0.08	-0.15	-1.13	-1.51	-1.60	-1.42	-1.58	-1.69	-1.78	-1.19
P49913	Cathelicidin antimicrobial peptide OS=Homo sapiens GN=CAMP PE=1 SV=1 - [CAMP_HUMAN]	-2.36	-2.07	-2.29	-2.00	-0.34	-0.06	1.22	1.51	0.99	0.70	0.89	1.18	3.63	3.26	3.18	3.09	3.02	2.00	1.93	3.57
P08311	Cathepsin G OS=Homo sapiens GN=CTSG PE=1 SV=2 - [CATG_HUMAN]	-1.62	-1.47	-1.70	-1.66	-0.12	-0.04	1.01	1.13	0.68	0.68	0.42	0.37	2.94	1.75	2.04	2.18	2.54	1.41	1.51	2.85
Q03135	Caveolin-1 OS=Homo sapiens GN=CAV1 PE=1 SV=4 - [CAV1_HUMAN]	-2.69	-2.66	-2.73	-2.76	-0.79	-0.71	-0.83	-0.81	-0.75	-0.81	-0.35	-0.36	2.00	2.26	2.34	1.99	1.94	1.91	1.90	2.02

P48509	CD151 antigen OS=Homo sapiens GN=CD151 PE=1 SV=3 - [CD151_HUMAN]	-2.62	-2.57	-2.25	-2.19	0.52	0.57	0.14	0.19	0.27	0.22	-0.14	-0.10	2.81	2.48	2.10	2.87	2.50	3.13	2.75	2.45
P16070	CD44 antigen OS=Homo sapiens GN=CD44 PE=1 SV=3 - [CD44_HUMAN]	-1.06	-0.85	-0.89	-0.74	0.50	0.51	0.30	0.32	0.40	0.47	0.93	0.94	1.15	1.73	1.71	1.40	1.17	1.61	1.20	0.98
P08962	CD63 antigen OS=Homo sapiens GN=CD63 PE=1 SV=2 - [CD63_HUMAN]	-0.69	-0.79	-1.30	-1.41	1.08	0.97	0.38	0.28	0.46	0.57	0.37	0.26	1.13	1.06	1.67	1.29	1.91	1.75	2.36	1.75
Q8N3J6	Cell adhesion molecule 2 OS=Homo sapiens GN=CADM2 PE=2 SV=1 - [CADM2_HUMAN]	1.66	1.62	2.00	2.14	0.09	0.04	0.54	0.77	0.50	0.44	0.37	0.48	-0.87	-1.22	-1.72	-1.40	-1.72	-1.83	-2.23	-1.43
Q8N126	Cell adhesion molecule 3 OS=Homo sapiens GN=CADM3 PE=1 SV=1 - [CADM3_HUMAN]	1.64	1.34	1.99	1.56	-0.53	-0.59	0.50	0.31	0.17	0.29	0.31	0.29	-0.95	-1.36	-1.56	-1.34	-1.59	-2.15	-2.40	-1.19
Q8NFZ8	Cell adhesion molecule 4 OS=Homo sapiens GN=CADM4 PE=1 SV=1 - [CADM4_HUMAN]	1.22	0.89	1.24	0.89	-0.05	-0.18	0.26	-0.05	-0.24	0.04	-0.14	-0.43	-0.96	-1.32	-1.30	-1.28	-1.14	-1.27	-1.30	-0.83
Q8N111	Cell cycle exit and neuronal differentiation protein 1 OS=Homo sapiens GN=CEND1 PE=2 SV=1 - [CEND_HUMAN]	1.96	1.83	2.28	2.41	-0.55	-0.55	1.22	1.10	0.39	0.46	0.07	0.11	-0.87	-1.73	-2.24	-1.40	-1.90	-2.31	-2.81	-1.29
Q9H305	Cell death-inducing p53-target protein 1 OS=Homo sapiens GN=CDIP1 PE=2 SV=1 - [CDIP1_HUMAN]	1.91	1.86	2.42	2.37	-0.06	-0.11	1.10	1.05	0.44	0.50	0.57	0.52	-0.76	-1.34	-1.85	-1.39	-1.89	-1.99	-2.50	-1.25
Q8WUJ3	Cell migration-inducing and hyaluronan-binding protein OS=Homo sapiens GN=CEMIP PE=1 SV=2 - [CEMIP_HUMAN]	-0.20	-0.33	-0.77	-0.67	2.64	2.65	1.35	1.40	1.70	1.83	0.97	0.86	1.76	1.21	1.68	2.21	2.70	3.01	3.50	2.23

P43121	Cell surface glycoprotein MUC18 OS=Homo sapiens GN=MCAM PE=1 SV=2 - [MUC18_HUMAN]	-1.24	-1.23	-1.70	-1.64	0.06	0.02	-0.19	-0.23	-0.13	-0.19	-0.07	0.04	1.42	1.49	1.74	1.61	1.74	1.81	1.85	1.58
P29373	Cellular retinoic acid-binding protein 2 OS=Homo sapiens GN=CRABP2 PE=1 SV=2 - [RABP2_HUMAN]	-1.48	-2.26	-1.24	-2.02	0.18	-0.59	0.29	-0.48	-0.17	0.61	0.21	-0.57	1.83	1.70	1.45	2.12	1.88	1.65	1.41	1.60
Q5SW79	Centrosomal protein of 170 kDa OS=Homo sapiens GN=CEP170 PE=1 SV=1 - [CE170_HUMAN]	1.40	1.40	1.33	1.43	0.10	0.27	0.30	0.45	0.31	0.08	0.20	0.38	-0.94	-1.06	-1.10	-1.24	-1.29	-1.37	-1.30	-0.85
Q9Y4F5	Centrosomal protein of 170 kDa protein B OS=Homo sapiens GN=CEP170B PE=1 SV=4 - [C170B_HUMAN]	1.81	1.57	1.78	1.53	-0.20	-0.35	0.21	0.05	-0.06	0.14	0.06	-0.03	-1.40	-1.65	-1.66	-1.50	-1.39	-1.83	-1.75	-1.29
Q96G23	Ceramide synthase 2 OS=Homo sapiens GN=CERS2 PE=1 SV=1 - [CERS2_HUMAN]	-1.11	-1.11	-1.38	-1.38	0.21	0.24	-0.08	-0.06	0.02	-0.12	0.01	-0.13	1.26	1.30	1.15	1.19	1.29	1.14	1.57	1.24
Q9NTU7	Cerebellin-4 OS=Homo sapiens GN=CBLN4 PE=1 SV=1 - [CBLN4_HUMAN]	-2.96	-3.01	-3.52	-3.57	-1.06	-1.12	-2.13	-2.19	-1.70	-1.64	-1.14	-1.21	0.88	1.82	2.37	1.35	1.91	1.88	2.44	1.45
P00450	Ceruloplasmin OS=Homo sapiens GN=CP PE=1 SV=1 - [CERU_HUMAN]	-2.04	-2.01	-1.86	-1.66	-0.53	-0.54	-0.71	-0.71	-0.56	-0.60	-0.41	-0.40	1.40	1.54	1.53	1.34	1.34	1.40	1.38	1.33
O00408	cGMP-dependent 3',5'-cyclic phosphodiesterase OS=Homo sapiens GN=PDE2A PE=1 SV=1 - [PDE2A_HUMAN]	1.90	1.90	1.81	1.79	0.01	0.06	0.73	0.70	0.22	0.46	0.50	0.26	-0.97	-1.10	-1.07	-1.11	-1.15	-1.84	-1.65	-0.88
O76074	cGMP-specific 3',5'-cyclic phosphodiesterase OS=Homo sapiens GN=PDE5A PE=1 SV=2 - [PDE5A_HUMAN]	-1.87	-1.52	-1.84	-1.99	-0.30	-0.23	-0.48	-0.58	-0.45	-0.36	-0.58	-0.74	1.45	1.61	1.37	1.54	1.58	1.52	1.75	1.48

P36222	Chitinase-3-like protein 1 OS=Homo sapiens GN=CHI3L1 PE=1 SV=2 - [CH3L1_HUMAN]	-0.12	-0.26	-0.48	-0.52	2.78	2.25	1.33	1.10	1.53	2.04	2.02	1.84	1.71	2.37	2.63	2.25	2.58	2.87	3.13	1.98
O00299	Chloride intracellular channel protein 1 OS=Homo sapiens GN=CLIC1 PE=1 SV=4 - [CLIC1_HUMAN]	-1.68	-1.61	-1.72	-1.70	-0.03	0.11	-0.14	-0.10	0.00	-0.10	-0.40	-0.23	1.62	1.38	1.45	1.76	1.57	1.78	1.70	1.43
Q96NY7	Chloride intracellular channel protein 6 OS=Homo sapiens GN=CLIC6 PE=2 SV=3 - [CLIC6_HUMAN]	-0.52	-0.88	-1.43	-1.60	1.05	0.84	1.10	1.09	0.56	0.70	0.28	0.26	2.06	1.11	1.86	1.58	2.17	1.71	1.97	2.58
Q9Y6A2	Cholesterol 24 hydroxylase OS=Homo sapiens GN=CYP46A1 PE=1 SV=1 - [CP46A_HUMAN]	2.17	2.57	1.94	2.33	-0.26	0.13	0.59	0.99	0.64	0.25	-0.05	0.34	-1.35	-2.22	-1.99	-1.90	-1.65	-2.45	-2.22	-1.24
Q9H2X0	Chordin OS=Homo sapiens GN=CHRD PE=1 SV=2 - [CHRD_HUMAN]	-0.24	-0.05	-0.66	-0.46	2.55	2.74	0.86	1.06	1.89	1.69	0.92	1.11	1.16	1.17	1.58	1.97	2.39	2.77	3.19	1.58
O95503	Chromobox protein homolog 6 OS=Homo sapiens GN=CBX6 PE=1 SV=1 - [CBX6_HUMAN]	-1.90	-1.87	-1.88	-1.85	0.83	0.86	1.30	1.33	0.51	0.49	-0.33	-0.30	3.26	1.58	1.55	2.42	2.40	2.72	2.70	3.24
O14578	Citron Rho-interacting kinase OS=Homo sapiens GN=CIT PE=1 SV=2 - [CTRO_HUMAN]	1.67	1.56	1.59	1.68	-0.04	0.01	0.51	0.67	0.39	0.34	0.03	0.14	-1.01	-1.45	-1.61	-1.30	-1.19	-1.39	-1.43	-1.13
P09496	Clathrin light chain A OS=Homo sapiens GN=CLTA PE=1 SV=1 - [CLCA_HUMAN]	1.75	1.77	1.84	1.82	-0.21	-0.19	0.60	0.62	0.26	0.21	0.27	0.26	-1.11	-1.53	-1.42	-1.49	-1.41	-2.02	-2.03	-1.12
P09497	Clathrin light chain B OS=Homo sapiens GN=CLTB PE=1 SV=1 - [CLCB_HUMAN]	1.21	1.06	1.30	1.25	-0.14	-0.24	0.23	0.20	0.02	0.07	-0.06	0.00	-0.89	-1.24	-1.29	-1.13	-1.22	-1.32	-1.59	-0.98
P78369	Claudin-10 OS=Homo sapiens GN=CLDN10 PE=1 SV=2 - [CLD10_HUMAN]	1.27	1.96	1.22	1.91	-0.45	0.24	0.00	0.69	0.73	0.05	-0.03	0.65	-1.21	-1.30	-1.25	-1.20	-1.14	-1.74	-1.68	-1.15

O75508	Claudin-11 OS=Homo sapiens GN=CLDN11 PE=1 SV=2 - [CLD11_HUMAN]	1.14	1.02	1.42	1.30	-0.37	-0.36	0.29	0.16	-0.04	-0.15	-0.14	-0.09	-0.86	-1.08	-1.27	-1.18	-1.46	-1.40	-1.68	-1.07
Q8IUQ0	Clavesin-1 OS=Homo sapiens GN=CLVS1 PE=1 SV=1 - [CLVS1_HUMAN]	1.90	1.90	1.88	1.88	0.49	0.49	0.74	0.74	0.32	0.33	0.04	0.04	-1.11	-1.85	-1.83	-1.55	-1.52	-1.43	-1.40	-1.07
Q5SYC1	Clavesin-2 OS=Homo sapiens GN=CLVS2 PE=2 SV=1 - [CLVS2_HUMAN]	1.47	1.71	1.75	2.11	-0.18	0.07	0.79	1.09	0.87	0.43	0.48	0.92	-0.95	-1.58	-1.42	-1.11	-1.46	-1.91	-1.91	-1.18
P10909	Clusterin OS=Homo sapiens GN=CLU PE=1 SV=1 - [CLUS_HUMAN]	-0.28	-0.43	-0.60	-0.74	3.02	2.92	2.02	1.91	2.29	2.35	2.01	1.88	2.43	2.42	2.60	2.78	3.04	3.36	3.62	2.71
P00740	Coagulation factor IX OS=Homo sapiens GN=F9 PE=1 SV=2 - [FA9_HUMAN]	-2.51	-2.46	-2.33	-2.18	-0.60	-0.39	-0.95	-1.01	-0.78	-0.72	-0.41	-0.26	1.50	2.04	1.92	1.71	1.51	1.66	1.61	1.30
P00742	Coagulation factor X OS=Homo sapiens GN=F10 PE=1 SV=2 - [FA10_HUMAN]	-1.66	-1.54	-1.83	-1.77	0.60	0.69	0.07	0.13	0.24	0.12	0.02	0.00	1.81	1.80	1.85	1.93	2.09	2.34	2.42	1.97
P00488	Coagulation factor XIII A chain OS=Homo sapiens GN=F13A1 PE=1 SV=4 - [F13A_HUMAN]	-1.57	-1.46	-1.60	-1.64	0.29	0.36	-0.27	-0.27	0.08	0.10	0.39	0.41	1.13	2.07	2.15	1.67	1.80	1.98	2.06	1.43
Q9P299	Coatomer subunit zeta-2 OS=Homo sapiens GN=COPZ2 PE=2 SV=1 - [COPZ2_HUMAN]	-1.99	-1.90	-2.33	-2.24	-0.07	0.02	-0.41	-0.32	-0.29	-0.37	-0.70	-0.61	1.63	1.30	1.63	1.64	1.99	1.90	2.25	1.98
P23528	Cofilin-1 OS=Homo sapiens GN=CFL1 PE=1 SV=3 - [COF1_HUMAN]	1.02	0.89	1.34	1.31	-0.20	-0.39	-0.17	-0.24	-0.51	-0.42	-0.65	-0.62	-1.04	-1.61	-1.93	-1.30	-1.67	-1.22	-1.59	-1.35
Q9Y281	Cofilin-2 OS=Homo sapiens GN=CFL2 PE=1 SV=1 - [COF2_HUMAN]	0.77	0.69	1.15	1.23	-0.45	-0.38	-0.21	-0.19	-0.28	-0.28	-0.52	-0.53	-0.91	-1.31	-1.72	-0.93	-1.33	-1.42	-1.53	-1.49
Q96JN2	Coiled-coil domain-containing protein 136 OS=Homo sapiens GN=CCDC136 PE=2 SV=3 - [CC136_HUMAN]	2.98	2.55	2.90	2.51	-0.06	-0.33	1.13	1.00	0.59	0.84	0.51	0.25	-1.68	-1.92	-1.66	-1.73	-1.60	-2.64	-2.59	-1.51

Q9NQR7	Coiled-coil domain-containing protein 177 OS=Homo sapiens GN=CCDC177 PE=2 SV=3 - [CC177_HUMAN]	1.93	1.79	1.55	1.48	-0.04	0.25	0.29	0.14	0.32	0.16	-0.34	-0.07	-2.18	-2.23	-1.46	-1.77	-1.15	-1.98	-1.80	-1.19
Q9BQI4	Coiled-coil domain-containing protein 3 OS=Homo sapiens GN=CCDC3 PE=2 SV=1 - [CCDC3_HUMAN]	0.00	-0.28	-0.03	-0.05	3.85	3.72	1.75	1.54	2.55	2.59	1.79	1.76	1.88	2.07	1.82	2.87	2.65	3.99	3.86	1.84
Q53HC0	Coiled-coil domain-containing protein 92 OS=Homo sapiens GN=CCDC92 PE=1 SV=2 - [CCD92_HUMAN]	1.38	1.40	1.32	1.34	-0.46	-0.44	-0.34	-0.32	-0.12	-0.13	-0.30	-0.28	-1.67	-1.67	-1.61	-1.48	-1.42	-1.86	-1.79	-1.60
P02462	Collagen alpha-1(IV) chain OS=Homo sapiens GN=COL4A1 PE=1 SV=3 - [CO4A1_HUMAN]	-1.28	-1.33	-1.86	-1.82	0.46	0.37	-0.63	-0.57	-0.10	-0.15	-0.30	-0.28	0.97	1.30	1.40	1.75	1.80	2.12	2.23	1.20
P12109	Collagen alpha-1(VI) chain OS=Homo sapiens GN=COL6A1 PE=1 SV=3 - [CO6A1_HUMAN]	-1.54	-1.61	-1.74	-1.76	-0.27	-0.25	-0.48	-0.49	-0.21	-0.21	0.21	0.23	1.16	1.94	1.96	1.44	1.52	1.35	1.48	1.30
P27658	Collagen alpha-1(VIII) chain OS=Homo sapiens GN=COL8A1 PE=1 SV=2 - [CO8A1_HUMAN]	-0.71	-0.89	-1.08	-0.99	2.16	1.81	1.06	0.71	0.72	1.14	0.66	0.25	1.98	1.64	1.80	2.26	2.41	3.26	3.44	2.24
Q03692	Collagen alpha-1(X) chain OS=Homo sapiens GN=COL10A1 PE=1 SV=2 - [COAA1_HUMAN]	-1.57	-1.58	-1.49	-1.50	2.61	2.59	1.59	1.58	1.59	1.61	1.03	1.01	3.22	2.60	2.52	3.21	3.13	4.16	4.08	3.15
Q99715	Collagen alpha-1(XII) chain OS=Homo sapiens GN=COL12A1 PE=1 SV=2 - [COCA1_HUMAN]	-2.55	-2.56	-2.84	-2.87	-1.00	-0.96	-1.18	-1.14	-0.93	-0.97	-0.75	-0.73	1.49	1.92	2.09	1.70	1.91	1.76	1.98	1.72

Q05707	Collagen alpha-1(XIV) chain OS=Homo sapiens GN=COL14A1 PE=1 SV=3 - [COEA1_HUMAN]	-3.06	-3.09	-3.32	-3.30	-0.98	-0.97	-1.13	-1.09	-1.18	-1.19	-1.43	-1.42	2.02	1.76	1.91	2.02	2.19	2.23	2.40	2.25
Q07092	Collagen alpha-1(XVI) chain OS=Homo sapiens GN=COL16A1 PE=1 SV=2 - [COGA1_HUMAN]	-2.96	-3.12	-3.09	-3.29	-1.00	-1.21	-1.65	-1.82	-1.57	-1.55	-1.58	-1.72	1.36	1.34	1.25	1.71	1.69	2.03	1.97	1.51
P39060	Collagen alpha-1(XVIII) chain OS=Homo sapiens GN=COL18A1 PE=1 SV=5 - [COIA1_HUMAN]	-2.35	-2.14	-2.50	-2.32	0.37	0.42	-0.15	-0.05	0.07	0.08	0.08	0.20	2.11	2.33	2.58	2.25	2.51	2.50	2.73	2.31
Q96P44	Collagen alpha-1(XXI) chain OS=Homo sapiens GN=COL21A1 PE=2 SV=1 - [COLA1_HUMAN]	-2.13	-2.14	-2.45	-2.63	-1.03	-1.34	-1.12	-1.41	-1.37	-1.11	-1.07	-1.28	1.60	1.51	1.57	1.57	1.59	1.53	1.34	1.51
P08572	Collagen alpha-2(IV) chain OS=Homo sapiens GN=COL4A2 PE=1 SV=4 - [CO4A2_HUMAN]	-2.22	-2.37	-2.36	-2.41	0.27	0.07	-0.78	-0.82	-0.42	-0.43	-0.50	-0.59	1.28	1.63	1.74	1.72	1.81	2.19	2.40	1.48
P12110	Collagen alpha-2(VI) chain OS=Homo sapiens GN=COL6A2 PE=1 SV=4 - [CO6A2_HUMAN]	-1.48	-1.44	-1.52	-1.48	-0.17	-0.16	-0.45	-0.40	-0.14	-0.16	0.23	0.26	1.15	1.70	1.87	1.28	1.52	1.22	1.34	1.19
P12111	Collagen alpha-3(VI) chain OS=Homo sapiens GN=COL6A3 PE=1 SV=5 - [CO6A3_HUMAN]	-1.90	-1.92	-1.96	-1.95	-0.24	-0.24	-0.56	-0.58	-0.24	-0.25	0.15	0.16	1.38	2.07	2.15	1.69	1.79	1.66	1.71	1.41
P29400	Collagen alpha-5(IV) chain OS=Homo sapiens GN=COL4A5 PE=1 SV=2 - [CO4A5_HUMAN]	-2.00	-2.11	-2.38	-2.49	-0.17	-0.28	-0.63	-0.74	-0.67	-0.55	-0.58	-0.70	1.42	1.43	1.80	1.48	1.87	1.82	2.20	1.81
Q14031	Collagen alpha-6(IV) chain OS=Homo sapiens GN=COL4A6 PE=2 SV=3 - [CO4A6_HUMAN]	-1.90	-1.66	-2.17	-2.25	-0.32	-0.40	-0.67	-0.54	-0.28	-0.55	-0.04	-0.07	1.29	1.87	2.18	1.38	1.65	1.73	1.84	1.68

Q5KU26	Collectin-12 OS=Homo sapiens GN=COLEC12 PE=1 SV=3 - [COL12_HUMAN]	-2.42	-2.35	-2.37	-2.36	-0.53	-0.46	-0.75	-0.72	-0.40	-0.44	-0.19	-0.09	1.66	2.17	2.07	1.94	1.92	1.84	1.77	1.65
P02745	Complement C1q subcomponent subunit A OS=Homo sapiens GN=C1QA PE=1 SV=2 - [C1QA_HUMAN]	-1.42	-1.38	-1.28	-1.24	1.04	1.07	0.37	0.41	0.74	0.70	1.07	1.10	1.84	2.49	2.23	2.15	1.81	2.44	2.19	1.65
P02747	Complement C1q subcomponent subunit C OS=Homo sapiens GN=C1QC PE=1 SV=3 - [C1QC_HUMAN]	-0.89	-0.69	-0.84	-0.87	1.03	1.02	0.40	0.32	0.67	0.46	0.80	0.77	1.17	1.69	1.64	1.46	1.30	1.75	1.65	1.13
Q9BXJ4	Complement C1q tumor necrosis factor related protein 3 OS=Homo sapiens GN=C1QTNF3 PE=1 SV=1 - [C1QT3_HUMAN]	-2.70	-2.51	-2.96	-2.77	-0.53	-0.35	-0.75	-0.56	-0.52	-0.70	-1.26	-1.08	2.01	1.44	1.70	2.03	2.30	2.15	2.41	2.28
Q9BXJ2	Complement C1q tumor necrosis factor related protein 7 OS=Homo sapiens GN=C1QTNF7 PE=1 SV=1 - [C1QT7_HUMAN]	-1.71	-1.68	-1.85	-1.82	1.04	1.07	-0.21	-0.17	0.34	0.30	0.32	0.35	1.56	2.04	2.17	2.05	2.19	2.73	2.87	1.71
P00736	Complement C1r subcomponent OS=Homo sapiens GN=C1R PE=1 SV=2 - [C1R_HUMAN]	-1.40	-1.42	-1.57	-1.63	0.56	0.57	-0.21	-0.27	0.27	0.24	0.67	0.70	1.45	2.27	2.22	1.73	1.73	1.83	2.13	1.30
P09871	Complement C1s subcomponent OS=Homo sapiens GN=C1S PE=1 SV=1 - [C1S_HUMAN]	-1.49	-1.35	-1.43	-1.33	0.23	0.39	-0.45	-0.22	0.14	-0.10	0.22	0.39	1.22	1.76	1.82	1.25	1.26	1.48	1.62	1.12
P01024	Complement C3 OS=Homo sapiens GN=C3 PE=1 SV=2 - [CO3_HUMAN]	-1.66	-1.69	-1.74	-1.71	-0.12	-0.08	-0.78	-0.76	-0.30	-0.30	0.11	0.16	1.09	1.93	1.88	1.53	1.49	1.67	1.67	1.03
P0C0L4	Complement C4-A OS=Homo sapiens GN=C4A PE=1 SV=2 - [CO4A_HUMAN]	-0.60	-0.64	-0.83	-1.04	1.51	1.46	0.81	0.77	1.02	1.07	0.80	0.75	1.46	1.39	1.58	1.69	1.88	2.08	2.27	1.65

P0C0L5	Complement C4-B OS=Homo sapiens GN=C4B PE=1 SV=2 - [CO4B_HUMAN]	-0.18	-0.23	-0.85	-0.67	2.42	2.29	1.41	1.32	1.70	1.73	1.23	1.35	1.71	1.92	1.94	2.11	2.38	2.54	2.64	2.07
Q9NPY3	Complement component C1q receptor OS=Homo sapiens GN=CD93 PE=1 SV=3 - [C1QR1_HUMAN]	-2.10	-1.94	-2.38	-2.21	-0.52	-0.36	-0.81	-0.65	-0.49	-0.65	-0.68	-0.52	1.34	1.43	1.70	1.49	1.76	1.57	1.84	1.63
P13671	Complement component C6 OS=Homo sapiens GN=C6 PE=1 SV=3 - [CO6_HUMAN]	-1.61	-1.55	-1.80	-1.80	0.52	0.36	-0.50	-0.50	-0.14	0.02	-0.05	-0.17	1.17	1.52	1.72	1.67	1.85	2.04	2.11	1.36
P10643	Complement component C7 OS=Homo sapiens GN=C7 PE=1 SV=2 - [CO7_HUMAN]	-0.25	-0.32	-0.51	-0.43	1.12	1.09	0.36	0.54	0.87	0.80	0.87	0.80	0.97	1.23	1.47	1.08	1.34	1.18	1.60	1.15
P07357	Complement component C8 alpha chain OS=Homo sapiens GN=C8A PE=1 SV=2 - [CO8A_HUMAN]	-1.85	-1.77	-1.65	-1.59	-0.29	-0.31	-0.65	-0.61	-0.38	-0.42	0.12	0.08	1.25	1.97	1.91	1.65	1.45	1.74	1.38	1.39
P07358	Complement component C8 beta chain OS=Homo sapiens GN=C8B PE=1 SV=3 - [CO8B_HUMAN]	-1.65	-1.72	-1.67	-1.85	0.24	0.10	-0.42	-0.56	-0.01	0.08	0.62	0.63	1.12	2.16	2.36	1.81	2.20	1.71	2.33	1.48
P07360	Complement component C8 gamma chain OS=Homo sapiens GN=C8G PE=1 SV=3 - [CO8G_HUMAN]	-1.39	-1.46	-1.27	-1.52	0.48	0.34	-0.37	-0.33	-0.12	-0.16	0.31	0.08	1.23	1.71	1.56	1.49	1.41	1.74	1.88	1.10
P08174	Complement decay-accelerating factor OS=Homo sapiens GN=CD55 PE=1 SV=4 - [DAF_HUMAN]	-1.28	-1.35	-1.33	-1.36	0.86	0.71	0.28	0.28	0.41	0.58	0.63	0.49	1.56	1.74	1.83	1.80	1.90	1.98	2.16	1.60
P00746	Complement factor D OS=Homo sapiens GN=CFD PE=1 SV=5 - [CFAD_HUMAN]	-0.86	-0.88	-1.00	-1.03	1.09	1.06	0.25	0.23	0.92	0.96	0.65	0.62	1.16	1.51	1.65	1.84	1.99	1.93	2.07	1.32

P08603	Complement factor H OS=Homo sapiens GN=CFH PE=1 SV=4 - [CFAH_HUMAN]	-1.81	-1.74	-1.90	-1.78	0.16	0.19	-0.47	-0.34	-0.03	-0.13	0.28	0.35	1.40	2.09	2.08	1.72	1.81	2.00	2.04	1.55
O14810	Complexin-1 OS=Homo sapiens GN=CPLX1 PE=1 SV=1 - [CPLX1_HUMAN]	1.85	2.02	2.25	2.23	-0.11	-0.45	0.48	0.43	-0.38	-0.32	-0.46	-0.52	-1.49	-2.31	-2.71	-1.94	-2.57	-2.17	-2.64	-1.90
Q6PUV4	Complexin-2 OS=Homo sapiens GN=CPLX2 PE=1 SV=2 - [CPLX2_HUMAN]	2.91	1.09	3.56	2.22	0.18	-0.70	1.00	0.25	-0.21	0.09	-0.48	-0.73	-1.66	-3.39	-4.05	-2.46	-3.11	-2.74	-3.40	-2.23
Q8WXI2	Connector enhancer of kinase suppressor of ras 2 OS=Homo sapiens GN=CNKSR2 PE=1 SV=1 - [CNKR2_HUMAN]	1.53	1.61	1.65	1.69	0.12	-0.27	0.80	0.50	0.46	0.29	0.34	0.30	-1.20	-1.49	-1.42	-1.70	-1.50	-2.08	-2.01	-1.06
Q12860	Contactin-1 OS=Homo sapiens GN=CNTN1 PE=1 SV=1 - [CNTN1_HUMAN]	1.61	1.67	1.77	1.71	0.19	0.10	0.59	0.60	0.41	0.42	0.28	0.31	-0.90	-1.45	-1.56	-1.28	-1.25	-1.64	-1.67	-1.06
Q02246	Contactin-2 OS=Homo sapiens GN=CNTN2 PE=1 SV=1 - [CNTN2_HUMAN]	1.74	1.56	1.62	1.53	0.41	0.32	0.48	0.34	0.46	0.53	0.10	0.12	-1.26	-1.34	-1.45	-1.18	-1.11	-1.30	-1.30	-1.28
P78357	Contactin-associated protein 1 OS=Homo sapiens GN=CNTNAP1 PE=1 SV=1 - [CNTP1_HUMAN]	2.53	2.41	2.54	2.57	0.11	0.05	0.81	0.76	0.55	0.45	0.46	0.56	-1.66	-1.79	-2.06	-1.80	-1.72	-2.37	-2.39	-1.80
Q9UHC6	Contactin-associated protein-like 2 OS=Homo sapiens GN=CNTNAP2 PE=1 SV=1 - [CNTP2_HUMAN]	1.34	1.42	1.67	1.60	0.31	0.27	0.34	0.37	0.31	0.26	0.25	0.17	-0.97	-1.26	-1.29	-1.07	-1.21	-1.07	-1.35	-1.04
Q96A23	Copine-4 OS=Homo sapiens GN=CPNE4 PE=2 SV=1 - [CPNE4_HUMAN]	2.39	2.32	2.58	2.29	0.13	0.11	0.65	0.91	0.54	0.52	0.43	0.70	-1.64	-1.83	-1.97	-1.91	-2.19	-2.54	-2.85	-1.90
Q9HCH3	Copine-5 OS=Homo sapiens GN=CPNE5 PE=1 SV=2 - [CPNE5_HUMAN]	2.09	1.86	2.49	2.31	-0.18	-0.37	0.47	0.25	0.01	0.24	0.44	0.24	-1.82	-1.42	-1.86	-1.82	-1.98	-2.48	-2.47	-1.73

Q8WZ74	Cortactin-binding protein 2 OS=Homo sapiens GN=CTTNBP2 PE=1 SV=1 - [CTTB2_HUMAN]	1.62	1.39	1.49	1.26	0.13	-0.11	0.16	-0.08	-0.17	0.07	0.06	-0.18	-1.41	-1.56	-1.43	-1.52	-1.39	-1.51	-1.37	-1.27
P12277	Creatine kinase B-type OS=Homo sapiens GN=CKB PE=1 SV=1 - [KCRB_HUMAN]	1.69	1.71	1.71	1.73	-0.19	-0.17	0.58	0.56	0.35	0.36	0.26	0.26	-0.97	-1.41	-1.43	-1.21	-1.20	-1.89	-1.91	-0.91
P12532	Creatine kinase U-type, mitochondrial OS=Homo sapiens GN=CKMT1A PE=1 SV=1 - [KCRU_HUMAN]	2.15	2.15	1.87	2.08	0.14	0.16	0.83	0.64	0.24	0.43	0.15	0.06	-1.23	-2.13	-2.00	-1.75	-1.66	-1.87	-1.82	-1.16
Q9BZC1	CUGBP Elav-like family member 4 OS=Homo sapiens GN=CELF4 PE=1 SV=1 - [CELF4_HUMAN]	1.60	1.76	2.27	2.43	-0.71	-0.56	0.60	0.76	-0.04	-0.19	-0.09	0.06	-0.95	-1.69	-2.36	-1.77	-2.43	-2.33	-3.00	-1.61
Q16281	Cyclic nucleotide-gated cation channel alpha-3 OS=Homo sapiens GN=CNGA3 PE=1 SV=2 - [CNGA3_HUMAN]	1.14	2.19	0.81	1.87	-0.48	0.57	-0.12	0.93	0.41	-0.64	-1.02	0.03	-1.21	-2.15	-1.83	-1.75	-1.42	-1.63	-1.31	-0.87
P50238	Cysteine-rich protein 1 OS=Homo sapiens GN=CRIP1 PE=1 SV=3 - [CRIP1_HUMAN]	-3.59	-3.46	-3.71	-3.58	-2.30	-2.18	-2.10	-1.78	-1.88	-2.12	-1.22	-1.10	1.58	2.22	2.41	1.68	1.88	1.27	1.40	1.79
Q9UPY5	Cystine/glutamate transporter OS=Homo sapiens GN=SLC7A11 PE=1 SV=1 - [XCT_HUMAN]	-1.45	-1.54	-1.05	-1.27	0.34	0.11	0.16	-0.07	-0.14	0.10	0.39	0.23	1.29	1.55	1.54	1.28	1.18	1.58	1.37	1.24
Q53TN4	Cytochrome b reductase 1 OS=Homo sapiens GN=CYBRD1 PE=1 SV=1 - [CYBR1_HUMAN]	-1.84	-1.62	-2.00	-1.81	-0.32	-0.31	0.09	0.05	-0.08	-0.06	0.29	0.33	1.93	2.42	2.22	1.88	1.92	1.81	1.59	1.97

P07919	Cytochrome b-c1 complex subunit 6, mitochondrial OS=Homo sapiens GN=UQCRH PE=1 SV=2 - [QCR6_HUMAN]	1.47	1.47	1.57	1.57	-0.15	0.04	0.66	0.66	0.19	0.21	0.22	0.35	-0.77	-1.23	-1.32	-1.20	-1.31	-1.56	-1.68	-0.87
P00167	Cytochrome b5 OS=Homo sapiens GN=CYB5A PE=1 SV=2 - [CYB5_HUMAN]	-1.80	-1.63	-1.73	-1.53	0.07	0.12	-0.69	-0.59	-0.16	-0.25	0.12	0.24	1.16	2.05	1.85	1.58	1.60	1.85	1.64	1.10
Q96BR5	Cytochrome c oxidase assembly factor 7 OS=Homo sapiens GN=COA7 PE=1 SV=2 - [COA7_HUMAN]	1.46	1.27	1.91	1.71	-0.10	-0.30	0.32	0.27	0.32	0.34	0.12	0.07	-1.09	-1.33	-1.78	-1.09	-1.53	-1.58	-2.03	-1.44
P14854	Cytochrome c oxidase subunit 6B1 OS=Homo sapiens GN=COX6B1 PE=1 SV=2 - [CX6B1_HUMAN]	1.55	1.87	1.61	2.00	0.01	0.28	0.52	0.74	0.49	0.22	0.25	0.37	-1.06	-1.34	-1.27	-1.33	-1.31	-1.30	-1.59	-1.01
P09669	Cytochrome c oxidase subunit 6C OS=Homo sapiens GN=COX6C PE=1 SV=2 - [COX6C_HUMAN]	1.18	1.41	1.73	1.71	-0.36	-0.28	0.45	0.70	0.47	0.31	0.22	0.33	-0.83	-1.01	-1.40	-1.15	-1.36	-1.77	-2.06	-0.99
O14548	Cytochrome c oxidase subunit 7A-related protein, mitochondrial OS=Homo sapiens GN=COX7A2L PE=1 SV=2 - [COX7R_HUMAN]	1.54	1.63	1.74	1.99	0.05	-0.05	0.40	0.49	0.12	-0.18	-0.03	0.15	-1.09	-1.33	-1.65	-1.60	-1.72	-1.50	-1.80	-1.39
P24310	Cytochrome c oxidase subunit 7A1, mitochondrial OS=Homo sapiens GN=COX7A1 PE=1 SV=2 - [CX7A1_HUMAN]	1.58	1.49	1.68	1.59	-0.01	-0.11	-0.16	-0.25	-0.29	-0.19	0.27	0.17	-1.68	-1.31	-1.42	-1.74	-1.84	-1.61	-1.71	-1.78
Q6UW02	Cytochrome P450 20A1 OS=Homo sapiens GN=CYP20A1 PE=2 SV=1 - [CP20A_HUMAN]	-2.46	-2.40	-2.36	-2.30	-0.02	0.04	-0.43	-0.37	-0.11	-0.17	0.02	0.07	2.09	2.48	2.38	2.32	2.23	2.42	2.33	2.00

O14576	Cytoplasmic dynein 1 intermediate chain 1 OS=Homo sapiens GN=DYNC1I1 PE=1 SV=2 - [DC1I1_HUMAN]	1.35	1.45	1.40	1.45	-0.23	-0.05	0.19	0.33	0.18	0.06	-0.15	-0.03	-1.01	-1.35	-1.38	-1.14	-1.09	-1.49	-1.43	-1.04
O00154	Cytosolic acyl coenzyme A thioester hydrolase OS=Homo sapiens GN=ACOT7 PE=1 SV=3 - [BACH_HUMAN]	2.55	2.33	2.51	2.32	0.10	0.04	1.02	0.74	0.47	0.66	0.02	0.08	-1.37	-2.24	-2.35	-1.69	-1.85	-2.24	-2.36	-1.42
Q5M775	Cytospin-B OS=Homo sapiens GN=SPECC1 PE=1 SV=1 - [CYTSB_HUMAN]	1.21	1.41	1.03	1.13	-0.31	-0.14	0.02	0.16	0.08	-0.05	-0.04	-0.13	-1.02	-1.25	-1.27	-1.18	-1.21	-1.31	-1.26	-0.90
P07585	Decorin OS=Homo sapiens GN=DCN PE=1 SV=1 - [PGS2_HUMAN]	-2.13	-2.13	-2.19	-2.23	-0.63	-0.67	-1.10	-1.17	-1.02	-0.93	-1.10	-1.15	1.01	1.14	1.14	1.26	1.23	1.64	1.65	1.11
Q8IZD9	Dedicator of cytokinesis protein 3 OS=Homo sapiens GN=DOCK3 PE=1 SV=1 - [DOCK3_HUMAN]	1.33	2.30	1.05	2.03	-0.50	0.47	0.05	1.02	0.84	-0.12	-0.34	0.62	-1.23	-1.67	-1.40	-1.43	-1.15	-1.85	-1.57	-0.94
Q96HP0	Dedicator of cytokinesis protein 6 OS=Homo sapiens GN=DOCK6 PE=1 SV=3 - [DOCK6_HUMAN]	-1.66	-1.51	-1.67	-1.76	-0.25	-0.18	-0.49	-0.66	-0.55	-0.45	-0.26	-0.36	0.89	1.41	1.44	1.24	1.25	1.47	1.35	1.22
Q92629	Delta-sarcoglycan OS=Homo sapiens GN=SGCD PE=1 SV=2 - [SGCD_HUMAN]	-2.30	-2.32	-2.23	-2.33	0.01	-0.15	-0.17	-0.33	-0.16	-0.09	0.04	-0.08	2.17	2.50	2.22	2.34	2.11	2.51	2.27	2.15
O43323	Desert hedgehog protein OS=Homo sapiens GN=DHH PE=1 SV=1 - [DHH_HUMAN]	-0.79	-0.35	-1.27	-0.83	1.21	1.64	0.34	0.78	1.03	0.59	0.79	1.22	1.19	1.58	2.06	1.42	1.90	1.99	2.46	1.68
P17661	Desmin OS=Homo sapiens GN=DES PE=1 SV=3 - [DESM_HUMAN]	-3.38	-3.31	-3.67	-3.59	-1.13	-1.06	-1.97	-1.93	-1.38	-1.43	-1.03	-1.01	1.58	2.41	2.75	2.05	2.39	2.46	2.61	1.80
Q02487	Desmocollin-2 OS=Homo sapiens GN=DSC2 PE=1 SV=1 - [DSC2_HUMAN]	-2.45	-2.42	-2.59	-2.66	-0.25	-0.27	-1.04	-1.09	-0.64	-0.74	-0.13	-0.13	1.24	2.02	2.38	1.71	1.89	2.04	2.34	1.53

Q14574	Desmocollin-3 OS=Homo sapiens GN=DSC3 PE=1 SV=3 - [DSC3_HUMAN]	-1.79	-1.95	-1.99	-2.14	-0.14	-0.28	-0.71	-0.76	-0.50	-0.32	-0.19	-0.27	1.15	1.54	1.72	1.49	1.51	1.55	1.74	1.46
Q14126	Desmoglein-2 OS=Homo sapiens GN=DSG2 PE=1 SV=2 - [DSG2_HUMAN]	-2.43	-2.49	-2.63	-2.52	-0.29	-0.36	-0.76	-0.82	-0.54	-0.52	-0.17	-0.21	1.65	2.09	2.23	1.83	1.92	1.91	2.22	1.72
P15924	Desmoplakin OS=Homo sapiens GN=DSP PE=1 SV=3 - [DESP_HUMAN]	-2.60	-2.57	-2.66	-2.68	-0.62	-0.66	-0.94	-0.95	-0.89	-0.88	-0.94	-0.97	1.62	1.69	1.72	1.70	1.83	1.86	1.99	1.83
P52824	Diacylglycerol kinase theta OS=Homo sapiens GN=DGKQ PE=1 SV=2 - [DGKQ_HUMAN]	1.62	1.55	1.57	1.50	0.13	0.05	0.48	0.41	0.14	0.22	-0.31	-0.38	-1.09	-1.92	-1.88	-1.37	-1.32	-1.51	-1.46	-1.03
Q9UBP4	Dickkopf-related protein 3 OS=Homo sapiens GN=DKK3 PE=1 SV=2 - [DKK3_HUMAN]	-0.46	-0.27	-0.58	-0.60	2.14	2.22	1.55	1.51	2.30	2.17	2.40	2.53	1.42	2.29	2.85	2.63	2.71	2.55	2.58	1.78
Q14194	Dihydropyrimidinase-related protein 1 OS=Homo sapiens GN=CRMP1 PE=1 SV=1 - [DPYL1_HUMAN]	1.45	1.67	1.84	1.96	-0.29	-0.20	0.34	0.53	0.34	0.17	0.18	0.23	-1.25	-1.51	-1.73	-1.44	-1.67	-1.74	-1.89	-1.52
O14531	Dihydropyrimidinase-related protein 4 OS=Homo sapiens GN=DPYSL4 PE=1 SV=2 - [DPYL4_HUMAN]	1.42	1.21	1.62	1.43	-0.24	-0.01	0.64	0.50	0.02	0.06	-0.35	-0.20	-1.06	-1.81	-2.05	-1.34	-1.53	-1.75	-2.05	-1.10
Q9BPU6	Dihydropyrimidinase-related protein 5 OS=Homo sapiens GN=DPYSL5 PE=1 SV=1 - [DPYL5_HUMAN]	2.04	2.24	2.09	2.29	-0.04	0.06	0.48	0.41	0.27	0.33	-0.22	0.00	-1.46	-2.36	-2.38	-1.68	-1.86	-1.93	-2.21	-1.53
P42658	Dipeptidyl aminopeptidase-like protein 6 OS=Homo sapiens GN=DPP6 PE=1 SV=2 - [DPP6_HUMAN]	1.39	1.56	1.60	1.78	-0.04	-0.11	0.31	0.55	0.36	0.24	0.12	0.21	-0.86	-1.18	-1.33	-1.09	-1.32	-1.82	-2.15	-1.14
P27487	Dipeptidyl peptidase 4 OS=Homo sapiens GN=DPP4 PE=1 SV=2 - [DPP4_HUMAN]	-1.91	-1.93	-1.91	-1.92	0.66	0.65	-0.79	-0.67	0.40	0.01	-0.18	-0.15	1.24	1.37	1.78	1.97	2.36	2.38	2.52	1.27

P98082	Disabled homolog 2 OS=Homo sapiens GN=DAB2 PE=1 SV=3 - [DAB2_HUMAN]	-0.97	-1.25	-1.32	-1.59	0.71	0.44	0.21	-0.07	0.28	0.56	0.50	0.22	1.24	1.48	1.82	1.56	1.91	1.67	2.01	1.59
P78536	Disintegrin and metalloproteinase domain-containing protein 17 OS=Homo sapiens GN=ADAM17 PE=1 SV=1 - [ADA17_HUMAN]	-1.54	-1.65	-1.41	-1.51	0.37	0.25	-0.28	-0.39	-0.08	0.04	0.66	0.55	1.31	2.21	2.07	1.61	1.48	1.89	1.75	1.19
Q9P0K1	Disintegrin and metalloproteinase domain-containing protein 22 OS=Homo sapiens GN=ADAM22 PE=1 SV=1 - [ADA22_HUMAN]	2.11	2.03	2.24	2.51	-0.13	0.10	0.83	0.94	0.52	0.53	0.01	0.24	-1.11	-1.93	-2.34	-1.40	-2.00	-2.10	-2.30	-1.50
O75077	Disintegrin and metalloproteinase domain-containing protein 23 OS=Homo sapiens GN=ADAM23 PE=1 SV=1 - [ADA23_HUMAN]	2.36	2.50	2.42	2.66	-0.11	0.11	0.70	1.01	0.59	0.52	0.36	0.37	-1.43	-1.76	-1.13	-1.76	-1.93	-2.11	-2.57	-1.53
Q15700	Disks large homolog 2 OS=Homo sapiens GN=DLG2 PE=1 SV=3 - [DLG2_HUMAN]	1.72	1.67	1.72	1.60	-0.15	-0.29	0.35	0.27	0.15	0.23	-0.10	-0.17	-1.11	-1.78	-2.14	-1.39	-1.56	-1.91	-1.98	-1.30
Q92796	Disks large homolog 3 OS=Homo sapiens GN=DLG3 PE=1 SV=2 - [DLG3_HUMAN]	1.83	1.36	1.74	1.66	0.04	0.11	0.25	0.29	0.10	0.40	0.12	0.17	-0.86	-1.41	-1.57	-1.45	-1.45	-1.43	-1.47	-0.98
P78352	Disks large homolog 4 OS=Homo sapiens GN=DLG4 PE=1 SV=3 - [DLG4_HUMAN]	1.83	1.92	1.92	1.99	-0.13	-0.13	0.57	0.50	0.22	0.25	0.05	0.14	-1.24	-1.80	-1.95	-1.67	-1.77	-2.19	-2.10	-1.38
O14490	Disks large-associated protein 1 OS=Homo sapiens GN=DLGAP1 PE=1 SV=1 - [DLGP1_HUMAN]	1.64	1.23	1.89	1.77	-0.33	-0.27	0.69	0.62	-0.21	0.08	-0.23	-0.67	-0.85	-1.83	-2.09	-1.38	-1.70	-1.66	-1.87	-1.14

Q9P1A6	Disks large-associated protein 2 OS=Homo sapiens GN=DLGAP2 PE=1 SV=4 - [DLGP2_HUMAN]	1.44	1.34	1.41	1.45	0.14	0.37	0.36	0.59	0.29	0.06	-0.21	-0.15	-1.16	-1.70	-1.82	-1.50	-1.36	-1.47	-1.33	-1.03
Q8TDJ6	DmX-like protein 2 OS=Homo sapiens GN=DMXL2 PE=1 SV=2 - [DMXL2_HUMAN]	1.28	1.59	1.36	1.34	-0.04	0.19	0.39	0.36	0.32	0.23	0.09	0.08	-0.97	-1.28	-1.21	-0.97	-1.06	-1.28	-1.24	-0.89
Q9UBS3	DnaJ homolog subfamily B member 9 OS=Homo sapiens GN=DNAJB9 PE=1 SV=1 - [DNJB9_HUMAN]	0.73	0.77	-0.20	-0.15	2.93	3.40	3.12	3.39	3.16	2.69	2.15	2.62	2.30	1.58	1.42	2.23	2.00	2.40	2.18	2.45
P39656	Dolichyl-diphosphooligosaccharide--protein glycosyltransferase 48 kDa subunit OS=Homo sapiens GN=DDOST PE=1 SV=4 - [OST48_HUMAN]	-1.71	-1.68	-1.32	-1.45	-0.11	-0.01	-0.31	-0.50	-0.31	-0.31	-0.22	-0.09	1.18	1.35	1.28	1.36	1.23	1.58	1.41	1.07
P04844	Dolichyl-diphosphooligosaccharide--protein glycosyltransferase subunit 2 OS=Homo sapiens GN=RPN2 PE=1 SV=3 - [RPN2_HUMAN]	-1.34	-1.23	-1.47	-1.20	0.17	0.26	-0.38	-0.32	0.04	-0.16	-0.07	0.04	0.88	1.18	1.21	1.16	1.20	1.28	1.45	1.14
P61803	Dolichyl-diphosphooligosaccharide--protein glycosyltransferase subunit DAD1 OS=Homo sapiens GN=DAD1 PE=1 SV=3 - [DAD1_HUMAN]	-1.32	-1.21	-1.98	-1.86	0.35	0.41	-0.42	-0.40	-0.09	-0.11	0.07	0.03	0.95	1.25	1.90	1.15	1.81	1.63	2.29	1.62
P46977	Dolichyl-diphosphooligosaccharide--protein glycosyltransferase subunit STT3A OS=Homo sapiens GN=STT3A PE=1 SV=2 - [STT3A_HUMAN]	-2.15	-2.21	-1.83	-1.88	0.05	0.12	-0.39	-0.44	-0.24	-0.21	-0.04	0.01	1.79	2.17	1.85	2.01	1.69	2.32	1.97	1.49

Q9P0K9	DOMON domain-containing protein FRRS1L OS=Homo sapiens GN=FRRS1L PE=2 SV=2 - [FRS1L_HUMAN]	1.60	1.93	1.77	2.10	0.34	0.66	0.51	0.84	0.87	0.54	0.48	0.80	-1.04	-1.12	-1.30	-1.03	-1.20	-1.28	-1.45	-1.20
Q02750	Dual specificity mitogen-activated protein kinase kinase 1 OS=Homo sapiens GN=MAP2K1 PE=1 SV=2 - [MP2K1_HUMAN]	1.76	1.83	2.24	2.26	0.60	0.41	0.93	0.87	0.62	0.59	0.38	0.47	-0.85	-1.38	-1.80	-1.16	-1.67	-1.34	-1.61	-1.28
P46734	Dual specificity mitogen-activated protein kinase kinase 3 OS=Homo sapiens GN=MAP2K3 PE=1 SV=2 - [MP2K3_HUMAN]	-1.31	-1.35	-1.44	-1.53	-0.15	-0.19	0.35	0.13	0.05	0.20	-0.02	-0.07	1.56	1.29	1.47	1.32	1.64	1.14	1.31	1.75
Q05193	Dynamin-1 OS=Homo sapiens GN=DNM1 PE=1 SV=2 - [DYN1_HUMAN]	2.07	2.07	2.19	2.12	-0.30	-0.26	0.75	0.81	0.44	0.39	0.23	0.27	-1.33	-1.77	-1.87	-1.67	-1.71	-2.36	-2.21	-1.28
Q9UQ16	Dynamin-3 OS=Homo sapiens GN=DNM3 PE=1 SV=4 - [DYN3_HUMAN]	1.86	1.80	2.01	2.02	-0.25	-0.19	0.67	0.69	0.39	0.44	0.11	0.13	-1.09	-1.81	-1.89	-1.45	-1.61	-2.14	-2.25	-1.21
Q5VV43	Dyslexia-associated protein KIAA0319 OS=Homo sapiens GN=KIAA0319 PE=1 SV=1 - [K0319_HUMAN]	-2.32	-2.46	-2.58	-2.72	-0.21	-0.36	-0.93	-1.07	-0.39	-0.25	-0.40	-0.54	1.44	1.93	2.18	2.10	2.37	2.09	2.35	1.72
Q09019	Dystrophia myotonica WD repeat-containing protein OS=Homo sapiens GN=DMWD PE=1 SV=3 - [DMWD_HUMAN]	1.20	1.23	1.06	1.10	-0.37	-0.34	0.20	0.23	0.05	0.02	-0.32	-0.29	-0.95	-1.51	-1.38	-1.15	-1.01	-1.59	-1.45	-0.80
Q32P44	Echinoderm microtubule-associated protein-like 3 OS=Homo sapiens GN=EML3 PE=1 SV=1 - [EMAL3_HUMAN]	-1.01	-0.96	-1.19	-0.93	0.09	0.44	-0.23	-0.06	0.22	-0.03	0.04	0.11	0.90	1.05	1.17	1.04	1.27	1.20	1.35	0.95

P49961	Ectonucleoside triphosphate diphosphohydrolase 1 OS=Homo sapiens GN=ENTPD1 PE=1 SV=1 - [ENTP1_HUMAN]	-2.32	-2.19	-2.36	-2.35	0.00	0.05	-0.44	-0.46	-0.29	-0.28	-0.08	-0.10	1.62	2.06	2.11	1.78	1.90	1.92	2.31	1.94
Q6UWR7	Ectonucleotide pyrophosphatase/phosphodiesterase family member 6 OS=Homo sapiens GN=ENPP6 PE=1 SV=2 - [ENPP6_HUMAN]	-1.41	-1.37	-1.36	-1.45	0.31	0.17	0.33	0.32	0.45	0.41	0.79	0.76	1.80	2.31	2.40	1.94	1.86	1.68	1.71	1.96
Q6NXP0	EF-hand calcium-binding domain-containing protein 12 OS=Homo sapiens GN=EFCAB12 PE=2 SV=1 - [EFC12_HUMAN]	2.08	1.56	1.94	1.42	0.15	-0.38	0.97	0.45	0.02	0.55	-0.39	-0.92	-1.05	-2.47	-2.34	-1.50	-1.36	-1.95	-1.82	-0.91
Q12805	EGF-containing fibulin-like extracellular matrix protein 1 OS=Homo sapiens GN=EFEMP1 PE=1 SV=2 - [FBLN3_HUMAN]	-0.96	-0.81	-1.43	-1.09	0.92	1.02	0.53	0.54	0.79	0.74	0.64	0.66	1.43	1.63	2.05	1.58	1.88	1.68	2.29	1.79
O95967	EGF-containing fibulin-like extracellular matrix protein 2 OS=Homo sapiens GN=EFEMP2 PE=1 SV=3 - [FBLN4_HUMAN]	-1.45	-1.33	-1.40	-1.30	1.22	1.25	0.40	0.47	0.75	0.65	0.83	0.70	1.91	1.99	2.22	2.04	2.12	2.58	2.70	1.98
Q9H4M9	EH domain-containing protein 1 OS=Homo sapiens GN=EHD1 PE=1 SV=2 - [EHD1_HUMAN]	-1.34	-1.40	-1.17	-1.27	0.18	0.12	0.41	0.36	0.18	0.22	0.09	0.04	1.79	1.45	1.36	1.51	1.41	1.46	1.35	1.64
Q9NZN3	EH domain-containing protein 3 OS=Homo sapiens GN=EHD3 PE=1 SV=2 - [EHD3_HUMAN]	1.09	0.99	1.55	1.61	0.01	-0.06	0.62	0.49	0.34	0.31	0.35	0.35	-0.80	-1.01	-1.50	-1.02	-1.34	-1.60	-1.92	-1.09
P15502	Elastin OS=Homo sapiens GN=ELN PE=1 SV=3 - [ELN_HUMAN]	-2.96	-3.07	-2.54	-2.45	0.81	0.81	-1.06	-1.14	-0.43	-0.49	-1.18	-1.24	2.15	1.82	1.17	2.75	2.08	3.98	3.41	1.44

Q14576	ELAV-like protein 3 OS=Homo sapiens GN=ELAVL3 PE=1 SV=3 - [ELAV3_HUMAN]	2.05	2.16	2.66	2.96	0.16	0.43	0.79	1.07	0.74	0.74	0.75	0.77	-0.92	-1.27	-2.18	-1.52	-2.18	-1.69	-2.58	-1.80
Q8N336	ELMO domain-containing protein 1 OS=Homo sapiens GN=ELMOD1 PE=2 SV=3 - [ELMD1_HUMAN]	1.72	1.73	1.92	1.93	-0.09	0.02	0.57	0.58	0.30	0.18	0.03	0.07	-1.10	-2.05	-2.31	-1.87	-2.11	-1.61	-1.80	-1.30
Q05639	Elongation factor 1-alpha 2 OS=Homo sapiens GN=EEF1A2 PE=1 SV=1 - [EF1A2_HUMAN]	2.14	2.25	2.62	2.60	-0.12	0.15	0.91	0.89	0.60	0.48	0.33	0.23	-1.53	-1.98	-2.24	-1.38	-1.83	-2.20	-2.21	-1.66
Q9Y6C2	EMILIN-1 OS=Homo sapiens GN=EMILIN1 PE=1 SV=2 - [EMIL1_HUMAN]	-1.88	-2.00	-2.13	-2.06	0.35	0.39	-0.34	-0.19	0.05	0.02	-0.05	-0.04	1.59	1.93	2.01	1.90	2.18	2.15	2.51	1.83
Q9BXX0	EMILIN-2 OS=Homo sapiens GN=EMILIN2 PE=1 SV=3 - [EMIL2_HUMAN]	-1.33	-1.37	-1.65	-1.55	1.27	1.21	0.02	0.13	0.58	0.54	0.35	0.45	1.68	1.79	1.87	2.04	2.28	2.59	2.85	1.75
P17813	Endoglin OS=Homo sapiens GN=ENG PE=1 SV=2 - [EGLN_HUMAN]	-2.18	-2.20	-2.47	-2.48	0.37	0.50	-0.20	-0.26	-0.13	-0.26	-0.21	-0.08	1.99	2.14	2.13	2.11	2.11	2.68	2.68	2.29
Q99962	Endophilin-A1 OS=Homo sapiens GN=SH3GL2 PE=1 SV=1 - [SH3G2_HUMAN]	1.03	1.47	1.02	1.45	-0.19	-0.07	0.07	0.16	-0.15	-0.33	-0.49	-0.32	-0.86	-1.63	-1.72	-1.37	-1.43	-1.21	-1.39	-1.11
Q99963	Endophilin-A3 OS=Homo sapiens GN=SH3GL3 PE=1 SV=1 - [SH3G3_HUMAN]	1.53	1.54	1.77	1.69	-0.38	-0.46	0.50	0.51	-0.05	-0.02	-0.48	-0.52	-0.98	-2.02	-2.29	-1.52	-1.83	-1.83	-2.12	-1.14
Q86YB7	Enoyl-CoA hydratase domain-containing protein 2, mitochondrial OS=Homo sapiens GN=ECHDC2 PE=2 SV=2 - [ECHD2_HUMAN]	-2.43	-1.97	-2.11	-1.65	-0.27	0.19	-0.33	0.14	0.26	-0.20	0.10	0.56	2.16	2.54	2.22	2.26	1.94	2.14	1.82	1.85

Q96DC8	Enoyl-CoA hydratase domain-containing protein 3, mitochondrial OS=Homo sapiens GN=ECHDC3 PE=1 SV=2 - [ECHD3_HUMAN]	-0.82	-0.72	-1.40	-1.45	0.99	0.94	1.39	1.38	1.35	1.30	1.45	1.43	2.11	2.27	2.99	2.11	2.73	1.90	2.48	2.81
Q8N5V2	Ephexin-1 OS=Homo sapiens GN=NGEF PE=2 SV=2 - [NGEF_HUMAN]	1.60	1.44	1.78	1.44	-0.29	-0.47	0.17	-0.27	-0.24	0.13	-0.29	-0.36	-1.38	-1.95	-1.95	-1.52	-1.73	-1.91	-1.92	-1.68
P29323	Ephrin type-B receptor 2 OS=Homo sapiens GN=EPHB2 PE=1 SV=5 - [EPHB2_HUMAN]	2.29	2.72	2.17	2.60	-0.32	0.11	0.55	0.98	0.50	0.08	0.12	0.55	-1.68	-2.16	-2.05	-2.18	-2.06	-2.62	-2.51	-1.56
Q9H6S3	Epidermal growth factor receptor kinase substrate 8-like protein 2 OS=Homo sapiens GN=EPS8L2 PE=1 SV=2 - [ES8L2_HUMAN]	-0.97	-0.99	-1.32	-1.34	0.27	0.25	-0.04	-0.06	0.14	0.17	0.10	0.07	0.98	1.07	1.42	1.17	1.53	1.22	1.58	1.34
Q9UHF1	Epidermal growth factor-like protein 7 OS=Homo sapiens GN=EGFL7 PE=1 SV=3 - [EGFL7_HUMAN]	-2.44	-2.56	-2.24	-2.35	0.20	0.33	-0.34	-0.33	-0.14	-0.09	0.23	0.36	1.99	2.56	2.63	2.28	2.35	2.41	2.60	2.08
Q99944	Epidermal growth factor-like protein 8 OS=Homo sapiens GN=EGFL8 PE=1 SV=1 - [EGFL8_HUMAN]	-1.70	-1.41	-1.77	-1.48	0.28	0.57	0.22	0.51	0.65	0.37	0.45	0.74	1.97	2.16	2.22	2.09	2.17	1.96	2.03	2.05
P07099	Epoxide hydrolase 1 OS=Homo sapiens GN=EPHX1 PE=1 SV=1 - [HYEP_HUMAN]	-1.84	-1.76	-1.98	-1.85	0.33	0.39	0.07	0.07	0.13	0.14	0.20	0.20	1.88	2.07	1.89	1.83	1.87	1.98	2.09	1.84
O43556	Epsilon-sarcoglycan OS=Homo sapiens GN=SGCE PE=1 SV=6 - [SGCE_HUMAN]	-1.65	-1.68	-1.61	-1.53	0.01	0.09	-0.04	0.05	0.00	-0.03	0.26	0.02	1.78	1.95	1.59	1.87	1.56	1.64	1.61	1.69
P24390	ER lumen protein retaining receptor 1 OS=Homo sapiens GN=KDELR1 PE=1 SV=1 - [ERD21_HUMAN]	-1.89	-1.75	-1.80	-1.66	0.02	0.16	-0.78	-0.64	-0.25	-0.39	-0.33	-0.20	1.16	1.56	1.47	1.53	1.45	1.89	1.80	1.08

O15083	ERC protein 2 OS=Homo sapiens GN=ERC2 PE=1 SV=3 - [ERC2_HUMAN]	2.30	2.10	2.23	2.03	-0.80	-0.77	0.88	0.69	0.58	0.52	-0.18	-0.16	-1.38	-2.14	-2.29	-2.15	-2.19	-2.55	-2.69	-1.44
Q8TAM6	Ermin OS=Homo sapiens GN=ERMN PE=2 SV=1 - [ERMIN_HUMAN]	2.41	2.61	2.54	2.93	-0.75	-0.75	0.47	0.46	0.05	0.15	0.19	0.04	-1.92	-2.33	-2.55	-2.35	-2.67	-2.04	-2.49	-1.95
P27105	Erythrocyte band 7 integral membrane protein OS=Homo sapiens GN=STOM PE=1 SV=3 - [STOM_HUMAN]	-1.31	-1.23	-1.52	-1.35	0.63	0.69	0.12	0.19	0.54	0.51	0.82	1.00	1.48	2.04	2.25	1.64	2.01	1.87	2.05	1.63
P16452	Erythrocyte membrane protein band 4.2 OS=Homo sapiens GN=EPB42 PE=1 SV=3 - [EPB42_HUMAN]	-1.80	-1.76	-1.93	-1.86	0.30	0.42	0.43	0.66	0.81	0.67	1.25	1.38	2.23	3.14	3.15	2.57	2.55	2.05	2.02	2.46
P41214	Eukaryotic translation initiation factor 2D OS=Homo sapiens GN=EIF2D PE=1 SV=3 - [EIF2D_HUMAN]	-1.61	-1.64	-2.19	-2.22	0.09	0.05	-0.52	-0.55	-0.03	0.01	0.23	0.20	1.15	1.85	2.42	1.65	2.23	1.68	2.26	1.74
Q15056	Eukaryotic translation initiation factor 4H OS=Homo sapiens GN=EIF4H PE=1 SV=5 - [IF4H_HUMAN]	1.38	1.26	1.20	1.28	-0.02	-0.04	0.37	0.44	0.15	-0.01	-0.18	-0.20	-0.83	-1.49	-1.51	-1.44	-1.15	-1.39	-1.33	-0.86
P43004	Excitatory amino acid transporter 2 OS=Homo sapiens GN=SLC1A2 PE=1 SV=2 - [EAA2_HUMAN]	2.43	2.38	2.65	2.73	0.02	0.08	1.33	1.37	0.65	0.66	0.17	0.26	-0.96	-2.37	-2.67	-1.72	-2.05	-2.70	-3.13	-1.15
Q16610	Extracellular matrix protein 1 OS=Homo sapiens GN=ECM1 PE=1 SV=2 - [ECM1_HUMAN]	-1.80	-1.82	-1.96	-1.94	-0.21	-0.21	-0.64	-0.57	-0.25	-0.36	0.19	0.24	1.35	1.92	2.21	1.72	1.85	1.63	1.69	1.40
O94769	Extracellular matrix protein 2 OS=Homo sapiens GN=ECM2 PE=2 SV=1 - [ECM2_HUMAN]	-1.48	-1.65	-2.24	-2.41	0.88	0.71	-0.24	-0.41	0.01	0.19	-0.11	-0.29	1.29	1.37	2.12	1.69	2.46	2.34	3.10	2.06

Q8IWU6	Extracellular sulfatase Sulf-1 OS=Homo sapiens GN=SULF1 PE=1 SV=1 - [SULF1_HUMAN]	-0.10	-0.01	-0.72	-0.81	3.49	3.30	1.17	1.09	2.21	2.31	1.65	1.54	1.66	2.04	2.63	2.86	3.30	3.99	4.43	2.27
Q8IWU5	Extracellular sulfatase Sulf-2 OS=Homo sapiens GN=SULF2 PE=1 SV=1 - [SULF2_HUMAN]	-0.19	0.00	-0.42	-0.06	3.09	3.86	2.30	2.39	2.92	2.16	1.81	1.99	2.44	2.00	2.06	2.84	2.73	3.72	3.62	2.52
Q8N461	F-box/LRR-repeat protein 16 OS=Homo sapiens GN=FBXL16 PE=1 SV=2 - [FXL16_HUMAN]	1.71	1.24	1.71	1.23	-0.38	-0.13	0.55	0.24	0.05	0.15	-0.28	-0.33	-0.94	-1.78	-1.78	-1.25	-1.24	-1.38	-1.38	-0.92
P05413	Fatty acid-binding protein, heart OS=Homo sapiens GN=FABP3 PE=1 SV=4 - [FABPH_HUMAN]	1.06	1.15	1.38	1.48	-0.73	-0.60	-0.04	-0.01	-0.59	-0.71	-0.81	-0.69	-1.04	-1.83	-2.16	-1.35	-1.76	-1.39	-1.82	-1.35
O00519	Fatty-acid amide hydrolase 1 OS=Homo sapiens GN=FAAH PE=1 SV=2 - [FAAH1_HUMAN]	1.57	1.53	1.67	1.64	0.20	0.20	0.53	0.50	0.30	0.34	-0.14	-0.15	-0.98	-2.08	-2.05	-1.20	-1.30	-1.75	-1.72	-1.08
Q86UX7	Fermitin family homolog 3 OS=Homo sapiens GN=FERMT3 PE=1 SV=1 - [URP2_HUMAN]	-0.45	-0.60	-0.88	-0.93	0.77	0.58	0.45	0.40	0.61	0.71	0.64	0.57	0.95	1.22	1.45	1.26	1.60	1.28	1.63	1.50
P02794	Ferritin heavy chain OS=Homo sapiens GN=FTH1 PE=1 SV=2 - [FRIH_HUMAN]	2.36	2.29	2.89	2.75	0.48	0.50	1.70	1.54	1.40	1.29	0.96	1.19	-0.92	-1.36	-1.73	-1.03	-1.27	-1.76	-2.18	-1.17
P35555	Fibrillin-1 OS=Homo sapiens GN=FBN1 PE=1 SV=3 - [FBN1_HUMAN]	-2.21	-2.16	-2.51	-2.46	0.37	0.45	0.12	0.18	0.13	0.09	-0.12	-0.07	2.43	2.11	2.36	2.39	2.61	2.55	2.84	2.69
Q8TAT2	Fibroblast growth factor-binding protein 3 OS=Homo sapiens GN=FGFBP3 PE=1 SV=1 - [FGFP3_HUMAN]	-0.87	-0.94	-0.82	-0.88	1.26	1.19	0.36	0.29	0.71	0.78	1.02	0.95	1.29	1.90	1.84	1.68	1.63	2.12	2.06	1.24
Q14314	Fibroleukin OS=Homo sapiens GN=FGL2 PE=1 SV=1 - [FGL2_HUMAN]	-1.26	-1.15	-1.42	-1.32	1.07	1.20	0.63	0.84	0.96	0.73	1.15	1.31	1.92	2.20	2.70	2.07	2.35	2.43	2.50	2.22

Q06828	Fibromodulin OS=Homo sapiens GN=FMOD PE=1 SV=2 - [FMOD_HUMAN]	-2.39	-2.48	-2.56	-2.76	-0.66	-0.68	-0.48	-0.60	-0.55	-0.56	-0.87	-0.85	2.13	1.63	1.84	1.89	2.27	1.87	2.31	2.18
P02751	Fibronectin OS=Homo sapiens GN=FN1 PE=1 SV=4 - [FINC_HUMAN]	-2.13	-2.13	-2.21	-2.25	1.01	1.05	-0.53	-0.50	0.18	0.14	-0.04	0.02	1.68	2.10	2.21	2.32	2.40	3.06	3.24	1.74
Q9BTV5	Fibronectin type III and SPRY domain-containing protein 1 OS=Homo sapiens GN=FSD1 PE=1 SV=1 - [FSD1_HUMAN]	1.62	1.81	1.59	1.58	-0.44	-0.38	0.78	0.83	0.24	0.00	-0.12	0.01	-0.78	-1.95	-2.06	-1.45	-1.57	-2.21	-1.97	-0.94
P23142	Fibulin-1 OS=Homo sapiens GN=FBLN1 PE=1 SV=4 - [FBLN1_HUMAN]	-1.13	-1.15	-1.44	-1.35	0.87	0.91	-0.10	-0.24	0.20	0.20	0.45	0.46	0.94	1.73	2.03	1.23	1.75	2.05	2.35	1.33
Q9UBX5	Fibulin-5 OS=Homo sapiens GN=FBLN5 PE=1 SV=1 - [FBLN5_HUMAN]	-1.48	-1.33	-1.83	-2.03	1.48	1.48	0.59	0.49	0.85	0.84	0.29	0.21	2.00	1.82	2.05	2.33	2.65	2.80	3.42	2.48
P21333	Filamin-A OS=Homo sapiens GN=FLNA PE=1 SV=4 - [FLNA_HUMAN]	-2.34	-2.27	-2.47	-2.42	-0.89	-0.86	-1.02	-0.98	-0.83	-0.87	-0.50	-0.45	1.30	1.85	2.06	1.47	1.65	1.44	1.60	1.55
O75369	Filamin-B OS=Homo sapiens GN=FLNB PE=1 SV=2 - [FLNB_HUMAN]	-1.79	-1.73	-1.86	-1.81	-0.61	-0.49	-0.81	-0.72	-0.47	-0.60	-0.17	-0.11	1.14	1.69	1.68	1.28	1.26	1.27	1.25	1.13
Q14315	Filamin-C OS=Homo sapiens GN=FLNC PE=1 SV=3 - [FLNC_HUMAN]	-1.93	-1.85	-2.05	-1.95	-0.18	-0.12	-0.76	-0.71	-0.35	-0.43	-0.12	-0.07	1.27	1.79	1.86	1.63	1.63	1.78	1.86	1.36
P14207	Folate receptor beta OS=Homo sapiens GN=FOLR2 PE=1 SV=4 - [FOLR2_HUMAN]	-2.00	-1.98	-2.12	-2.14	0.23	0.31	0.09	0.09	0.17	0.10	0.34	0.42	2.16	2.35	2.43	2.16	2.13	2.18	2.35	2.28
Q9NZ56	Formin-2 OS=Homo sapiens GN=FMN2 PE=1 SV=4 - [FMN2_HUMAN]	1.69	1.13	1.29	1.28	0.36	-0.11	0.31	-0.22	0.61	0.45	0.45	-0.29	-1.33	-1.18	-1.12	-1.15	-1.17	-1.23	-1.16	-1.17
Q5TD97	Four and a half LIM domains protein 5 OS=Homo sapiens GN=FHL5 PE=1 SV=1 - [FHL5_HUMAN]	-3.41	-3.33	-3.29	-3.16	-1.07	-1.06	-1.61	-1.44	-1.29	-1.45	-1.03	-0.94	1.94	2.22	2.29	2.10	1.91	2.43	2.17	1.77

O75084	Frizzled-7 OS=Homo sapiens GN=FZD7 PE=1 SV=2 - [FZD7_HUMAN]	-2.00	-1.70	-2.41	-2.24	0.82	1.11	0.19	0.49	0.57	0.28	0.22	0.29	2.24	1.77	2.31	2.31	2.70	2.80	2.91	2.37
Q9H461	Frizzled-8 OS=Homo sapiens GN=FZD8 PE=1 SV=1 - [FZD8_HUMAN]	-2.60	-3.14	-3.21	-3.76	0.99	0.43	0.57	0.02	-0.26	0.29	-0.01	-0.56	3.22	2.60	3.20	2.92	3.54	3.56	4.18	3.85
Q9BQS8	FYVE and coiled-coil domain-containing protein 1 OS=Homo sapiens GN=FYCO1 PE=1 SV=3 - [FYCO1_HUMAN]	-0.97	-0.89	-1.29	-1.32	0.09	0.25	-0.08	-0.10	0.00	0.04	0.27	0.29	0.94	1.24	1.55	1.05	1.47	1.12	1.39	1.22
Q7Z2K8	G protein-regulated inducer of neurite outgrowth 1 OS=Homo sapiens GN=GPRIN1 PE=2 SV=2 - [GRIN1_HUMAN]	0.97	0.80	1.36	1.15	-0.13	0.01	-0.29	-0.40	0.01	-0.01	-0.01	-0.15	-0.98	-1.37	-1.50	-1.30	-1.43	-1.24	-1.41	-1.07
Q96PE1	G-protein coupled receptor 124 OS=Homo sapiens GN=GPR124 PE=1 SV=2 - [GP124_HUMAN]	-1.19	-1.22	-1.59	-1.61	0.01	-0.02	-0.37	-0.39	-0.08	-0.05	0.19	0.16	0.88	1.39	1.78	1.17	1.57	1.19	1.58	1.28
P14867	Gamma-aminobutyric acid receptor subunit alpha-1 OS=Homo sapiens GN=GABRA1 PE=1 SV=3 - [GBRA1_HUMAN]	1.74	1.75	1.81	1.92	-0.27	-0.50	0.39	0.22	-0.41	-0.25	-0.17	-0.30	-1.50	-2.15	-2.31	-1.99	-2.16	-2.61	-2.47	-1.72
P47870	Gamma-aminobutyric acid receptor subunit beta-2 OS=Homo sapiens GN=GABRB2 PE=1 SV=2 - [GBRB2_HUMAN]	2.00	1.72	1.72	1.71	-0.47	-0.46	0.43	0.58	0.13	-0.05	-0.02	-0.03	-1.22	-1.90	-1.92	-1.74	-1.76	-2.18	-2.17	-1.23
P28472	Gamma-aminobutyric acid receptor subunit beta-3 OS=Homo sapiens GN=GABRB3 PE=1 SV=1 - [GBRB3_HUMAN]	1.67	1.75	1.50	1.59	-0.20	-0.12	-0.29	-0.21	0.29	0.21	-0.34	-0.26	-1.89	-2.00	-1.84	-1.63	-1.52	-1.88	-1.72	-1.77

O75899	Gamma-aminobutyric acid type B receptor subunit 2 OS=Homo sapiens GN=GABBR2 PE=1 SV=1 - [GABR2_HUMAN]	1.88	2.25	2.23	2.67	-0.39	0.25	0.62	1.02	0.87	0.27	-0.04	0.47	-1.13	-1.82	-2.01	-1.38	-1.62	-2.00	-2.47	-1.27
P09104	Gamma-enolase OS=Homo sapiens GN=ENO2 PE=1 SV=3 - [ENOG_HUMAN]	1.74	1.75	1.76	1.86	0.65	0.71	0.82	0.85	0.56	0.55	0.02	-0.02	-0.86	-1.59	-1.55	-1.09	-1.24	-1.19	-1.20	-0.91
O76070	Gamma-synuclein OS=Homo sapiens GN=SNCG PE=1 SV=2 - [SYUG_HUMAN]	0.69	0.63	1.13	1.01	-0.47	-0.45	-0.45	-0.02	-0.49	-0.27	-0.58	-0.53	-0.94	-1.23	-1.68	-1.08	-1.39	-1.04	-1.38	-1.09
Q8TB36	Ganglioside-induced differentiation-associated protein 1 OS=Homo sapiens GN=GDAP1 PE=1 SV=3 - [GDAP1_HUMAN]	1.67	1.00	1.99	1.36	-0.26	-0.55	0.67	0.17	-0.21	0.04	-0.05	-0.34	-0.97	-1.59	-2.04	-1.48	-1.53	-1.88	-2.20	-1.19
Q96MZ0	Ganglioside-induced differentiation-associated protein 1-like 1 OS=Homo sapiens GN=GDAP1L1 PE=2 SV=2 - [GD1L1_HUMAN]	2.51	2.77	2.72	2.91	-0.67	-0.39	1.27	1.38	0.82	0.57	0.24	0.55	-1.44	-2.12	-2.09	-1.89	-1.97	-2.82	-2.93	-1.37
Q9H488	GDP-fucose protein O-fucosyltransferase 1 OS=Homo sapiens GN=POFUT1 PE=1 SV=1 - [OFUT1_HUMAN]	-1.27	-1.15	-1.43	-1.29	0.14	0.15	0.01	-0.03	0.14	0.08	-0.03	-0.02	1.17	1.46	1.43	1.38	1.64	1.51	1.55	1.45
Q13630	GDP-L-fucose synthase OS=Homo sapiens GN=TSTA3 PE=1 SV=1 - [FCL_HUMAN]	-0.81	-0.89	-0.79	-0.88	0.49	0.47	0.40	0.41	0.47	0.44	0.38	0.37	1.05	1.27	1.27	1.35	1.39	1.35	1.33	1.16
Q9NQX3	Gephyrin OS=Homo sapiens GN=GPHN PE=1 SV=1 - [GEPH_HUMAN]	1.41	1.59	1.68	1.79	-0.16	0.01	0.54	0.52	0.21	0.12	0.06	0.08	-1.11	-1.48	-1.70	-1.47	-1.66	-1.63	-1.78	-1.26
Q05329	Glutamate decarboxylase 2 OS=Homo sapiens GN=GAD2 PE=1 SV=1 - [DCE2_HUMAN]	2.08	2.55	2.25	2.71	-0.08	-0.17	0.84	1.27	0.73	0.53	0.00	0.02	-1.18	-1.70	-1.91	-1.52	-1.69	-1.90	-2.16	-1.35

P42262	Glutamate receptor 2 OS=Homo sapiens GN=GRIA2 PE=1 SV=3 - [GRIA2_HUMAN]	1.77	1.24	2.12	1.67	-0.26	-0.10	0.64	0.48	0.28	0.31	-0.15	-0.19	-0.97	-1.47	-1.66	-0.97	-1.33	-1.36	-1.78	-1.15
P42263	Glutamate receptor 3 OS=Homo sapiens GN=GRIA3 PE=1 SV=2 - [GRIA3_HUMAN]	1.70	1.67	1.58	1.54	-0.41	-0.39	0.27	0.29	0.09	0.18	-0.09	-0.10	-1.33	-1.20	-1.05	-1.49	-1.41	-1.86	-1.80	-1.18
Q05586	Glutamate receptor ionotropic, NMDA 1 OS=Homo sapiens GN=GRIN1 PE=1 SV=1 - [NMDZ1_HUMAN]	2.14	1.91	2.27	1.95	-0.34	-0.11	0.28	0.65	0.30	0.51	0.59	0.08	-1.45	-1.59	-1.87	-1.51	-1.92	-1.67	-2.08	-1.65
O94925	Glutaminase kidney isoform, mitochondrial OS=Homo sapiens GN=GLS PE=1 SV=1 - [GLSK_HUMAN]	1.27	1.34	1.52	1.44	-0.40	-0.54	0.26	0.13	-0.10	0.02	-0.23	-0.37	-1.12	-1.78	-1.84	-1.36	-1.50	-1.79	-1.79	-1.30
P15104	Glutamine synthetase OS=Homo sapiens GN=GLUL PE=1 SV=4 - [GLNA_HUMAN]	1.71	1.39	1.88	1.65	0.35	0.22	0.58	0.30	0.20	0.25	0.08	-0.02	-1.10	-1.51	-1.62	-1.25	-1.49	-1.30	-1.50	-1.34
P22352	Glutathione peroxidase 3 OS=Homo sapiens GN=GPX3 PE=1 SV=2 - [GPX3_HUMAN]	-2.45	-2.28	-2.81	-2.70	0.22	0.29	-0.40	-0.35	0.10	0.14	0.64	0.65	1.96	3.18	3.52	2.58	2.86	2.61	3.05	2.48
P21695	Glycerol-3-phosphate dehydrogenase [NAD(+)], cytoplasmic OS=Homo sapiens GN=GPD1 PE=1 SV=4 - [GPDA_HUMAN]	1.70	1.67	1.94	1.63	0.34	0.29	0.73	0.57	0.49	0.44	0.04	-0.14	-1.04	-1.56	-1.61	-1.23	-1.18	-1.23	-1.34	-1.09
P41250	Glycine--tRNA ligase OS=Homo sapiens GN=GARS PE=1 SV=3 - [SYG_HUMAN]	1.25	1.34	1.45	1.60	0.04	0.20	0.46	0.52	0.45	0.19	0.02	0.17	-0.86	-1.28	-1.39	-0.89	-1.03	-1.12	-1.43	-0.88
O75487	Glypican-4 OS=Homo sapiens GN=GPC4 PE=1 SV=4 - [GPC4_HUMAN]	-1.10	-1.04	-1.42	-1.30	0.91	0.97	0.15	0.22	0.63	0.63	0.81	0.93	1.22	2.01	2.23	1.77	2.11	2.04	2.31	1.63
Q9Y625	Glypican-6 OS=Homo sapiens GN=GPC6 PE=1 SV=1 - [GPC6_HUMAN]	-2.47	-2.40	-2.70	-2.81	0.43	0.33	-0.56	-0.70	0.18	0.25	0.69	0.54	1.78	3.23	3.36	2.75	2.94	2.94	3.15	2.00

Q4V328	GRIP1-associated protein 1 OS=Homo sapiens GN=GRIPAP1 PE=1 SV=1 - [GRAP1_HUMAN]	1.05	1.12	1.09	1.07	-0.12	-0.16	0.11	0.14	0.01	-0.04	-0.01	-0.09	-0.93	-1.17	-1.17	-1.09	-1.12	-1.24	-1.27	-0.90
O60861	Growth arrest-specific protein 7 OS=Homo sapiens GN=GAS7 PE=1 SV=3 - [GAS7_HUMAN]	1.70	1.81	1.41	1.58	-0.44	-0.46	0.09	0.01	-0.21	-0.09	-0.49	-0.42	-1.62	-2.06	-1.86	-2.12	-1.73	-2.56	-2.36	-1.50
Q8TBN0	Guanine nucleotide exchange factor for Rab-3A OS=Homo sapiens GN=RAB3IL1 PE=1 SV=1 - [R3GEF_HUMAN]	-1.73	-2.03	-1.90	-2.19	0.42	0.11	0.22	-0.08	-0.34	-0.04	-0.13	-0.43	2.00	1.60	1.77	1.72	1.89	2.12	2.29	2.18
P63096	Guanine nucleotide-binding protein G(i) subunit alpha-1 OS=Homo sapiens GN=GNAI1 PE=1 SV=2 - [GNAI1_HUMAN]	1.66	1.69	1.65	1.55	-0.01	-0.12	0.49	0.54	0.25	0.37	0.27	0.20	-1.04	-1.32	-1.29	-1.18	-1.27	-1.60	-1.70	-1.00
P61952	Guanine nucleotide-binding protein G(I)/G(S)/G(O) subunit gamma-11 OS=Homo sapiens GN=GNG11 PE=1 SV=1 - [GBG11_HUMAN]	-1.71	-1.84	-1.60	-1.73	-0.32	-0.46	-0.49	-0.62	-0.51	-0.37	0.16	0.03	1.27	1.88	1.77	1.37	1.26	1.37	1.26	1.18
Q9P2W3	Guanine nucleotide-binding protein G(I)/G(S)/G(O) subunit gamma-13 OS=Homo sapiens GN=GNG13 PE=2 SV=1 - [GBG13_HUMAN]	2.74	2.86	2.62	2.86	0.60	0.47	1.30	1.25	0.88	1.02	0.76	0.62	-1.38	-1.78	-2.23	-1.56	-1.99	-1.96	-2.41	-1.55
P59768	Guanine nucleotide-binding protein G(I)/G(S)/G(O) subunit gamma-2 OS=Homo sapiens GN=GNG2 PE=1 SV=2 - [GBG2_HUMAN]	2.03	2.03	2.50	2.44	0.00	-0.11	1.06	0.73	0.49	0.69	0.64	0.52	-1.07	-1.50	-1.91	-1.31	-1.85	-2.05	-2.69	-1.55

P63215	Guanine nucleotide-binding protein G(I)/G(S)/G(O) subunit gamma-3 OS=Homo sapiens GN=GNG3 PE=2 SV=1 - [GBG3_HUMAN]	2.00	1.92	2.14	2.31	-0.25	-0.20	0.58	0.70	0.05	0.18	0.13	0.26	-1.16	-1.83	-2.07	-1.75	-1.90	-2.36	-2.44	-1.42
P50150	Guanine nucleotide-binding protein G(I)/G(S)/G(O) subunit gamma-4 OS=Homo sapiens GN=GNG4 PE=1 SV=1 - [GBG4_HUMAN]	1.63	1.71	1.69	1.77	-0.62	-0.55	0.68	0.76	0.40	0.32	0.21	0.28	-0.89	-1.42	-1.48	-1.28	-1.33	-2.27	-2.33	-0.94
O60262	Guanine nucleotide-binding protein G(I)/G(S)/G(O) subunit gamma-7 OS=Homo sapiens GN=GNG7 PE=1 SV=1 - [GBG7_HUMAN]	1.47	1.63	1.95	1.92	-0.12	-0.10	0.44	0.44	0.27	0.15	0.04	0.32	-1.00	-1.21	-1.73	-1.40	-1.59	-1.71	-2.04	-1.42
P62873	Guanine nucleotide-binding protein G(I)/G(S)/G(T) subunit beta-1 OS=Homo sapiens GN=GNB1 PE=1 SV=3 - [GBB1_HUMAN]	1.62	1.68	1.70	1.70	-0.03	0.00	0.51	0.57	0.22	0.25	0.28	0.33	-0.99	-1.51	-1.47	-1.30	-1.25	-1.50	-1.77	-1.14
P09471	Guanine nucleotide-binding protein G(o) subunit alpha OS=Homo sapiens GN=GNAO1 PE=1 SV=4 - [GNAO_HUMAN]	2.42	2.36	2.57	2.42	0.05	-0.08	1.07	1.00	0.54	0.61	0.45	0.34	-1.26	-1.99	-2.11	-1.76	-1.87	-2.51	-2.59	-1.36
P19086	Guanine nucleotide-binding protein G(z) subunit alpha OS=Homo sapiens GN=GNAZ PE=2 SV=3 - [GNAZ_HUMAN]	2.07	1.52	1.89	1.89	0.10	0.07	0.80	0.72	0.51	0.53	0.29	0.22	-1.09	-1.47	-1.50	-1.39	-1.45	-1.93	-1.76	-1.09
O14775	Guanine nucleotide-binding protein subunit beta-5 OS=Homo sapiens GN=GNB5 PE=1 SV=2 - [GBB5_HUMAN]	1.36	1.24	1.74	1.62	-0.08	-0.25	0.46	0.36	-0.07	0.12	-0.07	-0.17	-0.91	-1.10	-1.55	-1.39	-1.63	-1.55	-1.86	-1.42

P33402	Guanylate cyclase soluble subunit alpha-2 OS=Homo sapiens GN=GUCY1A2 PE=1 SV=1 - [GCYA2_HUMAN]	0.98	1.45	1.19	1.66	-0.30	0.00	0.11	0.57	0.32	0.03	-0.34	0.09	-1.15	-1.32	-1.53	-0.94	-1.13	-1.30	-1.51	-1.11
P00739	Haptoglobin-related protein OS=Homo sapiens GN=HPR PE=1 SV=2 - [HPTR_HUMAN]	-1.57	-1.55	-1.32	-1.31	-0.58	-0.62	-0.11	-0.15	-0.59	-0.54	-0.63	-0.68	1.20	1.93	1.22	1.66	1.34	1.78	1.26	1.06
Q7Z4H3	HD domain-containing protein 2 OS=Homo sapiens GN=HDDC2 PE=1 SV=1 - [HDDC2_HUMAN]	1.11	1.04	0.98	0.91	-0.40	-0.48	-0.02	-0.09	-0.34	-0.27	-0.39	-0.47	-1.07	-1.50	-1.37	-1.35	-1.21	-1.53	-1.40	-0.93
O43301	Heat shock 70 kDa protein 12A OS=Homo sapiens GN=HSPA12A PE=1 SV=2 - [HS12A_HUMAN]	1.15	1.16	1.38	1.48	-0.22	-0.19	0.26	0.36	0.01	0.00	-0.22	-0.21	-0.81	-1.22	-1.52	-1.18	-1.45	-1.42	-1.56	-0.92
O95757	Heat shock 70 kDa protein 4L OS=Homo sapiens GN=HSPA4L PE=1 SV=3 - [HS74L_HUMAN]	1.61	1.46	1.64	1.57	0.14	-0.01	0.57	0.50	0.21	0.31	0.09	-0.05	-1.09	-1.58	-1.71	-1.30	-1.50	-1.44	-1.62	-1.22
P04792	Heat shock protein beta-1 OS=Homo sapiens GN=HSPB1 PE=1 SV=2 - [HSPB1_HUMAN]	-1.84	-1.81	-1.87	-1.75	-0.41	-0.41	-0.81	-0.84	-0.44	-0.51	-0.24	-0.28	1.04	1.67	1.76	1.49	1.58	1.58	1.53	1.15
Q9UK76	Hematological and neurological expressed 1 protein OS=Homo sapiens GN=HN1 PE=1 SV=3 - [HN1_HUMAN]	1.50	1.32	1.28	1.28	-0.32	-0.56	-0.37	-0.52	-0.58	-0.66	-0.47	-0.82	-1.96	-2.08	-1.75	-2.18	-1.69	-2.03	-1.64	-1.53
P69905	Hemoglobin subunit alpha OS=Homo sapiens GN=HBA1 PE=1 SV=2 - [HBA_HUMAN]	-1.83	-1.74	-1.96	-1.88	0.25	0.32	-0.36	-0.27	-0.01	-0.07	0.13	0.24	1.55	1.94	2.08	1.92	1.93	2.10	2.20	1.68
P68871	Hemoglobin subunit beta OS=Homo sapiens GN=HBB PE=1 SV=2 - [HBB_HUMAN]	-2.13	-2.05	-2.18	-2.07	0.34	0.42	-0.33	-0.23	0.08	-0.03	0.14	0.21	1.88	2.30	2.30	2.17	2.23	2.45	2.49	1.94

P02042	Hemoglobin subunit delta OS=Homo sapiens GN=HBD PE=1 SV=2 - [HBD_HUMAN]	-1.90	-1.90	-2.06	-2.01	-0.13	-0.10	-0.22	-0.15	0.08	-0.01	0.47	0.48	1.77	2.36	2.44	1.94	2.11	1.79	1.90	1.83
P02100	Hemoglobin subunit epsilon OS=Homo sapiens GN=HBE1 PE=1 SV=2 - [HBE_HUMAN]	-1.64	-1.60	-1.67	-1.62	1.58	1.62	0.38	0.43	1.24	1.19	1.52	1.57	2.08	3.17	3.19	2.87	2.90	3.20	3.23	2.11
P05546	Heparin cofactor 2 OS=Homo sapiens GN=SERPIND1 PE=1 SV=3 - [HEP2_HUMAN]	-2.98	-2.96	-3.06	-3.06	-1.10	-1.10	-1.52	-1.57	-1.00	-1.17	-1.10	-1.05	1.68	1.98	2.09	2.06	2.00	2.14	1.94	1.59
P19367	Hexokinase-1 OS=Homo sapiens GN=HK1 PE=1 SV=3 - [HXK1_HUMAN]	1.71	1.75	1.77	1.83	0.06	0.13	0.64	0.70	0.32	0.30	0.14	0.13	-1.01	-1.51	-1.66	-1.37	-1.44	-1.54	-1.68	-1.00
Q9UM19	Hippocalcin-like protein 4 OS=Homo sapiens GN=HPCAL4 PE=2 SV=3 - [HPCL4_HUMAN]	1.78	1.74	2.13	2.02	-0.31	-0.19	0.79	0.56	0.22	0.08	0.48	0.29	-0.93	-1.48	-1.54	-1.29	-1.41	-2.08	-2.30	-1.11
P04196	Histidine-rich glycoprotein OS=Homo sapiens GN=HRG PE=1 SV=1 - [HRG_HUMAN]	-2.02	-2.03	-2.33	-2.00	0.13	0.39	0.01	0.16	0.35	0.16	0.74	0.89	2.24	2.24	2.98	2.08	2.39	1.65	2.35	2.45
Q96DB2	Histone deacetylase 11 OS=Homo sapiens GN=HDAC11 PE=1 SV=1 - [HDA11_HUMAN]	1.34	1.54	1.19	1.27	-0.50	-0.23	0.92	0.13	-0.29	-0.09	0.00	-0.43	-0.86	-1.60	-1.69	-1.62	-1.44	-1.71	-1.66	-0.93
Q86YM7	Homer protein homolog 1 OS=Homo sapiens GN=HOMER1 PE=1 SV=2 - [HOME1_HUMAN]	1.87	1.64	1.84	1.48	-0.01	-0.54	0.64	0.24	-0.05	0.28	-0.01	-0.20	-1.03	-1.83	-1.79	-1.62	-1.49	-1.63	-1.85	-1.04
Q9GZV7	Hyaluronan and proteoglycan link protein 2 OS=Homo sapiens GN=HAPLN2 PE=1 SV=1 - [HPLN2_HUMAN]	2.53	2.47	2.75	2.68	-0.02	-0.11	0.80	0.79	0.38	0.46	0.00	-0.25	-1.74	-2.57	-2.98	-1.98	-2.41	-2.60	-2.86	-1.88

Q86UW8	Hyaluronan and proteoglycan link protein 4 OS=Homo sapiens GN=HAPLN4 PE=2 SV=1 - [HPLN4_HUMAN]	1.92	1.79	1.73	1.61	0.06	0.10	0.68	0.78	0.46	0.37	0.41	0.28	-1.00	-1.59	-1.32	-1.52	-1.33	-1.71	-1.52	-0.94
P00492	Hypoxanthine-guanine phosphoribosyltransferase OS=Homo sapiens GN=HPRT1 PE=1 SV=2 - [HPRT_HUMAN]	1.75	1.63	1.75	1.73	0.00	-0.11	0.25	0.20	0.03	-0.11	-0.34	-0.24	-1.17	-1.40	-1.74	-1.17	-1.50	-1.20	-1.48	-1.37
P01859	Ig gamma-2 chain C region OS=Homo sapiens GN=IGHG2 PE=1 SV=2 - [IGHG2_HUMAN]	-2.80	-2.69	-2.63	-2.70	-0.27	-0.15	-1.26	-1.22	-1.12	-1.15	-1.25	-1.46	1.41	1.19	1.16	1.68	1.63	2.58	2.39	1.20
P01861	Ig gamma-4 chain C region OS=Homo sapiens GN=IGHG4 PE=1 SV=1 - [IGHG4_HUMAN]	-3.30	-3.33	-3.07	-3.00	-1.52	-1.46	-1.82	-1.73	-1.64	-1.61	-1.18	-1.25	1.40	1.76	1.73	1.40	1.39	1.58	1.47	1.35
P01742	Ig heavy chain V-I region EU OS=Homo sapiens PE=1 SV=1 - [HV101_HUMAN]	-1.29	-1.31	-1.39	-1.41	0.72	0.69	0.04	0.02	0.54	0.57	1.36	1.33	1.38	2.65	2.74	1.89	1.99	1.99	2.08	1.49
P01743	Ig heavy chain V-I region HG3 OS=Homo sapiens PE=4 SV=1 - [HV102_HUMAN]	-1.44	-1.29	-1.65	-1.50	0.20	0.34	-0.46	-0.32	0.03	-0.11	0.26	0.41	1.03	1.71	1.91	1.35	1.57	1.62	1.83	1.25
P06331	Ig heavy chain V-II region ARH-77 OS=Homo sapiens PE=4 SV=1 - [HV209_HUMAN]	-1.90	-1.69	-1.81	-1.60	-0.23	-0.03	-0.87	-0.67	0.00	-0.20	0.43	0.63	1.08	2.34	2.24	1.73	1.65	1.65	1.56	1.00
P01776	Ig heavy chain V-III region WAS OS=Homo sapiens PE=1 SV=1 - [HV315_HUMAN]	-1.99	-1.83	-2.06	-1.97	-0.27	-0.19	-1.08	-0.92	-0.41	-0.54	0.21	0.29	1.19	2.23	2.38	1.63	1.69	1.70	1.80	1.03
P04430	Ig kappa chain V-I region BAN OS=Homo sapiens PE=1 SV=1 - [KV122_HUMAN]	-2.07	-2.19	-2.30	-2.42	-0.56	-0.69	-0.29	-0.41	0.30	0.43	1.17	1.04	1.84	3.24	3.46	2.53	2.76	1.49	1.72	2.07
P04433	Ig kappa chain V-III region VG (Fragment) OS=Homo sapiens PE=1 SV=1 - [KV309_HUMAN]	-1.58	-1.64	-1.70	-1.76	-0.19	-0.25	-0.65	-0.70	-0.22	-0.15	0.41	0.34	0.99	1.99	2.11	1.46	1.58	1.37	1.49	1.12

P04211	Ig lambda chain V region 4A OS=Homo sapiens PE=4 SV=1 - [LV001_HUMAN]	-1.23	-1.09	-1.18	-1.03	0.56	0.70	0.14	0.28	0.08	-0.06	0.09	0.23	1.43	1.33	1.27	1.20	1.15	1.78	1.72	1.38
P0CG05	Ig lambda-2 chain C regions OS=Homo sapiens GN=IGLC2 PE=1 SV=1 - [LAC2_HUMAN]	-2.04	-1.98	-1.55	-1.49	-0.08	-0.03	-0.32	-0.27	0.15	0.10	0.81	0.86	1.77	2.85	2.36	2.17	1.68	1.94	1.45	1.29
P55899	IgG receptor FcRn large subunit p51 OS=Homo sapiens GN=FCGRT PE=1 SV=1 - [FCGRN_HUMAN]	-2.24	-2.15	-2.17	-2.07	0.36	0.45	-0.39	-0.30	-0.05	0.05	0.78	0.86	1.90	3.02	2.94	2.33	2.26	2.58	2.51	1.83
Q9Y5U9	Immediate early response 3-interacting protein 1 OS=Homo sapiens GN=IER3IP1 PE=1 SV=1 - [IR3IP_HUMAN]	-1.05	-1.39	-1.22	-1.56	0.42	0.08	0.16	-0.18	-0.05	0.30	0.09	-0.25	1.26	1.15	1.31	1.38	1.55	1.46	1.63	1.44
B9A064	Immunoglobulin lambda-like polypeptide 5 OS=Homo sapiens GN=IGLL5 PE=2 SV=2 - [IGLL5_HUMAN]	-1.56	-1.24	-1.21	-1.26	0.52	0.44	0.14	-0.03	0.38	0.66	1.69	1.54	1.55	3.15	2.97	2.08	1.81	1.72	1.69	1.37
Q96ID5	Immunoglobulin superfamily member 21 OS=Homo sapiens GN=IGSF21 PE=2 SV=1 - [IGS21_HUMAN]	1.64	1.58	1.58	1.52	0.18	0.14	0.36	0.25	0.07	0.42	0.30	0.11	-1.35	-1.47	-1.62	-1.15	-1.28	-1.49	-1.31	-1.33
Q969P0	Immunoglobulin superfamily member 8 OS=Homo sapiens GN=IGSF8 PE=1 SV=1 - [IGSF8_HUMAN]	1.58	1.58	1.72	1.70	0.07	-0.01	0.50	0.44	0.25	0.29	0.18	0.27	-1.29	-1.48	-1.49	-1.57	-1.62	-1.79	-1.75	-1.36
Q8N608	Inactive dipeptidyl peptidase 10 OS=Homo sapiens GN=DPP10 PE=1 SV=2 - [DPP10_HUMAN]	2.06	1.99	2.06	1.99	-0.02	-0.16	0.73	0.72	0.37	0.44	0.51	0.42	-1.15	-1.67	-1.73	-1.58	-1.58	-2.10	-2.12	-1.15
Q13308	Inactive tyrosine-protein kinase 7 OS=Homo sapiens GN=PTK7 PE=1 SV=2 - [PTK7_HUMAN]	-1.24	-1.24	-1.23	-1.64	0.11	0.05	-0.03	-0.21	-0.06	0.21	0.20	0.21	1.31	1.76	1.85	1.40	1.53	1.40	1.78	1.31

Q70UQ0	Inhibitor of nuclear factor kappa-B kinase-interacting protein OS=Homo sapiens GN=IKBIP PE=1 SV=1 - [IKIP_HUMAN]	-2.91	-2.90	-3.27	-3.27	-0.47	-0.47	-1.19	-1.19	-0.90	-0.90	-1.10	-1.10	1.77	1.81	2.17	2.04	2.41	2.42	2.78	2.15
Q15181	Inorganic pyrophosphatase OS=Homo sapiens GN=PPA1 PE=1 SV=2 - [IPYR_HUMAN]	0.81	0.59	1.46	1.22	-0.31	-0.45	0.04	-0.05	-0.28	-0.23	-0.50	-0.56	-0.77	-1.21	-1.80	-1.10	-1.47	-1.11	-1.83	-1.33
P29218	Inositol monophosphatase 1 OS=Homo sapiens GN=IMPA1 PE=1 SV=1 - [IMPA1_HUMAN]	1.89	1.93	2.05	2.10	0.13	0.26	0.52	0.52	0.33	0.09	-0.24	-0.17	-1.41	-2.03	-2.35	-1.58	-1.95	-1.68	-1.97	-1.65
P17936	Insulin-like growth factor-binding protein 3 OS=Homo sapiens GN=IGFBP3 PE=1 SV=2 - [IBP3_HUMAN]	-0.67	-0.66	-0.87	-1.00	2.73	2.88	1.15	1.39	1.70	1.70	0.94	1.00	2.15	1.68	2.01	2.65	3.04	3.63	4.02	2.39
Q16270	Insulin-like growth factor-binding protein 7 OS=Homo sapiens GN=IGFBP7 PE=1 SV=1 - [IBP7_HUMAN]	-1.95	-1.87	-1.86	-1.75	0.24	0.29	-0.66	-0.67	-0.28	-0.26	-0.63	-0.40	0.96	1.23	1.41	1.47	1.59	2.05	2.16	1.34
P56199	Integrin alpha-1 OS=Homo sapiens GN=ITGA1 PE=1 SV=2 - [ITA1_HUMAN]	-2.08	-1.98	-2.04	-1.99	0.05	0.11	-0.51	-0.46	-0.28	-0.38	-0.50	-0.38	1.59	1.60	1.57	1.85	1.68	2.12	2.10	1.59
P26006	Integrin alpha-3 OS=Homo sapiens GN=ITGA3 PE=1 SV=5 - [ITA3_HUMAN]	-2.35	-2.34	-2.36	-2.26	0.16	0.27	-0.49	-0.35	-0.13	-0.05	0.02	0.08	2.05	2.44	2.42	2.17	2.15	2.28	2.37	1.95
P08648	Integrin alpha-5 OS=Homo sapiens GN=ITGA5 PE=1 SV=2 - [ITA5_HUMAN]	-1.13	-1.11	-1.69	-1.32	1.35	1.35	0.11	0.17	0.72	0.74	0.76	0.96	1.26	1.72	2.33	1.74	2.20	2.12	2.71	1.61
P53708	Integrin alpha-8 OS=Homo sapiens GN=ITGA8 PE=1 SV=3 - [ITA8_HUMAN]	-1.89	-1.85	-2.25	-2.20	-0.21	-0.18	-0.83	-0.79	-0.29	-0.41	-0.22	-0.40	1.01	1.40	1.93	1.29	1.78	1.63	1.93	1.64

Q13797	Integrin alpha-9 OS=Homo sapiens GN=ITGA9 PE=1 SV=2 - [ITA9_HUMAN]	-0.89	-1.12	-1.69	-1.83	0.00	0.21	0.07	0.12	0.22	0.04	0.14	0.12	1.29	1.24	1.83	1.37	1.77	1.40	1.68	1.83
P08514	Integrin alpha-IIb OS=Homo sapiens GN=ITGA2B PE=1 SV=3 - [ITA2B_HUMAN]	-1.31	-1.07	-1.27	-1.03	1.17	1.33	0.75	0.83	1.00	0.91	1.22	1.32	1.79	2.28	2.43	2.07	2.16	2.41	2.41	2.00
P05556	Integrin beta-1 OS=Homo sapiens GN=ITGB1 PE=1 SV=2 - [ITB1_HUMAN]	-1.70	-1.66	-1.87	-1.79	-0.03	0.01	-0.43	-0.37	-0.25	-0.30	-0.10	-0.06	1.32	1.62	1.82	1.61	1.64	1.77	1.75	1.46
P05106	Integrin beta-3 OS=Homo sapiens GN=ITGB3 PE=1 SV=2 - [ITB3_HUMAN]	-1.47	-1.57	-1.26	-1.35	1.16	0.93	0.45	-0.03	0.25	0.73	0.89	0.66	1.34	2.50	2.28	2.20	1.99	2.70	2.49	1.56
P18084	Integrin beta-5 OS=Homo sapiens GN=ITGB5 PE=1 SV=1 - [ITB5_HUMAN]	-1.02	-0.93	-1.32	-1.03	0.59	0.74	-0.15	-0.08	0.49	0.18	0.18	0.18	0.93	1.12	1.65	1.54	1.54	1.65	1.90	1.24
Q13418	Integrin-linked protein kinase OS=Homo sapiens GN=ILK PE=1 SV=2 - [ILK_HUMAN]	-1.66	-1.71	-1.95	-1.93	-0.49	-0.39	-0.52	-0.44	-0.55	-0.57	-0.46	-0.32	1.36	1.28	1.44	1.25	1.36	1.29	1.39	1.57
P19823	Inter-alpha-trypsin inhibitor heavy chain H2 OS=Homo sapiens GN=ITIH2 PE=1 SV=2 - [ITIH2_HUMAN]	-1.53	-1.53	-1.93	-2.02	0.85	0.82	-0.31	-0.36	0.24	0.22	0.38	0.42	1.16	1.90	2.21	1.73	2.17	2.15	2.52	1.78
Q14624	Inter-alpha-trypsin inhibitor heavy chain H4 OS=Homo sapiens GN=ITIH4 PE=1 SV=4 - [ITIH4_HUMAN]	-1.53	-1.50	-1.70	-1.53	0.07	0.10	-0.52	-0.60	-0.04	-0.08	0.36	0.40	0.97	2.04	1.94	1.50	1.60	1.67	1.66	1.11
Q86UX2	Inter-alpha-trypsin inhibitor heavy chain H5 OS=Homo sapiens GN=ITIH5 PE=2 SV=2 - [ITIH5_HUMAN]	-1.56	-1.33	-1.96	-1.88	-0.21	-0.03	-0.53	-0.42	-0.29	-0.32	-0.30	-0.18	1.12	1.41	1.65	1.34	1.83	1.34	1.84	1.55
P13164	Interferon-induced transmembrane protein 1 OS=Homo sapiens GN=IFITM1 PE=1 SV=3 - [IFM1_HUMAN]	-3.07	-2.93	-3.14	-3.00	-0.01	0.13	-1.21	-1.07	-0.34	-0.48	0.19	0.33	1.92	3.27	3.33	2.63	2.70	3.05	3.11	2.00

Q9NZN1	Interleukin-1 receptor accessory protein-like 1 OS=Homo sapiens GN=IL1RAPL 1 PE=1 SV=2 - [IRPL1_HUMAN]	3.44	2.29	3.52	2.24	0.83	-0.05	1.71	0.59	0.68	1.52	1.17	0.33	-1.68	-1.95	-2.05	-1.89	-1.52	-2.62	-2.30	-1.59
Q9NWZ3	Interleukin-1 receptor-associated kinase 4 OS=Homo sapiens GN=IRAK4 PE=1 SV=1 - [IRAK4_HUMAN]	-1.11	-1.29	-1.72	-1.90	0.97	0.79	0.48	0.30	0.54	0.73	0.41	0.22	1.64	1.52	2.13	1.87	2.48	2.07	2.68	2.26
Q14116	Interleukin-18 OS=Homo sapiens GN=IL18 PE=1 SV=1 - [IL18_HUMAN]	-0.78	-0.72	-1.33	-1.26	1.27	1.33	1.17	1.23	1.20	1.14	0.39	0.45	2.01	1.18	1.72	1.96	2.50	2.04	2.58	2.56
Q14213	Interleukin-27 subunit beta OS=Homo sapiens GN=EBI3 PE=1 SV=2 - [IL27B_HUMAN]	-1.00	-0.63	-0.60	-0.23	2.06	2.42	0.91	1.28	1.61	1.25	0.87	1.23	1.97	1.88	1.47	2.28	1.88	3.05	2.64	1.57
Q6DN90	IQ motif and SEC7 domain-containing protein 1 OS=Homo sapiens GN=IQSEC1 PE=1 SV=1 - [IQEC1_HUMAN]	1.31	1.03	1.13	1.03	-0.17	-0.29	0.33	0.25	-0.04	0.19	0.06	-0.04	-0.92	-1.16	-1.06	-1.15	-1.09	-1.57	-1.47	-0.88
Q5JU85	IQ motif and SEC7 domain-containing protein 2 OS=Homo sapiens GN=IQSEC2 PE=1 SV=1 - [IQEC2_HUMAN]	1.74	1.56	1.52	1.79	-0.06	-0.31	0.36	0.29	0.28	0.16	0.22	0.12	-1.35	-1.68	-1.70	-1.53	-1.35	-1.99	-1.85	-1.34
P14923	Junction plakoglobin OS=Homo sapiens GN=JUP PE=1 SV=3 - [PLAK_HUMAN]	-1.89	-1.79	-2.12	-2.24	-0.12	-0.22	-0.62	-0.65	-0.41	-0.39	0.04	-0.06	1.60	1.96	2.29	1.71	1.87	1.81	2.21	1.84
Q96JJ6	Junctophilin-4 OS=Homo sapiens GN=JPH4 PE=1 SV=2 - [JPH4_HUMAN]	0.71	0.72	0.70	0.71	-0.99	-0.98	0.05	0.06	-0.63	-0.64	-1.30	-1.29	-0.77	-2.00	-2.00	-1.32	-1.31	-1.71	-1.70	-0.86
Q92876	Kallikrein-6 OS=Homo sapiens GN=KLK6 PE=1 SV=1 - [KLK6_HUMAN]	-1.03	-0.88	-1.15	-1.03	0.32	0.47	0.22	0.30	0.38	0.23	0.53	0.64	1.37	1.58	1.49	1.60	1.48	1.54	1.49	1.41

P29622	Kallistatin OS=Homo sapiens GN=SERPINA4 PE=1 SV=3 - [KAIN_HUMAN]	-1.20	-1.20	-1.14	-1.13	1.35	1.34	0.43	0.43	0.88	0.88	1.06	1.05	1.69	2.27	2.19	2.11	2.05	2.53	2.46	1.63
Q9BW62	Katanin p60 ATPase-containing subunit A-like 1 OS=Homo sapiens GN=KATNAL1 PE=1 SV=1 - [KATL1_HUMAN]	1.22	1.12	1.44	1.47	-0.25	-0.08	0.25	0.32	0.16	-0.02	-0.11	0.11	-0.75	-1.32	-1.56	-0.92	-1.34	-1.21	-1.56	-1.18
Q7Z4H8	KDEL motif-containing protein 2 OS=Homo sapiens GN=KDELC2 PE=1 SV=2 - [KDEL2_HUMAN]	-2.47	-2.54	-2.37	-2.44	0.08	0.01	-1.16	-1.23	-0.49	-0.47	0.13	0.08	1.37	2.62	2.38	1.97	1.74	2.47	2.22	1.15
Q5VTJ3	Kelch domain-containing protein 7A OS=Homo sapiens GN=KLHDC7A PE=2 SV=5 - [KLD7A_HUMAN]	-1.08	-1.04	-1.27	-1.26	0.47	0.51	0.05	0.06	0.25	0.28	0.37	0.31	1.15	1.13	1.22	1.23	1.55	1.53	1.72	1.35
P23276	Kell blood group glycoprotein OS=Homo sapiens GN=KEL PE=1 SV=2 - [KELL_HUMAN]	-1.28	-1.35	-1.91	-1.98	0.49	0.41	0.50	0.43	0.93	1.01	1.59	1.51	1.83	2.87	3.50	2.32	2.95	1.75	2.38	2.47
P05783	Keratin, type I cytoskeletal 18 OS=Homo sapiens GN=KRT18 PE=1 SV=2 - [K1C18_HUMAN]	-2.30	-1.96	-2.25	-1.91	0.62	0.96	-0.43	-0.09	0.43	0.09	0.84	1.17	1.92	3.14	3.09	2.42	2.38	2.90	2.85	1.88
Q14894	Ketimine reductase mu-crystallin OS=Homo sapiens GN=CRYM PE=1 SV=1 - [CRYM_HUMAN]	1.29	1.41	1.38	1.38	-0.22	-0.23	0.45	0.44	0.02	0.01	-0.20	-0.37	-0.89	-1.53	-1.48	-0.91	-0.96	-1.15	-1.61	-0.90
O75525	KH domain-containing, RNA-binding, signal transduction-associated protein 3 OS=Homo sapiens GN=KHDRBS3 PE=1 SV=1 - [KHDR3_HUMAN]	1.73	1.57	1.96	1.90	-0.44	-0.04	0.09	0.15	0.09	-0.03	-0.15	0.14	-1.58	-1.67	-1.57	-1.45	-1.36	-1.71	-1.60	-1.47

Q12840	Kinesin heavy chain isoform 5A OS=Homo sapiens GN=KIF5A PE=1 SV=2 - [KIF5A_HUMAN]	1.58	1.67	1.41	1.50	-0.10	-0.02	0.31	0.39	0.32	0.24	-0.13	-0.06	-1.22	-1.71	-1.55	-1.31	-1.14	-1.70	-1.53	-1.04
Q12756	Kinesin-like protein KIF1A OS=Homo sapiens GN=KIF1A PE=1 SV=2 - [KIF1A_HUMAN]	1.85	2.00	1.67	1.87	0.06	0.37	0.34	0.42	0.58	0.41	0.03	0.02	-1.26	-1.79	-1.73	-1.50	-1.48	-1.60	-1.47	-1.17
Q7Z4S6	Kinesin-like protein KIF21A OS=Homo sapiens GN=KIF21A PE=1 SV=2 - [KI21A_HUMAN]	1.30	1.39	1.48	1.57	-0.01	-0.02	0.43	0.52	0.25	0.17	0.21	0.13	-0.78	-1.15	-1.34	-1.07	-1.28	-1.30	-1.40	-0.93
P01042	Kininogen-1 OS=Homo sapiens GN=KNG1 PE=1 SV=2 - [KNG1_HUMAN]	-1.48	-1.60	-1.62	-1.69	0.47	0.45	-0.17	-0.38	0.00	0.11	0.37	0.29	1.30	1.83	1.91	1.63	1.77	2.01	2.13	1.33
Q63ZY3	KN motif and ankyrin repeat domain-containing protein 2 OS=Homo sapiens GN=KANK2 PE=1 SV=1 - [KANK2_HUMAN]	-2.06	-2.16	-2.49	-2.62	-0.25	-0.29	-0.33	-0.38	-0.37	-0.30	0.11	-0.03	1.83	2.21	2.61	1.94	2.34	1.89	2.45	2.32
Q9NS61	Kv channel-interacting protein 2 OS=Homo sapiens GN=KCNIP2 PE=1 SV=3 - [KCIP2_HUMAN]	2.37	2.16	2.49	2.28	0.84	0.63	1.03	0.82	0.28	0.50	0.50	0.29	-1.28	-1.86	-1.99	-1.84	-1.96	-1.54	-1.67	-1.40
Q08431	Lactadherin OS=Homo sapiens GN=MFGE8 PE=1 SV=2 - [MFGM_HUMAN]	-1.11	-0.89	-1.46	-1.27	2.28	2.16	1.33	1.32	1.49	1.43	0.52	0.62	2.06	1.62	2.22	2.33	3.09	2.92	3.73	2.82
Q16363	Laminin subunit alpha-4 OS=Homo sapiens GN=LAMA4 PE=1 SV=4 - [LAMA4_HUMAN]	-1.77	-1.76	-2.30	-2.27	-0.06	-0.12	-0.87	-0.88	-0.61	-0.55	-0.75	-0.71	1.24	1.21	1.52	1.40	1.69	1.74	2.11	1.54
O15230	Laminin subunit alpha-5 OS=Homo sapiens GN=LAMA5 PE=1 SV=8 - [LAMA5_HUMAN]	-1.98	-1.98	-2.08	-2.12	-0.20	-0.25	-0.76	-0.77	-0.61	-0.58	-0.74	-0.77	1.21	1.22	1.41	1.45	1.57	1.74	1.86	1.38
P07942	Laminin subunit beta-1 OS=Homo sapiens GN=LAMB1 PE=1 SV=2 - [LAMB1_HUMAN]	-1.97	-2.08	-2.15	-2.11	0.35	0.26	-0.57	-0.59	-0.36	-0.38	-0.39	-0.40	1.48	1.63	1.68	1.63	1.76	2.18	2.27	1.55

P11047	Laminin subunit gamma-1 OS=Homo sapiens GN=LAMC1 PE=1 SV=3 - [LAMC1_HUMAN]	-2.13	-2.18	-2.13	-2.13	-0.44	-0.45	-0.89	-0.91	-0.81	-0.76	-0.76	-0.81	1.26	1.23	1.33	1.32	1.35	1.67	1.66	1.30
Q8NBF6	Late secretory pathway protein AVL9 homolog OS=Homo sapiens GN=AVL9 PE=1 SV=1 - [AVL9_HUMAN]	1.57	1.93	1.40	1.76	-0.38	-0.02	0.32	0.68	0.54	0.18	0.12	0.47	-1.20	-1.45	-1.29	-1.36	-1.19	-1.96	-1.79	-1.02
Q14766	Latent-transforming growth factor beta-binding protein 1 OS=Homo sapiens GN=LTBP1 PE=1 SV=4 - [LTBP1_HUMAN]	-1.51	-1.72	-1.85	-1.81	0.71	0.70	0.16	0.06	0.29	0.28	0.27	0.16	1.79	1.65	1.97	1.80	2.09	2.27	2.61	2.12
Q14767	Latent-transforming growth factor beta-binding protein 2 OS=Homo sapiens GN=LTBP2 PE=1 SV=3 - [LTBP2_HUMAN]	-1.46	-1.46	-1.23	-1.24	1.33	1.44	0.28	0.40	0.77	0.78	0.49	0.48	1.68	2.22	1.82	2.27	2.30	2.78	2.79	2.08
Q8N2S1	Latent-transforming growth factor beta-binding protein 4 OS=Homo sapiens GN=LTBP4 PE=1 SV=2 - [LTBP4_HUMAN]	-1.88	-1.75	-2.19	-2.40	0.32	0.44	-0.36	-0.40	-0.32	-0.32	-0.55	-0.48	1.57	1.15	1.44	1.48	1.81	2.14	2.38	1.93
Q99538	Legumain OS=Homo sapiens GN=LGMN PE=1 SV=1 - [LGMN_HUMAN]	-2.31	-2.32	-2.46	-2.50	-0.34	-0.29	-0.69	-0.74	-0.49	-0.54	-0.15	-0.14	1.72	2.19	2.31	1.83	2.10	2.17	2.33	1.77
P29536	Leiomodin-1 OS=Homo sapiens GN=LMOD1 PE=1 SV=3 - [LMOD1_HUMAN]	-2.59	-2.66	-2.60	-2.65	-1.33	-1.51	-1.09	-1.16	-0.99	-0.84	-0.30	-0.26	1.53	2.36	2.40	1.89	1.80	1.80	1.62	1.50
Q9UIC8	Leucine carboxyl methyltransferase 1 OS=Homo sapiens GN=LCMT1 PE=1 SV=2 - [LCMT1_HUMAN]	1.97	2.07	2.16	2.29	0.37	0.11	0.63	0.94	0.30	0.44	0.30	0.11	-1.20	-1.72	-1.89	-1.68	-1.57	-1.80	-1.62	-1.31

O60299	Leucine zipper putative tumor suppressor 3 OS=Homo sapiens GN=LZTS3 PE=2 SV=1 - [LZTS3_HUMAN]	2.25	2.38	2.17	2.30	-0.66	-0.53	0.50	0.64	0.15	0.02	-0.40	-0.27	-1.69	-2.65	-2.57	-2.20	-2.12	-2.93	-2.85	-1.60
O95970	Leucine-rich glioma-inactivated protein 1 OS=Homo sapiens GN=LGI1 PE=1 SV=1 - [LGI1_HUMAN]	1.70	1.76	2.10	2.16	0.56	0.51	0.74	0.81	0.52	0.52	0.25	0.15	-1.01	-1.44	-1.96	-1.33	-1.59	-1.37	-1.50	-1.27
Q96II8	Leucine-rich repeat and calponin homology domain-containing protein 3 OS=Homo sapiens GN=LRCH3 PE=1 SV=2 - [LRCH3_HUMAN]	-1.27	-1.22	-1.40	-1.35	0.12	0.17	-0.02	0.03	-0.26	-0.31	0.29	0.34	1.30	1.57	1.69	0.99	1.12	1.38	1.50	1.44
Q8N145	Leucine-rich repeat LGI family member 3 OS=Homo sapiens GN=LGI3 PE=2 SV=1 - [LGI3_HUMAN]	2.19	2.29	2.49	2.57	-0.27	-0.01	0.40	0.48	0.06	0.06	-0.16	-0.16	-1.66	-2.42	-2.54	-1.99	-2.30	-2.48	-2.60	-1.95
Q8N6Y2	Leucine-rich repeat-containing protein 17 OS=Homo sapiens GN=LRRC17 PE=2 SV=1 - [LRC17_HUMAN]	-3.54	-3.30	-3.61	-3.60	-0.77	-0.79	-0.79	-0.78	-0.74	-0.81	-0.59	-0.49	2.23	3.07	3.08	2.48	2.79	2.60	3.00	2.83
Q16873	Leukotriene C4 synthase OS=Homo sapiens GN=LTC4S PE=1 SV=1 - [LTC4S_HUMAN]	-2.21	-2.10	-2.42	-2.31	-0.68	-0.57	-1.24	-1.13	-0.75	-0.86	-0.98	-0.88	1.03	1.23	1.44	1.38	1.60	1.51	1.72	1.24
P48059	LIM and senescent cell antigen-like-containing domain protein 1 OS=Homo sapiens GN=LIMS1 PE=1 SV=4 - [LIMS1_HUMAN]	-2.04	-2.06	-2.02	-1.93	-0.48	-0.51	-0.54	-0.47	-0.49	-0.68	-0.56	-0.51	1.57	1.64	1.40	1.54	1.46	1.70	1.50	1.46
Q7Z4I7	LIM and senescent cell antigen-like-containing domain protein 2 OS=Homo sapiens GN=LIMS2 PE=1 SV=1 - [LIMS2_HUMAN]	-2.03	-2.19	-1.84	-2.03	-0.09	-0.20	-0.70	-0.64	-0.49	-0.63	-0.57	-0.70	1.39	1.58	1.46	1.73	1.61	1.93	1.74	1.21

Q13449	Limbic system-associated membrane protein OS=Homo sapiens GN=LSAMP PE=1 SV=2 - [LSAMP_HUMAN]	1.96	1.80	2.14	1.94	0.15	0.07	0.75	0.75	0.40	0.45	0.34	0.31	-0.95	-1.58	-1.74	-1.38	-1.56	-1.76	-1.93	-1.14
Q9BU23	Lipase maturation factor 2 OS=Homo sapiens GN=LMF2 PE=1 SV=2 - [LMF2_HUMAN]	-1.24	-1.33	-1.36	-1.48	0.11	-0.02	-0.27	-0.49	0.03	0.23	0.01	-0.08	1.02	1.54	1.46	1.51	1.39	1.53	1.47	1.10
Q93052	Lipoma-preferred partner OS=Homo sapiens GN=LPP PE=1 SV=1 - [LPP_HUMAN]	-2.16	-2.25	-2.30	-2.31	-0.82	-0.87	-0.75	-0.76	-0.76	-0.73	-0.51	-0.50	1.35	1.57	1.84	1.39	1.58	1.33	1.52	1.61
O75334	Liprin-alpha-2 OS=Homo sapiens GN=PPFIA2 PE=1 SV=2 - [LIPA2_HUMAN]	1.51	1.41	1.58	1.79	0.18	-0.08	0.45	0.29	0.17	0.22	-0.02	0.03	-1.01	-1.61	-1.57	-1.41	-1.34	-1.41	-1.29	-1.10
O75145	Liprin-alpha-3 OS=Homo sapiens GN=PPFIA3 PE=1 SV=3 - [LIPA3_HUMAN]	1.87	1.96	2.01	2.09	-0.31	-0.21	0.46	0.66	0.33	0.32	0.19	0.24	-1.44	-1.88	-1.95	-1.70	-1.86	-2.02	-2.09	-1.44
Q86W92	Liprin-beta-1 OS=Homo sapiens GN=PPFIBP1 PE=1 SV=2 - [LIPB1_HUMAN]	-1.53	-1.45	-1.73	-1.81	0.06	0.06	-0.23	-0.28	-0.18	-0.17	-0.20	-0.18	1.22	1.26	1.52	1.33	1.51	1.44	1.57	1.56
P23141	Liver carboxylesterase 1 OS=Homo sapiens GN=CES1 PE=1 SV=2 - [EST1_HUMAN]	-1.51	-1.51	-2.17	-2.09	1.68	1.55	1.04	0.86	0.93	1.11	0.70	0.65	2.43	2.34	2.67	2.69	3.16	3.05	3.68	3.11
P28330	Long-chain specific acyl-CoA dehydrogenase, mitochondrial OS=Homo sapiens GN=ACADL PE=2 SV=2 - [ACADL_HUMAN]	-2.03	-1.95	-3.02	-2.88	-0.68	-0.52	-0.03	0.03	-0.11	-0.16	-0.07	0.05	2.19	2.23	2.73	2.04	2.20	1.58	2.00	2.56
Q9UKU0	Long-chain-fatty-acid--CoA ligase 6 OS=Homo sapiens GN=ACSL6 PE=2 SV=4 - [ACSL6_HUMAN]	1.85	1.71	2.06	1.86	-0.14	-0.15	0.80	0.56	0.33	0.48	0.29	0.19	-1.18	-1.65	-1.91	-1.49	-1.72	-2.20	-2.23	-1.47

P52569	Low affinity cationic amino acid transporter 2 OS=Homo sapiens GN=SLC7A2 PE=1 SV=2 - [CTR2_HUMAN]	-1.06	-1.17	-1.45	-1.56	1.00	0.89	0.49	0.38	0.55	0.67	0.74	0.62	1.61	1.80	2.19	1.75	2.15	2.04	2.43	2.00
P51884	Lumican OS=Homo sapiens GN=LUM PE=1 SV=2 - [LUM_HUMAN]	-1.96	-1.88	-2.01	-1.98	0.75	0.89	-0.33	-0.27	0.00	-0.10	-0.86	-0.68	1.58	1.09	1.18	1.80	1.82	2.76	2.63	1.62
Q9Y5Y7	Lymphatic vessel endothelial hyaluronic acid receptor 1 OS=Homo sapiens GN=LYVE1 PE=1 SV=2 - [LYVE1_HUMAN]	-1.87	-1.89	-1.86	-1.87	-0.19	-0.10	-0.68	-0.57	-0.12	-0.22	0.15	0.25	1.36	2.02	2.00	1.56	1.55	1.54	1.52	1.35
Q6P1A2	Lysophospholipid acyltransferase 5 OS=Homo sapiens GN=LPCAT3 PE=1 SV=1 - [MBOA5_HUMAN]	-1.93	-1.81	-1.63	-1.51	0.31	0.42	-0.42	-0.31	0.05	-0.06	0.05	0.16	1.56	1.99	1.68	1.90	1.60	2.22	1.93	1.27
Q643R3	Lysophospholipid acyltransferase LPCAT4 OS=Homo sapiens GN=LPCAT4 PE=1 SV=1 - [LPCT4_HUMAN]	1.20	1.21	1.27	1.28	-0.58	-0.57	0.18	0.19	-0.27	-0.27	-0.29	-0.29	-0.96	-1.48	-1.57	-1.44	-1.52	-1.79	-1.87	-1.03
P22897	Macrophage mannose receptor 1 OS=Homo sapiens GN=MRC1 PE=1 SV=1 - [MRC1_HUMAN]	-1.77	-1.78	-2.35	-2.32	-0.11	-0.18	-0.39	-0.33	-0.17	-0.25	-0.06	-0.01	1.49	1.67	2.32	2.01	1.92	1.78	2.24	1.90
P39900	Macrophage metalloelastase OS=Homo sapiens GN=MMP12 PE=1 SV=1 - [MMP12_HUMAN]	-2.56	-2.47	-2.38	-2.29	-0.07	0.02	-0.41	-0.32	-0.18	-0.27	0.05	0.14	2.20	2.61	2.43	2.32	2.15	2.48	2.30	2.04
Q9H0U3	Magnesium transporter protein 1 OS=Homo sapiens GN=MAGT1 PE=1 SV=1 - [MAGT1_HUMAN]	-1.33	-1.53	-1.63	-1.83	-0.05	-0.26	-0.25	-0.45	-0.26	-0.06	0.13	-0.07	1.14	1.47	1.76	1.30	1.61	1.26	1.56	1.45
Q14168	MAGUK p55 subfamily member 2 OS=Homo sapiens GN=MPP2 PE=1 SV=3 - [MPP2_HUMAN]	2.23	2.19	2.31	2.30	0.22	0.05	0.52	0.45	0.34	0.45	0.23	0.18	-1.65	-1.87	-2.01	-1.67	-1.56	-1.96	-2.02	-1.56

Q14728	Major facilitator superfamily domain-containing protein 10 OS=Homo sapiens GN=MFSD10 PE=2 SV=1 - [MFS10_HUMAN]	-1.71	-1.74	-1.92	-1.95	-0.27	-0.30	-0.63	-0.66	-0.38	-0.35	-0.30	-0.33	1.13	1.42	1.62	1.39	1.61	1.42	1.63	1.35
P40925	Malate dehydrogenase, cytoplasmic OS=Homo sapiens GN=MDH1 PE=1 SV=4 - [MDHC_HUMAN]	1.78	1.69	1.92	1.91	0.16	0.01	0.64	0.66	0.16	0.26	-0.20	-0.25	-1.07	-2.11	-2.36	-1.43	-1.72	-1.70	-1.99	-1.25
O00187	Mannan-binding lectin serine protease 2 OS=Homo sapiens GN=MASP2 PE=1 SV=4 - [MASP2_HUMAN]	-1.07	-1.08	-0.83	-0.84	1.33	1.32	0.17	0.16	0.41	0.42	0.49	0.48	1.29	1.57	1.32	1.52	1.28	2.38	2.14	1.06
Q9NR34	Mannosyl-oligosaccharide 1,2-alpha-mannosidase IC OS=Homo sapiens GN=MAN1C1 PE=2 SV=1 - [MA1C1_HUMAN]	-1.42	-1.34	-1.72	-1.64	0.05	0.13	-0.31	-0.23	0.17	0.10	0.10	0.18	1.16	1.52	1.82	1.54	1.85	1.45	1.75	1.47
Q8WXG6	MAP kinase-activating death domain protein OS=Homo sapiens GN=MADD PE=1 SV=2 - [MADD_HUMAN]	1.29	1.41	1.51	1.40	-0.14	-0.42	0.72	0.44	0.17	0.34	0.18	0.07	-0.83	-1.31	-1.13	-1.02	-0.96	-1.74	-1.63	-0.86
Q9H9H5	MAP6 domain-containing protein 1 OS=Homo sapiens GN=MAP6D1 PE=1 SV=1 - [MA6D1_HUMAN]	1.56	1.65	1.87	1.77	-0.09	-0.27	0.33	0.49	0.01	0.36	0.38	0.26	-1.25	-1.33	-1.50	-1.51	-1.42	-1.94	-1.99	-1.22
Q96T17	MAP7 domain-containing protein 2 OS=Homo sapiens GN=MAP7D2 PE=1 SV=2 - [MA7D2_HUMAN]	1.01	1.01	1.47	1.47	-0.02	-0.02	-0.44	-0.42	-0.39	-0.45	-0.56	-0.51	-1.37	-1.46	-1.93	-1.17	-1.76	-1.20	-1.51	-1.91
O00339	Matrilin-2 OS=Homo sapiens GN=MATN2 PE=1 SV=4 - [MATN2_HUMAN]	-1.88	-1.93	-1.92	-1.90	-0.45	-0.52	-0.57	-0.63	-0.65	-0.60	-0.37	-0.38	1.36	1.60	1.54	1.28	1.25	1.40	1.37	1.38

P08493	Matrix Gla protein OS=Homo sapiens GN=MGP PE=1 SV=2 - [MGP_HUMAN]	-2.35	-2.18	-2.60	-2.35	0.97	0.95	-0.92	-0.84	0.37	0.38	0.68	0.63	1.35	3.28	3.12	2.75	2.92	3.35	3.45	1.53
P50281	Matrix metalloproteinase-14 OS=Homo sapiens GN=MMP14 PE=1 SV=3 - [MMP14_HUMAN]	-1.39	-1.44	-1.61	-1.66	0.46	0.40	-0.12	-0.17	0.23	0.29	0.18	0.12	1.32	1.57	1.79	1.70	1.93	1.82	2.05	1.55
Q9H239	Matrix metalloproteinase-28 OS=Homo sapiens GN=MMP28 PE=2 SV=2 - [MMP28_HUMAN]	-0.63	-0.84	-0.77	-0.87	1.89	1.62	0.55	0.46	1.04	1.14	0.92	0.66	1.24	1.40	1.68	1.58	1.80	2.33	2.56	1.43
P43356	Melanoma-associated antigen 2 OS=Homo sapiens GN=MAGEA2 PE=1 SV=1 - [MAGA2_HUMAN]	-2.47	-2.82	-2.08	-2.43	0.09	-0.26	-0.18	-0.52	-0.37	-0.02	0.58	0.23	2.35	3.06	2.66	2.49	2.10	2.55	2.16	1.97
Q16853	Membrane primary amine oxidase OS=Homo sapiens GN=AOC3 PE=1 SV=3 - [AOC3_HUMAN]	-2.38	-2.52	-2.65	-2.66	-0.07	-0.02	-0.74	-0.89	-0.47	-0.41	-0.14	-0.14	1.71	2.43	2.50	1.96	2.22	2.48	2.68	1.97
O00264	Membrane-associated progesterone receptor component 1 OS=Homo sapiens GN=PGRMC1 PE=1 SV=3 - [PGRC1_HUMAN]	-1.50	-1.48	-1.60	-1.55	0.30	0.18	-0.15	-0.12	-0.13	-0.07	0.15	0.16	1.44	1.69	1.72	1.72	1.47	1.82	1.60	1.51
Q14416	Metabotropic glutamate receptor 2 OS=Homo sapiens GN=GRM2 PE=1 SV=2 - [GRM2_HUMAN]	2.83	2.79	2.55	2.52	-0.51	-0.55	1.17	1.14	0.82	0.86	0.55	0.51	-1.60	-2.27	-2.00	-1.93	-1.66	-3.35	-3.08	-1.32
P41594	Metabotropic glutamate receptor 5 OS=Homo sapiens GN=GRM5 PE=1 SV=2 - [GRM5_HUMAN]	1.73	1.57	1.70	1.48	-0.12	-0.09	0.30	0.13	0.20	0.43	0.25	0.18	-1.09	-1.35	-1.32	-1.20	-1.06	-1.30	-1.42	-0.80
P01033	Metalloproteinase inhibitor 1 OS=Homo sapiens GN=TIMP1 PE=1 SV=1 - [TIMP1_HUMAN]	-0.39	-0.40	-0.32	-0.33	1.64	1.62	0.63	0.63	0.98	0.99	0.93	0.91	1.08	1.32	1.24	1.41	1.34	2.01	1.94	1.01

P35625	Metalloproteinase inhibitor 3 OS=Homo sapiens GN=TIMP3 PE=1 SV=2 - [TIMP3_HUMAN]	-0.83	-0.86	-0.94	-0.99	2.94	2.93	0.79	0.83	1.75	1.77	0.99	1.02	1.56	1.75	1.85	2.60	2.74	3.66	3.88	1.79
Q9UJH8	Meteorin OS=Homo sapiens GN=METRN PE=2 SV=2 - [METRN_HUMAN]	-1.00	-0.98	-1.31	-1.45	1.63	1.50	0.73	0.62	0.86	1.04	0.57	0.58	1.79	1.51	1.95	2.05	2.51	2.52	2.98	2.26
Q6NT16	MFS-type transporter SLC18B1 OS=Homo sapiens GN=SLC18B1 PE=1 SV=1 - [S18B1_HUMAN]	2.09	1.96	1.85	1.75	0.08	0.05	0.86	0.67	0.43	0.51	0.41	0.21	-1.18	-1.67	-1.39	-1.45	-1.31	-2.23	-1.79	-0.88
P55001	Microfibrillar-associated protein 2 OS=Homo sapiens GN=MFAP2 PE=2 SV=1 - [MFAP2_HUMAN]	-2.41	-2.39	-1.88	-1.68	0.94	0.78	0.48	0.37	0.46	0.62	0.28	0.15	2.71	2.51	1.60	2.66	1.89	3.15	2.31	1.88
Q13361	Microfibrillar-associated protein 5 OS=Homo sapiens GN=MFAP5 PE=1 SV=1 - [MFAP5_HUMAN]	-1.95	-2.01	-2.10	-2.17	1.91	1.84	-0.42	-0.49	0.29	0.36	-0.74	-0.81	1.58	1.22	1.36	2.33	2.49	3.84	3.99	1.74
P78559	Microtubule-associated protein 1A OS=Homo sapiens GN=MAP1A PE=1 SV=6 - [MAP1A_HUMAN]	1.44	1.50	1.59	1.66	-0.26	-0.20	0.08	0.13	-0.07	-0.11	-0.14	-0.06	-1.27	-1.59	-1.74	-1.55	-1.66	-1.69	-1.93	-1.48
P46821	Microtubule-associated protein 1B OS=Homo sapiens GN=MAP1B PE=1 SV=2 - [MAP1B_HUMAN]	0.89	0.84	1.21	1.22	-0.46	-0.40	-0.03	0.01	-0.20	-0.24	-0.19	-0.18	-0.87	-1.05	-1.37	-1.07	-1.39	-1.31	-1.67	-1.18
P11137	Microtubule-associated protein 2 OS=Homo sapiens GN=MAP2 PE=1 SV=4 - [MTAP2_HUMAN]	1.48	1.47	1.83	1.77	-0.35	-0.42	0.35	0.28	-0.06	0.00	-0.37	-0.36	-1.14	-1.77	-2.15	-1.48	-1.76	-1.78	-2.10	-1.46
Q96JE9	Microtubule-associated protein 6 OS=Homo sapiens GN=MAP6 PE=1 SV=2 - [MAP6_HUMAN]	2.01	1.88	2.27	2.19	0.29	0.27	0.51	0.46	0.22	0.27	0.10	0.01	-1.45	-2.10	-2.32	-1.77	-2.04	-1.67	-2.06	-1.72

Q15555	Microtubule-associated protein RP/EB family member 2 OS=Homo sapiens GN=MAPRE2 PE=1 SV=1 - [MARE2_HUMAN]	1.18	0.90	1.59	1.41	-0.09	-0.41	0.42	0.31	-0.16	0.03	-0.16	-0.35	-0.76	-1.32	-1.61	-1.07	-1.64	-1.32	-1.76	-0.96
Q9Y2H9	Microtubule-associated serine/threonine-protein kinase 1 OS=Homo sapiens GN=MAST1 PE=1 SV=2 - [MAST1_HUMAN]	1.19	1.27	1.34	1.34	0.32	-0.09	0.25	0.17	-0.07	0.16	-0.22	-0.68	-1.11	-1.41	-1.57	-1.31	-1.37	-1.37	-1.44	-1.06
P21741	Midkine OS=Homo sapiens GN=MDK PE=1 SV=1 - [MK_HUMAN]	0.16	-0.34	-0.54	-0.52	2.42	2.62	2.52	2.36	2.35	2.57	2.28	2.07	3.02	2.99	3.11	3.12	3.14	3.10	3.36	3.46
P20774	Mimecan OS=Homo sapiens GN=OGN PE=1 SV=1 - [MIME_HUMAN]	-2.07	-2.12	-2.12	-2.02	0.34	0.37	-0.26	-0.25	-0.18	-0.17	-0.87	-0.86	1.86	1.24	1.22	2.01	1.96	2.45	2.44	1.81
Q8TCT9	Minor histocompatibility antigen H13 OS=Homo sapiens GN=HM13 PE=1 SV=1 - [HM13_HUMAN]	-1.28	-1.44	-1.60	-1.35	0.63	0.54	0.08	-0.23	-0.26	-0.08	0.11	-0.11	1.04	1.39	1.53	1.23	1.41	1.55	1.87	1.17
Q9H936	Mitochondrial glutamate carrier 1 OS=Homo sapiens GN=SLC25A22 PE=1 SV=1 - [GHC1_HUMAN]	1.37	1.41	1.42	1.34	-0.13	-0.15	0.39	0.45	0.10	0.13	-0.06	-0.02	-1.04	-1.34	-1.56	-1.38	-1.37	-1.52	-1.54	-1.12
Q96DA6	Mitochondrial import inner membrane translocase subunit TIM14 OS=Homo sapiens GN=DNAJC19 PE=1 SV=3 - [TIM14_HUMAN]	1.12	1.04	1.22	1.15	-0.29	-0.35	0.29	0.21	-0.16	-0.08	0.07	-0.10	-0.78	-1.23	-1.42	-1.27	-1.42	-1.38	-1.53	-0.93
O43615	Mitochondrial import inner membrane translocase subunit TIM44 OS=Homo sapiens GN=TIMM44 PE=1 SV=2 - [TIM44_HUMAN]	0.93	1.12	1.02	0.95	-0.14	-0.24	0.00	0.01	-0.09	-0.09	-0.25	-0.27	-1.12	-1.48	-1.25	-1.05	-1.03	-1.11	-1.11	-0.92

P53779	Mitogen-activated protein kinase 10 OS=Homo sapiens GN=MAPK10 PE=1 SV=2 - [MK10_HUMAN]	1.68	1.72	2.13	2.15	-0.28	-0.25	0.56	0.76	0.52	0.36	0.01	0.25	-0.99	-1.66	-1.95	-1.38	-1.81	-1.88	-2.14	-1.31
Q9NYL2	Mitogen-activated protein kinase kinase kinase MLT OS=Homo sapiens GN=ZAK PE=1 SV=3 - [MLTK_HUMAN]	-1.78	-1.69	-2.07	-1.98	-0.64	-0.56	-0.84	-0.75	-0.54	-0.62	-0.53	-0.45	0.99	1.25	1.54	1.19	1.49	1.12	1.41	1.29
O60669	Monocarboxylate transporter 2 OS=Homo sapiens GN=SLC16A7 PE=1 SV=2 - [MOT2_HUMAN]	1.24	1.33	1.15	1.24	-0.43	-0.35	-0.04	0.05	-0.26	-0.34	-0.07	0.01	-1.22	-1.31	-1.23	-1.55	-1.46	-1.69	-1.60	-1.13
Q6UB35	Monofunctional C1-tetrahydrofolate synthase, mitochondrial OS=Homo sapiens GN=MTHFD1L PE=1 SV=1 - [C1TM_HUMAN]	1.36	1.14	1.61	1.36	-0.14	-0.26	0.00	-0.14	-0.23	0.01	0.32	-0.03	-1.22	-1.04	-1.20	-1.33	-1.54	-1.21	-1.37	-1.33
Q13201	Multimerin-1 OS=Homo sapiens GN=MMRN1 PE=1 SV=3 - [MMRN1_HUMAN]	-0.40	-0.30	-0.62	-0.78	1.17	1.40	0.29	0.36	0.67	0.75	0.93	1.02	0.88	1.33	1.66	1.31	1.49	1.68	2.24	1.21
Q9H8L6	Multimerin-2 OS=Homo sapiens GN=MMRN2 PE=1 SV=2 - [MMRN2_HUMAN]	-1.35	-1.29	-1.46	-1.47	0.23	0.28	-0.54	-0.53	-0.18	-0.19	0.06	0.08	1.05	1.45	1.47	1.18	1.39	1.59	1.67	1.15
P11229	Muscarinic acetylcholine receptor M1 OS=Homo sapiens GN=CHRM1 PE=1 SV=2 - [ACM1_HUMAN]	1.77	1.63	2.40	2.26	-0.85	-0.99	0.35	0.21	0.39	0.54	0.07	-0.08	-1.36	-1.70	-2.33	-1.21	-1.83	-2.64	-3.26	-1.98
O00499	Myc box-dependent-interacting protein 1 OS=Homo sapiens GN=BIN1 PE=1 SV=1 - [BIN1_HUMAN]	1.52	1.46	1.68	1.74	-0.03	-0.05	0.35	0.38	0.00	-0.08	-0.26	-0.22	-1.10	-1.66	-1.83	-1.40	-1.69	-1.51	-1.75	-1.21
P02686	Myelin basic protein OS=Homo sapiens GN=MBP PE=1 SV=3 - [MBP_HUMAN]	2.23	2.17	2.33	2.30	-0.29	-0.29	0.31	0.28	-0.06	-0.09	-0.24	-0.29	-1.88	-2.47	-2.57	-2.21	-2.29	-2.49	-2.47	-2.11

P02689	Myelin P2 protein OS=Homo sapiens GN=PMP2 PE=1 SV=3 - [MYP2_HUMAN]	2.25	1.95	2.40	2.16	-0.01	-0.17	0.64	0.38	0.18	0.17	0.19	-0.12	-1.57	-1.97	-2.13	-2.13	-2.31	-2.06	-2.26	-1.59
O60487	Myelin protein zero-like protein 2 OS=Homo sapiens GN=MPZL2 PE=1 SV=1 - [MPZL2_HUMAN]	-1.87	-1.36	-1.53	-1.01	0.91	1.42	-0.03	0.49	0.64	0.14	0.50	1.01	1.90	2.38	2.03	2.04	1.70	2.77	2.42	1.56
P60201	Myelin proteolipid protein OS=Homo sapiens GN=PLP1 PE=1 SV=2 - [MYPR_HUMAN]	2.05	1.96	2.42	2.22	-0.53	-0.70	0.57	0.38	-0.04	0.07	-0.34	-0.41	-1.50	-2.38	-2.74	-2.10	-2.35	-2.65	-3.12	-1.78
P20916	Myelin-associated glycoprotein OS=Homo sapiens GN=MAG PE=1 SV=1 - [MAG_HUMAN]	2.59	2.77	2.64	2.75	0.07	0.15	0.83	0.75	0.46	0.55	0.10	0.09	-1.96	-2.60	-2.68	-2.08	-2.16	-2.36	-2.47	-1.92
Q13875	Myelin-associated oligodendrocyte basic protein OS=Homo sapiens GN=MOBP PE=2 SV=2 - [MOBP_HUMAN]	2.24	2.03	2.09	2.08	-0.85	-0.65	-0.12	-0.09	-0.35	-0.62	-0.83	-0.91	-2.07	-2.68	-2.54	-2.49	-2.27	-2.69	-2.74	-1.99
Q16653	Myelin-oligodendrocyte glycoprotein OS=Homo sapiens GN=MOG PE=1 SV=2 - [MOG_HUMAN]	2.74	2.40	2.70	2.29	-0.28	-0.43	0.77	0.55	0.16	0.38	0.33	0.04	-1.83	-2.35	-2.13	-2.07	-2.01	-2.65	-2.95	-1.69
Q15773	Myeloid leukemia factor 2 OS=Homo sapiens GN=MLF2 PE=1 SV=1 - [MLF2_HUMAN]	1.62	1.57	1.64	1.65	-0.47	-0.33	-0.14	-0.30	0.01	-0.18	-0.23	-0.47	-1.42	-1.70	-1.74	-1.63	-1.35	-1.83	-1.71	-1.55
Q9NZM1	Myoferlin OS=Homo sapiens GN=MYOF PE=1 SV=1 - [MYOF_HUMAN]	-2.06	-2.08	-2.15	-2.14	-0.05	-0.01	-0.44	-0.41	-0.20	-0.26	0.09	0.07	1.72	2.21	2.27	1.87	1.93	2.07	2.07	1.84
P12829	Myosin light chain 4 OS=Homo sapiens GN=MYL4 PE=1 SV=3 - [MYL4_HUMAN]	-2.20	-2.08	-2.45	-2.49	-0.20	-0.30	0.05	0.04	-0.10	-0.12	-0.02	-0.04	2.18	2.02	2.17	1.76	2.21	1.88	2.11	2.32
P35749	Myosin-11 OS=Homo sapiens GN=MYH11 PE=1 SV=3 - [MYH11_HUMAN]	-2.78	-2.80	-2.99	-3.01	-1.06	-1.05	-1.22	-1.27	-1.22	-1.19	-1.06	-1.07	1.64	1.76	1.95	1.64	1.86	1.84	1.95	1.82

Q13613	Myotubularin-related protein 1 OS=Homo sapiens GN=MTMR1 PE=1 SV=4 - [MTMR1_HUMAN]	1.51	1.45	1.37	1.31	-0.12	-0.18	0.19	0.13	0.16	0.23	0.20	0.13	-1.26	-1.31	-1.17	-1.23	-1.07	-1.60	-1.49	-1.04
Q9Y216	Myotubularin-related protein 7 OS=Homo sapiens GN=MTMR7 PE=1 SV=3 - [MTMR7_HUMAN]	1.50	1.80	1.45	1.76	0.17	0.48	0.46	0.77	0.23	-0.07	0.41	0.71	-0.98	-1.08	-1.04	-1.54	-1.49	-1.34	-1.29	-0.92
Q8N9F0	N-acetylaspartate synthetase OS=Homo sapiens GN=NAT8L PE=1 SV=3 - [NAT8L_HUMAN]	2.29	1.53	2.30	1.54	0.22	-0.54	0.81	0.05	-0.56	0.20	0.65	-0.12	-1.43	-1.64	-1.66	-2.06	-2.07	-2.09	-2.10	-1.43
Q9Y3Q0	N-acetylated-alpha-linked acidic dipeptidase 2 OS=Homo sapiens GN=NAALAD2 PE=1 SV=1 - [NALD2_HUMAN]	-1.37	-1.00	-1.64	-1.46	0.48	0.80	-0.08	0.13	0.36	0.15	0.35	0.67	1.35	1.73	2.07	1.51	1.75	1.79	2.04	1.61
Q8N987	N-terminal EF-hand calcium-binding protein 1 OS=Homo sapiens GN=NECAB1 PE=1 SV=1 - [NECA1_HUMAN]	1.19	1.58	1.59	1.64	-0.22	-0.15	-0.04	-0.06	0.04	-0.12	-0.17	0.05	-0.89	-1.36	-1.78	-1.28	-1.75	-1.55	-1.90	-1.79
Q96P71	N-terminal EF-hand calcium-binding protein 3 OS=Homo sapiens GN=NECAB3 PE=1 SV=2 - [NECA3_HUMAN]	1.90	2.01	1.72	1.83	0.06	0.16	0.45	0.56	0.41	0.31	0.24	0.35	-1.39	-1.65	-1.48	-1.56	-1.38	-1.86	-1.68	-1.20
Q8IXJ6	NAD-dependent protein deacetylase sirtuin-2 OS=Homo sapiens GN=SIRT2 PE=1 SV=2 - [SIR2_HUMAN]	2.40	2.63	2.59	2.54	-0.32	-0.42	0.77	0.83	0.31	0.20	-0.20	-0.07	-1.57	-2.44	-2.53	-2.17	-2.08	-2.81	-3.00	-1.65
O00483	NADH dehydrogenase [ubiquinone] 1 alpha subcomplex subunit 4 OS=Homo sapiens GN=NDUFA4 PE=1 SV=1 - [NDUA4_HUMAN]	1.32	1.32	1.59	1.66	-0.13	-0.12	0.36	0.51	0.21	0.23	0.03	0.04	-0.83	-1.19	-1.57	-1.10	-1.45	-1.40	-1.77	-1.10

Q9NRX3	NADH dehydrogenase [ubiquinone] 1 alpha subcomplex subunit 4-like 2 OS=Homo sapiens GN=NDUFA4L2 PE=2 SV=1 - [NUA4L_HUMAN]	-1.44	-1.37	-1.62	-1.55	-0.03	0.03	-0.42	-0.36	-0.10	-0.16	0.22	0.28	1.07	1.66	1.84	1.30	1.49	1.39	1.58	1.26
O43181	NADH dehydrogenase [ubiquinone] iron-sulfur protein 4, mitochondrial OS=Homo sapiens GN=NDUFS4 PE=1 SV=1 - [NDUS4_HUMAN]	1.56	1.39	1.69	1.63	0.23	0.05	0.03	0.13	0.05	0.15	0.46	0.20	-1.33	-1.22	-1.38	-1.43	-1.54	-1.40	-1.60	-1.27
O43920	NADH dehydrogenase [ubiquinone] iron-sulfur protein 5 OS=Homo sapiens GN=NDUFS5 PE=1 SV=3 - [NDUS5_HUMAN]	1.42	1.65	1.49	1.66	0.09	0.10	0.47	0.72	0.24	0.07	0.17	0.28	-0.90	-1.20	-1.09	-1.18	-1.06	-1.29	-1.39	-0.92
Q9NZQ3	NCK-interacting protein with SH3 domain OS=Homo sapiens GN=NCKIPSD PE=1 SV=1 - [SPN90_HUMAN]	1.48	1.71	1.25	1.57	0.34	0.33	0.32	0.51	0.30	0.15	0.34	0.30	-1.10	-1.29	-1.41	-1.36	-1.38	-1.49	-1.40	-0.81
Q15843	NEDD8 OS=Homo sapiens GN=NEDD8 PE=1 SV=1 - [NEDD8_HUMAN]	1.22	0.99	1.64	1.45	-0.40	-0.45	0.18	0.03	-0.24	-0.01	-0.11	-0.36	-1.07	-1.46	-1.76	-1.17	-1.53	-1.44	-1.81	-1.38
Q6UXI9	Nephronectin OS=Homo sapiens GN=NPNT PE=2 SV=3 - [NPNT_HUMAN]	-1.15	-1.27	-1.30	-1.24	1.01	1.16	-0.02	0.14	0.40	0.38	0.10	0.16	1.32	1.27	1.36	1.69	1.58	2.29	2.47	1.31
Q6ZMZ3	Nesprin-3 OS=Homo sapiens GN=SYNE3 PE=1 SV=2 - [SYNE3_HUMAN]	-1.26	-1.37	-1.38	-1.53	-0.05	0.02	-0.26	-0.21	-0.13	-0.06	-0.35	-0.25	1.15	1.10	1.29	1.41	1.63	1.35	1.36	1.41
O95631	Netrin-1 OS=Homo sapiens GN=NTN1 PE=1 SV=2 - [NET1_HUMAN]	0.40	0.65	0.45	0.84	2.70	3.05	1.64	1.89	2.48	2.21	2.48	2.58	1.23	2.00	1.99	1.96	1.80	2.51	2.51	1.40
Q9HB63	Netrin-4 OS=Homo sapiens GN=NTN4 PE=2 SV=2 - [NET4_HUMAN]	-0.83	-0.80	-1.17	-1.14	0.64	0.44	-0.60	-0.57	0.33	0.18	0.00	0.10	1.80	1.83	1.74	1.80	2.00	2.14	2.05	1.75

Q9ULJ8	Neurabin-1 OS=Homo sapiens GN=PPP1R9A PE=1 SV=2 - [NEB1_HUMAN]	0.71	1.52	0.42	1.13	-0.47	-0.29	-0.44	-0.47	-0.21	-0.28	-0.77	-0.87	-1.33	-1.47	-1.18	-1.60	-1.30	-1.82	-1.43	-1.53
O15394	Neural cell adhesion molecule 2 OS=Homo sapiens GN=NCAM2 PE=1 SV=2 - [NCAM2_HUMAN]	1.72	1.94	1.93	2.03	0.17	0.21	1.04	0.87	0.53	0.67	0.61	0.66	-0.88	-1.19	-1.38	-1.23	-1.39	-1.65	-1.54	-1.06
P32004	Neural cell adhesion molecule L1 OS=Homo sapiens GN=L1CAM PE=1 SV=2 - [L1CAM_HUMAN]	2.38	2.28	2.40	2.50	0.79	0.68	1.00	1.08	0.68	0.77	0.77	0.76	-1.06	-1.65	-1.70	-1.48	-1.56	-1.54	-1.57	-1.11
Q9NPD7	Neuritin OS=Homo sapiens GN=NRN1 PE=1 SV=1 - [NRN1_HUMAN]	1.14	1.12	1.44	1.43	0.04	0.02	0.05	0.03	-0.22	-0.20	-0.46	-0.48	-1.04	-1.60	-1.91	-1.31	-1.61	-1.11	-1.42	-1.34
Q8NFP9	Neurobeachin OS=Homo sapiens GN=NBEA PE=1 SV=3 - [NBEA_HUMAN]	1.19	1.27	1.22	1.25	-0.20	-0.14	0.09	0.19	0.01	-0.03	-0.18	-0.02	-0.94	-1.08	-1.21	-1.35	-1.07	-1.40	-1.41	-1.14
P61601	Neurocalcin-delta OS=Homo sapiens GN=NCALD PE=2 SV=2 - [NCALD_HUMAN]	1.30	1.58	1.30	1.73	-0.17	0.20	0.25	0.48	0.15	-0.04	0.16	0.23	-1.16	-1.37	-1.51	-1.37	-1.43	-1.51	-1.60	-1.28
Q9UBB6	Neurochondrin OS=Homo sapiens GN=NCDN PE=1 SV=1 - [NCDN_HUMAN]	2.64	2.54	2.64	2.56	0.11	0.11	1.10	0.94	0.55	0.65	0.24	0.09	-1.40	-2.40	-2.34	-1.91	-1.93	-2.43	-2.45	-1.29
P05408	Neuroendocrine protein 7B2 OS=Homo sapiens GN=SCG5 PE=1 SV=2 - [7B2_HUMAN]	1.58	1.85	1.23	1.63	0.01	-0.08	0.51	0.74	0.37	0.33	0.37	0.35	-0.96	-1.07	-1.14	-0.90	-1.03	-1.52	-1.45	-0.99
O94856	Neurofascin OS=Homo sapiens GN=NFASC PE=1 SV=4 - [NFASC_HUMAN]	1.59	1.65	1.68	1.75	-0.25	-0.31	0.43	0.45	0.09	0.16	-0.03	-0.06	-1.17	-1.68	-1.62	-1.46	-1.54	-1.83	-1.95	-1.23
P12036	Neurofilament heavy polypeptide OS=Homo sapiens GN=NEFH PE=1 SV=4 - [NFH_HUMAN]	2.15	2.07	2.25	2.43	-0.38	-0.50	0.53	0.57	0.15	0.25	-0.32	-0.31	-1.71	-2.73	-2.63	-2.39	-2.35	-2.45	-2.58	-2.01
P07196	Neurofilament light polypeptide OS=Homo sapiens GN=NEFL PE=1 SV=3 - [NFL_HUMAN]	2.35	2.37	2.48	2.58	-0.46	-0.43	0.52	0.54	0.10	0.04	-0.20	-0.14	-1.73	-2.54	-2.72	-2.41	-2.40	-2.42	-2.75	-1.94

P07197	Neurofilament medium polypeptide OS=Homo sapiens GN=NEFM PE=1 SV=3 - [NFM_HUMAN]	2.04	1.98	2.42	2.40	-0.46	-0.57	0.51	0.51	-0.06	-0.03	-0.20	-0.27	-1.43	-2.18	-2.48	-2.05	-2.37	-2.52	-2.81	-1.80
Q92686	Neurogranin OS=Homo sapiens GN=NRGN PE=1 SV=1 - [NEUG_HUMAN]	1.21	1.10	2.00	1.77	0.11	-0.11	-0.31	-0.52	0.20	-0.07	-0.08	-0.09	-1.88	-1.28	-2.04	-1.47	-1.84	-1.37	-1.84	-2.40
Q8NFZ4	Neuroligin-2 OS=Homo sapiens GN=NLGN2 PE=1 SV=1 - [NLGN2_HUMAN]	1.63	1.81	1.84	1.66	0.31	0.30	0.11	0.24	0.52	0.30	0.19	0.45	-1.73	-1.31	-1.59	-1.54	-1.37	-1.34	-1.40	-1.57
P84074	Neuron-specific calcium-binding protein hippocalcin OS=Homo sapiens GN=HPCA PE=1 SV=2 - [HPCA_HUMAN]	1.86	1.69	1.83	1.67	0.11	-0.06	0.57	0.40	0.24	0.41	-0.77	-0.94	-1.22	-2.63	-2.61	-1.42	-1.39	-1.77	-1.74	-1.42
P62166	Neuronal calcium sensor 1 OS=Homo sapiens GN=NCS1 PE=1 SV=2 - [NCS1_HUMAN]	1.79	1.78	2.16	2.18	0.47	0.45	0.92	0.94	0.59	0.61	0.46	0.30	-0.79	-1.23	-1.56	-1.39	-1.49	-1.19	-1.70	-1.23
Q7Z3B1	Neuronal growth regulator 1 OS=Homo sapiens GN=NEGR1 PE=1 SV=3 - [NEGR1_HUMAN]	1.64	1.43	1.84	1.78	0.41	0.36	0.64	0.47	0.11	0.25	0.35	0.21	-0.96	-1.19	-1.55	-1.50	-1.64	-1.30	-1.70	-1.26
P51674	Neuronal membrane glycoprotein M6-a OS=Homo sapiens GN=GPM6A PE=1 SV=2 - [GPM6A_HUMAN]	1.57	1.17	1.45	1.07	0.12	-0.21	0.63	0.39	0.27	0.39	0.44	0.20	-0.77	-1.16	-1.18	-1.26	-1.17	-1.80	-1.56	-0.86
Q9Y639	Neuroplastin OS=Homo sapiens GN=NPTN PE=1 SV=2 - [NPTN_HUMAN]	1.34	1.33	1.53	1.56	0.16	-0.01	0.53	0.60	0.24	0.33	0.12	0.19	-0.79	-1.21	-1.49	-1.15	-1.39	-1.24	-1.68	-1.04
Q9P121	Neurotrimin OS=Homo sapiens GN=NTM PE=1 SV=1 - [NTRI_HUMAN]	1.65	1.79	1.83	1.80	0.28	0.19	0.71	0.83	0.38	0.47	0.46	0.31	-0.97	-1.31	-1.32	-1.32	-1.34	-1.67	-1.52	-1.01
Q6PIU2	Neutral cholesterol ester hydrolase 1 OS=Homo sapiens GN=NCEH1 PE=1 SV=3 - [NCEH1_HUMAN]	1.71	1.63	1.90	1.84	-0.20	0.04	0.71	0.61	0.37	0.24	0.12	0.08	-0.97	-1.52	-1.70	-1.40	-1.46	-1.75	-2.01	-1.13

Q96TA1	Niban-like protein 1 OS=Homo sapiens GN=FAM129B PE=1 SV=3 - [NIBL1_HUMAN]	-1.94	-1.77	-1.91	-1.78	0.37	0.47	0.08	0.07	0.00	-0.05	-0.24	-0.09	1.91	1.84	1.81	1.97	1.95	2.18	1.99	1.99
P14543	Nidogen-1 OS=Homo sapiens GN=NID1 PE=1 SV=3 - [NID1_HUMAN]	-1.81	-1.76	-1.97	-1.97	-0.16	-0.16	-0.75	-0.73	-0.48	-0.49	-0.44	-0.38	1.03	1.34	1.57	1.32	1.49	1.51	1.66	1.27
Q13253	Noggin OS=Homo sapiens GN=NOG PE=1 SV=1 - [NOGG_HUMAN]	-1.11	-1.22	-1.22	-1.32	1.31	1.19	0.52	0.41	0.64	0.75	0.24	0.13	1.69	1.36	1.45	1.89	2.00	2.40	2.50	1.80
P10153	Non-secretory ribonuclease OS=Homo sapiens GN=RNASE2 PE=1 SV=2 - [RNAS2_HUMAN]	-1.73	-1.30	-1.39	-0.95	-0.18	0.25	-0.26	0.18	0.10	-0.33	0.46	0.89	1.53	2.20	1.85	1.44	1.09	1.53	1.19	1.19
Q9GZM8	Nuclear distribution protein nudE-like 1 OS=Homo sapiens GN=NDEL1 PE=1 SV=1 - [NDEL1_HUMAN]	1.59	0.80	1.57	0.94	-0.12	-0.41	0.39	-0.10	-0.33	0.24	-0.58	-0.44	-1.02	-1.22	-1.37	-1.25	-1.16	-1.68	-1.54	-0.87
Q8NI08	Nuclear receptor coactivator 7 OS=Homo sapiens GN=NCOA7 PE=1 SV=2 - [NCOA7_HUMAN]	1.69	1.85	1.75	2.03	0.24	0.49	0.60	0.75	0.71	0.57	0.46	0.65	-0.94	-1.21	-1.36	-1.15	-1.28	-1.49	-1.55	-1.15
Q9Y2C4	Nuclease EXOG, mitochondrial OS=Homo sapiens GN=EXOG PE=1 SV=2 - [EXOG_HUMAN]	1.31	1.37	1.40	1.68	0.33	0.47	0.42	0.66	0.56	0.24	0.30	0.61	-1.01	-1.13	-1.11	-1.31	-1.15	-1.47	-1.36	-0.97
O00746	Nucleoside diphosphate kinase, mitochondrial OS=Homo sapiens GN=NME4 PE=1 SV=1 - [NDKM_HUMAN]	1.82	1.87	1.75	1.80	-0.78	-0.73	-0.24	-0.19	-0.23	-0.27	-0.20	-0.15	-2.00	-2.01	-1.95	-2.06	-1.99	-2.61	-2.54	-1.93
Q6UWY5	Olfactomedin-like protein 1 OS=Homo sapiens GN=OLFML1 PE=1 SV=2 - [OLFL1_HUMAN]	-2.70	-2.73	-3.37	-3.25	-0.70	-0.68	-0.62	-0.59	-0.89	-0.85	-1.16	-1.23	2.01	1.75	2.15	1.94	2.43	2.25	2.58	2.70

Q9NRN5	Olfactomedin-like protein 3 OS=Homo sapiens GN=OLFML3 PE=2 SV=1 - [OLFL3_HUMAN]	-1.45	-1.64	-1.85	-1.97	1.23	1.40	0.22	0.32	0.49	0.47	-0.11	-0.11	2.03	1.59	1.80	2.35	2.61	3.33	3.64	2.39
P23515	Oligodendrocyte-myelin glycoprotein OS=Homo sapiens GN=OMG PE=1 SV=2 - [OMGP_HUMAN]	2.24	2.27	2.48	2.59	-0.14	-0.14	0.73	0.79	0.51	0.34	0.40	0.43	-1.35	-1.73	-2.07	-1.70	-1.84	-2.41	-2.80	-1.79
Q96PE5	Opalin OS=Homo sapiens GN=OPALIN PE=2 SV=1 - [OPALI_HUMAN]	2.24	1.78	2.32	1.90	-0.56	-0.35	0.70	0.26	-0.63	-0.05	-0.01	-0.34	-1.60	-1.91	-1.94	-2.23	-2.23	-2.83	-2.69	-1.82
Q14982	Opioid-binding protein/cell adhesion molecule OS=Homo sapiens GN=OPCML PE=1 SV=1 - [OPCM_HUMAN]	1.76	1.69	2.00	2.10	-0.03	-0.02	0.57	0.71	0.24	0.25	0.18	0.25	-0.97	-1.64	-1.99	-1.43	-1.56	-1.67	-1.92	-1.29
Q99983	Osteomodulin OS=Homo sapiens GN=OMD PE=1 SV=1 - [OMD_HUMAN]	-2.28	-2.30	-2.37	-2.39	-1.03	-1.08	-1.03	-1.08	-1.34	-1.29	-1.16	-1.19	1.39	1.14	1.21	1.31	1.41	1.48	1.58	1.50
Q86UD1	Out at first protein homolog OS=Homo sapiens GN=OAF PE=2 SV=1 - [OAF_HUMAN]	-0.85	-0.74	-1.14	-1.02	1.58	1.69	0.45	0.57	1.05	0.95	0.74	0.85	1.36	1.60	1.88	1.83	2.12	2.41	2.70	1.65
Q8N573	Oxidation resistance protein 1 OS=Homo sapiens GN=OXR1 PE=1 SV=2 - [OXR1_HUMAN]	1.21	1.47	1.58	1.49	0.20	0.10	0.46	0.57	0.34	0.36	-0.12	-0.10	-0.94	-1.43	-1.57	-0.88	-1.04	-1.54	-1.43	-1.05
Q8WX93	Palladin OS=Homo sapiens GN=PALLD PE=1 SV=3 - [PALLD_HUMAN]	-2.37	-2.43	-2.54	-2.50	-0.61	-0.59	-0.95	-1.09	-0.86	-0.85	-0.79	-0.76	1.40	1.62	1.75	1.55	1.70	1.69	1.95	1.52
O75781	Paralemmin-1 OS=Homo sapiens GN=PALM PE=1 SV=2 - [PALM_HUMAN]	1.79	1.69	1.74	1.72	0.10	0.06	0.37	0.34	0.19	0.06	-0.25	-0.17	-1.26	-1.79	-1.78	-1.58	-1.69	-1.75	-1.70	-1.27
Q8IXS6	Paralemmin-2 OS=Homo sapiens GN=PALM2 PE=2 SV=3 - [PALM2_HUMAN]	1.01	0.99	1.02	1.01	-0.43	-0.45	-0.09	-0.11	-0.04	-0.01	-0.10	-0.12	-1.04	-1.10	-1.12	-0.99	-1.00	-1.45	-1.47	-1.05

Q8WXF1	Paraspeckle component 1 OS=Homo sapiens GN=PSPC1 PE=1 SV=1 - [PSPC1_HUMAN]	0.73	0.84	1.03	0.88	-0.67	-0.57	-0.31	-0.28	-0.54	-0.68	-0.44	-0.46	-1.09	-1.39	-1.34	-1.40	-1.53	-1.22	-1.56	-1.28
P20472	Parvalbumin alpha OS=Homo sapiens GN=PVALB PE=1 SV=2 - [PRVA_HUMAN]	2.22	2.21	2.78	2.61	0.54	0.67	0.64	0.62	0.75	0.61	0.09	0.47	-1.33	-1.88	-2.69	-1.47	-1.98	-1.38	-2.10	-1.91
P49023	Paxillin OS=Homo sapiens GN=PXN PE=1 SV=3 - [PAXI_HUMAN]	-1.37	-1.70	-1.34	-1.43	-0.05	-0.15	-0.25	-0.25	-0.37	-0.21	-0.02	0.02	1.38	1.38	1.38	1.29	1.24	1.24	1.26	1.13
O00151	PDZ and LIM domain protein 1 OS=Homo sapiens GN=PDLIM1 PE=1 SV=4 - [PDLI1_HUMAN]	-2.22	-2.14	-2.12	-2.21	0.01	0.00	-0.46	-0.51	-0.41	-0.40	-0.43	-0.52	1.75	1.91	1.80	1.70	1.72	2.13	1.97	1.58
Q96HC4	PDZ and LIM domain protein 5 OS=Homo sapiens GN=PDLIM5 PE=1 SV=5 - [PDLI5_HUMAN]	-1.94	-1.83	-1.73	-1.73	-0.48	-0.51	-0.71	-0.76	-0.70	-0.72	-0.52	-0.53	1.22	1.38	1.30	1.28	1.15	1.46	1.34	1.06
Q9NR12	PDZ and LIM domain protein 7 OS=Homo sapiens GN=PDLIM7 PE=1 SV=1 - [PDLI7_HUMAN]	-3.11	-3.00	-3.32	-3.10	-1.04	-1.02	-1.44	-1.39	-1.08	-1.10	-1.09	-0.97	1.72	2.04	2.17	1.84	1.99	1.98	2.01	1.85
P26022	Pentraxin-related protein PTX3 OS=Homo sapiens GN=PTX3 PE=1 SV=3 - [PTX3_HUMAN]	-1.77	-1.70	-1.90	-1.83	1.41	1.48	1.94	2.01	2.08	2.01	2.67	2.74	3.76	4.45	4.58	3.81	3.95	3.16	3.30	3.91
Q96AY3	Peptidyl-prolyl cis-trans isomerase FKBP10 OS=Homo sapiens GN=FKBP10 PE=1 SV=1 - [FKB10_HUMAN]	-2.10	-2.34	-1.95	-2.00	0.19	0.11	-0.30	-0.45	-0.21	-0.20	0.10	-0.07	1.43	1.83	1.98	2.24	1.78	2.22	2.11	1.67
O75381	Peroxisomal membrane protein PEX14 OS=Homo sapiens GN=PEX14 PE=1 SV=1 - [PEX14_HUMAN]	1.44	1.44	1.88	1.89	0.12	0.05	0.37	0.38	0.33	0.24	0.42	0.44	-1.02	-0.99	-1.51	-1.11	-1.61	-1.32	-1.82	-1.42

Q8IYB4	PEX5-related protein OS=Homo sapiens GN=PEX5L PE=1 SV=2 - [PEX5R_HUMAN]	1.12	1.11	1.33	1.25	-0.21	-0.49	0.14	0.05	-0.16	-0.05	-0.18	-0.42	-0.93	-1.23	-1.50	-0.92	-0.97	-1.81	-1.39	-1.03
A5PKW4	PH and SEC7 domain-containing protein 1 OS=Homo sapiens GN=PSD PE=1 SV=2 - [PSD1_HUMAN]	1.79	1.64	1.75	1.61	0.20	0.04	0.06	-0.09	0.35	0.50	-0.26	-0.41	-1.68	-2.04	-2.01	-1.26	-1.22	-1.61	-1.58	-1.63
Q9NYI0	PH and SEC7 domain-containing protein 3 OS=Homo sapiens GN=PSD3 PE=1 SV=2 - [PSD3_HUMAN]	1.80	1.63	1.95	1.94	-0.31	-0.44	0.92	0.85	0.37	0.35	0.24	0.08	-0.76	-1.55	-1.73	-1.40	-1.63	-2.14	-2.40	-0.95
Q9C0D0	Phosphatase and actin regulator 1 OS=Homo sapiens GN=PHACTR1 PE=2 SV=3 - [PHAR1_HUMAN]	1.63	1.28	1.42	1.07	-0.62	-0.98	0.00	-0.35	-0.48	-0.13	-0.20	-0.56	-1.57	-1.83	-1.62	-1.73	-1.52	-2.27	-2.06	-1.36
P36871	Phosphoglucomutase-1 OS=Homo sapiens GN=PGM1 PE=1 SV=3 - [PGM1_HUMAN]	1.38	1.30	1.62	1.60	-0.06	0.01	0.48	0.50	0.13	0.23	-0.24	-0.20	-0.84	-1.60	-1.82	-1.13	-1.41	-1.31	-1.57	-1.01
Q15124	Phosphoglucomutase-like protein 5 OS=Homo sapiens GN=PGM5 PE=1 SV=2 - [PGM5_HUMAN]	-2.73	-2.68	-2.90	-2.69	-0.68	-0.58	-1.45	-1.22	-0.90	-1.06	-0.78	-0.73	1.49	2.05	2.10	1.94	1.96	2.14	2.11	1.58
Q6ZUJ8	Phosphoinositide 3-kinase adapter protein 1 OS=Homo sapiens GN=PIK3AP1 PE=1 SV=2 - [BCAP_HUMAN]	-0.95	-0.78	-0.36	-0.76	1.11	0.65	0.49	0.17	0.41	0.50	1.20	0.69	1.50	2.16	1.46	1.48	1.20	2.04	1.40	0.99
P55058	Phospholipid transfer protein OS=Homo sapiens GN=PLTP PE=1 SV=1 - [PLTP_HUMAN]	-0.40	-0.34	-0.88	-0.60	2.68	3.06	0.47	0.73	1.90	1.59	0.91	1.20	1.21	1.55	2.00	2.25	2.58	3.37	3.69	1.24
Q92561	Phytanoyl-CoA hydroxylase-interacting protein OS=Homo sapiens GN=PHYHIP PE=1 SV=1 - [PHYIP_HUMAN]	2.73	2.75	3.03	3.16	-0.14	0.13	1.29	1.38	0.67	0.67	0.70	0.49	-1.46	-2.27	-2.47	-2.08	-2.40	-2.91	-3.11	-1.69

Q96FC7	Phytanoyl-CoA hydroxylase-interacting protein-like OS=Homo sapiens GN=PHYHIPL PE=1 SV=3 - [PHIPL_HUMAN]	1.43	1.55	1.76	1.75	-0.44	-0.49	0.60	0.59	0.14	0.08	0.24	0.24	-0.93	-1.56	-1.49	-1.33	-1.38	-1.93	-1.65	-1.02
P36955	Pigment epithelium-derived factor OS=Homo sapiens GN=SERPINF1 PE=1 SV=4 - [PEDF_HUMAN]	-2.39	-2.44	-2.43	-2.44	-0.85	-0.86	-1.20	-1.18	-1.11	-1.09	-0.68	-0.67	1.18	1.64	1.71	1.25	1.25	1.62	1.57	1.27
Q7Z3Z3	Piwi-like protein 3 OS=Homo sapiens GN=PIWIL3 PE=2 SV=2 - [PIWL3_HUMAN]	-2.12	-0.93	-2.04	-0.84	-0.37	0.82	-0.97	0.23	0.51	-0.68	-0.65	0.53	1.21	1.47	1.38	1.48	1.39	1.74	1.65	1.13
Q5JTB6	Placenta-specific protein 9 OS=Homo sapiens GN=PLAC9 PE=2 SV=1 - [PLAC9_HUMAN]	-1.43	-1.66	-1.61	-1.85	0.63	0.40	-0.35	-0.47	-0.26	-0.03	-0.20	-0.31	1.14	1.49	1.66	1.43	1.61	2.05	2.23	1.44
Q99959	Plakophilin-2 OS=Homo sapiens GN=PKP2 PE=1 SV=2 - [PKP2_HUMAN]	-2.36	-2.21	-2.49	-2.58	-0.21	-0.24	-0.50	-0.52	-0.49	-0.41	-0.41	-0.47	1.89	1.91	2.15	1.95	2.10	2.12	2.31	2.07
P03952	Plasma kallikrein OS=Homo sapiens GN=KLKB1 PE=1 SV=1 - [KLKB1_HUMAN]	-1.15	-1.34	-1.57	-1.51	0.93	0.79	0.49	0.22	0.47	0.56	0.95	0.73	1.73	2.10	2.23	1.92	1.99	2.06	2.13	1.80
P20020	Plasma membrane calcium-transporting ATPase 1 OS=Homo sapiens GN=ATP2B1 PE=1 SV=3 - [AT2B1_HUMAN]	1.96	1.82	2.11	1.93	0.08	-0.06	0.61	0.52	0.12	0.30	0.24	0.24	-1.31	-1.67	-1.70	-1.57	-1.76	-1.87	-1.98	-1.46
Q01814	Plasma membrane calcium-transporting ATPase 2 OS=Homo sapiens GN=ATP2B2 PE=1 SV=2 - [AT2B2_HUMAN]	2.11	2.06	2.16	2.14	-0.07	-0.09	0.85	0.83	0.37	0.45	0.09	0.16	-1.28	-2.17	-2.18	-1.67	-1.89	-2.32	-2.38	-1.46
Q16720	Plasma membrane calcium-transporting ATPase 3 OS=Homo sapiens GN=ATP2B3 PE=1 SV=3 - [AT2B3_HUMAN]	2.31	2.01	2.47	2.38	-0.34	-0.52	0.94	0.99	0.51	0.48	0.36	0.28	-1.13	-1.75	-2.05	-1.70	-1.89	-2.47	-2.60	-1.34

P00747	Plasminogen OS=Homo sapiens GN=PLG PE=1 SV=2 - [PLMN_HUMAN]	-0.82	-0.86	-0.96	-1.09	0.55	0.48	0.18	0.27	0.79	0.79	1.75	1.70	1.09	2.40	2.61	1.61	1.87	1.39	1.67	1.37
P02775	Platelet basic protein OS=Homo sapiens GN=PPBP PE=1 SV=3 - [CXCL7_HUMAN]	-2.18	-2.33	-1.90	-1.97	-0.15	-0.31	-0.58	-0.73	-0.75	-0.44	-0.30	-0.45	1.65	1.89	1.60	1.79	1.26	2.01	1.73	1.38
P02776	Platelet factor 4 OS=Homo sapiens GN=PF4 PE=1 SV=2 - [PLF4_HUMAN]	-1.82	-1.84	-1.72	-1.75	0.21	0.18	-0.50	-0.52	-0.05	-0.02	0.38	0.35	1.37	2.20	2.10	1.82	1.74	2.00	1.91	1.29
P13224	Platelet glycoprotein Ib beta chain OS=Homo sapiens GN=GP1BB PE=1 SV=1 - [GP1BB_HUMAN]	-0.97	-1.17	-1.10	-1.06	0.63	0.51	0.65	0.54	0.89	0.84	1.23	1.13	1.64	2.67	2.36	1.96	2.06	1.57	1.47	1.57
P09619	Platelet-derived growth factor receptor beta OS=Homo sapiens GN=PDGFRB PE=1 SV=1 - [PGFRB_HUMAN]	-1.90	-1.93	-2.05	-2.04	0.66	0.61	0.20	0.14	0.28	0.34	0.31	0.33	2.04	2.10	2.36	2.19	2.38	2.44	2.55	2.24
Q9UF11	Pleckstrin homology domain-containing family B member 1 OS=Homo sapiens GN=PLEKHB1 PE=1 SV=1 - [PKHB1_HUMAN]	1.90	1.64	1.95	1.77	-0.30	-0.27	0.32	0.12	-0.04	0.01	-0.37	-0.77	-1.45	-2.06	-2.11	-1.98	-2.12	-2.29	-2.34	-1.56
Q86SQ0	Pleckstrin homology-like domain family B member 2 OS=Homo sapiens GN=PHLDB2 PE=1 SV=2 - [PHLB2_HUMAN]	-1.99	-1.90	-2.17	-2.12	-0.28	-0.30	-0.69	-0.73	-0.46	-0.46	-0.13	-0.12	1.24	1.58	1.74	1.38	1.58	1.56	1.81	1.46
P21246	Pleiotrophin OS=Homo sapiens GN=PTN PE=1 SV=1 - [PTN_HUMAN]	-1.93	-1.76	-2.48	-2.31	1.72	1.74	0.74	0.68	0.92	0.98	-0.22	-0.45	2.23	1.74	1.94	2.93	3.18	3.66	3.65	3.12
Q7Z5L7	Podocan OS=Homo sapiens GN=PODN PE=1 SV=2 - [PODN_HUMAN]	-1.62	-1.32	-2.16	-1.96	0.85	1.00	-0.59	-0.40	0.35	0.15	-0.25	-0.09	1.08	1.48	1.91	1.80	2.22	2.49	2.95	1.66

Q6NZI2	Polymerase I and transcript release factor OS=Homo sapiens GN=PTRF PE=1 SV=1 - [PTRF_HUMAN]	-2.54	-2.59	-2.65	-2.74	-0.43	-0.48	-0.80	-0.82	-0.64	-0.54	-0.26	-0.30	1.76	2.28	2.18	2.00	1.82	2.08	2.08	1.76
P26599	Polypyrimidine tract-binding protein 1 OS=Homo sapiens GN=PTBP1 PE=1 SV=1 - [PTBP1_HUMAN]	-1.32	-1.51	-1.05	-1.25	0.40	0.24	-0.16	-0.35	-0.12	0.04	0.02	-0.14	1.43	1.54	1.27	1.69	1.49	1.67	1.70	1.21
P16389	Potassium voltage-gated channel subfamily A member 2 OS=Homo sapiens GN=KCNA2 PE=1 SV=2 - [KCNA2_HUMAN]	1.74	2.48	1.86	2.66	0.20	0.23	0.92	1.05	0.65	0.33	0.52	0.58	-1.38	-1.89	-2.07	-1.79	-1.90	-1.77	-1.93	-1.55
P48547	Potassium voltage-gated channel subfamily C member 1 OS=Homo sapiens GN=KCNC1 PE=2 SV=1 - [KCNC1_HUMAN]	2.93	2.63	2.77	2.48	0.27	-0.03	1.50	1.20	0.76	1.06	0.67	0.37	-1.38	-2.25	-2.10	-1.84	-1.68	-2.68	-2.52	-1.21
Q14003	Potassium voltage-gated channel subfamily C member 3 OS=Homo sapiens GN=KCNC3 PE=1 SV=3 - [KCNC3_HUMAN]	2.10	2.24	2.17	2.31	-0.03	0.10	1.16	1.30	0.99	0.85	0.10	0.23	-0.88	-1.99	-2.07	-1.22	-1.29	-2.15	-2.22	-0.95
Q9NZV8	Potassium voltage-gated channel subfamily D member 2 OS=Homo sapiens GN=KCND2 PE=1 SV=2 - [KCND2_HUMAN]	2.10	2.17	1.93	2.00	-0.03	0.04	0.85	0.93	0.79	0.72	-0.19	-0.13	-1.19	-2.28	-2.12	-1.34	-1.17	-2.14	-1.98	-1.01
O43526	Potassium voltage-gated channel subfamily KQT member 2 OS=Homo sapiens GN=KCNQ2 PE=1 SV=2 - [KCNQ2_HUMAN]	1.25	1.03	1.31	1.24	0.12	-0.05	0.32	0.37	0.06	0.22	-0.02	-0.06	-0.93	-1.06	-1.13	-1.05	-1.23	-1.19	-1.38	-1.20

O43525	Potassium voltage-gated channel subfamily KQT member 3 OS=Homo sapiens GN=KCNQ3 PE=1 SV=2 - [KCNQ3_HUMAN]	1.47	1.67	1.43	1.63	0.29	0.48	-0.82	-0.63	0.09	-0.11	-1.07	-0.88	-2.24	-2.54	-2.51	-1.55	-1.51	-1.20	-1.16	-2.19
P61758	Prefoldin subunit 3 OS=Homo sapiens GN=VBP1 PE=1 SV=3 - [PFD3_HUMAN]	1.35	0.94	1.17	1.10	-0.10	-0.07	0.18	-0.12	-0.21	0.11	-0.20	-0.29	-1.11	-1.50	-1.38	-1.13	-1.03	-1.49	-1.28	-1.01
Q96IZ0	PRKC apoptosis WT1 regulator protein OS=Homo sapiens GN=PAWR PE=1 SV=1 - [PAWR_HUMAN]	-2.40	-2.45	-2.88	-2.89	-0.47	-0.43	-0.63	-0.66	-0.95	-0.98	-1.05	-1.06	2.01	1.92	1.88	1.98	1.89	2.26	2.29	2.29
P01303	Pro-neuropeptide Y OS=Homo sapiens GN=NPY PE=1 SV=1 - [NPY_HUMAN]	3.03	2.67	3.19	2.68	0.68	0.81	1.83	1.99	1.51	1.39	0.78	0.68	-0.94	-1.78	-1.99	-1.48	-1.68	-2.14	-2.34	-1.13
Q8N9I9	Probable E3 ubiquitin-protein ligase DTX3 OS=Homo sapiens GN=DTX3 PE=1 SV=2 - [DTX3_HUMAN]	1.01	0.77	1.30	1.18	-0.51	-0.58	0.18	0.05	-0.10	0.08	-0.11	-0.75	-0.77	-1.11	-1.40	-0.90	-1.19	-1.53	-1.82	-1.05
Q8TED1	Probable glutathione peroxidase 8 OS=Homo sapiens GN=GPX8 PE=1 SV=2 - [GPX8_HUMAN]	-1.84	-1.89	-1.83	-1.87	0.15	0.10	-0.19	-0.23	-0.02	0.04	0.27	0.22	1.71	2.11	2.09	1.91	1.90	1.98	1.96	1.70
Q9Y2Q0	Probable phospholipid-transporting ATPase IA OS=Homo sapiens GN=ATP8A1 PE=1 SV=1 - [AT8A1_HUMAN]	2.17	1.99	2.09	1.85	0.14	0.13	1.06	0.85	0.69	0.66	0.66	0.55	-0.92	-1.40	-1.30	-1.23	-1.16	-1.94	-1.90	-1.03
Q9H3G5	Probable serine carboxypeptidase CPVL OS=Homo sapiens GN=CPVL PE=1 SV=2 - [CPVL_HUMAN]	-2.55	-2.41	-2.12	-2.04	0.11	0.20	-0.52	-0.34	-0.04	-0.19	-0.11	0.00	1.95	2.13	2.06	2.37	2.09	2.55	2.43	1.78
P01210	Proenkephalin-A OS=Homo sapiens GN=PENK PE=1 SV=1 - [PENK_HUMAN]	-0.35	-0.71	-0.99	-1.34	1.93	1.73	0.66	0.31	2.00	2.36	3.62	3.26	1.07	3.98	4.60	2.74	3.38	2.42	3.05	1.71

P51888	Prolargin OS=Homo sapiens GN=PRELP PE=1 SV=1 - [PRELP_HUMAN]	-1.97	-2.02	-2.34	-2.35	0.17	0.20	-0.60	-0.52	-0.21	-0.22	-0.70	-0.67	1.56	1.42	1.72	1.81	2.16	2.28	2.57	1.86
Q99946	Proline-rich transmembrane protein 1 OS=Homo sapiens GN=PRRT1 PE=2 SV=2 - [PRRT1_HUMAN]	1.60	1.53	1.24	1.16	0.05	-0.03	-0.25	-0.32	-0.01	0.07	0.17	0.09	-1.79	-1.42	-1.07	-1.50	-1.14	-1.56	-1.20	-1.42
Q7Z6L0	Proline-rich transmembrane protein 2 OS=Homo sapiens GN=PRRT2 PE=1 SV=1 - [PRRT2_HUMAN]	2.51	2.42	2.76	2.54	0.10	-0.04	0.79	0.92	0.58	0.57	0.40	0.27	-1.62	-2.22	-2.29	-1.99	-2.25	-2.66	-2.73	-1.84
Q5FWE3	Proline-rich transmembrane protein 3 OS=Homo sapiens GN=PRRT3 PE=1 SV=3 - [PRRT3_HUMAN]	2.11	1.81	2.25	2.04	0.29	0.35	0.85	0.74	0.01	0.26	0.54	0.58	-0.87	-1.10	-1.21	-1.69	-1.81	-1.30	-1.41	-1.08
Q32P28	Prolyl 3-hydroxylase 1 OS=Homo sapiens GN=LEPRE1 PE=1 SV=2 - [P3H1_HUMAN]	-2.18	-2.16	-2.52	-2.53	-0.16	-0.05	-0.61	-0.62	-0.54	-0.56	-0.24	-0.01	1.82	1.95	2.58	1.77	2.05	2.25	2.35	1.98
P13674	Prolyl 4-hydroxylase subunit alpha-1 OS=Homo sapiens GN=P4HA1 PE=1 SV=2 - [P4HA1_HUMAN]	-1.43	-1.37	-1.59	-1.54	0.14	0.24	-0.40	-0.37	-0.24	-0.26	-0.19	-0.17	1.05	1.68	1.45	1.22	1.41	1.61	1.66	1.21
Q92824	Proprotein convertase subtilisin/kexin type 5 OS=Homo sapiens GN=PCSK5 PE=1 SV=4 - [PCSK5_HUMAN]	-0.81	-1.04	-0.57	-0.79	2.95	2.72	0.50	0.27	1.45	1.68	0.77	0.54	1.36	1.58	1.34	2.52	2.28	3.74	3.50	1.13
Q9UHG2	ProSAAS OS=Homo sapiens GN=PCSK1N PE=1 SV=1 - [PCSK1_HUMAN]	1.68	1.37	1.50	1.40	0.37	0.14	0.71	0.37	0.24	0.52	0.31	0.09	-1.04	-1.33	-1.27	-1.13	-1.18	-1.42	-1.34	-1.02
O15354	Prosaposin receptor GPR37 OS=Homo sapiens GN=GPR37 PE=1 SV=2 - [GPR37_HUMAN]	1.69	1.90	1.85	2.06	0.04	0.25	0.64	0.86	0.47	0.27	0.43	0.63	-0.99	-1.26	-1.43	-1.39	-1.55	-1.66	-1.83	-1.14
Q16647	Prostacyclin synthase OS=Homo sapiens GN=PTGIS PE=1 SV=1 - [PTGIS_HUMAN]	-2.43	-2.21	-2.68	-2.39	-0.11	-0.42	-0.73	-0.78	-0.78	-0.59	-0.72	-0.88	1.68	1.70	1.78	1.79	2.12	1.89	1.96	1.91

P41222	Prostaglandin-H2 D-isomerase OS=Homo sapiens GN=PTGDS PE=1 SV=1 - [PTGDS_HUMAN]	-0.65	-0.81	-0.61	-0.71	1.55	1.45	0.77	0.59	0.81	0.92	0.76	0.52	1.51	1.41	1.31	1.71	1.42	2.30	2.01	1.50
P11171	Protein 4.1 OS=Homo sapiens GN=EPB41 PE=1 SV=4 - [41_HUMAN]	-1.45	-1.50	-1.37	-1.38	0.31	0.28	0.46	0.42	0.54	0.58	1.31	1.26	1.67	2.60	2.69	1.97	2.11	1.79	1.81	1.84
Q9UPA5	Protein bassoon OS=Homo sapiens GN=BSN PE=2 SV=4 - [BSN_HUMAN]	1.75	1.79	1.77	1.91	-0.06	-0.05	0.37	0.24	0.21	0.13	-0.14	-0.21	-1.35	-1.97	-1.99	-1.57	-1.69	-1.85	-1.91	-1.38
O00622	Protein CYR61 OS=Homo sapiens GN=CYR61 PE=1 SV=1 - [CYR61_HUMAN]	-0.18	0.33	-0.36	-0.43	3.27	3.07	1.99	1.75	2.09	2.34	2.05	1.82	1.95	1.33	2.26	2.20	2.82	2.67	3.87	2.85
O60879	Protein diaphanous homolog 2 OS=Homo sapiens GN=DIAPH2 PE=1 SV=1 - [DIAP2_HUMAN]	-1.49	-1.29	-1.30	-0.98	0.02	0.26	0.11	0.36	0.22	-0.10	-0.12	0.12	1.55	1.16	1.19	1.32	1.32	1.26	1.53	1.36
Q14554	Protein disulfide-isomerase A5 OS=Homo sapiens GN=PDIA5 PE=1 SV=1 - [PDIA5_HUMAN]	-1.20	-1.20	-1.34	-1.33	0.36	0.36	-0.29	-0.28	0.05	0.05	0.12	0.12	0.97	1.33	1.46	1.28	1.42	1.55	1.68	1.11
Q86XD5	Protein FAM131B OS=Homo sapiens GN=FAM131B PE=1 SV=3 - [F131B_HUMAN]	1.55	1.45	1.70	1.80	0.39	0.42	0.59	0.54	0.52	0.50	0.31	0.37	-1.12	-1.07	-1.46	-0.96	-1.01	-1.26	-1.14	-1.14
Q6UX46	Protein FAM150B OS=Homo sapiens GN=FAM150B PE=2 SV=2 - [F150B_HUMAN]	-1.49	-1.61	-1.93	-2.04	0.18	0.06	-0.45	-0.56	-0.14	-0.02	0.10	-0.02	1.10	1.60	2.03	1.51	1.95	1.66	2.10	1.54
Q8IYM0	Protein FAM186B OS=Homo sapiens GN=FAM186B PE=2 SV=2 - [F186B_HUMAN]	-2.20	-2.26	-2.36	-2.42	0.29	0.23	-0.27	-0.32	0.05	0.12	0.23	0.17	1.99	2.44	2.60	2.34	2.51	2.47	2.63	2.16
Q8N5C1	Protein FAM26E OS=Homo sapiens GN=FAM26E PE=2 SV=1 - [FA26E_HUMAN]	-1.57	-1.52	-1.73	-1.68	0.78	0.83	-0.12	-0.07	0.27	0.23	0.21	0.25	1.51	1.78	1.93	1.83	1.99	2.33	2.49	1.67

Q9NUQ9	Protein FAM49B OS=Homo sapiens GN=FAM49B PE=1 SV=1 - [FA49B_HUMAN]	1.93	2.41	2.32	2.39	0.11	0.29	0.50	0.67	0.32	0.07	0.01	0.03	-1.73	-2.08	-2.05	-1.95	-2.15	-2.06	-2.45	-1.73
Q9UPV7	Protein KIAA1045 OS=Homo sapiens GN=KIAA1045 PE=1 SV=2 - [K1045_HUMAN]	1.78	1.84	1.69	1.74	-0.32	-0.36	0.40	0.53	0.26	0.03	-0.18	0.05	-1.15	-1.67	-1.66	-1.41	-1.44	-1.95	-2.11	-1.23
Q9BY11	Protein kinase C and casein kinase substrate in neurons protein 1 OS=Homo sapiens GN=PACSIN1 PE=1 SV=1 - [PACN1_HUMAN]	1.24	1.23	1.57	1.53	-0.03	0.04	-0.01	0.08	-0.14	-0.21	-0.33	-0.10	-1.02	-1.33	-1.63	-1.49	-1.71	-1.28	-1.62	-1.33
Q969G5	Protein kinase C delta-binding protein OS=Homo sapiens GN=PRKCDBP PE=1 SV=3 [PRDBP_HUMAN]	-2.42	-2.21	-2.60	-2.98	-0.84	-0.72	-1.10	-1.21	-0.89	-0.77	-0.28	-0.28	1.35	2.01	2.59	1.68	1.92	1.47	1.80	1.63
Q02156	Protein kinase C epsilon type OS=Homo sapiens GN=PRKCE PE=1 SV=1 - [KPCE_HUMAN]	1.53	1.61	1.51	1.62	0.08	0.00	0.45	0.41	0.27	0.29	0.23	0.09	-0.99	-1.38	-1.39	-1.34	-1.41	-1.67	-1.72	-1.09
Q99435	Protein kinase C-binding protein NELL2 OS=Homo sapiens GN=NELL2 PE=1 SV=1 - [NELL2_HUMAN]	-1.14	-0.90	-1.31	-1.12	0.53	0.64	0.46	0.48	0.94	0.90	1.69	1.81	1.53	2.75	3.01	1.97	2.35	1.62	1.72	1.74
O14910	Protein lin-7 homolog A OS=Homo sapiens GN=LIN7A PE=1 SV=2 - [LIN7A_HUMAN]	1.75	1.83	1.78	1.65	0.02	0.21	0.18	0.05	0.27	0.17	0.34	0.32	-1.35	-1.40	-1.33	-1.44	-1.37	-1.67	-1.69	-1.20
Q9BZQ8	Protein Niban OS=Homo sapiens GN=FAM129A PE=1 SV=1 - [NIBAN_HUMAN]	-1.78	-1.91	-2.07	-1.89	0.30	0.36	-0.30	-0.20	-0.19	-0.14	-0.17	-0.05	1.81	2.00	1.84	1.86	1.82	2.26	2.16	1.85
Q9UFN0	Protein NipSnap homolog 3A OS=Homo sapiens GN=NIPSNAP3A PE=1 SV=2 - [NPS3A_HUMAN]	1.60	1.91	1.95	2.25	-0.69	-0.39	0.01	0.31	-0.47	-0.77	-0.94	-0.42	-1.54	-2.21	-3.13	-2.35	-2.69	-2.31	-2.66	-1.88

Q8TAE6	Protein phosphatase 1 regulatory subunit 14C OS=Homo sapiens GN=PPP1R14C PE=1 SV=3 [PP14C_HUMAN]	0.94	0.93	1.73	1.72	-0.28	-0.29	0.06	0.05	-0.65	-0.64	-0.68	-0.69	-0.82	-1.62	-2.41	-1.55	-2.33	-1.23	-2.02	-1.61
Q6ZSY5	Protein phosphatase 1 regulatory subunit 3F OS=Homo sapiens GN=PPP1R3F PE=1 SV=3 - [PPR3F_HUMAN]	1.81	1.63	1.64	1.46	0.88	0.33	0.28	0.10	-0.01	0.18	0.22	0.03	-1.47	-1.59	-1.43	-1.60	-1.43	-1.35	-1.39	-1.30
Q9Y6V0	Protein piccolo OS=Homo sapiens GN=PCLO PE=1 SV=4 - [PCLO_HUMAN]	1.53	1.60	1.81	1.82	-0.15	-0.24	0.60	0.56	0.34	0.35	0.05	0.05	-1.00	-1.52	-1.85	-1.37	-1.67	-1.56	-1.88	-1.26
Q3SYG4	Protein PTHB1 OS=Homo sapiens GN=BBS9 PE=1 SV=1 - [PTHB1_HUMAN]	-0.96	-0.60	-0.99	-0.63	0.63	0.99	0.56	0.92	0.53	0.16	0.47	0.83	1.58	1.44	1.46	1.16	1.19	1.58	1.61	1.62
Q9GZN7	Protein rogdi homolog OS=Homo sapiens GN=ROGDI PE=1 SV=1 - [ROGDI_HUMAN]	1.28	1.24	1.50	1.46	-0.32	-0.36	0.44	0.41	-0.09	-0.04	-0.11	-0.15	-0.78	-1.39	-1.61	-1.30	-1.51	-1.61	-1.83	-0.99
Q7L099	Protein RUFY3 OS=Homo sapiens GN=RUFY3 PE=1 SV=1 - [RUFY3_HUMAN]	2.01	1.87	2.01	1.81	0.02	-0.05	0.79	0.60	0.34	0.43	0.21	0.15	-1.04	-1.45	-1.69	-1.46	-1.48	-1.88	-1.94	-1.24
P60903	Protein S100-A10 OS=Homo sapiens GN=S100A10 PE=1 SV=2 - [S10AA_HUMAN]	-2.23	-2.23	-2.13	-2.12	-0.40	-0.31	-0.86	-0.75	-0.49	-0.56	-0.12	-0.02	1.44	2.09	2.03	1.72	1.72	1.78	1.72	1.39
P31949	Protein S100-A11 OS=Homo sapiens GN=S100A11 PE=1 SV=2 - [S10AB_HUMAN]	-2.01	-1.80	-2.07	-1.97	-0.10	0.04	-0.50	-0.40	-0.20	-0.37	-0.04	0.06	1.58	2.05	2.05	1.73	1.79	1.97	2.02	1.66
P26447	Protein S100-A4 OS=Homo sapiens GN=S100A4 PE=1 SV=1 - [S10A4_HUMAN]	-2.41	-2.46	-2.43	-2.29	-0.03	0.05	-0.64	-0.55	-0.22	-0.29	-0.20	-0.11	1.84	2.08	2.11	2.14	2.03	2.33	2.38	1.77
P06703	Protein S100-A6 OS=Homo sapiens GN=S100A6 PE=1 SV=1 - [S10A6_HUMAN]	-2.33	-1.96	-2.52	-2.50	-0.16	-0.08	-0.47	-0.42	-0.31	-0.35	-0.32	-0.35	1.80	1.62	2.12	1.77	2.13	2.05	2.23	2.13

Q8N9R8	Protein SCAI OS=Homo sapiens GN=SCAI PE=1 SV=2 - [SCAI_HUMAN]	1.36	1.37	1.31	1.35	-0.15	-0.08	0.48	0.61	0.38	0.35	0.17	0.28	-0.83	-1.16	-1.28	-1.09	-1.23	-1.49	-1.73	-0.92
A6NL88	Protein shisa-7 OS=Homo sapiens GN=SHISA7 PE=2 SV=3 - [SHSA7_HUMAN]	1.50	1.28	1.41	1.19	-0.74	-0.97	0.07	-0.15	-0.72	-0.49	-0.64	-0.87	-1.38	-2.14	-2.05	-1.96	-1.86	-2.26	-2.17	-1.27
Q5TF21	Protein SOGA3 OS=Homo sapiens GN=SOGA3 PE=2 SV=1 - [SOGA3_HUMAN]	1.92	1.73	2.10	1.84	0.09	-0.01	1.02	0.55	0.42	0.53	0.38	0.34	-0.99	-1.39	-1.47	-1.34	-1.25	-2.00	-1.80	-1.15
Q9HCD6	Protein TANC2 OS=Homo sapiens GN=TANC2 PE=1 SV=3 - [TANC2_HUMAN]	1.29	1.04	1.67	1.34	-0.62	-0.82	0.32	0.07	-0.20	-0.16	-0.16	-0.42	-0.92	-1.45	-1.83	-1.42	-1.50	-1.93	-2.17	-1.28
P61619	Protein transport protein Sec61 subunit alpha isoform 1 OS=Homo sapiens GN=SEC61A1 PE=1 SV=2 - [S61A1_HUMAN]	-1.70	-1.64	-1.70	-1.70	0.07	0.01	-0.56	-0.55	-0.45	-0.40	-0.20	-0.25	1.20	1.62	1.78	1.51	1.52	1.77	1.89	1.21
Q9UPX0	Protein turtle homolog B OS=Homo sapiens GN=IGSF9B PE=2 SV=2 - [TUTLB_HUMAN]	1.88	1.65	1.71	1.48	0.02	-0.21	0.64	0.41	0.28	0.52	0.32	0.08	-1.18	-1.56	-1.39	-1.33	-1.16	-1.87	-1.70	-1.00
Q9H313	Protein tweety homolog 1 OS=Homo sapiens GN=TTYH1 PE=2 SV=1 - [TTYH1_HUMAN]	2.46	2.53	2.32	2.33	-0.52	-0.51	0.99	0.97	0.55	0.64	0.31	0.40	-1.41	-2.12	-1.92	-1.88	-1.68	-3.01	-2.87	-1.26
Q9UPW8	Protein unc-13 homolog A OS=Homo sapiens GN=UNC13A PE=2 SV=4 - [UN13A_HUMAN]	1.96	2.02	1.87	1.97	-0.05	0.34	0.66	0.80	0.62	0.69	0.07	0.30	-1.00	-1.52	-1.67	-1.37	-1.15	-2.03	-2.12	-1.11
Q93097	Protein Wnt-2b OS=Homo sapiens GN=WNT2B PE=1 SV=2 - [WNT2B_HUMAN]	-0.86	-1.08	-1.20	-1.43	1.47	1.24	1.30	1.09	1.08	1.10	0.84	0.74	2.27	1.93	1.84	2.26	2.34	2.51	2.66	2.62
P56705	Protein Wnt-4 OS=Homo sapiens GN=WNT4 PE=1 SV=4 - [WNT4_HUMAN]	-0.31	-0.45	-0.76	-1.03	2.68	2.46	1.51	1.62	1.84	1.96	1.32	1.23	2.21	1.65	2.22	2.33	2.91	2.90	3.73	2.47

Q9Y6F9	Protein Wnt-6 OS=Homo sapiens GN=WNT6 PE=1 SV=2 - [WNT6_HUMAN]	-1.33	-1.56	-1.18	-1.23	0.73	0.67	0.80	0.75	0.82	0.88	0.83	0.77	1.95	1.92	2.31	2.00	2.20	1.80	2.29	2.19
Q9UM07	Protein-arginine deiminase type-4 OS=Homo sapiens GN=PADI4 PE=1 SV=2 - [PADI4_HUMAN]	-1.54	-1.52	-1.71	-1.69	0.20	0.22	0.91	0.93	0.57	0.56	0.40	0.41	2.51	1.95	2.11	2.13	2.30	1.73	1.90	2.69
P21980	Protein-glutamine gamma-glutamyltransferase 2 OS=Homo sapiens GN=TGM2 PE=1 SV=2 - [TGM2_HUMAN]	-2.17	-2.11	-2.32	-2.28	-0.30	-0.25	-1.11	-1.06	-0.67	-0.60	-0.38	-0.33	0.98	1.70	1.95	1.49	1.64	1.86	2.06	1.30
O94851	Protein-methionine sulfoxide oxidase MICAL2 OS=Homo sapiens GN=MICAL2 PE=1 SV=1 - [MICA2_HUMAN]	-1.19	-1.30	-1.27	-1.38	0.22	0.11	-0.10	-0.21	-0.10	0.02	0.01	-0.10	1.14	1.21	1.29	1.24	1.32	1.40	1.48	1.23
P00734	Prothrombin OS=Homo sapiens GN=F2 PE=1 SV=2 - [THRB_HUMAN]	-2.58	-2.68	-2.63	-2.58	-1.28	-1.19	-1.61	-1.59	-1.29	-1.37	-0.85	-0.79	0.97	1.77	1.95	1.51	1.44	1.33	1.45	1.09
Q9HC56	Protocadherin-9 OS=Homo sapiens GN=PCDH9 PE=1 SV=2 - [PCDH9_HUMAN]	0.98	1.25	1.36	1.43	-0.34	-0.31	0.25	0.33	0.16	0.15	-0.05	0.05	-0.91	-1.42	-1.55	-1.07	-1.25	-1.24	-1.59	-1.10
Q96QE2	Proton myo-inositol cotransporter OS=Homo sapiens GN=SLC2A13 PE=1 SV=3 - [MYCT_HUMAN]	1.48	1.80	1.75	2.24	-0.09	0.14	0.52	0.91	0.44	0.27	0.01	0.45	-0.88	-1.44	-1.65	-1.20	-1.48	-1.59	-2.03	-1.02
P48539	Purkinje cell protein 4 OS=Homo sapiens GN=PCP4 PE=1 SV=3 - [PCP4_HUMAN]	1.55	1.73	1.75	1.93	0.00	0.17	-0.39	-0.22	0.13	-0.04	-0.68	-0.51	-1.89	-2.23	-2.44	-1.56	-1.76	-1.57	-1.77	-2.08
Q9Y315	Putative deoxyribose-phosphate aldolase OS=Homo sapiens GN=DERA PE=1 SV=2 - [DEOC_HUMAN]	-1.26	-1.19	-1.36	-1.19	0.07	0.15	-0.13	-0.08	0.09	0.00	0.46	0.50	1.08	1.59	1.81	1.34	1.28	1.20	1.26	1.33

O75061	Putative tyrosine-protein phosphatase auxilin OS=Homo sapiens GN=DNAJC6 PE=1 SV=3 - [AUXI_HUMAN]	1.61	1.37	1.81	1.64	-0.33	-0.15	0.44	0.46	0.15	0.16	0.07	0.02	-0.96	-1.66	-1.86	-1.47	-1.87	-1.85	-2.22	-1.35
P30613	Pyruvate kinase PKLR OS=Homo sapiens GN=PKLR PE=1 SV=2 - [KPYR_HUMAN]	-1.66	-1.68	-1.77	-1.76	-0.30	-0.30	-0.21	-0.26	-0.19	-0.27	-0.18	-0.23	1.49	1.42	1.49	1.52	1.49	1.34	1.47	1.70
Q9Y4G8	Rap guanine nucleotide exchange factor 2 OS=Homo sapiens GN=RAPGEF2 PE=1 SV=1 - [RPGF2_HUMAN]	1.11	1.40	1.35	1.48	-0.23	-0.07	0.22	0.41	0.33	0.07	-0.07	0.28	-1.01	-1.33	-1.57	-1.24	-1.47	-1.44	-1.74	-1.19
P47736	Rap1 GTPase-activating protein 1 OS=Homo sapiens GN=RAP1GAP PE=1 SV=2 [RPGP1_HUMAN]	2.04	1.89	1.91	1.76	-0.08	-0.23	0.73	0.58	0.23	0.34	0.24	0.14	-1.18	-1.92	-1.74	-1.74	-1.61	-2.11	-2.03	-1.02
P46940	Ras GTPase-activating-like protein IQGAP1 OS=Homo sapiens GN=IQGAP1 PE=1 SV=1 - [IQGA1_HUMAN]	-1.52	-1.48	-1.60	-1.54	-0.16	-0.13	-0.42	-0.43	-0.33	-0.33	-0.29	-0.23	1.10	1.27	1.27	1.22	1.25	1.31	1.38	1.14
Q13576	Ras GTPase-activating-like protein IQGAP2 OS=Homo sapiens GN=IQGAP2 PE=1 SV=4 - [IQGA2_HUMAN]	-1.10	-1.10	-1.14	-1.36	0.42	0.38	0.18	0.17	0.34	0.36	0.40	0.18	1.33	1.29	1.53	1.46	1.72	1.53	1.78	1.57
P15153	Ras-related C3 botulinum toxin substrate 2 OS=Homo sapiens GN=RAC2 PE=1 SV=1 - [RAC2_HUMAN]	-1.07	-0.68	-1.09	-0.69	0.65	1.04	0.80	1.19	0.92	0.53	0.06	0.45	1.92	1.14	1.15	1.63	1.65	1.70	1.72	1.95
P10301	Ras-related protein R-Ras OS=Homo sapiens GN=RRAS PE=1 SV=1 - [RRAS_HUMAN]	-1.86	-1.89	-1.96	-1.79	-0.14	-0.03	-0.57	-0.56	-0.34	-0.33	-0.13	-0.10	1.43	1.70	1.79	1.69	1.67	1.68	1.81	1.38

P20336	Ras-related protein Rab-3A OS=Homo sapiens GN=RAB3A PE=1 SV=1 - [RAB3A_HUMAN]	1.75	1.66	2.03	1.87	-0.23	-0.11	0.90	0.76	0.18	0.36	-0.12	-0.28	-0.82	-1.83	-2.33	-1.44	-1.69	-1.95	-2.25	-1.16
Q13972	Ras-specific guanine nucleotide-releasing factor 1 OS=Homo sapiens GN=RASGRF1 PE=1 SV=2 - [RGRF1_HUMAN]	1.80	1.93	1.94	2.33	-0.08	0.21	0.35	0.74	0.68	0.40	-0.10	0.18	-1.33	-1.69	-1.55	-1.39	-1.40	-1.92	-1.93	-1.53
Q96PV0	Ras/Rap GTPase-activating protein SynGAP OS=Homo sapiens GN=SYNGAP1 PE=1 SV=4 - [SYGP1_HUMAN]	2.06	2.06	2.05	2.02	-0.08	-0.27	0.66	0.60	0.24	0.36	0.21	0.02	-1.38	-2.13	-2.07	-1.87	-1.79	-2.12	-2.12	-1.39
O95294	RasGAP-activating-like protein 1 OS=Homo sapiens GN=RASAL1 PE=1 SV=3 - [RASL1_HUMAN]	1.77	1.79	1.64	1.66	-0.74	-0.73	-0.03	-0.01	0.07	0.06	-0.32	-0.31	-1.74	-2.09	-1.97	-1.68	-1.55	-2.53	-2.40	-1.61
Q9BRK0	Receptor expression-enhancing protein 2 OS=Homo sapiens GN=REEP2 PE=2 SV=2 - [REEP2_HUMAN]	1.80	1.62	1.76	1.59	-0.30	-0.49	0.44	0.26	-0.12	0.06	-0.06	-0.24	-1.30	-1.85	-1.83	-1.71	-1.67	-2.12	-2.09	-1.26
Q13546	Receptor-interacting serine/threonine-protein kinase 1 OS=Homo sapiens GN=RIPK1 PE=1 SV=3 - [RIPK1_HUMAN]	-1.51	-1.55	-1.58	-1.61	0.42	0.39	-0.05	-0.08	0.14	0.17	-0.21	-0.24	1.52	1.31	1.37	1.72	1.79	1.92	1.98	1.60
Q92932	Receptor-type tyrosine-protein phosphatase N2 OS=Homo sapiens GN=PTPRN2 PE=1 SV=2 - [PTPR2_HUMAN]	1.45	1.80	1.51	1.86	0.20	0.73	0.24	0.59	0.16	0.22	0.04	0.34	-1.09	-1.46	-1.32	-1.14	-1.10	-1.52	-1.62	-1.06
Q16849	Receptor-type tyrosine-protein phosphatase-like N OS=Homo sapiens GN=PTPRN PE=1 SV=1 - [PTPRN_HUMAN]	1.15	1.89	1.57	2.05	-0.36	0.38	0.67	1.08	0.78	0.04	0.05	0.37	-0.90	-1.03	-1.52	-1.23	-1.36	-1.64	-2.00	-1.38

Q9UJD0	Regulating synaptic membrane exocytosis protein 3 OS=Homo sapiens GN=RIMS3 PE=1 SV=1 - [RIMS3_HUMAN]	1.95	1.93	1.80	1.78	-0.19	0.02	0.21	0.24	0.11	0.13	0.03	-0.09	-1.44	-2.01	-1.83	-1.79	-1.63	-1.68	-1.94	-1.41
Q9H4X1	Regulator of cell cycle RGCC OS=Homo sapiens GN=RGCC PE=1 SV=1 - [RGCC_HUMAN]	-1.14	-1.42	-1.30	-0.95	1.14	1.05	0.83	1.09	0.96	0.76	0.68	0.93	2.15	1.83	1.94	2.03	1.95	2.37	2.29	2.12
P49758	Regulator of G protein signaling 6 OS=Homo sapiens GN=RGS6 PE=1 SV=5 - [RGS6_HUMAN]	1.56	1.64	1.82	1.93	-0.34	-0.27	0.26	0.38	0.06	0.02	0.00	0.08	-1.16	-1.55	-1.82	-1.40	-1.79	-1.52	-2.18	-1.46
P49802	Regulator of G protein signaling 7 OS=Homo sapiens GN=RGS7 PE=1 SV=3 - [RGS7_HUMAN]	1.71	1.56	2.17	2.02	0.11	-0.04	0.77	0.62	0.17	0.32	0.28	0.12	-0.95	-1.42	-1.89	-1.35	-1.81	-1.61	-2.07	-1.44
A5PLK6	Regulator of G protein signaling protein-like OS=Homo sapiens GN=RGSL1 PE=2 SV=1 - [RGSL_HUMAN]	-2.83	-2.83	-3.62	-3.62	0.24	0.23	-0.48	-0.48	-0.36	-0.36	-0.73	-0.74	2.40	2.10	2.88	2.50	3.30	3.05	3.84	3.20
Q8WUF5	RelA-associated inhibitor OS=Homo sapiens GN=PPP1R13L PE=1 SV=4 - [IASPP_HUMAN]	-1.05	-0.96	-1.50	-1.41	1.38	1.46	0.51	0.59	1.02	0.94	0.85	0.93	1.61	1.90	2.34	2.02	2.46	2.41	2.85	2.06
Q9NWS8	Required for meiotic nuclear division protein 1 homolog OS=Homo sapiens GN=RMND1 PE=1 SV=2 - [RMND1_HUMAN]	1.10	0.96	1.00	1.04	-0.22	-0.27	-0.31	-0.24	-0.19	-0.19	-0.29	-0.34	-1.09	-1.25	-1.35	-1.18	-1.16	-1.35	-1.22	-1.07
Q16799	Reticulon-1 OS=Homo sapiens GN=RTN1 PE=1 SV=1 - [RTN1_HUMAN]	1.20	1.23	1.49	1.58	-0.38	-0.26	0.24	0.38	0.09	-0.04	0.18	0.11	-0.86	-1.07	-1.38	-1.29	-1.53	-1.45	-1.88	-1.32
O95197	Reticulon-3 OS=Homo sapiens GN=RTN3 PE=1 SV=2 - [RTN3_HUMAN]	1.74	1.92	2.10	1.99	-0.24	-0.13	0.55	0.50	0.20	0.07	0.05	0.03	-1.23	-1.81	-2.04	-1.68	-1.91	-2.06	-2.28	-1.49

O94788	Retinal dehydrogenase 2 OS=Homo sapiens GN=ALDH1A2 PE=1 SV=3 - [AL1A2_HUMAN]	-1.62	-0.88	-2.11	-1.96	0.35	0.49	-0.20	-0.07	0.25	-0.07	-0.12	-0.03	1.15	1.36	1.79	1.49	2.01	1.51	2.35	1.98
Q9HB40	Retinoid-inducible serine carboxypeptidase OS=Homo sapiens GN=SCPEP1 PE=1 SV=1 - [RISC_HUMAN]	-1.78	-1.88	-1.92	-2.02	0.38	0.28	-0.46	-0.56	-0.06	0.05	0.33	0.22	1.37	2.11	2.25	1.86	2.01	2.14	2.29	1.53
O95980	Reversion-inducing cysteine-rich protein with Kazal motifs OS=Homo sapiens GN=RECK PE=1 SV=1 - [RECK_HUMAN]	-2.37	-2.37	-1.94	-1.71	-0.24	-0.02	-0.51	-0.22	-0.28	-0.26	0.04	0.26	1.66	2.15	1.98	2.12	1.48	1.88	1.68	1.57
Q68EM7	Rho GTPase-activating protein 17 OS=Homo sapiens GN=ARHGAP17 PE=1 SV=1 - [RHG17_HUMAN]	-1.61	-1.36	-1.83	-1.50	-0.30	-0.12	-0.64	-0.41	-0.46	-0.67	-0.76	-0.71	1.21	1.09	1.20	1.20	1.34	1.39	1.52	1.33
Q5T5U3	Rho GTPase-activating protein 21 OS=Homo sapiens GN=ARHGAP21 PE=1 SV=1 - [RHG21_HUMAN]	1.09	1.15	1.25	1.23	-0.16	-0.08	0.20	0.07	0.05	0.09	0.01	0.04	-1.02	-1.08	-1.21	-1.13	-1.17	-1.24	-1.28	-1.00
Q9UNA1	Rho GTPase-activating protein 26 OS=Homo sapiens GN=ARHGAP26 PE=1 SV=1 - [RHG26_HUMAN]	1.45	1.22	1.37	1.16	-0.12	-0.29	0.25	0.05	0.03	-0.03	-0.01	0.15	-1.21	-1.28	-1.23	-1.37	-1.28	-1.69	-1.61	-1.17
A7KAX9	Rho GTPase-activating protein 32 OS=Homo sapiens GN=ARHGAP32 PE=1 SV=1 - [RHG32_HUMAN]	1.68	1.42	1.52	1.40	0.15	0.09	0.16	0.30	0.23	0.50	0.25	-0.03	-1.31	-1.34	-1.34	-1.17	-0.96	-1.52	-1.33	-1.04
P98171	Rho GTPase-activating protein 4 OS=Homo sapiens GN=ARHGAP4 PE=1 SV=2 - [RHG04_HUMAN]	-1.20	-1.31	-2.65	-2.76	0.46	0.35	0.28	0.16	0.55	0.66	0.59	0.47	1.53	1.79	3.23	1.89	3.34	1.64	3.09	2.99

Q17R89	Rho GTPase-activating protein 44 OS=Homo sapiens GN=ARHGAP44 PE=1 SV=1 - [RHG44_HUMAN]	1.73	1.78	1.99	1.97	-0.49	-0.33	0.57	0.62	0.34	0.19	-0.27	-0.22	-1.11	-1.99	-2.31	-1.41	-1.71	-2.09	-2.31	-1.22
P62745	Rho-related GTP-binding protein RhoB OS=Homo sapiens GN=RHOB PE=1 SV=1 - [RHOB_HUMAN]	1.85	1.95	1.99	1.98	0.41	0.42	0.89	0.88	0.89	0.82	0.44	0.55	-0.91	-1.16	-1.55	-1.00	-1.14	-1.52	-1.65	-0.91
P34096	Ribonuclease 4 OS=Homo sapiens GN=RNASE4 PE=1 SV=3 - [RNAS4_HUMAN]	-0.60	-0.14	-0.41	-0.31	2.79	2.81	1.20	1.32	1.78	1.76	1.53	1.55	1.60	2.61	1.94	2.86	2.20	3.85	3.18	1.17
Q93091	Ribonuclease K6 OS=Homo sapiens GN=RNASE6 PE=1 SV=2 - [RNAS6_HUMAN]	-1.62	-1.30	-1.84	-1.51	1.11	1.43	0.61	0.94	1.41	1.09	1.40	1.72	2.29	3.03	3.24	2.74	2.96	2.71	2.93	2.51
O43251	RNA binding protein fox-1 homolog 2 OS=Homo sapiens GN=RBFOX2 PE=1 SV=3 - [RFOX2_HUMAN]	0.90	1.07	0.88	1.05	-0.72	-0.56	-0.32	-0.15	-0.21	-0.37	-0.62	-0.45	-1.17	-1.51	-1.50	-1.24	-1.22	-1.64	-1.62	-1.14
P29558	RNA-binding motif, single-stranded-interacting protein 1 OS=Homo sapiens GN=RBMS1 PE=1 SV=3 - [RBMS1_HUMAN]	-2.08	-1.96	-2.04	-1.91	-0.61	-0.50	-1.04	-0.92	-0.68	-0.80	-0.58	-0.46	1.09	1.51	1.46	1.31	1.27	1.45	1.40	1.06
P51513	RNA-binding protein Nova-1 OS=Homo sapiens GN=NOVA1 PE=1 SV=1 - [NOVA1_HUMAN]	2.87	2.04	2.51	1.68	0.90	0.06	1.29	0.46	0.54	1.38	1.27	0.44	-1.53	-1.59	-1.24	-1.47	-1.10	-1.99	-1.63	-1.16
Q9UNW9	RNA-binding protein Nova-2 OS=Homo sapiens GN=NOVA2 PE=1 SV=1 - [NOVA2_HUMAN]	1.25	0.68	1.62	1.13	-0.40	-0.64	-0.11	-0.51	-0.74	-0.43	-0.28	-0.77	-0.99	-1.18	-1.59	-1.27	-1.67	-1.38	-1.74	-1.44

A6NHQ2	rRNA/tRNA 2'-O-methyltransferase fibrillarin-like protein 1 OS=Homo sapiens GN=FBLL1 PE=3 SV=1 - [FBLL1_HUMAN]	1.61	1.77	1.90	2.05	0.09	0.24	0.79	0.94	0.50	0.36	0.50	0.64	-0.77	-1.11	-1.40	-1.23	-1.51	-1.54	-1.82	-1.05
Q92736	Ryanodine receptor 2 OS=Homo sapiens GN=RYR2 PE=1 SV=3 - [RYR2_HUMAN]	1.95	1.92	2.12	2.19	-0.25	-0.31	0.42	0.38	0.07	0.23	0.10	0.05	-1.59	-1.82	-2.07	-1.68	-1.86	-2.24	-2.49	-1.69
Q9NZJ4	Sacsin OS=Homo sapiens GN=SACS PE=1 SV=2 - [SACS_HUMAN]	0.85	0.59	1.00	0.58	-0.32	-0.76	0.18	-0.39	-0.50	-0.21	-0.32	-0.61	-0.87	-1.16	-1.32	-1.03	-1.18	-1.31	-1.35	-0.90
Q14714	Sarcospan OS=Homo sapiens GN=SSPN PE=2 SV=3 - [SSPN_HUMAN]	-1.31	-1.36	-1.32	-1.36	0.19	0.15	0.42	0.38	0.31	0.36	0.33	0.29	1.79	1.65	1.65	1.70	1.71	1.49	1.49	1.81
Q86VB7	Scavenger receptor cysteine-rich type 1 protein M130 OS=Homo sapiens GN=CD163 PE=1 SV=2 - [C163A_HUMAN]	-1.19	-1.12	-1.19	-1.10	0.70	0.78	-0.08	0.08	0.62	0.51	0.88	1.03	1.18	2.13	2.10	1.58	1.67	1.77	1.81	1.25
O76054	SEC14-like protein 2 OS=Homo sapiens GN=SEC14L2 PE=1 SV=1 - [S14L2_HUMAN]	1.55	1.46	1.35	1.32	0.34	0.24	0.45	0.39	0.34	0.34	0.32	0.23	-1.00	-1.32	-1.36	-1.03	-1.16	-1.23	-1.37	-0.81
Q9UDX3	SEC14-like protein 4 OS=Homo sapiens GN=SEC14L4 PE=2 SV=1 - [S14L4_HUMAN]	-1.21	-1.08	-1.78	-1.65	0.49	0.61	-0.03	0.10	0.53	0.41	0.42	0.55	1.24	1.64	2.20	1.65	2.22	1.68	2.25	1.81
Q92765	Secreted frizzled-related protein 3 OS=Homo sapiens GN=FRZB PE=1 SV=2 - [SFRP3_HUMAN]	0.13	0.44	-0.52	-0.01	3.33	2.68	1.55	1.49	1.84	2.30	1.30	1.19	1.93	1.16	1.41	2.38	2.42	3.36	3.40	1.97
P13521	Secretogranin-2 OS=Homo sapiens GN=SCG2 PE=1 SV=2 - [SCG2_HUMAN]	1.85	1.73	1.78	1.88	-0.11	-0.11	0.55	0.68	0.36	0.30	0.03	0.10	-1.20	-1.83	-1.82	-1.36	-1.46	-1.90	-2.10	-1.31

O15126	Secretory carrier-associated membrane protein 1 OS=Homo sapiens GN=SCAMP1 PE=1 SV=2 - [SCAM1_HUMAN]	1.10	1.37	1.50	1.57	0.13	0.05	0.37	0.16	0.05	-0.05	0.12	0.17	-0.99	-1.19	-1.42	-1.19	-1.34	-1.31	-1.35	-1.05
Q8TAC9	Secretory carrier-associated membrane protein 5 OS=Homo sapiens GN=SCAMP5 PE=1 SV=1 - [SCAM5_HUMAN]	1.93	1.77	2.31	2.07	-0.14	-0.33	0.76	0.74	0.41	0.31	0.20	0.14	-1.05	-1.93	-2.23	-1.56	-1.94	-2.12	-2.42	-1.37
Q96G97	Seipin OS=Homo sapiens GN=BSCL2 PE=1 SV=3 - [BSCL2_HUMAN]	1.26	1.65	1.16	1.55	-0.24	0.15	0.14	0.54	0.28	-0.11	-0.21	0.18	-1.06	-1.46	-1.37	-1.33	-1.23	-1.52	-1.42	-0.95
Q13214	Semaphorin-3B OS=Homo sapiens GN=SEMA3B PE=2 SV=1 - [SEM3B_HUMAN]	-0.71	-0.86	-0.98	-1.02	1.81	1.84	0.61	0.54	0.99	0.98	0.48	0.48	1.20	1.18	1.46	1.80	2.13	2.68	2.84	1.58
Q9NS98	Semaphorin-3G OS=Homo sapiens GN=SEMA3G PE=2 SV=1 - [SEM3G_HUMAN]	-1.01	-1.08	-1.09	-1.14	2.47	2.39	0.76	0.68	1.39	1.46	0.87	0.84	1.76	1.94	2.12	2.44	2.82	3.40	3.78	2.09
O43236	Septin-4 OS=Homo sapiens GN=SEPT4 PE=1 SV=1 - [SEPT4_HUMAN]	1.39	1.61	1.69	1.91	-0.23	0.06	0.51	0.74	0.29	0.22	0.19	0.22	-0.83	-1.40	-1.46	-1.27	-1.48	-1.67	-1.94	-1.15
Q99719	Septin-5 OS=Homo sapiens GN=SEPT5 PE=1 SV=1 - [SEPT5_HUMAN]	1.40	1.43	1.74	1.82	-0.25	-0.19	0.35	0.51	0.22	0.27	-0.09	0.08	-0.86	-1.37	-1.82	-1.23	-1.59	-1.65	-2.03	-1.12
Q14141	Septin-6 OS=Homo sapiens GN=SEPT6 PE=1 SV=4 - [SEPT6_HUMAN]	1.80	1.15	1.76	1.55	-0.25	-0.87	0.34	0.08	-0.05	0.31	-0.13	-0.16	-1.01	-1.27	-1.89	-1.14	-1.25	-1.45	-1.56	-1.56
Q92599	Septin-8 OS=Homo sapiens GN=SEPT8 PE=1 SV=4 - [SEPT8_HUMAN]	1.20	1.08	1.12	1.07	-0.09	-0.07	0.21	0.13	-0.01	0.07	-0.20	-0.25	-0.92	-1.22	-1.45	-1.13	-1.16	-1.36	-1.11	-0.90
O95084	Serine protease 23 OS=Homo sapiens GN=PRSS23 PE=1 SV=1 - [PRS23_HUMAN]	-1.24	-1.25	-1.30	-1.32	1.78	1.64	0.96	0.69	0.97	1.18	0.94	0.93	1.44	2.27	2.47	2.21	2.39	2.81	3.29	2.20

P83110	Serine protease HTRA3 OS=Homo sapiens GN=HTRA3 PE=1 SV=2 - [HTRA3_HUMAN]	-1.72	-1.58	-1.96	-1.82	2.57	2.70	0.49	0.63	1.50	1.37	0.76	0.90	2.27	2.49	2.72	3.12	3.36	4.28	4.51	2.51
Q9NP81	Serine--tRNA ligase, mitochondrial OS=Homo sapiens GN=SARS2 PE=1 SV=1 - [SYSM_HUMAN]	0.99	1.15	1.02	1.16	-0.62	-0.41	-0.17	-0.07	-0.34	-0.37	-0.16	-0.19	-1.17	-1.26	-1.31	-1.33	-1.30	-1.51	-1.42	-1.14
Q13188	Serine/threonine-protein kinase 3 OS=Homo sapiens GN=STK3 PE=1 SV=2 - [STK3_HUMAN]	-1.49	-1.35	-1.47	-1.33	0.08	0.21	0.04	-0.01	-0.25	-0.38	0.07	0.06	1.39	1.75	1.31	1.14	1.12	1.55	1.53	1.38
Q8TDC3	Serine/threonine-protein kinase BRSK1 OS=Homo sapiens GN=BRSK1 PE=1 SV=2 - [BRSK1_HUMAN]	2.68	2.74	1.75	1.51	0.14	0.18	0.61	0.57	0.26	0.23	-0.07	-0.15	-1.69	-1.90	-1.82	-2.38	-1.57	-2.33	-2.00	-1.08
Q8IWQ3	Serine/threonine-protein kinase BRSK2 OS=Homo sapiens GN=BRSK2 PE=1 SV=3 - [BRSK2_HUMAN]	1.50	1.67	2.36	2.14	0.03	0.13	0.62	0.54	0.01	0.05	0.24	0.36	-0.95	-1.44	-1.75	-1.58	-1.87	-1.65	-1.98	-1.52
Q96Q04	Serine/threonine-protein kinase LMTK3 OS=Homo sapiens GN=LMTK3 PE=2 SV=2 - [LMTK3_HUMAN]	1.49	1.26	1.60	1.37	0.03	-0.21	-0.18	-0.41	-0.30	-0.06	-0.10	-0.34	-1.61	-1.58	-1.70	-1.52	-1.63	-1.48	-1.59	-1.72
Q8TD19	Serine/threonine-protein kinase Nek9 OS=Homo sapiens GN=NEK9 PE=1 SV=2 - [NEK9_HUMAN]	-1.16	-1.62	-1.07	-1.12	0.34	0.00	0.10	-0.32	0.01	0.04	-0.14	0.02	1.31	1.64	1.27	1.54	1.15	1.40	1.24	1.07
Q13153	Serine/threonine-protein kinase PAK 1 OS=Homo sapiens GN=PAK1 PE=1 SV=2 - [PAK1_HUMAN]	1.15	1.13	1.43	1.11	-0.64	-1.00	-0.61	-0.55	-1.04	-0.88	-1.04	-1.32	-1.53	-2.18	-2.47	-2.00	-2.21	-1.87	-2.31	-1.65
O75914	Serine/threonine-protein kinase PAK 3 OS=Homo sapiens GN=PAK3 PE=1 SV=2 - [PAK3_HUMAN]	1.02	1.07	1.30	1.35	-0.26	-0.21	0.03	0.08	-0.44	-0.48	-0.91	-0.86	-0.94	-1.92	-2.21	-1.47	-1.75	-1.29	-1.58	-1.21

Q16537	Serine/threonine-protein phosphatase 2A 56 kDa regulatory subunit epsilon isoform OS=Homo sapiens GN=PPP2R5E PE=1 SV=1 - [2A5E_HUMAN]	1.65	1.49	1.68	1.52	0.23	0.31	0.81	0.64	0.26	0.43	-0.06	-0.12	-0.92	-1.60	-1.67	-1.19	-1.22	-1.40	-1.45	-0.81
Q08209	Serine/threonine-protein phosphatase 2B catalytic subunit alpha isoform OS=Homo sapiens GN=PPP3CA PE=1 SV=1 - [PP2BA_HUMAN]	1.83	1.74	1.96	1.93	0.29	0.29	0.69	0.57	0.37	0.38	0.31	0.21	-1.20	-1.55	-1.48	-1.30	-1.38	-1.48	-1.50	-1.25
P16298	Serine/threonine-protein phosphatase 2B catalytic subunit beta isoform OS=Homo sapiens GN=PPP3CB PE=1 SV=2 - [PP2BB_HUMAN]	1.29	1.05	1.31	1.33	0.37	0.23	0.40	0.29	0.09	0.23	0.01	0.09	-0.93	-1.54	-1.35	-1.05	-1.26	-1.27	-1.29	-0.95
Q96HS1	Serine/threonine-protein phosphatase PGAM5, mitochondrial OS=Homo sapiens GN=PGAM5 PE=1 SV=2 - [PGAM5_HUMAN]	1.74	1.63	1.68	1.62	0.20	0.01	0.18	0.28	0.11	0.28	0.22	0.17	-1.29	-1.54	-1.52	-1.45	-1.43	-1.58	-1.56	-1.28
P50454	Serpin H1 OS=Homo sapiens GN=SERPINH1 PE=1 SV=2 - [SERPH_HUMAN]	-2.39	-2.23	-2.46	-2.41	-0.48	-0.49	-1.17	-1.08	-1.04	-1.09	-0.94	-0.85	0.98	1.34	1.43	1.30	1.45	1.68	1.84	1.19
P35542	Serum amyloid A-4 protein OS=Homo sapiens GN=SAA4 PE=1 SV=2 - [SAA4_HUMAN]	-3.16	-3.07	-3.21	-3.13	0.01	0.09	-0.88	-0.88	-0.27	-0.35	-0.40	-0.45	2.36	2.56	2.60	2.81	2.46	3.15	2.80	2.02
P02743	Serum amyloid P-component OS=Homo sapiens GN=APCS PE=1 SV=2 - [SAMP_HUMAN]	-0.93	-1.01	-1.67	-1.76	1.69	1.59	1.06	0.96	1.00	1.04	0.38	0.26	2.05	1.28	2.01	2.44	2.72	2.71	3.31	2.55

O95810	Serum deprivation-response protein OS=Homo sapiens GN=SDPR PE=1 SV=3 - [SDPR_HUMAN]	-1.69	-1.95	-1.87	-1.85	-0.15	-0.19	-0.34	-0.29	-0.36	-0.29	-0.19	-0.23	1.53	1.68	1.65	1.63	1.52	1.61	1.66	1.50
P27169	Serum paraoxonase/arylesterase 1 OS=Homo sapiens GN=PON1 PE=1 SV=3 - [PON1_HUMAN]	-1.35	-1.64	-1.64	-1.66	1.23	1.05	0.73	0.76	0.97	0.79	1.32	1.31	2.52	3.03	2.99	2.72	2.76	2.92	2.78	2.56
Q9Y566	SH3 and multiple ankyrin repeat domains protein 1 OS=Homo sapiens GN=SHANK1 PE=1 SV=2 - [SHAN1_HUMAN]	1.74	1.25	1.75	1.26	-0.09	-0.59	0.74	0.25	-0.06	0.44	0.40	-0.09	-0.94	-1.33	-1.35	-1.27	-1.28	-1.85	-1.86	-0.94
Q9UPX8	SH3 and multiple ankyrin repeat domains protein 2 OS=Homo sapiens GN=SHANK2 PE=1 SV=3 - [SHAN2_HUMAN]	1.36	1.46	1.96	2.05	-0.15	-0.06	0.41	0.49	-0.04	-0.14	-0.17	0.04	-1.08	-1.57	-2.01	-1.47	-1.65	-1.65	-2.11	-1.50
Q9BYB0	SH3 and multiple ankyrin repeat domains protein 3 OS=Homo sapiens GN=SHANK3 PE=1 SV=3 - [SHAN3_HUMAN]	1.84	1.59	1.76	1.68	0.03	-0.05	0.00	-0.04	-0.07	0.03	-0.14	-0.47	-1.37	-1.81	-1.83	-1.67	-1.70	-1.70	-1.73	-1.41
Q5HYK7	SH3 domain-containing protein 19 OS=Homo sapiens GN=SH3D19 PE=1 SV=2 - [SH319_HUMAN]	-1.69	-1.80	-1.74	-1.85	0.34	0.23	0.09	-0.05	-0.06	0.05	-0.08	-0.15	1.52	1.69	1.66	1.57	1.67	1.70	1.91	1.75
Q9BQI5	SH3-containing GRB2-like protein 3-interacting protein 1 OS=Homo sapiens GN=SGIP1 PE=1 SV=2 - [SGIP1_HUMAN]	1.41	1.40	1.56	1.40	-0.01	0.01	0.02	0.20	0.11	0.05	-0.14	-0.02	-1.11	-1.43	-1.59	-0.96	-1.22	-1.24	-1.51	-1.27
Q92529	SHC-transforming protein 3 OS=Homo sapiens GN=SHC3 PE=1 SV=1 - [SHC3_HUMAN]	1.15	1.47	1.26	1.48	0.06	0.11	0.27	0.61	0.18	0.04	-0.03	0.02	-0.83	-1.43	-1.46	-1.43	-1.45	-1.37	-1.38	-0.84

A0MZ66	Shootin-1 OS=Homo sapiens GN=KIAA1598 PE=1 SV=4 - [SHOT1_HUMAN]	1.51	1.51	1.82	1.76	0.08	0.14	0.59	0.57	0.35	0.29	-0.02	0.01	-0.81	-1.49	-1.60	-1.25	-1.47	-1.48	-1.71	-1.03
Q9BZZ2	Sialoadhesin OS=Homo sapiens GN=SIGLEC1 PE=1 SV=2 - [SN_HUMAN]	-0.29	-0.21	-0.47	-0.46	0.62	0.28	0.06	-0.16	0.14	0.48	1.08	0.74	0.91	2.29	2.46	1.80	2.10	1.90	2.10	1.13
P67812	Signal peptidase complex catalytic subunit SEC11A OS=Homo sapiens GN=SEC11A PE=1 SV=1 - [SC11A_HUMAN]	-1.98	-2.01	-2.03	-2.00	-0.04	-0.05	-0.61	-0.61	-0.30	-0.42	-0.28	-0.19	1.42	1.75	1.75	1.65	1.64	1.98	1.97	1.42
Q8IWY4	Signal peptide, CUB and EGF-like domain-containing protein 1 OS=Homo sapiens GN=SCUBE1 PE=1 SV=3 - [SCUB1_HUMAN]	-1.32	-1.25	-0.90	-0.82	1.59	1.66	0.93	1.00	1.05	0.98	0.36	0.43	2.31	1.69	1.26	2.34	1.92	2.89	2.47	1.89
Q8IX30	Signal peptide, CUB and EGF-like domain-containing protein 3 OS=Homo sapiens GN=SCUBE3 PE=1 SV=1 - [SCUB3_HUMAN]	-0.72	-0.97	-1.02	-1.17	2.15	1.92	1.13	0.99	1.00	1.51	0.92	0.53	2.00	1.55	2.03	2.29	2.47	2.95	3.15	2.29
Q8ND83	SLAIN motif-containing protein 1 OS=Homo sapiens GN=SLAIN1 PE=1 SV=3 - [SLAI1_HUMAN]	0.64	1.14	0.92	1.42	-0.51	-0.01	-0.23	0.27	-0.09	-0.59	-0.33	0.16	-0.81	-0.97	-1.26	-1.20	-1.48	-1.17	-1.45	-1.09
O75094	Slit homolog 3 protein OS=Homo sapiens GN=SLIT3 PE=2 SV=3 - [SLIT3_HUMAN]	0.19	0.08	-0.27	-0.23	2.75	2.92	1.73	1.73	1.86	2.02	1.34	1.24	1.78	1.26	1.57	2.29	2.37	3.30	3.21	1.96
Q96EQ0	Small glutamine-rich tetratricopeptide repeat-containing protein beta OS=Homo sapiens GN=SGTB PE=1 SV=1 - [SGTB_HUMAN]	1.48	1.61	1.62	1.84	-0.22	-0.14	0.23	0.41	0.07	-0.01	-0.24	-0.24	-1.15	-1.73	-2.08	-1.51	-1.79	-1.66	-1.85	-1.32

Q8NHG7	Small VCP/p97-interacting protein OS=Homo sapiens GN=SVIP PE=3 SV=1 - [SVIP_HUMAN]	0.98	1.33	1.46	1.50	-0.63	-0.93	-0.12	-0.27	-0.33	-0.48	-0.16	-0.46	-0.96	-1.10	-1.62	-1.43	-1.60	-1.54	-2.12	-1.52
P53814	Smoothelin OS=Homo sapiens GN=SMTN PE=1 SV=7 - [SMTN_HUMAN]	-2.70	-2.63	-3.23	-3.28	-1.21	-1.14	-1.35	-1.36	-1.28	-1.30	-1.02	-1.04	1.24	1.52	2.23	1.52	2.21	1.67	2.19	1.96
P35498	Sodium channel protein type 1 subunit alpha OS=Homo sapiens GN=SCN1A PE=1 SV=2 - [SCN1A_HUMAN]	1.62	1.74	2.11	2.26	0.20	0.32	0.52	0.77	0.46	0.30	0.15	0.25	-1.18	-1.81	-2.12	-1.58	-1.92	-1.85	-2.27	-1.51
Q99250	Sodium channel protein type 2 subunit alpha OS=Homo sapiens GN=SCN2A PE=1 SV=3 - [SCN2A_HUMAN]	1.87	1.73	2.07	1.98	-0.48	-0.75	0.44	0.36	-0.09	-0.01	-0.16	-0.39	-1.28	-1.79	-2.38	-1.78	-2.23	-2.08	-2.53	-1.55
Q9NSD5	Sodium- and chloride-dependent GABA transporter 2 OS=Homo sapiens GN=SLC6A13 PE=1 SV=3 - [S6A13_HUMAN]	-1.78	-1.61	-1.92	-1.77	0.12	0.21	-0.10	0.00	0.00	0.03	-0.03	-0.10	1.68	1.82	2.08	1.78	2.05	1.88	2.13	2.05
P48066	Sodium- and chloride-dependent GABA transporter 3 OS=Homo sapiens GN=SLC6A11 PE=2 SV=1 - [S6A11_HUMAN]	2.28	2.28	2.26	2.26	-0.49	-0.49	0.50	0.50	0.11	0.11	-0.37	-0.38	-1.37	-2.33	-2.38	-1.85	-1.88	-2.54	-2.58	-1.53
Q9H1V8	Sodium-dependent neutral amino acid transporter SLC6A17 OS=Homo sapiens GN=SLC6A17 PE=2 SV=3 - [S6A17_HUMAN]	1.98	2.20	1.82	2.10	-0.28	-0.11	0.70	0.95	0.46	0.31	0.34	0.44	-1.10	-1.78	-1.85	-1.27	-1.25	-2.44	-2.39	-1.08
Q6U841	Sodium-driven chloride bicarbonate exchanger OS=Homo sapiens GN=SLC4A10 PE=2 SV=1 - [S4A10_HUMAN]	1.89	1.76	1.89	1.87	0.05	-0.13	0.48	0.55	0.12	0.29	0.30	0.05	-1.38	-2.23	-2.50	-1.70	-1.69	-1.91	-2.13	-1.46

P32418	Sodium/calcium exchanger 1 OS=Homo sapiens GN=SLC8A1 PE=1 SV=3 - [NAC1_HUMAN]	2.06	1.90	2.47	2.25	-0.04	-0.01	0.88	0.91	0.49	0.48	0.48	0.46	-1.08	-1.63	-1.98	-1.57	-1.76	-1.99	-2.36	-1.34
Q9UPR5	Sodium/calcium exchanger 2 OS=Homo sapiens GN=SLC8A2 PE=2 SV=2 - [NAC2_HUMAN]	2.14	1.76	2.34	2.03	0.14	-0.01	1.06	0.67	0.52	0.57	0.51	0.29	-0.97	-1.58	-1.81	-1.29	-1.45	-1.73	-2.05	-1.20
Q92581	Sodium/hydrogen exchanger 6 OS=Homo sapiens GN=SLC9A6 PE=1 SV=2 - [SL9A6_HUMAN]	1.67	1.68	1.66	1.70	0.13	-0.07	0.60	0.63	0.25	0.22	0.20	0.23	-0.99	-1.32	-1.17	-1.28	-1.12	-1.76	-1.83	-1.00
P53794	Sodium/myo-inositol cotransporter OS=Homo sapiens GN=SLC5A3 PE=3 SV=2 - [SC5A3_HUMAN]	-0.55	-0.73	-0.48	-0.65	1.10	0.93	0.63	0.45	0.70	0.88	0.64	0.47	1.23	1.20	1.12	1.46	1.39	1.64	1.56	1.16
P05023	Sodium/potassium-transporting ATPase subunit alpha-1 OS=Homo sapiens GN=ATP1A1 PE=1 SV=1 - [AT1A1_HUMAN]	1.83	1.91	2.03	2.08	-0.05	-0.01	0.73	0.81	0.33	0.28	0.18	0.19	-0.94	-1.68	-1.76	-1.51	-1.61	-1.94	-2.01	-1.15
P13637	Sodium/potassium-transporting ATPase subunit alpha-3 OS=Homo sapiens GN=ATP1A3 PE=1 SV=3 - [AT1A3_HUMAN]	1.98	1.90	2.13	2.02	-0.22	-0.08	0.72	0.81	0.43	0.36	0.08	0.21	-1.17	-1.76	-1.81	-1.54	-1.71	-2.00	-2.23	-1.26
P05026	Sodium/potassium-transporting ATPase subunit beta-1 OS=Homo sapiens GN=ATP1B1 PE=1 SV=1 - [AT1B1_HUMAN]	1.95	1.92	2.41	2.28	0.09	0.07	1.05	0.95	0.44	0.59	0.40	0.30	-0.88	-1.69	-2.06	-1.20	-1.73	-1.88	-2.20	-1.30
P54709	Sodium/potassium-transporting ATPase subunit beta-3 OS=Homo sapiens GN=ATP1B3 PE=1 SV=1 - [AT1B3_HUMAN]	-2.13	-2.15	-1.90	-2.08	0.17	0.23	0.05	0.09	0.02	-0.01	-0.03	0.04	2.32	2.11	2.12	2.24	1.98	2.30	2.38	2.23

Q9UI40	Sodium/potassium/calcium exchanger 2 OS=Homo sapiens GN=SLC24A2 PE=1 SV=1 - [NCKX2_HUMAN]	2.15	2.45	2.30	2.54	-0.40	-0.10	0.86	1.18	0.87	0.73	0.03	0.31	-1.07	-2.11	-2.22	-1.34	-1.41	-2.57	-2.75	-1.14
Q9H2X9	Solute carrier family 12 member 5 OS=Homo sapiens GN=SLC12A5 PE=2 SV=3 - [S12A5_HUMAN]	2.43	2.33	2.41	2.56	-0.06	-0.08	0.82	0.96	0.43	0.46	0.22	0.24	-1.36	-2.16	-2.08	-1.83	-1.94	-2.38	-2.49	-1.45
Q9UKG4	Solute carrier family 13 member 4 OS=Homo sapiens GN=SLC13A4 PE=2 SV=2 - [S13A4_HUMAN]	-2.45	-2.40	-2.39	-2.34	0.30	0.35	-0.06	-0.01	0.03	-0.02	0.01	0.06	2.45	2.47	2.40	2.46	2.41	2.73	2.68	2.40
P11166	Solute carrier family 2, facilitated glucose transporter member 1 OS=Homo sapiens GN=SLC2A1 PE=1 SV=2 - [GTR1_HUMAN]	-1.50	-1.51	-1.69	-1.63	0.14	-0.05	0.01	-0.09	0.35	0.41	0.98	0.98	1.56	2.47	2.62	1.73	1.95	1.54	1.57	1.82
Q4U2R8	Solute carrier family 22 member 6 OS=Homo sapiens GN=SLC22A6 PE=1 SV=1 - [S22A6_HUMAN]	-1.64	-1.85	-3.02	-3.06	0.18	0.26	0.03	0.04	0.00	-0.05	-0.24	-0.22	1.89	2.09	2.87	1.98	3.13	2.38	3.38	3.24
Q8TCC7	Solute carrier family 22 member 8 OS=Homo sapiens GN=SLC22A8 PE=1 SV=1 - [S22A8_HUMAN]	-2.54	-2.52	-2.53	-2.51	0.47	0.56	-0.02	-0.04	0.29	0.33	0.42	0.44	2.42	2.97	2.95	2.37	2.36	2.55	2.54	2.42
Q86XE0	Sorting nexin-32 OS=Homo sapiens GN=SNX32 PE=2 SV=1 - [SNX32_HUMAN]	2.68	1.73	2.34	1.39	0.88	-0.07	0.88	-0.07	-0.10	0.85	0.34	-0.62	-1.74	-2.33	-2.01	-1.79	-1.46	-1.81	-1.48	-1.40
Q9H4F8	SPARC-related modular calcium-binding protein 1 OS=Homo sapiens GN=SMOC1 PE=1 SV=1 - [SMOC1_HUMAN]	0.09	-0.06	-0.22	-0.40	2.07	2.06	1.85	2.29	2.05	1.99	2.05	2.05	2.32	2.44	2.70	2.38	2.74	2.21	2.64	2.68

P02549	Spectrin alpha chain, erythrocytic 1 OS=Homo sapiens GN=SPTA1 PE=1 SV=5 - [SPTA1_HUMAN]	-1.47	-1.35	-1.69	-1.57	0.32	0.38	0.48	0.55	0.69	0.64	1.25	1.33	1.90	2.69	2.92	2.10	2.30	1.74	1.93	2.22
P11277	Spectrin beta chain, erythrocytic OS=Homo sapiens GN=SPTB PE=1 SV=5 - [SPTB1_HUMAN]	-1.20	-1.10	-1.29	-1.27	0.42	0.45	0.47	0.51	0.76	0.75	1.36	1.39	1.73	2.51	2.60	1.95	2.02	1.50	1.64	1.83
O15020	Spectrin beta chain, non-erythrocytic 2 OS=Homo sapiens GN=SPTBN2 PE=1 SV=3 - [SPTN2_HUMAN]	1.59	1.66	1.84	1.80	-0.27	-0.29	0.48	0.47	0.23	0.17	0.04	0.07	-1.20	-1.69	-1.75	-1.49	-1.58	-2.02	-2.09	-1.22
Q9H254	Spectrin beta chain, non-erythrocytic 4 OS=Homo sapiens GN=SPTBN4 PE=1 SV=2 - [SPTN4_HUMAN]	1.45	1.56	1.71	1.57	0.12	0.15	0.61	0.59	0.23	0.40	0.16	0.24	-1.20	-1.38	-1.54	-1.23	-1.21	-1.45	-1.57	-1.05
Q96SI9	Spermatid perinuclear RNA-binding protein OS=Homo sapiens GN=STRBP PE=1 SV=1 - [STRBP_HUMAN]	2.08	1.61	1.69	1.22	0.41	-0.06	0.33	-0.13	-0.08	0.39	0.25	-0.23	-1.69	-1.82	-1.44	-1.66	-1.27	-1.68	-1.29	-1.29
Q9NY59	Sphingomyelin phosphodiesterase 3 OS=Homo sapiens GN=SMPD3 PE=1 SV=1 - [NSMA2_HUMAN]	1.49	1.42	1.55	1.48	0.41	0.33	0.18	0.10	0.06	0.14	0.22	0.14	-1.26	-1.26	-1.33	-1.32	-1.38	-1.10	-1.16	-1.31
Q9C0H9	SRC kinase signaling inhibitor 1 OS=Homo sapiens GN=SRCIN1 PE=1 SV=3 - [SRCN1_HUMAN]	1.59	1.52	1.82	1.75	-0.29	-0.33	0.40	0.41	0.13	0.09	-0.04	-0.02	-1.05	-1.67	-1.96	-1.43	-1.63	-1.84	-2.02	-1.37
Q9NY15	Stabilin-1 OS=Homo sapiens GN=STAB1 PE=1 SV=3 - [STAB1_HUMAN]	-1.41	-1.23	-1.54	-1.63	0.71	0.94	-0.34	-0.14	0.29	0.17	-0.02	0.11	1.19	1.45	1.61	1.68	1.88	2.29	2.56	1.52
P16949	Stathmin OS=Homo sapiens GN=STMN1 PE=1 SV=3 - [STMN1_HUMAN]	1.86	1.75	2.18	2.28	-0.38	-0.52	0.18	0.29	-0.39	-0.38	-0.83	-0.61	-1.36	-2.56	-2.88	-1.96	-2.23	-1.90	-2.38	-1.81

Q93045	Stathmin-2 OS=Homo sapiens GN=STMN2 PE=1 SV=3 - [STMN2_HUMAN]	1.50	1.67	1.78	1.95	0.19	0.35	0.29	0.46	0.36	0.20	-0.27	-0.10	-1.16	-1.76	-2.05	-1.27	-1.55	-1.33	-1.61	-1.42
Q7RTN6	STE20-related kinase adapter protein alpha OS=Homo sapiens GN=STRADA PE=1 SV=1 - [STRAA_HUMAN]	2.25	2.01	2.29	2.05	0.20	-0.04	1.08	0.85	0.40	0.64	0.79	0.55	-1.11	-1.46	-1.50	-1.58	-1.61	-2.07	-2.10	-1.14
Q9BX79	Stimulated by retinoic acid gene 6 protein homolog OS=Homo sapiens GN=STRA6 PE=1 SV=1 - [STRA6_HUMAN]	-2.35	-2.29	-2.47	-2.41	-0.72	-0.66	-0.95	-0.89	-0.69	-0.74	-0.66	-0.60	1.45	1.70	1.81	1.64	1.76	1.61	1.74	1.58
P48061	Stromal cell-derived factor 1 OS=Homo sapiens GN=CXCL12 PE=1 SV=1 - [SDF1_HUMAN]	-0.94	-0.67	-1.06	-0.73	3.22	3.60	1.16	1.50	2.42	2.14	1.09	1.62	1.93	1.98	2.41	3.39	3.14	4.42	4.17	2.15
P09238	Stromelysin-2 OS=Homo sapiens GN=MMP10 PE=1 SV=1 - [MMP10_HUMAN]	-0.66	-0.56	-0.50	-0.54	3.96	3.81	1.67	1.58	2.58	2.62	2.06	1.69	2.39	2.72	2.55	3.31	3.15	4.60	4.44	2.24
P51649	Succinate-semialdehyde dehydrogenase, mitochondrial OS=Homo sapiens GN=ALDH5A1 PE=1 SV=2 - [SSDH_HUMAN]	1.38	1.38	1.70	1.54	-0.38	-0.35	-0.48	-0.45	-0.62	-0.67	-0.47	-0.61	-1.73	-2.00	-2.29	-1.98	-2.26	-1.85	-2.00	-2.18
P55809	Succinyl-CoA:3-ketoacid coenzyme A transferase 1, mitochondrial OS=Homo sapiens GN=OXCT1 PE=1 SV=1 - [SCOT1_HUMAN]	1.35	1.26	1.39	1.23	-0.29	-0.25	0.33	0.35	-0.01	0.01	-0.13	-0.21	-0.85	-1.58	-1.71	-1.26	-1.37	-1.64	-1.72	-1.03
Q9Y6N5	Sulfide:quinone oxidoreductase, mitochondrial OS=Homo sapiens GN=SQRDL PE=1 SV=1 - [SQRD_HUMAN]	-1.95	-1.86	-1.86	-1.88	0.28	0.34	-0.17	-0.18	-0.05	-0.08	0.01	0.02	1.75	1.94	1.89	1.91	1.87	2.15	2.14	1.77

P50224	Sulfotransferase 1A3/1A4 OS=Homo sapiens GN=SULT1A3 PE=1 SV=1 - [ST1A3_HUMAN]	-2.19	-1.77	-2.06	-1.64	-0.53	-0.11	-1.09	-0.66	-0.59	-1.01	-0.59	-0.17	1.16	1.61	1.47	1.21	1.09	1.65	1.52	1.04
O95425	Supervillin OS=Homo sapiens GN=SVIL PE=1 SV=2 - [SVIL_HUMAN]	-1.74	-1.81	-2.26	-2.23	-0.13	-0.18	-0.46	-0.45	-0.33	-0.39	-0.15	-0.18	1.54	1.75	2.07	1.65	1.90	1.64	1.99	1.89
Q9UGT4	Sushi domain-containing protein 2 OS=Homo sapiens GN=SUSD2 PE=1 SV=1 - [SUSD2_HUMAN]	-1.64	-1.58	-1.86	-1.95	0.51	0.56	-0.34	-0.25	0.10	-0.01	0.17	0.22	1.31	1.78	2.19	1.66	2.07	2.31	2.32	1.59
O60279	Sushi domain-containing protein 5 OS=Homo sapiens GN=SUSD5 PE=1 SV=3 - [SUSD5_HUMAN]	-1.98	-2.04	-1.84	-1.90	0.39	0.33	0.58	0.52	0.21	0.28	0.26	0.19	2.62	2.24	2.10	2.29	2.15	2.36	2.22	2.48
P78539	Sushi repeat-containing protein SRPX OS=Homo sapiens GN=SRPX PE=2 SV=1 - [SRPX_HUMAN]	-0.59	-0.72	-0.73	-0.77	2.98	2.62	1.11	1.05	1.70	1.50	0.87	0.66	1.58	1.38	1.60	2.20	2.58	3.35	3.50	2.02
O60687	Sushi repeat-containing protein SRPX2 OS=Homo sapiens GN=SRPX2 PE=1 SV=1 - [SRPX2_HUMAN]	-1.57	-1.42	-1.68	-1.64	0.04	0.25	-0.37	-0.02	0.05	-0.29	-0.42	-0.22	1.26	1.50	1.65	1.31	1.63	1.77	1.71	1.68
Q8TER0	Sushi, nidogen and EGF-like domain-containing protein 1 OS=Homo sapiens GN=SNED1 PE=2 SV=2 - [SNED1_HUMAN]	-1.34	-1.26	-1.23	-1.25	1.04	1.30	0.43	0.61	0.86	0.79	0.74	0.97	1.87	2.08	2.03	2.17	2.11	2.57	2.07	1.83
P17600	Synapsin-1 OS=Homo sapiens GN=SYN1 PE=1 SV=3 - [SYN1_HUMAN]	1.43	1.35	1.70	1.57	0.10	0.04	0.56	0.49	0.15	0.29	-0.07	-0.13	-0.86	-1.41	-1.66	-1.16	-1.36	-1.44	-1.54	-1.02
O14994	Synapsin-3 OS=Homo sapiens GN=SYN3 PE=1 SV=2 - [SYN3_HUMAN]	1.78	1.71	2.09	1.99	-0.19	-0.08	0.71	0.51	0.36	0.33	0.08	0.00	-1.15	-1.81	-2.11	-1.57	-1.86	-2.06	-2.52	-1.32

Q7L0J3	Synaptic vesicle glycoprotein 2A OS=Homo sapiens GN=SV2A PE=1 SV=1 - [SV2A_HUMAN]	2.03	2.20	2.14	2.21	-0.15	-0.18	1.09	1.11	0.51	0.64	0.31	0.24	-0.77	-1.68	-1.96	-1.36	-1.69	-1.95	-2.04	-1.09
O43761	Synaptogyrin-3 OS=Homo sapiens GN=SYNGR3 PE=1 SV=2 - [SNG3_HUMAN]	1.33	1.35	1.11	1.07	-0.27	-0.14	0.21	0.21	0.27	0.40	0.08	0.21	-1.06	-1.64	-1.43	-1.29	-1.16	-1.68	-1.37	-0.83
Q9UMS6	Synaptopodin-2 OS=Homo sapiens GN=SYNPO2 PE=1 SV=2 - [SYNP2_HUMAN]	-2.21	-2.15	-2.60	-2.40	-0.96	-1.02	-1.25	-1.21	-1.03	-1.08	-0.65	-0.60	1.02	1.58	1.66	1.37	1.40	1.28	1.31	1.14
P60880	Synaptosomal-associated protein 25 OS=Homo sapiens GN=SNAP25 PE=1 SV=1 - [SNP25_HUMAN]	1.56	1.63	1.46	1.54	-0.28	-0.22	0.37	0.43	0.18	0.14	0.11	0.07	-1.20	-1.59	-1.51	-1.53	-1.49	-1.76	-1.73	-1.24
P21579	Synaptotagmin-1 OS=Homo sapiens GN=SYT1 PE=1 SV=1 - [SYT1_HUMAN]	1.49	1.53	1.72	1.62	0.09	0.03	0.66	0.50	0.30	0.39	0.23	0.16	-1.03	-1.33	-1.48	-1.20	-1.25	-1.49	-1.56	-1.03
Q8IV01	Synaptotagmin-12 OS=Homo sapiens GN=SYT12 PE=2 SV=1 - [SYT12_HUMAN]	2.17	2.05	2.05	1.93	0.10	-0.11	0.81	0.76	0.34	0.56	0.28	0.09	-1.18	-1.94	-1.81	-1.69	-1.52	-2.15	-2.05	-1.14
Q8N9I0	Synaptotagmin-2 OS=Homo sapiens GN=SYT2 PE=1 SV=2 - [SYT2_HUMAN]	2.43	2.48	2.27	2.58	0.13	0.23	1.39	1.47	1.16	1.16	0.70	0.67	-0.78	-1.92	-2.09	-1.34	-1.78	-2.14	-2.45	-1.08
Q9BQG1	Synaptotagmin-3 OS=Homo sapiens GN=SYT3 PE=2 SV=1 - [SYT3_HUMAN]	2.01	1.91	2.23	2.13	0.01	-0.09	0.75	0.66	0.49	0.59	0.41	0.31	-1.20	-1.59	-1.82	-1.39	-1.60	-2.02	-2.24	-1.41
Q96C24	Synaptotagmin-like protein 4 OS=Homo sapiens GN=SYTL4 PE=1 SV=2 - [SYTL4_HUMAN]	-1.09	-1.02	-1.04	-0.89	0.65	0.71	-0.02	0.13	0.67	0.62	0.53	0.59	1.34	1.28	1.34	1.66	1.73	1.72	2.08	1.08
P34741	Syndecan-2 OS=Homo sapiens GN=SDC2 PE=1 SV=2 - [SDC2_HUMAN]	-1.57	-1.68	-1.75	-1.85	2.81	2.70	1.61	1.50	1.62	1.73	1.26	1.14	3.24	2.83	3.00	3.33	3.51	4.37	4.54	3.42

O15079	Syntaphilin OS=Homo sapiens GN=SNPH PE=1 SV=2 - [SNPH_HUMAN]	2.62	2.55	2.94	2.88	0.28	0.08	1.41	1.35	0.54	0.76	0.60	0.32	-1.16	-1.99	-2.31	-1.72	-2.08	-1.94	-2.25	-1.46
Q16623	Syntaxin-1A OS=Homo sapiens GN=STX1A PE=1 SV=1 - [STX1A_HUMAN]	1.32	1.24	1.40	1.58	-0.35	-0.26	0.39	0.49	0.05	0.11	-0.21	-0.10	-0.86	-1.53	-1.77	-1.34	-1.60	-1.64	-1.90	-1.19
P61266	Syntaxin-1B OS=Homo sapiens GN=STX1B PE=1 SV=1 - [STX1B_HUMAN]	1.90	1.85	1.88	2.12	-0.03	-0.14	0.75	0.87	0.24	0.36	0.27	0.29	-1.08	-1.76	-2.02	-1.54	-1.61	-1.87	-1.94	-1.18
P61764	Syntaxin-binding protein 1 OS=Homo sapiens GN=STXBP1 PE=1 SV=1 - [STXB1_HUMAN]	1.90	1.87	2.12	2.08	-0.09	-0.07	0.76	0.72	0.20	0.36	0.20	0.21	-1.18	-1.68	-1.91	-1.59	-1.78	-1.99	-2.16	-1.32
Q5T5C0	Syntaxin-binding protein 5 OS=Homo sapiens GN=STXBP5 PE=1 SV=1 - [STXB5_HUMAN]	1.72	1.64	1.41	1.92	-0.40	-0.25	0.56	0.86	0.72	0.24	0.18	0.36	-0.85	-1.62	-1.88	-1.49	-1.13	-1.90	-2.19	-0.81
O00560	Syntenin-1 OS=Homo sapiens GN=SDCBP PE=1 SV=1 - [SDCB1_HUMAN]	-0.87	-0.66	-0.61	-0.39	1.39	1.32	0.61	0.69	0.80	0.80	0.95	1.02	1.05	1.52	1.32	1.17	1.23	1.42	1.70	0.99
Q13009	T-lymphoma invasion and metastasis-inducing protein 1 OS=Homo sapiens GN=TIAM1 PE=1 SV=2 - [TIAM1_HUMAN]	1.75	1.61	1.69	1.55	0.03	-0.11	-0.07	-0.21	-0.39	-0.24	-0.31	-0.46	-1.77	-2.05	-2.01	-1.96	-1.90	-1.73	-1.68	-1.70
Q7Z7G0	Target of Nesh SH3 OS=Homo sapiens GN=ABI3BP PE=1 SV=1 - [TARSH_HUMAN]	-2.27	-2.27	-2.65	-2.47	-0.69	-0.67	-0.25	-0.21	-0.33	-0.39	-0.46	-0.48	1.97	1.77	1.68	1.98	1.86	1.70	1.80	2.17
Q4KMP7	TBC1 domain family member 10B OS=Homo sapiens GN=TBC1D10B PE=1 SV=3 [TB10B_HUMAN]	1.27	1.15	1.39	1.30	-0.16	-0.28	0.12	0.13	-0.01	0.02	0.11	-0.01	-1.14	-1.22	-1.24	-1.18	-1.23	-1.41	-1.57	-1.01
Q92752	Tenascin-R OS=Homo sapiens GN=TNR PE=1 SV=3 - [TENR_HUMAN]	1.66	1.70	1.64	1.75	-0.03	0.01	0.58	0.70	0.46	0.39	0.21	0.32	-0.95	-1.38	-1.39	-1.30	-1.24	-1.59	-1.54	-0.95

P22105	Tenascin-X OS=Homo sapiens GN=TNXB PE=1 SV=3 - [TENX_HUMAN]	-2.01	-2.06	-2.35	-2.24	-0.27	-0.24	-1.01	-0.99	-0.76	-0.76	-0.92	-0.93	1.24	1.18	1.44	1.37	1.57	1.85	2.07	1.39
P05452	Tetranectin OS=Homo sapiens GN=CLEC3B PE=1 SV=3 - [TETN_HUMAN]	-2.15	-2.02	-1.85	-1.70	-0.23	0.05	-0.51	-0.24	-0.21	-0.34	-0.24	-0.10	1.38	1.91	1.92	1.69	1.70	1.76	1.77	1.54
Q8NG11	Tetraspanin-14 OS=Homo sapiens GN=TSPAN14 PE=1 SV=1 - [TSN14_HUMAN]	-1.21	-1.27	-1.28	-1.34	0.34	0.28	0.00	-0.06	-0.03	0.04	0.17	0.10	1.26	1.38	1.45	1.28	1.35	1.53	1.60	1.34
Q96SJ8	Tetraspanin-18 OS=Homo sapiens GN=TSPAN18 PE=2 SV=1 - [TSN18_HUMAN]	-2.92	-2.49	-3.30	-2.87	0.09	0.51	-0.33	0.10	0.13	-0.29	0.01	0.43	2.64	2.93	3.30	2.66	3.04	2.99	3.37	3.03
P41732	Tetraspanin-7 OS=Homo sapiens GN=TSPAN7 PE=1 SV=2 - [TSN7_HUMAN]	1.71	1.64	1.42	1.35	-0.72	-0.80	0.01	-0.05	0.00	0.07	-0.71	-0.78	-1.64	-2.41	-2.13	-1.61	-1.32	-2.45	-2.16	-1.34
Q8N584	Tetratricopeptide repeat protein 39C OS=Homo sapiens GN=TTC39C PE=2 SV=2 - [TT39C_HUMAN]	2.07	2.65	2.18	2.75	-0.23	0.34	1.12	1.69	0.97	0.40	0.07	0.64	-0.90	-1.99	-2.11	-1.64	-1.74	-2.32	-2.42	-0.99
Q92623	Tetratricopeptide repeat protein 9A OS=Homo sapiens GN=TTC9 PE=2 SV=3 - [TTC9A_HUMAN]	1.30	0.84	1.27	0.81	0.09	-0.38	-0.20	-0.67	-0.53	-0.06	-0.07	-0.54	-1.19	-1.37	-1.34	-1.33	-1.30	-1.23	-1.20	-1.03
Q8WY91	THAP domain-containing protein 4 OS=Homo sapiens GN=THAP4 PE=1 SV=2 - [THAP4_HUMAN]	3.38	2.53	3.39	2.55	1.50	0.65	1.98	1.13	1.36	2.21	1.82	0.97	-1.35	-1.55	-1.58	-1.14	-1.15	-1.89	-1.91	-1.35
Q8NBS9	Thioredoxin domain-containing protein 5 OS=Homo sapiens GN=TXNDC5 PE=1 SV=2 - [TXND5_HUMAN]	-1.39	-1.27	-1.44	-1.31	0.23	0.35	-0.17	-0.10	0.07	-0.11	0.04	0.14	1.22	1.40	1.49	1.31	1.41	1.57	1.65	1.25
P07204	Thrombomodulin OS=Homo sapiens GN=THBD PE=1 SV=2 - [TRBM_HUMAN]	-2.03	-2.42	-2.69	-3.08	-0.09	-0.48	-1.02	-1.41	-0.69	-0.29	0.30	-0.10	1.07	2.33	2.98	1.77	2.43	1.92	2.58	1.74

Q6ZMP0	Thrombospondin type-1 domain-containing protein 4 OS=Homo sapiens GN=THSD4 PE=2 SV=2 - [THSD4_HUMAN]	-1.61	-1.38	-1.38	-1.08	0.75	0.76	0.24	0.33	0.43	0.30	0.30	0.35	1.90	1.77	1.40	1.80	1.39	2.06	1.83	1.46
P07996	Thrombospondin-1 OS=Homo sapiens GN=THBS1 PE=1 SV=2 - [TSP1_HUMAN]	-1.47	-1.43	-1.73	-1.55	1.83	1.88	0.56	0.55	1.07	0.92	0.36	0.49	2.39	1.99	2.13	2.51	2.81	3.33	3.61	2.57
P21731	Thromboxane A2 receptor OS=Homo sapiens GN=TBXA2R PE=1 SV=3 - [TA2R_HUMAN]	-1.62	-1.76	-1.51	-1.65	0.18	0.04	-0.23	-0.37	-0.07	0.07	0.06	-0.08	1.45	1.69	1.57	1.73	1.62	1.78	1.67	1.35
P04216	Thy-1 membrane glycoprotein OS=Homo sapiens GN=THY1 PE=1 SV=2 - [THY1_HUMAN]	1.52	1.61	1.79	1.88	0.15	0.24	-0.17	-0.07	-0.12	-0.21	-0.06	0.03	-1.63	-1.57	-1.85	-1.70	-1.97	-1.38	-1.65	-1.89
Q96MW7	Tigger transposable element-derived protein 1 OS=Homo sapiens GN=TIGD1 PE=1 SV=1 - [TIGD1_HUMAN]	-2.71	-2.62	-2.32	-2.24	-0.01	0.07	-0.66	-0.57	-0.56	-0.65	-0.24	-0.16	2.11	2.47	2.08	2.09	1.71	2.68	2.29	1.73
Q07157	Tight junction protein ZO-1 OS=Homo sapiens GN=TJP1 PE=1 SV=3 - [ZO1_HUMAN]	-1.29	-1.47	-1.29	-1.43	-0.03	-0.01	-0.27	-0.33	-0.10	-0.10	0.25	0.13	0.98	1.48	1.57	1.15	1.26	1.16	1.35	1.14
Q15025	TNFAIP3-interacting protein 1 OS=Homo sapiens GN=TNIP1 PE=1 SV=2 - [TNIP1_HUMAN]	2.03	2.21	1.92	2.10	0.32	0.49	0.53	0.71	0.37	0.20	0.13	0.30	-1.45	-1.90	-1.80	-1.80	-1.69	-1.73	-1.62	-1.33
Q6ZVM7	TOM1-like protein 2 OS=Homo sapiens GN=TOM1L2 PE=1 SV=1 - [TM1L2_HUMAN]	1.28	1.43	1.37	1.40	0.08	0.21	0.26	0.29	0.05	0.06	-0.08	-0.06	-0.97	-1.36	-1.32	-1.29	-1.27	-1.25	-1.21	-1.03
Q9UKE5	TRAF2 and NCK-interacting protein kinase OS=Homo sapiens GN=TNIK PE=1 SV=1 - [TNIK_HUMAN]	1.48	1.56	1.40	1.40	-0.04	0.10	0.15	0.23	0.00	0.04	-0.03	0.11	-1.27	-1.55	-1.28	-1.30	-1.36	-1.70	-1.32	-1.12

P20062	Transcobalamin-2 OS=Homo sapiens GN=TCN2 PE=1 SV=3 - [TCO2_HUMAN]	-2.00	-2.14	-2.07	-2.21	0.53	0.38	-0.08	-0.22	0.14	0.29	0.27	0.12	1.97	2.27	2.33	2.31	2.38	2.51	2.58	2.05
O43294	Transforming growth factor beta-1-induced transcript 1 protein OS=Homo sapiens GN=TGFB1I1 PE=1 SV=2 - [TGFI1_HUMAN]	-2.57	-2.50	-2.68	-2.68	-1.19	-1.12	-1.20	-1.15	-1.10	-1.11	-0.87	-0.92	1.57	1.62	1.75	1.48	1.62	1.44	1.46	1.44
Q15582	Transforming growth factor-beta-induced protein ig-h3 OS=Homo sapiens GN=TGFBI PE=1 SV=1 - [BGH3_HUMAN]	-2.05	-1.84	-2.02	-2.06	1.22	1.29	0.21	0.27	0.48	0.48	0.31	0.34	2.09	2.08	2.05	2.25	2.43	2.87	2.91	2.18
Q01995	Transgelin OS=Homo sapiens GN=TAGLN PE=1 SV=4 - [TAGL_HUMAN]	-2.94	-2.90	-3.19	-3.09	-1.42	-1.35	-1.78	-1.84	-1.52	-1.63	-1.35	-1.38	1.32	1.53	1.68	1.36	1.50	1.57	1.70	1.36
Q9UI15	Transgelin-3 OS=Homo sapiens GN=TAGLN3 PE=1 SV=2 - [TAGL3_HUMAN]	1.35	1.38	1.57	1.61	0.16	0.15	-0.12	-0.15	-0.17	-0.22	-0.43	-0.45	-1.27	-1.70	-1.97	-1.50	-1.80	-1.26	-1.51	-1.64
P30536	Translocator protein OS=Homo sapiens GN=TSPO PE=1 SV=3 - [TSPOA_HUMAN]	-2.73	-2.64	-2.64	-2.55	-0.16	-0.08	-0.58	-0.49	-0.42	-0.51	-0.25	-0.16	2.20	2.48	2.39	2.25	2.16	2.55	2.46	2.12
P51571	Translocon-associated protein subunit delta OS=Homo sapiens GN=SSR4 PE=1 SV=1 - [SSRD_HUMAN]	-1.55	-1.41	-1.46	-1.32	0.34	0.11	-0.57	-0.43	-0.13	-0.26	-0.20	-0.06	0.98	1.12	1.27	1.32	1.23	1.51	1.42	0.96
Q14956	Transmembrane glycoprotein NMB OS=Homo sapiens GN=GPNMB PE=1 SV=2 - [GPNMB_HUMAN]	-1.81	-1.65	-1.62	-1.45	0.46	0.62	0.83	1.00	0.82	0.66	1.57	1.73	2.70	3.39	3.18	2.50	2.31	2.25	2.06	2.51
Q8TC26	Transmembrane protein 163 OS=Homo sapiens GN=TMEM163 PE=2 SV=1 - [TM163_HUMAN]	1.69	1.58	1.76	1.56	-0.22	-0.26	0.28	0.39	0.13	-0.14	-0.36	-0.20	-1.24	-2.04	-1.94	-1.78	-1.64	-2.07	-2.13	-1.11

P02766	Transthyretin OS=Homo sapiens GN=TTR PE=1 SV=1 - [TTHY_HUMAN]	-1.26	-1.17	-2.18	-2.13	0.47	0.65	0.43	0.37	0.35	0.44	0.23	0.22	1.59	1.48	2.44	1.89	2.65	1.80	2.77	2.57
P53007	Tricarboxylate transport protein, mitochondrial OS=Homo sapiens GN=SLC25A1 PE=1 SV=2 - [TXTP_HUMAN]	-1.94	-1.86	-1.91	-1.83	-0.03	-0.02	0.07	0.13	-0.06	-0.11	-0.02	-0.01	2.07	1.69	1.74	1.78	1.82	1.67	1.71	2.01
Q9H6F2	Trimeric intracellular cation channel type A OS=Homo sapiens GN=TMEM38A PE=1 SV=1 - [TM38A_HUMAN]	1.42	1.44	1.86	1.88	-0.35	-0.33	0.07	0.09	-0.16	-0.18	-0.38	-0.37	-1.29	-1.80	-2.24	-1.56	-2.00	-1.78	-2.22	-1.73
Q9H2D6	TRIO and F-actin-binding protein OS=Homo sapiens GN=TRIOBP PE=1 SV=3 - [TARA_HUMAN]	-1.64	-1.58	-1.89	-1.79	-0.31	-0.28	-0.67	-0.68	-0.33	-0.34	-0.11	-0.12	0.92	1.61	1.67	1.19	1.49	1.42	1.43	1.23
P60174	Triosephosphate isomerase OS=Homo sapiens GN=TPI1 PE=1 SV=3 - [TPIS_HUMAN]	1.30	1.24	1.66	1.61	-0.13	-0.18	0.26	0.23	-0.11	-0.11	-0.50	-0.52	-0.87	-1.72	-2.06	-1.27	-1.65	-1.37	-1.62	-1.23
Q9C040	Tripartite motif-containing protein 2 OS=Homo sapiens GN=TRIM2 PE=1 SV=1 - [TRIM2_HUMAN]	1.65	1.66	1.75	1.79	0.13	0.22	0.45	0.77	0.46	0.38	-0.01	0.18	-0.86	-1.58	-1.69	-1.21	-1.35	-1.09	-1.30	-1.18
Q96LD4	Tripartite motif-containing protein 47 OS=Homo sapiens GN=TRIM47 PE=1 SV=2 - [TRI47_HUMAN]	-1.02	-0.91	-1.38	-1.25	1.20	1.33	0.19	0.52	0.79	0.67	0.63	0.88	1.11	1.50	1.99	1.34	2.08	1.72	2.51	1.74
Q9NZR1	Tropomodulin-2 OS=Homo sapiens GN=TMOD2 PE=1 SV=1 - [TMOD2_HUMAN]	1.63	1.52	1.91	1.79	0.16	0.02	0.75	0.65	0.35	0.51	0.14	0.08	-0.75	-1.50	-1.80	-1.18	-1.67	-1.46	-1.73	-1.08
P09493	Tropomyosin alpha-1 chain OS=Homo sapiens GN=TPM1 PE=1 SV=2 - [TPM1_HUMAN]	-2.45	-2.57	-2.53	-2.49	-1.26	-1.13	-1.56	-1.47	-1.17	-1.35	-0.94	-0.86	1.14	1.74	1.65	1.37	1.33	1.47	1.35	1.09

Q15714	TSC22 domain family protein 1 OS=Homo sapiens GN=TSC22D1 PE=1 SV=3 - [T22D1_HUMAN]	1.27	1.48	1.46	1.34	0.23	-0.24	0.57	0.07	0.08	0.00	-0.40	-0.17	-1.21	-1.51	-1.66	-1.36	-1.25	-1.46	-1.37	-1.22
Q9NY65	Tubulin alpha-8 chain OS=Homo sapiens GN=TUBA8 PE=1 SV=1 - [TBA8_HUMAN]	2.22	2.42	2.53	2.71	-0.06	-0.19	0.64	1.01	0.57	0.42	0.00	0.16	-1.61	-2.18	-2.49	-1.87	-2.13	-2.59	-3.12	-1.75
Q9H4B7	Tubulin beta-1 chain OS=Homo sapiens GN=TUBB1 PE=1 SV=1 - [TBB1_HUMAN]	-0.81	-0.52	-1.01	-0.79	0.46	0.63	0.04	0.37	0.86	0.63	0.83	1.31	0.93	1.76	2.03	1.44	1.71	1.30	1.57	1.21
Q13885	Tubulin beta-2A chain OS=Homo sapiens GN=TUBB2A PE=1 SV=1 - [TBB2A_HUMAN]	1.34	1.52	1.86	1.76	-0.41	-0.48	0.12	0.22	-0.12	-0.05	-0.14	-0.18	-1.12	-1.24	-1.89	-1.38	-1.73	-1.68	-1.71	-1.46
Q13509	Tubulin beta-3 chain OS=Homo sapiens GN=TUBB3 PE=1 SV=2 - [TBB3_HUMAN]	1.31	1.39	1.77	1.79	-0.35	-0.36	0.32	0.37	0.20	0.07	-0.08	0.18	-1.09	-1.51	-1.77	-1.41	-1.66	-1.87	-2.04	-1.27
P04350	Tubulin beta-4A chain OS=Homo sapiens GN=TUBB4A PE=1 SV=2 - [TBB4A_HUMAN]	2.18	2.14	2.28	2.23	-0.14	-0.18	0.75	0.55	0.30	0.45	0.44	0.29	-1.43	-1.87	-2.10	-1.79	-1.87	-2.23	-2.43	-1.49
O94811	Tubulin polymerization promoting protein OS=Homo sapiens GN=TPPP PE=1 SV=1 - [TPPP_HUMAN]	1.68	1.80	1.77	1.96	0.03	-0.03	0.12	0.10	0.06	-0.01	-0.05	0.04	-1.54	-1.81	-2.00	-1.77	-1.74	-1.80	-1.76	-1.84
O75347	Tubulin-specific chaperone A OS=Homo sapiens GN=TBCA PE=1 SV=3 - [TBCA_HUMAN]	0.96	1.04	1.15	1.11	-0.59	-0.53	-0.37	-0.29	-0.40	-0.47	-1.08	-1.03	-1.22	-1.89	-2.20	-1.56	-1.57	-1.65	-1.70	-1.35
O95407	Tumor necrosis factor receptor superfamily member 6B OS=Homo sapiens GN=TNFRSF6B PE=1 SV=1 - [TNF6B_HUMAN]	0.03	-0.11	-0.28	-0.41	3.38	3.24	1.44	1.31	2.20	2.34	1.79	1.66	1.47	1.77	2.07	2.34	2.65	3.34	3.64	1.78

Q96PE3	Type I inositol 3,4-bisphosphate 4-phosphatase OS=Homo sapiens GN=INPP4A PE=1 SV=1 - [INP4A_HUMAN]	1.37	1.01	1.24	0.80	-0.20	-0.10	0.50	0.24	0.06	0.17	-0.21	-0.27	-0.99	-1.52	-1.38	-1.23	-0.98	-1.46	-1.31	-0.80
P54829	Tyrosine-protein phosphatase non-receptor type 5 OS=Homo sapiens GN=PTPN5 PE=1 SV=4 - [PTN5_HUMAN]	2.15	2.30	2.11	2.26	-0.14	0.01	0.61	0.76	0.47	0.32	-0.03	0.11	-1.49	-2.18	-2.14	-1.80	-1.75	-2.30	-2.26	-1.44
P78324	Tyrosine-protein phosphatase non-receptor type substrate 1 OS=Homo sapiens GN=SIRPA PE=1 SV=2 - [SHPS1_HUMAN]	1.61	1.52	1.80	1.71	0.07	-0.02	0.69	0.77	0.33	0.46	0.08	0.14	-0.78	-1.53	-1.66	-1.18	-1.31	-1.49	-1.57	-0.89
A6NCW0	Ubiquitin carboxyl-terminal hydrolase 17-like protein 3 OS=Homo sapiens GN=USP17L3 PE=3 SV=1 - [U17L3_HUMAN]	-1.76	-1.75	-1.79	-1.74	0.16	0.25	-0.12	-0.02	-0.21	-0.31	-0.16	-0.20	1.72	1.54	1.51	1.54	1.59	1.94	1.98	1.78
P09936	Ubiquitin carboxyl-terminal hydrolase isozyme L1 OS=Homo sapiens GN=UCHL1 PE=1 SV=2 - [UCHL1_HUMAN]	1.72	1.80	2.15	2.21	-0.10	-0.04	0.76	0.80	0.27	0.26	0.08	0.05	-0.99	-1.80	-2.11	-1.57	-1.89	-1.96	-2.30	-1.36
P68036	Ubiquitin-conjugating enzyme E2 L3 OS=Homo sapiens GN=UBE2L3 PE=1 SV=1 - [UB2L3_HUMAN]	0.65	0.70	1.03	0.97	-0.43	-0.26	-0.45	-0.33	-0.56	-0.57	-0.98	-0.81	-1.02	-1.57	-1.68	-1.34	-1.61	-1.40	-1.48	-1.37
Q96S82	Ubiquitin-like protein 7 OS=Homo sapiens GN=UBL7 PE=1 SV=2 - [UBL7_HUMAN]	1.41	1.25	1.34	1.19	0.12	-0.04	0.43	0.28	0.11	0.27	0.29	0.13	-0.92	-1.11	-1.06	-1.11	-1.04	-1.31	-1.25	-0.85
C9J069	Uncharacterized protein C9orf172 OS=Homo sapiens GN=C9orf172 PE=3 SV=1 - [CI172_HUMAN]	2.51	2.43	2.57	2.49	0.49	0.41	0.90	0.83	0.56	0.64	0.60	0.52	-1.55	-1.90	-1.97	-1.84	-1.89	-2.03	-2.09	-1.60

Q69YL0	Uncharacterized protein DKFZp762I1415 OS=Homo sapiens PE=4 SV=1 - [YC029_HUMAN]	-3.94	-4.27	-4.00	-4.33	-2.11	-2.44	-2.70	-3.03	-2.83	-2.50	-2.59	-2.92	1.29	1.36	1.41	1.47	1.54	1.82	1.87	1.36
O60268	Uncharacterized protein KIAA0513 OS=Homo sapiens GN=KIAA0513 PE=2 SV=1 - [K0513_HUMAN]	1.49	1.51	1.77	1.74	-0.39	-0.36	0.49	0.42	-0.09	-0.10	-0.08	-0.15	-0.96	-1.65	-1.98	-1.45	-1.81	-2.02	-2.33	-1.14
Q9UPP5	Uncharacterized protein KIAA1107 OS=Homo sapiens GN=KIAA1107 PE=1 SV=2 - [K1107_HUMAN]	1.76	1.69	1.74	1.67	0.33	0.30	0.05	0.16	0.18	0.21	0.43	0.53	-1.42	-1.43	-1.36	-1.65	-1.62	-1.40	-1.38	-1.56
Q6NV74	Uncharacterized protein KIAA1211-like OS=Homo sapiens GN=KIAA1211L PE=2 SV=3 - [K121L_HUMAN]	1.96	2.05	2.12	2.37	-0.06	-0.03	0.26	0.47	-0.28	-0.07	-0.21	-0.17	-1.42	-2.14	-2.47	-2.25	-2.55	-2.13	-2.35	-1.84
O00159	Unconventional myosin-Ic OS=Homo sapiens GN=MYO1C PE=1 SV=4 - [MYO1C_HUMAN]	-2.44	-2.35	-2.44	-2.36	-0.42	-0.44	-0.71	-0.76	-0.55	-0.58	-0.53	-0.59	1.63	1.88	1.92	1.81	1.78	1.93	1.85	1.65
Q9Y4I1	Unconventional myosin-Va OS=Homo sapiens GN=MYO5A PE=1 SV=2 - [MYO5A_HUMAN]	1.63	1.73	1.79	1.86	-0.14	-0.10	0.54	0.63	0.20	0.23	0.02	0.08	-1.07	-1.67	-1.84	-1.38	-1.67	-1.82	-2.04	-1.26
Q9BSU1	UPF0183 protein C16orf70 OS=Homo sapiens GN=C16orf70 PE=1 SV=1 - [CP070_HUMAN]	1.23	1.33	1.40	1.50	-0.38	-0.29	0.43	0.53	0.34	0.25	0.16	0.25	-0.75	-1.07	-1.25	-0.95	-1.13	-1.63	-1.81	-0.91
Q9BUW7	UPF0184 protein C9orf16 OS=Homo sapiens GN=C9orf16 PE=1 SV=1 - [CI016_HUMAN]	2.17	1.83	2.05	1.71	0.68	0.33	1.00	0.65	0.34	0.69	0.57	0.22	-1.12	-1.60	-1.49	-1.46	-1.33	-1.51	-1.39	-0.99
Q7Z3D6	UPF0317 protein C14orf159, mitochondrial OS=Homo sapiens GN=C14orf159 PE=1 SV=2 - [CN159_HUMAN]	0.58	0.56	0.56	0.53	-1.06	-1.10	-0.91	-0.75	-1.16	-1.00	-1.66	-1.79	-1.24	-2.07	-2.09	-1.60	-1.66	-1.44	-1.59	-1.29

Q9H993	UPF0364 protein C6orf211 OS=Homo sapiens GN=C6orf211 PE=1 SV=1 - [CF211_HUMAN]	1.99	2.05	2.18	2.25	0.40	0.46	1.04	1.10	0.54	0.48	0.27	0.33	-0.89	-1.71	-1.92	-1.48	-1.68	-1.61	-1.81	-1.09
Q5T6V5	UPF0553 protein C9orf64 OS=Homo sapiens GN=C9orf64 PE=1 SV=1 - [CI064_HUMAN]	1.12	1.08	0.98	0.94	-0.27	-0.32	0.06	0.02	-0.27	-0.23	-0.23	-0.28	-1.01	-1.34	-1.21	-1.32	-1.18	-1.41	-1.28	-0.86
Q6UX73	UPF0764 protein C16orf89 OS=Homo sapiens GN=C16orf89 PE=1 SV=2 - [CP089_HUMAN]	-1.24	-2.01	-0.70	-1.06	2.58	2.19	0.44	-0.10	0.55	0.80	1.12	0.70	1.74	2.36	1.82	2.08	1.54	3.80	3.26	1.20
Q92738	USP6 N-terminal-like protein OS=Homo sapiens GN=USP6NL PE=1 SV=3 - [US6NL_HUMAN]	-1.75	-2.10	-1.73	-2.07	-0.36	-0.71	-0.47	-0.81	-0.68	-0.33	-0.49	-0.84	1.23	1.27	1.23	1.45	1.43	1.38	1.35	1.24
P46939	Utrophin OS=Homo sapiens GN=UTRN PE=1 SV=2 - [UTRO_HUMAN]	-1.70	-1.75	-1.76	-1.82	-0.49	-0.52	-0.69	-0.71	-0.62	-0.57	-0.40	-0.43	1.02	1.29	1.44	1.17	1.26	1.15	1.25	1.17
Q9BZF9	Uveal autoantigen with coiled-coil domains and ankyrin repeats OS=Homo sapiens GN=UACA PE=1 SV=2 - [UACA_HUMAN]	-2.10	-2.08	-1.89	-1.94	-0.04	-0.11	-0.43	-0.43	-0.32	-0.27	-0.15	-0.17	1.66	1.90	1.75	1.79	1.61	1.90	1.80	1.59
Q93050	V-type proton ATPase 116 kDa subunit a isoform 1 OS=Homo sapiens GN=ATP6V0A1 PE=2 SV=3 - [VPP1_HUMAN]	1.55	1.52	1.59	1.65	-0.22	-0.06	0.56	0.56	0.32	0.21	0.12	0.05	-0.88	-1.32	-1.55	-1.21	-1.33	-1.61	-1.67	-0.95
P38606	V-type proton ATPase catalytic subunit A OS=Homo sapiens GN=ATP6V1A PE=1 SV=2 - [VATA_HUMAN]	1.54	1.62	1.64	1.74	0.15	0.16	0.67	0.72	0.41	0.43	0.26	0.26	-0.79	-1.22	-1.46	-1.12	-1.23	-1.52	-1.66	-0.92

P21281	V-type proton ATPase subunit B, brain isoform OS=Homo sapiens GN=ATP6V1B2 PE=1 SV=3 - [VATB2_HUMAN]	1.62	1.77	1.96	1.91	0.04	0.04	0.67	0.74	0.37	0.40	0.23	0.19	-1.07	-1.58	-1.77	-1.45	-1.57	-1.73	-2.05	-1.17
Q9Y5K8	V-type proton ATPase subunit D OS=Homo sapiens GN=ATP6V1D PE=1 SV=1 - [VATD_HUMAN]	1.46	1.40	1.44	1.35	0.13	0.15	0.66	0.64	0.56	0.31	0.08	0.45	-0.97	-1.59	-1.31	-1.15	-1.10	-1.62	-1.53	-0.82
P36543	V-type proton ATPase subunit E 1 OS=Homo sapiens GN=ATP6V1E1 PE=1 SV=1 - [VATE1_HUMAN]	1.59	1.48	1.80	1.67	-0.06	-0.01	0.70	0.65	0.31	0.37	0.28	0.22	-0.85	-1.30	-1.48	-1.15	-1.36	-1.64	-1.83	-0.93
Q16864	V-type proton ATPase subunit F OS=Homo sapiens GN=ATP6V1F PE=1 SV=2 - [VATF_HUMAN]	1.82	1.40	1.90	1.84	-0.13	0.02	0.69	0.54	0.46	0.23	0.19	0.30	-0.96	-1.09	-1.53	-1.07	-1.50	-1.41	-1.85	-1.09
O95670	V-type proton ATPase subunit G 2 OS=Homo sapiens GN=ATP6V1G2 PE=1 SV=1 - [VATG2_HUMAN]	2.88	2.40	2.31	2.11	-0.13	-0.26	0.87	0.38	0.38	0.54	0.54	0.03	-1.79	-2.35	-2.08	-2.32	-1.89	-1.81	-1.25	-1.21
P19320	Vascular cell adhesion protein 1 OS=Homo sapiens GN=VCAM1 PE=1 SV=1 - [VCAM1_HUMAN]	-1.08	-1.11	-1.49	-1.33	0.04	0.14	-0.44	-0.38	0.05	-0.09	0.65	0.73	0.93	2.09	2.12	1.28	1.34	1.22	1.39	1.02
P17948	Vascular endothelial growth factor receptor 1 OS=Homo sapiens GN=FLT1 PE=1 SV=2 - [VGFR1_HUMAN]	-0.38	-0.69	-0.57	-0.72	1.88	1.71	0.44	0.24	0.84	0.99	0.61	0.44	0.88	1.24	1.32	1.53	1.55	2.56	2.34	1.07
P35968	Vascular endothelial growth factor receptor 2 OS=Homo sapiens GN=KDR PE=1 SV=2 - [VGFR2_HUMAN]	-2.17	-1.53	-2.51	-2.35	0.25	0.41	0.19	0.42	0.15	0.02	0.03	0.25	2.12	1.91	1.98	1.92	2.30	2.40	2.74	2.73

P13611	Versican core protein OS=Homo sapiens GN=VCAN PE=1 SV=3 - [CSPG2_HUMAN]	1.43	1.61	1.60	1.86	0.32	0.42	0.55	0.75	0.49	0.38	0.39	0.39	-1.10	-1.35	-1.56	-1.26	-1.21	-1.29	-1.32	-1.19
P23763	Vesicle-associated membrane protein 1 OS=Homo sapiens GN=VAMP1 PE=1 SV=1 - [VAMP1_HUMAN]	2.52	2.88	2.90	3.04	0.14	0.37	1.50	2.06	1.16	1.00	0.82	1.20	-0.93	-1.75	-1.88	-1.49	-1.85	-2.79	-2.68	-0.85
O95183	Vesicle-associated membrane protein 5 OS=Homo sapiens GN=VAMP5 PE=1 SV=1 - [VAMP5_HUMAN]	-1.37	-1.48	-1.64	-1.63	0.21	0.32	-0.24	-0.14	0.13	0.07	0.35	0.29	1.49	1.84	1.55	1.61	1.73	1.89	1.83	1.40
Q9BV40	Vesicle-associated membrane protein 8 OS=Homo sapiens GN=VAMP8 PE=1 SV=1 - [VAMP8_HUMAN]	-1.88	-1.65	-2.23	-1.99	0.22	0.45	-0.08	0.15	0.26	0.04	0.67	0.90	1.86	2.56	2.90	1.95	2.30	2.09	2.43	2.21
P46459	Vesicle-fusing ATPase OS=Homo sapiens GN=NSF PE=1 SV=3 - [NSF_HUMAN]	1.66	1.62	2.02	1.96	-0.23	-0.29	0.74	0.69	0.28	0.27	0.11	0.15	-0.85	-1.56	-1.85	-1.34	-1.59	-1.95	-2.31	-1.19
Q9P2U7	Vesicular glutamate transporter 1 OS=Homo sapiens GN=SLC17A7 PE=1 SV=1 - [VGLU1_HUMAN]	1.69	1.78	1.53	1.66	-0.38	-0.47	0.66	0.61	0.33	0.38	-0.11	-0.04	-0.83	-1.62	-1.82	-1.51	-1.30	-1.84	-1.85	-0.97
P08670	Vimentin OS=Homo sapiens GN=VIM PE=1 SV=4 - [VIME_HUMAN]	-2.39	-2.36	-2.61	-2.63	0.10	0.12	-0.34	-0.30	-0.18	-0.21	-0.20	-0.20	2.05	2.21	2.45	2.23	2.47	2.42	2.67	2.31
P18206	Vinculin OS=Homo sapiens GN=VCL PE=1 SV=4 - [VINC_HUMAN]	-2.32	-2.29	-2.45	-2.36	-0.56	-0.52	-0.89	-0.89	-0.67	-0.71	-0.44	-0.44	1.52	1.91	1.99	1.76	1.79	1.83	1.89	1.64
P62760	Visinin-like protein 1 OS=Homo sapiens GN=VSNL1 PE=1 SV=2 - [VISL1_HUMAN]	1.96	2.20	2.50	2.70	0.19	0.26	0.80	0.97	0.38	0.46	0.46	0.40	-0.97	-1.61	-2.10	-1.65	-2.06	-1.72	-2.21	-1.55
P02774	Vitamin D-binding protein OS=Homo sapiens GN=GC PE=1 SV=1 - [VTDB_HUMAN]	-2.23	-2.12	-2.07	-2.09	-0.53	-0.49	-0.73	-0.69	-0.79	-0.72	-0.79	-0.80	1.45	1.27	1.25	1.44	1.30	1.54	1.43	1.46

Q9BQB6	Vitamin K epoxide reductase complex subunit 1 OS=Homo sapiens GN=VKORC1 PE=1 SV=1 - [VKOR1_HUMAN]	-1.74	-1.59	-2.16	-2.01	0.19	0.34	-0.56	-0.41	-0.09	-0.24	-0.07	0.08	1.24	1.68	2.09	1.53	1.96	1.91	2.33	1.67
P07225	Vitamin K-dependent protein S OS=Homo sapiens GN=PROS1 PE=1 SV=1 - [PROS_HUMAN]	-2.30	-2.19	-2.35	-2.26	-0.25	-0.20	-0.66	-0.55	-0.15	-0.20	0.50	0.59	1.71	2.80	2.86	2.08	2.12	2.03	1.94	1.78
P04004	Vitronectin OS=Homo sapiens GN=VTN PE=1 SV=1 - [VTNC_HUMAN]	-1.72	-1.66	-1.74	-1.68	1.83	1.88	1.19	1.17	1.19	1.19	0.69	0.62	2.58	2.42	2.47	2.81	2.96	3.44	3.43	2.84
Q02641	Voltage-dependent L-type calcium channel subunit beta-1 OS=Homo sapiens GN=CACNB1 PE=1 SV=3 - [CACB1_HUMAN]	1.89	1.52	2.07	1.70	0.21	-0.17	0.80	0.43	0.30	0.67	0.58	0.21	-1.04	-1.31	-1.49	-1.19	-1.36	-1.70	-1.88	-1.20
O00305	Voltage-dependent L-type calcium channel subunit beta-4 OS=Homo sapiens GN=CACNB4 PE=1 SV=2 - [CACB4_HUMAN]	1.34	1.56	1.60	1.82	-0.32	-0.13	0.50	0.51	0.29	0.05	-0.13	0.01	-0.82	-1.30	-1.69	-1.08	-1.46	-1.72	-1.99	-1.07
Q00975	Voltage-dependent N-type calcium channel subunit alpha-1B OS=Homo sapiens GN=CACNA1B PE=1 SV=1 [CAC1B_HUMAN]	1.98	1.92	1.80	1.73	0.38	0.30	0.73	0.57	0.62	0.78	0.42	0.28	-1.35	-1.68	-1.79	-1.32	-1.26	-1.67	-1.61	-1.30
O00555	Voltage-dependent P/Q-type calcium channel subunit alpha-1A OS=Homo sapiens GN=CACNA1A PE=1 SV=2 - [CAC1A_HUMAN]	1.15	1.11	1.12	1.10	-0.45	-0.45	-0.26	-0.26	-0.52	-0.52	-0.12	-0.12	-1.40	-1.35	-1.25	-1.60	-1.59	-1.58	-1.57	-1.30

Q15878	Voltage-dependent R-type calcium channel subunit alpha-1E OS=Homo sapiens GN=CACNA1E PE=1 SV=3 [CAC1E_HUMAN]	1.67	1.53	1.60	1.50	-0.24	-0.12	0.24	0.21	0.11	0.12	0.33	0.47	-1.29	-1.31	-1.09	-1.26	-1.21	-1.40	-1.54	-1.14
Q13303	Voltage-gated potassium channel subunit beta-2 OS=Homo sapiens GN=KCNAB2 PE=1 SV=2 - [KCAB2_HUMAN]	1.69	1.68	1.56	1.45	-0.32	-0.20	0.37	0.35	0.12	0.06	-0.29	-0.31	-1.38	-2.23	-2.07	-1.64	-1.57	-2.20	-2.13	-1.26
P04275	von Willebrand factor OS=Homo sapiens GN=VWF PE=1 SV=4 - [VWF_HUMAN]	-2.36	-2.28	-2.47	-2.36	-0.75	-0.74	-1.13	-1.10	-1.04	-1.00	-0.89	-0.89	1.16	1.26	1.46	1.19	1.35	1.35	1.57	1.32
Q96PQ0	VPS10 domain-containing receptor SorCS2 OS=Homo sapiens GN=SORCS2 PE=1 SV=3 - [SORC2_HUMAN]	2.12	1.83	1.76	1.48	0.46	0.17	0.64	0.36	0.23	0.52	0.43	0.14	-1.42	-1.68	-1.33	-1.57	-1.20	-1.67	-1.32	-1.06
Q5VU97	VWFA and cache domain-containing protein 1 OS=Homo sapiens GN=CACHD1 PE=2 SV=2 - [CAHD1_HUMAN]	-1.18	-0.90	-1.27	-0.99	0.33	0.60	-0.08	0.20	0.49	0.22	0.16	0.43	1.15	1.34	1.43	1.43	1.52	1.49	1.58	1.25
Q92558	Wiskott-Aldrich syndrome protein family member 1 OS=Homo sapiens GN=WASF1 PE=1 SV=1 - [WASF1_HUMAN]	2.34	2.62	2.29	2.52	-0.25	-0.08	0.77	1.06	0.52	0.38	0.10	0.24	-1.51	-2.48	-2.33	-1.85	-1.80	-2.61	-2.56	-1.39
Q96H79	Zinc finger CCCH-type antiviral protein 1-like OS=Homo sapiens GN=ZC3HAV1L PE=1 SV=2 - [ZCCHL_HUMAN]	-3.04	-2.66	-2.55	-2.17	-0.97	-0.60	-0.81	-0.43	-0.62	-0.99	-0.68	-0.30	2.29	2.37	1.87	2.08	1.59	2.05	1.56	1.81

Q8NEK5	Zinc finger protein 548 OS=Homo sapiens GN=ZNF548 PE=2 SV=2 - [ZN548_HUMAN]	-1.96	-2.16	-1.41	-1.60	0.33	0.13	0.14	-0.06	-0.57	-0.36	0.45	0.24	2.16	2.42	1.85	1.63	1.08	2.28	1.72	1.61
Q8N4Q0	Zinc-binding alcohol dehydrogenase domain-containing protein 2 OS=Homo sapiens GN=ZADH2 PE=1 SV=1 - [ZADH2_HUMAN]	0.89	0.70	1.01	0.82	-0.84	-1.04	-0.22	-0.41	-0.49	-0.29	-0.42	-0.62	-1.06	-1.31	-1.43	-1.16	-1.27	-1.75	-1.87	-1.16
